# Measuring the health-related Sustainable Development Goals in 188 countries: a baseline analysis from the Global Burden of Disease Study 2015

**DOI:** 10.1016/S0140-6736(16)31467-2

**Published:** 2016-10-08

**Authors:** GBD 2015 SDG Collaborators, Stephen S Lim, Kate Allen, Zulfiqar A Bhutta, Lalit Dandona, Mohammad H Forouzanfar, Nancy Fullman, Peter W Gething, Ellen M Goldberg, Simon I Hay, Mollie Holmberg, Yohannes Kinfu, Michael J Kutz, Heidi J Larson, Xiaofeng Liang, Alan D Lopez, Rafael Lozano, Claire R McNellan, Ali H Mokdad, Meghan D Mooney, Mohsen Naghavi, Helen E Olsen, David M Pigott, Joshua A Salomon, Theo Vos, Haidong Wang, Amanuel Alemu Abajobir, Kalkidan Hassen Abate, Cristiana Abbafati, Kaja M Abbas, Foad Abd-Allah, Abdishakur M Abdulle, Biju Abraham, Ibrahim Abubakar, Laith J Abu-Raddad, Niveen M E Abu-Rmeileh, Gebre Yitayih Abyu, Tom Achoki, Akindele Olupelumi Adebiyi, Isaac Akinkunmi Adedeji, Kossivi Agbelenko Afanvi, Ashkan Afshin, Arnav Agarwal, Anurag Agrawal, Aliasghar Ahmad Kiadaliri, Hamid Ahmadieh, Kedir Yimam Ahmed, Ali Shafqat Akanda, Rufus Olusola Akinyemi, Tomi F Akinyemiju, Nadia Akseer, Ziyad Al-Aly, Khurshid Alam, Uzma Alam, Deena Alasfoor, Fadia S AlBuhairan, Saleh Fahed Aldhahri, Robert William Aldridge, Zewdie Aderaw Alemu, Raghib Ali, Ala'a Alkerwi, Mohammad AB Alkhateeb, François Alla, Peter Allebeck, Christine Allen, Rajaa Al-Raddadi, Khalid A Altirkawi, Elena Alvarez Martin, Nelson Alvis-Guzman, Azmeraw T Amare, Alemayehu Amberbir, Adeladza Kofi Amegah, Heresh Amini, Walid Ammar, Stephen Marc Amrock, Hjalte H Andersen, Benjamin O Anderson, Gregory M Anderson, Carl Abelardo T Antonio, Palwasha Anwari, Johan Ärnlöv, Al Artaman, Hamid Asayesh, Rana Jawad Asghar, Suleman Atique, Euripide Frinel G Arthur Avokpaho, Ashish Awasthi, Beatriz Paulina Ayala Quintanilla, Peter Azzopardi, Umar Bacha, Alaa Badawi, Kalpana Balakrishnan, Amitava Banerjee, Aleksandra Barac, Ryan Barber, Suzanne L Barker-Collo, Till Bärnighausen, Lope H Barrero, Tonatiuh Barrientos-Gutierrez, Sanjay Basu, Tigist Assefa Bayou, Shahrzad Bazargan-Hejazi, Justin Beardsley, Neeraj Bedi, Ettore Beghi, Yannick Béjot, Michelle L Bell, Aminu K Bello, Derrick A Bennett, Isabela M Bensenor, Habib Benzian, Adugnaw Berhane, Eduardo Bernabé, Oscar Alberto Bernal, Balem Demtsu Betsu, Addisu Shunu Beyene, Neeraj Bhala, Samir Bhatt, Sibhatu Biadgilign, Kelly A Bienhoff, Boris Bikbov, Agnes Binagwaho, Donal Bisanzio, Espen Bjertness, Jed Blore, Rupert R A Bourne, Michael Brainin, Michael Brauer, Alexandra Brazinova, Nicholas J K Breitborde, David M Broday, Traolach S Brugha, Rachelle Buchbinder, Zahid A Butt, Leah E Cahill, Ismael Ricardo Campos-Nonato, Julio Cesar Campuzano, Hélène Carabin, Rosario Cárdenas, Juan Jesus Carrero, Austin Carter, Daniel Casey, Valeria Caso, Carlos A Castañeda-Orjuela, Jacqueline Castillo Rivas, Ferrán Catalá-López, Fiorella Cavalleri, Pedro Cecílio, Hsing-Yi Chang, Jung-Chen Chang, Fiona J Charlson, Xuan Che, Alan Zian Chen, Peggy Pei-Chia Chiang, Mirriam Chibalabala, Vesper Hichilombwe Chisumpa, Jee-Young Jasmine Choi, Rajiv Chowdhury, Hanne Christensen, Liliana G Ciobanu, Massimo Cirillo, Matthew M Coates, Megan Coggeshall, Aaron J Cohen, Graham S Cooke, Cyrus Cooper, Leslie Trumbull Cooper, Benjamin C Cowie, John A Crump, Solomon Abrha Damtew, Rakhi Dandona, Paul I Dargan, José das Neves, Adrian C Davis, Kairat Davletov, E Filipa de Castro, Diego De Leo, Louisa Degenhardt, Liana C Del Gobbo, Kebede Deribe, Sarah Derrett, Don C Des Jarlais, Aniruddha Deshpande, Gabrielle A deVeber, Subhojit Dey, Samath D Dharmaratne, Preet K Dhillon, Eric L Ding, E Ray Dorsey, Kerrie E Doyle, Tim R Driscoll, Leilei Duan, Manisha Dubey, Bruce Bartholow Duncan, Hedyeh Ebrahimi, Aman Yesuf Endries, Sergey Petrovich Ermakov, Holly E Erskine, Babak Eshrati, Alireza Esteghamati, Saman Fahimi, Talha A Farid, Carla Sofia e Sa Farinha, André Faro, Maryam S Farvid, Farshad Farzadfar, Valery L Feigin, Manuela Mendonca Felicio, Seyed-Mohammad Fereshtehnejad, Jefferson G Fernandes, Joao C Fernandes, Alize J Ferrari, Florian Fischer, Joseph R A Fitchett, Christina Fitzmaurice, Nataliya Foigt, Kyle Foreman, F Gerry R Fowkes, Elisabeth Barboza Franca, Richard C Franklin, Maya Fraser, Joseph Friedman, Joseph Frostad, Thomas Fürst, Belinda Gabbe, Alberto L Garcia-Basteiro, Teshome Gebre, Tsegaye Tewelde Gebrehiwot, Amanuel Tesfay Gebremedhin, Alemseged Aregay Gebru, Bradford D Gessner, Richard F Gillum, Ibrahim Abdelmageem Mohamed Ginawi, Ababi Zergaw Giref, Maurice Giroud, Melkamu Dedefo Gishu, William Godwin, Philimon Gona, Amador Goodridge, Sameer Vali Gopalani, Carolyn C Gotay, Atsushi Goto, Hebe N Gouda, Nicholas Graetz, Karen Fern Greenwell, Max Griswold, Yuming Guo, Rahul Gupta, Rajeev Gupta, Vipin Gupta, Reyna A Gutiérrez, Bishal Gyawali, Juanita A Haagsma, Annie Haakenstad, Nima Hafezi-Nejad, Demewoz Haile, Gessessew Bugssa Hailu, Yara A Halasa, Randah Ribhi Hamadeh, Samer Hamidi, Mouhanad Hammami, Graeme J Hankey, Hilda L Harb, Josep Maria Haro, Mohammad Sadegh Hassanvand, Rasmus Havmoeller, Ileana Beatriz Heredia-Pi, Hans W Hoek, Masako Horino, Nobuyuki Horita, H Dean Hosgood, Damian G Hoy, Aung Soe Htet, Guoqing Hu, Hsiang Huang, Kim Moesgaard Iburg, Bulat T Idrisov, Manami Inoue, Farhad Islami, Troy A Jacobs, Kathryn H Jacobsen, Nader Jahanmehr, Mihajlo B Jakovljevic, Peter James, Henrica A F M Jansen, Mehdi Javanbakht, Achala Upendra Jayatilleke, Sun Ha Jee, Panniyammakal Jeemon, Vivekanand Jha, Ying Jiang, Tariku Jibat, Ye Jin, Jost B Jonas, Zubair Kabir, Yogeshwar Kalkonde, Ritul Kamal, Haidong Kan, Amit Kandel, André Karch, Corine Kakizi Karema, Chante Karimkhani, Palitha Karunapema, Amir Kasaeian, Nicholas J Kassebaum, Anil Kaul, Norito Kawakami, Jeanne Françoise Kayibanda, Peter Njenga Keiyoro, Laura Kemmer, Andrew Haddon Kemp, Andre Pascal Kengne, Andre Keren, Chandrasekharan Nair Kesavachandran, Yousef Saleh Khader, Abdur Rahman Khan, Ejaz Ahmad Khan, Gulfaraz Khan, Young-Ho Khang, Tawfik Ahmed Muthafer Khoja, Ardeshir Khosravi, Jagdish Khubchandani, Christian Kieling, Cho-il Kim, Daniel Kim, Sungroul Kim, Yun Jin Kim, Ruth W Kimokoti, Niranjan Kissoon, Miia Kivipelto, Luke D Knibbs, Yoshihiro Kokubo, Dhaval Kolte, Soewarta Kosen, Georgios A Kotsakis, Parvaiz A Koul, Ai Koyanagi, Michael Kravchenko, Hans Krueger, Barthelemy Kuate Defo, Ricardo S Kuchenbecker, Ernst J Kuipers, Xie Rachel Kulikoff, Veena S Kulkarni, G Anil Kumar, Gene F Kwan, Hmwe H Kyu, Aparna Lal, Dharmesh Kumar Lal, Ratilal Lalloo, Hilton Lam, Qing Lan, Sinead M Langan, Anders Larsson, Dennis Odai Laryea, Asma Abdul Latif, Janet L Leasher, James Leigh, Mall Leinsalu, Janni Leung, Ricky Leung, Miriam Levi, Yichong Li, Yongmei Li, Margaret Lind, Shai Linn, Steven E Lipshultz, Patrick Y Liu, Shiwei Liu, Yang Liu, Belinda K Lloyd, Loon-Tzian Lo, Giancarlo Logroscino, Paulo A Lotufo, Robyn M Lucas, Raimundas Lunevicius, Mohammed Magdy Abd El Razek, Carlos Magis-Rodriguez, Mahdi Mahdavi, Marek Majdan, Azeem Majeed, Reza Malekzadeh, Deborah Carvalho Malta, Chabila C Mapoma, David Joel Margolis, Randall V Martin, Jose Martinez-Raga, Felix Masiye, Amanda J Mason-Jones, João Massano, Richard Matzopoulos, Bongani M Mayosi, John J McGrath, Martin McKee, Peter A Meaney, Alem Mehari, Alemayehu B Mekonnen, Yohannes Adama Melaku, Peter Memiah, Ziad A Memish, Walter Mendoza, Gert B M Mensink, Atte Meretoja, Tuomo J Meretoja, Yonatan Moges Mesfin, Francis Apolinary Mhimbira, Renata Micha, Ted R Miller, Edward J Mills, Mojde Mirarefin, Awoke Misganaw, Philip B Mitchell, Charles N Mock, Alireza Mohammadi, Shafiu Mohammed, Lorenzo Monasta, Jonathan de la Cruz Monis, Julio Cesar Montañez Hernandez, Marcella Montico, Maziar Moradi-Lakeh, Lidia Morawska, Rintaro Mori, Ulrich O Mueller, Michele E Murdoch, Brighton Murimira, Joseph Murray, Gudlavalleti Venkata Satyanarayana Murthy, Srinivas Murthy, Kamarul Imran Musa, Jean B Nachega, Gabriele Nagel, Kovin S Naidoo, Luigi Naldi, Vinay Nangia, Bruce Neal, Chakib Nejjari, Charles R Newton, John N Newton, Frida Namnyak Ngalesoni, Peter Nguhiu, Grant Nguyen, Quyen Le Nguyen, Muhammad Imran Nisar, Patrick Martial Nkamedjie Pete, Sandra Nolte, Marika Nomura, Ole F Norheim, Bo Norrving, Carla Makhlouf Obermeyer, Felix Akpojene Ogbo, In-Hwan Oh, Olanrewaju Oladimeji, Pedro R Olivares, Bolajoko Olubukunola Olusanya, Jacob Olusegun Olusanya, John Nelson Opio, Eyal Oren, Alberto Ortiz, Richard H Osborne, Erika Ota, Mayowa O Owolabi, Mahesh PA, Eun-Kee Park, Hye-Youn Park, Charles D Parry, Mahboubeh Parsaeian, Tejas Patel, Vikram Patel, Angel J Paternina Caicedo, Snehal T Patil, Scott B Patten, George C Patton, Deepak Paudel, João Mário Pedro, David M Pereira, Norberto Perico, Konrad Pesudovs, Max Petzold, Michael Robert Phillips, Frédéric B Piel, Julian David Pillay, Christine Pinho, Farhad Pishgar, Suzanne Polinder, Richie G Poulton, Farshad Pourmalek, Mostafa Qorbani, Rynaz H S Rabiee, Amir Radfar, Vafa Rahimi-Movaghar, Mahfuzar Rahman, Mohammad Hifz Ur Rahman, Sajjad Ur Rahman, Rajesh Kumar Rai, Sasa Rajsic, Murugesan Raju, Usha Ram, Saleem M Rana, Chhabi Lal Ranabhat, Kavitha Ranganathan, Puja C Rao, Amany H Refaat, Marissa B Reitsma, Giuseppe Remuzzi, Serge Resnikoff, Antonio L Ribeiro, Maria Jesus Rios Blancas, Hirbo Shore Roba, Bayard Roberts, Alina Rodriguez, David Rojas-Rueda, Luca Ronfani, Gholamreza Roshandel, Gregory A Roth, Dietrich Rothenbacher, Ambuj Roy, Nobhojit Roy, Ben Benasco Sackey, Rajesh Sagar, Muhammad Muhammad Saleh, Juan R Sanabria, Damian F Santomauro, Itamar S Santos, Rodrigo Sarmiento-Suarez, Benn Sartorius, Maheswar Satpathy, Miloje Savic, Monika Sawhney, Susan M Sawyer, Josef Schmidhuber, Maria Inês Schmidt, Ione J C Schneider, Aletta E Schutte, David C Schwebel, Soraya Seedat, Sadaf G Sepanlou, Edson E Servan-Mori, Katya Shackelford, Amira Shaheen, Masood Ali Shaikh, Teresa Shamah Levy, Rajesh Sharma, Jun She, Sara Sheikhbahaei, Jiabin Shen, Kevin N Sheth, Muki Shey, Peilin Shi, Kenji Shibuya, Mika Shigematsu, Min-Jeong Shin, Rahman Shiri, Kawkab Shishani, Ivy Shiue, Inga Dora Sigfusdottir, Naris Silpakit, Diego Augusto Santos Silva, Jonathan I Silverberg, Edgar P Simard, Shireen Sindi, Abhishek Singh, Gitanjali M Singh, Jasvinder A Singh, Om Prakash Singh, Prashant Kumar Singh, Vegard Skirbekk, Amber Sligar, Samir Soneji, Kjetil Søreide, Reed J D Sorensen, Joan B Soriano, Sergey Soshnikov, Luciano A Sposato, Chandrashekhar T Sreeramareddy, Hans-Christian Stahl, Jeffrey D Stanaway, Vasiliki Stathopoulou, Nadine Steckling, Nicholas Steel, Dan J Stein, Caitlyn Steiner, Heidi Stöckl, Saverio Stranges, Mark Strong, Jiandong Sun, Bruno F Sunguya, Patrick Sur, Soumya Swaminathan, Bryan L Sykes, Cassandra E I Szoeke, Rafael Tabarés-Seisdedos, Karen M Tabb, Roberto Tchio Talongwa, Mohammed Rasoul Tarawneh, Mohammad Tavakkoli, Bineyam Taye, Hugh R Taylor, Bemnet Amare Tedla, Worku Tefera, Teketo Kassaw Tegegne, Dejen Yemane Tekle, Girma Temam Shifa, Abdullah Sulieman Terkawi, Gizachew Assefa Tessema, J S Thakur, Alan J Thomson, Andrew L Thorne-Lyman, Amanda G Thrift, George D Thurston, Taavi Tillmann, Ruoyan Tobe-Gai, Marcello Tonelli, Roman Topor-Madry, Fotis Topouzis, Bach Xuan Tran, Zacharie Tsala Dimbuene, Abera Kenay Tura, Emin Murat Tuzcu, Stefanos Tyrovolas, Kingsley Nnanna Ukwaja, Eduardo A Undurraga, Chigozie Jesse Uneke, Olalekan A Uthman, Aaron van Donkelaar, Yuri Y Varakin, Tommi Vasankari, Ana Maria Nogales Vasconcelos, J Lennert Veerman, Narayanaswamy Venketasubramanian, Raj Kumar Verma, Francesco S Violante, Vasiliy Victorovich Vlassov, Patricia Volkow, Stein Emil Vollset, Gregory R Wagner, Mitchell T Wallin, Linhong Wang, Valentine Wanga, David A Watkins, Scott Weichenthal, Elisabete Weiderpass, Robert G Weintraub, Daniel J Weiss, Andrea Werdecker, Ronny Westerman, Harvey A Whiteford, James D Wilkinson, Charles Shey Wiysonge, Charles D A Wolfe, Ingrid Wolfe, Sungho Won, Anthony D Woolf, Shimelash Bitew Workie, Mamo Wubshet, Gelin Xu, Ajit Kumar Yadav, Bereket Yakob, Ayalnesh Zemene Yalew, Lijing L Yan, Yuichiro Yano, Mehdi Yaseri, Pengpeng Ye, Paul Yip, Naohiro Yonemoto, Seok-Jun Yoon, Mustafa Z Younis, Chuanhua Yu, Zoubida Zaidi, Maysaa El Sayed Zaki, Carlos Zambrana-Torrelio, Tomas Zapata, Elias Asfaw Zegeye, Yi Zhao, Maigeng Zhou, Sanjay Zodpey, David Zonies, Christopher J L Murray

## Abstract

**Background:**

In September, 2015, the UN General Assembly established the Sustainable Development Goals (SDGs). The SDGs specify 17 universal goals, 169 targets, and 230 indicators leading up to 2030. We provide an analysis of 33 health-related SDG indicators based on the Global Burden of Diseases, Injuries, and Risk Factors Study 2015 (GBD 2015).

**Methods:**

We applied statistical methods to systematically compiled data to estimate the performance of 33 health-related SDG indicators for 188 countries from 1990 to 2015. We rescaled each indicator on a scale from 0 (worst observed value between 1990 and 2015) to 100 (best observed). Indices representing all 33 health-related SDG indicators (health-related SDG index), health-related SDG indicators included in the Millennium Development Goals (MDG index), and health-related indicators not included in the MDGs (non-MDG index) were computed as the geometric mean of the rescaled indicators by SDG target. We used spline regressions to examine the relations between the Socio-demographic Index (SDI, a summary measure based on average income per person, educational attainment, and total fertility rate) and each of the health-related SDG indicators and indices.

**Findings:**

In 2015, the median health-related SDG index was 59·3 (95% uncertainty interval 56·8–61·8) and varied widely by country, ranging from 85·5 (84·2–86·5) in Iceland to 20·4 (15·4–24·9) in Central African Republic. SDI was a good predictor of the health-related SDG index (*r*^2^=0·88) and the MDG index (*r*^2^=0·92), whereas the non-MDG index had a weaker relation with SDI (*r*^2^=0·79). Between 2000 and 2015, the health-related SDG index improved by a median of 7·9 (IQR 5·0–10·4), and gains on the MDG index (a median change of 10·0 [6·7–13·1]) exceeded that of the non-MDG index (a median change of 5·5 [2·1–8·9]). Since 2000, pronounced progress occurred for indicators such as met need with modern contraception, under-5 mortality, and neonatal mortality, as well as the indicator for universal health coverage tracer interventions. Moderate improvements were found for indicators such as HIV and tuberculosis incidence, minimal changes for hepatitis B incidence took place, and childhood overweight considerably worsened.

**Interpretation:**

GBD provides an independent, comparable avenue for monitoring progress towards the health-related SDGs. Our analysis not only highlights the importance of income, education, and fertility as drivers of health improvement but also emphasises that investments in these areas alone will not be sufficient. Although considerable progress on the health-related MDG indicators has been made, these gains will need to be sustained and, in many cases, accelerated to achieve the ambitious SDG targets. The minimal improvement in or worsening of health-related indicators beyond the MDGs highlight the need for additional resources to effectively address the expanded scope of the health-related SDGs.

**Funding:**

Bill & Melinda Gates Foundation.

## Background

In September, 2015, the UN General Assembly adopted “Transforming our World: The 2030 Agenda for Sustainable Development”, a resolution outlining a new framework to form the cornerstone of the sustainable development agenda for the period leading up to 2030.^1^ This new framework replaced the Millennium Development Goal (MDG) framework that expired in 2015, establishing 17 universal goals and 169 targets referred to as the Sustainable Development Goals (SDGs). The SDGs substantially broaden the development agenda beyond the MDGs and are expected to frame UN member state policies over the next 15 years. To measure progress towards achieving the goals, the UN Statistical Commission created the Inter-Agency and Expert Group on Sustainable Development Goal Indicators (IAEG-SDGs) with a mandate to draft an indicator framework that aligns with the targets. The IAEG-SDGs announced a total of 230 indicators to measure achievement of the 169 targets.^2^ Health is a core dimension of the SDGs; goal 3 aims to “ensure healthy lives and promote wellbeing for all at all ages”. Health-related indicators—ie, indicators directly pertaining to health services, health outcomes, and environmental, occupational, behavioural, or metabolic risks with well established causal connections to health—are also present in ten of the other 16 goals.^3^, ^4^ Across these 11 goals, there are 28 health-related targets with a total of 47 health-related indicators.

**Research in context**

**Evidence before this study**

Since the adoption of the Sustainable Development Goals (SDGs) in September, 2015, demand to establish independent, robust avenues for monitoring progress for the SDGs has escalated. However, substantial challenges exist in undertaking comprehensive and comparable assessments of health-related SDG indicators to monitor and guide development agendas and health policy implementation.

**Added value of this study**

The Global Burden of Diseases, Injuries, and Risk Factors Study (GBD) features more than 1870 collaborators from 124 countries and three territories and provides an independent analytical platform through which levels of health-related SDG indicators can be assessed across geographies and over time in a comparable manner. Drawing from GBD, we provide the measurement of 33 of the 47 health-related SDG indicators and introduce an overall health-related SDG index for 188 countries from 1990 to 2015.

**Implications of all the available evidence**

GBD and its analytical framework allow detailed analyses of country-level performance across health-related SDG indicators and over time. This information can be used to identify high-performing and low-performing countries, inform policy decisions, guide resource allocation, and monitor progress towards the health-related SDGs. The varied historical progress in improving a subset of health-related SDG indicators and rising prevalence of risks such as child overweight underscores the complex health landscape the world faces in the SDG era.

The SDGs were developed through a highly consultative and iterative process that included multiple meetings with expert groups, civil society, and governments. However, the process of developing the SDGs and the accompanying goals, targets, and indicators has not been without its critics. In both scientific settings and the news media, the common refrain has been that the SDGs are a long list of vague goals that lack clear, realistic, and measurable targets and indicators,^5^, ^6^, ^7^, ^8^, ^9^, ^10^, ^11^ and that they are not accompanied by a clear theory of change^12^ articulating how the pieces fit together.^3^ In view of the potential importance of the SDGs in directing national policies and donor investments, there has also been intense debate about the selection of targets and indicators;^12^ despite the lengthy list, some think that the SDGs are missing key areas of development, ranging from prohibition of forced labour^13^ to improvement of mental health.^14^, ^15^, ^16^ Concerns have also been expressed about the feasibility of measuring the 230 proposed indicators.^5^, ^6^, ^17^ Indeed, measurement of countries' current status and progress towards meeting the SDG targets will be an enormous task and will require collective action across a range of national and international organisations, both governmental and non-governmental. The difficulties of measurement are also further compounded by persistent problems of data availability, quality, and comparability across a host of indicators.^4^, ^18^ Furthermore, measurement of development indicators is accompanied by a high potential for political entanglement, which can lead to distorted estimates.^19^, ^20^, ^21^, ^22^ Independent monitoring of the SDG indicators will be crucial if they are to be used to accurately evaluate progress to ensure accountability and drive national and international development agendas towards meeting the SDGs.^4^, ^23^, ^24^, ^25^, ^26^

Despite these concerns, increasing work has been done in the past decade to generate independent, comparable, valid, and consistent measurements of development indicators.^27^, ^28^, ^29^, ^30^, ^31^, ^32^ To measure progress on the SDGs, these existing efforts will need to be leveraged, particularly those that provide comparable assessments of health outcomes and risks across countries and over time. The Global Burden of Diseases, Injuries, and Risk Factors Study (GBD) is a primary example of such an initiative. GBD is an open, collaborative, independent study to comprehensively measure epidemiological levels and trends of disease and risk factor burden worldwide, with more than 1870 individual collaborators from 124 countries and three territories across the full range of development. GBD uses a highly standardised approach to overcome challenges of inconsistent coding and indicator definitions across countries, missing and conflicting data, and time lags in measurement and estimation. Of the 47 health-related indicators included as part of the SDGs, estimates for 33 indicators are presently included as part of GBD. The GBD study also has several mechanisms to ensure independence, including the GBD Scientific Council that meets regularly to review all methods and major data changes, and the Independent Advisory Committee that meets twice yearly to review GBD progress and provide recommendations for strengthening GBD estimates.^33^

In this analysis, while acknowledging the continued debate about the structure, selection, and construction of SDG indicators, we used the GBD study to assess the current status of these 33 health-related SDG indicators. With this baseline assessment, we developed and estimated a summary indicator for the health-related SDG indicators and documented historical trends for this summary indicator. With the GBD results, we identified countries with the largest improvements between 1990 and 2015 to inform roadmaps and provide a basis for monitoring the health-related SDG indicators.

## Methods

### Overview of GBD

GBD is an annual effort to measure the health of populations at regional, country, and selected subnational levels.^33^ GBD produces estimates of mortality and morbidity by cause, age, sex, and country for the period 1990 to the most recent year, reflecting all available data sources adjusted for bias. GBD also measures many health system characteristics, risk factor exposure, and mortality and morbidity attributable to these risks. In addition to providing highly detailed standardised information for many outcomes and risks, various summary measures are also computed, including disability-adjusted life-years (DALYs) and healthy life expectancy. For the present analysis, we used estimates from GBD 2015 to provide a baseline assessment for 188 countries. Further details on GBD 2015, which covers 1990–2015, are available elsewhere.^34^, ^35^, ^36^, ^37^, ^38^, ^39^

### Indicators, definitions, and measurement approach

We defined health-related SDG indicators as indicators for health services, health outcomes, and environmental, occupational, behavioural, and metabolic risks with well established causal connections to health. Many of the 47 health-related SDG indicators selected by the IAEG-SDGs are produced as part of GBD. Table 1 outlines the ten goals, corresponding to 21 health-related targets and 33 health-related indicators included in this present iteration of GBD. This table also outlines the definition of the indicator used in this analysis; detailed descriptions of the estimation methods and data sources are given in the methods appendix pp 10–311. For the 14 health-related indicators that were not included in this analysis, their prospects for measurement in future iterations of GBD are described in table 2.

Direct outputs of GBD that are health-related SDG indicators include mortality disaggregated by age (under-5 and neonatal) and cause (maternal, cardiovascular disease, cancer, diabetes, chronic respiratory diseases, road injuries, self-harm, unintentional poisonings, exposure to forces of nature, interpersonal violence, and collective violence and legal intervention [ie, deaths due to law enforcement actions, irrespective of their legality]), as well as disease incidence (HIV, malaria, tuberculosis, and hepatitis B) and prevalence (neglected tropical diseases). The GBD comparative risk assessment includes measurement of exposure prevalence included as health-related SDG indicators (under-5 stunting, wasting, and overweight; tobacco smoking; harmful alcohol use; intimate partner violence; unsafe water, sanitation, and hygiene; household air pollution; and ambient particulate matter pollution), as well as deaths or disease burden attributable to risk factors selected as health-related SDG indicators (unsafe water, sanitation, and hygiene; household air pollution and ambient particulate matter pollution; and occupational risks).

Underlying GBD outputs are a range of additional health determinants that contribute to the estimation of morbidity and mortality, for which data are systematically compiled and estimates are produced. For example, GBD comprehensively analyses data from household surveys on vaccine coverage and combines survey estimates with reported administrative data to produce time series of vaccine coverage for all countries from 1990 to 2015. Estimates of vaccine coverage are then included as predictors of vaccine-preventable morbidity and mortality in GBD. Additional health indicators produced as part of GBD and included as health-related SDG indicators in this analysis are: met need with modern contraception among women of reproductive age, adolescent birth rate, skilled birth attendance coverage, and universal health coverage (UHC) tracer interventions. For UHC tracer interventions, we developed an index based on the geometric mean of the coverage of a set of UHC tracer interventions: met need with modern contraception; antenatal care (one or more visits and four or more visits); skilled birth attendance coverage; in-facility delivery rates; vaccination coverage (three doses of diphtheria–pertussis–tetanus, measles vaccine, and three doses of oral polio vaccine or inactivated polio vaccine); tuberculosis case detection rate; coverage of antiretroviral therapy for populations living with HIV, and coverage of insecticide-treated nets for malaria-endemic countries.

For selected indicators proposed by the IAEG-SDGs, we made modifications to the definition for clarity or on the basis of the definition used in GBD (table 1). For example, Indicator 2.2.2 proposes a measure of malnutrition that combined prevalence of wasting and overweight among children under age 5 years. As childhood wasting and overweight have very different determinants, we opted to report them separately. For childhood overweight, we report prevalence in children aged 2–4 years, the definition used in GBD based on thresholds set by the International Obesity Task Force.^40^

Further details on the estimation and data sources used for all indicators, compliant with Guidelines for Accurate and Transparent Health Estimates Reporting (GATHER),^41^, ^42^ are included in the methods appendix pp 10–311.

### Health-related SDG, health-related MDG, and health-related non-MDG indices

To identify broad patterns and more easily track general progress, we developed an overall health-related SDG index that is a function of the 33 health-related SDG indicators (referred to as the health-related SDG index). We also constructed two related indices: one reflecting the SDG health-related indicators previously included in the MDG monitoring framework (referred to as the MDG index) and one reflecting SDG health-related indicators not included in the MDGs (referred to as the non-MDG index).

Three broad approaches can be used to create composite measures: normative, preference weighted, and statistical. Normative approaches combine each indicator based on first principles or an over-riding construct such as the contribution of each indicator to overall health. Preference-weighted approaches weight each indicator by expressed or elicited social preferences for the relative importance of different indicators. Statistical approaches seek to reduce a long set of variables or indicators into common components of variance using methods such as principal component analysis or factor analysis. In this case, because the SDGs reflect the collective vision of UN member states, we used a preference-weighted approach, assuming that each SDG target should be treated equally.

To combine indicators, we adopted methods used to construct the Human Development Index,^43^ which include rescaling each indicator on a scale from 0 to 100 and then combining indicators using the geometric mean. The geometric mean allows indicators with very high values to partly compensate for low values on other indicators (referred to as partial substitutability). In the methods appendix pp 312–13, we describe results from alternative index construction methods (ie, principal component analysis; the arithmetic mean across targets referred to as complete substitutability; and the minimum value across targets referred to as zero substitutability). Quantitative targets for each of the health-related SDG indicators are not universally specified. As a result, we rescaled each health-related SDG indicator on a scale from 0 to 100, with 0 being the lowest (worst) value observed and 100 being the highest (best) value observed over the time period 1990–2015. We log-transformed mortality and morbidity before rescaling. We then estimated the health-related SDG index by first computing the geometric mean of each rescaled health-related SDG indicator for a given target, followed by the geometric mean of resulting values across all SDG targets. To avoid problems with indicator values close to 0, when computing indices we applied a floor of one to all indicators. This analytic approach weights each of the health-related SDG targets equally. In addition to the health-related SDG index, we also used the same methods to construct an index that represents 14 health-related SDG indicators that were previously MDG indicators and an index representing 19 non-MDG indicators (table 1). Uncertainty in the indicator and indices values was computed using a simulation analysis.

### Relations between health-related SDG indicators and the Socio-demographic Index and healthy life expectancy

As part of GBD 2015, we assessed cause-specific disease burden and risk exposure along the development spectrum, providing context on expected changes as countries progress to higher levels of income per person, higher educational attainment, and lower fertility.^34^, ^37^, ^38^, ^39^ We conducted a similar analysis by examining the relations of the overall health-related SDG index and each of the individual health-related SDG indicators with the Socio-demographic Index (SDI), a summary measure of development that uses lag-distributed income per person, average educational attainment in the population over age 15 years, and the total fertility rate. The SDI was constructed using the same method for the Human Development Index and the health-related SDG index. Each of the three components was first rescaled on a 0–1 scale, with 0 being the lowest (worst) value observed in the time period 1980–2015 and 1 being the highest (best) value observed. SDI was then computed as the geometric mean of these three rescaled components. To capture average relations, we used a spline regression (ie, piecewise linear regression with so-called knots specifying the intersection between pieces) of the health-related SDG indicators and health-related SDG index on SDI using the full set of data by country from 1990 to 2015. We also compared the health-related SDG indicators with the GBD 2015 estimates of healthy life expectancy^38^ to explore the relation between the SDGs and overall health achievement for each country.

### Role of the funding source

The funder of the study had no role in study design, data collection, data analysis, data interpretation, or writing of the report. The corresponding author had full access to all the data in the study and had final responsibility for the decision to submit for publication.

## Results

Of the 33 health-related SDG indicators, 21 were associated with a defined target, with 18 of them having an absolute level and three having a target relative to 2015 levels (table 3). The proportion of countries already meeting targets linked to health-related SDG indicators in 2015, as specified by absolute levels to be achieved, ranged from more than 60% for two indicators (maternal mortality ratio and under-5 mortality) to 0% for nine indicators. For these nine indicators, all targets involved full elimination of diseases (eg, tuberculosis, HIV, and neglected tropical diseases), reducing prevalence of health outcomes or risk to 0% (eg, childhood overweight and intimate partner violence), or reaching 100% for intervention coverage or health service provision (eg, skilled birth attendance, met need with modern contraception, and UHC tracer interventions).

**Figure 1:**
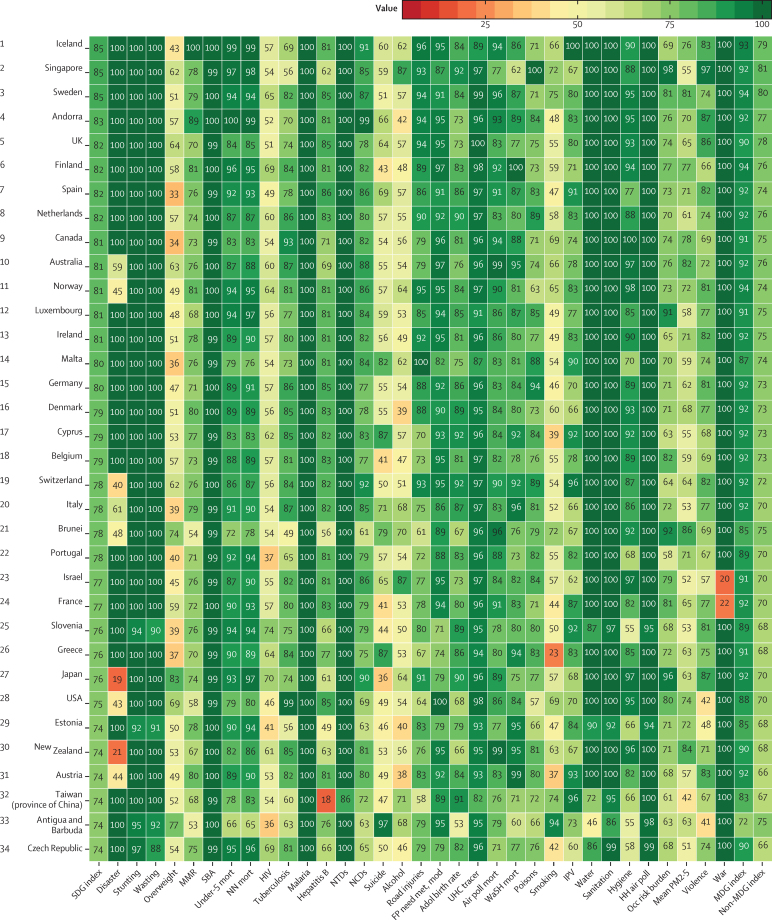

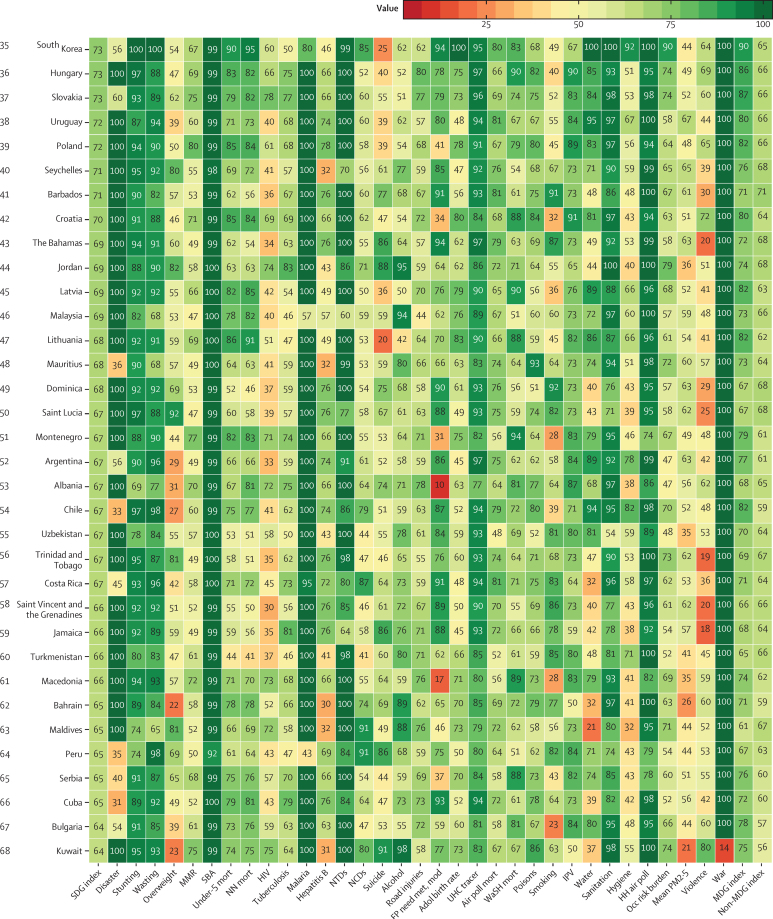

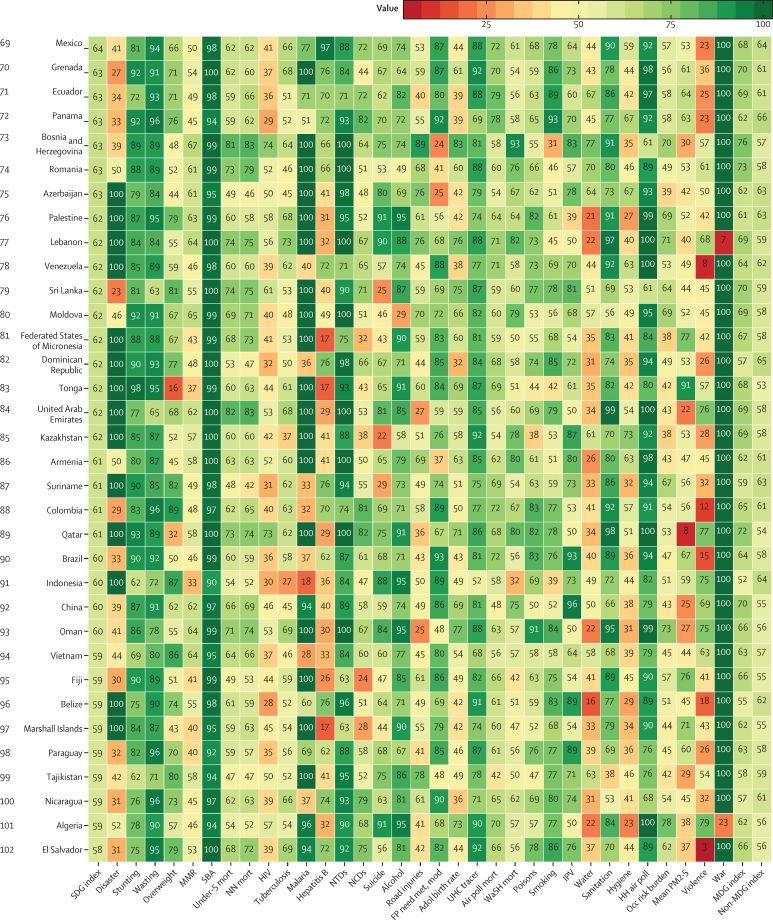

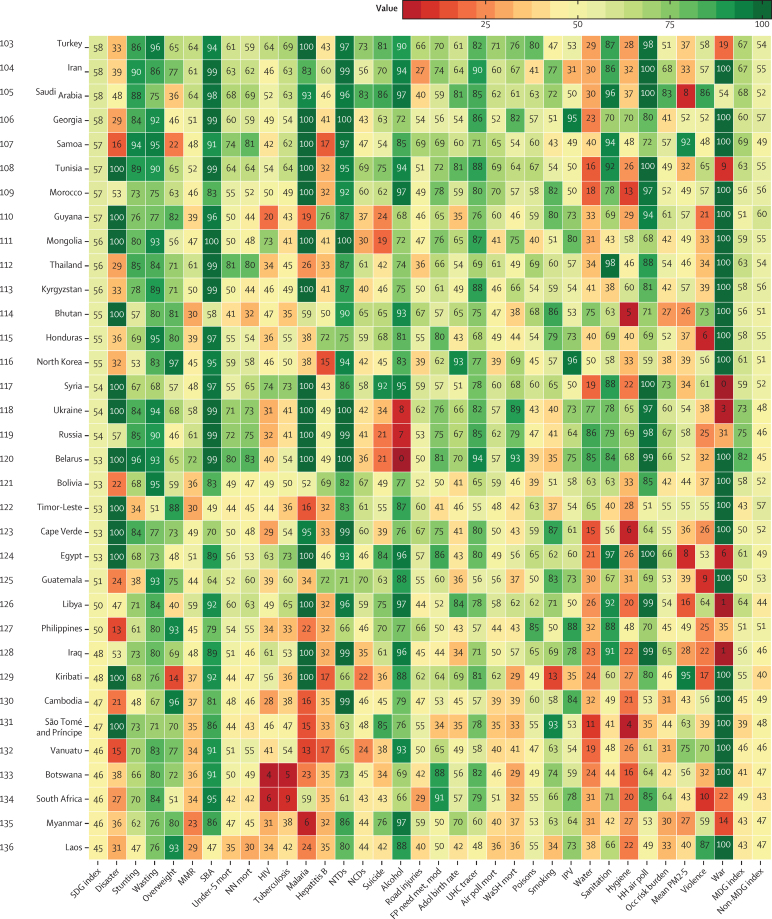

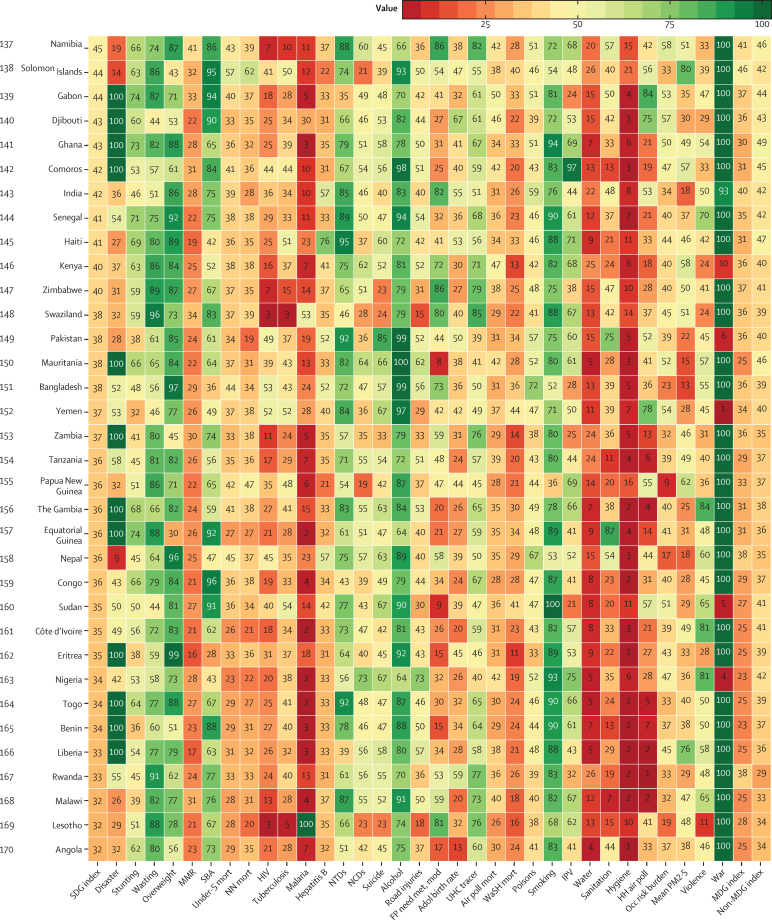

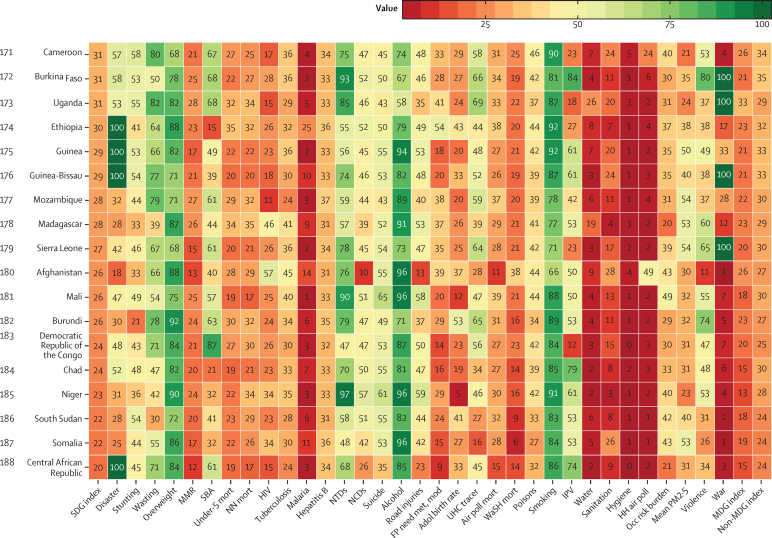
Performance of the health-related SDG index, MDG index, and non-MDG index, and 33 individual health-related indicators, by country, 2015 Countries are ranked by their health-related SDG index from highest to lowest. Indicators have been scaled from 0 to 100. Definitions of health-related SDG indicators are shown in table 1. SDG=Sustainable Development Goal. MDG=Millennium Development Goal. MMR=maternal mortality ratio. SBA=skilled birth attendance. Mort=mortality. NN mort=neonatal mortality. NTDs=neglected tropical diseases. NCDs=non-communicable diseases. FP need met, mod=family planning need met, modern contraception. Adol=adolescent. UHC=universal health coverage. Air poll mort=mortality attributable to air pollution. WaSH=water, sanitation, and hygiene. IPV=intimate partner violence. HH air poll=household air pollution. Occ risk burden=burden attributable to occupational risks. PM2·5=fine particulate matter smaller than 2·5 μm.

In 2015, the median health-related SDG index was 59·3 (95% uncertainty interval [UI] 56·8–61·8) across all 188 countries. This index was highest in Iceland (85·5, 84·2–86·5), Singapore (85·3, 84·1–86·3), and Sweden (85·3, 84·2–86·2) and lowest in the Central African Republic (20·4, 15·9–24·9), Somalia (21·6, 16·0–25·9), and South Sudan (22·5, 15·5–26·6; figure 1). Differences in the 95% UI range stem largely from differences in the availability and quality of underlying data sources for estimating individual indicators; for example, data were sparser for Somalia than they were for Sweden. Some patterns emerged contrary to what might have been expected. For example, the USA (74·9, 73·6–75·9) ranked 28th, driven by poorer performance on MDG indicators (eg, maternal mortality ratio) than other high-income countries^44^ and worse performance on non-MDG indicators—most notably, alcohol consumption, childhood overweight, and mortality due to interpersonal violence, self-harm, and unintentional poisoning. India (41·7, 39·7–43·7), despite rapid economic growth, was ranked 143rd, just below Comoros and Ghana.

**Figure 2:**
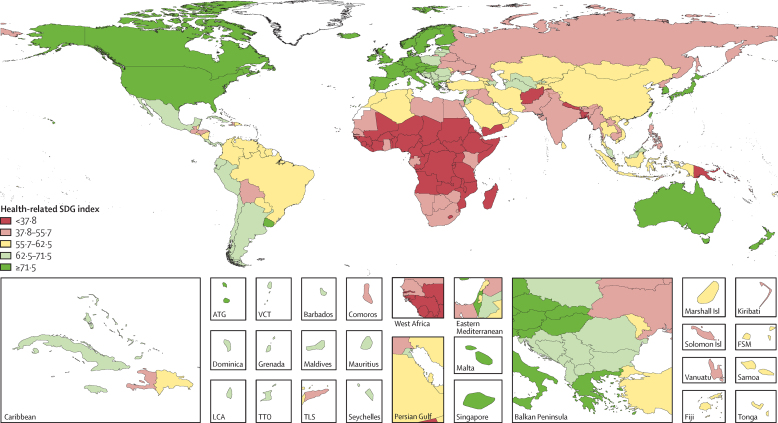
Map of health-related SDG index, by quintile, 2015 SDG=Sustainable Development Goal. ATG=Antigua and Barbuda. VCT=Saint Vincent and the Grenadines. LCA=Saint Lucia. TTO=Trinidad and Tobago. TLS=Timor-Leste. FSM=Federated States of Micronesia.

**Figure 3:**
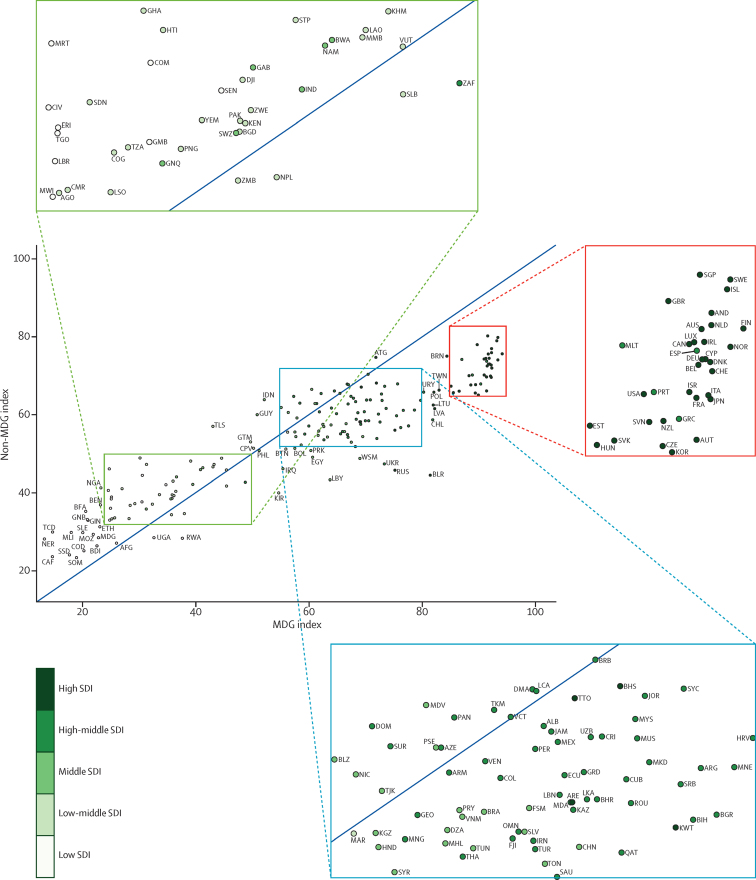
Non-MDG index versus MDG index, by country, 2015 The dark blue line shows the equivalence line, such that values that fall on this line are equivalent for both the MDG index and non-MDG index. Countries are abbreviated according to the ISO3 code. MDG=Millennium Development Goal. SDI=Socio-demographic Index.

Levels of the health-related SDG index were highly clustered (figure 2), with countries in the highest quintile (≥71·5) located mainly in western Europe, high-income North America, parts of Asia (Japan, South Korea, Singapore, and Brunei), and Australasia. The second highest quintile (62·5–71·5) included countries in southern Latin America, parts of eastern Europe, most of the Caribbean, and a subset of countries across other regions (eg, Mexico, Jordan, Azerbaijan, Malaysia, and Costa Rica), whereas countries in the middle quintile (55·7–62·5) were primarily located in South America; parts of east, central, and southeast Asia; and parts of North Africa and the Middle East. The countries in the fourth quintile (37·8–55·7) were mainly found in south and southeast Asia, southern sub-Saharan Africa, parts of North Africa and the Middle East, and parts of eastern Europe. Countries in western, eastern, and central sub-Saharan Africa, as well as a subset of other countries (eg, Afghanistan, Papua New Guinea, Yemen, and Nepal), dominated the lowest quintile (<37·8) of the health-related SDG index. Although the MDG index was correlated with the non-MDG index, country-level performance on these two indices varied considerably (figure 3). Performing well on the health-related MDG index did not guarantee good performance on the health-related non-MDG index. For example, the health-related MDG index in 2015 was similar for Indonesia (52·3, 49·8–54·6) and South Africa (48·9, 46·0–51·3), but Indonesia had a much higher non-MDG index (64·1, 62·0–66·6) than that of South Africa (42·9, 40·3–45·5). This difference for the non-MDG index was primarily driven by South Africa's lower performance for indicators such as childhood overweight, harmful alcohol use, and mortality due to self-harm and interpersonal violence.

**Figure 4:**
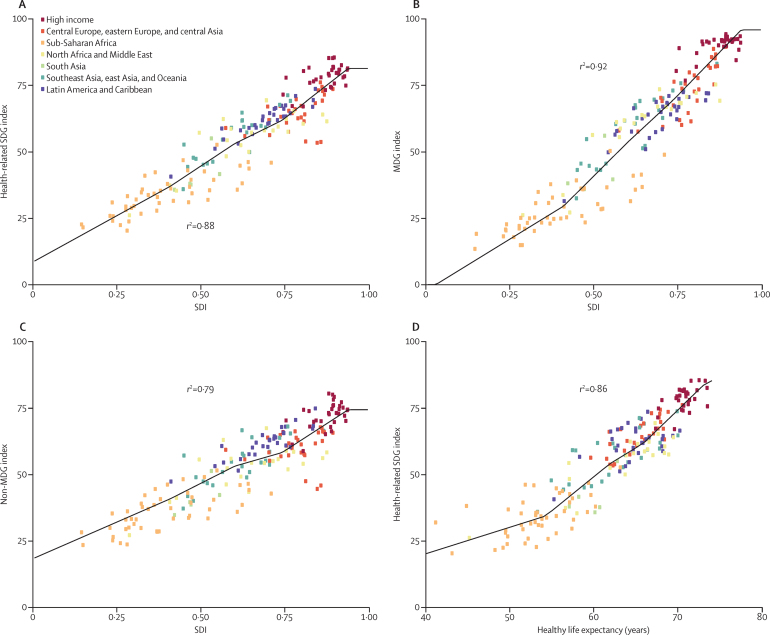
Relations (A) between the SDI and the health-related SDG index, (B) between the SDI and the MDG index, (C) between the SDI and the non-MDG index, and (D) between healthy life expectancy and the health-related SDG index, by country representing each of the seven GBD super regions, 2015 Each point represents a country and is colour coded according to the seven GBD super regions. The black lines were generated by spline regression. SDG=Sustainable Development Goal. SDI=Socio-demographic Index. MDG=Millennium Development Goal. GBD=Global Burden of Disease.

SDI was highly predictive of the overall health-related SDG index (*r*^2^=0·88) and MDG index (*r*^2^=0·92; figure 4). The non-MDG index was less well predicted by SDI (*r*^2^=0·79). This finding is reflective of the variable relations between individual health-related SDG indicators and SDI (results appendix pp 346–47). For instance, SDI was a poor predictor of mortality due to exposure to forces of nature, self-harm, interpersonal violence, and war (collective violence and legal intervention), as well as childhood overweight, intimate partner violence, and ambient particulate matter pollution. By contrast, SDI was highly predictive of maternal mortality ratio, under-5 mortality, and neonatal mortality, as well as mortality attributable to unsafe water, sanitation, and hygiene. Notably, the overall health-related SDG index also had a strong relation with healthy life expectancy (*r*^2^=0·86), a summary measure of population health.

**Figure 5:**
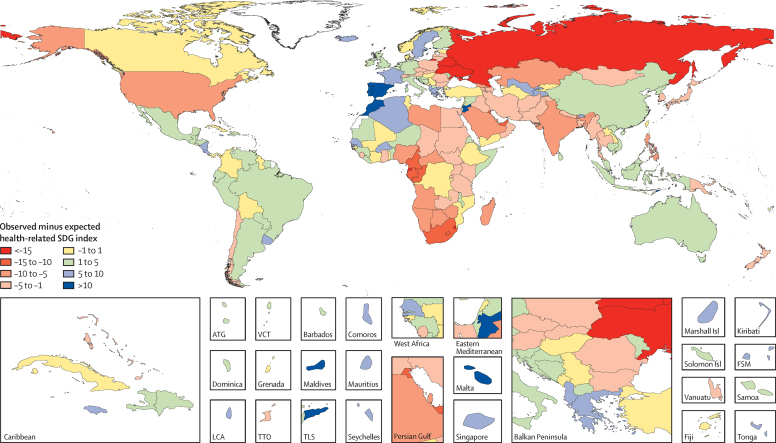
Map of observed health-related SDG index minus expected health-related SDG index, on the basis of SDI alone, 2015 The difference between the observed health-related SDG index and expected index (on the basis of SDI) reflects whether a country has a health-related SDG index above or below the expected level. Values for this difference are colour coded such that dark red reflects an observed health-related SDG index that is much lower than expected on the basis of SDI, and dark blue indicates that observed levels are much higher than expected on the basis of SDI. SDG=Sustainable Development Goal. SDI=Socio-demographic Index. ATG=Antigua and Barbuda. VCT=Saint Vincent and the Grenadines. LCA=Saint Lucia. TTO=Trinidad and Tobago. TLS=Timor-Leste. FSM=Federated States of Micronesia.

By subtracting expected levels for the health-related SDG index, on the basis of SDI alone, from observed levels (figure 5), we could identify potential geographical deviations well above or below expected values on the health-related SDG index. Countries that represent substantial deviations from the average might warrant further investigation to understand how and why they are underperforming or overperforming relative to the average. This deviation might be due, for example, to more or less efficient use of resources to improve health. Many countries in western Europe, Latin America, and parts of east and southeast Asia, as well as other countries such as Australia, recorded health-related SDG index levels that were higher than expected on the basis of SDI alone. Many of the countries with a health-related SDG index below expected levels on the basis of SDI were located in southern and central sub-Saharan Africa, eastern Europe and central Asia (eg, Belarus and Ukraine), North Africa and the Middle East, south Asia, and selected countries such as the USA.

**Figure 6:**
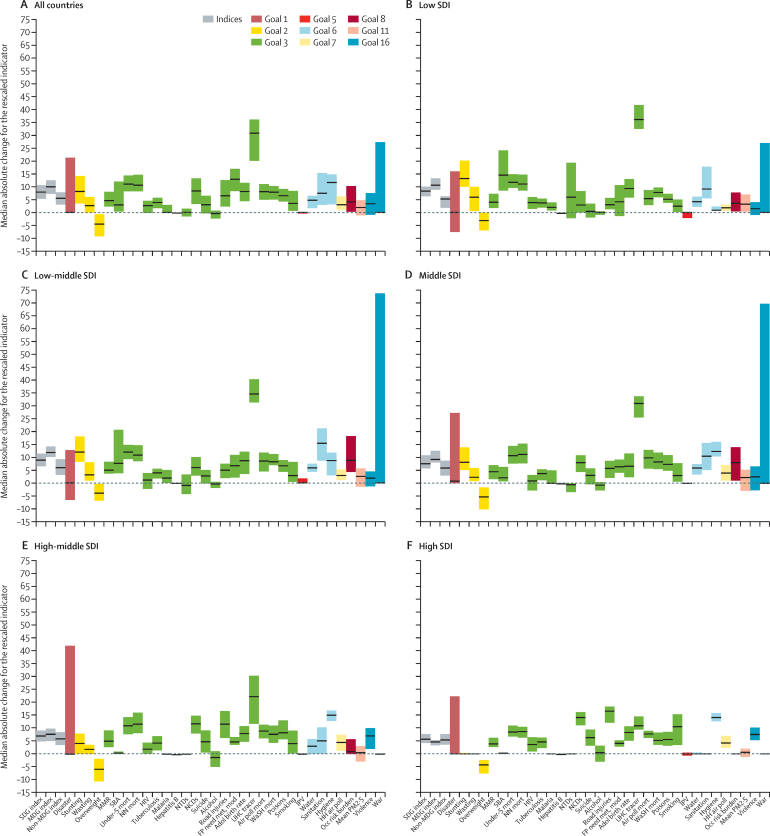
Median absolute change for health-related SDG index, MDG index, and 33 individual health-related SDG indicators (rescaled), (A) across all countries and in the (B) low-SDI quintile, (C) low-middle-SDI quintile, (D) middle-SDI quintile, (E) high-middle-SDI quintile, and (F) high-SDI quintile, 2000–15 Positive values indicate improvements between 2000–15, and negative values point to worsening performance for a given indicator during this time. Black stripes represent median absolute change and boxes represent IQR. Health-related indicators are colour coded according to the health-related goals they represent. Definitions of health-related SDG indicators are shown in table 1. SDG=Sustainable Development Goal. MDG=Millennium Development Goal. SDI=Socio-demographic Index. MMR=maternal mortality ratio. SBA=skilled birth attendance. Mort=mortality. NN mort=neonatal mortality. NTDs=neglected tropical diseases. NCDs=non-communicable diseases. FP need met, mod=family planning need met, modern contraception. Adol=adolescent. UHC=universal health coverage. Air poll mort=mortality attributable to air pollution. WaSH=water, sanitation, and hygiene. IPV=intimate partner violence. HH air poll=household air pollution. Occ risk burden=burden attributable to occupational risks. PM2·5=fine particulate matter smaller than 2·5 μm.

To provide a preliminary indication of potential trajectories in the next 15 years, we assessed absolute changes in the past 15 years for each of the 33 health-related SDG indicators and three summary indices (overall health-related SDG index, health-related MDG index, and non-MDG index). Overall, health-related SDG indicators largely improved since 2000, as summarised by the health-related SDG index; notably, gains in the health-related MDG index generally exceeded improvements in the non-MDG index (figure 6). Across countries, the most pronounced progress occurred for UHC tracer interventions, met need with modern contraception, hygiene, under-5 mortality, and neonatal mortality. Such striking gains for the indicator on UHC tracer interventions reflected the scale-up of antiretroviral therapy and coverage of insecticide-treated nets in malaria-endemic countries since the early 2000s.^31^, ^44^, ^45^ Of note, the relatively small improvement for the indicator on malaria incidence represents the large number of malaria-free countries in both 2000 and 2015.^46^ Health-related indicators covered by Target 3.3—which aims to end the epidemics of HIV, tuberculosis, malaria, and neglected tropical diseases, and to “combat hepatitis” by 2030—generally saw moderate progress (median absolute change of 2·7 [IQR −0·2 to 4·6] for HIV incidence and 3·9 [IQR 1·7 to 5·7] for tuberculosis incidence), although minimal changes occurred for hepatitis B incidence (−0·2 [–0·4 to −0·05]). In combination, these trends highlight the need for accelerated progress in order to meet Target 3.3. Substantial improvements occurred for childhood stunting (8·2 [3·5 to 14·2]) and, to a more modest extent, wasting (2·7 [0·0 to 6·0]), yet childhood overweight considerably worsened in the past 15 years (−4·5 [–9·2 to −0·7]). This trend occurred across SDI quintiles, emphasising the need for concerted policy attention to reverse this trend. Alcohol consumption worsened slightly in the past 15 years as well (−0·4 [–2·3 to 0·7]).

Between 2000 and 2015, distinct patterns for absolute changes in health-related SDG indicators surfaced across SDI quintiles (figure 6). While the indicator for UHC tracer interventions improved across all SDI quintiles, the most pronounced gains occurred in low-SDI and low-middle-SDI countries. Childhood stunting and wasting also improved at a faster pace for the low-SDI quintile than for other quintiles. Notably, mortality measures from the MDG agenda—maternal mortality ratio, under-5 mortality, and neonatal mortality—progressed at a similar pace across SDI quintiles. By contrast, mortality due to road injuries, non-communicable diseases (NCDs), and interpersonal violence declined faster in the higher-SDI quintiles than in the lower-SDI quintiles. Prevalence of smoking also had the largest reductions in countries in the high-SDI quintile.

**Figure 7:**
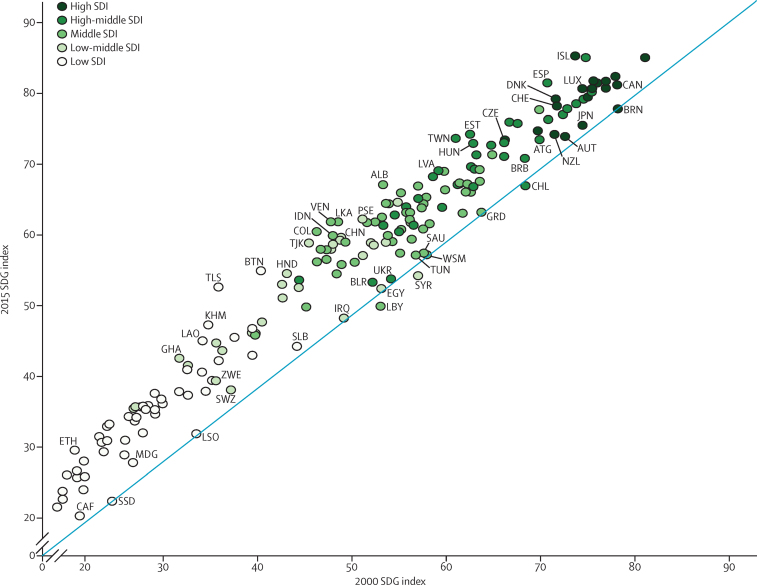
Health-related SDG index in 2015 versus 2000, by country The blue line shows the equivalence line, such that values that fall on this line are equivalent for both the health-related SDG index in 2000 and health-related SDG index in 2015. Only the top five and bottom five improvers in each SDI quintile, as determined by the absolute change from 2000 to 2015, are labelled; full results are shown in the results appendix. Countries are abbreviated according to the ISO3 code. SDI=Socio-demographic Index. SDG=Sustainable Development Goal.

Between 2000 and 2015, progress on the health-related SDG index (figure 7), as well as on individual health-related SDG indicators and on the MDG and non-MDG indices (results appendix p 6), was highly heterogeneous across geographies. Since 2000, the largest absolute improvements in the health-related SDG index occurred in Timor-Leste (18·5, 95% UI 16·2–20·8), Bhutan (16·2, 13·6–18·7), and Colombia (15·6, 14·1–16·8), whereas three countries—Libya, Syria, and Chile—experienced significant declines. Declines for the next two countries (Brunei and South Sudan) were between 0 and −0·5 and rounded to 0 in figure 7. Countries with the most pronounced gains for the health-related SDG index were found mainly in east, southeast, and central Asia, as well as parts of Latin America (eg, Venezuela and Honduras). Several countries in sub-Saharan Africa also recorded considerable gains in the health-related SDG index, including Rwanda, Ethiopia, Ghana, Namibia, and Angola.

To demonstrate the usefulness of these estimates for informing progress towards the SDGs, we also identified the geographies with the largest improvement in overall health-related SDG index between 2000 and 2015, stratified by SDI quintile classification in 2000. The five geographies were: Timor-Leste in the low-SDI quintile, Tajikistan in the low-middle-SDI quintile, Colombia in the middle-SDI quintile, Taiwan (province of China) in the middle-high-SDI quintile, and Iceland in the high-SDI quintile. Based on their gains for the health-related SDG index, these geographies could serve as case studies for understanding potential drivers of progress on the SDGs.

In Timor-Leste, changes in the health-related SDG index were largely driven by improvements in UHC tracer interventions, skilled birth attendance, met need with modern contraception, under-5 and neonatal mortality, childhood stunting, risk exposure to unsafe water and sanitation, and mortality from war or conflict. This overall improvement was despite worsening measures for childhood overweight, smoking prevalence, and alcohol use since 2000. Tajikistan recorded sizeable improvements across various health-related SDG indicators. Among indicators related to the MDGs, these included both measures of child mortality, childhood stunting, coverage of UHC tracer interventions, malaria incidence, and exposure to household air pollution. Improvements were also noted in mortality due to NCDs, interpersonal violence, and war or conflict, as well as mortality attributable to unsafe water, sanitation, and hygiene and to air pollution. However, several indicators either had minimal progress or worsened in Tajikistan, particularly childhood overweight and intimate partner violence. Colombia's most pronounced improvements since 2000 occurred for many of the non-MDG indicators, which included smoking prevalence and mortality rates due to NCDs, road injuries, interpersonal violence, and war. Sizeable improvements were also recorded for a subset of health-related MDG indicators—namely, coverage of UHC tracer interventions, adolescent birth rates, met need with modern contraception, and unsafe sanitation. Nonetheless, similar to other countries, Colombia had minimal progress in or worsened levels of alcohol consumption and hepatitis B incidence. In Taiwan, marked gains occurred for a subset of health-related SDG indicators previously associated with the MDG agenda (eg, adolescent birth rates and coverage of UHC tracer interventions); in parallel, Taiwan had considerable improvements for many non-MDG indicators, such as smoking prevalence and mortality due to NCDs, interpersonal violence, and road injuries. However, HIV and hepatitis B incidence worsened in Taiwan since 2000, and minimal progress occurred for ambient particulate matter pollution and several maternal and child health indicators. For Iceland, its progress on the health-related SDG health index was primarily driven by improvements in mortality due to NCDs and road injuries, smoking prevalence, adolescent birth rates, and both measures of child mortality. Similar to other countries, particularly those in the high-middle-SDI and high-SDI quintiles, Iceland had little progress in childhood overweight and worsening levels of alcohol consumption.

Further results are provided in the results appendix, and dynamic visualisations are available online.

## Discussion

### Summary of findings and implications

The ambitious SDG agenda is accompanied by numerous goals, targets, and indicators for tracking progress. Leading up to and following the UN SDG resolution^1^ in September, 2015, considerable debate surrounded the selection of indicators, including scepticism about the feasibility of their measurement.^5^, ^6^ In this study, we produced independent, highly standardised, and comparable estimates of 33 of the 47 health-related SDG indicators across 188 countries. To facilitate overall tracking, we also distilled these 33 health-related indicators into a health-related SDG index. Our findings show the wide range in this health-related SDG index in 2015, from 20·4 in Central African Republic to 85·5 in Iceland. Our historical analysis of these indicators also shows that progress can be achieved. Notable improvements were recorded for several health-related SDG indicators, particularly those that were also MDG indicators, such as under-5 mortality, met need with modern contraception, and childhood stunting. An index of the 14 MDG indicators that were included in the health-related SDG indicators had a median absolute change of 10·0 from 2000 to 2015, and larger reductions were generally found for countries at the lower end of the development spectrum. Our analysis also highlights the challenges associated with the expanded scope of the SDGs, with several of the non-MDG indicators having minimal improvements (eg, hepatitis B incidence) or worsening (eg, childhood overweight) between 2000 and 2015. This finding is further supported by the highly variable relation between the health-related MDG index and the health-related non-MDG index—good performance on the MDG index did not guarantee good performance on the non-MDG index. The overall health-related SDG index was well predicted by SDI; however, SDI was a variable predictor of the performance of individual health-related SDG indicators, particularly indicators that were not in the MDG agenda. Drawing from GBD, these findings provide a strong, comparable basis for monitoring the SDGs; furthermore, the independent nature of these results can enable accountability mechanisms for the multiple national and international, governmental, and non-governmental actors that must achieve progress on the SDGs.

These estimates also allow the identification of places that have made substantial progress on the health-related SDG indicators. These findings stand to strengthen the global evidence base of lessons learned for accelerating improvements in the health-related SDGs. The five geographies with the greatest improvement in the health-related SDG index between 2000 and 2015, stratified by SDI quintiles (Timor-Leste, Tajikistan, Colombia, Taiwan, and Iceland), have implemented a range of policies and interventions that might have contributed to their progress.

For instance, following acute conflict and violence during the late 1990s, Timor-Leste, in concert with the World Bank and other development partners, implemented a series of health sector rehabilitation and development projects in 2000 and 2001 to re-establish the country's health system and improve health service delivery to the poor.^47^, ^48^ In more recent years, health-care reform and financing have topped policy agendas in Timor-Leste,^49^ including the Ministry of Health's roll-out of a Basic Health Services Package and Hospital Services Package in 2007 under the pursuit of achieving UHC.^50^ Following almost a decade of civil conflict that severely disrupted health service provision, Tajikistan launched a series of health reforms beginning in the late 1990s^51^ and introduced a new benefits package for guaranteed health services in 2007.^52^ Moreover, after the civil war, the Tajik Government refocused policy attention for initiatives on particular diseases such as malaria;^53^ indeed, the country's multipronged malaria programme, which emphasises strong surveillance and cross-border activities with Afghanistan,^54^ has now brought Tajikistan close to eliminating the disease. Colombia, which experienced ongoing conflict and violence from the late 1980s to 2003,^55^ is globally recognised for its expansion of health insurance and services, especially to the poor. While Colombia's health system reforms began well before 2000 (the country approved its universal health insurance scheme in 1993),^56^ coverage increased substantially over time, as have the types of services covered by its insurance scheme (eg, cancer care).^57^ During the mid-1990s, Taiwan adopted a universal health insurance system,^58^ which is viewed as one of its most successful public entities. Taiwan also enacted many road safety laws between the mid-1990s and early 2000s, including mandatory helmet laws for motorcyclists in 1996 and an extension of seat belt laws to general roads in 2001.^59^ Iceland's tobacco control policies have been viewed as some of the world's most comprehensive,^60^ and the country's long-standing publicly funded health system provides UHC,^61^ a factor that might have contributed to its declines in NCD mortality.

Such progress also highlights important interactions between development goals and wider contextual factors, such as post-conflict experiences in Timor-Leste, Tajikistan, and Colombia, as well as a rebound in health following the dissolution of Soviet rule for Tajikistan. Furthermore, Taiwan's gains underscore the interplay between advancing economic development and deliberate investments in improving UHC. These vignettes highlight only a fraction of the possible learning for informing action towards improving the health-related SDGs. An important future area of work will be to understand in detail how these and other high-performing geographies have achieved substantial improvements in key SDG indicators.

Besides showing the feasibility and value of measuring many of the health-related SDGs, our findings also affirm concerns voiced during the SDG development process and following the UN resolution. One criticism of the SDGs was the incredibly ambitious nature of some of the targets,^5^ such as Target 3.3, which calls for the end of the epidemics of HIV, tuberculosis, and malaria by 2030. Our analysis of these indicators in the past 15 years suggests that a substantial change in the present trajectory of HIV and tuberculosis incidence will be needed to meet this target, and major technological leaps coupled with universal delivery are likely to be necessary. The vague nature of many of the SDG targets has also been a common criticism;^4^, ^8^, ^10^, ^62^ Of the 33 health-related SDG indicators in our study, we identified specific targets for only 21 of them. The absence of specific and attainable targets for SDG indicators, health related or otherwise, undermines the usefulness of the SDGs in driving development agendas, a limitation that can and should be addressed at this early stage of the SDG period.

Our analysis also represents a step towards producing a more cohesive understanding of the interactions between different SDG goals, targets, and indicators—a widely noted criticism.^3^, ^4^ We show the potential for quantifying these interactions by comparing the relations between education, income, and fertility—components of the SDI—and the 33 health-related SDG indicators and accompanying health-related SDG, MDG, and non-MDG indices. Although we acknowledge the ecological nature of this analysis and its usual caveats, SDI was a strong predictor of the overall health-related SDG and MDG indices, highlighting the general importance of income, education, and fertility, as well as intersectoral action for health-related development. However, SDI was a weaker predictor of the non-MDG index, particularly for indicators such as violence (intimate partner, interpersonal, and collective violence) and ambient particulate matter pollution. This finding shows that a sole focus on increasing income and education and decreasing fertility is probably insufficient to meet the SDG targets. It also raises questions about whether other common drivers, analogous to income, education, and fertility, can be determined and their relations with SDG indicators assessed. Combining this initial assessment of the 33 health-related SDG indicators with an expanded quantification and analysis of other potential drivers is an important future area of work that could help to create a more concise, cohesive, and actionable monitoring framework for the SDGs.

### Future directions for GBD monitoring efforts

In this report, we focused on measuring indicators proposed by the IAEG-SDGs. In future years, we plan to address three related sets of issues: first, improved assessment of the health-related SDG indicators measured at present; second, inclusion of the 14 currently excluded health-related indicators in the annualised GBD study; and third, potential expansion of indicators consistent with the framing of the targets. We address each of these issues in turn.

#### Improving the measurement of currently included health-related indicators

With the present analysis, we made several modifications that we believe improve several health-related indicators for the purposes of measuring progress towards each health-related SDG target. Future iterations are likely to incorporate further modifications to these and other indicators. First, as noted in the Methods section, rather than reporting on the combined prevalence of childhood wasting and overweight, we assessed and measured them separately. Our results support this decision, since they had divergent relations over time (ie, childhood wasting improved for most countries, whereas the prevalence of childhood overweight generally increased) and with SDI (ie, childhood wasting and overweight were negatively and positively correlated with SDI, respectively).

Second, the IAEG-SDGs' proposed indicator for harmful use of alcohol is the average national-level consumption per person in litres of pure alcohol. The health and non-health risks associated with harmful alcohol use are a function of not only average consumption at the population level but also use patterns (ie, amount consumed at a given time and frequency of consumption). For this analysis, we reported on the summary exposure value of harmful alcohol use, which takes into account the distribution of consumption and the prevalence of binge drinking.^39^

Third, we made two modifications to the measurement of disaster (Indicators 1.5.1, 11.5.1, and 13.1.2). For mortality attributable to disasters, we chose to report on the lagged 5 year average of disaster mortality. One of the corresponding health-related SDG targets (Target 1.5) is to “build the resilience of the poor and those in vulnerable situations and reduce their exposure and vulnerability to climate-related extreme events and other economic, social and environmental shocks and disasters”. Focusing solely on the observed mortality caused by natural disaster ignores the role of chance in the occurrence of natural disasters; for example, nations with weak resilience to natural disasters might not experience a natural disaster during a given period of time, whereas those with strong resilience might encounter them more frequently. Taking the moving average of disaster mortality lessens the contribution of chance in assessing progress for this indicator. Nonetheless, this adjustment cannot account for background risk of natural disasters, which varies by geography, and future research efforts could include developing a risk-standardised version of the indicator. For natural disasters, data on missing people and people affected by disaster are not readily available across countries and over time.

Fourth, for occupational health (Indicator 8.8.1), we reported on age-standardised all-cause DALY rate attributable to occupational risks rather than the suggested indicator, which is limited to fatal and non-fatal occupational injuries. This revision captures a wider set of occupational risks instead of only those that result in injuries, which supports the stated target of promotion of “safe and secure working environments for all workers” (Target 8.8). Fifth, for tobacco use prevalence (Indicator 3.a.1), GBD does not presently assess smokeless tobacco use. Furthermore, smokeless tobacco use has a notably different risk profile to smoked tobacco use,^63^ and thus it might warrant a subindicator akin to childhood malnutrition. Sixth, for clean fuels and technology (Indicator 7.1.2), we presently use a more limited definition that covers fuels used primarily for household cooking. Seventh, for homicide (Indicator 16.1.1), GBD does not measure this indicator by displacement or migratory status, and similarly for conflict-related deaths (Indicator 16.1.2), we do not measure deaths by displacement status or by more specified causes.

Eighth, data gaps also account for limitations in the estimation of the UHC tracer indicator (Indicator 3.8.1). We used a set of tracer interventions that were restricted to reproductive, maternal, and child health, as well as a subset of infectious diseases. There is a paucity of data for the coverage of NCD interventions in particular^64^, ^65^, ^66^, ^67^ and for higher-level care. Furthermore, the UHC tracer indicator only captures the use of interventions and not the quality of the intervention provided.^68^, ^69^ As more data become available on the delivery of interventions for NCDs and the modification of key risk factors, this information will be incorporated into revisions of the UHC tracer indicator. Finally, data gaps mean that, in this initial assessment, we have also not been able to include a measure of financial risk protection. Substantial investments are needed in this area to address data gaps to be able to track the central role of health system delivery in improving health.

#### Indicators not presently measured

Of the 14 health-related SDG indicators that were not included in this analysis, there are several that the GBD does not currently measure but that could be assessed in the future through GBD (table 2). These indicators include the coverage of treatment interventions for substance use disorders (Indicator 3.5.1), which would leverage the work on quantifying incidence, prevalence, and mortality for these conditions. Estimating the proportion of women and girls aged 15 years and older who are subjected to sexual violence by people other than an intimate partner (Indicator 5.2.2) would leverage work already undertaken by GBD on measuring prevalence of intimate partner violence. As part of GBD, we have also assembled a host of population-level data that would facilitate measurement of the coverage of health insurance or public health systems (Indicator 3.8.2), health worker density and distribution (Indicator 3.c.1), and completeness of death registration (Indicator 17.19.2). Other indicators are more difficult to measure because of data gaps or unclear definitions. For example, data sources to measure the proportion of people that feel safe walking alone around the area they live (Indicator 16.1.4) are not readily available for most countries.

#### Strengthening the indicators for selected targets

Various commentaries have pointed out the absence of indicators for key health outcomes and determinants. Proponents have argued for indicators for mental health that go beyond substance abuse disorders and suicide;^14^, ^15^, ^16^ other NCDs beyond cardiovascular diseases, cancer, diabetes, and chronic respiratory diseases;^70^ diseases related to ageing, including osteoarthritis and Alzheimer's disease;^71^, ^72^ non-fatal disorders that lead to substantial morbidity (eg, sensory disorders); and a host of major risk factors. Another example is Target 3.3, which aims to combat hepatitis—the indicator only tracks hepatitis B, although the data for hepatitis C monitoring are as robust as those for hepatitis B and a highly effective cure for hepatitis C is available. As shown in this report, our GBD collaboration provides the basis for measuring many of these indicators. The danger is that an exhaustive laundry list of indicators, a criticism already levelled at the present SDG list, would dilute the value of the SDGs in focusing attention on where it is most needed.

### Comparison with other assessments

There are several important similarities and differences between our assessment of the health-related SDGs and those produced by WHO^18^ and the Sustainable Development Solutions Network (SDSN).^17^ Like WHO, we focused on the health-related SDG indicators and did not cover indicators across all goals as SDSN does. With our focus on health, we covered 33 health-related indicators, compared with 21 by SDSN and 32 by WHO. Similar to SDSN, we produced a summary measure for the health-related SDG indicators included in the analysis. Most importantly, GBD uses standardised and internally consistent approaches to generate estimates across causes, risk factors, and underlying indicators. For example, we constrain the aggregation of cause-specific deaths to equal all-cause deaths. Furthermore, GBD also produces a complete set of estimates for 188 countries and for individual years from 1990 to 2015. By contrast, WHO and SDSN draw on disparate sources and methods for estimation and, as a result, report on an incomplete set of estimates by country. SDSN provides estimates for 149 countries, whereas estimates for health-related SDG indicators produced by WHO range from 194 countries for under-5 mortality and neonatal mortality to 109 countries for HIV incidence. WHO and SDSN also do not generate estimates for a consistent set of years. WHO reports 2015 estimates for only seven indicators and combines data from a range of years for ten indicators; for example, WHO combined skilled birth attendance estimates by country ranging from 2006 to 2014. SDSN reports 2015 estimates for only four indicators and combines data from a range of years for eight indicators; for example, country estimates for smoking prevalence from the SDSN report range from 2006 to 2013. Complete, consistent, comparable, and contemporary estimates of health-related SDG indicators are necessary to properly track progress on the SDGs.

### Limitations

This study has several limitations in addition to the ones we already described. First, all the limitations of GBD relevant to the 33 indicators used here apply.^34^, ^37^, ^38^, ^39^ Second, we tried to summarise the complexity of the 33 indicators using a summary measure for the health-related SDGs. Many approaches are available for developing summary measures. Since the SDGs are the outcome of a political consensus building process, we opted to use the stated targets as preferences of UN member states that have agreed to the SDG declaration. Our sensitivity analysis shows that using alternative weighting schemes produces broadly similar results (methods appendix pp 312–13). Our sensitivity analysis also highlights the limitation of the statistical approach (ie, principal component analysis) for constructing an index for this purpose, with the first principal component including both positive correlations with indicators such as maternal mortality ratio and negative correlations with indicators such as alcohol use. An alternative could be to weight each indicator by their contribution to healthy life expectancy. Third, we opted to construct the summary measure using the Human Development Index method of rescaling each component on a scale of 0 to 100, and then taking the geometric mean of the components. We chose to use the minimum and maximum observed values to rescale, as targets for all indicators are not clearly specified; however, the limitation of this approach is that minimum and maximum values might change in the future. In the next iteration of this analysis, we will use targets for all indicators and rescale them accordingly; to establish targets for indicators currently lacking explicit ones, we will determine plausible targets based on forecasts of trends through 2030. Fourth, a clear limitation, as highlighted by the UHC tracer indicator, is the need for broad investment in data systems in countries to properly assess progress on key health and development indicators such as the SDGs. As an example, there remains considerable uncertainty about levels and age patterns of mortality and the cause of death structure.^34^ Investments in high-quality vital registration systems and other related data collection systems, from censuses and household surveys to health management information systems, are crucial to the proper monitoring of progress towards the SDGs.

Our GBD collaboration aims to address several of the limitations noted above in future reporting of the health-related SDGs on an annual cycle. As noted, we will also leverage work that is underway to forecast country-specific disease burden, which will additionally provide information on the future trajectory of health-related SDG indicators based on historical trends and provide an explicit way to understand how those trajectories could be changed with different policy adoption. We will also address, in a staged manner, the absence of measures of geographical and socioeconomic inequality in the health-related SDG indicators.

## Conclusions

The measurement of 33 health-related SDG indicators presented here is the product of an extensive, open collaboration that represents many countries across a broad range of development. We invite others to join in this effort to produce an independent, robust basis for monitoring and assessing progress towards the SDGs. Independent measurement is a crucial component of accountability, but it is not the only component. These results should ideally be used as the basis for review and action at the country level. We hope that this collaboration is a major contribution to creating a culture of accountability for the SDGs. Other actors, especially governments, civil society organisations, donors, and global development institutions, need to participate in the process of using this information to enhance accountability through open and transparent review and action.

Correspondence to: Prof Christopher J L Murray, University of Washington, Institute for Health Metrics and Evaluation, 2301 5th Avenue, Suite 600, Seattle, WA 98121, USA **cjlm@uw.edu**

See **Online** for **infographic** http://www.thelancet.com/infographics/SDG

## Figures and Tables

**Table 1:** Health-related SDG goals and targets proposed by the Inter-Agency and Expert Group on SDG Indicators, and health-related SDG indicators used in this analysis

	**Health-related SDG indicator**	**Definition used in this analysis**	**Further details**	**Inclusion in MDG or non-MDG index**
**Goal 1: End poverty in all its forms everywhere**				
Target 1.5: By 2030, build the resilience of the poor and those in vulnerable situations and reduce their exposure and vulnerability to climate-related extreme events and other economic, social and environmental shocks, and disasters	Disaster (1.5.1; same as Indicators 11.5.1 and 13.1.2)	Age-standardised death rate due to exposure to forces of nature, per 100 000 population	Existing datasets do not comprehensively measure missing people and people affected by natural disasters. We revised this indicator to exposure to forces of nature and reported in age-standardised rates	Non-MDG
**Goal 2: End hunger, achieve food security and improved nutrition, and promote sustainable agriculture**				
Target 2.2: By 2030, end all forms of malnutrition, including achieving, by 2025, the internationally agreed targets on stunting and wasting in children under 5 years of age, and address the nutritional needs of adolescent girls, pregnant and lactating women, and older persons	Stunting (2.2.1)	Prevalence of stunting in children under age 5 years, %	Stunting is defined as below −2 SDs from the median height-for-age of the reference population. No indicator modifications required	MDG
Target 2.2 (as above)	Wasting (2.2.2a)	Prevalence of wasting in children under age 5 years, %	Wasting is defined as below −2 SDs from the median weight-for-height of the reference population. We separated reporting for indicator 2.2.2 into wasting (2.2.2a) and overweight (2.2.2b)	MDG
Target 2.2 (as above)	Overweight (2.2.2b)	Prevalence of overweight in children aged 2–4 years, %	We used the IOTF thresholds because the WHO cutoff at age 5 years can lead to an artificial shift in prevalence estimates when the analysis covers more age groups. Furthermore, considerably more studies use IOTF cutoffs than WHO cutoffs, which allowed us to build a larger database for estimating child overweight. We separated reporting for indicator 2.2.2 into wasting (2.2.2a) and overweight (2.2.2b)	Non-MDG
**Goal 3: Ensure healthy lives and promote wellbeing for all at all ages**				
Target 3.1: By 2030, reduce the global maternal mortality ratio to less than 70 per 100 000 livebirths	Maternal mortality ratio (3.1.1)	Maternal deaths per 100 000 livebirths	No indicator modifications required	MDG
Target 3.1 (as above)	Skilled birth attendance (3.1.2)	Proportion of births attended by skilled health personnel (doctors, nurses, midwives, or country-specific medical staff [eg, clinical officers]), %	No indicator modifications required	MDG
Target 3.2: By 2030, end preventable deaths of newborns and children under 5 years of age, with all countries aiming to reduce neonatal mortality to at least as low as 12 per 1000 livebirths and under-5 mortality to at least as low as 25 per 1000 livebirths	Under-5 mortality (3.2.1)	Probability of dying before age 5 years per 1000 livebirths	No indicator modifications required	MDG
Target 3.2 (as above)	Neonatal mortality (3.2.2)	Probability of dying during the first 28 days of life per 1000 livebirths	No indicator modifications required	MDG
Target 3.3: By 2030, end the epidemics of AIDS, tuberculosis, malaria, and neglected tropical diseases and combat hepatitis, water-borne diseases, and other communicable diseases	HIV (3.3.1)	Age-standardised rate of new HIV infections, per 1000 population	We revised this indicator to HIV incidence of all populations and reported in age-standardised rates	MDG
Target 3.3 (as above)	Tuberculosis (3.3.2)	Age-standardised rate of new and relapsed tuberculosis cases, per 1000 population	No indicator modifications required	MDG
Target 3.3 (as above)	Malaria (3.3.3)	Age-standardised rate of malaria cases, per 1000 population	No indicator modifications required	MDG
Target 3.3 (as above)	Hepatitis B (3.3.4)	Age-standardised rate of hepatitis B incidence, per 100 000 population	No indicator modifications required	Non-MDG
Target 3.3 (as above)	Neglected tropical diseases (3.3.5)	Age-standardised prevalence of neglected tropical diseases, per 100 000 population	People requiring interventions against neglected tropical diseases are not well defined; thus, we revised this indicator to the sum of the prevalence of 14 neglected tropical diseases currently measured in GBD: African trypanosomiasis, Chagas disease, cystic echinococcosis, cysticerosis, dengue, food-borne trematodiases, intestinal nematode infections, leishmaniasis, leprosy, lymphatic filariasis, onchocerciasis, rabies, schistosomiasis, and trachoma	Non-MDG
Target 3.4: By 2030, reduce by one-third premature mortality from NCDs through prevention and treatment, and promote mental health and wellbeing	NCDs (3.4.1)	Age-standardised death rate due to cardiovascular disease, cancer, diabetes, and chronic respiratory disease in populations aged 30–70 years, per 100 000 population	No indicator modifications required	Non-MDG
Target 3.4 (as above)	Suicide (3.4.2)	Age-standardised death rate due to self-harm, per 100 000 population	No indicator modifications required	Non-MDG
Target 3.5: Strengthen the prevention and treatment of substance abuse, including narcotic drug abuse and harmful use of alcohol	Alcohol (3.5.2)	Risk-weighted prevalence of alcohol consumption, as measured by the SEV for alcohol use, %	We revised this indicator to include six categories of alcohol consumption because national alcohol consumption per person does not capture the distribution of use. The SEV for alcohol use is based on two primary dimensions and subcategories of each: individual-level drinking (current drinkers, lifetime drinkers, lifetime abstainers, and alcohol consumption by current drinkers) and drinking patterns (binge drinkers and frequency of binge drinks). The SEV then weights these categories with their corresponding relative risks, which translates to a risk-weighted prevalence on a scale of 0% (no risk in the population) to 100% (the entire population experiences maximum risk associated with alcohol consumption)	Non-MDG
Target 3.6: By 2020, halve the number of global deaths and injuries from road traffic accidents	Road injuries (3.6.1)	Age-standardised death rate due to road traffic injuries, per 100 000 population	No indicator modifications required	Non-MDG
Target 3.7: By 2030, ensure universal access to sexual and reproductive health-care services, including for family planning, information and education, and the integration of reproductive health into national strategies and programmes	Family planning need met, modern contraception (3.7.1)	Proportion of women of reproductive age (15–49 years) who have their need for family planning satisfied with modern methods, % women aged 15–49 years	No indicator modifications required	MDG
Target 3.7 (as above)	Adolescent birth rate (3.7.2)	Birth rates for women aged 10–14 years and women aged 15–19 years, number of livebirths per 1000 women aged 10–14 years and women aged 15–19 years	No indicator modifications required	MDG
Target 3.8: Achieve universal health coverage, including financial risk protection, access to quality essential health-care services and access to safe, effective, quality, and affordable essential medicines and vaccines for all	Universal health coverage tracer (3.8.1)	Coverage of universal health coverage tracer interventions for prevention and treatment services, %	Tracer interventions included immunisation coverage (ie, coverage of three doses of diphtheria–pertussis–tetanus, measles vaccine, and three doses of oral polio vaccine or inactivated polio vaccine), met need with modern contraception, antenatal care coverage (one or more visits and four or more visits), skilled birth attendance, in-facility delivery rates, coverage of antiretroviral therapy for people living with HIV, tuberculosis case detection rate, and coverage of insecticide-treated nets in malaria-endemic countries	MDG
Target 3.9: By 2030, substantially reduce the number of deaths and illnesses from hazardous chemicals and air, water, and soil pollution and contamination	Air pollution mortality (3.9.1)	Age-standardised death rate attributable to household air pollution and ambient air pollution, per 100 000 population	No indicator modifications required	Non-MDG
Target 3.9 (as above)	WaSH mortality (3.9.2)	Age-standardised death rate attributable to unsafe WaSH, per 100 000 population	No indicator modifications required	Non-MDG
Target 3.9 (as above)	Poisons (3.9.3)	Age-standardised death rate due to unintentional poisonings, per 100 000 population	No indicator modifications required	Non-MDG
Target 3.a: Strengthen the implementation of the World Health Organization Framework Convention on Tobacco Control in all countries, as appropriate	Smoking (3.a.1)	Age-standardised prevalence of daily smoking in populations aged 10 years and older, % population aged 10 years and older	We revised this indicator to daily smoking because of data limitations regarding the systematic measurement of current smoking and to reflect populations aged 10 years and older	Non-MDG
**Goal 5: Achieve gender equality and empower all women and girls**				
Target 5.2: Eliminate all forms of violence against all women and girls in the public and private spheres, including trafficking and sexual and other types of exploitation	Intimate partner violence (5.2.1)	Age-standardised prevalence of women aged 15 years and older who experienced intimate partner violence, % women aged 15 years and older	Existing datasets do not comprehensively measure the status of ever-partnered women relative to never-partnered women; therefore, the denominator was revised to all women aged 15 years and older. Data on exposure to subtypes of violence are not systematically available across geographies and over time	Non-MDG
**Goal 6: Ensure availability and sustainable management of water and sanitation for all**				
Target 6.1: By 2030, achieve universal and equitable access to safe and affordable drinking water for all	Water (6.1.1)	Risk-weighted prevalence of populations using unsafe or unimproved water sources, as measured by the SEV for unsafe water, %	Different types of unsafe water sources have different relative risks associated with poor health outcomes; thus, we revised this indicator to SEV for water, which captures the relative risk of different types of unsafe water sources and then combines them into a risk-weighted prevalence on a scale of 0% (no risk in the population) to 100% (the entire population experiences maximum risk associated with unsafe water)	MDG
Target 6.2: By 2030, achieve access to adequate and equitable sanitation and hygiene for all and end open defecation, paying special attention to the needs of women and girls and those in vulnerable situations	Sanitation (6.2.1a)	Risk-weighted prevalence of populations using unsafe or unimproved sanitation, as measured by the SEV for unsafe sanitation, %	We separated reporting for indicator 6.2.1 into sanitation (6.2.1a) and hygiene (6.2.1b). We had three mutually exclusive, collectively exhaustive categories for sanitation at the household level: households with piped sanitation (with a sewer connection); households with improved sanitation without a sewer connection (pit latrine, ventilated improved latrine, pit latrine with slab, or composting toilet), as defined by the JMP; and households without improved sanitation (flush toilet that is not piped to sewer or septic tank, pit latrine without a slab or open pit, bucket, hanging toilet or hanging latrine, shared facilities, or no facilities), as defined by the JMP	MDG
Target 6.2 (as above)	Hygiene (6.2.1b)	Risk-weighted prevalence of populations with unsafe hygiene (no handwashing with soap), as measured by the SEV for unsafe hygiene, %	Safe hygiene practices were defined as handwashing with soap and water following toilet use or contact with excreta. We separated reporting for indicator 6.2.1 into sanitation (6.2.1a) and hygiene (6.2.1b)	Non-MDG
**Goal 7: Ensure access to affordable, reliable, sustainable, and modern energy for all**				
Target 7.1: By 2030, ensure universal access to affordable, reliable, and modern energy services	Household air pollution (7.1.2)	Risk-weighted prevalence of household air pollution, as measured by the SEV for household air pollution, %	Existing datasets do not comprehensively measure population use of clean fuels and technology for heating and lighting across geographies; thus, we revised this indicator to focus on exposure to clean (or unclean) fuels used for cooking	Non-MDG
**Goal 8: Promote sustained, inclusive, and sustainable economic growth, full and productive employment, and decent work for all**				
Target 8.8: Protect labour rights and promote safe and secure working environments for all workers, including migrant workers, in particular women migrants, and those in precarious employment	Occupational risk burden (8.8.1)	Age-standardised all-cause DALY rate attributable to occupational risks, per 100 000 population	We revised this indicator to the DALY rate attributable to occupational risks because DALYs combine measures of mortality and non-fatal outcomes into a singular summary measure, and occupational risks represent the full range of safety hazards that could be encountered in working environment	Non-MDG
**Goal 11: Make cities and human settlements inclusive, safe, resilient, and sustainable**				
Target 11.5: By 2030, significantly reduce the number of deaths and the number of people affected and substantially decrease the direct economic losses relative to global gross domestic product caused by disasters, including water-related disasters, with a focus on protecting the poor and people in vulnerable situations	Disaster (11.5.1; same as Indicators 1.5.1 and 13.1.2)	Age-standardised death rate due to exposure to forces of nature, per 100 000 population	Existing datasets do not comprehensively measure missing people and people affected by natural disasters; we revised this indicator to exposure to forces of nature and reported in age-standardised rates	Non-MDG
Target 11.6: By 2030, reduce the adverse per-capita environmental impact of cities, including by paying special attention to air quality and municipal and other waste management	Mean PM2·5 (11.6.2)	Population-weighted mean levels of PM2·5, μg/m^3^	No indicator modifications required	Non-MDG
**Goal 13: Take urgent action to combat climate change and its impacts**				
Target 13.1: Strengthen resilience and adaptive capacity to climate-related hazards and natural disasters in all countries	Disaster (13.1.2; same as Indicators 1.5.1 and 11.5.1)	Age-standardised death rate due to exposure to forces of nature, per 100 000 population	Existing datasets do not comprehensively measure missing people and people affected by natural disasters; we revised this indicator to exposure to forces of nature and reported in age-standardised rates	Non-MDG
**Goal 16: Promote peaceful and inclusive societies for sustainable development, provide access to justice for all, and build effective, accountable and inclusive institutions at all levels**				
Target 16.1: Significantly reduce all forms of violence and related death rates everywhere	Violence (16.1.1)	Age-standardised death rate due to interpersonal violence, per 100 000 population	Existing datasets do not comprehensively measure displacement and migratory status of victims of intentional homicide; we revised this indicator to deaths due to interpersonal violence (ie, homicide)	Non-MDG
Target 16.1 (as above)	War (16.1.2)	Age-standardised death rate due to collective violence and legal intervention, per 100 000 population	Existing datasets do not comprehensively measure the displacement status of deaths due to conflict; we revised this indicator to deaths due to collective violence and legal intervention (ie, war)	Non-MDG

Detailed descriptions of the data sources and methods used to estimate each health-related SDG indicator are in the methods appendix pp 10–311. SDG=Sustainable Development Goal. MDG=Millennium Development Goal. IOTF=International Obesity Task Force. GBD=Global Burden of Disease Study. NCDs=non-communicable diseases. SEV=summary exposure value. WaSH=water, sanitation, and hygiene. JMP=Joint Monitoring Program. DALY=disability-adjusted life-year. PM2·5=fine particulate matter smaller than 2·5 μm.

**Table 2:** Health-related SDG indicators (proposed by the Inter-Agency and Expert Group on SDG Indicators) excluded in the present analysis, and measurement needs and strategy for future reporting, by SDG target

	**Health-related SDG indicator**	**Measurement needs and strategy**
**Goal 3: Ensure healthy lives and promote wellbeing for all at all ages**		
Target 3.5: Strengthen the prevention and treatment of substance abuse, including narcotic drug abuse and harmful use of alcohol	3.5.1: Coverage of treatment interventions (pharmacological, psychosocial and rehabilitation and aftercare services) for substance use disorders	Prevalence of specific substance use disorders (opioid use disorders, cocaine use disorders, amphetamine use disorders, and cannabis use disorders), as well as alcohol use disorders, are presently estimated as part of GBD. Systematic reviews on coverage of specific interventions (eg, opioid substitution therapy) are in progress by GBD collaborators
Target 3.8: Achieve universal health coverage, including financial risk protection, access to quality essential health-care services and access to safe, effective, quality and affordable essential medicines and vaccines for all	3.8.2: Number of people covered by health insurance or a public health system per 1000 population	Omission of information on insurance depth and status of user fees within the public health system might limit the applications of this indicator. Construction of proxy measures of health-care use, for both outpatient and hospital care, by country and over time is feasible as part of future iterations of GBD and is likely to be an improved measurement strategy
Target 3.b: Support the research and development of vaccines and medicines for the communicable and non-communicable diseases that primarily affect developing countries, provide access to affordable essential medicines and vaccines, in accordance with the Doha Declaration on the TRIPS Agreement and Public Health, which affirms the right of developing countries to use to the full the provisions in TRIPS regarding flexibilities to protect public health, and, in particular, provide access to medicines for all	3.b.1: Proportion of the population with access to affordable medicines and vaccines on a sustainable basis. The recommended measure is percentage of health facilities with essential medicines and life-saving commodities in stock	Across all geographies and over time, comparable data on the stocking and stock-out rates of essential medicines and vaccines for all facility types (hospitals, primary care facilities, pharmacies, and other health-care outlets) and facility ownership (public, private, informal) are not available at present. In the absence of robust measures of stock-outs in both the public and private sectors across countries and over time, the measurement strategy for producing comparable results for this indicator is unclear. Furthermore, the proposed indicator stipulates measurement of not only access to medicines and vaccines, but also access to affordable medicines and vaccines. No comprehensive and comparable datasets on the status of essential medicine and vaccine affordability, in addition to their stocks, presently exist
Target 3.b (as above)	3.b.2: Total net official development assistance to the medical research and basic health sectors	DAH is currently assessed within a comprehensive, comparable analytical framework by source, channel, recipient country, and health focus area from 1990 to 2015; however, funding specifically for medical research (eg, research and development of vaccines and medicines, as described in Target 3.b) is not systematically available across source and recipient countries. Additionally, the appropriate assessment of country-level performance remains unclear (eg, whether countries that receive high levels of DAH for medical research are equivalent, in terms of indicator performance, to countries that disperse high levels of DAH for medical research)
Target 3.c: Substantially increase health financing and the recruitment, development, training and retention of the health workforce in developing countries, especially in least developed countries and small island developing States	3.c.1: Health worker density and distribution, as measured by number of health workers per 1000 population by cadre. Cadres include generalist medical practitioners, specialist medical practitioners (surgeons, anaesthetists, obstetricians, emergency medicine specialists, cardiologists, paediatricians, psychiatrists, ophthalmologists, gynaecologists, etc), nursing and midwifery professionals, and traditional and complementary medicine professionals, among others	A systematic analysis of population census data and Labour Force Surveys is possible as part of future iterations of GBD. The total quantity of individual health worker cadres that could be comparably assessed by geography by year will be a function of the availability of detailed International Labour Organization occupational codes across geographies and survey iteration
Target 3.d: Strengthen the capacity of all countries, in particular developing countries, for early warning, risk reduction and management of national and global health risks	3.d.1: International Health Regulations (IHR) capacity and health emergency preparedness. The WHO-recommended measure is the percentage of 13 core capacities that have been attained at a specific time (IHR core capacity index). The 13 core capacities are (1) national legislation, policy, and financing; (2) coordination and national focal point communications; (3) surveillance; (4) response; (5) preparedness; (6) risk communication; (7) human resources; (8) laboratory; (9) points of entry; (10) zoonotic events; (11) food safety; (12) chemical events; and (13) radionuclear emergencies	Comprehensive and comparable data for all components of the IHR core capacity index, for all geographies and over time, are not available at present. Specific core capacities, such as zoonotic events, could be assessed as part of future iterations of GBD; other core capacities, such as coordination and national focal point communications, have no clear measurement strategy beyond self-report from country representatives or secondary research on policy status and types of surveillance systems available, among others
**Goal 5: Achieve gender equality and empower all women and girls**		
Target 5.2: Eliminate all forms of violence against all women and girls in the public and private spheres, including trafficking and sexual and other types of exploitation	5.2.2: Proportion of women and girls aged 15 years and older subjected to sexual violence by persons other than an intimate partner in the previous 12 months, by age and place of occurrence	Prevalence of intimate partner violence among women and girls aged 15 years and older is currently estimated as part of GBD. An updated systematic review of the literature, data re-extraction, and analysis are needed to specifically quantify prevalence of sexual violence (separately or in addition to physical violence, or both) and by persons other than an intimate partner. Data availability by geography by year on the latter, sexual violence by persons other than intimate partners, might be limited
Target 5.6: Ensure universal access to sexual and reproductive health and reproductive rights as agreed in accordance with the Programme of Action of the International Conference on Population and Development and the Beijing Platform for Action and the outcome documents of their review conferences	5.6.1: Proportion of women aged 15–49 years who make their own informed decisions regarding sexual relations, contraceptive use, and reproductive health care	The proportion of women who make their own informed decisions regarding all three dimensions of this indicator—sexual relations, contraceptive use, and reproductive health care—are included in the Demographic and Health Survey (DHS) series. Data availablility for non-DHS countries is unclear. The feasibility of measuring this indicator as part of future iterations of GBD is under review at present
Target 5.6 (as above)	5.6.2: Number of countries with laws and regulations that guarantee women aged 15–49 access to sexual and reproductive health care, information, and education	Across all geographies and over time, comprehensive and comparable data documenting the status of laws and regulations regarding access to sexual and reproductive health care, information, and education do not exist at present. Compiling the past and current status of such laws and regulations might be possible; however, systematic assessment of their depth or intensity, enforcement, and effectiveness in guaranteeing access to reproductive health care, information, and education might be challenging across countries and over time
**Goal 6: Ensure availability and sustainable management of water and sanitation for all**		
Target 6.3: By 2030, improve water quality by reducing pollution, eliminating dumping and minimising release of hazardous chemicals and materials, halving the proportion of untreated waste water, and substantially increasing recycling and safe reuse globally	6.3.1: Proportion of waste water safely treated. UN Water defines this indicator as the proportion of waste water generated by both households (sewage and faecal sludge), as well as economic activities (based on ISIC categories) safely treated compared to total waste water generated both through households and economic activities. While the definition conceptually includes waste water generated from all economic activities, monitoring will focus on waste water generated from hazardous industries (as defined by relevant ISIC categories)	Across all geographies and over time, comprehensive and comparable data containing information on total waste water, as generated by both households and non-household entities (however they are to be defined), and waste water treatment status do not exist at present. UN Water suggests there will be sufficient data to generate estimates of global and regional levels of safely treated waste water by 2018; however, in the absence of more country-level data, it is difficult to determine the representativeness of such global and regional estimates
**Goal 16: Promote peaceful and inclusive societies for sustainable development, provide access to justice for all and build effective, accountable, and inclusive institutions at all levels**		
Target 16.1: Significantly reduce all forms of violence and related death rates everywhere	16.1.3: Proportion of population subjected to physical, psychological, or sexual violence in the previous 12 months	Prevalence of intimate partner violence among women and girls aged 15 years and older is currently estimated as part of GBD, as are the incidence and prevalence of interpersonal violence among all populations. An expanded systematic review of the literature and available data sources for all types of violence (physical, psychological, and sexual) for both men and women of all ages would be required for inclusion in future iterations of GBD
Target 16.1 (as above)	16.1.4: Proportion of people that feel safe walking alone around the area they live	Comprehensive data on reported safety, in general or walking alone near one's residence (or both), do not currently exist across geographies or over time. Substantive primary data collection is likely to be required
Target 16.2: End abuse, exploitations, trafficking and all forms of violence against and torture of children	16.2.3: Proportion of young women and men aged 18–29 years who experienced sexual violence by age 18	Prevalence of intimate partner violence among women and girls aged 15 years and older is estimated as part of GBD. An expanded systematic review and analysis of the literature and available data sources for both men and women, and for all types of sexual violence (ie, not limited to intimate partners) would be required. The feasibility of measuring this indicator as part of future iterations of GBD is under review at present
**Goal 17: Strengthen the means of implementation and revitalise the global partnership for sustainable development**		
Target 17.19: By 2030, build on existing initiatives to develop measurements of progress on sustainable development that complement gross domestic product, and support statistical capacity building in developing countries	17.19.2: Proportion of countries that (a) have conducted at least one population and housing census in the last 10 years; and (b) have achieved 100% birth registration and 80% death registration	For Indicator 17.19.2 (a), a comprehensive assessment of the availability and timing of population and housing censuses across all geographies is possible as part of GBD. For Indicator 17.19.2 (b), the systematic collation of vital registration data for all geographies is required; at present, vital registration data reported to WHO do not fully cover all geographies or years under analysis. Such data collation efforts would be required for both birth and death registration individually to determine completeness, with the latter viewed as more immediately feasible for future iterations of GBD

SDG=Sustainable Development Goal. GBD=Global Burden of Disease. TRIPS=Agreement on Trade-Related Aspects of Intellectual Property Rights. DAH=development assistance for health. IHR=International Health Regulations. DHS=Demographic and Health Survey. ISIC=International Standard Industrial Classification.

**Table 3:** Performance of health-related SDG indicators across all countries, 2015

	**Median (IQR)**	**Minimum**	**Maximum**	SDG target by 2030*	**Proportion of 188 countries achieving the SDG target in 2015**
Disaster (Indicator 1.5.1; same as Indicators 11.5.1 and 13.2.1)—age-standardised death rate due to exposure to forces of nature, per 100 000 population	0·0 (0·0–0·1)	0·0	7·5	Undefined	NA
Stunting (Indicator 2.2.1)—prevalence of stunting in children under age 5 years, %	12·5% (4·6–26·5)	0·0%	54·5%	Eliminate	16·5%
Wasting (Indicator 2.2.2a)—prevalence of wasting in children under age 5 years, %	3·6% (1·8–7·1)	0·0%	21·7%	Eliminate	16·5%
Overweight (Indicator 2.2.2b)—prevalence of overweight in children aged 2–4 years, %	23·1% (14·1–32·1)	2·6%	54·5%	Eliminate	0·0%
Maternal mortality ratio (Indicator 3.1.1)—maternal deaths per 100 000 livebirths	49·1 (15·2–239·1)	0·7	1073·9	<70 deaths per 100 000 livebirths	61·2%
Skilled birth attendance (Indicator 3.1.2)—proportion of births attended by skilled health personnel (doctors, nurses, midwives, or country-specific medical staff [eg, clinical officers]), %	98·1% (80·9–99·2)	20·6%	99·6%	100%	0·0%
Under-5 mortality (Indicator 3.2.1)—probability of dying before age 5 years per 1000 livebirths	17·5 (7·1–44·9)	1·9	130·5	At least as low as 25 deaths per 1000 livebirths	60·1%
Neonatal mortality (Indicator 3.2.2)—probability of dying during the first 28 days of life per 1000 livebirths	9·3 (3·5–21·0)	1·0	40·6	At least as low as 12 deaths per 1000 livebirths	57·5%
HIV (Indicator 3.3.1)—age-standardised rate of new HIV infections, per 1000 population	0·1 (0·0–0·4)	0·0	27·4	Eliminate	0·0%
Tuberculosis (Indicator 3.3.2)—age-standardised rate of new and relapsed tuberculosis cases, per 1000 population	0·6 (0·2–1·5)	0·0	26·1	Eliminate	0·0%
Malaria (Indicator 3.3.3)—age-standardised rate of malaria cases, per 1000 population	0·0 (0·0–18·5)	0·0	286·8	Eliminate	52·1%
Hepatitis B (Indicator 3.3.4)—age-standardised rate of hepatitis B incidence, per 100 000 population	18 38·6 (1070·4–2098·4)	444·5	2554·1	Undefined	NA
Neglected tropical diseases (Indicator 3.3.5)—age-standardised prevalence of neglected tropical diseases, per 100 000 population	14 474·0 (236·3–46 139·0)	9·8	119 695·4	Eliminate	0·0%
Non-communicable diseases (Indicator 3.4.1)—age-standardised death rate due to cardiovascular disease, cancer, diabetes, and chronic respiratory disease in populations aged 30–70 years, per 100 000 population	422·0 (291·4–552·5)	154·0	1442·5	Reduce by one-third	NA
Suicide (Indicator 3.4.2)—age-standardised death rate due to self-harm, per 100 000 population	10·3 (6·9–14·3)	2·2	34·0	Reduce by one-third	NA
Alcohol (Indicator 3.5.2)—risk-weighted prevalence of alcohol consumption, as measured by the SEV for alcohol use, %	7·8% (4·2–11·1)	0·7%	28·7%	Undefined	NA
Road injuries (Indicator 3.6.1)—age-standardised death rate due to road injuries, per 100 000 population	15·3 (9·7–23·2)	3·0	63·9	Reduce by half†	NA
Family planning need met, modern contraception (Indicator 3.7.1)—proportion of women of reproductive age (15–49 years) who have their need for family planning satisfied with modern methods, % women aged 15–49 years	72·4% (46·6–87·0)	15·8%	99·1%	100%	0·0%
Adolescent birth rate (Indicator 3.7.2)—birth rates for women aged 10–14 years and women aged 15–19 years, number of livebirths per 1000 women aged 10–14 years and women aged 15–19 years	22·9 (9·4–37·8)	1·1	102·6	Undefined	NA
Universal health coverage tracer (Indicator 3.8.1)—coverage of universal health coverage tracer interventions for prevention and treatment services, %	79·2% (64·9–88·1)	23·3%	94·6%	100%	0·0%
Air pollution mortality (Indicator 3.9.1)—age-standardised death rate attributable to household air pollution and ambient air pollution, per 100 000 population	74·9 (40·6–170·7)	9·0	427·3	Undefined	NA
WaSH mortality (Indicator 3.9.2)—age-standardised death rate attributable to unsafe WaSH, per 100 000 population	8·4 (2·4–44·2)	0·7	318·0	Undefined	NA
Poisons (Indicator 3.9.3)—age-standardised death rate due to unintentional poisonings, per 100 000 population	0·8 (0·4–2·0)	0·1	7·1	Undefined	NA
Smoking (Indicator 3.a.1)—age-standardised prevalence of daily smoking in populations aged 10 years and older, % population aged 10 years and older	11·0% (6·5–16·3)	0·7%	29·5%	Undefined	NA
Intimate partner violence (Indicator 5.2.1)—age-standardised prevalence of women aged 15 years and older who experienced intimate partner violence, % women aged 15 years and older	19·0% (13·7–25·7)	4·7%	44·6%	Eliminate	0·0%
Water (Indicator 6.1.1)—risk-weighted prevalence of populations using unsafe or unimproved water sources, as measured by the SEV for unsafe water, %	62·7% (21·2–83·0)	0·0%	98·4%	Eliminate	16·0%
Sanitation (Indicator 6.2.1a)—risk-weighted prevalence of populations using unsafe or unimproved sanitation, as measured by the SEV for unsafe sanitation, %	20·6% (3·6–57·5)	0·0%	96·4%	Eliminate	16·0%
Hygiene (Indicator 6.2.1b)—risk-weighted prevalence of populations with unsafe hygiene (no handwashing with soap), as measured by the SEV for unsafe hygiene, %	74·2% (60·5–94·1)	36·0%	99·7%	Eliminate	0·0%
Household air pollution (Indicator 7.1.2)—risk-weighted prevalence of household air pollution, as measured by the SEV for household air pollution, %	7·1% (0·3–36·0)	0·0%	73·6%	Eliminate	16·5%
Occupational risk burden (Indicator 8.8.1)—age-standardised all-cause DALY rate attributable to occupational risks, per 100 000 population	757·7 (552·7–999·2)	278·7	2148·3	Undefined	NA
Mean PM2·5 (Indicator 11.6.2)—population-weighted mean levels of PM2·5, μg/m^3^	21·7 (15·1–37·6)	3·4	107·3	Undefined	NA
Violence (Indicator 16.1.1)—age-standardised death rate due to interpersonal violence, per 100 000 population	3·7 (1·6–8·2)	0·4	58·3	Undefined	NA
War (Indicator 16.1.2)—age-standardised death rate due to collective violence and legal intervention, per 100 000 population	0·0 (0·0–0·0)	0·0	309·9	Undefined	NA

SDG=Sustainable Development Goal. NA=not applicable. SEV=summary exposure value. WaSH=water, sanitation, and hygiene. DALY=disability-adjusted life-year. PM2·5=fine particulate matter smaller than 2·5 μm in diameter.

* SDG targets without explicit achievement thresholds, such as “significantly reduce by 2030”, or with reduction-based thresholds, such as “reduce by one-third”, are reported as undefined.

† The target year for achieving indicator 3.6.1 is 2020.

## Supplementary Material

Supplementary appendices
