# Department of Error

**DOI:** 10.1016/S0140-6736(16)32610-1

**Published:** 2017-01-07

*GBD 2015 SDG Collaborators. Measuring the health-related Sustainable Development Goals in 188 countries: a baseline analysis from the Global Burden of Disease Study 2015.* Lancet *2016; **388:** 1813–50*—In this Article, Ubai Alsharif, Giorgia Giussani, Harish Gugnani, Sudha P Jayaraman, Thomas Truelsen, and João Vasco Santos should have been listed as authors. The affiliation details for Simon I Hay have been updated. The funding statement for Simon I Hay has been added. These corrections have been made to the online version as of Jan 5, 2017.

