# Supplementary material for: Measuring the health-related Sustainable Development Goals in 188 countries: a baseline analysis from the Global Burden of Disease Study 2015
Source: Lancet. 2016 Oct 8;388(10053):1813–50. doi: 10.1016/S0140-6736(16)31467-2 (PMC5055583; doi:10.1016/S0140-6736(16)31467-2)
Supplement: Supplementary appendices [file mmc1.pdf]

THE LANCET

Supplementary appendix

This appendix formed part of the original submission and has been peer reviewed. We post it as supplied by the authors.

Supplement to: GBD 2015 SDG Collaborators. Measuring the health-related Sustainable Development Goals in 188 countries: a baseline analysis from the Global Burden of Disease Study 2015. *Lancet* 2016; published online Sept 21. http://dx.doi.org/10.1016/S0140-6736(16)31467-2.

Methods Appendix to Measuring the health-related Sustainable Development Goals in 188 countries: An analysis from the Global Burden of Disease Study 2015

This methods appendix provides further methodological detail for the health-related Sustainable Development Goals (SDGs). The appendix is organized into broad sections following the structure of the main paper.

The supplementary results offer additional results tables and figures, as well as more detailed methodological figures.

Table of Contents

List of Methods Appendix Tables 2

List of Supplementary Results: Figures & Tables 3

Preamble 5

GATHER Statement 6

Part 1. Health-related SDG indicators 9
- Section 1. SDGs overview
- Section 2. Health-related SDGs
- Section 3. SDG Indicator write-ups

Part 2. Construction of the sensitivity analyses 287
- Section 1. Sensitivity analyses

Part 3. Comprehensive citation list 288

Part 4. Online tools and glossary of terms 853
- Section 1. Online tools
- Section 2. List of abbreviations

Supplementary Results .... *see additional document*

Methods Appendix: Tables

Methods Appendix Table 1. GATHER checklist of information that should be included in reports of global health estimates, with description of compliance and location of information for SDG Capstone

Methods Appendix Table 2. Comprehensive citation list

Supplementary Results

Figure 1. Levels of the health-related SDG index, MDG index, non-MDG index, and 33 individual health-related SDG indicators by country, 1990

Figure 2. Levels of the health-related SDG index, MDG index, non-MDG index, and 33 individual health-related SDG indicators by country, 2000

Figure 3. Map of health-related SDG index, by quintile, 1990

Figure 4. Map of health-related SDG index, by quintile, 2000

Figure 5. Absolute change in the health-related SDG index, MDG index, non-MDG index, and 33 individual health-related SDG indicators (rescaled), by country from 1990 to 2015

Figure 6. Absolute change in the health-related SDG index, MDG index, non-MDG index, and 33 individual health-related SDG indicators (rescaled), by country from 2000 to 2015

Figure 7a. Appendix Figure 7a. Comparison of health-related SDG index values using the arithmetic mean of targets versus the geometric mean, 2015.

Figure 7b. Comparison of health-related SDG index ranks by country using the arithmetic mean of targets versus the geometric mean, 2015.

Figure 8a. Comparison of health-related SDG index values using the geometric mean of the minimums across each target versus the standard geometric mean, 2015.

Figure 8b. Comparison of health-related SDG index ranks by country using the geometric mean of the minimums across each target versus the standard geometric mean, 2015.

Figure 9. Map of observed health-related SDG index minus expected health-related SDG index, on the basis of SDI alone, in 1990.

Figure 10. Map of observed health-related SDG index minus expected health-related SDG index, on the basis of SDI alone, in 2000.

Table 1a. Master country table w/ scaled values for each SDG health-related indicator by country for 1990, 2000, and 2015

Table 1b. Master country table w/ unscaled values for each SDG health-related indicator by country for 1990, 2000, and 2015

Table 2. Correlation coefficient with SDI of each composite index and health-related indicator, across all countries and years from 1990 to 2015, 5 year intervals.

Preamble

This appendix provides methodological detail, supplemental figures and tables, and more detailed results for the health-related Sustainable Development Goals (SDGs). The appendix is organized into broad sections following the structure of the main paper. This study complies with the Guidelines for Accurate and Transparent Health Estimates Reporting (GATHER) recommendations, and this appendix is more comprehensive and encyclopedic than previous Global Burden of Disease (GBD) appendices. It includes detailed tables, figures, indicator modeling write-ups and flowcharts, and information on data sourcing in an effort to maximize transparency in our estimation processes and provide a comprehensive account of analytical steps. Components of this document are the same as described in earlier GBD 2015 Capstone appendices but much more of this appendix are new text for the SDG Capstone. We intend this to be a living document, to be updated with each annual iteration of the Global Burden of Disease and in accordance with the 15 year timeline of the SDG cycle until their conclusion in 2030.

GATHER statement

This study complies with the Guidelines for Accurate and Transparent Health Estimates Reporting (GATHER) recommendations. We have documented the steps involved in our analytical procedures and detailed the data sources used in compliance with the Guidelines for Accurate and Transparent Health Estimates Reporting (GATHER). For additional GATHER reporting, please refer to Appendix Table 4 in Section 3.

Methods Appendix Table 1. GATHER checklist of information that should be included in reports of global health estimates, with description of compliance and location of information for SDG Capstone

#	GATHER checklist item	Description of compliance	Reference
**Objectives and funding**			
1	Define the indicators, populations, and time periods for which estimates were made.	Narrative provided in paper and appendix describing indicators, definitions, and populations.	Summary; Main text; Appendix Part 1. Sections 1-3; Supplementary Results
2	List the funding sources for the work.	Funding sources listed in paper.	Main text
**Data Inputs**			
For all data inputs from multiple sources that are synthesized as part of the study:			
3	Describe how the data were identified and how the data were accessed.	Narrative description of data seeking methodology provided.	Appendix Part 1. Sections 1-3
4	Specify the inclusion and exclusion criteria. Identify all ad-hoc exclusions.	Narrative about inclusion and exclusion criteria by data type provided.	Appendix Part 1. Sections 1-3
5	Provide information on all included data sources and their main characteristics. For each data source used, report reference information or contact name/institution, population represented, data collection method, year(s) of data collection, sex and age range, diagnostic criteria or measurement method, and sample size, as relevant.	List of all data sources provided in submission materials; interactive, online data source tool that provides metadata for data sources by component, geography, cause, risk, or impairment has been developed.	Appendix Part 3. Section 1.<br><br>http://ghdx.healthdata.org/<br><br>There is a forthcoming custom data source tool with additional information on data sourcing for GBD and SDG capstone publications.
6	Identify and describe any categories of input data that have potentially important biases (e.g., based on characteristics listed in item 5).	Summary of known biases by cause included in methodological appendix.	Appendix Part 1. Section 3
For data inputs that contribute to the analysis but were not synthesized as part of the study:			
7	Describe and give sources for any other data inputs.	Included in list of all data sources provided in submission materials, as well as online data source tool.	http://ghdx.healthdata.org/<br><br>There is a forthcoming custom data source tool with additional information on data sourcing for GBD and SDG capstone publications.
For all data inputs:			
8	Provide all data inputs in a file format from which data can be efficiently extracted (e.g., a spreadsheet as opposed to a PDF), including all relevant meta-data listed in item 5. For any data inputs that cannot be shared due to ethical or legal reasons, such	Downloads of input data will be available through online tools, including data visualization tools and data query tools. Input data not available in tools will be made available upon request.	Online data tools http://www.healthdata.org/results/data-visualizations; http://ghdx.healthdata.org/; http://ghdx.healthdata.org/gbd-data-tool

	as third-party ownership, provide a contact name or the name of the institution that retains the right to the data.		
**Data analysis**			
9	Provide a conceptual overview of the data analysis method. A diagram may be helpful.	Flow diagrams of the overall methodological processes, as well as cause-specific modelling processes have been provided.	Main text; Appendix Part 1. Section 3
10	Provide a detailed description of all steps of the analysis, including mathematical formulae. This description should cover, as relevant, data cleaning, data pre-processing, data adjustments and weighting of data sources, and mathematical or statistical model(s).	Flow diagrams and corresponding methodological write-ups for each cause and modelling processes have been provided.	Appendix Part 1. Section 3
11	Describe how candidate models were evaluated and how the final model(s) were selected.	Provided in the methodological write-ups.	Appendix Part 1. Section 3
12	Provide the results of an evaluation of model performance, if done, as well as the results of any relevant sensitivity analysis.	Provided in the methodological write-ups.	Appendix Part 2
13	Describe methods for calculating uncertainty of the estimates. State which sources of uncertainty were, and were not, accounted for in the uncertainty analysis.	Provided in the methodological write-ups.	Appendix Part 1. Section 3
14	State how analytic or statistical source code used to generate estimates can be accessed.	Access statement provided.	This will be available in an online repository that will be released upon publication of GBD 2015 Capstones.
**Results and Discussion**			
15	Provide published estimates in a file format from which data can be efficiently extracted.	GBD 2015 results will be made available through online data visualization tools, the Global Health Data Exchange, and the online data query tool (these tools are already available for GBD 2013 results).	Supplementary Results
16	Report a quantitative measure of the uncertainty of the estimates (e.g. uncertainty intervals).	Uncertainty intervals are provided with all results.	Main text; Supplementary Results
17	Interpret results in light of existing evidence. If updating a previous set of estimates, describe the reasons for changes in estimates.	Discussion of methodological changes between SDG rounds provided in the narrative of the paper and appendix.	Main text; Appendix Part 1. Section XX
18	Discuss limitations of the estimates. Include a discussion of any modelling assumptions or data limitations that affect interpretation of the estimates.	Discussion of limitations provided in the narrative of the main paper as well as in the methodological write-ups in the appendix.	Main text; Appendix Part 1. Section 3

Part 1. Health-related SDG indicators

Section 1. Sustainable Development Goals overview

In September 2015, the United Nations (UN) General Assembly established the Sustainable Development Goals (SDGs). The SDGs substantially broaden the development agenda beyond the MDGs and are expected to frame UN member state policies over the next 15 years. The SDGs specify 17 universal goals, 169 targets, and 230 indicators leading up to 2030. The SDGs substantially broaden the development agenda beyond the MDGs and are expected to frame UN member state policies over the next 15 years. We provide an analysis of 33 out of the 47 health-related SDG indicators based on data used and generated by the Global Burden of Diseases, Injuries and Risk Factors Study 2015 (GBD 2015).

Section 2. Health-related SDGs

Health is a core dimension of the SDGs; the third SDG aims to "ensure healthy lives and promote wellbeing for all at all ages." Health-related indicators are also present among ten of the other 16 goals. Across these 11 goals, there are 28 health-related targets with a total of 47 health-related indicators.

Of the 47 health-related indicators included as part of the SDGs, estimates for 33 indicators, using consistent approaches built on systematic efforts to compile all available data, are included as part of the GBD study. In this paper, while acknowledging the continued debate about the structure and choices of SDG indicators, we use the GBD study to provide an assessment of the current status of these 33 health-related SDG indicators, develop and compute a summary indicator of the health-related SDG indicators, document historical trends, identify high achievers to inform roadmaps and provide a basis for future monitoring of the health-related SDG indicators.

The GBD study is an annual effort to measure the health of populations at national, and selected sub-national levels, from 1990 to the most recent year of 2015. The GBD study produces estimates of mortality and morbidity by cause, age and sex as well as that attributable to a selected set of major risk factors. Many of the 47 health-related SDG indicators selected by the Inter-Agency and Expert Group on Sustainable Development Goal Indicators (IAEG-SDGs are produced as part of the GBD. Elsewhere in this appendix, we outline the 10 SDGs, corresponding 21 health-related targets, and 33 health-related indicators included in this iteration of the GBD and SDG reporting. Part 1. Section 3 of this appendix also further outlines the definition of each indicator used in analysis, as well as the estimation method and data sources.

Direct outputs of the GBD study that are health-related SDG indicators include mortality rates disaggregated by age (under-5 and neonatal) and cause (maternal, cardiovascular diseases, cancers, diabetes, chronic respiratory diseases, road traffic injuries, self-harm, unintentional poisonings, exposure to forces of nature, interpersonal violence, and collective violence and legal intervention) as well as measures of disease incidence (HIV/AIDS, malaria, tuberculosis [TB], hepatitis B) and prevalence (neglected tropical diseases [NTDs]). The GBD risk factor analysis includes measurement of exposure prevalence included as health-related SDG indicators (under-5 stunting, wasting and overweight; tobacco smoking; harmful alcohol use; intimate partner violence; unsafe water, sanitation, and hygiene [WaSH]; household air pollution; and ambient particulate matter) as well as deaths or disease burden attributable to risk factors selected as health-related SDG indicators (WaSH, household and ambient air pollution, and occupational risks).

As noted in the main text, for selected indicators proposed by the IAEG-SDGs, we made modifications to the definition for clarity and/or based on the definition used in GBD. For example, Indicator 2.2.2 proposes to measure of malnutrition that combined prevalence of wasting and overweight among children under 5. As childhood wasting and overweight have very different determinants, we have selected to report them separately. For childhood overweight, we report prevalence among children aged 2 to 4 years, the definition used in GBD based on thresholds set by the International Obesity Task Force (IOTF).

Further details on the estimation and data sources used for all indicators, compliant with Guidelines for Accurate and Transparent Health Estimates Reporting (GATHER), are included in Appendix Part 1. Section 3. Indicator-specific estimation and Appendix Part 4. Section 1. Comprehensive citation list.

Section 3. Indicator-specific estimation

The indicator-specific modeling write-ups follow the order of the SDG goals, targets and indicators proposed by the United Nations. In some cases, multiple indicators were addressed in a single write-up, for example natural disaster related-indicators (1.5.1, 11.5.1, and 13.1.2) are included in a single write-up along with war mortality (16.1.2).

The organization of this section is as follows:

Natural disasters (1.5.1, 11.5.1, 13.1.2)
Stunting (2.2.1)
Wasting (2.2.2a)
Overweight (2.2.2b)
Maternal mortality ratio (3.1.1.)
Skilled birth attendance (3.1.2, plus UHC [3.8.1])
Under-5 and neonatal mortality (3.2.1 & 3.3.2)
HIV incidence (3.3.1)
TB incidence (3.3.2)
Malaria incidence (3.3.3)
Hepatitis B incidence (3.3.4)
NTDs prevalence (3.3.5) – includes 14 individual NTDs
NCD mortality (3.4.1) - includes cardiovascular diseases, cancers, diabetes, and chronic respiratory diseases
Self-harm mortality (3.4.2)
Alcohol use (SEV) (3.5.2)
Road injury mortality (3.6.1)
Family planning need met with modern contraception (3.7.1)
Adolescent birth rates (3.7.2)

UHC – Universal Health Coverage: TB case detection, immunization, ANC1 and ANC4, skilled birth attendance, in-facility delivery rate, ART coverage, ITN coverage, and family planning need met with modern contraception (see also 3.7.1) (3.8.1)
UHC - TB case detection (3.8.1)
UHC - Immunization (3.8.1)
UHC - ANC1 and ANC4 (3.8.1)
UHC - In-facility delivery rate (3.8.1)
UHC - ART coverage (3.8.1)
UHC – Family planning need met with modern contraception (3.8.1, also 3.7.1)
UHC – ITN coverage
Deaths attributable to household air pollution and ambient air pollution (3.9.1)
Deaths attributable to WaSH (3.9.2)
Unintentional poisonings mortality (3.9.3)
Smoking prevalence (3.a.1)
Prevalence of intimate partner violence (5.2.1)
Water (SEV) (6.1.1.)
Sanitation (SEV) (6.2.1a)
Hygiene (SEV) (6.2.1b)
Household air pollution (SEV) (7.1.2)
DALY rates attributable to occupational risks (8.8.1)
Population-weighted PM2.5 (fine particulate matter) (11.6.2)
Interpersonal violence mortality (16.1.1)
War mortality (16.1.2)

Fatal Discontinuities SDG Capstone Appendix:

War mortality; natural disasters; and other fatal discontinuities

Flowchart

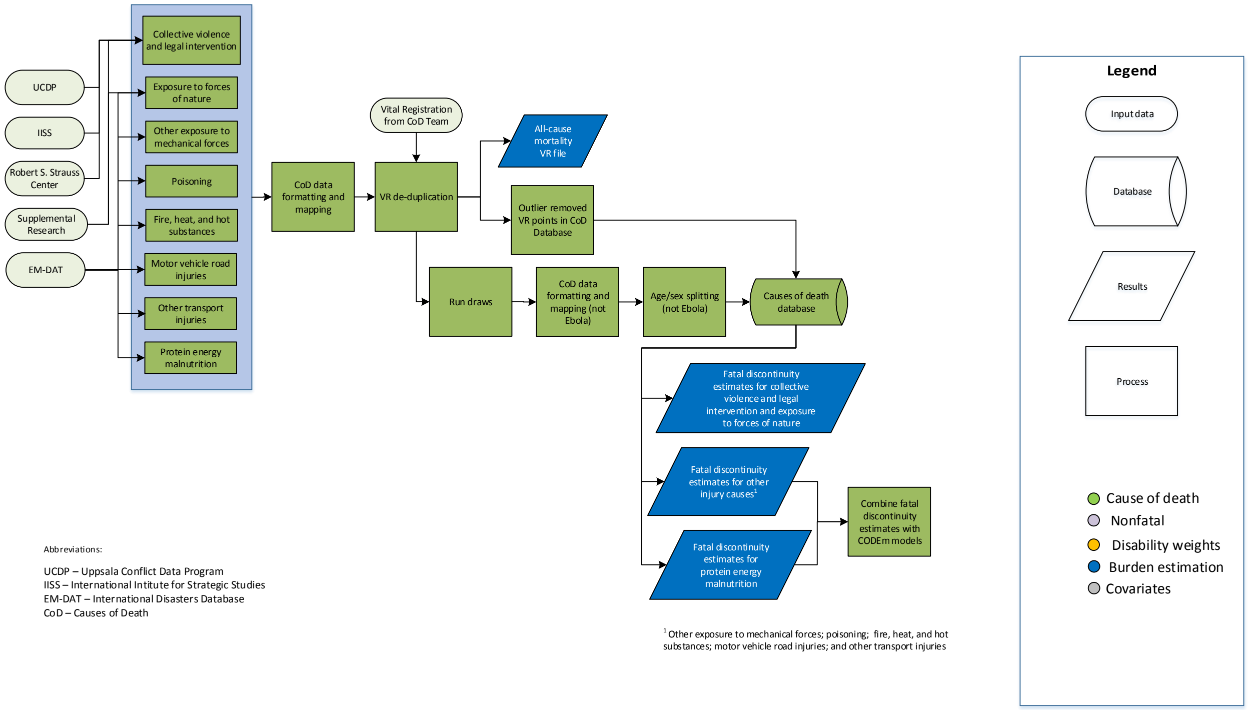

Input Data and Methodological Summary

Indicator definition

This modeling strategy encompasses indicators associated with natural disasters: 1.5.1, 11.5.1, 13.1.2; and war mortality: 16.1.2.

Indicator 1.5.1

As a component of SDG Goal 1. End poverty in all its forms everywhere, SDG Target 1.5., by 2030, build the resilience of the poor and those in vulnerable situations and reduce their exposure and vulnerability to climate-related extreme events and other economic, social and environmental shocks and disasters, is measured using SDG Health Index Indicator 1.5.1, number of deaths due to exposure to forces of nature per 100,000.

Indicator 11.5.1

As a component of SDG Goal 11. Make cities and human settlements inclusive, safe, resilient and sustainable, SDG Target 11.5, by 2030, significantly reduce the number of deaths and the number of people affected and substantially decrease the direct economic losses relative to global gross domestic product caused by disasters, including water-related disasters, with a focus on protecting the poor and

people in vulnerable situations, is measured using SDG Health Index Indicator 11.5.1, Number of deaths, missing persons and persons affected by disaster per 100,000 people.

Indicator 13.1.2

As a component of SDG Goal 13. Take urgent action to combat climate change and its impacts, SDG Target 13.1, strengthen resilience and adaptive capacity to climate-related hazards and natural disasters in all countries, is measured using SDG Health Index Indicator 13.1.2, number of deaths due to exposure to forces of nature per 100,000 people.

Indicator 16.1.2

As a component of SDG Goal 16. Promote peaceful and inclusive societies for sustainable development, provide access to justice for all and build effective, accountable and inclusive institutions at all levels, SDG Target 16.1, significantly reduce all forms of violence and related death rates everywhere, is measured using SDG Health Index Indicator 16.1.2, number of deaths due to collective violence and legal intervention (war) per 100,000.

Input data

Collective Violence and Legal Intervention

Data for collective violence and legal intervention comes from the Uppsala Conflict Data Program (UCDP), International Institute for Strategic Studies (IISS), and Robert S. Strauss Center for International Security and Law. The table below provides details about the various datasets we utilized from these sources, the dates they were last accessed, and the years for which we used the data provided.

Data source name	Date accessed	Years of data accessed	Type of data included
Uppsala Conflict Data Program[1]			
Battles	9/23/15	1989-2015	Armed conflict: incompatibility that concerns government and/or territory over which the use of armed force between the military forces of two parties, of which at least one is the government of a state, has resulted in at least 25 battle-related deaths each year
Non-state	11/4/15	1989-2015	The use of armed force between two organized armed groups, neither of which is the government of a state, which results in at least 25 battle-related deaths each year
One-sided	11/3/15	1989-2015	The use of armed force by the government of a state or by a formally organized group against civilians which results in at least 25 deaths in a year
Africa Georeferenced Event Dataset	11/4/15	1989-2008	UCDP battles; non-state; one-sided for African countries
PRIO Battles Deaths Dataset	9/23/15	1970-1989	Armed conflict (civil wars, etc.)
International Institute for Strategic Studies			
Armed Conflict Dataset	9/25/15	1997-Present	Insurgency, Inter-state, Intra-state
Robert S. Strauss Center For International Security And Law			
Armed Conflict Location and Event Dataset (ACLED)	9/15/15	1997-2015	Actions of opposition groups, governments, and militias across Africa, specifying the exact location and date of battle events, transfers of military control, headquarter establishment, civilian violence, and rioting
Social Conflict Analysis Database (SCAD)	9/15/15	1990-2015	Protests, riots, strikes, inter-communal conflict, government violence against civilians, and other forms of social conflict (covers Africa and Latin America)

Supplemental online research was conducted for recent conflicts where the databases above were not up-to-date. Where there was large variance in death estimates in recent years, we averaged estimates from all sources at the country-year level.

For country-years where multiple sources provided estimates, we prioritized sources in the following order: (1) country vital registration (VR) data, if death estimates were highest of all sources; (2) UCDP; (3) IISS; (4) country VR if death estimates were not the highest of all sources; (5) Strauss Center; (6) online supplemental research; and (7) combined average country data where applicable.

Exposure to Forces of Nature and Other Injury Causes

Data for disaster events which caused greater than 50 deaths due to exposure to forces of nature; poisonings; fire, heat, and hot substances; motor vehicle road injuries; other transport injuries; and other exposure to mechanical forces came from the Centre for Research on the Epidemiology of Disasters' International Disaster Database (EM-DAT). Data from EM-DAT were last accessed January 3, 2016. Supplemental online research was conducted for events where EM-DAT was not up-to-date.

For country-years where multiple sources provided estimates, we prioritized sources in the following order: (1) country VR data, if death estimates were highest of all sources; (2) EM-DAT; (3) country VR if death estimates were not the highest of all sources; (4) online supplemental research. Exceptions were made where it was clear that vital registration systems had been compromised by the event being measured. In those cases, such as in the United States following Hurricane Katrina in 2005, supplemental research was prioritized over VR data.

In locations where we produced estimates at the subnational level for GBD 2015, deaths due to all fatal discontinuity causes were assigned to the relevant subnational location(s) when that information could be obtained either through the data sources mentioned above or through additional online research. If no subnational location could be found, the deaths were split proportionally by population across all subnational locations.

A systematic literature review was not used to identify input data for fatal discontinuities, though some literature sources were identified through online supplemental research.

Modeling strategy

All input data for fatal discontinuity causes were run through the Causes of Death data formatting and mapping process detailed in Part 2.

VR de-duplication

For country-years where deaths due to fatal discontinuity causes were recorded in both VR and other utilized data sources, the higher of the two estimates were taken in the case of deaths due to war and collective violence and exposure to forces of nature.

For the other injury causes that also have a Cause of Death Ensemble model (CODEm), a process was established to avoid duplication of fatal discontinuity deaths in the two models. First, location-years with greater than 50 deaths due to the cause were identified. If these location-cause-years had VR death

estimates that were greater than 40% higher than the immediately surrounding years, the identified years were reviewed. Those that represented a true diversion from the trend of VR and could be linked to a specific fatal discontinuity event were marked as outliers in the VR data and the difference between the outlier year and the average of the surrounding years was included in the relevant cause in the fatal discontinuities database. The deaths from the identified events were subtracted from the all-cause VR estimates used in the all-cause mortality estimation process.

Uncertainty analysis for input and draw-level input to age-sex splitting

Uncertainty intervals (UIs) for deaths due to collective violence and legal intervention were generated using UCDP high and low death estimates. In cases where low and high estimates are not provided by the original source, regional average relative UI by type of fatal discontinuity is applied to the mean input that is available.

We assumed a normal distribution using the mean deaths and standard deviation based on high and low estimates. The standard deviation was capped at the mean divided by 1.96 in order to ensure that 95% of the 1,000 draws generated were greater than 0. Any negative draws were dropped from final calculations of means and uncertainty intervals.

Age-sex splitting

All compiled data were run through the causes of death age-sex splitting process detailed in Part 2.

Changes from GBD 2013

Only collective violence and legal intervention and exposure to forces of nature were modeled as fatal discontinuities (previously called mortality shocks) in GBD 2013. GBD 2015 also includes fatal discontinuity models for protein energy malnutrition (previously included as “famine” in exposure to forces of nature) and additional injury models (motor vehicle road injuries; other transport injuries; exposure to fire, heat, and hot substances; poisoning; and other exposure to mechanical forces). These new causes (unlike collective violence and legal intervention and exposure to forces of nature) are also modeled in CODEm and thus have two models (a shock model and a CODEm model) that are combined to produce the final estimates of deaths for these causes.

The VR de-duplication process and the use of the causes of death age-sex splitting process for fatal discontinuity causes were added in GBD 2015.

References

1. UCDP/PRIO Armed Conflict Dataset Codebook. Uppsala Conflict Data Program (UCDP); Centre for the Study of Civil Wars, International Peace Research Institute, Oslo (PRIO), 2013.

Childhood Undernutrition SDG Capstone Appendix

Flowchart

Childhood Undernutrition

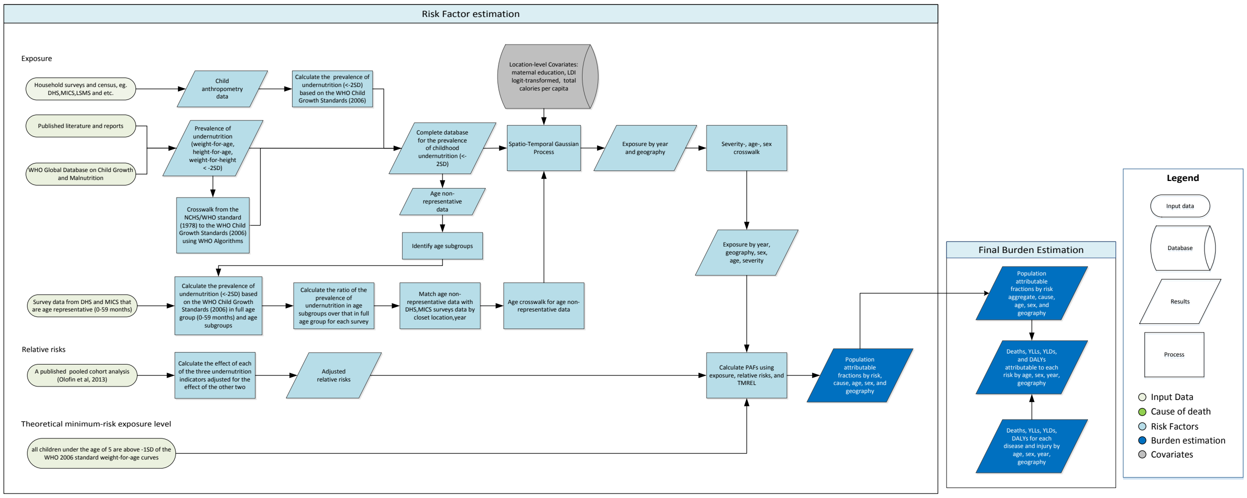

Input Data & Methodological Summary

Indicator definition

This modeling strategy encompasses indicators associated with child undernutrition: 2.2.1 and 2.2.2a.

Indicator 2.2.1

As a component of SDG Goal 2. End hunger, achieve food security, and improved nutrition, SDG Target 2.2, by 2030, end all forms of malnutrition, including achieving, by 2025, the internationally agreed targets on stunting and wasting in children under 5 years of age, and address the nutritional needs of adolescent girls, pregnant and lactating women and older persons, is measured using SDG Health Index Indicator 2.2.1, Prevalence of stunting among children under 5 (lower than two standard deviations from the median height for age of the reference population).

Indicator 2.2.2a

As a component of SDG Goal 2. End hunger, achieve food security, and improved nutrition, SDG Target 2.2, by 2030, end all forms of malnutrition, including achieving, by 2025, the internationally agreed targets on stunting and wasting in children under 5 years of age, and address the nutritional needs of adolescent girls, pregnant and lactating women and older persons, is measured using Indicator 2.2.2a, Prevalence of wasting among children under five (lower than two standard deviations from the median weight for height of the reference population).

Case definition

The exposure of childhood undernutrition was modeled by evaluating three anthropometric indicators which include underweight, wasting, and stunting. The definition of the three indicators are as follows:

Childhood underweight: Proportion of children aged 0 to 59 months in a given population who fall below 2 standard deviations (SD) of the WHO 2006 standard weight-for-age (wfa) curve. (1)

Childhood stunting: Proportion of children aged 0 to 59 months in a given population who fall below 2 standard deviations (SD) of the WHO 2006 height-for-age (hfa) curve.

Childhood wasting: Proportion of children aged 0 to 59 months in a given population who fall below 2 standard deviations (SD) of the WHO 2006 weight-for-height (wfh) curve.

Input data

There are two main inputs in the GBD 2015 undernutrition database—survey dataset and tabulated dataset. Survey dataset includes the standard multi-country or country-specific survey series such as: Reproductive and Health Surveys (RHS), Multiple Indicator Cluster Surveys (MICS), Demographic and Health Surveys (DHS), Living Standards Measurement Surveys (LSMS), China Health and Nutrition Survey (CHNS), and others. In the absence of survey data we used tabulated data from survey reports or published literature that have been extracted at IHME, downloaded from external databases or obtained from personal communication with external collaborators. The last update for tabulated dataset was conducted for GBD 2010. Tabulated data include survey reports or published literature from databases from UNICEF(2), the United Nations (UN) Statistics Division (3), and the WHO Global Database on Child Growth and Malnutrition(4).

Tabulated data based on the National Center for Health Statistics (NCHS)/WHO international growth reference (the NCHS reference) (5) were converted into data based on the World Health Organization (WHO) Child Growth Standards (the WHO 2006 standard) using WHO algorithms (6). Estimates that were not representative of all children under the age of 5 were adjusted based on age groups.

Modeling strategy

Exposure Estimate

To generate a complete time series of prevalence of childhood underweight, wasting, and stunting, we employed a three-step ST-GPR modeling strategy that uses linear regression, spatiotemporal regression and Gaussian Process Regression (GPR) which is specified in the main text of this manuscript. Identical strategies and covariates were used for each undernutrition indicator. A variety of combinations of socioeconomic and environmental covariates in different transformation format were tested by running mixed-effect models with exposure data to decide the inclusion and exclusion. The final list of covariates included in the childhood undernutrition models are mean years of education of women of reproductive age, log transformed lagged-distributed income and total caloric availability (kcal per capita), which remained the same as GBD 2013. Uncertainty in the estimates was based on the data variance, then calculated through ST-GPR.

The final step of exposure estimate is to calculate the distribution of undernutrition prevalence across different levels of severity and age- sex- groups. The levels of severity are defined as follows:

Severe: individuals less than 3SD below the median (<-3SD);

Moderate: individuals between 3SD and 2SD below the median (-3SD to -2SD);

Mild: individuals between 2SD and 1SD below the median (-2SD to -1SD).

In GBD 2013, prevalence of undernutrition in each of severity categories was predicted by applying a linear regression model of the prevalence of undernutrition in each of severity categories against the prevalence of undernutrition below -2SD of the reference median at global level using microdata from 179 DHS surveys. We assumed no difference in the prevalence of undernutrition at any severity level across age and sex among children under 5.

This strategy has experienced a major change in GBD 2015. We estimated the prevalence of undernutrition by GBD age-sex groups, assuming the distribution of undernutrition of different severity categories are difference across age and sex among children under 5. Using available microdata, we first created a pooled global database that consisted of binary indicators of undernutrition by GBD age-sex groups at individual level. Then we ran a logit regression model to predict the proportion of undernutrition outcome in most-detailed severity category (e.g. <-3SD) among the broader severity category (e.g. <-2SD) against the effects of age group and sex. We also took into account the covariance of the proportions among different age-sex groups by using variance-covariance matrix. Last, we applied the proportions by GBD age-sex group generated above onto our GPR estimates.

Theoretical minimum-risk exposure level

Theoretical minimum risk exposure levels (TMREL) for underweight, stunting, and wasting where all children under the age of 5 are above -1SD of the WHO 2006 standard weight-for-age, height-for-age, and weight-for-height curves respectively.

Relative risks

Relative risks (RRs) of risk-outcome pairs were extracted based on a study that conducted a pooled cohort analysis (7), which remained the same as GBD 2013. The final list of outcomes paired with childhood undernutrition risks included lower respiratory infections (LRIs), diarrhea, measles, and protein energy malnutrition (PEM). Originally in GBD 2013, upper respiratory infections (URIs) and otitis media were considered as analogies for LRI considering the similar pathological pathways they share. However, they were dropped from analysis in GBD 2015 due to the lack of evidence on the causal relationships with undernutrition risks. We also attributed 100% of PEM to childhood wasting and underweight but not stunting. A literature search was conducted for GBD 2015 searching for meta-analysis on the association of risk-outcome pairs published after January 1st, 2013, no updated results was found.

The RRs were adjusted using an optimization algorithm we developed at IHME for GBD 2013 that takes into account covariance between the three undernutrition indicators.

Body Mass Index SDG Capstone Appendix

Flowchart

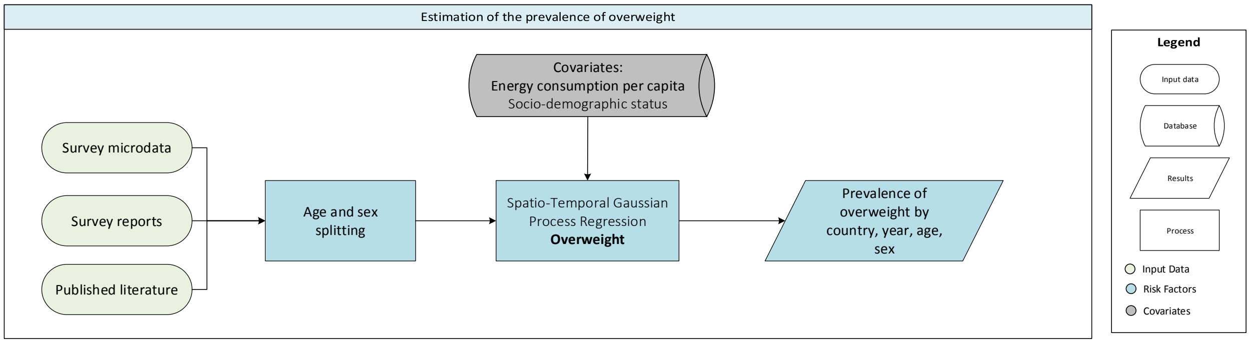

Input Data & Methodological Summary

Indicator definition

This modeling strategy encompasses SDG Health Index Indicators associated with childhood overweight: 2.2.2b.

Indicator 2.2.2b

As a component of SDG Goal 2. End hunger, achieve food security, and improved nutrition, SDG Target 2.2, by 2030, end all forms of malnutrition, including achieving, by 2025, the internationally agreed targets on stunting and wasting in children under 5 years of age, and address the nutritional needs of adolescent girls, pregnant and lactating women and older persons, is measured using SDG Health Index Indicator 2.2.2b, Prevalence of children aged 2 to 4 years with a body-mass index (BMI) exceeding the overweight cut-offs established by the International Obesity Task Force (IOTF) for each sex and by month of age.

Case definition

Exposure to overweight is defined using metrics related to national and subnational estimates of BMI. If a person has a BMI of greater than IOTF cutoff for each sex and age (in month), they are considered overweight.

Input Data

We searched Global Health Data Exchange (GHDx) database for individual level data from multi-country survey programs, national surveys, and longitudinal studies providing measured data on height and weight. Additionally we searched GHDx for published literature providing data on measured height and weight. We included surveys, reports, or studies that provided nationally or subnationally representative estimates of overweight. Sources were excluded if using alternative standards for defining child obesity

which were not comparable with the IOTF standard; utilizing alternative measurement methods (e.g., hydrodensitometry, MRI, CT, skin-fold thickness, and waist-circumference) to estimate the prevalence of overweight; or reporting data on children under the age of 2. We also excluded studies that did not select a random sample of the population; studies conducted among a particular population group (e.g., specific employment status, economic status, pregnant women, patient groups); studies that did not provide adequate details of the sampling method or the sample composition; and studies with a sample size of less than 100. Data points from surveys with high level of missingness caused by incomplete entries on height and weight (>15%) were also excluded. Report and literature data with age groups wider than the standard age groups or with data on both sexes combined were split using the approach used by Ng et al. Briefly, age-sex patterns were identified using sources with data on multiple age-sex groups and these patterns were applied to split aggregated report data. Uncertainty in the age-sex split was propagated by multiplying the standard error of the data by the square root of the number of splits performed.

Modeling strategy

We used a spatiotemporal Gaussian process regression (ST-GPR) model to estimate the prevalence of overweight among children aged 2 to 4 years. To borrow strength across age groups, we included overweight data from adults in modeling of the prevalence of overweight among children. The mean functions used in ST-GPR were estimated using the following hierarchical mixed-effects linear regressions, run separately by sex:

$$\mathrm{logit}(\mathrm{ow}_{\mathrm{c,a,t}}) = \beta_0 + \beta_1 \mathrm{energy}_{\mathrm{c,t}} + \beta_2\ \mathrm{SDS}_{\ \mathrm{c,t}} + \sum_{\mathrm{k}=3}^{19} \beta_{\mathrm{k}} \mathrm{I}_{\mathrm{A[a]}} + \alpha_{\mathrm{s}} + \alpha_{\mathrm{r}} + \alpha_{\mathrm{c}} + \epsilon_{\mathrm{c,a,t}}$$

where $\mathrm{energy}_{c,t}$ is a 10-year moving average of energy intake per capita; SDS (socio-demographic status) is a composite measure of social status combining economic, social, and demographic factors; $I_{A[a]}$ is an indicator variable for specific age group A that the overweight prevalence point $\mathrm{ow}_{c,a,t}$ is capturing, and α_s, α_r, and α_c are super region, region, and country-specific random effects. The estimated mean functions were then propagated through the ST-GPR framework to obtain 1,000 draws of overweight prevalence estimates.

Maternal Mortality SDG Capstone Appendix

Flowchart

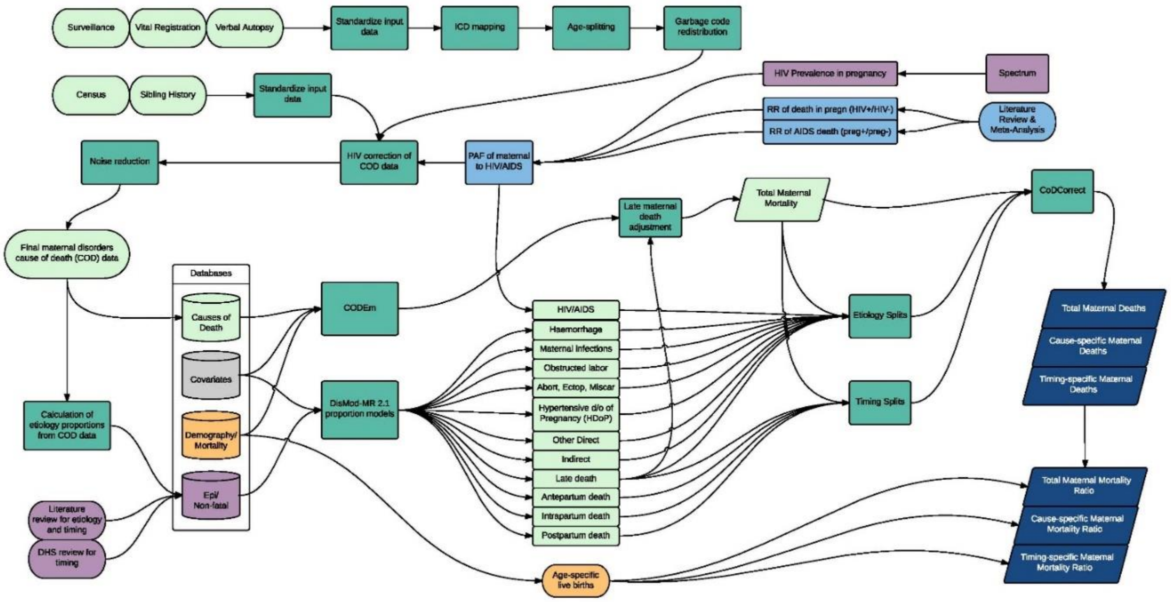

Input data & Methodological summary

Indicator definition

This modeling strategy encompassed the indicator associated with maternal mortality (3.1.1).

Indicator 3.1.1

As a component of SDG Goal 3. Ensure healthy lives and promote well-being for all at all ages, SDG Target 3.1, by 2030, reduce the global maternal mortality ratio to less than 70 per 100,000 live births, is measured using SDG Indicator 3.1.1, maternal mortality ratio (maternal deaths per 100,000 live births).

For the present analysis, we report on MMR estimates generated for women aged 15-49 to align with the proposed indicator definitions by the IAEG-SDGs. For the Global Burden of Disease Study 2015 (GBD 2015), MMR estimates are estimated for a wider age range (10-54 years); MMR results for women aged 15-49 are derived from broader maternal mortality analysis and thus we provide the data and methodological details on the broader definition for MMR.

Input Data

Maternal mortality data identification and processing

Appendix Figure 1a and 1b show the high-level view of data inputs, analytical steps, and outputs of the cause of death analysis frame. The complexity of the overall process can be usefully divided into three broad phases: data inputs on the event of death going into the cause of death database that are analyzed using CODEm; data inputs on precursors to death that are modeled through a variety of strategies; and the integration of these streams of analysis into a single set of cause of death estimates by age, sex, year, and geography with uncertainty through the CoDCorrect algorithm. The process for cancer and for HIV/AIDS is somewhat different, and described in more detail below.

Cause of death data identification

1. Overview of data types

The cause of death database contains six types of data sources: vital registration, verbal autopsy, cancer registry, police records, sibling history, surveillance, and survey/census. The highest-quality data will have detailed characteristics of each demographic group and detailed causes of death across the time series. Countries with complete vital registration systems are considered to be high-quality. For counties with incomplete vital registration systems, vital statistics for causes of death can be supplemented with other data types to provide cause-specific estimates.

2. ICD detail

A majority of the cause of death data is vital registration data obtained from either the WHO Mortality Database, country-specific mortality databases operated by official offices, provided by trusted country collaborators. It is considered to be the highest-quality data, as it is the most comprehensive. Each cause is coded directly to the most detailed cause of death when possible, whereas cause codes in International Classification of Diseases (ICD) tabulated data are coded to aggregated cause groups. The cause of death database contains 8,044 location-years of detailed data from 1980 to 2014, which includes underlying causes of deaths coded with 3-5 digit se codes, by country, year, sex, and age groups. Detail causes are coded to one of the following ICD detail coding systems: ICD8, ICD9, or ICD10. Each coding system has a similar cause hierarchy and cause list that has continually developed over time. ICD10 is the current standard and most exhaustive cause list. Within the cause lists 5-digit codes are truncated to 4-digit codes to condense the cause lists. Updates to ICD detail occur biannually as WHO releases new versions or as country collaborators provide additional data. Updates to data from WHO increasingly include ICD10 cause of death data, as it is the most current classification of cause of death, while updates to ICD8 and ICD9 detailed lists are less common. In the case of overlapping data, preference is given to data from pre-determined country collaborators, which is updated annually.

3. ICD tabulations lists

The ICD tabulation lists include the ICD8 List A (ICD8A), ICD9 Basic Tabulation List (BTL), ICD10 Mortality Tabulation, Russia Tabulation list, and India Medical Certification of Cause of Death (MCCD). These data sources make up 2,664 location-years from 1980 to 2014 in the cause of death database. All are condensed versions of the ICD9 and ICD10 detail lists, with some differences in the format of cause lists depending on the data source. ICD8A, ICD9 BTL, and ICD10 Mortality Tabulation cause of death are assigned to subtotal groups, referred to as chapters, and cause groups respective to ICD detail groups. Additionally ICD9 BTL includes ICD9 detail codes for some cancers and a custom tabulation scheme for

the former USSR countries. The Russia Tabulation lists and India Medical Certification of Causes of Death (MCCD) cause lists each have custom nomenclatures based on ICD detail cause codes.

Two of the drawbacks in data using tabulation lists are discrepancies in the accuracy of death counts and lack of detail to due to aggregated cause groups. There are instances where the sum of deaths in chapter subtotals are not equal to the sum of cause groups within the chapter. To account for any missing or duplicate deaths reported within the cause groupings, death counts are systematically adjusted, by calculating the differences between subtotals and sub-causes within the cause groups. Any differences are assigned to a remainder cause group. To account for the lack of cause code detail, select cause groups are disaggregated (Step 1.1) to create a complete cause list. Updates to ICD Tabulation lists obtained from WHO occur less frequently compared to ICD detailed lists, as more countries are reporting deaths in ICD detail. In instances of overlapping data, preference is given first to data from country collaborators' data from WHO, then preference to ICD detail data from WHO, before choosing to use ICD tabulation lists.

China DSP/ China CDC

The two primary sources of data for China are surveillance data from the China Disease Surveillance Points (DSP) system and vital registration collected by the Chinese Center for Disease Control and Prevention (CDC). In the China DSP data, deaths were reported across 145 disease surveillance points used from 1991 to 2003, and 161 points used from 2004 to 2007. While China DSP with ICD10 code is considered surveillance data, it provides national coverage and cause detail. Thus it receives similar processing and treatments to the China CDC vital registration from 2008 to 2012. From 2008 to 2012, all of the deaths and cause of death information from the Disease Surveillance Points system and other system points throughout China were collected and reported via the Mortality Registration and Reporting System, an online reporting system of the Chinese CDC. The deaths in these data are reported at the strata level, a metric that is specific to China. Counties are stratified by urban and rural classification, but definitions of urbanity vary across counties. In Step 7.1 we use a method developed to scale up deaths from strata level to the province level.

India CRS

The India Civil Registration System (CRS) is a continuous systematic recording of deaths and births. We consider these data to be similar to vital registration as the coverage is national. Usable cause-specific death data are in the form of a custom cause list and exist between 1983 and 1995, with 1983-1984 and 1986-1987 including deaths data specific to rural or urban locations. Due to the lack of complete India subnational locations we state-split the data after age-sex splitting to fill in data gaps (Step 1.2). Though this system is continuous we have not found any cause-specific data past 1995. The more recent years of the report only include data for all-cause mortality.

India MCCD

The India MCC) is the largest data source we have for India, with nationally representative data in a majority of the urban states and also union territories beginning in 1980. Deaths reported in this data source have been medically certified and are considered vital registration. The causes of death are reported in a tabulation list with a unique numbering scheme that conform to ICD9 and ICD10 detail codes, which must be disaggregated. Similar to the CRS, MCCD is state-split to fill in data gaps (Step 1.2 State Splitting); however, in these data state splitting occurs prior to age-sex splitting.

4. Verbal autopsy

VA coded to ICD 10 and VA coded to other lists

In countries without vital registration systems, verbal autopsy studies are a viable data source to inform cause of death. Data are obtained by trained interviewers who use a standardized questionnaire to ask relatives about the signs, symptoms, and demographic characteristics of recently deceased family members. Based on the answers to the questionnaires a cause of death is assigned.

Verbal autopsy (VA) data are highly heterogeneous: studies use different instruments, different cause lists from single causes to full ICD cause lists, different methods for assigning cause of death based on a completed verbal autopsy, different recall periods, and different age groups, quite apart from cultural differences in the interpretation of specific questions. The validity of cause of death must be considered when mapping to a GBD cause. Verbal autopsies are likely accurate in assigning cause of death to road injury or homicide, but less accurate for causes requiring medical certification, such as cardiovascular causes. Studies can also occur once in a particular country or as part of an extended network, such as INDEPTH. INDEPTH is a continuous surveillance source with several Demographic Surveillance Systems sites that collect data which is coded to ICD detail causes.

INTERVA modeled VA

In previous years, INTERVA modeled VA was excluded from our analysis. Verbal autopsies used in our analysis are non-INTERVA, as they use questionnaires and modules consistent with WHO standards. The Population Health Metrics Research Consortium (PHMRC) published a study that shows results of INTERVA-modeled VA are not compelling enough to be credible, thus we have decided to exclude data for all causes due to low validations with the exception of injuries in Sub-Saharan Africa.[1] We lack data in sub-Saharan Africa and use INTERVA to fill in gaps and stabilize injuries patterns.

India SCD and SRS

Deaths reported in verbal autopsy studies in rural Indian states can be accounted for in the Survey of Causes of Death (SCD) from 1980 to 1994 and in the Sample Registration System (SRS) post-1998 in urban and rural states. Data in the SCD were collected through a verbal autopsy survey from a sample of villages. To expand our estimates to more states and causes we used methods of state splitting post-mapping to GBD causes (Step 1.2). Like the SCD, SRS also records deaths in a sample of villages but also includes urban blocks in its sample. This survey is collected by the Registrar-General of India, and since 1999 SRS has merged with SCD to provide ongoing coverage on both urban and rural areas.[2] The maternal data reported in SRS are given in the maternal mortality ratio (MMR), which we convert to deaths by state and urbanity by using our estimates for the SRS population and neonatal mortality rates.

5. Other data types

Maternal mortality data

In locations with low-quality vital registration or no vital registration, maternal mortality metrics can be found in surveillance, surveys, census, and sibling history data sources. The best data have death counts due to maternal causes and the total number of deaths for women within the reproductive ages of 10 to 54 (previously 15 to 49) by year. If a data source is missing these components, it is necessary to create a complete cause list using live births and all-cause mortality deaths (Step 1.4). Though death counts is the preferred metric, maternal mortality (MM) is often measured using MMR, which is easily converted to

deaths using live births. An additional adjustment that must be applied to the China Maternal and Child Surveillance data is scaling data from the strata to the province level (Step 7).

Surveys and censuses reporting fraction of deaths due to selected injuries

Surveys and censuses are often used in countries with less developed VR systems, or in countries with adequate VR these data source are supplementary. Much like the VAs, the validity of cause of death is a concern due to lack of medical certification at the time of death. For these data sources we keep only causes related to maternal mortality and injuries. The remaining causes are accounted for as a remainder of total deaths in the sample size.

Police records

In most countries, police and crime reports are an important source of information for some types of injury deaths, notably road injuries and interpersonal violence. Our police data comes from reports on road traffic and crime trends. The police reports used in this analysis were obtained from published studies, national agencies, and institutional surveys such as the UN Crime Trends survey and United Nations Office on Drugs and Crime Global study on Homicides. We can assess whether police reports were likely to be complete and cover the entire country if police trends are close to trends seen in vital registration. Data are excluded in instances where police data for road traffic injuries are significantly lower than our vital registration. The threshold for exclusion is less than 80% of the cause fraction of the road traffic injuries in VR. Police data that meet our inclusion criteria and provide complete coverage are uploaded to the database for injuries causes.

6. Population-based cancer registries

Cancer registries with incidence

Data on cancer incidence was sought from individual population based cancer registries as well as from databases that include multiple registries, for example "Cancer Incidence in Five Continents" (CI5) (NID 133224), NORDCAN (NID: 113386), or EUREG (114368). Cancer registries were identified through the membership list of the International Association of Cancer Registries (IACR), through the GBD collaborator network, or through the GHDx. Registries were excluded if they were not representative of the coverage population, if they did not contain incidence data tabulated by cancer site, if the data were limited to years prior to 1980, if the source did not provide details on the population covered, or if the list of cancer sites included was not comprehensive.

Cancer registries with incidence and high quality mortality

In addition to incidence, some high-quality cancer registries also report cancer mortality data. These data were also extracted and used as inputs into the mortality-to-incidence (MI) ratio model.

General Modeling Strategy

Step 1. Standardize input data

The input data to the Cause of Death (CoD) database are received in various formats and must be standardized to run through central CoD machinery to then upload to the database. Raw data inputs come from data sources such as mortality databases, literature reviews, or reports. Usable data sources

must have a clear sample size of the number of deaths in the population and exhaustive cause lists. The complexity of the cleaning process varies drastically across data sources. For vital registration micro-data with the location, age, sex, year, and ICD-coded cause of every death, very little effort is necessary to standardize it into a consistent structure. Other sources may require weeks of careful review to accurately extract scans of hardcover cause of death reports into spreadsheets that can be transformed and standardized.

At this point, data are assigned source identifiers so that they can be linked to the Global Health Data Exchange (GHDx) and cited appropriately. Any aggregate age and sex categories are flagged for age-sex splitting. The methods of cause-of-death assignment and data collection are reviewed to determine which source type to assign; for example, we distinguish sibling history data from surveys with a verbal autopsy module. Only data at the most detailed level of the Global Burden of Disease location hierarchy are used. Documentation from the source is reviewed to determine if the population is representative of the location or only a subset of the population in that location. Data sources representing a subset of the population are flagged as non-representative; this flag is used by CODEm to increase the variance associated with such data points.

Finally, diagnostics are reviewed at this stage to avoid sending cleaning errors downstream. We review cause-specific deaths for each demographic group to ensure the data are reasonable. For example, it is unlikely that male breast cancer deaths are higher than female breast cancer or deaths from neonatal causes occur in age groups over one year. All deaths totals are compared with the sum of cause-specific deaths to ensure the observed deaths are accounted for and sample size is complete.

1.1 Disaggregation

Causes of death in tabulated vital registration data are condensed into aggregated groups, some of which can be mapped directly to GBD causes while other aggregated cause groups are not informative and cannot be mapped to GBD causes. To correct for this, aggregated causes were mapped and split onto multiple ICD9 and ICD10 detail causes, or targets, based on the ICD groupings within the aggregated causes. Both ICD9 and ICD10 detail codes serve as targets because they are the highest quality vital registration data and enabled the calculation of proportions used to split the aggregated cause data into detailed causes. The proportions of deaths from nearby countries within the super region were used to fill in data gaps as they were likely to have similar cause of death trends.

We determined the targets based on detail causes missing from the tabulated cause list. For example, in ICD9 BTL, the tabulated cause list includes a viral diseases group. In the hierarchy of causes, this group consists of measles, yellow fever, encephalitis, hepatitis, rabies, other infectious diseases, garbage code, and remainder of viral diseases. We did not consider this list to be an exhaustive list of viral diseases based on the range of ICD detail codes given in the ICD9 BTL documentation. To make the cause list exhaustive and inclusive of other viral diseases, we split the remainder of viral diseases group into: other meningitis, other infectious diseases, herpes, dengue, other neglected tropical diseases, and garbage code. After a list of targets was determined, the aggregated deaths were disaggregated to the target causes using ICD9 and ICD10 detail proportions generated at the super region level for the corresponding sex and age groups across all years in the time series. For example, in ICD9 detail data, 54.8% of deaths in males in Latin American and Caribbean within the target group for BTL Viral Diseases were designated "other meningitis", so 54.8% of deaths in the tabulated group, "remainder of viral diseases", were

assigned to "other meningitis" for any country within that particular super region. For any cause and demographic group where we lacked ICD detail, global proportions were used.

1.2 State splitting

Two important sources for cause of death estimation in India are the MCCD report, which reports medically certified deaths from health facilities in mostly urban areas [3], and the SCD, which collects information via verbal autopsy on about one-half of 1% of all rural deaths in India, based on populations living in about 1,300 primary health care centers spread throughout the country.[4] For both of these reports, data missingness impedes estimation of trends at the state level. We used a first-order, log-linear model of the four-way contingency table of deaths by sex, age, state, and year to estimate the missing state-years. We fit the model to all available data for MCCD and SCD separately for each cause, including state-specific all-age measurements and age-specific national measurements. From this, we produced estimates for each combination of sex, age, state, and year. We then used these estimates wherever the raw data did not include sex-, age-, state-specific death counts.

For MCCD, the model was fit separately for ICD10- and ICD9-based reports using the tabulated cause list present in the data. In the SCD report, the model was fit for each GBD cause in the data. As data from the SCD reports were relatively sparse, the pooling of like causes together led to an improved model fit.

1.3 Region-sex-cause to state-urbanicity-age-sex-cause algorithm

We also made use of the Special Survey of Deaths in 2001–2003 and 2004–2005, a representative, national and subnational verbal autopsy study under the Sample Registration System in India. Data for the top 10 causes of death were available by region and sex, but we require data by urban and rural state in India. To achieve this, we first split the regions into states in proportion to the number of GBD-estimated deaths in each state. We then determined the urbanicity of the resulting state-cause-deaths by applying the SRS distributions of urban and rural deaths by cause in India. We then used a relative-rate splitting algorithm that accounts for the population structure of urban and rural areas in the state. Finally, we applied the SRS all-India age distribution to the all-age urban-rural-state-data with a similar approach, accounting for the age-specific population structure of urban and rural areas in each state.

1.4 Calculate non-maternal deaths

In cases when maternal mortality metrics do not include both deaths due to maternal causes and deaths due to non-maternal causes for women of reproductive ages, live births, and all-cause mortality estimates can be used to calculate deaths. Many studies report maternal deaths as the MMR. MMR is the number of maternal deaths per 100,000 live births and can be used to calculate deaths when it has been derived from primary data and not estimated. Maternal deaths were calculated using MMR and live births, if live births were missing we substituted live birth estimates and used the following equation:

$$\text{Maternal deaths} = (\text{MMR}/100{,}000) * \text{Live births}$$

If a study was non-representative we extracted sample size and live births from that study. After maternal deaths were calculated, we used the difference from all-cause mortality estimates to determine non-maternal deaths.

A more accurate and data inclusive method of calculating maternal and non-maternal deaths incorporates coverage and splits deaths for a range of years into individual years. If there were live births in the study we adjusted the coverage.

Coverage = live births / GBD estimated live births

After coverage was calculated, totals deaths were scaled to be more representative. This gives a more accurate death count since the envelope assumes representative coverage. Using all-cause mortality as an all-cause total, non-maternal deaths were subsequently calculated.

Maternal envelope with coverage = maternal envelope * coverage

An additional adjustment can be applied to maternal data spanning over a range of consecutive years, which allows for more data inclusion. The years within specified year ranges are separated into individual years and total deaths within the year range were split between each individual year using the fixed proportions of maternal deaths from vital registration in that particular country. We only used vital registration to inform the proportions because it was both high quality and representative.

Step 2. Map to GBD cause list

In GBD 2015 we developed 411 maps to translate causes found in the input data to the GBD 2015 cause list. This included 40 maps for vital registration data, 279 for verbal autopsy data sources, and 92 for other data types. The largest and most universal maps used were those for ICD9 and ICD10 detail vital registration data. The input data causes varied from 3-4 digit ICD codes to custom cause lists with cause names such as cholera or hepatitis. Our mapping process made it possible to compare these various data sources across demographic groups.

Appendix Table 1 shows the ICD10-detail and ICD9-detail codes included in the mapping of each GBD cause.

2.1 India urban/rural splitting

Another source of data for urban and rural state estimation of cause of death trends in India is the CRS, from which we retrieved usable data over the period 1983-1995. From 1983-1987, data were available for the urban and rural populations of each state in the system. However, after 1988, only state-level data were available.

We can only use data at the most detailed location level that we estimated for cause of death models. As a result, in order to use of all years of the CRS data split state-level mortality into urban and rural state-level mortality. To do this, we assumed the same relative rate of cause-specific urban and rural mortality in 1988-1995 as was present in the data in 1983-1987. We applied the same algorithm that is used for age-sex splitting, modified for this purpose:

$$D_{s,u,y,a,x,c} = R_{s,u,c} N_{s,u,y,a,x} \frac{D_{s,,y,a,x,c}}{\sum_{u=urban}^{u=rural} (R_{s,u,c} N_{s,u,y,a,x})}$$

Where:

$D_{s,u,y,a,x,c}$ = Number of observed deaths in state s, urbanicity u (either rural or urban), year y, age a, sex x, and cause c

$R_{s,u,c}$ = Death rate in state s, urbanicity u, cause c from 1983-1987; data were not suitable for age or sex specific rates.

$N_{s,u,y,a,x}$ = GBD-estimated population in state s, urbanicity u, year y, age a, sex x

$D_{s,,y,a,x,c}$ = Number of observed deaths in state s, year y, age a, sex x, and cause c (the deaths that are being split)

The result was a full time series of CRS data for both rural and urban populations of each state in the CRS system.

2.2 State splitting

This step is described in step 1.2, above.

Step 3. Age-sex splitting

3.1 Generate global age-sex weights by cause

Different sources, particularly verbal autopsy studies, report deaths for a wide range of age-groups with varying intervals. For the analysis of causes of death, we mapped these different age intervals to the GBD standard set of age-groups. The approach to undertake this mapping was the same as in the prior two GBD studies, GBD 2013 and GBD 2010.

In the process of assembling a consolidated demographic database, perhaps the most impairing source of inconsistency is the aggregation of age groups. It is conventional to report such data in broad age groupings such as “0-4, 5-14, 15-49,” or to report data with both sexes together. The issue of comparability between age-sex groups arose when assembling the GBD cause of death database. The compiled database included 22 distinct tabulation formats for infants and 141 distinct tabulation formats for non-infants. We developed a tool, which we call age-sex splitting, that takes aggregated age groupings, and likewise the “both sexes combined” grouping, and divides them into what their constituent age groups would likely have been using respective cause-specific and country-specific age distributions. The analytical framework for the GBD includes three infant age categories: Early neonatal (0-6 days), late neonatal (7-27 days) and post neonatal (28 days to one year), and 17 non-infant age categories starting with age one to four years, then proceeding in five-year age groups until the terminal age group of 80+. We treat unknown ages and sexes in the same manner we treated the “all ages combined” age category and “both sexes combined” sex group. Through this process, we were able to directly compare all data sources on even terms.

The approach to age splitting is based on the following formula. The key assumption underlying this formula is that the relative risk of death by age group compared to a reference age group is invariant across populations. While this assumption is likely violated in specific cases, there is a strong biologically based pattern of the relative risk of death for a cause by age that is observed for most causes. The basic formula is as follows:

$$D_a = R_a N_a \left(\frac{D_a^{a+x}}{\sum_a^{a+x}(R_a N_a)}\right)$$

Where:

D_a= the number of deaths from a cause in age group *a*

R_a= the relative risk of death in age group *a* compared to a reference group

N_a= the country-year-sex-specific population in age group *a*

D_a^{a+x}= the number of deaths in the age group *a* to *a+x*

With the assumption of invariant relative risks of death by age with respect to a reference age group, this equation can be used, along with population distribution by age, to split an aggregate number of deaths for the age groups *a* to *a+x* into specific deaths for each age group within the aggregate interval.

In some cases, deaths are reported for an aggregate age group for both sexes combined. The task in this case is more complicated, but the same principle can be applied. In this case we assumed that the relative risks of death by and sex are constant.

$$D_{as} = R_{as}N_{as}\left(\frac{D_{as}^{a+x,s}}{\sum_a^{a+x}(R_{as}N_{as})}\right)$$

Where:

D_{as}= the number of deaths from a cause in age group *a,* sex *s*

R_{as}= the relative risk of death in age group *a* compared to a reference group for sex *s*

N_{as}= the country-year-sex-specific population in age group *a* for sex *s*

$D_a^{a+x,s}$= the number of deaths in the age group *a* to *a+x* for sex *s*

This equation can be used to split data aggregated over age and sex. The assumption, however, of invariant relative risks across age and sex is a stronger assumption. Fortunately, data pooled across sexes are less common in the published or unpublished cause of death data.

The relative risk of death in a particular age group for a given sex is derived from the global distribution of cause-specific mortality rates found in available vital registration data. Location-years from the following code systems are used, provided they report the requisite age- and sex-detail: ICD7, ICD8, ICD9 BTL, ICD10 tabulated, ICD9, and ICD10. Upon compiling these data, we mapped them to GBD causes, and aggregated up to cause level 3. This is the level at which a particular cause is split – that is, any daughter cause of a level 3 parent is split using the age distribution of that parent (so, chronic kidney disease due to diabetes would be split using the age pattern of chronic kidney disease).

We next adjusted separately for estimated adult and child vital registration completeness. Location-year-age-sex-specific deaths and population were then aggregated across all location-years, in order to produce cause-specific mortality rates by age and sex. These were used to determine the risk of death at any age relative to any reference age group.

Step 4. Correct age-sex violations

Occasionally, data sources will include deaths by a cause for which there is medical consensus that death is impossible for the sex and age. For example, there may be some number of deaths due to cervical cancer in males, or deaths due to maternal causes in ages under 10. We have constructed a conservative list of age-sex restrictions. When deaths violate these restrictions, we redistribute them proportionally onto all causes.

Step 5. Redistribution

A crucial aspect of enhancing the comparability of data for cause of death is to deal with uninformative, so-called garbage codes. Garbage codes are codes to which deaths were assigned that cannot or should not be considered as the underlying cause of death, for example: heart failure, ill-defined cancer site, senility, ill-defined external causes of injuries, and septicaemia. The methods for redistributing these

garbage-coded deaths were outlined in detail in Naghavi et al,[5] and the underlying algorithm for redistributing deaths assigned to these codes has not changed since GBD 2013.

5.1 Redistribute HIV-related garbage

The list of garbage codes known to be used to code deaths caused by HIV/AIDS can be found in Appendix Table 2.

Due to the disparate nature of HIV/AIDS mortality across space and time, dynamic redistribution of HIV/AIDS-related garbage codes was needed. To inform this redistribution, we generated target proportions for each garbage group by age band (Under 1 month, 1-59 months, 5-19 years, 20-49 years, 50-59 years, 60-69 years, 70-79 years, and 80+ years), 5-year time interval, and sex. The garbage groups will either target HIV or a remainder target. The allotment of deaths to either of these is based on the regional increase in the mortality rate of all codes in the group relative to the rates seen in 1980-1984 – an increase greater than 5% is assumed to be HIV/AIDS-related, and the proportion of those deaths exceeding 5% are redistributed to HIV/AIDS. Any increase ≤ 5% is then assigned to the remainder target.

5.2 Regress garbage codes versus non-garbage

As in GBD 2013, the statistical analysis used to determine proportions for garbage code redistribution for ill-defined cancer sites, ill-defined external causes of injury, unspecified stroke, heart failure, hypertension, and atherosclerosis was based on the approach outlined by Ahern et al.[6] For each redistribution package, we defined the "universe" of data as all deaths coded to either the package's garbage codes or the package's redistribution targets for each country, year, age, and sex. We then ran a regression based on the following equation, separately for each target group and sex:

$$TG_{crt} = \alpha + \beta_1 Gar_{crt} + \beta_2 Age_{crt} Gar_{crt} + \theta_r Gar_{crt} + \gamma_r + \varepsilon_{ct}$$

TG_{crt} = percentage of deaths within the given garbage code's universe which were coded to a given target group, by country

Gar_{crt} = percentage of deaths within the given garbage code's universe which were coded to a given set of garbage codes

α = constant

$\beta_1 =$ slope coefficient describing the association between Gar_{crt} and G_{crt}

$\beta_2 =$ slope coefficient describing the association between the interaction $Age_{crt}Gar_{crt}$ and G_{crt}

$\gamma_r =$ region specific random intercept (or super region if the random effect on region is not significant)

$\theta_r =$ region specific random slope (or super region if the random effect on region is not significant)

$\varepsilon_{ct} =$ standard error, normally distributed and calculated by bootstrapping

This regression was adjusted from GBD2013 to include fixed effects on the interaction of garbage and age to ensure smooth age patterns. We made this decision after investigating diagnostic visualizations that showed unlikely gaps between proportions assigned to different age groups.

Once proportions were produced for each country, sex, age, and target group, certain adjustments were made to conform our packages to the best medical evidence available. In some cases, we implemented restrictions on the proportions that the regressions could yield. For example, we did not allow any

redistribution onto Chagas disease outside of Latin America and the Caribbean, or suicide under the age of 15. In other cases, we capped the proportion for some targets to the level that would be produced from proportional redistribution; for example, hemoglobinopathies and hemolytic anemias were restricted to the level of proportional redistribution in the redistribution of left heart failure. Occasionally, further adjustments were made on a case-by-case basis per country, age, sex, and target group to suppress the impact of outliers based on existing epidemiological evidence and expert judgment.

5.3 VA anemia adjustment

To compensate for the over-representative cause fractions from anemia found in verbal autopsy studies, we redistributed these deaths based on the causal attribution of severe anemia from the GBD 2013 study. The proportions were country-year-age-sex specific.

Step 6. HIV/AIDS misclassification correction

In many location-years, certain causes of death known to be comorbid with HIV/AIDS (e.g., tuberculosis, other infectious diseases) are seen to have age-patterns that diverge from those observed in location-years without widespread HIV/AIDS epidemics, and are in fact more reflective of HIV/AIDS mortality trends. In order to identify these instances, a global relative age pattern is generated using all VR deaths in countries with observed HIV prevalence less than 1% using the following:

$$RR_{asc} = {}^{R_{asc}}/_{\bar{x}(R_{65sc}, R_{70sc}, R_{75sc})}$$

Where $RR_{asc}$is the relative death rate for age group a, sex s, and cause c; $R_{asc}$is the rate for that age group; and $\bar{x}(R_{65sc}, R_{70sc}, R_{75sc})$ is the mean of the rates in ages 65-69, 60-74, and 75-79 for that sex and cause. This is preferable to comparing mortality rates because we are able to isolate divergence in age pattern while accounting for varying levels of overall mortality by fixing death rates to age groups that are unlikely to be confounded by the presence of HIV. Expected deaths for an identified cause were then determined to be:

$$ED_{lyasc} = \bar{x}(R_{ly65sc}, R_{ly70sc}, R_{ly75sc}) * p_{lasc} * RR_{asc}$$

Where ED_{lasc} are deaths for location *l*, year *y*, age group *a*, sex *s*, and cause *c*; $\bar{x}(R_{l65sc}, R_{l70sc}, R_{l75sc})$ is the mean of the rates for ages 65-69, 60-74, and 75-79 for that location-year-sex-cause; p_{lasc} is the population for that location-year-age-sex-cause; and RR_{asc} is the global standard relative rate determined in the previous step for that age-sex-cause. The expected deaths remain attributed to that particular cause, while the difference between observed and expected are reallocated to HIV/AIDS.

Step 7. Scale strata to province

Over time, a higher proportion of deaths have been registered in China through the expansion of the DSP system and provincial and county efforts to increase cause of death registration. With the expansion of coverage, it is possible that province aggregates do not accurately represent the population distribution between urban and rural areas in each year. For this reason, we stratified the data preparation by urban and rural status for each county within each province. Stratification was based on the median level of urbanization across counties within each province as recorded in the 2010 China census. In the provinces of Tibet and Hainan, all counties were placed into one strata based on largely homogeneous urbanization levels within each province. This yielded a total of 62 analytical province-strata. Macao and Hong Kong were not included in this stratification system as the VR systems there are independent from that on the

mainland; no weighting scheme needs to be carried out in these complete VR systems with quality data on causes of death.

Within each province-strata, a larger proportion of deaths in-hospital might be reported than that of deaths outside of hospital because of the internet hospital reporting system. To avoid bias, we reweighted in-hospital and out-of-hospital deaths based on the age-sex-province-specific fraction of deaths in and out of hospital in the DSP system. DSP data have been used to establish these percentages because, in these communities, there is a concerted effort to identify all out-of-hospital deaths. Province-strata death rates are combined to produce overall province death rates by weighting each strata by population in each age-sex-year group. Province death rates are rescaled so that all-cause mortality equals the estimated death rate in each age-sex-year estimated in the life-table analysis. The Bayesian noise reduction algorithm was used to deal with zero counts and small number issues for rare causes.[7]

Step 8. Restrictions post-redistribution

Some causes of death can only be reliably assigned through an autopsy by a trained physician. For example, it is unlikely that a verbal autopsy would reliably distinguish between ischemic and hemorrhagic stroke.

In this step, it is ensured that the detail of the cause list at this point in the data prep process is reasonable given the detail of the original data source and the methods by which the cause of death was assigned. Two primary corrections are applied. First, any cause which is purely an artifact of the redistribution machinery targeting too detailed a cause is aggregated up to the parent cause. Second, a "bridge map" is applied over a certain set of sources to ensure that these sources do not contain causes which could not reliably be determined by the methodology. These two corrections are applied to ICD9-BTL, ICD10-tabulated, USSR tabulated ICD9, India MCCD reports, China-DSP-tabulated-ICD9, India SCD reports, and all verbal autopsy sources.

Step 9. Drop VR country years or mark as non-representative based on completeness

Lozano and colleagues[8] describe the negative impact that low-completeness VR data can have on cause of death modeling for the GBD 2010. In particular, in settings where a data source does not capture all deaths in a population, the cause composition of deaths captured might be different from those that are not. However, a completeness sensitivity test found that low-completeness VR data had little impact on the cause-specific mortality trends at the global level.

For GBD 2015, we investigated the impact of these data at the country and subnational levels using the more thorough diagnostic visualizations available to us. It was determined that these data produced unlikely trends in the models affected. Despite the minimal impact on global trends, better models were produced by eliminating or marking as non-representative data with extremely low completeness. VR completeness was estimated using death distribution methods (DDM) described in the all-cause mortality section of the appendix.

For this round, vital registration location-years with completeness below 50% were dropped, while location-years with completeness between 50-69% were marked as non-representative.

The following country-years were dropped from the database:

Location Years Below 50% Completeness	
**Location**	**Years Below 50% Completeness**
'Asir	1999 - 2012
Bahah	1999 - 2012
Eastern Province	1999 - 2003
Ha'il	1999 - 2012
Haiti	1981, 1999, 2002 - 2004
Iran	1980 - 1985, 1987
Jawf	1999 - 2012
Jizan	1999 - 2012
Makkah	1999 - 2005
Maranhão	1985
Najran	1999 - 2012
Northern Borders	1999 - 2012
Papua New Guinea	1980
Qassim	1999 - 2003
Riyadh	1999 - 2012
Tabuk	1999 - 2002
Turkey	1983, 1984, 1987 - 1995

Step 10. Cause aggregation

The cause list is organized in a top down hierarchical format containing 4 levels. The first group, or level 1, sums all causes. Following all cause-mortality are level 2 causes, which include 3 broad groupings of causes of deaths: communicable, maternal, neonatal, and nutritional diseases; non-communicable diseases; and injuries. Within those level 2 groupings are finer levels used for modeling. Level 3, or parent causes, are aggregated, meaning the mortality estimate for a parent cause in the hierarchy represents the sum of the causes under that rubric. Sub-causes within level 3 causes – level 4 – are more detailed. For example, the parent cause "intestinal infectious diseases" contains the 3 sub-causes: typhoid fever, paratyphoid fever, and other intestinal infectious diseases. Included in the parent cause estimate are deaths mapped directly to the parent and any level 4 sub-causes. In data where there was not enough information to assign a level 4 cause, we aggregated to the level 3 parent cause. Exceptions to aggregating the level 4 sub-causes to the parent are instances when certain sub-causes are not present. The United Nations Crime Trends police data only identifies homicides, aggregating homicides to injuries would not accurately represent all injuries.

Step 11. Remove shocks and HIV/AIDS maternal adjustments

For GBD 2015, CODEm models use an HIV/AIDS- and shock-free envelope, as described in the all-cause mortality section of the appendix. In order to be comparable, cause fractions must also be HIV/AIDS- and shock-free. Cause fractions were uploaded to the CoD database as the number of deaths due to the cause over an adjusted sample in which the number of deaths due to HIV/AIDS, collective violence and legal intervention, and exposure to forces of nature were removed.

11.1 Remove HIV/AIDS, shocks from denominator where HIV/AIDS in cause list

The first step to generate HIV- and shock-free cause fractions was to remove any deaths from the sample which were directly coded to HIV/AIDS, collective violence and legal intervention, or exposure to forces of nature. The resulting equation for a cause fraction uploaded to the database is simple:

$$CF_{l,t,a,x,c} = \frac{D_{l,t,a,x,c}}{D_{l,t,a,x} - D_{l,t,a,x,hiv} - D_{l,t,a,x,war} - D_{l,t,a,x,disaster}}$$

In this equation, $CF_{l,t,a,x,c}$ is the cause fraction for a location (l), year (t), age (a), sex (x), and cause (c), $D_{l,t,a,x,c}$ is the number of deaths observed in the sample for the same, $D_{l,t,a,x}$ is the total number of deaths observed in the sample in the location, year, age and sex, and $D_{l,t,a,x,hiv}$, $D_{l,t,a,x,hiv}$, and $D_{l,t,a,x,disaster}$ are the number of deaths observed in the sample for HIV/AIDS, collective violence and legal intervention, and exposure to forces of nature, respectively.

Cause fractions for HIV/AIDS and shock causes were also uploaded to the database for use in separate estimation processes described in the all-cause mortality section of the appendix. In this case, cause fractions followed the standard equation, with variables following the same explanation as above:

$$CF_{l,t,a,x,c} = \frac{D_{l,t,a,x,c}}{D_{l,t,a,x}}$$

11.2 Remove HIV/AIDS deaths from maternal mortality sources

HIV-free cause fractions were also uploaded for sources on mortality due to maternal causes. In these cases, the sample of all deaths observed in the study is likely to contain some amount of deaths due to HIV/AIDS and shocks, but the sample only includes cause information on maternal deaths. To account for the presence of HIV/AIDS and shocks in the entire sample, we assumed the same proportion of total deaths due to HIV/AIDS by location, age, sex, and year as provided from the estimation of HIV/AIDS and all-cause mortality described in the all-cause mortality section of the appendix.

Maternal mortality studies were only corrected for HIV/AIDS if the sample of total deaths was provided in the data source. Where sources only provided the maternal mortality rate, we applied the rate to the HIV- and shock-free envelope produced by the analysis described in the all-cause mortality section of the appendix and thus did not need to adjust cause fractions (CFs) at this point in the process.

Where a correction was applied, we applied the following equation:

$$CF_{l,t,a,x,mat} = D_{l,t,a,x,mat} * \frac{E[D_{l,t,a,x,hiv\_shock\_free}]}{E[D_{l,t,a,x}]}$$

In this equation, X is the resulting cause fraction due to maternal causes for the location (l), year (t), age (a), and sex (x); $D_{l,t,a,x,mat}$ is the number of observed deaths in the sample due to maternal causes, $E[D_{l,t,a,x}]$ is the GBD estimate of all-cause mortality in the location, year, age, and sex, and $E[D_{l,t,a,x,hiv\_shock\_free}]$ is the GBD estimate of HIV- and shock- free mortality in the location, year, age, and sex.

11.3 HIV/AIDS correction of sibling history, census, and survey data

As described in our analysis from GBD 2013, many studies have failed to find increased mortality in HIV-positive pregnant mothers, but those who have advanced HIV are known to have increased baseline mortality. Prior to GBD 2013, we did not distinguish between deaths in HIV+ women that were caused by pregnancy and those for which the pregnancy was incidental to their death. In order to more explicitly

quantify the contribution of pregnancy to death in HIV+ women, and therefore more accurately estimate the maternal death count, we completed two additional analyses for GBD 2013. First, we determined the population attributable fraction (PAF) of HIV/AIDS to pregnancy-related death. Second, we determined the proportion of pregnancy-related deaths in HIV-positive persons that are aggravated by pregnancy and are therefore by definition maternal deaths.

$$PAF = \frac{p(RR - 1)}{1 + p(RR - 1)}$$

Where PAF is the population attributable fraction, p denotes the prevalence of HIV in pregnancy, and RR is relative risk of mortality in HIV+ vs HIV- pregnant females.

To recap our analysis for GBD 2013, we used the paper published by Calvert and Ronsmans to identify sources[9] that could inform Step 1 of our HIV-correction analysis. We independently reviewed each of the component studies in Calvert and Ronsmans' review and extracted data directly, not from the systematic review paper. We only identified one additional study that was not used in Calvert and Ronsmans' analysis. We have, however, not used all the studies included in that review. Specific details are as follows: 1) Figueroa-Damian, et.al was excluded for not including any postpartum deaths at all. 2) In the case of Ryder, et al. and Zvandasara, et al. we excluded those deaths > 12 months after delivery. 3) We excluded the results from Chilongozi, et al. from the site that did not include any HIV-negative patients. 4) Leroy, et al. was not in the bibliography. We could not locate it for review so it was excluded. 5) Kourtis et al. was extracted with adjustment of the denominator based on the average number of hospitalizations per delivery in each group. 6) Ticconi, et al. was excluded for being both non-representative and including subgroup data from mothers with malaria infection. A total of 21 sources were included in our analysis of the increased mortality risk of HIV+ versus HIV- women in pregnancy.[9] We performed DerSimonian-Laird random effects meta-analysis to derive a pooled estimate of RR of death during pregnancy given HIV positivity.[10] The pooled effect size was 6·40 (95% uncertainty interval [UI] 3·98 − 10·29) which was then used to calculate an HIV PAF for each country, age group and year. In order to determine the proportion of those HIV-related deaths that were attributable to maternal causes, we performed a second systematic literature review. This time we sought evidence for the excess mortality risk of pregnancy in those women who are already HIV-positive. Most studies have failed to find such an effect, but most also did not stratify their study population by stage of HIV or antiretroviral therapy (ART) status. Only two studies did this stratification, with a pooled effect size of 1·13 (95% UI 0·73 − 1·77).

An updated literature review to inform the relative risk of mortality in pregnancy in HIV-positive versus HIV-negative women had 14 hits, but no usable sources. We completed this search on May 7, 2015, using the following two search strings:

> (""HIV""[Mesh] OR ""Acquired Immunodeficiency Syndrome""[Mesh]) AND (""Pregnancy""[Mesh] OR ""Postpartum Period""[Mesh]) AND ""Mortality""[Mesh]
>
> "HIV"(MeSH) AND ("pregnant"(Title/Abstract) OR "pregnancy"(Title/Abstract) OR "postpartum"(Title/Abstract) OR ""post partum""(Title/Abstract)) AND ("mortality"(Title/Abstract) OR "death"(Title/Abstract))"

Prevalence of HIV in pregnant women was calculated using UNAIDS' Spectrum model. Spectrum is a compartmental HIV progression model used to generate age-specific incidence, prevalence, and death rates from pre-calculated incidence curves and assumptions about intervention scale-up and local variation in epidemiology. For each location, we used UNAIDS' age-specific ratios of fertility in women

living with HIV to fertility in women not living with HIV. In most locations, this ratio is assumed to be greater than one in women aged 15-24 and less than one and decreasing as age increases beyond 24. Since Spectrum assumes fertile ages of 15-49, we used the ratio of HIV prevalence in pregnant women to HIV prevalence in the general population at either end of that range to extend estimates to age bands 10-14 and 50-54.

Unlike GBD 2013, when we applied the PAF correction to the envelope of maternal deaths predicted by CODEm, we instead applied country-year-age-group-specific PAF to maternal mortality input data prior to modeling in CODEm. This ensured that both the numerator and denominator of all cause fraction data were internally consistent in their exclusion of background HIV/AIDS mortality. The cause fractions for maternal deaths in sibling history, survey, and census data were therefore adjusted as follows:

$$CF_{l,t,a,x,mat_{adj}} = CF_{l,t,a,x,mat} * (1 - prop_{hiv_{l,t,a,x}})$$

$$prop_{hiv_{l,t,a,x}} = PAF_{l,t,a,x,hivpos} * (1 - \pi_{mat})$$

$$CF_{l,t,a,x,mat_{hiv}} = CF_{l,t,a,x,mat} * prop_{maternalhiv_{l,t,a,x}}$$

$$prop_{maternalhiv_{l,t,a,x}} = PAF_{l,t,a,x,hivpos} * \pi_{mat}$$

Where:

$\pi_{mat} = .13/1.13$ = The proportion of HIV/AIDS deaths during pregnancy that were exacerbated by the pregnancy.

$PAF_{l,t,a,x,hivpos}$ = The population-attributable fraction (PAF) that describes the percentage of all maternal deaths that were HIV-related for the location (l), year (t), age (a), and sex (x=Female)).

$CF_{l,t,a,x,mat}$ = The proportion of deaths due to all maternal causes before HIV/AIDS correction for the location, year, age, and sex.

$prop_{hiv_{l,t,a,x}}$ = The proportion of deaths in pregnancy for the location, year, age, and sex that are estimated to be incidental deaths due to HIV/AIDS, and therefore not a maternal cause of death.

$prop_{maternalhiv_{l,t,a,x}}$ =The proportion of deaths in pregnancy for the location, year, age, and sex that are estimated to be HIV-positive and maternal deaths which are aggravated by HIV/AIDS.

$CF_{l,t,a,x,mat_{adj}}$ = The proportion of deaths due to maternal causes after the adjustment for the location, year, age, and sex.

$CF_{l,t,a,x,mat_{hiv}}$ = The proportion of deaths due to maternal deaths aggravated by HIV/AIDS after the adjustment for the location, year, age, and sex.

11.4 HIV/AIDS correction of other maternal mortality data

Although there are a specific subset of codes in ICD-10 that correspond to HIV/AIDS deaths aggravated by pregnancy, these codes are sparsely used and unreliable. We therefore adapted the method above to also correct VR and VA sources for the systematic exclusion of HIV-related maternal deaths. This correction was calculated in the same manner, using the same input data as above, with the only

difference that HIV correction of VR and VA sources resulted in a net increase in maternal CF. Maternal deaths aggravated by HIV/AIDS are calculated as the following:

$$CF_{l,t,a,x,mat_{hivvr}} = CF_{l,t,a,x,matvr} * prop_{maternalhiv_{l,t,a,x}}$$

$$prop_{maternalhiv_{l,t,a,x}} = \frac{PAF_{l,t,a,x,hivpos} * \pi_{mat}}{1 - PAF_{l,t,a,x,hivpos} * \pi_{mat}}$$

Where all symbols are the same as described above.

Step 12. Noise reduction

To deal with problems of zero counts in vital registration, verbal autopsy, cancer registries, or sibling histories for a given age group in a given year, we use a Bayesian noise reduction algorithm. For this algorithm, we assume a normal prior and a normal data likelihood. We estimate the normal prior for a given country series of data by estimating a negative binomial for the fraction of deaths in each age group due to each respective cause with dummy variables for age and year. With two notable exceptions (detailed below), these regressions are country-specific, so borrowing strength over age is only within a data type in a country. The variance of the prior, τ^2, is estimated from the negative binomial regression, taking into account the variance-covariance matrix of the regression coefficients. For the data variance, we use the Wilson approximation which provides an estimate of σ^2 even in cases with a zero count of cause-specific deaths. The posterior estimate for each data point is:

$$Mean = \left(\frac{\tau^2}{\tau^2 + \sigma^2} X + \frac{\sigma^2}{\tau^2 + \sigma^2} \mu\right)$$

$$Variance = \left(\frac{\tau^2 \sigma^2}{\tau^2 + \sigma^2}\right)$$

Where X is the mean of the data and μ is the mean of the prior. This approach to noise reduction avoids the problem that zero counts in an *ln* rates model or a logit cause fraction model will be dropped from the regression and lead to upward bias in the estimates. This is particularly important in two settings: high-income countries with small numbers of cause-specific deaths, and in the analysis of sibling history data where for any given age group in any given year the number of deaths reported in the survey that are pregnancy-related or the number of deaths from all causes in that age group may be small.

Regarding the exceptions to the regression, the first is that country-years with populations under 1 million are pooled with the region data in order to prevent overdispersion and provide a stronger signal. Additionally, verbal autopsy data diverge from the above description in two ways. First, all data for a given super-region are pooled together and a study dummy variable is added, allowing for different studies and surveillance sites to borrow strength from one another within a super-region. Second, unless the data are part of a time series (e.g., Matlab), there is no year component to the regression.

Step 13. COD database and outlier identification

Death rates for different causes of death generally have a stable age pattern. In large populations, these patterns will not change very rapidly over time. We can assume a relatively stable pattern in death rates for all causes except for some epidemic diseases and specific types of injuries. Rare causes in large populations and prevalent causes in small populations usually have stochastic patterns. To correct for these stochastic patterns we implement a noise reduction process, explained in Step 12.

In vital registration data, we infrequently find one or more data points for specific geography/age/sex/years that lie very far from the stable pattern of death rates. In these situations, the model will usually ignore the data point(s). If the model fails to ignore these data, dramatic jumps or drops can occur in the death rates. When there is no logical explanation for variation in the death rates to this degree, we outlier the data point(s). The selection of data points to outlier occurs after data have been prepped for modeling, as well as during preliminary reviews of the models.

In non-vital-registration sources, data collection methods and data quality can vary widely from source to source. Where data points in each age-sex-geography-year are very sparse, extreme data points can have a bad effect on regional estimation. In these situations we investigate the study's methods and outlier lower-quality data points.

Identifying outliers in the cause of death data occurs prior to finalization of models for each cause. We do not automate the selection of outliers, but investigate the source of the offending data as well as reviewing other data sources for the same cause, geography, and year. Ultimately, outliers are identified based on the judgement of the modeler and senior faculty and are reversible to allow for decisions to be revisited in the future.

Modeling maternal mortality

2.1 Modeling Maternal disorders

CODEm models were informed by centrally prepped data stored in the CoD database. All data were corrected for incidental HIV deaths as described above. All data from all geographies were reviewed in CODEm models. Outliers were identified as those data where age patterns or temporal patterns were inconsistent with neighboring age groups or locations or where sparse data were predicting implausible overall temporal or age patterns for a given location. Overall maternal mortality was estimated with CODEm. Etiology-specific estimates were derived by multiplying the proportion outputs from DisMod-MR 2.1 by the total maternal deaths for that age-group, location, and year, which were scaled in relation to each other to equal one. HIV-related maternal deaths were estimated for all locations using the PAF approach described above for mortality data processing. Incidental HIV deaths during pregnancy were by definition excluded. DisMod-MR 2.1 is described in detail in Section 3.1.

2.2 CODEm

Overview of method

CODEm is a framework for modeling most cause-specific death rates in the GBD using five core principles: 1) Identify and use all the available data in the modeling process. Though data may vary in quality it all contains some signal of the true epidemiological process. 2) Develop a diverse set of plausible models to use for estimation. That is, build a number of models capturing well-documented associations to make estimates. 3) Assess the predictive validity of each plausible individual model and of an ensemble of models created from the pool of plausible models. 4) Choose the models and ensemble model with the best performance in the out-of-sample predictive validity tests.

For some causes, separate models were run for different age ranges when there was reason to believe that the relation between covariates and death rates might be different in different age ranges, for example, in children compared with adults. Separate models are developed for countries

with extensive, complete, and representative VR for every cause such that uncertainty can better reflect the more complete vital registration in these locations. A complete listing of countries with extensive, complete, and representative VR can be found in Appendix Table 3.

Model pool development

As many factors covary with a particular cause of death, a large range of plausible statistical models are developed for each cause. For the CODEm framework, four families of statistical models are developed using covariates (see 2x2 table in Foreman et al).[11] These are mixed effects linear models of the natural log of the death rate, mixed effects linear models of the logit of the cause fraction, spatiotemporal Gaussian process regression (ST-GPR) models of the log of the death rate, and ST-GPR of the logit of the cause fraction. All plausible relationships between covariates and relevant cause are identified, and all possible permutations of selected covariates are tested in linear models where the logit cause fraction or log death rate is the response variable. Because we test all permutations of covariates, multicollinearity between covariates may produce implausible signs on coefficients or unstable coefficients. All models where the sign on the coefficient is in the direction expected based on the literature and where the coefficient is statistically significant at $p < 0.05$ are retained. We run covariate selection for both cause fractions and death rates and then create both mixed effects only and ST models for each set of covariates. For a detailed explanation of the covariate selection algorithm see Foreman et al 2012.[1]

Testing model pool on 15% sample

The performance of all component models and ensembles is evaluated using out-of-sample predictive validity tests. Thirty percent of the data are excluded from the initial model fits, and half of that (15% of total) is used to evaluate and rank component models and then build ensembles. Data are held out from the analysis using the pattern of missingness for each cause in the cause of death database. Out-of-sample predictive validity testing is repeated until stable model results have been obtained. The out-of-sample performance tests include the root mean squared error of the log of the cause-specific death rate, the direction of the trend in the prediction compared to the data, and the validity of the 95% UI. For every model, we show the in-sample root mean squared error (RMSE) of the log death rates and the out-of-sample performance in the 15% of data not used in the model building process.

Ensemble development

After component models are ranked on their out-of-sample predictive validity they are weighted based on their ranking and each component model contributes a portion to the final estimate. How much each submodel contributes is a function of its relative ranking as well as the value of psi chosen, which dictates that distribution of rankings (see Foreman et al 2012 for the details of psi distribution).[1]

Testing ensembles

Using the second half of the holdout data (15% of total), the differently weighted ensembles and different values of psi are tested using the same predictive validity metrics as the component models. For every model, we show the in-sample RMSE of the log death rates and the out-of-sample performance in the 15% of data not used in the model building process. The ensemble with the best average trend and RMSE is chosen as the final ensemble weighting scheme.

Final estimation

After a model weighting scheme has been chosen, each model contributes a number of draws proportional to its weight such that 1,000 draws are created. The mean of the draws is used as the final estimate for the CODEm process and 95% UI are created from the 0.025 and 0.975 quantiles of the draws. The final assessment of ensemble model performance is the validity of the UIs; ideally, the 95% UI for a model would capture 95% of the data out-of-sample. Higher coverage suggests that UIs are too large and lower than 95% suggest UIs are too narrow.

3.1 Estimation of etiology and timing of maternal mortality DisMod-MR 2.1

Until GBD 2010, non-fatal estimates were based on a single data source on prevalence, incidence, remission, or a mortality risk selected by the researcher as most relevant to a particular geography and time. For GBD 2010, we set a more ambitious goal: to evaluate all available information on a disease that passes a minimum quality standard. That required a different analytical tool that would be able to pool disparate information presented in varying age groupings and from data sources using different methods. The DisMod-MR 1.0 tool used in GBD 2010 evaluated and pooled all available data, adjusted data for systematic bias associated with methods that varied from the reference and produced estimates by world regions with uncertainty intervals. For GBD 2013, the improved DisMod-MR 2.0 had increased computational speed allowing computations that were consistent between all disease parameters at the country rather than region level. The hundred-fold increase in speed of DisMod-MR 2.0 was partly due to a more efficient re-write of the code in C++ but also by changing to a model specification using log rates rather than a negative binomial model used in DisMod-MR 1.0. In cross-validation tests, the log rates specification worked as well or better than the negative binomial specification[12]. For GBD 2015, the computational engine (DisMod-MR 2.1) remained substantively unchanged but we re-wrote the 'wrapper' code that organized the flow of data and settings at each level of the analytical cascade. The sequence of estimation occurred at five levels: global, super-region, region, country, and, where applicable, subnational geographical units (see flow diagram of DisMod-MR 2.1 cascade, below). The super-region priors were generated at the global level with mixed-effects, non-linear regression using all available data; the super-region fit, in turn, informed the region fit, and so on down the cascade. The wrapper gave analysts the choice to branch the cascade in terms of time and sex at different levels depending on data density. The default used in most models was to branch by sex after the global fit but to retain all years of data until the lowest level in the cascade. For GBD 2015, we generated fits for the years 1990, 1995, 2000, 2005, 2010, and 2015.

In updating the 'wrapper,' we consolidated the code base into a single language, Python, to make the code more transparent and efficient and to better deal with subnational estimation. The computational engine is limited to three levels of random effects; we differentiated estimates at the super-region, region, and country level. In GBD 2013, the subnational units of China, Mexico, and the United Kingdom (UK) were treated as 'countries' such that a random effect was estimated for every geography with contributing data. However, the lack of a hierarchy between country and subnational units meant that the fit to country data contributed as much to the estimation of a subnational unit as the fits for all other countries in the region. We found inconsistency between the country fit and the aggregation of subnational estimates when the country's epidemiology varied from the average of the region. Adding an additional level of random effects required a prohibitively comprehensive rewrite of the underlying DisMod-MR engine. Instead, we added a fifth layer to the cascade, with subnational estimation informed

by the country fit and country covariates, plus an adjustment based on the average of the residuals between the subnational unit's available data and its prior. This mimicked the impact of a random effect on estimates between subnationals.

For GBD 2015 we improved how country covariates differentiate non-fatal estimates for diseases with sparse data. The coefficients for country covariates were re-estimated at each level of the cascade. For a given geography, country coefficients were calculated using both data and prior information available for that geography. In the absence of data, the coefficient of its parent geography was used, in order to utilize the predictive power of our covariates in data sparse situations.

Modeling age-specific fertility and live births

Data

For locations where the United Nations Population Division provides age-specific fertility rate (ASFR) for age groups 15-19, 20-24, 25-29, 30-34, 35-39, 40-44, 45-49 in their most recent update to the World Population Prospect (WPP), we start with their estimates for every five year time period (e.g. 1990-1995). We treat the given value as that of the midpoint year, so in the case of 1990-1995, we use the value for 1992. We then linearly interpolate in log space to generate values for the intervening years.

ASFR for locations not covered by the UNPOP

For locations not covered by UNPOP, including any subnational locations as well as countries such as Andorra, American Samoa, Bermuda, Northern Mariana Islands, and the United States (US) Virgin Islands, we took one of two approaches. If we could find relatively complete data for 1970's onwards, we would use those estimates. To address the small number of missing values in these datasets, we used a combination of linear mixed effects regression, simple linear interpolation, and 3-year rate of change extrapolation depending on the nature of values that were missing.

Linear mixed effects regression with age as categorical variable was applied to data when entire age groups were missing for a given location. Linear interpolation was applied to locations when missing ASFR values fell between years where ASFR was available. In locations where ASFR was missing for years where values did not fall between years where ASFR was available, but ASFR was present in years preceding or directly after the missing year, ASFR was calculated using annualized rate of change. Missing ASFR was interpolated based on the rate of change of ASFR of the 3 years preceding or following the missing year.

Secondly, in cases where there was little data or it did not cover most of the time period, we modeled ASFR using a database of fertility tables from the Human Fertility Database and from location-level surveys in the locations we were modeling. This process was as follows:

1. *Calculating empirical weights:* Using the database of tables, we created all possible pairs of tables. For each age category, we then calculated the difference between the two tables. These differences were then summed, producing a total difference for each pair of tables. We then created a series of indicator variables for each pair, indicating whether or not they were from the same country, region, or super-region, and how many years apart they were. We then average the difference for each category. So for example, we produced the mean difference for locations in the same super-region but not the same region or country that were 2 years apart in time. We then took the reciprocals of these differences to produce a weight, indicating how "close" a table is to another given their similarities in location and time.

2. *Fit model relating difference in TFR to difference in ASFR:* We again create all possible pairs of tables as in step 1. For each pair, we randomly select one table to be the predictor table. Then we fit the following model for each age group in 15-19, 20-24, 25-29, 30-34, 35-39, 40-44, and 45-49:

$$ASFR_1 - ASFR_2 = \beta_1(TFR_1 - TFR_2) + \beta_0$$

where table 1 is the table randomly designated as the predictor table.

3. *Produce a standard tables and predict:* Using the weights produced in the first step, we create a standard table from a selection of tables from the database. For each table, weights are calculated for all the other tables based on how far they are from the table in terms of year, and whether they are from the same country, region, or super-region. We then order by weight and take the first 300 tables. We then create an average table, weighted by weights calculated in step 1. This produces a standard table for each location-year. Due to the limited number of tables for many location, this can produce discontinuities from year to year. To prevent this, we applied a rolling mean over time to the standard, resulting in estimates that are relatively smooth over time. We then use this standard to predict the ASFR for each age group using the models produced in step 2 and the TFR for the country-year of interest:

$$ASFR_1 = \beta_1(TFR_1 - TFR_{standard}) + \beta_0 + ASFR_{standard}$$

where TFR_1 is the TFR in the location year where we are predicting ASFR.

Getting single year ASFR

Once we have five year ASFR values, we calculate single year ASFR using a spline and treating the ASFR values as midpoints for each age group. Though we do not use single-year ASFR for maternal calculations, they are used in other parts of the GBD, and so are incorporated in this process.

For high and low ages, we set fertility for 9 and below and 55 and above to 0, then used those in the interpolation. Because many sources do not have ages 10-14 and 50-54, which are necessary for our maternal estimates, we also extend our estimates to include these age groups. To do this, we created a linear interpolation between the value at age 15 and 0 at age 9 on the young side, and between the value at 50 and 0 at age 55. To these values, we then applied percentages of women who have gone through menarche[29–31] or have not gone through menopause[32–35], respectively. These values are given in the following tables:

age	Pre-menopause (%)	age	Post-menarche (%)
50	36	10	4
51	28	11	14
52	20	12	40
53	14	13	77
54	9	14	98

Because of the steep climb in fertility in the teen years, we made sure that our estimates in 10-14 were in-line with what we would expect by scaling them to the 15-19 age category. Using the mean of the ratios between 10-14 and 15-19 from the Indian DHS, the US census, and the Democratic Republic of the

Congo DHS, we scaled the 10, 11, 12, 13, and 14 ages so that their mean has this ratio with the mean of 15-19.

Scaling to births

To get our final ASFR estimates, we scale ASFR so that the total implied births from our ASFR estimates and the GBD populations is the same as the GBD births. GBD births are generally derived from the WPP 2015 Revision, and WHO, though for some locations we use location-specific sources. This scaling ensures consistency between our fertility results and the populations that are used in other parts of the GBD process. The exception to this is South Africa. There we used subnational estimates from UNAIDS, calculated live births implied by these, then used their sum for the South Africa national ASFR estimates. These values were then substituted into the GBD births.

To re-calculate five year age groups, we calculate the number of births in each five year age group and divide by the population in that age group. These are the final ASFR estimates used in our maternal mortality calculations.

References

1 Lozano R, Freeman MK, James SL, *et al.* Performance of InterVA for assigning causes of death to verbal autopsies: multisite validation study using clinical diagnostic gold standards. *Popul Health Metr* 2011; **9**: 50.

2 Office of the Registrar General and Census Commissioner. India Special Survey of Deaths. India, 2004.

3 Office of the Registrar General and Census Commissioner. India Medical Certification of Cause of Death Reports 1990-2010. India, 2014.

4 Office of the Registrar General and Census Commissioner. India Medical Certification of Cause of Death Reports 1980-1986. India.

5 Naghavi M, Makela S, Foreman K, O'Brien J, Pourmalek F, Lozano R. Algorithms for enhancing public health utility of national causes-of-death data. *Popul Health Metr* 2010; **8**: 9.

6 Ahern RM, Lozano R, Naghavi M, Foreman K, Gakidou E, Murray CJ. Improving the public health utility of global cardiovascular mortality data: the rise of ischemic heart disease. *Popul Health Metr* 2011; **9**: 8.

7 GBD 2013 Mortality and Causes of Death Collaborators. Global, regional, and national age-sex specific all-cause and cause-specific mortality for 240 causes of death, 1990-2013: a systematic analysis for the Global Burden of Disease Study 2013. *Lancet* 2015; **385**: 117–71.

8 Lozano R, Naghavi M, Foreman K, *et al.* Global and regional mortality from 235 causes of death for 20 age groups in 1990 and 2010: a systematic analysis for the Global Burden of Disease Study 2010. *Lancet* 2012; **380**: 2095–128.

9 Calvert C, Ronsmans C. The contribution of HIV to pregnancy-related mortality: a systematic review and meta-analysis. *AIDS* 2013; **27**: 1631–9.

10 DerSimonian R, Laird N. Meta-analysis in clinical trials. *Control Clin Trials* 1986; **7**: 177–88.

11 Foreman KJ, Lozano R, Lopez AD, Murray CJ. Modeling causes of death: an integrated approach using CODEm. *Popul Health Metr* 2012; **10**: 1.

12 Flaxman A, Vos T, Murray C. Integrated Meta-Regression Framework for Descriptive Epidemiology. University of Washington Press, 2014.

13 Hill K. Estimating census and death registration completeness. *Asian Pac Popul Forum* 1987; **1**: 8–13, 23–4.

14 Bennett NG, Horiuchi S. Estimating the completeness of death registration in a closed population. *Population Index* 1981; **47**: 207–21.

15 Brass W, Coale AJ. Methods of Analysis and Estimation. In: Brass W, ed. The Demography of Tropical Africa. Princeton: Princeton University Press, 1968.

16 Preston S, Coale AJ, Trussell J, Weinstein M. Estimating the completeness of reporting of adult deaths in populations that are approximately stable. *Popul Index* 1980; **46**: 179–202.

17 Preston S, Hill K. Estimating the completeness of death registration. *Population Studies* 1980; **34**: 349–66.

18 Murray CJL, Rajaratnam JK, Marcus J, Laakso T, Lopez AD. What can we conclude from death registration? Improved methods for evaluating completeness. *PLoS Med* 2010; **7**: e1000262.

19 Obermeyer Z, Rajaratnam JK, Park CH, *et al.* Measuring Adult Mortality Using Sibling Survival: A New Analytical Method and New Results for 44 Countries, 1974–2006. *PLOS Med* 2010; **7**: e1000260.

20 Gakidou E, King G. Death by survey: estimating adult mortality without selection bias from sibling survival data. *Demography* 2006; **43**: 569–85.

21 Masquelier B. Adult Mortality From Sibling Survival Data: A Reappraisal of Selection Biases. *Demography* 2012; **50**: 207–28.

22 Rogers RG, Crimmins EM, editors. Adult Mortality in Africa. In: International Handbook of Adult Mortality, 2011 edition. Dordrecht Netherlands ; New York: Springer, 2011: 151–70.

23 Lozano R, Wang H, Foreman KJ, *et al.* Progress towards Millennium Development Goals 4 and 5 on maternal and child mortality: an updated systematic analysis. *The Lancet* 2011; **378**: 1139–65.

24 Schnuelle P, Lorenz D, Trede M, Van Der Woude FJ. Impact of renal cadaveric transplantation on survival in end-stage renal failure: evidence for reduced mortality risk compared with hemodialysis during long-term follow-up. *J Am Soc Nephrol* 1998; **9**: 2135–41.

25 Brocklehurst P, French R. The association between maternal HIV infection and perinatal outcome: a systematic review of the literature and meta-analysis. *BJOG: An International Journal of Obstetrics and Gynaecology* 1998; **105**: 836–48.

26 Kim H-Y, Kasonde P, Mwiya M, *et al.* Pregnancy loss and role of infant HIV status on perinatal mortality among HIV-infected women. *BMC Pediatrics* 2012; **12**: 138.

27 256 © 2014 UU| B, 27 751 05 Uppsala | Tel 018-471 00 00018-471 00 00 | Org nr: 202100-2932 | VAT-nr: SE202100293201 | Contact | Registrar | Editor: Marie Allansson | About the WebSite | Last modified: Apr, 2016. UCDP/PRIO Armed Conflict Dataset - Uppsala University, Sweden. http://www.pcr.uu.se/research/ucdp/datasets/ucdp_prio_armed_conflict_dataset/ (accessed April 29, 2016).

28 UCDP/PRIO Armed Conflict Dataset Codebook. Uppsala Conflict Data Program (UCDP); Centre for the Study of Civil Wars, International Peace Research Institute, Oslo (PRIO), 2013.

29 Anderson SE, Must A. Interpreting the continued decline in the average age at menarche: results from two nationally representative surveys of U.S. girls studied 10 years apart. *J Pediatr* 2005; **147**: 753–60.

30 Demerath EW, Towne B, Chumlea WC, *et al.* Recent decline in age at menarche: the Fels Longitudinal Study. *Am J Hum Biol* 2004; **16**: 453–7.

31 Talma H, Schönbeck Y, van Dommelen P, Bakker B, van Buuren S, Hirasing RA. Trends in menarcheal age between 1955 and 2009 in the Netherlands. *PLoS ONE* 2013; **8**: e60056.

32 Greer W. Preprocessing histograms of age at menopause using the fast Fourier transform. *Maturitas* 2003; **44**: 267–77.

33 Greer W, Sandridge AL, Chehabeddine RS. The frequency distribution of age at natural menopause among Saudi Arabian women. *Maturitas* 2003; **46**: 263–72.

34 Luoto R, Kaprio J, Uutela A. Age at natural menopause and sociodemographic status in Finland. *Am J Epidemiol* 1994; **139**: 64–76.

35 Stanford JL, Hartge P, Brinton LA, Hoover RN, Brookmeyer R. Factors influencing the age at natural menopause. *J Chronic Dis* 1987; **40**: 995–1002.

Skilled Birth Attendance (SBA) Capstone Appendix

Flowchart

3.1.2 Skilled Birth Attendance Coverage Estimation Flowchart

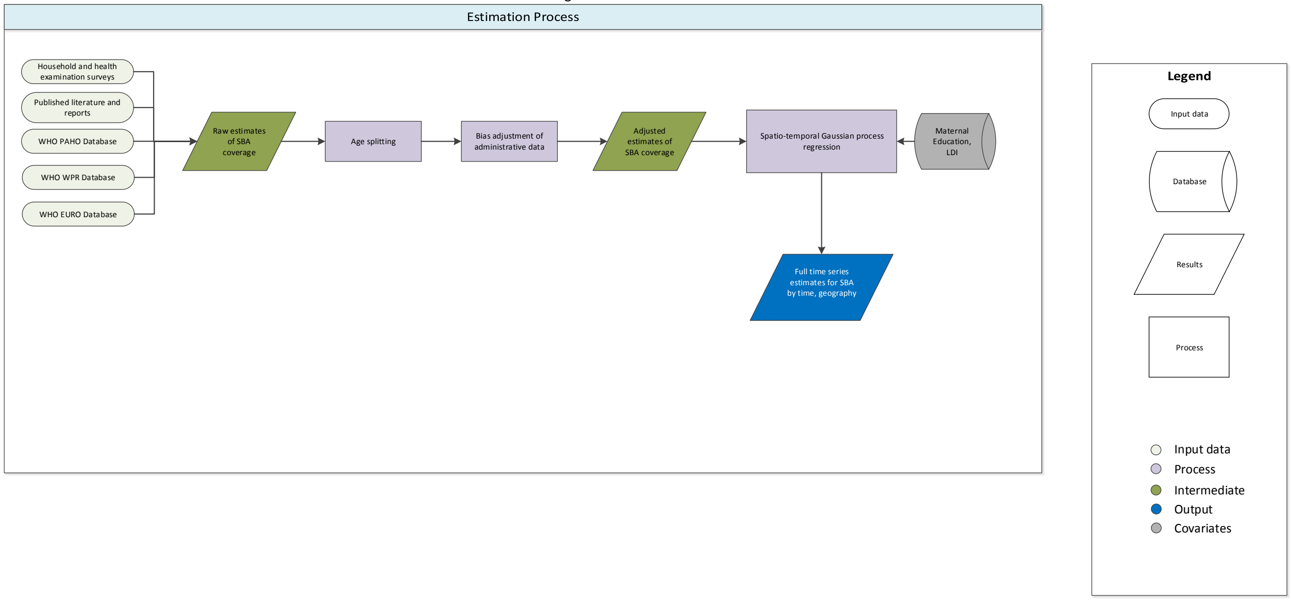

Input data & Methodological summary

Indicator definition

This modeling strategy encompassed the indicator associated with skilled birth attendance (3.1.2).

Indicator 3.1.2

As a component of SDG Goal 3. Ensure healthy lives and promote well-being for all at all ages, SDG Target 3.1, by 2030, reduce the global maternal mortality ratio to less than 70 per 100,000 live births, is measured using SDG Indicator 3.1.2, the proportion of births attended by skilled health personnel (doctors, nurses, and midwives). This indicator is also a component of the UHC tracer indicator.

Input data

For the present analysis, we used individual-level microdata from population health surveys and tabulated survey report data on skilled birth attendance (SBA). As defined by the World Health Organization (WHO), SBA reflects the proportion of births in a given year where a doctor, nurse, or midwife was present.[1]

Survey data which provided individual-level data, and specifically among female respondents, were identified and extracted. Major multi-country survey programs included in the analysis include the Demographic and Health Surveys (DHS),[2] Multiple Indicator Cluster Surveys (MICS),[3] Reproductive Health Surveys (RHS),[4] Living Standards Measurement Study (LSMS) surveys,[5] and World Health Surveys (WHS).[6] We also conducted a comprehensive search of the Global Health Data Exchange (GHDx),[7]as well as targeted internet searches and review of Ministry of Health websites, to identify national surveys and

other multi-country survey programs. In addition, we utilized tabulated report data from regional WHO databases, when available, including the PAHO, WHO WPR, and the WHO European Health for All databases.

We excluded all data sources that were not nationally representative or had high levels of missingness. We applied survey weights based on survey sampling frames whenever they were available to generate weighted national estimates of SBA coverage accompanied by estimates of standard error (SE). Estimates of SE, as well as sample sizes, were used to calculate uncertainty, as described below. Any point estimates with sample sizes less than 50 were reviewed to ensure that were not substantive outliers and would otherwise have an undue influence on our analysis.

Due to potential bias in recall, we limited our analysis to women who gave birth up to five years prior to the time of survey; due to data limitations, we used a limit of up to two years for some surveys. We also had to standardize the definition of "skilled health professional" across countries, which varied by differences in quality of training or health professional roles. For this analysis, doctors, nurses, and midwives were included as our foundational definition for SBA, and we extended this to include country-specific medical staff based on the number of years of training they received and/or their comparable ability to intervene in an emergency situation (eg, clinical officers). Care received during delivery by traditional health personnel was not considered a birth overseen by a skilled attendant.

Modeling strategy

Data processing

Age splitting

Most household surveys collection information on maternal and child health (MCH) indicators for children under 5 and/or mothers who gave birth within five years prior to the time of survey. To maximize data use for our model, we included SBA information for children aged 12 to 59 at the time of survey. Children younger than 12 months of age were excluded to minimize the influence of potentially censored observations. SBA coverage estimates were assigned to birth-cohort years based on a child's age prior to the time of survey: we used responses recorded for children aged 12 to 23 months for SBA coverage for one year prior to the time of survey, children aged 24 to 35 months for coverage two years prior to the time of survey, and so forth.

Age-specific estimates are easily computed from individual-level microdata, but many published reports and survey summaries present data in broader age aggregates (eg, SBA coverage for children aged 12 to 35 months). To standardize these age groups, we applied an age-splitting model used in the GBD study,[9] as well as analyses that generated smoking and obesity prevalence by age group.[10,11]

Using surveys with microdata as the reference, we used the following model to generate standardized age group-specific estimates for SBA:

$$\tilde{P}_{a,c,t,k} = P^{a+x}_{a,c,t,k} \frac{P_{a,c,t,j}}{P^{a+x}_{a,c,t,j}}$$

where $\tilde{P}_{a,c,k}$ is the adjusted estimate of coverage for target age group a in country c and year t of survey k; and $P_{a,c,k}^{a+x}$ is coverage reported from survey k, for country c in year t for the age group spanning age a to age $(a + x)$. The ratio of coverage between the target age group and broader age group from a survey j with microdata from the same country-year was used to split data from survey k. Surveys to be split were ideally matched with DHS or MICS surveys. If microdata were not available for the same year, ratios within five years of the survey that required age-splitting were applied.

Bias adjustments

Intervention coverage estimates based on administrative sources can be biased, yet the direction and magnitude of such biases are not universal. Some studies show that coverage estimates from administrative data source are systematically higher than those of survey-based estimates,[12] while other studies show that bias directionality is more heterogeneous.[13] Such biases may arise for a number of reasons, including discrepancies in the accurate reporting of services or interventions provided (eg, number of skilled attendants) and target population (eg, number of children born), as well as capturing these data in a timely manner from both public and private sector facilities and healthcare providers.

For SBA, we view individual-level data collected through population health surveys as the most accurate and least biased source of information, particularly for geographies with incomplete health information systems. We thus used SBA coverage estimates from household surveys to calculate country-specific adjustment factors:

$$logit\left(P_{s,\,c,t}\right) = \beta_0 + \beta_1 logit\left(\tilde{P}_{a,\,c,t}\right) + \sum_{k=2}^{2+B} \beta_k S_k + \varepsilon_{c,t}$$

where $P_{s,\,c,t}$ is the survey-based estimate for SBA coverage (s) in country c for year t; $\tilde{P}_{a,\,c,t}$ is the administrative estimate for coverage in country c in year t; S_k is a spline basis used to capture the secular trend in coverage; β_1 is the estimated adjustment factor used to correct for the administrative bias; and ε is the error term for country c in year t.

To quantify uncertainty for bias-adjusted estimates from the mixed-effects models described above, we calculated prediction error, $\widehat{PE}$, as follows:

$$\widehat{PE} = X^2 var(\hat{\beta})$$

where $var(\hat{\beta})$ is the variance for the estimated fixed-effects coefficient of the adjustment factor and X is the independent variable. Proper estimation of prediction errors is crucial as the data synthesis procedure, Gaussian process regression (GPR) (as described in the subsequent section), accounts for uncertainty from point estimates and bias adjustments when generating fitted values. More weight is given to data with less uncertainty. Prediction errors estimated from the bias adjustment were incorporated into the data variance and propagated through the GPR step to obtain estimates of SBA coverage and uncertainty intervals (UIs).

Trend estimation

We used a spatiotemporal Gaussian process regression (ST-GPR) to synthesize point estimates from multiple data sources and derive a complete time series for SBA coverage. This method has been used extensively in GBD and related studies, and accounts for uncertainty pertaining to each point estimate while borrowing strength across geographic space and time.[10, 11,15,16] Briefly, we assumed the Gaussian process was defined by a mean function *m*(•) and covariance function *Cov*(•).

We estimated the mean function using a two-step approach. Specifically, $m_c(t)$ can be expressed as:

$$m_c(t) = X\beta + h(r_{c,t})$$

where $X\beta$ is a linear model and $h(r_{c,t})$ is a smoothing function for the residuals; and $r_{c,t}$ is derived from the linear model. The following linear model was used for estimating SBA:

$$logit(P_{c,t}) = \beta_0 + \beta_1 medu_{c,t} + \beta_1 LDI_{c,t} + \alpha_c + \gamma_{R[c]} + \delta_c medu + \theta_{R[c]} medu + \varepsilon_{c,t}$$

where $P_{c,t}$ is SBA coverage for country c year t; $medu_{c,t}$ is the average years of education for women of reproductive age in country c and year t; $LDI_{c,t}$ is the lag-distributed income (LDI) in country c and year t; α_c and $\gamma_{R[c]}$ are country and region random intercepts, respectively. δ_c and $\theta_{R[c]}$ are country and region specific slope on education. These estimates were then run through ST-GPR, as documented elsewhere.[10]

Random draws of 1,000 samples were obtained from the distributions above for every country for a given vaccine. Ninety-five percent uncertainty intervals were calculated by taking the ordinal 25 and 975th draws from the sample distribution.

To assess the accuracy of our estimates in each bias adjustment step and in the modeling process, we performed cross-validation analyses by randomly holding out 20% of the sample and, if available, the corresponding administrative estimates for the given indicator of the same country and year, 10 separate times. We computed the average root mean squared errors (RMSE) across each country. Error in the bias adjustments was calculated as the mean difference between the adjusted administrative estimate for a given country, year, and corresponding survey-level estimates (which were considered the "gold-standard"); error in the modeling process was calculated as the difference between the modeled estimates and the sample data.

References

1 WHO Indicator and Measurement Registry (WHO IMR). WHO IMR. http://apps.who.int/gho/indicatorregistry/App_Main/indicator_registry.aspx (accessed Aug 11, 2015).

2 Measure DHS: Demographic and Health Surveys. http://www.measuredhs.com (accessed Aug 11, 2015).

3 UNICEF Stat. Monit. Multiple Indicator Cluster Survey (MICS). http://www.unicef.org/statistics/index_24302.html (accessed Aug 11, 2015).

4 Cent. Dis. Control Prev. Reproductive Health Surveys (RHS). http://www.cdc.gov/reproductivehealth/Global/surveys.htm (accessed Aug 11, 2015).

5 World Bank. Living Standard Measruement Studies (LSMS). http://go.worldbank.org/UK1ETMHBN0 (accessed Aug 11, 2015).

6 WHO Multi-Ctry. Stud. Data Arch. World Health Survey (WHS). http://apps.who.int/healthinfo/systems/surveydata/index.php/catalog/whs/about (accessed Aug 11, 2015).

7 IHME GHDx. Global Helath Data Exchange. http://ghdx.healthdata.org/ (accessed Aug 11, 2015).

8 WHO | WHO/UNICEF Joint Reporting Process. WHO. http://www.who.int/immunization/monitoring_surveillance/routine/reporting/reporting/en/ (accessed Aug 17, 2015).

9 Collaboration PS. Age-specific relevance of usual blood pressure to vascular mortality: a meta-analysis of individual data for one million adults in 61 prospective studies. *The Lancet* 2002; **360**: 1903–13.

10 Ng M, Freeman MK, Fleming TD, *et al.* Smoking Prevalence and Cigarette Consumption in 187 Countries, 1980-2012. *JAMA* 2014; **311**: 183.

11 Ng M, Fleming T, Robinson M, *et al.* Global, regional, and national prevalence of overweight and obesity in children and adults during 1980–2013: a systematic analysis for the Global Burden of Disease Study 2013. *The Lancet* 2014; **384**: 766–81.

12 Murray CJ, Shengelia B, Gupta N, Moussavi S, Tandon A, Thieren M. Validity of reported vaccination coverage in 45 countries. *The Lancet* 2003; **362**: 1022–7.

13 Lim SS, Stein DB, Charrow A, Murray CJ. Tracking progress towards universal childhood immunisation and the impact of global initiatives: a systematic analysis of three-dose diphtheria, tetanus, and pertussis immunisation coverage. *The Lancet* 2008; **372**: 2031–46.

HIV/AIDS SDG Capstone Appendix

Flowchart

HIV/AIDS

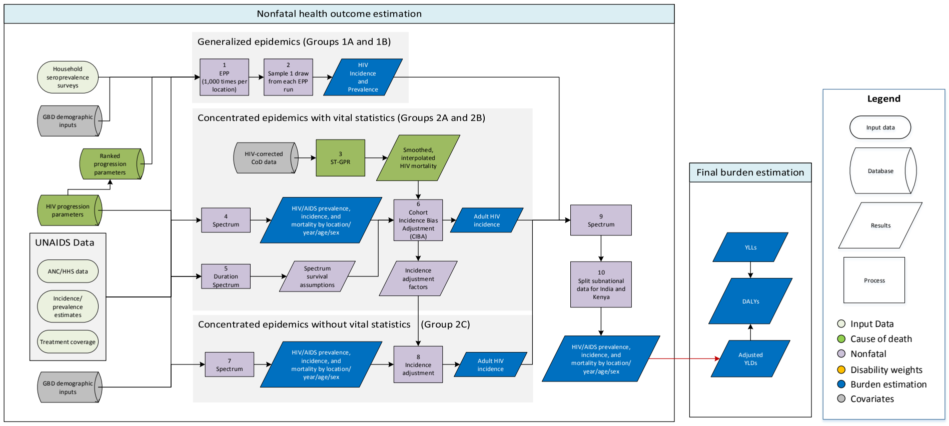

Input data & Methodological summary

Indicator definition

This modeling strategy encompassed the indicator associated with HIV incidence (3.3.1).

Indicator 3.3.1

As a component of SDG Goal 3. Ensure healthy lives and promote well-being for all at all ages, SDG Target 3.3, by 2030, end the epidemics of AIDS, tuberculosis, malaria and neglected tropical diseases and combat hepatitis, water-borne diseases and other communicable diseases, is measured using SDG Indicator 3.3.1, number of new HIV infections per 1,000.

Case definition

Infection with the human immunodeficiency virus (HIV) causes influenza-like symptoms during the acute period following infection and can lead to acquired immunodeficiency syndrome (AIDS) if untreated. HIV attacks the immune system of its host, leaving infected individuals more susceptible to opportunistic infections like tuberculosis. Although there are two different subtypes of HIV, HIV-1 and HIV-2, no distinction is made in our estimation process or presentation of results. For HIV, ICD 10 codes are B20-B24, C46-C469, D84.9; ICD 9 codes are 042-044, 112-118 (after 1980), 130 (after 1980), 136.3-136.8 (after 1980), 176.0-176.9 (after 1980), 279 (after 1980); and ICD9 BTL codes are B184-B185.

Input data

Model inputs

Household seroprevalence surveys

Geographically representative HIV seroprevalence survey results were used as inputs to the model for countries with generalized HIV epidemics where available.

GBD demographic inputs

Location-specific population, fertility, and HIV-free survival rates from GBD 2015 (see Part 1 for details on the generation of these data) and migration data from UNAIDS were used as inputs in modeling all locations.

UNAIDS data

Antenatal care, incidence, prevalence, and treatment coverage data from UNAIDS were used in modeling for all locations.

On-ART literature data

Data were identified by using search terms “HIV,” “mortality,” and “antiretroviral therapy” in PubMed searches across the literature. To be included, studies must include only HIV-positive people who receive antiretroviral therapy (ART) but who were ART-naïve prior to the study. In addition, studies must report either a duration-specific mortality proportion or a hazard ratio across age or sex, and must not include children.

For duration-specific survival data, studies must report uncertainty on mortality estimates or provide stratum-specific sample sizes and must include duration-specific data to allow for calculation of 0-6, 7-12, or 13-24 month conditional mortality. In addition, studies must either report separate mortality and loss-to-follow-up (LTFU) curves, be corrected for LTFU using vital registration (VR) data, or be conducted in a high-income setting. Finally, studies must report the percent of participants who are male, the median age of participants, and either specific data on the number of CD4 T lymphocytes (CD4 counts) or the median CD4 count used for the data.

Hazard ratio data for ages or sexes can only be used if the hazard ratios are controlled for other variables of interest (age, sex, and CD4 category).

Changes for GBD 2015

In GBD 2013, we identified 102 papers for extraction. For GBD 2015, we included 13 additional studies informing the duration-specific mortality estimation process and 26 studies informing the age and sex hazard ratio estimation process (some studies were used and counted in both). We also added one study to our LTFU analysis. In addition, we updated our data from the Antiretroviral Therapy Cohort Collaboration (ART-CC) with country-specific data pre- and post-2001 for enhanced use in estimating time trends for high-income countries. We excluded nine hazard ratio and four duration-specific mortality studies used in GBD 2013 which reported results on populations already present in other extracted studies. The inclusion of new ART-CC data necessitated the exclusion of four additional studies used in GBD 2013.

We also included on-ART cohort mortality data from 10 high-income nations with collaboration from ART-CC. These countries include Austria, Denmark, France, Germany, Italy, the Netherlands, Spain, Switzerland, the United Kingdom, and the United States. We excluded the US data because they were not fully representative of the complete with-HIV on-ART population at the time.

Off-ART literature data

In GBD 2013, to characterize uncertainty in the progression and death rates, we systematically reviewed the literature on mortality without ART. We searched terms related to pre-ART or ART-naive survival since seroconversion.[1] After screening, we identified 13 cohort studies that included the cohorts used by UNAIDS from which we extracted survival at each one-year point after infection. Screening for additional, recently published studies for GBD 2015 identified no new cohort studies for inclusion in this analysis.

Severity splits & disability weights

The basis of the GBD disability weight survey assessments are lay descriptions of sequelae highlighting major functional consequences and symptoms. The lay descriptions and disability weights (DWs) for HIV/AIDS severity levels are shown below.

Severity level	Lay description	DW (95% CI)
Symptomatic HIV	has weight loss, fatigue, and frequent infections.	0.274 (0.184-0.377)
AIDS with antiretroviral treatment	has occasional fevers and infections. The person takes daily medication that sometimes causes diarrhea.	0.078 (0.052-0.111)
AIDS without antiretroviral treatment	has severe weight loss, weakness, fatigue, cough and fever, and frequent infections, skin rashes, and diarrhea.	0.582 (0.406-0.743)

The proportion of people living with HIV/AIDS who are being treated with ART is an output of Spectrum, the compartmental model used to make consistent incidence, prevalence, and mortality estimates described below.

Modeling strategy

In GBD 2015, our general modeling strategy for estimating HIV incidence, prevalence, and mortality is similar in many ways to the strategy used in GBD 2013. In GBD 2015, we continue to use the Spectrum program rewritten in Python for GBD 2013 to facilitate faster and more flexible execution necessary for our more intensive computational needs. We made several changes to Spectrum's assumptions comparing to the Spectrum software used by UNAIDS. A key change in GBD 2015 is the application of Estimation and Projection Package (EPP) using an open-source computer program in R written by Jeffrey Eaton.[4] We ran EPP for all group 1 countries in order to produce incidence curves that were consistent with the demographic and epidemiological assumptions used in GBD 2015. This differed from GBD 2013, where we used the incidence curves provided by UNAIDS.

On-ART

First, we corrected reported probabilities of death for LTFU using an update of the approach developed by Verguet and colleagues.[5] Verguet and colleagues used tracing and follow-up studies to empirically estimate the relationship between death in LTFU and the rate of LTFU.

After extracting the survival data into duration-specific conditional mortality, we used DisMod-MR 2.0 to synthesize the data into estimates of conditional probability of death over initial CD4 count.[1] We modeled the data separately by duration and added a fixed effect on whether the study was conducted prior to 2002. Each analysis was conducted separately for high-income countries, GBD low-income countries outside of sub-Saharan Africa, and sub-Saharan Africa.

To create estimates of age-specific hazard ratios, we synthesized hazard ratio data in five broad age groups: 15-25, 25-35, 35-45, 45-55, and 55-100, and modeled the data using DisMod-MR 2.0.

To create estimates of sex-specific hazard ratios, we use the *metan* function in Stata to create estimates of relative risks separately by region, using female age groups as the reference group.

The age and sex hazard ratios were applied to the CD4-specific mortality rates, accounting for the distribution of ages and sexes in the mortality data. We then subtracted HIV-free mortality from the model life table process to calculate HIV-specific mortality, and used 1,000 draws from the posterior distribution for each age, sex, and CD4 category for conditional probabilities of death for 0-6 months, 7-12 months, and 13-24 months after initiation of ART as inputs into Spectrum.

Changes for GBD 2015

In GBD 2015, our primary methodological change was the analysis of on-ART mortality using a fixed effect on studies before/after 2002, only in the high-income region, to estimate conditional probability of death in DisMod-MR 2.0. By doing so, we incorporated changes over time in the quality of on-ART care, which may improve on-ART mortality. This change was also complemented by the inclusion of time-split data from ART-CC, which allowed us to incorporate this time trend across the large cohort. We then used the estimated post-2002 on-ART mortality through the rest of the on-ART estimation process.

Off-ART

Following UNAIDS assumptions, no-ART mortality is modeled as shown in the figure below.[1]

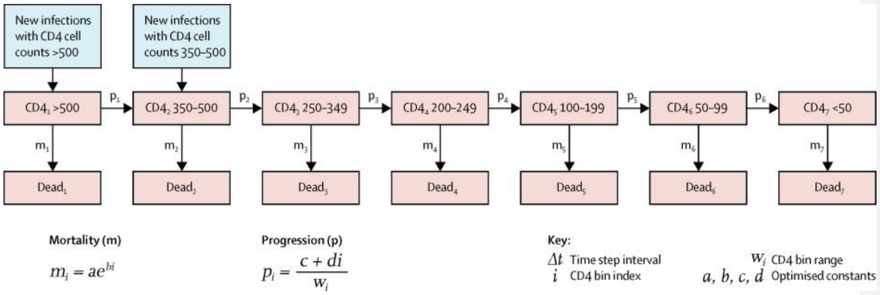

The death and progression rates between CD4 categories vary by age according to four age groups: 15–24 years, 25–34 years, 35–44 years, and 45 years or older. We modeled the logit of the conditional probability of death between years in these studies using the following formula:

$$logit\ (m_{ijk}) = \beta_0 + \sum_{i=1}^{4} \beta_{1i}\, a_i + \sum_{j=1}^{12} \beta_{2j} t_j + u_k + \varepsilon_{ijk}$$

In the formula, m is conditional probability of death from year t_j to t_{j+1}, a_i is an indicator variable for age group at seroconversion (15–24 years, 25–34 years, 35–44 years, and 45 years or older), t_j is an indicator variable of year since seroconversion, and u_K is a study-level random effect.

By sampling the variance-covariance matrix of the regression coefficients and the study-level random effect, we generated 1,000 survival curves for each age group that capture the systematic variation in survival across the available studies. For each of the 1,000 survival curves, we used a framework modeled after the UNAIDS optimization framework in which we find a set of progression and death rates that minimizes the sum of the squared errors for the fit to the survival curve.[6,7]

Burden estimation overview

UNAIDS uses two key analytical components in their epidemiological estimation. EPP is used to estimate incidence trajectories that are consistent with prevalence surveys and other prevalence measurements such as antenatal clinic serosurveillance. Spectrum is a compartmental HIV progression model used to generate age-specific incidence, prevalence, and death rates from the EPP incidence curves and assumptions about intervention scale-up and local variation in epidemiology.

For GBD 2013, we created an exact replica of Spectrum in Python. This enabled us to run thousands of iterations of the model at once on our computing cluster and allowed for more flexible input data structures. Additionally, in order to generate estimates with more realistic ranges of uncertainty than those in UNAIDS 2012, we adjusted all input data by uniformly sampled factors between 0.9 and 1.1. These changes, along with our new estimation of with- and without-ART mortality and CD4 progression parameters, persist into GBD 2015.

We have made several substantial improvements elsewhere in the process for GBD 2015. Of particular note, we have integrated EPP into the modeling process when feasible, enabling more robust and internally consistent incorporation of parameter uncertainty in generalized epidemics, and we have vastly improved the accuracy of the incidence adjustment used to fit Spectrum to high-quality VR data. Details of the impacts are included in the descriptions of the appropriate country strategies.

Due to the substantial differences in the quality and types of data available across different countries, we used three different methodologies to produce year-, age-, and sex-specific estimates of HIV incidence, prevalence, and mortality.

Countries with seroprevalence surveys and antenatal clinic data (Groups 1A and 1B)

We identified 43 countries – as well as 48 subnational locations from India, Kenya, Mozambique, and South Africa – with at least one geographically representative HIV seroprevalence survey. In order to ensure that our estimates of incidence and prevalence in these places were consistent with our estimates of HIV progression, we used a version of EPP written in R and C++ by Jeffrey Eaton to create new fits to the prevalence data in the UNAIDS files. By substituting in our own assumptions about HIV progression, we were able to ensure that the implied relationship between incidence and mortality/prevalence in EPP is similar to that in Spectrum.

In these locations, most of which experience generalized HIV epidemics, we expect estimates of HIV burden to exhibit substantial uncertainty. To reflect this, we induced a perfect correlation between the previously independent draws of HIV mortality with and without ART and CD4 progression. We paired the draws of the three parameter sets internally and with each other in the following way: we sorted without-ART mortality and CD4 progression internally by age (not CD4), meaning the highest draw of HIV mortality without ART for age a_i and CD4 category c_l will be paired with the highest draw of HIV mortality without ART for age a_k and CD4 category c_l. In the same way, we sorted with-ART mortality internally by age, sex, CD4 count at treatment initiation, and duration on treatment. After this sorting process, the lowest indexed draw of each parameter has the highest values and vice versa. This means that we will use the most extreme possible parameter sets in EPP and Spectrum and should see a commensurate expansion in the range of the uncertainty.

To ensure that this expanded uncertainty is replicated in EPP, we fit the model once for every set of paired draws of the progression parameters for every location. This means that the first iteration of EPP for Uganda sees the highest draws of all three sets of progression parameters. Such a procedure is necessary because EPP currently has no mechanism for incorporating uncertainty in any inputs except prevalence data. This process (Process 1 in the HIV/AIDS Estimation Flowchart), produced 1,000 sets of EPP output for each of the locations that make up the 47 countries in the group. Every set of EPP outputs contains 500 consistent draws of HIV incidence and prevalence in adults aged 15-49. In many cases, the algorithm used to fit EPP, incremental mixture importance sampling, failed, resulting in fewer than 1,000 sets of EPP results.

For every location in the group, we sampled one of the 500 incidence/prevalence draws from each of the sets of EPP results (Process 2 in the HIV/AIDS Estimation Flowchart). By sampling one draw from each set, we ensured that the distribution of progression parameters dictating the relationship between incidence and prevalence was exactly the same as the distribution of the sorted parameters generated in the previous step. In locations where not all 1,000 iterations of EPP fit successfully, we sampled one draw from every iteration that did succeed and then resampled with replacement from that set of draws. To maintain the link between the input progression draws and the resulting incidence and prevalence draws from EPP, we replaced any parameter draw associated with a failed run of EPP with the parameter draw that that failed draw was replaced with. At the end of this process, for every location in the set of 47 countries, we were left with 1,000 linked draws of adult incidence and prevalence and the exact progression parameters that generated those draws.

We then ran these results, along with the previously described demographic and HIV-specific inputs, through Spectrum to produce location-, year-, age-, and sex-specific estimates of HIV incidence, prevalence, and mortality (Process 9 in the HIV/AIDS Estimation Flowchart).

Countries with vital registration data (Group 2A and 2B)

VR is one of the highest-quality sources of data on HIV burden in many countries, so generating estimates that are consistent with these data, with necessary adjustment to account for any potential underreporting, is critical. We identified 116 countries – as well as 208 subnational locations from Brazil, China, Japan, Mexico, Saudi Arabia, Sweden, the United Kingdom, and the United States – with VR or sample registration systems (SRS) such as the Disease Surveillance Points (DSP) in China.

We imputed missing years of data to generate a complete time series for HIV from the estimated start year of the epidemic using spatiotemporal Gaussian process regression (ST-GPR). We analyzed mortality trends using ST-GPR starting in 1981, the year that HIV was first identified in the United States.[8] For ST-GPR, we adjusted the lambda (time weight) and GPR scale according to the completeness of vital registration data, based on whether a country had 10 or more years of complete VR data as analyzed by the Death Distribution Methods (DDM) model described in Part 1. We produced separate splines by country/age group, up to the peak year of death rate. We then ran a linear regression with random effects on region, age, and sex. Following this, we ran space-time residual smoothing, in which time, age, and space weights are used to inform smoothing of the residuals between data points and the linear regression estimate. From this process, we generated space-time estimates with the applied weights, along with the median absolute deviation (MAD) of the space-time estimates from the data. The MAD was calculated at various levels of the geographic hierarchy (e.g., subnational and national), and was added into the data variance term. The data variance and space-time estimates were then analyzed using GPR to return a final estimate of mortality along with uncertainty.

Although Spectrum produces HIV mortality estimates that are within the realm of possibility in most countries using the incidence curves provided in the UNAIDS 2012/2015 country files, it is a deterministic model that has not yet been integrated into an optimizable framework. Therefore, in order to "fit" it to VR data, we need to adjust input incidence. For GBD 2013, we used a process that assumed several different durations between HIV infection and HIV death and adjusted incidence based on death some number of years in the future. Although that method worked relatively well and substantially reduced the disconnect between Spectrum and the VR data, it required very rigid and unrealistic assumptions about these survival durations. For GBD 2015, we have improved the performance of this method, allowing Spectrum to fit to the VR data more closely.

To improve the fit of this process, we restructured Spectrum to add compartments that identify groups of people living with HIV by year of infection (Process 5 in the HIV/AIDS Estimation Flowchart). With this version of Spectrum we can output, among many other metrics, HIV deaths by year, age, sex, and infection cohort. This enables us to adjust incidence to fit to death much more precisely and without making any rigid assumptions about the time from HIV infection to HIV death.

We have incorporated these improvements into a cohort incidence bias adjustment (CIBA) process. First, we ran Spectrum normally to produce 1,000 draws of incidence, prevalence and mortality (Process 4 in the HIV/AIDS Estimation Flowchart). Then, by year, age, and sex, we took the ratio of VR deaths to Spectrum deaths to quantify the amount of bias in Spectrum. Using the mean duration data from the new version of Spectrum, for every year-, age-, and sex-specific infection cohort, we calculated the share of all HIV deaths observed over the course of the projection period in that cohort that would occur in each year after the year of infection. For example, projecting from 1970 through 2015, we identified the cohort of men infected in 1992 at the age of 16, calculated the total number of HIV deaths in that cohort in all subsequent years through the end of 2015, and divided the annual number of deaths by that total. This showed us the distribution of deaths among that cohort over the projection period. In the most extreme case (infections in 2014), we could only produce one point of that distribution (2015), so that single value is exactly 1.0; 100% of the deaths observed in that cohort occurred in 2015.

We then used these distributions of death to weigh the ratio of VR deaths to Spectrum deaths, meaning that ratios in the years where we expect the largest share of deaths were weighed most heavily. We then

multiplied the initial size of that cohort from the normal run of Spectrum by the sum of the combined ratios to get a new estimate of new cases in that year/age/sex combination.

We can write this method mathematically in the following way:

$$r_t = \frac{VR_t}{D_t}$$

$$\rho_t^{t-i} = \frac{d_t^{t-i}}{\sum_{k=t-i+1}^{n} d_k^{t-i}}$$

$$\alpha^{t-i} = \sum_{k=t-i+1}^{n} r_k * \rho_t^{t-i}$$

$$n_{\text{adjusted}}^{t-i} = \alpha^{t-i} * n^{t-i}$$

VR_t is the number of HIV/AIDS deaths in year t from ST-GPR, and D_t is the number of HIV/AIDS deaths from the first run of Spectrum. In the second equation, d_t^{t-i} is the number of HIV/AIDS deaths among members of infection cohort $t-i$ in year t, with $i \geq 1$, from the new, duration-tracking version of Spectrum, and n is final year of the projection. Therefore, ρ_t^{t-i} is the share of observed deaths in cohort $t-i$ that we expect to occur in year t. It follows that α^{t-i} is the weighted adjustment ratio described above, which we multiply by the estimated initial size of infection cohort $t-i$ as calculated in the first-stage Spectrum run to get the adjusted number of new cases, $n_{\text{adjusted}}^{t-i}$. This process is run separately for every sex and single-age pair.

CIBA (Process 6 in the HIV/AIDS Estimation Flowchart) allows ratios in each year after a given infection year to influence the final adjustment to incidence. The size of that influence is determined by the relative importance of that year in the cohort-year's distribution of deaths over time. The result is a new set of 1,000 draws of incidence and a set of 1,000 ratios of post-adjustment incidence to pre-adjustment incidence. We perform this adjustment using mean durations from the new version of Spectrum in order to try to shift the mean of the regular distribution of deaths.

Finally, to produce location-, year-, age-, and sex-specific estimates of HIV incidence, prevalence, and mortality, we ran the new estimates of incidence and all previously input data through Spectrum (Process 9 in the HIV/AIDS Estimation Flowchart).

Countries without survey data and vital registration data (Group 2C)

The remaining 24 countries – as well as nine subnational locations from China and Saudi Arabia – had neither geographically representative seroprevalence surveys nor reliable VR systems. To produce estimates of HIV burden in these countries, we assumed that Spectrum is similarly biased as in other Group 2 countries. This involved running Spectrum (Process 7 in the HIV/AIDS Estimation Flowchart), adjusting incidence using 1,000 adjustment ratios randomly sampled from the entire set of CIBA results (Process 8), and rerunning Spectrum using the new draws of adjusted incidence (Process 9). As above, the estimates of incidence, prevalence, and mortality were incorporated into the rest of the machinery via the reckoning process.

Originally, Cambodia, which does have a prevalence survey, was included in this group because we have not yet coded the machinery necessary to reproduce the Asian Epidemic Model used by UNAIDS to model prevalence and incidence in Southeast Asian countries.[9] The 2005 Demographic and Health (DHS) survey in Cambodia made clear that we were underestimating the burden due to HIV there by not using survey data during the modeling process. In order to more accurately represent the epidemic, we used the mortality profile from Thailand and scaled it by 80%, the ratio of estimated prevalence rate in Thailand in 2005 and the prevalence rate from the DHS survey in Cambodia. We then treated the scaled death series as VR data and added Cambodia to the 2B group that is run through CIBA.

Subnational splitting for India and Kenya

Spectrum results for India and Kenya subnational locations are modeled at higher levels of geography than our GBD locations. For example, Spectrum results for India are produced at the state level, while GBD 2015 estimates were produced at the state urban-rural level. Similarly, Spectrum is modeled at the province level, while we compute Kenyan subnational estimates for the 47 counties. To split the Spectrum results into more granular results for processing, we assign each GBD subnational unit to a Spectrum modeling unit. From this, we generate age/sex/year-specific proportions for population, HIV-specific death, and HIV-free mortality.

After this subnational splitting, results were incorporated into the all-cause mortality estimation machinery via the reckoning process described in Part 1.

HIV/AIDS resulting in other diseases

There are two Level 4 causes under the HIV/AIDS Level 3 cause in the GBD 2015 cause hierarchy. The modeling process for HIV/AIDS-tuberculosis is detailed in the capstone paper. We computed the number of people living with HIV resulting in other diseases by subtracting the number of people living with HIV/AIDS-tuberculosis from all people living with HIV/AIDS at the 1,000 draw level.

Commented [NF1]: Citation?

Commented [NF2]: Add reference list

Tuberculosis (TB) Incidence SDG Capstone Appendix

Flowchart

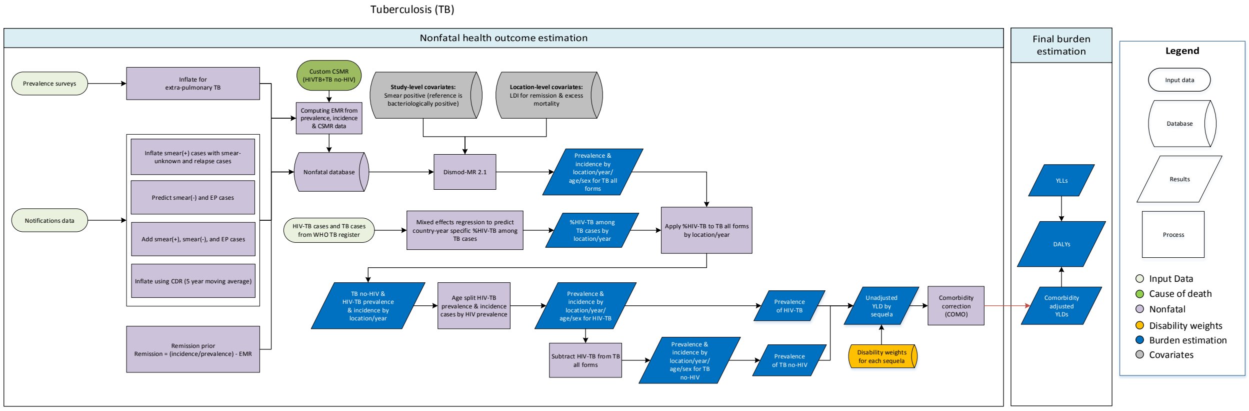

Input Data & Methodological Summary

Indicator definition

This modeling strategy encompassed the indicator associated with tuberculosis (TB) incidence (3.3.2).

Indicator 3.3.2

As a component of SDG Goal 3. Ensure healthy lives and promote well-being for all at all ages, SDG Target 3.3, by 2030, end the epidemics of AIDS, tuberculosis, malaria and neglected tropical diseases and combat hepatitis, water-borne diseases and other communicable diseases, is measured using SDG Indicator 3.3.2, number of new and relapsed TB cases per 1,000.

Case definition

TB is an infectious disease caused by *Mycobacterium tuberculosis.* The case definition includes all forms of TB including pulmonary TB and extrapulmonary TB which are bacteriologically confirmed or clinically diagnosed. For TB, the ICD 10 codes are A10-A14, A15-A19.9, B90-B90.9, K67.3, K93.0, M49.0, P37.0, Z03.0, Z11.1, Z20.1, Z23.2, and ICD 9 codes are 010-019.9, 137-137.9, 138.0, 138.9, 139.9, 320.4, 730.4-730.6, V01.1, V03.2, V12.01, V74.1. For HIV-TB, the ICD 10 code is B20.0.

Input data

For GBD 2015, input data included annual case notifications, data from prevalence surveys, and estimated cause-specific mortality (CSMR) of TB among HIV-positive and HIV-negative individuals. From these inputs, we calculated "priors" (expected values) on excess mortality and remission to give greater guidance to the model.

A systematic review done for GBD 2015 using the following PubMed search terms: (((tuberculosis[Title/Abstract]) OR TB[Title/Abstract]) OR Mycobacterium tuberculosis[Title/Abstract]) AND prevalence[Title/Abstract] Filters: Publication date from 2013/01/01 to 2015/12/31; Humans.

The exclusion criteria were:

1. Studies that were not population-based, e.g., hospital or clinic-based studies
2. Studies that did not provide primary data on epidemiological parameters, e.g., commentaries
3. Studies with a sample size of less than 150
4. Reviews

Modeling strategy

For GBD 2015, we used DisMod-MR 2.1, the GBD Bayesian meta-regression tool that adjusts for differences in methods between data sources and imposes consistency between data for different parameters.

Modeling TB incidence

We used the age- and sex-specific notifications in our analysis. There were age-specific missing data especially for younger age-groups in some countries. We imputed the missing age-groups for three forms of TB notifications (pulmonary smear-positive, pulmonary smear-negative, and extra-pulmonary). Smear-positive age-specific notifications were inflated with the proportion smear-unknown and relapsed cases only reported in the country-year data. Some countries reported only pulmonary smear-positive cases for selected years. Missing smear-negative and extrapulmonary cases were predicted from the adjusted smear-positive cases using a seemingly unrelated regression. All three types of notifications were added together and adjusted for undetected cases using WHO's estimates of country-year-specific case detection rates. We applied a 5-year moving average of case detection rates to avoid its fluctuation over time.

Modeling TB prevalence

Data from prevalence surveys reporting on pulmonary smear-positive TB and bacteriologically positive TB were included. We included a study covariate indicating whether it was bacteriologically positive TB (reference category) or smear-positive TB. We did not expect systematic bias between studies that used both symptoms and chest X-ray as screening methods and studies that used only one of the methods. We therefore did not adjust them for systematic bias but added more uncertainty to data points from studies that used only one of the screening methods. We also added more uncertainty to data points from subnational surveys. Because incidence data are for all forms of TB, we adjusted prevalence surveys to account for extrapulmonary cases. We predicted location-year-age-sex-specific proportions of extrapulmonary TB among all TB cases using data on the three forms of TB from the incidence data above and the lagged distributed income covariate from the IHME covariate database. We then computed the extrapulmonary inflation factor as *1+(proportion of extrapulmonary TB /(1- proportion of extrapulmonary TB))*, and applied it to data from prevalence surveys.

Modeling remission and excess mortality

We matched each prevalence data point and TB CSMR (TB and HIV-TB combined) by location, year, age, and sex to calculate excess mortality rate (EMR) using the function in DisMod. We also matched each

incidence data point and TB CSMR by location, year, age, and sex to calculate EMR for data-rich countries. We calculated remission using data from countries where both incidence and prevalence data were available. We matched incidence and prevalence data by location, year, age, and sex, and calculated remission as *remission=(incidence/prevalence)-EMR.* For data-rich countries, we assumed a remission of 2.0 (1.8-2.2). We ran two DisMod models: one where we used the calculated remission for low- and middle-income countries, and another where we applied the remission assumption for data-rich countries.

HIV-TB incidence and prevalence

The output from the DisMod model described above is for all forms of TB in HIV-negative and HIV-positive individuals. To separate out HIV-TB from all forms of TB, we first estimated the proportions of HIV-TB cases among all TB cases for all locations and years, using the adult HIV death rate covariate in a mixed effects regression. The input data for this regression (i.e., proportions of HIV-TB cases among all TB cases) were based on the number of TB cases recorded as HIV-positive and the number of TB cases with an HIV test result recorded in the WHO TB register. We applied the predicted location-year-specific proportions to TB incident and prevalent cases from DisMod, respectively, to generate HIV-TB incident and prevalent cases by location and year, which were then age-sex split based on the age-sex pattern of estimated HIV prevalence to generate location-year-age-sex-specific HIV-TB incident and prevalent cases.

Betas and exponentiated values (which can be interpreted as an odds ratio) from the two DisMod models are shown in the tables below:

Betas and exponentiated values from the model using remission calculated based on incidence and prevalence data

Covariate	Parameter	Beta (95% CI)	Exponentiated beta (95% CI)
Smear positive TB	Prevalence	-0.75 (-0.76 — -0.75)	0.47 (0.47 — 0.47)
Sex (male)	Prevalence	0.79 (0.68 — 0.89)	2.19 (1.98 — 2.44)
Sex (male)	Incidence	0.61 (0.59 — 0.63)	1.85 (1.81 — 1.88)
LDI (log-transformed)	Remission	0.12 (0.070 — 0.22)	1.13 (1.07 — 1.24)
LDI (log-transformed)	Excess mortality	-0.10 (-0.10 — -0.10)	0.90 (0.90 — 0.90)

Betas and exponentiated values from the model applying the remission assumption for data-rich countries

Covariate	Parameter	Beta (95% CI)	Exponentiated beta (95% CI)
Smear positive TB	Prevalence	-0.75 (-0.76 — -0.75)	0.47 (0.47 — 0.47)
Sex (male)	Prevalence	0.77 (0.68 — 0.86)	2.16 (1.97 — 2.36)
Sex (male)	Incidence	0.60 (0.60 — 0.61)	1.83 (1.82 — 1.84)
LDI (log-transformed)	Remission	0.091 (0.045 — 0.18)	1.10 (1.05 — 1.19)
LDI (log-transformed)	Excess mortality	-0.10 (-0.10 — -0.10)	0.90 (0.90 — 0.90)

Malaria SDG Capstone Appendix

Flowchart

Malaria

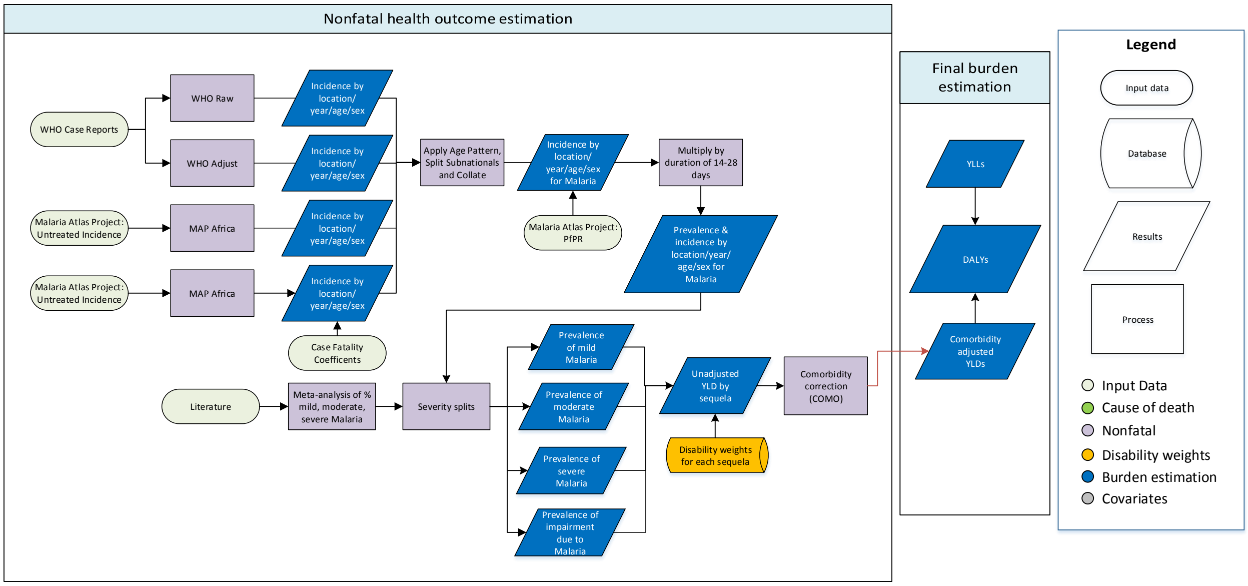

Input data and methodological summary

Indicator definition

This modeling strategy encompassed the indicator associated with malaria incidence (3.3.3).

Indicator 3.3.3

As a component of SDG Goal 3. Ensure healthy lives and promote well-being for all at all ages, SDG Target 3.3, by 2030, end the epidemics of AIDS, tuberculosis, malaria and neglected tropical diseases and combat hepatitis, water-borne diseases and other communicable diseases, is measured using SDG Indicator 3.3.3, malaria cases per 1,000.

Case definition

Malaria is an acute parasitic mosquito-borne disease. An individual with uncomplicated malaria experiences one to two weeks of persistent fever, chills/shivering, sweating, joint pains and headache. The individual will likely be lethargic and feverish, causing loss of daily function during the attack. Individuals with an untreated *P. falciparum* infection may develop severe malaria, which includes the symptoms of uncomplicated malaria plus potentially swelling, difficulty breathing, unconsciousness, and death. Rapid diagnostic test or microscopy are considered the gold-standard diagnostic approaches for the purposes of the Global Burden of Disease Study (GBD). The relevant ICD-10 codes are B50-B54.

Input data

Model inputs

For GBD 2015 a systematic review of malaria was not conducted. Updates to systematic reviews are performed on an ongoing schedule across all GBD causes and an update for will be performed in the next one to two iterations. However, as described below, GBD 2015 does feature a substantial estimation change relative to GBD 2013 through the integration of work by the Malaria Atlas Project (MAP).

Data for the malaria modeling process come from three main sources. For endemic countries in continental Africa, we use estimates from MAP (1). Specifically, we use spatiotemporal (ST) cubes of incidence in three broad age-bins (0-5, 5-14 and 15+). It should be noted that these incidence estimates differ slightly from those published previously. First, the cube was re-estimated using a newer antimalarial coverage covariate that includes all types of antimalarial drugs rather than just artemisinin-based combination therapy (ACT). This change improves estimates occurring before ACT rollout in the early 2000s and otherwise allows for the expansion of the initial period of interest (from 2000-2015 to 1990-2015). Second, we combined the incidence estimates with a measure of drug efficacy to produce estimates of untreated incidence that then serve as our quantity of interest for burden assignment. Ultimately, we use MAP data for the following countries: Angola, Benin, Botswana, Burkina Faso, Burundi, Cameroon, Central African Republic, Chad, Congo, Cote d'Ivoire, Democratic Republic of the Congo, Djibouti, Equatorial Guinea, Eritrea, Ethiopia, Gabon, Ghana, Guinea, Guinea-Bissau, Kenya (subnational), Liberia, Madagascar, Malawi, Mali, Mauritania, Mozambique, Namibia, Niger, Nigeria, Rwanda, Senegal, Sierra Leone, Somalia, South Sudan, Sudan, Swaziland, Tanzania, The Gambia, Togo, Uganda, Zambia, and Zimbabwe.

For most other countries with malaria transmission, we use confirmed cases reported in the 2013 World Malaria Report (WMR). These include: Afghanistan, Algeria, Argentina, Armenia, Azerbaijan, Bangladesh, Belize, Bhutan, Bolivia, Brazil (subnational), Cambodia, Cape Verde, China (subnational), Colombia, Comoros, Costa Rica, Dominican Republic, Ecuador, Egypt, El Salvador, Georgia, Guatemala, Guyana, Haiti, Honduras, Iran, Iraq, Kyrgyzstan, Laos, Malaysia, Mauritius, Mexico (subnational), Morocco, Nepal, Nicaragua, North Korea, Oman, Pakistan, Panama, Paraguay, Peru, Philippines, Sao Tome and Principe, Saudi Arabia (subnational), Solomon Islands, South Africa (subnational), South Korea, Sri Lanka, Suriname, Syria, Tajikistan, Thailand, Timor-Leste, Turkey, Turkmenistan, United Arab Emirates, Uzbekistan, Vanuatu, Venezuela, and Vietnam.

For the final data type, we used the GBD 2013 systematic review of studies with data on the clinical incidence of malaria. These studies were georeferenced so that the sites could be matched with spatially explicit covariates such as *Plasmodium falciparum* parasite rate (*Pf*PR). Data are standardized into four age categories: 0-4, 5-14, 15+ and all age. Countries whose results are estimated in this group include: India (subnational), Yemen, Indonesia, Myanmar and Papua New Guinea.

Severity splits

As in GBD 2013, we use a two-step process for determining malaria severity. For acute cases, severity splits for mild, moderate, and severe malaria were produced by analysis of Medical Expenditure Panel Survey data. These sequelae and their associated disability weights (DWs) are presented below.

Table 1. Severity level, lay description, and DW

Severity level	Lay description	DW (95% CI)
Mild	Has a low fever and mild discomfort but no difficulty with daily activities.	0.006 (0.002-0.012)
Moderate	Has a fever and aches and feels weak, which causes some difficulty with daily activities.	0.051 (0.032-0.074)
Severe	Has a high fever and pain and feels very weak, which causes great difficulty with daily activities.	0.133 (0.088-0.19)

To determine long-term neurological burden due to malaria, we use the work by Roca-Felter et al. (2008) that examined the number of uncomplicated cases that led to longer-term impairment. Analytically, this means multiplying incidence estimates (described in the section below) for persons under 20 by 0.00029 (0.000077-0.00057). This subset is then combined with excess mortality rates derived from all-cause mortality and standardized mortality ratios for neonatal encephalopathy (NE) in a DisMod model to produce prevalence estimates for all estimation years. Implicit in this process is an assumption that the disability and trend of impairment due to severe malaria follow NE. The subsequent severity splitting follows NE as well.

Modeling strategy

We stratify analysis of malaria morbidity into four different location sets: MAP Africa, WHO Raw, WHO Adjusted and Study Level.

MAP Africa:

For the subset of countries using data from MAP, we combine the broad age and sex untreated incidence estimates from the space-time cube with mortality estimates to generate incidence estimates by GBD standard age and sex groupings. As part of the mortality estimation process, we developed a regression model by age-bin where case fatality is a function of all-cause mortality and sex. More information can be found in the GBD 2015 mortality and causes of death paper. Using these models, we generated implied cases estimates where cases = death/case fatality for all age groups and both sexes. Subsequently, we scale the implied cases by age-bin so that they match the corresponding estimates from MAP. Although we carry forward the uncertainty from the MAP incidence estimates, we do not include the uncertainty of the age pattern.

WHO Raw:

Kyrgyzstan, Tajikistan, Belize, Panama, Iran, South Africa, China and Saudi Arabia, Tajikistan, Turkey, Azerbaijan, Uzbekistan, Georgia, South Korea, Argentina, Costa Rica, Armenia, Malaysia, Sri Lanka, Bhutan, Iraq, North Korea, Paraguay, Mexico, El Salvador, Ecuador, Cape Verde and Algeria all feature low case rates and are considered to have complete case reporting—particularly since the turn of the century. As such, our case rates are taken directly from those reported in the WMR. Because of systematic review cycles as well as the publication cycle of the WMR, we assume a flat trend of cases forward for the unmeasured years (e.g., 2014 and 2015). For some countries, data from the early 1990s were not reported. For these countries, cases were extrapolated via a mixed effects regression with reported cases as the outcome variable, year as the main predictor and random effects on location. The WMR does not provide information on the demographic characteristics of the malarial case, we apply the 5th percentile age pattern derived from the incidence estimates for MAP Africa to generate age- and sex-specific incidence estimates. In this case, the uncertainty in the broad age-bin pattern is taken into account.

In China, South Africa, and Saudi Arabia, we provide estimates at the subnational level, but the WMR only reports at the national level. Therefore, Chinese subnational estimates are derived from case reports from their malaria control program, while for South Africa and Saudi Arabia, we proportionally assign cases into the subnational geographies by MAP's 2010 world map of prevalence of *Pf*PR (2). This procedure is the assumption that the distribution of *Pf*PR is substantially similar to other malaria strains.

WHO Adjust:

For the remaining countries in the broader WHO grouping (see above) that feature either incomplete reporting systems or higher malaria burden, we adjust the WMR confirmed incidence rate upward via a proxy variable for health systems access. For Mexico, we replace the WHO estimates with subnational estimates from the national malaria control program.

First, we regress the confirmed incidence rate as a function of *Pf*PR and the interaction of health system access and malaria death rate with random effects on location. The regression is fit using all countries with complete or semi-complete case reports (e.g. not countries in MAP Africa or the study level approach) to derive stable relationships between *Pf*PR and the interaction term. Using the coefficients and associated uncertainty (fixed and random effects), we predict incidence rate with health system access set to the 95th percentile – thereby serving as a proxy for a more complete reporting system.

We apply the 5th percentile MAP Africa pattern to generate age- and sex-specific estimates. Although the uncertainty of the regression is captured, we do not capture uncertainty in the age and sex pattern.

Study Level:

For countries that feature a high malaria burden and poor case reporting infrastructure that are outside of Africa, we use an ordinary least squares regression with the natural log of incidence as the outcome. Predictors include the natural log of the malaria death rate, an indicator variable for Africa, the ratio between country and site level P *Pf*PR , interaction terms between each age bin and the associated log malaria death rate and an indicator variable on whether the data point relies on passive case detection. We use the 5th percentile age pattern derived from the MAP Africa estimates to distribute cases by age and sex.

Converting to prevalence

Once the separate incidence estimation procedures are complete, the results are combined and converted to prevalence by matching each draw with a draw of duration. Consistent with GBD 2013, we use a uniform distribution between 14 and 28 days for duration.

Comparison to GBD 2013

We have re-evaluated our location groupings for GBD 2013, moving several countries from the WHO adjustment category into the WHO Raw categories. Through the inclusion of the back-casting step, we account for the potential under-reporting that may have occurred pre-2000 in these reassigned countries.

GBD 2013 included an interpolation step to correct for jagged age-patterns as a correction for all-cause mortality during the age- and sex-splitting process. This step created overly smooth time and age trends and has since been removed through the inclusion of high- quality patterns from MAP. As such, we expect

more pronounced age patterns (higher in youth, less so in adults) and time trends (sharper declines since 2000) relative to GBD 2013.

The inclusion of MAP incidence estimates in general marks a major step forward in better understanding malarial burden in Africa. In general, we expect a slightly higher amount of disability relative to GBD 2013. Future GBD iterations will expand the geostatistical model currently used in Africa to the rest of the world.

In conjunction with changes in the mortality estimation, GBD 2015 burden due to malaria now features a fully consistent set of results via the integration of MAP incidence data into the modeling framework.

Acute Hepatitis B SDG Capstone Appendix:

Flowchart

Acute hepatitis B

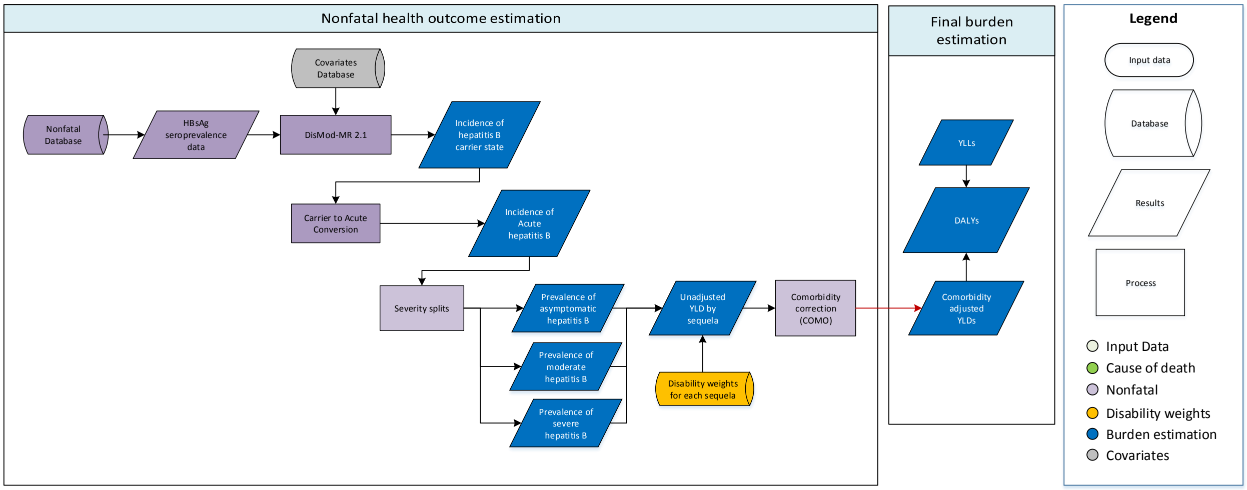

Input Data and Methodological Summary

Indicator definition

This modeling strategy encompassed the indicator associated with hepatitis B incidence (3.3.4).

Indicator 3.3.4

As a component of SDG Goal 3. Ensure healthy lives and promote well-being for all at all ages, SDG Target 3.3, by 2030, end the epidemics of AIDS, tuberculosis, malaria and neglected tropical diseases and combat hepatitis, water-borne diseases and other communicable diseases, is measured using SDG Indicator 3.3.4, hepatitis B incident cases per 100,000*).*

Case definition

We define acute hepatitis B as the period corresponding to initial infection with the hepatitis B virus, regardless of symptoms. It includes all ICD-10 codes under the heading B16 (Acute hepatitis B).

Input data

Model inputs

We use hepatitis B surface antigen (HBsAg) seroprevalence data from population-based studies and surveys for the incidence model.

Level	Prevalence
Data points	2,987
Studies	312
Locations	145
Regions	19

Updates to systematic reviews are performed on an ongoing schedule across all GBD causes.

Modeling strategy

We model the incidence of chronic HBsAg carriage using a full DisMod model of HBsAg seroprevalence. We then convert incidence of chronic carriage to total incidence of hepatitis B infection by dividing age-specific estimates of the incidence of chronic carriage by age-specific estimates of the probability of infection resulting in carriage based on Edmunds et al. (1993).

$$P(carrier \mid age \leq 6\ months) = 0.885$$

$$P(carrier \mid 6\ months \leq age < 25\ years) = e^{-0.645 \times age^{0.455}}$$

$$P(carrier \mid age \geq 25\ years) = e^{-0.645 \times 25^{0.455}} = 0.061$$

We then split symptomatic cases into moderate (73%) and severe (27%) severities based on data from McMahon et al. (1985).

Sequela	Description	Disability weight
Moderate	Has a fever and aches, and feels weak, which causes some difficulty with daily activities.	0.051 (0.032-0.074)
Severe	Has a high fever and pain, and feels very weak, which causes great difficulty with daily activities.	0.133 (0.088-0.19)
Asymptomatic	Infection with no apparent illness.	NA

Changes from GBD 2013 to GBD 2015

We have updated the severity splits, but the modeling strategy remains otherwise unchanged from GBD 2013.

Neglected Tropical Diseases (NTDs) SDG Capstone Appendix

African trypanosomiasis, Chagas disease, cystic echinococcosis, cysticerosis, dengue, food-borne trematodiases, intestinal nematode infections, leishmaniosis, leprosy, lymphatic filariasis, onchocerciasis, rabies, schistosomiasis, and trachoma

Indicator definition

This modeling strategy encompassed the indicator associated with neglected tropical disease prevalence (3.3.5).

Indicator 3.3.5

As a component of SDG Goal 3. Ensure healthy lives and promote well-being for all at all ages, SDG Target 3.3, by 2030, end the epidemics of AIDS, tuberculosis, malaria and neglected tropical diseases and combat hepatitis, water-borne diseases and other communicable diseases, is measured using SDG Indicator 3.3.3, prevalence of neglected tropical diseases.

Human African Trypanosomiasis (HAT) SDG Capstone Appendix

Flowchart

African trypanosomiasis

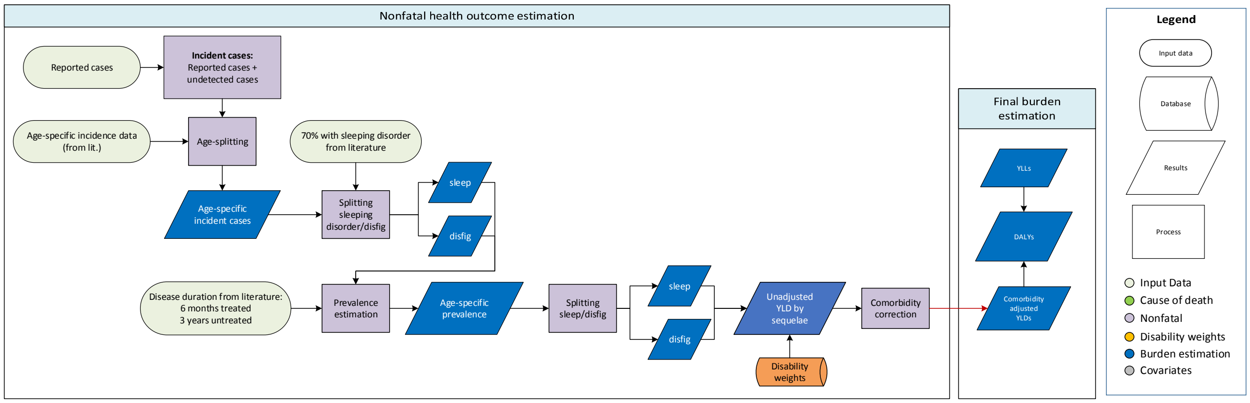

Input Data & Methodological Summary

Case Definition

Human African Trypanosomiasis (HAT), also known as sleeping sickness, is a vector-borne disease which is transmitted by the bite of the tsetse fly. It is caused by the parasite *Trypanosoma brucei* with two subspecies, namely *T.b. rhodesience* (makes up less than 5% of total HAT cases) and *T.b. gambiense*. Cases are diagnosed through laboratory methods which rest on finding the parasite in body fluid or tissue by microscopy. In highly endemic or epidemic areas where the likelihood of false positives in serological tests is deemed lower, a seropositive individual is considered affected even in the absence of parasitological confirmation. The ICD-10 code for HAT are B56.0, B56.1 and B56.9.

Input data

Model inputs

The input data for GBD 2015 included a) population at risk estimates from GBD 2010 ArcGIS analysis using geocoded case notifications for 2000 to 2009 [1] and population Count Grid estimates from Gridded Population of the World 3 [2, 3], b) population screened from 1997 to 2004 [4], c) historical data from GBD 2010 on total number of HAT cases reported [1, 4, 5], and d) cases reported annually to the WHO [6] – for Kenya, a study on cases reported subnationally [7] was used to split the national cases into five counties (HomaBay, Migori, Busia, Bungoma, Kakamega). A systematic review of literature was conducted in PubMed on 8/10/2016 using the following search string:

> ((African trypanosomiasis[Title/Abstract] AND incidence[Title/Abstract]) AND (“2009”[Date – Publication] : “2013”[Date – Publication])).

This yielded 72 studies of which only four met the inclusion criteria and were extracted. The inclusion criteria were:

1. Studies representative of the national population
2. Population-based studies
3. Studies with primary data on incidence
4. Studies of human African trypanosomiasis only (excluded studies on animal African trypanosimiasis)

The four studies extracted had national incidence data similar to the ones extracted from the WHO [6]. Therefore, only two studies with age-specific incidence data from active screening undertaken in the Democratic Republic of Congo [8] and Uganda [9] were used to inform age pattern for incidence and prevalence. Location-years with missing reported cases were excluded and five subnational locations for Kenya were added. The table below shows the number of studies included, and the number of countries or subnational units and GBD world regions represented.

	incidence
Studies	2
Countries/subnationals	34
GBD world regions	4

Severity splits/Sequelae

The basis of the GBD disability weight (DW) survey assessments are lay descriptions of sequelae highlighting major functional consequences and symptoms. The lay descriptions and disability weights for HAT sequelae due to HAT are shown below.

Sequela	Lay description	DW (95% CI)
Skin disfigurement, level 2	has a visible physical deformity that causes others to stare and comment. As a result, the person is worried and has trouble sleeping and concentrating	0.067 (0.044-0.096)
Motor plus cognitive impairments, severe	cannot move around without help, and cannot lift or hold objects, get dressed or sit upright. The person also has very low intelligence, speaks few words, and needs constant supervision and help with all daily activities	0.542 (0.37 – 0.702)

Modeling strategy

The non-fatal model for HAT involved estimating prevalence from incidence. First, a multi-level mixed-effects linear regression of natural log-transformed incidence rate (ratio of HAT cases reported to population at risk) on natural log-transformed screening coverage (ratio of number screened for HAT to population at risk), with country random effects, was performed. Gaps were then filled using exponential interpolation between years and extrapolation from 2014 to 2015 for reported cases; for screening coverage only extrapolation from 2014 to 2015 was done. Then 1,000 draws of mortality among treated cases were generated, assuming that 0.7% - 6.0% of all treated (reported) cases die [10, 11, 12].

Using the mean and variance-covariance matrix from the regression as parameters, a multivariate normal distribution was used to generate 1,000 draws of case detection rate (CDR), given the expected screening coverage. Undetected deaths were then estimated as the difference between the ratio of reported cases to CDR and reported cases (reported cases/CDR – reported cases). Estimates of incidence were obtained by adding the reported cases to the undetected cases. Without information on sex-specific incidence, equal incidence rates between both sexes was assumed. Finally, an age-pattern was applied to the incidence estimates using the incidence studies from DRC and Uganda [8, 9]. Assuming the same proportion in treated and untreated cases, the incidence estimates were then split into the two sequelae, skin disfigurement and sleeping disorder. This was done by generating 1,000 draws of the splitting proportion for the sequelae (70%-74% with sleeping disorder) based on a study that reported presence of symptoms at admission of patients in treatment centers [13] – draws were generated from a beta distribution with alpha parameter = 1884 and beta parameter = 649.

To compute prevalence of HAT, 1,000 draws of total duration of symptoms in untreated cases was generated from a normal distribution with mean = {ln(3) – 0.5 * sigma^2}, and standard deviation = sigma, where sigma = {ln(4.39)-ln(1.92))/(invnormal(0.975)*2)} – these parameters were based on a study of *T.b. gambiense* [14] which estimated an average duration of three years to untreated cases. An estimated duration of six months was applied to cases that received treatment, based on findings from a paper about *T.b. rhodesiense* in Uganda [11]. Prevalence was then estimated from the incident cases before applying age pattern. Prevalence of treated and untreated cases were summed up, assuming that untreated cases have been prevalent up to their death for a certain duration. For untreated cases, it was assumed that half the duration is spent with sleeping disorder (severe motor and cognitive impairment) and disfigurement [14]. Treated (i.e., reported) cases are assumed to have been prevalent for 0.5 years, and for the fraction of treated cases that present with sleeping disorder, it was assumed that this is present for half the total duration and that the rest of the duration is spent suffering from disfiguring skin disease. Treated cases that don't present with sleeping disorder were assigned disfigurement for the entire duration. Lastly, an age-pattern was applied to the prevalence estimates using the incidence studies from DRC and Uganda [8, 9].

Results from the model were assessed by visualizing time trends of incident and prevalent cases across locations and age (similar trends were applied in both sexes). Maps of the global distribution of HAT and the two sequelae were also generated. In addition, the estimated incident cases were compared with the cases reported to the WHO across time – as expected, the estimates from GBD 2015 were higher than the WHO numbers because we accounted for undetected cases.

Changes from GBD 2013 included: a) inclusion of new data on reported cases from WHO [6] (years 2013 and 2014 for 23 locations), b) inclusion of the following country (years) based on available historical data post-1980: Botswana (1983), Ethiopia (1980-1983), Guinea-Bissau (1980-1983, 1985-1987), Rwanda (1980, 1982-1988), and Sierra Leone (1981-1982), c) adding five subnational locations (out of 49) for Kenya, thusd) correcting the age-split proportion such that a 0.32/0.68 proportion was used for adults/kids –in GBD 2013, this proportion was 0.25/0.75 for adults/kids.

References

1. Simarro P, Cecchi G, Paone M, Franco J, Diarra A, Ruiz J, Fevre E, Courtin F, Mattioli R, Jannin J. The Atlas of human African Trypanosomiasis: a contribution to global mapping of neglected tropical diseases. International Journal of Health Geographics, 2010. 9:57
2. Center for International Earth Science Information Network (CIESIN), Columbia University; and Centro Internacional de Agricultura Tropical (CIAT). 2005. Gridded Population of the World, Version 3 (GPWv3): Population Count Grid. Palisades, NY: Socioeconomic Data and Applications Center (SEDAC), Columbia University. Available at http://sedac.ciesin.columbia.edu/gpw
3. Center for International Earth Science Information Network (CIESIN), Columbia University; United Nations Food and Agriculture Programme (FAO); and Centro Internacional de Agricultura Tropical (CIAT). 2005. Gridded Population of the World, Version 3 (GPWv3): Population Count Grid, Future Estimates. Palisades, NY: Socioeconomic Data and Applications Center (SEDAC), Columbia University. Available at http://sedac.ciesin.columbia.edu/gpw
4. WHO. Weekly epidemiological record. 2006, February 24. No. 8, 2006, 81. 69-80
5. WHO Department of Communicable Disease Surveillance and Response (CDS). WHO Report on Global Surveillance of Epidemic-prone Infectious Diseases. 2000. WHO/CDS/CSR/ISR/2000.1.
6. WHO Global Health Observatory Data Repository (http://apps.who.int/gho/data/node.main.A1635?lang=en). Accessed Sept. 2015
7. Ruto JJ, Karuga JW. Temporal and spatial epidemiology of sleeping sickness and use of geographical information system (GIS) in Kenya. J Vector Borne Dis 2009; 1. 18-25
8. Lutumba P, Makieya E, Shaw A, Meheus F, Boelaert M. Human African Trypanosomiasis in a Rural Community, Democratic Republic of Congo. Emerging Infectious Diseases, 2007. Vol 13: No.2, 248-54
9. Fevre E, Odiit M, Coleman P, Woolhouse M, and Welburn S. Estimating the burden of rhodesiense sleeping sickness during an outbreak in Serere, eastern Uganda. BMC Public Health 2008, 8:96
10. Balasegaram M Harris S, Checchi F, Ghorashian S, Hamel C, Karunakara U. Melarsoprol versus eflornithine for treating late-stage Gambian trypanosomiasis in the Republic of Congo. Bulletin of the World Health Organization 2006;84:783-791
11. Odiit M, Kansiime F, Enyaru JCK. Duration of symptoms and case fatality of sleeping sickness caused by Trypanosoma brucei rhodesiense in Tororo, Uganda. East African Medical Journal. December 1997. 792-5
12. Priotto G, Kasparian S, Mutombo W, Ngouama D, Ghorashian S, Arnold U, Ghabri S, Baudin E, Buard V, Kazadi-Kyanza S, Ilunga M, Mutangala W, Pohlig G, Schmid C, Karunakara U, Torreele E, Kande V. Nifurtimox-eflornithine combination therapy for second-stage African Trypanosoma brucei gambiense trypanosomiasis: a multicentre, randomised, phase III, non-inferiority trial. Lancet 2009; 374. 56-64
13. Blum J. Scmid C, Burri C. Clinical aspects of 2541 patients with second stage human African trypanosomiasis. Acta Tropica 97. 2006. 55-64
14. Checchi F, Filipe J, Haydon D, Chandramohan D, and Chappuis F. Estimates of the duration of early and late stage of gambiense sleeping sickness. BMC Infectious Diseases. 2008. 8:16

Chagas Disease SDG Capstone Appendix

Flowchart

Chagas disease

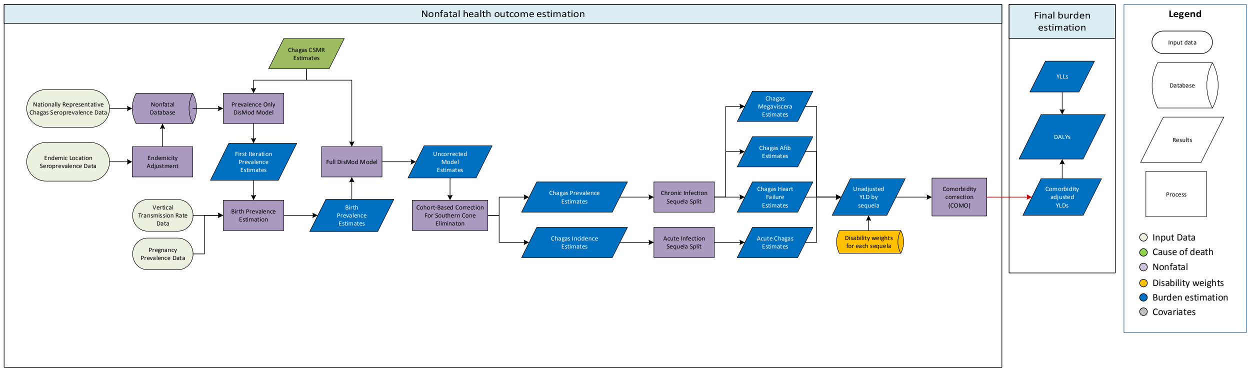

Input Data & Methodological Summary

Case definition

Chagas disease is defined by infection with the protozoa *Trypanosoma cruzi,* which is transmitted by *Triatominae* insect vectors (most common), blood transfusion, organ transplant, and congenital transmission. It includes an acute phase corresponding with the time of infection, and is typically asymptomatic. Chronic infection may be latent (i.e., asymptomatic), or result in cardiovascular or digestive sequelae. It includes all ICD-10 codes under the heading B57 (Chagas disease), with codes B57.0-B75.1 corresponding to the acute phase, B57.2 corresponding to chronic cardiovascular sequelae, and B57.3 corresponding to chronic digestive sequelae.

Input data

Model inputs

For GBD 2015 estimation, we used seroprevalence data to model Chagas. The table below illustrates the geographic distribution of model input data for the estimation process.

Level	Prevalence
Data points	407
Studies	56
Locations	20
Regions	4

We also use CSMR estimates in the modeling process, which will be addressed in further detail below.

Modeling strategy

We modeled Chagas disease using a full DisMod-MR 2.1 Bayesian meta-regression model incorporating seroprevalence data, as above, and CSMR estimates. We assume no remission. We eliminate all new infections, except those via vertical transmission, in Chile and Uruguay for years after the interruption of vector-based transmission. For non-endemic countries, we estimate the prevalence of imported chronic infections based on migration. For each non-endemic country, we estimate the total number of people infected with Chagas as the sum of the number of immigrants from each endemic country multiplied by the corresponding prevalence of Chagas in that endemic country.

We estimate five sequelae: symptomatic acute infection from incidence; and megaviscera, heart failure, atrial fibrillation, and chronic asymptomatic infection from prevalence. We assume that 5% of acute infections will be symptomatic. The proportion of chronic infections resulting in a given sequela varies by sex and age: the prevalence of megaviscera among those infected with Chagas ranges from 0% in children to nearly 10% among older adults; the prevalence of atrial fibrillation attributable to Chagas ranges from 0% among children to approximately 10% in men over 80 years of age; and the prevalence of heart failure attributable to Chagas among those who are infected ranges from 0% among young children to a maximum of 23% among men over 80 years of age.

Severity splits and disability weights

Sequela	Description	Disability Weight
Atrial fibrillation and flutter due to Chagas disease	Has periods of rapid and irregular heartbeats and occasional fainting.	0.224 (0.151-0.312)
Mild heart failure due to Chagas disease	Is short of breath and easily tires with moderate physical activity, such as walking uphill or more than a quarter-mile on level ground. The person feels comfortable at rest or during activities requiring less effort.	0.041 (0.026-0.062)
Moderate heart failure due to Chagas disease	Is short of breath and easily tires with minimal physical activity, such as walking only a short distance. The person feels comfortable at rest but avoids moderate activity.	0.072 (0.047-0.103)
Severe heart failure due to Chagas disease	Is short of breath and feels tired when at rest. The person avoids any physical activity, for fear of worsening the breathing problems.	0.179 (0.122-0.251)
Mild chronic digestive disease due to Chagas disease	Has some pain in the belly that causes nausea but does not interfere with daily activities.	0.011 (0.005-0.021)
Moderate chronic digestive disease due to Chagas disease	Has pain in the belly and feels nauseous. The person has difficulties with daily activities.	0.114 (0.078-0.159)

Acute Chagas disease	Has a fever and aches, and feels weak, which causes some difficulty with daily activities.	0.051 (0.032-0.074)
Asymptomatic Chagas disease	Latent Chagas infection (i.e., chronic infection with no apparent symptoms)	NA

Changes from GBD 2013 to GBD 2015

We have made no substantive changes in the modeling strategy for endemic countries from GBD 2013 for Chagas endemic countries. One notable improvement, however, is the estimation of Chagas disease among immigrants living in non-endemic countries which offers a more complete picture of Chagas' burden.

Cystic Echinococcosis SDG Capstone Appendix

Flowchart

Cystic echinococcosis

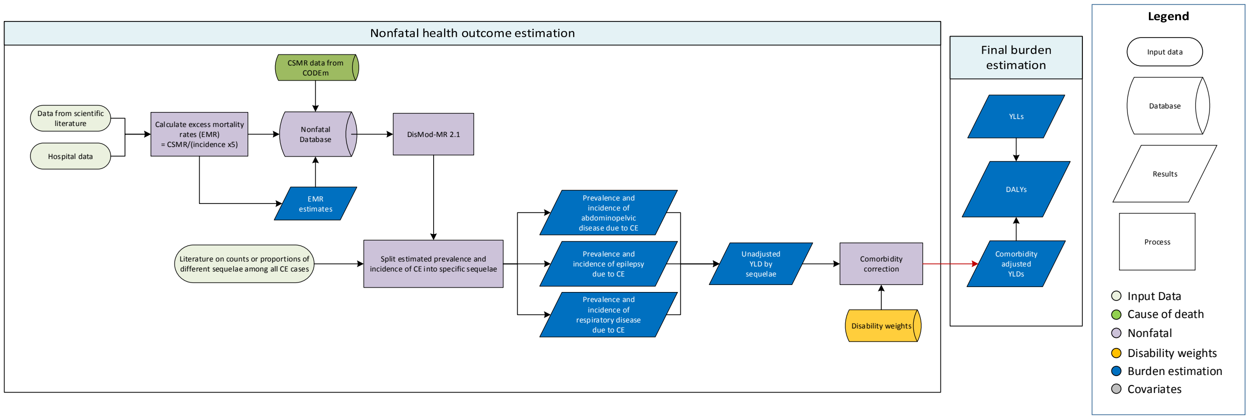

Input Data & Methodological Summary

Case definition

Ehinococcosis is a vector-borne disease caused by two species of tapeworm, *Echinococcus granulosis* (most common in sheep) which causes cystic echinococcosis (CE) and *E. multilocularis* (most common in foxes and wild dogs) which causes alveolar echinococcosis. Diagnosis is made by clinical findings, imaging, and serology. The ICD-10 codes for echinococcosis are B67-B67.9.

Input data

Model inputs

The nonfatal estimation for cystic echinococcosis (CE) focused on estimating incidence and prevalence of CE and its sequelae. A systematic review of literature was conducted in PubMed using the following search string:

> ("echinococcosis"[Title/Abstract] OR "hydatid disease"[Title/Abstract] OR "hydatidosis"[Title/Abstract] OR "echinococcal disease"[Title/Abstract] OR "Echinococcus granulosus infection"[Title/Abstract]) AND (“1990”[Date – Publication] : “2015”[Date – Publication]) AND (epidemiology OR incidence OR prevalence).

This yielded 1,619 studies of which 279 were included during the title/abstract screening. Following the full-text screening, 77 studies (32 incidence, 43 prevalence and 2 both) were included and extracted – studies were excluded because of one or more of the following reasons:

1. study not population-based
2. study does not have primary data on prevalence and/or incidence
3. study not in humans
4. study on sub-populations

5. review study

We combined the newly extracted studies with studies extracted during GBD 2013. The table below shows the number of studies finally included, and the number of countries or subnational units and GBD world regions represented.

	incidence	prevalence
Studies	47	58
Countries/subnationals	41	24
GBD world regions	12	8

Hospital data on incidence prepared by the GBD team was also used in the CE model. The table below shows the number of studies included in the hospital data, and the number of countries or subnational units and GBD world regions represented.

	incidence
Studies	38
Countries/subnationals	100
GBD world regions	8

Since we were interested in modeling symptomatic CE cases, we only used data on incidence of patients diagnosed by imaging techniques (mainly ultrasonography). Therefore we excluded prevalence data which were mostly from serological studies.

Two additional data sources that were used, including 1) data on echinococcosis endemicity (0=no cases/no data, 1=sporadic/mostly imported, 2=endemic/limited data, 3=highly endemic) provided by one of our echinococcosis collaborators, and 2) literature data on observed cases of abdominal, respiratory, and epileptic symptoms among echinococcosis cases [1].

Sequelae due to cystic echinoccocosis

The table below shows the sequelae due to echinococcosis and their associated disability weights.

Sequela	Lay description	DW (95% CI)
Chronic respiratory disease	has cough and shortness of breath after heavy physical activity, but is able to walk long distances and climb stairs.	0.019 (0.011-0.033)
Abdominal problems	has pain in the belly and feels nauseous. The person has difficulties with daily activities	0.114 (0.078 – 0.159)
Epilepsy	(Combined DW)	NA

Modeling strategy

DisMod MR was used to model the nonfatal burden of symptomatic cystic echinococcosis (CE) using only incidence data. Mortality estimates from the custom mortality model were used to inform the excess mortality parameter (CODEm estimates used as cause-specific mortality rate data). Estimates of

excess mortality rate were obtained and used to estimate prevalence (CSMR/EMR). A remission of 0.15-0.25 per case per year (duration 2 – 6.7 years, average 5 years) was assumed. The following steps were followed to estimate excess mortality rate: 1) create custom age groups for CE deaths at the 1,000 draw level; 2) calculate CSMR as CSMR=deaths/population at the 1,000 draw level – calculate mean CSMR, uncertainty interval, and standard error; and 3) calculate EMR as EMR=CSMR/(prevalence), where prevalence = (incidence*5) – standard error of EMR was calculated taking into consideration the standard errors of both prevalence and CSMR.

After running DisMod, a thousand draws of proportions for abdominal, respiratory and epileptic symptoms among echinococcosis cases, that add up to 1, were generated. Uncertainty in the splitting proportions was captured by drawing them from a Dirichlet distribution, informed by published data on cysts localization [1]. On average, the proportions of abdominal, respiratory, and epileptic symptoms due to echinococcosis were 0.8, 0.19, and 0.01, respectively. These proportions were used to split the prevalence and incidence from DisMod into the three sequelae.

Model evaluation was done by separately assessing the fit of the DisMod MR model and checking the estimates produced after estimating incidence and prevalence of sequelae due to cystic echinococcosis. Plots of time trends of incidence and prevalence across locations and age were used to evaluate the results. In addition, maps of the global distribution of incidence and prevalence were assessed across time.

Changes from GBD 2013 included: a) estimation of excess mortality rate using literature/hospital incidence and CSMR data, b) inclusion of echinococcosis endemicity in as a country-level covariate in DisMod, and c) assuming that remission is 0.15-0.25 instead of 0.1 to 1.0 based on discussions with GBD 2015 CE model reviewers (an average remission of five years was used to calculate prevalence of CE).

References

1. Eckert J, Deplazes P. Biological, Epidemiological, and Clinical Aspects of Echinococcosis, a Zoonosis of Increasing Concern. Clin Microbiol Rev, 2004; 17(1): 107-35

Cysticercosis SDG Capstone Appendix

Flowchart

Cysticercosis

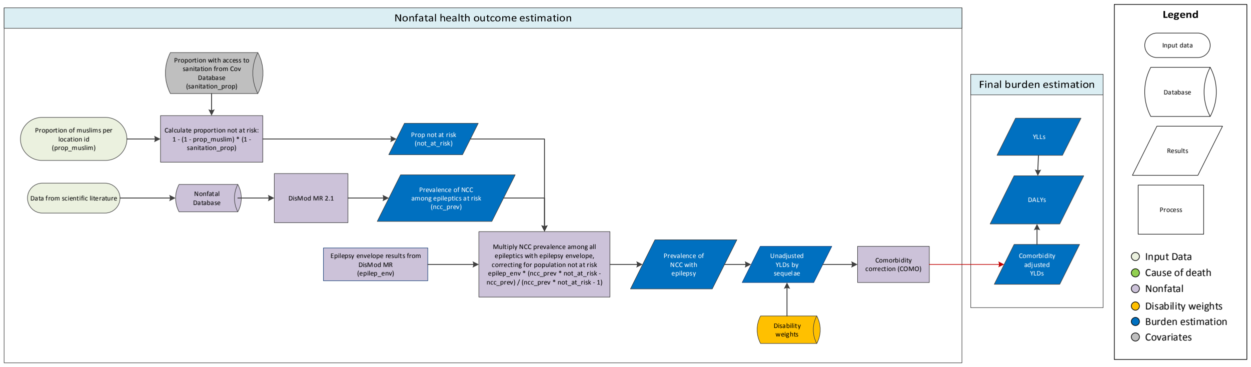

Input Data & Methodological Summary

Case Definition

Cysticercosis is a helminth disease caused by the pig tapeworm, *Taenia solium*, transmitted through the fecal oral route or by consumption of pork containing *T. solium* eggs. Diagnosis is made by Magnetic resonance imaging or CT brain scans for neurocysticercosis. The ICD-10 codes for cysticercosis are B69-B69.9.

Input data

Model inputs

The nonfatal estimation for cysticercosis focused on estimating prevalence of neurocysticercosis among epileptics at risk as well as the prevalence of neurocysticercosis with epilepsy. A systematic review of literature was conducted in PubMed using the following search string:

> ("cysticercosis"[Title/Abstract] OR "neurocysticercosis"[Title/Abstract] OR "cysticerciasis"[Title/Abstract] OR "Taenia solium"[Title/Abstract]) AND (“1990”[Date – Publication] : “2015”[Date – Publication]) AND (epidemiology OR prevalence)).

This yielded 1,038 studies of which 166 were included during the title/abstract screening. Following the full-text screening, 17 studies were included and extracted – studies were excluded because of one or more of the following reasons:

1. study not in epileptics
2. study not population-based
3. study does not have primary data on prevalence of neurocysticercosis among epileptics at risk
4. study not in humans (some studies were on cysticercosis in pigs)
5. study on comorbidities with neurocysticercosis (other than epilepsy)

6. study on sub-population, e.g., patients with neurological disorders
7. review study

We combined the newly extracted studies with studies extracted during GBD 2013. The table below shows the number of studies finally included, and the number of countries or subnational units and GBD world regions represented.

	prevalence
Studies	32
Countries/subnationals	23
GBD world regions	8

A study-level covariate was also created to indicate the type of diagnosis for each study, i.e., definitive or probable. Of the 77 rows of country-year-age-sex data, there were 15 rows with definitive diagnosis and 62 rows with probable diagnosis.

Three additional data sources that were used included 1) epilepsy envelope prevalence (from the epilepsy DisMod MR model), 2) proportion of the population with access to sanitation (from the GBD covariates database), and 3) proportion of the population that is Muslim (from the PEW Research Center [1].)(http://www.pewforum.org/2011/01/27/table-muslim-population-by-country/).

Modeling strategy

DisMod MR was used to model the prevalence (ONLY) of neurocysticercosis among epileptics at risk. In the model, pigs per capita and religion (binary, >50% Muslim) were used as country-level covariates. In addition, the prevalence of "definitive diagnosis" was crosswalked to that of "probable and definitive diagnosis" so as to not underestimate overall prevalence.

After running DisMod, we adjusted the fraction of people with epilepsy attributable to cysticercosis in endemic countries for the population at risk (based on the proportion of the population without access to sanitation and the proportion of the population that is Muslim). Predicted neurocysticercosis (NCC) prevalence among epileptics at risk such that Prevalence=P×(NM-N)/(NM-1), where P = prevalence of all-cause epilepsy in total population, N = proportion of NCC among epileptics at risk (non-Muslims without access to sanitation), and M = proportion of population not at risk of contracting NCC. It was assumed that the prevalence of epilepsy due to causes other than NCC is the same regardless of whether a population is at risk or not. It was also assumed that Muslims and non-Muslims have equal access to sanitation.

Model evaluation was done by separately assessing the fit of the DisMod MR model and checking the estimates produced after estimating prevalence of NCC with epilepsy. Plots of time trends of prevalence across locations and age were used to evaluate the results. In addition, maps of the global distribution of prevalence of NCC among epileptics at risk and prevalence of NCC with epilepsy were also assessed across time.

Other than using additional data extracted from literature, we updated the proportion of population with Muslim data by filling in subnational locations with national proportions – this was done due to lack of data on this covariate at the subnational level.

References:

1. "Table: Muslim Population by Country Pew Research Center, Washington, D.C." (March 26, 2014). http://www.pewforum.org/2011/01/27/table-muslim-population-by-country/

SDG Indicators Dengue Capstone Appendix

Flowchart

Dengue

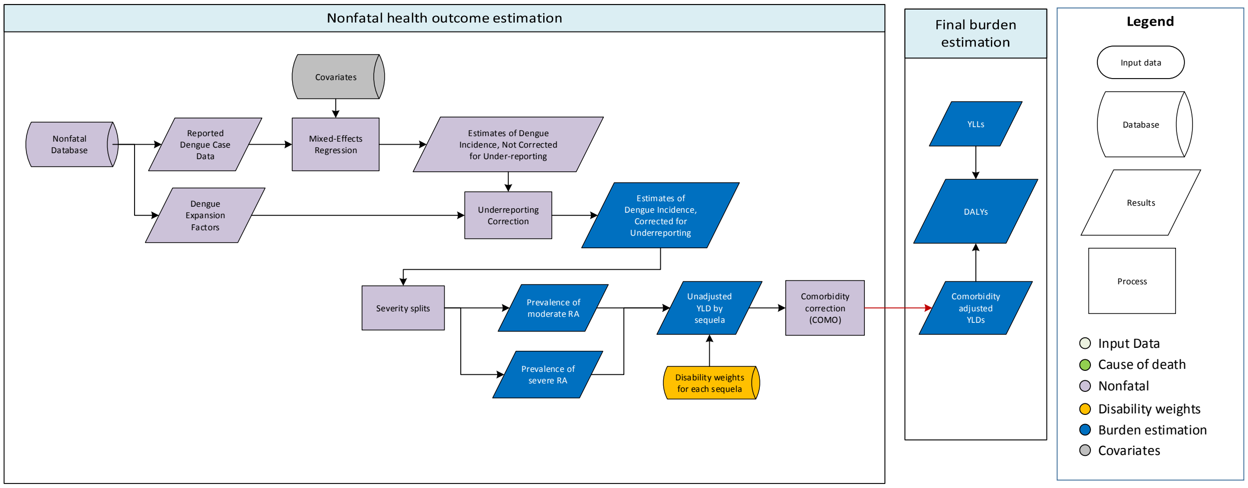

Input Data & Methodological Summary

Case definition

Dengue is mosquito-borne viral infection that causes febrile illness and, in severe cases, jaundice, hemorrhage, and death. It includes all ICD-10 codes under the heading A90 (Dengue fever [classical dengue]) and A91 (Dengue hemorrhagic fever).

Input data

Model inputs

For GBD 2015, we modeled dengue incidence based on officially reported cases. The table below illustrates the geographic distribution of data points used in our analysis.

Level	Incidence
Data points	2515
Studies	70
Locations	115
Regions	14

Updates to systematic reviews are performed on an ongoing schedule across all GBD causes, and an update for dengue fever will be performed in the next one to two iterations. While no systematic update was conducted, we did incorporate new expansion factor data that were provided by collaborators and have updated to the latest available case reports for GBD 2015.

Modeling strategy

We modeled dengue incidence using an improved variant of the methods used for GBD 2013, described by Stanaway et al. (2013). Briefly, we derive two dengue-specific covariates: first a variable to define the expected spatial distribution of the disease based on principal components analysis of dengue CSMR estimates and dengue transmission probability estimates (Bhatt et al. YEAR) and, second, a variable to define the country-specific trends, based on a mixed effects model of reported cases. We then estimate a mixed effects negative binomial model with number of reported cases as the dependent variable, fixed effects on the aforementioned spatial and temporal covariates, and random effects on location. These random effects are assumed to correspond to deviations in reporting completeness and, calibrating against published expansion factor data (i.e., estimates of the degree of underreporting), they are inflated to adjust for underreporting. The resulting incidence estimates are split into moderate (94.5%) and severe (5.5%) sequelae, based on the proportion of reported cases that were severe. We assume that 8.4% of symptomatic infections will produce post-acute chronic fatigue lasting an average of six months.

Severity splits and disability weights

Sequela	Description	Disability Weight
Moderate	Has a fever and aches, and feels weak, which causes some difficulty with daily activities.	0.051 (0.032-0.074)
Severe	Has a high fever and pain, and feels very weak, which causes great difficulty with daily activities.	0.133 (0.088-0.19)
Asymptomatic	Infection with no apparent illness.	NA

Changes from GBD 2013 to GBD 2015

The approach is largely the same as that used for GBD 2013. One notable change is the addition of the dengue trend covariate described above, which allows for dramatically improved trend estimates.

Foodborne Trematodiases SDG Capstone Appendix

Flowcharts

Clonorchiasis

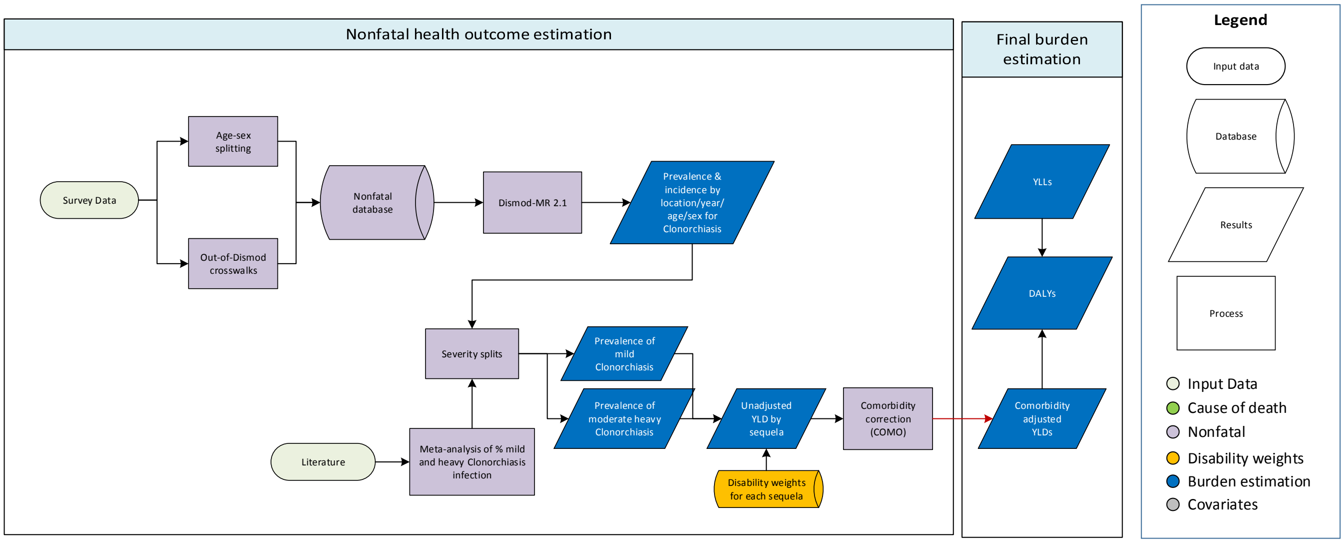

Fascioliasis

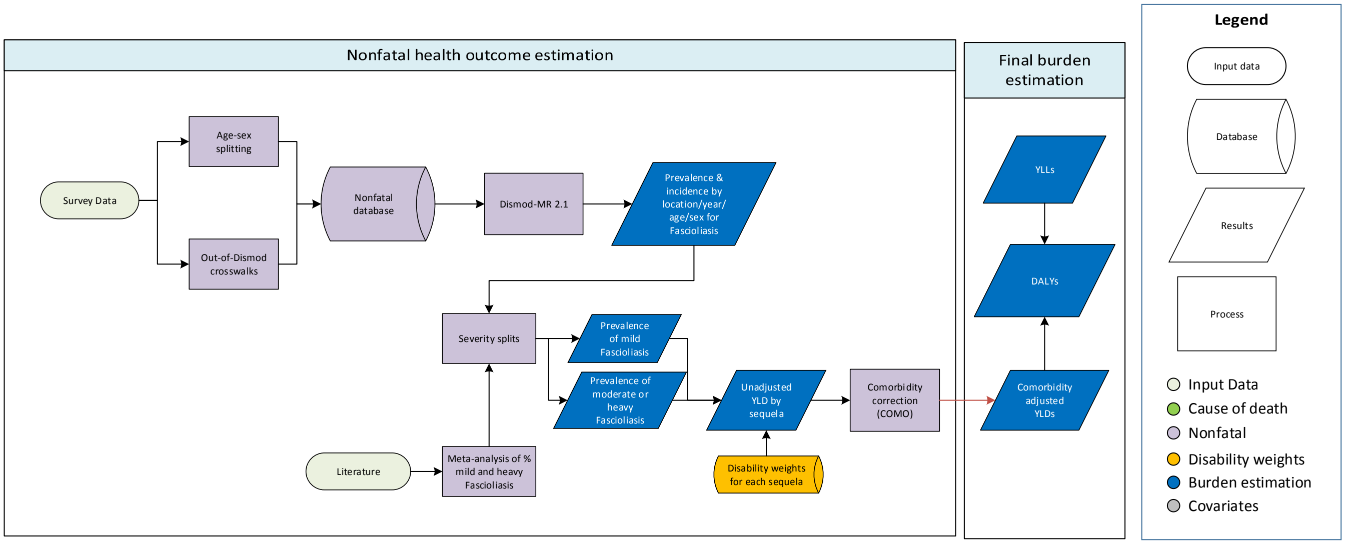

Intestinal fluke

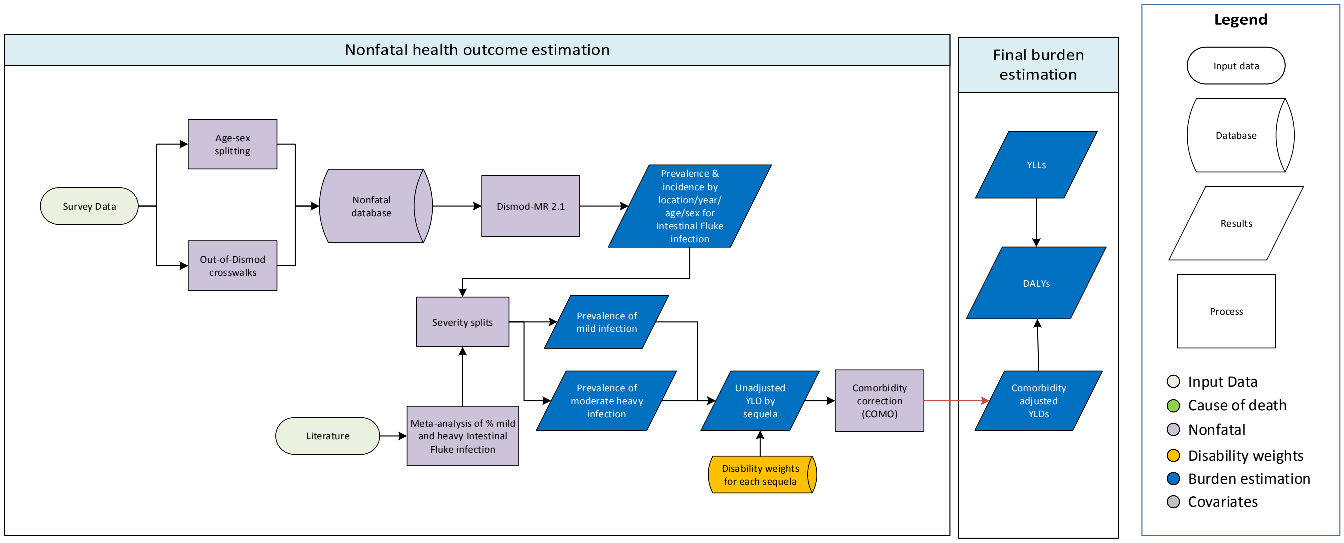

Opisthorchiasis

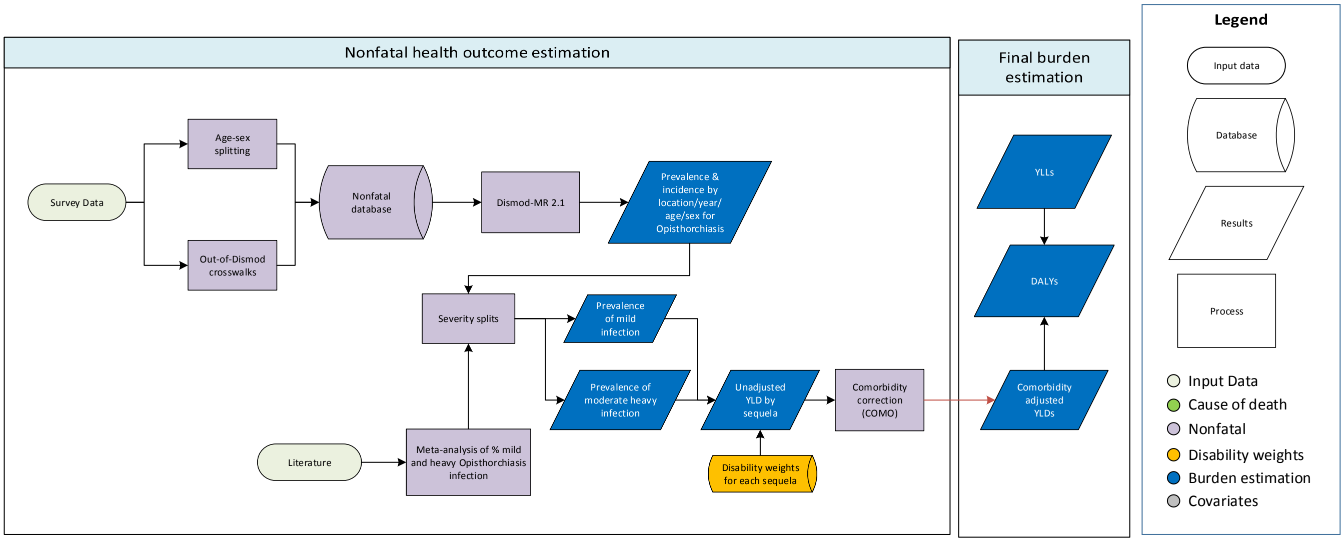

Paragonimiasis

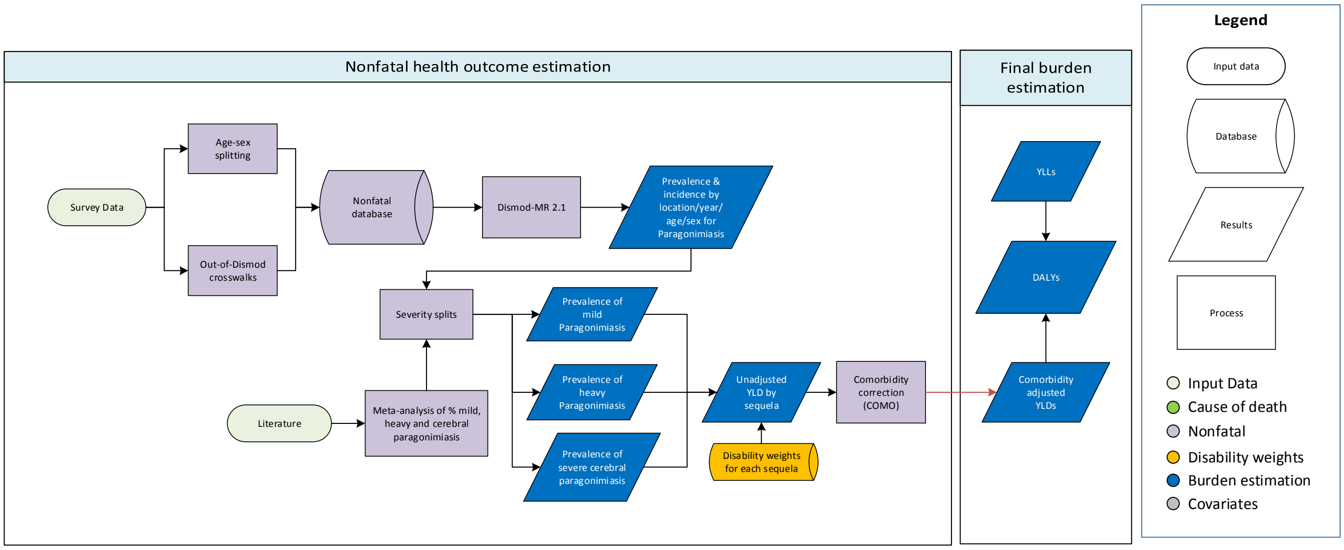

Input Data & Methodological Summary

Case definition

Human foodborne trematodiases (FBT) is defined as the infection with parasitic worms of the class trematoda, which are also known as flukes. Trematodes are transmitted via contaminated food and infection is highly related to food habits. Definitive hosts, including humans, become infected when ingesting viable metacercariae by consuming contaminated aquatic products (e.g. watercrest etc.). In the ICD-10, FBT are listed under code B66 [1].

FBT is subdivided into six types of FBT (see Table 1):

- Clonorchiasis
- Fascioliasis
- Intestinal fluke
- Opisthorchiasis
- Paragonimiasis (normal and cerebral infections)

Table 1. Subtypes of FBT

	Species of FBT	Also known as:	Carcinogen
1	Chlonorchiasis	(Chinese) Liver fluke	Associated with choliangiocarcinoma
2	Opisthorchiasis<br><br>(*O viverrini & O felineus*)	Liver fluke	Associated with choliangiocarcinoma<br><br>(*O viverrini*)

3	Fascioliasis	Liver fluke	No available evidence
4	Intenstinal fluke	Liver fluke	No available evidence
5	Paragonimiasis	Lung fluke	

Thresholds for heavy infection and duration by species of FBT

The majority of people infected with FBTs are asymptomatic. When symptoms do occur they are often non-specific. Among the clinical symptomatic group, severity is associated with worm burden, typically measured by fecal egg counts, and the duration of infection. The thresholds for heavy infection and duration by species of FBT are shown in Table 2. The clinical presentation of FBT depends on the target organs (liver, lung, or intestines). Clonorchiasis and opisthorchiasis patients may suffer from loss of appetite, fullness, indigestion, diarrhoea, pain in the right upper quadrant, lassitude, weight loss, ascites, and oedema.[2, 3] Cholangitis, obstructive jaundice, intra-abdominal mass, cholecystitis, and gallbladder or intrahepatic stones may occur as complications.[3, 4]

Table 2. Thresholds for heavy infection and duration by species of FBT

	Species of FBT	Case thresholds for heavy infection	Duration
1	Chlonorchiasis	10,000 eggs per g of feces	lifelong
2	Opisthorchiasis	10,000 eggs per g of feces	lifelong
3	Fascioliasis	1,000 eggs per g of faces	lifelong
4	Intenstinal fluke	1,000 eggs per g of faces	lifelong
5	Paragonimiasis	100 eggs per 5 ml sputum	lifelong
6	Cerebral paragonimiasis	Any infection of the brain with flukes and/or eggs of *Paragonimus* spp.	lifelong

Input data

Model inputs

For GBD 2010, the data came from the expert group and is the result of their analysis. The expert group analysis used the results of a systematic literature review performed by Furst et al. as a starting point for the analysis.[5] Furst et al. searched PubMed, WHOLIS, FAOBIB, Embase, CAB Abstracts, Literatura Latino Americana e do Caribe em Ciências de Saùde (LILACS), ISI Web of Science, BIOSIS preview, Science Direct, African Journals OnLine (AJOL), and the System for Information on Grey Literature in Europe (SIGLE), period Jan 1, 1980 to

Dec 31, 2008. The initial number of studies identified through the literature review was ~34,000 references. The literature review included extracted data from 181 studies. For GBD 2013 and GBD 2015 the search strategy was replicated to capture epidemiological studies published between 2008 and 2015.

Input data for the assessment of the total national number of infected people

Only studies that used countrywide surveys to estimate the national prevalence rates were included (or for China Province-wide surveys). Reason for choosing only national studies is that FBT shows a highly focal spatial distribution and local cross-sectional surveys would profoundly under- or overestimate true national prevalences. We decided not to model national and subnational together and get a coefficient on subnational, because there is not a one fits all relationship across the world. Infection is highly related to food habits and there are highly varying differences between national and sub-national prevalence rates. The final GBD 2015 dataset contained 29 prevalence studies from 17 countries. We used raw data from the selected studies as input for DisMod.

Prevalence intestinal fluke infection

Intestinal fluke is different from the other types of FBT, because there are several pathogens that fall under intestinal fluke infection. It can be caused by pathogens, such as Metagonimus spp., Echinostoma spp., Neodiplostomatidae.[6] When assessing the prevalence of intestinal fluke infection, we added the identified prevalence for each parasite species in order to obtain the overall prevalence of intestinal fluke infections. This approach may lead to a certain overestimation of the true prevalence, because people may be co-infected with more than one intestinal fluke species. There is no sufficient evidence about the proportion of co-infections, but the resulting overestimation of the true prevalence may be more than offset by the assumptions made in our previous modeling approach and the many challenges in generating the underlying epidemiological parameters (e.g., diagnostic inaccuracy in the detection of infections with the more than 50 intestinal fluke species). Also of note: the transmission source of intestinal fluke infections are species-specific and therefore vary. For instance, *Fasciolopsis buski* is usually transmitted by eating raw water plants with the infective parasite stage attached to the water plants, whereas Neodiplostomatidae are transmitted by eating undercooked and infested frogs, snakes, and tadpoles. Because of these different transmission pathways, the rate of co-infection might in fact be smaller than expected.

Input data to differentiate between asymptomatic and heavy infections

We estimated the proportion of heavily-infected among all infected in all available national and regional cross-sectional surveys. It is expected that heavy infection increases with age and there is data available on heavy infection by age group. We therefore decided to include age-dependent rates of heavy infection for clonorchiasis, opisthorchiasis, and intenstinal fluke infection. For (cerebral) paragonimiasis and fascioliasis there was not sufficient age-dependent data on high intensity FBT infection.

Modeling strategy

The GBD 2013 epidemiological modeling strategy for FBT made use DisMod-MR 2.0, a Bayesian meta-regression tool which built on GBD 2010's DisMod-MR. Updated characteristics of DisMod-MR 2.0 included the application of an offset lognormal rather than a negative binomial distribution. Dismod-MR 2.0 also executed its calculations in a cascade from global to country and (where applicable) from country to the subnational geographical unit, thereby

ensuring that estimates were consistent at all levels of the cascade. DisMod-MR 2.0 was used to estimate prevalence, by age, sex, year, and country for FBT.

We used a three-step process for the disease modeling of FBT. In the first step we used DisMod-MR to estimate assess the prevalence of FBT by age, sex, year, and country. In the second we differentiated between asymptomatic and heavy infections. MetaXL (a meta-analysis add in for Microsoft Excel) was used to estimate the proportion of heavy infected among all infected by age group for clonorchiasis, opisthorchiasis, and intenstinal fluke infection (see Table 3 and 4). These proportions were used to estimate the prevalence of heavy FBT infection.
The third step consisted of deselecting countries that have no autochtonous case reports of FBT (input 34,000 references from literature review).

Table 3. Percentage of high intensity infection by age group and type of FBT (based on 8 FBT prevalence studies)

Age	Clonorchiasis			Opisthorchiasis			Intestinal fluke infection		
category	Mean	Low	High	Mean	Low	High	Mean	Low	High
0-9	30%	17%	44%	10%	0%	29%	8%	3%	14%
10-19	15%	0%	43%	15%	0%	69%	11%	8%	14%
20-29	18%	10%	29%	16%	0%	52%	18%	15%	21%
30-39	17%	5%	34%	21%	0%	56%	22%	17%	28%
40-49	22%	13%	32%	28%	1%	68%	22%	13%	32%
50-59	18%	0%	49%	29%	0%	75%	17%	9%	28%
60+	32%	18%	47%	25%	0%	64%	15%	8%	23%

Table 4. Percentage of high intensity infection by type of FBT (based on 4 FBT prevalence studies)

Type of FBT	Mean	Low	High
Paragonimiasis	23%	0%	59%
Fascioliasis	19%	3%	41%

Cerebral paragonimiasis

It was assumed that 0.8% of cerebral involvement in paragonimiasis. This proportion was used to estimate the prevalence of cerebral paragonimiasis. This proportion is based on one study. The study was performed in Paju, South Korea. This is an area with 6,738 inhabitants and according to the survey, it was estimated that 29.6% of all individuals would react to intradermal test (= an immunological reaction indicating previous or current contact to the parasite). 25% of all "positive reactors" may have eggs in their sputum (= active infection with the parasite currently present in the human host). If these rates are applied to the community as a whole, the number of patients with active paragonimiasis would be at least 498 (=6,738*0.296*0.250). Furthermore, four cases of cerebral paragonimiasis were found in this community. Therefore, four out of 498 individuals with active paragonimus infection suffered from cerebral infection (=0.80%; 95% confidence interval 0.019%-1.587%).

Severity splits and disability weights

For GBD 2015, FBT was not split into health states with different severities. The table below shows the GBD 2015 disability weights that were used to calculate the burden of FBT in YLDs.

Table 5. Disability weights that were used to calculate FBT YLDs

Sequelae	Severity description	Health state name	Disability weight
Asymptomatic clonorchiasis	Clonorchiasis, currently without symptoms	N/A	0.000 (0.000-0.000)
Hheavy clonorchiasis	Abdominal pain and nausea reported as moderate	Abdominopelvic problem, moderate	0.114 (0.078-0.159)
Asymptomatic opisthorchiasis	Opisthorchiasis, currently without symptoms	N/A	0.000 (0.000-0.000)
Heavy opisthorchiasis	Abdominal pain and nausea reported as moderate	Abdominopelvic problem, moderate	0.114 (0.078-0.159)
Asymptomatic fascioliasis	Fascioliasis, currently without symptoms	N/A	0.000 (0.000-0.000)
Heavy fascioliasis	Abdominal pain and nausea reported as moderate	Abdominopelvic problem, moderate	0.114 (0.078-0.159)
Asymptomatic intestinal fluke infection	Intestinal fluke infection, currently without symptoms	N/A	0.000 (0.000-0.000)
Heavy intestinal fluke infection	Abdominal pain and nausea reported as moderate	Abdominopelvic problem, moderate	0.114 (0.078-0.159)
Asymptomatic paragonimiasis	Paragonimiasis, currently without symptoms	N/A	0.000 (0.000-0.000)
Heavy paragonimiasis	Cough, fever and weight loss	Tuberculosis, not HIV infected	0.333 (0.224-0.454)
Cerebral paragonimiasis	Epilepsy due to cerebral paragonimiasis	Epilepsy, less severe (seizures < once per month)	0.263 (0.173-0.367)
		Epilepsy, severe (seizures >= once per month	0.552 (0.375-0.710)

Note. N/A: not applicable

References

1. WHO. *International Statistical Classification of Diseases and Related Health Problems. 10th Revision. Version for 2007.* 2007 [cited 2009 October 14, 2009]; Available from: http://apps.who.int/classifications/apps/icd/icd10online/.
2. Rim, H.J., *Clonorchiasis: an update.* J Helminthol, 2005. **79**(3): p. 269-81.
3. Pungpak, S., et al., *Clinical features in severe opisthorchiasis viverrini.* Southeast Asian J Trop Med Public Health, 1985. **16**(3): p. 405-9.
4. Rim, H.J., *The current pathobiology and chemotherapy of clonorchiasis.* Korean J Parasitol, 1986. **24**(Suppl.): p. 1-141.
5. Furst, T., J. Keiser, and J. Utzinger, *Global burden of human food-borne trematodiasis: a systematic review and meta-analysis.* Lancet Infect Dis, 2012. **12**(3): p. 210-21.
6. Furst, T., et al., *Manifestation, diagnosis, and management of foodborne trematodiasis.* BMJ, 2012. **344**: p. e4093.

Ascariasis SDG Capstone Appendix

Flowchart

Ascariasis

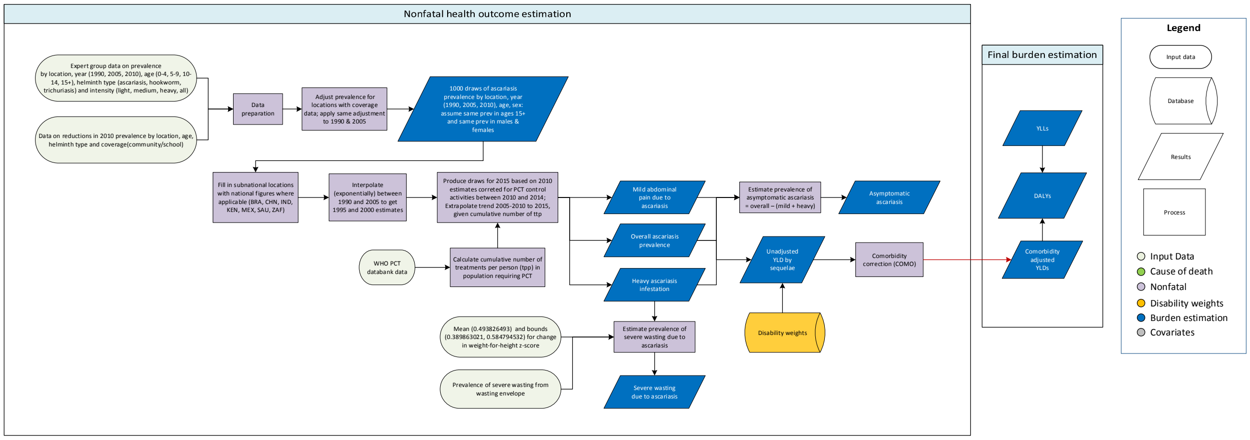

Case definition

Ascariasis is a helminth diseases caused by the parasitic roundworm, *Ascaris lumbricoides*. It is one of the three intestinal nematode infections (INI)/soil transmitted helminthiasis (STH) that we model in GBD. Diagnosis is made by microscopic exam of stool or by concentration procedures (recommended as eggs may be difficult to see). The ICD-10 codes for ascariasis are B77-B77.9.

Input data

Model inputs

Four different input data were used in the ascariasis nonfatal model. The first was prevalence data prepared by the expert group (EG) during GBD 2010 [1, 2]. They provided the data (mean, upper, lower) by location, year (1990, 2005, 2010), age (0-4, 5-9, 10-14, 15+ years), helminth type (ascariasis, hookworm disease, trichuriasis) and intensity of infection (light, medium, heavy, all). For the model, light infestation was not attributed any disability. The second data, also from the EG, was on reductions in prevalence in 2010, provided by location, age, helminth type, and coverage (community/school). The table below shows the number of countries or subnational units and GBD world regions represented in the data.

	prevalence
Countries/subnationals	163
GBD world regions	16

The third input data was from the WHO PCT Databank [3]. This data was downloaded from the source website and represented 121 locations and six GBD world regions. The last input data was 1,000 draws

of wasting envelope prevalence among children under 5 years – the methods used to generate estimates of wasting prevalence are detailed elsewhere (part of risk factors documentation). The table below shows the number of countries or subnational units and GBD world regions represented in the data.

	prevalence
Countries/subnationals	561
GBD world regions	21

Severity splits/Sequelae

The table below shows the list of sequelae due to ascariasis and the associated disability weights (DW). The sequelae were based on prevalence of medium and heavy infestation – medium infestation was assigned mild abdominopelvic problems; heavy infestation was assigned symptomatic worm infection; and light infestation was not attributed any disability.

Sequela	Lay description	DW
Mild abdominopelvic problems	has some pain in the belly that causes nausea but does not interfere with daily activities	0.011 (0.005-0.021)
Heavy infestation	has cramping pain and a bloated feeling in the belly	0.027 (0.015-0.043)
Severe wasting	is extremely skinny and has no energy	0.128 (0.082-0.183)
Asymptomatic ascariasis	N/A	N/A

Modeling strategy

In the estimation of morbidity due to ascariasis, the EG data was first prepared by formatting the location names to be consistent with the GBD 2015 location names and applying the 2010 prevalence to 1990 and 2005 for sub-Saharan Africa countries – estimates for these two years were missing. This was followed by using the data on reductions in 2010 prevalence to adjust the prevalence for locations with coverage data. After this adjustment, only data for medium infection, heavy infection, and all infection was retained.

Using the mean prevalence and the upper and lower bounds of the mean provided by the EG, 1,000 draws of prevalence were generated. This was done by multiplying the mean estimates by the exponent of random draws from a normal distribution with mean = 0 and standard deviation = sd, where sd = abs(abs(ln(upper)-ln(lower))/(invnormal(0.975)*2). These draws were created for all GBD age-groups, assuming the same prevalence in ages 15+ and same prevalence in males and females. Since the draws were only at the national level, subnational locations were filled with national figures where applicable (Brazil, China, India, Kenya, Mexico, Saudi Arabia, and South Africa).

To get 1995 and 2000 estimates, exponential interpolation of estimates between 1990 and 2005 was performed. The draws for 2015 were produced based on 2010 estimates corrected for PCT control activities between 2010 and 2014 – this was done by extrapolating the 2005-2010 trend to 2015, given cumulative number of treatments per person calculated using data from the WHO PCT Databank [3]. The 2005-2010 trend was applied to all intensities of infection. Prevalence was assumed to be zero for

the countries with missing input data and also in children younger than 28 days. The resulting estimates were 1,000 draws of ascariasis prevalence by GBD location, year, age, sex, and intensity level (mild, heavy, overall infection). To estimate the prevalence of asymptomatic ascariasis, prevalence of mild and heavy infestation was subtracted from the overall ascariasis prevalence.

The final step in the modeling process was to estimate the prevalence of severe wasting due to ascariasis in age groups 28-364 days and 1-4 years. This was done separately using 1,000 draws of prevalence of heavy infestation due to ascariasis and the wasting envelope prevalence. The initial step in determining prevalence of severe wasting due to ascariasis was generating 1,000 draws of change in weight-for-height z-score per heavy prevalent case from a random normal distribution with mean = 0.493826493 and standard deviation = 0.04972834 (calculated from upper and lower bounds of the mean estimate). The mean, upper and lower bounds were provided by a GBD collaborator who calculated them based on a published article [4]. The prevalence of severe wasting due to ascariasis was then obtained as a function of change in weight-for-height z-score (z_change) such that prevalence = p_wasting_env – Phi(Phi_inv(p_wasting_env) – z_change*p), where p_wasting_env = wasting envelope prevalence, Phi_inv is the inverse standard normal cumulative distribution function (cdf), and p = prevalence of heavy ascariasis infestation.

Model evaluation was done by plotting prevalence of overall ascariasis and that of each sequelae against year for each location and age group. Maps of the global distribution of total ascariasis prevalence and prevalence of sequelae due to ascariasis were also assessed across time and age. Since we used the same data and model as that used in GBD 2013, we compared GBD 2015 estimates for each sequela with those from GBD 2013 for each country and age. As expected, our estimates were very similar to those from GBD 2013.

The only change made from GBD 2013 modeling strategy was the incorporation of updated data from the WHO PCT databank [3] in the correction of estimates for MDA activities.

References:

1. Brooker S, Pullan R, Smith J, and Hotez P. Chapter: Intestinal nematodes. Cluster D: Communicable Diseases, Neglected Tropical Diseases Group. Global Burden of Diseases, Injuries, and Risk Factors Study. 2011 (4 July). 1-24
2. Brooker S & Smith JL. Impact of hookworm infection and deworming on anaemia in non-pregnant populations: a systematic review. Tropical Medicine and International Health. 2010. 15,7,776-795
3. WHO PCT Databank. 2015; http://www.who.int/neglected_diseases/preventive_chemotherapy/sth/en/
4. Hall A, Hewitt G, Tuffrey V, de Silva N. A review and meta-analysis of the impact of intestinal worms on child growth and nutrition. Maternal and Child Nutrition. 2008. 4. 118-236.

Hookworm Disease SDG Capstone Appendix

Flowchart

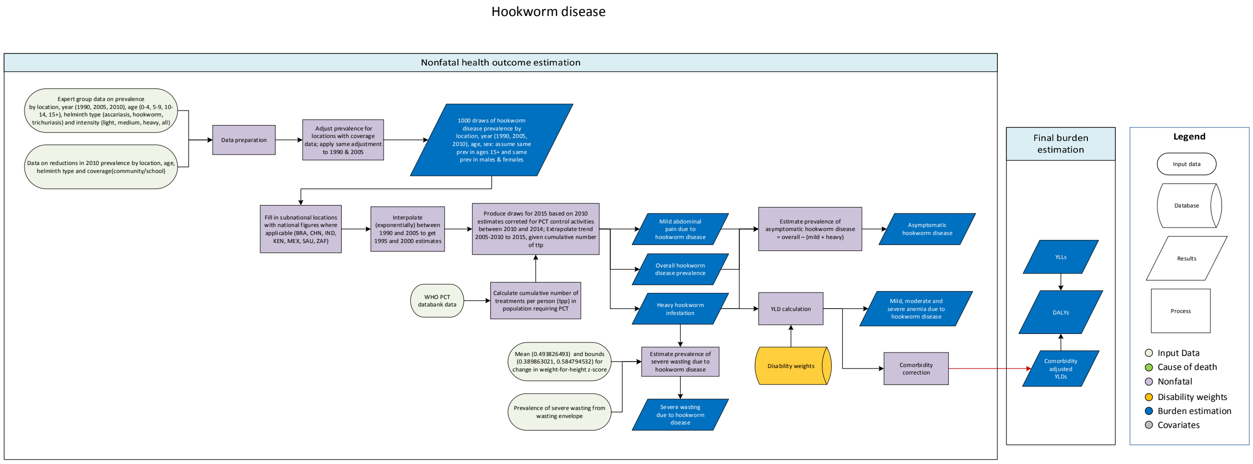

Case definition

Hookworm disease is a helminth disease caused by the parasitic roundworms, *Ancylostoma duodenale* and *Necator americanus*. It is one of the three intestinal nematode infections (INI)/soil transmitted helminthiasis (STH) that we model in GBD. Diagnosis is made by a microscopic exam of stool or by concentration procedures (recommended as eggs may be difficult to see). The ICD-10 codes for hookworm disease are B76-B76.9.

Input data

Model inputs

Four different input data were used in the hookworm disease nonfatal model. The first was prevalence data prepared by the expert group (EG) during GBD 2010 [1, 2]. They provided the data (mean, upper, lower) by location, year (1990, 2005, 2010), age (0-4, 5-9, 10-14, 15+ years), helminth type (ascariasis, hookworm disease, trichuriasis) and intensity of infection (light, medium, heavy, all). For the model, light infestation was not attributed any disability. The second data, also from the EG, was on reductions in prevalence in 2010, provided by location, age, helminth type, and coverage (community/school). The table below shows the number of countries or subnational units and GBD world regions represented in the data.

	prevalence
Countries/subnationals	163
GBD world regions	16

The third input data was from the WHO PCT Databank [3]. This data was downloaded from the source website and represented 121 locations and 6 GBD world regions. The last input data was 1,000 draws of wasting envelope prevalence among children under 5 years – the methods used to generate estimates of

wasting prevalence are detailed elsewhere (part of risk factors documentation). The table below shows the number of countries or subnational units and GBD world regions represented in the data.

	prevalence
Countries/subnationals	561
GBD world regions	21

Severity splits/Sequelae

The table below shows the list of sequelae due to hookworm disease and the associated disability weights (DW). The sequelae were based on prevalence of medium and heavy infestation – medium infestation was assigned mild abdominopelvic problems; heavy infestation was assigned symptomatic worm infection; light infestation was not attributed any disability.

Sequela	Lay description	DW
Mild abdominopelvic problems	has some pain in the belly that causes nausea but does not interfere with daily activities	0.011 (0.005-0.021)
Heavy infestation	has cramping pain and a bloated feeling in the belly	0.027 (0.015-0.044)
Severe wasting	is extremely skinny and has no energy	0.128 (0.082-0.183)
Asymptomatic hookworm disease	NA	NA
Mild anemia	feels slightly tired and weak at times, but this does not interfere with normal daily activities	0.004 (0.001-0.008)
Moderate anemia	feels moderate fatigue, weakness, and shortness of breath after exercise, making daily activities more difficult	0.052 (0.034-0.076)
Severe anemia	feels very weak, tired and short of breath, and has problems with activities that require physical effort or deep concentration	0.149 (0.101-0.210)

Modeling strategy

In the estimation of morbidity due to hookworm disease, the EG data was first prepared by formatting the location names to be consistent with the GBD 2015 location names and applying the 2010 prevalence to 1990 and 2005 for sub-Saharan Africa countries – estimates for these two years were missing. This was followed by using the data on reductions in 2010 prevalence to adjust the prevalence for locations with coverage data. After this adjustment, only data for medium infection, heavy infection, and all infection was retained.

Using the mean prevalence and the upper and lower bounds of the mean provided by the EG, 1,000 draws of prevalence were generated. This was done by multiplying the mean estimates by the exponent of random draws from a normal distribution with mean = 0 and standard deviation = sd, where sd = abs(abs(ln(upper)-ln(lower))/(invnormal(0.975)*2). These draws were created for all GBD age-groups, assuming the same prevalence in ages 15+ and same prevalence in males and females. Since the draws

were only at the national level, subnational locations were filled with national figures where applicable (Brazil, China, India, Kenya, Mexico, Saudi Arabia, and South Africa).

To get 1995 and 2000 estimates, exponential interpolation of estimates between 1990 and 2005 was performed. The draws for 2015 were produced based on 2010 estimates corrected for PCT control activities between 2010 and 2014 – this was done by extrapolating the 2005-2010 trend to 2015, given cumulative number of treatments per person calculated using data from the WHO PCT Databank [3]. The 2005-2010 trend was applied to all intensities of infection. Prevalence was assumed to be zero for the countries with missing input data and also in children younger than 28 days. The resulting estimates were 1,000 draws of hookworm disease prevalence by GBD location, year, age, sex, and intensity level (mild, heavy, overall infection). To estimate the prevalence of asymptomatic hookworm disease, prevalence of mild and heavy infestation was subtracted from the overall hookworm disease prevalence.

The final step in the modeling process was to estimate the prevalence of severe wasting due to hookworm disease in age groups 28-364 days and 1-4years. This was done separately using 1,000 draws of prevalence of heavy infestation due to hookworm disease and the wasting envelope prevalence. The initial step in determining prevalence of severe wasting due to hookworm disease was generating 1,000 draws of change in weight-for-height z-score per heavy prevalent case from a random normal distribution with mean = 0.493826493 and standard deviation = 0.04972834 (calculated from upper and lower bounds of the mean estimate). The mean, upper and lower bounds were provided by a GBD collaborator who calculated them based on a published article [4]. The prevalence of severe wasting due to hookworm disease was then obtained as a function of change in weight-for-height z-score (z_change) such that prevalence = p_wasting_env – Phi(Phi_inv(p_wasting_env) – z_change*p), where p_wasting_env = wasting envelope prevalence, Phi_inv is the inverse standard normal cumulative distribution function (cdf) and p = prevalence of heavy hookworm infestation. The burden of anemia due to hookworm disease was estimated (see anemia documentation for details).

Model evaluation was done by plotting prevalence of overall hookworm disease and that of each sequelae against year for each location and age group. Maps of the global distribution of total hookworm disease prevalence and prevalence of sequelae due to hookworm disease were also assessed across time and age. Since we used the same data and model as that used in GBD 2013, we compared GBD 2015 estimates for each sequela with those from GBD 2013 for each country and age. As expected, our estimates were very similar to those from GBD 2013.

The only change made from GBD 2013 modeling strategy was the incorporation of updated data from the WHO PCT databank [3] in the correction of estimates for MDA activities.

References:

1. Brooker S, Pullan R, Smith J, and Hotez P. Chapter: Intestinal nematodes. Cluster D: Communicable Diseases, Neglected Tropical Diseases Group. Global Burden of Diseases, Injuries, and Risk Factors Study. 2011 (4 July). 1-24
2. Brooker S & Smith JL. Impact of hookworm infection and deworming on anaemia in non-pregnant populations: a systematic review. Tropical Medicine and International Health. 2010. 15,7,776-795
3. WHO PCT Databank. 2015; http://www.who.int/neglected_diseases/preventive_chemotherapy/sth/en/

4. Hall A, Hewitt G, Tuffrey V, de Silva N. A review and meta-analysis of the impact of intestinal worms on child growth and nutrition. Maternal and Child Nutrition. 2008. 4. 118-236.

Trichuriasis SDG Capstone Appendix

Flowchart

Trichuriasis

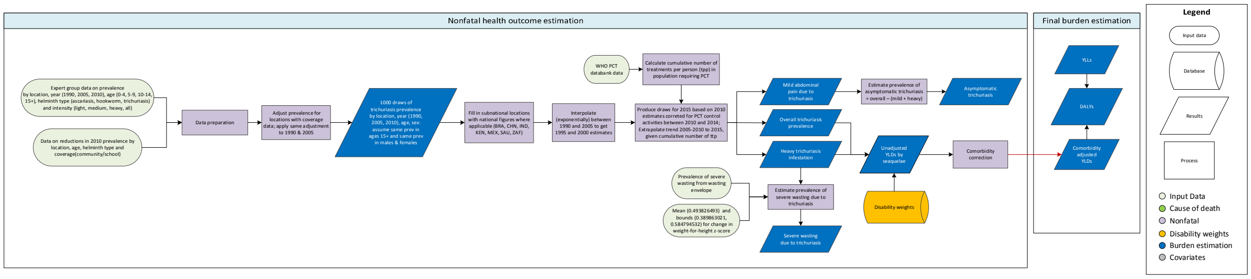

Case definitions

Trichuriasis is a helminth diseases caused by the parasitic roundworm *Trichuris trichiura*. It is one of the three intestinal nematode infections (INI)/soil transmitted helminthiasis (STH) that we model in GBD. Diagnosis is made by microscopic exam of stool or by concentration procedures (recommended as eggs may be difficult to see). The ICD-10 code for trichuriasis are B79.

Input data

Model inputs

Four different input data were used in the trichuriasis nonfatal model. The first was prevalence data prepared by the expert group (EG) during GBD 2010 [1, 2]. They provided the data (mean, upper, lower) by location, year (1990, 2005, 2010), age (0-4, 5-9, 10-14, 15+ years), helminth type (ascariasis, hookworm disease, trichuriasis) and intensity of infection (light, medium, heavy, all). For the model, light infestation was not attributed any disability. The second data, also from the EG, was on reductions in prevalence in 2010, provided by location, age, helminth type, and coverage (community/school). The table below shows the number of countries or subnational units and GBD world regions represented in the data.

	prevalence
Countries/subnationals	163
GBD world regions	16

The third input data was from the WHO PCT Databank [3]. This data was downloaded from the source website and represented 121 locations and 6 GBD world regions. The last input data was 1,000 draws of wasting envelope prevalence among children under 5 years – the methods used to generate estimates of wasting prevalence are detailed elsewhere (part of risk factors documentation). The table below shows the number of countries or subnational units and GBD world regions represented in the data.

	prevalence

Countries/subnationals	561
GBD world regions	21

Severity splits/Sequelae

The table below shows the list of sequelae due to trichuriasis and the associated disability weights (DW). The sequelae were based on prevalence of medium and heavy infestation – medium infestation was assigned mild abdominopelvic problems; heavy infestation was assigned symptomatic worm infection; light infestation was not attributed any disability.

Sequela	Lay description	DW (95% CI)
Mild abdominopelvic problems	has some pain in the belly that causes nausea but does not interfere with daily activities	0.011 (0.005-0.021)
Heavy infestation	has cramping pain and a bloated feeling in the belly	0.027 (0.015-0.044)
Severe wasting	is extremely skinny and has no energy	0.128 (0.082-0.183)
Asymptomatic trichuriasis	N/A	N/A

Modeling strategy

In the estimation of morbidity due to trichuriasis, the EG data was first prepared by formatting the location names to be consistent with the GBD 2015 location names and applying the 2010 prevalence to 1990 and 2005 for sub-Saharan Africa countries – estimates for these two years were missing. This was followed by using the data on reductions in 2010 prevalence to adjust the prevalence for locations with coverage data. After this adjustment, only data for medium infection, heavy infection, and all infection was retained.

Using the mean prevalence and the upper and lower bounds of the mean provided by the EG, 1,000 draws of prevalence were generated. This was done by multiplying the mean estimates by the exponent of random draws from a normal distribution with mean = 0 and standard deviation = sd, where sd = abs(abs(ln(upper)-ln(lower))/(invnormal(0.975)*2). These draws were created for all GBD age-groups, assuming the same prevalence in ages 15+ and same prevalence in males and females. Since the draws were only at the national level, subnational locations were filled with national figures where applicable (Brazil, China, India, Kenya, Mexico, Saudi Arabia, and South Africa).

To get 1995 and 2000 estimates, exponential interpolation of estimates between 1990 and 2005 was performed. The draws for 2015 were produced based on 2010 estimates corrected for PCT control activities between 2010 and 2014 – this was done by extrapolating the 2005-2010 trend to 2015, given the cumulative number of treatments per person calculated using data from the WHO PCT Databank [3]. The 2005-2010 trend was applied to all intensities of infection. Prevalence was assumed to be zero for the countries with missing input data and also in children younger than 28 days. The resulting estimates were 1,000 draws of trichuriasis prevalence by GBD location, year, age, sex, and intensity level (mild, heavy, overall infection). To estimate the prevalence of asymptomatic trichuriasis, prevalence of mild and heavy infestation was subtracted from the overall trichuriasis prevalence.

The final step in the modeling process was to estimate the prevalence of severe wasting due to trichuriasis in age groups 28-364 days and 1-4 years. This was done separately using 1,000 draws of prevalence of heavy infestation due to trichuriasis and the wasting envelope prevalence. The initial step in determining prevalence of severe wasting due to trichuriasis was generating 1,000 draws of change in weight-for-height z-score per heavy prevalent case from a random normal distribution with mean = 0. 493826493 and standard deviation = 0.04972834 (calculated from upper and lower bounds of the mean estimate). The mean, upper and lower bounds were provided by a GBD collaborator who calculated them based on a published article [4]. The prevalence of severe wasting due to trichuriasis was then obtained as a function of change in weight-for-height z-score (z_change) such that prevalence = p_wasting_env – Phi(Phi_inv(p_wasting_env) – z_change*p), where p_wasting_env = wasting envelope prevalence, Phi_inv is the inverse standard normal cumulative distribution function (cdf), and p = prevalence of heavy trichuriasis infestation.

Model evaluation was done by plotting prevalence of overall trichuriasis and that of each sequelae against year for each location and age group. Maps of the global distribution of total trichuriasis prevalence and prevalence of sequelae due to trichuriasis were also assessed across time and age. Since we used the same data and model as that used in GBD 2013, we compared GBD 2015 estimates for each sequela with those from GBD 2013 for each country and age. As expected, our estimates were very similar to those from GBD 2013.

The only change made from GBD 2013 modeling strategy was the incorporation of updated data from the WHO PCT databank [3] in the correction of estimates for MDA activities.

References:

1. Brooker S, Pullan R, Smith J, and Hotez P. Chapter: Intestinal nematodes. Cluster D: Communicable Diseases, Neglected Tropical Diseases Group. Global Burden of Diseases, Injuries, and Risk Factors Study. 2011 (4 July). 1-24
2. Brooker S & Smith JL. Impact of hookworm infection and deworming on anaemia in non-pregnant populations: a systematic review. Tropical Medicine and International Health. 2010. 15,7,776-795
3. WHO PCT Databank. 2015; http://www.who.int/neglected_diseases/preventive_chemotherapy/sth/en/
4. Hall A, Hewitt G, Tuffrey V, de Silva N. A review and meta-analysis of the impact of intestinal worms on child growth and nutrition. Maternal and Child Nutrition. 2008. 4. 118-236.

Cutaneous and mucocutaneous leishmaniasis SDG Capstone Appendix

Flowchart

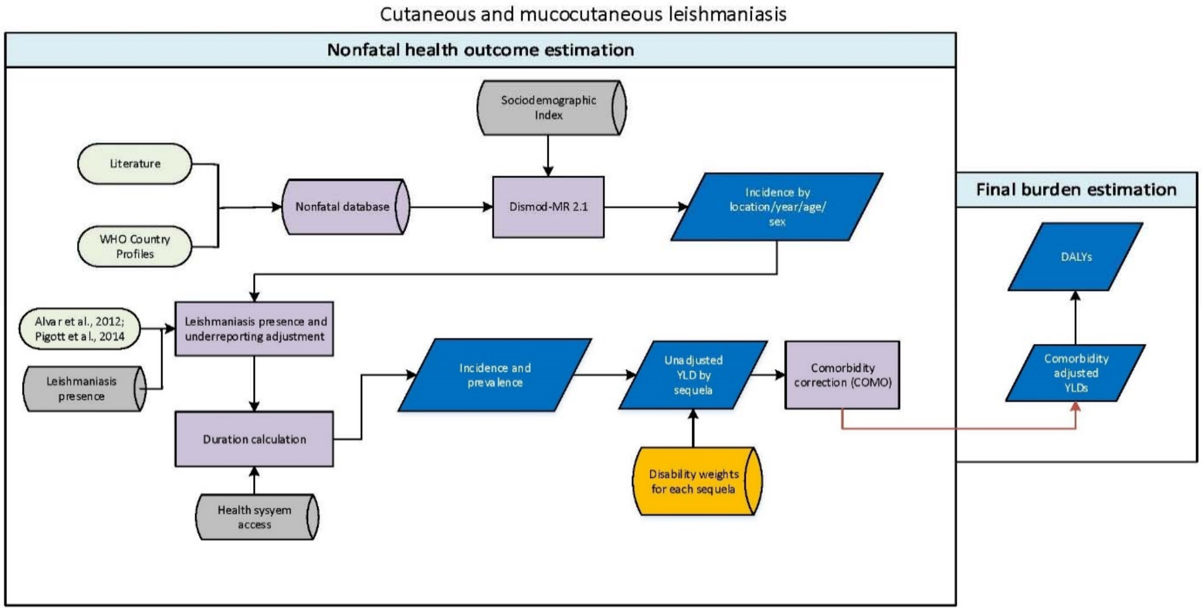

Input Data and Methodological Summary

Case Definition

Cutaneous leishmaniasis (CL) is the most common manifestation of disease caused by the *Leishmania* parasite, transmitted through the bite of phlebotomine sand flies. It causes the appearance of skin lesions, often beginning as papules or nodules and developing in/to ulcers, on parts of the body exposed to the bite of the sand fly. Mucocutaneous leishmaniasis (MCL) is a much more exceptional – and severe – presentation. Primarily isolated to Latin America, MCL infections can result in degradation of the mucous membranes, typically following an ulcerative sore from CL infection. Transmission varies by geographic region, as approximately 70 animal species have been identified as potential reservoir hosts of the parasite.

Input data

A systematic review of literature in the PubMed database was done on 17 July 2015 for prevalence and incidence data using the search term:

> (leishmaniasis[Title/Abstract] OR "visceral leishmaniasis"[Title/Abstract] OR kala-azar[Title/Abstract] OR "black fever"[Title/Abstract] OR "dumdum fever"[Title/Abstract] OR "cutaneous leishmaniasis"[Title/Abstract] OR "mucosal leishmaniasis"[Title/Abstract] OR

"mucocutanaeous leishmaniasis"[Title/Abstract] OR "oriental sore"[Title/Abstract] OR "tropical sore"[Title/Abstract] OR "chiclero ulcer"[Title/Abstract] OR "chiclero's ulcer"[Title/Abstract]) AND (“1990”[Date – Publication] : “2015”[Date – Publication]) AND (epidemiology OR prevalence OR incidence OR mortality OR fatality).

This search returned 3790 results, 258 of which passed title and abstract screening for CL and/or MCL. Upon full text review, 35 studies were selected – four reporting prevalence and 31 reporting incidence. For 66 countries, incidence from WHO country profiles were available.

Modeling strategy

The minimal amount of prevalence data conflicted with incidence where available, and thus was excluded from the model. No study level covariates were used. The sociodemographic index (SDI) was used as a country level covariate on the incidence data, with a floor of exp(-1) – as to allow a degree of regional and subnational variation while constraining the predictive power such that predictions in hypo-endemic countries with low SDI values and no data would not be unduly high.

In order to control for DisMod fitting values to locations known to be devoid of CL, we replace estimates in these locations with zeros. Then for locations with confirmed CL presence, we apply an underreporting factor reported in Alvar et al. In order to distinguish prevalence of acute cases and those that endure lifelong disability, we used a normalized version of the health system access (HSA) covariate such that 47.6% of cases with poor access to healthcare – defined as (cases * (1 - norm(HSA)]) – would progress to the lifelong stage. All acute cases were assumed a six month duration.

Visceral Leishmaniasis SDG Capstone Appendix

Flowchart

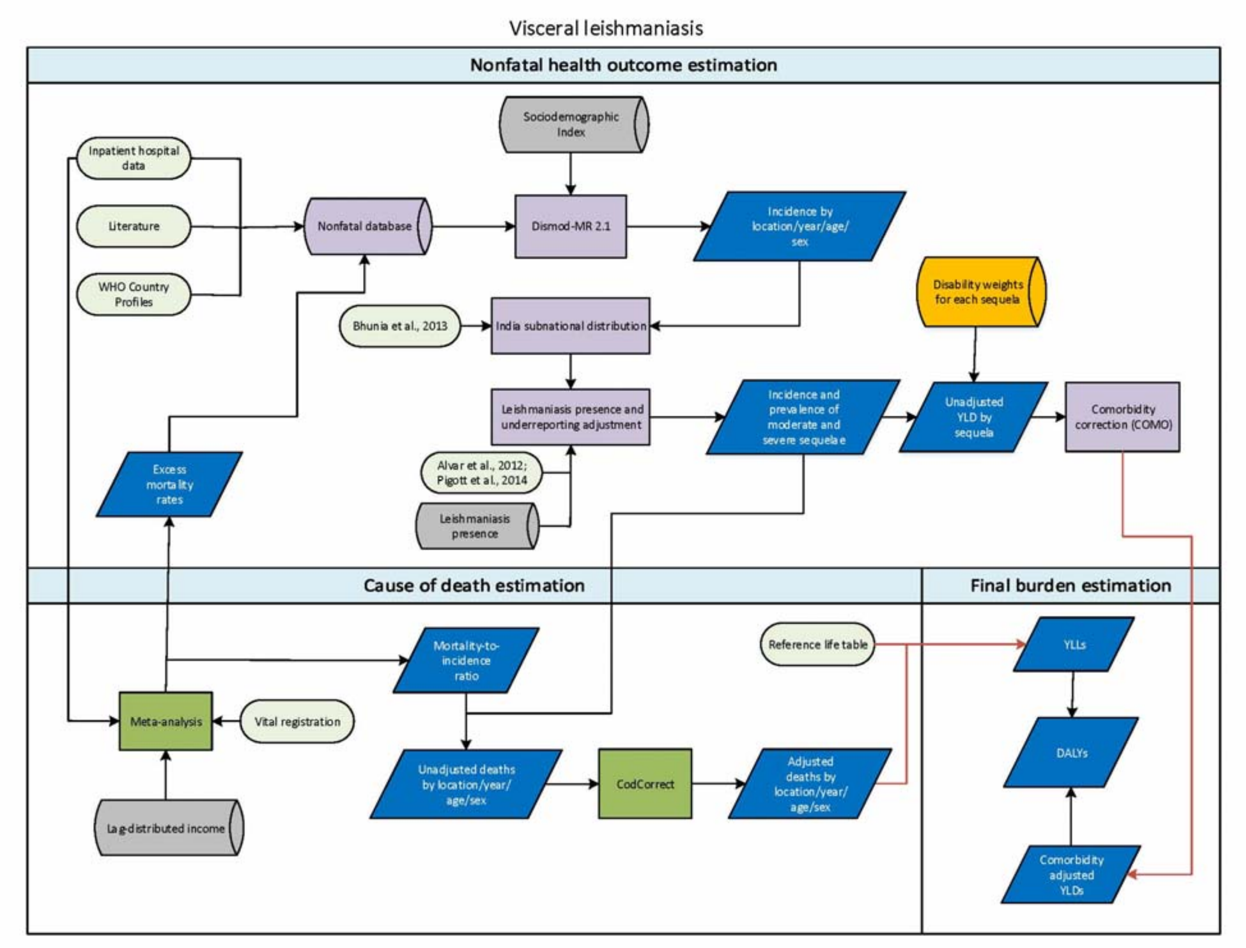

Input Data and Methodological Summary

Case Definition

Visceral leishmaniasis (VL) is the most serious manifestation of disease caused by the *Leishmania* parasite, transmitted through the bite of phlebotomine sand flies. Those infected typically present with fever, weight loss, anemia, leukopenia, thrombocytopenia, and enlargement of the spleen and liver. If left untreated, it can be fatal. Transmission varies by geographic region, as approximately 70 animal species have been identified as potential reservoir hosts of the parasite. The ICD9 code related to visceral leishmaniasis is 085.0, and the ICD10 code is B55.0.

Input data

A systematic review of literature in the PubMed database was done on 17 July 2015 for prevalence and incidence data using the search term:

> (leishmaniasis[Title/Abstract] OR "visceral leishmaniasis"[Title/Abstract] OR kala-azar[Title/Abstract] OR "black fever"[Title/Abstract] OR "dumdum fever"[Title/Abstract] OR "cutaneous leishmaniasis"[Title/Abstract] OR "mucosal leishmaniasis"[Title/Abstract] OR "mucocutanaeous leishmaniasis"[Title/Abstract] OR "oriental sore"[Title/Abstract] OR "tropical sore"[Title/Abstract] OR "chiclero ulcer"[Title/Abstract] OR "chiclero's ulcer"[Title/Abstract]) AND (“1990”[Date – Publication] : “2015”[Date – Publication]) AND (epidemiology OR prevalence OR incidence OR mortality OR fatality).

This search returned 3790 results, 274 of which passed title and abstract screening for VL. Upon full text review, 24 studies were selected – five reporting prevalence, 18 reporting exclusively incidence, and one reporting both.

Additionally, incidence data from WHO country profile reports were included for 50 countries, and inpatient hospital data from 75 unique locations was used in incidence estimation. In GBD 2013, we estimated country-year-specific MI ratios by running a linear regression of the logit of the MI ratio on the log of income per capita using vital registration and inpatient hospital data from Brazil and Spain, two countries in which we had both reliable mortality and incidence data at the national level. Those were used here. Assuming a duration of 3 months, excess mortality rates were calculated and used as well.

Modeling strategy

The minimal amount of prevalence data available conflicted with the relationship between incidence and excess mortality data, as well as the remission prior (set to 4 based on duration assumptions), and thus was excluded from the model. No study level covariates were used. The sociodemographic index (SDI) was used as a country level covariate on the incidence data, with a floor of exp(-1) – as to allow a degree of regional and subnational variation while constraining the predictive power such that predictions in hypo-endemic countries with low SDI values and no data would not be unduly high.

In order to best represent the documented distribution of VL in India, we used the national fit from the DisMod model and redistributed it amongst the Indian states based on data from Bhunia, et al. Further, in order to control for DisMod fitting values to locations known to be devoid of VL, we replace estimates in these locations with zeros. Then for locations with confirmed VL presence, we apply an underreporting factor reported in Alvar et al. Resultant incidence draws are then assumed to have a duration of three months, from which prevalence is calculated. Of those three months, three weeks are assumed to be spend with severe infection, and nine with moderate infection.

Leprosy Capstone Appendix

Flowchart

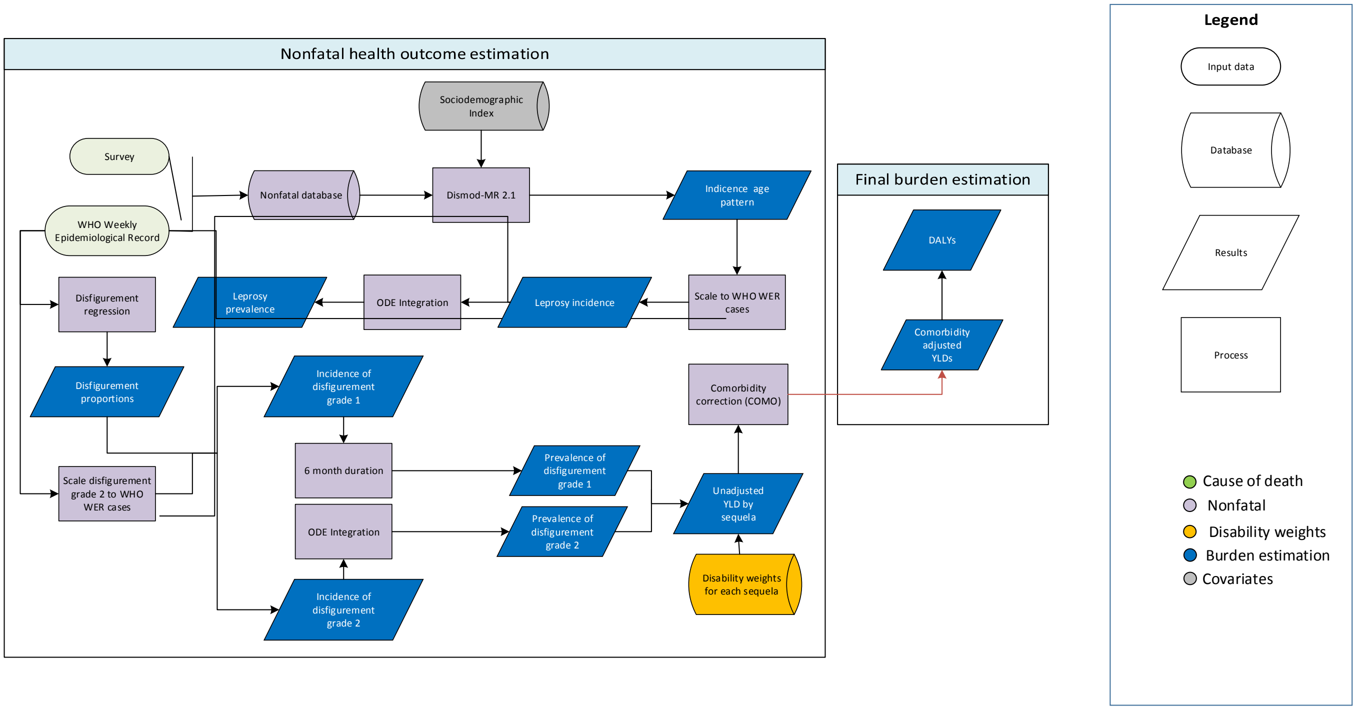

Input Data and Methodological Summary

Case Definition

Leprosy is a chronic bacterial infection caused by *Mycobacterium leprae*, primarily affecting the nervous system, skin, respiratory tract, and eyes. Transmission is facilitated through contact with fluid from the nose and mouth of an infected individual. Disability is associated with cases that develop to disfigurement, which is further subdivided into grade 1 and 2.

Sequela	Healthstate name	Healthstate description	Disability Weight (95% CI)
Disfigurement level 1 due to leprosy	Disfigurement, level 1	has a slight, visible physical deformity that others notice, which causes some worry and discomfort.	0.011 (0.005-0.021)
Disfigurement level 2 due to leprosy	Disfigurement, level 2	has a visible physical deformity that causes others to stare and comment. As a result, the person is worried and has trouble sleeping and concentrating.	0.067 (0.044-0.096)

Input data

Due to the cyclical nature of systematic review for GBD causes, no data collection was scheduled for GBD 2015. As such, leprosy will be a priority for the next iteration of the study.

Modeling strategy

All available leprosy incidence data was modeled using Dismod-MR 2.1. Following this, the age-sex specific incidence rates are scaled to match the reported incidence in the WHO Weekly Epidemiological Record (WER). Where multiple years are available for a particular country, missing years are interpolated/extrapolated in order to maintain a complete time series. Countries for which only one year is present, other years are assumed to be zero. We them stream out prevalence of leprosy as a function of time by integrating the ordinary differential equation

$$^{dp}/_{dt} = inc * (1 - prev) - prev * \ EMR$$

Where EMR = excess mortality rate, and is presumed to be zero for this condition.

In order to define the progression of leprosy incidence into disfigurement, we perform a generalized ordered logistic regression on WER data from Brazil, resulting in age-sex-specific probability of grade 2 disfigurement among incident cases of leprosy and grade 1 among incident cases of leprosy without grade 2 disfigurement. Then we use WER data to generate an envelope of grade 2, as was done for total leprosy. We are then able to take leprosy incidence, impose the age-sex-specific disfigurement proportions, and scale to the grade 2 envelope. From there, we apply the grade 1 proportions to the remaining leprosy cases that do not have grade 2 disability. For grade 1 prevalence, a duration of 6 months is applied. In deriving grade 2 prevalence, we use the same ODE integration function as described for total leprosy.

Lymphatic Filariasis Capstone Appendix

Flowchart

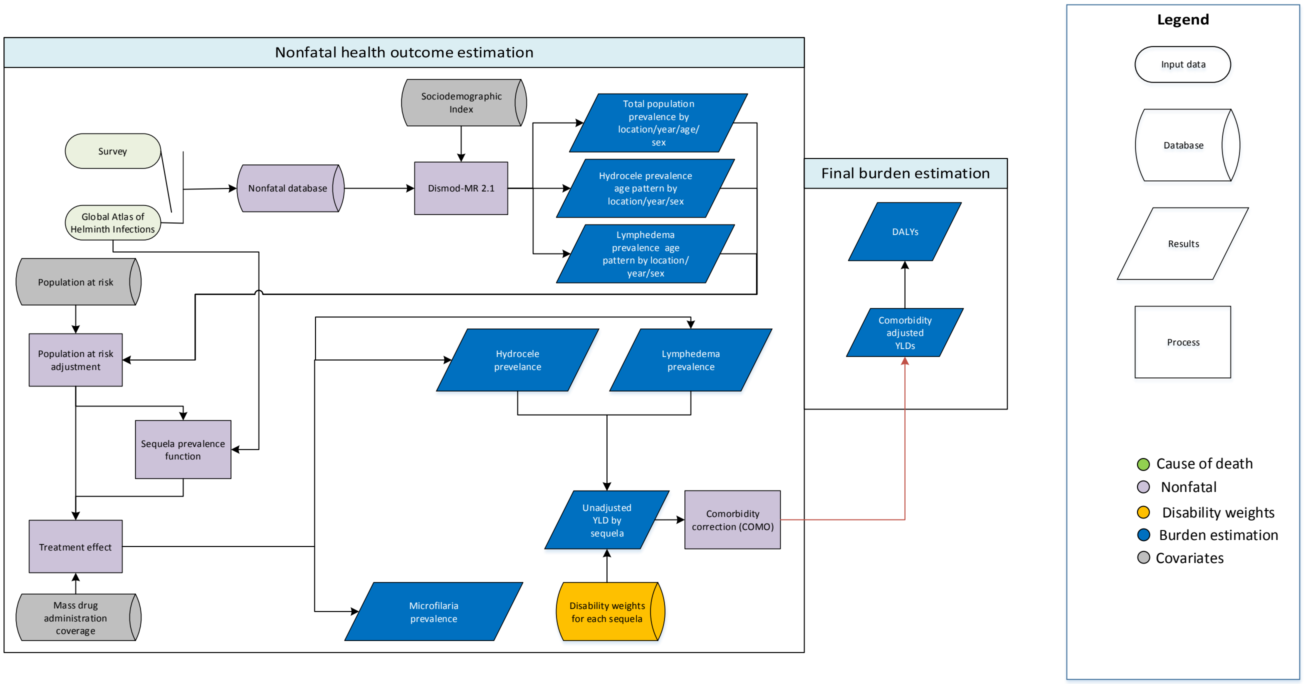

Input Data and Methodological Summary

Case Definition

Lymphatic filariasis (LF) is a neglected tropical disease spread in which threadlike nematodes invade the lymphatic system. The worms responsible – *Wuchereria bancrofti*, *Brugia malayi* and *Brugia timori* – are spread from human to human via mosquitoes. The most prominent clinical manifestations of LF are lymphedema (a swelling of the legs, also known in its more extreme manifestation as elephantiasis) and hydrocele (a collection of fluid in the sac around the testicles).

Input data

A systematic review of literature for GBD 2015 in the PubMed database was done on 10 August 2015 for prevalence and incidence data using the search ("lymphatic filariasis"[Title/Abstract] OR "filariasis"[Title/Abstract] OR "wuchereria"[Title/Abstract] OR "brugia"[Title/Abstract]) AND (“2013”[Date – Publication] : “2015”[Date – Publication]) AND (epidemiology OR incidence OR prevalence). This search returned 185 results, 121 of which passed title and abstract screening. Upon full text review, 37 studies were extracted for inclusion. Below is a summary of the geographic distribution of the data used.

Sequela	Data points	Regions	Countries	Subnational units
Prevalence of detectable microfilaria	1,552	10	40	28

Lymphedema due to lymphatic filariasis	511	10	25	15
Hydrocele due to lymphatic filariasis	265	8	22	12

Modeling strategy

Data on prevalence of microfilaria is modeled using Dismod-MR 2.1. Due to the fact that data is collected in endemic locations, we then scaled according to at-risk population in order to attain nationally representative values. We then use non-linear regression to estimate the reduction of microfilaria as a function of treatments per person. Using mass drug administration (MDA) coverage, we are then able to reduce the total estimated prevalence by exposing modeled total prevalence according to treatment efficacy.

For lymphedema and hydrocele, we incorporate survey data from the Global LF Atlas in a non-linear error-in-variables regression that determines the prevalence of lymphedema and hydrocele as functions of microfilaria prevalence, which is then applied to the total microfilaria Dismod model in order to attain an envelope of cases by location-year. Separately, all available prevalence data for these conditions is modeled in Dismod in order to determine an age-sex pattern.

In the estimation of lymphedema and hydrocele prevalence, we perform the same population at-risk correction that is done on microfilaria prevalence. For hydrocele prevalence after treatment, we take the value before MDA rollout in 2000 and reduce that by the same treatment efficacy function described for microfilaria prevalence, using dosage-reduction data specific to hydrocele along with the location-year specific MDA coverage. For lymphedema, we assume no new cases appear among treated individuals. As such, we reduce lymphedema prevalence in post-treatment years in accordance with MDA coverage.

Onchocerciasis SDG Capstone Appendix

Flowchart

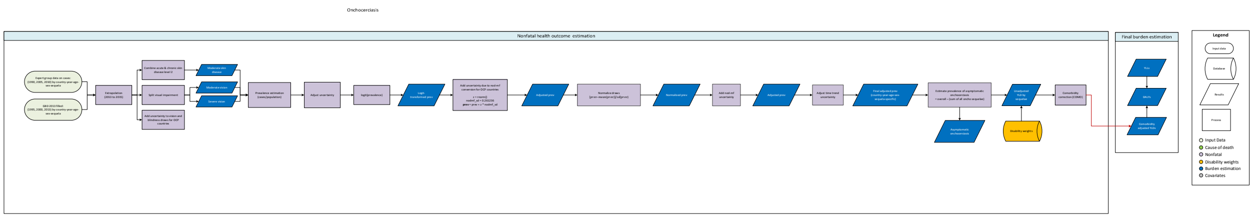

Input Data & Methodological Summary

Case definition

Onchocerciasis, also known as river blindness, is a helminth disease caused by a parasitic worm, *Onchocerca volvulus*, transmitted by repeated bites by *Similium* blackflies. Diagnosis is made by different methods including skin snip biopsy to identify larvae, surgical removal of nodules and exam for adult worms, slit lamp exam of anterior part of the eye where larvae or lesions caused by them are visible, and antibody tests (mostly useful to visitors to areas with parasites). The ICD-10 code for onchocerciasis is B73.

Input data

Model inputs

To model nonfatal outcomes due to onchocerciasis, prevalence data prepared by the expert group (EG) during GBD 2013 was used. These included 1,000 draws of infection and morbidity (visual impairment, blindness, and skin conditions) cases with confidence intervals for years 1990, 1995, 2000, 2005, 2010, and 2013 categorized by country, age, and sex. Details of materials and methods used by the EG to generate draws can be found elsewhere [1-5]. The data only represented African countries included in the African Programme for Onchocerciasis Control (APOC) – Angola, Burundi, Cameroon, Central African Republic, Chad, Congo, Democratic Republic of Congo, Ethiopia, Equatorial Guinea, Liberia, Malawi, Nigeria, Sudan, Tanzania, and Uganda – and the Onchocerciasis Control Programme (OCP) – Benin, Burkina Faso, Côte d'Ivoire, Ghana, Guinea Bissau, Guinea, Mali, Niger, Senegal, Sierra Leone, and Togo. The table below shows (by program) the number of countries and GBD world regions represented.

	APOC	OCP
Countries/subnationals	15	11
GBD world regions	3	1

We did not update the literature review for these data in GBD 2015. Updates to systematic reviews are performed on an ongoing schedule across all GBD causes, and an update for onchocerciasis will be performed in the next 1-2 iterations.

Severity splits/Sequelae

The table below shows the list of sequelae due to onchocerciasis, their lay descriptions, and the associated disability weights (DW).

Sequela	Lay description	DW
Moderate vision impairment	has vision problems that make it difficult to recognize faces or objects across a room	0.031 (0.019-0.049)
Severe vision impairment	has severe vision loss, which causes difficulty in daily activities, some emotional impact (for example worry), and some difficulty going outside the home without assistance	0.184 (0.125-0.258)
Blindness	is completely blind, which causes great difficulty in some daily activities, worry and anxiety, and great difficulty going outside the home without assistance	0.187 (0.124-0.260)
Mild skin disease	has a slight, visible physical deformity that is sometimes sore or itchy. Others notice the deformity, which causes some worry and discomfort	0.027 (0.015-0.042)
Mild skin disease without itch	has a slight, visible physical deformity that others notice, which causes some worry and discomfort	0.011 (0.005-0.021)
Moderate skin disease	has a visible physical deformity that is sore and itchy. Other people stare and comment, which causes the person to worry. The person has trouble sleeping and concentrating	0.188 (0.124-0.267)
Severe skin disease	has a visible physical deformity that is sore and itchy. Other people stare and comment, which causes the person to worry. The person has trouble sleeping and concentrating	0.188 (0.124-0.267)
Severe skin disease without itch	has an obvious physical deformity that makes others uncomfortable, which causes the person to avoid social contact, feel worried, sleep poorly, and think about suicide	0.405 (0.275-0.546)
Asymptomatic onchocerciasis	NA	NA

Modeling strategy

The nonfatal modeling for onchocerciasis included two major steps. In the first step, GBD 2013 prevalence was extrapolated to obtain GBD 2015 estimates. Acute skin disease level 2 and chronic skin disease level 2 were then summed up to create the “moderate skin disease” sequela. Within each of the OCP draws the number of cases with visual impairment and blindness was multiplied by a random value (the exponent of a normally distributed variable with mean zero and standard deviation 0.1) – this was

done to add some uncertainty to these estimates. Within each draw, the same randomly drawn value was applied to all country-year-age-sex. The other sequelae already had uncertainty quantified and were used as provided by the EG. Visual impairment was split into moderate and severe vision impairment by first multiplying the visual impairment estimates by a random value (from a normal distribution with mean 0.84 and standard deviation 0.0031) to generate moderate vision impairment, and then subtracting the resulting estimates from visual impairment to obtain estimates of severe vision impairment. Prevalence of sequelae was calculated by dividing the cases by the population.

The second step in modeling morbidity due to onchocerciasis was the adjustment of uncertainty in 1) conversion of nodule prevalence to microfilaria prevalence and 2) effects of mass treatment. To adjust for uncertainty in nodule to mf prevalence, the final draws from the first step were logit transformed, and then for OCP countries, we added uncertainty from a random value drawn from a normal distribution to the transformed estimates. The resulting estimates were then normalized and scaled using estimates published elsewhere [1]. To adjust for uncertainty due to MDA, the year when MDA with ivermectin started was set to 1990 and adjusted for some countries (1997 for Malawi; 1998 for Chad, Niger, and Tanzania; 1999 for Cameroon, Central African Republic, Equitorial Guinea, Liberia, Nigeria, and Uganda; 2001 for Congo, Ethiopia, and DRC; 2005 for Angola, Burundi, and South Sudan). Time trend uncertainty was then multiplied by the normalized prevalence estimates and then final prevalence was obtained by re-expanding the scaled normalized draws and adjusting the scale back from logit scale.

To estimate the prevalence of asymptomatic onchocerciasis, prevalence of morbidity (vision loss, blindness and skin conditions) was subtracted from the overall onchocerciasis prevalence – moderate vision impairment, severe vision impairment and blindness estimates were each multiplied by a factor of 8/33 before subtraction to account for cases that have concurring symptoms.

Model evaluation was done by separately assessing plots of time trends of prevalence across locations and age for each sequela. In addition, maps of the global distribution of total onchocerciasis prevalence and prevalence of sequelae due to onchocerciasis were also assessed across time. Since the modeling strategy was the same as that applied in GBD 2013, we compared our estimates with those from GBD 2013, and the correlation between the two was about 1.

We have made no substantive changes in the modeling strategy from GBD 2013.

References

1. Zouré HG, Noma M, Tekle AH, Amazigo UV, Diggle PJ, Giorgi E, Remme JH. The geographic distribution of onchocerciasis in the 20 participating countries of the African Programme for Onchocerciasis Control: (2) pre-control endemicity levels and estimated number infected. Parasites & Vectors. 2014. 7-326
2. Coffeng L, Stolk W, Hoerauf A, Habbema D, Bakker R, Hopkins A, de Vlas S. Elimination of African onchocerciasis: modeling the impact of increasing the frequency of ivermectin mass treatment. PLoS One. 2014. 9(12):e115886
3. Coffeng LE, Stolk WA, Zouré HG, Veerman JL, Agblewonu KB, Murdoch ME, Noma M, Fobi G, Richardus JH, Bundy DA, Habbema D, de Vlas SJ, Amazigo UV. African Programme For Onchocerciasis Control 1995-2015: model-estimated health impact and cost. PLoS Negl Trop Dis. 2013; 7(1): e2032

4. Murdoch ME, Asuzu MC, Hagan M, Makunde WH, Ngoumou P, Ogbuagu KF, Okello D, Ozoh G, Remme J. Onchocerciasis: the clinical and epidemiological burden of skin disease in Africa. Ann Trop Med Parasitol. 2002; 96(3): 283-96
5. Brieger WR, Awedoba AK, Eneanya CI, Hagan M, Ogbuagu KF, Okello DO, Ososanya OO, Ovuga EB, Noma M, Kale OO, Burnham GM, Remme JH. The effects of ivermectin on onchocercal skin disease and severe itching: results of a multicentre trial. Trop Med Int Health. 1998; 3(12): 951-61

Rabies SDG Capstone Appendix

Flowchart

Rabies

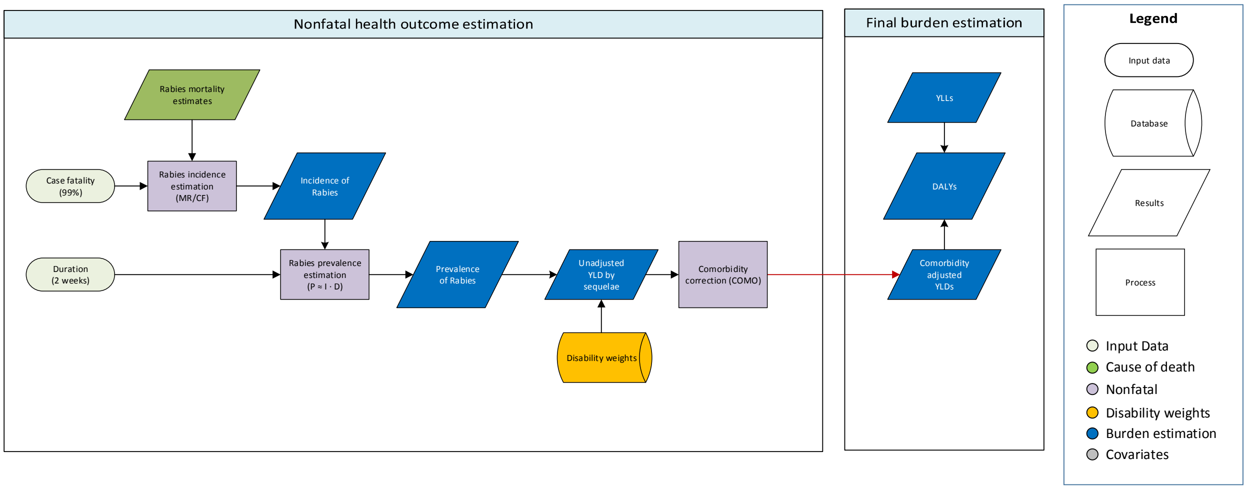

Input Data & Methodological Summary

Case definition

Rabies is a fatal viral infection, transmitted by animal bites. Without prophylactic vaccination the disease is almost universally fatal. The disease has a long incubation period (1-3 months), and early intervention with prophylactic vaccination is nearly 100% effective in preventing symptomatic disease. We model symptomatic infections, not including those infections in which intervention prevented the onset of symptomatic disease, corresponding to the ICD10 code A82.

Input data

Model inputs

As we derive our estimate of cases from our estimate of deaths, there are no incidence data used in the model. For GBD 2015, we modeled rabies mortality using all available data in the cause of death database. Data points were outliered if they reported an improbable number of rabies deaths (e.g., zero rabies deaths in a hyperendemic country) or if their inclusion in the model yielded distorted trends. In some cases multiple data sources for the same location differed dramatically both in their quality and reported rabies mortality (e.g., a verbal autopsy and vital registration source). In these cases the lower-quality data source was outliered.

Modeling strategy

We derive estimates of the number of symptomatic rabies infections (i.e., those not averted through prophylactic vaccination) based on rabies mortality estimates, assuming 99% case fatality. All cases are assumed to be severe.

We modeled rabies mortality using a two-model hybrid approach 1) a global CODEm model of all locations, using all data in the CoD database; and 2) a CODEm model restricted to data-rich countries. We have made two substantive changes in the modeling strategy from GBD 2013. First, we have changed from a single global model to the hybrid global/data rich model approach. Second, we conducted an exploratory analysis to determine the most predictive covariates for rabies and have updated the covariates used in the CODEm model accordingly.

Sequela description and DW

Sequela	Description	Disability Weight (95% CI)
Severe	Has a high fever and pain, and feels very weak, which causes great difficulty with daily activities.	0.133 (0.088-0.19)

Changes from GBD 2013 to GBD 2015

We have made no substantive changes in the modeling strategy from GBD 2013.

Schistosomiasis SDG Capstone Appendix

Flowchart

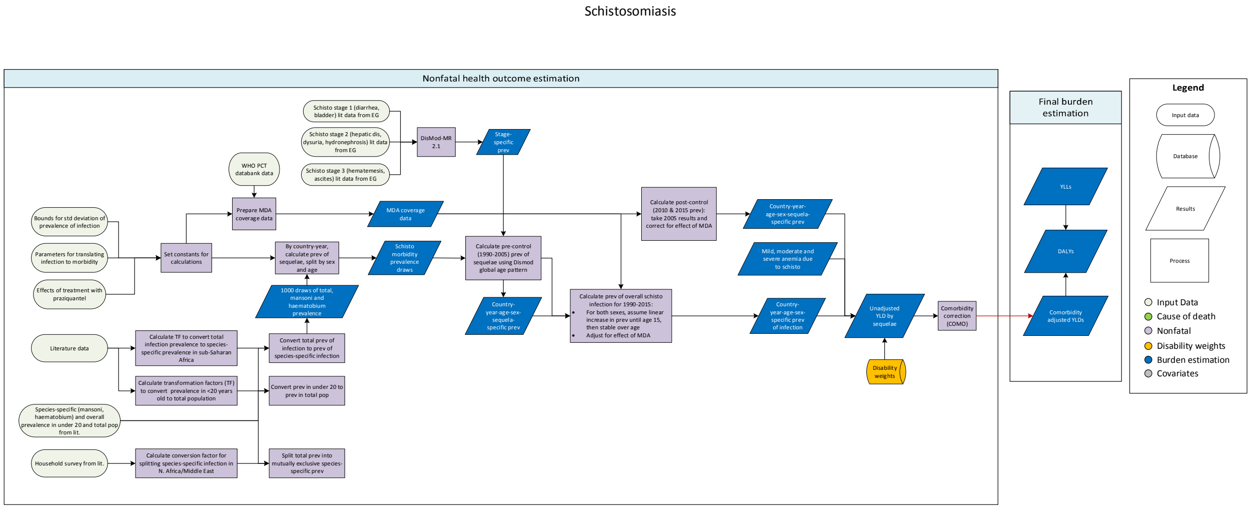

Input Data & Methodological Summary

Case definition

Schistosomiasis, also known as bilharzia or "snail fever," is a helminth disease caused by infection with five species of the parasite *Schistosoma* namely, *S. mansoni, S. japonicum, S. haematobium, S. mekongi, and S. intercalatuma*. The first three species cause the most infection and the last two rarely cause disease. Diagnosis is made by microscopic exam of stool or urine for parasite eggs. For less advanced infections, serologic techniques are used. The ICD-10 codes for schistosomiasis are B565-B65.9.

Input data

Model inputs

To model nonfatal outcomes due to schistosomiasis, data prepared by the expert group (EG) during GBD 2010 was used. To prepare the data, they compiled most recently published estimates of species-specific infection prevalence calculated using Bayesian geo-statistical models based on environmental predictors. Details of materials and methods used by the EG are referenced elsewhere [1-6]. Using citations compiled by the EG, a list of eight possible clinical sequelae and anemia sequelae were defined (mild infection, mild diarrhea, hematemesis (vomiting blood), hepatomegaly, ascites (build-up of fluid in the peritoneal cavity), dysuria (painful urination), bladder pathology, hydronephrosis (swelling of kidney due to build-up of urine in the kidney), mild anemia, moderate anemia, severe anemia).

Stage-specific prevalence data produced by the EG was also used as inputs for three separate single-parameter models in DisMod (stage 1: acute symptoms - diarrhea, active/mild schisto; stage 2: semi-acute/chronic symptoms - hepatomegaly, dysuria, and hydronephrosis; stage 3: long term chronic disease - haematemesis, ascites, and bladder pathology). The table below shows (by stage) the number

of literature studies included in GBD 2015, as well as the number of countries or subnational units and GBD world regions represented.

	Stage 1	Stage 2	Stage 3
Studies	76	40	10
Countries/subnationals	34	20	7
GBD world regions	8	8	5

Literature data on prevalence of infection by species (mansoni and haematobium) in under 20 and in the total population was also used [3-7] – for Brazil, this data was split into subnational using data from the Schostosomiasis Control Program (PCE) [8]. The table below shows the number of sources included, and the number of countries or subnational units and GBD world regions represented.

	prevalence
Sources	5
Countries/subnationals	94
GBD world regions	10

Additional literature sources were used to inform morbidity prevalence estimation. These are highlighted in modeling strategy section of this document.

We did not update the literature review for these data in GBD 2015. Updates to systematic reviews are performed on an ongoing schedule across all GBD causes, an update for schistosomiasis will be performed in the next 1-2 iterations.

Severity splits/Sequelae

The table below shows the list of clinical sequelae [8] (including mild, moderate, and severe anemia) due to schistosomiasis, their lay descriptions, and the associated disease stage and disability weights.

Clinical sequela	Lay description	Disease stage	Disability weights
Mild infection	has a low fever and mild discomfort , but no difficulty with daily activities	1	0.006 (0.002-0.012)
Mild diarrhea		1	0.056
Hepatomegaly	has some pain in the belly that causes nausea but does not interfere with daily activities	2	0.011 (0.005-0.021)
Dysuria	has some pain in the belly that causes nausea but does not interfere with daily activities	2	0.011 (0.005-0.021)
Hydronephrosis	has some pain in the belly that causes nausea but does not interfere with daily activities	2	0.011 (0.005-0.021)
Haematemesis	vomits blood and feels nauseous	3	0.325 (0.209-0.463)
Ascites	has pain in the belly and feels nauseous. The person has difficulties with daily activities	3	0.114 (0.078-0.159)

Bladder pathology	has some pain in the belly that causes nausea but does not interfere with daily activities	3	0.011 (0.005-0.021)
Mild anemia	feels slightly tired and weak at times, but this does not interfere with normal daily activities	NA	0.004 (0.001-0.008)
Moderate anemia	feels moderate fatigue, weakness, and shortness of breath after exercise, making daily activities more difficult	NA	0.052 (0.034-0.076)
Severe anemia	feels very weak, tired and short of breath, and has problems with activities that require physical effort or deep concentration	NA	0.149 (0.101-0.210)

Modeling strategy

The morbidity model for schistosomiasis involved six main steps. First, constants were set for calculations on 1) bounds for standard deviation of dispersion of logit prevalence of infection within countries (low=0.6, high=0.8), 2) parameters (a, b, c) for translating infection (x) to morbidity (y): y = (a + bx^c)/(1 + bx^c) – a [9-11], and 3) effects (mean, low, high) of treatment with praziquantel (PZQ) [12, 13]. Next, the conversion factor for splitting species-specific (mansoni, haematobium) infection in North Africa and Middle East was calculated, informed by literature data [14]. In the third step, literature data [4, 5] was used to calculate transformation factors for converting 1) prevalence in individuals under 20 years of age to total population, and 2) total infection prevalence to species-specific prevalence in sub-Saharan Africa.

Next, infection data was prepared by 1) splitting total prevalence in North Africa/Middle East into mutually exclusive species-specific prevalence, 2) converting prevalence in under 20 to prevalence in the total population, and 3) converting total prevalence of infection in sub-Saharan Africa to prevalence of species-specific infection. To generate 1,000 draws of prevalence, the prevalence estimates were scaled using values drawn from a standard normal distribution. Resulting estimates were split by age and sex, based on general pattern (infection: linear increase until age 15, then stable) or DisMod global age pattern for the three different stages of disease, each with a different pattern. All stage-specific DisMod models were run as proportion models and included year as a country-level covariate.

The final step was the prediction of post-control prevalence of infection and morbidity, adjusted for the impact of treatment, given cumulative number of PCT treatments per person at risk (draws of expected reductions in overall infection prevalence were obtained using data from the WHO PCT Databank [15]) and estimated species-specific efficacy, as reported in literature. For reversible sequelae, it was assumed that treatment effects are the same as for infection. For irreversible symptoms (advanced hepatic disease, ascites, hematemesis), it was assumed that incidence among the treated fraction of the population is zero and 10% of individuals with these conditions die each year due their schisto-related sequelae. The burden of anemia due to schistosomiasis was estimated (see anemia documentation for details).

Model evaluation was done by separately assessing the fit of the three stage-specific DisMod models and checking the final estimates produced after age-sex splits. Plots of time trends of prevalence across locations, and age were used to evaluate the results. In addition, maps of the global distribution of total schistosomiasis prevalence and prevalence of sequelae due to schistosomiasis were also assessed across time.

The main change made from GBD 2013 was splitting Brazil national data into subnational using data from the Schistosomiasis Control program [8]. In addition, newly updated data from the WHO PCT databank was downloaded and used in the model.

References:

1. Clements AC, Garba A, Sacko M, et al. Mapping the probability of schistosomiasis and associated uncertainty, West Africa. Emerg Infect Dis. 2008;14(10):1629-32
2. Center Clements AC, Moyeed R, Brooker S. Bayesian geostatistical prediction of the intensityof infection with Schistosoma mansoni in East Africa. Parasitology. 2006;133(Pt 6):711-9
3. Chitsulo L, Engels D, Montresor A, Savioli L. The global status of schistosomiasis and its control. Acta Trop. 2000;77(1):41-51
4. Schur N, Hurlimann E, Stensgaard AS, Chimfwembe K, Mushinge G, Simoonga C, Kabatereine NB, Kristensen TK, Utzinger J, Vounatsou P. Spatially explicit Schistosoma infection risk in eastern Africa using Bayesian geostatistical modelling. Acta Trop. (2011)
5. Schur N, Hurlimann E, Garba A, Traore MS, Ndir O, Ratard RC, Tchuente L-AT, Kristensen TK, Utzinger J, Vounatsou P. Geostatistical Model-Based Estimates of schistosomiasis Prevalence among Individuals Aged <20 Years in West Africa. PLoS Negl Trop Dis 2011;5(6): e1194
6. Steinmann P, Keiser J, Bos R, Tanner M, Utzinger J. schistosomiasis and water resources development: systematic review, meta-analysis, and estimates of people at risk. Lancet Infect Dis. 2006;6(7):411-25
7. Ministry of Health (China). China Schistosomiasis Prevalence National Sample Survey 2004
8. Secretariat of Health Surveillance, Ministry of Health (Brazil). Brazil DATASUS TABNET - Schistosomiasis Control Program (PCE)
9. van der Werf MJ, de Vlas SJ, Brooker S, et al. Quantification of clinical morbidity associated with schistosome infection in sub-Saharan Africa. Acta Trop. 2003;86(2-3):125-39
10. van der Werf MJ, de Vlas SJ, Looman CW, Nagelkerke NJ, Habbema JD, Engels D. Associating community prevalence of Schistosoma mansoni infection with prevalence of signs and symptoms. Acta Trop. 2002;82(2):127-37
11. van der Werf MJ, de Vlas SJ. Diagnosis of urinary schistosomiasis: A novel approach to compare bladder pathology measured by ultrasound and three methods for hematuria detection. Am. J. Trop. Med. Hyg. 2004;82:98-106
12. Danso-Appiah A, Utzinger J, Liu J, Olliaro P. Drugs for treating urinaty schistosomiasis (Review). The Cochrane Library. 2008(3); 1-72
13. Danso-Appiah A, Utzinger J, Liu J, Olliaro P. Drugs for treating urinaty schistosomiasis (Review). The Cochrane Library. 2013(2); 1-177
14. El-Khoby T, Galal N, Fenwick A, et al. The epidemiology of schistosomiasis in Egypt: summary findings in nine governorates. Am. J. Trop. Med. Hyg. 2000; 62: 88–99
15. WHO PCT Databank. 2015; http://www.who.int/neglected_diseases/preventive_chemotherapy/sch/en/

Non-communicable Diseases (NCDs) SDG Capstone Appendix

cardiovascular diseases, cancer except for liver cancer and non-melanom skin cancer, liver cancer, rheumatic heart disease, ischemic heart disease, cerebrovascular disease, ischemic stroke, hemorrhagic stroke, hypertensive heart disease, cardiomyopathy and myocarditis, other cardiovascular and circulatory diseases, chronic respiratory diseases, other chronic respiratory diseases, diabetes mellitus

Indicator definition

This modeling strategy encompasses the indicators associated with non-communicable disease mortality (3.3.5).

Indicator 3.4.1

As a component of SDG Goal 3. Ensure healthy lives and promote well-being for all at all ages, SDG Target 3.4, by 2030, reduce by one third premature mortality from NCDs through prevention and treatment and promote mental health and well-being, is measured using SDG Indicator 3.4.1, deaths due to cardiovascular disease, cancer, diabetes, and chronic respiratory disease among populations aged 30 to 70 per 100,000.

Cancers SDG Capstone Appendix

Input data and methodological summary for all cancers except for liver cancer and non-melanoma skin cancer

Flowchart

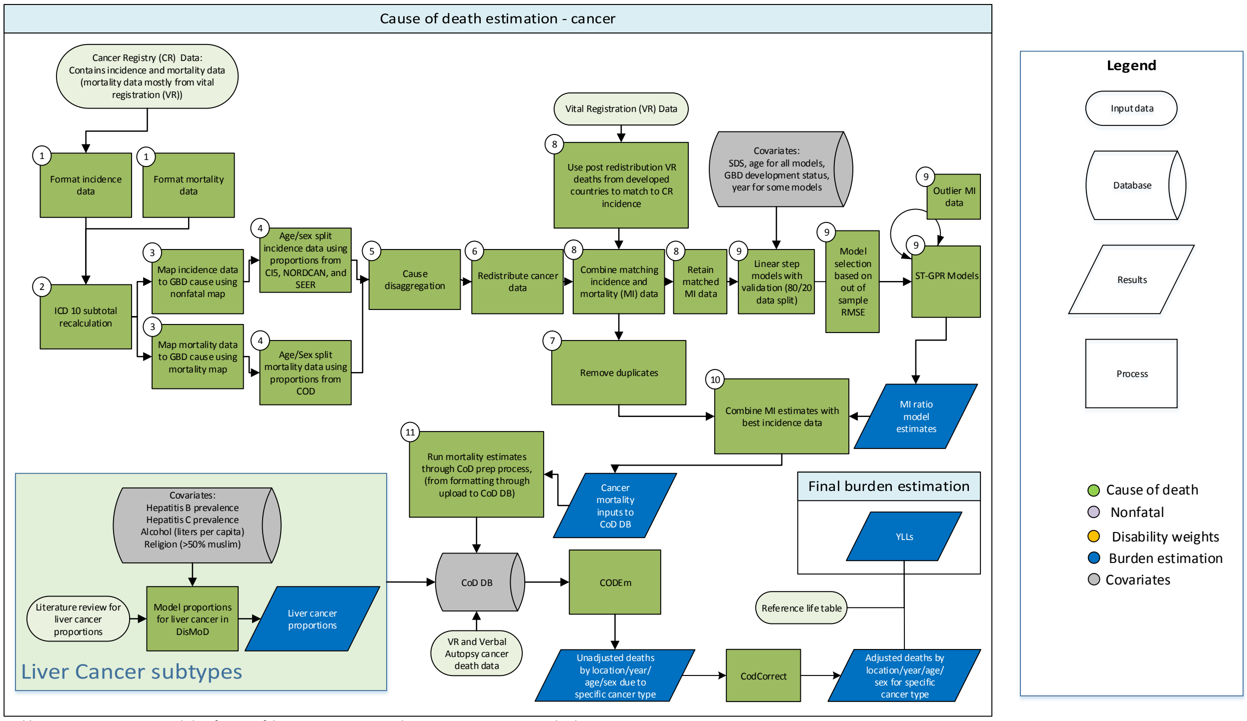

Abbreviations: ICD: International classification of diseases; CI5: Cancer Incidence in Five Continents, DB: database

Input Data & Methodological Summary

Input Data

The Cause of Death (COD) database contains multiple sources of cancer mortality data. These sources include vital registration, verbal autopsy, and cancer registry data. The cancer registry mortality estimates that are uploaded into the COD database stem from cancer registry incidence data that have been transformed to mortality estimates through the use of mortality-to-incidence (MI) ratios.

Data seeking processes

Cancer mortality data in the cause of death database other than cancer registry data

Sources for cancer mortality data other than cancer registry data are described in the COD database description (Part 2).

Cancer registry data

All cancer registry data used for GBD 2010 were also included for GBD 2013, and the majority of these data were also used for GBD 2015 unless superseded by newer data (see step 7 in flow chart and

below). Most new data were added based on availability and collaborator recommendation. Some new data were acquired and approved for GBD 2013 but were received after the deadline for adding new data to GBD 2013. More than half (56%) of the final incidence data and 35% of the final MI model input data came from the Cancer Incidence in Five Continents series (CI5).[1–10]

Cancer registry data were most often downloaded from a publicly available webpage or provided by collaborators. Most cancer registries only report cancer incidence. However, if a cancer registry also reported cancer mortality, mortality data were also extracted from the source to be used in the mortality-to-incidence estimation.

Inclusion and exclusion criteria

Only population-based cancer registries were included, and only those that included all cancers (no specialty registries), data for all age groups, and data for both sexes. Pathology-based cancer registries were included if they had a defined population. Hospital-based cancer registries were not included.

Cancer registry data were excluded from either the final incidence data input or the MI model input if a more detailed source (e.g., providing more detailed age or diagnostic groups) was available for the same population. Preference was given to registries with national coverage over those with only local coverage, except those from countries where the GBD study provides subnational estimates; thus some data were excluded because newly acquired national registry data could replace a regionally representative predecessor.

Data were excluded from the final incidence data input if the coverage population was unknown.

Bias of categories of input data

Cancer registry data can be biased in multiple ways. A high proportion of ill-defined cancer cases in the registry data requires redistribution of these cases to other cancers, which introduces a potential for bias. Changes between coding systems can lead to artificial differences in disease estimates; however, we adjust for this bias by mapping the different coding systems to the GBD causes. Underreporting of cancers that require advanced diagnostic techniques (e.g., leukemia and brain, pancreatic, and liver cancer) can be an issue in cancer registries from low-income countries. On the other hand, misclassification of metastatic sites as primary cancer can lead to overestimation of cancer sites that are common sites for metastases like brain or liver. Since many cancer registries are located in urban areas, the representativeness of the registry for the general population can also be problematic. The accuracy of mortality data reported in cancer registries usually depends on the quality of the vital registration system. If the vital registration system is incomplete or of poor quality, the mortality-to-incidence ratio can be biased to lower ratios.

Methods

Steps of analysis and data transformation processes

Cancer registry data went through multiple processing steps before integration with the COD database. First, the original data were transformed into standardized files, which included standardization of format, categorization, and registry names (#1 in flowchart).

Second, some cancer registries report individual codes as well as aggregated totals [e.g., C18, C19, and C20 are reported individually but the aggregated group of C18-C20 (colorectal cancer) is also reported in the registry data]. The data processing step “subtotal recalculation” (#2 in flowchart) verifies these totals and subtracts the values of any individual codes from the aggregates.

In the third step (#3 in the flowchart), cancer registry incidence data and cancer registry mortality data are mapped to GBD causes. A different map is used for incidence and for mortality data because of the assumption that there are no deaths for certain cancers. One example is basal cell carcinoma of the skin. In the cancer registry incidence data, basal cell carcinoma is mapped to non-melanoma skin cancer (basal cell carcinoma). However, if basal cell skin cancer is recorded in the cancer registry mortality data, the deaths are instead mapped to non-melanoma skin cancer (squamous cell carcinoma) under the assumption that they were indeed misclassified squamous cell skin cancers. Other examples are benign or in situ neoplasms. Benign or in situ neoplasms found in the cancer registry incidence dataset were simply dropped from that dataset. The same neoplasms reported in a cancer registry mortality dataset were mapped to the respective invasive cancer (e.g., melanoma in situ in the cancer registry incidence dataset was dropped from the dataset; melanoma in situ in the cancer registry mortality dataset was mapped to melanoma).

In the fourth data processing step (#4 in the flowchart) cancer registry data were standardized to the GBD age groups. Age-specific incidence rates were generated using CI5, SEER, and NORDCAN data, while age-specific mortality rates were generated from the CoD data through a method described in Part 2. Age-specific weights were then generated by applying the age-specific rates to a given registry population that required age-splitting to produce the expected number of cases/deaths for that registry by age. The expected number of cases/deaths for each sex, age, and cancer were then normalized to 1, creating final, age-specific proportions. These proportions were then applied to the total number of cases/deaths by sex and cancer to get the age-specific number of cases/deaths.

In the rare case that the cancer registry only contained data for both sexes combined, the now-age-specific cases/deaths were split and re-assigned to separate sexes using the same weights that are used for the age-splitting process. Starting from the expected number of deaths, proportions were generated by sex for each age (e.g., if for ages 15 to 19 years old there are six expected deaths for males and four expected deaths for females, then 60% of the combined-sex deaths for ages 15-19 years would be assigned to males and the remaining 40% would be assigned to females).

In the fifth step (#5 in the flowchart) data for cause entries that are aggregates of GBD causes were redistributed. Examples of these aggregated causes include some registries reporting ICD10 codes C00-C14 together as, “lip, oral cavity, and pharyngeal cancer.” These groups were broken down into sub-causes that could be mapped to single GBD causes. In this example, those include lip and oral cavity cancer (C00-C08), nasopharyngeal cancer (C11), cancer of other parts of the pharynx (C09-C10, C12-C13), and “Malignant neoplasm of other and ill-defined sites in the lip, oral cavity, and pharynx” (C14). To redistribute the data, weights were created using the same “rate-applied-to-population” method employed in age-sex splitting (see step four above). For the undefined code (C14 in the example) an “average all cancer” weight was used, which was generated by adding all cases from SEER/NORDCAN/CI5 and dividing the total by the combined population. Then, proportions were

generated by sub-cause for each aggregate cause as in the sex-splitting example above (see step four). The total number of cases from the aggregated group (C00-C14) was then recalculated for each subgroup and the undefined code (C14). C14 was then redistributed as a "garbage code" in step six. Distinct proportions were used for C44 (non-melanoma skin cancer) and C46 (Kaposi's sarcoma). Population data were not used to redistribute data for these ICD codes. Non-melanoma skin cancer processing is described under section "Input data and methodological summary for non-melanoma skin cancer (squamous-cell carcinoma)." C46 entries were redistributed as "other cancer," HIV, and C80 (other and unknown cancers) using proportions described in Part 2.

In the sixth step (#6 in the flowchart) unspecified codes ("garbage codes") were redistributed. Redistribution of cancer registry incidence and mortality data mirrored the process of the redistribution used in the cause of death database (Part 2).

In the seventh step (#7 in the flowchart) duplicate or redundant sources were removed from the processed cancer registry dataset. Duplicate sources were present if, for example, the cancer registry was part of the CI5 dataset but we also had data from the registry directly. Redundancies occurred and were removed as described in "Inclusion and Exclusion Criteria," where more detailed data were available, or when national registry data could replace regionally representative data. From here, two parallel selection processes were run to generate input data for the MI models and to generate incidence for final mortality estimation. Higher priority was given to registry data from the most standardized source when creating the final incidence input (generally CI5 data), whereas preference was given to registry data from sources with matching mortality and incidence for the MI model input (in order to reduce confounding due to oppositional input biases when matching the two data types).

In the eighth step (#8 in the flowchart) the processed incidence and mortality data from cancer registries were matched by cancer, age, sex, year, and location to generate MI ratios. Because some cancer registries do not report mortality data – even though high-quality vital registration system data are available to the registry's coverage area – processed vital registration mortality data from the CoD database were matched to the registry's incidence data for some countries. This was the case for certain registries in the following countries: Australia, Austria, Belgium, Bulgaria, Denmark, Estonia, Finland, Hungary, Iceland, Ireland, New Zealand, Norway, South Korea, and Switzerland.

The ninth step involved creating and selecting the MI models. All models were run separately by cancer, and the best model was selected from the following list (see Table below).

1. $\text{logit}\left(MI\ ratio_{c,a,s,t}\right) = \alpha + \beta_1 SDS_{c,t} + \sum_a^A \beta_2 I_a + \beta_3 I_s + \theta_c + \epsilon_{c,a,s,t}$
2. $\text{logit}\left(MI\ ratio_{c,a,s,t}\right) = \alpha + \beta_1 SDS_{c,t} + \sum_a^A \beta_2 I_a + \beta_3 I_s + \beta_4 t + \theta_c + \epsilon_{c,a,s,t}$
3. $\text{logit}\left(MI\ ratio_{c,a,s,t}\right) = \alpha + \beta_1 SDS_{c,t} + \sum_a^A \beta_2 I_a + \beta_3 I_s + \beta_4 DS + \theta_c + \epsilon_{c,a,s,t}$
4. $\text{logit}\left(MI\ ratio_{c,a,s,t}\right) = \alpha + \beta_1 SDS_{c,t} + \sum_a^A \beta_2 I_a + \beta_3 I_s + \beta_4 DS + \beta_5 t + \theta_c + \epsilon_{c,a,s,t}$
5. $\text{logit}\left(MI\ ratio_{c,a,s,t}\right) = \alpha + \beta_1 SDS_{c,t} + \sum_a^A \beta_2 I_a + \beta_3 I_s + \theta_c + \lambda_{SR}(SDS_{c,t}) + \beta_4 t + \epsilon_{c,a,s,t}$
6. $\text{logit}\left(MI\ ratio_{c,a,s,t}\right) = \alpha + \beta_1 SDS_{c,t} + \sum_a^A \beta_2 I_a + \beta_3 I_s + \theta_c + \lambda_{SR}(SDS_{c,t}) + \epsilon_{c,a,s,t}$
7. $\text{logit}\left(MI\ ratio_{c,a,s,t}\right) = \alpha + \beta_1 SDS_{c,t} + \sum_a^A \beta_2 I_a + \beta_3 I_s + \theta_c + \lambda_{SR}(SDS_{c,t}) + \beta_4 DS + \epsilon_{c,a,s,t}$
8. $\text{logit}\left(MI\ ratio_{c,a,s,t}\right) = \alpha + \beta_1 SDS_{c,t} + \sum_a^A \beta_2 I_a + \beta_3 I_s + \theta_c + \lambda_{SR}(SDS_{c,t}) +$
   $\beta_4 t + \beta_5 DS + \epsilon_{c,a,s,t}$

c: country; a: age group; t: time (years); s: sex
I: indicator variable
DS: binary variable for development status
θ_c: random effect by country (intercept)
$\lambda_{SR}(SDSc, t)$: random effect modifier between SDI and super-region (slope)
$\epsilon_{c,a,s,t}$: error term

Table: MI models

All models were tested at multiple stages before creating the final model output. Models were initiated with an SDI covariate (Socio-Demographic Index) and first tested using the complete input dataset (Part 4). If after that initial test the SDI covariate's coefficient was negative (as expected), the next step was to outlier any data point for which the residual from the prediction was greater than three times the MAD from the mean residual. Next, data were marked as outliers due to a random effect criterion: if the country-level random effect for a lower-income country was lower than the random effect for the USA, all data points for that country were marked as outliers. This process was run iteratively until all lower-income countries had country-level random effects greater than that of the USA. All data points marked outliers were dropped from the final dataset, and that dataset was used to create the final model predictions.

If the SDI coefficient was found to be positive (unexpected) after the initial SDI test, it was assumed to indicate an excess of unrealistic data in the input dataset. To remove these unrealistic data, SDI was temporarily removed from the model formula. The model proceeded as above without SDI until all unrealistic data points were removed and the SDI coefficient was found to be negative. Unrealistic data were marked as outliers using the same residual MAD and random effect methodology described above. Once SDI was established as negative (expected) the model proceeded as usual.

To select the best model formula, the initial model results were tested by comparing mean MI predictions and the mean root-mean-squared error (RMSE) values of 10 random samples of 80%/20% splits from the input dataset. Mean MI predictions were compared between developing and developed countries. Models were eliminated if the mean MI for developing countries was lower than the mean MI ratio for developed countries. For RMSE testing, the dataset was split into an 80% dataset for model development and a 20% dataset for model testing. The process was repeated 10 times. The best model for each cancer was selected based on the lowest mean out-of-sample RMSE from those models remaining after checking the mean MI. The table below contains the final models selected for each cancer.

**Cancer**	**Final model number (see numbering above)**
Ovarian cancer	1
Uterine cancer	1
Gallbladder cancer	1
Kidney cancer	1

Larynx cancer	1
Acute lymphoid leukemia	1
Chronic myeloid leukemia	1
Lip and oral cavity cancer	1
Pancreatic cancer	1
Hodgkin lymphoma	2
Acute myeloid leukemia	2
Chronic lymphoid leukemia	2
Malignant skin melanoma	2
Bladder cancer	3
Brain and nervous system cancer	3
Esophageal cancer	3
Tracheal, bronchus, and lung cancer	3
Mesothelioma	3
Multiple myeloma	3
Other cancer	3
Prostate cancer	4
Testicular cancer	4
Breast cancer	4
Colorectal cancer	4
Leukemia	4
Liver cancer	4
Non-Hodgkin lymphoma	4
Non-melanoma skin cancer (squamous cell carcinoma)	4
Stomach cancer	4
Nasopharynx cancer	6
Cervical cancer	7
Other pharynx cancer	8
Thyroid cancer	8

Table: Final model selections

Once the best models were selected, data points were manually outliered based on the results of the first run of the model algorithm. Data points were outliered if they clearly influenced the model in an unrealistic way. For example, a data point was marked as an outlier if it created a single-year, single-age-group spike in model predictions. This was mainly the case in countries with a small number of cases or deaths, or in age groups with small numbers of cases or deaths. Manual outliers were removed from the input dataset prior to initiating the second run of the model algorithm.

After best models were selected, all final outliers were dropped from the data input, and final linear predictions were created, the final linear predictions and residuals were used as input for space-time smoothing. Space-time smoothing is a spatiotemporal regression to smooth residuals over space, time, and age.[11–13] The weighted residuals were added to the linear model predictions and used as priors for

the third stage, a Gaussian process regression (GPR) implementing a Matern covariance function.[13–18] GPR is a nonparametric technique for interpolating non-linear trends that has been used extensively in the estimation of time series data. Final MI ratio predictions with 95% uncertainty intervals were obtained by back-transforming 1,000 draws from the posterior distribution.

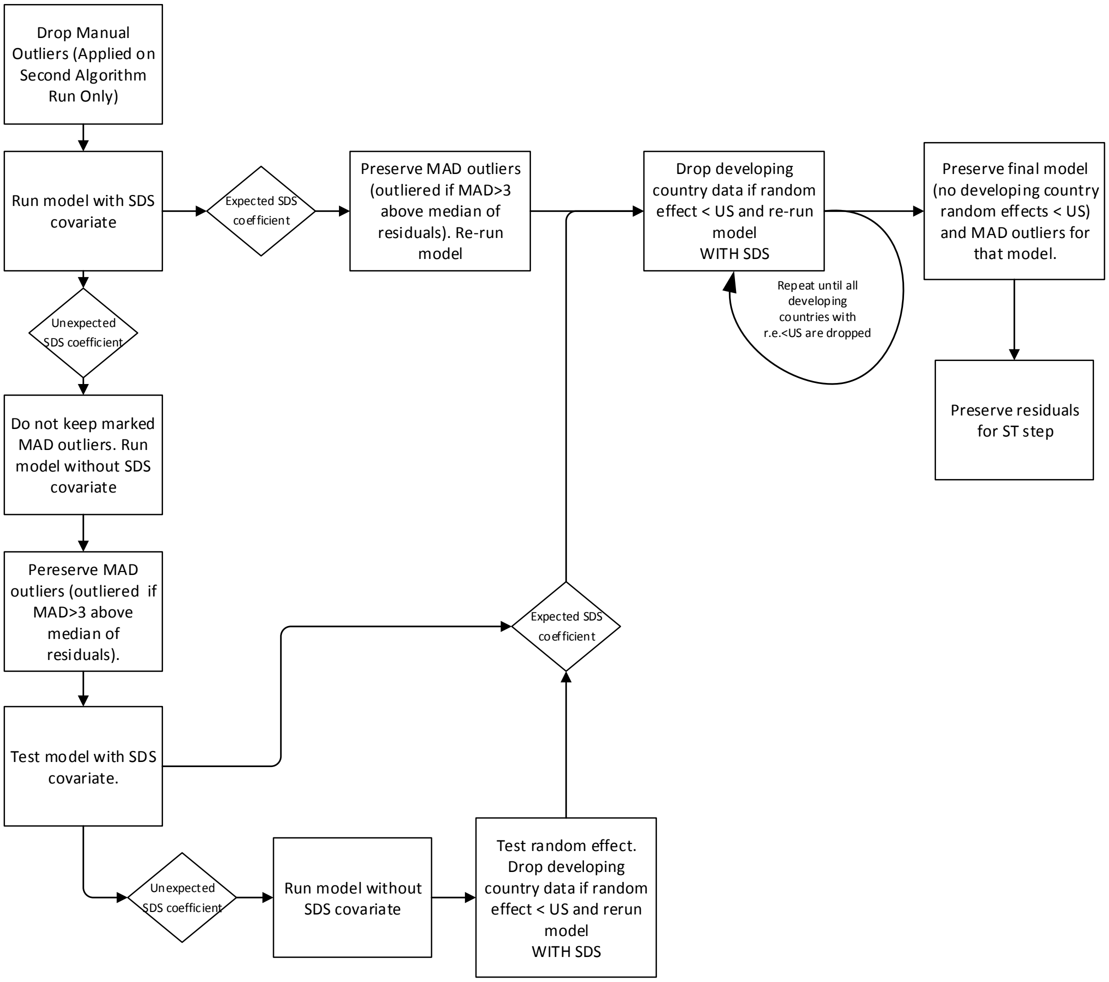

Figure 1: MI model estimation algorithm

Step 9 has undergone a revision compared to GBD 2010 and GBD 2013. In GBD 2010 and GBD 2013 only one model was used to predict all MI ratios, whereas for GBD 2015 we generated multiple models and chose a best model based on out-of-sample validation. Another major difference is that LDI (lagged distributed income) was used as a covariate in previous versions and was replaced by SDI for GBD 2015.

Final MI ratios were matched with the cancer registry incidence dataset in the ninth step (#10 in the flowchart) to generate mortality estimates (Incidence * Mortality/Incidence = Mortality). The final mortality estimates were then uploaded into the COD database (#11 in the flowchart).

After transforming cancer registry incidence data to mortality estimates, the modeling strategy followed the general CODEm process as described in Part 3.

Results

Interpretation of results

Cancer mortality estimates for GBD 2015 can differ from the GBD 2013 results for multiple reasons. First, compared to GBD 2013 more cancer mortality data were added to the cause of death database. Second, we added sources for cancer registry data, which were transformed into mortality estimates by using the MI ratio. Third, mapping of cancer ICD codes to the GBD cancer causes was updated slightly based on collaborator comments. One example is that mapping for the ICD10 code D46 (myelodysplastic syndrome) was changed from “other cancer” to “undefined cancer” for later redistribution to non-Hodgkin lymphoma and leukemia. The one major mapping change was the addition of subtypes for leukemia and non-melanoma skin cancer. Fourth, the method to redistribute undefined causes of death or undefined cancers changed compared to GBD 2013. Models for redistribution are now performed regionally rather than by super-region. Fifth, we updated and refined the mortality-to-incidence ratio estimation compared to GBD 2013. Whereas for GBD 2010 and GBD 2013 a single model was used to estimate the MI ratios for each location, by cancer, sex, and age, we developed multiple plausible models for GBD 2015 and chose the best model based on out-of-sample validation. Sixth, we reviewed the covariate inputs for the CODEm models and changed covariates when updated or improved covariates were available. Seventh, many covariates used in CODEm models were updated for GBD 2015 (Part 4).

The other group producing country-level cancer mortality estimates is the International Agency for Research on Cancer (IARC) with their GLOBOCAN database. Significantly different methods between the GBD study and GLOBOCAN can lead to differences in results. Whereas estimates in GLOBOCAN are based on the assumption that there are “In theory, […] as many methods as countries,”[19] the cancer estimation process for the GBD study follows a coherent, well-documented method for all cancers, which allows cross-validation of models as well as determination of uncertainty. Another major difference is the ability in the GBD study to adjust single cause estimates to the all-cause mortality, which is being determined independently. This also allows us to adjust individual causes of death to the all-cause mortality envelope which permits us to correct for the underdiagnosis of cancer in countries with inadequate diagnostic resources. Redistribution of a fraction of undefined causes of death to certain cancers is another methodical advantage the GBD study has over GLOBOCAN, and estimates for cancer mortality can therefore differ substantially in countries with a large proportion of undefined causes of deaths in their vital registration data or a large proportion of undefined cancer cases in their cancer registry data.

Limitations

There are certain limitations to consider when interpreting the GBD mortality cancer estimates. First, even though every effort is made to include the most recently available data for each country, data-seeking resources are not limitless and new data cannot always be accessed as soon as they are made available. It is therefore possible that the GBD study does not include all available data sources for cancer incidence or cancer mortality. Second, different redistribution methods can potentially change the cancer estimates substantially if the data sources used for the estimated location contain a large number of undefined causes; however, neglecting to account for these undefined deaths would likely

introduce an even greater bias in the disease estimates. Third, using mortality-to-incidence ratios to transform cancer registry incidence data to mortality estimates requires accurate MI ratios. For GBD 2015 the methodology to estimate MI ratios was improved with development of multiple different models and implementation of model cross-validation, but the method is still sensitive to underdiagnosis of cancer cases or underascertainment of cancer deaths. However, given that the majority of data used for the cancer mortality estimation come from vital registration data and not cancer registry data this is not a major limitation.

Non-melanoma skin cancer (squamous-cell carcinoma)

Data

Data seeking processes
The input data were identified and processed using the same methods as all other cancers described above.

Inclusion and exclusion criteria
Inclusion and exclusion criteria followed the same methods as described for other cancers (see above).

Bias of categories of input data
The potential biases of the input data are the same as for other cancers (see above).

Methods

Overall methodological process
The GBD produces estimates for non-melanoma skin cancer via two subgroups: non-melanoma skin cancer (basal cell carcinoma) and non-melanoma skin cancer (squamous cell carcinoma). While some cancer registries report non-melanoma skin cancer at the four- or five- digit level required to distinguish between the subtypes (e.g. "C44.01" vs. "C44.02", "173.01" vs. "173.02), most registries report these cancers at the three-digit level as "C44" or "173" ("Other and unspecified malignant neoplasm of skin"). Because of this, those incident cases that were reported at this three-digit level were split to "basal cell carcinoma" and "squamous cell carcinoma" based on proportions reported by Karagas et al during the cause disaggregation step (step #5 in the flowchart).[20] Since mortality estimates are produced for squamous cell carcinoma under the assumption that basal cell carcinoma causes almost no deaths, all mortalities reported as "C44" or "173" were simply mapped to the "squamous cell carcinoma" GBD cause. Apart from this additional step for some incident cases, the remainder of the cancer registry processing was the same as for other cancers as described above.

Steps of analysis and data transformation processes
Non-melanoma skin cancer (squamous cell carcinoma) mortality estimation followed the same steps as the other cancers (see flowchart and description above) except for step #5 in the flowchart as described above.

Model selection
The modeling strategy for non-melanoma skin cancer (squamous cell carcinoma) followed the general CODEm process.

Model performance and sensitivity

The modeling performance and sensitivity for non-melanoma skin cancer (squamous cell carcinoma) mirrored that of the general CODEm process.

Uncertainty intervals

Uncertainty was determined using standard CODEm methodology.

Results

Interpretation of results

Non-melanoma skin cancer mortality estimates are not available from other sources. GLOBOCAN, for example, does not report deaths due to non-melanoma skin cancer. Even though the data availability for non-melanoma skin cancer is poor, the fact that it is the most common incident cancer with rates expected to rise makes it a necessity to include the disease in the GBD framework.

[4–13]Limitations

Cancer registry data for non-melanoma skin cancer incidence have to be interpreted with caution due to a substantial amount of underreporting or rules that only the first non-melanoma skin cancer has to be registered. Many cancer registries therefore do not include non-melanoma skin cancers at all. For vital registration data we make the assumption that there are no deaths due to non-melanoma skin cancer (basal cell carcinoma), therefore all deaths attributed to basal cell carcinoma were included instead as squamous cell carcinoma. Based on collaborator recommendations we will test this assumption formally for GBD 2016.

References

1 Cancer Incidence in Five Continents.Vol I. Geneva, Switzerland: Union Internationale Contre le Cancer, 1966.

2 Cancer Incidence in Five Continents.Vol II. Geneva, Switzerland: Union Internationale Contre le Cancer, 1970.

3 Cancer Incidence in Five Continents.Vol III. Lyon, France: IARC, 1976.

4 Cancer Incidence in Five Continents.Vol IV. Lyon, France: IARC, 1982.

5 Cancer Incidence in Five Continents.Vol V. Lyon, France: IARC, 1987.

6 Cancer Incidence in Five Continents.Vol VI. Lyon, France: IARC, 1992.

7 Cancer Incidence in Five Continents.Vol VII. Lyon, France: IARC, 1997.

8 Cancer Incidence in Five Continents.Vol VIII. Lyon, France: IARC, 2002.

9 Cancer Incidence in Five Continents.Vol IX. Lyon, France: IARC, 2007.

10 Cancer Incidence in Five Continents.Vol X. Lyon, France: IARC, 2013.

11 Wang H, Liddell CA, Coates MM, *et al.* Global, regional, and national levels of neonatal, infant, and under-5 mortality during 1990-2013: a systematic analysis for the Global Burden of Disease Study 2013. *Lancet* 2014; **384**: 957–79.

12 Forouzanfar MH, Foreman KJ, Delossantos AM, *et al.* Breast and cervical cancer in 187 countries between 1980 and 2010: a systematic analysis. *Lancet* 2011; **378**: 1461–84.

13 Foreman KJ, Lozano R, Lopez AD, Murray CJ. Modeling causes of death: an integrated approach using CODEm. *Popul Health Metr* 2012; **10**: 1.

14 Murray CJL, Rosenfeld LC, Lim SS, *et al.* Global malaria mortality between 1980 and 2010: a systematic analysis. *Lancet* 2012; **379**: 413–31.

15 GBD 2013 Mortality and Causes of Death Collaborators. Global, regional, and national age-sex specific all-cause and cause-specific mortality for 240 causes of death, 1990-2013: a systematic analysis for the Global Burden of Disease Study 2013. *Lancet* 2015; **385**: 117–71.

16 Murray CJL, Ortblad KF, Guinovart C, *et al.* Global, regional, and national incidence and mortality for HIV, tuberculosis, and malaria during 1990-2013: a systematic analysis for the Global Burden of Disease Study 2013. *Lancet* 2014; **384**: 1005–70.

17 Rajaratnam JK, Marcus JR, Flaxman AD, *et al.* Neonatal, postneonatal, childhood, and under-5 mortality for 187 countries, 1970-2010: a systematic analysis of progress towards Millennium Development Goal 4. *Lancet* 2010; **375**: 1988–2008.

18 Rasmussen CE, Williams CKI. Gaussian processes for machine learning. Cambridge, Mass.: MIT Press, 2006.

19 International Agency for Research on Cancer, World Health Organization. GLOBOCAN estimated cancer incidence, mortality, and prevalence worldwide in 2012. Lyon, France: IARC, 2014 http://globocan.iarc.fr/Default.aspx (accessed April 19, 2016).

20 Karagas MR, Greenberg ER, Spencer SK, Stukel TA, Mott LA. Increase in incidence rates of basal cell and squamous cell skin cancer in New Hampshire, USA. New Hampshire Skin Cancer Study Group. *Int J Cancer* 1999; **81**: 555–9.

Liver Cancer SDG Capstone Appendix

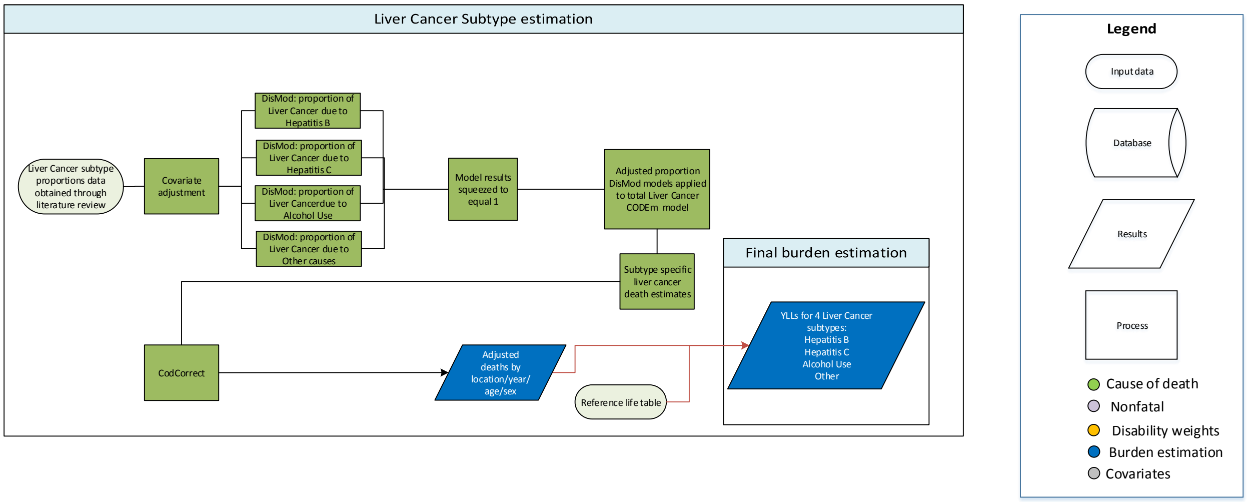

Input Data & Methodological Summary

Input data

Data seeking processes

The input data for the parent cause (liver cancer) were identified and processed using the same methods as all other cancers described above. To estimate the number of liver cancers for each of these sub-causes, DisMod-MR 2.1 was used to model the proportion of liver cancers due to the four subgroups. All publications used in GBD 2013 were included, and sources were supplemented with a systematic review of the published literature on the etiology of liver cancer. The literature search was performed in Pubmed on 8/13/15 with the following search string:

> (("Carcinoma, Hepatocellular"[Mesh] AND "etiology"[Subheading] AND ("epidemiology"[Subheading] OR "epidemiology"[All Fields] OR "epidemiology"[MeSH Terms])) AND ("Hepatitis B"[Mesh] OR "Hepatitis C"[Mesh] OR ("ethanol"[MeSH Terms] OR "ethanol"[All Fields] OR "alcohol"[All Fields] OR "alcohols"[MeSH Terms] OR "alcohols"[All Fields]) OR autoimmune[All Fields])).

The duration was restricted to articles published between 2013 and 2015. 385 articles were found, of which eight studies were added for liver cancer due to alcohol use (49 studies included from GBD 2013), 11 studies were added for liver cancer due to hepatitis B and C (99 studies included for GBD 2013), and 7 studies were added for liver cancer due to other causes (25 studies included for GBD 2013).

Inclusion and exclusion criteria

Articles were included if it was possible to identify at least one of the causes (alcohol, hepatitis B, hepatitis C, or “other causes”) as the only etiology.

Bias of categories of input data

The potential biases of the input data are the same as for other cancers (see above).

Modeling strategy

Overall methodological process

The modeling strategy for the parent cause "liver cancer" followed the general CODEm process. To estimate the fraction of liver cancer due to each etiology for each age-sex-geography-year DisMod-MR 2.1 was used.

Steps of analysis and data transformation processes

If the etiology was reported to be due to multiple causes, the cases due to multiple causes were split based on the proportion of cases in the individual etiologies reported in the publication. If cases were reported to be due to cryptogenic causes, these cases were removed from the denominator. A study covariate was used for publications that only assessed liver cancer in a cirrhotic population. The reference or "gold standard" that was used for crosswalking was the compilation of all studies that assessed the etiology of liver cancer in a general population. Smoothness (xi) was set at 0.1 to 0.3, heterogeneity (zeta) was set at 0.5 to 1. Time window for fit was 10 years, minimum coefficient of variation for global, super-region, region, and country was 0.4, 0.2, 0.1, and 0.1.

For liver cancer due to hepatitis C and hepatitis B, a prior value of 0 was set between age 0 and 0.01. For liver cancer due to alcohol a prior value of 0 was set for ages 0 to 5 years and a prior maximum value of 0.8 for ages older than 5.

For liver cancer due to hepatitis C, hepatitis C prevalence was used as a covariate with a predefined minimum of 0 and maximum of 10. Alcohol and hepatitis B prevalence were used as covariates with a pre-specified covariate of -1 to 0. A positive prior was set on the slope from age 0 to age 60.

For liver cancer due to hepatitis B, hepatitis B prevalence was used as a covariate with a predefined minimum of 0 and maximum of 10. Alcohol and hepatitis C prevalence were used as covariates with a pre-specified covariate of -1 to 0.

For liver cancer due to alcohol, alcohol (liters per capita) was used as a covariate with a predefined minimum of 0 and maximum of 10. Hepatitis B prevalence and hepatitis C prevalence were used as covariates with a pre-specified covariate of -1 to 0. A negative prior was set on the slope from age 0 to age 70.

For liver cancer due to other causes, hepatitis C prevalence, alcohol, and hepatitis B prevalence were used as covariates with a pre-specified covariate of -1 to 0.

To ensure coherent results between the cirrhosis and the liver cancer etiologies, the results from the liver cancer etiology models were transformed into covariates that were then used in the cirrhosis etiology models. The results from the cirrhosis etiology models were then used in the liver cancer proportion models.

The DisMod proportions for the underlying liver cancer etiologies were then squeezed to 100% and these final proportions were applied to the parent cause, "liver cancer," to derive the estimates for the

liver cancer etiologies.

Uncertainty intervals

Uncertainty was determined using standard DisMod methodology.

Results

Interpretation of results

Results for the liver cancer subtype mortality can differ from GBD 2013 for multiple reasons. First, additional sources were added based on an updated literature review. Second, to ensure consistency between cirrhosis and liver cancer etiologies the results from each model for cirrhosis and for liver cancer subtypes were converted into covariates and used in the respective other model. Third, DisMod methods were updated for GBD 2015 (Section 3).

Limitations

The etiological proportion models for the liver cancer sub-causes depend on the availability of data sources that inform DisMod about the local patterns of liver cancer etiologies as well as age patterns. Unfortunately, not many data sources provide data on the etiology of liver cancer by sex or by different age groups. Age patterns were therefore determined based on the assumption that there are no cases of liver cancer due to hepatitis B, hepatitis C, or alcohol in young age groups.

Cardiovascular Diseases SDG Capstone Appendix

Flowchart

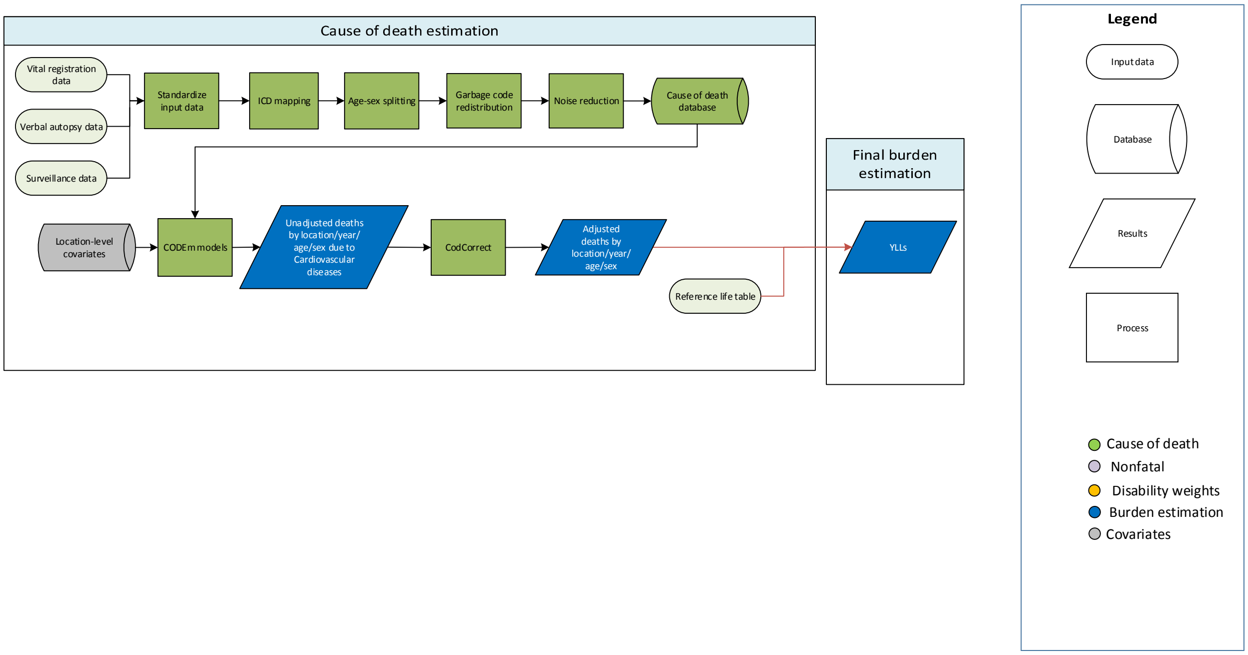

Input data

Vital registration, verbal autopsy, and surveillance data were used to model this cause. We outliered non-representative subnational verbal autopsies in a number of Indian states. We also outliered verbal autopsy data sources that were implausibly low in all age groups and ICD8 and ICD9 BTL data points that were inconsistent with the rest of the data and created implausible time trends.

Modeling strategy

We used a standard CODEm approach to model deaths from cardiovascular diseases. We have included two new variables, Socio-Demographic Index and the SEV scalar for rheumatic heart disease, as possible covariates for selection in the ensemble modeling process. Otherwise, there have been no substantive changes from the approach used in GBD 2013.

Rheumatic Heart Disease SDG Capstone Appendix

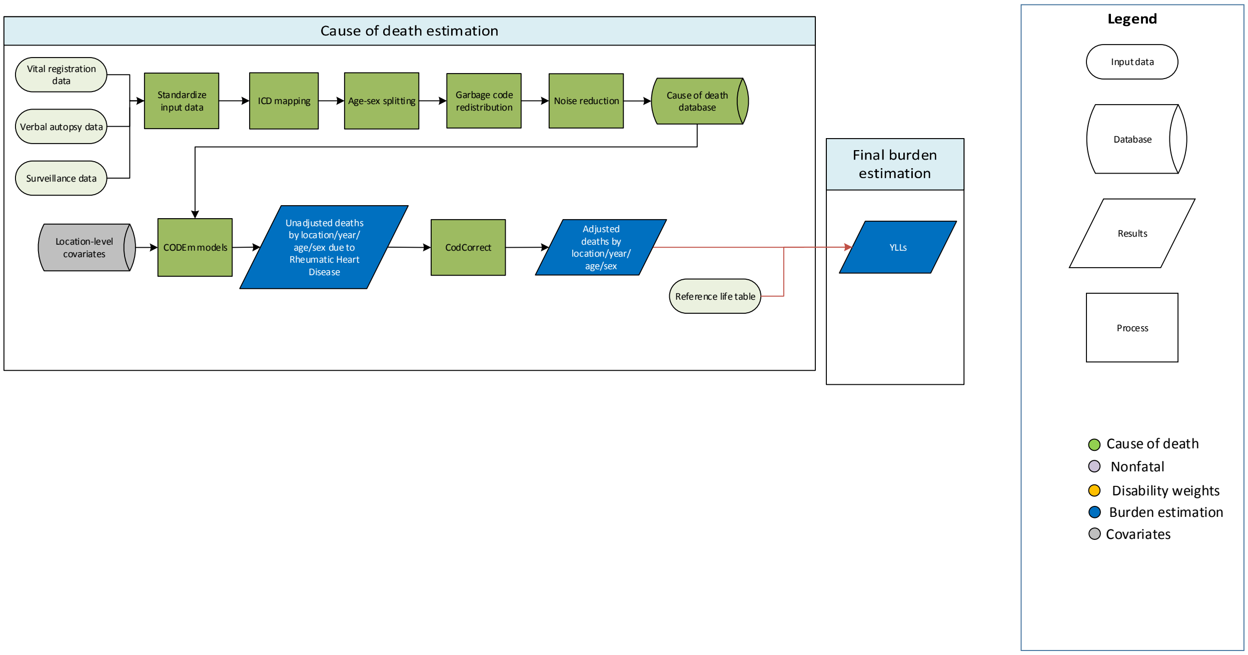

Input Data & Methodological Summary

Input data

Vital registration, verbal autopsy, and surveillance data were used to model rheumatic heart disease. We outliered ICD8 and ICD9 BTL data points which were inconsistent with the rest of the data and created implausible time trends. We also outliered data points which were too high after the redistribution process in a number of age groups.

Modeling strategy

We used a standard CODEm approach to model deaths from rheumatic heart disease. We have included two new variables, Socio-Demographic Index and the SEV scalar for rheumatic heart disease, as possible covariates for selection in the ensemble modeling process. Otherwise, there have been no substantive changes from the approach used in GBD 2013.

Ischemic Heart Disease SDG Capstone Appendix

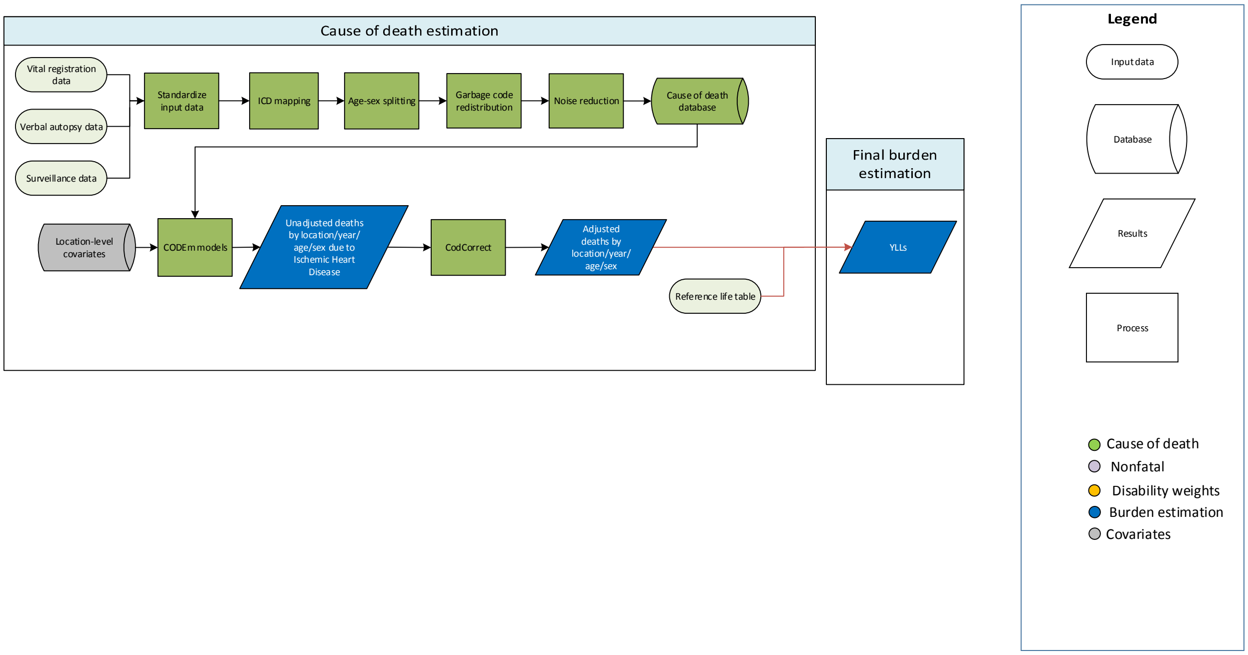

Input Data & Methodological Summary

Input data

Vital registration, verbal autopsy, and surveillance data were used to model ischemic heart disease. We outliered verbal autopsy data in countries and subnational locations where high-quality vital registration data were also available. We also outliered non-representative subnational verbal autopsy data points, ICD8 and ICD9 BTL data points which were inconsistent with the rest of the data and created implausible time trends, and data in a number of Indian states identified by experts as poor-quality.

Modeling strategy

We used a standard CODEm approach to model deaths from ischemic heart disease. We have included two new variables, Socio-Demographic Index and the SEV scalar for ischemic heart disease, as possible covariates for selection in the ensemble modeling process. Otherwise, there have been no substantive changes from the approach used in GBD 2013.

Cerebrovascular Disease SDG Capstone Appendix

Flowchart

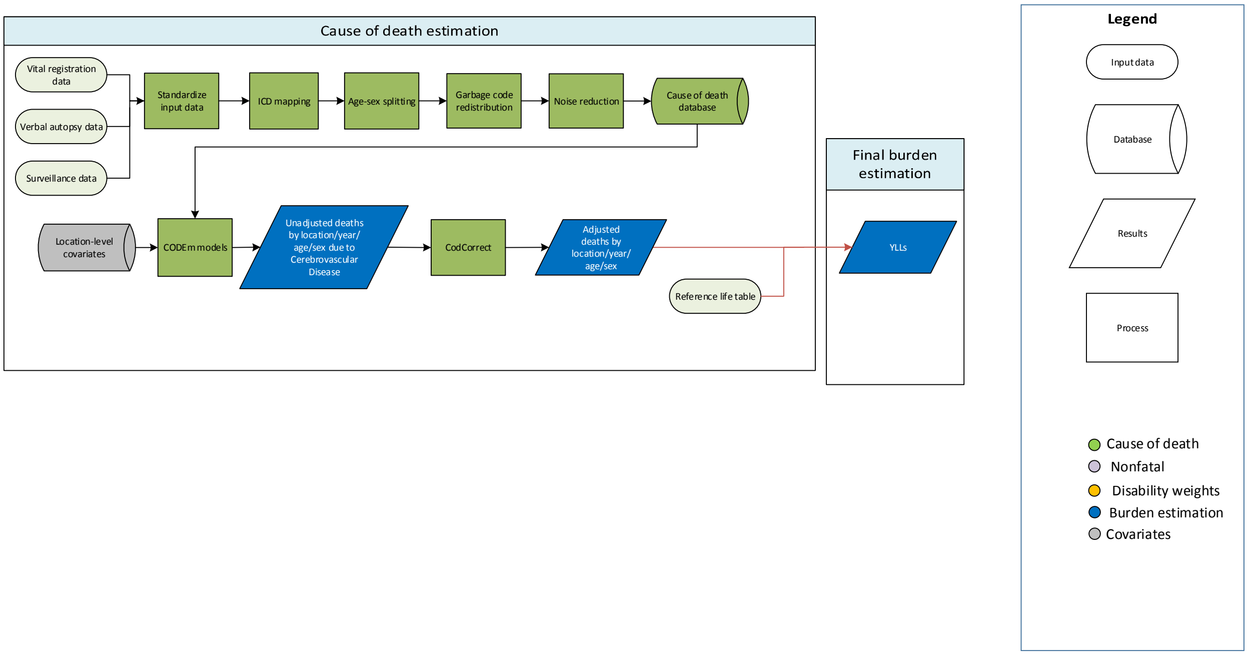

Input Data & Methodological Summary

Input data

Verbal autopsy and vital registration data were used to model this cause. We outliered non-representative subnational verbal autopsy data points. We reassigned deaths from verbal autopsy reports for cerebrovascular disease to the parent cardiovascular disease for both sexes for those under 20 years of age. We also outliered ICD8, ICD9 BTL, and ICD10 Tabulated data points which were inconsistent with the rest of the data and created implausible time trends. Data points from sources which were implausibly low in all age groups and data points that were causing the regional estimates to be improbably high were outliered.

Modeling strategy

We used a standard CODEm approach to model deaths from cerebrovascular disease. We have included two new variables, Socio-Demographic Index and the SEV scalar for cerebrovascular disease, as possible covariates for selection in the ensemble modeling process. Otherwise, there have been no substantive changes from the approach used in GBD 2013.

Ischemic Stroke SDG Capstone Appendix

Flowchart

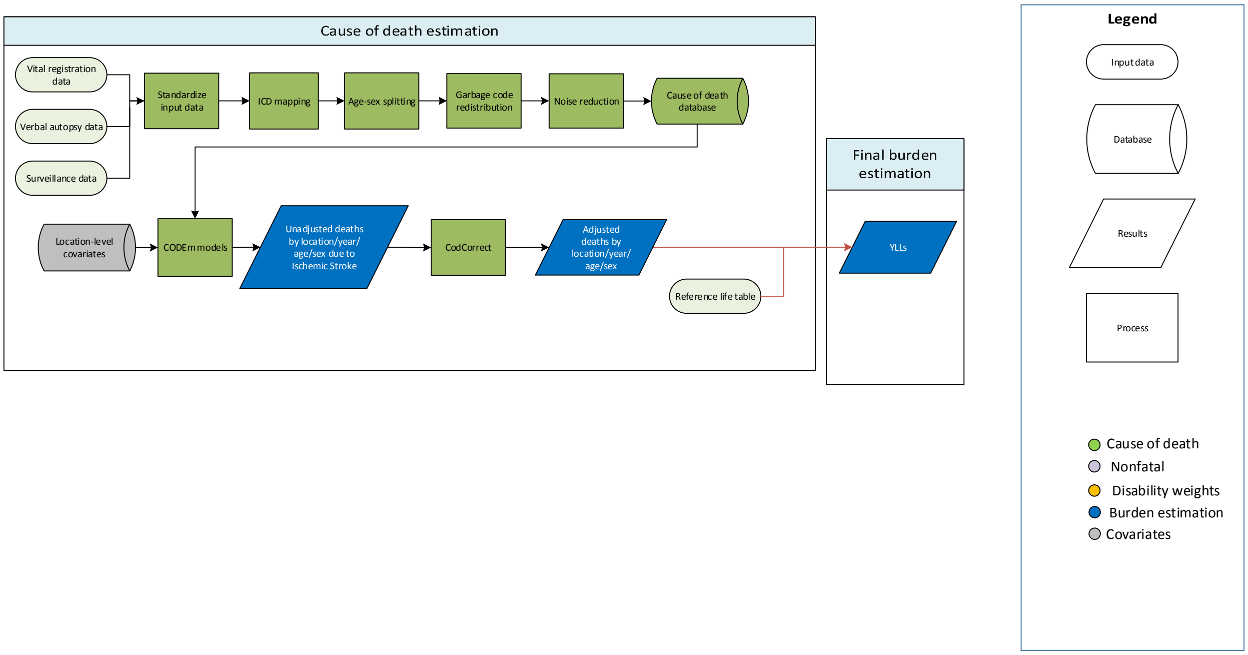

Input Data & Methodological Summary

Input data

Vital registration, verbal autopsy, and surveillance data were used to model ischemic stroke. We reassigned deaths from verbal autopsy reports for ischemic stroke to the parent cardiovascular disease for both sexes for those under 20 years of age. We outliered ICD8 data points which were inconsistent with the rest of the data and created implausible time trends.

Modeling strategy

We used a standard CODEm approach to model deaths from ischemic stroke. In locations with limited data on ischemic stroke, the subtype-specific deaths were estimated by squeezing both ischemic and hemorrhagic stroke to the overall cerebrovascular envelope. We have included two new variables, Socio-Demographic Index and the SEV scalar for ischemic stroke, as possible covariates for selection in the ensemble modeling process. Otherwise, there have been no substantive changes from the approach used in GBD 2013.

Hemorrhagic Stroke SDG Capstone Appendix

Flowchart

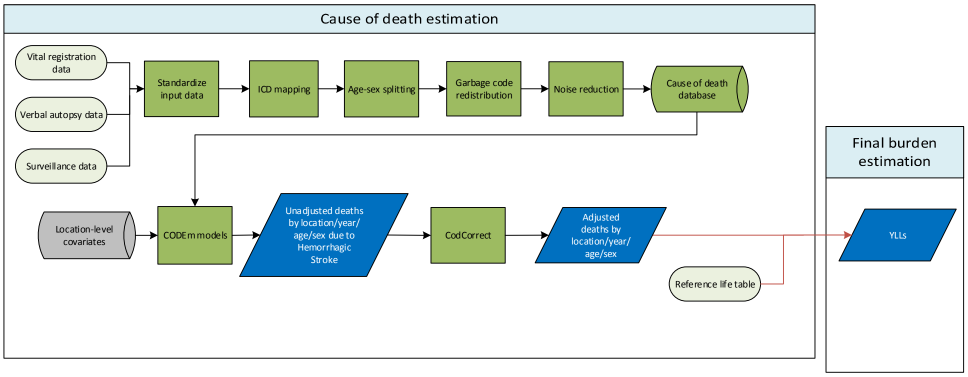

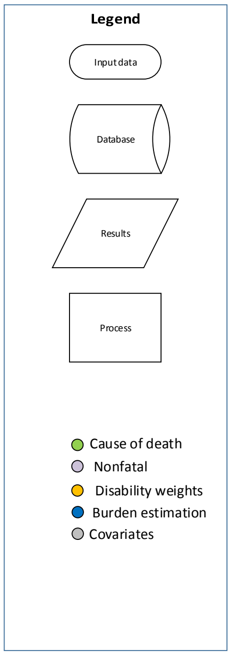

Input Data & Methodological Summary

Input data

Vital registration, verbal autopsy, and surveillance data were used to model hemorrhagic stroke. We reassigned deaths from verbal autopsy reports for hemorrhagic stroke to the parent cardiovascular disease for both sexes for those under 20 years of age. We outliered ICD8 data points which were inconsistent with the rest of the data and created implausible time trends.

Modeling strategy

We used a standard CODEm approach to model deaths from hemorrhagic stroke. In locations with limited data on hemorrhagic stroke, the subtype-specific deaths were estimated by squeezing both ischemic and hemorrhagic stroke to the overall cerebrovascular envelope. We have included two new variables, Socio-Demographic Index and the SEV scalar for hemorrhagic stroke, as possible covariates for selection in the ensemble modeling process. Otherwise, there have been no substantive changes from the approach used in GBD 2013.

Hypertensive Heart Disease SDG Capstone Appendix

Flowchart

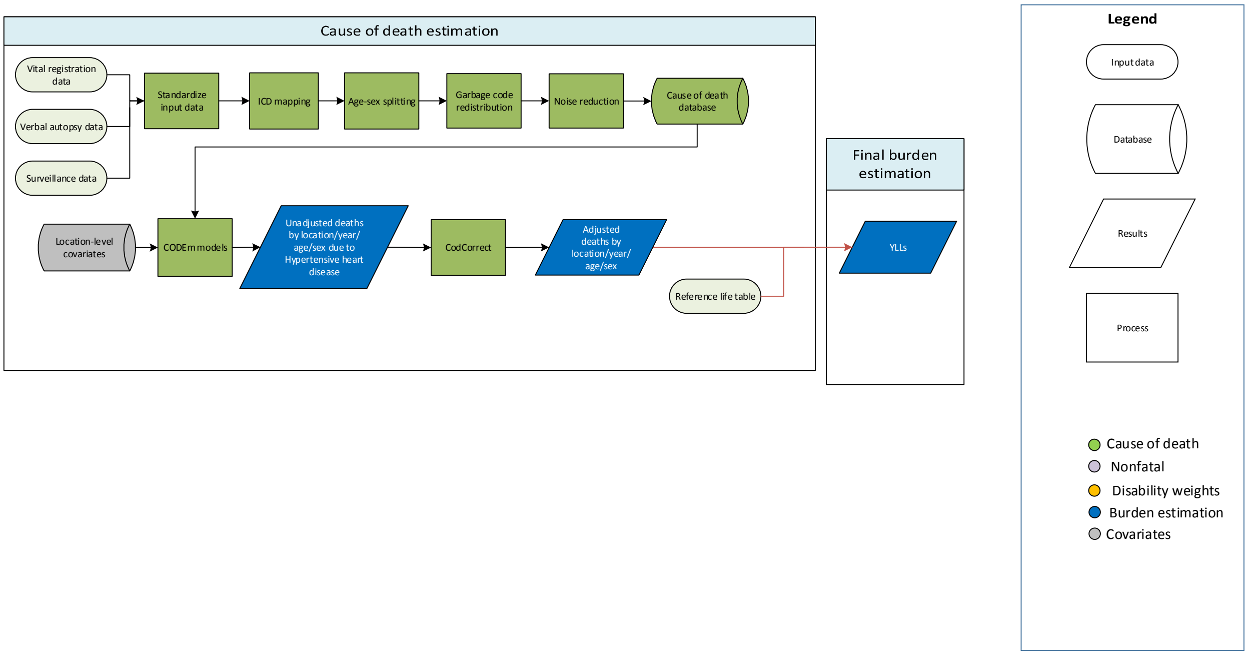

Input Data & Methodological Summary

Input data

Vital registration, verbal autopsy, and surveillance data were used to model hypertensive heart disease. We outliered ICD9 BTL data points, which were inconsistent with the rest of the data and created implausible time trends.

Modeling strategy

We used a standard CODEm approach to model deaths from hypertensive heart disease. We have included two new variables, Socio-Demographic Index and the SEV scalar for hypertensive heart disease, as possible covariates for selection in the ensemble modeling process. Otherwise, there have been no substantive changes from the approach used in GBD 2013.

Cardiomyopathy and Myocarditis SDG Capstone Appendix

Flowchart

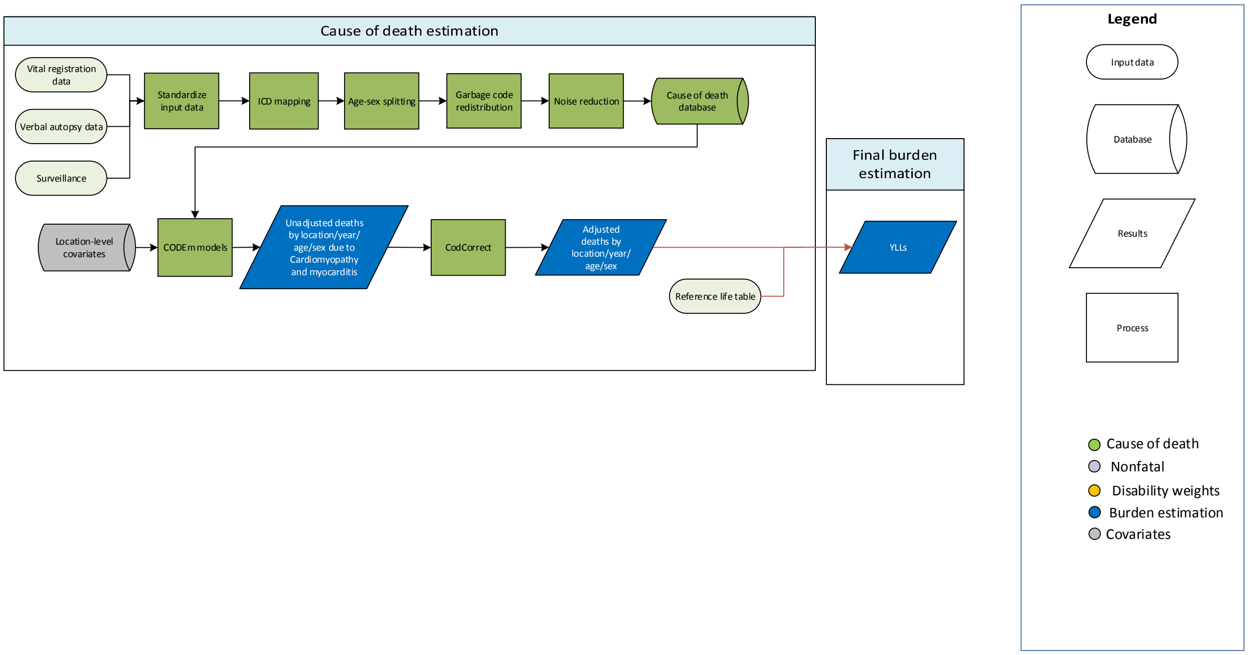

Input Data & Methodological Summary

Input data

Vital registration, verbal autopsy, and surveillance data were used to model cardiomyopathy and myocarditis. We outliered data points in Central Asia and Central and Eastern Europe due to implausibly high values which we attributed to variation in local coding practices. We also outliered ICD8 data points in countries where they were discontinuous with other data in the time series.

Modeling strategy

We used a standard CODEm approach to model deaths from cardiomyopathy and myocarditis. We have included two new variables, Socio-Demographic Index and the SEV scalar for cardiomyopathy and myocarditis, as possible covariates for selection in the ensemble modeling process. Otherwise, there have been no substantive changes from the approach used in GBD 2013. Finally, local differences in coding practices may explain some of the geographic variation that we see for deaths due to cardiomyopathy; we plan to explore this issue further in future iterations of GBD.

Other Cardiovascular and Circulatory Diseases SDG Capstone Appendix

Flowchart

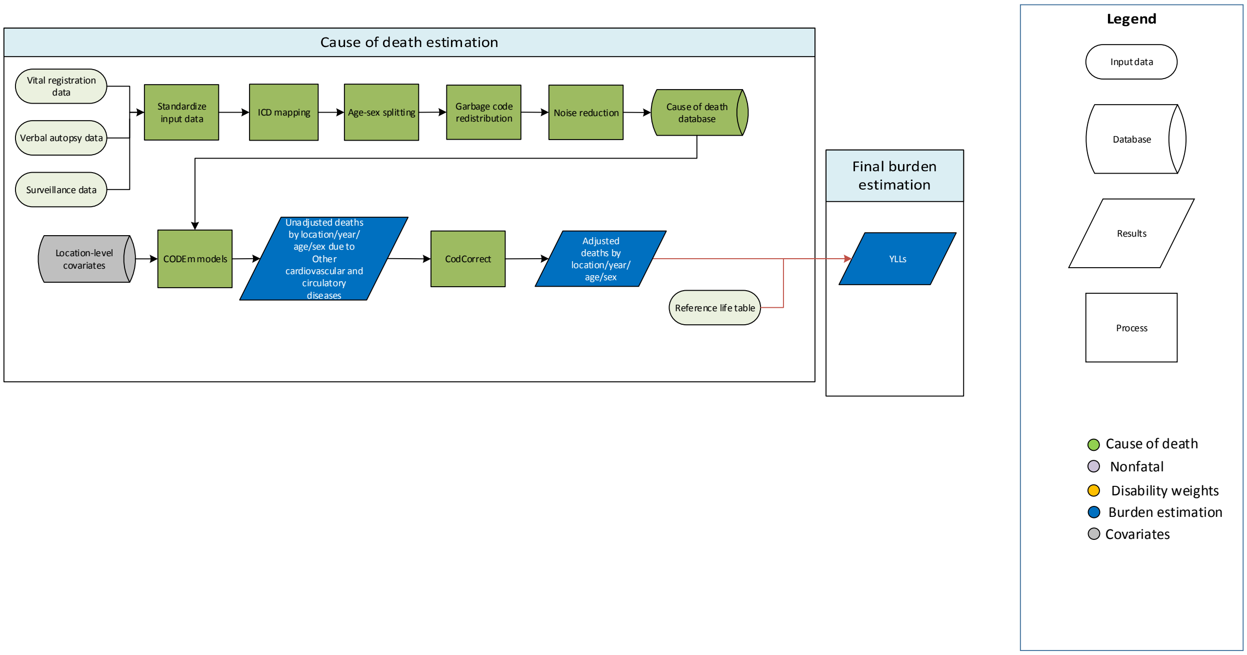

Input Data & Methodological Summary

Input data

Vital registration, verbal autopsy, and surveillance data were used to model other cardiovascular and circulatory diseases. We outliered ICD8 and ICD9 BTL data points that were inconsistent with the rest of the data and created implausible time trends. We also outliered ICD8 data points which were not nationally representative.

Modeling strategy

We used a standard CODEm approach to model deaths from other cardiovascular and circulatory diseases. We have included two new variables, Socio-Demographic Index and the SEV scalar for other cardiovascular and circulatory diseases, as possible covariates for selection in the ensemble modeling process. Otherwise, there have been no substantive changes from the approach used in GBD 2013.

Chronic Respiratory Diseases SDG Capstone Appendix

Flowchart

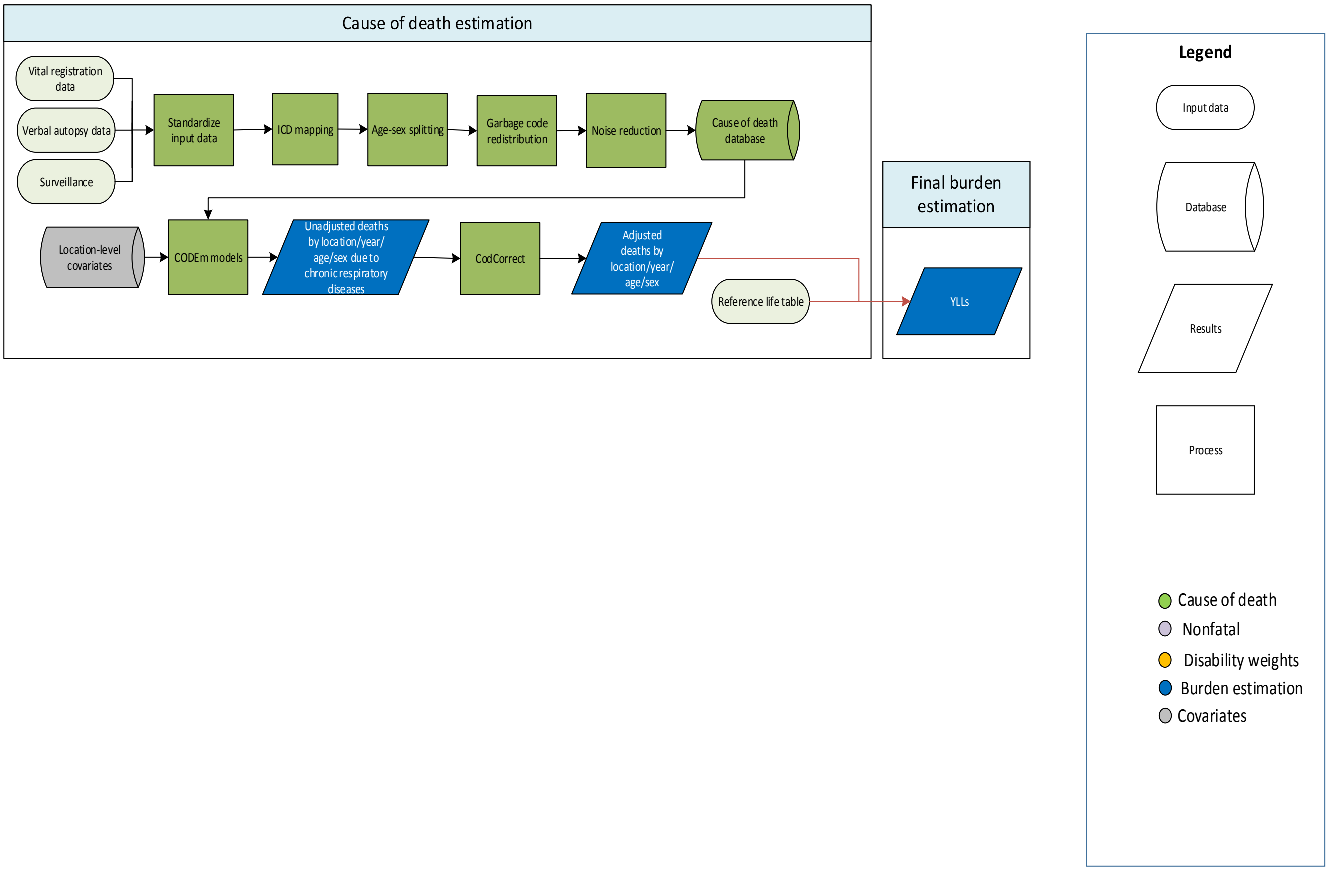

Input Data & Methodological Summary

Input data

Sources used to estimate chronic respiratory disease mortality included vital registration, verbal autopsy, and surveillance data from the cause of death (COD) database. Our outlier criteria excluded data points that (1) were implausibly high or low, (2) substantially conflicted with established age or temporal patterns, or (3) significantly conflicted with other data sources conducted from the same locations or locations with similar characteristics (i.e., Socio-Demographic Index).

For GBD 2015, there were two significant changes in the data preparation process that affect Chronic Respiratory Diseases and its children causes. First, the algorithm package that redistributes heart-failure-

related garbage codes has been updated to take into account the "side" of the heart failure – with right heart failure denoting an underlying respiratory disease. Second, verbal autopsy data are no longer used to inform children causes as they are thought to be unreliable below this cause level. Practically, this has a larger influence on the uncorrected children models than the parent Chronic Respiratory Diseases model discussed here.

Modeling strategy

The standard CODEm modeling approach was applied to estimate deaths due to chronic respiratory diseases. Chronic respiratory diseases served as the parent cause to chronic obstructive pulmonary disease, pneumoconiosis (including silicosis, asbestosis, coal worker's pneumoconiosis, other pneumoconiosis), asthma, interstitial lung disease and pulmonary sarcoidosis, and other chronic respiratory diseases. Functionally, this means the death estimates for Chronic Respiratory Diseases serve as an envelope into which the children causes are squeezed by the CodCorrect algorithm. This approach allows us to use a broader range of data – specifically verbal autopsy data – which cannot be accurately mapped to a cause further down in the hierarchy.

Separate models were conducted for male and female mortality, and the age range for both models was 0 to 80+ years. The same covariates from GBD 2013 were used, with the addition of the Socio-Demographic Index (SDI) covariate. Although all covariates in this model received updates for GBD 2015, cumulative cigarettes, smoking prevalence, and health systems access received the larger overhauls. The updates to the smoking-based covariates were particularly helpful in developing these models. Beyond changes in the underlying covariates, there were no substantial deviations from the GBD 2013 approach.

Other Chronic Respiratory Diseases SDG Capstone Appendix

Flowchart

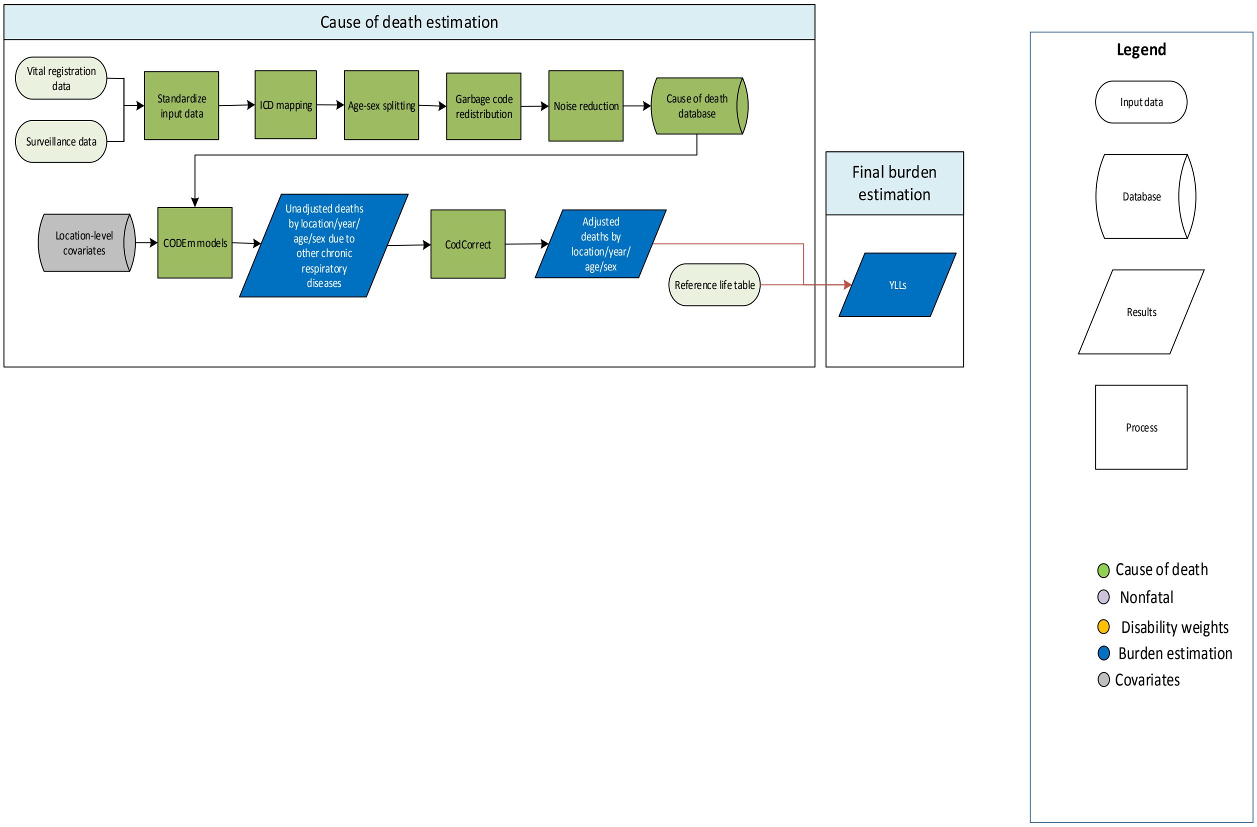

Input Data & Methodological Summary

Input data

Data used to estimate other chronic respiratory diseases included vital registration and surveillance data from the cause of death (COD) database. Our outlier criteria excluded data points that (1) were implausibly high or low, (2) substantially conflicted with established age or temporal patterns, or (3) significantly conflicted with other data sources conducted from the same locations or locations with similar characteristics (i.e., Socio-Demographic Index).

Modeling strategy

The standard CODEm modeling approach was applied to estimate deaths due to other chronic respiratory diseases. Separate models were conducted for male and female mortality, and the age range

for both models was 0 days to 80+ years. Like other respiratory causes, the mortality estimates from other chronic respiratory diseases were ultimately fit into the chronic respiratory envelope.

Besides general updates to the 2013 covariate set (specifically, the health systems and smoking-related ones), the modeling strategy remained unchanged from GBD 2013. For GBD 2015, we included two new covariates: the Socio-Demographic Index (SDI) covariate, and a standardized exposure variable (SEV) scalar (disease-specific values that reflect the combined effect of all GBD risks) for other respiratory diseases. However, as SEVs are essentially covariate/risk aggregate measures, no substantial changes were expected.

Diabetes Mellitus SDG Capstone Appendix

Flowchart

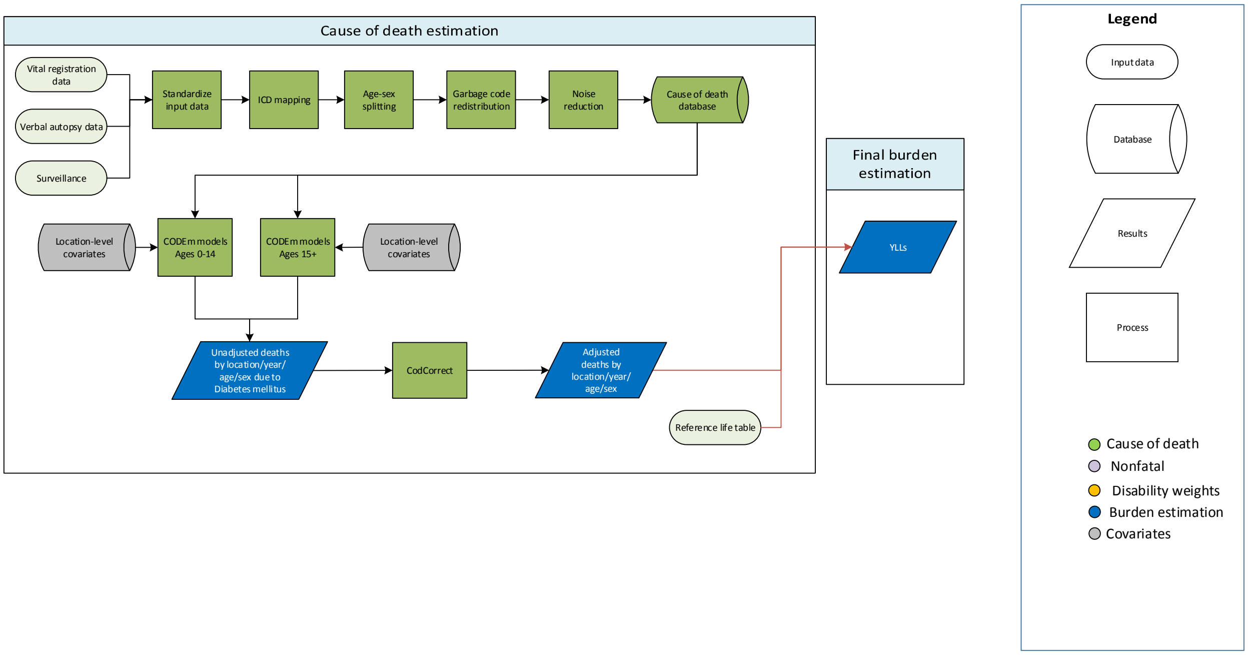

Input Data & Methodological Summary

Input data

Verbal Autopsy Data

We outliered VA data points in urban Indian states where high-quality vital registration data were also available. We also outliered data points where the VA data were implausible in all age groups as we determined that these data sources were unreliable.

Vital Registration Data

We outliered all data in four urban Indian states where the source of the data was unreliable according to expert opinion. We also outliered ICD9BTL data points which were inconsistent with the rest of the data series and created unlikely time trends.

Modeling strategy

We used a slight variation on the standard CODEm approach to model deaths from diabetes mellitus. Since deaths in younger age groups are almost exclusively due to Type 1 diabetes while deaths in older ages are primarily due to Type 2, we used two models to estimate overall diabetes deaths. The first is for deaths in 0-14 year olds; the second is for deaths in 15-80+ year olds. In previous iterations of GBD, we used a similar approach, but the two models had age ranges of 0-24 and 25-80+. This change was made

due to the increasing prevalence of Type 2 diabetes at younger ages, and thus the increasing likelihood that diabetes-related deaths in the 15-24 year age groups are due to Type 2 diabetes.

We have included two new variables, Socio-Demographic Index and the SEV scalar for diabetes mellitus, as possible covariates for selection in the ensemble modeling process for the model in older ages.

Alcohol Use SDG Capstone Appendix

Flowchart

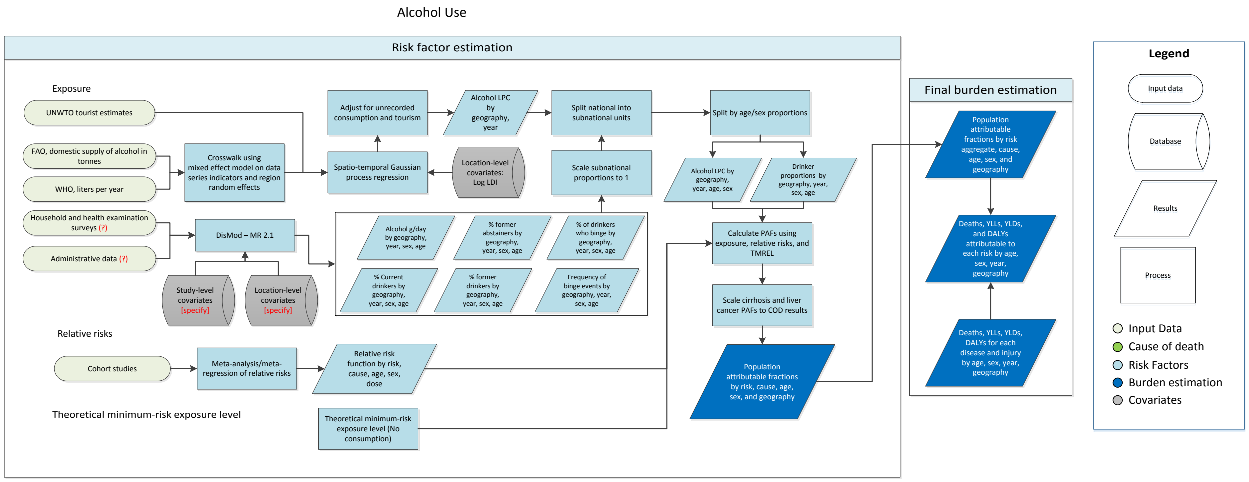

Input data and Methodological Summary

Indicator definition

This modeling strategy encompassed the indicator associated with alcohol use, as measured by the summary exposure value (SEV) (3.5.2).

Indicator 3.5.2

As a component of SDG Goal 3. Ensure healthy lives and promote well-being for all at all ages, SDG Target 3.5, by 2030, strengthen the prevention and treatment of substance abuse, including narcotic drug abuse and harmful use of alcohol, is measured using SDG Indicator 3.5.2, Risk-weighted prevalence of alcohol use.

Case Definition

The impact of alcohol consumption on morbidity and mortality can be largely described by two separate but related dimensions. The 1st dimension is the individual level drinking and consists of four indicators:

1. Current drinkers, defined as the proportion of individuals who have consumed at least one alcoholic beverage (or some approximation) in the last 12 months.
2. Former drinkers, defined as the proportion of individuals who have ever consumed an alcoholic beverage, but not in the last 12 months.
3. Lifetime abstainers, defined as the proportion of individuals who have never consumed an alcoholic beverage.
4. Alcohol consumption (in grams per day), defined as grams of alcohol consumed by current drinkers, per day, over a 12 month period.

The 2nd dimension of alcohol consumption relates to the pattern of drinking and consists of two indicators;

5. Binge drinkers, defined as the proportion of drinkers who have had a binge event in the past 12 months. A binge event was defined as consuming 60 grams of alcohol (approximately five drinks or more) in a single occasion for males and 48 grams of alcohol in a single occasion for females.
6. Binge times, defined as the proportion of drinking events that are binge amongst binge drinkers i.e. the proportion of days that a binger has a binge event.

Input data

For GBD 2013, a systematic review of the literature was conducted to capture population survey data on all six alcohol use indicators. In summary, the search was conducted in three stages involving electronic searches of the peer-reviewed literature via PubMed, the grey literature and, expert consultation. Updates to systematic reviews via PubMed are performed on an ongoing schedule across all GBD causes and risk factors, an update for alcohol use will be performed in the next 1-2 iterations. For GBD 2015, stages two and three of the literature review were conducted, prioritizing countries for which subnational estimates were generated. The Global Health Exchange (GHDx), IHME's online database of health-related data, was searched for population survey data containing participant-level information from which we could formulate the required alcohol use indicators. Data-sources were included if they captured a sample representative of the geographic location under study and contained variables that could be used to formulate any of the six alcohol use indicators. Relevant survey variables from each data-source were documented in a Microsoft Excel codebook and extracted using STATA 13.1. A total of 629 potential data-sources were available in GHDx across countries with subnational locations, out of which 127 data-sources (66,108 data-points) were included across all six indicators.

To generate estimates of alcohol consumption in grams per day, data from population surveys were used in combination with estimates of per capita consumption from the Food and Agriculture Organization (FAO) and the Global Information System on Alcohol and Health (GISAH database). Per capita consumption is an aggregate measure of recorded, unrecorded, and tourist per capita consumption of alcohol (UNWTO database) derived from sales, production, and other economic statistics. While population-based surveys provide accurate estimates of the prevalence of lifetime abstainers, former drinkers and current drinkers, they typically underestimate real alcohol consumption levels. As a result, the all-age, both-sex per capita consumption figures from the FAO and GISAH are considered to be a better estimate of overall volume of consumption. Per capita consumption, however, does not provide age- and sex-specific consumption estimates needed to compute alcohol-attributable burden of disease. Therefore, we use the age-sex pattern of consumption among drinkers modeled from the population survey data and the overall volume of consumption from FAO and GISAH to determine the total amount of alcohol consumed by country.

Modeling strategy

DisMod-MR 2.1 was used to estimate country-, year-, age- and sex-specific proportions of current drinkers, former drinkers, lifetime abstainers, binge drinkers, and binge times; and alcohol consumption in grams per day. We have made no substantive changes in the modeling strategy from GBD 2013. We ran single-parameter models for each alcohol use indicator and included a combination of the location- and study-level covariates in each model. An alcohol liters per capita location-level covariate was used for

all six indicators to assist in the predictive power of the models. Additionally, study-level covariates were used to accommodate for known sources of variability in the raw data. In the current drinkers, former drinkers, binge drinkers and binge times models, we included two covariates which adjusted estimates derived in past week and past month towards those derived in the past year respectively. Estimates derived in the past year were considered to be the gold standard given the previously outlined definition for each indicator. In the alcohol consumption model, we included a covariate which flagged estimates from one specific data-source — WHO's World Health Surveys — which were systematically higher than estimates derived from other sources due to differences in how alcohol use was captured in these surveys. If other data-points causing higher or lower modelled output were identified during the modelling process for a given indicator, the plausibility of these data points was assessed and the study methodology reviewed. Data points with methodological limitations were excluded as outliers.

A spatiotemporal Gaussian process regression was used to model total alcohol liters per capita.

Theoretical minimum-risk exposure level

For alcohol use, the theoretical minimum-risk exposure level (TMREL) was assumed to be no alcohol use or, in other words, 0 g/day of alcohol consumption.

Relative risks

The relative risks have not changed significantly since GBD 2013.

SDG Capstone Appendix: Road traffic injuries, interpersonal violence, unintentional poisoning and self-harm mortality

Flowchart

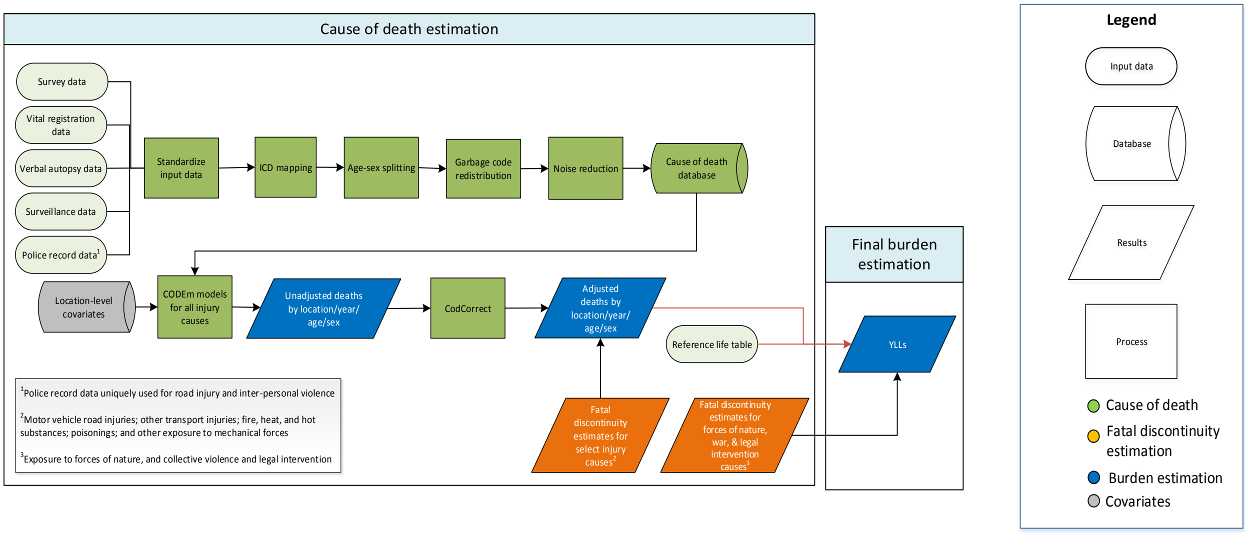

Input data & Methodological summary

Indicator definition

This modeling strategy encompassed the indicator associated with mortality due to self-harm (3.4.2), road injury (3.6.1), unintentional poisonings (3.9.3) and interpersonal violence (16.1.1).

Indicator 3.4.2

As a component of SDG Goal 3. Ensure healthy lives and promote well-being for all at all ages, SDG Target 3.4, reduce by one third premature mortality from NCDs through prevention and treatment and promote mental health and well-being, is measured using SDG Health Index Indicator 3.4.2, deaths due to self-harm per 100,000.

Indicator 3.6.1

As a component of SDG Goal 3. Ensure healthy lives and promote well-being for all at all ages, SDG Target 3.6, by 2030, halve the number of global deaths and injuries from road traffic accidents, is measured using SDG Health Index Indicator 3.6.1, number of deaths due to road injuries per 100,000.

Indicator 3.9.3

As a component of SDG Goal 3. Ensure healthy lives and promote well-being for all at all ages, SDG Target 3.9, by 2030, substantially reduce the number of deaths and illnesses from hazardous chemicals and air, water and soil pollution and contamination, is measured using SDG Indicator 3.9.3, deaths due to unintentional poisoning per 100,000.

Indicator 16.1.1

As a component of SDG Goal 16. Promote peaceful and inclusive societies for sustainable development, provide access to justice for all and build effective, accountable and inclusive institutions at all levels, SDG Target 16.1, by 2030, significantly reduce all forms of violence and related death rates everywhere, is measured using SDG Health Index Indicator 16.1.1, number of deaths due to interpersonal violence per 100,000.

Input data

In GBD 2015, we estimated mortality due to road traffic injury, interpersonal violence, self-harm and unintentional poisoning from vital registration (VR), verbal autopsy (VA), mortality surveillance, censuses, surveys, and police record data. Police and crime reports were data sources uniquely used for the estimation of deaths from road injury and interpersonal violence. The police data were collected from published studies, national agencies, and institutional surveys such as the United Nations Crime Trends Survey and the WHO Global Status Report on Road Safety Survey. For countries with VR data we did not use police records, except if the recorded number of road injury and interpersonal violence deaths from police records exceeded that in the VR.

Infrequently, data points were marked as outliers. Outlier criteria excluded data points that (1) were implausibly high or low relative to global or regional patterns, (2) substantially conflicted with established age or temporal patterns, or (3) significantly conflicted with other data sources conducted from the same locations or locations with similar characteristics (i.e., Socio-Demographic Index [SDI]).

Modeling strategy

Overview

In GBD 2015, the standard Cause of Death Ensemble model (CODEm) was applied to estimate deaths due to road injuries, unintentional poisonings, self-harm, and interpersonal violence.

GBD injury codes and categories

The International Classification of Diseases (ICD) was used to classify injuries because it is the standard diagnostic tool for epidemiology. In GBD, injury incidence and death are defined as ICD-9 codes E000-E999 and ICD-10 chapters V to Y.

There is one exception: deaths and cases of alcohol poisoning and drug overdoses are classified under drug and alcohol use disorders. We redistributed deaths coded as unspecified poisoning among the homicide, self-harm, unintentional poisoning, and drug use disorder causes. Upon close review of ICD-coded deaths for unspecified poisoning, we identified a specific age-pattern, in which the death rate spiked in ages 15–50 years. Noting this trend, we conducted further review of data and found that these deaths were likely due to drug use disorders. We then redistributed many unspecified poisoning deaths from unintentional poisoning to drug use disorders, and based on high-quality ICD-9 and ICD-10 data, re-assigned these deaths to the drug use disorder sub-causes.

Preparation of data

The preparation of cause of death (CoD) data includes age splitting, age-sex splitting, smoothing, and outlier detection. These steps are described in detail by Naghavi et al and Lozano et al.[1,2] The concept of "garbage codes" and redistribution of these codes was proposed in the GBD 1990.[3] Garbage codes are causes of death that should not be identified as specific underlying causes of death but have been entered as the underlying cause of death on death certificates. A classic example of these types of codes in injuries chapters are "Exposure to unspecified factor" (X59 in ICD-10 and E887 in ICD-9) and all undetermined intent codes (Y10-Y34 in ICD-10 and E980-E988 in ICD-9). Other examples of garbage codes in injuries are the coding of an injury death to intermediate codes like septicemia or peritonitis or as an ill-defined and unknown cause of mortality (R99). Approximately 2% of total deaths in countries with VR data are assigned to these three injury garbage code categories.

Splitting into sublevel causes

In countries with non-detail ICD code data, cause-of-injury categories were proportionally split into sublevel cause-of-injury categories. The sublevel cause-of-injury causes were created in the CoDCorrect process. One of the countries with non-detail ICD code data is South Africa, and in GBD 2013 the proportions of sublevel cause-of-injury were based on VR data. For GBD 2015 the proportions were based on the paper by Matzopoulos et al. 2015.[4]

Limitations and model assumptions

We added police data for road injuries and interpersonal violence to help predict level and age patterns in countries with sparse or absent cause of death data even though we know from countries with near-complete vital registration data that police records tend to underestimate the true level of deaths. However, we applied police data estimates in instances where reported deaths were higher than VR numbers.

Table – Injury Cause List

ID	Cause	Modeling Strategy	Covariate changes from GBD 2013
1.1	Road injuries	CODEm	+Cause-specific risk scalar for road injuries, SDI
1.1.a	Pedestrian road injuries	CODEm	+ Cause-specific risk scalar for pedestrian road injuries, SDS
1.1.b	Cyclist road injuries	CODEm	+ Cause-specific risk scalar for cyclist road injuries, SDI
1.1.c	Motorcyclist road injuries	CODEm	+ Cause-specific risk scalar for motorcyclist injuries, SDI
1.1.d	Motor vehicle road injuries	CODEm and fatal discontinuity estimation	+ Cause-specific risk scalar for motor vehicle road injuries, SDI
1.1.e	Other road injuries	CODEm	+ Cause-specific risk scalar for other road injuries, SDI
1.2	Other transport injuries	CODEm and fatal discontinuity estimation	+ Cause-specific risk scalar for other transport injuries, SDI
2.4	Poisonings	CODEm and fatal discontinuity estimation	+ Cause-specific risk scalar for poisonings, SDI
3.1	Self-harm	CODEm	+ Cause-specific risk scalar, SDI, major depressive disorder prevalence

3.2	Interpersonal violence	CODEm	+ Cause-specific risk scalar, SDI
3.2.a	Assault by firearm	CODEm	+ Cause-specific risk scalar for assault by firearm, SDI
3.2.b	Assault by sharp object	CODEm	+ Cause-specific risk scalar for assault by sharp object, SDI
3.2.c	Assault by other means	CODEm	+ Cause-specific risk scalar for assault by other means, SDI

References

1. Lozano R, Naghavi M, Foreman K, *et al.* Global and regional mortality from 235 causes of death for 20 age groups in 1990 and 2010: a systematic analysis for the Global Burden of Disease Study 2010. *The Lancet* 2012; **380**: 2095–128.

2. Global, regional, and national age–sex specific all-cause and cause-specific mortality for 240 causes of death, 1990–2013: a systematic analysis for the Global Burden of Disease Study 2013. *The Lancet* 2015; **385**: 117–71.

3. Murray CJL, Lopez AD, Harvard School of Public Health, World Health Organization, World Bank. The global burden of disease: a comprehensive assessment of mortality and disability from diseases, injuries, and risk factors in 1990 and projected to 2020. Cambridge, MA: Published by the Harvard School of Public Health on behalf of the World Health Organization and the World Bank : Distributed by Harvard University Press, 1996.

4. Matzopoulos R, Prinsloo M, Wyk VP, Gwebushe N, Mathews S, et al. Injury-related mortality in South Africa: a retrospective descriptive study of postmortem investigations. *Bull World Health Organ* 2015; **93**: 303–13.

Met Need for Family Planning with Modern Methods
SDG Capstone Appendix

Input Data & Methodological Summary

Indicator definition

This modeling strategy encompassed the indicator associated with the proportion of women aged 15 to 49 years with their family planning needs met with modern contraception methods (3.7.1). This indicator also is an individual component of Indicator 3.8.1, which is the composite indicator for universal health coverage (UHC) tracer interventions.

Indicator 3.7.1

As a component of SDG Goal 3. Ensure healthy lives and promote well-being for all at all ages, SDG Target 3.7, by 2030, ensure universal access to sexual and reproductive health-care services, including for family planning, information and education, and the integration of reproductive health into national strategies and programmes, is measured using SDG Indicator 3.7.1 proportion of women of reproductive age (15 to 49 years) who are sexually active and have their need for family planning satisfied with modern methods (ie, female and male sterilization, oral hormonal pills, intra-uterine devices (IUD), male condoms, injectables, implants [including Norplant], vaginal barrier methods, female condoms, and emergency contraception)

Input data

We defined modern contraception methods as the current use of male or female sterilization, male or female condoms, spermicide foam/jelly, oral contraceptive, diaphragms, implants, injections, or use of an IUD. Traditional contraception methods were defined as the current use of methods including withdrawal, period abstinence, the rhythm method, and lactational amenorrhea method (LAM).

Women between the ages of 15 and 49 who were fecund, sexually active, and did not wish to become pregnant within the next two years or longer were defined as having need for family planning. Of women with need for family planning, we defined met need with modern methods as women who currently use a modern method of contraception.

The present study used two primary types of input data in order to ultimately generate a time series of met need for family planning with modern methods: (1) individual-level microdata from which met need for family with planning with modern methods could be directly estimated; and (2) tabulated data from which met need with modern methods could be indirectly calculated based on reported estimates of modern contraception coverage, any contraception coverage, and unmet need for family planning. In addition, we updated a systematic review, originally conducted for the 2010 iteration of the Global Burden of Disease Study (GBD 2010), and data extraction process for modern contraception coverage to use as a covariate in our eventual model for estimating met need with modern methods.

Our primary data sources for met need with modern methods included multi-country survey series, such as Demographic and Health Surveys (DHS), Multiple Indicator Cluster Surveys (MICS), and Centers for Disease Control and Prevention Reproductive Health Surveys (CDC RHS). In addition, we extracted data

from the Performance Monitoring and Accountability 2020 (PMA2020) surveys, to which we were granted access. We originally sought a wider universe of population surveys, but our search was somewhat restricted to the survey series for which information on contraception use by method and marital status was readily women for all women of reproductive age. Notably, relatively few microdata sources were available for higher-income countries; subsequently, we heavily relied on tabulated data for these geographies.

The below table shows the number of studies included in the 2015 SDG Capstone paper.

Surveys	Contraception Methods	Unmet Need
DHS	232	214
MICS	114	3
CDC RHS	25	0
PMA2020	12	12
Country-specific	497	43

Among the surveys for which we had access to microdata, we applied survey weights based on survey sampling frames to generate weighted national estimates of met need accompanied by estimates of standard error (SE). In the absence of microdata or survey sampling information, we used survey sample sizes as a mechanism for informing uncertainty estimation.

For a number of our data sources, we could not directly estimate met need with modern methods from microdata or survey reports did not include tabulated estimates of met need with modern methods; instead, the latter would include information on prevalence of modern contraception use, prevalence of any contraception use, and prevalence of unmet need for family planning among women of reproductive age. Following the recommended analytic approach from DHS and Inter-agency Expert Group on the SDG Indicators (IAEG-SDGs)[1,2], we estimated met need with modern methods based on this formula:

$$Prev_{MetMod} = \frac{Prev_{Mod}}{Prev_{Any} + Prev_{Unmet}}$$

where $Prev_{MetMod}$ is the prevalence of met need with modern methods among women aged 15 to 49 years; $Prev_{Mod}$ is the prevalence of current modern contraception use among women aged 15 to 49 years; $Prev_{Any}$ is the prevalence of any contraception use among women aged 15 to 49 years; and $Prev_{Unmet}$ is the prevalence of women who have need for family planning but are not currently using any method of contraception (capturing unmet need). In future iterations of this analysis, we will prioritize gaining access to microdata to these surveys, so that we can directly estimate met need with modern methods from individual-level data.

For a subset of surveys, contraception use and met need was only reported for women who were currently or had ever been married. To predict the prevalence of modern contraceptive use for all women, we ran a regression on observations where we had the prevalence of modern contraceptive use for all women as well as married women by age group and geographic region. Using this relationship we were able to cross-walk modern contraceptive use prevalence of all women for countries where only married women were surveyed. We repeated this prediction with met need for family planning, running a regression on observations where we had data on all women and married women by age group and

geographic region to predict the met need of women in countries for which only data on married women were available. Individual data points were reviewed by country and outliered accordingly.

Modelling strategy

For the present analysis, we implemented a two-part modelling approach: (1) generate a time series of modern contraception use for each country; and (2) generate a time series of met need with modern methods using modern contraception prevalence as a model covariate. Spatiotemporal Gaussian process regression (ST-GPR), a model used widely within the GBD study to synthesize coherent trends and uncertainty from multiple sources of data, was used for each of these steps.

Modern contraception prevalence

Based on cross-walked prevalence estimates of modern contraception use, we applied ST-GPR to generate a time series of modern contraception prevalence by geography and age group from 1990 to 2015. First, a mixed-effect linear model was fit based on a fixed effect on age and covariates on educational attainment among women of reproductive age and income per capita, and random effects for countries, GBD regions, and GBD super-regions. We used the predictions from that first-stage model to calculate residuals which were then smoothed over space and time. GPR was then used to compute prevalence of modern contraceptive use and corresponding uncertainty.

Met need with modern methods

We leveraged the relationship between modern contraception prevalence and met need with modern contraption for modeling the latter as far more data sources had information on modern contraception prevalence than met need. In terms of ST-GPR for met need with modern contraception, the first stage included fitting a mixed-effect linear model with fixed effects on age, educational attainment, and income per capita; random effects for countries, GBD regions, and GBD super-regions; and modern contraception prevalence as a covariate. Smoothing over space time based on the residuals from the first-stage linear model then took place, followed by GPR to generate a cohesive time series of met need with modern contraception and uncertainty for all 188 countries and from 1990 to 2015.

References

1. Bradley, S. E. K., Croft, T. N. & Fishel, J. D. Revising Unmet Need for Family Planning: DHS Analytical Studies No. 25. 63 (2012).

2. United Nations Department of Economics and Social Affairs. *Goal 3: Ensure healthy lives and promote well-being for all at all ages*. (2015). at <https://sustainabledevelopment.un.org/sdg3>

Adolescent Birth Rates SDG Capstone Appendix

Flowchart

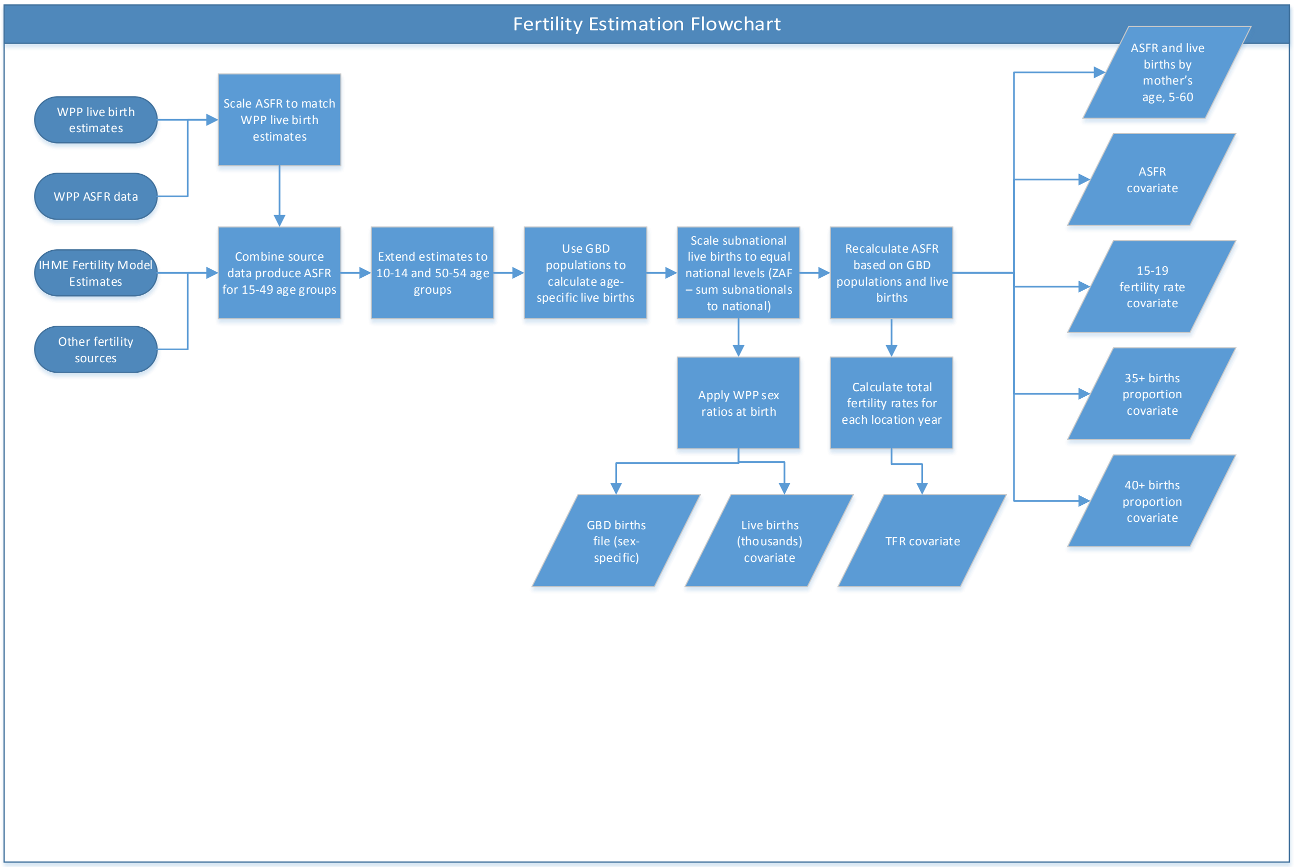

Input Data & Methodological Summary

Indicator definition

This modeling strategy encompassed the indicator associated with adolescent birth rates (3.7.2).

Indicator 3.7.2

As a component of SDG Goal 3. Ensure healthy lives and promote well-being for all at all ages, SDG Target 3.7, by 2030, ensure universal access to sexual and reproductive health-care services, including for family planning, information and education, and the integration of reproductive health into national strategies and programmes, is measured using SDG Indicator 3.7.2, birth rates (number of live births per 1,000 women) for women aged 10 to 14 years and women aged 15 to 19 years.

Input data

For locations where the United Nations Population (UNPOP) Division provides age-specific fertility rate (ASFR) for age groups 15-19, 20-24, 25-29, 30-34, 35-39, 40-44, 45-49 in their most recent update to the

World Population Prospect (WPP), we start with their estimates for every five year time period (e.g. 1990-1995). We treat the given value as that of the midpoint year, so in the case of 1990-1995, we use the value for 1992. We then linearly interpolate in log space to generate values for the intervening years.

Modeling Strategy

ASFR for locations not covered by the UNPOP

For locations not covered by UNPOP, including any subnational locations as well as countries such as Andorra, American Samoa, Bermuda, Northern Mariana Islands, and the United States (US) Virgin Islands, we took one of two approaches. If we could find relatively complete data for 1970's onwards, we would use those estimates. To address the small number of missing values in these datasets, we used a combination of linear mixed effects regression, simple linear interpolation, and 3-year rate of change extrapolation depending on the nature of values that were missing.

Linear mixed effects regression with age as categorical variable was applied to data when entire age groups were missing for a given location. Linear interpolation was applied to locations when missing ASFR values fell between years where ASFR was available. In locations where ASFR was missing for years where values did not fall between years where ASFR was available, but ASFR was present in years preceding or directly after the missing year, ASFR was calculated using annualized rate of change. Missing ASFR was interpolated based on the rate of change of ASFR of the 3 years preceding or following the missing year.

Secondly, in cases where there was little data or it did not cover most of the time period, we modeled ASFR using a database of fertility tables from the Human Fertility Database and from location-level surveys in the locations we were modeling. This process was as follows:

1. *Calculating empirical weights:* Using the database of tables, we created all possible pairs of tables. For each age category, we then calculated the difference between the two tables. These differences were then summed, producing a total difference for each pair of tables. We then created a series of indicator variables for each pair, indicating whether or not they were from the same country, region, or super-region, and how many years apart they were. We then average the difference for each category. So for example, we produced the mean difference for locations in the same super-region but not the same region or country that were 2 years apart in time. We then took the reciprocals of these differences to produce a weight, indicating how "close" a table is to another given their similarities in location and time.

2. *Fit model relating difference in total fertility rate (TFR) to difference in ASFR:* We again create all possible pairs of tables as in step 1. For each pair, we randomly select one table to be the predictor table. Then we fit the following model for each age group in 15-19, 20-24, 25-29, 30-34, 35-39, 40-44, and 45-49:

$$ASFR_1 - ASFR_2 = \beta_1(TFR_1 - TFR_2) + \beta_0$$

where table 1 is the table randomly designated as the predictor table.

3. *Produce a standard tables and predict:* Using the weights produced in the first step, we create a standard table from a selection of tables from the database. For each table, weights are calculated for all the other tables based on how far they are from the table in terms of year, and whether they are from the same country, region, or super-region. We then order by weight and

take the first 300 tables. We then create an average table, weighted by weights calculated in step 1. This produces a standard table for each location-year. Due to the limited number of tables for many location, this can produce discontinuities from year to year. To prevent this, we applied a rolling mean over time to the standard, resulting in estimates that are relatively smooth over time. We then use this standard to predict the ASFR for each age group using the models produced in step 2 and the TFR for the country-year of interest:

$$ASFR_1 = \beta_1(TFR_1 - TFR_{standard}) + \beta_0 + ASFR_{standard}$$

where TFR_1 is the TFR in the location year where we are predicting ASFR.

Getting single year ASFR and extending age groups

Once we have five year ASFR values, we calculate single year ASFR using a spline and treating the ASFR values as midpoints for each age group. Though we do not use single-year ASFR for maternal calculations, they are used in other parts of the GBD, and so are incorporated in this process.

For high and low ages, we set fertility for 9 and below and 55 and above to 0, then used those in the interpolation. Because many sources do not have ages 10-14 and 50-54, which are necessary for our maternal estimates, we also extend our estimates to include these age groups. To do this, we created a linear interpolation between the value at age 15 and 0 at age 9 on the young side, and between the value at 50 and 0 at age 55. To these values, we then applied percentages of women who have gone through puberty or have not gone through menopause, respectively. These values are given in the following tables:

age	percent through puberty
10	4
11	14
12	40
13	77
14	98

age	percent fertile
50	36
51	28
52	20
53	14
54	9

Because of the steep climb in fertility in the teen years, we made sure that our estimates in 10-14 were in-line with what we would expect by scaling them to the 15-19 age category. Using the mean of the ratios between 10-14 and 15-19 from the Indian Demographic and Health Survey (DHS), the US census, and the Democratic Republic of the Congo DHS, we scaled the 10, 11, 12, 13, and 14 ages so that their mean has this ratio with the mean of 15-19.

Scaling to births

To get our final ASFR estimates, we scale ASFR so that the total implied births from our ASFR estimates and the GBD populations is the same as the GBD births. GBD births are generally derived from the WPP 2015 Revision, and WHO, though for some locations we use location-specific sources. This scaling ensures consistency between our fertility results and the populations that are used in other parts of the GBD process. The exception to this is South Africa. There we used subnational estimates from UNAIDS,

calculated live births implied by these, then used their sum for the South Africa national ASFR estimates. These values were then substituted into the GBD births.

To re-calculate five year age groups, we calculate the number of births in each five year age group and divide by the population in that age group. These are the final ASFR estimates used in our maternal mortality calculations.

Universal Health Coverage (UHC) Tracer Indicator SDG Capstone Appendix

Summary of methodological approach

Indicator definition

This modeling strategy involves the construction of a composite indicator of universal health coverage (UHC) tracer interventions (Indicator 3.8.1), which include vaccination coverage (coverage of three doses of diphtheria-pertussis-tetanus [DPT3], measles vaccine, and three doses of the oral polio vaccine or inactivated polio vaccine); met need for modern contraception; antenatal care (ANC) coverage (one ANC visit [ANC1] and four ANC visits [ANC4]); skilled birth attendance (SBA); in-facility delivery rates; coverage of antiretroviral therapy (ART) among people living with HIV; tuberculosis (TB) case detection rates; and malaria intervention coverage (household ownership of insecticide-treated nets [ITNs]) in malaria-endemic countries.

Indicator 3.8.1

As a component of SDG Goal 3. Ensure healthy lives and promote well-being for all at all ages, SDG Target 3.8, achieve universal health coverage, including financial risk protection, access to quality essential health-care services and access to safe, effective, quality and affordable essential medicines and vaccines for all, is measured using SDG Indicator 3.8.1, UHC composite indicator based on the geometric mean of coverage of individual tracer interventions for prevention and treatment services.

UHC tracer indicator input data

Individual UHC tracer interventions serve as the input data for the composite UHC tracer indicator, and their write-ups are included in this portion of the appendix.

UHC tracer intervention	Main text	Appendix content
Diphtheria-pertussis-tetanus vaccination, three doses (DPT3)		
Measles vaccination		
Polio vaccination, three doses		
Met need with modern contraception methods		
Antenatal care, 1 visit (ANC1)		
Antenatal care, 4 visit (ANC4)		
Skilled birth attendance (SBA)		
In-facility delivery rate (IFD)		
Tuberculosis (TB) case detection rate		
Antiretroviral therapy (ART) coverage among people living with HIV		
Insecticide-treated net (ITN) coverage in malaria endemic countries		

In sum, each tracer intervention is estimated within the broader GBD study, with many used as covariates to inform cause-specific models. Most of the individual tracer interventions use population health survey microdata, or tabulated report data when microdata are not publicly available, as their primary input data

sources. For a subset of tracer interventions, including vaccination, TB case detection, and ITN coverages, administrative data sources are also used to supplement survey-based estimates.

Composite UHC tracer indicator modeling strategy

To construct the composite UHC tracer indicator, we used draw-level coverage estimates as computed as part of GBD 2015 and took the geometric mean of the 1,000 draws for each intervention and every geography-year under analysis. Ninety-five percent uncertainty intervals (95% UIs) were calculated by taking the 25th and 975th draws. There were two exceptions: (1) TB case detection rates, which were based on case detection reports from the World Health Organization (WHO) and interpolated to construct a full time series; and (2) ITN coverage, which was estimated separately for 40 malaria-endemic countries in sub-Saharan Africa and endemic countries outside of sub-Saharan Africa. More detail can be found in the following appendix section.

Upon calculating the geometric mean for each intervention and geography-year, we took the geometric mean of across the individual interventions to compute the composite UHC indicator for each geography from 1990 to 2015.

We tested two different modeling strategies. The first was taking the geometric mean of each individual tracer intervention to directly compute the composite UHC tracer indicator. The second was grouping a subset of interventions – those related to maternal health (ANC1, ANC4, SBA, IFD, and met need with modern contraception) and vaccination (measles, DPT3, and polio) – and taking the geometric means of each subgroup. The UHC tracer indicator composite was then based on the geometric mean of five interventions: maternal health indicators, vaccine indicators, TB case detection, ART coverage, and ITN coverage. Our final strategy was first approach – taking the geometric mean of each tracer intervention – as the individual interventions within vaccination and particularly maternal health represent different modes of health service delivery and responsiveness to population health needs.

Models were evaluated by expert review and tracer interventions for which poor data quality or availability had large effects on the composite UHC tracer indicator were excluded; as more data become available or standardized data processing steps can be evaluated and implemented, we aim to include a broader range of UHC tracer interventions. Using the same data, geometric means generally result in lower point estimates than arithmetic means. For the present study, we preferred using the geometric mean for calculating the composite UHC tracer indicator to prevent the undue influence of any one particular intervention.

This is the first time that the composite UHC tracer intervention indicator has been calculated. As the annual GBD study expands its analyses to include more interventions and types of prevention or treatment services, we will also extend the number of UHC tracer interventions and services encompassed by the composite indicator.

UHC – ART SDG Capstone Appendix

Indicator definition

This modeling strategy encompassed the indicator associated with universal health coverage (3.8.1), specifically antiretroviral therapy (ART) coverage.

Indicator 3.8.1

As a component of SDG Goal 3. Ensure healthy lives and promote well-being for all ages, SDG Target 3.8, achieve universal health coverage, including financial risk protection, access to quality essential health-care services and access to safe, effective, quality and affordable essential medicines and vaccines for all, is measured using SDG Indicator 3.8.1, UHC composite indicator based on the geometric mean of coverage of tracer interventions for prevention and treatment services.

Input data

ART Coverage Data

We define ART coverage as the percentage of individuals living with HIV who are receiving ART. This can be broken into two components: the numerator is the number of people receiving ART and the denominator is the number of people living with HIV. Location-, year-, and sex-specific data on the number of individuals receiving ART or the percentage of HIV positive individuals receiving ART were extracted from UNAIDS country files. Whether or not coverage is reported as a count or a percentage varies by year for a given location and sex. This data was estimated by UNAIDS using facility data reported to the WHO by ministries of health as well as data reported by non-profit organizations, private companies, and insurance companies (Cite: Estimation of antiretroviral therapy coverage: methodology and trends. Mary Mahy et al.).

Modelling strategy

Full details of the modelling strategy can be found in the GBD 2015 HIV paper published in Lancet HIV. Spectrum, the compartmental model used for estimation of HIV burden, takes ART coverage as an input which informs the initiation of treatment by sub-group within Spectrum. We report the number of individuals found to be on treatment after running Spectrum so that our estimates of the number people receiving treatment are consistent with our estimates of the number of people living with HIV

UHC Tracer Indicator - Skilled Birth Attendance Capstone Appendix

Flowchart

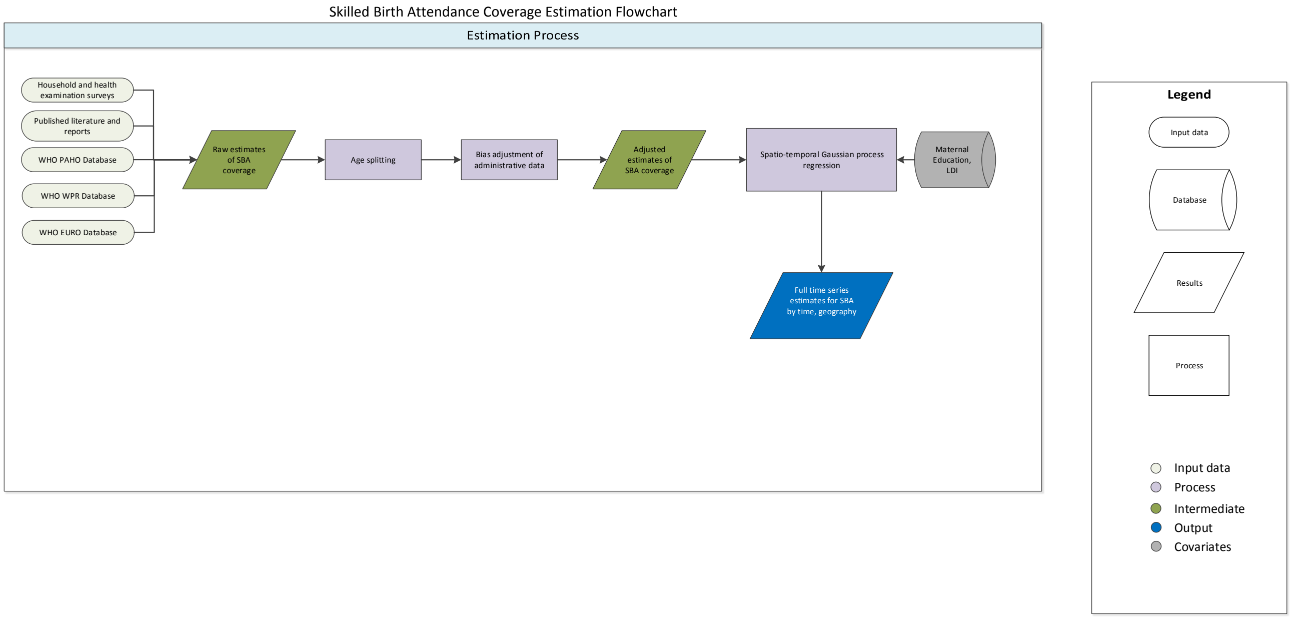

Input data & Methodological summary

Indicator definition

This modeling strategy pertains to the composite universal health coverage (UHC) tracer indicator (Indicator 3.8.1) and specifically the estimation of skilled birth attendance (SBA).

Indicator 3.8.1

As a component of SDG Goal 3. Ensure healthy lives and promote well-being for all at all ages, SDG Target 3.8, achieve universal health coverage, including financial risk protection, access to quality essential health-care services and access to safe, effective, quality and affordable essential medicines and vaccines for all, is measured using SDG Indicator 3.8.1, as SBA. Note that SBA is also represented by Indicator 3.1.2.

Input data

For the present analysis, we used individual-level microdata from population health surveys and tabulated survey report data on skilled birth attendance (SBA). As defined by the World Health Organization (WHO), SBA reflects the proportion of births in a given year where a doctor, nurse, or midwife was present.[1]

Survey data which provided individual-level data, and specifically among female respondents, were identified and extracted. Major multi-country survey programs included in the analysis include the Demographic and Health Surveys (DHS),[2] Multiple Indicator Cluster Surveys (MICS),[3] Reproductive Health Surveys (RHS),[4] Living Standards Measurement Study (LSMS) surveys,[5] and World Health Surveys (WHS).[6]

We also conducted a comprehensive search of the Global Health Data Exchange (GHDx),[7]as well as targeted internet searches and review of Ministry of Health websites, to identify national surveys and other multi-country survey programs. In addition, we utilized tabulated report data from regional WHO databases, when available, including the PAHO, WHO WPR, and the WHO European Health for All databases.

We excluded all data sources that were not nationally representative or had high levels of missingness. We applied survey weights based on survey sampling frames whenever they were available to generate weighted national estimates of SBA coverage accompanied by estimates of standard error (SE). Estimates of SE, as well as sample sizes, were used to calculate uncertainty, as described below. Any point estimates with sample sizes less than 50 were reviewed to ensure that were not substantive outliers and would otherwise have an undue influence on our analysis.

Due to potential bias in recall, we limited our analysis to women who gave birth up to five years prior to the time of survey; due to data limitations, we used a limit of up to two years for some surveys. We also had to standardize the definition of "skilled health professional" across countries, which varied by differences in quality of training or health professional roles. For this analysis, doctors, nurses, and midwives were included as our foundational definition for SBA, and we extended this to include country-specific medical staff based on the number of years of training they received and/or their comparable ability to intervene in an emergency situation (eg, clinical officers). Care received during delivery by traditional health personnel was not considered a birth overseen by a skilled attendant.

Modeling strategy

Data processing

Age splitting

Most household surveys collection information on maternal and child health (MCH) indicators for children under 5 and/or mothers who gave birth within five years prior to the time of survey. To maximize data use for our model, we included SBA information for children aged 12 to 59 at the time of survey. Children younger than 12 months of age were excluded to minimize the influence of potentially censored observations. SBA coverage estimates were assigned to birth-cohort years based on a child's age prior to the time of survey: we used responses recorded for children aged 12 to 23 months for SBA coverage for one year prior to the time of survey, children aged 24 to 35 months for coverage two years prior to the time of survey, and so forth.

Age-specific estimates are easily computed from individual-level microdata, but many published reports and survey summaries present data in broader age aggregates (eg, SBA coverage for children aged 12 to 35 months). To standardize these age groups, we applied an age-splitting model used in the GBD study,[9] as well as analyses that generated smoking and obesity prevalence by age group.[10,11]

Using surveys with microdata as the reference, we used the following model to generate standardized age group-specific estimates for SBA:

$$\tilde{P}_{a,c,t,k} = P_{a,c,t,k}^{a+x} \frac{P_{a,c,t,j}}{P_{a,c,t,j}^{a+x}}$$

where $\tilde{P}_{a,c,k}$ is the adjusted estimate of coverage for target age group a in country c and year t of survey k; and $P_{a,c,k}^{a+x}$ is coverage reported from survey k, for country c in year t for the age group spanning age a to age $(a + x)$. The ratio of coverage between the target age group and broader age group from a survey j with microdata from the same country-year was used to split data from survey k. Surveys to be split were ideally matched with DHS or MICS surveys. If microdata were not available for the same year, ratios within five years of the survey that required age-splitting were applied.

Bias adjustments

Intervention coverage estimates based on administrative sources can be biased, yet the direction and magnitude of such biases are not universal. Some studies show that coverage estimates from administrative data source are systematically higher than those of survey-based estimates,[12] while other studies show that bias directionality is more heterogeneous.[13] Such biases may arise for a number of reasons, including discrepancies in the accurate reporting of services or interventions provided (eg, number of skilled attendants) and target population (eg, number of children born), as well as capturing these data in a timely manner from both public and private sector facilities and healthcare providers.

For SBA, we view individual-level data collected through population health surveys as the most accurate and least biased source of information, particularly for geographies with incomplete health information systems. We thus used SBA coverage estimates from household surveys to calculate country-specific adjustment factors:

$$logit\left(P_{s,\,c,t}\right) = \beta_0 + \beta_1 logit\left(\tilde{P}_{a,\,c,t}\right) + \sum_{k=2}^{2+B} \beta_k S_k + \varepsilon_{c,t}$$

where $P_{s,\,c,t}$ is the survey-based estimate for SBA coverage (s) in country c for year t; $\tilde{P}_{a,\,c,t}$ is the administrative estimate for coverage in country c in year t; S_k is a spline basis used to capture the secular trend in coverage; β_1 is the estimated adjustment factor used to correct for the administrative bias; and ε is the error term for country c in year t.

To quantify uncertainty for bias-adjusted estimates from the mixed-effects models described above, we calculated prediction error, $\widehat{PE}$, as follows:

$$\widehat{PE} = X^2 var(\hat{\beta})$$

where $var(\hat{\beta})$ is the variance for the estimated fixed-effects coefficient of the adjustment factor and X is the independent variable. Proper estimation of prediction errors is crucial as the data synthesis procedure, Gaussian process regression (GPR) (as described in the subsequent section), accounts for uncertainty from point estimates and bias adjustments when generating fitted values. More weight is given to data with less uncertainty. Prediction errors estimated from the bias adjustment were incorporated into the data variance and propagated through the GPR step to obtain estimates of SBA coverage and uncertainty intervals (UIs).

Trend estimation

We used a spatiotemporal Gaussian process regression (ST-GPR) to synthesize point estimates from multiple data sources and derive a complete time series for SBA coverage. This method has been used extensively in GBD and related studies, and accounts for uncertainty pertaining to each point estimate while borrowing strength across geographic space and time.[10, 11,15,16] Briefly, we assumed the Gaussian process was defined by a mean function *m*(•) and covariance function *Cov*(•).

We estimated the mean function using a two-step approach. Specifically, $m_c(t)$ can be expressed as:

$$m_c(t) = X\beta + h(r_{c,t})$$

where $X\beta$ is a linear model and $h(r_{c,t})$ is a smoothing function for the residuals; and $r_{c,t}$ is derived from the linear model. The following linear model was used for estimating SBA:

$$logit(P_{c,t}) = \beta_0 + \beta_1 medu_{c,t} + \beta_1 LDI_{c,t} + \alpha_c + \gamma_{R[c]} + \delta_c medu + \theta_{R[c]} medu + \varepsilon_{c,t}$$

where $P_{c,t}$ is SBA coverage for country c year t; $medu_{c,t}$ is the average years of education for women of reproductive age in country c and year t; $LDI_{c,t}$ is the lag-distributed income in country c and year $t;$ α_c and $\gamma_{R[c]}$ are country and region random intercepts, respectively. δ_c and $\theta_{R[c]}$ are country and region specific slope on education. These estimates were then run through ST-GPR, as documented elsewhere.[10]

Random draws of 1,000 samples were obtained from the distributions above for every country for a given vaccine. Ninety-five percent uncertainty intervals were calculated by taking the ordinal 25 and 975th draws from the sample distribution.

To assess the accuracy of our estimates in each bias adjustment step and in the modeling process, we performed cross-validation analyses by randomly holding out 20% of the sample and, if available, the corresponding administrative estimates for the given indicator of the same country and year, 10 separate times. We computed the average root mean squared errors (RMSE) across each country. Error in the bias adjustments was calculated as the mean difference between the adjusted administrative estimate for a given country, year, and corresponding survey-level estimates (which were considered the "gold-standard"); error in the modeling process was calculated as the difference between the modeled estimates and the sample data.

References

1 WHO Indicator and Measurement Registry (WHO IMR). WHO IMR. http://apps.who.int/gho/indicatorregistry/App_Main/indicator_registry.aspx (accessed Aug 11, 2015).

2 Measure DHS: Demographic and Health Surveys. http://www.measuredhs.com (accessed Aug 11, 2015).

3 UNICEF Stat. Monit. Multiple Indicator Cluster Survey (MICS). http://www.unicef.org/statistics/index_24302.html (accessed Aug 11, 2015).

4 Cent. Dis. Control Prev. Reproductive Health Surveys (RHS). http://www.cdc.gov/reproductivehealth/Global/surveys.htm (accessed Aug 11, 2015).

5 World Bank. Living Standard Measruement Studies (LSMS). http://go.worldbank.org/UK1ETMHBN0 (accessed Aug 11, 2015).

6 WHO Multi-Ctry. Stud. Data Arch. World Health Survey (WHS). http://apps.who.int/healthinfo/systems/surveydata/index.php/catalog/whs/about (accessed Aug 11, 2015).

7 IHME GHDx. Global Helath Data Exchange. http://ghdx.healthdata.org/ (accessed Aug 11, 2015).

8 WHO | WHO/UNICEF Joint Reporting Process. WHO. http://www.who.int/immunization/monitoring_surveillance/routine/reporting/reporting/en/ (accessed Aug 17, 2015).

9 Collaboration PS. Age-specific relevance of usual blood pressure to vascular mortality: a meta-analysis of individual data for one million adults in 61 prospective studies. *The Lancet* 2002; **360**: 1903–13.

10 Ng M, Freeman MK, Fleming TD, *et al.* Smoking Prevalence and Cigarette Consumption in 187 Countries, 1980-2012. *JAMA* 2014; **311**: 183.

11 Ng M, Fleming T, Robinson M, *et al.* Global, regional, and national prevalence of overweight and obesity in children and adults during 1980–2013: a systematic analysis for the Global Burden of Disease Study 2013. *The Lancet* 2014; **384**: 766–81.

12 Murray CJ, Shengelia B, Gupta N, Moussavi S, Tandon A, Thieren M. Validity of reported vaccination coverage in 45 countries. *The Lancet* 2003; **362**: 1022–7.

13 Lim SS, Stein DB, Charrow A, Murray CJ. Tracking progress towards universal childhood immunisation and the impact of global initiatives: a systematic analysis of three-dose diphtheria, tetanus, and pertussis immunisation coverage. *The Lancet* 2008; **372**: 2031–46.

Tuberculosis Case Detection SDG Capstone Appendix

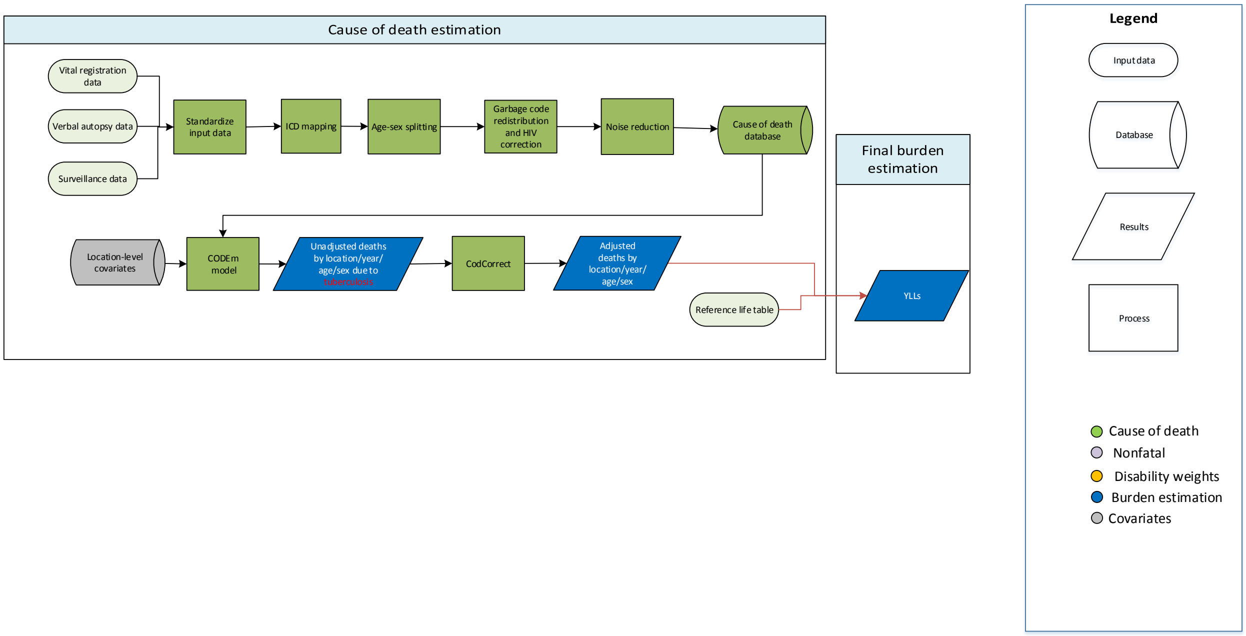

Input Data & Methodological Summary

Indicator definition

This modeling strategy encompassed the indicator associated with universal health care – ITN ownership/TB case detection/immunization/ANC1 and ANC4/in-facility delivery rate/skilled birth attendant/ART coverage/modern contraceptive coverage (3.8.1).

Indicator 3.8.1

As a component of SDG Goal 3. Ensure healthy lives and promote well-being for all ages, SDG Target 3.8, achieve universal health coverage, including financial risk protection, access to quality essential health-care services and access to safe, effective, quality and affordable essential medicines and vaccines for all, is measured using SDG Indicator 3.8.1.

Input data

Input data for modeling tuberculosis mortality among HIV-negative individuals include vital registration, verbal autopsy, and surveillance data from the World Health Organization (WHO). Vital registration data were adjusted for garbage coding (including ill-defined codes, and the use of intermediate causes) following GBD algorithms and misclassified HIV deaths (i.e., HIV deaths being assigned to other underlying causes of death such as tuberculosis or diarrhea because of stigma or misdiagnosis). This correction was done based on examining changes in the age pattern of diseases over time.

Verbal autopsy data in countries with age-standardized HIV prevalence greater than 5% were removed because of a high probability of misclassification, as verbal autopsy studies have poor validity in distinguishing HIV deaths from HIV-TB deaths. We also outliered data that were largely conflicting with the majority of data from other studies conducted either in the same or different countries (with similar sociodemographic characteristics) in the same region.

Modeling strategy

A general CODEm modeling strategy was used.

UHC – ANC 1/ANC4 Capstone Appendix

Flowchart

3.8.1 UHC – ANC1/ANC4 Coverage Estimation Flowchart

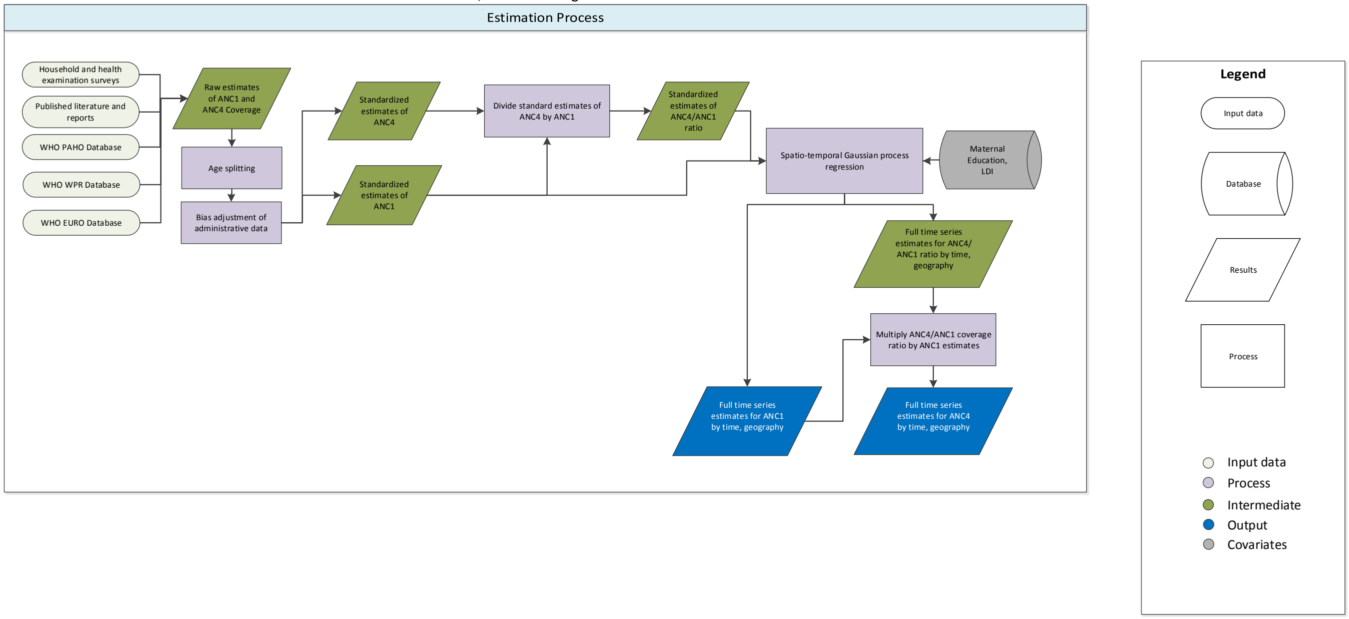

Input data & Methodological summary

Indicator definition

This modeling strategy encompassed the indicator associated with universal health care – ITN ownership/TB case detection/immunization/ANC1 and ANC4/in-facility delivery rate/skilled birth attendant/ART coverage/modern contraceptive coverage (3.8.1).

Indicator 3.8.1

As a component of SDG Goal 3, ensure healthy lives and promote well-being for all at all ages, SDG Target 3.8, achieve universal health coverage, including financial risk protection, access to quality essential health-care services and access to safe, effective, quality and affordable essential medicines and vaccines for all, is measured using SDG Indicator 3.8.1, coverage of tracer interventions for prevention and treatment services.

Input data

Our study included data from household level surveys as well as administrative reports of the proportion of skilled antenatal care attendance of at least one (ANC1) or at least four visits (ANC4) for a given birth, where skilled is defined by the WHO as visits where a doctor, nurse, or trained midwife was present.[1] Survey data which provided person-level information on utilization of maternal health services were identified and extracted. Major multi-country survey programs included in the analysis include the Demographic and Health Surveys (DHS)[2], the Multiple Indicator Cluster Surveys (MICS)[3], the Reproductive

Health Surveys (RHS)[4], the Living Standards Measurement Studies[5], and the World Health Surveys (WHS)[6]. In additional, a comprehensive search was performed on the Global Health Data Exchange (GHDx)[7], as well as a targeted Google search and a search on the websites of national ministries of health, to identify national surveys and smaller multi-country surveys.

A comprehensive database of administrative estimates of maternal health service coverage were not available. Instead, we utilized administrative estimates from regional WHO databases, when available, including the PAHO, WHO WPR, and the WHO European Health for All databases.

Sources that were not nationally representative were excluded, as were data from sources with high levels of missingness for a given indicator. Survey weights and clustering methodology were applied, when available, to obtain weighted national estimates of coverage with accurate estimates of standard errors. In addition to prevalence estimates, we captured information on sample size and standard errors, which were utilized in subsequent analytical steps to capture uncertainty from the data. All data points with sample sizes less than 50 were carefully reviewed to ensure that small samples would not influence the accuracy of analysis. Such points with extreme coverage levels were excluded. We utilize a total of 8,406 data points in our estimation process.

Modeling strategy

Data processing

Age Splitting

Household-level surveys typically collect information about MCH indicators for children under 5 years of age or mothers who have given birth at most 5 years prior to the time of survey. For the sake of utilizing as much data as available, we incorporated estimates for births 0-59 months prior to the survey for analysis. For each indicator, estimates were assigned to a given birth cohort year based on the birth age prior to the time of interview—we used the responses recorded for children aged 12-23 months to estimate coverage 1 year prior to the survey, 24-35 months to estimate coverage 2 years prior to the survey, and so forth.

While information aggregated to these specific age ranges was easily extracted from surveys with person-level data, many published reports and summaries of surveys presented data in broader age groups. We disaggregated these data into the age grouping of interest in this study by applying a splitting model previously used in the Global Burden of Diseases, Injuries, and Risk Factors Study (GBD)[9], as well as in a studies estimating global smoking[10] and obesity prevalence[11].

Using surveys that provided person-level data as references, the following model was applied on estimates with the broader age groups. Specifically, let $\tilde{P}_{a,c,k}$ be the adjusted estimate of coverage for a given indicator for the target age group a in country c and year t of survey k. To disaggregate data that were reported in a broader age group, the following formula was used:

$$\tilde{P}_{a,c,t,k} = P^{a+x}_{a,c,t,k} \frac{P_{a,c,t,j}}{P^{a+x}_{a,c,t,j}}$$

Where $P^{a+x}_{a,c,k}$ denotes the coverage reported from survey k, for country c in year t, but of the age group spanning age a to age $(a + x)$. The ratio of coverage between the age group of interest and the broader age group from a survey j with person-level data from the same country and year was used to split data from survey k. Surveys to be split were ideally matched with DHS or MICS surveys. If person-level data was not available for the same year, data within 5 years to be split was used.

Bias adjustments

Administrative estimates of SBA are most typically produced using data gathered from supply-side registries. The quality and accuracy of the data therefore depends on the completeness of the nation's health information system.[8] Previous studies have reported that administrative reports of MCH coverage indicators tend to be biased.[8,12,13]

To reduce the impact of these biases on the final results, we performed adjustments on administrative data to account for overall systematic error. Using mixed effects models, we compared administrative data and survey data to derive appropriate adjustment ratios:

$$logit\left(P_{s,\,c,t}\right) = \beta_0 + \beta_1 logit\left(\tilde{P}_{a,\,c,t}\right) + \varepsilon_{c,t}$$

where $P_{s,\,c,t}$ is the survey-based coverage for a specific indicator for country c in year t, $\tilde{P}_{a,\,c,t}$ is the administrative coverage for country *c* in year *t*, β_1 is the estimated adjustment factor used to correct for the administrative bias.

Trend estimation

We used a spatiotemporal Gaussian process regression (ST-GPR) to synthesize information from the various data sources in order to derive a complete time series for each indicator for all countries. This method has been used extensively in other studies to combine information from different sources, taking into account uncertainty for each data point as well as to interpolate nonlinear trends by borrowing strength across geographic space and time. [9-11] Briefly, we assumed the Gaussian process was defined by a mean function *m*(•) and covariance function *Cov*(•). The mean function was estimated using a mixed-effects linear regression as specified below:

$$logit(P_{c,t}) = \beta_0 + \beta_1 medu_{c,t} + +\beta_2 gdp_{ct} + \tau_t + \alpha_c + \gamma_{R[c]} + \varepsilon_{c,t} \qquad (1)$$

where $P_{c,t}$ is the estimated coverage of SBA in country c and year t, $medu_{c,t}$ is the average years of education for women of reproductive age, α_c and $\gamma_{R[c]}$ are country and region random intercept respectively, and τ_t is a random intercept on year. The estimates were then run through ST-GPR, as documented in Ng et. al (2014). [10] Random draws of 1,000 samples were obtained from the distributions above for every country for a given indicator. The final estimated prevalence for each country was the mean of the draws. In addition, 95% uncertainty intervals were calculated by taking the 2.5 and 97.5 percentile of the sample distribution.

To assess the accuracy of our estimates in each bias adjustment step and in the modeling process, we performed cross-validation by randomly holding out 20% of the sample and, if available, the corresponding administrative estimates for the given indicator of the same country and year, 10 separate times. We computed the average root mean squared errors (RMSE) across each country by indicator. Error in the bias adjustments was calculated as the mean difference between the adjusted administrative estimate for a given country, year and corresponding survey-level estimates (which were considered the "gold-standard"); error in the modeling process was calculated as the difference between the modeled estimates and the sample data.

References

1 WHO Indicator and Measurement Registry (WHO IMR). WHO IMR. http://apps.who.int/gho/indicatorregistry/App_Main/indicator_registry.aspx (accessed Aug 11, 2015).

2 Measure DHS: Demographic and Health Surveys. http://www.measuredhs.com (accessed Aug 11, 2015).

3 UNICEF Stat. Monit. Multiple Indicator Cluster Survey (MICS). http://www.unicef.org/statistics/index_24302.html (accessed Aug 11, 2015).

4 Cent. Dis. Control Prev. Reproductive Health Surveys (RHS). http://www.cdc.gov/reproductivehealth/Global/surveys.htm (accessed Aug 11, 2015).

5 World Bank. Living Standard Measruement Studies (LSMS). http://go.worldbank.org/UK1ETMHBN0 (accessed Aug 11, 2015).

6 WHO Multi-Ctry. Stud. Data Arch. World Health Survey (WHS). http://apps.who.int/healthinfo/systems/surveydata/index.php/catalog/whs/about (accessed Aug 11, 2015).

7 IHME GHDx. Global Helath Data Exchange. http://ghdx.healthdata.org/ (accessed Aug 11, 2015).

8 WHO | WHO/UNICEF Joint Reporting Process. WHO. http://www.who.int/immunization/monitoring_surveillance/routine/reporting/reporting/en/ (accessed Aug 17, 2015).

9 Collaboration PS. Age-specific relevance of usual blood pressure to vascular mortality: a meta-analysis of individual data for one million adults in 61 prospective studies. *The Lancet* 2002; **360**: 1903–13.

10 Ng M, Freeman MK, Fleming TD, *et al.* Smoking Prevalence and Cigarette Consumption in 187 Countries, 1980-2012. *JAMA* 2014; **311**: 183.

11 Ng M, Fleming T, Robinson M, *et al.* Global, regional, and national prevalence of overweight and obesity in children and adults during 1980–2013: a systematic analysis for the Global Burden of Disease Study 2013. *The Lancet* 2014; **384**: 766–81.

12 Murray CJ, Shengelia B, Gupta N, Moussavi S, Tandon A, Thieren M. Validity of reported vaccination coverage in 45 countries. *The Lancet* 2003; **362**: 1022–7.

13 Lim SS, Stein DB, Charrow A, Murray CJ. Tracking progress towards universal childhood immunisation and the impact of global initiatives: a systematic analysis of three-dose diphtheria, tetanus, and pertussis immunisation coverage. *The Lancet* 2008; **372**: 2031–46.

UHC – In-facility Birth Rate (IFD) Capstone Appendix

Input data & methodological summary

3.8.1 UHC – In Facility Birth Rate Coverage Estimation Flowchart

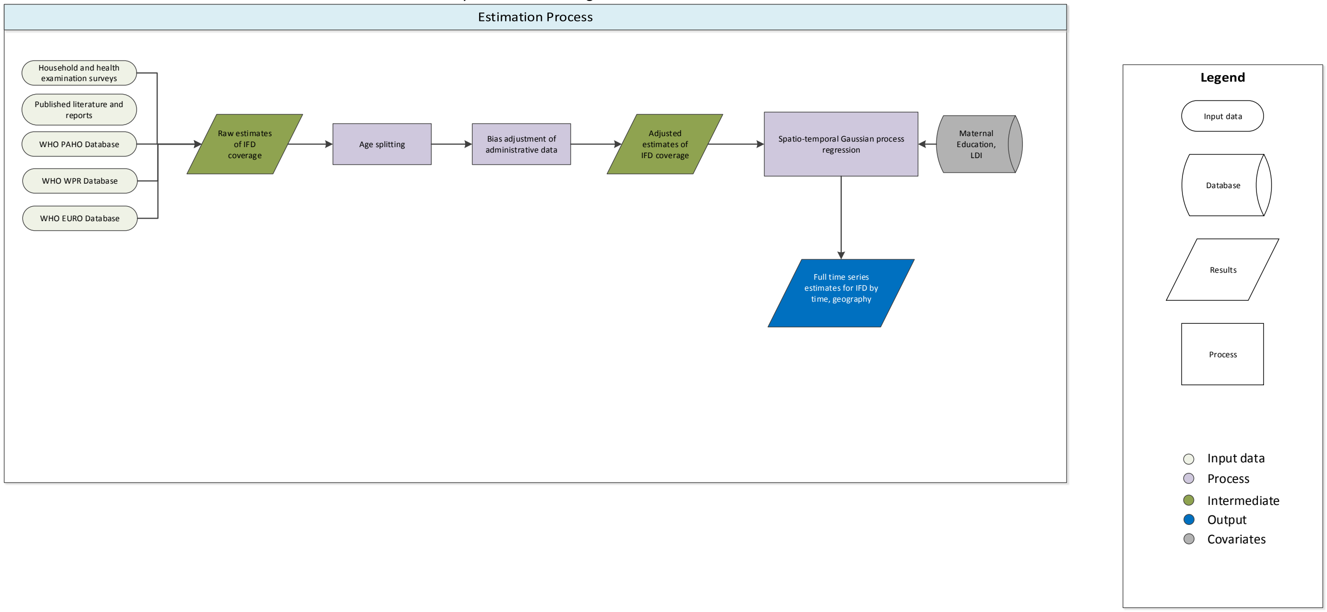

Indicator definition

Indicator definition This modeling strategy encompassed the indicator associated with universal health care – ITN ownership/TB case detection/immunization/ANC1 and ANC4/in-fertility birth rate/ART coverage/modern contraceptive coverage (3.8.1).

Indicator 3.8.1

As a component of SDG Goal 3. Ensure healthy lives and promote well-being for all at all ages, SDG Target 3.8, achieve universal health coverage, including financial risk protection, access to quality essential health-care services and access to safe, effective, quality and affordable essential medicines and vaccines for all, is measured using SDG Indicator 3.8.1, in-facility birth rate (IFD).

Input data

Our study included data from household level surveys as well as administrative reports of in-facility delivery (IFD), defined by the WHO as the proportion of births in a given year delivered in a health facility.[1] Survey data which provided person-level information were identified and extracted. Major multi-country survey programs included in the analysis include the Demographic and Health Surveys (DHS)[2], the Multiple Indicator Cluster Surveys (MICS)[3], the Reproductive Health Surveys (RHS)[4], the Living Standards Measurement Studies[5], and the World Health Surveys (WHS)[6]. In additional, a comprehensive search was performed on the Global Health Data Exchange (GHDx)[7], as well as a targeted Google search and a search on the websites of national ministries of health, to identify national surveys and smaller multi-country

surveys. In addition we utilized administrative estimates from regional WHO databases, when available, including the PAHO, WHO WPR, and the WHO European Health for All databases.

Sources that were not nationally representative were excluded, as were data from sources with high levels of missingness. Survey weights and clustering methodology were applied, when available, to obtain weighted national estimates of coverage with accurate estimates of standard errors. In addition to prevalence estimates, we captured information on sample size and standard errors, which were utilized in subsequent analytical steps to capture uncertainty from the data. All data points with sample sizes less than 50 were carefully reviewed to ensure that small samples would not influence the accuracy of analysis. Such points with extreme coverage levels were excluded. In total, we utilized 8,406 datapoints in our model.

Modeling strategy

Data processing

Age Splitting

Household-level surveys typically collect information about MCH indicators for children under 5 years of age or mothers who have given birth at most 5 years prior to the time of survey. For the sake of utilizing as much data as available, we incorporated estimates for births 0-59 months prior to the survey for analysis. For each indicator, estimates were assigned to a given birth cohort year based on the birth age prior to the time of interview—we used the responses recorded for children aged 12-23 months to estimate coverage 1 year prior to the survey, 24-35 months to estimate coverage 2 years prior to the survey, and so forth.

While information aggregated to these specific age ranges was easily extracted from surveys with person-level data, many published reports and summaries of surveys presented data in broader age groups. We disaggregated these data into the age grouping of interest in this study by applying a splitting model previously used in the Global Burden of Diseases, Injuries, and Risk Factors Study (GBD)[9], as well as in a studies estimating global smoking[10] and obesity prevalence[11].

Using surveys that provided person-level data as references, the following model was applied on estimates with the broader age groups. Specifically, let $\tilde{P}_{a,c,k}$ be the adjusted estimate of coverage for a given indicator for the target age group a in country c and year t of survey k. To disaggregate data that were reported in a broader age group, the following formula was used:

$$\tilde{P}_{a,c,t,k} = P^{a+x}_{a,c,t,k} \frac{P_{a,c,t,j}}{P^{a+x}_{a,c,t,j}}$$

Where $P^{a+x}_{a,c,k}$ denotes the coverage reported from survey k, for country c in year t, but of the age group spanning age a to age $(a + x)$. The ratio of coverage between the age group of interest and the broader age group from a survey j with person-level data from the same country and year was used to split data

from survey k. Surveys to be split were ideally matched with DHS or MICS surveys. If person-level data was not available for the same year, data within 5 years to be split was used.

Bias adjustments

Administrative estimates of IFD are most typically produced using data gathered from supply-side registries. The quality and accuracy of the data therefore depends on the completeness of the nation's health information system.[8] Previous studies have reported that administrative reports of MCH coverage indicators tend to be biased.[8,12,13]

To reduce the impact of these biases on the final results, we performed adjustments on administrative data to account for overall systematic error. Using mixed effects models, we compared administrative data and survey data to derive appropriate adjustment ratios:

$$logit\left(P_{s,\,c,t}\right) = \beta_0 + \beta_1 logit\left(\tilde{P}_{a,\,c,t}\right) + \varepsilon_{c,t}$$

where $P_{s,\,c,t}$ is the survey-based coverage for a specific indicator for country c in year t, $\tilde{P}_{a,\,c,t}$ is the administrative coverage for country *c* in year *t,* β_1 is the estimated adjustment factor used to correct for the administrative bias.

Trend estimation

We used a spatiotemporal Gaussian process regression (ST-GPR) to synthesize information from the various data sources in order to derive a complete time series for each indicator for all countries. This method has been used extensively in other studies to combine information from different sources, taking into account uncertainty for each data point as well as to interpolate nonlinear trends by borrowing strength across geographic space and time. [9-11] Briefly, we assumed the Gaussian process was defined by a mean function *m*(•) and covariance function *Cov*(•). The mean function was estimated using a mixed-effects linear regression as specified below:

$$logit(P_{c,t}) = \beta_0 + \beta_1 medu_{c,t} + +\beta_2 gdp_{ct} + \tau_t + \alpha_c + \gamma_{R[c]} + \varepsilon_{c,t} \qquad (1)$$

where $P_{c,t}$ is the estimated coverage of IFD in country c and year t, $medu_{c,t}$ is the average years of education for women of reproductive age, α_c and $\gamma_{R[c]}$ are country and region random intercept respectively, and τ_t is a random intercept on year. The estimates were then run through ST-GPR, as documented in Ng et. al (2014). [10] Random draws of 1,000 samples were obtained from the distributions above for every country for a given indicator. The final estimated prevalence for each country was the

mean of the draws. In addition, 95% uncertainty intervals were calculated by taking the 2.5 and 97.5 percentile of the sample distribution.

To assess the accuracy of our estimates in each bias adjustment step and in the modeling process, we performed cross-validation by randomly holding out 20% of the sample and, if available, the corresponding administrative estimates for the given indicator of the same country and year, 10 separate times. We computed the average root mean squared errors (RMSE) across each country by indicator. Error in the bias adjustments was calculated as the mean difference between the adjusted administrative estimate for a given country, year and corresponding survey-level estimates (which were considered the "gold-standard"); error in the modeling process was calculated as the difference between the modeled estimates and the sample data.

References

1 WHO Indicator and Measurement Registry (WHO IMR). WHO IMR. http://apps.who.int/gho/indicatorregistry/App_Main/indicator_registry.aspx (accessed Aug 11, 2015).

2 Measure DHS: Demographic and Health Surveys. http://www.measuredhs.com (accessed Aug 11, 2015).

3 UNICEF Stat. Monit. Multiple Indicator Cluster Survey (MICS). http://www.unicef.org/statistics/index_24302.html (accessed Aug 11, 2015).

4 Cent. Dis. Control Prev. Reproductive Health Surveys (RHS). http://www.cdc.gov/reproductivehealth/Global/surveys.htm (accessed Aug 11, 2015).

5 World Bank. Living Standard Measruement Studies (LSMS). http://go.worldbank.org/UK1ETMHBN0 (accessed Aug 11, 2015).

6 WHO Multi-Ctry. Stud. Data Arch. World Health Survey (WHS). http://apps.who.int/healthinfo/systems/surveydata/index.php/catalog/whs/about (accessed Aug 11, 2015).

7 IHME GHDx. Global Helath Data Exchange. http://ghdx.healthdata.org/ (accessed Aug 11, 2015).

8 WHO | WHO/UNICEF Joint Reporting Process. WHO. http://www.who.int/immunization/monitoring_surveillance/routine/reporting/reporting/en/ (accessed Aug 17, 2015).

9 Collaboration PS. Age-specific relevance of usual blood pressure to vascular mortality: a meta-analysis of individual data for one million adults in 61 prospective studies. *The Lancet* 2002; **360**: 1903–13.

10 Ng M, Freeman MK, Fleming TD, *et al.* Smoking Prevalence and Cigarette Consumption in 187 Countries, 1980-2012. *JAMA* 2014; **311**: 183.

11 Ng M, Fleming T, Robinson M, *et al.* Global, regional, and national prevalence of overweight and obesity in children and adults during 1980–2013: a systematic analysis for the Global Burden of Disease Study 2013. *The Lancet* 2014; **384**: 766–81.

12 Murray CJ, Shengelia B, Gupta N, Moussavi S, Tandon A, Thieren M. Validity of reported vaccination coverage in 45 countries. *The Lancet* 2003; **362**: 1022–7.

13 Lim SS, Stein DB, Charrow A, Murray CJ. Tracking progress towards universal childhood immunisation and the impact of global initiatives: a systematic analysis of three-dose diphtheria, tetanus, and pertussis immunisation coverage. *The Lancet* 2008; **372**: 2031–46.

UHC Indicator: Modern Contraceptive Coverage

Input data

For the 2015 SDG Capstone paper, we built off a systematic review conducted for the 2010 GBD, focusing on data pertaining to modern contraceptive use prevalence and unmet need. In large, we limited our data search to survey series containing variables on contraceptive use by method, marital status and sexual activity for which microdata is readily accessible: the Demographic and Health Surveys (DHS), Multiple Indicator Cluster Surveys (MICS) and CDC Reproductive Health Surveys (CDC RHS). In addition we included the data from the Gates Foundation's Performance Monitoring and Accountability 2020 (PMA2020) surveys to which we had been granted access.

From the DHS and PMA2020 surveys we also extracted variables related to calculated unmet need.

The below table shows the number of studies included in the 2015 SDG Capstone paper.

		Contraception Methods	Unmet Need
DHS		232	175
MICS		114	0
CDC RHS		25	0
PMA2020		15	15
Country-specific		497	0

Modeling Strategy

For the purposes of our analysis modern contraceptive use was a binary variable defined as the current use of male/female sterilization, male/female condom, spermicide foam/jelly, oral contraceptive, diaphragms, implants, injections or use of an IUD. Unmet need was a binary variable defined as fecund, sexually active women ages 15–49 who are not using contraception and do not wish to become pregnant at all (unmet need for limiting) or within the next two years (unmet need for spacing).

Since the definition for unmet need has changed over time in DHS surveys, we re-computed the variable for DHS surveys prior to 2010 using a program provided by DHS for this exact purpose (http://dhsprogram.com/topics/upload/Stata-Revised-unmet-need-variable-general.zip).

Following data extraction we constructed prevalence rates for modern contraceptive use and unmet need by calculating survey-weighted means of the variables across countries and age groups. The age groups we defined were ages 15-19, 20-24, 25-29, 30-39, 40-49.

As some surveys asked contraception use questions only of women who were currently or had ever been married, we needed to cross-walk the prevalence of contraception use between women of different marital status. To accomplish this first used a spatio-temporal regression with the education and lagged-distributed income covariates to generate estimates of the proportion of women currently married and the proportion of women ever married across time and space. We then cross-walked modern

contraceptive prevalence for all women against the estimates of women ever married and currently married.

After cross-walking the data we graphed modern contraception use prevalence across time and space and identified outliers from the prevalence rate trends. These outliers were dropped. The final step to estimate prevalence of modern contraceptive use was to use the cross-walked prevalence data, sans outliers, as an input to space-time Guassian Process Regression (ST-GPR) to estimate prevalence for all relevant GBD years, age groups and countries. First, a mixed effect linear model was fit using a fixed effect on age, education and lagged-distributed income covariates, and random effects for countries, regions, and super-regions. The predictions from that model were used to calculate residuals that were smoothed over GBD space-time. Finally, GPR was used to generate estimates of modern contraceptive prevalence for all necessary units. These estimates were population-weighted to collapse to country years and used as an input for the UHC model.

For more information on modern contraceptive coverage, please refer to Indicator 3.7.1.

UHC Tracer Indicator – Immunization Capstone Appendix

Flowchart

3.8.1 UHC – Immunization Coverage Estimation Flowchart

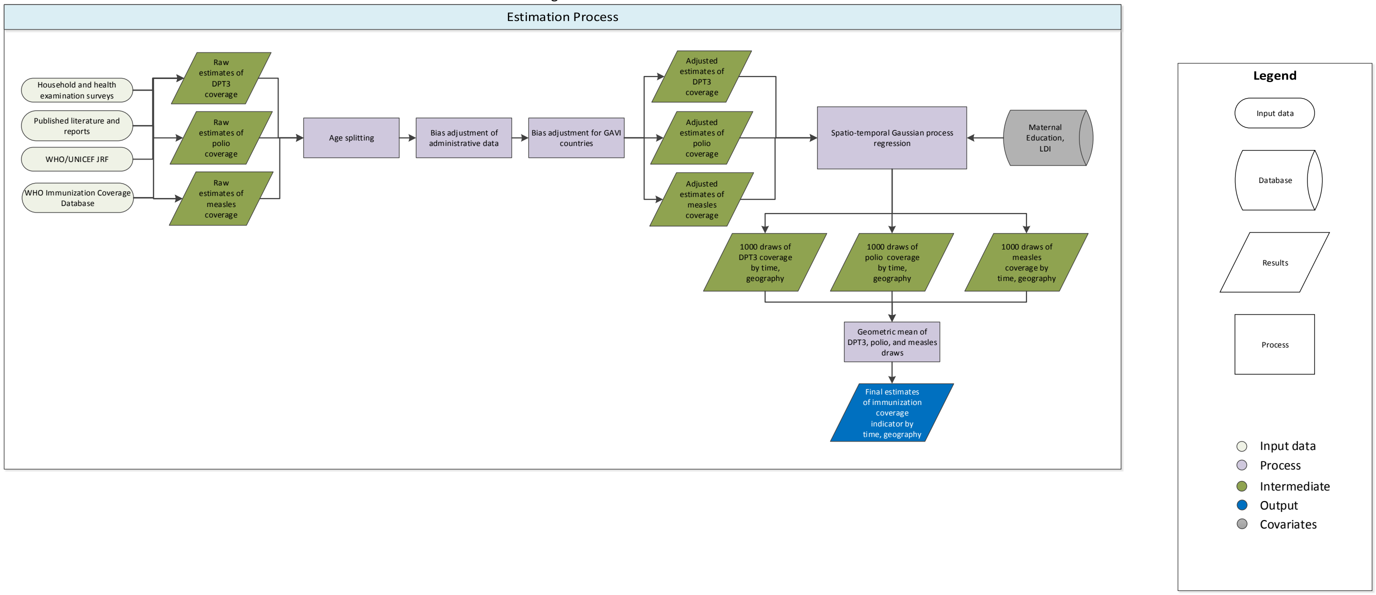

Input data & Methodological summary

Indicator definition

This modeling strategy pertains to the composite universal health coverage (UHC) tracer indicator (Indicator 3.8.1) and specifically the estimation of immunization coverage for diphtheria-pertussis-tetanus (DPT3), measles vaccine, and three doses of the oral polio vaccine or inactivated polio vaccine (OPV3 or IPV3).

Indicator 3.8.1

As a component of SDG Goal 3. Ensure healthy lives and promote well-being for all at all ages, SDG Target 3.8, achieve universal health coverage, including financial risk protection, access to quality essential health-care services and access to safe, effective, quality and affordable essential medicines and vaccines for all, is measured using SDG Indicator 3.8.1, three measures of immunization coverage: DPT3, measles, and polio (OPV3 and/or IPV3) among children aged 12 to 23 months.

Input data

The present study used data from household level surveys as well as administrative reports of immunization coverage. Survey data which provided person-level information on immunization were identified and extracted. Major multi-country survey programs included in the analysis include the Demographic and Health Surveys (DHS),[2] Multiple Indicator Cluster Surveys (MICS),[3] Reproductive Health Surveys (RHS),[4] Living Standards Measurement Study (LSMS) surveys,[5], and World Health Surveys (WHS).[6] We also conducted a comprehensive search of the Global Health Data Exchange (GHDx),[7]as well as

targeted internet searches and review of Ministry of Health websites, to identify national surveys and other multi-country survey programs.

Administrative estimates of immunization coverage were obtained from the Joint Reporting Process (JRF),[8] through which the World Health Organization (WHO) and UNICEF collates annual estimates of immunization coverage reported UN member states. These immunization coverage estimates are separate from those synthesized by WHO, and are calculated by dividing the number of doses of a given vaccine delivered to the target population (ie, children aged 12 to 23) by the number of individuals in that target population.

We excluded all data sources that were not nationally representative or had high levels of missingness. We applied survey weights based on survey sampling frames whenever they were available to generate weighted national estimates of vaccination coverage accompanied by estimates of standard error (SE). Estimates of SE, as well as sample sizes, were used to calculate uncertainty, as described below. Any point estimates with sample sizes less than 50 were reviewed to ensure that were not substantive outliers and would otherwise have an undue influence on our analysis.

Modeling strategy

Data processing

Age splitting

Most household surveys collection information on maternal and child health (MCH) indicators for children under 5 and/or mothers who gave birth within five years prior to the time of survey. To maximize data use for our model, we included immunization data for children aged 12 to 59 at the time of survey. Children younger than 12 months of age were excluded to minimize the influence of potentially censored observations. For each vaccine, coverage estimates were assigned to birth-cohort years based on a child's age prior to the time of survey: we used responses recorded for children aged 12 to 23 months for immunization coverage for one year prior to the time of survey, children aged 24 to 35 months for coverage two years prior to the time of survey, and so forth.

Age-specific estimates are easily computed from individual-level microdata, but many published reports and survey summaries present data in broader age aggregates (eg, DPT3 coverage for children aged 12 to 35 months). To standardize these age groups, we applied an age-splitting model used in the GBD study,[9] as well as analyses that generated smoking and obesity prevalence by age group.[10,11]

Using surveys with microdata as the reference, we used the following model to generate standardized age group-specific estimates of immunization coverage:

$$\tilde{P}_{a,c,t,k} = P^{a+x}_{a,c,t,k} \frac{P_{a,c,t,j}}{P^{a+x}_{a,c,t,j}}$$

where $\tilde{P}_{a,c,k}$ is the adjusted estimate of coverage for target age group a in country c and year t of survey k; and $P^{a+x}_{a,c,k}$ is coverage reported from survey k, for country c in year t for the age group spanning age a to age $(a + x)$. The ratio of coverage between the target age group and broader age group from a survey j with microdata from the same country-year was used to split data from survey k. Surveys to be split

were ideally matched with DHS or MICS surveys. If microdata were not available for the same year, ratios within five years of the survey that required age-splitting were applied.

Bias adjustments

Intervention coverage estimates based on administrative sources can be biased, yet the direction and magnitude of such biases are not universal. Some studies show that immunization coverage estimates from administrative data source are systematically higher than those of survey-based estimates,[12] while other studies show that bias directionality is more heterogeneous.[13] Such biases may arise for a number of reasons, including discrepancies in the accurate reporting of services or interventions provided (eg, number of vaccine doses administered) and target population (eg, number of children in need of vaccines), as well as capturing these data in a timely manner from both public and private sector facilities and healthcare providers.

For immunization coverage, we view individual-level data collected through population health surveys as the most accurate and least biased source of information of vaccination coverage, particularly for geographies with incomplete health information systems. We thus used vaccination coverage estimates from household surveys to calculate country-specific adjustment factors:

$$logit\left(P_{s,\,c,t}\right) = \beta_0 + \beta_1 logit\left(\tilde{P}_{a,\,c,t}\right) + \sum_{k=2}^{2+B} \beta_k S_k + \varepsilon_{c,t}$$

where $P_{s,\,c,t}$ is the survey-based estimate for immunization coverage (s) in country c for year t; $\tilde{P}_{a,\,c,t}$ is the administrative estimate for coverage in country c in year t; S_k is a spline basis used to capture the secular trend in coverage; β_1 is the estimated adjustment factor used to correct for the administrative bias; and ε is the error term for country c in year t.

Administrative estimates of immunization also may be subject to an additional bias from participation in performance-based health system support programs, such as the Global Alliance for Vaccines Initiative Immunization Services Support Program (Gavi ISS). It has previously been demonstrated that administrative estimates from participant countries are biased linearly with the number of year enrolled in the program.[14] To correct for this bias, we performed an additional bias adjustment on immunization coverage:

$$logit(P_{s,c,t}) = \beta_0 + \beta_1 logit(P_{a,c,t}) + \beta_2 T_{c,t}^{g} + \alpha_c + \varepsilon_{c,t}$$

where $P_{s,t}$is the survey-based estimate for immunization coverage (s) for country c in year t; $P_{a,t}$is the corresponding administrative coverage, T_t^g is the number of years of enrollment in the Gavi ISS program by year t; α_c is the country-specific random intercept to capture country-specific variation; β_2 is the estimated adjustment factor used to correct for the GAVI bias by the number of years of participation; and ε is the error term for country c in year t.

To quantify uncertainty for bias-adjusted estimates from the mixed-effects models described above, we calculated prediction error, $\widehat{PE}$, as follows:

$$\widehat{PE} = X^2 var(\hat{\beta})$$

where $var(\hat{\beta})$ is the variance for the estimated fixed-effects coefficient of the adjustment factor and X is the independent variable. Proper estimation of prediction errors is crucial as the data synthesis procedure, Gaussian process regression (GPR) (as described in the subsequent section), accounts for uncertainty from point estimates and bias adjustments when generating fitted values. More weight is given to data with less uncertainty. Prediction errors estimated from the bias adjustment were incorporated into the data variance and propagated through the GPR step to obtain estimates of coverage and uncertainty intervals (UIs).

Trend estimation

We used a spatiotemporal Gaussian process regression (ST-GPR) to synthesize point estimates from multiple data sources and derive a complete time series for each vaccine. This method has been used extensively GBD and related studies, and accounts for uncertainty pertaining to each point estimate while borrowing strength across geographic space and time.[10, 11,15,16] Briefly, we assumed the Gaussian process was defined by a mean function *m*(•) and covariance function *Cov*(•).

We estimated the mean function using a two-step approach. Specifically, $m_c(t)$ can be expressed as:

$$m_c(t) = X\beta + h(r_{c,t})$$

where $X\beta$ is a linear model and $h(r_{c,t})$ is a smoothing function for the residuals; and $r_{c,t}$ is derived from the linear model. The following linear model was used for the immunization indicators:

$$logit(P_{c,t}) = \beta_0 + \beta_1 medu_{c,t} + \beta_1 LDI_{c,t} + \alpha_c + \gamma_{R[c]} + \delta_c medu + \theta_{R[c]} medu + \varepsilon_{c,t}$$

where $P_{c,t}$ is vaccination coverage for country c year t; $medu_{c,t}$ is the average years of education for women of reproductive age in country c and year t; $LDI_{c,t}$ is the lag-distributed income in country c and year t; α_c and $\gamma_{R[c]}$ are country and region random intercepts, respectively. δ_c and $\theta_{R[c]}$ are country and region specific slope on education. These estimates were then run through ST-GPR, as documented elsewhere.[10]

Random draws of 1,000 samples were obtained from the distributions above for every country for a given vaccine. Ninety-five percent uncertainty intervals were calculated by taking the 25 and 975th draws from the sample distribution.

To assess the accuracy of our estimates in each bias adjustment step and in the modeling process, we performed cross-validation analyses by randomly holding out 20% of the sample and, if available, the corresponding administrative estimates for the given indicator of the same country and year, 10 separate times. We computed the average root mean squared errors (RMSE) across each country by vaccine. Error in the bias adjustments was calculated as the mean difference between the adjusted administrative estimate for a given country, year, and corresponding survey-level estimates (which were considered the "gold-standard"); error in the modeling process was calculated as the difference between the modeled estimates and the sample data.

References

1 WHO Indicator and Measurement Registry (WHO IMR). WHO IMR. http://apps.who.int/gho/indicatorregistry/App_Main/indicator_registry.aspx (accessed Aug 11, 2015).

2 Measure DHS: Demographic and Health Surveys. http://www.measuredhs.com (accessed Aug 11, 2015).

3 UNICEF Stat. Monit. Multiple Indicator Cluster Survey (MICS). http://www.unicef.org/statistics/index_24302.html (accessed Aug 11, 2015).

4 Cent. Dis. Control Prev. Reproductive Health Surveys (RHS). http://www.cdc.gov/reproductivehealth/Global/surveys.htm (accessed Aug 11, 2015).

5 World Bank. Living Standard Measruement Studies (LSMS). http://go.worldbank.org/UK1ETMHBN0 (accessed Aug 11, 2015).

6 WHO Multi-Ctry. Stud. Data Arch. World Health Survey (WHS). http://apps.who.int/healthinfo/systems/surveydata/index.php/catalog/whs/about (accessed Aug 11, 2015).

7 IHME GHDx. Global Helath Data Exchange. http://ghdx.healthdata.org/ (accessed Aug 11, 2015).

8 WHO | WHO/UNICEF Joint Reporting Process. WHO. http://www.who.int/immunization/monitoring_surveillance/routine/reporting/reporting/en/ (accessed Aug 17, 2015).

9 Collaboration PS. Age-specific relevance of usual blood pressure to vascular mortality: a meta-analysis of individual data for one million adults in 61 prospective studies. *The Lancet* 2002; **360**: 1903–13.

10 Ng M, Freeman MK, Fleming TD, *et al.* Smoking Prevalence and Cigarette Consumption in 187 Countries, 1980-2012. *JAMA* 2014; **311**: 183.

11 Ng M, Fleming T, Robinson M, *et al.* Global, regional, and national prevalence of overweight and obesity in children and adults during 1980–2013: a systematic analysis for the Global Burden of Disease Study 2013. *The Lancet* 2014; **384**: 766–81.

12 Murray CJ, Shengelia B, Gupta N, Moussavi S, Tandon A, Thieren M. Validity of reported vaccination coverage in 45 countries. *The Lancet* 2003; **362**: 1022–7.

13 Haddad S, Bicaba A, Feletto M, Fournier P, Zunzunegui MV. Heterogeneity in the validity of administrative-based estimates of immunization coverage across health districts in Burkina Faso: implications for measurement, monitoring and planning. *Health Policy Plan* 2010; **25**: 393–405.

14 Lim SS, Stein DB, Charrow A, Murray CJ. Tracking progress towards universal childhood immunisation and the impact of global initiatives: a systematic analysis of three-dose diphtheria, tetanus, and pertussis immunisation coverage. *The Lancet* 2008; **372**: 2031–46.

15 Rasmussen CE, Williams CKI. Gaussian processes for machine learning. Cambridge, Mass.: MIT Press, 2006 http://www.books24x7.com/marc.asp?bookid=12939 (accessed Aug 17, 2015).

16 Vasudevan S, Ramos F, Nettleton E, Durrant-Whyte H. Heteroscedastic Gaussian processes for data fusion in large scale terrain modeling. In: 2010 IEEE International Conference on Robotics and Automation (ICRA). 2010: 3452–9.

UHC Tracer Indicator – Insecticide-Treated Net (ITN) Coverage Capstone Appendix

Input data & Methodological summary

Indicator definition

This modeling strategy pertains to the composite universal health coverage (UHC) tracer indicator (Indicator 3.8.1) and specifically the estimation of insecticide-treated net (ITN) coverage among malaria-endemic countries.

Indicator 3.8.1

As a component of SDG Goal 3. Ensure healthy lives and promote well-being for all at all ages, SDG Target 3.8, achieve universal health coverage, including financial risk protection, access to quality essential health-care services and access to safe, effective, quality and affordable essential medicines and vaccines for all, is measured using SDG Indicator 3.8.1, which includes ITN coverage among malaria-endemic countries.

Countries which eliminated malaria prior to 2000 (the year at which ITNs became more widely available outside of ITN trials) or have been classified as at least pre-elimination according to the World Health Organization (WHO)[1] had 100% ITN coverage applied for all years of analysis; this applied to 115 of the 188 countries in the present study. This analytic decision was made because these countries have largely used other interventions to reduce their malaria burdens or fully eliminate the disease,[2] and in the cases where ITNs are distributed in these countries, their ownership and use is highly focal and seasonal.

Input data

ITNs were defined as either (1) a traditional ITN, which is treated with an insecticide designed to last up to one year and then needs retreatment at least every year thereafter to remain effective; or (2) a long-lasting insecticide-treated net (LLIN), which is impregnated with a type of insecticide meant to be effective for three to five years. However, since most household surveys combine LLINs received over three years ago as "more than 36 months [old]" rather than offering a more precise age measurement, LLINs obtained more than three years ago are no considered "active" ITNs.[3–5]

Among the 73 malaria-endemic countries for which ITN coverage was estimated, three types of data sources were used to inform ITN models: (1) ITN delivery records provided by net manufacturers and reported to WHO; (2) ITN distribution records reported to WHO from national malaria control programs; and (3) ITN ownership and use accounts derived from household surveys. The latter were extracted from multi-country survey series, including the Demographic and Health Surveys (DHS), Multiple Indicator Cluster Surveys (MICS), and Malaria Indicator Surveys (MIS), as well as country-specific surveys. ITN coverage estimates were calculated from survey microdata whenever possible; in the absence of microdata, we used nationally representative survey reports of ITN coverage.

We excluded all data sources that were not nationally representative or only included coverage estimates of nets that were not treated with insecticide. We applied survey weights based on survey sampling

frames whenever they were available to generate weighted national estimates of ITN coverage accompanied by estimates of standard error (SE).

Estimates of populations at risk (PAR) for malaria were provided from the Malaria Atlas Project (MAP); these originated from the WHO and earlier iterations of the World Malaria Report. Among malaria-endemic countries and those not classified as at least pre-elimination, PAR ranged from 0.1 to 1.0.

Modeling strategy

Drawing from a Bayesian compartmental "stock-and-flow" model originally developed by Flaxman and colleagues,[3] collaborators from MAP have honed these methods for estimating ITN coverage in sub-Saharan Africa over time.[5,6] This modeling approach has been used to generate annual estimate of ITN coverage for the WHO's annual World Malaria Report since 2009.[1,7–12] In brief, this model uses the relationships between net supply (from manufacturer deliveries), distribution (from national programs), and ownership by households (from population surveys), and tracks volumes of nets based on their "stock" or "flow" status throughout the delivery chain. Parameters on net discard rates at each stage were previously informed by continuously updated literature reviews; however, a substantive model improvement led by Bhatt and colleagues has been the development of a country-by-country loss function derived within the stock-and-flow model.[1,6,12]

Estimates of ITN coverage outside of sub-Saharan Africa (33 countries in the present analysis) using updated methods from MAP are currently in progress; thus we use previously generated results for ITN coverage for countries outside of sub-Saharan Africa for this study.

Sub-Saharan Africa

For 40 countries, we used national-level estimates of ITN coverage generated by Bhatt and colleagues from 2000 to 2015.[5,6] Five hundred draws of ITN coverage were provided for each geography-year, which resulted the duplication of these draws to create a total of 1,000 for uncertainty estimation. While this may have led to underestimated uncertainty for some geography-years, it is also possible that the duplicated draws represented a wider range of potential values (ie, an additional 500 draws all could have been within the minimum and maximum original draws) and thus uncertainty could have been overestimated. Future analyses will include the full 1,000 draws for these countries in sub-Saharan Africa.

Outside of sub-Saharan Africa

For 33 countries, we used national-level estimates of ITN coverage that were calculated for the GBD covariates database; the full 1,000 posterior draws also were available. These estimates spanned from 2000 to 2014, so we used 2014 estimates for 2015. This analytic necessity has the potential to underestimate ITN coverage (ie, if a country conducted a distribution campaign between 2014 and 2015) or overestimate coverage in the absence of continued or heightened net distribution in 2015.

Adjusting for minimum ITN coverage thresholds and population at risk

Prior to estimating ITN coverage for each geography-year, we applied two data adjustments. First, we replaced all draws of ITN coverage to 0.01 on a scale of 0 to 1 (1% on a scale of 0% to 100%) before 2000. This minimum was used to reflect the relatively minimal availability of ITNs before 2000, and to provide greater model stability (ie, analyses conducted in logit space require the replacement of zeros with a

small, non-zero value). In addition, we replaced all draws lower than 0.01 (1%) after 2000 to 0.01 (1%). Second, we divided all draws of ITN coverage by PAR, effectively weighting each draw for a geography's relative malaria risk (and thus need for ITNs). We replaced any draws that exceeded 1 (100%) with 1 (100%).

For each geography-year under analysis, ITN coverage estimates were generated by taking the mean of 1,000 draws of the posterior distribution, and the ordinal 25th and 975th draws of the distribution provided 95% uncertainty intervals (UIs).

References

1 World Health Organization (WHO). World Malaria Report 2015. Geneva, Switzerland: WHO, 2015.

2 Cotter C, Sturrock HJ, Hsiang MS, *et al.* The changing epidemiology of malaria elimination: new strategies for new challenges. *The Lancet* 2013; **382**: 900–11.

3 Flaxman AD, Fullman N, Otten MW Jr, *et al.* Rapid scaling up of insecticide-treated bed net coverage in Africa and its relationship with development assistance for health: a systematic synthesis of supply, distribution, and household survey data. *PLoS Med* 2010; **7**: e1000328.

4 Fullman N, Burstein R, Lim SS, Medlin C, Gakidou E. Nets, spray or both? The effectiveness of insecticide-treated nets and indoor residual spraying in reducing malaria morbidity and child mortality in sub-Saharan Africa. *Malar J* 2013; **12**: 62.

5 Bhatt S, Weiss DJ, Cameron E, *et al.* The effect of malaria control on Plasmodium falciparum in Africa between 2000 and 2015. *Nature* 2015; **526**: 207–11.

6 Bhatt S, Weiss DJ, Mappin B, *et al.* Coverage and system efficiencies of insecticide-treated nets in Africa from 2000 to 2017. *eLife* 2015; **4**. DOI:10.7554/eLife.09672.

7 World Health Organization (WHO). World Malaria Report 2009. Geneva, Switzerland: WHO, 2009.

8 World Health Organization (WHO). World Malaria Report 2010. Geneva, Switzerland: WHO, 2010.

9 World Health Organization (WHO). World Malaria Report 2011. Geneva, Switzerland: WHO, 2011.

10 World Health Organization (WHO). World Malaria Report 2012. Geneva, Switzerland: WHO, 2012.

11 World Health Organization (WHO). World Malaria Report 2013. Geneva, Switzerland: WHO, 2013.

12 World Health Organization (WHO). World Malaria Report 2014. WHO, 2014.

SDG Indicator: Deaths Attributable to Household Air Pollution and Ambient Air Pollution

Household Air Pollution SDG Capstone Appendix

Flowchart

Household Air Pollution from Solid Fuels

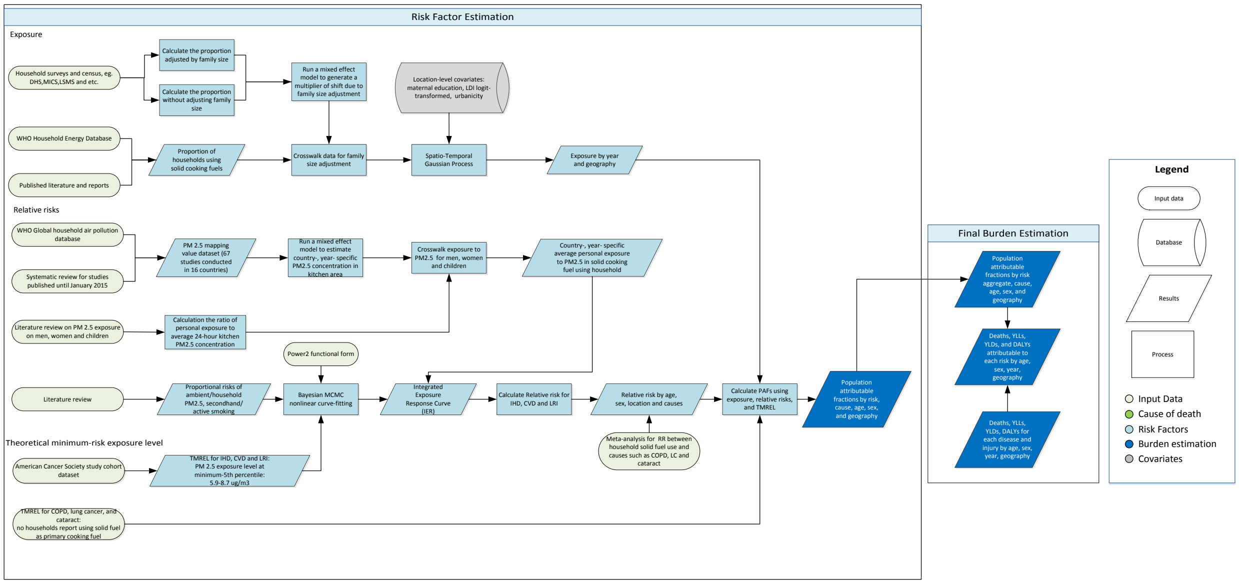

Input data and Methodological Summary

Indicator definition

This modeling strategy encompassed the indicator associated with deaths attributable to household air pollution (3.9.1).

Indicator 3.9.1

As a component of SDG Goal 3. Ensure healthy lives and promote well-being for all at all ages, SDG Target 3.9, by 2030, substantially reduce the number of deaths and illnesses from hazardous chemicals and air, water and soil pollution and contamination, is measured using SDG Indicator 3.9.1, deaths attributable to household air pollution and ambient air pollution per 100,000 [PAF estimate].

Case Definition

Exposure to household air pollution from solid fuels (HAP) is defined as the proportion of households using solid cooking fuels. The definition of solid fuel in our analysis includes coal, wood, charcoal, dung, and agricultural residues.

Input data

Data were extracted from the standard multi-country survey series such as Demographic and Health Surveys (DHS), Living Standards Measurement Surveys (LSMS), Multiple Indicator Cluster Surveys (MICS), and World Health Surveys (WHS), as well as country-specific survey series such as Kenya Welfare Monitoring Survey and South Africa General Household Survey. To fill the gaps of data in surveys and censuses, we also downloaded and updated HAP estimates from WHO Energy Database and extracted from literature through systematic review done in IHME. Each nationally or sub-nationally representative data point provided an estimate for the percentage of households using solid cooking fuels. Estimates for the usage of solid fuels for non-cooking purpose were excluded, i.e. primary fuels for lighting. The database, with estimates from 1980 to 2015, contained 685 studies from 150 countries. Updates to systematic reviews are performed on an ongoing schedule across all GBD causes and risk factors, an update for household air pollution will be performed in the next 1-2 iterations.

Modeling strategy

Household air pollution was modeled at household level using a three-step modeling strategy ST-GPR that uses linear regression, spatiotemporal regression and Gaussian Process Regression (GPR). The first step is a mixed-effect linear regression of logit-transformed proportion of households using solid cooking fuels. The linear model contains maternal education and proportion of population living in urban areas as covariates and has nested random effect by country, GBD region, and GBD super region respectively. The full ST-GPR process is specified elsewhere in this appendix.

Compare with GBD 2013, we have made changes in terms of the covariates utilized in the linear model. In GBD 2013, year represented the only fixed effect in the robust linear model. While in GBD 2015, we switched to average years of maternal education and proportion of population living in urban areas as predictors for HAP.

Theoretical minimum-risk exposure level

For outcomes where we extracted RR based on direct epidemiological evidence i.e. COPD, lung cancer, and cataract, TMREL was defined such that no households would report using solid fuel as their primary cooking fuel. For outcomes that utilize evidence based on the Integrated Exposure Response (IER), the TMREL is defined as uniform distribution between 33.3 and 41.9 ug/m^3. TMREL for household air pollution did not change from GBD 2013.

Relative risks

The disease-outcomes paired with household air pollution has not changed since GBD 2013. The sources of relative risks (RRs) used depend on the disease outcomes. RRs for COPD, lung cancer and cataract paired with household air pollution rely on direct epidemiological evidence i.e. meta-analyses. The RRs from meta-analyses remain unchanged after a systematic review on meta-analysis for the risk-outcome pairs published after Jan 1st, 2014. RRs for IHD, stroke and LRI are generated from the IERcurves. The IER curves are updated to reflect the newly updated data and utilization of a new method that specified elsewhere.

PM2.5 mapping value

The relative risk estimates describing the association of HAP with outcomes including Ischemic Heart

Disease (IHD), cardiovascular disease (CVD), and lower respiratory infections (LRI) were derived from the IER curves. This is done by first estimating the crosswalk values that map household use of solid fuel to PM2.5 exposure because the IER curve measures exposure using PM2.5. This step of the analysis relied on 67 studies conducted in 16 countries to generate the PM2.5 mapping values, which remain the same sources as GBD 2013. The PM2.5 exposure was then cross-walked to men, women and children by generating the ratio of personal exposure to average 24-hour kitchen PM2.5 concentration based on a study after the literature review in GBD 2013.

Ambient Particulate Matter Pollution Capstone SDG Appendix

Flowchart

Ambient PM2.5

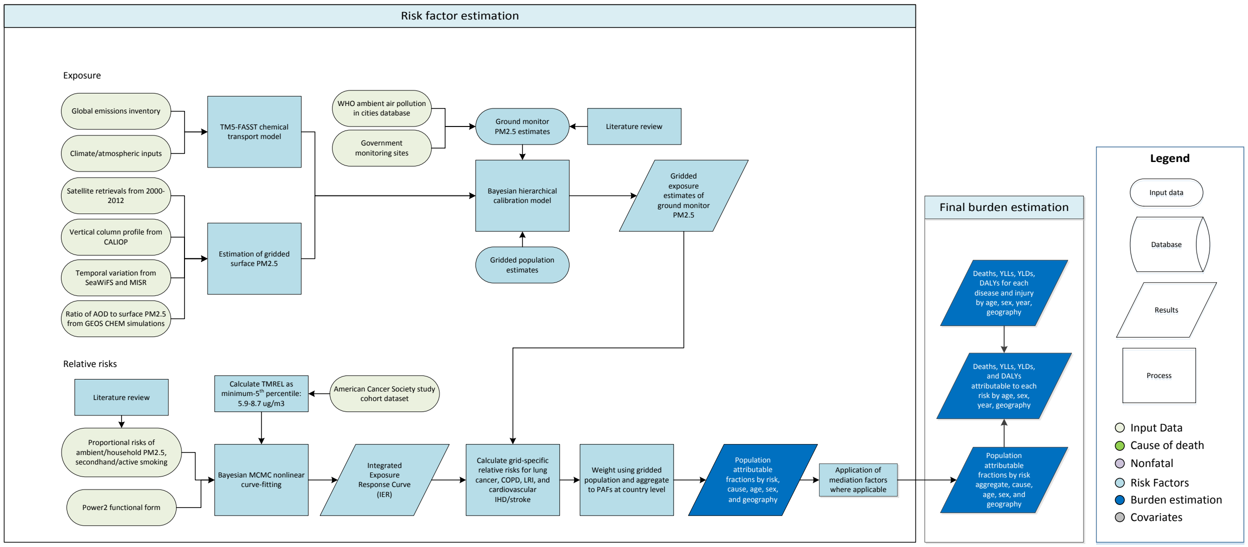

Input Data & Methodological Summary

Indicator definition

This modeling strategy encompassed the indicator associated with deaths attributable to ambient air pollution (3.9.1).

Indicator 3.9.1

As a component of SDG Goal 3. Ensure healthy lives and promote well-being for all at all ages, SDG Target 3.9, by 2030, substantially reduce the number of deaths and illnesses from hazardous chemicals and air, water and soil pollution and contamination, is measured using SDG Indicator 3.9.1, deaths attributable to household air pollution and ambient air pollution per 100,000 [PAF estimate].

Case definition

Exposure to ambient air pollution is defined as the population-weighted annual average mass concentration of particles with an aerodynamic diameter less than 2.5 micrometers ($PM_{2.5}$) in a cubic meter of air. This measurement is reported in µg/m^3.

Input Data

The data to estimate exposure to ambient air pollution is drawn from estimates of annual concentration of $PM_{2.5}$ – generated using satellite observations of aerosols in the atmosphere. To correct for bias in the satellite modeling approach, a spatially-varying flexible framework is used to combine modeled

concentrations with observations from ground-level monitoring of particles in more than 75 countries. All input data for GBD2015 was updated as follows:

Updated $PM_{2.5}$ ground measurement database

For the GBD2015 update we updated the database of annual average PM measurements to include more recent data and to incorporate additional locations where measurement data have become available. To facilitate this we collaborated with WHO and contributed to their recently released WHO Air Pollution in Cities database. We then used disaggregated (monitor-specific values and not the city averages that are reported by WHO) measurements from this database with additional site-specific information (e.g. all monitors in a city, monitor geo coordinates, monitor site type) such as that included in the GBD2013 database. In total measurements of concentrations of PM_{10} and $PM_{2.5}$ were retrieved from 6,003 ground monitors with the majority contributing measurements from 2014 (as there is a lag in reporting measurements, little data from 2015 were available). Where data were not available for 2014 (2760 monitors), data was used from 2015 (18 monitors), 2013 (2155), 2012 (564), 2011 (60), 2010 (375), 2009 (49), 2008 (21) and 2006 (1). For locations with only PM_{10} measurements, $PM_{2.5}$ measurements were estimated from PM_{10}. This was done by a locally derived conversion factor ($PM_{2.5}/PM_{10}$ ratio) estimated as population-weighted averages of location-specific conversion factors for the country. Location-specific conversion factors were estimated as the mean ratio of $PM_{2.5}$ to PM_{10} of stations for the same year. If national conversion factors were not available, regional ones were used, which were obtained by averaging country-specific conversion factors.

Updated satellite-based estimates

The updated satellite-based estimates are described in detail in van Donkelaar et al. 2016[1]. These estimates (~11 x 11 km resolution at the equator) combine aerosol optical depth retrievals from multiple satellites with the GEOS Chem chemical transport model and land use information.

Updated population data

A comprehensive set of population data on a high-resolution grid was obtained from the Gridded Population of the World (GPW v4) database. These data are provided on a 0.0417°×0.0417° resolution. To aggregate these estimates of population to each 0.1°×0.1° grid cell, the central 3 × 3 population cells were summed. As this accounted for a resolution higher than necessary, the same was done four other times, offset by one cell in a North, South, East and West direction. The average of five quantities was used as the aggregated population estimate for each cell. Estimates of population for 2000, 2005, 2010, 2015 and 2020 were extracted from GPW version 4 and estimates for 1990 and 1995 were extracted from GPW version 3 as described previously for GBD2013[3].

Modeling Strategy

The methodology used to estimate the burden of ambient particulate matter pollution has seen significant changes since GBD2013.
The GBD2010 and GBD2013 estimates both used a single global function to calibrate the mean of the chemical transport model and satellite-based estimates to available ground measurements. In both instances the approach taken was recognized at the time to be a compromise between what could be easily implemented under tight timeframes and one that most efficiently utilized all of the data sources. In particular, the GBD2013 exposure estimates were known to underestimate ground measurements in specific locations (see discussion in Brauer et al., 2015[2]) such that it would be desirable to allow measurements to make a larger contribution to the final estimates where they were available. Therefore, for GBD2015 we implemented a Bayesian Hierarchical modelling approach using Integrated Nested Laplace Approximations (INLA) in which the satellite-based estimates, ground measurements and land use information are combined in a spatially varying flexible framework. Formal external evaluation using ground measurements was conducted and shown to lead to improved predictions of ground measurements in all super regions compared to GBD2013 estimates and an alternative geographically-weighted regression approach. Further, based on the external evaluation analyses, addition of the TM5 chemical transport model estimates of PM2.5 annual average did not improve the estimates and these were therefore not included.

Bayesian hierarchical models (BHM) provide an extremely useful and flexible framework in which to model complex relationships and dependencies in data. Uncertainty can also be propagated through the model allowing uncertainty arising from different components, both data sources and models, to be propagated through the models into estimates of uncertainty associated with the final estimates. In the hierarchical modeling approach coefficients associated with satellite-based estimates were estimated for each country. Where data were insufficient within a country, information can be `borrowed' from a higher aggregation (region) and if enough information is still not available from an even higher level (super-region). Individual country level estimates were therefore based on a combination of information from the country, its region and super-region.

All modelling was performed on the log-scale with the unit of measurement being a grid cell. The model was constructed with the inclusion of all variables assessed statistically, based on model fit (DIC, a measure of the relative quality of a model and closely related to that of AIC but for Bayesian models) and predictive ability. The hierarchical structure was applied to the intercept and slope terms with all modelling on the log scale. The model was of the form:

$$\log(PM2.5_i) = \beta_0 + \beta_1 \log SAT_i + other\ variables + \varepsilon_i$$

where *i* denotes the grid cell. The following sets of variables were considering in developing the models:

Continuous explanatory variables:

- (SAT) Estimate of $PM_{2.5}$ (in μgm^{-3}) for 2014 from satellite remote sensing on the log-scale.
- (CTM) Estimate of $PM_{2.5}$ (in μgm^{-3}) for 2014 from chemical transport models on the log-scale.
- Estimate of population for 2014 on the log-scale.

- (SNAOC) Estimate of the sum of sulfate, nitrate, ammonium and organic carbon as estimated from GEOS Chem
- (DST) Estimate of compositional concentrations for mineral dust from GEOS Chem
- (EDxDU) The log of the elevation difference between the elevation at the ground measurement location and the mean elevation within the GEOS Chem simulation grid cell multiplied by the inverse distance to the nearest urban land surface

Discrete explanatory variables:

- Binary variable indicting whether exact location of ground measurement is known
- Binary variable indicting whether exact type of ground monitor is known
- Binary variable indicting whether ground measurement is $PM_{2.5}$ or converted from PM_{10}

Random Effects:

- Grid cell random effects on the intercept to allow for multiple ground monitors in a grid cell.
- Country-region-super-region hierarchical random effects for the intercept
- Country-region-super-region hierarchical random effects for the satellite remote sensing term.
- Country-region-super-region hierarchical random effects for the coefficient associated with the difference between estimates from CTM and SAT.
- Country-region-super-region hierarchical random effects for the coefficient log(POP)
- Country level random effects for intercept, satellite and difference between CTM and SAT are independent and identically distributed.
- Country level random effects for population uses a neighbourhood structure allowing specific borrowing of information from neighbouring countries.
- All region random effects are assumed to be independent and identically distributed.
- All super-region random effects are assumed to be independent and identically distributed.

Interactions*:*

- Interactions between the binary variables and the effects of log(SAT) and log(CTM/SAT)

Due to both the complexity of the models and the size of the data, notably the number of spatial predictions that are required in this setting, recently developed techniques that perform 'approximate' Bayesian inference based on integrated nested Laplace approximations (INLA) have been developed as a computationally attractive alternative to Markov Chain Monte Carlo methods. Computation was performed using the R interface to the INLA computational engine (R-INLA) with the size of the task of fitting the models and performing predictions for each of the ca. 1.4 million grid cells requiring the use of a high performance computing cluster (HPC) with high memory nodes. As in GBD2010 and GBD2013 the spatial model was built combining the different data sources for a single year (2014, corresponds to the most recent measurement data). The spatially-varying functions from this model were then applied to the satellite-based estimates from all other years - in other words assuming that the spatial relationship between the different data sources does not change over time. This is undoubtedly a simplification but to do otherwise would require assembling multi-year measurement databases which is not feasible given

current data availability and computational constraints. As the spatial model was built using the most recently available (2014) measurement and satellite-based estimates, 2015 estimates were based on extrapolation. Instead of extrapolating using an exponential model based on a 1-year trend as in GBD2013, splines based on a 5 year trend (2010-2014) were fit and applied to the 2014 grid-cell values to estimate levels for 2015. This reduced the likelihood of 2015 estimates being overly influenced by meteorological events in a specific year and to better represent the duration of exposure relevant to the epidemiologic studies included in the integrated exposure-response functions.

Model Evaluation

Model evaluation and comparison was performed by fitting models on a training set and predicting exposures at locations for which measurements were known (the validation set). The selection of the training (20%) and validation (80%) set consisted of taking a random sample of the total number of sites measuring PM2.5 (or having a value converted from PM10 measurements). Sampling was performed using sampling probabilities based on the cross-tabulation of PM2.5 categories (0-24.9, 25-49.9, 50-74.9, 75-99.9, 100+ µg/m3) and super-regions. The resulting hold-out evaluation data set was a sample of 20% of the sites that have the same distribution over PM2.5 categories and super-regions as the entire set of sites.

This process was used to generate multiple training and validation set combinations, allowing for example cross-validation to be performed. In the evaluation, 25 sets of training/validation data were used. The following models were considered in the evaluation phase:

(A) The GBD2013 model, using a simple linear regression with a fused estimate of SAT and CTM together with interactions with three binary variables representing whether the measurement was converted from PM10 and whether the exact site type and location is known.
(B) A hierarchical model with SAT, the TM5 CTM estimates, population and the three binary variables described above
(C) A hierarchical model with SAT, population, SNAOC, DST, EDxDU, population and the three binary variables
    - Estimate of population for 2014 on the log-scale.
    - Estimate of the sum of sulfate, nitrate, ammonium and organic carbon as estimated from GEOS Chem
    - Estimate of compositional concentrations for mineral dust from GEOS Chem
    - The log of the elevation difference between the elevation at the ground measurement location and the mean elevation within the GEOS Chem simulation grid cell multiplied by the inverse distance to the nearest urban land surface

For each training/evaluation set combination, model fit and prediction accuracy were evaluated for each of the 25 training/evaluation set combinations with the following metrics:

Model fit

- R^2
- DIC

Predictive accuracy

- R^2 arising from a linear regression of predicted vs actual measurements at each location
- RMSE – root mean squared error

- WRMSE – weighted (by population) root mean squared error
- MSE – mean square error
- MAE – mean absolute error

This evaluation indicated the final model improved predictions of ground measurements in all super regions compared to GBD2013 estimates (median global R^2 [population-weighted RMSE] 0.82 (12.1 μg/m^3), 0.60 [13.5 μg.m^3] for GBD2015 and GBD2013, respectively).

Error! Reference source not found. shows the RMSE (median from the 25 runs) for each of the three models, by super-region. The accuracy of the prediction varies between super-regions, with lower errors being observed in areas where there are more monitoring sites. In each of the super-regions, the largest errors are seen for model A which are considerably higher than those for models B and C, with model C showing a small improvement over B (except in super-region 5, North Afirca/Middle East).

Figure 2 shows scatter plots of the observed and predicted measurements using the three models for each super-region. The predicted measurements are the median values over those obtained from the 25 training sets. Predictions from the two Bayesian hierarchical models (B&C) match the observed values more closely than the linear model (A) with much less spread around a straight line (with slope one and zero intercept, shown in red). In Central Europe and Sub-Saharan Africa it is noticeable that, in addition to reduced spread, models B&C are much better at predicting higher values. The same patterns of results in predictive ability were seen when looking at regions and individual countries. In all cases, model C performed better than model B with both being considerable better than model A.

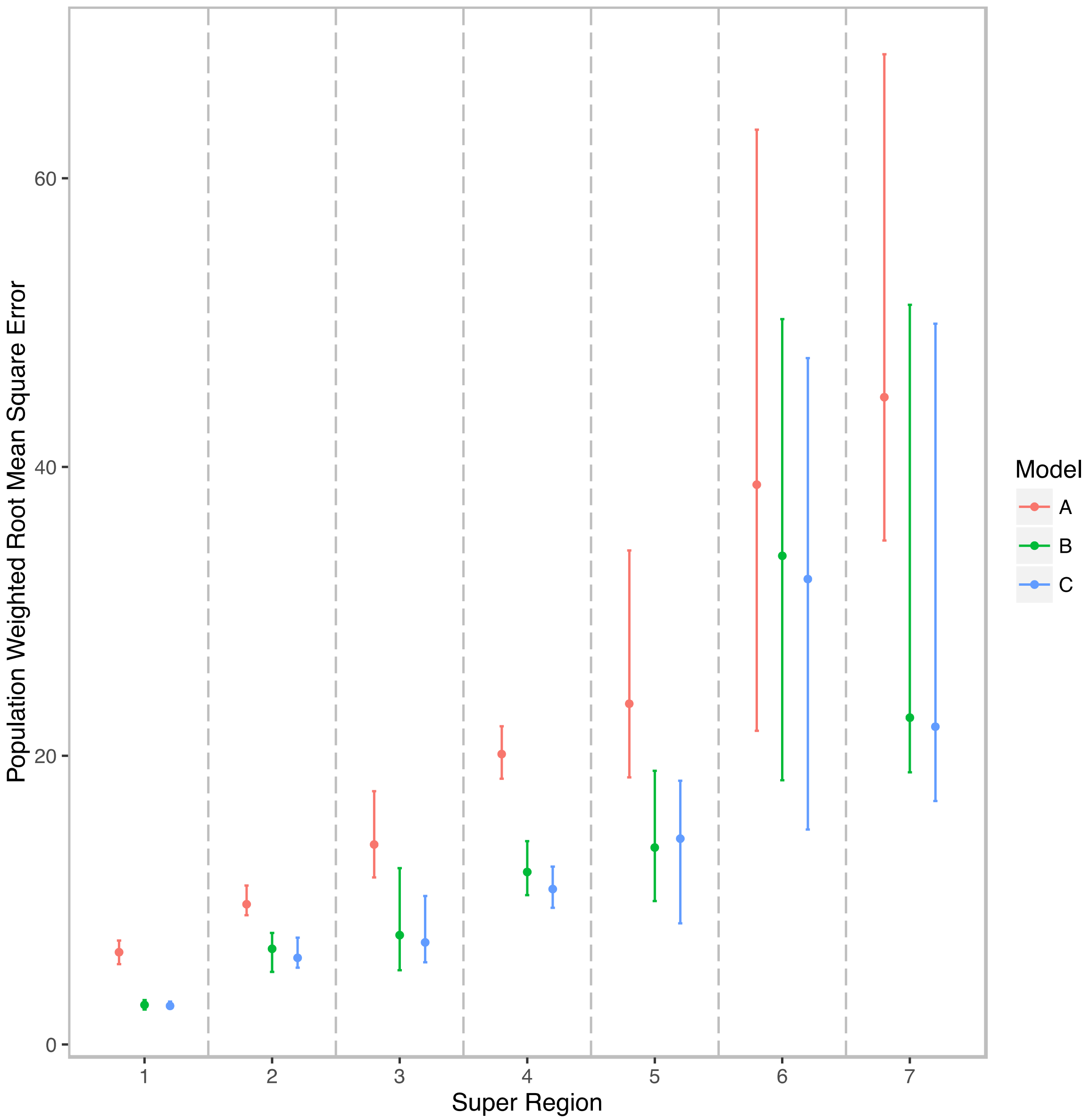

Figure 1: Comparison of RMSE from three models by super-region. Dots denote the median of the distribution from 25 training/evaluation sets and the vertical lines the range of values. Super-regions are 1: high income, 2: Central Europe, Eastern Europe, Central Asia, 3: Latin America and Caribbean, 4: Southeast Asia, East Asia and Oceania, 5: North Africa / Middle East, 6: Sub-Saharan Africa, 7: South Asia.

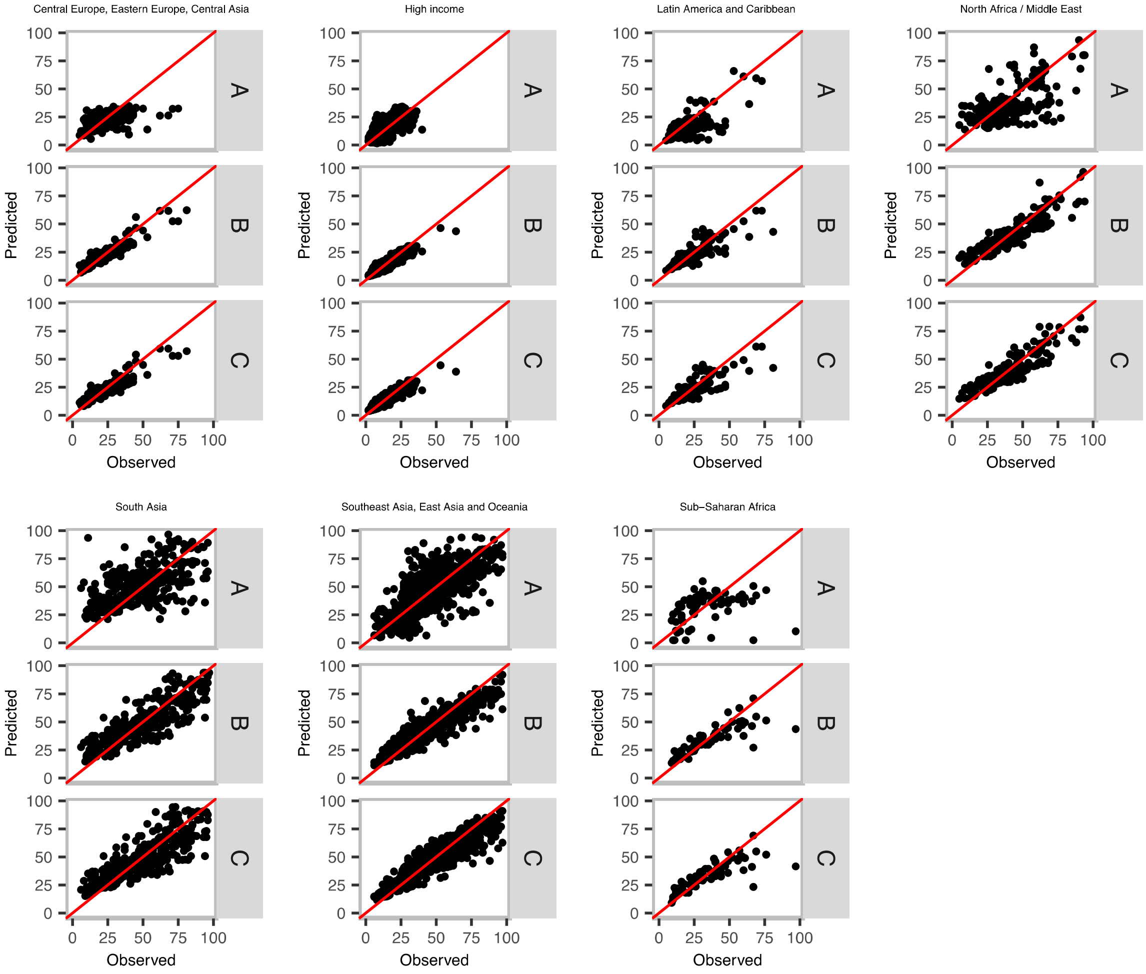

Figure 2: Comparison of observed and predicted measurements using three different models, by super-region. The red line has slope one and intercept zero.

Overall, the best model in terms of model fit and predictive ability was one with the following components:

- Estimates of $PM_{2.5}$ (in μgm^{-3}) from satellite remote sensing (SAT), population, and information on the GEOS Chem simulated chemical composition, elevation and distance to urban land use (SNAOC, DST and EDxDU).
- Binary variables indicting whether exact location and type of ground measurement is known, and whether the measurement was $PM_{2.5}$ or converted from PM_{10}.
- Interactions between the binary variables and the effects of estimates from satellite remote sensing.
- Grid cell random effects on the intercept to allow for multiple ground monitors in a grid cell.
- Country-region-super-region hierarchical random effects for intercepts, satellite remote sensing and population terms.
- Country level random effects for population using a neighbourhood structure allowing specific borrowing of information from neighbouring countries.

Relative Risk

Relative risks are generated using integrated exposure-response functions (IER) that are fit to available epidemiologic data using a Bayesian MCMC approach and a modified power function. The IER are estimated based on published relative risks for long-term exposure to ambient PM2.5, household air pollution, second-hand smoking, and active (cigarette) smoking. The concentration of particulate matter for each type of exposure is estimated based on literature values and used to map the relative risks to a curve generated for the entire range of exposure from these sources. The input data for this curve fitting process has been updated since GBD2013, adding new studies that estimate exposure at finer spatial scales, including studies of within-city exposure that focus on traffic-related air pollution. In addition, changes were made to the curve-fitting process. In order to account for differences in study design, temporal patterns of exposure and other differences among the studies of the different sources of PM2.5, a source-specific heterogeneity parameter was added to the IER. This resulted in much wider, and, in our view, more realistic, uncertainty intervals for the burden estimates, by propagating through the entire process the current uncertainty regarding the mechanisms and magnitude of health impacts of exposure to PM2.5 from diverse sources.

IER Functional Form

Data Likelihood

$$\log(RR_i) \sim \mathcal{N}\left(\mu_i, \sqrt{\sigma_i^2 + \delta_{source_i}}\right)$$

Model

$$\mu_i = \log\left(\frac{1+\alpha \times \left(1 - e^{-\beta \times (exposure_i - TMREL)^\gamma}\right)}{1+\alpha \times \left(1 - e^{-\beta \times (counterfactual_i - TMREL)^\gamma}\right)}\right)$$

Data

RR_i : measured relative risk for data point i

σ_i : variance of data point i based on study information

$source_i$: exposure source type (outdoor/household air pollution, secondhand/active smoking)

$TMREL$: theoretical minimum risk exposure level

$exposure_i$: measured exposure for data point i

$counterfactual_i$: counterfactual exposure for data point i

Priors

$$\alpha \sim \Gamma(1.0, 0.01)$$
$$\beta \sim \Gamma(1.0, 0.01)$$
$$\gamma \sim \Gamma(1.0, 0.01)$$
$$\delta \sim \Gamma(1.0, 0.01)$$

We also modified the way in which age-specific IER for IHD and stroke were estimated. In accordance with previously published work on other cardiovascular risk factors, the impact of air pollution on cardiovascular health is known to vary with age. To account for this phenomenon, age-specific RRs were based on a log-linear model of RR as a function of age, where the intercept (RR=1) is forced to age 110. In GBD2010 and GBD2013 the age for a relative risk estimate from a given study was estimated as the median age at death or disease incidence in that study. However, this may not accurately represent the age distribution of the entire study population so we re-estimated this variable as the mean age at enrollment + half of the average follow-up time to better represent the average age of the study population during the period of follow-up.. When compared to GBD2013, this change produced RRs that were generally lower for younger age groups, given that median age at event tends to produce a higher anchor age than average age during follow-up.

The relative risks are generated on the grid-level based on estimated exposure, and then applied to generate PAFs. These PAFs are aggregated using the grid-level population to create population-weighted national estimates of attributable burden, using the following formula:

PM2.5 Aggregation Formula

$$PAF_{A,\ C,\ L} = \frac{\sum\ ((RR_{A,\ C} - 1)\ *\ Pop_i)}{\sum(\ RR_{A,\ C}\ *\ Pop_i)}$$

A = age group

C = cause

L = location

i = grid

$RR_{A,\ C} =$ *grid-level RR based on* $PM_{2.5}$ *and given age/cause IER curve*

TMREL

The TMREL for ambient PM is estimated using a uniform distribution between the minimum and 5th percentile of exposure observed in the studies used to generate the GBD estimates. This estimate was updated for GBD2015 as new studies were added to the analysis and studies used previously were updated through continued follow-up. The newer estimates included several large studies that included exposure at lower levels of PM2.5. As a result, the TMREL for GBD2015 was ~U(2.4, 5.9), lower than GBD2013’s distribution ~U(5.9, 8.7), which had the effect, all things being equal, of increasing the estimated attributable burden relative to the GBD2013 estimate

References

(1) van Donkelaar, A.; Martin, R. V; Brauer, M.; Hsu, N. C.; Kahn, R. A.; Levy, R. C.; Lyapustin, A.; Sayer, A. M.; Winker, D. M. Global Estimates of Fine Particulate Matter using a Combined Geophysical-Statistical Method with Information from Satellites, Models, and Monitors. *Environ. Sci. Technol.* **2016**, *50* (7), 3762–3772.

(2) Brauer, M.; Freedman, G.; Frostad, J.; van Donkelaar, A.; Martin, R. V; Dentener, F.; Van Dingenen, R.; Estep, K.; Amini, H.; Apte, J. S.; et al. Ambient Air Pollution Exposure Estimation for the Global Burden of Disease 2013. *Environ. Sci. Technol.* **2015**, *50* (1), 79–88.

(3) Brauer, M.; Amann, M.; Burnett, R. T.; Cohen, A.; Dentener, F.; Ezzati, M.; Henderson, S. B.; Krzyzanowski, M.; Martin, R. V; Van Dingenen, R.; et al. Exposure assessment for estimation of the global burden of disease attributable to outdoor air pollution. *Environ. Sci. Technol.* **2012**, *46* (2), 652–660.

Deaths attributable to WaSH SDG Capstone Appendix

Input data & Methodological Summary

Indicator definition

This modeling strategy encompassed the indicator associated with deaths attributable to unsafe water, sanitation, and hygiene (WaSH) (3.9.2).

For GBD 2015, the WaSH category is an aggregate of the risk estimates for water (6.1.1), hygiene (6.2.1b) and sanitation (6.2.1a). These are modeled independently and then aggregated together to generate the overall risk estimates for deaths attributable to WaSH.

Indicator 3.9.2

As a component of SDG Goal 3. Ensure healthy lives and promote well-being for all at all ages, SDG Target 3.9 by 2030, substantially reduce the number of deaths and illnesses from hazardous chemicals and air, water and soil pollution and contamination, is measured using SDG Health Index Indicator 3.9.2, deaths attributable to unsafe WaSH per 100,000 [PAF estimate].

Input data

This indicator includes estimates made independently from unsafe hygiene, unsafe sanitation, and unsafe water risk factors. For both water and sanitation, nationally representative surveys and censuses are the primary source for input data. For hygiene, a combination of data from nationally representative surveys and epidemiological studies are extracted and used as input data.

Modeling strategy

Each of the three risks that make up WaSH are modelled using a three step process that includes linear regression, spatial-temporal smoothing, and Gaussian Process Regression (ST-GPR). In modeling water and sanitation exposure, socio-demographic index was used as a fixed effect, with random effects placed at geographic locations including GBD 2015-specific super regions and regions. The final step of modelling was GPR, which incorporated source-specific uncertainty estimates and produced full times series of each risk estimate for each location.

We estimate the PAF and burden of each of the 3 WaSH categories independently. This means that the calculation for PAF of all three is simply aggregating the PAF of each using this equation: 1 –((1-PAF)(1-PAF)(1-PAF)).

For additional information on the three risk models that are used to estimate Indicator 3.9.2, please refer to the write-ups for water, sanitation and hygiene in this appendix.

Unsafe Water SDG Capstone Appendix

Flowchart

Unsafe Drinking Water

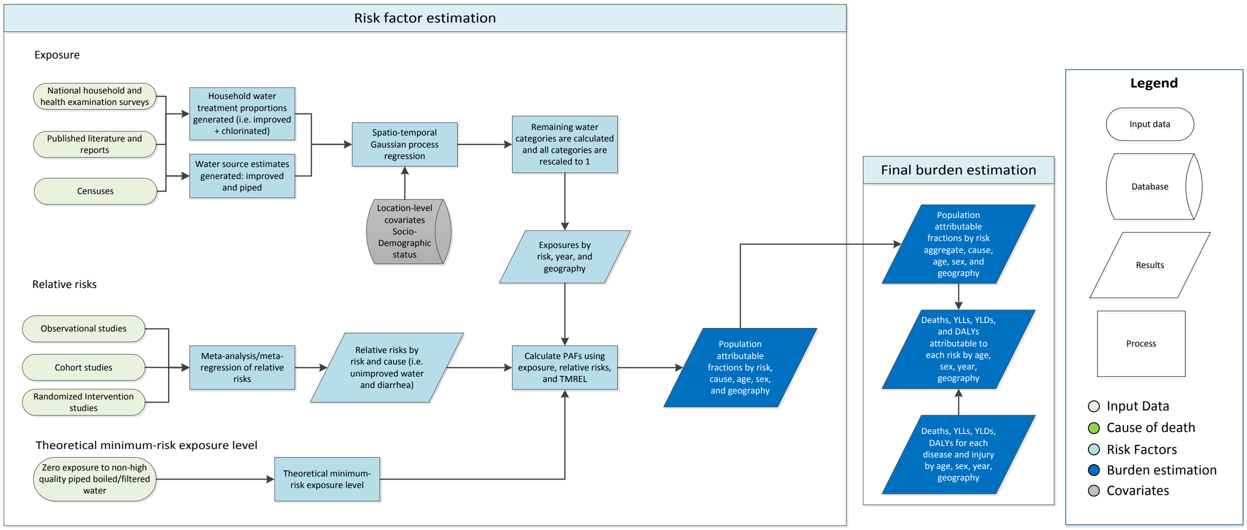

Input data and Methodological Summary

Case Definition

For GBD 2015, exposure to unsafe water is defined based on reported primary water source used by the household and use of household water treatment (HWT) to improve the quality of drinking water before consumption. Water sources were defined as improved based on the JMP designation (The WHO), which includes piped water as improved water, and households with access to piped water connection to the house, yard, or plot were defined as having access to piped water supply. Solar treatment, chlorine treatment, boiling, or the use of filters were all assumed to be effective point-of-use household water treatments, and based on effect sizes published by Wolf et al. (2014) boiling or filtering was the most effective form of water treatment.

Input Data

The search for usable household surveys and censuses was conducted using the Global Health Data Exchange (GHDx) database. All surveys through December 2015 that provide household level micro-data on water source were added. Tabulated and report data was lower priority and was only updated when time permitted. HWT input data was limited to two large survey series (DHS and MICS) due to time constraints. An update to HWT input data is a top priority for estimating exposure to unsafe water in future iterations.

Modeling Strategy

Water source data is modeled in two distinct categories: household prevalence of improved water and household proportion of piped water within improved population in order to prevent the population with access to piped water from exceeding the population with access to improved water (which includes piped). HWT is modeled in 6 distinct categories based on the 3 water treatment categories (filtered/boiled, solar/chlorine, or untreated) and 2 water source categories (piped or improved). We have made no substantive changes in the modeling strategy from GBD 2013. By year and geography, each of the above categories are modeled using a 3-step modeling scheme of mixed effect linear regression followed by spatio-temporal Gaussian process regression (ST-GPR), which outputs full time series estimates for each GBD 2015 location. Socio-demographic status (SDS), an index metric that includes a measure of education and income level, was used as a fixed effect in the linear regression since it proved to have significant coefficients. Random effects were placed at GBD 2015 region and super-region levels.

The process of vetting and validating models was accomplished primarily through an examination of ST-GPR scatter plots by GBD 2015 location from 1990-2015. Any unfitting data points were re-inspected for error at the level of extraction and survey implementation, and subsequently excluded from analysis if deemed appropriate. In addition to SDS, a number of different potential fixed effects were considered, including lag-distributed income and urbanicity, but SDS proved to be the strongest predictor of unsafe water. Uncertainty in the estimates was initially formed based on standard deviation by survey, then propagated through ST-GPR modeling by means of confidence intervals around each data point that reflect the point-estimate specific variance.

Once models are fully vetted, full time series outputs from ST-GPR modeling are then converted from proportion to prevalence by year and geography and then rescaled to form 9 mutually exclusive categories that sum up to 1. The table below provides the final result of this rescaling.

Category	*Definition*
Unimproved, no HWT	Proportion of households that use unimproved source, and *do not* use any HWT to purify their drinking water.
Unimproved, chlorine/solar	Proportion of households that use unimproved source, and solar or chlorine treatment to purify their drinking water.
Unimproved, boil/filter	Proportion of households that use unimproved source, and boil or filter to purify their drinking water.
Improved water except piped, no HWT	Proportion of households that use improved sources other than piped water supply, and *do not* use any HWT to purify their drinking water.
Improved water except piped, chlorine/solar	Proportion of households that use improved sources other than piped water supply, and use solar or chlorine treatment to purify their drinking water.
Improved water except piped, boil/filter	Proportion of households that use improved sources other than piped water supply, and boil/filter their drinking water.
Piped water, no boil/filter	Proportion of households that use piped water supply, and *do not* use any HWT to purify their drinking water

Piped water, chlorine/solar	Proportion of households that use piped water supply, and *use* solar or chlorine water treatment to purify their drinking water.
Piped water, boil/filter	Proportion of households that use piped water supply, and boil or filter to purify their drinking water

Due to the nature of modeling piped water exposure as a proportion of total improved water access, we are limited in only using sources for piped water that also include total improved water values. It should be noted that high-income countries are assumed to have risk of unsafe water which could lead to an underestimate of unsafe water health burden in these countries. Another limitation in our analysis is the paucity of data on HWT. The inclusion of more location-specific data on water treatment utilization at the household level can greatly improve our estimates in future iterations. High-income countries were assumed to have 0 risk of unsafe water, and the TMREL was applied to these countries.

Theoretical minimum-risk exposure level

The theoretical minimum-risk exposure level for unsafe water is defined as all households have access to high quality piped water that has been boiled or filtered before drinking. This exposure level is applied to all households in high-income countries, as well as households in countries in Southern Latin America region or Eastern Europe region that report piped water source and filtered or boiled water treatment.

Relative risks

GBD 2015 employ the same relative risks for unsafe water as was done for GBD 2013. There are 3 adverse health outcomes paired with unsafe water that comprise of diarrheal diseases, typhoid fever, and paratyphoid fever. A meta-analysis by Wolf et al. 2014 provides relative risk evidence for the relationship between unsafe water and diarrheal diseases. Wolf et al. 2014 publish relative risk values for water-source interventions and point-of-use treatment interventions separately so the combined effect of a source intervention and point-of-use intervention is assumed to be multiplicative in order to match GBD 2015 exposure definitions. In the absence of better data, the relative risk for typhoid and paratyphoid fevers were assumed to be the same as the relative risk for diarrheal disease. Furthermore, it is assumed that there is a difference in piped water quality between Eastern Europe and Southern Latin America compared to rest of the developing world. As a result, we use effect sizes that are region-specific. The implication of this assumption is that no household in developing countries have access to high-quality piped water (TMREL). Please refer to appendix tables for more information on relative risk values and citations.

References

1. "Improved and Unimproved Water Sources and Sanitation Facilities."*WHO / UNICEF Joint Monitoring Programme: Wat/san Categories*. The WHO/UNICEF, n.d. Web. 08 June 2016

2. Wolf, Jennyfer, Annette Prüss-Ustün, Oliver Cumming, Jamie Bartram, Sophie Bonjour, Sandy Cairncross, Thomas Clasen, John M. Colford, Valerie Curtis, Jennifer De France, Lorna Fewtrell, Matthew C. Freeman, Bruce Gordon, Paul R. Hunter, Aurelie Jeandron, Richard B. Johnston, Daniel Mäusezahl, Colin Mathers, Maria Neira, and Julian P. T. Higgins. "Systematic Review: Assessing the Impact of Drinking Water and Sanitation on Diarrhoeal Disease in Low- and Middle-income Settings: Systematic Review and Meta-regression." Trop Med Int Health Tropical Medicine & International Health 19.8 (2014): 928-42. Web.

Unsafe Sanitation SDG Capstone Appendix

Flowchart

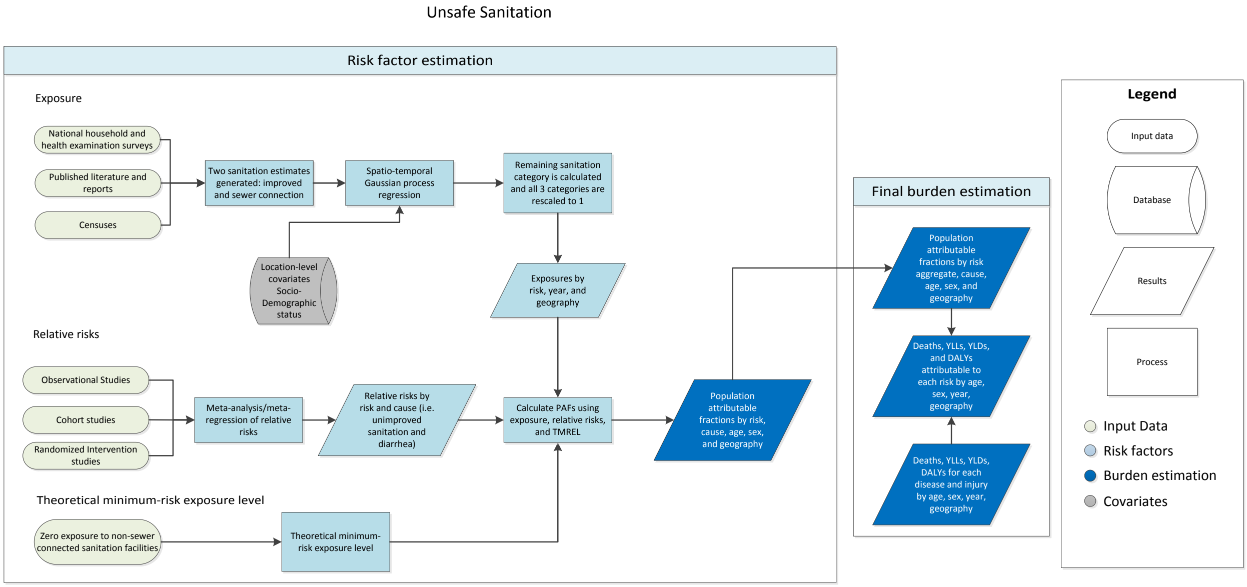

Input data and Methodological Summary

Case Definition

Exposure to unsafe sanitation were defined based on the primary toilet type used by households. Improved facilities are defined as such based on JMP designation (The WHO). Sewer connection toilets included flush toilets or any toilet with connection to the sewer or septic tank.

Input Data

The search for usable household surveys and censuses was conducted using the Global Health Data Exchange (GHDx) database. Searches were conducted from October 2015 to December 2015, with the final search household level micro-data on toilet type conducted on December 15, 2015. Due to the organized nature of the GHDx, the only search term used was “unsafe sanitation”, which yielded just under 1400 results, of which 795 were extracted and used as inputs for modeling. Tabulated and report data was lower priority and was only updated when time permitted.

Modeling

There were no substantive changes in the modeling process from GBD 2015. Two distinct models are produced from sanitation data: prevalence of households with improved sanitation and the proportion of households with a sewer connection over the total improved sanitation population. Prevalence of households with a sewer connection is modeling with improved sanitation prevalence as the denominator in order to prevent the population with access to sewer connection from exceeding the population with access to improved sanitation. By each geography-year, both models are generated using a 3-step modeling scheme of mixed effect linear regression followed by spatio-temporal Gaussian process regression (ST-GPR), which outputs full time series estimates for each GBD 2015 location. Socio-

demographic status (SDS), an index metric that includes a measure of education and income level, was used as a fixed effect in the linear regression since it proved to have significant coefficients. Random effects were placed at GBD 2015 region and super-region levels.

The process of vetting and validating models was accomplished primarily through an examination of ST-GPR scatter plots by GBD 2015 location from 1990-2015. Any unfitting data points were re-inspected for error at the level of extraction and survey implementation, and subsequently excluded from analysis if deemed appropriate. In addition to SDS, a number of different potential fixed effects were considered, including lag-distributed income and urbanicity, but SDS proved to be the strongest predictor of unsafe sanitation. Uncertainty in the estimates was initially formed based on standard deviation by survey, then propagated through ST-GPR modeling by means of confidence intervals around each data point that reflect the point-estimate specific variance.

Once models are fully vetted, full time series outputs from ST-GPR modeling are then converted from proportion to prevalence by year and geography and then rescaled to form 3 mutually exclusive categories that sum up to 1. The table below provides the final result of this rescaling.

Category	*Definition*
Unimproved sanitation	Proportion of households that use unimproved sanitation facilities.
Improved sanitation, excluding sewer	Proportion of households that use improved sanitation facilities except those with sewer connection.
Sanitation facilities with sewer connection	Proportion of households that use toilet facilities with sewer connection.

Due to the nature of modeling sanitation with sewer connection as a proportion of total improved sanitation access, we are limited in only using sources for sewer connection that also include total improved sanitation values. It should be noted that high-income countries are assumed to have risk of unsafe sanitation which could lead to an underestimate of unsafe sanitation health burden in these countries. Another limitation that extends to the other two risk factors that comprise WaSH (unsafe water and unsafe hygiene) and can be improved upon in future iterations is taking into account covariance of access to water, sanitation and handwashing facilities. Currently, all three components of WaSH are modeled independently, which may lead to an overestimation of the burden of WaSH factors. High-income countries were assumed to have 0 risk of unsafe sanitation and the TMREL was applied to these countries.

Theoretical minimum-risk exposure level

The theoretical minimum-risk exposure level for unsafe sanitation was defined as all households have access to a sanitation facility with sewer connection. Since it is assumed that all households in high-income countries have access to sewer-connected sanitation, this counterfactual exposure level is applied to all households in high-income countries.

Relative risks

GBD 2015 employ the same relative risks for unsafe water as was done for GBD 2013. Three adverse health outcomes are paired with unsafe sanitation, which comprise of diarrheal diseases, typhoid fever, and paratyphoid fever. A meta-analysis by Wolf et al. 2014 provides relative risk evidence for the relationship between unsafe sanitation and diarrheal diseases. In the absence of better data, the relative risk for typhoid and paratyphoid fevers were assumed to be the same as the relative risk for diarrheal disease. Please refer to appendix tables for more information on relative risk values and citations.

References

1. "Improved and Unimproved Water Sources and Sanitation Facilities."*WHO / UNICEF Joint Monitoring Programme: Wat/san Categories*. The WHO/UNICEF, n.d. Web. 08 June 2016
2. Wolf, Jennyfer, Annette Prüss-Ustün, Oliver Cumming, Jamie Bartram, Sophie Bonjour, Sandy Cairncross, Thomas Clasen, John M. Colford, Valerie Curtis, Jennifer De France, Lorna Fewtrell, Matthew C. Freeman, Bruce Gordon, Paul R. Hunter, Aurelie Jeandron, Richard B. Johnston, Daniel Mäusezahl, Colin Mathers, Maria Neira, and Julian P. T. Higgins. "Systematic Review: Assessing the Impact of Drinking Water and Sanitation on Diarrhoeal Disease in Low- and Middle-income Settings: Systematic Review and Meta-regression." Trop Med Int Health Tropical Medicine & International Health 19.8 (2014): 928-42. Web.

Unsafe Hygiene SDG Capstone Appendix

Flowchart

Unsafe Handwashing

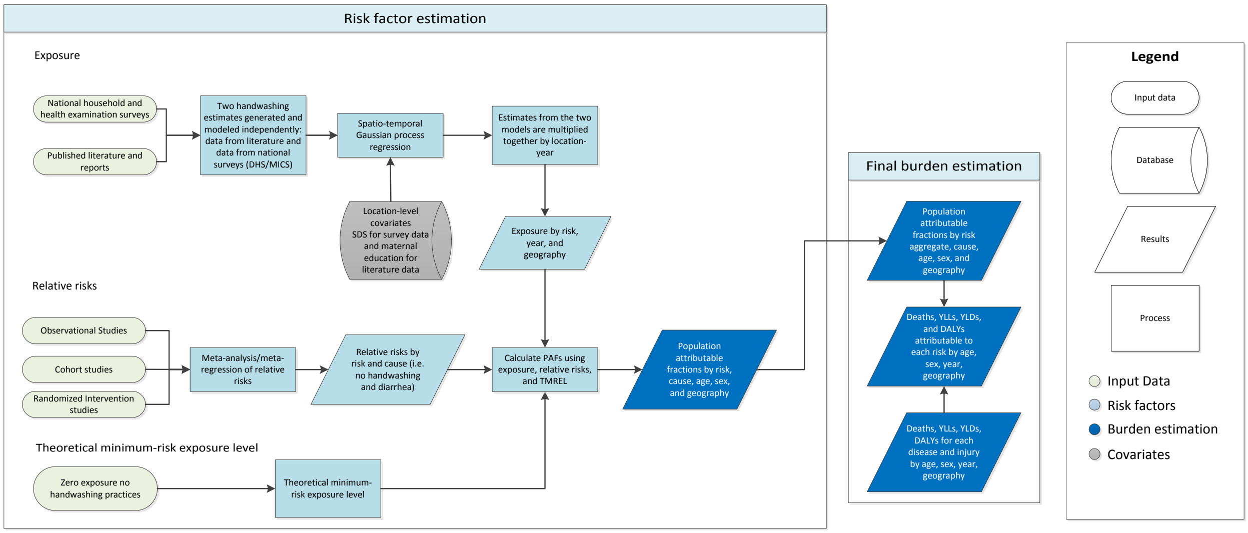

Input data and Methodological Summary

Case Definition

Unsafe hygiene is composed of global handwashing practices. Handwashing is defined as the observed prevalence of handwashing with soap and water after using a toilet or after contact with excreta, including children's excreta. We estimate the burden of unsafe handwashing in both developed and developing settings.

Input Data

There were two main sources that were used in our estimation of handwashing practices, estimates from scientific literature and estimates from household survey series. Relevant literature on handwashing prevalence was gathered from a meta-analysis published recently by Freeman et al. (2014). Since water and soap availability data is very limited, only country-specific Demographic Health Surveys (DHS) and Malaria Indicator Survey Series (MICS) conducted after 2006 were able to be used as input data.

Modeling Strategy

Input data from scientific literature and input data from household survey series were modeled independently. Data from literature primarily measured a population's handwashing practices under ideal conditions, such as when water and soap was readily available. Additionally, these estimates from literature would likely be susceptible to acquiescence bias. Alternatively, data from DHS and MICS only provide insight into the availability of water, soap, and washing stations, which, alone, does not indicate how often a person may wash their hands after contact with excreta. Thus, after modeling data from

literature and data from surveys independently, these values were multiplied together by location-year in order to gain a more accurate representation of true handwashing prevalence.

Other than modeling literature data and survey data independently, we have made no substantive changes in the modeling strategy from GBD 2013. By year and location, estimates are generated using a 3-step modeling scheme of mixed effect linear regression followed by spatio-temporal Gaussian process regression (ST-GPR). When modeling survey input data, socio-demographic status (SDS), an index metric that includes a measure of education and income level, proved to have the most significant coefficient and was used as a fixed effect in the linear regression. To better inform our model that used scientific literature as input data, maternal education or average years of education for women ages 15-54, was implemented as a fixed effect in the linear regression. For both models, random effects were placed at GBD 2015 region and super-region levels.

The process of vetting and validating models was accomplished primarily through an examination of ST-GPR scatter plots by GBD 2015 location from 1990-2015. Any unfitting data points were re-inspected for error at the level of extraction and survey implementation, and subsequently excluded from analysis if deemed appropriate. In addition to SDS, a number of different potential fixed effects were considered, including lag-distributed income and urbanicity, but SDS and maternal education proved to be the strongest predictors of handwashing practices for their respective models. As mentioned above, once models were adequately vetted, full time series outputs from each of the models were multiplied together at each location-year.

A considerable limitation for when estimating handwashing practices for over 190 independent locations around the world is data sparseness. Even when data is published on handwashing prevalence, the definition is often altered from the GBD 2015 standard definition or it may only pertain to certain populations (such as hospital patients) and lacks representativeness at the geographic scale we require. The incorporation of questions about soap and water availability in DHS and MICS has added much-needed information but there remains a large data gap that must be filled if we are to become more certain in handwashing estimates.

Theoretical minimum-risk exposure level

The theoretical minimum-risk exposure level for unsafe hygiene is defined as all households engaging in handwashing with soap practices after any contact with excreta, including children's excreta.

Relative risks

GBD 2015 use the same relative risks for unsafe hygiene as was done for GBD 2013. There are 3 adverse health outcomes paired with unsafe hygiene that include diarrheal diseases, typhoid fever, and paratyphoid fever. A meta-analysis by Freeman et al. 2014 provides relative risk evidence for the relationship between unsafe hygiene and diarrheal diseases. In the absence of adequate data, the relative risk for typhoid and paratyphoid fevers were assumed to be the same as the relative risk for diarrheal disease based on analogous transmission pathways (feco-oral pathway). Please refer to appendix tables for more information on relative risk values and citations.

References

1. Freeman, M. C., Stocks, M. E., Cumming, O., Jeandron, A., Higgins, J. P., Wolf, J., Curtis, V. (2014). Systematic review: Hygiene and health: Systematic review of handwashing practices worldwide and update of health effects. *Trop Med Int Health Tropical Medicine & International Health, 19*(8), 906-916. doi:10.1111/tmi.12339

Smoking SDG Capstone Appendix

Flowchart

Tobacco Smoke

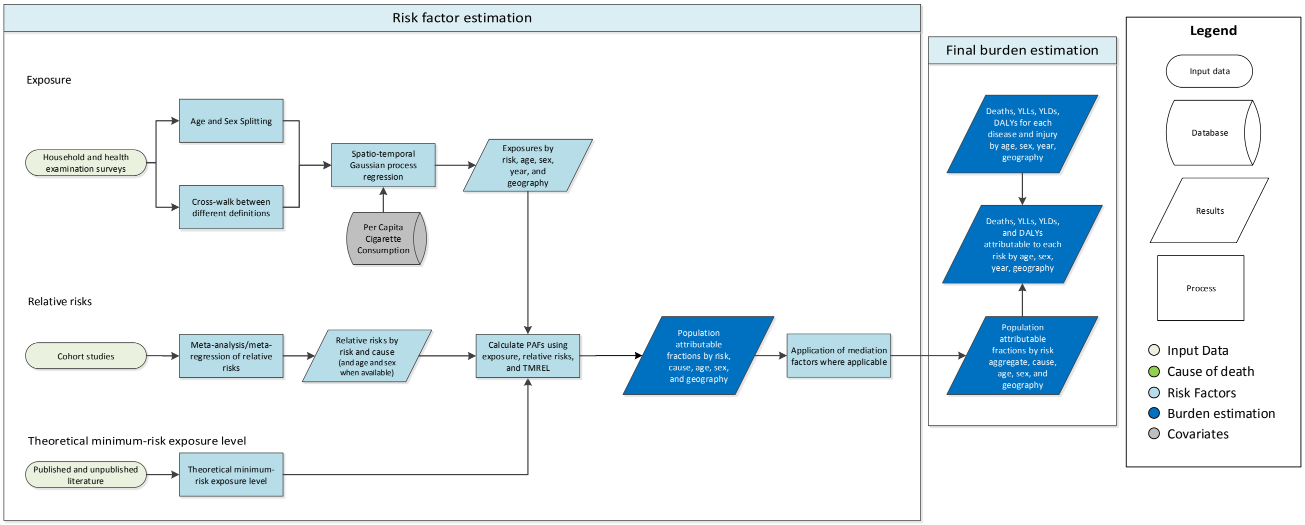

Input Data and Methodological Summary

Indicator definition

This modeling strategy encompassed the SDG health-related indicator associated with smoking prevalence (3.a.1).

Indicator 3.a.1

As a component of SDG Goal 3. Ensure healthy lives and promote well-being for all at all ages, SDG Target 3.a, strengthen the implementation of the World Health Organization Framework Convention on Tobacco Control in all countries, as appropriate, is measured using SDG Indicator 3.a.1, age-standardized prevalence of current tobacco use (smoking) among populations aged 18 and older.

Definition

We used the Smoking Impact Ratio (SIR) for modeling burden attributable to smoking for cancers, chronic obstructive pulmonary disease (COPD), interstitial lung disease, other chronic respiratory diseases, and pneumoconiosis. SIR is the population lung cancer mortality in excess of lung cancer mortality among never-smokers, relative to excess lung-cancer mortality observed in a known reference group of smokers. Currently, SIR is adjusted to account for differences in baseline never-smoker lung cancer mortality across geography, age, and sex, but not for differences across time.

We used 5-year lagged smoking prevalence, for modeling burden attributable to smoking for cardiovascular diseases, TB, diabetes, lower respiratory infections, asthma, cataracts, macular

degeneration, fractures, rheumatoid arthritis, and peptic ulcer disease. Smoking is a dichotomous exposure defined as current daily use of smoked tobacco.

A full list of outcomes included in GBD 2015 and their exposure definition is available in the table below.

Outcome	Exposure
Atrial fibrillation and flutter	5-year lagged smoking prevalence
Aortic aneurysm	5-year lagged smoking prevalence
Hypertensive heart disease	5-year lagged smoking prevalence
Ischemic heart disease	5-year lagged smoking prevalence
Other cardiovascular and circulatory diseases	5-year lagged smoking prevalence
Peripheral vascular disease	5-year lagged smoking prevalence
Hemorrhagic stroke	5-year lagged smoking prevalence
Ischemic stroke	5-year lagged smoking prevalence
Diabetes	5-year lagged smoking prevalence
Lower respiratory infections	5-year lagged smoking prevalence
Asthma	5-year lagged smoking prevalence
Tuberculosis	5-year lagged smoking prevalence
Peptic ulcer disease*	5-year lagged smoking prevalence
Rheumatoid arthritis*	5-year lagged smoking prevalence
Cataract*	5-year lagged smoking prevalence
Macular degeneration*	5-year lagged smoking prevalence
Hip fracture*	5-year lagged smoking prevalence
Non-hip fracture*	5-year lagged smoking prevalence
Bladder cancer	Smoking Impact Ratio (SIR)
Colon and rectum cancer	Smoking Impact Ratio (SIR)
Esophageal cancer	Smoking Impact Ratio (SIR)
Kidney cancer	Smoking Impact Ratio (SIR)
Leukemia	Smoking Impact Ratio (SIR)
Liver cancer	Smoking Impact Ratio (SIR)
Tracheal, bronchus, and lung cancer	Smoking Impact Ratio (SIR)
Lip and oral cavity cancer	Smoking Impact Ratio (SIR)
Nasopharynx cancer	Smoking Impact Ratio (SIR)
Pancreatic cancer	Smoking Impact Ratio (SIR)
Stomach cancer	Smoking Impact Ratio (SIR)
Larynx cancer*	Smoking Impact Ratio (SIR)
Chronic obstructive pulmonary disease	Smoking Impact Ratio (SIR)
Interstitial lung disease and pulmonary sarcoidosis	Smoking Impact Ratio (SIR)
Other chronic respiratory diseases	Smoking Impact Ratio (SIR)
Pneumoconiosis	Smoking Impact Ratio (SIR)

* New outcome in GBD 2015

Input data

Consistent with GBD 2013, we used nationally representative survey data to estimate smoking prevalence. Survey and report data identified in the Global Health Data Exchange (GHDx), the WHO InfoBase, and the International Smoking Statistics (ISS) Database.

Inclusion Criteria

- Nationally representative
- Report current use of any of the following frequency-type combinations:
    - Daily use of smoked tobacco
    - Any use (both daily and occasional) of smoked tobacco
    - Daily use of cigarettes
    - Any use (both daily and occasional) of cigarettes
    - Daily use of any tobacco (both smoked and smokeless)
    - Any use (both daily and occasional) of any tobacco (both smoked and smokeless)
    - Daily use of any tobacco excluding cigarettes
- Report data within the time period of January 1, 1980 – December 31, 2015 for any geography estimated in the GBD framework
- Smoking prevalence reported among individuals ages 10+

Global Health Data Exchange (GHDx)

Sources were identified through a systematic search of the GHDx.

- Search Terms (Keywords): Tobacco Use
- Time Period: January 1, 1980 – December 31, 2015
- Data Type: Survey OR Report
- Search Date: February 16, 2016

Out of 3,912 sources identified in the GHDx, 2,664 sources were included.

WHO InfoBase and International Smoking Statistics (ISS) Database

An effort was made to replace database-derived estimates used in GBD 2013 with original extractions from primary data sources.

Outliers

Throughout the modeling process, data were assessed for bias and outliers were flagged. A data point was flagged as a candidate outlier if it was not consistent with the majority of other data points in a country with respect to level, age-pattern, sex-pattern, or temporal trend. In data-scarce countries, data points were also compared to data from other countries in a region. Candidate outliers were scrutinized for potential sources of bias and were ultimately excluded if the point or source was deemed to not be representative.

Modeling Strategy

Data Extraction

When possible, we extracted individual smoking status for all available frequency-type categories (listed above) from person-level microdata and collapsed these data to produce prevalence estimates in the standard GBD 5-year age-sex groups. If microdata were unavailable we extracted the most granular age-sex groups available from survey reports. Any available measures of uncertainty were extracted, including standard error, confidence or uncertainty intervals, and sample size.

Data Preparation: Crosswalking

Regressions to crosswalk other frequency-type categories to the gold-standard definition of daily use of smoked tobacco were estimated in the form:

$$\mathrm{p_{daily-smoked,k}} = \beta_1 \mathrm{p_{i,k}} + \epsilon_\mathrm{k}$$

where $p_{daily\text{-}smoked,k}$ is the prevalence of daily smoking reported in survey *k*, and $p_{i,k}$ is the prevalence of an alternative frequency-type combination *i* also reported in survey *k*. Consistent with previous GBD smoking crosswalks, the intercept was omitted from the regression. The estimated regression coefficient β_1 was used to crosswalk alternative frequency-type categories to the gold-standard daily smoking definition in sources only providing the alternative category. Predication error at the data-point level was used to propagate uncertainty and was calculated using the following equation:

$$\mathrm{PE_k} = \sigma_\epsilon^2 + \mathrm{X_k^2 var}(\hat{\beta})$$

Compared to the separate frequency and type crosswalks used in GBD 2013, the combined frequency-type crosswalk used in GBD 2015 represents an improvement because patterns in frequency that may vary by type and patterns in type that may vary by frequency are captured.

Data Preparation: Age and Sex Splitting

Report data provided in age groups wider than the standard GBD 5-year age groups or as both sexes combined were split using the approach used in Ng et al. Briefly, age-sex patterns were identified using sources with data on multiple age-sex groups and these patterns were applied to split aggregated report data. Uncertainty in the age-sex split was propagated by multiplying the standard error of the data (including the predication error of the crosswalk) by the square root of the number of splits performed.

Modeling: Linear Model

After data preparation, the dataset consisted of prevalence estimates of daily smoked tobacco use in standard GBD country-year-age-sex groups. The mean function used in ST-GPR was estimated using the following hierarchical mixed-effects linear regression, run separately by sex:

$$\mathrm{logit}(\mathrm{p_{c,a,t}}) = \beta_0 + \beta_1 \mathrm{CPC_{c,t}} + \sum_{\mathrm{k=2}}^{16} \beta_\mathrm{k} \mathrm{I_{A[a]}} + \alpha_\mathrm{s} + \alpha_\mathrm{r} + \alpha_\mathrm{c} + \epsilon_\mathrm{c,a,t}$$

where $CPC_{c,t}$ is the annual tobacco consumption per capita covariate, $I_{A[a]}$ is a dummy variable indicating specific age group A that the prevalence point $p_{c,a,t}$ is capturing, and α_s, α_r, and α_c are super region, region, and country-specific random effects.

Modeling: Spatio-Temporal Gaussian Process Regression (ST-GPR)

The estimated mean function was then propagated through the ST-GPR framework to obtain 1,000 draws of smoking prevalence estimates for each location, year, age, and sex. Parameter selection for the ST-GPR hyper-parameters were selected through out-of-sample cross-validation using the strategy described elsewhere in this appendix.

Smoking Impact Ratio Estimation

We have made no substantive changes in the SIR estimation strategy from GBD 2013. The only change in input data for estimating never-smoker lung-cancer mortality was to update data from the China Kadoorie Biobank prospective cohort to include follow-up through 2014. Country-year-age-sex specific lung cancer mortality rates are derived from GBD 2015 Cause of Death estimation and detailed in that Capstone's appendix. The formula for calculating SIR is:

$$SIR = \frac{C_{LC} - N_{LC}}{S^*_{LC} - N^*_{LC}} \times \frac{N^*_{LC}}{N_{LC}}$$

C_{LC}: age-sex-specific lung cancer mortality rate in the population of interest
N_{LC}: age-sex-specific lung cancer mortality rate of never-smokers in the population of interest
S^*_{LC}: age-sex-specific lung cancer mortality rate for life-long smokers in a reference population
N^*_{LC}: age-sex-specific lung cancer mortality rate for never smokers in the reference population

Theoretical minimum-risk exposure level

The theoretical minimum-risk exposure level is that no one in the population smokes tobacco; that is, the smoking impact ratio is zero and smoking prevalence is zero.

Relative risk

We have made no substantive updates to relative risks for outcomes included in GBD 2013. The following outcomes using 5-year lagged smoking prevalence as the exposure were added in GBD 2015: peptic ulcer disease, rheumatoid arthritis, cataracts, macular degeneration, hip fracture, and non-hip fracture. Larynx cancer was the only new outcome added using SIR as the exposure. Relative risks for rheumatoid arthritis, cataracts, and macular degeneration were derived from recent published meta-analyses. We performed out own meta-analyses of prospective cohort studies to derive relative risks for peptic ulcer disease, hip fracture, and non-hip fracture. We used Kontis et al.'s re-analysis of CPS-II smokers for the relative risk of larynx cancer.

Intimate Partner Violence SDG Capstone Appendix

Flowchart

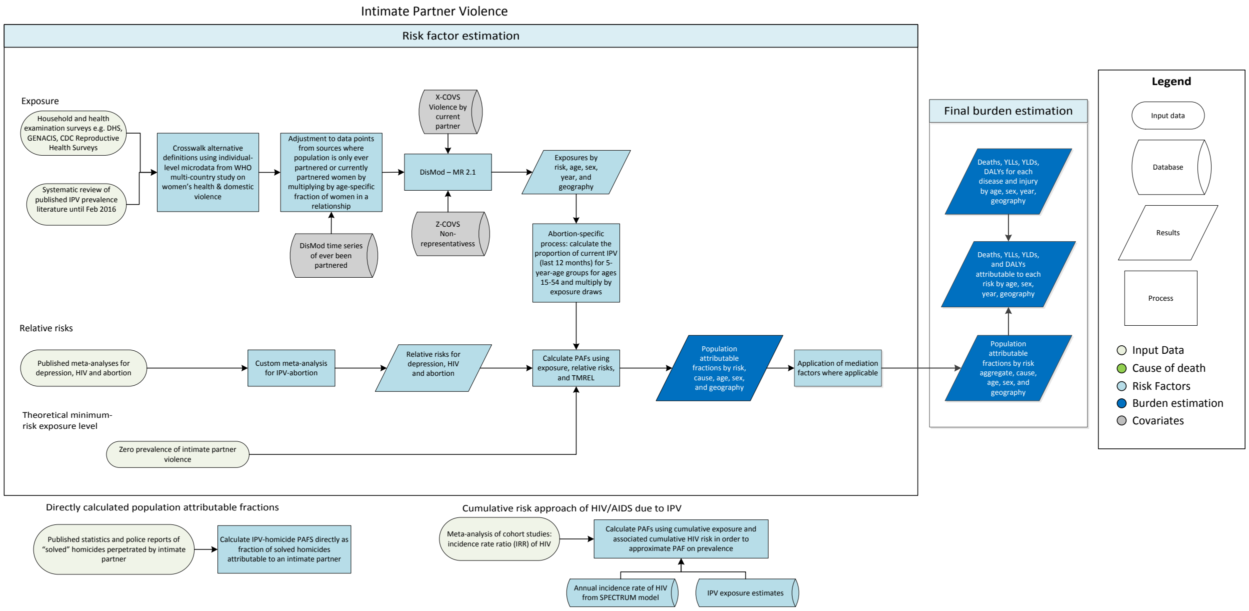

Input Data & Methodological Summary

Indicator definition

This modeling strategy encompassed the SDG health-related indicator associated with prevalence of intimate partner violence (5.2.1).

Indicator 5.2.1

As a component of SDG Goal 5. Eliminate all forms of violence against all women and girls in the public and private spheres, including trafficking and sexual and other types of exploitation, SDG Target 5.2 eliminate all forms of violence against all women and girls in the public and private spheres, including trafficking and sexual and other types of exploitation, is measured using SDG Indicator 5.2.1, Prevalence of women aged 15 years and older who experienced intimate partner violence.

Case Definition

The case definition for intimate partner violence (IPV) is ever experienced one or more acts of physical and/or sexual violence by a current or former intimate partner since the age of 15 years. Estimated in females only because IPV is more common in females and there is more evidence quantifying the associated risk for health outcomes.

- Physical violence is defined as: being slapped or having something thrown at you that could hurt you, being pushed or shoved, being hit with a fist or something else that could hurt, being kicked, dragged, or beaten up, being choked or burnt on purpose, and/or being threatened with or actually having a gun, knife, or other weapon used on you.

- Sexual violence is defined as: being physically forced to have intercourse when you did not want to, having sexual intercourse because you were afraid of what your partner might do, and/or being forced to do something that you found humiliating or degrading (the definition of humiliating and degrading may vary across studies depending on the regional and cultural setting).
- Intimate partner is defined as: a partner to whom you are married or with whom you cohabit. In countries where people date, dating partners will also be considered (a partner with whom you have an intimate (sexual) relationship with but are not married to or cohabiting).

Input data

A systematic review of the intimate partner violence prevalence literature was conducted in Pubmed for anything published between November 2014 and February 2016. The following search terms were used to conduct the systematic review:

((("health surveys"[MeSH Terms] AND prevalence[Title/Abstract]) OR ("sentinel surveillance"[MeSH Terms] AND prevalence[Title/Abstract]) OR ("prevalence"[Title/Abstract] AND cross sectional studies[MeSH Terms])) AND (abuse, sexual[MeSH Terms] OR domestic violence[MeSH Terms] OR abuse, partner[MeSH Terms] OR abuse, spousal[MeSH Terms] OR rape[MeSH Terms]) NOT ("comment"[Publication Type] OR "letter"[Publication Type] OR "editorial"[Publication Type]))

This query produced 92 results, and of these, 33 data points were extracted for 13 different countries. In addition to literature, we supplemented this data with surveys tagged with “intimate partner violence” in the GHDx. Some of the big survey series that were updated or newly added include: all new Demographic and Health surveys, the National Youth Risk Behavior Survey, the Gender, Alcohol and Culture International Study (GENACIS), the CDC Reproductive Health Surveys, Mexican National Addiction Survey, USA Collaborative Psychiatric Epidemiology Surveys, and the Brazil National Alcohol and Drug Survey.

We get the proportion of solved homicides that were perpetrated by an intimate partner from crime statistics and police reports. For GBD 2013, the main source of these crime statistics and police reports came from an IPV-homicide systematic review in the Lancet in 2013.

In GBD 2015, an updated systematic review was done for IPV homicide sources in PubMed through April 2016. The query used for this Pubmed search was:

((IPV[All Fields] OR ("intimate partner violence"[MeSH Terms] OR ("intimate"[All Fields] AND "partner"[All Fields] AND "violence"[All Fields]) OR "intimate partner violence"[All Fields])) AND (("homicide"[MeSH Terms] OR "homicide"[All Fields]) OR femicide[All Fields])) AND ("2013/01/01"[PDAT] : "3000/12/31"[PDAT])

These literature sources were supplemented with sources from the GHDx that were tagged with Intimate partner violence AND Homicide.

Modeling strategy

For GBD 2015, we use three distinct approaches to estimate burden attributable to IPV, including 1) the traditional exposure and relative risk to PAF method for depression, suicide and abortion; 2) the direct

PAF approach for estimating the proportion of homicides that are perpetrated by an intimate partner; and 3) a cumulative risk approach for estimating the burden of HIV/AIDS attributable to IPV.

Estimating attributable burden to IPV for depression, suicide and abortion

Before upload to DisMod, we first adjust data with variable recall periods (previous 12 months versus lifetime), type of violence (sexual, physical, or both) and severity (severe only versus all levels). To convert data to our gold standard definition of ever having experienced any IPV, we use data from the WHO multi-country violence against women surveys to construct crosswalk regressions. The dependent variable in each of these regression was ever any IPV (gold standard), while the key independent variable was one of the 11 alternative metrics of IPV that were represented in our dataset:

1. Physical IPV in the past 12 months
2. Sexual IPV in the past 12 months
3. Severe IPV in the past 12 months
4. Severe physical IPV in the past 12 months
5. Severe sexual IPV in the past 12 months
6. Any IPV (physical and/or sexual) in the past 12 months
7. Ever any physical IPV
8. Ever any sexual IPV
9. Ever any severe IPV
10. Ever severe physical IPV
11. Ever severe sexual IPV

For alternate metrics 1-6 there is likely to be a relationship between current exposure and age. For these metrics we included a series of age dummies:

$$logit(GSait) = \beta + \beta 1 logit(ALTait) + \beta 2 Ia + \varepsilon$$

For alternate metrics 7-11, we ran the following regression:

$$logit(GSit) = \beta 0 + \beta 1 logit(ALTit) + \varepsilon$$

where GS refers to the gold standard metric of IPV prevalence, ALT is the alternate metric of IPV prevalence, *Ia* refers to the complete set of age-group indicators, *a* refers to an age-group, *i* refers to a country, and *t* refers to year. We included age-group indicators in the first six regressions because we expected the prevalence of recent IPV to vary by age. Using the intercepts, coefficients, and variance-covariance matrix from each of these eleven regressions, we were able to convert all of the alternate metrics of IPV prevalence in our dataset to estimates of "ever any IPV". We eliminated observations based on alternate metrics of IPV which came from studies that also provided estimates of IPV based on the gold standard definition (i.e. duplicates).

After applying crosswalks to the alternate metrics of IPV in the manner described above, we made an additional adjustment to the subset of our data that was based on only ever-partnered, currently partnered women currently married women or ever married women. To adjust these values so that they reflected IPV prevalence in the entire female population, regardless of partnered status, we multiplied estimates from these studies by the age-specific fraction of women who had ever been partnered.

An updated time series was generated in GBD 2015 using MICS and DHS data in a single parameter DisMod model to reflect the most recent data on proportion of women that have ever been partnered. This revised time series was used to adjust values for surveys with restricted partner status to reflect the prevalence among all women in the population.

After these pre-DisMod crosswalks and adjustments, a single-parameter prevalence model was run in DisMod with age mesh points at 0 14 15 20 30 40 50 60 80 & 100. A study-level covariate fixed effect (x-cov) was used to adjust data points where the survey question used to calculate prevalence only asked about violence perpetrated by the woman's spouse. A study-level fixed effect on integrand variance (z-cov) to indicate whether a study was nationally representative or not was used to account for the heterogeneity introduced by studies that are not generalizable to the entire population.

We tried using alcohol liters per capita, prevalence of binge drinking, and prevalence of male binge drinking in the GBD 2015 model as national-level fixed effects, but they were not significant so they were ultimately dropped.

Direct PAF for female homicides

The burden of homicides attributable to intimate partner violence is modeled as a direct PAF.

Input data all fed into a single-parameter proportion DisMod model, which has age mesh points at 0 10 20 45 & 100. The model has a study-level covariate fixed effect on integrand value (x-cov) for sources just including police reported homicides. We also included a study-level fixed effect on integrant variance (z-cov) to indicate whether a study was nationally representative or not.

In GBD 2015, we added prevalence of binge drinking to the model as a country-level covariate.

Cumulative risk approach for PAF of HIV/AIDS due to IPV

The third and final modelling approach that we used to assess burden attributable to intimate partner violence was a cumulative risk approach to measure the burden of HIV/AIDS attributable to IPV.

The approach itself remained the same in GBD 2015, but included updated intimate partner violence exposure numbers from the DisMod model described above, as well as revised HIV incidence numbers.

From the literature we have information on the incidence rate ratio (IRR) of HIV incidence from two cohort studies (Jewkes et al, Lancet 2010 & Kouyoumdijian, et al AIDS 2013). As we measure burden based on deaths and prevalence, we need to be able to quantify attributable fractions on prevalence and death rather than incidence. To get a PAF on prevalence we need to consider the history of exposure to IPV and the accumulated associated risk of incident HIV due to IPV, relative to the overall risk of HIV at the population level. The ratio of cumulative IPV-attributable HIV incidence to total HIV incidence is an approximation of the relevant PAF on HIV prevalence and we will assume this PAF can also applied to mortality.

$$\frac{Cumulative\ HIV\ incidence\ due\ to\ IPV}{Cumulative\ HIV\ incidence\ overall} = \frac{1 - \prod_{a=o}^{a=n}\left(1 - PAF_{ay} * I_{ay}\right)}{1 - \prod_{a=o}^{a=n}(1 - I_{ay})}$$

Where:

I = annual incidence rate of HIV

a = age (15-84)

y = year (1980-2013)

$$PAF_{HIV\ incidence} = \frac{[Prevalence\ of\ IPV]_{ay} * (IRR-1)}{[Prevalence\ of\ IPV]_{ay} * (IRR-1)+1}$$

Theoretical minimum-risk exposure level

The theoretical minimum-risk exposure level is zero exposure to intimate partner violence, as defined above.

Relative risks

We estimate burden attributable to IPV for abortion, depression, suicide, interpersonal violence (i.e. homicide) and HIV incidence. We have added HIV as an outcome for GBD 2013 in response to bolstered causal evidence from a second prospective study published in 2013 (Kouyoumdjian, 2013). We use a pooled incidence rate ratio (IRR) of 1.59 (95% CI 1.3-1.94) from a meta-analysis of the two available prospective studies as of date.

The relative risks for depression and suicide come from a systematic review of longitudinal studies assessing intimate partner violence and incident depressive symptoms and suicide attempts. For the relative risk for IPV-abortion, we ran a custom meta-analysis in GBD 2013 that we continued to use in GBD 2015. An important methodological note with IPV-abortion is that we must apply the pooled relative risk for abortion to the current prevalence of IPV (in the previous 12 months), rather than lifetime prevalence. This is because the relevant exposure for abortion would be recent IPV, and because the case definition for all but one of the RR component studies was physical or sexual IPV in the past year.

Unsafe Water SDG Capstone Appendix

Flowchart

Unsafe Drinking Water

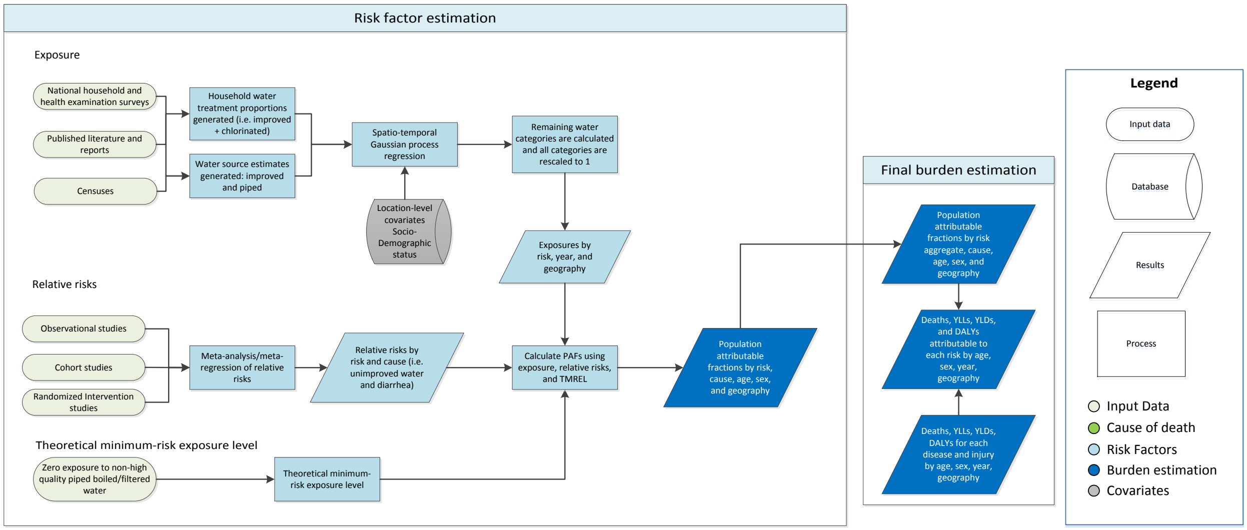

Input data and Methodological Summary

Indicator definition

This modeling strategy encompassed the indicator associated with water (SEV) (6.1.1).

Indicator 6.1.1

As a component of SDG Goal 6. Ensure availability and sustainable management of water and sanitation for all, SDG Target 6.1, by 2030, achieve universal and equitable access to safe and affordable drinking water for all, is measured using SDG Indicator 6.1.1, risk-weighted prevalence of population using unsafe/unimproved water sources.

Case Definition

For GBD 2015, exposure to unsafe water is defined based on reported primary water source used by the household and use of household water treatment (HWT) to improve the quality of drinking water before consumption. Water sources were defined as improved based on the JMP designation (The WHO), which includes piped water as improved water, and households with access to piped water connection to the house, yard, or plot were defined as having access to piped water supply. Solar treatment, chlorine treatment, boiling, or the use of filters were all assumed to be effective point-of-use household water treatments, and based on effect sizes published by Wolf et al. (2014) boiling or filtering was the most effective form of water treatment.

Input Data

The search for usable household surveys and censuses was conducted using the Global Health Data Exchange (GHDx) database. All surveys through December 2015 that provide household level micro-data on water source were added. Tabulated and report data was lower priority and was only updated when

time permitted. HWT input data was limited to two large survey series (DHS and MICS) due to time constraints. An update to HWT input data is a top priority for estimating exposure to unsafe water in future iterations.

Modeling Strategy

Water source data is modeled in two distinct categories: household prevalence of improved water and household proportion of piped water within improved population in order to prevent the population with access to piped water from exceeding the population with access to improved water (which includes piped). HWT is modeled in 6 distinct categories based on the 3 water treatment categories (filtered/boiled, solar/chlorine, or untreated) and 2 water source categories (piped or improved). We have made no substantive changes in the modeling strategy from GBD 2013. By year and geography, each of the above categories are modeled using a 3-step modeling scheme of mixed effect linear regression followed by spatio-temporal Gaussian process regression (ST-GPR), which outputs full time series estimates for each GBD 2015 location. Socio-demographic status (SDS), an index metric that includes a measure of education and income level, was used as a fixed effect in the linear regression since it proved to have significant coefficients. Random effects were placed at GBD 2015 region and super-region levels.

The process of vetting and validating models was accomplished primarily through an examination of ST-GPR scatter plots by GBD 2015 location from 1990-2015. Any unfitting data points were re-inspected for error at the level of extraction and survey implementation, and subsequently excluded from analysis if deemed appropriate. In addition to SDS, a number of different potential fixed effects were considered, including lag-distributed income and urbanicity, but SDS proved to be the strongest predictor of unsafe water. Uncertainty in the estimates was initially formed based on standard deviation by survey, then propagated through ST-GPR modeling by means of confidence intervals around each data point that reflect the point-estimate specific variance.

Once models are fully vetted, full time series outputs from ST-GPR modeling are then converted from proportion to prevalence by year and geography and then rescaled to form 9 mutually exclusive categories that sum up to 1. The table below provides the final result of this rescaling.

Category	*Definition*
Unimproved, no HWT	Proportion of households that use unimproved source, and *do not* use any HWT to purify their drinking water.
Unimproved, chlorine/solar	Proportion of households that use unimproved source, and solar or chlorine treatment to purify their drinking water.
Unimproved, boil/filter	Proportion of households that use unimproved source, and boil or filter to purify their drinking water.
Improved water except piped, no HWT	Proportion of households that use improved sources other than piped water supply, and *do not* use any HWT to purify their drinking water.

Improved water except piped, chlorine/solar	Proportion of households that use improved sources other than piped water supply, and use solar or chlorine treatment to purify their drinking water.
Improved water except piped, boil/filter	Proportion of households that use improved sources other than piped water supply, and boil/filter their drinking water.
Piped water, no boil/filter	Proportion of households that use piped water supply, and *do not* use any HWT to purify their drinking water
Piped water, chlorine/solar	Proportion of households that use piped water supply, and *use* solar or chlorine water treatment to purify their drinking water.
Piped water, boil/filter	Proportion of households that use piped water supply, and boil or filter to purify their drinking water

Due to the nature of modeling piped water exposure as a proportion of total improved water access, we are limited in only using sources for piped water that also include total improved water values. It should be noted that high-income countries are assumed to have risk of unsafe water which could lead to an underestimate of unsafe water health burden in these countries. Another limitation in our analysis is the paucity of data on HWT. The inclusion of more location-specific data on water treatment utilization at the household level can greatly improve our estimates in future iterations. High-income countries were assumed to have 0 risk of unsafe water, and the TMREL was applied to these countries.

Theoretical minimum-risk exposure level

The theoretical minimum-risk exposure level for unsafe water is defined as all households have access to high quality piped water that has been boiled or filtered before drinking. This exposure level is applied to all households in high-income countries, as well as households in countries in Southern Latin America region or Eastern Europe region that report piped water source and filtered or boiled water treatment.

Relative risks

GBD 2015 employ the same relative risks for unsafe water as was done for GBD 2013. There are 3 adverse health outcomes paired with unsafe water that comprise of diarrheal diseases, typhoid fever, and paratyphoid fever. A meta-analysis by Wolf et al. 2014 provides relative risk evidence for the relationship between unsafe water and diarrheal diseases. Wolf et al. 2014 publish relative risk values for water-source interventions and point-of-use treatment interventions separately so the combined effect of a source intervention and point-of-use intervention is assumed to be multiplicative in order to match GBD 2015 exposure definitions. In the absence of better data, the relative risk for typhoid and paratyphoid fevers were assumed to be the same as the relative risk for diarrheal disease. Furthermore, it is assumed that there is a difference in piped water quality between Eastern Europe and Southern Latin America compared to rest of the developing world. As a result, we use effect sizes that are region-specific. The implication of this assumption is that no household in developing countries have access to high-quality piped water (TMREL). Please refer to appendix tables for more information on relative risk values and citations.

References

1. "Improved and Unimproved Water Sources and Sanitation Facilities."*WHO / UNICEF Joint Monitoring Programme: Wat/san Categories*. The WHO/UNICEF, n.d. Web. 08 June 2016
2. Wolf, Jennyfer, Annette Prüss-Ustün, Oliver Cumming, Jamie Bartram, Sophie Bonjour, Sandy Cairncross, Thomas Clasen, John M. Colford, Valerie Curtis, Jennifer De France, Lorna Fewtrell, Matthew C. Freeman, Bruce Gordon, Paul R. Hunter, Aurelie Jeandron, Richard B. Johnston, Daniel Mäusezahl, Colin Mathers, Maria Neira, and Julian P. T. Higgins. "Systematic Review: Assessing the Impact of Drinking Water and Sanitation on Diarrhoeal Disease in Low- and Middle-income Settings: Systematic Review and Meta-regression." Trop Med Int Health Tropical Medicine & International Health 19.8 (2014): 928-42. Web.

Unsafe Sanitation SDG Capstone Appendix

Flowchart

Unsafe Sanitation

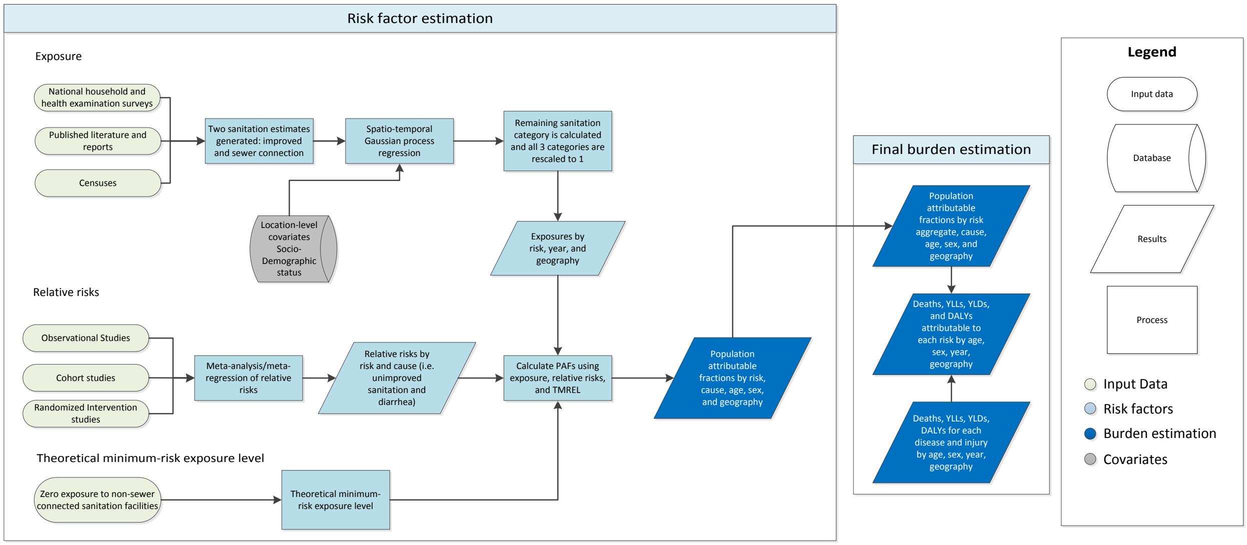

Input data and Methodological Summary

Indicator definition

This modeling strategy encompassed the indicator associated with sanitation (SEV) (6.2.1a).

Indicator 6.2.1a

As a component of SDG Goal 6. Ensure availability and sustainable management of water and sanitation for all, SDG Target 6.2, by 2030, achieve access to adequate and equitable sanitation and hygiene for all and end open defecation, paying special attention to the needs of women and girls and those in vulnerable situations, is measured using SDG Indicator 6.2.1a, risk-weighted prevalence of population using unsafe sanitation practices.

Case Definition

Exposure to unsafe sanitation were defined based on the primary toilet type used by households. Improved facilities are defined as such based on JMP designation (The WHO). Sewer connection toilets included flush toilets or any toilet with connection to the sewer or septic tank.

Input Data

The search for usable household surveys and censuses was conducted using the Global Health Data Exchange (GHDx) database. Searches were conducted from October 2015 to December 2015, with the final search household level micro-data on toilet type conducted on December 15, 2015. Due to the organized nature of the GHDx, the only search term used was “unsafe sanitation”, which yielded just

under 1400 results, of which 795 were extracted and used as inputs for modeling. Tabulated and report data was lower priority and was only updated when time permitted.

Modeling

There were no substantive changes in the modeling process from GBD 2015. Two distinct models are produced from sanitation data: prevalence of households with improved sanitation and the proportion of households with a sewer connection over the total improved sanitation population. Prevalence of households with a sewer connection is modeling with improved sanitation prevalence as the denominator in order to prevent the population with access to sewer connection from exceeding the population with access to improved sanitation. By each geography-year, both models are generated using a 3-step modeling scheme of mixed effect linear regression followed by spatio-temporal Gaussian process regression (ST-GPR), which outputs full time series estimates for each GBD 2015 location. Socio-demographic status (SDS), an index metric that includes a measure of education and income level, was used as a fixed effect in the linear regression since it proved to have significant coefficients. Random effects were placed at GBD 2015 region and super-region levels.

The process of vetting and validating models was accomplished primarily through an examination of ST-GPR scatter plots by GBD 2015 location from 1990-2015. Any unfitting data points were re-inspected for error at the level of extraction and survey implementation, and subsequently excluded from analysis if deemed appropriate. In addition to SDS, a number of different potential fixed effects were considered, including lag-distributed income and urbanicity, but SDS proved to be the strongest predictor of unsafe sanitation. Uncertainty in the estimates was initially formed based on standard deviation by survey, then propagated through ST-GPR modeling by means of confidence intervals around each data point that reflect the point-estimate specific variance.

Once models are fully vetted, full time series outputs from ST-GPR modeling are then converted from proportion to prevalence by year and geography and then rescaled to form 3 mutually exclusive categories that sum up to 1. The table below provides the final result of this rescaling.

Category	*Definition*
Unimproved sanitation	Proportion of households that use unimproved sanitation facilities.
Improved sanitation, excluding sewer	Proportion of households that use improved sanitation facilities except those with sewer connection.
Sanitation facilities with sewer connection	Proportion of households that use toilet facilities with sewer connection.

Due to the nature of modeling sanitation with sewer connection as a proportion of total improved sanitation access, we are limited in only using sources for sewer connection that also include total improved sanitation values. It should be noted that high-income countries are assumed to have risk of unsafe sanitation which could lead to an underestimate of unsafe sanitation health burden in these countries. Another limitation that extends to the other two risk factors that comprise WaSH (unsafe water and unsafe hygiene) and can be improved upon in future iterations is taking into account

covariance of access to water, sanitation and handwashing facilities. Currently, all three components of WaSH are modeled independently, which may lead to an overestimation of the burden of WaSH factors. High-income countries were assumed to have 0 risk of unsafe sanitation and the TMREL was applied to these countries.

Theoretical minimum-risk exposure level

The theoretical minimum-risk exposure level for unsafe sanitation was defined as all households have access to a sanitation facility with sewer connection. Since it is assumed that all households in high-income countries have access to sewer-connected sanitation, this counterfactual exposure level is applied to all households in high-income countries.

Relative risks

GBD 2015 employ the same relative risks for unsafe water as was done for GBD 2013. Three adverse health outcomes are paired with unsafe sanitation, which comprise of diarrheal diseases, typhoid fever, and paratyphoid fever. A meta-analysis by Wolf et al. 2014 provides relative risk evidence for the relationship between unsafe sanitation and diarrheal diseases. In the absence of better data, the relative risk for typhoid and paratyphoid fevers were assumed to be the same as the relative risk for diarrheal disease. Please refer to appendix tables for more information on relative risk values and citations.

References

1. "Improved and Unimproved Water Sources and Sanitation Facilities."*WHO / UNICEF Joint Monitoring Programme: Wat/san Categories*. The WHO/UNICEF, n.d. Web. 08 June 2016
2. Wolf, Jennyfer, Annette Prüss-Ustün, Oliver Cumming, Jamie Bartram, Sophie Bonjour, Sandy Cairncross, Thomas Clasen, John M. Colford, Valerie Curtis, Jennifer De France, Lorna Fewtrell, Matthew C. Freeman, Bruce Gordon, Paul R. Hunter, Aurelie Jeandron, Richard B. Johnston, Daniel Mäusezahl, Colin Mathers, Maria Neira, and Julian P. T. Higgins. "Systematic Review: Assessing the Impact of Drinking Water and Sanitation on Diarrhoeal Disease in Low- and Middle-income Settings: Systematic Review and Meta-regression." Trop Med Int Health Tropical Medicine & International Health 19.8 (2014): 928-42. Web.

Unsafe Hygiene Capstone Appendix

Flowchart

Unsafe Handwashing

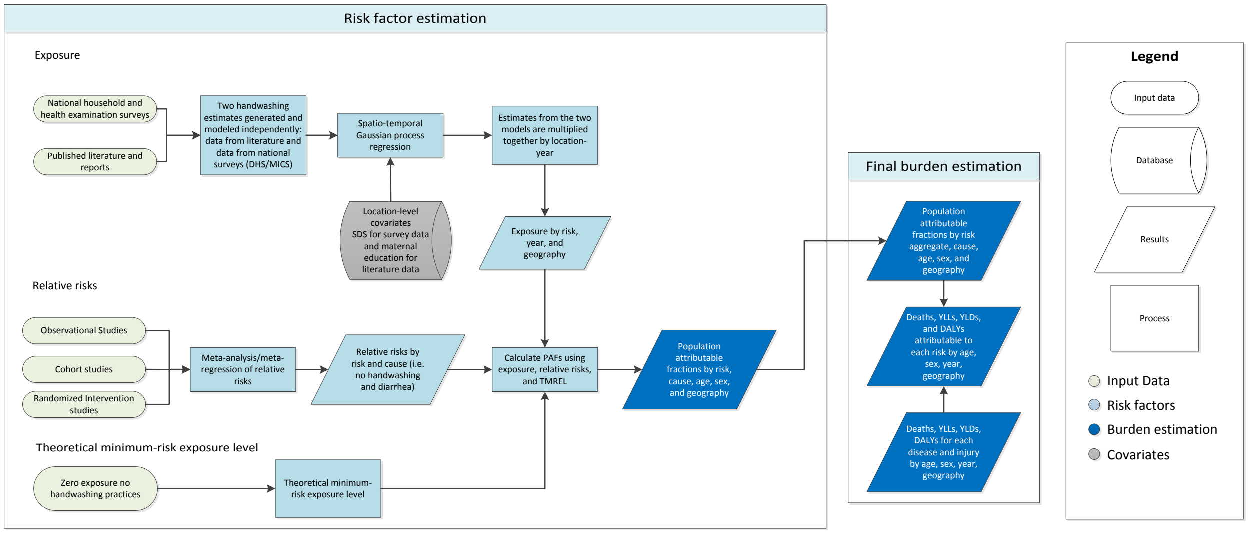

Input data and Methodological Summary

Indicator definition

This modeling strategy encompassed the indicator associated with hygiene [handwashing] (SEV) (6.2.1b).

Indicator 6.2.1b

As a component of SDG Goal 6. Ensure availability and sustainable management of water and sanitation for all, SDG Target 6.2, by 2030, achieve access to adequate and equitable sanitation and hygiene for all and end open defecation, paying special attention to the needs of women and girls and those in vulnerable situations, is measured using SDG Indicator 6.2.1b, risk-weighted prevalence of population with unsafe hygiene (no handwashing with soap).

Case Definition

Unsafe hygiene is composed of global handwashing practices. Handwashing is defined as the observed prevalence of handwashing with soap and water after using a toilet or after contact with excreta, including children's excreta. We estimate the burden of unsafe handwashing in both developed and developing settings.

Input Data

There were two main sources that were used in our estimation of handwashing practices, estimates from scientific literature and estimates from household survey series. Relevant literature on handwashing prevalence was gathered from a meta-analysis published recently by Freeman et al. (2014). Since water

and soap availability data is very limited, only country-specific Demographic Health Surveys (DHS) and Malaria Indicator Survey Series (MICS) conducted after 2006 were able to be used as input data.

Modeling Strategy

Input data from scientific literature and input data from household survey series were modeled independently. Data from literature primarily measured a population's handwashing practices under ideal conditions, such as when water and soap was readily available. Additionally, these estimates from literature would likely be susceptible to acquiescence bias. Alternatively, data from DHS and MICS only provide insight into the availability of water, soap, and washing stations, which, alone, does not indicate how often a person may wash their hands after contact with excreta. Thus, after modeling data from literature and data from surveys independently, these values were multiplied together by location-year in order to gain a more accurate representation of true handwashing prevalence.

Other than modeling literature data and survey data independently, we have made no substantive changes in the modeling strategy from GBD 2013. By year and location, estimates are generated using a 3-step modeling scheme of mixed effect linear regression followed by spatio-temporal Gaussian process regression (ST-GPR). When modeling survey input data, socio-demographic status (SDS), an index metric that includes a measure of education and income level, proved to have the most significant coefficient and was used as a fixed effect in the linear regression. To better inform our model that used scientific literature as input data, maternal education or average years of education for women ages 15-54, was implemented as a fixed effect in the linear regression. For both models, random effects were placed at GBD 2015 region and super-region levels.

The process of vetting and validating models was accomplished primarily through an examination of ST-GPR scatter plots by GBD 2015 location from 1990-2015. Any unfitting data points were re-inspected for error at the level of extraction and survey implementation, and subsequently excluded from analysis if deemed appropriate. In addition to SDS, a number of different potential fixed effects were considered, including lag-distributed income and urbanicity, but SDS and maternal education proved to be the strongest predictors of handwashing practices for their respective models. As mentioned above, once models were adequately vetted, full time series outputs from each of the models were multiplied together at each location-year.

A considerable limitation for when estimating handwashing practices for over 190 independent locations around the world is data sparseness. Even when data is published on handwashing prevalence, the definition is often altered from the GBD 2015 standard definition or it may only pertain to certain populations (such as hospital patients) and lacks representativeness at the geographic scale we require. The incorporation of questions about soap and water availability in DHS and MICS has added much-needed information but there remains a large data gap that must be filled if we are to become more certain in handwashing estimates.

Theoretical minimum-risk exposure level

The theoretical minimum-risk exposure level for unsafe hygiene is defined as all households engaging in handwashing with soap practices after any contact with excreta, including children's excreta.

Relative risks

GBD 2015 use the same relative risks for unsafe hygiene as was done for GBD 2013. There are 3 adverse health outcomes paired with unsafe hygiene that include diarrheal diseases, typhoid fever, and paratyphoid fever. A meta-analysis by Freeman et al. 2014 provides relative risk evidence for the relationship between unsafe hygiene and diarrheal diseases. In the absence of adequate data, the relative risk for typhoid and paratyphoid fevers were assumed to be the same as the relative risk for diarrheal disease based on analogous transmission pathways (feco-oral pathway). Please refer to appendix tables for more information on relative risk values and citations.

References

1. Freeman, M. C., Stocks, M. E., Cumming, O., Jeandron, A., Higgins, J. P., Wolf, J., Curtis, V. (2014). Systematic review: Hygiene and health: Systematic review of handwashing practices worldwide and update of health effects. *Trop Med Int Health Tropical Medicine & International Health, 19*(8), 906-916. doi:10.1111/tmi.12339

Household Air Pollution SDG Capstone Appendix

Flowchart

Household Air Pollution from Solid Fuels

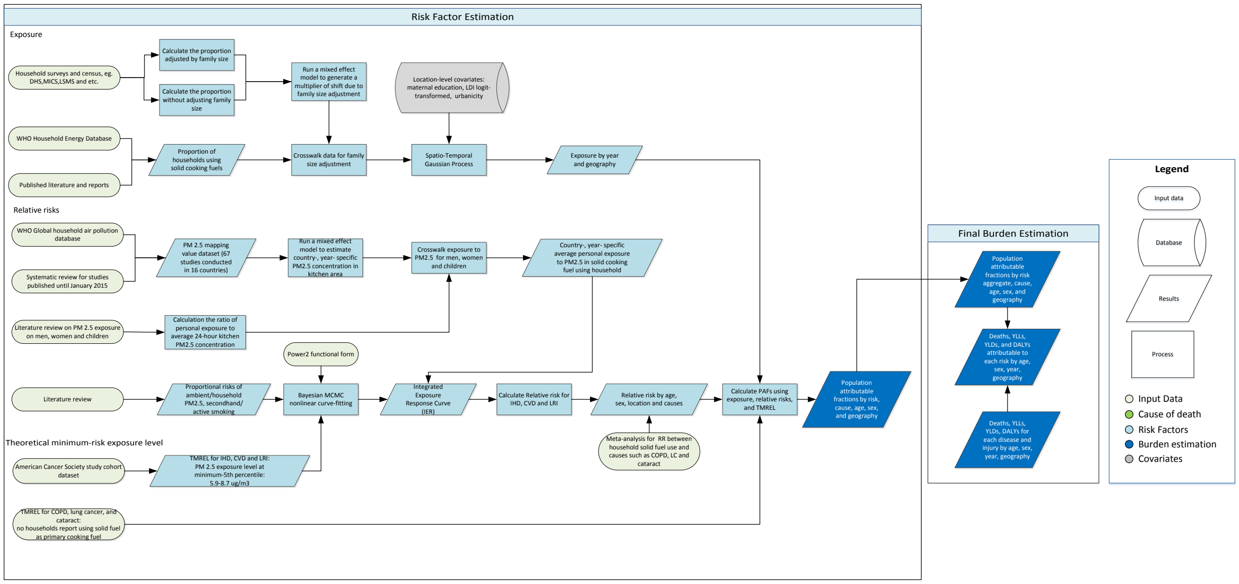

Input Data & Methodological Summary

Indicator definition

This modeling strategy encompassed the indicator associated with risk weighted prevalence of population using unsafe cooking fuel (7.1.2).

Indicator 3.9.1

As a component of SDG Goal 7. Ensure access to affordable, reliable, sustainable, and modern energy for all, SDG Target 7.1, by 2030, ensure universal access to affordable, reliable and modern energy services, is measured using SDG Health Index Indicator 7.1.2, risk weighted prevalence of population using unsafe cooking fuel, which comes from household air pollution (HAP).

Case definition

Exposure to household air pollution from solid fuels (HAP) is defined as the proportion of households using solid cooking fuels. The definition of solid fuel in our analysis includes coal, wood, charcoal, dung, and agricultural residues.

Input data

Data were extracted from the standard multi-country survey series such as Demographic and Health Surveys (DHS), Living Standards Measurement Surveys (LSMS), Multiple Indicator Cluster Surveys (MICS), and World Health Surveys (WHS), as well as country-specific survey series such as Kenya Welfare Monitoring Survey and South Africa General Household Survey. To fill the gaps of data in surveys and censuses, we also downloaded and updated HAP estimates from WHO Energy Database and extracted

from literature through systematic review done in IHME. Each nationally or sub-nationally representative data point provided an estimate for the percentage of households using solid cooking fuels. Estimates for the usage of solid fuels for non-cooking purpose were excluded, i.e. primary fuels for lighting. The database, with estimates from 1980 to 2015, contained 685 studies from 150 countries. Updates to systematic reviews are performed on an ongoing schedule across all GBD causes and risk factors, an update for household air pollution will be performed in the next 1-2 iterations.

Modeling strategy

Household air pollution was modeled at household level using a three-step modeling strategy ST-GPR that uses linear regression, spatiotemporal regression and Gaussian Process Regression (GPR). The first step is a mixed-effect linear regression of logit-transformed proportion of households using solid cooking fuels. The linear model contains maternal education and proportion of population living in urban areas as covariates and has nested random effect by country, GBD region, and GBD super region respectively. The full ST-GPR process is specified elsewhere in this appendix.

Compared with GBD 2013, we have made changes in terms of the covariates utilized in the linear model. A variety of combinations of socioeconomic and environmental covariates in different transformation format were tested by running mixed-effect models with exposure data. The final list of covariates included in the exposure model are maternal education and the proportion of population living in urban area.

Theoretical minimum-risk exposure level

For outcomes where we extracted RR based on direct epidemiological evidence i.e. COPD, lung cancer, and cataract, TMREL was defined such that no households would report using solid fuel as their primary cooking fuel. For outcomes that utilize evidence based on the Integrated Exposure Response (IER), the TMREL is defined as uniform distribution between 33.3 and 41.9 ug/m^3. TMREL for household air pollution did not change from GBD 2013.

Relative risks

The disease-outcomes paired with household air pollution has not changed since GBD 2013. The list of outcomes paired with household air pollution has not changed since GBD 2013, which included lower respiratory infections (LRI), stroke, Ischemic Heart Disease (IHD), chronic obstructive pulmonary disease (COPD), lung cancer and cataract. The relative risks of all outcomes but not cataract were generated by using the integrated exposure-response functions (IER). The relative risks for cataract were extracted from a meta-analysis paper (1). The IER curves are updated to reflect the newly updated data and utilization of a new method that specified elsewhere.

PM2.5 mapping value

The relative risk estimates describing the association of HAP with outcomes including Ischemic Heart Disease (IHD), cardiovascular disease (CVD), and lower respiratory infections (LRI) were derived from the IER curves. This is done by first estimating the crosswalk values that map household use of solid fuel to PM2.5 exposure because the IER curve measures exposure using PM2.5. This step of the analysis relied on 67 studies conducted in 16 countries to generate the PM2.5 mapping values, which remain the same sources as GBD 2013. The PM2.5 exposure was then cross-walked to men, women and children by

generating the ratio of personal exposure to average 24-hour kitchen PM2.5 concentration based on a study after the literature review in GBD 2013.

References

1. Smith KR, Bruce N, Balakrishnan K, Adair-Rohani H, Balmes J, Chafe Z, et al. Millions Dead: How Do We Know and What Does It Mean? Methods Used in the Comparative Risk Assessment of Household Air Pollution. Annu Rev Public Health. 2014;35(1):185–206.

Alcohol Capstone Appendix

Flowchart

Alcohol Use

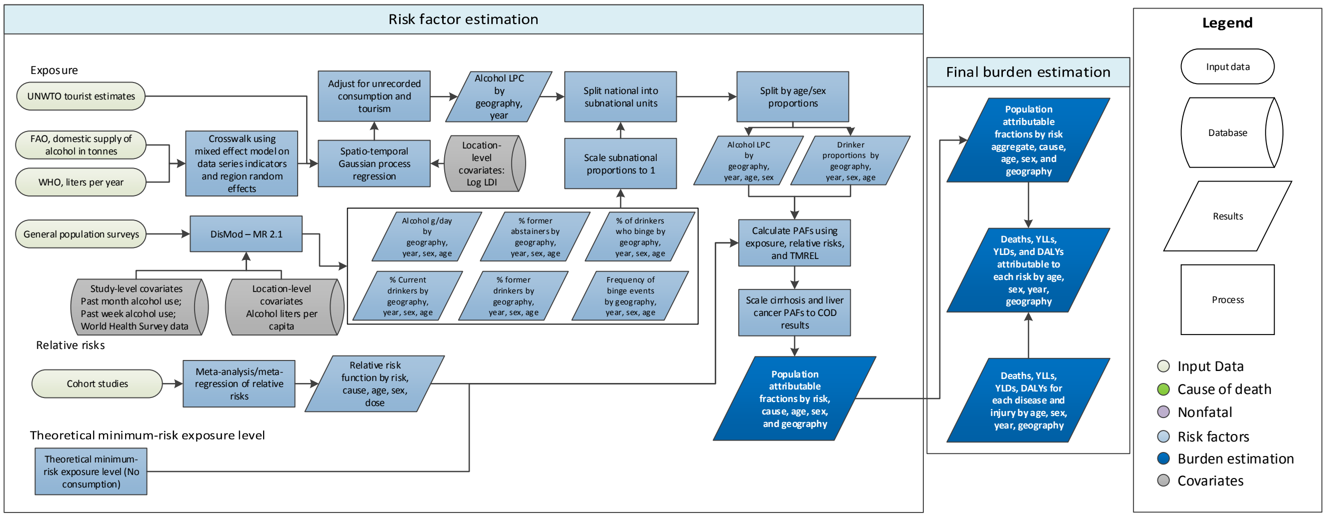

Input Data and Methodological Summary

Exposure

Case definition

The impact of alcohol consumption on morbidity and mortality can be largely described by two separate but related dimensions. The 1st dimension is the individual level drinking and consists of four indicators;

1. Current drinkers, defined as the proportion of individuals who have consumed at least one alcoholic beverage (or some approximation) in the last 12 months.
2. Former drinkers, defined as the proportion of individuals who have ever consumed an alcoholic beverage, but not in the last 12 months.
3. Lifetime abstainers, defined as the proportion of individuals who have never consumed an alcoholic beverage.
4. Alcohol consumption (in grams per day), defined as grams of alcohol consumed by current drinkers, per day, over a 12 month period.

The 2nd dimension of alcohol consumption relates to the pattern of drinking and consists of two indicators;

5. Binge drinkers, defined as the proportion of drinkers who have had a binge event in the past 12 months. A binge event was defined as consuming 60 grams of alcohol (approximately five drinks or more) in a single occasion for males and 48 grams of alcohol in a single occasion for females.
6. Binge times, defined as the proportion of drinking events that are binge amongst binge drinkers i.e. the proportion of days that a binger has a binge event.

Input data

For GBD 2013, a systematic review of the literature was conducted to capture population survey data on all six alcohol use indicators. In summary, the search was conducted in three stages involving electronic searches of the peer-reviewed literature via PubMed, the grey literature and, expert consultation. Updates to systematic reviews via PubMed are performed on an ongoing schedule across all GBD causes and risk factors, an update for alcohol use will be performed in the next 1-2 iterations. For GBD 2015, stages two and three of the literature review were conducted, prioritizing countries for which subnational estimates were generated. The Global Health Exchange (GHDx), IHME's online database of health-related data, was searched for population survey data containing participant-level information from which we could formulate the required alcohol use indicators. Data-sources were included if they captured a sample representative of the geographic location under study and contained variables that could be used to formulate any of the six alcohol use indicators. Relevant survey variables from each data-source were documented in a Microsoft Excel codebook and extracted using STATA 13.1. A total of 629 potential data-sources were available in GhDx across countries with subnational locations, out of which 127 data-sources (66,108 data-points) were included across all six indicators.

To generate estimates of alcohol consumption in grams per day, data from population surveys were used in combination with estimates of per capita consumption from the Food and Agriculture Organization (FAO) [1] and the Global Information System on Alcohol and Health (GISAH database [2]) Per capita consumption is an aggregate measure of recorded, unrecorded, and tourist per capita consumption of alcohol (UNWTO database [3]) derived from sales, production, and other economic statistics. While population-based surveys provide accurate estimates of the prevalence of lifetime abstainers, former drinkers and current drinkers, they typically underestimate real alcohol consumption levels. As a result, the all-age, both-sex per capita consumption figures from the FAO and GISAH are considered to be a better estimate of overall volume of consumption. Per capita consumption, however, does not provide age- and sex-specific consumption estimates needed to compute alcohol-attributable burden of disease. Therefore, we use the age-sex pattern of consumption among drinkers modeled from the population survey data and the overall volume of consumption from FAO and GISAH to determine the total amount of alcohol consumed by country.

To generate estimates of alcohol consumption in liter per capita, raw inputs were obtained from FAOSTAT [1] and WHO GISAH database [2]. To provide more stable time trends in the model, FAO sales data was transformed to a lagged 5-year average. FAO data was used when WHO data wasn't available. Otherwise, FAO and WHO data was adjusted (crosswalked) by running a mixed effect model on the log average of the data with indicators for the FAO and WHO data series, as well as random effects on super region, region, country, and time. Each data point was adjusted by the predicted betas on super-region and region.

$$Log\ Average\ Data = D + (Super\ Region\ |D, Region\ |\ D, Country\ |D, Year|\ D)$$

$$Transformed\ data = \ data\ *\ e^{\widehat{\beta_1} + \widehat{\beta_3}}$$

Where D = Indicator variable for data source

To generate uncertainty, a Lowess model was run on the adjusted data and the standard deviation between the difference of the Lowess smoothed model and the adjusted data points was used for data points missing uncertainty.

Unrecorded consumption was incorporated into the alcohol LPC data using estimates provided by the WHO [4]. WHO estimates were only reported for the years 1990, 2005, and 2010 so for missing years, estimates were interpolated. For years outside this range, unrecorded estimates were carried forward or backwards from the closest year. Unrecorded consumption estimates were reported in liters per capita so estimates were added to adjusted data points to account for unrecorded consumption.

Tourism data was obtained through the UNWTO [4]. A crosswalk was applied across different tourist categories, similar to the one used for FAO and WHO data, to estimate tourist proportions for a given country. Tourism consumption was incorporated after modeling unadjusted alcohol LPC as outlined below.

Data Preparation & Modeling strategy

DisMod-MR 2.1 was used to estimate country-, year-, age- and sex-specific proportions of current drinkers, former drinkers, lifetime abstainers, binge drinkers, and binge times; and alcohol consumption as a continuous variable in grams per day. We have made no substantive changes in the modeling strategy from GBD 2013. We ran single-parameter models for each alcohol use indicator and included a combination of location- and study-level covariates in each model. An alcohol liters per capita location-level covariate was used for all six indicators to assist in the predictive power of the models. Additionally, study-level covariates were used to accommodate for known sources of variability in the raw data. In the current drinkers, former drinkers, binge drinkers and binge times models, we included two covariates which adjusted estimates derived in the past week and past month towards those derived in the past year respectively. Estimates derived in the past year were considered to be the gold standard given the previously outlined definition for each indicator.

In the alcohol consumption model, we included a separate study-level covariate flagging data points derived from The World Health Organization’s World Health Surveys (WHS) conducted across multiple countries. There was considerable variability in estimates derived from the WHS which may have been influenced by methodological differences in how alcohol use was captured. This study-level covariate looked for unsystematic bias between data-points and added more uncertainty onto those from the WHS. If other data-points causing higher or lower modelled output were identified during the modelling process for a given indicator, the plausibility of these data points was assessed and the study methodology reviewed. Data points with methodological limitations, for instance those derived from survey items not entirely representatively of the alcohol use indicators required, with small sample sizes, or derived from samples not entirely representative of the general population were excluded.

A spatial-temporal Gaussian process regression was used to model total alcohol in liters per capita (see appendix, section 2). Parameters and a random effect model for the prior were chosen using out-of-sample cross validation. This produced estimates of alcohol LPC for a complete time series for the years 1980-2015 by country.

Alcohol LPC was adjusted for each country hosting tourists using the following equations:

$$Alcohol\ LPC_H = Unadjusted\ Alcohol\ LPC_H + Alcohol\ LPC_{Consumption\ abroad} - Alcohol\ LPC_{Tourist\ consumption}$$

$$Alcohol\ LPC_{Consumption\ abroad} = \frac{\sum_V Proportion\ of\ tourists_{H,V} * Unadjusted\ Alcohol\ LPC_H * \frac{Average\ length\ of\ stay_{H,V}}{365} * Tourist\ Population_V}{Population_H}$$

$$Alcohol\ LPC_{Tourist\ consumption} = \frac{\sum_V Proportion\ of\ tourists_V * Unadjusted\ Alcohol\ LPC_V * \frac{Average\ length\ of\ stay_V}{365} * Tourist\ Population_H}{Population_H}$$

Where H = Host country, V = Visiting country

Or, in other words, alcohol LPC was adjusted by adding in the per capita rate of consumption abroad and subtracting the per capita rate of tourist consumption domestically.

After adjusting alcohol LPC by tourist consumption and unrecorded consumption for all location/years reported, sex-specific and age-specific estimates were generated by incorporating estimates modeled in Dismod for percentage of current drinkers within a location/year/sex/age, as well as consumption trends modeled in Dismod g/day by location/year/sex/age, using the following equations.

$$Proportion\ of\ total\ consumption_{l,y,s,a} = \frac{Alcohol\ \ g/day_{l,y,s,a} * Population_{l,y,s,a} * \%\ Current\ drinkers_{l,y,s,a}}{\sum_{s,a} Alcohol\ \ g/day_{l,y,s,a} * Population_{l,y,s,a} * \%\ Current\ drinkers_{l,y,s,a}}$$

$$Alcohol\ LPC_{l,y,s,a} = \frac{Alcohol\ LPC_{l,y} * Population_{l,y} * Proportion\ of\ total\ consumption_{l,y,s,a}}{Population_{l,y,s,a}}$$

Where L = location, Y = Year, S = Sex, A = Age

A similar scalar was applied so that total subnational consumption equaled national consumption.

Theoretical minimum-risk exposure level

For alcohol use, the theoretical minimum-risk exposure level (TMREL) was assumed to be no alcohol use, i.e. 0 g/day of alcohol consumption. This diverges from the definition of other theoretical minimum-risk exposure level of risks because, for some alcohol-use relative risks, there's a preventative effect for low levels of consumption. However, due to the modeling of alcohol relative risks outlined below, it was found that 0 g/day provided the most consistency between the definition of alcohol-use TMREL and other GBD risk's TMREL. This is an area of improvement for future GBD iterations. Current research suggests that the preventative effect noted in studies may be due to issues in estimating abstainer populations. [5-7] If this is the case, a TMREL of 0 would still be valid.

Relative risk

Relative risks were derived for each GBD cause by mapping functions to the dose-response relationships found in meta-analysis. [11-22] Due to data availability, for high levels of consumption, uncertainty in

the relative risk functions increases greatly. To minimize the uncertainty of these measures, relative risks were estimated up to the 90th percentile of exposures in men (85 g/day) and the 95th percentile of exposures in women (60 g/day). For exposures beyond this, the associated relative risk was carried forward from these chosen percentile exposure levels. Though a dose-response relationship is evident at higher levels of exposure, the shape of the relative risk function is highly uncertain for higher levels of exposure both due to a lack of observations at these exposure levels, as well as confounding variables affecting estimation of the relative risk of these populations. Thusly, our relative risk estimates are likely an underestimate for the top 10% of male exposures and 5% of female exposures. For exact relative risks used, see appendix section 4.

Population Attributable Fraction

For chronic conditions, PAF was defined as

$$PAF(x) = \frac{P_A + P_F * RR_F + \int_0^{150} P(x) * RR_C(x)\,dx - 1}{P_A + P_F * RR_F + \int_0^{150} P(x) * RR_C(x)\,dx} \qquad P(x) = P_C * \frac{\Gamma(k,\theta)}{\int_{0.1}^{150} \Gamma(k,\theta)}$$

where:

x = alcohol consumption in g/day
P_A = Prevalence of lifetime abstainers
P_F = Prevalence of former drinkers
P(x) = Prevalence of alcohol consumption
RR_F = Relative risk of former drinkers
RR_C (x) = Relative risk function for drinkers

$$k = \frac{\bar{x}^2}{\sigma(\bar{x})^2}$$

$$\theta = \frac{\sigma(\bar{x})^2}{\bar{x}^2}$$

A thousand draws were taken of PAFs to generate uncertainty. The gamma distribution was used to estimate individual level variation within drinking populations [8-9]. Binge drinkers were not taken into account for chronic causes since the pattern of drinking has not been found to be an indicator of most outcomes [10].

For non-chronic conditions, such as injuries, binge drinking was accounted for in the model since patterns of drinking is significant.

$$PAF(x) = \frac{P_A + P_F + P_C + P_{C+B} * RR_{C+B}(x) - 1}{P_A + P_F + P_C + P_{C+B} * RR_{C+B}(x)} \qquad RR_{C+B}(x) = P_D * P_{D+B} * (RR_{crude}(x) - 1) + 1$$

where:

P_{C+B} = Prevalence of current drinkers who binge
RR_{C+B} = Relative risk of current drinkers who binge
RR_{crude} = Relative risk for a given mean level of consumption

P_D = Proportion of a day that is a binge event
P_{D+B} = Proportion of all days where a binge event occurs

The estimated PAF draws were then used to estimate YLL, YLDs, and DALYs, as per the other risk factors (see appendix section 2).

References

[1] Food and Agriculture Organization of the United Nations. FAOSTAT Statistics Database.

[2] World Health Organization (WHO). WHO Global Health Observatory - Recorded adult per capita alcohol consumption, Total per country. Geneva, Switzerland: World Health Organization (WHO).

[3] UN World Tourism Organization (UNWTO). UN World Tourism Organization Compendium of Tourism Statistics 2015 [Electronic]. Madrid, Spain: UN World Tourism Organization (UNWTO), 2016.

[4] World Health Organization (WHO). WHO Global Health Observatory – Unrecorded consumption by country. Geneva, Switzerland: World Health Organization (WHO).

[5] Rehm, J., et al. "Are lifetime abstainers the best control group in alcohol epidemiology? On the stability and validity of reported lifetime abstention."American journal of epidemiology 168.8 (2008): 866-871.

[6] Chikritzhs, Tanya, Kaye Fillmore, and T. I. M. Stockwell. "A healthy dose of scepticism: four good reasons to think again about protective effects of alcohol on coronary heart disease." Drug and alcohol review 28, no. 4 (2009): 441-444.

[7] Jackson, Rod, Joanna Broad, Jennie Connor, and Susan Wells. "Alcohol and ischaemic heart disease: probably no free lunch." The Lancet 366, no. 9501 (2005): 1911-1912.

[8] Kehoe, Tara, Gerrit Gmel, Kevin D. Shield, Gerhard Gmel, and Jürgen Rehm. "Determining the best population-level alcohol consumption model and its impact on estimates of alcohol-attributable harms." Population health metrics 10, no. 1 (2012): 1.

[9] Rehm, Jürgen, Tara Kehoe, Gerrit Gmel, Fred Stinson, Bridget Grant, and Gerhard Gmel. "Statistical modeling of volume of alcohol exposure for epidemiological studies of population health: the US example." Population Health Metrics 8, no. 1 (2010): 1.

[10] Rehm, Jürgen, Robin Room, Kathryn Graham, Maristela Monteiro, Gerhard Gmel, and Christopher T. Sempos. "The relationship of average volume of alcohol consumption and patterns of drinking to burden of disease: an overview." Addiction 98, no. 9 (2003): 1209-1228.

[11] Roerecke M, Rehm J. Alcohol consumption and the risk for morbidity and mortality of ischemic heart disease - A systemic review and meta-analysis. Toronto, Canada: Centre for Addiction and Mental Health; 2011

[12] Bagnardi V, Blangiardo M, La Vecchia C, Corrao G. A meta-analysis of alcohol drinking and cancer risk. Br J Cancer. 2001; 85(11): 1700-5.

[13] Corrao G, Bagnardi V, Zambon A, La Vecchia C. A meta-analysis of alcohol consumption and the risk of 15 diseases. Prev Med. 2004; 38(5): 613-9.

[14] Samokhvalov AV, Irving HM, Rehm J. Alcohol consumption as a risk factor for pneumonia: a systematic review and meta-analysis. Epidemiol Infect. 2010; 138(12): 1789-95.

[15] Samokhvalov AV, Irving H, Mohapatra S, Rehm J. Alcohol consumption, unprovoked seizures, and epilepsy: a systematic review and meta-analysis. Epilepsia. 2010; 51(7): 1177-84.

[16] Samokhvalov AV, Irving HM, Rehm J. Alcohol consumption as a risk factor for atrial fibrillation: a systematic review and meta-analysis. Eur J Cardiovasc Prev Rehabil. 2010; 17(6): 706-12.

[17] Rehm J, Samokhvalov AV, Neuman MG, Room R, Parry C, Lönnroth K, Patra J, Poznyak V, Popova S. The association between alcohol use, alcohol use disorders and tuberculosis (TB). A systematic review. BMC Public Health. 2009; 450.

[18] Lönnroth K, Williams BG, Stadlin S, Jaramillo E, Dye C. Alcohol use as a risk factor for tuberculosis - a systematic review. BMC Public Health. 2008; 289.

[19] Roerecke M, Rehm J. Alcohol consumption and the risk for morbidity and mortality of ischemic heart disease - A systemic review and meta-analysis. Toronto, Canada: Centre for Addiction and Mental Health; 2011.

[20] Rehm J, Taylor B, Mohapatra S, Irving H, Baliunas D, Patra J, Roerecke M.. Alcohol as a risk factor for liver cirrhosis: a systematic review and meta-analysis. Drug Alcohol Rev. 2010; 29(4): 437-45.

[21] Patra J, Taylor B, Irving H, Roerecke M, Baliunas D, Mohapatra S, Rehm J. Alcohol consumption and the risk of morbidity and mortality for different stroke types--a systematic review and meta-analysis. BMC Public Health. 2010; 258.

[22] Taylor B, Irving HM, Kanteres F, Room R, Borges G, Cherpitel C, Greenfield T, Rehm J. The more you drink, the harder you fall: a systematic review and meta-analysis of how acute alcohol consumption and injury or collision risk increase together. Drug Alcohol Depend. 2010; 110(1-2): 108-16.

Occupational Exposures & Risks SDG Capstone Appendix

Flowchart

Occupational Risk Factors
(except abestos and injuries)

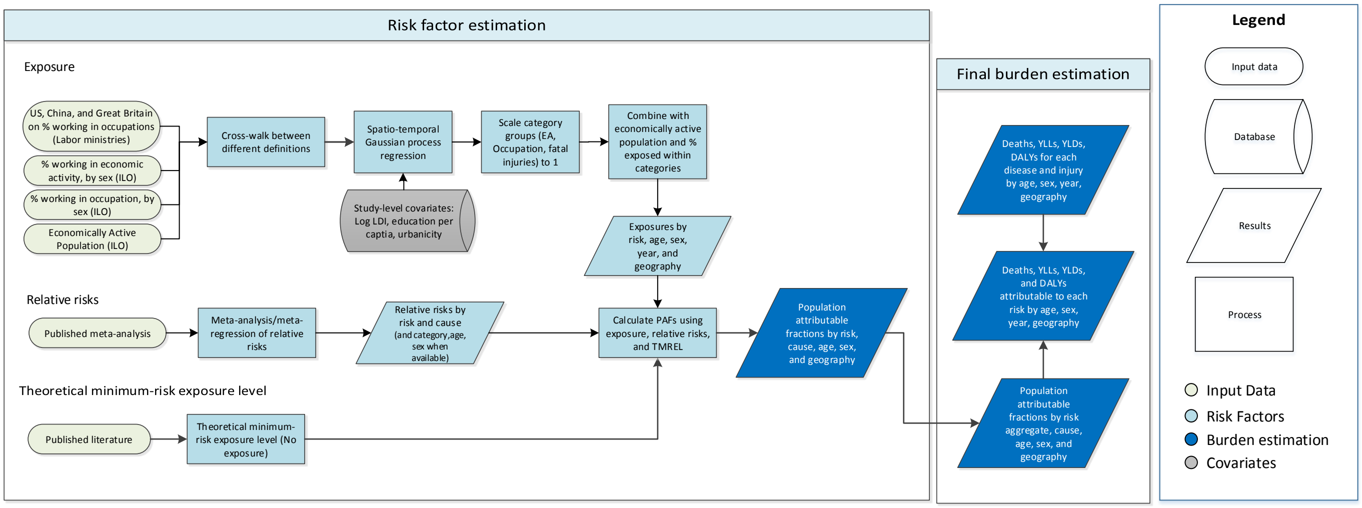

Input Data & Methodological Summary

Indicator definition

This modeling strategy encompassed the indicator associated with DALY rates attributable to occupational risks (8.8.1).

Indicator 8.8.1

As a component of SDG Goal 8. Promote sustained, inclusive and sustainable economic growth, full and productive employment and decent work for all, SDG Target 8.8, protect labour rights and promote safe and secure working environments for all workers, including migrant workers, in particular women migrants, and those in precarious employment, is measured using SDG Indicator 8.8.1, age-standardised all-cause DALY rates (per 100,000) attributable to occupational risks.

Occupational Asbestos SDG Capstone Appendix

Input Data & Methodological Summary

Case definition

We define exposure to occupational asbestos as the cumulative exposure to asbestos using mesothelioma death rate in a smoking impact ratio analogue. This definition assumes that all exposure to mesothelioma in the population is occupational. We estimate the burden of occupational asbestos for both sexes for ages 15 and above in both developed and developing countries.

Input data

The following were the data inputs required for the estimation of cumulative exposure to occupational asbestos in GBD 2013.

1. Mortality rate due to mesothelioma in 1990, 2005, 2010, and 2013

Cause-specific mortality rates for mesothelioma, C_{LC} by country, age, and sex were generated by causes of death models for GBD 2013[1].

2. Mortality rate due to mesothelioma in a population not exposed to asbestos

We calculated the background mortality of mesothelioma, N_{LC}, from the model used by Lin et al[2]. Using the uncertainty around the coefficients, we created 1,000 draws of the mortality due to mesothelioma if there was no asbestos consumption in a country. The mean value for background mortality is 0.73 and 0.47 deaths per million males and females, respectively.

3. Mortality rate due to mesothelioma in a population highly exposed to asbestos

We found the mortality rate for highly exposed individuals from asbestos workers, C^*_{LC} from Goodman et al[3]. We used all studies that reported both the number of person-years followed and the number of cases of mesothelioma and found the death rate of all individuals included in the studies. The death rate for highly exposed individuals is 226 per million people.

Modeling strategy

The approach used to estimate the burden of cancer caused by occupational exposure to asbestos was different than the approach used for all other occupational carcinogens. This approach, analogous to the

---

[1] Naghavi, M & GBD Mortality and Causes of Death Collaborators. (2014). Global, regional, and national levels of age-sex specific all-cause and cause-specific mortality for 240 causes of death, 1990-2013: a systematic analysis for the Global Burden of Disease Study 2013. *Lancet 2014*. In press.

[2] Lin R-T, Takahashi K, Karjalainen A, *et al.* Ecological association between asbestos-related diseases and historical asbestos consumption: an international analysis. *Lancet* 2007; **369**: 844–9.

[3] Goodman M, Morgan RW, Ray R, Malloy CD, Zhao K. Cancer in asbestos-exposed occupational cohorts: a meta-analysis. *Cancer Causes Control* 1999; **10**: 453–65.

Peto-Lopez Smoking Impact Ratio (SIR)[4], uses mesothelioma deaths as a marker for exposure to asbestos in order to take into account issues of latency and risk accumulation.

For asbestos, the excess lung cancer deaths analogous measurement for a population is the excess deaths due to mesothelioma observed in that population divided by the excess deaths in a population that is heavily exposed to asbestos. The asbestos impact ratio (AIR) gives a measurement for the exposure level of a population to asbestos. We then use the AIR and relative risks to calculate the Population Attributable Fraction (PAF) for each cause related to asbestos. Formally, the AIR is defined as:

$$AIR = \frac{C_{LC} - N_{LC}}{C^{*}_{LC} - N_{LC}}$$

Where:

C_{LC} = (country-sex-specific) mesothelioma mortality rate in the study population
N_{LC} = mesothelioma mortality rate in a population not exposed to asbestos
C^{*}_{LC} = mesothelioma mortality rate in a population highly exposed to asbestos

Relative Risks

Relative risks for occupational asbestos were searched for and provided by the expert groups (same as GBD 2010). Based on expert group recommendation, we updated the relative risks for lung cancer with a recent meta-analysis which was unavailable during GBD 2010. *Table 1* outlines the relative risk estimates along with the outcomes used to estimate the burden attributable to occupational exposure of asbestos. These numbers have remained unchanged since GBD 2010.

Outcome	Sex	Age	High Exposure RR (95% CI)	Low Exposure RR (95% CI)	RR application	Source
Larynx cancer	Both	15-79	1.38 (1.17- 1.60)	1	Mortality and Morbidity	IOM, 2006[5]
Lung cancer (males)	Both	15-79	2.27 (1.67-2.85)	1.65 (1.50-1.79)	Mortality and Morbidity	Lenters et al, 2011
Lung cancer (females)	Both	15-79	1.86 (1.56-2.15)	1.52 (1.46-1.58)	Mortality and Morbidity	Lenters et al, 2011
Mesothelioma	Both	15-79	38.4 (13.5- 110)	20.7 (6.5-65.5)	Mortality and Morbidity	Rake et al 2009[6]
Ovarian cancer	Female	15-79	1.77 (1.37- 2.28)	1	Mortality and Morbidity	Camargo et al, 2011[7]

Table 1: Relative risks for occupational asbestos used in GBD 2013.

[4] Peto R, Lopez AD, Boreham J, Thun M, Heath C Jr. Mortality from tobacco in developed countries: indirect estimation from national vital statistics. *Lancet* 1992; **339**: 1268–78.

[5] Institute of Medicine of the National Academies (IOM), Asbestos: selected cancers. 2006, The National Academies Press: Washington, DC.

[6] Rake C, Gilham C, Hatch J, Darnton A, Hodgson J, Peto J. Occupational, domestic and environmental mesothelioma risks in the British population: a case-control study. *Br. J. Cancer* 2009; **100**(7): 1175–1183.

[7] Camargo MC, Stayner LT, Straif K, Reina M, Al-Alem U, Demers PA, et al. Occupational exposure to asbestos and ovarian cancer: a meta-analysis. *Environ. Health Perspect.* 2011; **119**(9): 1211–1217.

Theoretical Minimum-Risk Exposure Level

TMREL draws for occupational asbestos were prepped to feed into the central PAF calculate using the following code:

"J:\WORK\2013\05_risk\01_database\02_data\occ\03_tmred\code\fix_occ_asbestos_tmred.do"

Attributable Burden Estimation

Population attributable fractions (PAFs) for occupational asbestos were calculated centrally using the following formula similar to all occupational carcinogens. The following equation which takes into account exposure distribution (P_i); relative risks i.e. measure of effect on outcome associated with each level of exposure (RR_i); and the counterfactual level of risk exposure (TMREL) was used to calculate PAFs centrally.

$$PAF = \frac{\sum_{i=1}^{n} P_i(RR_i - 1)}{\sum_{i=1}^{n} P_i(RR_i - 1) + 1}$$

GBD 2010 vs. GBD 2013

- Mesothelioma was not part of cause list in GBD 2010. As a result, cause specific mortality rate for mesothelioma for all country years had to be modeled in GBD 2010 separately. In GDB 2013, mesothelioma was added to the cause list. As a result, causes of death output of cause-specific mortality rates of mesothelioma for all country years were used in GBD 2013.
- Updated relative risks for lung cancer associated with occupational asbestos based on a newly published meta-analysis.

Occupational Asthmagens SDG Capstone Appendix

Input data & Methodological summary

Case definition

Exposure to occupational asthmagens is defined as the proportion of the population exposed based on distribution of the population in eight occupational groups. The burden of occupational asthmagens is only estimated for ages 15 and older. We model the burden of occupational asthmagens in both developed and developing countries.

Input Data

Description of data identification and prep

The primary data type used to estimate exposure to occupational asthmagens is data on economic activity by country obtained from the ILO database. Data in the ILO database are primarily from censuses and national labor force surveys. The database was supplemented with subnational data for UK and China. Data processing involved sex-splitting estimate of industry groups to generate sex-specific estimates and crosswalking between different ISIC versions (classification systems used by ILO.)

Modeling strategy

1. Calculate a time series of the percentage of the workforce working in 8 occupational groups. This step is modeled using ST-GPR. This first step of generating the distribution of the labor force across occupational groups is analogous to all other occupational risks (except injuries and asbestos). The ST-GPR model is run separately for each economic activity by sex for ages 15 and above.
2. Run an additional spacetime model to further subdivide the 7th occupational group by economic activities. This was done to match the exposure categories in the reported relative risks.
3. Rescale GPR output such that the proportion of individuals employed in all economic activity groups add up to 1 i.e 100% of the labor force.
4. Calculate the population level exposure to all occupational asthmagens using proportion of the population that is economically active population, proportion that is employed in each economic activity/occupation group, and the prevalence of asthmagen exposure in each economic activity based on data provided the expert group.

   Exposure group j = % of pop that is economically active * % of economically active in economic activity i * % of occupation/economic activity i that is exposed to level j

5. Prep and save draws of occupational asthmagens to feed into the PAF calculator.

Relative Risks

The relative risks and the outcomes associated with occupational asthmagens was systematically searched for and generated by the occupational risk expert group in GBD 2010. For GBD 2013, there were

no major updates made to relative risks used in GBD 2010. The table below outlines the relative risk values used for occupational asthmagens in GBD 2013 provided by the expert group.

		Males			Females	
	**RR**	**95% LL**	**95% UL**	**RR**	**95% LL**	**95% UL**
Background	1			1		
Administrative	1			1		
Technical	1.05	0.98	1.12	1.06	1.03	1.1
Sales	1.14	1.05	1.23	1.13	1.08	1.18
Agriculture	1.5	1.11	2.03	1.5	1.11	2.03
Mining	1.95	1.58	2.40	1.95	1.58	2.40
Transport	1.31	1.22	1.40	1.22	1.13	1.31
Manufacturing	1.56	1.47	1.65	1.33	1.27	1.39
Services	1.53	1.42	1.66	1.41	1.35	1.46

All RRs except for that for agricultural occupations came from a study by Karjalainen and co-workers. This is the only comprehensive national population study of incident asthma. Relative risks for agricultural occupations were based on a study by Kogevinas and co-workers, with an inverse variance-weighted estimate obtained using the separate estimates for “farmers” and “agricultural” workers provided in the paper. There were limited updates made to the relative risk draw files used for occupational asthmagens in GBD 2013 compared to 2010. Relative risk draws for occupational asthmagens were centrally migrated from GBD 2010 infrastructure to GBD 2013. As a result, code files that prep RR draws from scratch do not exist for all relative risks in the table above.

Theoretical Minimum-Risk Exposure Level

The theoretical minimum risk exposure level is defined as no occupational exposure to asthmagens. TMREL for occupational asthmagens remained unchanged since GBD 2010. Code files that prep TMREL draws for occupational asthmagens from scratch do not exist because they were centrally migrated from GBD 2010 to GBD 2013 infrastructure.

Attributable Burden Estimation

Population Attributable Fractions (PAFs) were calculated centrally using input draws of exposure, relative risks, and theoretical minimum level prepared by the modeler (Astha). The following equation which takes into account exposure distribution (P_i); relative risks i.e. measure of effect on outcome associated with each level of exposure (RR_i); and the counterfactual level of risk exposure (TMREL) was used to calculate PAFs centrally.

$$PAF = \frac{\sum_{i=1}^{n} P_i(RR_i - 1)}{\sum_{i=1}^{n} P_i(RR_i - 1) + 1}$$

GBD 2010 vs. GBD 2013

- Data updates included using economic activity data from newly downloaded ILO database and additional of subnational data for China and the UK.
- Methodology from GBD 2010 has remained unchanged

Occupational Carcinogens SDG Capstone Appendix

Input data & Methodological summary

Case definition

Exposure to occupational carcinogens is defined as the proportion of people ever exposed (taking into account worker turnover) to each carcinogen (listed below) based on the distribution of the population in nine economic activity groups. The burden of occupational carcinogens is only estimated for ages 15 and older. We model the burden of occupational carcinogens in both developed and developing countries.

Input data

Description of data identification and prep

The primary data type used to estimate exposure to occupational carcinogens is data on economic activity by country obtained from the ILO database. Data in the ILO database are primarily from censuses and national labor force surveys. The database was supplemented with subnational data for UK and China. Data processing involved sex-splitting estimate of industry groups to generate sex-specific estimates and crosswalking between different ISIC versions (classification systems used by ILO.)

Data on prevalence of carcinogens by industry was estimated using data from the CAREX database. This was provided by the expert group for GBD 2010. The proportion of workers within an industry who were exposed to each carcinogen was distributed between “high” and “low” exposure. This was done to account for varying levels of exposure to occupational carcinogens within each industry. The carcinogen prevalence were distributed 10:90 (high:low) for developed regions and 50:50 (high:low) for developing countries. This assumption was made based on the methodology devised by the expert group in GBD 2010 and also for GBD 2013. Occupational turnover factors used in the analysis were provided by the expert group for use in GBD 2010 and were used as is for GBD 2013. See notes on future iterations for more information on turnover factors.

Modeling strategy

6. Calculate a time series of the percentage of the workforce working in 9 economic activity groups. This step is modeled using ST-GPR. This first step of generating the distribution of the labor force across 9 economic activities is used for occupational carcinogens as well as particulates, noise, ergonomic factors and asthmagens. The ST-GPR model is run separately for each economic activity by sex for ages 15 and above.
7. Rescale GPR output such that the proportion of individuals employed in all economic activity groups add up to 1 i.e 100% of the labor force.
8. Calculate the population level exposure to all occupational carcinogens (except asbestos) using proportion of the population that is economically active population, proportion that is employed in each economic activity, turnover rates for each economic activity, prevalence of carcinogen exposure in each economic activity based on CAREX, and exposure partition factor (EPF).

Industry	Covariates
Agriculture, Hunting, Forestry and Fishing	1) Proportion of the population living in levels of population density (150-300, 300-500, 500-1,000 and over 1,000 people per square km);<br>2) Proportion of population living in bands of latitude (15-30, 30-45 and over 45 degrees of latitude);<br>3) Proportion of the population living in levels of altitude (100-500, 500-1500 and over 1500 meters of elevation);<br>4) mean years of education of the population by sex;<br>5) Lagged-distributed income in 2005 international dollars (a smoothed measure of gross domestic product).
Mining and Quarrying<br><br>Wholesale and Retail Trade and Restaurants and Hotels	1) Proportion of the population living in levels of population density (150-300, 300-500, 500-1,000 and over 1,000 people per square km);<br>2) Proportion of the population living in levels of altitude (100-500, 500-1500 and over 1500 meters of elevation);<br>3) number of 4 wheel vehicles per capita;<br>4) mean years of education of the population by sex;<br>5) Lagged-distributed income in 2005 international dollars (a smoothed measure of gross domestic product).
Manufacturing<br><br>Electricity, Gas and Water<br><br>Transport, Storage and Communication	1) Proportion of the population living in levels of population density (150-300, 300-500, 500-1,000 and over 1,000 people per square km);<br>2) Proportion of population living in bands of latitude (15-30, 30-45 and over 45 degrees of latitude);<br>3) Proportion of the population living in levels of altitude (100-500, 500-1500 and over 1500 meters of elevation);<br>4) number of 4 wheel vehicles per capita;<br>5) mean years of education of the population by sex;<br>6) Lagged-distributed income in 2005 international dollars (a smoothed measure of gross domestic product).
Construction	1) Proportion of the population living in levels of population density (150-300, 300-500, 500-1,000 and over 1,000 people per square km);<br>2) Proportion of the population living in levels of altitude (100-500, 500-1500 and over 1500 meters of elevation);<br>3) mean years of education of the population by sex;<br>4) Lagged-distributed income in 2005 international dollars (a smoothed measure of gross domestic product).
Financing, Insurance, Real Estate and Business Services<br><br>Community, Social and Personal Services	1) Proportion of the population living in levels of population density (150-300, 300-500, 500-1,000 and over 1,000 people per square km);<br>2) mean years of education of the population by sex;<br>3) Lagged-distributed income in 2005 international dollars (a smoothed measure of gross domestic product).

9. Prep and save draws of occupational carcinogens (except asbestos) into the PAF calculator.

Table below outlines the country-level covariates used to model the 9 economic activities.

Relative Risks

The relative risks and the outcomes associated with each carcinogen was systematically searched for and generated by the occupational risk expert group in GBD 2010. For GBD 2013, there were n omajor updates made to relative risks used in GBD 2010. We added RR for kidney cancer associated with occupational trichloroethylene to the list of relative risks used in GBD 2013 based on IARC's reclassification of tricholorethylene as a Group 1 carcinogen. Relative risks outline for each carcinogen-cancer pair outlined in the table below were extracted from a single key study or meta-analysis.

Carcinogen	Outcome	RR (high)	LL (high)	UL (high)	RR (low)	LL (low)	UL (low)	Source
Arsenic	Trachea, bronchus, and lung cancers	2.05	1.43	2.85	1	1	1	Lee-Feldstein 1986
Benzene	Leukemia	2.62	1.57	4.39	1.64	1.10	2.39	Khalade et al, 2010
Beryllium	Trachea, bronchus, and lung cancers	1.17	1.08	1.28	1	1	1	Schubauer-Berigan et al, 2011
Cadmium	Trachea, bronchus, and lung cancers	1.19	1.09	1.29	1	1	1	Verougstraete et al, 2003
Chromium VI	Trachea, bronchus, and lung cancers	1.18	1.12	1.25	1	1	1	Cole & Rodu, 2005
Diesel engine exhaust	Trachea, bronchus, and lung cancers	1.47	1.29	1.67	1	1	1	Lipsett & Campelman, 1999
Second hand smoke	Trachea, bronchus, and lung cancers	1.24	1.18	1.29	1	1	1	Stayner et al, 2007
Formaldehyde	Leukemia	1.47	1.19	1.83	1	1	1	Collins and Lineker, 2004
Formaldehyde	Nasopharynx cancer	2.1	1.05	4.21	1	1	1	Hauptmann et al, 2004
Nickel	Trachea, bronchus, and lung cancers	2.1	1.3	3.2	1	1	1	Grimsrud et al 2005
PAHs	Trachea, bronchus, and lung cancers	1.31	1.16	1.48	1	1	1	Armstrong et al, 2004
Silica	Trachea, bronchus, and lung cancers	1.32	1.24	1.41	1	1	1	Kurihara & Wada, 2004
Sulpuric acid	Larynx cancer	4.28	2.13	8.58	1.91	0.97	3.78	Soskolne et al, 1992
Trichloroethylene	Kidney cancer	1.24	1.06	1.45	1	1	1	Kelsh et al, 2010

There were limited updates made to the relative risk draw files used for occupational carcinogens in GBD 2013 compared to 2010. Relative risk draws were centrally migrated from GBD 2010 infrastructure to GBD 2013. As a result, code files that prep RR draws from scratch do not exist for all relative risks in the table above.

Theoretical Minimum-Risk Exposure Level

The theoretical minimum risk exposure level is defined as no occupational exposure to carcinogens. TMREL draws were prepped by the modeler (Astha). TMREL for all carcinogens remained largely unchanged since GBD 2010. Code files that prep TMREL draws for occupational carcinogens from scratch do not exist because they were centrally migrated from GBD 2010 to GBD 2013 infrastructure.

Attributable Burden Estimation

Population Attributable Fractions (PAFs) were calculated centrally by Stan Biryukov (Data Specialist) using input draws of exposure, relative risks, and theoretical minimum level prepared by the modeler (Astha). The following equation which takes into account exposure distribution (P_i); relative risks i.e. measure of effect on outcome associated with each level of exposure (RR_i); and the counterfactual level of risk exposure (TMREL) was used to calculate PAFs centrally.

$$PAF = \frac{\sum_{i=1}^{n} P_i(RR_i - 1)}{\sum_{i=1}^{n} P_i(RR_i - 1) + 1}$$

GBD 2010 vs. GBD 2013

- Both groups 1 and 2A carcinogens, as classified by IARC, were analyzed in GBD 2010 and only group 1 was reported. In GBD 2013, we generated estimates for only those carcinogens that we reported in GBD 2010 which includes group 1 carcinogens.
- Based on the reclassification of tricholorethylene from group 2A carcinogen to group 1 we included it to the list of occupational carcinogens that were analyzed in GBD 2013.
- Data updates included using economic activity data from newly downloaded ILO database and additional of subnational data for China and the UK.

Occupational Injuries SDG Capstone Appendix

Input data & Methodological summary

Case definition

Proportion of injuries (fatal or non-fatal) attributed to occupational exposure estimated based on rates of fatal injuries by country-year as reported to the ILO database. The burden of occupational injuries is only estimated for ages 15 and older. We model the burden of occupational injuries in both developed and developing countries.

Input Data

Description of data identification and prep

The primary data type used to estimate the burden of occupational injuries is data on rates of fatal injury reported by economic activity and country year from the ILO database. This was updated in GBD 2013 by using a newly downloaded ILO database.

Modeling strategy

1. Data on fatal injury rates reported by economic activity and country-year from the ILO database is prepped for modeling.
2. Based on a linear regression using fatal injury data from the ILO database generate a time series of fatal injury rates for all country years for all 9 economic activities (EA). Also, generate a full time series using a similar model for the total rate of fatal injuries for all country years.
3. Rescale EA-specific fatal injury rate such that it adds up to the total fatal injury rate modeled for that country year. This is done to ensure that the EA-specific rates are add up to the reported total rate of fatal injuries.
4. Generate injury counts using modeled rates of fatal injuries for all economic activity groups for all country years. The following equation was used to generate injury counts.

   *# of injuries by industry for each age = % of workforce in industry * economically active population by age * rate of injuries / 100,000 people*

5. Calculate injury PAFs using the following equation. The denominator in the PAF equation, total unintentional injuries was estimated by summing across injury deaths estimated by GBD causes of death models:

   PAF = (total occupational injuries - theoretical minimum) / total unintentional injuries

   Where, total occupational injuries = injury count calculated from step 4, theoretical minimum is assumed to be zero i.e. all occupational injuries can be avoided, total unintentional injuries = sum of injury deaths in each cause that is selected by economic activity. NOTE: Although, the PAF estimated

above is for fatal injuries given that we don't have a good way of estimating non-fatal occupational injury PAFs we assume that YLL PAFs = YLD PAFs.

6. Finally, calculate and save draws of occupational injury PAFs to feed into the GBD infrastructure.

Relative Risks

There are no relative risk estimates for occupational injuries because PAFs are estimated directly.

Theoretical Minimum-Risk Exposure Level

The theoretical minimum risk exposure level is defined as 0 injury deaths per 1,000,000 person-years.

Attributable Burden Estimation

Population Attributable Fractions (PAFs) for occupational injuries are estimated directly by the modeler (Astha). PAF is calculated using the equation and code files outlined in step 5 of the modeling strategy section. The PAF estimation uses GBD outputs from causes of death. Therefore, occupational injury PAFs should be calculated using the finalized cause of death numbers.

GBD 2010 vs. GBD 2013

- Data updates included using fatal injury data from newly downloaded ILO database.
- For GBD 2010 rates of fatal injuries were modeled using ST-GPR in 3 broad groups combining economic activities (agriculture, industry, and service). In GBD 2013, we changed this to model rates of fatal injury rates by detailed groups i.e. 9 economic activities using a simple linear regression.
- Use of occupation group and injury type matrix to exclude implausible injury type and occupation pairing e.g. snake bite deaths among clerical workers etc. In GBD 2010, we assumed that all unintentional injuries were included in the denominator of the PAF calculation. However, in GBD 2013, we calculated PAFs for each economic activity separately. Only the injury types marked by a "x" in the matrix outlined in the above table contributed to the denominator of the PAF equation for the respective economic activity
- For GBD 2013, we estimated the non-fatal burden of occupational injuries which wasn't done in GBD 2010. We don't have data sources estimating non-fatal injuries as a result we assumed that the burden of non-fatal would be equal to the fatal burden of occupational injuries i.e. YLL PAFs = YLD PAFs for occupational injuries.
- TMREL changed from 5 injury deaths per 1,000,000 person-years, based on rates of clerical workers in the US to 0 injury deaths per 1,000,000 person-years. This was done to be consistent with the new definition of TMREL for GBD based on the risk consultative meeting in winter 2015.

Occupational Ergonomic Factors SDG Capstone Appendix

Input data & Methodological Summary

Case definition

Exposure to occupational ergonomic factors is defined as the proportion of the population exposed based on distribution of the population in occupation groups. Occupation groups of the population was used as a proxy because there aren't direct measures of exposure to occupational ergonomic factors associated with low back pain. The burden of occupational ergonomic factors is only estimated for ages 15 and older. We model the burden of occupational ergonomic factors in both developed and developing countries.

Data inputs

Description of data identification and prep

The primary data type used to estimate exposure to occupational ergonomic factors is data on occupationl groups by country obtained from the ILO database. Data in the ILO database are primarily from censuses and national labor force surveys. The database was supplemented with subnational data for UK and China. Data processing involved sex-splitting estimate of industry groups to generate sex-specific estimates and crosswalking between different ISIC versions (classification systems used by ILO.)

Modeling strategy

The modeling strategy for occupational exposure to ergonomic factors associated with low back pain has remained unchanged since GBD 2010[8]. The strategy used to estimate exposure has been summarized below.

There are few direct measurements of exposure to ergonomic factors in the workplace. We estimated risk of exposure to occupational ergonomic factors by using the composition of a country's workforce in seven different occupational groups. This work has been published since GBD 2010[9]. The number of workers in a particular occupation group was determined by multiplying the percentage of the workforce in the occupation group by the percentage of economically active people. The steps involved in estimating exposure are outlined below.

7. Calculate a time series of the percentage of the workforce working in 8 occupational groups. This step is modeled using ST-GPR. This first step of generating the distribution of the labor force across occupational groups is analogous to all other occupational risks (except injuries and asbestos). The ST-GPR model is run separately for each economic activity by sex for ages 15 and above.

---

[8] Lim SS, et al. A comparative risk assessment of burden of disease and injury attributable to 67 risk factors and risk factor clusters in 21 regions, 1990-2010: a systematic analysis for the Global Burden of Disease Study 2010. *Lancet 2012*; **380**: 2224-60.

[9] Driscoll T, Jacklyn G, Orchard J, Passmore E, Vos T, Freedman G, Lim S, Punnett L. The global burden of occupationally related low back pain: estimates from the Global Burden of Disease 2010 study. Ann Rheum Dis. 2014 Jun;73(6):975-81. doi: 10.1136/annrheumdis-2013-204631. Epub 2014 Mar 24.

8. Rescale GPR output such that the proportion of individuals employed in all economic activity groups add up to 1 i.e. 100% of the labor force.
9. Calculate the population level exposure to all occupational ergonomic factors using proportion of the population that is economically active, and proportion that is employed in each occupation group.

$$P_{c,y,a,s,i} = EAP_{c,y,a,s,i} \, X \; EAC_{c,y,a,s}$$

Where:

$P_{c,y,a,s,i}$ = Prevalence of occupational exposure to ergonomic factors associated with low back pain;
$EAP_{c,y,a,s,i}$ = Proportion of the population that is economically active, by industry, country, year, age, and sex;
$EAC_{c,y,a,s}$ = Proportion of the population that is economically active, by country, year, age, and sex;
c = Country;
y = Year;
a = GBD age group;
s = Sex;
i = classification of occupation (7 types).

10. Prep and save draws of exposure to feed into the PAF calculator.

Relative Risks

The relative risks and the outcomes associated with occupational ergonomic factors was systematically searched for and generated by the occupational risk expert group in GBD 2010. For GBD 2013, there were no major updates made to relative risks used in GBD 2010. The table below outlines the relative risk values used for occupational ergonomic factors in GBD 2013 provided by the expert group which has been published since GBD 2010[2].

	**RR (95% CI)**
Background	1
Professional, technical, and related workers	1.17 (1.06-1.28)
Administrative and managerial workers	1.20 (0.96-1.50)
Clerical and related workers	1
Sales workers	1.21 (1.01-1.44)
Service workers	1.47 (1.38-1.57)
Agriculture, animal husbandry and forestry workers, fishermen, and hunters	3.73 (2.61-5.33)
Production and related workers	1.54 (1.41-1.68)
Transport equipment operators and laborers	1.54 (1.41-1.68)

Relative risk draws for occupational ergonomic factors were centrally migrated from GBD 2010 infrastructure to GBD 2013. As a result, code files that prep RR draws from scratch do not exist for all relative risks in the table above.

Theoretical Minimum-Risk Exposure Level

The theoretical minimum risk exposure level is defined as no occupational exposure to asthmagens. TMREL for occupational asthmagens remained unchanged since GBD 2010. Code files that prep TMREL

draws for occupational carcinogens from scratch do not exist because they were centrally migrated from GBD 2010 to GBD 2013 infrastructure.

Attributable Burden Estimation

Population Attributable Fractions (PAFs) were calculated centrally by ***Stan Biryukov (Data Specialist)*** using input draws of exposure, relative risks, and theoretical minimum level prepared by the modeler (Astha). The following equation which takes into account exposure distribution (P_i); relative risks i.e. measure of effect on outcome associated with each level of exposure (RR_i); and the counterfactual level of risk exposure (TMREL) was used to calculate PAFs centrally.

$$PAF = \frac{\sum_{i=1}^{n} P_i(RR_i - 1)}{\sum_{i=1}^{n} P_i(RR_i - 1) + 1}$$

GBD 2010 vs. GBD 2013

- Data updates included using data on occupational groups from newly downloaded ILO database and additional of subnational data for China and the UK.
- Methodology remained unchanged since GBD 2010

Occupational Noise SDG Capstone Appendix

Input data & Methodological summary

Case definition

Definition of occupational noise as a risk factor is determined by noise levels in decibels (dB) which is associated with varying levels of severity in hearing loss (see Table 1.)

Occupational exposure and outcome pairs

Exposure (dB)	Outcome
Low (85-90dB)	Mild hearing loss
High (90dB +)	Moderate, moderately severe, severe, profound, and complete hearing loss

In the absence of direct exposure data however, exposure to occupational noise is defined based on the proportion of the population exposed based on the distribution of the population in nine economic activities. The burden of occupational noise is only estimated for ages 15 and older. We model the burden of occupational ergonomic factors in both developed and developing countries.

Input Data

Economically Active Population by Industry

One of the primary inputs in our exposure model is the composition of a country's workforce. We generated a complete time series of estimates of the proportion of economically active population ($EAP_{c,y,a,s}$) by industry, country, year, age, and sex from 1980-2013 using spacetime GPR. Data on economic activity by country and industry over time was obtained from the International Labor Organization[10]. ILO data are primarily from censuses and national labor force surveys; these were also supplemented with data from the National Bureau of Statistics of China and the 1991 and 2001 India Censuses. For countries which did not provide sex specific estimates of industry groups to the ILO but did provide total estimates, the regional average of male to female participation in the industry was applied in order to determine sex specific estimates.

Economically Active Population

The proportion of the population that is economically active was based on estimates produced by the ILO[3]. ILO estimates are produced separately by age for males and females for all but 10 countries between 1990 and 2013. We used the regional average as an estimate for the EAP in these countries. Because there were no estimates for 70-80 year olds, the 65-70 estimates were carried forward. The data

[10] International Labor Organization (ILO). ILOSTAT Database. http://www.ilo.org/ilostat/. Accessed: Sept 16, 2013

for the population aged younger than 15 was too sparse and this age range was excluded. This data was provided by the expert group.

Prevalence of Noise Exposure by Industry

The exposure levels by industry were assumed to vary by industry and the country's development status. Australian national data on noise exposure in various industries (sampled across a range of tasks) provided the basis for the mean and standard deviation of noise exposure in each industry[4]. This information was in turn used to estimate the proportion of workers exposed at low levels of noise (85-90db) and high levels of noise (90+db). No account was made of the use of hearing protection, except for the mining industry, where mean exposure levels were decreased by 3dB to take this into account. These proportions were used for developed countries for 1990, 2005, 2010, and 2013. This data was provided by the expert group. The prevalence of noise exposure by industry were modified for developing countries, to take into account the likely higher exposure levels in developing countries due to the less extensive use of noise controls. For developing countries, the mean exposure was estimated to be three dB higher (double the noise level compared to developing countries) [1]. Table 2 shows the exposure levels for both high and low exposure by country's development status. This data was provided by the expert group.

Prevalence of exposure by industry (per 100,000 workers)

Industry	Developed, 90+ dB	Developed, 85-90 dB	Developing, 90+ dB	Developing, 85-90 dB
Agriculture, Hunting, Forestry and Fishing	0.180	0.144	0.261	0.167
Mining and Quarrying	0.393	0.294	0.572	0.254
Manufacturing	0.106	0.245	0.233	0.322
Electricity, Gas and Water	0.204	0.123	0.274	0.138
Construction	0.251	0.194	0.362	0.210
Wholesale and Retail Trade and Restaurants and Hotels	0	0.018	0.001	0.231
Transport, Storage and Communication	0.079	0.202	0.180	0.287
Financing, Insurance, Real Estate and Business Services	0	0.031	0.004	0.230
Community, Social and Personal Services	0.09	0.131	0.159	0.176

Modeling strategy

The modeling strategy for exposure to occupational noise has remained unchanged since GBD 2010. The strategy used to generate exposure estimates for occupational noise has been summarized below.

1. Calculate a time series of the percentage of the workforce working in 9 economic activities/industries. This step is modeled using ST-GPR. This first step of generating the distribution of the labor force across economic activities is the same for a number of other occupational risks. The ST-GPR model is run separately for each economic activity by sex for ages 15 and above.
2. Rescale GPR output such that the proportion of individuals employed in all economic activity groups add up to 1 i.e. 100% of the labor force.

3. Calculate the population level exposure to occupational noise by industry using proportion of the population that is economically active, proportion that is employed in each economic activity group, and prevalence of noise exposure associated with each economic activity/industry. Population exposure is then determined as the sum of each industry's exposure for that country, age, and sex group. This equates to:

$$P_{c,a,s,j,t} = \sum_{i=1}^{9} (EAP_{i,s,j,t} \; X \; CP_{i,c} \;\; X \;\; EAC_{i,s,j,t})$$

$P_{c,a,s,j,t}$ = Prevalence of exposure to occupational noise, by country, year, age, sex, and level of exposure;
$EAP_{i,s,j,t}$ = Proportion of the population that is economically active, by industry, country, year, age, and sex;
$CP_{i,c}$ = Prevalence of noise exposure by industry, and exposure level;
$EAC_{c,y,a,s}$ = Proportion of the population that is economically active, by country, year, age, and sex;
c = exposure level (high or low);
a = 5-year age group;
s = sex;
j = country;
t = year;
i = Industry.

4. Prep and save draws of exposure to feed into the PAF calculator.

Relative Risks

Relative risks for occupational noise were estimated by occupational risk expert group for GBD 2010. See expert group documentation from GBD 2010 on details of how these were estimated. The tables below list RRs by age and noise exposure level.

Relative risk by age and noise exposure level (41db or more)

	High exposure (>90dB)			Low exposure (85-90dB)		
Age	RR	LL	UL	RR	LL	UL
15-19	7.96	4.74	13.37	2.92	1.74	4.91
20-24	7.96	4.74	13.37	2.92	1.74	4.91
25-34	6.63	4.74	9.28	3.42	2.45	4.79
35-44	5.93	4.24	8.29	3.79	2.71	5.30
45-54	5.55	3.94	7.81	3.88	2.75	5.46
55-64	3.56	2.53	5.02	2.66	1.89	3.75
65-74	2.14	1.62	2.84	1.80	1.35	2.39
75-84	1.29	1.07	1.56	1.22	1.01	1.47
85+	1.00	1.00	1.22	1.00	1.00	1.22

Relative risk by age and noise exposure level (25db or more)

	High exposure (>90dB)			Low exposure (85-90dB)		
Age	RR	LL	UL	RR	LL	UL
15-19	7.29	4.91	10.82	2.74	1.85	4.07
20-24	7.29	4.91	10.82	2.74	1.85	4.07
25-34	5.44	4.32	6.86	2.91	2.31	3.66
35-44	3.07	2.71	3.48	2.17	1.91	2.46
45-54	2.55	2.36	2.77	1.99	1.83	2.15

55-64	1.85	1.71	2.00	1.55	1.43	1.68
65-74	1.45	1.37	1.52	1.30	1.23	1.37
75-84	1.13	1.05	1.21	1.09	1.02	1.17
85+	1.00	1.00	1.07	1.00	1.00	1.07

The underlying sources for these RR estimates are:

Agrawal Y, Platz E, Niparko J. Prevalence of hearing loss and differences by demographic characteristics among US adults. Data from the National Health and Nutrition Examination Survey, 1999-2004. Archives of Internal Medicine 2008;168(14):1522-1530.

Davis A. The prevalence of hearing impairment and reported hearing disability among adults in Great Britain. International Journal of Epidemiology 1989,18: 911-917.

Wilson D, Walsh P, Sanchez L, Davis A, Taylor A, Tucker G, Meagher I. The epidemiology of hearing impairment in an Australian adult population. International Journal of Epidemiology 1999;28:247-252.

The relative risks used to assess the attributable burden of occupational exposure to noise levels that are associated with hearing loss has remained unchanged since GBD 2010. The relative risk draws were migrated centrally from GBD 2010 to GBD 2013 infrastructure. As a result, code that preps RR draws from scratch do not exist for occupational noise.

Theoretical Minimum-Risk Exposure Level

The TMREL for occupational noise is defined as all individuals are exposed to background noise levels. This definition of TMREL has remained unchanged since GBD 2010. The TMREL draws were migrated centrally from GBD 2010 to GBD 2013 infrastructure. As a result, prep code for this step does not exist.

Attributable Burden Estimation

Population Attributable Fractions (PAFs) were calculated centrally by ***Stan Biryukov (Data Specialist)*** using input draws of exposure, relative risks, and theoretical minimum level prepared by the modeler (Astha). The following equation which takes into account exposure distribution (P_i); relative risks i.e. measure of effect on outcome associated with each level of exposure (RR_i); and the counterfactual level of risk exposure (TMREL) was used to calculate PAFs centrally.

$$PAF = \frac{\sum_{i=1}^{n} P_i(RR_i - 1)}{\sum_{i=1}^{n} P_i(RR_i - 1) + 1}$$

Occupational Particulates SDG Capstone Appendix

Input data & Methodological Summary

Case definition

The exposure definition for occupational particulates is a proxy measure based on industry data which were grouped to be consistent with exposure data in studies that provide relative risk information. COPD is the only outcome associated with occupational particulates exposure. Burden of occupational particulates is estimated for age groups 15 and beyond in both developing and developed countries.

Input Data

Economically Active Population by Industry ($EAP_{c,y,a,s}$)

Data on economic activity by country and industry over time was obtained from the International Labor Organization[11]. ILO data are primarily from censuses and national labor force surveys; these were also supplemented with data from the National Bureau of Statistics of China and the 1991 and 2001 India Censuses. For countries which did not provide sex specific estimates of industry groups to the ILO but did provide total estimates, the regional average of male to female participation in the industry was applied in order to determine sex specific estimates. These numbers were downloaded directly from the ILO database.

Economically Active Population ($EAC_{c,y,a,s}$)

The proportion of the population that is economically active was based on estimates produced by the ILO[2]. ILO estimates are produced separately by age for males and females for all but 10 countries between 1990 and 2013. We used the regional average as an estimate for the EAP in these countries. Because there were no estimates for 70-80 year olds, the 65-70 estimates were carried forward. The data for the population aged younger than 15 was too sparse and this age range was excluded. These numbers were provided to us by the expert group.

Exposure Partition Factor (EPF_{level})

The exposure levels vary by industry. We assumed that the exposure levels by industry also varied by a country's development status[4]. Table 2 shows the exposure levels for both high and low exposure by country's development status. These numbers were provided by the expert group.

Economic Activity	Developed, high	Developed, low	Developing, high	Developing, low
Agriculture, Hunting, Forestry and Fishing	0.1	0.5	0.1	0.7
Mining and Quarrying	0.1	0.7	0.4	0.4
Manufacturing	0.1	0.7	0.4	0.4
Electricity, Gas and Water	0.1	0.5	0.1	0.7
Construction	0.1	0.7	0.4	0.4

[11] International Labor Organization (ILO). ILOSTAT Database. http://www.ilo.org/ilostat/. Accessed: Sept 16, 2013

Wholesale and Retail Trade and Restaurants and Hotels	0	0.05	0	0.1
Transport, Storage and Communication	0.1	0.5	0.1	0.7
Financing, Insurance, Real Estate and Business Services	0	0.05	0	0.1
Community, Social and Personal Services	0	0.05	0	0.1

Modeling strategy

The modeling strategy for occupational exposure to occupational particulates associated with COPD has remained unchanged since GBD 2010[12]. The modeling strategy has been summarized below.

There are few direct measurements of exposure to particulates, gasses and fumes in the workplace. We estimated exposure to occupational particulates using the composition of a country's workforce in nine different industries[13]. Exposure to particulates in a particular industry was determined by multiplying the percentage of the workforce in the industry by the percentage of economically active people and by the prevalence of exposure to particulates in the industry. Population exposure is then determined as the sum of each industry's exposure for that country, age and sex group.

Formally, this is given by:

$$P_{c,y,a,s,level} = \sum_{i=1}^{9} EAP_{c,y,a,s,i} \ X \ EAC_{c,y,a,s} \ X \ EPF_l$$

$P_{c,y,a,s,level}$ = Prevalence of exposure to occupational particulates, by country, year, age, sex, and level of exposure;
$EAP_{c,y,a,s,i}$ = Proportion of the population that is economically active, by industry, country, year, age, and sex;
$EAC_{c,y,a,s}$ = Proportion of the population that is economically active, by country, year, age, and sex;
EPF_{level} = Exposure partition factor, by level of exposure;
c = Country;
y = Year;
a = 5-year age group;
s = Sex;
l = Level of exposure (high or low)
i = Industry (9 types).

Relative Risks

Relative risks for occupational particulates were estimated by the occupational risk expert group by conducting a systematic review of international literature and meta-analysis of relevant studies. The table below outlines the effect sizes associated with occupational particulates and COPD used in GBD 2013.

Relative risk by age and particulates exposure level

[12] Lim SS, et al. A comparative risk assessment of burden of disease and injury attributable to 67 risk factors and risk factor clusters in 21 regions, 1990-2010: a systematic analysis for the Global Burden of Disease Study 2010. *Lancet 2012*; **380**: 2224-60.
[13] Driscoll T, et al. The global burden of non-malignant respiratory disease due to occupational airborne exposures. *American Journal of Industrial Medicine 2005; 48*(6): 432-445.

		High exposure			Low exposure		
Outcome	Age	RR	LL	UL	RR	LL	UL
COPD	15-80	2.31	1.45	3.73	1.44	1.07	1.95

The relative risks used for occupational particulates remained unchanged since GBD 2010. Draws of relative risks for occupational particulates were migrated centrally from GBD 2010 to GBD 2013 infrastructure. As a result, code that preps relative risks from scratch do not exist.

Theoretical Minimum-Risk Exposure Level

The TMREL for occupational particulates is defined as no occupational exposure to particulates, gases, and fumes.

Attributable Burden Estimation

Population Attributable Fractions (PAFs) were calculated centrally using input draws of exposure, relative risks, and theoretical minimum level prepared by the modeler (Astha). The following equation which takes into account exposure distribution (P_i); relative risks i.e. measure of effect on outcome associated with each level of exposure (RR_i); and the counterfactual level of risk exposure (TMREL) was used to calculate PAFs centrally.

$$PAF = \frac{\sum_{i=1}^{n} P_i(RR_i - 1)}{\sum_{i=1}^{n} P_i(RR_i - 1) + 1}$$

Ambient Particulate Matter Pollution SDG Capstone Appendix

Flowchart

Ambient PM2.5

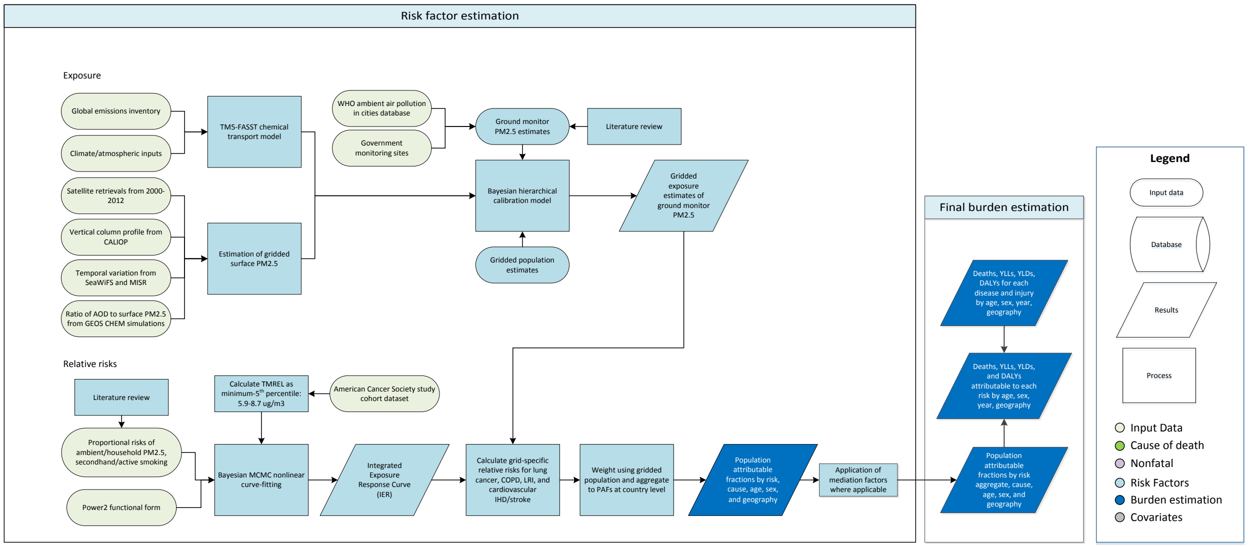

Input Data & Methodological Summary

Indicator definition

This modeling strategy encompassed the indicator associated with population-weighted PM2.5 (fine particulate matter) (11.6.2)

Indicator 11.6.2

As a component of SDG Goal 11. Make cities and human settlements inclusive, safe, resilient and sustainable, SDG Target 11.6, by 2030, reduce the adverse per capita environmental impact of cities, including by paying special attention to air quality and municipal and other waste management, is measured using SDG Indicator 11.6.2, Population-weighted mean levels of fine particulate matter (PM2.5).

Case definition

Exposure to ambient air pollution is defined as the population-weighted annual average mass concentration of particles with an aerodynamic diameter less than 2.5 micrometers ($PM_{2.5}$) in a cubic meter of air. This measurement is reported in $\mu g/m^3$.

Input data

The data to estimate exposure to ambient air pollution is drawn from estimates of annual concentration of $PM_{2.5}$ – generated using satellite observations of aerosols in the atmosphere. To correct for bias in the satellite modeling approach, a spatially-varying flexible framework is used to combine modeled concentrations with observations from ground-level monitoring of particles in more than 75 countries. All input data for GBD2015 was updated as follows:

Updated $PM_{2.5}$ ground measurement database

For the GBD2015 update we updated the database of annual average PM measurements to include more recent data and to incorporate additional locations where measurement data have become available. To facilitate this we collaborated with WHO and contributed to their recently released WHO Air Pollution in Cities database. We then used disaggregated (monitor-specific values and not the city averages that are reported by WHO) measurements from this database with additional site-specific information (e.g. all monitors in a city, monitor geo coordinates, monitor site type) such as that included in the GBD2013 database. In total measurements of concentrations of PM_{10} and $PM_{2.5}$ were retrieved from 6,003 ground monitors with the majority contributing measurements from 2014 (as there is a lag in reporting measurements, little data from 2015 were available). Where data were not available for 2014 (2760 monitors), data was used from 2015 (18 monitors), 2013 (2155), 2012 (564), 2011 (60), 2010 (375), 2009 (49), 2008 (21) and 2006 (1). For locations with only PM_{10} measurements, $PM_{2.5}$ measurements were estimated from PM_{10}. This was done by a locally derived conversion factor ($PM_{2.5}/PM_{10}$ ratio) estimated as population-weighted averages of location-specific conversion factors for the country. Location-specific conversion factors were estimated as the mean ratio of $PM_{2.5}$ to PM_{10} of stations for the same year. If national conversion factors were not available, regional ones were used, which were obtained by averaging country-specific conversion factors.

Updated satellite-based estimates

The updated satellite-based estimates are described in detail in van Donkelaar et al. 2016[1]. These estimates (~11 x 11 km resolution at the equator) combine aerosol optical depth retrievals from multiple satellites with the GEOS Chem chemical transport model and land use information.

Updated population data

A comprehensive set of population data on a high-resolution grid was obtained from the Gridded Population of the World (GPW v4) database. These data are provided on a 0.0417°×0.0417° resolution. To aggregate these estimates of population to each 0.1°×0.1° grid cell, the central 3 × 3 population cells were summed. As this accounted for a resolution higher than necessary, the same was done four other times, offset by one cell in a North, South, East and West direction. The average of five quantities was used as the aggregated population estimate for each cell. Estimates of population for 2000, 2005, 2010, 2015 and 2020 were extracted from GPW version 4 and estimates for 1990 and 1995 were extracted from GPW version 3 as described previously for GBD2013[3].

Modeling strategy

The methodology used to estimate the burden of ambient particulate matter pollution has seen significant changes since GBD2013.

The GBD2010 and GBD2013 estimates both used a single global function to calibrate the mean of the chemical transport model and satellite-based estimates to available ground measurements. In both instances the approach taken was recognized at the time to be a compromise between what could be easily implemented under tight timeframes and one that most efficiently utilized all of the data sources. In particular, the GBD2013 exposure estimates were known to underestimate ground measurements in specific locations (see discussion in Brauer et al., 2015[2]) such that it would be desirable to allow measurements to make a larger contribution to the final estimates where they were available. Therefore, for GBD2015 we implemented a Bayesian Hierarchical modelling approach using Integrated Nested Laplace Approximations (INLA) in which the satellite-based estimates, ground measurements and land use information are combined in a spatially varying flexible framework. Formal external evaluation using ground measurements was conducted and shown to lead to improved predictions of ground measurements in all super regions compared to GBD2013 estimates and an alternative geographically-weighted regression approach. Further, based on the external evaluation analyses, addition of the TM5 chemical transport model estimates of PM2.5 annual average did not improve the estimates and these were therefore not included.

Bayesian hierarchical models (BHM) provide an extremely useful and flexible framework in which to model complex relationships and dependencies in data. Uncertainty can also be propagated through the model allowing uncertainty arising from different components, both data sources and models, to be propagated through the models into estimates of uncertainty associated with the final estimates. In the hierarchical modeling approach coefficients associated with satellite-based estimates were estimated for each country. Where data were insufficient within a country, information can be `borrowed' from a higher aggregation (region) and if enough information is still not available from an even higher level (super-region). Individual country level estimates were therefore based on a combination of information from the country, its region and super-region.

All modelling was performed on the log-scale with the unit of measurement being a grid cell. The model was constructed with the inclusion of all variables assessed statistically, based on model fit (DIC, a measure of the relative quality of a model and closely related to that of AIC but for Bayesian models) and predictive ability. The hierarchical structure was applied to the intercept and slope terms with all modelling on the log scale. The model was of the form

$$\log(PM2.5_i) = \beta_0 + \beta_1 \log SAT_i + other\ variables + \varepsilon_i$$

where *i* denotes the grid cell. The following sets of variables were considering in developing the models:

Continuous explanatory variables:

- (SAT) Estimate of $PM_{2.5}$ (in μgm^{-3}) for 2014 from satellite remote sensing on the log-scale.
- (CTM) Estimate of $PM_{2.5}$ (in μgm^{-3}) for 2014 from chemical transport models on the log-scale.

- Estimate of population for 2014 on the log-scale.
- (SNAOC) Estimate of the sum of sulfate, nitrate, ammonium and organic carbon as estimated from GEOS Chem
- (DST) Estimate of compositional concentrations for mineral dust from GEOS Chem
- (EDxDU) The log of the elevation difference between the elevation at the ground measurement location and the mean elevation within the GEOS Chem simulation grid cell multiplied by the inverse distance to the nearest urban land surface

Discrete explanatory variables:

- Binary variable indicting whether exact location of ground measurement is known
- Binary variable indicting whether exact type of ground monitor is known
- Binary variable indicting whether ground measurement is $PM_{2.5}$ or converted from PM_{10}

Random Effects:

- Grid cell random effects on the intercept to allow for multiple ground monitors in a grid cell.
- Country-region-super-region hierarchical random effects for the intercept
- Country-region-super-region hierarchical random effects for the satellite remote sensing term.
- Country-region-super-region hierarchical random effects for the coefficient associated with the difference between estimates from CTM and SAT.
- Country-region-super-region hierarchical random effects for the coefficient log(POP)
- Country level random effects for intercept, satellite and difference between CTM and SAT are independent and identically distributed.
- Country level random effects for population uses a neighbourhood structure allowing specific borrowing of information from neighbouring countries.
- All region random effects are assumed to be independent and identically distributed.
- All super-region random effects are assumed to be independent and identically distributed.

Interactions*:*

- Interactions between the binary variables and the effects of log(SAT) and log(CTM/SAT)

Due to both the complexity of the models and the size of the data, notably the number of spatial predictions that are required in this setting, recently developed techniques that perform 'approximate' Bayesian inference based on integrated nested Laplace approximations (INLA) have been developed as a computationally attractive alternative to Markov Chain Monte Carlo methods. Computation was performed using the R interface to the INLA computational engine (R-INLA) with the size of the task of fitting the models and performing predictions for each of the ca. 1.4 million grid cells requiring the use of a high performance computing cluster (HPC) with high memory nodes. As in GBD2010 and GBD2013 the spatial model was built combining the different data sources for a single year (2014, corresponds to the most recent measurement data). The spatially-varying functions from this model were then applied to the satellite-based estimates from all other years - in other words assuming that the spatial relationship between the different data sources does not change over time. This is undoubtedly a simplification but to

do otherwise would require assembling multi-year measurement databases which is not feasible given current data availability and computational constraints. As the spatial model was built using the most recently available (2014) measurement and satellite-based estimates, 2015 estimates were based on extrapolation. Instead of extrapolating using an exponential model based on a 1-year trend as in GBD2013, splines based on a 5 year trend (2010-2014) were fit and applied to the 2014 grid-cell values to estimate levels for 2015. This reduced the likelihood of 2015 estimates being overly influenced by meteorological events in a specific year and to better represent the duration of exposure relevant to the epidemiologic studies included in the integrated exposure-response functions.

Model Evaluation

Model evaluation and comparison was performed by fitting models on a training set and predicting exposures at locations for which measurements were known (the validation set). The selection of the training (20%) and validation (80%) set consisted of taking a random sample of the total number of sites measuring PM2.5 (or having a value converted from PM10 measurements). Sampling was performed using sampling probabilities based on the cross-tabulation of PM2.5 categories (0-24.9, 25-49.9, 50-74.9, 75-99.9, 100+ µg/m3) and super-regions. The resulting hold-out evaluation data set was a sample of 20% of the sites that have the same distribution over PM2.5 categories and super-regions as the entire set of sites.

This process was used to generate multiple training and validation set combinations, allowing for example cross-validation to be performed. In the evaluation, 25 sets of training/validation data were used. The following models were considered in the evaluation phase:

(A) The GBD2013 model, using a simple linear regression with a fused estimate of SAT and CTM together with interactions with three binary variables representing whether the measurement was converted from PM10 and whether the exact site type and location is known.
(B) A hierarchical model with SAT, the TM5 CTM estimates, population and the three binary variables described above
(C) A hierarchical model with SAT, population, SNAOC, DST, EDxDU, population and the three binary variables
    - Estimate of population for 2014 on the log-scale.
    - Estimate of the sum of sulfate, nitrate, ammonium and organic carbon as estimated from GEOS Chem
    - Estimate of compositional concentrations for mineral dust from GEOS Chem
    - The log of the elevation difference between the elevation at the ground measurement location and the mean elevation within the GEOS Chem simulation grid cell multiplied by the inverse distance to the nearest urban land surface

For each training/evaluation set combination, model fit and prediction accuracy were evaluated for each of the 25 training/evaluation set combinations with the following metrics:

Model fit
- R^2
- DIC

Predictive accuracy
- R^2 arising from a linear regression of predicted vs actual measurements at each location

- RMSE – root mean squared error
- WRMSE – weighted (by population) root mean squared error
- MSE – mean square error
- MAE – mean absolute error

This evaluation indicated the final model improved predictions of ground measurements in all super regions compared to GBD2013 estimates (median global R^2 [population-weighted RMSE] 0.82 (12.1 $\mu g/m^3$), 0.60 [13.5 $\mu g.m^3$] for GBD2015 and GBD2013, respectively).

Error! Reference source not found. shows the RMSE (median from the 25 runs) for each of the three models, by super-region. The accuracy of the prediction varies between super-regions, with lower errors being observed in areas where there are more monitoring sites. In each of the super-regions, the largest errors are seen for model A which are considerably higher than those for models B and C, with model C showing a small improvement over B (except in super-region 5, North Afirca/Middle East).

Figure 2 shows scatter plots of the observed and predicted measurements using the three models for each super-region. The predicted measurements are the median values over those obtained from the 25 training sets. Predictions from the two Bayesian hierarchical models (B&C) match the observed values more closely than the linear model (A) with much less spread around a straight line (with slope one and zero intercept, shown in red). In Central Europe and Sub-Saharan Africa it is noticeable that, in addition to reduced spread, models B&C are much better at predicting higher values. The same patterns of results in predictive ability were seen when looking at regions and individual countries. In all cases, model C performed better than model B with both being considerable better than model A.

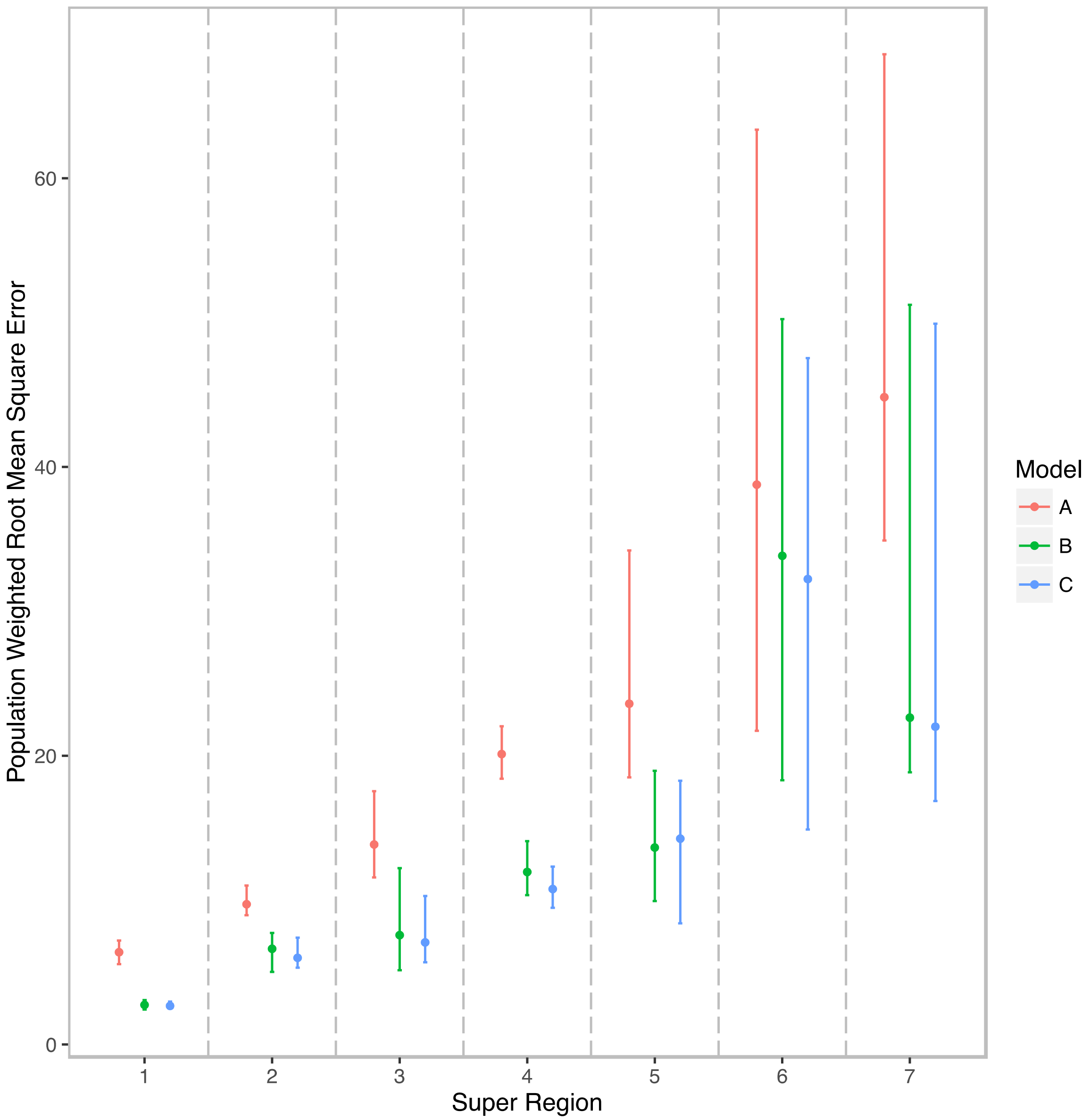

Figure 1: Comparison of RMSE from three models by super-region. Dots denote the median of the distribution from 25 training/evaluation sets and the vertical lines the range of values. Super-regions are 1: high income, 2: Central Europe, Eastern Europe, Central Asia, 3: Latin America and Caribbean, 4: Southeast Asia, East Asia and Oceania, 5: North Africa / Middle East, 6: Sub-Saharan Africa, 7: South Asia.

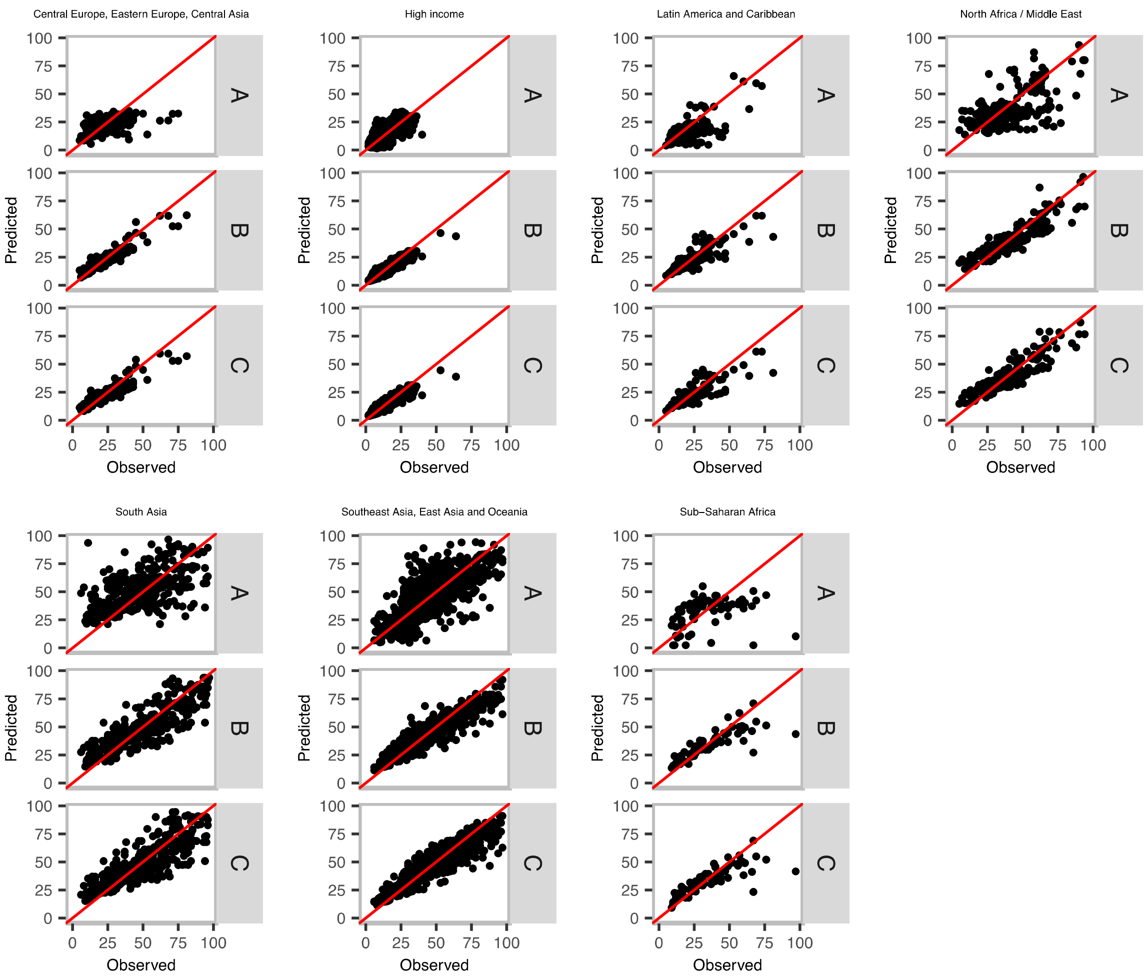

Figure 2: Comparison of observed and predicted measurements using three different models, by super-region. The red line has slope one and intercept zero.

Overall, the best model in terms of model fit and predictive ability was one with the following components:

- Estimates of $PM_{2.5}$ (in μgm^{-3}) from satellite remote sensing (SAT), population, and information on the GEOS Chem simulated chemical composition, elevation and distance to urban land use (SNAOC, DST and EDxDU).
- Binary variables indicting whether exact location and type of ground measurement is known, and whether the measurement was $PM_{2.5}$ or converted from PM_{10}.
- Interactions between the binary variables and the effects of estimates from satellite remote sensing.
- Grid cell random effects on the intercept to allow for multiple ground monitors in a grid cell.
- Country-region-super-region hierarchical random effects for intercepts, satellite remote sensing and population terms.
- Country level random effects for population using a neighbourhood structure allowing specific borrowing of information from neighbouring countries.

Relative Risk

Relative risks are generated using integrated exposure-response functions (IER) that are fit to available epidemiologic data using a Bayesian MCMC approach and a modified power function. The IER are estimated based on published relative risks for long-term exposure to ambient PM2.5, household air pollution, second-hand smoking, and active (cigarette) smoking. The concentration of particulate matter for each type of exposure is estimated based on literature values and used to map the relative risks to a curve generated for the entire range of exposure from these sources. The input data for this curve fitting process has been updated since GBD2013, adding new studies that estimate exposure at finer spatial scales, including studies of within-city exposure that focus on traffic-related air pollution. In addition, changes were made to the curve-fitting process. In order to account for differences in study design, temporal patterns of exposure and other differences among the studies of the different sources of PM2.5, a source-specific heterogeneity parameter was added to the IER. This resulted in much wider, and, in our view, more realistic, uncertainty intervals for the burden estimates, by propagating through the entire process the current uncertainty regarding the mechanisms and magnitude of health impacts of exposure to PM2.5 from diverse sources.

IER Functional Form

Data Likelihood

$$\log(RR_i) \sim \mathcal{N}\left(\mu_i, \sqrt{\sigma_i^2 + \delta_{source_i}}\right)$$

Model

$$\mu_i = \log\left(\frac{1+\alpha \times \left(1-e^{-\beta \times (exposure_i - TMREL)^{\gamma}}\right)}{1+\alpha \times \left(1-e^{-\beta \times (counterfactual_i - TMREL)^{\gamma}}\right)}\right)$$

Data

RR_i : measured relative risk for data point i
σ_i : variance of data point i based on study information
$source_i$: exposure source type (outdoor/household air pollution, secondhand/active smoking)
$TMREL$: theoretical minimum risk exposure level
$exposure_i$: measured exposure for data point i
$counterfactual_i$: counterfactual exposure for data point i

Priors

$$\alpha \sim \Gamma(1.0, 0.01)$$
$$\beta \sim \Gamma(1.0, 0.01)$$
$$\gamma \sim \Gamma(1.0, 0.01)$$
$$\delta \sim \Gamma(1.0, 0.01)$$

We also modified the way in which age-specific IER for IHD and stroke were estimated. In accordance with previously published work on other cardiovascular risk factors, the impact of air pollution on cardiovascular health is known to vary with age. To account for this phenomenon, age-specific RRs were based on a log-linear model of RR as a function of age, where the intercept (RR=1) is forced to age 110. In

GBD2010 and GBD2013 the age for a relative risk estimate from a given study was estimated as the median age at death or disease incidence in that study. However, this may not accurately represent the age distribution of the entire study population so we re-estimated this variable as the mean age at enrollment + half of the average follow-up time to better represent the average age of the study population during the period of follow-up.. When compared to GBD2013, this change produced RRs that were generally lower for younger age groups, given that median age at event tends to produce a higher anchor age than average age during follow-up.

The relative risks are generated on the grid-level based on estimated exposure, and then applied to generate PAFs. These PAFs are aggregated using the grid-level population to create population-weighted national estimates of attributable burden, using the following formula:

PM2.5 Aggregation Formula

$$PAF_{A,\ C,\ L} = \frac{\sum\ ((RR_{A,\ C} - 1)\ *\ Pop_i)}{\sum(\ RR_{A,\ C}\ *\ Pop_i)}$$

A = age group

C = cause

L = location

i = grid

$RR_{A,\ C}$ = *grid-level RR based on* $PM_{2.5}$ *and given age/cause IER curve*

TMREL

The TMREL for ambient PM is estimated using a uniform distribution between the minimum and 5th percentile of exposure observed in the studies used to generate the GBD estimates. This estimate was updated for GBD2015 as new studies were added to the analysis and studies used previously were updated through continued follow-up. The newer estimates included several large studies that included exposure at lower levels of PM2.5. As a result, the TMREL for GBD2015 was ~U(2.4, 5.9), lower than GBD2013's distribution ~U(5.9, 8.7), which had the effect, all things being equal, of increasing the estimated attributable burden relative to the GBD 2013 estimates.

References

(1) van Donkelaar, A.; Martin, R. V; Brauer, M.; Hsu, N. C.; Kahn, R. A.; Levy, R. C.; Lyapustin, A.; Sayer, A. M.; Winker, D. M. Global Estimates of Fine Particulate Matter using a Combined Geophysical-

Statistical Method with Information from Satellites, Models, and Monitors. *Environ. Sci. Technol.* **2016**, *50* (7), 3762–3772.

(2) Brauer, M.; Freedman, G.; Frostad, J.; van Donkelaar, A.; Martin, R. V; Dentener, F.; Van Dingenen, R.; Estep, K.; Amini, H.; Apte, J. S.; et al. Ambient Air Pollution Exposure Estimation for the Global Burden of Disease 2013. *Environ. Sci. Technol.* **2015**, *50* (1), 79–88.

(3) Brauer, M.; Amann, M.; Burnett, R. T.; Cohen, A.; Dentener, F.; Ezzati, M.; Henderson, S. B.; Krzyzanowski, M.; Martin, R. V; Van Dingenen, R.; et al. Exposure assessment for estimation of the global burden of disease attributable to outdoor air pollution. *Environ. Sci. Technol.* **2012**, *46* (2), 652–660.

Part 2. SDG index sensitivity analyses

In this analysis we have constructed indices that represent overall performance on: the health-related SDG indicators (referred to as the SDG index); the indicators that were previous MDG indicators (MDG index); and indicators that are newly added compared to the MDGs (non-MDG index). These indices were constructed by first rescaling each health-related SDG indicator on a 0 to 100 scale with 0 being the lowest (worst) value observed and 100 being the highest (best) value observed over the time period 1990 to 2015. The health-related SDG index was then computed by first determining the geometric mean of each rescaled health-related SDG indicator for a given target and then taking the geometric mean of the resulting values across the targets. This approach weights each of the health-related SDG targets equally and assumes partial substitutability with high values on one target partly compensating for low values on another target.

In addition to this approach to constructing the index, we used three alternative construction methods, namely: (i) principal component analysis (PCA); (ii) arithmetic mean across the health-related targets; and (iii) minimum value across the health-related targets. For each of the alternative methods we first rescaled the individual health-related indicators as described above. The PCA identified five principal components with eigenvalues greater than one. The first principal component, however, included both positively, eg maternal mortality ratio, and negatively correlated indicators, eg. alcohol use. Having a negative correlation on some components is not in line with the goals of the SDG targets and we do not compare the results of the PCA further.

For the second approach, we first take the arithmetic mean of indicator values for a given target and then the arithmetic mean of the resulting values across the targets. In contrast to the approach using the geometric mean, this approach assumes complete substitutability whereby poor performance on a target is linearly compensated for by better performance on another target. The resulting index using this approach was highly correlated with the approach using the geometric mean both in terms of 2015 values of the health-related SDG index (Appendix Figure 7a; Pearson correlation coefficient = 0.99, $p<0.0001$) as well as the corresponding country ranks (Appendix Figure 7b; Spearman's rank correlation coefficient=0.99, $p<0.0001$).

For the third approach, we first take the geometric mean of indicator values for a given target then determine the minimum of the resulting values across the health-related targets. This approach adopts what is called zero substitutability, ie better performance on one target in no way compensates for poor performance on another target. The resulting index using this approach was also well correlated with the approach using the geometric mean both in terms of 2015 values of the health-related SDG index (Appendix Figure 8a; Pearson correlation coefficient = 0.88, $p<0.0001$) and country rank (Appendix Figure 8b; Spearman's rank correlation coefficient=0.91, $p<0.0001$), although not as highly as the approach taking the arithmetic mean.

Part 3. Comprehensive citation list

Methods Appendix Table 2. Comprehensive Citation List

Citation
"Bangladesh MMS Study 2010", http://hdl.handle.net/1902.29/11389 UNF:5:jLAuvSAfyJ74D1rNQTLSww== MEASURE Evaluation [Distributor] V6 [Version]
[A survey on weight and height of children (1 month-7 years) and plotting of growth curves (1 month-18 years) in Taiwan, 1987-1988] as it appears in World Health Organization (WHO). WHO Global Database on Child Growth and Malnutrition - Historical. Geneva, Switzerland: World Health Organization (WHO).
[Changes in tobacco smoking patterns in Poland in the years 1976, 1986 and 1990] as it appears in P.N. Lee Statistics and Computing Ltd. International Mortality and Smoking Statistics Version 4.04. Sutton, United Kingdom: P.N. Lee Statistics and Computing Ltd, 2009.
[Child malnutrition in the Mexican rural setting] as it appears in World Health Organization (WHO). WHO Global Database on Child Growth and Malnutrition - Historical. Geneva, Switzerland: World Health Organization (WHO).
[Desarrollo físico y nutrición de preescolares habaneros según nuevos patrones de crecimiento de la OMS] as it appears in World Health Organization (WHO). WHO Global Database on Child Growth and Malnutrition. Geneva, Switzerland: World Health Organization (WHO).
[Growth and nutritional status of children from 0 to 11, State of Paraiba (Brazilian Northeast)] as it appears in World Health Organization (WHO). WHO Global Database on Child Growth and Malnutrition - Historical. Geneva, Switzerland: World Health Organization (WHO).
[Longitudinal study of children born in Pelotas, RS, Brazil in 1982. Methodology and preliminary results] as it appears in World Health Organization (WHO). WHO Global Database on Child Growth and Malnutrition - Historical. Geneva, Switzerland: World Health Organization (WHO).
[Nutritional and feeding status of preschool children in the semi-arid region of Bahia (Brazil): I. Anthropometric assessment] as it appears in World Health Organization (WHO). WHO Global Database on Child Growth and Malnutrition - Historical. Geneva, Switzerland: World Health Organization (WHO).
[Nutritional status of 0-to-59-month-old children in urban and rural areas of Cameroon] as it appears in World Health Organization (WHO). WHO Global Database on Child Growth and Malnutrition. Geneva, Switzerland: World Health Organization (WHO).
[Nutritional status of children under 6, according to land tenure, in rural areas of the State of Pernambuco, Northeast of Brazil] as it appears in World Health Organization (WHO). WHO Global Database on Child Growth and Malnutrition - Historical. Geneva, Switzerland: World Health Organization (WHO).
[Politico-administrative distribution of nutritional status according to a height census of 1st grade school children in Panama] as it appears in World Health Organization (WHO). WHO Global Database on Child Growth and Malnutrition - Historical. Geneva, Switzerland: World Health Organization (WHO).
[Risk factors for malnutrition in 0-59-month-old infants in 2 districts of Niger] as it appears in World Health Organization (WHO). WHO Global Database on Child Growth and Malnutrition - Historical. Geneva, Switzerland: World Health Organization (WHO).
[Smoking habits of young Israeli soldiers] as it appears in P.N. Lee Statistics and Computing Ltd. International Mortality and Smoking Statistics Version 4.04. Sutton, United Kingdom: P.N. Lee Statistics and Computing Ltd, 2009.
[Spanish cross-sectional growth study 2008. Part II. Height, weight and body mass index values from birth to adulthood] as it appears in World Health Organization (WHO). WHO Global Database on Child Growth and Malnutrition. Geneva, Switzerland: World Health Organization (WHO).
[Surveillance of the nutritional status of the population in Kinshasa, Zaire (1991-1994)] as it appears in World Health Organization (WHO). WHO Global Database on Child Growth and Malnutrition - Historical. Geneva, Switzerland: World Health Organization (WHO).
A Development Monitoring Service at the Local Level: Monitoring Change in Kerala: The First Five Years as it appears in World Health Organization (WHO). WHO Global Database on Child Growth and Malnutrition - Historical. Geneva, Switzerland: World Health Organization (WHO).
A mass survey of diabetes mellitus in a population of 300,000 in 14 provinces and municipalities in China (author's transl). Zhonghua Nei Ke Za Zhi. 1981; 20(11): 678-83.
A Nutritional Surveillance Report for the Lao People's Democratic Republic as it appears in World Health Organization (WHO). WHO Global Database on Child Growth and Malnutrition - Historical. Geneva, Switzerland: World Health Organization (WHO).
A study of the association between improved sanitation facilities and children's height in Lesotho as it appears in World Health Organization (WHO). WHO Global Database on Child Growth and Malnutrition - Historical. Geneva, Switzerland: World Health Organization (WHO).
AA al-Nuaim; EA Bamgboye; KA al-Rubeaan; Y al-Mazrou. Overweight and obesity in Saudi Arabian adult population, role of socio-demographic variables. J Community Health. 1997; 22(3): 211-23.
Aa I, Grove MA, Haugsjå AH, Hinderaker SG. High maternal mortality estimated by the sisterhood method in a rural area of Mali. BMC Pregnancy Childbirth. 2011; 11(1): 56.
Aamodt G, Stene LC, Njolstad PR, Sovik O, Joner G. Spatiotemporal trends and age-period-cohort modeling of the incidence of type 1 diabetes among children aged Diabetes Care. 2007; 30(4): 884-9.
Aarhus University, Addiction Switzerland Research Institute, Alcohol Research Group, Public Health Institute, Centre for Addiction and Mental Health (Canada), Centre for Alcohol Policy Research, Turning Point Alcohol and Drug Centre (Australia), Kettil Bruun Society for Social and Epidemiological Research on Alcohol, University of North Dakota. Belize Gender, Alcohol and Culture: An International Study (GENACIS) 2005.
Aarhus University, Addiction Switzerland Research Institute, Alcohol Research Group, Public Health Institute, Centre for Addiction and Mental Health (Canada), Centre for Alcohol Policy Research, Turning Point Alcohol and Drug Centre (Australia), Kettil Bruun Society for Social and Epidemiological Research on Alcohol, University of North Dakota. Brazil - Botucatu Gender, Alcohol and Culture: An International Study (GENACIS) 2001-2002.
Aarhus University, Addiction Switzerland Research Institute, Alcohol Research Group, Public Health Institute, Centre for Addiction and Mental Health (Canada), Centre for Alcohol Policy Research, Turning Point Alcohol and Drug Centre (Australia), Kettil Bruun Society for Social and Epidemiological Research on Alcohol, University of North Dakota. Canada Gender, Alcohol and Culture: An International Study (GENACIS) 2004.

Appendix: Citation List

Citation
Aarhus University, Addiction Switzerland Research Institute, Alcohol Research Group, Public Health Institute, Centre for Addiction and Mental Health (Canada), Centre for Alcohol Policy Research, Turning Point Alcohol and Drug Centre (Australia), Kettil Bruun Society for Social and Epidemiological Research on Alcohol, University of North Dakota. Costa Rica Gender, Alcohol and Culture: An International Study (GENACIS) 2003.
Aarhus University, Addiction Switzerland Research Institute, Alcohol Research Group, Public Health Institute, Centre for Addiction and Mental Health (Canada), Centre for Alcohol Policy Research, Turning Point Alcohol and Drug Centre (Australia), Kettil Bruun Society for Social and Epidemiological Research on Alcohol, University of North Dakota. Denmark Gender, Alcohol and Culture: An International Study (GENACIS) 2003.
Aarhus University, Addiction Switzerland Research Institute, Alcohol Research Group, Public Health Institute, Centre for Addiction and Mental Health (Canada), Centre for Alcohol Policy Research, Turning Point Alcohol and Drug Centre (Australia), Kettil Bruun Society for Social and Epidemiological Research on Alcohol, University of North Dakota. France Gender, Alcohol and Culture: An International Study (GENACIS) 1999.
Aarhus University, Addiction Switzerland Research Institute, Alcohol Research Group, Public Health Institute, Centre for Addiction and Mental Health (Canada), Centre for Alcohol Policy Research, Turning Point Alcohol and Drug Centre (Australia), Kettil Bruun Society for Social and Epidemiological Research on Alcohol, University of North Dakota. Germany Gender, Alcohol and Culture: An International Study (GENACIS) 2000.
Aarhus University, Addiction Switzerland Research Institute, Alcohol Research Group, Public Health Institute, Centre for Addiction and Mental Health (Canada), Centre for Alcohol Policy Research, Turning Point Alcohol and Drug Centre (Australia), Kettil Bruun Society for Social and Epidemiological Research on Alcohol, University of North Dakota. Iceland Gender, Alcohol and Culture: An International Study (GENACIS) 2001.
Aarhus University, Addiction Switzerland Research Institute, Alcohol Research Group, Public Health Institute, Centre for Addiction and Mental Health (Canada), Centre for Alcohol Policy Research, Turning Point Alcohol and Drug Centre (Australia), Kettil Bruun Society for Social and Epidemiological Research on Alcohol, University of North Dakota. India - Karnataka Gender, Alcohol and Culture: An International Study (GENACIS) 2003.
Aarhus University, Addiction Switzerland Research Institute, Alcohol Research Group, Public Health Institute, Centre for Addiction and Mental Health (Canada), Centre for Alcohol Policy Research, Turning Point Alcohol and Drug Centre (Australia), Kettil Bruun Society for Social and Epidemiological Research on Alcohol, University of North Dakota. Japan Gender, Alcohol and Culture: An International Study (GENACIS) 2001.
Aarhus University, Addiction Switzerland Research Institute, Alcohol Research Group, Public Health Institute, Centre for Addiction and Mental Health (Canada), Centre for Alcohol Policy Research, Turning Point Alcohol and Drug Centre (Australia), Kettil Bruun Society for Social and Epidemiological Research on Alcohol, University of North Dakota. Mexico Gender, Alcohol and Culture: An International Study (GENACIS) 1998.
Aarhus University, Addiction Switzerland Research Institute, Alcohol Research Group, Public Health Institute, Centre for Addiction and Mental Health (Canada), Centre for Alcohol Policy Research, Turning Point Alcohol and Drug Centre (Australia), Kettil Bruun Society for Social and Epidemiological Research on Alcohol, University of North Dakota. New Zealand Gender, Alcohol and Culture: An International Study (GENACIS) 2007.
Aarhus University, Addiction Switzerland Research Institute, Alcohol Research Group, Public Health Institute, Centre for Addiction and Mental Health (Canada), Centre for Alcohol Policy Research, Turning Point Alcohol and Drug Centre (Australia), Kettil Bruun Society for Social and Epidemiological Research on Alcohol, University of North Dakota. Sri Lanka Gender, Alcohol and Culture: An International Study (GENACIS) 2002.
Aarhus University, Addiction Switzerland Research Institute, Alcohol Research Group, Public Health Institute, Centre for Addiction and Mental Health (Canada), Centre for Alcohol Policy Research, Turning Point Alcohol and Drug Centre (Australia), Kettil Bruun Society for Social and Epidemiological Research on Alcohol, University of North Dakota. Sweden Gender, Alcohol and Culture: An International Study (GENACIS) 2002.
Aarhus University, Addiction Switzerland Research Institute, Alcohol Research Group, Public Health Institute, Centre for Addiction and Mental Health (Canada), Centre for Alcohol Policy Research, Turning Point Alcohol and Drug Centre (Australia), Kettil Bruun Society for Social and Epidemiological Research on Alcohol, University of North Dakota. United States Gender, Alcohol and Culture: An International Study (GENACIS) 2000.
Aarhus University, Addiction Switzerland Research Institute, Alcohol Research Group, Public Health Institute, Centre for Addiction and Mental Health (Canada), Centre for Alcohol Policy Research, Turning Point Alcohol and Drug Centre (Australia), Kettil Bruun Society for Social and Epidemiological Research on Alcohol, University of North Dakota. Uruguay Gender, Alcohol and Culture: An International Study (GENACIS) 2004.
Aarhus University, Addiction Switzerland Research Institute, Alcohol Research Group, Public Health Institute, Centre for Addiction and Mental Health (Canada), Centre for Alcohol Policy Research, Turning Point Alcohol and Drug Centre (Australia), Kettil Bruun Society for Social and Epidemiological Research on Alcohol, University of North Dakota. Argentina - Buenos Aires Gender, Alcohol and Culture: An International Study (GENACIS) 2003.
Aarhus University, Addiction Switzerland Research Institute, Alcohol Research Group, Public Health Institute, Centre for Addiction and Mental Health (Canada), Centre for Alcohol Policy Research, Turning Point Alcohol and Drug Centre (Australia), Kettil Bruun Society for Social and Epidemiological Research on Alcohol, University of North Dakota. Australia - Victoria Gender, Alcohol and Culture: An International Study (GENACIS) 2007.
Aarhus University, Addiction Switzerland Research Institute, Alcohol Research Group, Public Health Institute, Centre for Addiction and Mental Health (Canada), Centre for Alcohol Policy Research, Turning Point Alcohol and Drug Centre (Australia), Kettil Bruun Society for Social and Epidemiological Research on Alcohol, University of North Dakota. Australia Gender, Alcohol and Culture: An International Study (GENACIS) 1994.

Appendix: Citation List

Citation
Aarhus University, Addiction Switzerland Research Institute, Alcohol Research Group, Public Health Institute, Centre for Addiction and Mental Health (Canada), Centre for Alcohol Policy Research, Turning Point Alcohol and Drug Centre (Australia), Kettil Bruun Society for Social and Epidemiological Research on Alcohol, University of North Dakota. Austria Gender, Alcohol and Culture: An International Study (GENACIS) 1993.
Aarhus University, Addiction Switzerland Research Institute, Alcohol Research Group, Public Health Institute, Centre for Addiction and Mental Health (Canada), Centre for Alcohol Policy Research, Turning Point Alcohol and Drug Centre (Australia), Kettil Bruun Society for Social and Epidemiological Research on Alcohol, University of North Dakota. Czech Republic Gender, Alcohol and Culture: An International Study (GENACIS) 2002.
Aarhus University, Addiction Switzerland Research Institute, Alcohol Research Group, Public Health Institute, Centre for Addiction and Mental Health (Canada), Centre for Alcohol Policy Research, Turning Point Alcohol and Drug Centre (Australia), Kettil Bruun Society for Social and Epidemiological Research on Alcohol, University of North Dakota. Hungary Gender, Alcohol and Culture: An International Study (GENACIS) 2001.
Aarhus University, Addiction Switzerland Research Institute, Alcohol Research Group, Public Health Institute, Centre for Addiction and Mental Health (Canada), Centre for Alcohol Policy Research, Turning Point Alcohol and Drug Centre (Australia), Kettil Bruun Society for Social and Epidemiological Research on Alcohol, University of North Dakota. Ireland Gender, Alcohol and Culture: An International Study (GENACIS) 2002.
Aarhus University, Addiction Switzerland Research Institute, Alcohol Research Group, Public Health Institute, Centre for Addiction and Mental Health (Canada), Centre for Alcohol Policy Research, Turning Point Alcohol and Drug Centre (Australia), Kettil Bruun Society for Social and Epidemiological Research on Alcohol, University of North Dakota. Israel Gender, Alcohol and Culture: An International Study (GENACIS) 2001.
Aarhus University, Addiction Switzerland Research Institute, Alcohol Research Group, Public Health Institute, Centre for Addiction and Mental Health (Canada), Centre for Alcohol Policy Research, Turning Point Alcohol and Drug Centre (Australia), Kettil Bruun Society for Social and Epidemiological Research on Alcohol, University of North Dakota. Italy - Tuscany Gender, Alcohol and Culture: An International Study (GENACIS) 2001-2002.
Aarhus University, Addiction Switzerland Research Institute, Alcohol Research Group, Public Health Institute, Centre for Addiction and Mental Health (Canada), Centre for Alcohol Policy Research, Turning Point Alcohol and Drug Centre (Australia), Kettil Bruun Society for Social and Epidemiological Research on Alcohol, University of North Dakota. Kazakhstan Gender, Alcohol and Culture: An International Study (GENACIS) 2002-2003.
Aarhus University, Addiction Switzerland Research Institute, Alcohol Research Group, Public Health Institute, Centre for Addiction and Mental Health (Canada), Centre for Alcohol Policy Research, Turning Point Alcohol and Drug Centre (Australia), Kettil Bruun Society for Social and Epidemiological Research on Alcohol, University of North Dakota. Netherlands - Limburg Gender, Alcohol and Culture: An International Study (GENACIS) 1999.
Aarhus University, Addiction Switzerland Research Institute, Alcohol Research Group, Public Health Institute, Centre for Addiction and Mental Health (Canada), Centre for Alcohol Policy Research, Turning Point Alcohol and Drug Centre (Australia), Kettil Bruun Society for Social and Epidemiological Research on Alcohol, University of North Dakota. Nicaragua Gender, Alcohol and Culture: An International Study (GENACIS) 2005.
Aarhus University, Addiction Switzerland Research Institute, Alcohol Research Group, Public Health Institute, Centre for Addiction and Mental Health (Canada), Centre for Alcohol Policy Research, Turning Point Alcohol and Drug Centre (Australia), Kettil Bruun Society for Social and Epidemiological Research on Alcohol, University of North Dakota. Nigeria Gender, Alcohol and Culture: An International Study (GENACIS) 2003.
Aarhus University, Addiction Switzerland Research Institute, Alcohol Research Group, Public Health Institute, Centre for Addiction and Mental Health (Canada), Centre for Alcohol Policy Research, Turning Point Alcohol and Drug Centre (Australia), Kettil Bruun Society for Social and Epidemiological Research on Alcohol, University of North Dakota. Norway Gender, Alcohol and Culture: An International Study (GENACIS) 1999.
Aarhus University, Addiction Switzerland Research Institute, Alcohol Research Group, Public Health Institute, Centre for Addiction and Mental Health (Canada), Centre for Alcohol Policy Research, Turning Point Alcohol and Drug Centre (Australia), Kettil Bruun Society for Social and Epidemiological Research on Alcohol, University of North Dakota. Peru - Lima and Ayacucho Gender, Alcohol and Culture: An International Study (GENACIS) 2005.
Aarhus University, Addiction Switzerland Research Institute, Alcohol Research Group, Public Health Institute, Centre for Addiction and Mental Health (Canada), Centre for Alcohol Policy Research, Turning Point Alcohol and Drug Centre (Australia), Kettil Bruun Society for Social and Epidemiological Research on Alcohol, University of North Dakota. Spain Gender, Alcohol and Culture: An International Study (GENACIS) 2003.
Aarhus University, Addiction Switzerland Research Institute, Alcohol Research Group, Public Health Institute, Centre for Addiction and Mental Health (Canada), Centre for Alcohol Policy Research, Turning Point Alcohol and Drug Centre (Australia), Kettil Bruun Society for Social and Epidemiological Research on Alcohol, University of North Dakota. United States Gender, Alcohol and Culture: An International Study (GENACIS) 1995-1996.
Aarhus University, Addiction Switzerland Research Institute, Alcohol Research Group, Public Health Institute, Centre for Addiction and Mental Health (Canada), Centre for Alcohol Policy Research, Turning Point Alcohol and Drug Centre (Australia), Kettil Bruun Society for Social and Epidemiological Research on Alcohol, University of North Dakota. United States Gender, Alcohol and Culture: An International Study (GENACIS) 2001.
Aaron R, Joseph A, Abraham S, Muliyil J, George K, Prasad J, Minz S, Abraham VJ, Bose A. Suicides in young people in rural southern India. Lancet. 2004; 363(9415): 1117-8.

Appendix: Citation List

Citation
Abate A, Degarege A, Erko B. Community knowledge, attitude and practice about malaria in a low endemic setting of Shewa Robit Town, northeastern Ethiopia. BMC Public Health. 2013; 13(1): 312. As it appears in Malaria Atlas Project. Malaria Atlas Project Plasmodium Falciparum Parasite Rate Database. Oxford, United Kingdom: Malaria Atlas Project.
Abbas SM, Alam AY, Majid A. To determine the probable causes of death in an urban slum community of Pakistan among adults 18 years and above by verbal autopsy. J Pak Med Assoc. 2011; 61(3): 235-8.
Abbas ZG, Lutale JK, Bakker K, Baker N, Archibald LK. The 'Step by Step' Diabetic Foot Project in Tanzania: a model for improving patient outcomes in less-developed countries. Int Wound J. 2011; 8(2): 169-75.
Abbott CA, Carrington AL, Ashe H, Bath S, Every LC, Griffiths J, Hann AW, Hussein A, Jackson N, Johnson KE, Ryder CH, Torkington R, Van Ross ERE, Whalley AM, Widdows P, Williamson S, Boulton AJM, North-West Diabetes Foot Care Study. The North-West Diabetes Foot Care Study: incidence of, and risk factors for, new diabetic foot ulceration in a community-based patient cohort. Diabet Med. 2002; 19(5): 377-84.
Abdalmaula GH, Barbadoro P, Marigliano A, Illuminati D, Di Stanislao F, D'Errico MM, Prospero E. Human visceral leishmaniasis: a picture from Italy. J Infect Public Health. 2013; 6(6): 465-72.
Abdel-Hady E-S, Mashaly A-M, Sherief LS, Hassan M, Al-Gohary A, Farag MK, El-Khoeriby F. Why do mothers die in Dakahlia, Egypt? J Obstet Gynaecol Res. 2007; 33(3): 283-7.
Abdella N, Al Arouj M, Al Nakhi A, Al Assoussi A, Moussa M. Non-insulin-dependent diabetes in Kuwait: prevalence rates and associated risk factors. Diabetes Res Clin Pract. 1998; 42(3): 187-96.
Abdellatif MZ, El-Mabrouk K, Ewis AA. An epidemiological study of cutaneous leishmaniasis in Al-jabal Al-gharbi, Libya. Korean J Parasitol. 2013; 51(1): 75-84.
Abdel-Rahman TA, Collins KJ, Doré C. Oxylog studies of energy expenditure and schistosomiasis in the Sudan. J Trop Med Hyg. 1990; 93(6): 365-71.
Abdel-Wahab MF, Esmat G, Narooz SI, Yosery A, Struewing JP, Strickland GT. Sonographic studies of schoolchildren in a village endemic for Schistosoma mansoni. Trans R Soc Trop Med Hyg. 1990; 84(1): 69-73.
Abdel-Wahab MF, Esmat G, Ramzy I, Narooz S, Medhat E, Ibrahim M, El-Boraey Y, Strickland GT. The epidemiology of schistosomiasis in Egypt: Fayoum Governorate. Am J Trop Med Hyg. 2000; 62 Suppl 2: 55-64.
Abdi J, Akhoundi B, Mohebali M, Ghaderipour A, Kakoee Z, Najafi F. Seroepidemiological survey of human visceral leishmaniasis in ilam province, west of iran in 2013. Iran J Parasitol. 2015; 10(1): 56-61.
Abdi J, Taherikalani M, Asadolahi K, Emaneini M. Echinococcosis/Hydatidosis in Ilam province, Western Iran. Iran J Parasitol. 2013; 8(3): 417-22.
Abdi-Rad I, Khoshkalam M, Farrokh-Islamlou H-R. The prevalence at birth of overt congenital anomalies in Urmia, Northwestern Iran. Arch Iran Med. 2008; 11(2): 148-51.
Abdul Mujeeb S, Aamir K, Mehmood K. Seroprevalence of HBV, HCV and HIV infections among college going first time voluntary blood donors. J Pak Med Assoc. 2000; 50(8): 269-70.
Abdul Mujeeb S, Nanan D, Sabir S, Altaf A, Kadir M. Hepatitis B and C infection in first-time blood donors in Karachi--a possible subgroup for sentinel surveillance. East Mediterr Health J. 2006; 12(6): 735-41.
Abdulla S, Gemperli A, Mukasa O, Armstrong Schellenberg JRM, Lengeler C, Vounatsou P, Smith T. Spatial effects of the social marketing of insecticide-treated nets on malaria morbidity. Trop Med Int Health. 2005; 10(1): 11-8. As it appears in Malaria Atlas Project. Malaria Atlas Project Plasmodium Falciparum Parasite Rate Database. Oxford, United Kingdom: Malaria Atlas Project.
Abdullah MR. Malaria and Malaria Control in Jeli, Peninsular Malaysia [dissertation]. Liverpool, United Kingdom: University of Liverpool, 1998. As it appears in Malaria Atlas Project. Malaria Atlas Project Plasmodium Falciparum Parasite Rate Database. Oxford, United Kingdom: Malaria Atlas Project.
Abdullah S, Adazu K, Masanja H, Diallo D, Hodgson A, Ilboudo-Sanogo E, Nhacolo A, Owusu-Agyei S, Thompson R, Smith T, Binka FN. Patterns of age-specific mortality in children in endemic areas of sub-Saharan Africa. Am J Trop Med Hyg. 2007; 77(6 Suppl): 99-105.
Abdul-Rahim HF, Abu-Rmeileh NM, Husseini A, Holmboe-Ottesen G, Jervell J, Bjertness E. Obesity and selected co-morbidities in an urban Palestinian population. Int J Obes Relat Metab Disord. 2001; 25(11): 1736-40.
Abdul-Rahim HF, Husseini A, Bjertness E, Giacaman R, Gordon NH, Jervell J. The metabolic syndrome in the West Bank population: an urban-rural comparison. Diabetes Care. 2001; 24(2): 275-9.
Abdul-Rahim HF, Husseini A, Giacaman R, Jervell J, Bjertness E. Diabetes mellitus in an urban Palestinian population: prevalence and associated factors. East Mediterr Health J. 2001; 7(1-2): 67-78.
Abdur Rab M, Freeman TW, Rahim S, Durrani N, Simon-Taha A, Rowland M. High altitude epidemic malaria in Bamian province, central Afghanistan. East Mediterr Health J. 2003; 9(3): 232-9. As it appears in Malaria Atlas Project. Malaria Atlas Project Plasmodium Falciparum Parasite Rate Database. Oxford, United Kingdom: Malaria Atlas Project.
Abebe SM, Berhane Y, Worku A, Assefa A. Diabetes mellitus in North West Ethiopia: a community based study. BMC Public Health. 2014; 97.
Abedi F, Madani H, Asadi A, Nejatizadeh A. Significance of blood-related high-risk behaviors and horizontal transmission of hepatitis B virus in Iran. Arch Virol. 2011; 156(4): 629-35.
Abeya SG, Afework MF, Yalew AW. Intimate partner violence against women in western Ethiopia: prevalence, patterns, and associated factors. BMC Public Health. 2011; 913.
Abiaka C, Olusi S, Al-Awadhi A. Reference ranges of copper and zinc and the prevalence of their deficiencies in an Arab population aged 15-80 years. Biol Trace Elem Res. 2003; 91(1): 33-43.
Abose T. Re-orientation and Definition of the Role of Malaria Vector-control in Ethiopia. Geneva, Switzerland: World Health Organization, 1998. (WHO/MAL/98.1085) As it appears in Malaria Atlas Project. Malaria Atlas Project Plasmodium Falciparum Parasite Rate Database. Oxford, United Kingdom: Malaria Atlas Project.

Appendix: Citation List

Citation
Abouchadi S, Belghiti Alaoui A, Meski FZ, De Brouwere V. Implementing a maternal mortality surveillance system in Morocco - challenges and opportunities. Trop Med Int Health. 2013; 18(3): 357-65.
Abougalambou SSI, Abougalambou AS. Explorative study on diabetes neuropathy among type II diabetic patients in Universiti Sains Malaysia Hospital. Diabetes Metab Syndr. 2012; 6(3): 167–72.
Abraham TM, Pencina KM, Pencina MJ, Fox CS. Trends in diabetes incidence: the Framingham Heart Study. Diabetes Care. 2015; 38(3): 482-7.
Abrantes MM, Lamounier JA, Colosimo EA. PrevalÃªncia de sobrepeso e obesidade nas regiÃµes Nordeste e Sudeste do Brasil. Rev Assoc Med Bras. 2003; 49(2): 162-6.
Abt Associates Inc., Kenya National Bureau of Statistics, Ministry of Health (Kenya). Kenya Household Health Expenditure and Utilization Survey 2007. Nairobi, Kenya: Kenya National Bureau of Statistics.
Abt Associates Inc., National Center for Health Statistics (NCHS), Centers for Disease Control and Prevention (CDC), National Center for Immunization and Respiratory Diseases, CDC (NCIRD). United States National Immunization Survey 1995-1996. Hyattsville, United States: National Center for Health Statistics (NCHS), Centers for Disease Control and Prevention (CDC).
Abt Associates Inc., National Center for Health Statistics (NCHS), Centers for Disease Control and Prevention (CDC), National Center for Immunization and Respiratory Diseases, CDC (NCIRD). United States National Immunization Survey 1996-1997. Hyattsville, United States: National Center for Health Statistics (NCHS), Centers for Disease Control and Prevention (CDC).
Abt Associates Inc., National Center for Health Statistics (NCHS), Centers for Disease Control and Prevention (CDC), National Center for Immunization and Respiratory Diseases, CDC (NCIRD). United States National Immunization Survey 1996-1998. Hyattsville, United States: National Center for Health Statistics (NCHS), Centers for Disease Control and Prevention (CDC).
Abt Associates Inc., National Center for Health Statistics (NCHS), Centers for Disease Control and Prevention (CDC), National Center for Immunization and Respiratory Diseases, CDC (NCIRD). United States National Immunization Survey 1998-1999. Hyattsville, United States: National Center for Health Statistics (NCHS), Centers for Disease Control and Prevention (CDC).
Abt Associates Inc., National Center for Health Statistics (NCHS), Centers for Disease Control and Prevention (CDC), National Center for Immunization and Respiratory Diseases, CDC (NCIRD). United States National Immunization Survey 1999-2000. Hyattsville, United States: National Center for Health Statistics (NCHS), Centers for Disease Control and Prevention (CDC).
Abt Associates Inc., National Center for Health Statistics (NCHS), Centers for Disease Control and Prevention (CDC), National Center for Immunization and Respiratory Diseases, CDC (NCIRD). United States National Immunization Survey 2000-2001. Hyattsville, United States: National Center for Health Statistics (NCHS), Centers for Disease Control and Prevention (CDC).
Abt Associates Inc., National Center for Health Statistics (NCHS), Centers for Disease Control and Prevention (CDC), National Center for Immunization and Respiratory Diseases, CDC (NCIRD). United States National Immunization Survey 2001-2002. Hyattsville, United States: National Center for Health Statistics (NCHS), Centers for Disease Control and Prevention (CDC).
Abt Associates Inc., National Center for Health Statistics (NCHS), Centers for Disease Control and Prevention (CDC), National Center for Immunization and Respiratory Diseases, CDC (NCIRD). United States National Immunization Survey 2003-2004. Hyattsville, United States: National Center for Health Statistics (NCHS), Centers for Disease Control and Prevention (CDC).
Abu Dhabi General Authority for Health Services, Centers for Disease Control and Prevention (CDC), Dubai Health Authority, Ministry of Education (United Arab Emirates), Ministry of Health (United Arab Emirates), World Health Organization (WHO). United Arab Emirates Global School-Based Student Health Survey 2005. Geneva, Switzerland: World Health Organization (WHO).
Abu Dhabi General Authority for Health Services, Centers for Disease Control and Prevention (CDC), Dubai Health Authority, Ministry of Education (United Arab Emirates), Ministry of Health (United Arab Emirates), World Health Organization (WHO). United Arab Emirates Global School-Based Student Health Survey 2010.
Abu Habib N, Lie RT, Oneko O, Shao J, Bergsjø P, Daltveit AK. Sociodemographic characteristics and perinatal mortality among singletons in North East Tanzania: a registry-based study. J Epidemiol Community Health. 2008; 62: 960-965.
Abu Sayeed M, Mahtab H, Akter Khanam P, Abul Ahsan K, Banu A, Rashid AN, Azad Khan AK. Diabetes and impaired fasting glycemia in the tribes of Khagrachari hill tracts of Bangladesh. Diabetes Care. 2004; 27(5): 1054-9.
Abu Sham'a R a. H, Darwazah AK, Kufri FH, Yassin IH, Torok NI. MetS and cardiovascular risk factors among Palestinians of East Jerusalem. East Mediterr Health J. 2009; 15(6): 1464–73.
Abusheikha N, Akagbosu F, Marcus S, Lass A, Cousins C, Brinsden P. Viral screening and assisted conception treatment--the Bourn Hall experience. J Assist Reprod Genet. 1999; 16(6): 337-9.
Academy of Preventive Medicine (Kazakhstan), Macro International, Inc. Kazakhstan Demographic and Health Survey 1999. Calverton, United States: Macro International, Inc.
Aché A, Matos AJ. Interrupting Chagas disease transmission in Venezuela. Rev Inst Med Trop Sao Paulo. 2001; 43(1): 37-43.
Acheson ED, Gardner MJ, Pippard EC, Grime LP. Mortality Of Two Groups Of Women Who Manufactured Gas Masks From Chrysotile And Crocidolite Asbestos: A 40-Year Follow-Up. Br J Ind Med. 1982; 39(4): 344-8 as it appears in Camargo MC, Stayner LT, Straif K, Reina M, Al-Alem U, Demers PA, Landrigan PJ. Occupational exposure to asbestos and ovarian cancer: a meta-analysis. Environ Health Perspect. 2011; 119(9): 1211-7.
Achidi EA, Apinjoh TO, Mbunwe E, Besingi R, Yafi C, Wenjighe Awah N, Ajua A, Anchang JK. Febrile status, malarial parasitaemia and gastro-intestinal helminthiases in schoolchildren resident at different altitudes, in south-western Cameroon. Ann Trop Med Parasitol. 2008; 102(2): 103-18. As it appears in Malaria Atlas Project. Malaria Atlas Project Plasmodium Falciparum Parasite Rate Database. Oxford, United Kingdom: Malaria Atlas Project.
Achwan WA, Muttaqin Z, Zakaria E, Depamede SA, Mulyanto, Sumoharjo S, Tsuda F, Takahashi K, Abe N, Mishiro S. Epidemiology of hepatitis B, C, and E viruses and human immunodeficiency virus infections in Tahuna, Sangihe-Talaud Archipelago, Indonesia. Intervirology. 2007; 50(6): 408-11.

Appendix: Citation List

Citation
ACIL Australia Pty Ltd., Australian National University, Ministry of Health (Timor-Leste), National Statistics Directorate (Timor-Leste), University of Newcastle (Australia). Timor-Leste Demographic and Health Survey 2003. Newcastle, Australia: University of Newcastle (Australia).
Ackerman IN, Osborne RH. Obesity and increased burden of hip and knee joint disease in Australia: results from a national survey. BMC Musculoskelet Disord. 2012; 13(1): 254.
Ackerson LK, Kawachi I, Barbeau EM, Subramanian SV. Effects of individual and proximate educational context on intimate partner violence: a population-based study of women in India. Am J Public Health. 2008; 98(3): 507-14.
ACNielsen Bangladesh, Directorate General of Health Services, Ministry of Health and Family Welfare (Bangladesh), United Nations Children's Fund (UNICEF), World Health Organization (WHO). Bangladesh EPI Coverage Evaluation Survey 2007-2008.
ACNielsen Bangladesh, Directorate General of Health Services, Ministry of Health and Family Welfare (Bangladesh), United Nations Children's Fund (UNICEF), World Health Organization (WHO). Bangladesh EPI Coverage Evaluation Survey 2009.
ACNielsen Bangladesh, Directorate General of Health Services, Ministry of Health and Family Welfare (Bangladesh), United Nations Children's Fund (UNICEF), World Health Organization (WHO). Bangladesh EPI Coverage Evaluation Survey 2010.
ACNielsen Bangladesh, Directorate General of Health Services, Ministry of Health and Family Welfare (Bangladesh), United Nations Children's Fund (UNICEF), World Health Organization (WHO). Bangladesh EPI Coverage Evaluation Survey 2013.
ACNielsen, Directorate General of Health Services, Ministry of Health and Family Welfare (Bangladesh), United Nations Children's Fund (UNICEF), World Health Organization (WHO). Bangladesh EPI Coverage Evaluation Survey 2005.
Acosta-Cazares B, Escobedo-de la Pena J. High burden of cardiovascular disease risk factors in Mexico: An epidemic of ischemic heart disease that may be on its way? Am Heart J. 2010; 160(2): 230–6.
Acosta-Jamett G, Cleaveland S, Cunningham AA, Bronsvoort BM deC, Craig PS. Echinococcus granulosus infection in humans and livestock in the Coquimbo region, north-central Chile. Vet Parasitol. 2010; 169(1-2): 102-10.
Acquaye JK, Mingle JA. Hepatitis B viral markers in Ghanaian pregnant women. West Afr J Med. 1994; 13(3): 134-7.
Action on Smoking and Health Foundation (Thailand), Centers for Disease Control and Prevention (CDC), Faculty of Public Health at Mahidol University (Thailand), Health Systems Research Institute (Thailand), Ministry of Public Health (Thailand), National Statistical Office (Thailand), Tobacco Control Research and Knowledge Management Center (Thailand), World Health Organization (WHO). Thailand Global Adult Tobacco Survey 2009. Atlanta, United States: Centers for Disease Control and Prevention (CDC), 2011.
Action on Smoking and Health Foundation (Thailand), Centers for Disease Control and Prevention (CDC), Faculty of Public Health at Mahidol University (Thailand), Health Systems Research Institute (Thailand), Ministry of Public Health (Thailand), National Statistical Office (Thailand), Tobacco Control Research and Knowledge Management Center (Thailand), World Health Organization (WHO). Thailand Global Adult Tobacco Survey 2011. Atlanta, United States: Centers for Disease Control and Prevention (CDC).
Acute and chronic undernutrition in Swaziland as it appears in World Health Organization (WHO). WHO Global Database on Child Growth and Malnutrition. Geneva, Switzerland: World Health Organization (WHO).
Acute malnutrition and high childhood mortality related to diarrhea. Lessons from the 1991 Kurdish refugee crisis as it appears in World Health Organization (WHO). WHO Global Database on Child Growth and Malnutrition - Historical. Geneva, Switzerland: World Health Organization (WHO).
Adam Z. Iron Supplementation and Malaria: A Randomised, Placebo-controlled Field Trial in Rural Ethiopia [dissertation]. London, United Kingdom: London School of Hygiene and Tropical Medicine, 1996. As it appears in Malaria Atlas Project. Malaria Atlas Project Plasmodium Falciparum Parasite Rate Database. Oxford, United Kingdom: Malaria Atlas Project.
Adamo B, Stroffolini T, Sagliocca L, Simonetti A, Iadanza F, Fossi E, Tancredi F, Mele A. Ad hoc survey of hepatitis B vaccination campaign in newborns of HBsAg positive mothers and in 12-year-old subjects in southern Italy. Vaccine. 1998; 16(8): 775-7.
Adams RJ, Appleton S, Wilson DH, Taylor AW, Dal Grande E, Chittleborough C, Gill T, Ruffin R. Population comparison of two clinical approaches to the metabolic syndrome: implications of the new International Diabetes Federation consensus definition. Diabetes Care. 2005; 28(11): 2777-9.
Adane AA, Ayele TA, Ararsa LG, Bitew BD, Zeleke BM. Adverse birth outcomes among deliveries at Gondar University Hospital, Northwest Ethiopia. BMC Pregnancy Childbirth. 2014; 14: 90.
Adazu K, Lindblade KA, Rosen DH, Odhiambo F, Ofware P, Kwach J, Van Eijk AM, Decock KM, Amornkul P, Karanja D, Vulule JM, Slutsker L. Health and demographic surveillance in rural western Kenya: a platform for evaluating interventions to reduce morbidity and mortality from infectious diseases. Am J Trop Med Hyg. 2005; 73(6): 1151-8.
Addis Ababa University, Bill and Melinda Gates Foundation (BMGF), Central Statistical Agency (Ethiopia), Johns Hopkins Bloomberg School of Public Health, Ministry of Health (Ethiopia). Ethiopia Performance, Monitoring, and Accountability Survey, Round 1 2014. Baltimore, United States: Johns Hopkins Bloomberg School of Public Health.
Addis Ababa University, Bill and Melinda Gates Foundation (BMGF), Central Statistical Agency (Ethiopia), Johns Hopkins Bloomberg School of Public Health, Ministry of Health (Ethiopia). Ethiopia Performance, Monitoring, and Accountability Survey, Round 2 2014. Baltimore, United States: Johns Hopkins Bloomberg School of Public Health.
Addis Ababa University, Bill and Melinda Gates Foundation (BMGF), Central Statistical Agency (Ethiopia), Johns Hopkins Bloomberg School of Public Health, Ministry of Health (Ethiopia). Ethiopia Performance, Monitoring, and Accountability Survey, Round 3 2015. Baltimore, United States: Johns Hopkins Bloomberg School of Public Health.
Addis Ababa University, Ethiopian Public Health Association, Johns Hopkins University (JHU), Ministry of Health (Ethiopia). Ethiopia National Blindness and Low Vision Survey 2005-2006.
Addis Ababa University, Program for Appropriate Technology in Health (PATH), UmeÃ¥ University, World Health Organization (WHO). Ethiopia WHO Multi-country Study on Women's Health and Domestic Violence Against Women 2002.
Adebajo AO, Smith DJ, Hazleman BL, Wreghitt TG. Seroepidemiological associations between tuberculosis, malaria, hepatitis B, and AIDS in West Africa. J Med Virol. 1994; 42(4): 366-8.

Appendix: Citation List

Citation
Adegoke AA, Campbell M, Ogundeji MO, Lawoyin T, Thomson AM. Place of birth or place of death: An evaluation of 1139 maternal deaths in Nigeria. Midwifery. 2013; 29(11): e115-21.
Adegoke AA, Campbell M, Ogundeji MO, Lawoyin TO, Thomson AM. Community Study of Maternal Mortality in South West Nigeria: How Applicable is the Sisterhood Method. Matern Child Health J. 2013; 17(2): 319-29.
Adel A, Boughoufalah A, Saegerman C, De Deken R, Bouchene Z, Soukehal A, Berkvens D, Boelaert M. Epidemiology of visceral leishmaniasis in Algeria: an update. PLoS One. 2014; 9(6): e99207.
Adel ET, Marie-FranÃ§oise R-C, Mahmud Salaheddin M, Najeeb E, Ahmed AM, Ibrahim B, Gerard L. Nutritional status of under-five children in libya; a national population-based survey. Libyan J Med. 2008; 3(1): 13-9.
Adeleke SI, Asani MO, Belonwu RO, Gwarzo GD, Farouk ZL. Childhood diabetes mellitus in Kano, North West, Nigeria. Niger J Med. 2010; 19(2): 145-7.
Ademowo OG, Falusi AG, Mewoyeka OO. Prevalence of asymptomatic parasitaemia in an urban and rural community in south western Nigeria. Cent Afr J Med. 1995; 41(1): 18-21. As it appears in Malaria Atlas Project. Malaria Atlas Project Plasmodium Falciparum Parasite Rate Database. Oxford, United Kingdom: Malaria Atlas Project.
Adhikari P, Haldar JP. Prevalence of bancroftian filariasis in Burdwan district, West Bengal: II. Vector and microfilariae density in colliery and non-colliery areas. J Commun Dis. 1995; 27(3): 181-5.
Adhikari R, Bhusal K. Surveillance of lymphatic filariasis in selected districts of Nepal. J Inst Med. 2008; 35-40. As it appears in London School of Hygiene and Tropical Medicine. Global Atlas of Helminth Infections - Lymphatic Filiariasis.
Adimora GN, Odetunde IO. Perinatal mortality in University of Nigeria Teaching Hospital (UNTH) Enugu at the end of the last millennium. Niger J Clin Pract. 2007; 10(1): 19-23.
Adish AA, Esrey SA, Gyorkos TW, Johns T. Risk factors for iron deficiency anaemia in preschool children in northern Ethiopia. Public Health Nutr. 1999; 2(3): 243-52. As it appears in Malaria Atlas Project. Malaria Atlas Project Plasmodium Falciparum Parasite Rate Database. Oxford, United Kingdom: Malaria Atlas Project.
Aditama TY. Prevalence of tuberculosis in Indonesia, Singapore, Brunei Darussalam and the Philippines. Tubercle. 1991; 72(4): 255-60.
Adja AM. Mission Report of the Entomological and Parasitological Survey from July 2002 in Soubré, Southwest of Côte d'Ivoire. Bouaké, Côte d'Ivoire: Pierre-Richet Institute, 2002. As it appears in Malaria Atlas Project. Malaria Atlas Project Plasmodium Falciparum Parasite Rate Database. Oxford, United Kingdom: Malaria Atlas Project.
Administrative Department of Science, Techonology, and Innovation (Colombia), Center for Development Projects, Pontifical Xavierian University, Ministry of Social Protection (Colombia), Specialized Information Systems. Colombia National Health Survey 2007-2008.
Adungo. Kenya Plasmodium Falciparum Parasite Rate Data, Adungo 1992. As it appears in Malaria Atlas Project. Malaria Atlas Project Plasmodium Falciparum Parasite Rate Database. Oxford, United Kingdom: Malaria Atlas Project.
Adventist Development and Relief Agency (ADRA), Azerbaijan Ministry of Health, State Statistical Committee of Azerbaijan, and Centers for Disease Control and Prevention (CDC). (2003) Azerbaijan Reproductive Health Survey 2001. Atlanta, United States: Centers for Disease Control and Prevention (CDC).
Aekplakorn W, Kongsakon R. Intimate partner violence among women in slum communities in Bangkok, Thailand. Singapore Med J. 2007; 48(8): 763-8.
Ae-Ngibise KA, Masanja H, Kellerman R, Owusu-Agyei S. Risk factors for injury mortality in rural Tanzania: a secondary data analysis. BMJ Open. 2012; 2(6): e001721.
Afari E. In Vivo and In Vitro Plasmodium Falciparum Sensitivity to Chloroquine and In Vitro Response of Plasmodium Falciparum to Amodiaquine, Quinine and Sulfadoxine/pyrimethamine. Legon, Ghana: Epidemiology Unit, Noguchi Memorial Institute for Medical Research, 1988. As it appears in Malaria Atlas Project. Malaria Atlas Project Plasmodium Falciparum Parasite Rate Database. Oxford, United Kingdom: Malaria Atlas Project.
Afari EA, Akanmori BD, Nakano T, Ofori-Adjei D. Plasmodium falciparum: sensitivity to chloroquine in vivo in three ecological zones in Ghana. Trans R Soc Trop Med Hyg. 1992; 86(3): 231-2. As it appears in Malaria Atlas Project. Malaria Atlas Project Plasmodium Falciparum Parasite Rate Database. Oxford, United Kingdom: Malaria Atlas Project.
Afari EA, Appawu M, Dunyo S, Baffoe-Wilmot A, Nkrumah FK. Malaria infection, morbidity and transmission in two ecological zones Southern Ghana. Afr J Health Sci. 1995; 2(2): 312-5. As it appears in Malaria Atlas Project. Malaria Atlas Project Plasmodium Falciparum Parasite Rate Database. Oxford, United Kingdom: Malaria Atlas Project.
Afari EA, Dunyo S, Appawu M, Nkrumah FK. In vivo seasonal assessment of Plasmodium falciparum sensitivity to chloroquine in two different malaria endemic communities in Southern Ghana. Afr J Health Sci. 1994; 1(3): 112-5. As it appears in Malaria Atlas Project. Malaria Atlas Project Plasmodium Falciparum Parasite Rate Database. Oxford, United Kingdom: Malaria Atlas Project.
Afghanistan - Panjshir Valley and Shamali Plains Nutritional and Mortality Survey 2002 as it appears in World Health Organization (WHO). WHO Global Database on Child Growth and Malnutrition - Historical. Geneva, Switzerland: World Health Organization (WHO).
Afghanistan - Badakshan Children Under-5 Nutrition Survey 2000 as it appears in World Health Organization (WHO). WHO Global Database on Child Growth and Malnutrition - Historical. Geneva, Switzerland: World Health Organization (WHO).
Afghanistan - Faizabad City Nutritional and Mortality Survey 2000 as it appears in World Health Organization (WHO). WHO Global Database on Child Growth and Malnutrition - Historical. Geneva, Switzerland: World Health Organization (WHO).
Afghanistan - Herat City Nutritional and Mortality Survey 2000 as it appears in World Health Organization (WHO). WHO Global Database on Child Growth and Malnutrition - Historical. Geneva, Switzerland: World Health Organization (WHO).
Afghanistan - Jalalabad City and Internally Displaced People Camps Comparative Malnutrition Survey 1996 as it appears in World Health Organization (WHO). WHO Global Database on Child Growth and Malnutrition - Historical. Geneva, Switzerland: World Health Organization (WHO).
Afghanistan - Jalalabad City Nutritional and Mortality Survey 2000 as it appears in World Health Organization (WHO). WHO Global Database on Child Growth and Malnutrition - Historical. Geneva, Switzerland: World Health Organization (WHO).

Appendix: Citation List

Citation
Afghanistan - Kabul Baseline Survey on the Nutritional Status of Children Under-5 1990 as it appears in World Health Organization (WHO). WHO Global Database on Child Growth and Malnutrition - Historical. Geneva, Switzerland: World Health Organization (WHO).
Afghanistan - Kabul City Nutritional and Mortality Survey 2000 as it appears in World Health Organization (WHO). WHO Global Database on Child Growth and Malnutrition - Historical. Geneva, Switzerland: World Health Organization (WHO).
Afghanistan - Kabul City Nutritional and Mortality Survey 2001 as it appears in World Health Organization (WHO). WHO Global Database on Child Growth and Malnutrition - Historical. Geneva, Switzerland: World Health Organization (WHO).
Afghanistan - Kabul Nutrition Survey 2000 as it appears in World Health Organization (WHO). WHO Global Database on Child Growth and Malnutrition - Historical. Geneva, Switzerland: World Health Organization (WHO).
Afghanistan - Kohistan District Nutritional and Food Security Survey 2002 as it appears in World Health Organization (WHO). WHO Global Database on Child Growth and Malnutrition - Historical. Geneva, Switzerland: World Health Organization (WHO).
Afghanistan - Laghman Nutritional Survey 1999 as it appears in World Health Organization (WHO). WHO Global Database on Child Growth and Malnutrition - Historical. Geneva, Switzerland: World Health Organization (WHO).
Afghanistan - Mazar-e Sharif City Nutritional and Mortality Survey 2000 as it appears in World Health Organization (WHO). WHO Global Database on Child Growth and Malnutrition - Historical. Geneva, Switzerland: World Health Organization (WHO).
Afghanistan - Panjshir Valley and Shamali Plains Nutritional Survey 2001 as it appears in World Health Organization (WHO). WHO Global Database on Child Growth and Malnutrition - Historical. Geneva, Switzerland: World Health Organization (WHO).
Afghanistan - Samangan Nutrition Survey 2002 as it appears in World Health Organization (WHO). WHO Global Database on Child Growth and Malnutrition - Historical. Geneva, Switzerland: World Health Organization (WHO).
Afghanistan - Sang Charak District Nutritional Anthropometric Survey in Children Under-5 2002 as it appears in World Health Organization (WHO). WHO Global Database on Child Growth and Malnutrition - Historical. Geneva, Switzerland: World Health Organization (WHO).
Afghanistan National Risk and Vulnerability Assessment 2005 as it appears in World Health Organization (WHO). WHO Household Energy Database. Geneva, Switzerland: World Health Organization (WHO), 2010.
Afghanistan National Survey of EPI Coverage 1999
Afghanistan Nutrition Survey 1996 as it appears in World Health Organization (WHO). WHO Global Database on Child Growth and Malnutrition - Historical. Geneva, Switzerland: World Health Organization (WHO).
Afghanistan Nutritional Anthropometry, Health, Food Security, and Agriculture Assessment 2001 as it appears in World Health Organization (WHO). WHO Global Database on Child Growth and Malnutrition - Historical. Geneva, Switzerland: World Health Organization (WHO).
Aflatoonian MR, Sharifi I, Hakimi Parizi M, Fekri AR, Aflatoonian B, Sharifi M, Khosravi A, Khamesipour A, Sharifi H. A prospective cohort study of cutaneous leishmaniasis risk and opium addiction in south eastern Iran. PLoS One. 2014; 9(2): e89043.
African Pest and Environment Management Foundation. Uganda Plasmodium Falciparum Parasite Rate Data, African Pest and Environment Management Foundation 1994. As it appears in Malaria Atlas Project. Malaria Atlas Project Plasmodium Falciparum Parasite Rate Database. Oxford, United Kingdom: Malaria Atlas Project.
African Population and Health Research Center, INDEPTH. Kenya - Nairobi Urban Health and Demographic Surveillance System.
Aga Khan University, All India Institute of Medical Sciences, Centre for Chronic Disease Control (India), Emory University, Madras Diabetes Research Foundation, National Institute of Mental Health and Neuro Sciences (India), Public Health Foundation of India. Center for Cardio-Metabolic Risk Reduction in South Asia Study 2010-2011. [Unpublished].
Aga Khan University, Central Statistics Organization (Afghanistan), Ministry of Public Health (Afghanistan), United Nations Children's Fund (UNICEF). Afghanistan National Nutrition Survey 2013.
Aga Khan University, Ministry of National Health Services, Regulations & Coordination (Pakistan), Ministry of Planning and Development (Pakistan), Pakistan Medical Research Council, United Nations Children's Fund (UNICEF). Pakistan National Nutrition Survey 2011.
AGB Attwood, Ministry of Agriculture, Nature Management, and Fisheries (Netherlands), Ministry of Welfare, Health, and Culture (Netherlands), TNO Nutrition. Netherlands Dutch National Food Consumption Survey 1987-1988.
AGB Fresh Foods, Ministry of Agriculture, Nature Management, and Fisheries (Netherlands), TNO Nutrition. Netherlands Dutch National Food Consumption Survey 1992.
Agbo K, Gayibor HA, Kessie K, Gnamey K. Anticorps et densite parasitaire chez les enfants de 0-3 ans sous chimioprophylaxie antipalustre a Korbongou (Nord-Togo). Afrique. Medicale. 1990; 29(284): 196-8. As it appears in Malaria Atlas Project. Malaria Atlas Project Plasmodium Falciparum Parasite Rate Database. Oxford, United Kingdom: Malaria Atlas Project.
Agdamag DM, Kageyama S, Alesna ET, Solante RM, Leaño PS, Heredia AML, Abellanosa-Tac-An IP, Vibal ET, Jereza LD, Ichimura H. Rapid spread of hepatitis C virus among injecting-drug users in the Philippines: Implications for HIV epidemics. J Med Virol. 2005; 77(2): 221-6.
Agency for Statistics (Bosnia and Herzegovina), Birks Sinclair and Associates, LTD, Federal Office of Statistics (Federation of Bosnia and Herzegovina), Independent Bureau for Humanitarian Issues (IBHI), Institute for Social and Economic Research, University of Essex, Institute of Statistics (Republic of Srpska). Bosnia and Herzegovina Living Standards Measurement Survey 2004-2005.
Agency for Statistics (Bosnia and Herzegovina), Federal Ministry of Health (Bosnia and Herzegovina), Ministry of Health and Social Welfare (Republic of Srpska), Public Health Institute of Federation of Bosnia and Herzegovina, United Nations Children's Fund (UNICEF), United Nations Entity for Gender Equality and the Empowerment of Women (UN Women). Bosnia and Herzegovina Multiple Indicator Cluster Survey 2011-2012.
Agency for Statistics (Bosnia and Herzegovina), Institute of Statistics (Republic of Srpska), Federal Office of Statistics (Bosnia and Herzegovina), Independent Bureau for Humanitarian Issues (IBHI), Birks Sinclair and Associates, LTD, Institute for Social and Economic Research, University of Essex. Bosnia and Herzegovina Living Standards Measurement Survey 2002. Washington, DC, United States: World Bank (WB).

Appendix: Citation List

Citation
Agency for Statistics (Bosnia and Herzegovina), Institute of Statistics (Republic of Srpska), Federal Office of Statistics (Bosnia and Herzegovina), Independent Bureau for Humanitarian Issues (IBHI), Birks Sinclair and Associates, LTD, Institute for Social and Economic Research, University of Essex. Bosnia and Herzegovina Living Standards Measurement Survey 2003. Washington, DC, United States: World Bank (WB).
Agency for Statistics (Bosnia and Herzegovina), Institute of Statistics (Republic of Srpska), Federal Office of Statistics (Bosnia and Herzegovina), Swedish International Development Agency (SIDA), UK Department for International Development (DFID), United Nations Development Programme (UNDP), European Commission (EC), Government of Japan, World Bank (WB). Bosnia and Herzegovina Living Standards Measurement Survey 2001. Washington, DC, United States: World Bank (WB).
Agency for Statistics (Bosnia and Herzegovina), Ministry of Health (Federation of Bosnia and Herzegovina), Ministry of Health and Social Welfare (Republic of Srpska), United Nations Children's Fund (UNICEF). Bosnia and Herzegovina Multiple Indicator Cluster Survey 2000. New York, United States: United Nations Children's Fund (UNICEF).
Agency of the Republic of Kazakhstan on Statistics and United Nations Children's Fund (UNICEF). Kazakhstan Multiple Indicator Cluster Survey 2006. New York, United States: United Nations Children's Fund (UNICEF).
Agency of the Republic of Kazakhstan on Statistics, United Nations Children's Fund (UNICEF). Kazakhstan Multiple Indicator Cluster Survey 2010-2011. New York, United States: United Nations Children's Fund (UNICEF), 2013.
Agency of the Republic of Kazakhstan on Statistics, World Bank. Kazakhstan Living Standards Measurement Survey 1996. Washington DC, United States: World Bank.
Agency of the Republic of Kazakhstan on Statistics. Kazakhstan Census 2009.
Agency on Statistics Under the President of the Republic of Tajikistan, European Commission (EC), Federal Statistical Office (Germany), ICON-INSTITUTE Consulting Group. Tajikistan Survey on Infant, Child, and Maternal Mortality 2010.
Aggarwal AN, Gupta D, Agarwal R, Sethi S, Thakur JS, Anjinappa SM, Chadha VK, Kumar R, Sharma M, Behera D, Jindal SK. Prevalence of pulmonary tuberculosis among adults in a north Indian district. PLoS One. 2015; 10(2): e0117363.
Agi PI, Ebenezer A. Observations on Filarial Infection in Amassoma Community in the Niger Delta, Nigeria. Niger J Appl Sci Environ Manag. 2009; 13(1). As it appears in London School of Hygiene and Tropical Medicine. Global Atlas of Helminth Infections - Lymphatic Filiariasis.
Agnamey PP, Leroy G, Kouamouo J, Brasseur P. [Sensitivity in vivo and in vitro of Plasmodium falciparum to chloroquine and amodiaquine in Bangangte (west Cameroon)]. Bull Soc Pathol Exot. 1995; 88(4): 149-51. As it appears in Malaria Atlas Project. Malaria Atlas Project Plasmodium Falciparum Parasite Rate Database. Oxford, United Kingdom: Malaria Atlas Project.
Agoh C. Contribution to the Evaluation of the Sensitivity of Plasmodium falciparum to Chloroquine in Greater Abidjan (Côte d'Ivoire). Abidjan, Côte d'Ivoire: Faculty of Pharmacy, National University of Côte d'Ivoire, 1994. As it appears in Malaria Atlas Project. Malaria Atlas Project Plasmodium Falciparum Parasite Rate Database. Oxford, United Kingdom: Malaria Atlas Project.
Agrawal RP, Ola V, Bishnoi P, Gothwal S, Sirohi P, Agrawal R. Prevalence of micro and macrovascular complications and their risk factors in type-2 diabetes mellitus. J Assoc Physicians India. 2014; 62(6): 504–8.
Aguiar-Santos AM, Medeiros Z, Bonfim C, Rocha AC, Brandão E, Miranda T, Oliveira P, Sarinho ESC. Epidemiological assessment of neglected diseases in children: lymphatic filariasis and soil-transmitted helminthiasis. J Pediatr (Rio J). 2013; 89(3): 250-5.
Aguilar V HM, Abad-Franch F, Racines V J, Paucar C A. Epidemiology of Chagas disease in Ecuador. A brief review. Mem Inst Oswaldo Cruz. 1999; 94(Suppl 1): 387-93.
Aguilar-Salinas CA, Rojas R, Gómez-Pérez FJ, García E, Valles V, Ríos-Torres JM, Franco A, Olaiz G, Sepúlveda J, Rull JA. Prevalence and characteristics of early-onset type 2 diabetes in Mexico. Am J Med. 2002; 113(7): 569-74.
Ahmad AF, Ngui R, Muhammad Aidil R, Lim YA, Rohela M. Current status of parasitic infections among Pangkor Island community in Peninsular Malaysia. Trop Biomed. 2014; 31.0(4): 836-43.
Ahmad K, Khan MD, Qureshi MB, Munami S, Shah RA, Rasheed H, Jamali B, Baluch A, Khan MA. Prevalence and causes of blindness and low vision in a rural setting in Pakistan. Ophthalmic Epidemiol. 2005; 12(1): 19-23.
Ahmad Z. Maternal mortality in an obstetric unit. J Pak Med Assoc. 1985; 35(8): 243-8.
Ahmadi NA, Hamidi M. A retrospective analysis of human cystic echinococcosis in Hamedan province, an endemic region of Iran. Ann Trop Med Parasitol. 2008; 102(7): 603-9.
Ahmed F, Waslien C, Al-Sumaie MA, Prakash P, Allafi A. Trends and risk factors of hyperglycemia and diabetes among Kuwaiti adults: National Nutrition Surveillance Data from 2002 to 2009. BMC Public Health. 2013; 103.
Ahmed K, Muhammad Z, Qayum I. Prevalence of cutaneous manifestations of diabetes mellitus. J Ayub Med Coll Abbottabad. 2009; 21(2): 76-9.
Ahti TM, MÃ¤kivaara LA, Luukkaala T, Hakama M, Laurikka JO. Lifestyle factors and varicose veins: does cross-sectional design result in underestimate of the risk?. Phlebology. 2010; 25(4): 201-6.
Aichi Cancer Center Research Institute. Japan Aichi Cancer Registry Report 2006.
Aimoni C, Bianchini C, Borin M, Ciorba A, Fellin R, Martini A, Scanelli G, Volpato S. Diabetes, cardiovascular risk factors and idiopathic sudden sensorineural hearing loss: a case-control study. Audiol Neurootol. 2010; 15(2): 111-5.
Ajdukovic M, Ogresta J, Rusac S. Family Violence and Health Among Elderly in Croatia. J Aggress Maltreat Trauma. 2009; 18(3): 261-79.
Ajero CMU, Nwoke BEB, Nwanjo HU, Oze G, Okafor MC, Nwosu D, Anyaehie B, Uloneme GC. Bancroftian Filariasis in the Niger Delta Area of Eastern Nigeria. Res J Med Sci. 2007; 1(2): 113-7. As it appears in London School of Hygiene and Tropical Medicine. Global Atlas of Helminth Infections - Lymphatic Filiariasis.
Ajlouni K, Jaddou H, Batieha A. Diabetes and impaired glucose tolerance in Jordan: prevalence and associated risk factors. J Intern Med. 1998; 244(4): 317-23.
Ajlouni K, Jaddou H, Batieha A. Obesity in Jordan. Int J Obes Relat Metab Disord. 1998; 22(7): 624-8.
Akbar N, Basuki B, Mulyanto, Garabrant DH, Sulaiman A, Noer HM. Ethnicity, socioeconomic status, transfusions and risk of hepatitis B and hepatitis C infection. J Gastroenterol Hepatol. 1997; 12(11): 752-7.

Appendix: Citation List

Citation
Akbar SM, Onji M, Kanaoka M, Horiike N, Michitaka K, Masumoto T, Nonaka T, Kanda K, Kajino K, Kumamoto I. The seroepidemiology of hepatitis A and B in a Japanese town. Asia Pac J Public Health. 1992; 6(2): 26-9.
Akcam FZ, Uskun E, Avsar K, Songur Y. Hepatitis B virus and hepatitis C virus seroprevalence in rural areas of the southwestern region of Turkey. Int J Infect Dis. 2009; 13(2): 274-84.
Akenji TN, Ntonifor NN, Kimbi HK, Abongwa EL, Ching JK, Ndukum MB, Anong DN, Nkwescheu A, Songmbe M, Boyo MG, Ndamukong KN, Titanji VPK. The epidemiology of malaria in Bolifamba, a rural community on the eastern slopes of Mount Cameroon: seasonal variation in the parasitological indices of transmission. Ann Trop Med Parasitol. 2005; 99(3): 221-7. As it appears in Malaria Atlas Project. Malaria Atlas Project Plasmodium Falciparum Parasite Rate Database. Oxford, United Kingdom: Malaria Atlas Project.
Akgün S, Çolak M, Bakar C. Identifying and verifying causes of death in Turkey: National verbal autopsy survey. Public Health. 2012; 126(2): 150-8.
Akhwale WS, Lum JK, Kaneko A, Eto H, Obonyo C, Björkman A, Kobayakawa T. Anemia and malaria at different altitudes in the western highlands of Kenya. Acta Trop. 2004; 91(2): 167-75. As it appears in Malaria Atlas Project. Malaria Atlas Project Plasmodium Falciparum Parasite Rate Database. Oxford, United Kingdom: Malaria Atlas Project.
Akogbeto M, Modiano D, Bosman A. Malaria transmission in the lagoon area of Cotonou, Benin. Parassitologia. 1992; 34(1-3): 147-54. As it appears in Malaria Atlas Project. Malaria Atlas Project Plasmodium Falciparum Parasite Rate Database. Oxford, United Kingdom: Malaria Atlas Project.
Akogun OB. Filariasis in Gongola State Nigeria. I: Clinical and parasitological studies in Mutum-Biyu District. Angew Parasitol. 1992; 33(3): 125-31. As it appears in London School of Hygiene and Tropical Medicine. Global Atlas of Helminth Infections - Lymphatic Filiariasis.
Akpogheneta OJ, Duah NO, Tetteh KKA, Dunyo S, Lanar DE, Pinder M, Conway DJ. Duration of naturally acquired antibody responses to blood-stage Plasmodium falciparum is age dependent and antigen specific. Infect Immun. 2008; 76(4): 1748-55. As it appears in Malaria Atlas Project. Malaria Atlas Project Plasmodium Falciparum Parasite Rate Database. Oxford, United Kingdom: Malaria Atlas Project.
Akter S, Rahman MM, Abe SK, Sultana P. Prevalence of diabetes and prediabetes and their risk factors among Bangladeshi adults: a nationwide survey. Bull World Health Organ. 2014; 92(3): 204–13.
Al Aboud F, Al Aboud K. Leprosy in Saudi Arabia. Lepr Rev. 2007; 78(4): 405-8.
Al Rakaf MS, Kurdi AM, Ammari AN, Al Hashem AM, Shoukri MM, Garne E, Majeed-Saidan MA. Patterns of folic acid use in pregnant Saudi women and prevalence of neural tube defects - Results from a nested case-control study. Prev Med Rep. 2015; 2: 572–6.
Al Riyami AA, Afifi M. Smoking in Oman: prevalence and characteristics of smokers. East Mediterr Health J. 2004; 10(4-5): 600â€“9.
Al Zenki S, Al Omirah H, Al Hooti S, Al Hamad N, Jackson RT, Rao A, Al Jahmah N, Al Obaid I, Al Ghanim J, Al Somaie M, Zaghloul S, Al Othman A. High prevalence of metabolic syndrome among Kuwaiti adults--a wake-up call for public health intervention. Int J Environ Res Public Health. 2012; 9(5): 1984-96.
Alabi O, Doctor HV, Jumare A, Sahabi N, Abdulwahab A, Findley SE, Abubakar SD. Health & demographic surveillance system profile: the Nahuche Health and Demographic Surveillance System, Northern Nigeria (Nahuche HDSS). Int J Epidemiol. 2014; 43(6): 1770-80.
Alarouj M, Bennakhi A, Alnesef Y, Sharifi M, Elkum N. Diabetes and associated cardiovascular risk factors in the State of Kuwait: the first national survey. Int J Clin Pract. 2013; 67(1): 89-96.
Alauddin M. Maternal mortality in rural Bangladesh: the Tangail District. Stud Fam Plann. 1986; 17(1): 13-21.
Alavi A, Sanjari M, Haghdoost A, Sibbald RG. Common foot examination features of 247 Iranian patients with diabetes. Int Wound J. 2009; 6(2): 117-22.
Alavian SM, Tabatabaei SV, Nourizad S, Mansouri F, Khademi N, Amini Kafi-abad S, Gharehbaghian A, Abolghasemi H. Seroepidemiology of HBV Infection in Kermanshah- West of Iran; a Population Based Study. Jundishapur J Microbiol. 2012; 5(4): 564-9.
Alavinia S, Arzamani K, Reihani M, Jafari J. Some epidemiological aspects of cutaneous leishmaniasis in northern Khorasan province, Iran. Iran J Arthropod Borne Dis. 2009; 3(2): 50-4.
Al-Baghli NA, Al-Ghamdi AJ, Al-Turki KA, Al Elq AH, El-Zubaier AG, Bahnassy A. Prevalence of diabetes mellitus and impaired fasting glucose levels in the Eastern Province of Saudi Arabia: results of a screening campaign. Singapore Med J. 2010; 51(12): 923-30.
Al-Baghli NA, Al-Ghamdi AJ, Al-Turki KA, El-Zubaier AG, Al-Ameer MM, Al-Baghli FA. Overweight and obesity in the eastern province of Saudi Arabia. Saudi Med J. 2008; 29(9): 1319-25.
Albania Institute of Public Health (IPH), Ministry of Health (Albania), National Institute of Statistics (Albania), and Centers for Disease Control and Prevention. (2005) Albania Reproductive Health Survey 2001. Atlanta, United States: Centers for Disease Control and Prevention (CDC).
Albania Institute of Statistics (INSTAT). Albania Vital Registration - Causes of Death 1993-2010. Albania: Albania Institute of Statistics (INSTAT).
Albania National Nutrition Survey 1996-1998 as it appears in World Health Organization (WHO). WHO Global Database on Child Growth and Malnutrition. Geneva, Switzerland: World Health Organization (WHO).
Albania Vital Registration - Deaths 1987 ICD9 as it appears in World Health Organization (WHO). WHO Mortality Database Version July 2012. Geneva, Switzerland: World Health Organization (WHO), 2012.
Albania Vital Registration - Deaths 1988 ICD9 as it appears in World Health Organization (WHO). WHO Mortality Database Version July 2012. Geneva, Switzerland: World Health Organization (WHO), 2012.
Albania Vital Registration - Deaths 1989 ICD9 as it appears in World Health Organization (WHO). WHO Mortality Database Version July 2012. Geneva, Switzerland: World Health Organization (WHO), 2012.
Albania Vital Registration - Deaths 1992 ICD9 as it appears in World Health Organization (WHO). WHO Mortality Database Version November 2015. Geneva, Switzerland: World Health Organization (WHO), 2015.
Albania Vital Registration - Deaths 1993 ICD9 as it appears in World Health Organization (WHO). WHO Mortality Database Version November 2015. Geneva, Switzerland: World Health Organization (WHO), 2015.
Albania Vital Registration - Deaths 1994 ICD9 as it appears in World Health Organization (WHO). WHO Mortality Database Version November 2015. Geneva, Switzerland: World Health Organization (WHO), 2015.

Appendix: Citation List

Citation
Albania Vital Registration - Deaths 1995 ICD9 as it appears in World Health Organization (WHO). WHO Mortality Database Version November 2015. Geneva, Switzerland: World Health Organization (WHO), 2015.
Albania Vital Registration - Deaths 1996 ICD9 as it appears in World Health Organization (WHO). WHO Mortality Database Version November 2015. Geneva, Switzerland: World Health Organization (WHO), 2015.
Albania Vital Registration - Deaths 1997 ICD9 as it appears in World Health Organization (WHO). WHO Mortality Database Version November 2015. Geneva, Switzerland: World Health Organization (WHO), 2015.
Albania Vital Registration - Deaths 1998 ICD9 as it appears in World Health Organization (WHO). WHO Mortality Database Version November 2015. Geneva, Switzerland: World Health Organization (WHO), 2015.
Albania Vital Registration - Deaths 1999 ICD9 as it appears in World Health Organization (WHO). WHO Mortality Database Version November 2015. Geneva, Switzerland: World Health Organization (WHO), 2015.
Albania Vital Registration - Deaths 2000 ICD9 as it appears in World Health Organization (WHO). WHO Mortality Database Version November 2015. Geneva, Switzerland: World Health Organization (WHO), 2015.
Albania Vital Registration - Deaths 2001 ICD9 as it appears in World Health Organization (WHO). WHO Mortality Database Version November 2015. Geneva, Switzerland: World Health Organization (WHO), 2015.
Albania Vital Registration - Deaths 2002 ICD9 as it appears in World Health Organization (WHO). WHO Mortality Database Version July 2012. Geneva, Switzerland: World Health Organization (WHO), 2012.
Albania Vital Registration - Deaths 2003 ICD9 as it appears in World Health Organization (WHO). WHO Mortality Database Version July 2012. Geneva, Switzerland: World Health Organization (WHO), 2012.
Albania Vital Registration - Deaths 2004 ICD9 as it appears in World Health Organization (WHO). WHO Mortality Database Version July 2012. Geneva, Switzerland: World Health Organization (WHO), 2012.
Albania Vital Registration - Deaths 2005 ICD9 as it appears in World Health Organization (WHO). WHO Mortality Database Version November 2015. Geneva, Switzerland: World Health Organization (WHO), 2015.
Albania Vital Registration - Deaths 2006 ICD9 as it appears in World Health Organization (WHO). WHO Mortality Database Version November 2015. Geneva, Switzerland: World Health Organization (WHO), 2015.
Albania Vital Registration - Deaths 2007 ICD9 as it appears in World Health Organization (WHO). WHO Mortality Database Version November 2015. Geneva, Switzerland: World Health Organization (WHO), 2015.
Albania Vital Registration - Deaths 2008 ICD9 as it appears in World Health Organization (WHO). WHO Mortality Database Version November 2015. Geneva, Switzerland: World Health Organization (WHO), 2015.
Albania Vital Registration - Deaths 2009 ICD9 as it appears in World Health Organization (WHO). WHO Mortality Database Version November 2015. Geneva, Switzerland: World Health Organization (WHO), 2015.
Albarracin-Veizaga H, de Carvalho ME, Nascimento EM, Rodrigues VL, Casanova C, Barata JM. Chagas disease in an area of recent occupation in Cochabamba, Bolivia. Rev Saude Publica. 1999; 33(3): 230–6.
Alberti K. A Malaria Assessment in Damot Gale Woreda, Wolayita Zone, Ethiopia. Paris, France: Epicentre, 2003. As it appears in Malaria Atlas Project. Malaria Atlas Project Plasmodium Falciparum Parasite Rate Database. Oxford, United Kingdom: Malaria Atlas Project.
Albin M, Jakobsson K, Attewell R, Johansson L, Welinder H. Mortality And Cancer Morbidity In Cohorts Of Asbestos Cement Workers And Referents. Br J Ind Med. 1990; 47(9): 602-10 as it appears in Lenters V, Vermeulen R, Dogger S, Stayner L, Portengen L, Burdorf A, Heederik D. A meta-analysis of asbestos and lung cancer: is better quality exposure assessment associated with steeper slopes of the exposure-response relationships?. Environ Health Perspect. 2011; 119(11): 1547-55.
Albuquerque LC, Mendonça IR, Cardoso PN, Baldaçara LR, Borges MR, Borges Jda C, Pranchevicius MC. HIV/AIDS-related visceral leishmaniasis: a clinical and epidemiological description of visceral leishmaniasis in northern Brazil. Rev Soc Bras Med Trop. 2014; 47(1): 38-46.
Albuquerque MF, Marzochi MC, Sabroza PC, Braga MC, Padilha T, Silva MC, Silva MR, Schindler HC, Maciel MA, Souza W. Bancroftian filariasis in two urban areas of Recife, Brazil: pre-control observations on infection and disease. Trans R Soc Trop Med Hyg. 1995; 89(4): 373-7. As it appears in London School of Hygiene and Tropical Medicine. Global Atlas of Helminth Infections - Lymphatic Filiariasis.
Alcohol and Drug Information Center (ADIC) (Sri Lanka). Sri Lanka Sentinel Tobacco Use Prevalence Survey 2001.
Alcohol and Drug Information Centre (ADIC) (Sri Lanka), National Institute of Social Development (NISD) (Sri Lanka). Sri Lanka Spot Survey on Alcohol and Tobacco Trends, July 2010.
Alcohol and Drug Information Centre (ADIC) (Sri Lanka), National Institute of Social Development (NISD) (Sri Lanka). Sri Lanka Spot Survey on Tobacco Trends, December 2012.
Alcohol and Drug Information Centre (ADIC) (Sri Lanka), National Institute of Social Development (NISD) (Sri Lanka). Sri Lanka Spot Survey on Tobacco Trends, July 2012.
Alcohol and Drug Information Centre (ADIC) (Ukraine), Institute of Cardiology (Ukraine). Tobacco or Health in Ukraine. Alcohol and Drug Information Centre (ADIC) (Ukraine), 1999.
Alcohol Research Group, Public Health Institute. India - Goa Alcohol Use Study 2004-2008.
Al-Daghri NM, Al-Attas OS, Alokail MS, Alkharfy KM, Yousef M, Sabico SL, Chrousos GP. Diabetes mellitus type 2 and other chronic non-communicable diseases in the central region, Saudi Arabia (Riyadh cohort 2): a decade of an epidemic. BMC Med. 2011; 76.
Al-Daghri NM, Al-Attas OS, Alokail MS, Alkharfy KM, Sabico SLB, Chrousos GP. Decreasing prevalence of the full metabolic syndrome but a persistently high prevalence of dyslipidemia among adult Arabs. PLoS One. 2010; 5(8): e12159.
Alemayehu T, Ye-ebiyo Y, Ghebreyesus TA, Witten KH, Bosman A, Teklehaimanot A. Malaria, schistosomiasis, and intestinal helminths in relation to microdams in Tigray, northern Ethiopia. Parassitologia. 1998; 40(3): 259-67. As it appears in Malaria Atlas Project. Malaria Atlas Project Plasmodium Falciparum Parasite Rate Database. Oxford, United Kingdom: Malaria Atlas Project.
Alemu A, Tsegaye W, Golassa L, Abebe G. Urban malaria and associated risk factors in Jimma town, south-west Ethiopia. Malar J. 2011; 10(1): 173. As it appears in Malaria Atlas Project. Malaria Atlas Project Plasmodium Falciparum Parasite Rate Database. Oxford, United Kingdom: Malaria Atlas Project.

Appendix: Citation List

Citation
Alexander ND. Wuchereria bancrofti infection and disease in a rural area of Papua New Guinea. P N G Med J. 2000; 43(3-4): 166-71.
Alexandre CO, Camargo LM, Mattei D, Ferreira MU, Katzin AM, Camargo EP, da Silva LH. Humoral immune response to the 72 kDa heat shock protein from Plasmodium falciparum in populations at hypoendemic areas of malaria in western Brazilian Amazon. Acta Trop. 1997; 64(3-4): 155-66. As it appears in Malaria Atlas Project. Malaria Atlas Project Plasmodium Falciparum Parasite Rate Database. Oxford, United Kingdom: Malaria Atlas Project.
al-Faleh FZ, Ramia S, Arif M, Ayoola EA, al-Rashed RS, al-Jeffry M, Hossain A, el-Hazmi M. Profile of hepatitis C virus and the possible modes of transmission of the virus in the Gizan area of Saudi Arabia: a community-based study. Ann Trop Med Parasitol. 1995; 89(4): 431-7.
Alger J, Andrade H, Pang L, Krogstad D. Recurrent Malaria Infection in Honduras. 970757. Honduras: UNDP/World Bank/WHO, 2001. As it appears in Malaria Atlas Project. Malaria Atlas Project Plasmodium Falciparum Parasite Rate Database. Oxford, United Kingdom: Malaria Atlas Project.
Algeria - Setif Cancer Registry 1986-1989 - CI5 as it appears in Parkin DM, International Agency for Research on Cancer, International Association of Cancer Registries. Cancer Incidence in Five Continents. Vol. I to VIII. Lyon, France, IARCPress, 2005.
Algeria - Setif Cancer Registry 1990-1993 - CI5 as it appears in Parkin DM, International Agency for Research on Cancer, International Association of Cancer Registries. Cancer Incidence in Five Continents. Vol. I to VIII. Lyon, France, IARCPress, 2005.
Algeria - Setif Cancer Registry 1998-2002 - CI5 as it appears in Curado MP, Edwards B, Shin HR, Storm H, Ferlay J, Heanue M and Boyle P, eds (2007). Cancer Incidence in Five Continents, Vol. IX Periodic Data (electronic version). Lyon, IARC.http://ci5.iarc.fr
Algeria - Setif Cancer Registry 2003-2007 - CI5 as it appears in Forman D, Bray F, Brewster DH, Gombe Mbalawa C, Kohler B, Piñeros M, Steliarova-Foucher E, Swaminathan R and Ferlay J, eds (2013). Cancer Incidence in Five Continents, Vol. X Summary Database (electronic version). Lyon, IARC. http://ci5.iarc.fr
Algeria - Tindouf Nutrition Survey 2002 as it appears in World Health Organization (WHO). WHO Global Database on Child Growth and Malnutrition - Historical. Geneva, Switzerland: World Health Organization (WHO).
Algeria Maternal and Child Health Survey 1992 as it appears in World Health Organization (WHO). WHO Global Database on Child Growth and Malnutrition. Geneva, Switzerland: World Health Organization (WHO).
Algeria Multiple Indicator Cluster Survey 1995 as it appears in World Health Organization (WHO). WHO Global Database on Child Growth and Malnutrition - Historical. Geneva, Switzerland: World Health Organization (WHO).
Algeria Multiple Indicator Cluster Survey 2000 as it appears in World Health Organization (WHO). WHO Global Database on Child Growth and Malnutrition. Geneva, Switzerland: World Health Organization (WHO).
Algeria Multiple Indicator Cluster Survey 2006 as it appears in World Health Organization (WHO). WHO Global Database on Child Growth and Malnutrition. Geneva, Switzerland: World Health Organization (WHO).
Algeria Multiple Indicator Cluster Survey 2006 as it appears in World Health Organization (WHO). WHO Household Energy Database. Geneva, Switzerland: World Health Organization (WHO), 2010.
Algeria National Immunization Coverage Survey 1986.
Algeria National Immunization Coverage Survey 1987.
Algeria National Immunization Coverage Survey 1989.
Algeria Strategies to Fight Anemia and Growth Retardation in Saharawi Refugee Children as it appears in World Health Organization (WHO). WHO Global Database on Child Growth and Malnutrition - Historical. Geneva, Switzerland: World Health Organization (WHO).
Algeria Tumour Registry of Algiers 1993-1997 - CI5 as it appears in Parkin DM, International Agency for Research on Cancer, International Association of Cancer Registries. Cancer Incidence in Five Continents. Vol. I to VIII. Lyon, France, IARCPress, 2005.
Algeria Vital Registration Death Data 1950 as it appears in United Nations Statistics Division (UNSD). United Nations Demographic Yearbook - Historical Supplement 1997. New York City, United States: United Nations Statistics Division (UNSD).
Algeria Vital Registration Death Data 1951 as it appears in United Nations Statistics Division (UNSD). United Nations Demographic Yearbook - Historical Supplement 1997. New York City, United States: United Nations Statistics Division (UNSD).
Algeria Vital Registration Death Data 1952 as it appears in United Nations Statistics Division (UNSD). United Nations Demographic Yearbook - Historical Supplement 1997. New York City, United States: United Nations Statistics Division (UNSD).
Algeria Vital Registration Death Data 1953 as it appears in United Nations Statistics Division (UNSD). United Nations Demographic Yearbook - Historical Supplement 1997. New York City, United States: United Nations Statistics Division (UNSD).
Algeria Vital Registration Death Data 1954 as it appears in United Nations Statistics Division (UNSD). United Nations Demographic Yearbook - Historical Supplement 1997. New York City, United States: United Nations Statistics Division (UNSD).
Algeria Vital Registration Death Data 1955 as it appears in United Nations Statistics Division (UNSD). United Nations Demographic Yearbook - Historical Supplement 1997. New York City, United States: United Nations Statistics Division (UNSD).
Algeria Vital Registration Death Data 1956 as it appears in United Nations Statistics Division (UNSD). United Nations Demographic Yearbook - Historical Supplement 1997. New York City, United States: United Nations Statistics Division (UNSD).
Algeria Vital Registration Death Data 1964 as it appears in United Nations Statistics Division (UNSD). United Nations Demographic Yearbook - Historical Supplement 1997. New York City, United States: United Nations Statistics Division (UNSD).
Algeria Vital Registration Death Data 1965 as it appears in United Nations Statistics Division (UNSD). United Nations Demographic Yearbook - Historical Supplement 1997. New York City, United States: United Nations Statistics Division (UNSD).
Algeria Vital Registration Death Data 1980 as it appears in United Nations Statistics Division (UNSD). United Nations Demographic Yearbook - Historical Supplement 1997. New York City, United States: United Nations Statistics Division (UNSD).
Algeria Vital Registration Death Data 1981 as it appears in United Nations Statistics Division (UNSD). United Nations Demographic Yearbook - Historical Supplement 1997. New York City, United States: United Nations Statistics Division (UNSD).
Algeria Vital Registration Death Data 1982 as it appears in United Nations Statistics Division (UNSD). United Nations Demographic Yearbook - Historical Supplement 1997. New York City, United States: United Nations Statistics Division (UNSD).
Algeria Vital Registration Death Data 1985 as it appears in United Nations Statistics Division (UNSD). United Nations Demographic Yearbook - Historical Supplement 1997. New York City, United States: United Nations Statistics Division (UNSD).

Appendix: Citation List

Citation
Algeria Vital Registration Death Data 1986 as it appears in United Nations Statistics Division (UNSD). United Nations Demographic Yearbook - Historical Supplement 1997. New York City, United States: United Nations Statistics Division (UNSD).
Algeria Vital Registration Death Data 1998 as it appears in United Nations Statistics Division (UNSD). United Nations Demographic Yearbook 2000. New York City, United States: United Nations Statistics Division (UNSD), 2002.
Al-Habori M, Al-Mamari M, Al-Meeri A. Type II diabetes mellitus and impaired glucose tolerance in Yemen: prevalence, associated metabolic changes and risk factors. Diabetes Res Clin Pract. 2004; 65(3): 275-81.
Al-Herbish AS, El-Mouzan MI, Al-Salloum AA, Al-Qurachi MM, Al-Omar AA. Prevalence of type 1 diabetes mellitus in Saudi Arabian children and adolescents. Saudi Med J. 2008; 29(9): 1285-8.
Ali AA, Yassin K, Omer R. Domestic violence against women in Eastern Sudan. BMC Public Health. 2014; 14: 1136.
Ali AAA, Adam I. Anaemia and stillbirth in Kassala Hospital, Eastern Sudan. J Trop Pediatr. 2011; 57(1): 62-4.
Ali AAA, Adam I. Lack of antenatal care, education, and high maternal mortality in Kassala hospital, eastern Sudan during 2005-2009. J Matern Fetal Neonatal Med. 2011; 24(8): 1077-8.
Ali AAA, Elgessim ME, Taha E, Adam GK. Factors associated with perinatal mortality in Kassala, Eastern Sudan: a community-based study 2010-2011. J Trop Pediatr. 2014; 60(1): 79-82.
Alikor CA, Emem-Chioma PC. EPIDEMIOLOGY OF DIABETES AND IMPAIRED FASTING GLUCOSE IN A RURAL COMMUNITY OF NIGERIAN NIGER DELTA REGION. Niger J Med. 2015; 24(2): 114–24.
Alikor EA, Erhabor ON. Seroprevalence of hepatitis B surface antigenaemia in children in a tertiary health institution in the Niger Delta of Nigeria. Niger J Med. 2007; 16(3): 250-1.
Alilio M, National Institute for Medical Research (Tanzania). Tanzania Plasmodium Falciparum Parasite Rate Data, M. Alilio, National Institute for Medical Research 1990. As it appears in Malaria Atlas Project. Malaria Atlas Project Plasmodium Falciparum Parasite Rate Database. Oxford, United Kingdom: Malaria Atlas Project.
AlimentaciÃ³n del menor de 18 meses: relaciÃ³n con el estado nutricional as it appears in World Health Organization (WHO). WHO Global Database on Child Growth and Malnutrition - Historical. Geneva, Switzerland: World Health Organization (WHO).
Alizadeh AHM, Ranjbar M, Ansari S, MirArab A, Alavian SM, Mohammad K, Adibi P, Sadri GH, Keramat F, Ardalan A, Arabi M, Gharekhani S, Ataei A, Amraei GR, Hosseinzadeh M, Hatami S, Zali M. Seroprevalence of hepatitis B in Nahavand, Islamic Republic of Iran. East Mediterr Health J. 2006; 12(5): 528-37.
Alkadi HO, Al-Maktari MT, Nooman MA. Chloroquine-Resistant Plasmodium falciparum Local Strain in Taiz Governorate, Republic of Yemen. Chemotherapy. 2006; 52(4): 166-70. As it appears in Malaria Atlas Project. Malaria Atlas Project Plasmodium Falciparum Parasite Rate Database. Oxford, United Kingdom: Malaria Atlas Project.
Alkan ML, Maayan S, Belmaker I, Arbeli Y, Mani N, Ben-Yshai F. Serological markers for hepatitis B and treponemal infection among HIV carriers from Ethiopia. Isr J Med Sci. 1993; 29(6-7): 390-2.
Al-Kassimi FA, Abdullah AK, Al-Hajjaj MS, Al-Orainey IO, Bamgboye EA, Chowdhury MNH. Nationwide community survey of tuberculosis epidemiology in Saudi Arabia. Tuber Lung Dis. 1993; 74(4): 254-60.
Alkerwi A ’a, Donneau A-F, Sauvageot N, Lair M-L, Scheen A, Albert A, Guillaume M. Prevalence of the metabolic syndrome in Luxembourg according to the Joint Interim Statement definition estimated from the ORISCAV-LUX study. BMC Public Health. 2011; 11(1): 4.
Alkerwi A, Pagny S, Lair M-L, Delagardelle C, Beissel J. Level of unawareness and management of diabetes, hypertension, and dyslipidemia among adults in Luxembourg: findings from ORISCAV-LUX study. PLoS One. 2013; 8(3): e57920.
Allal-Elasmi M, Feki M, Zayani Y, Hsairi M, Haj Taieb S, Jemaa R, Sanhaji H, Omar S, Mebazaa A, Kaabachi N. Prehypertension among adults in Great Tunis region (Tunisia): A population-based study. Pathol Biol. 2012; 60(3): 174–9.
Allal-Elasmi M, Haj Taieb S, Hsairi M, Zayani Y, Omar S, Sanhaji H, Jemaa R, Feki M, Elati J, Mebazaa A, Kaabachi N. The metabolic syndrome: prevalence, main characteristics and association with socio-economic status in adults living in Great Tunis. Diabetes Metab. 2010; 36(3): 204–8.
Al-Lawati JA, Jousilahti PJ. Prevalence and 10-year secular trend of obesity in Oman. Saudi Med J. 2004; 25(3): 346-51.
Al-Lawati JA, Mohammed AJ, Al-Hinai HQ, Jousilahti P. Prevalence of the metabolic syndrome among Omani adults. Diabetes Care. 2003; 26(6): 1781-5.
Allen SJ, Otoo LN, Cooke GA, O'Donnell A, Greenwood BM. Sensitivity of Plasmodium falciparum to chlorproguanil in Gambian children after five years of continuous chemoprophylaxis. Trans R Soc Trop Med Hyg. 1990; 84(2): 218. As it appears in Malaria Atlas Project. Malaria Atlas Project Plasmodium Falciparum Parasite Rate Database. Oxford, United Kingdom: Malaria Atlas Project.
Allwood PB, Division of Environmental Health, Minnesota Department of Health, Minnesota Food Safety Partnership. Handwashing among public restroom users at the Minnesota State Fair. Minnesota Department of Health. 2004.
al-Mahroos F, McKeigue PM. High prevalence of diabetes in Bahrainis. Associations with ethnicity and raised plasma cholesterol. Diabetes Care. 1998; 21(6): 936-42.
Al-Mahroos F, Al-Roomi K. Diabetic neuropathy, foot ulceration, peripheral vascular disease and potential risk factors among patients with diabetes in Bahrain: a nationwide primary care diabetes clinic-based study. Ann Saudi Med. 2007; 27(1): 25-31.
Almajwal AM, Al-Baghli NA, Batterham MJ, Williams PG, Al-Turki KA, Al-Ghamdi AJ. Performance of body mass index in predicting diabetes and hypertension in the Eastern Province of Saudi Arabia. Ann Saudi Med. 2009; 29(6): 437–45.
Al-Maktari M, Bassiouny H, Al-Hamd Z, Assabri AM, El-Massry EG, Shatat HZ. Malaria status in Al-Hodeidah Governorate, Yemen: malariometric parasitic survey and chloroquine resistance P. falciparum local strain. J Egypt Soc Parasitol. 2003; 33(2): 361-72. As it appears in Malaria Atlas Project. Malaria Atlas Project Plasmodium Falciparum Parasite Rate Database. Oxford, United Kingdom: Malaria Atlas Project.
Al-Maktari MT. Malaria Situation in Zabid District, Al-Hodeidah Governorate, Republic of Yemen [Master's thesis]. Sana'a, Yemen: Sana'a University, 1995. As it appears in Malaria Atlas Project. Malaria Atlas Project Plasmodium Falciparum Parasite Rate Database. Oxford, United Kingdom: Malaria Atlas Project.
al-Mannai A, Dickerson JW, Morgan JB, Khalfan H. Obesity in Bahraini adults. J R Soc Health. 1996; 116(1): 30-40.

Appendix: Citation List

Citation
Almaraz MC, González-Romero S, Bravo M, Caballero FF, Palomo MJ, Vallejo R, Esteva I, Calleja F, Soriguer F. Incidence of lower limb amputations in individuals with and without diabetes mellitus in Andalusia (Spain) from 1998 to 2006. Diabetes Res Clin Pract. 2012; 95(3): 399-405.
Al-Maskari F, El-Sadig M. Prevalence of risk factors for diabetic foot complications. BMC Fam Pract. 2007; 8(1): 59.
Al-Mendalawi MD, Abduljabbar MA, Aljubeh JM, Amalraj A, Cherian MP. Incidence trends of childhood type 1 diabetes in eastern Saudi Arabia. Saudi Med J. 2010; 31(9): 1074-1075.
al-Mohdzar SA, Haque E, Abdullah WA. Changes of perinatal statistics in a semiurban setup between two time periods in Malaysia. Asia Oceania J Obstet Gynaecol. 1993; 19(4): 401-5.
Al-Moslih MI, Al-Huraibi MA. Prevalence of hepatitis C virus among patients with liver disease in the Republic of Yemen. East Mediterr Health J. 2001; 7(4-5): 771-8.
Al-Nassiri K, Raja'a Y. Hepatitis B infection in Yemenis in Sana'a: pattern and risk factors. East Mediterr Health J. 2000; 7(1-2): 147-52.
Al-Nozha M, Al-Kanhal A, Al-Othaimeen A, Al-Mohaeza A, Osman A, Al-Shammery A, El-Shabrawy M, King Abdulaziz City for Science and Technology (KACST). Evaluation of the Nutritional Status of the People of Saudi Arabia. Riyadh, Saudi Arabia: King Abdulaziz City for Science and Technology (KACST), 1991.
Al-Nozha MM, Al-Maatouq MA, Al-Mazrou YY, Al-Harthi SS, Arafah MR, Khalil MZ, Khan NB, Al-Khadra A, Al-Marzouki K, Nouh MS, Abdullah M, Attas O, Al-Shahid MS, Al-Mobeireek A. Diabetes mellitus in Saudi Arabia. Saudi Med J. 2004; 25(11): 1603-10.
Al-Nozha MM, Al-Mazrou YY, Al-Maatouq MA, Arafah MR, Khalil MZ, Khan NB, Al-Marzouki K, Abdullah MA, Al-Khadra AH, Al-Harthi SS, Al-Shahid MS, Al-Mobeireek A, Nouh MS. Obesity in Saudi Arabia. Saudi Med J. 2005; 26(5): 824-9.
al-Nuaim AR, al-Rubeaan K, al-Mazrou Y, al-Attas O, al-Daghari N, Khoja T. High prevalence of overweight and obesity in Saudi Arabia. Int J Obes Relat Metab Disord. 1996; 20(6): 547-52.
Alonso PL, Lindsay SW, Armstrong JRM, de Francisco A, Shenton FC, Greenwood BM, Conteh M, Cham K, Hill AG, David PH, Fegan G, Hall AJ. The effect of insecticide-treated bed nets on mortality of Gambian children. Lancet. 1991; 337(8756): 1499-502.
Alonso PL, Smith T, Schellenberg JR, Masanja H, Mwankusye S, Urassa H, Bastos de Azevedo I, Chongela J, Kobero S, Menendez C. Randomised trial of efficacy of SPf66 vaccine against Plasmodium falciparum malaria in children in southern Tanzania. Lancet. 1994; 344(8931): 1175-81. As it appears in Malaria Atlas Project. Malaria Atlas Project Plasmodium Falciparum Parasite Rate Database. Oxford, United Kingdom: Malaria Atlas Project.
Al-Othaimeen AI, Al-Nozha M, Osman AK. Obesity: an emerging problem in Saudi Arabia. Analysis of data from the National Nutrition Survey. East Mediterr Health J. 2007; 13(2): 441-8.
Al-Rubeaan K, Al Derwish M, Ouizi S, Youssef AM, Subhani SN, Ibrahim HM, Alamri BN. Diabetic foot complications and their risk factors from a large retrospective cohort study. PLoS One. 2015; 10(5): e0124446.
Al-Rubeaan K, Al-Manaa H, Khoja T, Ahmad N, Al-Sharqawi A, Siddiqui K, AlNaqeb D, Aburisheh K, Youssef A, Al-Batil A, Al-Otaibi M, Ghamdi AA. The Saudi Abnormal Glucose Metabolism and Diabetes Impact Study (SAUDI-DM). Ann Saudi Med. 2014; 34(6): 465â€“75.
Al-Rubeaan K. National surveillance for type 1, type 2 diabetes and prediabetes among children and adolescents: a population-based study (SAUDI-DM). J Epidemiol Community Health. 2015; nan.
Al-Taiar A, Assabri A, Al-Habori M, Azazy A, Algabri A, Alganadi M, Whitty CJM, Jaffar S. Socioeconomic and environmental factors important for acquiring non-severe malaria in children in Yemen: a case-control study. Trans R Soc Trop Med Hyg. 2009; 103(1): 72-8. As it appears in Malaria Atlas Project. Malaria Atlas Project Plasmodium Falciparum Parasite Rate Database. Oxford, United Kingdom: Malaria Atlas Project.
Alten B, Caglar S, Simsek F, Kaynas S. Effect of insecticide-treated bednets for malaria control in Southeast Anatolia-Turkey. J Vector Ecol. 2003; 28(1): 97-107. As it appears in Malaria Atlas Project. Malaria Atlas Project Plasmodium Falciparum Parasite Rate Database. Oxford, United Kingdom: Malaria Atlas Project.
Al-Traif I, Ali A, Dafalla M, Al-Tamimi W, Qassem L. Prevalence of hepatitis delta antibody among HBsAG carriers in Saudi Arabia. Ann Saudi Med. 2004; 24(5): 343-4.
Alvarado-Mora MV, Gutierrez Fernandez MF, Gomes-Gouvêa MS, de Azevedo Neto RS, Carrilho FJ, Pinho JRR. Hepatitis B (HBV), Hepatitis C (HCV) and Hepatitis Delta (HDV) Viruses in the Colombian Population-How Is the Epidemiological Situation? PLoS One. 2011; 6(4): e18888.
Alvarez J, Pavao J, Mack KP, Chow JM, Baumrind N, Kimerling R. Lifetime interpersonal violence and self-reported chlamydia trachomatis diagnosis among California women. J Womens Health (Larchmt). 2009; 18(1): 57-63.
Alvarez-Torices JC, Franch-Nadal J, Alvarez-Guisasola F, Hernandez-Mejia R, Cueto-Espinar A. Self-reported height and weight and prevalence of obesity. Study in a Spanish population. Int J Obes Relat Metab Disord. 1993; 17(11): 663-7.
Alves FP, Durlacher RR, Menezes MJ, Krieger H, Silva LHP, Camargo EP. High prevalence of asymptomatic Plasmodium vivax and Plasmodium falciparum infections in native Amazonian populations. Am J Trop Med Hyg. 2002; 66(6): 641-8. As it appears in Malaria Atlas Project. Malaria Atlas Project Plasmodium Falciparum Parasite Rate Database. Oxford, United Kingdom: Malaria Atlas Project.
Alves J, Roque AL, Cravo P, Valdez T, Jelinek T, do Rosário VE, Arez AP. Epidemiological characterization of Plasmodium falciparum in the Republic of Cabo Verde: implications for potential large-scale re-emergence of malaria. Malar J. 2006; 5(1): 1-8. As it appears in Malaria Atlas Project. Malaria Atlas Project Plasmodium Falciparum Parasite Rate Database. Oxford, United Kingdom: Malaria Atlas Project.
Alves SV. Maternal mortality in Pernambuco, Brazil: what has changed in ten years? Reprod Health Matters. 2007; 15(30): 134-44.
Amagana D. Anti-TRAP (Thrombospodin Related Adhesive Protein) Immune Response and Malaria Morbidity in an Area of Hyperendemic Malaria in Mali (West Africa) [dissertation]. Rome, Italy: Sapienza University of Rome, 1997. As it appears in Malaria Atlas Project. Malaria Atlas Project Plasmodium Falciparum Parasite Rate Database. Oxford, United Kingdom: Malaria Atlas Project.
Amaral C de A, Portela MC, Muniz PT, Farias E dos S, Araujo TS de, Souza OF de. Association of handgrip strength with self-reported diseases in adults in Rio Branco, Acre State, Brazil: a population-based study. Cad Saude Publica. 2015; 31(6): 1313â€“25.
Amaral Carvalho Hospital (Brazil), National Cancer Institute (Brazil), Secretary of Municipal Health of Jau (Brazil). Brazil - Jahú BasePopWeb Database - Population Based Cancer Registry (RCBP Jahú) Statistics. Rio de Janeiro, Brazil: National Cancer Institute (Brazil).

Appendix: Citation List

Citation
Amarin Z, Khader Y, Okour A, Jaddou H, Al-Qutob R. National maternal mortality ratio for Jordan, 2007-2008. Int J Gynaecol Obstet. 2010; 111(2): 152-6.
Amaro JL, Macharelli CA, Yamamoto H, Kawano PR, Padovani CV, Agostinho AD. Prevalence and risk factors for urinary and fecal incontinence in Brazilian women. Int Braz J Urol. 2009; 35(5): 592-598.
AMATEM (Turkey), Plaza Ltd. Research, World Health Organization (WHO). Turkey WHO Multi-country Survey Study on Health and Health System Responsiveness 2000-2001. Geneva, Switzerland: World Health Organization (WHO).
Amazigo UO, Chime AB. Hepatitis-B virus infection in rural and urban populations of eastern Nigeria: prevalence of serological markers. East Afr Med J. 1990; 67(8): 539-44.
Amazonas State Foundation Oncology Control Center (FCECON), National Cancer Institute (Brazil). Brazil - Manaus BasePopWeb Database - Population Based Cancer Registry (RCBP Manaus) Statistics. Rio de Janeiro, Brazil: National Cancer Institute (Brazil).
Ameneshewa B. The Behavior and Biology of Anopheles Arabiensis in Relation to Epidemiology and Control of Malaria in Ethiopia [dissertation]. Liverpool, United Kingdom: University of Liverpool, 1995. As it appears in Malaria Atlas Project. Malaria Atlas Project Plasmodium Falciparum Parasite Rate Database. Oxford, United Kingdom: Malaria Atlas Project.
American Samoa Vital Registration Death Data 1952 as it appears in United Nations Statistics Division (UNSD). United Nations Demographic Yearbook - Historical Supplement 1997. New York City, United States: United Nations Statistics Division (UNSD).
American Samoa Vital Registration Death Data 1953 as it appears in United Nations Statistics Division (UNSD). United Nations Demographic Yearbook - Historical Supplement 1997. New York City, United States: United Nations Statistics Division (UNSD).
American Samoa Vital Registration Death Data 1954 as it appears in United Nations Statistics Division (UNSD). United Nations Demographic Yearbook - Historical Supplement 1997. New York City, United States: United Nations Statistics Division (UNSD).
American Samoa Vital Registration Death Data 1955 as it appears in United Nations Statistics Division (UNSD). United Nations Demographic Yearbook - Historical Supplement 1997. New York City, United States: United Nations Statistics Division (UNSD).
American Samoa Vital Registration Death Data 1956 as it appears in United Nations Statistics Division (UNSD). United Nations Demographic Yearbook - Historical Supplement 1997. New York City, United States: United Nations Statistics Division (UNSD).
American Samoa Vital Registration Death Data 1957 as it appears in United Nations Statistics Division (UNSD). United Nations Demographic Yearbook - Historical Supplement 1997. New York City, United States: United Nations Statistics Division (UNSD).
American Samoa Vital Registration Death Data 1958 as it appears in United Nations Statistics Division (UNSD). United Nations Demographic Yearbook - Historical Supplement 1997. New York City, United States: United Nations Statistics Division (UNSD).
American Samoa Vital Registration Death Data 1959 as it appears in United Nations Statistics Division (UNSD). United Nations Demographic Yearbook - Historical Supplement 1997. New York City, United States: United Nations Statistics Division (UNSD).
American Samoa Vital Registration Death Data 1960 as it appears in United Nations Statistics Division (UNSD). United Nations Demographic Yearbook - Historical Supplement 1997. New York City, United States: United Nations Statistics Division (UNSD).
American Samoa Vital Registration Death Data 1961 as it appears in United Nations Statistics Division (UNSD). United Nations Demographic Yearbook - Historical Supplement 1997. New York City, United States: United Nations Statistics Division (UNSD).
American Samoa Vital Registration Death Data 1962 as it appears in United Nations Statistics Division (UNSD). United Nations Demographic Yearbook - Historical Supplement 1997. New York City, United States: United Nations Statistics Division (UNSD).
American Samoa Vital Registration Death Data 1963 as it appears in United Nations Statistics Division (UNSD). United Nations Demographic Yearbook - Historical Supplement 1997. New York City, United States: United Nations Statistics Division (UNSD).
American Samoa Vital Registration Death Data 1964 as it appears in United Nations Statistics Division (UNSD). United Nations Demographic Yearbook - Historical Supplement 1997. New York City, United States: United Nations Statistics Division (UNSD).
American Samoa Vital Registration Death Data 1965 as it appears in United Nations Statistics Division (UNSD). United Nations Demographic Yearbook - Historical Supplement 1997. New York City, United States: United Nations Statistics Division (UNSD).
American Samoa Vital Registration Death Data 1966 as it appears in United Nations Statistics Division (UNSD). United Nations Demographic Yearbook - Historical Supplement 1997. New York City, United States: United Nations Statistics Division (UNSD).
American Samoa Vital Registration Death Data 1967 as it appears in United Nations Statistics Division (UNSD). United Nations Demographic Yearbook - Historical Supplement 1997. New York City, United States: United Nations Statistics Division (UNSD).
American Samoa Vital Registration Death Data 1968 as it appears in United Nations Statistics Division (UNSD). United Nations Demographic Yearbook - Historical Supplement 1997. New York City, United States: United Nations Statistics Division (UNSD).
American Samoa Vital Registration Death Data 1969 as it appears in United Nations Statistics Division (UNSD). United Nations Demographic Yearbook - Historical Supplement 1997. New York City, United States: United Nations Statistics Division (UNSD).
American Samoa Vital Registration Death Data 1970 as it appears in United Nations Statistics Division (UNSD). United Nations Demographic Yearbook - Historical Supplement 1997. New York City, United States: United Nations Statistics Division (UNSD).
American Samoa Vital Registration Death Data 1971 as it appears in United Nations Statistics Division (UNSD). United Nations Demographic Yearbook - Historical Supplement 1997. New York City, United States: United Nations Statistics Division (UNSD).
American Samoa Vital Registration Death Data 1972 as it appears in United Nations Statistics Division (UNSD). United Nations Demographic Yearbook - Historical Supplement 1997. New York City, United States: United Nations Statistics Division (UNSD).
American Samoa Vital Registration Death Data 1973 as it appears in United Nations Statistics Division (UNSD). United Nations Demographic Yearbook - Historical Supplement 1997. New York City, United States: United Nations Statistics Division (UNSD).
American Samoa Vital Registration Death Data 1976 as it appears in United Nations Statistics Division (UNSD). United Nations Demographic Yearbook - Historical Supplement 1997. New York City, United States: United Nations Statistics Division (UNSD).
American Samoa Vital Registration Death Data 1982 as it appears in United Nations Statistics Division (UNSD). United Nations Demographic Yearbook - Historical Supplement 1997. New York City, United States: United Nations Statistics Division (UNSD).
American Samoa Vital Registration Death Data 1984 as it appears in United Nations Statistics Division (UNSD). United Nations Demographic Yearbook - Historical Supplement 1997. New York City, United States: United Nations Statistics Division (UNSD).

Appendix: Citation List

Citation
American Samoa Vital Registration Death Data 1985 as it appears in United Nations Statistics Division (UNSD). United Nations Demographic Yearbook - Historical Supplement 1997. New York City, United States: United Nations Statistics Division (UNSD).
American Samoa Vital Registration Death Data 1986 as it appears in United Nations Statistics Division (UNSD). United Nations Demographic Yearbook - Historical Supplement 1997. New York City, United States: United Nations Statistics Division (UNSD).
American Samoa Vital Registration Death Data 1987 as it appears in United Nations Statistics Division (UNSD). United Nations Demographic Yearbook - Historical Supplement 1997. New York City, United States: United Nations Statistics Division (UNSD).
American Samoa Vital Registration Death Data 1988 as it appears in United Nations Statistics Division (UNSD). United Nations Demographic Yearbook - Historical Supplement 1997. New York City, United States: United Nations Statistics Division (UNSD).
American Samoa Vital Registration Death Data 1989 as it appears in United Nations Statistics Division (UNSD). United Nations Demographic Yearbook - Historical Supplement 1997. New York City, United States: United Nations Statistics Division (UNSD).
American Samoa Vital Registration Death Data 1990 as it appears in United Nations Statistics Division (UNSD). United Nations Demographic Yearbook - Historical Supplement 1997. New York City, United States: United Nations Statistics Division (UNSD).
American Samoa Vital Registration Death Data 1991 as it appears in United Nations Statistics Division (UNSD). United Nations Demographic Yearbook - Historical Supplement 1997. New York City, United States: United Nations Statistics Division (UNSD).
American Samoa Vital Registration Death Data 1992 as it appears in United Nations Statistics Division (UNSD). United Nations Demographic Yearbook - Historical Supplement 1997. New York City, United States: United Nations Statistics Division (UNSD).
American Samoa Vital Registration Death Data 1993 as it appears in United Nations Statistics Division (UNSD). United Nations Demographic Yearbook - Historical Supplement 1997. New York City, United States: United Nations Statistics Division (UNSD).
American University of Beirut, World Health Organization (WHO). Lebanon STEPS Noncommunicable Disease Risk Factors Survey 2008-2009.
Amin L, Shah BR, Bierman AS, Lipscombe LL, Wu CF, Feig DS, Booth GL. Gender differences in the impact of poverty on health: disparities in risk of diabetes-related amputation. Diabet Med. 2014; 31(11): 1410-7.
Amin R. Immunization coverage and child mortality in two rural districts of Sierra Leone. Soc Sci Med. 1996; 42(11): 1599-604.
Amin TT, Al-Mohammed HI, Kaliyadan F, Mohammed BS. Cutaneous leishmaniasis in Al Hassa, Saudi Arabia: epidemiological trends from 2000 to 2010. Asian Pac J Trop Med. 2013; 6(8): 667-72.
Amini M, Afshin-Nia F, Bashardoost N, Aminorroaya A, Shahparian M, Kazemi M. Prevalence and risk factors of diabetes mellitus in the Isfahan city population (aged 40 or over) in 1993. Diabetes Res Clin Pract. 1997; 38(3): 185-90.
Amini S, Mahmoodi MF, Andalibi S, Solati AA. Seroepidemiology of hepatitis B, delta and human immunodeficiency virus infections in Hamadan province, Iran: a population based study. J Trop Med Hyg. 1993; 96(5): 277-87.
Amirudin R, Akil H, Akahane Y, Suzuki H. Hepatitis B and C virus infection in Ujung Pandang, Indonesia. Gastroenterol Jpn. 1991; 26(3): 184-8.
Amowitz LL, Kim G, Reis C, Asher JL, Iacopino V. Human rights abuses and concerns about women's health and human rights in southern Iraq. JAMA. 2004; 291(12): 1471-9.
Analysis of Changes in the Physical Development Occuring in the Children in Havana Between 1972-1993 as it appears in World Health Organization (WHO). WHO Global Database on Child Growth and Malnutrition - Historical. Geneva, Switzerland: World Health Organization (WHO).
Analytical and Information Center of the Ministry of Health of Uzbekistan, Macro International, Inc, Ministry of Macroeconomics and Statistics (Uzbekistan). Uzbekistan Special Demographic and Health Survey 2002. Calverton, United States: Macro International, Inc.
Analytical and Information Center of the Ministry of Health of Uzbekistan, Macro International, Inc, Ministry of Macroeconomics and Statistics (Uzbekistan). Uzbekistan Special Demographic and Health Survey 2002. Calverton, United States: Macro International, Inc.
Ancel P-Y, Goffinet F, EPIPAGE-2 Writing Group, Kuhn P, Langer B, Matis J, Hernandorena X, Chabanier P, Joly-Pedespan L, Lecomte B, Vendittelli F, Dreyfus M, Guillois B, Burguet A, Sagot P, Sizun J, Beuchee A, Rouget F, Favreau A, Saliba E, Bednarek N, Morville P, Thiriez G, Marpeau L, Marret S, Kayem G, Durrmeyer X, Granier M, Baud O, Jarreau P-H, Mitanchez D, Boileau P, Boulot P, Cambonie G, Daude H, Bédu A, Mons F, Fresson J, Vieux R, Alberge C, Alberge C, Arnaud C, Vayssiere C, Truffert P, Pierrat V, Subtil D, D'Ercole C, Gire C, Simeoni U, Bongain A, Sentilhes L, Roze J-C, Gondry J, Leke A, Deiber M, Claris O, Picaud J-C, Ego A, Debillon T, Poulichet A, Coline E, Favre A, Flechelles O, Samperiz S, Ramful D, Branger B, Benhammou V, Foix-L'Helias L, Marchand-Martin L, Kaminski M. Survival and morbidity of preterm children born at 22 through 34 weeks' gestation in France in 2011: results of the EPIPAGE-2 cohort study. JAMA Pediatr. 2015; 169(3): 230–8.
Andargie G, Berhane Y, Worku A, Kebede Y. Predictors of perinatal mortality in rural population of Northwest Ethiopia: a prospective longitudinal study. BMC Public Health. 2013; 13: 168.
Andersen BR, Westergaard HB, Bødker B, Weber T, Møller M, Sørensen JL. Maternal mortality in Denmark, 1985-1994. Eur J Obstet Gynecol Reprod Biol. 2009; 142(2): 124-8.
Andersen E, Jones T, Purnomo, Masbar S, Wiady I, Tirtolusumo S, Bangs M, Charoenvit Y, Gunawan S, Hoffwan S. Assessment of age-dependent immunity to malaria in transmigrants. Am J Trop Med Hyg. 1997; 56(6): 647-9. As it appears in Malaria Atlas Project. Malaria Atlas Project Plasmodium Falciparum Parasite Rate Database. Oxford, United Kingdom: Malaria Atlas Project.
Andersgaard AB, Langhoff-Roos J, Øian P. Direct maternal deaths in Norway 1976-1995. Acta Obstet Gynecol Scand. 2008; 87(8): 856-61.
Anderson JL, Warren CA, Perez E, Louis RI, Phillips S, Wheeler J, Cole M, Misra R. Gender and ethnic differences in hand hygiene practices among college students. Am J Infect Control. 2008; 36(5): 361-8.
Anderson NH, Sadler LC, McKinlay CJD, McCowan LME. INTERGROWTH-21st vs customized birthweight standards for identification of perinatal mortality and morbidity. Am J Obstet Gynecol. 2016; 214(4): 509e1–7.
Anderson SR, Righarts A, Maguire H. Surveillance of antenatal infections--HIV, hepatitis B, syphilis and rubella susceptibility in London. Commun Dis Public Health. 2004; 7(4): 251-7.
Andersson N, Ho-Foster A, Mitchell S, Scheepers E, Goldstein S. Risk factors for domestic physical violence: national cross-sectional household surveys in eight southern African countries. BMC Womens Health. 2007; 7: 11.

Appendix: Citation List

Citation
Andjelkovich DA, Janszen DB, Brown MH, Richardson RB, Miller FJ. Mortality Of Iron Foundry Workers: Iv. Analysis Of A Subcohort Exposed To Formaldehyde. J Occup Environ Med. 1995; 37(7): 826-37 as it appears in Collins JJ, Lineker GA. A review and meta-analysis of formaldehyde exposure and leukemia. Regul Toxicol Pharmacol. 2004; 40(2): 81-91.
Andorra Vital Registration Death Data 1950 as it appears in United Nations Statistics Division (UNSD). United Nations Demographic Yearbook - Historical Supplement 1997. New York City, United States: United Nations Statistics Division (UNSD).
Andorra Vital Registration Death Data 1951 as it appears in United Nations Statistics Division (UNSD). United Nations Demographic Yearbook - Historical Supplement 1997. New York City, United States: United Nations Statistics Division (UNSD).
Andorra Vital Registration Death Data 1952 as it appears in United Nations Statistics Division (UNSD). United Nations Demographic Yearbook - Historical Supplement 1997. New York City, United States: United Nations Statistics Division (UNSD).
Andorra Vital Registration Death Data 1953 as it appears in United Nations Statistics Division (UNSD). United Nations Demographic Yearbook - Historical Supplement 1997. New York City, United States: United Nations Statistics Division (UNSD).
Andorra Vital Registration Death Data 1954 as it appears in United Nations Statistics Division (UNSD). United Nations Demographic Yearbook - Historical Supplement 1997. New York City, United States: United Nations Statistics Division (UNSD).
Andreeva TI, Krasovsky KS. Changes in smoking prevalence in Ukraine in 2001-5. Tob Control. 2007; 16(3): 202-6.
Andrews, Gary R., and George C. Myers. Australian [Adelaide] Longitudinal Study of Aging, Waves 1-5 [1992-1997]. ICPSR06707-v3. Ann Arbor, MI: Inter-university Consortium for Political and Social Research [distributor], 2000. doi:10.3886/ICPSR06707.v3
Andriamangatiana-Rason MD, Lepers JP, Raharimalala L, Fontenille D, Coulanges P. [Parasitologic and serologic study of a population of schoolchildren in a village of the Highland Plateaux of Madagascar. Arch Inst Pasteur Madagascar. 1990; 57(1): 75-91. As it appears in Malaria Atlas Project. Malaria Atlas Project Plasmodium Falciparum Parasite Rate Database. Oxford, United Kingdom: Malaria Atlas Project.
Anemia and undernutrition among preschool children in Uttar Pradesh, India as it appears in World Health Organization (WHO). WHO Global Database on Child Growth and Malnutrition - Historical. Geneva, Switzerland: World Health Organization (WHO).
Ang LW, Tey SH, Cutter J, James L, Goh KT. Seroprevalence of hepatitis B virus infection among children and adolescents in Singapore, 2008-2010. J Med Virol. 2013; 85(4): 583-8.
Angola - Lunda Norte Anthropometric Nutritional Survey in Cafunfo 1995 as it appears in World Health Organization (WHO). WHO Global Database on Child Growth and Malnutrition - Historical. Geneva, Switzerland: World Health Organization (WHO).
Angola Investigation Report on Nutrition 2007 as it appears in World Health Organization (WHO). WHO Global Database on Child Growth and Malnutrition. Geneva, Switzerland: World Health Organization (WHO).
Angola Nutritional Surveillance and Progress in N'Dalatando as it appears in World Health Organization (WHO). WHO Global Database on Child Growth and Malnutrition - Historical. Geneva, Switzerland: World Health Organization (WHO).
Anjana RM, Pradeepa R, Deepa M, Datta M, Sudha V, Unnikrishnan R, Bhansali A, Joshi SR, Joshi PP, Yajnik CS, Dhandhania VK, Nath LM, Das AK, Rao PV, Madhu SV, Shukla DK, Kaur T, Priya M, Nirmal E, Parvathi SJ, Subhashini S, Subashini R, Ali MK, Mohan V. Prevalence of diabetes and prediabetes (impaired fasting glucose and/or impaired glucose tolerance) in urban and rural India: phase I results of the Indian Council of Medical Research-INdia DIABetes (ICMR-INDIAB) study. Diabetologia. 2011; 54(12): 3022-7.
Anokute CC. Epidemiologic studies of diabetes mellitus in Saudi Arabia--Part I--Screening of 3158 males in King Saud University. J R Soc Health. 1990; 110(6): 201-3.
Anonymous. [Results on integrated malaria prevention measures in Early Health Care in 4 locations at Phu Quoc district]. J Vector Borne Dis. 1995; 1: 7-12. As it appears in Malaria Atlas Project. Malaria Atlas Project Plasmodium Falciparum Parasite Rate Database. Oxford, United Kingdom: Malaria Atlas Project.
Anosike JC, Nwoke BE, Ajayi EG, Onwuliri CO, Okoro OU, Oku EE, Asor JE, Amajuoyi OU, Ikpeama CA, Ogbusu FI, Meribe CO. Lymphatic filariasis among the Ezza people of Ebonyi State, eastern Nigeria. Ann Agric Environ Med. 2005; 12(2): 181-6. As it appears in London School of Hygiene and Tropical Medicine. Global Atlas of Helminth Infections - Lymphatic Filiariasis.
Anosike JC, Onwuliri CO. Studies on filariasis in Bauchi State, Nigeria. II. The prevalence of human filariasis in Darazo Local Government area. Appl Parasitol. 1994; 35(4): 242-50. As it appears in London School of Hygiene and Tropical Medicine. Global Atlas of Helminth Infections - Lymphatic Filiariasis.
Anosike JC, Onwuliri COE, Onwuliri VA. Human filariasis in Dass local government area of Bauchi state, Nigeria. Trop Ecol. 2003; 44(2): 217-27. As it appears in London School of Hygiene and Tropical Medicine. Global Atlas of Helminth Infections - Lymphatic Filiariasis.
Anosike JC. The status of human filariasis in north-western zone of Bauchi State, Nigeria. Appl Parasitol. 1994; 35(2): 133-40.
Anosike JC. The status of human filariasis in north-western zone of Bauchi State, Nigeria. Appl Parasitol. 1994; 35(2): 133-40. As it appears in London School of Hygiene and Tropical Medicine. Global Atlas of Helminth Infections - Lymphatic Filiariasis.
Anothay O, Pongvongsa T. Childhood malaria in the Lao People's Democratic Republic. Bull World Health Organ. 1998; 76(1): 29-34. As it appears in Malaria Atlas Project. Malaria Atlas Project Plasmodium Falciparum Parasite Rate Database. Oxford, United Kingdom: Malaria Atlas Project.
Ansara DL, Hindin MJ. Perpetration of Intimate Partner Aggression by Men and Women in the Philippines Prevalence and Associated Factors. J Interpers Violence. 2009; 24(9): 1579-90.
Ansari M, Sharma Y, Roy A, Biswas S, Sharma P. Epidemiologic investigations of a malaria outbreak in northern Delhi area. J Am Mosq Control Assoc. 2001; 17(4): 216-20. As it appears in Malaria Atlas Project. Malaria Atlas Project Plasmodium Falciparum Parasite Rate Database. Oxford, United Kingdom: Malaria Atlas Project.
Ansari-Moghaddam A, Ostovaneh MR, Sharifi Mood B, Sanei-Moghaddam E, Modabbernia A, Poustchi H. Seroprevalence of hepatitis B surface antigen and anti hepatitis C antibody in zahedan city, iran: a population-based study. Hepat Mon. 2012; 12(9): e6618.
Ansel Vishal L, Nazeer Y, Ravishankaran R, Mahalakshmi N, Kaliraj P. Evaluation of rapid blood sample collection in the detection of circulating filarial antigens for epidemiological survey by rWbSXP-1 capture assay. PLoS One. 2014; 9.0(7): e102260.
Anteneh A, Araya T, Misganaw A. Factors associated with place of death in Addis Ababa, Ethiopia. BMC Palliat Care. 2013; 12(14): 14.
Anthropometric assessment of the nutritional status of preschool-age children in Cape Verde as it appears in World Health Organization (WHO). WHO Global Database on Child Growth and Malnutrition. Geneva, Switzerland: World Health Organization (WHO).

Appendix: Citation List

Citation
Anthropometric assessment of young children attending a health center in Sana'a as it appears in World Health Organization (WHO). WHO Global Database on Child Growth and Malnutrition - Historical. Geneva, Switzerland: World Health Organization (WHO).
Anthropometric Characteristics of a Group of Socio-Economically Privileged Children in Brazzaville as it appears in World Health Organization (WHO). WHO Global Database on Child Growth and Malnutrition - Historical. Geneva, Switzerland: World Health Organization (WHO).
Anthropometric study of Ethiopian pre-school children as it appears in World Health Organization (WHO). WHO Global Database on Child Growth and Malnutrition - Historical. Geneva, Switzerland: World Health Organization (WHO).
Antigua and Barbuda Living Conditions and Household Budget Survey 2005-2006 as it appears in World Health Organization (WHO). WHO Household Energy Database. Geneva, Switzerland: World Health Organization (WHO), 2010.
Antigua and Barbuda Population and Housing Census 2001 as it appears in World Health Organization (WHO). WHO Household Energy Database. Geneva, Switzerland: World Health Organization (WHO), 2010.
Antigua and Barbuda Vital Registration - Deaths 1961 ICD7 as it appears in World Health Organization (WHO). WHO Mortality Database Version July 2012. Geneva, Switzerland: World Health Organization (WHO), 2012.
Antigua and Barbuda Vital Registration - Deaths 1962 ICD7 as it appears in World Health Organization (WHO). WHO Mortality Database Version July 2012. Geneva, Switzerland: World Health Organization (WHO), 2012.
Antigua and Barbuda Vital Registration - Deaths 1963 ICD7 as it appears in World Health Organization (WHO). WHO Mortality Database Version July 2012. Geneva, Switzerland: World Health Organization (WHO), 2012.
Antigua and Barbuda Vital Registration - Deaths 1964 ICD7 as it appears in World Health Organization (WHO). WHO Mortality Database Version July 2012. Geneva, Switzerland: World Health Organization (WHO), 2012.
Antigua and Barbuda Vital Registration - Deaths 1966 ICD7 as it appears in World Health Organization (WHO). WHO Mortality Database Version July 2012. Geneva, Switzerland: World Health Organization (WHO), 2012.
Antigua and Barbuda Vital Registration - Deaths 1969 ICD8 as it appears in World Health Organization (WHO). WHO Mortality Database Version May 2013. Geneva, Switzerland: World Health Organization (WHO), 2013.
Antigua and Barbuda Vital Registration - Deaths 1970 ICD8 as it appears in World Health Organization (WHO). WHO Mortality Database Version May 2013. Geneva, Switzerland: World Health Organization (WHO), 2013.
Antigua and Barbuda Vital Registration - Deaths 1971 ICD8 as it appears in World Health Organization (WHO). WHO Mortality Database Version May 2013. Geneva, Switzerland: World Health Organization (WHO), 2013.
Antigua and Barbuda Vital Registration - Deaths 1972 ICD8 as it appears in World Health Organization (WHO). WHO Mortality Database Version May 2013. Geneva, Switzerland: World Health Organization (WHO), 2013.
Antigua and Barbuda Vital Registration - Deaths 1973 ICD8 as it appears in World Health Organization (WHO). WHO Mortality Database Version May 2013. Geneva, Switzerland: World Health Organization (WHO), 2013.
Antigua and Barbuda Vital Registration - Deaths 1974 ICD8 as it appears in World Health Organization (WHO). WHO Mortality Database Version May 2013. Geneva, Switzerland: World Health Organization (WHO), 2013.
Antigua and Barbuda Vital Registration - Deaths 1975 ICD8 as it appears in World Health Organization (WHO). WHO Mortality Database Version May 2013. Geneva, Switzerland: World Health Organization (WHO), 2013.
Antigua and Barbuda Vital Registration - Deaths 1976 ICD8 as it appears in World Health Organization (WHO). WHO Mortality Database Version May 2013. Geneva, Switzerland: World Health Organization (WHO), 2013.
Antigua and Barbuda Vital Registration - Deaths 1977 ICD8 as it appears in World Health Organization (WHO). WHO Mortality Database Version May 2013. Geneva, Switzerland: World Health Organization (WHO), 2013.
Antigua and Barbuda Vital Registration - Deaths 1978 ICD8 as it appears in World Health Organization (WHO). WHO Mortality Database Version May 2013. Geneva, Switzerland: World Health Organization (WHO), 2013.
Antigua and Barbuda Vital Registration - Deaths 1983 ICD9 as it appears in World Health Organization (WHO). WHO Mortality Database Version February 2014. Geneva, Switzerland: World Health Organization (WHO), 2014.
Antigua and Barbuda Vital Registration - Deaths 1985 ICD9 as it appears in World Health Organization (WHO). WHO Mortality Database Version November 2015. Geneva, Switzerland: World Health Organization (WHO), 2015.
Antigua and Barbuda Vital Registration - Deaths 1986 ICD9 as it appears in World Health Organization (WHO). WHO Mortality Database Version February 2014. Geneva, Switzerland: World Health Organization (WHO), 2014.
Antigua and Barbuda Vital Registration - Deaths 1987 ICD9 as it appears in World Health Organization (WHO). WHO Mortality Database Version November 2015. Geneva, Switzerland: World Health Organization (WHO), 2015.
Antigua and Barbuda Vital Registration - Deaths 1988 ICD9 as it appears in World Health Organization (WHO). WHO Mortality Database Version February 2014. Geneva, Switzerland: World Health Organization (WHO), 2014.
Antigua and Barbuda Vital Registration - Deaths 1989 ICD9 as it appears in World Health Organization (WHO). WHO Mortality Database Version November 2015. Geneva, Switzerland: World Health Organization (WHO), 2015.
Antigua and Barbuda Vital Registration - Deaths 1990 ICD9 as it appears in World Health Organization (WHO). WHO Mortality Database Version February 2014. Geneva, Switzerland: World Health Organization (WHO), 2014.
Antigua and Barbuda Vital Registration - Deaths 1991 ICD9 as it appears in World Health Organization (WHO). WHO Mortality Database Version November 2015. Geneva, Switzerland: World Health Organization (WHO), 2015.
Antigua and Barbuda Vital Registration - Deaths 1992 ICD9 as it appears in World Health Organization (WHO). WHO Mortality Database Version February 2014. Geneva, Switzerland: World Health Organization (WHO), 2014.
Antigua and Barbuda Vital Registration - Deaths 1993 ICD9 as it appears in World Health Organization (WHO). WHO Mortality Database Version November 2015. Geneva, Switzerland: World Health Organization (WHO), 2015.
Antigua and Barbuda Vital Registration - Deaths 1994 ICD9 as it appears in World Health Organization (WHO). WHO Mortality Database Version February 2014. Geneva, Switzerland: World Health Organization (WHO), 2014.

Appendix: Citation List

Citation
Antigua and Barbuda Vital Registration - Deaths 1995 ICD9 as it appears in World Health Organization (WHO). WHO Mortality Database Version November 2015. Geneva, Switzerland: World Health Organization (WHO), 2015.
Antigua and Barbuda Vital Registration - Deaths 1998 ICD10 as it appears in World Health Organization (WHO). WHO Mortality Database Version November 2015. Geneva, Switzerland: World Health Organization (WHO), 2015.
Antigua and Barbuda Vital Registration - Deaths 1999 ICD10 as it appears in World Health Organization (WHO). WHO Mortality Database Version November 2015. Geneva, Switzerland: World Health Organization (WHO), 2015.
Antigua and Barbuda Vital Registration - Deaths 2000 ICD10 as it appears in World Health Organization (WHO). WHO Mortality Database Version November 2015. Geneva, Switzerland: World Health Organization (WHO), 2015.
Antigua and Barbuda Vital Registration - Deaths 2001 ICD10 as it appears in World Health Organization (WHO). WHO Mortality Database Version November 2015. Geneva, Switzerland: World Health Organization (WHO), 2015.
Antigua and Barbuda Vital Registration - Deaths 2002 ICD10 as it appears in World Health Organization (WHO). WHO Mortality Database Version November 2015. Geneva, Switzerland: World Health Organization (WHO), 2015.
Antigua and Barbuda Vital Registration - Deaths 2003 ICD10 as it appears in World Health Organization (WHO). WHO Mortality Database Version November 2015. Geneva, Switzerland: World Health Organization (WHO), 2015.
Antigua and Barbuda Vital Registration - Deaths 2004 ICD10 as it appears in World Health Organization (WHO). WHO Mortality Database Version November 2015. Geneva, Switzerland: World Health Organization (WHO), 2015.
Antigua and Barbuda Vital Registration - Deaths 2005 ICD10 as it appears in World Health Organization (WHO). WHO Mortality Database Version November 2015. Geneva, Switzerland: World Health Organization (WHO), 2015.
Antigua and Barbuda Vital Registration - Deaths 2006 ICD10 as it appears in World Health Organization (WHO). WHO Mortality Database Version November 2015. Geneva, Switzerland: World Health Organization (WHO), 2015.
Antigua and Barbuda Vital Registration - Deaths 2007 ICD10 as it appears in World Health Organization (WHO). WHO Mortality Database Version November 2015. Geneva, Switzerland: World Health Organization (WHO), 2015.
Antigua and Barbuda Vital Registration - Deaths 2008 ICD10 as it appears in World Health Organization (WHO). WHO Mortality Database Version November 2015. Geneva, Switzerland: World Health Organization (WHO), 2015.
Antigua and Barbuda Vital Registration - Deaths 2009 ICD10 as it appears in World Health Organization (WHO). WHO Mortality Database Version November 2015. Geneva, Switzerland: World Health Organization (WHO), 2015.
Antigua and Barbuda Vital Registration - Deaths 2012 ICD10 as it appears in World Health Organization (WHO). WHO Mortality Database Version November 2015. Geneva, Switzerland: World Health Organization (WHO), 2015.
Antigua and Barbuda Vital Registration - Deaths 2013 ICD10 as it appears in World Health Organization (WHO). WHO Mortality Database Version November 2015. Geneva, Switzerland: World Health Organization (WHO), 2015.
Antigua and Barbuda Vital Registration Death Data 1950 as it appears in United Nations Statistics Division (UNSD). United Nations Demographic Yearbook - Historical Supplement 1997. New York City, United States: United Nations Statistics Division (UNSD).
Antigua and Barbuda Vital Registration Death Data 1951 as it appears in United Nations Statistics Division (UNSD). United Nations Demographic Yearbook - Historical Supplement 1997. New York City, United States: United Nations Statistics Division (UNSD).
Antigua and Barbuda Vital Registration Death Data 1952 as it appears in United Nations Statistics Division (UNSD). United Nations Demographic Yearbook - Historical Supplement 1997. New York City, United States: United Nations Statistics Division (UNSD).
Antigua and Barbuda Vital Registration Death Data 1953 as it appears in United Nations Statistics Division (UNSD). United Nations Demographic Yearbook - Historical Supplement 1997. New York City, United States: United Nations Statistics Division (UNSD).
Antigua and Barbuda Vital Registration Death Data 1954 as it appears in United Nations Statistics Division (UNSD). United Nations Demographic Yearbook - Historical Supplement 1997. New York City, United States: United Nations Statistics Division (UNSD).
Antigua and Barbuda Vital Registration Death Data 1955 as it appears in United Nations Statistics Division (UNSD). United Nations Demographic Yearbook - Historical Supplement 1997. New York City, United States: United Nations Statistics Division (UNSD).
Antigua and Barbuda Vital Registration Death Data 1956 as it appears in United Nations Statistics Division (UNSD). United Nations Demographic Yearbook - Historical Supplement 1997. New York City, United States: United Nations Statistics Division (UNSD).
Antigua and Barbuda Vital Registration Death Data 1957 as it appears in United Nations Statistics Division (UNSD). United Nations Demographic Yearbook - Historical Supplement 1997. New York City, United States: United Nations Statistics Division (UNSD).
Antigua and Barbuda Vital Registration Death Data 1958 as it appears in United Nations Statistics Division (UNSD). United Nations Demographic Yearbook - Historical Supplement 1997. New York City, United States: United Nations Statistics Division (UNSD).
Antigua and Barbuda Vital Registration Death Data 1959 as it appears in United Nations Statistics Division (UNSD). United Nations Demographic Yearbook - Historical Supplement 1997. New York City, United States: United Nations Statistics Division (UNSD).
Antigua and Barbuda Vital Registration Death Data 1960 as it appears in United Nations Statistics Division (UNSD). United Nations Demographic Yearbook - Historical Supplement 1997. New York City, United States: United Nations Statistics Division (UNSD).
Antigua and Barbuda Vital Registration Death Data 1965 as it appears in United Nations Statistics Division (UNSD). United Nations Demographic Yearbook - Historical Supplement 1997. New York City, United States: United Nations Statistics Division (UNSD).
Anumudu C, Afolami M, Igwe C, Nwagwu M, Keshinro O. Nutritional anaemia and malaria in pre-school and school age children. Ann Afr Med. 2008; 7(1): 11. As it appears in Malaria Atlas Project. Malaria Atlas Project Plasmodium Falciparum Parasite Rate Database. Oxford, United Kingdom: Malaria Atlas Project.
Anumudu CI, Adepoju A, Adediran M, Adeoye O, Kassim A, Oyewole I, Nwuba RI. Malaria prevalence and treatment seeking behaviour of young Nigerian adults. Ann Afr Med. 2007; 5(2): 82-8. As it appears in Malaria Atlas Project. Malaria Atlas Project Plasmodium Falciparum Parasite Rate Database. Oxford, United Kingdom: Malaria Atlas Project.
Anwar Z, Djamil H, Pardede N, Ismail R. The pattern of the causes of death in children in rural swampy area of South Sumatra, Indonesia. Paediatr Indones. 1987; 27(6-May): 93-8.
Aoki M, Hisamichi S, Tominaga S, eds. Smoking and Health 1987. Proceedings of the 6th World Conference on Smoking and Health. World Conference on Smoking and Health; 1987 Nov 9-12; Tokyo, Japan. Amsterdam, The Netherlands: Excerpta Medica, 1988.

Appendix: Citation List

Citation
Aoyagi K, Kusano Y, Takamura N, Abe Y, Osaki M, Une H. Obesity and cardiovascular risk factors among men and women aged 40 years and older in a rural area of Japan. J Physiol Anthropol. 2006; 25(6): 371-5.
Apea-Kubi KA, Yamaguchi S, Sakyi B, Ofori-Adjei D. HTLV-1 and other viral sexually transmitted infections in antenatal and gynaecological patients in Ghana. West Afr J Med. 2006; 25(1): 17-21.
Apt W, Zulantay I, Arnello M, Oddó D, González S, Rodríguez J, Kemmerling U, Truyens C, Carlier Y. Congenital infection by Trypanosoma cruzi in an endemic area of Chile: a multidisciplinary study. Trans R Soc Trop Med Hyg. 2013; 107(2): 98-104.
Apt W, Zulantay I, Solari A, Ortiz S, Oddo D, Corral G, Truyens C, Carlier Y. Vertical transmission of Trypanosoma cruzi in the Province of Choapa, IV Region, Chile: Preliminary Report (2005-2008). Biol Res. 2010; 43(3): 269-74.
Aquino JA, Pegado KA, Barros LP, Machado LFA. Soroprevalência de infecções por vírus da hepatite B e vírus da hepatite C em indivíduos do Estado do Pará. Rev Soc Bras Med Trop. 2008; 334-7.
Arab Fund for Economic and Social Development (AFESD), Arab Gulf Program for Development (AGFUND), Central Statistical Organization (Qatar), Council of Health Ministers of GCC States, Ministry of Public Health (Qatar), United Nations Children's Fund (UNICEF), United Nations Population Fund (UNFPA), United Nations Statistics Division (UNSD), World Health Organization (WHO). Qatar Family Health Survey 1998.
Arab Fund for Economic and Social Development (AFESD), Arab Gulf Program for Development (AGFUND), Gulf-Co-operation Council (GCC), Ministry of Health (Saudi Arabia), United Nations Children's Fund (UNICEF), United Nations Population Fund (UNFPA), United Nations Statistics Division (UNSD), World Health Organization (WHO). Saudi Arabia Family Health Survey 1996-1997.
Arab Gulf Program for Development (AGFUND), Council of Health Ministers of GCC States, Ministry of Health (Saudi Arabia), United Nations Children's Fund (UNICEF), United Nations Population Fund (UNFPA), World Health Organization (WHO). Saudi Arabia Child Health Survey 1987-1988.
Arab M. Diabetes mellitus in Egypt. World Health Stat Q. 1992; 45(4): 334-7.
Arabshahi S, Lahmann PH, Williams GM, van der Pols JC. Predictors of change in weight and waist circumference: 15-year longitudinal study in Australian adults. Eur J Clin Nutr. 2014; 68(3): 309â€“15.
Arap Siongok TK, Mahmoud AA, Ouma JH, Warren KS, Muller AS, Handa AK, Houser HB. Morbidity in Schistosomiasis mansoni in relation to intensity of infection: study of a community in Machakos, Kenya. Am J Trop Med Hyg. 1976; 25(2): 273-84.
Araújo AB, Castagno VD, Gallina T, Berne MEA. Prevalence of Chagas disease among pregnant women in the southern region of Rio Grande do Sul. Rev Soc Bras Med Trop. 2009; 42(6): 732-3.
Araújo Filho A, Salomão SR, Berezovsky A, Cinoto RW, Morales PHA, Santos FRG, Belfort R Jr. Prevalence of visual impairment, blindness, ocular disorders and cataract surgery outcomes in low-income elderly from a metropolitan region of São Paulo-Brazil. Arq Bras Oftalmol. 2008; 71(2): 246-53.
Araya T, Tensou B, Davey G, Berhane Y. La vigilancia de entierros detectó una reducción significativa en las muertes relacionadas con el VIH en Addis Ababa, Etiopía. Trop Med Int Health. 2011; 16(12): 1483-9.
Archibald CP, Mak JW, Mathias RG, Selvajothi S. Antibodies to Plasmodium falciparum in an indigenous population from a malaria endemic area of Malaysia. Acta Trop. 1990; 48(2): 149-57. As it appears in Malaria Atlas Project. Malaria Atlas Project Plasmodium Falciparum Parasite Rate Database. Oxford, United Kingdom: Malaria Atlas Project.
Archibong EI, Sobande AA, Asindi AA. Antenatal intrauterine fetal death: a prospective study in a tertiary hospital in south-western Saudi Arabia. J Obstet Gynaecol. 2003; 23(2): 170-3.
Ardabil University of Medical Sciences, Digestive Diseases Research Center (Iran), International Agency for Research on Cancer (IARC). Iran - Ardabil Cancer Registry Extracts 1985-2008.
Arez AP, Pinto J, Pålsson K, Snounou G, Jaenson TG, do Rosário VE. Transmission of Mixed Plasmodium Species and Plasmodium Falciparum Genotypes. Am J Trop Med Hyg. 2003; 68(2): 161-8. As it appears in Malaria Atlas Project. Malaria Atlas Project Plasmodium Falciparum Parasite Rate Database. Oxford, United Kingdom: Malaria Atlas Project.
Argentina - Bahia Blanca Cancer Registry 1993-1997 - CI5 as it appears in Parkin DM, International Agency for Research on Cancer, International Association of Cancer Registries. Cancer Incidence in Five Continents. Vol. I to VIII. Lyon, France, IARCPress, 2005.
Argentina - Bahia Blanca Cancer Registry 1998-2002 - CI5 as it appears in Curado MP, Edwards B, Shin HR, Storm H, Ferlay J, Heanue M and Boyle P, eds (2007). Cancer Incidence in Five Continents, Vol. IX Periodic Data (electronic version). Lyon, IARC.http://ci5.iarc.fr
Argentina - Bahia Blanca Cancer Registry 2003-2007 - CI5 as it appears in Forman D, Bray F, Brewster DH, Gombe Mbalawa C, Kohler B, Piñeros M, Steliarova-Foucher E, Swaminathan R and Ferlay J, eds (2013). Cancer Incidence in Five Continents, Vol. X Summary Database (electronic version). Lyon, IARC. http://ci5.iarc.fr
Argentina - Concordia Cancer Registry 1990-1994 - CI5 as it appears in Parkin DM, International Agency for Research on Cancer, International Association of Cancer Registries. Cancer Incidence in Five Continents. Vol. I to VIII. Lyon, France, IARCPress, 2005.
Argentina - Concordia Cancer Registry 1993-1997 - CI5 as it appears in Parkin DM, International Agency for Research on Cancer, International Association of Cancer Registries. Cancer Incidence in Five Continents. Vol. I to VIII. Lyon, France, IARCPress, 2005.
Argentina - Cordoba Cancer Registry 2003-2007 - CI5 as it appears in Forman D, Bray F, Brewster DH, Gombe Mbalawa C, Kohler B, Piñeros M, Steliarova-Foucher E, Swaminathan R and Ferlay J, eds (2013). Cancer Incidence in Five Continents, Vol. X Summary Database (electronic version). Lyon, IARC. http://ci5.iarc.fr
Argentina - Mendoza Cancer Registry 2003-2007 - CI5 as it appears in Forman D, Bray F, Brewster DH, Gombe Mbalawa C, Kohler B, Piñeros M, Steliarova-Foucher E, Swaminathan R and Ferlay J, eds (2013). Cancer Incidence in Five Continents, Vol. X Summary Database (electronic version). Lyon, IARC. http://ci5.iarc.fr
Argentina - Tierra del Fuego Cancer Registry 2004-2007 - CI5 as it appears in Forman D, Bray F, Brewster DH, Gombe Mbalawa C, Kohler B, Piñeros M, Steliarova-Foucher E, Swaminathan R and Ferlay J, eds (2013). Cancer Incidence in Five Continents, Vol. X Summary Database (electronic version). Lyon, IARC. http://ci5.iarc.fr
Argentina Childhood Living Conditions as it appears in World Health Organization (WHO). WHO Global Database on Child Growth and Malnutrition - Historical. Geneva, Switzerland: World Health Organization (WHO).

Appendix: Citation List

Citation
Argentina Observatory on Drugs, Secretariat for Programming Drug Abuse Prevention and the Fight against Drug Trafficking (SEDRONAR). Argentina National Survey of High School Students 2005.
Argentina Observatory on Drugs, Secretariat for Programming Drug Abuse Prevention and the Fight against Drug Trafficking (SEDRONAR). Argentina National Survey of High School Students 2007.
Argentina Observatory on Drugs, Secretariat for Programming Drug Abuse Prevention and the Fight against Drug Trafficking (SEDRONAR). Argentina National Survey of High School Students 2009.
Argentina Observatory on Drugs, Secretariat for Programming Drug Abuse Prevention and the Fight against Drug Trafficking (SEDRONAR). Argentina National Survey of High School Students 2011.
Argentina Vital Registration - Deaths 2006 ICD10 as it appears in World Health Organization (WHO). WHO Mortality Database Version November 2015. Geneva, Switzerland: World Health Organization (WHO), 2015.
Argentina Vital Registration - Deaths 1968 ICD7 as it appears in World Health Organization (WHO). WHO Mortality Database Version July 2012. Geneva, Switzerland: World Health Organization (WHO), 2012.
Argentina Vital Registration - Deaths 1969 ICD8 as it appears in World Health Organization (WHO). WHO Mortality Database Version May 2013. Geneva, Switzerland: World Health Organization (WHO), 2013.
Argentina Vital Registration - Deaths 1970 ICD8 as it appears in World Health Organization (WHO). WHO Mortality Database Version May 2013. Geneva, Switzerland: World Health Organization (WHO), 2013.
Argentina Vital Registration - Deaths 1977 ICD8 as it appears in World Health Organization (WHO). WHO Mortality Database Version May 2013. Geneva, Switzerland: World Health Organization (WHO), 2013.
Argentina Vital Registration - Deaths 1978 ICD8 as it appears in World Health Organization (WHO). WHO Mortality Database Version May 2013. Geneva, Switzerland: World Health Organization (WHO), 2013.
Argentina Vital Registration - Deaths 1979 ICD9 as it appears in World Health Organization (WHO). WHO Mortality Database Version November 2015. Geneva, Switzerland: World Health Organization (WHO), 2015.
Argentina Vital Registration - Deaths 1980 ICD9 as it appears in World Health Organization (WHO). WHO Mortality Database Version February 2014. Geneva, Switzerland: World Health Organization (WHO), 2014.
Argentina Vital Registration - Deaths 1981 ICD9 as it appears in World Health Organization (WHO). WHO Mortality Database Version November 2015. Geneva, Switzerland: World Health Organization (WHO), 2015.
Argentina Vital Registration - Deaths 1982 ICD9 as it appears in World Health Organization (WHO). WHO Mortality Database Version February 2014. Geneva, Switzerland: World Health Organization (WHO), 2014.
Argentina Vital Registration - Deaths 1983 ICD9 as it appears in World Health Organization (WHO). WHO Mortality Database Version November 2015. Geneva, Switzerland: World Health Organization (WHO), 2015.
Argentina Vital Registration - Deaths 1984 ICD9 as it appears in World Health Organization (WHO). WHO Mortality Database Version February 2014. Geneva, Switzerland: World Health Organization (WHO), 2014.
Argentina Vital Registration - Deaths 1985 ICD9 as it appears in World Health Organization (WHO). WHO Mortality Database Version February 2014. Geneva, Switzerland: World Health Organization (WHO), 2014.
Argentina Vital Registration - Deaths 1986 ICD9 as it appears in World Health Organization (WHO). WHO Mortality Database Version February 2014. Geneva, Switzerland: World Health Organization (WHO), 2014.
Argentina Vital Registration - Deaths 1987 ICD9 as it appears in World Health Organization (WHO). WHO Mortality Database Version February 2014. Geneva, Switzerland: World Health Organization (WHO), 2014.
Argentina Vital Registration - Deaths 1988 ICD9 as it appears in World Health Organization (WHO). WHO Mortality Database Version February 2014. Geneva, Switzerland: World Health Organization (WHO), 2014.
Argentina Vital Registration - Deaths 1989 ICD9 as it appears in World Health Organization (WHO). WHO Mortality Database Version February 2014. Geneva, Switzerland: World Health Organization (WHO), 2014.
Argentina Vital Registration - Deaths 1990 ICD9 as it appears in World Health Organization (WHO). WHO Mortality Database Version February 2014. Geneva, Switzerland: World Health Organization (WHO), 2014.
Argentina Vital Registration - Deaths 1991 ICD9 as it appears in World Health Organization (WHO). WHO Mortality Database Version February 2014. Geneva, Switzerland: World Health Organization (WHO), 2014.
Argentina Vital Registration - Deaths 1992 ICD9 as it appears in World Health Organization (WHO). WHO Mortality Database Version February 2014. Geneva, Switzerland: World Health Organization (WHO), 2014.
Argentina Vital Registration - Deaths 1993 ICD9 as it appears in World Health Organization (WHO). WHO Mortality Database Version February 2014. Geneva, Switzerland: World Health Organization (WHO), 2014.
Argentina Vital Registration - Deaths 1994 ICD9 as it appears in World Health Organization (WHO). WHO Mortality Database Version February 2014. Geneva, Switzerland: World Health Organization (WHO), 2014.
Argentina Vital Registration - Deaths 1995 ICD9 as it appears in World Health Organization (WHO). WHO Mortality Database Version February 2014. Geneva, Switzerland: World Health Organization (WHO), 2014.
Argentina Vital Registration - Deaths 1996 ICD9 as it appears in World Health Organization (WHO). WHO Mortality Database Version February 2014. Geneva, Switzerland: World Health Organization (WHO), 2014.
Argentina Vital Registration - Deaths 1997 ICD10 as it appears in World Health Organization (WHO). WHO Mortality Database Version November 2015. Geneva, Switzerland: World Health Organization (WHO), 2015.
Argentina Vital Registration - Deaths 1998 ICD10 as it appears in World Health Organization (WHO). WHO Mortality Database Version November 2015. Geneva, Switzerland: World Health Organization (WHO), 2015.
Argentina Vital Registration - Deaths 1999 ICD10 as it appears in World Health Organization (WHO). WHO Mortality Database Version November 2015. Geneva, Switzerland: World Health Organization (WHO), 2015.
Argentina Vital Registration - Deaths 2000 ICD10 as it appears in World Health Organization (WHO). WHO Mortality Database Version November 2015. Geneva, Switzerland: World Health Organization (WHO), 2015.

Appendix: Citation List

Citation
Argentina Vital Registration - Deaths 2001 ICD10 as it appears in World Health Organization (WHO). WHO Mortality Database Version November 2015. Geneva, Switzerland: World Health Organization (WHO), 2015.
Argentina Vital Registration - Deaths 2002 ICD10 as it appears in World Health Organization (WHO). WHO Mortality Database Version November 2015. Geneva, Switzerland: World Health Organization (WHO), 2015.
Argentina Vital Registration - Deaths 2003 ICD10 as it appears in World Health Organization (WHO). WHO Mortality Database Version November 2015. Geneva, Switzerland: World Health Organization (WHO), 2015.
Argentina Vital Registration - Deaths 2004 ICD10 as it appears in World Health Organization (WHO). WHO Mortality Database Version November 2015. Geneva, Switzerland: World Health Organization (WHO), 2015.
Argentina Vital Registration - Deaths 2005 ICD10 as it appears in World Health Organization (WHO). WHO Mortality Database Version November 2015. Geneva, Switzerland: World Health Organization (WHO), 2015.
Argentina Vital Registration - Deaths 2007 ICD10 as it appears in World Health Organization (WHO). WHO Mortality Database Version November 2015. Geneva, Switzerland: World Health Organization (WHO), 2015.
Argentina Vital Registration - Deaths 2008 ICD10 as it appears in World Health Organization (WHO). WHO Mortality Database Version November 2015. Geneva, Switzerland: World Health Organization (WHO), 2015.
Argentina Vital Registration - Deaths 2009 ICD10 as it appears in World Health Organization (WHO). WHO Mortality Database Version November 2015. Geneva, Switzerland: World Health Organization (WHO), 2015.
Argentina Vital Registration - Deaths 2010 ICD10 as it appears in World Health Organization (WHO). WHO Mortality Database Version November 2015. Geneva, Switzerland: World Health Organization (WHO), 2015.
Argentina Vital Registration - Deaths 2011 ICD10 as it appears in World Health Organization (WHO). WHO Mortality Database Version November 2015. Geneva, Switzerland: World Health Organization (WHO), 2015.
Argentina Vital Registration - Deaths 2012 ICD10 as it appears in World Health Organization (WHO). WHO Mortality Database Version November 2015. Geneva, Switzerland: World Health Organization (WHO), 2015.
Argentina Vital Registration - Deaths 2013 ICD10 as it appears in World Health Organization (WHO). WHO Mortality Database Version November 2015. Geneva, Switzerland: World Health Organization (WHO), 2015.
Argentina Vital Registration Death Data 1950 as it appears in United Nations Statistics Division (UNSD). United Nations Demographic Yearbook - Historical Supplement 1997. New York City, United States: United Nations Statistics Division (UNSD).
Argentina Vital Registration Death Data 1951 as it appears in United Nations Statistics Division (UNSD). United Nations Demographic Yearbook - Historical Supplement 1997. New York City, United States: United Nations Statistics Division (UNSD).
Argentina Vital Registration Death Data 1952 as it appears in United Nations Statistics Division (UNSD). United Nations Demographic Yearbook - Historical Supplement 1997. New York City, United States: United Nations Statistics Division (UNSD).
Argentina Vital Registration Death Data 1953 as it appears in United Nations Statistics Division (UNSD). United Nations Demographic Yearbook - Historical Supplement 1997. New York City, United States: United Nations Statistics Division (UNSD).
Argentina Vital Registration Death Data 1954 as it appears in United Nations Statistics Division (UNSD). United Nations Demographic Yearbook - Historical Supplement 1997. New York City, United States: United Nations Statistics Division (UNSD).
Argentina Vital Registration Death Data 1955 as it appears in United Nations Statistics Division (UNSD). United Nations Demographic Yearbook - Historical Supplement 1997. New York City, United States: United Nations Statistics Division (UNSD).
Argentina Vital Registration Death Data 1956 as it appears in United Nations Statistics Division (UNSD). United Nations Demographic Yearbook - Historical Supplement 1997. New York City, United States: United Nations Statistics Division (UNSD).
Argentina Vital Registration Death Data 1957 as it appears in United Nations Statistics Division (UNSD). United Nations Demographic Yearbook - Historical Supplement 1997. New York City, United States: United Nations Statistics Division (UNSD).
Argentina Vital Registration Death Data 1958 as it appears in United Nations Statistics Division (UNSD). United Nations Demographic Yearbook - Historical Supplement 1997. New York City, United States: United Nations Statistics Division (UNSD).
Argentina Vital Registration Death Data 1959 as it appears in United Nations Statistics Division (UNSD). United Nations Demographic Yearbook - Historical Supplement 1997. New York City, United States: United Nations Statistics Division (UNSD).
Argentina Vital Registration Death Data 1960 as it appears in United Nations Statistics Division (UNSD). United Nations Demographic Yearbook - Historical Supplement 1997. New York City, United States: United Nations Statistics Division (UNSD).
Argentina Vital Registration Death Data 1961 as it appears in United Nations Statistics Division (UNSD). United Nations Demographic Yearbook - Historical Supplement 1997. New York City, United States: United Nations Statistics Division (UNSD).
Argentina Vital Registration Death Data 1962 as it appears in United Nations Statistics Division (UNSD). United Nations Demographic Yearbook - Historical Supplement 1997. New York City, United States: United Nations Statistics Division (UNSD).
Argentina Vital Registration Death Data 1963 as it appears in United Nations Statistics Division (UNSD). United Nations Demographic Yearbook - Historical Supplement 1997. New York City, United States: United Nations Statistics Division (UNSD).
Argentina Vital Registration Death Data 1964 as it appears in United Nations Statistics Division (UNSD). United Nations Demographic Yearbook - Historical Supplement 1997. New York City, United States: United Nations Statistics Division (UNSD).
Argentina Vital Registration Death Data 1965 as it appears in United Nations Statistics Division (UNSD). United Nations Demographic Yearbook - Historical Supplement 1997. New York City, United States: United Nations Statistics Division (UNSD).
Argentina Vital Registration Death Data 1966 as it appears in United Nations Statistics Division (UNSD). United Nations Demographic Yearbook - Historical Supplement 1997. New York City, United States: United Nations Statistics Division (UNSD).
Argentina Vital Registration Death Data 1967 as it appears in United Nations Statistics Division (UNSD). United Nations Demographic Yearbook - Historical Supplement 1997. New York City, United States: United Nations Statistics Division (UNSD).
Argentina Vital Registration Death Data 1976 as it appears in United Nations Statistics Division (UNSD). United Nations Demographic Yearbook - Historical Supplement 1997. New York City, United States: United Nations Statistics Division (UNSD).
Argentina Vital Registration Death Data 2006 as it appears in United Nations Statistics Division (UNSD). United Nations Demographic Yearbook 2006. New York City, United States: United Nations Statistics Division (UNSD), 2008.

Appendix: Citation List

Citation
Argentina Vital Registration Death Data 2011 as it appears in United Nations Statistics Division (UNSD). United Nations Demographic Yearbook 2012. New York City, United States: United Nations Statistics Division (UNSD), 2013.
Arguillas MO, Domingo EO, Tsuda F, Mayumi M, Suzuki H. Seroepidemiology of hepatitis C virus infection in the Philippines: a preliminary study and comparison with hepatitis B virus infection among blood donors, medical personnel, and patient groups in Davao, Philippines. Gastroenterol Jpn. 1991; 170-5.
Arieta CEL, de Oliveira DF, Lupinacci AP de C, Novaes P, Paccola M, Jose NK, Limburg H. Cataract remains an important cause of blindness in Campinas, Brazil. Ophthalmic Epidemiol. 2009; 16(1): 58-63.
Arinola O. Complement factors and circulating immune complexes in children with urinary schistosomiasis and asymptomatic malaria. Afr J Med Med Sci. 2005; 34(1): 9-13. As it appears in Malaria Atlas Project. Malaria Atlas Project Plasmodium Falciparum Parasite Rate Database. Oxford, United Kingdom: Malaria Atlas Project.
Aristides Maltez Hospital (Brazil), Bahia League Against Cancer (Brazil), National Cancer Institute (Brazil). Brazil - Salvador BasePopWeb Database - Population Based Cancer Registry (RCBP Salvador) Statistics. Rio de Janeiro, Brazil: National Cancer Institute (Brazil).
Armenia Health and Nutritional Status of Children and Women 1998 as it appears in World Health Organization (WHO). WHO Global Database on Child Growth and Malnutrition. Geneva, Switzerland: World Health Organization (WHO).
Armenia Integrated Living Conditions Survey 1996 as it appears in World Health Organization (WHO). WHO Household Energy Database. Geneva, Switzerland: World Health Organization (WHO), 2010.
Armenia Integrated Living Conditions Survey 2004 as it appears in World Health Organization (WHO). WHO Household Energy Database. Geneva, Switzerland: World Health Organization (WHO), 2010.
Armenia Integrated Living Conditions Survey 2005 as it appears in World Health Organization (WHO). WHO Household Energy Database. Geneva, Switzerland: World Health Organization (WHO), 2010.
Armenia Integrated Living Conditions Survey 2006 as it appears in World Health Organization (WHO). WHO Household Energy Database. Geneva, Switzerland: World Health Organization (WHO), 2010.
Armenia Integrated Living Conditions Survey 2007 as it appears in World Health Organization (WHO). WHO Household Energy Database. Geneva, Switzerland: World Health Organization (WHO), 2010.
Armenia Integrated Survey of Living Standards 2001 as it appears in World Health Organization (WHO). WHO Household Energy Database. Geneva, Switzerland: World Health Organization (WHO), 2010.
Armenia Integrated Survey of Living Standards 2002 as it appears in World Health Organization (WHO). WHO Household Energy Database. Geneva, Switzerland: World Health Organization (WHO), 2010.
Armenia Integrated Survey of Living Standards 2003 as it appears in World Health Organization (WHO). WHO Household Energy Database. Geneva, Switzerland: World Health Organization (WHO), 2010.
Armenia Millennium Development Goal Indicators as it appears in World Health Organization (WHO). WHO Household Energy Database. Geneva, Switzerland: World Health Organization (WHO), 2010.
Armenia Vital Registration - Deaths 1981 ICD9 as it appears in World Health Organization (WHO). WHO Mortality Database Version November 2015. Geneva, Switzerland: World Health Organization (WHO), 2015.
Armenia Vital Registration - Deaths 1982 ICD9 as it appears in World Health Organization (WHO). WHO Mortality Database Version November 2015. Geneva, Switzerland: World Health Organization (WHO), 2015.
Armenia Vital Registration - Deaths 1985 ICD9 as it appears in World Health Organization (WHO). WHO Mortality Database Version November 2015. Geneva, Switzerland: World Health Organization (WHO), 2015.
Armenia Vital Registration - Deaths 1986 ICD9 as it appears in World Health Organization (WHO). WHO Mortality Database Version November 2015. Geneva, Switzerland: World Health Organization (WHO), 2015.
Armenia Vital Registration - Deaths 1987 ICD9 as it appears in World Health Organization (WHO). WHO Mortality Database Version November 2015. Geneva, Switzerland: World Health Organization (WHO), 2015.
Armenia Vital Registration - Deaths 1988 ICD9 as it appears in World Health Organization (WHO). WHO Mortality Database Version November 2015. Geneva, Switzerland: World Health Organization (WHO), 2015.
Armenia Vital Registration - Deaths 1989 ICD9 as it appears in World Health Organization (WHO). WHO Mortality Database Version November 2015. Geneva, Switzerland: World Health Organization (WHO), 2015.
Armenia Vital Registration - Deaths 1990 ICD9 as it appears in World Health Organization (WHO). WHO Mortality Database Version November 2015. Geneva, Switzerland: World Health Organization (WHO), 2015.
Armenia Vital Registration - Deaths 1991 ICD9 as it appears in World Health Organization (WHO). WHO Mortality Database Version November 2015. Geneva, Switzerland: World Health Organization (WHO), 2015.
Armenia Vital Registration - Deaths 1992 ICD9 as it appears in World Health Organization (WHO). WHO Mortality Database Version November 2015. Geneva, Switzerland: World Health Organization (WHO), 2015.
Armenia Vital Registration - Deaths 1993 ICD9 as it appears in World Health Organization (WHO). WHO Mortality Database Version November 2015. Geneva, Switzerland: World Health Organization (WHO), 2015.
Armenia Vital Registration - Deaths 1994 ICD9 as it appears in World Health Organization (WHO). WHO Mortality Database Version November 2015. Geneva, Switzerland: World Health Organization (WHO), 2015.
Armenia Vital Registration - Deaths 1995 ICD9 as it appears in World Health Organization (WHO). WHO Mortality Database Version November 2015. Geneva, Switzerland: World Health Organization (WHO), 2015.
Armenia Vital Registration - Deaths 1996 ICD9 as it appears in World Health Organization (WHO). WHO Mortality Database Version November 2015. Geneva, Switzerland: World Health Organization (WHO), 2015.
Armenia Vital Registration - Deaths 1997 ICD9 as it appears in World Health Organization (WHO). WHO Mortality Database Version November 2015. Geneva, Switzerland: World Health Organization (WHO), 2015.
Armenia Vital Registration - Deaths 1998 ICD9 as it appears in World Health Organization (WHO). WHO Mortality Database Version November 2015. Geneva, Switzerland: World Health Organization (WHO), 2015.

Appendix: Citation List

Citation
Armenia Vital Registration - Deaths 1999 ICD9 as it appears in World Health Organization (WHO). WHO Mortality Database Version November 2015. Geneva, Switzerland: World Health Organization (WHO), 2015.
Armenia Vital Registration - Deaths 2000 ICD9 as it appears in World Health Organization (WHO). WHO Mortality Database Version November 2015. Geneva, Switzerland: World Health Organization (WHO), 2015.
Armenia Vital Registration - Deaths 2001 ICD9 as it appears in World Health Organization (WHO). WHO Mortality Database Version November 2015. Geneva, Switzerland: World Health Organization (WHO), 2015.
Armenia Vital Registration - Deaths 2002 ICD9 as it appears in World Health Organization (WHO). WHO Mortality Database Version November 2015. Geneva, Switzerland: World Health Organization (WHO), 2015.
Armenia Vital Registration - Deaths 2003 ICD9 as it appears in World Health Organization (WHO). WHO Mortality Database Version November 2015. Geneva, Switzerland: World Health Organization (WHO), 2015.
Armenia Vital Registration - Deaths 2006 ICD10 as it appears in World Health Organization (WHO). WHO Mortality Database Version November 2015. Geneva, Switzerland: World Health Organization (WHO), 2015.
Armenia Vital Registration - Deaths 2008 ICD10 as it appears in World Health Organization (WHO). WHO Mortality Database Version November 2015. Geneva, Switzerland: World Health Organization (WHO), 2015.
Armenia Vital Registration - Deaths 2009 ICD10 as it appears in World Health Organization (WHO). WHO Mortality Database Version November 2015. Geneva, Switzerland: World Health Organization (WHO), 2015.
Armenia Vital Registration - Deaths 2010 ICD10 as it appears in World Health Organization (WHO). WHO Mortality Database Version November 2015. Geneva, Switzerland: World Health Organization (WHO), 2015.
Armenia Vital Registration - Deaths 2011 ICD10 as it appears in World Health Organization (WHO). WHO Mortality Database Version November 2015. Geneva, Switzerland: World Health Organization (WHO), 2015.
Armenia Vital Registration - Deaths 2012 ICD10 as it appears in World Health Organization (WHO). WHO Mortality Database Version November 2015. Geneva, Switzerland: World Health Organization (WHO), 2015.
Armenia Vital Registration Death Data 2004 as it appears in United Nations Statistics Division (UNSD). United Nations Demographic Yearbook 2004. New York City, United States: United Nations Statistics Division (UNSD), 2007.
Armenia Vital Registration Death Data 2007 as it appears in United Nations Statistics Division (UNSD). United Nations Demographic Yearbook 2007. New York City, United States: United Nations Statistics Division (UNSD), 2009.
Armstrong B, Tremblay C, Baris D, Thériault G. Lung cancer mortality and polynuclear aromatic hydrocarbons: a case-cohort study of aluminum production workers in Arvida, Quebec, Canada. Am J Epidemiol. 1994; 139(3): 250-62 as it appears in Armstrong B, Hutchinson E, Unwin J, Fletcher T. Lung cancer risk after exposure to polycyclic aromatic hydrocarbons: a review and meta-analysis. Environ. Health Perspect. 2004; 112(9): 970–978.
Armstrong SA, Gangam N, Chipman ML, Rootman DS. The prevalence of positive hepatitis B, hepatitis C, and HIV serology in cornea donors prescreened by medical and social history in Ontario, Canada. Cornea. 1997; 16(5): 512-6.
Arpi ML, Italia S, Motta RM, Raimondo M, Mancuso M, Tomaselli L, Squatrito S, Vigneri R, Purrello F. Incidence of type I diabetes in the district of Catania, Sicily. Acta Diabetol. 1994; 31(1): 37-9.
Arroyo P, Loria A, FernÃ¡ndez V, Flegal KM, Kuri-Morales P, Olaiz G, Tapia-Conyer R. Prevalence of pre-obesity and obesity in urban adult Mexicans in comparison with other large surveys. Obes Res. 2000; 8(2): 179-85.
Arruda ME, Zimmerman RH, Souza RM, Oliveira-Ferreira J. Prevalence and level of antibodies to the circumsporozoite protein of human malaria parasites in five states of the Amazon region of Brazil. Mem Inst Oswaldo Cruz. 2007; 102(3): 367-72. As it appears in Malaria Atlas Project. Malaria Atlas Project Plasmodium Falciparum Parasite Rate Database. Oxford, United Kingdom: Malaria Atlas Project.
Arslan O, Çepni MS, Etiler N. Spatial analysis of perinatal mortality rates with geographic information systems in Kocaeli, Turkey. Public Health. 2013; 127(4): 369-79.
Arthur FKN, Adu-Frimpong M, Osei-Yeboah J, Mensah FO, Owusu L. The prevalence of metabolic syndrome and its predominant components among pre-and postmenopausal Ghanaian women. BMC Res Notes. 2013; 6: 446.
Arunachalam N, Mariappan T, Vijayakumar KN, Sabesan S, Panicker KN. Mattancherry urban agglomeration, a diminishing focus of lymphatic filariasis in Kerala. J Commun Dis. 1996; 28(3): 168-70.
Asakura T, Tachibana K, Watanabe S, Teshima D, Ikeda M, Tokudome S. Concomitant carriage of hepatitis B virus and human T-lymphotropic virus type I among blood donors in Kitakyushu, Japan. J Infect. 1991; 23(1): 33-7.
Aschner P, King H, Triana de Torrado M, Rodriguez BM. Glucose intolerance in Colombia. A population-based survey in an urban community. Diabetes Care. 1993; 16(1): 90-3.
Asfour MG, Lambourne A, Soliman A, Al-Behlani S, Al-Asfoor D, Bold A, Mahtab H, King H. High prevalence of diabetes mellitus and impaired glucose tolerance in the Sultanate of Oman: results of the 1991 national survey. Diabet Med. 1995; 12(12): 1122-5.
Asghar S, Khan AKA, Ali SMK, Sayeed MA, Bhowmik B, Diep ML, Shi Z, Hussain A. Incidence of diabetes in Asian-Indian subjects: a five year follow-up study from Bangladesh. Prim Care Diabetes. 2011; 5(2): 117-24.
Ashford R, Craig P, Oppenheimer S. Polyparasitism on the Kenya coast. 2. Spatial heterogeneity in parasite distributions. Ann Trop Med Parasitol. 1993; 87(3): 283-93. As it appears in Malaria Atlas Project. Malaria Atlas Project Plasmodium Falciparum Parasite Rate Database. Oxford, United Kingdom: Malaria Atlas Project.
Ashford RW, Craig PS, Oppenheimer SJ. Polyparasitism on the Kenya coast. 1. Prevalence, and association between parasitic infections. Ann Trop Med Parasitol. 1992; 86(6): 671-9. As it appears in Malaria Atlas Project. Malaria Atlas Project Plasmodium Falciparum Parasite Rate Database. Oxford, United Kingdom: Malaria Atlas Project.
Ashok S, Ramu M, Deepa R, Mohan V. Prevalence of neuropathy in type 2 diabetic patients attending a diabetes centre in South India. J Assoc Physicians India. 2002; 546–50.
Ashraf H, Alam NH, Rothermundt C, Brooks A, Bardhan P, Hossain L, Salam MA, Hassan MS, Beglinger C, Gyr N. Prevalence and risk factors of hepatitis B and C virus infections in an impoverished urban community in Dhaka, Bangladesh. BMC Infect Dis. 2010; 10(1): 208.

Appendix: Citation List

Citation
Ashton RA, Kefyalew T, Tesfaye G, Pullan RL, Yadeta D, Reithinger R, Kolaczinski JH, Brooker S. School-based surveys of malaria in Oromia Regional State, Ethiopia: a rapid survey method for malaria in low transmission settings. Malar J. 2011; 10(1): 25. As it appears in Malaria Atlas Project. Malaria Atlas Project Plasmodium Falciparum Parasite Rate Database. Oxford, United Kingdom: Malaria Atlas Project.
Ashton RA, Kyabayinze DJ, Opio T, Auma A, Edwards T, Matwale G, Onapa A, Brooker S, Kolaczinski JH. The impact of mass drug administration and long-lasting insecticidal net distribution on Wuchereria bancrofti infection in humans and mosquitoes: an observational study in northern Uganda. Parasit Vectors. 2011; 134. As it appears in London School of Hygiene and Tropical Medicine. Global Atlas of Helminth Infections - Lymphatic Filiariasis.
Ashwell MJ, Cossart YE. An autopsy survey of hepatitis B in Sydney. Pathology. 1995; 27(1): 43-7.
Asih PB, Rozi IE, Herdiana H, Pratama NR, Hidayati AP, Marantina SS, Kosasih S, Chand K, Wangsamuda S, Rusdy FA, Sumiwi ME, Imran A, Yuniarti T, Sianturi T, Nur J, Asnita A, Bukhari B, Barussanah C, Yani M, Ainun C, Jamil K, Mariam C, Sengkerij SP, Laihad FJ, Hawley W, Syafruddin D. The baseline distribution of malaria in the initial phase of elimination in Sabang Municipality, Aceh Province, Indonesia. Malar J. 2012; 11(1): 291. As it appears in Malaria Atlas Project. Malaria Atlas Project Plasmodium Falciparum Parasite Rate Database. Oxford, United Kingdom: Malaria Atlas Project.
Asiki G, Baisley K, Newton R, Marions L, Seeley J, Kamali A, Smedman L. Adverse pregnancy outcomes in rural Uganda (1996-2013): trends and associated factors from serial cross sectional surveys. BMC Pregnancy Childbirth. 2015; 15: 279.
Asmae H, Fatima A, Hajiba F, Mbarek K, Khadija B, Mohamed R, Faiza S. Coexistence of Leishmania tropica and Leishmania infantum in Sefrou province, Morocco. Acta Trop. 2013; 94-99.
Asnani MR, McCaw-Binns AM, Reid ME. Excess risk of maternal death from sickle cell disease in Jamaica: 1998-2007. PLoS One. 2011; 6(10): e26281.
Asociación Demográfica Salvadoreña (ADS), Division of Reproductive Health-Centers for Disease Control and Prevention (CDC). (2004) El Salvador Reproductive Health Survey 2002-2003. San Salvador, El Salvador: ADS.
Asociación Demográfica Salvadoreña (ADS), Division of Reproductive Health-Centers for Disease Control and Prevention (CDC). (2009) El Salvador Reproductive Health Survey 2008. San Salvador, El Salvador: ADS.
Aspinall S, Joubert JJ, Evans AC, Joseph S, Steele AD, Lecatsas G. Prevalence of hepatitis B in !Kung (San) children from Bushmanland, Namibia. Ann Trop Paediatr. 1994; 14(2): 163-7.
Assabari. Yemen Plasmodium Falciparum Parasite Rate Data, Assabari 1997. As it appears in Malaria Atlas Project. Malaria Atlas Project Plasmodium Falciparum Parasite Rate Database. Oxford, United Kingdom: Malaria Atlas Project.
Assefa N, Berhane Y, Worku A. Pregnancy rates and pregnancy loss in Eastern Ethiopia. Acta Obstet Gynecol Scand. 2013; 92(6): 642-7.
Assessment of Nutritional Status and Associated Factors of Under-Fives in Dar es Salaam Region from 9-23 September 1991 as it appears in World Health Organization (WHO). WHO Global Database on Child Growth and Malnutrition - Historical. Geneva, Switzerland: World Health Organization (WHO).
Assessment of Nutritional Status and Infant Mortality in the Continental Region of Equatorial Guinea as it appears in World Health Organization (WHO). WHO Global Database on Child Growth and Malnutrition - Historical. Geneva, Switzerland: World Health Organization (WHO).
Assessment of the Nutritional Status of Children Under 5 in the Gaza Strip as it appears in World Health Organization (WHO). WHO Global Database on Child Growth and Malnutrition - Historical. Geneva, Switzerland: World Health Organization (WHO).
Assessments of the nutritional status of children on the Nembi Plateau in 1978 and 1980 as it appears in World Health Organization (WHO). WHO Global Database on Child Growth and Malnutrition - Historical. Geneva, Switzerland: World Health Organization (WHO).
Assis AM, Barreto ML, Prado MS, Reis MG, Parraga IM, Blanton RE. Schistosoma mansoni infection and nutritional status in schoolchildren: a randomized, double-blind trial in northeastern Brazil. Am J Clin Nutr. 1998; 68(6): 1247-53.
Assis AMO, Prado MS, Barreto ML, Reis MG, Conceição Pinheiro SM, Parraga IM, Blanton RE. Childhood stunting in Northeast Brazil: the role of Schistosoma mansoni infection and inadequate dietary intake. Eur J Clin Nutr. 2004; 58(7): 1022-9.
Associates for Community and Population Research (ACPR), International Centre for Diarrhoeal Disease Research (Bangladesh), MEASURE Evaluation Project, Carolina Population Center, University of North Carolina, National Institute of Population Research and Training (NIPORT). Bangladesh Urban Health Survey 2006.
Associates for Community and Population Research (ACPR), International Centre for Diarrhoeal Disease Research, Bangladesh (ICDDR,B), Johns Hopkins University (JHU), Mitra and Associates, National Institute of Population Research and Training (NIPORT), ORC Macro. Bangladesh Demographic and Health Survey - Maternal Mortality Data.
Associates for Community and Population Research (ACPR), International Centre for Diarrhoeal Disease Research, Bangladesh (ICDDR,B), Johns Hopkins University (JHU), Mitra and Associates, National Institute of Population Research and Training (NIPORT), ORC Macro. Bangladesh Special Demographic and Health Survey 2001. Calverton, United States: ORC Macro.
Associates for Community and Population Research (ACPR), Johns Hopkins University (JHU), Macro International, Inc, Mitra and Associates, National Institute of Population Research and Training (NIPORT). Bangladesh Demographic and Health Survey - Complete Birth History Data.
Association for Family Welfare of Guatemala (APROFAM) and Division of Reproductive Health-Centers for Disease Control and Prevention (CDC). Guatemala Contraceptive Prevalence Survey 1978-1979. Guatemala City, Guatemala. 1979.
Association of Nordic Cancer Registries (ANCR). Denmark NORDCAN Cancer Incidence Data Tables, Age-Specific by Countries. Copenhagen, Denmark: Association of Nordic Cancer Registries (ANCR).
Association of Nordic Cancer Registries (ANCR). Finland NORDCAN Cancer Incidence Data Tables, Age-Specific by Countries. Copenhagen, Denmark: Association of Nordic Cancer Registries (ANCR).
Association of Nordic Cancer Registries (ANCR). Iceland NORDCAN Cancer Incidence Data Tables, Age-Specific by Countries. Copenhagen, Denmark: Association of Nordic Cancer Registries (ANCR).
Association of Nordic Cancer Registries (ANCR). Norway NORDCAN Cancer Incidence Data Tables, Age-Specific by Countries. Copenhagen, Denmark: Association of Nordic Cancer Registries (ANCR).

Appendix: Citation List

Citation
Association of Nordic Cancer Registries (ANCR). Sweden NORDCAN Cancer Incidence Data Tables, Age-Specific by Countries. Copenhagen, Denmark: Association of Nordic Cancer Registries (ANCR).
Association of Nordic Cancer Registries (ANCR). Sweden NORDCAN Cancer Mortality Data Tables, Age-Specific by Countries. Copenhagen, Denmark: Association of Nordic Cancer Registries (ANCR).
Association to Fight Cancer in Goiás (Brazil), National Cancer Institute (Brazil). Brazil - Goiânia BasePopWeb Database - Population Based Cancer Registry (RCBP Goiânia) Statistics. Rio de Janeiro, Brazil: National Cancer Institute (Brazil).
Astagneau P, Lang T, Delarocque E, Jeannee E, Salem G. Arterial hypertension in urban Africa: an epidemiological study on a representative sample of Dakar inhabitants in Senegal. J Hypertens. 1992; 10(9): 1095-101.
Asulin Y, McCann TJ, McCarty CW, Hage RW, Rooney PJ, Macpherson CNL. Cancer incidence and mortality in Grenada 1990-2000. West Indian Med J. 2004; 53(6): 368-73.
Asuzu MC, Johnson OO, Owoaje ET, Kaufman JS, Rotimi C, Cooper RS. The Idikan adult mortality study. Afr J Med Med Sci. 2000; 29(2): 115-8.
Atabek ME, Kart H, Erkul I. Prevalence of hepatitis A, B, C and E virus in adolescents with type-1 diabetes mellitus. Int J Adolesc Med Health. 2003; 15(2): 133-7.
Atanasova MV, Haydouchka IA, Zlatev SP, Stoilova YD, Iliev YT, Mateva NG. Prevalence of antibodies against hepatitis C virus and hepatitis B coinfection in healthy population in Bulgaria. A seroepidemiological study. Minerva Gastroenterol Dietol. 2004; 50(1): 89-96.
Atangana J, Fomena A, Tamesse J, Fondjo E. [Agricultural activities and epidemiology of malaria in Soudano-Sahelian zone in Cameroon]. Bull Soc Pathol Exot. 2012; 105(1): 23-29. As it appears in Malaria Atlas Project. Malaria Atlas Project Plasmodium Falciparum Parasite Rate Database. Oxford, United Kingdom: Malaria Atlas Project.
Atkinson SH, Rockett K, Sirugo G, Bejon PA, Fulford A, O'Connell MA, Bailey R, Kwiatkowski DP, Prentice AM. Seasonal childhood anaemia in West Africa is associated with the haptoglobin 2-2 genotype. PLoS Med. 2006; 3(5): e172. As it appears in Malaria Atlas Project. Malaria Atlas Project Plasmodium Falciparum Parasite Rate Database. Oxford, United Kingdom: Malaria Atlas Project.
Atta A, Atia MM, Mostafa AM. Prevalence of microfilaria versus intradermal test in El-Koren, Sharkia G., Egypt. J Egypt Soc Parasitol. 1984; 14(1): 251-7. As it appears in London School of Hygiene and Tropical Medicine. Global Atlas of Helminth Infections - Lymphatic Filiariasis.
Aubry P, Niel L, Niyongabo T, Kerguelen S, Larouze B. Seroprevalence of hepatitis E virus in an adult urban population from Burundi. Am J Trop Med Hyg. 1997; 57(3): 272-3.
Auckland UniServices, University of Auckland (New Zealand), Health Research Council of New Zealand, Ministry of Health (New Zealand), National Research Bureau Ltd (New Zealand), World Health Organization (WHO). New Zealand Mental Health Survey 2003-2004.
Audibert M, Josseran R, Josse R, Adjidji A. Irrigation, schistosomiasis, and malaria in the Logone Valley, Cameroon. Am J Trop Med Hyg. 1990; 42(6): 550-60. As it appears in Malaria Atlas Project. Malaria Atlas Project Plasmodium Falciparum Parasite Rate Database. Oxford, United Kingdom: Malaria Atlas Project.
Auer H, Aspock H. Echinococcosis in Austria. Zentralbl Bakteriol. 1990; 272(4): 498-508.
Aunger R, Schmidt WP, Ranpura A, Coombes Y, Maina PM, Matiko CN, Curtis V. Three kinds of psychological determinants for hand-washing behaviour in Kenya. Soc Sci Med. 2010; 70(3): 383-91.
Australasian Association of Cancer Registries, Australian Institute of Health and Welfare. Australia Cancer Incidence and Mortality Books 2012. Canberra, Australia: Australian Institute of Health and Welfare, 2012.
Australia - Capital Territory Cancer Registry 1978-1982 - CI5 as it appears in Parkin DM, International Agency for Research on Cancer, International Association of Cancer Registries. Cancer Incidence in Five Continents. Vol. I to VIII. Lyon, France, IARCPress, 2005.
Australia - Capital Territory Cancer Registry 1983-1987 - CI5 as it appears in Parkin DM, International Agency for Research on Cancer, International Association of Cancer Registries. Cancer Incidence in Five Continents. Vol. I to VIII. Lyon, France, IARCPress, 2005.
Australia - Capital Territory Cancer Registry 1993-1997 - CI5 as it appears in Parkin DM, International Agency for Research on Cancer, International Association of Cancer Registries. Cancer Incidence in Five Continents. Vol. I to VIII. Lyon, France, IARCPress, 2005.
Australia - New South Wales Cancer Registry 1978-1982 - CI5 as it appears in Parkin DM, International Agency for Research on Cancer, International Association of Cancer Registries. Cancer Incidence in Five Continents. Vol. I to VIII. Lyon, France, IARCPress, 2005.
Australia - New South Wales Cancer Registry 1983-1987 - CI5 as it appears in Parkin DM, International Agency for Research on Cancer, International Association of Cancer Registries. Cancer Incidence in Five Continents. Vol. I to VIII. Lyon, France, IARCPress, 2005.
Australia - New South Wales Cancer Registry 1993-1997 - CI5 as it appears in Parkin DM, International Agency for Research on Cancer, International Association of Cancer Registries. Cancer Incidence in Five Continents. Vol. I to VIII. Lyon, France, IARCPress, 2005.
Australia - Queensland Cancer Registry 1982 - CI5 as it appears in Parkin DM, International Agency for Research on Cancer, International Association of Cancer Registries. Cancer Incidence in Five Continents. Vol. I to VIII. Lyon, France, IARCPress, 2005.
Australia - Queensland Cancer Registry 1993 - CI5 as it appears in Parkin DM, International Agency for Research on Cancer, International Association of Cancer Registries. Cancer Incidence in Five Continents. Vol. I to VIII. Lyon, France, IARCPress, 2005.
Australia - Queensland Cancer Registry 1994 - CI5 as it appears in Parkin DM, International Agency for Research on Cancer, International Association of Cancer Registries. Cancer Incidence in Five Continents. Vol. I to VIII. Lyon, France, IARCPress, 2005.
Australia - Queensland Cancer Registry 1995 - CI5 as it appears in Parkin DM, International Agency for Research on Cancer, International Association of Cancer Registries. Cancer Incidence in Five Continents. Vol. I to VIII. Lyon, France, IARCPress, 2005.
Australia - Queensland Cancer Registry 1996 - CI5 as it appears in Parkin DM, International Agency for Research on Cancer, International Association of Cancer Registries. Cancer Incidence in Five Continents. Vol. I to VIII. Lyon, France, IARCPress, 2005.
Australia - Queensland Cancer Registry 1997 - CI5 as it appears in Parkin DM, International Agency for Research on Cancer, International Association of Cancer Registries. Cancer Incidence in Five Continents. Vol. I to VIII. Lyon, France, IARCPress, 2005.
Australia - South Australia Cancer Registry 1978-1982 - CI5 as it appears in Parkin DM, International Agency for Research on Cancer, International Association of Cancer Registries. Cancer Incidence in Five Continents. Vol. I to VIII. Lyon, France, IARCPress, 2005.

Appendix: Citation List

Citation
Australia - South Australia Cancer Registry 1980 - CI5 as it appears in Ferlay J, Parkin DM, Curado MP, Bray F, Edwards B, Shin HR and Forman D. Cancer Incidence in Five Continents, Volumes I to IX: IARC CancerBase No. 9 [Internet]. Lyon, France: International Agency for Research on Cancer; 2010. Available from: http://ci5.iarc.fr
Australia - South Australia Cancer Registry 1981 - CI5 as it appears in Ferlay J, Parkin DM, Curado MP, Bray F, Edwards B, Shin HR and Forman D. Cancer Incidence in Five Continents, Volumes I to IX: IARC CancerBase No. 9 [Internet]. Lyon, France: International Agency for Research on Cancer; 2010. Available from: http://ci5.iarc.fr
Australia - South Australia Cancer Registry 1983-1987 - CI5 as it appears in Parkin DM, International Agency for Research on Cancer, International Association of Cancer Registries. Cancer Incidence in Five Continents. Vol. I to VIII. Lyon, France, IARCPress, 2005.
Australia - South Australia Cancer Registry 1988-1992 - CI5 as it appears in Parkin DM, International Agency for Research on Cancer, International Association of Cancer Registries. Cancer Incidence in Five Continents. Vol. I to VIII. Lyon, France, IARCPress, 2005.
Australia - South Australia Cancer Registry 1993-1997 - CI5 as it appears in Parkin DM, International Agency for Research on Cancer, International Association of Cancer Registries. Cancer Incidence in Five Continents. Vol. I to VIII. Lyon, France, IARCPress, 2005.
Australia - South Australia Cancer Registry 1998-2002 - CI5 as it appears in Curado MP, Edwards B, Shin HR, Storm H, Ferlay J, Heanue M and Boyle P, eds (2007). Cancer Incidence in Five Continents, Vol. IX Periodic Data (electronic version). Lyon, IARC.http://ci5.iarc.fr
Australia - Tasmania Cancer Registry 1978-1982 - CI5 as it appears in Parkin DM, International Agency for Research on Cancer, International Association of Cancer Registries. Cancer Incidence in Five Continents. Vol. I to VIII. Lyon, France, IARCPress, 2005.
Australia - Tasmania Cancer Registry 1980 - CI5 as it appears in Ferlay J, Parkin DM, Curado MP, Bray F, Edwards B, Shin HR and Forman D. Cancer Incidence in Five Continents, Volumes I to IX: IARC CancerBase No. 9 [Internet]. Lyon, France: International Agency for Research on Cancer; 2010. Available from: http://ci5.iarc.fr
Australia - Tasmania Cancer Registry 1981 - CI5 as it appears in Ferlay J, Parkin DM, Curado MP, Bray F, Edwards B, Shin HR and Forman D. Cancer Incidence in Five Continents, Volumes I to IX: IARC CancerBase No. 9 [Internet]. Lyon, France: International Agency for Research on Cancer; 2010. Available from: http://ci5.iarc.fr
Australia - Tasmania Cancer Registry 1983-1987 - CI5 as it appears in Parkin DM, International Agency for Research on Cancer, International Association of Cancer Registries. Cancer Incidence in Five Continents. Vol. I to VIII. Lyon, France, IARCPress, 2005.
Australia - Tasmania Cancer Registry 1988-1992 - CI5 as it appears in Parkin DM, International Agency for Research on Cancer, International Association of Cancer Registries. Cancer Incidence in Five Continents. Vol. I to VIII. Lyon, France, IARCPress, 2005.
Australia - Tasmania Cancer Registry 1993-1997 - CI5 as it appears in Parkin DM, International Agency for Research on Cancer, International Association of Cancer Registries. Cancer Incidence in Five Continents. Vol. I to VIII. Lyon, France, IARCPress, 2005.
Australia - Tasmania Cancer Registry 1998-2002 - CI5 as it appears in Curado MP, Edwards B, Shin HR, Storm H, Ferlay J, Heanue M and Boyle P, eds (2007). Cancer Incidence in Five Continents, Vol. IX Periodic Data (electronic version). Lyon, IARC.http://ci5.iarc.fr
Australia - Tasmania Cancer Registry 2001 - CI5 as it appears in Ferlay J, Parkin DM, Curado MP, Bray F, Edwards B, Shin HR and Forman D. Cancer Incidence in Five Continents, Volumes I to IX: IARC CancerBase No. 9 [Internet]. Lyon, France: International Agency for Research on Cancer; 2010. Available from: http://ci5.iarc.fr
Australia - Tasmania Cancer Registry 2002 - CI5 as it appears in Ferlay J, Parkin DM, Curado MP, Bray F, Edwards B, Shin HR and Forman D. Cancer Incidence in Five Continents, Volumes I to IX: IARC CancerBase No. 9 [Internet]. Lyon, France: International Agency for Research on Cancer; 2010. Available from: http://ci5.iarc.fr
Australia - Victoria Cancer Registry 1988-1992 - CI5 as it appears in Parkin DM, International Agency for Research on Cancer, International Association of Cancer Registries. Cancer Incidence in Five Continents. Vol. I to VIII. Lyon, France, IARCPress, 2005.
Australia - Western Australia Cancer Registry 1982 - CI5 as it appears in Parkin DM, International Agency for Research on Cancer, International Association of Cancer Registries. Cancer Incidence in Five Continents. Vol. I to VIII. Lyon, France, IARCPress, 2005.
Australia - Western Australia Cancer Registry 1988-1992 - CI5 as it appears in Parkin DM, International Agency for Research on Cancer, International Association of Cancer Registries. Cancer Incidence in Five Continents. Vol. I to VIII. Lyon, France, IARCPress, 2005.
Australia - Western Australia Cancer Registry 1998-2002 - CI5 as it appears in Curado MP, Edwards B, Shin HR, Storm H, Ferlay J, Heanue M and Boyle P, eds (2007). Cancer Incidence in Five Continents, Vol. IX Periodic Data (electronic version). Lyon, IARC.http://ci5.iarc.fr
Australia National Campaign Against Drug Abuse Social Issues Survey 1988 as it appears in P.N. Lee Statistics and Computing Ltd. International Mortality and Smoking Statistics Version 4.04. Sutton, United Kingdom: P.N. Lee Statistics and Computing Ltd, 2009.
Australia National Campaign Against Drug Abuse Social Issues Survey 1993 as it appears in P.N. Lee Statistics and Computing Ltd. International Mortality and Smoking Statistics Version 4.04. Sutton, United Kingdom: P.N. Lee Statistics and Computing Ltd, 2009.
Australia National Drug Strategy Household Survey 1998 as it appears in P.N. Lee Statistics and Computing Ltd. International Mortality and Smoking Statistics Version 4.04. Sutton, United Kingdom: P.N. Lee Statistics and Computing Ltd, 2009.
Australia National Health Survey 1989-1990 as it appears in P.N. Lee Statistics and Computing Ltd. International Mortality and Smoking Statistics Version 4.04. Sutton, United Kingdom: P.N. Lee Statistics and Computing Ltd, 2009.
Australia National Nutrition Survey 1995-1996 as it appears in World Health Organization (WHO). WHO Global Database on Child Growth and Malnutrition. Geneva, Switzerland: World Health Organization (WHO).
Australia National Survey of Mental Health and Wellbeing 1997 as it appears in P.N. Lee Statistics and Computing Ltd. International Mortality and Smoking Statistics Version 4.04. Sutton, United Kingdom: P.N. Lee Statistics and Computing Ltd, 2009.
Australia National Tobacco Campaign Evaluation Survey 1997 as it appears in P.N. Lee Statistics and Computing Ltd. International Mortality and Smoking Statistics Version 4.04. Sutton, United Kingdom: P.N. Lee Statistics and Computing Ltd, 2009.
Australia National Tobacco Campaign Evaluation Survey 1998 as it appears in P.N. Lee Statistics and Computing Ltd. International Mortality and Smoking Statistics Version 4.04. Sutton, United Kingdom: P.N. Lee Statistics and Computing Ltd, 2009.
Australia National Tobacco Campaign Evaluation Survey 1999 as it appears in P.N. Lee Statistics and Computing Ltd. International Mortality and Smoking Statistics Version 4.04. Sutton, United Kingdom: P.N. Lee Statistics and Computing Ltd, 2009.
Australia National Tobacco Campaign Evaluation Survey 2000 as it appears in P.N. Lee Statistics and Computing Ltd. International Mortality and Smoking Statistics Version 4.04. Sutton, United Kingdom: P.N. Lee Statistics and Computing Ltd, 2009.

Appendix: Citation List

Citation
Australia Schools Health And Fitness Survey 1985 as it appears in P.N. Lee Statistics and Computing Ltd. International Mortality and Smoking Statistics Version 4.04. Sutton, United Kingdom: P.N. Lee Statistics and Computing Ltd, 2009.
Australia Vital Registration - Deaths 2006 ICD10 as it appears in World Health Organization (WHO). WHO Mortality Database Version November 2015. Geneva, Switzerland: World Health Organization (WHO), 2015.
Australia Vital Registration - Deaths 1950 ICD7 as it appears in World Health Organization (WHO). WHO Mortality Database Version July 2012. Geneva, Switzerland: World Health Organization (WHO), 2012.
Australia Vital Registration - Deaths 1951 ICD7 as it appears in World Health Organization (WHO). WHO Mortality Database Version July 2012. Geneva, Switzerland: World Health Organization (WHO), 2012.
Australia Vital Registration - Deaths 1952 ICD7 as it appears in World Health Organization (WHO). WHO Mortality Database Version July 2012. Geneva, Switzerland: World Health Organization (WHO), 2012.
Australia Vital Registration - Deaths 1953 ICD7 as it appears in World Health Organization (WHO). WHO Mortality Database Version July 2012. Geneva, Switzerland: World Health Organization (WHO), 2012.
Australia Vital Registration - Deaths 1954 ICD7 as it appears in World Health Organization (WHO). WHO Mortality Database Version July 2012. Geneva, Switzerland: World Health Organization (WHO), 2012.
Australia Vital Registration - Deaths 1955 ICD7 as it appears in World Health Organization (WHO). WHO Mortality Database Version July 2012. Geneva, Switzerland: World Health Organization (WHO), 2012.
Australia Vital Registration - Deaths 1956 ICD7 as it appears in World Health Organization (WHO). WHO Mortality Database Version July 2012. Geneva, Switzerland: World Health Organization (WHO), 2012.
Australia Vital Registration - Deaths 1957 ICD7 as it appears in World Health Organization (WHO). WHO Mortality Database Version July 2012. Geneva, Switzerland: World Health Organization (WHO), 2012.
Australia Vital Registration - Deaths 1958 ICD7 as it appears in World Health Organization (WHO). WHO Mortality Database Version July 2012. Geneva, Switzerland: World Health Organization (WHO), 2012.
Australia Vital Registration - Deaths 1959 ICD7 as it appears in World Health Organization (WHO). WHO Mortality Database Version July 2012. Geneva, Switzerland: World Health Organization (WHO), 2012.
Australia Vital Registration - Deaths 1960 ICD7 as it appears in World Health Organization (WHO). WHO Mortality Database Version July 2012. Geneva, Switzerland: World Health Organization (WHO), 2012.
Australia Vital Registration - Deaths 1961 ICD7 as it appears in World Health Organization (WHO). WHO Mortality Database Version July 2012. Geneva, Switzerland: World Health Organization (WHO), 2012.
Australia Vital Registration - Deaths 1962 ICD7 as it appears in World Health Organization (WHO). WHO Mortality Database Version July 2012. Geneva, Switzerland: World Health Organization (WHO), 2012.
Australia Vital Registration - Deaths 1963 ICD7 as it appears in World Health Organization (WHO). WHO Mortality Database Version July 2012. Geneva, Switzerland: World Health Organization (WHO), 2012.
Australia Vital Registration - Deaths 1964 ICD7 as it appears in World Health Organization (WHO). WHO Mortality Database Version July 2012. Geneva, Switzerland: World Health Organization (WHO), 2012.
Australia Vital Registration - Deaths 1965 ICD7 as it appears in World Health Organization (WHO). WHO Mortality Database Version July 2012. Geneva, Switzerland: World Health Organization (WHO), 2012.
Australia Vital Registration - Deaths 1966 ICD7 as it appears in World Health Organization (WHO). WHO Mortality Database Version July 2012. Geneva, Switzerland: World Health Organization (WHO), 2012.
Australia Vital Registration - Deaths 1967 ICD7 as it appears in World Health Organization (WHO). WHO Mortality Database Version July 2012. Geneva, Switzerland: World Health Organization (WHO), 2012.
Australia Vital Registration - Deaths 1968 ICD8 as it appears in World Health Organization (WHO). WHO Mortality Database Version May 2013. Geneva, Switzerland: World Health Organization (WHO), 2013.
Australia Vital Registration - Deaths 1969 ICD8 as it appears in World Health Organization (WHO). WHO Mortality Database Version May 2013. Geneva, Switzerland: World Health Organization (WHO), 2013.
Australia Vital Registration - Deaths 1970 ICD8 as it appears in World Health Organization (WHO). WHO Mortality Database Version May 2013. Geneva, Switzerland: World Health Organization (WHO), 2013.
Australia Vital Registration - Deaths 1971 ICD8 as it appears in World Health Organization (WHO). WHO Mortality Database Version May 2013. Geneva, Switzerland: World Health Organization (WHO), 2013.
Australia Vital Registration - Deaths 1972 ICD8 as it appears in World Health Organization (WHO). WHO Mortality Database Version May 2013. Geneva, Switzerland: World Health Organization (WHO), 2013.
Australia Vital Registration - Deaths 1973 ICD8 as it appears in World Health Organization (WHO). WHO Mortality Database Version May 2013. Geneva, Switzerland: World Health Organization (WHO), 2013.
Australia Vital Registration - Deaths 1974 ICD8 as it appears in World Health Organization (WHO). WHO Mortality Database Version May 2013. Geneva, Switzerland: World Health Organization (WHO), 2013.
Australia Vital Registration - Deaths 1975 ICD8 as it appears in World Health Organization (WHO). WHO Mortality Database Version May 2013. Geneva, Switzerland: World Health Organization (WHO), 2013.
Australia Vital Registration - Deaths 1976 ICD8 as it appears in World Health Organization (WHO). WHO Mortality Database Version May 2013. Geneva, Switzerland: World Health Organization (WHO), 2013.
Australia Vital Registration - Deaths 1977 ICD8 as it appears in World Health Organization (WHO). WHO Mortality Database Version May 2013. Geneva, Switzerland: World Health Organization (WHO), 2013.
Australia Vital Registration - Deaths 1978 ICD8 as it appears in World Health Organization (WHO). WHO Mortality Database Version May 2013. Geneva, Switzerland: World Health Organization (WHO), 2013.
Australia Vital Registration - Deaths 1979 ICD9 as it appears in World Health Organization (WHO). WHO Mortality Database Version November 2015. Geneva, Switzerland: World Health Organization (WHO), 2015.

Appendix: Citation List

Citation
Australia Vital Registration - Deaths 1980 ICD9 as it appears in World Health Organization (WHO). WHO Mortality Database Version July 2012. Geneva, Switzerland: World Health Organization (WHO), 2012.
Australia Vital Registration - Deaths 1981 ICD9 as it appears in World Health Organization (WHO). WHO Mortality Database Version July 2012. Geneva, Switzerland: World Health Organization (WHO), 2012.
Australia Vital Registration - Deaths 1982 ICD9 as it appears in World Health Organization (WHO). WHO Mortality Database Version July 2012. Geneva, Switzerland: World Health Organization (WHO), 2012.
Australia Vital Registration - Deaths 1983 ICD9 as it appears in World Health Organization (WHO). WHO Mortality Database Version July 2012. Geneva, Switzerland: World Health Organization (WHO), 2012.
Australia Vital Registration - Deaths 1984 ICD9 as it appears in World Health Organization (WHO). WHO Mortality Database Version July 2012. Geneva, Switzerland: World Health Organization (WHO), 2012.
Australia Vital Registration - Deaths 1985 ICD9 as it appears in World Health Organization (WHO). WHO Mortality Database Version July 2012. Geneva, Switzerland: World Health Organization (WHO), 2012.
Australia Vital Registration - Deaths 1986 ICD9 as it appears in World Health Organization (WHO). WHO Mortality Database Version July 2012. Geneva, Switzerland: World Health Organization (WHO), 2012.
Australia Vital Registration - Deaths 1987 ICD9 as it appears in World Health Organization (WHO). WHO Mortality Database Version July 2012. Geneva, Switzerland: World Health Organization (WHO), 2012.
Australia Vital Registration - Deaths 1988 ICD9 as it appears in World Health Organization (WHO). WHO Mortality Database Version July 2012. Geneva, Switzerland: World Health Organization (WHO), 2012.
Australia Vital Registration - Deaths 1989 ICD9 as it appears in World Health Organization (WHO). WHO Mortality Database Version July 2012. Geneva, Switzerland: World Health Organization (WHO), 2012.
Australia Vital Registration - Deaths 1990 ICD9 as it appears in World Health Organization (WHO). WHO Mortality Database Version July 2012. Geneva, Switzerland: World Health Organization (WHO), 2012.
Australia Vital Registration - Deaths 1991 ICD9 as it appears in World Health Organization (WHO). WHO Mortality Database Version July 2012. Geneva, Switzerland: World Health Organization (WHO), 2012.
Australia Vital Registration - Deaths 1992 ICD9 as it appears in World Health Organization (WHO). WHO Mortality Database Version July 2012. Geneva, Switzerland: World Health Organization (WHO), 2012.
Australia Vital Registration - Deaths 1993 ICD9 as it appears in World Health Organization (WHO). WHO Mortality Database Version July 2012. Geneva, Switzerland: World Health Organization (WHO), 2012.
Australia Vital Registration - Deaths 1994 ICD9 as it appears in World Health Organization (WHO). WHO Mortality Database Version July 2012. Geneva, Switzerland: World Health Organization (WHO), 2012.
Australia Vital Registration - Deaths 1995 ICD9 as it appears in World Health Organization (WHO). WHO Mortality Database Version July 2012. Geneva, Switzerland: World Health Organization (WHO), 2012.
Australia Vital Registration - Deaths 1996 ICD9 as it appears in World Health Organization (WHO). WHO Mortality Database Version July 2012. Geneva, Switzerland: World Health Organization (WHO), 2012.
Australia Vital Registration - Deaths 1997 ICD9 as it appears in World Health Organization (WHO). WHO Mortality Database Version July 2012. Geneva, Switzerland: World Health Organization (WHO), 2012.
Australia Vital Registration - Deaths 1998 ICD10 as it appears in World Health Organization (WHO). WHO Mortality Database Version November 2015. Geneva, Switzerland: World Health Organization (WHO), 2015.
Australia Vital Registration - Deaths 1999 ICD10 as it appears in World Health Organization (WHO). WHO Mortality Database Version November 2015. Geneva, Switzerland: World Health Organization (WHO), 2015.
Australia Vital Registration - Deaths 2000 ICD10 as it appears in World Health Organization (WHO). WHO Mortality Database Version November 2015. Geneva, Switzerland: World Health Organization (WHO), 2015.
Australia Vital Registration - Deaths 2001 ICD10 as it appears in World Health Organization (WHO). WHO Mortality Database Version November 2015. Geneva, Switzerland: World Health Organization (WHO), 2015.
Australia Vital Registration - Deaths 2002 ICD10 as it appears in World Health Organization (WHO). WHO Mortality Database Version November 2015. Geneva, Switzerland: World Health Organization (WHO), 2015.
Australia Vital Registration - Deaths 2003 ICD10 as it appears in World Health Organization (WHO). WHO Mortality Database Version November 2015. Geneva, Switzerland: World Health Organization (WHO), 2015.
Australia Vital Registration - Deaths 2004 ICD10 as it appears in World Health Organization (WHO). WHO Mortality Database Version November 2015. Geneva, Switzerland: World Health Organization (WHO), 2015.
Australia Vital Registration - Deaths 2007 ICD10 as it appears in World Health Organization (WHO). WHO Mortality Database Version November 2015. Geneva, Switzerland: World Health Organization (WHO), 2015.
Australia Vital Registration - Deaths 2008 ICD10 as it appears in World Health Organization (WHO). WHO Mortality Database Version November 2015. Geneva, Switzerland: World Health Organization (WHO), 2015.
Australia Vital Registration - Deaths 2009 ICD10 as it appears in World Health Organization (WHO). WHO Mortality Database Version November 2015. Geneva, Switzerland: World Health Organization (WHO), 2015.
Australia Vital Registration - Deaths 2010 ICD10 as it appears in World Health Organization (WHO). WHO Mortality Database Version November 2015. Geneva, Switzerland: World Health Organization (WHO), 2015.
Australia Vital Registration - Deaths 2011 ICD10 as it appears in World Health Organization (WHO). WHO Mortality Database Version November 2015. Geneva, Switzerland: World Health Organization (WHO), 2015.
Australian Agency for International Development (AusAID), Department of Health (Tokelau), Fiji School of Medicine, World Health Organization (WHO). Tokelau STEPS Noncommunicable Disease Risk Factors Survey 2005.

Appendix: Citation List

Citation
Australian Agency for International Development (AusAID), Fiji School of Medicine, Menzies Center for Population Health Research, University of Tasmania (Australia), Ministry of Health (Fiji), World Health Organization (WHO). Fiji STEPS Noncommunicable Disease Risk Factors Survey 2002.
Australian Bureau of Statistics, Australian Health Survey Reference Group, Roy Morgan Research, University of Western Australia. Australia Child and Adolescent Survey of Mental Health and Wellbeing 2013-2014.
Australian Bureau of Statistics, Department of Health and Family Services (Australia). Australia National Nutrition Survey 1995-1996. Canberra, Australia: Australian Bureau of Statistics.
Australian Bureau of Statistics. Australia National Health Survey 2001. Canberra, Australia: Australian Bureau of Statistics.
Australian Bureau of Statistics. Australia National Health Survey 2004-2005.
Australian Bureau of Statistics. Australia National Health Survey 2007-2008. Canberra, Australia: Australian Bureau of Statistics.
Australian Bureau of Statistics. Australia National Health Survey 2011-2013.
Australian Bureau of Statistics. Australia National Survey of Mental Health and Wellbeing 2007. Canberra, Australia: Australian Bureau of Statistics, 2007.
Australian Bureau of Statistics. Australia Vital Registration - Deaths 2005.
Australian Bureau of Statistics. Australia Vital Statistics - Deaths 2014. Canberra, Australia: Australian Bureau of Statistics.
Australian Bureau of Statistics. Australia Women's Safety Survey 1996.
Australian Bureau of Statistics.Australia National Health Survey 1995. Canberra, Australia:Australian Bureau of Statistics.
Australian Institute of Health and Welfare, Department of Health and Ageing (Australia), Roy Morgan Research, Social Research Centre (Australia). Australia National Drug Strategy Household Survey 2007.
Australian Institute of Health and Welfare, National Heart Foundation (Australia). Australia Risk Factor Prevalence Survey 1994.
Australian Institute of Health and Welfare, National Perinatal Epidemiology and Statistics Unit (NPESU), University of New South Wales. Australia's Mothers and Babies 1997. Canberra, Australia: Australian Institute of Health and Welfare, 1999.
Australian Institute of Health and Welfare, National Perinatal Epidemiology and Statistics Unit (NPESU), University of New South Wales. Australia's Mothers and Babies 1998. Canberra, Australia: Australian Institute of Health and Welfare, 2000.
Australian Institute of Health and Welfare, National Perinatal Epidemiology and Statistics Unit (NPESU), University of New South Wales. Australia's Mothers and Babies 1999. Canberra, Australia: Australian Institute of Health and Welfare, 2001.
Australian Institute of Health and Welfare, National Perinatal Epidemiology and Statistics Unit (NPESU), University of New South Wales. Australia's Mothers and Babies 2000. Canberra, Australia: Australian Institute of Health and Welfare, 2003.
Australian Institute of Health and Welfare, National Perinatal Epidemiology and Statistics Unit (NPESU), University of New South Wales. Australia's Mothers and Babies 2001. Canberra, Australia: Australian Institute of Health and Welfare, 2004.
Australian Institute of Health and Welfare, National Perinatal Epidemiology and Statistics Unit (NPESU), University of New South Wales. Australia's Mothers and Babies 2002. Canberra, Australia: Australian Institute of Health and Welfare, 2004.
Australian Institute of Health and Welfare, National Perinatal Epidemiology and Statistics Unit (NPESU), University of New South Wales. Australia's Mothers and Babies 2003. Canberra, Australia: Australian Institute of Health and Welfare, 2005.
Australian Institute of Health and Welfare, National Perinatal Epidemiology and Statistics Unit (NPESU), University of New South Wales. Australia's Mothers and Babies 2004. Canberra, Australia: Australian Institute of Health and Welfare, 2006.
Australian Institute of Health and Welfare, National Perinatal Epidemiology and Statistics Unit (NPESU), University of New South Wales. Australia's Mothers and Babies 2005. Canberra, Australia: Australian Institute of Health and Welfare, 2007.
Australian Institute of Health and Welfare, National Perinatal Epidemiology and Statistics Unit (NPESU), University of New South Wales. Australia's Mothers and Babies 2006. Canberra, Australia: Australian Institute of Health and Welfare, 2008.
Australian Institute of Health and Welfare, National Perinatal Epidemiology and Statistics Unit (NPESU), University of New South Wales. Australia's Mothers and Babies 2007. Canberra, Australia: Australian Institute of Health and Welfare, 2009.
Australian Institute of Health and Welfare, National Perinatal Epidemiology and Statistics Unit (NPESU), University of New South Wales. Australia's Mothers and Babies 2008. Canberra, Australia: Australian Institute of Health and Welfare, 2010.
Australian Institute of Health and Welfare, National Perinatal Epidemiology and Statistics Unit (NPESU), University of New South Wales. Australia's Mothers and Babies 2009. Canberra, Australia: Australian Institute of Health and Welfare, 2011.
Australian Institute of Health and Welfare, National Perinatal Epidemiology and Statistics Unit (NPESU), University of New South Wales. Australia's Mothers and Babies 2010. Canberra, Australia: Australian Institute of Health and Welfare, 2012.
Australian Institute of Health and Welfare, National Perinatal Epidemiology and Statistics Unit (NPESU), University of New South Wales. Australia's Mothers and Babies 2011. Canberra, Australia: Australian Institute of Health and Welfare, 2013.
Australian Institute of Health and Welfare, National Perinatal Epidemiology and Statistics Unit (NPESU), University of New South Wales. Australia's Mothers and Babies 2012. Canberra, Australia: Australian Institute of Health and Welfare, 2014.
Australian Institute of Health and Welfare, National Perinatal Epidemiology and Statistics Unit (NPESU), University of New South Wales. Australia's Mothers and Babies In Brief 2013. Canberra, Australia: Australian Institute of Health and Welfare, 2015.
Australian Institute of Health and Welfare, Roy Morgan Research. Australia National Drug Strategy Household Survey 2010.
Australian Institute of Health and Welfare. Australia Maternal Deaths 1997-1999. Canberra, Australia: Australian Institute of Health and Welfare, 2004.
Australian Institute of Health and Welfare. Australia Maternal Deaths 2000-2002. Canberra, Australia: Australian Institute of Health and Welfare, 2006.
Australian Institute of Health and Welfare. Australia Maternal Deaths 2006-2010. Canberra, Australia: Australian Institute of Health and Welfare, 2014.
Australian Institute of Health and Welfare. Australia Maternal Deaths 2008-2012. Canberra, Australia: Australian Institute of Health and Welfare, 2015.
Australian Institute of Health and Welfare. Australia National Drug Strategy Household Survey 2001.

Appendix: Citation List

Citation
Australian Institute of Health and Welfare. Australia National Drug Strategy Household Survey 2004.
Australian Institute of Health and Welfare. Australian Maternal Deaths 2003-2005. Canberra, Australia: Australian Institute of Health and Welfare, 2008.
Austria - Tyrol Cancer Registry 1998-2002 - CI5 as it appears in Curado MP, Edwards B, Shin HR, Storm H, Ferlay J, Heanue M and Boyle P, eds (2007). Cancer Incidence in Five Continents, Vol. IX Periodic Data (electronic version). Lyon, IARC.http://ci5.iarc.fr
Austria - Vorarlberg Cancer Registry 1998-2002 - CI5 as it appears in Curado MP, Edwards B, Shin HR, Storm H, Ferlay J, Heanue M and Boyle P, eds (2007). Cancer Incidence in Five Continents, Vol. IX Periodic Data (electronic version). Lyon, IARC.http://ci5.iarc.fr
Austria Cancer Registry 1998-2002 - CI5 as it appears in Curado MP, Edwards B, Shin HR, Storm H, Ferlay J, Heanue M and Boyle P, eds (2007). Cancer Incidence in Five Continents, Vol. IX Periodic Data (electronic version). Lyon, IARC.http://ci5.iarc.fr
Austria Microcensus 1991 as it appears in P.N. Lee Statistics and Computing Ltd. International Mortality and Smoking Statistics Version 4.04. Sutton, United Kingdom: P.N. Lee Statistics and Computing Ltd, 2009.
Austria Smoking Habits: Results of the Microcensus 1997 as it appears in P.N. Lee Statistics and Computing Ltd. International Mortality and Smoking Statistics Version 4.04. Sutton, United Kingdom: P.N. Lee Statistics and Computing Ltd, 2009.
Austria Vital Registration - Deaths 1955 ICD7 as it appears in World Health Organization (WHO). WHO Mortality Database Version July 2012. Geneva, Switzerland: World Health Organization (WHO), 2012.
Austria Vital Registration - Deaths 1956 ICD7 as it appears in World Health Organization (WHO). WHO Mortality Database Version July 2012. Geneva, Switzerland: World Health Organization (WHO), 2012.
Austria Vital Registration - Deaths 1957 ICD7 as it appears in World Health Organization (WHO). WHO Mortality Database Version July 2012. Geneva, Switzerland: World Health Organization (WHO), 2012.
Austria Vital Registration - Deaths 1958 ICD7 as it appears in World Health Organization (WHO). WHO Mortality Database Version July 2012. Geneva, Switzerland: World Health Organization (WHO), 2012.
Austria Vital Registration - Deaths 1959 ICD7 as it appears in World Health Organization (WHO). WHO Mortality Database Version July 2012. Geneva, Switzerland: World Health Organization (WHO), 2012.
Austria Vital Registration - Deaths 1960 ICD7 as it appears in World Health Organization (WHO). WHO Mortality Database Version July 2012. Geneva, Switzerland: World Health Organization (WHO), 2012.
Austria Vital Registration - Deaths 1961 ICD7 as it appears in World Health Organization (WHO). WHO Mortality Database Version July 2012. Geneva, Switzerland: World Health Organization (WHO), 2012.
Austria Vital Registration - Deaths 1962 ICD7 as it appears in World Health Organization (WHO). WHO Mortality Database Version July 2012. Geneva, Switzerland: World Health Organization (WHO), 2012.
Austria Vital Registration - Deaths 1963 ICD7 as it appears in World Health Organization (WHO). WHO Mortality Database Version July 2012. Geneva, Switzerland: World Health Organization (WHO), 2012.
Austria Vital Registration - Deaths 1964 ICD7 as it appears in World Health Organization (WHO). WHO Mortality Database Version July 2012. Geneva, Switzerland: World Health Organization (WHO), 2012.
Austria Vital Registration - Deaths 1965 ICD7 as it appears in World Health Organization (WHO). WHO Mortality Database Version July 2012. Geneva, Switzerland: World Health Organization (WHO), 2012.
Austria Vital Registration - Deaths 1966 ICD7 as it appears in World Health Organization (WHO). WHO Mortality Database Version July 2012. Geneva, Switzerland: World Health Organization (WHO), 2012.
Austria Vital Registration - Deaths 1967 ICD7 as it appears in World Health Organization (WHO). WHO Mortality Database Version July 2012. Geneva, Switzerland: World Health Organization (WHO), 2012.
Austria Vital Registration - Deaths 1968 ICD7 as it appears in World Health Organization (WHO). WHO Mortality Database Version July 2012. Geneva, Switzerland: World Health Organization (WHO), 2012.
Austria Vital Registration - Deaths 1969 ICD8 as it appears in World Health Organization (WHO). WHO Mortality Database Version May 2013. Geneva, Switzerland: World Health Organization (WHO), 2013.
Austria Vital Registration - Deaths 1970 ICD8 as it appears in World Health Organization (WHO). WHO Mortality Database Version May 2013. Geneva, Switzerland: World Health Organization (WHO), 2013.
Austria Vital Registration - Deaths 1971 ICD8 as it appears in World Health Organization (WHO). WHO Mortality Database Version May 2013. Geneva, Switzerland: World Health Organization (WHO), 2013.
Austria Vital Registration - Deaths 1972 ICD8 as it appears in World Health Organization (WHO). WHO Mortality Database Version May 2013. Geneva, Switzerland: World Health Organization (WHO), 2013.
Austria Vital Registration - Deaths 1973 ICD8 as it appears in World Health Organization (WHO). WHO Mortality Database Version May 2013. Geneva, Switzerland: World Health Organization (WHO), 2013.
Austria Vital Registration - Deaths 1974 ICD8 as it appears in World Health Organization (WHO). WHO Mortality Database Version May 2013. Geneva, Switzerland: World Health Organization (WHO), 2013.
Austria Vital Registration - Deaths 1975 ICD8 as it appears in World Health Organization (WHO). WHO Mortality Database Version May 2013. Geneva, Switzerland: World Health Organization (WHO), 2013.
Austria Vital Registration - Deaths 1976 ICD8 as it appears in World Health Organization (WHO). WHO Mortality Database Version May 2013. Geneva, Switzerland: World Health Organization (WHO), 2013.
Austria Vital Registration - Deaths 1977 ICD8 as it appears in World Health Organization (WHO). WHO Mortality Database Version May 2013. Geneva, Switzerland: World Health Organization (WHO), 2013.
Austria Vital Registration - Deaths 1978 ICD8 as it appears in World Health Organization (WHO). WHO Mortality Database Version May 2013. Geneva, Switzerland: World Health Organization (WHO), 2013.
Austria Vital Registration - Deaths 1979 ICD8 as it appears in World Health Organization (WHO). WHO Mortality Database Version May 2013. Geneva, Switzerland: World Health Organization (WHO), 2013.

Appendix: Citation List

Citation
Austria Vital Registration - Deaths 1980 ICD9 as it appears in World Health Organization (WHO). WHO Mortality Database Version July 2012. Geneva, Switzerland: World Health Organization (WHO), 2012.
Austria Vital Registration - Deaths 1981 ICD9 as it appears in World Health Organization (WHO). WHO Mortality Database Version July 2012. Geneva, Switzerland: World Health Organization (WHO), 2012.
Austria Vital Registration - Deaths 1982 ICD9 as it appears in World Health Organization (WHO). WHO Mortality Database Version July 2012. Geneva, Switzerland: World Health Organization (WHO), 2012.
Austria Vital Registration - Deaths 1983 ICD9 as it appears in World Health Organization (WHO). WHO Mortality Database Version February 2014. Geneva, Switzerland: World Health Organization (WHO), 2014.
Austria Vital Registration - Deaths 1984 ICD9 as it appears in World Health Organization (WHO). WHO Mortality Database Version July 2012. Geneva, Switzerland: World Health Organization (WHO), 2012.
Austria Vital Registration - Deaths 1985 ICD9 as it appears in World Health Organization (WHO). WHO Mortality Database Version July 2012. Geneva, Switzerland: World Health Organization (WHO), 2012.
Austria Vital Registration - Deaths 1986 ICD9 as it appears in World Health Organization (WHO). WHO Mortality Database Version July 2012. Geneva, Switzerland: World Health Organization (WHO), 2012.
Austria Vital Registration - Deaths 1987 ICD9 as it appears in World Health Organization (WHO). WHO Mortality Database Version July 2012. Geneva, Switzerland: World Health Organization (WHO), 2012.
Austria Vital Registration - Deaths 1988 ICD9 as it appears in World Health Organization (WHO). WHO Mortality Database Version July 2012. Geneva, Switzerland: World Health Organization (WHO), 2012.
Austria Vital Registration - Deaths 1989 ICD9 as it appears in World Health Organization (WHO). WHO Mortality Database Version July 2012. Geneva, Switzerland: World Health Organization (WHO), 2012.
Austria Vital Registration - Deaths 1990 ICD9 as it appears in World Health Organization (WHO). WHO Mortality Database Version July 2012. Geneva, Switzerland: World Health Organization (WHO), 2012.
Austria Vital Registration - Deaths 1991 ICD9 as it appears in World Health Organization (WHO). WHO Mortality Database Version July 2012. Geneva, Switzerland: World Health Organization (WHO), 2012.
Austria Vital Registration - Deaths 1992 ICD9 as it appears in World Health Organization (WHO). WHO Mortality Database Version July 2012. Geneva, Switzerland: World Health Organization (WHO), 2012.
Austria Vital Registration - Deaths 1993 ICD9 as it appears in World Health Organization (WHO). WHO Mortality Database Version July 2012. Geneva, Switzerland: World Health Organization (WHO), 2012.
Austria Vital Registration - Deaths 1994 ICD9 as it appears in World Health Organization (WHO). WHO Mortality Database Version July 2012. Geneva, Switzerland: World Health Organization (WHO), 2012.
Austria Vital Registration - Deaths 1995 ICD9 as it appears in World Health Organization (WHO). WHO Mortality Database Version July 2012. Geneva, Switzerland: World Health Organization (WHO), 2012.
Austria Vital Registration - Deaths 1996 ICD9 as it appears in World Health Organization (WHO). WHO Mortality Database Version July 2012. Geneva, Switzerland: World Health Organization (WHO), 2012.
Austria Vital Registration - Deaths 1997 ICD9 as it appears in World Health Organization (WHO). WHO Mortality Database Version July 2012. Geneva, Switzerland: World Health Organization (WHO), 2012.
Austria Vital Registration - Deaths 1998 ICD9 as it appears in World Health Organization (WHO). WHO Mortality Database Version July 2012. Geneva, Switzerland: World Health Organization (WHO), 2012.
Austria Vital Registration - Deaths 1999 ICD9 as it appears in World Health Organization (WHO). WHO Mortality Database Version July 2012. Geneva, Switzerland: World Health Organization (WHO), 2012.
Austria Vital Registration - Deaths 2000 ICD9 as it appears in World Health Organization (WHO). WHO Mortality Database Version July 2012. Geneva, Switzerland: World Health Organization (WHO), 2012.
Austria Vital Registration - Deaths 2001 ICD9 as it appears in World Health Organization (WHO). WHO Mortality Database Version July 2012. Geneva, Switzerland: World Health Organization (WHO), 2012.
Austria Vital Registration - Deaths 2002 ICD10 as it appears in World Health Organization (WHO). WHO Mortality Database Version November 2015. Geneva, Switzerland: World Health Organization (WHO), 2015.
Austria Vital Registration - Deaths 2003 ICD10 as it appears in World Health Organization (WHO). WHO Mortality Database Version November 2015. Geneva, Switzerland: World Health Organization (WHO), 2015.
Austria Vital Registration - Deaths 2004 ICD10 as it appears in World Health Organization (WHO). WHO Mortality Database Version November 2015. Geneva, Switzerland: World Health Organization (WHO), 2015.
Austria Vital Registration - Deaths 2005 ICD10 as it appears in World Health Organization (WHO). WHO Mortality Database Version November 2015. Geneva, Switzerland: World Health Organization (WHO), 2015.
Austria Vital Registration - Deaths 2006 ICD10 as it appears in World Health Organization (WHO). WHO Mortality Database Version November 2015. Geneva, Switzerland: World Health Organization (WHO), 2015.
Austria Vital Registration - Deaths 2007 ICD10 as it appears in World Health Organization (WHO). WHO Mortality Database Version November 2015. Geneva, Switzerland: World Health Organization (WHO), 2015.
Austria Vital Registration - Deaths 2008 ICD10 as it appears in World Health Organization (WHO). WHO Mortality Database Version November 2015. Geneva, Switzerland: World Health Organization (WHO), 2015.
Austria Vital Registration - Deaths 2009 ICD10 as it appears in World Health Organization (WHO). WHO Mortality Database Version November 2015. Geneva, Switzerland: World Health Organization (WHO), 2015.
Austria Vital Registration - Deaths 2010 ICD10 as it appears in World Health Organization (WHO). WHO Mortality Database Version November 2015. Geneva, Switzerland: World Health Organization (WHO), 2015.
Austria Vital Registration - Deaths 2011 ICD10 as it appears in World Health Organization (WHO). WHO Mortality Database Version November 2015. Geneva, Switzerland: World Health Organization (WHO), 2015.

Appendix: Citation List

Citation
Austria Vital Registration - Deaths 2012 ICD10 as it appears in World Health Organization (WHO). WHO Mortality Database Version November 2015. Geneva, Switzerland: World Health Organization (WHO), 2015.
Austria Vital Registration - Deaths 2013 ICD10 as it appears in World Health Organization (WHO). WHO Mortality Database Version November 2015. Geneva, Switzerland: World Health Organization (WHO), 2015.
Austria Vital Registration - Deaths 2014 ICD10 as it appears in World Health Organization (WHO). WHO Mortality Database Version November 2015. Geneva, Switzerland: World Health Organization (WHO), 2015.
Austrian Cancer Registry. Austria Cancer Incidence (New Cases per Year) by Site and Gender 1983-2010. Vienna, Austria: Statistics Austria.
Austrian Central Statistical Office, Minnesota Population Center. Austria Population Census and Building and Housing Census 1981 from the Integrated Public Use Microdata Series, International: [Machine-readable database]. Minneapolis: University of Minnesota.
Austrian Central Statistical Office, Minnesota Population Center. Austria Population Census and Building and Housing Census 1991 from the Integrated Public Use Microdata Series, International: [Machine-readable database]. Minneapolis: University of Minnesota.
Austrian Central Statistical Office. Austria Smoking Habits: Results of the Microcensus 1997. Vienna, Austria: Austrian Central Statistical Office, 1999.
Autonomous Women's Center, World Health Organization (WHO). Serbia and Montenegro WHO Multi-country Study on Women's Health and Domestic Violence Against Women 2003.
Awah N, Kimbi H, Ndamukong K, Mbuh J. The Epidemiology and Consequences of Malaria Infection in Primary School Children in the Muea Area and its Environs. In: Multilateral Initiative on Malaria Conference 2005 Abstracts; 2005 Nov 13-15; Yaoundé, Cameroon. Amsterdam, Netherlands: Elsevier; 2005. As it appears in Malaria Atlas Project. Malaria Atlas Project Plasmodium Falciparum Parasite Rate Database. Oxford, United Kingdom: Malaria Atlas Project.
Awoleke JO, Adanikin AI. Baird-Pattinson Aetiological Classification and Phases of Delay Contributing to Stillbirths in a Nigerian Tertiary Hospital. J Pregnancy. 2016; 2016: 1703809.
Ayah R, Joshi MD, Wanjiru R, Njau EK, Otieno CF, Njeru EK, Mutai KK. A population-based survey of prevalence of diabetes and correlates in an urban slum community in Nairobi, Kenya. BMC Public Health. 2013; 13: 371.
Ayles H, Schaap A, Nota A, Sismanidis C, Tembwe R, De Haas P, Muyoyeta M, Beyers N, Peter Godfrey-Faussett for the ZAMSTAR Study Team. Prevalence of tuberculosis, HIV and respiratory symptoms in two Zambian communities: implications for tuberculosis control in the era of HIV. PLoS One. 2009; 4(5): e5602.
Azad K, Barnett S, Banerjee B, Shaha S, Khan K, Rego AR, Barua S, Flatman D, Pagel C, Prost A, Ellis M, Costello A. Effect of scaling up women's groups on birth outcomes in three rural districts in Bangladesh: a cluster-randomised controlled trial. Lancet. 2010; 375(9721): 1193-202.
Azadbakht L, Esmaillzadeh A. Dietary and non-dietary determinants of central adiposity among Tehrani women. Public Health Nutr. 2008; 11(5): 528-34.
Azerbaijan Internally Displaced Persons Living in the Southern Camps and Surrounding Areas 1999 as it appears in World Health Organization (WHO). WHO Global Database on Child Growth and Malnutrition - Historical. Geneva, Switzerland: World Health Organization (WHO).
Azerbaijan Living Standards Measurement Survey 1995 as it appears in World Health Organization (WHO). WHO Household Energy Database. Geneva, Switzerland: World Health Organization (WHO), 2010.
Azerbaijan Ministry of Health, GroundWork LLC, State Statistical Committee of Azerbaijan, United Nations Children's Fund (UNICEF). Azerbaijan Nutrition Survey 2013.
Azerbaijan Ministry of Health, United Nations Children's Fund (UNICEF). Azerbaijan National Immunization Programme Evaluation 1999.
Azerbaijan Ministry of Health. Azerbaijan Demographic and Health Survey 2011.
Azerbaijan Reproductive Health Survey 2001 as it appears in World Health Organization (WHO). WHO Global Database on Child Growth and Malnutrition. Geneva, Switzerland: World Health Organization (WHO).
Azerbaijan Vital Registration - Deaths 1981 ICD9 as it appears in World Health Organization (WHO). WHO Mortality Database Version November 2015. Geneva, Switzerland: World Health Organization (WHO), 2015.
Azerbaijan Vital Registration - Deaths 1982 ICD9 as it appears in World Health Organization (WHO). WHO Mortality Database Version November 2015. Geneva, Switzerland: World Health Organization (WHO), 2015.
Azerbaijan Vital Registration - Deaths 1985 ICD9 as it appears in World Health Organization (WHO). WHO Mortality Database Version November 2015. Geneva, Switzerland: World Health Organization (WHO), 2015.
Azerbaijan Vital Registration - Deaths 1986 ICD9 as it appears in World Health Organization (WHO). WHO Mortality Database Version November 2015. Geneva, Switzerland: World Health Organization (WHO), 2015.
Azerbaijan Vital Registration - Deaths 1987 ICD9 as it appears in World Health Organization (WHO). WHO Mortality Database Version November 2015. Geneva, Switzerland: World Health Organization (WHO), 2015.
Azerbaijan Vital Registration - Deaths 1988 ICD9 as it appears in World Health Organization (WHO). WHO Mortality Database Version November 2015. Geneva, Switzerland: World Health Organization (WHO), 2015.
Azerbaijan Vital Registration - Deaths 1989 ICD9 as it appears in World Health Organization (WHO). WHO Mortality Database Version November 2015. Geneva, Switzerland: World Health Organization (WHO), 2015.
Azerbaijan Vital Registration - Deaths 1990 ICD9 as it appears in World Health Organization (WHO). WHO Mortality Database Version November 2015. Geneva, Switzerland: World Health Organization (WHO), 2015.
Azerbaijan Vital Registration - Deaths 1991 ICD9 as it appears in World Health Organization (WHO). WHO Mortality Database Version November 2015. Geneva, Switzerland: World Health Organization (WHO), 2015.
Azerbaijan Vital Registration - Deaths 1992 ICD9 as it appears in World Health Organization (WHO). WHO Mortality Database Version November 2015. Geneva, Switzerland: World Health Organization (WHO), 2015.
Azerbaijan Vital Registration - Deaths 1993 ICD9 as it appears in World Health Organization (WHO). WHO Mortality Database Version November 2015. Geneva, Switzerland: World Health Organization (WHO), 2015.

Appendix: Citation List

Citation
Azerbaijan Vital Registration - Deaths 1994 ICD9 as it appears in World Health Organization (WHO). WHO Mortality Database Version November 2015. Geneva, Switzerland: World Health Organization (WHO), 2015.
Azerbaijan Vital Registration - Deaths 1995 ICD9 as it appears in World Health Organization (WHO). WHO Mortality Database Version November 2015. Geneva, Switzerland: World Health Organization (WHO), 2015.
Azerbaijan Vital Registration - Deaths 1996 ICD9 as it appears in World Health Organization (WHO). WHO Mortality Database Version November 2015. Geneva, Switzerland: World Health Organization (WHO), 2015.
Azerbaijan Vital Registration - Deaths 1997 ICD9 as it appears in World Health Organization (WHO). WHO Mortality Database Version November 2015. Geneva, Switzerland: World Health Organization (WHO), 2015.
Azerbaijan Vital Registration - Deaths 1998 ICD9 as it appears in World Health Organization (WHO). WHO Mortality Database Version November 2015. Geneva, Switzerland: World Health Organization (WHO), 2015.
Azerbaijan Vital Registration - Deaths 1999 ICD9 as it appears in World Health Organization (WHO). WHO Mortality Database Version November 2015. Geneva, Switzerland: World Health Organization (WHO), 2015.
Azerbaijan Vital Registration - Deaths 2000 ICD9 as it appears in World Health Organization (WHO). WHO Mortality Database Version November 2015. Geneva, Switzerland: World Health Organization (WHO), 2015.
Azerbaijan Vital Registration - Deaths 2001 ICD10 as it appears in World Health Organization (WHO). WHO Mortality Database Version November 2015. Geneva, Switzerland: World Health Organization (WHO), 2015.
Azerbaijan Vital Registration - Deaths 2002 ICD10 as it appears in World Health Organization (WHO). WHO Mortality Database Version November 2015. Geneva, Switzerland: World Health Organization (WHO), 2015.
Azerbaijan Vital Registration - Deaths 2003 ICD10 as it appears in World Health Organization (WHO). WHO Mortality Database Version November 2015. Geneva, Switzerland: World Health Organization (WHO), 2015.
Azerbaijan Vital Registration - Deaths 2004 ICD10 as it appears in World Health Organization (WHO). WHO Mortality Database Version November 2015. Geneva, Switzerland: World Health Organization (WHO), 2015.
Azerbaijan Vital Registration - Deaths 2007 ICD10 as it appears in World Health Organization (WHO). WHO Mortality Database Version November 2015. Geneva, Switzerland: World Health Organization (WHO), 2015.
Azerbaijan Vital Registration Death Data 2005 as it appears in TransMonEE 2013 Database, UNICEF Regional Office for CEE/CIS.
Azerbaijan Vital Registration Death Data 2006 as it appears in TransMonEE 2013 Database, UNICEF Regional Office for CEE/CIS.
Azimi-Nezhad M, Ghayour-Mobarhan M, Parizadeh MR, Safarian M, Esmaeili H, Parizadeh SM, Khodaee G, Hosseini J, Abasalti Z, Hassankhani B, Ferns G. Prevalence of type 2 diabetes mellitus in Iran and its relationship with gender, urbanisation, education, marital status and occupation. Singapore Med J. 2008; 49(7): 571-6.
Ba F. Malaria in the Mesoendemic Area: Relationships between Transmission, Infection and Malaria Morbidity in Ndiop (Senegal) [dissertation]. Dakar, Senegal: Cheikh Anta Diop University, 2000. As it appears in Malaria Atlas Project. Malaria Atlas Project Plasmodium Falciparum Parasite Rate Database. Oxford, United Kingdom: Malaria Atlas Project.
BÃ¶rsch-Supan, A. (2013). Survey of Health, Ageing and Retirement in Europe (SHARE) Wave 1. Release version: 2.6.0. SHARE-ERIC. Data set. DOI: 10.6103/SHARE.w1.260
BÃ¶rsch-Supan, A. (2013). Survey of Health, Ageing and Retirement in Europe (SHARE) Wave 2. Release version: 2.6.0. SHARE-ERIC. Data set. DOI: 10.6103/SHARE.w2.260
BÃ¶rsch-Supan, A. (2013). Survey of Health, Ageing and Retirement in Europe (SHARE) Wave 4. Release version: 1.1.1. SHARE-ERIC. Data set. DOI: 10.6103/SHARE.w4.111
BÃ¶rsch-Supan, A. (2015). Survey of Health, Ageing and Retirement in Europe (SHARE) Wave 5. Release version: 1.0.0. SHARE-ERIC. Data set. DOI: 10.6103/SHARE.w5.100
Baba M, Davis WA, Norman PE, Davis TM. Temporal changes in the prevalence and associates of foot ulceration in type 2 diabetes: the Fremantle Diabetes Study. J Diabetes Complicat. 2015; 29(3): 356-61.
Babiker H, Lines J, Hill W, Walliker D. Population structure of Plasmodium falciparum in villages with different malaria endemicity in east Africa. Am J Trop Med Hyg. 1997; 56(2): 141-7. As it appears in Malaria Atlas Project. Malaria Atlas Project Plasmodium Falciparum Parasite Rate Database. Oxford, United Kingdom: Malaria Atlas Project.
Babu BV, Acharya AS, Mallick G, Jangid PK, Nayak AN, Satyanarayana K. Lymphatic filariasis in Khurda district of Orissa, India: an epidemiological study. Southeast Asian J Trop Med Public Health. 2001; 32(2): 240-3.
Babu CS, Satishchandra P, Sinha S, Subbakrishna DK. Co-morbidities in people living with epilepsy: hospital based case-control study from a resource-poor setting. Epilepsy Res. 2009; 86(2-3): 146-52.
Babuadze G, Alvar J, Argaw D, de Koning HP, Iosava M, Kekelidze M, Tsertsvadze N, Tsereteli D, Chakhunashvili G, Mamatsashvili T, Beria N, Kalandadze I, Ejov M, Imnadze P. Epidemiology of visceral leishmaniasis in Georgia. PLoS Negl Trop Dis. 2014; 8(3): e2725.
Bada AS, Olatunji PO, Adewuyi JO, Iseniyi JO, Onile BA. Hepatitis B surface antigenaemia in Ilorin, Kwara State, Nigeria. Cent Afr J Med. 1996; 42(5): 139-41.
Baddoura R, Wehbeh-Chidiac C. Prevalence of tobacco use among the adult Lebanese population. East Mediterr Health J. 2001; 7(4-5): 819-28.
Bader E, Alhaj AM, Hussan AA, Adam I. Malaria and stillbirth in Omdurman Maternity Hospital, Sudan. Int J Gynaecol Obstet. 2010; 109(2): 144-6.
Badr IA, Saif AM, Al-Rajhi AA. Changing patterns of visual loss in the Eastern Province, Kingdom of Saudi Arabia. Saudi J Ophthalmol. 2004; 18: SI56-SI64.
Baecke JA, Burema J, Frijters JE, Hautvast JG, van der Wiel-Wetzels WA. Obesity in young Dutch adults: I, socio-demographic variables and body mass index. Int J Obes (Lond). 1983; 7(1): 1-12.
Baena-Diez JM, Elosua R, Cano JF, Masia R, Sala J, Marrugat J, Schroder H. Waist circumference and impaired fasting glucose screening in a Mediterranean population. Diabetes Res Clin Pract. 2009; 86(2): e12–4.
Bah E, Parkin DM, Hall AJ, Jack AD, Whittle H. Cancer in the Gambia: 1988-97. Br J Cancer. 2001; 84(9): 1207-14.

Appendix: Citation List

Citation
Bahamas Department of Statistics. Bahamas Population and Housing Census 1990.
Bahamas Department of Statistics. Bahamas Population and Housing Census 2000.
Bahamas Department of Statistics. Bahamas Vital Statistics 2009. Nassau, The Bahamas: Bahamas Department of Statistics.
Bahamas Department of Statistics. Bahamas Vital Statistics Report 2010. Nassau, The Bahamas: Bahamas Department of Statistics.
Bahamas Department of Statistics. Bahamas Vital Statistics Report 2012. Nassau, The Bahamas: Bahamas Department of Statistics.
Bahamas Vital Registration - Deaths 1972 ICD8 as it appears in World Health Organization (WHO). WHO Mortality Database Version May 2013. Geneva, Switzerland: World Health Organization (WHO), 2013.
Bahamas Vital Registration - Deaths 1974 ICD8 as it appears in World Health Organization (WHO). WHO Mortality Database Version May 2013. Geneva, Switzerland: World Health Organization (WHO), 2013.
Bahamas Vital Registration - Deaths 1975 ICD8 as it appears in World Health Organization (WHO). WHO Mortality Database Version May 2013. Geneva, Switzerland: World Health Organization (WHO), 2013.
Bahamas Vital Registration - Deaths 1976 ICD8 as it appears in World Health Organization (WHO). WHO Mortality Database Version May 2013. Geneva, Switzerland: World Health Organization (WHO), 2013.
Bahamas Vital Registration - Deaths 1977 ICD8 as it appears in World Health Organization (WHO). WHO Mortality Database Version May 2013. Geneva, Switzerland: World Health Organization (WHO), 2013.
Bahamas Vital Registration - Deaths 1979 ICD8 as it appears in World Health Organization (WHO). WHO Mortality Database Version May 2013. Geneva, Switzerland: World Health Organization (WHO), 2013.
Bahamas Vital Registration - Deaths 1980 ICD9 as it appears in World Health Organization (WHO). WHO Mortality Database Version February 2014. Geneva, Switzerland: World Health Organization (WHO), 2014.
Bahamas Vital Registration - Deaths 1981 ICD9 as it appears in World Health Organization (WHO). WHO Mortality Database Version November 2015. Geneva, Switzerland: World Health Organization (WHO), 2015.
Bahamas Vital Registration - Deaths 1983 ICD9 as it appears in World Health Organization (WHO). WHO Mortality Database Version November 2015. Geneva, Switzerland: World Health Organization (WHO), 2015.
Bahamas Vital Registration - Deaths 1984 ICD9 as it appears in World Health Organization (WHO). WHO Mortality Database Version February 2014. Geneva, Switzerland: World Health Organization (WHO), 2014.
Bahamas Vital Registration - Deaths 1985 ICD9 as it appears in World Health Organization (WHO). WHO Mortality Database Version November 2015. Geneva, Switzerland: World Health Organization (WHO), 2015.
Bahamas Vital Registration - Deaths 1987 ICD9 as it appears in World Health Organization (WHO). WHO Mortality Database Version February 2014. Geneva, Switzerland: World Health Organization (WHO), 2014.
Bahamas Vital Registration - Deaths 1993 ICD9 as it appears in World Health Organization (WHO). WHO Mortality Database Version November 2015. Geneva, Switzerland: World Health Organization (WHO), 2015.
Bahamas Vital Registration - Deaths 1994 ICD9 as it appears in World Health Organization (WHO). WHO Mortality Database Version February 2014. Geneva, Switzerland: World Health Organization (WHO), 2014.
Bahamas Vital Registration - Deaths 1995 ICD9 as it appears in World Health Organization (WHO). WHO Mortality Database Version November 2015. Geneva, Switzerland: World Health Organization (WHO), 2015.
Bahamas Vital Registration - Deaths 1996 ICD9 as it appears in World Health Organization (WHO). WHO Mortality Database Version November 2015. Geneva, Switzerland: World Health Organization (WHO), 2015.
Bahamas Vital Registration - Deaths 1997 ICD9 as it appears in World Health Organization (WHO). WHO Mortality Database Version November 2015. Geneva, Switzerland: World Health Organization (WHO), 2015.
Bahamas Vital Registration - Deaths 1998 ICD9 as it appears in World Health Organization (WHO). WHO Mortality Database Version November 2015. Geneva, Switzerland: World Health Organization (WHO), 2015.
Bahamas Vital Registration - Deaths 1999 ICD10 as it appears in World Health Organization (WHO). WHO Mortality Database Version November 2015. Geneva, Switzerland: World Health Organization (WHO), 2015.
Bahamas Vital Registration - Deaths 2000 ICD10 as it appears in World Health Organization (WHO). WHO Mortality Database Version November 2015. Geneva, Switzerland: World Health Organization (WHO), 2015.
Bahamas Vital Registration - Deaths 2001 ICD10 as it appears in World Health Organization (WHO). WHO Mortality Database Version November 2015. Geneva, Switzerland: World Health Organization (WHO), 2015.
Bahamas Vital Registration - Deaths 2002 ICD10 as it appears in World Health Organization (WHO). WHO Mortality Database Version November 2015. Geneva, Switzerland: World Health Organization (WHO), 2015.
Bahamas Vital Registration - Deaths 2003 ICD10 as it appears in World Health Organization (WHO). WHO Mortality Database Version November 2015. Geneva, Switzerland: World Health Organization (WHO), 2015.
Bahamas Vital Registration - Deaths 2004 ICD10 as it appears in World Health Organization (WHO). WHO Mortality Database Version November 2015. Geneva, Switzerland: World Health Organization (WHO), 2015.
Bahamas Vital Registration - Deaths 2005 ICD10 as it appears in World Health Organization (WHO). WHO Mortality Database Version November 2015. Geneva, Switzerland: World Health Organization (WHO), 2015.
Bahamas Vital Registration - Deaths 2006 ICD10 as it appears in World Health Organization (WHO). WHO Mortality Database Version November 2015. Geneva, Switzerland: World Health Organization (WHO), 2015.
Bahamas Vital Registration - Deaths 2007 ICD10 as it appears in World Health Organization (WHO). WHO Mortality Database Version November 2015. Geneva, Switzerland: World Health Organization (WHO), 2015.
Bahamas Vital Registration - Deaths 2008 ICD10 as it appears in World Health Organization (WHO). WHO Mortality Database Version November 2015. Geneva, Switzerland: World Health Organization (WHO), 2015.
Bahamas Vital Registration - Deaths 2009 ICD10 as it appears in World Health Organization (WHO). WHO Mortality Database Version November 2015. Geneva, Switzerland: World Health Organization (WHO), 2015.

Appendix: Citation List

Citation
Bahamas Vital Registration - Deaths 2010 ICD10 as it appears in World Health Organization (WHO). WHO Mortality Database Version November 2015. Geneva, Switzerland: World Health Organization (WHO), 2015.
Bahamas Vital Registration - Deaths 2011 ICD10 as it appears in World Health Organization (WHO). WHO Mortality Database Version November 2015. Geneva, Switzerland: World Health Organization (WHO), 2015.
Bahamas Vital Registration - Deaths 2012 ICD10 as it appears in World Health Organization (WHO). WHO Mortality Database Version November 2015. Geneva, Switzerland: World Health Organization (WHO), 2015.
Bahíllo MP, Hermoso F, Ochoa C, García-Fernández JA, Rodrigo J, Marugán JM, de la Torre S, Manzano F, Lema T, García-Velázquez J, Castilla-León Childhood Type 1 Diabetes Epidemiology Study Group. Incidence and prevalence of type 1 diabetes in children aged Pediatr Diabetes. 2007; 8(6): 369-73.
Bahrain Cancer Registry 1998-2002 - CI5 as it appears in Curado MP, Edwards B, Shin HR, Storm H, Ferlay J, Heanue M and Boyle P, eds (2007). Cancer Incidence in Five Continents, Vol. IX Periodic Data (electronic version). Lyon, IARC.http://ci5.iarc.fr
Bahrain Child Health Survey 1989 as it appears in World Health Organization (WHO). WHO Global Database on Child Growth and Malnutrition. Geneva, Switzerland: World Health Organization (WHO).
Bahrain Population and Housing Census 1971 as it appears in United Nations Statistics Division (UNSD). United Nations Demographic Yearbook. New York City, United States: United Nations Statistics Division (UNSD).
Bahrain Population and Housing Census 1991 as it appears in United Nations Statistics Division (UNSD). United Nations Demographic Yearbook - Historical Supplement 1997. New York City, United States: United Nations Statistics Division (UNSD).
Bahrain Population, Housing, Buildings, and Establishments Census 2001 as it appears in United Nations Statistics Division (UNSD). United Nations Demographic Yearbook 2001. New York City, United States: United Nations Statistics Division (UNSD), 2003.
Bahrain Review of the EPI 1980.
Bahrain Vital Registration - Deaths 1985 ICD9 as it appears in World Health Organization (WHO). WHO Mortality Database Version November 2015. Geneva, Switzerland: World Health Organization (WHO), 2015.
Bahrain Vital Registration - Deaths 1987 ICD9 as it appears in World Health Organization (WHO). WHO Mortality Database Version November 2015. Geneva, Switzerland: World Health Organization (WHO), 2015.
Bahrain Vital Registration - Deaths 1988 ICD9 as it appears in World Health Organization (WHO). WHO Mortality Database Version November 2015. Geneva, Switzerland: World Health Organization (WHO), 2015.
Bahrain Vital Registration - Deaths 1997 ICD10 as it appears in World Health Organization (WHO). WHO Mortality Database Version November 2015. Geneva, Switzerland: World Health Organization (WHO), 2015.
Bahrain Vital Registration - Deaths 1998 ICD10 as it appears in World Health Organization (WHO). WHO Mortality Database Version November 2015. Geneva, Switzerland: World Health Organization (WHO), 2015.
Bahrain Vital Registration - Deaths 1999 ICD10 as it appears in World Health Organization (WHO). WHO Mortality Database Version November 2015. Geneva, Switzerland: World Health Organization (WHO), 2015.
Bahrain Vital Registration - Deaths 2000 ICD10 as it appears in World Health Organization (WHO). WHO Mortality Database Version November 2015. Geneva, Switzerland: World Health Organization (WHO), 2015.
Bahrain Vital Registration - Deaths 2001 ICD10 as it appears in World Health Organization (WHO). WHO Mortality Database Version November 2015. Geneva, Switzerland: World Health Organization (WHO), 2015.
Bahrain Vital Registration - Deaths 2002 ICD10 as it appears in World Health Organization (WHO). WHO Mortality Database Version November 2015. Geneva, Switzerland: World Health Organization (WHO), 2015.
Bahrain Vital Registration - Deaths 2003 ICD10 as it appears in World Health Organization (WHO). WHO Mortality Database Version November 2015. Geneva, Switzerland: World Health Organization (WHO), 2015.
Bahrain Vital Registration - Deaths 2004 ICD10 as it appears in World Health Organization (WHO). WHO Mortality Database Version November 2015. Geneva, Switzerland: World Health Organization (WHO), 2015.
Bahrain Vital Registration - Deaths 2005 ICD10 as it appears in World Health Organization (WHO). WHO Mortality Database Version November 2015. Geneva, Switzerland: World Health Organization (WHO), 2015.
Bahrain Vital Registration - Deaths 2006 ICD10 as it appears in World Health Organization (WHO). WHO Mortality Database Version November 2015. Geneva, Switzerland: World Health Organization (WHO), 2015.
Bahrain Vital Registration - Deaths 2007 ICD10 as it appears in World Health Organization (WHO). WHO Mortality Database Version November 2015. Geneva, Switzerland: World Health Organization (WHO), 2015.
Bahrain Vital Registration - Deaths 2008 ICD10 as it appears in World Health Organization (WHO). WHO Mortality Database Version November 2015. Geneva, Switzerland: World Health Organization (WHO), 2015.
Bahrain Vital Registration - Deaths 2009 ICD10 as it appears in World Health Organization (WHO). WHO Mortality Database Version November 2015. Geneva, Switzerland: World Health Organization (WHO), 2015.
Bahrain Vital Registration - Deaths 2010 ICD10 as it appears in World Health Organization (WHO). WHO Mortality Database Version November 2015. Geneva, Switzerland: World Health Organization (WHO), 2015.
Bahrain Vital Registration - Deaths 2011 ICD10 as it appears in World Health Organization (WHO). WHO Mortality Database Version November 2015. Geneva, Switzerland: World Health Organization (WHO), 2015.
Bahrain Vital Registration - Deaths 2012 ICD10 as it appears in World Health Organization (WHO). WHO Mortality Database Version November 2015. Geneva, Switzerland: World Health Organization (WHO), 2015.
Bahrain Vital Registration - Deaths 2013 ICD10 as it appears in World Health Organization (WHO). WHO Mortality Database Version November 2015. Geneva, Switzerland: World Health Organization (WHO), 2015.
Bahrain Vital Registration Death Data 2012 as it appears in United Nations Statistics Division (UNSD). United Nations Demographic Yearbook 2012. New York City, United States: United Nations Statistics Division (UNSD), 2013.
Baiden F, Hodgson A, Adjuik M, Adongo P, Ayaga B, Binka F. Trend and causes of neonatal mortality in the Kassena-Nankana district of northern Ghana, 1995-2002. Trop Med Int Health. 2006; 11(4): 532-9.

Appendix: Citation List

Citation
Baird J, Jones T, Danudirgo E, Annis B, Bangs M, Basri H, Punomo, Masbar S. Age-dependent acquired protection against Plasmodium falciparum in people having two years exposure to hyperendemic malaria. Am J Trop Med Hyg. 1991; 45(1): 65-76. As it appears in Malaria Atlas Project. Malaria Atlas Project Plasmodium Falciparum Parasite Rate Database. Oxford, United Kingdom: Malaria Atlas Project.
Baird J, Punomo, Masbar S. Plasmodium ovale in Indonesia. Southeast Asian J Trop Med Public Health. 1990; 21(4): 541-4. As it appears in Malaria Atlas Project. Malaria Atlas Project Plasmodium Falciparum Parasite Rate Database. Oxford, United Kingdom: Malaria Atlas Project.
Baird J, Wiady I, Fryauff D, Sutanihardja M, Leksana B, Widjaya H, Kysdarmanto, Subianto B. In vivo resistance to chloroquine by Plasmodium vivax and Plasmodium falciparum at Nabire, Irian Jaya, Indonesia. Am J Trop Med Hyg. 1997; 56(6): 627-31. As it appears in Malaria Atlas Project. Malaria Atlas Project Plasmodium Falciparum Parasite Rate Database. Oxford, United Kingdom: Malaria Atlas Project.
Baird JK, Purnomo, Basri H, Bangs MJ, Andersen EM, Jones TR, Masbar S, Harjosuwarno S, Subianto B, Arbani PR. Age-Specific Prevalence of Plasmodium falciparum Among Six Populations with Limited Histories of Exposure to Endemic Malaria. Am J Trop Med Hyg. 1993; 49(6): 707-19. As it appears in Malaria Atlas Project. Malaria Atlas Project Plasmodium Falciparum Parasite Rate Database. Oxford, United Kingdom: Malaria Atlas Project.
Bakalis S, Akolekar R, Gallo DM, Poon LC, Nicolaides KH. Umbilical and fetal middle cerebral artery Doppler at 30-34 weeks' gestation in the prediction of adverse perinatal outcome. Ultrasound Obstet Gynecol. 2015; 45(4): 409–20.
Baker CK, Norris FH, Diaz DMV, Perilla JL, Murphy AD, Hill EG. Violence and PTSD in Mexico: gender and regional differences. Soc Psychiatry Psychiatr Epidemiol. 2005; 40(7): 519-28.
Baker IDI Heart and Diabetes Institute, Imperial College London, Ministry of Health and Quality of Life (Mauritius), National Public Health Institute (Finland), UmeÃ¥ University. Mauritius Noncommunicable Disease Survey 2009.
Bakhshi E, Eshraghian MR, Mohammad K, Foroushani AR, Zeraati H, Fotouhi A, Siassi F, Seifi B. Sociodemographic and smoking associated with obesity in adult women in Iran: results from the National Health Survey. J Public Health (Oxf). 2008; 30(4): 429-35.
Bakir TM, Kurbaan KM, al Fawaz I, Ramia S. Infection with hepatitis viruses (B and C) and human retroviruses (HTLV-1 and HIV) in Saudi children receiving cycled cancer chemotherapy. J Trop Pediatr. 1995; 41(4): 206-9.
Balagopal P, Kamalamma N, Patel TG, Misra R. A community-based participatory diabetes prevention and management intervention in rural India using community health workers. Diabetes Educ. 2012; 38(6): 822-34.
Balanda KP, Buckley CM, Barron SJ, Fahy LE, Madden JM, Harrington JM, Perry IJ, Kearney PM. Prevalence of diabetes in the Republic of Ireland: results from the National Health Survey (SLAN) 2007. PLoS One. 2013; 8(10): e78406.
Bald, NM, Diallo I, Bald, MD, Barry IS, Kaba L, Diallo MM, Kak, A, Camara A, Bah D, Barry MM, Sangar,-Bah M, Maugendre D. Diabetes and impaired fasting glucose in rural and urban populations in Futa Jallon (Guinea): prevalence and associated risk factors. Diabetes Metab. 2007; 33(2): 114-20.
Baldé MC, Camara M, Barry AO, Sow S, Sidibb CT, Lamah O, Lodi OE, Camara SK, Condé N, Bah H. [Malaria prevalence survey in 24 Guinean villages in the Kindia area]. Bull Soc Pathol Exot. 2001; 94(2): 192-4. As it appears in Malaria Atlas Project. Malaria Atlas Project Plasmodium Falciparum Parasite Rate Database. Oxford, United Kingdom: Malaria Atlas Project.
Baldo V, Floreani A, Menegon T, Grella P, Paternoster DM, Trivello R. Hepatitis C virus, hepatitis B virus and human immunodeficiency virus infection in pregnant women in North-East Italy: a seroepidemiological study. Eur J Epidemiol. 2000; 16(1): 87-91.
Balew A, Brhane M, Nigussie M, Rigat S, Tibebu Y, Sisay F, Tamirat G. Prevalence of P. Falciparum and P. Vivax and its Associated Risk Factors in Harar, Eastern Ethiopia. Nairobi, Kenya, 2009. As it appears in Malaria Atlas Project. Malaria Atlas Project Plasmodium Falciparum Parasite Rate Database. Oxford, United Kingdom: Malaria Atlas Project.
Baliraine FN, Afrane YA, Amenya DA, Bonizzoni M, Menge DM, Zhou G, Zhong D, Vardo-Zalik AM, Githeko AK, Yan G. High Prevalence of Asymptomatic Plasmodium falciparum Infections in a Highland Area of Western Kenya: A Cohort Study. J Infect Dis. 2009; 200(1): 66-74. As it appears in Malaria Atlas Project. Malaria Atlas Project Plasmodium Falciparum Parasite Rate Database. Oxford, United Kingdom: Malaria Atlas Project.
Balkew M. Studies on the Bionomics and Molecular Insecticide Resistance of Anophelines in Gorgora, North-west Ethiopia [dissertation]. Khartoum, Sudan: University of Khartoum, 2008. As it appears in Malaria Atlas Project. Malaria Atlas Project Plasmodium Falciparum Parasite Rate Database. Oxford, United Kingdom: Malaria Atlas Project.
Balogun WO, Adeleye JO, Akinlade KS, Kuti M, Otegbayo JA. Low prevalence of hepatitis-C viral seropositivity among patients with type-2 diabetes mellitus in a tertiary hospital. J Natl Med Assoc. 2006; 98(11): 1805-8.
Baltazar JC, Ancheta CA, Aban IB, Fernando RE, Baquilod MM. Prevalence and correlates of diabetes mellitus and impaired glucose tolerance among adults in Luzon, Philippines. Diabetes Res Clin Pract. 2004; 64(2): 107-15.
Bamia C, Trichopoulou A, Lenas D, Trichopoulos D. Tobacco smoking in relation to body fat mass and distribution in a general population sample. Int J Obes Relat Metab Disord. 2004; 28(8): 1091â€“6.
Banajeh SM, Al-Rabee AM, Al-Arashi IH. Burden of perinatal conditions in Yemen: A 12-year hospital-based study. East Mediterr Health J. 2005; 11(4): 680-9.
Banda LT, Parkin DM, Dzamalala CP, Liomba NG. Cancer incidence in Blantyre, Malawi 1994-1998. Trop Med Int Health. 2001; 6(4): 296-304.
Banerjee A, Chakravarty R, Mondal PN, Chakraborty MS. Hepatitis B virus genotype D infection among antenatal patients attending a maternity hospital in Calcutta, India: assessment of infectivity status. Southeast Asian J Trop Med Public Health. 2005; 36(1): 203-6.
Bang AT, Bang RA, Baitule SB, Reddy MH, Deshmukh MD. Effect of home-based neonatal care and management of sepsis on neonatal mortality: field trial in rural India. Lancet. 1999; 354(9194): 1955-61.
Bangladesh Bureau of Statistics, CDC Foundation, Centers for Disease Control and Prevention (CDC), Ministry of Health and Family Welfare (Bangladesh), National Institute of Population Research and Training (NIPORT), National Institute of Preventive and Social Medicine, University of Dhaka (Bangladesh), World Health Organization (WHO). Bangladesh Global Adult Tobacco Survey 2009.
Bangladesh Bureau of Statistics, Government of Bangladesh, Ministry of Planning (Bangladesh), United Nations Children's Fund (UNICEF). Bangladesh Multiple Indicator Cluster Survey 2012-2013. New York, United States: United Nations Children's Fund (UNICEF), 2015.
Bangladesh Bureau of Statistics, Ministry of Planning (Bangladesh). Bangladesh Welfare Monitoring Survey 2009.

Appendix: Citation List

Citation
Bangladesh Bureau of Statistics, Mitra and Associates, United Nations Children's Fund (UNICEF). Bangladesh Multiple Indicator Cluster Survey 2006. New York, United States: United Nations Children's Fund (UNICEF).
Bangladesh Bureau of Statistics, United Nations Children's Fund (UNICEF). Achieving the Mid-Decade Goals for Children in Bangladesh 1996.
Bangladesh Bureau of Statistics, United Nations Children's Fund (UNICEF). Bangladesh Multiple Indicator Cluster Survey 1996.
Bangladesh Bureau of Statistics. Bangladesh Population and Housing Census 1981.
Bangladesh Bureau of Statistics. Bangladesh Population and Housing Census 1991.
Bangladesh Bureau of Statistics. Bangladesh Population and Housing Census 2001.
Bangladesh Bureau of Statistics. Bangladesh Sample Vital Registration System 2010.
Bangladesh Bureau of Statistics. Bangladesh Sample Vital Registration System 2012.
Bangladesh Bureau of Statistics. Bangladesh Sample Vital Registration System 2013.
Bangladesh Bureau of Statistics. Bangladesh Sample Vital Registration System 2014.
Bangladesh Bureau of Statistics. Bangladesh Smoking, Tobacco, and Health Survey 1995.
Bangladesh Child Nutrition Status Survey 1989-1990 as it appears in World Health Organization (WHO). WHO Global Database on Child Growth and Malnutrition. Geneva, Switzerland: World Health Organization (WHO).
Bangladesh Child Nutrition Survey 1992 as it appears in World Health Organization (WHO). WHO Global Database on Child Growth and Malnutrition. Geneva, Switzerland: World Health Organization (WHO).
Bangladesh Child Nutrition Survey 1995-1996 as it appears in World Health Organization (WHO). WHO Global Database on Child Growth and Malnutrition. Geneva, Switzerland: World Health Organization (WHO).
Bangladesh Draft Report on Sentinel Tobacco Use Prevalence Study.
Bangladesh EPI Coverage Evaluation Survey 2006
Bangladesh Handbook on Environmental Statistics 2005 as it appears in World Health Organization (WHO). WHO Household Energy Database. Geneva, Switzerland: World Health Organization (WHO), 2010.
Bangladesh Health and Nutritional Surveillance for Development as it appears in World Health Organization (WHO). WHO Global Database on Child Growth and Malnutrition - Historical. Geneva, Switzerland: World Health Organization (WHO).
Bangladesh in Facts and Figures: Annual Report of the Nutritional Surveillance Project 2003 as it appears in World Health Organization (WHO). WHO Global Database on Child Growth and Malnutrition - Historical. Geneva, Switzerland: World Health Organization (WHO).
Bangladesh in Facts and Figures: Annual Report of the Nutritional Surveillance Project 2004 as it appears in World Health Organization (WHO). WHO Global Database on Child Growth and Malnutrition - Historical. Geneva, Switzerland: World Health Organization (WHO).
Bangladesh Nutritional Blindness Study 1982-1983 as it appears in World Health Organization (WHO). WHO Global Database on Child Growth and Malnutrition. Geneva, Switzerland: World Health Organization (WHO).
Bangladesh Nutritional Surveillance for Disaster Preparedness and Prevention of Nutritional Blindness: Seasonality of Nutritional Status as it appears in World Health Organization (WHO). WHO Global Database on Child Growth and Malnutrition - Historical. Geneva, Switzerland: World Health Organization (WHO).
Bangladesh Nutritional Surveillance Project Data on Rural National Using the WHO Child Growth Standards 1998 as it appears in World Health Organization (WHO). WHO Global Database on Child Growth and Malnutrition. Geneva, Switzerland: World Health Organization (WHO).
Bangladesh Nutritional Surveillance Project Data on Urban Poor Using the WHO Child Growth Standards 1991 as it appears in World Health Organization (WHO). WHO Global Database on Child Growth and Malnutrition. Geneva, Switzerland: World Health Organization (WHO).
Bangladesh Nutritional Surveillance Project Data on Urban Poor Using the WHO Child Growth Standards 1992 as it appears in World Health Organization (WHO). WHO Global Database on Child Growth and Malnutrition. Geneva, Switzerland: World Health Organization (WHO).
Bangladesh Nutritional Surveillance Project Data on Urban Poor Using the WHO Child Growth Standards 1993 as it appears in World Health Organization (WHO). WHO Global Database on Child Growth and Malnutrition. Geneva, Switzerland: World Health Organization (WHO).
Bangladesh Nutritional Surveillance Project Data on Urban Poor Using the WHO Child Growth Standards 1994 as it appears in World Health Organization (WHO). WHO Global Database on Child Growth and Malnutrition. Geneva, Switzerland: World Health Organization (WHO).
Bangladesh Nutritional Surveillance Project Data on Urban Poor Using the WHO Child Growth Standards 1995 as it appears in World Health Organization (WHO). WHO Global Database on Child Growth and Malnutrition. Geneva, Switzerland: World Health Organization (WHO).
Bangladesh Nutritional Surveillance Project Data on Urban Poor Using the WHO Child Growth Standards 1996 as it appears in World Health Organization (WHO). WHO Global Database on Child Growth and Malnutrition. Geneva, Switzerland: World Health Organization (WHO).
Bangladesh Nutritional Surveillance Project Data on Urban Poor Using the WHO Child Growth Standards 1997 as it appears in World Health Organization (WHO). WHO Global Database on Child Growth and Malnutrition. Geneva, Switzerland: World Health Organization (WHO).
Bangladesh Nutritional Surveillance Project Data on Urban Poor Using the WHO Child Growth Standards 1998 as it appears in World Health Organization (WHO). WHO Global Database on Child Growth and Malnutrition. Geneva, Switzerland: World Health Organization (WHO).
Bangladesh Nutritional Surveillance Project Data on Urban Poor Using the WHO Child Growth Standards 2001 as it appears in World Health Organization (WHO). WHO Global Database on Child Growth and Malnutrition. Geneva, Switzerland: World Health Organization (WHO).
Bangladesh Nutritional Surveillance Project Data on Urban Poor Using the WHO Child Growth Standards 2003 as it appears in World Health Organization (WHO). WHO Global Database on Child Growth and Malnutrition. Geneva, Switzerland: World Health Organization (WHO).
Bangladesh Nutritional Surveillance Project Data on Urban Poor Using the WHO Child Growth Standards 2006 as it appears in World Health Organization (WHO). WHO Global Database on Child Growth and Malnutrition. Geneva, Switzerland: World Health Organization (WHO).
Bangladesh Nutritional Surveillance Project National Rural Data Using the WHO Child Growth Standards 1992 as it appears in World Health Organization (WHO). WHO Global Database on Child Growth and Malnutrition. Geneva, Switzerland: World Health Organization (WHO).
Bangladesh Nutritional Surveillance Project National Rural Data Using the WHO Child Growth Standards 1996 as it appears in World Health Organization (WHO). WHO Global Database on Child Growth and Malnutrition. Geneva, Switzerland: World Health Organization (WHO).

Appendix: Citation List

Citation
Bangladesh Nutritional Surveillance Project National Rural Data Using the WHO Child Growth Standards 1999 as it appears in World Health Organization (WHO). WHO Global Database on Child Growth and Malnutrition. Geneva, Switzerland: World Health Organization (WHO).
Bangladesh Nutritional Surveillance Project National Rural Data Using the WHO Child Growth Standards 2001 as it appears in World Health Organization (WHO). WHO Global Database on Child Growth and Malnutrition. Geneva, Switzerland: World Health Organization (WHO).
Bangladesh Nutritional Surveillance Project National Rural Data Using the WHO Child Growth Standards 2002 as it appears in World Health Organization (WHO). WHO Global Database on Child Growth and Malnutrition. Geneva, Switzerland: World Health Organization (WHO).
Bangladesh Nutritional Surveillance Project National Rural Data Using the WHO Child Growth Standards 2004 as it appears in World Health Organization (WHO). WHO Global Database on Child Growth and Malnutrition. Geneva, Switzerland: World Health Organization (WHO).
Bangladesh Nutritional Surveillance Project Rural Data Using the WHO Child Growth Standards 2005 as it appears in World Health Organization (WHO). WHO Global Database on Child Growth and Malnutrition. Geneva, Switzerland: World Health Organization (WHO).
Bangladesh Population and Housing Census 2011.
Bangladesh Progress toward the Achievement of the Goals for the 1990's, 1994.
Bangladesh Progress toward the Achievement of the Goals for the 1990's, 1995.
Bangladesh Rural Advancement Committee (BRAC), Damien Foundation, International Centre for Diarrhoeal Disease Research (Bangladesh), KNCV Tuberculosis Foundation, World Health Organization (WHO). Bangladesh Tuberculosis Disease-cum-Infection Prevalence Survey 2007-2009.
Bangladesh Rural Advancement Committee (BRAC), Research Triangle Institute, Inc. (RTI), University of Dhaka, World Health Organization Regional Office for South-East Asia (SEARO). Impact of Tobacco-Related Illness in Bangladesh. New Delhi, India: World Health Organization Regional Office for South-East Asia (SEARO), 2007.
Bangs M. Tanzania Plasmodium Falciparum Parasite Rate Data, Personal Communication with M. Bangs 2009. As it appears in Malaria Atlas Project. Malaria Atlas Project Plasmodium Falciparum Parasite Rate Database. Oxford, United Kingdom: Malaria Atlas Project.
Bano N, Chaudhri R, Yasmeen L, Shafi F, Ejaz L. A study of maternal mortality in 8 principal hospitals in Pakistan in 2009. Int J Gynaecol Obstet. 2011; 114(3): 255-9.
Bansal D, Gudala K, Muthyala H, Esam HP, Nayakallu R, Bhansali A. Prevalence and risk factors of development of peripheral diabetic neuropathy in type 2 diabetes mellitus in a tertiary care setting. J Diabetes Investig. 2014; 5(6): 714–21.
Banu S, Rahman MT, Uddin MKM, Khatun R, Ahmed T, Rahman MM, Husain MA, van Leth F. Epidemiology of tuberculosis in an urban slum of Dhaka City, Bangladesh. PLoS One. 2013; 8(10): e77721.
Baqui AH, Choi Y, Williams EK, Arifeen SE, Mannan I, Darmstadt GL, Black RE. Levels, timing, and etiology of stillbirths in Sylhet district of Bangladesh. BMC Pregnancy Childbirth. 2011; 11: 25.
Bar ME, Damborsky MP, Oscherov EB, Alvarez BM, Mizdraji G, Avalos G. [Household infestation by triatomines and human seroprevalence in Empedrado Department, Corrientes, Argentina]. Cad Saude Publica. 1997; 13(2): 305–12.
Bar ME, Damborsky MP, Oscherov EB, Wisnivesky-Colli C. [Epidemiology of Chagas disease in San Roque, Corrientes. Triatomine infestation and human seroprevalence]. Medicina (B Aires). 2005; 65(2): 97–102.
Bar ME, Oscherov EB, Pieri Damborsky M, Borda M. Epidemiology of American trypanosomiasis in the North of Corrientes province, Argentina. Medicina (B Aires). 2010; 70(2): 133-8.
Baragatti M, Fournet F, Henry M-C, Assi S, Ouedraogo H, Rogier C, Salem G. Social and environmental malaria risk factors in urban areas of Ouagadougou, Burkina Faso. Malar J. 2009; 8(1): 13. As it appears in Malaria Atlas Project. Malaria Atlas Project Plasmodium Falciparum Parasite Rate Database. Oxford, United Kingdom: Malaria Atlas Project.
Baragou S, Djibril M, Atta B, Damorou F, Pio M, Balogou A. Prevalence of cardiovascular risk factors in an urban area of Togo: a WHO STEPS-wise approach in Lome, Togo. Cardiovasc J Afr. 2012; 23(6): 309-12.
Barat P, Valade A, Brosselin P, Alberti C, Maurice-Tison S, L,vy-Marchal C. The growing incidence of type 1 diabetes in children: the 17-year French experience in Aquitaine. Diabetes Metab. 2008; 34(6 Pt 1): 601-5.
Barata RA, Peixoto JC, Tanure A, Gomes ME, Apolinário EC, Bodevan EC, de Araújo HS, Dias ES, Pinheiro Ada C. Epidemiology of visceral leishmaniasis in a reemerging focus of intense transmission in Minas Gerais State, Brazil. Biomed Res Int. 2013; 405083.
Barbados National Health and Nutrition Survey 1981 as it appears in World Health Organization (WHO). WHO Global Database on Child Growth and Malnutrition. Geneva, Switzerland: World Health Organization (WHO).
Barbados Statistical Service, Caribbean Community (CARICOM) Secretariat, Barbados Population and Housing Census 2000.
Barbados Statistical Service, Caribbean Community (CARICOM) Secretariat. Barbados Population and Housing Census 1980.
Barbados Statistical Service, Caribbean Food and Nutrition Institute, Pan American Health Organization, Ministry of Health (Barbados). Barbados Food Consumption and Anthropometric Survey 2000-2001.
Barbados Statistical Service, United Nations Children's Fund (UNICEF). Barbados Multiple Indicator Cluster Survey 2012. New York, United States: United Nations Children's Fund (UNICEF).
Barbados Statistical Service. Barbados Population and Housing Census 1970.
Barbados Statistical Service. Barbados Population and Housing Census 1990.
Barbados Vital Registration - Deaths 1955 ICD7 as it appears in World Health Organization (WHO). WHO Mortality Database Version July 2012. Geneva, Switzerland: World Health Organization (WHO), 2012.
Barbados Vital Registration - Deaths 1956 ICD7 as it appears in World Health Organization (WHO). WHO Mortality Database Version July 2012. Geneva, Switzerland: World Health Organization (WHO), 2012.
Barbados Vital Registration - Deaths 1957 ICD7 as it appears in World Health Organization (WHO). WHO Mortality Database Version July 2012. Geneva, Switzerland: World Health Organization (WHO), 2012.
Barbados Vital Registration - Deaths 1958 ICD7 as it appears in World Health Organization (WHO). WHO Mortality Database Version July 2012. Geneva, Switzerland: World Health Organization (WHO), 2012.
Barbados Vital Registration - Deaths 1959 ICD7 as it appears in World Health Organization (WHO). WHO Mortality Database Version July 2012. Geneva, Switzerland: World Health Organization (WHO), 2012.

Appendix: Citation List

Citation
Barbados Vital Registration - Deaths 1960 ICD7 as it appears in World Health Organization (WHO). WHO Mortality Database Version July 2012. Geneva, Switzerland: World Health Organization (WHO), 2012.
Barbados Vital Registration - Deaths 1961 ICD7 as it appears in World Health Organization (WHO). WHO Mortality Database Version July 2012. Geneva, Switzerland: World Health Organization (WHO), 2012.
Barbados Vital Registration - Deaths 1962 ICD7 as it appears in World Health Organization (WHO). WHO Mortality Database Version July 2012. Geneva, Switzerland: World Health Organization (WHO), 2012.
Barbados Vital Registration - Deaths 1963 ICD7 as it appears in World Health Organization (WHO). WHO Mortality Database Version July 2012. Geneva, Switzerland: World Health Organization (WHO), 2012.
Barbados Vital Registration - Deaths 1964 ICD7 as it appears in World Health Organization (WHO). WHO Mortality Database Version July 2012. Geneva, Switzerland: World Health Organization (WHO), 2012.
Barbados Vital Registration - Deaths 1965 ICD7 as it appears in World Health Organization (WHO). WHO Mortality Database Version July 2012. Geneva, Switzerland: World Health Organization (WHO), 2012.
Barbados Vital Registration - Deaths 1966 ICD7 as it appears in World Health Organization (WHO). WHO Mortality Database Version July 2012. Geneva, Switzerland: World Health Organization (WHO), 2012.
Barbados Vital Registration - Deaths 1967 ICD7 as it appears in World Health Organization (WHO). WHO Mortality Database Version July 2012. Geneva, Switzerland: World Health Organization (WHO), 2012.
Barbados Vital Registration - Deaths 1968 ICD8 as it appears in World Health Organization (WHO). WHO Mortality Database Version May 2013. Geneva, Switzerland: World Health Organization (WHO), 2013.
Barbados Vital Registration - Deaths 1969 ICD8 as it appears in World Health Organization (WHO). WHO Mortality Database Version May 2013. Geneva, Switzerland: World Health Organization (WHO), 2013.
Barbados Vital Registration - Deaths 1970 ICD8 as it appears in World Health Organization (WHO). WHO Mortality Database Version May 2013. Geneva, Switzerland: World Health Organization (WHO), 2013.
Barbados Vital Registration - Deaths 1971 ICD8 as it appears in World Health Organization (WHO). WHO Mortality Database Version May 2013. Geneva, Switzerland: World Health Organization (WHO), 2013.
Barbados Vital Registration - Deaths 1972 ICD8 as it appears in World Health Organization (WHO). WHO Mortality Database Version May 2013. Geneva, Switzerland: World Health Organization (WHO), 2013.
Barbados Vital Registration - Deaths 1973 ICD8 as it appears in World Health Organization (WHO). WHO Mortality Database Version May 2013. Geneva, Switzerland: World Health Organization (WHO), 2013.
Barbados Vital Registration - Deaths 1974 ICD8 as it appears in World Health Organization (WHO). WHO Mortality Database Version May 2013. Geneva, Switzerland: World Health Organization (WHO), 2013.
Barbados Vital Registration - Deaths 1975 ICD8 as it appears in World Health Organization (WHO). WHO Mortality Database Version May 2013. Geneva, Switzerland: World Health Organization (WHO), 2013.
Barbados Vital Registration - Deaths 1976 ICD8 as it appears in World Health Organization (WHO). WHO Mortality Database Version May 2013. Geneva, Switzerland: World Health Organization (WHO), 2013.
Barbados Vital Registration - Deaths 1977 ICD8 as it appears in World Health Organization (WHO). WHO Mortality Database Version May 2013. Geneva, Switzerland: World Health Organization (WHO), 2013.
Barbados Vital Registration - Deaths 1978 ICD8 as it appears in World Health Organization (WHO). WHO Mortality Database Version May 2013. Geneva, Switzerland: World Health Organization (WHO), 2013.
Barbados Vital Registration - Deaths 1979 ICD9 as it appears in World Health Organization (WHO). WHO Mortality Database Version November 2015. Geneva, Switzerland: World Health Organization (WHO), 2015.
Barbados Vital Registration - Deaths 1980 ICD9 as it appears in World Health Organization (WHO). WHO Mortality Database Version February 2014. Geneva, Switzerland: World Health Organization (WHO), 2014.
Barbados Vital Registration - Deaths 1981 ICD9 as it appears in World Health Organization (WHO). WHO Mortality Database Version February 2014. Geneva, Switzerland: World Health Organization (WHO), 2014.
Barbados Vital Registration - Deaths 1982 ICD9 as it appears in World Health Organization (WHO). WHO Mortality Database Version February 2014. Geneva, Switzerland: World Health Organization (WHO), 2014.
Barbados Vital Registration - Deaths 1983 ICD9 as it appears in World Health Organization (WHO). WHO Mortality Database Version February 2014. Geneva, Switzerland: World Health Organization (WHO), 2014.
Barbados Vital Registration - Deaths 1984 ICD9 as it appears in World Health Organization (WHO). WHO Mortality Database Version February 2014. Geneva, Switzerland: World Health Organization (WHO), 2014.
Barbados Vital Registration - Deaths 1985 ICD9 as it appears in World Health Organization (WHO). WHO Mortality Database Version February 2014. Geneva, Switzerland: World Health Organization (WHO), 2014.
Barbados Vital Registration - Deaths 1986 ICD9 as it appears in World Health Organization (WHO). WHO Mortality Database Version February 2014. Geneva, Switzerland: World Health Organization (WHO), 2014.
Barbados Vital Registration - Deaths 1987 ICD9 as it appears in World Health Organization (WHO). WHO Mortality Database Version February 2014. Geneva, Switzerland: World Health Organization (WHO), 2014.
Barbados Vital Registration - Deaths 1988 ICD9 as it appears in World Health Organization (WHO). WHO Mortality Database Version February 2014. Geneva, Switzerland: World Health Organization (WHO), 2014.
Barbados Vital Registration - Deaths 1989 ICD9 as it appears in World Health Organization (WHO). WHO Mortality Database Version February 2014. Geneva, Switzerland: World Health Organization (WHO), 2014.
Barbados Vital Registration - Deaths 1990 ICD9 as it appears in World Health Organization (WHO). WHO Mortality Database Version February 2014. Geneva, Switzerland: World Health Organization (WHO), 2014.
Barbados Vital Registration - Deaths 1991 ICD9 as it appears in World Health Organization (WHO). WHO Mortality Database Version February 2014. Geneva, Switzerland: World Health Organization (WHO), 2014.

Appendix: Citation List

Citation
Barbados Vital Registration - Deaths 1992 ICD9 as it appears in World Health Organization (WHO). WHO Mortality Database Version February 2014. Geneva, Switzerland: World Health Organization (WHO), 2014.
Barbados Vital Registration - Deaths 1993 ICD9 as it appears in World Health Organization (WHO). WHO Mortality Database Version February 2014. Geneva, Switzerland: World Health Organization (WHO), 2014.
Barbados Vital Registration - Deaths 1994 ICD9 as it appears in World Health Organization (WHO). WHO Mortality Database Version February 2014. Geneva, Switzerland: World Health Organization (WHO), 2014.
Barbados Vital Registration - Deaths 1995 ICD9 as it appears in World Health Organization (WHO). WHO Mortality Database Version February 2014. Geneva, Switzerland: World Health Organization (WHO), 2014.
Barbados Vital Registration - Deaths 2000 ICD10 as it appears in World Health Organization (WHO). WHO Mortality Database Version November 2015. Geneva, Switzerland: World Health Organization (WHO), 2015.
Barbados Vital Registration - Deaths 2001 ICD10 as it appears in World Health Organization (WHO). WHO Mortality Database Version November 2015. Geneva, Switzerland: World Health Organization (WHO), 2015.
Barbados Vital Registration - Deaths 2002 ICD10 as it appears in World Health Organization (WHO). WHO Mortality Database Version November 2015. Geneva, Switzerland: World Health Organization (WHO), 2015.
Barbados Vital Registration - Deaths 2003 ICD10 as it appears in World Health Organization (WHO). WHO Mortality Database Version November 2015. Geneva, Switzerland: World Health Organization (WHO), 2015.
Barbados Vital Registration - Deaths 2004 ICD10 as it appears in World Health Organization (WHO). WHO Mortality Database Version November 2015. Geneva, Switzerland: World Health Organization (WHO), 2015.
Barbados Vital Registration - Deaths 2005 ICD10 as it appears in World Health Organization (WHO). WHO Mortality Database Version November 2015. Geneva, Switzerland: World Health Organization (WHO), 2015.
Barbados Vital Registration - Deaths 2006 ICD10 as it appears in World Health Organization (WHO). WHO Mortality Database Version November 2015. Geneva, Switzerland: World Health Organization (WHO), 2015.
Barbados Vital Registration - Deaths 2007 ICD10 as it appears in World Health Organization (WHO). WHO Mortality Database Version November 2015. Geneva, Switzerland: World Health Organization (WHO), 2015.
Barbados Vital Registration - Deaths 2008 ICD10 as it appears in World Health Organization (WHO). WHO Mortality Database Version November 2015. Geneva, Switzerland: World Health Organization (WHO), 2015.
Barbados Vital Registration - Deaths 2009 ICD10 as it appears in World Health Organization (WHO). WHO Mortality Database Version November 2015. Geneva, Switzerland: World Health Organization (WHO), 2015.
Barbados Vital Registration - Deaths 2010 ICD10 as it appears in World Health Organization (WHO). WHO Mortality Database Version November 2015. Geneva, Switzerland: World Health Organization (WHO), 2015.
Barbados Vital Registration - Deaths 2011 ICD10 as it appears in World Health Organization (WHO). WHO Mortality Database Version November 2015. Geneva, Switzerland: World Health Organization (WHO), 2015.
Barbados Vital Registration - Deaths 2012 ICD10 as it appears in World Health Organization (WHO). WHO Mortality Database Version November 2015. Geneva, Switzerland: World Health Organization (WHO), 2015.
Barbados Vital Registration Death Data 1950 as it appears in United Nations Statistics Division (UNSD). United Nations Demographic Yearbook - Historical Supplement 1997. New York City, United States: United Nations Statistics Division (UNSD).
Barbados Vital Registration Death Data 1951 as it appears in United Nations Statistics Division (UNSD). United Nations Demographic Yearbook - Historical Supplement 1997. New York City, United States: United Nations Statistics Division (UNSD).
Barbados Vital Registration Death Data 1952 as it appears in United Nations Statistics Division (UNSD). United Nations Demographic Yearbook - Historical Supplement 1997. New York City, United States: United Nations Statistics Division (UNSD).
Barbados Vital Registration Death Data 1953 as it appears in United Nations Statistics Division (UNSD). United Nations Demographic Yearbook - Historical Supplement 1997. New York City, United States: United Nations Statistics Division (UNSD).
Barbados Vital Registration Death Data 1954 as it appears in United Nations Statistics Division (UNSD). United Nations Demographic Yearbook - Historical Supplement 1997. New York City, United States: United Nations Statistics Division (UNSD).
Barbagallo CM, Cavera G, Sapienza M, Noto D, Cefalù AB, Pagano M, Montalto G, Notarbartolo A, Averna MR. Prevalence of overweight and obesity in a rural southern Italy population and relationships with total and cardiovascular mortality: the Ventimiglia di Sicilia project. Int J Obes (Lond). 2001; 25(2): 185-90.
Barbosa DS, Belo VS, Rangel ME, Werneck GL. Spatial analysis for identification of priority areas for surveillance and control in a visceral leishmaniasis endemic area in Brazil. Acta Trop. 2014; 56-62.
Barcel¢ A, Daroca MC, Ribera R, Duarte E, Zapata A, Vohra M. Diabetes in Bolivia. Rev Panam Salud Publica. 2001; 10(5): 318-23.
Barcus MJ, Elyazar IRF, Marwoto H, Richie TL, Basri H, Wiady I, Fryauff DJ, Maguire JD, Bangs MJ, Baird JK. Primary infection by Plasmodium falciparum or P. vivax in a cohort of Javanese migrants to Indonesian Papua. Ann Trop Med Parasitol. 2003; 97(6): 565-74. As it appears in Malaria Atlas Project. Malaria Atlas Project Plasmodium Falciparum Parasite Rate Database. Oxford, United Kingdom: Malaria Atlas Project.
Barcus MJ. Indonesia Plasmodium Falciparum Parasite Rate Data, Personal Communication with M.J. Barcus 2001. As it appears in Malaria Atlas Project. Malaria Atlas Project Plasmodium Falciparum Parasite Rate Database. Oxford, United Kingdom: Malaria Atlas Project.
Barger B, Maiga H, Traore OB, Tekete M, Tembine I, Dara A, Traore ZI, Gantt S, Doumbo OK, Djimde AA. Intermittent preventive treatment using artemisinin-based combination therapy reduces malaria morbidity among school-aged children in Mali. Trop Med Int Health. 2009; 14(7): 784-91. As it appears in Malaria Atlas Project. Malaria Atlas Project Plasmodium Falciparum Parasite Rate Database. Oxford, United Kingdom: Malaria Atlas Project.
Barnett S, Nair N, Tripathy P, Borghi J, Rath S, Costello A. A prospective key informant surveillance system to measure maternal mortality -- findings from indigenous populations in Jharkhand and Orissa, India. BMC Pregnancy Childbirth. 2008; 8(6): 6.

Appendix: Citation List

Citation
Barnish G, Maude G, Bockarie M, Erunkulu O, Dumbaya M, Greenwood B. Malaria in a rural area of Sierra Leone. II. Parasitological and related results from pre- and post-rains clinical surveys. Ann Trop Med Parasitol. 1993; 87(2): 137-48. As it appears in Malaria Atlas Project. Malaria Atlas Project Plasmodium Falciparum Parasite Rate Database. Oxford, United Kingdom: Malaria Atlas Project.
Barnish G, Maude GH, Bockarie MJ, Eggelte TA, Greenwood BM, Ceesay S. Malaria in a rural area of Sierra Leone. I. Initial results. Ann Trop Med Parasitol. 1993; 87(2): 125-36.
Barodji W, Nurisa I, Sumardi S, Sutopo T. Kepadatan vektor dan penderita malaria di Desa Waiklibang, Kecamatan Tanjung Bunga, Flores Timur Sebelum dan Sesudah Gempa Bumi. Cermin Dunia Kedokteran. 1996; 106: 15-8. As it appears in Malaria Atlas Project. Malaria Atlas Project Plasmodium Falciparum Parasite Rate Database. Oxford, United Kingdom: Malaria Atlas Project.
Barodji W, Sumardi, Mujiono. Use of Permethrine Impregnated Bednets by East Flores. Bull Health Res. 1994; 22(4): 30-44. As it appears in Malaria Atlas Project. Malaria Atlas Project Plasmodium Falciparum Parasite Rate Database. Oxford, United Kingdom: Malaria Atlas Project.
Bar-On H, Friedlander Y, Kidron M, Kark JD. Serum glucose and insulin characteristics and prevalence of diabetes mellitus and impaired glucose tolerance in the adult Jewish population of Jerusalem. Nutr Metab Cardiovasc Dis. 1992; 2: 75-8.
Barria von-Bischhoffhausen F, Silva JC, Limburg H, Castillo DL, Martínez RL, Muñoz RD, Salinas AE, Vegas IF, Werner SM, Riquelme LA. Analysis of barriers, coverage and postoperative outcomes of cataract surgery determined by quick survey avoidable blindness in the VIII region, Chile. Arch Ophthalmol. 2007; 64(1/2): 69-87.
Barros FC, Victora CG, Barros AJD, Santos IS, Albernaz E, Matijasevich A, Domingues MR, Sclowitz IKT, Hallal PC, Silveira MF, Vaughan JP. The challenge of reducing neonatal mortality in middle-income countries: findings from three Brazilian birth cohorts in 1982, 1993, and 2004. Lancet. 2005; 365(9462): 847-54.
Barry AE, Schultz L, Senn N, Nale J, Kiniboro B, Siba PM, Mueller I, Reeder JC. High Levels of Genetic Diversity of Plasmodium falciparum Populations in Papua New Guinea despite Variable Infection Prevalence. Am J Trop Med Hyg. 2013; 88(4): 718-25. As it appears in Malaria Atlas Project. Malaria Atlas Project Plasmodium Falciparum Parasite Rate Database. Oxford, United Kingdom: Malaria Atlas Project.
Bart PA, Jacquier P, Zuber PL, Lavanchy D, Frei PC. Seroprevalence of HBV (anti-HBc, HBsAg and anti-HBs) and HDV infections among 9006 women at delivery. Liver. 1996; 16(2): 110-6.
Bartlett LA, Mawji S, Whitehead S, Crouse C, Dalil S, Ionete D, Salama P. Where giving birth is a forecast of death: maternal mortality in four districts of Afghanistan, 1999-2002. Lancet. 2005; 365(9462): 864-70.
Barton EN, King SD, Douglas LL. The seroprevalence of hepatitis and retroviral infection in Jamaican haemodialysis patients. West Indian Med J. 1998; 47(3): 105-7.
Bartra C, Mittal P, Adak T, Sharma V. Malaria investigation in District Jodhpur, Rajasthan, during the summer season. Indian J Malariol. 1998; 36(3-4): 75-80. As it appears in Malaria Atlas Project. Malaria Atlas Project Plasmodium Falciparum Parasite Rate Database. Oxford, United Kingdom: Malaria Atlas Project.
Baruah J, Kusre G, Bora R. Pattern of Gross Congenital Malformations in a Tertiary Referral Hospital in Northeast India. Indian J Pediatr. 2015; 82(10): 917–22.
Barutwanayo M, Coosemans M, Delacollette C, Bisore S, Mpitabakana P, Seruzingo D. [Campaign against malaria vectors in the framework of a rural development project in Burundi]. Ann Soc Belg Med Trop. 1990; 71(Suppl 1): 113-25. As it appears in Malaria Atlas Project. Malaria Atlas Project Plasmodium Falciparum Parasite Rate Database. Oxford, United Kingdom: Malaria Atlas Project.
Bashour H, Abdulsalam A, Jabr A, Cheikha S, Tabbaa M, Lahham M, Dihman R, Khadra M, Campbell OMR. Maternal mortality in Syria: causes, contributing factors and preventability. Trop Med Int Health. 2009; 14(9): 1122-7.
Basic Support for Institutionalizing Child Survival (BASICS), Centers for Disease Control and Prevention (CDC), Johns Hopkins University (JHU), USAID. Nigeria Integrated Baseline Survey 1995.
Basic Support for Institutionalizing Child Survival (BASICS), Save the Children USA. Guinea - Mandiana Mortality Study 1998-1999.
Basile KC. Prevalence of wife rape and other intimate partner sexual coercion in a nationally representative sample of women. Violence Vict. 2002; 17(5): 511-24.
Basit A, Danish Alvi SF, Fawwad A, Ahmed K, Yakoob Ahmedani M, Hakeem R. Temporal changes in the prevalence of diabetes, impaired fasting glucose and its associated risk factors in the rural area of Baluchistan. Diabetes Res Clin Pract. 2011; 94(3): 456-62.
Basit A, Hydrie MZ, Ahmed K, Hakeem R. Prevalence of diabetes, impaired fasting glucose and associated risk factors in a rural area of Baluchistan province according to new ADA criteria. J Pak Med Assoc. 2002; 52(8): 357-60.
Basit A, Hydrie MZ, Hakeem R, Ahmedani MY, Masood Q. Frequency of chronic complications of type II diabetes. J Coll Physicians Surg Pak. 2004; 14(2): 79-83.
Baskent University, Ministry of Health (Turkey), State Institute of Statistics (Turkey). Turkey Verbal Autopsy Survey 2003.
Bassaw B, Roopnarinesingh S, Sirjusingh A. An audit of perinatal mortality. West Indian Med J. 2001; 50(1): 42-6.
Baudon D, Robert V, Darriet F, Huerre M. [Impact of building a dam on the transmission of malaria. Malaria survey conducted in southeast Mauritania]. Bull Soc Pathol Exot. 1986; 79(1): 123-9. As it appears in Malaria Atlas Project. Malaria Atlas Project Plasmodium Falciparum Parasite Rate Database. Oxford, United Kingdom: Malaria Atlas Project.
Baul MK, Manjusha. Maternal mortality--a ten-year study. J Indian Med Assoc. 2004; 102(1): 18-9, 25.
Bawah AA, Binka FN. How many years of life could be saved if malaria were eliminated from a hyperendemic area of northern Ghana? Am J Trop Med Hyg. 2007; 77(6 Suppl): 145-52.
Bayero University Kano, Bill and Melinda Gates Foundation (BMGF), Center for Research, Evaluation, and Resource Development (CRERD), Federal Ministry of Health (Nigeria), Johns Hopkins Bloomberg School of Public Health, Lagos State Ministry of Health (Nigeria), National Bureau of Statistics (Nigeria), National Population Commission of Nigeria. Nigeria - Lagos Performance, Monitoring, and Accountability Survey, Round 1 2014. Baltimore, United States: Johns Hopkins Bloomberg School of Public Health.
Bayero University Kano, Bill and Melinda Gates Foundation (BMGF), Center for Research, Evaluation, and Resource Development (CRERD), Johns Hopkins Bloomberg School of Public Health, Ministry of Health (Nigeria), National Bureau of Statistics (Nigeria), National Population Commission of Nigeria. Nigeria - Kaduna Performance, Monitoring, and Accountability Survey, Round 1 2014. Baltimore, United States: Johns Hopkins Bloomberg School of Public Health.

Appendix: Citation List

Citation
Bayindir Cevik A, Metin Karaaslan M, Kocan S, Pekmezci H, Baydur Sahin S, Kirbas A, Ayaz T. Prevalence and screening for risk factors of type 2 diabetes in Rize, Nourtheast Turkey: findings from a population-based study. Prim Care Diabetes. 2015; nan.
Beck F, Guignard R, Richard JB, Wilquin JL, Peretti-Watel P. Recent Increase in smoking in France: main results of the Health Barometer. Bull Epidemiol Hebd (Paris). 2011; 230-3.
Beckers K, Schaad UB, Heininger U. Compliance with antenatal screening for hepatitis B surface antigen carrier status in pregnant women and consecutive procedures in exposed newborns. Eur J Pediatr. 2004; 163(11): 654-7.
Befidi-Mengue RN, Ratard RC, Beltran G, D'Alessandro A, Rice J, Befidi-Mengue R, Kouemeni LE, Cline BL. Impact of Schistosoma haematobium infection and of praziquantel treatment on anaemia of primary school children in Bertoua, Cameroon. J Trop Med Hyg. 1993; 9(8): 225-30.
Beghi E, Monticelli ML. Diabetic polyneuropathy in the elderly. Prevalence and risk factors in two geographic areas of Italy. Italian General Practitioner Study Group (IGPSG). Acta Neurol Scand. 1997; 96(4): 223-8.
Beier J, Oster C, Onyango F, Bales J, Sherwood J, Perkins P, Chumo D, Koech dv, Whitmire R, Roberts C. Plasmodium falciparum incidence relative to entomologic inoculation rates at a site proposed for testing malaria vaccines in western Kenya. Am J Trop Med Hyg. 1994; 50(5): 529-36. As it appears in Malaria Atlas Project. Malaria Atlas Project Plasmodium Falciparum Parasite Rate Database. Oxford, United Kingdom: Malaria Atlas Project.
Bejon P, Lusingu J, Olotu A, Leach A, Lievens M, Vekemans J, Mshamu S, Lang T, Gould J, Dubois MC, Demoitié MA, Stallaert JF, Vansadia P, Carter T, Njuguna P, Awuondo KO, Malabeja A, Abdul O, Gesase S, Mturi N, Drakeley CJ, Savarese B, Villafana T, Ballou WR, Cohen J, Riley EM, Lemnge MM, Marsh K, von Seidlein L. Efficacy of RTS,S/AS01E Vaccine against Malaria in Children 5 to 17 Months of Age. N Engl J Med. 2008; 359(24): 2521-32. As it appears in Malaria Atlas Project. Malaria Atlas Project Plasmodium Falciparum Parasite Rate Database. Oxford, United Kingdom: Malaria Atlas Project.
Bejon P, Mwacharo J, Kai O, Mwangi T, Milligan P, Todryk S, Keating S, Lang T, Lowe B, Gikonyo C, Molyneux C, Fegan G, Gilbert SC, Peshu N, Marsh K, Hill AVS. A Phase 2b Randomised Trial of the Candidate Malaria Vaccines FP9 ME-TRAP and MVA ME-TRAP among Children in Kenya. PLoS Clin Trials. 2006; 1(6): e29. As it appears in Malaria Atlas Project. Malaria Atlas Project Plasmodium Falciparum Parasite Rate Database. Oxford, United Kingdom: Malaria Atlas Project.
Belarus Cancer Registry 1983-1987 - CI5 as it appears in Parkin DM, International Agency for Research on Cancer, International Association of Cancer Registries. Cancer Incidence in Five Continents. Vol. I to VIII. Lyon, France, IARCPress, 2005.
Belarus Cancer Registry 1988-1992 - CI5 as it appears in Parkin DM, International Agency for Research on Cancer, International Association of Cancer Registries. Cancer Incidence in Five Continents. Vol. I to VIII. Lyon, France, IARCPress, 2005.
Belarus Cancer Registry 1993-1997 - CI5 as it appears in Parkin DM, International Agency for Research on Cancer, International Association of Cancer Registries. Cancer Incidence in Five Continents. Vol. I to VIII. Lyon, France, IARCPress, 2005.
Belarus Cancer Registry 1998-2002 - CI5 as it appears in Curado MP, Edwards B, Shin HR, Storm H, Ferlay J, Heanue M and Boyle P, eds (2007). Cancer Incidence in Five Continents, Vol. IX Periodic Data (electronic version). Lyon, IARC.http://ci5.iarc.fr
Belarus Cancer Registry 2003-2007 - CI5 as it appears in Forman D, Bray F, Brewster DH, Gombe Mbalawa C, Kohler B, Piñeros M, Steliarova-Foucher E, Swaminathan R and Ferlay J, eds (2013). Cancer Incidence in Five Continents, Vol. X Summary Database (electronic version). Lyon, IARC. http://ci5.iarc.fr
Belarus Vital Registration - Deaths 1981 ICD9 as it appears in World Health Organization (WHO). WHO Mortality Database Version November 2015. Geneva, Switzerland: World Health Organization (WHO), 2015.
Belarus Vital Registration - Deaths 1982 ICD9 as it appears in World Health Organization (WHO). WHO Mortality Database Version November 2015. Geneva, Switzerland: World Health Organization (WHO), 2015.
Belarus Vital Registration - Deaths 1985 ICD9 as it appears in World Health Organization (WHO). WHO Mortality Database Version November 2015. Geneva, Switzerland: World Health Organization (WHO), 2015.
Belarus Vital Registration - Deaths 1986 ICD9 as it appears in World Health Organization (WHO). WHO Mortality Database Version November 2015. Geneva, Switzerland: World Health Organization (WHO), 2015.
Belarus Vital Registration - Deaths 1987 ICD9 as it appears in World Health Organization (WHO). WHO Mortality Database Version November 2015. Geneva, Switzerland: World Health Organization (WHO), 2015.
Belarus Vital Registration - Deaths 1988 ICD9 as it appears in World Health Organization (WHO). WHO Mortality Database Version November 2015. Geneva, Switzerland: World Health Organization (WHO), 2015.
Belarus Vital Registration - Deaths 1989 ICD9 as it appears in World Health Organization (WHO). WHO Mortality Database Version November 2015. Geneva, Switzerland: World Health Organization (WHO), 2015.
Belarus Vital Registration - Deaths 1990 ICD9 as it appears in World Health Organization (WHO). WHO Mortality Database Version November 2015. Geneva, Switzerland: World Health Organization (WHO), 2015.
Belarus Vital Registration - Deaths 1991 ICD9 as it appears in World Health Organization (WHO). WHO Mortality Database Version November 2015. Geneva, Switzerland: World Health Organization (WHO), 2015.
Belarus Vital Registration - Deaths 1992 ICD9 as it appears in World Health Organization (WHO). WHO Mortality Database Version November 2015. Geneva, Switzerland: World Health Organization (WHO), 2015.
Belarus Vital Registration - Deaths 1993 ICD9 as it appears in World Health Organization (WHO). WHO Mortality Database Version November 2015. Geneva, Switzerland: World Health Organization (WHO), 2015.
Belarus Vital Registration - Deaths 1994 ICD9 as it appears in World Health Organization (WHO). WHO Mortality Database Version November 2015. Geneva, Switzerland: World Health Organization (WHO), 2015.
Belarus Vital Registration - Deaths 1995 ICD9 as it appears in World Health Organization (WHO). WHO Mortality Database Version November 2015. Geneva, Switzerland: World Health Organization (WHO), 2015.
Belarus Vital Registration - Deaths 1997 ICD9 as it appears in World Health Organization (WHO). WHO Mortality Database Version November 2015. Geneva, Switzerland: World Health Organization (WHO), 2015.

Appendix: Citation List

Citation
Belarus Vital Registration - Deaths 1998 ICD9 as it appears in World Health Organization (WHO). WHO Mortality Database Version November 2015. Geneva, Switzerland: World Health Organization (WHO), 2015.
Belarus Vital Registration - Deaths 1999 ICD9 as it appears in World Health Organization (WHO). WHO Mortality Database Version November 2015. Geneva, Switzerland: World Health Organization (WHO), 2015.
Belarus Vital Registration - Deaths 2000 ICD9 as it appears in World Health Organization (WHO). WHO Mortality Database Version November 2015. Geneva, Switzerland: World Health Organization (WHO), 2015.
Belarus Vital Registration - Deaths 2001 ICD9 as it appears in World Health Organization (WHO). WHO Mortality Database Version November 2015. Geneva, Switzerland: World Health Organization (WHO), 2015.
Belarus Vital Registration - Deaths 2002 ICD10 as it appears in World Health Organization (WHO). WHO Mortality Database Version November 2015. Geneva, Switzerland: World Health Organization (WHO), 2015.
Belarus Vital Registration - Deaths 2003 ICD10 as it appears in World Health Organization (WHO). WHO Mortality Database Version November 2015. Geneva, Switzerland: World Health Organization (WHO), 2015.
Belarus Vital Registration - Deaths 2007 ICD10 as it appears in World Health Organization (WHO). WHO Mortality Database Version November 2015. Geneva, Switzerland: World Health Organization (WHO), 2015.
Belarus Vital Registration - Deaths 2008 ICD10 as it appears in World Health Organization (WHO). WHO Mortality Database Version November 2015. Geneva, Switzerland: World Health Organization (WHO), 2015.
Belarus Vital Registration - Deaths 2009 ICD10 as it appears in World Health Organization (WHO). WHO Mortality Database Version November 2015. Geneva, Switzerland: World Health Organization (WHO), 2015.
Belarus Vital Registration - Deaths 2011 ICD10 as it appears in World Health Organization (WHO). WHO Mortality Database Version November 2015. Geneva, Switzerland: World Health Organization (WHO), 2015.
Belarus Vital Registration Death Data 2011 as it appears in United Nations Statistics Division (UNSD). United Nations Demographic Yearbook 2012. New York City, United States: United Nations Statistics Division (UNSD), 2013.
Belarusian State University, Concluzia-Prim Center for Survey Methodology (Moldova), Institute for Advanced Studies (Austria), London School of Hygiene and Tropical Medicine, University of Aberdeen. Belarus Health in Times of Transition Household Survey 2010.
Belfki H, Ben Ali S, Aounallah-Skhiri H, Traissac P, Bougatef S, Maire B, Delpeuch F, Achour N, Ben Romdhane H. Prevalence and determinants of the metabolic syndrome among Tunisian adults: results of the Transition and Health Impact in North Africa (TAHINA) project. Public Health Nutr. 2013; 16(4): 582–90.
Belgian Cancer Registry. Belgium Cancer Registry - Incidence. Brussels, Belgium: Belgian Cancer Registry.
Belgium - Antwerp Cancer Registry 1998-2001 - CI5 as it appears in Curado MP, Edwards B, Shin HR, Storm H, Ferlay J, Heanue M and Boyle P, eds (2007). Cancer Incidence in Five Continents, Vol. IX Periodic Data (electronic version). Lyon, IARC.http://ci5.iarc.fr
Belgium - Flanders Cancer Registry 1997-1998 - CI5 as it appears in Parkin DM, International Agency for Research on Cancer, International Association of Cancer Registries. Cancer Incidence in Five Continents. Vol. I to VIII. Lyon, France, IARCPress, 2005.
Belgium - Flanders Cancer Registry 1998-2001 - CI5 as it appears in Curado MP, Edwards B, Shin HR, Storm H, Ferlay J, Heanue M and Boyle P, eds (2007). Cancer Incidence in Five Continents, Vol. IX Periodic Data (electronic version). Lyon, IARC.http://ci5.iarc.fr
Belgium - Flanders Cancer Registry 1998-2002 - CI5 as it appears in Curado MP, Edwards B, Shin HR, Storm H, Ferlay J, Heanue M and Boyle P, eds (2007). Cancer Incidence in Five Continents, Vol. IX Periodic Data (electronic version). Lyon, IARC.http://ci5.iarc.fr
Belgium - Limburg Cancer Registry 1997-1998 - CI5 as it appears in Parkin DM, International Agency for Research on Cancer, International Association of Cancer Registries. Cancer Incidence in Five Continents. Vol. I to VIII. Lyon, France, IARCPress, 2005.
Belgium General Population Census 1961 as it appears in United Nations Statistics Division (UNSD). United Nations Demographic Yearbook. New York City, United States: United Nations Statistics Division (UNSD).
Belgium General Population Census 1970 as it appears in United Nations Statistics Division (UNSD). United Nations Demographic Yearbook. New York City, United States: United Nations Statistics Division (UNSD).
Belgium Vital Registration - Deaths 1980 ICD9 as it appears in World Health Organization (WHO). WHO Mortality Database Version November 2015. Geneva, Switzerland: World Health Organization (WHO), 2015.
Belgium Vital Registration - Deaths 1981 ICD9 as it appears in World Health Organization (WHO). WHO Mortality Database Version November 2015. Geneva, Switzerland: World Health Organization (WHO), 2015.
Belgium Vital Registration - Deaths 1982 ICD9 as it appears in World Health Organization (WHO). WHO Mortality Database Version November 2015. Geneva, Switzerland: World Health Organization (WHO), 2015.
Belgium Vital Registration - Deaths 1983 ICD9 as it appears in World Health Organization (WHO). WHO Mortality Database Version November 2015. Geneva, Switzerland: World Health Organization (WHO), 2015.
Belgium Vital Registration - Deaths 1984 ICD9 as it appears in World Health Organization (WHO). WHO Mortality Database Version November 2015. Geneva, Switzerland: World Health Organization (WHO), 2015.
Belgium Vital Registration - Deaths 1985 ICD9 as it appears in World Health Organization (WHO). WHO Mortality Database Version November 2015. Geneva, Switzerland: World Health Organization (WHO), 2015.
Belgium Vital Registration - Deaths 1986 ICD9 as it appears in World Health Organization (WHO). WHO Mortality Database Version November 2015. Geneva, Switzerland: World Health Organization (WHO), 2015.
Belgium Vital Registration - Deaths 1987 ICD9 as it appears in World Health Organization (WHO). WHO Mortality Database Version November 2015. Geneva, Switzerland: World Health Organization (WHO), 2015.
Belgium Vital Registration - Deaths 1988 ICD9 as it appears in World Health Organization (WHO). WHO Mortality Database Version November 2015. Geneva, Switzerland: World Health Organization (WHO), 2015.
Belgium Vital Registration - Deaths 1989 ICD9 as it appears in World Health Organization (WHO). WHO Mortality Database Version November 2015. Geneva, Switzerland: World Health Organization (WHO), 2015.
Belgium Vital Registration - Deaths 1990 ICD9 as it appears in World Health Organization (WHO). WHO Mortality Database Version November 2015. Geneva, Switzerland: World Health Organization (WHO), 2015.

Appendix: Citation List

Citation
Belgium Vital Registration - Deaths 1991 ICD9 as it appears in World Health Organization (WHO). WHO Mortality Database Version November 2015. Geneva, Switzerland: World Health Organization (WHO), 2015.
Belgium Vital Registration - Deaths 1992 ICD9 as it appears in World Health Organization (WHO). WHO Mortality Database Version November 2015. Geneva, Switzerland: World Health Organization (WHO), 2015.
Belgium Vital Registration - Deaths 1993 ICD9 as it appears in World Health Organization (WHO). WHO Mortality Database Version November 2015. Geneva, Switzerland: World Health Organization (WHO), 2015.
Belgium Vital Registration - Deaths 1994 ICD9 as it appears in World Health Organization (WHO). WHO Mortality Database Version November 2015. Geneva, Switzerland: World Health Organization (WHO), 2015.
Belgium Vital Registration - Deaths 1995 ICD9 as it appears in World Health Organization (WHO). WHO Mortality Database Version November 2015. Geneva, Switzerland: World Health Organization (WHO), 2015.
Belgium Vital Registration - Deaths 1996 ICD9 as it appears in World Health Organization (WHO). WHO Mortality Database Version November 2015. Geneva, Switzerland: World Health Organization (WHO), 2015.
Belgium Vital Registration - Deaths 1997 ICD9 as it appears in World Health Organization (WHO). WHO Mortality Database Version November 2015. Geneva, Switzerland: World Health Organization (WHO), 2015.
Belgium Vital Registration - Deaths 1998 ICD10 as it appears in World Health Organization (WHO). WHO Mortality Database Version November 2015. Geneva, Switzerland: World Health Organization (WHO), 2015.
Belgium Vital Registration - Deaths 1999 ICD10 as it appears in World Health Organization (WHO). WHO Mortality Database Version November 2015. Geneva, Switzerland: World Health Organization (WHO), 2015.
Belgium Vital Registration - Deaths 2004 ICD10 as it appears in World Health Organization (WHO). WHO Mortality Database Version November 2015. Geneva, Switzerland: World Health Organization (WHO), 2015.
Belgium Vital Registration - Deaths 2000 ICD10 as it appears in World Health Organization (WHO). WHO Mortality Database Version November 2015. Geneva, Switzerland: World Health Organization (WHO), 2015.
Belgium Vital Registration - Deaths 2001 ICD10 as it appears in World Health Organization (WHO). WHO Mortality Database Version November 2015. Geneva, Switzerland: World Health Organization (WHO), 2015.
Belgium Vital Registration - Deaths 2002 ICD10 as it appears in World Health Organization (WHO). WHO Mortality Database Version November 2015. Geneva, Switzerland: World Health Organization (WHO), 2015.
Belgium Vital Registration - Deaths 2003 ICD10 as it appears in World Health Organization (WHO). WHO Mortality Database Version November 2015. Geneva, Switzerland: World Health Organization (WHO), 2015.
Belgium Vital Registration - Deaths 2005 ICD10 as it appears in World Health Organization (WHO). WHO Mortality Database Version November 2015. Geneva, Switzerland: World Health Organization (WHO), 2015.
Belgium Vital Registration - Deaths 2006 ICD10 as it appears in World Health Organization (WHO). WHO Mortality Database Version November 2015. Geneva, Switzerland: World Health Organization (WHO), 2015.
Belgium Vital Registration - Deaths 2007 ICD10 as it appears in World Health Organization (WHO). WHO Mortality Database Version November 2015. Geneva, Switzerland: World Health Organization (WHO), 2015.
Belgium Vital Registration - Deaths 2008 ICD10 as it appears in World Health Organization (WHO). WHO Mortality Database Version November 2015. Geneva, Switzerland: World Health Organization (WHO), 2015.
Belgium Vital Registration - Deaths 2009 ICD10 as it appears in World Health Organization (WHO). WHO Mortality Database Version November 2015. Geneva, Switzerland: World Health Organization (WHO), 2015.
Belgium Vital Registration - Deaths 2010 ICD10 as it appears in World Health Organization (WHO). WHO Mortality Database Version November 2015. Geneva, Switzerland: World Health Organization (WHO), 2015.
Belgium Vital Registration - Deaths 2011 ICD10 as it appears in World Health Organization (WHO). WHO Mortality Database Version November 2015. Geneva, Switzerland: World Health Organization (WHO), 2015.
Belgium Vital Registration - Deaths 2012 ICD10 as it appears in World Health Organization (WHO). WHO Mortality Database Version November 2015. Geneva, Switzerland: World Health Organization (WHO), 2015.
Belgium Vital Registration Death Data 2010 as it appears in United Nations Statistics Division (UNSD). United Nations Demographic Yearbook 2012. New York City, United States: United Nations Statistics Division (UNSD), 2013.
Belhassen-Garcia M, Romero-Alegria A, Velasco-Tirado V, Alonso-Sardon M, Lopez-Bernus A, Alvela-Suarez L, del Villar LP, Carpio-Perez A, Galindo-Perez I, Cordero-Sanchez M, Pardo-Lledias J. Study of hydatidosis-attributed mortality in endemic area. PLoS One. 2014; 9(3): e91342.
Belize Central Statistical Office (CSO), Belize Family Life Association, Division of Reproductive Health-Centers for Disease Control and Prevention (CDC), Ministry of Health (Belize). Belize Family Health Survey 1991. Atlanta, United States: Centers for Disease Control and Prevention (CDC).
Belize Central Statistical Office. Belize Family Health Survey 1999. Belmopan, Belize: Belize Central Statistical Office.
Belize Central Statistical Office. Belize Population and Housing Census 2000.
Belize Population and Housing Census 2000 as it appears in United Nations Statistics Division (UNSD). United Nations Demographic Yearbook 2009-2010. New York City, United States: United Nations Statistics Division (UNSD), 2011.
Belize Population and Housing Census 2000 as it appears in World Health Organization (WHO). WHO Household Energy Database. Geneva, Switzerland: World Health Organization (WHO), 2010.
Belize Vital Registration - Deaths 1981 ICD9 as it appears in World Health Organization (WHO). WHO Mortality Database Version November 2015. Geneva, Switzerland: World Health Organization (WHO), 2015.
Belize Vital Registration - Deaths 1964 ICD7 as it appears in World Health Organization (WHO). WHO Mortality Database Version July 2012. Geneva, Switzerland: World Health Organization (WHO), 2012.
Belize Vital Registration - Deaths 1965 ICD7 as it appears in World Health Organization (WHO). WHO Mortality Database Version July 2012. Geneva, Switzerland: World Health Organization (WHO), 2012.

Appendix: Citation List

Citation
Belize Vital Registration - Deaths 1966 ICD7 as it appears in World Health Organization (WHO). WHO Mortality Database Version July 2012. Geneva, Switzerland: World Health Organization (WHO), 2012.
Belize Vital Registration - Deaths 1967 ICD7 as it appears in World Health Organization (WHO). WHO Mortality Database Version July 2012. Geneva, Switzerland: World Health Organization (WHO), 2012.
Belize Vital Registration - Deaths 1968 ICD8 as it appears in World Health Organization (WHO). WHO Mortality Database Version May 2013. Geneva, Switzerland: World Health Organization (WHO), 2013.
Belize Vital Registration - Deaths 1969 ICD8 as it appears in World Health Organization (WHO). WHO Mortality Database Version May 2013. Geneva, Switzerland: World Health Organization (WHO), 2013.
Belize Vital Registration - Deaths 1970 ICD8 as it appears in World Health Organization (WHO). WHO Mortality Database Version May 2013. Geneva, Switzerland: World Health Organization (WHO), 2013.
Belize Vital Registration - Deaths 1971 ICD8 as it appears in World Health Organization (WHO). WHO Mortality Database Version May 2013. Geneva, Switzerland: World Health Organization (WHO), 2013.
Belize Vital Registration - Deaths 1972 ICD8 as it appears in World Health Organization (WHO). WHO Mortality Database Version May 2013. Geneva, Switzerland: World Health Organization (WHO), 2013.
Belize Vital Registration - Deaths 1973 ICD8 as it appears in World Health Organization (WHO). WHO Mortality Database Version May 2013. Geneva, Switzerland: World Health Organization (WHO), 2013.
Belize Vital Registration - Deaths 1974 ICD8 as it appears in World Health Organization (WHO). WHO Mortality Database Version May 2013. Geneva, Switzerland: World Health Organization (WHO), 2013.
Belize Vital Registration - Deaths 1975 ICD8 as it appears in World Health Organization (WHO). WHO Mortality Database Version May 2013. Geneva, Switzerland: World Health Organization (WHO), 2013.
Belize Vital Registration - Deaths 1976 ICD8 as it appears in World Health Organization (WHO). WHO Mortality Database Version May 2013. Geneva, Switzerland: World Health Organization (WHO), 2013.
Belize Vital Registration - Deaths 1977 ICD8 as it appears in World Health Organization (WHO). WHO Mortality Database Version May 2013. Geneva, Switzerland: World Health Organization (WHO), 2013.
Belize Vital Registration - Deaths 1978 ICD8 as it appears in World Health Organization (WHO). WHO Mortality Database Version May 2013. Geneva, Switzerland: World Health Organization (WHO), 2013.
Belize Vital Registration - Deaths 1979 ICD8 as it appears in World Health Organization (WHO). WHO Mortality Database Version May 2013. Geneva, Switzerland: World Health Organization (WHO), 2013.
Belize Vital Registration - Deaths 1980 ICD9 as it appears in World Health Organization (WHO). WHO Mortality Database Version November 2015. Geneva, Switzerland: World Health Organization (WHO), 2015.
Belize Vital Registration - Deaths 1982 ICD9 as it appears in World Health Organization (WHO). WHO Mortality Database Version November 2015. Geneva, Switzerland: World Health Organization (WHO), 2015.
Belize Vital Registration - Deaths 1983 ICD9 as it appears in World Health Organization (WHO). WHO Mortality Database Version November 2015. Geneva, Switzerland: World Health Organization (WHO), 2015.
Belize Vital Registration - Deaths 1984 ICD9 as it appears in World Health Organization (WHO). WHO Mortality Database Version November 2015. Geneva, Switzerland: World Health Organization (WHO), 2015.
Belize Vital Registration - Deaths 1986 ICD9 as it appears in World Health Organization (WHO). WHO Mortality Database Version November 2015. Geneva, Switzerland: World Health Organization (WHO), 2015.
Belize Vital Registration - Deaths 1987 ICD9 as it appears in World Health Organization (WHO). WHO Mortality Database Version February 2014. Geneva, Switzerland: World Health Organization (WHO), 2014.
Belize Vital Registration - Deaths 1989 ICD9 as it appears in World Health Organization (WHO). WHO Mortality Database Version February 2014. Geneva, Switzerland: World Health Organization (WHO), 2014.
Belize Vital Registration - Deaths 1990 ICD9 as it appears in World Health Organization (WHO). WHO Mortality Database Version February 2014. Geneva, Switzerland: World Health Organization (WHO), 2014.
Belize Vital Registration - Deaths 1991 ICD9 as it appears in World Health Organization (WHO). WHO Mortality Database Version February 2014. Geneva, Switzerland: World Health Organization (WHO), 2014.
Belize Vital Registration - Deaths 1993 ICD9 as it appears in World Health Organization (WHO). WHO Mortality Database Version February 2014. Geneva, Switzerland: World Health Organization (WHO), 2014.
Belize Vital Registration - Deaths 1994 ICD9 as it appears in World Health Organization (WHO). WHO Mortality Database Version February 2014. Geneva, Switzerland: World Health Organization (WHO), 2014.
Belize Vital Registration - Deaths 1995 ICD9 as it appears in World Health Organization (WHO). WHO Mortality Database Version February 2014. Geneva, Switzerland: World Health Organization (WHO), 2014.
Belize Vital Registration - Deaths 1996 ICD9 as it appears in World Health Organization (WHO). WHO Mortality Database Version February 2014. Geneva, Switzerland: World Health Organization (WHO), 2014.
Belize Vital Registration - Deaths 1997 ICD10 as it appears in World Health Organization (WHO). WHO Mortality Database Version November 2015. Geneva, Switzerland: World Health Organization (WHO), 2015.
Belize Vital Registration - Deaths 1998 ICD10 as it appears in World Health Organization (WHO). WHO Mortality Database Version November 2015. Geneva, Switzerland: World Health Organization (WHO), 2015.
Belize Vital Registration - Deaths 1999 ICD10 as it appears in World Health Organization (WHO). WHO Mortality Database Version November 2015. Geneva, Switzerland: World Health Organization (WHO), 2015.
Belize Vital Registration - Deaths 2000 ICD10 as it appears in World Health Organization (WHO). WHO Mortality Database Version November 2015. Geneva, Switzerland: World Health Organization (WHO), 2015.
Belize Vital Registration - Deaths 2001 ICD10 as it appears in World Health Organization (WHO). WHO Mortality Database Version November 2015. Geneva, Switzerland: World Health Organization (WHO), 2015.

Appendix: Citation List

Citation
Belize Vital Registration - Deaths 2002 ICD10 as it appears in World Health Organization (WHO). WHO Mortality Database Version November 2015. Geneva, Switzerland: World Health Organization (WHO), 2015.
Belize Vital Registration - Deaths 2003 ICD10 as it appears in World Health Organization (WHO). WHO Mortality Database Version November 2015. Geneva, Switzerland: World Health Organization (WHO), 2015.
Belize Vital Registration - Deaths 2004 ICD10 as it appears in World Health Organization (WHO). WHO Mortality Database Version November 2015. Geneva, Switzerland: World Health Organization (WHO), 2015.
Belize Vital Registration - Deaths 2005 ICD10 as it appears in World Health Organization (WHO). WHO Mortality Database Version November 2015. Geneva, Switzerland: World Health Organization (WHO), 2015.
Belize Vital Registration - Deaths 2006 ICD10 as it appears in World Health Organization (WHO). WHO Mortality Database Version November 2015. Geneva, Switzerland: World Health Organization (WHO), 2015.
Belize Vital Registration - Deaths 2007 ICD10 as it appears in World Health Organization (WHO). WHO Mortality Database Version November 2015. Geneva, Switzerland: World Health Organization (WHO), 2015.
Belize Vital Registration - Deaths 2008 ICD10 as it appears in World Health Organization (WHO). WHO Mortality Database Version November 2015. Geneva, Switzerland: World Health Organization (WHO), 2015.
Belize Vital Registration - Deaths 2009 ICD10 as it appears in World Health Organization (WHO). WHO Mortality Database Version November 2015. Geneva, Switzerland: World Health Organization (WHO), 2015.
Belize Vital Registration - Deaths 2010 ICD10 as it appears in World Health Organization (WHO). WHO Mortality Database Version November 2015. Geneva, Switzerland: World Health Organization (WHO), 2015.
Belize Vital Registration - Deaths 2011 ICD10 as it appears in World Health Organization (WHO). WHO Mortality Database Version November 2015. Geneva, Switzerland: World Health Organization (WHO), 2015.
Belize Vital Registration - Deaths 2012 ICD10 as it appears in World Health Organization (WHO). WHO Mortality Database Version November 2015. Geneva, Switzerland: World Health Organization (WHO), 2015.
Belize Vital Registration - Deaths 2013 ICD10 as it appears in World Health Organization (WHO). WHO Mortality Database Version November 2015. Geneva, Switzerland: World Health Organization (WHO), 2015.
Belize Vital Registration Death Data 1950 as it appears in United Nations Statistics Division (UNSD). United Nations Demographic Yearbook - Historical Supplement 1997. New York City, United States: United Nations Statistics Division (UNSD).
Belize Vital Registration Death Data 1951 as it appears in United Nations Statistics Division (UNSD). United Nations Demographic Yearbook - Historical Supplement 1997. New York City, United States: United Nations Statistics Division (UNSD).
Belize Vital Registration Death Data 1952 as it appears in United Nations Statistics Division (UNSD). United Nations Demographic Yearbook - Historical Supplement 1997. New York City, United States: United Nations Statistics Division (UNSD).
Belize Vital Registration Death Data 1953 as it appears in United Nations Statistics Division (UNSD). United Nations Demographic Yearbook - Historical Supplement 1997. New York City, United States: United Nations Statistics Division (UNSD).
Belize Vital Registration Death Data 1954 as it appears in United Nations Statistics Division (UNSD). United Nations Demographic Yearbook - Historical Supplement 1997. New York City, United States: United Nations Statistics Division (UNSD).
Belize Vital Registration Death Data 1955 as it appears in United Nations Statistics Division (UNSD). United Nations Demographic Yearbook - Historical Supplement 1997. New York City, United States: United Nations Statistics Division (UNSD).
Belize Vital Registration Death Data 1956 as it appears in United Nations Statistics Division (UNSD). United Nations Demographic Yearbook - Historical Supplement 1997. New York City, United States: United Nations Statistics Division (UNSD).
Belize Vital Registration Death Data 1957 as it appears in United Nations Statistics Division (UNSD). United Nations Demographic Yearbook - Historical Supplement 1997. New York City, United States: United Nations Statistics Division (UNSD).
Belize Vital Registration Death Data 1958 as it appears in United Nations Statistics Division (UNSD). United Nations Demographic Yearbook - Historical Supplement 1997. New York City, United States: United Nations Statistics Division (UNSD).
Belize Vital Registration Death Data 1959 as it appears in United Nations Statistics Division (UNSD). United Nations Demographic Yearbook - Historical Supplement 1997. New York City, United States: United Nations Statistics Division (UNSD).
Belize Vital Registration Death Data 1960 as it appears in United Nations Statistics Division (UNSD). United Nations Demographic Yearbook - Historical Supplement 1997. New York City, United States: United Nations Statistics Division (UNSD).
Belize Vital Registration Death Data 1961 as it appears in United Nations Statistics Division (UNSD). United Nations Demographic Yearbook - Historical Supplement 1997. New York City, United States: United Nations Statistics Division (UNSD).
Belize Vital Registration Death Data 1962 as it appears in United Nations Statistics Division (UNSD). United Nations Demographic Yearbook - Historical Supplement 1997. New York City, United States: United Nations Statistics Division (UNSD).
Belize Vital Registration Death Data 1963 as it appears in United Nations Statistics Division (UNSD). United Nations Demographic Yearbook - Historical Supplement 1997. New York City, United States: United Nations Statistics Division (UNSD).
Belize Vital Registration Death Data 1985 as it appears in United Nations Statistics Division (UNSD). United Nations Demographic Yearbook - Historical Supplement 1997. New York City, United States: United Nations Statistics Division (UNSD).
Belize Vital Registration Death Data 1988 as it appears in United Nations Statistics Division (UNSD). United Nations Demographic Yearbook - Historical Supplement 1997. New York City, United States: United Nations Statistics Division (UNSD).
Belize Vital Registration Death Data 1992 as it appears in United Nations Statistics Division (UNSD). United Nations Demographic Yearbook - Historical Supplement 1997. New York City, United States: United Nations Statistics Division (UNSD).
Bell JS, Ouédraogo M, Ganaba R, Sombié I, Byass P, Baggaley RF, Filippi V, Fitzmaurice AE, Graham WJ. The epidemiology of pregnancy outcomes in rural Burkina Faso. Trop Med Int Health. 2008; 13 Suppl 1: 31-43.
Belo AC. Prevalence of hepatitis B virus markers in surgeons in Lagos, Nigeria. East Afr Med J. 2000; 77(5): 283-5.
Beltranena F, Casasola K, Silva JC, Limburg H. Cataract blindness in 4 regions of Guatemala: results of a population-based survey. Ophthalmology. 2007; 114(8): 1558-63.

Appendix: Citation List

Citation
BEMFAM, Division of Reproductive Health-Centers for Disease Control and Prevention (CDC). (1984) Brazil - Amazonas State Family Planning/Maternal and Child Health Survey 1982. Rio de Janeiro, BEMFAM.
Ben Romdhane H, Ben Ali S, Aissi W, Traissac P, Aounallah-Skhiri H, Bougatef S, Maire B, Delpeuch F, Achour N. Prevalence of diabetes in Northern African countries: the case of Tunisia. BMC Public Health. 2014; 86.
Ben Romdhane H, Skhiri H, Bougatef S, Ennigrou S, Gharbi D, Chahed MK, Achour N. Hypertension prevalence, awareness, treatment and control: results from a community based survey. Tunis Med. 2005; 83(Suppl 5): 41-6.
Benallal K, Gassen B, Bouiba L, Depaquit J, Harrat Z. Entomological investigation following the resurgence of human visceral leishmaniasis in southern Algeria. Acta Trop. 2013; 128(3): 518-21.
Bener A, Zirie M, Musallam M, Khader YS, Al-Hamaq AOAA. Prevalence of metabolic syndrome according to Adult Treatment Panel III and International Diabetes Federation criteria: a population-based study. Metab Syndr Relat Disord. 2009; 7(3): 221-9.
Benin - Borgou Epidemiological Investigation Report for the Sub-Prefecture of Tchaourou 1991 as it appears in World Health Organization (WHO). WHO Global Database on Child Growth and Malnutrition - Historical. Geneva, Switzerland: World Health Organization (WHO).
Benin - Epidemiological Investigation Report in the District of Agbangnizoun as it appears in World Health Organization (WHO). WHO Global Database on Child Growth and Malnutrition - Historical. Geneva, Switzerland: World Health Organization (WHO).
Benin - Zou Nutritional Status Survey in the Urban Area of Abomey 1990 as it appears in World Health Organization (WHO). WHO Global Database on Child Growth and Malnutrition - Historical. Geneva, Switzerland: World Health Organization (WHO).
Benin EPI Evaluation of Immunisation Coverage and Social Mobilisation 1991.
Benin External EPI Review 2001.
Benin External Review of the Extended Programme on Immunisation 2008.
Benin General Census of Population and Housing 1992 as it appears in United Nations Statistics Division (UNSD). United Nations Demographic Yearbook. New York City, United States: United Nations Statistics Division (UNSD).
Benin National Immunization Coverage Survey 1995.
Benito A. Epidemiological Bases of Malaria in Equatorial Guinea. Equatorial Guinea: Cooperation Espagnole Guinee Equatoriale, 1992. As it appears in Malaria Atlas Project. Malaria Atlas Project Plasmodium Falciparum Parasite Rate Database. Oxford, United Kingdom: Malaria Atlas Project.
Benner C, Carabin H, Sanchez-Serrano LP, Budke CM, Carmena D. Analysis of the economic impact of cystic echinococcosis in Spain. Bull World Health Organ. 2010; 88(1): 49-57.
Bennis I, De Brouwere V, Ameur B, El Idrissi Laamrani A, Chichaoui S, Hamid S, Boelaert M. Control of cutaneous leishmaniasis caused by Leishmania major in south-eastern Morocco. Trop Med Int Health. 2015; nan.
Berdasco A. Body mass index values in the Cuban adult population. Eur J Clin Nutr. 1994; S155-164.
Bere E, Westersjo JH. Nature trips and traditional methods for food procurement in relation to weight status. Scand J Public Health. 2013; 41(2): 180-4.
Bereczky S, Liljander A, Rooth I, Faraja L, Granath F, Montgomery SM, Färnert A. Multiclonal asymptomatic Plasmodium falciparum infections predict a reduced risk of malaria disease in a Tanzanian population. Microbes Infect. 2007; 9(1): 103-10. As it appears in Malaria Atlas Project. Malaria Atlas Project Plasmodium Falciparum Parasite Rate Database. Oxford, United Kingdom: Malaria Atlas Project.
Berg CJ, Atrash HK, Koonin LM, Tucker M. Pregnancy-related mortality in the United States, 1987-1990. Obstet Gynecol. 1996; 88(2): 161-7.
Berg CJ, Callaghan WM, Syverson C, Henderson Z. Pregnancy-related mortality in the United States, 1998 to 2005. Obstet Gynecol. 2010; 116(6): 1302-9.
Berg CJ, Chang J, Callaghan WM, Whitehead SJ. Pregnancy-related mortality in the United States, 1991-1997. Obstet Gynecol. 2003; 101(2): 289-96.
Berger B, Stenström G, Sundkvist G. Incidence, prevalence, and mortality of diabetes in a large population. A report from the Skaraborg Diabetes Registry. Diabetes Care. 1999; 22(5): 773-8.
Berger J, Manz A. Cancer Of The Stomach And The Colon-Rectum Among Workers In A Coke Gas Plant. Am J Ind Med. 1992; 22(6): 825-34 as it appears in Armstrong B, Hutchinson E, Unwin J, Fletcher T. Lung cancer risk after exposure to polycyclic aromatic hydrocarbons: a review and meta-analysis. Environ. Health Perspect. 2004; 112(9): 970–978.
Bergsjø P, Vangen S, Lie RT, Lyatuu R, Lie-Nielsen E, Oneko O. Recording of maternal deaths in an East African university hospital. Acta Obstet Gynecol Scand. 2010; 89(6): 789-93.
Berhan Y, Waernbaum I, Lind T, Mollsten A, Dahlquist G. Thirty years of prospective nationwide incidence of childhood type 1 diabetes: the accelerating increase by time tends to level off in Sweden. Diabetes. 2011; 60(2): 577-81.
Berhane Y, Hogberg U. Prolonged Labour in Rural Ethiopia: A Community Based Study. Afr J Reprod Health. 1999; 3(2): 33-9.
Berhane Y, Worku A, Bejiga A, Adamu L, Alemayehu W, Bedri A, Haile Z, Ayalew A, Adamu Y, Gebre T, Kebede TD, West E, West S. Prevalence and causes of blindness and Low Vision in Ethiopia. Ethiop J Health Dev. 2008; 21(3): 204-10.
Berhe N, Gundersen SG, Abebe F, Birrie H, Medhin G, Gemetchu T. Praziquantel side effects and efficacy related to Schistosoma mansoni egg loads and morbidity in primary school children in north-east Ethiopia. Acta Trop. 1999; 72(1): 53-63.
Berhe, Gebretsadik, Enqueselassie, Fikre, Hailu, Elena, Mekonnen, Wondale, Teklu, Tsigemariam, Gebretsadik, Ataklti, Berhe, Rezene, Haile, Tweodros, Aseffa, Abraham. Population-based prevalence survey of tuberculosis in the Tigray region of Ethiopia. BMC Infect Dis. 2013; 448.
Bermuda Cancer Registry 1983-1987 - CI5 as it appears in Parkin DM, International Agency for Research on Cancer, International Association of Cancer Registries. Cancer Incidence in Five Continents. Vol. I to VIII. Lyon, France, IARCPress, 2005.
Bermuda Diabetes Association, Bermuda Hospitals Board (BHB), Caribbean Public Health Agency (CARPHA), World Health Organization (WHO). Bermuda STEPS Noncommunicable Disease Risk Factors Survey 2013-2014.
Bermuda Population and Housing Census 1960 as it appears in United Nations Statistics Division (UNSD). United Nations Demographic Yearbook. New York City, United States: United Nations Statistics Division (UNSD).

Appendix: Citation List

Citation
Bermuda Vital Registration - Deaths 1964 ICD7 as it appears in World Health Organization (WHO). WHO Mortality Database Version July 2012. Geneva, Switzerland: World Health Organization (WHO), 2012.
Bermuda Vital Registration - Deaths 1965 ICD7 as it appears in World Health Organization (WHO). WHO Mortality Database Version July 2012. Geneva, Switzerland: World Health Organization (WHO), 2012.
Bermuda Vital Registration - Deaths 1967 ICD7 as it appears in World Health Organization (WHO). WHO Mortality Database Version July 2012. Geneva, Switzerland: World Health Organization (WHO), 2012.
Bermuda Vital Registration - Deaths 1968 ICD8 as it appears in World Health Organization (WHO). WHO Mortality Database Version May 2013. Geneva, Switzerland: World Health Organization (WHO), 2013.
Bermuda Vital Registration - Deaths 1969 ICD8 as it appears in World Health Organization (WHO). WHO Mortality Database Version May 2013. Geneva, Switzerland: World Health Organization (WHO), 2013.
Bermuda Vital Registration - Deaths 1970 ICD8 as it appears in World Health Organization (WHO). WHO Mortality Database Version May 2013. Geneva, Switzerland: World Health Organization (WHO), 2013.
Bermuda Vital Registration - Deaths 1971 ICD8 as it appears in World Health Organization (WHO). WHO Mortality Database Version May 2013. Geneva, Switzerland: World Health Organization (WHO), 2013.
Bermuda Vital Registration - Deaths 1972 ICD8 as it appears in World Health Organization (WHO). WHO Mortality Database Version May 2013. Geneva, Switzerland: World Health Organization (WHO), 2013.
Bermuda Vital Registration - Deaths 1973 ICD8 as it appears in World Health Organization (WHO). WHO Mortality Database Version May 2013. Geneva, Switzerland: World Health Organization (WHO), 2013.
Bermuda Vital Registration - Deaths 1974 ICD8 as it appears in World Health Organization (WHO). WHO Mortality Database Version May 2013. Geneva, Switzerland: World Health Organization (WHO), 2013.
Bermuda Vital Registration - Deaths 1975 ICD8 as it appears in World Health Organization (WHO). WHO Mortality Database Version May 2013. Geneva, Switzerland: World Health Organization (WHO), 2013.
Bermuda Vital Registration - Deaths 1976 ICD8 as it appears in World Health Organization (WHO). WHO Mortality Database Version May 2013. Geneva, Switzerland: World Health Organization (WHO), 2013.
Bermuda Vital Registration - Deaths 1977 ICD8 as it appears in World Health Organization (WHO). WHO Mortality Database Version May 2013. Geneva, Switzerland: World Health Organization (WHO), 2013.
Bermuda Vital Registration - Deaths 1978 ICD8 as it appears in World Health Organization (WHO). WHO Mortality Database Version May 2013. Geneva, Switzerland: World Health Organization (WHO), 2013.
Bermuda Vital Registration - Deaths 1980 ICD9 as it appears in World Health Organization (WHO). WHO Mortality Database Version July 2012. Geneva, Switzerland: World Health Organization (WHO), 2012.
Bermuda Vital Registration - Deaths 1983 ICD9 as it appears in World Health Organization (WHO). WHO Mortality Database Version July 2012. Geneva, Switzerland: World Health Organization (WHO), 2012.
Bermuda Vital Registration - Deaths 1984 ICD9 as it appears in World Health Organization (WHO). WHO Mortality Database Version July 2012. Geneva, Switzerland: World Health Organization (WHO), 2012.
Bermuda Vital Registration - Deaths 1985 ICD9 as it appears in World Health Organization (WHO). WHO Mortality Database Version July 2012. Geneva, Switzerland: World Health Organization (WHO), 2012.
Bermuda Vital Registration - Deaths 1986 ICD9 as it appears in World Health Organization (WHO). WHO Mortality Database Version July 2012. Geneva, Switzerland: World Health Organization (WHO), 2012.
Bermuda Vital Registration - Deaths 1987 ICD9 as it appears in World Health Organization (WHO). WHO Mortality Database Version July 2012. Geneva, Switzerland: World Health Organization (WHO), 2012.
Bermuda Vital Registration - Deaths 1988 ICD9 as it appears in World Health Organization (WHO). WHO Mortality Database Version July 2012. Geneva, Switzerland: World Health Organization (WHO), 2012.
Bermuda Vital Registration - Deaths 1989 ICD9 as it appears in World Health Organization (WHO). WHO Mortality Database Version July 2012. Geneva, Switzerland: World Health Organization (WHO), 2012.
Bermuda Vital Registration - Deaths 1990 ICD9 as it appears in World Health Organization (WHO). WHO Mortality Database Version July 2012. Geneva, Switzerland: World Health Organization (WHO), 2012.
Bermuda Vital Registration - Deaths 1991 ICD9 as it appears in World Health Organization (WHO). WHO Mortality Database Version July 2012. Geneva, Switzerland: World Health Organization (WHO), 2012.
Bermuda Vital Registration - Deaths 1992 ICD9 as it appears in World Health Organization (WHO). WHO Mortality Database Version July 2012. Geneva, Switzerland: World Health Organization (WHO), 2012.
Bermuda Vital Registration - Deaths 1993 ICD9 as it appears in World Health Organization (WHO). WHO Mortality Database Version July 2012. Geneva, Switzerland: World Health Organization (WHO), 2012.
Bermuda Vital Registration - Deaths 1994 ICD9 as it appears in World Health Organization (WHO). WHO Mortality Database Version November 2015. Geneva, Switzerland: World Health Organization (WHO), 2015.
Bermuda Vital Registration - Deaths 1995 ICD9 as it appears in World Health Organization (WHO). WHO Mortality Database Version November 2015. Geneva, Switzerland: World Health Organization (WHO), 2015.
Bermuda Vital Registration - Deaths 1996 ICD10 as it appears in World Health Organization (WHO). WHO Mortality Database Version November 2015. Geneva, Switzerland: World Health Organization (WHO), 2015.
Bermuda Vital Registration - Deaths 1997 ICD10 as it appears in World Health Organization (WHO). WHO Mortality Database Version November 2015. Geneva, Switzerland: World Health Organization (WHO), 2015.
Bermuda Vital Registration - Deaths 1998 ICD10 as it appears in World Health Organization (WHO). WHO Mortality Database Version November 2015. Geneva, Switzerland: World Health Organization (WHO), 2015.
Bermuda Vital Registration - Deaths 1999 ICD10 as it appears in World Health Organization (WHO). WHO Mortality Database Version November 2015. Geneva, Switzerland: World Health Organization (WHO), 2015.

Appendix: Citation List

Citation
Bermuda Vital Registration - Deaths 2000 ICD10 as it appears in World Health Organization (WHO). WHO Mortality Database Version November 2015. Geneva, Switzerland: World Health Organization (WHO), 2015.
Bermuda Vital Registration - Deaths 2001 ICD10 as it appears in World Health Organization (WHO). WHO Mortality Database Version November 2015. Geneva, Switzerland: World Health Organization (WHO), 2015.
Bermuda Vital Registration - Deaths 2002 ICD10 as it appears in World Health Organization (WHO). WHO Mortality Database Version November 2015. Geneva, Switzerland: World Health Organization (WHO), 2015.
Bermuda Vital Registration - Deaths 2003 ICD10 as it appears in World Health Organization (WHO). WHO Mortality Database Version November 2015. Geneva, Switzerland: World Health Organization (WHO), 2015.
Bermuda Vital Registration - Deaths 2004 ICD10 as it appears in World Health Organization (WHO). WHO Mortality Database Version November 2015. Geneva, Switzerland: World Health Organization (WHO), 2015.
Bermuda Vital Registration - Deaths 2005 ICD10 as it appears in World Health Organization (WHO). WHO Mortality Database Version November 2015. Geneva, Switzerland: World Health Organization (WHO), 2015.
Bermuda Vital Registration - Deaths 2006 ICD10 as it appears in World Health Organization (WHO). WHO Mortality Database Version November 2015. Geneva, Switzerland: World Health Organization (WHO), 2015.
Bermuda Vital Registration - Deaths 2007 ICD10 as it appears in World Health Organization (WHO). WHO Mortality Database Version November 2015. Geneva, Switzerland: World Health Organization (WHO), 2015.
Bermuda Vital Registration - Deaths 2008 ICD10 as it appears in World Health Organization (WHO). WHO Mortality Database Version November 2015. Geneva, Switzerland: World Health Organization (WHO), 2015.
Bermuda Vital Registration - Deaths 2009 ICD10 as it appears in World Health Organization (WHO). WHO Mortality Database Version November 2015. Geneva, Switzerland: World Health Organization (WHO), 2015.
Bermuda Vital Registration - Deaths 2010 ICD10 as it appears in World Health Organization (WHO). WHO Mortality Database Version November 2015. Geneva, Switzerland: World Health Organization (WHO), 2015.
Bermuda Vital Registration - Deaths 2011 ICD10 as it appears in World Health Organization (WHO). WHO Mortality Database Version November 2015. Geneva, Switzerland: World Health Organization (WHO), 2015.
Bermuda Vital Registration - Deaths 2012 ICD10 as it appears in World Health Organization (WHO). WHO Mortality Database Version November 2015. Geneva, Switzerland: World Health Organization (WHO), 2015.
Bermuda Vital Registration - Deaths 2013 ICD10 as it appears in World Health Organization (WHO). WHO Mortality Database Version November 2015. Geneva, Switzerland: World Health Organization (WHO), 2015.
Bern C, Joshi AB, Jha SN, Das ML, Hightower A, Thakur GD, Bista MB. Factors associated with visceral leishmaniasis in Nepal: bed-net use is strongly protective. Am J Trop Med Hyg. 2000; 63(3-4): 184-8.
Bernhard P, Makunde RW, Magnussen P, Lemnge MM. Genital manifestations and reproductive health in female residents of a Wuchereria bancrofti-endemic area in Tanzania. Trans R Soc Trop Med Hyg. 2000; 94(4): 409-12. As it appears in London School of Hygiene and Tropical Medicine. Global Atlas of Helminth Infections - Lymphatic Filiariasis.
Berraho M, Nejjari C, Raherison C, El Achhab Y, Tachfouti N, Serhier Z, Dartigues JF, Barberger-Gateau P. Body mass index, disability, and 13-year mortality in older French adults. J Aging Health. 2010; 22(1): 68-83.
Berrang-Ford L, Lundine J, Breau S. Conflict and human African trypanosomiasis. Soc Sci Med. 2011; 72(3): 398-407.
Berry G, de Klerk NH, Reid A, Ambrosini GL, Fritschi L, Olsen NJ, Merler E, Musk AW. Malignant pleural and peritoneal mesotheliomas in former miners and millers of crocidolite at Wittenoom, Western Australia. Occup Environ Med. 2004; 61(4): e14 as it appears in Lenters V, Vermeulen R, Dogger S, Stayner L, Portengen L, Burdorf A, Heederik D. A meta-analysis of asbestos and lung cancer: is better quality exposure assessment associated with steeper slopes of the exposure-response relationships?. Environ Health Perspect. 2011; 119(11): 1547-55.
Berry G, Newhouse ML, Wagner JC. Mortality From All Cancers Of Asbestos Factory Workers In East London 1933-80. Occup Environ Med. 2000; 57(11): 782-5 as it appears in Camargo MC, Stayner LT, Straif K, Reina M, Al-Alem U, Demers PA, Landrigan PJ. Occupational exposure to asbestos and ovarian cancer: a meta-analysis. Environ Health Perspect. 2011; 119(9): 1211-7.
Berry G, Newhouse ML. Mortality Of Workers Manufacturing Friction Materials Using Asbestos. Br J Ind Med. 1983; 40(1): 1-7 as it appears in Lenters V, Vermeulen R, Dogger S, Stayner L, Portengen L, Burdorf A, Heederik D. A meta-analysis of asbestos and lung cancer: is better quality exposure assessment associated with steeper slopes of the exposure-response relationships?. Environ Health Perspect. 2011; 119(11): 1547-55.
Berta Vanrullen I, Volatier J-L, Bertaut A, Dufour A, Dallongeville J. Characteristics of energy intake under-reporting in French adults. Br J Nutr. 2014; 111(7): 1292â€“302.
Bertherat E, Georges-Courbot MC, Nabias R, Georges AJ, Renaut A. Seroprevalence of four sexually transmitted diseases in a semi-urban population of Gabon. Int J STD AIDS. 1998; 9(1): 31-6.
Bertolini DA, Pinho JRR, Saraceni CP, Moreira RC, Granato CFH, Carrilho FJ. Prevalence of serological markers of hepatitis B virus in pregnant women from Paraná State, Brazil. Braz J Med Biol Res. 2006; 39(8): 1083-90.
Bertoni AG, Krop JS, Anderson GF, Brancati FL. Diabetes-related morbidity and mortality in a national sample of U.S. elders. Diabetes Care. 2002; 25(3): 471-5.
Better health data with a portable microcomputer at the periphery: an anthropometric survey in Cape Verde as it appears in World Health Organization (WHO). WHO Global Database on Child Growth and Malnutrition. Geneva, Switzerland: World Health Organization (WHO).
Betts PR, Logatchov M, Volkov I, Murphy H, Dombrowskaya N, Borzikh S, Ivanova I, Twyman S, Vartan J. An assessment of paediatric diabetes care in three centres in Russia and in Southampton, UK. The Paediatric Teams in Moscow, Tula, Tambov, Southampton. Diabet Med. 1999; 16(9): 772-8.
Beuria MK, Bal MS, Mandal NN, Das MK. Age-dependent prevalence of asymptomatic amicrofilaraemic individuals in a Wuchereria bancrofti-endemic region of India. Trans R Soc Trop Med Hyg. 2003; 97(3): 297-8.

Appendix: Citation List

Citation
Beutels M, Van Damme P, Aelvoet W, Desmyter J, Dondeyne F, Goilav C, Mak R, Muylle L, Pierard D, Stroobant A, Van Loock F, Waumans P, Vranckx R. Prevalence of hepatitis A, B and C in the Flemish population. Eur J Epidemiol. 1997; 13(3): 275-80.
Bhagyalaxmi A, Atul T, Shikha J. Prevalence of risk factors of non-communicable diseases in a District of Gujarat, India. J Health Popul Nutr. 2013; 31(1): 78-85.
Bhalla P, Garg S, Kakkar M, Sharma VK. Community-based study of hepatitis B markers in women of reproductive age. Indian J Gastroenterol. 2003; 22(1): 33-4.
Bhandari N, Mazumder S, Taneja S, Sommerfelt H, Strand TA. Effect of implementation of Integrated Management of Neonatal and Childhood Illness (IMNCI) programme on neonatal and infant mortality: cluster randomised controlled trial. BMJ. 2012; 344: e1634.
Bhansali A, Dhandania VK, Deepa M, Anjana RM, Joshi SR, Joshi PP, Madhu SV, Rao PV, Subashini R, Sudha V, Unnikrishnan R, Das AK, Shukla DK, Kaur T, Mohan V, Pradeepa R. Prevalence of and risk factors for hypertension in urban and rural India: the ICMR-INDIAB study. J Hum Hypertens. 2015; 29(3): 204–9.
Bharati DR, Pal R, Kar S, Rekha R, Yamuna TV, Basu M. Prevalence and determinants of diabetes mellitus in Puducherry, South India. J Pharm Bioallied Sci. 2011; 3(4): 513-8.
Bharati DR, Pal R, Rekha R, Yamuna TV. Evaluation of the burden of type 2 diabetes mellitus in population of Puducherry, South India. Diabetes Metab Syndr. 2011; 5(1): 12-6.
Bharati S, Pal M, Bhattacharya BN, Bharati P. Prevalence and causes of chronic energy deficiency and obesity in Indian women. Hum Biol. 2007; 79(4): 395-412.
Bhat J, Rao VG, Gopi PG, Yadav R, Selvakumar N, Tiwari B, Gadge V, Bhondeley MK, Wares F. Prevalence of pulmonary tuberculosis amongst the tribal population of Madhya Pradesh, central India. Int J Epidemiol. 2009; 38(4): 1026-32.
Bhat PN, Navaneetham K, Rajan SI. Maternal mortality in India: estimates from a regression model. Stud Fam Plann. 1995; 26(4): 217-32.
Bhatia JC. Levels and causes of maternal mortality in southern India. Stud Fam Plann. 1993; 24(5): 310-8.
Bhatt S, Weiss DJ, Cameron E, Bisanzio D, Mappin B, Dalrymple U, Battle KE, Moyes CL, Henry A, Eckhoff PA, Wenger EA, Briët O, Penny MA, Smith TA, Bennett A, Yukich J, Eisele TP, Griffin JT, Fergus CA, Lynch M, Lindgren F, Cohen JM, Murray CLJ, Smith DL, Hay SI, Cibulskis RE, Gething PW. The effect of malaria control on Plasmodium falciparum in Africa between 2000 and 2015. Nature. 2015; 526: 207-11.
Bhatta CP, Thapa B, Rana BB. Seroprevalence of hepatitis "B" in Kathmandu Medical College Teaching Hospital (KMCTH). Kathmandu Univ Med J. 2003; 1(2): 113-6.
Bhattacharya P, Chandra P-K, Datta S, Banerjee A, Chakraborty S, Rajendran K, Basu S-K, Bhattacharya S-K, Chakravarty R. Significant increase in HBV, HCV, HIV and syphilis infections among blood donors in West Bengal, Eastern India 2004-2005: exploratory screening reveals high frequency of occult HBV infection. World J Gastroenterol. 2007; 13(27): 3730-3.
Bhattarai A, Ali AS, Kachur SP, Mårtensson A, Abbas AK, Khatib R, Al-Mafazy AW, Ramsan M, Rotllant G, Gerstenmaier JF, Molteni F, Abdulla S, Montgomery SM, Kaneko A, Björkman A. Impact of Artemisinin-Based Combination Therapy and Insecticide-Treated Nets on Malaria Burden in Zanzibar. PLoS Med. 2007; 4(11): e309. As it appears in Malaria Atlas Project. Malaria Atlas Project Plasmodium Falciparum Parasite Rate Database. Oxford, United Kingdom: Malaria Atlas Project.
Bhowmik B, Afsana F, My Diep L, Binte Munir S, Wright E, Mahmood S, Khan AKA, Hussain A. Increasing prevalence of type 2 diabetes in a rural bangladeshi population: a population based study for 10 years. Diabetes Metab. 2013; 37(1): 46-53.
Bhowmik B, Munir SB, Ahmed KR, Siddiquee T, Diep LM, Wright E, Hassan Z, Debnath PR, Mahtab H, Azad Khan AK, Hussain A. Anthropometric indices of obesity and type 2 diabetes in Bangladeshi population: Chandra Rural Diabetes Study (CRDS). Obes Res Clin Pract. 2014; 8(3): e201–98.
Bhumiratana A, Koyadun S, Suvannadabba S, Karnjanopas K, Rojanapremsuk J, Buddhirakkul P, Tantiwattanasup W. Field trial of the ICT filariasis for diagnosis of Wuchereria bancrofti infections in an endemic population of Thailand. Southeast Asian J Trop Med Public Health. 1999; 30(3): 562-8. As it appears in London School of Hygiene and Tropical Medicine. Global Atlas of Helminth Infections - Lymphatic Filiariasis.
Bhumiratana A, Wattanakull B, Koyadun S, Suvannadabba S, Rojanapremsuk J, Tantiwattanasup W. Relationship between male hydrocele and infection prevalences in clustered communities with uncertain transmission of Wuchereria bancrofti on the Thailand-Myanmar border. Southeast Asian J Trop Med Public Health. 2002; 33(1): 7-17. As it appears in London School of Hygiene and Tropical Medicine. Global Atlas of Helminth Infections - Lymphatic Filiariasis.
Bhutan Living Standards Survey 2003 as it appears in World Health Organization (WHO). WHO Household Energy Database. Geneva, Switzerland: World Health Organization (WHO), 2010.
Bhutan Living Standards Survey 2007 as it appears in World Health Organization (WHO). WHO Household Energy Database. Geneva, Switzerland: World Health Organization (WHO), 2010.
Bhutan National Nutrition Survey 1985-1988 as it appears in World Health Organization (WHO). WHO Global Database on Child Growth and Malnutrition. Geneva, Switzerland: World Health Organization (WHO).
Bhutan Population and Housing Census 2005 as it appears in United Nations Statistics Division (UNSD). United Nations Demographic Yearbook 2006. New York City, United States: United Nations Statistics Division (UNSD), 2008.
Bhutta ZA, Memon ZA, Soofi S, Salat MS, Cousens S, Martines J. Implementing community-based perinatal care: results from a pilot study in rural Pakistan. Bull World Health Organ. 2008; 86(6): 452-9.
Biancardi MA, Conca Moreno M, Torres N, Pepe C, Altcheh J, Freilij H. [Seroprevalence of Chagas disease in 17 rural communities of "Monte Impenetrable", Chaco Province]. Medicina (B Aires). 2003; 63(2): 125–129.
Bielefeld University, Federal Ministry of Family Affairs, Senior Citizens, Women and Youth (Germany), Institute for Applied Social Sciences (INFAS). Germany Health, Well-Being and Personal Safety of Women 2003.
Bigoga JD, Manga L, Titanji VP, Coetzee M, Leke RG. Malaria vectors and transmission dynamics in coastal south-western Cameroon. Malar J. 2007; 6(1): 5. As it appears in Malaria Atlas Project. Malaria Atlas Project Plasmodium Falciparum Parasite Rate Database. Oxford, United Kingdom: Malaria Atlas Project.

Appendix: Citation List

Citation
Bill and Melinda Gates Foundation (BMGF), Ghana Health Service, Ghana Statistical Service, Johns Hopkins Bloomberg School of Public Health, Kwame Nkrumah University of Science and Technology (KNUST), University for Development Studies (Ghana). Ghana Performance, Monitoring, and Accountability Survey, Round 2 2014. Baltimore, United States: Johns Hopkins Bloomberg School of Public Health.
Bill and Melinda Gates Foundation (BMGF), Ghana Health Service, Ghana Statistical Service, Johns Hopkins Bloomberg School of Public Health, Kwame Nkrumah University of Science and Technology (KNUST), University for Development Studies (Ghana). Ghana Performance, Monitoring, and Accountability Survey, Round 3 2014. Baltimore, United States: Johns Hopkins Bloomberg School of Public Health.
Bill and Melinda Gates Foundation (BMGF), Ghana Health Service, Ghana Statistical Service, Johns Hopkins Bloomberg School of Public Health, Kwame Nkrumah University of Science and Technology (KNUST), University for Development Studies (Ghana). Ghana Performance, Monitoring, and Accountability Survey, Round 4 2015. Baltimore, United States: Johns Hopkins Bloomberg School of Public Health.
Bill and Melinda Gates Foundation (BMGF), International Center for Reproductive Health (Kenya), Johns Hopkins Bloomberg School of Public Health, Kenya National Bureau of Statistics, Ministry of Health (Kenya), National Council for Population and Development (Kenya). Kenya Performance, Monitoring, and Accountability Survey, Round 2 2014. Baltimore, United States: Johns Hopkins Bloomberg School of Public Health.
Bill and Melinda Gates Foundation (BMGF), International Center for Reproductive Health (Kenya), Johns Hopkins Bloomberg School of Public Health, Kenya National Bureau of Statistics, Ministry of Health (Kenya), National Council for Population and Development (Kenya). Kenya Performance, Monitoring, and Accountability Survey, Round 3 2015. Baltimore, United States: Johns Hopkins Bloomberg School of Public Health.
Bill and Melinda Gates Foundation (BMGF), Johns Hopkins Bloomberg School of Public Health, Makerere University School of Public Health (MUSPH), Uganda Bureau of Statistics. Uganda Performance, Monitoring, and Accountability Survey, Round 2 2015. Baltimore, United States: Johns Hopkins Bloomberg School of Public Health.
Bill and Melinda Gates Foundation (BMGF), Johns Hopkins Bloomberg School of Public Health, Makerere University School of Public Health (MUSPH), Uganda Bureau of Statistics. Uganda Performance, Monitoring, and Accountability Survey, Round 3 2015. Baltimore, United States: Johns Hopkins Bloomberg School of Public Health.
Bill and Melinda Gates Foundation (BMGF), Johns Hopkins Bloomberg School of Public Health, National Institute of Statistics (Niger). Niger - Niamey Performance, Monitoring, and Accountability Survey, Round 1 2015. Baltimore, United States: Johns Hopkins Bloomberg School of Public Health.
Bill and Melinda Gates Foundation (BMGF), Johns Hopkins Bloomberg School of Public Health, Uganda Bureau of Statistics. Uganda Performance, Monitoring, and Accountability Survey, Round 1 2014. Baltimore, United States: Johns Hopkins Bloomberg School of Public Health.
Bin Ghouth AS. Malaria in Hajjar Valley: School Malariometry, March 2001. Baghdad, Iraq: Arab Board for Medical Specializations in Community Medicine, 2002. As it appears in Malaria Atlas Project. Malaria Atlas Project Plasmodium Falciparum Parasite Rate Database. Oxford, United Kingdom: Malaria Atlas Project.
Binh TQ, Phuong PT, Nhung BT, Tung DD. Metabolic syndrome among a middle-aged population in the Red River Delta region of Vietnam. BMC Endocr Disord. 2014; 14: 77.
Binka FN, Kubaje A, Adjuik M, Williams LA, Lengeler C, Maude GH, Armah GE, Kajihara B, Adiamah JH, Smith PG. Impact of permethrin impregnated bednets on child mortality in Kassena-Nankana district, Ghana: a randomized controlled trial. Trop Med Int Health. 1996; 1(2): 147-54.
Biran A, Schmidt WP, Wright R, Jones T, Seshadri M, Isaac P, Nathan NA, Hall P, McKenna J, Granger S, Bidinger P, Curtis V. The effect of a soap promotion and hygiene education campaign on handwashing behaviour in rural India: a cluster randomised trial. Trop Med Int Health. 2009; 14(10): 1303-14.
Biran A, Schmidt WP, Zeleke L, Emukule H, Khay H, Parker J, Peprah D. Hygiene and sanitation practices amongst residents of three long-term refugee camps in Thailand, Ethiopia and Kenya. Trop Med Int Health. 2012; 17(9): 113-41.
Biran A, Tabyshalieva A, Salmorbekova Z. Formative research for hygiene promotion in Kyrgyzstan. Health Policy Plan. 2005; 20(4): 213-21.
Births and Deaths Registry (Ghana). Ghana - Accra Births and Deaths Registry - Deaths 2000-2007.
Biswas S, Seth RK, Tyagi PK, Sharma SK, Dash AP. Naturally Acquired Immunity and Reduced Susceptibility to falciparum Malaria in Two Subpopulations of Endemic Eastern India. Scand J Immunol. 2008; 67(2): 177-84. As it appears in Malaria Atlas Project. Malaria Atlas Project Plasmodium Falciparum Parasite Rate Database. Oxford, United Kingdom: Malaria Atlas Project.
Bjerregaard-Andersen M, Hansen L, da Silva LI, Joaquím LC, Hennild DE, Christiansen L, Aaby P, Benn CS, Christensen K, Sodemann M, Jensen DM, Beck-Nielsen H. Risk of metabolic syndrome and diabetes among young twins and singletons in Guinea-bissau. Diabetes Care. 2013; 36(11): 3549-56.
Björkman A, Willcox M, Marbiah N, Payne D. Susceptibility of Plasmodium falciparum to different doses of quinine in vivo and to quinine and quinidine in vitro in relation to chloroquine in Liberia. Bull World Health Organ. 1991; 69(4): 459-65. As it appears in Malaria Atlas Project. Malaria Atlas Project Plasmodium Falciparum Parasite Rate Database. Oxford, United Kingdom: Malaria Atlas Project.
Blampain-Azzibrouck G, Lekoulou F, Snounou G, Ravollet JC, Ntoumi F. Short communication: Plasmodium falciparum and P. malariae infections in isolates from sickle cell gene carriers living in a hyperendemic area of Gabon. Trop Med Int Health. 1999; 4(12): 872-4. As it appears in Malaria Atlas Project. Malaria Atlas Project Plasmodium Falciparum Parasite Rate Database. Oxford, United Kingdom: Malaria Atlas Project.
Blencowe H, Cousens S, Jassir FB, Say L, Chou D, Mathers C, Hogan D, Shiekh S, Qureshi ZU, You D, Lawn JE; Lancet Stillbirth Epidemiology Investigator Group. National, regional, and worldwide estimates of stillbirth rates in 2015, with trends from 2000: a systematic analysis. Lancet Glob Health. 2016; 4(2): e98-108.

Appendix: Citation List

Citation

Blocher J, Schmutzhard E, Wilkins PP, Gupton PN, Schaffert M, Auer H, Gotwald T, Matuja W, Winkler AS. A cross-sectional study of people with epilepsy and neurocysticercosis in Tanzania: clinical characteristics and diagnostic approaches. PLoS Negl Trop Dis. 2011; 5(6): e1185.

Bloemen LJ, Youk A, Bradley TD, Bodner KM, Marsh G. Lymphohaematopoietic cancer risk among chemical workers exposed to benzene. Occup Environ Med. 2004; 61(3): 270-4 as it appears in Khalade A, Jaakkola MS, Pukkala E, Jaakkola JJK. Exposure to benzene at work and the risk of leukemia: a systematic review and meta-analysis. Environ Health 2010; 9: 31.

Bloland PB, Boriga DA, Ruebush TK, McCormick JB, Roberts JM, Oloo AJ, Hawley W, Lal A, Nahlen B, Campbell CC. Longitudinal cohort study of the epidemiology of malaria infections in an area of intense malaria transmission II. Descriptive epidemiology of malaria infection and disease among children. Am J Trop Med Hyg. 1999; 60(4): 641-8. As it appears in Malaria Atlas Project. Malaria Atlas Project Plasmodium Falciparum Parasite Rate Database. Oxford, United Kingdom: Malaria Atlas Project.

Bloomberg Initiative to Reduce Tobacco Use, Bloomberg Philanthropies, CDC Foundation, Centers for Disease Control and Prevention (CDC), Global Tobacco Surveillance System, Johns Hopkins Bloomberg School of Public Health, Kiev International Institute of Sociology, Ministry of Health (Ukraine), National University of Kyiv-Mohyla Academy, Research Triangle Institute, Inc. (RTI). Ukraine Global Adult Tobacco Survey 2009-2010. Atlanta, United States: Centers for Disease Control and Prevention (CDC).

Bloomberg Initiative to Reduce Tobacco Use, CDC Foundation, Centers for Disease Control and Prevention (CDC), Central Statistical Office (Poland), Johns Hopkins Bloomberg School of Public Health, Medical University of Warsaw, Ministry of Health (Poland), Pentor Research International, WHO Regional Office for Europe (EURO-WHO). Poland Global Adult Tobacco Survey 2009-2010. Warsaw, Poland: Ministry of Health (Poland).

Bloomberg Philanthropies, CDC Foundation, Centers for Disease Control and Prevention (CDC), General Statistics Office (Viet Nam), Hanoi Medical University, Ministry of Health (Viet Nam), World Health Organization (WHO). Vietnam Global Adult Tobacco Survey 2010.

Bo S, Durazzo M, Guidi S, Carello M, Sacerdote C, Silli B, Rosato R, Cassader M, Gentile L, Pagano G. Dietary magnesium and fiber intakes and inflammatory and metabolic indicators in middle-aged subjects from a population-based cohort. Am J Clin Nutr. 2006; 84(5): 1062-9.

Bobak M, McKee M, Rose R, Marmot M. Alcohol consumption in a national sample of the Russian population. Addiction. 1999; 94(6): 857â€“66.

Bockarie MJ, Tavul L, Kastens W, Michael E, Kazura JW. Impact of untreated bednets on prevalence of Wuchereria bancrofti transmitted by Anopheles farauti in Papua New Guinea. Med Vet Entomol. 2002; 16(1): 116-9.

Bocquier A, Cortaredona S, Nauleau S, Jardin M, Verger P. Prevalence of treated diabetes: Geographical variations at the small-area level and their association with area-level characteristics. A multilevel analysis in Southeastern France. Diabetes Metab. 2011; 37(1): 39-46.

Bødker B, Hvidman L, Weber T, Møller M, Aarre A, Nielsen KM, Sørensen JL. Maternal deaths in Denmark 2002-2006. Acta Obstet Gynecol Scand. 2009; 88(5): 556-62.

Bødker R, Kisinza W, Malima R, Msangeni H, Lindsay S. Resurgence of Malaria in the Usambara Mountains, Tanzania, An Epidemic of Drug-Resistant Parasites. Glob Change Hum Health. 2000; 1(2): 134-53. As it appears in Malaria Atlas Project. Malaria Atlas Project Plasmodium Falciparum Parasite Rate Database. Oxford, United Kingdom: Malaria Atlas Project.

Bødker R. Variation in Malaria Risk in the Usambara Mountains, Tanzania [dissertation]. Copenhagen, Denmark: University of Copenhagen, 2000. As it appears in Malaria Atlas Project. Malaria Atlas Project Plasmodium Falciparum Parasite Rate Database. Oxford, United Kingdom: Malaria Atlas Project.

Boffetta P, Agudo A, Ahrens W, Benhamou E, Benhamou S, Darby SC, Ferro G, Fortes C, Gonzalez CA, Jöckel KH, Krauss M, Kreienbrock L, Kreuzer M, Mendes A, Merletti F, Nyberg F, Pershagen G, Pohlabeln H, Riboli E, Schmid G, Simonato L, Trédaniel J, Whitley E, Wichmann HE, Winck C, Zambon P, Saracci R. Multicenter case-control study of exposure to environmental tobacco smoke and lung cancer in Europe. J Natl Cancer Inst. 1998; 90(19): 1440-50 as it appears in Stayner L, Bena J, Sasco AJ, Smith R, Steenland K, Kreuzer M, et al. Lung cancer risk and workplace exposure to environmental tobacco smoke. Am J Public Health 2007; 97(3): 545–551.

Boffetta P, Ahrens W, Nyberg F, Mukeria A, Brüske-Hohlfeld I, Fortes C, Constantinescu V, Simonato L, Batura-Gabryel H, Lea S, Gaborieau VR, Benhamou S. Exposure To Environmental Tobacco Smoke And Risk Of Adenocarcinoma Of The Lung. Int J Cancer. 1999; 83(5): 635-9 as it appears in Stayner L, Bena J, Sasco AJ, Smith R, Steenland K, Kreuzer M, et al. Lung cancer risk and workplace exposure to environmental tobacco smoke. Am J Public Health 2007; 97(3): 545–551.

Bogaerts J, Ahmed J, Akhter N, Begum N, Rahman M, Nahar S, Van Ranst M, Verhaegen J. Sexually transmitted infections among married women in Dhaka, Bangladesh: unexpected high prevalence of herpes simplex type 2 infection. Sex Transm Infect. 2001; 77(2): 114-9.

Boisier P, Rabarijaona L, Piollet M, Roux JF, Zeller HG. Hepatitis B virus infection in general population in Madagascar: evidence for different epidemiological patterns in urban and in rural areas. Epidemiol Infect. 1996; 117(1): 133-7.

Boivin MJ, Giordani B, Ndanga K, Maky MM, Manzeki KM, Ngunu N, Muamba K. Effects of treatment for intestinal parasites and malaria on the cognitive abilities of schoolchildren in Zaire, Africa. Health Psychol. 1993; 12(3): 220-6. As it appears in Malaria Atlas Project. Malaria Atlas Project Plasmodium Falciparum Parasite Rate Database. Oxford, United Kingdom: Malaria Atlas Project.

Bolivia Household Survey 2003-2004 as it appears in World Health Organization (WHO). WHO Household Energy Database. Geneva, Switzerland: World Health Organization (WHO), 2010.

Bolivia Household Survey 2007 as it appears in World Health Organization (WHO). WHO Household Energy Database. Geneva, Switzerland: World Health Organization (WHO), 2010.

Bolivia Multiple Indicator Cluster Survey 1996 as it appears in World Health Organization (WHO). WHO Global Database on Child Growth and Malnutrition - Historical. Geneva, Switzerland: World Health Organization (WHO).

Bolivia National Employment Survey 1997 as it appears in World Health Organization (WHO). WHO Household Energy Database. Geneva, Switzerland: World Health Organization (WHO), 2010.

Bolivia National Immunization Coverage Survey 1987.

Bolivia National Immunization Coverage Survey 1990.

Bolivia National Nutrition Survey 1981 as it appears in World Health Organization (WHO). WHO Global Database on Child Growth and Malnutrition. Geneva, Switzerland: World Health Organization (WHO).

Appendix: Citation List

Citation
Bolivia National Survey of Population and Housing 1988 as it appears in United Nations Statistics Division (UNSD). United Nations Demographic Yearbook Special Census Topic, 2000 Round. New York City, United States: United Nations Statistics Division (UNSD).
Bolivia Survey of Coverage by MR (15-39 Years) and MMR (Children 12-23 Months) 2006.
Bolivia Vital Registration - Deaths 2000 ICD10 as it appears in World Health Organization (WHO). WHO Mortality Database Version November 2015. Geneva, Switzerland: World Health Organization (WHO), 2015.
Bolivia Vital Registration - Deaths 2001 ICD10 as it appears in World Health Organization (WHO). WHO Mortality Database Version November 2015. Geneva, Switzerland: World Health Organization (WHO), 2015.
Bolivia Vital Registration - Deaths 2002 ICD10 as it appears in World Health Organization (WHO). WHO Mortality Database Version November 2015. Geneva, Switzerland: World Health Organization (WHO), 2015.
Bolivia Vital Registration - Deaths 2003 ICD10 as it appears in World Health Organization (WHO). WHO Mortality Database Version November 2015. Geneva, Switzerland: World Health Organization (WHO), 2015.
Bolivia Vital Registration Death Data 1951 as it appears in United Nations Statistics Division (UNSD). United Nations Demographic Yearbook - Historical Supplement 1997. New York City, United States: United Nations Statistics Division (UNSD).
Bolivia Vital Registration Death Data 1952 as it appears in United Nations Statistics Division (UNSD). United Nations Demographic Yearbook - Historical Supplement 1997. New York City, United States: United Nations Statistics Division (UNSD).
Bolivia Vital Registration Death Data 1953 as it appears in United Nations Statistics Division (UNSD). United Nations Demographic Yearbook - Historical Supplement 1997. New York City, United States: United Nations Statistics Division (UNSD).
Bolivia Vital Registration Death Data 1954 as it appears in United Nations Statistics Division (UNSD). United Nations Demographic Yearbook - Historical Supplement 1997. New York City, United States: United Nations Statistics Division (UNSD).
Bolivia Vital Registration Death Data 1955 as it appears in United Nations Statistics Division (UNSD). United Nations Demographic Yearbook - Historical Supplement 1997. New York City, United States: United Nations Statistics Division (UNSD).
Bolivia Vital Registration Death Data 1956 as it appears in United Nations Statistics Division (UNSD). United Nations Demographic Yearbook - Historical Supplement 1997. New York City, United States: United Nations Statistics Division (UNSD).
Bolivia Vital Registration Death Data 1957 as it appears in United Nations Statistics Division (UNSD). United Nations Demographic Yearbook - Historical Supplement 1997. New York City, United States: United Nations Statistics Division (UNSD).
Bolivia Vital Registration Death Data 1958 as it appears in United Nations Statistics Division (UNSD). United Nations Demographic Yearbook - Historical Supplement 1997. New York City, United States: United Nations Statistics Division (UNSD).
Bolivia Vital Registration Death Data 1965 as it appears in United Nations Statistics Division (UNSD). United Nations Demographic Yearbook - Historical Supplement 1997. New York City, United States: United Nations Statistics Division (UNSD).
Bolivia Vital Registration Death Data 1966 as it appears in United Nations Statistics Division (UNSD). United Nations Demographic Yearbook - Historical Supplement 1997. New York City, United States: United Nations Statistics Division (UNSD).
Bolivia Vital Registration Death Data 1976 as it appears in United Nations Statistics Division (UNSD). United Nations Demographic Yearbook - Historical Supplement 1997. New York City, United States: United Nations Statistics Division (UNSD).
Bolivia Vital Registration Death Data 1977 as it appears in United Nations Statistics Division (UNSD). United Nations Demographic Yearbook - Historical Supplement 1997. New York City, United States: United Nations Statistics Division (UNSD).
Bombelli M, Facchetti R, Fodri D, Brambilla G, Sega R, Grassi G, Mancia G. Impact of body mass index and waist circumference on the cardiovascular risk and all-cause death in a general population: data from the PAMELA study. Nutr Metab Cardiovasc Dis. 2013; 23(7): 650–6.
Bonaldi C, Vernay M, Roudier C, Salanave B, Oleko A, Malon A, Castetbon K, Fagot-Campagna A. A first national prevalence estimate of diagnosed and undiagnosed diabetes in France in 18- to 74-year-old individuals: the French Nutrition and Health Survey 2006/2007. Diabet Med. 2011; 28(5): 583–9.
Bonfim C, Lessa F, Oliveira C o, Evangelista MJ, do Espírito Santo M, Meireles E, Pereira JC, Medeiros Z. [The occurrence and distribution of lymphatic filariasis in Greater Metropolitan Recife: the case of an endemic area in Jaboatão dos Guararapes, Pernambuco, Brazil]. Cad Saude Publica. 2003; 19(5): 1497-505. As it appears in London School of Hygiene and Tropical Medicine. Global Atlas of Helminth Infections - Lymphatic Filiariasis.
Bongiorno MR, Pistone G, Aricò G. Hepatitis B and hepatitis C virus infections in dermatological patients in west Sicily: a seroepidemiological study. J Eur Acad Dermatol Venereol. 2002; 16(1): 43-6.
Bonizzoni M, Afrane Y, Baliraine FN, Amenya DA, Githeko AK, Yan G. Genetic structure of Plasmodium falciparum populations between lowland and highland sites and antimalarial drug resistance in Western Kenya. Infect Genet Evol. 2009; 9(5): 806-12. As it appears in Malaria Atlas Project. Malaria Atlas Project Plasmodium Falciparum Parasite Rate Database. Oxford, United Kingdom: Malaria Atlas Project.
Bonora E, Kiechl S, Willeit J, Oberhollenzer F, Egger G, Meigs JB, Bonadonna RC, Muggeo M, Bruneck study. Population-based incidence rates and risk factors for type 2 diabetes in white individuals: the Bruneck study. Diabetes. 2004; 53(7): 1782-9.
Bonura F, Sorgi M, Perna AM, Puccio G, Tramuto F, Cajozzo C, Romano N, Vitale F. Pregnant women as a sentinel population to target and implement hepatitis B virus (HBV) vaccine coverage: a three-year survey in Palermo, Sicily. Vaccine. 2005; 23(25): 3243-6.
Borchgrevink CP, Cha J, Kim S. Hand washing practices in a college town environment. J Environ Health. 2013; 75(9): 18-24.
Borges-Pereira J, Sarquis O, Zauza PL, Britto C, Lima MM. [Epidemiology of Chagas disease in four rural localities in Jaguaruana, State of Ceará: seroprevalence of infection, parasitemia and clinical characteristics]. Rev Soc Bras Med Trop. 2008; 41(4): 345–51.
Borissova AM, Shinkov A, Kovatcheva R, Vlahov J, Dakovska L, Todorov T. Changes in the prevalence of diabetes mellitus in bulgaria (2006-2012). Clin Med Insights Endocrinol Diabetes. 2015; 8: 41-5.
Boronat M, Saavedra P, Perez-Martin N, Lopez-Madrazo MJ, Rodriguez-Perez C, Novoa FJ. High levels of lipoprotein(a) are associated with a lower prevalence of diabetes with advancing age: results of a cross-sectional epidemiological survey in Gran Canaria, Spain. Cardiovasc Diabetol. 2012; 11: 81.
Boronat M, Varillas VF, Saavedra P, Su rez V, Bosch E, Carrillo A, N¢voa FJ. Diabetes mellitus and impaired glucose regulation in the Canary Islands (Spain): prevalence and associated factors in the adult population of Telde, Gran Canaria. Diabet Med. 2006; 23(2): 148-55.

Appendix: Citation List

Citation
Borssén B, Bergenheim T, Lithner F. The epidemiology of foot lesions in diabetic patients aged 15-50 years. Diabet Med. 1990; 7(5): 438-44.
Bosman A, Modiano D, Voglino M, Pizzi L, Bartoloni P, Kandia Diallo I, De Giorgi F. Malaria transmission in a central area of Futa Djalon (Guinea): results of a parasitological survey during the 1989 rainy season. Parassitologia. 1992; 34(1-3): 135-42. As it appears in Malaria Atlas Project. Malaria Atlas Project Plasmodium Falciparum Parasite Rate Database. Oxford, United Kingdom: Malaria Atlas Project.
Bosnia and Herzegovina Vital Registration - Deaths 1991 ICD9 as it appears in World Health Organization (WHO). WHO Mortality Database Version February 2014. Geneva, Switzerland: World Health Organization (WHO), 2014.
Bosnia and Herzegovina Vital Registration - Deaths 2011 ICD10 as it appears in World Health Organization (WHO). WHO Mortality Database Version November 2015. Geneva, Switzerland: World Health Organization (WHO), 2015.
Bosnia and Herzegovina Vital Registration Death Data 1998 as it appears in United Nations Statistics Division (UNSD). United Nations Demographic Yearbook 2003. New York City, United States: United Nations Statistics Division (UNSD), 2006.
Bosnia and Herzegovina Vital Registration Death Data 2003 as it appears in United Nations Statistics Division (UNSD). United Nations Demographic Yearbook 2003. New York City, United States: United Nations Statistics Division (UNSD), 2006.
Bosnia and Herzegovina Vital Registration Death Data 2004 as it appears in United Nations Statistics Division (UNSD). United Nations Demographic Yearbook 2004. New York City, United States: United Nations Statistics Division (UNSD), 2007.
Bosnia and Herzegovina Vital Registration Death Data 2006 as it appears in United Nations Statistics Division (UNSD). United Nations Demographic Yearbook 2006. New York City, United States: United Nations Statistics Division (UNSD), 2008.
Bosnia and Herzegovina Vital Registration Death Data 2007 as it appears in United Nations Statistics Division (UNSD). United Nations Demographic Yearbook 2007. New York City, United States: United Nations Statistics Division (UNSD), 2009.
Bosnia and Herzegovina Vital Registration Death Data 2009 as it appears in United Nations Statistics Division (UNSD). United Nations Demographic Yearbook 2009-2010. New York City, United States: United Nations Statistics Division (UNSD), 2011.
Bosnia and Herzegovina Vital Statistics - Deaths 2000 as it appears in TransMonEE 2013 Database, UNICEF Regional Office for CEE/CIS.
Bosnia and Herzegovina Vital Statistics - Deaths 2001 as it appears in TransMonEE 2013 Database, UNICEF Regional Office for CEE/CIS.
Bosnia and Herzegovina Vital Statistics - Deaths 2002 as it appears in TransMonEE 2013 Database, UNICEF Regional Office for CEE/CIS.
Bosompem KM, Bentum IA, Otchere J, Anyan WK, Brown CA, Osada Y, Takeo S, Kojima S, Ohta N. Infant schistosomiasis in Ghana: a survey in an irrigation community. Trop Med Int Health. 2004; 9(8): 917-22.
Boström S, Giusti P, Arama C, Persson J-O, Dara V, Traore B, Dolo A, Doumbo O, Troye-Blomberg M. Changes in the levels of cytokines, chemokines and malaria-specific antibodies in response to Plasmodium falciparum infection in children living in sympatry in Mali. Malar J. 2012; 11(1): 109. As it appears in Malaria Atlas Project. Malaria Atlas Project Plasmodium Falciparum Parasite Rate Database. Oxford, United Kingdom: Malaria Atlas Project.
Botswana Demographic Survey 2006 as it appears in World Health Organization (WHO). WHO Household Energy Database. Geneva, Switzerland: World Health Organization (WHO), 2010.
Botswana EPI Evaluation 1987.
Botswana Family Health Survey 1996 as it appears in World Health Organization (WHO). WHO Global Database on Child Growth and Malnutrition. Geneva, Switzerland: World Health Organization (WHO).
Botswana Multiple Indicator Cluster Survey 2000 as it appears in World Health Organization (WHO). WHO Global Database on Child Growth and Malnutrition. Geneva, Switzerland: World Health Organization (WHO).
Botswana Population and Housing Census 1991 as it appears in United Nations Statistics Division (UNSD). United Nations Demographic Yearbook Special Census Topic, 2000 Round. New York City, United States: United Nations Statistics Division (UNSD).
Botswana Population and Housing Census 1991 as it appears in World Health Organization (WHO). WHO Household Energy Database. Geneva, Switzerland: World Health Organization (WHO), 2010.
Botswana Population and Housing Census 2001 as it appears in World Health Organization (WHO). WHO Household Energy Database. Geneva, Switzerland: World Health Organization (WHO), 2010.
Boudin C, Olivier M, Molez J, Chiron J, Ambroise-Thomas P. High human malarial infectivity to laboratory-bred Anopheles gambiae in a village in Burkina Faso. Am J Trop Med Hyg. 1993; 48(5): 700-6. As it appears in Malaria Atlas Project. Malaria Atlas Project Plasmodium Falciparum Parasite Rate Database. Oxford, United Kingdom: Malaria Atlas Project.
Boudin C, Robert V, Carnevale P, Ambroise-Thomas P. Epidemiology of Plasmodium falciparum in a rice field and a savanna area in Burkina Faso. Comparative study on the acquired immunoprotection in native populations. Acta Trop. 1992; 51(2): 103-11. As it appears in Malaria Atlas Project. Malaria Atlas Project Plasmodium Falciparum Parasite Rate Database. Oxford, United Kingdom: Malaria Atlas Project.
Boudin C, Robert V, Verhave JP, Carnevale P, Ambroise-Thomas P. Plasmodium falciparum and P. malariae epidemiology in a West African village. Bull World Health Organ. 1991; 69(2): 199-205. As it appears in Malaria Atlas Project. Malaria Atlas Project Plasmodium Falciparum Parasite Rate Database. Oxford, United Kingdom: Malaria Atlas Project.
Bouguerra R, Alberti H, Salem LB, Rayana CB, Atti JE, Gaigi S, Slama CB, Zouari B, Alberti K. The global diabetes pandemic: the Tunisian experience. Eur J Clin Nutr. 2007; 61(2): 160-5.
Bouma M, Rowland M. Failure of passive zooprophylaxis: cattle ownership in Pakistan is associated with a higher prevalence of malaria. Trans R Soc Trop Med Hyg. 1995; 89(4): 351-3. As it appears in Malaria Atlas Project. Malaria Atlas Project Plasmodium Falciparum Parasite Rate Database. Oxford, United Kingdom: Malaria Atlas Project.
Bouma MJ, Parvez SD, Nesbit R, Winkler AM. Malaria control using permethrin applied to tents of nomadic Afghan refugees in northern Pakistan. Bull World Health Organ. 1996; 74(4): 413-21. As it appears in Malaria Atlas Project. Malaria Atlas Project Plasmodium Falciparum Parasite Rate Database. Oxford, United Kingdom: Malaria Atlas Project.
Bourdel-Marchasson I, Dubroca B, Manciet G, Decamps A, Emeriau JP, Dartigues JF. Prevalence of diabetes and effect on quality of life in older French living in the community: the PAQUID Epidemiological Survey. J Am Geriatr Soc. 1997; 45(3): 295-301.
Bourdel-Marchasson I, Helmer C, Barberger-Gateau P, Peuchant E, Février B, Ritchie K, Dartigues JF. Characteristics of undiagnosed diabetes in community-dwelling French elderly: the 3C study. Diabetes Res Clin Pract. 2007; 76(2): 257-64.

Appendix: Citation List

Citation
Boureima SH. Sondage Paludométrique dans les différentes zones épidémiologiques du Niger en période de saison des pluies [Malarial survey in different epidemiological zones in Niger during the rainy season]. Malar Infect Dis Afr. 1995; 2: 12-5. As it appears in Malaria Atlas Project. Malaria Atlas Project Plasmodium Falciparum Parasite Rate Database. Oxford, United Kingdom: Malaria Atlas Project.
Bouvier P, Rougemont A, Breslow N, Doumbo O, Delley V, Dicko A, Diakite M, Mauris A, Robert CF. Seasonality and Malaria in a West African Village: Does High Parasite Density Predict Fever Incidence? Am J Epidemiol. 1997; 145(9): 850-7. As it appears in Malaria Atlas Project. Malaria Atlas Project Plasmodium Falciparum Parasite Rate Database. Oxford, United Kingdom: Malaria Atlas Project.
Bouvier-Colle MH, Ouedraogo C, Dumont A, Vangeenderhuysen C, Salanave B, Decam C. Maternal mortality in West Africa. Rates, causes and substandard care from a prospective survey. Acta Obstet Gynecol Scand. 2001; 80(2): 113-9.
Bouvier-Colle M-H, Saucedo M, Deneux-Tharaux C, CNEMM. [The confidential enquiries into maternal deaths, 1996-2006 in France: what consequences for the obstetrical care?]. J Gynecol Obstet Biol Reprod (Paris). 2011; 40(2): 87-102.
Bouvier-Colle MH, Varnoux N, Costes P, Hatton F. Reasons for the underreporting of maternal mortality in France, as indicated by a survey of all deaths among women of childbearing age. Int J Epidemiol. 1991; 20(3): 717-21.
Bovet P, Chiolero A, Shamlaye C, Paccaud F. Prevalence of overweight in the Seychelles: 15 year trends and association with socio-economic status. Obes Rev. 2008; 9(6): 511-7.
Bovet P. Distribution of blood pressure, body mass index and smoking habits in the urban population of Dar es Salaam, Tanzania, and associations with socioeconomic status. Int J Epidemiol. 2002; 31(1): 240-7.
Bowman NM, Kawai V, Levy MZ, Cornejo del Carpio JG, Cabrera L, Delgado F, Malaga F, Cordova Benzaquen E, Pinedo VV, Steurer F, Seitz AE, Gilman RH, Bern C. Chagas disease transmission in periurban communities of Arequipa, Peru. Clin Infect Dis. 2008; 46(12): 1822-8.
Bowry TR, Pade J, Omari M, Chemtai A. A pilot study of hepatitis B virus seroepidemiology suggests widespread immunosuppression in the nomadic inhabitants of Turkana District of Kenya. East Afr Med J. 1985; 62(7): 501-6.
Boyd A, Won KY, McClintock SK, Donovan CV, Laney SJ, Williams SA, Pilotte N, Streit TG, Beau de Rochars MVE, Lammie PJ. A community-based study of factors associated with continuing transmission of lymphatic filariasis in Leogane, Haiti. PLoS Negl Trop Dis. 2010; 4(3): e640. As it appears in London School of Hygiene and Tropical Medicine. Global Atlas of Helminth Infections - Lymphatic Filiariasis.
Boylan EA, McNulty BA, Walton J, Flynn A, Nugent AP, Gibney MJ. The prevalence and trends in overweight and obesity in Irish adults between 1990 and 2011. Public Health Nutr. 2014; 17(11): 2389â€“97.
Boylan S, Welch A, Pikhart H, Malyutina S, Pajak A, Kubinova R, Bragina O, Simonova G, Stepaniak U, Gilis-Januszewska A, Milla L, Peasey A, Marmot M, Bobak M. Dietary habits in three Central and Eastern European countries: the HAPIEE study. BMC Public Health. 2009; 439.
Brabin L, Brabin BJ, Dimitrakakis M, Gust I. Factors affecting the prevalence of infection with hepatitis B virus among non-pregnant women in the Alexishafen area of Papua New Guinea. Ann Trop Med Parasitol. 1989; 83(4): 365-74.
Brabin L, Verhoeff FH, Kazembe P, Brabin BJ, Chimsuku L, Broadhead R. Improving antenatal care for pregnant adolescents in southern Malawi. Acta Obstet Gynecol Scand. 1998; 77(4): 402-9.
Bracebridge S, Irwin D, Millership S. Prevention of perinatal hepatitis B transmission in a health authority area: an audit. Commun Dis Public Health. 2004; 7(2): 138-41.
Braddick MR, Reilly WJ. Human hydatid disease leading to hospital admission in Scotland 1968-1989. Health Bull (Edinb). 1993; 51(2): 80-5.
Bradshaw D, Dorrington R. Maternal mortality ratio-trends in the vital registration data. S Afr J Obstet Gynaecol. 2012; 18(2): 38-42.
Brady-West DC, Buchner LM. Retrospective audit of blood donation at a hospital-based blood centre. Implications for blood product supply and safety. West Indian Med J. 2000; 49(3): 226-8.
Braga C, de Albuquerque MF, Schindler H, Rezende A, Maciel A, Silva MC, Furtado A, de Carvalho AB, Lapa T, Ximenes RA. [Epidemiological pattern of lymphatic filariasis in children living in endemic areas]. J Pediatr (Rio J). 1997; 73(2): 95-100.
Braga C, Dourado I, Ximenes R, Miranda J, Alexander N. Bancroftian filariasis in an endemic area of Brazil: differences between genders during puberty. Rev Soc Bras Med Trop. 2005; 38(3): 224-8. As it appears in London School of Hygiene and Tropical Medicine. Global Atlas of Helminth Infections - Lymphatic Filiariasis.
Braga C, Dourado MI, Ximenes RA de A, Alves L, Brayner F, Rocha A, Alexander N. Field evaluation of the whole blood immunochromatographic test for rapid bancroftian filariasis diagnosis in the northeast of Brazil. Rev Inst Med Trop Sao Paulo. 2003; 45(3): 125-9. As it appears in London School of Hygiene and Tropical Medicine. Global Atlas of Helminth Infections - Lymphatic Filiariasis.
Braga C, Ximenes RA, Albuquerque M, Souza WV, Miranda J, Brayner F, Alves L, Silva L, Dourado I. [Evaluation of a social and environmental indicator used in the identification of lymphatic filariasis transmission in urban centers]. Cad Saude Publica. 2001; 17(5): 1211-8. As it appears in London School of Hygiene and Tropical Medicine. Global Atlas of Helminth Infections - Lymphatic Filiariasis.
Braga WSM, Castilho M da C, Borges FG, Martinho AC de S, Rodrigues IS, Azevedo EP de, Scazufca M, Menezes PR. Prevalence of hepatitis B virus infection and carriage after nineteen years of vaccination program in the Western Brazilian Amazon. Rev Soc Bras Med Trop. 2012; 45(1): 13-7.
Bragonier R, Reyburn H, Nasveld P, Edstein M, Auliffe A. Rainy-season prevalence of malaria in Bobonaro district, East Timor. Ann Trop Med Parasitol. 2002; 96(7): 739-43. As it appears in Malaria Atlas Project. Malaria Atlas Project Plasmodium Falciparum Parasite Rate Database. Oxford, United Kingdom: Malaria Atlas Project.
Branca F, Pastore G, Rossi L, Sette S, Stojanovska Ancevska B, Janeva N, Kolevska L, Peova S, Muratovska O, Venovska K. Multiple Indicators Cluster Survey in FYR Macedonia With Micronutrient Component. Rome, Italy: National Institute of Nutrition, 2000.
Branch O, Casapia WM, Gamboa DV, Hernandez JN, Alava FF, Roncal N, Alvarez E, Perez EJ, Gotuzzo E. Clustered local transmission and asymptomatic Plasmodium falciparum and Plasmodium vivax malaria infections in a recently emerged, hypoendemic Peruvian Amazon community. Malar J. 2005; 4(1): 27. As it appears in Malaria Atlas Project. Malaria Atlas Project Plasmodium Falciparum Parasite Rate Database. Oxford, United Kingdom: Malaria Atlas Project.
Brandão E, Bonfim C, Cabral D, Lima JL, Aguiar-Santos AM, Maciel A, Medeiros Z. Mapping of Wuchereria bancrofti infection in children and adolescents in an endemic area of Brazil. Acta Trop. 2011; 120(1-2): 151-4.

Appendix: Citation List

Citation
Brandicourt O, Carme B, Gay F, Turk P, Getnilini M. Widespread in vitro resistance to chloroquine of Plasmodium falciparum in the Congo, 1987. Trop Med Parasitol. 1991; 42(1): 55-9. As it appears in Malaria Atlas Project. Malaria Atlas Project Plasmodium Falciparum Parasite Rate Database. Oxford, United Kingdom: Malaria Atlas Project.
Bratina NU, Tahirovic H, Battelino T, Krzisnik C. Incidence of childhood-onset Type I diabetes in Slovenia and the Tuzia region (Bosnia and Herzegovina) in the period 1990-1998. Diabetologia. 2001; B27-31.
Bravo Tobar I, Parra F, Nello Pérez C, Rodríguez-Bonfante C, Useche F, Bonfante-Cabarcas R. Prevalence of Trypanosoma cruzi antibodies and inflammatory markers in uncompensated heart failure. Rev Soc Bras Med Trop. 2011; 44(6): 691-6.
Brazil - Aracaju Cancer Registry 2003-2006 - CI5 as it appears in Forman D, Bray F, Brewster DH, Gombe Mbalawa C, Kohler B, Piñeros M, Steliarova-Foucher E, Swaminathan R and Ferlay J, eds (2013). Cancer Incidence in Five Continents, Vol. X Summary Database (electronic version). Lyon, IARC. http://ci5.iarc.fr
Brazil - Belem Cancer Registry 1989 - CI5 as it appears in Parkin DM, International Agency for Research on Cancer, International Association of Cancer Registries. Cancer Incidence in Five Continents. Vol. I to VIII. Lyon, France, IARCPress, 2005.
Brazil - Belem Cancer Registry 1989-1991 - CI5 as it appears in Parkin DM, International Agency for Research on Cancer, International Association of Cancer Registries. Cancer Incidence in Five Continents. Vol. I to VIII. Lyon, France, IARCPress, 2005.
Brazil - Belo Horizonte Cancer Registry 2003-2005 - CI5 as it appears in Forman D, Bray F, Brewster DH, Gombe Mbalawa C, Kohler B, Piñeros M, Steliarova-Foucher E, Swaminathan R and Ferlay J, eds (2013). Cancer Incidence in Five Continents, Vol. X Summary Database (electronic version). Lyon, IARC. http://ci5.iarc.fr
Brazil - Brasilia Cancer Registry 1998 - CI5 as it appears in Curado MP, Edwards B, Shin HR, Storm H, Ferlay J, Heanue M and Boyle P, eds (2007). Cancer Incidence in Five Continents, Vol. IX Periodic Data (electronic version). Lyon, IARC.http://ci5.iarc.fr
Brazil - Brasilia Cancer Registry 1998-2001 - CI5 as it appears in Curado MP, Edwards B, Shin HR, Storm H, Ferlay J, Heanue M and Boyle P, eds (2007). Cancer Incidence in Five Continents, Vol. IX Periodic Data (electronic version). Lyon, IARC.http://ci5.iarc.fr
Brazil - Cuiaba Cancer Registry 2000 - CI5 as it appears in Curado MP, Edwards B, Shin HR, Storm H, Ferlay J, Heanue M and Boyle P, eds (2007). Cancer Incidence in Five Continents, Vol. IX Periodic Data (electronic version). Lyon, IARC.http://ci5.iarc.fr
Brazil - Cuiaba Cancer Registry 2000-2002 - CI5 as it appears in Curado MP, Edwards B, Shin HR, Storm H, Ferlay J, Heanue M and Boyle P, eds (2007). Cancer Incidence in Five Continents, Vol. IX Periodic Data (electronic version). Lyon, IARC.http://ci5.iarc.fr
Brazil - Cuiaba Cancer Registry 2003-2006 - CI5 as it appears in Forman D, Bray F, Brewster DH, Gombe Mbalawa C, Kohler B, Piñeros M, Steliarova-Foucher E, Swaminathan R and Ferlay J, eds (2013). Cancer Incidence in Five Continents, Vol. X Summary Database (electronic version). Lyon, IARC. http://ci5.iarc.fr
Brazil - Fortaleza Cancer Registry 1978-1982 - CI5 as it appears in Parkin DM, International Agency for Research on Cancer, International Association of Cancer Registries. Cancer Incidence in Five Continents. Vol. I to VIII. Lyon, France, IARCPress, 2005.
Brazil - Fortaleza Cancer Registry 2003-2006 - CI5 as it appears in Forman D, Bray F, Brewster DH, Gombe Mbalawa C, Kohler B, Piñeros M, Steliarova-Foucher E, Swaminathan R and Ferlay J, eds (2013). Cancer Incidence in Five Continents, Vol. X Summary Database (electronic version). Lyon, IARC. http://ci5.iarc.fr
Brazil - Goiania Cancer Registry 1988 - CI5 as it appears in Ferlay J, Parkin DM, Curado MP, Bray F, Edwards B, Shin HR and Forman D. Cancer Incidence in Five Continents, Volumes I to IX: IARC CancerBase No. 9 [Internet]. Lyon, France: International Agency for Research on Cancer; 2010. Available from: http://ci5.iarc.fr
Brazil - Goiania Cancer Registry 1988-1989 - CI5 as it appears in Parkin DM, International Agency for Research on Cancer, International Association of Cancer Registries. Cancer Incidence in Five Continents. Vol. I to VIII. Lyon, France, IARCPress, 2005.
Brazil - Goiania Cancer Registry 1989-1991 - CI5 as it appears in Ferlay J, Parkin DM, Curado MP, Bray F, Edwards B, Shin HR and Forman D. Cancer Incidence in Five Continents, Volumes I to IX: IARC CancerBase No. 9 [Internet]. Lyon, France: International Agency for Research on Cancer; 2010. Available from: http://ci5.iarc.fr
Brazil - Goiania Cancer Registry 1990 - CI5 as it appears in Ferlay J, Parkin DM, Curado MP, Bray F, Edwards B, Shin HR and Forman D. Cancer Incidence in Five Continents, Volumes I to IX: IARC CancerBase No. 9 [Internet]. Lyon, France: International Agency for Research on Cancer; 2010. Available from: http://ci5.iarc.fr
Brazil - Goiania Cancer Registry 1990-1992 - CI5 as it appears in Ferlay J, Parkin DM, Curado MP, Bray F, Edwards B, Shin HR and Forman D. Cancer Incidence in Five Continents, Volumes I to IX: IARC CancerBase No. 9 [Internet]. Lyon, France: International Agency for Research on Cancer; 2010. Available from: http://ci5.iarc.fr
Brazil - Goiania Cancer Registry 1990-1993 - CI5 as it appears in Parkin DM, International Agency for Research on Cancer, International Association of Cancer Registries. Cancer Incidence in Five Continents. Vol. I to VIII. Lyon, France, IARCPress, 2005.
Brazil - Goiania Cancer Registry 1991 - CI5 as it appears in Ferlay J, Parkin DM, Curado MP, Bray F, Edwards B, Shin HR and Forman D. Cancer Incidence in Five Continents, Volumes I to IX: IARC CancerBase No. 9 [Internet]. Lyon, France: International Agency for Research on Cancer; 2010. Available from: http://ci5.iarc.fr
Brazil - Goiania Cancer Registry 1993 - CI5 as it appears in Ferlay J, Parkin DM, Curado MP, Bray F, Edwards B, Shin HR and Forman D. Cancer Incidence in Five Continents, Volumes I to IX: IARC CancerBase No. 9 [Internet]. Lyon, France: International Agency for Research on Cancer; 2010. Available from: http://ci5.iarc.fr
Brazil - Goiania Cancer Registry 1994 - CI5 as it appears in Ferlay J, Parkin DM, Curado MP, Bray F, Edwards B, Shin HR and Forman D. Cancer Incidence in Five Continents, Volumes I to IX: IARC CancerBase No. 9 [Internet]. Lyon, France: International Agency for Research on Cancer; 2010. Available from: http://ci5.iarc.fr
Brazil - Goiania Cancer Registry 1995 - CI5 as it appears in Ferlay J, Parkin DM, Curado MP, Bray F, Edwards B, Shin HR and Forman D. Cancer Incidence in Five Continents, Volumes I to IX: IARC CancerBase No. 9 [Internet]. Lyon, France: International Agency for Research on Cancer; 2010. Available from: http://ci5.iarc.fr
Brazil - Goiania Cancer Registry 1996 - CI5 as it appears in Ferlay J, Parkin DM, Curado MP, Bray F, Edwards B, Shin HR and Forman D. Cancer Incidence in Five Continents, Volumes I to IX: IARC CancerBase No. 9 [Internet]. Lyon, France: International Agency for Research on Cancer; 2010. Available from: http://ci5.iarc.fr

Appendix: Citation List

Citation
Brazil - Goiania Cancer Registry 1997 - CI5 as it appears in Ferlay J, Parkin DM, Curado MP, Bray F, Edwards B, Shin HR and Forman D. Cancer Incidence in Five Continents, Volumes I to IX: IARC CancerBase No. 9 [Internet]. Lyon, France: International Agency for Research on Cancer; 2010. Available from: http://ci5.iarc.fr
Brazil - Goiania Cancer Registry 1998-2001 - CI5 as it appears in Ferlay J, Parkin DM, Curado MP, Bray F, Edwards B, Shin HR and Forman D. Cancer Incidence in Five Continents, Volumes I to IX: IARC CancerBase No. 9 [Internet]. Lyon, France: International Agency for Research on Cancer; 2010. Available from: http://ci5.iarc.fr
Brazil - Goiania Cancer Registry 1998-2002 - CI5 as it appears in Ferlay J, Parkin DM, Curado MP, Bray F, Edwards B, Shin HR and Forman D. Cancer Incidence in Five Continents, Volumes I to IX: IARC CancerBase No. 9 [Internet]. Lyon, France: International Agency for Research on Cancer; 2010. Available from: http://ci5.iarc.fr
Brazil - Goiania Cancer Registry 1999 - CI5 as it appears in Ferlay J, Parkin DM, Curado MP, Bray F, Edwards B, Shin HR and Forman D. Cancer Incidence in Five Continents, Volumes I to IX: IARC CancerBase No. 9 [Internet]. Lyon, France: International Agency for Research on Cancer; 2010. Available from: http://ci5.iarc.fr
Brazil - Goiania Cancer Registry 1999-2002 - CI5 as it appears in Curado MP, Edwards B, Shin HR, Storm H, Ferlay J, Heanue M and Boyle P, eds (2007). Cancer Incidence in Five Continents, Vol. IX Periodic Data (electronic version). Lyon, IARC.http://ci5.iarc.fr
Brazil - Goiania Cancer Registry 2001 - CI5 as it appears in Ferlay J, Parkin DM, Curado MP, Bray F, Edwards B, Shin HR and Forman D. Cancer Incidence in Five Continents, Volumes I to IX: IARC CancerBase No. 9 [Internet]. Lyon, France: International Agency for Research on Cancer; 2010. Available from: http://ci5.iarc.fr
Brazil - Goiania Cancer Registry 2002 - CI5 as it appears in Ferlay J, Parkin DM, Curado MP, Bray F, Edwards B, Shin HR and Forman D. Cancer Incidence in Five Continents, Volumes I to IX: IARC CancerBase No. 9 [Internet]. Lyon, France: International Agency for Research on Cancer; 2010. Available from: http://ci5.iarc.fr
Brazil - Goiania Cancer Registry 2003-2007 - CI5 as it appears in Forman D, Bray F, Brewster DH, Gombe Mbalawa C, Kohler B, Piñeros M, Steliarova-Foucher E, Swaminathan R and Ferlay J, eds (2013). Cancer Incidence in Five Continents, Vol. X Summary Database (electronic version). Lyon, IARC. http://ci5.iarc.fr
Brazil - Hearts of Brazil Survey 2004
Brazil - Porto Alegre Cancer Registry 1979-1982 - CI5 as it appears in Parkin DM, International Agency for Research on Cancer, International Association of Cancer Registries. Cancer Incidence in Five Continents. Vol. I to VIII. Lyon, France, IARCPress, 2005.
Brazil - Porto Alegre Cancer Registry 1987 - CI5 as it appears in Parkin DM, International Agency for Research on Cancer, International Association of Cancer Registries. Cancer Incidence in Five Continents. Vol. I to VIII. Lyon, France, IARCPress, 2005.
Brazil - Porto Alegre Cancer Registry 1990-1992 - CI5 as it appears in Parkin DM, International Agency for Research on Cancer, International Association of Cancer Registries. Cancer Incidence in Five Continents. Vol. I to VIII. Lyon, France, IARCPress, 2005.
Brazil - Porto Alegre Cancer Registry 1991-1995 - CI5 as it appears in Parkin DM, International Agency for Research on Cancer, International Association of Cancer Registries. Cancer Incidence in Five Continents. Vol. I to VIII. Lyon, France, IARCPress, 2005.
Brazil - Sao Paulo Cancer Registry 1978 - CI5 as it appears in Parkin DM, International Agency for Research on Cancer, International Association of Cancer Registries. Cancer Incidence in Five Continents. Vol. I to VIII. Lyon, France, IARCPress, 2005.
Brazil - Sao Paulo Cancer Registry 1998-2002 - CI5 as it appears in Curado MP, Edwards B, Shin HR, Storm H, Ferlay J, Heanue M and Boyle P, eds (2007). Cancer Incidence in Five Continents, Vol. IX Periodic Data (electronic version). Lyon, IARC.http://ci5.iarc.fr
Brazil - Sao Paulo Cancer Registry 2000-2002 - CI5 as it appears in Curado MP, Edwards B, Shin HR, Storm H, Ferlay J, Heanue M and Boyle P, eds (2007). Cancer Incidence in Five Continents, Vol. IX Periodic Data (electronic version). Lyon, IARC.http://ci5.iarc.fr
Brazil - Sao Paulo Cancer Registry 2003-2007 - CI5 as it appears in Forman D, Bray F, Brewster DH, Gombe Mbalawa C, Kohler B, Piñeros M, Steliarova-Foucher E, Swaminathan R and Ferlay J, eds (2013). Cancer Incidence in Five Continents, Vol. X Summary Database (electronic version). Lyon, IARC. http://ci5.iarc.fr
Brazil - Training Report of the Germano Sinval Faria Training Unit as it appears in World Health Organization (WHO). WHO Global Database on Child Growth and Malnutrition - Historical. Geneva, Switzerland: World Health Organization (WHO).
Brazil National Demographic and Health Survey of Children and Women 2006-2007 as it appears in World Health Organization (WHO). WHO Global Database on Child Growth and Malnutrition. Geneva, Switzerland: World Health Organization (WHO).
Brazil National Survey on Health and Nutrition 1989 as it appears in World Health Organization (WHO). WHO Global Database on Child Growth and Malnutrition - Historical. Geneva, Switzerland: World Health Organization (WHO).
Brazil Vital Registration - Deaths 1981 ICD9 as it appears in World Health Organization (WHO). WHO Mortality Database Version November 2015. Geneva, Switzerland: World Health Organization (WHO), 2015.
Brazil Vital Registration - Deaths 1982 ICD9 as it appears in World Health Organization (WHO). WHO Mortality Database Version November 2015. Geneva, Switzerland: World Health Organization (WHO), 2015.
Brazil Vital Registration - Deaths 1983 ICD9 as it appears in World Health Organization (WHO). WHO Mortality Database Version November 2015. Geneva, Switzerland: World Health Organization (WHO), 2015.
Brazil Vital Registration - Deaths 1984 ICD9 as it appears in World Health Organization (WHO). WHO Mortality Database Version November 2015. Geneva, Switzerland: World Health Organization (WHO), 2015.
Brazil Vital Registration - Deaths 1985 ICD9 as it appears in World Health Organization (WHO). WHO Mortality Database Version November 2015. Geneva, Switzerland: World Health Organization (WHO), 2015.
Brazil Vital Registration - Deaths 1986 ICD9 as it appears in World Health Organization (WHO). WHO Mortality Database Version November 2015. Geneva, Switzerland: World Health Organization (WHO), 2015.
Brazil Vital Registration - Deaths 1987 ICD9 as it appears in World Health Organization (WHO). WHO Mortality Database Version November 2015. Geneva, Switzerland: World Health Organization (WHO), 2015.
Brazil Vital Registration - Deaths 1988 ICD9 as it appears in World Health Organization (WHO). WHO Mortality Database Version November 2015. Geneva, Switzerland: World Health Organization (WHO), 2015.

Appendix: Citation List

Citation
Brazil Vital Registration - Deaths 1989 ICD9 as it appears in World Health Organization (WHO). WHO Mortality Database Version November 2015. Geneva, Switzerland: World Health Organization (WHO), 2015.
Brazil Vital Registration - Deaths 1990 ICD9 as it appears in World Health Organization (WHO). WHO Mortality Database Version November 2015. Geneva, Switzerland: World Health Organization (WHO), 2015.
Brazil Vital Registration - Deaths 1991 ICD9 as it appears in World Health Organization (WHO). WHO Mortality Database Version November 2015. Geneva, Switzerland: World Health Organization (WHO), 2015.
Brazil Vital Registration - Deaths 1992 ICD9 as it appears in World Health Organization (WHO). WHO Mortality Database Version November 2015. Geneva, Switzerland: World Health Organization (WHO), 2015.
Brazil Vital Registration - Deaths 1993 ICD9 as it appears in World Health Organization (WHO). WHO Mortality Database Version November 2015. Geneva, Switzerland: World Health Organization (WHO), 2015.
Brazil Vital Registration - Deaths 1994 ICD9 as it appears in World Health Organization (WHO). WHO Mortality Database Version November 2015. Geneva, Switzerland: World Health Organization (WHO), 2015.
Brazil Vital Registration - Deaths 1995 ICD9 as it appears in World Health Organization (WHO). WHO Mortality Database Version November 2015. Geneva, Switzerland: World Health Organization (WHO), 2015.
Brazil Vital Registration - Deaths 1996 ICD10 as it appears in World Health Organization (WHO). WHO Mortality Database Version November 2015. Geneva, Switzerland: World Health Organization (WHO), 2015.
Brazil Vital Registration - Deaths 1997 ICD10 as it appears in World Health Organization (WHO). WHO Mortality Database Version November 2015. Geneva, Switzerland: World Health Organization (WHO), 2015.
Brazil Vital Registration - Deaths 1998 ICD10 as it appears in World Health Organization (WHO). WHO Mortality Database Version November 2015. Geneva, Switzerland: World Health Organization (WHO), 2015.
Brazil Vital Registration - Deaths 1999 ICD10 as it appears in World Health Organization (WHO). WHO Mortality Database Version November 2015. Geneva, Switzerland: World Health Organization (WHO), 2015.
Brazil Vital Registration - Deaths 2000 ICD10 as it appears in World Health Organization (WHO). WHO Mortality Database Version November 2015. Geneva, Switzerland: World Health Organization (WHO), 2015.
Brazil Vital Registration - Deaths 2001 ICD10 as it appears in World Health Organization (WHO). WHO Mortality Database Version November 2015. Geneva, Switzerland: World Health Organization (WHO), 2015.
Brazil Vital Registration - Deaths 2002 ICD10 as it appears in World Health Organization (WHO). WHO Mortality Database Version November 2015. Geneva, Switzerland: World Health Organization (WHO), 2015.
Brazil Vital Registration - Deaths 2003 ICD10 as it appears in World Health Organization (WHO). WHO Mortality Database Version November 2015. Geneva, Switzerland: World Health Organization (WHO), 2015.
Brazil Vital Registration - Deaths 2004 ICD10 as it appears in World Health Organization (WHO). WHO Mortality Database Version November 2015. Geneva, Switzerland: World Health Organization (WHO), 2015.
Brazil Vital Registration - Deaths 2005 ICD10 as it appears in World Health Organization (WHO). WHO Mortality Database Version November 2015. Geneva, Switzerland: World Health Organization (WHO), 2015.
Brazil Vital Registration - Deaths 2006 ICD10 as it appears in World Health Organization (WHO). WHO Mortality Database Version November 2015. Geneva, Switzerland: World Health Organization (WHO), 2015.
Brazil Vital Registration - Deaths 2007 ICD10 as it appears in World Health Organization (WHO). WHO Mortality Database Version November 2015. Geneva, Switzerland: World Health Organization (WHO), 2015.
Brazil Vital Registration - Deaths 2008 ICD10 as it appears in World Health Organization (WHO). WHO Mortality Database Version November 2015. Geneva, Switzerland: World Health Organization (WHO), 2015.
Brazil Vital Registration - Deaths 2009 ICD10 as it appears in World Health Organization (WHO). WHO Mortality Database Version November 2015. Geneva, Switzerland: World Health Organization (WHO), 2015.
Brazil Vital Registration - Deaths 2010 ICD10 as it appears in World Health Organization (WHO). WHO Mortality Database Version November 2015. Geneva, Switzerland: World Health Organization (WHO), 2015.
Brazil Vital Registration Death Data 1974 as it appears in United Nations Statistics Division (UNSD). United Nations Demographic Yearbook - Historical Supplement 1997. New York City, United States: United Nations Statistics Division (UNSD).
Brazil Vital Registration Death Data 1975 as it appears in United Nations Statistics Division (UNSD). United Nations Demographic Yearbook - Historical Supplement 1997. New York City, United States: United Nations Statistics Division (UNSD).
Brazil Vital Registration Death Data 1976 as it appears in United Nations Statistics Division (UNSD). United Nations Demographic Yearbook - Historical Supplement 1997. New York City, United States: United Nations Statistics Division (UNSD).
Brazil Vital Registration Death Data 1977 as it appears in United Nations Statistics Division (UNSD). United Nations Demographic Yearbook - Historical Supplement 1997. New York City, United States: United Nations Statistics Division (UNSD).
Brazil Vital Registration Death Data 1978 as it appears in United Nations Statistics Division (UNSD). United Nations Demographic Yearbook - Historical Supplement 1997. New York City, United States: United Nations Statistics Division (UNSD).
Brazil Vital Registration Death Data 1979 as it appears in United Nations Statistics Division (UNSD). United Nations Demographic Yearbook - Historical Supplement 1997. New York City, United States: United Nations Statistics Division (UNSD).
Brazil Vital Registration Death Data 1980 as it appears in United Nations Statistics Division (UNSD). United Nations Demographic Yearbook - Historical Supplement 1997. New York City, United States: United Nations Statistics Division (UNSD).
Brazil Vital Registration Death Data 2011 as it appears in United Nations Statistics Division (UNSD). United Nations Demographic Yearbook 2012. New York City, United States: United Nations Statistics Division (UNSD), 2013.
Brazilian Center for Analysis and Planning (CEBRAP), Ministry of Health (Brazil). Brazil National Demographic and Health Survey of Children and Women 2006-2007. Rio de Janeiro, Brazil: Ministry of Health (Brazil).
Brazilian Center of Information on Psychotropic Drugs (CEBRID), National Anti-Drug Secretariat (Brazil). Brazil Household Survey About the Use of Psychotropic Drugs 2005.

Appendix: Citation List

Citation
Brazilian Institute of Geography and Statistics (IBGE) and Minnesota Population Center. Brazil General Census 1991 from the Integrated Public Use Microdata Series, International: [Machine-readable database]. Minneapolis: University of Minnesota.
Brazilian Institute of Geography and Statistics (IBGE), Ministry of Education (Brazil), Ministry of Health (Brazil), Ministry of Planning, Budget, and Management (Brazil), Secretariat of Health Surveillance, Ministry of Health (Brazil). Brazil National Survey of School Health 2012.
Brazilian Institute of Geography and Statistics (IBGE), Minnesota Population Center. Brazil General Census 1970 from the Integrated Public Use Microdata Series, International: [Machine-readable database]. Minneapolis: University of Minnesota.
Brazilian Institute of Geography and Statistics (IBGE), Minnesota Population Center. Brazil General Census 1980 from the Integrated Public Use Microdata Series, International: [Machine-readable database]. Minneapolis: University of Minnesota.
Brazilian Institute of Geography and Statistics (IBGE), Minnesota Population Center. Brazil General Census 2000 from the Integrated Public Use Microdata Series, International: [Machine-readable database]. Minneapolis: University of Minnesota.
Brazilian Institute of Geography and Statistics (IBGE), National Census Service (Brazil), Minnesota Population Center. Brazil General Census 1960 from the Integrated Public Use Microdata Series, International: [Machine-readable database]. Minneapolis: University of Minnesota.
Brazilian Institute of Geography and Statistics (IBGE), World Bank (WB). Brazil Living Standards Measurement Survey 1996-1997. Washington DC, United States: World Bank (WB).
Brazilian Institute of Geography and Statistics (IBGE). Brazil Civil Registry Statistics - Live births and Fetal deaths by residence of mother 2005. Rio de Janeiro, Brazil: Brazilian Institute of Geography and Statistics (IBGE).
Brazilian Institute of Geography and Statistics (IBGE). Brazil Civil Registry Statistics - Live births and Fetal deaths by residence of mother 2006. Rio de Janeiro, Brazil: Brazilian Institute of Geography and Statistics (IBGE).
Brazilian Institute of Geography and Statistics (IBGE). Brazil Civil Registry Statistics - Live births and Fetal deaths by residence of mother 2007. Rio de Janeiro, Brazil: Brazilian Institute of Geography and Statistics (IBGE).
Brazilian Institute of Geography and Statistics (IBGE). Brazil Civil Registry Statistics - Live births and Fetal deaths by residence of mother 2008. Rio de Janeiro, Brazil: Brazilian Institute of Geography and Statistics (IBGE).
Brazilian Institute of Geography and Statistics (IBGE). Brazil Civil Registry Statistics - Live births and Fetal deaths by residence of mother 2009. Rio de Janeiro, Brazil: Brazilian Institute of Geography and Statistics (IBGE).
Brazilian Institute of Geography and Statistics (IBGE). Brazil Civil Registry Statistics - Live births and Fetal deaths by residence of mother 2010. Rio de Janeiro, Brazil: Brazilian Institute of Geography and Statistics (IBGE).
Brazilian Institute of Geography and Statistics (IBGE). Brazil Civil Registry Statistics - Live births and Fetal deaths by residence of mother 2011. Rio de Janeiro, Brazil: Brazilian Institute of Geography and Statistics (IBGE).
Brazilian Institute of Geography and Statistics (IBGE). Brazil Civil Registry Statistics - Live births and Fetal deaths by residence of mother 2012. Rio de Janeiro, Brazil: Brazilian Institute of Geography and Statistics (IBGE).
Brazilian Institute of Geography and Statistics (IBGE). Brazil Civil Registry Statistics - Live births and Fetal deaths by residence of mother 2013. Rio de Janeiro, Brazil: Brazilian Institute of Geography and Statistics (IBGE).
Brazilian Institute of Geography and Statistics (IBGE). Brazil Civil Registry Statistics - Live births and Fetal deaths by residence of mother 2014. Rio de Janeiro, Brazil: Brazilian Institute of Geography and Statistics (IBGE).
Brazilian Institute of Geography and Statistics (IBGE). Brazil Consumer Expenditure Survey 2002-2003. Rio de Janeiro, Brazil: Brazilian Institute of Geography and Statistics (IBGE).
Brazilian Institute of Geography and Statistics (IBGE). Brazil National Household Sample Survey 1992.
Brazilian Institute of Geography and Statistics (IBGE). Brazil National Household Sample Survey 1993. Rio de Janeiro, Brazil: Brazilian Institute of Geography and Statistics (IBGE).
Brazilian Institute of Geography and Statistics (IBGE). Brazil National Household Sample Survey 1995. Rio de Janeiro, Brazil: Brazilian Institute of Geography and Statistics (IBGE).
Brazilian Institute of Geography and Statistics (IBGE). Brazil National Household Sample Survey 1996. Rio de Janeiro, Brazil: Brazilian Institute of Geography and Statistics (IBGE).
Brazilian Institute of Geography and Statistics (IBGE). Brazil National Household Sample Survey 1997. Rio de Janeiro, Brazil: Brazilian Institute of Geography and Statistics (IBGE).
Brazilian Institute of Geography and Statistics (IBGE). Brazil National Household Sample Survey 1998. Rio de Janeiro, Brazil: Brazilian Institute of Geography and Statistics (IBGE).
Brazilian Institute of Geography and Statistics (IBGE). Brazil National Household Sample Survey 1999. Rio de Janeiro, Brazil: Brazilian Institute of Geography and Statistics (IBGE).
Brazilian Institute of Geography and Statistics (IBGE). Brazil National Household Sample Survey 2001. Rio de Janeiro, Brazil: Brazilian Institute of Geography and Statistics (IBGE).
Brazilian Institute of Geography and Statistics (IBGE). Brazil National Household Sample Survey 2002. Rio de Janeiro, Brazil: Brazilian Institute of Geography and Statistics (IBGE).
Brazilian Institute of Geography and Statistics (IBGE). Brazil National Household Sample Survey 2003. Rio de Janeiro, Brazil: Brazilian Institute of Geography and Statistics (IBGE).
Brazilian Institute of Geography and Statistics (IBGE). Brazil National Household Sample Survey 2004. Rio de Janeiro, Brazil: Brazilian Institute of Geography and Statistics (IBGE).
Brazilian Institute of Geography and Statistics (IBGE). Brazil National Household Sample Survey 2005. Rio de Janeiro, Brazil: Brazilian Institute of Geography and Statistics (IBGE).
Brazilian Institute of Geography and Statistics (IBGE). Brazil National Household Sample Survey 2006. Rio de Janeiro, Brazil: Brazilian Institute of Geography and Statistics (IBGE).
Brazilian Institute of Geography and Statistics (IBGE). Brazil National Household Sample Survey 2007. Rio de Janeiro, Brazil: Brazilian Institute of Geography and Statistics (IBGE).

Appendix: Citation List

Citation
Brazilian Institute of Geography and Statistics (IBGE). Brazil National Household Sample Survey 2008. Rio de Janeiro, Brazil: Brazilian Institute of Geography and Statistics (IBGE).
Brazilian Institute of Geography and Statistics (IBGE). Brazil National Household Sample Survey 2009.
Brazilian Institute of Geography and Statistics. Brazil Census 2000.
Brazilian Society for Family Welfare (BEMFAM), Macro International, Inc. Brazil Demographic and Health Survey - Maternal Mortality Data.
Brazilian Society for Family Welfare (BEMFAM), Macro International, Inc. Brazil Demographic and Health Survey 1991. Calverton, United States: Macro International, Inc.
Brazilian Society for Family Welfare (BEMFAM), Macro International, Inc. Brazil Demographic and Health Survey 1996. Calverton, United States: Macro International, Inc.
Brazilian Society for Family Welfare (BEMFAM), Westinghouse; Institute for Resource Development. Brazil Demographic and Health Survey 1986. Columbia, United States: Westinghouse; Institute for Resource Development.
Brenière SF, Bosseno MF, Noireau F, Yacsik N, Liegeard P, Aznar C, Hontebeyrie M. Integrate study of a Bolivian population infected by Trypanosoma cruzi, the agent of Chagas disease. Mem Inst Oswaldo Cruz. 2002; 97(3): 289–95.
Brevi A, Naldi L, Cainelli T, Parazzini F. Prevalence and awareness of hepatitis B virus carrier status in Italy. Genitourin Med. 1993; 69(3): 241.
Brian G, Ramke J, Maher L, Page A, Szetu J. The prevalence of diabetes among adults aged 40 years and over in Fiji. N Z Med J. 2010; 123(1327): 68-75.
Brian G, Ramke J, Page A, Maher L, Szetu J, Qalo Qoqonokana M. The association of diabetes and BMI among Melanesian and Indian Fijians aged ≥ 40 years. Br J Nutr. 2011; 105(10): 1539–45.
Brieger W, Sesay H, Adesina H, Mosanya M, Ogunlade P, Ayodele J, Orisasona S. Urban malaria treatment behaviour in the context of low levels of malaria transmission in Lagos, Nigeria. Afr J Med Med Sci. 2000; 30(Suppl): 7-15. As it appears in Malaria Atlas Project. Malaria Atlas Project Plasmodium Falciparum Parasite Rate Database. Oxford, United Kingdom: Malaria Atlas Project.
Brito CR do N, Sampaio GHF, Câmara ACJ da, Nunes DF, Azevedo PRM de, Chiari E, Galvão LM da C. Seroepidemiology of Trypanosoma cruzi infection in the semiarid rural zone of the State of Rio Grande do Norte, Brazil. Rev Soc Bras Med Trop. 2012; 45(3): 346–52.
Brito LL, Barreto ML, Silva RDCR, Assis AMO, Reis MG, Parraga IM, Blanton RE. Moderate- and low-intensity co-infections by intestinal helminths and Schistosoma mansoni, dietary iron intake, and anemia in Brazilian children. Am J Trop Med Hyg. 2006; 75(5): 939-44.
Brito LL, Barreto ML, Silva Rde C, Assis AM, Reis MG, Parraga I, Blanton RE. [Risk factors for iron-deficiency anemia in children and adolescents with intestinal helminthic infections]. Rev Panam Salud Publica. 2003; 14(6): 422-31.
Brohall G, Behre CJ, Hulthe J, Wikstrand J, Fagerberg B. Prevalence of diabetes and impaired glucose tolerance in 64-year-old Swedish women: experiences of using repeated oral glucose tolerance tests. Diabetes Care. 2006; 29(2): 363-7.
Brooker S, Akhwale W, Pullan R, Estambale B, Clarke SE, Snow RW, Hotez PJ. Epidemiology of Plasmodium-Helminth Co-Infection in Africa: Populations at Risk, Potential Impact on Anemia, and Prospects for Combining Control. Am J Trop Med Hyg. 2007; 77(Suppl 6): 88-98. As it appears in Malaria Atlas Project. Malaria Atlas Project Plasmodium Falciparum Parasite Rate Database. Oxford, United Kingdom: Malaria Atlas Project.
Brooker SJ, Pullan RL, Gitonga CW, Ashton RA, Kolaczinski JH, Kabatereine NB, Snow RW. Plasmodium-Helminth Coinfection and Its Sources of Heterogeneity Across East Africa. J Infect Dis. 2012; 205(5): 841-52. As it appears in Malaria Atlas Project. Malaria Atlas Project Plasmodium Falciparum Parasite Rate Database. Oxford, United Kingdom: Malaria Atlas Project.
Brooks SE, Hanchard B, Wolff C, Samuels E, Allen J. Age-specific incidence of cancer in Kingston and St. Andrew, Jamaica, 1988-1992. West Indian Med J. 1995; 44(3): 102-5.
Brooks SE, Wolff C. Age- specific incidence of cancer in Kingston and St. Andrew, Jamaica. Part I: 1978-1982. West Indian Med J. 1991; 40(3): 127-8.
Brown L, Thurman T, Bloem J, Kendall C. Sexual violence in Lesotho. Stud Fam Plann. 2006; 37(4): 269-80.
Brown P, Breguet G, Smallwood L, Ney R, Moerdowo RM, Gerety RJ. Serologic markers of hepatitis A and B in the population of Bali, Indonesia. Am J Trop Med Hyg. 1985; 34(3): 616-9.
Brule Ville AssociÃ©s (BVA), French Committee for Health Education (CFES). France Youth Health Barometer 1997.
Brundu D, Piseddu T, Stegel G, Masu G, Ledda S, Masala G. Retrospective study of human cystic echinococcosis in Italy based on the analysis of hospital discharge records between 2001 and 2012. Acta Trop. 2014; 91-6.
Brunei Darussalam Vital Registration - Deaths 1996 ICD10 as it appears in World Health Organization (WHO). WHO Mortality Database Version November 2015. Geneva, Switzerland: World Health Organization (WHO), 2015.
Brunei Darussalam Vital Registration - Deaths 1997 ICD10 as it appears in World Health Organization (WHO). WHO Mortality Database Version November 2015. Geneva, Switzerland: World Health Organization (WHO), 2015.
Brunei Darussalam Vital Registration - Deaths 1998 ICD10 as it appears in World Health Organization (WHO). WHO Mortality Database Version November 2015. Geneva, Switzerland: World Health Organization (WHO), 2015.
Brunei Darussalam Vital Registration - Deaths 1999 ICD10 as it appears in World Health Organization (WHO). WHO Mortality Database Version November 2015. Geneva, Switzerland: World Health Organization (WHO), 2015.
Brunei Darussalam Vital Registration - Deaths 2000 ICD10 as it appears in World Health Organization (WHO). WHO Mortality Database Version November 2015. Geneva, Switzerland: World Health Organization (WHO), 2015.
Brunei Darussalam Vital Registration - Deaths 2001 ICD10 as it appears in World Health Organization (WHO). WHO Mortality Database Version November 2015. Geneva, Switzerland: World Health Organization (WHO), 2015.
Brunei Darussalam Vital Registration - Deaths 2002 ICD10 as it appears in World Health Organization (WHO). WHO Mortality Database Version November 2015. Geneva, Switzerland: World Health Organization (WHO), 2015.
Brunei Darussalam Vital Registration - Deaths 2003 ICD10 as it appears in World Health Organization (WHO). WHO Mortality Database Version November 2015. Geneva, Switzerland: World Health Organization (WHO), 2015.
Brunei Darussalam Vital Registration - Deaths 2004 ICD10 as it appears in World Health Organization (WHO). WHO Mortality Database Version November 2015. Geneva, Switzerland: World Health Organization (WHO), 2015.

Appendix: Citation List

Citation
Brunei Darussalam Vital Registration - Deaths 2005 ICD10 as it appears in World Health Organization (WHO). WHO Mortality Database Version November 2015. Geneva, Switzerland: World Health Organization (WHO), 2015.
Brunei Darussalam Vital Registration - Deaths 2006 ICD10 as it appears in World Health Organization (WHO). WHO Mortality Database Version November 2015. Geneva, Switzerland: World Health Organization (WHO), 2015.
Brunei Darussalam Vital Registration - Deaths 2007 ICD10 as it appears in World Health Organization (WHO). WHO Mortality Database Version November 2015. Geneva, Switzerland: World Health Organization (WHO), 2015.
Brunei Darussalam Vital Registration - Deaths 2008 ICD10 as it appears in World Health Organization (WHO). WHO Mortality Database Version November 2015. Geneva, Switzerland: World Health Organization (WHO), 2015.
Brunei Darussalam Vital Registration - Deaths 2009 ICD10 as it appears in World Health Organization (WHO). WHO Mortality Database Version November 2015. Geneva, Switzerland: World Health Organization (WHO), 2015.
Brunei Darussalam Vital Registration - Deaths 2010 ICD10 as it appears in World Health Organization (WHO). WHO Mortality Database Version November 2015. Geneva, Switzerland: World Health Organization (WHO), 2015.
Brunei Darussalam Vital Registration - Deaths 2011 ICD10 as it appears in World Health Organization (WHO). WHO Mortality Database Version November 2015. Geneva, Switzerland: World Health Organization (WHO), 2015.
Brunei Darussalam Vital Registration - Deaths 2012 ICD10 as it appears in World Health Organization (WHO). WHO Mortality Database Version November 2015. Geneva, Switzerland: World Health Organization (WHO), 2015.
Brunei Darussalam Vital Registration - Deaths 2013 ICD10 as it appears in World Health Organization (WHO). WHO Mortality Database Version November 2015. Geneva, Switzerland: World Health Organization (WHO), 2015.
Brunei Darussalam Vital Registration Death Data 1950 as it appears in United Nations Statistics Division (UNSD). United Nations Demographic Yearbook - Historical Supplement 1997. New York City, United States: United Nations Statistics Division (UNSD).
Brunei Darussalam Vital Registration Death Data 1951 as it appears in United Nations Statistics Division (UNSD). United Nations Demographic Yearbook - Historical Supplement 1997. New York City, United States: United Nations Statistics Division (UNSD).
Brunei Darussalam Vital Registration Death Data 1952 as it appears in United Nations Statistics Division (UNSD). United Nations Demographic Yearbook - Historical Supplement 1997. New York City, United States: United Nations Statistics Division (UNSD).
Brunei Darussalam Vital Registration Death Data 1953 as it appears in United Nations Statistics Division (UNSD). United Nations Demographic Yearbook - Historical Supplement 1997. New York City, United States: United Nations Statistics Division (UNSD).
Brunei Darussalam Vital Registration Death Data 1954 as it appears in United Nations Statistics Division (UNSD). United Nations Demographic Yearbook - Historical Supplement 1997. New York City, United States: United Nations Statistics Division (UNSD).
Brunei Darussalam Vital Registration Death Data 1955 as it appears in United Nations Statistics Division (UNSD). United Nations Demographic Yearbook - Historical Supplement 1997. New York City, United States: United Nations Statistics Division (UNSD).
Brunei Darussalam Vital Registration Death Data 1956 as it appears in United Nations Statistics Division (UNSD). United Nations Demographic Yearbook - Historical Supplement 1997. New York City, United States: United Nations Statistics Division (UNSD).
Brunei Darussalam Vital Registration Death Data 1957 as it appears in United Nations Statistics Division (UNSD). United Nations Demographic Yearbook - Historical Supplement 1997. New York City, United States: United Nations Statistics Division (UNSD).
Brunei Darussalam Vital Registration Death Data 1958 as it appears in United Nations Statistics Division (UNSD). United Nations Demographic Yearbook - Historical Supplement 1997. New York City, United States: United Nations Statistics Division (UNSD).
Brunei Darussalam Vital Registration Death Data 1959 as it appears in United Nations Statistics Division (UNSD). United Nations Demographic Yearbook - Historical Supplement 1997. New York City, United States: United Nations Statistics Division (UNSD).
Brunei Darussalam Vital Registration Death Data 1964 as it appears in United Nations Statistics Division (UNSD). United Nations Demographic Yearbook - Historical Supplement 1997. New York City, United States: United Nations Statistics Division (UNSD).
Brunei Darussalam Vital Registration Death Data 1965 as it appears in United Nations Statistics Division (UNSD). United Nations Demographic Yearbook - Historical Supplement 1997. New York City, United States: United Nations Statistics Division (UNSD).
Brunei Darussalam Vital Registration Death Data 1966 as it appears in United Nations Statistics Division (UNSD). United Nations Demographic Yearbook - Historical Supplement 1997. New York City, United States: United Nations Statistics Division (UNSD).
Brunei Darussalam Vital Registration Death Data 1967 as it appears in United Nations Statistics Division (UNSD). United Nations Demographic Yearbook - Historical Supplement 1997. New York City, United States: United Nations Statistics Division (UNSD).
Brunei Darussalam Vital Registration Death Data 1968 as it appears in United Nations Statistics Division (UNSD). United Nations Demographic Yearbook - Historical Supplement 1997. New York City, United States: United Nations Statistics Division (UNSD).
Brunei Darussalam Vital Registration Death Data 1969 as it appears in United Nations Statistics Division (UNSD). United Nations Demographic Yearbook - Historical Supplement 1997. New York City, United States: United Nations Statistics Division (UNSD).
Brunei Darussalam Vital Registration Death Data 1970 as it appears in United Nations Statistics Division (UNSD). United Nations Demographic Yearbook - Historical Supplement 1997. New York City, United States: United Nations Statistics Division (UNSD).
Brunei Darussalam Vital Registration Death Data 1971 as it appears in United Nations Statistics Division (UNSD). United Nations Demographic Yearbook - Historical Supplement 1997. New York City, United States: United Nations Statistics Division (UNSD).
Brunei Darussalam Vital Registration Death Data 1972 as it appears in United Nations Statistics Division (UNSD). United Nations Demographic Yearbook - Historical Supplement 1997. New York City, United States: United Nations Statistics Division (UNSD).
Brunei Darussalam Vital Registration Death Data 1973 as it appears in United Nations Statistics Division (UNSD). United Nations Demographic Yearbook - Historical Supplement 1997. New York City, United States: United Nations Statistics Division (UNSD).
Brunei Darussalam Vital Registration Death Data 1974 as it appears in United Nations Statistics Division (UNSD). United Nations Demographic Yearbook - Historical Supplement 1997. New York City, United States: United Nations Statistics Division (UNSD).
Brunei Darussalam Vital Registration Death Data 1975 as it appears in United Nations Statistics Division (UNSD). United Nations Demographic Yearbook - Historical Supplement 1997. New York City, United States: United Nations Statistics Division (UNSD).
Brunei Darussalam Vital Registration Death Data 1976 as it appears in United Nations Statistics Division (UNSD). United Nations Demographic Yearbook - Historical Supplement 1997. New York City, United States: United Nations Statistics Division (UNSD).

Appendix: Citation List

Citation
Brunei Darussalam Vital Registration Death Data 1977 as it appears in United Nations Statistics Division (UNSD). United Nations Demographic Yearbook - Historical Supplement 1997. New York City, United States: United Nations Statistics Division (UNSD).
Brunei Darussalam Vital Registration Death Data 1978 as it appears in United Nations Statistics Division (UNSD). United Nations Demographic Yearbook - Historical Supplement 1997. New York City, United States: United Nations Statistics Division (UNSD).
Brunei Darussalam Vital Registration Death Data 1982 as it appears in United Nations Statistics Division (UNSD). United Nations Demographic Yearbook - Historical Supplement 1997. New York City, United States: United Nations Statistics Division (UNSD).
Brunei Darussalam Vital Registration Death Data 1983 as it appears in United Nations Statistics Division (UNSD). United Nations Demographic Yearbook - Historical Supplement 1997. New York City, United States: United Nations Statistics Division (UNSD).
Brunei Darussalam Vital Registration Death Data 1984 as it appears in United Nations Statistics Division (UNSD). United Nations Demographic Yearbook - Historical Supplement 1997. New York City, United States: United Nations Statistics Division (UNSD).
Brunei Darussalam Vital Registration Death Data 1985 as it appears in United Nations Statistics Division (UNSD). United Nations Demographic Yearbook - Historical Supplement 1997. New York City, United States: United Nations Statistics Division (UNSD).
Brunei Darussalam Vital Registration Death Data 1986 as it appears in United Nations Statistics Division (UNSD). United Nations Demographic Yearbook - Historical Supplement 1997. New York City, United States: United Nations Statistics Division (UNSD).
Brunei Darussalam Vital Registration Death Data 1987 as it appears in United Nations Statistics Division (UNSD). United Nations Demographic Yearbook - Historical Supplement 1997. New York City, United States: United Nations Statistics Division (UNSD).
Brunei Darussalam Vital Registration Death Data 1988 as it appears in United Nations Statistics Division (UNSD). United Nations Demographic Yearbook - Historical Supplement 1997. New York City, United States: United Nations Statistics Division (UNSD).
Brunei Darussalam Vital Registration Death Data 1989 as it appears in United Nations Statistics Division (UNSD). United Nations Demographic Yearbook - Historical Supplement 1997. New York City, United States: United Nations Statistics Division (UNSD).
Brunei Darussalam Vital Registration Death Data 1990 as it appears in United Nations Statistics Division (UNSD). United Nations Demographic Yearbook - Historical Supplement 1997. New York City, United States: United Nations Statistics Division (UNSD).
Brunei Darussalam Vital Registration Death Data 1991 as it appears in United Nations Statistics Division (UNSD). United Nations Demographic Yearbook - Historical Supplement 1997. New York City, United States: United Nations Statistics Division (UNSD).
Brunei Darussalam Vital Registration Death Data 1992 as it appears in United Nations Statistics Division (UNSD). United Nations Demographic Yearbook - Historical Supplement 1997. New York City, United States: United Nations Statistics Division (UNSD).
Brunei Darussalam Vital Registration Death Data 2003 as it appears in United Nations Statistics Division (UNSD). United Nations Demographic Yearbook 2003. New York City, United States: United Nations Statistics Division (UNSD), 2006.
Brunei Darussalam Vital Registration Death Data 2004 as it appears in United Nations Statistics Division (UNSD). United Nations Demographic Yearbook 2004. New York City, United States: United Nations Statistics Division (UNSD), 2007.
Brunei Population Census 1960 as it appears in United Nations Statistics Division (UNSD). United Nations Demographic Yearbook. New York City, United States: United Nations Statistics Division (UNSD).
Bruno G, Maule M, Biggeri A, Ledda A, Mannu C, Merletti F, Songini M, Sardinian Group for Diabetes Epidemiology. More than 20 years of registration of type 1 diabetes in Sardinian children: temporal variations of incidence with age, period of diagnosis, and year of birth. Diabetes. 2013; 62(10): 3542-6.
Bruno G, Maule M, Merletti F, Novelli G, Falorni A, Iannilli A, Iughetti L, Altobelli E, d'Annunzio G, Piffer S, Pozzilli P, Iafusco D, Songini M, Roncarolo F, Toni S, Carle F, Cherubini V. Age-period-cohort analysis of 1990-2003 incidence time trends of childhood diabetes in Italy: the RIDI study. Diabetes. 2010; 59(9): 2281-7.
Bruno G, Merletti F, Biggeri A, Cerutti F, Grosso N, De Salvia A, Vitali E, Pagano G. Increasing trend of type I diabetes in children and young adults in the province of Turin (Italy). Analysis of age, period and birth cohort effects from 1984 to 1996. Diabetologia. 2001; 44(1): 22-5.
Brutus L, Schneider D, Postigo J, Delgado W, Mollinedo S, Chippaux J-P. Evidence of congenital transmission of Trypanosoma cruzi in a vector-free area of Bolivia. Trans R Soc Trop Med Hyg. 2007; 101(11): 1159–60.
Brutus L, Schneider D, Postigo J, Romero M, Santalla J, Chippaux JP. Congenital Chagas disease: diagnostic and clinical aspects in an area without vectorial transmission, Bermejo, Bolivia. Acta Trop. 2008; 106(3): 195-9.
Brutus L, Watier L, Briand V, Hanitrasoamampionana V, Razanatsoarilala H, Cot M. Parasitic co-infections: does Ascaris lumbricoides protect against Plasmodium falciparum infection? Am J Trop Med Hyg. 2006; 75(2): 194-8. As it appears in Malaria Atlas Project. Malaria Atlas Project Plasmodium Falciparum Parasite Rate Database. Oxford, United Kingdom: Malaria Atlas Project.
Brutus L, Watier L, Hanitrasoamampionana V, Razanatsoarilala H, Cot M. Confirmation of the protective effect of Ascaris lumbricoides on Plasmodium falciparum infection: results of a randomized trial in Madagascar. Am J Trop Med Hyg. 2007; 77(6): 1091-5. As it appears in Malaria Atlas Project. Malaria Atlas Project Plasmodium Falciparum Parasite Rate Database. Oxford, United Kingdom: Malaria Atlas Project.
Bruun C, Siersma V, Guassora AD, Holstein P, de Fine Olivarius N. Amputations and foot ulcers in patients newly diagnosed with type 2 diabetes mellitus and observed for 19 years. The role of age, gender and co-morbidity. Diabet Med. 2013; 30(8): 964-72.
Bsrat A, Berhe N, Balkew M, Yohannes M, Teklu T, Gadisa E, Medhin G, Abera A. Epidemiological study of cutaneous leishmaniasis in Saesie Tsaeda-emba district, eastern Tigray, northern Ethiopia. Parasit Vectors. 2015; 149.
Buch H, Vinding T, La Cour M, Appleyard M, Jensen GB, Nielsen NV. Prevalence and causes of visual impairment and blindness among 9980 Scandinavian adults: the Copenhagen City Eye Study. Ophthalmology. 2004; 111(1): 53-61.
Buch H, Vinding T, Nielsen NV. Prevalence and causes of visual impairment according to World Health Organization and United States criteria in an aged, urban Scandinavian population: the Copenhagen City Eye Study. Ophthalmology. 2001; 108(12): 2347-57.
Buchmueller TC, Johar M. Obesity and health expenditures: evidence from Australia. Econ Hum Biol. 2015; 17: 42â€“58.
Buchwald S, Kocher T, Biffar R, Harb A, Holtfreter B, Meisel P. Tooth loss and periodontitis by socio-economic status and inflammation in a longitudinal population-based study. J Clin Periodontol. 2013; 40(3): 203-11.
Buck AA, Anderson RI, MacRae AA. Epidemiology of poly-parasitism IV Combined effects on the state of health. Trop Med Parasitol. 1978; 29(3): 253-68.

Appendix: Citation List

Citation
Buck C, Reid DD. Cancer In Coking Plant Workers. Br J Ind Med. 1956; 13(4): 265-9 as it appears in Armstrong B, Hutchinson E, Unwin J, Fletcher T. Lung cancer risk after exposure to polycyclic aromatic hydrocarbons: a review and meta-analysis. Environ. Health Perspect. 2004; 112(9): 970–978.
Buckland G, Salas-SalvadÃ³ J, Roure E, BullÃ³ M, Serra-Majem L. Sociodemographic risk factors associated with metabolic syndrome in a Mediterranean population. Public Health Nutr. 2008; 11(12): 1372-8.
Buckland GG, Salas-SalvadÃ³ J, Serra-Majem L, Castell C, CabrÃ© J, Salleras-SanmartÃ- L. Increase in metabolic syndrome as defined by ATPIII from 1992-1993 to 2002-2003 in a Mediterranean population. Nutr Rev. 2009; S117-125.
Budge PJ, Dorkenoo AM, Sodahlon YK, Fasuyi OB, Mathieu E. Ongoing surveillance for lymphatic filariasis in Togo: assessment of alternatives and nationwide reassessment of transmission status. Am J Trop Med Hyg. 2014; 90.0(1): 89-95.
Bulgaria Cancer Registry 1998-2002 - CI5 as it appears in Curado MP, Edwards B, Shin HR, Storm H, Ferlay J, Heanue M and Boyle P, eds (2007). Cancer Incidence in Five Continents, Vol. IX Periodic Data (electronic version). Lyon, IARC.http://ci5.iarc.fr
Bulgaria Census 1965 as it appears in United Nations Statistics Division (UNSD). United Nations Demographic Yearbook. New York City, United States: United Nations Statistics Division (UNSD).
Bulgaria Census 1975 as it appears in United Nations Statistics Division (UNSD). United Nations Demographic Yearbook - Historical Supplement 1997. New York City, United States: United Nations Statistics Division (UNSD).
Bulgaria Vital Registration - Deaths 1965 ICD7 as it appears in World Health Organization (WHO). WHO Mortality Database Version July 2012. Geneva, Switzerland: World Health Organization (WHO), 2012.
Bulgaria Vital Registration - Deaths 1966 ICD7 as it appears in World Health Organization (WHO). WHO Mortality Database Version July 2012. Geneva, Switzerland: World Health Organization (WHO), 2012.
Bulgaria Vital Registration - Deaths 1967 ICD7 as it appears in World Health Organization (WHO). WHO Mortality Database Version July 2012. Geneva, Switzerland: World Health Organization (WHO), 2012.
Bulgaria Vital Registration - Deaths 1968 ICD8 as it appears in World Health Organization (WHO). WHO Mortality Database Version May 2013. Geneva, Switzerland: World Health Organization (WHO), 2013.
Bulgaria Vital Registration - Deaths 1969 ICD8 as it appears in World Health Organization (WHO). WHO Mortality Database Version May 2013. Geneva, Switzerland: World Health Organization (WHO), 2013.
Bulgaria Vital Registration - Deaths 1970 ICD8 as it appears in World Health Organization (WHO). WHO Mortality Database Version May 2013. Geneva, Switzerland: World Health Organization (WHO), 2013.
Bulgaria Vital Registration - Deaths 1971 ICD8 as it appears in World Health Organization (WHO). WHO Mortality Database Version May 2013. Geneva, Switzerland: World Health Organization (WHO), 2013.
Bulgaria Vital Registration - Deaths 1972 ICD8 as it appears in World Health Organization (WHO). WHO Mortality Database Version May 2013. Geneva, Switzerland: World Health Organization (WHO), 2013.
Bulgaria Vital Registration - Deaths 1973 ICD8 as it appears in World Health Organization (WHO). WHO Mortality Database Version May 2013. Geneva, Switzerland: World Health Organization (WHO), 2013.
Bulgaria Vital Registration - Deaths 1974 ICD8 as it appears in World Health Organization (WHO). WHO Mortality Database Version May 2013. Geneva, Switzerland: World Health Organization (WHO), 2013.
Bulgaria Vital Registration - Deaths 1975 ICD8 as it appears in World Health Organization (WHO). WHO Mortality Database Version May 2013. Geneva, Switzerland: World Health Organization (WHO), 2013.
Bulgaria Vital Registration - Deaths 1976 ICD8 as it appears in World Health Organization (WHO). WHO Mortality Database Version May 2013. Geneva, Switzerland: World Health Organization (WHO), 2013.
Bulgaria Vital Registration - Deaths 1977 ICD8 as it appears in World Health Organization (WHO). WHO Mortality Database Version May 2013. Geneva, Switzerland: World Health Organization (WHO), 2013.
Bulgaria Vital Registration - Deaths 1978 ICD8 as it appears in World Health Organization (WHO). WHO Mortality Database Version May 2013. Geneva, Switzerland: World Health Organization (WHO), 2013.
Bulgaria Vital Registration - Deaths 1979 ICD8 as it appears in World Health Organization (WHO). WHO Mortality Database Version May 2013. Geneva, Switzerland: World Health Organization (WHO), 2013.
Bulgaria Vital Registration - Deaths 1980 ICD9 as it appears in World Health Organization (WHO). WHO Mortality Database Version February 2014. Geneva, Switzerland: World Health Organization (WHO), 2014.
Bulgaria Vital Registration - Deaths 1981 ICD9 as it appears in World Health Organization (WHO). WHO Mortality Database Version February 2014. Geneva, Switzerland: World Health Organization (WHO), 2014.
Bulgaria Vital Registration - Deaths 1982 ICD9 as it appears in World Health Organization (WHO). WHO Mortality Database Version February 2014. Geneva, Switzerland: World Health Organization (WHO), 2014.
Bulgaria Vital Registration - Deaths 1983 ICD9 as it appears in World Health Organization (WHO). WHO Mortality Database Version February 2014. Geneva, Switzerland: World Health Organization (WHO), 2014.
Bulgaria Vital Registration - Deaths 1984 ICD9 as it appears in World Health Organization (WHO). WHO Mortality Database Version February 2014. Geneva, Switzerland: World Health Organization (WHO), 2014.
Bulgaria Vital Registration - Deaths 1985 ICD9 as it appears in World Health Organization (WHO). WHO Mortality Database Version February 2014. Geneva, Switzerland: World Health Organization (WHO), 2014.
Bulgaria Vital Registration - Deaths 1986 ICD9 as it appears in World Health Organization (WHO). WHO Mortality Database Version February 2014. Geneva, Switzerland: World Health Organization (WHO), 2014.
Bulgaria Vital Registration - Deaths 1987 ICD9 as it appears in World Health Organization (WHO). WHO Mortality Database Version February 2014. Geneva, Switzerland: World Health Organization (WHO), 2014.
Bulgaria Vital Registration - Deaths 1988 ICD9 as it appears in World Health Organization (WHO). WHO Mortality Database Version February 2014. Geneva, Switzerland: World Health Organization (WHO), 2014.

Appendix: Citation List

Citation
Bulgaria Vital Registration - Deaths 1989 ICD9 as it appears in World Health Organization (WHO). WHO Mortality Database Version February 2014. Geneva, Switzerland: World Health Organization (WHO), 2014.
Bulgaria Vital Registration - Deaths 1990 ICD9 as it appears in World Health Organization (WHO). WHO Mortality Database Version July 2012. Geneva, Switzerland: World Health Organization (WHO), 2012.
Bulgaria Vital Registration - Deaths 1991 ICD9 as it appears in World Health Organization (WHO). WHO Mortality Database Version July 2012. Geneva, Switzerland: World Health Organization (WHO), 2012.
Bulgaria Vital Registration - Deaths 1992 ICD9 as it appears in World Health Organization (WHO). WHO Mortality Database Version July 2012. Geneva, Switzerland: World Health Organization (WHO), 2012.
Bulgaria Vital Registration - Deaths 1993 ICD9 as it appears in World Health Organization (WHO). WHO Mortality Database Version July 2012. Geneva, Switzerland: World Health Organization (WHO), 2012.
Bulgaria Vital Registration - Deaths 1994 ICD9 as it appears in World Health Organization (WHO). WHO Mortality Database Version July 2012. Geneva, Switzerland: World Health Organization (WHO), 2012.
Bulgaria Vital Registration - Deaths 1995 ICD9 as it appears in World Health Organization (WHO). WHO Mortality Database Version July 2012. Geneva, Switzerland: World Health Organization (WHO), 2012.
Bulgaria Vital Registration - Deaths 1996 ICD9 as it appears in World Health Organization (WHO). WHO Mortality Database Version July 2012. Geneva, Switzerland: World Health Organization (WHO), 2012.
Bulgaria Vital Registration - Deaths 1997 ICD9 as it appears in World Health Organization (WHO). WHO Mortality Database Version July 2012. Geneva, Switzerland: World Health Organization (WHO), 2012.
Bulgaria Vital Registration - Deaths 1998 ICD9 as it appears in World Health Organization (WHO). WHO Mortality Database Version July 2012. Geneva, Switzerland: World Health Organization (WHO), 2012.
Bulgaria Vital Registration - Deaths 1999 ICD9 as it appears in World Health Organization (WHO). WHO Mortality Database Version July 2012. Geneva, Switzerland: World Health Organization (WHO), 2012.
Bulgaria Vital Registration - Deaths 2000 ICD9 as it appears in World Health Organization (WHO). WHO Mortality Database Version July 2012. Geneva, Switzerland: World Health Organization (WHO), 2012.
Bulgaria Vital Registration - Deaths 2001 ICD9 as it appears in World Health Organization (WHO). WHO Mortality Database Version July 2012. Geneva, Switzerland: World Health Organization (WHO), 2012.
Bulgaria Vital Registration - Deaths 2002 ICD9 as it appears in World Health Organization (WHO). WHO Mortality Database Version July 2012. Geneva, Switzerland: World Health Organization (WHO), 2012.
Bulgaria Vital Registration - Deaths 2003 ICD9 as it appears in World Health Organization (WHO). WHO Mortality Database Version July 2012. Geneva, Switzerland: World Health Organization (WHO), 2012.
Bulgaria Vital Registration - Deaths 2004 ICD9 as it appears in World Health Organization (WHO). WHO Mortality Database Version July 2012. Geneva, Switzerland: World Health Organization (WHO), 2012.
Bulgaria Vital Registration - Deaths 2005 ICD10 as it appears in World Health Organization (WHO). WHO Mortality Database Version November 2015. Geneva, Switzerland: World Health Organization (WHO), 2015.
Bulgaria Vital Registration - Deaths 2006 ICD10 as it appears in World Health Organization (WHO). WHO Mortality Database Version November 2015. Geneva, Switzerland: World Health Organization (WHO), 2015.
Bulgaria Vital Registration - Deaths 2007 ICD10 as it appears in World Health Organization (WHO). WHO Mortality Database Version November 2015. Geneva, Switzerland: World Health Organization (WHO), 2015.
Bulgaria Vital Registration - Deaths 2008 ICD10 as it appears in World Health Organization (WHO). WHO Mortality Database Version November 2015. Geneva, Switzerland: World Health Organization (WHO), 2015.
Bulgaria Vital Registration - Deaths 2009 ICD10 as it appears in World Health Organization (WHO). WHO Mortality Database Version November 2015. Geneva, Switzerland: World Health Organization (WHO), 2015.
Bulgaria Vital Registration - Deaths 2010 ICD10 as it appears in World Health Organization (WHO). WHO Mortality Database Version November 2015. Geneva, Switzerland: World Health Organization (WHO), 2015.
Bulgaria Vital Registration - Deaths 2011 ICD10 as it appears in World Health Organization (WHO). WHO Mortality Database Version November 2015. Geneva, Switzerland: World Health Organization (WHO), 2015.
Bulgaria Vital Registration - Deaths 2012 ICD10 as it appears in World Health Organization (WHO). WHO Mortality Database Version November 2015. Geneva, Switzerland: World Health Organization (WHO), 2015.
Bulgarian National Cancer Registry. Bulgaria Cancer Incidence Report 2004. Sofia, Bulgaria: "Avis 24" Ltd, 2007.
Bulgarian National Cancer Registry. Bulgaria Cancer Incidence Report 2005. Sofia, Bulgaria: "Avis 24" Ltd, 2008.
Bulgarian National Cancer Registry. Bulgaria Cancer Incidence Report 2006. Sofia, Bulgaria: "Avis 24" Ltd, 2008.
Bulgarian National Cancer Registry. Bulgaria Cancer Incidence Report 2007. Sofia, Bulgaria: "Avis 24" Ltd, 2009.
Bulgarian National Cancer Registry. Bulgaria Cancer Incidence Report 2008. Sofia, Bulgaria: "Avis 24" Ltd, 2010.
Bulgarian National Cancer Registry. Bulgaria Cancer Incidence Report 2009. Sofia, Bulgaria: "Avis 24" Ltd, 2011.
Bulgarian National Cancer Registry. Bulgaria Cancer Incidence Report 2010. Sofia, Bulgaria: "Avis 24" Ltd, 2012.
Burattini MN, Massad E, Coutinho FA, Baruzzi RG. Malaria prevalence amongst Brazilian Indians assessed by a new mathematical model. Epidemiol Infect. 1993; 111(3): 525-37. As it appears in Malaria Atlas Project. Malaria Atlas Project Plasmodium Falciparum Parasite Rate Database. Oxford, United Kingdom: Malaria Atlas Project.
Burazeri G, Roshi E, Jewkes R, Jordan S, Bjegovic V, Laaser U. Factors associated with spousal physical violence in Albania: cross sectional study. BMJ. 2005; 331(7510): 197-201.
Burchard L, Cáceres J, Sagua H, Inés Bahamonde M, Neira I, Araya J, Goycolea M. [Current human and canine seroprevalence of Chagasic infection in San Pedro de Atacama County, II Region of Antofagasta, Chile, 1995]. Bol Chil Parasitol. 1996; 51(3-4): 76–9.
Bureau of Health Promotion, Department of Health (Taiwan), Centers for Disease Control and Prevention (CDC), World Health Organization (WHO). Taiwan Global School-Based Student Health Survey 2012.

Appendix: Citation List

Citation
Bureau of Health Promotion, Department of Health (Taiwan). Taiwan Tobacco Control Annual Report 2009. New Taipei City, Taiwan: Bureau of Health Promotion, Department of Health (Taiwan), 2009.
Bureau of Labor Statistics (United States). United States National Longitudinal Survey of Youth 1997. Washington, D.C., United States: Bureau of Labor Statistics (United States).
Bureau of Policy and Strategy, Ministry of Public Health (Thailand). Thailand Health Profile 2005-2007. Bangkok, Thailand: Ministry of Public Health (Thailand).
Bureau of Policy and Strategy, Ministry of Public Health (Thailand). Thailand Public Health Statistics 2009. Bangkok, Thailand: Bureau of Policy and Strategy, Ministry of Public Health (Thailand).
Bureau of Policy and Strategy, Ministry of Public Health (Thailand). Thailand Public Health Statistics 2010. Bangkok, Thailand: Bureau of Policy and Strategy, Ministry of Public Health (Thailand).
Bureau of Policy and Strategy, Ministry of Public Health (Thailand). Thailand Public Health Statistics 2011. Bangkok, Thailand: Bureau of Policy and Strategy, Ministry of Public Health (Thailand).
Bureau of Statistics (Guyana), ICF Macro, Ministry of Health (Guyana). Guyana Demographic and Health Survey 2009. Calverton, United States: ICF Macro, 2011.
Bureau of Statistics (Guyana), International Statistical Institute. Guyana World Fertility Survey 1975. Voorburg, Netherlands: International Statistical Institute.
Bureau of Statistics (Guyana), Ministry of Health (Guyana), United Nations Children's Fund (UNICEF). Guyana Multiple Indicator Cluster Survey 2014.
Bureau of Statistics (Guyana), United Nations Children's Fund (UNICEF). Guyana Multiple Indicator Cluster Survey 2000. New York, United States: United Nations Children's Fund (UNICEF)
Bureau of Statistics (Guyana), World Bank. Guyana Living Standards Measurement Survey 1992-1993.
Bureau of Statistics (Guyana). Guyana Population and Housing Census 1991.
Bureau of Statistics (Guyana). Guyana Population and Housing Census 2002-2003 - CARICOM. Turkeyen, Guyana: Caribbean Community (CARICOM) Secretariat, 2009
Bureau of Statistics (Lesotho), ICF Macro, Ministry of Health and Social Welfare (Lesotho). Lesotho Demographic and Health Survey - Maternal Mortality Data.
Bureau of Statistics (Lesotho), International Statistical Institute. Lesotho World Fertility Survey 1977. Voorburg, Netherlands: International Statistical Institute.
Bureau of Statistics (Lesotho), Macro International, Inc, Ministry of Health and Social Welfare (Lesotho). Lesotho Demographic and Health Survey 2004-2005. Calverton, United States: Macro International, Inc.
Bureau of Statistics (Lesotho), Ministry of Health (Lesotho), United Nations Children's Fund (UNICEF). Lesotho Multiple Indicator Cluster Survey 1996.
Bureau of Statistics (Lesotho), United Nations Children's Fund (UNICEF). Lesotho Multiple Indicator Cluster Survey 2000. New York, United States: United Nations Children's Fund (UNICEF).
Bureau of Statistics (Lesotho), United Nations Population Fund (UNFPA). Lesotho Demographic Survey 2001.
Bureau of Statistics (Lesotho), World Bank. Lesotho Core Welfare Indicators Questionnaire 2002.
Bureau of Statistics (Lesotho). Lesotho Demographic Survey 2011.
Bureau of Statistics (Lesotho). Lesotho Population and Housing Census 1986.
Bureau of Statistics (Lesotho). Lesotho Population and Housing Census 1996.
Bureau of Statistics (Lesotho). Lesotho Population and Housing Census 2006.
Bureau of Statistics (Liberia), United Nations Children's Fund (UNICEF). Liberia Multiple Indicator Cluster Survey 1995.
Bureau of Statistics (Punjab), United Nations Children's Fund (UNICEF), United Nations Development Programme (UNDP). Pakistan - Punjab Multiple Indicator Cluster Survey 2011. New York, United States: United Nations Children's Fund (UNICEF), 2013.
Bureau of Statistics (Punjab), United Nations Children's Fund (UNICEF). Pakistan - Punjab Multiple Indicator Cluster Survey 2014. New York, United States: United Nations Children's Fund (UNICEF), 2015.
Bureau of Statistics (Tanzania), Macro International, Inc, Ministry of Health (Tanzania). Tanzania Demographic and Health Survey 1991-1992. Calverton, United States: Macro International, Inc.
Bureau of Statistics (Tanzania), Macro International, Inc, Planning Commission (Tanzania). Tanzania Demographic and Health Survey 1996. Calverton, United States: Macro International, Inc.
Bureau of Statistics (Tanzania), Macro International, Inc. Tanzania Demographic and Health Survey 1999. Calverton, United States: Macro International, Inc.
Bureau of Statistics (Tanzania), Minnesota Population Center. Tanzania Population Census 1988 from the Integrated Public Use Microdata Series, International: [Machine-readable database]. Minneapolis: University of Minnesota.
Bureau of Statistics (Tanzania), Oxford Policy Management. Tanzania Household Budget Survey 2000-2001. Dar es Salaam, Tanzania: Bureau of Statistics (Tanzania).
Bureau of Statistics (Tanzania). Tanzania Household Budget Survey 1991-1992. Dar es Salaam, Tanzania: Bureau of Statistics (Tanzania).
Burke JP, Williams K, Haffner SM, Villalpando CG, Stern MP. Elevated incidence of type 2 diabetes in San Antonio, Texas, compared with that of Mexico City, Mexico. Diabetes Care. 2001; 24(9): 1573-8.
Burke L, Suswardany DL, Michener K, Mazurki S, Adair T, Elmiyati C, Rao C. Utility of local health registers in measuring perinatal mortality: a case study in rural Indonesia. BMC Pregnancy Childbirth. 2011; 11: 20.
Burkina Faso Core Welfare Indicators Questionnaire Survey 2007 as it appears in United Nations Children's Fund (UNICEF). UNICEF Childinfo - Nutritional Status. New York, United States: United Nations Children's Fund (UNICEF).
Burkina Faso Demographic Survey 1991.
Burkina Faso EPI Review 2003.

Appendix: Citation List

Citation
Burkina Faso EPI Review 2009.
Burkina Faso General Population Census 1985 as it appears in United Nations Statistics Division (UNSD). United Nations Demographic Yearbook - Historical Supplement 1997. New York City, United States: United Nations Statistics Division (UNSD).
Burkina Faso Global Fund Household Survey 2008
Burkina Faso Human Development Report on the Private Sector 2007 as it appears in World Health Organization (WHO). WHO Household Energy Database. Geneva, Switzerland: World Health Organization (WHO), 2010.
Burkina Faso National Nutrition Survey 2009 as it appears in World Health Organization (WHO). WHO Global Database on Child Growth and Malnutrition. Geneva, Switzerland: World Health Organization (WHO).
Burkot TR, Garner P, Paru R, Dagoro H, Barnes A, McDougall S, Wirtz RA, Campbell G, Spark R. Effects of untreated bed nets on the transmission of Plasmodium falciparum, P. vivax and Wuchereria bancrofti in Papua New Guinea. Trans R Soc Trop Med Hyg. 1990; 84(6): 773-9. As it appears in Malaria Atlas Project. Malaria Atlas Project Plasmodium Falciparum Parasite Rate Database. Oxford, United Kingdom: Malaria Atlas Project.
Burns M. Base-line Malaria Prevalence Monitoring Results in Largo and Tobanda Refugee Camps, Sierra Leone. Sierra Leone, 2006. As it appears in Malaria Atlas Project. Malaria Atlas Project Plasmodium Falciparum Parasite Rate Database. Oxford, United Kingdom: Malaria Atlas Project.
Burns MR. Malaria: A Baseline Assessment for Somalia May-July 2002. A Report of a Short Term Consultancy Assignment for the World Health Organization, Eastern Mediterranean Regional Office, Roll Back Malaria, Complex Emergency Network Coordination Group. Geneva, Switzerland: World Health Organization; 2002. As it appears in Malaria Atlas Project. Malaria Atlas Project Plasmodium Falciparum Parasite Rate Database. Oxford, United Kingdom: Malaria Atlas Project.
Burundi - Bubanza Food Survey in North and Central Imbo 1985 as it appears in World Health Organization (WHO). WHO Global Database on Child Growth and Malnutrition - Historical. Geneva, Switzerland: World Health Organization (WHO).
Burundi - Bururi Nutritional Survey in Bututsi 1985 as it appears in World Health Organization (WHO). WHO Global Database on Child Growth and Malnutrition - Historical. Geneva, Switzerland: World Health Organization (WHO).
Burundi Immunization Coverage Survey for Children 12-23 Months 1989.
Burundi Institute of Statistics and Economic Studies, ICF International, Ministry of Public Health and the Fight against AIDS (Burundi). Burundi Demographic and Health Survey 2010-2011. Fairfax, United States: ICF International, 2012.
Burundi Institute of Statistics and Economic Studies, ICF Macro, Ministry of Public Health and the Fight against AIDS (Burundi), National Institute of Public Health (Burundi). Burundi Malaria Indicator Survey 2012-2013. Calverton, United States: ICF Macro, 2013.
Burundi Institute of Statistics and Economic Studies, United Nations Children's Fund (UNICEF). Burundi Multiple Indicator Cluster Survey 2000. New York, United States: United Nations Children's Fund (UNICEF).
Burundi Institute of Statistics and Economic Studies. Burundi Priority Survey 1998-1999.
Burundi National Nutrition Survey 2005 as it appears in World Health Organization (WHO). WHO Global Database on Child Growth and Malnutrition. Geneva, Switzerland: World Health Organization (WHO).
Burundi National Nutritional Surveillance Survey of Populations Subject to Food Aid 1995 as it appears in World Health Organization (WHO). WHO Global Database on Child Growth and Malnutrition - Historical. Geneva, Switzerland: World Health Organization (WHO).
Burundi Population and Housing Census 1990 as it appears in United Nations Statistics Division (UNSD). United Nations Demographic Yearbook - Historical Supplement 1997. New York City, United States: United Nations Statistics Division (UNSD).
Burundi Priority Survey 1998-1999 as it appears in World Health Organization (WHO). WHO Household Energy Database. Geneva, Switzerland: World Health Organization (WHO), 2010.
Burundi Routine Immunization Coverage Survey and Post Measles Campaign Survey 2012.
Burundi Survey on Indicators of Development 2002 as it appears in World Health Organization (WHO). WHO Household Energy Database. Geneva, Switzerland: World Health Organization (WHO), 2010.
Busselton Population Medical Research Institute (Australia). Australia - Busselton Health Study 1981.
Bustinduy AL, Parraga IM, Thomas C, Mungai PL, Mutuku F, Muchiri EM, Kitron U, King CH. Anemia, impaired growth and exercise intolerence in Kenyan children: the role of schistosomiasis and polyparasitism [Abstract]. In Abstract Book. American Society of Tropical Medicine and Hygiene 59th Annual Meeting; 2010 Nov 3-7; Atlanta, United States. Am J Trop Med Hyg. 2010; 83(Suppl 5): 318.
Bustinduy AL, Parraga IM, Thomas CL, Mungai PL, Mutuku F, Muchiri EM, Kitron U, King CH. Impact of polyparasitic infections on anemia and undernutrition among Kenyan children living in a Schistosoma haematobium-endemic area. Am J Trop Med Hyg. 2013; 88(3): 433-40. As it appears in Malaria Atlas Project. Malaria Atlas Project Plasmodium Falciparum Parasite Rate Database. Oxford, United Kingdom: Malaria Atlas Project.
Bustinduy AL, Parraga IM, Thomas CL, Mungai PL, Mutuku F, Muchiri EM, Kitron U, King CH. Impact of polyparasitic infections on anemia and undernutrition among Kenyan children living in a Schistosoma haematobium-endemic area. Am J Trop Med Hyg. 2013; 88(3): 433-40.
Bustos MD, Saul A, Salazar NP, Gomes M. Profile of Morong, Bataan, an area of low malaria endemicity in the Philippines. Acta Trop. 1997; 63(4): 195-207. As it appears in Malaria Atlas Project. Malaria Atlas Project Plasmodium Falciparum Parasite Rate Database. Oxford, United Kingdom: Malaria Atlas Project.
Bustos P, da Silva AAM, Amigo H, Bettiol H, Barbieri MA. Metabolic syndrome in young adults from two socioeconomic Latin American settings. Nutr Metab Cardiovasc Dis. 2007; 17(8): 581-9.
Bwonya JE. Evaluation of Malaria Control in Kisumu Municipality, Kenya: A Case Study [Master's thesis]. Nairobi, Kenya: University of Nairobi, 1987. As it appears in Malaria Atlas Project. Malaria Atlas Project Plasmodium Falciparum Parasite Rate Database. Oxford, United Kingdom: Malaria Atlas Project.
Byanju R, Gupta R. Lymphatic Filariasis: Epidemiological Analysis of the Situation in Salyantar VDC of Dhading District, Nepal. J Nat Hist Mus. 2012; 136-45. As it appears in London School of Hygiene and Tropical Medicine. Global Atlas of Helminth Infections - Lymphatic Filiariasis.

Appendix: Citation List

Citation
Byaruhanga RN. Improving healthcare by perinatal mortality audit and feedback. Trop Doct. 2000; 30(2): 94-7.
Bye T, Romundstad PR, Ronneberg A, Hilt B. Health Survey Of Former Workers In A Norwegian Coke Plant: Part 2. Cancer Incidence And Cause Specific Mortality. Occup Environ Med. 1998; 55(9): 622-6 as it appears in Armstrong B, Hutchinson E, Unwin J, Fletcher T. Lung cancer risk after exposure to polycyclic aromatic hydrocarbons: a review and meta-analysis. Environ. Health Perspect. 2004; 112(9): 970–978.
Byrraju Foundation, Centre for Chronic Disease Control (India), Cooperative for Assistance and Relief Everywhere (CARE), School of Population Health, University of Queensland (Australia). India - Andhra Pradesh Rural Health Initiative Mortality Surveillance 2003-2007.
C Manes, N Papazoglou, E Sossidou, K Soulis, D Milarakis, A Satsoglou, A Sakallerou. Prevalence of diabetic neuropathy and foot ulceration: identification of potential risk factors – a population-based study. Wounds. 2002; 14(Pt 1): 11-15.
CÃ´te d'Ivoire Living Standards Measurement Survey 1985-1986 as it appears in World Health Organization (WHO). WHO Household Energy Database. Geneva, Switzerland: World Health Organization (WHO), 2010.
CÃ´te d'Ivoire Living Standards Measurement Survey 1986-1987 as it appears in World Health Organization (WHO). WHO Household Energy Database. Geneva, Switzerland: World Health Organization (WHO), 2010.
CÃ´te d'Ivoire Living Standards Measurement Survey 1987-1988 as it appears in World Health Organization (WHO). WHO Household Energy Database. Geneva, Switzerland: World Health Organization (WHO), 2010.
CÃ´te d'Ivoire Living Standards Measurement Survey 1988-1989 as it appears in World Health Organization (WHO). WHO Household Energy Database. Geneva, Switzerland: World Health Organization (WHO), 2010.
Cabezas-Cerrato J. The prevalence of clinical diabetic polyneuropathy in Spain: a study in primary care and hospital clinic groups. Neuropathy Spanish Study Group of the Spanish Diabetes Society (SDS). Diabetologia. 1998; 41(11): 1263-9.
Cai L, Liu A, Zhang L, Li S, Wang P. Prevalence, awareness, treatment, and control of hypertension among adults in Beijing, China. Clin Exp Hypertens. 2012; 34(1): 45–52.
Calderón-Garcidueñas AL, Martínez-Salazar G, Fernández-Díaz H, Cerda-Flores RM. [Hospital maternal mortality: causes and consistency between clinical and autopsy diagnosis at the Northeastern Medical Center of the IMSS, Mexico]. Ginecol Obstet Mex. 2002; 70: 95-102.
California Center for Population Research (CCPR), University of California Los Angeles (UCLA), Center for Research and Teaching in Economics (CIDE) (Mexico), National Institute of Public Health (Mexico), Universidad Iberoamericana. Mexico Family Life Survey 2005-2006.
Calis JCJ, Phiri KS, Faragher EB, Brabin BJ, Bates I, Cuevas LE, de Haan RJ, Phiri AI, Malange P, Khoka M, Hulshof PJM, van Lieshout L, Beld MGHM, Teo YY, Rockett KA, Richardson A, Kwiatkowski DP, Molyneux ME, van Hensbroek MB. Severe Anemia in Malawian Children. N Engl J Med. 2008; 358(9): 888-99. As it appears in Malaria Atlas Project. Malaria Atlas Project Plasmodium Falciparum Parasite Rate Database. Oxford, United Kingdom: Malaria Atlas Project.
Callegari-Jacques SM, Salzano FM, Weimer TA, Franco MH, Mestriner MA, Hutz MH, Schüler L. The Wai Wai Indians of South America: history and genetics. Ann Hum Biol. 1996; 23(3): 189-201. As it appears in Malaria Atlas Project. Malaria Atlas Project Plasmodium Falciparum Parasite Rate Database. Oxford, United Kingdom: Malaria Atlas Project.
Calma CL, Neghina AM, Moldovan R, Dumitrascu V, Marincu I, Neghina R. Cystic echinococcosis in Arad County, Romania. Vector Borne Zoonotic Dis. 2012; 12(4): 333-5.
Calma CL, Neghina AM, Vlaicu B, Neghina R. Cystic echinococcosis in the human population of a western Romanian county, 2004-2010. Clin Microbiol Infect. 2011; 17(11): 1731-4.
Calori G, Gallus G, Garancini P, Repetto F, Micossi P. Identification of the cohort of type 1 diabetes presenting in Lombardy in 1983-84: a validated assessment. Diabet Med. 1990; 7(7): 595-9.
Calzada JE, Pineda V, Garisto JD, Samudio F, Santamaria AM, Saldaña A. Human trypanosomiasis in the eastern region of the Panama Province: new endemic areas for Chagas disease. Am J Trop Med Hyg. 2010; 82(4): 580-2.
Camargo LM, dal Colletto GM, Ferreira MU, Gurgel S de M, Escobar AL, Marques A, Krieger H, Camargo EP, da Silva LH. Hypoendemic malaria in Rondonia (Brazil, western Amazon region): seasonal variation and risk groups in an urban locality. Am J Trop Med Hyg. 1996; 55(1): 32-8. As it appears in Malaria Atlas Project. Malaria Atlas Project Plasmodium Falciparum Parasite Rate Database. Oxford, United Kingdom: Malaria Atlas Project.
Camargo LM, Ferreira MU, Krieger H, De Camargo EP, Da Silva LP. Unstable hypoendemic malaria in Rondonia (western Amazon region, Brazil): epidemic outbreaks and work-associated incidence in an agro-industrial rural settlement. Am J Trop Med Hyg. 1994; 51(1): 16-25. As it appears in Malaria Atlas Project. Malaria Atlas Project Plasmodium Falciparum Parasite Rate Database. Oxford, United Kingdom: Malaria Atlas Project.
Camargo LM, Noronha E, Salcedo JM, Dutra AP, Krieger H, Pereira da Silva LH, Camargo EP. The epidemiology of malaria in Rondonia (Western Amazon region, Brazil): study of a riverine population. Acta Trop. 1999; 72(1): 1-11. As it appears in Malaria Atlas Project. Malaria Atlas Project Plasmodium Falciparum Parasite Rate Database. Oxford, United Kingdom: Malaria Atlas Project.
Cambodia Anthropometric Survey 2008 - National Institute of Statistics as it appears in World Health Organization (WHO). WHO Global Database on Child Growth and Malnutrition. Geneva, Switzerland: World Health Organization (WHO).
Cambodia National Micronutrient Survey 2000 as it appears in World Health Organization (WHO). WHO Global Database on Child Growth and Malnutrition - Historical. Geneva, Switzerland: World Health Organization (WHO).
Cambodia Nutritional Status Survey in 12 Villages 1993-1994 as it appears in World Health Organization (WHO). WHO Global Database on Child Growth and Malnutrition - Historical. Geneva, Switzerland: World Health Organization (WHO).
Cambodia Vaccine Coverage Survey of Children 12-23 Months of Age 1995.
Cameroon Household Survey 2001 as it appears in World Health Organization (WHO). WHO Household Energy Database. Geneva, Switzerland: World Health Organization (WHO), 2010.
Cameroon Household Survey 2004 as it appears in World Health Organization (WHO). WHO Household Energy Database. Geneva, Switzerland: World Health Organization (WHO), 2010.
Cameroon Immunization Coverage Survey November-December 1990.
Cameroon National Evaluation of Immunization Coverage among Children Aged 12 to 23 Months 2005.

Appendix: Citation List

Citation
Cammarano G, Crosignani P, Berrino H, Berra G. Additional Follow-Up Of Cancer Mortality Among Workers In A Thermoelectric Power Plant. Scand J Work Environ Health. 1986; 12(6): 631-2 as it appears in Armstrong B, Hutchinson E, Unwin J, Fletcher T. Lung cancer risk after exposure to polycyclic aromatic hydrocarbons: a review and meta-analysis. Environ. Health Perspect. 2004; 112(9): 970–978.
Campbell GH, Collins FH, Brandling-Bennett AD, Schwartz IK, Roberts JM. Age-specific prevalence of antibody to a synthetic peptide of the circumsporozoite protein of Plasmodium falciparum in children from three villages in Kenya. Am J Trop Med Hyg. 1987; 37(2): 220-4. As it appears in Malaria Atlas Project. Malaria Atlas Project Plasmodium Falciparum Parasite Rate Database. Oxford, United Kingdom: Malaria Atlas Project.
Campbell O, Gipson R, Issa AH, Matta N, El Deeb B, El Mohandes A, Alwen A, Mansour E. National maternal mortality ratio in Egypt halved between 1992-93 and 2000. Bull World Health Organ. 2005; 83(6): 462-71.
Campos H, Mata L, Siles X, Vives M, Ordovas JM, Schaefer EJ. Prevalence of cardiovascular risk factors in rural and urban Costa Rica. Circulation. 1992; 85(2): 648-58.
Canada - Alberta Cancer Registry 1978-1982 - CI5 as it appears in Parkin DM, International Agency for Research on Cancer, International Association of Cancer Registries. Cancer Incidence in Five Continents. Vol. I to VIII. Lyon, France, IARCPress, 2005.
Canada - Alberta Cancer Registry 1981 - CI5 as it appears in Ferlay J, Parkin DM, Curado MP, Bray F, Edwards B, Shin HR and Forman D. Cancer Incidence in Five Continents, Volumes I to IX: IARC CancerBase No. 9 [Internet]. Lyon, France: International Agency for Research on Cancer; 2010. Available from: http://ci5.iarc.fr
Canada - Alberta Cancer Registry 1982 - CI5 as it appears in Ferlay J, Parkin DM, Curado MP, Bray F, Edwards B, Shin HR and Forman D. Cancer Incidence in Five Continents, Volumes I to IX: IARC CancerBase No. 9 [Internet]. Lyon, France: International Agency for Research on Cancer; 2010. Available from: http://ci5.iarc.fr
Canada - Alberta Cancer Registry 1983 - CI5 as it appears in Ferlay J, Parkin DM, Curado MP, Bray F, Edwards B, Shin HR and Forman D. Cancer Incidence in Five Continents, Volumes I to IX: IARC CancerBase No. 9 [Internet]. Lyon, France: International Agency for Research on Cancer; 2010. Available from: http://ci5.iarc.fr
Canada - Alberta Cancer Registry 1984 - CI5 as it appears in Ferlay J, Parkin DM, Curado MP, Bray F, Edwards B, Shin HR and Forman D. Cancer Incidence in Five Continents, Volumes I to IX: IARC CancerBase No. 9 [Internet]. Lyon, France: International Agency for Research on Cancer; 2010. Available from: http://ci5.iarc.fr
Canada - Alberta Cancer Registry 1986 - CI5 as it appears in Ferlay J, Parkin DM, Curado MP, Bray F, Edwards B, Shin HR and Forman D. Cancer Incidence in Five Continents, Volumes I to IX: IARC CancerBase No. 9 [Internet]. Lyon, France: International Agency for Research on Cancer; 2010. Available from: http://ci5.iarc.fr
Canada - Alberta Cancer Registry 1988 - CI5 as it appears in Ferlay J, Parkin DM, Curado MP, Bray F, Edwards B, Shin HR and Forman D. Cancer Incidence in Five Continents, Volumes I to IX: IARC CancerBase No. 9 [Internet]. Lyon, France: International Agency for Research on Cancer; 2010. Available from: http://ci5.iarc.fr
Canada - Alberta Cancer Registry 1988-1992 - CI5 as it appears in Parkin DM, International Agency for Research on Cancer, International Association of Cancer Registries. Cancer Incidence in Five Continents. Vol. I to VIII. Lyon, France, IARCPress, 2005.
Canada - Alberta Cancer Registry 1991 - CI5 as it appears in Ferlay J, Parkin DM, Curado MP, Bray F, Edwards B, Shin HR and Forman D. Cancer Incidence in Five Continents, Volumes I to IX: IARC CancerBase No. 9 [Internet]. Lyon, France: International Agency for Research on Cancer; 2010. Available from: http://ci5.iarc.fr
Canada - Alberta Cancer Registry 1992 - CI5 as it appears in Ferlay J, Parkin DM, Curado MP, Bray F, Edwards B, Shin HR and Forman D. Cancer Incidence in Five Continents, Volumes I to IX: IARC CancerBase No. 9 [Internet]. Lyon, France: International Agency for Research on Cancer; 2010. Available from: http://ci5.iarc.fr
Canada - Alberta Cancer Registry 1993 - CI5 as it appears in Ferlay J, Parkin DM, Curado MP, Bray F, Edwards B, Shin HR and Forman D. Cancer Incidence in Five Continents, Volumes I to IX: IARC CancerBase No. 9 [Internet]. Lyon, France: International Agency for Research on Cancer; 2010. Available from: http://ci5.iarc.fr
Canada - Alberta Cancer Registry 1996 - CI5 as it appears in Ferlay J, Parkin DM, Curado MP, Bray F, Edwards B, Shin HR and Forman D. Cancer Incidence in Five Continents, Volumes I to IX: IARC CancerBase No. 9 [Internet]. Lyon, France: International Agency for Research on Cancer; 2010. Available from: http://ci5.iarc.fr
Canada - Alberta Cancer Registry 1997 - CI5 as it appears in Ferlay J, Parkin DM, Curado MP, Bray F, Edwards B, Shin HR and Forman D. Cancer Incidence in Five Continents, Volumes I to IX: IARC CancerBase No. 9 [Internet]. Lyon, France: International Agency for Research on Cancer; 2010. Available from: http://ci5.iarc.fr
Canada - Alberta Cancer Registry 1998 - CI5 as it appears in Ferlay J, Parkin DM, Curado MP, Bray F, Edwards B, Shin HR and Forman D. Cancer Incidence in Five Continents, Volumes I to IX: IARC CancerBase No. 9 [Internet]. Lyon, France: International Agency for Research on Cancer; 2010. Available from: http://ci5.iarc.fr
Canada - Alberta Cancer Registry 2001 - CI5 as it appears in Ferlay J, Parkin DM, Curado MP, Bray F, Edwards B, Shin HR and Forman D. Cancer Incidence in Five Continents, Volumes I to IX: IARC CancerBase No. 9 [Internet]. Lyon, France: International Agency for Research on Cancer; 2010. Available from: http://ci5.iarc.fr
Canada - Alberta Cancer Registry 2002 - CI5 as it appears in Ferlay J, Parkin DM, Curado MP, Bray F, Edwards B, Shin HR and Forman D. Cancer Incidence in Five Continents, Volumes I to IX: IARC CancerBase No. 9 [Internet]. Lyon, France: International Agency for Research on Cancer; 2010. Available from: http://ci5.iarc.fr
Canada - British Columbia Cancer Registry 1978-1982 - CI5 as it appears in Parkin DM, International Agency for Research on Cancer, International Association of Cancer Registries. Cancer Incidence in Five Continents. Vol. I to VIII. Lyon, France, IARCPress, 2005.
Canada - British Columbia Cancer Registry 1981 - CI5 as it appears in Ferlay J, Parkin DM, Curado MP, Bray F, Edwards B, Shin HR and Forman D. Cancer Incidence in Five Continents, Volumes I to IX: IARC CancerBase No. 9 [Internet]. Lyon, France: International Agency for Research on Cancer; 2010. Available from: http://ci5.iarc.fr

Appendix: Citation List

Citation
Canada - British Columbia Cancer Registry 1982 - CI5 as it appears in Ferlay J, Parkin DM, Curado MP, Bray F, Edwards B, Shin HR and Forman D. Cancer Incidence in Five Continents, Volumes I to IX: IARC CancerBase No. 9 [Internet]. Lyon, France: International Agency for Research on Cancer; 2010. Available from: http://ci5.iarc.fr
Canada - British Columbia Cancer Registry 1983 - CI5 as it appears in Ferlay J, Parkin DM, Curado MP, Bray F, Edwards B, Shin HR and Forman D. Cancer Incidence in Five Continents, Volumes I to IX: IARC CancerBase No. 9 [Internet]. Lyon, France: International Agency for Research on Cancer; 2010. Available from: http://ci5.iarc.fr
Canada - British Columbia Cancer Registry 1983-1987 - CI5 as it appears in Parkin DM, International Agency for Research on Cancer, International Association of Cancer Registries. Cancer Incidence in Five Continents. Vol. I to VIII. Lyon, France, IARCPress, 2005.
Canada - British Columbia Cancer Registry 1986 - CI5 as it appears in Ferlay J, Parkin DM, Curado MP, Bray F, Edwards B, Shin HR and Forman D. Cancer Incidence in Five Continents, Volumes I to IX: IARC CancerBase No. 9 [Internet]. Lyon, France: International Agency for Research on Cancer; 2010. Available from: http://ci5.iarc.fr
Canada - British Columbia Cancer Registry 1987 - CI5 as it appears in Ferlay J, Parkin DM, Curado MP, Bray F, Edwards B, Shin HR and Forman D. Cancer Incidence in Five Continents, Volumes I to IX: IARC CancerBase No. 9 [Internet]. Lyon, France: International Agency for Research on Cancer; 2010. Available from: http://ci5.iarc.fr
Canada - British Columbia Cancer Registry 1988 - CI5 as it appears in Ferlay J, Parkin DM, Curado MP, Bray F, Edwards B, Shin HR and Forman D. Cancer Incidence in Five Continents, Volumes I to IX: IARC CancerBase No. 9 [Internet]. Lyon, France: International Agency for Research on Cancer; 2010. Available from: http://ci5.iarc.fr
Canada - British Columbia Cancer Registry 1988-1992 - CI5 as it appears in Parkin DM, International Agency for Research on Cancer, International Association of Cancer Registries. Cancer Incidence in Five Continents. Vol. I to VIII. Lyon, France, IARCPress, 2005.
Canada - British Columbia Cancer Registry 1991 - CI5 as it appears in Ferlay J, Parkin DM, Curado MP, Bray F, Edwards B, Shin HR and Forman D. Cancer Incidence in Five Continents, Volumes I to IX: IARC CancerBase No. 9 [Internet]. Lyon, France: International Agency for Research on Cancer; 2010. Available from: http://ci5.iarc.fr
Canada - British Columbia Cancer Registry 1992 - CI5 as it appears in Ferlay J, Parkin DM, Curado MP, Bray F, Edwards B, Shin HR and Forman D. Cancer Incidence in Five Continents, Volumes I to IX: IARC CancerBase No. 9 [Internet]. Lyon, France: International Agency for Research on Cancer; 2010. Available from: http://ci5.iarc.fr
Canada - British Columbia Cancer Registry 1993 - CI5 as it appears in Ferlay J, Parkin DM, Curado MP, Bray F, Edwards B, Shin HR and Forman D. Cancer Incidence in Five Continents, Volumes I to IX: IARC CancerBase No. 9 [Internet]. Lyon, France: International Agency for Research on Cancer; 2010. Available from: http://ci5.iarc.fr
Canada - British Columbia Cancer Registry 1993-1997 - CI5 as it appears in Parkin DM, International Agency for Research on Cancer, International Association of Cancer Registries. Cancer Incidence in Five Continents. Vol. I to VIII. Lyon, France, IARCPress, 2005.
Canada - British Columbia Cancer Registry 1996 - CI5 as it appears in Ferlay J, Parkin DM, Curado MP, Bray F, Edwards B, Shin HR and Forman D. Cancer Incidence in Five Continents, Volumes I to IX: IARC CancerBase No. 9 [Internet]. Lyon, France: International Agency for Research on Cancer; 2010. Available from: http://ci5.iarc.fr
Canada - British Columbia Cancer Registry 1997 - CI5 as it appears in Ferlay J, Parkin DM, Curado MP, Bray F, Edwards B, Shin HR and Forman D. Cancer Incidence in Five Continents, Volumes I to IX: IARC CancerBase No. 9 [Internet]. Lyon, France: International Agency for Research on Cancer; 2010. Available from: http://ci5.iarc.fr
Canada - British Columbia Cancer Registry 1998 - CI5 as it appears in Ferlay J, Parkin DM, Curado MP, Bray F, Edwards B, Shin HR and Forman D. Cancer Incidence in Five Continents, Volumes I to IX: IARC CancerBase No. 9 [Internet]. Lyon, France: International Agency for Research on Cancer; 2010. Available from: http://ci5.iarc.fr
Canada - British Columbia Cancer Registry 2001 - CI5 as it appears in Ferlay J, Parkin DM, Curado MP, Bray F, Edwards B, Shin HR and Forman D. Cancer Incidence in Five Continents, Volumes I to IX: IARC CancerBase No. 9 [Internet]. Lyon, France: International Agency for Research on Cancer; 2010. Available from: http://ci5.iarc.fr
Canada - British Columbia Cancer Registry 2002 - CI5 as it appears in Ferlay J, Parkin DM, Curado MP, Bray F, Edwards B, Shin HR and Forman D. Cancer Incidence in Five Continents, Volumes I to IX: IARC CancerBase No. 9 [Internet]. Lyon, France: International Agency for Research on Cancer; 2010. Available from: http://ci5.iarc.fr
Canada - Manitoba Cancer Registry 1978-1982 - CI5 as it appears in Parkin DM, International Agency for Research on Cancer, International Association of Cancer Registries. Cancer Incidence in Five Continents. Vol. I to VIII. Lyon, France, IARCPress, 2005.
Canada - Manitoba Cancer Registry 1981 - CI5 as it appears in Ferlay J, Parkin DM, Curado MP, Bray F, Edwards B, Shin HR and Forman D. Cancer Incidence in Five Continents, Volumes I to IX: IARC CancerBase No. 9 [Internet]. Lyon, France: International Agency for Research on Cancer; 2010. Available from: http://ci5.iarc.fr
Canada - Manitoba Cancer Registry 1982 - CI5 as it appears in Ferlay J, Parkin DM, Curado MP, Bray F, Edwards B, Shin HR and Forman D. Cancer Incidence in Five Continents, Volumes I to IX: IARC CancerBase No. 9 [Internet]. Lyon, France: International Agency for Research on Cancer; 2010. Available from: http://ci5.iarc.fr
Canada - Manitoba Cancer Registry 1983-1987 - CI5 as it appears in Parkin DM, International Agency for Research on Cancer, International Association of Cancer Registries. Cancer Incidence in Five Continents. Vol. I to VIII. Lyon, France, IARCPress, 2005.
Canada - Manitoba Cancer Registry 1983-1992 - CI5 as it appears in Ferlay J, Parkin DM, Curado MP, Bray F, Edwards B, Shin HR and Forman D. Cancer Incidence in Five Continents, Volumes I to IX: IARC CancerBase No. 9 [Internet]. Lyon, France: International Agency for Research on Cancer; 2010. Available from: http://ci5.iarc.fr
Canada - Manitoba Cancer Registry 1986 - CI5 as it appears in Ferlay J, Parkin DM, Curado MP, Bray F, Edwards B, Shin HR and Forman D. Cancer Incidence in Five Continents, Volumes I to IX: IARC CancerBase No. 9 [Internet]. Lyon, France: International Agency for Research on Cancer; 2010. Available from: http://ci5.iarc.fr
Canada - Manitoba Cancer Registry 1987 - CI5 as it appears in Ferlay J, Parkin DM, Curado MP, Bray F, Edwards B, Shin HR and Forman D. Cancer Incidence in Five Continents, Volumes I to IX: IARC CancerBase No. 9 [Internet]. Lyon, France: International Agency for Research on Cancer; 2010. Available from: http://ci5.iarc.fr

Appendix: Citation List

Citation
Canada - Manitoba Cancer Registry 1988-1992 - CI5 as it appears in Parkin DM, International Agency for Research on Cancer, International Association of Cancer Registries. Cancer Incidence in Five Continents. Vol. I to VIII. Lyon, France, IARCPress, 2005.
Canada - Manitoba Cancer Registry 1989 - CI5 as it appears in Ferlay J, Parkin DM, Curado MP, Bray F, Edwards B, Shin HR and Forman D. Cancer Incidence in Five Continents, Volumes I to IX: IARC CancerBase No. 9 [Internet]. Lyon, France: International Agency for Research on Cancer; 2010. Available from: http://ci5.iarc.fr
Canada - Manitoba Cancer Registry 1991 - CI5 as it appears in Ferlay J, Parkin DM, Curado MP, Bray F, Edwards B, Shin HR and Forman D. Cancer Incidence in Five Continents, Volumes I to IX: IARC CancerBase No. 9 [Internet]. Lyon, France: International Agency for Research on Cancer; 2010. Available from: http://ci5.iarc.fr
Canada - Manitoba Cancer Registry 1992 - CI5 as it appears in Ferlay J, Parkin DM, Curado MP, Bray F, Edwards B, Shin HR and Forman D. Cancer Incidence in Five Continents, Volumes I to IX: IARC CancerBase No. 9 [Internet]. Lyon, France: International Agency for Research on Cancer; 2010. Available from: http://ci5.iarc.fr
Canada - Manitoba Cancer Registry 1993-1997 - CI5 as it appears in Parkin DM, International Agency for Research on Cancer, International Association of Cancer Registries. Cancer Incidence in Five Continents. Vol. I to VIII. Lyon, France, IARCPress, 2005.
Canada - Manitoba Cancer Registry 1994 - CI5 as it appears in Ferlay J, Parkin DM, Curado MP, Bray F, Edwards B, Shin HR and Forman D. Cancer Incidence in Five Continents, Volumes I to IX: IARC CancerBase No. 9 [Internet]. Lyon, France: International Agency for Research on Cancer; 2010. Available from: http://ci5.iarc.fr
Canada - Manitoba Cancer Registry 1996 - CI5 as it appears in Ferlay J, Parkin DM, Curado MP, Bray F, Edwards B, Shin HR and Forman D. Cancer Incidence in Five Continents, Volumes I to IX: IARC CancerBase No. 9 [Internet]. Lyon, France: International Agency for Research on Cancer; 2010. Available from: http://ci5.iarc.fr
Canada - Manitoba Cancer Registry 1997 - CI5 as it appears in Ferlay J, Parkin DM, Curado MP, Bray F, Edwards B, Shin HR and Forman D. Cancer Incidence in Five Continents, Volumes I to IX: IARC CancerBase No. 9 [Internet]. Lyon, France: International Agency for Research on Cancer; 2010. Available from: http://ci5.iarc.fr
Canada - Manitoba Cancer Registry 1998-2002 - CI5 as it appears in Curado MP, Edwards B, Shin HR, Storm H, Ferlay J, Heanue M and Boyle P, eds (2007). Cancer Incidence in Five Continents, Vol. IX Periodic Data (electronic version). Lyon, IARC.http://ci5.iarc.fr
Canada - Manitoba Cancer Registry 1999 - CI5 as it appears in Ferlay J, Parkin DM, Curado MP, Bray F, Edwards B, Shin HR and Forman D. Cancer Incidence in Five Continents, Volumes I to IX: IARC CancerBase No. 9 [Internet]. Lyon, France: International Agency for Research on Cancer; 2010. Available from: http://ci5.iarc.fr
Canada - Manitoba Cancer Registry 2001 - CI5 as it appears in Ferlay J, Parkin DM, Curado MP, Bray F, Edwards B, Shin HR and Forman D. Cancer Incidence in Five Continents, Volumes I to IX: IARC CancerBase No. 9 [Internet]. Lyon, France: International Agency for Research on Cancer; 2010. Available from: http://ci5.iarc.fr
Canada - Manitoba Cancer Registry 2002 - CI5 as it appears in Ferlay J, Parkin DM, Curado MP, Bray F, Edwards B, Shin HR and Forman D. Cancer Incidence in Five Continents, Volumes I to IX: IARC CancerBase No. 9 [Internet]. Lyon, France: International Agency for Research on Cancer; 2010. Available from: http://ci5.iarc.fr
Canada - Maritime Provinces Cancer Registry 1978-1982 - CI5 as it appears in Parkin DM, International Agency for Research on Cancer, International Association of Cancer Registries. Cancer Incidence in Five Continents. Vol. I to VIII. Lyon, France, IARCPress, 2005.
Canada - Maritime Provinces Cancer Registry 1983-1987 - CI5 as it appears in Parkin DM, International Agency for Research on Cancer, International Association of Cancer Registries. Cancer Incidence in Five Continents. Vol. I to VIII. Lyon, France, IARCPress, 2005.
Canada - New Brunswick Cancer Registry 1978-1982 - CI5 as it appears in Parkin DM, International Agency for Research on Cancer, International Association of Cancer Registries. Cancer Incidence in Five Continents. Vol. I to VIII. Lyon, France, IARCPress, 2005.
Canada - New Brunswick Cancer Registry 1981 - CI5 as it appears in Ferlay J, Parkin DM, Curado MP, Bray F, Edwards B, Shin HR and Forman D. Cancer Incidence in Five Continents, Volumes I to IX: IARC CancerBase No. 9 [Internet]. Lyon, France: International Agency for Research on Cancer; 2010. Available from: http://ci5.iarc.fr
Canada - New Brunswick Cancer Registry 1982 - CI5 as it appears in Ferlay J, Parkin DM, Curado MP, Bray F, Edwards B, Shin HR and Forman D. Cancer Incidence in Five Continents, Volumes I to IX: IARC CancerBase No. 9 [Internet]. Lyon, France: International Agency for Research on Cancer; 2010. Available from: http://ci5.iarc.fr
Canada - New Brunswick Cancer Registry 1983-1986 - CI5 as it appears in Parkin DM, International Agency for Research on Cancer, International Association of Cancer Registries. Cancer Incidence in Five Continents. Vol. I to VIII. Lyon, France, IARCPress, 2005.
Canada - New Brunswick Cancer Registry 1983-1987 - CI5 as it appears in Ferlay J, Parkin DM, Curado MP, Bray F, Edwards B, Shin HR and Forman D. Cancer Incidence in Five Continents, Volumes I to IX: IARC CancerBase No. 9 [Internet]. Lyon, France: International Agency for Research on Cancer; 2010. Available from: http://ci5.iarc.fr
Canada - New Brunswick Cancer Registry 1984 - CI5 as it appears in Ferlay J, Parkin DM, Curado MP, Bray F, Edwards B, Shin HR and Forman D. Cancer Incidence in Five Continents, Volumes I to IX: IARC CancerBase No. 9 [Internet]. Lyon, France: International Agency for Research on Cancer; 2010. Available from: http://ci5.iarc.fr
Canada - New Brunswick Cancer Registry 1987 - CI5 as it appears in Ferlay J, Parkin DM, Curado MP, Bray F, Edwards B, Shin HR and Forman D. Cancer Incidence in Five Continents, Volumes I to IX: IARC CancerBase No. 9 [Internet]. Lyon, France: International Agency for Research on Cancer; 2010. Available from: http://ci5.iarc.fr
Canada - New Brunswick Cancer Registry 1988-1992 - CI5 as it appears in Parkin DM, International Agency for Research on Cancer, International Association of Cancer Registries. Cancer Incidence in Five Continents. Vol. I to VIII. Lyon, France, IARCPress, 2005.
Canada - New Brunswick Cancer Registry 1989 - CI5 as it appears in Ferlay J, Parkin DM, Curado MP, Bray F, Edwards B, Shin HR and Forman D. Cancer Incidence in Five Continents, Volumes I to IX: IARC CancerBase No. 9 [Internet]. Lyon, France: International Agency for Research on Cancer; 2010. Available from: http://ci5.iarc.fr
Canada - New Brunswick Cancer Registry 1991 - CI5 as it appears in Ferlay J, Parkin DM, Curado MP, Bray F, Edwards B, Shin HR and Forman D. Cancer Incidence in Five Continents, Volumes I to IX: IARC CancerBase No. 9 [Internet]. Lyon, France: International Agency for Research on Cancer; 2010. Available from: http://ci5.iarc.fr

Appendix: Citation List

Citation
Canada - New Brunswick Cancer Registry 1992 - CI5 as it appears in Ferlay J, Parkin DM, Curado MP, Bray F, Edwards B, Shin HR and Forman D. Cancer Incidence in Five Continents, Volumes I to IX: IARC CancerBase No. 9 [Internet]. Lyon, France: International Agency for Research on Cancer; 2010. Available from: http://ci5.iarc.fr
Canada - New Brunswick Cancer Registry 1993-1997 - CI5 as it appears in Parkin DM, International Agency for Research on Cancer, International Association of Cancer Registries. Cancer Incidence in Five Continents. Vol. I to VIII. Lyon, France, IARCPress, 2005.
Canada - New Brunswick Cancer Registry 1994 - CI5 as it appears in Ferlay J, Parkin DM, Curado MP, Bray F, Edwards B, Shin HR and Forman D. Cancer Incidence in Five Continents, Volumes I to IX: IARC CancerBase No. 9 [Internet]. Lyon, France: International Agency for Research on Cancer; 2010. Available from: http://ci5.iarc.fr
Canada - New Brunswick Cancer Registry 1996 - CI5 as it appears in Ferlay J, Parkin DM, Curado MP, Bray F, Edwards B, Shin HR and Forman D. Cancer Incidence in Five Continents, Volumes I to IX: IARC CancerBase No. 9 [Internet]. Lyon, France: International Agency for Research on Cancer; 2010. Available from: http://ci5.iarc.fr
Canada - New Brunswick Cancer Registry 1997 - CI5 as it appears in Ferlay J, Parkin DM, Curado MP, Bray F, Edwards B, Shin HR and Forman D. Cancer Incidence in Five Continents, Volumes I to IX: IARC CancerBase No. 9 [Internet]. Lyon, France: International Agency for Research on Cancer; 2010. Available from: http://ci5.iarc.fr
Canada - New Brunswick Cancer Registry 1998-2002 - CI5 as it appears in Curado MP, Edwards B, Shin HR, Storm H, Ferlay J, Heanue M and Boyle P, eds (2007). Cancer Incidence in Five Continents, Vol. IX Periodic Data (electronic version). Lyon, IARC.http://ci5.iarc.fr
Canada - New Brunswick Cancer Registry 1999 - CI5 as it appears in Ferlay J, Parkin DM, Curado MP, Bray F, Edwards B, Shin HR and Forman D. Cancer Incidence in Five Continents, Volumes I to IX: IARC CancerBase No. 9 [Internet]. Lyon, France: International Agency for Research on Cancer; 2010. Available from: http://ci5.iarc.fr
Canada - New Brunswick Cancer Registry 2001 - CI5 as it appears in Ferlay J, Parkin DM, Curado MP, Bray F, Edwards B, Shin HR and Forman D. Cancer Incidence in Five Continents, Volumes I to IX: IARC CancerBase No. 9 [Internet]. Lyon, France: International Agency for Research on Cancer; 2010. Available from: http://ci5.iarc.fr
Canada - New Brunswick Cancer Registry 2002 - CI5 as it appears in Ferlay J, Parkin DM, Curado MP, Bray F, Edwards B, Shin HR and Forman D. Cancer Incidence in Five Continents, Volumes I to IX: IARC CancerBase No. 9 [Internet]. Lyon, France: International Agency for Research on Cancer; 2010. Available from: http://ci5.iarc.fr
Canada - Newfoundland and Labrador Cancer Registry 1978-1982 - CI5 as it appears in Parkin DM, International Agency for Research on Cancer, International Association of Cancer Registries. Cancer Incidence in Five Continents. Vol. I to VIII. Lyon, France, IARCPress, 2005.
Canada - Newfoundland and Labrador Cancer Registry 1983-1987 - CI5 as it appears in Parkin DM, International Agency for Research on Cancer, International Association of Cancer Registries. Cancer Incidence in Five Continents. Vol. I to VIII. Lyon, France, IARCPress, 2005.
Canada - Newfoundland and Labrador Cancer Registry 1988-1992 - CI5 as it appears in Parkin DM, International Agency for Research on Cancer, International Association of Cancer Registries. Cancer Incidence in Five Continents. Vol. I to VIII. Lyon, France, IARCPress, 2005.
Canada - Newfoundland and Labrador Cancer Registry 1993-1997 - CI5 as it appears in Parkin DM, International Agency for Research on Cancer, International Association of Cancer Registries. Cancer Incidence in Five Continents. Vol. I to VIII. Lyon, France, IARCPress, 2005.
Canada - Newfoundland Cancer Registry 1981 - CI5 as it appears in Ferlay J, Parkin DM, Curado MP, Bray F, Edwards B, Shin HR and Forman D. Cancer Incidence in Five Continents, Volumes I to IX: IARC CancerBase No. 9 [Internet]. Lyon, France: International Agency for Research on Cancer; 2010. Available from: http://ci5.iarc.fr
Canada - Newfoundland Cancer Registry 1982 - CI5 as it appears in Ferlay J, Parkin DM, Curado MP, Bray F, Edwards B, Shin HR and Forman D. Cancer Incidence in Five Continents, Volumes I to IX: IARC CancerBase No. 9 [Internet]. Lyon, France: International Agency for Research on Cancer; 2010. Available from: http://ci5.iarc.fr
Canada - Newfoundland Cancer Registry 1983 - CI5 as it appears in Ferlay J, Parkin DM, Curado MP, Bray F, Edwards B, Shin HR and Forman D. Cancer Incidence in Five Continents, Volumes I to IX: IARC CancerBase No. 9 [Internet]. Lyon, France: International Agency for Research on Cancer; 2010. Available from: http://ci5.iarc.fr
Canada - Newfoundland Cancer Registry 1984 - CI5 as it appears in Ferlay J, Parkin DM, Curado MP, Bray F, Edwards B, Shin HR and Forman D. Cancer Incidence in Five Continents, Volumes I to IX: IARC CancerBase No. 9 [Internet]. Lyon, France: International Agency for Research on Cancer; 2010. Available from: http://ci5.iarc.fr
Canada - Newfoundland Cancer Registry 1986 - CI5 as it appears in Ferlay J, Parkin DM, Curado MP, Bray F, Edwards B, Shin HR and Forman D. Cancer Incidence in Five Continents, Volumes I to IX: IARC CancerBase No. 9 [Internet]. Lyon, France: International Agency for Research on Cancer; 2010. Available from: http://ci5.iarc.fr
Canada - Newfoundland Cancer Registry 1987 - CI5 as it appears in Ferlay J, Parkin DM, Curado MP, Bray F, Edwards B, Shin HR and Forman D. Cancer Incidence in Five Continents, Volumes I to IX: IARC CancerBase No. 9 [Internet]. Lyon, France: International Agency for Research on Cancer; 2010. Available from: http://ci5.iarc.fr
Canada - Newfoundland Cancer Registry 1988 - CI5 as it appears in Ferlay J, Parkin DM, Curado MP, Bray F, Edwards B, Shin HR and Forman D. Cancer Incidence in Five Continents, Volumes I to IX: IARC CancerBase No. 9 [Internet]. Lyon, France: International Agency for Research on Cancer; 2010. Available from: http://ci5.iarc.fr
Canada - Newfoundland Cancer Registry 1989 - CI5 as it appears in Ferlay J, Parkin DM, Curado MP, Bray F, Edwards B, Shin HR and Forman D. Cancer Incidence in Five Continents, Volumes I to IX: IARC CancerBase No. 9 [Internet]. Lyon, France: International Agency for Research on Cancer; 2010. Available from: http://ci5.iarc.fr
Canada - Newfoundland Cancer Registry 1991 - CI5 as it appears in Ferlay J, Parkin DM, Curado MP, Bray F, Edwards B, Shin HR and Forman D. Cancer Incidence in Five Continents, Volumes I to IX: IARC CancerBase No. 9 [Internet]. Lyon, France: International Agency for Research on Cancer; 2010. Available from: http://ci5.iarc.fr
Canada - Newfoundland Cancer Registry 1992 - CI5 as it appears in Ferlay J, Parkin DM, Curado MP, Bray F, Edwards B, Shin HR and Forman D. Cancer Incidence in Five Continents, Volumes I to IX: IARC CancerBase No. 9 [Internet]. Lyon, France: International Agency for Research on Cancer; 2010. Available from: http://ci5.iarc.fr

Appendix: Citation List

Citation
Canada - Newfoundland Cancer Registry 1993 - CI5 as it appears in Ferlay J, Parkin DM, Curado MP, Bray F, Edwards B, Shin HR and Forman D. Cancer Incidence in Five Continents, Volumes I to IX: IARC CancerBase No. 9 [Internet]. Lyon, France: International Agency for Research on Cancer; 2010. Available from: http://ci5.iarc.fr
Canada - Newfoundland Cancer Registry 1994 - CI5 as it appears in Ferlay J, Parkin DM, Curado MP, Bray F, Edwards B, Shin HR and Forman D. Cancer Incidence in Five Continents, Volumes I to IX: IARC CancerBase No. 9 [Internet]. Lyon, France: International Agency for Research on Cancer; 2010. Available from: http://ci5.iarc.fr
Canada - Newfoundland Cancer Registry 1996 - CI5 as it appears in Ferlay J, Parkin DM, Curado MP, Bray F, Edwards B, Shin HR and Forman D. Cancer Incidence in Five Continents, Volumes I to IX: IARC CancerBase No. 9 [Internet]. Lyon, France: International Agency for Research on Cancer; 2010. Available from: http://ci5.iarc.fr
Canada - Newfoundland Cancer Registry 1997 - CI5 as it appears in Ferlay J, Parkin DM, Curado MP, Bray F, Edwards B, Shin HR and Forman D. Cancer Incidence in Five Continents, Volumes I to IX: IARC CancerBase No. 9 [Internet]. Lyon, France: International Agency for Research on Cancer; 2010. Available from: http://ci5.iarc.fr
Canada - Newfoundland Cancer Registry 1998 - CI5 as it appears in Ferlay J, Parkin DM, Curado MP, Bray F, Edwards B, Shin HR and Forman D. Cancer Incidence in Five Continents, Volumes I to IX: IARC CancerBase No. 9 [Internet]. Lyon, France: International Agency for Research on Cancer; 2010. Available from: http://ci5.iarc.fr
Canada - Newfoundland Cancer Registry 1999 - CI5 as it appears in Ferlay J, Parkin DM, Curado MP, Bray F, Edwards B, Shin HR and Forman D. Cancer Incidence in Five Continents, Volumes I to IX: IARC CancerBase No. 9 [Internet]. Lyon, France: International Agency for Research on Cancer; 2010. Available from: http://ci5.iarc.fr
Canada - Newfoundland Cancer Registry 2001 - CI5 as it appears in Ferlay J, Parkin DM, Curado MP, Bray F, Edwards B, Shin HR and Forman D. Cancer Incidence in Five Continents, Volumes I to IX: IARC CancerBase No. 9 [Internet]. Lyon, France: International Agency for Research on Cancer; 2010. Available from: http://ci5.iarc.fr
Canada - Newfoundland Cancer Registry 2002 - CI5 as it appears in Ferlay J, Parkin DM, Curado MP, Bray F, Edwards B, Shin HR and Forman D. Cancer Incidence in Five Continents, Volumes I to IX: IARC CancerBase No. 9 [Internet]. Lyon, France: International Agency for Research on Cancer; 2010. Available from: http://ci5.iarc.fr
Canada - Northwest Territories and Yukon Cancer Registry 1978-1982 - CI5 as it appears in Parkin DM, International Agency for Research on Cancer, International Association of Cancer Registries. Cancer Incidence in Five Continents. Vol. I to VIII. Lyon, France, IARCPress, 2005.
Canada - Northwest Territories and Yukon Cancer Registry 1983-1987 - CI5 as it appears in Parkin DM, International Agency for Research on Cancer, International Association of Cancer Registries. Cancer Incidence in Five Continents. Vol. I to VIII. Lyon, France, IARCPress, 2005.
Canada - Nova Scotia Cancer Registry 1978-1982 - CI5 as it appears in Parkin DM, International Agency for Research on Cancer, International Association of Cancer Registries. Cancer Incidence in Five Continents. Vol. I to VIII. Lyon, France, IARCPress, 2005.
Canada - Nova Scotia Cancer Registry 1981 - CI5 as it appears in Ferlay J, Parkin DM, Curado MP, Bray F, Edwards B, Shin HR and Forman D. Cancer Incidence in Five Continents, Volumes I to IX: IARC CancerBase No. 9 [Internet]. Lyon, France: International Agency for Research on Cancer; 2010. Available from: http://ci5.iarc.fr
Canada - Nova Scotia Cancer Registry 1982 - CI5 as it appears in Ferlay J, Parkin DM, Curado MP, Bray F, Edwards B, Shin HR and Forman D. Cancer Incidence in Five Continents, Volumes I to IX: IARC CancerBase No. 9 [Internet]. Lyon, France: International Agency for Research on Cancer; 2010. Available from: http://ci5.iarc.fr
Canada - Nova Scotia Cancer Registry 1983 - CI5 as it appears in Ferlay J, Parkin DM, Curado MP, Bray F, Edwards B, Shin HR and Forman D. Cancer Incidence in Five Continents, Volumes I to IX: IARC CancerBase No. 9 [Internet]. Lyon, France: International Agency for Research on Cancer; 2010. Available from: http://ci5.iarc.fr
Canada - Nova Scotia Cancer Registry 1983-1987 - CI5 as it appears in Parkin DM, International Agency for Research on Cancer, International Association of Cancer Registries. Cancer Incidence in Five Continents. Vol. I to VIII. Lyon, France, IARCPress, 2005.
Canada - Nova Scotia Cancer Registry 1984 - CI5 as it appears in Ferlay J, Parkin DM, Curado MP, Bray F, Edwards B, Shin HR and Forman D. Cancer Incidence in Five Continents, Volumes I to IX: IARC CancerBase No. 9 [Internet]. Lyon, France: International Agency for Research on Cancer; 2010. Available from: http://ci5.iarc.fr
Canada - Nova Scotia Cancer Registry 1986 - CI5 as it appears in Ferlay J, Parkin DM, Curado MP, Bray F, Edwards B, Shin HR and Forman D. Cancer Incidence in Five Continents, Volumes I to IX: IARC CancerBase No. 9 [Internet]. Lyon, France: International Agency for Research on Cancer; 2010. Available from: http://ci5.iarc.fr
Canada - Nova Scotia Cancer Registry 1987 - CI5 as it appears in Ferlay J, Parkin DM, Curado MP, Bray F, Edwards B, Shin HR and Forman D. Cancer Incidence in Five Continents, Volumes I to IX: IARC CancerBase No. 9 [Internet]. Lyon, France: International Agency for Research on Cancer; 2010. Available from: http://ci5.iarc.fr
Canada - Nova Scotia Cancer Registry 1988 - CI5 as it appears in Ferlay J, Parkin DM, Curado MP, Bray F, Edwards B, Shin HR and Forman D. Cancer Incidence in Five Continents, Volumes I to IX: IARC CancerBase No. 9 [Internet]. Lyon, France: International Agency for Research on Cancer; 2010. Available from: http://ci5.iarc.fr
Canada - Nova Scotia Cancer Registry 1988-1992 - CI5 as it appears in Parkin DM, International Agency for Research on Cancer, International Association of Cancer Registries. Cancer Incidence in Five Continents. Vol. I to VIII. Lyon, France, IARCPress, 2005.
Canada - Nova Scotia Cancer Registry 1989 - CI5 as it appears in Ferlay J, Parkin DM, Curado MP, Bray F, Edwards B, Shin HR and Forman D. Cancer Incidence in Five Continents, Volumes I to IX: IARC CancerBase No. 9 [Internet]. Lyon, France: International Agency for Research on Cancer; 2010. Available from: http://ci5.iarc.fr
Canada - Nova Scotia Cancer Registry 1991 - CI5 as it appears in Ferlay J, Parkin DM, Curado MP, Bray F, Edwards B, Shin HR and Forman D. Cancer Incidence in Five Continents, Volumes I to IX: IARC CancerBase No. 9 [Internet]. Lyon, France: International Agency for Research on Cancer; 2010. Available from: http://ci5.iarc.fr

Appendix: Citation List

Citation
Canada - Nova Scotia Cancer Registry 1992 - CI5 as it appears in Ferlay J, Parkin DM, Curado MP, Bray F, Edwards B, Shin HR and Forman D. Cancer Incidence in Five Continents, Volumes I to IX: IARC CancerBase No. 9 [Internet]. Lyon, France: International Agency for Research on Cancer; 2010. Available from: http://ci5.iarc.fr
Canada - Nova Scotia Cancer Registry 1993 - CI5 as it appears in Ferlay J, Parkin DM, Curado MP, Bray F, Edwards B, Shin HR and Forman D. Cancer Incidence in Five Continents, Volumes I to IX: IARC CancerBase No. 9 [Internet]. Lyon, France: International Agency for Research on Cancer; 2010. Available from: http://ci5.iarc.fr
Canada - Nova Scotia Cancer Registry 1993-1997 - CI5 as it appears in Parkin DM, International Agency for Research on Cancer, International Association of Cancer Registries. Cancer Incidence in Five Continents. Vol. I to VIII. Lyon, France, IARCPress, 2005.
Canada - Nova Scotia Cancer Registry 1994 - CI5 as it appears in Ferlay J, Parkin DM, Curado MP, Bray F, Edwards B, Shin HR and Forman D. Cancer Incidence in Five Continents, Volumes I to IX: IARC CancerBase No. 9 [Internet]. Lyon, France: International Agency for Research on Cancer; 2010. Available from: http://ci5.iarc.fr
Canada - Nova Scotia Cancer Registry 1996 - CI5 as it appears in Ferlay J, Parkin DM, Curado MP, Bray F, Edwards B, Shin HR and Forman D. Cancer Incidence in Five Continents, Volumes I to IX: IARC CancerBase No. 9 [Internet]. Lyon, France: International Agency for Research on Cancer; 2010. Available from: http://ci5.iarc.fr
Canada - Nova Scotia Cancer Registry 1997 - CI5 as it appears in Ferlay J, Parkin DM, Curado MP, Bray F, Edwards B, Shin HR and Forman D. Cancer Incidence in Five Continents, Volumes I to IX: IARC CancerBase No. 9 [Internet]. Lyon, France: International Agency for Research on Cancer; 2010. Available from: http://ci5.iarc.fr
Canada - Nova Scotia Cancer Registry 1998 - CI5 as it appears in Ferlay J, Parkin DM, Curado MP, Bray F, Edwards B, Shin HR and Forman D. Cancer Incidence in Five Continents, Volumes I to IX: IARC CancerBase No. 9 [Internet]. Lyon, France: International Agency for Research on Cancer; 2010. Available from: http://ci5.iarc.fr
Canada - Nova Scotia Cancer Registry 1999 - CI5 as it appears in Ferlay J, Parkin DM, Curado MP, Bray F, Edwards B, Shin HR and Forman D. Cancer Incidence in Five Continents, Volumes I to IX: IARC CancerBase No. 9 [Internet]. Lyon, France: International Agency for Research on Cancer; 2010. Available from: http://ci5.iarc.fr
Canada - Nova Scotia Cancer Registry 2001 - CI5 as it appears in Ferlay J, Parkin DM, Curado MP, Bray F, Edwards B, Shin HR and Forman D. Cancer Incidence in Five Continents, Volumes I to IX: IARC CancerBase No. 9 [Internet]. Lyon, France: International Agency for Research on Cancer; 2010. Available from: http://ci5.iarc.fr
Canada - Nova Scotia Cancer Registry 2002 - CI5 as it appears in Ferlay J, Parkin DM, Curado MP, Bray F, Edwards B, Shin HR and Forman D. Cancer Incidence in Five Continents, Volumes I to IX: IARC CancerBase No. 9 [Internet]. Lyon, France: International Agency for Research on Cancer; 2010. Available from: http://ci5.iarc.fr
Canada - Ontario Cancer Registry 1978-1982 - CI5 as it appears in Parkin DM, International Agency for Research on Cancer, International Association of Cancer Registries. Cancer Incidence in Five Continents. Vol. I to VIII. Lyon, France, IARCPress, 2005.
Canada - Ontario Cancer Registry 1981 - CI5 as it appears in Ferlay J, Parkin DM, Curado MP, Bray F, Edwards B, Shin HR and Forman D. Cancer Incidence in Five Continents, Volumes I to IX: IARC CancerBase No. 9 [Internet]. Lyon, France: International Agency for Research on Cancer; 2010. Available from: http://ci5.iarc.fr
Canada - Ontario Cancer Registry 1982 - CI5 as it appears in Ferlay J, Parkin DM, Curado MP, Bray F, Edwards B, Shin HR and Forman D. Cancer Incidence in Five Continents, Volumes I to IX: IARC CancerBase No. 9 [Internet]. Lyon, France: International Agency for Research on Cancer; 2010. Available from: http://ci5.iarc.fr
Canada - Ontario Cancer Registry 1983 - CI5 as it appears in Ferlay J, Parkin DM, Curado MP, Bray F, Edwards B, Shin HR and Forman D. Cancer Incidence in Five Continents, Volumes I to IX: IARC CancerBase No. 9 [Internet]. Lyon, France: International Agency for Research on Cancer; 2010. Available from: http://ci5.iarc.fr
Canada - Ontario Cancer Registry 1983-1987 - CI5 as it appears in Parkin DM, International Agency for Research on Cancer, International Association of Cancer Registries. Cancer Incidence in Five Continents. Vol. I to VIII. Lyon, France, IARCPress, 2005.
Canada - Ontario Cancer Registry 1984 - CI5 as it appears in Ferlay J, Parkin DM, Curado MP, Bray F, Edwards B, Shin HR and Forman D. Cancer Incidence in Five Continents, Volumes I to IX: IARC CancerBase No. 9 [Internet]. Lyon, France: International Agency for Research on Cancer; 2010. Available from: http://ci5.iarc.fr
Canada - Ontario Cancer Registry 1986 - CI5 as it appears in Ferlay J, Parkin DM, Curado MP, Bray F, Edwards B, Shin HR and Forman D. Cancer Incidence in Five Continents, Volumes I to IX: IARC CancerBase No. 9 [Internet]. Lyon, France: International Agency for Research on Cancer; 2010. Available from: http://ci5.iarc.fr
Canada - Ontario Cancer Registry 1987 - CI5 as it appears in Ferlay J, Parkin DM, Curado MP, Bray F, Edwards B, Shin HR and Forman D. Cancer Incidence in Five Continents, Volumes I to IX: IARC CancerBase No. 9 [Internet]. Lyon, France: International Agency for Research on Cancer; 2010. Available from: http://ci5.iarc.fr
Canada - Ontario Cancer Registry 1988 - CI5 as it appears in Ferlay J, Parkin DM, Curado MP, Bray F, Edwards B, Shin HR and Forman D. Cancer Incidence in Five Continents, Volumes I to IX: IARC CancerBase No. 9 [Internet]. Lyon, France: International Agency for Research on Cancer; 2010. Available from: http://ci5.iarc.fr
Canada - Ontario Cancer Registry 1988-1992 - CI5 as it appears in Parkin DM, International Agency for Research on Cancer, International Association of Cancer Registries. Cancer Incidence in Five Continents. Vol. I to VIII. Lyon, France, IARCPress, 2005.
Canada - Ontario Cancer Registry 1989 - CI5 as it appears in Ferlay J, Parkin DM, Curado MP, Bray F, Edwards B, Shin HR and Forman D. Cancer Incidence in Five Continents, Volumes I to IX: IARC CancerBase No. 9 [Internet]. Lyon, France: International Agency for Research on Cancer; 2010. Available from: http://ci5.iarc.fr
Canada - Ontario Cancer Registry 1991 - CI5 as it appears in Ferlay J, Parkin DM, Curado MP, Bray F, Edwards B, Shin HR and Forman D. Cancer Incidence in Five Continents, Volumes I to IX: IARC CancerBase No. 9 [Internet]. Lyon, France: International Agency for Research on Cancer; 2010. Available from: http://ci5.iarc.fr

Appendix: Citation List

Citation
Canada - Ontario Cancer Registry 1992 - CI5 as it appears in Ferlay J, Parkin DM, Curado MP, Bray F, Edwards B, Shin HR and Forman D. Cancer Incidence in Five Continents, Volumes I to IX: IARC CancerBase No. 9 [Internet]. Lyon, France: International Agency for Research on Cancer; 2010. Available from: http://ci5.iarc.fr
Canada - Ontario Cancer Registry 1993 - CI5 as it appears in Ferlay J, Parkin DM, Curado MP, Bray F, Edwards B, Shin HR and Forman D. Cancer Incidence in Five Continents, Volumes I to IX: IARC CancerBase No. 9 [Internet]. Lyon, France: International Agency for Research on Cancer; 2010. Available from: http://ci5.iarc.fr
Canada - Ontario Cancer Registry 1993-1997 - CI5 as it appears in Parkin DM, International Agency for Research on Cancer, International Association of Cancer Registries. Cancer Incidence in Five Continents. Vol. I to VIII. Lyon, France, IARCPress, 2005.
Canada - Ontario Cancer Registry 1994 - CI5 as it appears in Ferlay J, Parkin DM, Curado MP, Bray F, Edwards B, Shin HR and Forman D. Cancer Incidence in Five Continents, Volumes I to IX: IARC CancerBase No. 9 [Internet]. Lyon, France: International Agency for Research on Cancer; 2010. Available from: http://ci5.iarc.fr
Canada - Ontario Cancer Registry 1996 - CI5 as it appears in Ferlay J, Parkin DM, Curado MP, Bray F, Edwards B, Shin HR and Forman D. Cancer Incidence in Five Continents, Volumes I to IX: IARC CancerBase No. 9 [Internet]. Lyon, France: International Agency for Research on Cancer; 2010. Available from: http://ci5.iarc.fr
Canada - Ontario Cancer Registry 1997 - CI5 as it appears in Ferlay J, Parkin DM, Curado MP, Bray F, Edwards B, Shin HR and Forman D. Cancer Incidence in Five Continents, Volumes I to IX: IARC CancerBase No. 9 [Internet]. Lyon, France: International Agency for Research on Cancer; 2010. Available from: http://ci5.iarc.fr
Canada - Ontario Cancer Registry 1998 - CI5 as it appears in Ferlay J, Parkin DM, Curado MP, Bray F, Edwards B, Shin HR and Forman D. Cancer Incidence in Five Continents, Volumes I to IX: IARC CancerBase No. 9 [Internet]. Lyon, France: International Agency for Research on Cancer; 2010. Available from: http://ci5.iarc.fr
Canada - Ontario Cancer Registry 1999 - CI5 as it appears in Ferlay J, Parkin DM, Curado MP, Bray F, Edwards B, Shin HR and Forman D. Cancer Incidence in Five Continents, Volumes I to IX: IARC CancerBase No. 9 [Internet]. Lyon, France: International Agency for Research on Cancer; 2010. Available from: http://ci5.iarc.fr
Canada - Ontario Cancer Registry 2001 - CI5 as it appears in Ferlay J, Parkin DM, Curado MP, Bray F, Edwards B, Shin HR and Forman D. Cancer Incidence in Five Continents, Volumes I to IX: IARC CancerBase No. 9 [Internet]. Lyon, France: International Agency for Research on Cancer; 2010. Available from: http://ci5.iarc.fr
Canada - Ontario Cancer Registry 2002 - CI5 as it appears in Ferlay J, Parkin DM, Curado MP, Bray F, Edwards B, Shin HR and Forman D. Cancer Incidence in Five Continents, Volumes I to IX: IARC CancerBase No. 9 [Internet]. Lyon, France: International Agency for Research on Cancer; 2010. Available from: http://ci5.iarc.fr
Canada - Prince Edward Island Cancer Registry 1983 - CI5 as it appears in Ferlay J, Parkin DM, Curado MP, Bray F, Edwards B, Shin HR and Forman D. Cancer Incidence in Five Continents, Volumes I to IX: IARC CancerBase No. 9 [Internet]. Lyon, France: International Agency for Research on Cancer; 2010. Available from: http://ci5.iarc.fr
Canada - Prince Edward Island Cancer Registry 1984 - CI5 as it appears in Ferlay J, Parkin DM, Curado MP, Bray F, Edwards B, Shin HR and Forman D. Cancer Incidence in Five Continents, Volumes I to IX: IARC CancerBase No. 9 [Internet]. Lyon, France: International Agency for Research on Cancer; 2010. Available from: http://ci5.iarc.fr
Canada - Prince Edward Island Cancer Registry 1986 - CI5 as it appears in Ferlay J, Parkin DM, Curado MP, Bray F, Edwards B, Shin HR and Forman D. Cancer Incidence in Five Continents, Volumes I to IX: IARC CancerBase No. 9 [Internet]. Lyon, France: International Agency for Research on Cancer; 2010. Available from: http://ci5.iarc.fr
Canada - Prince Edward Island Cancer Registry 1987 - CI5 as it appears in Ferlay J, Parkin DM, Curado MP, Bray F, Edwards B, Shin HR and Forman D. Cancer Incidence in Five Continents, Volumes I to IX: IARC CancerBase No. 9 [Internet]. Lyon, France: International Agency for Research on Cancer; 2010. Available from: http://ci5.iarc.fr
Canada - Prince Edward Island Cancer Registry 1988 - CI5 as it appears in Ferlay J, Parkin DM, Curado MP, Bray F, Edwards B, Shin HR and Forman D. Cancer Incidence in Five Continents, Volumes I to IX: IARC CancerBase No. 9 [Internet]. Lyon, France: International Agency for Research on Cancer; 2010. Available from: http://ci5.iarc.fr
Canada - Prince Edward Island Cancer Registry 1989 - CI5 as it appears in Ferlay J, Parkin DM, Curado MP, Bray F, Edwards B, Shin HR and Forman D. Cancer Incidence in Five Continents, Volumes I to IX: IARC CancerBase No. 9 [Internet]. Lyon, France: International Agency for Research on Cancer; 2010. Available from: http://ci5.iarc.fr
Canada - Prince Edward Island Cancer Registry 1991 - CI5 as it appears in Ferlay J, Parkin DM, Curado MP, Bray F, Edwards B, Shin HR and Forman D. Cancer Incidence in Five Continents, Volumes I to IX: IARC CancerBase No. 9 [Internet]. Lyon, France: International Agency for Research on Cancer; 2010. Available from: http://ci5.iarc.fr
Canada - Prince Edward Island Cancer Registry 1992 - CI5 as it appears in Ferlay J, Parkin DM, Curado MP, Bray F, Edwards B, Shin HR and Forman D. Cancer Incidence in Five Continents, Volumes I to IX: IARC CancerBase No. 9 [Internet]. Lyon, France: International Agency for Research on Cancer; 2010. Available from: http://ci5.iarc.fr
Canada - Prince Edward Island Cancer Registry 1993 - CI5 as it appears in Ferlay J, Parkin DM, Curado MP, Bray F, Edwards B, Shin HR and Forman D. Cancer Incidence in Five Continents, Volumes I to IX: IARC CancerBase No. 9 [Internet]. Lyon, France: International Agency for Research on Cancer; 2010. Available from: http://ci5.iarc.fr
Canada - Prince Edward Island Cancer Registry 1994 - CI5 as it appears in Ferlay J, Parkin DM, Curado MP, Bray F, Edwards B, Shin HR and Forman D. Cancer Incidence in Five Continents, Volumes I to IX: IARC CancerBase No. 9 [Internet]. Lyon, France: International Agency for Research on Cancer; 2010. Available from: http://ci5.iarc.fr
Canada - Prince Edward Island Cancer Registry 1996 - CI5 as it appears in Ferlay J, Parkin DM, Curado MP, Bray F, Edwards B, Shin HR and Forman D. Cancer Incidence in Five Continents, Volumes I to IX: IARC CancerBase No. 9 [Internet]. Lyon, France: International Agency for Research on Cancer; 2010. Available from: http://ci5.iarc.fr

Appendix: Citation List

Citation
Canada - Prince Edward Island Cancer Registry 1997 - CI5 as it appears in Ferlay J, Parkin DM, Curado MP, Bray F, Edwards B, Shin HR and Forman D. Cancer Incidence in Five Continents, Volumes I to IX: IARC CancerBase No. 9 [Internet]. Lyon, France: International Agency for Research on Cancer; 2010. Available from: http://ci5.iarc.fr
Canada - Prince Edward Island Cancer Registry 1998 - CI5 as it appears in Ferlay J, Parkin DM, Curado MP, Bray F, Edwards B, Shin HR and Forman D. Cancer Incidence in Five Continents, Volumes I to IX: IARC CancerBase No. 9 [Internet]. Lyon, France: International Agency for Research on Cancer; 2010. Available from: http://ci5.iarc.fr
Canada - Prince Edward Island Cancer Registry 1999 - CI5 as it appears in Ferlay J, Parkin DM, Curado MP, Bray F, Edwards B, Shin HR and Forman D. Cancer Incidence in Five Continents, Volumes I to IX: IARC CancerBase No. 9 [Internet]. Lyon, France: International Agency for Research on Cancer; 2010. Available from: http://ci5.iarc.fr
Canada - Prince Edward Island Cancer Registry 2001 - CI5 as it appears in Ferlay J, Parkin DM, Curado MP, Bray F, Edwards B, Shin HR and Forman D. Cancer Incidence in Five Continents, Volumes I to IX: IARC CancerBase No. 9 [Internet]. Lyon, France: International Agency for Research on Cancer; 2010. Available from: http://ci5.iarc.fr
Canada - Prince Edward Island Cancer Registry 2002 - CI5 as it appears in Ferlay J, Parkin DM, Curado MP, Bray F, Edwards B, Shin HR and Forman D. Cancer Incidence in Five Continents, Volumes I to IX: IARC CancerBase No. 9 [Internet]. Lyon, France: International Agency for Research on Cancer; 2010. Available from: http://ci5.iarc.fr
Canada - Quebec Cancer Registry 1978-1981 - CI5 as it appears in Parkin DM, International Agency for Research on Cancer, International Association of Cancer Registries. Cancer Incidence in Five Continents. Vol. I to VIII. Lyon, France, IARCPress, 2005.
Canada - Quebec Cancer Registry 1983-1987 - CI5 as it appears in Parkin DM, International Agency for Research on Cancer, International Association of Cancer Registries. Cancer Incidence in Five Continents. Vol. I to VIII. Lyon, France, IARCPress, 2005.
Canada - Quebec Cancer Registry 1988-1992 - CI5 as it appears in Parkin DM, International Agency for Research on Cancer, International Association of Cancer Registries. Cancer Incidence in Five Continents. Vol. I to VIII. Lyon, France, IARCPress, 2005.
Canada - Quebec Cancer Registry 1993-1997 - CI5 as it appears in Parkin DM, International Agency for Research on Cancer, International Association of Cancer Registries. Cancer Incidence in Five Continents. Vol. I to VIII. Lyon, France, IARCPress, 2005.
Canada - Saskatchewan Cancer Registry 1978-1982 - CI5 as it appears in Parkin DM, International Agency for Research on Cancer, International Association of Cancer Registries. Cancer Incidence in Five Continents. Vol. I to VIII. Lyon, France, IARCPress, 2005.
Canada - Saskatchewan Cancer Registry 1981 - CI5 as it appears in Ferlay J, Parkin DM, Curado MP, Bray F, Edwards B, Shin HR and Forman D. Cancer Incidence in Five Continents, Volumes I to IX: IARC CancerBase No. 9 [Internet]. Lyon, France: International Agency for Research on Cancer; 2010. Available from: http://ci5.iarc.fr
Canada - Saskatchewan Cancer Registry 1982 - CI5 as it appears in Ferlay J, Parkin DM, Curado MP, Bray F, Edwards B, Shin HR and Forman D. Cancer Incidence in Five Continents, Volumes I to IX: IARC CancerBase No. 9 [Internet]. Lyon, France: International Agency for Research on Cancer; 2010. Available from: http://ci5.iarc.fr
Canada - Saskatchewan Cancer Registry 1983 - CI5 as it appears in Ferlay J, Parkin DM, Curado MP, Bray F, Edwards B, Shin HR and Forman D. Cancer Incidence in Five Continents, Volumes I to IX: IARC CancerBase No. 9 [Internet]. Lyon, France: International Agency for Research on Cancer; 2010. Available from: http://ci5.iarc.fr
Canada - Saskatchewan Cancer Registry 1983-1987 - CI5 as it appears in Parkin DM, International Agency for Research on Cancer, International Association of Cancer Registries. Cancer Incidence in Five Continents. Vol. I to VIII. Lyon, France, IARCPress, 2005.
Canada - Saskatchewan Cancer Registry 1984 - CI5 as it appears in Ferlay J, Parkin DM, Curado MP, Bray F, Edwards B, Shin HR and Forman D. Cancer Incidence in Five Continents, Volumes I to IX: IARC CancerBase No. 9 [Internet]. Lyon, France: International Agency for Research on Cancer; 2010. Available from: http://ci5.iarc.fr
Canada - Saskatchewan Cancer Registry 1986 - CI5 as it appears in Ferlay J, Parkin DM, Curado MP, Bray F, Edwards B, Shin HR and Forman D. Cancer Incidence in Five Continents, Volumes I to IX: IARC CancerBase No. 9 [Internet]. Lyon, France: International Agency for Research on Cancer; 2010. Available from: http://ci5.iarc.fr
Canada - Saskatchewan Cancer Registry 1987 - CI5 as it appears in Ferlay J, Parkin DM, Curado MP, Bray F, Edwards B, Shin HR and Forman D. Cancer Incidence in Five Continents, Volumes I to IX: IARC CancerBase No. 9 [Internet]. Lyon, France: International Agency for Research on Cancer; 2010. Available from: http://ci5.iarc.fr
Canada - Saskatchewan Cancer Registry 1988 - CI5 as it appears in Ferlay J, Parkin DM, Curado MP, Bray F, Edwards B, Shin HR and Forman D. Cancer Incidence in Five Continents, Volumes I to IX: IARC CancerBase No. 9 [Internet]. Lyon, France: International Agency for Research on Cancer; 2010. Available from: http://ci5.iarc.fr
Canada - Saskatchewan Cancer Registry 1988-1992 - CI5 as it appears in Parkin DM, International Agency for Research on Cancer, International Association of Cancer Registries. Cancer Incidence in Five Continents. Vol. I to VIII. Lyon, France, IARCPress, 2005.
Canada - Saskatchewan Cancer Registry 1989 - CI5 as it appears in Ferlay J, Parkin DM, Curado MP, Bray F, Edwards B, Shin HR and Forman D. Cancer Incidence in Five Continents, Volumes I to IX: IARC CancerBase No. 9 [Internet]. Lyon, France: International Agency for Research on Cancer; 2010. Available from: http://ci5.iarc.fr
Canada - Saskatchewan Cancer Registry 1991 - CI5 as it appears in Ferlay J, Parkin DM, Curado MP, Bray F, Edwards B, Shin HR and Forman D. Cancer Incidence in Five Continents, Volumes I to IX: IARC CancerBase No. 9 [Internet]. Lyon, France: International Agency for Research on Cancer; 2010. Available from: http://ci5.iarc.fr
Canada - Saskatchewan Cancer Registry 1992 - CI5 as it appears in Ferlay J, Parkin DM, Curado MP, Bray F, Edwards B, Shin HR and Forman D. Cancer Incidence in Five Continents, Volumes I to IX: IARC CancerBase No. 9 [Internet]. Lyon, France: International Agency for Research on Cancer; 2010. Available from: http://ci5.iarc.fr
Canada - Saskatchewan Cancer Registry 1993 - CI5 as it appears in Ferlay J, Parkin DM, Curado MP, Bray F, Edwards B, Shin HR and Forman D. Cancer Incidence in Five Continents, Volumes I to IX: IARC CancerBase No. 9 [Internet]. Lyon, France: International Agency for Research on Cancer; 2010. Available from: http://ci5.iarc.fr
Canada - Saskatchewan Cancer Registry 1993-1997 - CI5 as it appears in Parkin DM, International Agency for Research on Cancer, International Association of Cancer Registries. Cancer Incidence in Five Continents. Vol. I to VIII. Lyon, France, IARCPress, 2005.

Appendix: Citation List

Citation
Canada - Saskatchewan Cancer Registry 1994 - CI5 as it appears in Ferlay J, Parkin DM, Curado MP, Bray F, Edwards B, Shin HR and Forman D. Cancer Incidence in Five Continents, Volumes I to IX: IARC CancerBase No. 9 [Internet]. Lyon, France: International Agency for Research on Cancer; 2010. Available from: http://ci5.iarc.fr
Canada - Saskatchewan Cancer Registry 1996 - CI5 as it appears in Ferlay J, Parkin DM, Curado MP, Bray F, Edwards B, Shin HR and Forman D. Cancer Incidence in Five Continents, Volumes I to IX: IARC CancerBase No. 9 [Internet]. Lyon, France: International Agency for Research on Cancer; 2010. Available from: http://ci5.iarc.fr
Canada - Saskatchewan Cancer Registry 1997 - CI5 as it appears in Ferlay J, Parkin DM, Curado MP, Bray F, Edwards B, Shin HR and Forman D. Cancer Incidence in Five Continents, Volumes I to IX: IARC CancerBase No. 9 [Internet]. Lyon, France: International Agency for Research on Cancer; 2010. Available from: http://ci5.iarc.fr
Canada - Saskatchewan Cancer Registry 1998 - CI5 as it appears in Ferlay J, Parkin DM, Curado MP, Bray F, Edwards B, Shin HR and Forman D. Cancer Incidence in Five Continents, Volumes I to IX: IARC CancerBase No. 9 [Internet]. Lyon, France: International Agency for Research on Cancer; 2010. Available from: http://ci5.iarc.fr
Canada - Saskatchewan Cancer Registry 1999 - CI5 as it appears in Ferlay J, Parkin DM, Curado MP, Bray F, Edwards B, Shin HR and Forman D. Cancer Incidence in Five Continents, Volumes I to IX: IARC CancerBase No. 9 [Internet]. Lyon, France: International Agency for Research on Cancer; 2010. Available from: http://ci5.iarc.fr
Canada - Saskatchewan Cancer Registry 2001 - CI5 as it appears in Ferlay J, Parkin DM, Curado MP, Bray F, Edwards B, Shin HR and Forman D. Cancer Incidence in Five Continents, Volumes I to IX: IARC CancerBase No. 9 [Internet]. Lyon, France: International Agency for Research on Cancer; 2010. Available from: http://ci5.iarc.fr
Canada - Saskatchewan Cancer Registry 2002 - CI5 as it appears in Ferlay J, Parkin DM, Curado MP, Bray F, Edwards B, Shin HR and Forman D. Cancer Incidence in Five Continents, Volumes I to IX: IARC CancerBase No. 9 [Internet]. Lyon, France: International Agency for Research on Cancer; 2010. Available from: http://ci5.iarc.fr
Canada Cancer Registry 1978-1982 - CI5 as it appears in Parkin DM, International Agency for Research on Cancer, International Association of Cancer Registries. Cancer Incidence in Five Continents. Vol. I to VIII. Lyon, France, IARCPress, 2005.
Canada Cancer Registry 1984 - CI5 as it appears in Parkin DM, International Agency for Research on Cancer, International Association of Cancer Registries. Cancer Incidence in Five Continents. Vol. I to VIII. Lyon, France, IARCPress, 2005.
Canada Cancer Registry 1988-1992 - CI5 as it appears in Parkin DM, International Agency for Research on Cancer, International Association of Cancer Registries. Cancer Incidence in Five Continents. Vol. I to VIII. Lyon, France, IARCPress, 2005.
Canada Cancer Registry 1989 - CI5 as it appears in Parkin DM, International Agency for Research on Cancer, International Association of Cancer Registries. Cancer Incidence in Five Continents. Vol. I to VIII. Lyon, France, IARCPress, 2005.
Canada Cancer Registry 1993-1997 - CI5 as it appears in Parkin DM, International Agency for Research on Cancer, International Association of Cancer Registries. Cancer Incidence in Five Continents. Vol. I to VIII. Lyon, France, IARCPress, 2005.
Canada Cancer Registry 1994 - CI5 as it appears in Parkin DM, International Agency for Research on Cancer, International Association of Cancer Registries. Cancer Incidence in Five Continents. Vol. I to VIII. Lyon, France, IARCPress, 2005.
Canada Cancer Registry 1998-2002 - CI5 as it appears in Curado MP, Edwards B, Shin HR, Storm H, Ferlay J, Heanue M and Boyle P, eds (2007). Cancer Incidence in Five Continents, Vol. IX Periodic Data (electronic version). Lyon, IARC.http://ci5.iarc.fr
Canada Cancer Registry 1999 - CI5 as it appears in Curado MP, Edwards B, Shin HR, Storm H, Ferlay J, Heanue M and Boyle P, eds (2007). Cancer Incidence in Five Continents, Vol. IX Periodic Data (electronic version). Lyon, IARC.http://ci5.iarc.fr
Canada Fitness Survey 1981 as it appears in P.N. Lee Statistics and Computing Ltd. International Mortality and Smoking Statistics Version 4.04. Sutton, United Kingdom: P.N. Lee Statistics and Computing Ltd, 2009.
Canada General Social Survey 1985 as it appears in P.N. Lee Statistics and Computing Ltd. International Mortality and Smoking Statistics Version 4.04. Sutton, United Kingdom: P.N. Lee Statistics and Computing Ltd, 2009.
Canada General Social Survey 1991 as it appears in P.N. Lee Statistics and Computing Ltd. International Mortality and Smoking Statistics Version 4.04. Sutton, United Kingdom: P.N. Lee Statistics and Computing Ltd, 2009.
Canada General Social Survey 1996 as it appears in P.N. Lee Statistics and Computing Ltd. International Mortality and Smoking Statistics Version 4.04. Sutton, United Kingdom: P.N. Lee Statistics and Computing Ltd, 2009.
Canada Health Promotion Survey 1985 as it appears in P.N. Lee Statistics and Computing Ltd. International Mortality and Smoking Statistics Version 4.04. Sutton, United Kingdom: P.N. Lee Statistics and Computing Ltd, 2009.
Canada Health Promotion Survey 1990 as it appears in P.N. Lee Statistics and Computing Ltd. International Mortality and Smoking Statistics Version 4.04. Sutton, United Kingdom: P.N. Lee Statistics and Computing Ltd, 2009.
Canada Labor Force Survey Supplement 1981 as it appears in P.N. Lee Statistics and Computing Ltd. International Mortality and Smoking Statistics Version 4.04. Sutton, United Kingdom: P.N. Lee Statistics and Computing Ltd, 2009.
Canada Labor Force Survey Supplement 1983 as it appears in P.N. Lee Statistics and Computing Ltd. International Mortality and Smoking Statistics Version 4.04. Sutton, United Kingdom: P.N. Lee Statistics and Computing Ltd, 2009.
Canada Labor Force Survey Supplement 1986 as it appears in P.N. Lee Statistics and Computing Ltd. International Mortality and Smoking Statistics Version 4.04. Sutton, United Kingdom: P.N. Lee Statistics and Computing Ltd, 2009.
Canada National Alcohol and Other Drugs Survey 1989 as it appears in P.N. Lee Statistics and Computing Ltd. International Mortality and Smoking Statistics Version 4.04. Sutton, United Kingdom: P.N. Lee Statistics and Computing Ltd, 2009.
Canada Smoking Survey 1994 as it appears in P.N. Lee Statistics and Computing Ltd. International Mortality and Smoking Statistics Version 4.04. Sutton, United Kingdom: P.N. Lee Statistics and Computing Ltd, 2009.
Canada Vital Registration - Deaths 1950 ICD7 as it appears in World Health Organization (WHO). WHO Mortality Database Version July 2012. Geneva, Switzerland: World Health Organization (WHO), 2012.
Canada Vital Registration - Deaths 1951 ICD7 as it appears in World Health Organization (WHO). WHO Mortality Database Version July 2012. Geneva, Switzerland: World Health Organization (WHO), 2012.

Appendix: Citation List

Citation
Canada Vital Registration - Deaths 1952 ICD7 as it appears in World Health Organization (WHO). WHO Mortality Database Version July 2012. Geneva, Switzerland: World Health Organization (WHO), 2012.
Canada Vital Registration - Deaths 1953 ICD7 as it appears in World Health Organization (WHO). WHO Mortality Database Version July 2012. Geneva, Switzerland: World Health Organization (WHO), 2012.
Canada Vital Registration - Deaths 1954 ICD7 as it appears in World Health Organization (WHO). WHO Mortality Database Version July 2012. Geneva, Switzerland: World Health Organization (WHO), 2012.
Canada Vital Registration - Deaths 1955 ICD7 as it appears in World Health Organization (WHO). WHO Mortality Database Version July 2012. Geneva, Switzerland: World Health Organization (WHO), 2012.
Canada Vital Registration - Deaths 1956 ICD7 as it appears in World Health Organization (WHO). WHO Mortality Database Version July 2012. Geneva, Switzerland: World Health Organization (WHO), 2012.
Canada Vital Registration - Deaths 1957 ICD7 as it appears in World Health Organization (WHO). WHO Mortality Database Version July 2012. Geneva, Switzerland: World Health Organization (WHO), 2012.
Canada Vital Registration - Deaths 1958 ICD7 as it appears in World Health Organization (WHO). WHO Mortality Database Version July 2012. Geneva, Switzerland: World Health Organization (WHO), 2012.
Canada Vital Registration - Deaths 1959 ICD7 as it appears in World Health Organization (WHO). WHO Mortality Database Version July 2012. Geneva, Switzerland: World Health Organization (WHO), 2012.
Canada Vital Registration - Deaths 1960 ICD7 as it appears in World Health Organization (WHO). WHO Mortality Database Version July 2012. Geneva, Switzerland: World Health Organization (WHO), 2012.
Canada Vital Registration - Deaths 1961 ICD7 as it appears in World Health Organization (WHO). WHO Mortality Database Version July 2012. Geneva, Switzerland: World Health Organization (WHO), 2012.
Canada Vital Registration - Deaths 1962 ICD7 as it appears in World Health Organization (WHO). WHO Mortality Database Version July 2012. Geneva, Switzerland: World Health Organization (WHO), 2012.
Canada Vital Registration - Deaths 1963 ICD7 as it appears in World Health Organization (WHO). WHO Mortality Database Version July 2012. Geneva, Switzerland: World Health Organization (WHO), 2012.
Canada Vital Registration - Deaths 1964 ICD7 as it appears in World Health Organization (WHO). WHO Mortality Database Version July 2012. Geneva, Switzerland: World Health Organization (WHO), 2012.
Canada Vital Registration - Deaths 1965 ICD7 as it appears in World Health Organization (WHO). WHO Mortality Database Version July 2012. Geneva, Switzerland: World Health Organization (WHO), 2012.
Canada Vital Registration - Deaths 1966 ICD7 as it appears in World Health Organization (WHO). WHO Mortality Database Version July 2012. Geneva, Switzerland: World Health Organization (WHO), 2012.
Canada Vital Registration - Deaths 1967 ICD7 as it appears in World Health Organization (WHO). WHO Mortality Database Version July 2012. Geneva, Switzerland: World Health Organization (WHO), 2012.
Canada Vital Registration - Deaths 1968 ICD7 as it appears in World Health Organization (WHO). WHO Mortality Database Version July 2012. Geneva, Switzerland: World Health Organization (WHO), 2012.
Canada Vital Registration - Deaths 1969 ICD8 as it appears in World Health Organization (WHO). WHO Mortality Database Version May 2013. Geneva, Switzerland: World Health Organization (WHO), 2013.
Canada Vital Registration - Deaths 1970 ICD8 as it appears in World Health Organization (WHO). WHO Mortality Database Version May 2013. Geneva, Switzerland: World Health Organization (WHO), 2013.
Canada Vital Registration - Deaths 1971 ICD8 as it appears in World Health Organization (WHO). WHO Mortality Database Version May 2013. Geneva, Switzerland: World Health Organization (WHO), 2013.
Canada Vital Registration - Deaths 1972 ICD8 as it appears in World Health Organization (WHO). WHO Mortality Database Version May 2013. Geneva, Switzerland: World Health Organization (WHO), 2013.
Canada Vital Registration - Deaths 1973 ICD8 as it appears in World Health Organization (WHO). WHO Mortality Database Version May 2013. Geneva, Switzerland: World Health Organization (WHO), 2013.
Canada Vital Registration - Deaths 1974 ICD8 as it appears in World Health Organization (WHO). WHO Mortality Database Version May 2013. Geneva, Switzerland: World Health Organization (WHO), 2013.
Canada Vital Registration - Deaths 1975 ICD8 as it appears in World Health Organization (WHO). WHO Mortality Database Version May 2013. Geneva, Switzerland: World Health Organization (WHO), 2013.
Canada Vital Registration - Deaths 1976 ICD8 as it appears in World Health Organization (WHO). WHO Mortality Database Version May 2013. Geneva, Switzerland: World Health Organization (WHO), 2013.
Canada Vital Registration - Deaths 1977 ICD8 as it appears in World Health Organization (WHO). WHO Mortality Database Version May 2013. Geneva, Switzerland: World Health Organization (WHO), 2013.
Canada Vital Registration - Deaths 1978 ICD8 as it appears in World Health Organization (WHO). WHO Mortality Database Version May 2013. Geneva, Switzerland: World Health Organization (WHO), 2013.
Canada Vital Registration - Deaths 1979 ICD9 as it appears in World Health Organization (WHO). WHO Mortality Database Version November 2015. Geneva, Switzerland: World Health Organization (WHO), 2015.
Canada Vital Registration - Deaths 1980 ICD9 as it appears in World Health Organization (WHO). WHO Mortality Database Version February 2014. Geneva, Switzerland: World Health Organization (WHO), 2014.
Canada Vital Registration - Deaths 1981 ICD9 as it appears in World Health Organization (WHO). WHO Mortality Database Version February 2014. Geneva, Switzerland: World Health Organization (WHO), 2014.
Canada Vital Registration - Deaths 1982 ICD9 as it appears in World Health Organization (WHO). WHO Mortality Database Version February 2014. Geneva, Switzerland: World Health Organization (WHO), 2014.
Canada Vital Registration - Deaths 1983 ICD9 as it appears in World Health Organization (WHO). WHO Mortality Database Version February 2014. Geneva, Switzerland: World Health Organization (WHO), 2014.

Appendix: Citation List

Citation
Canada Vital Registration - Deaths 1984 ICD9 as it appears in World Health Organization (WHO). WHO Mortality Database Version February 2014. Geneva, Switzerland: World Health Organization (WHO), 2014.
Canada Vital Registration - Deaths 1985 ICD9 as it appears in World Health Organization (WHO). WHO Mortality Database Version February 2014. Geneva, Switzerland: World Health Organization (WHO), 2014.
Canada Vital Registration - Deaths 1986 ICD9 as it appears in World Health Organization (WHO). WHO Mortality Database Version July 2012. Geneva, Switzerland: World Health Organization (WHO), 2012.
Canada Vital Registration - Deaths 1987 ICD9 as it appears in World Health Organization (WHO). WHO Mortality Database Version July 2012. Geneva, Switzerland: World Health Organization (WHO), 2012.
Canada Vital Registration - Deaths 1988 ICD9 as it appears in World Health Organization (WHO). WHO Mortality Database Version July 2012. Geneva, Switzerland: World Health Organization (WHO), 2012.
Canada Vital Registration - Deaths 1989 ICD9 as it appears in World Health Organization (WHO). WHO Mortality Database Version July 2012. Geneva, Switzerland: World Health Organization (WHO), 2012.
Canada Vital Registration - Deaths 1990 ICD9 as it appears in World Health Organization (WHO). WHO Mortality Database Version July 2012. Geneva, Switzerland: World Health Organization (WHO), 2012.
Canada Vital Registration - Deaths 1991 ICD9 as it appears in World Health Organization (WHO). WHO Mortality Database Version July 2012. Geneva, Switzerland: World Health Organization (WHO), 2012.
Canada Vital Registration - Deaths 1992 ICD9 as it appears in World Health Organization (WHO). WHO Mortality Database Version July 2012. Geneva, Switzerland: World Health Organization (WHO), 2012.
Canada Vital Registration - Deaths 1993 ICD9 as it appears in World Health Organization (WHO). WHO Mortality Database Version July 2012. Geneva, Switzerland: World Health Organization (WHO), 2012.
Canada Vital Registration - Deaths 1994 ICD9 as it appears in World Health Organization (WHO). WHO Mortality Database Version July 2012. Geneva, Switzerland: World Health Organization (WHO), 2012.
Canada Vital Registration - Deaths 1995 ICD9 as it appears in World Health Organization (WHO). WHO Mortality Database Version July 2012. Geneva, Switzerland: World Health Organization (WHO), 2012.
Canada Vital Registration - Deaths 1996 ICD9 as it appears in World Health Organization (WHO). WHO Mortality Database Version July 2012. Geneva, Switzerland: World Health Organization (WHO), 2012.
Canada Vital Registration - Deaths 1997 ICD9 as it appears in World Health Organization (WHO). WHO Mortality Database Version July 2012. Geneva, Switzerland: World Health Organization (WHO), 2012.
Canada Vital Registration - Deaths 1998 ICD9 as it appears in World Health Organization (WHO). WHO Mortality Database Version July 2012. Geneva, Switzerland: World Health Organization (WHO), 2012.
Canada Vital Registration - Deaths 1999 ICD9 as it appears in World Health Organization (WHO). WHO Mortality Database Version July 2012. Geneva, Switzerland: World Health Organization (WHO), 2012.
Canada Vital Registration - Deaths 2000 ICD10 as it appears in World Health Organization (WHO). WHO Mortality Database Version November 2015. Geneva, Switzerland: World Health Organization (WHO), 2015.
Canada Vital Registration - Deaths 2001 ICD10 as it appears in World Health Organization (WHO). WHO Mortality Database Version November 2015. Geneva, Switzerland: World Health Organization (WHO), 2015.
Canada Vital Registration - Deaths 2002 ICD10 as it appears in World Health Organization (WHO). WHO Mortality Database Version November 2015. Geneva, Switzerland: World Health Organization (WHO), 2015.
Canada Vital Registration - Deaths 2003 ICD10 as it appears in World Health Organization (WHO). WHO Mortality Database Version November 2015. Geneva, Switzerland: World Health Organization (WHO), 2015.
Canada Vital Registration - Deaths 2004 ICD10 as it appears in World Health Organization (WHO). WHO Mortality Database Version November 2015. Geneva, Switzerland: World Health Organization (WHO), 2015.
Canada Vital Registration - Deaths 2005 ICD10 as it appears in World Health Organization (WHO). WHO Mortality Database Version November 2015. Geneva, Switzerland: World Health Organization (WHO), 2015.
Canada Vital Registration - Deaths 2006 ICD10 as it appears in World Health Organization (WHO). WHO Mortality Database Version November 2015. Geneva, Switzerland: World Health Organization (WHO), 2015.
Canada Vital Registration - Deaths 2007 ICD10 as it appears in World Health Organization (WHO). WHO Mortality Database Version November 2015. Geneva, Switzerland: World Health Organization (WHO), 2015.
Canada Vital Registration - Deaths 2008 ICD10 as it appears in World Health Organization (WHO). WHO Mortality Database Version November 2015. Geneva, Switzerland: World Health Organization (WHO), 2015.
Canada Vital Registration - Deaths 2009 ICD10 as it appears in World Health Organization (WHO). WHO Mortality Database Version November 2015. Geneva, Switzerland: World Health Organization (WHO), 2015.
Canada Vital Registration - Deaths 2010 ICD10 as it appears in World Health Organization (WHO). WHO Mortality Database Version November 2015. Geneva, Switzerland: World Health Organization (WHO), 2015.
Canada Vital Registration - Deaths 2011 ICD10 as it appears in World Health Organization (WHO). WHO Mortality Database Version November 2015. Geneva, Switzerland: World Health Organization (WHO), 2015.
Canadian Institute for Health Information (CIHI). Canada Discharge Abstract Database 1994-1998.
Canadian Institute for Health Information (CIHI). Canada Discharge Abstract Database 1998-2003.
Canadian Institute for Health Information (CIHI). Canada Discharge Abstract Database 2003-2008.
Canadian Institute for Health Information (CIHI). Canada Discharge Abstract Database 2008-2010.
Canadian International Development Agency (CIDA), Caribbean Community (CARICOM) Secretariat. Dominica Population and Housing Census 2001.
Canadian Society for International Health, Ministry of Health (Croatia). Croatia Adult Health Survey 2003.

Appendix: Citation List

Citation
Canavan RJ, Unwin NC, Kelly WF, Connolly VM. Diabetes- and nondiabetes-related lower extremity amputation incidence before and after the introduction of better organized diabetes foot care: continuous longitudinal monitoring using a standard method. Diabetes Care. 2008; 31(3): 459-63.
Cancer Association of Namibia, Namibian Cancer Registry. Namibia Cancer Registry Report 2000-2005. Windhoek, Namibia: Cancer Association of Namibia, 2009.
Cancer Control Department, Ministry of Health (Turkey). Turkey Active Cancer Registration System 8 Provinces Incidence 2007.
Cancer Registry of Republic of Slovenia, Institute of Oncology Ljubljana (Slovenia). Slovenia Cancer 2009. Ljubljana, Slovenia: Cancer Registry of Republic of Slovenia, 2013.
Cancer Registry of Republic of Slovenia. Slovenia Cancer 2007. Ljubljana, Slovenia: Cancer Registry of Republic of Slovenia, 2010.
Cancer Registry of Republic of Slovenia. Slovenia Cancer 2008. Ljubljana, Slovenia: Cancer Registry of Republic of Slovenia, 2011.
Cancer Registry of Republic of Slovenia. Slovenia Cancer Incidence 2003. Ljubljana, Slovenia: Cancer Registry of Republic of Slovenia, 2006.
Cancer Registry of Republic of Slovenia. Slovenia Cancer Incidence 2004. Ljubljana, Slovenia: Cancer Registry of Republic of Slovenia, 2007.
Cancer Registry of Republic of Slovenia. Slovenia Cancer Incidence 2006. Ljubljana, Slovenia: Cancer Registry of Republic of Slovenia, 2009.
Cancrini G, Bartoloni A, Guglielmetti P, Roselli M, Pereira L. Malaria parasitological indices in the Cordillera Province. Ann Trop Med Parasitol. 1992; 86(3): 217-23. As it appears in Malaria Atlas Project. Malaria Atlas Project Plasmodium Falciparum Parasite Rate Database. Oxford, United Kingdom: Malaria Atlas Project.
Candotti D, Mundy C, Kadewele G, Nkhoma W, Bates I, Allain JP. Serological and molecular screening for viruses in blood donors from Ntcheu, Malawi: high prevalence of HIV-1 subtype C and of markers of hepatitis B and C viruses. J Med Virol. 2001; 65(1): 1-5.
Candrilli SD, Davis KL, Kan HJ, Lucero MA, Rousculp MD. Prevalence and the associated burden of illness of symptoms of diabetic peripheral neuropathy and diabetic retinopathy. J Diabet Complications. 2007; 21(5): 306-14.
Canh, T. Monitoring the Chemoresistance of Plasmodium Falciparum to Amino-4 Quinoleines. Bangui, Central African Republic: Faculty of Health Sciences, University of Bangui, 1990. As it appears in Malaria Atlas Project. Malaria Atlas Project Plasmodium Falciparum Parasite Rate Database. Oxford, United Kingdom: Malaria Atlas Project.
Cano J, Berzosa P, Lucio A de, Descalzo MA, Bobuakasi L, Nzambo S, Ondo M, Buatiche JN, Nseng G, Benito A. Transmission of malaria and genotypic variability of Plasmodium falciparum on the Island of Annobon (Equatorial Guinea). Malar J. 2007; 6(1): 141. As it appears in Malaria Atlas Project. Malaria Atlas Project Plasmodium Falciparum Parasite Rate Database. Oxford, United Kingdom: Malaria Atlas Project.
Cantwell R, Clutton-Brock T, Cooper G, Dawson A, Drife J, Garrod D, Harper A, Hulbert D, Lucas S, McClure J, Millward-Sadler H, Neilson J, Nelson-Piercy C, Norman J, O'Herlihy C, Oates M, Shakespeare J, de Swiet M, Williamson C, Beale V, Knight M, Lennox C, Miller A, Parmar D, Rogers J, Springett A. Saving Mothers' Lives: Reviewing maternal deaths to make motherhood safer: 2006-2008. The Eighth Report of the Confidential Enquiries into Maternal Deaths in the United Kingdom. BJOG. 2011; 1-203.
Cao WC, Xu JF, Ren ZX. Epidemiological surveillance of filariasis after its control in Shandong Province, China. Southeast Asian J Trop Med Public Health. 1994; 25(4): 714-8.
Capacent Gallup. Iceland Prevalence of Smoking 2008.
Cape Verde - Poverty Reduction and Growth Strategy Paper II as it appears in World Health Organization (WHO). WHO Household Energy Database. Geneva, Switzerland: World Health Organization (WHO), 2010.
Cape Verde Core Welfare Indicator Questionnaire Survey 2006 as it appears in United Nations Children's Fund (UNICEF). UNICEF Childinfo - Nutritional Status. New York, United States: United Nations Children's Fund (UNICEF).
Cape Verde National Document for International Conference on Nutrition 1992 as it appears in World Health Organization (WHO). WHO Global Database on Child Growth and Malnutrition - Historical. Geneva, Switzerland: World Health Organization (WHO).
Cape Verde National Statistics Institute (INE) Division of Reproductive Health-Centers for Disease Control and Prevention (CDC). (2000) Cape Verde Reproductive Health Survey 1998. Atlanta, United States: Centers for Disease Control and Prevention (CDC).
Cape Verde Survey on DTP3 and Measles Coverage 2009.
Cape Verde Survey on DTP3 and Measles Coverage 2010.
Cape Verde Vital Registration - Deaths 1980 ICD8 as it appears in World Health Organization (WHO). WHO Mortality Database Version May 2013. Geneva, Switzerland: World Health Organization (WHO), 2013.
Cape Verde Vital Registration - Deaths 2011 ICD10 as it appears in World Health Organization (WHO). WHO Mortality Database Version November 2015. Geneva, Switzerland: World Health Organization (WHO), 2015.
Cape Verde Vital Registration - Deaths 2012 ICD10 as it appears in World Health Organization (WHO). WHO Mortality Database Version November 2015. Geneva, Switzerland: World Health Organization (WHO), 2015.
Cape Verde Vital Registration Death Data 1955 as it appears in United Nations Statistics Division (UNSD). United Nations Demographic Yearbook - Historical Supplement 1997. New York City, United States: United Nations Statistics Division (UNSD).
Cape Verde Vital Registration Death Data 1956 as it appears in United Nations Statistics Division (UNSD). United Nations Demographic Yearbook - Historical Supplement 1997. New York City, United States: United Nations Statistics Division (UNSD).
Cape Verde Vital Registration Death Data 1957 as it appears in United Nations Statistics Division (UNSD). United Nations Demographic Yearbook - Historical Supplement 1997. New York City, United States: United Nations Statistics Division (UNSD).
Cape Verde Vital Registration Death Data 1959 as it appears in United Nations Statistics Division (UNSD). United Nations Demographic Yearbook - Historical Supplement 1997. New York City, United States: United Nations Statistics Division (UNSD).
Cape Verde Vital Registration Death Data 1960 as it appears in United Nations Statistics Division (UNSD). United Nations Demographic Yearbook - Historical Supplement 1997. New York City, United States: United Nations Statistics Division (UNSD).
Cape Verde Vital Registration Death Data 1966 as it appears in United Nations Statistics Division (UNSD). United Nations Demographic Yearbook - Historical Supplement 1997. New York City, United States: United Nations Statistics Division (UNSD).
Cape Verde Vital Registration Death Data 1967 as it appears in United Nations Statistics Division (UNSD). United Nations Demographic Yearbook - Historical Supplement 1997. New York City, United States: United Nations Statistics Division (UNSD).

Appendix: Citation List

Citation
Cape Verde Vital Registration Death Data 1968 as it appears in United Nations Statistics Division (UNSD). United Nations Demographic Yearbook - Historical Supplement 1997. New York City, United States: United Nations Statistics Division (UNSD).
Cape Verde Vital Registration Death Data 1969 as it appears in United Nations Statistics Division (UNSD). United Nations Demographic Yearbook - Historical Supplement 1997. New York City, United States: United Nations Statistics Division (UNSD).
Cape Verde Vital Registration Death Data 1970 as it appears in United Nations Statistics Division (UNSD). United Nations Demographic Yearbook - Historical Supplement 1997. New York City, United States: United Nations Statistics Division (UNSD).
Cape Verde Vital Registration Death Data 1971 as it appears in United Nations Statistics Division (UNSD). United Nations Demographic Yearbook - Historical Supplement 1997. New York City, United States: United Nations Statistics Division (UNSD).
Cape Verde Vital Registration Death Data 1972 as it appears in United Nations Statistics Division (UNSD). United Nations Demographic Yearbook - Historical Supplement 1997. New York City, United States: United Nations Statistics Division (UNSD).
Cape Verde Vital Registration Death Data 1973 as it appears in United Nations Statistics Division (UNSD). United Nations Demographic Yearbook - Historical Supplement 1997. New York City, United States: United Nations Statistics Division (UNSD).
Cape Verde Vital Registration Death Data 1974 as it appears in United Nations Statistics Division (UNSD). United Nations Demographic Yearbook - Historical Supplement 1997. New York City, United States: United Nations Statistics Division (UNSD).
Cape Verde Vital Registration Death Data 1975 as it appears in United Nations Statistics Division (UNSD). United Nations Demographic Yearbook - Historical Supplement 1997. New York City, United States: United Nations Statistics Division (UNSD).
Cape Verde Vital Registration Death Data 1979 as it appears in United Nations Statistics Division (UNSD). United Nations Demographic Yearbook - Historical Supplement 1997. New York City, United States: United Nations Statistics Division (UNSD).
Cape Verde Vital Registration Death Data 1983 as it appears in United Nations Statistics Division (UNSD). United Nations Demographic Yearbook - Historical Supplement 1997. New York City, United States: United Nations Statistics Division (UNSD).
Cape Verde Vital Registration Death Data 1984 as it appears in United Nations Statistics Division (UNSD). United Nations Demographic Yearbook - Historical Supplement 1997. New York City, United States: United Nations Statistics Division (UNSD).
Cape Verde Vital Registration Death Data 1985 as it appears in United Nations Statistics Division (UNSD). United Nations Demographic Yearbook - Historical Supplement 1997. New York City, United States: United Nations Statistics Division (UNSD).
Cape Verde Vital Registration Death Data 1990 as it appears in United Nations Statistics Division (UNSD). United Nations Demographic Yearbook - Historical Supplement 1997. New York City, United States: United Nations Statistics Division (UNSD).
Cape Verde Vital Registration Death Data 1991 as it appears in United Nations Statistics Division (UNSD). United Nations Demographic Yearbook - Historical Supplement 1997. New York City, United States: United Nations Statistics Division (UNSD).
Cappuccio FP, Micah FB, Emmett L, Kerry SM, Antwi S, Martin-Peprah R, Phillips RO, Plange-Rhule J, Eastwood JB. Prevalence, detection, management, and control of hypertension in Ashanti, West Africa. Hypertension. 2004; 43(5): 1017-22.
Carabin H, Millogo A, Praet N, Hounton S, Tarnagda Z, Ganaba R, Dorny P, Nitima P, Cowan LD, Evaluation du Fardeau Economique de la Cysticercose Au Burkina Faso (EFECAB). Seroprevalence to the antigens of Taenia solium cysticercosis among residents of three villages in Burkina Faso: a cross-sectional study. PLoS Negl Trop Dis. 2009; 3(11): e555.
Carba DB, Bas IN, Gultiano SA, Lee NR, Adair LS. Waist circumference and the risk of hypertension and prediabetes among Filipino women. Eur J Nutr. 2013; 52(2): 825-32.
Cardiological Society of India. India - Kerala Coronary Artery Disease Risk Factors Prevalence Study 2011.
Cardoso EJ, Valdéz GC, Campos AC, de la Luz Sanchez R, Mendoza CR, Hernández AP, Ramírez MH, Habana JR, González EB, Matzumura PD, Carlier Y. Maternal fetal transmission of Trypanosoma cruzi: a problem of public health little studied in Mexico. Exp Parasitol. 2012; 131(4): 425-32.
Cardwell CR, Carson DJ, Patterson CC. Secular trends, disease maps and ecological analyses of the incidence of childhood onset Type 1 diabetes in Northern Ireland, 1989-2003. Diabet Med. 2007; 24(3): 289-95.
CARE International, Department of Statistics and National Accounts (Niger), Macro International, Inc. Niger Demographic and Health Survey - Maternal Mortality Data.
CARE International, Macro International, Inc. Niger Demographic and Health Survey 1998. Calverton, United States: Macro International, Inc.
Caremani M, Maestrini R, Occhini U, Sassoli S, Accorsi A, Giorgio A, Filice C. Echographic epidemiology of cystic hydatid disease in Italy. Eur J Epidemiol. 1993; 9(4): 401-4.
Caribbean Community (CARICOM) Secretariat, Belize Central Statistical Office. Belize Population and Housing Census 1980.
Caribbean Community (CARICOM) Secretariat, Central Statistical Office (Trinidad and Tobago), Family Development and Children's Research Centre, University of the West Indies (FDCRC), Sir Arthur Lewis Institute of Social and Economic Studies, University of the West Indies (SALISES), United Nations Economic Commission for Latin America and the Caribbean (ECLAC), United Nations Population Fund (UNFPA). Trinidad and Tobago Population and Housing Census 2011.
Caribbean Community (CARICOM) Secretariat, Central Statistical Office (Trinidad and Tobago). Trinidad and Tobago Population and Housing Census 2000.
Caribbean Community (CARICOM) Secretariat, Central Statistical Office of Saint Lucia. Saint Lucia Population and Housing Census 2001.
Caribbean Community (CARICOM) Secretariat, Central Statistical Office, Ministry of Finance (Dominica). Dominica Population and Housing Census 1981.
Caribbean Community (CARICOM) Secretariat, St. Vincent and Grenadines Population and Housing Census 1980.
Caribbean Community (CARICOM) Secretariat. Grenada Population and Housing Census 1981.
Caribbean Community (CARICOM) Secretariat. Grenada Population and Housing Census 2001.
Caribbean Community (CARICOM) Secretariat. Population and Housing Census of the Commonwealth Caribbean 1990-1991. Georgetown, Guyana: Caribbean Community (CARICOM) Secretariat, 2007.

Appendix: Citation List

Citation
Caribbean Epidemiology Centre (CAREC), Central Statistical Office (Trinidad and Tobago), Ministry of Health (Trinidad and Tobago), Pan American Health Organization (PAHO), University of the West Indies. Trinidad and Tobago STEPS Noncommunicable Disease Risk Factors Survey 2011.
Caribbean Epidemiology Centre (CAREC), Ministry of Health and Social Development (Virgin Islands, British), Pan American Health Organization (PAHO), World Health Organization (WHO). Virgin Islands, British STEPS Noncommunicable Disease Risk Factors Survey 2009.
Carme B, Ndounga M, Kissila AM, Mbitsi A, Samba G, Baya T. Resultats de l'enquete menee en 1996 a Brazzaville de la chimioresistance du Plasmodium falciparum. Malaria and Infectious Diseases in Africa. 1997; 7: 53-60. As it appears in Malaria Atlas Project. Malaria Atlas Project Plasmodium Falciparum Parasite Rate Database. Oxford, United Kingdom: Malaria Atlas Project.
Carmena D, Sanchez-Serrano LP, Barbero-Martinez I. Echinococcus granulosus infection in Spain. Zoonoses Public Health. 2008; 55(3): 156-65.
Carneiro I, Hill N. China Plasmodium Falciparum Parasite Rate Data, Personal Communication with I. Carneiro and N. Hill 2009. As it appears in Malaria Atlas Project. Malaria Atlas Project Plasmodium Falciparum Parasite Rate Database. Oxford, United Kingdom: Malaria Atlas Project.
Carnevale P, Robert V, Boudin C, Halna JM, Pazart L, Gazin P, Richard A, Mouchet J. [Control of malaria using mosquito nets impregnated with pyrethroids in Burkina Faso]. Bull Soc Pathol Exot. 1988; 81(5): 832-46. As it appears in Malaria Atlas Project. Malaria Atlas Project Plasmodium Falciparum Parasite Rate Database. Oxford, United Kingdom: Malaria Atlas Project.
Carolina Population Center, University of North Carolina at Chapel Hill, Chinese Center for Disease Control and Prevention (CCDC). China Health and Nutrition Survey 1989-2011. Chapel Hill, United States: Carolina Population Center, University of North Carolina at Chapel Hill.
Carolina Population Center, University of North Carolina at Chapel Hill, Chinese Center for Disease Control and Prevention (CCDC). China Health and Nutrition Survey 2000.
Carolina Population Center, University of North Carolina at Chapel Hill, Department of Statistics (Jordan). Jordan Demographic Survey 1981.
Carolina Population Center, University of North Carolina at Chapel Hill, Institute of Sociology, Russian Academy of Sciences, National Research University Higher School of Economics (Russia), ZAO Demoscope. Russia Longitudinal Monitoring Survey of HSE, Round I 1992.
Carolina Population Center, University of North Carolina at Chapel Hill, Institute of Sociology, Russian Academy of Sciences, National Research University Higher School of Economics (Russia), ZAO Demoscope. Russia Longitudinal Monitoring Survey of HSE, Round II 1992-1993.
Carolina Population Center, University of North Carolina at Chapel Hill, Institute of Sociology, Russian Academy of Sciences, National Research University Higher School of Economics (Russia), ZAO Demoscope. Russia Longitudinal Monitoring Survey of HSE, Round III 1993.
Carolina Population Center, University of North Carolina at Chapel Hill, Office of Population Studies, University of San Carlos (Philippines). Philippines - Cebu Longitudinal Health and Nutrition Survey 2002-2003. Chapel Hill, United States: Carolina Population Center, University of North Carolina at Chapel Hill.
Carolina Population Center, University of North Carolina at Chapel Hill, Office of Population Studies, University of San Carlos (Philippines). Philippines - Cebu Longitudinal Health and Nutrition Survey 2004-2006. Chapel Hill, United States: Carolina Population Center, University of North Carolina at Chapel Hill.
Carrara GC, Petrarca V, Niang M, Coluzzi M. Anopheles pharoensis and transmission of Plasmodium falciparum in the Senegal River delta, West Africa. Med Vet Entomol. 1990; 4(4): 421-4. As it appears in Malaria Atlas Project. Malaria Atlas Project Plasmodium Falciparum Parasite Rate Database. Oxford, United Kingdom: Malaria Atlas Project.
Carrasco E, P¸rez-Bravo F, Dorman J, Mondrag¢n A, Santos JL. Increasing incidence of type 1 diabetes in population from Santiago of Chile: trends in a period of 18 years (1986-2003). Diabetes Metab Res Rev. 2006; 22(1): 34-7.
Carrasco E, Perez-Bravo F, Santos JL, Lopez G, Calvillan M, Wolff C, Garcia de los Rios M. One of the lowest validated incidence rates of insulin dependent diabetes mellitus in the Americas: Santiago, Chile. Diabetes Res Clin Pract. 1996; S153-7.
Carter AO, Saadi HF, Reed RL, Dunn EV. Assessment of obesity, lifestyle, and reproductive health needs of female citizens of Al Ain, United Arab Emirates. J Health Popul Nutr. 2004; 22(1): 75-83.
Carvalho EOC de, Rosa JA da, de Carvalho AA, Chaves HCO, Souza EA de, Ostermayer AL, Camargo LMA de. Study on Chagas disease occurrence in the municipality of Monte Negro, State of Rondônia, Brazilian Amazon. Rev Soc Bras Med Trop. 2011; 44(6): 703-7.
Carvalho ME de, Ferreira MU, Souza MRD de, Ninomia RT, Matos GF, Camargo LMA, Ferreira CS, Carvalho ME de, Ferreira MU, Souza MRD de, Ninomia RT, Matos GF, Camargo LMA, Ferreira CS. Malaria seroepidemiology: comparison between indirect fluorescent antibody test and enzyme immunoassay using bloodspot eluates. Mem Inst Oswaldo Cruz. 1992; 87(2): 205-8. As it appears in Malaria Atlas Project. Malaria Atlas Project Plasmodium Falciparum Parasite Rate Database. Oxford, United Kingdom: Malaria Atlas Project.
Carvalho ME de, Glasser CM, Ciaravolo RM de C, Etzel A, Santos LA dos, Ferreira CS. Sorologia da malaria vivax no foco Aldeia dos Indios, Municipio de Peruibe, Estado de Sao Paulo, 1984 a 1986. Cad Saude Publica. 1988; 4(3): 276-92. As it appears in Malaria Atlas Project. Malaria Atlas Project Plasmodium Falciparum Parasite Rate Database. Oxford, United Kingdom: Malaria Atlas Project.
Carvalho TA, Queiroz MG, Cardoso GL, Diniz IG, Silva AN, Pinto AY, Guerreiro JF. Plasmodium vivax infection in Anajás, State of Pará: no differential resistance profile among Duffy-negative and Duffy-positive individuals. Malar J. 2012; 11(1): 430. As it appears in Malaria Atlas Project. Malaria Atlas Project Plasmodium Falciparum Parasite Rate Database. Oxford, United Kingdom: Malaria Atlas Project.
Casablanca Ministry of Health (Morocco), Faculty of Medicine and Pharmacy of Casablanca (Morocco), Ibn Rochd University Hospital (Morocco), Lalla Salma Association to Fight Against Cancer (Morocco), National Institute of Oncology Sidi Mohamed Ben Abdellah Rabat (Morocco). Morocco - Cancer Registry of Greater Casablanca 2004. Rabat, Morocco: Lalla Salma Association to Fight Against Cancer (Morocco), 2007.
Case, A. 2003. Agincourt Integrated Family Survey 2002. [dataset] Version 1. Cape Town: DataFirst [distributor].

Appendix: Citation List

Citation
Cassava and Child Health Among Sakata: A Nutritional Study of an Ethnic Group in Northern Bandundu Region in Zaire as it appears in World Health Organization (WHO). WHO Global Database on Child Growth and Malnutrition - Historical. Geneva, Switzerland: World Health Organization (WHO).
Castellanos LMDJ, Vasquez JCO, David V. Mortalidad de Mujeres en Edad Reproductiva y Mortalidad Materna. Tegucigalpa, Honduras: Facultad de Ciencias Médicas de la Universidad Nacional Autónoma de Honduras (UNAH); 1990.
Castetbon K, Vernay M, Malon A, Salanave B, Deschamps V, Roudier C, Oleko A, Szego E, Hercberg S. Dietary intake, physical activity and nutritional status in adults: the French nutrition and health survey (ENNS, 2006-2007). Br J Nutr. 2009; 102(5): 733–43.
Casu A, Pascutto C, Bernardinelli L, Songini M. Type 1 diabetes among sardinian children is increasing: the Sardinian diabetes register for children aged 0-14 years (1989-1999). Diabetes Care. 2004; 27(7): 1623-9.
Catholic University of Leuven, Organization for Economic Co-operation and Development (OECD), United Nations Population Division. Mortality in Developing Countries: Tome I Data Bank. Organization for Economic Co-operation and Development (OECD), 1980.
Cattand P, Desjeux P, Guzmán MG, Jannin J, Kroeger A, Medici A, Musgrove P, Nathan MB, Shaw A, Schofield CJ. Tropical Diseases Lacking Adequate Control Measures: Dengue, Leishmaniasis, and African Trypanosomiasis. In: Jamison DT, Breman JG, Measham AR, et al., editors. Disease Control Priorities in Developing Countries. New York, United States: Oxford University Press; 2006. 451-66.
Cavalcante IJ, Vale MR. Epidemiological aspects of visceral leishmaniasis (kala-azar) in Ceará in the period 2007 to 2011. Rev Bras Epidemiol. 2014; 17(4): 911-24.
Cayetano Heredia University, Flora Tristan Center of Peruvian Women, World Health Organization (WHO). Peru WHO Multi-country Study on Women's Health and Domestic Violence Against Women 2000.
CBG Health Research Ltd., Ministry of Health (New Zealand), University of Wollongong. New Zealand Health Survey 2011-2012. Wellington, New Zealand: Ministry of Health (New Zealand).
CBG Health Research Ltd., Ministry of Health (New Zealand). New Zealand Health Survey 2012-2013. Wellington, New Zealand: Ministry of Health (New Zealand).
CBG Health Research Ltd., Ministry of Health (New Zealand). New Zealand Health Survey 2013-2014. Wellington, New Zealand: Ministry of Health (New Zealand).
CDC Foundation, Centers for Disease Control and Prevention (CDC), Department of Health (Philippines), Johns Hopkins Bloomberg School of Public Health, National Statistics Office (Philippines), Research Triangle Institute, Inc. (RTI), World Health Organization (WHO). Philippines Global Adult Tobacco Survey 2009. Atlanta, United States: Centers for Disease Control and Prevention (CDC).
CDC Foundation, Centers for Disease Control and Prevention (CDC), Global Tobacco Surveillance System, Hacettepe University, Johns Hopkins Bloomberg School of Public Health, Ministry of Health (Turkey), Turkish Statistical Institute, World Health Organization (WHO). Turkey Global Adult Tobacco Survey 2008.
CDC Foundation, Centers for Disease Control and Prevention (CDC), Hacettepe University, Johns Hopkins Bloomberg School of Public Health, Ministry of Health (Turkey), Research Triangle Institute, Inc. (RTI), Turkish Statistical Institute, World Health Organization (WHO). Turkey Global Adult Tobacco Survey 2012.
CDC Foundation, Centers for Disease Control and Prevention (CDC), Johns Hopkins Bloomberg School of Public Health, Ministry of Health (Nigeria), National Bureau of Statistics (Nigeria), World Health Organization (WHO). Nigeria Global Adult Tobacco Survey 2012.
CDC Foundation, Centers for Disease Control and Prevention (CDC), Johns Hopkins Bloomberg School of Public Health, Ministry of Health (Uganda), Research Triangle Institute, Inc. (RTI), Uganda Bureau of Statistics, World Health Organization (WHO). Uganda Global Adult Tobacco Survey 2013.
CDC Foundation, Centers for Disease Control and Prevention (CDC), Ministry of Health (Argentina), National Institute of Statistics and Censuses (Argentina), Pan American Health Organization (PAHO). Argentina Global Adult Tobacco Survey 2012. Atlanta, United States: Centers for Disease Control and Prevention (CDC), 2013.
CDC Retro-CI, Ministry of the Fight Against AIDS (Côte d'Ivoire), National Institute of Statistics (Côte d'Ivoire), ORC Macro. Côte d'Ivoire AIDS Indicator Survey 2005. Calverton, United States: ORC Macro.
Cecatti JG, Faúndes A, Surita FG. Maternal mortality in Campinas: evolution, under-registration and avoidance. Sao Paulo Med J. 1999; 117(1): 5-12.
Ceesay MM, Morgan MW, Kamanda MO, Willoughby VR, Lisk DR. Prevalence of diabetes in rural and urban populations in southern Sierra Leone: a preliminary survey. Trop Med Int Health. 1997; 2(3): 272-7.
Ceesay SM, Prentice AM, Cole TJ, Foord F, Weaver LT, Poskitt EM, Whitehead RG. Effects on birth weight and perinatal mortality of maternal dietary supplements in rural Gambia: 5 year randomised controlled trial. BMJ. 1997; 315(7111): 786-90.
Census Bureau (Chad), Macro International, Inc, National Institute of Statistical, Economic and Demographic Studies (Chad). Chad Demographic and Health Survey - Maternal Mortality Data.
Census Bureau (Chad), Macro International, Inc, National Institute of Statistical, Economic and Demographic Studies (Chad). Chad Demographic and Health Survey 1996-1997. Calverton, United States: Macro International, Inc.
Census Commissioner (Fiji). Fiji Population and Housing Census 1956.
Census Commissioner (Seychelles). Seychelles Population and Housing Census 1971.
Census Commissioner (Seychelles). Seychelles Population and Housing Census 1977.
Center for Addiction Medicine, National Institute of Mental Health and Neurosciences (India), World Health Organization (WHO). India - Karnataka Unrecorded Alcohol Consumption Survey 2001-2002.
Center for Addiction Medicine, National Institute of Mental Health and Neurosciences (India). India Alcohol Misuse Epidemiological Survey 2011-2012.
Center for Demographic Research, New Economic School (Russia). Russia Mortality Data 2012. [Unpublished].
Center for Demographic Research, New Economic School (Russia). Russia Mortality Rates by Region, Age, Sex, and Cause of Death 1989-1998. Moscow, Russia: Center for Demographic Research, New Economic School (Russia), 2014-02-12. http://demogr.nes.ru/index.php/ru/demogr_indicat/data

Appendix: Citation List

Citation
Center for Demographic Research, New Economic School (Russia). Russia Mortality Rates by Region, Age, Sex, and Cause of Death 1999-2005. Moscow, Russia: Center for Demographic Research, New Economic School (Russia).
Center for Demographic Research, New Economic School (Russia). Russia Mortality Rates by Region, Age, Sex, and Cause of Death 2011-2013. Moscow, Russia: Center for Demographic Research, New Economic School (Russia).
Center for Demographic Research, New Economic School (Russia). Russia Population Data 1989-2011. [Unpublished].
Center for Demographic Research, New Economic School (Russia). Russia Vital Registration - Deaths 2001.
Center for Demographic Research, New Economic School (Russia). Russia Vital Registration - Deaths 2002.
Center for Demographic Research, New Economic School (Russia). Russia Vital Registration - Deaths 2003.
Center for Demographic Research, New Economic School (Russia). Russia Vital Registration - Deaths 2004.
Center for Demographic Research, New Economic School (Russia). Russia Vital Registration - Deaths 2005.
Center for Demographic Research, New Economic School (Russia). Russia Vital Registration - Deaths 2006.
Center for Demographic Research, New Economic School (Russia). Russia Vital Registration - Deaths 2007.
Center for Demographic Research, New Economic School (Russia). Russia Vital Registration - Deaths 2008.
Center for Demographic Research, New Economic School (Russia). Russia Vital Registration - Deaths 2009.
Center for Demographic Research, New Economic School (Russia). Russia Vital Registration - Deaths 2010.
Center for Demographic Research, New Economic School (Russia). Russia Vital Registration - Deaths 2011.
Center for Demographic Research, New Economic School (Russia). Russia Vital Registration - Deaths 2012.
Center for Demographic Research, New Economic School (Russia). Russia Vital Registration - Deaths 2013.
Center for Demographic Research, New Economic School (Russia). Russia Vital Registration - Deaths 2014.
Center for Disease Control and Prevention, Ministry of Health and Medical Education (Iran). Iran National Cancer Registry Report 2003-2004.
Center for Disease Control and Prevention, Ministry of Health and Medical Education (Iran). Iran National Cancer Registry Report 2005-2006.
Center for Disease Control and Prevention, Ministry of Health and Medical Education (Iran). Iran National Cancer Registry Report 2006-2007.
Center for Health Policies and Services (Romania). Knowledge, Attitudes and Practices of the General Population Regarding Tobacco Use and Related Legislation. Bucharest, Romania: Center for Health Policies and Services (Romania), 2007.
Center for Human Resource Research, Ohio State University, Korea Labor Institute (South Korea), RAND Corporation, Statistics Netherlands. Korea, South Longitudinal Study of Ageing 2006. Seoul, South Korea: Korea Labor Institute (South Korea).
Center for Population and Policy Studies, Gadjah Mada University (Indonesia), RAND Corporation, SurveyMETER. Indonesia Family Life Survey 2007-2008. Santa Monica, United States: RAND Corporation.
Center for Population and Policy Studies, Gadjah Mada University (Indonesia), RAND Corporation. Indonesia Family Life Survey 2000. Santa Monica, United States: RAND Corporation.
Center for Population Studies and Responsible Parenthood (CEPAR) (El Salvador), Westinghouse; Institute for Resource Development. Ecuador Demographic and Health Survey 1987. Columbia, United States: Westinghouse; Institute for Resource Development.
Center for Research and Teaching in Economics (CIDE) (Mexico), Duke University, National Institute of Public Health (Mexico), Universidad Iberoamericana, University of California, Los Angeles (UCLA). Mexico Family Life Survey 2008-2013.
Center for Research in Human Development (CRDH), Cheikh Anta Diop University, Hospital Aristide Le Dantec, ICF Macro, Ministry of Health and Prevention (Senegal), National Agency of Statistics and Demography (Senegal). Senegal Demographic and Health Survey - Maternal Mortality Data.
Center for Research in Human Development (CRDH), Cheikh Anta Diop University, Hospital Aristide Le Dantec, ICF Macro, National Agency of Statistics and Demography (Senegal). Senegal Demographic and Health Survey 2010-2011. Calverton, United States: ICF Macro.
Center for Research on Environment Health and Population Activities (Nepal), Ministry of Health (Nepal), New ERA, Options UK, Safe Motherhood Network Federation (Nepal). Nepal Maternal Mortality and Morbidity Study 2008-2009.
Center for Scientific and Technological Information, Oswaldo Cruz Foundation and World Health Organization (WHO). Brazil World Health Survey 2003. Geneva, Switzerland: World Health Organization (WHO), 2005.
Center for Social and Demographic Studies (CESDEM), ICF International, Ministry of Public Health and Social Assistance (Dominican Republic). Dominican Republic Demographic and Health Survey 2013. Fairfax, United States: ICF International, 2014.
Center for Social and Demographic Studies (CESDEM), Macro International, Inc, National Council for Population and Family (Dominican Republic), National Planning Office (Dominican Republic), Profamilia. Dominican Republic Demographic and Health Survey - Maternal Mortality Data.
Center for Social and Demographic Studies (CESDEM), Macro International, Inc, National Planning Office (Dominican Republic), Profamilia. Dominican Republic Demographic and Health Survey 1996. Calverton, United States: Macro International, Inc.
Center for Social and Demographic Studies (CESDEM), Macro International, Inc. Dominican Republic Demographic and Health Survey 1999. Calverton, United States: Macro International, Inc.
Center for Social and Demographic Studies (CESDEM), Macro International, Inc. Dominican Republic Demographic and Health Survey 2002. Calverton, United States: Macro International, Inc.
Center for Social and Demographic Studies (CESDEM), Macro International, Inc. Dominican Republic Demographic and Health Survey 2007. Calverton, United States: Macro International, Inc.
Center for Social and Demographic Studies (CESDEM), United Nations Children's Fund (UNICEF). Dominican Republic Multiple Indicator Cluster Survey 2000. New York, United States: United Nations Children's Fund (UNICEF).
Center for Sociological Studies, Lomonosov Moscow State University, Concluzia-Prim Center for Survey Methodology (Moldova), Institute for Advanced Studies (Austria), London School of Hygiene and Tropical Medicine, University of Aberdeen. Russia Health in Times of Transition Household Survey 2010.

Appendix: Citation List

Citation
Center for Studies of Population and Social Development (CEPAR) (Ecuador) and Division of Reproductive Health-Centers for Disease Control and Prevention (CDC). (2005) Ecuador Reproductive Health Survey 2004. Quito, Ecuador: CEPAR.
Center for Studies of Population and Social Development (CEPAR) (Ecuador), Division of Reproductive Health-Centers for Disease Control and Prevention (CDC). Ecuador Reproductive Health Survey 1999. Atlanta, United States: Centers for Disease Control and Prevention (CDC), 2001.
Center for Studies of Population and Social Development (CEPAR), Division of Reproductive Health-Centers for Disease Control and Prevention (CDC). (1995) Ecuador Reproductive Health Survey 1994. Atlanta, United States: Centers for Disease Control and Prevention (CDC).
Center for Studies of Population and Social Development (CEPAR), Ecuador Ministry of Health (MSP), Division of Reproductive Health-Centers for Disease Control and Prevention (CDC). English Language Report (1992) Ecuador Family Planning/Maternal and Child Health Survey 1989. Atlanta, United States: Centers for Disease Control and Prevention (CDC).
Center for Studies of Population and Social Development (Ecuador), Division of Reproductive Health, Centers for Disease Control and Prevention (CDC), Ecuador Ministry of Health (MSP), National Institute of Statistics and Censuses (Ecuador). Ecuador Reproductive Health Survey - Maternal Mortality Data.
Center for Study of Public Opinion (Kazakhstan), Concluzia-Prim Center for Survey Methodology (Moldova), Institute for Advanced Studies (Austria), London School of Hygiene and Tropical Medicine, University of Aberdeen. Kazakhstan Health in Times of Transition Household Survey 2010.
Center for the Study of State and Society (CEDES), Ministry of Health and Environment (Argentina), National Institute of Statistics and Censuses (Argentina). Argentina National Survey of Risk Factors 2005.
Center of Public Health Sibiu (Romania). Attitudes and Behaviors for a Healthy Lifestyle 2005.
Center of Public Health Sibiu (Romania). Romania Knowledge, Attitudes and Practices About Tobacco Use 2003.
CentERdata. Netherlands Dutch National Bank Household Survey 1995. Tillburg, Netherlands: CentERdata.
CentERdata. Netherlands Dutch National Bank Household Survey 1996. Tillburg, Netherlands: CentERdata.
CentERdata. Netherlands Dutch National Bank Household Survey 1997. Tillburg, Netherlands: CentERdata.
CentERdata. Netherlands Dutch National Bank Household Survey 1998. Tillburg, Netherlands: CentERdata.
CentERdata. Netherlands Dutch National Bank Household Survey 1999. Tillburg, Netherlands: CentERdata.
CentERdata. Netherlands Dutch National Bank Household Survey 2000. Tillburg, Netherlands: CentERdata.
CentERdata. Netherlands Dutch National Bank Household Survey 2001. Tillburg, Netherlands: CentERdata.
CentERdata. Netherlands Dutch National Bank Household Survey 2002. Tillburg, Netherlands: CentERdata.
CentERdata. Netherlands Dutch National Bank Household Survey 2003. Tillburg, Netherlands: CentERdata.
CentERdata. Netherlands Dutch National Bank Household Survey 2004. Tillburg, Netherlands: CentERdata.
CentERdata. Netherlands Dutch National Bank Household Survey 2005. Tillburg, Netherlands: CentERdata.
CentERdata. Netherlands Dutch National Bank Household Survey 2006. Tillburg, Netherlands: CentERdata.
CentERdata. Netherlands Dutch National Bank Household Survey 2007. Tillburg, Netherlands: CentERdata.
CentERdata. Netherlands Dutch National Bank Household Survey 2008. Tillburg, Netherlands: CentERdata.
CentERdata. Netherlands Dutch National Bank Household Survey 2009. Tillburg, Netherlands: CentERdata.
CentERdata. Netherlands Dutch National Bank Household Survey 2010. Tilburg, Netherlands: CentERdata.
CentERdata. Netherlands Dutch National Bank Household Survey 2011. Tilburg, Netherlands: CentERdata.
CentERdata. Netherlands Dutch National Bank Household Survey 2012. Tilburg, Netherlands: CentERdata.
CentERdata. Netherlands Dutch National Bank Household Survey 2013. Tilburg, Netherlands: CentERdata.
CentERdata. Netherlands Dutch National Bank Household Survey 2014.
Centers for Disease Control and Prevention (CDC) and World Health Organization (WHO). Albania Global Youth Tobacco Survey 2004. United States: Centers for Disease Control and Prevention (CDC), 2004.
Centers for Disease Control and Prevention (CDC) and World Health Organization (WHO). Antigua and Barbuda Global Youth Tobacco Survey 2000. United States: Centers for Disease Control and Prevention (CDC), 2000.
Centers for Disease Control and Prevention (CDC) and World Health Organization (WHO). Argentina Global Youth Tobacco Survey 2000. Atlanta, United States: Centers for Disease Control and Prevention (CDC).
Centers for Disease Control and Prevention (CDC) and World Health Organization (WHO). Argentina Global Youth Tobacco Survey 2007. United States: Centers for Disease Control and Prevention (CDC), 2007.
Centers for Disease Control and Prevention (CDC) and World Health Organization (WHO). Armenia Global Youth Tobacco Survey 2004. United States: Centers for Disease Control and Prevention (CDC), 2004.
Centers for Disease Control and Prevention (CDC) and World Health Organization (WHO). Bahamas Global Youth Tobacco Survey 2000. United States: Centers for Disease Control and Prevention (CDC), 2000.
Centers for Disease Control and Prevention (CDC) and World Health Organization (WHO). Bahamas Global Youth Tobacco Survey 2004. United States: Centers for Disease Control and Prevention (CDC), 2004.
Centers for Disease Control and Prevention (CDC) and World Health Organization (WHO). Bahrain Global Youth Tobacco Survey 2002. United States: Centers for Disease Control and Prevention (CDC), 2002.
Centers for Disease Control and Prevention (CDC) and World Health Organization (WHO). Barbados Global Youth Tobacco Survey 2002. United States: Centers for Disease Control and Prevention (CDC), 2002.
Centers for Disease Control and Prevention (CDC) and World Health Organization (WHO). Belarus Global Youth Tobacco Survey 2003. Atlanta, United States: Centers for Disease Control and Prevention (CDC).
Centers for Disease Control and Prevention (CDC) and World Health Organization (WHO). Belize Global Youth Tobacco Survey 2003. Atlanta, United States: Centers for Disease Control and Prevention (CDC).

Appendix: Citation List

Citation
Centers for Disease Control and Prevention (CDC) and World Health Organization (WHO). Bhutan Global Youth Tobacco Survey 2004. Atlanta, United States: Centers for Disease Control and Prevention (CDC).
Centers for Disease Control and Prevention (CDC) and World Health Organization (WHO). Bhutan Global Youth Tobacco Survey 2006. United States: Centers for Disease Control and Prevention (CDC), 2006.
Centers for Disease Control and Prevention (CDC) and World Health Organization (WHO). Bosnia and Herzegovina Global Youth Tobacco Survey 2003. United States: Centers for Disease Control and Prevention (CDC), 2003.
Centers for Disease Control and Prevention (CDC) and World Health Organization (WHO). Botswana Global Youth Tobacco Survey 2001. United States: Centers for Disease Control and Prevention (CDC), 2001.
Centers for Disease Control and Prevention (CDC) and World Health Organization (WHO). Bulgaria Global Youth Tobacco Survey 2002. Atlanta, United States: Centers for Disease Control and Prevention (CDC).
Centers for Disease Control and Prevention (CDC) and World Health Organization (WHO). Cambodia Global Youth Tobacco Survey 2003. United States: Centers for Disease Control and Prevention (CDC), 2003.
Centers for Disease Control and Prevention (CDC) and World Health Organization (WHO). Cape Verde Global Youth Tobacco Survey 2007. United States: Centers for Disease Control and Prevention (CDC), 2007.
Centers for Disease Control and Prevention (CDC) and World Health Organization (WHO). Comoros Global Youth Tobacco Survey 2007. United States: Centers for Disease Control and Prevention (CDC), 2007.
Centers for Disease Control and Prevention (CDC) and World Health Organization (WHO). Congo Global Youth Tobacco Survey 2006. United States: Centers for Disease Control and Prevention (CDC), 2006.
Centers for Disease Control and Prevention (CDC) and World Health Organization (WHO). Costa Rica Global Youth Tobacco Survey 1999. United States: Centers for Disease Control and Prevention (CDC), 1999.
Centers for Disease Control and Prevention (CDC) and World Health Organization (WHO). Costa Rica Global Youth Tobacco Survey 2002. Atlanta, United States: Centers for Disease Control and Prevention (CDC).
Centers for Disease Control and Prevention (CDC) and World Health Organization (WHO). Croatia Global Youth Tobacco Survey 2003. Atlanta, United States: Centers for Disease Control and Prevention (CDC).
Centers for Disease Control and Prevention (CDC) and World Health Organization (WHO). Croatia Global Youth Tobacco Survey 2007. United States: Centers for Disease Control and Prevention (CDC), 2007.
Centers for Disease Control and Prevention (CDC) and World Health Organization (WHO). Cyprus Global Youth Tobacco Survey 2005. United States: Centers for Disease Control and Prevention (CDC), 2005.
Centers for Disease Control and Prevention (CDC) and World Health Organization (WHO). Czech Republic Global Youth Tobacco Survey 2002. Atlanta, United States: Centers for Disease Control and Prevention (CDC).
Centers for Disease Control and Prevention (CDC) and World Health Organization (WHO). Czech Republic Global Youth Tobacco Survey 2007. United States: Centers for Disease Control and Prevention (CDC), 2007.
Centers for Disease Control and Prevention (CDC) and World Health Organization (WHO). Dominica Global Youth Tobacco Survey 2000. Atlanta, United States: Centers for Disease Control and Prevention (CDC).
Centers for Disease Control and Prevention (CDC) and World Health Organization (WHO). Dominican Republic Global Youth Tobacco Survey 2004. Atlanta, United States: Centers for Disease Control and Prevention (CDC).
Centers for Disease Control and Prevention (CDC) and World Health Organization (WHO). Egypt Global Youth Tobacco Survey 2001. Atlanta, United States: Centers for Disease Control and Prevention (CDC).
Centers for Disease Control and Prevention (CDC) and World Health Organization (WHO). Egypt Global Youth Tobacco Survey 2005. Atlanta, United States: Centers for Disease Control and Prevention (CDC).
Centers for Disease Control and Prevention (CDC) and World Health Organization (WHO). El Salvador Global Youth Tobacco Survey 2003. Atlanta, United States: Centers for Disease Control and Prevention (CDC).
Centers for Disease Control and Prevention (CDC) and World Health Organization (WHO). Eritrea Global Youth Tobacco Survey 2006. Atlanta, United States: Centers for Disease Control and Prevention (CDC).
Centers for Disease Control and Prevention (CDC) and World Health Organization (WHO). Estonia Global Youth Tobacco Survey 2003. Atlanta, United States: Centers for Disease Control and Prevention (CDC).
Centers for Disease Control and Prevention (CDC) and World Health Organization (WHO). Estonia Global Youth Tobacco Survey 2007. United States: Centers for Disease Control and Prevention (CDC), 2007.
Centers for Disease Control and Prevention (CDC) and World Health Organization (WHO). Federated States of Micronesia Global Youth Tobacco Survey 2007. United States: Centers for Disease Control and Prevention (CDC), 2007.
Centers for Disease Control and Prevention (CDC) and World Health Organization (WHO). Fiji Global Youth Tobacco Survey 1999. United States: Centers for Disease Control and Prevention (CDC), 1999.
Centers for Disease Control and Prevention (CDC) and World Health Organization (WHO). Fiji Global Youth Tobacco Survey 2005. Atlanta, United States: Centers for Disease Control and Prevention (CDC).
Centers for Disease Control and Prevention (CDC) and World Health Organization (WHO). Georgia Global Youth Tobacco Survey 2002. Atlanta, United States: Centers for Disease Control and Prevention (CDC).
Centers for Disease Control and Prevention (CDC) and World Health Organization (WHO). Ghana Global Youth Tobacco Survey 2006. Atlanta, United States: Centers for Disease Control and Prevention (CDC).
Centers for Disease Control and Prevention (CDC) and World Health Organization (WHO). Greece Global Youth Tobacco Survey 2005. United States: Centers for Disease Control and Prevention (CDC), 2005.
Centers for Disease Control and Prevention (CDC) and World Health Organization (WHO). Guyana Global Youth Tobacco Survey 2000. United States: Centers for Disease Control and Prevention (CDC), 2000.
Centers for Disease Control and Prevention (CDC) and World Health Organization (WHO). Guyana Global Youth Tobacco Survey 2004. Atlanta, United States: Centers for Disease Control and Prevention (CDC).

Appendix: Citation List

Citation
Centers for Disease Control and Prevention (CDC) and World Health Organization (WHO). Haiti Global Youth Tobacco Survey 2005. Atlanta, United States: Centers for Disease Control and Prevention (CDC).
Centers for Disease Control and Prevention (CDC) and World Health Organization (WHO). Hungary Global Youth Tobacco Survey 2002. Atlanta, United States: Centers for Disease Control and Prevention (CDC).
Centers for Disease Control and Prevention (CDC) and World Health Organization (WHO). India Global Youth Tobacco Survey 2006. Atlanta, United States: Centers for Disease Control and Prevention (CDC).
Centers for Disease Control and Prevention (CDC) and World Health Organization (WHO). Indonesia Global Youth Tobacco Survey 2006. Atlanta, United States: Centers for Disease Control and Prevention (CDC).
Centers for Disease Control and Prevention (CDC) and World Health Organization (WHO). Iran, Islamic Rep. Global Youth Tobacco Survey 2003. Atlanta, United States: Centers for Disease Control and Prevention (CDC).
Centers for Disease Control and Prevention (CDC) and World Health Organization (WHO). Islamic Republic of Iran Global Youth Tobacco Survey 2007. United States: Centers for Disease Control and Prevention (CDC), 2007.
Centers for Disease Control and Prevention (CDC) and World Health Organization (WHO). Jamaica Global Youth Tobacco Survey 2000. Atlanta, United States: Centers for Disease Control and Prevention (CDC).
Centers for Disease Control and Prevention (CDC) and World Health Organization (WHO). Jamaica Global Youth Tobacco Survey 2006. Atlanta, United States: Centers for Disease Control and Prevention (CDC).
Centers for Disease Control and Prevention (CDC) and World Health Organization (WHO). Jordan Global Youth Tobacco Survey 1999. United States: Centers for Disease Control and Prevention (CDC), 1999.
Centers for Disease Control and Prevention (CDC) and World Health Organization (WHO). Jordan Global Youth Tobacco Survey 2003. Atlanta, United States: Centers for Disease Control and Prevention (CDC).
Centers for Disease Control and Prevention (CDC) and World Health Organization (WHO). Jordan Global Youth Tobacco Survey 2007. Atlanta, United States: Centers for Disease Control and Prevention (CDC).
Centers for Disease Control and Prevention (CDC) and World Health Organization (WHO). Kazakhstan Global Youth Tobacco Survey 2004. Atlanta, United States: Centers for Disease Control and Prevention (CDC).
Centers for Disease Control and Prevention (CDC) and World Health Organization (WHO). Korea, South Global Youth Tobacco Survey 2005. United States: Centers for Disease Control and Prevention (CDC), 2005.
Centers for Disease Control and Prevention (CDC) and World Health Organization (WHO). Kuwait Global Youth Tobacco Survey 2001. United States: Centers for Disease Control and Prevention (CDC), 2001.
Centers for Disease Control and Prevention (CDC) and World Health Organization (WHO). Kuwait Global Youth Tobacco Survey 2005. United States: Centers for Disease Control and Prevention (CDC), 2005.
Centers for Disease Control and Prevention (CDC) and World Health Organization (WHO). Latvia Global Youth Tobacco Survey 2002. Atlanta, United States: Centers for Disease Control and Prevention (CDC).
Centers for Disease Control and Prevention (CDC) and World Health Organization (WHO). Latvia Global Youth Tobacco Survey 2007. United States: Centers for Disease Control and Prevention (CDC), 2007.
Centers for Disease Control and Prevention (CDC) and World Health Organization (WHO). Lebanon Global Youth Tobacco Survey 2001. United States: Centers for Disease Control and Prevention (CDC), 2001.
Centers for Disease Control and Prevention (CDC) and World Health Organization (WHO). Lesotho Global Youth Tobacco Survey 2002. Atlanta, United States: Centers for Disease Control and Prevention (CDC).
Centers for Disease Control and Prevention (CDC) and World Health Organization (WHO). Libyan Arab Jamahiriya Global Youth Tobacco Survey 2003. United States: Centers for Disease Control and Prevention (CDC), 2003.
Centers for Disease Control and Prevention (CDC) and World Health Organization (WHO). Libyan Arab Jamahiriya Global Youth Tobacco Survey 2007. United States: Centers for Disease Control and Prevention (CDC), 2007.
Centers for Disease Control and Prevention (CDC) and World Health Organization (WHO). Lithuania Global Youth Tobacco Survey 2001. United States: Centers for Disease Control and Prevention (CDC), 2001.
Centers for Disease Control and Prevention (CDC) and World Health Organization (WHO). Lithuania Global Youth Tobacco Survey 2005. Atlanta, United States: Centers for Disease Control and Prevention (CDC).
Centers for Disease Control and Prevention (CDC) and World Health Organization (WHO). Macedonia Global Youth Tobacco Survey 2003. United States: Centers for Disease Control and Prevention (CDC), 2003.
Centers for Disease Control and Prevention (CDC) and World Health Organization (WHO). Malawi Global Youth Tobacco Survey 2001. Atlanta, United States: Centers for Disease Control and Prevention (CDC).
Centers for Disease Control and Prevention (CDC) and World Health Organization (WHO). Malaysia Global Youth Tobacco Survey 2003. United States: Centers for Disease Control and Prevention (CDC), 2003.
Centers for Disease Control and Prevention (CDC) and World Health Organization (WHO). Maldives Global Youth Tobacco Survey 2004. Atlanta, United States: Centers for Disease Control and Prevention (CDC).
Centers for Disease Control and Prevention (CDC) and World Health Organization (WHO). Maldives Global Youth Tobacco Survey 2007. United States: Centers for Disease Control and Prevention (CDC), 2007.
Centers for Disease Control and Prevention (CDC) and World Health Organization (WHO). Mauritania Global Youth Tobacco Survey 2006. Atlanta, United States: Centers for Disease Control and Prevention (CDC).
Centers for Disease Control and Prevention (CDC) and World Health Organization (WHO). Mauritius Global Youth Tobacco Survey 2003. Atlanta, United States: Centers for Disease Control and Prevention (CDC).
Centers for Disease Control and Prevention (CDC) and World Health Organization (WHO). Moldova Global Youth Tobacco Survey 2004. Atlanta, United States: Centers for Disease Control and Prevention (CDC).
Centers for Disease Control and Prevention (CDC) and World Health Organization (WHO). Mongolia Global Youth Tobacco Survey 2007. United States: Centers for Disease Control and Prevention (CDC), 2007.

Appendix: Citation List

Citation
Centers for Disease Control and Prevention (CDC) and World Health Organization (WHO). Montenegro Global Youth Tobacco Survey 2004. United States: Centers for Disease Control and Prevention (CDC), 2004.
Centers for Disease Control and Prevention (CDC) and World Health Organization (WHO). Morocco Global Youth Tobacco Survey 2001. United States: Centers for Disease Control and Prevention (CDC), 2001.
Centers for Disease Control and Prevention (CDC) and World Health Organization (WHO). Morocco Global Youth Tobacco Survey 2006. Atlanta, United States: Centers for Disease Control and Prevention (CDC).
Centers for Disease Control and Prevention (CDC) and World Health Organization (WHO). Myanmar Global Youth Tobacco Survey 2004. Atlanta, United States: Centers for Disease Control and Prevention (CDC).
Centers for Disease Control and Prevention (CDC) and World Health Organization (WHO). Namibia Global Youth Tobacco Survey 2004. Atlanta, United States: Centers for Disease Control and Prevention (CDC).
Centers for Disease Control and Prevention (CDC) and World Health Organization (WHO). Nepal Global Youth Tobacco Survey 2001. United States: Centers for Disease Control and Prevention (CDC), 2001.
Centers for Disease Control and Prevention (CDC) and World Health Organization (WHO). Nepal Global Youth Tobacco Survey 2007. United States: Centers for Disease Control and Prevention (CDC), 2007.
Centers for Disease Control and Prevention (CDC) and World Health Organization (WHO). New Zealand Global Youth Tobacco Survey 2007. United States: Centers for Disease Control and Prevention (CDC), 2007.
Centers for Disease Control and Prevention (CDC) and World Health Organization (WHO). Nicaragua Global Youth Tobacco Survey 2003. United States: Centers for Disease Control and Prevention (CDC), 2003.
Centers for Disease Control and Prevention (CDC) and World Health Organization (WHO). Niger Global Youth Tobacco Survey 2001. United States: Centers for Disease Control and Prevention (CDC), 2001.
Centers for Disease Control and Prevention (CDC) and World Health Organization (WHO). Oman Global Youth Tobacco Survey 2002. United States: Centers for Disease Control and Prevention (CDC), 2002.
Centers for Disease Control and Prevention (CDC) and World Health Organization (WHO). Pakistan Global Youth Tobacco Survey 2003. Atlanta, United States: Centers for Disease Control and Prevention (CDC).
Centers for Disease Control and Prevention (CDC) and World Health Organization (WHO). Panama Global Youth Tobacco Survey 2002. Atlanta, United States: Centers for Disease Control and Prevention (CDC).
Centers for Disease Control and Prevention (CDC) and World Health Organization (WHO). Peru Global Youth Tobacco Survey 2007. Atlanta, United States: Centers for Disease Control and Prevention (CDC).
Centers for Disease Control and Prevention (CDC) and World Health Organization (WHO). Philippines Global Youth Tobacco Survey 2004. Atlanta, United States: Centers for Disease Control and Prevention (CDC).
Centers for Disease Control and Prevention (CDC) and World Health Organization (WHO). Philippines Global Youth Tobacco Survey 2007. United States: Centers for Disease Control and Prevention (CDC), 2007.
Centers for Disease Control and Prevention (CDC) and World Health Organization (WHO). Poland Global Youth Tobacco Survey 2003. United States: Centers for Disease Control and Prevention (CDC), 2003.
Centers for Disease Control and Prevention (CDC) and World Health Organization (WHO). Qatar Global Youth Tobacco Survey 2004. Atlanta, United States: Centers for Disease Control and Prevention (CDC).
Centers for Disease Control and Prevention (CDC) and World Health Organization (WHO). Qatar Global Youth Tobacco Survey 2007. United States: Centers for Disease Control and Prevention (CDC), 2007.
Centers for Disease Control and Prevention (CDC) and World Health Organization (WHO). Romania Global Youth Tobacco Survey 2004. Atlanta, United States: Centers for Disease Control and Prevention (CDC).
Centers for Disease Control and Prevention (CDC) and World Health Organization (WHO). Russia Global Youth Tobacco Survey 1999. United States: Centers for Disease Control and Prevention (CDC), 1999.
Centers for Disease Control and Prevention (CDC) and World Health Organization (WHO). Russia Global Youth Tobacco Survey 2004. Atlanta, United States: Centers for Disease Control and Prevention (CDC).
Centers for Disease Control and Prevention (CDC) and World Health Organization (WHO). Saint Lucia Global Youth Tobacco Survey 2000. United States: Centers for Disease Control and Prevention (CDC), 2000.
Centers for Disease Control and Prevention (CDC) and World Health Organization (WHO). Saint Vincent and the Grenadines Global Youth Tobacco Survey 2000. United States: Centers for Disease Control and Prevention (CDC), 2000.
Centers for Disease Control and Prevention (CDC) and World Health Organization (WHO). Saint Vincent and the Grenadines Global Youth Tobacco Survey 2007. United States: Centers for Disease Control and Prevention (CDC), 2007.
Centers for Disease Control and Prevention (CDC) and World Health Organization (WHO). Samoa Global Youth Tobacco Survey 2007. Atlanta, United States: Centers for Disease Control and Prevention (CDC).
Centers for Disease Control and Prevention (CDC) and World Health Organization (WHO). Saudi Arabia Global Youth Tobacco Survey 2007. United States: Centers for Disease Control and Prevention (CDC), 2007.
Centers for Disease Control and Prevention (CDC) and World Health Organization (WHO). Senegal Global Youth Tobacco Survey 2002. Atlanta, United States: Centers for Disease Control and Prevention (CDC).
Centers for Disease Control and Prevention (CDC) and World Health Organization (WHO). Senegal Global Youth Tobacco Survey 2007. United States: Centers for Disease Control and Prevention (CDC), 2007.
Centers for Disease Control and Prevention (CDC) and World Health Organization (WHO). Seychelles Global Youth Tobacco Survey 2002. Atlanta, United States: Centers for Disease Control and Prevention (CDC).
Centers for Disease Control and Prevention (CDC) and World Health Organization (WHO). Seychelles Global Youth Tobacco Survey 2007. United States: Centers for Disease Control and Prevention (CDC), 2007.
Centers for Disease Control and Prevention (CDC) and World Health Organization (WHO). Singapore Global Youth Tobacco Survey 2000. United States: Centers for Disease Control and Prevention (CDC), 2000.

Appendix: Citation List

Citation
Centers for Disease Control and Prevention (CDC) and World Health Organization (WHO). Slovakia Global Youth Tobacco Survey 2003. Atlanta, United States: Centers for Disease Control and Prevention (CDC).
Centers for Disease Control and Prevention (CDC) and World Health Organization (WHO). Slovakia Global Youth Tobacco Survey 2007. United States: Centers for Disease Control and Prevention (CDC), 2007.
Centers for Disease Control and Prevention (CDC) and World Health Organization (WHO). Slovenia Global Youth Tobacco Survey 2003. Atlanta, United States: Centers for Disease Control and Prevention (CDC).
Centers for Disease Control and Prevention (CDC) and World Health Organization (WHO). Slovenia Global Youth Tobacco Survey 2007. United States: Centers for Disease Control and Prevention (CDC), 2007.
Centers for Disease Control and Prevention (CDC) and World Health Organization (WHO). South Africa Global Youth Tobacco Survey 1999. Atlanta, United States: Centers for Disease Control and Prevention (CDC).
Centers for Disease Control and Prevention (CDC) and World Health Organization (WHO). South Africa Global Youth Tobacco Survey 2002. Atlanta, United States: Centers for Disease Control and Prevention (CDC).
Centers for Disease Control and Prevention (CDC) and World Health Organization (WHO). Sri Lanka Global Youth Tobacco Survey 1999. United States: Centers for Disease Control and Prevention (CDC), 1999.
Centers for Disease Control and Prevention (CDC) and World Health Organization (WHO). Sudan Global Youth Tobacco Survey 2001. United States: Centers for Disease Control and Prevention (CDC), 2001.
Centers for Disease Control and Prevention (CDC) and World Health Organization (WHO). Sudan Global Youth Tobacco Survey 2005. Atlanta, United States: Centers for Disease Control and Prevention (CDC).
Centers for Disease Control and Prevention (CDC) and World Health Organization (WHO). Suriname Global Youth Tobacco Survey 2004. Atlanta, United States: Centers for Disease Control and Prevention (CDC).
Centers for Disease Control and Prevention (CDC) and World Health Organization (WHO). Swaziland Global Youth Tobacco Survey 2001. United States: Centers for Disease Control and Prevention (CDC), 2001.
Centers for Disease Control and Prevention (CDC) and World Health Organization (WHO). Swaziland Global Youth Tobacco Survey 2005. Atlanta, United States: Centers for Disease Control and Prevention (CDC).
Centers for Disease Control and Prevention (CDC) and World Health Organization (WHO). Syria Global Youth Tobacco Survey 2007. United States: Centers for Disease Control and Prevention (CDC), 2007.
Centers for Disease Control and Prevention (CDC) and World Health Organization (WHO). Syria, AR Global Youth Tobacco Survey 2002. Atlanta, United States: Centers for Disease Control and Prevention (CDC).
Centers for Disease Control and Prevention (CDC) and World Health Organization (WHO). Taiwan, Province of China-Junior High Global Youth Tobacco Survey 2004. United States: Centers for Disease Control and Prevention (CDC), 2004.
Centers for Disease Control and Prevention (CDC) and World Health Organization (WHO). Taiwan, Province of China-Senior High Global Youth Tobacco Survey 2006. United States: Centers for Disease Control and Prevention (CDC), 2006.
Centers for Disease Control and Prevention (CDC) and World Health Organization (WHO). Tajikistan Global Youth Tobacco Survey 2004. Atlanta, United States: Centers for Disease Control and Prevention (CDC).
Centers for Disease Control and Prevention (CDC) and World Health Organization (WHO). Timor-Leste Global Youth Tobacco Survey 2006. Atlanta, United States: Centers for Disease Control and Prevention (CDC).
Centers for Disease Control and Prevention (CDC) and World Health Organization (WHO). Togo Global Youth Tobacco Survey 2002. United States: Centers for Disease Control and Prevention (CDC), 2002.
Centers for Disease Control and Prevention (CDC) and World Health Organization (WHO). Togo Global Youth Tobacco Survey 2007. United States: Centers for Disease Control and Prevention (CDC), 2007.
Centers for Disease Control and Prevention (CDC) and World Health Organization (WHO). Trinidad and Tobago Global Youth Tobacco Survey 2007. Atlanta, United States: Centers for Disease Control and Prevention (CDC).
Centers for Disease Control and Prevention (CDC) and World Health Organization (WHO). Tunisia Global Youth Tobacco Survey 2001. United States: Centers for Disease Control and Prevention (CDC), 2001.
Centers for Disease Control and Prevention (CDC) and World Health Organization (WHO). Turkey Global Youth Tobacco Survey 2003. Atlanta, United States: Centers for Disease Control and Prevention (CDC).
Centers for Disease Control and Prevention (CDC) and World Health Organization (WHO). Uganda Global Youth Tobacco Survey 2007. Atlanta, United States: Centers for Disease Control and Prevention (CDC).
Centers for Disease Control and Prevention (CDC) and World Health Organization (WHO). Ukraine Global Youth Tobacco Survey 1999. United States: Centers for Disease Control and Prevention (CDC), 1999.
Centers for Disease Control and Prevention (CDC) and World Health Organization (WHO). Ukraine Global Youth Tobacco Survey 2005. United States: Centers for Disease Control and Prevention (CDC), 2005.
Centers for Disease Control and Prevention (CDC) and World Health Organization (WHO). United Arab Emirates Global Youth Tobacco Survey 2002. United States: Centers for Disease Control and Prevention (CDC), 2002.
Centers for Disease Control and Prevention (CDC) and World Health Organization (WHO). United Arab Emirates Global Youth Tobacco Survey 2005. Atlanta, United States: Centers for Disease Control and Prevention (CDC).
Centers for Disease Control and Prevention (CDC) and World Health Organization (WHO). Uruguay Global Youth Tobacco Survey 2007. Atlanta, United States: Centers for Disease Control and Prevention (CDC).
Centers for Disease Control and Prevention (CDC) and World Health Organization (WHO). Vanuatu Global Youth Tobacco Survey 2007. Atlanta, United States: Centers for Disease Control and Prevention (CDC).
Centers for Disease Control and Prevention (CDC) and World Health Organization (WHO). Venezuela, RB Global Youth Tobacco Survey 1999. Atlanta, United States: Centers for Disease Control and Prevention (CDC).
Centers for Disease Control and Prevention (CDC) and World Health Organization (WHO). Viet Nam Global Youth Tobacco Survey 2007. Atlanta, United States: Centers for Disease Control and Prevention (CDC).

Appendix: Citation List

Citation
Centers for Disease Control and Prevention (CDC) and World Health Organization (WHO). Yemen Global Youth Tobacco Survey 2002. Atlanta, United States: Centers for Disease Control and Prevention (CDC).
Centers for Disease Control and Prevention (CDC), Central Agency for Public Mobilization and Statistics (Egypt), Ministry of Health and Population (Egypt), World Health Organization (WHO). Egypt Global Adult Tobacco Survey 2009. Atlanta, United States: Centers for Disease Control and Prevention (CDC).
Centers for Disease Control and Prevention (CDC), Central Board of Secondary Education (India), World Health Organization (WHO). India Global School-Based Student Health Survey 2007. Geneva, Switzerland: World Health Organization (WHO).
Centers for Disease Control and Prevention (CDC), Central Bureau of Statistics (Kenya), Macro International, Inc, Ministry of Health (Kenya), National Council for Population and Development (Kenya). Kenya Demographic and Health Survey 2003. Calverton, United States: Macro International, Inc.
Centers for Disease Control and Prevention (CDC), Central Statistical Office (Zambia), Food Security, Health and Nutrition Information System (Zambia), Food and Agriculture Organization of the United Nations (FAO), United Nations Children's Fund (UNICEF). Zambia Multiple Indicator Cluster Survey 1995.
Centers for Disease Control and Prevention (CDC), Clinton Health Access Initiative (CHAI), Global Health Group, UCSF Global Health Sciences, London School of Hygiene and Tropical Medicine, Ministry of Health (Swaziland), South African Medical Research Council, Swaziland Central Statistical Office, World Health Organization (WHO). Swaziland Malaria Indicator Survey 2010. As it appears in Malaria Atlas Project. Malaria Atlas Project Plasmodium Falciparum Parasite Rate Database. Oxford, United Kingdom: Malaria Atlas Project.
Centers for Disease Control and Prevention (CDC), Department of Health (Papua New Guinea), United Nations Children's Fund (UNICEF), University of Papua New Guinea. Papua New Guinea National Nutrition Survey 2005.
Centers for Disease Control and Prevention (CDC), Department of Hygiene and Epidemiology, University of Thessaly (Greece), MRB Hellas, National School of Public Health (Greece), World Health Organization (WHO). Greece Global Adult Tobacco Survey 2013.
Centers for Disease Control and Prevention (CDC), Ghana Statistical Service, Government of Japan, Ministry of Health (Ghana), Navrongo Health Research Centre, USAID, United Nations Children's Fund (UNICEF), United Nations Population Fund (UNFPA). Ghana Multiple Indicator Cluster Survey 2011. New York, United States: United Nations Children's Fund (UNICEF), 2013.
Centers for Disease Control and Prevention (CDC), Global Tobacco Surveillance System, Ministry of Health (Mexico), National Council Against Addictions (Mexico), National Institute of Public Health (Mexico), Pan American Health Organization (PAHO), WHO Framework Convention on Tobacco Control. Mexico Global Adult Tobacco Survey 2009.
Centers for Disease Control and Prevention (CDC), Government of Niger, United Nations Children's Fund (UNICEF), United Nations Population Fund (UNFPA). Niger Multiple Indicator Cluster Survey 1996.
Centers for Disease Control and Prevention (CDC), Haiti Child Health Institute (CHI), Haitian Institute of Statistics and Informatics, Macro International, Inc. Haiti Demographic and Health Survey 2012. Fairfax, United States: ICF International, 2013.
Centers for Disease Control and Prevention (CDC), ICF Macro, Kenya Medical Research Institute (KEMRI), Kenya National Bureau of Statistics, Measure DHS, Ministry of Public Health and Sanitation (Kenya), Population Services International (PSI), President's Malaria Initiative (PMI), United Nations Children's Fund (UNICEF), Walter Reed Project, World Health Organization (WHO). Kenya Malaria Indicator Survey 2010. Nairobi, Kenya: Kenya National Bureau of Statistics.
Centers for Disease Control and Prevention (CDC), ICF Macro, Kenya Medical Research Institute (KEMRI), Kenya National Bureau of Statistics, Ministry of Public Health and Sanitation (Kenya), National AIDS and STI Control Program (Kenya), National Aids Control Council (NACC), National Coordinating Agency for Population and Development (Kenya). Kenya Demographic and Health Survey - Maternal Mortality Data.
Centers for Disease Control and Prevention (CDC), ICF Macro, Ministry of Health (Uganda), Uganda Bureau of Statistics, Uganda Viral Research Institute. Uganda AIDS Indicator Survey 2011. Calverton, United States: ICF Macro.
Centers for Disease Control and Prevention (CDC), INDEPTH, International Vaccine Institute. Bangladesh - Abhoynagar, Mirsarai, and Kamalapur Health and Demographic Surveillance System.
Centers for Disease Control and Prevention (CDC), Institute of Nutrition of Central America and Panama, Pan American Health Organization (PAHO). El Salvador - Santa Tecla Diabetes, Hypertension, and Chronic Disease Risk Factors Survey 2004.
Centers for Disease Control and Prevention (CDC), Institute of Public Health (Macedonia), Joint United Nations Program on HIV/AIDS (UNAIDS), United Nations Children's Fund (UNICEF), United Nations Educational, Scientific and Cultural Organization (UNESCO), World Health Organization (WHO). Macedonia Global School-Based Student Health Survey 2007. Geneva, Switzerland: World Health Organization (WHO).
Centers for Disease Control and Prevention (CDC), Johns Hopkins Bloomberg School of Public Health, Kenya National Bureau of Statistics, Ministry of Health (Kenya), Research Triangle Institute, Inc. (RTI), World Health Organization (WHO). Kenya Global Adult Tobacco Survey 2014.
Centers for Disease Control and Prevention (CDC), Johns Hopkins Bloomberg School of Public Health, Latin American Center for Human Economy, Ministry of Public Health (Uruguay), National Institute of Statistics (Uruguay), Pan American Health Organization (PAHO), Research Triangle Institute, Inc. (RTI), World Health Organization (WHO). Uruguay Global Adult Tobacco Survey 2009.
Centers for Disease Control and Prevention (CDC), Johns Hopkins Bloomberg School of Public Health, Ministry of Development Planning and Statistics (Qatar), Supreme Council of Health (Qatar), World Health Organization (WHO). Qatar Global Adult Tobacco Survey 2013.
Centers for Disease Control and Prevention (CDC), Johns Hopkins Bloomberg School of Public Health, Ministry of Health and Social Action (Senegal), National Agency of Statistics and Demography (Senegal), Research Triangle Institute, Inc. (RTI), World Health Organization (WHO). Senegal Global Adult Tobacco Survey 2013.
Centers for Disease Control and Prevention (CDC), Johns Hopkins Bloomberg School of Public Health, Ministry of Public Health (Cameroon), National Institute of Statistics (Cameroon), World Health Organization (WHO). Cameroon Global Adult Tobacco Survey 2013.
Centers for Disease Control and Prevention (CDC), Johns Hopkins Bloomberg School of Public Health, Pakistan Bureau of Statistics, Research Triangle Institute, Inc. (RTI), World Health Organization (WHO). Pakistan Global Adult Tobacco Survey 2014.

Appendix: Citation List

Citation
Centers for Disease Control and Prevention (CDC), Johns Hopkins Bloomberg School of Public Health, World Health Organization (WHO). Kazakhstan Global Adult Tobacco Survey 2014.
Centers for Disease Control and Prevention (CDC), Joint United Nations Program on HIV/AIDS (UNAIDS), Ministry of Education (Tajikistan), Ministry of Health (Tajikistan), United Nations Children's Fund (UNICEF), United Nations Educational, Scientific and Cultural Organization (UNESCO), World Health Organization (WHO). Tajikistan Global School-Based Student Health Survey 2006 . Geneva, Switzerland: World Health Organization (WHO).
Centers for Disease Control and Prevention (CDC), Joint United Nations Program on HIV/AIDS (UNAIDS), Ministry of Education and Sports (Uganda), Ministry of Health (Uganda), United Nations Children's Fund (UNICEF), United Nations Educational, Scientific and Cultural Organization (UNESCO), World Health Organization (WHO). Uganda Global School-Based Student Health Survey 2003 . Geneva, Switzerland: World Health Organization (WHO).
Centers for Disease Control and Prevention (CDC), Joint United Nations Program on HIV/AIDS (UNAIDS), Ministry of Education and Vocational Training (Tanzania), Ministry of Health and Social Welfare (Tanzania), United Nations Children's Fund (UNICEF), United Nations Educational, Scientific and Cultural Organization (UNESCO), World Health Organization (WHO). Tanzania - Dar es Salaam Global School-Based Student Health Survey 2006. Geneva, Switzerland: World Health Organization (WHO).
Centers for Disease Control and Prevention (CDC), Joint United Nations Program on HIV/AIDS (UNAIDS), Ministry of Education, Women Affairs, and Culture (Tonga), Ministry of Health (Tonga), United Nations Children's Fund (UNICEF), United Nations Educational, Scientific and Cultural Organization (UNESCO), World Health Organization (WHO). Tonga Global School-Based Student Health Survey 2010. Geneva, Switzerland: World Health Organization (WHO).
Centers for Disease Control and Prevention (CDC), Joint United Nations Program on HIV/AIDS (UNAIDS), Ministry of Health (Cook Islands), United Nations Children's Fund (UNICEF), United Nations Educational, Scientific and Cultural Organization (UNESCO), World Health Organization (WHO). Cook Islands Global School-Based Student Health Survey 2011. Geneva, Switzerland: World Health Organization (WHO).
Centers for Disease Control and Prevention (CDC), Joint United Nations Program on HIV/AIDS (UNAIDS), Ministry of Health (Djibouti), Ministry of National and Higher Education (Djibouti), United Nations Children's Fund (UNICEF), United Nations Educational, Scientific and Cultural Organization (UNESCO), World Health Organization (WHO). Djibouti Global School-Based Student Health Survey 2007. Geneva, Switzerland: World Health Organization (WHO).
Centers for Disease Control and Prevention (CDC), Joint United Nations Program on HIV/AIDS (UNAIDS), Ministry of Health (Fiji), United Nations Children's Fund (UNICEF), United Nations Educational, Scientific and Cultural Organization (UNESCO), World Health Organization (WHO). Fiji Global School-Based Student Health Survey 2010. Geneva, Switzerland: World Health Organization (WHO).
Centers for Disease Control and Prevention (CDC), Joint United Nations Program on HIV/AIDS (UNAIDS), Ministry of Health (Morocco), United Nations Children's Fund (UNICEF), United Nations Educational, Scientific and Cultural Organization (UNESCO), World Health Organization (WHO). Morocco Global School-Based Student Health Survey 2010-2011. Geneva, Switzerland: World Health Organization (WHO), 2013.
Centers for Disease Control and Prevention (CDC), Joint United Nations Program on HIV/AIDS (UNAIDS), Ministry of Health (St. Lucia), United Nations Children's Fund (UNICEF), United Nations Educational, Scientific and Cultural Organization (UNESCO), World Health Organization (WHO). Saint Lucia Global School-Based Student Health Survey 2007. Geneva, Switzerland: World Health Organization (WHO).
Centers for Disease Control and Prevention (CDC), Joint United Nations Program on HIV/AIDS (UNAIDS), Ministry of Health and the Environment (St. Vincent and the Grenadines), United Nations Children's Fund (UNICEF), United Nations Educational, Scientific and Cultural Organization (UNESCO), World Health Organization (WHO). Saint Vincent and the Grenadines Global School-Based Student Health Survey 2007. Geneva, Switzerland: World Health Organization (WHO).
Centers for Disease Control and Prevention (CDC), Joint United Nations Program on HIV/AIDS (UNAIDS), Ministry of Public Health (Benin), United Nations Children's Fund (UNICEF), United Nations Educational, Scientific and Cultural Organization (UNESCO), World Health Organization (WHO). Benin Global School-Based Student Health Survey 2009. Geneva, Switzerland: World Health Organization (WHO).
Centers for Disease Control and Prevention (CDC), Joint United Nations Program on HIV/AIDS (UNAIDS), Pakistan Medical Research Council, United Nations Children's Fund (UNICEF), United Nations Educational, Scientific and Cultural Organization (UNESCO), World Health Organization (WHO). Pakistan Global School-Based Student Health Survey 2009. Geneva, Switzerland: World Health Organization (WHO).
Centers for Disease Control and Prevention (CDC), Joint United Nations Program on HIV/AIDS (UNAIDS), United Nations Children's Fund (UNICEF), United Nations Educational, Scientific and Cultural Organization (UNESCO), World Health Organization (WHO). Algeria Global School-Based Student Health Survey 2010. Geneva, Switzerland: World Health Organization (WHO).
Centers for Disease Control and Prevention (CDC), Joint United Nations Program on HIV/AIDS (UNAIDS), United Nations Children's Fund (UNICEF), United Nations Educational, Scientific and Cultural Organization (UNESCO), World Health Organization (WHO). Costa Rica Global School-Based Student Health Survey 2009. Geneva, Switzerland: World Health Organization (WHO).
Centers for Disease Control and Prevention (CDC), Joint United Nations Program on HIV/AIDS (UNAIDS), United Nations Children's Fund (UNICEF), United Nations Educational, Scientific and Cultural Organization (UNESCO), World Health Organization (WHO). Guyana Global School-Based Student Health Survey 2004 . Geneva, Switzerland: World Health Organization (WHO).
Centers for Disease Control and Prevention (CDC), Joint United Nations Program on HIV/AIDS (UNAIDS), United Nations Children's Fund (UNICEF), United Nations Educational, Scientific and Cultural Organization (UNESCO), World Health Organization (WHO). Myanmar Global School-Based Student Health Survey 2007. Geneva, Switzerland: World Health Organization (WHO).
Centers for Disease Control and Prevention (CDC), Kenya Medical Research Institute (KEMRI). Kenya KEMRI/CDC Health and Demographic Surveillance System.

Appendix: Citation List

Citation
Centers for Disease Control and Prevention (CDC), MEASURE Evaluation Project, Carolina Population Center, University of North Carolina, Ministry of Health (Mozambique), National Statistics Institute (Mozambique), US Census Bureau. Mozambique National Survey on the Causes of Death 2007-2008.
Centers for Disease Control and Prevention (CDC), Ministry of Education (Argentina), Ministry of Health (Argentina), Pan American Health Organization (PAHO), Public Opinion, Services, and Markets (OPSM), World Health Organization (WHO). Argentina Global School-Based Student Health Survey 2007.
Centers for Disease Control and Prevention (CDC), Ministry of Education (Indonesia), Ministry of Health (Indonesia), World Health Organization (WHO). Indonesia Global School-Based Student Health Survey 2007.
Centers for Disease Control and Prevention (CDC), Ministry of Education (Jordan), Ministry of Health (Jordan), World Health Organization (WHO). Jordan Global School-Based Student Health Survey 2007. Geneva, Switzerland: World Health Organization (WHO).
Centers for Disease Control and Prevention (CDC), Ministry of Education (Syria), Ministry of Health (Syria), World Health Organization (WHO). Syria Global School-Based Student Health Survey 2010-2011. Geneva, Switzerland: World Health Organization (WHO), 2013.
Centers for Disease Control and Prevention (CDC), Ministry of Education (Trinidad and Tobago), Ministry of Health (Trinidad and Tobago), World Health Organization (WHO). Trinidad and Tobago Global School-Based Student Health Survey 2007. Geneva, Switzerland: World Health Organization (WHO).
Centers for Disease Control and Prevention (CDC), Ministry of Education and Training (Swaziland), Ministry of Health (Swaziland), World Health Organization (WHO). Swaziland Global Youth Tobacco Survey 2009. Atlanta, United States: Centers for Disease Control and Prevention (CDC).
Centers for Disease Control and Prevention (CDC), Ministry of Health (Belize), Pan American Health Organization (PAHO). Belize Diabetes, Hypertension, and Noncommunicable Disease Risk Factors Survey 2005-2006.
Centers for Disease Control and Prevention (CDC), Ministry of Health (Indonesia), National Institute of Health Research and Development (NIHRD), Ministry of Health (Indonesia), Statistics Indonesia, World Health Organization (WHO). Indonesia Global Adult Tobacco Survey 2011. Geneva, Switzerland: World Health Organization (WHO), 2014.
Centers for Disease Control and Prevention (CDC), Ministry of Health (Iraq), World Health Organization (WHO). Iraq Global School-Based Student Health Survey 2012. Geneva, Switzerland: World Health Organization (WHO), 2014.
Centers for Disease Control and Prevention (CDC), Ministry of Health (Jordan), World Health Organization (WHO). Jordan STEPS Noncommunicable Disease Risk Factors Survey 2007.
Centers for Disease Control and Prevention (CDC), Ministry of Health (Kuwait), WHO Regional Office for the Eastern Mediterranean. Kuwait Nutrition Surveillance System.
Centers for Disease Control and Prevention (CDC), Ministry of Health (Malaysia), World Health Organization (WHO). Malaysia Global School-Based Student Health Survey 2012.
Centers for Disease Control and Prevention (CDC), Ministry of Health (Morocco), World Health Organization (WHO). Morocco Global School-Based Student Health Survey 2006. Geneva, Switzerland: World Health Organization (WHO).
Centers for Disease Control and Prevention (CDC), Ministry of Health (Nicaragua), Pan American Health Organization (PAHO). Nicaragua - Managua Diabetes, Hypertension, and Non-Communicable Disease Risk Factors Survey 2003.
Centers for Disease Control and Prevention (CDC), Ministry of Health (Oman), United Nations Children's Fund (UNICEF), WHO Regional Office for the Eastern Mediterranean. Oman Food Fortification Study 2004. Muscat, Oman: Ministry of Health (Oman).
Centers for Disease Control and Prevention (CDC), Ministry of Health (Panama), World Health Organization (WHO). Panama Global Adult Tobacco Survey 2013.
Centers for Disease Control and Prevention (CDC), Ministry of Health (Romania), National Institute of Public Health (Romania), National Institute of Statistics (Romania), TOTEM Communication, WHO Regional Office for Europe (EURO-WHO). Romania Global Adult Tobacco Survey 2011. Bucharest, Romania: Ministry of Health (Romania), 2013.
Centers for Disease Control and Prevention (CDC), Ministry of Health (Rwanda), National Institute of Statistics of Rwanda, Rwanda Biomedical Center, World Health Organization (WHO). Rwanda STEPS Noncommunicable Disease Risk Factor Survey 2012-2013.
Centers for Disease Control and Prevention (CDC), Ministry of Health (Togo), Office of Scientific and Technical Research Overseas (ORSTOM) (France), United States Agency for International Development (USAID). Togo Malaria and Anemia Among Children 0-12 Years in Afagnan 1991-1992. As it appears in Malaria Atlas Project. Malaria Atlas Project Plasmodium Falciparum Parasite Rate Database. Oxford, United Kingdom: Malaria Atlas Project.
Centers for Disease Control and Prevention (CDC), Ministry of Health (Vanuatu), World Health Organization (WHO). Vanuatu Global School-Based Student Health Survey 2011.
Centers for Disease Control and Prevention (CDC), Ministry of Health and Population (Egypt), World Health Organization (WHO). Egypt Global School-Based Student Health Survey 2011-2012. Geneva, Switzerland: World Health Organization (WHO), 2014.
Centers for Disease Control and Prevention (CDC), Ministry of Health and Prevention (Senegal), World Health Organization (WHO). Senegal Global School-Based Student Health Survey 2005. Geneva, Switzerland: World Health Organization (WHO).
Centers for Disease Control and Prevention (CDC), Ministry of Health and Quality of Life (Mauritius), World Health Organization (WHO). Mauritius Global School-Based Student Health Survey 2007.
Centers for Disease Control and Prevention (CDC), Ministry of Public Health (Tunisia), World Health Organization (WHO). Tunisia Global School-Based Student Health Survey 2008. Geneva, Switzerland: World Health Organization (WHO).
Centers for Disease Control and Prevention (CDC), Ministry of Public Health (Uruguay), World Health Organization (WHO). Uruguay Global School-Based Student Health Survey 2006. Geneva, Switzerland: World Health Organization (WHO).
Centers for Disease Control and Prevention (CDC), Ministry of Public Health (Uruguay), World Health Organization (WHO). Uruguay Global School-Based Student Health Survey 2012.
Centers for Disease Control and Prevention (CDC), National Institute of Justice (United States), Schulman, Ronca and Bucuvalas Inc. (SRBI). United States National Violence Against Women Survey 1995-1996.

Appendix: Citation List

Citation
Centers for Disease Control and Prevention (CDC), National Statistics Institute (Guinea-Bissau), United Nations Children's Fund (UNICEF). Guinea-Bissau Multiple Indicator Cluster Survey 2010.
Centers for Disease Control and Prevention (CDC), ORC Macro. Reproductive, Maternal and Child Health in Eastern Europe and Eurasia: A Comparative Report. Atlanta, United States: Centers for Disease Control and Prevention (CDC), 2003.
Centers for Disease Control and Prevention (CDC), Public Health Institute, Ministry of Health (Mongolia), World Health Organization (WHO). Mongolia Global School-Based Student Health Survey 2010.
Centers for Disease Control and Prevention (CDC), Secretariat of Health and Environment (Libya), World Health Organization (WHO). Libya Global School-Based Student Health Survey 2007. Geneva, Switzerland: World Health Organization (WHO).
Centers for Disease Control and Prevention (CDC), Supreme Council of Health (Qatar), World Health Organization (WHO). Qatar Global School-Based Student Health Survey 2011-2012. Geneva, Switzerland: World Health Organization (WHO), 2014.
Centers for Disease Control and Prevention (CDC), United Nations Children's Fund (UNICEF). Afghanistan - Badghis Nutrition and Health Survey 2002.
Centers for Disease Control and Prevention (CDC), World Health Organization (WHO). Afghanistan Global School-Based Student Health Survey 2014.
Centers for Disease Control and Prevention (CDC), World Health Organization (WHO). Albania Global Youth Tobacco Survey 2009. Atlanta, United States: Centers for Disease Control and Prevention (CDC), 2013.
Centers for Disease Control and Prevention (CDC), World Health Organization (WHO). Antigua and Barbuda Global Youth Tobacco Survey 2009. Atlanta, United States: Centers for Disease Control and Prevention (CDC), 2013.
Centers for Disease Control and Prevention (CDC), World Health Organization (WHO). Bahamas Global Youth Tobacco Survey 2009. Atlanta, United States: Centers for Disease Control and Prevention (CDC).
Centers for Disease Control and Prevention (CDC), World Health Organization (WHO). Belize Global Youth Tobacco Survey 2008. Atlanta, United States: Centers for Disease Control and Prevention (CDC).
Centers for Disease Control and Prevention (CDC), World Health Organization (WHO). Bolivia Global Youth Tobacco Survey 2012. Atlanta, United States: Centers for Disease Control and Prevention (CDC).
Centers for Disease Control and Prevention (CDC), World Health Organization (WHO). Bosnia and Herzegovina - Republika Srpska Global Youth Tobacco Survey 2008. Atlanta, United States: Centers for Disease Control and Prevention (CDC).
Centers for Disease Control and Prevention (CDC), World Health Organization (WHO). Bosnia and Herzegovina Global Youth Tobacco Survey 2008. Atlanta, United States: Centers for Disease Control and Prevention (CDC).
Centers for Disease Control and Prevention (CDC), World Health Organization (WHO). Botswana Global Youth Tobacco Survey 2008. Atlanta, United States: Centers for Disease Control and Prevention (CDC).
Centers for Disease Control and Prevention (CDC), World Health Organization (WHO). Bulgaria Global Youth Tobacco Survey 2008. Atlanta, United States: Centers for Disease Control and Prevention (CDC).
Centers for Disease Control and Prevention (CDC), World Health Organization (WHO). Burundi Global Youth Tobacco Survey 2008. Atlanta, United States: Centers for Disease Control and Prevention (CDC).
Centers for Disease Control and Prevention (CDC), World Health Organization (WHO). CÃ´te d'Ivoire Global Youth Tobacco Survey 2009. Atlanta, United States: Centers for Disease Control and Prevention (CDC).
Centers for Disease Control and Prevention (CDC), World Health Organization (WHO). Cambodia Global Youth Tobacco Survey 2010. Atlanta, United States: Centers for Disease Control and Prevention (CDC).
Centers for Disease Control and Prevention (CDC), World Health Organization (WHO). Congo, Rep. Global Youth Tobacco Survey 2009. Atlanta, United States: Centers for Disease Control and Prevention (CDC).
Centers for Disease Control and Prevention (CDC), World Health Organization (WHO). Costa Rica Global Youth Tobacco Survey 2008. Atlanta, United States: Centers for Disease Control and Prevention (CDC).
Centers for Disease Control and Prevention (CDC), World Health Organization (WHO). Cuba Global Youth Tobacco Survey 2010. Atlanta, United States: Centers for Disease Control and Prevention (CDC).
Centers for Disease Control and Prevention (CDC), World Health Organization (WHO). Czech Republic Global Youth Tobacco Survey 2011. Atlanta, United States: Centers for Disease Control and Prevention (CDC), 2013.
Centers for Disease Control and Prevention (CDC), World Health Organization (WHO). Djibouti Global Youth Tobacco Survey 2009. Atlanta, United States: Centers for Disease Control and Prevention (CDC).
Centers for Disease Control and Prevention (CDC), World Health Organization (WHO). Dominican Republic Global Youth Tobacco Survey 2011. Atlanta, United States: Centers for Disease Control and Prevention (CDC).
Centers for Disease Control and Prevention (CDC), World Health Organization (WHO). Egypt Global Youth Tobacco Survey 2009. Atlanta, United States: Centers for Disease Control and Prevention (CDC), 2013.
Centers for Disease Control and Prevention (CDC), World Health Organization (WHO). El Salvador Global Youth Tobacco Survey 2009. Atlanta, United States: Centers for Disease Control and Prevention (CDC).
Centers for Disease Control and Prevention (CDC), World Health Organization (WHO). Fiji Global Youth Tobacco Survey 2009. Atlanta, United States: Centers for Disease Control and Prevention (CDC), 2013.
Centers for Disease Control and Prevention (CDC), World Health Organization (WHO). Georgia Global Youth Tobacco Survey 2008. Atlanta, United States: Centers for Disease Control and Prevention (CDC).
Centers for Disease Control and Prevention (CDC), World Health Organization (WHO). Ghana Global Youth Tobacco Survey 2009. Atlanta, United States: Centers for Disease Control and Prevention (CDC).
Centers for Disease Control and Prevention (CDC), World Health Organization (WHO). Grenada Global Youth Tobacco Survey 2009. Atlanta, United States: Centers for Disease Control and Prevention (CDC).
Centers for Disease Control and Prevention (CDC), World Health Organization (WHO). Guam Global Youth Tobacco Survey 2011. Atlanta, United States: Centers for Disease Control and Prevention (CDC), 2013.

Appendix: Citation List

Citation
Centers for Disease Control and Prevention (CDC), World Health Organization (WHO). Guatemala Global Youth Tobacco Survey 2008. Atlanta, United States: Centers for Disease Control and Prevention (CDC).
Centers for Disease Control and Prevention (CDC), World Health Organization (WHO). Guinea Global Youth Tobacco Survey 2008. Atlanta, United States: Centers for Disease Control and Prevention (CDC).
Centers for Disease Control and Prevention (CDC), World Health Organization (WHO). Guyana Global Youth Tobacco Survey 2010. Atlanta, United States: Centers for Disease Control and Prevention (CDC).
Centers for Disease Control and Prevention (CDC), World Health Organization (WHO). Hungary Global Youth Tobacco Survey 2008. Atlanta, United States: Centers for Disease Control and Prevention (CDC).
Centers for Disease Control and Prevention (CDC), World Health Organization (WHO). India Global Youth Tobacco Survey 2009. Atlanta, United States: Centers for Disease Control and Prevention (CDC), 2013.
Centers for Disease Control and Prevention (CDC), World Health Organization (WHO). Indonesia Global Youth Tobacco Survey 2009. Atlanta, United States: Centers for Disease Control and Prevention (CDC), 2013.
Centers for Disease Control and Prevention (CDC), World Health Organization (WHO). Italy Global Youth Tobacco Survey 2010. Atlanta, United States: Centers for Disease Control and Prevention (CDC), 2013.
Centers for Disease Control and Prevention (CDC), World Health Organization (WHO). Jamaica Global Youth Tobacco Survey 2010. Atlanta, United States: Centers for Disease Control and Prevention (CDC).
Centers for Disease Control and Prevention (CDC), World Health Organization (WHO). Jordan Global Youth Tobacco Survey 2009. Atlanta, United States: Centers for Disease Control and Prevention (CDC), 2013.
Centers for Disease Control and Prevention (CDC), World Health Organization (WHO). Kazakhstan Global Youth Tobacco Survey 2009. Atlanta, United States: Centers for Disease Control and Prevention (CDC).
Centers for Disease Control and Prevention (CDC), World Health Organization (WHO). Kiribati Global School-Based Student Health Survey 2011. Geneva, Switzerland: World Health Organization (WHO), 2014.
Centers for Disease Control and Prevention (CDC), World Health Organization (WHO). Kiribati Global Youth Tobacco Survey 2009. Atlanta, United States: Centers for Disease Control and Prevention (CDC), 2013.
Centers for Disease Control and Prevention (CDC), World Health Organization (WHO). Korea, South Global Youth Tobacco Survey 2008. Atlanta, United States: Centers for Disease Control and Prevention (CDC).
Centers for Disease Control and Prevention (CDC), World Health Organization (WHO). Kuwait Global School-Based Student Health Survey 2011. Geneva, Switzerland: World Health Organization (WHO), 2014.
Centers for Disease Control and Prevention (CDC), World Health Organization (WHO). Kyrgyzstan Global Youth Tobacco Survey 2004. Atlanta, United States: Centers for Disease Control and Prevention (CDC).
Centers for Disease Control and Prevention (CDC), World Health Organization (WHO). Kyrgyzstan Global Youth Tobacco Survey 2008. Atlanta, United States: Centers for Disease Control and Prevention (CDC).
Centers for Disease Control and Prevention (CDC), World Health Organization (WHO). Laos Global Youth Tobacco Survey 2011. Atlanta, United States: Centers for Disease Control and Prevention (CDC), 2013.
Centers for Disease Control and Prevention (CDC), World Health Organization (WHO). Libya Global Youth Tobacco Survey 2010.
Centers for Disease Control and Prevention (CDC), World Health Organization (WHO). Lithuania Global Youth Tobacco Survey 2009. Atlanta, United States: Centers for Disease Control and Prevention (CDC).
Centers for Disease Control and Prevention (CDC), World Health Organization (WHO). Macedonia Global Youth Tobacco Survey 2008. Atlanta, United States: Centers for Disease Control and Prevention (CDC).
Centers for Disease Control and Prevention (CDC), World Health Organization (WHO). Madagascar Global Youth Tobacco Survey 2008. Atlanta, United States: Centers for Disease Control and Prevention (CDC).
Centers for Disease Control and Prevention (CDC), World Health Organization (WHO). Malawi Global Youth Tobacco Survey 2009. Atlanta, United States: Centers for Disease Control and Prevention (CDC), 2013.
Centers for Disease Control and Prevention (CDC), World Health Organization (WHO). Malaysia Global Youth Tobacco Survey 2009. Atlanta, United States: Centers for Disease Control and Prevention (CDC).
Centers for Disease Control and Prevention (CDC), World Health Organization (WHO). Maldives Global Youth Tobacco Survey 2011. Atlanta, United States: Centers for Disease Control and Prevention (CDC).
Centers for Disease Control and Prevention (CDC), World Health Organization (WHO). Mali Global Youth Tobacco Survey 2008. Atlanta, United States: Centers for Disease Control and Prevention (CDC).
Centers for Disease Control and Prevention (CDC), World Health Organization (WHO). Mauritania Global Youth Tobacco Survey 2009. Atlanta, United States: Centers for Disease Control and Prevention (CDC).
Centers for Disease Control and Prevention (CDC), World Health Organization (WHO). Mauritius Global School-Based Student Health Survey 2011. Geneva, Switzerland: World Health Organization (WHO), 2014.
Centers for Disease Control and Prevention (CDC), World Health Organization (WHO). Mauritius Global Youth Tobacco Survey 2008. Atlanta, United States: Centers for Disease Control and Prevention (CDC).
Centers for Disease Control and Prevention (CDC), World Health Organization (WHO). Mexico Global Youth Tobacco Survey 2011. Atlanta, United States: Centers for Disease Control and Prevention (CDC).
Centers for Disease Control and Prevention (CDC), World Health Organization (WHO). Moldova Global Youth Tobacco Survey 2008. Atlanta, United States: Centers for Disease Control and Prevention (CDC).
Centers for Disease Control and Prevention (CDC), World Health Organization (WHO). Montenegro Global Youth Tobacco Survey 2008. Atlanta, United States: Centers for Disease Control and Prevention (CDC).
Centers for Disease Control and Prevention (CDC), World Health Organization (WHO). Morocco Global Youth Tobacco Survey 2010. Atlanta, United States: Centers for Disease Control and Prevention (CDC), 2013.

Appendix: Citation List

Citation
Centers for Disease Control and Prevention (CDC), World Health Organization (WHO). Myanmar Global Youth Tobacco Survey 2011. Atlanta, United States: Centers for Disease Control and Prevention (CDC).
Centers for Disease Control and Prevention (CDC), World Health Organization (WHO). Namibia Global Youth Tobacco Survey 2008. Atlanta, United States: Centers for Disease Control and Prevention (CDC), 2013.
Centers for Disease Control and Prevention (CDC), World Health Organization (WHO). Nepal Global Youth Tobacco Survey 2011. Atlanta, United States: Centers for Disease Control and Prevention (CDC), 2013.
Centers for Disease Control and Prevention (CDC), World Health Organization (WHO). New Caledonia Global Youth Tobacco Survey 2010. Atlanta, United States: Centers for Disease Control and Prevention (CDC), 2013.
Centers for Disease Control and Prevention (CDC), World Health Organization (WHO). New Zealand Global Youth Tobacco Survey 2008. Atlanta, United States: Centers for Disease Control and Prevention (CDC).
Centers for Disease Control and Prevention (CDC), World Health Organization (WHO). New Zealand Global Youth Tobacco Survey 2010. Atlanta, United States: Centers for Disease Control and Prevention (CDC), 2013.
Centers for Disease Control and Prevention (CDC), World Health Organization (WHO). Niger Global Youth Tobacco Survey 2009. Atlanta, United States: Centers for Disease Control and Prevention (CDC), 2013.
Centers for Disease Control and Prevention (CDC), World Health Organization (WHO). Oman Global Youth Tobacco Survey 2010. Atlanta, United States: Centers for Disease Control and Prevention (CDC), 2013.
Centers for Disease Control and Prevention (CDC), World Health Organization (WHO). Panama Global Youth Tobacco Survey 2008. Atlanta, United States: Centers for Disease Control and Prevention (CDC).
Centers for Disease Control and Prevention (CDC), World Health Organization (WHO). Paraguay Global Youth Tobacco Survey 2008. Atlanta, United States: Centers for Disease Control and Prevention (CDC).
Centers for Disease Control and Prevention (CDC), World Health Organization (WHO). Philippines Global School-Based Student Health Survey 2003 . Geneva, Switzerland: World Health Organization (WHO).
Centers for Disease Control and Prevention (CDC), World Health Organization (WHO). Philippines Global School-Based Student Health Survey 2007. Geneva, Switzerland: World Health Organization (WHO).
Centers for Disease Control and Prevention (CDC), World Health Organization (WHO). Romania Global Youth Tobacco Survey 2009. Atlanta, United States: Centers for Disease Control and Prevention (CDC).
Centers for Disease Control and Prevention (CDC), World Health Organization (WHO). Rwanda Global Youth Tobacco Survey 2008. Atlanta, United States: Centers for Disease Control and Prevention (CDC).
Centers for Disease Control and Prevention (CDC), World Health Organization (WHO). Saint Lucia Global Youth Tobacco Survey 2011. Atlanta, United States: Centers for Disease Control and Prevention (CDC).
Centers for Disease Control and Prevention (CDC), World Health Organization (WHO). Saint Vincent and the Grenadines Global Youth Tobacco Survey 2011. Atlanta, United States: Centers for Disease Control and Prevention (CDC).
Centers for Disease Control and Prevention (CDC), World Health Organization (WHO). Sao Tome and Principe Global Youth Tobacco Survey 2010. Atlanta, United States: Centers for Disease Control and Prevention (CDC).
Centers for Disease Control and Prevention (CDC), World Health Organization (WHO). Saudi Arabia Global Youth Tobacco Survey 2010. Atlanta, United States: Centers for Disease Control and Prevention (CDC), 2013.
Centers for Disease Control and Prevention (CDC), World Health Organization (WHO). Serbia Global Youth Tobacco Survey 2008. Atlanta, United States: Centers for Disease Control and Prevention (CDC).
Centers for Disease Control and Prevention (CDC), World Health Organization (WHO). Slovenia Global Youth Tobacco Survey 2011. Atlanta, United States: Centers for Disease Control and Prevention (CDC).
Centers for Disease Control and Prevention (CDC), World Health Organization (WHO). Solomon Islands Global School-Based Student Health Survey 2011.
Centers for Disease Control and Prevention (CDC), World Health Organization (WHO). South Africa Global Youth Tobacco Survey 2011. Atlanta, United States: Centers for Disease Control and Prevention (CDC), 2013.
Centers for Disease Control and Prevention (CDC), World Health Organization (WHO). Sri Lanka Global Youth Tobacco Survey 2011. Atlanta, United States: Centers for Disease Control and Prevention (CDC).
Centers for Disease Control and Prevention (CDC), World Health Organization (WHO). Sudan Global School-Based Student Health Survey 2011-2012.
Centers for Disease Control and Prevention (CDC), World Health Organization (WHO). Sudan Global Youth Tobacco Survey 2009. Atlanta, United States: Centers for Disease Control and Prevention (CDC).
Centers for Disease Control and Prevention (CDC), World Health Organization (WHO). Suriname Global Youth Tobacco Survey 2009. Atlanta, United States: Centers for Disease Control and Prevention (CDC).
Centers for Disease Control and Prevention (CDC), World Health Organization (WHO). Syria Global Youth Tobacco Survey 2010.
Centers for Disease Control and Prevention (CDC), World Health Organization (WHO). Thailand Global Youth Tobacco Survey 2005. Atlanta, United States: Centers for Disease Control and Prevention (CDC).
Centers for Disease Control and Prevention (CDC), World Health Organization (WHO). Tonga Global Youth Tobacco Survey 2010. Atlanta, United States: Centers for Disease Control and Prevention (CDC).
Centers for Disease Control and Prevention (CDC), World Health Organization (WHO). Trinidad and Tobago Global School-Based Student Health Survey 2011.
Centers for Disease Control and Prevention (CDC), World Health Organization (WHO). Trinidad and Tobago Global Youth Tobacco Survey 2011. Atlanta, United States: Centers for Disease Control and Prevention (CDC).
Centers for Disease Control and Prevention (CDC), World Health Organization (WHO). Tunisia Global Youth Tobacco Survey 2010.
Centers for Disease Control and Prevention (CDC), World Health Organization (WHO). Uganda Global Youth Tobacco Survey 2011. Atlanta, United States: Centers for Disease Control and Prevention (CDC).

Appendix: Citation List

Citation
Centers for Disease Control and Prevention (CDC), World Health Organization (WHO). Ukraine Global Youth Tobacco Survey 2011. Atlanta, United States: Centers for Disease Control and Prevention (CDC).
Centers for Disease Control and Prevention (CDC), World Health Organization (WHO). United States National Youth Tobacco Survey 2000. Atlanta, United States: Centers for Disease Control and Prevention (CDC).
Centers for Disease Control and Prevention (CDC), World Health Organization (WHO). Venezuela Global Youth Tobacco Survey 2010. Atlanta, United States: Centers for Disease Control and Prevention (CDC).
Centers for Disease Control and Prevention (CDC), World Health Organization (WHO). Vietnam Global School-Based Student Health Survey 2012-2013.
Centers for Disease Control and Prevention (CDC), World Health Organization (WHO). Yemen Global Youth Tobacco Survey 2008. Atlanta, United States: Centers for Disease Control and Prevention (CDC).
Centers for Disease Control and Prevention (CDC), World Health Organization (WHO). Zambia Global Youth Tobacco Survey 2011. Atlanta, United States: Centers for Disease Control and Prevention (CDC), 2013.
Centers for Disease Control and Prevention (CDC). Azerbaijan Health and Nutrition Survey of Internally Displaced and Resident Population 1996.
Centers for Disease Control and Prevention (CDC). Lymphatic Filariasis in the Americas: An Epidemiologic History. Atlanta, United States: Centers for Disease Control and Prevention (CDC), 2002. As it appears in London School of Hygiene and Tropical Medicine. Global Atlas of Helminth Infections - Lymphatic Filiariasis.
Centers for Disease Control and Prevention (CDC). Summary of Notifiable Diseases - United States 1995. MMWR Morb Mortal Wkly Rep. 1996; 44(53): 1-87.
Centers for Disease Control and Prevention (CDC). Summary of Notifiable Diseases - United States 2000. MMWR Morb Mortal Wkly Rep. 2002; 49(53): 1-102.
Centers for Disease Control and Prevention (CDC). Summary of Notifiable Diseases - United States 2006. MMWR Morb Mortal Wkly Rep. 2007; 55(53): 1-94.
Centers for Disease Control and Prevention (CDC). Summary of Notifiable Diseases - United States 2010. MMWR Morb Mortal Wkly Rep. 2012; 59(53): 1-110. Erratum in: MMWR Morb Mortal Wkly Rep. 2012; 61(29): 562.
Centers for Disease Control and Prevention (CDC). Summary of Notifiable Diseases - United States 2012. MMWR Morb Mortal Wkly Rep. 2014; 61(53): 1-121.
Centers for Disease Control and Prevention (CDC). Summary of Notifiable Infectious Diseases and Conditions - United States, 2013. MMWR Morb Mortal Wkly Rep. 62(53): 1-119.
Centers for Disease Control and Prevention (CDC). Tobacco use among adults -- United States, 2005. MMWR Morb Mortal Wkly Rep. 2006; 55(42): 1145-8.
Centers for Disease Control and Prevention (CDC). United States Behavioral Risk Factor Surveillance System 1984. Atlanta, Georgia: CDC, US Department of Health and Human Services
Centers for Disease Control and Prevention (CDC). United States Behavioral Risk Factor Surveillance System 1985. Atlanta, Georgia: CDC, US Department of Health and Human Services.
Centers for Disease Control and Prevention (CDC). United States Behavioral Risk Factor Surveillance System 1989. Atlanta, Georgia: CDC, US Department of Health and Human Services.
Centers for Disease Control and Prevention (CDC). United States Behavioral Risk Factor Surveillance System 1990. Atlanta, Georgia: CDC, US Department of Health and Human Services.
Centers for Disease Control and Prevention (CDC). United States Behavioral Risk Factor Surveillance System 1991. Atlanta, Georgia: CDC, US Department of Health and Human Services.
Centers for Disease Control and Prevention (CDC). United States Behavioral Risk Factor Surveillance System 1992. Atlanta, Georgia: CDC, US Department of Health and Human Services.
Centers for Disease Control and Prevention (CDC). United States Behavioral Risk Factor Surveillance System 1993. Atlanta, Georgia: CDC, US Department of Health and Human Services.
Centers for Disease Control and Prevention (CDC). United States Behavioral Risk Factor Surveillance System 1994. Atlanta, Georgia: CDC, US Department of Health and Human Services.
Centers for Disease Control and Prevention (CDC). United States Behavioral Risk Factor Surveillance System 1995. Atlanta, Georgia: CDC, US Department of Health and Human Services.
Centers for Disease Control and Prevention (CDC). United States Behavioral Risk Factor Surveillance System 1996. Atlanta, Georgia: CDC, US Department of Health and Human Services.
Centers for Disease Control and Prevention (CDC). United States Behavioral Risk Factor Surveillance System 1997. Atlanta, Georgia: CDC, US Department of Health and Human Services.
Centers for Disease Control and Prevention (CDC). United States Behavioral Risk Factor Surveillance System 1998. Atlanta, Georgia: CDC, US Department of Health and Human Services.
Centers for Disease Control and Prevention (CDC). United States Behavioral Risk Factor Surveillance System 2003. Atlanta, Georgia: CDC, US Department of Health and Human Services.
Centers for Disease Control and Prevention (CDC). United States Behavioral Risk Factor Surveillance System 2004. Atlanta, Georgia: CDC, US Department of Health and Human Services.
Centers for Disease Control and Prevention (CDC). United States Behavioral Risk Factor Surveillance System 2005. Atlanta, Georgia: CDC, US Department of Health and Human Services.
Centers for Disease Control and Prevention (CDC). United States Behavioral Risk Factor Surveillance System 2006. Atlanta, Georgia: CDC, US Department of Health and Human Services.

Appendix: Citation List

Citation
Centers for Disease Control and Prevention (CDC). United States Behavioral Risk Factor Surveillance System 2008. Atlanta, Georgia: CDC, US Department of Health and Human Services.
Centers for Disease Control and Prevention (CDC). United States Behavioral Risk Factor Surveillance System 2010. Atlanta, United States: Centers for Disease Control and Prevention (CDC).
Centers for Disease Control and Prevention (CDC). United States Behavioral Risk Factor Surveillance System 2011. Atlanta, Georgia: CDC, US Department of Health and Human Services.
Centers for Disease Control and Prevention (CDC). United States Behavioral Risk Factor Surveillance System 2012. Atlanta, Georgia: CDC, US Department of Health and Human Services, 2013.
Centers for Disease Control and Prevention (CDC). United States Behavioral Risk Factor Surveillance System 2013. Atlanta, United States: Centers for Disease Control and Prevention (CDC), 2014.
Centers for Disease Control and Prevention (CDC). United States Behavioral Risk Factor Surveillance System 2014. Atlanta, United States: Centers for Disease Control and Prevention (CDC), 2015.
Centers for Disease Control and Prevention (CDC). United States Behavioral Risk Factor Surveillance System; 1988. Atlanta, Georgia: CDC, US Department of Health and Human Services.
Centers for Disease Control and Prevention (CDC). United States Behavioral Risk Factor Surveillance System1986. Atlanta, Georgia: CDC, US Department of Health and Human Services.
Centers for Disease Control and Prevention (CDC). United States Behavioral Risk Factor Surveillance System1987. Atlanta, Georgia: CDC, US Department of Health and Human Services.
Centers for Disease Control and Prevention (CDC). United States Behavioral Risk Factor Surveillance System1999. Atlanta, Georgia: CDC, US Department of Health and Human Services.
Centers for Disease Control and Prevention (CDC). United States National Youth Risk Behavior Survey 1991. Atlanta, United States: Centers for Disease Control and Prevention (CDC).
Centers for Disease Control and Prevention (CDC). United States National Youth Risk Behavior Survey 1993. Atlanta, United States: Centers for Disease Control and Prevention (CDC).
Centers for Disease Control and Prevention (CDC). United States National Youth Risk Behavior Survey 1995. Atlanta, United States: Centers for Disease Control and Prevention (CDC), 1996.
Centers for Disease Control and Prevention (CDC). United States National Youth Risk Behavior Survey 1997. Atlanta, United States: Centers for Disease Control and Prevention (CDC), 1998.
Centers for Disease Control and Prevention (CDC). United States National Youth Risk Behavior Survey 1999. Atlanta, United States: Centers for Disease Control and Prevention (CDC), 2000.
Centers for Disease Control and Prevention (CDC). United States National Youth Risk Behavior Survey 2001. Atlanta, United States: Centers for Disease Control and Prevention (CDC), 2002.
Centers for Disease Control and Prevention (CDC). United States National Youth Risk Behavior Survey 2003. Atlanta, United States: Centers for Disease Control and Prevention (CDC), 2004.
Centers for Disease Control and Prevention (CDC). United States National Youth Risk Behavior Survey 2005. Atlanta, United States: Centers for Disease Control and Prevention (CDC), 2006.
Centers for Disease Control and Prevention (CDC). United States National Youth Risk Behavior Survey 2007. Atlanta, United States: Centers for Disease Control and Prevention (CDC), 2008.
Centers for Disease Control and Prevention (CDC). United States National Youth Risk Behavior Survey 2009. Atlanta, United States: Centers for Disease Control and Prevention (CDC), 2010.
Centers for Disease Control and Prevention (CDC). United States National Youth Risk Behavior Survey 2011. Atlanta, United States: Centers for Disease Control and Prevention (CDC), 2012.
Centers for Disease Control and Prevention (CDC). United States National Youth Risk Behavior Survey 2013. Atlanta, United States: Centers for Disease Control and Prevention (CDC), 2014.
Centers for Disease Control and Prevention (CDC). United States National Youth Tobacco Survey 1999. Atlanta, United States: Centers for Disease Control and Prevention (CDC).
Centers for Disease Control and Prevention (CDC). United States National Youth Tobacco Survey 2002. Atlanta, United States: Centers for Disease Control and Prevention (CDC).
Centers for Disease Control and Prevention (CDC). United States National Youth Tobacco Survey 2004. Atlanta, United States: Centers for Disease Control and Prevention (CDC).
Centers for Disease Control and Prevention (CDC). United States National Youth Tobacco Survey 2006. Atlanta, United States: Centers for Disease Control and Prevention (CDC).
Centers for Disease Control and Prevention (CDC). United States National Youth Tobacco Survey 2012. Atlanta, United States: Centers for Disease Control and Prevention (CDC).
Centers for Disease Control and Prevention (CDC). USA National Health Interview Survey 1997
Centers for Disease Control, R.O.C (Taiwan). Taiwan Tuberculosis Cases by Age Group, Sex, and Place of Living. Taipei, Taiwan: Centers for Disease Control, R.O.C (Taiwan).
Central Administration of Statistics (Lebanon), League of Arab States, Ministry of Social Affairs (Lebanon), Pan Arab Project for Family Health (PAPFAM). Lebanon Family Health Survey 2004.
Central Administration of Statistics (Lebanon), United Nations Children's Fund (UNICEF). Lebanon Multiple Indicator Cluster Survey 2000. New York, United States: United Nations Children's Fund (UNICEF).
Central Administration of Statistics (Lebanon). Lebanon Multiple Indicator Cluster Survey 2009.
Central African Institute of Statistics, Economic and Social Studies (ICASEES) (Central African Republic), ICF International. Central African Republic Multiple Indicator Cluster Survey 2010-2011. Fairfax, United States: ICF International, 2013.

Appendix: Citation List

Citation
Central African Republic EPI Review 1993.
Central African Republic Immunization Coverage Survey February 1991.
Central African Republic Nutrition Survey 1995 as it appears in World Health Organization (WHO). WHO Global Database on Child Growth and Malnutrition. Geneva, Switzerland: World Health Organization (WHO).
Central African Republic Population and Housing Census 1975 as it appears in United Nations Statistics Division (UNSD). United Nations Demographic Yearbook - Historical Supplement 1997. New York City, United States: United Nations Statistics Division (UNSD).
Central African Republic Priority Survey on Household Living Conditions 1992-1993 as it appears in World Health Organization (WHO). WHO Household Energy Database. Geneva, Switzerland: World Health Organization (WHO), 2010.
Central Agency for Public Mobilisation and Statistics (CAPMAS) (Egypt), International Statistical Institute. Egypt World Fertility Survey 1980. Voorburg, Netherlands: International Statistical Institute.
Central Agency for Public Mobilization and Statistics (Egypt), League of Arab States. Egypt Maternal and Child Health Survey 1991.
Central Agency for Public Mobilization and Statistics (Egypt), Minnesota Population Center. Egypt General Census for Population, Housing, and Establishments 2006 - IPUMS. University of Minnesota, 2011.
Central Agency for Public Mobilization and Statistics (Egypt), Minnesota Population Center. Egypt Population, Housing, and Establishment Census 1996 - IPUMS. University of Minnesota.
Central Bank of Nigeria, National Bureau of Statistics (Nigeria), Nigerian Communications Commission (NCC). Nigeria General Household Survey 2007. Abuja, Nigeria: National Bureau of Statistics (Nigeria).
Central Bank of Nigeria, National Bureau of Statistics (Nigeria), Nigerian Communications Commission (NCC). Nigeria General Household Survey 2008.
Central Board of Health (Zambia), Central Statistical Office (Zambia), Macro International, Inc, Ministry of Health (Zambia), University of Zambia. Zambia Demographic and Health Survey - Maternal Mortality Data.
Central Board of Health (Zambia), Central Statistical Office (Zambia), Macro International, Inc. Zambia Demographic and Health Survey 2001-2002. Calverton, United States: Macro International, Inc.
Central Bureau of Health Intelligence (India). India National Health Profile 2013. New Delhi, India: Central Bureau of Health Intelligence (India), 2014.
Central Bureau of Statistics (CBS) (Kenya), Minnesota Population Center. Kenya Population and Housing Census 1989 from the Integrated Public Use Microdata Series, International: [Machine-readable database]. Minneapolis: University of Minnesota.
Central Bureau of Statistics (CBS) (Kenya), Minnesota Population Center. Kenya Population and Housing Census 1999 from the Integrated Public Use Microdata Series, International: [Machine-readable database]. Minneapolis: University of Minnesota.
Central Bureau of Statistics (Ghana), International Statistical Institute. Ghana World Fertility Survey 1979-1980. International Statistical Institute.
Central Bureau of Statistics (Indonesia), International Statistical Institute. Indonesia World Fertility Survey 1976. Voorburg, Netherlands: International Statistical Institute.
Central Bureau of Statistics (Indonesia), Macro International, Inc, Ministry of Health (Indonesia), National Family Planning Coordinating Board (Indonesia). Indonesia Demographic and Health Survey 1991. Calverton, United States: Macro International, Inc.
Central Bureau of Statistics (Indonesia), Macro International, Inc, Ministry of Health (Indonesia), National Family Planning Coordinating Board (Indonesia). Indonesia Demographic and Health Survey 1994. Calverton, United States: Macro International, Inc.
Central Bureau of Statistics (Indonesia), Macro International, Inc, Ministry of Health (Indonesia), National Family Planning Coordinating Board (Indonesia). Indonesia Demographic and Health Survey 1997. Calverton, United States: Macro International, Inc.
Central Bureau of Statistics (Indonesia), Ministry of Health (Indonesia), United Nations Children's Fund (UNICEF). Indonesia National Socioeconomic Survey 1995.
Central Bureau of Statistics (Indonesia), Ministry of Health (Indonesia), United Nations Children's Fund (UNICEF). Indonesia National Socioeconomic Survey 1996.
Central Bureau of Statistics (Indonesia), Ministry of Health (Indonesia), United Nations Children's Fund (UNICEF). Indonesia National Socioeconomic Survey 1997.
Central Bureau of Statistics (Indonesia), Ministry of Health (Indonesia), World Bank. Indonesia National Socioeconomic Survey 1998.
Central Bureau of Statistics (Indonesia), Ministry of Health (Indonesia), World Bank. Indonesia National Socioeconomic Survey 1999.
Central Bureau of Statistics (Indonesia), Ministry of Health (Indonesia), World Bank. Indonesia National Socioeconomic Survey 2000.
Central Bureau of Statistics (Indonesia), Ministry of Health (Indonesia), World Bank. Indonesia National Socioeconomic Survey 2001.
Central Bureau of Statistics (Indonesia), National Family Planning Coordinating Board (Indonesia), Westinghouse; Institute for Resource Development. Indonesia Demographic and Health Survey 1987. Columbia, United States: Westinghouse; Institute for Resource Development.
Central Bureau of Statistics (Indonesia), United Nations Children's Fund (UNICEF). Indonesia Multiple Indicator Cluster Survey 1995.
Central Bureau of Statistics (Indonesia). Indonesia Intercensal Population Survey 1976 .
Central Bureau of Statistics (Indonesia). Indonesia Intercensal Population Survey 1985.
Central Bureau of Statistics (Indonesia). Indonesia Intercensal Population Survey 1995.
Central Bureau of Statistics (Indonesia). Indonesia National Socioeconomic Survey 1992.
Central Bureau of Statistics (Indonesia). Indonesia National Socioeconomic Survey 1993.
Central Bureau of Statistics (Indonesia). Indonesia National Socioeconomic Survey 1994.
Central Bureau of Statistics (Indonesia). Indonesia Population and Housing Census 1971.
Central Bureau of Statistics (Israel), Ministry of Health (Israel). Israel Health Survey 2009.
Central Bureau of Statistics (Israel), Minnesota Population Center. Israel Census of Population and Housing 1983 from the Integrated Public Use Microdata Series, International: [Machine-readable database]. Minneapolis: University of Minnesota.

Appendix: Citation List

Citation
Central Bureau of Statistics (Israel). Israel Stillbirths 2013, Israel Live Births 2014, and Israel Statistical Abstract 2015. Jerusalem, Israel: Central Bureau of Statistics (Israel).
Central Bureau of Statistics (Kenya), International Statistical Institute. Kenya World Fertility Survey 1977-1978. Voorburg, Netherlands: International Statistical Institute.
Central Bureau of Statistics (Kenya), Macro International, Inc, National Council for Population Development (NCPD). Kenya Demographic and Health Survey 1993. Calverton, United States: Macro International, Inc.
Central Bureau of Statistics (Kenya), Macro International, Inc, National Council for Population Development (NCPD). Kenya Demographic and Health Survey 1998. Calverton, United States: Macro International, Inc.
Central Bureau of Statistics (Kenya), UK Department for International Development (DFID), United States Agency for International Development (USAID), European Union (EU), Danish International Development Agency (DANIDA), World Bank (WB), United Nations Development Programme (UNDP). Kenya Integrated Household Budget Survey 2005-2006. Nairobi, Kenya: Central Bureau of Statistics (Kenya).
Central Bureau of Statistics (Kenya), United Nations Children's Fund (UNICEF). Kenya Multiple Indicator Cluster Survey 2000. New York, United States: United Nations Children's Fund (UNICEF).
Central Bureau of Statistics (Kenya). Kenya Welfare Monitoring Survey III 1997. Nairobi, Kenya: Kenya National Bureau of Statistics.
Central Bureau of Statistics (Namibia), National Planning Commission (Namibia). Namibia Household Income and Expenditure Survey 2003-2004. Windhoek, Namibia: Central Bureau of Statistics (Namibia), National Planning Commission (Namibia).
Central Bureau of Statistics (Nepal), Minnesota Population Center. Nepal National Population Census 2001 from the Integrated Public Use Microdata Series, International: [Machine-readable database]. Minneapolis: University of Minnesota.
Central Bureau of Statistics (Nepal), United Nations Children's Fund (UNICEF). Nepal Multiple Indicator Cluster Survey 2014. New York, United States: United Nations Children's Fund (UNICEF), 2015.
Central Bureau of Statistics (Nepal). Nepal Census 2011.
Central Bureau of Statistics (North Korea), Institute of Children's Nutrition (North Korea), United Nations Children's Fund (UNICEF). Korea, North Multiple Indicator Cluster Survey 2009.
Central Bureau of Statistics (North Korea), United Nations Children's Fund (UNICEF), World Food Programme (WFP). Korea, North Nutrition Assessment 2004.
Central Bureau of Statistics (North Korea), United Nations Children's Fund (UNICEF). Korea, North Multiple Indicator Cluster Survey 2000.
Central Bureau of Statistics (North Korea). Korea, North Population Census 2008.
Central Bureau of Statistics (Sudan), Federal Ministry of Health (Sudan), Government of Sudan, Ministry of Health (South Sudan), Southern Sudan Centre for Census, Statistics and Evaluation. Sudan - South Multiple Indicator Cluster Survey 2010. New York, United States: United Nations Children's Fund (UNICEF), 2015.
Central Bureau of Statistics (Sudan), Federal Ministry of Health (Sudan), United Nations Children's Fund (UNICEF). Sudan Multiple Indicator Cluster Survey 2014.
Central Bureau of Statistics (Sudan), Ministry of Health (South Sudan). Sudan - North Multiple Indicator Cluster Survey 2010. New York, United States: United Nations Children's Fund (UNICEF), 2015.
Central Bureau of Statistics (Syria), International Statistical Institute. Syria World Fertility Survey 1978. International Statistical Institute.
Central Bureau of Statistics (Syria), League of Arab States. Syria Family Health Survey 2001.
Central Bureau of Statistics (Syria), League of Arab States. Syria Maternal and Child Health Survey 1993.
Central Bureau of the Census (Cameroon), Ministry of Economic Affairs and Planning (Cameroon). Cameroon Population and Housing Census 1976.
Central Bureau of the Census (Niger). Niger General Population Census 1988.
Central Bureau of the Census and Population Studies (Cameroon), ICF International, Ministry of Economy, Planning and Regional Development (Cameroon), National Institute of Statistics (Cameroon), Pasteur Center of Cameroon. Cameroon Demographic and Health Survey - Maternal Mortality Data.
Central Bureau of the Census and Population Studies (Cameroon), Macro International, Inc. Cameroon Demographic and Health Survey 1998. Calverton, United States: Macro International, Inc.
Central Census Bureau (Mali), Minnesota Population Center. Mali Census 2009 from the Integrated Public Use Microdata Series, International: [Machine-readable database]. Minneapolis: University of Minnesota.
Central Census Bureau (Mali), Minnesota Population Center. Mali General Population and Housing Census 1987 from the Integrated Public Use Microdata Series, International: [Machine-readable database]. Minneapolis: University of Minnesota.
Central Census Bureau (Mali), Minnesota Population Center. Mali General Population and Housing Census 1998 from the Integrated Public Use Microdata Series, International: [Machine-readable database]. Minneapolis: University of Minnesota.
Central Census Bureau (Mali), National Institute of Statistics (INSTAT) (Mali). Mali Population and Housing Census 2009.
Central Department of Statistics and Information (Saudi Arabia). Saudi Arabia Demographic Research Bulletin 2001. Riyadh, Saudi Arabia: Central Department of Statistics and Information (Saudi Arabia).
Central Department of Statistics and Information (Saudi Arabia). Saudi Arabia Demographic Research Bulletin 2007. Riyadh, Saudi Arabia: Central Department of Statistics and Information (Saudi Arabia).
Central Department of Statistics and Information (Saudi Arabia). Saudi Arabia Population and Housing Census 1992. Riyadh, Saudi Arabia: Central Department of Statistics and Information (Saudi Arabia).
Central Department of Statistics and Information (Saudi Arabia). Saudi Arabia Population and Housing Census 2004. Riyadh, Saudi Arabia: Central Department of Statistics and Information (Saudi Arabia).
Central Office of Statistics and Information (Venezuela), International Statistical Institute. Venezuela World Fertility Survey 1977. Voorburg, Netherlands: International Statistical Institute.

Appendix: Citation List

Citation
Central Office of Statistics and Information (Venezuela), Minnesota Population Center. Venezuela Population and Housing Census 1981 from the Integrated Public Use Microdata Series, International: [Machine-readable database]. Minneapolis: University of Minnesota.
Central Office of Statistics and Information (Venezuela), Minnesota Population Center. Venezuela Population and Housing Census 1990 from the Integrated Public Use Microdata Series, International: [Machine-readable database]. Minneapolis: University of Minnesota.
Central Office of the Census (Comoros). Comoros Population and Housing Census 1980.
Central Organization for Statistics and Information Technology (Iraq), Kurdistan Regional Statistics Office, Ministry of Health (Iraq), United Nations Children's Fund (UNICEF). Iraq Multiple Cluster Indicator Survey - Complete Birth History Data.
Central Organization for Statistics and Information Technology (Iraq), Kurdistan Regional Statistics Office, Ministry of Health (Iraq), United Nations Children's Fund (UNICEF). Iraq Multiple Indicator Cluster Survey 2011. New York, United States: United Nations Children's Fund (UNICEF), 2013.
Central Organization for Statistics and Information Technology (Iraq), Ministry of Health (Iraq), United Nations Children's Fund (UNICEF), World Health Organization (WHO). Iraq Child and Maternal Mortality Survey 1999.
Central Organization for Statistics and Information Technology (Iraq), Ministry of Health (Iraq), World Health Organization (WHO). Iraq STEPS Noncommunicable Disease Risk Factors Survey 2006.
Central Organization for Statistics and Information Technology (Iraq), The Fafo Research Foundation. Iraq Multiple Indicator Rapid Assessment 2004.
Central Organization for Statistics and Information Technology (Iraq). Iraq Living Conditions Survey 2004.
Central Planning Organization (Yemen), International Statistical Institute. Yemen World Fertility Survey 1979. Voorburg, Netherlands: International Statistical Institute.
Central Planning Unit (Belize). British Honduras Population and Housing Census 1970.
Central Statistical Agency (Ethiopia), Government of Ethiopia, United Nations Population Fund (UNFPA), United Nations Development Programme (UNDP). Ethiopia Population and Housing Census 2007. Addis Ababa, Ethiopia: Central Statistical Agency (Ethiopia).
Central Statistical Agency (Ethiopia), ICF Macro, Ministry of Health (Ethiopia), Population and Housing Census Commissions Office (PHCCO). Ethiopia Demographic and Health Survey - Maternal Mortality Data.
Central Statistical Agency (Ethiopia), ICF Macro, Ministry of Health (Ethiopia). Ethiopia Demographic and Health Survey 2010-2011. Calverton, United States: ICF Macro.
Central Statistical Agency (Ethiopia), ORC Macro. Ethiopia Demographic and Health Survey 2000. Calverton, United States: ORC Macro, 2001.
Central Statistical Agency (Ethiopia), United Nations Population Fund (UNFPA), United States Agency for International Development (USAID). Ethiopia Population and Housing Census 1994.
Central Statistical Agency (Ethiopia), World Bank. Ethiopia Living Standards Measurement Study - Integrated Survey on Agriculture 2013-2014. Washington DC, United States: World Bank, 2015.
Central Statistical Agency (Ethiopia). Ethiopia Health and Nutrition Survey 1998.
Central Statistical Agency (Ethiopia). Ethiopia National Fertility and Family Survey 1990-1991. Addis Ababa, Ethiopia: Central Statistical Agency (Ethiopia).
Central Statistical Agency (Ethiopia). Ethiopia Welfare Monitoring Survey 1996.
Central Statistical Agency (Ethiopia). Ethiopia Welfare Monitoring Survey 1998.
Central Statistical Agency (Ethiopia). Ethiopia Welfare Monitoring Survey 2004.
Central Statistical Bureau (Kuwait), Ministry of Health (Kuwait). Kuwait Annual Bulletin for Vital Statistics - Births and Deaths 2005. Kuwait City, Kuwait: Central Statistical Bureau (Kuwait).
Central Statistical Bureau (Kuwait), Ministry of Health (Kuwait). Kuwait Annual Bulletin for Vital Statistics - Births and Deaths 2006. Kuwait City, Kuwait: Central Statistical Bureau (Kuwait).
Central Statistical Bureau (Kuwait), Ministry of Health (Kuwait). Kuwait Annual Bulletin for Vital Statistics - Births and Deaths 2007. Kuwait City, Kuwait: Central Statistical Bureau (Kuwait).
Central Statistical Bureau (Kuwait), Ministry of Health (Kuwait). Kuwait Annual Bulletin for Vital Statistics - Births and Deaths 2008. Kuwait City, Kuwait: Central Statistical Bureau (Kuwait).
Central Statistical Bureau (Kuwait), Ministry of Health (Kuwait). Kuwait Annual Bulletin for Vital Statistics - Births and Deaths 2009. Kuwait City, Kuwait: Central Statistical Bureau (Kuwait).
Central Statistical Bureau (Kuwait), Ministry of Health (Kuwait). Kuwait Annual Bulletin for Vital Statistics - Births and Deaths 2010. Kuwait City, Kuwait: Central Statistical Bureau (Kuwait).
Central Statistical Bureau (Kuwait), Ministry of Health (Kuwait). Kuwait Annual Bulletin for Vital Statistics - Births and Deaths 2011. Kuwait City, Kuwait: Central Statistical Bureau (Kuwait).
Central Statistical Bureau (Kuwait). Kuwait Annual Bulletin for Vital Statistics - Births and Deaths 2012.
Central Statistical Bureau (Latvia), Eurostat. Latvia European Health Interview Survey 2008.
Central Statistical Office (Grenada). Grenada Core Welfare Indicators Questionnaire Survey 2005.
Central Statistical Office (Grenada). Grenada Population and Housing Census 1991.
Central Statistical Office (Mauritius). Mauritius Population and Housing Census 1983.
Central Statistical Office (Poland). Poland Demographic Yearbook 2007. Warsaw, Poland: Central Statistical Office (Poland), 2007.
Central Statistical Office (Poland). Poland Health Interview Survey 1996.
Central Statistical Office (Swaziland), Macro International, Inc. Swaziland Demographic and Health Survey 2006-2007. Calverton, United States: Macro International, Inc.
Central Statistical Office (Swaziland), United Nations Children's Fund (UNICEF), United Nations Educational, Scientific and Cultural Organization (UNESCO), United Nations Population Fund (UNFPA). Swaziland Multiple Indicator Cluster Survey 2014.

Appendix: Citation List

Citation
Central Statistical Office (Swaziland), United Nations Children's Fund (UNICEF). Swaziland Multiple Indicator Cluster Survey 2000. New York, United States: United Nations Children's Fund (UNICEF).
Central Statistical Office (Swaziland), United Nations Children's Fund (UNICEF). Swaziland Multiple Indicator Cluster Survey 2010. New York, United States: United Nations Children's Fund (UNICEF).
Central Statistical Office (Swaziland), United Nations Department for Technical Cooperation and Development (UNDTCD). Swaziland Population Census 1986.
Central Statistical Office (Trinidad and Tobago) and United Nations Children's Fund (UNICEF). Trinidad and Tobago Multiple Indicator Cluster Survey 2006. New York, United States: United Nations Children's Fund (UNICEF).
Central Statistical Office (Trinidad and Tobago), International Statistical Institute. Trinidad and Tobago World Fertility Survey 1977. Voorburg, Netherlands: International Statistical Institute.
Central Statistical Office (Trinidad and Tobago), United Nations Children's Fund (UNICEF). Trinidad and Tobago Multiple Indicator Cluster Survey 2000. New York, United States: United Nations Children's Fund (UNICEF).
Central Statistical Office (Trinidad and Tobago). Trinidad and Tobago Population and Housing Census 1990.
Central Statistical Office (Zambia), Food Security, Health and Nutrition Information System (Zambia), United Nations Children's Fund (UNICEF). Zambia Multiple Indicator Cluster Survey 1999. New York, United States: United Nations Children's Fund (UNICEF).
Central Statistical Office (Zambia), ICF International, Ministry of Health (Zambia), Tropical Diseases Research Centre, University Teaching Hospital (Zambia), University of Zambia. Zambia Demographic and Health Survey 2013-2014. Fairfax, United States: ICF International, 2015.
Central Statistical Office (Zambia), London School of Hygiene and Tropical Medicine. Zambia Living Conditions Monitoring Survey 1998. Lusaka, Zambia: Central Statistical Office (Zambia).
Central Statistical Office (Zambia), Macro International, Inc, Ministry of Health (Zambia). Zambia Demographic and Health Survey 1996-1997. Calverton, United States: Macro International, Inc.
Central Statistical Office (Zambia), Macro International, Inc, University of Zambia. Zambia Demographic and Health Survey 1992. Calverton, United States: Macro International, Inc.
Central Statistical Office (Zambia), Macro International, Inc. Zambia Demographic and Health Survey 2007. Calverton, United States: Macro International, Inc.
Central Statistical Office (Zambia), Minnesota Population Center. Zambia Census 1990 from the Integrated Public Use Microdata Series, International: [Machine-readable database]. Minneapolis: University of Minnesota.
Central Statistical Office (Zambia), Minnesota Population Center. Zambia Census 2000 from the Integrated Public Use Microdata Series, International: [Machine-readable database]. Minneapolis: University of Minnesota.
Central Statistical Office (Zambia), Minnesota Population Center. Zambia Census 2010 from the Integrated Public Use Microdata Series, International: [Machine-readable database]. Minneapolis: University of Minnesota.
Central Statistical Office (Zambia). Zambia Census of Population and Housing 1980.
Central Statistical Office (Zambia). Zambia Census of Population and Housing 2010.
Central Statistical Office (Zambia). Zambia Global Fund Household Survey 2008. Lusaka, Zambia: Central Statistical Office (Zambia).
Central Statistical Office (Zambia). Zambia Household Health Coverage Survey 2008. Lusaka, Zambia: Central Statistical Office (Zambia).
Central Statistical Office (Zambia). Zambia Living Conditions Monitoring Survey 1996. Lusaka, Zambia: Central Statistical Office (Zambia).
Central Statistical Office (Zambia). Zambia Living Conditions Monitoring Survey 2002-2003. Lusaka, Zambia: Central Statistical Office (Zambia).
Central Statistical Office (Zambia). Zambia Living Conditions Monitoring Survey 2004-2005. Lusaka, Zambia: Central Statistical Office (Zambia).
Central Statistical Office (Zambia). Zambia Living Conditions Monitoring Survey 2010.
Central Statistical Office (Zimbabwe), Macro International, Inc. Zimbabwe Demographic and Health Survey 1994. Calverton, United States: Macro International, Inc.
Central Statistical Office (Zimbabwe), Macro International, Inc. Zimbabwe Demographic and Health Survey 1999. Calverton, United States: Macro International, Inc.
Central Statistical Office (Zimbabwe), Macro International, Inc. Zimbabwe Demographic and Health Survey 2005-2006. Calverton, United States: Macro International, Inc.
Central Statistical Office (Zimbabwe), Macro Systems, Inc.; Institute for Resource Development. Zimbabwe Demographic and Health Survey 1988-1989. Columbia, United States: Macro Systems, Inc.
Central Statistical Office (Zimbabwe). Zimbabwe Multiple Indicator Monitoring Survey 2009. New York, United States: United Nations Children's Fund (UNICEF).
Central Statistical Office (Zimbabwe). Zimbabwe Population and Housing Census 1982.
Central Statistical Office (Zimbabwe). Zimbabwe Population and Housing Census 1992.
Central Statistical Office (Zimbabwe). Zimbabwe Population and Housing Census 2002.
Central Statistical Office of Saint Lucia, Minnesota Population Center. Saint Lucia Population and Housing Census 1991 from the Integrated Public Use Microdata Series, International: [Machine-readable database]. Minneapolis: University of Minnesota.
Central Statistical Office of Saint Lucia, Minnesota Population Center. Saint Lucia Population Census 1980 from the Integrated Public Use Microdata Series, International: Version 6.0 [Machine-readable database]. Minneapolis: University of Minnesota.
Central Statistical Office of Saint Lucia. Saint Lucia Population and Housing Census 2010.
Central Statistical Office, Ministry of Finance (Dominica). Dominica Population and Housing Census 1970.
Central Statistical Office, Ministry of Finance (Dominica). Dominica Population and Housing Census 1991.
Central Statistical Organization (Iraq), Minnesota Population Center. Iraq Population and Housing Census 1997 from the Integrated Public Use Microdata Series, International: [Machine-readable database]. Minneapolis: University of Minnesota.

Appendix: Citation List

Citation
Central Statistical Organization (Iraq), United Nations Children's Fund (UNICEF). Iraq Multiple Indicator Cluster Survey 2000. New York, United States: United Nations Children's Fund (UNICEF).
Central Statistical Organization (Qatar), Council of Health Ministers of GCC States, Ministry of Public Health (Qatar). Qatar Child Health Survey 1987.
Central Statistical Organization (Qatar), Ministry of Public Health (Qatar). Qatar Vital Statistics Annual Bulletin 1984.
Central Statistical Organization (Qatar), Ministry of Public Health (Qatar). Qatar Vital Statistics Annual Bulletin 1985.
Central Statistical Organization (Qatar). Qatar Annual Statistical Abstract 1990. Doha, Qatar: Central Statistical Organization (Qatar), 1991.
Central Statistical Organization (Qatar). Qatar Annual Statistical Abstract 1991. Doha, Qatar: Central Statistical Organization (Qatar), 1992.
Central Statistical Organization (Qatar). Qatar Annual Statistical Abstract 1992. Doha, Qatar: Central Statistical Organization (Qatar), 1993.
Central Statistical Organization (Qatar). Qatar Annual Statistical Abstract 1993. Doha, Qatar: Central Statistical Organization (Qatar), 1994. and Hamad Medical Corporation (Qatar), Ministry of Public Health (Qatar). Qatar Annual Health Report 2002. Doha, Qatar: Hamad Medical Corporation (Qatar), 2003.
Central Statistical Organization (Qatar). Qatar Annual Statistical Abstract 1994. Doha, Qatar: Central Statistical Organization (Qatar), 1995. and Hamad Medical Corporation (Qatar), Ministry of Public Health (Qatar). Qatar Annual Health Report 2002. Doha, Qatar: Hamad Medical Corporation (Qatar), 2003.
Central Statistical Organization (Qatar). Qatar Annual Statistical Abstract 1995. Doha, Qatar: Central Statistical Organization (Qatar), 1996. and Hamad Medical Corporation (Qatar), Ministry of Public Health (Qatar). Qatar Annual Health Report 2002. Doha, Qatar: Hamad Medical Corporation (Qatar), 2003.
Central Statistical Organization (Qatar). Qatar Annual Statistical Abstract 1996. Doha, Qatar: Central Statistical Organization (Qatar), 1997. and Hamad Medical Corporation (Qatar), Ministry of Public Health (Qatar). Qatar Annual Health Report 2002. Doha, Qatar: Hamad Medical Corporation (Qatar), 2003.
Central Statistical Organization (Qatar). Qatar Annual Statistical Abstract 1997. Doha, Qatar: Central Statistical Organization (Qatar), 1998. and Hamad Medical Corporation (Qatar), Ministry of Public Health (Qatar). Qatar Annual Health Report 2002. Doha, Qatar: Hamad Medical Corporation (Qatar), 2003.
Central Statistical Organization (Yemen), ICF International, Ministry of Public Health and Population (Yemen). Yemen Demographic and Health Survey 2013. Fairfax, United States: ICF International, 2015.
Central Statistical Organization (Yemen), League of Arab States, Macro International, Inc. Yemen Demographic and Health Survey 1991-1992. Calverton, United States: Macro International, Inc.
Central Statistical Organization (Yemen), League of Arab States, Ministry of Public Health and Population (Yemen), Pan Arab Project for Family Health (PAPFAM). Yemen Family Health Survey 2003.
Central Statistical Organization (Yemen), Macro International, Inc. Yemen Demographic and Health Survey 1997. Calverton, United States: Macro International, Inc.
Central Statistical Organization (Yemen). Yemen Household Budget Survey 2005-2006. Sana'a, Yemen: Central Statistical Organization (Yemen).
Central Statistical Service (South Africa), Minnesota Population Center. South Africa Census 1996 from the Integrated Public Use Microdata Series, International: [Machine-readable database]. Minneapolis: University of Minnesota.
Central Statistical Service (South Africa). South Africa October Household Survey 1993.
Central Statistical Service (South Africa). South Africa October Household Survey 1997.
Central Statistics Department (Gambia), United Nations Children's Fund (UNICEF). Gambia Multiple Indicator Cluster Survey 2000. New York, United States: United Nations Children's Fund (UNICEF).
Central Statistics Department (Gambia). Gambia Household Education and Health Survey 1993-1994. Banjul, Gambia: Central Statistics Department (Gambia).
Central Statistics Department (Gambia). Gambia Population and Housing Census 1983.
Central Statistics Office (Afghanistan), UNICEF Afghanistan Country Office, German Technical Cooperation Agency (GTZ). Afghanistan Multiple Indicator Cluster Survey 2000. New York, United States: United Nations Children's Fund (UNICEF).
Central Statistics Office (Botswana), Macro Systems, Inc.; Institute for Resource Development, Ministry of Health (Botswana). Botswana Demographic and Health Survey 1988. Columbia, United States: Macro Systems, Inc.; Institute for Resource Development.
Central Statistics Office (Botswana), Ministry of Health (Botswana). Botswana Health Statistics Report 2009. Gaborone, Botswana: Central Statistics Office (Botswana), 2012.
Central Statistics Office (Botswana), National AIDS Coordinating Agency (Botswana). Botswana AIDS Impact Survey 2008. Gaborone, Botswana: Central Statistics Office (Botswana).
Central Statistics Office (Botswana), United Nations Children's Fund (UNICEF). Botswana Multiple Indicator Cluster Survey 2000. 2015.
Central Statistics Office (Botswana), United Nations Population Fund (UNFPA). Botswana Population and Housing Census 2011. Gaborone, Botswana: Central Statistics Office (Botswana).
Central Statistics Office (Botswana). Botswana AIDS Impact Survey 2004. Gaborone, Botswana: Central Statistics Office (Botswana).
Central Statistics Office (Botswana). Botswana Demographic Survey 2006. Gaborone, Botswana: Central Statistics Office (Botswana).
Central Statistics Office (Botswana). Botswana Family Health Survey 1996. Gaborone, Botswana: Central Statistics Office (Botswana).
Central Statistics Office (Botswana). Botswana Family Health Survey 2007-2008. Gaborone, Botswana: Central Statistics Office (Botswana), 2009.
Central Statistics Office (Botswana). Botswana Household Income and Expenditure Survey 1993-1995. Gaborone, Botswana: Central Statistics Office (Botswana).
Central Statistics Office (Botswana). Botswana Household Income and Expenditure Survey 2002-2003. Gaborone, Botswana: Central Statistics Office (Botswana).
Central Statistics Office (Botswana). Botswana Population and Housing Census 1981.

Appendix: Citation List

Citation
Central Statistics Office (Botswana). Botswana Population and Housing Census 2001.
Central Statistics Office (Botswana). Botswana Stats Brief Maternal Mortality Ratio 2005-2009. Gaborone, Botswana: Central Statistics Office (Botswana), 2010.
Central Statistics Office (Botswana). Botswana Stats Brief Maternal Mortality Ratio 2007-2011. Gaborone, Botswana: Central Statistics Office (Botswana), 2012.
Central Statistics Office (Botswana). Botswana Vital Statistics Report 2011. Gaborone, Botswana: Central Statistics Office (Botswana).
Central Statistics Office (Botswana). Botswana Vital Statistics Report 2012. Gaborone, Botswana: Central Statistics Office (Botswana).
Central Statistics Office (Mauritius). Mauritius Population and Housing Census 1990.
Central Statistics Office (Mauritius). Mauritius Population and Housing Census 2000.
Central Statistics Office (Namibia), Macro International, Inc, Ministry of Health and Social Services (Namibia), National Planning Commission (Namibia). Namibia Demographic and Health Survey - Maternal Mortality Data.
Central Statistics Office (Namibia), Macro International, Inc, Ministry of Health and Social Services (Namibia). Namibia Demographic and Health Survey 1992. Calverton, United States: Macro International, Inc.
Central Statistics Office (Sierra Leone), Government of Sierra Leone, United Nations Children's Fund (UNICEF). Sierra Leone Multiple Indicator Cluster Survey 1995.
Central Statistics Office (Sierra Leone), United Nations Children's Fund (UNICEF). Sierra Leone Multiple Indicator Cluster Survey 2000. New York, United States: United Nations Children's Fund (UNICEF).
Central Statistics Office (Sierra Leone). Sierra Leone Census 2004.
Central Statistics Office (Sierra Leone). Sierra Leone Population and Housing Census 1985.
Central Statistics Organization (Afghanistan), European Union (EU), Ministry of Rural Rehabilitation and Development (Afghanistan). Afghanistan National Risk and Vulnerability Assessment 2005.
Central Statistics Organization (Afghanistan), ICF Macro, Indian Institute of Health Management Research (IIHMR), Ministry of Public Health (Afghanistan), World Health Organization Regional Office for the Eastern Mediterranean (EMRO-WHO). Afghanistan Special Demographic and Health Survey 2010. Calverton, United States: ICF Macro.
Central Statistics Organization (Afghanistan), ICON-INSTITUTE Consulting Group. Afghanistan National Risk and Vulnerability Assessment 2011-2012.
Central Statistics Organization (Afghanistan), Ministry of Rural Rehabilitation and Development (Afghanistan). Afghanistan National Risk and Vulnerability Assessment 2007-2008.
Central Statistics Organization (Afghanistan), United Nations Children's Fund (UNICEF). Afghanistan Multiple Indicator Cluster Survey 2003.
Central Statistics Organization (Afghanistan), United Nations Children's Fund (UNICEF). Afghanistan Multiple Indicator Cluster Survey 2010-2011. New York, United States: United Nations Children's Fund (UNICEF), 2013.
Central Statistics Organization (Bahrain), Ministry of Health (Bahrain). Bahrain Child Health Survey 1989.
Central Statistics Organization (Bahrain). Bahrain Population, Housing, Buildings, and Establishments Census 2001.
Centre for Bhutan Studies and GNH Research. Bhutan Gross National Happiness Survey 2010.
Centre for Disease Prevention and Control (Latvia), National Institute for Health and Welfare (Finland), Riga StradiÅ†Å¡ University. Latvia Health Behavior Among the Adult Population 2012.
Centre for Drug Research, University of Amsterdam. Netherlands Licit and Illicit Drug Use Survey 1997.
Centre for Economic Reform and Transformation (CERT), University of Edinburgh, Economics Education and Research Consortium (EERC) (Ukraine), Institute for the Study of Labor (IZA), Kiev International Institute of Sociology, Rhine-Westphalia Institute for Economic Research (RWI). Ukraine Longitudinal Monitoring Survey 2003. Bonn, Germany: Institute for the Study of Labor (IZA).
Centre for Economic Reform and Transformation (CERT), University of Edinburgh, Economics Education and Research Consortium (EERC) (Ukraine), Institute for the Study of Labor (IZA), Kiev International Institute of Sociology, Rhine-Westphalia Institute for Economic Research (RWI). Ukraine Longitudinal Monitoring Survey 2004. Bonn, Germany: Institute for the Study of Labor (IZA).
Centre for Economic Reform and Transformation (CERT), University of Edinburgh, Economics Education and Research Consortium (EERC) (Ukraine), Institute for the Study of Labor (IZA), Kiev International Institute of Sociology, Rhine-Westphalia Institute for Economic Research (RWI). Ukraine Longitudinal Monitoring Survey 2007. Bonn, Germany: Institute for the Study of Labor (IZA).
Centre for Health Promotion Studies, National University of Ireland, Galway, Health Promotion Unit, Department of Health and Children (Ireland). Ireland Survey of Lifestyle Attitudes and Nutrition 1998. Dublin, Ireland: Health Promotion Unit, Department of Health and Children (Ireland).
Centre for International Health and Development, Institute of Child Health (CIHD), Food and Nutrition Security Analysis Unit (Somalia), Ministry of Health (Somalia), Ministry of Health and Labor (Somaliland), Puntland Ministry of Health (Somalia), United Nations Children's Fund (UNICEF), World Food Programme (WFP), World Health Organization (WHO). Somalia National Micronutrient and Anthropometric Survey 2009.
Centre for Physical Activity and Health, University of Sydney (Australia), Department of Health and Social Affairs (Micronesia), Fiji School of Medicine, Micronesia Human Resources Development Center, Pohnpei State Department of Health Services, World Health Organization (WHO). Micronesia - Pohnpei STEPS Noncommunicable Disease Risk Factors Survey 2002.
Centre for Research and Documentation in Health Economics (CREDES) (France). France Survey of Health and Welfare 2002.
Centre for the Study of African Economies (CSAE), Ghana Statistical Service. Ghana Urban Household Panel Survey 2006. Oxford, United Kingdom: Centre for the Study of African Economies (CSAE).
Centre for Vision Research, University of Sydney. Australia - Blue Mountains Eye Study 1992-1994. As it appears in Bourne R, Global Burden of Disease Vision Loss Expert Group. Vision Loss Database - Survey Data on Vision Loss by Severity and Etiology. [Unpublished].

Appendix: Citation List

Citation
Centre of Health Economics (Latvia), Lithuanian University of Health Sciences, National Institute for Health Development (Estonia), National Institute for Health and Welfare (Finland). Social Determinants of Health Behaviors Finbalt Health Monitor 1998-2008. Helsinki, Finland: National Institute for Health and Welfare (Finland), 2011.
Centre of Health Economics (Latvia), National Institute for Health and Welfare (Finland). Latvia Health Behavior Among the Adult Population 2010.
Centre of Health Economics (Latvia). Latvia Health Behavior Among the Adult Population 2008.
Cetinkaya F, Gürses N, Öztürk F. Hepatitis B seroprevalence among children in a Turkish hospital. J Hosp Infect. 1995; 29(3): 217-9.
Chad - N'Djamena Nutrition Survey 1995 as it appears in World Health Organization (WHO). WHO Global Database on Child Growth and Malnutrition - Historical. Geneva, Switzerland: World Health Organization (WHO).
Chad National Immunization Coverage Survey 1990.
Chad Vaccination Coverage Survey 2012.
Chadha VK. Tuberculosis epidemiology in India: a review. Int J Tuberc Lung Dis. 2005; 9(10): 1072-82.
Chadijah S, Labajto S, Garijto T, Wijaya Y, Udin Y. Efektivitas diagnosis mikroskopis malaria di puskesmas Donggala, puskesmas Lembasada, dan puskesmas Kulawai, Provinsi Sulawesi Tengah. Jurnal Ekologi Kesehatan. 2006; 5(1): 385-94. As it appears in Malaria Atlas Project. Malaria Atlas Project Plasmodium Falciparum Parasite Rate Database. Oxford, United Kingdom: Malaria Atlas Project.
Chahkandi T, Taheri F, Kazemi T, Bijari B. The Prevalence of Diabetes and Prediabetes Among Elementary School Children in Birjand. Iran J Pediatr. 2015; 25(1): e183.
Chai J-Y, Lee S-H, Choi S-Y, Lee J-S, Yong T-S, Park K-J, Yang K-A, Lee K-H, Park M-J, Park H-R, Kim M-J, Rim H-J. A survey of Brugia malayi infection on the Heugsan Islands, Korea. Korean J Parasitol. 2003; 41(1): 69-73.
Chakma T, Rao PV, Pall S, Kaushal LS, Datta M, Tiwary RS. Survey of pulmonary tuberculosis in a primitive tribe of Madhya Pradesh. Indian J Tuberc. 1996; 43: 85-89.
Chalumeau M, Salanave B, Bouvier-Colle MH, de Bernis L, Prual A, Bréart G. Risk factors for perinatal mortality in West Africa: a population-based study of 20326 pregnancies. MOMA group. Acta Paediatr. 2000; 89(9): 1115-21.
Cham M, Vangen S, Sundby J. Maternal deaths in rural Gambia. Glob Public Health. 2007; 2(4): 359-72.
Chamay Weber C, Haller DM, Narring F. Is there a role for primary care physicians' screening of excessive weight and eating concerns in adolescence?. J Pediatr. 2010; 157(1): 32-5.
Chamberlin J, Bryan JP, Jones DL, Reyes L, Hakre S. Seroprevalence of hepatitis B virus among school-age children in the Stann Creek District of Belize, Central America. Am J Trop Med Hyg. 1996; 55(4): 452-5.
Chambon R, Lemardeley P, Boudin C, Ringwald P, Chandenier J. [Surveillance of the in vivo sensitivity of Plasmodium falciparum to antimalarial agents: the results of initial tests of the OCEAC Malaria Network]. Med Trop (Mars). 1997; 57(4): 357-60. As it appears in Malaria Atlas Project. Malaria Atlas Project Plasmodium Falciparum Parasite Rate Database. Oxford, United Kingdom: Malaria Atlas Project.
Champetier de Ribes G, Ranaivoson G, Rakotoherisoa E, Rakotoson JD, Andriamahefazafy B. [A malaria epidemic in the south of Madagascar?]. Arch Inst Pasteur Madagascar. 1994; 61(2): 66-9. As it appears in Malaria Atlas Project. Malaria Atlas Project Plasmodium Falciparum Parasite Rate Database. Oxford, United Kingdom: Malaria Atlas Project.
Chand G, Barde PV, Singh N. Emergence of new foci of filariasis in Madhya Pradesh, India. Trans R Soc Trop Med Hyg. 2013; 107(7): 462-4.
Chand G, Pandey GD, Tiwary RS. Prevalence of Wuchereria bancrofti infection among the tribals of Panna district of Madhya Pradesh. J Commun Dis. 1996; 28(4): 304-7.
Chand G, Roy SK, Tiwary RS. Malaria epidemic in Lamta PHC of Balaghat district – a rice cultivating ecosystem. J Commun Dis. 1997; 29(2): 179-81. As it appears in Malaria Atlas Project. Malaria Atlas Project Plasmodium Falciparum Parasite Rate Database. Oxford, United Kingdom: Malaria Atlas Project.
Chanda E, Hemingway J, Kleinschmidt I, Rehman AM, Ramdeen V, Phiri FN, Coetzer S, Mthembu D, Shinondo CJ, Chizema-Kawesha E, Kamuliwo M, Mukonka V, Baboo KS, Coleman M. Insecticide Resistance and the Future of Malaria Control in Zambia. PLoS One. 2011; 6(9): e24336. As it appears in Malaria Atlas Project. Malaria Atlas Project Plasmodium Falciparum Parasite Rate Database. Oxford, United Kingdom: Malaria Atlas Project.
Chandenier J, Ndounga M, Carme B, Gay F, Mbitsi A, Hayette MP, Stanghellini A, Ossoh JO, Baudon D, Zitsamélé RC. [Drug sensitivity of Plasmodium falciparum in vivo and in vitro in Brazzaville (Congo)]. Sante. 1995; 5(1): 25-9. As it appears in Malaria Atlas Project. Malaria Atlas Project Plasmodium Falciparum Parasite Rate Database. Oxford, United Kingdom: Malaria Atlas Project.
Chandiwana SK. Human bilharziasis in a peri-urban area in Zimbabwe with special reference to its relationship to malnutrition in school children. Cent Afr J Med. 1983; 29(2): 23-6.
Chandra G, Chatterjee SN, Das S, Sarkar N. Lymphatic filariasis in the coastal areas of Digha, West Bengal, India. Trop Doct. 2007; 37(3): 136-9.
Chandra G, Hati AK. Filariasis survey in a rural area of West Bengal. J Commun Dis. 1996; 28(3): 206-8.
Chandramohan D, Carnerio I, Cox J. Tanzania Plasmodium Falciparum Parasite Rate Data, Personal Communication with D. Chandramohan, I. Carneiro, and J. Cox 2005. As it appears in Malaria Atlas Project. Malaria Atlas Project Plasmodium Falciparum Parasite Rate Database. Oxford, United Kingdom: Malaria Atlas Project.
Chandrasena TGAN, Premaratna R, Abeyewickrema W, de Silva NR. Evaluation of the ICT whole-blood antigen card test to detect infection due to Wuchereria bancrofti in Sri Lanka. Trans R Soc Trop Med Hyg. 2002; 96(1): 60-3. As it appears in London School of Hygiene and Tropical Medicine. Global Atlas of Helminth Infections - Lymphatic Filiariasis.
Chandy H, Heng YV, Samol H, Husum H. Comparing two survey methods for estimating maternal and perinatal mortality in rural Cambodia. Women Birth. 2008; 21(1): 9-12.
Chang H-C, Yen C-J, Lee Y-C, Chiu T-Y, Jan C-F. Seroprevalence of hepatitis B viral markers among freshmen--20 years after mass hepatitis B vaccination program in Taiwan. J Formos Med Assoc. 2007; 106(7): 513-9.
Chang J, Elam-Evans LD, Berg CJ, Herndon J, Flowers L, Seed KA, Syverson CJ, Centers for Disease Control and Prevention (CDC). Pregnancy-related Mortality Surveillance - United States 1991-1999. MMWR Surveill Summ. 2003; 52(2): 1-8.

Appendix: Citation List

Citation
Chang MS, Chan KL, Ho BC. Control of Mansonia mosquitos, vectors of brugian filariasis in Sarawak, Malaysia. Southeast Asian J Trop Med Public Health. 1993; 93-104. As it appears in London School of Hygiene and Tropical Medicine. Global Atlas of Helminth Infections - Lymphatic Filiariasis.
Chapman BA, Burt MJ, Frampton CM, Collett JA, Yeo KH, Wilkinson ID, Cook HB, Barclay MJ, Ross AG, George PM. The prevalence of viral hepatitis (HAV, HBV and HCV) in the Christchurch community. N Z Med J. 2000; 113(1118): 394-6.
Charafoudine H, Pesson B. [Bancroft's filariasis in Anjouan (Comoro Islands)]. Bull Soc Pathol Exot Filiales. 1986; 79(2): 229-36. As it appears in London School of Hygiene and Tropical Medicine. Global Atlas of Helminth Infections - Lymphatic Filiariasis.
Charles M, Ejskjaer N, Witte DR, Borch-Johnsen K, Lauritzen T, Sandbaek A. Prevalence of neuropathy and peripheral arterial disease and the impact of treatment in people with screen-detected type 2 diabetes: the ADDITION-Denmark study. Diabetes Care. 2011; 34(10): 2244–9.
Charles University, International Consortium of Psychiatric Epidemiology, World Health Organization (WHO). Czech Republic Sample Survey of Mental Health 1998-1999.
Charlton KE, Lambert EV, Kreft J. Physical activity, change in blood pressure and predictors of mortality in older South Africans--a 2-year follow-up study. S Afr Med J. 1997; 87(9): 1124-30.
Charlwood JD. United Nations High Commission for Refugees Programme and Technical Support Section, East Sudan Medical Entomology Mission, 18th August-19th September 1997. Khartoum, Sudan: United Nations High Commission for Refugees, 1997. As it appears in Malaria Atlas Project. Malaria Atlas Project Plasmodium Falciparum Parasite Rate Database. Oxford, United Kingdom: Malaria Atlas Project.
Chau N, Bertrand JP, Mur JM, Figueredo A, Patris A, Moulin JJ, Pham QT. Mortality In Retired Coke Oven Plant Workers. Br J Ind Med. 1993; 50(2): 127-35 as it appears in Armstrong B, Hutchinson E, Unwin J, Fletcher T. Lung cancer risk after exposure to polycyclic aromatic hydrocarbons: a review and meta-analysis. Environ. Health Perspect. 2004; 112(9): 970–978.
Chaudhuri V, Nanu A, Panda SK, Chand P. Evaluation of serologic screening of blood donors in India reveals a lack of correlation between anti-HBc titer and PCR-amplified HBV DNA. Transfusion. 2003; 43(10): 1442-8.
Checchi F, Nyasulu P, Chandramohan D, Roberts B. Rates and causes of death in Chiradzulu District, Malawi, 2008: a key informant study. Trop Med Int Health. 2011; 16(3): 375-8.
Checkoway H, Hughes JM, Weill H, Seixas NS, Demers PA. Crystalline Silica Exposure, Radiological Silicosis, And Lung Cancer Mortality In Diatomaceous Earth Industry Workers. Thorax. 1999; 54(1): 56-9 as it appears in Kurihara N, Wada O. Silicosis and smoking strongly increase lung cancer risk in silica-exposed workers. Ind Health 2004; 42(3): 303–314.
Cheikh Anta Diop University, ICF International, National Agency of Statistics and Demography (Senegal). Senegal Continuous Demographic and Health Survey 2014. Fairfax, United States: ICF International, 2015.
Chen C-H, Yang P-M, Huang G-T, Lee H-S, Sung J-L, Sheu J-C. Estimation of seroprevalence of hepatitis B virus and hepatitis C virus in Taiwan from a large-scale survey of free hepatitis screening participants. J Formos Med Assoc. 2007; 106(2): 148-55.
Chen G, Lin W, Shen F, Iloeje UH, London WT, Evans AA. Chronic hepatitis B virus infection and mortality from non-liver causes: results from the Haimen City cohort study. Int J Epidemiol. 2005; 34(1): 132-7.
Chen H, Wu X, Wei M, Eichner JE, Fan Y, Zhang Z, Lei C, Stone DU, Yang J. Changes in the prevalence of visual impairment due to blinding trachoma in Sichuan province, China: a comparative study between 1987 and 2006. Ophthalmic Epidemiol. 2012; 19(1): 29-37.
Chen HD, Shaw CK, Tseng WP, Chen HI, Lee ML. Prevalence of diabetes mellitus and impaired glucose tolerance in Aborigines and Chinese in eastern Taiwan. Diabetes Res Clin Pract. 1997; 38(3): 199-205.
Chen H-F, Li C-Y, Chen P, See T-T, Lee H-Y. Seroprevalence of hepatitis B and C in type 2 diabetic patients. J Chin Med Assoc. 2006; 69(4): 146-52.
Chen K-H, Seow K-M, Chen L-R. The role of preterm placental calcification on assessing risks of stillbirth. Placenta. 2015; 36(9): 1039–44.
Chen KT, Chen CJ, Gregg EW, Engelgau MM, Narayan KM. Prevalence of type 2 diabetes mellitus in Taiwan: ethnic variation and risk factors. Diabetes Res Clin Pract. 2001; 51(1): 59-66.
Chen KT, Chen CJ, Gregg EW, Williamson DF, Narayan KM. High prevalence of impaired fasting glucose and type 2 diabetes mellitus in Penghu Islets, Taiwan: evidence of a rapidly emerging epidemic? Diabetes Res Clin Pract. 1999; 44(1): 59-69.
Chen N-H, Chuang L-P, Yang C-T, Kushida CA, Hsu S-C, Wang P-C, Lin S-W, Chou Y-T, Chen R-S, Li H-Y, Lai S-C. The prevalence of restless legs syndrome in Taiwanese adults. Psychiatry Clin Neurosci. 2010; 64(2): 170-8.
Chen S-J, Cheng C-Y, Li A-F, Peng K-L, Chou P, Chiou S-H, Hsu W-M. Prevalence and associated risk factors of myopic maculopathy in elderly Chinese: the Shihpai eye study. Invest Ophthalmol Vis Sci. 2012; 53(8): 4868-73.
Cherian A, Syam UK, Sreevidya D, Jayaraman T, Oommen A, Rajshekhar V, Radhakrishnan K, Thomas SV. Low seroprevalence of systemic cysticercosis among patients with epilepsy in Kerala--South India. J Infect Public Health. 2014; 7(4): 271-6.
Cherry N, Shaikh K, McDonald C, Chowdhury Z. Stillbirth in rural Bangladesh: arsenic exposure and other etiological factors: a report from Gonoshasthaya Kendra. Bull World Health Organ. 2008; 86(3): 172-7.
Cherry NM, Burgess GL, Turner S, Mcdonald JC. Crystalline Silica And Risk Of Lung Cancer In The Potteries. Occup Environ Med. 1998; 55(11): 779-85 as it appears in Kurihara N, Wada O. Silicosis and smoking strongly increase lung cancer risk in silica-exposed workers. Ind Health 2004; 42(3): 303–314.
Chesnais CB, Missamou F, Pion SD, Bopda J, Louya F, Majewski AC, Fischer PU, Weil GJ, Boussinesq M. A case study of risk factors for lymphatic filariasis in the Republic of Congo. Parasit Vectors. 2014; 300.
Chesnais CB, Missamou F, Pion SD, Bopda J, Louya F, Majewski AC, Weil GJ, Boussinesq M. Semi-quantitative scoring of an immunochromatographic test for circulating filarial antigen. Am J Trop Med Hyg. 2013; 89.0(5): 916-8.
Cheumaga B. Comparative Malariometric Study in Areas Rural (Bandoumka and Bapoudeu) and Urban (Ville de Bafang) in the Department of Haut-Kam. Yaoundé, Cameroon: University of Yaoundé, 1987. As it appears in Malaria Atlas Project. Malaria Atlas Project Plasmodium Falciparum Parasite Rate Database. Oxford, United Kingdom: Malaria Atlas Project.
Cheun H-I, Kong Y, Cho S-H, Lee J-S, Chai J-Y, Lee J-S, Lee J-K, Kim T-S. Successful control of lymphatic filariasis in the Republic of Korea. Korean J Parasitol. 2009; 47(4): 323-35.

Appendix: Citation List

Citation
Cheun H-I, Lee J-S, Cho S-H, Kong Y, Kim T-S. Elimination of lymphatic filariasis in the Republic of Korea: an epidemiological survey of formerly endemic areas, 2002-2006. Trop Med Int Health. 2009; 14(4): 445-9.
Chhetri MR, Chapman RS. Prevalence and determinants of diabetes among the elderly population in the Kathmandu Valley of Nepal. Nepal Med Coll J. 2009; 11(1): 34-8.
Chhotray GP, Mohapatra M, Acharya AS, Ranjit MR. A clinico-epidemiological perspective of lymphatic filariasis in Satyabadi block of Puri district, Orissa. Indian J Med Res. 2001; 114: 65-71.
Chhotray GP, Ranjit MR, Khuntia HK, Acharya AS. Precontrol observations on lymphatic filariasis and geo-helminthiases in two coastal districts of rural Orrisa. Indian J Med Res. 2005; 122(5): 388-94.
Chi BH, Vwalika B, Killam WP, Wamalume C, Giganti MJ, Mbewe R, Stringer EM, Chintu NT, Putta NB, Liu KC, Chibwesha CJ, Rouse DJ, Stringer JSA. Implementation of the Zambia Electronic Perinatal Record System for comprehensive prenatal and delivery care. Int J Gynaecol Obstet. 2011; 113(2): 131-6.
Chiang F, Kuper H, Lindfield R, Keenan T, Seyam N, Magauran D, Khalilia N, Batta H, Abdeen Z, Sargent N. Rapid assessment of avoidable blindness in the Occupied Palestinian Territories. PLoS One. 2010; 5(7): e11854.
Chiang P-H, Liu C-L, Lin M-H, Peng L-N, Chen L-K, Chen J-D, Hou S-M. Survival benefits of metabolic syndrome among older men aged 75 years and over in Taiwan. J Nutr Health Aging. 2012; 16(6): 520–4.
Chiaramonte M, Floreani A, Naccarato R. Hepatitis B virus infection in homes for the aged. J Med Virol. 1982; 9(4): 247-55.
Chiaramonte M, Floreani A, Silvan C, Zampieri L, Trivello R, Renzulli G, Moschen M, Naccarato R. Hepatitis A and hepatitis B virus infection in children and adolescents in north-east Italy. J Med Virol. 1983; 12(3): 179-86.
Chief Medical Office of Greenland. Greenland Vital Registration - Deaths 1995. [Unpublished].
Chief Medical Office of Greenland. Greenland Vital Registration - Deaths 1996. [Unpublished].
Chief Medical Office of Greenland. Greenland Vital Registration - Deaths 1997. [Unpublished].
Chief Medical Office of Greenland. Greenland Vital Registration - Deaths 1998. [Unpublished].
Chief Medical Office of Greenland. Greenland Vital Registration - Deaths 1999. [Unpublished].
Chief Medical Office of Greenland. Greenland Vital Registration - Deaths 2000. [Unpublished].
Chief Medical Office of Greenland. Greenland Vital Registration - Deaths 2001. [Unpublished].
Chief Medical Office of Greenland. Greenland Vital Registration - Deaths 2002. [Unpublished].
Chief Medical Office of Greenland. Greenland Vital Registration - Deaths 2003. [Unpublished].
Chief Medical Office of Greenland. Greenland Vital Registration - Deaths 2004. [Unpublished].
Chief Medical Office of Greenland. Greenland Vital Registration - Deaths 2005. [Unpublished].
Chief Medical Office of Greenland. Greenland Vital Registration - Deaths 2006. [Unpublished].
Chief Medical Office of Greenland. Greenland Vital Registration - Deaths 2007. [Unpublished].
Chief Medical Office of Greenland. Greenland Vital Registration - Deaths 2008. [Unpublished].
Chief Medical Office of Greenland. Greenland Vital Registration - Deaths 2009. [Unpublished].
Chief Medical Office of Greenland. Greenland Vital Registration - Deaths 2010. [Unpublished].
Chief Medical Office of Greenland. Greenland Vital Registration - Deaths 2011. [Unpublished].
Chief Medical Office of Greenland. Greenland Vital Registration - Deaths 2012. [Unpublished].
Chief Medical Office of Greenland. Greenland Vital Registration - Deaths 2013. [Unpublished].
Chiguzo AN. Malaria Situation Analysis in Korogwe District. 1996. As it appears in Malaria Atlas Project. Malaria Atlas Project Plasmodium Falciparum Parasite Rate Database. Oxford, United Kingdom: Malaria Atlas Project.
Chihana M, Floyd S, Molesworth A, Crampin AC, Kayuni N, Price A, Zaba B, Jahn A, Mvula H, Dube A, Ngwira B, Glynn JR, French N. Adult mortality and probable cause of death in rural northern Malawi in the era of HIV treatment. Trop Med Int Health. 2012; 17(8): E74-83.
Chikwem JO, Mohammed I, Okara GC, Ukwandu NC, Ola TO. Prevalence of transmissible blood infections among blood donors at the University of Maiducuri Teaching Hospital, Maiduguri, Nigeria. East Afr Med J. 1997; 74(4): 213-6.
Child health and household water supply: a longitudinal study of growth and its environmental determinants in rural Malawi as it appears in World Health Organization (WHO). WHO Global Database on Child Growth and Malnutrition - Historical. Geneva, Switzerland: World Health Organization (WHO).
Child health in Rwanda as it appears in World Health Organization (WHO). WHO Global Database on Child Growth and Malnutrition - Historical. Geneva, Switzerland: World Health Organization (WHO).
Child Population Groups from 0-5 Years of Age: Percentage of Children Below -2 SDs of the NCHS Reference in Thailand as it appears in World Health Organization (WHO). WHO Global Database on Child Growth and Malnutrition - Historical. Geneva, Switzerland: World Health Organization (WHO).
Child Population Groups from 0-5 Years of Age: Percentage of Children Below -2 SDs of the NCHS Reference in Zaire as it appears in World Health Organization (WHO). WHO Global Database on Child Growth and Malnutrition - Historical. Geneva, Switzerland: World Health Organization (WHO).
Childhood malnutrition in the Western Province of the Solomon Islands as it appears in World Health Organization (WHO). WHO Global Database on Child Growth and Malnutrition - Historical. Geneva, Switzerland: World Health Organization (WHO).
Children and Women in Eritrea: 1994 as it appears in World Health Organization (WHO). WHO Global Database on Child Growth and Malnutrition. Geneva, Switzerland: World Health Organization (WHO).
Children of Russia 2000-2001 as it appears in World Health Organization (WHO). WHO Global Database on Child Growth and Malnutrition. Geneva, Switzerland: World Health Organization (WHO).
Children's Hospital Zagreb, National Institute of Health (Italy), United Nations Children's Fund (UNICEF). Croatia Multiple Indicator Cluster Survey 1996.
Chile - Valdivia Cancer Registry 1998-2002 - CI5 as it appears in Curado MP, Edwards B, Shin HR, Storm H, Ferlay J, Heanue M and Boyle P, eds (2007). Cancer Incidence in Five Continents, Vol. IX Periodic Data (electronic version). Lyon, IARC.http://ci5.iarc.fr

Appendix: Citation List

Citation
Chile - Valdivia Cancer Registry 2003-2007 - CI5 as it appears in Forman D, Bray F, Brewster DH, Gombe Mbalawa C, Kohler B, Piñeros M, Steliarova-Foucher E, Swaminathan R and Ferlay J, eds (2013). Cancer Incidence in Five Continents, Vol. X Summary Database (electronic version). Lyon, IARC. http://ci5.iarc.fr
Chile Annual Nutrition Monitoring Newsletter 1998 as it appears in World Health Organization (WHO). WHO Global Database on Child Growth and Malnutrition. Geneva, Switzerland: World Health Organization (WHO).
Chile Annual Nutrition Monitoring Newsletter 1999 as it appears in World Health Organization (WHO). WHO Global Database on Child Growth and Malnutrition. Geneva, Switzerland: World Health Organization (WHO).
Chile Food Nutrition Situation 1984-1987 as it appears in World Health Organization (WHO). WHO Global Database on Child Growth and Malnutrition. Geneva, Switzerland: World Health Organization (WHO).
Chile Ministry of Health Child Underweight Data 2006 as it appears in United Nations Children's Fund (UNICEF). UNICEF Childinfo - Nutritional Status. New York, United States: United Nations Children's Fund (UNICEF).
Chile National Health Service System 1994 as it appears in World Health Organization (WHO). WHO Global Database on Child Growth and Malnutrition. Geneva, Switzerland: World Health Organization (WHO).
Chile National Health Service System 2003-2004 as it appears in World Health Organization (WHO). WHO Global Database on Child Growth and Malnutrition. Geneva, Switzerland: World Health Organization (WHO).
Chile National Health Service System 2006 as it appears in World Health Organization (WHO). WHO Global Database on Child Growth and Malnutrition. Geneva, Switzerland: World Health Organization (WHO).
Chile National Health Service System 2007 as it appears in World Health Organization (WHO). WHO Global Database on Child Growth and Malnutrition. Geneva, Switzerland: World Health Organization (WHO).
Chile National Health Service System 2008 as it appears in World Health Organization (WHO). WHO Global Database on Child Growth and Malnutrition. Geneva, Switzerland: World Health Organization (WHO).
Chile National Survey of Breastfeeding 2005, Chile Survey of Children Served by the National Kindergarten Board 2005-2006 and Chile Survey of State Preschool Institutions 2005-2006 as it appears in World Health Organization (WHO). WHO Global Database on Child Growth and Malnutrition. Geneva, Switzerland: World Health Organization (WHO).
Chile Nutritional Status of the Child Population 1986 as it appears in World Health Organization (WHO). WHO Global Database on Child Growth and Malnutrition. Geneva, Switzerland: World Health Organization (WHO).
Chile Vital Registration - Deaths 1954 ICD7 as it appears in World Health Organization (WHO). WHO Mortality Database Version July 2012. Geneva, Switzerland: World Health Organization (WHO), 2012.
Chile Vital Registration - Deaths 1955 ICD7 as it appears in World Health Organization (WHO). WHO Mortality Database Version July 2012. Geneva, Switzerland: World Health Organization (WHO), 2012.
Chile Vital Registration - Deaths 1956 ICD7 as it appears in World Health Organization (WHO). WHO Mortality Database Version July 2012. Geneva, Switzerland: World Health Organization (WHO), 2012.
Chile Vital Registration - Deaths 1957 ICD7 as it appears in World Health Organization (WHO). WHO Mortality Database Version July 2012. Geneva, Switzerland: World Health Organization (WHO), 2012.
Chile Vital Registration - Deaths 1958 ICD7 as it appears in World Health Organization (WHO). WHO Mortality Database Version July 2012. Geneva, Switzerland: World Health Organization (WHO), 2012.
Chile Vital Registration - Deaths 1959 ICD7 as it appears in World Health Organization (WHO). WHO Mortality Database Version July 2012. Geneva, Switzerland: World Health Organization (WHO), 2012.
Chile Vital Registration - Deaths 1960 ICD7 as it appears in World Health Organization (WHO). WHO Mortality Database Version July 2012. Geneva, Switzerland: World Health Organization (WHO), 2012.
Chile Vital Registration - Deaths 1961 ICD7 as it appears in World Health Organization (WHO). WHO Mortality Database Version July 2012. Geneva, Switzerland: World Health Organization (WHO), 2012.
Chile Vital Registration - Deaths 1962 ICD7 as it appears in World Health Organization (WHO). WHO Mortality Database Version July 2012. Geneva, Switzerland: World Health Organization (WHO), 2012.
Chile Vital Registration - Deaths 1963 ICD7 as it appears in World Health Organization (WHO). WHO Mortality Database Version July 2012. Geneva, Switzerland: World Health Organization (WHO), 2012.
Chile Vital Registration - Deaths 1964 ICD7 as it appears in World Health Organization (WHO). WHO Mortality Database Version July 2012. Geneva, Switzerland: World Health Organization (WHO), 2012.
Chile Vital Registration - Deaths 1965 ICD7 as it appears in World Health Organization (WHO). WHO Mortality Database Version July 2012. Geneva, Switzerland: World Health Organization (WHO), 2012.
Chile Vital Registration - Deaths 1966 ICD7 as it appears in World Health Organization (WHO). WHO Mortality Database Version July 2012. Geneva, Switzerland: World Health Organization (WHO), 2012.
Chile Vital Registration - Deaths 1967 ICD7 as it appears in World Health Organization (WHO). WHO Mortality Database Version July 2012. Geneva, Switzerland: World Health Organization (WHO), 2012.
Chile Vital Registration - Deaths 1968 ICD8 as it appears in World Health Organization (WHO). WHO Mortality Database Version May 2013. Geneva, Switzerland: World Health Organization (WHO), 2013.
Chile Vital Registration - Deaths 1969 ICD8 as it appears in World Health Organization (WHO). WHO Mortality Database Version May 2013. Geneva, Switzerland: World Health Organization (WHO), 2013.
Chile Vital Registration - Deaths 1970 ICD8 as it appears in World Health Organization (WHO). WHO Mortality Database Version May 2013. Geneva, Switzerland: World Health Organization (WHO), 2013.
Chile Vital Registration - Deaths 1971 ICD8 as it appears in World Health Organization (WHO). WHO Mortality Database Version May 2013. Geneva, Switzerland: World Health Organization (WHO), 2013.
Chile Vital Registration - Deaths 1972 ICD8 as it appears in World Health Organization (WHO). WHO Mortality Database Version May 2013. Geneva, Switzerland: World Health Organization (WHO), 2013.

Appendix: Citation List

Citation
Chile Vital Registration - Deaths 1973 ICD8 as it appears in World Health Organization (WHO). WHO Mortality Database Version May 2013. Geneva, Switzerland: World Health Organization (WHO), 2013.
Chile Vital Registration - Deaths 1974 ICD8 as it appears in World Health Organization (WHO). WHO Mortality Database Version May 2013. Geneva, Switzerland: World Health Organization (WHO), 2013.
Chile Vital Registration - Deaths 1975 ICD8 as it appears in World Health Organization (WHO). WHO Mortality Database Version May 2013. Geneva, Switzerland: World Health Organization (WHO), 2013.
Chile Vital Registration - Deaths 1976 ICD8 as it appears in World Health Organization (WHO). WHO Mortality Database Version May 2013. Geneva, Switzerland: World Health Organization (WHO), 2013.
Chile Vital Registration - Deaths 1977 ICD8 as it appears in World Health Organization (WHO). WHO Mortality Database Version May 2013. Geneva, Switzerland: World Health Organization (WHO), 2013.
Chile Vital Registration - Deaths 1978 ICD8 as it appears in World Health Organization (WHO). WHO Mortality Database Version May 2013. Geneva, Switzerland: World Health Organization (WHO), 2013.
Chile Vital Registration - Deaths 1979 ICD8 as it appears in World Health Organization (WHO). WHO Mortality Database Version May 2013. Geneva, Switzerland: World Health Organization (WHO), 2013.
Chile Vital Registration - Deaths 1980 ICD9 as it appears in World Health Organization (WHO). WHO Mortality Database Version February 2014. Geneva, Switzerland: World Health Organization (WHO), 2014.
Chile Vital Registration - Deaths 1981 ICD9 as it appears in World Health Organization (WHO). WHO Mortality Database Version February 2014. Geneva, Switzerland: World Health Organization (WHO), 2014.
Chile Vital Registration - Deaths 1982 ICD9 as it appears in World Health Organization (WHO). WHO Mortality Database Version February 2014. Geneva, Switzerland: World Health Organization (WHO), 2014.
Chile Vital Registration - Deaths 1983 ICD9 as it appears in World Health Organization (WHO). WHO Mortality Database Version February 2014. Geneva, Switzerland: World Health Organization (WHO), 2014.
Chile Vital Registration - Deaths 1984 ICD9 as it appears in World Health Organization (WHO). WHO Mortality Database Version February 2014. Geneva, Switzerland: World Health Organization (WHO), 2014.
Chile Vital Registration - Deaths 1985 ICD9 as it appears in World Health Organization (WHO). WHO Mortality Database Version November 2015. Geneva, Switzerland: World Health Organization (WHO), 2015.
Chile Vital Registration - Deaths 1986 ICD9 as it appears in World Health Organization (WHO). WHO Mortality Database Version February 2014. Geneva, Switzerland: World Health Organization (WHO), 2014.
Chile Vital Registration - Deaths 1987 ICD9 as it appears in World Health Organization (WHO). WHO Mortality Database Version February 2014. Geneva, Switzerland: World Health Organization (WHO), 2014.
Chile Vital Registration - Deaths 1988 ICD9 as it appears in World Health Organization (WHO). WHO Mortality Database Version February 2014. Geneva, Switzerland: World Health Organization (WHO), 2014.
Chile Vital Registration - Deaths 1989 ICD9 as it appears in World Health Organization (WHO). WHO Mortality Database Version February 2014. Geneva, Switzerland: World Health Organization (WHO), 2014.
Chile Vital Registration - Deaths 1990 ICD9 as it appears in World Health Organization (WHO). WHO Mortality Database Version February 2014. Geneva, Switzerland: World Health Organization (WHO), 2014.
Chile Vital Registration - Deaths 1991 ICD9 as it appears in World Health Organization (WHO). WHO Mortality Database Version November 2015. Geneva, Switzerland: World Health Organization (WHO), 2015.
Chile Vital Registration - Deaths 1992 ICD9 as it appears in World Health Organization (WHO). WHO Mortality Database Version February 2014. Geneva, Switzerland: World Health Organization (WHO), 2014.
Chile Vital Registration - Deaths 1993 ICD9 as it appears in World Health Organization (WHO). WHO Mortality Database Version November 2015. Geneva, Switzerland: World Health Organization (WHO), 2015.
Chile Vital Registration - Deaths 1994 ICD9 as it appears in World Health Organization (WHO). WHO Mortality Database Version November 2015. Geneva, Switzerland: World Health Organization (WHO), 2015.
Chile Vital Registration - Deaths 1995 ICD9 as it appears in World Health Organization (WHO). WHO Mortality Database Version November 2015. Geneva, Switzerland: World Health Organization (WHO), 2015.
Chile Vital Registration - Deaths 1996 ICD9 as it appears in World Health Organization (WHO). WHO Mortality Database Version November 2015. Geneva, Switzerland: World Health Organization (WHO), 2015.
Chile Vital Registration - Deaths 1997 ICD10 as it appears in World Health Organization (WHO). WHO Mortality Database Version November 2015. Geneva, Switzerland: World Health Organization (WHO), 2015.
Chile Vital Registration - Deaths 1998 ICD10 as it appears in World Health Organization (WHO). WHO Mortality Database Version November 2015. Geneva, Switzerland: World Health Organization (WHO), 2015.
Chile Vital Registration - Deaths 1999 ICD10 as it appears in World Health Organization (WHO). WHO Mortality Database Version November 2015. Geneva, Switzerland: World Health Organization (WHO), 2015.
Chile Vital Registration - Deaths 2000 ICD10 as it appears in World Health Organization (WHO). WHO Mortality Database Version November 2015. Geneva, Switzerland: World Health Organization (WHO), 2015.
Chile Vital Registration - Deaths 2001 ICD10 as it appears in World Health Organization (WHO). WHO Mortality Database Version November 2015. Geneva, Switzerland: World Health Organization (WHO), 2015.
Chile Vital Registration - Deaths 2002 ICD10 as it appears in World Health Organization (WHO). WHO Mortality Database Version November 2015. Geneva, Switzerland: World Health Organization (WHO), 2015.
Chile Vital Registration - Deaths 2003 ICD10 as it appears in World Health Organization (WHO). WHO Mortality Database Version November 2015. Geneva, Switzerland: World Health Organization (WHO), 2015.
Chile Vital Registration - Deaths 2004 ICD10 as it appears in World Health Organization (WHO). WHO Mortality Database Version November 2015. Geneva, Switzerland: World Health Organization (WHO), 2015.

Appendix: Citation List

Citation
Chile Vital Registration - Deaths 2005 ICD10 as it appears in World Health Organization (WHO). WHO Mortality Database Version November 2015. Geneva, Switzerland: World Health Organization (WHO), 2015.
Chile Vital Registration - Deaths 2006 ICD10 as it appears in World Health Organization (WHO). WHO Mortality Database Version November 2015. Geneva, Switzerland: World Health Organization (WHO), 2015.
Chile Vital Registration - Deaths 2007 ICD10 as it appears in World Health Organization (WHO). WHO Mortality Database Version November 2015. Geneva, Switzerland: World Health Organization (WHO), 2015.
Chile Vital Registration - Deaths 2008 ICD10 as it appears in World Health Organization (WHO). WHO Mortality Database Version November 2015. Geneva, Switzerland: World Health Organization (WHO), 2015.
Chile Vital Registration - Deaths 2009 ICD10 as it appears in World Health Organization (WHO). WHO Mortality Database Version November 2015. Geneva, Switzerland: World Health Organization (WHO), 2015.
Chile Vital Registration - Deaths 2010 ICD10 as it appears in World Health Organization (WHO). WHO Mortality Database Version November 2015. Geneva, Switzerland: World Health Organization (WHO), 2015.
Chile Vital Registration - Deaths 2011 ICD10 as it appears in World Health Organization (WHO). WHO Mortality Database Version November 2015. Geneva, Switzerland: World Health Organization (WHO), 2015.
Chile Vital Registration - Deaths 2012 ICD10 as it appears in World Health Organization (WHO). WHO Mortality Database Version November 2015. Geneva, Switzerland: World Health Organization (WHO), 2015.
Chile Vital Registration - Deaths 2013 ICD10 as it appears in World Health Organization (WHO). WHO Mortality Database Version November 2015. Geneva, Switzerland: World Health Organization (WHO), 2015.
Chile Vital Registration Death Data 1950 as it appears in United Nations Statistics Division (UNSD). United Nations Demographic Yearbook - Historical Supplement 1997. New York City, United States: United Nations Statistics Division (UNSD).
Chile Vital Registration Death Data 1951 as it appears in United Nations Statistics Division (UNSD). United Nations Demographic Yearbook - Historical Supplement 1997. New York City, United States: United Nations Statistics Division (UNSD).
Chile Vital Registration Death Data 1952 as it appears in United Nations Statistics Division (UNSD). United Nations Demographic Yearbook - Historical Supplement 1997. New York City, United States: United Nations Statistics Division (UNSD).
Chile Vital Registration Death Data 1953 as it appears in United Nations Statistics Division (UNSD). United Nations Demographic Yearbook - Historical Supplement 1997. New York City, United States: United Nations Statistics Division (UNSD).
Chile Vital Registration Death Data 2010 as it appears in United Nations Statistics Division (UNSD). United Nations Demographic Yearbook 2012. New York City, United States: United Nations Statistics Division (UNSD), 2013.
Chilima DM, Ismail SJ. Anthropometric characteristics of older people in rural Malawi. Eur J Clin Nutr. 1998; 52(9): 643-9.
Chin CY, Pengal S. Cardiovascular disease risk in a semirural community in Malaysia. Asia Pac J Public Health. 2009; 21(4): 410-20.
China - Beijing Cancer Registry 1993-1997 - CI5 as it appears in Parkin DM, International Agency for Research on Cancer, International Association of Cancer Registries. Cancer Incidence in Five Continents. Vol. I to VIII. Lyon, France, IARCPress, 2005.
China - Jiashan Cancer Registry 1993-1997 - CI5 as it appears in Parkin DM, International Agency for Research on Cancer, International Association of Cancer Registries. Cancer Incidence in Five Continents. Vol. I to VIII. Lyon, France, IARCPress, 2005.
China - Macao Vital Registration Death Data 2000 as it appears in United Nations Statistics Division (UNSD). United Nations Demographic Yearbook 2003. New York City, United States: United Nations Statistics Division (UNSD), 2006.
China - Macao Vital Registration Death Data 2003 as it appears in United Nations Statistics Division (UNSD). United Nations Demographic Yearbook 2003. New York City, United States: United Nations Statistics Division (UNSD), 2006.
China - Macao Vital Registration Death Data 2004 as it appears in United Nations Statistics Division (UNSD). United Nations Demographic Yearbook 2004. New York City, United States: United Nations Statistics Division (UNSD), 2007.
China - Macao Vital Registration Death Data 2006 as it appears in United Nations Statistics Division (UNSD). United Nations Demographic Yearbook 2006. New York City, United States: United Nations Statistics Division (UNSD), 2008.
China - Macao Vital Registration Death Data 2007 as it appears in United Nations Statistics Division (UNSD). United Nations Demographic Yearbook 2007. New York City, United States: United Nations Statistics Division (UNSD), 2009.
China - Macao Vital Registration Death Data 2008 as it appears in United Nations Statistics Division (UNSD). United Nations Demographic Yearbook 2008. New York City, United States: United Nations Statistics Division (UNSD), 2010.
China - Macao Vital Registration Death Data 2009 as it appears in United Nations Statistics Division (UNSD). United Nations Demographic Yearbook 2009-2010. New York City, United States: United Nations Statistics Division (UNSD), 2011.
China - Nutritional Status of Children Aged 0-5 Years Old in China (2008) - National (26 Nutrition Surveillance Sites from Rural Areas) as it appears in World Health Organization (WHO). WHO Global Database on Child Growth and Malnutrition. Geneva, Switzerland: World Health Organization (WHO).
China - Nutritional Status of Children Aged 0-5 Years Old in China (2010) - National (38 Nutrition Surveillance Sites from 25 Provinces) as it appears in World Health Organization (WHO). WHO Global Database on Child Growth and Malnutrition. Geneva, Switzerland: World Health Organization (WHO).
China - Shanghai Cancer Registry 1978-1982 - CI5 as it appears in Parkin DM, International Agency for Research on Cancer, International Association of Cancer Registries. Cancer Incidence in Five Continents. Vol. I to VIII. Lyon, France, IARCPress, 2005.
China - Shanghai Cancer Registry 1983-1987 - CI5 as it appears in Parkin DM, International Agency for Research on Cancer, International Association of Cancer Registries. Cancer Incidence in Five Continents. Vol. I to VIII. Lyon, France, IARCPress, 2005.
China - Shanghai Cancer Registry 1984 - CI5 as it appears in Parkin DM, International Agency for Research on Cancer, International Association of Cancer Registries. Cancer Incidence in Five Continents. Vol. I to VIII. Lyon, France, IARCPress, 2005.
China - Shanghai Cancer Registry 1988 - CI5 as it appears in Ferlay J, Parkin DM, Curado MP, Bray F, Edwards B, Shin HR and Forman D. Cancer Incidence in Five Continents, Volumes I to IX: IARC CancerBase No. 9 [Internet]. Lyon, France: International Agency for Research on Cancer; 2010. Available from: http://ci5.iarc.fr

Appendix: Citation List

Citation
China - Shanghai Cancer Registry 1988-1992 - CI5 as it appears in Parkin DM, International Agency for Research on Cancer, International Association of Cancer Registries. Cancer Incidence in Five Continents. Vol. I to VIII. Lyon, France, IARCPress, 2005.
China - Shanghai Cancer Registry 1989 - CI5 as it appears in Ferlay J, Parkin DM, Curado MP, Bray F, Edwards B, Shin HR and Forman D. Cancer Incidence in Five Continents, Volumes I to IX: IARC CancerBase No. 9 [Internet]. Lyon, France: International Agency for Research on Cancer; 2010. Available from: http://ci5.iarc.fr
China - Shanghai Cancer Registry 1993-1997 - CI5 as it appears in Parkin DM, International Agency for Research on Cancer, International Association of Cancer Registries. Cancer Incidence in Five Continents. Vol. I to VIII. Lyon, France, IARCPress, 2005.
China - Shanghai Cancer Registry 1998-2002 - CI5 as it appears in Curado MP, Edwards B, Shin HR, Storm H, Ferlay J, Heanue M and Boyle P, eds (2007). Cancer Incidence in Five Continents, Vol. IX Periodic Data (electronic version). Lyon, IARC.http://ci5.iarc.fr
China - Tianjin Cancer Registry 1981-1982 - CI5 as it appears in Parkin DM, International Agency for Research on Cancer, International Association of Cancer Registries. Cancer Incidence in Five Continents. Vol. I to VIII. Lyon, France, IARCPress, 2005.
China - Tianjin Cancer Registry 1983-1987 - CI5 as it appears in Parkin DM, International Agency for Research on Cancer, International Association of Cancer Registries. Cancer Incidence in Five Continents. Vol. I to VIII. Lyon, France, IARCPress, 2005.
China - Tianjin Cancer Registry 1985 - CI5 as it appears in Parkin DM, International Agency for Research on Cancer, International Association of Cancer Registries. Cancer Incidence in Five Continents. Vol. I to VIII. Lyon, France, IARCPress, 2005.
China - Tianjin Cancer Registry 1988-1992 - CI5 as it appears in Parkin DM, International Agency for Research on Cancer, International Association of Cancer Registries. Cancer Incidence in Five Continents. Vol. I to VIII. Lyon, France, IARCPress, 2005.
China - Tianjin Cancer Registry 1993-1997 - CI5 as it appears in Parkin DM, International Agency for Research on Cancer, International Association of Cancer Registries. Cancer Incidence in Five Continents. Vol. I to VIII. Lyon, France, IARCPress, 2005.
China Data Center, University of Michigan, National Bureau of Statistics of China. China Census 2000 - China Data Center.
China National Growth and Development Survey of Children 1987 as it appears in World Health Organization (WHO). WHO Global Database on Child Growth and Malnutrition - Historical. Geneva, Switzerland: World Health Organization (WHO).
China National Maternal and Child Health Surveillance System Under-5 Mortality 1996-2013 - MCHS. [Unpublished].
China Nutritional Status of Children Survey 1992 as it appears in World Health Organization (WHO). WHO Global Database on Child Growth and Malnutrition - Historical. Geneva, Switzerland: World Health Organization (WHO).
China Pilot Survey of the Food and Nutrition Surveillance System 1995 as it appears in World Health Organization (WHO). WHO Global Database on Child Growth and Malnutrition - Historical. Geneva, Switzerland: World Health Organization (WHO).
China Population Information and Research Center, National Bureau of Statistics of China. China In-Depth Fertility Sample Survey 1987.
China Population Information and Research Center, National Bureau of Statistics of China. China National Population Census 1982 - China Data Center.
Chinese Center for Disease Control and Prevention (CCDC), Ministry of Health (China). China Disease Surveillance Points 2005.
Chinese Center for Disease Control and Prevention (CCDC), Ministry of Health (China). China Disease Surveillance Points 2006.
Chinese Center for Disease Control and Prevention (CCDC), Ministry of Health (China). China Disease Surveillance Points 2007.
Chinese Center for Disease Control and Prevention (CCDC), Ministry of Health (China). China Disease Surveillance Points 2008.
Chinese Center for Disease Control and Prevention (CCDC), Ministry of Health (China). China Disease Surveillance Points 2009.
Chinese Center for Disease Control and Prevention (CCDC), Ministry of Health (China). China Disease Surveillance Points 2010.
Chinese Center for Disease Control and Prevention (CCDC), Ministry of Health (China). China National Injury Surveillance System 2006 - China CDC. [Unpublished].
Chinese Center for Disease Control and Prevention (CCDC), Ministry of Health (China). China National Injury Surveillance System 2007 - China CDC. [Unpublished].
Chinese Center for Disease Control and Prevention (CCDC), Ministry of Health (China). China National Injury Surveillance System 2008 - China CDC. [Unpublished].
Chinese Center for Disease Control and Prevention (CCDC), Ministry of Health (China). China National Injury Surveillance System 2009 - China CDC. [Unpublished].
Chinese Center for Disease Control and Prevention (CCDC), Ministry of Health (China). China National Injury Surveillance System 2010 - China CDC. [Unpublished].
Chinese Center for Disease Control and Prevention (CCDC), Ministry of Health (China). China National Injury Surveillance System 2011 - China CDC. [Unpublished].
Chinese Center for Disease Control and Prevention (CCDC), Ministry of Health (China). China National Injury Surveillance System 2012 - China CDC. [Unpublished].
Chinese Center for Disease Control and Prevention (CCDC), Ministry of Health (China). China National Injury Surveillance System 2013. [Unpublished].
Chinese Center for Disease Control and Prevention (CCDC), Ministry of Health (China). China National Injury Surveillance System 2014. [Unpublished].
Chinese Center for Disease Control and Prevention (CCDC). China Chronic Disease and Risk Factor Surveillance 2010. [Unpublished].
Chinese Center for Disease Control and Prevention (CCDC). China Chronic Disease and Risk Factor Surveillance 2013.
Chinese Center for Disease Control and Prevention (CCDC). China Disease Surveillance Points 1991 - China CDC.
Chinese Center for Disease Control and Prevention (CCDC). China Disease Surveillance Points 1992 - China CDC.
Chinese Center for Disease Control and Prevention (CCDC). China Disease Surveillance Points 1993 - China CDC.
Chinese Center for Disease Control and Prevention (CCDC). China Disease Surveillance Points 1994 - China CDC.
Chinese Center for Disease Control and Prevention (CCDC). China Disease Surveillance Points 1995 - China CDC.
Chinese Center for Disease Control and Prevention (CCDC). China Disease Surveillance Points 1996 - China CDC.
Chinese Center for Disease Control and Prevention (CCDC). China Disease Surveillance Points 1997 - China CDC.
Chinese Center for Disease Control and Prevention (CCDC). China Disease Surveillance Points 1998 - China CDC.

Appendix: Citation List

Citation
Chinese Center for Disease Control and Prevention (CCDC). China Disease Surveillance Points 1999 - China CDC.
Chinese Center for Disease Control and Prevention (CCDC). China Disease Surveillance Points 2000 - China CDC.
Chinese Center for Disease Control and Prevention (CCDC). China Disease Surveillance Points 2001 - China CDC.
Chinese Center for Disease Control and Prevention (CCDC). China Disease Surveillance Points 2002 - China CDC.
Chinese Center for Disease Control and Prevention (CCDC). China Disease Surveillance Points 2004 - China CDC.
Chinese Center for Disease Control and Prevention (CCDC). China Disease Surveillance Points 2005 - China CDC.
Chinese Center for Disease Control and Prevention (CCDC). China Disease Surveillance Points 2006 - China CDC.
Chinese Center for Disease Control and Prevention (CCDC). China Disease Surveillance Points 2007 - China CDC.
Chinese Center for Disease Control and Prevention (CCDC). China Disease Surveillance Points and Information System for Disease Control and Prevention Deaths 2013.
Chinese Center for Disease Control and Prevention (CCDC). China Disease Surveillance Points and Information System for Disease Control and Prevention Deaths 2014.
Chinese Center for Disease Control and Prevention (CCDC). China Injury Comprehensive Surveillance Study 2009-2011 - China CDC. [Unpublished].
Chinese Center for Disease Control and Prevention (CCDC). China Mortality Registration and Reporting System 2008.
Chinese Center for Disease Control and Prevention (CCDC). China Mortality Registration and Reporting System 2009.
Chinese Center for Disease Control and Prevention (CCDC). China Mortality Registration and Reporting System 2010.
Chinese Center for Disease Control and Prevention (CCDC). China Mortality Registration and Reporting System 2011.
Chinese Center for Disease Control and Prevention (CCDC). China Mortality Registration and Reporting System 2012.
Chinese Center for Disease Control and Prevention (CCDC). China National Tuberculosis Prevalence Survey 1990.
Chinese Center for Disease Control and Prevention (CCDC). China National Tuberculosis Prevalence Survey 2000.
Chinese Center for Disease Control and Prevention (CCDC). China National Tuberculosis Prevalence Survey 2010.
Chinese Center for Disease Control and Prevention (CCDC). China National Tuberculosis Survey 1984-1985.
Chinese Center for Disease Control and Prevention (CCDC). China Notifiable Infectious Diseases 2004.
Chinese Center for Disease Control and Prevention (CCDC). China Notifiable Infectious Diseases 2004-2012. [Unpublished].
Chinese Center for Disease Control and Prevention (CCDC). China Notifiable Infectious Diseases 2005.
Chinese Center for Disease Control and Prevention (CCDC). China Notifiable Infectious Diseases 2006.
Chinese Center for Disease Control and Prevention (CCDC). China Notifiable Infectious Diseases 2007.
Chinese Center for Disease Control and Prevention (CCDC). China Notifiable Infectious Diseases 2008.
Chinese Center for Disease Control and Prevention (CCDC). China Notifiable Infectious Diseases 2009.
Chinese Center for Disease Control and Prevention (CCDC). China Notifiable Infectious Diseases 2010.
Chinese Center for Disease Control and Prevention (CCDC). China Notifiable Infectious Diseases 2011.
Chinese Center for Disease Control and Prevention (CCDC). China Notifiable Infectious Diseases 2012.
Chinese Center for Disease Control and Prevention (CCDC). China Notifiable Infectious Diseases 2013 - China CDC.
Chinese Center for Disease Control and Prevention (CCDC). China Notifiable Infectious Diseases 2014 - China CDC.
Chiquete E, Sánchez LV, Becerra G, Quintero A, Maldonado M, Panduro A. Performance of the serologic and molecular screening of blood donations for the hepatitis B and C viruses in a Mexican Transfusion Center. Ann Hepatol. 2005; 4(4): 275-8.
Chittleborough CR, Baldock KL, Phillips PJ, Taylor AW, North West Adelaide Health Study Team. Achievement of management targets associated with incident and long-term diagnosed diabetes among a representative population sample. Diabetes Res Clin Pract. 2010; 88(3): 322–7.
Chiu HC, Chang HY, Mau LW, Lee TK, Liu HW. Height, weight, and body mass index of elderly persons in Taiwan. J Gerontol A Biol Sci Med Sci. 2000; 55(11): M684-690.
Chiwanga FS, Njelekela MA. Diabetic foot: prevalence, knowledge, and foot self-care practices among diabetic patients in Dar es Salaam, Tanzania - a cross-sectional study. J Foot Ankle Res. 2015; 20.
Chizzolini C, Dupont A, Akue JP, Kaufmann MH, Verdini AS, Pessi A, Del Giudice G. Natural antibodies against three distinct and defined antigens of Plasmodium falciparum in residents of a mesoendemic area in Gabon. Am J Trop Med Hyg. 1988; 39(2): 150-6. As it appears in Malaria Atlas Project. Malaria Atlas Project Plasmodium Falciparum Parasite Rate Database. Oxford, United Kingdom: Malaria Atlas Project.
Chlabicz S, Bonifatiuk I, Radziwon P. Prevalence of hepatitis C virus antibodies among blood donors in north-eastern Poland. Hepatol Res. 2005; 33(3): 206-10.
Cho H. Racial Differences in the Prevalence of Intimate Partner Violence Against Women and Associated Factors. J Interpers Violence. 2012; 27(2): 344-63.
Cho NH. Diabetes burden and prevention in Korea and the Western Pacific Region. Diabetes Res Clin Pract. 2014; S282-7.
Cho SY, Kang SY, Lee JB. Metagonimiasis in Korea. Arzneimittelforschung. 1984; 34(9B): 1211-3.
Chong JW, Craig ME, Cameron FJ, Clarke CF, Rodda CP, Donath SM, Werther GA. Marked increase in type 1 diabetes mellitus incidence in children aged 0-14 yr in Victoria, Australia, from 1999 to 2002. Pediatr Diabetes. 2007; 8(2): 67-73.
Chongsrisawat V, Thawornsuk N, Theamboonlers A, Louisirirotchanakul S, Poovorawan Y. Hepatitis B virus DNA in unusual serological profiles of hepatitis B surface antigen-positive sera. Viral Immunol. 2006; 19(4): 623-9.
Chongsuvivatwong V, Impat A, Tayakkanonta K. A survey of neonatal tetanus and perinatal mortality in southern Muslim communities in Thailand. Southeast Asian J Trop Med Public Health. 1993; 24(4): 654-8.
Cho-Ngwa F, Amambua AN, Ambele MA, Titanji VPK. Evidence for the exacerbation of lymphedema of geochemical origin, podoconiosis, by onchocerciasis. J Infect Public Health. 2009; 2(4): 198-203.
Chou P, Chen HH, Hsiao KJ. Community-based epidemiological study on diabetes in Pu-Li, Taiwan. Diabetes Care. 1992; 15(1): 81-9.
Chou P, Liao MJ, Kuo HS, Hsiao KJ, Tsai ST. A population survey on the prevalence of diabetes in Kin-Hu, Kinmen. Diabetes Care. 1994; 17(9): 1055-8.

Appendix: Citation List

Citation
Choudhury N, Ramesh V, Saraswat S, Naik S. Effectiveness of mandatory transmissible diseases screening in Indian blood donors. Indian J Med Res. 1995; 229-32.
Chowdhury A, Santra A, Chakravorty R, Banerji A, Pal S, Dhali GK, Datta S, Banerji S, Manna B, Chowdhury SR, Bhattacharya SK, Mazumder DG. Community-based epidemiology of hepatitis B virus infection in West Bengal, India: prevalence of hepatitis B e antigen-negative infection and associated viral variants. J Gastroenterol Hepatol. 2005; 20(11): 1712-20.
Chowdhury R, Mondal D, Chowdhury V, Faria S, Alvar J, Nabi SG, Boelaert M, Dash AP. How far are we from visceral leishmaniasis elimination in Bangladesh? An assessment of epidemiological surveillance data. PLoS Negl Trop Dis. 2014; 8(8): e3020.
Christian Health Association of Liberia, Ministry of Health and Social Welfare (Liberia), United Nations Children's Fund (UNICEF). Liberia National Nutrition Survey 1999-2000.
Christian Medical College, Vellore (India), MRC Epidemiology Resource Center, University of Southampton. India - Vellore Birth Cohort Study 1998-2002.
Christian P, Katz J, Wu L, Kimbrough-Pradhan E, Khatry SK, LeClerq SC, West KP Jr. Risk factors for pregnancy-related mortality: a prospective study in rural Nepal. Public Health. 2008; 122(2): 161-72.
Christiana O, Olajumoke M, Oyetunde S. Lymphatic filariasis and associated morbidities in rural communities of Ogun State, Southwestern Nigeria. Travel Med Infect Dis. 2014; 12.0(1): 95-101.
Chronic Disease Research Centre, University of the West Indies, Ministry of Health (Barbados). Barbados Health of the Nation Survey 2011.
Chrystie I, Sumner D, Palmer S, Kenney A, Banatvala J. Screening of pregnant women for evidence of current hepatitis B infection: selective or universal? Health Trends. 1992; 24(1): 13-5.
Chu BK, Deming M, Biritwum NK, Bougma WR, Dorkenoo AM, El-Setouhy M, Fischer PU, Gass K, Gonzalez de Peña M, Mercado-Hernandez L, Kyelem D, Lammie PJ, Flueckiger RM, Mwingira UJ, Noordin R, Offei Owusu I, Ottesen EA, Pavluck A, Pilotte N, Rao RU, Samarasekera D, Schmaedick MA, Settinayake S, Simonsen PE, Supali T, Taleo F, Torres M, Weil GJ, Won KY. Transmission assessment surveys (TAS) to define endpoints for lymphatic filariasis mass drug administration: a multicenter evaluation. PLoS Negl Trop Dis. 2013; 7.0(12): e2584.
Chu BK, Gass K, Batcho W, 'Ake M, Dorkenoo AM, Adjinacou E, Mafi ', Addiss DG. Pilot assessment of soil-transmitted helminthiasis in the context of transmission assessment surveys for lymphatic filariasis in Benin and Tonga. PLoS Negl Trop Dis. 2014; 8.0(2): e2708.
Chuit R, Subias E, Pérez AC, Paulone I, Wisnivesky-Colli C, Segura EL. Usefulness of serology for the evaluation of Trypanosoma cruzi transmission in endemic areas of Chagas' disease. Rev Soc Bras Med Trop. 1989; 22(3): 119–124.
Chulalongkorn University, Institute of Population Studies (Thailand), Westinghouse; Institute for Resource Development. Thailand Demographic and Health Survey 1987. Columbia, United States: Westinghouse; Institute for Resource Development.
Chulalongkorn University, Ministry of Public Health (Thailand). An Economic Analysis of Tobacco Control in Thailand. Washington DC, United States: World Bank, 2003.
Chumpitazi BFF, Peyron F, Simon J, Boudin C, Sheick-Zakiuddin I, Picot S, Ambroise-Thomas P. Longitudinal survey in an endemic region of plasma soluble interleukin-2 receptor and antibody levels in Plasmodium falciparum malaria. J Clin Microbiol. 1990; 28(7): 1545-50. As it appears in Malaria Atlas Project. Malaria Atlas Project Plasmodium Falciparum Parasite Rate Database. Oxford, United Kingdom: Malaria Atlas Project.
Chunsuttiwat S, Biggs BA, Maynard J, Thamapalo S, Laoboripat S, Bovornsin S, Charanasri U, Pinyowiwat W, Kunasol P. Integration of hepatitis B vaccination into the expanded programme on immunization in Chonburi and Chiangmai provinces, Thailand. Vaccine. 1997; 15(6-7): 769-74.
Chuuk Department of Health Services (Micronesia), Department of Health and Social Affairs (Micronesia), World Health Organization (WHO). Micronesia - Chuuk STEPS Noncommunicable Disease Risk Factors Survey 2006.
Chuwa JKM. Tanzania Plasmodium Falciparum Parasite Rate Data, J.K.M. Chuwa, M. Med, Muhimbili University, 1991. As it appears in Malaria Atlas Project. Malaria Atlas Project Plasmodium Falciparum Parasite Rate Database. Oxford, United Kingdom: Malaria Atlas Project.
CIET International, United Nations Children's Fund (UNICEF). Afghanistan Multiple Indicator Baseline Survey 1997.
Cigarette smoking and educational level among young Israelis upon release from military service in 1988--a public health challenge as it appears in P.N. Lee Statistics and Computing Ltd. International Mortality and Smoking Statistics Version 4.04. Sutton, United Kingdom: P.N. Lee Statistics and Computing Ltd, 2009.
Cinek O, Sumnik Z, Vavrinec J. Continuing increase in incidence of childhood-onset type 1 diabetes in the Czech Republic 1990-2001. Eur J Pediatr. 2003; 162(6): 428-9.
Cisneros-Castolo M, Hernández-Ruiz L, Ibarra-Robles IE, Fernández-Gárate RH, Escobedo-De La Peña J. Prevalence of hepatitis B virus infection and related risk factors in a rural community of Mexico. Am J Trop Med Hyg. 2001; 65(6): 759-63.
Cissé B, Sokhna C, Boulanger D, Milet J, Bâ EH, Richardson K, Hallett R, Sutherland C, Simondon K, Simondon F, Alexander N, Gaye O, Targett G, Lines J, Greenwood B, Trape J-F. Seasonal intermittent preventive treatment with artesunate and sulfadoxine-pyrimethamine for prevention of malaria in Senegalese children: a randomised, placebo-controlled, double-blind trial. Lancet. 2006; 367(9511): 659-67. As it appears in Malaria Atlas Project. Malaria Atlas Project Plasmodium Falciparum Parasite Rate Database. Oxford, United Kingdom: Malaria Atlas Project.
Civil Registry and Identification Service (Chile), Ministry of Health (Chile). Chile Vital Statistics Yearbook 2013. 2015.
Clarke SE, Bøgh C, Brown RC, Pinder M, Walraven GE, Lindsay SW. Do untreated bednets protect against malaria? Trans R Soc Trop Med Hyg. 2001; 95(5): 457-62. As it appears in Malaria Atlas Project. Malaria Atlas Project Plasmodium Falciparum Parasite Rate Database. Oxford, United Kingdom: Malaria Atlas Project.
Clarke SE, Brooker S, Njagi JK, Njau E, Estambale B, Muchiri E, Magnussen P. Malaria morbidity among school children living in two areas of contrasting transmission in western Kenya. Am J Trop Med Hyg. 2004; 71(6): 732-8. As it appears in Malaria Atlas Project. Malaria Atlas Project Plasmodium Falciparum Parasite Rate Database. Oxford, United Kingdom: Malaria Atlas Project.

Appendix: Citation List

Citation
Clarke SE, Jukes MCH, Njagi JK, Khasakhala L, Cundill B, Otido J, Crudder C, Estambale BBA, Brooker S. Effect of intermittent preventive treatment of malaria on health and education in schoolchildren: a cluster-randomised, double-blind, placebo-controlled trial. Lancet. 2008; 372(9633): 127-38. As it appears in Malaria Atlas Project. Malaria Atlas Project Plasmodium Falciparum Parasite Rate Database. Oxford, United Kingdom: Malaria Atlas Project.
Cliffe S, Black D, Bryant J, Sullivan E. Maternal deaths in New South Wales, Australia: a data linkage project. Aust N Z J Obstet Gynaecol. 2008; 48(3): 255-60.
Clift A, Morgan C, Anderson D, Toole M. Alarming levels of hepatitis B virus detected among rural Tibetans. Trop Doct. 2004; 34(3): 156-7.
Clin B, Morlais F, Dubois B, Guizard AV, Desoubeaux N, Marquignon MF, Raffaelli C, Paris C, Galateau-salle F, Launoy G, Letourneux M. Occupational Asbestos Exposure And Digestive Cancers - A Cohort Study. Ailment Pharmacol Ther. 2009; 30(4): 364-74 as it appears in Camargo MC, Stayner LT, Straif K, Reina M, Al-Alem U, Demers PA, Landrigan PJ. Occupational exposure to asbestos and ovarian cancer: a meta-analysis. Environ Health Perspect. 2011; 119(9): 1211-7.
Cocco PL, Carta P, Belli S, Picchiri GF, Flore MV. Mortality Of Sardinian Lead And Zinc Miners: 1960-88. Occup Environ Med. 1960; 51(10): 674-82 as it appears in Kurihara N, Wada O. Silicosis and smoking strongly increase lung cancer risk in silica-exposed workers. Ind Health 2004; 42(3): 303–314.
Cockburn N, Steven D, Lecuona K, Joubert F, Rogers G, Cook C, Polack S. Prevalence, Causes and Socio-Economic Determinants of Vision Loss in Cape Town, South Africa. Atashili J, editor. PLoS One. 2012; 7(2): e30718.
Cockram CS, Woo J, Lau E, Chan JC, Chan AY, Lau J, Swaminathan R, Donnan SP. The prevalence of diabetes mellitus and impaired glucose tolerance among Hong Kong Chinese adults of working age. Diabetes Res Clin Pract. 1993; 21(1): 67-73.
Coggan C, Hooper R, Adams B. Self-reported injury rates in New Zealand. N Z Med J. 2002; 115(61): U167.
Coggon D, Harris EC, Poole J, Palmer KT. Extended Follow-Up Of A Cohort Of British Chemical Workers Exposed To Formaldehyde. J Natl Cancer Inst. 2003; 95(21): 1608-15 as it appears in Collins JJ, Lineker GA. A review and meta-analysis of formaldehyde exposure and leukemia. Regul Toxicol Pharmacol. 2004; 40(2): 81-91.
Coghlan B, Brennan RJ, Ngoy P, Dofara D, Otto B, Clements M, Stewart T. Mortality in the Democratic Republic of Congo: a nationwide survey. Lancet. 2006; 367(9504): 44-51.
Cohen DL, Neil HA, Thorogood M, Mann JI. A population-based study of the incidence of complications associated with type 2 diabetes in the elderly. Diabet Med. 1991; 8(10): 928-33.
Coimbra Júnior CE, Santos RV, Yoshida CF, Baptista ML, Flowers NM, do Valle AC. Hepatitis B epidemiology and cultural practices in Amerindian populations of Amazonia: the Tupí-Mondé and the Xavánte from Brazil. Soc Sci Med. 1996; 42(12): 1735-43.
Coker AL, Davis KE, Arias I, Desai S, Sanderson M, Brandt HM, Smith PH. Physical and mental health effects of intimate partner violence for men and women. Am J Prev Med. 2002; 23(4): 260-8.
Colagiuri S, Colagiuri R, Na'ati S, Muimuiheata S, Hussain Z, Palu T. The prevalence of diabetes in the Kingdom of Tonga. Diabetes Care. 2002; 25(2): 1378-83.
Coleman RE, Maneechai N, Ponlawat A, Kumpitak C, Rachapaew N, Miller RS, Sattabongkot J. Short report: Failure of the OptiMAL rapid malaria test as a tool for the detection of asymptomatic malaria in an area of Thailand endemic for Plasmodium falciparum. Am J Trop Med Hyg. 2002; 67(6): 563-5. As it appears in Malaria Atlas Project. Malaria Atlas Project Plasmodium Falciparum Parasite Rate Database. Oxford, United Kingdom: Malaria Atlas Project.
Coll M, Borrell C, Villabi J, Goicoechea J. PrÃ©valence du tabagisme en Andorre: DonnÃ©es de rÃ©fÃ©rence pour l'Ã©valuation des interventions. Rev Epidemiol Sante Publique. 2000; 305â€“8.
Coll-Cárdenas R, Espinoza-Gómez F, Maldonado-Rodríguez A, Reyes-López PA, Huerta-Viera M, Rojas-Larios F. Active transmission of human chagas disease in Colima Mexico. Mem Inst Oswaldo Cruz. 2004; 99(4): 363–8.
Collins JJ, Ireland B, Buckley CF, Shepperly D. Lymphohaematopoeitic cancer mortality among workers with benzene exposure. Occup Environ Med. 2003; 60(9): 676-9 as it appears in Khalade A, Jaakkola MS, Pukkala E, Jaakkola JJK. Exposure to benzene at work and the risk of leukemia: a systematic review and meta-analysis. Environ Health 2010; 9: 31.
Collins VR, Dowse GK, Toelupe PM, Imo TT, Aloaina FL, Spark RA, Zimmet PZ. Increasing prevalence of NIDDM in the Pacific island population of Western Samoa over a 13-year period. Diabetes Care. 1994; 17(4): 288-96.
Colombia - Cali Cancer Registry 1982-1986 - CI5 as it appears in Parkin DM, International Agency for Research on Cancer, International Association of Cancer Registries. Cancer Incidence in Five Continents. Vol. I to VIII. Lyon, France, IARCPress, 2005.
Colombia - Cali Cancer Registry 1983 - CI5 as it appears in Ferlay J, Parkin DM, Curado MP, Bray F, Edwards B, Shin HR and Forman D. Cancer Incidence in Five Continents, Volumes I to IX: IARC CancerBase No. 9 [Internet]. Lyon, France: International Agency for Research on Cancer; 2010. Available from: http://ci5.iarc.fr
Colombia - Cali Cancer Registry 1984 - CI5 as it appears in Ferlay J, Parkin DM, Curado MP, Bray F, Edwards B, Shin HR and Forman D. Cancer Incidence in Five Continents, Volumes I to IX: IARC CancerBase No. 9 [Internet]. Lyon, France: International Agency for Research on Cancer; 2010. Available from: http://ci5.iarc.fr
Colombia - Cali Cancer Registry 1985 - CI5 as it appears in Ferlay J, Parkin DM, Curado MP, Bray F, Edwards B, Shin HR and Forman D. Cancer Incidence in Five Continents, Volumes I to IX: IARC CancerBase No. 9 [Internet]. Lyon, France: International Agency for Research on Cancer; 2010. Available from: http://ci5.iarc.fr
Colombia - Cali Cancer Registry 1986 - CI5 as it appears in Ferlay J, Parkin DM, Curado MP, Bray F, Edwards B, Shin HR and Forman D. Cancer Incidence in Five Continents, Volumes I to IX: IARC CancerBase No. 9 [Internet]. Lyon, France: International Agency for Research on Cancer; 2010. Available from: http://ci5.iarc.fr
Colombia - Cali Cancer Registry 1987 - CI5 as it appears in Ferlay J, Parkin DM, Curado MP, Bray F, Edwards B, Shin HR and Forman D. Cancer Incidence in Five Continents, Volumes I to IX: IARC CancerBase No. 9 [Internet]. Lyon, France: International Agency for Research on Cancer; 2010. Available from: http://ci5.iarc.fr
Colombia - Cali Cancer Registry 1987-1991 - CI5 as it appears in Parkin DM, International Agency for Research on Cancer, International Association of Cancer Registries. Cancer Incidence in Five Continents. Vol. I to VIII. Lyon, France, IARCPress, 2005.

Appendix: Citation List

Citation
Colombia - Cali Cancer Registry 1988 - CI5 as it appears in Ferlay J, Parkin DM, Curado MP, Bray F, Edwards B, Shin HR and Forman D. Cancer Incidence in Five Continents, Volumes I to IX: IARC CancerBase No. 9 [Internet]. Lyon, France: International Agency for Research on Cancer; 2010. Available from: http://ci5.iarc.fr
Colombia - Cali Cancer Registry 1989 - CI5 as it appears in Ferlay J, Parkin DM, Curado MP, Bray F, Edwards B, Shin HR and Forman D. Cancer Incidence in Five Continents, Volumes I to IX: IARC CancerBase No. 9 [Internet]. Lyon, France: International Agency for Research on Cancer; 2010. Available from: http://ci5.iarc.fr
Colombia - Cali Cancer Registry 1990 - CI5 as it appears in Ferlay J, Parkin DM, Curado MP, Bray F, Edwards B, Shin HR and Forman D. Cancer Incidence in Five Continents, Volumes I to IX: IARC CancerBase No. 9 [Internet]. Lyon, France: International Agency for Research on Cancer; 2010. Available from: http://ci5.iarc.fr
Colombia - Cali Cancer Registry 1991 - CI5 as it appears in Ferlay J, Parkin DM, Curado MP, Bray F, Edwards B, Shin HR and Forman D. Cancer Incidence in Five Continents, Volumes I to IX: IARC CancerBase No. 9 [Internet]. Lyon, France: International Agency for Research on Cancer; 2010. Available from: http://ci5.iarc.fr
Colombia - Cali Cancer Registry 1992 - CI5 as it appears in Ferlay J, Parkin DM, Curado MP, Bray F, Edwards B, Shin HR and Forman D. Cancer Incidence in Five Continents, Volumes I to IX: IARC CancerBase No. 9 [Internet]. Lyon, France: International Agency for Research on Cancer; 2010. Available from: http://ci5.iarc.fr
Colombia - Cali Cancer Registry 1992-1996 - CI5 as it appears in Parkin DM, International Agency for Research on Cancer, International Association of Cancer Registries. Cancer Incidence in Five Continents. Vol. I to VIII. Lyon, France, IARCPress, 2005.
Colombia - Cali Cancer Registry 1993 - CI5 as it appears in Ferlay J, Parkin DM, Curado MP, Bray F, Edwards B, Shin HR and Forman D. Cancer Incidence in Five Continents, Volumes I to IX: IARC CancerBase No. 9 [Internet]. Lyon, France: International Agency for Research on Cancer; 2010. Available from: http://ci5.iarc.fr
Colombia - Cali Cancer Registry 1994 - CI5 as it appears in Ferlay J, Parkin DM, Curado MP, Bray F, Edwards B, Shin HR and Forman D. Cancer Incidence in Five Continents, Volumes I to IX: IARC CancerBase No. 9 [Internet]. Lyon, France: International Agency for Research on Cancer; 2010. Available from: http://ci5.iarc.fr
Colombia - Cali Cancer Registry 1995 - CI5 as it appears in Ferlay J, Parkin DM, Curado MP, Bray F, Edwards B, Shin HR and Forman D. Cancer Incidence in Five Continents, Volumes I to IX: IARC CancerBase No. 9 [Internet]. Lyon, France: International Agency for Research on Cancer; 2010. Available from: http://ci5.iarc.fr
Colombia - Cali Cancer Registry 1996 - CI5 as it appears in Ferlay J, Parkin DM, Curado MP, Bray F, Edwards B, Shin HR and Forman D. Cancer Incidence in Five Continents, Volumes I to IX: IARC CancerBase No. 9 [Internet]. Lyon, France: International Agency for Research on Cancer; 2010. Available from: http://ci5.iarc.fr
Colombia - Cali Cancer Registry 1997 - CI5 as it appears in Ferlay J, Parkin DM, Curado MP, Bray F, Edwards B, Shin HR and Forman D. Cancer Incidence in Five Continents, Volumes I to IX: IARC CancerBase No. 9 [Internet]. Lyon, France: International Agency for Research on Cancer; 2010. Available from: http://ci5.iarc.fr
Colombia - Cali Cancer Registry 1998 - CI5 as it appears in Ferlay J, Parkin DM, Curado MP, Bray F, Edwards B, Shin HR and Forman D. Cancer Incidence in Five Continents, Volumes I to IX: IARC CancerBase No. 9 [Internet]. Lyon, France: International Agency for Research on Cancer; 2010. Available from: http://ci5.iarc.fr
Colombia - Cali Cancer Registry 1998-2002 - CI5 as it appears in Curado MP, Edwards B, Shin HR, Storm H, Ferlay J, Heanue M and Boyle P, eds (2007). Cancer Incidence in Five Continents, Vol. IX Periodic Data (electronic version). Lyon, IARC.http://ci5.iarc.fr
Colombia - Cali Cancer Registry 1999 - CI5 as it appears in Ferlay J, Parkin DM, Curado MP, Bray F, Edwards B, Shin HR and Forman D. Cancer Incidence in Five Continents, Volumes I to IX: IARC CancerBase No. 9 [Internet]. Lyon, France: International Agency for Research on Cancer; 2010. Available from: http://ci5.iarc.fr
Colombia - Cali Cancer Registry 2000 - CI5 as it appears in Ferlay J, Parkin DM, Curado MP, Bray F, Edwards B, Shin HR and Forman D. Cancer Incidence in Five Continents, Volumes I to IX: IARC CancerBase No. 9 [Internet]. Lyon, France: International Agency for Research on Cancer; 2010. Available from: http://ci5.iarc.fr
Colombia - Cali Cancer Registry 2001 - CI5 as it appears in Ferlay J, Parkin DM, Curado MP, Bray F, Edwards B, Shin HR and Forman D. Cancer Incidence in Five Continents, Volumes I to IX: IARC CancerBase No. 9 [Internet]. Lyon, France: International Agency for Research on Cancer; 2010. Available from: http://ci5.iarc.fr
Colombia - Cali Cancer Registry 2002 - CI5 as it appears in Ferlay J, Parkin DM, Curado MP, Bray F, Edwards B, Shin HR and Forman D. Cancer Incidence in Five Continents, Volumes I to IX: IARC CancerBase No. 9 [Internet]. Lyon, France: International Agency for Research on Cancer; 2010. Available from: http://ci5.iarc.fr
Colombia Integrated National Adaptation Plan. Colombia Plasmodium Falciparum Parasite Rate Data, Personal Communication with Colombia Integrated National Adaptation Plan 2008. As it appears in Malaria Atlas Project. Malaria Atlas Project Plasmodium Falciparum Parasite Rate Database. Oxford, United Kingdom: Malaria Atlas Project.
Colombia Vital Registration - Deaths 1953 ICD7 as it appears in World Health Organization (WHO). WHO Mortality Database Version July 2012. Geneva, Switzerland: World Health Organization (WHO), 2012.
Colombia Vital Registration - Deaths 1954 ICD7 as it appears in World Health Organization (WHO). WHO Mortality Database Version July 2012. Geneva, Switzerland: World Health Organization (WHO), 2012.
Colombia Vital Registration - Deaths 1955 ICD7 as it appears in World Health Organization (WHO). WHO Mortality Database Version July 2012. Geneva, Switzerland: World Health Organization (WHO), 2012.
Colombia Vital Registration - Deaths 1956 ICD7 as it appears in World Health Organization (WHO). WHO Mortality Database Version July 2012. Geneva, Switzerland: World Health Organization (WHO), 2012.
Colombia Vital Registration - Deaths 1957 ICD7 as it appears in World Health Organization (WHO). WHO Mortality Database Version July 2012. Geneva, Switzerland: World Health Organization (WHO), 2012.
Colombia Vital Registration - Deaths 1958 ICD7 as it appears in World Health Organization (WHO). WHO Mortality Database Version July 2012. Geneva, Switzerland: World Health Organization (WHO), 2012.

Appendix: Citation List

Citation
Colombia Vital Registration - Deaths 1959 ICD7 as it appears in World Health Organization (WHO). WHO Mortality Database Version July 2012. Geneva, Switzerland: World Health Organization (WHO), 2012.
Colombia Vital Registration - Deaths 1960 ICD7 as it appears in World Health Organization (WHO). WHO Mortality Database Version July 2012. Geneva, Switzerland: World Health Organization (WHO), 2012.
Colombia Vital Registration - Deaths 1961 ICD7 as it appears in World Health Organization (WHO). WHO Mortality Database Version July 2012. Geneva, Switzerland: World Health Organization (WHO), 2012.
Colombia Vital Registration - Deaths 1962 ICD7 as it appears in World Health Organization (WHO). WHO Mortality Database Version July 2012. Geneva, Switzerland: World Health Organization (WHO), 2012.
Colombia Vital Registration - Deaths 1963 ICD7 as it appears in World Health Organization (WHO). WHO Mortality Database Version July 2012. Geneva, Switzerland: World Health Organization (WHO), 2012.
Colombia Vital Registration - Deaths 1964 ICD7 as it appears in World Health Organization (WHO). WHO Mortality Database Version July 2012. Geneva, Switzerland: World Health Organization (WHO), 2012.
Colombia Vital Registration - Deaths 1965 ICD7 as it appears in World Health Organization (WHO). WHO Mortality Database Version July 2012. Geneva, Switzerland: World Health Organization (WHO), 2012.
Colombia Vital Registration - Deaths 1966 ICD7 as it appears in World Health Organization (WHO). WHO Mortality Database Version July 2012. Geneva, Switzerland: World Health Organization (WHO), 2012.
Colombia Vital Registration - Deaths 1967 ICD7 as it appears in World Health Organization (WHO). WHO Mortality Database Version July 2012. Geneva, Switzerland: World Health Organization (WHO), 2012.
Colombia Vital Registration - Deaths 1968 ICD8 as it appears in World Health Organization (WHO). WHO Mortality Database Version May 2013. Geneva, Switzerland: World Health Organization (WHO), 2013.
Colombia Vital Registration - Deaths 1969 ICD8 as it appears in World Health Organization (WHO). WHO Mortality Database Version May 2013. Geneva, Switzerland: World Health Organization (WHO), 2013.
Colombia Vital Registration - Deaths 1970 ICD8 as it appears in World Health Organization (WHO). WHO Mortality Database Version May 2013. Geneva, Switzerland: World Health Organization (WHO), 2013.
Colombia Vital Registration - Deaths 1972 ICD8 as it appears in World Health Organization (WHO). WHO Mortality Database Version May 2013. Geneva, Switzerland: World Health Organization (WHO), 2013.
Colombia Vital Registration - Deaths 1974 ICD8 as it appears in World Health Organization (WHO). WHO Mortality Database Version May 2013. Geneva, Switzerland: World Health Organization (WHO), 2013.
Colombia Vital Registration - Deaths 1975 ICD8 as it appears in World Health Organization (WHO). WHO Mortality Database Version May 2013. Geneva, Switzerland: World Health Organization (WHO), 2013.
Colombia Vital Registration - Deaths 1976 ICD8 as it appears in World Health Organization (WHO). WHO Mortality Database Version May 2013. Geneva, Switzerland: World Health Organization (WHO), 2013.
Colombia Vital Registration - Deaths 1977 ICD8 as it appears in World Health Organization (WHO). WHO Mortality Database Version May 2013. Geneva, Switzerland: World Health Organization (WHO), 2013.
Colombia Vital Registration - Deaths 1979 ICD9 as it appears in World Health Organization (WHO). WHO Mortality Database Version November 2015. Geneva, Switzerland: World Health Organization (WHO), 2015.
Colombia Vital Registration - Deaths 1984 ICD9 as it appears in World Health Organization (WHO). WHO Mortality Database Version November 2015. Geneva, Switzerland: World Health Organization (WHO), 2015.
Colombia Vital Registration - Deaths 1985 ICD9 as it appears in World Health Organization (WHO). WHO Mortality Database Version November 2015. Geneva, Switzerland: World Health Organization (WHO), 2015.
Colombia Vital Registration - Deaths 1986 ICD9 as it appears in World Health Organization (WHO). WHO Mortality Database Version November 2015. Geneva, Switzerland: World Health Organization (WHO), 2015.
Colombia Vital Registration - Deaths 1987 ICD9 as it appears in World Health Organization (WHO). WHO Mortality Database Version November 2015. Geneva, Switzerland: World Health Organization (WHO), 2015.
Colombia Vital Registration - Deaths 1988 ICD9 as it appears in World Health Organization (WHO). WHO Mortality Database Version November 2015. Geneva, Switzerland: World Health Organization (WHO), 2015.
Colombia Vital Registration - Deaths 1989 ICD9 as it appears in World Health Organization (WHO). WHO Mortality Database Version November 2015. Geneva, Switzerland: World Health Organization (WHO), 2015.
Colombia Vital Registration - Deaths 1990 ICD9 as it appears in World Health Organization (WHO). WHO Mortality Database Version November 2015. Geneva, Switzerland: World Health Organization (WHO), 2015.
Colombia Vital Registration - Deaths 1991 ICD9 as it appears in World Health Organization (WHO). WHO Mortality Database Version November 2015. Geneva, Switzerland: World Health Organization (WHO), 2015.
Colombia Vital Registration - Deaths 1992 ICD9 as it appears in World Health Organization (WHO). WHO Mortality Database Version November 2015. Geneva, Switzerland: World Health Organization (WHO), 2015.
Colombia Vital Registration - Deaths 1993 ICD9 as it appears in World Health Organization (WHO). WHO Mortality Database Version November 2015. Geneva, Switzerland: World Health Organization (WHO), 2015.
Colombia Vital Registration - Deaths 1994 ICD9 as it appears in World Health Organization (WHO). WHO Mortality Database Version November 2015. Geneva, Switzerland: World Health Organization (WHO), 2015.
Colombia Vital Registration - Deaths 1995 ICD9 as it appears in World Health Organization (WHO). WHO Mortality Database Version November 2015. Geneva, Switzerland: World Health Organization (WHO), 2015.
Colombia Vital Registration - Deaths 1996 ICD9 as it appears in World Health Organization (WHO). WHO Mortality Database Version November 2015. Geneva, Switzerland: World Health Organization (WHO), 2015.
Colombia Vital Registration - Deaths 1997 ICD10 as it appears in World Health Organization (WHO). WHO Mortality Database Version November 2015. Geneva, Switzerland: World Health Organization (WHO), 2015.

Appendix: Citation List

Citation
Colombia Vital Registration - Deaths 1998 ICD10 as it appears in World Health Organization (WHO). WHO Mortality Database Version November 2015. Geneva, Switzerland: World Health Organization (WHO), 2015.
Colombia Vital Registration - Deaths 1999 ICD10 as it appears in World Health Organization (WHO). WHO Mortality Database Version November 2015. Geneva, Switzerland: World Health Organization (WHO), 2015.
Colombia Vital Registration - Deaths 2000 ICD10 as it appears in World Health Organization (WHO). WHO Mortality Database Version November 2015. Geneva, Switzerland: World Health Organization (WHO), 2015.
Colombia Vital Registration - Deaths 2001 ICD10 as it appears in World Health Organization (WHO). WHO Mortality Database Version November 2015. Geneva, Switzerland: World Health Organization (WHO), 2015.
Colombia Vital Registration - Deaths 2002 ICD10 as it appears in World Health Organization (WHO). WHO Mortality Database Version November 2015. Geneva, Switzerland: World Health Organization (WHO), 2015.
Colombia Vital Registration - Deaths 2004 ICD10 as it appears in World Health Organization (WHO). WHO Mortality Database Version November 2015. Geneva, Switzerland: World Health Organization (WHO), 2015.
Colombia Vital Registration - Deaths 2005 ICD10 as it appears in World Health Organization (WHO). WHO Mortality Database Version November 2015. Geneva, Switzerland: World Health Organization (WHO), 2015.
Colombia Vital Registration - Deaths 2006 ICD10 as it appears in World Health Organization (WHO). WHO Mortality Database Version November 2015. Geneva, Switzerland: World Health Organization (WHO), 2015.
Colombia Vital Registration - Deaths 2007 ICD10 as it appears in World Health Organization (WHO). WHO Mortality Database Version November 2015. Geneva, Switzerland: World Health Organization (WHO), 2015.
Colombia Vital Registration - Deaths 2008 ICD10 as it appears in World Health Organization (WHO). WHO Mortality Database Version November 2015. Geneva, Switzerland: World Health Organization (WHO), 2015.
Colombia Vital Registration - Deaths 2009 ICD10 as it appears in World Health Organization (WHO). WHO Mortality Database Version November 2015. Geneva, Switzerland: World Health Organization (WHO), 2015.
Colombia Vital Registration - Deaths 2010 ICD10 as it appears in World Health Organization (WHO). WHO Mortality Database Version November 2015. Geneva, Switzerland: World Health Organization (WHO), 2015.
Colombia Vital Registration - Deaths 2011 ICD10 as it appears in World Health Organization (WHO). WHO Mortality Database Version November 2015. Geneva, Switzerland: World Health Organization (WHO), 2015.
Colombia Vital Registration - Deaths 2012 ICD10 as it appears in World Health Organization (WHO). WHO Mortality Database Version November 2015. Geneva, Switzerland: World Health Organization (WHO), 2015.
Colombia Vital Registration Death Data 1950 as it appears in United Nations Statistics Division (UNSD). United Nations Demographic Yearbook - Historical Supplement 1997. New York City, United States: United Nations Statistics Division (UNSD).
Colombia Vital Registration Death Data 1951 as it appears in United Nations Statistics Division (UNSD). United Nations Demographic Yearbook - Historical Supplement 1997. New York City, United States: United Nations Statistics Division (UNSD).
Colombia Vital Registration Death Data 1952 as it appears in United Nations Statistics Division (UNSD). United Nations Demographic Yearbook - Historical Supplement 1997. New York City, United States: United Nations Statistics Division (UNSD).
Colombia Vital Registration Death Data 1971 as it appears in United Nations Statistics Division (UNSD). United Nations Demographic Yearbook - Historical Supplement 1997. New York City, United States: United Nations Statistics Division (UNSD).
Colombia Vital Registration Death Data 1973 as it appears in United Nations Statistics Division (UNSD). United Nations Demographic Yearbook - Historical Supplement 1997. New York City, United States: United Nations Statistics Division (UNSD).
Colombia Vital Registration Death Data 2011 as it appears in United Nations Statistics Division (UNSD). United Nations Demographic Yearbook 2011. New York City, United States: United Nations Statistics Division (UNSD), 2012.
Colombian Family Welfare Institute, Ministry of Social Protection (Colombia), National Institute of Health (Colombia), Profamilia. Colombia National Survey of the Nutritional Situation 2010.
Colombian Family Welfare Institute, National Institute of Health (Colombia), Pan American Health Organization (PAHO), Profamilia, Universidad de Antioquia (Colombia). Colombia National Survey of the Nutritional Situation 2004-2005.
Combourieu I. Results of the Malariometric Surveys Conducted in Djoum and Ngaoundère in Cameroon [dissertation]. Bordeaux, France: University of Bordeaux,1989. As it appears in Malaria Atlas Project. Malaria Atlas Project Plasmodium Falciparum Parasite Rate Database. Oxford, United Kingdom: Malaria Atlas Project.
Commission of the European Communities (2012): Eurobarometer 27 (Mar-May 1987). Faits et Opinions, Paris. GESIS Data Archive, Cologne. ZA1712 Data file Version 1.0.1, doi:10.4232/1.10884
Commission of the European Communities (2012): Eurobarometer 29 (Mar-Apr 1988). Faits et Opinions, Paris. GESIS Data Archive, Cologne. ZA1714 Data file Version 1.0.1, doi:10.4232/1.10886
Commission of the European Communities (2012): Eurobarometer 31A (Jun-Jul 1989). Faits et Opinions, Paris. GESIS Data Archive, Cologne. ZA1751 Data file Version 1.0.1, doi:10.4232/1.10889
Commission of the European Communities (2012): Eurobarometer 32 (Oct-Nov 1989). INRA, Brussels. GESIS Data Archive, Cologne. ZA1752 Data file Version 1.1.0, doi:10.4232/1.10890
Commission of the European Communities (2012): Eurobarometer 32 (Oct-Nov 1989). INRA, Brussels. GESIS Data Archive, Cologne. ZA1752 Data file Version 1.1.0,doi:10.4232/1.10890
Commission of the European Communities (2012): Eurobarometer 34.1 (Nov 1990). INRA, Brussels. GESIS Data Archive, Cologne. ZA1961 Data file Version 1.0.1, doi:10.4232/1.10893
Commission of the European Communities (2012): Eurobarometer 36 (Oct-Nov 1991). INRA, Brussels. GESIS Data Archive, Cologne. ZA2081 Data file Version 1.1.0, doi:10.4232/1.10848
Commission of the European Communities (2012): Eurobarometer 38.0 (Sep-Oct 1992). INRA, Brussels. GESIS Data Archive, Cologne. ZA2294 Data file Version 1.1.0, doi:10.4232/1.10903
Committee for Health and Social Insurance (Libya), League of Arab States. Libya Maternal and Child Health Survey 1995.

Appendix: Citation List

Citation
Commonwealth Department of Health and Family Services. Australia Survey of Contraception Use and Ease 1995. Canberra, Australia: Australian Social Science Data Archive, The Australian National University, 2004.
Community Water and Sanitation Agency (Ghana), Global Public-Private Partnership for Handwashing with Soap (PPPHW), London School of Hygiene and Tropical Medicine, Research International. What Motivates Handwashing in Ghana? A Re-analysis of the Results of the Formative Research.
Comoros Demographic and Health Survey 1996 as it appears in World Health Organization (WHO). WHO Global Database on Child Growth and Malnutrition - Historical. Geneva, Switzerland: World Health Organization (WHO).
Comoros Household Survey 2004 as it appears in World Health Organization (WHO). WHO Household Energy Database. Geneva, Switzerland: World Health Organization (WHO), 2010.
Comoros Immunization Coverage Survey November 2007.
Comoros Immunization Coverage Survey October 2010.
Comoros Post Measles Campaign and Routine Immunization Coverage Survey 2013.
Comparison of estimates of under-nutrition for pre-school rural Pakistani children based on the WHO standard and the National Center for Health Statistics (NCHS) reference as it appears in World Health Organization (WHO). WHO Global Database on Child Growth and Malnutrition. Geneva, Switzerland: World Health Organization (WHO).
Comparison of Portuguese prevalence of childhood overweight and obesity using WHO child growth standards, CDC 2000 growth charts, IOTF criteria and NCHS growth charts as it appears in World Health Organization (WHO). WHO Global Database on Child Growth and Malnutrition. Geneva, Switzerland: World Health Organization (WHO).
Comparison of the growth standards between Saudi and American children aged 0-5 years as it appears in World Health Organization (WHO). WHO Global Database on Child Growth and Malnutrition. Geneva, Switzerland: World Health Organization (WHO).
Comparison of the prevalence of shortness, underweight, and overweight among US children aged 0 to 59 months by using the CDC 2000 and the WHO 2006 growth charts as it appears in World Health Organization (WHO). WHO Global Database on Child Growth and Malnutrition. Geneva, Switzerland: World Health Organization (WHO).
Comprehensive Care Youth Foundation (FUNDAINIL), National Anti-Drug Office (Venezuela). Venezuela National Study of Drug Consumption in the General Population 2011.
Concluzia-Prim Center for Survey Methodology (Moldova), East-Ukranian Foundation For Social Research, Institute for Advanced Studies (Austria), London School of Hygiene and Tropical Medicine, University of Aberdeen. Ukraine Health in Times of Transition Household Survey 2010.
Concluzia-Prim Center for Survey Methodology (Moldova), Independent Sociology and Information Service (OPINIA) (Moldova), Institute for Advanced Studies (Austria), London School of Hygiene and Tropical Medicine, University of Aberdeen. Moldova Health in Times of Transition Household Survey 2010.
Concluzia-Prim Center for Survey Methodology (Moldova), Institute for Advanced Studies (Austria), International Centre for Sociological, Political and Social Psychological Research (Kyrgyzstan), London School of Hygiene and Tropical Medicine, University of Aberdeen. Kyrgyzstan Health in Times of Transition Household Survey 2011.
Concluzia-Prim Center for Survey Methodology (Moldova), Institute for Advanced Studies (Austria), London School of Hygiene and Tropical Medicine, University of Aberdeen. Azerbaijan Health in Times of Transition Household Survey 2010.
Congo EPI Review 1987.
Congo EPI Review 1988.
Congo External EPI Review 2010.
Congo International EPI Review 1990.
Congo National Survey on the Nutritional Status of Preschool Age Children 1987 as it appears in World Health Organization (WHO). WHO Global Database on Child Growth and Malnutrition. Geneva, Switzerland: World Health Organization (WHO).
Congo, DR Immunization Coverage Survey 2012.
Congo, DR Study of Mothers Motivators on Attendance and Non-Attendance at the Pre-School Consultation as it appears in World Health Organization (WHO). WHO Global Database on Child Growth and Malnutrition - Historical. Geneva, Switzerland: World Health Organization (WHO).
Consistent improvement in the nutritional status of Colombian children between 1965 and 1989 as it appears in World Health Organization (WHO). WHO Global Database on Child Growth and Malnutrition. Geneva, Switzerland: World Health Organization (WHO).
Consonni D, Pesatori AC, Tironi A, Bernucci I, Zocchetti C, Bertazzi PA. Mortality study in an Italian oil refinery: extension of the follow-up. Am J Ind Med. 1999; 35(3): 287-94 as it appears in Khalade A, Jaakkola MS, Pukkala E, Jaakkola JJK. Exposure to benzene at work and the risk of leukemia: a systematic review and meta-analysis. Environ Health 2010; 9: 31.
Consumer Safety Institute (Netherlands). Netherlands Injury Surveillance System 1998. [Unpublished].
Consumer Safety Institute (Netherlands). Netherlands Injury Surveillance System 1999. [Unpublished].
Consumer Safety Institute (Netherlands). Netherlands Injury Surveillance System 2000. [Unpublished].
Consumer Safety Institute (Netherlands). Netherlands Injury Surveillance System 2001. [Unpublished].
Consumer Safety Institute (Netherlands). Netherlands Injury Surveillance System 2002. [Unpublished].
Consumer Safety Institute (Netherlands). Netherlands Injury Surveillance System 2003. [Unpublished].
Consumer Safety Institute (Netherlands). Netherlands Injury Surveillance System 2004. [Unpublished].
Consumer Safety Institute (Netherlands). Netherlands Injury Surveillance System 2005. [Unpublished].
Consumer Safety Institute (Netherlands). Netherlands Injury Surveillance System 2006. [Unpublished].
Consumer Safety Institute (Netherlands). Netherlands Injury Surveillance System 2007. [Unpublished].
Consumer Safety Institute (Netherlands). Netherlands Injury Surveillance System 2008. [Unpublished].
Consumer Safety Institute (Netherlands). Netherlands Injury Surveillance System 2009. [Unpublished].
Consumer Safety Institute (Netherlands). Netherlands Injury Surveillance System 2010. [Unpublished].

Appendix: Citation List

Citation
Consumer Safety Institute (Netherlands). Netherlands Injury Surveillance System 2011. [Unpublished].
Consumer Safety Institute (Netherlands). Netherlands Injury Surveillance System 2012. [Unpublished].
Control of Project Management in Africa. Senegal - Dakar Draft Report: Public-Private Partnership Initiative for Handwashing - Situation Benchmark. Dakar, Senegal: Control of Project Management in Africa, 2005.
Cook JA, Baker ST, Warren KS, Jordan P. A controlled study of morbidity of schistosomiasis mansoni in St. Lucian children, based on quantitative egg excretion. Am J Trop Med Hyg. 1974; 23(4): 625-33.
Cook JA, Jordan P, Woodstock L, Pilgrim V. A controlled trial of hycanthone and placebo in schistosomiasis mansoni in St. Lucia. Ann Trop Med Parasitol. 1977; 71(2): 197-202.
Cooney RM, Flanagan KP, Zehyle E. Review of surgical management of cystic hydatid disease in a resource limited setting: Turkana, Kenya. Eur J Gastroenterol Hepatol. 2004; 16(11): 1233-6.
Cooper R, Rotimi C, Ataman S, McGee D, Osotimehin B, Kadiri S, Muna W, Kingue S, Fraser H, Forrester T, Bennett F, Wilks R. The prevalence of hypertension in seven populations of West African origin. Am J Public Health. 1997; 87(2): 160-8.
Cooppan RM, Schutte CH, Mayet FG, Dingle CE, Van Deventer JM, Mosese PG. Morbidity from urinary schistosomiasis in relation to intensity of infection in the Natal Province of South Africa. Am J Trop Med Hyg. 1986; 35(4): 765-76.
Co-ordination Action on Human Rights Violations (CAHRV). Comparative Reanalysis of Prevalence of Violence Against Women and Health Impact Data in Europe - Obstacles and Possible Solutions. OsnabrÃ¼ck, Germany: Co-ordination Action on Human Rights Violations (CAHRV), 2006.(CAHRV-Report 2006. Co-ordination Action on Human Rights Violations funded through the European Commission, 6th Framework Programme, Project No. 506348).
Corbett EL, Butterworth AE, Fulford AJ, Ouma JH, Sturrock RF. Nutritional status of children with schistosomiasis mansoni in two different areas of Machakos District, Kenya. Trans R Soc Trop Med Hyg. 1992; 86(3): 266-73.
Corrao G, Zambon A, Bagnardi V, Aricò S, Loguercio C, D'Amicis A, Collaborative SIDECIR Group. Nutrient intakes, nutritional patterns and the risk of liver cirrhosis: an explorative case-control study. Eur J Epidemiol. 2004; 19(9): 861-9.
Correa AM, Carmona Fonseca J, Alcaraz Lopez GM. Malaria entre la población Tule (Kuna) del resguardo Caimán Nuevo (Turbo y Necoclí; Antioquia, Colombia), 2003-2004. Investiga Educa Enferm. 2005; 23(2): 16-33. As it appears in Malaria Atlas Project. Malaria Atlas Project Plasmodium Falciparum Parasite Rate Database. Oxford, United Kingdom: Malaria Atlas Project.
Correia RA, Araújo HC, Furtado BMA, Bonfim C. [Epidemiological features of maternal deaths occurred in Recife, PE, Brazil (2000-2006)]. Rev Bras Enferm. 2011; 64(1): 91-7.
Cortez-Dias N, Martins S, Belo A, Fiuza M. Prevalence, management and control of diabetes mellitus and associated risk factors in primary health care in Portugal. Rev Port Cardiol. 2010; 29(4): 509-37.
COSEP-Consulting Ltd., Consaúde Ltd., ICF International, National Malaria Control (Angola), President's Malaria Initiative (PMI). Angola Malaria Indicator Survey 2011. Fairfax, United States: ICF International.
COSEP-Consulting Ltd., Consaúde Ltd., ICF International, National Malaria Control (Angola), President's Malaria Initiative (PMI). Angola Malaria Indicator Survey 2011. Fairfax, United States: ICF International. As it appears in Malaria Atlas Project. Malaria Atlas Project Plasmodium Falciparum Parasite Rate Database. Oxford, United Kingdom: Malaria Atlas Project.
COSEP-Consulting Ltd., Consaúde Ltd., Macro International, Inc, Ministry of Health (Angola). Angola Malaria Indicator Survey 2006-2007. Calverton, United States: Macro International, Inc.
COSEP-Consulting Ltd., Consaúde Ltd., Macro International, Inc, Ministry of Health (Angola). Angola Malaria Indicator Survey 2006-2007. Calverton, United States: Macro International, Inc. As it appears in Malaria Atlas Project. Malaria Atlas Project Plasmodium Falciparum Parasite Rate Database. Oxford, United Kingdom: Malaria Atlas Project.
Costa B, Barrio F, Cabre J-J, Pinol J-L, Cos F-X, Sole C, Bolibar B, Castell C, Lindstrom J, Barengo N, Tuomilehto J, DE-PLAN-CAT Research Group. Shifting from glucose diagnostic criteria to the new HbA(1c) criteria would have a profound impact on prevalence of diabetes among a high-risk Spanish population. Diabet Med. 2011; 28(10): 1234–7.
Costa Rica Cancer Registry 1980 - CI5 as it appears in Parkin DM, International Agency for Research on Cancer, International Association of Cancer Registries. Cancer Incidence in Five Continents. Vol. I to VIII. Lyon, France, IARCPress, 2005.
Costa Rica Cancer Registry 1980-1982 - CI5 as it appears in Parkin DM, International Agency for Research on Cancer, International Association of Cancer Registries. Cancer Incidence in Five Continents. Vol. I to VIII. Lyon, France, IARCPress, 2005.
Costa Rica Cancer Registry 1981 - CI5 as it appears in Ferlay J, Parkin DM, Curado MP, Bray F, Edwards B, Shin HR and Forman D. Cancer Incidence in Five Continents, Volumes I to IX: IARC CancerBase No. 9 [Internet]. Lyon, France: International Agency for Research on Cancer; 2010. Available from: http://ci5.iarc.fr
Costa Rica Cancer Registry 1982 - CI5 as it appears in Ferlay J, Parkin DM, Curado MP, Bray F, Edwards B, Shin HR and Forman D. Cancer Incidence in Five Continents, Volumes I to IX: IARC CancerBase No. 9 [Internet]. Lyon, France: International Agency for Research on Cancer; 2010. Available from: http://ci5.iarc.fr
Costa Rica Cancer Registry 1983 - CI5 as it appears in Ferlay J, Parkin DM, Curado MP, Bray F, Edwards B, Shin HR and Forman D. Cancer Incidence in Five Continents, Volumes I to IX: IARC CancerBase No. 9 [Internet]. Lyon, France: International Agency for Research on Cancer; 2010. Available from: http://ci5.iarc.fr
Costa Rica Cancer Registry 1984 - CI5 as it appears in Parkin DM, International Agency for Research on Cancer, International Association of Cancer Registries. Cancer Incidence in Five Continents. Vol. I to VIII. Lyon, France, IARCPress, 2005.
Costa Rica Cancer Registry 1984-1987 - CI5 as it appears in Parkin DM, International Agency for Research on Cancer, International Association of Cancer Registries. Cancer Incidence in Five Continents. Vol. I to VIII. Lyon, France, IARCPress, 2005.
Costa Rica Cancer Registry 1985 - CI5 as it appears in Ferlay J, Parkin DM, Curado MP, Bray F, Edwards B, Shin HR and Forman D. Cancer Incidence in Five Continents, Volumes I to IX: IARC CancerBase No. 9 [Internet]. Lyon, France: International Agency for Research on Cancer; 2010. Available from: http://ci5.iarc.fr

Appendix: Citation List

Citation
Costa Rica Cancer Registry 1986 - CI5 as it appears in Ferlay J, Parkin DM, Curado MP, Bray F, Edwards B, Shin HR and Forman D. Cancer Incidence in Five Continents, Volumes I to IX: IARC CancerBase No. 9 [Internet]. Lyon, France: International Agency for Research on Cancer; 2010. Available from: http://ci5.iarc.fr
Costa Rica Cancer Registry 1987 - CI5 as it appears in Ferlay J, Parkin DM, Curado MP, Bray F, Edwards B, Shin HR and Forman D. Cancer Incidence in Five Continents, Volumes I to IX: IARC CancerBase No. 9 [Internet]. Lyon, France: International Agency for Research on Cancer; 2010. Available from: http://ci5.iarc.fr
Costa Rica Cancer Registry 1988 - CI5 as it appears in Ferlay J, Parkin DM, Curado MP, Bray F, Edwards B, Shin HR and Forman D. Cancer Incidence in Five Continents, Volumes I to IX: IARC CancerBase No. 9 [Internet]. Lyon, France: International Agency for Research on Cancer; 2010. Available from: http://ci5.iarc.fr
Costa Rica Cancer Registry 1988-1992 - CI5 as it appears in Parkin DM, International Agency for Research on Cancer, International Association of Cancer Registries. Cancer Incidence in Five Continents. Vol. I to VIII. Lyon, France, IARCPress, 2005.
Costa Rica Cancer Registry 1989 - CI5 as it appears in Parkin DM, International Agency for Research on Cancer, International Association of Cancer Registries. Cancer Incidence in Five Continents. Vol. I to VIII. Lyon, France, IARCPress, 2005.
Costa Rica Cancer Registry 1990 - CI5 as it appears in Ferlay J, Parkin DM, Curado MP, Bray F, Edwards B, Shin HR and Forman D. Cancer Incidence in Five Continents, Volumes I to IX: IARC CancerBase No. 9 [Internet]. Lyon, France: International Agency for Research on Cancer; 2010. Available from: http://ci5.iarc.fr
Costa Rica Cancer Registry 1991 - CI5 as it appears in Ferlay J, Parkin DM, Curado MP, Bray F, Edwards B, Shin HR and Forman D. Cancer Incidence in Five Continents, Volumes I to IX: IARC CancerBase No. 9 [Internet]. Lyon, France: International Agency for Research on Cancer; 2010. Available from: http://ci5.iarc.fr
Costa Rica Cancer Registry 1992 - CI5 as it appears in Ferlay J, Parkin DM, Curado MP, Bray F, Edwards B, Shin HR and Forman D. Cancer Incidence in Five Continents, Volumes I to IX: IARC CancerBase No. 9 [Internet]. Lyon, France: International Agency for Research on Cancer; 2010. Available from: http://ci5.iarc.fr
Costa Rica Cancer Registry 1993 - CI5 as it appears in Ferlay J, Parkin DM, Curado MP, Bray F, Edwards B, Shin HR and Forman D. Cancer Incidence in Five Continents, Volumes I to IX: IARC CancerBase No. 9 [Internet]. Lyon, France: International Agency for Research on Cancer; 2010. Available from: http://ci5.iarc.fr
Costa Rica Cancer Registry 1993-1997 - CI5 as it appears in Parkin DM, International Agency for Research on Cancer, International Association of Cancer Registries. Cancer Incidence in Five Continents. Vol. I to VIII. Lyon, France, IARCPress, 2005.
Costa Rica Cancer Registry 1994 - CI5 as it appears in Parkin DM, International Agency for Research on Cancer, International Association of Cancer Registries. Cancer Incidence in Five Continents. Vol. I to VIII. Lyon, France, IARCPress, 2005.
Costa Rica Cancer Registry 1995 - CI5 as it appears in Ferlay J, Parkin DM, Curado MP, Bray F, Edwards B, Shin HR and Forman D. Cancer Incidence in Five Continents, Volumes I to IX: IARC CancerBase No. 9 [Internet]. Lyon, France: International Agency for Research on Cancer; 2010. Available from: http://ci5.iarc.fr
Costa Rica Cancer Registry 1996 - CI5 as it appears in Ferlay J, Parkin DM, Curado MP, Bray F, Edwards B, Shin HR and Forman D. Cancer Incidence in Five Continents, Volumes I to IX: IARC CancerBase No. 9 [Internet]. Lyon, France: International Agency for Research on Cancer; 2010. Available from: http://ci5.iarc.fr
Costa Rica Cancer Registry 1997 - CI5 as it appears in Ferlay J, Parkin DM, Curado MP, Bray F, Edwards B, Shin HR and Forman D. Cancer Incidence in Five Continents, Volumes I to IX: IARC CancerBase No. 9 [Internet]. Lyon, France: International Agency for Research on Cancer; 2010. Available from: http://ci5.iarc.fr
Costa Rica Cancer Registry 1998 - CI5 as it appears in Ferlay J, Parkin DM, Curado MP, Bray F, Edwards B, Shin HR and Forman D. Cancer Incidence in Five Continents, Volumes I to IX: IARC CancerBase No. 9 [Internet]. Lyon, France: International Agency for Research on Cancer; 2010. Available from: http://ci5.iarc.fr
Costa Rica Cancer Registry 1998-2002 - CI5 as it appears in Curado MP, Edwards B, Shin HR, Storm H, Ferlay J, Heanue M and Boyle P, eds (2007). Cancer Incidence in Five Continents, Vol. IX Periodic Data (electronic version). Lyon, IARC.http://ci5.iarc.fr
Costa Rica Cancer Registry 1999 - CI5 as it appears in Curado MP, Edwards B, Shin HR, Storm H, Ferlay J, Heanue M and Boyle P, eds (2007). Cancer Incidence in Five Continents, Vol. IX Periodic Data (electronic version). Lyon, IARC.http://ci5.iarc.fr
Costa Rica Cancer Registry 2000 - CI5 as it appears in Ferlay J, Parkin DM, Curado MP, Bray F, Edwards B, Shin HR and Forman D. Cancer Incidence in Five Continents, Volumes I to IX: IARC CancerBase No. 9 [Internet]. Lyon, France: International Agency for Research on Cancer; 2010. Available from: http://ci5.iarc.fr
Costa Rica Cancer Registry 2001 - CI5 as it appears in Ferlay J, Parkin DM, Curado MP, Bray F, Edwards B, Shin HR and Forman D. Cancer Incidence in Five Continents, Volumes I to IX: IARC CancerBase No. 9 [Internet]. Lyon, France: International Agency for Research on Cancer; 2010. Available from: http://ci5.iarc.fr
Costa Rica Cancer Registry 2002 - CI5 as it appears in Ferlay J, Parkin DM, Curado MP, Bray F, Edwards B, Shin HR and Forman D. Cancer Incidence in Five Continents, Volumes I to IX: IARC CancerBase No. 9 [Internet]. Lyon, France: International Agency for Research on Cancer; 2010. Available from: http://ci5.iarc.fr
Costa Rica Cancer Registry 2003-2007 - CI5 as it appears in Forman D, Bray F, Brewster DH, Gombe Mbalawa C, Kohler B, Piñeros M, Steliarova-Foucher E, Swaminathan R and Ferlay J, eds (2013). Cancer Incidence in Five Continents, Vol. X Summary Database (electronic version). Lyon, IARC. http://ci5.iarc.fr
Costa Rica Change in Stunting in High Prevalence Cantons as it appears in World Health Organization (WHO). WHO Global Database on Child Growth and Malnutrition - Historical. Geneva, Switzerland: World Health Organization (WHO).
Costa Rica National Height Census of Schoolchildren in First Grade 1989 as it appears in World Health Organization (WHO). WHO Global Database on Child Growth and Malnutrition - Historical. Geneva, Switzerland: World Health Organization (WHO).
Costa Rica National Nutrition Survey 1996 as it appears in World Health Organization (WHO). WHO Global Database on Child Growth and Malnutrition. Geneva, Switzerland: World Health Organization (WHO).

Appendix: Citation List

Citation
Costa Rica National Nutrition Survey 2008-2009 as it appears in World Health Organization (WHO). WHO Global Database on Child Growth and Malnutrition. Geneva, Switzerland: World Health Organization (WHO).
Costa Rica National Population and Housing Census 1973 as it appears in World Health Organization (WHO). WHO Household Energy Database. Geneva, Switzerland: World Health Organization (WHO), 2010.
Costa Rica National Population and Housing Census 1984 as it appears in World Health Organization (WHO). WHO Household Energy Database. Geneva, Switzerland: World Health Organization (WHO), 2010.
Costa Rica Permanent Household Survey 2000 as it appears in World Health Organization (WHO). WHO Household Energy Database. Geneva, Switzerland: World Health Organization (WHO), 2010.
Costa Rica Social Security Institute and Division of Reproductive Health-Centers for Disease Control and Prevention (CDC). Costa Rica 1993 Reproductive Health Survey. Atlanta, United States: Centers for Disease Control and Prevention (CDC).
Costa Rica Social Security Institute. Costa Rica Young Adult Reproductive Health Survey 1990-1991. San Jose, Costa Rica. 1991.
Costa Rica Vital Registration - Deaths 1956 ICD7 as it appears in World Health Organization (WHO). WHO Mortality Database Version July 2012. Geneva, Switzerland: World Health Organization (WHO), 2012.
Costa Rica Vital Registration - Deaths 1957 ICD7 as it appears in World Health Organization (WHO). WHO Mortality Database Version July 2012. Geneva, Switzerland: World Health Organization (WHO), 2012.
Costa Rica Vital Registration - Deaths 1958 ICD7 as it appears in World Health Organization (WHO). WHO Mortality Database Version July 2012. Geneva, Switzerland: World Health Organization (WHO), 2012.
Costa Rica Vital Registration - Deaths 1959 ICD7 as it appears in World Health Organization (WHO). WHO Mortality Database Version July 2012. Geneva, Switzerland: World Health Organization (WHO), 2012.
Costa Rica Vital Registration - Deaths 1960 ICD7 as it appears in World Health Organization (WHO). WHO Mortality Database Version July 2012. Geneva, Switzerland: World Health Organization (WHO), 2012.
Costa Rica Vital Registration - Deaths 1961 ICD7 as it appears in World Health Organization (WHO). WHO Mortality Database Version July 2012. Geneva, Switzerland: World Health Organization (WHO), 2012.
Costa Rica Vital Registration - Deaths 1962 ICD7 as it appears in World Health Organization (WHO). WHO Mortality Database Version July 2012. Geneva, Switzerland: World Health Organization (WHO), 2012.
Costa Rica Vital Registration - Deaths 1963 ICD7 as it appears in World Health Organization (WHO). WHO Mortality Database Version July 2012. Geneva, Switzerland: World Health Organization (WHO), 2012.
Costa Rica Vital Registration - Deaths 1964 ICD7 as it appears in World Health Organization (WHO). WHO Mortality Database Version July 2012. Geneva, Switzerland: World Health Organization (WHO), 2012.
Costa Rica Vital Registration - Deaths 1965 ICD7 as it appears in World Health Organization (WHO). WHO Mortality Database Version July 2012. Geneva, Switzerland: World Health Organization (WHO), 2012.
Costa Rica Vital Registration - Deaths 1966 ICD7 as it appears in World Health Organization (WHO). WHO Mortality Database Version July 2012. Geneva, Switzerland: World Health Organization (WHO), 2012.
Costa Rica Vital Registration - Deaths 1967 ICD7 as it appears in World Health Organization (WHO). WHO Mortality Database Version July 2012. Geneva, Switzerland: World Health Organization (WHO), 2012.
Costa Rica Vital Registration - Deaths 1968 ICD8 as it appears in World Health Organization (WHO). WHO Mortality Database Version May 2013. Geneva, Switzerland: World Health Organization (WHO), 2013.
Costa Rica Vital Registration - Deaths 1969 ICD8 as it appears in World Health Organization (WHO). WHO Mortality Database Version May 2013. Geneva, Switzerland: World Health Organization (WHO), 2013.
Costa Rica Vital Registration - Deaths 1970 ICD8 as it appears in World Health Organization (WHO). WHO Mortality Database Version May 2013. Geneva, Switzerland: World Health Organization (WHO), 2013.
Costa Rica Vital Registration - Deaths 1971 ICD8 as it appears in World Health Organization (WHO). WHO Mortality Database Version May 2013. Geneva, Switzerland: World Health Organization (WHO), 2013.
Costa Rica Vital Registration - Deaths 1972 ICD8 as it appears in World Health Organization (WHO). WHO Mortality Database Version May 2013. Geneva, Switzerland: World Health Organization (WHO), 2013.
Costa Rica Vital Registration - Deaths 1973 ICD8 as it appears in World Health Organization (WHO). WHO Mortality Database Version May 2013. Geneva, Switzerland: World Health Organization (WHO), 2013.
Costa Rica Vital Registration - Deaths 1974 ICD8 as it appears in World Health Organization (WHO). WHO Mortality Database Version May 2013. Geneva, Switzerland: World Health Organization (WHO), 2013.
Costa Rica Vital Registration - Deaths 1975 ICD8 as it appears in World Health Organization (WHO). WHO Mortality Database Version May 2013. Geneva, Switzerland: World Health Organization (WHO), 2013.
Costa Rica Vital Registration - Deaths 1976 ICD8 as it appears in World Health Organization (WHO). WHO Mortality Database Version May 2013. Geneva, Switzerland: World Health Organization (WHO), 2013.
Costa Rica Vital Registration - Deaths 1977 ICD8 as it appears in World Health Organization (WHO). WHO Mortality Database Version May 2013. Geneva, Switzerland: World Health Organization (WHO), 2013.
Costa Rica Vital Registration - Deaths 1978 ICD8 as it appears in World Health Organization (WHO). WHO Mortality Database Version May 2013. Geneva, Switzerland: World Health Organization (WHO), 2013.
Costa Rica Vital Registration - Deaths 1979 ICD8 as it appears in World Health Organization (WHO). WHO Mortality Database Version May 2013. Geneva, Switzerland: World Health Organization (WHO), 2013.
Costa Rica Vital Registration - Deaths 1980 ICD9 as it appears in World Health Organization (WHO). WHO Mortality Database Version November 2015. Geneva, Switzerland: World Health Organization (WHO), 2015.
Costa Rica Vital Registration - Deaths 1981 ICD9 as it appears in World Health Organization (WHO). WHO Mortality Database Version November 2015. Geneva, Switzerland: World Health Organization (WHO), 2015.

Appendix: Citation List

Citation
Costa Rica Vital Registration - Deaths 1982 ICD9 as it appears in World Health Organization (WHO). WHO Mortality Database Version November 2015. Geneva, Switzerland: World Health Organization (WHO), 2015.
Costa Rica Vital Registration - Deaths 1983 ICD9 as it appears in World Health Organization (WHO). WHO Mortality Database Version November 2015. Geneva, Switzerland: World Health Organization (WHO), 2015.
Costa Rica Vital Registration - Deaths 1984 ICD9 as it appears in World Health Organization (WHO). WHO Mortality Database Version November 2015. Geneva, Switzerland: World Health Organization (WHO), 2015.
Costa Rica Vital Registration - Deaths 1985 ICD9 as it appears in World Health Organization (WHO). WHO Mortality Database Version November 2015. Geneva, Switzerland: World Health Organization (WHO), 2015.
Costa Rica Vital Registration - Deaths 1986 ICD9 as it appears in World Health Organization (WHO). WHO Mortality Database Version November 2015. Geneva, Switzerland: World Health Organization (WHO), 2015.
Costa Rica Vital Registration - Deaths 1987 ICD9 as it appears in World Health Organization (WHO). WHO Mortality Database Version November 2015. Geneva, Switzerland: World Health Organization (WHO), 2015.
Costa Rica Vital Registration - Deaths 1988 ICD9 as it appears in World Health Organization (WHO). WHO Mortality Database Version November 2015. Geneva, Switzerland: World Health Organization (WHO), 2015.
Costa Rica Vital Registration - Deaths 1989 ICD9 as it appears in World Health Organization (WHO). WHO Mortality Database Version November 2015. Geneva, Switzerland: World Health Organization (WHO), 2015.
Costa Rica Vital Registration - Deaths 1990 ICD9 as it appears in World Health Organization (WHO). WHO Mortality Database Version November 2015. Geneva, Switzerland: World Health Organization (WHO), 2015.
Costa Rica Vital Registration - Deaths 1991 ICD9 as it appears in World Health Organization (WHO). WHO Mortality Database Version February 2014. Geneva, Switzerland: World Health Organization (WHO), 2014.
Costa Rica Vital Registration - Deaths 1992 ICD9 as it appears in World Health Organization (WHO). WHO Mortality Database Version February 2014. Geneva, Switzerland: World Health Organization (WHO), 2014.
Costa Rica Vital Registration - Deaths 1993 ICD9 as it appears in World Health Organization (WHO). WHO Mortality Database Version February 2014. Geneva, Switzerland: World Health Organization (WHO), 2014.
Costa Rica Vital Registration - Deaths 1994 ICD9 as it appears in World Health Organization (WHO). WHO Mortality Database Version February 2014. Geneva, Switzerland: World Health Organization (WHO), 2014.
Costa Rica Vital Registration - Deaths 1995 ICD9 as it appears in World Health Organization (WHO). WHO Mortality Database Version February 2014. Geneva, Switzerland: World Health Organization (WHO), 2014.
Costa Rica Vital Registration - Deaths 1996 ICD9 as it appears in World Health Organization (WHO). WHO Mortality Database Version February 2014. Geneva, Switzerland: World Health Organization (WHO), 2014.
Costa Rica Vital Registration - Deaths 1997 ICD10 as it appears in World Health Organization (WHO). WHO Mortality Database Version November 2015. Geneva, Switzerland: World Health Organization (WHO), 2015.
Costa Rica Vital Registration - Deaths 1998 ICD10 as it appears in World Health Organization (WHO). WHO Mortality Database Version November 2015. Geneva, Switzerland: World Health Organization (WHO), 2015.
Costa Rica Vital Registration - Deaths 1999 ICD10 as it appears in World Health Organization (WHO). WHO Mortality Database Version November 2015. Geneva, Switzerland: World Health Organization (WHO), 2015.
Costa Rica Vital Registration - Deaths 2000 ICD10 as it appears in World Health Organization (WHO). WHO Mortality Database Version November 2015. Geneva, Switzerland: World Health Organization (WHO), 2015.
Costa Rica Vital Registration - Deaths 2001 ICD10 as it appears in World Health Organization (WHO). WHO Mortality Database Version November 2015. Geneva, Switzerland: World Health Organization (WHO), 2015.
Costa Rica Vital Registration - Deaths 2002 ICD10 as it appears in World Health Organization (WHO). WHO Mortality Database Version November 2015. Geneva, Switzerland: World Health Organization (WHO), 2015.
Costa Rica Vital Registration - Deaths 2003 ICD10 as it appears in World Health Organization (WHO). WHO Mortality Database Version November 2015. Geneva, Switzerland: World Health Organization (WHO), 2015.
Costa Rica Vital Registration - Deaths 2004 ICD10 as it appears in World Health Organization (WHO). WHO Mortality Database Version November 2015. Geneva, Switzerland: World Health Organization (WHO), 2015.
Costa Rica Vital Registration - Deaths 2005 ICD10 as it appears in World Health Organization (WHO). WHO Mortality Database Version November 2015. Geneva, Switzerland: World Health Organization (WHO), 2015.
Costa Rica Vital Registration - Deaths 2006 ICD10 as it appears in World Health Organization (WHO). WHO Mortality Database Version November 2015. Geneva, Switzerland: World Health Organization (WHO), 2015.
Costa Rica Vital Registration - Deaths 2007 ICD10 as it appears in World Health Organization (WHO). WHO Mortality Database Version November 2015. Geneva, Switzerland: World Health Organization (WHO), 2015.
Costa Rica Vital Registration - Deaths 2008 ICD10 as it appears in World Health Organization (WHO). WHO Mortality Database Version November 2015. Geneva, Switzerland: World Health Organization (WHO), 2015.
Costa Rica Vital Registration - Deaths 2009 ICD10 as it appears in World Health Organization (WHO). WHO Mortality Database Version November 2015. Geneva, Switzerland: World Health Organization (WHO), 2015.
Costa Rica Vital Registration - Deaths 2010 ICD10 as it appears in World Health Organization (WHO). WHO Mortality Database Version November 2015. Geneva, Switzerland: World Health Organization (WHO), 2015.
Costa Rica Vital Registration - Deaths 2011 ICD10 as it appears in World Health Organization (WHO). WHO Mortality Database Version November 2015. Geneva, Switzerland: World Health Organization (WHO), 2015.
Costa Rica Vital Registration - Deaths 2012 ICD10 as it appears in World Health Organization (WHO). WHO Mortality Database Version November 2015. Geneva, Switzerland: World Health Organization (WHO), 2015.
Costa Rica Vital Registration - Deaths 2013 ICD10 as it appears in World Health Organization (WHO). WHO Mortality Database Version November 2015. Geneva, Switzerland: World Health Organization (WHO), 2015.

Appendix: Citation List

Citation
Costa Rica Vital Registration Death Data 1950 as it appears in United Nations Statistics Division (UNSD). United Nations Demographic Yearbook - Historical Supplement 1997. New York City, United States: United Nations Statistics Division (UNSD).
Costa Rica Vital Registration Death Data 1952 as it appears in United Nations Statistics Division (UNSD). United Nations Demographic Yearbook - Historical Supplement 1997. New York City, United States: United Nations Statistics Division (UNSD).
Costa Rica Vital Registration Death Data 1953 as it appears in United Nations Statistics Division (UNSD). United Nations Demographic Yearbook - Historical Supplement 1997. New York City, United States: United Nations Statistics Division (UNSD).
Costa Rica Vital Registration Death Data 1954 as it appears in United Nations Statistics Division (UNSD). United Nations Demographic Yearbook - Historical Supplement 1997. New York City, United States: United Nations Statistics Division (UNSD).
Costa Rica Vital Registration Death Data 1955 as it appears in United Nations Statistics Division (UNSD). United Nations Demographic Yearbook - Historical Supplement 1997. New York City, United States: United Nations Statistics Division (UNSD).
Costa Rican Demographic Association, Ministry of Health (Costa Rica), United Nations Children's Fund (UNICEF). Costa Rica Multiple Indicator Cluster Survey 2011. New York, United States: United Nations Children's Fund (UNICEF), 2013.
Costa Rican Demographic Association, Westinghouse Health Systems, Inc.. Costa Rica Contraceptive Prevalence Survey 1981.
Costa Rican Demographic Association. Costa Rica Family Planning/Maternal and Child Health Survey 1986. San Jose, Costa Rica: Costa Rican Demographic Association, 1987.
Costantini AS, Benvenuti A, Vineis P, Kriebel D, Tumino R, Ramazzotti V, Rodella S, Stagnaro E, Crosignani P, Amadori D, Mirabelli D, Sommani L, Belletti I, Troschel L, Romeo L, Miceli G, Tozzi GA, Mendico I, Maltoni SA, Miligi L. Risk of leukemia and multiple myeloma associated with exposure to benzene and other organic solvents: evidence from the Italian Multicenter Case-control study. Am J Ind Med. 2008; 51(11): 803-11 as it appears in Khalade A, Jaakkola MS, Pukkala E, Jaakkola JJK. Exposure to benzene at work and the risk of leukemia: a systematic review and meta-analysis. Environ Health 2010; 9: 31.
Costantino JP, Redmond CK, Bearden A. Occupationally Related Cancer Risk Among Coke Oven Workers: 30 Years Of Follow-Up. J Occup Environ Med. 1995; 37(5): 597-604 as it appears in Armstrong B, Hutchinson E, Unwin J, Fletcher T. Lung cancer risk after exposure to polycyclic aromatic hydrocarbons: a review and meta-analysis. Environ. Health Perspect. 2004; 112(9): 970–978.
Costello J, Castellan RM, Swecker GS, Kullman GJ. Mortality Of A Cohort Of U.S. Workers Employed In The Crushed Stone Industry, 1940-1980. Am J Ind Med. 1980; 27(5): 625-40 as it appears in Kurihara N, Wada O. Silicosis and smoking strongly increase lung cancer risk in silica-exposed workers. Ind Health 2004; 42(3): 303–314.
Costello J, Graham WGB. Vermont Granite Workers' Mortality Study. Am J Ind Med. 1988; 13(4): 483-97 as it appears in Kurihara N, Wada O. Silicosis and smoking strongly increase lung cancer risk in silica-exposed workers. Ind Health 2004; 42(3): 303–314.
Cot M, Brutus L, Le Goff G, Rajaonarivelo V, Raveloson A. [The campaign against malaria in central western Madagascar: comparison of lambda-cyhalothrin and DDT house spraying. Parasite. 2001; 8(4): 309-16. As it appears in Malaria Atlas Project. Malaria Atlas Project Plasmodium Falciparum Parasite Rate Database. Oxford, United Kingdom: Malaria Atlas Project.
Côte d'Ivoire External EPI Review 2001.
Côte d'Ivoire External EPI Review 2010.
Côte d'Ivoire Immunization Coverage Survey 1987.
Côte d'Ivoire Immunization Coverage Survey Feburary 1991.
Côte d'Ivoire Vaccination Coverage Survey 2013.
Cote d'Ivoire - Nutritional Survey of a Population of Liberian Refugees in the Prefecture of Tabou 1994 as it appears in World Health Organization (WHO). WHO Global Database on Child Growth and Malnutrition - Historical. Geneva, Switzerland: World Health Organization (WHO).
Coulibaly YI, Dao S, Traore AK, Diallo A, Sacko M, Traoré SF. [Presence and risk of transmission of Wuchereria bancrofti is a reality in rural Mali: the case of the town of Bariambani in the Cirle of Kati]. Mali Med. 2006; 21(1): 12-7. As it appears in London School of Hygiene and Tropical Medicine. Global Atlas of Helminth Infections - Lymphatic Filiariasis.
Coulibaly YI, Dembele B, Diallo AA, Konaté S, Dolo H, Coulibaly SY, Doumbia SS, Soumaoro L, Coulibaly ME, Bockarie MJ, Molyneux D, Nutman TB, Klion AD, Toure YT, Traore SF. The Impact of Six Annual Rounds of Mass Drug Administration on Wuchereria bancrofti Infections in Humans and in Mosquitoes in Mali. Am J Trop Med Hyg. 2015; 93.0(2): 356-60.
Coulibaly YI, Dembele B, Diallo AA, Kristensen S, Konate S, Dolo H, Dicko I, Sangare MB, Keita F, Boatin BA, Traore AK, Nutman TB, Klion AD, Touré YT, Traore SF. Wuchereria bancrofti transmission pattern in southern Mali prior to and following the institution of mass drug administration. Parasit Vectors. 2013; 6.0(1): 247.
Council of Census Coordinator (Mozambique). Mozambique Population and Housing Census 1980.
Council of Health Ministers of GCC States, Ministry of Health (Kuwait), United Nations Statistics Division (UNSD). Kuwait Family Health Survey 1996.
Council of Health Ministers of GCC States, Ministry of Health (Kuwait), World Health Organization (WHO). Kuwait World Health Survey 2008-2010.
Council of Health Ministers of GCC States, Ministry of Health (Kuwait). Kuwait Child Health Survey 1987.
Council of Health Ministers of GCC States, Ministry of Health (Oman). Oman Family Health Survey 1995.
Council of Health Ministers of GCC States, Ministry of Health (United Arab Emirates). United Arab Emirates Family Health Survey 1995.
Country Indicators for Foreign Policy (CIFP). Mali: International Courses in French Speaking Country. Ottawa, Canada: Country Indicators for Foreign Policy (CIFP), 1998. As it appears in Malaria Atlas Project. Malaria Atlas Project Plasmodium Falciparum Parasite Rate Database. Oxford, United Kingdom: Malaria Atlas Project.
Country Mortality Data 1980-1999 as it appears in United Nations Statistics Division (UNSD). United Nations Demographic Yearbook 2004. New York City, United States: United Nations Statistics Division (UNSD), 2007.
Countrywide Integrated Noncommunicable Diseases Intervention Programme (CINDI), Kyrgyzstan Program of Ministry of Health and National Statistic Center. Kyrgyzstan CINDI Highlights 2002.

Appendix: Citation List

Citation
Coura JR, Naranjo MA, Willcox HP. Chagas' disease in the Brazilian Amazon. II. A serological survey. Rev Inst Med Trop Sao Paulo. 1995; 37(2): 103–7.
Coutinho CF de S, Souza-Santos R, Teixeira NFD, Georg I, Gomes TF, Boia MN, dos Reis NB, Maia A de O, Lima MM. An entomoepidemiological investigation of Chagas disease in the state of Ceará, Northeast Region of Brazil. Cad Saude Publica. 2014; 30(4): 785–93.
Coutinho E, Barbosa FS, Barbosa JM, Pessoa P, Pinto RF, Oliveira PA, Rodrigues BA. Inquérito clínico-nutricional e antropométrico preliminar, em áreas endêmicas de esquistossomose mansônica, no Nordeste do Brasil. Rev Soc Bras Med Trop. 1972; 6: 211-36.
Coutinho EM, Abath FG, Barbosa CS, Domingues AL, Melo MC, Montenegro SM, Lucena MA, Romani SA, Souza WV, Coutinho AD. Factors involved in Schistosoma mansoni infection in rural areas of northeast Brazil. Mem Inst Oswaldo Cruz. 1997; 92(5): 707-15.
Cox J. Remote Sensing as a Tool for Malaria Stratification in Cambodia: A Feasibility Study in Ratanakiri. London, United Kingdom: London School of Hygiene && Tropical Medicine, 2005. As it appears in Malaria Atlas Project. Malaria Atlas Project Plasmodium Falciparum Parasite Rate Database. Oxford, United Kingdom: Malaria Atlas Project.
Cox MJ, Kum DE, Tavul L, Narara A, Raiko A, Baisor M, Alpers MP, Medley GF, Day KP. Dynamics of malaria parasitaemia associated with febrile illness in children from a rural area of Madang, Papua New Guinea. Trans R Soc Trop Med Hyg. 1994; 88(2): 191-7. As it appears in Malaria Atlas Project. Malaria Atlas Project Plasmodium Falciparum Parasite Rate Database. Oxford, United Kingdom: Malaria Atlas Project.
Craig ME, Femia G, Broyda V, Lloyd M, Howard NJ. Type 2 diabetes in Indigenous and non-Indigenous children and adolescents in New South Wales. Med J Aust. 2007; 186(10): 497-9.
Craig ME, Howard NJ, Silink M, Chan A. The rising incidence of childhood type 1 diabetes in New South Wales, Australia. J Pediatr Endocrinol Metab. 2000; 13(4): 363-72.
Crawford F, McCowan C, Dimitrov BD, Woodburn J, Wylie GH, Booth E, Leese GP, Bekker HL, Kleijnen J, Fahey T. The risk of foot ulceration in people with diabetes screened in community settings: findings from a cohort study. QJM. 2011; 104(5): 403-10.
Crestini MM. Malaria Control Trial Using Lambda-cyhalothrin Treated Nets in Yanomami Communities in Amazonas State, Venezuela [dissertation]. London, United Kingdom: London School of Hygiene and Tropical Medicine, 2004. As it appears in Malaria Atlas Project. Malaria Atlas Project Plasmodium Falciparum Parasite Rate Database. Oxford, United Kingdom: Malaria Atlas Project.
Crestini MM. Venezuela Plasmodium Falciparum Parasite Rate Data, Personal Communication with M. Magris-Crestini 2006. As it appears in Malaria Atlas Project. Malaria Atlas Project Plasmodium Falciparum Parasite Rate Database. Oxford, United Kingdom: Malaria Atlas Project.
Croatia - Growth Monitoring of Preschool Children: An Overall Report 1993-1996 as it appears in World Health Organization (WHO). WHO Global Database on Child Growth and Malnutrition. Geneva, Switzerland: World Health Organization (WHO).
Croatia Cancer Registry 1988-1991 - CI5 as it appears in Parkin DM, International Agency for Research on Cancer, International Association of Cancer Registries. Cancer Incidence in Five Continents. Vol. I to VIII. Lyon, France, IARCPress, 2005.
Croatia Cancer Registry 1993-1997 - CI5 as it appears in Parkin DM, International Agency for Research on Cancer, International Association of Cancer Registries. Cancer Incidence in Five Continents. Vol. I to VIII. Lyon, France, IARCPress, 2005.
Croatia Cancer Registry 1998-2002 - CI5 as it appears in Curado MP, Edwards B, Shin HR, Storm H, Ferlay J, Heanue M and Boyle P, eds (2007). Cancer Incidence in Five Continents, Vol. IX Periodic Data (electronic version). Lyon, IARC.http://ci5.iarc.fr
Croatia Cancer Registry 2003-2007 - CI5 as it appears in Forman D, Bray F, Brewster DH, Gombe Mbalawa C, Kohler B, Piñeros M, Steliarova-Foucher E, Swaminathan R and Ferlay J, eds (2013). Cancer Incidence in Five Continents, Vol. X Summary Database (electronic version). Lyon, IARC. http://ci5.iarc.fr
Croatia Vital Registration - Deaths 1991 ICD9 as it appears in World Health Organization (WHO). WHO Mortality Database Version July 2012. Geneva, Switzerland: World Health Organization (WHO), 2012.
Croatia Vital Registration - Deaths 1992 ICD9 as it appears in World Health Organization (WHO). WHO Mortality Database Version July 2012. Geneva, Switzerland: World Health Organization (WHO), 2012.
Croatia Vital Registration - Deaths 1993 ICD9 as it appears in World Health Organization (WHO). WHO Mortality Database Version July 2012. Geneva, Switzerland: World Health Organization (WHO), 2012.
Croatia Vital Registration - Deaths 1994 ICD9 as it appears in World Health Organization (WHO). WHO Mortality Database Version July 2012. Geneva, Switzerland: World Health Organization (WHO), 2012.
Croatia Vital Registration - Deaths 1995 ICD10 as it appears in World Health Organization (WHO). WHO Mortality Database Version November 2015. Geneva, Switzerland: World Health Organization (WHO), 2015.
Croatia Vital Registration - Deaths 1996 ICD10 as it appears in World Health Organization (WHO). WHO Mortality Database Version November 2015. Geneva, Switzerland: World Health Organization (WHO), 2015.
Croatia Vital Registration - Deaths 1997 ICD10 as it appears in World Health Organization (WHO). WHO Mortality Database Version November 2015. Geneva, Switzerland: World Health Organization (WHO), 2015.
Croatia Vital Registration - Deaths 1998 ICD10 as it appears in World Health Organization (WHO). WHO Mortality Database Version November 2015. Geneva, Switzerland: World Health Organization (WHO), 2015.
Croatia Vital Registration - Deaths 1999 ICD10 as it appears in World Health Organization (WHO). WHO Mortality Database Version November 2015. Geneva, Switzerland: World Health Organization (WHO), 2015.
Croatia Vital Registration - Deaths 2000 ICD10 as it appears in World Health Organization (WHO). WHO Mortality Database Version November 2015. Geneva, Switzerland: World Health Organization (WHO), 2015.
Croatia Vital Registration - Deaths 2001 ICD10 as it appears in World Health Organization (WHO). WHO Mortality Database Version November 2015. Geneva, Switzerland: World Health Organization (WHO), 2015.
Croatia Vital Registration - Deaths 2002 ICD10 as it appears in World Health Organization (WHO). WHO Mortality Database Version November 2015. Geneva, Switzerland: World Health Organization (WHO), 2015.
Croatia Vital Registration - Deaths 2003 ICD10 as it appears in World Health Organization (WHO). WHO Mortality Database Version November 2015. Geneva, Switzerland: World Health Organization (WHO), 2015.

Appendix: Citation List

Citation
Croatia Vital Registration - Deaths 2004 ICD10 as it appears in World Health Organization (WHO). WHO Mortality Database Version November 2015. Geneva, Switzerland: World Health Organization (WHO), 2015.
Croatia Vital Registration - Deaths 2005 ICD10 as it appears in World Health Organization (WHO). WHO Mortality Database Version November 2015. Geneva, Switzerland: World Health Organization (WHO), 2015.
Croatia Vital Registration - Deaths 2006 ICD10 as it appears in World Health Organization (WHO). WHO Mortality Database Version November 2015. Geneva, Switzerland: World Health Organization (WHO), 2015.
Croatia Vital Registration - Deaths 2007 ICD10 as it appears in World Health Organization (WHO). WHO Mortality Database Version November 2015. Geneva, Switzerland: World Health Organization (WHO), 2015.
Croatia Vital Registration - Deaths 2008 ICD10 as it appears in World Health Organization (WHO). WHO Mortality Database Version November 2015. Geneva, Switzerland: World Health Organization (WHO), 2015.
Croatia Vital Registration - Deaths 2009 ICD10 as it appears in World Health Organization (WHO). WHO Mortality Database Version November 2015. Geneva, Switzerland: World Health Organization (WHO), 2015.
Croatia Vital Registration - Deaths 2010 ICD10 as it appears in World Health Organization (WHO). WHO Mortality Database Version November 2015. Geneva, Switzerland: World Health Organization (WHO), 2015.
Croatia Vital Registration - Deaths 2011 ICD10 as it appears in World Health Organization (WHO). WHO Mortality Database Version November 2015. Geneva, Switzerland: World Health Organization (WHO), 2015.
Croatia Vital Registration - Deaths 2012 ICD10 as it appears in World Health Organization (WHO). WHO Mortality Database Version November 2015. Geneva, Switzerland: World Health Organization (WHO), 2015.
Croatia Vital Registration - Deaths 2013 ICD10 as it appears in World Health Organization (WHO). WHO Mortality Database Version November 2015. Geneva, Switzerland: World Health Organization (WHO), 2015.
Croatian Bureau of Statistics. Croatia Statistical Yearbook 2013. Zagreb, Croatia: Croatian Bureau of Statistics, 2013.
Croatian National Cancer Registry, Croatian National Institute of Public Health. Croatia - Cancer Incidence in Croatia 2009.
Croatian National Cancer Registry, Croatian National Institute of Public Health. Croatia - Cancer Incidence in Croatia 2010. Zagreb, Croatia: Croatian National Institute of Public Health.
Croatian National Cancer Registry, European Network of Cancer Registries. Croatia EUREG Cancer Incidence Tables, Rates by Age.
Croatian National Institute of Public Health. Croatia Health Service Yearbook 2014. Zagreb, Croatia: Croatian National Institute of Public Health, 2015.
Crompton P, Ventura AM, de Souza JM, Santos E, Strickland GT, Silbergeld E. Assessment of Mercury Exposure and Malaria in a Brazilian Amazon Riverine Community. Environ Res. 2002; 90(2): 69-75. As it appears in Malaria Atlas Project. Malaria Atlas Project Plasmodium Falciparum Parasite Rate Database. Oxford, United Kingdom: Malaria Atlas Project.
Crompton PD, Traore B, Kayentao K, Doumbo S, Ongoiba A, Diakite SAS, Krause MA, Doumtabe D, Kone Y, Weiss G, Huang C-Y, Doumbia S, Guindo A, Fairhurst RM, Miller LH, Pierce SK, Doumbo OK. Sickle Cell Trait is Associated with a Delayed Onset of Malaria: Implications for Time-to-Event Analysis in Clinical Studies of Malaria. J Infect Dis. 2008; 198(9): 1265-75. As it appears in Malaria Atlas Project. Malaria Atlas Project Plasmodium Falciparum Parasite Rate Database. Oxford, United Kingdom: Malaria Atlas Project.
Crook SE, Baptista A. The effect of permethrin-impregnated wall-curtains on malaria transmission and morbidity in the suburbs of Maputo, Mozambique. Trop Geogr Med. 1995; 47(2): 64-7. As it appears in Malaria Atlas Project. Malaria Atlas Project Plasmodium Falciparum Parasite Rate Database. Oxford, United Kingdom: Malaria Atlas Project.
Cross J, Basaca-Sevilla V. Intestinal Parasitic Infections in Southeast Asia. Southeast Asian J Trop Med Public Health. 1981; 12(2): 262-74.
Cross JH, Basaca-Sevilla V, United States Naval Medical Research Unit No. 2. Biomedical Surveys in the Philippines. Manila, Philippines: United States Naval Medical Research Unit No. 2, 1984.
Croxson SC, Burden AC, Bodington M, Botha JL. The prevalence of diabetes in elderly people. Diabet Med. 1991; 8(1): 28-31.
Cuba - Villa Clara Cancer Registry 1995-1997 - CI5 as it appears in Parkin DM, International Agency for Research on Cancer, International Association of Cancer Registries. Cancer Incidence in Five Continents. Vol. I to VIII. Lyon, France, IARCPress, 2005.
Cuba - Villa Clara Cancer Registry 2004-2007 - CI5 as it appears in Forman D, Bray F, Brewster DH, Gombe Mbalawa C, Kohler B, Piñeros M, Steliarova-Foucher E, Swaminathan R and Ferlay J, eds (2013). Cancer Incidence in Five Continents, Vol. X Summary Database (electronic version). Lyon, IARC. http://ci5.iarc.fr
Cuba Cancer Registry 1986 - CI5 as it appears in Parkin DM, International Agency for Research on Cancer, International Association of Cancer Registries. Cancer Incidence in Five Continents. Vol. I to VIII. Lyon, France, IARCPress, 2005.
Cuba Cancer Registry 1986-1987 - CI5 as it appears in Parkin DM, International Agency for Research on Cancer, International Association of Cancer Registries. Cancer Incidence in Five Continents. Vol. I to VIII. Lyon, France, IARCPress, 2005.
Cuba Census of Population and Dwellings 2002 as it appears in World Health Organization (WHO). WHO Household Energy Database. Geneva, Switzerland: World Health Organization (WHO), 2010.
Cuba Evaluation Figures for the Millennium Development Goals, Update 2006-2008 as it appears in World Health Organization (WHO). WHO Household Energy Database. Geneva, Switzerland: World Health Organization (WHO), 2010.
Cuba National Survey of the Nutrition and Food Hygiene Institute 2005 as it appears in United Nations Children's Fund (UNICEF). UNICEF Childinfo - Nutritional Status. New York, United States: United Nations Children's Fund (UNICEF).
Cuba Nutrition Situation of the Country 1988 as it appears in World Health Organization (WHO). WHO Global Database on Child Growth and Malnutrition - Historical. Geneva, Switzerland: World Health Organization (WHO).
Cuba Population and Housing Census 1981 as it appears in United Nations Statistics Division (UNSD). United Nations Demographic Yearbook - Historical Supplement 1997. New York City, United States: United Nations Statistics Division (UNSD).
Cuba Vital Registration - Deaths 1959 ICD7 as it appears in World Health Organization (WHO). WHO Mortality Database Version July 2012. Geneva, Switzerland: World Health Organization (WHO), 2012.
Cuba Vital Registration - Deaths 1964 ICD7 as it appears in World Health Organization (WHO). WHO Mortality Database Version July 2012. Geneva, Switzerland: World Health Organization (WHO), 2012.

Appendix: Citation List

Citation
Cuba Vital Registration - Deaths 1965 ICD7 as it appears in World Health Organization (WHO). WHO Mortality Database Version July 2012. Geneva, Switzerland: World Health Organization (WHO), 2012.
Cuba Vital Registration - Deaths 1968 ICD8 as it appears in World Health Organization (WHO). WHO Mortality Database Version May 2013. Geneva, Switzerland: World Health Organization (WHO), 2013.
Cuba Vital Registration - Deaths 1969 ICD8 as it appears in World Health Organization (WHO). WHO Mortality Database Version May 2013. Geneva, Switzerland: World Health Organization (WHO), 2013.
Cuba Vital Registration - Deaths 1970 ICD8 as it appears in World Health Organization (WHO). WHO Mortality Database Version May 2013. Geneva, Switzerland: World Health Organization (WHO), 2013.
Cuba Vital Registration - Deaths 1971 ICD8 as it appears in World Health Organization (WHO). WHO Mortality Database Version May 2013. Geneva, Switzerland: World Health Organization (WHO), 2013.
Cuba Vital Registration - Deaths 1972 ICD8 as it appears in World Health Organization (WHO). WHO Mortality Database Version May 2013. Geneva, Switzerland: World Health Organization (WHO), 2013.
Cuba Vital Registration - Deaths 1973 ICD8 as it appears in World Health Organization (WHO). WHO Mortality Database Version May 2013. Geneva, Switzerland: World Health Organization (WHO), 2013.
Cuba Vital Registration - Deaths 1974 ICD8 as it appears in World Health Organization (WHO). WHO Mortality Database Version May 2013. Geneva, Switzerland: World Health Organization (WHO), 2013.
Cuba Vital Registration - Deaths 1975 ICD8 as it appears in World Health Organization (WHO). WHO Mortality Database Version May 2013. Geneva, Switzerland: World Health Organization (WHO), 2013.
Cuba Vital Registration - Deaths 1976 ICD8 as it appears in World Health Organization (WHO). WHO Mortality Database Version May 2013. Geneva, Switzerland: World Health Organization (WHO), 2013.
Cuba Vital Registration - Deaths 1977 ICD8 as it appears in World Health Organization (WHO). WHO Mortality Database Version May 2013. Geneva, Switzerland: World Health Organization (WHO), 2013.
Cuba Vital Registration - Deaths 1978 ICD8 as it appears in World Health Organization (WHO). WHO Mortality Database Version May 2013. Geneva, Switzerland: World Health Organization (WHO), 2013.
Cuba Vital Registration - Deaths 1979 ICD9 as it appears in World Health Organization (WHO). WHO Mortality Database Version November 2015. Geneva, Switzerland: World Health Organization (WHO), 2015.
Cuba Vital Registration - Deaths 1980 ICD9 as it appears in World Health Organization (WHO). WHO Mortality Database Version February 2014. Geneva, Switzerland: World Health Organization (WHO), 2014.
Cuba Vital Registration - Deaths 1981 ICD9 as it appears in World Health Organization (WHO). WHO Mortality Database Version February 2014. Geneva, Switzerland: World Health Organization (WHO), 2014.
Cuba Vital Registration - Deaths 1982 ICD9 as it appears in World Health Organization (WHO). WHO Mortality Database Version February 2014. Geneva, Switzerland: World Health Organization (WHO), 2014.
Cuba Vital Registration - Deaths 1983 ICD9 as it appears in World Health Organization (WHO). WHO Mortality Database Version February 2014. Geneva, Switzerland: World Health Organization (WHO), 2014.
Cuba Vital Registration - Deaths 1984 ICD9 as it appears in World Health Organization (WHO). WHO Mortality Database Version February 2014. Geneva, Switzerland: World Health Organization (WHO), 2014.
Cuba Vital Registration - Deaths 1985 ICD9 as it appears in World Health Organization (WHO). WHO Mortality Database Version February 2014. Geneva, Switzerland: World Health Organization (WHO), 2014.
Cuba Vital Registration - Deaths 1986 ICD9 as it appears in World Health Organization (WHO). WHO Mortality Database Version February 2014. Geneva, Switzerland: World Health Organization (WHO), 2014.
Cuba Vital Registration - Deaths 1987 ICD9 as it appears in World Health Organization (WHO). WHO Mortality Database Version February 2014. Geneva, Switzerland: World Health Organization (WHO), 2014.
Cuba Vital Registration - Deaths 1988 ICD9 as it appears in World Health Organization (WHO). WHO Mortality Database Version February 2014. Geneva, Switzerland: World Health Organization (WHO), 2014.
Cuba Vital Registration - Deaths 1989 ICD9 as it appears in World Health Organization (WHO). WHO Mortality Database Version February 2014. Geneva, Switzerland: World Health Organization (WHO), 2014.
Cuba Vital Registration - Deaths 1990 ICD9 as it appears in World Health Organization (WHO). WHO Mortality Database Version February 2014. Geneva, Switzerland: World Health Organization (WHO), 2014.
Cuba Vital Registration - Deaths 1991 ICD9 as it appears in World Health Organization (WHO). WHO Mortality Database Version February 2014. Geneva, Switzerland: World Health Organization (WHO), 2014.
Cuba Vital Registration - Deaths 1992 ICD9 as it appears in World Health Organization (WHO). WHO Mortality Database Version February 2014. Geneva, Switzerland: World Health Organization (WHO), 2014.
Cuba Vital Registration - Deaths 1993 ICD9 as it appears in World Health Organization (WHO). WHO Mortality Database Version February 2014. Geneva, Switzerland: World Health Organization (WHO), 2014.
Cuba Vital Registration - Deaths 1994 ICD9 as it appears in World Health Organization (WHO). WHO Mortality Database Version February 2014. Geneva, Switzerland: World Health Organization (WHO), 2014.
Cuba Vital Registration - Deaths 1995 ICD9 as it appears in World Health Organization (WHO). WHO Mortality Database Version February 2014. Geneva, Switzerland: World Health Organization (WHO), 2014.
Cuba Vital Registration - Deaths 1996 ICD9 as it appears in World Health Organization (WHO). WHO Mortality Database Version February 2014. Geneva, Switzerland: World Health Organization (WHO), 2014.
Cuba Vital Registration - Deaths 1997 ICD9 as it appears in World Health Organization (WHO). WHO Mortality Database Version July 2012. Geneva, Switzerland: World Health Organization (WHO), 2012.
Cuba Vital Registration - Deaths 1998 ICD9 as it appears in World Health Organization (WHO). WHO Mortality Database Version July 2012. Geneva, Switzerland: World Health Organization (WHO), 2012.

Appendix: Citation List

Citation
Cuba Vital Registration - Deaths 1999 ICD9 as it appears in World Health Organization (WHO). WHO Mortality Database Version July 2012. Geneva, Switzerland: World Health Organization (WHO), 2012.
Cuba Vital Registration - Deaths 2000 ICD9 as it appears in World Health Organization (WHO). WHO Mortality Database Version July 2012. Geneva, Switzerland: World Health Organization (WHO), 2012.
Cuba Vital Registration - Deaths 2001 ICD10 as it appears in World Health Organization (WHO). WHO Mortality Database Version November 2015. Geneva, Switzerland: World Health Organization (WHO), 2015.
Cuba Vital Registration - Deaths 2002 ICD10 as it appears in World Health Organization (WHO). WHO Mortality Database Version November 2015. Geneva, Switzerland: World Health Organization (WHO), 2015.
Cuba Vital Registration - Deaths 2003 ICD10 as it appears in World Health Organization (WHO). WHO Mortality Database Version November 2015. Geneva, Switzerland: World Health Organization (WHO), 2015.
Cuba Vital Registration - Deaths 2004 ICD10 as it appears in World Health Organization (WHO). WHO Mortality Database Version November 2015. Geneva, Switzerland: World Health Organization (WHO), 2015.
Cuba Vital Registration - Deaths 2005 ICD10 as it appears in World Health Organization (WHO). WHO Mortality Database Version November 2015. Geneva, Switzerland: World Health Organization (WHO), 2015.
Cuba Vital Registration - Deaths 2006 ICD10 as it appears in World Health Organization (WHO). WHO Mortality Database Version November 2015. Geneva, Switzerland: World Health Organization (WHO), 2015.
Cuba Vital Registration - Deaths 2007 ICD10 as it appears in World Health Organization (WHO). WHO Mortality Database Version November 2015. Geneva, Switzerland: World Health Organization (WHO), 2015.
Cuba Vital Registration - Deaths 2008 ICD10 as it appears in World Health Organization (WHO). WHO Mortality Database Version November 2015. Geneva, Switzerland: World Health Organization (WHO), 2015.
Cuba Vital Registration - Deaths 2009 ICD10 as it appears in World Health Organization (WHO). WHO Mortality Database Version November 2015. Geneva, Switzerland: World Health Organization (WHO), 2015.
Cuba Vital Registration - Deaths 2010 ICD10 as it appears in World Health Organization (WHO). WHO Mortality Database Version November 2015. Geneva, Switzerland: World Health Organization (WHO), 2015.
Cuba Vital Registration - Deaths 2011 ICD10 as it appears in World Health Organization (WHO). WHO Mortality Database Version November 2015. Geneva, Switzerland: World Health Organization (WHO), 2015.
Cuba Vital Registration - Deaths 2012 ICD10 as it appears in World Health Organization (WHO). WHO Mortality Database Version November 2015. Geneva, Switzerland: World Health Organization (WHO), 2015.
Cuba Vital Registration - Deaths 2013 ICD10 as it appears in World Health Organization (WHO). WHO Mortality Database Version November 2015. Geneva, Switzerland: World Health Organization (WHO), 2015.
Cuba Vital Registration Death Data 1960 as it appears in United Nations Statistics Division (UNSD). United Nations Demographic Yearbook - Historical Supplement 1997. New York City, United States: United Nations Statistics Division (UNSD).
Cuba Vital Registration Death Data 1961 as it appears in United Nations Statistics Division (UNSD). United Nations Demographic Yearbook - Historical Supplement 1997. New York City, United States: United Nations Statistics Division (UNSD).
Cuba Vital Registration Death Data 1962 as it appears in United Nations Statistics Division (UNSD). United Nations Demographic Yearbook - Historical Supplement 1997. New York City, United States: United Nations Statistics Division (UNSD).
Cuba Vital Registration Death Data 1963 as it appears in United Nations Statistics Division (UNSD). United Nations Demographic Yearbook - Historical Supplement 1997. New York City, United States: United Nations Statistics Division (UNSD).
Cubbins LA, Vannoy D. Socioeconomic resources, gender traditionalism, and wife abuse in urban Russian couples. J Marriage Fam. 2005; 67(1): 37-52.
Cucunubá ZM, Flórez AC, Cárdenas A, Pavía P, Montilla M, Aldana R, Villamizar K, Ríos LC, Nicholls RS, Puerta CJ. Prevalence and risk factors for Chagas disease in pregnant women in Casanare, Colombia. Am J Trop Med Hyg. 2012; 87(5): 837-42.
Cucunubá ZM, Guerra AP, Rahirant SJ, Rivera JA, Cortés LJ, Nicholls RS. Asymptomatic Plasmodium spp. infection in Tierralta, Colombia. Mem Inst Oswaldo Cruz. 2008; 103(7): 668-73. As it appears in Malaria Atlas Project. Malaria Atlas Project Plasmodium Falciparum Parasite Rate Database. Oxford, United Kingdom: Malaria Atlas Project.
Cugati S, Wang JJ, Rochtchina E, Mitchell P. Ten-year incidence of diabetes in older Australians: the Blue Mountains Eye Study. Med J Aust. 2007; 186(3): 131-5.
Cumberbatch CG, Younger NO, Ferguson TS, McFarlane SR, Francis DK, Wilks RJ, Tulloch-Reid MK. Reported hours of sleep, diabetes prevalence and glucose control in jamaican adults: analysis from the Jamaica lifestyle survey 2007-2008. Int J Endocrinol. 2011; 716214.
Cunha L, Plouzeau C, Ingrand P, Gudo JPS, Ingrand I, Mondlane J, Beauchant M, Agius G. Use of replacement blood donors to study the epidemiology of major blood-borne viruses in the general population of Maputo, Mozambique. J Med Virol. 2007; 79(12): 1832-40.
Cunningham R, Northwood JL, Kelly CD, Boxall EH, Andrews NJ. Routine antenatal screening for hepatitis B using pooled sera: validation and review of 10 years experience. J Clin Pathol. 1998; 51(5): 392-5.
Cunningham-Myrie C, Younger-Coleman N, Tulloch-Reid M, McFarlane S, Francis D, Ferguson T, Gordon-Strachan G, Wilks R. Diabetes mellitus in Jamaica: sex differences in burden, risk factors, awareness, treatment and control in a developing country. Trop Med Int Health. 2013; 18(11): 1365-78.
Cuong TQ, Dibley MJ, Bowe S, Hanh TT, Loan TT. Obesity in adults: an emerging problem in urban areas of Ho Chi Minh City, Vietnam. Eur J Clin Nutr. 2007; 61(5): 673-81.
Curado MP, Edwards B, Shin HR, Storm H, Ferlay J, Heanue M and Boyle P, eds (2007). Cancer Incidence in Five Continents, Vol. IX Periodic Data (electronic version). Lyon, IARC. http://ci5.iarc.fr
Currie, C. and Corbett, J., Scottish Schools Adolescent Lifestyle and Substance Use Survey, 2002 [computer file]. Colchester, Essex: UK Data Archive [distributor], June 2005. SN: 5195, http://dx.doi.org/10.5255/UKDA-SN-5195-1.
Currie, C. and Corbett, J., Scottish Schools Adolescent Lifestyle and Substance Use Survey, 2004 [computer file]. Colchester, Essex: UK Data Archive [distributor], September 2005. SN: 5239, http://dx.doi.org/10.5255/UKDA-SN-5239-1.

Appendix: Citation List

Citation
Curtale F, Nabil M, el Wakeel A, Shamy MY. Anaemia and intestinal parasitic infections among school age children in Behera Governorate, Egypt Behera Survey Team. J Trop Pediatr. 1998; 44(6): 323-8.
Curtis V, Kanki B, Cousens S, Diallo I, Kpozehouen A, SangarÃ© M, Nikiema M. Evidence of behaviour change following a hygiene promotion programme in Burkina Faso. Bull World Health Organ. 2001; 79(6): 518-27.
Cyprus Cancer Registry 1998-2002 - CI5 as it appears in Curado MP, Edwards B, Shin HR, Storm H, Ferlay J, Heanue M and Boyle P, eds (2007). Cancer Incidence in Five Continents, Vol. IX Periodic Data (electronic version). Lyon, IARC.http://ci5.iarc.fr
Cyprus Cancer Registry 2003-2007 - CI5 as it appears in Forman D, Bray F, Brewster DH, Gombe Mbalawa C, Kohler B, Piñeros M, Steliarova-Foucher E, Swaminathan R and Ferlay J, eds (2013). Cancer Incidence in Five Continents, Vol. X Summary Database (electronic version). Lyon, IARC. http://ci5.iarc.fr
Cyprus Census 1960 as it appears in United Nations Statistics Division (UNSD). United Nations Demographic Yearbook. New York City, United States: United Nations Statistics Division (UNSD).
Cyprus Census 1992 as it appears in United Nations Statistics Division (UNSD). United Nations Demographic Yearbook - Historical Supplement 1997. New York City, United States: United Nations Statistics Division (UNSD).
Cyprus Microcensus 1973 as it appears in United Nations Statistics Division (UNSD). United Nations Demographic Yearbook - Historical Supplement 1997. New York City, United States: United Nations Statistics Division (UNSD).
Cyprus Vital Registration - Deaths 1999 ICD10 as it appears in World Health Organization (WHO). WHO Mortality Database Version November 2015. Geneva, Switzerland: World Health Organization (WHO), 2015.
Cyprus Vital Registration - Deaths 2000 ICD10 as it appears in World Health Organization (WHO). WHO Mortality Database Version November 2015. Geneva, Switzerland: World Health Organization (WHO), 2015.
Cyprus Vital Registration - Deaths 2004 ICD10 as it appears in World Health Organization (WHO). WHO Mortality Database Version November 2015. Geneva, Switzerland: World Health Organization (WHO), 2015.
Cyprus Vital Registration - Deaths 2005 ICD10 as it appears in World Health Organization (WHO). WHO Mortality Database Version November 2015. Geneva, Switzerland: World Health Organization (WHO), 2015.
Cyprus Vital Registration - Deaths 2006 ICD10 as it appears in World Health Organization (WHO). WHO Mortality Database Version November 2015. Geneva, Switzerland: World Health Organization (WHO), 2015.
Cyprus Vital Registration - Deaths 2007 ICD10 as it appears in World Health Organization (WHO). WHO Mortality Database Version November 2015. Geneva, Switzerland: World Health Organization (WHO), 2015.
Cyprus Vital Registration - Deaths 2008 ICD10 as it appears in World Health Organization (WHO). WHO Mortality Database Version November 2015. Geneva, Switzerland: World Health Organization (WHO), 2015.
Cyprus Vital Registration - Deaths 2009 ICD10 as it appears in World Health Organization (WHO). WHO Mortality Database Version November 2015. Geneva, Switzerland: World Health Organization (WHO), 2015.
Cyprus Vital Registration - Deaths 2010 ICD10 as it appears in World Health Organization (WHO). WHO Mortality Database Version November 2015. Geneva, Switzerland: World Health Organization (WHO), 2015.
Cyprus Vital Registration - Deaths 2011 ICD10 as it appears in World Health Organization (WHO). WHO Mortality Database Version November 2015. Geneva, Switzerland: World Health Organization (WHO), 2015.
Cyprus Vital Registration - Deaths 2012 ICD10 as it appears in World Health Organization (WHO). WHO Mortality Database Version November 2015. Geneva, Switzerland: World Health Organization (WHO), 2015.
Cyprus Vital Registration Death Data 1974 as it appears in United Nations Statistics Division (UNSD). United Nations Demographic Yearbook - Historical Supplement 1997. New York City, United States: United Nations Statistics Division (UNSD).
Cyprus Vital Registration Death Data 1975 as it appears in United Nations Statistics Division (UNSD). United Nations Demographic Yearbook - Historical Supplement 1997. New York City, United States: United Nations Statistics Division (UNSD).
Cyprus Vital Registration Death Data 1976 as it appears in United Nations Statistics Division (UNSD). United Nations Demographic Yearbook - Historical Supplement 1997. New York City, United States: United Nations Statistics Division (UNSD).
Cyprus Vital Registration Death Data 1977 as it appears in United Nations Statistics Division (UNSD). United Nations Demographic Yearbook - Historical Supplement 1997. New York City, United States: United Nations Statistics Division (UNSD).
Cyprus Vital Registration Death Data 1978 as it appears in United Nations Statistics Division (UNSD). United Nations Demographic Yearbook - Historical Supplement 1997. New York City, United States: United Nations Statistics Division (UNSD).
Cyprus Vital Registration Death Data 1979 as it appears in United Nations Statistics Division (UNSD). United Nations Demographic Yearbook - Historical Supplement 1997. New York City, United States: United Nations Statistics Division (UNSD).
Cyprus Vital Registration Death Data 1980 as it appears in United Nations Statistics Division (UNSD). United Nations Demographic Yearbook - Historical Supplement 1997. New York City, United States: United Nations Statistics Division (UNSD).
Cyprus Vital Registration Death Data 1981 as it appears in United Nations Statistics Division (UNSD). United Nations Demographic Yearbook - Historical Supplement 1997. New York City, United States: United Nations Statistics Division (UNSD).
Cyprus Vital Registration Death Data 1982 as it appears in United Nations Statistics Division (UNSD). United Nations Demographic Yearbook - Historical Supplement 1997. New York City, United States: United Nations Statistics Division (UNSD).
Cyprus Vital Registration Death Data 1983 as it appears in United Nations Statistics Division (UNSD). United Nations Demographic Yearbook - Historical Supplement 1997. New York City, United States: United Nations Statistics Division (UNSD).
Cyprus Vital Registration Death Data 1984 as it appears in United Nations Statistics Division (UNSD). United Nations Demographic Yearbook - Historical Supplement 1997. New York City, United States: United Nations Statistics Division (UNSD).
Cyprus Vital Registration Death Data 1985 as it appears in United Nations Statistics Division (UNSD). United Nations Demographic Yearbook - Historical Supplement 1997. New York City, United States: United Nations Statistics Division (UNSD).
Cyprus Vital Registration Death Data 1986 as it appears in United Nations Statistics Division (UNSD). United Nations Demographic Yearbook - Historical Supplement 1997. New York City, United States: United Nations Statistics Division (UNSD).

Appendix: Citation List

Citation
Cyprus Vital Registration Death Data 1987 as it appears in United Nations Statistics Division (UNSD). United Nations Demographic Yearbook - Historical Supplement 1997. New York City, United States: United Nations Statistics Division (UNSD).
Cyprus Vital Registration Death Data 1988 as it appears in United Nations Statistics Division (UNSD). United Nations Demographic Yearbook - Historical Supplement 1997. New York City, United States: United Nations Statistics Division (UNSD).
Cyprus Vital Registration Death Data 1989 as it appears in United Nations Statistics Division (UNSD). United Nations Demographic Yearbook - Historical Supplement 1997. New York City, United States: United Nations Statistics Division (UNSD).
Cyprus Vital Registration Death Data 1990 as it appears in United Nations Statistics Division (UNSD). United Nations Demographic Yearbook - Historical Supplement 1997. New York City, United States: United Nations Statistics Division (UNSD).
Cyprus Vital Registration Death Data 1991 as it appears in United Nations Statistics Division (UNSD). United Nations Demographic Yearbook - Historical Supplement 1997. New York City, United States: United Nations Statistics Division (UNSD).
Cyprus Vital Registration Death Data 1992 as it appears in United Nations Statistics Division (UNSD). United Nations Demographic Yearbook - Historical Supplement 1997. New York City, United States: United Nations Statistics Division (UNSD).
Cyprus Vital Registration Death Data 1993 as it appears in United Nations Statistics Division (UNSD). United Nations Demographic Yearbook - Historical Supplement 1997. New York City, United States: United Nations Statistics Division (UNSD).
Cyprus Vital Registration Death Data 1994 as it appears in United Nations Statistics Division (UNSD). United Nations Demographic Yearbook - Historical Supplement 1997. New York City, United States: United Nations Statistics Division (UNSD).
Cyprus Vital Registration Death Data 1995 as it appears in United Nations Statistics Division (UNSD). United Nations Demographic Yearbook - Historical Supplement 1997. New York City, United States: United Nations Statistics Division (UNSD).
Cyprus Vital Registration Death Data 1996 as it appears in United Nations Statistics Division (UNSD). United Nations Demographic Yearbook - Historical Supplement 1997. New York City, United States: United Nations Statistics Division (UNSD).
Cyprus Vital Registration Death Data 2001 as it appears in United Nations Statistics Division (UNSD). United Nations Demographic Yearbook 2001. New York City, United States: United Nations Statistics Division (UNSD), 2003.
Cyprus Vital Registration Death Data 2002 as it appears in United Nations Statistics Division (UNSD). United Nations Demographic Yearbook 2002. New York City, United States: United Nations Statistics Division (UNSD), 2005.
Cyprus Vital Registration Death Data 2003 as it appears in United Nations Statistics Division (UNSD). United Nations Demographic Yearbook 2003. New York City, United States: United Nations Statistics Division (UNSD), 2006.
Czech National Cancer Registry. Czech Republic Cancer Incidence Report 2003. Prague, Czech Republic: Institute of Health Information and Statistics of the Czech Republic, 2006.
Czech National Cancer Registry. Czech Republic Cancer Incidence Report 2004. Prague, Czech Republic: Institute of Health Information and Statistics of the Czech Republic, 2007.
Czech National Cancer Registry. Czech Republic Cancer Incidence Report 2006. Prague, Czech Republic: Institute of Health Information and Statistics of the Czech Republic, 2009.
Czech National Cancer Registry. Czech Republic Cancer Incidence Report 2007. Prague, Czech Republic: Institute of Health Information and Statistics of the Czech Republic, 2010.
Czech National Cancer Registry. Czech Republic Cancer Incidence Report 2008. Prague, Czech Republic: Institute of Health Information and Statistics of the Czech Republic, 2011.
Czech National Cancer Registry. Czech Republic Cancer Incidence Report 2009. Prague, Czech Republic: Institute of Health Information and Statistics of the Czech Republic, 2012.
Czech National Cancer Registry. Czech Republic Incidence of Malignant Neoplasms 2010. Prague, Czech Republic: Institute of Health Information and Statistics of the Czech Republic, 2013.
Czech Republic Cancer Registry 1983 - CI5 as it appears in Ferlay J, Parkin DM, Curado MP, Bray F, Edwards B, Shin HR and Forman D. Cancer Incidence in Five Continents, Volumes I to IX: IARC CancerBase No. 9 [Internet]. Lyon, France: International Agency for Research on Cancer; 2010. Available from: http://ci5.iarc.fr
Czech Republic Cancer Registry 1984 - CI5 as it appears in Ferlay J, Parkin DM, Curado MP, Bray F, Edwards B, Shin HR and Forman D. Cancer Incidence in Five Continents, Volumes I to IX: IARC CancerBase No. 9 [Internet]. Lyon, France: International Agency for Research on Cancer; 2010. Available from: http://ci5.iarc.fr
Czech Republic Cancer Registry 1985 - CI5 as it appears in Ferlay J, Parkin DM, Curado MP, Bray F, Edwards B, Shin HR and Forman D. Cancer Incidence in Five Continents, Volumes I to IX: IARC CancerBase No. 9 [Internet]. Lyon, France: International Agency for Research on Cancer; 2010. Available from: http://ci5.iarc.fr
Czech Republic Cancer Registry 1986 - CI5 as it appears in Ferlay J, Parkin DM, Curado MP, Bray F, Edwards B, Shin HR and Forman D. Cancer Incidence in Five Continents, Volumes I to IX: IARC CancerBase No. 9 [Internet]. Lyon, France: International Agency for Research on Cancer; 2010. Available from: http://ci5.iarc.fr
Czech Republic Cancer Registry 1987 - CI5 as it appears in Ferlay J, Parkin DM, Curado MP, Bray F, Edwards B, Shin HR and Forman D. Cancer Incidence in Five Continents, Volumes I to IX: IARC CancerBase No. 9 [Internet]. Lyon, France: International Agency for Research on Cancer; 2010. Available from: http://ci5.iarc.fr
Czech Republic Cancer Registry 1988 - CI5 as it appears in Ferlay J, Parkin DM, Curado MP, Bray F, Edwards B, Shin HR and Forman D. Cancer Incidence in Five Continents, Volumes I to IX: IARC CancerBase No. 9 [Internet]. Lyon, France: International Agency for Research on Cancer; 2010. Available from: http://ci5.iarc.fr
Czech Republic Cancer Registry 1988-1992 - CI5 as it appears in Parkin DM, International Agency for Research on Cancer, International Association of Cancer Registries. Cancer Incidence in Five Continents. Vol. I to VIII. Lyon, France, IARCPress, 2005.
Czech Republic Cancer Registry 1989 - CI5 as it appears in Ferlay J, Parkin DM, Curado MP, Bray F, Edwards B, Shin HR and Forman D. Cancer Incidence in Five Continents, Volumes I to IX: IARC CancerBase No. 9 [Internet]. Lyon, France: International Agency for Research on Cancer; 2010. Available from: http://ci5.iarc.fr

Appendix: Citation List

Citation
Czech Republic Cancer Registry 1990 - CI5 as it appears in Parkin DM, International Agency for Research on Cancer, International Association of Cancer Registries. Cancer Incidence in Five Continents. Vol. I to VIII. Lyon, France, IARCPress, 2005.
Czech Republic Cancer Registry 1991 - CI5 as it appears in Ferlay J, Parkin DM, Curado MP, Bray F, Edwards B, Shin HR and Forman D. Cancer Incidence in Five Continents, Volumes I to IX: IARC CancerBase No. 9 [Internet]. Lyon, France: International Agency for Research on Cancer; 2010. Available from: http://ci5.iarc.fr
Czech Republic Cancer Registry 1992 - CI5 as it appears in Ferlay J, Parkin DM, Curado MP, Bray F, Edwards B, Shin HR and Forman D. Cancer Incidence in Five Continents, Volumes I to IX: IARC CancerBase No. 9 [Internet]. Lyon, France: International Agency for Research on Cancer; 2010. Available from: http://ci5.iarc.fr
Czech Republic Cancer Registry 1993 - CI5 as it appears in Ferlay J, Parkin DM, Curado MP, Bray F, Edwards B, Shin HR and Forman D. Cancer Incidence in Five Continents, Volumes I to IX: IARC CancerBase No. 9 [Internet]. Lyon, France: International Agency for Research on Cancer; 2010. Available from: http://ci5.iarc.fr
Czech Republic Cancer Registry 1993-1997 - CI5 as it appears in Parkin DM, International Agency for Research on Cancer, International Association of Cancer Registries. Cancer Incidence in Five Continents. Vol. I to VIII. Lyon, France, IARCPress, 2005.
Czech Republic Cancer Registry 1994 - CI5 as it appears in Ferlay J, Parkin DM, Curado MP, Bray F, Edwards B, Shin HR and Forman D. Cancer Incidence in Five Continents, Volumes I to IX: IARC CancerBase No. 9 [Internet]. Lyon, France: International Agency for Research on Cancer; 2010. Available from: http://ci5.iarc.fr
Czech Republic Cancer Registry 1995 - CI5 as it appears in Parkin DM, International Agency for Research on Cancer, International Association of Cancer Registries. Cancer Incidence in Five Continents. Vol. I to VIII. Lyon, France, IARCPress, 2005.
Czech Republic Cancer Registry 1996 - CI5 as it appears in Ferlay J, Parkin DM, Curado MP, Bray F, Edwards B, Shin HR and Forman D. Cancer Incidence in Five Continents, Volumes I to IX: IARC CancerBase No. 9 [Internet]. Lyon, France: International Agency for Research on Cancer; 2010. Available from: http://ci5.iarc.fr
Czech Republic Cancer Registry 1997 - CI5 as it appears in Ferlay J, Parkin DM, Curado MP, Bray F, Edwards B, Shin HR and Forman D. Cancer Incidence in Five Continents, Volumes I to IX: IARC CancerBase No. 9 [Internet]. Lyon, France: International Agency for Research on Cancer; 2010. Available from: http://ci5.iarc.fr
Czech Republic Cancer Registry 1998 - CI5 as it appears in Ferlay J, Parkin DM, Curado MP, Bray F, Edwards B, Shin HR and Forman D. Cancer Incidence in Five Continents, Volumes I to IX: IARC CancerBase No. 9 [Internet]. Lyon, France: International Agency for Research on Cancer; 2010. Available from: http://ci5.iarc.fr
Czech Republic Cancer Registry 1998-2002 - CI5 as it appears in Ferlay J, Parkin DM, Curado MP, Bray F, Edwards B, Shin HR and Forman D. Cancer Incidence in Five Continents, Volumes I to IX: IARC CancerBase No. 9 [Internet]. Lyon, France: International Agency for Research on Cancer; 2010. Available from: http://ci5.iarc.fr
Czech Republic Cancer Registry 1999 - CI5 as it appears in Ferlay J, Parkin DM, Curado MP, Bray F, Edwards B, Shin HR and Forman D. Cancer Incidence in Five Continents, Volumes I to IX: IARC CancerBase No. 9 [Internet]. Lyon, France: International Agency for Research on Cancer; 2010. Available from: http://ci5.iarc.fr
Czech Republic Cancer Registry 2000 - CI5 as it appears in Ferlay J, Parkin DM, Curado MP, Bray F, Edwards B, Shin HR and Forman D. Cancer Incidence in Five Continents, Volumes I to IX: IARC CancerBase No. 9 [Internet]. Lyon, France: International Agency for Research on Cancer; 2010. Available from: http://ci5.iarc.fr
Czech Republic Cancer Registry 2001 - CI5 as it appears in Ferlay J, Parkin DM, Curado MP, Bray F, Edwards B, Shin HR and Forman D. Cancer Incidence in Five Continents, Volumes I to IX: IARC CancerBase No. 9 [Internet]. Lyon, France: International Agency for Research on Cancer; 2010. Available from: http://ci5.iarc.fr
Czech Republic Cancer Registry 2002 - CI5 as it appears in Ferlay J, Parkin DM, Curado MP, Bray F, Edwards B, Shin HR and Forman D. Cancer Incidence in Five Continents, Volumes I to IX: IARC CancerBase No. 9 [Internet]. Lyon, France: International Agency for Research on Cancer; 2010. Available from: http://ci5.iarc.fr
Czech Republic Cancer Registry 2003-2007 - CI5 as it appears in Forman D, Bray F, Brewster DH, Gombe Mbalawa C, Kohler B, Piñeros M, Steliarova-Foucher E, Swaminathan R and Ferlay J, eds (2013). Cancer Incidence in Five Continents, Vol. X Summary Database (electronic version). Lyon, IARC. http://ci5.iarc.fr
Czech Republic Statistical Office, World Health Organization (WHO) Collaborating Center for Perinatal Medicine/Institute for the Care of Mother and Child, Prague, Centers for Disease Control and Prevention (CDC). Czech Republic Reproductive Health Survey 1993. Atlanta, United States: Centers for Disease Control and Prevention (CDC), 1995.
Czech Republic Vital Registration - Deaths 1993 ICD9 as it appears in World Health Organization (WHO). WHO Mortality Database Version July 2012. Geneva, Switzerland: World Health Organization (WHO), 2012.
Czech Republic Vital Registration - Deaths 1994 ICD10 as it appears in World Health Organization (WHO). WHO Mortality Database Version November 2015. Geneva, Switzerland: World Health Organization (WHO), 2015.
Czech Republic Vital Registration - Deaths 1995 ICD10 as it appears in World Health Organization (WHO). WHO Mortality Database Version November 2015. Geneva, Switzerland: World Health Organization (WHO), 2015.
Czech Republic Vital Registration - Deaths 1996 ICD10 as it appears in World Health Organization (WHO). WHO Mortality Database Version November 2015. Geneva, Switzerland: World Health Organization (WHO), 2015.
Czech Republic Vital Registration - Deaths 1997 ICD10 as it appears in World Health Organization (WHO). WHO Mortality Database Version November 2015. Geneva, Switzerland: World Health Organization (WHO), 2015.
Czech Republic Vital Registration - Deaths 1998 ICD10 as it appears in World Health Organization (WHO). WHO Mortality Database Version November 2015. Geneva, Switzerland: World Health Organization (WHO), 2015.
Czech Republic Vital Registration - Deaths 1999 ICD10 as it appears in World Health Organization (WHO). WHO Mortality Database Version November 2015. Geneva, Switzerland: World Health Organization (WHO), 2015.
Czech Republic Vital Registration - Deaths 2000 ICD10 as it appears in World Health Organization (WHO). WHO Mortality Database Version November 2015. Geneva, Switzerland: World Health Organization (WHO), 2015.

Appendix: Citation List

Citation
Czech Republic Vital Registration - Deaths 2001 ICD10 as it appears in World Health Organization (WHO). WHO Mortality Database Version November 2015. Geneva, Switzerland: World Health Organization (WHO), 2015.
Czech Republic Vital Registration - Deaths 2002 ICD10 as it appears in World Health Organization (WHO). WHO Mortality Database Version November 2015. Geneva, Switzerland: World Health Organization (WHO), 2015.
Czech Republic Vital Registration - Deaths 2003 ICD10 as it appears in World Health Organization (WHO). WHO Mortality Database Version November 2015. Geneva, Switzerland: World Health Organization (WHO), 2015.
Czech Republic Vital Registration - Deaths 2004 ICD10 as it appears in World Health Organization (WHO). WHO Mortality Database Version November 2015. Geneva, Switzerland: World Health Organization (WHO), 2015.
Czech Republic Vital Registration - Deaths 2005 ICD10 as it appears in World Health Organization (WHO). WHO Mortality Database Version November 2015. Geneva, Switzerland: World Health Organization (WHO), 2015.
Czech Republic Vital Registration - Deaths 2006 ICD10 as it appears in World Health Organization (WHO). WHO Mortality Database Version November 2015. Geneva, Switzerland: World Health Organization (WHO), 2015.
Czech Republic Vital Registration - Deaths 2007 ICD10 as it appears in World Health Organization (WHO). WHO Mortality Database Version November 2015. Geneva, Switzerland: World Health Organization (WHO), 2015.
Czech Republic Vital Registration - Deaths 2008 ICD10 as it appears in World Health Organization (WHO). WHO Mortality Database Version November 2015. Geneva, Switzerland: World Health Organization (WHO), 2015.
Czech Republic Vital Registration - Deaths 2009 ICD10 as it appears in World Health Organization (WHO). WHO Mortality Database Version November 2015. Geneva, Switzerland: World Health Organization (WHO), 2015.
Czech Republic Vital Registration - Deaths 2010 ICD10 as it appears in World Health Organization (WHO). WHO Mortality Database Version November 2015. Geneva, Switzerland: World Health Organization (WHO), 2015.
Czech Republic Vital Registration - Deaths 2011 ICD10 as it appears in World Health Organization (WHO). WHO Mortality Database Version November 2015. Geneva, Switzerland: World Health Organization (WHO), 2015.
Czech Republic Vital Registration - Deaths 2012 ICD10 as it appears in World Health Organization (WHO). WHO Mortality Database Version November 2015. Geneva, Switzerland: World Health Organization (WHO), 2015.
Czech Republic Vital Registration - Deaths 2013 ICD10 as it appears in World Health Organization (WHO). WHO Mortality Database Version November 2015. Geneva, Switzerland: World Health Organization (WHO), 2015.
Czechoslovakia - Czech Republic Vital Registration - Deaths 1986 ICD9 as it appears in World Health Organization (WHO). WHO Mortality Database Version July 2012. Geneva, Switzerland: World Health Organization (WHO), 2012.
Czechoslovakia - Czech Republic Vital Registration - Deaths 1987 ICD9 as it appears in World Health Organization (WHO). WHO Mortality Database Version July 2012. Geneva, Switzerland: World Health Organization (WHO), 2012.
Czechoslovakia - Czech Republic Vital Registration - Deaths 1988 ICD9 as it appears in World Health Organization (WHO). WHO Mortality Database Version July 2012. Geneva, Switzerland: World Health Organization (WHO), 2012.
Czechoslovakia - Czech Republic Vital Registration - Deaths 1989 ICD9 as it appears in World Health Organization (WHO). WHO Mortality Database Version July 2012. Geneva, Switzerland: World Health Organization (WHO), 2012.
Czechoslovakia - Czech Republic Vital Registration - Deaths 1990 ICD9 as it appears in World Health Organization (WHO). WHO Mortality Database Version July 2012. Geneva, Switzerland: World Health Organization (WHO), 2012.
Czechoslovakia - Czech Republic Vital Registration - Deaths 1991 ICD9 as it appears in World Health Organization (WHO). WHO Mortality Database Version July 2012. Geneva, Switzerland: World Health Organization (WHO), 2012.
Czechoslovakia - Czech Republic Vital Registration - Deaths 1992 ICD9 as it appears in World Health Organization (WHO). WHO Mortality Database Version July 2012. Geneva, Switzerland: World Health Organization (WHO), 2012.
Da Rocha EM, Fontes G, Brito AC, Silva TR, Medeiros Z, Antunes CM. [Bancroftian filariasis in urban areas of Alagoas State, Northeast Brazil: study in the general population]. Rev Soc Bras Med Trop. 2000; 33(6): 545-51.
Da Villa G, Andjaparidze A, Cauletti M, Franco E, Roggendorf M, Sepe A, Zaratti L. Viral hepatitis in the Bhutanese population: preliminary results of a seroepidemiological investigation. Res Virol. 1997; 148(2): 115-7.
Dachlan YP, Yotopranoto S, Sutanto BV, Santoso SHB, Widodo AS, Kusmartisnawati, Sutanto A, Gerudug IKK, Takagi M, Tsuda Y, Tanabe K, Kawamoto F, Yoshinaga K, Kanbara H. Malaria Endemic Patterns on Lombok and Sumbawa Islands, Indonesia. Trop Med Health. 2005; 33(2): 105-13. As it appears in Malaria Atlas Project. Malaria Atlas Project Plasmodium Falciparum Parasite Rate Database. Oxford, United Kingdom: Malaria Atlas Project.
Dadzie S, Boakye D, Asoala V, Koram K, Kiszewski A, Appawu M. A community-wide study of malaria reduction: evaluating efficacy and user-acceptance of a low-cost repellent in northern Ghana. Am J Trop Med Hyg. 2013; 88(2): 309-14. As it appears in Malaria Atlas Project. Malaria Atlas Project Plasmodium Falciparum Parasite Rate Database. Oxford, United Kingdom: Malaria Atlas Project.
Dahiru T, Jibo A, Hassan AA, Mande AT. Prevalence of diabetes in a semi-urban community in Northern Nigeria. Niger J Med. 2008; 17(4): 414-6.
Dahlquist GG, Nystrom L, Patterson CC. Incidence of type 1 diabetes in Sweden among individuals aged 0-34 years, 1983-2007: an analysis of time trends. Diabetes Care. 2011; 34(8): 1754-9.
Dalal K, Rahman F, Jansson B. Wife abuse in rural Bangladesh. J Biosoc Sci. 2009; 41(5): 561-73.
Dalekos GN, Zervou E, Merkouropoulos MH, Tsianos EV. Prevalence of hepatitis B and C viruses infection in chronic alcoholics with or without liver disease in Ioannina, Greece: low incidence of HCV infection. Eur J Epidemiol. 1996; 12(1): 21-5.
D'Alessandro U, Olaleye BO, McGuire W, Langerock P, Bennett S, Aikins MK, Thomson MC, Cham MK, Cham BA, Greenwood BM. Mortality and morbidity from malaria in Gambian children after introduction of an impregnated bednet programme. Lancet. 1995; 345(8948): 479-83.

Appendix: Citation List

Citation
D'Alessandro U, Olaleye BO, McGuire W, Langerock P, Bennett S, Aikins MK, Thomson MC, Cham MK, Cham BA, Greenwood BM. Mortality and morbidity from malaria in Gambian children after introduction of an impregnated bednet programme. Lancet. 1995; 345(8948): 479-83. As it appears in Malaria Atlas Project. Malaria Atlas Project Plasmodium Falciparum Parasite Rate Database. Oxford, United Kingdom: Malaria Atlas Project.
Dal-Ré R, Aguilar L, Coronel P. Current prevalence of hepatitis B, A and C in a healthy Spanish population. A seroepidemiological study. Infection. 1991; 19(6): 409-13.
Damale NKR, Lassey AT, Bekoe V. Hepatitis B virus seroprevalence among parturients in Accra, Ghana. Int J Gynaecol Obstet. 2005; 90(3): 240-1.
Damien GB, Djènontin A, Rogier C, Corbel V, Bangana SB, Chandre F, Akogbéto M, Kindé-Gazard D, Massougbodji A, Henry M-C. Malaria infection and disease in an area with pyrethroid-resistant vectors in southern Benin. Malar J. 2010; 9(1): 380. As it appears in Malaria Atlas Project. Malaria Atlas Project Plasmodium Falciparum Parasite Rate Database. Oxford, United Kingdom: Malaria Atlas Project.
Danaei G, Finucane MM, Lu Y, Singh GM, Cowan MJ, Paciorek CJ, Lin JK, Farzadfar F, Khang YH, Stevens GA, Rao M, Ali MK, Riley LM, Robinson CA, Ezzati M, Global Burden of Metabolic Risk Factors of Chronic Diseases Collaborating Group (Blood Glucose). National, regional, and global trends in fasting plasma glucose and diabetes prevalence since 1980: systematic analysis of health examination surveys and epidemiological studies with 370 country-years and 2.7 million participants. Lancet. 2011; 378(9785): 31-40.
Dang T, Christophel E. [Survey on malaria in Na Hang, Tuyen Quang province in Oct 1994]. J Vector Borne Dis. 1996; 2: 3-10. As it appears in Malaria Atlas Project. Malaria Atlas Project Plasmodium Falciparum Parasite Rate Database. Oxford, United Kingdom: Malaria Atlas Project.
Dang T, Van Thai P. [Evaluation on outbreak of malaria in Hmong and Dao ethnic minority people in Yen Huong commune, Ham Yen district, Tuyen Quang province in 1997]. J Vector Borne Dis. 1998; 1: 6-10. As it appears in Malaria Atlas Project. Malaria Atlas Project Plasmodium Falciparum Parasite Rate Database. Oxford, United Kingdom: Malaria Atlas Project.
Daniel CR, Prabhakaran D, Kapur K, Graubard BI, Devasenapathy N, Ramakrishnan L, George PS, Shetty H, Ferrucci LM, Yurgalevitch S, Chatterjee N, Reddy KS, Rastogi T, Gupta PC, Mathew A, Sinha R. A cross-sectional investigation of regional patterns of diet and cardio-metabolic risk in India. Open Nutr J. 2011; 12.
Danish Cancer Society, Danish Health and Medicines Authority, Danish Heart Foundation, Danish Lung Association, Ramboll. Denmark Monitoring Smoking Habits in the Danish Population 2003.
Danish Cancer Society, Danish Health and Medicines Authority, Danish Heart Foundation, Danish Lung Association, Ramboll. Denmark Monitoring Smoking Habits in the Danish Population 2004.
Danish Cancer Society, Danish Health and Medicines Authority, Danish Heart Foundation, Danish Lung Association, Ramboll. Denmark Monitoring Smoking Habits in the Danish Population 2005.
Danish Cancer Society, Danish Health and Medicines Authority, Danish Heart Foundation, Danish Lung Association, Ramboll. Denmark Monitoring Smoking Habits in the Danish Population 2006.
Danish Cancer Society, Danish Health and Medicines Authority, Danish Heart Foundation, Danish Lung Association, TNS Gallup. Denmark Monitoring Smoking Habits in the Danish Population 2007.
Danish Cancer Society, Danish Health and Medicines Authority, Danish Heart Foundation, Danish Lung Association, TNS Gallup. Denmark Monitoring Smoking Habits in the Danish Population 2008.
Danish Cancer Society, Danish Health and Medicines Authority, Danish Heart Foundation, Danish Lung Association, TNS Gallup. Denmark Monitoring Smoking Habits in the Danish Population 2009.
Danish Cancer Society, Danish Health and Medicines Authority, Danish Heart Foundation, Danish Lung Association, TNS Gallup. Denmark Monitoring Smoking Habits in the Danish Population 2010.
Danish Cancer Society, Danish Health and Medicines Authority, Danish Heart Foundation, Danish Lung Association, TNS Gallup. Denmark Monitoring Smoking Habits in the Danish Population 2011.
Danish Cancer Society, Danish Health and Medicines Authority, Danish Heart Foundation, Danish Lung Association, TNS Gallup. Denmark Monitoring Smoking Habits in the Danish Population 2012.
Danish Cancer Society, Danish Health and Medicines Authority, Danish Heart Foundation, Danish Lung Association, TNS Gallup. Denmark Monitoring Smoking Habits in the Danish Population 2013.
Danish Health and Medicines Authority. Denmark Alcohol, Drugs and Tobacco Statistics 2003. Copenhagen, Denmark: Danish Health and Medicines Authority, 2004.
Danish National Institute of Public Health. Denmark Health Interview Survey 1986-1987.
Danish National Institute of Public Health. Denmark Health Interview Survey 1994.
Danish National Institute of Public Health. Denmark Health Interview Survey 2000.
Danish National Institute of Public Health. Denmark Health Interview Survey 2005.
Danish National Institute of Public Health. Denmark Health Interview Survey 2010.
Danish National Institute of Public Health. Denmark Health Interview Survey Database - Overweight and Underweight.
Danish National Institute of Public Health. Denmark National Health Examination Survey 2007-2008.
Danso-Appiah A, De Vlas SJ, Bosompem KM, Habbema JDF. Determinants of health-seeking behaviour for schistosomiasis-related symptoms in the context of integrating schistosomiasis control within the regular health services in Ghana. Trop Med Int Health. 2004; 9(7): 784-94.
Daoud W. [Epidemiologic study of malaria in the foothill area of the Taez region of the Arabic Republic of Yemen]. Bull Soc Pathol Exot. 1988; 81(3): 351-9. As it appears in Malaria Atlas Project. Malaria Atlas Project Plasmodium Falciparum Parasite Rate Database. Oxford, United Kingdom: Malaria Atlas Project.
Daramola OOM, George AO, Ogunbiyi AO, Otegbayo JA. Hepatitis B virus in Nigerians with lichen planus. West Afr J Med. 2004; 23(2): 104-6.

Appendix: Citation List

Citation
D'Argenio P, Esposito D, Mele A, Ortolani G, Adamo B, Rapicetta M, Forte P, Pisani A, Soldo L, Sarrecchia B. Decline in the exposure to hepatitis A and B infections in children in Naples, Italy. Public Health. 1989; 103(5): 385-9.
Das D, Kumar S, Sahoo PK, Dash AP. A survey of bancroftian filariasis for microfilariae & circulating antigenaemia in two villages of Madhya Pradesh. Indian J Med Res. 2005; 121(6): 771-5.
Das NG, Bhuyan M, Das SC. Entomological and epidemiological studies on malaria in Rajmahal range, Bihar. Indian J Malariol. 2000; 37(3/4): 88-96. As it appears in Malaria Atlas Project. Malaria Atlas Project Plasmodium Falciparum Parasite Rate Database. Oxford, United Kingdom: Malaria Atlas Project.
Das PK, Das LK, Parida SK, Patra KP, Jambulingam P. Lambdacyhalothrin treated bed nets as an alternative method of malaria control in tribal villages of Koraput District, Orissa State, India. Southeast Asian J Trop Med Public Health. 1993; 24(3): 513-21. As it appears in Malaria Atlas Project. Malaria Atlas Project Plasmodium Falciparum Parasite Rate Database. Oxford, United Kingdom: Malaria Atlas Project.
Das PK, Harris VK, Shoma B, Bose YN, Annie S. Trend of hepatitis B virus infection in southern Indian blood donors. Indian J Gastroenterol. 1999; 18(4): 182.
Das S, Bapat U, More NS, Alcock G, Fernandez A, Osrin D. Nutritional status of young children in Mumbai slums: a follow-up anthropometric study. Open Nutr J. 2012; 100.
Das VNR, Siddiqui NA, Kumar N, Verma N, Verma RB, Dinesh DS, Kar SK, Das P. A pilot study on the status of lymphatic filariasis in a rural community of Bihar. J Commun Dis. 2006; 38(2): 169-75. As it appears in London School of Hygiene and Tropical Medicine. Global Atlas of Helminth Infections - Lymphatic Filiariasis.
Dash S, Ramasankaram K, Ukey U, Naidu N. Study of causes of deaths among the insured population of a north easternv district of Andhra Pradesh. Natl J Res Community Med. 2014; 3(3): 274-81.
Dassanayake AS, Kasturiratne A, Rajindrajith S, Kalubowila U, Chakrawarthi S, De Silva AP, Makaya M, Mizoue T, Kato N, Wickremasinghe AR, de Silva HJ. Prevalence and risk factors for non-alcoholic fatty liver disease among adults in an urban Sri Lankan population. J Gastroenterol Hepatol. 2009; 24(7): 1284-8.
Datta M, Gopi PG, Appegowda BN, Bhima Rao KR, Gopalan BN. Tuberculosis in North Arcot District of Tamil Nadu - a sample survey. Indian J Tuberc. 2000; 47(3): 147-54.
Davaalkham D, Ojima T, Nymadawa P, Tsend N, Lkhagvasuren T, Wiersma S, Uehara R, Watanabe M, Oki I, Nakamura Y. Seroepidemiology of hepatitis B virus infection among children in Mongolia: results of a nationwide survey. Pediatr Int. 2007; 49(3): 368-74.
Davey A, Lele U, Elias MF, Dore GA, Siegler IC, Johnson MA, Hausman DB, Tenover JL, Poon LW. Diabetes mellitus in centenarians. J Am Geriatr Soc. 2012; 60(3): 468-73.
Davies CA, Vandelanotte C, Duncan MJ, van Uffelen JGZ. Associations of physical activity and screen-time on health related quality of life in adults. Prev Med. 2012; 55(1): 46-9.
Davies M, Brophy S, Williams R, Taylor A. The prevalence, severity, and impact of painful diabetic peripheral neuropathy in type 2 diabetes. Diabetes Care. 2006; 29(7): 1518–22.
Davis JC, Clark TD, Kemble SK, Talemwa N, Njama-Meya D, Staedke SG, Dorsey G. Longitudinal study of urban malaria in a cohort of Ugandan children: description of study site, census and recruitment. Malar J. 2006; 5(1): 18. As it appears in Malaria Atlas Project. Malaria Atlas Project Plasmodium Falciparum Parasite Rate Database. Oxford, United Kingdom: Malaria Atlas Project.
Davis TM, Yeap BB, Davis WA, Bruce DG. Lipid-lowering therapy and peripheral sensory neuropathy in type 2 diabetes: the Fremantle Diabetes Study. Diabetologia. 2008; 51(4): 562-6.
Davis WA, Norman PE, Bruce DG, Davis TM. Predictors, consequences and costs of diabetes-related lower extremity amputation complicating type 2 diabetes: the Fremantle Diabetes Study. Diabetologia. 2006; 49(11): 2634-41.
Dawar M, Patrick DM, Bigham M, Cook D, Krajden M, Ng H. Impact of universal preadolescent vaccination against hepatitis B on antenatal seroprevalence of hepatitis B markers in British Columbia women. CMAJ. 2003; 168(6): 703-4.
Dazza MC, Trebucq A, Gaudebout C, Jarretou A, Le Hesran JY, Josse R, Delaporte E, Bréchot C, Larouze B. Population-based study of serum hepatitis B virus DNA in Gabon. Trans R Soc Trop Med Hyg. 1993; 87(5): 539-40.
de Abreu D, Guessous I, Gaspoz J-M, Marques-Vidal P. Compliance with the Swiss Society for Nutritionâ€™s dietary recommendations in the population of Geneva, Switzerland: a 10-year trend study (1999-2009). J Acad Nutr Diet. 2014; 114(5): 774â€“80.
De Andrade AL, Zicker F, Luquetti AO, Oliveira RM, Silva SA, Souza JM, Martelli CM. Surveillance of Trypanosoma cruzi transmission by serological screening of schoolchildren. Bull World Health Organ. 1992; 70(5): 625–9.
De Andrade FB, de FranÃ§a Caldas A Jr, Kitoko PM. Relationship between oral health, nutrient intake and nutritional status in a sample of Brazilian elderly people. Gerodontology. 2009; 26(1): 40-5.
De Arruda ME, Aragaki C, Gagliardi F, Haile RW. A seroprevalence and descriptive epidemiological study of malaria among Indian tribes of the Amazon basin of Brazil. Ann Trop Med Parasitol. 1996; 90(2): 135-43. As it appears in Malaria Atlas Project. Malaria Atlas Project Plasmodium Falciparum Parasite Rate Database. Oxford, United Kingdom: Malaria Atlas Project.
De Beaufort CE, Cecchi-Tenerini R, Clerc R, Hilaire M, Schwartz F, Manciaux M. [Incidence of insulin-dependent diabetes in six to sixteen year old school children in Lorraine]. Arch Fr Pediatr. 1991; 48(3): 228.
De Beaufort CE, Michel G, Glaesener G. The incidence of type 1 (insulin-dependent) diabetes mellitus in subjects aged 0-19 years in Luxembourg: a retrospective study from 1977 to 1986. Diabetologia. 1988; 31(10): 758-61.
de Freitas SN, Caiaffa WT, CÃ©sar CC, Faria VA, do Nascimento RM, Coelho GL. Nutritional Risk in the Urban Population of Ouro Preto, Southeastern Region of Brazil: The Ouro Preto Heart Study. Arq Bras Cardiol. 2007; 88(2): 191-9.
de Klerk NH, Musk AW. Silica, Compensated Silicosis, And Lung Cancer In Western Australian Goldminers. Occup Environ Med. 1998; 55(4): 243-8 as it appears in Kurihara N, Wada O. Silicosis and smoking strongly increase lung cancer risk in silica-exposed workers. Ind Health 2004; 42(3): 303–314.
de León AC, Coello SD, González DA, Díaz BB, Rodríguez JC, Hernández AG, Aguirre-Jaime A, Pérez Mdel C. Impaired fasting glucose, ancestry and waist-to-height ratio: main predictors of incident diagnosed diabetes in the Canary Islands. Diabet Med. 2012; 29(3): 399-403.

Appendix: Citation List

Citation
De Lima e Costa MF, Leite ML, Rocha RS, de Almeida Magalhães MH, Katz N. Anthropometric measures in relation to Schistosomiasis mansoni and socioeconomic variables. Int J Epidemiol. 1988; 17(4): 880-6.
De Lima e Costa MF, Rocha RS, Colley D, Gazzinelli G, Katz N. Validity of selected clinical signs and symptoms in diagnosis of Schistosoma mansoni infection. Rev Inst Med Trop Sao Paulo. 1991; 33(1): 12-7.
De Lima e Costa MF, Rocha RS, Katz N. Schistosomiasis morbidity and its relation to the Schistosoma mansoni egg count in an hyperendemic area in the State of Minas Gerais. Rev Inst Med Trop Sao Paulo. 1985; 27(2): 66-75.
De Lorenzo A, Bedogni G, Andreoli A, Kandil S, el-Hefni S, Brancati A. Assessment of body hydration in subjects with schistosomiasis. Ann Hum Biol. 1997; 24(4): 315-2.
De Nicola L, Donfrancesco C, Minutolo R, Lo Noce C, Palmieri L, De Curtis A, Iacoviello L, Zoccali C, Gesualdo L, Conte G, Vanuzzo D, Giampaoli S, ANMCO-SIN Research Group. Prevalence and cardiovascular risk profile of chronic kidney disease in Italy: results of the 2008-12 National Health Examination Survey. Nephrol Dial Transplant. 2015; 30(5): 806–14.
De Pablos-Velasco PL, Martínez-Martín FJ, Rodríguez-Pérez F, Anía BJ, Losada A, Betancor P. Prevalence and determinants of diabetes mellitus and glucose intolerance in a Canarian Caucasian population - comparison of the 1997 ADA and the 1985 WHO criteria. The Guía Study. Diabet Med. 2001; 18(3): 235-41.
De Rochars MB, Kanjilal S, Direny AN, Radday J, Lafontant JG, Mathieu E, Rheingans RD, Haddix AC, Streit TG, Beach MJ, Addiss DG, Lammie PJ. The Leogane, Haiti demonstration project: decreased microfilaremia and program costs after three years of mass drug administration. Am J Trop Med Hyg. 2005; 73(5): 888-94. As it appears in London School of Hygiene and Tropical Medicine. Global Atlas of Helminth Infections - Lymphatic Filiariasis.
De Sereday MS, Gonzalez C, Giorgini D, De Loredo L, Braguinsky J, Cobeñas C, Libman C, Tesone C. Prevalence of diabetes, obesity, hypertension and hyperlipidemia in the central area of Argentina. Diabetes Metab. 2004; 30(4): 335-9.
de Sonnaville JJ, Colly LP, Wijkel D, Heine RJ. The prevalence and determinants of foot ulceration in type II diabetic patients in a primary health care setting. Diabetes Res Clin Pract. 1997; 35(2-3): 149-56.
de Souza DK, Sesay S, Moore MG, Ansumana R, Narh CA, Kollie K, Rebollo MP, Koudou BG, Koroma JB, Bolay FK, Boakye DA, Bockarie MJ. No evidence for lymphatic filariasis transmission in big cities affected by conflict related rural-urban migration in Sierra Leone and Liberia. PLoS Negl Trop Dis. 2014; 8.0(2): e2700.
De Souza NCN, Botelho CAO, Honer MR. Retrospective study of a pioneer antenatal screening program with 8,477 pregnant women in Brazil. Clin Exp Obstet Gynecol. 2004; 31(3): 217-20.
De Wit LM, van Straten A, van Herten M, Penninx BWJH, Cuijpers P. Depression and body mass index, a u-shaped association. BMC Public Health. 2009; 14.
de Wytt CN, Jackson RV, Hockings GI, Joyner JM, Strakosch CR. Polyneuropathy in Australian outpatients with type II diabetes mellitus. J Diabetes Complicat. 1999; 13(2): 74–8.
De Zwaan M, Gruss B, MÃ¼ller A, Philipsen A, Graap H, Martin A, Glaesmer H, Hilbert A. Association between obesity and adult attention-deficit/hyperactivity disorder in a German community-based sample. Obes Facts. 2011; 4(3): 204-11.
DECODE Study Group. Age- and sex-specific prevalences of diabetes and impaired glucose regulation in 13 European cohorts. Diabetes Care. 2003; 26(1): 61-9.
Deedwania PC, Gupta R, Sharma KK, Achari V, Gupta B, Maheshwari A, Gupta A. High prevalence of metabolic syndrome among urban subjects in India: a multisite study. Diabetes Metab Syndr. 2014; 8(3): 156–61.
Deepa M, Farooq S, Deepa R, Manjula D, Mohan V. Prevalence and significance of generalized and central body obesity in an urban Asian Indian population in Chennai, India (CURES: 47). Eur J Clin Nutr. 2009; 63(2): 259-67.
Defay R, Delcourt C, Ranvier M, Lacroux A, Papoz L. Relationships between physical activity, obesity and diabetes mellitus in a French elderly population: the POLA study. Pathologies Oculaires lieÃ©s Ã¡ l' Age. Int J Obes Relat Metab Disord. 2001; 25(4): 512-8.
Degertekin H, Tuzcu A, Yalçin K. Horizontal transmission of HBV infection among students in Turkey. Public Health. 2000; 114(5): 411-2.
Del Bruto OH, Noboa CA. Late-onset epilepsy in Ecuador: aetiology and clinical features in 225 patients. Trop Geogr Neurol. 1991; 1: 31–4.
Del Brutto OH, Del Brutto VJ. Reduced percentage of neurocysticercosis cases among patients with late-onset epilepsy in the new millennium. Clin Neurol Neurosurg. 2012; 114(9): 1254-6.
Del Nero L, Lamizana L, Nebie I, Sare S, Bougouma L, Pietra V. In vivo sensitivity of Plasmodium falciparum to halofantrine hydrochloride in Burkina Faso. Am J Trop Med Hyg. 1994; 50(1): 102-6. As it appears in Malaria Atlas Project. Malaria Atlas Project Plasmodium Falciparum Parasite Rate Database. Oxford, United Kingdom: Malaria Atlas Project.
Del Nero L, Lamizana L, Pietra V, Nebi I. Sensitivity to antimalarial drugs by Plasmodium falciparum in Goundry, Oubritenga province, Burkina Faso. Parassitologia. 1994; 36(3): 287-93. As it appears in Malaria Atlas Project. Malaria Atlas Project Plasmodium Falciparum Parasite Rate Database. Oxford, United Kingdom: Malaria Atlas Project.
Del Pino N, Martínez Peralta L, Pampuro S, Pimentel E, Libonatti O. HTLV-I/II seroprevalence and coinfection with other pathogens in blood donors in Buenos Aires. J Acquir Immune Defic Syndr. 1994; 7(2): 206-7.
Delacollette C, Barutwanayo M, Mpitabakana P. Epidémiologie du paludisme au Burundi. Med Afr Noire. 1990; 37(12): 718-21. As it appears in Malaria Atlas Project. Malaria Atlas Project Plasmodium Falciparum Parasite Rate Database. Oxford, United Kingdom: Malaria Atlas Project.
Delacollette C, Barutwanayo M. Mortality and morbidity at young ages in a stable hyperendemic malaria region, community Nyanza-Lac, Imbo South, Burundi. Bull Soc Pathol Exot. 1993; 86(5): 373-9.
Delacollette C, Van der Stuyft P, Molima K, Delacollette-Lebrun C, Wery M. Etude de la mortalité globale et de la mortalité liée au paludisme dans le Kivu montagneux, Zaïre. Rev Epidemiol Sante Publique. 1989; 37(2): 161-6.
Delacollette C, Van der Stuyft P, Molima K, Hendrix L, Wéry M. [Malaria index according to age and seasons in the health region of Katana, in mountainous Kivu, Zaire]. Ann Soc Belg Med Trop. 1990; 70(4): 263-8. As it appears in Malaria Atlas Project. Malaria Atlas Project Plasmodium Falciparum Parasite Rate Database. Oxford, United Kingdom: Malaria Atlas Project.

Appendix: Citation List

Citation
Delcourt C, Vauzelle-Kervroedan F, Cathelineau G, Papoz L. Low prevalence of long-term complications in non-insulin-dependent diabetes mellitus in France: a multicenter study. CODIAB-INSERM-ZENECA Pharma Study Group. J Diabetes Complicat. 1998; 12(2): 88-95.
Delfini LF. Assignment Report on Malaria Control in Yemen. 14 September–25 November 1985. Cairo, Egypt: World Health Organization Regional Office for the Eastern Mediterranean (EMRO-WHO), 1986. As it appears in Malaria Atlas Project. Malaria Atlas Project Plasmodium Falciparum Parasite Rate Database. Oxford, United Kingdom: Malaria Atlas Project.
Delgado S, Castillo Neyra R, Quispe Machaca VR, Ancca Juárez J, Chou Chu L, Verastegui MR, Moscoso Apaza GM, Bocángel CD, Tustin AW, Sterling CR, Comrie AC, Náquira C, Cornejo del Carpio JG, Gilman RH, Bern C, Levy MZ. A history of chagas disease transmission, control, and re-emergence in peri-rural La Joya, Peru. PLoS Negl Trop Dis. 2011; 5(2): e970.
Dell L, Teta MJ. Mortality Among Workers At A Plastics Manufacturing And Research And Development Facility: 1946-1988. Am J Ind Med. 1988; 28(3): 373-84 as it appears in Collins JJ, Lineker GA. A review and meta-analysis of formaldehyde exposure and leukemia. Regul Toxicol Pharmacol. 2004; 40(2): 81-91.
Delley V, Bouvier P, Breslow N, Doumbo O, Sagara I, Diakite M, Mauris A, Dolo A, Rougemont A. What does a single determination of malaria parasite density mean? A longitudinal survey in Mali. Trop Med Int Health. 2000; 5(6): 404-12. As it appears in Malaria Atlas Project. Malaria Atlas Project Plasmodium Falciparum Parasite Rate Database. Oxford, United Kingdom: Malaria Atlas Project.
Deloron P, Campbell GH, Brandling-Bennett D, Roberts JM, Schwartz IK, Odera JS, Lal AA, Osanga CO, de la Cruz V, McCutchan TM. Antibodies to Plasmodium falciparum ring-infected erythrocyte surface antigen and P. falciparum and P. malariae circumsporozoite proteins: seasonal prevalence in Kenyan villages. Am J Trop Med Hyg. 1989; 41(4): 395-9. As it appears in Malaria Atlas Project. Malaria Atlas Project Plasmodium Falciparum Parasite Rate Database. Oxford, United Kingdom: Malaria Atlas Project.
Deloron P, Cot M. Antibodies to the ring-infected erythrocyte surface antigen and the circumsporozoite protein of Plasmodium falciparum in a rural community from Burkina Faso. Trans R Soc Trop Med Hyg. 1990; 84(2): 191-5. As it appears in Malaria Atlas Project. Malaria Atlas Project Plasmodium Falciparum Parasite Rate Database. Oxford, United Kingdom: Malaria Atlas Project.
Deloron P, Duverseau Y, Zevallos-Ipenza A, Magloire R, Stanfill P, Nguyen-Dinh P. Antibodies to Pf155, a major antigen of Plasmodium falciparum: seroepidemiological studies in Haiti. Bull World Health Organ. 1987; 65(3): 339-44. As it appears in Malaria Atlas Project. Malaria Atlas Project Plasmodium Falciparum Parasite Rate Database. Oxford, United Kingdom: Malaria Atlas Project.
Dembele H. Malaria and Pregnancy, Seasonality and Relationship with Anemia and Low Birth Weight in Bougoula-Hameau (Sikasso Mali) [dissertation]. Bamako, Mali: National School of Medicine and Pharmacy of Mali, 1995. As it appears in Malaria Atlas Project. Malaria Atlas Project Plasmodium Falciparum Parasite Rate Database. Oxford, United Kingdom: Malaria Atlas Project.
Demir A, Kalyoncu A, Selcuk T, Artvinli M, Sahin A. Prevalence of Asthma, Allergy, and Respiratory Symptoms in Hasançelebi/Hekimhan/Malatya in Eastern Turkey. Turk Respir J. 2001; 2(2): 29-34.
Demirel Y, Duran B, Toktamis A, Erden O, Cetin M. Seroprevalence of syphilis, hepatitis B and C, and human immunodeficiency virus infections among women. Saudi Med J. 2004; 25(12): 2037-8.
Demirtürk N, Demirdal T, Toprak D, Altindiş M, Aktepe OC. Hepatitis B and C virus in West-Central Turkey: seroprevalence in healthy individuals admitted to a university hospital for routine health checks. Turk J Gastroenterol. 2006; 17(4): 267-72.
Demographic Research Unit (Togo), Department of Statistics (Togo), Macro International, Inc, Ministry of Public Health, Social Affairs and the Status of Women (Togo). Togo Demographic and Health Survey - Maternal Mortality Data.
Demographic Research Unit (Togo), Department of Statistics (Togo), Macro Systems, Inc.; Institute for Resource Development, Ministry of Public Health, Social Affairs and the Status of Women (Togo). Togo Demographic and Health Survey 1988. Columbia, United States: Macro Systems, Inc.
Deneux-Tharaux C, Berg C, Bouvier-Colle M-H, Gissler M, Harper M, Nannini A, Alexander S, Wildman K, Breart G, Buekens P. Underreporting of pregnancy-related mortality in the United States and Europe. Obstet Gynecol. 2005; 106(4): 684-92.
Denis F, Ranger-Rogez S, Alain S, Mounier M, Debrock C, Wagner A, Delpeyroux C, Tabaste JL, Aubard Y, Preux P-M. Screening of pregnant women for hepatitis B markers in a French Provincial University Hospital (Limoges) during 15 years. Eur J Epidemiol. 2004; 19(10): 973-8.
Denmark Cancer Registry 1970 - CI5 as it appears in Ferlay J, Parkin DM, Curado MP, Bray F, Edwards B, Shin HR and Forman D. Cancer Incidence in Five Continents, Volumes I to IX: IARC CancerBase No. 9 [Internet]. Lyon, France: International Agency for Research on Cancer; 2010. Available from: http://ci5.iarc.fr
Denmark Cancer Registry 1971 - CI5 as it appears in Ferlay J, Parkin DM, Curado MP, Bray F, Edwards B, Shin HR and Forman D. Cancer Incidence in Five Continents, Volumes I to IX: IARC CancerBase No. 9 [Internet]. Lyon, France: International Agency for Research on Cancer; 2010. Available from: http://ci5.iarc.fr
Denmark Cancer Registry 1972 - CI5 as it appears in Ferlay J, Parkin DM, Curado MP, Bray F, Edwards B, Shin HR and Forman D. Cancer Incidence in Five Continents, Volumes I to IX: IARC CancerBase No. 9 [Internet]. Lyon, France: International Agency for Research on Cancer; 2010. Available from: http://ci5.iarc.fr
Denmark Cancer Registry 1973 - CI5 as it appears in Ferlay J, Parkin DM, Curado MP, Bray F, Edwards B, Shin HR and Forman D. Cancer Incidence in Five Continents, Volumes I to IX: IARC CancerBase No. 9 [Internet]. Lyon, France: International Agency for Research on Cancer; 2010. Available from: http://ci5.iarc.fr
Denmark Cancer Registry 1974 - CI5 as it appears in Ferlay J, Parkin DM, Curado MP, Bray F, Edwards B, Shin HR and Forman D. Cancer Incidence in Five Continents, Volumes I to IX: IARC CancerBase No. 9 [Internet]. Lyon, France: International Agency for Research on Cancer; 2010. Available from: http://ci5.iarc.fr
Denmark Cancer Registry 1975 - CI5 as it appears in Ferlay J, Parkin DM, Curado MP, Bray F, Edwards B, Shin HR and Forman D. Cancer Incidence in Five Continents, Volumes I to IX: IARC CancerBase No. 9 [Internet]. Lyon, France: International Agency for Research on Cancer; 2010. Available from: http://ci5.iarc.fr
Denmark Cancer Registry 1976 - CI5 as it appears in Ferlay J, Parkin DM, Curado MP, Bray F, Edwards B, Shin HR and Forman D. Cancer Incidence in Five Continents, Volumes I to IX: IARC CancerBase No. 9 [Internet]. Lyon, France: International Agency for Research on Cancer; 2010. Available from: http://ci5.iarc.fr

Appendix: Citation List

Citation
Denmark Cancer Registry 1977 - CI5 as it appears in Ferlay J, Parkin DM, Curado MP, Bray F, Edwards B, Shin HR and Forman D. Cancer Incidence in Five Continents, Volumes I to IX: IARC CancerBase No. 9 [Internet]. Lyon, France: International Agency for Research on Cancer; 2010. Available from: http://ci5.iarc.fr
Denmark Cancer Registry 1978 - CI5 as it appears in Ferlay J, Parkin DM, Curado MP, Bray F, Edwards B, Shin HR and Forman D. Cancer Incidence in Five Continents, Volumes I to IX: IARC CancerBase No. 9 [Internet]. Lyon, France: International Agency for Research on Cancer; 2010. Available from: http://ci5.iarc.fr
Denmark Cancer Registry 1978-1982 - CI5 as it appears in Parkin DM, International Agency for Research on Cancer, International Association of Cancer Registries. Cancer Incidence in Five Continents. Vol. I to VIII. Lyon, France, IARCPress, 2005.
Denmark Cancer Registry 1979 - CI5 as it appears in Ferlay J, Parkin DM, Curado MP, Bray F, Edwards B, Shin HR and Forman D. Cancer Incidence in Five Continents, Volumes I to IX: IARC CancerBase No. 9 [Internet]. Lyon, France: International Agency for Research on Cancer; 2010. Available from: http://ci5.iarc.fr
Denmark Cancer Registry 1980 - CI5 as it appears in Ferlay J, Parkin DM, Curado MP, Bray F, Edwards B, Shin HR and Forman D. Cancer Incidence in Five Continents, Volumes I to IX: IARC CancerBase No. 9 [Internet]. Lyon, France: International Agency for Research on Cancer; 2010. Available from: http://ci5.iarc.fr
Denmark Cancer Registry 1981 - CI5 as it appears in Ferlay J, Parkin DM, Curado MP, Bray F, Edwards B, Shin HR and Forman D. Cancer Incidence in Five Continents, Volumes I to IX: IARC CancerBase No. 9 [Internet]. Lyon, France: International Agency for Research on Cancer; 2010. Available from: http://ci5.iarc.fr
Denmark Cancer Registry 1982 - CI5 as it appears in Ferlay J, Parkin DM, Curado MP, Bray F, Edwards B, Shin HR and Forman D. Cancer Incidence in Five Continents, Volumes I to IX: IARC CancerBase No. 9 [Internet]. Lyon, France: International Agency for Research on Cancer; 2010. Available from: http://ci5.iarc.fr
Denmark Cancer Registry 1983 - CI5 as it appears in Ferlay J, Parkin DM, Curado MP, Bray F, Edwards B, Shin HR and Forman D. Cancer Incidence in Five Continents, Volumes I to IX: IARC CancerBase No. 9 [Internet]. Lyon, France: International Agency for Research on Cancer; 2010. Available from: http://ci5.iarc.fr
Denmark Cancer Registry 1983-1987 - CI5 as it appears in Parkin DM, International Agency for Research on Cancer, International Association of Cancer Registries. Cancer Incidence in Five Continents. Vol. I to VIII. Lyon, France, IARCPress, 2005.
Denmark Cancer Registry 1984 - CI5 as it appears in Ferlay J, Parkin DM, Curado MP, Bray F, Edwards B, Shin HR and Forman D. Cancer Incidence in Five Continents, Volumes I to IX: IARC CancerBase No. 9 [Internet]. Lyon, France: International Agency for Research on Cancer; 2010. Available from: http://ci5.iarc.fr
Denmark Cancer Registry 1985 - CI5 as it appears in Ferlay J, Parkin DM, Curado MP, Bray F, Edwards B, Shin HR and Forman D. Cancer Incidence in Five Continents, Volumes I to IX: IARC CancerBase No. 9 [Internet]. Lyon, France: International Agency for Research on Cancer; 2010. Available from: http://ci5.iarc.fr
Denmark Cancer Registry 1986 - CI5 as it appears in Ferlay J, Parkin DM, Curado MP, Bray F, Edwards B, Shin HR and Forman D. Cancer Incidence in Five Continents, Volumes I to IX: IARC CancerBase No. 9 [Internet]. Lyon, France: International Agency for Research on Cancer; 2010. Available from: http://ci5.iarc.fr
Denmark Cancer Registry 1987 - CI5 as it appears in Ferlay J, Parkin DM, Curado MP, Bray F, Edwards B, Shin HR and Forman D. Cancer Incidence in Five Continents, Volumes I to IX: IARC CancerBase No. 9 [Internet]. Lyon, France: International Agency for Research on Cancer; 2010. Available from: http://ci5.iarc.fr
Denmark Cancer Registry 1988 - CI5 as it appears in Ferlay J, Parkin DM, Curado MP, Bray F, Edwards B, Shin HR and Forman D. Cancer Incidence in Five Continents, Volumes I to IX: IARC CancerBase No. 9 [Internet]. Lyon, France: International Agency for Research on Cancer; 2010. Available from: http://ci5.iarc.fr
Denmark Cancer Registry 1988-1992 - CI5 as it appears in Parkin DM, International Agency for Research on Cancer, International Association of Cancer Registries. Cancer Incidence in Five Continents. Vol. I to VIII. Lyon, France, IARCPress, 2005.
Denmark Cancer Registry 1989 - CI5 as it appears in Ferlay J, Parkin DM, Curado MP, Bray F, Edwards B, Shin HR and Forman D. Cancer Incidence in Five Continents, Volumes I to IX: IARC CancerBase No. 9 [Internet]. Lyon, France: International Agency for Research on Cancer; 2010. Available from: http://ci5.iarc.fr
Denmark Cancer Registry 1990 - CI5 as it appears in Ferlay J, Parkin DM, Curado MP, Bray F, Edwards B, Shin HR and Forman D. Cancer Incidence in Five Continents, Volumes I to IX: IARC CancerBase No. 9 [Internet]. Lyon, France: International Agency for Research on Cancer; 2010. Available from: http://ci5.iarc.fr
Denmark Cancer Registry 1991 - CI5 as it appears in Ferlay J, Parkin DM, Curado MP, Bray F, Edwards B, Shin HR and Forman D. Cancer Incidence in Five Continents, Volumes I to IX: IARC CancerBase No. 9 [Internet]. Lyon, France: International Agency for Research on Cancer; 2010. Available from: http://ci5.iarc.fr
Denmark Cancer Registry 1992 - CI5 as it appears in Ferlay J, Parkin DM, Curado MP, Bray F, Edwards B, Shin HR and Forman D. Cancer Incidence in Five Continents, Volumes I to IX: IARC CancerBase No. 9 [Internet]. Lyon, France: International Agency for Research on Cancer; 2010. Available from: http://ci5.iarc.fr
Denmark Cancer Registry 1993 - CI5 as it appears in Ferlay J, Parkin DM, Curado MP, Bray F, Edwards B, Shin HR and Forman D. Cancer Incidence in Five Continents, Volumes I to IX: IARC CancerBase No. 9 [Internet]. Lyon, France: International Agency for Research on Cancer; 2010. Available from: http://ci5.iarc.fr
Denmark Cancer Registry 1993-1997 - CI5 as it appears in Ferlay J, Parkin DM, Curado MP, Bray F, Edwards B, Shin HR and Forman D. Cancer Incidence in Five Continents, Volumes I to IX: IARC CancerBase No. 9 [Internet]. Lyon, France: International Agency for Research on Cancer; 2010. Available from: http://ci5.iarc.fr
Denmark Cancer Registry 1994 - CI5 as it appears in Ferlay J, Parkin DM, Curado MP, Bray F, Edwards B, Shin HR and Forman D. Cancer Incidence in Five Continents, Volumes I to IX: IARC CancerBase No. 9 [Internet]. Lyon, France: International Agency for Research on Cancer; 2010. Available from: http://ci5.iarc.fr

Appendix: Citation List

Citation
Denmark Cancer Registry 1995 - CI5 as it appears in Ferlay J, Parkin DM, Curado MP, Bray F, Edwards B, Shin HR and Forman D. Cancer Incidence in Five Continents, Volumes I to IX: IARC CancerBase No. 9 [Internet]. Lyon, France: International Agency for Research on Cancer; 2010. Available from: http://ci5.iarc.fr
Denmark Cancer Registry 1996 - CI5 as it appears in Ferlay J, Parkin DM, Curado MP, Bray F, Edwards B, Shin HR and Forman D. Cancer Incidence in Five Continents, Volumes I to IX: IARC CancerBase No. 9 [Internet]. Lyon, France: International Agency for Research on Cancer; 2010. Available from: http://ci5.iarc.fr
Denmark Cancer Registry 1997 - CI5 as it appears in Ferlay J, Parkin DM, Curado MP, Bray F, Edwards B, Shin HR and Forman D. Cancer Incidence in Five Continents, Volumes I to IX: IARC CancerBase No. 9 [Internet]. Lyon, France: International Agency for Research on Cancer; 2010. Available from: http://ci5.iarc.fr
Denmark Cancer Registry 1998 - CI5 as it appears in Ferlay J, Parkin DM, Curado MP, Bray F, Edwards B, Shin HR and Forman D. Cancer Incidence in Five Continents, Volumes I to IX: IARC CancerBase No. 9 [Internet]. Lyon, France: International Agency for Research on Cancer; 2010. Available from: http://ci5.iarc.fr
Denmark Cancer Registry 1998-2002 - CI5 as it appears in Ferlay J, Parkin DM, Curado MP, Bray F, Edwards B, Shin HR and Forman D. Cancer Incidence in Five Continents, Volumes I to IX: IARC CancerBase No. 9 [Internet]. Lyon, France: International Agency for Research on Cancer; 2010. Available from: http://ci5.iarc.fr
Denmark Cancer Registry 1999 - CI5 as it appears in Ferlay J, Parkin DM, Curado MP, Bray F, Edwards B, Shin HR and Forman D. Cancer Incidence in Five Continents, Volumes I to IX: IARC CancerBase No. 9 [Internet]. Lyon, France: International Agency for Research on Cancer; 2010. Available from: http://ci5.iarc.fr
Denmark Cancer Registry 2000 - CI5 as it appears in Ferlay J, Parkin DM, Curado MP, Bray F, Edwards B, Shin HR and Forman D. Cancer Incidence in Five Continents, Volumes I to IX: IARC CancerBase No. 9 [Internet]. Lyon, France: International Agency for Research on Cancer; 2010. Available from: http://ci5.iarc.fr
Denmark Cancer Registry 2001 - CI5 as it appears in Ferlay J, Parkin DM, Curado MP, Bray F, Edwards B, Shin HR and Forman D. Cancer Incidence in Five Continents, Volumes I to IX: IARC CancerBase No. 9 [Internet]. Lyon, France: International Agency for Research on Cancer; 2010. Available from: http://ci5.iarc.fr
Denmark Cancer Registry 2002 - CI5 as it appears in Ferlay J, Parkin DM, Curado MP, Bray F, Edwards B, Shin HR and Forman D. Cancer Incidence in Five Continents, Volumes I to IX: IARC CancerBase No. 9 [Internet]. Lyon, France: International Agency for Research on Cancer; 2010. Available from: http://ci5.iarc.fr
Denmark Eurobarometer 32: The Single European Market, Drugs, Alcohol, and Cancer 1989 as it appears in P.N. Lee Statistics and Computing Ltd. International Mortality and Smoking Statistics Version 4.04. Sutton, United Kingdom: P.N. Lee Statistics and Computing Ltd, 2009.
Denmark Health Interview Survey 1991 as it appears in P.N. Lee Statistics and Computing Ltd. International Mortality and Smoking Statistics Version 4.04. Sutton, United Kingdom: P.N. Lee Statistics and Computing Ltd, 2009.
Denmark Health Interview Survey 1998 as it appears in P.N. Lee Statistics and Computing Ltd. International Mortality and Smoking Statistics Version 4.04. Sutton, United Kingdom: P.N. Lee Statistics and Computing Ltd, 2009.
Denmark Health Interview Survey 1999 as it appears in P.N. Lee Statistics and Computing Ltd. International Mortality and Smoking Statistics Version 4.04. Sutton, United Kingdom: P.N. Lee Statistics and Computing Ltd, 2009.
Denmark Health Interview Survey 2001 as it appears in P.N. Lee Statistics and Computing Ltd. International Mortality and Smoking Statistics Version 4.04. Sutton, United Kingdom: P.N. Lee Statistics and Computing Ltd, 2009.
Denmark Longitudinal Health Behavior Survey 1990 as it appears in P.N. Lee Statistics and Computing Ltd. International Mortality and Smoking Statistics Version 4.04. Sutton, United Kingdom: P.N. Lee Statistics and Computing Ltd, 2009.
Denmark Vital Registration - Deaths 2007 ICD10 as it appears in World Health Organization (WHO). WHO Mortality Database Version November 2015. Geneva, Switzerland: World Health Organization (WHO), 2015.
Denmark Vital Registration - Deaths 1951 ICD7 as it appears in World Health Organization (WHO). WHO Mortality Database Version July 2012. Geneva, Switzerland: World Health Organization (WHO), 2012.
Denmark Vital Registration - Deaths 1952 ICD7 as it appears in World Health Organization (WHO). WHO Mortality Database Version July 2012. Geneva, Switzerland: World Health Organization (WHO), 2012.
Denmark Vital Registration - Deaths 1953 ICD7 as it appears in World Health Organization (WHO). WHO Mortality Database Version July 2012. Geneva, Switzerland: World Health Organization (WHO), 2012.
Denmark Vital Registration - Deaths 1954 ICD7 as it appears in World Health Organization (WHO). WHO Mortality Database Version July 2012. Geneva, Switzerland: World Health Organization (WHO), 2012.
Denmark Vital Registration - Deaths 1955 ICD7 as it appears in World Health Organization (WHO). WHO Mortality Database Version July 2012. Geneva, Switzerland: World Health Organization (WHO), 2012.
Denmark Vital Registration - Deaths 1956 ICD7 as it appears in World Health Organization (WHO). WHO Mortality Database Version July 2012. Geneva, Switzerland: World Health Organization (WHO), 2012.
Denmark Vital Registration - Deaths 1957 ICD7 as it appears in World Health Organization (WHO). WHO Mortality Database Version July 2012. Geneva, Switzerland: World Health Organization (WHO), 2012.
Denmark Vital Registration - Deaths 1958 ICD7 as it appears in World Health Organization (WHO). WHO Mortality Database Version July 2012. Geneva, Switzerland: World Health Organization (WHO), 2012.
Denmark Vital Registration - Deaths 1959 ICD7 as it appears in World Health Organization (WHO). WHO Mortality Database Version July 2012. Geneva, Switzerland: World Health Organization (WHO), 2012.
Denmark Vital Registration - Deaths 1960 ICD7 as it appears in World Health Organization (WHO). WHO Mortality Database Version July 2012. Geneva, Switzerland: World Health Organization (WHO), 2012.
Denmark Vital Registration - Deaths 1961 ICD7 as it appears in World Health Organization (WHO). WHO Mortality Database Version July 2012. Geneva, Switzerland: World Health Organization (WHO), 2012.

Appendix: Citation List

Citation
Denmark Vital Registration - Deaths 1962 ICD7 as it appears in World Health Organization (WHO). WHO Mortality Database Version July 2012. Geneva, Switzerland: World Health Organization (WHO), 2012.
Denmark Vital Registration - Deaths 1963 ICD7 as it appears in World Health Organization (WHO). WHO Mortality Database Version July 2012. Geneva, Switzerland: World Health Organization (WHO), 2012.
Denmark Vital Registration - Deaths 1964 ICD7 as it appears in World Health Organization (WHO). WHO Mortality Database Version July 2012. Geneva, Switzerland: World Health Organization (WHO), 2012.
Denmark Vital Registration - Deaths 1965 ICD7 as it appears in World Health Organization (WHO). WHO Mortality Database Version July 2012. Geneva, Switzerland: World Health Organization (WHO), 2012.
Denmark Vital Registration - Deaths 1966 ICD7 as it appears in World Health Organization (WHO). WHO Mortality Database Version July 2012. Geneva, Switzerland: World Health Organization (WHO), 2012.
Denmark Vital Registration - Deaths 1967 ICD7 as it appears in World Health Organization (WHO). WHO Mortality Database Version July 2012. Geneva, Switzerland: World Health Organization (WHO), 2012.
Denmark Vital Registration - Deaths 1968 ICD7 as it appears in World Health Organization (WHO). WHO Mortality Database Version July 2012. Geneva, Switzerland: World Health Organization (WHO), 2012.
Denmark Vital Registration - Deaths 1969 ICD8 as it appears in World Health Organization (WHO). WHO Mortality Database Version May 2013. Geneva, Switzerland: World Health Organization (WHO), 2013.
Denmark Vital Registration - Deaths 1970 ICD8 as it appears in World Health Organization (WHO). WHO Mortality Database Version May 2013. Geneva, Switzerland: World Health Organization (WHO), 2013.
Denmark Vital Registration - Deaths 1971 ICD8 as it appears in World Health Organization (WHO). WHO Mortality Database Version May 2013. Geneva, Switzerland: World Health Organization (WHO), 2013.
Denmark Vital Registration - Deaths 1972 ICD8 as it appears in World Health Organization (WHO). WHO Mortality Database Version May 2013. Geneva, Switzerland: World Health Organization (WHO), 2013.
Denmark Vital Registration - Deaths 1973 ICD8 as it appears in World Health Organization (WHO). WHO Mortality Database Version May 2013. Geneva, Switzerland: World Health Organization (WHO), 2013.
Denmark Vital Registration - Deaths 1974 ICD8 as it appears in World Health Organization (WHO). WHO Mortality Database Version May 2013. Geneva, Switzerland: World Health Organization (WHO), 2013.
Denmark Vital Registration - Deaths 1975 ICD8 as it appears in World Health Organization (WHO). WHO Mortality Database Version May 2013. Geneva, Switzerland: World Health Organization (WHO), 2013.
Denmark Vital Registration - Deaths 1976 ICD8 as it appears in World Health Organization (WHO). WHO Mortality Database Version May 2013. Geneva, Switzerland: World Health Organization (WHO), 2013.
Denmark Vital Registration - Deaths 1977 ICD8 as it appears in World Health Organization (WHO). WHO Mortality Database Version May 2013. Geneva, Switzerland: World Health Organization (WHO), 2013.
Denmark Vital Registration - Deaths 1978 ICD8 as it appears in World Health Organization (WHO). WHO Mortality Database Version May 2013. Geneva, Switzerland: World Health Organization (WHO), 2013.
Denmark Vital Registration - Deaths 1979 ICD8 as it appears in World Health Organization (WHO). WHO Mortality Database Version May 2013. Geneva, Switzerland: World Health Organization (WHO), 2013.
Denmark Vital Registration - Deaths 1980 ICD8 as it appears in World Health Organization (WHO). WHO Mortality Database Version May 2013. Geneva, Switzerland: World Health Organization (WHO), 2013.
Denmark Vital Registration - Deaths 1981 ICD8 as it appears in World Health Organization (WHO). WHO Mortality Database Version May 2013. Geneva, Switzerland: World Health Organization (WHO), 2013.
Denmark Vital Registration - Deaths 1982 ICD8 as it appears in World Health Organization (WHO). WHO Mortality Database Version May 2013. Geneva, Switzerland: World Health Organization (WHO), 2013.
Denmark Vital Registration - Deaths 1983 ICD8 as it appears in World Health Organization (WHO). WHO Mortality Database Version May 2013. Geneva, Switzerland: World Health Organization (WHO), 2013.
Denmark Vital Registration - Deaths 1984 ICD8 as it appears in World Health Organization (WHO). WHO Mortality Database Version May 2013. Geneva, Switzerland: World Health Organization (WHO), 2013.
Denmark Vital Registration - Deaths 1985 ICD8 as it appears in World Health Organization (WHO). WHO Mortality Database Version May 2013. Geneva, Switzerland: World Health Organization (WHO), 2013.
Denmark Vital Registration - Deaths 1986 ICD8 as it appears in World Health Organization (WHO). WHO Mortality Database Version May 2013. Geneva, Switzerland: World Health Organization (WHO), 2013.
Denmark Vital Registration - Deaths 1987 ICD8 as it appears in World Health Organization (WHO). WHO Mortality Database Version May 2013. Geneva, Switzerland: World Health Organization (WHO), 2013.
Denmark Vital Registration - Deaths 1988 ICD8 as it appears in World Health Organization (WHO). WHO Mortality Database Version May 2013. Geneva, Switzerland: World Health Organization (WHO), 2013.
Denmark Vital Registration - Deaths 1989 ICD8 as it appears in World Health Organization (WHO). WHO Mortality Database Version May 2013. Geneva, Switzerland: World Health Organization (WHO), 2013.
Denmark Vital Registration - Deaths 1990 ICD8 as it appears in World Health Organization (WHO). WHO Mortality Database Version May 2013. Geneva, Switzerland: World Health Organization (WHO), 2013.
Denmark Vital Registration - Deaths 1991 ICD8 as it appears in World Health Organization (WHO). WHO Mortality Database Version May 2013. Geneva, Switzerland: World Health Organization (WHO), 2013.
Denmark Vital Registration - Deaths 1992 ICD8 as it appears in World Health Organization (WHO). WHO Mortality Database Version May 2013. Geneva, Switzerland: World Health Organization (WHO), 2013.
Denmark Vital Registration - Deaths 1993 ICD8 as it appears in World Health Organization (WHO). WHO Mortality Database Version May 2013. Geneva, Switzerland: World Health Organization (WHO), 2013.

Appendix: Citation List

Citation
Denmark Vital Registration - Deaths 1994 ICD10 as it appears in World Health Organization (WHO). WHO Mortality Database Version November 2015. Geneva, Switzerland: World Health Organization (WHO), 2015.
Denmark Vital Registration - Deaths 1995 ICD10 as it appears in World Health Organization (WHO). WHO Mortality Database Version November 2015. Geneva, Switzerland: World Health Organization (WHO), 2015.
Denmark Vital Registration - Deaths 1996 ICD10 as it appears in World Health Organization (WHO). WHO Mortality Database Version November 2015. Geneva, Switzerland: World Health Organization (WHO), 2015.
Denmark Vital Registration - Deaths 1997 ICD10 as it appears in World Health Organization (WHO). WHO Mortality Database Version November 2015. Geneva, Switzerland: World Health Organization (WHO), 2015.
Denmark Vital Registration - Deaths 1998 ICD10 as it appears in World Health Organization (WHO). WHO Mortality Database Version November 2015. Geneva, Switzerland: World Health Organization (WHO), 2015.
Denmark Vital Registration - Deaths 1999 ICD10 as it appears in World Health Organization (WHO). WHO Mortality Database Version November 2015. Geneva, Switzerland: World Health Organization (WHO), 2015.
Denmark Vital Registration - Deaths 2000 ICD10 as it appears in World Health Organization (WHO). WHO Mortality Database Version November 2015. Geneva, Switzerland: World Health Organization (WHO), 2015.
Denmark Vital Registration - Deaths 2001 ICD10 as it appears in World Health Organization (WHO). WHO Mortality Database Version November 2015. Geneva, Switzerland: World Health Organization (WHO), 2015.
Denmark Vital Registration - Deaths 2002 ICD10 as it appears in World Health Organization (WHO). WHO Mortality Database Version November 2015. Geneva, Switzerland: World Health Organization (WHO), 2015.
Denmark Vital Registration - Deaths 2003 ICD10 as it appears in World Health Organization (WHO). WHO Mortality Database Version November 2015. Geneva, Switzerland: World Health Organization (WHO), 2015.
Denmark Vital Registration - Deaths 2004 ICD10 as it appears in World Health Organization (WHO). WHO Mortality Database Version November 2015. Geneva, Switzerland: World Health Organization (WHO), 2015.
Denmark Vital Registration - Deaths 2005 ICD10 as it appears in World Health Organization (WHO). WHO Mortality Database Version November 2015. Geneva, Switzerland: World Health Organization (WHO), 2015.
Denmark Vital Registration - Deaths 2006 ICD10 as it appears in World Health Organization (WHO). WHO Mortality Database Version November 2015. Geneva, Switzerland: World Health Organization (WHO), 2015.
Denmark Vital Registration - Deaths 2008 ICD10 as it appears in World Health Organization (WHO). WHO Mortality Database Version November 2015. Geneva, Switzerland: World Health Organization (WHO), 2015.
Denmark Vital Registration - Deaths 2009 ICD10 as it appears in World Health Organization (WHO). WHO Mortality Database Version November 2015. Geneva, Switzerland: World Health Organization (WHO), 2015.
Denmark Vital Registration - Deaths 2010 ICD10 as it appears in World Health Organization (WHO). WHO Mortality Database Version November 2015. Geneva, Switzerland: World Health Organization (WHO), 2015.
Denmark Vital Registration - Deaths 2011 ICD10 as it appears in World Health Organization (WHO). WHO Mortality Database Version November 2015. Geneva, Switzerland: World Health Organization (WHO), 2015.
Denmark Vital Registration - Deaths 2012 ICD10 as it appears in World Health Organization (WHO). WHO Mortality Database Version November 2015. Geneva, Switzerland: World Health Organization (WHO), 2015.
Denoeud L, Fievet N, Aubouy A, Ayemonna P, Kiniffo R, Massougbodji A, Cot M. Is chloroquine chemoprophylaxis still effective to prevent low birth weight? Results of a study in Benin. Malar J. 2007; 6: 27.
Dent AE, Chelimo K, Sumba PO, Spring MD, Crabb BS, Moormann AM, Tisch DJ, Kazura JW. Temporal stability of naturally acquired immunity to Merozoite Surface Protein-1 in Kenyan Adults. Malar J. 2009; 8(1): 162. As it appears in Malaria Atlas Project. Malaria Atlas Project Plasmodium Falciparum Parasite Rate Database. Oxford, United Kingdom: Malaria Atlas Project.
Department of Applied Research for Development (Madagascar), ICF Macro, National Institute of Statistics (Madagascar). Madagascar Demographic and Health Survey - Maternal Mortality Data.
Department of Applied Research for Development (Madagascar), Macro International, Inc. Madagascar Demographic and Health Survey 1992. Calverton, United States: Macro International, Inc.
Department of Biological Anthropology, University of Cambridge (UK), Institute of Epidemiology, Disease Control & Research (Bangladesh), National Centre for Control of Rheumatic Fever & Heart Diseases (Bangladesh), National Institute of Preventive and Social Medicine, University of Dhaka (Bangladesh), World Health Organization (WHO). Bangladesh STEPS Noncommunicable Disease Risk Factors Survey 2002.
Department of Census and Statistics (Sri Lanka), International Statistical Institute. Sri Lanka World Fertility Survey 1975. Voorburg, Netherlands: International Statistical Institute.
Department of Census and Statistics (Sri Lanka), Ministry of Health, Nutrition and Welfare (Sri Lanka). Sri Lanka Demographic and Health Survey 2000. Colombo, Sri Lanka: Department of Census and Statistics (Sri Lanka), 2008.
Department of Census and Statistics (Sri Lanka), Westinghouse; Institute for Resource Development. Sri Lanka Demographic and Health Survey 1987. Columbia, United States: Westinghouse; Institute for Resource Development.
Department of Census and Statistics (Sri Lanka). Sri Lanka Demographic and Health Survey 1993.
Department of Census and Statistics (Sri Lanka). Sri Lanka Demographic and Health Survey 2006-2007.
Department of Census and Statistics (Sri Lanka). Sri Lanka Household Income and Expenditure Survey 2009-2010.
Department of Census and Statistics (Sri Lanka). Sri Lanka Population and Housing Census 1971.
Department of Census and Statistics (Sri Lanka). Sri Lanka Population and Housing Census 1981.
Department of Census and Statistics (Sri Lanka). Sri Lanka Population and Housing Census 2001.
Department of Economic Planning and Development (Brunei Darussalam). Brunei Darussalam Population and Housing Census 2001.
Department of Economics, University of Chile, Ministry of Planning (Chile). Chile National Socioeconomic Characterization Survey 1990.
Department of Economics, University of Chile, Ministry of Planning (Chile). Chile National Socioeconomic Characterization Survey 1992.

Appendix: Citation List

Citation
Department of Economics, University of Chile, Ministry of Planning (Chile). Chile National Socioeconomic Characterization Survey 1998.
Department of Epidemiology and Public Health, University College London, National Centre for Social Research (NatCen). United Kingdom Health Survey for England 2013-2014 - HSCIC.
Department of Epidemiology and Public Health, University College London, Scottish Centre for Social Research (ScotCen), University of Glasgow. United Kingdom - Scottish Health Survey 2012 - Scottish Government.
Department of Health (American Samoa), Monash University (Australia), World Health Organization (WHO). American Samoa STEPS Noncommunicable Disease Risk Factors Survey 2004.
Department of Health (Ireland), Economic and Social Research Institute (ESRI) (Ireland). Ireland Smoking and Drinking Among Young People 1993.
Department of Health (Papua New Guinea). Papua New Guinea National Immunization Coverage Survey 2005-2006.
Department of Health (Philippines), Department of Science and Technology (Philippines), Food and Nutrition Research Institute, Department of Science and Technology (Philippines), National Statistics Office (Philippines). Philippines Updating of Nutritional Status of Filipino Children and Other Population Groups 2011.
Department of Health (Philippines), Macro International, Inc, National Statistics Office (Philippines). Philippines Demographic and Health Survey - Maternal Mortality Data.
Department of Health (Philippines), Macro International, Inc, National Statistics Office (Philippines). Philippines Demographic and Health Survey 1998. Calverton, United States: Macro International, Inc.
Department of Health (Philippines), University of the Philippines, Manila. Philippines Baseline Behavioural Risk Factor Survey 2001.
Department of Health (Philippines). Philippines Vital Statistics - Deaths 1979-2000.
Department of Health (Philippines). Philippines Vital Statistics - Deaths 2001-2005.
Department of Health (South Africa), Human Sciences Research Council, World Health Organization (WHO). South Africa WHO Study on Global AGEing and Adult Health 2007-2008. Geneva, Switzerland: World Health Organization (WHO).
Department of Health (South Africa), Macro International, Inc, South African Medical Research Council. South Africa Demographic and Health Survey 1998. Calverton, United States: Macro International, Inc.
Department of Health (South Africa), Macro International, Inc, South African Medical Research Council. South Africa Demographic and Health Survey 2003-2004.
Department of Health (South Africa). South Africa Malaria Control Programme Monthly Report: Period 01-05-95 to 31-05-95. Jozini, South Africa: Department of Health (South Africa), 1995. As it appears in Malaria Atlas Project. Malaria Atlas Project Plasmodium Falciparum Parasite Rate Database. Oxford, United Kingdom: Malaria Atlas Project.
Department of Health (Taiwan). Taiwan Annual Statistical Summary of Causes of Death 1994. Taipei City, Taiwan: Department of Health (Taiwan).
Department of Health (Taiwan). Taiwan Statistics of Causes of Death 1995. Taipei City, Taiwan: Department of Health (Taiwan).
Department of Health (Taiwan). Taiwan Statistics of Causes of Death 1996. Taipei City, Taiwan: Department of Health (Taiwan).
Department of Health (Taiwan). Taiwan Statistics of Causes of Death 1997. Taipei City, Taiwan: Department of Health (Taiwan).
Department of Health (Taiwan). Taiwan Statistics of Causes of Death 1998. Taipei City, Taiwan: Department of Health (Taiwan).
Department of Health (Taiwan). Taiwan Statistics of Causes of Death 1999. Taipei City, Taiwan: Department of Health (Taiwan).
Department of Health (Taiwan). Taiwan Statistics of Causes of Death 2000. Taipei City, Taiwan: Department of Health (Taiwan).
Department of Health (Taiwan). Taiwan Statistics of Causes of Death 2001. Taipei City, Taiwan: Department of Health (Taiwan).
Department of Health (Taiwan). Taiwan Statistics of Causes of Death 2002. Taipei City, Taiwan: Department of Health (Taiwan).
Department of Health (Taiwan). Taiwan Statistics of Causes of Death 2003. Taipei City, Taiwan: Department of Health (Taiwan).
Department of Health (Taiwan). Taiwan Statistics of Causes of Death 2004. Taipei City, Taiwan: Department of Health (Taiwan).
Department of Health (Taiwan). Taiwan Statistics of Causes of Death 2005. Taipei City, Taiwan: Department of Health (Taiwan).
Department of Health (Taiwan). Taiwan Statistics of Causes of Death 2006. Taipei City, Taiwan: Department of Health (Taiwan).
Department of Health (Taiwan). Taiwan Statistics of Causes of Death 2008. Taipei City, Taiwan: Department of Health (Taiwan).
Department of Health (Taiwan). Taiwan Statistics of Causes of Death 2009.
Department of Health (Taiwan). Taiwan Statistics of Causes of Death 2010. Taipei City, Taiwan: Department of Health (Taiwan).
Department of Health (Taiwan). Taiwan Statistics of Causes of Death 2011. Taipei City, Taiwan: Department of Health (Taiwan).
Department of Health (Taiwan). Taiwan Statistics of Causes of Death 2012. Taipei City, Taiwan: Department of Health (Taiwan).
Department of Health (Taiwan). Taiwan Vital Statistics - Deaths 2007.
Department of Health and Ageing (Australia), World Health Organization (WHO). Australia World Health Survey 2003. Geneva, Switzerland: World Health Organization (WHO), 2005.
Department of Health Information, Ministry for Health (Malta). Malta National Health Interview Survey 2002.
Department of Home Affairs (South Africa), Statistics South Africa. South Africa Vital Registration - Causes of Death 1997-2005. Pretoria, South Africa: Statistics South Africa.
Department of Home Affairs (South Africa), Statistics South Africa. South Africa Vital Registration - Causes of Death 2006. Pretoria, South Africa: Statistics South Africa.
Department of Home Affairs (South Africa), Statistics South Africa. South Africa Vital Registration - Causes of Death 2007. Pretoria, South Africa: Statistics South Africa.
Department of Home Affairs (South Africa), Statistics South Africa. South Africa Vital Registration - Causes of Death 2008. Pretoria, South Africa: Statistics South Africa.
Department of Home Affairs (South Africa), Statistics South Africa. South Africa Vital Registration - Causes of Death 2009. Pretoria, South Africa: Statistics South Africa.
Department of Home Affairs (South Africa), Statistics South Africa. South Africa Vital Registration - Causes of Death 2010. Pretoria, South Africa: Statistics South Africa.

Appendix: Citation List

Citation
Department of Home Affairs (South Africa), Statistics South Africa. South Africa Vital Registration - Causes of Death 2011. Pretoria, South Africa: Statistics South Africa.
Department of Home Affairs (South Africa), Statistics South Africa. South Africa Vital Registration - Causes of Death 2012. Pretoria, South Africa: Statistics South Africa.
Department of Home Affairs (South Africa), Statistics South Africa. South Africa Vital Registration - Causes of Death 2013. Pretoria, South Africa: Statistics South Africa.
Department of Ophthalmology and Visual Sciences, University of Wisconsin-Madison. United States - Wisconsin Beaver Dam Eye Study 1988-1990. As it appears in Bourne R, Global Burden of Disease Vision Loss Expert Group. Vision Loss Database - Survey Data on Vision Loss by Severity and Etiology. [Unpublished].
Department of Public Health, Pontifical Catholic University of Chile, Institute of Nutrition and Food Technology (INTA), University of Chile, Ministry of Health (Chile), Pan American Health Organization (PAHO). Chile National Health Survey 2003.
Department of Pulmonary Medicine, Postgraduate Institute of Medical Education & Research (India). India Study on Epidemiology of Asthma, Respiratory Symptoms and Chronic Bronchitis (INSEARCH) 2007-2009.
Department of Pulmonary Medicine, Postgraduate Institute of Medical Education & Research (India). India Study on Epidemiology of Asthma, Respiratory Symptoms and Chronic Bronchitis 2004-2005.
Department of Statistics (Côte d'Ivoire), International Statistical Institute. Côte d'Ivoire World Fertility Survey 1980-1981. Voorburg, Netherlands: International Statistical Institute.
Department of Statistics (Côte d'Ivoire), World Bank (WB). Côte d'Ivoire Living Standards Measurement Survey 1985-1986. Washington, DC, United States: World Bank (WB)
Department of Statistics (Côte d'Ivoire), World Bank (WB). Côte d'Ivoire Living Standards Measurement Survey 1986-1987. Washington, DC, United States: World Bank (WB)
Department of Statistics (Côte d'Ivoire). Côte d'Ivoire Demographic Survey 1978-1979.
Department of Statistics (Côte d'Ivoire). Côte d'Ivoire Living Standards Measurement Survey 1987-1988. Washington, DC, United States: World Bank (WB).
Department of Statistics (Côte d'Ivoire). Côte d'Ivoire Living Standards Measurement Survey 1988-1989. Washington, DC, United States: World Bank (WB).
Department of Statistics (Jamaica), Minnesota Population Center. Jamaica Population Census 1982 from the Integrated Public Use Microdata Series, International: [Machine-readable database]. Minneapolis: University of Minnesota, 2011.
Department of Statistics (Jordan), ICF International. Jordan Demographic and Health Survey 2012. Fairfax, United States: ICF International, 2013.
Department of Statistics (Jordan), ICF Macro. Jordan Interim Demographic and Health Survey 2009. Calverton, United States: ICF Macro, 2010.
Department of Statistics (Jordan), Macro International, Inc, Ministry of Planning (Jordan). Jordan Demographic and Health Survey - Maternal Mortality Data.
Department of Statistics (Jordan), Macro International, Inc. Jordan Demographic and Health Survey 1997. Calverton, United States: Macro International, Inc.
Department of Statistics (Jordan), Macro International, Inc. Jordan Demographic and Health Survey 2002. Calverton, United States: Macro International, Inc.
Department of Statistics (Jordan), Macro International, Inc. Jordan Demographic and Health Survey 2007. Calverton, United States: Macro International, Inc.
Department of Statistics (Jordan), Macro International, Inc.; Institute for Resource Development, Ministry of Planning (Jordan). Jordan Demographic and Health Survey 1990. Calverton, United States: Macro International, Inc.
Department of Statistics (Jordan), Minnesota Population Center. Jordan Population and Housing Census 2004 from the Integrated Public Use Microdata Series, International: [Machine-readable database]. Minneapolis: University of Minnesota.
Department of Statistics (Jordan), United Nations, US Census Bureau. Jordan Population and Housing Census 1979. Amman, Jordan: Department of Statistics (Jordan).
Department of Statistics (Jordan), United States Census Bureau. Jordan Annual Fertility Survey 1999.
Department of Statistics (Laos). Laos Expenditure and Consumption Survey 2007-2008. Vientiane, Laos: Department of Statistics (Laos).
Department of Statistics (Malaysia), Minnesota Population Center. Malaysia Population and Housing Census 1970 from the Integrated Public Use Microdata Series, International: [Machine-readable database]. Minneapolis: University of Minnesota.
Department of Statistics (Malaysia), Minnesota Population Center. Malaysia Population and Housing Census 1980 from the Integrated Public Use Microdata Series, International: [Machine-readable database]. Minneapolis: University of Minnesota.
Department of Statistics (Malaysia), Minnesota Population Center. Malaysia Population and Housing Census 1991 from the Integrated Public Use Microdata Series, International: [Machine-readable database]. Minneapolis: University of Minnesota.
Department of Statistics (Malaysia), Minnesota Population Center. Malaysia Population and Housing Census 2000 from the Integrated Public Use Microdata Series, International: [Machine-readable database]. Minneapolis: University of Minnesota.
Department of Statistics (Malaysia). Malaysia - Peninsular Vital Statistics - Deaths 1998.
Department of Statistics (Malaysia). Vital Statistics: Peninsular Malaysia 1980. Kuala Lumpur, Malaysia: Department of Statistics (Malaysia), 1983.
Department of Statistics (Malaysia). Vital Statistics: Peninsular Malaysia 1981. Kuala Lumpur, Malaysia: Department of Statistics (Malaysia), 1983.
Department of Statistics (Malaysia). Vital Statistics: Peninsular Malaysia 1982. Kuala Lumpur, Malaysia: Department of Statistics (Malaysia), 1984.

Appendix: Citation List

Citation
Department of Statistics (Mexico), International Statistical Institute. Mexico World Fertility Survey 1976-1977. Voorburg, Netherlands: International Statistical Institute.
Department of Statistics (Mexico). Mexico General Population and Housing Census 1980.
Department of Statistics (Rwanda), United Nations Children's Fund (UNICEF). Rwanda Multiple Indicator Cluster Survey 2000. New York, United States: United Nations Children's Fund (UNICEF).
Department of Statistics (Sao Tome and Principe). Sao Tome and Principe Population and Housing Census 1981.
Department of Statistics (Senegal), International Statistical Institute. Senegal World Fertility Survey 1978. Voorburg, Netherlands: International Statistical Institute.
Department of Statistics (Senegal), Westinghouse; Institute for Resource Development. Senegal Demographic and Health Survey 1986. Columbia, United States: Westinghouse; Institute for Resource Development.
Department of Statistics (Sudan), International Statistical Institute, Ministry of National Planning (North Sudan). Sudan World Fertility Survey 1978-1979. International Statistical Institute.
Department of Statistics (Togo), Macro International, Inc. Togo Demographic and Health Survey 1998. Calverton, United States: Macro International, Inc.
Department of Statistics and Accounting, Ministery of the Economy and Finance (Cameroon) and United Nations Children's Fund (UNICEF). Cameroon Multiple Indicator Cluster Survey 2000. New York, United States: United Nations Children's Fund (UNICEF).
Department of Statistics and Census (Panama), Minnesota Population Center. Panama Population and Housing Census 1960 from the Integrated Public Use Microdata Series, International: [Machine-readable database]. Minneapolis: University of Minnesota.
Department of Statistics and Census (Panama), Minnesota Population Center. Panama Population and Housing Census 1980 from the Integrated Public Use Microdata Series, International: [Machine-readable database]. Minneapolis: University of Minnesota.
Department of Statistics and Census (Panama), Minnesota Population Center. Panama Population and Housing Census 1990 from the Integrated Public Use Microdata Series, International: [Machine-readable database]. Minneapolis: University of Minnesota.
Department of Statistics and Census (Panama), Minnesota Population Center. Panama Population and Housing Census 2000 from the Integrated Public Use Microdata Series, International: [Machine-readable database]. Minneapolis: University of Minnesota.
Department of Statistics and Censuses (Costa Rica), International Statistical Institute. Costa Rica World Fertility Survey 1976. Voorburg, Netherlands: International Statistical Institute.
Department of Statistics and Censuses (Costa Rica), Minnesota Population Center. Costa Rica National Population and Housing Census 1973 from the Integrated Public Use Microdata Series, International: [Machine-readable database]. Minneapolis: University of Minnesota.
Department of Statistics and Censuses (Costa Rica), Minnesota Population Center. Costa Rica National Population and Housing Census 1984 from the Integrated Public Use Microdata Series, International: [Machine-readable database]. Minneapolis: University of Minnesota.
Department of Statistics and Censuses (Honduras), Latin American and Caribbean Demographic Center (CELADE). Honduras National Demographic Survey 1970-1972.
Department of Statistics and Censuses (Honduras), Latin American and Caribbean Demographic Center (CELADE). Honduras National Demographic Survey 1983-1984.
Department of Statistics and Censuses (Honduras). Honduras Population and Housing Census 1988.
Department of Statistics and Censuses (Paraguay), International Statistical Institute. Paraguay World Fertility Survey 1979. Voorburg, Netherlands: International Statistical Institute.
Department of Statistics and Censuses (Paraguay). Paraguay Population and Housing Census 1982.
Department of Statistics and Demographic Studies (Djibouti), League of Arab States, Ministry of Health (Djibouti), Pan Arab Project for Family Health (PAPFAM). Djibouti Family Health Survey 2002.
Department of Statistics and Demographic Studies (Djibouti), League of Arab States, Ministry of Health (Djibouti), Pan Arab Project for Family Health (PAPFAM). Djibouti Family Health Survey 2012.
Department of Statistics and Health Information, Ministry of Health (Chile), National Institute of Statistics (Chile). Chile Vital Statistics - Deaths 2010. Santiago, Chile: Department of Statistics and Health Information, Ministry of Health (Chile).
Department of Statistics and National Accounts (Mauritania), International Statistical Institute. Mauritania World Fertility Survey 1981. Voorburg, Netherlands: International Statistical Institute.
Department of Statistics and National Accounts (Mauritania). Mauritania Population and Housing Census 1988.
Department of Statistics and National Accounts (Niger), Macro International, Inc. Niger Demographic and Health Survey 1992. Calverton, United States: Macro International, Inc.
Department of Statistics and National Accounts (Niger), Macro International, Inc. Niger Demographic and Health Survey 2006. Calverton, United States: Macro International, Inc.
Department of Statistics and National Accounts (Niger), United Nations Department of Economic and Social Development. Niger Continuous Survey on Economic and Social Conditions 1994. Niamey, Niger: Department of Statistics and National Accounts (Niger).
Department of Statistics and National Accounts (Niger), United Nations Department of Economic and Social Development. Niger Continuous Survey on Economic and Social Conditions 1995-1996. Niamey, Niger: Department of Statistics and National Accounts (Niger).
Department of Statistics, Ministry of Finance (Antigua and Barbuda). Antigua and Barbuda Population and Housing Census 1991.
Department of Statistics, Surveys and Censuses (Paraguay). Paraguay Integrated Household Survey 1997-1998. Asunción, Paraguay: Department of Statistics, Surveys and Censuses (Paraguay).
Department of Statistics, Surveys and Censuses (Paraguay). Paraguay Integrated Household Survey 2000-2001. Asunción, Paraguay: Department of Statistics, Surveys and Censuses (Paraguay).
Department of Statistics, Surveys and Censuses (Paraguay). Paraguay Permanent Household Survey 2009. Asuncin, Paraguay: Department of Statistics, Surveys and Censuses (Paraguay)
Department of Statistics, Surveys and Censuses (Paraguay). Paraguay Population and Housing Census 1992.
Department of Statistics, Surveys and Censuses (Paraguay). Paraguay Population and Housing Census 2002.

Appendix: Citation List

Citation
Derakhshan A, Sardarinia M, Khalili D, Momenan AA, Azizi F, Hadaegh F. Sex specific incidence rates of type 2 diabetes and its risk factors over 9 years of follow-up: Tehran Lipid and Glucose Study. PLoS One. 2014; 9(7): e102563.
Deribew A, Tessema F, Girma B. Determinants of under-five mortality in Gilgel Gibe Field Research Center, Southwest Ethiopia. Ethiop J Health Dev. 2007; 21(2): 117-24.
DeRoo LA, Vlastos AT, Mock P, Vlastos G, Morabia A. Comparison of women's breast cancer risk factors in Geneva, Switzerland and Shanghai, China. Prev Med. 2010; 51(6): 497-501.
Desai, Sonalde, Reeve Vanneman, and National Council of Applied Economic Research, New Delhi. India Human Development Survey (IHDS), 2005. ICPSR22626-v8. Ann Arbor, MI: Inter-university Consortium for Political and Social Research [distributor], 2010-06-29. doi:10.3886/ICPSR22626.v8.
Desjeux P. Leishmaniasis. Public health aspects and control. Clin Dermatol. 1996; 14(5): 417-23.
Desjeux P. Leishmaniasis: current situation and new perspectives. Comp Immunol Microbiol Infect Dis. 2004; 27(5): 305-18.
Desjeux P. The increase in risk factors for leishmaniasis worldwide. Trans R Soc Trop Med Hyg. 2001; 95(3): 239-43.
Deterioration in the nutritional status of young children and their mothers in Brazzaville, Congo, following the 1994 devaluation of the CFA franc as it appears in World Health Organization (WHO). WHO Global Database on Child Growth and Malnutrition - Historical. Geneva, Switzerland: World Health Organization (WHO).
Determinants of child nutrition and mortality in north-west Uganda as it appears in World Health Organization (WHO). WHO Global Database on Child Growth and Malnutrition - Historical. Geneva, Switzerland: World Health Organization (WHO).
Dev V, Phookan S, Sharma VP, Dash AP, Anand SP. Malaria parasite burden and treatment seeking behavior in ethnic communities of Assam, Northeastern India. J Infect. 2006; 52(2): 131-9. As it appears in Malaria Atlas Project. Malaria Atlas Project Plasmodium Falciparum Parasite Rate Database. Oxford, United Kingdom: Malaria Atlas Project.
Dev V, Raghavendra K, Barman K, Phookan S, Dash AP. Wash-resistance and field efficacy of Olyset net, a permethrin-incorporated long-lasting insecticidal netting, against Anopheles minimus-transmitted malaria in Assam, Northeastern India. Vector Borne Zoonotic Dis. 2010; 10(4): 403-10. As it appears in Malaria Atlas Project. Malaria Atlas Project Plasmodium Falciparum Parasite Rate Database. Oxford, United Kingdom: Malaria Atlas Project.
Dev V. Malaria survey in Tarajulie tea estate and adjoining hamlets in Sonitpur District, Assam. Indian J Malariol. 1996; 33(1): 21-9. As it appears in Malaria Atlas Project. Malaria Atlas Project Plasmodium Falciparum Parasite Rate Database. Oxford, United Kingdom: Malaria Atlas Project.
Develoux M, Chegou A, Prual A, Olivar M. Malaria in the oasis of Bilma, Republic of Niger. Trans R Soc Trop Med Hyg. 1994; 88(6): 644. As it appears in Malaria Atlas Project. Malaria Atlas Project Plasmodium Falciparum Parasite Rate Database. Oxford, United Kingdom: Malaria Atlas Project.
Dewi R, Marwoto A, Nalim S, Sekartuti E, Tijitra E. Penelitian malaria di Kecamatan Teluk Dalam, Nias, Sumatera Utara. Cermin Dunia Kedokteran. 1996; 106: 5-9. As it appears in Malaria Atlas Project. Malaria Atlas Project Plasmodium Falciparum Parasite Rate Database. Oxford, United Kingdom: Malaria Atlas Project.
Deyessa N, Kassaye M, Demeke B, Taffa N. Magnitude, type and outcomes of physical violence against married women in Butajira, southern Ethiopia. Ethiop Med J. 1998; 36(2): 83-92.
Dhungana RR, Devkota S, Khanal MK, Gurung Y, Giri RK, Parajuli RK, Adhikari A, Joshi S, Hada B, Shayami A. Prevalence of cardiovascular health risk behaviors in a remote rural community of Sindhuli district, Nepal. BMC Cardiovasc Disord. 2014; 14: 92.
Dia I, Konate L, Samb B, Sarr J-B, Diop A, Rogerie F, Faye M, Riveau G, Remoue F, Diallo M, Fontenille D. Bionomics of malaria vectors and relationship with malaria transmission and epidemiology in three physiographic zones in the Senegal River Basin. Acta Trop. 2008; 105(2): 145-53. As it appears in Malaria Atlas Project. Malaria Atlas Project Plasmodium Falciparum Parasite Rate Database. Oxford, United Kingdom: Malaria Atlas Project.
Diabetic and Cancer Society of Maldives (DCSM), Indhira Gandhi Memorial Hospital (Maldives), Ministry of Health (Maldives), World Health Organization (WHO). Maldives - MalÃ© STEPS Noncommunicable Disease Risk Factors Survey 2004.
Diallo A, Ndam NT, Moussiliou A, Dos Santos S, Ndonky A, Borderon M, Oliveau S, Lalou R, Le Hesran J-Y. Asymptomatic Carriage of Plasmodium in Urban Dakar: The Risk of Malaria Should Not Be Underestimated. PLoS One. 2012; 7(2): e31100. As it appears in Malaria Atlas Project. Malaria Atlas Project Plasmodium Falciparum Parasite Rate Database. Oxford, United Kingdom: Malaria Atlas Project.
Diallo AH, Meda N, Sommerfelt H, Traore GS, Cousens S, Tylleskar T. The high burden of infant deaths in rural Burkina Faso: a prospective community-based cohort study. BMC Public Health. 2012; 12(739).
Diallo S, Konate L, Ndir O, Dieng T, Dieng Y, Bah IB, Faye O, Gaye O. [Malaria in the central health district of Dakar (Senegal). Entomological, parasitological and clinical data]. Sante. 2000; 10(3): 221-9. As it appears in Malaria Atlas Project. Malaria Atlas Project Plasmodium Falciparum Parasite Rate Database. Oxford, United Kingdom: Malaria Atlas Project.
Diallo S, Ndir O, Faye O, Diop BM, Dieng Y, Bah IB, Dieng T, Gaye O, Konate L, Faye O. [Malaria in the southern sanitary district of Dakar (Senegal). 1. Parasitemia and malarial attacks]. Bull Soc Pathol Exot. 1998; 91(3): 208-13. As it appears in Malaria Atlas Project. Malaria Atlas Project Plasmodium Falciparum Parasite Rate Database. Oxford, United Kingdom: Malaria Atlas Project.
Dias CM, Graca MJ. National Health Survey in Portugal: History, Methods and Some Results. Lisbon, Portugal: National Health Institute Doutor Ricardo Jorge (INSA), 2000.
Diawara L. Malaria in the Forest Area of Tai (Côte d'Ivoire): Entomological Transmission Indices and Parasite Prevalence Among Children in the Villages of Zapobly and Gahably. Abidjan, Côte d'Ivoire: Faculty of Science and Technology, National University of Côte d'Ivoire, 1996. As it appears in Malaria Atlas Project. Malaria Atlas Project Plasmodium Falciparum Parasite Rate Database. Oxford, United Kingdom: Malaria Atlas Project.
Diaz T, Achi R. Infectious diseases in a Nicaraguan refugee camp in Costa Rica. Trop Doct. 1989; 19(1): 14-7. As it appears in Malaria Atlas Project. Malaria Atlas Project Plasmodium Falciparum Parasite Rate Database. Oxford, United Kingdom: Malaria Atlas Project.

Appendix: Citation List

Citation
Dicko A, Sagara I, Sissoko MS, Guindo O, Diallo AI, Kone M, Toure OB, Sacko M, Doumbo OK. Impact of intermittent preventive treatment with sulphadoxine-pyrimethamine targeting the transmission season on the incidence of clinical malaria in children in Mali. Malar J. 2008; 7(1): 123. As it appears in Malaria Atlas Project. Malaria Atlas Project Plasmodium Falciparum Parasite Rate Database. Oxford, United Kingdom: Malaria Atlas Project.
Dicko A, WHO-PEEM, WARDA. Mali - Niono Parasitological and Clinical Survey 1995. As it appears in Malaria Atlas Project. Malaria Atlas Project Plasmodium Falciparum Parasite Rate Database. Oxford, United Kingdom: Malaria Atlas Project.
Dieng AB. Epidemiological Situation of Malaria in Children in Sahelian Area During a Transmission Season. Study of Family Similarities [Master's thesis]. Paris, France: Pierre and Marie Curie University,2002. As it appears in Malaria Atlas Project. Malaria Atlas Project Plasmodium Falciparum Parasite Rate Database. Oxford, United Kingdom: Malaria Atlas Project.
Dieng T, Dieng Y, Ndiaye JL, Mboup N, Bah IB, Faye B, Ndiaye D, Faye O, Ndir O, Gaye O. Epidemiological, Parasitological and Clinical Aspects of Malaria in Louga (Senegal). Presented at: 5th MIM Pan African Malaria Conference; 2009 Nov 2-6; Nairobi, Kenya. As it appears in Malaria Atlas Project. Malaria Atlas Project Plasmodium Falciparum Parasite Rate Database. Oxford, United Kingdom: Malaria Atlas Project.
Diet & nutrition situation in rural India as it appears in World Health Organization (WHO). WHO Global Database on Child Growth and Malnutrition - Historical. Geneva, Switzerland: World Health Organization (WHO).
Diet & nutrition situation in rural India as it appears in World Health Organization (WHO). WHO Global Database on Child Growth and Malnutrition. Geneva, Switzerland: World Health Organization (WHO).
Dietary and Nutritional Status of Chinese Population (1992 National Nutrition Survey) as it appears in World Health Organization (WHO). WHO Global Database on Child Growth and Malnutrition - Historical. Geneva, Switzerland: World Health Organization (WHO).
Dietary and Nutritional Status of Chinese Population (1992 National Nutrition Survey) as it appears in World Health Organization (WHO). WHO Global Database on Child Growth and Malnutrition. Geneva, Switzerland: World Health Organization (WHO).
Dietary pattern and state of nutrition among children in drought-prone areas of southern Ethiopia as it appears in World Health Organization (WHO). WHO Global Database on Child Growth and Malnutrition - Historical. Geneva, Switzerland: World Health Organization (WHO).
Differential correlates of nutritional status in Kinshasa, Zaire as it appears in World Health Organization (WHO). WHO Global Database on Child Growth and Malnutrition - Historical. Geneva, Switzerland: World Health Organization (WHO).
Dijivoh C, Massougbodji A, Turk P, Fayomi E, Gay F, Danis M. [Low levels of chloroquine resistance of Plasmodium falciparum in the province of Zou in Benin]. Bull Soc Pathol Exot. 1988; 81(3): 332-7. As it appears in Malaria Atlas Project. Malaria Atlas Project Plasmodium Falciparum Parasite Rate Database. Oxford, United Kingdom: Malaria Atlas Project.
Dijkema MBA, Mallant SF, Gehring U, van den Hurk K, Alssema M, van Strien RT, Fischer PH, Nijpels G, Stehouwer CDA, Hoek G, Dekker JM, Brunekreef B. Long-term exposure to traffic-related air pollution and type 2 diabetes prevalence in a cross-sectional screening-study in the Netherlands. Environ Health. 2011; 10: 76.
Dilek, Demir C, Bay A, Akdeniz H, Öner AF. Seropositivity rates of HBsAg, anti-HCV, anti-HIV and VDRL in blood donors in Eastern Turkey. Turk J Haematol. 2007; 24(1): 4-7.
Ding L, Song A, Dai M, Xu M, Sun W, Xu B, Sun J, Wang T, Xu Y, Lu J, Wang W, Bi Y, Ning G. Serum lipoprotein (a) concentrations are inversely associated with T2D, prediabetes, and insulin resistance in a middle-aged and elderly Chinese population. J Lipid Res. 2015; 56(4): 920–6.
Dionadji M, Boy B, Mouanodji M, Batakao G. [Prevalence of diabetes mellitus in rural areas in Chad]. Med Trop (Mars). 2010; 70(4): 414-5.
Diop A. Epidemiology of Malaria in the Saloum Delta (Fatick, Senegal): Role of Anopheles Melas in Transmission [dissertation]. Dakar, Senegal: Faculy of Science and Technology, Cheikh Anta Diop University, 1998. As it appears in Malaria Atlas Project. Malaria Atlas Project Plasmodium Falciparum Parasite Rate Database. Oxford, United Kingdom: Malaria Atlas Project.
Diosque P, Padilla AM, Cimino RO, Cardozo RM, Negrette OS, Marco JD, Zacca R, Meza C, Juarez A, Rojo H, Rey R, Corrales RM, Nasser JR, Basombrío MA. Chagas disease in rural areas of Chaco Province, Argentina: epidemiologic survey in humans, reservoirs, and vectors. Am J Trop Med Hyg. 2004; 71(5): 590–3.
Directorate for Economic Planning (Bosnia and Herzegovina), Federal Office of Statistics (Federation of Bosnia and Herzegovina), Institute of Statistics (Republic of Srpska), Ministry of Health (Federation of Bosnia and Herzegovina), Ministry of Health and Social Welfare (Republic of Srpska), Public Health Institute of Federation of Bosnia and Herzegovina, United Nations Children's Fund (UNICEF). Bosnia and Herzegovina Multiple Indicator Cluster Survey 2006. New York, United States: United Nations Children's Fund (UNICEF).
Directorate for Health and Social Affairs (Norway), Norwegian Institute for Alcohol and Drug Research (SIRUS). Norwegian Tobacco Statistics 1973-2006. Oslo, Norway: Norwegian Institute for Alcohol and Drug Research (SIRUS), 2007.
Directorate General of Epidemiology, Ministry of Health (Mexico). Mexico Cases of Respiratory Tuberculosis by Age Groups 1990.
Directorate General of Epidemiology, Ministry of Health (Mexico). Mexico Cases of Respiratory Tuberculosis by Age Groups 2012.
Directorate General of Health Services, Ministry of Health and Family Welfare (Bangladesh), United Nations Children's Fund (UNICEF), World Health Organization (WHO). Bangladesh EPI Coverage Evaluation Survey 1991.
Directorate General of Health Services, Ministry of Health and Family Welfare (Bangladesh), United Nations Children's Fund (UNICEF), World Health Organization (WHO). Bangladesh EPI Coverage Evaluation Survey 1992.
Directorate General of Health Services, Ministry of Health and Family Welfare (Bangladesh), United Nations Children's Fund (UNICEF), World Health Organization (WHO). Bangladesh EPI Coverage Evaluation Survey 1993.
Directorate General of Health Services, Ministry of Health and Family Welfare (Bangladesh), United Nations Children's Fund (UNICEF), World Health Organization (WHO). Bangladesh EPI Coverage Evaluation Survey 1994.
Directorate General of Health Services, Ministry of Health and Family Welfare (Bangladesh), United Nations Children's Fund (UNICEF), World Health Organization (WHO). Bangladesh EPI Coverage Evaluation Survey 1995.
Directorate General of Health Services, Ministry of Health and Family Welfare (Bangladesh), United Nations Children's Fund (UNICEF), World Health Organization (WHO). Bangladesh EPI Coverage Evaluation Survey 1997.

Appendix: Citation List

Citation
Directorate General of Health Services, Ministry of Health and Family Welfare (Bangladesh), United Nations Children's Fund (UNICEF), World Health Organization (WHO). Bangladesh EPI Coverage Evaluation Survey 1998.
Directorate General of Health Services, Ministry of Health and Family Welfare (Bangladesh), United Nations Children's Fund (UNICEF), World Health Organization (WHO). Bangladesh EPI Coverage Evaluation Survey 2000.
Directorate General of Health Services, Ministry of Health and Family Welfare (Bangladesh), United Nations Children's Fund (UNICEF), World Health Organization (WHO). Bangladesh EPI Coverage Evaluation Survey 2001.
Directorate General of Health Services, Ministry of Health and Family Welfare (Bangladesh), United Nations Children's Fund (UNICEF), World Health Organization (WHO). Bangladesh EPI Coverage Evaluation Survey 2002.
Directorate General of Health Services, Ministry of Health and Family Welfare (Bangladesh), United Nations Children's Fund (UNICEF), World Health Organization (WHO). Bangladesh EPI Coverage Evaluation Survey 2003.
Directorate General of Health Services, Ministry of Health and Family Welfare (Bangladesh). Bangladesh Health Bulletin 2009. Dhaka, Bangladesh: Directorate General of Health Services, Ministry of Health and Family Welfare (Bangladesh).
Directorate General of Health Services, Ministry of Health and Family Welfare (Bangladesh). Bangladesh Health Bulletin 2012. Dhaka, Bangladesh: Directorate General of Health Services, Ministry of Health and Family Welfare (Bangladesh).
Directorate General of Health Services, Ministry of Health and Family Welfare (Bangladesh). Bangladesh Health Bulletin 2013. Dhaka, Bangladesh: Directorate General of Health Services, Ministry of Health and Family Welfare (Bangladesh), 2014.
Directorate General of Health Services, Ministry of Health and Family Welfare (Bangladesh). Bangladesh Health Bulletin 2014. Dhaka, Bangladesh: Directorate General of Health Services, Ministry of Health and Family Welfare (Bangladesh), 2014.
Directorate General of Health-Duhok (Iraq), Kurdistan Regional Government (Iraq), Ministry of Health (Iraq), World Health Organization (WHO). Iraq - DahÅ«k STEPS Noncommunicable Disease Risk Factors Survey 2003-2004.
Directorate General of National Statistics (Oman). Oman Sociodemographic Survey 1975.
Directorate General of National Statistics (Oman). Oman Sociodemographic Survey 1977-1979.
Directorate General of Planning and Statistics (Guinea), Ministry of the Interior and Security (Guinea), Minnesota Population Center. Guinea General Census of Population and Housing 1983 from the Integrated Public Use Microdata Series, International: [Machine-readable database]. Minneapolis: University of Minnesota.
Directorate General of Statistics and National Accounting (Togo), United Nations Children's Fund (UNICEF). Togo Multiple Indicator Cluster Survey 2006. New York, United States: United Nations Children's Fund (UNICEF).
Directorate General of Statistics and National Accounting (Togo), United Nations Children's Fund (UNICEF). Togo Multiple Indicator Cluster Survey 2010. New York, United States: United Nations Children's Fund (UNICEF).
Directorate General of Statistics and National Accounts (Togo), ICF International, Ministry of Health (Togo). Togo Demographic and Health Survey 2013-2014. Fairfax, United States: ICF International, 2015.
Directorate of Censuses and Surveys (Cape Verde). Cape Verde Population and Housing Census 1980.
Directorate of Economics and Statistics and Office of Chief Registrar Births and Deaths (Delhi Territory, India). India - Delhi Medical Certification of Cause of Death Report 2012.
Directorate of Economics and Statistics and Office of Chief Registrar Births and Deaths (Delhi Territory, India). India - Delhi Medical Certification of Cause of Death Report 2013.
Directorate of Economics and Statistics and Office of Chief Registrar Births and Deaths (Delhi Territory, India). India - Delhi Report on Medical Certification of Cause of Deaths 2011.
Directorate of Forecasting and Statistics (Senegal), Minnesota Population Center. Senegal General Population and Housing Census 2002 from the Integrated Public Use Microdata Series, International: [Machine-readable database]. Minneapolis: University of Minnesota.
Directorate of Forecasting and Statistics, Ministry of the Economy, Finance and Planning (Senegal), Macro International, Inc. Senegal Demographic and Health Survey 1992-1993. Calverton, United States: Macro International, Inc.
Directorate of Forecasting and Statistics, Ministry of the Economy, Finance and Planning (Senegal), Macro International, Inc. Senegal Demographic and Health Survey 1997. Calverton, United States: Macro International, Inc.
Directorate of Forecasting and Statistics, Ministry of the Economy, Finance and Planning (Senegal), Ministry of Economics and Finance (Senegal), United Nations Children's Fund (UNICEF). Senegal Multiple Indicator Cluster Survey 2000. New York, United States: United Nations Children's Fund (UNICEF).
Directorate of Health (Iceland), Public Health Institute of Iceland, University of Iceland. Iceland Survey of Health and Wellbeing 2007.
Directorate of Statistics and Census (Panama), Ministry of Economy and Finance (Panama), World Bank. Panama Living Standard Measurement Survey 2003. Washington DC, United States: World Bank.
Directorate of Statistics and Census (Panama), Ministry of Economy and Finance (Panama), World Bank. Panama Living Standard Measurement Survey 2008. Washington DC, United States: World Bank.
Directorate of Statistics of the High Commission for Planning (Morocco), World Bank. Morocco Living Standards Measurement Survey 1990-1991.
Diro E, Lynen L, Gebregziabiher B, Assefa A, Lakew W, Belew Z, Hailu A, Boelaert M, van Griensven J. Clinical aspects of paediatric visceral leishmaniasis in North-west Ethiopia. Trop Med Int Health. 2015; 20(1): 8-16.
Dissanayake S. Microfilaraemia, serum antibody and development of clinical disease in microfilaraemic subjects infected with Wuchereria bancrofti and treated with diethylcarbamazine citrate. Trans R Soc Trop Med Hyg. 1989; 83(3): 384-8. As it appears in London School of Hygiene and Tropical Medicine. Global Atlas of Helminth Infections - Lymphatic Filiariasis.
DivisÃ£o de Censos e InquÃ©ritos, RÃ©publica de Cabo Verde. Cape Verde Population and Housing Census 1990. DivisÃ£o de Censos e InquÃ©ritos, 1990.
Division of Reproductive Health, Centers for Disease Control and Prevention (CDC), El Salvador Demographic Association (ADS). El Salvador Reproductive Health Survey - Maternal Mortality Data.

Appendix: Citation List

Citation
Division of Reproductive Health, Centers for Disease Control and Prevention (CDC), Georgia Ministry of Labor, Health and Social Affairs, National Center for Disease Control and Public Health (Georgia), National Statistics Office of Georgia. Georgia Reproductive Health Survey 2010-2011.
Division of Reproductive Health, Centers for Disease Control and Prevention (CDC), Ministry of Health (Uganda). Uganda AIDS Indicator Survey 2004-2005. Calverton, United States: Macro International, Inc.
Division of Reproductive Health, Centers for Disease Control and Prevention (CDC). Russia Reproductive Health Survey 1999.
Division of Reproductive Health-Centers for Disease Control and Prevention (CDC) and Kiev International Institute of Sociology. (2001) Ukraine Reproductive Health Survey 1999. Atlanta, United States: Centers for Disease Control and Prevention (CDC).
Division of Reproductive Health-Centers for Disease Control and Prevention (CDC) and Ministry of Health. (1989) Swaziland Family Planning/Maternal and Child Health Survey 1988. Atlanta, United States: Centers for Disease Control and Prevention (CDC).
Division of Reproductive Health-Centers for Disease Control and Prevention (CDC) and Moldova Ministry of Health. (1998) Moldova Reproductive Health Survey 1997. Atlanta, United States: Centers for Disease Control and Prevention (CDC).
Division of Reproductive Health-Centers for Disease Control and Prevention (CDC) and Romanian Association of Public Health and Health Management. (2001) Romania Reproductive Health Survey 1999. Atlanta, United States: Centers for Disease Control and Prevention (CDC).
Division of Reproductive Health-Centers for Disease Control and Prevention (CDC), National Institute for Development Information (Nicaragua). Nicaragua Reproductive Health Survey 2006-2007. Managua, Nicaragua: National Institute for Development Information (Nicaragua).
Division of Reproductive Health-Centers for Disease Control and Prevention (CDC). (2005): Paraguay Reproductive Health Survey 2004. Asunción, Paraguay, Paraguayan Center for Population Studies (CEPEP).
Division of Reproductive Health-Centers for Disease Control and Prevention (CDC). El Salvador Family Planning/Maternal and Child Health Survey 1988. Final English Language Report. Atlanta, United States: Centers for Disease Control and Prevention (CDC).
Division of Reproductive Health-Centers for Disease Control and Prevention (CDC). Paraguay Contraceptive Prevalence Survey 1998. Atlanta, United States: Centers for Disease Control and Prevention (CDC).
Division of Reproductive Health-Centers for Disease Control and Prevention (CDC). Paraguay Reproductive Health Survey 1995-1996. Asunción, Paraguay, Paraguayan Center for Population Studies (CEPEP).
Division of Statistics and Economic Studies (Central African Republic), Macro International, Inc. Central African Republic Demographic and Health Survey 1994-1995. Calverton, United States: Macro International, Inc.
Division of Statistics and Economic Studies (Central African Republic), Ministry of Economy, Planning and International Cooperation (Central African Republic). Central African Republic General Population Census 1988.
Divison Garrote JA, Masso Orozco J, Carrion Valero L, Lopez Abril J, Carbayo Herencia JA, Artigao Rodenas LM, Gil Guillen V, Grupo de Enfermedades Vasculares de Albacete (GEVA). [Trends in prevalence of risk factors and global cardiovascular risk in general population of albacete, Spain (1992-94 a 2004-06)]. Rev Esp Salud Publica. 2011; 85(3): 275–84.
Dixit V, Kurup AV, Gupta AK, Kataria OM, Prasad GB. Bancroftian filariasis in south east Madhya Pradesh: Pre-control epidemilogical observations. Indian J Clin Biochem. 1997; 12(Suppl 1): 39-43.
Dixon RA, Pinikahana JP. Malaria and proximity to irrigation projects: A parasitaemia prevalence study from Sri Lanka. Mosq Borne Dis Bull. 1994; 11(4): 116-21. As it appears in Malaria Atlas Project. Malaria Atlas Project Plasmodium Falciparum Parasite Rate Database. Oxford, United Kingdom: Malaria Atlas Project.
DjÃ¤rv T, Wikman A, Nordenstedt H, Johar A, Lagergren J, Lagergren P. Physical activity, obesity and gastroesophageal reflux disease in the general population. World J Gastroenterol. 2012; 18(28): 3710-4.
Djaman JA, Kauffy PC, Yavo W, Basco LK, Kone M. [In vivo evaluation of sulfadoxine-pyrimethamine efficacy during uncomplicated falciparum malaria in children of Yopougon (Abidjan, Côte d'Ivoire)]. Bull Soc Pathol Exot. 2004; 97(3): 180-2. As it appears in Malaria Atlas Project. Malaria Atlas Project Plasmodium Falciparum Parasite Rate Database. Oxford, United Kingdom: Malaria Atlas Project.
Djibouti Household Energy Survey 2004 as it appears in World Health Organization (WHO). WHO Household Energy Database. Geneva, Switzerland: World Health Organization (WHO), 2010.
Djibouti Household Survey - Social Indicators 1996 as it appears in World Health Organization (WHO). WHO Global Database on Child Growth and Malnutrition. Geneva, Switzerland: World Health Organization (WHO).
Djibouti Household Survey - Social Indicators 1996 as it appears in World Health Organization (WHO). WHO Household Energy Database. Geneva, Switzerland: World Health Organization (WHO), 2010.
Djibouti Intercensal Demographic Survey 1991.
Djibouti Survey of Nutrition and Risk Factors for Cardiovascular Diseases 1995 as it appears in World Health Organization (WHO). WHO Global Database on Child Growth and Malnutrition - Historical. Geneva, Switzerland: World Health Organization (WHO).
Djoufack J. Study of Seasonal Malaria Morbidity in the Urban Emonbo Quarter of Yaounde. Yaoundé, Cameroon: Faculty of Medicine and Biomedical Sciences, University of Yaoundé, 1997. As it appears in Malaria Atlas Project. Malaria Atlas Project Plasmodium Falciparum Parasite Rate Database. Oxford, United Kingdom: Malaria Atlas Project.
Do HTP, Geleijnse JM, Le MB, Kok FJ, Feskens EJM. National prevalence and associated risk factors of hypertension and prehypertension among Vietnamese adults. Am J Hypertens. 2015; 28(1): 89â€"97.
Doan HN, Nguyen DT, Tran TU, Le DC. [Some remarks on immuno-response and resistant P. falciparum in Khanh Nam – a highly endemic malaria area]. J Vector Borne Dis. 1995; 1: 13-8. As it appears in Malaria Atlas Project. Malaria Atlas Project Plasmodium Falciparum Parasite Rate Database. Oxford, United Kingdom: Malaria Atlas Project.
Dobrodeeva LK, Kornienko EB, Petrenya NN, Lutfalieva GT, Schegoleva LS, Demeneva LV, Duberman BL, Tkachev AV, Chiba H, Senoo H, Ito K, Mizoguchi E, Yoshida S, Tajima K. A unique seroepidemiological pattern of HBV, HCV and HTLV-I in Nenets and Komi in northwestern Russia. Asian Pac J Cancer Prev. 2005; 6(3): 342-5.
Doctor HV, Weinreb AA. Estimation of AIDS adult mortality by verbal autopsy in rural Malawi. AIDS. 2003; 17(17): 2509-13.

Appendix: Citation List

Citation
Doctors Without Borders (MSF), Faculty of Public Health at Mahidol University (Thailand). Cambodia Plasmodium Falciparum Parasite Rate Data, Personal Communication with SMRU MSF-B, Médecins Sans Frontières 2008. As it appears in Malaria Atlas Project. Malaria Atlas Project Plasmodium Falciparum Parasite Rate Database. Oxford, United Kingdom: Malaria Atlas Project.
Doctors Without Borders (MSF), Faculty of Public Health at Mahidol University (Thailand). Cambodia Plasmodium Falciparum Parasite Rate Data, Personal Communication with SMRU MSF-H, Médecins Sans Frontières 2005. As it appears in Malaria Atlas Project. Malaria Atlas Project Plasmodium Falciparum Parasite Rate Database. Oxford, United Kingdom: Malaria Atlas Project.
Dodoo D, Aikins A, Kusi KA, Lamptey H, Remarque E, Milligan P, Bosomprah S, Chilengi R, Osei YD, Akanmori BD, Theisen M. Cohort study of the association of antibody levels to AMA1, MSP119, MSP3 and GLURP with protection from clinical malaria in Ghanaian children. Malar J. 2008; 7(1): 142. As it appears in Malaria Atlas Project. Malaria Atlas Project Plasmodium Falciparum Parasite Rate Database. Oxford, United Kingdom: Malaria Atlas Project.
Dodoo D, Omer FM, Todd J, Akanmori BD, Koram KA, Riley EM. Absolute Levels and Ratios of Proinflammatory and Anti-inflammatory Cytokine Production In Vitro Predict Clinical Immunity to Plasmodium falciparum Malaria. J Infect Dis. 2002; 185(7): 971-9. As it appears in Malaria Atlas Project. Malaria Atlas Project Plasmodium Falciparum Parasite Rate Database. Oxford, United Kingdom: Malaria Atlas Project.
Dogan N, Toprak D, Demir S. Hypertension prevalence and risk factors among adult population in Afyonkarahisar region: a cross-sectional research. Anatol J Cardiol. 2012; 12(1): 47–52.
Dogara MM, Nock HI, Agbede RIS, Ndams SI, Joseph KK. Prevalence Of Lymphatic Filariasis In Three Villages In Kano State, Nigeria. Internet J Trop Med. 2012; 8(1). As it appears in London School of Hygiene and Tropical Medicine. Global Atlas of Helminth Infections - Lymphatic Filiariasis.
Doi H, Kaneko A, Panjaitan W, Ishii A. Chemotherapeutic malaria control operation by single dose of Fansidar plus primaquine in North Sumatra, Indonesia. Southeast Asian J Trop Med Public Health. 1989; 20(3): 341-9. As it appears in Malaria Atlas Project. Malaria Atlas Project Plasmodium Falciparum Parasite Rate Database. Oxford, United Kingdom: Malaria Atlas Project.
Doll R, Vessey MP, Beasley RWR, Buckley AR, Fear EC, Fisher REW, Gammon EJ, Gunn W, Hughes GO, Lee K, Norman-Smith B. Mortality Of Gasworkers--Final Report Of A Prospective Study. Br J Ind Med. 1972; 29(4): 394-406 as it appears in Armstrong B, Hutchinson E, Unwin J, Fletcher T. Lung cancer risk after exposure to polycyclic aromatic hydrocarbons: a review and meta-analysis. Environ. Health Perspect. 2004; 112(9): 970–978.
Dolo A, Camara F, Poudiougou B, Toure A, Kouriba B, Bagayogo M, Sangare D, Diallo M, Bosman A, Modiano D, Toure Y, Doumbo O. [Epidemiology of malaria in a village of Sudanese savannah area in Mali (Bancoumana). 2 Entomo-parasitological and clinical study]. Bull Soc Pathol Exot. 2003; 96(4): 308-12. As it appears in Malaria Atlas Project. Malaria Atlas Project Plasmodium Falciparum Parasite Rate Database. Oxford, United Kingdom: Malaria Atlas Project.
Domarle O, Migot-Nabias F, Mvoukani JL, Lu CY, Nabias R, Mayombo J, Tiga H, Deloron P. Factors influencing resistance to reinfection with Plasmodium falciparum. Am J Trop Med Hyg. 1999; 61(6): 926-31. As it appears in Malaria Atlas Project. Malaria Atlas Project Plasmodium Falciparum Parasite Rate Database. Oxford, United Kingdom: Malaria Atlas Project.
Domingo EO, Tiu E, Peters PA, Warren KS, Mahmoud AA, Houser HB. Morbidity in schistosomiasis japonica in relation to intensity of infection: study of a community in Leyte, Philippines. Am J Trop Med Hyg. 1980; 29(5): 858-67.
Domínguez A, Bruguera M, Vidal J, Plans P, Salleras L. Changes in the seroepidemiology of hepatitis B infection in Catalonia 1989-1996. Vaccine. 2000; 18(22): 2345-50.
Dominica Vital Registration - Deaths 1961 ICD7 as it appears in World Health Organization (WHO). WHO Mortality Database Version July 2012. Geneva, Switzerland: World Health Organization (WHO), 2012.
Dominica Vital Registration - Deaths 1962 ICD7 as it appears in World Health Organization (WHO). WHO Mortality Database Version July 2012. Geneva, Switzerland: World Health Organization (WHO), 2012.
Dominica Vital Registration - Deaths 1967 ICD7 as it appears in World Health Organization (WHO). WHO Mortality Database Version July 2012. Geneva, Switzerland: World Health Organization (WHO), 2012.
Dominica Vital Registration - Deaths 1968 ICD7 as it appears in World Health Organization (WHO). WHO Mortality Database Version July 2012. Geneva, Switzerland: World Health Organization (WHO), 2012.
Dominica Vital Registration - Deaths 1969 ICD8 as it appears in World Health Organization (WHO). WHO Mortality Database Version May 2013. Geneva, Switzerland: World Health Organization (WHO), 2013.
Dominica Vital Registration - Deaths 1970 ICD8 as it appears in World Health Organization (WHO). WHO Mortality Database Version May 2013. Geneva, Switzerland: World Health Organization (WHO), 2013.
Dominica Vital Registration - Deaths 1971 ICD8 as it appears in World Health Organization (WHO). WHO Mortality Database Version May 2013. Geneva, Switzerland: World Health Organization (WHO), 2013.
Dominica Vital Registration - Deaths 1972 ICD8 as it appears in World Health Organization (WHO). WHO Mortality Database Version May 2013. Geneva, Switzerland: World Health Organization (WHO), 2013.
Dominica Vital Registration - Deaths 1973 ICD8 as it appears in World Health Organization (WHO). WHO Mortality Database Version May 2013. Geneva, Switzerland: World Health Organization (WHO), 2013.
Dominica Vital Registration - Deaths 1974 ICD8 as it appears in World Health Organization (WHO). WHO Mortality Database Version May 2013. Geneva, Switzerland: World Health Organization (WHO), 2013.
Dominica Vital Registration - Deaths 1975 ICD8 as it appears in World Health Organization (WHO). WHO Mortality Database Version May 2013. Geneva, Switzerland: World Health Organization (WHO), 2013.
Dominica Vital Registration - Deaths 1976 ICD8 as it appears in World Health Organization (WHO). WHO Mortality Database Version May 2013. Geneva, Switzerland: World Health Organization (WHO), 2013.
Dominica Vital Registration - Deaths 1977 ICD8 as it appears in World Health Organization (WHO). WHO Mortality Database Version May 2013. Geneva, Switzerland: World Health Organization (WHO), 2013.
Dominica Vital Registration - Deaths 1978 ICD8 as it appears in World Health Organization (WHO). WHO Mortality Database Version May 2013. Geneva, Switzerland: World Health Organization (WHO), 2013.

Appendix: Citation List

Citation
Dominica Vital Registration - Deaths 1979 ICD9 as it appears in World Health Organization (WHO). WHO Mortality Database Version November 2015. Geneva, Switzerland: World Health Organization (WHO), 2015.
Dominica Vital Registration - Deaths 1980 ICD9 as it appears in World Health Organization (WHO). WHO Mortality Database Version February 2014. Geneva, Switzerland: World Health Organization (WHO), 2014.
Dominica Vital Registration - Deaths 1981 ICD9 as it appears in World Health Organization (WHO). WHO Mortality Database Version February 2014. Geneva, Switzerland: World Health Organization (WHO), 2014.
Dominica Vital Registration - Deaths 1982 ICD9 as it appears in World Health Organization (WHO). WHO Mortality Database Version February 2014. Geneva, Switzerland: World Health Organization (WHO), 2014.
Dominica Vital Registration - Deaths 1983 ICD9 as it appears in World Health Organization (WHO). WHO Mortality Database Version February 2014. Geneva, Switzerland: World Health Organization (WHO), 2014.
Dominica Vital Registration - Deaths 1984 ICD9 as it appears in World Health Organization (WHO). WHO Mortality Database Version November 2015. Geneva, Switzerland: World Health Organization (WHO), 2015.
Dominica Vital Registration - Deaths 1985 ICD9 as it appears in World Health Organization (WHO). WHO Mortality Database Version November 2015. Geneva, Switzerland: World Health Organization (WHO), 2015.
Dominica Vital Registration - Deaths 1986 ICD9 as it appears in World Health Organization (WHO). WHO Mortality Database Version November 2015. Geneva, Switzerland: World Health Organization (WHO), 2015.
Dominica Vital Registration - Deaths 1987 ICD9 as it appears in World Health Organization (WHO). WHO Mortality Database Version November 2015. Geneva, Switzerland: World Health Organization (WHO), 2015.
Dominica Vital Registration - Deaths 1988 ICD9 as it appears in World Health Organization (WHO). WHO Mortality Database Version November 2015. Geneva, Switzerland: World Health Organization (WHO), 2015.
Dominica Vital Registration - Deaths 1989 ICD9 as it appears in World Health Organization (WHO). WHO Mortality Database Version November 2015. Geneva, Switzerland: World Health Organization (WHO), 2015.
Dominica Vital Registration - Deaths 1990 ICD9 as it appears in World Health Organization (WHO). WHO Mortality Database Version November 2015. Geneva, Switzerland: World Health Organization (WHO), 2015.
Dominica Vital Registration - Deaths 1991 ICD9 as it appears in World Health Organization (WHO). WHO Mortality Database Version November 2015. Geneva, Switzerland: World Health Organization (WHO), 2015.
Dominica Vital Registration - Deaths 1992 ICD9 as it appears in World Health Organization (WHO). WHO Mortality Database Version November 2015. Geneva, Switzerland: World Health Organization (WHO), 2015.
Dominica Vital Registration - Deaths 1993 ICD9 as it appears in World Health Organization (WHO). WHO Mortality Database Version November 2015. Geneva, Switzerland: World Health Organization (WHO), 2015.
Dominica Vital Registration - Deaths 1994 ICD9 as it appears in World Health Organization (WHO). WHO Mortality Database Version November 2015. Geneva, Switzerland: World Health Organization (WHO), 2015.
Dominica Vital Registration - Deaths 1995 ICD9 as it appears in World Health Organization (WHO). WHO Mortality Database Version November 2015. Geneva, Switzerland: World Health Organization (WHO), 2015.
Dominica Vital Registration - Deaths 1996 ICD9 as it appears in World Health Organization (WHO). WHO Mortality Database Version February 2014. Geneva, Switzerland: World Health Organization (WHO), 2014.
Dominica Vital Registration - Deaths 1997 ICD9 as it appears in World Health Organization (WHO). WHO Mortality Database Version February 2014. Geneva, Switzerland: World Health Organization (WHO), 2014.
Dominica Vital Registration - Deaths 1998 ICD9 as it appears in World Health Organization (WHO). WHO Mortality Database Version February 2014. Geneva, Switzerland: World Health Organization (WHO), 2014.
Dominica Vital Registration - Deaths 1999 ICD9 as it appears in World Health Organization (WHO). WHO Mortality Database Version February 2014. Geneva, Switzerland: World Health Organization (WHO), 2014.
Dominica Vital Registration - Deaths 2000 ICD9 as it appears in World Health Organization (WHO). WHO Mortality Database Version July 2012. Geneva, Switzerland: World Health Organization (WHO), 2012.
Dominica Vital Registration - Deaths 2001 ICD10 as it appears in World Health Organization (WHO). WHO Mortality Database Version November 2015. Geneva, Switzerland: World Health Organization (WHO), 2015.
Dominica Vital Registration - Deaths 2002 ICD10 as it appears in World Health Organization (WHO). WHO Mortality Database Version November 2015. Geneva, Switzerland: World Health Organization (WHO), 2015.
Dominica Vital Registration - Deaths 2003 ICD10 as it appears in World Health Organization (WHO). WHO Mortality Database Version November 2015. Geneva, Switzerland: World Health Organization (WHO), 2015.
Dominica Vital Registration - Deaths 2004 ICD10 as it appears in World Health Organization (WHO). WHO Mortality Database Version November 2015. Geneva, Switzerland: World Health Organization (WHO), 2015.
Dominica Vital Registration - Deaths 2005 ICD10 as it appears in World Health Organization (WHO). WHO Mortality Database Version November 2015. Geneva, Switzerland: World Health Organization (WHO), 2015.
Dominica Vital Registration - Deaths 2006 ICD10 as it appears in World Health Organization (WHO). WHO Mortality Database Version November 2015. Geneva, Switzerland: World Health Organization (WHO), 2015.
Dominica Vital Registration - Deaths 2007 ICD10 as it appears in World Health Organization (WHO). WHO Mortality Database Version November 2015. Geneva, Switzerland: World Health Organization (WHO), 2015.
Dominica Vital Registration - Deaths 2008 ICD10 as it appears in World Health Organization (WHO). WHO Mortality Database Version November 2015. Geneva, Switzerland: World Health Organization (WHO), 2015.
Dominica Vital Registration - Deaths 2009 ICD10 as it appears in World Health Organization (WHO). WHO Mortality Database Version November 2015. Geneva, Switzerland: World Health Organization (WHO), 2015.
Dominica Vital Registration - Deaths 2010 ICD10 as it appears in World Health Organization (WHO). WHO Mortality Database Version November 2015. Geneva, Switzerland: World Health Organization (WHO), 2015.

Appendix: Citation List

Citation
Dominica Vital Registration - Deaths 2011 ICD10 as it appears in World Health Organization (WHO). WHO Mortality Database Version November 2015. Geneva, Switzerland: World Health Organization (WHO), 2015.
Dominica Vital Registration - Deaths 2012 ICD10 as it appears in World Health Organization (WHO). WHO Mortality Database Version November 2015. Geneva, Switzerland: World Health Organization (WHO), 2015.
Dominica Vital Registration - Deaths 2013 ICD10 as it appears in World Health Organization (WHO). WHO Mortality Database Version November 2015. Geneva, Switzerland: World Health Organization (WHO), 2015.
Dominica Vital Registration Death Data 1950 as it appears in United Nations Statistics Division (UNSD). United Nations Demographic Yearbook - Historical Supplement 1997. New York City, United States: United Nations Statistics Division (UNSD).
Dominica Vital Registration Death Data 1951 as it appears in United Nations Statistics Division (UNSD). United Nations Demographic Yearbook - Historical Supplement 1997. New York City, United States: United Nations Statistics Division (UNSD).
Dominica Vital Registration Death Data 1952 as it appears in United Nations Statistics Division (UNSD). United Nations Demographic Yearbook - Historical Supplement 1997. New York City, United States: United Nations Statistics Division (UNSD).
Dominica Vital Registration Death Data 1953 as it appears in United Nations Statistics Division (UNSD). United Nations Demographic Yearbook - Historical Supplement 1997. New York City, United States: United Nations Statistics Division (UNSD).
Dominica Vital Registration Death Data 1954 as it appears in United Nations Statistics Division (UNSD). United Nations Demographic Yearbook - Historical Supplement 1997. New York City, United States: United Nations Statistics Division (UNSD).
Dominica Vital Registration Death Data 1955 as it appears in United Nations Statistics Division (UNSD). United Nations Demographic Yearbook - Historical Supplement 1997. New York City, United States: United Nations Statistics Division (UNSD).
Dominica Vital Registration Death Data 1956 as it appears in United Nations Statistics Division (UNSD). United Nations Demographic Yearbook - Historical Supplement 1997. New York City, United States: United Nations Statistics Division (UNSD).
Dominica Vital Registration Death Data 1957 as it appears in United Nations Statistics Division (UNSD). United Nations Demographic Yearbook - Historical Supplement 1997. New York City, United States: United Nations Statistics Division (UNSD).
Dominica Vital Registration Death Data 1958 as it appears in United Nations Statistics Division (UNSD). United Nations Demographic Yearbook - Historical Supplement 1997. New York City, United States: United Nations Statistics Division (UNSD).
Dominica Vital Registration Death Data 1959 as it appears in United Nations Statistics Division (UNSD). United Nations Demographic Yearbook - Historical Supplement 1997. New York City, United States: United Nations Statistics Division (UNSD).
Dominica Vital Registration Death Data 1960 as it appears in United Nations Statistics Division (UNSD). United Nations Demographic Yearbook - Historical Supplement 1997. New York City, United States: United Nations Statistics Division (UNSD).
Dominica Vital Registration Death Data 1963 as it appears in United Nations Statistics Division (UNSD). United Nations Demographic Yearbook - Historical Supplement 1997. New York City, United States: United Nations Statistics Division (UNSD).
Dominica Vital Registration Death Data 1966 as it appears in United Nations Statistics Division (UNSD). United Nations Demographic Yearbook - Historical Supplement 1997. New York City, United States: United Nations Statistics Division (UNSD).
Dominican Republic Multiple Indicator Cluster Survey 2006 as it appears in World Health Organization (WHO). WHO Household Energy Database. Geneva, Switzerland: World Health Organization (WHO), 2010.
Dominican Republic National Multipurpose Household Survey 2006 as it appears in World Health Organization (WHO). WHO Global Database on Child Growth and Malnutrition. Geneva, Switzerland: World Health Organization (WHO).
Dominican Republic Population and Housing Census 2002 as it appears in World Health Organization (WHO). WHO Household Energy Database. Geneva, Switzerland: World Health Organization (WHO), 2010.
Dominican Republic Vital Registration - Deaths 1956 ICD7 as it appears in World Health Organization (WHO). WHO Mortality Database Version July 2012. Geneva, Switzerland: World Health Organization (WHO), 2012.
Dominican Republic Vital Registration - Deaths 1957 ICD7 as it appears in World Health Organization (WHO). WHO Mortality Database Version July 2012. Geneva, Switzerland: World Health Organization (WHO), 2012.
Dominican Republic Vital Registration - Deaths 1958 ICD7 as it appears in World Health Organization (WHO). WHO Mortality Database Version July 2012. Geneva, Switzerland: World Health Organization (WHO), 2012.
Dominican Republic Vital Registration - Deaths 1959 ICD7 as it appears in World Health Organization (WHO). WHO Mortality Database Version July 2012. Geneva, Switzerland: World Health Organization (WHO), 2012.
Dominican Republic Vital Registration - Deaths 1960 ICD7 as it appears in World Health Organization (WHO). WHO Mortality Database Version July 2012. Geneva, Switzerland: World Health Organization (WHO), 2012.
Dominican Republic Vital Registration - Deaths 1961 ICD7 as it appears in World Health Organization (WHO). WHO Mortality Database Version July 2012. Geneva, Switzerland: World Health Organization (WHO), 2012.
Dominican Republic Vital Registration - Deaths 1962 ICD7 as it appears in World Health Organization (WHO). WHO Mortality Database Version July 2012. Geneva, Switzerland: World Health Organization (WHO), 2012.
Dominican Republic Vital Registration - Deaths 1963 ICD7 as it appears in World Health Organization (WHO). WHO Mortality Database Version July 2012. Geneva, Switzerland: World Health Organization (WHO), 2012.
Dominican Republic Vital Registration - Deaths 1965 ICD7 as it appears in World Health Organization (WHO). WHO Mortality Database Version July 2012. Geneva, Switzerland: World Health Organization (WHO), 2012.
Dominican Republic Vital Registration - Deaths 1966 ICD7 as it appears in World Health Organization (WHO). WHO Mortality Database Version July 2012. Geneva, Switzerland: World Health Organization (WHO), 2012.
Dominican Republic Vital Registration - Deaths 1967 ICD7 as it appears in World Health Organization (WHO). WHO Mortality Database Version July 2012. Geneva, Switzerland: World Health Organization (WHO), 2012.
Dominican Republic Vital Registration - Deaths 1968 ICD8 as it appears in World Health Organization (WHO). WHO Mortality Database Version May 2013. Geneva, Switzerland: World Health Organization (WHO), 2013.
Dominican Republic Vital Registration - Deaths 1969 ICD8 as it appears in World Health Organization (WHO). WHO Mortality Database Version May 2013. Geneva, Switzerland: World Health Organization (WHO), 2013.

Appendix: Citation List

Citation
Dominican Republic Vital Registration - Deaths 1970 ICD8 as it appears in World Health Organization (WHO). WHO Mortality Database Version May 2013. Geneva, Switzerland: World Health Organization (WHO), 2013.
Dominican Republic Vital Registration - Deaths 1971 ICD8 as it appears in World Health Organization (WHO). WHO Mortality Database Version May 2013. Geneva, Switzerland: World Health Organization (WHO), 2013.
Dominican Republic Vital Registration - Deaths 1972 ICD8 as it appears in World Health Organization (WHO). WHO Mortality Database Version May 2013. Geneva, Switzerland: World Health Organization (WHO), 2013.
Dominican Republic Vital Registration - Deaths 1973 ICD8 as it appears in World Health Organization (WHO). WHO Mortality Database Version May 2013. Geneva, Switzerland: World Health Organization (WHO), 2013.
Dominican Republic Vital Registration - Deaths 1974 ICD8 as it appears in World Health Organization (WHO). WHO Mortality Database Version May 2013. Geneva, Switzerland: World Health Organization (WHO), 2013.
Dominican Republic Vital Registration - Deaths 1975 ICD8 as it appears in World Health Organization (WHO). WHO Mortality Database Version May 2013. Geneva, Switzerland: World Health Organization (WHO), 2013.
Dominican Republic Vital Registration - Deaths 1976 ICD8 as it appears in World Health Organization (WHO). WHO Mortality Database Version May 2013. Geneva, Switzerland: World Health Organization (WHO), 2013.
Dominican Republic Vital Registration - Deaths 1977 ICD8 as it appears in World Health Organization (WHO). WHO Mortality Database Version May 2013. Geneva, Switzerland: World Health Organization (WHO), 2013.
Dominican Republic Vital Registration - Deaths 1978 ICD8 as it appears in World Health Organization (WHO). WHO Mortality Database Version May 2013. Geneva, Switzerland: World Health Organization (WHO), 2013.
Dominican Republic Vital Registration - Deaths 1979 ICD8 as it appears in World Health Organization (WHO). WHO Mortality Database Version May 2013. Geneva, Switzerland: World Health Organization (WHO), 2013.
Dominican Republic Vital Registration - Deaths 1980 ICD9 as it appears in World Health Organization (WHO). WHO Mortality Database Version February 2014. Geneva, Switzerland: World Health Organization (WHO), 2014.
Dominican Republic Vital Registration - Deaths 1981 ICD9 as it appears in World Health Organization (WHO). WHO Mortality Database Version February 2014. Geneva, Switzerland: World Health Organization (WHO), 2014.
Dominican Republic Vital Registration - Deaths 1982 ICD9 as it appears in World Health Organization (WHO). WHO Mortality Database Version February 2014. Geneva, Switzerland: World Health Organization (WHO), 2014.
Dominican Republic Vital Registration - Deaths 1983 ICD9 as it appears in World Health Organization (WHO). WHO Mortality Database Version February 2014. Geneva, Switzerland: World Health Organization (WHO), 2014.
Dominican Republic Vital Registration - Deaths 1984 ICD9 as it appears in World Health Organization (WHO). WHO Mortality Database Version February 2014. Geneva, Switzerland: World Health Organization (WHO), 2014.
Dominican Republic Vital Registration - Deaths 1985 ICD9 as it appears in World Health Organization (WHO). WHO Mortality Database Version February 2014. Geneva, Switzerland: World Health Organization (WHO), 2014.
Dominican Republic Vital Registration - Deaths 1986 ICD9 as it appears in World Health Organization (WHO). WHO Mortality Database Version February 2014. Geneva, Switzerland: World Health Organization (WHO), 2014.
Dominican Republic Vital Registration - Deaths 1987 ICD9 as it appears in World Health Organization (WHO). WHO Mortality Database Version February 2014. Geneva, Switzerland: World Health Organization (WHO), 2014.
Dominican Republic Vital Registration - Deaths 1988 ICD9 as it appears in World Health Organization (WHO). WHO Mortality Database Version February 2014. Geneva, Switzerland: World Health Organization (WHO), 2014.
Dominican Republic Vital Registration - Deaths 1989 ICD9 as it appears in World Health Organization (WHO). WHO Mortality Database Version February 2014. Geneva, Switzerland: World Health Organization (WHO), 2014.
Dominican Republic Vital Registration - Deaths 1990 ICD9 as it appears in World Health Organization (WHO). WHO Mortality Database Version July 2012. Geneva, Switzerland: World Health Organization (WHO), 2012.
Dominican Republic Vital Registration - Deaths 1991 ICD9 as it appears in World Health Organization (WHO). WHO Mortality Database Version February 2014. Geneva, Switzerland: World Health Organization (WHO), 2014.
Dominican Republic Vital Registration - Deaths 1992 ICD9 as it appears in World Health Organization (WHO). WHO Mortality Database Version February 2014. Geneva, Switzerland: World Health Organization (WHO), 2014.
Dominican Republic Vital Registration - Deaths 1994 ICD9 as it appears in World Health Organization (WHO). WHO Mortality Database Version November 2015. Geneva, Switzerland: World Health Organization (WHO), 2015.
Dominican Republic Vital Registration - Deaths 1995 ICD9 as it appears in World Health Organization (WHO). WHO Mortality Database Version November 2015. Geneva, Switzerland: World Health Organization (WHO), 2015.
Dominican Republic Vital Registration - Deaths 1996 ICD10 as it appears in World Health Organization (WHO). WHO Mortality Database Version November 2015. Geneva, Switzerland: World Health Organization (WHO), 2015.
Dominican Republic Vital Registration - Deaths 1997 ICD10 as it appears in World Health Organization (WHO). WHO Mortality Database Version November 2015. Geneva, Switzerland: World Health Organization (WHO), 2015.
Dominican Republic Vital Registration - Deaths 1998 ICD10 as it appears in World Health Organization (WHO). WHO Mortality Database Version November 2015. Geneva, Switzerland: World Health Organization (WHO), 2015.
Dominican Republic Vital Registration - Deaths 1999 ICD10 as it appears in World Health Organization (WHO). WHO Mortality Database Version November 2015. Geneva, Switzerland: World Health Organization (WHO), 2015.
Dominican Republic Vital Registration - Deaths 2000 ICD10 as it appears in World Health Organization (WHO). WHO Mortality Database Version November 2015. Geneva, Switzerland: World Health Organization (WHO), 2015.
Dominican Republic Vital Registration - Deaths 2001 ICD10 as it appears in World Health Organization (WHO). WHO Mortality Database Version November 2015. Geneva, Switzerland: World Health Organization (WHO), 2015.
Dominican Republic Vital Registration - Deaths 2002 ICD10 as it appears in World Health Organization (WHO). WHO Mortality Database Version November 2015. Geneva, Switzerland: World Health Organization (WHO), 2015.

Appendix: Citation List

Citation
Dominican Republic Vital Registration - Deaths 2003 ICD10 as it appears in World Health Organization (WHO). WHO Mortality Database Version November 2015. Geneva, Switzerland: World Health Organization (WHO), 2015.
Dominican Republic Vital Registration - Deaths 2004 ICD10 as it appears in World Health Organization (WHO). WHO Mortality Database Version November 2015. Geneva, Switzerland: World Health Organization (WHO), 2015.
Dominican Republic Vital Registration - Deaths 2005 ICD10 as it appears in World Health Organization (WHO). WHO Mortality Database Version November 2015. Geneva, Switzerland: World Health Organization (WHO), 2015.
Dominican Republic Vital Registration - Deaths 2006 ICD10 as it appears in World Health Organization (WHO). WHO Mortality Database Version November 2015. Geneva, Switzerland: World Health Organization (WHO), 2015.
Dominican Republic Vital Registration - Deaths 2007 ICD10 as it appears in World Health Organization (WHO). WHO Mortality Database Version November 2015. Geneva, Switzerland: World Health Organization (WHO), 2015.
Dominican Republic Vital Registration - Deaths 2008 ICD10 as it appears in World Health Organization (WHO). WHO Mortality Database Version November 2015. Geneva, Switzerland: World Health Organization (WHO), 2015.
Dominican Republic Vital Registration - Deaths 2009 ICD10 as it appears in World Health Organization (WHO). WHO Mortality Database Version November 2015. Geneva, Switzerland: World Health Organization (WHO), 2015.
Dominican Republic Vital Registration - Deaths 2010 ICD10 as it appears in World Health Organization (WHO). WHO Mortality Database Version November 2015. Geneva, Switzerland: World Health Organization (WHO), 2015.
Dominican Republic Vital Registration - Deaths 2011 ICD10 as it appears in World Health Organization (WHO). WHO Mortality Database Version November 2015. Geneva, Switzerland: World Health Organization (WHO), 2015.
Dominican Republic Vital Registration - Deaths 2012 ICD10 as it appears in World Health Organization (WHO). WHO Mortality Database Version November 2015. Geneva, Switzerland: World Health Organization (WHO), 2015.
Dominican Republic Vital Registration Death Data 1950 as it appears in United Nations Statistics Division (UNSD). United Nations Demographic Yearbook - Historical Supplement 1997. New York City, United States: United Nations Statistics Division (UNSD).
Dominican Republic Vital Registration Death Data 1951 as it appears in United Nations Statistics Division (UNSD). United Nations Demographic Yearbook - Historical Supplement 1997. New York City, United States: United Nations Statistics Division (UNSD).
Dominican Republic Vital Registration Death Data 1952 as it appears in United Nations Statistics Division (UNSD). United Nations Demographic Yearbook - Historical Supplement 1997. New York City, United States: United Nations Statistics Division (UNSD).
Dominican Republic Vital Registration Death Data 1953 as it appears in United Nations Statistics Division (UNSD). United Nations Demographic Yearbook - Historical Supplement 1997. New York City, United States: United Nations Statistics Division (UNSD).
Dominican Republic Vital Registration Death Data 1954 as it appears in United Nations Statistics Division (UNSD). United Nations Demographic Yearbook - Historical Supplement 1997. New York City, United States: United Nations Statistics Division (UNSD).
Dominican Republic Vital Registration Death Data 1955 as it appears in United Nations Statistics Division (UNSD). United Nations Demographic Yearbook - Historical Supplement 1997. New York City, United States: United Nations Statistics Division (UNSD).
Dominican Republic Vital Registration Death Data 1964 as it appears in United Nations Statistics Division (UNSD). United Nations Demographic Yearbook - Historical Supplement 1997. New York City, United States: United Nations Statistics Division (UNSD).
Dominican Republic Vital Registration Death Data 2007 as it appears in United Nations Statistics Division (UNSD). United Nations Demographic Yearbook 2007. New York City, United States: United Nations Statistics Division (UNSD), 2009.
Dominican Republic Vital Registration Death Data 2008 as it appears in United Nations Statistics Division (UNSD). United Nations Demographic Yearbook 2008. New York City, United States: United Nations Statistics Division (UNSD), 2010.
Dominican Republic Vital Registration Death Data 2009 as it appears in United Nations Statistics Division (UNSD). United Nations Demographic Yearbook 2009-2010. New York City, United States: United Nations Statistics Division (UNSD), 2011.
Dominican Republic Vital Registration Death Data 2010 as it appears in United Nations Statistics Division (UNSD). United Nations Demographic Yearbook 2011. New York City, United States: United Nations Statistics Division (UNSD), 2012.
Donati S, Senatore S, Ronconi A, Regional maternal mortality working group. Maternal mortality in Italy: a record-linkage study. BJOG. 2011; 118(7): 872-9.
Donato F, Monarca S, Marchionna G, Rossi A, Cicioni C, Chiesa R, Colin D, Boffetta P. Mortality From Cancer And Chronic Respiratory Diseases Among Workers Who Manufacture Carbon Electrodes. Occup Environ Med. 2000; 57(7): 484-7 as it appears in Armstrong B, Hutchinson E, Unwin J, Fletcher T. Lung cancer risk after exposure to polycyclic aromatic hydrocarbons: a review and meta-analysis. Environ. Health Perspect. 2004; 112(9): 970–978.
Dong D, Xu G, Sun Y, Hu P. Lung cancer among workers exposed to silica dust in Chinese refractory plants. Scand J Work Environ Health. 1995; 69-72 as it appears in Kurihara N, Wada O. Silicosis and smoking strongly increase lung cancer risk in silica-exposed workers. Ind Health 2004; 42(3): 303–314.
Dong Y, Gao W, Nan H, Yu H, Li F, Duan W, Wang Y, Sun B, Qian R, Tuomilehto J, Qiao Q. Prevalence of Type 2 diabetes in urban and rural Chinese populations in Qingdao, China. Diabet Med. 2005; 22(10): 1427-33.
Dongre A, Singh A, Deshmukh P, Garg B. A community based cross sectional study on feasibility of lay interviewers in ascertaining causes of adult deaths by using verbal autopsy in rural Wardha. Online J Health Allied Sci. 2008; 7(4): 4.
Dono Brahim B. Evaluation of Endemic Malaria and Vector Control Practices in the Bongor Prefecture of Mayo-Kebbi (Republic of Chad). Yaoundé, Cameroon: Organization for Coordination in the Fight Against Endemic Diseases in Central Africa (OCEAC), 1988. As it appears in Malaria Atlas Project. Malaria Atlas Project Plasmodium Falciparum Parasite Rate Database. Oxford, United Kingdom: Malaria Atlas Project.
Dorfman SF. Maternal mortality in New York City, 1981-1983. Obstet Gynecol. 1990; 76(3 Pt 1): 317-23.
Dorkenoo AM, Sodahlon YK, Bronzan RN, Yakpa K, Sossou E, Ouro-Medeli A, Teko M, Seim A, Mathieu E. [Lymphatic filariasis transmission assessment survey in schools three years after stopping mass drug treatment with albendazole and ivermectine in the 7 endemic districts in Togo]. Bull Soc Pathol Exot. 2015; 108.0(3): 181-7.

Appendix: Citation List

Citation
Dorynska A, Polak M, Kozela M, Szafraniec K, Piotrowski W, Bielecki W, Drygas W, Kozakiewicz K, Piwonski J, Tykarski A, Zdrojewski T, Pajak A. Cardiovascular disease (CVD) risk factors in Krakow and in the whole Poland adult population. Results from the WOBASZ study and Polish arm of the HAPIEE project. Przegl Epidemiol. 2015; 69(1): 79–86.
Dos Santos JI, Lopes MA, Deliège-Vasconcelos E, Couto-Fernandez JC, Patel BN, Barreto ML, Ferreira Júnior OC, Galvão-Castro B. Seroprevalence of HIV, HTLV-I/II and other perinatally-transmitted pathogens in Salvador, Bahia. Rev Inst Med Trop Sao Paulo. 1995; 37(4): 343-8.
Dos Santos MM, Amaral S, Harmen SP, Joseph HM, Fernandes JL, Counahan ML. The prevalence of common skin infections in four districts in Timor-Leste: a cross sectional survey. BMC Infect Dis. 2010; 61.
Dossou-Yovo J, Ouattara A, Doannio JM, Diarrassouba S, Chauvancy G. [Malaria surveys in a humid savannah region in Côte d'Ivoire]. Med Trop (Mars). 1998; 58(1): 51-6. As it appears in Malaria Atlas Project. Malaria Atlas Project Plasmodium Falciparum Parasite Rate Database. Oxford, United Kingdom: Malaria Atlas Project.
Doumbo O, Koita O, Traore SF, Sangare O, Coulibaly A, Robert V, Soula G, Quilici M, Toure YT. Les aspects parasitologiques de l'épidémiologie du paludisme dans le Sahara malien. Med Afr Noire. 1991; 38(2): 103-9. As it appears in Malaria Atlas Project. Malaria Atlas Project Plasmodium Falciparum Parasite Rate Database. Oxford, United Kingdom: Malaria Atlas Project.
Doumbo O, Traore S, Sow Y, Dembele M, Soula G, Coulibaly A, Dolo A, Sangare O, Koita O, Pichard E. [Impact of curtains and blankets impregnated with permethrin on the malarial indicators and the number of malarial attacks per child in a village in an area hyperendemic for malaria on the Malian savannah (preliminary results of the first year study)]. Bull Soc Pathol Exot. 1991; 84(5): 761-74. As it appears in Malaria Atlas Project. Malaria Atlas Project Plasmodium Falciparum Parasite Rate Database. Oxford, United Kingdom: Malaria Atlas Project.
Dounias G, Kypraiou E, Rachiotis G, Tsovili E, Kostopoulos S. Prevalence of hepatitis B virus markers in municipal solid waste workers in Keratsini (Greece). Occup Med (Lond). 2005; 55(1): 60-3.
Doupis J, Tentolouris N, Mastrokostopoulos A, Kokkinos A, Doupis C, Zdrava A, Kafantogias A. Prevalence of type 2 diabetes in the southwest Albanian adult population. Rural Remote Health. 2007; 7(2): 744.
Dowell SF, Davis HL, Holt EA, Ruff AJ, Kissinger PJ, Bijoux J, Boulos R, Boulos C, Halsey NA. The utility of verbal autopsies for identifying HIV-1-related deaths in Haitian children. AIDS. 1993; 7(9): 1255-9.
Downs JA, Mguta C, Kaatano GM, Mitchell KB, Kinung'hi S, Simplice H, Kalluvya SE, Changalucha JM, Johnson WD Jr, Fitzgerald DW. Female urogenital schistosomiasis in Tanzania's Lake Zone region: a highly-sporadic distribution [Abstract]. Am J Trop Med Hyg. 2010; 83(Suppl 5): 211-82.
Dowse GK, Gareeboo H, Alberti KG, Zimmet P, Tuomilehto J, Purran A, Fareed D, Chitson P, Collins VR. Changes in population cholesterol concentrations and other cardiovascular risk factor levels after five years of the non-communicable disease intervention programme in Mauritius. Mauritius Non-communicable Disease Study Group. BMJ. 1995; 311(7015): 1255â€“9.
Dowse GK, Gareeboo H, Zimmet PZ, Alberti KG, Tuomilehto J, Fareed D, Brissonnette LG, Finch CF. High prevalence of NIDDM and impaired glucose tolerance in Indian, Creole, and Chinese Mauritians. Mauritius Noncommunicable Disease Study Group. Diabetes. 1990; 39(3): 390-6.
Dowse GK, Zimmet PZ, Gareeboo H, George K, Alberti MM, Tuomilehto J, Finch CF, Chitson P, Tulsidas H. Abdominal obesity and physical inactivity as risk factors for NIDDM and impaired glucose tolerance in Indian, Creole, and Chinese Mauritians. Diabetes Care. 1991; 14(4): 271-82.
DOXA. Italy Tobacco Use Survey 2006.
DOXA. Italy Tobacco Use Survey 2007.
DOXA. Italy Tobacco Use Survey 2008.
DOXA. Italy Tobacco Use Survey 2009.
DOXA. Italy Tobacco Use Survey 2010.
Dozie IN, Chukwuocha UM, Onwuliri CO, Nwoke BE. Malaria Among Asymptomatic School Children in Ezinihitte Local Government Area of Imo State, Nigeria. 2008. As it appears in Malaria Atlas Project. Malaria Atlas Project Plasmodium Falciparum Parasite Rate Database. Oxford, United Kingdom: Malaria Atlas Project.
Dr. Elizabeth Quamina Cancer Registry, Ministry of Health (Trinidad and Tobago). Trinidad and Tobago Cancer Incidence 1995-2006.
Drakeley CJ, Carneiro I, Reyburn H, Malima R, Lusingu JPA, Cox J, Theander TG, Nkya WMMM, Lemnge MM, Riley EM. Altitude-Dependent and -Independent Variations in Plasmodium falciparum Prevalence in Northeastern Tanzania. J Infect Dis. 2005; 191(10): 1589-98. As it appears in Malaria Atlas Project. Malaria Atlas Project Plasmodium Falciparum Parasite Rate Database. Oxford, United Kingdom: Malaria Atlas Project.
Drakeley CJ. Gambia Plasmodium Falciparum Parasite Rate Data, Personal Communication with C.J. Drakeley 2008. As it appears in Malaria Atlas Project. Malaria Atlas Project Plasmodium Falciparum Parasite Rate Database. Oxford, United Kingdom: Malaria Atlas Project.
Drankiewicz D, Dundes L. Handwashing among female college students. Am J Infect Control. 2003; 31(2): 67-71.
Drivsholm T, Ibsen H, Schroll M, Davidsen M, Borch-Johnsen K. Increasing prevalence of diabetes mellitus and impaired glucose tolerance among 60-year-old Danes. Diabet Med. 2001; 18(2): 126-32.
Droumaguet C, Balkau B, Simon D, Caces E, Tichet J, Charles MA, Eschwege E, DESIR Study Group. Use of HbA1c in predicting progression to diabetes in French men and women: data from an Epidemiological Study on the Insulin Resistance Syndrome (DESIR). Diabetes Care. 2006; 29(7): 1619–25.
Duarte EC, Gyorkos TW, Pang L, Abrahamowicz M. Epidemiology of malaria in a hypoendemic Brazilian Amazon migrant population: a cohort study. Am J Trop Med Hyg. 2004; 70(3): 229-37. As it appears in Malaria Atlas Project. Malaria Atlas Project Plasmodium Falciparum Parasite Rate Database. Oxford, United Kingdom: Malaria Atlas Project.
Dubey ML, Sharma SK, Ganguly NK, Mahajan RC. Seroepidemiological study of malaria in a rural population of Chandigarh. Indian J Malariol. 1989; 26(4): 187-90. As it appears in Malaria Atlas Project. Malaria Atlas Project Plasmodium Falciparum Parasite Rate Database. Oxford, United Kingdom: Malaria Atlas Project.

Appendix: Citation List

Citation
Duboz P, Boetsch G, Gueye L, Macia E. Hypertension prevalence, awareness, treatment and control in Dakar (Senegal). J Hum Hypertens. 2014; 28(8): 489â€“93.
Duboz P, Chapuis-Lucciani N, Boëtsch G, Gueye L. Prevalence of diabetes and associated risk factors in a Senegalese urban (Dakar) population. Diabetes Metab. 2012; 38(4): 332-6.
Duc Son LN, Kusama K, Hung NT, Loan TT, Chuyen NV, Kunii D, Sakai T, Yamamoto S. Prevalence and risk factors for diabetes in Ho Chi Minh City, Vietnam. Diabet Med. 2004; 21(4): 371-6.
Ducorps M, Baleynaud S, Mayaudon H, Castagne C, Bauduceau B. A prevalence survey of diabetes in Mauritania. Diabetes Care. 1996; 19(7): 761-3.
Duda RB, Anarfi JK, Adanu RMK, Seffah J, Darko R, Hill AG. The health of the “older women” in Accra, Ghana: results of the Women’s Health Study of Accra. J Cross Cult Gerontol. 2011; 26(3): 299–314.
Dudarev AA, Dorofeyev VM, Dushkina EV, Alloyarov PR, Chupakhin VS, Sladkova YN, Kolesnikova TA, Fridman KB, Nilsson LM, Evengard B. Food and water security issues in Russia III: food- and waterborne diseases in the Russian Arctic, Siberia and the Far East, 2000-2011. Int J Circumpolar Health. 2013; 21856.
Duerksen R, Limburg H, Carron JE, Foster A. Cataract blindness in Paraguay – results of a national survey. Ophthalmic Epidemiol. 2003; 10(5): 349-57.
Dugee O, Khor GL, Lye M-S, Luvsannyam L, Janchiv O, Jamyan B, Esa N. Association of major dietary patterns with obesity risk among Mongolian men and women. Asia Pac J Clin Nutr. 2009; 18(3): 433-40.
Dulal S, Sapkota YD. Prevalence of blindness and visual impairment and its causes among people aged 50 years and above in Karnali Zone, Nepal. Nepal J Ophthalmol. 2012; 4(2).
Dunstan DW, Zimmet PZ, Welborn TA, De Courten MP, Cameron AJ, Sicree RA, Dwyer T, Colagiuri S, Jolley D, Knuiman M, Atkins R, Shaw JE. The rising prevalence of diabetes and impaired glucose tolerance: the Australian Diabetes, Obesity and Lifestyle Study. Diabetes Care. 2002; 25(5): 829-34.
Dunyo SK, Appawu M, Nkrumah FK, Baffoe-Wilmot A, Pedersen EM, Simonsen PE. Lymphatic filariasis on the coast of Ghana. Trans R Soc Trop Med Hyg. 1996; 90(6): 634-8. As it appears in London School of Hygiene and Tropical Medicine. Global Atlas of Helminth Infections - Lymphatic Filiariasis.
Duong TH, Nguyen PH, Henley K, Peters M. Risk factors for hepatitis B infection in rural Vietnam. Asian Pac J Cancer Prev. 2009; 10(1): 97-102.
Dupont A, Delaporte E, Jego JM, Schrijvers D, Merlin M, Josse R. Prevalence of hepatitis B antigen among randomized representative urban and rural populations in Gabon. Ann Soc Belg Med Trop. 1988; 68(2): 157-8.
Durukan T, Öndero?lu L, Deren Ö, Saygan-Karamürsel B, Erdem G, Oram O, Gülsevin M, Yurdakök S, Özkutlu S, Çeliker A, Kale G, Çaglar M, Güçer S, Bulun A, Talim B, Büyükpamukçu N, Çiftçi A, Tunçbilek E, Balci S, Alanay Y. Perinatal mortality rate - hospital based study during 1998-2001 at Hacettepe University. J Perinat Med. 2005; 31(5): 435-40.
Dutch Hospital Data. Netherlands National Medical Registry 1998. [Unpublished].
Dutch Hospital Data. Netherlands National Medical Registry 1999. [Unpublished].
Dutch Hospital Data. Netherlands National Medical Registry 2000. [Unpublished].
Dutch Hospital Data. Netherlands National Medical Registry 2001. [Unpublished].
Dutch Hospital Data. Netherlands National Medical Registry 2002. [Unpublished].
Dutch Hospital Data. Netherlands National Medical Registry 2003. [Unpublished].
Dutch Hospital Data. Netherlands National Medical Registry 2004. [Unpublished].
Dutch Hospital Data. Netherlands National Medical Registry 2005. [Unpublished].
Dutch Hospital Data. Netherlands National Medical Registry 2006. [Unpublished].
Dutch Hospital Data. Netherlands National Medical Registry 2007. [Unpublished].
Dutch Hospital Data. Netherlands National Medical Registry 2008. [Unpublished].
Dutch Hospital Data. Netherlands National Medical Registry 2009. [Unpublished].
Dutch Hospital Data. Netherlands National Medical Registry 2010. [Unpublished].
Dutch Hospital Data. Netherlands National Medical Registry 2011. [Unpublished].
Dutch Hospital Data. Netherlands National Medical Registry 2012. [Unpublished].
Duthe G, Pison G. Adult mortality in a rural area of Senegal: Non-communicable diseases have a large impact in Mlomp. Demographic Res. 2008; 19(37): 1419-48.
Duverseau Y, Molez JF, Zevallos-Ipenza A. One-time Study of Malaria Morbidity in Bellevue. In: Molez JF, ed. Malaria Epidemiology and Hemoglobinopathies in Haiti: Public Health Research in the Caribbean. End-of-study Report. Port-au-Prince, Haiti: Office of Scientific and Technical Research Overseas (ORSTOM), 1988. As it appears in Malaria Atlas Project. Malaria Atlas Project Plasmodium Falciparum Parasite Rate Database. Oxford, United Kingdom: Malaria Atlas Project.
Duvigneaud N, Wijndaele K, Matton L, Deriemaeker P, Philippaerts R, Lefevre J, Thomis M, Duquet W. Socio-economic and lifestyle factors associated with overweight in Flemish adult men and women. BMC Public Health. 2007; 23.
Dyck PJ, Kratz KM, Karnes JL, Litchy WJ, Klein R, Pach JM, Wilson DM, O'Brien PC, Melton LJ 3rd, Service FJ. The prevalence by staged severity of various types of diabetic neuropathy, retinopathy, and nephropathy in a population-based cohort: the Rochester Diabetic Neuropathy Study. Neurology. 1993; 43(4): 817-24.
Dysoley L, Kaneko A, Eto H, Mita T, Socheat D, Björkman A, Kobayakawa T. Changing patterns of forest malaria among the mobile adult male population in Chumkiri District, Cambodia. Acta Trop. 2008; 106(3): 207-12. As it appears in Malaria Atlas Project. Malaria Atlas Project Plasmodium Falciparum Parasite Rate Database. Oxford, United Kingdom: Malaria Atlas Project.
Dzodzomenyo M, Dunyo SK, Ahorlu CK, Coker WZ, Appawu MA, Pedersen EM, Simonsen PE. Bancroftian filariasis in an irrigation project community in southern Ghana. Trop Med Int Health. 1999; 4(1): 13-8. As it appears in London School of Hygiene and Tropical Medicine. Global Atlas of Helminth Infections - Lymphatic Filiariasis.

Appendix: Citation List

Citation
Earth Institute, Columbia University, Millennium Promise. Baseline Report: Millenium Research Village Sauri, Kenya. New York City, United States: Earth Institute, Columbia University, 2007. As it appears in Malaria Atlas Project. Malaria Atlas Project Plasmodium Falciparum Parasite Rate Database. Oxford, United Kingdom: Malaria Atlas Project.
Earth Institute, Columbia University, United Nations Development Programme (UNDP). Annual Report for Pampaida, Nigeria, Millennium Village. Year 1: February 2006 - February 2007. New York, United States: United Nations Development Programme (UNDP). As it appears in Malaria Atlas Project. Malaria Atlas Project Plasmodium Falciparum Parasite Rate Database. Oxford, United Kingdom: Malaria Atlas Project.
Earth Institute, Columbia University. Annual Report for Koraro, Ethiopia: Millennium Research Village July 2005 to June 2006. New York City, United States: Earth Institute, Columbia University, 2006. As it appears in Malaria Atlas Project. Malaria Atlas Project Plasmodium Falciparum Parasite Rate Database. Oxford, United Kingdom: Malaria Atlas Project.
Earth Trends: The Environmental Information Portal as it appears in World Health Organization (WHO). WHO Household Energy Database. Geneva, Switzerland: World Health Organization (WHO), 2010.
Ebenezer A, Amadi EC, Agi PI. Studies on the microfilaria, antigenemia and clinical signs of bancroftian filariasis in Epie creek communities, Niger Delta, Nigeria. Int Res J Microbiol. 2011; 2(9): 370-4. As it appears in London School of Hygiene and Tropical Medicine. Global Atlas of Helminth Infections - Lymphatic Filiariasis.
Eberle F. Evaluation In Vivo of the Chemical Sensitivity of Plasmodium Falciparum to Chloroquine in a School in Dschang. OCEAC Technical Document. Yaounde, Cameroon: Organization for Coordination in the Fight Against Endemic Diseases in Central Africa (OCEAC), 1990. As it appears in Malaria Atlas Project. Malaria Atlas Project Plasmodium Falciparum Parasite Rate Database. Oxford, United Kingdom: Malaria Atlas Project.
Ecevit A, Oguz SS, Tarcan A, Yazici C, Dilmen U. The changing pattern of perinatal mortality and causes of death in central Anatolian region of Turkey. J Matern Fetal Neonatal Med. 2012; 25(9): 1738-41.
Echimane AK, Ahnoux AA, Adoubi I, Hien S, M'Bra K, D'Horpock A, Diomande M, Anongba D, Mensah-Adoh I, Parkin DM. Cancer Incidence in Abidjan, Ivory Coast 1995-1997. Cancer. 2000; 89(3): 653-63.
Echouffo-Tcheugui JB, Dzudie A, Epacka ME, Choukem SP, Doualla MS, Luma H, Kengne AP. Prevalence and determinants of undiagnosed diabetes in an urban sub-Saharan African population. Prim Care Diabetes. 2012; 6(3): 229–34.
Eckhardt CL, Torheim LE, Monterrubio E, Barquera S, Ruel MT. The overlap of overweight and anaemia among women in three countries undergoing the nutrition transition. Eur J Clin Nutr. 2008; 62(2): 238-46.
Economic and Social Research Institute (ESRI) (Ireland), Health Promotion Unit, Department of Health and Children (Ireland), National University of Ireland, Galway, Royal College of Surgeons in Ireland (RCSI), University College Cork. Ireland Survey of Lifestyle Attitudes and Nutrition 2007. Dublin, Ireland: Health Promotion Unit, Department of Health and Children (Ireland).
Economic and Social Research Institute (ESRI) (Ireland), National Crime Council (Ireland). Ireland National Study of Domestic Abuse 2003.
Economic and Social Research Institute (ESRI) (Ireland), Women's Aid (Ireland). Ireland Making the Links Study 1995.
Economic Development Initiatives (EDI), Muhimbili University of Health and Allied Sciences (Tanzania), Rockwool Foundation Research Unit. Tanzania - Kagera Living Standards Measurement Study 2010. Washington DC, United States: World Bank.
Economic Development Initiatives (EDI), World Bank (WB). Tanzania Core Welfare Indicators Questionnaire Survey 2005. Bukoba, Tanzania: Economic Development Initiatives (EDI).
Economic Development Initiatives (EDI), World Bank (WB). Tanzania Core Welfare Indicators Questionnaire Survey 2006-2007. Bukoba, Tanzania: Economic Development Initiatives (EDI).
Economic Development Initiatives (EDI). Tanzania Mainland Truck Roads and Zanzibar Rural Roads Activities Impact Evaluation 2009. Economic Development Initiatives (EDI), 2010.
Economic Policy, Planning and Statistics Office (Marshall Islands), Secretariat of the Pacific Community (SPC). Marshall Islands Census 2011.
Economic Policy, Planning and Statistics Office (Marshall Islands), Secretariat of the Pacific Community (SPC). Marshall Islands Demographic and Health Survey 2007.
Economic Policy, Planning and Statistics Office (Marshall Islands). Marshall Islands Community Survey 2006.
Ecuador - Cuenca Tumor Registry 2003-2007 - CI5 as it appears in Forman D, Bray F, Brewster DH, Gombe Mbalawa C, Kohler B, Piñeros M, Steliarova-Foucher E, Swaminathan R and Ferlay J, eds (2013). Cancer Incidence in Five Continents, Vol. X Summary Database (electronic version). Lyon, IARC. http://ci5.iarc.fr
Ecuador - Quito Cancer Registry 1985 - CI5 as it appears in Ferlay J, Parkin DM, Curado MP, Bray F, Edwards B, Shin HR and Forman D. Cancer Incidence in Five Continents, Volumes I to IX: IARC CancerBase No. 9 [Internet]. Lyon, France: International Agency for Research on Cancer; 2010. Available from: http://ci5.iarc.fr
Ecuador - Quito Cancer Registry 1985-1987 - CI5 as it appears in Parkin DM, International Agency for Research on Cancer, International Association of Cancer Registries. Cancer Incidence in Five Continents. Vol. I to VIII. Lyon, France, IARCPress, 2005.
Ecuador - Quito Cancer Registry 1986 - CI5 as it appears in Ferlay J, Parkin DM, Curado MP, Bray F, Edwards B, Shin HR and Forman D. Cancer Incidence in Five Continents, Volumes I to IX: IARC CancerBase No. 9 [Internet]. Lyon, France: International Agency for Research on Cancer; 2010. Available from: http://ci5.iarc.fr
Ecuador - Quito Cancer Registry 1987 - CI5 as it appears in Ferlay J, Parkin DM, Curado MP, Bray F, Edwards B, Shin HR and Forman D. Cancer Incidence in Five Continents, Volumes I to IX: IARC CancerBase No. 9 [Internet]. Lyon, France: International Agency for Research on Cancer; 2010. Available from: http://ci5.iarc.fr
Ecuador - Quito Cancer Registry 1988 - CI5 as it appears in Ferlay J, Parkin DM, Curado MP, Bray F, Edwards B, Shin HR and Forman D. Cancer Incidence in Five Continents, Volumes I to IX: IARC CancerBase No. 9 [Internet]. Lyon, France: International Agency for Research on Cancer; 2010. Available from: http://ci5.iarc.fr
Ecuador - Quito Cancer Registry 1989 - CI5 as it appears in Ferlay J, Parkin DM, Curado MP, Bray F, Edwards B, Shin HR and Forman D. Cancer Incidence in Five Continents, Volumes I to IX: IARC CancerBase No. 9 [Internet]. Lyon, France: International Agency for Research on Cancer; 2010. Available from: http://ci5.iarc.fr

Appendix: Citation List

Citation
Ecuador - Quito Cancer Registry 1990 - CI5 as it appears in Ferlay J, Parkin DM, Curado MP, Bray F, Edwards B, Shin HR and Forman D. Cancer Incidence in Five Continents, Volumes I to IX: IARC CancerBase No. 9 [Internet]. Lyon, France: International Agency for Research on Cancer; 2010. Available from: http://ci5.iarc.fr
Ecuador - Quito Cancer Registry 1991 - CI5 as it appears in Ferlay J, Parkin DM, Curado MP, Bray F, Edwards B, Shin HR and Forman D. Cancer Incidence in Five Continents, Volumes I to IX: IARC CancerBase No. 9 [Internet]. Lyon, France: International Agency for Research on Cancer; 2010. Available from: http://ci5.iarc.fr
Ecuador - Quito Cancer Registry 1992 - CI5 as it appears in Ferlay J, Parkin DM, Curado MP, Bray F, Edwards B, Shin HR and Forman D. Cancer Incidence in Five Continents, Volumes I to IX: IARC CancerBase No. 9 [Internet]. Lyon, France: International Agency for Research on Cancer; 2010. Available from: http://ci5.iarc.fr
Ecuador - Quito Cancer Registry 1993 - CI5 as it appears in Ferlay J, Parkin DM, Curado MP, Bray F, Edwards B, Shin HR and Forman D. Cancer Incidence in Five Continents, Volumes I to IX: IARC CancerBase No. 9 [Internet]. Lyon, France: International Agency for Research on Cancer; 2010. Available from: http://ci5.iarc.fr
Ecuador - Quito Cancer Registry 1993-1997 - CI5 as it appears in Parkin DM, International Agency for Research on Cancer, International Association of Cancer Registries. Cancer Incidence in Five Continents. Vol. I to VIII. Lyon, France, IARCPress, 2005.
Ecuador - Quito Cancer Registry 1994 - CI5 as it appears in Ferlay J, Parkin DM, Curado MP, Bray F, Edwards B, Shin HR and Forman D. Cancer Incidence in Five Continents, Volumes I to IX: IARC CancerBase No. 9 [Internet]. Lyon, France: International Agency for Research on Cancer; 2010. Available from: http://ci5.iarc.fr
Ecuador - Quito Cancer Registry 1995 - CI5 as it appears in Ferlay J, Parkin DM, Curado MP, Bray F, Edwards B, Shin HR and Forman D. Cancer Incidence in Five Continents, Volumes I to IX: IARC CancerBase No. 9 [Internet]. Lyon, France: International Agency for Research on Cancer; 2010. Available from: http://ci5.iarc.fr
Ecuador - Quito Cancer Registry 1996 - CI5 as it appears in Ferlay J, Parkin DM, Curado MP, Bray F, Edwards B, Shin HR and Forman D. Cancer Incidence in Five Continents, Volumes I to IX: IARC CancerBase No. 9 [Internet]. Lyon, France: International Agency for Research on Cancer; 2010. Available from: http://ci5.iarc.fr
Ecuador - Quito Cancer Registry 1997 - CI5 as it appears in Ferlay J, Parkin DM, Curado MP, Bray F, Edwards B, Shin HR and Forman D. Cancer Incidence in Five Continents, Volumes I to IX: IARC CancerBase No. 9 [Internet]. Lyon, France: International Agency for Research on Cancer; 2010. Available from: http://ci5.iarc.fr
Ecuador - Quito Cancer Registry 1998 - CI5 as it appears in Ferlay J, Parkin DM, Curado MP, Bray F, Edwards B, Shin HR and Forman D. Cancer Incidence in Five Continents, Volumes I to IX: IARC CancerBase No. 9 [Internet]. Lyon, France: International Agency for Research on Cancer; 2010. Available from: http://ci5.iarc.fr
Ecuador - Quito Cancer Registry 1998-2002 - CI5 as it appears in Curado MP, Edwards B, Shin HR, Storm H, Ferlay J, Heanue M and Boyle P, eds (2007). Cancer Incidence in Five Continents, Vol. IX Periodic Data (electronic version). Lyon, IARC.http://ci5.iarc.fr
Ecuador - Quito Cancer Registry 1999 - CI5 as it appears in Ferlay J, Parkin DM, Curado MP, Bray F, Edwards B, Shin HR and Forman D. Cancer Incidence in Five Continents, Volumes I to IX: IARC CancerBase No. 9 [Internet]. Lyon, France: International Agency for Research on Cancer; 2010. Available from: http://ci5.iarc.fr
Ecuador - Quito Cancer Registry 2000 - CI5 as it appears in Ferlay J, Parkin DM, Curado MP, Bray F, Edwards B, Shin HR and Forman D. Cancer Incidence in Five Continents, Volumes I to IX: IARC CancerBase No. 9 [Internet]. Lyon, France: International Agency for Research on Cancer; 2010. Available from: http://ci5.iarc.fr
Ecuador - Quito Cancer Registry 2001 - CI5 as it appears in Ferlay J, Parkin DM, Curado MP, Bray F, Edwards B, Shin HR and Forman D. Cancer Incidence in Five Continents, Volumes I to IX: IARC CancerBase No. 9 [Internet]. Lyon, France: International Agency for Research on Cancer; 2010. Available from: http://ci5.iarc.fr
Ecuador - Quito Cancer Registry 2002 - CI5 as it appears in Ferlay J, Parkin DM, Curado MP, Bray F, Edwards B, Shin HR and Forman D. Cancer Incidence in Five Continents, Volumes I to IX: IARC CancerBase No. 9 [Internet]. Lyon, France: International Agency for Research on Cancer; 2010. Available from: http://ci5.iarc.fr
Ecuador - Quito Cancer Registry 2003-2007 - CI5 as it appears in Forman D, Bray F, Brewster DH, Gombe Mbalawa C, Kohler B, Piñeros M, Steliarova-Foucher E, Swaminathan R and Ferlay J, eds (2013). Cancer Incidence in Five Continents, Vol. X Summary Database (electronic version). Lyon, IARC. http://ci5.iarc.fr
Ecuador Living Conditions Survey 2005-2006 as it appears in World Health Organization (WHO). WHO Household Energy Database. Geneva, Switzerland: World Health Organization (WHO), 2010.
Ecuador Living Standards Measurement Survey 1998 as it appears in World Health Organization (WHO). WHO Global Database on Child Growth and Malnutrition. Geneva, Switzerland: World Health Organization (WHO).
Ecuador Population and Housing Census 1990 as it appears in World Health Organization (WHO). WHO Household Energy Database. Geneva, Switzerland: World Health Organization (WHO), 2010.
Ecuador Population and Housing Census 2001 as it appears in World Health Organization (WHO). WHO Household Energy Database. Geneva, Switzerland: World Health Organization (WHO), 2010.
Ecuador Rapid Assessment for Avoidable Blindness Survey 2009. [Unpublished]. As it appears in Bourne R, Global Burden of Disease Vision Loss Expert Group. Vision Loss Database - Survey Data on Vision Loss by Severity and Etiology. [Unpublished].
Ecuador Vital Registration - Deaths 1961 ICD7 as it appears in World Health Organization (WHO). WHO Mortality Database Version July 2012. Geneva, Switzerland: World Health Organization (WHO), 2012.
Ecuador Vital Registration - Deaths 1963 ICD7 as it appears in World Health Organization (WHO). WHO Mortality Database Version July 2012. Geneva, Switzerland: World Health Organization (WHO), 2012.
Ecuador Vital Registration - Deaths 1964 ICD7 as it appears in World Health Organization (WHO). WHO Mortality Database Version July 2012. Geneva, Switzerland: World Health Organization (WHO), 2012.
Ecuador Vital Registration - Deaths 1965 ICD7 as it appears in World Health Organization (WHO). WHO Mortality Database Version July 2012. Geneva, Switzerland: World Health Organization (WHO), 2012.

Appendix: Citation List

Citation
Ecuador Vital Registration - Deaths 1966 ICD7 as it appears in World Health Organization (WHO). WHO Mortality Database Version July 2012. Geneva, Switzerland: World Health Organization (WHO), 2012.
Ecuador Vital Registration - Deaths 1967 ICD7 as it appears in World Health Organization (WHO). WHO Mortality Database Version July 2012. Geneva, Switzerland: World Health Organization (WHO), 2012.
Ecuador Vital Registration - Deaths 1968 ICD8 as it appears in World Health Organization (WHO). WHO Mortality Database Version May 2013. Geneva, Switzerland: World Health Organization (WHO), 2013.
Ecuador Vital Registration - Deaths 1969 ICD8 as it appears in World Health Organization (WHO). WHO Mortality Database Version May 2013. Geneva, Switzerland: World Health Organization (WHO), 2013.
Ecuador Vital Registration - Deaths 1970 ICD8 as it appears in World Health Organization (WHO). WHO Mortality Database Version May 2013. Geneva, Switzerland: World Health Organization (WHO), 2013.
Ecuador Vital Registration - Deaths 1971 ICD8 as it appears in World Health Organization (WHO). WHO Mortality Database Version May 2013. Geneva, Switzerland: World Health Organization (WHO), 2013.
Ecuador Vital Registration - Deaths 1972 ICD8 as it appears in World Health Organization (WHO). WHO Mortality Database Version May 2013. Geneva, Switzerland: World Health Organization (WHO), 2013.
Ecuador Vital Registration - Deaths 1973 ICD8 as it appears in World Health Organization (WHO). WHO Mortality Database Version May 2013. Geneva, Switzerland: World Health Organization (WHO), 2013.
Ecuador Vital Registration - Deaths 1974 ICD8 as it appears in World Health Organization (WHO). WHO Mortality Database Version May 2013. Geneva, Switzerland: World Health Organization (WHO), 2013.
Ecuador Vital Registration - Deaths 1975 ICD8 as it appears in World Health Organization (WHO). WHO Mortality Database Version May 2013. Geneva, Switzerland: World Health Organization (WHO), 2013.
Ecuador Vital Registration - Deaths 1977 ICD8 as it appears in World Health Organization (WHO). WHO Mortality Database Version May 2013. Geneva, Switzerland: World Health Organization (WHO), 2013.
Ecuador Vital Registration - Deaths 1978 ICD8 as it appears in World Health Organization (WHO). WHO Mortality Database Version May 2013. Geneva, Switzerland: World Health Organization (WHO), 2013.
Ecuador Vital Registration - Deaths 1979 ICD9 as it appears in World Health Organization (WHO). WHO Mortality Database Version November 2015. Geneva, Switzerland: World Health Organization (WHO), 2015.
Ecuador Vital Registration - Deaths 1980 ICD9 as it appears in World Health Organization (WHO). WHO Mortality Database Version November 2015. Geneva, Switzerland: World Health Organization (WHO), 2015.
Ecuador Vital Registration - Deaths 1981 ICD9 as it appears in World Health Organization (WHO). WHO Mortality Database Version November 2015. Geneva, Switzerland: World Health Organization (WHO), 2015.
Ecuador Vital Registration - Deaths 1982 ICD9 as it appears in World Health Organization (WHO). WHO Mortality Database Version November 2015. Geneva, Switzerland: World Health Organization (WHO), 2015.
Ecuador Vital Registration - Deaths 1983 ICD9 as it appears in World Health Organization (WHO). WHO Mortality Database Version November 2015. Geneva, Switzerland: World Health Organization (WHO), 2015.
Ecuador Vital Registration - Deaths 1984 ICD9 as it appears in World Health Organization (WHO). WHO Mortality Database Version November 2015. Geneva, Switzerland: World Health Organization (WHO), 2015.
Ecuador Vital Registration - Deaths 1985 ICD9 as it appears in World Health Organization (WHO). WHO Mortality Database Version November 2015. Geneva, Switzerland: World Health Organization (WHO), 2015.
Ecuador Vital Registration - Deaths 1986 ICD9 as it appears in World Health Organization (WHO). WHO Mortality Database Version November 2015. Geneva, Switzerland: World Health Organization (WHO), 2015.
Ecuador Vital Registration - Deaths 1987 ICD9 as it appears in World Health Organization (WHO). WHO Mortality Database Version November 2015. Geneva, Switzerland: World Health Organization (WHO), 2015.
Ecuador Vital Registration - Deaths 1988 ICD9 as it appears in World Health Organization (WHO). WHO Mortality Database Version November 2015. Geneva, Switzerland: World Health Organization (WHO), 2015.
Ecuador Vital Registration - Deaths 1989 ICD9 as it appears in World Health Organization (WHO). WHO Mortality Database Version November 2015. Geneva, Switzerland: World Health Organization (WHO), 2015.
Ecuador Vital Registration - Deaths 1990 ICD9 as it appears in World Health Organization (WHO). WHO Mortality Database Version November 2015. Geneva, Switzerland: World Health Organization (WHO), 2015.
Ecuador Vital Registration - Deaths 1991 ICD9 as it appears in World Health Organization (WHO). WHO Mortality Database Version November 2015. Geneva, Switzerland: World Health Organization (WHO), 2015.
Ecuador Vital Registration - Deaths 1992 ICD9 as it appears in World Health Organization (WHO). WHO Mortality Database Version November 2015. Geneva, Switzerland: World Health Organization (WHO), 2015.
Ecuador Vital Registration - Deaths 1993 ICD9 as it appears in World Health Organization (WHO). WHO Mortality Database Version November 2015. Geneva, Switzerland: World Health Organization (WHO), 2015.
Ecuador Vital Registration - Deaths 1994 ICD9 as it appears in World Health Organization (WHO). WHO Mortality Database Version November 2015. Geneva, Switzerland: World Health Organization (WHO), 2015.
Ecuador Vital Registration - Deaths 1995 ICD9 as it appears in World Health Organization (WHO). WHO Mortality Database Version November 2015. Geneva, Switzerland: World Health Organization (WHO), 2015.
Ecuador Vital Registration - Deaths 1996 ICD9 as it appears in World Health Organization (WHO). WHO Mortality Database Version July 2012. Geneva, Switzerland: World Health Organization (WHO), 2012.
Ecuador Vital Registration - Deaths 1997 ICD10 as it appears in World Health Organization (WHO). WHO Mortality Database Version November 2015. Geneva, Switzerland: World Health Organization (WHO), 2015.
Ecuador Vital Registration - Deaths 1998 ICD10 as it appears in World Health Organization (WHO). WHO Mortality Database Version November 2015. Geneva, Switzerland: World Health Organization (WHO), 2015.

Appendix: Citation List

Citation
Ecuador Vital Registration - Deaths 1999 ICD10 as it appears in World Health Organization (WHO). WHO Mortality Database Version November 2015. Geneva, Switzerland: World Health Organization (WHO), 2015.
Ecuador Vital Registration - Deaths 2000 ICD10 as it appears in World Health Organization (WHO). WHO Mortality Database Version November 2015. Geneva, Switzerland: World Health Organization (WHO), 2015.
Ecuador Vital Registration - Deaths 2001 ICD10 as it appears in World Health Organization (WHO). WHO Mortality Database Version November 2015. Geneva, Switzerland: World Health Organization (WHO), 2015.
Ecuador Vital Registration - Deaths 2002 ICD10 as it appears in World Health Organization (WHO). WHO Mortality Database Version November 2015. Geneva, Switzerland: World Health Organization (WHO), 2015.
Ecuador Vital Registration - Deaths 2003 ICD10 as it appears in World Health Organization (WHO). WHO Mortality Database Version November 2015. Geneva, Switzerland: World Health Organization (WHO), 2015.
Ecuador Vital Registration - Deaths 2004 ICD10 as it appears in World Health Organization (WHO). WHO Mortality Database Version November 2015. Geneva, Switzerland: World Health Organization (WHO), 2015.
Ecuador Vital Registration - Deaths 2005 ICD10 as it appears in World Health Organization (WHO). WHO Mortality Database Version November 2015. Geneva, Switzerland: World Health Organization (WHO), 2015.
Ecuador Vital Registration - Deaths 2006 ICD10 as it appears in World Health Organization (WHO). WHO Mortality Database Version November 2015. Geneva, Switzerland: World Health Organization (WHO), 2015.
Ecuador Vital Registration - Deaths 2007 ICD10 as it appears in World Health Organization (WHO). WHO Mortality Database Version November 2015. Geneva, Switzerland: World Health Organization (WHO), 2015.
Ecuador Vital Registration - Deaths 2008 ICD10 as it appears in World Health Organization (WHO). WHO Mortality Database Version November 2015. Geneva, Switzerland: World Health Organization (WHO), 2015.
Ecuador Vital Registration - Deaths 2009 ICD10 as it appears in World Health Organization (WHO). WHO Mortality Database Version November 2015. Geneva, Switzerland: World Health Organization (WHO), 2015.
Ecuador Vital Registration - Deaths 2010 ICD10 as it appears in World Health Organization (WHO). WHO Mortality Database Version November 2015. Geneva, Switzerland: World Health Organization (WHO), 2015.
Ecuador Vital Registration - Deaths 2011 ICD10 as it appears in World Health Organization (WHO). WHO Mortality Database Version November 2015. Geneva, Switzerland: World Health Organization (WHO), 2015.
Ecuador Vital Registration - Deaths 2012 ICD10 as it appears in World Health Organization (WHO). WHO Mortality Database Version November 2015. Geneva, Switzerland: World Health Organization (WHO), 2015.
Ecuador Vital Registration - Deaths 2013 ICD10 as it appears in World Health Organization (WHO). WHO Mortality Database Version November 2015. Geneva, Switzerland: World Health Organization (WHO), 2015.
Ecuador Vital Registration Death Data 1954 as it appears in United Nations Statistics Division (UNSD). United Nations Demographic Yearbook - Historical Supplement 1997. New York City, United States: United Nations Statistics Division (UNSD).
Ecuador Vital Registration Death Data 1955 as it appears in United Nations Statistics Division (UNSD). United Nations Demographic Yearbook - Historical Supplement 1997. New York City, United States: United Nations Statistics Division (UNSD).
Ecuador Vital Registration Death Data 1956 as it appears in United Nations Statistics Division (UNSD). United Nations Demographic Yearbook - Historical Supplement 1997. New York City, United States: United Nations Statistics Division (UNSD).
Ecuador Vital Registration Death Data 1957 as it appears in United Nations Statistics Division (UNSD). United Nations Demographic Yearbook - Historical Supplement 1997. New York City, United States: United Nations Statistics Division (UNSD).
Ecuador Vital Registration Death Data 1958 as it appears in United Nations Statistics Division (UNSD). United Nations Demographic Yearbook - Historical Supplement 1997. New York City, United States: United Nations Statistics Division (UNSD).
Ecuador Vital Registration Death Data 1959 as it appears in United Nations Statistics Division (UNSD). United Nations Demographic Yearbook - Historical Supplement 1997. New York City, United States: United Nations Statistics Division (UNSD).
Ecuador Vital Registration Death Data 1960 as it appears in United Nations Statistics Division (UNSD). United Nations Demographic Yearbook - Historical Supplement 1997. New York City, United States: United Nations Statistics Division (UNSD).
Ecuador Vital Registration Death Data 1962 as it appears in United Nations Statistics Division (UNSD). United Nations Demographic Yearbook - Historical Supplement 1997. New York City, United States: United Nations Statistics Division (UNSD).
Ecuador Vital Registration Death Data 1976 as it appears in United Nations Statistics Division (UNSD). United Nations Demographic Yearbook - Historical Supplement 1997. New York City, United States: United Nations Statistics Division (UNSD).
Ecuadorian Professional Training Service (SECAP), World Bank. Ecuador Living Standards Measurement Survey 1994. Washington DC, United States: World Bank.
Edling C, Jarvholm B, Andersson L, Axelson O. Mortality And Cancer Incidence Among Workers In An Abrasive Manufacturing Industry. Br J Ind Med. 1987; 44(1): 57-9 as it appears in Collins JJ, Lineker GA. A review and meta-analysis of formaldehyde exposure and leukemia. Regul Toxicol Pharmacol. 2004; 40(2): 81-91.
Edmond KM, Quigley MA, Zandoh C, Danso S, Hurt C, Owusu Agyei S, Kirkwood BR. Aetiology of stillbirths and neonatal deaths in rural Ghana: implications for health programming in developing countries. Paediatr Perinat Epidemiol. 2008; 22(5): 430-7.
Edmonds M, Boulton A, Buckenham T, Every N, Foster A, Freeman D, Gadsby R, Gibby O, Knowles A, Pooke M, Tovey F, Unwin N, Wolfe J. Report of the Diabetic Foot and Amputation Group. Diabet Med. 1996; 13(9 Suppl 4): S27-42.
Eduardo Mondlane University (Mozambique), Manhica Health Research Center (CISM), Ministry of Health (Mozambique). Mozambique Main Causes of Reported Death Study 2001.
Eglit T, Rajasalu T, Lember M. Prevalence of diabetes and impaired glucose regulation in Estonia. Diabet Med. 2011; 28(4): 504-5.
Egwang TG, Apio B, Riley E, Okello D. Plasmodium falciparum malariometric indices in Apac district, northern Uganda. East Afr Med J. 2000; 77(8): 413-6. As it appears in Malaria Atlas Project. Malaria Atlas Project Plasmodium Falciparum Parasite Rate Database. Oxford, United Kingdom: Malaria Atlas Project.

Appendix: Citation List

Citation
Egypt - Gharbiah Cancer Registry 1999-2002 - CI5 as it appears in Curado MP, Edwards B, Shin HR, Storm H, Ferlay J, Heanue M and Boyle P, eds (2007). Cancer Incidence in Five Continents, Vol. IX Periodic Data (electronic version). Lyon, IARC.http://ci5.iarc.fr
Egypt - Gharbiah Cancer Registry 2003-2007 - CI5 as it appears in Forman D, Bray F, Brewster DH, Gombe Mbalawa C, Kohler B, Piñeros M, Steliarova-Foucher E, Swaminathan R and Ferlay J, eds (2013). Cancer Incidence in Five Continents, Vol. X Summary Database (electronic version). Lyon, IARC. http://ci5.iarc.fr
Egypt National Cluster Survey for Vaccination Coverage 1984.
Egypt National Survey for Assessment of Vitamin A Status 1995 as it appears in World Health Organization (WHO). WHO Global Database on Child Growth and Malnutrition - Historical. Geneva, Switzerland: World Health Organization (WHO).
Egypt Nutrition Status Survey 1980 as it appears in World Health Organization (WHO). WHO Global Database on Child Growth and Malnutrition - Historical. Geneva, Switzerland: World Health Organization (WHO).
Egypt Population and Housing Census 1976 as it appears in United Nations Statistics Division (UNSD). United Nations Demographic Yearbook - Historical Supplement 1997. New York City, United States: United Nations Statistics Division (UNSD).
Egypt Vital Registration - Deaths 1954 ICD7 as it appears in World Health Organization (WHO). WHO Mortality Database Version July 2012. Geneva, Switzerland: World Health Organization (WHO), 2012.
Egypt Vital Registration - Deaths 1955 ICD7 as it appears in World Health Organization (WHO). WHO Mortality Database Version July 2012. Geneva, Switzerland: World Health Organization (WHO), 2012.
Egypt Vital Registration - Deaths 1956 ICD7 as it appears in World Health Organization (WHO). WHO Mortality Database Version July 2012. Geneva, Switzerland: World Health Organization (WHO), 2012.
Egypt Vital Registration - Deaths 1957 ICD7 as it appears in World Health Organization (WHO). WHO Mortality Database Version July 2012. Geneva, Switzerland: World Health Organization (WHO), 2012.
Egypt Vital Registration - Deaths 1958 ICD7 as it appears in World Health Organization (WHO). WHO Mortality Database Version July 2012. Geneva, Switzerland: World Health Organization (WHO), 2012.
Egypt Vital Registration - Deaths 1959 ICD7 as it appears in World Health Organization (WHO). WHO Mortality Database Version July 2012. Geneva, Switzerland: World Health Organization (WHO), 2012.
Egypt Vital Registration - Deaths 1960 ICD7 as it appears in World Health Organization (WHO). WHO Mortality Database Version July 2012. Geneva, Switzerland: World Health Organization (WHO), 2012.
Egypt Vital Registration - Deaths 1961 ICD7 as it appears in World Health Organization (WHO). WHO Mortality Database Version July 2012. Geneva, Switzerland: World Health Organization (WHO), 2012.
Egypt Vital Registration - Deaths 1962 ICD7 as it appears in World Health Organization (WHO). WHO Mortality Database Version July 2012. Geneva, Switzerland: World Health Organization (WHO), 2012.
Egypt Vital Registration - Deaths 1963 ICD7 as it appears in World Health Organization (WHO). WHO Mortality Database Version July 2012. Geneva, Switzerland: World Health Organization (WHO), 2012.
Egypt Vital Registration - Deaths 1964 ICD7 as it appears in World Health Organization (WHO). WHO Mortality Database Version July 2012. Geneva, Switzerland: World Health Organization (WHO), 2012.
Egypt Vital Registration - Deaths 1965 ICD7 as it appears in World Health Organization (WHO). WHO Mortality Database Version July 2012. Geneva, Switzerland: World Health Organization (WHO), 2012.
Egypt Vital Registration - Deaths 1966 ICD7 as it appears in World Health Organization (WHO). WHO Mortality Database Version July 2012. Geneva, Switzerland: World Health Organization (WHO), 2012.
Egypt Vital Registration - Deaths 1967 ICD7 as it appears in World Health Organization (WHO). WHO Mortality Database Version July 2012. Geneva, Switzerland: World Health Organization (WHO), 2012.
Egypt Vital Registration - Deaths 1970 ICD8 as it appears in World Health Organization (WHO). WHO Mortality Database Version May 2013. Geneva, Switzerland: World Health Organization (WHO), 2013.
Egypt Vital Registration - Deaths 1971 ICD8 as it appears in World Health Organization (WHO). WHO Mortality Database Version May 2013. Geneva, Switzerland: World Health Organization (WHO), 2013.
Egypt Vital Registration - Deaths 1972 ICD8 as it appears in World Health Organization (WHO). WHO Mortality Database Version May 2013. Geneva, Switzerland: World Health Organization (WHO), 2013.
Egypt Vital Registration - Deaths 1973 ICD8 as it appears in World Health Organization (WHO). WHO Mortality Database Version May 2013. Geneva, Switzerland: World Health Organization (WHO), 2013.
Egypt Vital Registration - Deaths 1974 ICD8 as it appears in World Health Organization (WHO). WHO Mortality Database Version May 2013. Geneva, Switzerland: World Health Organization (WHO), 2013.
Egypt Vital Registration - Deaths 1975 ICD8 as it appears in World Health Organization (WHO). WHO Mortality Database Version May 2013. Geneva, Switzerland: World Health Organization (WHO), 2013.
Egypt Vital Registration - Deaths 1976 ICD8 as it appears in World Health Organization (WHO). WHO Mortality Database Version May 2013. Geneva, Switzerland: World Health Organization (WHO), 2013.
Egypt Vital Registration - Deaths 1977 ICD8 as it appears in World Health Organization (WHO). WHO Mortality Database Version May 2013. Geneva, Switzerland: World Health Organization (WHO), 2013.
Egypt Vital Registration - Deaths 1978 ICD8 as it appears in World Health Organization (WHO). WHO Mortality Database Version May 2013. Geneva, Switzerland: World Health Organization (WHO), 2013.
Egypt Vital Registration - Deaths 1979 ICD8 as it appears in World Health Organization (WHO). WHO Mortality Database Version May 2013. Geneva, Switzerland: World Health Organization (WHO), 2013.
Egypt Vital Registration - Deaths 1980 ICD9 as it appears in World Health Organization (WHO). WHO Mortality Database Version November 2015. Geneva, Switzerland: World Health Organization (WHO), 2015.
Egypt Vital Registration - Deaths 1987 ICD9 as it appears in World Health Organization (WHO). WHO Mortality Database Version November 2015. Geneva, Switzerland: World Health Organization (WHO), 2015.

Appendix: Citation List

Citation
Egypt Vital Registration - Deaths 1991 ICD9 as it appears in World Health Organization (WHO). WHO Mortality Database Version November 2015. Geneva, Switzerland: World Health Organization (WHO), 2015.
Egypt Vital Registration - Deaths 1992 ICD9 as it appears in World Health Organization (WHO). WHO Mortality Database Version November 2015. Geneva, Switzerland: World Health Organization (WHO), 2015.
Egypt Vital Registration - Deaths 2000 ICD10 as it appears in World Health Organization (WHO). WHO Mortality Database Version November 2015. Geneva, Switzerland: World Health Organization (WHO), 2015.
Egypt Vital Registration - Deaths 2001 ICD10 as it appears in World Health Organization (WHO). WHO Mortality Database Version November 2015. Geneva, Switzerland: World Health Organization (WHO), 2015.
Egypt Vital Registration - Deaths 2002 ICD10 as it appears in World Health Organization (WHO). WHO Mortality Database Version November 2015. Geneva, Switzerland: World Health Organization (WHO), 2015.
Egypt Vital Registration - Deaths 2003 ICD10 as it appears in World Health Organization (WHO). WHO Mortality Database Version November 2015. Geneva, Switzerland: World Health Organization (WHO), 2015.
Egypt Vital Registration - Deaths 2004 ICD10 as it appears in World Health Organization (WHO). WHO Mortality Database Version November 2015. Geneva, Switzerland: World Health Organization (WHO), 2015.
Egypt Vital Registration - Deaths 2005 ICD10 as it appears in World Health Organization (WHO). WHO Mortality Database Version November 2015. Geneva, Switzerland: World Health Organization (WHO), 2015.
Egypt Vital Registration - Deaths 2006 ICD10 as it appears in World Health Organization (WHO). WHO Mortality Database Version November 2015. Geneva, Switzerland: World Health Organization (WHO), 2015.
Egypt Vital Registration - Deaths 2007 ICD10 as it appears in World Health Organization (WHO). WHO Mortality Database Version November 2015. Geneva, Switzerland: World Health Organization (WHO), 2015.
Egypt Vital Registration - Deaths 2008 ICD10 as it appears in World Health Organization (WHO). WHO Mortality Database Version November 2015. Geneva, Switzerland: World Health Organization (WHO), 2015.
Egypt Vital Registration - Deaths 2009 ICD10 as it appears in World Health Organization (WHO). WHO Mortality Database Version November 2015. Geneva, Switzerland: World Health Organization (WHO), 2015.
Egypt Vital Registration - Deaths 2010 ICD10 as it appears in World Health Organization (WHO). WHO Mortality Database Version November 2015. Geneva, Switzerland: World Health Organization (WHO), 2015.
Egypt Vital Registration - Deaths 2011 ICD10 as it appears in World Health Organization (WHO). WHO Mortality Database Version November 2015. Geneva, Switzerland: World Health Organization (WHO), 2015.
Egypt Vital Registration - Deaths 2012 ICD10 as it appears in World Health Organization (WHO). WHO Mortality Database Version November 2015. Geneva, Switzerland: World Health Organization (WHO), 2015.
Egypt Vital Registration - Deaths 2013 ICD10 as it appears in World Health Organization (WHO). WHO Mortality Database Version November 2015. Geneva, Switzerland: World Health Organization (WHO), 2015.
Egypt Vital Registration Death Data 1950 as it appears in United Nations Statistics Division (UNSD). United Nations Demographic Yearbook - Historical Supplement 1997. New York City, United States: United Nations Statistics Division (UNSD).
Egypt Vital Registration Death Data 1951 as it appears in United Nations Statistics Division (UNSD). United Nations Demographic Yearbook - Historical Supplement 1997. New York City, United States: United Nations Statistics Division (UNSD).
Egypt Vital Registration Death Data 1952 as it appears in United Nations Statistics Division (UNSD). United Nations Demographic Yearbook - Historical Supplement 1997. New York City, United States: United Nations Statistics Division (UNSD).
Egypt Vital Registration Death Data 1953 as it appears in United Nations Statistics Division (UNSD). United Nations Demographic Yearbook - Historical Supplement 1997. New York City, United States: United Nations Statistics Division (UNSD).
Egypt Vital Registration Death Data 1968 as it appears in United Nations Statistics Division (UNSD). United Nations Demographic Yearbook - Historical Supplement 1997. New York City, United States: United Nations Statistics Division (UNSD).
Egypt Vital Registration Death Data 1969 as it appears in United Nations Statistics Division (UNSD). United Nations Demographic Yearbook - Historical Supplement 1997. New York City, United States: United Nations Statistics Division (UNSD).
Egypt Vital Registration Death Data 1981 as it appears in United Nations Statistics Division (UNSD). United Nations Demographic Yearbook - Historical Supplement 1997. New York City, United States: United Nations Statistics Division (UNSD).
Egypt Vital Registration Death Data 1983 as it appears in United Nations Statistics Division (UNSD). United Nations Demographic Yearbook - Historical Supplement 1997. New York City, United States: United Nations Statistics Division (UNSD).
Egypt Vital Registration Death Data 1984 as it appears in United Nations Statistics Division (UNSD). United Nations Demographic Yearbook - Historical Supplement 1997. New York City, United States: United Nations Statistics Division (UNSD).
Egypt Vital Registration Death Data 1985 as it appears in United Nations Statistics Division (UNSD). United Nations Demographic Yearbook - Historical Supplement 1997. New York City, United States: United Nations Statistics Division (UNSD).
Egypt Vital Registration Death Data 1986 as it appears in United Nations Statistics Division (UNSD). United Nations Demographic Yearbook - Historical Supplement 1997. New York City, United States: United Nations Statistics Division (UNSD).
Egypt Vital Registration Death Data 1988 as it appears in United Nations Statistics Division (UNSD). United Nations Demographic Yearbook - Historical Supplement 1997. New York City, United States: United Nations Statistics Division (UNSD).
Egypt Vital Registration Death Data 1989 as it appears in United Nations Statistics Division (UNSD). United Nations Demographic Yearbook - Historical Supplement 1997. New York City, United States: United Nations Statistics Division (UNSD).
Egypt Vital Registration Death Data 1990 as it appears in United Nations Statistics Division (UNSD). United Nations Demographic Yearbook - Historical Supplement 1997. New York City, United States: United Nations Statistics Division (UNSD).
Egypt Vital Registration Death Data 1998 as it appears in United Nations Statistics Division (UNSD). United Nations Demographic Yearbook 2000. New York City, United States: United Nations Statistics Division (UNSD), 2002.
Egypt Vital Registration Death Data 1999 as it appears in United Nations Statistics Division (UNSD). United Nations Demographic Yearbook 2004. New York City, United States: United Nations Statistics Division (UNSD), 2007.

Appendix: Citation List

Citation
Egyptian Smoking Prevention Research Institute (ESPRI), World Health Organization Regional Office for the Eastern Mediterranean (EMRO-WHO). Egypt Tobacco Use in Shisha: Studies on Waterpipe Smoking. Geneva, Switzerland: World Health Organization (WHO), 2006.
Ehehalt S, Blumenstock G, Willasch AM, Hub R, Ranke MB, Neu A. Continuous rise in incidence of childhood Type 1 diabetes in Germany. Diabet Med. 2008; 25(6): 755-7.
Ehrhardt S, Burchard GD, Mantel C, Cramer JP, Kaiser S, Kubo M, Otchwemah RN, Bienzle U, Mockenhaupt FP. Malaria, Anemia, and Malnutrition in African Children-Defining Intervention Priorities. J Infect Dis. 2006; 194(1): 108-14. As it appears in Malaria Atlas Project. Malaria Atlas Project Plasmodium Falciparum Parasite Rate Database. Oxford, United Kingdom: Malaria Atlas Project.
EiÃµsdÃ³ttir SP, KristjÃ¡nsson AL, SigfÃºsdÃ³ttir ID, Garber CE, Allegrante JP. Trends in body mass index among Icelandic adolescents and young adults from 1992 to 2007. Int J Environ Res Public Health. 2010; 7(5): 2191-207.
Eidsdottir ST, Kristjansson AL, Sigfusdottir ID, Garber CE, Allegrante JP. Association between higher BMI and depressive symptoms in Icelandic adolescents: the mediational function of body image. Eur J Public Health. 2014; 24(6): 888â€“92.
Eisele TP, Keating J, Bennett A, Londono B, Johnson D, Lafontant C, Krogstad DJ. Prevalence of Plasmodium falciparum Infection in Rainy Season, Artibonite Valley, Haiti, 2006. Emerg Infect Dis. 2007; 13(10): 1494-6. As it appears in Malaria Atlas Project. Malaria Atlas Project Plasmodium Falciparum Parasite Rate Database. Oxford, United Kingdom: Malaria Atlas Project.
Eisele TP, Miller JM, Moonga HB, Hamainza B, Hutchinson P, Keating J. Malaria Infection and Anemia Prevalence in Zambia's Luangwa District: An Area of Near-Universal Insecticide-Treated Mosquito Net Coverage. Am J Trop Med Hyg. 2011; 84(1): 152-7. As it appears in Malaria Atlas Project. Malaria Atlas Project Plasmodium Falciparum Parasite Rate Database. Oxford, United Kingdom: Malaria Atlas Project.
Eisikovits Z, Winstok Z, Fishman G. The First Israeli National Survey on Domestic Violence. Violence Against Women. 2004; 10(7): 729-48.
Ejezie GC, Akpan IF. Human ecology and parasitic infections. 1. The effect of occupation on the prevalence of parasitic infections in Calabar, Nigeria. J Hyg Epidemiol Microbiol Immunol. 1992; 36(2): 161-7. As it appears in Malaria Atlas Project. Malaria Atlas Project Plasmodium Falciparum Parasite Rate Database. Oxford, United Kingdom: Malaria Atlas Project.
Ejezie GC, Onyezili NI, Okeke GC, Enwonwu CO. Ijanikin: a study of environmental health in a rural Nigerian community. J Hyg Epidemiol Microbiol Immunol. 1987; 31(2): 163-72. As it appears in Malaria Atlas Project. Malaria Atlas Project Plasmodium Falciparum Parasite Rate Database. Oxford, United Kingdom: Malaria Atlas Project.
Ekanem EE, Asindi AA, Okoi OU. Community-based surveillance of paediatric deaths in Cross River State, Nigeria. Trop Geogr Med. 1994; 46(5): 305-8.
EkbÃ¤ck G, NÃ¤slund I, Montgomery SM, Ordell S. Self-perceived oral health and obesity among 65 years old in two Swedish counties. Swed Dent J. 2010; 34(4): 207-15.
Ekelund U, Neovius M, LinnÃ© Y, Brage S, Wareham NJ, RÃ¶ssner S. Associations between physical activity and fat mass in adolescents: the Stockholm Weight Development Study. Am J Clin Nutr. 2005; 81(2): 355-60.
Ekpenyong EA, Eyo JE. Malaria control and treatment strategies among school children in semi-urban tropical communities. West Indian Med J. 2008; 57(5): 456-61. As it appears in Malaria Atlas Project. Malaria Atlas Project Plasmodium Falciparum Parasite Rate Database. Oxford, United Kingdom: Malaria Atlas Project.
El Kariem MAA. Epidemiology of Malaria in Displaced Population South Khartoum [Master's thesis]. Khartoum, Sudan: University of Khartoum,1998. As it appears in Malaria Atlas Project. Malaria Atlas Project Plasmodium Falciparum Parasite Rate Database. Oxford, United Kingdom: Malaria Atlas Project.
El Karim MA, Collins KJ, Dore C. Energy expenditure of agricultural workers in an area of endemic schistosomiasis in the Sudan. Br J Ind Med. 1987; 44(1): 64-7.
el Mahdi EM, Abdel Rahman Iel M, Mukhtar Sel D. Pattern of diabetes mellitus in the Sudan. Trop Geogr Med. 1989; 41(4): 353-7.
El Mubarak. Malaria Epidemiology in Sudan. 1991. As it appears in Malaria Atlas Project. Malaria Atlas Project Plasmodium Falciparum Parasite Rate Database. Oxford, United Kingdom: Malaria Atlas Project.
El Safi S. Studies on Anopheles Arabiensis and the Impact of Microclimatic Conditions on Malaria Transmission in the Gezira Area [dissertation]. Khartoum, Sudan: University of Khartoum,1992. As it appears in Malaria Atlas Project. Malaria Atlas Project Plasmodium Falciparum Parasite Rate Database. Oxford, United Kingdom: Malaria Atlas Project.
El Salvador Assessment of Nutritional Food Situation 1988 as it appears in World Health Organization (WHO). WHO Global Database on Child Growth and Malnutrition. Geneva, Switzerland: World Health Organization (WHO).
El Salvador Demographic Association (ADS), Division of Reproductive Health-Centers for Disease Control and Prevention (CDC). El Salvador Family Planning/Maternal and Child Survey 1993. Atlanta, United States: Centers for Disease Control and Prevention (CDC).
El Salvador Demographic Association (ADS), Division of Reproductive Health-Centers for Disease Control and Prevention (CDC). El Salvador Reproductive Health Survey 1998. Atlanta, United States: Centers for Disease Control and Prevention (CDC).
El Salvador Final Report on the Baseline Evaluation of the National Nutrition Education Program as it appears in World Health Organization (WHO). WHO Global Database on Child Growth and Malnutrition - Historical. Geneva, Switzerland: World Health Organization (WHO).
El Salvador Multipurpose Household Survey 2000 as it appears in World Health Organization (WHO). WHO Household Energy Database. Geneva, Switzerland: World Health Organization (WHO), 2010.
El Salvador Multipurpose Household Survey 2007 as it appears in World Health Organization (WHO). WHO Household Energy Database. Geneva, Switzerland: World Health Organization (WHO), 2010.
El Salvador Population and Housing Census 1971 as it appears in United Nations Statistics Division (UNSD). United Nations Demographic Yearbook. New York City, United States: United Nations Statistics Division (UNSD).
El Salvador Reproductive Health Survey 2008 as it appears in World Health Organization (WHO). WHO Global Database on Child Growth and Malnutrition. Geneva, Switzerland: World Health Organization (WHO).
El Salvador Vital Registration - Deaths 1950 ICD7 as it appears in World Health Organization (WHO). WHO Mortality Database Version July 2012. Geneva, Switzerland: World Health Organization (WHO), 2012.
El Salvador Vital Registration - Deaths 1951 ICD7 as it appears in World Health Organization (WHO). WHO Mortality Database Version July 2012. Geneva, Switzerland: World Health Organization (WHO), 2012.

Appendix: Citation List

Citation
El Salvador Vital Registration - Deaths 1952 ICD7 as it appears in World Health Organization (WHO). WHO Mortality Database Version July 2012. Geneva, Switzerland: World Health Organization (WHO), 2012.
El Salvador Vital Registration - Deaths 1953 ICD7 as it appears in World Health Organization (WHO). WHO Mortality Database Version July 2012. Geneva, Switzerland: World Health Organization (WHO), 2012.
El Salvador Vital Registration - Deaths 1954 ICD7 as it appears in World Health Organization (WHO). WHO Mortality Database Version July 2012. Geneva, Switzerland: World Health Organization (WHO), 2012.
El Salvador Vital Registration - Deaths 1955 ICD7 as it appears in World Health Organization (WHO). WHO Mortality Database Version July 2012. Geneva, Switzerland: World Health Organization (WHO), 2012.
El Salvador Vital Registration - Deaths 1956 ICD7 as it appears in World Health Organization (WHO). WHO Mortality Database Version July 2012. Geneva, Switzerland: World Health Organization (WHO), 2012.
El Salvador Vital Registration - Deaths 1957 ICD7 as it appears in World Health Organization (WHO). WHO Mortality Database Version July 2012. Geneva, Switzerland: World Health Organization (WHO), 2012.
El Salvador Vital Registration - Deaths 1958 ICD7 as it appears in World Health Organization (WHO). WHO Mortality Database Version July 2012. Geneva, Switzerland: World Health Organization (WHO), 2012.
El Salvador Vital Registration - Deaths 1959 ICD7 as it appears in World Health Organization (WHO). WHO Mortality Database Version July 2012. Geneva, Switzerland: World Health Organization (WHO), 2012.
El Salvador Vital Registration - Deaths 1960 ICD7 as it appears in World Health Organization (WHO). WHO Mortality Database Version July 2012. Geneva, Switzerland: World Health Organization (WHO), 2012.
El Salvador Vital Registration - Deaths 1961 ICD7 as it appears in World Health Organization (WHO). WHO Mortality Database Version July 2012. Geneva, Switzerland: World Health Organization (WHO), 2012.
El Salvador Vital Registration - Deaths 1962 ICD7 as it appears in World Health Organization (WHO). WHO Mortality Database Version July 2012. Geneva, Switzerland: World Health Organization (WHO), 2012.
El Salvador Vital Registration - Deaths 1963 ICD7 as it appears in World Health Organization (WHO). WHO Mortality Database Version July 2012. Geneva, Switzerland: World Health Organization (WHO), 2012.
El Salvador Vital Registration - Deaths 1964 ICD7 as it appears in World Health Organization (WHO). WHO Mortality Database Version July 2012. Geneva, Switzerland: World Health Organization (WHO), 2012.
El Salvador Vital Registration - Deaths 1965 ICD7 as it appears in World Health Organization (WHO). WHO Mortality Database Version July 2012. Geneva, Switzerland: World Health Organization (WHO), 2012.
El Salvador Vital Registration - Deaths 1966 ICD7 as it appears in World Health Organization (WHO). WHO Mortality Database Version July 2012. Geneva, Switzerland: World Health Organization (WHO), 2012.
El Salvador Vital Registration - Deaths 1967 ICD7 as it appears in World Health Organization (WHO). WHO Mortality Database Version July 2012. Geneva, Switzerland: World Health Organization (WHO), 2012.
El Salvador Vital Registration - Deaths 1968 ICD8 as it appears in World Health Organization (WHO). WHO Mortality Database Version May 2013. Geneva, Switzerland: World Health Organization (WHO), 2013.
El Salvador Vital Registration - Deaths 1969 ICD8 as it appears in World Health Organization (WHO). WHO Mortality Database Version May 2013. Geneva, Switzerland: World Health Organization (WHO), 2013.
El Salvador Vital Registration - Deaths 1970 ICD8 as it appears in World Health Organization (WHO). WHO Mortality Database Version May 2013. Geneva, Switzerland: World Health Organization (WHO), 2013.
El Salvador Vital Registration - Deaths 1971 ICD8 as it appears in World Health Organization (WHO). WHO Mortality Database Version May 2013. Geneva, Switzerland: World Health Organization (WHO), 2013.
El Salvador Vital Registration - Deaths 1972 ICD8 as it appears in World Health Organization (WHO). WHO Mortality Database Version May 2013. Geneva, Switzerland: World Health Organization (WHO), 2013.
El Salvador Vital Registration - Deaths 1973 ICD8 as it appears in World Health Organization (WHO). WHO Mortality Database Version May 2013. Geneva, Switzerland: World Health Organization (WHO), 2013.
El Salvador Vital Registration - Deaths 1974 ICD8 as it appears in World Health Organization (WHO). WHO Mortality Database Version May 2013. Geneva, Switzerland: World Health Organization (WHO), 2013.
El Salvador Vital Registration - Deaths 1981 ICD9 as it appears in World Health Organization (WHO). WHO Mortality Database Version November 2015. Geneva, Switzerland: World Health Organization (WHO), 2015.
El Salvador Vital Registration - Deaths 1982 ICD9 as it appears in World Health Organization (WHO). WHO Mortality Database Version November 2015. Geneva, Switzerland: World Health Organization (WHO), 2015.
El Salvador Vital Registration - Deaths 1983 ICD9 as it appears in World Health Organization (WHO). WHO Mortality Database Version November 2015. Geneva, Switzerland: World Health Organization (WHO), 2015.
El Salvador Vital Registration - Deaths 1984 ICD9 as it appears in World Health Organization (WHO). WHO Mortality Database Version February 2014. Geneva, Switzerland: World Health Organization (WHO), 2014.
El Salvador Vital Registration - Deaths 1990 ICD9 as it appears in World Health Organization (WHO). WHO Mortality Database Version November 2015. Geneva, Switzerland: World Health Organization (WHO), 2015.
El Salvador Vital Registration - Deaths 1991 ICD9 as it appears in World Health Organization (WHO). WHO Mortality Database Version November 2015. Geneva, Switzerland: World Health Organization (WHO), 2015.
El Salvador Vital Registration - Deaths 1992 ICD9 as it appears in World Health Organization (WHO). WHO Mortality Database Version November 2015. Geneva, Switzerland: World Health Organization (WHO), 2015.
El Salvador Vital Registration - Deaths 1993 ICD9 as it appears in World Health Organization (WHO). WHO Mortality Database Version February 2014. Geneva, Switzerland: World Health Organization (WHO), 2014.
El Salvador Vital Registration - Deaths 1995 ICD9 as it appears in World Health Organization (WHO). WHO Mortality Database Version November 2015. Geneva, Switzerland: World Health Organization (WHO), 2015.

Appendix: Citation List

Citation
El Salvador Vital Registration - Deaths 1996 ICD9 as it appears in World Health Organization (WHO). WHO Mortality Database Version November 2015. Geneva, Switzerland: World Health Organization (WHO), 2015.
El Salvador Vital Registration - Deaths 1997 ICD10 as it appears in World Health Organization (WHO). WHO Mortality Database Version November 2015. Geneva, Switzerland: World Health Organization (WHO), 2015.
El Salvador Vital Registration - Deaths 1998 ICD10 as it appears in World Health Organization (WHO). WHO Mortality Database Version November 2015. Geneva, Switzerland: World Health Organization (WHO), 2015.
El Salvador Vital Registration - Deaths 1999 ICD10 as it appears in World Health Organization (WHO). WHO Mortality Database Version November 2015. Geneva, Switzerland: World Health Organization (WHO), 2015.
El Salvador Vital Registration - Deaths 2000 ICD10 as it appears in World Health Organization (WHO). WHO Mortality Database Version November 2015. Geneva, Switzerland: World Health Organization (WHO), 2015.
El Salvador Vital Registration - Deaths 2001 ICD10 as it appears in World Health Organization (WHO). WHO Mortality Database Version November 2015. Geneva, Switzerland: World Health Organization (WHO), 2015.
El Salvador Vital Registration - Deaths 2002 ICD10 as it appears in World Health Organization (WHO). WHO Mortality Database Version November 2015. Geneva, Switzerland: World Health Organization (WHO), 2015.
El Salvador Vital Registration - Deaths 2003 ICD10 as it appears in World Health Organization (WHO). WHO Mortality Database Version November 2015. Geneva, Switzerland: World Health Organization (WHO), 2015.
El Salvador Vital Registration - Deaths 2004 ICD10 as it appears in World Health Organization (WHO). WHO Mortality Database Version November 2015. Geneva, Switzerland: World Health Organization (WHO), 2015.
El Salvador Vital Registration - Deaths 2005 ICD10 as it appears in World Health Organization (WHO). WHO Mortality Database Version November 2015. Geneva, Switzerland: World Health Organization (WHO), 2015.
El Salvador Vital Registration - Deaths 2006 ICD10 as it appears in World Health Organization (WHO). WHO Mortality Database Version November 2015. Geneva, Switzerland: World Health Organization (WHO), 2015.
El Salvador Vital Registration - Deaths 2007 ICD10 as it appears in World Health Organization (WHO). WHO Mortality Database Version November 2015. Geneva, Switzerland: World Health Organization (WHO), 2015.
El Salvador Vital Registration - Deaths 2008 ICD10 as it appears in World Health Organization (WHO). WHO Mortality Database Version November 2015. Geneva, Switzerland: World Health Organization (WHO), 2015.
El Salvador Vital Registration - Deaths 2009 ICD10 as it appears in World Health Organization (WHO). WHO Mortality Database Version November 2015. Geneva, Switzerland: World Health Organization (WHO), 2015.
El Salvador Vital Registration - Deaths 2010 ICD10 as it appears in World Health Organization (WHO). WHO Mortality Database Version November 2015. Geneva, Switzerland: World Health Organization (WHO), 2015.
El Salvador Vital Registration - Deaths 2011 ICD10 as it appears in World Health Organization (WHO). WHO Mortality Database Version November 2015. Geneva, Switzerland: World Health Organization (WHO), 2015.
El Salvador Vital Registration - Deaths 2012 ICD10 as it appears in World Health Organization (WHO). WHO Mortality Database Version November 2015. Geneva, Switzerland: World Health Organization (WHO), 2015.
El Salvador Vital Registration Death Data 1975 as it appears in United Nations Statistics Division (UNSD). United Nations Demographic Yearbook - Historical Supplement 1997. New York City, United States: United Nations Statistics Division (UNSD).
El Salvador Vital Registration Death Data 1976 as it appears in United Nations Statistics Division (UNSD). United Nations Demographic Yearbook - Historical Supplement 1997. New York City, United States: United Nations Statistics Division (UNSD).
El Salvador Vital Registration Death Data 1977 as it appears in United Nations Statistics Division (UNSD). United Nations Demographic Yearbook - Historical Supplement 1997. New York City, United States: United Nations Statistics Division (UNSD).
El Salvador Vital Registration Death Data 1978 as it appears in United Nations Statistics Division (UNSD). United Nations Demographic Yearbook - Historical Supplement 1997. New York City, United States: United Nations Statistics Division (UNSD).
El Salvador Vital Registration Death Data 1979 as it appears in United Nations Statistics Division (UNSD). United Nations Demographic Yearbook - Historical Supplement 1997. New York City, United States: United Nations Statistics Division (UNSD).
El Salvador Vital Registration Death Data 1980 as it appears in United Nations Statistics Division (UNSD). United Nations Demographic Yearbook - Historical Supplement 1997. New York City, United States: United Nations Statistics Division (UNSD).
El Salvador Vital Registration Death Data 1985 as it appears in United Nations Statistics Division (UNSD). United Nations Demographic Yearbook - Historical Supplement 1997. New York City, United States: United Nations Statistics Division (UNSD).
El Salvador Vital Registration Death Data 1986 as it appears in United Nations Statistics Division (UNSD). United Nations Demographic Yearbook - Historical Supplement 1997. New York City, United States: United Nations Statistics Division (UNSD).
El Salvador Vital Registration Death Data 1987 as it appears in United Nations Statistics Division (UNSD). United Nations Demographic Yearbook - Historical Supplement 1997. New York City, United States: United Nations Statistics Division (UNSD).
El Salvador Vital Registration Death Data 1988 as it appears in United Nations Statistics Division (UNSD). United Nations Demographic Yearbook - Historical Supplement 1997. New York City, United States: United Nations Statistics Division (UNSD).
El Salvador Vital Registration Death Data 1989 as it appears in United Nations Statistics Division (UNSD). United Nations Demographic Yearbook - Historical Supplement 1997. New York City, United States: United Nations Statistics Division (UNSD).
El Salvador Vital Registration Death Data 1994 as it appears in United Nations Statistics Division (UNSD). United Nations Demographic Yearbook - Historical Supplement 1997. New York City, United States: United Nations Statistics Division (UNSD).
El Salvador Vital Registration Death Data 2011 as it appears in United Nations Statistics Division (UNSD). United Nations Demographic Yearbook 2012. New York City, United States: United Nations Statistics Division (UNSD), 2013.
El Sayed BB.Transmission and Risk Factors of Urban and Peri-urban Malaria in Khartoum State, Sudan [dissertation]. Khartoum, Sudan: University of Khartoum, 1998. As it appears in Malaria Atlas Project. Malaria Atlas Project Plasmodium Falciparum Parasite Rate Database. Oxford, United Kingdom: Malaria Atlas Project.

Appendix: Citation List

Citation
El Sheikh NMH. Epidemiology of Malaria in Displaced Population South Khartoum. Prevalence, Environmental and Entomological Aspects [Master's thesis]. Khartoum, Sudan: University of Khartoum, 1998. As it appears in Malaria Atlas Project. Malaria Atlas Project Plasmodium Falciparum Parasite Rate Database. Oxford, United Kingdom: Malaria Atlas Project.
El Tayeb RA. Deltamethrin-treated Sudanese Thobs, a Control Method for Malaria in an Endemic Region. Arusha, Tanzania, 2002. As it appears in Malaria Atlas Project. Malaria Atlas Project Plasmodium Falciparum Parasite Rate Database. Oxford, United Kingdom: Malaria Atlas Project.
El Yamani LE. Immuno-epidemiological Study of Malaria in Rahad River Area, Eastern Sudan [Master's thesis]. Khartoum, Sudan: University of Khartoum, 2002. As it appears in Malaria Atlas Project. Malaria Atlas Project Plasmodium Falciparum Parasite Rate Database. Oxford, United Kingdom: Malaria Atlas Project.
Elbagir MN, Eltom MA, Elmahadi EM, Kadam IM, Berne C. A high prevalence of diabetes mellitus and impaired glucose tolerance in the Danagla community in northern Sudan. Diabet Med. 1998; 15(2): 164-9.
Elbagir MN, Eltom MA, Elmahadi EM, Kadam IM, Berne C. A population-based study of the prevalence of diabetes and impaired glucose tolerance in adults in northern Sudan. Diabetes Care. 1996; 19(10): 1126-8.
Elbagir MN, Eltom MA, Mahadi EO, Berne C. Pattern of long-term complications in Sudanese insulin-treated diabetic patients. Diabetes Res Clin Pract. 1995; 30(1): 59-67.
Elefsiniotis IS, Glynou I, Brokalaki H, Magaziotou I, Pantazis KD, Fotiou A, Liosis G, Kada H, Saroglou G. Serological and virological profile of chronic HBV infected women at reproductive age in Greece. A two-year single center study. Eur J Obstet Gynecol Reprod Biol. 2007; 132(2): 200-3.
Elefsiniotis IS, Glynou I, Pantazis KD, Fotos NV, Magaziotou I, Kada H. Prevalence of chronic HBV infection among 13,581 women at reproductive age in Greece. A prospective single center study. J Clin Virol. 2005; 32(2): 179-80.
Eleftheriou A, Teloni F, Ioannou P. HDV infection in Cyprus. Prog Clin Biol Res. 1992; 277-85.
Elhassan EM, Mirghani OA, Adam I. High maternal mortality and stillbirth in the Wad Medani Hospital, Central Sudan, 2003-2007. Trop Doct. 2009; 39(4): 238-9.
El-Hazmi MA, Al-Swailem A, Warsy AS, Al-Sudairy F, Sulaimani R, Al-Swailem A, Al-Meshari A. The prevalence of diabetes mellitus and impaired glucose tolerance in the population of Riyadh. Ann Saudi Med. 1995; 15(6): 598-601.
El-Hazmi MA, Warsy AS, Al-Swailem AR, Al-Swailem AM, Sulaimani R, Al-Meshari AA. Diabetes mellitus and impaired glucose tolerance in Saudi Arabia. Ann Saudi Med. 1996; 16(4): 381-5.
El-Hazmi MM. Prevalence of HBV, HCV, HIV-1, 2 and HTLV-I/II infections among blood donors in a teaching hospital in the Central region of Saudi Arabia. Saudi Med J. 2004; 25(1): 26-33.
Elissa N, Migot-Nabias F, Luty A, Renaut A, Touré F, Vaillant M, Lawoko M, Yangari P, Mayombo J, Lekoulou F, Tshipamba P, Moukagni R, Millet P, Deloron P. Relationship between entomological inoculation rate, Plasmodium falciparum prevalence rate, and incidence of malaria attack in rural Gabon. Acta Trop. 2003; 85(3): 355-61. As it appears in Malaria Atlas Project. Malaria Atlas Project Plasmodium Falciparum Parasite Rate Database. Oxford, United Kingdom: Malaria Atlas Project.
El-Khoby T, Galal N, Fenwick A, Barakat R, El-Hawey A, Nooman Z, Habib M, Abdel-Wahab F, Gabr NS, Hammam HM, Hussein MH, Mikhail NN, Cline BL, Strickland GT. The epidemiology of schistosomiasis in Egypt: summary findings in nine governorates. Am J Trop Med Hyg. 2000; 62 Suppl 2: 88-99.
Ellis M, Azad K, Banerjee B, Shaha SK, Prost A, Rego AR, Barua S, Costello A, Barnett S. Intrapartum-Related Stillbirths and Neonatal Deaths in Rural Bangladesh: A Prospective, Community-Based Cohort Study. Pediatrics. 2011; 127(5): e1182-90.
Ellman R, Maxwell C, Finch R, Shayo D. Malaria and anaemia at different altitudes in the Muheza district of Tanzania: childhood morbidity in relation to level of exposure to infection. Ann Trop Med Parasitol. 1998; 92(7): 741-53. As it appears in Malaria Atlas Project. Malaria Atlas Project Plasmodium Falciparum Parasite Rate Database. Oxford, United Kingdom: Malaria Atlas Project.
Elmamoun NA. On the Epidemiology of Malaria in the Kordofan [dissertation]. Khartoum, Sudan: University of Khartoum, 1993. As it appears in Malaria Atlas Project. Malaria Atlas Project Plasmodium Falciparum Parasite Rate Database. Oxford, United Kingdom: Malaria Atlas Project.
El-Sayed B, El-Zaki S-E, Babiker H, Gadalla N, Ageep T, Mansour F, Baraka O, Milligan P, Babiker A. A Randomized Open-Label Trial of Artesunate- Sulfadoxine-Pyrimethamine with or without Primaquine for Elimination of Sub-Microscopic P. falciparum Parasitaemia and Gametocyte Carriage in Eastern Sudan. PLoS One. 2007; 2(12): e1311. As it appears in Malaria Atlas Project. Malaria Atlas Project Plasmodium Falciparum Parasite Rate Database. Oxford, United Kingdom: Malaria Atlas Project.
Elshafei M, Gamra H, Khandekar R, Al Hashimi M, Pai A, Ahmed MF. Prevalence and determinants of diabetic retinopathy among persons ≥ 40 years of age with diabetes in Qatar: a community-based survey. Eur J Ophthalmol. 2011; 21(1): 39-47.
Elsharawy MA, Hassan K, Alawad N, Kredees A, Almulhim A. Screening of diabetic foot in surgical inpatients: a hospital-based study in saudi arabia. Int J Angiol. 2012; 21(4): 213-6.
Elyazar IRFE, Baird JK. Indonesia Plasmodium Falciparum Parasite Rate Data, Personal Communication with I.R.F.E. Elyazar and J.K. Baird 2006. As it appears in Malaria Atlas Project. Malaria Atlas Project Plasmodium Falciparum Parasite Rate Database. Oxford, United Kingdom: Malaria Atlas Project.
El-Zanaty and Associates, ICF International, Ministry of Health and Population (Egypt), National Population Council (Egypt). Egypt Special Demographic and Health Survey 2015. Fairfax, United States: ICF International, 2015.
El-Zanaty and Associates, ICF International, Ministry of Health and Population (Egypt). Egypt Demographic and Health Survey 2014. Fairfax, United States: ICF International, 2015.
El-Zanaty and Associates, Macro International, Inc, Ministry of Health and Population (Egypt), National Population Council (Egypt). Egypt Demographic and Health Survey 2005. Calverton, United States: Macro International, Inc.
El-Zanaty and Associates, Macro International, Inc, Ministry of Health and Population (Egypt), National Population Council (Egypt). Egypt Interim Demographic and Health Survey 2003. Calverton, United States: Macro International, Inc.

Appendix: Citation List

Citation
El-Zanaty and Associates, Macro International, Inc, Ministry of Health and Population (Egypt). Egypt Demographic and Health Survey 2008. Calverton, United States: Macro International, Inc, 2009.
El-Zanaty and Associates, Macro International, Inc. Egypt Interim Demographic and Health Survey 1997-1998.
El-Zanaty and Associates, Macro International, Inc. Egypt Interim Demographic and Health Survey 1998. Calverton, United States: Macro International, Inc.
El-Ziny MA, Salem NA, El-Hawary AK, Chalaby NM, Elsharkawy AA. Epidemiology of childhood type 1 diabetes mellitus in Nile Delta, northern Egypt - a retrospective study. J Clin Res Pediatr Endocrinol. 2014; 6(1): 9-15.
Encuesta nutricional en ninos de 2 anos de la provincia de Misiones. I. Indicadores antropometricos as it appears in World Health Organization (WHO). WHO Global Database on Child Growth and Malnutrition - Historical. Geneva, Switzerland: World Health Organization (WHO).
Eneanya CI. Seasonal variation in malaria episodes among residents in a semi-urban community in South-East Nigeria. Niger J Parasitol. 1998; 19: 39-43. As it appears in Malaria Atlas Project. Malaria Atlas Project Plasmodium Falciparum Parasite Rate Database. Oxford, United Kingdom: Malaria Atlas Project.
Energy Statistics of Non-OECD Countries 1996-1997 as it appears in World Health Organization (WHO). WHO Household Energy Database. Geneva, Switzerland: World Health Organization (WHO), 2010.
Energy, Modernity and the Fight Against Poverty as it appears in World Health Organization (WHO). WHO Household Energy Database. Geneva, Switzerland: World Health Organization (WHO), 2010.
Enevold A, Lusingu JP, Mmbando B, Alifrangis M, Lemnge MM, Bygbjerg IC, Theander TG, Vestergaard LS. Reduced Risk of Uncomplicated Malaria Episodes in Children with Alpha+-Thalassemia in Northeastern Tanzania. Am J Trop Med Hyg. 2008; 78(5): 714-20. As it appears in Malaria Atlas Project. Malaria Atlas Project Plasmodium Falciparum Parasite Rate Database. Oxford, United Kingdom: Malaria Atlas Project.
Engelaer FM, Koopman JJE, Bodegom D van, Eriksson UK, Westendorp RGJ. Determinants of epidemiologic transition in rural Africa: the role of socioeconomic status and drinking water source. Trans R Soc Trop Med Hyg. 2014; 108(6): 372-9.
Engelbrecht F, Tögel E, Beck H-P, Enwezor F, Oettli A, Felger I. Analysis of Plasmodium falciparum infections in a village community in Northern Nigeria: determination of msp2 genotypes and parasite-specific IgG responses. Acta Trop. 2000; 74(1): 63-71. As it appears in Malaria Atlas Project. Malaria Atlas Project Plasmodium Falciparum Parasite Rate Database. Oxford, United Kingdom: Malaria Atlas Project.
English M, Muhoro A, Aluda M, Were S, Ross A, Peshu N. Outcome of Delivery and Cause-Specific Mortality and Severe Morbidity in Early Infancy: A Kenyan District Hospital Birth Cohort. Am J Trop Med Hyg. 2003; 69(2): 228-32.
Enosse S, Magnussen P, Abacassamo F, Gómez-Olivé X, Rønn AM, Thompson R, Alifrangis M. Rapid increase of Plasmodium falciparum dhfr/dhps resistant haplotypes, after the adoption of sulphadoxine-pyrimethamine as first line treatment in 2002, in southern Mozambique. Malar J. 2008; 7(1): 115. As it appears in Malaria Atlas Project. Malaria Atlas Project Plasmodium Falciparum Parasite Rate Database. Oxford, United Kingdom: Malaria Atlas Project.
Enterline PE, Hartley J, Henderson V. Asbestos And Cancer: A Cohort Followed Up To Death. Br J Ind Med. 1987; 44(6): 396-401 as it appears in Lenters V, Vermeulen R, Dogger S, Stayner L, Portengen L, Burdorf A, Heederik D. A meta-analysis of asbestos and lung cancer: is better quality exposure assessment associated with steeper slopes of the exposure-response relationships?. Environ Health Perspect. 2011; 119(11): 1547-55.
Environmental Health Project (United States), Ministry of Health (Peru), PRISMA (Peru), Swiss Agency for Development and Cooperation, United States Agency for International Development (USAID), Water and Sanitation Program (WSP), World Bank. Peru Behavioral Study of Handwashing with Soap in Peri-urban and Rural Areas 2004.
Environmental Health Project (United States). First Year Summary Report: Development of a Community-based Environmental Management Program for Malaria Control in Kampala and Jinja, Uganda. Arlington, United States: Environmental Health Project (United States), 2003. As it appears in Malaria Atlas Project. Malaria Atlas Project Plasmodium Falciparum Parasite Rate Database. Oxford, United Kingdom: Malaria Atlas Project.
Enyong P. Prevalence of Malaria in Two Villages in the Southwest Province in Cameroon. Khartoum, Sudan: Medical Institute of Medicinal Plants, Ministry of Scientific Research, 1990. As it appears in Malaria Atlas Project. Malaria Atlas Project Plasmodium Falciparum Parasite Rate Database. Oxford, United Kingdom: Malaria Atlas Project.
EPI Coverage Situation in Women and Children of Afghanistan, Report of Post NID's, Routine Coverage and Acceleration Campaign Survey in Afghanistan 1999.
Epidemiological Analysis of Predictors of Childhood Malnutrition and Mortality in Southwest Uganda as it appears in World Health Organization (WHO). WHO Global Database on Child Growth and Malnutrition - Historical. Geneva, Switzerland: World Health Organization (WHO).
Epidemiological Study of the Current Nutritional Demographic Status of Two Populations of the Gao Region (Mali) as it appears in World Health Organization (WHO). WHO Global Database on Child Growth and Malnutrition - Historical. Geneva, Switzerland: World Health Organization (WHO).
Epidemiological Transition and Health Impact in North Africa (TAHINA), Ministry of Health and Population (Algeria), National Institute of Public Health (Algeria). Algeria - Study of Causes of Death, TAHINA 2002. Alger, Algeria: National Institute of Public Health (Algeria), 2008.
Equatorial Guinea National Immunization Coverage Survey 1991.
Equatorial Guinea National Immunization Coverage Survey 1994.
Erbas T, Ertas M, Yucel A, Keskinaslan A, Senocak M, TURNEP Study Group. Prevalence of peripheral neuropathy and painful peripheral neuropathy in Turkish diabetic patients. J Clin Neurophysiol. 2011; 28(1): 51–5.
Erdem G. Perinatal mortality in Turkey. Paediatr Perinat Epidemiol. 2003; 17(1): 17-21.
Erden S, Büyüköztürk S, Calangu S, Yilmaz G, Palanduz S, Badur S. A study of serological markers of hepatitis B and C viruses in Istanbul, Turkey. Med Princ Pract. 2003; 12(3): 184-8.

Appendix: Citation List

Citation
Erem C, Yildiz R, Kavgaci H, Karahan C, Deger O, Can G, Telatar M. Prevalence of diabetes, obesity and hypertension in a Turkish population (Trabzon city). Diabetes Res Clin Pract. 2001; 54(3): 203-8.
Erhart A, Ngo DT, Phan VK, Ta TT, Van Overmeir C, Speybroeck N, Obsomer V, Le XH, Le KT, Coosemans M, D'Alessandro U. Epidemiology of forest malaria in central Vietnam: a large scale cross-sectional survey. Malar J. 2005; 4: 58. As it appears in Malaria Atlas Project. Malaria Atlas Project Plasmodium Falciparum Parasite Rate Database. Oxford, United Kingdom: Malaria Atlas Project.
Erhart A, Thang ND, Bien TH, Tung NM, Hung NQ, Hung LX, Tuy TQ, Speybroeck N, Cong LD, Coosemans M, D'Alessandro U. Malaria epidemiology in a rural area of the Mekong Delta: a prospective community-based study. Trop Med Int Health. 2004; 9(10): 1081-90. As it appears in Malaria Atlas Project. Malaria Atlas Project Plasmodium Falciparum Parasite Rate Database. Oxford, United Kingdom: Malaria Atlas Project.
Erhart A, Thang ND, Hung NQ, Toi LV, Hung LX, Tuy TQ, Cong LD, Speybroeck N, Coosemans M, D'Alessandro U. Forest malaria in Vietnam: a challenge for control. Am J Trop Med Hyg. 2004; 70(2): 110-8. As it appears in Malaria Atlas Project. Malaria Atlas Project Plasmodium Falciparum Parasite Rate Database. Oxford, United Kingdom: Malaria Atlas Project.
Eriksson H, Welin L, Wilhelmsen L, Larsson B, Ohlson LO, SvÃ¤rdsudd K, Tibblin G. Metabolic disturbances in hypertension: results from the population study "men born in 1913.". J Intern Med. 1992; 232(5): 389-95.
Eriksson J, Reimert CM, Kabatereine NB, Kazibwe F, Ireri E, Kadzo H, Eltahir HB, Mohamed AO, Vennervald BJ, Venge P. The 434(G>C) polymorphism within the coding sequence of Eosinophil Cationic Protein (ECP) correlates with the natural course of Schistosoma mansoni infection. Int J Parasitol. 2007; 37(12): 1359-66.
Eritrea - Nutritional Baseline Survey for the Integrated Food Security Program in Gash and Setit 1994 as it appears in World Health Organization (WHO). WHO Global Database on Child Growth and Malnutrition - Historical. Geneva, Switzerland: World Health Organization (WHO).
Ernest E, Nonga HE, Kynsieri N, Cleaveland S. A retrospective survey of human hydatidosis based on hospital records during the period 1990-2003 in Ngorongoro, Tanzania. Zoonoses Public Health. 2010; 57(7-8): e124-129.
Ertekin V, Selimoğlu MA, Altinkaynak S. Sero-epidemiology of hepatitis B infection in an urban paediatric population in Turkey. Public Health. 2003; 117(1): 49-53.
Eschwege E, Basdevant A, Crine A, Moisan C, Charles M-A. Type 2 diabetes mellitus in France in 2012: results from the ObEpi survey. Diabetes Metab. 2015; 41(1): 55â€“61.
Escobedo J, Buitron LV, Velasco MF, Ramirez JC, Hernandez R, Macchia A, Pellegrini F, Schargrodsky H, Boissonnet C, Champagne BM. High prevalence of diabetes and impaired fasting glucose in urban Latin America: the CARMELA Study. Diabet Med. 2009; 26(9): 864-71.
Esmaillzadeh A, Mirmiran P, Azizi F. Comparative evaluation of anthropometric measures to predict cardiovascular risk factors in Tehranian adult women. Public Health Nutr. 2006; 9(1): 61-9.
ESPAD Report 1995: Alcohol and Other Drug Use Among Students in 26 European Countries as it appears in P.N. Lee Statistics and Computing Ltd. International Mortality and Smoking Statistics Version 4.04. Sutton, United Kingdom: P.N. Lee Statistics and Computing Ltd, 2009.
ESPAD Report 1999: Alcohol and Other Drug Use Among Students in 30 European Countries as it appears in P.N. Lee Statistics and Computing Ltd. International Mortality and Smoking Statistics Version 4.04. Sutton, United Kingdom: P.N. Lee Statistics and Computing Ltd, 2009.
Espelt A, Goday A, Franch J, Borrell C. Validity of self-reported diabetes in health interview surveys for measuring social inequalities in the prevalence of diabetes. J Epidemiol Community Health. 2012; 66(7): e15.
Espinel M. Ecuador Plasmodium Falciparum Parasite Rate Data, Personal Communication with M. Espinel 2008. As it appears in Malaria Atlas Project. Malaria Atlas Project Plasmodium Falciparum Parasite Rate Database. Oxford, United Kingdom: Malaria Atlas Project.
Esposito F, Lombardi S, Modiano D, Zavala F, Reeme J, Lamizana L, Coluzzi M, Nussenzweig RS. Prevalence and levels of antibodies to the circumsporozoite protein of Plasmodium falciparum in an endemic area and their relationship to resistance against malaria infection. Trans R Soc Trop Med Hyg. 1988; 82(6): 827-32. As it appears in Malaria Atlas Project. Malaria Atlas Project Plasmodium Falciparum Parasite Rate Database. Oxford, United Kingdom: Malaria Atlas Project.
Esscher A, Högberg U, Haglund B, Essën B. Maternal mortality in Sweden 1988-2007: more deaths than officially reported. Acta Obstet Gynecol Scand. 2013; 92(1): 40-6.
Esser MB, Gururaj G, Rao GN, Jernigan DH, Murthy P, Jayarajan D, Lakshmanan S, Benegal V, Collaborators Group on Epidemiological Study of Patterns and Consequences of Alcohol Misuse in India. Harms to Adults from Othersâ€™ Heavy Drinking in Five Indian States. Alcohol Alcohol. 2016; 51(2): 177â€“85.
Estado nutricional de prÃ©-escolares de comunidades rurais do nordeste brasileiro as it appears in World Health Organization (WHO). WHO Global Database on Child Growth and Malnutrition - Historical. Geneva, Switzerland: World Health Organization (WHO).
Estado nutricional de preescolares y las mujeres en Mexico: resultados de una encuesta probabilistica nacional as it appears in World Health Organization (WHO). WHO Global Database on Child Growth and Malnutrition. Geneva, Switzerland: World Health Organization (WHO).
Estambale BB, Simonsen PE, Knight R, Bwayo JJ. Bancroftian filariasis in Kwale District of Kenya. I. Clinical and parasitological survey in an endemic community. Ann Trop Med Parasitol. 1994; 88(2): 145-51. As it appears in London School of Hygiene and Tropical Medicine. Global Atlas of Helminth Infections - Lymphatic Filiariasis.
Esteban JJN, Martínez MS, Navalón PG, Serrano OP, Patiño JRC, Purón MEC, Martínez-Vizcaíno V. Visual impairment and quality of life: gender differences in the elderly in Cuenca, Spain. Qual Life Res. 2008; 17(1): 37-45.
Estonia Cancer Registry 1980 - CI5 as it appears in Ferlay J, Parkin DM, Curado MP, Bray F, Edwards B, Shin HR and Forman D. Cancer Incidence in Five Continents, Volumes I to IX: IARC CancerBase No. 9 [Internet]. Lyon, France: International Agency for Research on Cancer; 2010. Available from: http://ci5.iarc.fr

Appendix: Citation List

Citation
Estonia Cancer Registry 1981 - CI5 as it appears in Ferlay J, Parkin DM, Curado MP, Bray F, Edwards B, Shin HR and Forman D. Cancer Incidence in Five Continents, Volumes I to IX: IARC CancerBase No. 9 [Internet]. Lyon, France: International Agency for Research on Cancer; 2010. Available from: http://ci5.iarc.fr
Estonia Cancer Registry 1982 - CI5 as it appears in Ferlay J, Parkin DM, Curado MP, Bray F, Edwards B, Shin HR and Forman D. Cancer Incidence in Five Continents, Volumes I to IX: IARC CancerBase No. 9 [Internet]. Lyon, France: International Agency for Research on Cancer; 2010. Available from: http://ci5.iarc.fr
Estonia Cancer Registry 1983 - CI5 as it appears in Ferlay J, Parkin DM, Curado MP, Bray F, Edwards B, Shin HR and Forman D. Cancer Incidence in Five Continents, Volumes I to IX: IARC CancerBase No. 9 [Internet]. Lyon, France: International Agency for Research on Cancer; 2010. Available from: http://ci5.iarc.fr
Estonia Cancer Registry 1983-1987 - CI5 as it appears in Parkin DM, International Agency for Research on Cancer, International Association of Cancer Registries. Cancer Incidence in Five Continents. Vol. I to VIII. Lyon, France, IARCPress, 2005.
Estonia Cancer Registry 1984 - CI5 as it appears in Ferlay J, Parkin DM, Curado MP, Bray F, Edwards B, Shin HR and Forman D. Cancer Incidence in Five Continents, Volumes I to IX: IARC CancerBase No. 9 [Internet]. Lyon, France: International Agency for Research on Cancer; 2010. Available from: http://ci5.iarc.fr
Estonia Cancer Registry 1985 - CI5 as it appears in Ferlay J, Parkin DM, Curado MP, Bray F, Edwards B, Shin HR and Forman D. Cancer Incidence in Five Continents, Volumes I to IX: IARC CancerBase No. 9 [Internet]. Lyon, France: International Agency for Research on Cancer; 2010. Available from: http://ci5.iarc.fr
Estonia Cancer Registry 1986 - CI5 as it appears in Ferlay J, Parkin DM, Curado MP, Bray F, Edwards B, Shin HR and Forman D. Cancer Incidence in Five Continents, Volumes I to IX: IARC CancerBase No. 9 [Internet]. Lyon, France: International Agency for Research on Cancer; 2010. Available from: http://ci5.iarc.fr
Estonia Cancer Registry 1987 - CI5 as it appears in Ferlay J, Parkin DM, Curado MP, Bray F, Edwards B, Shin HR and Forman D. Cancer Incidence in Five Continents, Volumes I to IX: IARC CancerBase No. 9 [Internet]. Lyon, France: International Agency for Research on Cancer; 2010. Available from: http://ci5.iarc.fr
Estonia Cancer Registry 1988 - CI5 as it appears in Ferlay J, Parkin DM, Curado MP, Bray F, Edwards B, Shin HR and Forman D. Cancer Incidence in Five Continents, Volumes I to IX: IARC CancerBase No. 9 [Internet]. Lyon, France: International Agency for Research on Cancer; 2010. Available from: http://ci5.iarc.fr
Estonia Cancer Registry 1988-1992 - CI5 as it appears in Parkin DM, International Agency for Research on Cancer, International Association of Cancer Registries. Cancer Incidence in Five Continents. Vol. I to VIII. Lyon, France, IARCPress, 2005.
Estonia Cancer Registry 1989 - CI5 as it appears in Ferlay J, Parkin DM, Curado MP, Bray F, Edwards B, Shin HR and Forman D. Cancer Incidence in Five Continents, Volumes I to IX: IARC CancerBase No. 9 [Internet]. Lyon, France: International Agency for Research on Cancer; 2010. Available from: http://ci5.iarc.fr
Estonia Cancer Registry 1990 - CI5 as it appears in Ferlay J, Parkin DM, Curado MP, Bray F, Edwards B, Shin HR and Forman D. Cancer Incidence in Five Continents, Volumes I to IX: IARC CancerBase No. 9 [Internet]. Lyon, France: International Agency for Research on Cancer; 2010. Available from: http://ci5.iarc.fr
Estonia Cancer Registry 1991 - CI5 as it appears in Ferlay J, Parkin DM, Curado MP, Bray F, Edwards B, Shin HR and Forman D. Cancer Incidence in Five Continents, Volumes I to IX: IARC CancerBase No. 9 [Internet]. Lyon, France: International Agency for Research on Cancer; 2010. Available from: http://ci5.iarc.fr
Estonia Cancer Registry 1992 - CI5 as it appears in Ferlay J, Parkin DM, Curado MP, Bray F, Edwards B, Shin HR and Forman D. Cancer Incidence in Five Continents, Volumes I to IX: IARC CancerBase No. 9 [Internet]. Lyon, France: International Agency for Research on Cancer; 2010. Available from: http://ci5.iarc.fr
Estonia Cancer Registry 1993 - CI5 as it appears in Ferlay J, Parkin DM, Curado MP, Bray F, Edwards B, Shin HR and Forman D. Cancer Incidence in Five Continents, Volumes I to IX: IARC CancerBase No. 9 [Internet]. Lyon, France: International Agency for Research on Cancer; 2010. Available from: http://ci5.iarc.fr
Estonia Cancer Registry 1993-1997 - CI5 as it appears in Parkin DM, International Agency for Research on Cancer, International Association of Cancer Registries. Cancer Incidence in Five Continents. Vol. I to VIII. Lyon, France, IARCPress, 2005.
Estonia Cancer Registry 1994 - CI5 as it appears in Ferlay J, Parkin DM, Curado MP, Bray F, Edwards B, Shin HR and Forman D. Cancer Incidence in Five Continents, Volumes I to IX: IARC CancerBase No. 9 [Internet]. Lyon, France: International Agency for Research on Cancer; 2010. Available from: http://ci5.iarc.fr
Estonia Cancer Registry 1995 - CI5 as it appears in Ferlay J, Parkin DM, Curado MP, Bray F, Edwards B, Shin HR and Forman D. Cancer Incidence in Five Continents, Volumes I to IX: IARC CancerBase No. 9 [Internet]. Lyon, France: International Agency for Research on Cancer; 2010. Available from: http://ci5.iarc.fr
Estonia Cancer Registry 1996 - CI5 as it appears in Ferlay J, Parkin DM, Curado MP, Bray F, Edwards B, Shin HR and Forman D. Cancer Incidence in Five Continents, Volumes I to IX: IARC CancerBase No. 9 [Internet]. Lyon, France: International Agency for Research on Cancer; 2010. Available from: http://ci5.iarc.fr
Estonia Cancer Registry 1997 - CI5 as it appears in Ferlay J, Parkin DM, Curado MP, Bray F, Edwards B, Shin HR and Forman D. Cancer Incidence in Five Continents, Volumes I to IX: IARC CancerBase No. 9 [Internet]. Lyon, France: International Agency for Research on Cancer; 2010. Available from: http://ci5.iarc.fr
Estonia Cancer Registry 1998 - CI5 as it appears in Ferlay J, Parkin DM, Curado MP, Bray F, Edwards B, Shin HR and Forman D. Cancer Incidence in Five Continents, Volumes I to IX: IARC CancerBase No. 9 [Internet]. Lyon, France: International Agency for Research on Cancer; 2010. Available from: http://ci5.iarc.fr
Estonia Cancer Registry 1998-2002 - CI5 as it appears in Curado MP, Edwards B, Shin HR, Storm H, Ferlay J, Heanue M and Boyle P, eds (2007). Cancer Incidence in Five Continents, Vol. IX Periodic Data (electronic version). Lyon, IARC.http://ci5.iarc.fr

Appendix: Citation List

Citation
Estonia Cancer Registry 1999 - CI5 as it appears in Ferlay J, Parkin DM, Curado MP, Bray F, Edwards B, Shin HR and Forman D. Cancer Incidence in Five Continents, Volumes I to IX: IARC CancerBase No. 9 [Internet]. Lyon, France: International Agency for Research on Cancer; 2010. Available from: http://ci5.iarc.fr
Estonia Cancer Registry 2000 - CI5 as it appears in Ferlay J, Parkin DM, Curado MP, Bray F, Edwards B, Shin HR and Forman D. Cancer Incidence in Five Continents, Volumes I to IX: IARC CancerBase No. 9 [Internet]. Lyon, France: International Agency for Research on Cancer; 2010. Available from: http://ci5.iarc.fr
Estonia Cancer Registry 2001 - CI5 as it appears in Ferlay J, Parkin DM, Curado MP, Bray F, Edwards B, Shin HR and Forman D. Cancer Incidence in Five Continents, Volumes I to IX: IARC CancerBase No. 9 [Internet]. Lyon, France: International Agency for Research on Cancer; 2010. Available from: http://ci5.iarc.fr
Estonia Cancer Registry 2002 - CI5 as it appears in Ferlay J, Parkin DM, Curado MP, Bray F, Edwards B, Shin HR and Forman D. Cancer Incidence in Five Continents, Volumes I to IX: IARC CancerBase No. 9 [Internet]. Lyon, France: International Agency for Research on Cancer; 2010. Available from: http://ci5.iarc.fr
Estonia Cancer Registry 2003-2007 - CI5 as it appears in Forman D, Bray F, Brewster DH, Gombe Mbalawa C, Kohler B, Piñeros M, Steliarova-Foucher E, Swaminathan R and Ferlay J, eds (2013). Cancer Incidence in Five Continents, Vol. X Summary Database (electronic version). Lyon, IARC. http://ci5.iarc.fr
Estonia Census 1989 as it appears in United Nations Statistics Division (UNSD). United Nations Demographic Yearbook - Historical Supplement 1997. New York City, United States: United Nations Statistics Division (UNSD).
Estonia Vital Registration - Deaths 1981 ICD9 as it appears in World Health Organization (WHO). WHO Mortality Database Version November 2015. Geneva, Switzerland: World Health Organization (WHO), 2015.
Estonia Vital Registration - Deaths 1982 ICD9 as it appears in World Health Organization (WHO). WHO Mortality Database Version November 2015. Geneva, Switzerland: World Health Organization (WHO), 2015.
Estonia Vital Registration - Deaths 1985 ICD9 as it appears in World Health Organization (WHO). WHO Mortality Database Version November 2015. Geneva, Switzerland: World Health Organization (WHO), 2015.
Estonia Vital Registration - Deaths 1986 ICD9 as it appears in World Health Organization (WHO). WHO Mortality Database Version November 2015. Geneva, Switzerland: World Health Organization (WHO), 2015.
Estonia Vital Registration - Deaths 1987 ICD9 as it appears in World Health Organization (WHO). WHO Mortality Database Version November 2015. Geneva, Switzerland: World Health Organization (WHO), 2015.
Estonia Vital Registration - Deaths 1988 ICD9 as it appears in World Health Organization (WHO). WHO Mortality Database Version November 2015. Geneva, Switzerland: World Health Organization (WHO), 2015.
Estonia Vital Registration - Deaths 1989 ICD9 as it appears in World Health Organization (WHO). WHO Mortality Database Version November 2015. Geneva, Switzerland: World Health Organization (WHO), 2015.
Estonia Vital Registration - Deaths 1990 ICD9 as it appears in World Health Organization (WHO). WHO Mortality Database Version November 2015. Geneva, Switzerland: World Health Organization (WHO), 2015.
Estonia Vital Registration - Deaths 1991 ICD9 as it appears in World Health Organization (WHO). WHO Mortality Database Version November 2015. Geneva, Switzerland: World Health Organization (WHO), 2015.
Estonia Vital Registration - Deaths 1992 ICD9 as it appears in World Health Organization (WHO). WHO Mortality Database Version November 2015. Geneva, Switzerland: World Health Organization (WHO), 2015.
Estonia Vital Registration - Deaths 1993 ICD9 as it appears in World Health Organization (WHO). WHO Mortality Database Version November 2015. Geneva, Switzerland: World Health Organization (WHO), 2015.
Estonia Vital Registration - Deaths 1994 ICD9 as it appears in World Health Organization (WHO). WHO Mortality Database Version November 2015. Geneva, Switzerland: World Health Organization (WHO), 2015.
Estonia Vital Registration - Deaths 1995 ICD9 as it appears in World Health Organization (WHO). WHO Mortality Database Version November 2015. Geneva, Switzerland: World Health Organization (WHO), 2015.
Estonia Vital Registration - Deaths 1996 ICD9 as it appears in World Health Organization (WHO). WHO Mortality Database Version November 2015. Geneva, Switzerland: World Health Organization (WHO), 2015.
Estonia Vital Registration - Deaths 1997 ICD10 as it appears in World Health Organization (WHO). WHO Mortality Database Version November 2015. Geneva, Switzerland: World Health Organization (WHO), 2015.
Estonia Vital Registration - Deaths 1998 ICD10 as it appears in World Health Organization (WHO). WHO Mortality Database Version November 2015. Geneva, Switzerland: World Health Organization (WHO), 2015.
Estonia Vital Registration - Deaths 1999 ICD10 as it appears in World Health Organization (WHO). WHO Mortality Database Version November 2015. Geneva, Switzerland: World Health Organization (WHO), 2015.
Estonia Vital Registration - Deaths 2000 ICD10 as it appears in World Health Organization (WHO). WHO Mortality Database Version November 2015. Geneva, Switzerland: World Health Organization (WHO), 2015.
Estonia Vital Registration - Deaths 2001 ICD10 as it appears in World Health Organization (WHO). WHO Mortality Database Version November 2015. Geneva, Switzerland: World Health Organization (WHO), 2015.
Estonia Vital Registration - Deaths 2002 ICD10 as it appears in World Health Organization (WHO). WHO Mortality Database Version November 2015. Geneva, Switzerland: World Health Organization (WHO), 2015.
Estonia Vital Registration - Deaths 2003 ICD10 as it appears in World Health Organization (WHO). WHO Mortality Database Version November 2015. Geneva, Switzerland: World Health Organization (WHO), 2015.
Estonia Vital Registration - Deaths 2004 ICD10 as it appears in World Health Organization (WHO). WHO Mortality Database Version November 2015. Geneva, Switzerland: World Health Organization (WHO), 2015.
Estonia Vital Registration - Deaths 2005 ICD10 as it appears in World Health Organization (WHO). WHO Mortality Database Version November 2015. Geneva, Switzerland: World Health Organization (WHO), 2015.

Appendix: Citation List

Citation
Estonia Vital Registration - Deaths 2006 ICD10 as it appears in World Health Organization (WHO). WHO Mortality Database Version November 2015. Geneva, Switzerland: World Health Organization (WHO), 2015.
Estonia Vital Registration - Deaths 2007 ICD10 as it appears in World Health Organization (WHO). WHO Mortality Database Version November 2015. Geneva, Switzerland: World Health Organization (WHO), 2015.
Estonia Vital Registration - Deaths 2008 ICD10 as it appears in World Health Organization (WHO). WHO Mortality Database Version November 2015. Geneva, Switzerland: World Health Organization (WHO), 2015.
Estonia Vital Registration - Deaths 2009 ICD10 as it appears in World Health Organization (WHO). WHO Mortality Database Version November 2015. Geneva, Switzerland: World Health Organization (WHO), 2015.
Estonia Vital Registration - Deaths 2010 ICD10 as it appears in World Health Organization (WHO). WHO Mortality Database Version November 2015. Geneva, Switzerland: World Health Organization (WHO), 2015.
Estonia Vital Registration - Deaths 2011 ICD10 as it appears in World Health Organization (WHO). WHO Mortality Database Version November 2015. Geneva, Switzerland: World Health Organization (WHO), 2015.
Estonia Vital Registration - Deaths 2012 ICD10 as it appears in World Health Organization (WHO). WHO Mortality Database Version November 2015. Geneva, Switzerland: World Health Organization (WHO), 2015.
Estonian Centre for Health Education and Promotion. Estonia Health Behavior Among the Adult Population 1994.
Estonian Centre for Health Education and Promotion. Estonia Health Behavior Among the Adult Population 1996.
Estonian Centre for Health Education and Promotion. Estonia Health Behavior Among the Adult Population 1998.
Estonian Centre for Health Education and Promotion. Estonia Health Behavior Among the Adult Population 2000.
Estonian Centre for Health Education and Promotion. Estonia Health Behavior Among the Adult Population 2002.
Estonian Centre for Health Education and Promotion. Estonia Health Behaviour Among the Estonian Adult Population Obesity Estimates 1990, 1992, 1994, 1996.
Etang J, Chouaibou M, Toto JC, Faye O, Manga L, Samé-Ekobo A, Awono-Ambene P, Simard F. A preliminary test of the protective efficacy of permethrin-treated bed nets in an area of Anopheles gambiae metabolic resistance to pyrethroids in north Cameroon. Trans R Soc Trop Med Hyg. 2007; 101(9): 881-4. As it appears in Malaria Atlas Project. Malaria Atlas Project Plasmodium Falciparum Parasite Rate Database. Oxford, United Kingdom: Malaria Atlas Project.
Etard JF, Kodio B, Traoré S. Assessment of maternal mortality and late maternal mortality among a cohort of pregnant women in Bamako, Mali. Br J Obstet Gynaecol. 1999; 106(1): 60-5.
Etard JF, Le Hesran JY, Diallo A, Diallo JP, Ndiaye JL, Delaunay V. Childhood mortality and probable causes of death using verbal autopsy in Niakhar, Senegal, 1989-2000. Int J Epidemiol. 2004; 33(6): 1286-92.
Eteng MU. Effect of Plasmodium falciparum parasitaemia on some haematological parameters in adolescent and adult Nigerian HbAA and HbAS blood genotypes. Cent Afr J Med. 2002; 48(11-12): 129-32. As it appears in Malaria Atlas Project. Malaria Atlas Project Plasmodium Falciparum Parasite Rate Database. Oxford, United Kingdom: Malaria Atlas Project.
Ethiopia Immunization Coverage Survey 2012.
Ethiopia National EPI Coverage Survey 2001.
Ethiopia National Rural Nutrition Survey 1982-1983 as it appears in World Health Organization (WHO). WHO Global Database on Child Growth and Malnutrition - Historical. Geneva, Switzerland: World Health Organization (WHO).
Ethiopia Welfare Monitoring Survey 1996 as it appears in World Health Organization (WHO). WHO Household Energy Database. Geneva, Switzerland: World Health Organization (WHO), 2010.
Ethiopia Welfare Monitoring Survey 1998 as it appears in World Health Organization (WHO). WHO Household Energy Database. Geneva, Switzerland: World Health Organization (WHO), 2010.
Ethiopia Welfare Monitoring Survey 2000 as it appears in World Health Organization (WHO). WHO Household Energy Database. Geneva, Switzerland: World Health Organization (WHO), 2010.
Ethiopia Welfare Monitoring Survey 2004 as it appears in World Health Organization (WHO). WHO Household Energy Database. Geneva, Switzerland: World Health Organization (WHO), 2010.
Ethiopia WHO/UNICEF Joint Nutrition Programme Annual Report 1985 as it appears in World Health Organization (WHO). WHO Global Database on Child Growth and Malnutrition - Historical. Geneva, Switzerland: World Health Organization (WHO).
Ethiopian Administration for Returnees and Refugees (ARRA), United Nations High Commissioner for Refugees (UNHCR), Centre for International Child Health, Institute of Child Health. Micronutrient Nutrition Survey: Fugnido and Kebribeya Refugee Camps, Ethiopia, 2001. London, United Kingdom, Institute of Child Health, 2001. As it appears in Malaria Atlas Project. Malaria Atlas Project Plasmodium Falciparum Parasite Rate Database. Oxford, United Kingdom: Malaria Atlas Project.
Ethiopian Health and Nutrition Research Center, Ministry of Health (Ethiopia), World Health Organization (WHO). Ethiopia Tuberculosis Prevalence Survey 2010-2011.
Ethiopian Health and Nutrition Research Center. Ethiopia Global Fund Household Survey 2008.
Etukumana EA, Puepet FH, Obadofin MO. Risk factors for diabetes mellitus among rural adults in Nigeria. Niger J Med. 2014; 23(3): 213–9.
European Commission (2012): Eurobarometer 41.0 (Mar-May 1994). INRA, Brussels. GESIS Data Archive, Cologne. ZA2490 Data file Version 1.1.0, doi:10.4232/1.10909
European Commission (2012): Eurobarometer 43.0 (Mar-Apr 1995). INRA, Brussels. GESIS Data Archive, Cologne. ZA2636 Data file Version 1.0.1, doi:10.4232/1.10912
European Commission (2012): Eurobarometer 44.3 (Feb-Apr 1996). INRA, Brussels. GESIS Data Archive, Cologne. ZA2829 Data file Version 1.0.1, doi:10.4232/1.10920
European Commission (2012): Eurobarometer 52.1 (Nov-Dec 1999). INRA, Brussels; NSD, Bergen. GESIS Data Archive, Cologne. ZA3205 Data file Version 2.0.0, doi:10.4232/1.11377
European Commission (2012): Eurobarometer 57.2OVR (Apr-Jun 2002). European Opinion Research Group (EORG), Brussels. GESIS Data Archive, Cologne. ZA3641 Data file Version 1.0.1, doi:10.4232/1.10951

Appendix: Citation List

Citation
European Commission (2012): Eurobarometer 58.2 (Oct-Dec 2002). European Opinion Research Group (EORG), Brussels. GESIS Data Archive, Cologne. ZA3886 Data file Version 1.0.1, doi:10.4232/1.10954
European Commission (2012): Eurobarometer 59.0 (Jan-Feb 2003). European Opinion Research Group (EORG), Brussels. GESIS Data Archive, Cologne. ZA3903 Data file Version 1.0.1, doi:10.4232/1.11352
European Commission (2012): Eurobarometer 64.1 (Sep-Oct 2005). TNS OPINION SOCIAL, Brussels [Producer]. GESIS Data Archive, Cologne. ZA4413 Data file Version 1.1.0, doi:10.4232/1.10969
European Commission (2012): Eurobarometer 64.3 (Nov-Dec 2005). TNS OPINION SOCIAL, Brussels [Producer]. GESIS Data Archive, Cologne. ZA4415 Data file Version 1.0.1, doi:10.4232/1.10971
European Commission (2012): Eurobarometer 66.2 (Oct-Nov 2006). TNS OPINION SOCIAL, Brussels [Producer]. GESIS Data Archive, Cologne. ZA4527 Data file Version 1.0.1, doi:10.4232/1.10981
European Commission (2012): Eurobarometer 72.3 (Oct 2009). TNS OPINION SOCIAL, Brussels [Producer]. GESIS Data Archive, Cologne. ZA4977 Data file Version 2.0.0, doi:10.4232/1.11140
European Commission (2012): Eurobarometer 72.3 (Oct 2009). TNS OPINION SOCIAL, Brussels [Producer]. GESIS Data Archive, Cologne. ZA4977 Data file Version 2.0.0,doi:10.4232/1.11140
European Commission (2012): Eurobarometer 77.1 (2012). TNS OPINION SOCIAL, Brussels [Producer]. GESIS Data Archive, Cologne. ZA5597 Data file Version 2.0.0, doi:10.4232/1.11481
European Institute for Crime Prevention and Control, affiliated with the United Nations (HEUNI), United Nations Office on Drugs and Crime (UNODC), Statistics Canada, United Nations Interregional Crime and Justice Research Institute (UNICRI). International Violence Against Women Surveys (IVAWS) Data 2002-2005. As provided by the Global Burden of Disease Child Sexual Abuse and Intimate Partner Violence Expert Group. [Unpublished].
European Monitoring Centre for Drugs and Drug Addiction (EMCDDA), European School Survey Project on Alcohol and Other Drugs (ESPAD), Pompidou Group, Council of Europe, Swedish Council for Information on Alcohol and Other Drugs (CAN). ESPAD Report 2007: Substance Use Among Students in 35 European Countries. Stockholm, Sweden: Swedish Council for Information on Alcohol and Other Drugs (CAN), 2009.
European Monitoring Centre for Drugs and Drug Addiction (EMCDDA), European School Survey Project on Alcohol and Other Drugs (ESPAD), Pompidou Group, Council of Europe, Swedish Council for Information on Alcohol and Other Drugs (CAN). ESPAD Report 2011: Substance Use Among Students in 36 European Countries. Stockholm, Sweden: Swedish Council for Information on Alcohol and Other Drugs (CAN), 2012.
European Network of Cancer Registries, Slovakia National Cancer Registry. Slovakia EUREG Cancer Incidence and Mortality Tables. Lyon, France: International Agency for Research on Cancer (IARC).
European Network of Cancer Registries. Latvia EUREG Cancer Incidence and Mortality Tables. Lyon, France: International Agency for Research on Cancer (IARC).
European Network of Cancer Registries. Malta EUREG Cancer Incidence and Mortality Tables. Lyon, France: International Agency for Research on Cancer (IARC).
European School Survey Project on Alcohol and Other Drugs (ESPAD), Pompidou Group, Council of Europe, Swedish Council for Information on Alcohol and Other Drugs (CAN). Alcohol and Drug Use Among European 17-18 Year Old Students: Data from the ESPAD Project. Stockholm, Sweden: Swedish Council for Information on Alcohol and Other Drugs (CAN), 2007.
European School Survey Project on Alcohol and Other Drugs (ESPAD), Pompidou Group, Council of Europe, Swedish Council for Information on Alcohol and Other Drugs (CAN). ESPAD Report 2003: Alcohol and Other Drug Use Among Students in 35 European Countries. Stockholm, Sweden: Swedish Council for Information on Alcohol and Other Drugs (CAN), 2004.
European Union (EU), Government of Albania, Institute of Statistics (Albania). Albania Census 2011.
European Union Agency for Fundamental Rights. European Union Violence Against Women Study 2012.
EURO-PERISTAT, EuroNeoStat, European Surveillance of Congenital Anomalies (EUROCAT), Netherlands Organisation for Applied Scientific Research (TNO), Surveillance of Cerebral Palsy in Europe (SCPE). European Perinatal Health Report 2004. EURO-PERISTAT, 2008.
Eurostat, Hungarian Central Statistical Office (HCSO). Hungary European Health Interview Survey 2009.
Eurostat, Institute of Health Information and Statistics of the Czech Republic. Czech Republic European Health Interview Survey 2008.
Eurostat, Ministry for Health, the Elderly, and Community Care (Malta), Ministry for Social Policy (Malta). Malta European Health Interview Survey 2008.
Eurostat, Ministry of Health (Bulgaria), National Statistical Institute of Bulgaria. Bulgaria European Health Interview Survey 2008.
Eurostat, National Institute of Statistics (Romania). Romania European Health Interview Survey 2008.
Eurostat. Eurostat Tobacco Use Prevalence 1999.
Eurostat. Slovenia European Health Interview Survey 2007.
EvaluaciÃ³n del estado nutricional de la poblaciÃ³n de niÃ±os de 9 a 24 meses de edad, residentes en los partidos del Gran Buenos Aires as it appears in World Health Organization (WHO). WHO Global Database on Child Growth and Malnutrition - Historical. Geneva, Switzerland: World Health Organization (WHO).
Evaluation of drought-related acute undernutrition--Mauritania, 1983 as it appears in World Health Organization (WHO). WHO Global Database on Child Growth and Malnutrition - Historical. Geneva, Switzerland: World Health Organization (WHO).
Evaluation of the Nutritional Status of Preschool Children in the Region Lambarene as it appears in World Health Organization (WHO). WHO Global Database on Child Growth and Malnutrition - Historical. Geneva, Switzerland: World Health Organization (WHO).
Evaluation of the Prevalence of Malnutrition in the Departments of Maradi, Tahoua, and Zinder in Late November Through Early December 1985 as it appears in World Health Organization (WHO). WHO Global Database on Child Growth and Malnutrition - Historical. Geneva, Switzerland: World Health Organization (WHO).
Evaluation of Vitamin A and Iron Deficiencies in CÃ´te d'Ivoire: Final Report as it appears in World Health Organization (WHO). WHO Global Database on Child Growth and Malnutrition. Geneva, Switzerland: World Health Organization (WHO).

Appendix: Citation List

Citation

Evaluation of Vitamin A Status of Children Aged 6-59 Months in the Northwest of CÃ´te d'Ivoire as it appears in World Health Organization (WHO). WHO Global Database on Child Growth and Malnutrition - Historical. Geneva, Switzerland: World Health Organization (WHO).

Evanoff BA, Gustavsson P, Hogstedt C. Mortality And Incidence Of Cancer In A Cohort Of Swedish Chimney Sweeps: An Extended Follow Up Study. Br J Ind Med. 1993; 50(5): 450-9 as it appears in Armstrong B, Hutchinson E, Unwin J, Fletcher T. Lung cancer risk after exposure to polycyclic aromatic hydrocarbons: a review and meta-analysis. Environ. Health Perspect. 2004; 112(9): 970–978.

Evaristo-Neto AD, Foss-Freitas MC, Foss MC. Prevalence of diabetes mellitus and impaired glucose tolerance in a rural community of Angola. Diabetol Metab Syndr. 2010; 63.

Evertsen J, Alemzadeh R, Wang X. Increasing incidence of pediatric type 1 diabetes mellitus in Southeastern Wisconsin: relationship with body weight at diagnosis. PLoS One. 2009; 4(9): e6873.

Ezeamama AE, Friedman JF, Acosta LP, Bellinger DC, Langdon GC, Manalo DL, Olveda RM, Kurtis JD, McGarvey ST. Helminth infection and cognitive impairment among Filipino children. Am J Trop Med Hyg. 2005; 72(5): 540-8.

Ezedinachi EN, Ejezie GC, Usanga EA, Gemade EI, Ikpatt NW, Alaribe AA. New trends in chloroquine efficacy in the treatment of malaria: chloroquine-resistant Plasmodium falciparum in Anambra and Benue States of Nigeria. Cent Afr J Med. 1991; 37(6): 180-6. As it appears in Malaria Atlas Project. Malaria Atlas Project Plasmodium Falciparum Parasite Rate Database. Oxford, United Kingdom: Malaria Atlas Project.

Ezeoma IT, Abioye-Kuteyi EA, Oladeji AO. Body build and blood pressure in a rural Nigerian community. Niger Postgrad Med J. 2001; 8(3): 140-4.

Faeh D, William J, Tappy L, Ravussin E, Bovet P. Prevalence, awareness and control of diabetes in the Seychelles and relationship with excess body weight. BMC Public Health. 2007; 163.

Fahimfar N, Khalili D, Mohebi R, Azizi F, Hadaegh F. Risk factors for ischemic stroke; results from 9 years of follow-up in a population based cohort of Iran. BMC Neurol. 2012; 12: 117.

Faiman R, Abbasi I, Jaffe C, Motro Y, Nasereddin A, Schnur LF, Torem M, Pratlong F, Dedet JP, Warburg A. A newly emerged cutaneous leishmaniasis focus in northern Israel and two new reservoir hosts of Leishmania major. PLoS Negl Trop Dis. 2013; 7(2): e2058.

Fakhfakh R, Hsairi M, Maalej M, Achour N, Nacef T. Tobacco use in Tunisia: behaviour and awareness. Bull World Health Organ. 2002; 80(5): 350â€“6.

Fakhrzadeh H, Ghaderpanahi M, Sharifi F, Badamchizade Z, Mirarefin M, Larijani B. Increased risk of chronic kidney disease in elderly with metabolic syndrome and high levels of C-reactive protein: Kahrizak Elderly Study. Kidney Blood Press Res. 2009; 32(6): 457-63.

Falade CO, Nash O, Akingbola TS, Michael OS, Olojede F, Ademowo OG. Blood banking in a malaria-endemic area: evaluating the problem posed by malarial parasitaemias. Ann Trop Med Parasitol. 2009; 103(5): 383-92. As it appears in Malaria Atlas Project. Malaria Atlas Project Plasmodium Falciparum Parasite Rate Database. Oxford, United Kingdom: Malaria Atlas Project.

Family Health Development Division, Ministry of Health (Malaysia). Malaysia Adult Nutrition Survey 2002-2003.

Family Health International, Honduras Family Planning Association (ASHONPLAFA), Management Sciences for Health (MSH), Ministry of Health (Honduras). Honduras National Survey of Maternal and Child Health 1984.

Family Planning and MCH Project, Ministry of Health and Population (Nepal), International Statistical Institute. Nepal World Fertility Survey 1976. Voorburg, Netherlands: International Statistical Institute.

Family Planning Association (Trinidad and Tobago), Westinghouse; Institute for Resource Development. Trinidad and Tobago Demographic and Health Survey 1987. Columbia, United States: Westinghouse; Institute for Resource Development.

Famine in southern Sudan as it appears in World Health Organization (WHO). WHO Global Database on Child Growth and Malnutrition - Historical. Geneva, Switzerland: World Health Organization (WHO).

Famodu AA, Awodu OA. Anthropometric indices as determinants of haemorheological cardiovascular disease risk factors in Nigerian adults living in a semi-urban community. Clin Hemorheol Microcirc. 2009; 43(4): 335-44.

Fan JG, Cai XB, Li L, Li XJ, Dai F, Zhu J. Alcohol consumption and metabolic syndrome among Shanghai adults: a randomized multistage stratified cluster sampling investigation. World J Gastroenterol. 2008; 14(15): 2418-24.

Fan S-Y, Huang C-L, Liu Y, Li L-L, Shi X-H. [Epidemiology study of clonorchiasis in Futian district of Shenzhen]. J Trop Med. 2008; 1: 75-76.

Fano V, Pezzotti P, Gnavi R, Bontempi K, Miceli M, Pagnozzi E, Giarrizzo ML, Fortino A. The role of socio-economic factors on prevalence and health outcomes of persons with diabetes in Rome, Italy. Eur J Public Health. 2013; 23(6): 991-7.

Faraj C, Adlaoui E, Rhajaou M, Lyagoubi M. Estimation of malaria transmission in high-risk provinces of Morocco. East Mediterr Health J. 2003; 9(4): 542-7. As it appears in Malaria Atlas Project. Malaria Atlas Project Plasmodium Falciparum Parasite Rate Database. Oxford, United Kingdom: Malaria Atlas Project.

Fargues P, Nassour O, National Institute for Demographic Studies (France), Sahel Institute. Twelve Years of Urban Mortality in the Sahel. Levels, Trends, Seasons, and Causes of Mortality in Bamako, 1974-1985. Paris, France: National Institute for Demographic Studies (France), 1988.

Farid MA. Malaria in Socotra Island, Democratic Yemen. Cairo, Egypt: World Health Organization Regional Office for the Eastern Mediterranean (EMRO-WHO); 1988. As it appears in Malaria Atlas Project. Malaria Atlas Project Plasmodium Falciparum Parasite Rate Database. Oxford, United Kingdom: Malaria Atlas Project.

Faris R, Ramzy RM, Gad AM, Weil GJ, Buck AA. Community diagnosis of Bancroftian filariasis. Trans R Soc Trop Med Hyg. 1993; 87(6): 659-61. As it appears in London School of Hygiene and Tropical Medicine. Global Atlas of Helminth Infections - Lymphatic Filiariasis.

Farnert A, Snounou G, Rooth I, Bjorkman A. Daily dynamics of Plasmodium falciparum subpopulations in asymptomatic children in a holoendemic area. Am J Trop Med Hyg. 1997; 56(5): 538-47. As it appears in Malaria Atlas Project. Malaria Atlas Project Plasmodium Falciparum Parasite Rate Database. Oxford, United Kingdom: Malaria Atlas Project.

Farooq M, Samaan SA, Nielsen J. Assessment of severity of disease caused by Schistosoma haematobium and S. Bull World Health Organ. 1966; 35(3): 389-404.

Appendix: Citation List

Citation
Farzadfar F, Murray CJL, Gakidou E, Bossert T, Namdaritabar H, Alikhani S, Moradi G, Delavari A, Jamshidi H, Ezzati M. Effectiveness of diabetes and hypertension management by rural primary health-care workers (Behvarz workers) in Iran: a nationally representative observational study. Lancet. 2012; 379(9810): 47–54.
Fässler M, Zimmermann R, QuackLötscher KC. Maternal mortality in Switzerland 1995-2004. Swiss Med Wkly. 2010; 140(1-2): 25-30.
Fathy FM, El-Kasah F, El-Ahwal AM. Emerging cutaneous leishmaniasis in Sirte-Libya: epidemiology, recognition and management. J Egypt Soc Parasitol. 2009; 39(3): 881-905.
Fatmi Z, Hadden WC, Razzak JA, Qureshi HI, Hyder AA, Pappas G. Incidence, patterns and severity of reported unintentional injuries in Pakistan for persons five years and older: results of the National Health Survey of Pakistan 1990-94. BMC Public Health. 2007; 152.
Fatusic J, Hudic I, Fatusic Z. Perinatal mortality during fifteen-year period in Tuzla Canton, Bosnia and Herzegovina. Med Arch. 2012; 66(4): 258-61.
Fauveau V, Koenig MA, Chakraborty J, Chowdhury AI. Causes of maternal mortality in rural Bangladesh, 1976-85. Bull World Health Organ. 1988; 66(5): 643-51.
Fauveau V, Wojtyniak B, Chakraborty J, Sarder AM, Briend A. The effect of maternal and child health and family planning services on mortality: Is prevention enough?. BMJ. 1990; 301(6743): 103–7.
Fauveau VA. The Lao People's Democratic Republic: maternal mortality and female mortality: determining causes of deaths. World Health Stat Q. 1995; 48(1): 44-6.
Fava C, Sjogren M, Montagnana M, Danese E, Almgren P, Engstrom G, Nilsson P, Hedblad B, Guidi GC, Minuz P, Melander O. Prediction of blood pressure changes over time and incidence of hypertension by a genetic risk score in Swedes. Hypertension. 2013; 61(2): 319–26.
Faye O, Bâ M, N'Dir O, Gaye O, Ousmane F, Dieng T, Bah IB, Dieng Y, Diallo S. [Endemic parasitoses in the villages surrounding the Saloum fossil valley, Senegal]. Dakar Med. 1998; 43(1): 104-8. As it appears in Malaria Atlas Project. Malaria Atlas Project Plasmodium Falciparum Parasite Rate Database. Oxford, United Kingdom: Malaria Atlas Project.
Faye O, Diallo S, Gaye O, Faye O, Mouchet J. [Evaluation de l'efficacité du fénitrothion (Sumithion PM40) sur la densité du vecteur et la prévalence du paludisme à Pout (Thiès, Sénégal)]. Ann Soc Belg Med Trop. 1992; 72(2): 103-12. As it appears in Malaria Atlas Project. Malaria Atlas Project Plasmodium Falciparum Parasite Rate Database. Oxford, United Kingdom: Malaria Atlas Project.
Faye O, Diop A, Gaye O, Diop BM, Bah IB, Dieng T, Dieng Y, N'Dir O, Diallo S. [Evaluation of parasitic risks for the population bordering on the Mbeubeuss public waste disposal, Dakar]. Dakar Med. 1998; 43(1): 90-4. As it appears in Malaria Atlas Project. Malaria Atlas Project Plasmodium Falciparum Parasite Rate Database. Oxford, United Kingdom: Malaria Atlas Project.
Faye O, Fontenille D, Gaye O, Sy N, Molez JF, Konate L, Hebrard G, Herve JP, Trouillet J, Diallo S. [Malaria and rice growing in the Senegal River delta (Senegal)]. Ann Soc Belg Med Trop. 1995; 75(3): 179-89. As it appears in Malaria Atlas Project. Malaria Atlas Project Plasmodium Falciparum Parasite Rate Database. Oxford, United Kingdom: Malaria Atlas Project.
Faye O, Gaye O, Fontenille D, Hébrard G, Konate L, Sy N, Hervé JP, Touré Y, Diallo S, Molez JF. [Drought and malaria decrease in the Niayes area of Senegal]. Sante. 1995; 5(5): 299-305. As it appears in Malaria Atlas Project. Malaria Atlas Project Plasmodium Falciparum Parasite Rate Database. Oxford, United Kingdom: Malaria Atlas Project.
Faye O, Gaye O, Hervé JP, Diack PA, Diallo S. [Malaria in the Saharan region of Senegal. 2. Parasitological indices]. Ann Soc Belg Med Trop. 1993; 73(1): 31-6. As it appears in Malaria Atlas Project. Malaria Atlas Project Plasmodium Falciparum Parasite Rate Database. Oxford, United Kingdom: Malaria Atlas Project.
Faye O, N'Dir B, Corréa J, Faye O, N'Dir O, Gaye O, Bah IB, Dieng T, Dieng Y, Diallo S. [Evaluation of parasitic risks related to the revitalization of the Ferlo fossil valley (Senegal)]. Dakar Med. 1998; 43(2): 183-7. As it appears in Malaria Atlas Project. Malaria Atlas Project Plasmodium Falciparum Parasite Rate Database. Oxford, United Kingdom: Malaria Atlas Project.
Fazizi F, Esmaillzadeh A, Mirmiran FP. Obesity and cardiovascular disease risk factors in Tehran adults: a population-based study. East Mediterr Health J. 2004; 10(6): 887-97.
Fedele D, Comi G, Coscelli C, Cucinotta D, Feldman EL, Ghirlanda G, Greene DA, Negrin P, Santeusanio F. A multicenter study on the prevalence of diabetic neuropathy in Italy. Italian Diabetic Neuropathy Committee. Diabetes Care. 1997; 20(5): 836-43.
Federal Bureau of Statistics (Pakistan) and World Bank. Pakistan Living Standards Measurement Survey 1991. Islamabad, Pakistan: Federal Bureau of Statistics (Pakistan).
Federal Bureau of Statistics (Pakistan). Pakistan Demographic Survey 1991.
Federal Bureau of Statistics (Pakistan). Pakistan Demographic Survey 1993-1994.
Federal Bureau of Statistics (Pakistan). Pakistan Demographic Survey 1995-1996.
Federal Bureau of Statistics (Pakistan). Pakistan Demographic Survey 1997.
Federal Bureau of Statistics (Pakistan). Pakistan Demographic Survey 1999.
Federal Bureau of Statistics (Pakistan). Pakistan Demographic Survey 2005. Islamabad, Pakistan: Federal Bureau of Statistics (Pakistan).
Federal Bureau of Statistics (Pakistan). Pakistan Demographic Survey 2006.
Federal Bureau of Statistics (Pakistan). Pakistan Integrated Household Survey 1998-1999. Islamabad, Pakistan: Federal Bureau of Statistics (Pakistan).
Federal Bureau of Statistics (Pakistan). Pakistan Integrated Household Survey 2001-2002. Islamabad, Pakistan: Federal Bureau of Statistics (Pakistan).
Federal Bureau of Statistics (Pakistan). Pakistan Social and Living Standards Measurement Survey 2004-2005. Islamabad, Pakistan: Federal Bureau of Statistics (Pakistan).
Federal Bureau of Statistics (Pakistan). Pakistan Social and Living Standards Measurement Survey 2005-2006. Islamabad, Pakistan: Federal Bureau of Statistics (Pakistan).
Federal Bureau of Statistics (Pakistan). Pakistan Social and Living Standards Measurement Survey 2007-2008. Islamabad, Pakistan: Federal Bureau of Statistics (Pakistan).
Federal Centre for Health Education (BZGA) (Germany). Germany Youth Drug Use in Germany 1997.
Federal Centre for Health Education (BZGA) (Germany). Germany Youth Drug Use in Germany 2001.

Appendix: Citation List

Citation
Federal Centre for Health Education (BZGA) (Germany). Germany Youth Drug Use in Germany 2004.
Federal Environment Agency (Germany), Federal Institute for Drugs and Medical Devices (Germany), Max Planck Institute of Psychiatry, Robert Koch Institute. Germany National Health Interview and Examination Survey 1997-1999. Berlin, Germany: Robert Koch Institute, 2000.
Federal Ministry of Food, Agriculture, and Consumer Protection (Germany), Max Rubner Institute. Germany National Nutrition Survey II 2005-2007. Max Rubner Institute.
Federal Ministry of Health (Austria), Statistics Austria. Austria Hospital Inpatient Discharges 1989-1992.
Federal Ministry of Health (Austria), Statistics Austria. Austria Hospital Inpatient Discharges 1993-1997.
Federal Ministry of Health (Austria), Statistics Austria. Austria Hospital Inpatient Discharges 1998-2002.
Federal Ministry of Health (Austria), Statistics Austria. Austria Hospital Inpatient Discharges 2003-2007.
Federal Ministry of Health (Austria). Austria Nutrition Report 2008.
Federal Ministry of Health (Germany), Institute for Therapy and Health Research (IFT). Germany Population Survey on the Consumption of Psychoative Substances in the German Adult Population 2000.
Federal Ministry of Health (Sudan), Central Bureau of Statistics (Sudan), United Nations Children's Fund (UNICEF). Sudan Multiple Indicator Cluster Survey, North Sudan 2000. New York, United States: United Nations Children's Fund (UNICEF).
Federal Ministry of Health (Sudan), League of Arab States. Sudan Maternal and Child Health Survey 1992-1993.
Federal Ministry of Health (Sudan), World Health Organization (WHO). Sudan - Khartoum STEPS Noncommunicable Disease Risk Factors Survey 2005-2006.
Federal Ministry of Health and Central Bureau of Statistics, Sudan Household and Health Survey - 2, 2012, National report. Khartoum, Republic of Sudan: Federal Ministry of Health and Central Bureau of Statistics.
Federal Ministry of Health and Social Security (Germany), Institute for Therapy and Health Research (IFT). Germany Epidemiological Survey of Substance Abuse Among Adults 2003.
Federal Office of Public Health (Switzerland). Switzerland PERMA Study 1974-1989.
Federal Office of Statistics (Bosnia and Herzegovina). Bosnia and Herzegovina Statistical Yearbook 2004. Sarajevo, Bosnia and Herzegovina: Federal Office of Statistics (Bosnia and Herzegovina), 2004.
Federal Office of Statistics (Bosnia and Herzegovina). Bosnia and Herzegovina Statistical Yearbook 2010. Sarajevo, Bosnia and Herzegovina: Federal Office of Statistics (Bosnia and Herzegovina), 2010.
Federal Office of Statistics (Nigeria), Macro International, Inc, National Population Commission of Nigeria, UK Department for International Development (DFID), United Nations Children's Fund (UNICEF), United Nations Population Fund (UNFPA). Nigeria Demographic and Health Survey - Maternal Mortality Data.
Federal Office of Statistics (Nigeria), Macro International, Inc.; Institute for Resource Development. Nigeria Demographic and Health Survey 1990. Calverton, United States: Macro International, Inc.
Federal Office of Statistics (Nigeria), United Nations Children's Fund (UNICEF). Nigeria Multiple Indicator Cluster Survey 1995.
Federal Office of Statistics (Nigeria). Nigeria Living Standards Survey 2003-2004.
Federal Statistical Office (Germany). Germany Microcensus 1995.
Federal Statistical Office (Germany). Germany Microcensus 2003.
Federal Statistical Office (Germany). Germany Microcensus 2005.
Federal Statistical Office (Germany). Germany Statistical Yearbook 2006. Wiesbaden, Germany: Federal Statistical Office (Germany), 2006.
Federal Statistical Office (Germany). Germany Statistical Yearbook 2007. Wiesbaden, Germany: Federal Statistical Office (Germany), 2007.
Federal Statistical Office (Germany). Germany Statistical Yearbook 2010. Wiesbaden, Germany: Federal Statistical Office (Germany), 2010.
Federal Statistical Office (Switzerland), MIS Trend SA. Switzerland Health Survey 2007.
Federal Statistical Office (Switzerland), National Institute for Cancer Epidemiology and Registration (Switzerland). Switzerland Statistics of Cancer Incidence 1983-2007. Zurich, Switzerland: National Institute for Cancer Epidemiology and Registration (Switzerland), 2010.
Federal Statistical Office (Switzerland). Switzerland Body Mass Index by Age, Sex, and Language Region. NeuchÃ¢tel, Switzerland: Federal Statistical Office (Switzerland).
Federal Statistical Office (Switzerland). Switzerland Federal Population Census 1960.
Federal Statistical Office (Switzerland). Switzerland Health Survey 1993.
Federal Statistical Office (Switzerland). Switzerland Health Survey 1997.
Federal Statistical Office (Switzerland). Switzerland Health Survey 2002.
Federal Statistical Office (Switzerland). Switzerland Health Survey 2012.
Federated States of Micronesia Division of Statistics, Secretariat of the Pacific Community (SPC), United States Census Bureau. Micronesia Population and Housing Census 2000.
Federated States of Micronesia Division of Statistics. Micronesia Census 1994 - Pacificweb.org.
Feeding Practices in Infants and Young Children in Rangoon Division 1980-1981 as it appears in World Health Organization (WHO). WHO Global Database on Child Growth and Malnutrition - Historical. Geneva, Switzerland: World Health Organization (WHO).
Feinsod FM, Faris R, Gad A, el Said S, Soliman BA, Abd-el Azem IS, Saah AJ. Clinical manifestations of Wuchereria bancrofti filariasis in an endemic village in the Nile Delta. Ann Soc Belg Med Trop. 1987; 67(3): 259-65. As it appears in London School of Hygiene and Tropical Medicine. Global Atlas of Helminth Infections - Lymphatic Filiariasis.
Félix-Redondo FJ, Baena-Díez JM, Grau M, Tormo MÁ, Fernández-Bergés D. Prevalence of obesity and cardiovascular risk in the general population of a health area in Extremadura (Spain): the Hermex study. Endocrinol Nutr. 2012; 59(3): 160–8.
Feng ML, Li SG, Wu ZY, Yin CH, Zhang XJ, Zhai JG, Chen J, Han SF, Zhang XX, Shang YL, Yan XC. [An epidemiological survey on paragonimiasis in Jin Miaopu township in Shanxi province]. Chin J Prev Med. 2007; 41(Suppl): 131-33.
Feng XL, Zhu J, Zhang L, Song L, Hipgrave D, Guo S, Ronsmans C, Guo Y, Yang Q. Socio-economic disparities in maternal mortality in China between 1996 and 2006. BJOG. 2010; 117(12): 1527-36.

Appendix: Citation List

Citation
Feresu SA, Harlow SD, Welch K, Gillespie BW. Incidence of and socio-demographic risk factors for stillbirth, preterm birth and low birthweight among Zimbabwean women. Paediatr Perinat Epidemiol. 2004; 18(2): 154-63.
Ferguson TS, Tulloch-Reid MK, Younger NOM, Wright-Pascoe RA, Boyne MS, McFarlane SR, Francis DK, Wilks RJ. Diabetic foot complications among patients attending a specialist diabetes clinic in Jamaica: prevalence and associated factors. West Indian Med J. 2013; 62(3): 216–23.
Ferguson TS, Younger N, Tulloch-Reid MK, Lawrence-Wright MB, Forrester TE, Cooper RS, Van den Broeck J, Wilks RJ. Progression from prehypertension to hypertension in a Jamaican cohort: incident hypertension and its predictors. West Indian Med J. 2010; 59(5): 486–93.
Ferguson TS, Younger NOM, Tulloch-Reid MK, Wright MBL, Ward EM, Ashley DE, Wilks RJ. Prevalence of prehypertension and its relationship to risk factors for cardiovascular disease in Jamaica: analysis from a cross-sectional survey. BMC Cardiovasc Disord. 2008; 8: 20.
Fernando DJ, Siribaddana S, de Silva D. Impaired glucose tolerance and diabetes mellitus in a suburban Sri Lankan community. Postgrad Med J. 1994; 70(823): 347-9.
Fernando DJ. The prevalence of neuropathic foot ulceration in Sri Lankan diabetic patients. Ceylon Med J. 1996; 41(3): 96-8.
Fernando SD, Paranavitane SR, Rajakaruna J, Weerasinghe S, Silva D, Wickremasinghe AR. The health and nutritional status of school children in two rural communities in Sri Lanka. Trop Med Int Health. 2000; 5(6): 450-2. As it appears in Malaria Atlas Project. Malaria Atlas Project Plasmodium Falciparum Parasite Rate Database. Oxford, United Kingdom: Malaria Atlas Project.
Ferrer A, Padrós G, Formiga F, Rojas-Farreras S, Perez JM, Pujol R. Diabetes mellitus: prevalence and effect of morbidities in the oldest old. The Octabaix study. J Am Geriatr Soc. 2012; 60(3): 462-7.
Ferrer JF, Del Pino N, Esteban E, Sherman MP, Dube S, Dube DK, Basombrio MA, Pimentel E, Segovia A, Quirulas S, Poiesz BJ. High Rate of Infection with the Human T-Cell Leukemia Retrovirus Type II in Four Indian Populations of Argentina. Virology. 1993; 197(2): 576-84.
Fèvre EM, Odiit M, Coleman PG, Woolhouse MEJ, Welburn SC. Estimating the burden of rhodesiense sleeping sickness during an outbreak in Serere, eastern Uganda. BMC Public Health. 2008; 8: 96.
Fezeu L, Minkoulou E, Balkau B, Kengne A-P, Awah P, Unwin N, Alberti GKMM, Mbanya J-C. Association between socioeconomic status and adiposity in urban Cameroon. Int J Epidemiol. 2006; 35(1): 105-11.
Field appraisal of the nutritional status of preschool children and their mothers and the investigation of its determinants in rural Korea as it appears in World Health Organization (WHO). WHO Global Database on Child Growth and Malnutrition - Historical. Geneva, Switzerland: World Health Organization (WHO).
Field, J. et al. , National Survey of Sexual Attitudes and Lifestyles, 1990 [computer file]. Colchester, Essex: UK Data Archive [distributor], August 1995. SN: 3434, http://dx.doi.org/10.5255/UKDA-SN-3434-1
Figueroa JP, Ward E, Walters C, Ashley DE, Wilks RJ. High risk health behaviours among adult Jamaicans. West Indian Med J. 2005; 54(1): 70â€“6.
Fiji Bureau of Statistics, International Statistical Institute. Fiji World Fertility Survey 1974. Voorburg, Netherlands: International Statistical Institute.
Fiji Immunization Coverage Survey Report 2005.
Fiji National EPI Survey 1999.
Fiji National Immunisation Coverage Survey 2008.
Fiji National Nutrition Survey 1993 as it appears in World Health Organization (WHO). WHO Global Database on Child Growth and Malnutrition. Geneva, Switzerland: World Health Organization (WHO).
Fiji Population and Housing Census 1966 as it appears in United Nations Statistics Division (UNSD). United Nations Demographic Yearbook - Historical Supplement 1997. New York City, United States: United Nations Statistics Division (UNSD).
Fiji School of Medicine, Menzies Center for Population Health Research, University of Tasmania (Australia), Ministry of Health (Marshall Islands), World Health Organization (WHO). Marshall Islands STEPS Noncommunicable Disease Risk Factors Survey 2002.
Fiji School of Medicine, Ministry of Health and Medical Services (Solomon Islands), World Health Organization (WHO). Solomon Islands STEPS Noncommunicable Disease Risk Factors Survey 2005-2006.
Fiji Vital Registration - Deaths 1999 ICD9 as it appears in World Health Organization (WHO). WHO Mortality Database Version November 2015. Geneva, Switzerland: World Health Organization (WHO), 2015.
Fiji Vital Registration - Deaths 1978 ICD8 as it appears in World Health Organization (WHO). WHO Mortality Database Version May 2013. Geneva, Switzerland: World Health Organization (WHO), 2013.
Fiji Vital Registration - Deaths 2001 ICD10 as it appears in World Health Organization (WHO). WHO Mortality Database Version November 2015. Geneva, Switzerland: World Health Organization (WHO), 2015.
Fiji Vital Registration - Deaths 2002 ICD10 as it appears in World Health Organization (WHO). WHO Mortality Database Version November 2015. Geneva, Switzerland: World Health Organization (WHO), 2015.
Fiji Vital Registration - Deaths 2003 ICD10 as it appears in World Health Organization (WHO). WHO Mortality Database Version November 2015. Geneva, Switzerland: World Health Organization (WHO), 2015.
Fiji Vital Registration - Deaths 2004 ICD10 as it appears in World Health Organization (WHO). WHO Mortality Database Version November 2015. Geneva, Switzerland: World Health Organization (WHO), 2015.
Fiji Vital Registration - Deaths 2005 ICD10 as it appears in World Health Organization (WHO). WHO Mortality Database Version November 2015. Geneva, Switzerland: World Health Organization (WHO), 2015.
Fiji Vital Registration - Deaths 2006 ICD10 as it appears in World Health Organization (WHO). WHO Mortality Database Version November 2015. Geneva, Switzerland: World Health Organization (WHO), 2015.
Fiji Vital Registration - Deaths 2007 ICD10 as it appears in World Health Organization (WHO). WHO Mortality Database Version November 2015. Geneva, Switzerland: World Health Organization (WHO), 2015.
Fiji Vital Registration - Deaths 2008 ICD10 as it appears in World Health Organization (WHO). WHO Mortality Database Version November 2015. Geneva, Switzerland: World Health Organization (WHO), 2015.

Appendix: Citation List

Citation
Fiji Vital Registration - Deaths 2009 ICD10 as it appears in World Health Organization (WHO). WHO Mortality Database Version November 2015. Geneva, Switzerland: World Health Organization (WHO), 2015.
Fiji Vital Registration - Deaths 2011 ICD10 as it appears in World Health Organization (WHO). WHO Mortality Database Version November 2015. Geneva, Switzerland: World Health Organization (WHO), 2015.
Fiji Vital Registration - Deaths 2012 ICD10 as it appears in World Health Organization (WHO). WHO Mortality Database Version November 2015. Geneva, Switzerland: World Health Organization (WHO), 2015.
Fiji Vital Registration Death Data 1950 as it appears in United Nations Statistics Division (UNSD). United Nations Demographic Yearbook - Historical Supplement 1997. New York City, United States: United Nations Statistics Division (UNSD).
Fiji Vital Registration Death Data 1951 as it appears in United Nations Statistics Division (UNSD). United Nations Demographic Yearbook - Historical Supplement 1997. New York City, United States: United Nations Statistics Division (UNSD).
Fiji Vital Registration Death Data 1952 as it appears in United Nations Statistics Division (UNSD). United Nations Demographic Yearbook - Historical Supplement 1997. New York City, United States: United Nations Statistics Division (UNSD).
Fiji Vital Registration Death Data 1953 as it appears in United Nations Statistics Division (UNSD). United Nations Demographic Yearbook - Historical Supplement 1997. New York City, United States: United Nations Statistics Division (UNSD).
Fiji Vital Registration Death Data 1954 as it appears in United Nations Statistics Division (UNSD). United Nations Demographic Yearbook - Historical Supplement 1997. New York City, United States: United Nations Statistics Division (UNSD).
Fiji Vital Registration Death Data 1955 as it appears in United Nations Statistics Division (UNSD). United Nations Demographic Yearbook - Historical Supplement 1997. New York City, United States: United Nations Statistics Division (UNSD).
Fiji Vital Registration Death Data 1956 as it appears in United Nations Statistics Division (UNSD). United Nations Demographic Yearbook - Historical Supplement 1997. New York City, United States: United Nations Statistics Division (UNSD).
Fiji Vital Registration Death Data 1957 as it appears in United Nations Statistics Division (UNSD). United Nations Demographic Yearbook - Historical Supplement 1997. New York City, United States: United Nations Statistics Division (UNSD).
Fiji Vital Registration Death Data 1958 as it appears in United Nations Statistics Division (UNSD). United Nations Demographic Yearbook - Historical Supplement 1997. New York City, United States: United Nations Statistics Division (UNSD).
Fiji Vital Registration Death Data 1959 as it appears in United Nations Statistics Division (UNSD). United Nations Demographic Yearbook - Historical Supplement 1997. New York City, United States: United Nations Statistics Division (UNSD).
Fiji Vital Registration Death Data 1960 as it appears in United Nations Statistics Division (UNSD). United Nations Demographic Yearbook - Historical Supplement 1997. New York City, United States: United Nations Statistics Division (UNSD).
Fiji Vital Registration Death Data 1961 as it appears in United Nations Statistics Division (UNSD). United Nations Demographic Yearbook - Historical Supplement 1997. New York City, United States: United Nations Statistics Division (UNSD).
Fiji Vital Registration Death Data 1962 as it appears in United Nations Statistics Division (UNSD). United Nations Demographic Yearbook - Historical Supplement 1997. New York City, United States: United Nations Statistics Division (UNSD).
Fiji Vital Registration Death Data 1963 as it appears in United Nations Statistics Division (UNSD). United Nations Demographic Yearbook - Historical Supplement 1997. New York City, United States: United Nations Statistics Division (UNSD).
Fiji Vital Registration Death Data 1964 as it appears in United Nations Statistics Division (UNSD). United Nations Demographic Yearbook - Historical Supplement 1997. New York City, United States: United Nations Statistics Division (UNSD).
Fiji Vital Registration Death Data 1965 as it appears in United Nations Statistics Division (UNSD). United Nations Demographic Yearbook - Historical Supplement 1997. New York City, United States: United Nations Statistics Division (UNSD).
Fiji Vital Registration Death Data 1967 as it appears in United Nations Statistics Division (UNSD). United Nations Demographic Yearbook - Historical Supplement 1997. New York City, United States: United Nations Statistics Division (UNSD).
Fiji Vital Registration Death Data 1968 as it appears in United Nations Statistics Division (UNSD). United Nations Demographic Yearbook - Historical Supplement 1997. New York City, United States: United Nations Statistics Division (UNSD).
Fiji Vital Registration Death Data 1969 as it appears in United Nations Statistics Division (UNSD). United Nations Demographic Yearbook - Historical Supplement 1997. New York City, United States: United Nations Statistics Division (UNSD).
Fiji Vital Registration Death Data 1970 as it appears in United Nations Statistics Division (UNSD). United Nations Demographic Yearbook - Historical Supplement 1997. New York City, United States: United Nations Statistics Division (UNSD).
Fiji Vital Registration Death Data 1971 as it appears in United Nations Statistics Division (UNSD). United Nations Demographic Yearbook - Historical Supplement 1997. New York City, United States: United Nations Statistics Division (UNSD).
Fiji Vital Registration Death Data 1972 as it appears in United Nations Statistics Division (UNSD). United Nations Demographic Yearbook - Historical Supplement 1997. New York City, United States: United Nations Statistics Division (UNSD).
Fiji Vital Registration Death Data 1973 as it appears in United Nations Statistics Division (UNSD). United Nations Demographic Yearbook - Historical Supplement 1997. New York City, United States: United Nations Statistics Division (UNSD).
Fiji Vital Registration Death Data 1974 as it appears in United Nations Statistics Division (UNSD). United Nations Demographic Yearbook - Historical Supplement 1997. New York City, United States: United Nations Statistics Division (UNSD).
Fiji Vital Registration Death Data 1975 as it appears in United Nations Statistics Division (UNSD). United Nations Demographic Yearbook - Historical Supplement 1997. New York City, United States: United Nations Statistics Division (UNSD).
Fiji Vital Registration Death Data 1976 as it appears in United Nations Statistics Division (UNSD). United Nations Demographic Yearbook - Historical Supplement 1997. New York City, United States: United Nations Statistics Division (UNSD).
Fiji Vital Registration Death Data 1977 as it appears in United Nations Statistics Division (UNSD). United Nations Demographic Yearbook - Historical Supplement 1997. New York City, United States: United Nations Statistics Division (UNSD).
Fiji Vital Registration Death Data 1979 as it appears in United Nations Statistics Division (UNSD). United Nations Demographic Yearbook - Historical Supplement 1997. New York City, United States: United Nations Statistics Division (UNSD).
Fiji Vital Registration Death Data 1980 as it appears in United Nations Statistics Division (UNSD). United Nations Demographic Yearbook - Historical Supplement 1997. New York City, United States: United Nations Statistics Division (UNSD).

Appendix: Citation List

Citation
Fiji Vital Registration Death Data 1981 as it appears in United Nations Statistics Division (UNSD). United Nations Demographic Yearbook - Historical Supplement 1997. New York City, United States: United Nations Statistics Division (UNSD).
Fiji Vital Registration Death Data 1982 as it appears in United Nations Statistics Division (UNSD). United Nations Demographic Yearbook - Historical Supplement 1997. New York City, United States: United Nations Statistics Division (UNSD).
Fiji Vital Registration Death Data 1983 as it appears in United Nations Statistics Division (UNSD). United Nations Demographic Yearbook - Historical Supplement 1997. New York City, United States: United Nations Statistics Division (UNSD).
Fiji Vital Registration Death Data 1984 as it appears in United Nations Statistics Division (UNSD). United Nations Demographic Yearbook - Historical Supplement 1997. New York City, United States: United Nations Statistics Division (UNSD).
Fiji Vital Registration Death Data 1985 as it appears in United Nations Statistics Division (UNSD). United Nations Demographic Yearbook - Historical Supplement 1997. New York City, United States: United Nations Statistics Division (UNSD).
Fiji Vital Registration Death Data 1986 as it appears in United Nations Statistics Division (UNSD). United Nations Demographic Yearbook - Historical Supplement 1997. New York City, United States: United Nations Statistics Division (UNSD).
Fiji Vital Registration Death Data 1987 as it appears in United Nations Statistics Division (UNSD). United Nations Demographic Yearbook - Historical Supplement 1997. New York City, United States: United Nations Statistics Division (UNSD).
Fikree FF, Midhet F, Sadruddin S, Berendes WH. Maternal mortality in different Pakistani sites: ratios, clinical causes and determiniants. Acta Obstet Gynecol Scand. 1997; 76(7): 637-645.
Filippidis FT, Vardavas CI, Loukopoulou A, Behrakis P, Connolly GN, Tountas Y. Prevalence and determinants of tobacco use among adults in Greece: 4 year trends. Eur J Public Health. 2013; 23(5): 772-6.
Fillinger U, Ndenga B, Githeko A, Lindsay SW. Integrated malaria vector control with microbial larvicides and insecticide-treated nets in western Kenya: a controlled trial. Bull World Health Organ. 2009; 87(9): 655-65. As it appears in Malaria Atlas Project. Malaria Atlas Project Plasmodium Falciparum Parasite Rate Database. Oxford, United Kingdom: Malaria Atlas Project.
Fillinger U. Kenya Plasmodium Falciparum Parasite Rate Data, Personal Communication with U. Fillinger 2006. As it appears in Malaria Atlas Project. Malaria Atlas Project Plasmodium Falciparum Parasite Rate Database. Oxford, United Kingdom: Malaria Atlas Project.
Finland Adolescent Health and Lifestyle Survey 1981 as it appears in P.N. Lee Statistics and Computing Ltd. International Mortality and Smoking Statistics Version 4.04. Sutton, United Kingdom: P.N. Lee Statistics and Computing Ltd, 2009.
Finland Adolescent Health and Lifestyle Survey 1983 as it appears in P.N. Lee Statistics and Computing Ltd. International Mortality and Smoking Statistics Version 4.04. Sutton, United Kingdom: P.N. Lee Statistics and Computing Ltd, 2009.
Finland Adolescent Health and Lifestyle Survey 1985 as it appears in P.N. Lee Statistics and Computing Ltd. International Mortality and Smoking Statistics Version 4.04. Sutton, United Kingdom: P.N. Lee Statistics and Computing Ltd, 2009.
Finland Adolescent Health and Lifestyle Survey 1987 as it appears in P.N. Lee Statistics and Computing Ltd. International Mortality and Smoking Statistics Version 4.04. Sutton, United Kingdom: P.N. Lee Statistics and Computing Ltd, 2009.
Finland Adolescent Health and Lifestyle Survey 1989 as it appears in P.N. Lee Statistics and Computing Ltd. International Mortality and Smoking Statistics Version 4.04. Sutton, United Kingdom: P.N. Lee Statistics and Computing Ltd, 2009.
Finland Adolescent Health and Lifestyle Survey 1991 as it appears in P.N. Lee Statistics and Computing Ltd. International Mortality and Smoking Statistics Version 4.04. Sutton, United Kingdom: P.N. Lee Statistics and Computing Ltd, 2009.
Finland Adolescent Health and Lifestyle Survey 1993 as it appears in P.N. Lee Statistics and Computing Ltd. International Mortality and Smoking Statistics Version 4.04. Sutton, United Kingdom: P.N. Lee Statistics and Computing Ltd, 2009.
Finland Adolescent Health and Lifestyle Survey 1995 as it appears in P.N. Lee Statistics and Computing Ltd. International Mortality and Smoking Statistics Version 4.04. Sutton, United Kingdom: P.N. Lee Statistics and Computing Ltd, 2009.
Finland Adolescent Health and Lifestyle Survey 1997 as it appears in P.N. Lee Statistics and Computing Ltd. International Mortality and Smoking Statistics Version 4.04. Sutton, United Kingdom: P.N. Lee Statistics and Computing Ltd, 2009.
Finland Adolescent Health and Lifestyle Survey 1999 as it appears in P.N. Lee Statistics and Computing Ltd. International Mortality and Smoking Statistics Version 4.04. Sutton, United Kingdom: P.N. Lee Statistics and Computing Ltd, 2009.
Finland Cancer Registry 1970 - CI5 as it appears in Ferlay J, Parkin DM, Curado MP, Bray F, Edwards B, Shin HR and Forman D. Cancer Incidence in Five Continents, Volumes I to IX: IARC CancerBase No. 9 [Internet]. Lyon, France: International Agency for Research on Cancer; 2010. Available from: http://ci5.iarc.fr
Finland Cancer Registry 1971 - CI5 as it appears in Ferlay J, Parkin DM, Curado MP, Bray F, Edwards B, Shin HR and Forman D. Cancer Incidence in Five Continents, Volumes I to IX: IARC CancerBase No. 9 [Internet]. Lyon, France: International Agency for Research on Cancer; 2010. Available from: http://ci5.iarc.fr
Finland Cancer Registry 1972 - CI5 as it appears in Ferlay J, Parkin DM, Curado MP, Bray F, Edwards B, Shin HR and Forman D. Cancer Incidence in Five Continents, Volumes I to IX: IARC CancerBase No. 9 [Internet]. Lyon, France: International Agency for Research on Cancer; 2010. Available from: http://ci5.iarc.fr
Finland Cancer Registry 1973 - CI5 as it appears in Ferlay J, Parkin DM, Curado MP, Bray F, Edwards B, Shin HR and Forman D. Cancer Incidence in Five Continents, Volumes I to IX: IARC CancerBase No. 9 [Internet]. Lyon, France: International Agency for Research on Cancer; 2010. Available from: http://ci5.iarc.fr
Finland Cancer Registry 1974 - CI5 as it appears in Ferlay J, Parkin DM, Curado MP, Bray F, Edwards B, Shin HR and Forman D. Cancer Incidence in Five Continents, Volumes I to IX: IARC CancerBase No. 9 [Internet]. Lyon, France: International Agency for Research on Cancer; 2010. Available from: http://ci5.iarc.fr
Finland Cancer Registry 1975 - CI5 as it appears in Ferlay J, Parkin DM, Curado MP, Bray F, Edwards B, Shin HR and Forman D. Cancer Incidence in Five Continents, Volumes I to IX: IARC CancerBase No. 9 [Internet]. Lyon, France: International Agency for Research on Cancer; 2010. Available from: http://ci5.iarc.fr
Finland Cancer Registry 1976 - CI5 as it appears in Ferlay J, Parkin DM, Curado MP, Bray F, Edwards B, Shin HR and Forman D. Cancer Incidence in Five Continents, Volumes I to IX: IARC CancerBase No. 9 [Internet]. Lyon, France: International Agency for Research on Cancer; 2010. Available from: http://ci5.iarc.fr

Appendix: Citation List

Citation
Finland Cancer Registry 1977 - CI5 as it appears in Ferlay J, Parkin DM, Curado MP, Bray F, Edwards B, Shin HR and Forman D. Cancer Incidence in Five Continents, Volumes I to IX: IARC CancerBase No. 9 [Internet]. Lyon, France: International Agency for Research on Cancer; 2010. Available from: http://ci5.iarc.fr
Finland Cancer Registry 1977-1981 - CI5 as it appears in Parkin DM, International Agency for Research on Cancer, International Association of Cancer Registries. Cancer Incidence in Five Continents. Vol. I to VIII. Lyon, France, IARCPress, 2005.
Finland Cancer Registry 1978 - CI5 as it appears in Ferlay J, Parkin DM, Curado MP, Bray F, Edwards B, Shin HR and Forman D. Cancer Incidence in Five Continents, Volumes I to IX: IARC CancerBase No. 9 [Internet]. Lyon, France: International Agency for Research on Cancer; 2010. Available from: http://ci5.iarc.fr
Finland Cancer Registry 1979 - CI5 as it appears in Ferlay J, Parkin DM, Curado MP, Bray F, Edwards B, Shin HR and Forman D. Cancer Incidence in Five Continents, Volumes I to IX: IARC CancerBase No. 9 [Internet]. Lyon, France: International Agency for Research on Cancer; 2010. Available from: http://ci5.iarc.fr
Finland Cancer Registry 1980 - CI5 as it appears in Ferlay J, Parkin DM, Curado MP, Bray F, Edwards B, Shin HR and Forman D. Cancer Incidence in Five Continents, Volumes I to IX: IARC CancerBase No. 9 [Internet]. Lyon, France: International Agency for Research on Cancer; 2010. Available from: http://ci5.iarc.fr
Finland Cancer Registry 1981 - CI5 as it appears in Ferlay J, Parkin DM, Curado MP, Bray F, Edwards B, Shin HR and Forman D. Cancer Incidence in Five Continents, Volumes I to IX: IARC CancerBase No. 9 [Internet]. Lyon, France: International Agency for Research on Cancer; 2010. Available from: http://ci5.iarc.fr
Finland Cancer Registry 1982 - CI5 as it appears in Ferlay J, Parkin DM, Curado MP, Bray F, Edwards B, Shin HR and Forman D. Cancer Incidence in Five Continents, Volumes I to IX: IARC CancerBase No. 9 [Internet]. Lyon, France: International Agency for Research on Cancer; 2010. Available from: http://ci5.iarc.fr
Finland Cancer Registry 1982-1986 - CI5 as it appears in Parkin DM, International Agency for Research on Cancer, International Association of Cancer Registries. Cancer Incidence in Five Continents. Vol. I to VIII. Lyon, France, IARCPress, 2005.
Finland Cancer Registry 1983 - CI5 as it appears in Ferlay J, Parkin DM, Curado MP, Bray F, Edwards B, Shin HR and Forman D. Cancer Incidence in Five Continents, Volumes I to IX: IARC CancerBase No. 9 [Internet]. Lyon, France: International Agency for Research on Cancer; 2010. Available from: http://ci5.iarc.fr
Finland Cancer Registry 1984 - CI5 as it appears in Ferlay J, Parkin DM, Curado MP, Bray F, Edwards B, Shin HR and Forman D. Cancer Incidence in Five Continents, Volumes I to IX: IARC CancerBase No. 9 [Internet]. Lyon, France: International Agency for Research on Cancer; 2010. Available from: http://ci5.iarc.fr
Finland Cancer Registry 1985 - CI5 as it appears in Ferlay J, Parkin DM, Curado MP, Bray F, Edwards B, Shin HR and Forman D. Cancer Incidence in Five Continents, Volumes I to IX: IARC CancerBase No. 9 [Internet]. Lyon, France: International Agency for Research on Cancer; 2010. Available from: http://ci5.iarc.fr
Finland Cancer Registry 1986 - CI5 as it appears in Ferlay J, Parkin DM, Curado MP, Bray F, Edwards B, Shin HR and Forman D. Cancer Incidence in Five Continents, Volumes I to IX: IARC CancerBase No. 9 [Internet]. Lyon, France: International Agency for Research on Cancer; 2010. Available from: http://ci5.iarc.fr
Finland Cancer Registry 1987 - CI5 as it appears in Ferlay J, Parkin DM, Curado MP, Bray F, Edwards B, Shin HR and Forman D. Cancer Incidence in Five Continents, Volumes I to IX: IARC CancerBase No. 9 [Internet]. Lyon, France: International Agency for Research on Cancer; 2010. Available from: http://ci5.iarc.fr
Finland Cancer Registry 1987-1992 - CI5 as it appears in Parkin DM, International Agency for Research on Cancer, International Association of Cancer Registries. Cancer Incidence in Five Continents. Vol. I to VIII. Lyon, France, IARCPress, 2005.
Finland Cancer Registry 1988 - CI5 as it appears in Ferlay J, Parkin DM, Curado MP, Bray F, Edwards B, Shin HR and Forman D. Cancer Incidence in Five Continents, Volumes I to IX: IARC CancerBase No. 9 [Internet]. Lyon, France: International Agency for Research on Cancer; 2010. Available from: http://ci5.iarc.fr
Finland Cancer Registry 1989 - CI5 as it appears in Ferlay J, Parkin DM, Curado MP, Bray F, Edwards B, Shin HR and Forman D. Cancer Incidence in Five Continents, Volumes I to IX: IARC CancerBase No. 9 [Internet]. Lyon, France: International Agency for Research on Cancer; 2010. Available from: http://ci5.iarc.fr
Finland Cancer Registry 1990 - CI5 as it appears in Ferlay J, Parkin DM, Curado MP, Bray F, Edwards B, Shin HR and Forman D. Cancer Incidence in Five Continents, Volumes I to IX: IARC CancerBase No. 9 [Internet]. Lyon, France: International Agency for Research on Cancer; 2010. Available from: http://ci5.iarc.fr
Finland Cancer Registry 1991 - CI5 as it appears in Ferlay J, Parkin DM, Curado MP, Bray F, Edwards B, Shin HR and Forman D. Cancer Incidence in Five Continents, Volumes I to IX: IARC CancerBase No. 9 [Internet]. Lyon, France: International Agency for Research on Cancer; 2010. Available from: http://ci5.iarc.fr
Finland Cancer Registry 1992 - CI5 as it appears in Ferlay J, Parkin DM, Curado MP, Bray F, Edwards B, Shin HR and Forman D. Cancer Incidence in Five Continents, Volumes I to IX: IARC CancerBase No. 9 [Internet]. Lyon, France: International Agency for Research on Cancer; 2010. Available from: http://ci5.iarc.fr
Finland Cancer Registry 1993 - CI5 as it appears in Ferlay J, Parkin DM, Curado MP, Bray F, Edwards B, Shin HR and Forman D. Cancer Incidence in Five Continents, Volumes I to IX: IARC CancerBase No. 9 [Internet]. Lyon, France: International Agency for Research on Cancer; 2010. Available from: http://ci5.iarc.fr
Finland Cancer Registry 1993-1997 - CI5 as it appears in Ferlay J, Parkin DM, Curado MP, Bray F, Edwards B, Shin HR and Forman D. Cancer Incidence in Five Continents, Volumes I to IX: IARC CancerBase No. 9 [Internet]. Lyon, France: International Agency for Research on Cancer; 2010. Available from: http://ci5.iarc.fr
Finland Cancer Registry 1994 - CI5 as it appears in Ferlay J, Parkin DM, Curado MP, Bray F, Edwards B, Shin HR and Forman D. Cancer Incidence in Five Continents, Volumes I to IX: IARC CancerBase No. 9 [Internet]. Lyon, France: International Agency for Research on Cancer; 2010. Available from: http://ci5.iarc.fr

Appendix: Citation List

Citation
Finland Cancer Registry 1995 - CI5 as it appears in Ferlay J, Parkin DM, Curado MP, Bray F, Edwards B, Shin HR and Forman D. Cancer Incidence in Five Continents, Volumes I to IX: IARC CancerBase No. 9 [Internet]. Lyon, France: International Agency for Research on Cancer; 2010. Available from: http://ci5.iarc.fr
Finland Cancer Registry 1996 - CI5 as it appears in Ferlay J, Parkin DM, Curado MP, Bray F, Edwards B, Shin HR and Forman D. Cancer Incidence in Five Continents, Volumes I to IX: IARC CancerBase No. 9 [Internet]. Lyon, France: International Agency for Research on Cancer; 2010. Available from: http://ci5.iarc.fr
Finland Cancer Registry 1997 - CI5 as it appears in Ferlay J, Parkin DM, Curado MP, Bray F, Edwards B, Shin HR and Forman D. Cancer Incidence in Five Continents, Volumes I to IX: IARC CancerBase No. 9 [Internet]. Lyon, France: International Agency for Research on Cancer; 2010. Available from: http://ci5.iarc.fr
Finland Cancer Registry 1998 - CI5 as it appears in Ferlay J, Parkin DM, Curado MP, Bray F, Edwards B, Shin HR and Forman D. Cancer Incidence in Five Continents, Volumes I to IX: IARC CancerBase No. 9 [Internet]. Lyon, France: International Agency for Research on Cancer; 2010. Available from: http://ci5.iarc.fr
Finland Cancer Registry 1998-2002 - CI5 as it appears in Ferlay J, Parkin DM, Curado MP, Bray F, Edwards B, Shin HR and Forman D. Cancer Incidence in Five Continents, Volumes I to IX: IARC CancerBase No. 9 [Internet]. Lyon, France: International Agency for Research on Cancer; 2010. Available from: http://ci5.iarc.fr
Finland Cancer Registry 1999 - CI5 as it appears in Ferlay J, Parkin DM, Curado MP, Bray F, Edwards B, Shin HR and Forman D. Cancer Incidence in Five Continents, Volumes I to IX: IARC CancerBase No. 9 [Internet]. Lyon, France: International Agency for Research on Cancer; 2010. Available from: http://ci5.iarc.fr
Finland Cancer Registry 2000 - CI5 as it appears in Ferlay J, Parkin DM, Curado MP, Bray F, Edwards B, Shin HR and Forman D. Cancer Incidence in Five Continents, Volumes I to IX: IARC CancerBase No. 9 [Internet]. Lyon, France: International Agency for Research on Cancer; 2010. Available from: http://ci5.iarc.fr
Finland Cancer Registry 2001 - CI5 as it appears in Ferlay J, Parkin DM, Curado MP, Bray F, Edwards B, Shin HR and Forman D. Cancer Incidence in Five Continents, Volumes I to IX: IARC CancerBase No. 9 [Internet]. Lyon, France: International Agency for Research on Cancer; 2010. Available from: http://ci5.iarc.fr
Finland Cancer Registry 2002 - CI5 as it appears in Ferlay J, Parkin DM, Curado MP, Bray F, Edwards B, Shin HR and Forman D. Cancer Incidence in Five Continents, Volumes I to IX: IARC CancerBase No. 9 [Internet]. Lyon, France: International Agency for Research on Cancer; 2010. Available from: http://ci5.iarc.fr
Finland Cancer Registry 2003-2007 - CI5 as it appears in Forman D, Bray F, Brewster DH, Gombe Mbalawa C, Kohler B, Piñeros M, Steliarova-Foucher E, Swaminathan R and Ferlay J, eds (2013). Cancer Incidence in Five Continents, Vol. X Summary Database (electronic version). Lyon, IARC. http://ci5.iarc.fr
Finland Health Behavior and Health Among the Adult Population 1980 as it appears in P.N. Lee Statistics and Computing Ltd. International Mortality and Smoking Statistics Version 4.04. Sutton, United Kingdom: P.N. Lee Statistics and Computing Ltd, 2009.
Finland Health Behavior and Health Among the Adult Population 1981 as it appears in P.N. Lee Statistics and Computing Ltd. International Mortality and Smoking Statistics Version 4.04. Sutton, United Kingdom: P.N. Lee Statistics and Computing Ltd, 2009.
Finland Health Behavior and Health Among the Adult Population 1983 as it appears in P.N. Lee Statistics and Computing Ltd. International Mortality and Smoking Statistics Version 4.04. Sutton, United Kingdom: P.N. Lee Statistics and Computing Ltd, 2009.
Finland Health Behavior and Health Among the Adult Population 1984 as it appears in P.N. Lee Statistics and Computing Ltd. International Mortality and Smoking Statistics Version 4.04. Sutton, United Kingdom: P.N. Lee Statistics and Computing Ltd, 2009.
Finland Health Behavior and Health Among the Adult Population 1985 as it appears in P.N. Lee Statistics and Computing Ltd. International Mortality and Smoking Statistics Version 4.04. Sutton, United Kingdom: P.N. Lee Statistics and Computing Ltd, 2009.
Finland Health Behavior and Health Among the Adult Population 1987 as it appears in P.N. Lee Statistics and Computing Ltd. International Mortality and Smoking Statistics Version 4.04. Sutton, United Kingdom: P.N. Lee Statistics and Computing Ltd, 2009.
Finland Health Behavior and Health Among the Adult Population 1988 as it appears in P.N. Lee Statistics and Computing Ltd. International Mortality and Smoking Statistics Version 4.04. Sutton, United Kingdom: P.N. Lee Statistics and Computing Ltd, 2009.
Finland Health Behavior and Health Among the Adult Population 1989 as it appears in P.N. Lee Statistics and Computing Ltd. International Mortality and Smoking Statistics Version 4.04. Sutton, United Kingdom: P.N. Lee Statistics and Computing Ltd, 2009.
Finland Health Behavior and Health Among the Adult Population 1991 as it appears in P.N. Lee Statistics and Computing Ltd. International Mortality and Smoking Statistics Version 4.04. Sutton, United Kingdom: P.N. Lee Statistics and Computing Ltd, 2009.
Finland Health Behavior and Health Among the Adult Population 1992 as it appears in P.N. Lee Statistics and Computing Ltd. International Mortality and Smoking Statistics Version 4.04. Sutton, United Kingdom: P.N. Lee Statistics and Computing Ltd, 2009.
Finland Health Behavior and Health Among the Adult Population 1994 as it appears in P.N. Lee Statistics and Computing Ltd. International Mortality and Smoking Statistics Version 4.04. Sutton, United Kingdom: P.N. Lee Statistics and Computing Ltd, 2009.
Finland Health Behavior and Health Among the Adult Population 1995 as it appears in P.N. Lee Statistics and Computing Ltd. International Mortality and Smoking Statistics Version 4.04. Sutton, United Kingdom: P.N. Lee Statistics and Computing Ltd, 2009.
Finland Health Behavior and Health Among the Adult Population 1997 as it appears in P.N. Lee Statistics and Computing Ltd. International Mortality and Smoking Statistics Version 4.04. Sutton, United Kingdom: P.N. Lee Statistics and Computing Ltd, 2009.
Finland Health Behavior and Health Among the Finnish Elderly Population 1985 as it appears in P.N. Lee Statistics and Computing Ltd. International Mortality and Smoking Statistics Version 4.04. Sutton, United Kingdom: P.N. Lee Statistics and Computing Ltd, 2009.
Finland Health Behavior and Health Among the Finnish Elderly Population 1987 as it appears in P.N. Lee Statistics and Computing Ltd. International Mortality and Smoking Statistics Version 4.04. Sutton, United Kingdom: P.N. Lee Statistics and Computing Ltd, 2009.
Finland Health Behavior and Health Among the Finnish Elderly Population 1989 as it appears in P.N. Lee Statistics and Computing Ltd. International Mortality and Smoking Statistics Version 4.04. Sutton, United Kingdom: P.N. Lee Statistics and Computing Ltd, 2009.
Finland Health Behavior and Health Among the Finnish Elderly Population 1993 as it appears in P.N. Lee Statistics and Computing Ltd. International Mortality and Smoking Statistics Version 4.04. Sutton, United Kingdom: P.N. Lee Statistics and Computing Ltd, 2009.

Appendix: Citation List

Citation
Finland Health Behavior and Health Among the Finnish Elderly Population 1995 as it appears in P.N. Lee Statistics and Computing Ltd. International Mortality and Smoking Statistics Version 4.04. Sutton, United Kingdom: P.N. Lee Statistics and Computing Ltd, 2009.
Finland Health Behavior and Health Among the Finnish Elderly Population 1997 as it appears in P.N. Lee Statistics and Computing Ltd. International Mortality and Smoking Statistics Version 4.04. Sutton, United Kingdom: P.N. Lee Statistics and Computing Ltd, 2009.
Finland Health Behavior and Health Among the Finnish Elderly Population 1999 as it appears in P.N. Lee Statistics and Computing Ltd. International Mortality and Smoking Statistics Version 4.04. Sutton, United Kingdom: P.N. Lee Statistics and Computing Ltd, 2009.
Finland Vital Registration - Deaths 1952 ICD7 as it appears in World Health Organization (WHO). WHO Mortality Database Version July 2012. Geneva, Switzerland: World Health Organization (WHO), 2012.
Finland Vital Registration - Deaths 1953 ICD7 as it appears in World Health Organization (WHO). WHO Mortality Database Version July 2012. Geneva, Switzerland: World Health Organization (WHO), 2012.
Finland Vital Registration - Deaths 1954 ICD7 as it appears in World Health Organization (WHO). WHO Mortality Database Version July 2012. Geneva, Switzerland: World Health Organization (WHO), 2012.
Finland Vital Registration - Deaths 1955 ICD7 as it appears in World Health Organization (WHO). WHO Mortality Database Version July 2012. Geneva, Switzerland: World Health Organization (WHO), 2012.
Finland Vital Registration - Deaths 1956 ICD7 as it appears in World Health Organization (WHO). WHO Mortality Database Version July 2012. Geneva, Switzerland: World Health Organization (WHO), 2012.
Finland Vital Registration - Deaths 1957 ICD7 as it appears in World Health Organization (WHO). WHO Mortality Database Version July 2012. Geneva, Switzerland: World Health Organization (WHO), 2012.
Finland Vital Registration - Deaths 1958 ICD7 as it appears in World Health Organization (WHO). WHO Mortality Database Version July 2012. Geneva, Switzerland: World Health Organization (WHO), 2012.
Finland Vital Registration - Deaths 1959 ICD7 as it appears in World Health Organization (WHO). WHO Mortality Database Version July 2012. Geneva, Switzerland: World Health Organization (WHO), 2012.
Finland Vital Registration - Deaths 1960 ICD7 as it appears in World Health Organization (WHO). WHO Mortality Database Version July 2012. Geneva, Switzerland: World Health Organization (WHO), 2012.
Finland Vital Registration - Deaths 1961 ICD7 as it appears in World Health Organization (WHO). WHO Mortality Database Version July 2012. Geneva, Switzerland: World Health Organization (WHO), 2012.
Finland Vital Registration - Deaths 1962 ICD7 as it appears in World Health Organization (WHO). WHO Mortality Database Version July 2012. Geneva, Switzerland: World Health Organization (WHO), 2012.
Finland Vital Registration - Deaths 1963 ICD7 as it appears in World Health Organization (WHO). WHO Mortality Database Version July 2012. Geneva, Switzerland: World Health Organization (WHO), 2012.
Finland Vital Registration - Deaths 1964 ICD7 as it appears in World Health Organization (WHO). WHO Mortality Database Version July 2012. Geneva, Switzerland: World Health Organization (WHO), 2012.
Finland Vital Registration - Deaths 1965 ICD7 as it appears in World Health Organization (WHO). WHO Mortality Database Version July 2012. Geneva, Switzerland: World Health Organization (WHO), 2012.
Finland Vital Registration - Deaths 1966 ICD7 as it appears in World Health Organization (WHO). WHO Mortality Database Version July 2012. Geneva, Switzerland: World Health Organization (WHO), 2012.
Finland Vital Registration - Deaths 1967 ICD7 as it appears in World Health Organization (WHO). WHO Mortality Database Version July 2012. Geneva, Switzerland: World Health Organization (WHO), 2012.
Finland Vital Registration - Deaths 1968 ICD7 as it appears in World Health Organization (WHO). WHO Mortality Database Version July 2012. Geneva, Switzerland: World Health Organization (WHO), 2012.
Finland Vital Registration - Deaths 1969 ICD8 as it appears in World Health Organization (WHO). WHO Mortality Database Version May 2013. Geneva, Switzerland: World Health Organization (WHO), 2013.
Finland Vital Registration - Deaths 1970 ICD8 as it appears in World Health Organization (WHO). WHO Mortality Database Version May 2013. Geneva, Switzerland: World Health Organization (WHO), 2013.
Finland Vital Registration - Deaths 1971 ICD8 as it appears in World Health Organization (WHO). WHO Mortality Database Version May 2013. Geneva, Switzerland: World Health Organization (WHO), 2013.
Finland Vital Registration - Deaths 1972 ICD8 as it appears in World Health Organization (WHO). WHO Mortality Database Version May 2013. Geneva, Switzerland: World Health Organization (WHO), 2013.
Finland Vital Registration - Deaths 1973 ICD8 as it appears in World Health Organization (WHO). WHO Mortality Database Version May 2013. Geneva, Switzerland: World Health Organization (WHO), 2013.
Finland Vital Registration - Deaths 1974 ICD8 as it appears in World Health Organization (WHO). WHO Mortality Database Version May 2013. Geneva, Switzerland: World Health Organization (WHO), 2013.
Finland Vital Registration - Deaths 1975 ICD8 as it appears in World Health Organization (WHO). WHO Mortality Database Version May 2013. Geneva, Switzerland: World Health Organization (WHO), 2013.
Finland Vital Registration - Deaths 1976 ICD8 as it appears in World Health Organization (WHO). WHO Mortality Database Version May 2013. Geneva, Switzerland: World Health Organization (WHO), 2013.
Finland Vital Registration - Deaths 1977 ICD8 as it appears in World Health Organization (WHO). WHO Mortality Database Version May 2013. Geneva, Switzerland: World Health Organization (WHO), 2013.
Finland Vital Registration - Deaths 1978 ICD8 as it appears in World Health Organization (WHO). WHO Mortality Database Version May 2013. Geneva, Switzerland: World Health Organization (WHO), 2013.
Finland Vital Registration - Deaths 1979 ICD8 as it appears in World Health Organization (WHO). WHO Mortality Database Version May 2013. Geneva, Switzerland: World Health Organization (WHO), 2013.
Finland Vital Registration - Deaths 1980 ICD8 as it appears in World Health Organization (WHO). WHO Mortality Database Version May 2013. Geneva, Switzerland: World Health Organization (WHO), 2013.

Appendix: Citation List

Citation
Finland Vital Registration - Deaths 1981 ICD8 as it appears in World Health Organization (WHO). WHO Mortality Database Version May 2013. Geneva, Switzerland: World Health Organization (WHO), 2013.
Finland Vital Registration - Deaths 1982 ICD8 as it appears in World Health Organization (WHO). WHO Mortality Database Version May 2013. Geneva, Switzerland: World Health Organization (WHO), 2013.
Finland Vital Registration - Deaths 1983 ICD8 as it appears in World Health Organization (WHO). WHO Mortality Database Version May 2013. Geneva, Switzerland: World Health Organization (WHO), 2013.
Finland Vital Registration - Deaths 1984 ICD8 as it appears in World Health Organization (WHO). WHO Mortality Database Version May 2013. Geneva, Switzerland: World Health Organization (WHO), 2013.
Finland Vital Registration - Deaths 1985 ICD8 as it appears in World Health Organization (WHO). WHO Mortality Database Version May 2013. Geneva, Switzerland: World Health Organization (WHO), 2013.
Finland Vital Registration - Deaths 1986 ICD8 as it appears in World Health Organization (WHO). WHO Mortality Database Version May 2013. Geneva, Switzerland: World Health Organization (WHO), 2013.
Finland Vital Registration - Deaths 1987 ICD9 as it appears in World Health Organization (WHO). WHO Mortality Database Version November 2015. Geneva, Switzerland: World Health Organization (WHO), 2015.
Finland Vital Registration - Deaths 1988 ICD9 as it appears in World Health Organization (WHO). WHO Mortality Database Version November 2015. Geneva, Switzerland: World Health Organization (WHO), 2015.
Finland Vital Registration - Deaths 1989 ICD9 as it appears in World Health Organization (WHO). WHO Mortality Database Version November 2015. Geneva, Switzerland: World Health Organization (WHO), 2015.
Finland Vital Registration - Deaths 1990 ICD9 as it appears in World Health Organization (WHO). WHO Mortality Database Version November 2015. Geneva, Switzerland: World Health Organization (WHO), 2015.
Finland Vital Registration - Deaths 1991 ICD9 as it appears in World Health Organization (WHO). WHO Mortality Database Version November 2015. Geneva, Switzerland: World Health Organization (WHO), 2015.
Finland Vital Registration - Deaths 1992 ICD9 as it appears in World Health Organization (WHO). WHO Mortality Database Version November 2015. Geneva, Switzerland: World Health Organization (WHO), 2015.
Finland Vital Registration - Deaths 1993 ICD9 as it appears in World Health Organization (WHO). WHO Mortality Database Version November 2015. Geneva, Switzerland: World Health Organization (WHO), 2015.
Finland Vital Registration - Deaths 1994 ICD9 as it appears in World Health Organization (WHO). WHO Mortality Database Version November 2015. Geneva, Switzerland: World Health Organization (WHO), 2015.
Finland Vital Registration - Deaths 1995 ICD9 as it appears in World Health Organization (WHO). WHO Mortality Database Version November 2015. Geneva, Switzerland: World Health Organization (WHO), 2015.
Finland Vital Registration - Deaths 1996 ICD10 as it appears in World Health Organization (WHO). WHO Mortality Database Version November 2015. Geneva, Switzerland: World Health Organization (WHO), 2015.
Finland Vital Registration - Deaths 1997 ICD10 as it appears in World Health Organization (WHO). WHO Mortality Database Version November 2015. Geneva, Switzerland: World Health Organization (WHO), 2015.
Finland Vital Registration - Deaths 1998 ICD10 as it appears in World Health Organization (WHO). WHO Mortality Database Version November 2015. Geneva, Switzerland: World Health Organization (WHO), 2015.
Finland Vital Registration - Deaths 1999 ICD10 as it appears in World Health Organization (WHO). WHO Mortality Database Version November 2015. Geneva, Switzerland: World Health Organization (WHO), 2015.
Finland Vital Registration - Deaths 2000 ICD10 as it appears in World Health Organization (WHO). WHO Mortality Database Version November 2015. Geneva, Switzerland: World Health Organization (WHO), 2015.
Finland Vital Registration - Deaths 2001 ICD10 as it appears in World Health Organization (WHO). WHO Mortality Database Version November 2015. Geneva, Switzerland: World Health Organization (WHO), 2015.
Finland Vital Registration - Deaths 2002 ICD10 as it appears in World Health Organization (WHO). WHO Mortality Database Version November 2015. Geneva, Switzerland: World Health Organization (WHO), 2015.
Finland Vital Registration - Deaths 2003 ICD10 as it appears in World Health Organization (WHO). WHO Mortality Database Version November 2015. Geneva, Switzerland: World Health Organization (WHO), 2015.
Finland Vital Registration - Deaths 2004 ICD10 as it appears in World Health Organization (WHO). WHO Mortality Database Version November 2015. Geneva, Switzerland: World Health Organization (WHO), 2015.
Finland Vital Registration - Deaths 2005 ICD10 as it appears in World Health Organization (WHO). WHO Mortality Database Version November 2015. Geneva, Switzerland: World Health Organization (WHO), 2015.
Finland Vital Registration - Deaths 2006 ICD10 as it appears in World Health Organization (WHO). WHO Mortality Database Version November 2015. Geneva, Switzerland: World Health Organization (WHO), 2015.
Finland Vital Registration - Deaths 2007 ICD10 as it appears in World Health Organization (WHO). WHO Mortality Database Version November 2015. Geneva, Switzerland: World Health Organization (WHO), 2015.
Finland Vital Registration - Deaths 2008 ICD10 as it appears in World Health Organization (WHO). WHO Mortality Database Version November 2015. Geneva, Switzerland: World Health Organization (WHO), 2015.
Finland Vital Registration - Deaths 2009 ICD10 as it appears in World Health Organization (WHO). WHO Mortality Database Version November 2015. Geneva, Switzerland: World Health Organization (WHO), 2015.
Finland Vital Registration - Deaths 2010 ICD10 as it appears in World Health Organization (WHO). WHO Mortality Database Version November 2015. Geneva, Switzerland: World Health Organization (WHO), 2015.
Finland Vital Registration - Deaths 2011 ICD10 as it appears in World Health Organization (WHO). WHO Mortality Database Version November 2015. Geneva, Switzerland: World Health Organization (WHO), 2015.
Finland Vital Registration - Deaths 2012 ICD10 as it appears in World Health Organization (WHO). WHO Mortality Database Version November 2015. Geneva, Switzerland: World Health Organization (WHO), 2015.

Appendix: Citation List

Citation
Finland Vital Registration - Deaths 2013 ICD10 as it appears in World Health Organization (WHO). WHO Mortality Database Version November 2015. Geneva, Switzerland: World Health Organization (WHO), 2015.
Firmann M, Marques-Vidal P, Paccaud F, Mooser V, Rodondi N, Waeber G, Vollenweider P. Prevalence, treatment and control of dyslipidaemia in Switzerland: still a long way to go. Eur J Cardiovasc Prev Rehabil. 2010; 17(6): 682–7.
Firmo JO, Lima Costa MF, Guerra HL, Rocha RS. Urban schistosomiasis: morbidity, sociodemographic characteristics and water contact patterns predictive of infection. Int J Epidemiol. 1996; 25(6): 1292-300.
First RHOSA Nutrition Survey: Anthropometric Assessment of Nutritional Status in Black Under-fives in Rural South Africa as it appears in World Health Organization (WHO). WHO Global Database on Child Growth and Malnutrition - Historical. Geneva, Switzerland: World Health Organization (WHO).
Fischer P, Djuardi Y, Ismid IS, Rückert P, Bradley M, Supali T. Long-lasting reduction of Brugia timori microfilariae following a single dose of diethylcarbamazine combined with albendazole. Trans R Soc Trop Med Hyg. 2003; 97(4): 446-8. As it appears in London School of Hygiene and Tropical Medicine. Global Atlas of Helminth Infections - Lymphatic Filiariasis.
Fischer P, Supali T, Wibowo H, Bonow I, Williams SA. Detection of DNA of nocturnally periodic Brugia malayi in night and day blood samples by a polymerase chain reaction-ELISA-based method using an internal control DNA. Am J Trop Med Hyg. 2000; 62(2): 291-6. As it appears in London School of Hygiene and Tropical Medicine. Global Atlas of Helminth Infections - Lymphatic Filiariasis.
Flicker L, McCaul KA, Hankey GJ, Jamrozik K, Brown WJ, Byles JE, Almeida OP. Body mass index and survival in men and women aged 70 to 75. J Am Geriatr Soc. 2010; 58(2): 234-41.
Flohr C. The links between gut worms, malaria, and atopic dermatitis: a study in rural Vietnam. J Invest Dermatol. 2005; 125: 594. As it appears in Malaria Atlas Project. Malaria Atlas Project Plasmodium Falciparum Parasite Rate Database. Oxford, United Kingdom: Malaria Atlas Project.
Florey LS. Evidence of Plasmodium Species Interactions in an Endemic Population in Coastal Kenya. New Orleans, United States of America: 2008. As it appears in Malaria Atlas Project. Malaria Atlas Project Plasmodium Falciparum Parasite Rate Database. Oxford, United Kingdom: Malaria Atlas Project.
Fondjo E. Parasitological Study of Malaria Transmission in the Nkolbison and Nkolbikok Neighborhoods. Yaoundé, Cameroon: Organization for Coordination in the Fight Against Endemic Diseases in Central Africa (OCEAC), 1991. As it appears in Malaria Atlas Project. Malaria Atlas Project Plasmodium Falciparum Parasite Rate Database. Oxford, United Kingdom: Malaria Atlas Project.
Fondjo E. Study of the Behavior of the An Gambiae Complex and the Transmission of Malaria in Two Ecological Aspects of Mali and Cameroon [dissertation]. Bamako, Mali: Higher Institute of Training and Applied Research, University of Bamako, 1996. As it appears in Malaria Atlas Project. Malaria Atlas Project Plasmodium Falciparum Parasite Rate Database. Oxford, United Kingdom: Malaria Atlas Project.
Fonseca Eda S, D'Andrea LA, Taniguchi HH, Hiramoto RM, Tolezano JE, Guimarães RB. Spatial epidemiology of American cutaneous leishmaniasis in a municipality of west São Paulo State, Brazil. J Vector Borne Dis. 2014; 51(4): 271-5.
Fontes CJF. Parasitological Survey of the Population of Guariba-Colniza for Identification of Individuals with Asymptomatic Infection by Plasmodium, Colniza, Mato Grosso. Cuiabá, Brazil: State Program for Malaria Control, Coordination of Epidemiological Surveillance, Ministry of Health, Mato Grosso, 2005. As it appears in Malaria Atlas Project. Malaria Atlas Project Plasmodium Falciparum Parasite Rate Database. Oxford, United Kingdom: Malaria Atlas Project.
Fontes G, Braun RF, Fraiha Neto H, Vieira JBF, Padilha SS, Rocha RC, da Rocha EMM. [Lymphatic filariasis in Belém, Pará State, North of Brazil and the perspective of elimination]. Rev Soc Bras Med Trop. 2005; 38(2): 131-6. As it appears in London School of Hygiene and Tropical Medicine. Global Atlas of Helminth Infections - Lymphatic Filiariasis.
Food and Agriculture Organization of the United Nations (FAO), United Nations Children's Fund (UNICEF). Somalia Plasmodium Falciparum Parasite Rate Data, Personal Communication with FAO Somalia and UNICEF Somalia 2009. As it appears in Malaria Atlas Project. Malaria Atlas Project Plasmodium Falciparum Parasite Rate Database. Oxford, United Kingdom: Malaria Atlas Project.
Food and Agriculture Organization of the United Nations (FAO), World Health Organization (WHO). Joint WHO/FAO Workshop on Foodborne Trematode Infections in Asia. Manila, Philippines: World Health Organization Regional Office for the Western Pacific (WPRO-WHO), 2004.
Food and Agriculture Organization of the United Nations (FAO). FAOSTAT Food Balance Sheets, October 2014. Rome, Italy: Food and Agriculture Organization of the United Nations (FAO).
Food and Nutrition Research Institute, Department of Science and Technology (Philippines). Philippines National Nutrition Survey 1998.
Food and Nutrition Research Institute, Department of Science and Technology (Philippines). Philippines National Nutrition Survey 2003.
Food and Nutrition Research Institute, Department of Science and Technology (Philippines). Philippines National Nutrition Survey 2013-2014.
Food and Nutrition Services, Ministry of Health (Israel), Israel Center for Disease Control (ICDC). Israel National Health and Nutrition Survey 1999-2001.
Forastiere F, Pupp N, Magliola E, Valesini S, Tidei F, Perucci CA. Respiratory Cancer Mortality Among Workers Employed In Thermoelectric Power Plants. Scand J Work Environ Health. 1989; 15(6): 383-6 as it appears in Armstrong B, Hutchinson E, Unwin J, Fletcher T. Lung cancer risk after exposure to polycyclic aromatic hydrocarbons: a review and meta-analysis. Environ. Health Perspect. 2004; 112(9): 970–978.
Forga Llenas L, Goni Iriarte MJ, Cambra Contin K, Ibanez Beroiz B, Chueca Guendulain M, Berrade Zubiri S. Incidence and temporal trends of childhood type 1 diabetes between 1975 and 2012 in Navarre (Spain). Gac Sanit. 2015; 29(1): 51-4.
Forouhi NG, Luan J, Hennings S, Wareham NJ. Incidence of Type 2 diabetes in England and its association with baseline impaired fasting glucose: the Ely study 1990-2000. Diabet Med. 2007; 24(2): 200-7.
Forrester T, Wilks R, Bennett F, McFarlane-Anderson N, McGee D, Cooper R, Fraser H. Obesity in the Caribbean. Ciba Found Symp. 1996; 17-36.
Forsyth D, Bradley D. Irreversible damage by schistosoma haematobium in schoolchildren. Lancet. 1964; 2(7352): 169-7.

Appendix: Citation List

Citation
Forsyth DM, Bradley DJ. The consequences of Bilharziasis. Medical and public health importance in North-west Tanzania. Bull World Health Organ. 1966; 34(5): 715-35.
Forsyth DM. Anaemia in Zanzibar. Trans R Soc Trop Med Hyg. 1970; 64(4): 601-6.
Fortney JA, Susanti I, Gadalla S, Saleh S, Rogers SM, Potts M. Reproductive Mortality in Two Developing Countries. Am J Public Health. 1986; 76(2): 134–8.
Foster C, Rotimi C, Fraser H, Sundarum C, Liao Y, Gibson E, Holder Y, Hoyos M, Mellanson-King R. Hypertension, diabetes, and obesity in Barbados: findings from a recent population-based survey. Ethn Dis. 1993; 3(4): 404-12.
Foyaca-Sibat H, Cowan LD, Carabin H, Targonska I, Anwary MA, Serrano-Oca-a G, Krecek RC, Willingham AL 3rd. Accuracy of serological testing for the diagnosis of prevalent neurocysticercosis in outpatients with epilepsy, Eastern Cape Province, South Africa. PLoS Negl Trop Dis. 2009; 3(12): e562.
France - Bas Rhin Cancer Registry 1980 - CI5 as it appears in Ferlay J, Parkin DM, Curado MP, Bray F, Edwards B, Shin HR and Forman D. Cancer Incidence in Five Continents, Volumes I to IX: IARC CancerBase No. 9 [Internet]. Lyon, France: International Agency for Research on Cancer; 2010. Available from: http://ci5.iarc.fr
France - Bas Rhin Cancer Registry 1981 - CI5 as it appears in Ferlay J, Parkin DM, Curado MP, Bray F, Edwards B, Shin HR and Forman D. Cancer Incidence in Five Continents, Volumes I to IX: IARC CancerBase No. 9 [Internet]. Lyon, France: International Agency for Research on Cancer; 2010. Available from: http://ci5.iarc.fr
France - Bas Rhin Cancer Registry 1982 - CI5 as it appears in Ferlay J, Parkin DM, Curado MP, Bray F, Edwards B, Shin HR and Forman D. Cancer Incidence in Five Continents, Volumes I to IX: IARC CancerBase No. 9 [Internet]. Lyon, France: International Agency for Research on Cancer; 2010. Available from: http://ci5.iarc.fr
France - Bas Rhin Cancer Registry 1983 - CI5 as it appears in Ferlay J, Parkin DM, Curado MP, Bray F, Edwards B, Shin HR and Forman D. Cancer Incidence in Five Continents, Volumes I to IX: IARC CancerBase No. 9 [Internet]. Lyon, France: International Agency for Research on Cancer; 2010. Available from: http://ci5.iarc.fr
France - Bas Rhin Cancer Registry 1984 - CI5 as it appears in Ferlay J, Parkin DM, Curado MP, Bray F, Edwards B, Shin HR and Forman D. Cancer Incidence in Five Continents, Volumes I to IX: IARC CancerBase No. 9 [Internet]. Lyon, France: International Agency for Research on Cancer; 2010. Available from: http://ci5.iarc.fr
France - Bas Rhin Cancer Registry 1986 - CI5 as it appears in Ferlay J, Parkin DM, Curado MP, Bray F, Edwards B, Shin HR and Forman D. Cancer Incidence in Five Continents, Volumes I to IX: IARC CancerBase No. 9 [Internet]. Lyon, France: International Agency for Research on Cancer; 2010. Available from: http://ci5.iarc.fr
France - Bas Rhin Cancer Registry 1987 - CI5 as it appears in Ferlay J, Parkin DM, Curado MP, Bray F, Edwards B, Shin HR and Forman D. Cancer Incidence in Five Continents, Volumes I to IX: IARC CancerBase No. 9 [Internet]. Lyon, France: International Agency for Research on Cancer; 2010. Available from: http://ci5.iarc.fr
France - Bas Rhin Cancer Registry 1988 - CI5 as it appears in Ferlay J, Parkin DM, Curado MP, Bray F, Edwards B, Shin HR and Forman D. Cancer Incidence in Five Continents, Volumes I to IX: IARC CancerBase No. 9 [Internet]. Lyon, France: International Agency for Research on Cancer; 2010. Available from: http://ci5.iarc.fr
France - Bas Rhin Cancer Registry 1989 - CI5 as it appears in Ferlay J, Parkin DM, Curado MP, Bray F, Edwards B, Shin HR and Forman D. Cancer Incidence in Five Continents, Volumes I to IX: IARC CancerBase No. 9 [Internet]. Lyon, France: International Agency for Research on Cancer; 2010. Available from: http://ci5.iarc.fr
France - Bas Rhin Cancer Registry 1991 - CI5 as it appears in Ferlay J, Parkin DM, Curado MP, Bray F, Edwards B, Shin HR and Forman D. Cancer Incidence in Five Continents, Volumes I to IX: IARC CancerBase No. 9 [Internet]. Lyon, France: International Agency for Research on Cancer; 2010. Available from: http://ci5.iarc.fr
France - Bas Rhin Cancer Registry 1992 - CI5 as it appears in Ferlay J, Parkin DM, Curado MP, Bray F, Edwards B, Shin HR and Forman D. Cancer Incidence in Five Continents, Volumes I to IX: IARC CancerBase No. 9 [Internet]. Lyon, France: International Agency for Research on Cancer; 2010. Available from: http://ci5.iarc.fr
France - Bas Rhin Cancer Registry 1993 - CI5 as it appears in Ferlay J, Parkin DM, Curado MP, Bray F, Edwards B, Shin HR and Forman D. Cancer Incidence in Five Continents, Volumes I to IX: IARC CancerBase No. 9 [Internet]. Lyon, France: International Agency for Research on Cancer; 2010. Available from: http://ci5.iarc.fr
France - Bas Rhin Cancer Registry 1994-1997 - CI5 as it appears in Ferlay J, Parkin DM, Curado MP, Bray F, Edwards B, Shin HR and Forman D. Cancer Incidence in Five Continents, Volumes I to IX: IARC CancerBase No. 9 [Internet]. Lyon, France: International Agency for Research on Cancer; 2010. Available from: http://ci5.iarc.fr
France - Bas Rhin Cancer Registry 1996 - CI5 as it appears in Ferlay J, Parkin DM, Curado MP, Bray F, Edwards B, Shin HR and Forman D. Cancer Incidence in Five Continents, Volumes I to IX: IARC CancerBase No. 9 [Internet]. Lyon, France: International Agency for Research on Cancer; 2010. Available from: http://ci5.iarc.fr
France - Bas Rhin Cancer Registry 1997 - CI5 as it appears in Ferlay J, Parkin DM, Curado MP, Bray F, Edwards B, Shin HR and Forman D. Cancer Incidence in Five Continents, Volumes I to IX: IARC CancerBase No. 9 [Internet]. Lyon, France: International Agency for Research on Cancer; 2010. Available from: http://ci5.iarc.fr
France - Bas Rhin Cancer Registry 1998 - CI5 as it appears in Ferlay J, Parkin DM, Curado MP, Bray F, Edwards B, Shin HR and Forman D. Cancer Incidence in Five Continents, Volumes I to IX: IARC CancerBase No. 9 [Internet]. Lyon, France: International Agency for Research on Cancer; 2010. Available from: http://ci5.iarc.fr
France - Bas Rhin Cancer Registry 1999 - CI5 as it appears in Ferlay J, Parkin DM, Curado MP, Bray F, Edwards B, Shin HR and Forman D. Cancer Incidence in Five Continents, Volumes I to IX: IARC CancerBase No. 9 [Internet]. Lyon, France: International Agency for Research on Cancer; 2010. Available from: http://ci5.iarc.fr
France - Bas Rhin Cancer Registry 2001 - CI5 as it appears in Ferlay J, Parkin DM, Curado MP, Bray F, Edwards B, Shin HR and Forman D. Cancer Incidence in Five Continents, Volumes I to IX: IARC CancerBase No. 9 [Internet]. Lyon, France: International Agency for Research on Cancer; 2010. Available from: http://ci5.iarc.fr

Appendix: Citation List

Citation
France - Bas Rhin Cancer Registry 2002 - CI5 as it appears in Ferlay J, Parkin DM, Curado MP, Bray F, Edwards B, Shin HR and Forman D. Cancer Incidence in Five Continents, Volumes I to IX: IARC CancerBase No. 9 [Internet]. Lyon, France: International Agency for Research on Cancer; 2010. Available from: http://ci5.iarc.fr
France - Bas Rhin Cancer Registry 2003-2007 - CI5 as it appears in Forman D, Bray F, Brewster DH, Gombe Mbalawa C, Kohler B, Piñeros M, Steliarova-Foucher E, Swaminathan R and Ferlay J, eds (2013). Cancer Incidence in Five Continents, Vol. X Summary Database (electronic version). Lyon, IARC. http://ci5.iarc.fr
France - Bas-Rhin Cancer Registry 1993-1997 - CI5 as it appears in Parkin DM, International Agency for Research on Cancer, International Association of Cancer Registries. Cancer Incidence in Five Continents. Vol. I to VIII. Lyon, France, IARCPress, 2005.
France - Bas-Rhin Cancer Registry 1998-2002 - CI5 as it appears in Curado MP, Edwards B, Shin HR, Storm H, Ferlay J, Heanue M and Boyle P, eds (2007). Cancer Incidence in Five Continents, Vol. IX Periodic Data (electronic version). Lyon, IARC.http://ci5.iarc.fr
France - Calvados Cancer Registry 1980 - CI5 as it appears in Ferlay J, Parkin DM, Curado MP, Bray F, Edwards B, Shin HR and Forman D. Cancer Incidence in Five Continents, Volumes I to IX: IARC CancerBase No. 9 [Internet]. Lyon, France: International Agency for Research on Cancer; 2010. Available from: http://ci5.iarc.fr
France - Calvados Cancer Registry 1981 - CI5 as it appears in Ferlay J, Parkin DM, Curado MP, Bray F, Edwards B, Shin HR and Forman D. Cancer Incidence in Five Continents, Volumes I to IX: IARC CancerBase No. 9 [Internet]. Lyon, France: International Agency for Research on Cancer; 2010. Available from: http://ci5.iarc.fr
France - Calvados Cancer Registry 1982 - CI5 as it appears in Ferlay J, Parkin DM, Curado MP, Bray F, Edwards B, Shin HR and Forman D. Cancer Incidence in Five Continents, Volumes I to IX: IARC CancerBase No. 9 [Internet]. Lyon, France: International Agency for Research on Cancer; 2010. Available from: http://ci5.iarc.fr
France - Calvados Cancer Registry 1983 - CI5 as it appears in Ferlay J, Parkin DM, Curado MP, Bray F, Edwards B, Shin HR and Forman D. Cancer Incidence in Five Continents, Volumes I to IX: IARC CancerBase No. 9 [Internet]. Lyon, France: International Agency for Research on Cancer; 2010. Available from: http://ci5.iarc.fr
France - Calvados Cancer Registry 1983-1987 - CI5 as it appears in Parkin DM, International Agency for Research on Cancer, International Association of Cancer Registries. Cancer Incidence in Five Continents. Vol. I to VIII. Lyon, France, IARCPress, 2005.
France - Calvados Cancer Registry 1984 - CI5 as it appears in Ferlay J, Parkin DM, Curado MP, Bray F, Edwards B, Shin HR and Forman D. Cancer Incidence in Five Continents, Volumes I to IX: IARC CancerBase No. 9 [Internet]. Lyon, France: International Agency for Research on Cancer; 2010. Available from: http://ci5.iarc.fr
France - Calvados Cancer Registry 1985 - CI5 as it appears in Ferlay J, Parkin DM, Curado MP, Bray F, Edwards B, Shin HR and Forman D. Cancer Incidence in Five Continents, Volumes I to IX: IARC CancerBase No. 9 [Internet]. Lyon, France: International Agency for Research on Cancer; 2010. Available from: http://ci5.iarc.fr
France - Calvados Cancer Registry 1986 - CI5 as it appears in Ferlay J, Parkin DM, Curado MP, Bray F, Edwards B, Shin HR and Forman D. Cancer Incidence in Five Continents, Volumes I to IX: IARC CancerBase No. 9 [Internet]. Lyon, France: International Agency for Research on Cancer; 2010. Available from: http://ci5.iarc.fr
France - Calvados Cancer Registry 1987 - CI5 as it appears in Ferlay J, Parkin DM, Curado MP, Bray F, Edwards B, Shin HR and Forman D. Cancer Incidence in Five Continents, Volumes I to IX: IARC CancerBase No. 9 [Internet]. Lyon, France: International Agency for Research on Cancer; 2010. Available from: http://ci5.iarc.fr
France - Calvados Cancer Registry 1988 - CI5 as it appears in Ferlay J, Parkin DM, Curado MP, Bray F, Edwards B, Shin HR and Forman D. Cancer Incidence in Five Continents, Volumes I to IX: IARC CancerBase No. 9 [Internet]. Lyon, France: International Agency for Research on Cancer; 2010. Available from: http://ci5.iarc.fr
France - Calvados Cancer Registry 1988-1992 - CI5 as it appears in Parkin DM, International Agency for Research on Cancer, International Association of Cancer Registries. Cancer Incidence in Five Continents. Vol. I to VIII. Lyon, France, IARCPress, 2005.
France - Calvados Cancer Registry 1989 - CI5 as it appears in Ferlay J, Parkin DM, Curado MP, Bray F, Edwards B, Shin HR and Forman D. Cancer Incidence in Five Continents, Volumes I to IX: IARC CancerBase No. 9 [Internet]. Lyon, France: International Agency for Research on Cancer; 2010. Available from: http://ci5.iarc.fr
France - Calvados Cancer Registry 1990 - CI5 as it appears in Ferlay J, Parkin DM, Curado MP, Bray F, Edwards B, Shin HR and Forman D. Cancer Incidence in Five Continents, Volumes I to IX: IARC CancerBase No. 9 [Internet]. Lyon, France: International Agency for Research on Cancer; 2010. Available from: http://ci5.iarc.fr
France - Calvados Cancer Registry 1991 - CI5 as it appears in Ferlay J, Parkin DM, Curado MP, Bray F, Edwards B, Shin HR and Forman D. Cancer Incidence in Five Continents, Volumes I to IX: IARC CancerBase No. 9 [Internet]. Lyon, France: International Agency for Research on Cancer; 2010. Available from: http://ci5.iarc.fr
France - Calvados Cancer Registry 1992 - CI5 as it appears in Ferlay J, Parkin DM, Curado MP, Bray F, Edwards B, Shin HR and Forman D. Cancer Incidence in Five Continents, Volumes I to IX: IARC CancerBase No. 9 [Internet]. Lyon, France: International Agency for Research on Cancer; 2010. Available from: http://ci5.iarc.fr
France - Calvados Cancer Registry 1993 - CI5 as it appears in Ferlay J, Parkin DM, Curado MP, Bray F, Edwards B, Shin HR and Forman D. Cancer Incidence in Five Continents, Volumes I to IX: IARC CancerBase No. 9 [Internet]. Lyon, France: International Agency for Research on Cancer; 2010. Available from: http://ci5.iarc.fr
France - Calvados Cancer Registry 1993-1997 - CI5 as it appears in Parkin DM, International Agency for Research on Cancer, International Association of Cancer Registries. Cancer Incidence in Five Continents. Vol. I to VIII. Lyon, France, IARCPress, 2005.
France - Calvados Cancer Registry 1994 - CI5 as it appears in Ferlay J, Parkin DM, Curado MP, Bray F, Edwards B, Shin HR and Forman D. Cancer Incidence in Five Continents, Volumes I to IX: IARC CancerBase No. 9 [Internet]. Lyon, France: International Agency for Research on Cancer; 2010. Available from: http://ci5.iarc.fr
France - Calvados Cancer Registry 1996 - CI5 as it appears in Ferlay J, Parkin DM, Curado MP, Bray F, Edwards B, Shin HR and Forman D. Cancer Incidence in Five Continents, Volumes I to IX: IARC CancerBase No. 9 [Internet]. Lyon, France: International Agency for Research on Cancer; 2010. Available from: http://ci5.iarc.fr

Appendix: Citation List

Citation
France - Calvados Cancer Registry 1997 - CI5 as it appears in Ferlay J, Parkin DM, Curado MP, Bray F, Edwards B, Shin HR and Forman D. Cancer Incidence in Five Continents, Volumes I to IX: IARC CancerBase No. 9 [Internet]. Lyon, France: International Agency for Research on Cancer; 2010. Available from: http://ci5.iarc.fr
France - Calvados Cancer Registry 1998 - CI5 as it appears in Ferlay J, Parkin DM, Curado MP, Bray F, Edwards B, Shin HR and Forman D. Cancer Incidence in Five Continents, Volumes I to IX: IARC CancerBase No. 9 [Internet]. Lyon, France: International Agency for Research on Cancer; 2010. Available from: http://ci5.iarc.fr
France - Calvados Cancer Registry 1998-2002 - CI5 as it appears in Curado MP, Edwards B, Shin HR, Storm H, Ferlay J, Heanue M and Boyle P, eds (2007). Cancer Incidence in Five Continents, Vol. IX Periodic Data (electronic version). Lyon, IARC.http://ci5.iarc.fr
France - Calvados Cancer Registry 1999 - CI5 as it appears in Ferlay J, Parkin DM, Curado MP, Bray F, Edwards B, Shin HR and Forman D. Cancer Incidence in Five Continents, Volumes I to IX: IARC CancerBase No. 9 [Internet]. Lyon, France: International Agency for Research on Cancer; 2010. Available from: http://ci5.iarc.fr
France - Calvados Cancer Registry 2001 - CI5 as it appears in Ferlay J, Parkin DM, Curado MP, Bray F, Edwards B, Shin HR and Forman D. Cancer Incidence in Five Continents, Volumes I to IX: IARC CancerBase No. 9 [Internet]. Lyon, France: International Agency for Research on Cancer; 2010. Available from: http://ci5.iarc.fr
France - Calvados Cancer Registry 2002 - CI5 as it appears in Ferlay J, Parkin DM, Curado MP, Bray F, Edwards B, Shin HR and Forman D. Cancer Incidence in Five Continents, Volumes I to IX: IARC CancerBase No. 9 [Internet]. Lyon, France: International Agency for Research on Cancer; 2010. Available from: http://ci5.iarc.fr
France - Calvados Cancer Registry 2003-2007 - CI5 as it appears in Forman D, Bray F, Brewster DH, Gombe Mbalawa C, Kohler B, Piñeros M, Steliarova-Foucher E, Swaminathan R and Ferlay J, eds (2013). Cancer Incidence in Five Continents, Vol. X Summary Database (electronic version). Lyon, IARC. http://ci5.iarc.fr
France - Doubs Cancer Registry 1980 - CI5 as it appears in Ferlay J, Parkin DM, Curado MP, Bray F, Edwards B, Shin HR and Forman D. Cancer Incidence in Five Continents, Volumes I to IX: IARC CancerBase No. 9 [Internet]. Lyon, France: International Agency for Research on Cancer; 2010. Available from: http://ci5.iarc.fr
France - Doubs Cancer Registry 1981 - CI5 as it appears in Ferlay J, Parkin DM, Curado MP, Bray F, Edwards B, Shin HR and Forman D. Cancer Incidence in Five Continents, Volumes I to IX: IARC CancerBase No. 9 [Internet]. Lyon, France: International Agency for Research on Cancer; 2010. Available from: http://ci5.iarc.fr
France - Doubs Cancer Registry 1982 - CI5 as it appears in Ferlay J, Parkin DM, Curado MP, Bray F, Edwards B, Shin HR and Forman D. Cancer Incidence in Five Continents, Volumes I to IX: IARC CancerBase No. 9 [Internet]. Lyon, France: International Agency for Research on Cancer; 2010. Available from: http://ci5.iarc.fr
France - Doubs Cancer Registry 1983 - CI5 as it appears in Ferlay J, Parkin DM, Curado MP, Bray F, Edwards B, Shin HR and Forman D. Cancer Incidence in Five Continents, Volumes I to IX: IARC CancerBase No. 9 [Internet]. Lyon, France: International Agency for Research on Cancer; 2010. Available from: http://ci5.iarc.fr
France - Doubs Cancer Registry 1983-1987 - CI5 as it appears in Parkin DM, International Agency for Research on Cancer, International Association of Cancer Registries. Cancer Incidence in Five Continents. Vol. I to VIII. Lyon, France, IARCPress, 2005.
France - Doubs Cancer Registry 1984 - CI5 as it appears in Ferlay J, Parkin DM, Curado MP, Bray F, Edwards B, Shin HR and Forman D. Cancer Incidence in Five Continents, Volumes I to IX: IARC CancerBase No. 9 [Internet]. Lyon, France: International Agency for Research on Cancer; 2010. Available from: http://ci5.iarc.fr
France - Doubs Cancer Registry 1985 - CI5 as it appears in Ferlay J, Parkin DM, Curado MP, Bray F, Edwards B, Shin HR and Forman D. Cancer Incidence in Five Continents, Volumes I to IX: IARC CancerBase No. 9 [Internet]. Lyon, France: International Agency for Research on Cancer; 2010. Available from: http://ci5.iarc.fr
France - Doubs Cancer Registry 1986 - CI5 as it appears in Ferlay J, Parkin DM, Curado MP, Bray F, Edwards B, Shin HR and Forman D. Cancer Incidence in Five Continents, Volumes I to IX: IARC CancerBase No. 9 [Internet]. Lyon, France: International Agency for Research on Cancer; 2010. Available from: http://ci5.iarc.fr
France - Doubs Cancer Registry 1987 - CI5 as it appears in Ferlay J, Parkin DM, Curado MP, Bray F, Edwards B, Shin HR and Forman D. Cancer Incidence in Five Continents, Volumes I to IX: IARC CancerBase No. 9 [Internet]. Lyon, France: International Agency for Research on Cancer; 2010. Available from: http://ci5.iarc.fr
France - Doubs Cancer Registry 1988 - CI5 as it appears in Ferlay J, Parkin DM, Curado MP, Bray F, Edwards B, Shin HR and Forman D. Cancer Incidence in Five Continents, Volumes I to IX: IARC CancerBase No. 9 [Internet]. Lyon, France: International Agency for Research on Cancer; 2010. Available from: http://ci5.iarc.fr
France - Doubs Cancer Registry 1988-1992 - CI5 as it appears in Parkin DM, International Agency for Research on Cancer, International Association of Cancer Registries. Cancer Incidence in Five Continents. Vol. I to VIII. Lyon, France, IARCPress, 2005.
France - Doubs Cancer Registry 1989 - CI5 as it appears in Ferlay J, Parkin DM, Curado MP, Bray F, Edwards B, Shin HR and Forman D. Cancer Incidence in Five Continents, Volumes I to IX: IARC CancerBase No. 9 [Internet]. Lyon, France: International Agency for Research on Cancer; 2010. Available from: http://ci5.iarc.fr
France - Doubs Cancer Registry 1990 - CI5 as it appears in Ferlay J, Parkin DM, Curado MP, Bray F, Edwards B, Shin HR and Forman D. Cancer Incidence in Five Continents, Volumes I to IX: IARC CancerBase No. 9 [Internet]. Lyon, France: International Agency for Research on Cancer; 2010. Available from: http://ci5.iarc.fr
France - Doubs Cancer Registry 1991 - CI5 as it appears in Ferlay J, Parkin DM, Curado MP, Bray F, Edwards B, Shin HR and Forman D. Cancer Incidence in Five Continents, Volumes I to IX: IARC CancerBase No. 9 [Internet]. Lyon, France: International Agency for Research on Cancer; 2010. Available from: http://ci5.iarc.fr
France - Doubs Cancer Registry 1992 - CI5 as it appears in Ferlay J, Parkin DM, Curado MP, Bray F, Edwards B, Shin HR and Forman D. Cancer Incidence in Five Continents, Volumes I to IX: IARC CancerBase No. 9 [Internet]. Lyon, France: International Agency for Research on Cancer; 2010. Available from: http://ci5.iarc.fr

Appendix: Citation List

Citation
France - Doubs Cancer Registry 1993 - CI5 as it appears in Ferlay J, Parkin DM, Curado MP, Bray F, Edwards B, Shin HR and Forman D. Cancer Incidence in Five Continents, Volumes I to IX: IARC CancerBase No. 9 [Internet]. Lyon, France: International Agency for Research on Cancer; 2010. Available from: http://ci5.iarc.fr
France - Doubs Cancer Registry 1993-1997 - CI5 as it appears in Parkin DM, International Agency for Research on Cancer, International Association of Cancer Registries. Cancer Incidence in Five Continents. Vol. I to VIII. Lyon, France, IARCPress, 2005.
France - Doubs Cancer Registry 1995 - CI5 as it appears in Ferlay J, Parkin DM, Curado MP, Bray F, Edwards B, Shin HR and Forman D. Cancer Incidence in Five Continents, Volumes I to IX: IARC CancerBase No. 9 [Internet]. Lyon, France: International Agency for Research on Cancer; 2010. Available from: http://ci5.iarc.fr
France - Doubs Cancer Registry 1996 - CI5 as it appears in Ferlay J, Parkin DM, Curado MP, Bray F, Edwards B, Shin HR and Forman D. Cancer Incidence in Five Continents, Volumes I to IX: IARC CancerBase No. 9 [Internet]. Lyon, France: International Agency for Research on Cancer; 2010. Available from: http://ci5.iarc.fr
France - Doubs Cancer Registry 1997 - CI5 as it appears in Ferlay J, Parkin DM, Curado MP, Bray F, Edwards B, Shin HR and Forman D. Cancer Incidence in Five Continents, Volumes I to IX: IARC CancerBase No. 9 [Internet]. Lyon, France: International Agency for Research on Cancer; 2010. Available from: http://ci5.iarc.fr
France - Doubs Cancer Registry 1998 - CI5 as it appears in Ferlay J, Parkin DM, Curado MP, Bray F, Edwards B, Shin HR and Forman D. Cancer Incidence in Five Continents, Volumes I to IX: IARC CancerBase No. 9 [Internet]. Lyon, France: International Agency for Research on Cancer; 2010. Available from: http://ci5.iarc.fr
France - Doubs Cancer Registry 1998-2002 - CI5 as it appears in Curado MP, Edwards B, Shin HR, Storm H, Ferlay J, Heanue M and Boyle P, eds (2007). Cancer Incidence in Five Continents, Vol. IX Periodic Data (electronic version). Lyon, IARC.http://ci5.iarc.fr
France - Doubs Cancer Registry 1999 - CI5 as it appears in Ferlay J, Parkin DM, Curado MP, Bray F, Edwards B, Shin HR and Forman D. Cancer Incidence in Five Continents, Volumes I to IX: IARC CancerBase No. 9 [Internet]. Lyon, France: International Agency for Research on Cancer; 2010. Available from: http://ci5.iarc.fr
France - Doubs Cancer Registry 2001 - CI5 as it appears in Ferlay J, Parkin DM, Curado MP, Bray F, Edwards B, Shin HR and Forman D. Cancer Incidence in Five Continents, Volumes I to IX: IARC CancerBase No. 9 [Internet]. Lyon, France: International Agency for Research on Cancer; 2010. Available from: http://ci5.iarc.fr
France - Doubs Cancer Registry 2002 - CI5 as it appears in Ferlay J, Parkin DM, Curado MP, Bray F, Edwards B, Shin HR and Forman D. Cancer Incidence in Five Continents, Volumes I to IX: IARC CancerBase No. 9 [Internet]. Lyon, France: International Agency for Research on Cancer; 2010. Available from: http://ci5.iarc.fr
France - Doubs Cancer Registry 2003-2007 - CI5 as it appears in Forman D, Bray F, Brewster DH, Gombe Mbalawa C, Kohler B, Piñeros M, Steliarova-Foucher E, Swaminathan R and Ferlay J, eds (2013). Cancer Incidence in Five Continents, Vol. X Summary Database (electronic version). Lyon, IARC. http://ci5.iarc.fr
France - Haut Rhin Cancer Registry 2003-2007 - CI5 as it appears in Forman D, Bray F, Brewster DH, Gombe Mbalawa C, Kohler B, Piñeros M, Steliarova-Foucher E, Swaminathan R and Ferlay J, eds (2013). Cancer Incidence in Five Continents, Vol. X Summary Database (electronic version). Lyon, IARC. http://ci5.iarc.fr
France - Herault Cancer Registry 1988-1992 - CI5 as it appears in Parkin DM, International Agency for Research on Cancer, International Association of Cancer Registries. Cancer Incidence in Five Continents. Vol. I to VIII. Lyon, France, IARCPress, 2005.
France - Herault Cancer Registry 1998-2002 - CI5 as it appears in Curado MP, Edwards B, Shin HR, Storm H, Ferlay J, Heanue M and Boyle P, eds (2007). Cancer Incidence in Five Continents, Vol. IX Periodic Data (electronic version). Lyon, IARC.http://ci5.iarc.fr
France - Herault Cancer Registry 2003-2007 - CI5 as it appears in Forman D, Bray F, Brewster DH, Gombe Mbalawa C, Kohler B, Piñeros M, Steliarova-Foucher E, Swaminathan R and Ferlay J, eds (2013). Cancer Incidence in Five Continents, Vol. X Summary Database (electronic version). Lyon, IARC. http://ci5.iarc.fr
France - Isere Cancer Registry 1983-1987 - CI5 as it appears in Parkin DM, International Agency for Research on Cancer, International Association of Cancer Registries. Cancer Incidence in Five Continents. Vol. I to VIII. Lyon, France, IARCPress, 2005.
France - Isere Cancer Registry 1988-1992 - CI5 as it appears in Parkin DM, International Agency for Research on Cancer, International Association of Cancer Registries. Cancer Incidence in Five Continents. Vol. I to VIII. Lyon, France, IARCPress, 2005.
France - Isere Cancer Registry 1993-1997 - CI5 as it appears in Parkin DM, International Agency for Research on Cancer, International Association of Cancer Registries. Cancer Incidence in Five Continents. Vol. I to VIII. Lyon, France, IARCPress, 2005.
France - Isere Cancer Registry 1998-2002 - CI5 as it appears in Curado MP, Edwards B, Shin HR, Storm H, Ferlay J, Heanue M and Boyle P, eds (2007). Cancer Incidence in Five Continents, Vol. IX Periodic Data (electronic version). Lyon, IARC.http://ci5.iarc.fr
France - Isere Cancer Registry 2003-2007 - CI5 as it appears in Forman D, Bray F, Brewster DH, Gombe Mbalawa C, Kohler B, Piñeros M, Steliarova-Foucher E, Swaminathan R and Ferlay J, eds (2013). Cancer Incidence in Five Continents, Vol. X Summary Database (electronic version). Lyon, IARC. http://ci5.iarc.fr
France - Loire Atlantique Cancer Registry 2000 - CI5 as it appears in Curado MP, Edwards B, Shin HR, Storm H, Ferlay J, Heanue M and Boyle P, eds (2007). Cancer Incidence in Five Continents, Vol. IX Periodic Data (electronic version). Lyon, IARC.http://ci5.iarc.fr
France - Loire Atlantique Cancer Registry 2003-2007 - CI5 as it appears in Forman D, Bray F, Brewster DH, Gombe Mbalawa C, Kohler B, Piñeros M, Steliarova-Foucher E, Swaminathan R and Ferlay J, eds (2013). Cancer Incidence in Five Continents, Vol. X Summary Database (electronic version). Lyon, IARC. http://ci5.iarc.fr
France - Manche Cancer Registry 1994-1997 - CI5 as it appears in Parkin DM, International Agency for Research on Cancer, International Association of Cancer Registries. Cancer Incidence in Five Continents. Vol. I to VIII. Lyon, France, IARCPress, 2005.
France - Manche Cancer Registry 1995 - CI5 as it appears in Parkin DM, International Agency for Research on Cancer, International Association of Cancer Registries. Cancer Incidence in Five Continents. Vol. I to VIII. Lyon, France, IARCPress, 2005.
France - Manche Cancer Registry 1998-2002 - CI5 as it appears in Curado MP, Edwards B, Shin HR, Storm H, Ferlay J, Heanue M and Boyle P, eds (2007). Cancer Incidence in Five Continents, Vol. IX Periodic Data (electronic version). Lyon, IARC.http://ci5.iarc.fr

Appendix: Citation List

Citation
France - Manche Cancer Registry 2000 - CI5 as it appears in Curado MP, Edwards B, Shin HR, Storm H, Ferlay J, Heanue M and Boyle P, eds (2007). Cancer Incidence in Five Continents, Vol. IX Periodic Data (electronic version). Lyon, IARC.http://ci5.iarc.fr
France - Manche Cancer Registry 2003-2007 - CI5 as it appears in Forman D, Bray F, Brewster DH, Gombe Mbalawa C, Kohler B, Piñeros M, Steliarova-Foucher E, Swaminathan R and Ferlay J, eds (2013). Cancer Incidence in Five Continents, Vol. X Summary Database (electronic version). Lyon, IARC. http://ci5.iarc.fr
France - Martinique Cancer Registry 1981-1982 - CI5 as it appears in Parkin DM, International Agency for Research on Cancer, International Association of Cancer Registries. Cancer Incidence in Five Continents. Vol. I to VIII. Lyon, France, IARCPress, 2005.
France - Martinique Cancer Registry 1985 - CI5 as it appears in Parkin DM, International Agency for Research on Cancer, International Association of Cancer Registries. Cancer Incidence in Five Continents. Vol. I to VIII. Lyon, France, IARCPress, 2005.
France - Martinique Cancer Registry 1993-1995 - CI5 as it appears in Parkin DM, International Agency for Research on Cancer, International Association of Cancer Registries. Cancer Incidence in Five Continents. Vol. I to VIII. Lyon, France, IARCPress, 2005.
France - Martinique Cancer Registry 1994 - CI5 as it appears in Parkin DM, International Agency for Research on Cancer, International Association of Cancer Registries. Cancer Incidence in Five Continents. Vol. I to VIII. Lyon, France, IARCPress, 2005.
France - Martinique Cancer Registry 1995 - CI5 as it appears in Parkin DM, International Agency for Research on Cancer, International Association of Cancer Registries. Cancer Incidence in Five Continents. Vol. I to VIII. Lyon, France, IARCPress, 2005.
France - Martinique Cancer Registry 2000 - CI5 as it appears in Curado MP, Edwards B, Shin HR, Storm H, Ferlay J, Heanue M and Boyle P, eds (2007). Cancer Incidence in Five Continents, Vol. IX Periodic Data (electronic version). Lyon, IARC.http://ci5.iarc.fr
France - Martinique Cancer Registry 2003-2007 - CI5 as it appears in Forman D, Bray F, Brewster DH, Gombe Mbalawa C, Kohler B, Piñeros M, Steliarova-Foucher E, Swaminathan R and Ferlay J, eds (2013). Cancer Incidence in Five Continents, Vol. X Summary Database (electronic version). Lyon, IARC. http://ci5.iarc.fr
France - Somme Cancer Registry 1983-1984 - CI5 as it appears in Parkin DM, International Agency for Research on Cancer, International Association of Cancer Registries. Cancer Incidence in Five Continents. Vol. I to VIII. Lyon, France, IARCPress, 2005.
France - Somme Cancer Registry 1988-1992 - CI5 as it appears in Parkin DM, International Agency for Research on Cancer, International Association of Cancer Registries. Cancer Incidence in Five Continents. Vol. I to VIII. Lyon, France, IARCPress, 2005.
France - Somme Cancer Registry 1993-1997 - CI5 as it appears in Parkin DM, International Agency for Research on Cancer, International Association of Cancer Registries. Cancer Incidence in Five Continents. Vol. I to VIII. Lyon, France, IARCPress, 2005.
France - Somme Cancer Registry 1998-2002 - CI5 as it appears in Curado MP, Edwards B, Shin HR, Storm H, Ferlay J, Heanue M and Boyle P, eds (2007). Cancer Incidence in Five Continents, Vol. IX Periodic Data (electronic version). Lyon, IARC.http://ci5.iarc.fr
France - Somme Cancer Registry 2003-2007 - CI5 as it appears in Forman D, Bray F, Brewster DH, Gombe Mbalawa C, Kohler B, Piñeros M, Steliarova-Foucher E, Swaminathan R and Ferlay J, eds (2013). Cancer Incidence in Five Continents, Vol. X Summary Database (electronic version). Lyon, IARC. http://ci5.iarc.fr
France - Tarn Cancer Registry 1983 - CI5 as it appears in Ferlay J, Parkin DM, Curado MP, Bray F, Edwards B, Shin HR and Forman D. Cancer Incidence in Five Continents, Volumes I to IX: IARC CancerBase No. 9 [Internet]. Lyon, France: International Agency for Research on Cancer; 2010. Available from: http://ci5.iarc.fr
France - Tarn Cancer Registry 1983-1987 - CI5 as it appears in Parkin DM, International Agency for Research on Cancer, International Association of Cancer Registries. Cancer Incidence in Five Continents. Vol. I to VIII. Lyon, France, IARCPress, 2005.
France - Tarn Cancer Registry 1984 - CI5 as it appears in Ferlay J, Parkin DM, Curado MP, Bray F, Edwards B, Shin HR and Forman D. Cancer Incidence in Five Continents, Volumes I to IX: IARC CancerBase No. 9 [Internet]. Lyon, France: International Agency for Research on Cancer; 2010. Available from: http://ci5.iarc.fr
France - Tarn Cancer Registry 1985 - CI5 as it appears in Ferlay J, Parkin DM, Curado MP, Bray F, Edwards B, Shin HR and Forman D. Cancer Incidence in Five Continents, Volumes I to IX: IARC CancerBase No. 9 [Internet]. Lyon, France: International Agency for Research on Cancer; 2010. Available from: http://ci5.iarc.fr
France - Tarn Cancer Registry 1986 - CI5 as it appears in Ferlay J, Parkin DM, Curado MP, Bray F, Edwards B, Shin HR and Forman D. Cancer Incidence in Five Continents, Volumes I to IX: IARC CancerBase No. 9 [Internet]. Lyon, France: International Agency for Research on Cancer; 2010. Available from: http://ci5.iarc.fr
France - Tarn Cancer Registry 1987 - CI5 as it appears in Ferlay J, Parkin DM, Curado MP, Bray F, Edwards B, Shin HR and Forman D. Cancer Incidence in Five Continents, Volumes I to IX: IARC CancerBase No. 9 [Internet]. Lyon, France: International Agency for Research on Cancer; 2010. Available from: http://ci5.iarc.fr
France - Tarn Cancer Registry 1988 - CI5 as it appears in Ferlay J, Parkin DM, Curado MP, Bray F, Edwards B, Shin HR and Forman D. Cancer Incidence in Five Continents, Volumes I to IX: IARC CancerBase No. 9 [Internet]. Lyon, France: International Agency for Research on Cancer; 2010. Available from: http://ci5.iarc.fr
France - Tarn Cancer Registry 1988-1992 - CI5 as it appears in Parkin DM, International Agency for Research on Cancer, International Association of Cancer Registries. Cancer Incidence in Five Continents. Vol. I to VIII. Lyon, France, IARCPress, 2005.
France - Tarn Cancer Registry 1989 - CI5 as it appears in Ferlay J, Parkin DM, Curado MP, Bray F, Edwards B, Shin HR and Forman D. Cancer Incidence in Five Continents, Volumes I to IX: IARC CancerBase No. 9 [Internet]. Lyon, France: International Agency for Research on Cancer; 2010. Available from: http://ci5.iarc.fr
France - Tarn Cancer Registry 1990 - CI5 as it appears in Ferlay J, Parkin DM, Curado MP, Bray F, Edwards B, Shin HR and Forman D. Cancer Incidence in Five Continents, Volumes I to IX: IARC CancerBase No. 9 [Internet]. Lyon, France: International Agency for Research on Cancer; 2010. Available from: http://ci5.iarc.fr
France - Tarn Cancer Registry 1991 - CI5 as it appears in Ferlay J, Parkin DM, Curado MP, Bray F, Edwards B, Shin HR and Forman D. Cancer Incidence in Five Continents, Volumes I to IX: IARC CancerBase No. 9 [Internet]. Lyon, France: International Agency for Research on Cancer; 2010. Available from: http://ci5.iarc.fr

Appendix: Citation List

Citation
France - Tarn Cancer Registry 1992 - CI5 as it appears in Ferlay J, Parkin DM, Curado MP, Bray F, Edwards B, Shin HR and Forman D. Cancer Incidence in Five Continents, Volumes I to IX: IARC CancerBase No. 9 [Internet]. Lyon, France: International Agency for Research on Cancer; 2010. Available from: http://ci5.iarc.fr
France - Tarn Cancer Registry 1993-1995 - CI5 as it appears in Ferlay J, Parkin DM, Curado MP, Bray F, Edwards B, Shin HR and Forman D. Cancer Incidence in Five Continents, Volumes I to IX: IARC CancerBase No. 9 [Internet]. Lyon, France: International Agency for Research on Cancer; 2010. Available from: http://ci5.iarc.fr
France - Tarn Cancer Registry 1993-1997 - CI5 as it appears in Parkin DM, International Agency for Research on Cancer, International Association of Cancer Registries. Cancer Incidence in Five Continents. Vol. I to VIII. Lyon, France, IARCPress, 2005.
France - Tarn Cancer Registry 1994 - CI5 as it appears in Ferlay J, Parkin DM, Curado MP, Bray F, Edwards B, Shin HR and Forman D. Cancer Incidence in Five Continents, Volumes I to IX: IARC CancerBase No. 9 [Internet]. Lyon, France: International Agency for Research on Cancer; 2010. Available from: http://ci5.iarc.fr
France - Tarn Cancer Registry 1995 - CI5 as it appears in Ferlay J, Parkin DM, Curado MP, Bray F, Edwards B, Shin HR and Forman D. Cancer Incidence in Five Continents, Volumes I to IX: IARC CancerBase No. 9 [Internet]. Lyon, France: International Agency for Research on Cancer; 2010. Available from: http://ci5.iarc.fr
France - Tarn Cancer Registry 1996 - CI5 as it appears in Ferlay J, Parkin DM, Curado MP, Bray F, Edwards B, Shin HR and Forman D. Cancer Incidence in Five Continents, Volumes I to IX: IARC CancerBase No. 9 [Internet]. Lyon, France: International Agency for Research on Cancer; 2010. Available from: http://ci5.iarc.fr
France - Tarn Cancer Registry 1997 - CI5 as it appears in Ferlay J, Parkin DM, Curado MP, Bray F, Edwards B, Shin HR and Forman D. Cancer Incidence in Five Continents, Volumes I to IX: IARC CancerBase No. 9 [Internet]. Lyon, France: International Agency for Research on Cancer; 2010. Available from: http://ci5.iarc.fr
France - Tarn Cancer Registry 1998-2002 - CI5 as it appears in Curado MP, Edwards B, Shin HR, Storm H, Ferlay J, Heanue M and Boyle P, eds (2007). Cancer Incidence in Five Continents, Vol. IX Periodic Data (electronic version). Lyon, IARC.http://ci5.iarc.fr
France - Tarn Cancer Registry 1999 - CI5 as it appears in Ferlay J, Parkin DM, Curado MP, Bray F, Edwards B, Shin HR and Forman D. Cancer Incidence in Five Continents, Volumes I to IX: IARC CancerBase No. 9 [Internet]. Lyon, France: International Agency for Research on Cancer; 2010. Available from: http://ci5.iarc.fr
France - Tarn Cancer Registry 2000 - CI5 as it appears in Ferlay J, Parkin DM, Curado MP, Bray F, Edwards B, Shin HR and Forman D. Cancer Incidence in Five Continents, Volumes I to IX: IARC CancerBase No. 9 [Internet]. Lyon, France: International Agency for Research on Cancer; 2010. Available from: http://ci5.iarc.fr
France - Tarn Cancer Registry 2001 - CI5 as it appears in Ferlay J, Parkin DM, Curado MP, Bray F, Edwards B, Shin HR and Forman D. Cancer Incidence in Five Continents, Volumes I to IX: IARC CancerBase No. 9 [Internet]. Lyon, France: International Agency for Research on Cancer; 2010. Available from: http://ci5.iarc.fr
France - Tarn Cancer Registry 2002 - CI5 as it appears in Ferlay J, Parkin DM, Curado MP, Bray F, Edwards B, Shin HR and Forman D. Cancer Incidence in Five Continents, Volumes I to IX: IARC CancerBase No. 9 [Internet]. Lyon, France: International Agency for Research on Cancer; 2010. Available from: http://ci5.iarc.fr
France - Tarn Cancer Registry 2003-2007 - CI5 as it appears in Forman D, Bray F, Brewster DH, Gombe Mbalawa C, Kohler B, Piñeros M, Steliarova-Foucher E, Swaminathan R and Ferlay J, eds (2013). Cancer Incidence in Five Continents, Vol. X Summary Database (electronic version). Lyon, IARC. http://ci5.iarc.fr
France - Vendee Cancer Registry 1998-2002 - CI5 as it appears in Curado MP, Edwards B, Shin HR, Storm H, Ferlay J, Heanue M and Boyle P, eds (2007). Cancer Incidence in Five Continents, Vol. IX Periodic Data (electronic version). Lyon, IARC.http://ci5.iarc.fr
France - Vendee Cancer Registry 2003-2007 - CI5 as it appears in Forman D, Bray F, Brewster DH, Gombe Mbalawa C, Kohler B, Piñeros M, Steliarova-Foucher E, Swaminathan R and Ferlay J, eds (2013). Cancer Incidence in Five Continents, Vol. X Summary Database (electronic version). Lyon, IARC. http://ci5.iarc.fr
France Adult Health Barometer 1993 as it appears in P.N. Lee Statistics and Computing Ltd. International Mortality and Smoking Statistics Version 4.04. Sutton, United Kingdom: P.N. Lee Statistics and Computing Ltd, 2009.
France CFES Adolescent Health Survey 1981 as it appears in P.N. Lee Statistics and Computing Ltd. International Mortality and Smoking Statistics Version 4.04. Sutton, United Kingdom: P.N. Lee Statistics and Computing Ltd, 2009.
France CFES Adolescent Health Survey 1983 as it appears in P.N. Lee Statistics and Computing Ltd. International Mortality and Smoking Statistics Version 4.04. Sutton, United Kingdom: P.N. Lee Statistics and Computing Ltd, 2009.
France CFES Adolescent Health Survey 1984 as it appears in P.N. Lee Statistics and Computing Ltd. International Mortality and Smoking Statistics Version 4.04. Sutton, United Kingdom: P.N. Lee Statistics and Computing Ltd, 2009.
France CFES Adolescent Health Survey 1994 as it appears in P.N. Lee Statistics and Computing Ltd. International Mortality and Smoking Statistics Version 4.04. Sutton, United Kingdom: P.N. Lee Statistics and Computing Ltd, 2009.
France CFES Adult Health Survey 1986 as it appears in P.N. Lee Statistics and Computing Ltd. International Mortality and Smoking Statistics Version 4.04. Sutton, United Kingdom: P.N. Lee Statistics and Computing Ltd, 2009.
France CFES Adult Health Survey 1989 as it appears in P.N. Lee Statistics and Computing Ltd. International Mortality and Smoking Statistics Version 4.04. Sutton, United Kingdom: P.N. Lee Statistics and Computing Ltd, 2009.
France CFES Adult Health Survey 1992 as it appears in P.N. Lee Statistics and Computing Ltd. International Mortality and Smoking Statistics Version 4.04. Sutton, United Kingdom: P.N. Lee Statistics and Computing Ltd, 2009.
France CFES Adult Health Survey 1995 as it appears in P.N. Lee Statistics and Computing Ltd. International Mortality and Smoking Statistics Version 4.04. Sutton, United Kingdom: P.N. Lee Statistics and Computing Ltd, 2009.
France CFES Adult Health Survey 1999 as it appears in P.N. Lee Statistics and Computing Ltd. International Mortality and Smoking Statistics Version 4.04. Sutton, United Kingdom: P.N. Lee Statistics and Computing Ltd, 2009.
France CFES Campaign Evaluation Survey 1988 as it appears in P.N. Lee Statistics and Computing Ltd. International Mortality and Smoking Statistics Version 4.04. Sutton, United Kingdom: P.N. Lee Statistics and Computing Ltd, 2009.

Appendix: Citation List

Citation
France CFES Youth and Tobacco Survey 1980 as it appears in P.N. Lee Statistics and Computing Ltd. International Mortality and Smoking Statistics Version 4.04. Sutton, United Kingdom: P.N. Lee Statistics and Computing Ltd, 2009.
France CFES Youth and Tobacco Survey 1990 as it appears in P.N. Lee Statistics and Computing Ltd. International Mortality and Smoking Statistics Version 4.04. Sutton, United Kingdom: P.N. Lee Statistics and Computing Ltd, 2009.
France Decennial Survey on Health and Medical Care 1980-1981 as it appears in P.N. Lee Statistics and Computing Ltd. International Mortality and Smoking Statistics Version 4.04. Sutton, United Kingdom: P.N. Lee Statistics and Computing Ltd, 2009.
France Decennial Survey on Health and Medical Care 1991-1992 as it appears in P.N. Lee Statistics and Computing Ltd. International Mortality and Smoking Statistics Version 4.04. Sutton, United Kingdom: P.N. Lee Statistics and Computing Ltd, 2009.
France Eurobarometer 32: The Single European Market, Drugs, Alcohol, and Cancer 1989 as it appears in P.N. Lee Statistics and Computing Ltd. International Mortality and Smoking Statistics Version 4.04. Sutton, United Kingdom: P.N. Lee Statistics and Computing Ltd, 2009.
France Eurobarometer 38: European Court of Justice, Passive Smoking, And Consumer Issues 1992 as it appears in P.N. Lee Statistics and Computing Ltd. International Mortality and Smoking Statistics Version 4.04. Sutton, United Kingdom: P.N. Lee Statistics and Computing Ltd, 2009.
France Survey of Living Conditions of People in Disadvantaged Situations 1986-1987 as it appears in P.N. Lee Statistics and Computing Ltd. International Mortality and Smoking Statistics Version 4.04. Sutton, United Kingdom: P.N. Lee Statistics and Computing Ltd, 2009.
France Vital Registration - Deaths 1950 ICD7 as it appears in World Health Organization (WHO). WHO Mortality Database Version July 2012. Geneva, Switzerland: World Health Organization (WHO), 2012.
France Vital Registration - Deaths 1952 ICD7 as it appears in World Health Organization (WHO). WHO Mortality Database Version July 2012. Geneva, Switzerland: World Health Organization (WHO), 2012.
France Vital Registration - Deaths 1953 ICD7 as it appears in World Health Organization (WHO). WHO Mortality Database Version July 2012. Geneva, Switzerland: World Health Organization (WHO), 2012.
France Vital Registration - Deaths 1954 ICD7 as it appears in World Health Organization (WHO). WHO Mortality Database Version July 2012. Geneva, Switzerland: World Health Organization (WHO), 2012.
France Vital Registration - Deaths 1955 ICD7 as it appears in World Health Organization (WHO). WHO Mortality Database Version July 2012. Geneva, Switzerland: World Health Organization (WHO), 2012.
France Vital Registration - Deaths 1956 ICD7 as it appears in World Health Organization (WHO). WHO Mortality Database Version July 2012. Geneva, Switzerland: World Health Organization (WHO), 2012.
France Vital Registration - Deaths 1957 ICD7 as it appears in World Health Organization (WHO). WHO Mortality Database Version July 2012. Geneva, Switzerland: World Health Organization (WHO), 2012.
France Vital Registration - Deaths 1958 ICD7 as it appears in World Health Organization (WHO). WHO Mortality Database Version July 2012. Geneva, Switzerland: World Health Organization (WHO), 2012.
France Vital Registration - Deaths 1959 ICD7 as it appears in World Health Organization (WHO). WHO Mortality Database Version July 2012. Geneva, Switzerland: World Health Organization (WHO), 2012.
France Vital Registration - Deaths 1960 ICD7 as it appears in World Health Organization (WHO). WHO Mortality Database Version July 2012. Geneva, Switzerland: World Health Organization (WHO), 2012.
France Vital Registration - Deaths 1961 ICD7 as it appears in World Health Organization (WHO). WHO Mortality Database Version July 2012. Geneva, Switzerland: World Health Organization (WHO), 2012.
France Vital Registration - Deaths 1962 ICD7 as it appears in World Health Organization (WHO). WHO Mortality Database Version July 2012. Geneva, Switzerland: World Health Organization (WHO), 2012.
France Vital Registration - Deaths 1963 ICD7 as it appears in World Health Organization (WHO). WHO Mortality Database Version July 2012. Geneva, Switzerland: World Health Organization (WHO), 2012.
France Vital Registration - Deaths 1964 ICD7 as it appears in World Health Organization (WHO). WHO Mortality Database Version July 2012. Geneva, Switzerland: World Health Organization (WHO), 2012.
France Vital Registration - Deaths 1965 ICD7 as it appears in World Health Organization (WHO). WHO Mortality Database Version July 2012. Geneva, Switzerland: World Health Organization (WHO), 2012.
France Vital Registration - Deaths 1966 ICD7 as it appears in World Health Organization (WHO). WHO Mortality Database Version July 2012. Geneva, Switzerland: World Health Organization (WHO), 2012.
France Vital Registration - Deaths 1967 ICD7 as it appears in World Health Organization (WHO). WHO Mortality Database Version July 2012. Geneva, Switzerland: World Health Organization (WHO), 2012.
France Vital Registration - Deaths 1968 ICD8 as it appears in World Health Organization (WHO). WHO Mortality Database Version May 2013. Geneva, Switzerland: World Health Organization (WHO), 2013.
France Vital Registration - Deaths 1969 ICD8 as it appears in World Health Organization (WHO). WHO Mortality Database Version May 2013. Geneva, Switzerland: World Health Organization (WHO), 2013.
France Vital Registration - Deaths 1970 ICD8 as it appears in World Health Organization (WHO). WHO Mortality Database Version May 2013. Geneva, Switzerland: World Health Organization (WHO), 2013.
France Vital Registration - Deaths 1971 ICD8 as it appears in World Health Organization (WHO). WHO Mortality Database Version May 2013. Geneva, Switzerland: World Health Organization (WHO), 2013.
France Vital Registration - Deaths 1972 ICD8 as it appears in World Health Organization (WHO). WHO Mortality Database Version May 2013. Geneva, Switzerland: World Health Organization (WHO), 2013.
France Vital Registration - Deaths 1973 ICD8 as it appears in World Health Organization (WHO). WHO Mortality Database Version May 2013. Geneva, Switzerland: World Health Organization (WHO), 2013.
France Vital Registration - Deaths 1974 ICD8 as it appears in World Health Organization (WHO). WHO Mortality Database Version May 2013. Geneva, Switzerland: World Health Organization (WHO), 2013.

Appendix: Citation List

Citation
France Vital Registration - Deaths 1975 ICD8 as it appears in World Health Organization (WHO). WHO Mortality Database Version May 2013. Geneva, Switzerland: World Health Organization (WHO), 2013.
France Vital Registration - Deaths 1976 ICD8 as it appears in World Health Organization (WHO). WHO Mortality Database Version May 2013. Geneva, Switzerland: World Health Organization (WHO), 2013.
France Vital Registration - Deaths 1977 ICD8 as it appears in World Health Organization (WHO). WHO Mortality Database Version May 2013. Geneva, Switzerland: World Health Organization (WHO), 2013.
France Vital Registration - Deaths 1978 ICD8 as it appears in World Health Organization (WHO). WHO Mortality Database Version May 2013. Geneva, Switzerland: World Health Organization (WHO), 2013.
France Vital Registration - Deaths 1979 ICD9 as it appears in World Health Organization (WHO). WHO Mortality Database Version November 2015. Geneva, Switzerland: World Health Organization (WHO), 2015.
France Vital Registration - Deaths 1980 ICD9 as it appears in World Health Organization (WHO). WHO Mortality Database Version July 2012. Geneva, Switzerland: World Health Organization (WHO), 2012.
France Vital Registration - Deaths 1981 ICD9 as it appears in World Health Organization (WHO). WHO Mortality Database Version July 2012. Geneva, Switzerland: World Health Organization (WHO), 2012.
France Vital Registration - Deaths 1982 ICD9 as it appears in World Health Organization (WHO). WHO Mortality Database Version July 2012. Geneva, Switzerland: World Health Organization (WHO), 2012.
France Vital Registration - Deaths 1983 ICD9 as it appears in World Health Organization (WHO). WHO Mortality Database Version July 2012. Geneva, Switzerland: World Health Organization (WHO), 2012.
France Vital Registration - Deaths 1984 ICD9 as it appears in World Health Organization (WHO). WHO Mortality Database Version July 2012. Geneva, Switzerland: World Health Organization (WHO), 2012.
France Vital Registration - Deaths 1985 ICD9 as it appears in World Health Organization (WHO). WHO Mortality Database Version July 2012. Geneva, Switzerland: World Health Organization (WHO), 2012.
France Vital Registration - Deaths 1986 ICD9 as it appears in World Health Organization (WHO). WHO Mortality Database Version July 2012. Geneva, Switzerland: World Health Organization (WHO), 2012.
France Vital Registration - Deaths 1987 ICD9 as it appears in World Health Organization (WHO). WHO Mortality Database Version July 2012. Geneva, Switzerland: World Health Organization (WHO), 2012.
France Vital Registration - Deaths 1988 ICD9 as it appears in World Health Organization (WHO). WHO Mortality Database Version July 2012. Geneva, Switzerland: World Health Organization (WHO), 2012.
France Vital Registration - Deaths 1989 ICD9 as it appears in World Health Organization (WHO). WHO Mortality Database Version July 2012. Geneva, Switzerland: World Health Organization (WHO), 2012.
France Vital Registration - Deaths 1990 ICD9 as it appears in World Health Organization (WHO). WHO Mortality Database Version July 2012. Geneva, Switzerland: World Health Organization (WHO), 2012.
France Vital Registration - Deaths 1991 ICD9 as it appears in World Health Organization (WHO). WHO Mortality Database Version July 2012. Geneva, Switzerland: World Health Organization (WHO), 2012.
France Vital Registration - Deaths 1992 ICD9 as it appears in World Health Organization (WHO). WHO Mortality Database Version July 2012. Geneva, Switzerland: World Health Organization (WHO), 2012.
France Vital Registration - Deaths 1993 ICD9 as it appears in World Health Organization (WHO). WHO Mortality Database Version July 2012. Geneva, Switzerland: World Health Organization (WHO), 2012.
France Vital Registration - Deaths 1994 ICD9 as it appears in World Health Organization (WHO). WHO Mortality Database Version July 2012. Geneva, Switzerland: World Health Organization (WHO), 2012.
France Vital Registration - Deaths 1995 ICD9 as it appears in World Health Organization (WHO). WHO Mortality Database Version July 2012. Geneva, Switzerland: World Health Organization (WHO), 2012.
France Vital Registration - Deaths 1996 ICD9 as it appears in World Health Organization (WHO). WHO Mortality Database Version July 2012. Geneva, Switzerland: World Health Organization (WHO), 2012.
France Vital Registration - Deaths 1997 ICD9 as it appears in World Health Organization (WHO). WHO Mortality Database Version July 2012. Geneva, Switzerland: World Health Organization (WHO), 2012.
France Vital Registration - Deaths 1998 ICD9 as it appears in World Health Organization (WHO). WHO Mortality Database Version July 2012. Geneva, Switzerland: World Health Organization (WHO), 2012.
France Vital Registration - Deaths 1999 ICD9 as it appears in World Health Organization (WHO). WHO Mortality Database Version July 2012. Geneva, Switzerland: World Health Organization (WHO), 2012.
France Vital Registration - Deaths 2000 ICD10 as it appears in World Health Organization (WHO). WHO Mortality Database Version November 2015. Geneva, Switzerland: World Health Organization (WHO), 2015.
France Vital Registration - Deaths 2001 ICD10 as it appears in World Health Organization (WHO). WHO Mortality Database Version November 2015. Geneva, Switzerland: World Health Organization (WHO), 2015.
France Vital Registration - Deaths 2002 ICD10 as it appears in World Health Organization (WHO). WHO Mortality Database Version November 2015. Geneva, Switzerland: World Health Organization (WHO), 2015.
France Vital Registration - Deaths 2003 ICD10 as it appears in World Health Organization (WHO). WHO Mortality Database Version November 2015. Geneva, Switzerland: World Health Organization (WHO), 2015.
France Vital Registration - Deaths 2004 ICD10 as it appears in World Health Organization (WHO). WHO Mortality Database Version November 2015. Geneva, Switzerland: World Health Organization (WHO), 2015.
France Vital Registration - Deaths 2005 ICD10 as it appears in World Health Organization (WHO). WHO Mortality Database Version November 2015. Geneva, Switzerland: World Health Organization (WHO), 2015.
France Vital Registration - Deaths 2006 ICD10 as it appears in World Health Organization (WHO). WHO Mortality Database Version November 2015. Geneva, Switzerland: World Health Organization (WHO), 2015.

Appendix: Citation List

Citation
France Vital Registration - Deaths 2007 ICD10 as it appears in World Health Organization (WHO). WHO Mortality Database Version November 2015. Geneva, Switzerland: World Health Organization (WHO), 2015.
France Vital Registration - Deaths 2008 ICD10 as it appears in World Health Organization (WHO). WHO Mortality Database Version November 2015. Geneva, Switzerland: World Health Organization (WHO), 2015.
France Vital Registration - Deaths 2009 ICD10 as it appears in World Health Organization (WHO). WHO Mortality Database Version November 2015. Geneva, Switzerland: World Health Organization (WHO), 2015.
France Vital Registration - Deaths 2010 ICD10 as it appears in World Health Organization (WHO). WHO Mortality Database Version November 2015. Geneva, Switzerland: World Health Organization (WHO), 2015.
France Vital Registration - Deaths 2011 ICD10 as it appears in World Health Organization (WHO). WHO Mortality Database Version November 2015. Geneva, Switzerland: World Health Organization (WHO), 2015.
France Vital Registration Death Data 2011 as it appears in United Nations Statistics Division (UNSD). United Nations Demographic Yearbook 2012. New York City, United States: United Nations Statistics Division (UNSD), 2013.
Franco F, Chellini E, Seniori Costantini A, Gioia A, Carra G, Paolinelli F, Martelli C, Vigotti M. Mortality in the coke oven plant of Carrara, Italy. Med Lav . 1993; 84(6): 443-7 as it appears in Armstrong B, Hutchinson E, Unwin J, Fletcher T. Lung cancer risk after exposure to polycyclic aromatic hydrocarbons: a review and meta-analysis. Environ. Health Perspect. 2004; 112(9): 970–978.
Franklin GM, Kahn LB, Baxter J, Marshall JA, Hamman RF. Sensory neuropathy in non-insulin-dependent diabetes mellitus. The San Luis Valley Diabetes Study. Am J Epidemiol. 1990; 131(4): 633-43.
Fraser M, Taleo G, Taleo F, Yaviong J, Amos M, Babu M, Kalkoa M. Evaluation of the program to eliminate lymphatic filariasis in Vanuatu following two years of mass drug administration implementation: results and methodologic approach. Am J Trop Med Hyg. 2005; 73(4): 753-8. As it appears in London School of Hygiene and Tropical Medicine. Global Atlas of Helminth Infections - Lymphatic Filiariasis.
Fraser-Hurt N, Felger I, Edoh D, Steiger S, Mashaka M, Masanja H, Smith T, Mbena F, Beck H-P. Effect of insecticide-treated bed nets on haemoglobin values, prevalence and multiplicity of infection with Plasmodium falciparum in a randomized controlled trial in Tanzania. Trans R Soc Trop Med Hyg. 1999; 93(Suppl 1): 47-51. As it appears in Malaria Atlas Project. Malaria Atlas Project Plasmodium Falciparum Parasite Rate Database. Oxford, United Kingdom: Malaria Atlas Project.
Frederiksen P, Jensen KE, Kjaer SK. Sociodemographic factors and risk-taking behaviour during adolescence and obesity among more than 40 000 Danes. Public Health Nutr. 2014; 17(1): 162â€“9.
Free University of Brussels (ULB), Ghent University, International Agency for Research on Cancer (IARC), Limburg University Center, Scientific Institute of Public Health (IPH) (Belgium), Statistics Belgium. Belgium Food Consumption Survey 2004.
Freeman TW, Chandiwana SK. A retrospective analysis of data from the 1991 national malaria prevalence survey in Zimbabwe. Malar Infect Dis Afr. 1996; 4: 19-24. As it appears in Malaria Atlas Project. Malaria Atlas Project Plasmodium Falciparum Parasite Rate Database. Oxford, United Kingdom: Malaria Atlas Project.
French Institute for Public Health Surveillance (INVS), French National Health Insurance Fund for Salaried Workers (CNAMTS), National Conservatory of Arts and Trades (CNAM) (France), University of Paris 13. France National Nutrition and Health Survey 2006-2007.
French Institute of Health and Medical Research (INSERM), Kantar Health, Roche (France). France National Survey of Overweight and Obesity 2009.
French Institute of Health and Medical Research (INSERM), Kantar Health, Roche (France). France National Survey of Overweight and Obesity 2012.
French Institute of Health and Medical Research (INSERM), Roche (France), TNS Sofres (France). France National Survey of Overweight and Obesity 2006.
French Institute of Health and Medical Research (INSERM). France Three-City Cohort Study 1999-2001.
French Monitoring Centre for Drugs and Drug Addiction. France Survey on Health and Consumption During the Day of Defense Preparation 2000.
French Monitoring Centre for Drugs and Drug Addiction. France Survey on Health and Consumption During the Day of Defense Preparation 2001.
French Monitoring Centre for Drugs and Drug Addiction. France Survey on Health and Consumption During the Day of Defense Preparation 2002.
French Monitoring Centre for Drugs and Drug Addiction. France Survey on Health and Consumption During the Day of Defense Preparation 2003.
French Monitoring Centre for Drugs and Drug Addiction. France Survey on Health and Consumption During the Day of Defense Preparation 2005.
French Monitoring Centre for Drugs and Drug Addiction. France Survey on Health and Consumption During the Day of Defense Preparation 2008.
French Monitoring Centre for Drugs and Drug Addiction. France Survey on Health and Consumption During the Day of Defense Preparation 2014.
Friedman JF, Kanzaria HK, Acosta LP, Langdon GC, Manalo DL, Wu H, Olveda RM, McGarvey ST, Kurtis JD. Relationship between Schistosoma japonicum and nutritional status among children and young adults in Leyte, the Philippines. Am J Trop Med Hyg. 2005; 72(5): 527-33.
Friis H, Mwaniki D, Omondi B, Muniu E, Magnussen P, Geissler W, Thiong'o F, Michaelsen KF. Serum retinol concentrations and Schistosoma mansoni, intestinal helminths, and malarial parasitemia: a cross-sectional study in Kenyan preschool and primary school children. Am J Clin Nutr. 1997; 66(3): 665-71. As it appears in Malaria Atlas Project. Malaria Atlas Project Plasmodium Falciparum Parasite Rate Database. Oxford, United Kingdom: Malaria Atlas Project.
Friis K, Lyng JI, Lasgaard M, Larsen FB. Energy drink consumption and the relation to socio-demographic factors and health behaviour among young adults in Denmark. A population-based study. Eur J Public Health. 2014; 24(5): 840â€“4.
Frisk F, Hakeberg M, Ahlqwist M, Bengtsson C. Endodontic variables and coronary heart disease. Acta Odontol Scand. 2003; 61(5): 257-62.

Appendix: Citation List

Citation
Fryauff D, Gomez-Saladin E, Purnomo, Sumawinata I, Sutamihardja MA, Tuti S, Subianto B, Richie TL. Comparative performance of the ParaSight F test for detection of Plasmodium falciparum in malaria-immune and nonimmune populations in Irian Jaya, Indonesia. Bull World Health Organ. 1997; 75(6): 547-52. As it appears in Malaria Atlas Project. Malaria Atlas Project Plasmodium Falciparum Parasite Rate Database. Oxford, United Kingdom: Malaria Atlas Project.
Fryauff DJ, Baird JK, Candraikusuma D, Masbar S, Sutamihardja MA, Leksana B, Tuti S, Marwoto H, Richie T, Romzan A. Survey of in Vivo Sensitivity to Chloroquine by Plasmodium falciparum and P. vivax in Lombok, Indonesia. Am J Trop Med Hyg. 1997; 56(2): 241-4. As it appears in Malaria Atlas Project. Malaria Atlas Project Plasmodium Falciparum Parasite Rate Database. Oxford, United Kingdom: Malaria Atlas Project.
Fryauff DJ, Leksana B, Masbar S, Wiady I, Sismadi P, Susanti AI, Nagesha HS, Atmosoedjono S, Bangs MJ, Baird JK. The drug sensitivity and transmission dynamics of human malaria on Nias Island, North Sumatra, Indonesia. Ann Trop Med Parasitol. 2002; 96(5): 447-62. As it appears in Malaria Atlas Project. Malaria Atlas Project Plasmodium Falciparum Parasite Rate Database. Oxford, United Kingdom: Malaria Atlas Project.
Fryauff DJ, Purnomo S, Sutamihardja MA, Elyazar IRS, Susanti I, Krisin, Subianto B, Marwoto H. Performance of the OptiMAL assay for detection and identification of malaria infections in asymptomatic residents of Irian Jaya. Am J Trop Med Hyg. 2000; 63(3-4): 139-45. As it appears in Malaria Atlas Project. Malaria Atlas Project Plasmodium Falciparum Parasite Rate Database. Oxford, United Kingdom: Malaria Atlas Project.
Fryauff DJ, Sumawinata I, Purnomo, Richie TL, Tjitra E, Bangs MJ, Kadir A, Ingkokusumo G. In vivo responses to antimalarials by Plasmodium falciparum and Plasmodium vivax from isolated Gag Island off northwest Irian Jaya, Indonesia. Am J Trop Med Hyg. 1999; 60(4): 542-6. As it appears in Malaria Atlas Project. Malaria Atlas Project Plasmodium Falciparum Parasite Rate Database. Oxford, United Kingdom: Malaria Atlas Project.
Fryauff DJ, Tuti S, Mardi A, Masbar S, Patipelohi R, Leksana B, Kain KC, Bangs MJ, Richie TL, Baird JK. Chloroquine-resistant Plasmodium vivax in transmigration settlements of West Kalimantan, Indonesia. Am J Trop Med Hyg. 1998; 59(4): 513-8. As it appears in Malaria Atlas Project. Malaria Atlas Project Plasmodium Falciparum Parasite Rate Database. Oxford, United Kingdom: Malaria Atlas Project.
Fu Q, Li SZ, Wu WP, Hou YY, Zhang S, Feng Y, Zhang LP, Tang LH. Endemic characteristics of infantile visceral leishmaniasis in the People's Republic of China. Parasit Vectors. 2013; 143.
Fuh JL, Wang SJ, Hwu CM, Lu SR. Glucose tolerance status and cognitive impairment in early middle-aged women. Diabet Med. 2007; 24(7): 788-91.
Fujii Y, Kaneko S, Nzou SM, Mwau M, Njenga SM, Tanigawa C, Kimotho J, Mwangi AW, Kiche I, Matsumoto S, Niki M, Osada-Oka M, Ichinose Y, Inoue M, Itoh M, Tachibana H, Ishii K, Tsuboi T, Yoshida LM, Mondal D, Haque R, Hamano S, Changoma M, Hoshi T, Kamo K-I, Karama M, Miura M, Hirayama K. Serological surveillance development for tropical infectious diseases using simultaneous microsphere-based multiplex assays and finite mixture models. PLoS Negl Trop Dis. 2014; 8(7): e3040.
Fukuizumi K, Sata M, Suzuki H, Nakano H, Tanikawa K. Hepatitis C virus seroconversion rate in a hyperendemic area of HCV in Japan: a prospective study. Scand J Infect Dis. 1997; 29(4): 345-7.
Fulford AJ, Mbugua GG, Ouma JH, Kariuki HC, Sturrock RF, Butterworth AE. Differences in the rate of hepatosplenomegaly due to Schistosoma mansoni infection between two areas in Machakos District, Kenya. Trans R Soc Trop Med Hyg. 1991; 85(4): 481-8. As it appears in Malaria Atlas Project. Malaria Atlas Project Plasmodium Falciparum Parasite Rate Database. Oxford, United Kingdom: Malaria Atlas Project.
Furusyo N, Hayashi J, Ariyama I, Sawayama Y, Etoh Y, Kashiwagi S. Lower hepatitis G virus infection prevalence compared to hepatitis B and C virus infection prevalences. Dig Dis Sci. 2000; 45(1): 188-95.
Furusyo N, Hayashi J, Kakuda K, Sawayama Y, Ariyama I, Eddie R, Kashiwagi S. Markedly high seroprevalence of hepatitis B virus infection in comparison to hepatitis C virus and human T lymphotropic virus type-1 infections in selected Solomon Islands populations. Am J Trop Med Hyg. 1999; 61(1): 85-91.
GÃ³mez-Cabello A, Pedrero-Chamizo R, Olivares PR, HernÃ¡ndez-Perera R, RodrÃ-guez-Marroyo JA, Mata E, Aznar S, Villa JG, Espino-TorÃ³n L, Gusi N, GonzÃ¡lez-Gross M, CasajÃºs JA, Ara I, Vicente-RodrÃ-guez G, EXERNET Study Group. Sitting time increases the overweight and obesity risk independently of walking time in elderly people from Spain. Maturitas. 2012; 73(4): 337-43.
Gabon National Immunization Coverage Survey 1986.
Gabon National Immunization Coverage Survey 1987.
Gabon National Immunization Coverage Survey 1988.
Gabon National Immunization Coverage Survey 1989.
Gabon National Immunization Coverage Survey 1990.
Gabon National Immunization Coverage Survey 1991.
Gaffo AL, Guillén-Pinto D, Campos-Olazábal P, Burneo JG. [Cysticercosis as the main cause of partial seizures in children in Peru]. Rev Neurol. 2004; 39(10): 924–6.
Gajalakshmi V, Peto R, Kanaka S, Balasubramanian S. Verbal autopsy of 48 000 adult deaths attributable to medical causes in Chennai (formerly Madras), India. BMC Public Health. 2002; 2: 7.
Gajalakshmi V, Peto R, Kanimozhi VC, Whitlock G, Veeramani D. Cohort Profile: the Chennai prospective study of mortality among 500,000 adults in Tamil Nadu, South India. Int J Epidemiol. 2007; 36(6): 1190-5.
Gakidou E, Mallinger L, Abbott-Klafter J, Guerrero R, Villalpando S, Ridaura RL, Aekplakorn W, Naghavi M, Lim S, Lozano R, Murray CJ. Management of diabetes and associated cardiovascular risk factors in seven countries: a comparison of data from national health examination surveys. Bull World Health Organ. 2011; 89(3): 172-83.
Gallup International, World Bank (WB). Bulgaria Living Standards Measurement Survey 1995. Washington, DC, United States: World Bank (WB).
Gallup International, World Bank (WB). Bulgaria Living Standards Measurement Survey 1997. Washington, DC, United States: World Bank (WB).

Appendix: Citation List

Citation
Gallup Poll. Iceland Prevalence of Smoking 2005.
Gallus S, Colombo P, Scarpino V, Zuccaro P, Apolone G, La Vecchia C. Smoking in Italy, 2002. Tumori. 2002; 88(6): 453-6.
Gallus S, Odone A, Lugo A, Bosetti C, Colombo P, Zuccaro P, La Vecchia C. Overweight and obesity prevalence and determinants in Italy: an update to 2010. Eur J Nutr. 2013; 52(2): 677-85.
Gambia Bureau of Statistics (GBOS), ICF International, Ministry of Health and Social Welfare (Gambia). Gambia Demographic and Health Survey 2013. Fairfax, United States: ICF International, 2015.
Gambia Bureau of Statistics (GBOS), United Nations Children's Fund (UNICEF). Gambia Multiple Indicator Cluster Survey 2005-2006. New York, United States: United Nations Children's Fund (UNICEF).
Gambia Bureau of Statistics (GBOS), United Nations Children's Fund (UNICEF). Gambia Multiple Indicator Cluster Survey 2010.
Gambia Cancer Registry 1987-1989 - CI5 as it appears in Parkin DM, International Agency for Research on Cancer, International Association of Cancer Registries. Cancer Incidence in Five Continents. Vol. I to VIII. Lyon, France, IARCPress, 2005.
Gambia Cancer Registry 1997-1998 - CI5 as it appears in Parkin DM, International Agency for Research on Cancer, International Association of Cancer Registries. Cancer Incidence in Five Continents. Vol. I to VIII. Lyon, France, IARCPress, 2005.
Gambia EPI Coverage Evaluation Survey 1992.
Gambia Immunization Coverage Survey 2002.
Gambia Immunization Survey 2005.
Gambia Immunization Survey 2006.
Gambia National Household Poverty Survey 1998 as it appears in World Health Organization (WHO). WHO Household Energy Database. Geneva, Switzerland: World Health Organization (WHO), 2010.
Gambia Population and Housing Census 1973 as it appears in United Nations Statistics Division (UNSD). United Nations Demographic Yearbook. New York City, United States: United Nations Statistics Division (UNSD).
Gamboa-León R, Ramirez-Gonzalez C, Pacheco-Tucuch FS, O'Shea M, Rosecrans K, Pippitt J, Dumonteil E, Buekens P. Seroprevalence of Trypanosoma cruzi Among Mothers and Children in Rural Mayan Communities and Associated Reproductive Outcomes. Am J Trop Med Hyg. 2014; 91(2): 348–53.
Gandacu D, Glazer Y, Anis E, Karakis I, Warshavsky B, Slater P, Grotto I. Resurgence of cutaneous leishmaniasis in Israel, 2001-2012. Emerg Infect Dis. 2014; 20(10): 1605-11.
Gangaidzo IT, Moyo VM, Khumalo H, Saungweme T, Gomo Z, Rouault T, Gordeuk VR. Hepatitis C virus in Zimbabwe. Cent Afr J Med. 1997; 43(5): 122-5.
Ganju SA, Goel A. Sero-surveillance of HIV, HBV and HCV infections in antenatal and STD clinic attendees. J Commun Dis. 2004; 36(1): 60-2.
Ganyaglo GYK, Hill WC. A 6-year (2004-2009) review of maternal mortality at the Eastern Regional Hospital, Koforidua, Ghana. Semin Perinatol. 2012; 36(1): 79-83.
Gao WG, Dong YH, Pang ZC, Nan HR, Zhang L, Wang SJ, Ren J, Ning F, Qiao Q. Increasing trend in the prevalence of Type 2 diabetes and pre-diabetes in the Chinese rural and urban population in Qingdao, China. Diabet Med. 2009; 26(12): 1220-7.
Garancini MP, Calori G, Manara E, Izzo A, Ebbli E, Galli L, Boari L, Gallus G. An Italian population-based study of the prevalence of diabetes: some methodological aspects. Diabete Metab. 1993; 19(1 Pt 2): 116-20.
Garap JN, Sheeladevi S, Shamanna BR, Nirmalan PK, Brian G, Williams C. Blindness and vision impairment in the elderly of Papua New Guinea. Clin Experiment Ophthalmol. 2006; 34(4): 335-41.
Garba JA, Umar S. Aetiology of maternal mortality using verbal autopsy at Sokoto, North-Western Nigeria. Afr J Prim Health Care Fam Med. 2013; 5(1): 6 pages.
Garba M, Nayama M, Alio AP, Holloway ML, Hamisu BS, Salihu HM. Maternal mortality in Niger: a retrospective study in a high risk maternity. Afr J Med Med Sci. 2011; 40(4): 393-7.
Garbutt C, Simmons G, Patrick D, Miller T. The public hand hygiene practices of New Zealanders: a national survey. N Z Med J. 2007; 120(1265): U2810.
Garces RG, Sobel HL, Pabellon JA, Lopez JM Jr, de Quiroz Castro M, Nyunt-U S. A comparison of vital registration and reproductive-age mortality survey in Bukidnon, Philippines, 2008. Int J Gynaecol Obstet. 2012; 119(2): 121-4.
Garcez MR, Pereira JL, Fontanelli M de M, Marchioni DML, Fisberg RM. Prevalence of dyslipidemia according to the nutritional status in a representative sample of Sao Paulo. Arq Bras Cardiol. 2014; 103(6): 476â€“84.
Garcia-Fulgueiras A, Tormo MJ, Rodriguez T, Perez-Flores D, Chirlaque D, Navarro C. Prevalence of hepatitis B and C markers in the south-east of Spain: an unlinked community-based serosurvey of 2,203 adults. Scand J Infect Dis. 1996; 28(1): 17-20.
Garcia-Noval J, Allan JC, Fletes C, Moreno E, de Mata F, Alvarez R-T, de Alfaro HS, Yurrita P, Higueros-Morales H, Mencos F, Craig PS. Epidemiology of Taenia solium taeniasis and cysticercosis in two rural Guatemalan communities. Am J Trop Med Hyg. 1996; 55(3): 282.
Gardella F, Assi S, Simon F, Bogreau H, Eggelte T, Ba F, Foumane V, Henry M-C, Kientega PT, Basco L, Trape J-F, Lalou R, Martelloni M, Desbordes M, Baragatti M, Briolant S, Almeras L, Pradines B, Fusai T, Rogier C. Antimalarial drug use in general populations of tropical Africa. Malar J. 2008; 7(1): 124. As it appears in Malaria Atlas Project. Malaria Atlas Project Plasmodium Falciparum Parasite Rate Database. Oxford, United Kingdom: Malaria Atlas Project.
Gardete-Correia L, Boavida JM, Raposo JF, Mesquita AC, Fona C, Carvalho R, Massano-Cardoso S. First diabetes prevalence study in Portugal: PREVADIAB study. Diabet Med. 2010; 27(8): 879-81.
Gardner MJ, Winter PD, Pannett B, Powell CA. Follow Up Study Of Workers Manufacturing Chrysotile Asbestos Cement Products. Br J Ind Med. 1986; 43(11): 726-32 as it appears in Camargo MC, Stayner LT, Straif K, Reina M, Al-Alem U, Demers PA, Landrigan PJ. Occupational exposure to asbestos and ovarian cancer: a meta-analysis. Environ Health Perspect. 2011; 119(9): 1211-7.
Gardon J, Eberle F, Louis JP, Cheringou H, Trebucq A, Hengy C. Evaluation de la chloroquino-sensibilite de Plasmodium falciparum au Cameroun. Med Afr Noire. 1991; 38(4): 270-2. As it appears in Malaria Atlas Project. Malaria Atlas Project Plasmodium Falciparum Parasite Rate Database. Oxford, United Kingdom: Malaria Atlas Project.

Appendix: Citation List

Citation
Garenne M, Maire B, Fontaine O, Dieng K, Briend A. Risques de deces associes a differents etats nutritionnels chez l'enfant d'age prescolaire: Etude realisee a Niakhar (Senegal), 1983-1986 [Risk of Death Associated with Different Nutritional States in Children of Preschool age: Study Conducted in Niakhar (Senegal) 1983-1986]. Paris, France: Population and Development Research Center (CEPED) (France); 2000.
Garenne M, Mbaye K, Bah MD, Correa P. Risk factors for maternal mortality: a case-control study in Dakar hospitals (Senegal). Afr J Reprod Health. 1997; 1(1): 14-24.
Garenne M, Sauerborn R, Nougtara A, Borchert M, Benzler J, Diesfeld J. Direct and indirect estimates of maternal mortality in rural Burkina Faso. Stud Fam Plann. 1997; 28(1): 54-61.
Garenne M, Willie D, Maire B, Fontaine O, Eeckels R, Briend A, Van den Broeck J. Incidence and duration of severe wasting in two African populations. Public Health Nutr. 2009; 12(11): 1974-82.
Garfinkel L, Auerbach O, Joubert L. Involuntary smoking and lung cancer: a case-control study. J Natl Cancer Inst. 1985; 75(3): 463-9 as it appears in Stayner L, Bena J, Sasco AJ, Smith R, Steenland K, Kreuzer M, et al. Lung cancer risk and workplace exposure to environmental tobacco smoke. Am J Public Health 2007; 97(3): 545–551.
Garg S, Mathur DR, Garg DK. Comparison of seropositivity of HIV, HBV, HCV and syphilis in replacement and voluntary blood donors in western India. Indian J Pathol Microbiol. 2001; 44(4): 409-12.
Garjito TA, Jastal, Rosmini, Srikandi Y, Sasono PMD. Study to Determine the Risk Factors for Transmission (Transmission Dynamics) of Malaria in Palolo Subdistrict, Donggala Regency, Central Sulawesi Province. Jakarta, Indonesia: Agency for Health Research and Development, Ministry of Health (Indonesia), 2004. As it appears in Malaria Atlas Project. Malaria Atlas Project Plasmodium Falciparum Parasite Rate Database. Oxford, United Kingdom: Malaria Atlas Project.
Garmendia ML, Alonso FT, Kain J, Uauy R, Corvalan C. Alarming weight gain in women of a post-transitional country. Public Health Nutr. 2014; 17(3): 667â€“73.
Garrib A, Herbst AJ, Hosegood V, Newell M-L. Injury mortality in rural South Africa 2000 - 2007: rates and associated factors. Trop Med Int Health. 2011; 16(4): 439-46.
Garrib A, Jaffar S, Knight S, Bradshaw D, Bennish ML. Rates and causes of child mortality in an area of high HIV prevalence in rural South Africa. Trop Med Int Health. 2006; 11(12): 1841-8.
Gartner CE, Barendregt JJ, Hall WD. Predicting the future prevalence of cigarette smoking in Australia: how low can we go and by when?. Tob Control. 2009; 18(3): 183-9.
Gasarasi DB, Premji ZG, Mujinja PG, Mpembeni R. Acute adenolymphangitis due to bancroftian filariasis in Rufiji district, south east Tanzania. Acta Trop. 2000; 75(1): 19-28. As it appears in London School of Hygiene and Tropical Medicine. Global Atlas of Helminth Infections - Lymphatic Filiariasis.
Gascon J, Merlos A, Madrenys N, Torres JM, Bada JL. Epidemiology of malaria in Nyarutovu (Rwanda): a clinical, parasitological and serological study. Trans R Soc Trop Med Hyg. 1988; 82(2): 222. As it appears in Malaria Atlas Project. Malaria Atlas Project Plasmodium Falciparum Parasite Rate Database. Oxford, United Kingdom: Malaria Atlas Project.
Gatling W, Budd S, Walters D, Mullee MA, Goddard JR, Hill RD. Evidence of an increasing prevalence of diagnosed diabetes mellitus in the Poole area from 1983 to 1996. Diabet Med. 1998; 15(12): 1015-21.
Gauchan E, Malla T, Basnet S, Rao KS. Variability of presentations and CT-scan findings in children with neurocysticercosis. Kathmandu Univ Med J. 2011; 9(34): 17-21.
Gautret P, Barreto M, Méndez F, Zorrilla G, Carrasquilla G. High prevalence of malaria in a village of the Colombian Pacific Coast. Mem Inst Oswaldo Cruz. 1995; 90(5): 559-60. As it appears in Malaria Atlas Project. Malaria Atlas Project Plasmodium Falciparum Parasite Rate Database. Oxford, United Kingdom: Malaria Atlas Project.
Gaye O, Bah IB, Diallo S, Faye O, Baudon D. [Malaria morbidity in rural and urban areas in Senegal]. Med Trop (Mars). 1989; 49(1): 59-62. As it appears in Malaria Atlas Project. Malaria Atlas Project Plasmodium Falciparum Parasite Rate Database. Oxford, United Kingdom: Malaria Atlas Project.
Gazin P, Freier C, Turk P, Gineste B, Carnevale P. [Malaria in employees of an African industrial enterprise (Bobo Dioulasso, Burkina Faso)]. Ann Soc Belg Med Trop. 1988; 68(4): 285-92. As it appears in Malaria Atlas Project. Malaria Atlas Project Plasmodium Falciparum Parasite Rate Database. Oxford, United Kingdom: Malaria Atlas Project.
Gazin P, Louis JP, Mulder L, Eberle F, Jambou R, Moyroud, Hengy C. [Evaluation of Plasmodium falciparum susceptibility to chloroquine and amodiaquine using a simplified vivo, 7-day test in southern Cameroon]. Med Trop (Mars). 1990; 50(1): 27-31. As it appears in Malaria Atlas Project. Malaria Atlas Project Plasmodium Falciparum Parasite Rate Database. Oxford, United Kingdom: Malaria Atlas Project.
Gazin P, Robert V, Carnevale P. Le paludisme urbain à Bobo-Dioulasso (Burkina Faso) / 2 : Les indices paludologiques. Cahiers ORSTOM Entomol Medicale Parasitol. 1987; 25(1): 27-31. As it appears in Malaria Atlas Project. Malaria Atlas Project Plasmodium Falciparum Parasite Rate Database. Oxford, United Kingdom: Malaria Atlas Project.
Gazin P, Robert V, Cot M, Carnevale P. Plasmodium falciparum incidence and patency in a high seasonal transmission area of Burkina Faso. Trans R Soc Trop Med Hyg. 1988; 82(1): 50-5. As it appears in Malaria Atlas Project. Malaria Atlas Project Plasmodium Falciparum Parasite Rate Database. Oxford, United Kingdom: Malaria Atlas Project.
Gazin P, Robert V, Cot M, Simon J, Halna JM, Darriet F, Legrand D, Carnevale P, Ambroise-Thomas P. [Malaria in Oudalan, a Sahelian region of Burkina Faso]. Ann Soc Belg Med Trop. 1988; 68(3): 255-64. As it appears in Malaria Atlas Project. Malaria Atlas Project Plasmodium Falciparum Parasite Rate Database. Oxford, United Kingdom: Malaria Atlas Project.
Gazin P. Malaria in Burkina Faso: Epidemiological Study of Transmission, Parasitological Indices, Morbidity, and Lethality [dissertation]. Montpellier, France: University of Montpellier I, 1990. As it appears in Malaria Atlas Project. Malaria Atlas Project Plasmodium Falciparum Parasite Rate Database. Oxford, United Kingdom: Malaria Atlas Project.
Gbakima AA, Sahr F. Filariasis in the Kaiyamba Chiefdom, Moyamba District Sierra Leone: an epidemiological and clinical study. Public Health. 1996; 110(3): 169-74. As it appears in London School of Hygiene and Tropical Medicine. Global Atlas of Helminth Infections - Lymphatic Filiariasis.

Appendix: Citation List

Citation
Gbakima AA. Inland valley swamp rice development: malaria, schistosomiasis, onchocerciasis in south central Sierra Leone. Public Health. 1994; 108(2): 149-57. As it appears in Malaria Atlas Project. Malaria Atlas Project Plasmodium Falciparum Parasite Rate Database. Oxford, United Kingdom: Malaria Atlas Project.
Gbary AR, Guiguemdé TR, Ouedraogo JB. [Emergence of chloroquine-resistant malaria in West Africa: the case of Sokode (Togo)]. Trop Med Parasitol. 1988; 39(2): 142-4. As it appears in Malaria Atlas Project. Malaria Atlas Project Plasmodium Falciparum Parasite Rate Database. Oxford, United Kingdom: Malaria Atlas Project.
Gebel K, Ding D, Bauman AE. Volume and intensity of physical activity in a large population-based cohort of middle-aged and older Australians: prospective relationships with weight gain, and physical function. Prev Med. 2014; 60: 131â€“3.
Geiger C, Agustar HK, Compaoré G, Coulibaly B, Sié A, Becher H, Lanzer M, Jänisch T. Declining malaria parasite prevalence and trends of asymptomatic parasitaemia in a seasonal transmission setting in north-western Burkina Faso between 2000 and 2009-2012. Malar J. 2013; 12(1): 27. As it appears in Malaria Atlas Project. Malaria Atlas Project Plasmodium Falciparum Parasite Rate Database. Oxford, United Kingdom: Malaria Atlas Project.
Gelaye B, Revilla L, Lopez T, Sanchez S, Williams MA. Prevalence of metabolic syndrome and its relationship with leisure time physical activity among Peruvian adults. Eur J Clin Invest. 2009; 39(10): 891–8.
Gemmy Cheung CM, Li X, Cheng C-Y, Zheng Y, Mitchell P, Wang JJ, Jonas JB, Nangia V, Wong TY. Prevalence and risk factors for age-related macular degeneration in Indians: a comparative study in Singapore and India. Am J Ophthalmol. 2013; 155(4): 764–73.
General Administration of Statistics and Censuses (El Salvador), Ministry of Health (El Salvador), United Nations Children's Fund (UNICEF). El Salvador Multiple Indicator Cluster Survey 2014.
General Bureau of Statistics (Suriname), Ministry of Planning and Development Cooperation (Suriname), Ministry of Social Affairs and Housing (Suriname), United Nations Children's Fund (UNICEF). Suriname Multiple Indicator Cluster Survey 2010. New York, United States: United Nations Children's Fund (UNICEF), 2013.
General Bureau of Statistics (Suriname), Pan American Health Organization (PAHO), United Nations Children's Fund (UNICEF), United Nations Development Programme (UNDP). Suriname Multiple Indicator Cluster Survey 1999-2000. New York, United States: United Nations Children's Fund (UNICEF).
General Bureau of Statistics (Suriname). Suriname Census 2004.
General Directorate of Statistics (Gabon), ICF International, Ministry of Economy, Employment and Sustainable Development (Gabon), Ministry of Health (Gabon). Gabon Demographic and Health Survey - Maternal Mortality Data.
General Directorate of Statistics (Gabon), ICF International, Ministry of Economy, Employment and Sustainable Development (Gabon), Ministry of Health (Gabon). Gabon Demographic and Health Survey 2012. Fairfax, United States: ICF International, 2013.
General Directorate of Statistics and Economic Studies (Gabon), Macro International, Inc. Gabon Demographic and Health Survey 2000-2001. Calverton, United States: Macro International, Inc.
General Directorate of Statistics and Forecasting (Comoros), ICF International. Comoros Demographic and Health Survey 2012-2013. Fairfax, United States: ICF International, 2014.
General Information Authority (Libya). Libya Vital Statistics 2006. Tripoli, Libya: General Information Authority (Libya).
General Information Authority (Libya). Libya Vital Statistics 2007. Tripoli, Libya: General Information Authority (Libya).
General Information Authority (Libya). Libya Vital Statistics 2008. Tripoli, Libya: General Information Authority (Libya).
General Register Office for Scotland, Northern Ireland Statistics and Research Agency (NISRA), Office for National Statistics (United Kingdom). United Kingdom All-Cause Mortality Data 1950-2012. [Unpublished].
General Secretariat for Development Planning (Qatar), Ministry of Public Health (Qatar). Qatar Vital Statistics Annual Bulletin 2000.
General Secretariat for Development Planning (Qatar), Ministry of Public Health (Qatar). Qatar Vital Statistics Annual Bulletin 2002.
General Secretariat for Development Planning (Qatar), Ministry of Public Health (Qatar). Qatar Vital Statistics Annual Bulletin 2003.
General Statistical Office (Suriname), United Nations Children's Fund (UNICEF). Suriname Multiple Indicator Cluster Survey 2006. New York, United States: United Nations Children's Fund (UNICEF).
General Statistics Office (Viet Nam), Macro International, Inc, National Institute of Hygiene and Epidemiology (Viet Nam). Vietnam AIDS Indicator Survey 2005. Calverton, United States: Macro International, Inc.
General Statistics Office (Viet Nam), Macro International, Inc. Vietnam Demographic and Health Survey 2002. Calverton, United States: Macro International, Inc.
General Statistics Office (Viet Nam), Ministry of Health (Viet Nam). Vietnam National Health Survey 2001-2002.
General Statistics Office (Viet Nam), Minnesota Population Center. Viet Nam Population and Housing Census 1999 from the Integrated Public Use Microdata Series, International: [Machine-readable database]. Minneapolis: University of Minnesota.
General Statistics Office (Viet Nam), Minnesota Population Center. Viet Nam Population and Housing Census 2009 from the Integrated Public Use Microdata Series, International: [Machine-readable database]. Minneapolis: University of Minnesota.
General Statistics Office (Viet Nam), Minnesota Population Center. Viet Nam Population Census 1989 from the Integrated Public Use Microdata Series, International: [Machine-readable database]. Minneapolis: University of Minnesota.
General Statistics Office (Viet Nam), United Nations Children's Fund (UNICEF). Vietnam Multiple Indicator Cluster Survey 1996.
General Statistics Office (Viet Nam), United Nations Children's Fund (UNICEF). Vietnam Multiple Indicator Cluster Survey 2000. New York, United States: United Nations Children's Fund (UNICEF).
General Statistics Office (Viet Nam), United Nations Children's Fund (UNICEF). Vietnam Multiple Indicator Cluster Survey 2006. New York, United States: United Nations Children's Fund (UNICEF).
General Statistics Office (Viet Nam), United Nations Children's Fund (UNICEF). Vietnam Multiple Indicator Cluster Survey 2010-2011. New York, United States: United Nations Children's Fund (UNICEF).
General Statistics Office (Viet Nam), United Nations Children's Fund (UNICEF). Vietnam Multiple Indicator Cluster Survey 2013-2014. New York, United States: United Nations Children's Fund (UNICEF), 2015.

Appendix: Citation List

Citation
General Statistics Office (Viet Nam), United Nations Development Programme (UNDP), World Bank (WB). Viet Nam Living Standards Measurement Survey 2002. General Statistical Office, World Bank.
General Statistics Office (Viet Nam), United Nations Development Programme (UNDP), World Bank (WB). Viet Nam Living Standards Measurement Survey 2004. General Statistical Office, World Bank.
General Statistics Office (Viet Nam), United Nations Development Programme (UNDP), World Bank. Vietnam Living Standards Measurement Survey 2006.
General Statistics Office (Viet Nam), United Nations Population Fund (UNFPA). Vietnam Population and Housing Census 2009.
General Statistics Office (Viet Nam), United Nations Population Fund (UNFPA). Vietnam Population Change and Family Planning Survey 2011.
General Statistics Office (Viet Nam), World Bank. Vietnam Living Standards Measurement Survey 1997-1998. Washington DC, United States: World Bank.
General Statistics Office (Viet Nam). Population Change and Family Planning Survey 2007. Hanoi, Viet Nam: General Statistics Office (Viet Nam), 2008.
General Statistics Office (Viet Nam). Viet Nam Population Change and Family Planning Survey 2008. Hanoi, Viet Nam: General Statistics Office (Viet Nam).
General Statistics Office (Viet Nam). Vietnam Population Change and Family Planning Survey 2006.
General Statistics Office (Viet Nam). Vietnam Population Change and Family Planning Survey 2013.
Georgia Center for Disease Control (NCDC), Georgia Ministry of Labor, Health and Social Affairs (MOLHSA), Division of Reproductive Health-Centers for Disease Control and Prevention (CDC). (2001) Georgia Reproductive Health Survey 1999-2000. Atlanta, United States: Centers for Disease Control and Prevention (CDC).
Georgia Center for Disease Control (NCDC), Georgian Ministry of Labor Health and Social Affairs (MOLHSA), Division of Reproductive Health, Centers for Disease Control and Prevention (CDC). Georgia Reproductive Health Survey 2005. Atlanta, United States: Centers for Disease Control and Prevention (CDC).
Georgia Immunization Programme Evaluation 1999.
Georgia National Nutrition Survey 2009 as it appears in World Health Organization (WHO). WHO Global Database on Child Growth and Malnutrition. Geneva, Switzerland: World Health Organization (WHO).
Georgia Vital Registration - Deaths 1981 ICD9 as it appears in World Health Organization (WHO). WHO Mortality Database Version November 2015. Geneva, Switzerland: World Health Organization (WHO), 2015.
Georgia Vital Registration - Deaths 1982 ICD9 as it appears in World Health Organization (WHO). WHO Mortality Database Version November 2015. Geneva, Switzerland: World Health Organization (WHO), 2015.
Georgia Vital Registration - Deaths 1985 ICD9 as it appears in World Health Organization (WHO). WHO Mortality Database Version November 2015. Geneva, Switzerland: World Health Organization (WHO), 2015.
Georgia Vital Registration - Deaths 1986 ICD9 as it appears in World Health Organization (WHO). WHO Mortality Database Version November 2015. Geneva, Switzerland: World Health Organization (WHO), 2015.
Georgia Vital Registration - Deaths 1987 ICD9 as it appears in World Health Organization (WHO). WHO Mortality Database Version November 2015. Geneva, Switzerland: World Health Organization (WHO), 2015.
Georgia Vital Registration - Deaths 1988 ICD9 as it appears in World Health Organization (WHO). WHO Mortality Database Version November 2015. Geneva, Switzerland: World Health Organization (WHO), 2015.
Georgia Vital Registration - Deaths 1989 ICD9 as it appears in World Health Organization (WHO). WHO Mortality Database Version November 2015. Geneva, Switzerland: World Health Organization (WHO), 2015.
Georgia Vital Registration - Deaths 1990 ICD9 as it appears in World Health Organization (WHO). WHO Mortality Database Version November 2015. Geneva, Switzerland: World Health Organization (WHO), 2015.
Georgia Vital Registration - Deaths 1991 ICD9 as it appears in World Health Organization (WHO). WHO Mortality Database Version November 2015. Geneva, Switzerland: World Health Organization (WHO), 2015.
Georgia Vital Registration - Deaths 1992 ICD9 as it appears in World Health Organization (WHO). WHO Mortality Database Version November 2015. Geneva, Switzerland: World Health Organization (WHO), 2015.
Georgia Vital Registration - Deaths 1994 ICD9 as it appears in World Health Organization (WHO). WHO Mortality Database Version November 2015. Geneva, Switzerland: World Health Organization (WHO), 2015.
Georgia Vital Registration - Deaths 1995 ICD9 as it appears in World Health Organization (WHO). WHO Mortality Database Version November 2015. Geneva, Switzerland: World Health Organization (WHO), 2015.
Georgia Vital Registration - Deaths 1996 ICD9 as it appears in World Health Organization (WHO). WHO Mortality Database Version November 2015. Geneva, Switzerland: World Health Organization (WHO), 2015.
Georgia Vital Registration - Deaths 1997 ICD9 as it appears in World Health Organization (WHO). WHO Mortality Database Version November 2015. Geneva, Switzerland: World Health Organization (WHO), 2015.
Georgia Vital Registration - Deaths 1998 ICD10 as it appears in World Health Organization (WHO). WHO Mortality Database Version November 2015. Geneva, Switzerland: World Health Organization (WHO), 2015.
Georgia Vital Registration - Deaths 1999 ICD10 as it appears in World Health Organization (WHO). WHO Mortality Database Version November 2015. Geneva, Switzerland: World Health Organization (WHO), 2015.
Georgia Vital Registration - Deaths 2000 ICD10 as it appears in World Health Organization (WHO). WHO Mortality Database Version November 2015. Geneva, Switzerland: World Health Organization (WHO), 2015.
Georgia Vital Registration - Deaths 2001 ICD10 as it appears in World Health Organization (WHO). WHO Mortality Database Version November 2015. Geneva, Switzerland: World Health Organization (WHO), 2015.
Georgia Vital Registration - Deaths 2004 ICD10 as it appears in World Health Organization (WHO). WHO Mortality Database Version November 2015. Geneva, Switzerland: World Health Organization (WHO), 2015.

Appendix: Citation List

Citation
Georgia Vital Registration - Deaths 2005 ICD10 as it appears in World Health Organization (WHO). WHO Mortality Database Version November 2015. Geneva, Switzerland: World Health Organization (WHO), 2015.
Georgia Vital Registration - Deaths 2006 ICD10 as it appears in World Health Organization (WHO). WHO Mortality Database Version November 2015. Geneva, Switzerland: World Health Organization (WHO), 2015.
Georgia Vital Registration - Deaths 2007 ICD10 as it appears in World Health Organization (WHO). WHO Mortality Database Version November 2015. Geneva, Switzerland: World Health Organization (WHO), 2015.
Georgia Vital Registration - Deaths 2009 ICD10 as it appears in World Health Organization (WHO). WHO Mortality Database Version November 2015. Geneva, Switzerland: World Health Organization (WHO), 2015.
Georgia Vital Registration - Deaths 2010 ICD10 as it appears in World Health Organization (WHO). WHO Mortality Database Version November 2015. Geneva, Switzerland: World Health Organization (WHO), 2015.
Georgia Vital Registration - Deaths 2011 ICD10 as it appears in World Health Organization (WHO). WHO Mortality Database Version November 2015. Geneva, Switzerland: World Health Organization (WHO), 2015.
Georgia Vital Registration - Deaths 2012 ICD10 as it appears in World Health Organization (WHO). WHO Mortality Database Version November 2015. Geneva, Switzerland: World Health Organization (WHO), 2015.
Georgia Vital Registration - Deaths 2013 ICD10 as it appears in World Health Organization (WHO). WHO Mortality Database Version November 2015. Geneva, Switzerland: World Health Organization (WHO), 2015.
Georgia Vital Registration - Deaths 2014 ICD10 as it appears in World Health Organization (WHO). WHO Mortality Database Version November 2015. Geneva, Switzerland: World Health Organization (WHO), 2015.
Georgia Vital Registration Death Data 2003 as it appears in United Nations Statistics Division (UNSD). United Nations Demographic Yearbook 2004. New York City, United States: United Nations Statistics Division (UNSD), 2007.
Georgia Vital Registration Death Data 2008 as it appears in United Nations Statistics Division (UNSD). United Nations Demographic Yearbook 2009-2010. New York City, United States: United Nations Statistics Division (UNSD), 2011.
Georgia Vital Registration Death Data 2012 as it appears in United Nations Statistics Division (UNSD). United Nations Demographic Yearbook 2012. New York City, United States: United Nations Statistics Division (UNSD), 2013.
Georgia Vital Statistics - Deaths 2002 as it appears in TransMonEE 2013 Database, UNICEF Regional Office for CEE/CIS.
Germani D, Belli S, Bruno C, Grignoli M, Nesti M, Pirastu R, Comba P. Cohort Mortality Study Of Women Compensated For Asbestosis In Italy. Am J Ind Med. 1999; 36(1): 129-34 as it appears in Camargo MC, Stayner LT, Straif K, Reina M, Al-Alem U, Demers PA, Landrigan PJ. Occupational exposure to asbestos and ovarian cancer: a meta-analysis. Environ Health Perspect. 2011; 119(9): 1211-7.
Germany - Hamburg Cancer Registry 1978-1979 - CI5 as it appears in Parkin DM, International Agency for Research on Cancer, International Association of Cancer Registries. Cancer Incidence in Five Continents. Vol. I to VIII. Lyon, France, IARCPress, 2005.
Germany - Saarland Cancer Registry 1978-1982 - CI5 as it appears in Parkin DM, International Agency for Research on Cancer, International Association of Cancer Registries. Cancer Incidence in Five Continents. Vol. I to VIII. Lyon, France, IARCPress, 2005.
Germany - Saarland Cancer Registry 1983-1987 - CI5 as it appears in Parkin DM, International Agency for Research on Cancer, International Association of Cancer Registries. Cancer Incidence in Five Continents. Vol. I to VIII. Lyon, France, IARCPress, 2005.
Germany - Saarland Cancer Registry 1988-1992 - CI5 as it appears in Parkin DM, International Agency for Research on Cancer, International Association of Cancer Registries. Cancer Incidence in Five Continents. Vol. I to VIII. Lyon, France, IARCPress, 2005.
Germany - Saarland Cancer Registry 1993-1997 - CI5 as it appears in Parkin DM, International Agency for Research on Cancer, International Association of Cancer Registries. Cancer Incidence in Five Continents. Vol. I to VIII. Lyon, France, IARCPress, 2005.
Germany Eurobarometer 38: European Court of Justice, Passive Smoking, And Consumer Issues 1992 as it appears in P.N. Lee Statistics and Computing Ltd. International Mortality and Smoking Statistics Version 4.04. Sutton, United Kingdom: P.N. Lee Statistics and Computing Ltd, 2009.
Germany Microcensus 1992 as it appears in P.N. Lee Statistics and Computing Ltd. International Mortality and Smoking Statistics Version 4.04. Sutton, United Kingdom: P.N. Lee Statistics and Computing Ltd, 2009.
Germany Microcensus 1999 as it appears in P.N. Lee Statistics and Computing Ltd. International Mortality and Smoking Statistics Version 4.04. Sutton, United Kingdom: P.N. Lee Statistics and Computing Ltd, 2009.
Germany Microcensus 2003 as it appears in P.N. Lee Statistics and Computing Ltd. International Mortality and Smoking Statistics Version 4.04. Sutton, United Kingdom: P.N. Lee Statistics and Computing Ltd, 2009.
Germany Telephone Health Survey 2002-2003 as it appears in P.N. Lee Statistics and Computing Ltd. International Mortality and Smoking Statistics Version 4.04. Sutton, United Kingdom: P.N. Lee Statistics and Computing Ltd, 2009.
Germany Vital Registration - Deaths 1991 ICD9 as it appears in World Health Organization (WHO). WHO Mortality Database Version November 2015. Geneva, Switzerland: World Health Organization (WHO), 2015.
Germany Vital Registration - Deaths 1992 ICD9 as it appears in World Health Organization (WHO). WHO Mortality Database Version November 2015. Geneva, Switzerland: World Health Organization (WHO), 2015.
Germany Vital Registration - Deaths 1993 ICD9 as it appears in World Health Organization (WHO). WHO Mortality Database Version November 2015. Geneva, Switzerland: World Health Organization (WHO), 2015.
Germany Vital Registration - Deaths 1994 ICD9 as it appears in World Health Organization (WHO). WHO Mortality Database Version November 2015. Geneva, Switzerland: World Health Organization (WHO), 2015.
Germany Vital Registration - Deaths 1995 ICD9 as it appears in World Health Organization (WHO). WHO Mortality Database Version November 2015. Geneva, Switzerland: World Health Organization (WHO), 2015.
Germany Vital Registration - Deaths 1996 ICD9 as it appears in World Health Organization (WHO). WHO Mortality Database Version November 2015. Geneva, Switzerland: World Health Organization (WHO), 2015.
Germany Vital Registration - Deaths 1997 ICD9 as it appears in World Health Organization (WHO). WHO Mortality Database Version November 2015. Geneva, Switzerland: World Health Organization (WHO), 2015.

Appendix: Citation List

Citation
Germany Vital Registration - Deaths 1998 ICD10 as it appears in World Health Organization (WHO). WHO Mortality Database Version November 2015. Geneva, Switzerland: World Health Organization (WHO), 2015.
Germany Vital Registration - Deaths 2000 ICD10 as it appears in World Health Organization (WHO). WHO Mortality Database Version November 2015. Geneva, Switzerland: World Health Organization (WHO), 2015.
Germany Vital Registration - Deaths 2003 ICD10 as it appears in World Health Organization (WHO). WHO Mortality Database Version November 2015. Geneva, Switzerland: World Health Organization (WHO), 2015.
Germany Vital Registration - Deaths 2004 ICD10 as it appears in World Health Organization (WHO). WHO Mortality Database Version November 2015. Geneva, Switzerland: World Health Organization (WHO), 2015.
Germany Vital Registration - Deaths 2005 ICD10 as it appears in World Health Organization (WHO). WHO Mortality Database Version November 2015. Geneva, Switzerland: World Health Organization (WHO), 2015.
Germany Vital Registration - Deaths 2006 ICD10 as it appears in World Health Organization (WHO). WHO Mortality Database Version November 2015. Geneva, Switzerland: World Health Organization (WHO), 2015.
Germany Vital Registration - Deaths 1999 ICD10 as it appears in World Health Organization (WHO). WHO Mortality Database Version November 2015. Geneva, Switzerland: World Health Organization (WHO), 2015.
Germany Vital Registration - Deaths 2001 ICD10 as it appears in World Health Organization (WHO). WHO Mortality Database Version November 2015. Geneva, Switzerland: World Health Organization (WHO), 2015.
Germany Vital Registration - Deaths 2002 ICD10 as it appears in World Health Organization (WHO). WHO Mortality Database Version November 2015. Geneva, Switzerland: World Health Organization (WHO), 2015.
Germany Vital Registration - Deaths 2007 ICD10 as it appears in World Health Organization (WHO). WHO Mortality Database Version November 2015. Geneva, Switzerland: World Health Organization (WHO), 2015.
Germany Vital Registration - Deaths 2008 ICD10 as it appears in World Health Organization (WHO). WHO Mortality Database Version November 2015. Geneva, Switzerland: World Health Organization (WHO), 2015.
Germany Vital Registration - Deaths 2009 ICD10 as it appears in World Health Organization (WHO). WHO Mortality Database Version November 2015. Geneva, Switzerland: World Health Organization (WHO), 2015.
Germany Vital Registration - Deaths 2010 ICD10 as it appears in World Health Organization (WHO). WHO Mortality Database Version November 2015. Geneva, Switzerland: World Health Organization (WHO), 2015.
Germany Vital Registration - Deaths 2011 ICD10 as it appears in World Health Organization (WHO). WHO Mortality Database Version November 2015. Geneva, Switzerland: World Health Organization (WHO), 2015.
Germany Vital Registration - Deaths 2012 ICD10 as it appears in World Health Organization (WHO). WHO Mortality Database Version November 2015. Geneva, Switzerland: World Health Organization (WHO), 2015.
Germany Vital Registration - Deaths 2013 ICD10 as it appears in World Health Organization (WHO). WHO Mortality Database Version November 2015. Geneva, Switzerland: World Health Organization (WHO), 2015.
Gessoni G, Manoni F. Prevalence of anti-hepatitis C virus antibodies among teenagers in the Venetian area: a seroepidemiological study. Eur J Med. 1993; 2(2): 79-82.
Getahun H. Marriage through abduction ('Telefa') in rural north west Ethiopia. Ethiop Med J. 2001; 39(2): 105-12.
GfK, Netherlands Nutrition Centre Foundation, TNO Nutrition. Netherlands National Food Consumption Survey 1997-1998.
Ghaem Maralani H, Tai BC, Wong TY, Tai ES, Li J, Wang JJ, Mitchell P. The prognostic role of body mass index on mortality amongst the middle-aged and elderly: a competing risk analysis. Diabetes Res Clin Pract. 2014; 103(1): 42â€“50.
Ghana Core Welfare Indicators Survey 1997 as it appears in World Health Organization (WHO). WHO Household Energy Database. Geneva, Switzerland: World Health Organization (WHO), 2010.
Ghana EPI Cluster Survey 2012.
Ghana Health Service, Ghana Statistical Service, ICF International. Ghana Demographic and Health Survey 2014. Fairfax, United States: ICF International, 2015.
Ghana Health Service, Ghana Statistical Service, Macro International, Inc. Ghana Special Demographic and Health Survey 2007-2008. Calverton, United States: Macro International, Inc, 2010.
Ghana Health Service, Ministry of Health (Ghana), University of Ghana, World Health Organization (WHO). Ghana WHO Study on Global AGEing and Adult Health 2007-2008.
Ghana Health Service, World Health Organization (WHO). Ghana - Greater Accra Region STEPS Noncommunicable Disease Risk Factors Survey 2006.
Ghana Progress/Utilization Report on the EPI and Safe Motherhood Program 1990.
Ghana Progress/Utilization Report on the EPI and Safe Motherhood Program 1994.
Ghana Statistical Service, Macro International, Inc, Ministry of Health (Ghana). Ghana Demographic and Health Survey 2008. Calverton, United States: Macro International, Inc.
Ghana Statistical Service, Macro International, Inc. Ghana Demographic and Health Survey - Maternal Mortality Data.
Ghana Statistical Service, Macro International, Inc. Ghana Demographic and Health Survey 1993-1994. Calverton, United States: Macro International, Inc.
Ghana Statistical Service, Macro International, Inc. Ghana Demographic and Health Survey 1998-1999. Calverton, United States: Macro International, Inc.
Ghana Statistical Service, Macro International, Inc. Ghana Demographic and Health Survey 2003. Calverton, United States: Macro International, Inc.
Ghana Statistical Service, Macro Systems, Inc.; Institute for Resource Development. Ghana Demographic and Health Survey 1988. Columbia, United States: Macro Systems, Inc.
Ghana Statistical Service, Minnesota Population Center. Ghana Census 2010 from the Integrated Public Use Microdata Series, International: [Machine-readable database]. Minneapolis: University of Minnesota.

Appendix: Citation List

Citation
Ghana Statistical Service, Minnesota Population Center. Ghana Population and Housing Census 2000 from the Integrated Public Use Microdata Series, International: [Machine-readable database]. Minneapolis: University of Minnesota.
Ghana Statistical Service, World Bank. Ghana Core Welfare Indicators Survey 1997.
Ghana Statistical Service. Ghana Child Labor Survey 2001. Accra, Ghana Statistical Service.
Ghana Statistical Service. Ghana Living Standards Measurement Survey 2005-2006. Accra, Ghana Statistical Service.
Ghana Statistical Service. Ghana Living Standards Survey 1998-1999.
Ghana VAST Study Team. Vitamin A supplementation in northern Ghana: effects on clinic attendances, hospital admissions, and child mortality. Lancet. 1993; 342(8862): 7-12.
Gharbi M, Akrout M, Zouari B. Prevalence and risk factors of non-insulin-dependent diabetes mellitus in the rural and urban population of Tunisia. Rev Epidemiol Sante Publique. 2002; 50(4): 349-55.
Ghasemi A, Zahediasl S, Syedmoradi L, Azizi F. Association between serum nitric oxide metabolites and hypertension in a general population. Int Angiol. 2011; 30(4): 380–7.
Ghazizadeh A. Domestic violence: a crosssectional study in an Iranian city. East Mediterr Health J. 2005; 11(5-6): 880-7.
Ghebreyesus TA, Haile M, Getachew A, Alemayehu T, Witten KH, Medhin A, Yohannes M, Asgedom Y, Ye-ebiyo Y, Lindsay SW, Byass P. Pilot studies on the possible effects on malaria of small-scale irrigation dams in Tigray regional state, Ethiopia. J Public Health. 1998; 20(2): 238-40. As it appears in Malaria Atlas Project. Malaria Atlas Project Plasmodium Falciparum Parasite Rate Database. Oxford, United Kingdom: Malaria Atlas Project.
Ghorbani A, Ziaee A, Esmailzadehha N, Javadi H. Association between health-related quality of life and impaired glucose metabolism in Iran: the Qazvin Metabolic Diseases Study. Diabet Med. 2014; 31(6): 754–8.
Ghosh SK, Yadav RS, Das BS, Sharma VP. Influence of nutritional and haemoglobin status on malaria infection in children. Indian J Pediatr. 1995; 62(3): 321-6. As it appears in Malaria Atlas Project. Malaria Atlas Project Plasmodium Falciparum Parasite Rate Database. Oxford, United Kingdom: Malaria Atlas Project.
Giannouli P, Zervas I, Armeni E, Koundi K, Spyropoulou A, Alexandrou A, Kazani A, Areti A, Creatsa M, Lambrinoudaki I. Determinants of quality of life in Greek middle-age women: a population survey. Maturitas. 2012; 71(2): 154-61.
Giboda M, Pholsena K, Hongvanthong B, Gutvirth J, Rubik I. Malariometric survey in Keoudom District, Laos: sensitivity of Plasmodium falciparum to anti-malarials and automedication with chloroquine. Southeast Asian J Trop Med Public Health. 1992; 23(3): 383-8. As it appears in Malaria Atlas Project. Malaria Atlas Project Plasmodium Falciparum Parasite Rate Database. Oxford, United Kingdom: Malaria Atlas Project.
Giday A, Tadesse B. Prevalence and determinants of hypertension in rural and urban areas of southern Ethiopia. Ethiop Med J. 2011; 49(2): 139–47.
Gidding HF, Warlow M, MacIntyre CR, Backhouse J, Gilbert GL, Quinn HE, McIntyre PB. The impact of a new universal infant and school-based adolescent hepatitis B vaccination program in Australia. Vaccine. 2007; 25(51): 8637-41.
Giha HA, Nasr A, Iriemenam NC, Balogun HA, Arnot D, Theander TG, Troye-Blomberg M, Berzins K, ElGhazali G. Age-dependent association between IgG2 and IgG3 subclasses to Pf332-C231 antigen and protection from malaria, and induction of protective antibodies by sub-patent malaria infections, in Daraweesh. Vaccine. 2010; 28(7): 1732-9. As it appears in Malaria Atlas Project. Malaria Atlas Project Plasmodium Falciparum Parasite Rate Database. Oxford, United Kingdom: Malaria Atlas Project.
Gil J, Mora T. The determinants of misreporting weight and height: The role of social norms. Econ Hum Biol. 2011; 9(1): 78-91.
Gilani SI, Leon DA. Prevalence and sociodemographic determinants of tobacco use among adults in Pakistan: findings of a nationwide survey conducted in 2012. Popul Health Metr. 2013; 11(1): 16.
Gilbert NL, Dare OK, Libman MD, Muchaal PK, Ogden NH. Hospitalization for trichinellosis and echinococcosis in Canada, 2001-2005: the tip of the iceberg? Can J Public Health. 2010; 101(4): 337-40.
Gill CJ, Phiri-Mazala G, Guerina NG, Kasimba J, Mulenga C, MacLeod WB, Waitolo N, Knapp AB, Mirochnick M, Mazimba A, Fox MP, Sabin L, Seidenberg P, Simon JL, Hamer DH. Effect of training traditional birth attendants on neonatal mortality (Lufwanyama Neonatal Survival Project): randomised controlled study. BMJ. 2011; 342: d346.
Gill HK, Yadav SB, Ramesh V, Bhatia E. A prospective study of prevalence and association of peripheral neuropathy in Indian patients with newly diagnosed type 2 diabetes mellitus. J Postgrad Med. 2014; 60(3): 270–5.
Gill TK, Hill CL, Shanahan EM, Taylor AW, Appleton SL, Grant JF, Shi Z, Dal Grande E, Price K, Adams RJ. Vitamin D levels in an Australian population. BMC Public Health. 2014; 14: 1001.
Gilmore AB, McKee M, Rose R. Prevalence and determinants of smoking in Belarus: a national household survey, 2000. Eur J Epidemiol. 2001; 17(3): 245â€“53.
Gilmore AB, McKee M, Telishevska M, Rose R. Epidemiology of smoking in Ukraine, 2000. Prev Med. 2001; 33(5): 453-61.
Gilsing AMJ, Weijenberg MP, Hughes LAE, Ambergen T, Dagnelie PC, Goldbohm RA, Brandt PA van den, Schouten LJ. Longitudinal changes in BMI in older adults are associated with meat consumption differentially, by type of meat consumed. J Nutr. 2012; 142(2): 340-9.
Girardin O, Dao D, Koudou BG, Essé C, Cissé G, Yao T, N'Goran EK, Tschannen AB, Bordmann G, Lehmann B, Nsabimana C, Keiser J, Killeen GF, Singer BH, Tanner M, Utzinger J. Opportunities and limiting factors of intensive vegetable farming in malaria endemic Côte d'Ivoire. Acta Trop. 2004; 89(2): 109-23. As it appears in Malaria Atlas Project. Malaria Atlas Project Plasmodium Falciparum Parasite Rate Database. Oxford, United Kingdom: Malaria Atlas Project.
Giri BR, Sharma KP, Chapagai RN, Palzom D. Diabetes and hypertension in urban bhutanese men and women. Indian J Community Med. 2013; 38(3): 138-43.
Gissler M, Berg C, Bouvier-Colle M-H, Buekens P. Methods for identifying pregnancy-associated deaths: population-based data from Finland 1987-2000. Paediatr Perinat Epidemiol. 2004; 18(6): 448-55.
Gissler M, Deneux-Tharaux C, Alexander S, Berg CJ, Bouvier-Colle M-H, Harper M, Nannini A, Bréart G, Buekens P. Pregnancy-related deaths in four regions of Europe and the United States in 1999-2000: characterisation of unreported deaths. Eur J Obstet Gynecol Reprod Biol. 2007; 133(2): 179-85.

Appendix: Citation List

Citation
Gissler M, Kauppila R, Meriläinen J, Toukomaa H, Hemminki E. Pregnancy-associated deaths in Finland 1987-1994--definition problems and benefits of record linkage. Acta Obstet Gynecol Scand. 1997; 76(7): 651-7.
Gitonga CW, Karanja PN, Kihara J, Mwanje M, Juma E, Snow RW, Noor AM, Brooker S. Implementing school malaria surveys in Kenya: towards a national surveillance system. Malar J. 2010; 9: 306. As it appears in Malaria Atlas Project. Malaria Atlas Project Plasmodium Falciparum Parasite Rate Database. Oxford, United Kingdom: Malaria Atlas Project.
Gkolfinopoulou K, Bitsolas N, Patrinos S, Veneti L, Marka A, Dougas G, Pervanidou D, Detsis M, Triantafillou E, Georgakopoulou T, Billinis C, Kremastinou J, Hadjichristodoulou C. Epidemiology of human leishmaniasis in Greece, 1981-2011. Euro Surveill. 2013; 18(29): 20532.
Glasgow KW, Schabas R, Williams DC, Wallace E, Nalezyty LA. A population-based hepatitis B seroprevalence and risk factor study in a northern Ontario town. Can J Public Health. 1997; 88(2): 87-90.
Glass DC, Gray CN, Jolley DJ, Gibbons C, Sim MR, Fritschi L, Adams GG, Bisby JA, Manuell R. Leukemia risk associated with low-level benzene exposure. Epidemiology. 2003; 14(5): 569-77 as it appears in Khalade A, Jaakkola MS, Pukkala E, Jaakkola JJK. Exposure to benzene at work and the risk of leukemia: a systematic review and meta-analysis. Environ Health 2010; 9: 31.
Gliksman MD, Dwyer T, Wlodarczyk J, Pierce JP. Cigarette smoking in Australian schoolchildren. Med J Aust. 1989; 150(2): 81â€“4.
Global Alliance for Vaccines and Immunization (GAVI). Togo EPI Review 2012-2013.
Global Alliance for Vaccines and Immunization (GAVI). Togo External EPI Review 2001.
Global Fund to Fight Aids Tuberculosis and Malaria (GFATM), ICF International, National Center for Endemic Diseases (CNE) (Sao Tome and Principe), National Institute of Statistics (Sao Tome and Principe), United Nations Children's Fund (UNICEF), United Nations Development Programme (UNDP). Sao Tome and Principe Multiple Indicator Cluster Survey 2014. New York, United States: United Nations Children's Fund (UNICEF), 2016.
Global Fund to Fight Aids Tuberculosis and Malaria (GFATM). Haiti Global Fund Household Survey 2008.
Global Lower Extremity Amputation Study Group. Epidemiology of lower extremity amputation in centres in Europe, North America and East Asia. The Global Lower Extremity Amputation Study Group. Br J Surg. 2000; 87(3): 328-37.
Glumer C, Jorgensen T, Borch-Johnsen K, Inter99 study. Prevalences of diabetes and impaired glucose regulation in a Danish population: the Inter99 study. Diabetes Care. 2003; 26(8): 2335–40.
Glynn JR, Calvert C, Price A, Chihana M, Kachiwanda L, Mboma S, Zaba B, Crampin AC. Measuring causes of adult mortality in rural northern Malawi over a decade of change. Glob Health Action. 2014; 7.
Gnémé A, Guelbéogo WM, Riehle MM, Tiono AB, Diarra A, Kabré GB, Sagnon N, Vernick KD. Plasmodium species occurrence, temporal distribution and interaction in a child-aged population in rural Burkina Faso. Malar J. 2013; 12(1): 67. As it appears in Malaria Atlas Project. Malaria Atlas Project Plasmodium Falciparum Parasite Rate Database. Oxford, United Kingdom: Malaria Atlas Project.
Goel D, Dhanai JS, Agarwal A, Mehlotra V, Saxena V. Neurocysticercosis and its impact on crude prevalence rate of epilepsy in an Indian community. Neurol India. 2011; 59(1): 37-40.
Gogos CA, Fouka KP, Nikiforidis G, Avgeridis K, Sakellaropoulos G, Bassaris H, Maniatis A, Skoutelis A. Prevalence of hepatitis B and C virus infection in the general population and selected groups in South-Western Greece. Eur J Epidemiol. 2003; 18(6): 551-7.
Goh KT, Chan YW, Wong LY, Kong KH, Oon CJ, Guan R. The prevalence of hepatitis B virus markers in dental personnel in Singapore. Trans R Soc Trop Med Hyg. 1988; 82(6): 908-10.
Goh KT, Kong KH, Heng BH, Oon CJ. Seroepidemiology of hepatitis A and hepatitis B virus infection in a Gurkha community in Singapore. J Med Virol. 1993; 41(2): 146-9.
Goh KT. Prevention and control of hepatitis B virus infection in Singapore. Ann Acad Med Singapore. 1997; 26(5): 671-81.
Gokcel A, Ozsahin AK, Sezgin N, Karakose H, Ertorer ME, Akbaba M, Baklaci N, Sengul A, Guvener N. High prevalence of diabetes in Adana, a southern province of Turkey. Diabetes Care. 2003; 26(11): 3031-4.
Goldney RD, Dunn KI, Air TM, Dal Grande E, Taylor AW. Relationships between body mass index, mental health, and suicidal ideation: population perspective using two methods. Aust N Z J Psychiatry. 2009; 43(7): 652-8.
Goldsmith R, Nahmias J, Schantz P, Peleg H, Shtamler B, el-On J. Resurgence of hydatid disease (echinococcosis) in communities in northern Israel. Trans R Soc Trop Med Hyg. 1991; 85(1): 98-100.
Golozar A, Khademi H, Kamangar F, Poutschi H, Islami F, Abnet CC, Freedman ND, Taylor PR, Pharoah P, Boffetta P, Brennan PJ, Dawsey SM, Malekzadeh R, Etemadi A. Diabetes mellitus and its correlates in an Iranian adult population. PLoS One. 2011; 6(10): e26725.
Gomes A de C, Paula MB de, Duarte AMR de C, Lima MA, Malafronte R dos S, Mucci LF, Gotlieb SLD, Natal D. Epidemiological and ecological aspects related to malaria in the area of influence of the lake at Porto Primavera dam, in western Sao Paulo State, Brazil. Rev Inst Med Trop Sao Paulo. 2008; 50(5): 287-95. As it appears in Malaria Atlas Project. Malaria Atlas Project Plasmodium Falciparum Parasite Rate Database. Oxford, United Kingdom: Malaria Atlas Project.
Gomes C dos S, Maciel ACC, Freire A do NF, Moreira M de A, Ribeiro M de O, Guerra RO. Depressive symptoms and functional decline in an elderly sample of urban center in Northeastern Brazil. Arch Gerontol Geriatr. 2014; 58(2): 214â€“8.
Gomes MC, Ventura MT, Nunes RS. How many maternal deaths are there in Portugal? J Matern Fetal Neonatal Med. 2012; 25(10): 1975-9.
Gomez-Barroso D, Herrador Z, San Martin JV, Gherasim A, Aguado M, Romero-Mate A, Molina L, Aparicio P, Benito A. Spatial distribution and cluster analysis of a leishmaniasis outbreak in the south-western Madrid region, Spain, September 2009 to April 2013. Euro Surveill. 2015; 20(7): 11-20.
Gomez-Huelgas R, Mancera-Romero J, Bernal-Lopez MR, Jansen-Chaparro S, Baca-Osorio AJ, Toledo E, Perez-Gonzalez R, Guijarro-Merino R, Tinahones FJ, Martinez-Gonzalez MA. Prevalence of cardiovascular risk factors in an urban adult population from southern Spain. IMAP Study. Int J Clin Pract. 2011; 65(1): 35–40.
Goncalves A, Ferrinho P, Dias F. The epidemiology of malaria in Prabis, Guinea-Bissau. Mem Inst Oswaldo Cruz. 1996; 91(1): 11-7. As it appears in Malaria Atlas Project. Malaria Atlas Project Plasmodium Falciparum Parasite Rate Database. Oxford, United Kingdom: Malaria Atlas Project.
Gong C, Meng X, Jiang Y, Wang X, Cui H, Chen X. Trends in childhood type 1 diabetes mellitus incidence in Beijing from 1995 to 2010: a retrospective multicenter study based on hospitalization data. Diabetes Technol Ther. 2015; 17(3): 159-65.

Appendix: Citation List

Citation
Gonlugur U, Ozcelik S, Gonlugur TE, Arici S, Celiksoz A, Elagoz S, Cevit R. The retrospective annual surgical incidence of cystic echinococcosis in Sivas, Turkey. Zoonoses Public Health. 2009; 56(5): 209-14.
Gonzlez L, Roses A, Alomar P, del Valle JM, Garau A, Ferrer P, Maim- M, Llinares R, Blanco I, Lardinois R. The maternal-infant center in the control of hepatitis B. Acta Obstet Gynecol Scand. 1988; 67(5): 421-7.
GonzÃ¡lez-Molero I, Rojo-MartÃnez G, Morcillo S, Gutierrez C, Rubio E, PÃ©rez-Valero V, Esteva I, Ruiz de Adana MS, Almaraz MC, Colomo N, Olveira G, Soriguer F. Hypovitaminosis D and incidence of obesity: a prospective study. Eur J Clin Nutr. 2013; 67(6): 680-2.
González JM, Olano V, Vergara J, Arvalo-Herrera M, Carrasquilla G, Herrera S, López JA. Unstable, low-level transmission of malaria on the Colombian Pacific Coast. Ann Trop Med Parasitol. 1997; 91(4): 349-58. As it appears in Malaria Atlas Project. Malaria Atlas Project Plasmodium Falciparum Parasite Rate Database. Oxford, United Kingdom: Malaria Atlas Project.
Gonzalez MA, Kroeger A, Meyer R. Malaria in Nicaragua - A Fight to Win? Managua, Nicaragua: Ministry of Health, 1996. As it appears in Malaria Atlas Project. Malaria Atlas Project Plasmodium Falciparum Parasite Rate Database. Oxford, United Kingdom: Malaria Atlas Project.
González-Cerón L, Rodríguez MH. An enzyme-linked immunosorbent assay using detergent-soluble Plasmodium vivax antigen for seroepidemiological surveys. Trans R Soc Trop Med Hyg. 1991; 85(3): 358-61. As it appears in Malaria Atlas Project. Malaria Atlas Project Plasmodium Falciparum Parasite Rate Database. Oxford, United Kingdom: Malaria Atlas Project.
González-Villalpando C, Dávila-Cervantes CA, Zamora-Macorra M, Trejo-Valdivia B, González-Villalpando ME. Incidence of type 2 diabetes in Mexico: results of the Mexico City Diabetes Study after 18 years of follow-up. Salud Publica Mex. 2014; 56(1): 11-7.
Gopi PG, Subramani R, Radhakrishna S, Kolappan C, Sadacharam K, Devi TS, Frieden TR, Narayanan PR. A baseline survey of the prevalence of tuberculosis in a community in south India at the commencement of a DOTS programme. Int J Tuberc Lung Dis. 2003; 7(12): 1154-62.
Gopinath B, Flood VM, Wang JJ, Burlutsky G, Mitchell P. Lower dairy products and calcium intake is associated with adverse retinal vascular changes in older adults. Nutr Metab Cardiovasc Dis. 2014; 24(2): 155â€“61.
Gopinath S, Ortqvist E, Norgren S, Green A, Sanjeevi CB. Variations in incidence of type 1 diabetes in different municipalities of stockholm. Ann N Y Acad Sci. 2008; 200-7.
Goswami D, Rathore AM, Batra S, Dubey C, Tyagi S, Wadhwa L. Facility-based review of 296 maternal deaths at a tertiary centre in India: Could they be prevented? J Obstet Gynaecol Res. 2013; 39(12): 1569-79.
Goudar SS, Goco N, Somannavar MS, Vernekar SS, Mallapur AA, Moore JL, Wallace DD, Sloan NL, Patel A, Hibberd PL, Koso-Thomas M, McClure EM, Goldenberg RL. Institutional deliveries and perinatal and neonatal mortality in Southern and Central India. Reprod Health. 2015; 12(Suppl 2): S13.
Gould IT, Perner MS, Santini MS, Saavedra SB, Bezzi G, Maglianese MI, Antman JG, Gutiérrez JA, Salomón OD. [Visceral leishmaniasis in Argentina. Cases notification and distribution of vectors (2006-2012)]. Medicina (B Aires). 2013; 73(2): 104-10.
Gourdy P, Ruidavets JB, Ferrieres J, Ducimetiere P, Amouyel P, Arveiler D, Cottel D, Lamamy N, Bingham A, Hanaire-Broutin H; MONICA Study. Prevalence of type 2 diabetes and impaired fasting glucose in the middle-aged population of three French regions - the MONICA study 1995-97. Diabetes Metab. 2001; 27(3): 347-58.
Government Delegation for the National Plan on Drugs (Spain), Ministry of Health, Social Services and Equality (Spain). Spain Household Survey on Alcohol and Drugs 2013.
Government Delegation for the National Plan on Drugs (Spain), Ministry of Health, Social Services and Equality (Spain). Spain Survey on Drug Use in Secondary Schools 2002.
Government Delegation for the National Plan on Drugs (Spain). Spain Household Survey on Alcohol and Drugs 1997.
Government Delegation for the National Plan on Drugs (Spain). Spain Household Survey on Alcohol and Drugs 1999.
Government Delegation for the National Plan on Drugs (Spain). Spain Household Survey on Alcohol and Drugs 2001.
Government Delegation for the National Plan on Drugs (Spain). Spain Household Survey on Alcohol and Drugs 2003-2004.
Government Delegation for the National Plan on Drugs (Spain). Spain Survey on Drug Use in Secondary Schools 1998.
Government Delegation for the National Plan on Drugs (Spain). Spain Survey on Drug Use in Secondary Schools 2000.
Government of Balochistan (Pakistan), United Nations Children's Fund (UNICEF). Pakistan - Balochistan Multiple Indicator Cluster Survey 2010. New York, United States: United Nations Children's Fund (UNICEF).
Government of Belize. From Girls to Women: Growing up Healthy in Belize. Belmopan, Belize: Government of Belize, 1997.
Government of Bhutan, United Nations Children's Fund (UNICEF), World Health Organization (WHO). Bhutan EPI Coverage Evaluation Survey 1991.
Government of Bhutan, United Nations Children's Fund (UNICEF), World Health Organization (WHO). Bhutan National EPI Coverage Evaluation Survey 2002.
Government of Bhutan, United Nations Children's Fund (UNICEF), World Health Organization (WHO). Bhutan National EPI Coverage Survey 2008-2009.
Government of Central African Republic, United Nations Children's Fund (UNICEF), World Health Organization (WHO). Central African Republic External EPI Review 2002.
Government of Guinea. Guinea EPI External Review 2000.
Government of Guyana, Joint United Nations Program on HIV/AIDS (UNAIDS). Guyana Global AIDS Response Progress Report 2012. Geneva, Switzerland: Joint United Nations Program on HIV/AIDS (UNAIDS), 2012.
Government of India. India - Andhra Pradesh and Odisha Mortality Survey 2005-2006.
Government of Japan, Inter-American Development Bank (IDB), Latin American and Caribbean Demographic Center (CELADE), National Institute of Statistics and Censuses (Nicaragua), UK Department for International Development (DFID), United Nations Development Programme (UNDP), United Nations Economic Commission for Latin America and the Caribbean (ECLAC), United Nations Population Fund (UNFPA), United States Census Bureau. Nicaragua Population and Housing Census 2005.

Appendix: Citation List

Citation
Government of Kenya, United Nations Children's Fund (UNICEF), University of Nairobi. Kenya National Micronutrient Survey 1994. As it appears in Malaria Atlas Project. Malaria Atlas Project Plasmodium Falciparum Parasite Rate Database. Oxford, United Kingdom: Malaria Atlas Project.
Government of Lesotho, Irish Aid, United Nations Children's Fund (UNICEF). Lesotho National Nutrition and EPI Cluster Survey 2002.
Government of Lesotho. Lesotho Post SIAs and Routine Immunization Coverage Survey 2013.
Government of Mongolia, National Statistical Office of Mongolia, United Nations Children's Fund (UNICEF), United Nations Population Fund (UNFPA). Mongolia Multiple Indicator Cluster Survey 2013.
Government of Myanmar, United Nations Children's Fund (UNICEF), World Health Organization (WHO). Myanmar EPI Review 1986-1987.
Government of Niger, Macro International, Inc, United Nations Children's Fund (UNICEF). Niger Multiple Indicator Cluster Survey 2000. New York, United States: United Nations Children's Fund (UNICEF).
Government of Rwanda, World Health Organization (WHO). Rwanda Integrated Post Measles-Rubella Campaign and Routine Immunization Coverage Evaluation Survey 2013.
Government of Senegal, United Nations Children's Fund (UNICEF). Senegal Multiple Indicator Cluster Survey 1996.
Government of Sierra Leone. Sierra Leone Routine Immunization Coverage Survey 2013.
Government of Togo, People's Republic of China, United Nations Children's Fund (UNICEF), United Nations Development Programme (UNDP), United Nations Population Fund (UNFPA). Togo Population and Housing Census 2010. Lomé, Togo: Directorate General of Statistics and National Accounts (Togo).
Government of Yemen Arab Republic, World Health Organization (WHO). Yemen Arab Republic Joint Government/WHO/Unicef EPI Coverage Review 1987.
Grabauskas V, Petkeviciene J, Klumbiene J, Vaisvalavicius V. The prevalence of overweight and obesity in relation to social and behavioural factors (Lithuanian health behaviour monitoring). Medicina (Kaunas). 2003; 39(12): 1223â€"30.
Graciani A, Leon-Munoz LM, Guallar-Castillon P, Rodriguez-Artalejo F, Banegas JR. Cardiovascular health in a southern Mediterranean European country: a nationwide population-based study. Circ Cardiovasc Qual Outcomes. 2013; 6(1): 90–8.
Gramiccia M, Scalone A, Di Muccio T, Orsini S, Fiorentino E, Gradoni L. The burden of visceral leishmaniasis in Italy from 1982 to 2012: a retrospective analysis of the multi-annual epidemic that occurred from 1989 to 2009. Euro Surveill. 2013; 18(29): 20535.
Grande ED, Hickling J, Taylor A, Woollacott T. Domestic violence in South Australia: a population survey of males and females. Aust N Z J Public Health. 2003; 27(5): 543-50.
Grandinetti A, Kaholokula JK, Theriault AG, Mor JM, Chang HK, Waslien C. Prevalence of diabetes and glucose intolerance in an ethnically diverse rural community of Hawaii. Ethn Dis. 2007; 17(2): 250-5.
Graner S, Klingberg-Allvin M, Phuc HD, Huong DL, Krantz G, Mogren I. Adverse perinatal and neonatal outcomes and their determinants in rural Vietnam 1999-2005. Paediatr Perinat Epidemiol. 2010; 24(6): 535-45.
Granja AC, Machungo F, Bergstrom S. Avoidability of maternal death in Mozambique: audit and retrospective risk assessment in 106 consecutive cases. Afr J Health Sci. 2000; 7(3-4): 83-7.
Granja AC, Machungo F, Gomes A, Bergström S. Adolescent maternal mortality in Mozambique. J Adolesc Health. 2001; 28(4): 303-6.
Grauslund J. Long-term mortality and retinopathy in type 1 diabetes. Acta Ophthalmol. 2010; 1-14.
Gray GC, Kassira EN, Rodier GR, Myers MC, Calamaio CA, Gregory M, Nagi MA, Kamal K, Botros BA, Soliman AK, Hassan NF, Gregory R, Arunkumar BK, Cope A, Hyams KC. Remote village survey for agents causing hepatosplenic disease in the Republic of Yemen. Trop Doct. 1999; 29(4): 212-9. As it appears in Malaria Atlas Project. Malaria Atlas Project Plasmodium Falciparum Parasite Rate Database. Oxford, United Kingdom: Malaria Atlas Project.
Greece Vital Registration - Deaths 1956 ICD7 as it appears in World Health Organization (WHO). WHO Mortality Database Version July 2012. Geneva, Switzerland: World Health Organization (WHO), 2012.
Greece Vital Registration - Deaths 1957 ICD7 as it appears in World Health Organization (WHO). WHO Mortality Database Version July 2012. Geneva, Switzerland: World Health Organization (WHO), 2012.
Greece Vital Registration - Deaths 1958 ICD7 as it appears in World Health Organization (WHO). WHO Mortality Database Version July 2012. Geneva, Switzerland: World Health Organization (WHO), 2012.
Greece Vital Registration - Deaths 1959 ICD7 as it appears in World Health Organization (WHO). WHO Mortality Database Version July 2012. Geneva, Switzerland: World Health Organization (WHO), 2012.
Greece Vital Registration - Deaths 1960 ICD7 as it appears in World Health Organization (WHO). WHO Mortality Database Version July 2012. Geneva, Switzerland: World Health Organization (WHO), 2012.
Greece Vital Registration - Deaths 1961 ICD7 as it appears in World Health Organization (WHO). WHO Mortality Database Version July 2012. Geneva, Switzerland: World Health Organization (WHO), 2012.
Greece Vital Registration - Deaths 1962 ICD7 as it appears in World Health Organization (WHO). WHO Mortality Database Version July 2012. Geneva, Switzerland: World Health Organization (WHO), 2012.
Greece Vital Registration - Deaths 1963 ICD7 as it appears in World Health Organization (WHO). WHO Mortality Database Version July 2012. Geneva, Switzerland: World Health Organization (WHO), 2012.
Greece Vital Registration - Deaths 1964 ICD7 as it appears in World Health Organization (WHO). WHO Mortality Database Version July 2012. Geneva, Switzerland: World Health Organization (WHO), 2012.
Greece Vital Registration - Deaths 1965 ICD7 as it appears in World Health Organization (WHO). WHO Mortality Database Version July 2012. Geneva, Switzerland: World Health Organization (WHO), 2012.
Greece Vital Registration - Deaths 1966 ICD7 as it appears in World Health Organization (WHO). WHO Mortality Database Version July 2012. Geneva, Switzerland: World Health Organization (WHO), 2012.
Greece Vital Registration - Deaths 1967 ICD7 as it appears in World Health Organization (WHO). WHO Mortality Database Version July 2012. Geneva, Switzerland: World Health Organization (WHO), 2012.

Appendix: Citation List

Citation
Greece Vital Registration - Deaths 1968 ICD8 as it appears in World Health Organization (WHO). WHO Mortality Database Version May 2013. Geneva, Switzerland: World Health Organization (WHO), 2013.
Greece Vital Registration - Deaths 1969 ICD8 as it appears in World Health Organization (WHO). WHO Mortality Database Version May 2013. Geneva, Switzerland: World Health Organization (WHO), 2013.
Greece Vital Registration - Deaths 1970 ICD8 as it appears in World Health Organization (WHO). WHO Mortality Database Version May 2013. Geneva, Switzerland: World Health Organization (WHO), 2013.
Greece Vital Registration - Deaths 1971 ICD8 as it appears in World Health Organization (WHO). WHO Mortality Database Version May 2013. Geneva, Switzerland: World Health Organization (WHO), 2013.
Greece Vital Registration - Deaths 1972 ICD8 as it appears in World Health Organization (WHO). WHO Mortality Database Version May 2013. Geneva, Switzerland: World Health Organization (WHO), 2013.
Greece Vital Registration - Deaths 1973 ICD8 as it appears in World Health Organization (WHO). WHO Mortality Database Version May 2013. Geneva, Switzerland: World Health Organization (WHO), 2013.
Greece Vital Registration - Deaths 1974 ICD8 as it appears in World Health Organization (WHO). WHO Mortality Database Version May 2013. Geneva, Switzerland: World Health Organization (WHO), 2013.
Greece Vital Registration - Deaths 1975 ICD8 as it appears in World Health Organization (WHO). WHO Mortality Database Version May 2013. Geneva, Switzerland: World Health Organization (WHO), 2013.
Greece Vital Registration - Deaths 1976 ICD8 as it appears in World Health Organization (WHO). WHO Mortality Database Version May 2013. Geneva, Switzerland: World Health Organization (WHO), 2013.
Greece Vital Registration - Deaths 1977 ICD8 as it appears in World Health Organization (WHO). WHO Mortality Database Version May 2013. Geneva, Switzerland: World Health Organization (WHO), 2013.
Greece Vital Registration - Deaths 1978 ICD8 as it appears in World Health Organization (WHO). WHO Mortality Database Version May 2013. Geneva, Switzerland: World Health Organization (WHO), 2013.
Greece Vital Registration - Deaths 1979 ICD9 as it appears in World Health Organization (WHO). WHO Mortality Database Version November 2015. Geneva, Switzerland: World Health Organization (WHO), 2015.
Greece Vital Registration - Deaths 1980 ICD9 as it appears in World Health Organization (WHO). WHO Mortality Database Version November 2015. Geneva, Switzerland: World Health Organization (WHO), 2015.
Greece Vital Registration - Deaths 1981 ICD9 as it appears in World Health Organization (WHO). WHO Mortality Database Version November 2015. Geneva, Switzerland: World Health Organization (WHO), 2015.
Greece Vital Registration - Deaths 1982 ICD9 as it appears in World Health Organization (WHO). WHO Mortality Database Version November 2015. Geneva, Switzerland: World Health Organization (WHO), 2015.
Greece Vital Registration - Deaths 1983 ICD9 as it appears in World Health Organization (WHO). WHO Mortality Database Version November 2015. Geneva, Switzerland: World Health Organization (WHO), 2015.
[truncated: 761,197 more chars]
