# Supplementary material for: Measuring the health-related Sustainable Development Goals in 188 countries: a baseline analysis from the Global Burden of Disease Study 2015
Source: Lancet. 2016 Oct 8;388(10053):1813–50. doi: 10.1016/S0140-6736(16)31467-2 (PMC5055583; doi:10.1016/S0140-6736(16)31467-2)
Supplement: Supplementary appendices [file mmc2.pdf]

THE LANCET

Supplementary appendix

This appendix formed part of the original submission and has been peer reviewed. We post it as supplied by the authors.

Supplement to: GBD 2015 SDG Collaborators. Measuring the health-related Sustainable Development Goals in 188 countries: a baseline analysis from the Global Burden of Disease Study 2015. *Lancet* 2016; published online Sept 21. http://dx.doi.org/10.1016/S0140-6736(16)31467-2.

Supplementary Results to Measuring the health-related Sustainable Development Goals in 188 countries: An analysis from the Global Burden of Disease Study 2015

The supplementary results offer additional results tables and figures, as well as more detailed methodological figures.

List of Supplementary Results: Figures & Tables

Figure 1. Performance for the health-related SDG index, MDG index, and non-MDG index, and 33 individual health-related indicators by country, 1990, page 1

Figure 2. Performance for the health-related SDG index, MDG index, and non-MDG index, and 33 individual health-related indicators by country, 2000, page 2

Figure 3. Map of health-related SDG index, by quintile, 1990, page 3

Figure 4. Map of health-related SDG index, by quintile, 2000, page 4

Figure 5. Absolute change in the health-related SDG index, health-related MDG index, and 33 individual health-related SDG indicators (rescaled), by country from 1990 to 2015, page 5

Figure 6. Absolute change in the health-related SDG index, health-related MDG index, and 33 individual health-related SDG indicators (rescaled), by country from 2000 to 2015, page 6

Figure 7a. Appendix Figure 7a. Comparison of health-related SDG index values using the arithmetic mean of targets versus the geometric mean, 2015, page 7

Figure 7b. Comparison of health-related SDG index ranks by country using the arithmetic mean of targets versus the geometric mean, 2015, page 8

Figure 8a. Comparison of health-related SDG index values using the geometric mean of the minimums across each target versus the standard geometric mean, 2015, page 9

Figure 8b. Comparison of health-related SDG index ranks by country using the geometric mean of the minimums across each target versus the standard geometric mean, 2015, page 10

Figure 9. Map of observed health-related SDG index minus expected health-related SDG index, on the basis of SDI alone, in 1990, page 11

Figure 10. Map of observed health-related SDG index minus expected health-related SDG index, on the basis of SDI alone, in 2000, page 12

Table 1a. Master country table w/ scaled values for each SDG health-related indicator by country for 1990, 2000, and 2015, page 13

Table 1b. Master country table w/ unscaled values for each SDG health-related indicator by country for 1990, 2000, and 2015, page 175

Table 2. Correlation coefficient with SDI of each composite index and health-related indicator, across all countries and years from 1990 to 2015, 5 year intervals, page 346

Figure 1. Performance for the health-related SDG index, MDG index, and non-MDG index, and 33 individual health-related indicators by country, 1990

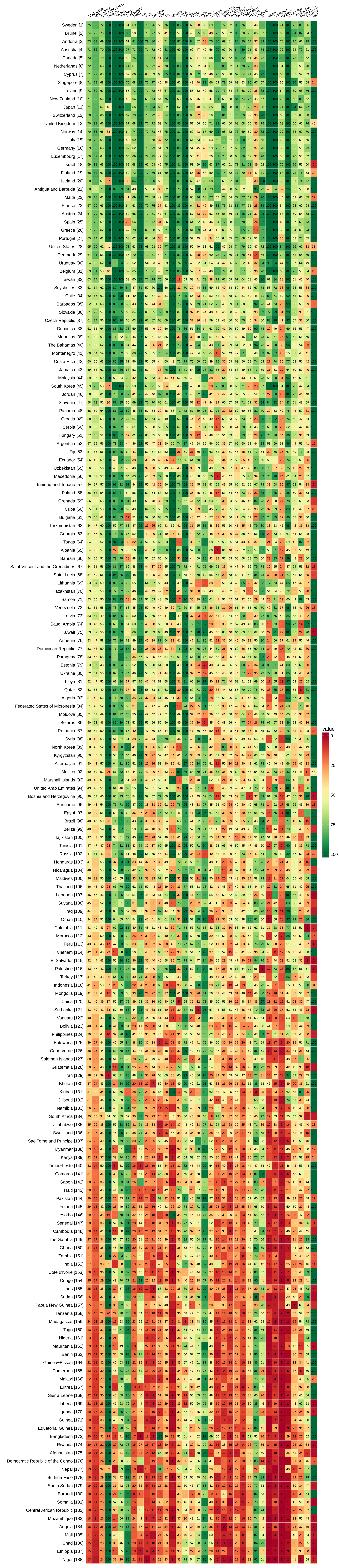

Figure 2. Performance for the health-related SDG index, MDG index, and non-MDG index, and 33 individual health-related indicators by country, 2000

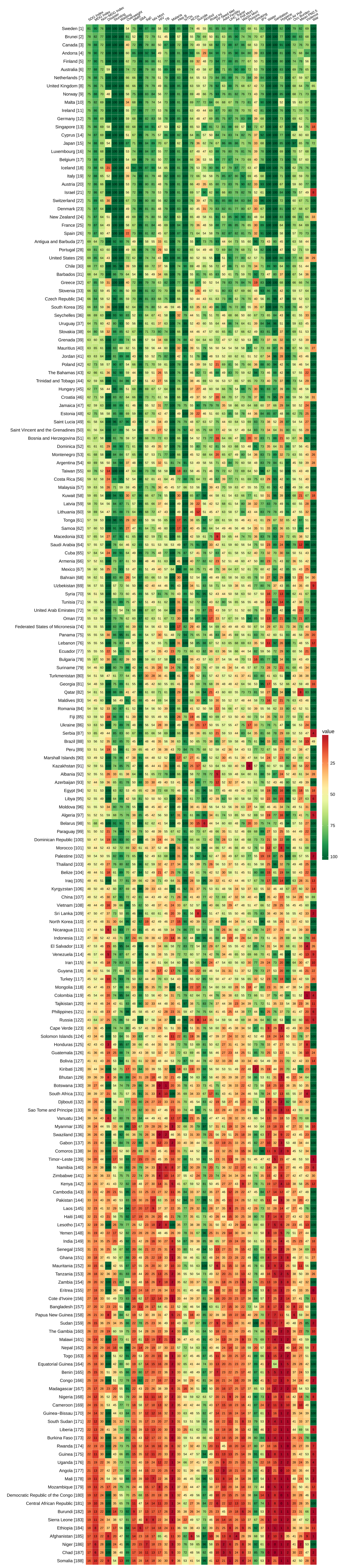

Figure 3. Map of health−related SDG index, by quintile, 1990.

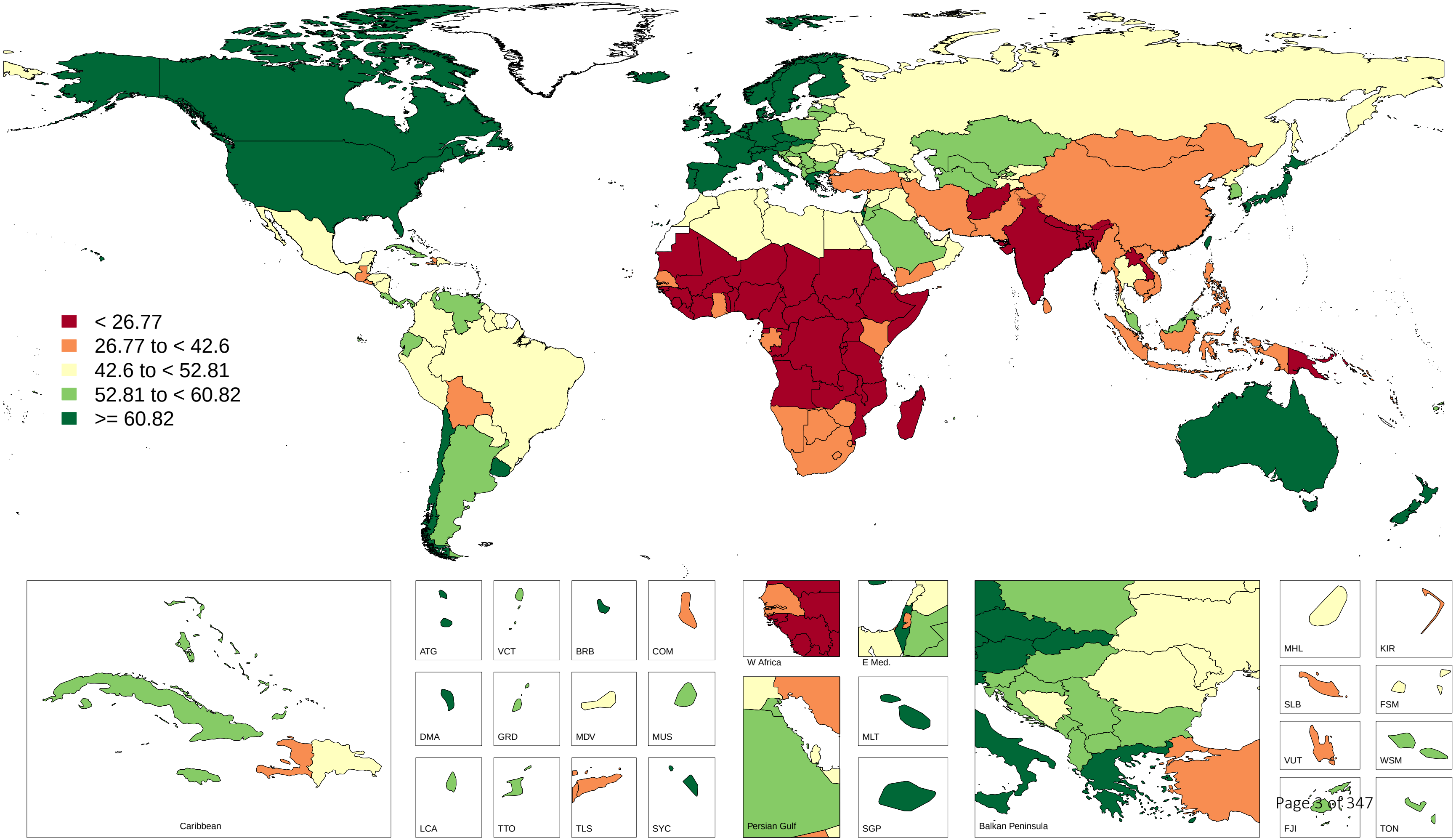

Figure 4. Map of health−related SDG index, by quintile, 2000.

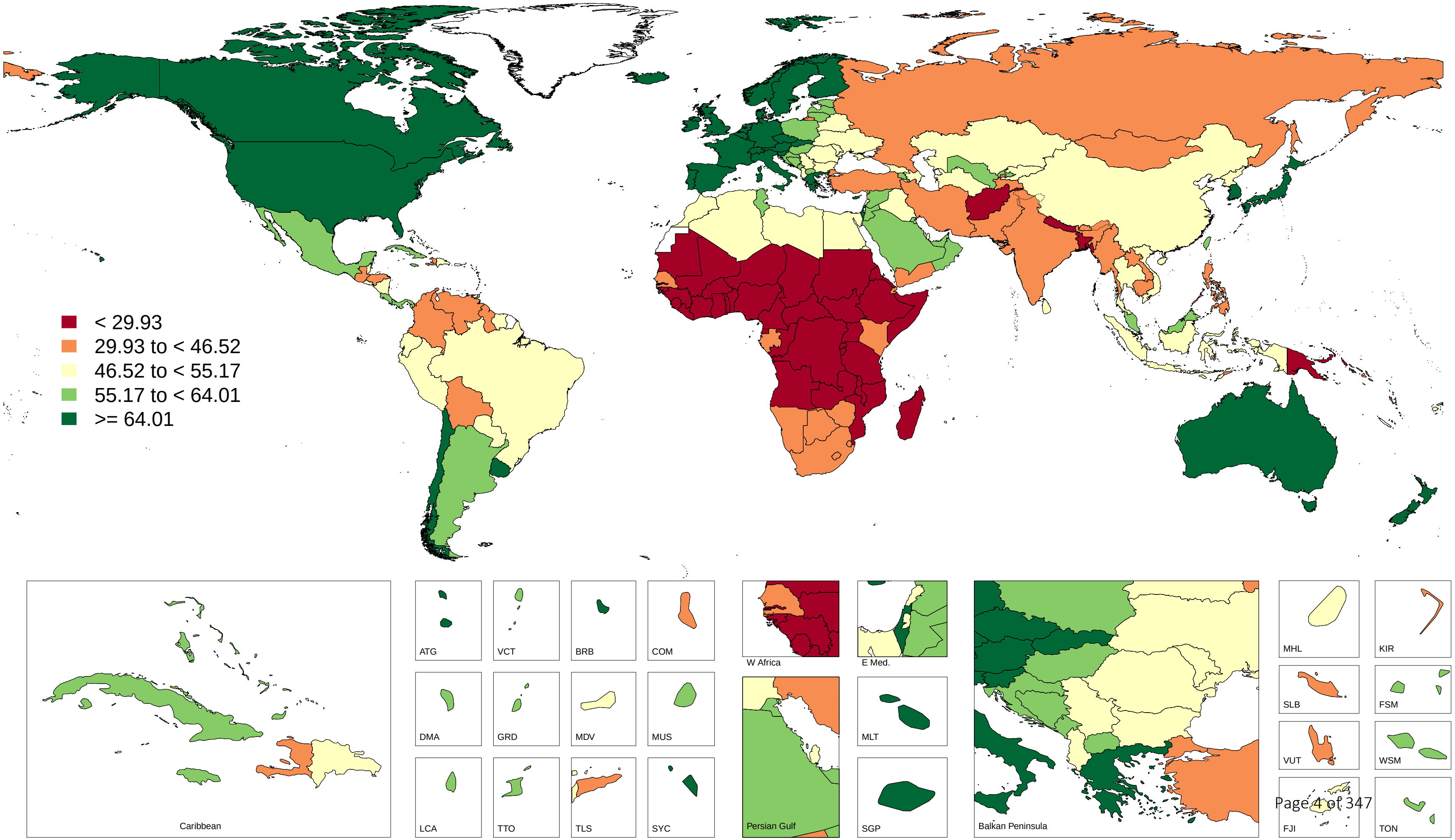

Figure 5. Absolute change in the health−related SDG index, health−related MDG index, health−related non−MDG index, and 33 individual health−related SDG indicators (rescaled), by country from 1990 to 2015.

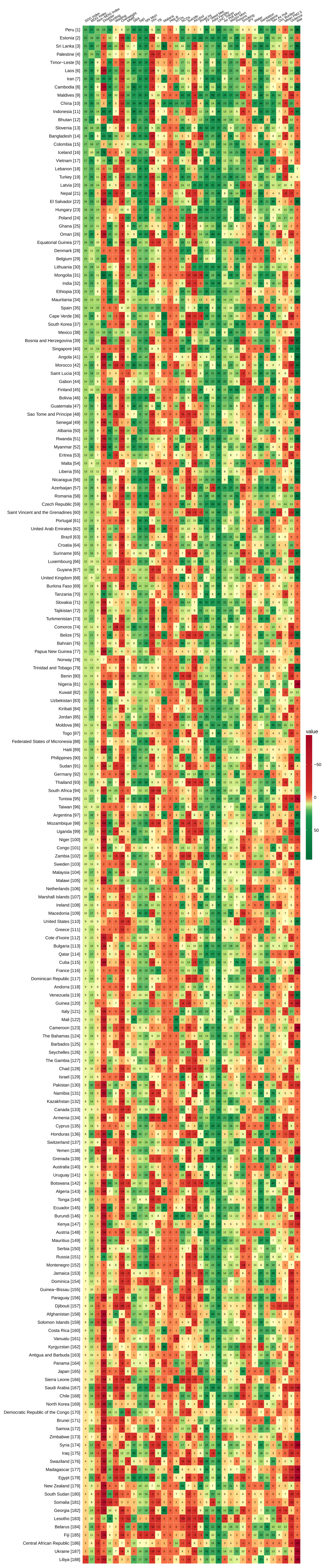

Figure 6. Absolute change in the health−related SDG index, health−related MDG index, health−related non−MDG index, and 33 individual health−related SDG indicators (rescaled), by country from 2000 to 2015.

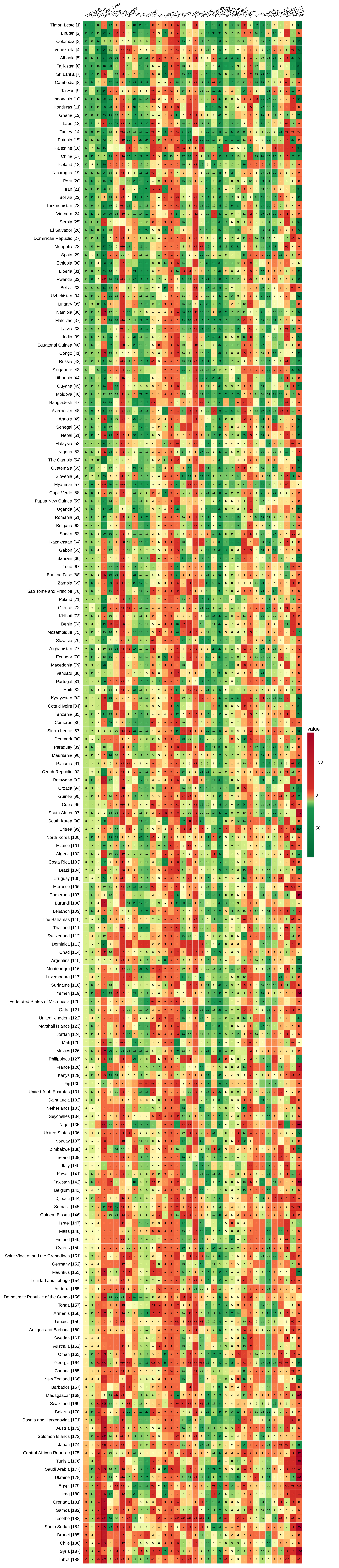

Figure 7a. Comparison of health-related SDG index values using the arithmetic mean of targets versus the geometric mean, in 2015

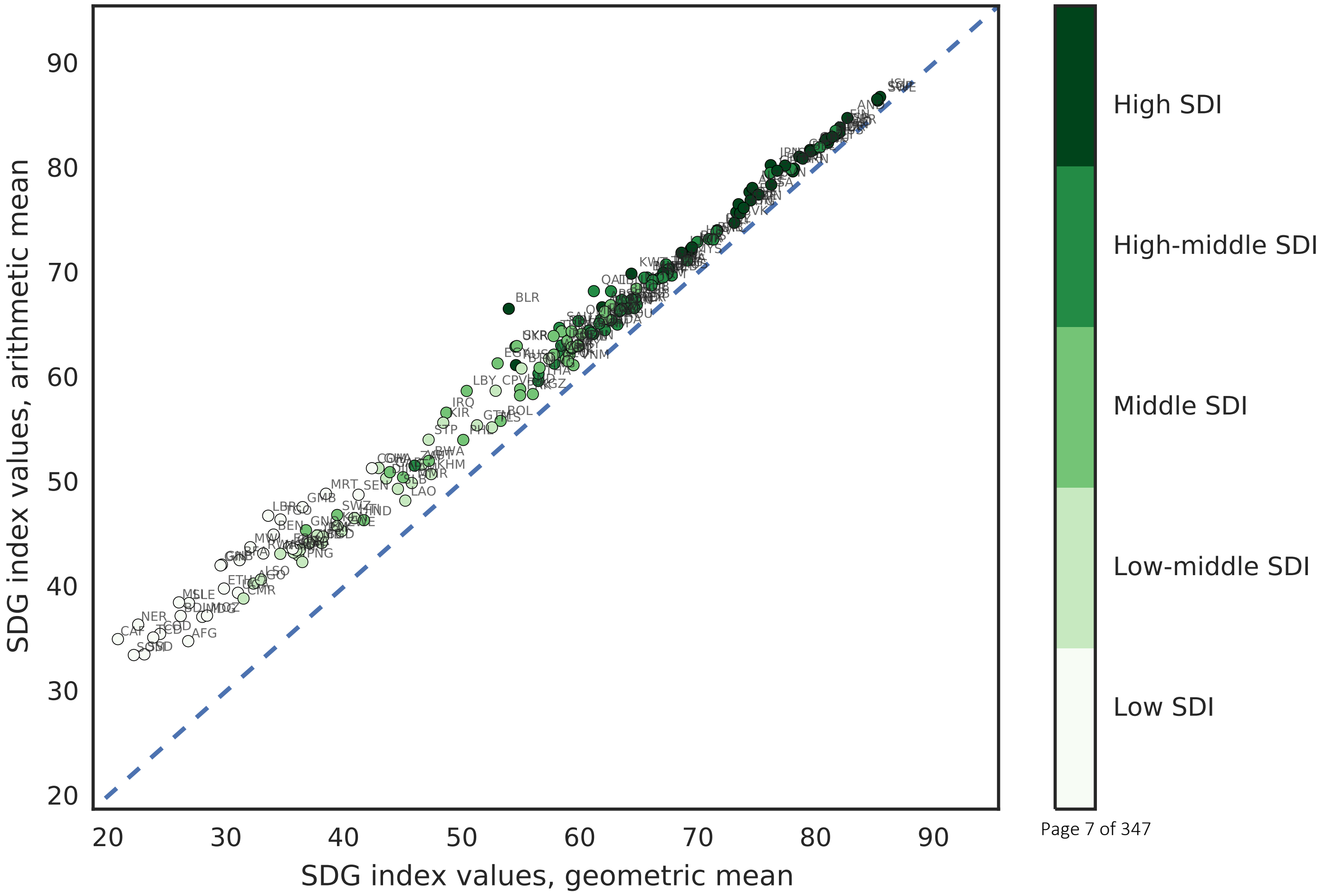

Figure 7b. Comparison of health-related SDG index ranks by country using the arithmetic mean of targets versus the geometric mean, 2015.

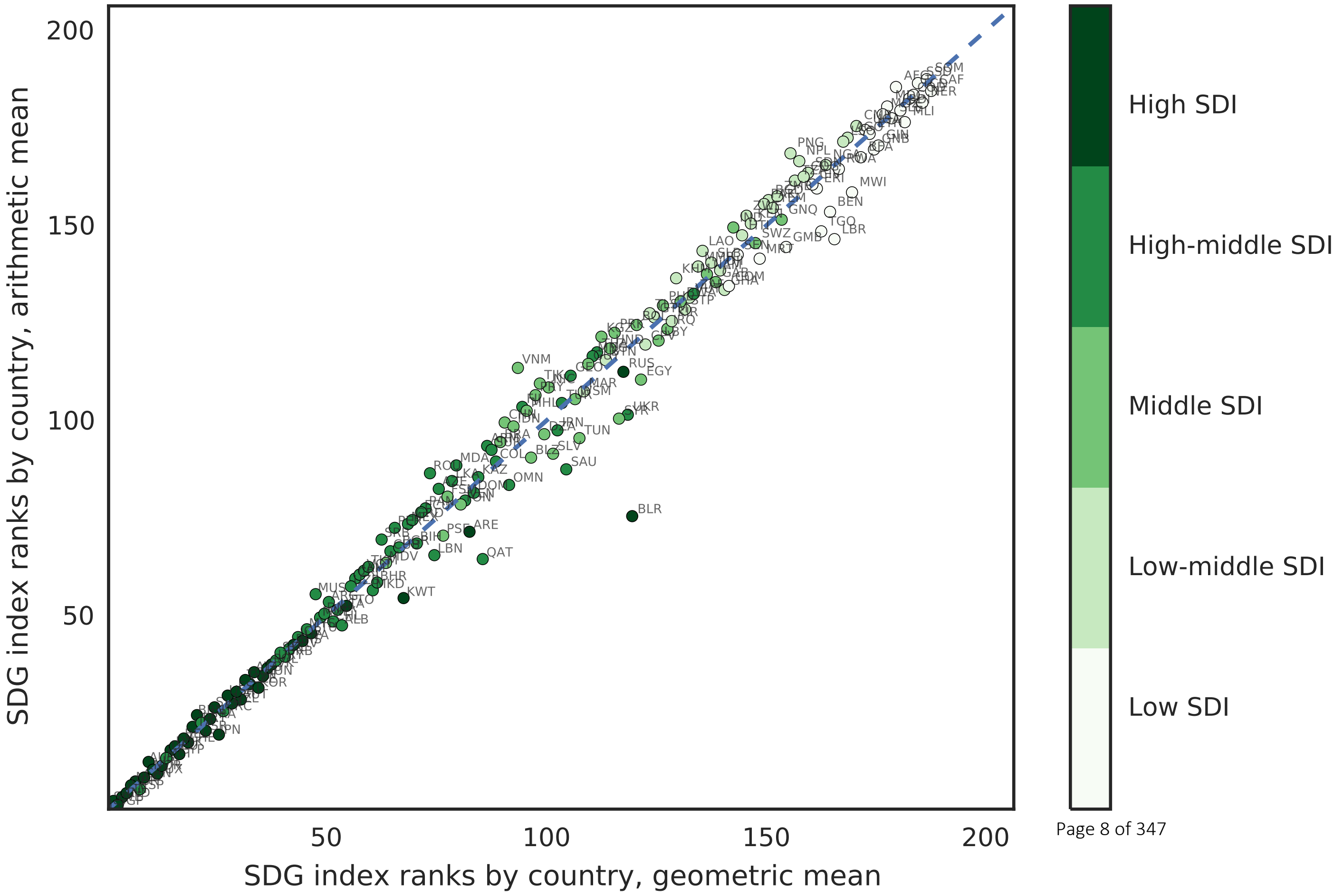

Figure 8a. Comparison of health-related SDG index values using the geometric mean of the minimums across each target versus the standard geometric mean, in 2015

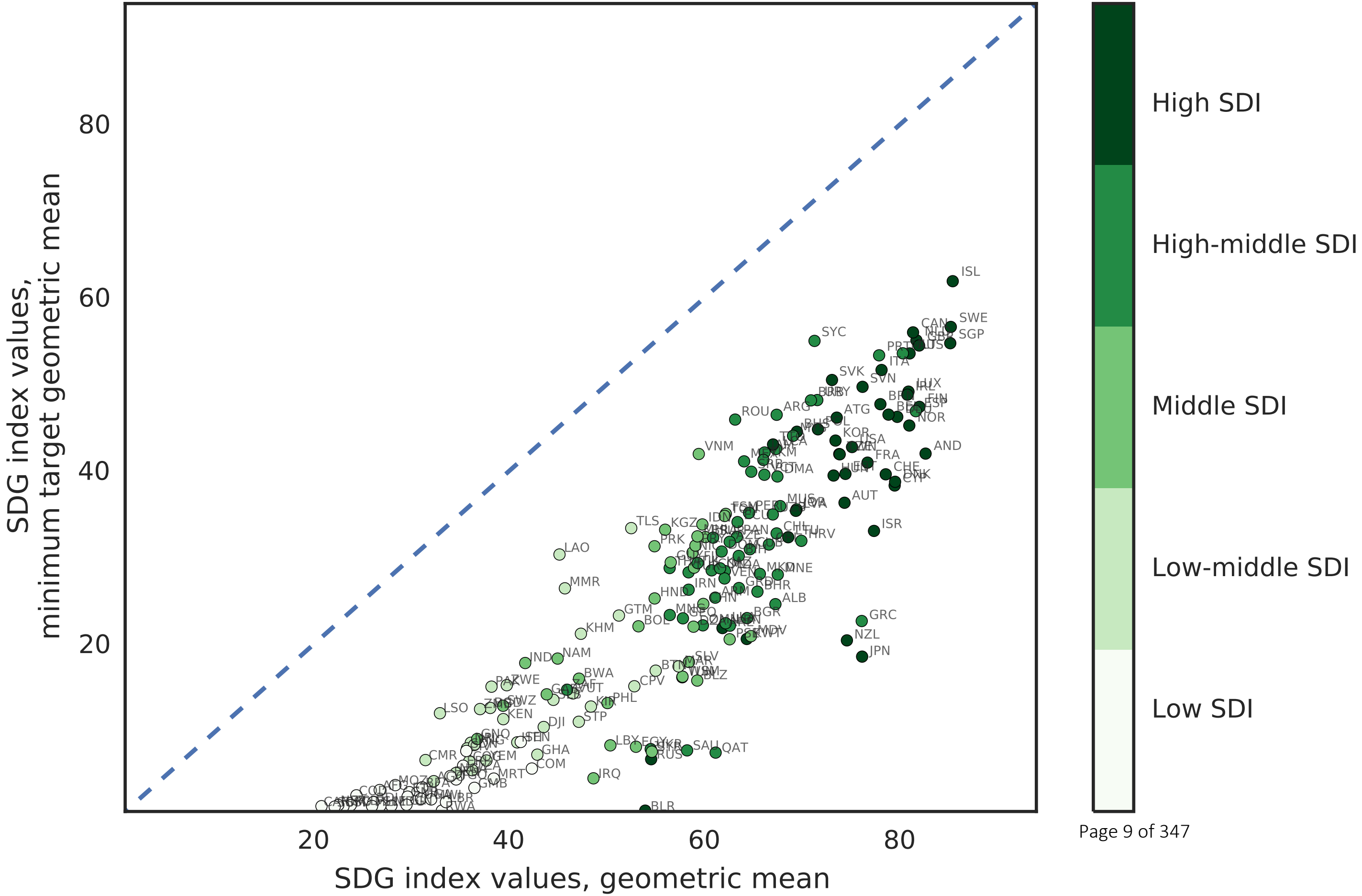

Figure 8b. Comparison of health-related SDG index ranks by country using the geometric mean of the minimums across each target versus the standard geometric mean, in 2015

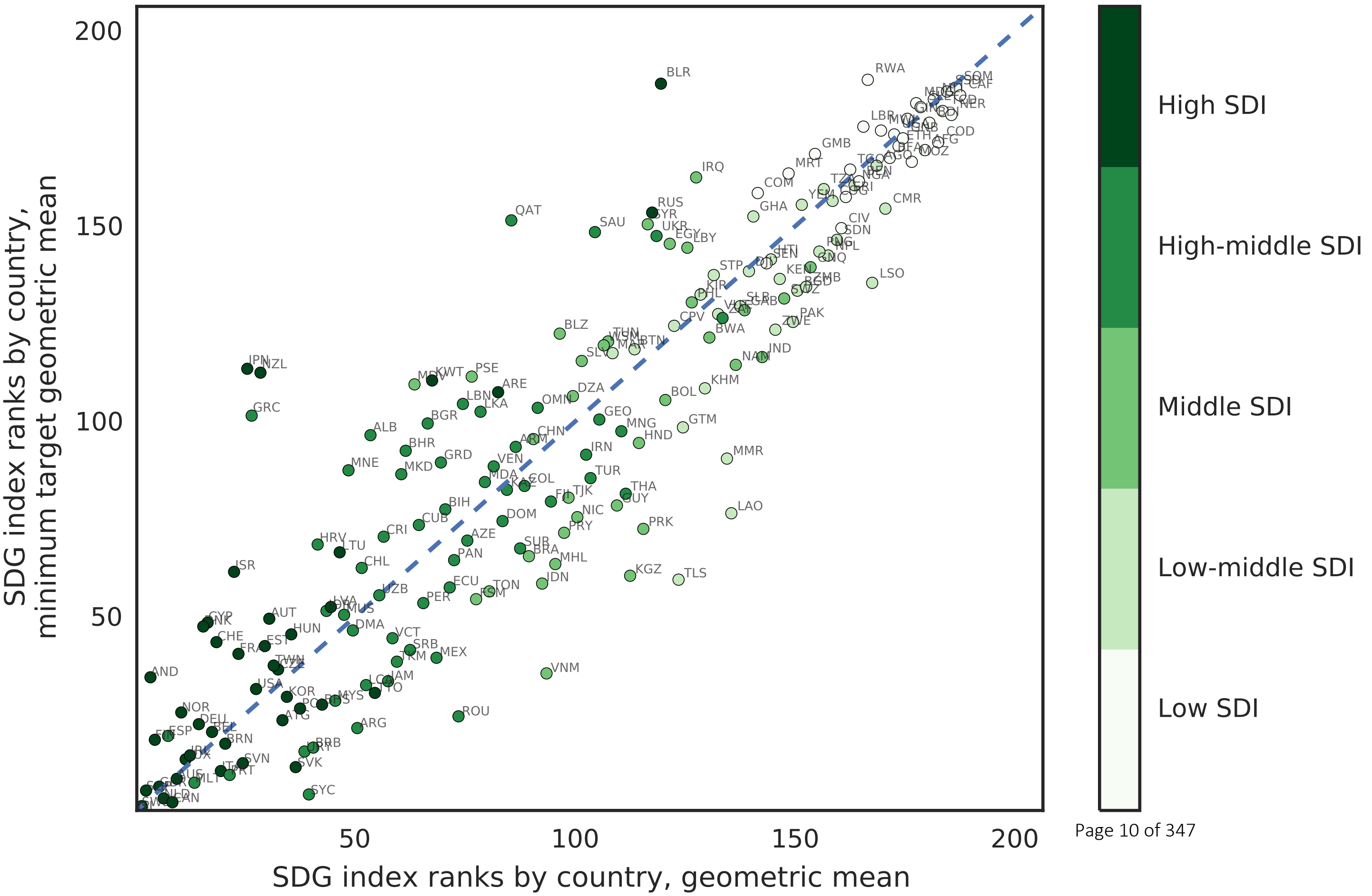

Figure 9. Map of observed health−related SDG index minus expected health−related SDG index, on the basis of SDI alone, in 1990.

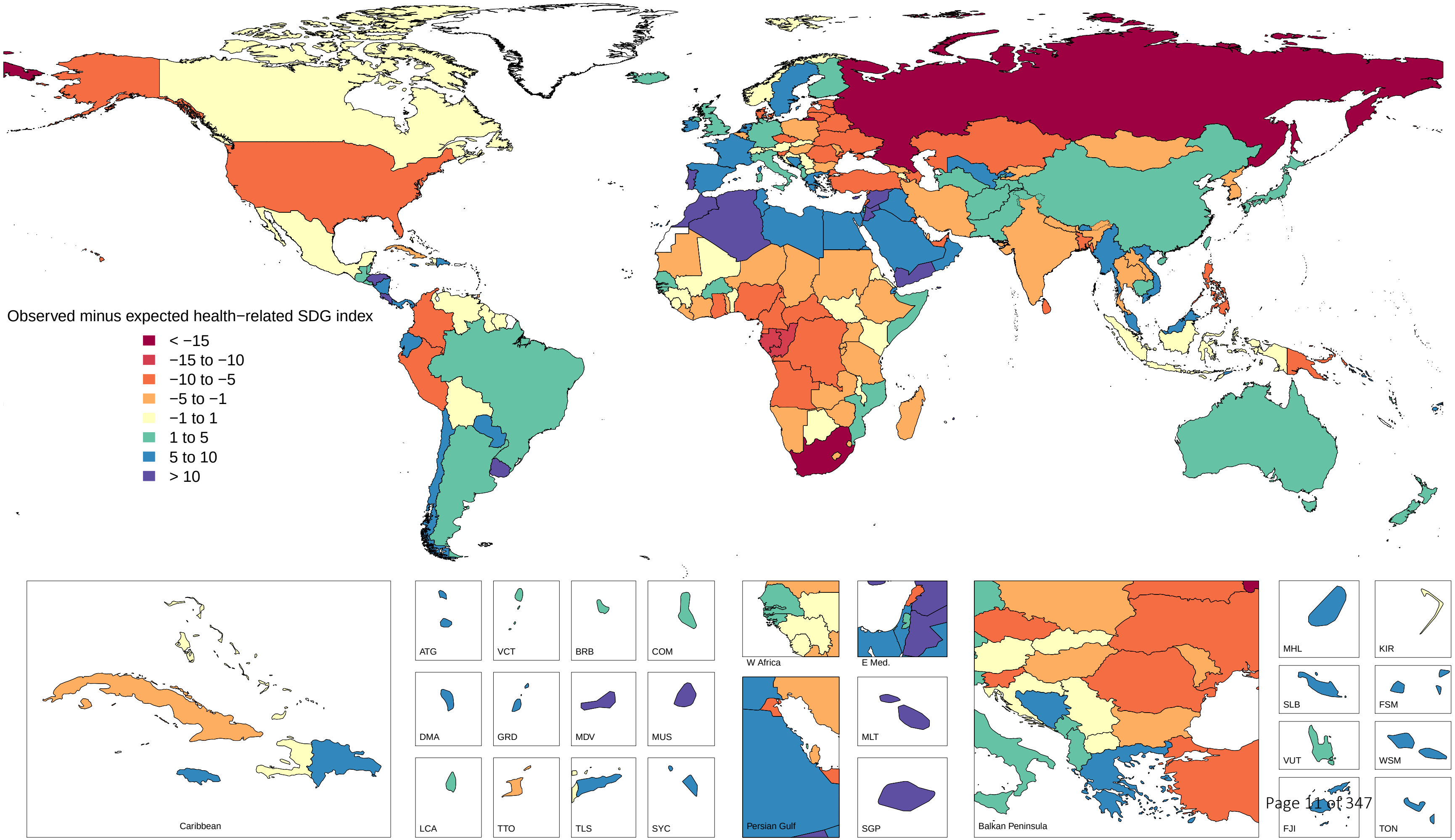

Figure 10. Map of observed health−related SDG index minus expected health−related SDG index, on the basis of SDI alone, in 2000.

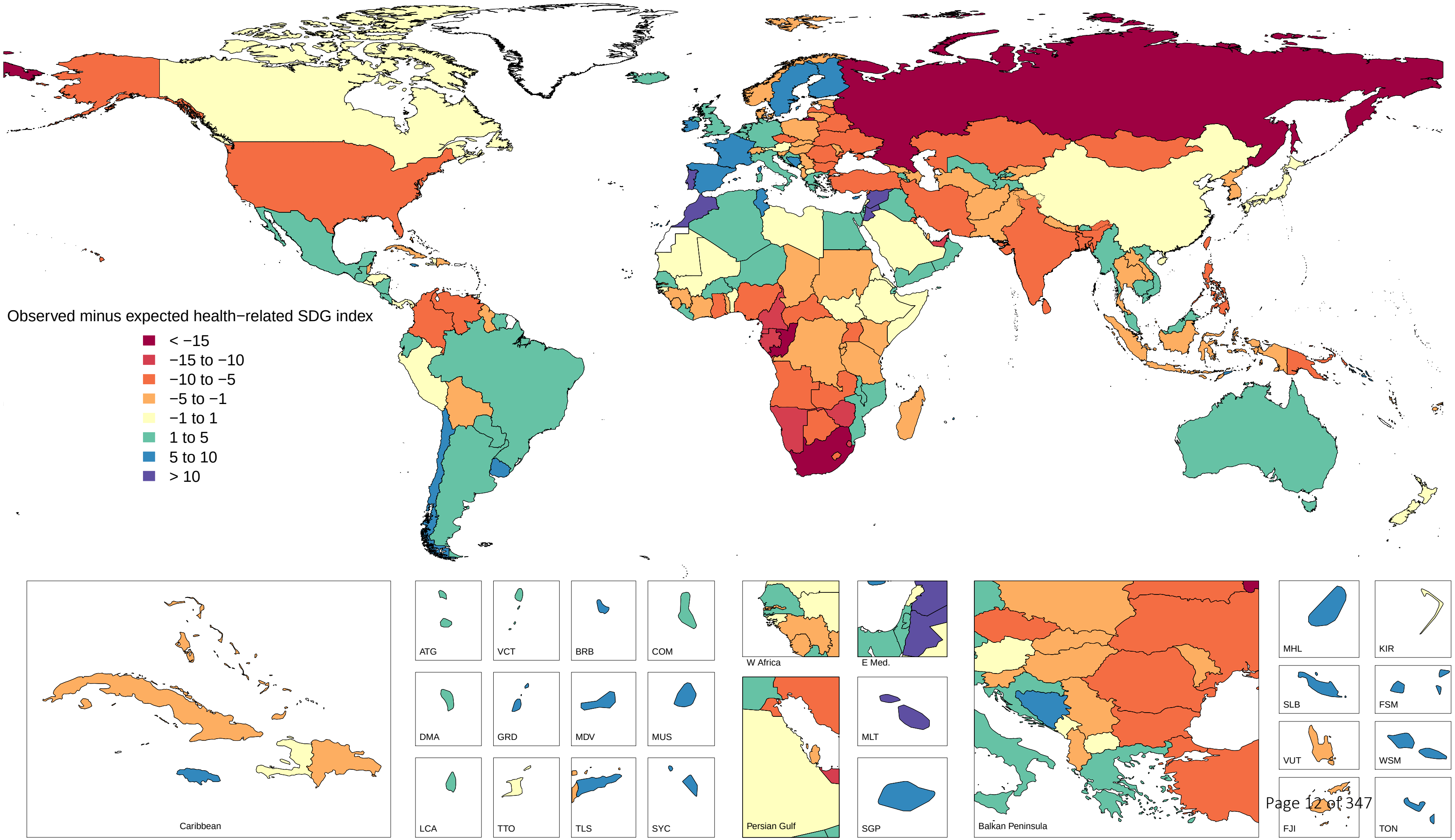

Table 1a. Scaled values for each SDG health-related indicator and three summary indicators by country for 1990, 2000, and 2015

Location	Geometric mean of all indicators			Geometric mean of all indicators with corresponding MDG indicator			Geometric mean of all indicators without corresponding MDG indicator			Indicator 1.5.1: Age-standardised death rate due to exposure to forces of nature (per 100,000 population)		
	1990	2000	2015	1990	2000	2015	1990	2000	2015	1990	2000	2015
Canada	73.12 (72.22-74.03)	77.96 (76.94-78.92)	81.40 (80.19-82.33)	82.28 (81.67-82.90)	88.18 (87.55-88.87)	91.12 (90.28-92.02)	69.52 (67.93-71.07)	71.96 (69.19-74.06)	74.57 (71.02-77.01)	100.00 (100.00-100.00)	100.00 (100.00-100.00)	100.00 (100.00-100.00)
United States	65.42 (63.75-66.71)	69.24 (67.56-70.55)	74.92 (73.63-75.95)	78.93 (78.44-79.46)	85.22 (84.68-85.76)	88.44 (87.92-88.95)	62.24 (60.06-63.98)	64.37 (62.16-66.03)	70.28 (68.56-71.66)	41.42 (32.28-49.77)	43.33 (34.29-50.11)	42.97 (33.96-50.09)
Australia	73.17 (72.35-74.02)	76.72 (75.66-77.73)	80.93 (79.75-81.92)	82.22 (81.54-82.89)	88.30 (87.59-89.00)	91.85 (91.17-92.44)	70.26 (69.03-71.48)	72.31 (70.86-73.61)	75.85 (73.84-77.49)	100.00 (100.00-100.00)	58.70 (47.17-68.78)	58.85 (47.32-68.36)
New Zealand	70.75 (69.73-71.73)	71.07 (69.56-72.57)	74.42 (73.25-75.52)	80.20 (79.60-80.87)	86.67 (85.98-87.35)	89.60 (88.91-90.30)	66.41 (64.37-68.26)	64.41 (61.93-66.60)	67.98 (66.04-69.67)	100.00 (100.00-100.00)	51.07 (41.40-60.65)	20.65 (17.44-24.00)
Brunei	73.56 (72.36-74.63)	78.03 (77.03-79.03)	78.00 (76.81-79.30)	76.68 (75.72-77.56)	81.77 (80.56-82.71)	84.53 (83.74-85.27)	73.03 (71.42-74.58)	76.56 (75.10-77.87)	75.30 (73.43-77.16)	100.00 (100.00-100.00)	100.00 (100.00-100.00)	47.92 (40.48-58.36)
Japan	70.54 (68.89-72.31)	74.16 (72.18-75.89)	75.71 (73.57-78.87)	82.06 (81.04-82.94)	88.26 (87.31-89.14)	92.37 (91.77-92.88)	67.37 (65.28-69.80)	69.45 (66.96-71.58)	69.85 (67.15-73.93)	45.93 (31.98-70.15)	54.10 (40.64-68.62)	18.78 (10.87-38.67)
Singapore	71.09 (69.71-72.80)	74.51 (72.88-76.64)	85.26 (84.06-86.34)	79.26 (78.28-80.19)	86.44 (85.68-87.17)	91.75 (91.18-92.26)	67.53 (65.64-69.67)	68.95 (66.75-71.70)	80.51 (78.66-82.25)	100.00 (100.00-100.00)	57.59 (50.70-67.10)	100.00 (100.00-100.00)
South Korea	58.74 (57.14-60.46)	65.54 (64.01-67.16)	73.24 (72.06-74.62)	75.49 (74.32-76.62)	82.78 (81.03-83.86)	90.11 (89.50-90.75)	52.88 (50.90-55.04)	58.63 (56.71-60.60)	65.30 (63.33-67.25)	26.56 (19.80-36.99)	35.67 (28.26-46.66)	56.02 (49.00-65.64)
Andorra	73.47 (71.78-75.29)	77.77 (76.29-79.32)	82.59 (81.16-84.06)	83.02 (80.38-84.34)	89.64 (88.35-90.63)	92.43 (91.13-93.62)	69.45 (66.90-72.12)	72.19 (69.76-74.59)	77.24 (74.65-79.62)	100.00 (100.00-100.00)	100.00 (100.00-100.00)	100.00 (100.00-100.00)
Austria	67.07 (65.93-68.19)	72.24 (71.10-73.27)	74.12 (72.56-75.49)	78.51 (77.41-79.53)	86.47 (85.55-87.34)	91.55 (90.77-92.30)	62.82 (60.76-64.73)	65.82 (63.40-67.72)	66.37 (63.62-68.68)	100.00 (100.00-100.00)	100.00 (100.00-100.00)	43.55 (36.16-51.06)
Belgium	63.48 (62.17-65.05)	73.46 (72.36-74.48)	78.73 (77.58-79.79)	81.03 (80.31-81.70)	87.64 (86.84-88.33)	91.66 (91.02-92.30)	57.52 (55.45-59.58)	67.22 (64.99-69.01)	72.78 (70.61-74.59)	40.44 (33.52-48.23)	100.00 (100.00-100.00)	100.00 (100.00-100.00)

Table 1a. Scaled values for each SDG health-related indicator and three summary indicators by country for 1990, 2000, and 2015												
	**Geometric mean of all indicators**			**Geometric mean of all indicators with corresponding MDG indicator**			**Geometric mean of all indicators without corresponding MDG indicator**			**Indicator 1.5.1: Age-standardised death rate due to exposure to forces of nature (per 100,000 population)**		
**Location**	**1990**	**2000**	**2015**	**1990**	**2000**	**2015**	**1990**	**2000**	**2015**	**1990**	**2000**	**2015**
Cyprus	71.20 (70.11-72.31)	74.27 (73.16-75.26)	79.37 (78.14-80.46)	79.45 (78.45-80.40)	86.55 (85.60-87.44)	92.08 (91.16-93.20)	67.86 (65.59-69.89)	68.76 (66.50-70.56)	73.28 (70.91-75.20)	100.00 (100.00-100.00)	100.00 (100.00-100.00)	100.00 (100.00-100.00)
Denmark	63.89 (62.17-65.50)	71.21 (69.99-72.30)	79.40 (78.03-80.75)	81.01 (80.21-81.83)	87.36 (86.49-88.20)	92.36 (91.54-93.14)	58.12 (55.64-60.53)	64.48 (61.87-66.40)	73.03 (70.12-75.37)	100.00 (100.00-100.00)	100.00 (100.00-100.00)	100.00 (100.00-100.00)
Finland	68.40 (67.18-69.71)	76.72 (75.80-77.59)	81.91 (80.77-82.87)	83.96 (83.36-84.62)	89.87 (89.20-90.57)	94.31 (93.54-95.02)	62.69 (60.75-64.65)	71.09 (69.40-72.62)	75.90 (73.51-77.68)	100.00 (100.00-100.00)	100.00 (100.00-100.00)	100.00 (100.00-100.00)
France	67.22 (66.07-68.50)	70.38 (69.18-71.65)	76.52 (75.31-77.78)	78.89 (78.23-79.53)	86.92 (86.28-87.57)	91.54 (90.82-92.16)	62.73 (60.90-64.53)	63.54 (61.41-65.51)	69.96 (67.60-71.93)	100.00 (100.00-100.00)	49.50 (40.41-58.09)	100.00 (100.00-100.00)
Germany	69.06 (68.04-70.00)	74.74 (73.74-75.67)	79.67 (78.47-80.68)	79.77 (79.02-80.53)	88.02 (87.34-88.74)	91.88 (91.22-92.52)	65.40 (63.80-66.91)	69.06 (67.11-70.64)	73.25 (70.25-75.33)	100.00 (100.00-100.00)	100.00 (100.00-100.00)	100.00 (100.00-100.00)
Greece	66.54 (65.04-67.89)	67.02 (65.37-68.52)	75.96 (74.23-77.44)	77.16 (75.76-78.34)	85.33 (84.20-86.34)	90.52 (89.74-91.19)	62.08 (59.42-64.31)	59.24 (56.00-61.73)	68.16 (63.89-71.04)	100.00 (100.00-100.00)	31.39 (26.65-36.39)	100.00 (100.00-100.00)
Iceland	68.24 (66.72-70.24)	73.35 (72.08-74.37)	85.50 (84.15-86.54)	82.87 (82.15-83.55)	88.25 (87.46-88.96)	93.35 (92.57-94.17)	61.20 (57.85-64.23)	66.01 (62.98-68.17)	79.26 (75.79-81.88)	26.79 (20.18-40.77)	20.96 (17.92-23.78)	100.00 (100.00-100.00)
Ireland	71.04 (70.02-71.97)	75.07 (74.18-75.94)	80.73 (79.55-81.74)	80.04 (79.26-80.77)	86.46 (85.77-87.17)	92.00 (91.46-92.52)	67.21 (64.90-68.93)	69.93 (68.26-71.30)	74.75 (72.00-76.83)	100.00 (100.00-100.00)	100.00 (100.00-100.00)	100.00 (100.00-100.00)
Israel	68.61 (67.47-69.76)	72.00 (70.97-73.00)	77.21 (75.97-78.39)	80.52 (79.82-81.21)	86.05 (85.30-86.80)	91.13 (90.50-91.78)	64.78 (62.83-66.42)	66.54 (64.37-68.40)	70.46 (67.56-72.77)	100.00 (100.00-100.00)	100.00 (100.00-100.00)	100.00 (100.00-100.00)
Italy	69.06 (67.96-70.19)	72.48 (71.12-73.70)	78.03 (76.55-79.38)	79.04 (77.84-80.18)	87.63 (86.78-88.54)	92.25 (91.43-92.98)	64.55 (62.19-66.55)	64.81 (61.71-67.25)	70.18 (66.48-72.94)	100.00 (100.00-100.00)	52.03 (42.31-60.97)	61.29 (49.16-70.99)
Luxembourg	68.77 (67.68-69.95)	74.13 (73.08-75.18)	80.87 (79.57-81.93)	81.60 (80.82-82.40)	88.04 (87.25-88.84)	91.41 (90.64-92.15)	63.90 (62.02-65.70)	67.69 (65.51-69.49)	74.73 (71.78-76.94)	100.00 (100.00-100.00)	100.00 (100.00-100.00)	100.00 (100.00-100.00)

Appendix Table 1a. Scaled values for each SDG health-related indicator and three summary indicators by country for 1990, 2000, and 2015

Location	Geometric mean of all indicators			Geometric mean of all indicators with corresponding MDG indicator			Geometric mean of all indicators without corresponding MDG indicator			Indicator 1.5.1: Age-standardised death rate due to exposure to forces of nature (per 100,000 population)		
	1990	2000	2015	1990	2000	2015	1990	2000	2015	1990	2000	2015
Malta	67.62 (65.98-69.53)	75.17 (73.64-76.40)	80.38 (78.87-81.79)	78.37 (77.28-79.42)	81.93 (80.87-82.96)	87.18 (86.37-87.97)	61.95 (58.74-65.07)	69.47 (65.18-72.35)	74.45 (70.10-77.47)	100.00 (100.00-100.00)	100.00 (100.00-100.00)	100.00 (100.00-100.00)
Netherlands	71.85 (70.79-72.89)	75.78 (74.80-76.71)	81.66 (80.59-82.64)	81.62 (80.86-82.31)	87.62 (86.87-88.33)	92.42 (91.79-93.01)	68.33 (66.68-69.81)	70.79 (69.11-72.38)	76.19 (73.96-78.06)	100.00 (100.00-100.00)	100.00 (100.00-100.00)	100.00 (100.00-100.00)
Norway	69.54 (68.23-70.69)	75.21 (74.02-76.24)	80.88 (79.52-82.01)	82.69 (82.01-83.37)	88.35 (87.56-89.06)	93.55 (92.91-94.12)	64.93 (62.97-66.59)	69.75 (67.67-71.51)	74.33 (71.53-76.49)	38.39 (32.45-44.01)	48.14 (39.57-55.86)	45.46 (37.48-53.62)
Portugal	65.45 (64.32-66.66)	69.40 (68.15-70.58)	77.89 (76.57-79.01)	73.76 (72.56-74.79)	81.39 (80.37-82.39)	89.03 (88.15-89.90)	61.67 (59.79-63.63)	62.93 (60.30-65.05)	70.46 (66.96-72.80)	100.00 (100.00-100.00)	60.27 (48.40-69.21)	100.00 (100.00-100.00)
Spain	66.90 (64.22-68.41)	70.29 (67.46-71.88)	81.69 (80.13-82.81)	77.55 (76.48-78.54)	86.86 (86.13-87.55)	91.57 (90.86-92.17)	59.43 (51.30-63.02)	60.06 (51.58-63.83)	74.00 (68.91-77.00)	100.00 (100.00-100.00)	46.89 (38.57-54.87)	100.00 (100.00-100.00)
Sweden	75.22 (74.27-76.11)	81.03 (80.09-81.88)	85.26 (84.16-86.21)	82.66 (81.98-83.31)	89.79 (89.10-90.50)	93.54 (92.92-94.10)	72.13 (70.11-73.74)	76.22 (73.94-78.12)	80.10 (77.35-82.08)	100.00 (100.00-100.00)	100.00 (100.00-100.00)	100.00 (100.00-100.00)
Switzerland	70.35 (69.41-71.34)	71.34 (70.17-72.47)	78.41 (77.20-79.65)	82.14 (81.40-82.88)	89.25 (88.54-89.95)	92.47 (91.69-93.25)	65.82 (64.16-67.34)	64.65 (62.87-66.27)	72.24 (70.18-74.16)	100.00 (100.00-100.00)	30.26 (25.63-35.23)	39.83 (33.28-46.46)
United Kingdom	70.04 (68.53-71.56)	75.36 (73.98-76.61)	81.97 (80.86-83.01)	81.05 (80.48-81.61)	86.41 (85.91-86.91)	89.89 (89.42-90.36)	66.46 (64.34-68.63)	71.13 (69.29-72.79)	78.25 (76.58-79.80)	100.00 (100.00-100.00)	100.00 (100.00-100.00)	100.00 (100.00-100.00)
Argentina	56.79 (55.35-58.48)	60.43 (58.61-62.53)	67.31 (65.18-68.78)	58.51 (56.13-60.64)	69.34 (67.93-70.55)	76.83 (75.48-77.98)	56.29 (53.38-58.90)	56.05 (51.80-59.40)	61.36 (54.93-64.65)	100.00 (100.00-100.00)	49.93 (42.57-60.19)	56.41 (49.42-66.00)
Chile	62.48 (61.40-63.58)	67.88 (66.54-69.02)	67.14 (64.22-68.98)	66.21 (64.70-67.59)	76.96 (75.99-77.85)	82.03 (81.24-82.76)	60.78 (58.57-62.74)	62.68 (59.00-65.10)	58.94 (47.80-62.87)	100.00 (100.00-100.00)	100.00 (100.00-100.00)	33.00 (25.72-43.93)
Uruguay	63.81 (62.68-64.97)	64.23 (63.02-65.53)	71.54 (70.08-72.67)	68.16 (66.53-69.59)	74.88 (73.71-75.96)	80.44 (79.26-81.45)	62.36 (60.32-64.16)	59.53 (56.97-61.57)	66.05 (61.64-68.64)	100.00 (100.00-100.00)	41.62 (34.09-52.48)	100.00 (100.00-100.00)

Appendix Table 1a. Scaled values for each SDG health-related indicator and three summary indicators by country for 1990, 2000, and 2015

Location	Geometric mean of all indicators			Geometric mean of all indicators with corresponding MDG indicator			Geometric mean of all indicators without corresponding MDG indicator			Indicator 1.5.1: Age-standardised death rate due to exposure to forces of nature (per 100,000 population)		
	1990	2000	2015	1990	2000	2015	1990	2000	2015	1990	2000	2015
Belarus	50.31 (48.79-51.78)	51.08 (49.31-52.88)	53.48 (52.23-54.76)	63.41 (60.74-65.92)	67.75 (66.61-68.87)	81.60 (80.28-82.76)	46.42 (44.48-48.32)	45.92 (43.58-48.32)	44.70 (43.05-46.35)	100.00 (100.00-100.00)	100.00 (100.00-100.00)	100.00 (100.00-100.00)
Estonia	52.51 (48.74-54.98)	61.79 (60.35-63.37)	74.44 (73.18-75.59)	67.40 (64.84-69.48)	75.07 (73.28-76.39)	85.27 (83.54-86.57)	47.86 (43.30-50.98)	56.39 (54.19-58.43)	67.57 (65.36-69.59)	100.00 (100.00-100.00)	58.20 (44.90-79.26)	100.00 (100.00-100.00)
Latvia	52.87 (51.20-54.62)	58.32 (56.87-59.95)	69.27 (67.98-70.48)	64.34 (61.82-66.45)	69.52 (67.66-71.13)	82.13 (80.25-83.74)	49.24 (47.06-51.44)	54.27 (52.13-56.46)	62.74 (60.75-64.60)	100.00 (100.00-100.00)	56.21 (43.23-77.38)	100.00 (100.00-100.00)
Lithuania	53.22 (51.51-54.93)	57.72 (55.94-59.68)	68.43 (67.18-69.74)	64.49 (61.42-66.85)	69.44 (66.62-71.50)	82.38 (80.23-83.94)	49.90 (47.92-52.04)	53.74 (51.47-56.28)	61.81 (59.86-63.62)	100.00 (100.00-100.00)	47.27 (36.21-67.71)	100.00 (100.00-100.00)
Moldova	51.07 (49.07-53.05)	51.37 (49.91-52.76)	62.02 (60.05-63.81)	57.05 (52.47-60.70)	55.37 (53.05-57.46)	69.16 (65.41-72.74)	49.41 (46.91-51.78)	50.38 (48.24-52.27)	58.43 (56.20-60.56)	100.00 (100.00-100.00)	34.15 (29.26-38.67)	46.38 (41.09-52.96)
Russia	46.75 (44.54-48.98)	43.01 (40.85-45.48)	53.79 (48.87-56.96)	61.22 (58.47-63.53)	63.74 (62.20-65.19)	75.36 (73.13-77.18)	42.25 (39.57-44.88)	37.15 (34.57-39.97)	45.98 (40.42-49.96)	31.07 (23.92-41.89)	24.85 (18.28-35.06)	57.22 (50.30-66.76)
Ukraine	52.33 (50.89-53.83)	53.11 (51.81-54.52)	53.95 (51.32-56.39)	61.24 (58.48-63.62)	62.91 (61.37-64.38)	73.49 (71.72-75.12)	49.88 (47.96-51.78)	50.27 (48.27-52.19)	47.61 (44.41-50.49)	100.00 (100.00-100.00)	100.00 (100.00-100.00)	100.00 (100.00-100.00)
Albania	54.21 (51.40-56.89)	52.23 (49.24-54.60)	67.29 (64.72-69.46)	48.49 (42.80-53.70)	55.07 (49.36-59.16)	67.58 (62.65-71.55)	57.35 (54.14-60.11)	50.72 (47.50-53.58)	64.97 (60.48-68.18)	100.00 (100.00-100.00)	25.93 (21.45-30.97)	100.00 (100.00-100.00)
Bosnia and Herzegovina	48.96 (46.65-50.98)	60.98 (58.96-62.77)	63.25 (61.17-65.34)	56.55 (51.87-60.69)	66.87 (62.81-70.12)	76.38 (73.00-78.78)	46.14 (43.63-48.79)	57.75 (55.02-60.16)	56.94 (53.82-59.70)	100.00 (100.00-100.00)	100.00 (100.00-100.00)	38.55 (30.02-55.61)
Bulgaria	54.83 (52.12-56.90)	54.77 (52.53-56.64)	64.16 (62.08-66.02)	64.71 (61.69-67.32)	67.18 (64.14-69.52)	77.76 (74.69-79.93)	48.15 (40.19-52.29)	49.57 (46.44-52.39)	57.37 (53.91-60.28)	100.00 (100.00-100.00)	30.25 (25.64-34.62)	54.04 (41.62-73.60)
Croatia	57.63 (55.63-59.40)	61.90 (59.98-63.75)	69.84 (67.96-71.45)	65.11 (61.36-68.06)	70.84 (67.31-73.73)	79.90 (76.33-82.61)	54.82 (52.37-57.08)	57.97 (55.68-60.24)	63.94 (61.17-66.41)	100.00 (100.00-100.00)	100.00 (100.00-100.00)	100.00 (100.00-100.00)

Appendix Table 1a. Scaled values for each SDG health-related indicator and three summary indicators by country for 1990, 2000, and 2015

Location	Geometric mean of all indicators			Geometric mean of all indicators with corresponding MDG indicator			Geometric mean of all indicators without corresponding MDG indicator			Indicator 1.5.1: Age-standardised death rate due to exposure to forces of nature (per 100,000 population)		
	1990	2000	2015	1990	2000	2015	1990	2000	2015	1990	2000	2015
Czech Republic	61.01 (59.44-62.40)	65.62 (63.91-67.34)	73.63 (72.00-75.00)	74.23 (71.71-76.11)	83.83 (81.29-85.61)	89.55 (86.97-91.13)	56.21 (53.88-58.31)	58.16 (55.83-60.41)	65.83 (63.31-68.01)	100.00 (100.00-100.00)	51.91 (38.70-71.54)	100.00 (100.00-100.00)
Hungary	57.22 (55.37-58.92)	62.14 (60.33-63.93)	73.14 (71.46-74.49)	68.06 (65.46-70.11)	76.83 (74.10-78.85)	85.68 (82.91-87.48)	51.79 (48.64-54.38)	55.54 (52.97-58.02)	65.92 (63.23-68.19)	100.00 (100.00-100.00)	44.47 (34.43-62.84)	100.00 (100.00-100.00)
Macedonia	55.85 (53.02-57.92)	56.98 (54.52-58.94)	65.52 (63.40-67.35)	57.19 (51.08-61.29)	65.28 (59.68-69.21)	73.79 (69.75-77.06)	56.70 (54.21-59.06)	54.31 (51.80-56.46)	61.87 (59.16-64.26)	100.00 (100.00-100.00)	26.93 (22.42-32.26)	100.00 (100.00-100.00)
Montenegro	60.39 (58.27-62.31)	60.59 (58.71-62.23)	67.37 (65.31-69.06)	63.57 (59.60-67.16)	68.13 (64.17-71.24)	78.80 (74.86-81.86)	59.19 (56.44-61.49)	57.61 (55.23-59.90)	61.45 (58.75-63.79)	100.00 (100.00-100.00)	100.00 (100.00-100.00)	100.00 (100.00-100.00)
Poland	55.65 (53.88-57.59)	62.49 (60.56-64.36)	71.52 (69.94-73.05)	62.78 (59.09-65.84)	73.20 (69.53-76.09)	82.24 (79.31-84.78)	53.59 (51.11-55.83)	58.18 (55.61-60.57)	66.03 (63.43-68.32)	100.00 (100.00-100.00)	57.44 (44.20-76.91)	100.00 (100.00-100.00)
Romania	50.31 (48.48-52.16)	53.52 (51.78-55.13)	63.00 (61.23-64.85)	53.95 (50.33-57.08)	59.18 (56.20-61.91)	72.72 (69.30-75.58)	50.34 (47.97-52.69)	51.74 (49.33-53.90)	58.37 (55.67-60.90)	100.00 (100.00-100.00)	33.03 (26.30-41.12)	50.47 (38.67-64.81)
Serbia	57.55 (55.39-59.47)	52.86 (50.80-54.84)	64.59 (62.69-66.52)	59.70 (55.16-63.49)	64.92 (60.62-68.18)	75.65 (71.98-78.46)	56.85 (54.30-59.08)	48.77 (46.21-51.18)	59.98 (57.57-62.44)	100.00 (100.00-100.00)	44.09 (36.23-52.31)	40.13 (30.75-58.36)
Slovakia	61.02 (59.49-62.54)	64.12 (62.47-65.88)	72.89 (71.03-74.59)	71.90 (68.94-74.04)	79.90 (77.01-81.88)	86.70 (84.03-88.33)	56.63 (54.52-58.73)	57.59 (54.99-59.95)	66.30 (63.57-68.73)	100.00 (100.00-100.00)	31.89 (24.36-44.86)	60.10 (45.34-80.86)
Slovenia	58.07 (56.29-60.03)	66.12 (64.54-67.61)	76.14 (74.46-77.48)	72.66 (70.03-74.59)	81.81 (78.87-83.71)	88.76 (85.68-90.41)	52.42 (49.84-54.87)	58.73 (56.41-60.92)	67.89 (64.86-70.46)	36.43 (27.92-53.88)	44.56 (36.12-54.66)	100.00 (100.00-100.00)
Armenia	52.80 (49.94-55.15)	56.64 (53.83-58.82)	61.02 (57.82-63.29)	47.20 (42.23-51.47)	51.83 (46.46-55.49)	62.01 (55.16-66.10)	57.57 (55.11-59.92)	60.90 (58.58-62.82)	60.98 (58.58-63.23)	100.00 (100.00-100.00)	100.00 (100.00-100.00)	49.90 (40.34-61.94)
Azerbaijan	49.98 (47.67-51.98)	52.11 (50.42-53.53)	62.70 (61.02-64.39)	41.81 (38.29-45.01)	43.53 (41.18-45.73)	61.54 (58.92-63.95)	56.95 (54.00-59.51)	58.99 (56.78-61.05)	63.12 (60.50-65.81)	100.00 (100.00-100.00)	35.66 (29.32-44.92)	100.00 (100.00-100.00)

Location	Geometric mean of all indicators			Geometric mean of all indicators with corresponding MDG indicator			Geometric mean of all indicators without corresponding MDG indicator			Indicator 1.5.1: Age-standardised death rate due to exposure to forces of nature (per 100,000 population)		
	1990	2000	2015	1990	2000	2015	1990	2000	2015	1990	2000	2015
Georgia	54.34 (50.63-56.90)	54.11 (50.02-56.60)	57.60 (54.15-60.06)	46.53 (39.22-50.81)	47.94 (39.38-51.97)	60.15 (52.41-65.02)	60.89 (58.20-63.41)	59.43 (56.83-61.79)	57.35 (54.66-59.68)	100.00 (100.00-100.00)	100.00 (100.00-100.00)	28.83 (23.64-36.77)
Kazakhstan	53.17 (51.29-54.89)	52.26 (50.82-53.82)	61.53 (59.67-63.34)	55.32 (52.40-57.92)	59.00 (56.75-60.92)	69.32 (66.63-71.72)	53.27 (50.96-55.34)	50.64 (48.80-52.65)	57.79 (55.48-60.07)	100.00 (100.00-100.00)	100.00 (100.00-100.00)	100.00 (100.00-100.00)
Kyrgyzstan	50.09 (48.03-51.99)	47.68 (45.64-49.66)	55.99 (54.08-57.62)	45.49 (41.97-48.89)	50.47 (47.63-52.83)	57.68 (54.56-60.44)	53.52 (51.08-55.80)	48.13 (45.60-50.57)	55.81 (53.59-57.82)	100.00 (100.00-100.00)	41.68 (34.42-51.01)	33.43 (27.52-42.28)
Mongolia	41.25 (39.01-43.29)	44.97 (43.00-46.98)	56.37 (53.90-58.61)	37.29 (32.92-41.08)	46.71 (42.98-49.69)	59.41 (55.05-63.19)	44.38 (42.00-46.58)	45.58 (43.13-47.88)	55.28 (52.69-57.79)	15.14 (12.11-19.30)	22.78 (18.68-28.63)	100.00 (100.00-100.00)
Tajikistan	47.20 (45.21-49.18)	44.09 (42.05-45.97)	59.04 (57.10-60.88)	43.00 (39.73-46.23)	42.94 (40.78-45.20)	58.05 (55.19-60.63)	50.70 (48.05-53.45)	45.97 (43.38-48.36)	59.41 (56.79-61.86)	100.00 (100.00-100.00)	23.98 (19.71-30.43)	42.38 (34.51-53.54)
Turkmenistan	54.46 (52.36-56.43)	54.20 (52.02-56.10)	66.14 (63.94-68.04)	47.32 (43.54-51.08)	50.68 (46.62-53.98)	64.66 (60.22-68.12)	59.75 (57.88-61.57)	57.63 (55.34-59.67)	66.35 (63.72-68.61)	100.00 (100.00-100.00)	47.32 (38.56-58.36)	100.00 (100.00-100.00)
Uzbekistan	55.97 (54.27-57.67)	56.08 (54.40-57.46)	67.10 (65.40-68.71)	52.65 (49.75-55.49)	56.60 (54.06-58.65)	70.35 (67.24-73.15)	56.13 (53.49-58.45)	54.99 (52.77-57.03)	64.02 (61.65-66.41)	100.00 (100.00-100.00)	100.00 (100.00-100.00)	100.00 (100.00-100.00)
Colombia	43.28 (41.82-44.63)	44.94 (43.36-46.44)	60.65 (58.77-62.23)	48.89 (45.81-51.11)	53.52 (50.33-55.95)	65.03 (61.42-67.99)	43.32 (41.79-45.06)	44.03 (42.46-45.93)	60.52 (58.97-61.86)	26.77 (22.78-31.04)	20.22 (17.10-23.41)	28.74 (24.40-33.15)
Costa Rica	60.26 (58.05-62.06)	59.05 (56.84-60.76)	66.56 (64.17-68.32)	55.87 (51.54-58.92)	62.39 (57.73-65.50)	71.01 (66.21-74.14)	63.02 (60.88-65.00)	58.14 (55.72-60.11)	64.07 (60.59-66.44)	100.00 (100.00-100.00)	23.80 (20.05-27.55)	45.45 (37.61-52.57)
El Salvador	42.01 (39.91-43.89)	46.53 (44.64-48.36)	58.15 (55.70-60.34)	43.60 (40.10-46.13)	52.76 (49.31-55.52)	66.45 (62.10-70.09)	43.28 (41.01-45.57)	45.74 (43.70-47.81)	55.92 (53.41-58.25)	100.00 (100.00-100.00)	14.54 (12.10-17.02)	31.07 (26.42-35.91)
Guatemala	37.92 (35.92-39.51)	41.20 (39.19-42.81)	51.25 (49.12-53.25)	28.03 (25.27-30.27)	36.49 (33.24-38.82)	49.85 (45.96-53.24)	46.10 (43.93-48.23)	45.68 (43.74-47.49)	53.27 (51.00-55.38)	100.00 (100.00-100.00)	18.69 (15.76-21.73)	23.52 (20.53-25.77)

Table title: **Appendix Table 1a. Scaled values for each SDG health-related indicator and three summary indicators by country for 1990, 2000, and 2015**

Appendix Table 1a. Scaled values for each SDG health-related indicator and three summary indicators by country for 1990, 2000, and 2015

Location	Geometric mean of all indicators			Geometric mean of all indicators with corresponding MDG indicator			Geometric mean of all indicators without corresponding MDG indicator			Indicator 1.5.1: Age-standardised death rate due to exposure to forces of nature (per 100,000 population)		
	1990	2000	2015	1990	2000	2015	1990	2000	2015	1990	2000	2015
Honduras	46.58 (44.60-48.30)	41.66 (39.38-43.46)	54.71 (51.84-57.20)	35.43 (32.89-37.70)	43.28 (41.02-45.34)	57.85 (54.87-60.16)	55.21 (52.59-57.51)	43.30 (40.25-45.85)	54.61 (50.74-57.77)	100.00 (100.00-100.00)	3.14 (1.18-4.18)	36.37 (30.60-42.23)
Mexico	49.62 (47.65-51.27)	56.48 (55.01-57.83)	64.01 (62.34-65.35)	50.48 (48.74-51.93)	59.86 (57.99-61.37)	68.41 (66.04-70.20)	50.64 (47.84-53.09)	56.24 (53.85-58.31)	63.61 (61.59-65.33)	25.65 (18.00-34.62)	25.44 (18.23-31.77)	41.32 (30.71-50.36)
Nicaragua	46.12 (44.14-47.74)	46.73 (44.85-48.30)	58.86 (56.59-61.05)	37.37 (34.42-39.81)	44.35 (41.29-46.77)	56.51 (52.43-59.84)	52.76 (50.35-54.81)	49.67 (47.61-51.43)	60.83 (58.40-62.94)	100.00 (100.00-100.00)	6.26 (4.73-7.75)	30.79 (26.22-35.37)
Panama	57.88 (56.21-59.46)	55.20 (53.51-56.78)	63.35 (61.35-65.24)	50.44 (47.45-52.83)	54.70 (51.42-57.24)	62.31 (58.86-65.39)	63.57 (61.73-65.47)	57.70 (55.87-59.49)	65.70 (63.69-67.65)	100.00 (100.00-100.00)	30.14 (25.40-35.36)	32.60 (27.62-36.82)
Venezuela	52.92 (51.27-54.44)	46.50 (44.66-48.05)	62.04 (59.86-63.92)	50.98 (47.90-53.30)	56.75 (53.70-59.33)	64.14 (60.41-67.17)	55.49 (53.62-57.31)	44.43 (42.29-46.31)	61.96 (59.40-64.24)	100.00 (100.00-100.00)	4.75 (3.06-6.02)	100.00 (100.00-100.00)
Bolivia	39.78 (37.04-41.86)	41.13 (39.45-43.48)	53.18 (51.15-55.10)	30.70 (29.35-32.07)	40.89 (39.70-42.09)	57.67 (55.66-59.52)	46.94 (42.41-49.97)	42.84 (40.22-45.67)	51.60 (48.76-54.16)	100.00 (100.00-100.00)	20.48 (14.51-30.00)	22.28 (16.05-32.12)
Ecuador	56.00 (54.56-57.36)	54.78 (53.29-56.50)	63.37 (61.68-65.05)	48.78 (47.28-50.32)	54.68 (53.47-55.82)	68.81 (67.60-70.04)	59.15 (56.72-61.16)	55.09 (52.72-57.47)	60.76 (58.31-63.13)	100.00 (100.00-100.00)	21.58 (15.45-31.30)	34.31 (26.96-45.29)
Peru	42.69 (41.21-44.02)	52.55 (51.03-54.10)	64.64 (63.06-66.22)	40.05 (38.57-41.43)	50.82 (49.76-51.88)	67.08 (65.41-68.65)	45.08 (42.78-47.06)	53.69 (51.19-56.08)	63.01 (60.62-65.27)	16.78 (11.46-25.48)	19.43 (13.63-28.74)	35.37 (27.98-46.36)
Antigua and Barbuda	67.85 (65.92-69.62)	69.43 (67.55-71.08)	73.66 (71.85-75.36)	62.30 (59.26-64.76)	64.46 (61.71-66.83)	71.99 (69.40-74.38)	71.41 (68.71-73.84)	72.78 (69.72-75.23)	74.98 (72.24-77.36)	100.00 (100.00-100.00)	100.00 (100.00-100.00)	100.00 (100.00-100.00)
The Bahamas	60.65 (58.93-62.34)	62.30 (60.39-64.14)	69.49 (67.55-71.34)	58.77 (55.88-61.31)	66.47 (63.88-68.61)	72.08 (68.94-74.64)	62.44 (60.05-64.57)	60.87 (58.45-63.32)	68.38 (65.61-70.72)	100.00 (100.00-100.00)	35.82 (28.40-46.81)	100.00 (100.00-100.00)
Barbados	62.27 (60.23-64.14)	67.81 (65.81-69.65)	71.02 (69.10-72.94)	60.65 (57.75-63.08)	63.83 (61.01-66.13)	70.62 (67.43-73.27)	63.36 (60.57-66.03)	69.73 (67.08-72.22)	70.65 (67.82-73.09)	100.00 (100.00-100.00)	100.00 (100.00-100.00)	100.00 (100.00-100.00)

Appendix Table 1a. Scaled values for each SDG health-related indicator and three summary indicators by country for 1990, 2000, and 2015

Location	Geometric mean of all indicators			Geometric mean of all indicators with corresponding MDG indicator			Geometric mean of all indicators without corresponding MDG indicator			Indicator 1.5.1: Age-standardised death rate due to exposure to forces of nature (per 100,000 population)		
	1990	2000	2015	1990	2000	2015	1990	2000	2015	1990	2000	2015
Belize	47.67 (42.29-50.18)	48.10 (42.90-50.56)	59.16 (54.08-61.77)	38.20 (28.63-42.14)	43.82 (33.95-47.81)	55.25 (43.89-59.94)	53.85 (51.31-56.45)	51.44 (48.94-53.98)	62.10 (59.62-64.36)	100.00 (100.00-100.00)	17.67 (12.19-26.60)	100.00 (100.00-100.00)
Cuba	55.28 (53.51-57.12)	56.67 (54.85-58.47)	64.59 (62.80-66.39)	60.85 (57.54-63.41)	64.02 (61.26-66.34)	72.44 (69.39-74.59)	53.48 (50.94-55.95)	54.07 (51.57-56.61)	60.41 (57.76-62.99)	100.00 (100.00-100.00)	24.28 (17.78-34.42)	31.18 (24.02-42.01)
Dominica	60.83 (58.45-62.76)	60.64 (58.71-62.51)	67.55 (65.45-69.30)	55.23 (51.37-58.56)	60.89 (57.49-63.74)	66.92 (63.56-69.92)	63.59 (60.62-66.08)	60.98 (58.28-63.60)	68.11 (65.53-70.59)	100.00 (100.00-100.00)	28.56 (21.61-39.19)	100.00 (100.00-100.00)
Dominican Republic	52.68 (50.48-54.34)	50.47 (47.91-52.54)	61.93 (59.99-63.48)	43.23 (39.72-45.54)	47.21 (42.87-49.82)	57.47 (53.86-60.18)	58.84 (56.28-61.10)	53.61 (51.09-56.21)	64.95 (62.61-66.97)	100.00 (100.00-100.00)	18.72 (13.05-27.88)	100.00 (100.00-100.00)
Grenada	55.50 (53.25-57.76)	63.04 (60.91-64.73)	63.39 (61.30-65.76)	52.70 (48.77-55.98)	60.39 (56.87-63.31)	69.94 (66.48-72.88)	57.53 (54.61-60.42)	64.80 (62.28-67.07)	61.02 (58.35-64.16)	100.00 (100.00-100.00)	100.00 (100.00-100.00)	26.70 (20.10-40.64)
Guyana	44.70 (42.77-46.51)	46.02 (44.23-47.85)	56.71 (54.62-58.66)	35.65 (32.80-37.95)	39.57 (37.06-41.78)	51.02 (48.17-53.53)	51.54 (48.96-53.91)	51.07 (48.42-53.44)	60.26 (57.13-63.02)	100.00 (100.00-100.00)	56.41 (43.38-77.57)	100.00 (100.00-100.00)
Haiti	29.95 (27.12-32.41)	32.33 (29.15-34.86)	40.75 (36.58-43.53)	15.86 (12.65-18.43)	20.58 (16.36-23.43)	31.47 (24.34-35.31)	43.38 (39.74-46.43)	42.60 (39.21-45.85)	47.48 (43.82-50.83)	100.00 (100.00-100.00)	20.58 (14.60-30.12)	27.01 (20.21-37.49)
Jamaica	59.28 (57.49-60.92)	61.88 (60.26-63.26)	66.23 (64.43-67.95)	53.28 (50.38-55.65)	58.80 (56.26-60.76)	68.03 (65.24-70.59)	62.05 (59.38-64.47)	63.13 (60.72-65.30)	64.50 (61.80-67.10)	100.00 (100.00-100.00)	100.00 (100.00-100.00)	100.00 (100.00-100.00)
Saint Lucia	53.67 (51.70-55.59)	61.50 (59.78-63.14)	67.40 (65.39-69.30)	49.02 (45.29-52.13)	57.54 (54.30-60.35)	67.14 (63.74-70.17)	56.04 (53.48-58.75)	63.86 (61.27-66.03)	67.97 (65.25-70.10)	100.00 (100.00-100.00)	100.00 (100.00-100.00)	100.00 (100.00-100.00)
Saint Vincent and the Grenadines	53.70 (51.37-55.87)	61.32 (59.29-63.15)	66.26 (64.33-68.20)	51.11 (47.48-54.31)	56.37 (53.16-59.19)	65.64 (62.17-68.37)	55.59 (52.48-58.55)	63.67 (60.77-66.24)	65.75 (63.00-68.37)	100.00 (100.00-100.00)	100.00 (100.00-100.00)	100.00 (100.00-100.00)
Suriname	48.84 (46.44-50.87)	54.23 (51.85-55.98)	60.95 (58.82-62.85)	42.62 (39.25-45.16)	46.43 (43.07-48.58)	58.55 (55.12-61.25)	54.09 (51.08-56.82)	60.02 (56.96-62.57)	63.23 (60.48-65.54)	100.00 (100.00-100.00)	100.00 (100.00-100.00)	100.00 (100.00-100.00)

Appendix Table 1a. Scaled values for each SDG health-related indicator and three summary indicators by country for 1990, 2000, and 2015

Location	Geometric mean of all indicators			Geometric mean of all indicators with corresponding MDG indicator			Geometric mean of all indicators without corresponding MDG indicator			Indicator 1.5.1: Age-standardised death rate due to exposure to forces of nature (per 100,000 population)		
	1990	2000	2015	1990	2000	2015	1990	2000	2015	1990	2000	2015
Trinidad and Tobago	55.68 (54.10-57.07)	62.16 (60.65-63.58)	67.01 (65.32-68.62)	56.60 (53.85-58.76)	58.80 (56.30-60.91)	69.41 (66.59-71.83)	57.23 (55.16-59.05)	65.64 (63.50-67.46)	67.35 (65.12-69.30)	100.00 (100.00-100.00)	100.00 (100.00-100.00)	100.00 (100.00-100.00)
Brazil	47.85 (46.30-49.40)	52.76 (50.68-54.59)	60.10 (58.22-61.89)	47.05 (44.95-48.70)	55.85 (53.71-57.61)	64.06 (61.88-65.97)	49.92 (47.56-52.16)	52.16 (49.41-54.57)	57.62 (54.59-60.29)	32.73 (22.79-44.29)	35.28 (26.16-43.28)	32.58 (22.76-45.87)
Paraguay	52.52 (50.53-54.14)	50.90 (49.30-52.25)	59.08 (57.29-60.73)	44.52 (41.45-47.14)	50.40 (47.77-52.56)	62.62 (59.94-64.99)	57.91 (55.33-60.16)	52.37 (50.15-54.18)	57.76 (55.06-59.96)	100.00 (100.00-100.00)	21.43 (18.71-23.15)	31.60 (27.17-34.13)
China	41.15 (39.46-42.77)	47.62 (46.15-49.05)	59.84 (58.58-61.26)	40.06 (37.81-42.05)	51.76 (50.10-53.24)	69.67 (68.18-71.10)	39.47 (36.36-42.66)	45.43 (43.15-47.52)	54.63 (52.60-56.64)	37.02 (30.94-43.72)	30.13 (24.74-36.49)	38.96 (33.20-45.60)
North Korea	50.15 (46.83-53.13)	47.13 (42.93-50.71)	54.67 (51.80-57.73)	46.02 (38.67-50.90)	45.38 (37.31-50.82)	60.50 (55.95-64.61)	52.26 (48.48-55.59)	48.11 (43.70-51.60)	51.05 (46.66-55.10)	100.00 (100.00-100.00)	30.71 (23.58-41.50)	31.52 (24.34-42.37)
Taiwan	63.33 (61.58-64.81)	60.21 (58.29-62.22)	73.85 (72.04-75.56)	73.91 (70.54-76.47)	76.37 (73.42-78.50)	83.15 (80.19-85.18)	56.21 (53.64-58.52)	52.10 (48.87-54.93)	66.56 (62.77-69.46)	100.00 (100.00-100.00)	14.45 (9.62-22.52)	100.00 (100.00-100.00)
Cambodia	27.80 (24.65-30.10)	33.02 (31.03-34.80)	47.44 (45.58-49.33)	13.53 (10.19-16.27)	21.04 (18.54-23.18)	44.73 (42.08-47.23)	41.29 (38.69-43.90)	42.29 (39.60-44.67)	49.08 (46.64-51.70)	100.00 (100.00-100.00)	20.19 (15.60-27.21)	21.42 (16.45-29.65)
Indonesia	41.46 (39.92-43.09)	46.71 (45.29-48.15)	60.08 (58.29-61.89)	28.73 (26.89-30.57)	38.41 (36.67-40.01)	52.28 (49.76-54.59)	49.85 (47.48-52.35)	52.03 (49.77-54.13)	64.12 (61.80-66.57)	36.68 (27.98-53.57)	41.52 (33.44-51.40)	100.00 (100.00-100.00)
Laos	24.80 (22.53-27.09)	32.40 (30.51-34.21)	45.18 (43.23-47.18)	12.91 (10.84-15.40)	22.61 (20.79-24.28)	43.40 (41.04-45.52)	38.12 (34.84-41.27)	41.32 (38.25-44.07)	47.47 (44.66-50.03)	100.00 (100.00-100.00)	32.46 (24.86-47.37)	30.57 (24.07-39.59)
Malaysia	59.21 (57.43-60.64)	58.94 (57.31-60.53)	69.18 (67.46-70.76)	55.62 (52.28-58.11)	63.24 (60.16-65.56)	72.97 (70.32-75.12)	60.20 (58.12-62.09)	56.48 (54.37-58.64)	65.57 (62.89-67.94)	100.00 (100.00-100.00)	26.01 (20.51-34.73)	100.00 (100.00-100.00)
Maldives	45.74 (40.41-48.49)	53.84 (48.44-56.32)	64.80 (61.64-67.24)	33.39 (25.23-37.61)	45.49 (35.27-49.87)	60.65 (53.92-64.73)	55.03 (52.29-57.88)	59.64 (57.17-61.92)	66.76 (64.15-69.28)	100.00 (100.00-100.00)	100.00 (100.00-100.00)	100.00 (100.00-100.00)

Appendix Table 1a. Scaled values for each SDG health-related indicator and three summary indicators by country for 1990, 2000, and 2015

Location	Geometric mean of all indicators			Geometric mean of all indicators with corresponding MDG indicator			Geometric mean of all indicators without corresponding MDG indicator			Indicator 1.5.1: Age-standardised death rate due to exposure to forces of nature (per 100,000 population)		
	1990	2000	2015	1990	2000	2015	1990	2000	2015	1990	2000	2015
Mauritius	60.72 (59.01-62.13)	62.87 (61.02-64.50)	67.77 (66.03-69.54)	58.31 (55.06-61.10)	64.54 (61.36-66.97)	73.08 (70.43-75.18)	60.64 (58.28-62.75)	61.12 (58.60-63.55)	63.91 (61.14-66.56)	100.00 (100.00-100.00)	100.00 (100.00-100.00)	36.15 (27.57-49.36)
Myanmar	32.70 (29.25-35.66)	35.91 (32.51-39.05)	45.67 (42.31-49.09)	17.86 (14.96-20.40)	24.41 (21.29-27.24)	43.20 (39.61-46.54)	44.04 (39.81-48.28)	43.68 (39.42-47.86)	46.85 (42.78-50.92)	100.00 (100.00-100.00)	55.10 (42.25-73.96)	36.07 (28.96-45.83)
Philippines	39.25 (37.64-40.82)	43.79 (42.01-45.44)	49.97 (48.00-51.97)	35.81 (33.15-38.07)	41.19 (38.20-43.34)	51.38 (47.93-54.12)	43.56 (41.42-45.48)	47.51 (45.51-49.48)	51.04 (48.72-53.38)	10.01 (6.82-15.39)	23.03 (17.17-33.08)	13.42 (9.70-19.84)
Sri Lanka	40.85 (38.73-43.76)	47.29 (45.37-49.43)	62.03 (59.70-64.21)	40.25 (37.00-43.06)	50.31 (47.39-52.99)	70.13 (66.31-73.19)	41.58 (38.87-45.64)	46.62 (44.22-49.46)	58.69 (55.84-61.27)	32.80 (24.49-48.75)	36.67 (28.33-53.03)	22.64 (18.58-26.71)
Seychelles	62.59 (60.59-64.27)	65.54 (63.68-67.20)	71.29 (69.27-73.07)	64.04 (60.51-66.64)	69.39 (66.60-71.83)	75.82 (72.83-77.98)	61.70 (59.41-64.06)	63.33 (60.66-65.72)	68.17 (65.52-70.70)	100.00 (100.00-100.00)	100.00 (100.00-100.00)	100.00 (100.00-100.00)
Thailand	45.70 (43.82-47.37)	49.12 (47.17-50.86)	56.31 (54.01-58.39)	43.18 (39.80-45.74)	51.98 (48.16-54.53)	62.81 (58.69-65.78)	48.67 (46.66-50.70)	49.32 (47.21-51.28)	53.80 (51.36-56.50)	23.64 (17.93-33.82)	27.14 (21.40-35.74)	29.01 (22.32-41.33)
Timor-Leste	32.35 (29.63-34.71)	34.14 (32.51-35.91)	52.80 (50.69-54.86)	14.05 (11.37-16.59)	20.11 (18.08-22.07)	43.19 (40.28-45.75)	49.58 (46.42-52.62)	44.42 (41.75-47.16)	57.26 (54.33-60.17)	100.00 (100.00-100.00)	100.00 (100.00-100.00)	100.00 (100.00-100.00)
Vietnam	42.41 (40.46-44.39)	47.51 (45.78-49.27)	59.44 (57.14-61.77)	31.45 (29.11-33.42)	44.05 (42.04-45.71)	62.79 (60.48-64.91)	48.06 (45.26-50.87)	48.77 (46.29-51.34)	57.20 (54.09-60.21)	29.16 (22.09-41.94)	15.70 (11.69-22.44)	43.87 (33.46-61.63)
Federated States of Micronesia	51.07 (46.24-55.40)	55.27 (50.89-59.16)	61.94 (56.43-65.72)	48.19 (43.77-51.75)	54.90 (51.09-58.15)	66.72 (62.77-69.84)	51.74 (45.38-57.39)	54.64 (47.78-59.93)	57.94 (50.88-63.28)	100.00 (100.00-100.00)	100.00 (100.00-100.00)	100.00 (100.00-100.00)
Fiji	56.68 (54.35-59.01)	53.26 (51.28-55.23)	59.23 (56.88-61.45)	55.13 (51.31-58.10)	59.04 (55.62-61.77)	65.75 (62.41-68.60)	56.47 (53.31-59.38)	50.23 (47.39-53.01)	55.34 (52.14-58.54)	100.00 (100.00-100.00)	18.24 (15.89-19.78)	29.59 (25.55-31.85)
Kiribati	36.74 (33.18-40.24)	38.93 (35.75-42.57)	47.85 (43.73-51.27)	38.08 (33.29-41.70)	44.19 (40.11-47.75)	54.82 (50.61-58.74)	33.62 (26.93-39.33)	34.00 (27.61-39.89)	40.21 (32.47-46.24)	100.00 (100.00-100.00)	100.00 (100.00-100.00)	100.00 (100.00-100.00)

Appendix Table 1a. Scaled values for each SDG health-related indicator and three summary indicators by country for 1990, 2000, and 2015

Location	Geometric mean of all indicators			Geometric mean of all indicators with corresponding MDG indicator			Geometric mean of all indicators without corresponding MDG indicator			Indicator 1.5.1: Age-standardised death rate due to exposure to forces of nature (per 100,000 population)		
	1990	2000	2015	1990	2000	2015	1990	2000	2015	1990	2000	2015
Marshall Islands	49.34 (46.22-52.00)	52.54 (49.42-55.12)	59.13 (55.76-61.96)	42.56 (37.69-46.27)	49.44 (45.41-52.63)	61.79 (57.50-65.16)	51.07 (46.36-55.12)	52.21 (46.90-56.39)	54.93 (48.80-59.53)	100.00 (100.00-100.00)	100.00 (100.00-100.00)	100.00 (100.00-100.00)
Papua New Guinea	24.67 (20.94-29.55)	26.42 (21.93-30.43)	36.01 (29.70-40.59)	17.89 (14.72-20.85)	20.99 (17.55-23.72)	32.51 (28.82-36.04)	28.74 (23.50-35.89)	29.55 (23.64-35.18)	37.31 (28.90-43.39)	100.00 (100.00-100.00)	7.59 (6.07-8.54)	31.99 (27.39-34.60)
Samoa	52.99 (49.94-55.63)	57.05 (53.96-59.70)	57.37 (54.07-59.99)	54.74 (50.13-58.34)	60.07 (56.06-63.20)	69.16 (65.38-72.05)	49.61 (41.39-54.50)	52.69 (44.90-57.78)	49.02 (40.09-54.18)	100.00 (100.00-100.00)	100.00 (100.00-100.00)	16.46 (14.28-17.89)
Solomon Islands	38.06 (33.26-41.69)	42.77 (38.13-46.55)	44.40 (39.58-48.03)	26.49 (22.90-29.65)	34.05 (30.57-37.14)	45.60 (41.50-49.02)	43.65 (37.39-48.88)	46.10 (40.03-51.36)	41.97 (36.15-46.71)	100.00 (100.00-100.00)	100.00 (100.00-100.00)	13.79 (11.75-15.14)
Tonga	54.30 (51.58-56.71)	57.35 (54.68-59.56)	61.76 (57.80-64.76)	54.50 (50.01-58.18)	59.10 (55.01-62.20)	67.65 (63.64-71.06)	51.74 (46.48-55.72)	53.30 (46.07-57.91)	53.19 (43.25-59.75)	100.00 (100.00-100.00)	100.00 (100.00-100.00)	100.00 (100.00-100.00)
Vanuatu	40.42 (34.59-44.63)	37.82 (33.03-41.78)	46.33 (41.58-49.88)	30.36 (23.66-34.30)	34.49 (27.96-38.09)	45.58 (40.32-49.27)	45.97 (38.44-51.35)	39.91 (33.63-44.74)	46.06 (40.25-50.72)	100.00 (100.00-100.00)	5.74 (4.15-6.53)	14.53 (12.42-15.89)
Afghanistan	19.79 (15.88-23.16)	17.49 (14.18-20.61)	26.20 (21.58-29.55)	11.42 (9.06-14.53)	12.86 (10.51-16.15)	26.16 (20.43-29.80)	28.17 (21.31-33.08)	22.27 (17.16-26.86)	27.30 (21.49-31.40)	100.00 (100.00-100.00)	8.03 (6.01-11.14)	18.20 (14.63-24.27)
Algeria	51.19 (47.04-54.07)	51.21 (48.00-53.31)	58.74 (55.72-61.40)	42.64 (34.33-47.21)	52.21 (46.49-55.79)	61.90 (55.56-66.28)	55.94 (52.58-58.83)	50.97 (47.92-53.73)	56.06 (52.69-59.24)	100.00 (100.00-100.00)	58.77 (46.24-74.19)	52.11 (41.41-66.80)
Bahrain	53.87 (50.79-56.29)	56.10 (53.22-58.82)	65.33 (61.88-67.95)	54.89 (48.43-58.52)	61.60 (55.78-65.27)	70.72 (65.05-74.29)	51.41 (46.69-54.85)	51.40 (46.80-55.19)	58.69 (50.81-63.53)	100.00 (100.00-100.00)	100.00 (100.00-100.00)	100.00 (100.00-100.00)
Egypt	48.59 (43.19-51.14)	52.04 (48.22-54.44)	52.58 (49.07-55.90)	39.49 (29.59-43.23)	51.44 (43.24-55.30)	60.81 (52.44-65.18)	54.80 (52.33-57.35)	52.66 (50.32-54.98)	49.32 (46.75-53.08)	100.00 (100.00-100.00)	100.00 (100.00-100.00)	100.00 (100.00-100.00)
Iran	37.91 (34.42-40.51)	46.03 (43.09-48.51)	58.08 (54.69-60.96)	39.38 (32.93-43.47)	53.65 (48.43-57.48)	66.97 (61.49-70.68)	39.19 (36.05-42.00)	44.54 (41.72-47.27)	55.15 (51.54-58.38)	2.46 (0.23-4.17)	18.34 (14.75-24.43)	38.63 (31.31-50.79)

Appendix Table 1a. Scaled values for each SDG health-related indicator and three summary indicators by country for 1990, 2000, and 2015

Location	Geometric mean of all indicators			Geometric mean of all indicators with corresponding MDG indicator			Geometric mean of all indicators without corresponding MDG indicator			Indicator 1.5.1: Age-standardised death rate due to exposure to forces of nature (per 100,000 population)		
	1990	2000	2015	1990	2000	2015	1990	2000	2015	1990	2000	2015
Iraq	44.27 (40.27-46.69)	47.93 (44.49-50.25)	48.40 (45.18-50.92)	40.04 (31.72-43.85)	44.80 (38.16-48.12)	55.56 (49.51-59.03)	47.24 (43.95-50.36)	50.81 (47.53-53.68)	46.38 (43.16-49.54)	100.00 (100.00-100.00)	100.00 (100.00-100.00)	52.58 (41.75-67.33)
Jordan	58.41 (55.80-60.58)	62.85 (60.75-64.56)	69.43 (67.50-71.20)	56.01 (51.18-59.53)	62.95 (58.89-65.90)	73.54 (70.39-75.88)	60.70 (58.03-63.19)	63.89 (61.28-66.18)	67.55 (65.17-69.84)	100.00 (100.00-100.00)	100.00 (100.00-100.00)	100.00 (100.00-100.00)
Kuwait	52.86 (50.19-55.12)	58.73 (55.86-61.06)	64.08 (61.24-66.77)	58.80 (53.06-62.39)	65.47 (59.97-69.14)	75.35 (70.41-78.92)	49.95 (47.04-52.95)	54.16 (50.15-57.61)	56.28 (49.95-60.50)	100.00 (100.00-100.00)	100.00 (100.00-100.00)	100.00 (100.00-100.00)
Lebanon	45.55 (41.11-48.49)	55.16 (50.94-58.03)	62.37 (59.23-65.02)	46.86 (37.04-52.98)	54.63 (45.15-60.00)	68.52 (62.45-72.99)	46.49 (43.48-49.24)	55.53 (52.14-58.49)	59.07 (55.59-62.58)	100.00 (100.00-100.00)	100.00 (100.00-100.00)	100.00 (100.00-100.00)
Libya	51.88 (48.22-54.47)	51.96 (48.97-54.36)	50.08 (47.45-52.53)	47.24 (40.24-51.56)	55.25 (49.36-59.24)	63.89 (58.22-68.14)	52.25 (48.87-55.11)	49.03 (45.93-51.84)	43.54 (40.28-46.77)	100.00 (100.00-100.00)	100.00 (100.00-100.00)	47.43 (37.82-62.30)
Morocco	43.23 (38.73-45.93)	50.01 (44.91-52.67)	57.26 (52.74-60.09)	33.26 (26.06-37.16)	44.16 (33.25-48.20)	56.39 (47.28-60.97)	49.62 (46.38-52.63)	52.39 (49.21-55.47)	55.76 (51.98-59.02)	100.00 (100.00-100.00)	42.79 (34.54-55.99)	52.89 (41.98-67.68)
Palestine	41.69 (36.89-46.08)	49.97 (46.34-52.78)	62.42 (58.93-65.48)	46.63 (35.50-51.71)	54.30 (48.01-58.20)	61.23 (54.81-65.45)	41.75 (38.59-47.56)	49.60 (45.65-52.77)	63.13 (59.17-67.11)	100.00 (100.00-100.00)	54.77 (42.48-72.37)	100.00 (100.00-100.00)
Oman	43.89 (38.12-49.42)	55.38 (50.12-58.84)	59.57 (56.35-62.35)	33.83 (30.59-41.20)	53.48 (43.45-58.87)	66.09 (59.45-70.36)	52.01 (43.78-58.16)	56.28 (51.83-60.27)	55.92 (52.00-59.20)	100.00 (100.00-100.00)	100.00 (100.00-100.00)	40.55 (32.57-51.86)
Qatar	51.31 (47.44-55.07)	53.99 (49.69-57.58)	60.63 (56.72-65.00)	55.40 (49.49-59.16)	61.17 (56.03-65.12)	72.16 (66.84-75.69)	49.13 (44.51-53.96)	50.65 (45.06-55.49)	54.15 (48.82-59.62)	100.00 (100.00-100.00)	100.00 (100.00-100.00)	100.00 (100.00-100.00)
Saudi Arabia	52.86 (49.31-55.56)	56.71 (53.91-59.39)	57.61 (54.27-61.61)	46.83 (39.72-50.43)	55.29 (49.79-58.56)	68.36 (63.80-71.38)	56.12 (53.45-59.23)	56.77 (54.00-59.99)	52.06 (47.96-57.12)	100.00 (100.00-100.00)	100.00 (100.00-100.00)	47.52 (29.54-73.05)
Sudan	24.74 (22.48-27.64)	26.16 (23.48-29.11)	35.46 (31.33-38.64)	11.87 (9.80-15.06)	15.10 (12.23-18.96)	27.14 (20.80-31.54)	37.10 (33.41-40.39)	35.68 (32.02-38.88)	41.29 (37.42-44.86)	100.00 (100.00-100.00)	29.26 (23.57-36.36)	49.75 (38.19-63.40)

Appendix Table 1a. Scaled values for each SDG health-related indicator and three summary indicators by country for 1990, 2000, and 2015

Location	Geometric mean of all indicators			Geometric mean of all indicators with corresponding MDG indicator			Geometric mean of all indicators without corresponding MDG indicator			Indicator 1.5.1: Age-standardised death rate due to exposure to forces of nature (per 100,000 population)		
	**1990**	**2000**	**2015**	**1990**	**2000**	**2015**	**1990**	**2000**	**2015**	**1990**	**2000**	**2015**
Syria	50.24 (44.89-52.94)	56.08 (50.86-58.65)	54.40 (50.54-56.61)	41.82 (31.55-46.16)	50.50 (40.52-54.61)	58.79 (49.69-63.30)	54.62 (51.61-57.27)	58.33 (55.46-60.97)	52.47 (50.16-54.74)	100.00 (100.00-100.00)	100.00 (100.00-100.00)	100.00 (100.00-100.00)
Tunisia	46.77 (41.69-49.46)	55.83 (49.44-58.52)	57.33 (51.23-60.23)	46.74 (34.92-51.77)	55.10 (40.53-60.13)	63.43 (49.83-68.91)	47.19 (44.38-49.76)	55.93 (52.73-58.58)	54.52 (51.29-57.54)	24.09 (19.52-32.48)	100.00 (100.00-100.00)	100.00 (100.00-100.00)
Turkey	41.47 (38.00-43.62)	45.38 (42.78-47.19)	58.14 (55.50-60.43)	42.55 (35.44-46.03)	52.05 (46.07-55.38)	67.08 (61.05-70.91)	42.96 (40.38-45.52)	44.09 (41.95-46.16)	54.44 (52.04-57.19)	36.27 (29.07-46.73)	9.71 (7.13-13.31)	33.36 (27.34-44.26)
United Arab Emirates	49.14 (44.89-52.60)	55.58 (52.49-58.42)	61.55 (58.36-64.54)	49.06 (42.54-53.62)	59.74 (54.53-63.62)	69.26 (64.59-73.42)	50.59 (45.03-54.88)	54.91 (50.67-58.25)	58.44 (53.69-62.38)	100.00 (100.00-100.00)	100.00 (100.00-100.00)	100.00 (100.00-100.00)
Yemen	29.45 (25.47-33.01)	30.79 (26.72-34.38)	37.48 (32.70-40.95)	15.09 (12.41-18.28)	18.98 (15.80-22.63)	33.75 (26.24-37.98)	42.26 (36.41-47.14)	40.38 (34.26-45.05)	39.77 (34.48-43.97)	100.00 (100.00-100.00)	22.49 (18.29-30.09)	53.23 (42.17-68.23)
Bangladesh	20.07 (17.32-22.63)	27.14 (23.27-30.45)	37.74 (33.27-40.73)	9.71 (7.10-12.28)	20.29 (15.58-23.40)	35.97 (26.52-40.22)	31.08 (28.03-33.96)	32.28 (27.44-36.25)	38.79 (35.22-42.19)	18.66 (15.65-22.10)	22.93 (19.28-26.89)	51.93 (42.05-61.52)
Bhutan	36.79 (33.79-39.76)	38.81 (36.67-40.87)	55.10 (52.64-57.60)	24.18 (19.38-28.48)	35.91 (32.07-39.20)	56.08 (53.43-58.62)	43.33 (39.83-46.86)	38.97 (35.97-41.83)	51.43 (47.84-54.67)	100.00 (100.00-100.00)	8.49 (6.64-10.46)	100.00 (100.00-100.00)
India	26.63 (24.36-28.63)	30.79 (28.80-32.50)	41.70 (39.68-43.77)	17.56 (15.07-19.66)	24.22 (21.60-26.19)	39.64 (36.55-42.08)	33.01 (30.23-36.05)	34.97 (32.38-37.45)	42.39 (39.88-44.93)	35.37 (19.55-76.80)	24.64 (16.86-33.63)	36.09 (26.68-53.12)
Nepal	19.46 (16.88-22.11)	25.59 (22.77-27.69)	35.84 (32.62-38.40)	7.12 (5.46-9.09)	20.11 (15.38-22.43)	38.15 (31.43-41.65)	32.22 (28.18-35.34)	29.37 (26.50-31.85)	34.89 (31.83-37.74)	32.73 (27.57-38.77)	16.40 (13.68-19.55)	8.83 (6.95-10.85)
Pakistan	29.46 (26.08-31.75)	32.74 (29.10-34.95)	38.04 (34.02-40.42)	16.23 (12.41-18.64)	23.56 (17.74-26.35)	36.00 (26.89-39.54)	40.86 (38.10-44.00)	39.84 (37.14-42.64)	39.71 (36.69-42.72)	100.00 (100.00-100.00)	27.97 (23.65-32.92)	27.90 (23.59-32.85)
Botswana	38.62 (29.45-44.53)	38.23 (20.89-46.74)	46.23 (28.74-53.43)	26.87 (20.05-29.87)	26.54 (21.20-29.91)	41.41 (35.68-45.04)	44.19 (31.88-52.12)	43.70 (20.89-55.43)	46.62 (25.30-55.76)	100.00 (100.00-100.00)	100.00 (100.00-100.00)	37.64 (29.42-49.34)

Appendix Table 1a. Scaled values for each SDG health-related indicator and three summary indicators by country for 1990, 2000, and 2015

Location	Geometric mean of all indicators			Geometric mean of all indicators with corresponding MDG indicator			Geometric mean of all indicators without corresponding MDG indicator			Indicator 1.5.1: Age-standardised death rate due to exposure to forces of nature (per 100,000 population)		
	1990	2000	2015	1990	2000	2015	1990	2000	2015	1990	2000	2015
Lesotho	28.80 (25.50-32.06)	31.71 (27.31-35.23)	32.02 (22.35-38.81)	18.64 (14.92-22.49)	19.21 (14.98-22.19)	28.37 (21.75-31.87)	34.62 (30.86-38.30)	38.85 (34.04-43.00)	33.60 (21.82-42.57)	16.44 (12.38-22.62)	100.00 (100.00-100.00)	29.09 (22.63-38.36)
Namibia	36.32 (32.30-38.88)	33.90 (29.74-36.84)	44.88 (40.29-48.53)	26.13 (20.28-29.18)	27.66 (21.86-30.35)	41.00 (35.81-44.34)	42.67 (39.36-45.68)	37.57 (33.19-41.55)	46.14 (40.46-50.96)	100.00 (100.00-100.00)	100.00 (100.00-100.00)	18.55 (14.01-25.65)
South Africa	35.40 (33.06-37.45)	38.18 (35.93-40.40)	45.99 (44.15-47.77)	38.00 (34.16-40.37)	39.20 (36.21-41.33)	48.92 (45.91-51.34)	34.21 (31.20-36.81)	37.22 (34.44-40.03)	42.92 (40.26-45.49)	53.60 (29.00-65.85)	21.24 (17.91-25.23)	26.59 (22.51-30.47)
Swaziland	34.11 (29.92-38.39)	35.51 (30.38-39.96)	38.23 (28.16-45.87)	26.41 (22.10-31.35)	25.92 (20.57-29.18)	35.76 (26.91-39.68)	38.09 (33.37-42.63)	40.24 (33.76-46.15)	38.67 (25.93-48.70)	100.00 (100.00-100.00)	100.00 (100.00-100.00)	32.45 (25.44-42.65)
Zimbabwe	35.16 (31.26-38.36)	33.86 (27.16-37.66)	39.53 (34.25-43.61)	29.20 (22.75-32.11)	29.56 (24.97-32.00)	36.64 (31.62-39.84)	37.80 (33.61-42.07)	35.85 (26.85-41.04)	40.61 (34.91-45.55)	100.00 (100.00-100.00)	32.53 (26.50-41.96)	30.72 (24.64-38.58)
Benin	22.52 (20.53-25.06)	24.94 (22.17-27.49)	33.83 (29.18-38.04)	11.64 (9.37-14.66)	14.86 (11.82-17.78)	23.33 (17.77-27.51)	30.64 (28.84-32.81)	31.02 (28.52-33.42)	37.14 (32.11-41.77)	100.00 (100.00-100.00)	50.99 (38.83-66.35)	100.00 (100.00-100.00)
Burkina Faso	19.14 (17.87-20.56)	21.91 (20.30-24.18)	31.05 (27.20-34.99)	8.19 (7.11-9.40)	10.78 (9.18-13.24)	20.72 (16.91-25.46)	29.07 (27.35-30.83)	30.06 (28.43-31.97)	35.46 (31.66-39.07)	100.00 (100.00-100.00)	100.00 (100.00-100.00)	57.59 (48.18-68.02)
Cameroon	22.25 (20.13-24.85)	23.89 (21.65-26.66)	31.11 (27.28-34.66)	12.43 (10.58-15.22)	14.54 (12.56-17.57)	25.84 (20.18-30.07)	29.86 (27.03-33.11)	30.67 (27.71-33.86)	33.80 (29.72-37.92)	100.00 (100.00-100.00)	52.84 (44.39-65.09)	57.01 (47.05-67.99)
Cape Verde	38.11 (34.44-41.58)	42.97 (38.18-46.55)	52.73 (47.10-55.63)	26.05 (20.89-31.12)	35.62 (27.37-40.53)	50.37 (39.51-55.00)	43.74 (40.22-47.26)	45.34 (41.26-49.35)	51.64 (48.19-55.04)	100.00 (100.00-100.00)	100.00 (100.00-100.00)	100.00 (100.00-100.00)
Chad	15.29 (14.02-16.68)	17.01 (15.81-18.27)	23.90 (20.88-27.02)	4.89 (4.14-5.86)	6.42 (5.80-7.06)	14.87 (12.30-18.48)	28.34 (26.49-30.32)	27.96 (25.82-30.26)	30.16 (25.99-34.13)	100.00 (100.00-100.00)	100.00 (100.00-100.00)	52.12 (42.60-63.35)
Cote d'Ivoire	26.03 (23.58-28.87)	27.16 (23.67-29.93)	35.43 (30.81-39.43)	13.00 (10.85-15.81)	17.65 (13.82-20.70)	24.72 (18.96-28.84)	35.77 (32.76-38.71)	33.20 (30.06-36.31)	40.85 (35.97-45.19)	100.00 (100.00-100.00)	49.84 (41.11-60.38)	48.66 (39.99-59.86)

Appendix Table 1a. Scaled values for each SDG health-related indicator and three summary indicators by country for 1990, 2000, and 2015

Location	Geometric mean of all indicators			Geometric mean of all indicators with corresponding MDG indicator			Geometric mean of all indicators without corresponding MDG indicator			Indicator 1.5.1: Age-standardised death rate due to exposure to forces of nature (per 100,000 population)		
	1990	2000	2015	1990	2000	2015	1990	2000	2015	1990	2000	2015
The Gambia	27.24 (23.02-31.00)	25.86 (22.01-28.87)	35.93 (30.89-39.95)	16.77 (13.56-20.49)	21.94 (17.35-25.95)	30.66 (23.96-35.90)	33.97 (28.00-37.70)	28.09 (22.96-31.16)	37.90 (31.23-42.26)	100.00 (100.00-100.00)	18.50 (13.41-24.34)	100.00 (100.00-100.00)
Ghana	26.93 (24.59-29.72)	29.84 (26.92-32.48)	42.72 (37.14-47.17)	13.14 (11.49-15.92)	18.28 (15.07-21.44)	30.31 (23.46-35.27)	37.70 (34.13-41.18)	37.40 (34.36-40.26)	49.13 (44.13-53.90)	100.00 (100.00-100.00)	44.77 (36.74-54.13)	100.00 (100.00-100.00)
Guinea	20.48 (18.91-22.05)	21.53 (20.02-23.56)	29.47 (25.91-32.70)	8.22 (6.91-9.58)	11.25 (9.92-13.45)	21.13 (16.31-24.81)	33.05 (31.19-35.11)	29.83 (28.02-31.76)	33.16 (30.12-36.27)	100.00 (100.00-100.00)	100.00 (100.00-100.00)	100.00 (100.00-100.00)
Guinea-Bissau	22.45 (17.35-26.00)	23.81 (18.14-27.87)	29.03 (19.93-34.68)	11.43 (9.48-13.81)	13.53 (11.33-16.63)	21.02 (16.78-26.00)	31.84 (23.58-36.82)	31.76 (22.97-36.96)	33.38 (22.11-39.36)	100.00 (100.00-100.00)	100.00 (100.00-100.00)	100.00 (100.00-100.00)
Liberia	20.63 (18.55-22.98)	22.16 (20.48-24.33)	33.39 (29.46-36.97)	12.95 (10.48-16.28)	13.45 (11.35-16.56)	25.10 (20.25-29.97)	25.60 (22.81-28.17)	27.70 (25.93-29.62)	36.26 (32.91-39.22)	100.00 (100.00-100.00)	40.71 (30.81-53.94)	100.00 (100.00-100.00)
Mali	17.08 (15.93-18.46)	19.46 (18.06-21.23)	25.96 (23.12-28.82)	7.14 (6.16-8.41)	10.78 (9.43-12.93)	18.17 (14.75-21.85)	27.30 (25.96-28.66)	26.50 (24.96-28.15)	30.05 (27.38-32.73)	100.00 (100.00-100.00)	54.08 (43.23-65.27)	47.14 (38.49-57.82)
Mauritania	23.02 (20.76-25.78)	29.82 (26.28-33.18)	37.99 (33.08-42.33)	11.42 (9.48-14.44)	15.38 (12.42-18.74)	24.89 (19.47-30.30)	33.16 (29.71-36.66)	40.87 (35.19-44.60)	46.33 (40.36-50.84)	100.00 (100.00-100.00)	100.00 (100.00-100.00)	100.00 (100.00-100.00)
Niger	12.54 (11.35-14.35)	17.03 (15.77-19.15)	22.78 (20.56-25.34)	4.12 (3.41-4.98)	6.41 (5.85-7.06)	13.43 (11.24-16.80)	23.68 (21.64-28.10)	27.82 (25.36-32.23)	28.37 (25.80-30.80)	100.00 (100.00-100.00)	100.00 (100.00-100.00)	31.09 (25.12-37.64)
Nigeria	23.22 (21.22-25.61)	24.28 (22.63-26.71)	34.46 (30.70-37.70)	10.38 (9.09-12.38)	12.31 (10.90-14.79)	23.40 (18.72-27.40)	35.98 (32.15-39.81)	35.01 (32.25-37.92)	41.50 (38.01-44.57)	100.00 (100.00-100.00)	52.28 (42.75-62.83)	41.80 (33.36-50.18)
Sao Tome and Principe	33.64 (29.71-36.98)	37.86 (33.67-41.23)	46.92 (41.23-51.37)	22.30 (17.41-26.38)	27.54 (21.38-31.81)	39.27 (29.58-44.44)	39.85 (36.33-43.15)	42.43 (39.13-45.69)	48.34 (43.37-53.06)	100.00 (100.00-100.00)	100.00 (100.00-100.00)	100.00 (100.00-100.00)
Senegal	27.84 (24.94-30.40)	30.71 (27.49-32.96)	41.10 (36.02-45.23)	16.34 (13.18-18.99)	21.35 (16.63-24.22)	34.90 (26.43-38.92)	36.28 (33.62-39.31)	36.04 (33.61-38.31)	42.30 (37.58-47.01)	100.00 (100.00-100.00)	24.66 (18.62-31.45)	54.40 (45.82-65.39)

Appendix Table 1a. Scaled values for each SDG health-related indicator and three summary indicators by country for 1990, 2000, and 2015												
	**Geometric mean of all indicators**			**Geometric mean of all indicators with corresponding MDG indicator**			**Geometric mean of all indicators without corresponding MDG indicator**			**Indicator 1.5.1: Age-standardised death rate due to exposure to forces of nature (per 100,000 population)**		
**Location**	**1990**	**2000**	**2015**	**1990**	**2000**	**2015**	**1990**	**2000**	**2015**	**1990**	**2000**	**2015**
Sierra Leone	21.62 (19.44-24.31)	18.58 (16.94-20.47)	26.78 (23.81-30.05)	10.52 (8.83-13.17)	11.41 (10.03-13.85)	20.20 (17.22-24.69)	30.29 (26.29-34.18)	23.73 (21.38-26.04)	30.00 (27.06-33.00)	100.00 (100.00-100.00)	34.24 (24.30-41.75)	41.76 (34.37-52.06)
Togo	23.48 (21.82-25.52)	25.13 (23.13-27.69)	34.35 (30.04-38.35)	12.76 (11.16-15.24)	14.99 (13.22-18.30)	25.25 (20.46-30.38)	32.03 (29.91-34.43)	32.39 (29.64-35.04)	38.65 (34.39-42.86)	100.00 (100.00-100.00)	100.00 (100.00-100.00)	100.00 (100.00-100.00)
Burundi	18.47 (14.38-21.89)	18.61 (16.43-20.77)	25.83 (22.40-29.26)	11.24 (8.91-14.11)	12.67 (10.34-15.70)	22.71 (18.32-27.64)	23.37 (17.50-27.88)	22.22 (19.79-24.33)	26.60 (23.22-29.69)	100.00 (100.00-100.00)	100.00 (100.00-100.00)	29.99 (23.85-36.13)
Comoros	30.87 (25.83-34.71)	34.17 (30.26-36.85)	42.38 (37.17-46.13)	20.02 (15.05-23.28)	21.43 (16.03-24.50)	30.73 (23.07-35.11)	36.27 (30.76-41.42)	39.41 (35.95-42.79)	44.67 (39.58-49.53)	100.00 (100.00-100.00)	100.00 (100.00-100.00)	100.00 (100.00-100.00)
Djibouti	36.71 (32.33-40.58)	37.87 (32.19-42.44)	43.13 (36.68-48.23)	23.08 (18.08-27.16)	25.78 (19.74-29.82)	36.15 (27.01-41.40)	44.20 (39.67-48.62)	43.30 (35.96-49.23)	43.21 (36.19-49.24)	100.00 (100.00-100.00)	100.00 (100.00-100.00)	100.00 (100.00-100.00)
Eritrea	22.02 (19.66-24.79)	27.19 (23.85-30.27)	34.81 (28.89-39.83)	10.25 (8.28-12.97)	17.90 (14.29-20.91)	25.28 (19.73-29.78)	32.48 (29.15-36.05)	33.15 (29.08-37.16)	39.14 (32.02-45.66)	100.00 (100.00-100.00)	100.00 (100.00-100.00)	100.00 (100.00-100.00)
Ethiopia	14.71 (13.01-16.62)	18.35 (16.98-20.02)	29.70 (26.00-32.95)	6.08 (4.69-7.67)	8.29 (7.20-9.83)	23.22 (18.14-26.81)	23.84 (21.41-26.27)	27.04 (25.00-28.96)	31.51 (28.00-35.07)	100.00 (100.00-100.00)	36.68 (28.55-44.15)	100.00 (100.00-100.00)
Kenya	32.37 (30.63-33.91)	33.44 (31.73-34.99)	39.57 (37.74-41.36)	22.07 (20.36-23.50)	25.39 (23.86-26.80)	36.31 (34.13-38.28)	37.21 (34.37-39.80)	36.91 (34.09-39.56)	39.50 (36.86-42.11)	100.00 (100.00-100.00)	41.36 (29.87-59.08)	36.71 (27.93-47.31)
Madagascar	23.86 (21.98-25.78)	24.75 (22.95-26.79)	27.94 (24.85-30.70)	13.23 (11.01-15.38)	16.53 (14.76-18.33)	22.97 (18.79-25.90)	28.45 (25.37-31.65)	27.60 (25.02-30.47)	28.69 (25.00-32.17)	100.00 (100.00-100.00)	23.26 (18.43-26.99)	27.66 (23.21-31.91)
Malawi	22.19 (19.87-24.33)	25.83 (22.74-28.39)	32.15 (28.63-34.86)	11.28 (9.00-13.32)	14.05 (11.12-15.97)	24.96 (19.49-27.97)	28.96 (26.40-31.73)	31.53 (28.05-34.55)	33.21 (29.91-36.34)	100.00 (100.00-100.00)	100.00 (100.00-100.00)	26.00 (20.59-32.05)
Mozambique	17.77 (16.58-19.40)	19.36 (17.93-21.07)	28.16 (24.01-31.86)	7.85 (6.64-8.99)	11.13 (10.22-12.84)	22.06 (17.49-25.33)	25.94 (24.17-28.34)	24.53 (22.48-26.61)	29.56 (25.32-33.91)	100.00 (100.00-100.00)	17.41 (14.23-21.14)	32.15 (27.27-37.51)

Appendix Table 1a. Scaled values for each SDG health-related indicator and three summary indicators by country for 1990, 2000, and 2015

Location	Geometric mean of all indicators			Geometric mean of all indicators with corresponding MDG indicator			Geometric mean of all indicators without corresponding MDG indicator			Indicator 1.5.1: Age-standardised death rate due to exposure to forces of nature (per 100,000 population)		
	1990	2000	2015	1990	2000	2015	1990	2000	2015	1990	2000	2015
Rwanda	20.03 (18.19-21.79)	21.90 (19.98-23.70)	33.06 (29.92-35.74)	17.66 (15.06-20.04)	19.43 (17.08-21.68)	37.80 (34.23-41.02)	21.15 (18.88-23.09)	22.93 (20.43-25.19)	28.55 (25.06-31.76)	100.00 (100.00-100.00)	100.00 (100.00-100.00)	54.85 (41.93-65.81)
Somalia	18.19 (13.55-22.08)	16.42 (12.23-20.14)	21.67 (15.76-25.93)	10.97 (8.59-13.81)	10.32 (7.74-13.36)	19.09 (14.73-22.80)	25.17 (18.43-30.16)	22.36 (16.67-27.11)	23.57 (16.92-28.39)	100.00 (100.00-100.00)	8.61 (6.45-11.72)	24.60 (18.28-29.63)
South Sudan	18.97 (14.68-22.34)	22.45 (17.26-26.50)	22.50 (15.52-26.65)	9.81 (7.50-12.47)	11.60 (9.23-14.50)	17.86 (12.71-22.34)	25.98 (19.10-30.38)	29.82 (22.24-34.52)	24.29 (16.56-28.82)	100.00 (100.00-100.00)	100.00 (100.00-100.00)	27.56 (16.45-32.62)
Tanzania	24.38 (22.78-26.08)	28.01 (25.98-29.83)	36.27 (32.41-39.79)	16.06 (14.20-17.78)	18.30 (16.69-19.85)	29.40 (26.69-32.09)	28.46 (26.36-30.78)	32.29 (29.39-35.10)	37.45 (32.41-42.07)	19.82 (15.76-24.83)	36.42 (30.56-42.02)	57.56 (45.65-69.28)
Uganda	20.50 (18.80-22.25)	21.31 (19.59-23.01)	30.80 (27.51-34.11)	15.51 (13.25-17.45)	18.84 (17.04-20.55)	32.78 (29.97-35.35)	23.28 (21.31-25.50)	22.37 (20.12-24.75)	28.70 (24.56-32.83)	100.00 (100.00-100.00)	35.87 (23.34-44.01)	53.07 (41.11-63.34)
Zambia	26.75 (24.11-29.43)	27.87 (25.51-30.00)	36.93 (32.88-40.68)	18.54 (16.07-20.72)	19.79 (17.68-21.50)	35.88 (33.10-38.33)	31.43 (27.54-35.41)	31.50 (28.02-34.57)	34.60 (29.46-39.29)	100.00 (100.00-100.00)	100.00 (100.00-100.00)	100.00 (100.00-100.00)
Angola	17.57 (11.80-21.52)	21.04 (14.88-25.39)	31.62 (20.29-37.68)	10.02 (8.06-12.51)	13.39 (10.75-16.46)	25.34 (19.94-30.66)	24.51 (15.37-30.47)	26.87 (18.08-32.35)	33.54 (20.27-39.81)	100.00 (100.00-100.00)	41.75 (34.21-52.60)	32.15 (24.93-43.04)
Central African Republic	18.18 (16.24-20.11)	18.90 (14.87-22.58)	20.42 (15.40-24.91)	9.01 (7.65-10.48)	10.17 (8.18-12.84)	14.84 (11.83-19.03)	26.18 (23.20-28.83)	25.94 (19.58-31.07)	23.81 (16.91-29.01)	100.00 (100.00-100.00)	100.00 (100.00-100.00)	100.00 (100.00-100.00)
Congo	25.41 (22.28-28.82)	24.82 (21.63-27.68)	35.60 (30.07-39.81)	16.95 (13.52-20.80)	18.30 (14.41-21.86)	28.58 (22.10-33.07)	29.39 (26.05-32.61)	27.78 (24.69-30.61)	37.01 (31.77-41.38)	100.00 (100.00-100.00)	100.00 (100.00-100.00)	43.48 (35.95-54.24)
Democratic Republic of the Congo	19.66 (17.51-22.10)	19.31 (17.55-21.39)	24.12 (21.28-27.38)	12.11 (9.99-14.96)	12.39 (10.71-15.22)	20.40 (17.25-24.84)	24.24 (21.61-26.76)	23.61 (21.28-25.75)	25.34 (22.18-28.56)	100.00 (100.00-100.00)	100.00 (100.00-100.00)	48.30 (40.88-58.71)
Equatorial Guinea	20.22 (12.44-25.15)	25.02 (16.86-30.45)	35.85 (24.22-41.75)	12.58 (10.50-14.40)	17.60 (14.52-21.94)	31.42 (23.48-37.18)	26.33 (14.91-33.89)	30.02 (19.00-36.58)	36.07 (24.16-42.50)	100.00 (100.00-100.00)	100.00 (100.00-100.00)	100.00 (100.00-100.00)

Appendix Table 1a. Scaled values for each SDG health-related indicator and three summary indicators by country for 1990, 2000, and 2015												
	**Geometric mean of all indicators**			**Geometric mean of all indicators with corresponding MDG indicator**			**Geometric mean of all indicators without corresponding MDG indicator**			**Indicator 1.5.1: Age-standardised death rate due to exposure to forces of nature (per 100,000 population)**		
**Location**	**1990**	**2000**	**2015**	**1990**	**2000**	**2015**	**1990**	**2000**	**2015**	**1990**	**2000**	**2015**
Gabon	30.26 (26.61-33.56)	34.57 (30.58-37.53)	43.80 (38.75-47.66)	19.87 (15.37-23.42)	23.19 (17.67-26.48)	36.76 (27.83-40.79)	35.93 (31.90-39.75)	40.14 (36.54-43.62)	44.27 (38.92-48.66)	100.00 (100.00-100.00)	100.00 (100.00-100.00)	100.00 (100.00-100.00)

Location	Appendix Table 1a. Scaled values for each SDG health-related indicator and three summary indicators by country for 1990, 2000, and 2015											
	**Indicator 2.2.1: Prevalence of stunting among children under 5 years of age**			**Indicator 2.2.2a: Prevalence of wasting among children under 5 years of age**			**Indicator 2.2.2b: Prevalence of overweight among children aged 2 to 4 years**			**Indicator 3.1.1: Maternal mortality ratio (maternal deaths per 100,000 live births)**		
	**1990**	**2000**	**2015**	**1990**	**2000**	**2015**	**1990**	**2000**	**2015**	**1990**	**2000**	**2015**
Canada	100.00 (100.00-100.00)	100.00 (100.00-100.00)	100.00 (100.00-100.00)	100.00 (100.00-100.00)	100.00 (100.00-100.00)	100.00 (100.00-100.00)	57.53 (43.21-69.96)	40.11 (23.18-55.58)	33.67 (17.23-49.97)	75.32 (72.72-78.09)	72.48 (69.63-75.17)	73.23 (70.89-75.67)
United States	100.00 (100.00-100.00)	100.00 (100.00-100.00)	100.00 (100.00-100.00)	100.00 (100.00-100.00)	100.00 (100.00-100.00)	100.00 (100.00-100.00)	78.21 (73.07-82.99)	72.20 (66.27-77.57)	68.66 (60.05-76.29)	62.78 (60.38-65.65)	62.39 (59.94-65.35)	57.63 (55.01-60.80)
Australia	100.00 (100.00-100.00)	100.00 (100.00-100.00)	100.00 (100.00-100.00)	100.00 (100.00-100.00)	100.00 (100.00-100.00)	100.00 (100.00-100.00)	76.89 (68.41-84.17)	73.81 (68.76-78.76)	62.85 (49.10-73.92)	72.50 (69.66-75.47)	72.35 (69.52-75.06)	76.05 (73.21-79.12)
New Zealand	100.00 (100.00-100.00)	100.00 (100.00-100.00)	100.00 (100.00-100.00)	100.00 (100.00-100.00)	100.00 (100.00-100.00)	100.00 (100.00-100.00)	47.88 (31.29-63.52)	49.41 (32.92-64.38)	53.32 (40.02-65.47)	66.24 (63.06-69.41)	68.76 (65.75-71.92)	66.86 (63.89-70.15)
Brunei	100.00 (100.00-100.00)	100.00 (100.00-100.00)	100.00 (100.00-100.00)	100.00 (100.00-100.00)	100.00 (100.00-100.00)	100.00 (100.00-100.00)	86.23 (80.25-91.30)	81.11 (72.70-87.87)	73.96 (64.08-81.99)	49.65 (45.50-54.27)	51.70 (47.77-55.82)	54.25 (50.16-58.39)
Japan	100.00 (100.00-100.00)	100.00 (100.00-100.00)	100.00 (100.00-100.00)	100.00 (100.00-100.00)	100.00 (100.00-100.00)	100.00 (100.00-100.00)	90.35 (87.76-92.88)	87.07 (82.77-90.74)	83.35 (76.95-88.76)	66.00 (63.69-68.54)	70.54 (68.38-72.89)	74.35 (72.16-76.56)
Singapore	100.00 (100.00-100.00)	100.00 (100.00-100.00)	100.00 (100.00-100.00)	100.00 (100.00-100.00)	100.00 (100.00-100.00)	100.00 (100.00-100.00)	76.44 (67.21-84.29)	69.11 (58.09-78.64)	61.54 (49.17-72.64)	68.48 (65.71-71.28)	67.66 (64.98-70.48)	77.69 (74.88-80.45)
South Korea	100.00 (100.00-100.00)	100.00 (100.00-100.00)	100.00 (100.00-100.00)	100.00 (100.00-100.00)	100.00 (100.00-100.00)	100.00 (100.00-100.00)	67.96 (56.98-77.36)	62.43 (50.44-72.83)	54.06 (40.12-65.66)	57.52 (54.54-60.93)	63.99 (61.24-67.08)	67.24 (64.23-70.47)
Andorra	100.00 (100.00-100.00)	100.00 (100.00-100.00)	100.00 (100.00-100.00)	100.00 (100.00-100.00)	100.00 (100.00-100.00)	100.00 (100.00-100.00)	61.15 (44.14-75.63)	59.69 (43.58-73.53)	56.51 (38.75-71.61)	81.19 (75.66-89.99)	86.29 (80.16-95.60)	88.90 (83.23-95.39)
Austria	100.00 (100.00-100.00)	100.00 (100.00-100.00)	100.00 (100.00-100.00)	100.00 (100.00-100.00)	100.00 (100.00-100.00)	100.00 (100.00-100.00)	58.71 (41.19-74.20)	52.68 (33.18-69.21)	49.45 (28.94-66.30)	67.52 (64.97-70.31)	72.87 (70.36-75.37)	79.82 (77.25-82.25)
Belgium	100.00 (100.00-100.00)	100.00 (100.00-100.00)	100.00 (100.00-100.00)	100.00 (100.00-100.00)	100.00 (100.00-100.00)	100.00 (100.00-100.00)	59.48 (42.40-73.70)	54.19 (35.12-69.46)	56.85 (38.76-71.44)	65.31 (62.72-68.22)	69.28 (66.76-71.95)	73.41 (70.82-76.02)

	Appendix Table 1a. Scaled values for each SDG health-related indicator and three summary indicators by country for 1990, 2000, and 2015											
	**Indicator 2.2.1: Prevalence of stunting among children under 5 years of age**			**Indicator 2.2.2a: Prevalence of wasting among children under 5 years of age**			**Indicator 2.2.2b: Prevalence of overweight among children aged 2 to 4 years**			**Indicator 3.1.1: Maternal mortality ratio (maternal deaths per 100,000 live births)**		
**Location**	**1990**	**2000**	**2015**	**1990**	**2000**	**2015**	**1990**	**2000**	**2015**	**1990**	**2000**	**2015**
Cyprus	100.00 (100.00-100.00)	100.00 (100.00-100.00)	100.00 (100.00-100.00)	100.00 (100.00-100.00)	100.00 (100.00-100.00)	100.00 (100.00-100.00)	51.70 (33.79-67.21)	50.95 (32.82-67.36)	52.78 (35.32-68.17)	65.76 (61.48-69.80)	67.04 (63.06-70.98)	76.61 (72.87-80.21)
Denmark	100.00 (100.00-100.00)	100.00 (100.00-100.00)	100.00 (100.00-100.00)	100.00 (100.00-100.00)	100.00 (100.00-100.00)	100.00 (100.00-100.00)	56.42 (38.23-72.00)	49.39 (29.57-66.75)	50.50 (30.86-66.89)	69.57 (66.96-72.49)	75.83 (72.97-78.53)	79.66 (77.70-81.77)
Finland	100.00 (100.00-100.00)	100.00 (100.00-100.00)	100.00 (100.00-100.00)	100.00 (100.00-100.00)	100.00 (100.00-100.00)	100.00 (100.00-100.00)	59.36 (41.18-74.38)	62.44 (46.26-75.92)	58.09 (40.42-71.75)	72.10 (69.47-74.88)	72.97 (70.18-75.85)	81.00 (78.11-83.77)
France	100.00 (100.00-100.00)	100.00 (100.00-100.00)	100.00 (100.00-100.00)	100.00 (100.00-100.00)	100.00 (100.00-100.00)	100.00 (100.00-100.00)	64.21 (48.26-77.55)	58.23 (40.47-72.22)	58.84 (41.25-73.11)	62.78 (59.92-65.83)	67.24 (64.53-70.14)	72.20 (69.14-75.44)
Germany	100.00 (100.00-100.00)	100.00 (100.00-100.00)	100.00 (100.00-100.00)	100.00 (100.00-100.00)	100.00 (100.00-100.00)	100.00 (100.00-100.00)	66.76 (51.67-79.66)	59.06 (42.47-73.70)	47.18 (27.22-64.68)	61.52 (58.97-64.63)	68.10 (65.47-70.84)	71.24 (68.62-74.05)
Greece	100.00 (100.00-100.00)	100.00 (100.00-100.00)	100.00 (100.00-100.00)	100.00 (100.00-100.00)	100.00 (100.00-100.00)	100.00 (100.00-100.00)	47.35 (28.60-64.69)	39.67 (19.38-57.29)	36.68 (15.31-56.04)	69.87 (67.08-72.61)	71.77 (69.14-74.36)	69.77 (66.88-72.80)
Iceland	100.00 (100.00-100.00)	100.00 (100.00-100.00)	100.00 (100.00-100.00)	100.00 (100.00-100.00)	100.00 (100.00-100.00)	100.00 (100.00-100.00)	37.52 (17.66-56.19)	42.84 (22.77-61.21)	42.59 (22.60-61.58)	83.86 (81.33-86.35)	93.95 (91.39-96.41)	99.94 (100.00-100.00)
Ireland	100.00 (100.00-100.00)	100.00 (100.00-100.00)	100.00 (100.00-100.00)	100.00 (100.00-100.00)	100.00 (100.00-100.00)	100.00 (100.00-100.00)	55.45 (35.81-71.30)	55.13 (41.59-66.01)	51.15 (31.28-67.75)	73.39 (70.11-76.39)	77.20 (74.17-80.11)	78.12 (74.85-81.12)
Israel	100.00 (100.00-100.00)	100.00 (100.00-100.00)	100.00 (100.00-100.00)	100.00 (100.00-100.00)	100.00 (100.00-100.00)	100.00 (100.00-100.00)	62.16 (44.82-76.35)	55.58 (37.16-70.93)	45.00 (23.32-63.66)	67.82 (65.24-70.66)	72.17 (69.59-74.83)	75.57 (72.78-78.44)
Italy	100.00 (100.00-100.00)	100.00 (100.00-100.00)	100.00 (100.00-100.00)	100.00 (100.00-100.00)	100.00 (100.00-100.00)	100.00 (100.00-100.00)	47.99 (28.80-65.59)	38.62 (19.35-56.73)	38.81 (19.64-57.72)	68.97 (66.43-71.58)	73.58 (70.98-76.30)	79.47 (76.86-82.10)
Luxembourg	100.00 (100.00-100.00)	100.00 (100.00-100.00)	100.00 (100.00-100.00)	100.00 (100.00-100.00)	100.00 (100.00-100.00)	100.00 (100.00-100.00)	64.82 (48.29-78.74)	53.22 (34.80-68.99)	47.56 (28.01-64.07)	69.06 (66.30-71.91)	74.13 (71.30-76.78)	68.26 (65.31-71.41)

Location	Appendix Table 1a. Scaled values for each SDG health-related indicator and three summary indicators by country for 1990, 2000, and 2015											
	Indicator 2.2.1: Prevalence of stunting among children under 5 years of age			Indicator 2.2.2a: Prevalence of wasting among children under 5 years of age			Indicator 2.2.2b: Prevalence of overweight among children aged 2 to 4 years			Indicator 3.1.1: Maternal mortality ratio (maternal deaths per 100,000 live births)		
	1990	2000	2015	1990	2000	2015	1990	2000	2015	1990	2000	2015
Malta	100.00 (100.00-100.00)	100.00 (100.00-100.00)	100.00 (100.00-100.00)	100.00 (100.00-100.00)	100.00 (100.00-100.00)	100.00 (100.00-100.00)	41.28 (19.85-59.02)	33.95 (13.01-53.88)	36.10 (16.23-55.81)	68.64 (65.79-71.68)	68.26 (65.53-71.17)	75.71 (72.93-78.32)
Netherlands	100.00 (100.00-100.00)	100.00 (100.00-100.00)	100.00 (100.00-100.00)	100.00 (100.00-100.00)	100.00 (100.00-100.00)	100.00 (100.00-100.00)	73.77 (60.45-83.83)	65.80 (50.83-78.73)	57.22 (38.68-72.06)	66.94 (64.09-69.81)	65.89 (63.03-68.79)	74.27 (71.51-77.09)
Norway	100.00 (100.00-100.00)	100.00 (100.00-100.00)	100.00 (100.00-100.00)	100.00 (100.00-100.00)	100.00 (100.00-100.00)	100.00 (100.00-100.00)	63.78 (47.39-77.72)	58.41 (42.17-72.81)	48.63 (29.50-66.14)	73.84 (71.04-76.67)	75.31 (72.50-78.13)	80.70 (78.10-82.84)
Portugal	100.00 (100.00-100.00)	100.00 (100.00-100.00)	100.00 (100.00-100.00)	100.00 (100.00-100.00)	100.00 (100.00-100.00)	100.00 (100.00-100.00)	63.07 (47.47-76.94)	48.92 (29.80-66.10)	39.90 (20.65-56.67)	61.50 (58.68-64.73)	65.77 (63.17-68.62)	70.56 (67.80-73.33)
Spain	100.00 (100.00-100.00)	100.00 (100.00-100.00)	100.00 (100.00-100.00)	100.00 (100.00-100.00)	100.00 (100.00-100.00)	100.00 (100.00-100.00)	24.90 (3.78-44.93)	22.48 (2.59-43.46)	33.03 (12.21-52.23)	66.50 (63.81-69.21)	72.61 (70.18-75.24)	76.26 (73.68-78.82)
Sweden	100.00 (100.00-100.00)	100.00 (100.00-100.00)	100.00 (100.00-100.00)	100.00 (100.00-100.00)	100.00 (100.00-100.00)	100.00 (100.00-100.00)	60.90 (44.39-75.07)	54.15 (36.07-70.43)	51.46 (32.35-67.66)	69.13 (66.61-71.87)	74.76 (72.51-77.16)	79.45 (77.29-81.73)
Switzerland	100.00 (100.00-100.00)	100.00 (100.00-100.00)	100.00 (100.00-100.00)	100.00 (100.00-100.00)	100.00 (100.00-100.00)	100.00 (100.00-100.00)	67.03 (51.50-80.79)	66.83 (50.74-79.85)	61.66 (44.02-75.59)	73.32 (70.64-76.12)	72.59 (69.81-75.44)	75.92 (73.15-78.78)
United Kingdom	100.00 (100.00-100.00)	100.00 (100.00-100.00)	100.00 (100.00-100.00)	100.00 (100.00-100.00)	100.00 (100.00-100.00)	100.00 (100.00-100.00)	67.14 (54.41-77.84)	66.09 (59.70-72.08)	64.48 (54.05-73.43)	68.14 (65.84-70.67)	65.69 (63.42-68.39)	70.21 (67.85-72.70)
Argentina	73.12 (62.82-81.82)	84.11 (78.87-88.67)	90.50 (85.49-94.39)	92.53 (86.68-96.42)	94.13 (89.11-96.99)	95.79 (91.74-98.28)	43.93 (21.79-62.46)	37.16 (11.41-58.21)	28.96 (5.89-50.07)	45.90 (42.56-49.98)	48.19 (44.93-52.09)	49.21 (45.55-53.60)
Chile	87.92 (83.50-91.46)	95.29 (93.99-96.32)	97.17 (95.46-98.27)	98.38 (96.85-99.29)	98.20 (96.94-99.08)	98.01 (95.88-99.20)	51.18 (30.96-70.06)	39.39 (16.64-60.72)	27.09 (0.82-50.62)	48.66 (45.29-52.51)	58.54 (55.41-61.86)	60.30 (57.05-63.54)
Uruguay	78.11 (73.24-82.54)	80.07 (74.76-84.91)	86.83 (80.26-92.02)	94.02 (89.67-96.73)	92.75 (87.78-96.17)	93.88 (88.39-97.25)	58.11 (37.88-74.46)	50.31 (28.95-68.11)	38.62 (15.48-59.78)	52.08 (48.79-55.80)	55.67 (52.78-59.46)	59.82 (56.76-63.21)

Appendix Table 1a. Scaled values for each SDG health-related indicator and three summary indicators by country for 1990, 2000, and 2015

Location	Indicator 2.2.1: Prevalence of stunting among children under 5 years of age			Indicator 2.2.2a: Prevalence of wasting among children under 5 years of age			Indicator 2.2.2b: Prevalence of overweight among children aged 2 to 4 years			Indicator 3.1.1: Maternal mortality ratio (maternal deaths per 100,000 live births)		
	1990	2000	2015	1990	2000	2015	1990	2000	2015	1990	2000	2015
Belarus	89.47 (84.53-93.13)	91.22 (87.49-94.10)	96.36 (94.45-97.70)	88.90 (72.97-96.82)	90.64 (78.72-96.88)	93.28 (83.06-97.94)	70.75 (60.33-79.14)	71.68 (62.09-79.89)	65.40 (54.18-75.15)	52.74 (49.20-56.63)	51.71 (48.33-55.52)	71.78 (68.26-75.36)
Estonia	79.90 (71.57-87.10)	84.67 (78.12-89.64)	92.19 (88.15-95.08)	83.99 (61.34-95.49)	88.00 (71.28-96.28)	91.42 (78.67-97.71)	70.48 (60.10-79.23)	68.81 (58.60-77.68)	50.19 (36.05-62.91)	51.92 (48.49-55.77)	58.61 (55.71-61.99)	78.11 (75.37-81.02)
Latvia	83.59 (76.87-89.40)	83.82 (76.73-89.29)	92.33 (88.66-95.15)	86.35 (66.89-95.91)	86.92 (67.25-96.21)	91.68 (80.37-97.42)	64.16 (53.51-74.44)	71.38 (61.87-79.50)	54.56 (41.73-67.09)	49.77 (46.43-53.54)	57.18 (54.08-60.70)	66.17 (63.34-69.41)
Lithuania	81.94 (74.46-88.07)	85.09 (78.34-90.20)	92.27 (88.64-95.04)	84.33 (62.90-95.44)	87.57 (68.87-96.64)	91.27 (77.40-97.66)	72.90 (63.83-80.82)	73.46 (64.63-81.51)	58.69 (46.86-69.22)	54.76 (51.38-58.42)	63.65 (60.76-66.61)	69.06 (66.90-71.69)
Moldova	81.02 (72.82-86.97)	79.66 (72.56-85.65)	91.93 (88.90-94.08)	76.68 (43.99-93.30)	78.86 (54.39-92.22)	91.38 (81.27-96.66)	75.60 (66.91-82.79)	77.96 (71.02-83.89)	66.95 (56.89-75.92)	49.65 (46.22-53.51)	55.35 (52.18-58.93)	64.56 (61.34-68.02)
Russia	71.41 (63.95-77.64)	74.98 (67.25-82.05)	85.46 (78.17-90.55)	86.15 (72.03-94.29)	86.08 (68.15-95.07)	89.62 (73.49-96.70)	50.57 (41.18-59.77)	59.25 (48.93-68.57)	46.22 (31.92-57.97)	47.60 (44.01-51.63)	49.27 (45.89-53.23)	61.38 (58.59-64.40)
Ukraine	68.72 (57.82-78.21)	70.39 (65.03-75.29)	83.79 (77.46-89.44)	86.80 (67.44-96.08)	89.41 (79.91-95.25)	93.96 (83.99-98.41)	74.37 (64.88-81.96)	78.40 (70.22-85.11)	68.40 (58.06-76.94)	49.22 (45.79-52.99)	48.28 (44.94-52.24)	58.28 (55.02-62.00)
Albania	26.84 (12.92-40.30)	32.58 (24.55-40.31)	68.64 (59.53-76.90)	62.79 (46.12-76.71)	61.00 (49.70-72.25)	76.85 (65.21-84.93)	47.69 (29.65-62.71)	37.58 (21.01-53.18)	30.54 (11.95-47.32)	56.20 (52.64-60.26)	63.84 (60.51-67.19)	70.18 (65.64-74.83)
Bosnia and Herzegovina	66.13 (55.37-75.27)	81.15 (77.24-84.61)	89.38 (85.74-92.26)	72.61 (59.04-82.80)	77.97 (71.01-84.22)	89.28 (84.76-93.08)	69.92 (57.45-80.22)	59.02 (46.28-70.65)	47.75 (31.13-61.02)	53.63 (49.85-57.80)	57.44 (53.23-61.89)	66.53 (62.44-70.50)
Bulgaria	85.48 (79.88-89.78)	85.59 (80.52-89.71)	91.11 (87.38-93.94)	83.07 (73.84-89.66)	82.03 (73.59-88.48)	85.42 (78.42-91.00)	17.29 (0.00-36.05)	39.12 (20.82-54.07)	38.88 (19.57-55.13)	51.14 (48.09-54.92)	49.54 (46.31-53.44)	61.20 (58.04-64.53)
Croatia	80.22 (72.84-86.20)	83.27 (76.95-88.45)	90.61 (86.42-93.62)	81.95 (72.85-88.84)	82.33 (73.35-89.22)	87.69 (81.79-92.57)	67.02 (54.81-77.30)	63.88 (50.00-75.55)	46.06 (29.53-60.88)	63.87 (61.10-66.88)	65.57 (62.72-68.41)	70.64 (68.04-73.41)

Appendix Table 1a. Scaled values for each SDG health-related indicator and three summary indicators by country for 1990, 2000, and 2015

Location	Indicator 2.2.1: Prevalence of stunting among children under 5 years of age			Indicator 2.2.2a: Prevalence of wasting among children under 5 years of age			Indicator 2.2.2b: Prevalence of overweight among children aged 2 to 4 years			Indicator 3.1.1: Maternal mortality ratio (maternal deaths per 100,000 live births)		
	1990	2000	2015	1990	2000	2015	1990	2000	2015	1990	2000	2015
Czech Republic	95.31 (94.28-96.23)	96.10 (95.24-96.82)	97.30 (96.08-98.20)	90.12 (86.92-93.15)	85.99 (81.78-89.85)	88.34 (82.49-92.88)	60.09 (45.41-73.01)	58.67 (43.64-71.81)	54.48 (39.50-67.68)	62.77 (60.00-65.79)	69.79 (67.10-72.49)	75.19 (72.49-77.93)
Hungary	95.56 (93.99-96.91)	95.81 (93.98-97.25)	97.17 (95.97-98.10)	86.51 (79.91-91.63)	86.32 (78.78-91.71)	88.21 (82.55-92.82)	36.87 (19.25-53.09)	50.55 (33.39-64.13)	47.30 (30.45-61.56)	61.37 (58.21-64.46)	67.79 (65.23-70.68)	69.35 (66.36-72.34)
Macedonia	84.92 (79.19-89.28)	86.78 (83.67-89.23)	93.59 (91.57-95.25)	89.27 (83.55-93.06)	90.79 (87.24-93.84)	92.99 (89.94-95.53)	63.81 (49.24-75.95)	61.38 (46.83-73.79)	56.54 (40.87-69.08)	63.03 (59.76-66.38)	64.89 (61.91-67.86)	71.52 (68.12-74.72)
Montenegro	79.82 (71.78-86.05)	83.78 (78.41-88.26)	87.52 (83.60-90.85)	81.55 (72.54-88.45)	83.91 (76.35-90.32)	89.99 (85.87-93.39)	55.88 (40.71-69.00)	57.22 (42.88-70.40)	44.48 (30.97-56.25)	67.73 (63.52-71.94)	65.18 (61.00-69.39)	76.56 (72.12-80.92)
Poland	88.04 (83.41-91.90)	90.41 (86.52-93.59)	94.32 (91.98-96.21)	87.21 (80.16-91.96)	86.97 (80.15-92.01)	90.40 (85.37-94.27)	53.58 (37.30-66.65)	53.79 (38.36-67.83)	49.99 (33.37-64.78)	54.62 (51.66-58.09)	66.36 (63.75-69.19)	79.70 (77.10-82.43)
Romania	82.81 (79.21-86.02)	79.96 (77.51-82.27)	87.83 (82.93-91.64)	88.75 (85.00-92.00)	87.50 (84.48-90.70)	89.37 (83.93-93.60)	65.23 (52.31-77.05)	60.88 (46.81-73.40)	51.59 (35.94-66.42)	39.50 (35.70-44.08)	52.22 (49.07-55.88)	61.41 (57.94-64.83)
Serbia	82.42 (76.05-87.65)	84.73 (80.07-88.50)	91.36 (89.10-93.29)	81.97 (73.03-89.30)	82.84 (74.66-88.79)	87.46 (82.88-91.37)	55.94 (40.51-69.17)	60.22 (46.39-73.26)	64.89 (56.12-72.52)	60.56 (54.89-65.41)	66.66 (63.65-69.70)	68.23 (65.05-71.30)
Slovakia	85.68 (79.69-90.08)	87.71 (82.43-91.64)	93.30 (90.41-95.48)	85.37 (78.11-91.02)	84.96 (77.79-90.91)	89.27 (83.42-93.56)	60.49 (46.07-72.92)	62.24 (47.51-73.26)	61.71 (48.02-73.34)	63.53 (60.22-66.88)	66.51 (63.60-69.66)	74.67 (71.55-77.68)
Slovenia	87.12 (81.42-91.21)	90.12 (86.16-93.33)	93.74 (91.09-95.61)	85.13 (77.92-90.97)	85.83 (78.86-91.33)	89.58 (84.45-93.70)	55.69 (41.12-68.39)	50.34 (35.77-63.91)	39.11 (23.28-53.66)	68.69 (65.75-71.56)	68.69 (66.06-71.62)	76.37 (73.47-79.16)
Armenia	72.03 (63.37-79.20)	73.30 (68.19-77.81)	80.13 (74.15-84.63)	86.25 (74.60-93.86)	86.96 (79.66-92.31)	87.21 (76.89-94.12)	63.06 (49.68-74.22)	61.22 (51.82-69.44)	45.04 (29.89-58.22)	49.23 (45.45-53.38)	50.23 (46.63-54.10)	58.26 (54.40-62.18)
Azerbaijan	63.87 (54.16-71.84)	65.01 (59.62-70.07)	79.15 (72.55-84.54)	81.47 (65.88-91.66)	80.76 (70.73-88.53)	83.74 (68.99-92.68)	80.50 (72.63-87.23)	78.36 (70.45-84.62)	44.41 (29.24-59.13)	52.23 (48.89-56.26)	49.74 (46.08-53.46)	60.72 (56.60-65.05)

Appendix Table 1a. Scaled values for each SDG health-related indicator and three summary indicators by country for 1990, 2000, and 2015

Location	Indicator 2.2.1: Prevalence of stunting among children under 5 years of age			Indicator 2.2.2a: Prevalence of wasting among children under 5 years of age			Indicator 2.2.2b: Prevalence of overweight among children aged 2 to 4 years			Indicator 3.1.1: Maternal mortality ratio (maternal deaths per 100,000 live births)		
	1990	2000	2015	1990	2000	2015	1990	2000	2015	1990	2000	2015
Georgia	74.37 (66.55-80.83)	75.38 (69.96-79.86)	84.34 (79.61-88.61)	86.19 (73.52-93.65)	89.07 (81.84-94.06)	91.78 (84.89-96.33)	66.09 (53.72-76.61)	60.81 (48.25-71.85)	46.12 (29.71-60.77)	51.55 (48.03-55.57)	55.25 (51.86-59.06)	51.38 (47.58-55.44)
Kazakhstan	72.38 (64.88-78.92)	74.30 (68.69-79.28)	85.39 (81.09-88.97)	82.04 (68.50-91.21)	85.33 (75.76-91.91)	86.73 (76.77-93.45)	71.68 (61.67-80.41)	75.40 (67.67-82.00)	52.25 (38.07-64.95)	46.13 (42.69-50.09)	46.55 (43.10-50.57)	57.15 (53.73-60.81)
Kyrgyzstan	56.32 (46.23-65.21)	60.25 (52.62-67.59)	78.36 (73.62-82.36)	86.67 (75.62-94.01)	87.20 (77.95-93.39)	89.16 (81.87-93.96)	63.71 (51.03-74.14)	69.20 (58.78-77.58)	71.09 (62.59-77.93)	45.56 (41.97-49.65)	46.01 (42.38-50.02)	49.72 (45.88-54.06)
Mongolia	53.29 (45.47-60.95)	57.28 (52.10-62.17)	79.76 (74.30-84.62)	89.31 (80.96-94.63)	87.56 (79.71-92.68)	93.45 (87.55-96.93)	58.33 (43.07-71.05)	65.75 (53.54-75.90)	55.91 (41.52-68.88)	33.34 (28.62-38.71)	33.01 (28.35-38.13)	47.31 (42.68-52.12)
Tajikistan	49.93 (38.60-60.45)	42.01 (37.21-47.09)	61.54 (53.89-68.29)	61.41 (30.79-80.89)	61.25 (43.91-74.95)	70.68 (50.18-84.66)	79.05 (70.22-86.28)	82.80 (75.16-89.49)	80.00 (73.27-86.21)	44.65 (40.73-48.89)	47.59 (44.09-51.84)	57.58 (53.24-61.70)
Turkmenistan	58.75 (48.43-67.96)	61.39 (54.08-67.87)	80.48 (74.60-85.77)	77.11 (58.23-89.62)	77.11 (61.62-87.06)	82.66 (66.94-92.37)	67.68 (55.90-77.38)	64.38 (52.38-75.25)	46.64 (32.11-60.23)	46.51 (42.78-50.76)	45.03 (41.05-49.33)	60.98 (57.35-64.68)
Uzbekistan	48.17 (37.54-59.15)	56.69 (48.96-63.74)	78.11 (71.36-83.84)	70.88 (47.34-85.58)	71.59 (52.88-83.84)	83.73 (68.16-92.63)	48.34 (33.64-60.85)	56.13 (44.66-66.62)	54.80 (39.85-67.50)	48.71 (45.09-52.78)	49.74 (46.28-53.79)	57.24 (53.06-61.43)
Colombia	67.45 (63.92-70.59)	73.63 (70.71-76.08)	82.98 (79.78-85.75)	92.14 (88.77-94.93)	94.90 (92.66-96.68)	95.77 (93.20-97.61)	84.83 (78.76-89.69)	83.82 (79.69-87.38)	89.19 (82.59-94.14)	40.03 (35.91-44.93)	42.74 (39.11-47.19)	48.31 (44.54-52.43)
Costa Rica	85.78 (82.79-88.39)	89.09 (86.88-91.13)	93.27 (91.82-94.51)	94.70 (91.67-96.96)	95.42 (92.64-97.34)	96.33 (94.04-97.91)	60.94 (43.85-76.28)	52.41 (31.26-69.78)	41.74 (19.82-61.59)	55.75 (52.57-59.23)	53.67 (50.59-57.31)	58.17 (54.65-61.85)
El Salvador	50.35 (45.23-55.22)	65.21 (60.20-69.87)	74.78 (70.72-78.76)	93.51 (91.01-95.64)	95.12 (93.32-96.75)	95.09 (92.35-97.19)	85.41 (78.68-90.82)	83.66 (78.05-87.90)	79.13 (67.70-87.80)	35.99 (31.73-40.89)	48.73 (44.96-52.96)	52.56 (48.46-57.25)
Guatemala	13.85 (6.33-20.47)	25.23 (19.72-31.50)	37.59 (29.48-45.67)	90.40 (85.90-93.99)	87.81 (83.19-91.85)	93.09 (89.02-96.10)	81.37 (74.93-86.58)	73.84 (68.83-78.42)	75.46 (63.49-84.81)	35.40 (31.19-40.40)	39.49 (35.62-43.84)	44.45 (39.46-49.87)

Appendix Table 1a. Scaled values for each SDG health-related indicator and three summary indicators by country for 1990, 2000, and 2015

Location	Indicator 2.2.1: Prevalence of stunting among children under 5 years of age			Indicator 2.2.2a: Prevalence of wasting among children under 5 years of age			Indicator 2.2.2b: Prevalence of overweight among children aged 2 to 4 years			Indicator 3.1.1: Maternal mortality ratio (maternal deaths per 100,000 live births)		
	1990	2000	2015	1990	2000	2015	1990	2000	2015	1990	2000	2015
Honduras	36.95 (32.12-42.05)	49.15 (43.81-54.61)	68.98 (64.17-73.18)	92.65 (90.61-94.72)	94.79 (92.97-96.39)	95.49 (93.27-97.14)	90.94 (84.24-95.89)	87.19 (82.26-91.23)	80.14 (70.68-86.99)	33.29 (28.59-38.73)	38.10 (32.57-44.20)	39.46 (32.98-46.11)
Mexico	61.20 (58.58-63.71)	72.74 (70.81-74.49)	80.87 (79.52-82.15)	82.23 (78.09-86.68)	92.79 (91.12-94.55)	93.72 (92.28-95.26)	52.65 (33.07-68.38)	53.37 (32.75-69.44)	66.47 (52.87-78.36)	44.32 (41.03-48.42)	46.64 (43.47-50.60)	49.63 (46.91-53.42)
Nicaragua	56.83 (51.05-62.26)	63.02 (58.82-67.03)	76.47 (72.02-80.38)	89.69 (84.28-93.69)	92.67 (89.91-95.12)	96.08 (93.73-97.90)	77.21 (66.23-86.55)	76.68 (70.61-82.01)	73.13 (58.33-83.55)	42.25 (38.12-46.70)	40.34 (36.41-44.77)	45.24 (40.87-50.04)
Panama	80.99 (78.35-83.45)	85.93 (83.24-88.41)	92.23 (90.48-93.71)	91.88 (88.41-94.56)	95.33 (93.30-97.00)	96.11 (93.81-97.77)	82.18 (71.78-90.11)	81.29 (70.32-89.96)	76.07 (62.73-86.81)	45.24 (41.09-49.70)	46.03 (42.40-50.27)	45.12 (40.81-50.10)
Venezuela	72.93 (70.36-75.34)	73.83 (71.55-75.91)	84.99 (82.17-87.46)	86.87 (83.76-90.24)	86.78 (83.28-90.52)	89.26 (83.01-93.84)	62.95 (44.09-77.61)	66.52 (48.89-81.25)	59.06 (40.20-74.82)	45.39 (41.92-49.47)	47.21 (43.87-51.32)	46.20 (41.88-50.91)
Bolivia	40.57 (35.12-45.77)	52.61 (47.96-57.16)	67.94 (62.64-73.01)	92.88 (89.39-95.58)	93.60 (90.81-95.96)	94.99 (91.78-97.14)	64.36 (57.85-70.37)	60.91 (54.42-66.78)	58.91 (50.08-66.53)	22.56 (16.80-28.46)	30.80 (25.84-36.43)	35.68 (29.46-41.89)
Ecuador	45.64 (39.52-51.30)	55.68 (50.91-60.38)	71.86 (67.03-76.35)	91.55 (87.16-94.60)	90.63 (86.70-94.06)	93.47 (89.60-96.40)	77.07 (70.70-82.22)	75.61 (69.69-80.53)	70.62 (64.33-76.40)	37.56 (33.65-42.46)	44.27 (40.49-48.59)	48.84 (44.43-53.00)
Peru	46.55 (40.69-52.00)	54.56 (49.81-59.07)	74.08 (70.55-77.50)	93.08 (90.13-95.51)	95.52 (93.73-97.01)	97.95 (96.94-98.68)	62.60 (55.38-68.93)	60.81 (54.13-66.86)	69.06 (63.48-74.70)	32.75 (28.07-38.25)	39.11 (35.06-43.95)	49.94 (45.36-54.97)
Antigua and Barbuda	89.13 (85.82-91.87)	91.95 (89.43-94.05)	94.70 (92.89-96.10)	89.41 (83.50-93.96)	90.10 (84.26-94.36)	92.30 (87.85-95.63)	80.75 (72.75-86.95)	75.73 (66.84-83.32)	77.48 (68.62-84.47)	48.46 (44.58-52.61)	49.34 (45.31-53.68)	53.24 (49.41-57.33)
The Bahamas	89.44 (86.18-92.11)	92.26 (89.74-94.44)	93.79 (91.71-95.56)	89.60 (83.55-94.01)	90.44 (84.52-94.50)	91.43 (86.17-95.06)	63.66 (51.87-74.10)	58.83 (46.98-69.78)	60.32 (47.40-70.95)	44.41 (40.19-48.87)	44.26 (39.78-48.98)	49.42 (45.00-54.19)
Barbados	87.52 (83.57-90.44)	85.53 (81.27-89.07)	90.46 (87.71-92.77)	88.46 (81.67-93.54)	78.93 (67.03-87.98)	81.78 (72.79-88.78)	65.25 (54.54-74.55)	64.28 (54.15-73.71)	57.48 (45.09-67.77)	43.02 (39.05-47.41)	53.97 (50.26-57.97)	52.59 (48.20-56.52)

Appendix Table 1a. Scaled values for each SDG health-related indicator and three summary indicators by country for 1990, 2000, and 2015

Location	Indicator 2.2.1: Prevalence of stunting among children under 5 years of age			Indicator 2.2.2a: Prevalence of wasting among children under 5 years of age			Indicator 2.2.2b: Prevalence of overweight among children aged 2 to 4 years			Indicator 3.1.1: Maternal mortality ratio (maternal deaths per 100,000 live births)		
	1990	2000	2015	1990	2000	2015	1990	2000	2015	1990	2000	2015
Belize	48.44 (36.99-58.35)	61.04 (52.34-69.14)	74.94 (69.37-80.01)	87.26 (79.19-92.89)	89.00 (82.59-93.40)	89.63 (84.83-93.57)	75.16 (66.86-82.39)	70.45 (60.76-79.34)	74.37 (65.20-81.98)	50.05 (46.52-54.41)	46.59 (42.65-50.85)	54.81 (50.29-59.09)
Cuba	87.31 (83.67-90.41)	89.08 (86.80-91.16)	89.39 (86.69-91.69)	89.99 (84.54-94.11)	90.97 (87.22-94.01)	92.35 (88.93-95.19)	61.75 (49.57-72.11)	64.15 (53.22-74.21)	48.99 (35.18-61.74)	48.80 (45.41-52.78)	48.78 (45.34-52.78)	51.55 (48.03-55.49)
Dominica	82.86 (77.68-87.20)	88.46 (85.12-91.43)	92.49 (89.87-94.44)	88.44 (81.90-93.50)	89.86 (83.46-94.25)	91.85 (86.48-95.21)	77.90 (69.80-84.56)	70.99 (60.44-79.63)	69.01 (57.90-78.21)	59.19 (55.73-62.76)	61.06 (57.61-64.73)	52.67 (48.51-57.27)
Dominican Republic	71.34 (66.94-75.73)	83.69 (81.07-86.12)	90.14 (87.88-92.06)	91.56 (88.63-94.22)	92.83 (90.58-94.95)	92.67 (89.38-95.29)	86.61 (82.81-89.71)	79.63 (74.83-83.62)	76.78 (70.37-82.31)	41.68 (37.71-46.25)	46.93 (43.43-51.00)	48.44 (44.14-53.03)
Grenada	81.67 (76.55-86.28)	87.42 (83.45-90.61)	92.15 (89.75-94.15)	87.61 (79.76-93.24)	88.86 (82.23-93.84)	91.43 (86.25-95.19)	80.86 (73.50-87.16)	74.28 (65.23-81.96)	71.36 (61.63-80.04)	48.84 (45.06-53.08)	55.57 (52.01-59.25)	54.20 (50.57-58.29)
Guyana	69.77 (62.75-76.33)	76.96 (72.92-80.57)	75.63 (69.75-80.77)	60.56 (41.89-75.58)	60.81 (47.62-73.16)	77.10 (64.52-86.30)	87.56 (82.08-92.09)	83.79 (77.40-88.95)	82.25 (76.12-87.84)	36.68 (32.30-41.95)	34.20 (29.57-39.51)	39.40 (34.12-44.79)
Haiti	44.38 (37.53-51.01)	55.90 (49.60-61.76)	68.93 (62.82-74.26)	75.66 (65.68-83.31)	75.49 (66.09-82.77)	80.37 (71.07-87.27)	89.97 (85.86-93.54)	92.10 (89.23-94.50)	89.12 (84.96-92.94)	17.10 (10.69-23.94)	17.18 (10.89-24.07)	19.03 (11.13-26.97)
Jamaica	80.60 (77.12-83.41)	87.80 (86.19-89.31)	91.58 (88.74-93.60)	85.88 (80.85-90.33)	88.94 (85.48-92.20)	88.67 (82.05-93.21)	68.46 (58.23-77.61)	60.83 (48.93-71.27)	58.66 (45.76-69.55)	51.81 (47.50-56.43)	45.69 (41.30-50.30)	49.22 (44.47-54.67)
Saint Lucia	93.58 (91.65-95.25)	95.60 (94.13-96.72)	97.02 (96.04-97.79)	84.62 (75.72-91.19)	86.62 (79.00-92.42)	88.44 (82.25-93.17)	92.08 (87.96-95.49)	90.49 (85.99-94.35)	91.58 (87.76-94.88)	48.05 (44.10-52.15)	42.57 (38.58-46.97)	47.04 (42.77-51.48)
Saint Vincent and the Grenadines	81.20 (75.25-85.70)	87.39 (83.77-90.58)	92.40 (90.08-94.37)	87.45 (79.99-92.87)	89.04 (82.13-93.77)	91.83 (86.86-95.38)	66.18 (54.29-75.60)	55.97 (42.33-67.21)	50.54 (37.38-63.24)	47.99 (43.65-52.35)	53.99 (50.48-57.94)	51.87 (47.73-56.09)
Suriname	74.85 (67.92-80.62)	79.50 (75.13-83.27)	89.82 (86.98-92.24)	77.72 (64.95-86.93)	79.00 (69.61-86.08)	85.21 (77.06-91.18)	90.02 (85.37-93.90)	88.41 (83.18-92.78)	81.95 (74.91-87.62)	46.67 (42.66-51.38)	42.46 (38.06-47.23)	49.12 (44.54-54.07)

Location	Appendix Table 1a. Scaled values for each SDG health-related indicator and three summary indicators by country for 1990, 2000, and 2015											
	**Indicator 2.2.1: Prevalence of stunting among children under 5 years of age**			**Indicator 2.2.2a: Prevalence of wasting among children under 5 years of age**			**Indicator 2.2.2b: Prevalence of overweight among children aged 2 to 4 years**			**Indicator 3.1.1: Maternal mortality ratio (maternal deaths per 100,000 live births)**		
	**1990**	**2000**	**2015**	**1990**	**2000**	**2015**	**1990**	**2000**	**2015**	**1990**	**2000**	**2015**
Trinidad and Tobago	90.58 (88.23-92.60)	91.23 (89.08-93.09)	95.03 (93.31-96.41)	82.11 (74.05-88.79)	83.65 (76.81-89.35)	87.46 (80.21-92.65)	90.95 (86.95-94.30)	86.35 (80.31-91.25)	80.58 (73.07-86.43)	43.96 (40.55-48.31)	46.68 (43.09-50.97)	49.40 (45.14-53.85)
Brazil	71.02 (68.78-73.36)	81.79 (80.06-83.37)	90.30 (89.39-91.18)	91.15 (88.03-93.80)	84.71 (79.95-89.19)	91.66 (88.45-94.15)	79.96 (72.10-86.46)	70.04 (61.14-77.69)	49.63 (28.47-67.65)	42.58 (39.29-47.06)	44.79 (41.47-48.84)	45.86 (42.47-50.10)
Paraguay	72.52 (68.16-76.24)	74.15 (68.40-79.12)	82.02 (76.58-86.65)	97.12 (95.32-98.45)	95.96 (92.09-98.17)	95.53 (89.83-98.44)	79.18 (74.16-83.42)	73.65 (60.30-84.99)	69.75 (53.34-82.13)	35.38 (30.98-40.52)	39.10 (34.82-44.00)	40.39 (34.97-45.83)
China	49.87 (43.99-55.31)	67.46 (62.56-71.71)	86.69 (84.01-89.00)	86.51 (81.79-90.40)	91.45 (88.41-94.08)	91.18 (87.51-94.02)	72.41 (65.43-78.70)	72.03 (64.28-78.97)	61.67 (47.18-73.11)	38.70 (34.67-43.37)	42.46 (38.96-46.80)	62.18 (59.13-65.45)
North Korea	30.17 (1.26-58.83)	29.70 (17.43-43.18)	52.99 (26.26-75.26)	79.91 (49.21-95.32)	64.21 (48.18-77.26)	82.84 (57.87-94.88)	95.12 (90.93-98.52)	95.13 (91.04-98.46)	96.81 (93.40-99.49)	40.76 (32.67-48.70)	41.51 (33.40-48.72)	44.87 (38.07-52.11)
Taiwan	100.00 (100.00-100.00)	100.00 (100.00-100.00)	100.00 (100.00-100.00)	100.00 (100.00-100.00)	100.00 (100.00-100.00)	100.00 (100.00-100.00)	52.54 (35.32-67.66)	46.90 (29.59-62.68)	52.42 (35.31-66.34)	60.25 (54.86-65.65)	64.26 (59.35-68.89)	67.59 (61.90-72.74)
Cambodia	11.13 (2.93-20.17)	20.66 (13.44-28.47)	47.57 (40.51-54.68)	50.30 (28.40-68.21)	50.61 (34.70-64.74)	67.04 (54.69-78.22)	97.12 (93.36-99.93)	95.13 (92.64-97.24)	95.68 (91.49-98.75)	22.56 (17.27-28.66)	20.87 (15.35-27.20)	37.12 (30.39-43.13)
Indonesia	22.71 (15.49-29.82)	40.90 (36.22-45.34)	61.54 (55.61-67.44)	64.58 (49.06-78.05)	70.66 (59.90-79.98)	71.59 (56.62-82.49)	90.14 (82.54-95.27)	87.24 (81.61-91.57)	86.53 (78.54-92.46)	22.16 (17.03-28.20)	24.10 (18.86-29.88)	33.07 (26.76-39.86)
Laos	19.52 (12.25-26.60)	29.01 (22.46-35.63)	47.37 (40.61-53.64)	57.12 (39.04-71.87)	54.49 (36.61-68.88)	76.49 (64.93-85.25)	95.55 (91.20-98.69)	94.29 (89.23-98.07)	92.70 (86.29-97.11)	15.64 (8.96-22.47)	16.97 (10.62-23.47)	29.38 (22.77-36.22)
Malaysia	57.74 (51.20-64.14)	70.73 (67.01-74.30)	81.73 (78.27-84.84)	53.92 (32.66-71.39)	58.73 (41.46-72.81)	67.51 (50.68-80.00)	68.08 (53.10-81.46)	58.66 (39.06-73.97)	53.13 (34.39-69.90)	42.03 (37.98-46.38)	44.84 (41.10-48.91)	46.65 (41.75-51.94)
Maldives	44.81 (38.30-51.37)	55.94 (50.73-61.08)	73.96 (69.49-78.06)	55.21 (36.84-70.33)	48.68 (30.98-66.01)	64.94 (47.48-77.88)	93.28 (87.58-97.61)	91.44 (85.02-96.07)	81.35 (71.33-88.85)	32.57 (26.18-38.55)	41.41 (36.62-46.28)	51.88 (46.48-57.32)

Appendix Table 1a. Scaled values for each SDG health-related indicator and three summary indicators by country for 1990, 2000, and 2015

Location	Indicator 2.2.1: Prevalence of stunting among children under 5 years of age			Indicator 2.2.2a: Prevalence of wasting among children under 5 years of age			Indicator 2.2.2b: Prevalence of overweight among children aged 2 to 4 years			Indicator 3.1.1: Maternal mortality ratio (maternal deaths per 100,000 live births)		
	1990	2000	2015	1990	2000	2015	1990	2000	2015	1990	2000	2015
Mauritius	71.02 (66.79-75.03)	82.65 (79.59-85.33)	89.59 (87.39-91.58)	52.08 (31.70-68.36)	59.53 (41.24-74.45)	68.23 (52.24-81.00)	68.24 (52.95-80.82)	62.47 (44.29-77.11)	56.94 (37.25-72.61)	45.43 (41.85-49.87)	51.37 (47.90-55.23)	49.46 (45.95-53.44)
Myanmar	16.02 (9.27-22.81)	32.96 (26.41-39.37)	62.50 (56.49-68.24)	60.60 (47.14-72.79)	66.16 (55.42-75.99)	76.32 (65.06-85.32)	96.33 (92.17-99.44)	94.71 (89.79-98.39)	79.76 (69.42-88.65)	11.36 (2.44-21.30)	12.93 (4.08-22.41)	23.04 (13.36-32.11)
Philippines	38.87 (35.13-43.01)	47.01 (43.09-50.83)	61.22 (55.25-66.58)	75.62 (69.63-81.98)	76.40 (70.05-82.66)	79.50 (69.25-87.24)	98.03 (94.38-100.00)	97.51 (93.84-99.85)	93.28 (86.73-97.73)	40.80 (36.79-45.48)	44.91 (41.38-49.07)	45.00 (40.71-49.77)
Sri Lanka	56.51 (51.76-61.17)	72.60 (69.09-75.89)	80.58 (77.22-83.69)	49.80 (35.70-63.49)	50.16 (35.54-64.09)	63.36 (46.81-76.92)	86.30 (76.69-93.31)	85.11 (75.51-92.56)	80.66 (68.63-89.23)	44.07 (40.17-48.55)	46.17 (42.70-50.17)	54.72 (48.74-60.61)
Seychelles	89.31 (87.19-91.14)	92.60 (91.09-94.01)	95.48 (94.47-96.35)	89.23 (84.22-93.50)	89.95 (84.54-94.20)	91.74 (87.16-95.20)	84.05 (74.12-91.71)	82.52 (72.44-90.67)	80.46 (68.80-89.36)	46.81 (42.09-51.85)	52.39 (48.18-56.83)	54.77 (50.20-59.22)
Thailand	66.17 (62.35-69.98)	76.40 (72.90-79.68)	85.10 (81.87-87.68)	76.26 (66.92-84.72)	79.81 (70.49-87.19)	83.71 (75.12-90.10)	89.77 (82.27-95.22)	82.41 (71.65-90.83)	71.41 (55.43-83.79)	51.85 (48.16-55.57)	55.61 (51.49-59.98)	60.83 (55.03-67.31)
Timor-Leste	4.86 (0.00-13.41)	16.61 (10.13-23.88)	33.53 (25.44-41.19)	50.81 (26.87-69.35)	50.46 (31.78-67.22)	51.02 (29.17-68.21)	92.29 (85.93-97.08)	93.26 (87.72-97.25)	87.97 (79.90-93.82)	16.01 (8.94-23.35)	22.26 (15.46-29.98)	29.57 (22.70-38.12)
Vietnam	13.14 (7.35-18.89)	39.34 (35.41-43.34)	68.82 (63.89-73.39)	68.14 (59.19-77.01)	66.45 (59.26-74.95)	80.33 (71.47-87.31)	98.25 (95.14-100.00)	96.32 (93.18-98.70)	85.85 (76.35-92.93)	50.19 (44.37-55.89)	54.82 (49.54-59.96)	63.86 (57.21-70.09)
Federated States of Micronesia	80.35 (76.96-83.56)	83.11 (80.19-85.97)	87.56 (85.21-89.71)	84.74 (75.56-91.20)	87.49 (79.92-92.81)	88.44 (81.10-93.44)	64.61 (45.02-80.48)	65.66 (46.12-80.59)	66.59 (47.28-82.08)	37.32 (27.94-46.39)	38.98 (29.38-49.37)	42.69 (32.54-51.61)
Fiji	83.65 (80.57-86.32)	86.32 (83.64-88.65)	89.96 (88.12-91.65)	85.42 (77.56-91.51)	88.39 (81.30-93.23)	88.92 (82.57-93.51)	63.60 (44.91-79.50)	50.93 (27.98-69.95)	51.48 (29.70-71.85)	41.04 (35.30-47.30)	38.67 (33.69-43.95)	40.77 (35.25-46.99)
Kiribati	51.80 (45.59-57.74)	58.02 (51.88-63.90)	68.36 (63.35-72.97)	65.84 (49.07-78.40)	71.34 (55.73-82.99)	75.70 (61.98-86.14)	18.53 (0.00-42.80)	16.96 (0.00-38.83)	13.76 (0.00-37.45)	28.49 (22.56-35.12)	33.47 (28.11-39.44)	37.13 (30.96-43.42)

Appendix Table 1a. Scaled values for each SDG health-related indicator and three summary indicators by country for 1990, 2000, and 2015

Location	Indicator 2.2.1: Prevalence of stunting among children under 5 years of age			Indicator 2.2.2a: Prevalence of wasting among children under 5 years of age			Indicator 2.2.2b: Prevalence of overweight among children aged 2 to 4 years			Indicator 3.1.1: Maternal mortality ratio (maternal deaths per 100,000 live births)		
	1990	2000	2015	1990	2000	2015	1990	2000	2015	1990	2000	2015
Marshall Islands	74.81 (70.49-78.73)	77.70 (73.77-80.79)	84.24 (81.24-86.80)	82.84 (72.80-90.08)	86.04 (78.33-91.94)	86.94 (79.28-92.66)	50.70 (27.84-70.35)	46.76 (22.48-66.59)	42.76 (18.23-63.99)	39.04 (33.22-44.83)	37.68 (30.73-46.55)	39.55 (32.85-46.17)
Papua New Guinea	30.45 (22.48-37.73)	37.65 (30.81-44.21)	50.76 (43.76-56.82)	81.55 (70.39-89.53)	84.08 (75.98-90.23)	85.66 (77.56-91.55)	61.57 (40.99-77.83)	62.07 (42.67-78.75)	70.85 (53.03-83.81)	18.50 (9.01-27.03)	19.06 (10.07-28.08)	21.52 (11.60-30.21)
Samoa	88.23 (85.97-90.05)	90.72 (89.25-92.03)	93.78 (92.58-94.81)	93.42 (89.55-96.14)	94.58 (91.82-96.59)	94.86 (91.81-97.02)	27.72 (1.55-52.87)	27.09 (3.24-50.49)	22.22 (0.00-46.22)	46.24 (39.19-53.39)	47.21 (40.07-56.03)	48.32 (41.22-55.11)
Solomon Islands	50.77 (45.33-56.20)	53.40 (47.20-59.34)	63.33 (57.34-68.19)	76.70 (66.69-84.57)	83.80 (76.02-90.19)	86.11 (79.00-91.77)	59.74 (39.85-78.11)	54.60 (30.92-73.08)	42.80 (18.40-63.32)	28.23 (18.77-37.83)	29.97 (20.97-39.95)	31.52 (21.68-40.96)
Tonga	96.96 (96.42-97.51)	97.61 (97.15-98.02)	98.35 (98.01-98.67)	94.29 (91.33-96.60)	94.78 (91.79-96.96)	95.39 (92.53-97.34)	35.25 (11.20-57.14)	28.85 (4.18-51.40)	16.20 (0.00-41.35)	31.65 (25.10-37.81)	32.23 (27.18-37.81)	37.21 (30.32-44.13)
Vanuatu	62.46 (57.49-67.12)	62.21 (57.49-66.73)	69.50 (64.91-74.01)	77.46 (65.16-86.46)	80.15 (70.99-87.40)	82.82 (73.26-89.83)	78.81 (65.37-90.11)	77.83 (64.10-88.90)	77.40 (62.09-89.17)	31.78 (23.11-41.08)	32.01 (22.74-42.63)	33.93 (25.03-42.13)
Afghanistan	29.54 (13.91-44.04)	19.85 (8.92-29.98)	33.07 (16.99-49.56)	40.52 (9.26-64.32)	47.47 (32.54-62.27)	65.72 (43.52-81.36)	81.79 (72.79-88.97)	91.58 (85.34-96.16)	87.70 (80.58-93.59)	14.34 (5.62-23.00)	13.91 (5.64-22.60)	13.30 (4.42-22.96)
Algeria	70.67 (62.40-77.28)	67.85 (61.29-73.67)	77.74 (67.85-85.50)	77.62 (70.05-85.02)	75.03 (68.19-82.00)	89.62 (82.15-94.25)	81.53 (72.26-88.43)	76.07 (65.51-84.61)	57.27 (42.72-69.76)	35.02 (29.90-40.58)	37.70 (32.63-43.17)	45.64 (40.69-50.60)
Bahrain	79.26 (73.85-84.00)	83.11 (76.41-88.34)	89.02 (83.67-92.85)	77.96 (71.78-84.15)	80.37 (69.41-88.57)	84.12 (73.68-91.64)	28.26 (9.42-45.72)	26.03 (9.20-42.66)	21.59 (2.87-39.30)	48.40 (43.22-53.03)	53.51 (48.70-57.89)	58.16 (52.87-63.07)
Egypt	49.71 (41.93-56.78)	62.91 (57.31-68.56)	68.46 (60.53-75.35)	85.95 (79.98-91.23)	81.74 (74.63-87.84)	73.35 (60.10-84.02)	60.15 (52.62-68.25)	52.91 (43.18-61.42)	47.94 (33.79-61.68)	35.76 (31.34-40.67)	45.32 (41.40-49.86)	51.45 (47.10-56.02)
Iran	60.13 (48.30-70.62)	79.49 (73.95-84.14)	90.19 (85.09-93.69)	71.07 (55.36-82.87)	82.63 (76.62-87.62)	85.85 (76.10-92.50)	86.47 (79.62-91.79)	80.57 (72.03-87.23)	76.82 (67.31-84.69)	47.94 (42.38-53.66)	54.20 (50.07-58.50)	60.50 (54.83-65.55)

Appendix Table 1a. Scaled values for each SDG health-related indicator and three summary indicators by country for 1990, 2000, and 2015

Location	Indicator 2.2.1: Prevalence of stunting among children under 5 years of age			Indicator 2.2.2a: Prevalence of wasting among children under 5 years of age			Indicator 2.2.2b: Prevalence of overweight among children aged 2 to 4 years			Indicator 3.1.1: Maternal mortality ratio (maternal deaths per 100,000 live births)		
	1990	2000	2015	1990	2000	2015	1990	2000	2015	1990	2000	2015
Iraq	60.14 (51.13-68.23)	55.55 (47.46-63.45)	72.73 (63.39-79.70)	85.41 (80.58-89.97)	76.59 (70.09-83.15)	79.61 (69.60-87.38)	57.12 (42.26-69.49)	81.34 (72.77-87.83)	68.96 (57.36-78.72)	35.61 (29.88-41.67)	38.90 (33.09-45.24)	47.62 (40.46-54.65)
Jordan	73.41 (65.65-80.17)	81.05 (75.17-85.71)	87.77 (82.69-91.54)	86.40 (77.74-92.33)	89.31 (83.42-93.77)	90.18 (84.13-94.56)	79.28 (72.97-84.66)	87.84 (83.53-91.51)	81.68 (75.16-87.34)	40.69 (35.94-45.88)	43.12 (38.37-48.21)	58.36 (53.89-62.80)
Kuwait	86.28 (81.05-90.35)	93.78 (92.30-95.07)	94.94 (92.53-96.72)	88.96 (81.71-94.10)	93.08 (89.43-95.84)	93.24 (88.39-96.40)	42.81 (26.87-57.23)	30.44 (12.63-46.84)	22.71 (4.19-38.20)	69.48 (66.14-72.56)	66.96 (64.10-69.79)	75.46 (71.63-79.32)
Lebanon	71.04 (60.15-79.29)	76.38 (69.92-82.11)	84.49 (77.94-89.95)	85.12 (75.80-91.63)	83.23 (75.63-89.02)	83.67 (72.88-91.32)	57.06 (42.56-69.82)	49.17 (35.70-60.98)	55.21 (40.54-67.47)	53.77 (47.65-59.67)	57.24 (51.91-62.72)	64.16 (57.59-70.50)
Libya	60.64 (50.08-70.98)	64.00 (53.16-72.50)	71.35 (60.23-79.90)	84.33 (76.03-90.71)	88.68 (84.05-92.97)	83.85 (73.62-91.11)	36.69 (18.15-51.57)	41.93 (25.68-56.29)	40.13 (23.46-54.91)	57.16 (52.29-62.19)	58.47 (53.73-63.24)	59.08 (53.61-64.52)
Morocco	53.42 (44.59-61.83)	61.92 (53.28-69.76)	72.97 (62.36-81.81)	87.92 (84.27-91.24)	71.84 (62.32-80.23)	74.81 (58.69-86.26)	70.41 (64.36-75.90)	69.02 (61.36-75.96)	62.71 (48.61-74.25)	24.85 (18.39-31.39)	31.98 (26.27-37.94)	45.53 (38.38-52.22)
Palestine	76.16 (66.31-83.91)	81.77 (75.32-87.27)	86.84 (83.09-90.17)	87.42 (78.27-93.37)	88.88 (83.28-93.22)	94.67 (92.78-96.45)	77.34 (67.17-85.15)	73.37 (64.52-81.11)	78.88 (71.14-85.14)	56.18 (50.05-62.44)	64.88 (61.08-68.49)	63.38 (57.90-67.96)
Oman	63.94 (55.38-71.38)	78.73 (73.09-83.35)	86.36 (80.82-90.96)	64.86 (53.50-75.29)	76.25 (68.88-82.55)	77.54 (64.73-87.29)	64.26 (51.75-75.26)	61.78 (48.26-73.43)	55.48 (40.23-67.62)	54.36 (47.56-60.84)	59.92 (54.53-64.71)	63.80 (58.78-68.48)
Qatar	84.73 (77.64-90.25)	88.09 (82.27-92.47)	93.36 (90.21-95.92)	83.80 (71.44-91.50)	85.91 (75.63-92.47)	88.83 (81.01-94.17)	36.82 (19.11-52.92)	41.15 (22.32-56.99)	31.92 (14.68-49.42)	46.22 (40.37-51.89)	47.04 (41.46-52.42)	57.71 (52.60-62.83)
Saudi Arabia	65.44 (60.56-70.10)	77.70 (74.15-81.11)	87.71 (85.44-89.57)	90.12 (86.87-92.99)	63.70 (52.14-74.65)	74.92 (66.97-82.23)	53.57 (38.21-66.53)	45.78 (29.95-59.69)	35.67 (18.32-50.74)	60.14 (55.89-64.11)	62.15 (59.07-65.25)	63.71 (60.46-66.94)
Sudan	37.88 (25.66-48.58)	33.78 (23.34-44.67)	49.77 (39.61-59.13)	51.18 (34.83-65.86)	34.99 (22.11-50.45)	44.45 (31.76-58.45)	87.37 (80.87-92.59)	85.95 (78.69-91.59)	80.86 (71.49-87.87)	19.88 (13.09-27.38)	22.27 (14.80-30.90)	26.81 (19.63-35.01)

	Appendix Table 1a. Scaled values for each SDG health-related indicator and three summary indicators by country for 1990, 2000, and 2015											
**Location**	**Indicator 2.2.1: Prevalence of stunting among children under 5 years of age**			**Indicator 2.2.2a: Prevalence of wasting among children under 5 years of age**			**Indicator 2.2.2b: Prevalence of overweight among children aged 2 to 4 years**			**Indicator 3.1.1: Maternal mortality ratio (maternal deaths per 100,000 live births)**		
	**1990**	**2000**	**2015**	**1990**	**2000**	**2015**	**1990**	**2000**	**2015**	**1990**	**2000**	**2015**
Syria	51.05 (40.09-61.08)	59.57 (53.74-64.86)	66.68 (56.74-75.99)	66.76 (51.78-78.44)	72.64 (64.94-80.46)	68.24 (49.52-81.53)	61.11 (46.86-73.01)	60.26 (46.31-71.65)	57.07 (42.02-69.49)	37.67 (32.65-42.95)	44.52 (39.36-49.54)	48.43 (43.61-52.97)
Tunisia	74.25 (67.08-80.34)	80.97 (76.58-84.96)	88.91 (84.71-92.32)	80.91 (72.19-87.99)	88.54 (83.85-92.63)	90.29 (84.72-94.38)	80.80 (74.74-85.93)	74.59 (64.58-82.76)	65.36 (51.92-76.04)	42.60 (37.64-47.34)	47.21 (42.67-52.07)	52.14 (46.61-58.03)
Turkey	64.24 (53.86-72.62)	74.63 (68.67-79.66)	86.35 (80.70-90.67)	86.29 (79.98-91.37)	92.62 (90.11-94.95)	95.73 (92.88-97.69)	81.75 (75.06-87.46)	77.73 (71.53-83.21)	65.34 (53.58-75.34)	40.14 (34.73-45.66)	49.76 (45.12-54.18)	63.57 (59.93-67.42)
United Arab Emirates	64.24 (52.79-74.33)	73.31 (64.22-80.97)	77.34 (68.26-85.08)	46.47 (20.39-66.24)	54.00 (31.36-71.85)	64.59 (40.49-80.86)	68.81 (57.43-78.21)	74.30 (66.67-81.51)	68.42 (56.75-79.49)	55.09 (49.10-61.03)	58.40 (53.88-63.18)	62.12 (55.26-68.93)
Yemen	25.09 (14.94-34.78)	17.47 (8.56-25.44)	32.20 (22.01-42.48)	48.69 (31.45-64.26)	51.94 (37.63-65.40)	46.02 (27.73-63.00)	79.90 (73.38-85.79)	81.71 (73.29-88.52)	77.18 (66.31-85.16)	22.63 (14.91-32.45)	22.75 (13.87-31.86)	26.39 (15.59-35.58)
Bangladesh	11.80 (4.96-18.76)	22.61 (17.24-27.99)	48.01 (42.74-53.25)	40.60 (23.82-57.64)	51.40 (38.35-64.59)	56.36 (42.55-69.29)	95.73 (92.39-98.23)	97.90 (96.03-99.39)	96.87 (94.39-99.00)	17.02 (11.22-23.82)	20.29 (14.39-26.78)	28.70 (22.35-35.16)
Bhutan	24.47 (14.05-34.74)	36.01 (29.89-42.07)	57.24 (49.77-64.42)	83.98 (76.60-89.90)	85.87 (80.59-90.46)	80.00 (71.77-86.80)	91.90 (87.20-95.83)	88.72 (82.74-93.65)	80.91 (71.69-87.71)	19.51 (12.22-27.63)	24.17 (17.37-31.59)	29.76 (22.00-38.02)
India	8.62 (3.29-14.05)	20.49 (15.68-25.56)	45.64 (41.75-49.42)	37.70 (25.89-52.57)	45.06 (34.62-58.41)	50.93 (40.94-62.74)	90.39 (86.52-93.58)	92.06 (88.93-94.69)	85.99 (81.19-90.30)	19.47 (14.15-25.31)	21.41 (16.02-27.38)	28.40 (23.25-34.00)
Nepal	1.46 (0.00-9.12)	16.03 (8.52-23.46)	45.37 (38.17-52.25)	68.81 (56.78-79.04)	66.28 (56.11-76.52)	64.35 (52.01-75.16)	97.47 (94.77-99.60)	97.75 (95.63-99.46)	95.65 (93.19-97.81)	18.38 (11.50-26.07)	24.05 (18.23-31.13)	25.18 (18.03-33.04)
Pakistan	25.21 (19.41-31.19)	39.76 (33.91-45.45)	37.51 (30.10-45.38)	42.94 (27.75-57.85)	53.31 (38.19-67.37)	61.33 (47.61-73.80)	81.61 (76.15-86.29)	83.11 (74.82-89.24)	84.63 (78.97-89.18)	22.46 (16.60-29.10)	19.17 (13.47-25.73)	24.01 (17.79-30.81)
Botswana	42.05 (32.82-51.07)	54.37 (48.15-60.24)	66.39 (59.40-73.01)	65.01 (40.18-81.75)	74.42 (62.38-83.60)	80.21 (62.75-90.33)	84.00 (72.62-91.96)	78.87 (64.78-88.74)	72.08 (55.28-83.55)	37.80 (19.99-52.95)	28.86 (2.30-45.59)	35.93 (9.30-50.34)

Appendix Table 1a. Scaled values for each SDG health-related indicator and three summary indicators by country for 1990, 2000, and 2015

Location	Indicator 2.2.1: Prevalence of stunting among children under 5 years of age			Indicator 2.2.2a: Prevalence of wasting among children under 5 years of age			Indicator 2.2.2b: Prevalence of overweight among children aged 2 to 4 years			Indicator 3.1.1: Maternal mortality ratio (maternal deaths per 100,000 live births)		
	**1990**	**2000**	**2015**	**1990**	**2000**	**2015**	**1990**	**2000**	**2015**	**1990**	**2000**	**2015**
Lesotho	18.73 (8.61-29.27)	25.50 (17.91-32.76)	51.44 (44.52-58.36)	79.04 (63.30-89.75)	78.62 (69.43-85.71)	88.37 (82.13-92.87)	81.17 (67.94-89.47)	77.18 (67.30-85.38)	77.54 (66.74-86.05)	31.77 (23.71-41.25)	26.32 (18.21-34.35)	20.84 (5.47-32.38)
Namibia	47.77 (40.30-55.37)	54.89 (48.60-60.78)	66.44 (60.85-71.54)	66.49 (47.47-79.52)	65.85 (50.70-78.27)	74.44 (59.21-85.19)	90.92 (85.48-95.04)	93.47 (89.85-96.49)	87.50 (81.63-92.17)	32.13 (25.65-39.35)	25.59 (17.84-34.19)	41.25 (27.92-50.93)
South Africa	55.84 (51.73-59.81)	56.30 (52.30-60.43)	69.58 (66.60-72.43)	69.41 (57.21-79.22)	71.12 (60.72-80.71)	84.24 (78.37-89.48)	51.69 (32.69-66.62)	56.62 (43.49-68.33)	50.60 (30.32-66.43)	34.72 (30.12-40.09)	34.94 (30.44-40.24)	34.45 (29.27-40.34)
Swaziland	45.68 (36.72-54.70)	45.81 (38.08-53.04)	59.13 (51.45-66.55)	95.72 (92.31-98.07)	91.77 (87.73-95.02)	95.77 (92.16-97.91)	62.62 (42.68-77.12)	66.17 (51.38-78.00)	73.37 (59.22-84.46)	37.94 (29.37-46.55)	36.43 (27.18-43.77)	34.14 (16.94-47.50)
Zimbabwe	55.60 (50.13-61.20)	51.23 (45.58-56.52)	59.43 (54.29-64.83)	87.94 (80.13-93.36)	74.93 (59.25-85.35)	88.73 (81.40-93.74)	82.49 (76.02-88.19)	74.70 (65.89-81.70)	86.70 (80.68-91.23)	30.63 (24.86-36.81)	22.49 (13.30-29.69)	27.05 (12.79-37.92)
Benin	36.16 (24.96-46.45)	38.87 (31.23-46.82)	35.51 (25.49-45.51)	59.26 (35.69-77.09)	64.57 (50.74-76.76)	59.62 (38.68-75.45)	93.04 (88.02-96.56)	86.48 (81.13-90.81)	50.90 (36.12-64.24)	18.67 (11.25-26.58)	19.96 (12.97-28.25)	22.74 (7.67-35.67)
Burkina Faso	37.70 (30.14-45.09)	34.17 (27.70-40.49)	53.27 (44.81-61.36)	42.04 (15.87-64.61)	34.35 (7.39-57.81)	50.30 (23.77-69.27)	92.24 (88.04-95.44)	85.47 (80.83-89.66)	77.83 (68.60-85.88)	22.47 (16.25-29.51)	21.25 (14.36-28.73)	24.92 (13.84-37.46)
Cameroon	44.98 (35.69-53.62)	45.16 (37.06-52.32)	57.64 (48.30-65.51)	81.49 (71.80-89.17)	77.47 (67.88-85.76)	80.48 (69.16-88.74)	77.91 (69.74-84.63)	71.57 (62.69-78.93)	68.27 (56.58-78.02)	20.65 (14.67-27.42)	17.86 (10.96-25.37)	20.91 (8.01-33.11)
Cape Verde	68.07 (61.31-74.38)	74.24 (68.11-79.52)	84.43 (79.53-88.08)	77.55 (65.64-86.15)	74.11 (58.29-84.67)	77.02 (62.24-87.14)	84.96 (76.12-90.98)	79.93 (69.88-87.95)	72.85 (60.20-82.55)	41.16 (36.23-46.07)	44.82 (37.49-50.56)	48.88 (43.43-53.64)
Chad	33.01 (21.75-43.93)	37.59 (29.82-45.16)	47.76 (38.00-56.79)	45.82 (18.82-67.61)	47.54 (28.25-64.32)	46.94 (21.45-67.25)	93.70 (89.74-96.95)	88.58 (83.43-92.66)	82.50 (72.92-89.70)	19.96 (12.48-27.49)	16.86 (9.35-25.27)	20.08 (4.53-34.80)
Cote d'Ivoire	57.54 (49.73-64.83)	49.44 (40.69-57.26)	55.78 (46.30-64.08)	68.19 (53.91-79.33)	73.50 (61.55-83.13)	72.45 (57.41-83.82)	82.48 (77.17-87.15)	83.06 (77.04-88.06)	82.53 (75.68-87.97)	19.77 (13.45-26.75)	15.93 (8.12-23.58)	20.78 (8.72-34.34)

	Appendix Table 1a. Scaled values for each SDG health-related indicator and three summary indicators by country for 1990, 2000, and 2015											
	**Indicator 2.2.1: Prevalence of stunting among children under 5 years of age**			**Indicator 2.2.2a: Prevalence of wasting among children under 5 years of age**			**Indicator 2.2.2b: Prevalence of overweight among children aged 2 to 4 years**			**Indicator 3.1.1: Maternal mortality ratio (maternal deaths per 100,000 live births)**		
**Location**	**1990**	**2000**	**2015**	**1990**	**2000**	**2015**	**1990**	**2000**	**2015**	**1990**	**2000**	**2015**
The Gambia	51.62 (42.55-60.47)	59.57 (52.94-65.70)	67.76 (61.81-73.32)	56.63 (31.29-75.85)	59.05 (41.46-73.61)	65.91 (51.06-78.71)	80.49 (71.12-87.77)	74.55 (63.31-83.10)	81.61 (72.21-88.65)	19.69 (6.26-33.15)	20.41 (13.56-28.09)	24.40 (14.02-34.03)
Ghana	45.17 (38.59-51.83)	50.11 (42.90-56.55)	72.76 (67.83-77.16)	68.95 (59.25-78.43)	67.43 (56.04-77.16)	82.02 (74.18-88.35)	94.57 (91.77-97.10)	88.19 (83.93-91.90)	88.25 (82.61-92.49)	20.11 (11.74-28.78)	19.53 (12.50-26.89)	27.70 (13.23-39.17)
Guinea	48.72 (39.35-57.28)	43.47 (37.34-50.00)	53.45 (45.34-61.46)	58.98 (38.60-74.28)	66.41 (55.51-76.28)	66.28 (51.50-79.34)	83.90 (75.81-90.28)	84.76 (79.04-89.33)	81.63 (72.22-89.13)	14.36 (7.27-21.97)	14.80 (8.07-22.14)	17.35 (6.75-29.68)
Guinea-Bissau	42.09 (31.09-52.77)	43.86 (35.82-51.53)	53.70 (44.87-62.62)	62.95 (40.86-78.92)	63.36 (47.87-76.08)	77.05 (63.12-86.78)	87.97 (80.39-93.38)	83.93 (75.87-90.26)	70.82 (58.65-81.04)	21.69 (5.20-38.90)	21.25 (3.18-39.94)	21.16 (0.00-38.44)
Liberia	46.92 (37.54-55.96)	37.58 (29.42-45.47)	54.03 (46.08-61.71)	68.26 (49.93-81.22)	71.51 (59.57-81.27)	77.48 (66.19-86.28)	72.09 (58.33-82.25)	80.22 (70.82-86.84)	79.27 (69.59-87.06)	13.91 (5.55-22.84)	15.46 (7.85-23.69)	17.21 (5.63-28.11)
Mali	46.02 (37.29-54.15)	38.94 (30.55-47.24)	49.08 (39.51-57.72)	51.26 (29.76-67.97)	49.90 (32.29-65.36)	54.19 (34.57-72.05)	94.88 (91.59-97.68)	87.75 (83.25-91.54)	74.93 (62.31-84.46)	16.80 (10.78-23.91)	18.67 (12.48-25.31)	24.90 (14.55-36.59)
Mauritania	18.35 (8.84-27.54)	42.07 (34.05-49.68)	66.31 (58.19-73.40)	48.59 (27.91-65.32)	55.46 (34.04-70.48)	65.47 (46.62-80.05)	88.91 (82.40-93.80)	86.92 (81.64-90.98)	84.33 (74.99-90.86)	13.13 (6.16-20.41)	16.92 (10.16-25.90)	22.22 (10.11-34.60)
Niger	31.40 (22.74-39.75)	23.64 (16.40-31.49)	36.26 (26.80-45.59)	29.12 (6.68-50.12)	41.45 (24.05-57.52)	41.95 (17.40-61.89)	94.64 (91.00-97.28)	94.62 (91.37-97.33)	90.16 (84.91-94.24)	21.18 (13.91-28.80)	20.18 (13.19-27.74)	23.90 (13.35-35.19)
Nigeria	23.59 (15.41-31.95)	29.14 (22.60-35.95)	52.83 (45.65-59.58)	51.23 (31.01-67.43)	54.81 (40.82-68.50)	57.71 (39.89-71.95)	87.88 (84.25-91.04)	78.66 (71.77-84.56)	73.22 (62.49-81.69)	20.65 (10.37-31.79)	20.09 (12.69-27.59)	27.77 (14.23-37.23)
Sao Tome and Principe	53.18 (44.27-61.62)	58.46 (51.26-64.85)	73.43 (66.18-79.29)	78.76 (66.36-87.44)	78.35 (68.41-86.14)	70.85 (53.29-84.19)	79.53 (69.55-87.48)	76.72 (67.37-84.49)	70.32 (58.66-79.80)	29.69 (23.02-36.91)	27.52 (21.61-33.59)	35.07 (23.34-46.68)
Senegal	51.85 (44.92-58.54)	58.30 (51.46-65.00)	70.62 (64.83-75.24)	75.80 (67.24-83.19)	67.20 (55.91-77.32)	74.66 (65.29-82.84)	91.42 (87.66-94.53)	92.38 (87.84-95.80)	92.47 (88.10-96.00)	20.13 (13.78-27.39)	19.67 (13.39-26.61)	22.08 (6.50-34.84)

Appendix Table 1a. Scaled values for each SDG health-related indicator and three summary indicators by country for 1990, 2000, and 2015

Location	Indicator 2.2.1: Prevalence of stunting among children under 5 years of age			Indicator 2.2.2a: Prevalence of wasting among children under 5 years of age			Indicator 2.2.2b: Prevalence of overweight among children aged 2 to 4 years			Indicator 3.1.1: Maternal mortality ratio (maternal deaths per 100,000 live births)		
	1990	2000	2015	1990	2000	2015	1990	2000	2015	1990	2000	2015
Sierra Leone	40.43 (33.78-47.03)	37.88 (30.54-45.00)	45.69 (37.91-53.00)	69.03 (61.04-77.26)	57.13 (44.44-69.66)	66.83 (54.70-77.27)	85.67 (77.49-91.85)	81.23 (72.80-88.32)	67.82 (55.43-78.38)	24.09 (12.20-35.22)	17.39 (10.53-24.92)	15.25 (5.18-26.79)
Togo	43.10 (36.48-48.86)	50.60 (44.25-56.54)	63.62 (57.74-68.70)	76.76 (63.99-86.26)	62.36 (44.18-76.95)	77.05 (64.78-86.36)	95.49 (92.68-98.03)	90.79 (85.99-94.51)	88.26 (80.78-93.68)	20.84 (14.22-27.72)	20.06 (12.87-28.34)	26.86 (15.89-37.83)
Burundi	20.88 (10.31-31.32)	13.85 (6.67-20.69)	20.95 (7.22-34.46)	76.58 (63.63-85.86)	72.94 (64.56-80.66)	77.75 (61.10-88.86)	94.76 (90.29-98.12)	92.46 (85.60-97.18)	91.80 (85.69-96.57)	9.03 (0.00-20.87)	9.45 (2.28-17.31)	23.67 (10.55-35.59)
Comoros	37.73 (23.79-52.49)	24.17 (17.29-30.69)	52.98 (42.92-62.35)	84.87 (79.01-89.89)	52.40 (39.23-65.58)	56.98 (31.96-74.64)	71.79 (57.68-83.79)	59.65 (44.01-72.54)	61.09 (39.77-76.87)	17.99 (6.37-30.44)	20.06 (13.54-26.72)	31.29 (20.16-41.99)
Djibouti	58.49 (52.94-63.45)	56.35 (50.55-61.76)	60.44 (46.71-71.83)	58.61 (46.44-70.20)	40.64 (22.46-58.38)	44.50 (0.08-74.61)	86.52 (76.53-93.88)	77.32 (62.68-87.55)	53.21 (33.01-70.73)	24.07 (12.93-35.36)	20.59 (6.37-33.60)	21.61 (5.59-34.65)
Eritrea	7.76 (0.00-19.12)	36.42 (28.90-43.29)	38.27 (21.99-53.62)	60.88 (39.73-76.44)	45.66 (28.11-62.69)	58.97 (20.84-82.76)	95.17 (90.28-98.68)	95.80 (92.50-98.39)	99.25 (96.41-100.00)	12.65 (5.30-20.16)	16.60 (5.70-28.56)	15.60 (0.00-31.85)
Ethiopia	15.04 (0.00-30.83)	17.07 (10.95-23.70)	40.96 (29.46-52.25)	54.63 (19.45-79.02)	56.30 (44.88-68.79)	63.98 (42.00-80.20)	95.39 (90.32-99.00)	94.18 (90.05-97.34)	87.55 (78.63-94.16)	12.98 (6.22-20.50)	13.81 (6.93-20.59)	23.03 (10.86-34.77)
Kenya	39.01 (34.81-43.09)	43.44 (39.90-46.99)	62.63 (60.14-65.21)	73.86 (68.05-80.39)	72.41 (66.90-79.09)	86.36 (83.47-89.65)	79.57 (69.62-87.90)	81.85 (74.53-87.87)	84.22 (76.61-89.83)	23.29 (17.61-29.67)	19.09 (13.00-25.74)	24.52 (18.63-30.97)
Madagascar	16.68 (6.26-26.93)	20.00 (12.59-27.26)	32.93 (17.54-48.36)	53.06 (25.83-72.08)	55.13 (35.11-70.91)	39.31 (0.00-72.42)	90.19 (84.44-94.67)	91.02 (86.63-94.65)	87.00 (77.56-94.14)	22.96 (17.40-29.46)	21.78 (14.56-34.93)	26.01 (12.65-39.98)
Malawi	18.37 (8.88-27.60)	19.20 (12.24-25.92)	38.76 (30.07-47.08)	78.16 (64.68-87.73)	73.28 (61.74-82.66)	82.19 (72.74-89.57)	61.60 (48.36-72.95)	60.98 (50.73-70.52)	76.71 (67.49-84.31)	19.04 (13.10-26.08)	17.26 (9.09-24.69)	31.26 (19.80-42.63)
Mozambique	14.22 (0.18-27.74)	28.19 (20.85-35.63)	43.92 (31.39-56.32)	66.49 (41.31-83.41)	75.24 (66.20-83.43)	79.02 (64.46-89.16)	82.31 (70.98-90.78)	75.68 (66.97-83.12)	70.57 (57.39-80.87)	24.21 (17.62-31.72)	24.39 (18.02-32.82)	26.74 (11.34-39.69)

Appendix Table 1a. Scaled values for each SDG health-related indicator and three summary indicators by country for 1990, 2000, and 2015

Location	Indicator 2.2.1: Prevalence of stunting among children under 5 years of age			Indicator 2.2.2a: Prevalence of wasting among children under 5 years of age			Indicator 2.2.2b: Prevalence of overweight among children aged 2 to 4 years			Indicator 3.1.1: Maternal mortality ratio (maternal deaths per 100,000 live births)		
	1990	2000	2015	1990	2000	2015	1990	2000	2015	1990	2000	2015
Rwanda	19.77 (9.40-30.22)	28.79 (21.83-35.32)	44.58 (36.47-52.78)	71.89 (53.28-85.42)	72.50 (60.03-82.75)	90.60 (84.46-94.81)	77.65 (69.69-84.48)	75.35 (68.24-81.90)	62.29 (47.26-74.84)	15.42 (7.95-23.13)	13.41 (6.58-20.68)	24.40 (12.49-36.08)
Somalia	56.97 (41.97-69.46)	54.34 (48.97-59.29)	43.84 (28.75-57.85)	56.39 (19.73-79.56)	13.36 (0.00-36.78)	54.81 (23.48-78.70)	83.38 (72.07-91.82)	88.73 (80.35-95.21)	86.01 (74.91-93.67)	15.16 (0.00-36.01)	15.68 (0.01-36.85)	17.06 (0.36-36.02)
South Sudan	31.18 (13.20-47.13)	31.13 (20.88-41.53)	53.95 (42.25-64.46)	42.37 (0.00-73.36)	32.46 (8.72-52.47)	29.91 (0.00-62.45)	72.90 (56.87-85.40)	73.61 (59.61-84.89)	72.29 (55.65-84.37)	19.91 (2.95-38.52)	20.65 (2.58-39.52)	19.73 (0.00-37.20)
Tanzania	27.22 (19.21-34.66)	29.91 (23.57-36.73)	44.87 (31.16-56.99)	70.03 (52.89-82.52)	80.59 (71.41-87.71)	81.10 (65.90-90.81)	78.20 (71.91-83.76)	82.60 (77.71-87.10)	81.58 (72.26-89.43)	18.15 (12.00-25.13)	19.42 (12.70-26.42)	26.42 (10.66-39.13)
Uganda	31.88 (25.00-38.71)	34.79 (28.68-40.64)	55.01 (44.49-64.24)	86.14 (77.52-92.41)	73.27 (57.64-83.96)	82.19 (68.57-91.54)	78.32 (71.58-83.95)	77.64 (71.94-82.76)	82.08 (72.70-89.06)	24.49 (18.23-30.93)	21.98 (15.44-28.49)	28.30 (15.02-40.80)
Zambia	26.74 (14.83-38.24)	20.71 (13.10-28.12)	40.86 (29.44-51.41)	83.47 (71.68-90.93)	82.30 (75.14-88.50)	79.53 (66.33-88.74)	70.39 (58.27-80.22)	59.19 (48.88-68.58)	44.75 (24.28-62.44)	23.88 (17.84-30.14)	21.59 (14.38-28.64)	29.80 (17.28-41.25)
Angola	18.21 (6.45-30.07)	27.48 (17.84-37.12)	61.88 (52.52-70.23)	74.44 (59.54-84.99)	70.44 (57.91-80.37)	80.08 (67.63-87.95)	86.34 (78.46-92.54)	80.23 (69.89-88.09)	56.10 (37.37-71.25)	17.34 (2.08-36.75)	18.71 (2.01-39.18)	22.53 (2.15-40.14)
Central African Republic	34.75 (22.89-44.93)	35.43 (24.79-44.77)	45.27 (34.54-56.17)	69.55 (55.86-80.59)	64.53 (49.91-76.12)	70.52 (55.99-82.11)	76.67 (66.43-84.86)	77.50 (67.44-85.84)	83.60 (73.96-91.07)	11.28 (4.03-18.68)	11.04 (0.00-25.81)	11.66 (0.00-30.18)
Congo	43.27 (31.46-53.83)	50.76 (40.20-60.03)	65.82 (57.85-72.80)	69.77 (54.61-82.01)	72.30 (59.11-82.79)	78.96 (68.55-86.74)	77.33 (64.70-86.46)	78.48 (68.08-86.38)	83.88 (75.18-90.22)	21.34 (12.23-29.82)	16.02 (8.03-23.71)	20.86 (7.47-33.84)
Democratic Republic of the Congo	28.90 (17.53-39.58)	30.02 (19.81-39.31)	42.83 (32.71-51.58)	59.74 (40.73-74.85)	54.53 (37.04-69.60)	70.66 (57.26-81.44)	58.73 (39.28-73.44)	70.23 (57.47-80.77)	84.07 (74.77-90.72)	23.60 (14.86-33.89)	25.24 (18.83-32.05)	20.63 (10.26-32.12)
Equatorial Guinea	18.93 (7.41-30.43)	41.75 (32.46-50.16)	74.21 (67.67-80.11)	77.91 (66.36-86.92)	80.49 (72.72-87.14)	88.40 (81.35-93.31)	69.17 (54.53-81.00)	59.87 (43.46-72.67)	30.21 (8.77-48.78)	13.34 (0.00-33.68)	18.79 (2.03-38.15)	25.76 (4.48-40.35)

	Appendix Table 1a. Scaled values for each SDG health-related indicator and three summary indicators by country for 1990, 2000, and 2015											
	**Indicator 2.2.1: Prevalence of stunting among children under 5 years of age**			**Indicator 2.2.2a: Prevalence of wasting among children under 5 years of age**			**Indicator 2.2.2b: Prevalence of overweight among children aged 2 to 4 years**			**Indicator 3.1.1: Maternal mortality ratio (maternal deaths per 100,000 live births)**		
**Location**	**1990**	**2000**	**2015**	**1990**	**2000**	**2015**	**1990**	**2000**	**2015**	**1990**	**2000**	**2015**
Gabon	55.94 (44.85-65.48)	62.23 (54.11-69.81)	74.49 (67.34-80.09)	82.55 (73.01-89.51)	84.44 (77.53-90.17)	86.85 (80.52-92.16)	80.91 (71.12-88.79)	78.08 (69.59-85.01)	71.13 (59.89-80.46)	25.67 (19.15-32.29)	22.03 (14.72-28.91)	32.83 (21.32-42.72)

Location	Appendix Table 1a. Scaled values for each SDG health-related indicator and three summary indicators by country for 1990, 2000, and 2015											
	**Indicator 3.1.2: Proportion of births attended by skilled health personnel (doctors, nurses, midwives, or country-specific medical staff [e.g., clinical officers])**			**Indicator 3.2.1: Under-5 mortality rate (probability of dying before the age of 5 per 1,000 live births)**			**Indicator 3.2.2: Neonatal mortality rate (probability of dying during the first 28 days of life per 1,000 live births)**			**Indicator 3.3.1: Age-standardised rate of new HIV infections (per 1,000 population)**		
	**1990**	**2000**	**2015**	**1990**	**2000**	**2015**	**1990**	**2000**	**2015**	**1990**	**2000**	**2015**
Canada	98.52 (97.89-99.02)	99.05 (98.65-99.40)	99.38 (99.06-99.64)	73.11 (70.16-76.55)	78.89 (75.99-82.04)	82.52 (79.61-84.99)	73.68 (71.03-76.38)	79.77 (77.29-82.33)	83.46 (81.16-85.11)	41.49 (37.51-46.03)	50.13 (45.38-54.52)	53.85 (44.82-64.93)
United States	98.49 (97.92-98.99)	98.42 (97.87-98.91)	99.22 (98.83-99.53)	67.61 (64.62-71.09)	73.69 (70.75-77.03)	79.35 (76.48-82.20)	68.11 (65.43-70.83)	74.39 (71.77-77.02)	80.29 (78.04-82.33)	36.39 (34.60-38.20)	42.67 (40.71-44.73)	45.59 (42.89-48.34)
Australia	99.01 (98.70-99.27)	99.19 (98.93-99.42)	99.54 (99.36-99.71)	70.70 (67.70-74.15)	78.57 (75.62-81.68)	86.88 (84.00-89.30)	71.76 (69.07-74.62)	79.68 (77.09-82.33)	88.50 (86.43-90.30)	49.02 (44.01-53.31)	55.27 (48.32-62.07)	59.74 (52.24-66.78)
New Zealand	98.91 (98.60-99.16)	99.19 (98.95-99.41)	99.57 (99.40-99.73)	68.06 (65.18-71.36)	75.42 (72.70-78.50)	81.69 (78.78-84.17)	73.71 (71.14-76.35)	80.22 (78.06-82.60)	86.44 (84.30-88.08)	53.98 (50.31-57.25)	55.28 (48.82-61.63)	61.10 (53.14-68.96)
Brunei	99.18 (99.04-99.30)	99.19 (99.05-99.30)	99.29 (99.16-99.42)	69.63 (67.29-72.96)	72.21 (69.98-74.84)	72.15 (68.75-75.02)	77.09 (74.23-80.14)	78.58 (76.10-81.18)	77.55 (74.46-79.89)	53.39 (43.93-68.09)	51.25 (39.03-59.57)	54.10 (46.93-62.94)
Japan	99.12 (98.99-99.25)	99.18 (99.05-99.32)	99.28 (99.15-99.41)	78.96 (76.04-82.12)	84.14 (81.13-87.12)	93.35 (90.26-95.32)	83.79 (81.14-86.47)	88.92 (86.46-91.43)	97.50 (95.44-98.87)	69.00 (67.31-70.60)	69.94 (68.27-71.61)	69.56 (67.66-71.45)
Singapore	98.75 (98.59-98.91)	99.17 (99.03-99.30)	99.42 (99.30-99.54)	74.86 (71.89-78.01)	88.09 (85.21-90.86)	97.32 (93.94-99.09)	76.95 (74.17-79.84)	90.82 (88.43-93.20)	98.17 (96.04-99.38)	46.09 (41.28-50.32)	47.31 (45.31-49.25)	54.14 (50.21-57.53)
South Korea	98.68 (98.51-98.84)	99.16 (99.03-99.30)	99.42 (99.29-99.53)	66.40 (61.74-70.42)	76.16 (73.10-79.36)	90.49 (86.30-94.52)	70.91 (63.63-75.83)	80.38 (77.69-83.02)	94.92 (91.38-98.52)	52.79 (50.45-54.85)	52.92 (45.52-57.92)	60.34 (57.36-63.03)
Andorra	99.54 (99.39-99.67)	99.61 (99.48-99.75)	99.69 (99.57-99.82)	79.11 (75.87-82.39)	92.21 (89.22-95.27)	99.54 (96.50-100.00)	80.00 (76.48-83.50)	93.64 (90.46-96.38)	99.16 (96.14-100.00)	44.30 (18.14-58.62)	49.03 (34.15-58.64)	51.83 (38.00-67.56)
Austria	99.14 (98.95-99.32)	99.36 (99.20-99.52)	99.57 (99.43-99.71)	70.61 (67.60-74.03)	80.40 (77.23-83.53)	88.89 (85.38-90.80)	70.98 (68.14-73.62)	81.50 (78.67-84.27)	90.06 (87.56-91.45)	46.62 (37.46-65.05)	48.00 (39.79-57.02)	52.98 (44.53-61.99)
Belgium	99.10 (98.90-99.29)	99.31 (99.13-99.47)	99.49 (99.34-99.64)	69.91 (67.00-73.22)	79.20 (76.30-82.30)	87.50 (84.31-89.59)	71.46 (68.74-73.97)	80.76 (78.25-83.20)	89.42 (86.99-90.74)	45.08 (39.28-50.13)	50.28 (42.70-57.09)	56.57 (49.72-64.17)

Location	Appendix Table 1a. Scaled values for each SDG health-related indicator and three summary indicators by country for 1990, 2000, and 2015											
	**Indicator 3.1.2: Proportion of births attended by skilled health personnel (doctors, nurses, midwives, or country-specific medical staff [e.g., clinical officers])**			**Indicator 3.2.1: Under-5 mortality rate (probability of dying before the age of 5 per 1,000 live births)**			**Indicator 3.2.2: Neonatal mortality rate (probability of dying during the first 28 days of life per 1,000 live births)**			**Indicator 3.3.1: Age-standardised rate of new HIV infections (per 1,000 population)**		
	**1990**	**2000**	**2015**	**1990**	**2000**	**2015**	**1990**	**2000**	**2015**	**1990**	**2000**	**2015**
Cyprus	98.89 (98.66-99.10)	99.19 (99.00-99.37)	99.39 (99.22-99.54)	64.73 (61.89-68.40)	75.68 (72.71-78.85)	83.48 (81.27-85.25)	62.85 (60.04-65.73)	74.96 (72.18-77.62)	83.30 (80.17-85.32)	59.69 (50.37-68.22)	56.76 (47.63-66.05)	61.75 (51.72-81.76)
Denmark	99.25 (99.06-99.42)	99.44 (99.26-99.59)	99.55 (99.40-99.68)	71.57 (68.44-75.14)	80.79 (77.85-83.74)	88.81 (85.04-90.81)	70.93 (68.06-73.87)	80.67 (78.22-83.36)	89.09 (85.98-90.80)	43.92 (38.90-48.86)	47.55 (41.23-55.59)	56.43 (48.31-66.71)
Finland	99.17 (98.97-99.35)	99.36 (99.20-99.51)	99.58 (99.44-99.71)	76.63 (73.56-80.05)	85.83 (82.58-89.01)	95.71 (90.79-98.32)	76.01 (73.04-79.22)	86.07 (83.31-88.92)	95.13 (91.62-97.04)	61.14 (55.51-66.35)	60.92 (53.71-68.28)	69.12 (60.02-79.55)
France	99.09 (98.89-99.29)	99.30 (99.13-99.47)	99.53 (99.38-99.67)	72.15 (69.15-75.50)	81.02 (77.98-84.06)	90.23 (85.39-93.02)	74.83 (72.14-77.62)	83.86 (81.31-86.44)	93.04 (88.92-95.39)	36.81 (34.22-39.15)	46.04 (41.44-49.90)	56.64 (50.86-61.59)
Germany	99.07 (98.87-99.28)	99.31 (99.14-99.48)	99.50 (99.35-99.65)	71.44 (68.49-74.81)	81.55 (78.51-84.54)	88.86 (86.38-90.29)	73.01 (70.30-75.65)	83.37 (80.94-85.80)	90.79 (87.39-92.62)	51.04 (46.49-54.98)	58.05 (51.83-63.63)	57.09 (49.65-65.10)
Greece	98.77 (98.52-98.99)	99.04 (98.82-99.22)	99.28 (99.09-99.44)	69.07 (66.07-72.61)	78.74 (75.81-81.69)	90.03 (86.98-92.11)	67.68 (64.99-70.31)	77.63 (75.12-80.09)	89.46 (87.22-90.78)	55.06 (53.19-56.86)	62.71 (60.97-64.30)	63.89 (60.71-66.50)
Iceland	99.16 (98.95-99.34)	99.32 (99.15-99.48)	99.50 (99.35-99.64)	77.73 (75.14-80.31)	86.76 (84.96-88.48)	98.54 (94.63-100.00)	79.95 (77.64-82.42)	89.56 (87.43-91.31)	99.49 (97.07-100.00)	49.82 (43.13-55.64)	53.97 (47.95-59.34)	57.20 (48.95-68.04)
Ireland	98.61 (98.31-98.88)	99.15 (98.96-99.34)	99.47 (99.32-99.60)	70.86 (67.96-74.60)	76.83 (73.80-79.84)	88.96 (85.64-90.94)	71.53 (68.78-74.16)	77.23 (74.55-79.92)	90.28 (87.84-91.63)	45.58 (40.95-49.38)	53.19 (47.10-58.26)	57.34 (51.51-62.54)
Israel	99.08 (98.89-99.27)	99.31 (99.13-99.47)	99.46 (99.31-99.60)	66.23 (63.34-69.72)	75.68 (72.72-78.94)	87.37 (84.22-89.47)	67.94 (65.13-70.85)	77.65 (75.06-80.33)	90.03 (87.73-91.39)	48.90 (42.76-54.69)	53.11 (46.31-59.83)	55.50 (48.96-62.97)
Italy	99.09 (98.88-99.27)	99.27 (99.08-99.43)	99.39 (99.22-99.53)	70.86 (67.89-74.14)	80.97 (77.97-84.09)	91.05 (86.70-93.34)	68.28 (65.62-71.01)	79.28 (76.69-81.90)	89.67 (86.19-91.64)	36.88 (28.50-44.80)	47.99 (41.07-54.73)	53.63 (46.23-61.05)
Luxembourg	98.87 (98.63-99.09)	99.26 (99.08-99.44)	99.55 (99.41-99.69)	71.68 (68.88-74.78)	83.67 (81.49-85.95)	94.36 (90.70-96.00)	74.92 (72.50-77.39)	87.16 (85.16-89.19)	97.26 (94.76-98.34)	47.50 (40.62-53.73)	55.23 (48.01-62.23)	56.23 (48.60-65.01)

Location	Appendix Table 1a. Scaled values for each SDG health-related indicator and three summary indicators by country for 1990, 2000, and 2015											
	**Indicator 3.1.2: Proportion of births attended by skilled health personnel (doctors, nurses, midwives, or country-specific medical staff [e.g., clinical officers])**			**Indicator 3.2.1: Under-5 mortality rate (probability of dying before the age of 5 per 1,000 live births)**			**Indicator 3.2.2: Neonatal mortality rate (probability of dying during the first 28 days of life per 1,000 live births)**			**Indicator 3.3.1: Age-standardised rate of new HIV infections (per 1,000 population)**		
	**1990**	**2000**	**2015**	**1990**	**2000**	**2015**	**1990**	**2000**	**2015**	**1990**	**2000**	**2015**
Malta	98.05 (97.69-98.39)	98.62 (98.35-98.87)	99.09 (98.89-99.28)	70.62 (67.79-73.79)	76.23 (74.10-78.85)	78.63 (75.13-81.12)	68.22 (65.72-70.74)	73.93 (71.49-76.17)	76.14 (73.13-78.05)	48.66 (42.50-53.63)	53.73 (48.53-57.97)	53.50 (47.28-60.32)
Netherlands	99.02 (98.82-99.22)	99.29 (99.12-99.46)	99.52 (99.37-99.66)	72.49 (69.47-75.89)	77.98 (75.01-81.28)	87.06 (83.63-89.07)	72.33 (69.65-75.11)	77.86 (75.19-80.39)	87.48 (84.80-89.02)	40.29 (35.25-44.52)	51.11 (45.52-55.64)	59.87 (52.89-66.03)
Norway	99.36 (99.21-99.52)	99.56 (99.42-99.70)	99.70 (99.57-99.83)	72.02 (69.05-75.35)	82.85 (79.79-85.84)	93.51 (90.04-95.27)	73.09 (70.26-76.07)	84.39 (81.81-87.07)	94.90 (92.58-96.12)	48.45 (41.59-54.76)	56.11 (48.95-63.36)	63.70 (55.90-72.10)
Portugal	98.63 (98.36-98.87)	99.06 (98.85-99.25)	99.38 (99.23-99.53)	63.34 (60.51-66.94)	76.11 (73.36-79.13)	91.89 (88.98-94.09)	64.16 (61.35-66.97)	78.16 (75.76-80.62)	94.27 (92.30-95.70)	29.98 (24.99-35.30)	28.53 (22.98-33.96)	36.63 (30.09-44.43)
Spain	98.59 (98.32-98.84)	98.98 (98.75-99.20)	99.28 (99.09-99.44)	71.08 (68.11-74.35)	81.08 (78.06-84.10)	91.74 (88.26-93.54)	71.87 (69.23-74.49)	82.09 (79.55-84.59)	92.67 (90.27-93.86)	31.21 (26.84-35.29)	44.57 (40.54-47.96)	48.92 (42.78-54.62)
Sweden	99.24 (99.05-99.41)	99.39 (99.22-99.56)	99.57 (99.43-99.71)	76.14 (73.07-79.44)	87.22 (84.29-90.19)	94.24 (90.65-95.88)	75.60 (72.89-78.42)	87.75 (85.15-90.25)	94.10 (91.69-95.25)	49.71 (45.31-53.71)	57.83 (52.65-62.90)	64.85 (59.19-71.69)
Switzerland	99.27 (99.10-99.44)	99.41 (99.26-99.56)	99.57 (99.43-99.71)	72.50 (69.47-75.99)	79.76 (76.85-82.77)	86.47 (82.76-88.59)	72.48 (69.76-75.11)	80.05 (77.41-82.61)	86.99 (83.72-88.88)	40.13 (34.98-46.24)	55.77 (49.26-62.15)	56.36 (48.46-66.96)
United Kingdom	98.99 (98.76-99.19)	99.25 (99.07-99.43)	99.47 (99.33-99.63)	70.87 (68.03-74.20)	77.96 (75.11-81.04)	83.78 (80.82-86.09)	71.84 (69.12-74.53)	78.79 (76.41-81.13)	84.80 (82.54-86.29)	53.06 (51.10-54.96)	49.46 (47.05-51.89)	51.33 (49.24-53.39)
Argentina	97.58 (96.99-98.09)	98.58 (98.26-98.87)	98.87 (98.61-99.12)	50.02 (47.41-54.50)	56.97 (54.20-61.10)	65.66 (62.89-68.99)	46.88 (44.21-49.57)	54.53 (51.83-57.30)	66.13 (63.76-68.23)	32.79 (31.29-34.32)	31.54 (30.19-32.98)	33.21 (31.23-35.74)
Chile	98.90 (98.58-99.18)	99.17 (98.92-99.40)	99.34 (99.14-99.54)	57.93 (55.09-61.93)	68.65 (65.78-72.13)	75.02 (72.19-77.66)	57.30 (54.67-59.98)	71.67 (69.27-74.18)	77.15 (74.97-78.90)	38.77 (33.03-41.61)	36.54 (34.22-38.42)	41.06 (39.11-42.85)
Uruguay	99.03 (98.74-99.30)	99.19 (98.94-99.42)	99.31 (99.09-99.53)	54.07 (51.30-58.40)	61.18 (58.31-65.22)	70.92 (65.80-74.93)	52.73 (49.84-55.53)	61.42 (58.57-64.34)	73.10 (68.10-76.42)	42.86 (30.81-48.75)	37.43 (32.94-40.77)	40.01 (36.25-43.92)

Appendix Table 1a. Scaled values for each SDG health-related indicator and three summary indicators by country for 1990, 2000, and 2015

Location	Indicator 3.1.2: Proportion of births attended by skilled health personnel (doctors, nurses, midwives, or country-specific medical staff [e.g., clinical officers])			Indicator 3.2.1: Under-5 mortality rate (probability of dying before the age of 5 per 1,000 live births)			Indicator 3.2.2: Neonatal mortality rate (probability of dying during the first 28 days of life per 1,000 live births)			Indicator 3.3.1: Age-standardised rate of new HIV infections (per 1,000 population)		
	1990	2000	2015	1990	2000	2015	1990	2000	2015	1990	2000	2015
Belarus	99.24 (99.08-99.40)	99.09 (98.91-99.26)	99.48 (99.34-99.61)	56.21 (52.62-60.73)	61.81 (58.14-66.14)	80.42 (75.98-83.04)	55.54 (50.96-60.51)	62.29 (57.27-67.53)	82.77 (77.14-86.77)	55.92 (46.97-63.53)	40.71 (35.64-44.95)	39.67 (35.42-43.68)
Estonia	99.20 (99.03-99.35)	99.20 (99.05-99.36)	99.54 (99.41-99.67)	59.41 (56.30-63.37)	67.06 (63.79-70.95)	90.19 (85.53-95.28)	59.91 (56.70-63.44)	70.15 (66.75-73.58)	93.74 (89.07-98.17)	60.54 (58.28-62.57)	41.99 (38.35-45.10)	41.11 (37.64-44.20)
Latvia	99.28 (99.12-99.43)	99.17 (99.00-99.32)	99.54 (99.41-99.68)	58.86 (55.99-63.03)	64.68 (61.21-68.47)	82.27 (76.38-88.75)	58.67 (55.57-62.00)	66.46 (62.89-69.93)	85.08 (78.49-91.90)	44.87 (39.94-51.01)	37.46 (33.83-40.01)	41.94 (37.36-47.08)
Lithuania	99.26 (99.10-99.41)	99.17 (99.00-99.33)	99.54 (99.40-99.67)	63.58 (60.63-67.45)	67.97 (64.95-71.55)	86.37 (83.88-87.90)	66.52 (63.37-70.02)	71.91 (69.09-75.13)	90.58 (86.99-92.67)	53.09 (48.66-63.07)	46.62 (42.98-49.22)	51.15 (46.27-57.47)
Moldova	99.35 (99.20-99.49)	99.09 (98.91-99.25)	99.44 (99.29-99.58)	48.83 (44.54-54.31)	47.76 (43.44-53.38)	69.08 (61.61-76.29)	47.35 (43.02-52.28)	45.43 (40.85-50.21)	71.21 (62.25-78.79)	47.09 (36.61-54.15)	39.52 (36.10-42.36)	40.44 (36.21-44.16)
Russia	99.30 (99.14-99.44)	99.17 (99.01-99.34)	99.49 (99.36-99.62)	55.81 (53.04-59.88)	57.11 (54.31-61.09)	72.43 (69.57-75.65)	54.97 (52.08-57.93)	56.46 (53.65-59.40)	74.88 (72.33-77.40)	44.83 (39.95-53.40)	33.70 (30.08-36.55)	31.57 (27.18-35.25)
Ukraine	99.39 (99.24-99.53)	99.15 (98.97-99.30)	99.39 (99.23-99.53)	55.47 (51.03-60.86)	55.52 (50.59-60.93)	71.13 (63.49-79.25)	54.66 (49.62-60.28)	53.76 (47.97-59.77)	73.32 (63.89-82.50)	41.22 (36.33-44.37)	28.24 (25.11-30.54)	31.07 (29.01-33.05)
Albania	98.24 (97.93-98.52)	98.30 (97.99-98.56)	99.08 (98.89-99.26)	42.50 (38.96-47.72)	51.17 (47.19-55.95)	66.59 (60.04-72.06)	59.53 (56.25-62.85)	64.75 (61.08-68.63)	80.67 (72.93-85.39)	75.11 (69.80-80.34)	72.17 (68.22-75.37)	72.37 (68.46-76.86)
Bosnia and Herzegovina	97.96 (97.62-98.30)	99.06 (98.86-99.23)	99.37 (99.20-99.53)	58.52 (55.69-62.51)	68.42 (65.38-72.23)	81.34 (77.61-84.98)	57.24 (54.28-60.28)	70.07 (67.30-72.83)	82.78 (79.26-86.45)	77.02 (66.93-90.42)	72.25 (68.81-74.72)	73.68 (69.99-77.43)
Bulgaria	99.21 (99.05-99.39)	99.14 (98.95-99.32)	99.43 (99.26-99.57)	58.59 (55.73-62.62)	59.16 (56.23-63.14)	72.75 (66.49-77.68)	59.64 (56.66-62.52)	59.91 (56.89-62.93)	76.10 (70.27-80.94)	63.14 (54.83-85.39)	57.04 (52.00-60.94)	58.53 (51.83-65.77)
Croatia	99.24 (99.05-99.40)	99.18 (99.00-99.34)	99.37 (99.20-99.51)	66.43 (63.54-70.17)	72.70 (69.66-76.22)	84.95 (81.45-88.40)	64.14 (61.17-67.16)	70.81 (68.03-73.66)	83.92 (80.53-87.29)	60.34 (57.31-62.87)	61.01 (59.03-62.82)	68.53 (65.32-73.24)

	Appendix Table 1a. Scaled values for each SDG health-related indicator and three summary indicators by country for 1990, 2000, and 2015											
Location	Indicator 3.1.2: Proportion of births attended by skilled health personnel (doctors, nurses, midwives, or country-specific medical staff [e.g., clinical officers])			Indicator 3.2.1: Under-5 mortality rate (probability of dying before the age of 5 per 1,000 live births)			Indicator 3.2.2: Neonatal mortality rate (probability of dying during the first 28 days of life per 1,000 live births)			Indicator 3.3.1: Age-standardised rate of new HIV infections (per 1,000 population)		
	**1990**	**2000**	**2015**	**1990**	**2000**	**2015**	**1990**	**2000**	**2015**	**1990**	**2000**	**2015**
Czech Republic	99.21 (99.03-99.39)	99.21 (99.04-99.38)	99.37 (99.22-99.52)	66.61 (63.75-70.33)	81.43 (78.36-84.58)	94.98 (91.08-96.60)	67.12 (64.26-69.99)	82.74 (79.90-85.41)	96.08 (93.45-97.23)	67.53 (65.02-69.93)	68.48 (65.72-71.09)	69.18 (65.39-74.17)
Hungary	99.19 (99.02-99.36)	99.19 (99.01-99.37)	99.37 (99.20-99.52)	59.98 (57.03-63.88)	69.20 (66.28-72.71)	83.12 (76.77-87.64)	54.79 (51.77-57.72)	67.34 (64.51-70.22)	82.06 (75.66-86.97)	53.09 (51.57-54.60)	56.72 (55.14-58.28)	65.82 (63.18-68.62)
Macedonia	95.16 (94.47-95.83)	97.96 (97.63-98.30)	98.96 (98.75-99.16)	45.98 (43.13-50.85)	61.83 (58.73-65.75)	71.13 (63.15-78.50)	39.03 (36.32-41.74)	58.54 (55.32-61.76)	69.75 (60.68-78.00)	76.60 (71.75-82.00)	72.75 (68.11-76.75)	73.25 (66.58-81.89)
Montenegro	99.21 (99.03-99.38)	99.07 (98.90-99.26)	99.35 (99.19-99.51)	57.90 (51.87-64.57)	56.75 (53.32-61.38)	82.20 (75.74-88.08)	54.82 (47.52-63.31)	52.60 (48.87-56.53)	82.86 (75.99-89.23)	75.33 (66.65-94.69)	71.36 (64.40-78.67)	70.94 (62.12-85.67)
Poland	99.14 (98.96-99.32)	99.23 (99.06-99.39)	99.49 (99.35-99.63)	59.06 (56.24-62.98)	71.16 (68.18-74.53)	84.80 (78.32-89.34)	53.94 (51.20-56.70)	69.71 (67.04-72.42)	84.24 (77.91-88.98)	58.03 (55.18-60.63)	58.97 (55.89-61.98)	60.87 (57.23-66.40)
Romania	99.11 (98.92-99.30)	98.82 (98.59-99.04)	99.14 (98.94-99.32)	46.94 (44.39-51.73)	53.99 (51.16-58.19)	73.49 (68.13-77.50)	49.52 (46.78-52.24)	55.96 (53.26-58.71)	78.83 (74.27-82.40)	55.70 (52.81-58.00)	50.34 (48.31-52.24)	51.78 (48.67-56.45)
Serbia	99.04 (98.84-99.22)	98.90 (98.68-99.09)	99.23 (99.06-99.40)	53.31 (50.18-57.46)	65.25 (61.97-69.05)	74.77 (71.26-78.46)	50.21 (46.80-53.69)	66.07 (62.68-69.47)	75.50 (72.20-78.46)	58.95 (56.36-62.28)	57.54 (47.24-61.39)	57.43 (54.86-59.58)
Slovakia	99.20 (99.01-99.37)	99.20 (99.00-99.37)	99.45 (99.30-99.59)	63.40 (60.32-67.19)	70.54 (67.49-74.09)	79.46 (75.52-82.09)	64.79 (61.75-67.90)	73.18 (70.53-76.01)	81.61 (78.06-83.88)	79.10 (68.07-98.66)	80.23 (75.45-84.74)	77.73 (72.16-83.49)
Slovenia	99.22 (99.02-99.38)	99.21 (99.02-99.38)	99.36 (99.20-99.52)	69.85 (66.60-73.54)	81.28 (78.23-84.66)	93.94 (90.19-97.52)	69.83 (66.80-73.01)	81.71 (78.69-84.67)	94.25 (91.31-97.13)	65.31 (58.45-73.21)	69.78 (65.87-73.43)	74.00 (69.11-78.82)
Armenia	97.43 (95.74-98.58)	97.98 (97.22-98.62)	99.53 (99.08-99.86)	39.77 (36.77-45.13)	48.15 (44.52-52.89)	62.54 (58.32-67.04)	38.65 (35.09-42.49)	46.16 (42.33-49.95)	63.27 (56.93-69.81)	80.93 (70.29-86.15)	60.60 (53.43-65.79)	52.40 (48.29-56.20)
Azerbaijan	91.74 (86.96-94.93)	88.38 (86.74-89.93)	94.91 (92.14-96.95)	28.70 (25.68-34.30)	33.38 (30.20-39.13)	49.21 (44.96-53.79)	26.28 (22.49-30.01)	30.30 (26.56-33.99)	45.76 (40.93-50.97)	57.65 (46.81-68.39)	49.31 (43.44-53.58)	50.23 (46.36-54.78)

	Appendix Table 1a. Scaled values for each SDG health-related indicator and three summary indicators by country for 1990, 2000, and 2015											
	**Indicator 3.1.2: Proportion of births attended by skilled health personnel (doctors, nurses, midwives, or country-specific medical staff [e.g., clinical officers])**			**Indicator 3.2.1: Under-5 mortality rate (probability of dying before the age of 5 per 1,000 live births)**			**Indicator 3.2.2: Neonatal mortality rate (probability of dying during the first 28 days of life per 1,000 live births)**			**Indicator 3.3.1: Age-standardised rate of new HIV infections (per 1,000 population)**		
**Location**	**1990**	**2000**	**2015**	**1990**	**2000**	**2015**	**1990**	**2000**	**2015**	**1990**	**2000**	**2015**
Georgia	97.51 (95.92-98.53)	97.73 (97.07-98.28)	99.43 (99.09-99.70)	42.46 (39.29-47.46)	45.14 (42.14-50.08)	59.62 (55.75-63.79)	40.28 (36.87-43.51)	42.14 (38.72-45.55)	58.56 (53.30-64.54)	70.78 (59.82-77.53)	60.06 (57.89-62.39)	49.05 (45.29-52.60)
Kazakhstan	99.10 (98.50-99.55)	99.09 (98.64-99.44)	99.57 (99.17-99.88)	43.58 (40.47-48.75)	45.39 (41.96-50.45)	59.72 (55.50-64.24)	42.60 (39.20-46.15)	43.53 (39.83-47.27)	59.58 (53.60-66.15)	48.88 (43.46-52.60)	41.03 (37.10-44.12)	42.43 (38.69-46.32)
Kyrgyzstan	98.69 (97.69-99.32)	98.46 (97.60-99.08)	99.25 (98.65-99.66)	33.22 (30.84-38.63)	38.77 (36.37-44.11)	49.59 (46.91-53.10)	27.49 (24.67-30.75)	32.74 (29.89-35.97)	43.78 (40.80-46.93)	51.60 (44.28-57.80)	43.03 (37.95-47.45)	45.84 (41.02-49.89)
Mongolia	96.85 (95.36-98.00)	98.39 (97.94-98.77)	99.55 (99.16-99.87)	26.62 (24.14-32.63)	35.07 (32.23-40.98)	50.24 (46.17-54.55)	27.45 (23.98-31.34)	34.57 (31.06-38.19)	48.40 (43.45-52.55)	72.75 (58.04-86.83)	69.70 (65.55-72.84)	72.70 (67.60-77.12)
Tajikistan	92.28 (87.24-95.68)	90.37 (86.27-93.52)	93.87 (91.11-96.18)	26.26 (23.50-32.43)	31.66 (28.77-37.75)	47.17 (42.53-52.69)	28.21 (24.33-32.19)	32.74 (29.14-36.31)	46.52 (41.45-51.30)	57.42 (47.19-71.38)	44.43 (39.02-48.32)	50.16 (44.66-55.28)
Turkmenistan	96.23 (93.82-98.01)	97.14 (95.50-98.33)	99.25 (98.46-99.76)	25.63 (22.09-32.21)	29.81 (25.07-36.08)	43.54 (38.55-48.68)	24.83 (19.92-29.57)	27.88 (22.06-33.15)	40.52 (34.80-46.44)	61.81 (59.90-63.63)	35.73 (30.49-40.02)	36.57 (30.69-42.22)
Uzbekistan	97.91 (96.72-98.79)	98.35 (97.51-99.02)	99.55 (99.06-99.91)	38.79 (35.73-44.39)	41.76 (37.99-47.34)	53.24 (48.93-57.80)	38.20 (34.51-41.78)	40.22 (36.19-44.39)	51.43 (46.16-57.25)	55.25 (43.77-68.01)	44.49 (39.16-48.81)	57.55 (52.84-61.45)
Colombia	81.45 (79.58-83.33)	87.93 (86.62-89.17)	97.38 (95.99-98.39)	47.53 (44.58-52.08)	53.37 (50.34-58.04)	61.51 (55.97-66.24)	51.42 (48.36-54.55)	56.43 (53.05-59.88)	65.40 (58.37-71.81)	42.69 (37.73-46.18)	40.13 (37.29-42.27)	39.58 (35.97-43.35)
Costa Rica	98.69 (97.79-99.32)	98.44 (96.92-99.37)	99.51 (98.86-99.95)	56.60 (53.36-60.84)	61.57 (58.65-65.23)	70.64 (64.74-75.26)	54.84 (50.85-58.83)	61.43 (56.46-65.68)	72.08 (65.79-76.53)	43.71 (40.55-46.59)	41.38 (38.33-44.46)	44.73 (41.84-48.00)
El Salvador	97.66 (96.68-98.47)	99.28 (98.88-99.62)	99.81 (99.38-100.00)	37.48 (34.34-42.67)	49.46 (45.65-54.22)	67.98 (61.68-73.14)	39.82 (35.88-43.94)	50.27 (46.26-54.51)	72.23 (64.37-77.19)	39.85 (36.01-42.92)	34.14 (30.81-37.18)	39.42 (36.09-42.96)
Guatemala	27.84 (24.31-30.98)	42.81 (39.56-46.22)	63.81 (50.77-75.85)	28.69 (26.53-34.76)	38.07 (35.87-43.33)	51.78 (47.60-56.17)	43.66 (40.09-47.57)	50.12 (47.07-53.56)	59.99 (56.01-63.20)	33.43 (31.32-35.17)	32.10 (28.61-35.14)	39.11 (35.06-42.75)

	**Appendix Table 1a. Scaled values for each SDG health-related indicator and three summary indicators by country for 1990, 2000, and 2015**											
	**Indicator 3.1.2: Proportion of births attended by skilled health personnel (doctors, nurses, midwives, or country-specific medical staff [e.g., clinical officers])**			**Indicator 3.2.1: Under-5 mortality rate (probability of dying before the age of 5 per 1,000 live births)**			**Indicator 3.2.2: Neonatal mortality rate (probability of dying during the first 28 days of life per 1,000 live births)**			**Indicator 3.3.1: Age-standardised rate of new HIV infections (per 1,000 population)**		
**Location**	**1990**	**2000**	**2015**	**1990**	**2000**	**2015**	**1990**	**2000**	**2015**	**1990**	**2000**	**2015**
Honduras	44.04 (34.67-52.77)	66.22 (58.32-73.20)	96.91 (94.93-98.27)	36.84 (34.46-42.15)	45.09 (42.45-49.81)	54.81 (51.92-58.76)	37.29 (34.40-40.44)	44.48 (41.61-47.47)	53.87 (49.51-57.34)	35.01 (32.66-37.00)	35.20 (33.27-37.78)	36.30 (33.14-39.71)
Mexico	78.81 (74.04-83.13)	91.03 (89.25-92.65)	97.90 (95.76-99.17)	41.68 (39.02-46.49)	51.04 (48.16-54.99)	62.01 (56.77-66.44)	39.90 (36.67-43.41)	48.83 (45.55-52.38)	61.86 (55.18-67.90)	39.74 (38.21-41.25)	40.28 (38.95-41.70)	41.16 (39.66-42.70)
Nicaragua	69.58 (60.59-77.74)	90.57 (87.51-93.17)	96.91 (93.65-98.76)	33.98 (31.71-39.57)	45.49 (42.94-49.94)	61.95 (57.72-65.84)	34.84 (32.03-37.59)	45.27 (42.41-48.00)	63.49 (58.47-67.74)	54.98 (52.01-57.75)	46.03 (43.03-48.59)	39.23 (34.15-44.48)
Panama	85.58 (76.72-92.09)	90.11 (87.34-92.47)	94.43 (90.67-96.98)	50.72 (47.51-55.10)	54.16 (51.70-57.85)	59.49 (54.08-64.30)	53.65 (50.02-57.54)	56.74 (53.26-60.03)	62.36 (55.75-68.89)	39.46 (34.36-43.30)	29.72 (26.92-32.04)	29.46 (25.48-33.23)
Venezuela	94.62 (90.67-97.31)	97.50 (96.02-98.67)	98.13 (96.37-99.22)	50.13 (47.48-54.62)	55.76 (53.04-59.86)	60.07 (57.32-63.69)	49.37 (46.43-52.25)	55.09 (52.17-57.97)	59.69 (56.96-61.90)	42.10 (34.84-47.55)	38.03 (34.80-40.37)	39.48 (35.54-44.22)
Bolivia	41.44 (38.45-44.31)	60.53 (58.34-62.72)	83.02 (77.90-87.18)	22.00 (20.22-28.12)	32.22 (30.08-37.78)	49.36 (45.28-53.82)	20.12 (16.66-23.81)	30.16 (27.05-33.41)	46.56 (42.28-50.46)	54.26 (50.85-57.16)	48.21 (45.57-50.18)	49.34 (46.82-51.68)
Ecuador	86.43 (82.86-89.65)	95.32 (93.31-96.81)	98.29 (97.00-99.12)	39.34 (36.84-44.71)	46.62 (44.28-50.82)	59.14 (55.61-62.82)	49.25 (46.34-52.29)	54.21 (51.71-57.06)	65.72 (61.88-68.76)	40.24 (35.80-43.11)	35.17 (32.85-36.94)	35.76 (33.24-38.28)
Peru	62.44 (57.64-67.08)	65.20 (60.78-69.70)	91.59 (89.24-93.82)	29.57 (27.45-35.51)	45.86 (43.25-50.64)	61.38 (58.69-64.76)	32.13 (28.86-35.58)	46.80 (43.74-49.85)	63.84 (59.16-67.49)	36.64 (35.25-38.16)	38.25 (36.32-39.85)	42.61 (40.11-44.94)
Antigua and Barbuda	98.34 (97.24-99.13)	99.11 (98.50-99.57)	99.55 (99.13-99.86)	58.11 (53.32-62.94)	58.09 (54.07-62.81)	65.54 (58.83-71.30)	55.95 (49.97-63.13)	55.29 (49.54-61.21)	64.84 (55.63-71.64)	35.53 (28.47-43.57)	32.77 (27.43-36.87)	36.31 (29.67-42.16)
The Bahamas	98.35 (97.25-99.15)	99.07 (98.45-99.54)	99.43 (98.93-99.78)	47.55 (40.40-55.12)	58.63 (52.84-64.93)	61.52 (50.84-69.84)	36.42 (27.10-46.22)	50.92 (43.48-59.29)	54.38 (38.81-66.64)	24.36 (21.06-27.06)	26.45 (24.79-28.07)	33.83 (30.19-37.13)
Barbados	97.36 (95.75-98.57)	98.27 (97.21-98.99)	99.49 (99.01-99.84)	51.73 (44.75-59.07)	56.17 (49.48-62.35)	61.53 (51.44-70.22)	43.80 (34.91-53.52)	49.06 (39.84-58.53)	55.74 (42.00-67.62)	33.63 (29.32-37.75)	33.97 (31.12-36.51)	35.91 (31.67-40.70)

Appendix Table 1a. Scaled values for each SDG health-related indicator and three summary indicators by country for 1990, 2000, and 2015

Location	Indicator 3.1.2: Proportion of births attended by skilled health personnel (doctors, nurses, midwives, or country-specific medical staff [e.g., clinical officers])			Indicator 3.2.1: Under-5 mortality rate (probability of dying before the age of 5 per 1,000 live births)			Indicator 3.2.2: Neonatal mortality rate (probability of dying during the first 28 days of life per 1,000 live births)			Indicator 3.3.1: Age-standardised rate of new HIV infections (per 1,000 population)		
	1990	2000	2015	1990	2000	2015	1990	2000	2015	1990	2000	2015
Belize	80.95 (78.88-83.07)	93.85 (91.68-95.60)	97.63 (96.47-98.50)	43.71 (39.36-49.05)	51.89 (46.88-57.38)	60.73 (52.55-67.21)	40.43 (35.77-45.09)	48.98 (43.50-54.59)	59.35 (47.71-69.29)	38.98 (30.06-47.25)	21.48 (17.66-24.89)	27.73 (24.96-30.53)
Cuba	98.78 (97.92-99.37)	98.98 (98.29-99.48)	99.59 (99.17-99.88)	64.07 (61.19-67.67)	73.20 (70.27-76.49)	79.10 (76.90-81.63)	65.18 (62.35-67.97)	75.19 (72.56-77.75)	80.84 (78.73-82.92)	55.61 (49.73-60.64)	48.40 (44.11-53.21)	42.67 (38.69-46.48)
Dominica	97.13 (95.35-98.39)	98.53 (97.66-99.21)	99.42 (98.97-99.78)	52.54 (47.63-57.94)	53.30 (48.65-58.58)	51.76 (43.88-58.93)	49.17 (42.89-55.93)	49.39 (43.76-55.27)	46.43 (37.13-56.07)	38.90 (31.36-47.63)	34.92 (29.62-38.61)	37.41 (31.02-42.66)
Dominican Republic	94.19 (93.20-95.10)	98.62 (98.30-98.89)	99.55 (99.24-99.80)	35.05 (32.56-41.01)	44.69 (41.77-49.59)	52.53 (48.43-56.83)	29.40 (26.28-32.59)	39.18 (35.81-42.59)	47.06 (42.27-51.14)	26.38 (24.53-28.62)	23.80 (21.83-25.82)	31.93 (29.69-34.17)
Grenada	97.98 (96.59-98.91)	99.02 (98.34-99.49)	99.55 (99.13-99.86)	51.10 (42.60-60.06)	56.65 (50.20-63.91)	61.96 (51.38-71.14)	47.63 (37.62-59.62)	53.77 (45.45-62.99)	60.06 (46.15-72.10)	38.79 (31.92-48.07)	34.23 (27.72-38.33)	37.15 (30.26-43.22)
Guyana	84.53 (78.55-89.88)	89.52 (86.85-91.81)	95.83 (94.16-97.18)	37.80 (35.39-43.14)	42.78 (40.26-47.43)	50.37 (45.63-55.26)	31.32 (28.13-34.68)	36.46 (33.22-39.53)	44.13 (38.78-48.99)	29.76 (23.46-35.92)	17.07 (13.67-20.75)	20.33 (17.19-23.69)
Haiti	15.64 (12.02-19.30)	18.67 (15.89-21.58)	42.49 (35.96-49.34)	15.84 (13.62-22.96)	25.16 (22.37-31.72)	35.68 (29.38-42.39)	15.63 (11.83-19.50)	26.06 (22.21-29.65)	34.55 (27.77-40.60)	15.35 (13.71-16.92)	20.48 (18.33-23.18)	24.65 (21.31-28.29)
Jamaica	93.55 (91.91-94.87)	98.07 (97.38-98.62)	99.42 (98.91-99.79)	50.73 (45.05-56.83)	55.17 (49.80-61.45)	59.12 (51.17-66.29)	47.00 (40.47-54.25)	51.75 (44.31-59.97)	56.08 (45.66-66.20)	33.30 (26.01-37.70)	31.41 (28.83-33.44)	35.25 (31.87-38.55)
Saint Lucia	97.80 (96.42-98.86)	98.71 (97.89-99.29)	99.46 (98.96-99.80)	48.45 (41.04-56.32)	57.00 (50.41-64.36)	60.09 (48.72-70.03)	45.02 (36.44-55.31)	54.71 (46.32-64.86)	57.92 (43.14-71.27)	39.12 (33.35-45.22)	34.33 (29.33-37.21)	39.37 (33.08-44.09)
Saint Vincent and the Grenadines	97.73 (96.25-98.77)	98.86 (98.16-99.40)	99.40 (98.89-99.74)	43.53 (37.94-49.35)	47.86 (42.84-53.92)	55.48 (47.25-62.97)	36.77 (29.72-43.92)	41.14 (35.28-47.18)	49.74 (39.08-60.19)	31.98 (25.30-41.35)	27.08 (21.44-30.69)	30.46 (24.02-36.05)
Suriname	90.34 (85.30-94.17)	93.65 (90.38-96.10)	98.25 (97.14-99.07)	38.24 (35.50-43.17)	40.96 (38.15-45.81)	48.22 (44.05-52.74)	32.89 (29.53-36.17)	35.02 (31.41-38.62)	41.88 (36.76-46.29)	32.39 (24.99-38.83)	25.84 (23.10-28.37)	30.87 (27.21-34.12)

Location	Appendix Table 1a. Scaled values for each SDG health-related indicator and three summary indicators by country for 1990, 2000, and 2015											
	**Indicator 3.1.2: Proportion of births attended by skilled health personnel (doctors, nurses, midwives, or country-specific medical staff [e.g., clinical officers])**			**Indicator 3.2.1: Under-5 mortality rate (probability of dying before the age of 5 per 1,000 live births)**			**Indicator 3.2.2: Neonatal mortality rate (probability of dying during the first 28 days of life per 1,000 live births)**			**Indicator 3.3.1: Age-standardised rate of new HIV infections (per 1,000 population)**		
	**1990**	**2000**	**2015**	**1990**	**2000**	**2015**	**1990**	**2000**	**2015**	**1990**	**2000**	**2015**
Trinidad and Tobago	98.73 (98.05-99.24)	98.91 (98.10-99.46)	99.63 (99.21-99.92)	50.13 (46.58-54.78)	50.60 (47.74-54.59)	58.05 (49.55-65.65)	41.76 (37.39-45.99)	41.79 (36.99-45.80)	50.97 (39.80-61.97)	29.55 (25.53-32.87)	27.26 (24.48-29.85)	34.50 (31.22-37.45)
Brazil	84.18 (79.74-88.01)	97.35 (95.66-98.48)	99.26 (98.06-99.87)	37.78 (34.91-43.18)	48.10 (44.54-53.44)	60.11 (55.42-64.37)	35.99 (32.79-39.14)	45.73 (42.14-49.79)	59.02 (53.40-64.00)	34.62 (32.69-36.31)	34.82 (33.21-36.35)	36.01 (34.58-37.43)
Paraguay	67.07 (63.74-70.03)	78.62 (74.87-81.99)	91.77 (84.85-96.20)	47.26 (44.43-52.15)	50.17 (47.21-54.46)	59.03 (54.47-63.26)	45.07 (41.98-48.31)	47.54 (44.34-51.01)	57.38 (51.87-62.27)	46.35 (38.40-49.48)	38.52 (35.82-41.07)	35.28 (30.50-39.59)
China	63.05 (38.95-83.12)	80.99 (70.67-88.77)	96.83 (92.87-98.97)	36.42 (31.86-43.12)	44.38 (40.18-49.73)	66.36 (60.45-72.62)	36.02 (30.77-41.20)	42.91 (38.23-47.34)	68.91 (60.42-76.01)	65.56 (62.98-67.65)	49.11 (47.19-50.97)	46.36 (44.31-48.30)
North Korea	91.13 (76.49-97.72)	91.70 (79.21-97.91)	94.55 (85.63-98.75)	37.52 (22.79-51.84)	19.18 (0.00-42.65)	58.78 (45.23-72.11)	35.68 (19.90-51.69)	42.47 (27.28-59.98)	57.85 (41.83-74.85)	65.81 (50.94-70.09)	49.17 (40.09-53.02)	45.94 (39.18-50.39)
Taiwan	96.57 (90.60-99.31)	98.28 (94.83-99.73)	99.26 (97.57-100.00)	72.46 (69.53-75.74)	72.90 (69.86-76.27)	78.10 (70.66-83.11)	78.46 (75.63-81.38)	78.06 (75.26-81.04)	82.56 (74.22-89.32)	75.28 (69.58-78.72)	58.19 (54.44-61.41)	54.35 (49.71-58.77)
Cambodia	17.76 (11.85-24.05)	33.14 (28.88-37.08)	80.69 (75.51-84.85)	20.76 (18.77-27.02)	24.84 (22.78-31.06)	48.03 (45.21-51.65)	18.79 (15.68-22.06)	22.75 (19.87-25.85)	45.69 (42.66-48.90)	30.91 (23.14-35.25)	26.87 (23.93-29.71)	28.11 (24.08-32.04)
Indonesia	34.08 (32.20-35.99)	65.35 (64.10-66.61)	89.66 (87.73-91.30)	27.82 (25.81-33.62)	38.58 (36.26-43.65)	53.55 (50.15-57.68)	28.44 (25.23-31.73)	37.97 (35.07-40.89)	52.00 (48.42-55.08)	65.59 (57.26-68.00)	41.98 (34.48-44.99)	30.42 (26.51-33.13)
Laos	10.31 (4.76-16.50)	19.51 (15.59-23.68)	46.87 (38.99-55.00)	11.09 (8.66-18.08)	16.65 (14.09-23.38)	35.01 (30.21-40.06)	2.56 (0.00-7.06)	9.44 (5.52-13.65)	29.86 (23.84-35.40)	63.30 (56.07-65.39)	37.44 (29.11-40.85)	33.89 (30.48-36.13)
Malaysia	92.77 (89.53-95.24)	96.99 (95.71-98.09)	99.62 (99.26-99.88)	60.12 (57.28-64.14)	71.34 (68.33-74.76)	77.95 (74.14-80.61)	62.59 (59.30-65.78)	76.16 (73.23-79.05)	81.81 (78.39-84.22)	37.70 (30.88-43.19)	36.43 (34.59-38.02)	40.15 (38.19-42.02)
Maldives	72.23 (63.44-80.52)	87.67 (82.75-91.45)	98.89 (98.28-99.41)	32.59 (30.18-38.14)	45.33 (42.79-50.07)	65.96 (62.95-69.03)	32.54 (29.22-35.83)	44.35 (41.25-47.57)	69.26 (64.10-72.80)	66.71 (63.31-69.02)	69.04 (65.72-71.70)	72.11 (68.37-75.29)

Location	Appendix Table 1a. Scaled values for each SDG health-related indicator and three summary indicators by country for 1990, 2000, and 2015											
	**Indicator 3.1.2: Proportion of births attended by skilled health personnel (doctors, nurses, midwives, or country-specific medical staff [e.g., clinical officers])**			**Indicator 3.2.1: Under-5 mortality rate (probability of dying before the age of 5 per 1,000 live births)**			**Indicator 3.2.2: Neonatal mortality rate (probability of dying during the first 28 days of life per 1,000 live births)**			**Indicator 3.3.1: Age-standardised rate of new HIV infections (per 1,000 population)**		
	**1990**	**2000**	**2015**	**1990**	**2000**	**2015**	**1990**	**2000**	**2015**	**1990**	**2000**	**2015**
Mauritius	96.81 (95.16-98.06)	98.81 (98.11-99.33)	99.84 (99.61-100.00)	54.61 (51.89-58.71)	58.98 (56.34-62.63)	64.45 (61.47-67.83)	50.61 (47.77-53.40)	55.75 (53.05-58.42)	62.74 (59.88-65.08)	53.62 (50.87-55.85)	43.92 (40.98-46.20)	41.34 (38.14-43.96)
Myanmar	48.65 (38.42-59.14)	66.54 (60.58-71.91)	85.82 (81.03-89.87)	22.25 (18.36-28.80)	29.41 (25.09-36.19)	47.48 (42.31-52.21)	20.78 (15.68-26.09)	27.65 (22.24-32.97)	44.89 (39.14-49.91)	29.87 (9.65-38.01)	26.13 (18.35-29.45)	31.28 (27.55-33.67)
Philippines	48.29 (45.20-51.35)	58.23 (56.12-60.17)	78.81 (75.58-81.89)	36.40 (34.16-41.86)	45.01 (42.58-49.76)	53.94 (50.85-57.97)	39.48 (36.21-43.01)	46.70 (43.53-49.94)	55.05 (51.66-58.15)	45.42 (35.09-54.83)	41.79 (36.66-46.22)	33.81 (26.01-40.65)
Sri Lanka	92.57 (90.80-94.21)	98.92 (98.37-99.33)	99.94 (99.76-100.00)	42.66 (40.25-47.71)	60.62 (57.82-64.49)	74.10 (68.98-77.97)	38.10 (35.03-41.32)	59.97 (56.75-63.15)	75.10 (70.24-79.12)	60.88 (52.89-64.55)	61.14 (54.81-64.10)	61.31 (56.59-65.93)
Seychelles	81.36 (73.16-87.48)	92.90 (89.12-95.61)	98.05 (96.97-98.90)	59.25 (56.51-62.88)	64.47 (61.68-67.75)	68.89 (64.45-72.40)	60.30 (56.85-64.11)	67.09 (63.79-70.44)	71.50 (66.69-74.64)	100.00 (100.00-100.00)	40.89 (34.07-45.89)	40.97 (32.18-49.61)
Thailand	75.60 (71.46-79.46)	96.44 (95.35-97.38)	99.33 (99.01-99.63)	50.46 (48.05-54.44)	62.31 (60.09-65.65)	80.59 (76.11-83.38)	43.90 (40.66-46.90)	59.34 (55.51-62.39)	80.24 (75.98-83.21)	24.90 (20.90-28.24)	31.72 (28.03-34.80)	34.11 (30.32-37.46)
Timor-Leste	5.39 (0.50-10.60)	15.05 (10.19-20.55)	49.26 (39.79-57.90)	15.41 (12.84-22.15)	22.63 (19.47-28.76)	44.34 (36.25-52.52)	18.56 (14.60-22.47)	26.12 (21.87-30.20)	45.04 (37.27-53.53)	55.38 (16.41-69.10)	44.81 (26.16-56.06)	44.41 (30.91-55.26)
Vietnam	64.73 (57.16-72.35)	82.15 (79.17-84.76)	94.83 (92.92-96.44)	39.57 (37.08-44.67)	50.30 (47.89-54.68)	64.46 (61.87-67.60)	37.47 (34.53-40.56)	47.91 (44.95-50.85)	65.63 (61.14-68.89)	65.48 (48.24-70.46)	36.58 (24.37-42.80)	37.35 (31.03-41.76)
Federated States of Micronesia	86.45 (76.89-93.12)	92.91 (88.26-96.07)	98.62 (97.09-99.56)	42.88 (37.80-48.90)	53.67 (48.21-59.19)	67.71 (60.66-73.76)	46.80 (42.00-52.01)	56.32 (50.44-63.31)	73.25 (64.22-79.30)	45.33 (23.81-52.65)	43.33 (30.89-48.82)	40.80 (33.01-47.02)
Fiji	97.89 (95.75-99.24)	98.70 (97.39-99.55)	99.43 (98.73-99.87)	51.27 (45.10-56.89)	49.82 (45.11-55.02)	49.16 (39.84-57.74)	56.85 (51.12-62.66)	54.61 (49.66-59.61)	52.87 (44.53-61.71)	53.21 (42.04-60.23)	49.08 (44.90-52.92)	43.98 (39.24-48.77)
Kiribati	78.34 (64.60-88.48)	84.07 (74.08-90.66)	91.68 (85.64-96.01)	27.92 (23.57-33.95)	33.40 (29.45-39.69)	44.38 (33.23-54.44)	34.90 (29.98-39.62)	38.94 (34.73-43.50)	47.22 (36.81-57.54)	54.98 (44.36-60.58)	55.17 (52.50-57.42)	55.66 (50.01-61.90)

Appendix Table 1a. Scaled values for each SDG health-related indicator and three summary indicators by country for 1990, 2000, and 2015

Location	Indicator 3.1.2: Proportion of births attended by skilled health personnel (doctors, nurses, midwives, or country-specific medical staff [e.g., clinical officers])			Indicator 3.2.1: Under-5 mortality rate (probability of dying before the age of 5 per 1,000 live births)			Indicator 3.2.2: Neonatal mortality rate (probability of dying during the first 28 days of life per 1,000 live births)			Indicator 3.3.1: Age-standardised rate of new HIV infections (per 1,000 population)		
	1990	2000	2015	1990	2000	2015	1990	2000	2015	1990	2000	2015
Marshall Islands	83.64 (73.01-91.16)	90.54 (85.53-94.54)	95.48 (92.55-97.63)	41.79 (35.34-49.00)	44.10 (36.32-52.46)	58.74 (49.50-68.45)	47.36 (41.70-53.78)	48.26 (41.00-56.06)	62.56 (51.33-75.65)	54.89 (19.60-73.12)	52.50 (28.73-65.88)	44.88 (33.76-55.94)
Papua New Guinea	55.12 (46.43-62.93)	52.08 (45.35-58.72)	64.92 (50.49-78.01)	26.12 (22.70-33.06)	30.28 (25.19-35.54)	41.54 (32.18-50.43)	36.74 (32.76-41.49)	39.35 (34.30-43.78)	47.30 (39.03-54.78)	37.38 (32.60-42.21)	31.38 (28.99-33.78)	34.78 (32.25-37.23)
Samoa	83.96 (73.76-91.66)	90.87 (85.17-94.95)	90.98 (84.76-95.43)	57.04 (46.91-65.13)	63.82 (54.19-73.63)	73.60 (61.86-83.37)	63.14 (51.12-75.58)	70.76 (57.37-81.91)	80.74 (67.20-91.45)	47.76 (24.03-56.60)	45.63 (29.87-52.21)	42.22 (33.01-49.91)
Solomon Islands	74.55 (60.24-84.91)	83.48 (76.61-89.33)	95.08 (91.54-97.60)	40.89 (36.22-46.96)	46.67 (41.18-52.60)	57.32 (50.49-63.52)	48.57 (44.15-53.15)	52.21 (47.34-57.40)	61.97 (54.12-70.46)	41.72 (20.79-47.43)	40.31 (29.28-44.35)	40.58 (32.47-46.37)
Tonga	84.07 (74.50-91.27)	93.43 (90.99-95.44)	98.88 (97.76-99.57)	51.81 (44.87-59.30)	53.01 (45.99-60.71)	59.66 (50.43-67.70)	55.34 (48.42-64.17)	55.92 (48.68-65.51)	63.06 (51.75-74.31)	65.51 (54.80-72.30)	55.24 (51.15-58.89)	44.33 (37.33-52.95)
Vanuatu	80.93 (68.86-89.39)	84.38 (77.41-89.97)	90.73 (84.46-95.04)	43.67 (38.62-50.34)	43.96 (38.87-49.39)	51.20 (44.08-57.06)	49.86 (45.45-54.72)	49.11 (44.71-53.38)	54.53 (47.22-61.35)	41.14 (16.64-46.64)	40.96 (30.27-45.20)	41.03 (32.55-47.61)
Afghanistan	22.93 (15.59-30.74)	22.58 (16.09-28.97)	39.62 (32.23-46.62)	14.12 (12.19-21.40)	18.14 (16.05-24.23)	28.33 (24.94-32.85)	11.98 (7.59-16.82)	16.97 (13.18-21.26)	28.53 (24.13-33.26)	64.93 (36.51-79.04)	65.26 (49.91-73.94)	56.82 (45.54-69.22)
Algeria	73.72 (65.35-80.64)	85.32 (80.27-89.66)	94.44 (92.10-96.29)	36.89 (34.11-42.26)	44.91 (41.98-49.62)	54.40 (50.06-58.52)	34.29 (31.10-37.49)	41.86 (38.38-45.41)	51.69 (45.81-56.57)	61.21 (58.65-63.92)	56.35 (53.93-58.96)	56.77 (51.23-60.99)
Bahrain	91.23 (87.61-94.13)	96.77 (95.38-97.94)	99.24 (98.79-99.60)	55.66 (52.73-59.86)	65.49 (62.19-69.44)	77.63 (73.79-81.53)	52.17 (48.99-55.29)	65.67 (62.29-69.17)	77.89 (74.13-81.78)	54.89 (46.79-60.93)	53.07 (48.36-56.41)	51.69 (44.80-59.88)
Egypt	36.66 (34.65-38.63)	64.89 (63.36-66.46)	88.87 (86.34-91.04)	28.39 (26.05-33.94)	41.56 (38.61-46.69)	55.52 (50.23-60.44)	25.10 (22.00-28.31)	38.29 (35.06-41.61)	52.91 (46.96-58.75)	76.37 (71.40-79.87)	71.96 (68.77-74.64)	62.84 (57.26-68.30)
Iran	82.17 (76.36-87.25)	94.46 (92.33-96.22)	98.71 (98.11-99.21)	30.33 (26.70-36.26)	43.74 (39.18-49.37)	62.97 (57.98-68.32)	27.75 (23.65-31.97)	39.85 (34.73-45.15)	62.46 (55.33-69.91)	55.61 (52.60-57.78)	50.56 (47.83-52.87)	46.11 (44.04-48.16)

Appendix Table 1a. Scaled values for each SDG health-related indicator and three summary indicators by country for 1990, 2000, and 2015

Location	Indicator 3.1.2: Proportion of births attended by skilled health personnel (doctors, nurses, midwives, or country-specific medical staff [e.g., clinical officers])			Indicator 3.2.1: Under-5 mortality rate (probability of dying before the age of 5 per 1,000 live births)			Indicator 3.2.2: Neonatal mortality rate (probability of dying during the first 28 days of life per 1,000 live births)			Indicator 3.3.1: Age-standardised rate of new HIV infections (per 1,000 population)		
	**1990**	**2000**	**2015**	**1990**	**2000**	**2015**	**1990**	**2000**	**2015**	**1990**	**2000**	**2015**
Iraq	52.65 (42.99-61.75)	66.20 (58.71-72.33)	89.10 (85.91-91.79)	37.15 (34.79-42.52)	41.96 (39.49-47.08)	50.76 (47.73-54.83)	31.63 (28.80-34.81)	36.01 (33.35-38.87)	46.32 (42.25-49.63)	81.75 (71.87-88.99)	71.15 (65.09-76.15)	60.95 (52.62-72.86)
Jordan	91.94 (90.77-93.05)	98.48 (98.19-98.74)	99.59 (99.39-99.78)	47.36 (44.85-52.04)	53.12 (50.29-57.50)	62.65 (60.31-65.91)	45.97 (42.89-49.10)	51.51 (48.28-54.65)	63.38 (59.17-66.60)	81.56 (72.80-89.94)	75.01 (68.07-80.40)	74.21 (65.34-86.76)
Kuwait	87.08 (82.68-90.57)	95.65 (94.21-96.89)	99.04 (98.62-99.40)	60.99 (57.41-64.78)	65.67 (62.54-69.74)	73.80 (69.66-78.19)	62.96 (59.05-66.58)	67.27 (64.17-70.88)	75.17 (71.29-79.48)	66.03 (58.70-71.19)	74.26 (69.65-77.49)	74.72 (67.84-82.42)
Lebanon	97.33 (96.25-98.17)	98.95 (98.51-99.32)	99.94 (99.76-100.00)	45.68 (41.69-51.14)	55.26 (50.58-60.94)	74.05 (68.82-77.51)	43.24 (39.01-47.79)	52.39 (46.70-58.63)	75.23 (69.11-80.12)	50.81 (28.13-59.84)	55.49 (42.61-61.21)	55.77 (46.27-63.52)
Libya	70.02 (61.78-78.12)	80.90 (74.57-86.14)	91.74 (88.59-94.18)	42.38 (39.69-47.22)	50.20 (47.46-53.58)	59.57 (54.35-64.40)	42.76 (39.48-46.15)	50.01 (46.91-53.37)	62.51 (55.50-68.83)	51.20 (32.64-56.71)	50.02 (37.36-55.38)	49.28 (38.62-56.09)
Morocco	27.27 (24.62-30.09)	61.18 (58.59-63.71)	82.66 (77.61-86.79)	31.68 (29.44-37.31)	41.28 (39.16-45.75)	54.66 (50.23-59.11)	27.46 (24.42-30.68)	37.43 (34.98-40.04)	51.65 (46.61-56.11)	59.89 (57.35-61.83)	51.84 (48.99-54.10)	49.56 (46.94-51.79)
Palestine	95.45 (93.56-96.92)	98.09 (97.37-98.67)	98.62 (98.06-99.04)	43.53 (40.28-48.54)	51.86 (48.57-56.61)	59.76 (54.70-65.24)	40.36 (36.74-43.97)	48.99 (45.28-52.88)	57.84 (51.47-64.58)	74.05 (68.27-79.36)	63.40 (59.07-66.76)	57.89 (52.08-63.65)
Oman	93.45 (90.91-95.53)	97.15 (95.88-98.11)	99.22 (98.79-99.55)	42.13 (38.99-47.00)	62.02 (59.15-65.94)	71.21 (67.83-75.21)	40.53 (36.55-43.97)	63.37 (59.22-67.55)	73.98 (70.60-77.44)	63.86 (60.21-67.38)	50.70 (47.04-54.06)	53.43 (50.57-56.81)
Qatar	95.15 (93.25-96.83)	98.16 (97.29-98.79)	99.77 (99.54-99.97)	55.20 (49.19-61.61)	61.07 (54.51-67.51)	73.00 (67.09-79.37)	53.28 (46.42-60.77)	60.44 (51.95-69.00)	74.21 (67.64-80.99)	64.26 (53.65-73.95)	71.12 (64.76-76.05)	73.44 (65.84-82.87)
Saudi Arabia	86.58 (82.69-89.50)	93.40 (91.38-95.02)	97.57 (96.47-98.40)	40.21 (36.57-44.17)	53.50 (49.52-57.38)	67.51 (61.71-71.67)	37.60 (33.25-41.80)	51.01 (45.41-56.34)	68.65 (59.91-74.21)	54.54 (38.42-60.93)	53.19 (46.03-57.18)	52.06 (46.19-56.78)
Sudan	68.29 (64.34-72.30)	78.41 (72.91-83.49)	90.84 (87.50-93.74)	17.94 (15.65-24.75)	24.67 (22.16-31.27)	36.42 (31.22-41.98)	15.95 (11.70-20.42)	22.83 (18.72-27.07)	33.88 (27.85-38.78)	39.62 (33.42-46.84)	36.28 (33.45-39.61)	39.60 (34.23-45.99)

Appendix Table 1a. Scaled values for each SDG health-related indicator and three summary indicators by country for 1990, 2000, and 2015

Location	Indicator 3.1.2: Proportion of births attended by skilled health personnel (doctors, nurses, midwives, or country-specific medical staff [e.g., clinical officers])			Indicator 3.2.1: Under-5 mortality rate (probability of dying before the age of 5 per 1,000 live births)			Indicator 3.2.2: Neonatal mortality rate (probability of dying during the first 28 days of life per 1,000 live births)			Indicator 3.3.1: Age-standardised rate of new HIV infections (per 1,000 population)		
	1990	2000	2015	1990	2000	2015	1990	2000	2015	1990	2000	2015
Syria	75.62 (68.59-81.69)	89.42 (86.55-91.79)	97.18 (96.14-97.99)	41.93 (39.56-46.06)	55.83 (53.27-59.36)	54.78 (48.83-61.52)	43.68 (40.64-46.71)	57.03 (54.26-59.96)	65.13 (61.12-68.40)	78.06 (75.00-80.32)	81.25 (78.72-83.64)	74.49 (70.18-79.56)
Tunisia	74.48 (71.28-77.62)	92.25 (90.43-93.83)	98.98 (98.54-99.34)	38.88 (36.49-44.30)	50.94 (48.55-54.44)	63.96 (60.01-67.52)	37.19 (34.13-40.23)	48.55 (45.93-51.32)	64.37 (59.46-67.91)	65.57 (61.29-68.77)	61.20 (57.53-64.65)	53.96 (48.94-58.81)
Turkey	73.77 (71.08-76.36)	82.17 (80.36-83.98)	94.08 (92.11-95.67)	31.57 (28.74-37.20)	43.97 (40.64-49.25)	60.63 (54.99-65.49)	28.10 (24.80-31.40)	40.49 (36.84-43.88)	59.23 (51.96-65.74)	84.81 (78.45-91.01)	69.95 (65.96-73.89)	64.25 (58.66-71.10)
United Arab Emirates	96.78 (95.35-97.89)	99.16 (98.68-99.53)	99.85 (99.65-100.00)	53.57 (44.50-62.97)	66.58 (58.33-75.54)	81.62 (72.32-89.11)	51.37 (40.41-64.73)	67.23 (55.58-77.55)	83.17 (73.25-91.39)	56.87 (33.24-66.37)	55.63 (38.52-65.72)	52.79 (39.14-66.00)
Yemen	32.26 (29.49-35.08)	28.19 (24.50-31.70)	49.02 (41.02-56.49)	18.97 (17.37-25.59)	27.73 (25.60-33.79)	37.24 (33.85-42.32)	16.08 (12.94-19.50)	25.74 (22.66-28.82)	37.92 (34.56-40.76)	49.01 (33.36-53.18)	48.44 (42.54-50.96)	52.28 (45.30-57.25)
Bangladesh	0.89 (0.00-4.55)	11.16 (7.49-15.14)	36.46 (22.66-52.05)	17.53 (16.02-24.14)	27.95 (25.93-33.67)	43.61 (41.66-48.36)	3.61 (0.00-7.56)	16.57 (13.60-20.26)	34.35 (31.28-37.39)	100.00 (100.00-100.00)	64.15 (58.75-66.27)	53.22 (51.02-55.20)
Bhutan	17.98 (3.42-39.98)	30.96 (12.72-50.97)	57.52 (37.82-75.71)	19.99 (17.99-26.14)	28.35 (26.17-34.33)	41.20 (38.26-45.97)	6.64 (2.04-11.25)	17.51 (13.49-21.54)	31.90 (27.68-35.64)	52.46 (27.61-63.18)	47.80 (34.25-55.01)	46.99 (36.60-56.65)
India	28.20 (17.15-41.43)	41.51 (34.40-49.88)	75.28 (61.06-86.74)	21.20 (19.48-27.41)	27.97 (26.01-33.89)	39.09 (36.30-43.99)	8.35 (4.75-12.08)	15.95 (12.69-19.61)	28.04 (23.90-31.86)	40.35 (38.74-41.98)	29.68 (28.45-30.95)	35.64 (34.01-37.22)
Nepal	2.14 (0.00-8.10)	14.50 (10.11-19.02)	47.27 (32.85-61.02)	17.68 (16.04-24.18)	28.78 (26.78-34.62)	44.73 (41.95-49.34)	5.14 (1.47-9.22)	18.84 (15.78-22.22)	37.07 (33.78-39.78)	81.29 (62.43-86.45)	37.47 (23.85-46.10)	45.01 (38.71-49.41)
Pakistan	12.74 (9.57-15.71)	30.25 (25.84-34.30)	61.15 (52.29-69.41)	22.11 (20.29-28.37)	26.37 (24.44-32.30)	33.89 (31.38-39.16)	5.28 (2.09-8.99)	10.40 (7.33-13.74)	19.42 (15.95-22.76)	68.54 (49.91-73.34)	63.45 (53.77-67.63)	49.23 (39.44-57.69)
Botswana	80.23 (76.07-83.83)	85.62 (78.65-91.17)	90.72 (84.93-94.91)	37.14 (34.46-42.66)	33.69 (30.94-39.02)	50.20 (45.68-54.68)	37.68 (34.33-41.01)	37.92 (34.19-41.48)	48.73 (43.23-53.49)	5.81 (5.10-6.56)	0.48 (0.00-1.16)	3.94 (2.80-5.19)

Appendix Table 1a. Scaled values for each SDG health-related indicator and three summary indicators by country for 1990, 2000, and 2015

Location	Indicator 3.1.2: Proportion of births attended by skilled health personnel (doctors, nurses, midwives, or country-specific medical staff [e.g., clinical officers])			Indicator 3.2.1: Under-5 mortality rate (probability of dying before the age of 5 per 1,000 live births)			Indicator 3.2.2: Neonatal mortality rate (probability of dying during the first 28 days of life per 1,000 live births)			Indicator 3.3.1: Age-standardised rate of new HIV infections (per 1,000 population)		
	1990	2000	2015	1990	2000	2015	1990	2000	2015	1990	2000	2015
Lesotho	44.96 (34.93-55.83)	52.49 (48.90-56.05)	66.75 (59.38-73.45)	26.10 (24.02-32.25)	22.95 (20.88-29.03)	28.05 (24.33-34.17)	17.56 (14.37-21.10)	17.55 (14.59-20.89)	19.78 (14.71-24.29)	12.30 (11.15-13.62)	2.02 (1.27-2.71)	2.96 (1.99-3.91)
Namibia	68.52 (65.17-71.55)	78.55 (76.36-80.53)	85.98 (80.44-90.39)	31.23 (28.57-37.09)	33.66 (30.86-38.93)	43.02 (38.84-47.69)	28.82 (25.12-32.70)	32.60 (28.98-36.21)	38.70 (33.22-43.91)	13.76 (12.87-14.67)	3.33 (2.64-4.03)	7.34 (6.30-8.45)
South Africa	87.13 (83.11-90.97)	91.11 (88.78-92.89)	94.52 (91.44-96.85)	29.05 (26.14-34.74)	30.80 (28.00-36.23)	42.11 (38.13-47.08)	29.10 (25.54-32.91)	33.49 (30.22-37.23)	41.97 (37.29-46.66)	24.45 (23.17-25.80)	3.67 (3.09-4.28)	5.74 (4.87-6.66)
Swaziland	74.40 (65.58-81.91)	76.16 (72.81-79.23)	82.89 (77.21-87.39)	31.60 (29.10-37.12)	25.65 (23.41-31.77)	36.75 (33.19-41.33)	36.36 (33.20-39.51)	35.77 (32.79-38.90)	39.46 (35.81-43.41)	7.15 (6.08-8.25)	0.12 (0.00-0.49)	2.71 (1.98-3.67)
Zimbabwe	70.61 (67.54-73.64)	73.79 (71.51-75.96)	66.68 (59.60-73.25)	33.04 (30.86-38.65)	29.49 (27.32-35.39)	36.76 (34.07-41.89)	34.44 (31.25-37.69)	34.66 (31.32-38.17)	34.67 (31.08-37.86)	3.59 (2.89-4.37)	3.76 (2.64-4.84)	7.50 (5.29-9.55)
Benin	70.61 (63.50-77.22)	79.54 (76.81-82.09)	88.29 (84.89-91.24)	11.60 (10.39-18.71)	17.07 (15.27-23.66)	29.50 (27.04-35.23)	13.60 (8.68-18.66)	19.75 (15.05-24.56)	30.82 (26.61-35.02)	25.74 (21.48-29.76)	22.48 (20.78-24.30)	27.16 (24.32-29.73)
Burkina Faso	37.47 (31.22-44.25)	42.99 (38.98-47.07)	68.01 (60.01-75.51)	8.50 (7.15-15.74)	11.93 (10.45-18.65)	22.16 (19.89-28.23)	13.12 (8.03-18.41)	17.35 (12.35-22.54)	27.28 (22.49-31.93)	15.40 (13.24-17.52)	22.31 (20.14-24.77)	28.12 (25.53-30.88)
Cameroon	62.09 (59.08-65.29)	58.21 (55.77-60.70)	66.79 (60.70-72.26)	17.58 (15.98-24.14)	17.46 (15.78-24.07)	26.55 (23.35-32.72)	19.59 (15.02-24.39)	18.46 (13.98-23.19)	25.35 (20.84-29.92)	16.34 (13.69-18.85)	13.43 (12.10-14.75)	16.69 (14.04-19.29)
Cape Verde	47.74 (35.25-59.17)	56.97 (45.01-68.09)	69.91 (58.37-79.69)	35.92 (33.71-41.26)	40.83 (37.90-45.58)	50.13 (47.64-53.71)	34.94 (31.92-38.01)	39.13 (36.04-41.91)	47.58 (44.68-50.58)	28.04 (24.58-31.78)	29.00 (26.33-31.73)	28.80 (21.11-33.99)
Chad	11.00 (6.03-16.79)	16.48 (12.35-20.24)	21.07 (13.87-29.76)	9.19 (7.80-16.27)	11.24 (9.66-18.30)	18.60 (15.84-24.80)	10.44 (5.62-15.47)	13.39 (8.65-18.17)	20.97 (15.45-25.87)	21.24 (17.65-24.75)	16.90 (15.19-18.70)	23.39 (19.89-26.97)
Cote d'Ivoire	46.96 (40.32-53.36)	62.08 (58.51-65.68)	61.87 (54.68-68.82)	15.75 (14.26-22.43)	16.96 (15.32-23.40)	25.67 (23.20-31.59)	10.78 (6.83-15.21)	12.29 (8.27-16.55)	20.71 (16.68-24.77)	14.68 (12.57-16.75)	14.38 (12.73-16.03)	17.85 (15.25-20.62)

Appendix Table 1a. Scaled values for each SDG health-related indicator and three summary indicators by country for 1990, 2000, and 2015

Location	Indicator 3.1.2: Proportion of births attended by skilled health personnel (doctors, nurses, midwives, or country-specific medical staff [e.g., clinical officers])			Indicator 3.2.1: Under-5 mortality rate (probability of dying before the age of 5 per 1,000 live births)			Indicator 3.2.2: Neonatal mortality rate (probability of dying during the first 28 days of life per 1,000 live births)			Indicator 3.3.1: Age-standardised rate of new HIV infections (per 1,000 population)		
	1990	2000	2015	1990	2000	2015	1990	2000	2015	1990	2000	2015
The Gambia	51.46 (39.84-61.99)	54.34 (47.00-61.37)	58.96 (47.47-68.77)	21.71 (19.81-28.22)	27.58 (25.44-33.55)	40.60 (38.23-44.56)	21.07 (16.48-26.28)	26.49 (22.35-31.11)	37.88 (34.44-41.70)	35.28 (32.00-38.45)	22.04 (20.09-24.02)	27.47 (23.57-32.06)
Ghana	39.46 (35.80-43.28)	48.06 (45.04-51.24)	65.04 (58.43-71.67)	19.90 (18.14-26.25)	24.59 (22.64-30.55)	36.48 (34.19-41.80)	17.28 (12.66-22.13)	21.89 (17.57-26.57)	32.37 (28.38-36.41)	20.52 (18.36-22.68)	18.77 (17.17-20.34)	24.73 (22.24-27.55)
Guinea	33.21 (26.05-40.58)	34.71 (31.08-37.94)	49.19 (42.16-56.08)	6.34 (5.02-13.87)	12.24 (10.78-19.19)	22.50 (20.09-27.37)	2.84 (0.00-8.37)	10.99 (7.44-16.08)	21.89 (18.07-26.50)	24.68 (20.11-28.62)	20.31 (18.72-22.02)	23.45 (20.65-26.76)
Guinea-Bissau	35.43 (24.50-47.37)	36.87 (30.02-44.56)	38.61 (27.32-49.80)	8.43 (6.74-15.64)	11.72 (9.73-18.86)	19.71 (16.69-25.90)	7.97 (1.94-14.65)	12.15 (6.29-18.59)	20.44 (14.93-26.16)	29.06 (25.82-31.74)	16.47 (15.06-17.77)	18.18 (15.47-20.75)
Liberia	44.33 (32.50-56.81)	38.67 (31.56-45.83)	62.96 (57.08-68.75)	6.66 (5.10-14.05)	13.24 (11.87-20.06)	31.30 (29.11-36.93)	5.13 (0.55-9.88)	13.40 (9.49-17.67)	31.70 (28.22-35.15)	22.42 (19.18-25.50)	19.92 (18.03-22.00)	26.30 (22.27-30.66)
Mali	23.66 (18.67-28.91)	39.26 (35.07-43.55)	57.02 (49.18-64.26)	6.35 (5.21-13.68)	10.16 (8.63-17.05)	18.65 (15.83-24.85)	1.91 (0.00-7.28)	7.30 (4.46-12.38)	17.12 (12.77-22.07)	23.79 (20.41-27.60)	21.91 (19.82-24.05)	25.35 (22.29-29.19)
Mauritania	51.88 (43.21-59.47)	54.59 (51.60-57.33)	64.14 (55.05-72.84)	22.46 (20.56-28.46)	25.88 (24.07-31.79)	36.66 (34.36-41.96)	17.12 (12.79-21.76)	20.38 (16.45-24.89)	30.88 (26.69-34.78)	35.45 (16.21-41.57)	29.77 (19.62-34.17)	38.70 (35.03-40.97)
Niger	11.33 (7.43-15.38)	15.18 (11.52-18.72)	31.60 (24.19-39.55)	0.64 (0.00-8.33)	6.87 (5.38-14.11)	21.71 (19.23-27.78)	9.32 (3.57-15.98)	18.43 (12.88-24.75)	33.85 (28.89-39.29)	27.91 (25.59-30.32)	22.91 (21.05-24.91)	34.03 (30.14-37.68)
Nigeria	33.06 (27.85-38.28)	37.51 (32.94-41.98)	42.88 (36.80-49.12)	8.48 (6.97-15.71)	11.27 (9.56-18.35)	23.48 (21.06-28.25)	8.52 (2.95-14.98)	10.98 (5.75-17.03)	22.46 (17.90-27.92)	19.83 (16.18-23.54)	17.59 (15.93-19.31)	19.98 (17.32-22.98)
Sao Tome and Principe	78.94 (70.07-85.66)	81.59 (77.19-85.83)	85.55 (80.29-89.71)	22.95 (21.25-28.85)	29.80 (27.94-34.71)	44.03 (40.74-48.87)	25.13 (20.84-29.88)	30.68 (26.88-34.61)	42.90 (39.29-46.41)	58.28 (56.44-60.03)	46.90 (44.87-48.70)	45.93 (43.99-47.92)
Senegal	43.64 (38.90-48.25)	65.54 (61.53-69.03)	75.24 (69.79-80.04)	16.43 (14.96-23.18)	20.80 (18.98-27.05)	37.71 (35.75-42.95)	17.98 (12.90-24.04)	22.33 (17.62-27.76)	37.85 (34.14-41.99)	31.21 (28.91-33.17)	25.11 (23.40-26.78)	29.37 (26.27-33.92)

Appendix Table 1a. Scaled values for each SDG health-related indicator and three summary indicators by country for 1990, 2000, and 2015

Location	Indicator 3.1.2: Proportion of births attended by skilled health personnel (doctors, nurses, midwives, or country-specific medical staff [e.g., clinical officers])			Indicator 3.2.1: Under-5 mortality rate (probability of dying before the age of 5 per 1,000 live births)			Indicator 3.2.2: Neonatal mortality rate (probability of dying during the first 28 days of life per 1,000 live births)			Indicator 3.3.1: Age-standardised rate of new HIV infections (per 1,000 population)		
	**1990**	**2000**	**2015**	**1990**	**2000**	**2015**	**1990**	**2000**	**2015**	**1990**	**2000**	**2015**
Sierra Leone	40.36 (29.05-51.54)	40.02 (33.94-45.94)	61.09 (53.98-67.56)	4.80 (3.56-12.50)	8.22 (7.03-15.41)	19.96 (18.06-25.12)	3.69 (0.00-8.79)	8.08 (4.25-12.89)	21.33 (17.69-25.35)	31.27 (25.52-38.73)	22.23 (20.31-24.04)	25.74 (21.70-31.79)
Togo	42.96 (38.42-47.49)	52.50 (48.48-56.68)	67.04 (59.10-73.61)	16.39 (14.71-23.02)	19.85 (18.05-26.08)	29.45 (27.38-35.18)	16.09 (10.96-21.28)	19.68 (14.98-24.50)	27.33 (23.06-31.50)	20.88 (18.89-22.82)	15.05 (13.41-16.73)	24.65 (20.97-28.38)
Burundi	17.56 (9.91-26.15)	37.33 (27.60-47.37)	63.26 (48.56-75.95)	12.46 (10.38-19.24)	13.42 (11.20-20.17)	29.94 (23.93-36.32)	16.09 (10.81-21.76)	16.77 (11.61-22.82)	32.08 (25.21-37.90)	14.54 (12.28-17.18)	17.08 (15.39-18.95)	23.84 (20.98-26.53)
Comoros	51.21 (37.14-64.44)	69.23 (62.98-74.89)	84.39 (77.09-89.84)	20.35 (18.31-27.03)	27.62 (24.23-33.54)	41.47 (34.70-48.10)	14.23 (10.29-18.09)	22.10 (17.25-26.97)	36.12 (28.23-43.14)	65.03 (41.82-71.56)	45.12 (30.74-52.83)	43.50 (33.97-51.73)
Djibouti	75.33 (60.19-87.47)	79.94 (70.82-86.98)	90.14 (82.70-94.92)	21.71 (19.72-28.27)	23.78 (21.65-30.07)	33.12 (29.72-38.67)	25.48 (21.12-30.43)	27.10 (22.86-31.78)	34.65 (30.17-38.80)	22.90 (17.24-28.66)	21.64 (19.04-24.37)	25.47 (21.57-29.28)
Eritrea	12.51 (7.11-18.81)	24.17 (20.96-27.54)	28.11 (12.86-46.50)	15.96 (13.94-22.31)	18.38 (9.67-26.80)	32.64 (28.43-38.37)	22.37 (17.59-27.53)	26.71 (18.74-33.82)	36.35 (31.17-40.66)	26.10 (21.83-29.99)	26.35 (23.66-28.90)	31.16 (27.57-34.92)
Ethiopia	3.90 (0.00-10.92)	6.40 (2.28-10.83)	14.94 (5.95-25.89)	9.09 (7.62-16.10)	16.60 (15.02-23.14)	34.80 (32.39-40.21)	3.75 (0.00-8.61)	14.04 (10.36-18.57)	32.15 (28.64-35.64)	15.69 (13.66-18.08)	17.72 (15.89-19.73)	26.15 (23.68-28.96)
Kenya	47.92 (42.35-53.34)	48.09 (43.68-52.77)	51.54 (35.57-66.89)	25.93 (23.92-31.95)	26.80 (24.67-32.60)	38.29 (35.96-43.45)	28.61 (25.06-32.35)	30.27 (26.94-33.81)	37.70 (34.38-40.84)	8.18 (7.52-8.88)	14.10 (13.11-15.16)	15.50 (14.00-17.01)
Madagascar	54.90 (51.02-58.78)	43.01 (39.60-46.67)	43.66 (30.82-56.63)	16.61 (14.40-23.06)	22.81 (20.28-29.30)	33.57 (29.28-39.29)	20.59 (16.12-25.43)	26.38 (22.26-30.76)	35.28 (30.14-39.53)	30.51 (8.19-45.32)	38.40 (23.47-48.71)	46.41 (35.23-58.13)
Malawi	55.52 (48.29-62.75)	61.41 (56.88-65.85)	76.10 (66.06-84.96)	6.82 (5.04-13.75)	13.29 (11.32-20.18)	28.14 (24.88-32.61)	8.61 (3.59-14.22)	19.37 (14.78-24.33)	30.52 (25.69-35.17)	6.62 (5.70-7.63)	6.99 (5.65-8.25)	13.05 (11.07-16.18)
Mozambique	41.52 (31.18-52.39)	45.96 (43.51-48.24)	61.12 (53.13-68.71)	7.82 (6.56-15.20)	14.54 (13.05-21.27)	29.48 (27.24-35.22)	6.99 (2.94-11.44)	17.27 (13.40-21.36)	32.13 (28.56-35.33)	16.13 (14.59-17.67)	8.28 (7.31-9.26)	11.07 (9.15-13.31)

Location	Appendix Table 1a. Scaled values for each SDG health-related indicator and three summary indicators by country for 1990, 2000, and 2015											
	**Indicator 3.1.2: Proportion of births attended by skilled health personnel (doctors, nurses, midwives, or country-specific medical staff [e.g., clinical officers])**			**Indicator 3.2.1: Under-5 mortality rate (probability of dying before the age of 5 per 1,000 live births)**			**Indicator 3.2.2: Neonatal mortality rate (probability of dying during the first 28 days of life per 1,000 live births)**			**Indicator 3.3.1: Age-standardised rate of new HIV infections (per 1,000 population)**		
	**1990**	**2000**	**2015**	**1990**	**2000**	**2015**	**1990**	**2000**	**2015**	**1990**	**2000**	**2015**
Rwanda	27.08 (18.63-36.07)	32.38 (25.87-39.23)	77.16 (65.79-85.94)	14.96 (12.92-21.83)	14.40 (12.42-21.07)	33.10 (28.96-38.74)	17.10 (12.58-22.62)	15.70 (11.13-20.87)	32.90 (27.62-37.74)	12.73 (11.23-14.19)	15.69 (14.21-17.33)	23.55 (21.17-25.99)
Somalia	29.70 (13.42-49.48)	27.98 (16.59-41.68)	31.96 (17.16-49.07)	10.75 (9.11-17.96)	14.33 (12.49-20.88)	21.84 (18.67-27.91)	13.57 (8.71-18.75)	17.86 (13.49-22.71)	25.72 (20.82-30.38)	35.75 (30.32-40.68)	29.68 (27.23-31.91)	34.17 (30.32-37.65)
South Sudan	36.27 (17.12-57.31)	35.39 (20.59-52.23)	41.19 (27.80-54.18)	9.32 (7.53-16.28)	17.01 (15.20-23.87)	22.88 (19.36-27.75)	13.08 (8.69-17.77)	22.97 (18.97-27.45)	28.74 (23.83-33.51)	25.17 (18.68-32.54)	20.14 (17.30-23.23)	22.95 (19.74-26.77)
Tanzania	53.68 (50.38-56.78)	44.24 (41.08-47.41)	56.01 (44.30-66.81)	14.86 (13.42-21.63)	20.11 (18.33-26.49)	34.88 (32.24-40.18)	18.39 (14.43-22.63)	24.63 (20.99-28.57)	36.39 (32.77-39.85)	10.95 (9.90-12.11)	12.53 (11.06-14.01)	16.71 (14.43-18.99)
Uganda	36.62 (32.67-40.64)	40.00 (36.05-43.46)	68.17 (59.48-76.02)	13.14 (11.84-20.15)	18.51 (16.73-24.95)	31.90 (29.73-37.52)	18.72 (14.44-23.37)	23.63 (19.53-28.02)	33.75 (30.30-37.32)	7.22 (5.68-8.82)	11.56 (10.38-12.76)	14.94 (12.63-17.71)
Zambia	50.11 (44.51-55.22)	48.50 (44.75-52.44)	73.52 (66.44-79.88)	13.02 (11.01-19.75)	15.99 (13.72-22.68)	33.02 (29.72-37.30)	20.50 (15.96-25.27)	26.07 (21.45-31.11)	37.78 (34.06-41.89)	6.12 (5.16-6.99)	6.91 (5.80-8.02)	11.22 (9.88-12.78)
Angola	36.79 (28.28-45.76)	43.82 (40.02-47.54)	72.71 (65.71-79.09)	10.42 (7.64-17.65)	14.80 (11.90-21.84)	29.42 (24.07-36.32)	17.11 (10.58-23.90)	21.61 (16.06-27.39)	34.63 (28.13-40.85)	27.02 (22.68-30.97)	20.04 (18.33-21.65)	21.07 (18.18-23.97)
Central African Republic	48.25 (42.82-53.60)	46.68 (42.39-51.16)	60.62 (53.85-67.58)	11.87 (9.42-18.41)	13.56 (10.50-20.75)	19.32 (13.71-26.19)	10.76 (5.26-16.24)	13.65 (8.05-19.48)	17.49 (9.63-24.11)	11.03 (8.84-13.07)	11.30 (9.90-12.84)	15.49 (12.77-18.01)
Congo	91.67 (87.78-94.52)	92.29 (90.06-94.14)	95.63 (94.10-96.88)	25.70 (23.37-31.88)	22.36 (19.69-28.45)	36.38 (32.32-41.16)	31.87 (27.09-36.93)	27.29 (22.21-32.38)	37.58 (32.79-42.95)	13.14 (11.52-14.84)	16.17 (14.59-17.91)	19.20 (16.57-22.53)
Democratic Republic of the Congo	65.71 (56.01-74.44)	68.35 (63.43-72.52)	86.67 (83.92-89.07)	13.33 (10.85-20.04)	15.34 (12.83-22.16)	27.05 (22.09-33.86)	19.13 (13.86-24.45)	20.49 (15.25-25.59)	30.05 (24.42-35.85)	18.29 (16.19-20.78)	18.76 (17.10-20.52)	26.49 (21.33-30.90)
Equatorial Guinea	37.62 (27.31-48.84)	67.25 (59.14-75.37)	92.01 (88.53-94.78)	12.80 (10.28-19.68)	14.32 (11.25-21.20)	27.21 (21.27-33.70)	14.29 (8.58-20.45)	15.30 (9.73-21.27)	27.07 (19.82-33.40)	22.78 (17.21-26.88)	15.81 (14.38-17.51)	20.61 (16.78-23.77)

Appendix Table 1a. Scaled values for each SDG health-related indicator and three summary indicators by country for 1990, 2000, and 2015

Location	Indicator 3.1.2: Proportion of births attended by skilled health personnel (doctors, nurses, midwives, or country-specific medical staff [e.g., clinical officers])			Indicator 3.2.1: Under-5 mortality rate (probability of dying before the age of 5 per 1,000 live births)			Indicator 3.2.2: Neonatal mortality rate (probability of dying during the first 28 days of life per 1,000 live births)			Indicator 3.3.1: Age-standardised rate of new HIV infections (per 1,000 population)		
	1990	2000	2015	1990	2000	2015	1990	2000	2015	1990	2000	2015
Gabon	85.09 (80.65-88.70)	88.42 (86.82-89.87)	94.50 (93.06-95.68)	28.10 (25.87-34.15)	31.30 (29.19-37.30)	40.30 (37.57-44.14)	27.24 (23.59-31.70)	29.77 (26.38-33.66)	36.94 (33.61-40.54)	18.96 (17.16-20.75)	11.96 (10.62-13.27)	18.50 (15.21-22.06)

Location	Appendix Table 1a. Scaled values for each SDG health-related indicator and three summary indicators by country for 1990, 2000, and 2015											
	Indicator 3.3.2: Age-standardised rate of new and relapsed TB cases (per 1,000 population)			Indicator 3.3.3: Age-standaridsed rate of malaria cases (per 1,000 population)			Indicator 3.3.4: Age-standardised rate of hepatitis B incident cases (per 100,000 population)			Indicator 3.3.5: Age-standardised prevalence rate of neglected tropical diseases (NTDs) (per 100,000 population)		
	1990	2000	2015	1990	2000	2015	1990	2000	2015	1990	2000	2015
Canada	83.40 (81.86-84.86)	86.84 (84.97-88.60)	92.56 (90.58-94.23)	100.00 (100.00-100.00)	100.00 (100.00-100.00)	100.00 (100.00-100.00)	72.02 (65.51-76.81)	71.95 (65.68-77.37)	71.25 (64.11-76.94)	99.99 (99.99-100.00)	99.99 (99.99-100.00)	100.00 (99.99-100.00)
United States	77.17 (73.74-80.34)	92.94 (91.71-94.02)	99.18 (97.81-100.00)	100.00 (100.00-100.00)	100.00 (100.00-100.00)	100.00 (100.00-100.00)	85.47 (80.11-88.95)	85.64 (80.66-89.16)	85.22 (79.73-89.09)	99.98 (99.98-99.99)	99.97 (99.97-99.98)	99.98 (99.97-99.98)
Australia	82.64 (79.30-85.84)	88.59 (86.93-90.26)	87.09 (83.61-89.90)	100.00 (100.00-100.00)	100.00 (100.00-100.00)	100.00 (100.00-100.00)	68.99 (62.40-74.27)	69.21 (62.52-75.06)	68.76 (61.44-74.72)	99.97 (99.95-99.98)	99.98 (99.96-99.98)	99.98 (99.96-99.99)
New Zealand	74.77 (71.92-77.54)	81.74 (79.41-83.96)	85.19 (82.57-87.71)	100.00 (100.00-100.00)	100.00 (100.00-100.00)	100.00 (100.00-100.00)	63.22 (56.41-68.90)	63.28 (56.37-69.55)	63.27 (55.38-69.79)	99.99 (99.99-100.00)	100.00 (99.99-100.00)	100.00 (99.99-100.00)
Brunei	41.16 (39.69-42.68)	45.29 (43.76-46.74)	49.10 (47.34-50.70)	100.00 (100.00-100.00)	100.00 (100.00-100.00)	100.00 (100.00-100.00)	56.51 (47.00-65.14)	56.82 (46.73-65.76)	56.36 (46.19-65.92)	99.88 (99.84-99.91)	99.89 (99.85-99.91)	99.84 (99.79-99.89)
Japan	61.62 (59.01-64.67)	67.08 (64.19-70.42)	74.22 (72.61-75.81)	100.00 (100.00-100.00)	100.00 (100.00-100.00)	100.00 (100.00-100.00)	61.91 (54.82-68.03)	61.76 (54.27-68.12)	61.40 (53.25-68.21)	99.96 (99.95-99.97)	99.96 (99.95-99.97)	99.96 (99.95-99.97)
Singapore	48.35 (46.38-50.42)	52.68 (50.73-54.61)	56.42 (54.06-59.03)	100.00 (100.00-100.00)	100.00 (100.00-100.00)	100.00 (100.00-100.00)	61.73 (54.55-67.75)	61.81 (54.45-68.63)	61.76 (53.86-68.20)	99.98 (99.97-99.99)	99.98 (99.97-99.99)	99.94 (99.90-99.97)
South Korea	33.72 (31.86-35.58)	46.05 (44.37-47.87)	50.12 (48.25-52.19)	51.52 (44.04-68.87)	59.17 (57.40-61.75)	80.05 (78.03-82.90)	46.02 (38.33-53.39)	45.87 (36.05-54.29)	46.43 (37.13-54.64)	98.60 (98.33-98.85)	98.67 (98.40-98.90)	98.58 (98.29-98.84)
Andorra	70.91 (66.87-75.00)	70.87 (66.78-75.08)	70.26 (65.78-75.08)	100.00 (100.00-100.00)	100.00 (100.00-100.00)	100.00 (100.00-100.00)	81.45 (71.46-88.55)	81.42 (70.91-89.12)	81.23 (69.98-89.04)	99.98 (99.98-99.99)	99.99 (99.98-99.99)	99.97 (99.96-99.98)
Austria	67.21 (64.43-69.91)	75.65 (73.65-77.71)	81.94 (79.29-84.23)	100.00 (100.00-100.00)	100.00 (100.00-100.00)	100.00 (100.00-100.00)	81.50 (71.53-88.61)	81.45 (70.96-89.14)	81.27 (69.99-89.08)	99.99 (99.99-99.99)	99.99 (99.99-99.99)	99.99 (99.99-99.99)
Belgium	71.59 (68.64-74.33)	76.78 (74.85-78.63)	81.18 (78.91-83.46)	100.00 (100.00-100.00)	100.00 (100.00-100.00)	100.00 (100.00-100.00)	83.49 (78.27-87.32)	83.57 (78.35-87.47)	83.35 (77.60-87.76)	99.99 (99.99-99.99)	99.99 (99.99-99.99)	99.99 (99.99-100.00)

	Appendix Table 1a. Scaled values for each SDG health-related indicator and three summary indicators by country for 1990, 2000, and 2015											
	**Indicator 3.3.2: Age-standardised rate of new and relapsed TB cases (per 1,000 population)**			**Indicator 3.3.3: Age-standaridsed rate of malaria cases (per 1,000 population)**			**Indicator 3.3.4: Age-standardised rate of hepatitis B incident cases (per 100,000 population)**			**Indicator 3.3.5: Age-standardised prevalence rate of neglected tropical diseases (NTDs) (per 100,000 population)**		
**Location**	**1990**	**2000**	**2015**	**1990**	**2000**	**2015**	**1990**	**2000**	**2015**	**1990**	**2000**	**2015**
Cyprus	85.26 (80.79-90.26)	85.85 (81.83-90.28)	85.44 (80.95-90.14)	100.00 (100.00-100.00)	100.00 (100.00-100.00)	100.00 (100.00-100.00)	81.85 (75.98-85.79)	81.76 (76.39-85.97)	81.73 (75.70-86.20)	99.99 (99.99-99.99)	99.99 (99.99-99.99)	99.99 (99.99-99.99)
Denmark	76.70 (73.77-79.65)	79.13 (77.13-81.40)	85.19 (82.45-87.98)	100.00 (100.00-100.00)	100.00 (100.00-100.00)	100.00 (100.00-100.00)	82.99 (77.35-86.84)	83.06 (77.56-87.05)	82.72 (76.63-87.10)	99.99 (99.99-99.99)	99.99 (99.99-99.99)	99.99 (99.99-99.99)
Finland	69.17 (66.45-71.74)	76.65 (74.40-78.89)	83.53 (80.45-86.31)	100.00 (100.00-100.00)	100.00 (100.00-100.00)	100.00 (100.00-100.00)	81.45 (71.44-88.58)	81.39 (70.88-89.10)	81.18 (69.94-89.04)	100.00 (99.99-100.00)	100.00 (100.00-100.00)	100.00 (100.00-100.00)
France	52.81 (50.11-55.16)	68.64 (66.63-70.65)	79.61 (77.13-81.94)	100.00 (100.00-100.00)	100.00 (100.00-100.00)	100.00 (100.00-100.00)	83.43 (78.20-87.14)	83.52 (78.48-87.49)	83.37 (77.43-87.72)	99.99 (99.99-99.99)	99.99 (99.99-99.99)	99.99 (99.99-99.99)
Germany	69.35 (66.47-72.03)	78.43 (75.72-81.07)	86.36 (83.52-89.04)	100.00 (100.00-100.00)	100.00 (100.00-100.00)	100.00 (100.00-100.00)	85.00 (79.94-88.62)	85.12 (80.18-88.76)	84.75 (79.06-88.80)	99.99 (99.99-99.99)	99.99 (99.99-99.99)	99.99 (99.99-100.00)
Greece	71.02 (68.34-73.59)	81.80 (80.42-83.23)	83.66 (81.17-86.13)	100.00 (100.00-100.00)	100.00 (100.00-100.00)	100.00 (100.00-100.00)	77.15 (71.08-81.71)	77.10 (71.00-81.96)	77.16 (70.20-82.26)	99.99 (99.99-99.99)	99.99 (99.99-100.00)	100.00 (99.99-100.00)
Iceland	66.97 (63.66-70.45)	65.21 (62.18-68.40)	69.11 (65.21-73.61)	100.00 (100.00-100.00)	100.00 (100.00-100.00)	100.00 (100.00-100.00)	81.35 (71.38-88.44)	81.36 (70.89-89.05)	81.30 (70.04-89.06)	99.99 (99.99-99.99)	99.99 (99.99-100.00)	99.99 (99.99-99.99)
Ireland	67.31 (64.73-69.83)	75.54 (73.59-77.29)	79.83 (77.43-82.39)	100.00 (100.00-100.00)	100.00 (100.00-100.00)	100.00 (100.00-100.00)	81.42 (71.49-88.48)	81.41 (70.93-89.12)	81.39 (70.27-89.12)	99.99 (99.99-99.99)	99.99 (99.99-99.99)	99.99 (99.99-99.99)
Israel	75.29 (72.13-78.33)	79.15 (77.23-80.96)	82.11 (79.21-84.95)	100.00 (100.00-100.00)	100.00 (100.00-100.00)	100.00 (100.00-100.00)	81.43 (71.47-88.50)	81.49 (70.97-89.18)	81.27 (70.03-89.04)	99.93 (99.92-99.94)	99.94 (99.92-99.95)	99.95 (99.94-99.96)
Italy	71.92 (69.01-74.62)	81.67 (79.19-84.05)	87.17 (84.56-89.85)	100.00 (100.00-100.00)	100.00 (100.00-100.00)	100.00 (100.00-100.00)	82.74 (77.30-86.60)	82.75 (77.49-86.86)	82.44 (76.39-86.92)	99.99 (99.98-99.99)	99.99 (99.99-99.99)	99.98 (99.98-99.99)
Luxembourg	73.87 (70.68-76.99)	77.06 (73.76-80.47)	76.63 (72.30-81.11)	100.00 (100.00-100.00)	100.00 (100.00-100.00)	100.00 (100.00-100.00)	81.40 (71.40-88.51)	81.39 (70.92-89.08)	81.19 (69.88-89.06)	99.99 (99.99-99.99)	99.99 (99.99-99.99)	99.99 (99.99-99.99)

Location	Appendix Table 1a. Scaled values for each SDG health-related indicator and three summary indicators by country for 1990, 2000, and 2015											
	**Indicator 3.3.2: Age-standardised rate of new and relapsed TB cases (per 1,000 population)**			**Indicator 3.3.3: Age-standaridsed rate of malaria cases (per 1,000 population)**			**Indicator 3.3.4: Age-standardised rate of hepatitis B incident cases (per 100,000 population)**			**Indicator 3.3.5: Age-standardised prevalence rate of neglected tropical diseases (NTDs) (per 100,000 population)**		
	**1990**	**2000**	**2015**	**1990**	**2000**	**2015**	**1990**	**2000**	**2015**	**1990**	**2000**	**2015**
Malta	76.53 (72.61-80.51)	72.96 (69.69-76.35)	72.96 (69.13-77.09)	100.00 (100.00-100.00)	100.00 (100.00-100.00)	100.00 (100.00-100.00)	81.51 (71.52-88.55)	81.40 (70.88-89.12)	81.11 (70.00-89.04)	99.99 (99.99-99.99)	99.99 (99.99-99.99)	99.99 (99.99-99.99)
Netherlands	70.10 (67.33-72.77)	79.09 (76.68-81.32)	86.21 (84.00-88.48)	100.00 (100.00-100.00)	100.00 (100.00-100.00)	100.00 (100.00-100.00)	82.99 (77.71-86.82)	82.69 (77.03-86.78)	82.62 (76.64-87.21)	99.99 (99.99-99.99)	99.99 (99.99-99.99)	99.99 (99.99-99.99)
Norway	74.68 (71.82-77.58)	76.74 (74.31-79.23)	81.40 (78.83-84.24)	100.00 (100.00-100.00)	100.00 (100.00-100.00)	100.00 (100.00-100.00)	81.35 (71.33-88.46)	81.34 (70.84-89.04)	81.12 (69.86-89.02)	99.99 (99.99-99.99)	99.99 (99.99-99.99)	99.99 (99.99-99.99)
Portugal	50.66 (48.26-53.04)	49.93 (47.78-52.01)	64.69 (62.61-66.59)	100.00 (100.00-100.00)	100.00 (100.00-100.00)	100.00 (100.00-100.00)	81.58 (71.68-88.61)	81.50 (71.00-89.20)	81.27 (70.04-89.06)	99.63 (99.54-99.71)	99.64 (99.55-99.72)	99.66 (99.57-99.73)
Spain	55.66 (53.08-58.00)	66.91 (64.79-68.93)	77.65 (75.63-79.47)	100.00 (100.00-100.00)	100.00 (100.00-100.00)	100.00 (100.00-100.00)	86.58 (81.78-89.86)	86.68 (81.67-90.24)	86.06 (80.70-90.00)	99.96 (99.96-99.97)	99.96 (99.95-99.97)	99.94 (99.93-99.95)
Sweden	75.83 (72.78-78.98)	81.52 (77.93-84.91)	82.30 (78.26-86.54)	100.00 (100.00-100.00)	100.00 (100.00-100.00)	100.00 (100.00-100.00)	85.21 (80.05-88.71)	85.30 (80.21-88.94)	84.85 (79.19-88.86)	99.99 (99.99-99.99)	99.99 (99.99-99.99)	99.99 (99.99-99.99)
Switzerland	73.51 (70.67-76.46)	81.84 (79.73-84.01)	83.85 (80.97-86.76)	100.00 (100.00-100.00)	100.00 (100.00-100.00)	100.00 (100.00-100.00)	82.72 (77.01-86.69)	82.76 (77.29-86.83)	82.41 (76.47-86.75)	99.98 (99.98-99.99)	99.98 (99.98-99.99)	99.98 (99.98-99.99)
United Kingdom	73.71 (70.09-77.31)	80.86 (78.84-82.80)	74.37 (70.17-78.38)	100.00 (100.00-100.00)	100.00 (100.00-100.00)	100.00 (100.00-100.00)	85.28 (80.19-88.80)	85.43 (80.07-89.07)	84.69 (79.17-88.83)	100.00 (100.00-100.00)	100.00 (100.00-100.00)	100.00 (100.00-100.00)
Argentina	49.77 (47.70-51.86)	50.94 (49.05-52.86)	58.72 (56.79-60.64)	66.25 (64.31-69.14)	80.96 (79.13-83.84)	100.00 (100.00-100.00)	73.97 (67.66-78.83)	73.96 (67.39-79.14)	73.68 (66.63-79.07)	87.29 (84.85-89.38)	90.81 (89.13-92.23)	90.79 (88.96-92.30)
Chile	51.35 (49.63-53.31)	56.23 (54.62-58.04)	61.90 (60.01-63.80)	100.00 (100.00-100.00)	100.00 (100.00-100.00)	100.00 (100.00-100.00)	73.92 (67.14-78.85)	73.92 (67.46-78.96)	73.68 (66.78-79.00)	85.48 (81.75-88.53)	89.87 (87.81-91.69)	86.46 (82.88-89.62)
Uruguay	58.90 (56.90-60.83)	63.04 (61.34-64.80)	67.96 (66.20-69.88)	100.00 (100.00-100.00)	100.00 (100.00-100.00)	100.00 (100.00-100.00)	73.95 (67.18-78.85)	73.94 (67.44-78.99)	73.72 (66.82-79.05)	99.32 (99.15-99.45)	99.40 (99.25-99.52)	99.65 (99.57-99.72)

	Appendix Table 1a. Scaled values for each SDG health-related indicator and three summary indicators by country for 1990, 2000, and 2015											
	Indicator 3.3.2: Age-standardised rate of new and relapsed TB cases (per 1,000 population)			Indicator 3.3.3: Age-standaridsed rate of malaria cases (per 1,000 population)			Indicator 3.3.4: Age-standardised rate of hepatitis B incident cases (per 100,000 population)			Indicator 3.3.5: Age-standardised prevalence rate of neglected tropical diseases (NTDs) (per 100,000 population)		
**Location**	**1990**	**2000**	**2015**	**1990**	**2000**	**2015**	**1990**	**2000**	**2015**	**1990**	**2000**	**2015**
Belarus	56.18 (54.41-58.06)	53.83 (52.27-55.36)	54.40 (52.42-56.19)	100.00 (100.00-100.00)	100.00 (100.00-100.00)	100.00 (100.00-100.00)	48.92 (41.30-56.20)	48.95 (39.46-57.19)	48.75 (39.53-57.20)	99.97 (99.96-99.98)	99.98 (99.97-99.98)	99.98 (99.97-99.98)
Estonia	49.86 (47.71-52.12)	46.70 (44.81-48.79)	56.13 (53.63-58.44)	100.00 (100.00-100.00)	100.00 (100.00-100.00)	100.00 (100.00-100.00)	48.89 (41.29-56.15)	48.98 (39.46-57.23)	48.74 (39.47-57.18)	99.98 (99.97-99.98)	99.98 (99.98-99.98)	99.98 (99.97-99.98)
Latvia	47.14 (45.08-49.30)	43.90 (42.10-45.93)	54.08 (51.97-56.34)	100.00 (100.00-100.00)	100.00 (100.00-100.00)	100.00 (100.00-100.00)	48.91 (41.31-56.17)	48.97 (39.46-57.22)	48.72 (39.43-57.19)	99.98 (99.97-99.98)	99.98 (99.98-99.99)	99.98 (99.98-99.99)
Lithuania	43.39 (41.43-45.70)	43.12 (41.34-45.16)	47.20 (45.06-49.27)	100.00 (100.00-100.00)	100.00 (100.00-100.00)	100.00 (100.00-100.00)	48.89 (41.26-56.12)	48.96 (39.49-57.20)	48.72 (39.48-57.22)	99.97 (99.97-99.98)	99.98 (99.97-99.98)	99.98 (99.97-99.98)
Moldova	51.37 (49.23-53.60)	46.97 (45.14-48.98)	48.38 (46.29-50.59)	100.00 (100.00-100.00)	100.00 (100.00-100.00)	100.00 (100.00-100.00)	48.95 (41.31-56.18)	48.97 (39.53-57.21)	48.72 (39.49-57.19)	99.94 (99.93-99.95)	99.95 (99.94-99.96)	99.97 (99.97-99.98)
Russia	41.41 (39.41-43.44)	36.72 (34.76-38.74)	41.43 (39.58-43.55)	100.00 (100.00-100.00)	100.00 (100.00-100.00)	100.00 (100.00-100.00)	48.91 (41.28-56.19)	48.97 (39.49-57.22)	48.78 (39.49-57.19)	99.38 (99.25-99.49)	99.38 (99.26-99.49)	99.37 (99.24-99.48)
Ukraine	44.45 (42.41-46.47)	39.10 (37.25-41.14)	40.73 (38.84-42.80)	100.00 (100.00-100.00)	100.00 (100.00-100.00)	100.00 (100.00-100.00)	48.94 (41.35-56.21)	48.98 (39.48-57.24)	48.77 (39.53-57.20)	99.79 (99.75-99.83)	99.80 (99.76-99.84)	99.80 (99.75-99.84)
Albania	76.22 (73.32-79.32)	77.71 (74.47-81.06)	74.88 (71.09-79.21)	100.00 (100.00-100.00)	100.00 (100.00-100.00)	100.00 (100.00-100.00)	66.41 (54.63-76.14)	66.44 (54.69-75.88)	66.26 (53.17-76.36)	99.99 (99.99-99.99)	99.99 (99.99-99.99)	99.99 (99.99-99.99)
Bosnia and Herzegovina	60.35 (58.92-61.71)	62.72 (61.00-64.39)	63.56 (60.90-66.00)	100.00 (100.00-100.00)	100.00 (100.00-100.00)	100.00 (100.00-100.00)	66.48 (54.55-76.23)	66.41 (54.68-75.99)	66.18 (53.09-76.27)	99.97 (99.96-99.98)	99.98 (99.98-99.98)	99.98 (99.98-99.99)
Bulgaria	62.64 (59.91-65.23)	57.64 (55.09-60.38)	63.44 (61.37-65.84)	100.00 (100.00-100.00)	100.00 (100.00-100.00)	100.00 (100.00-100.00)	63.38 (56.36-69.24)	63.46 (56.31-69.90)	63.31 (55.14-69.93)	99.98 (99.97-99.98)	99.98 (99.97-99.98)	99.98 (99.98-99.99)
Croatia	49.36 (47.22-51.48)	56.13 (54.06-58.26)	69.27 (66.82-71.62)	100.00 (100.00-100.00)	100.00 (100.00-100.00)	100.00 (100.00-100.00)	66.16 (59.48-71.70)	66.06 (59.06-72.38)	65.90 (58.24-72.33)	99.98 (99.98-99.98)	99.98 (99.98-99.98)	99.98 (99.98-99.99)

Location	Appendix Table 1a. Scaled values for each SDG health-related indicator and three summary indicators by country for 1990, 2000, and 2015											
	**Indicator 3.3.2: Age-standardised rate of new and relapsed TB cases (per 1,000 population)**			**Indicator 3.3.3: Age-standaridsed rate of malaria cases (per 1,000 population)**			**Indicator 3.3.4: Age-standardised rate of hepatitis B incident cases (per 100,000 population)**			**Indicator 3.3.5: Age-standardised prevalence rate of neglected tropical diseases (NTDs) (per 100,000 population)**		
	**1990**	**2000**	**2015**	**1990**	**2000**	**2015**	**1990**	**2000**	**2015**	**1990**	**2000**	**2015**
Czech Republic	68.77 (66.18-71.33)	74.88 (72.90-76.85)	80.51 (77.48-83.36)	100.00 (100.00-100.00)	100.00 (100.00-100.00)	100.00 (100.00-100.00)	66.52 (54.64-76.23)	66.45 (54.73-76.00)	66.20 (53.07-76.26)	99.99 (99.98-99.99)	99.99 (99.99-99.99)	99.99 (99.98-99.99)
Hungary	55.88 (53.75-58.05)	63.56 (61.66-65.56)	74.71 (71.85-77.17)	100.00 (100.00-100.00)	100.00 (100.00-100.00)	100.00 (100.00-100.00)	66.54 (54.84-76.26)	66.49 (54.75-76.03)	66.25 (53.10-76.31)	99.98 (99.97-99.98)	99.98 (99.98-99.99)	99.99 (99.98-99.99)
Macedonia	55.74 (54.29-57.21)	60.79 (59.40-62.18)	67.90 (65.55-70.14)	100.00 (100.00-100.00)	100.00 (100.00-100.00)	100.00 (100.00-100.00)	66.46 (54.61-76.19)	66.42 (54.66-75.92)	66.21 (53.15-76.30)	99.98 (99.98-99.98)	99.99 (99.98-99.99)	99.99 (99.98-99.99)
Montenegro	74.12 (71.14-77.23)	76.68 (73.33-80.14)	74.19 (70.72-77.97)	100.00 (100.00-100.00)	100.00 (100.00-100.00)	100.00 (100.00-100.00)	66.48 (54.58-76.22)	66.46 (54.74-76.00)	66.22 (53.15-76.30)	99.98 (99.97-99.98)	99.98 (99.98-99.98)	99.98 (99.98-99.99)
Poland	52.47 (50.44-54.60)	60.78 (58.91-62.60)	67.62 (65.46-69.62)	100.00 (100.00-100.00)	100.00 (100.00-100.00)	100.00 (100.00-100.00)	78.02 (71.83-82.44)	78.17 (72.19-82.74)	77.66 (71.03-82.74)	99.98 (99.98-99.99)	99.99 (99.99-99.99)	99.99 (99.99-99.99)
Romania	43.98 (42.00-46.06)	39.10 (37.25-41.09)	46.27 (44.21-48.29)	100.00 (100.00-100.00)	100.00 (100.00-100.00)	100.00 (100.00-100.00)	66.50 (54.61-76.19)	66.45 (54.70-76.02)	66.22 (53.13-76.29)	99.96 (99.96-99.97)	99.98 (99.97-99.98)	99.98 (99.98-99.98)
Serbia	65.49 (63.52-67.39)	68.69 (66.45-71.04)	69.65 (67.24-72.23)	100.00 (100.00-100.00)	100.00 (100.00-100.00)	100.00 (100.00-100.00)	66.50 (54.56-76.20)	66.46 (54.78-75.98)	66.23 (53.14-76.31)	99.94 (99.93-99.95)	99.96 (99.95-99.97)	99.98 (99.97-99.98)
Slovakia	70.33 (67.58-73.04)	74.38 (71.46-77.31)	76.80 (72.72-81.09)	100.00 (100.00-100.00)	100.00 (100.00-100.00)	100.00 (100.00-100.00)	66.53 (54.77-76.25)	66.48 (54.76-76.03)	66.24 (53.09-76.31)	99.99 (99.98-99.99)	99.99 (99.99-99.99)	99.99 (99.99-99.99)
Slovenia	60.79 (58.47-63.11)	69.71 (67.57-71.96)	75.42 (72.33-78.51)	100.00 (100.00-100.00)	100.00 (100.00-100.00)	100.00 (100.00-100.00)	66.53 (54.58-76.28)	66.44 (54.74-75.99)	66.17 (53.07-76.25)	99.97 (99.97-99.98)	99.98 (99.98-99.99)	99.99 (99.98-99.99)
Armenia	64.12 (62.09-66.09)	62.79 (61.07-64.46)	60.23 (57.96-62.36)	33.21 (26.02-52.45)	100.00 (100.00-100.00)	100.00 (100.00-100.00)	41.32 (31.77-50.46)	41.33 (29.71-52.18)	40.97 (30.26-51.51)	99.51 (99.28-99.67)	99.75 (99.69-99.81)	99.55 (99.39-99.66)
Azerbaijan	40.44 (38.89-41.95)	40.14 (38.52-41.54)	44.69 (42.76-46.40)	38.94 (31.65-56.28)	53.33 (51.55-55.73)	100.00 (100.00-100.00)	41.33 (31.71-50.51)	41.25 (29.81-52.16)	40.82 (30.30-51.26)	97.39 (96.10-98.27)	98.84 (98.55-99.14)	98.19 (97.26-98.77)

Location	Appendix Table 1a. Scaled values for each SDG health-related indicator and three summary indicators by country for 1990, 2000, and 2015											
	**Indicator 3.3.2: Age-standardised rate of new and relapsed TB cases (per 1,000 population)**			**Indicator 3.3.3: Age-standaridsed rate of malaria cases (per 1,000 population)**			**Indicator 3.3.4: Age-standardised rate of hepatitis B incident cases (per 100,000 population)**			**Indicator 3.3.5: Age-standardised prevalence rate of neglected tropical diseases (NTDs) (per 100,000 population)**		
	**1990**	**2000**	**2015**	**1990**	**2000**	**2015**	**1990**	**2000**	**2015**	**1990**	**2000**	**2015**
Georgia	49.01 (47.20-50.72)	54.57 (52.95-56.22)	53.84 (51.84-55.59)	34.77 (27.51-51.56)	64.53 (62.65-67.29)	100.00 (100.00-100.00)	41.33 (31.74-50.51)	41.25 (29.73-52.22)	40.84 (30.29-51.27)	99.58 (99.36-99.71)	99.78 (99.73-99.83)	99.62 (99.44-99.73)
Kazakhstan	31.84 (29.90-33.80)	31.38 (29.60-33.25)	36.83 (34.77-38.87)	100.00 (100.00-100.00)	100.00 (100.00-100.00)	100.00 (100.00-100.00)	41.31 (31.62-50.62)	41.25 (29.71-52.24)	40.88 (30.34-51.37)	86.47 (82.16-90.01)	91.69 (89.82-93.45)	87.66 (82.77-91.67)
Kyrgyzstan	46.56 (44.88-48.19)	43.86 (42.33-45.38)	49.16 (47.63-50.68)	34.48 (27.53-51.06)	96.03 (95.27-97.07)	100.00 (100.00-100.00)	41.30 (31.60-50.56)	41.20 (29.77-52.25)	40.84 (30.29-51.34)	85.86 (80.33-89.95)	91.77 (89.92-93.57)	87.31 (82.22-91.06)
Mongolia	37.21 (35.58-38.68)	38.73 (37.17-40.14)	40.73 (39.13-42.31)	100.00 (100.00-100.00)	100.00 (100.00-100.00)	100.00 (100.00-100.00)	41.01 (33.60-48.90)	40.87 (31.49-49.78)	40.94 (30.82-49.68)	99.77 (99.73-99.81)	99.84 (99.81-99.87)	99.83 (99.79-99.86)
Tajikistan	48.91 (47.14-50.63)	48.24 (46.67-49.70)	52.42 (50.53-54.20)	33.40 (28.13-42.24)	30.48 (29.00-32.22)	100.00 (100.00-100.00)	41.26 (31.67-50.41)	41.17 (29.76-52.18)	40.79 (30.14-51.27)	94.21 (92.20-95.81)	96.76 (96.01-97.50)	94.86 (92.65-96.43)
Turkmenistan	42.62 (40.90-44.25)	40.80 (39.15-42.41)	45.76 (44.16-47.54)	55.96 (49.49-69.44)	100.00 (100.00-100.00)	100.00 (100.00-100.00)	41.29 (31.62-50.49)	41.20 (29.75-52.25)	40.86 (30.32-51.39)	98.11 (97.30-98.72)	98.99 (98.75-99.24)	98.23 (97.40-98.81)
Uzbekistan	48.75 (47.15-50.23)	45.75 (44.21-47.17)	50.06 (48.47-51.75)	48.94 (41.02-69.96)	95.07 (94.16-96.33)	100.00 (100.00-100.00)	42.95 (35.23-50.94)	42.98 (33.52-51.53)	42.99 (33.30-51.43)	99.84 (99.79-99.88)	99.91 (99.89-99.92)	99.85 (99.80-99.88)
Colombia	52.21 (50.55-54.00)	54.48 (53.03-56.11)	63.22 (61.57-65.05)	29.60 (27.27-32.59)	31.36 (29.70-33.32)	31.92 (30.24-34.08)	70.79 (63.83-76.23)	70.73 (63.90-76.40)	69.78 (62.16-76.00)	73.80 (68.42-78.14)	79.15 (75.14-82.53)	74.29 (69.13-78.69)
Costa Rica	60.40 (58.77-62.05)	63.59 (62.01-65.26)	73.20 (71.29-75.07)	47.52 (45.82-49.80)	44.54 (42.96-46.57)	95.18 (94.37-96.38)	71.91 (57.09-82.75)	72.30 (58.31-83.11)	72.25 (57.37-83.31)	79.24 (73.97-83.65)	88.17 (85.41-90.77)	79.92 (74.75-84.08)
El Salvador	57.72 (56.59-58.85)	66.03 (64.65-67.32)	69.08 (66.53-71.49)	34.60 (33.00-36.46)	57.81 (55.92-60.51)	93.91 (92.94-95.39)	72.02 (57.05-82.85)	72.42 (58.44-83.33)	72.43 (57.77-83.50)	78.40 (73.60-82.57)	83.44 (80.32-86.27)	92.22 (89.90-94.07)
Guatemala	34.20 (32.56-35.98)	46.66 (44.96-48.38)	59.70 (57.56-61.88)	25.24 (21.88-30.73)	31.60 (29.59-33.99)	34.10 (32.30-36.36)	71.97 (57.05-82.81)	72.39 (58.45-83.32)	72.35 (57.47-83.38)	55.30 (46.94-62.28)	62.71 (56.08-68.63)	70.99 (64.68-76.10)

	Appendix Table 1a. Scaled values for each SDG health-related indicator and three summary indicators by country for 1990, 2000, and 2015											
	Indicator 3.3.2: Age-standardised rate of new and relapsed TB cases (per 1,000 population)			Indicator 3.3.3: Age-standaridsed rate of malaria cases (per 1,000 population)			Indicator 3.3.4: Age-standardised rate of hepatitis B incident cases (per 100,000 population)			Indicator 3.3.5: Age-standardised prevalence rate of neglected tropical diseases (NTDs) (per 100,000 population)		
**Location**	**1990**	**2000**	**2015**	**1990**	**2000**	**2015**	**1990**	**2000**	**2015**	**1990**	**2000**	**2015**
Honduras	49.46 (48.20-50.59)	50.33 (49.21-51.45)	55.43 (54.08-56.74)	36.29 (33.68-39.68)	38.19 (36.27-40.51)	37.95 (36.35-40.13)	71.93 (57.06-82.75)	72.31 (58.38-83.18)	72.27 (57.40-83.30)	67.75 (60.53-73.97)	78.01 (73.36-82.21)	74.63 (68.43-79.59)
Mexico	51.89 (50.06-53.89)	56.54 (54.44-58.69)	65.63 (64.10-67.23)	29.07 (27.21-31.26)	64.13 (62.22-67.03)	76.78 (74.90-79.77)	99.08 (97.04-100.00)	99.14 (97.13-100.00)	96.82 (93.98-98.97)	85.92 (83.36-88.04)	88.39 (86.31-90.16)	88.23 (86.14-90.11)
Nicaragua	52.01 (50.87-53.18)	58.89 (57.76-59.99)	65.53 (63.66-67.30)	28.05 (24.75-33.32)	34.15 (32.26-36.49)	36.64 (34.73-39.02)	73.96 (67.68-78.72)	73.92 (67.83-79.01)	73.93 (67.00-79.50)	82.38 (78.44-85.75)	86.43 (83.84-88.89)	93.24 (90.90-95.08)
Panama	47.64 (46.35-48.96)	50.55 (49.28-51.84)	52.01 (50.71-53.22)	56.26 (54.43-58.78)	48.14 (46.50-50.32)	51.49 (49.74-53.75)	71.91 (57.12-82.75)	72.29 (58.32-83.08)	72.25 (57.38-83.29)	71.70 (66.04-76.49)	93.73 (92.49-94.87)	93.19 (91.17-95.02)
Venezuela	48.68 (47.01-50.49)	54.80 (53.35-56.45)	61.97 (60.22-63.57)	39.00 (36.72-41.84)	39.32 (37.33-41.80)	40.08 (38.42-42.41)	71.92 (57.10-82.74)	72.31 (58.30-83.13)	72.28 (57.40-83.33)	69.15 (63.66-73.96)	71.60 (66.69-76.05)	70.72 (63.98-76.82)
Bolivia	38.13 (36.89-39.35)	44.26 (43.03-45.60)	49.92 (48.39-51.31)	52.42 (49.98-55.51)	52.97 (50.88-55.59)	52.24 (50.28-54.99)	69.56 (62.41-75.18)	69.63 (62.44-75.56)	69.50 (61.94-75.71)	80.04 (75.96-83.84)	87.03 (84.42-89.24)	82.38 (78.59-85.68)
Ecuador	36.38 (35.07-37.70)	43.14 (41.77-44.52)	51.47 (49.85-52.84)	24.10 (22.78-25.81)	22.56 (21.35-24.18)	70.71 (68.82-73.62)	69.56 (62.46-75.21)	69.63 (62.44-75.57)	69.50 (61.94-75.71)	65.44 (58.42-71.64)	72.63 (67.59-77.17)	71.22 (64.13-76.78)
Peru	27.93 (26.66-29.23)	38.47 (37.11-39.75)	47.02 (44.92-48.85)	41.61 (38.95-45.07)	42.86 (40.95-45.22)	42.59 (40.66-45.09)	69.61 (62.51-75.23)	69.65 (62.47-75.59)	69.49 (61.95-75.71)	76.98 (72.14-81.30)	83.85 (80.76-86.88)	84.42 (80.40-87.67)
Antigua and Barbuda	62.15 (59.54-64.60)	61.12 (58.72-63.58)	62.64 (59.03-66.20)	100.00 (100.00-100.00)	100.00 (100.00-100.00)	100.00 (100.00-100.00)	76.03 (69.83-80.64)	76.13 (70.06-80.83)	75.95 (69.23-80.89)	99.77 (99.68-99.84)	99.82 (99.73-99.87)	99.70 (99.59-99.79)
The Bahamas	52.23 (50.80-53.61)	54.74 (53.27-56.07)	62.61 (60.50-64.65)	100.00 (100.00-100.00)	100.00 (100.00-100.00)	100.00 (100.00-100.00)	76.00 (69.79-80.60)	76.06 (69.84-80.74)	75.89 (69.21-80.87)	99.85 (99.82-99.88)	99.89 (99.87-99.92)	99.86 (99.82-99.89)
Barbados	66.93 (64.20-69.39)	66.03 (63.65-68.54)	66.80 (63.18-70.43)	100.00 (100.00-100.00)	100.00 (100.00-100.00)	100.00 (100.00-100.00)	76.04 (69.83-80.59)	76.05 (69.82-80.76)	75.91 (69.21-80.88)	99.86 (99.83-99.89)	99.86 (99.82-99.89)	99.74 (99.63-99.81)

Appendix Table 1a. Scaled values for each SDG health-related indicator and three summary indicators by country for 1990, 2000, and 2015

Location	Indicator 3.3.2: Age-standardised rate of new and relapsed TB cases (per 1,000 population)			Indicator 3.3.3: Age-standaridsed rate of malaria cases (per 1,000 population)			Indicator 3.3.4: Age-standardised rate of hepatitis B incident cases (per 100,000 population)			Indicator 3.3.5: Age-standardised prevalence rate of neglected tropical diseases (NTDs) (per 100,000 population)		
	1990	2000	2015	1990	2000	2015	1990	2000	2015	1990	2000	2015
Belize	53.14 (51.94-54.36)	47.36 (46.04-48.82)	52.05 (50.65-53.39)	18.94 (17.62-20.49)	25.30 (23.97-26.96)	59.77 (57.95-62.40)	75.94 (69.68-80.56)	75.98 (69.68-80.69)	75.86 (69.21-80.80)	85.23 (81.26-88.66)	91.82 (89.98-93.64)	95.91 (94.72-96.93)
Cuba	75.44 (72.18-78.43)	77.02 (74.75-79.30)	79.29 (76.63-82.00)	100.00 (100.00-100.00)	100.00 (100.00-100.00)	100.00 (100.00-100.00)	75.94 (69.65-80.55)	75.97 (69.65-80.68)	75.81 (69.21-80.79)	84.52 (81.80-87.08)	86.56 (84.14-88.64)	84.46 (81.46-87.04)
Dominica	56.37 (55.00-57.68)	56.55 (55.21-57.84)	58.59 (56.83-60.25)	100.00 (100.00-100.00)	100.00 (100.00-100.00)	100.00 (100.00-100.00)	75.98 (69.77-80.61)	76.01 (69.77-80.69)	75.86 (69.21-80.86)	84.59 (80.60-88.12)	99.85 (99.82-99.88)	99.89 (99.84-99.92)
Dominican Republic	41.13 (39.70-42.51)	44.12 (42.82-45.48)	49.79 (48.34-51.26)	34.35 (32.77-36.47)	35.41 (33.50-37.80)	35.58 (33.57-38.03)	75.94 (69.69-80.57)	75.99 (69.75-80.68)	75.86 (69.21-80.84)	97.97 (95.73-99.12)	97.98 (95.74-99.13)	97.96 (95.72-99.13)
Grenada	69.32 (67.43-71.31)	68.39 (66.45-70.48)	67.51 (64.56-70.20)	100.00 (100.00-100.00)	100.00 (100.00-100.00)	100.00 (100.00-100.00)	76.00 (69.79-80.58)	76.01 (69.76-80.74)	75.84 (69.18-80.83)	61.70 (54.50-68.38)	87.74 (85.41-89.71)	83.56 (79.34-87.11)
Guyana	47.96 (46.66-49.21)	42.15 (40.84-43.55)	43.10 (41.82-44.43)	17.24 (15.04-20.15)	16.70 (14.45-19.71)	19.18 (17.79-20.85)	76.00 (69.80-80.63)	75.99 (69.68-80.73)	75.82 (69.20-80.85)	81.41 (76.11-85.76)	90.04 (87.72-92.10)	87.37 (83.91-90.23)
Haiti	41.53 (40.22-42.82)	44.93 (43.71-46.21)	50.80 (49.54-51.95)	19.08 (16.90-21.97)	20.60 (18.73-22.89)	22.73 (21.28-24.45)	76.03 (69.77-80.66)	76.05 (69.86-80.72)	75.90 (69.19-80.86)	60.63 (53.75-66.69)	76.91 (72.61-80.61)	95.09 (93.86-96.16)
Jamaica	76.46 (73.98-78.95)	77.08 (74.90-79.31)	80.98 (77.35-84.72)	100.00 (100.00-100.00)	100.00 (100.00-100.00)	100.00 (100.00-100.00)	76.01 (69.60-80.62)	75.97 (69.70-80.84)	75.63 (68.94-80.74)	71.33 (66.00-75.94)	75.38 (70.83-79.14)	63.94 (57.30-69.70)
Saint Lucia	49.83 (48.08-51.60)	48.57 (46.82-50.38)	56.75 (54.67-58.92)	100.00 (100.00-100.00)	100.00 (100.00-100.00)	100.00 (100.00-100.00)	76.00 (69.82-80.66)	76.04 (69.86-80.68)	75.89 (69.23-80.86)	55.00 (46.22-62.00)	78.51 (74.51-82.10)	77.21 (71.35-81.68)
Saint Vincent and the Grenadines	54.96 (53.15-56.71)	51.53 (49.77-53.38)	55.91 (53.62-58.23)	100.00 (100.00-100.00)	100.00 (100.00-100.00)	100.00 (100.00-100.00)	75.98 (69.75-80.59)	75.96 (69.63-80.70)	75.82 (69.22-80.79)	72.39 (65.93-77.94)	91.67 (89.92-93.28)	84.99 (80.91-88.38)
Suriname	60.50 (58.84-62.14)	58.28 (56.90-59.76)	61.79 (59.68-63.78)	25.64 (22.03-31.54)	24.18 (20.20-30.83)	33.42 (30.37-37.86)	75.94 (69.63-80.54)	75.97 (69.59-80.73)	75.85 (69.21-80.86)	94.76 (93.58-95.74)	95.57 (94.53-96.42)	94.38 (92.94-95.55)

Location	Appendix Table 1a. Scaled values for each SDG health-related indicator and three summary indicators by country for 1990, 2000, and 2015											
	Indicator 3.3.2: Age-standardised rate of new and relapsed TB cases (per 1,000 population)			Indicator 3.3.3: Age-standaridsed rate of malaria cases (per 1,000 population)			Indicator 3.3.4: Age-standardised rate of hepatitis B incident cases (per 100,000 population)			Indicator 3.3.5: Age-standardised prevalence rate of neglected tropical diseases (NTDs) (per 100,000 population)		
	1990	2000	2015	1990	2000	2015	1990	2000	2015	1990	2000	2015
Trinidad and Tobago	55.61 (53.61-57.66)	55.62 (53.79-57.56)	62.00 (59.73-64.25)	100.00 (100.00-100.00)	100.00 (100.00-100.00)	100.00 (100.00-100.00)	75.98 (69.76-80.59)	76.00 (69.69-80.73)	75.87 (69.23-80.85)	97.60 (97.03-98.09)	98.21 (97.85-98.53)	97.53 (96.95-98.04)
Brazil	53.87 (51.46-55.97)	55.51 (53.04-57.58)	58.19 (56.68-59.92)	32.60 (29.31-37.74)	37.54 (35.76-39.74)	37.33 (35.56-39.62)	62.81 (55.93-68.75)	62.62 (55.22-68.91)	61.67 (53.64-68.36)	86.26 (83.49-88.69)	91.44 (89.83-92.86)	86.68 (83.97-89.06)
Paraguay	54.49 (53.21-55.72)	54.82 (53.54-56.02)	55.93 (54.58-57.19)	64.23 (57.35-78.12)	66.92 (59.96-80.73)	69.24 (62.26-82.88)	62.43 (55.47-68.48)	62.43 (55.22-68.70)	62.14 (54.00-68.77)	86.80 (83.83-89.30)	90.87 (88.92-92.50)	87.76 (85.14-90.11)
China	40.32 (38.13-42.22)	42.29 (40.31-44.12)	44.88 (43.21-46.37)	67.41 (64.34-71.01)	71.06 (69.12-73.98)	93.77 (92.76-95.20)	5.27 (0.00-17.77)	26.39 (14.73-36.35)	39.75 (29.50-48.54)	64.61 (58.20-70.07)	86.24 (83.68-88.36)	88.66 (86.43-90.45)
North Korea	46.99 (45.25-48.54)	45.67 (44.22-47.03)	49.98 (48.19-51.59)	34.02 (31.53-36.92)	26.56 (25.24-28.08)	37.88 (36.40-39.73)	15.57 (8.85-26.22)	15.47 (2.50-27.07)	15.00 (0.00-26.90)	84.65 (78.87-88.93)	90.12 (88.11-92.02)	93.91 (90.94-95.81)
Taiwan	55.91 (53.83-57.84)	57.56 (54.98-59.97)	59.90 (57.49-62.22)	100.00 (100.00-100.00)	100.00 (100.00-100.00)	100.00 (100.00-100.00)	18.32 (10.34-29.10)	18.44 (4.60-30.25)	17.87 (4.28-29.98)	59.19 (51.72-65.61)	83.08 (80.02-85.65)	85.87 (82.92-88.44)
Cambodia	31.31 (30.00-32.60)	32.11 (30.81-33.53)	37.89 (36.60-39.21)	9.99 (5.97-16.90)	10.68 (6.66-17.60)	16.23 (12.32-22.89)	35.70 (28.71-43.55)	35.89 (25.76-44.93)	35.25 (24.48-44.77)	70.25 (63.77-75.66)	84.31 (81.14-86.98)	99.47 (99.33-99.58)
Indonesia	20.04 (18.95-21.17)	23.44 (22.23-24.62)	26.79 (25.56-27.98)	11.76 (7.58-16.00)	13.51 (8.67-18.44)	18.50 (11.72-25.18)	36.15 (28.15-44.65)	36.32 (25.73-45.81)	36.10 (25.01-46.16)	67.74 (61.63-73.10)	81.62 (78.17-84.60)	83.97 (80.28-87.06)
Laos	33.29 (32.08-34.54)	37.15 (35.89-38.42)	42.00 (40.57-43.35)	20.11 (17.87-23.09)	21.89 (20.10-23.98)	23.51 (22.16-25.26)	34.89 (27.68-43.53)	35.11 (23.50-45.13)	35.41 (23.53-45.65)	69.27 (62.18-75.17)	77.06 (72.53-81.23)	80.31 (75.88-83.91)
Malaysia	44.27 (42.99-45.53)	45.38 (44.11-46.64)	46.41 (44.76-48.05)	40.94 (36.05-48.06)	45.45 (43.85-47.58)	56.72 (54.91-59.26)	57.40 (50.35-63.72)	57.16 (49.50-64.33)	56.55 (48.36-63.67)	60.20 (52.97-66.24)	65.68 (59.35-71.32)	59.78 (52.29-66.21)
Maldives	47.40 (46.20-48.66)	53.84 (52.40-55.24)	57.78 (55.59-59.78)	100.00 (100.00-100.00)	100.00 (100.00-100.00)	100.00 (100.00-100.00)	31.94 (16.89-47.22)	32.11 (16.30-49.83)	31.75 (15.72-49.28)	99.71 (99.44-99.82)	99.77 (99.56-99.86)	99.74 (99.54-99.83)

Location	Appendix Table 1a. Scaled values for each SDG health-related indicator and three summary indicators by country for 1990, 2000, and 2015											
	**Indicator 3.3.2: Age-standardised rate of new and relapsed TB cases (per 1,000 population)**			**Indicator 3.3.3: Age-standaridsed rate of malaria cases (per 1,000 population)**			**Indicator 3.3.4: Age-standardised rate of hepatitis B incident cases (per 100,000 population)**			**Indicator 3.3.5: Age-standardised prevalence rate of neglected tropical diseases (NTDs) (per 100,000 population)**		
	**1990**	**2000**	**2015**	**1990**	**2000**	**2015**	**1990**	**2000**	**2015**	**1990**	**2000**	**2015**
Mauritius	54.56 (52.34-57.06)	59.76 (57.46-62.22)	58.91 (56.12-61.68)	100.00 (100.00-100.00)	100.00 (100.00-100.00)	100.00 (100.00-100.00)	31.96 (16.97-47.60)	32.06 (16.24-49.89)	31.71 (15.74-49.16)	99.26 (98.60-99.60)	99.31 (98.68-99.65)	99.28 (98.62-99.62)
Myanmar	31.67 (30.30-33.03)	33.75 (32.41-35.09)	38.25 (36.82-39.56)	1.85 (0.00-5.36)	2.53 (0.42-5.99)	6.01 (3.11-9.67)	32.17 (17.14-47.71)	32.21 (16.50-50.14)	31.85 (15.85-49.41)	60.12 (51.91-66.96)	68.46 (62.32-74.10)	85.72 (82.34-88.89)
Philippines	24.46 (22.97-26.01)	27.76 (26.23-29.35)	33.24 (31.91-34.68)	20.82 (19.18-22.82)	22.81 (21.32-24.72)	22.31 (20.55-24.46)	31.41 (24.33-39.73)	31.33 (19.18-41.35)	31.55 (20.21-41.51)	55.72 (47.76-62.77)	59.26 (52.04-65.61)	66.49 (59.89-72.35)
Sri Lanka	40.30 (38.63-42.25)	46.23 (44.62-47.95)	52.72 (51.01-54.43)	17.69 (16.58-19.15)	19.79 (18.60-21.20)	100.00 (100.00-100.00)	39.13 (31.49-46.98)	39.32 (29.60-47.99)	39.58 (29.72-48.42)	77.43 (72.25-81.52)	91.37 (89.56-92.99)	90.49 (87.98-92.65)
Seychelles	55.69 (53.85-57.57)	58.34 (56.25-60.41)	57.18 (53.85-60.32)	100.00 (100.00-100.00)	100.00 (100.00-100.00)	100.00 (100.00-100.00)	31.95 (16.93-47.59)	32.01 (16.09-49.78)	31.52 (15.45-49.02)	69.63 (62.19-75.67)	79.46 (75.15-83.91)	69.57 (62.51-75.62)
Thailand	39.45 (38.14-40.75)	42.09 (40.64-43.52)	45.18 (43.74-46.64)	23.61 (21.32-26.64)	26.58 (25.16-28.48)	26.18 (24.49-28.29)	33.34 (25.35-42.15)	33.74 (22.52-43.37)	33.31 (22.55-42.59)	77.18 (71.78-81.47)	88.24 (85.88-90.45)	86.80 (83.54-89.36)
Timor-Leste	34.38 (33.07-35.67)	35.17 (33.70-36.53)	35.94 (34.24-37.64)	14.61 (13.25-16.24)	15.77 (14.48-17.50)	15.94 (14.43-17.90)	31.91 (16.82-47.47)	31.95 (16.02-49.74)	31.62 (15.62-48.93)	82.07 (77.01-85.90)	88.28 (85.59-90.68)	83.11 (77.92-86.98)
Vietnam	37.46 (36.23-38.77)	41.12 (39.93-42.40)	45.54 (44.31-46.77)	21.99 (17.89-29.17)	24.23 (20.24-30.95)	28.22 (24.85-33.65)	33.31 (25.52-42.27)	33.29 (22.80-43.20)	32.89 (21.24-42.59)	63.30 (56.55-68.97)	67.32 (61.47-72.44)	84.48 (80.91-87.81)
Federated States of Micronesia	49.24 (47.97-50.48)	52.92 (51.60-54.30)	53.31 (51.42-55.24)	100.00 (100.00-100.00)	100.00 (100.00-100.00)	100.00 (100.00-100.00)	16.91 (0.94-33.80)	17.39 (0.47-36.62)	16.93 (0.29-36.82)	74.30 (67.91-79.26)	81.97 (78.25-85.47)	74.61 (66.69-80.82)
Fiji	51.97 (50.63-53.20)	55.04 (53.77-56.25)	58.69 (57.00-60.26)	100.00 (100.00-100.00)	100.00 (100.00-100.00)	100.00 (100.00-100.00)	25.84 (17.65-35.47)	25.70 (14.46-36.66)	25.72 (13.66-36.12)	61.14 (53.72-67.47)	70.24 (64.42-75.19)	63.09 (55.58-69.83)
Kiribati	28.24 (27.06-29.55)	31.52 (30.26-32.80)	35.59 (34.17-37.01)	100.00 (100.00-100.00)	100.00 (100.00-100.00)	100.00 (100.00-100.00)	17.16 (1.23-34.29)	17.57 (0.79-36.68)	17.19 (0.89-36.87)	59.27 (51.49-65.92)	63.23 (56.87-69.02)	66.42 (59.96-71.84)

	Appendix Table 1a. Scaled values for each SDG health-related indicator and three summary indicators by country for 1990, 2000, and 2015											
	**Indicator 3.3.2: Age-standardised rate of new and relapsed TB cases (per 1,000 population)**			**Indicator 3.3.3: Age-standaridsed rate of malaria cases (per 1,000 population)**			**Indicator 3.3.4: Age-standardised rate of hepatitis B incident cases (per 100,000 population)**			**Indicator 3.3.5: Age-standardised prevalence rate of neglected tropical diseases (NTDs) (per 100,000 population)**		
**Location**	**1990**	**2000**	**2015**	**1990**	**2000**	**2015**	**1990**	**2000**	**2015**	**1990**	**2000**	**2015**
Marshall Islands	47.46 (46.08-48.82)	51.62 (50.37-52.89)	53.81 (52.00-55.59)	100.00 (100.00-100.00)	100.00 (100.00-100.00)	100.00 (100.00-100.00)	16.90 (0.97-33.92)	17.33 (0.36-36.79)	16.94 (0.38-36.86)	61.93 (53.95-68.24)	67.23 (61.47-72.34)	62.91 (55.64-69.13)
Papua New Guinea	48.11 (46.79-49.46)	47.42 (46.06-48.67)	48.22 (46.77-49.70)	5.02 (2.52-8.26)	4.57 (2.20-7.82)	5.84 (3.10-9.37)	20.61 (12.02-30.82)	20.90 (9.21-31.87)	20.79 (8.32-31.75)	50.04 (40.45-58.29)	55.45 (47.13-62.57)	54.42 (45.80-61.63)
Samoa	54.67 (53.21-56.03)	59.62 (58.14-61.12)	62.19 (59.81-64.35)	100.00 (100.00-100.00)	100.00 (100.00-100.00)	100.00 (100.00-100.00)	16.90 (1.24-34.18)	17.20 (0.00-36.81)	16.82 (0.16-36.72)	96.22 (95.00-97.09)	97.11 (96.11-97.80)	97.46 (96.73-98.10)
Solomon Islands	39.58 (38.28-40.89)	44.46 (43.19-45.74)	49.78 (48.55-51.10)	9.79 (7.77-12.30)	10.84 (9.16-12.88)	11.62 (10.27-13.23)	22.01 (14.65-31.73)	21.78 (9.65-32.63)	22.29 (9.52-33.50)	73.15 (66.68-78.80)	80.92 (76.83-84.63)	73.61 (67.27-78.82)
Tonga	63.25 (60.39-65.95)	65.32 (62.66-67.92)	61.42 (58.13-64.47)	100.00 (100.00-100.00)	100.00 (100.00-100.00)	100.00 (100.00-100.00)	17.03 (1.38-34.01)	17.37 (0.35-36.91)	17.06 (0.59-36.86)	90.73 (82.76-93.81)	94.79 (92.05-96.23)	93.04 (90.84-94.81)
Vanuatu	43.45 (42.22-44.79)	47.96 (46.77-49.20)	53.70 (52.47-54.91)	10.94 (8.79-13.77)	11.74 (9.83-14.11)	12.73 (11.28-14.47)	16.86 (0.96-33.53)	17.30 (0.34-36.52)	16.99 (0.59-36.77)	95.73 (94.56-96.70)	96.16 (95.11-97.03)	65.02 (58.54-71.31)
Afghanistan	38.86 (37.34-40.16)	41.39 (40.01-42.69)	45.10 (43.71-46.48)	11.58 (7.44-19.00)	11.14 (7.01-18.51)	14.30 (10.10-21.94)	31.70 (10.27-52.72)	30.50 (8.94-53.10)	31.34 (9.34-54.29)	73.91 (67.02-79.60)	81.69 (77.97-85.15)	76.08 (69.33-82.18)
Algeria	45.38 (44.09-46.78)	50.30 (49.02-51.72)	54.12 (52.45-55.76)	51.13 (41.70-90.62)	96.81 (96.18-97.67)	95.53 (94.73-96.68)	31.77 (10.30-52.91)	30.62 (9.13-53.15)	31.51 (9.66-54.45)	90.28 (84.88-93.53)	91.28 (85.97-94.37)	90.40 (85.09-93.63)
Bahrain	53.96 (52.37-55.58)	57.74 (55.93-59.48)	66.06 (63.40-68.45)	100.00 (100.00-100.00)	100.00 (100.00-100.00)	100.00 (100.00-100.00)	30.89 (8.61-52.25)	29.74 (7.37-52.72)	30.17 (6.69-53.55)	99.86 (99.83-99.89)	99.90 (99.87-99.92)	99.86 (99.82-99.89)
Egypt	61.95 (60.44-63.50)	68.16 (66.49-70.01)	73.47 (70.93-75.91)	68.82 (61.85-82.50)	69.61 (62.63-83.22)	100.00 (100.00-100.00)	46.36 (39.44-53.30)	46.24 (37.60-54.08)	46.36 (37.32-54.43)	87.80 (80.42-91.84)	89.44 (82.00-93.31)	93.19 (91.65-95.02)
Iran	59.88 (58.36-61.46)	64.39 (62.73-66.08)	62.86 (60.88-64.88)	37.67 (35.97-39.67)	54.30 (52.46-56.69)	82.55 (80.77-85.30)	59.85 (52.56-65.89)	60.32 (52.77-67.04)	60.06 (52.05-66.82)	98.52 (98.22-98.78)	98.77 (98.55-98.97)	98.52 (98.17-98.81)

Location	Appendix Table 1a. Scaled values for each SDG health-related indicator and three summary indicators by country for 1990, 2000, and 2015											
	**Indicator 3.3.2: Age-standardised rate of new and relapsed TB cases (per 1,000 population)**			**Indicator 3.3.3: Age-standaridsed rate of malaria cases (per 1,000 population)**			**Indicator 3.3.4: Age-standardised rate of hepatitis B incident cases (per 100,000 population)**			**Indicator 3.3.5: Age-standardised prevalence rate of neglected tropical diseases (NTDs) (per 100,000 population)**		
	**1990**	**2000**	**2015**	**1990**	**2000**	**2015**	**1990**	**2000**	**2015**	**1990**	**2000**	**2015**
Iraq	44.10 (42.72-45.51)	48.64 (47.37-50.10)	53.08 (51.66-54.56)	53.60 (51.55-56.14)	64.05 (61.84-67.01)	100.00 (100.00-100.00)	31.82 (10.36-52.74)	30.71 (9.31-53.20)	31.52 (9.48-54.44)	99.33 (99.14-99.49)	99.41 (99.25-99.54)	99.38 (99.21-99.52)
Jordan	78.06 (74.78-81.23)	82.48 (79.24-86.07)	82.80 (78.34-87.19)	100.00 (100.00-100.00)	100.00 (100.00-100.00)	100.00 (100.00-100.00)	42.05 (34.37-49.87)	42.43 (33.39-50.91)	42.56 (33.03-51.10)	88.63 (85.85-90.85)	91.40 (89.74-92.90)	85.63 (81.92-88.74)
Kuwait	48.92 (46.56-51.30)	55.48 (53.43-57.54)	64.20 (61.82-66.44)	100.00 (100.00-100.00)	100.00 (100.00-100.00)	100.00 (100.00-100.00)	31.45 (9.56-52.72)	29.59 (6.91-52.66)	30.90 (8.06-53.97)	99.96 (99.95-99.97)	99.99 (99.99-99.99)	99.99 (99.99-99.99)
Lebanon	63.78 (62.00-65.46)	70.79 (69.09-72.66)	72.77 (69.97-75.66)	100.00 (100.00-100.00)	100.00 (100.00-100.00)	100.00 (100.00-100.00)	31.98 (10.81-52.78)	30.77 (9.36-53.29)	31.55 (9.90-54.51)	99.86 (99.83-99.89)	99.90 (99.87-99.92)	99.86 (99.83-99.89)
Libya	58.24 (56.22-60.09)	63.25 (61.26-65.29)	64.96 (62.58-67.41)	100.00 (100.00-100.00)	100.00 (100.00-100.00)	100.00 (100.00-100.00)	31.39 (9.46-52.78)	30.39 (8.39-52.96)	31.55 (9.67-54.54)	90.68 (86.61-93.41)	95.87 (92.50-97.78)	95.92 (92.63-97.88)
Morocco	40.40 (39.05-41.74)	45.55 (44.29-46.81)	48.62 (47.29-49.96)	68.93 (61.96-82.60)	100.00 (100.00-100.00)	100.00 (100.00-100.00)	31.87 (10.55-52.93)	30.78 (9.32-53.22)	31.64 (9.74-54.47)	93.81 (92.40-95.09)	95.62 (94.64-96.52)	92.11 (90.05-93.90)
Palestine	75.08 (70.21-80.31)	68.38 (64.23-72.53)	67.65 (63.25-72.01)	100.00 (100.00-100.00)	100.00 (100.00-100.00)	100.00 (100.00-100.00)	31.77 (10.32-52.90)	30.64 (9.27-53.18)	31.49 (9.63-54.48)	94.23 (92.13-95.75)	96.32 (95.53-97.07)	94.53 (92.66-96.01)
Oman	60.98 (59.01-62.92)	66.92 (64.68-69.13)	68.66 (65.72-71.71)	71.81 (64.85-85.15)	71.68 (64.72-85.04)	100.00 (100.00-100.00)	30.96 (8.82-52.56)	29.72 (7.29-52.97)	29.74 (5.85-53.40)	99.47 (99.31-99.59)	99.68 (99.61-99.75)	99.55 (99.37-99.66)
Qatar	61.43 (58.73-64.02)	61.27 (58.40-64.26)	62.02 (59.59-64.58)	100.00 (100.00-100.00)	100.00 (100.00-100.00)	100.00 (100.00-100.00)	29.88 (7.35-51.86)	28.85 (5.61-52.56)	29.05 (4.46-53.08)	99.86 (99.82-99.89)	99.90 (99.87-99.92)	99.86 (99.82-99.89)
Saudi Arabia	50.02 (47.75-52.29)	56.49 (54.07-58.56)	62.54 (60.02-65.37)	40.28 (38.52-42.35)	52.77 (50.96-55.26)	92.94 (91.84-94.59)	48.04 (41.54-55.00)	48.80 (40.32-56.50)	45.51 (35.91-53.56)	96.41 (95.78-96.99)	96.70 (96.11-97.20)	95.69 (94.92-96.37)
Sudan	48.53 (47.24-49.85)	49.28 (48.02-50.60)	53.80 (52.42-55.14)	11.65 (9.10-14.26)	10.18 (8.05-12.62)	13.95 (12.12-15.89)	41.82 (34.29-49.81)	42.62 (33.23-51.25)	42.25 (32.23-50.97)	64.49 (54.90-72.69)	68.19 (58.93-76.08)	76.58 (69.07-82.24)

	Appendix Table 1a. Scaled values for each SDG health-related indicator and three summary indicators by country for 1990, 2000, and 2015											
	**Indicator 3.3.2: Age-standardised rate of new and relapsed TB cases (per 1,000 population)**			**Indicator 3.3.3: Age-standaridsed rate of malaria cases (per 1,000 population)**			**Indicator 3.3.4: Age-standardised rate of hepatitis B incident cases (per 100,000 population)**			**Indicator 3.3.5: Age-standardised prevalence rate of neglected tropical diseases (NTDs) (per 100,000 population)**		
**Location**	**1990**	**2000**	**2015**	**1990**	**2000**	**2015**	**1990**	**2000**	**2015**	**1990**	**2000**	**2015**
Syria	77.71 (74.49-80.78)	75.66 (72.11-79.01)	72.66 (69.44-76.12)	69.34 (62.36-82.97)	70.01 (63.03-83.57)	100.00 (100.00-100.00)	43.62 (35.81-51.31)	43.29 (33.60-52.14)	43.24 (33.77-51.76)	84.98 (81.61-87.96)	89.05 (86.96-90.81)	85.61 (82.13-88.49)
Tunisia	54.94 (53.66-56.25)	62.15 (61.01-63.15)	63.56 (61.77-65.15)	100.00 (100.00-100.00)	100.00 (100.00-100.00)	100.00 (100.00-100.00)	31.81 (10.27-52.89)	30.71 (9.27-53.21)	31.60 (9.79-54.51)	94.65 (93.63-95.62)	95.70 (94.89-96.38)	94.76 (93.80-95.61)
Turkey	55.44 (53.95-56.89)	62.53 (60.75-64.22)	68.58 (66.05-71.24)	52.98 (46.70-64.66)	53.96 (52.23-56.35)	100.00 (100.00-100.00)	42.63 (35.30-49.98)	42.78 (33.03-51.24)	42.53 (32.56-51.00)	96.12 (95.06-97.00)	97.54 (97.01-98.04)	96.55 (95.63-97.31)
United Arab Emirates	55.97 (54.20-57.84)	63.81 (61.80-65.88)	68.26 (65.01-71.82)	61.59 (54.82-75.46)	100.00 (100.00-100.00)	100.00 (100.00-100.00)	29.93 (7.39-52.00)	28.51 (5.19-52.84)	29.17 (5.25-52.89)	99.86 (99.82-99.89)	99.89 (99.87-99.92)	99.86 (99.81-99.89)
Yemen	43.34 (42.02-44.67)	46.01 (44.68-47.24)	51.77 (50.37-53.12)	25.56 (16.02-35.74)	26.01 (16.54-35.92)	27.99 (17.35-39.09)	38.89 (31.36-47.19)	39.36 (28.84-48.74)	39.65 (29.47-48.74)	74.22 (62.89-81.73)	78.34 (67.48-85.28)	84.08 (79.08-87.70)
Bangladesh	36.09 (34.76-37.41)	41.26 (39.96-42.49)	43.01 (41.57-44.43)	17.71 (13.79-24.39)	21.77 (18.15-27.60)	23.61 (20.50-28.54)	52.24 (44.68-58.79)	52.40 (44.25-59.94)	52.43 (43.36-60.01)	60.53 (53.57-66.79)	66.05 (59.85-71.47)	71.93 (65.86-77.12)
Bhutan	29.97 (28.46-31.46)	31.95 (30.27-33.46)	35.39 (33.49-37.10)	18.13 (16.82-19.67)	21.43 (20.16-23.03)	58.94 (57.13-61.52)	49.28 (41.93-56.21)	49.27 (40.82-57.13)	49.71 (40.96-57.64)	89.53 (86.22-92.33)	94.31 (92.98-95.57)	90.12 (86.35-92.87)
India	24.79 (22.98-26.57)	26.95 (24.98-28.79)	33.54 (31.94-35.09)	8.34 (5.74-11.73)	8.47 (5.75-11.81)	10.42 (7.01-14.37)	57.25 (50.25-63.58)	57.64 (49.93-64.36)	57.37 (49.07-64.39)	82.61 (79.47-85.26)	86.77 (84.32-88.82)	85.35 (82.65-87.68)
Nepal	26.55 (25.40-27.76)	30.37 (29.12-31.68)	34.88 (33.46-36.28)	22.53 (18.06-30.95)	22.37 (17.91-30.75)	23.29 (18.85-31.56)	57.49 (50.24-63.81)	57.36 (49.26-64.60)	57.10 (48.91-64.41)	63.67 (56.68-69.93)	72.06 (66.58-77.03)	74.52 (68.67-79.50)
Pakistan	31.68 (30.47-32.96)	34.93 (33.71-36.24)	37.11 (35.84-38.34)	18.71 (14.97-24.74)	18.03 (14.28-24.13)	18.54 (14.89-24.43)	51.87 (44.55-58.63)	52.22 (43.90-59.79)	52.19 (43.04-59.67)	90.66 (87.77-92.90)	93.82 (92.49-95.06)	91.52 (88.72-93.68)
Botswana	11.85 (10.80-12.95)	1.10 (0.43-1.85)	4.91 (4.05-5.68)	20.63 (17.62-23.62)	20.23 (17.18-23.25)	22.95 (18.81-27.36)	34.96 (23.55-46.89)	35.14 (21.66-48.19)	34.86 (22.17-48.79)	72.02 (64.31-78.26)	77.82 (70.66-83.33)	72.84 (64.77-78.95)

Location	Appendix Table 1a. Scaled values for each SDG health-related indicator and three summary indicators by country for 1990, 2000, and 2015											
	**Indicator 3.3.2: Age-standardised rate of new and relapsed TB cases (per 1,000 population)**			**Indicator 3.3.3: Age-standaridsed rate of malaria cases (per 1,000 population)**			**Indicator 3.3.4: Age-standardised rate of hepatitis B incident cases (per 100,000 population)**			**Indicator 3.3.5: Age-standardised prevalence rate of neglected tropical diseases (NTDs) (per 100,000 population)**		
	**1990**	**2000**	**2015**	**1990**	**2000**	**2015**	**1990**	**2000**	**2015**	**1990**	**2000**	**2015**
Lesotho	18.07 (16.76-19.39)	8.00 (7.21-8.83)	4.69 (4.01-5.46)	100.00 (100.00-100.00)	100.00 (100.00-100.00)	100.00 (100.00-100.00)	35.01 (23.66-46.96)	35.21 (21.83-48.22)	34.88 (21.97-48.83)	66.37 (59.52-72.68)	76.62 (71.50-81.17)	66.26 (59.32-72.74)
Namibia	15.05 (13.97-16.18)	6.37 (5.55-7.37)	10.28 (9.35-11.17)	7.83 (4.25-11.61)	7.63 (4.13-11.37)	11.28 (7.24-17.61)	37.00 (29.30-45.53)	36.96 (27.26-46.00)	37.19 (27.14-46.69)	86.73 (83.02-89.84)	91.98 (90.17-93.81)	87.88 (84.03-90.81)
South Africa	20.91 (19.07-23.02)	9.66 (7.92-11.36)	9.00 (7.12-10.61)	46.04 (41.51-52.57)	100.00 (100.00-100.00)	59.07 (57.24-61.68)	34.76 (27.22-42.61)	35.17 (24.95-44.83)	34.88 (24.01-44.50)	60.46 (50.84-67.90)	69.01 (60.56-75.35)	60.77 (50.42-68.25)
Swaziland	14.60 (13.35-15.84)	2.36 (1.65-3.17)	3.14 (2.36-3.93)	46.89 (43.00-51.69)	46.23 (42.48-50.84)	53.18 (38.14-69.14)	35.08 (23.57-46.96)	35.21 (22.08-48.21)	34.88 (22.06-48.77)	43.00 (26.60-55.97)	52.75 (37.59-64.73)	46.25 (32.45-57.99)
Zimbabwe	19.28 (18.07-20.62)	10.21 (9.13-11.38)	15.03 (14.15-15.99)	14.42 (12.43-16.54)	14.43 (12.45-16.62)	13.80 (9.57-17.92)	36.51 (28.63-45.05)	36.66 (26.77-45.88)	37.16 (26.86-46.11)	48.26 (30.62-62.27)	55.43 (38.03-68.91)	65.25 (55.00-73.14)
Benin	37.54 (36.22-39.02)	36.45 (35.11-37.84)	40.26 (38.94-41.49)	3.56 (1.54-6.47)	3.17 (1.31-5.92)	2.85 (1.77-4.69)	32.94 (21.59-45.25)	33.19 (19.40-46.17)	32.76 (19.93-46.54)	50.11 (27.22-67.27)	59.51 (36.91-76.50)	77.77 (65.70-85.59)
Burkina Faso	32.04 (30.79-33.36)	31.44 (30.15-32.81)	36.50 (35.21-37.77)	0.63 (0.04-2.41)	0.44 (0.00-2.27)	1.72 (0.31-4.00)	32.95 (21.56-45.15)	33.19 (19.45-46.17)	32.77 (19.87-46.74)	53.02 (38.77-65.60)	58.75 (44.81-70.86)	92.74 (86.70-95.37)
Cameroon	35.03 (33.58-36.42)	31.85 (30.53-33.27)	35.76 (34.41-37.13)	3.01 (1.65-5.16)	2.29 (1.14-4.25)	3.89 (2.28-6.34)	34.34 (27.03-42.58)	34.62 (24.49-43.99)	33.81 (23.40-43.46)	25.53 (10.34-38.34)	40.50 (27.36-51.42)	75.17 (69.44-79.91)
Cape Verde	47.64 (46.35-48.86)	51.05 (49.67-52.30)	53.74 (52.05-55.42)	17.51 (11.35-30.65)	50.59 (48.73-52.99)	94.70 (93.79-96.01)	32.87 (21.28-45.37)	33.17 (19.22-46.06)	32.79 (19.84-46.66)	98.72 (98.37-99.01)	99.07 (98.86-99.25)	99.01 (98.69-99.26)
Chad	33.22 (32.05-34.48)	30.58 (29.33-31.96)	33.09 (31.81-34.37)	5.55 (2.28-9.19)	5.27 (2.16-8.71)	7.12 (2.42-12.33)	32.98 (21.66-45.20)	33.22 (19.59-46.05)	32.77 (20.00-46.61)	66.54 (53.62-76.67)	72.48 (60.10-81.76)	70.19 (57.40-80.32)
Cote d'Ivoire	31.59 (30.18-32.99)	28.98 (27.70-30.33)	34.41 (33.14-35.67)	1.04 (0.23-3.02)	0.91 (0.16-2.92)	1.71 (0.74-3.71)	33.06 (21.62-45.57)	33.28 (19.76-46.23)	32.79 (19.99-46.73)	30.91 (12.87-45.68)	43.89 (28.27-56.45)	72.97 (63.52-79.65)

	Appendix Table 1a. Scaled values for each SDG health-related indicator and three summary indicators by country for 1990, 2000, and 2015											
	**Indicator 3.3.2: Age-standardised rate of new and relapsed TB cases (per 1,000 population)**			**Indicator 3.3.3: Age-standaridsed rate of malaria cases (per 1,000 population)**			**Indicator 3.3.4: Age-standardised rate of hepatitis B incident cases (per 100,000 population)**			**Indicator 3.3.5: Age-standardised prevalence rate of neglected tropical diseases (NTDs) (per 100,000 population)**		
**Location**	**1990**	**2000**	**2015**	**1990**	**2000**	**2015**	**1990**	**2000**	**2015**	**1990**	**2000**	**2015**
The Gambia	38.71 (37.47-39.90)	38.89 (37.64-40.14)	40.82 (39.43-42.15)	4.82 (2.76-6.90)	4.43 (2.44-6.45)	15.13 (11.33-18.87)	32.98 (21.59-45.48)	33.19 (19.39-45.95)	32.74 (19.84-46.56)	82.35 (72.73-89.19)	84.58 (74.93-91.27)	82.60 (72.86-89.22)
Ghana	33.96 (32.66-35.37)	33.45 (32.00-34.96)	39.23 (37.95-40.56)	1.74 (0.60-3.69)	1.39 (0.42-3.45)	2.91 (1.22-5.25)	34.95 (26.97-43.13)	34.84 (24.20-44.79)	34.65 (23.57-44.29)	51.52 (33.90-67.03)	57.50 (40.42-72.55)	79.28 (68.32-86.39)
Guinea	35.81 (34.56-37.11)	33.08 (31.71-34.50)	36.36 (35.13-37.57)	1.97 (0.66-4.02)	1.59 (0.45-3.60)	1.57 (0.88-3.30)	32.98 (21.59-45.16)	33.22 (19.57-46.06)	32.78 (20.01-46.63)	44.15 (25.88-59.91)	53.95 (36.87-68.43)	55.91 (41.09-68.97)
Guinea-Bissau	34.92 (33.65-36.25)	31.57 (30.30-32.95)	29.66 (28.43-30.91)	2.72 (0.73-4.88)	2.68 (0.73-4.84)	9.77 (5.34-14.43)	32.97 (21.63-45.21)	33.21 (19.51-46.07)	32.76 (19.97-46.59)	56.66 (35.24-71.02)	63.06 (42.06-76.79)	74.05 (52.86-87.17)
Liberia	28.85 (27.68-30.11)	29.92 (28.59-31.19)	31.60 (30.06-33.01)	2.21 (0.22-4.83)	1.61 (0.00-3.83)	2.88 (0.92-5.24)	32.98 (21.66-45.22)	33.23 (19.63-46.04)	32.79 (20.00-46.62)	12.32 (0.00-36.40)	24.65 (10.13-47.42)	38.58 (25.42-54.46)
Mali	34.97 (33.70-36.26)	35.90 (34.75-37.21)	39.65 (38.34-40.95)	1.66 (0.64-3.45)	1.53 (0.52-3.34)	1.33 (0.73-3.09)	32.97 (21.62-45.24)	33.23 (19.66-46.09)	32.80 (20.01-46.61)	40.47 (25.27-54.28)	46.35 (31.28-59.68)	89.70 (80.56-93.10)
Mauritania	35.22 (33.93-36.48)	37.21 (35.91-38.47)	42.59 (41.25-43.93)	11.13 (7.09-15.99)	10.02 (6.28-14.92)	12.98 (9.44-17.44)	33.00 (21.59-45.16)	33.24 (19.59-46.12)	32.79 (20.00-46.58)	73.63 (63.65-82.78)	75.02 (65.23-84.05)	81.90 (75.16-87.99)
Niger	32.72 (31.49-33.96)	32.48 (31.25-33.74)	35.30 (33.96-36.62)	3.37 (0.91-6.54)	3.03 (0.67-6.11)	2.66 (0.68-5.43)	32.95 (21.31-45.44)	33.19 (19.37-46.14)	32.75 (19.81-46.56)	73.15 (66.03-79.80)	76.02 (69.07-82.09)	96.62 (92.92-97.83)
Nigeria	36.50 (35.22-37.85)	36.90 (35.62-38.16)	37.51 (35.91-39.05)	1.89 (0.90-3.81)	1.64 (0.73-3.58)	2.50 (1.18-4.51)	33.54 (25.68-42.25)	33.34 (21.89-43.25)	33.31 (22.62-42.94)	43.06 (26.65-56.48)	49.64 (34.86-62.11)	55.66 (43.58-65.93)
Sao Tome and Principe	44.89 (43.64-46.14)	46.03 (44.78-47.29)	47.45 (45.94-48.86)	15.22 (13.86-17.01)	15.26 (13.91-17.04)	15.23 (13.66-17.21)	32.96 (21.56-45.26)	33.22 (19.59-46.04)	32.77 (19.93-46.67)	62.51 (54.23-69.59)	73.52 (67.18-79.16)	63.01 (54.79-70.69)
Senegal	29.27 (28.05-30.64)	30.53 (29.20-31.91)	32.83 (31.59-34.08)	4.02 (2.54-5.98)	3.67 (2.25-5.63)	10.88 (9.17-12.75)	32.98 (21.68-45.24)	33.19 (19.33-46.00)	32.75 (19.86-46.57)	76.92 (71.39-81.56)	80.00 (75.22-84.08)	89.25 (85.76-91.68)

Location	Appendix Table 1a. Scaled values for each SDG health-related indicator and three summary indicators by country for 1990, 2000, and 2015											
	**Indicator 3.3.2: Age-standardised rate of new and relapsed TB cases (per 1,000 population)**			**Indicator 3.3.3: Age-standaridsed rate of malaria cases (per 1,000 population)**			**Indicator 3.3.4: Age-standardised rate of hepatitis B incident cases (per 100,000 population)**			**Indicator 3.3.5: Age-standardised prevalence rate of neglected tropical diseases (NTDs) (per 100,000 population)**		
	**1990**	**2000**	**2015**	**1990**	**2000**	**2015**	**1990**	**2000**	**2015**	**1990**	**2000**	**2015**
Sierra Leone	36.18 (34.97-37.46)	34.38 (33.17-35.68)	36.46 (35.19-37.69)	2.00 (0.31-4.56)	1.44 (0.12-3.89)	2.00 (0.68-4.69)	33.21 (24.83-41.88)	33.56 (23.02-43.31)	33.89 (23.02-43.78)	12.59 (0.00-39.92)	21.62 (6.12-48.60)	77.91 (66.93-82.39)
Togo	39.25 (37.99-40.62)	37.70 (36.43-39.08)	41.46 (40.18-42.71)	1.23 (0.14-3.37)	1.19 (0.14-3.26)	1.77 (1.05-3.51)	32.97 (21.61-45.21)	33.18 (19.31-46.02)	32.76 (19.89-46.71)	63.51 (46.67-76.72)	66.99 (50.61-79.91)	92.17 (85.07-94.45)
Burundi	26.61 (25.31-27.92)	25.60 (24.38-26.89)	34.45 (33.22-35.69)	1.94 (0.44-4.21)	1.76 (0.48-3.86)	6.38 (4.58-8.67)	35.12 (27.33-43.54)	34.99 (24.79-44.62)	35.28 (24.78-44.99)	31.12 (0.00-54.14)	38.22 (8.01-60.51)	79.02 (71.01-83.86)
Comoros	37.59 (36.26-38.94)	40.55 (39.31-41.80)	44.10 (42.72-45.57)	9.57 (8.23-11.19)	9.92 (8.66-11.59)	10.31 (8.97-12.02)	31.34 (15.37-47.64)	31.43 (14.96-48.94)	31.03 (14.90-48.19)	59.93 (51.26-67.21)	70.50 (64.63-75.79)	66.91 (59.43-73.31)
Djibouti	35.80 (34.37-37.16)	33.30 (31.99-34.61)	33.98 (32.58-35.35)	25.40 (16.22-35.30)	23.05 (14.86-32.19)	30.14 (19.95-43.49)	31.34 (15.25-47.69)	31.43 (15.08-48.92)	31.03 (14.94-48.20)	65.78 (37.75-82.97)	70.41 (43.39-86.81)	66.29 (38.66-83.56)
Eritrea	34.13 (32.88-35.49)	34.26 (32.86-35.66)	36.62 (35.33-37.89)	12.07 (10.23-14.24)	12.08 (10.25-14.24)	17.63 (10.55-24.34)	31.36 (15.30-47.65)	31.44 (15.01-49.04)	31.04 (14.97-48.26)	56.23 (33.40-75.56)	61.34 (39.01-80.38)	63.86 (45.81-79.11)
Ethiopia	23.84 (22.65-25.16)	23.72 (22.48-25.18)	31.82 (30.48-33.18)	11.08 (8.19-14.58)	11.12 (8.22-14.63)	24.85 (22.62-27.23)	35.23 (27.81-43.65)	35.95 (25.74-44.94)	35.87 (25.37-45.20)	51.90 (41.36-61.73)	59.38 (49.21-68.00)	54.80 (44.09-63.85)
Kenya	38.21 (35.93-40.38)	30.90 (28.82-32.83)	36.80 (34.77-39.05)	4.95 (3.75-6.85)	4.52 (3.42-6.38)	6.89 (3.97-10.11)	41.97 (35.07-49.26)	41.44 (32.61-49.87)	41.37 (31.77-49.72)	63.91 (52.06-74.30)	67.04 (55.44-77.27)	75.07 (66.24-81.89)
Madagascar	37.47 (36.06-38.96)	39.30 (37.93-40.73)	41.28 (39.72-42.84)	5.96 (4.17-8.30)	5.58 (3.92-7.83)	9.27 (7.69-11.07)	31.35 (15.33-47.66)	31.45 (15.04-49.00)	31.04 (14.99-48.21)	7.55 (0.00-26.97)	16.27 (7.01-35.07)	56.61 (46.90-66.31)
Malawi	27.78 (26.51-29.10)	20.75 (19.70-21.93)	28.19 (26.92-29.55)	1.54 (0.51-3.34)	1.60 (0.57-3.41)	3.85 (2.53-5.72)	36.92 (28.92-45.63)	36.48 (25.21-46.18)	36.90 (26.20-46.51)	40.61 (21.89-56.65)	47.20 (30.47-62.34)	87.08 (78.09-91.18)
Mozambique	32.08 (30.69-33.48)	25.07 (23.95-26.37)	23.75 (22.52-24.95)	0.88 (0.34-2.67)	0.82 (0.31-2.64)	2.80 (1.74-4.82)	36.81 (29.02-44.92)	36.94 (26.02-46.64)	36.90 (25.87-46.34)	29.67 (14.38-42.83)	33.06 (18.27-45.75)	58.80 (47.29-66.71)

Location	Appendix Table 1a. Scaled values for each SDG health-related indicator and three summary indicators by country for 1990, 2000, and 2015											
	**Indicator 3.3.2: Age-standardised rate of new and relapsed TB cases (per 1,000 population)**			**Indicator 3.3.3: Age-standaridsed rate of malaria cases (per 1,000 population)**			**Indicator 3.3.4: Age-standardised rate of hepatitis B incident cases (per 100,000 population)**			**Indicator 3.3.5: Age-standardised prevalence rate of neglected tropical diseases (NTDs) (per 100,000 population)**		
	**1990**	**2000**	**2015**	**1990**	**2000**	**2015**	**1990**	**2000**	**2015**	**1990**	**2000**	**2015**
Rwanda	29.52 (28.20-30.87)	28.22 (26.90-29.48)	39.83 (38.48-41.18)	6.51 (2.46-12.05)	6.39 (2.45-11.82)	12.88 (9.28-16.33)	31.37 (15.28-47.60)	31.50 (15.38-49.40)	31.11 (14.95-48.39)	47.69 (18.61-66.59)	56.78 (28.04-75.23)	60.53 (43.78-71.39)
Somalia	32.31 (31.04-33.66)	30.28 (29.05-31.54)	30.50 (29.37-31.77)	9.35 (6.81-11.60)	9.13 (6.63-11.33)	11.14 (7.98-14.54)	35.53 (28.66-43.57)	36.05 (26.67-45.07)	35.68 (25.32-44.92)	46.02 (31.73-58.74)	52.73 (39.44-64.42)	47.77 (33.44-60.08)
South Sudan	28.45 (27.00-29.77)	26.57 (25.32-27.91)	28.02 (26.72-29.34)	4.12 (2.06-7.33)	3.40 (1.66-6.20)	6.30 (2.91-9.94)	31.35 (15.29-47.65)	31.45 (15.03-48.96)	31.04 (15.00-48.20)	50.19 (38.70-60.92)	52.99 (41.65-62.58)	57.61 (47.09-66.88)
Tanzania	29.05 (27.84-30.27)	25.24 (24.05-26.55)	29.02 (27.77-30.28)	2.81 (1.43-4.68)	2.41 (1.26-4.19)	6.64 (5.21-8.46)	35.91 (28.58-43.85)	35.74 (25.36-45.20)	35.27 (24.03-44.61)	47.87 (37.09-57.14)	54.68 (44.82-62.92)	71.42 (63.72-77.24)
Uganda	26.94 (25.63-28.30)	21.99 (20.88-23.30)	29.16 (27.88-30.52)	1.18 (0.38-3.12)	1.16 (0.38-3.11)	5.35 (3.35-8.08)	33.46 (25.41-42.12)	33.54 (22.77-43.05)	33.00 (22.23-42.39)	57.26 (44.76-67.48)	65.79 (54.97-74.42)	85.46 (79.92-89.21)
Zambia	25.51 (24.11-26.96)	18.39 (17.32-19.57)	23.99 (22.82-25.21)	2.52 (1.39-4.29)	2.15 (1.14-3.88)	4.85 (3.61-6.81)	35.30 (28.32-43.24)	35.29 (25.36-44.48)	35.07 (24.44-44.22)	55.14 (43.83-65.81)	61.75 (51.75-70.98)	57.03 (46.11-66.96)
Angola	24.01 (22.73-25.22)	25.26 (24.10-26.52)	27.73 (26.45-28.98)	4.57 (2.64-7.20)	3.71 (2.04-6.09)	6.60 (4.52-9.31)	32.19 (20.57-44.44)	32.19 (18.82-45.11)	31.96 (19.26-45.35)	44.01 (23.54-59.17)	51.07 (31.56-65.69)	51.12 (33.81-64.01)
Central African Republic	23.45 (22.34-24.71)	20.16 (19.10-21.39)	24.16 (23.04-25.30)	1.79 (0.30-4.39)	1.31 (0.14-3.80)	3.01 (0.68-6.54)	34.06 (26.07-42.59)	34.06 (23.70-43.78)	34.00 (23.46-43.68)	54.38 (44.26-62.73)	62.11 (53.04-69.28)	68.06 (61.52-73.78)
Congo	25.26 (24.05-26.55)	26.76 (25.46-28.05)	32.86 (31.44-34.18)	3.11 (1.50-5.16)	2.44 (1.02-4.36)	4.29 (2.39-6.62)	33.75 (25.74-42.44)	34.42 (23.95-43.80)	34.25 (23.66-43.86)	40.41 (23.05-54.77)	49.59 (31.93-62.75)	43.02 (25.37-56.60)
Democratic Republic of the Congo	28.07 (26.85-29.32)	27.83 (26.59-29.13)	29.58 (28.03-31.07)	1.09 (0.40-3.07)	1.03 (0.36-3.01)	3.33 (2.35-5.19)	32.20 (20.62-44.40)	32.20 (18.80-45.11)	31.96 (19.26-45.35)	35.49 (17.13-50.15)	45.24 (27.85-58.42)	46.55 (31.62-58.53)
Equatorial Guinea	30.18 (28.92-31.40)	27.93 (26.74-29.24)	27.60 (26.18-28.98)	1.75 (0.81-3.87)	1.78 (0.85-3.90)	2.29 (0.30-5.24)	32.18 (20.50-44.94)	32.18 (18.85-45.02)	31.94 (19.26-45.42)	57.09 (48.87-64.13)	65.40 (58.92-71.13)	60.73 (52.93-67.25)

	Appendix Table 1a. Scaled values for each SDG health-related indicator and three summary indicators by country for 1990, 2000, and 2015											
	**Indicator 3.3.2: Age-standardised rate of new and relapsed TB cases (per 1,000 population)**			**Indicator 3.3.3: Age-standaridsed rate of malaria cases (per 1,000 population)**			**Indicator 3.3.4: Age-standardised rate of hepatitis B incident cases (per 100,000 population)**			**Indicator 3.3.5: Age-standardised prevalence rate of neglected tropical diseases (NTDs) (per 100,000 population)**		
**Location**	**1990**	**2000**	**2015**	**1990**	**2000**	**2015**	**1990**	**2000**	**2015**	**1990**	**2000**	**2015**
Gabon	25.85 (24.60-27.14)	22.61 (21.30-23.77)	28.02 (26.68-29.32)	2.40 (1.06-4.46)	2.07 (0.93-4.06)	4.85 (1.82-9.08)	33.16 (25.31-41.53)	33.27 (22.95-42.94)	33.32 (22.42-43.08)	34.06 (14.28-50.76)	39.76 (20.84-55.64)	34.69 (15.19-51.27)

Location	Appendix Table 1a. Scaled values for each SDG health-related indicator and three summary indicators by country for 1990, 2000, and 2015											
	**Indicator 3.4.1: Death rate due to cardiovascular disease, cancer, diabetes, and chronic respiratory disease among populations aged 30 to 70 (per 100,000 population)**			**Indicator 3.4.2: Age-standardised death rate due to self-harm (per 100,000 population)**			**Indicator 3.5.2: Risk-weighted prevalence of alcohol consumption, as measured by the summary exposure value (SEV) for alcohol use**			**Indicator 3.6.1: Age-standardised death rate due to road injuries (per 100,000 population)**		
	**1990**	**2000**	**2015**	**1990**	**2000**	**2015**	**1990**	**2000**	**2015**	**1990**	**2000**	**2015**
Canada	60.37 (55.77-64.26)	69.74 (64.90-73.14)	82.16 (76.29-85.41)	46.77 (42.82-53.62)	49.20 (45.30-55.62)	53.68 (49.54-59.61)	56.51 (54.05-58.57)	59.24 (57.24-61.15)	56.21 (53.38-59.22)	57.53 (53.67-61.58)	67.76 (64.24-71.34)	78.86 (75.48-82.08)
United States	52.37 (47.98-56.68)	59.54 (55.10-63.44)	69.39 (64.58-72.88)	48.72 (44.95-55.26)	52.15 (48.46-58.08)	49.49 (45.56-55.84)	53.47 (50.93-55.88)	54.63 (52.10-57.10)	53.94 (49.89-58.77)	50.69 (46.66-55.25)	54.98 (51.05-59.18)	64.03 (60.54-67.53)
Australia	59.60 (55.11-63.44)	74.36 (68.98-77.63)	87.50 (80.89-90.53)	47.12 (43.19-53.99)	48.64 (44.70-55.33)	55.05 (51.09-60.49)	48.91 (46.07-51.59)	58.30 (56.07-60.53)	53.78 (50.50-57.25)	56.00 (51.87-60.16)	67.12 (63.53-70.63)	79.17 (75.83-82.25)
New Zealand	52.64 (48.19-56.93)	64.93 (60.27-68.69)	80.89 (74.40-84.30)	45.82 (41.90-52.99)	47.50 (43.51-54.23)	53.26 (49.23-58.91)	52.93 (50.44-55.18)	57.97 (55.99-59.95)	55.98 (53.08-59.07)	46.94 (42.57-51.89)	61.06 (57.25-64.99)	75.65 (71.93-78.98)
Brunei	47.58 (42.90-52.39)	55.28 (50.66-59.59)	61.39 (55.85-66.01)	75.12 (68.49-80.77)	79.26 (74.25-83.33)	78.88 (74.23-83.04)	66.04 (61.51-69.89)	68.98 (65.19-72.58)	69.98 (67.04-73.25)	40.46 (34.31-47.06)	49.57 (45.07-54.73)	60.95 (56.19-65.77)
Japan	72.77 (67.61-76.08)	79.11 (73.33-82.28)	89.92 (82.91-92.99)	40.34 (36.54-47.92)	36.00 (32.22-44.39)	36.47 (32.53-45.02)	62.52 (60.69-64.17)	61.50 (59.62-63.43)	64.06 (61.04-67.03)	65.21 (61.78-68.76)	73.88 (70.79-76.84)	90.64 (88.36-92.79)
Singapore	48.36 (44.03-52.93)	64.53 (59.84-68.31)	85.24 (78.75-88.88)	39.83 (35.71-47.85)	49.50 (45.50-55.89)	58.54 (54.32-63.70)	88.14 (87.03-89.24)	88.34 (87.15-89.40)	86.52 (85.08-87.80)	65.62 (61.91-69.27)	80.66 (77.55-83.50)	93.31 (90.41-96.07)
South Korea	45.85 (41.34-50.79)	62.56 (58.03-66.35)	84.67 (77.57-88.94)	49.77 (45.84-55.92)	34.78 (30.61-43.41)	25.17 (20.38-35.97)	67.71 (65.33-69.82)	63.13 (61.07-64.88)	61.82 (59.27-64.34)	28.22 (22.44-35.38)	39.79 (34.90-45.96)	61.51 (56.87-65.97)
Andorra	84.05 (79.87-87.98)	93.13 (89.86-96.10)	99.49 (95.96-100.00)	62.09 (53.48-77.61)	65.79 (56.67-79.96)	66.32 (56.84-76.30)	27.66 (19.99-39.07)	28.80 (23.83-34.73)	42.22 (35.59-50.73)	74.73 (66.74-86.93)	84.39 (77.05-94.26)	94.03 (88.13-99.71)
Austria	57.42 (53.11-61.44)	66.37 (61.57-69.99)	80.44 (74.47-83.71)	33.02 (28.93-41.89)	39.86 (35.88-47.76)	49.22 (45.25-55.70)	32.40 (28.43-36.67)	35.26 (31.35-39.60)	38.18 (31.11-46.61)	52.89 (48.79-57.33)	65.18 (61.58-68.78)	83.36 (80.06-86.56)
Belgium	57.41 (53.06-61.47)	65.72 (61.18-69.39)	76.87 (71.76-80.66)	37.06 (33.08-45.30)	35.67 (31.75-44.13)	40.60 (36.09-48.62)	49.03 (46.45-51.35)	52.52 (48.93-56.11)	46.72 (40.44-54.14)	48.94 (44.88-53.82)	55.25 (51.24-59.57)	72.88 (69.29-76.23)

Location	Appendix Table 1a. Scaled values for each SDG health-related indicator and three summary indicators by country for 1990, 2000, and 2015											
	**Indicator 3.4.1: Death rate due to cardiovascular disease, cancer, diabetes, and chronic respiratory disease among populations aged 30 to 70 (per 100,000 population)**			**Indicator 3.4.2: Age-standardised death rate due to self-harm (per 100,000 population)**			**Indicator 3.5.2: Risk-weighted prevalence of alcohol consumption, as measured by the summary exposure value (SEV) for alcohol use**			**Indicator 3.6.1: Age-standardised death rate due to road injuries (per 100,000 population)**		
	**1990**	**2000**	**2015**	**1990**	**2000**	**2015**	**1990**	**2000**	**2015**	**1990**	**2000**	**2015**
Cyprus	62.84 (58.13-66.51)	63.81 (59.19-67.64)	82.78 (76.62-86.08)	81.89 (77.45-85.50)	80.71 (75.81-84.24)	87.01 (82.50-90.54)	53.89 (50.85-57.24)	56.88 (53.55-60.47)	57.31 (52.27-63.36)	46.03 (41.19-51.66)	49.64 (45.51-54.36)	70.28 (66.88-73.77)
Denmark	50.30 (45.82-54.82)	59.90 (55.57-63.88)	77.59 (71.81-80.99)	27.96 (23.74-38.32)	44.83 (40.64-51.91)	54.74 (50.54-60.44)	35.12 (31.32-39.15)	30.53 (25.90-35.04)	38.96 (30.69-48.96)	59.97 (56.14-63.93)	69.57 (66.12-72.84)	87.82 (84.61-90.71)
Finland	55.03 (50.67-59.26)	68.89 (64.26-72.42)	82.10 (76.09-85.54)	24.61 (20.57-35.62)	32.22 (28.15-41.56)	42.54 (37.99-50.11)	48.26 (45.29-51.34)	48.15 (45.00-51.29)	47.61 (42.12-53.90)	59.10 (55.23-63.10)	73.15 (69.88-76.41)	88.89 (85.81-91.85)
France	64.22 (59.71-67.89)	69.74 (64.90-73.15)	78.77 (73.31-82.07)	30.94 (26.78-40.66)	36.39 (32.39-44.91)	41.12 (36.65-48.70)	39.70 (36.50-42.54)	48.20 (45.23-50.74)	52.62 (48.20-57.34)	48.83 (44.56-53.75)	58.83 (54.92-62.90)	77.88 (75.05-80.63)
Germany	53.71 (49.29-57.94)	64.35 (59.92-68.10)	77.36 (71.98-80.55)	44.65 (40.72-51.85)	48.06 (44.11-54.72)	54.55 (50.54-60.18)	41.57 (38.51-44.28)	47.09 (44.30-49.53)	54.02 (50.17-58.24)	58.99 (55.05-62.90)	69.02 (65.56-72.47)	87.58 (84.76-90.15)
Greece	63.54 (58.90-67.25)	68.29 (63.57-71.87)	74.72 (69.33-78.22)	82.85 (78.99-85.87)	87.44 (83.82-90.14)	87.17 (83.04-90.13)	43.64 (40.28-46.76)	50.31 (46.95-53.24)	52.58 (46.52-59.17)	46.90 (42.46-51.82)	52.07 (48.11-56.65)	66.78 (62.96-70.34)
Iceland	65.25 (60.47-69.08)	75.81 (70.28-79.38)	91.35 (84.55-95.20)	46.52 (42.41-53.45)	53.05 (48.85-58.99)	59.57 (55.19-64.47)	69.08 (66.09-72.39)	69.84 (67.28-72.58)	62.12 (56.11-69.31)	68.27 (64.75-71.89)	80.46 (77.48-83.45)	96.15 (93.38-98.84)
Ireland	55.05 (49.85-59.73)	62.59 (57.93-66.69)	82.13 (74.77-85.88)	51.62 (47.32-57.91)	49.20 (45.31-55.48)	56.19 (52.00-61.72)	48.63 (45.71-51.43)	44.40 (41.53-47.23)	49.09 (44.52-54.17)	58.37 (53.95-62.84)	68.55 (64.96-72.16)	92.26 (89.34-94.96)
Israel	58.48 (54.18-62.56)	68.56 (63.80-72.07)	86.00 (79.17-89.36)	58.81 (54.90-63.88)	56.59 (52.57-62.13)	64.51 (60.35-68.94)	89.79 (88.86-90.71)	89.69 (88.83-90.57)	86.92 (84.72-89.28)	63.38 (59.53-67.02)	61.86 (57.86-65.75)	76.88 (73.29-80.07)
Italy	60.72 (56.10-64.56)	72.13 (67.08-75.42)	84.90 (78.16-87.95)	62.66 (58.96-67.45)	66.43 (62.70-70.65)	70.92 (67.05-74.72)	50.17 (46.99-53.30)	56.22 (52.99-59.28)	68.43 (62.67-74.64)	53.05 (49.07-57.39)	58.10 (54.27-62.12)	74.78 (71.28-78.17)
Luxembourg	53.69 (49.22-57.90)	66.85 (62.58-70.58)	84.27 (77.72-87.86)	40.73 (36.65-48.48)	48.34 (44.38-54.89)	59.07 (54.87-64.12)	42.58 (39.73-45.12)	46.97 (44.25-49.52)	52.62 (47.94-57.80)	50.16 (45.85-54.90)	63.45 (59.78-67.44)	85.11 (82.01-87.84)

	Appendix Table 1a. Scaled values for each SDG health-related indicator and three summary indicators by country for 1990, 2000, and 2015											
	**Indicator 3.4.1: Death rate due to cardiovascular disease, cancer, diabetes, and chronic respiratory disease among populations aged 30 to 70 (per 100,000 population)**			**Indicator 3.4.2: Age-standardised death rate due to self-harm (per 100,000 population)**			**Indicator 3.5.2: Risk-weighted prevalence of alcohol consumption, as measured by the summary exposure value (SEV) for alcohol use**			**Indicator 3.6.1: Age-standardised death rate due to road injuries (per 100,000 population)**		
**Location**	**1990**	**2000**	**2015**	**1990**	**2000**	**2015**	**1990**	**2000**	**2015**	**1990**	**2000**	**2015**
Malta	60.20 (55.32-64.34)	69.32 (64.21-73.36)	84.49 (77.94-88.15)	75.61 (71.53-79.19)	77.34 (73.36-80.89)	82.14 (78.27-85.50)	67.70 (65.49-69.96)	71.51 (69.03-74.10)	62.24 (56.90-68.10)	77.09 (73.94-80.35)	83.80 (80.91-86.75)	99.93 (99.15-100.00)
Netherlands	57.55 (53.19-61.52)	63.74 (59.34-67.52)	79.78 (74.15-83.54)	51.59 (47.84-57.69)	54.50 (50.76-60.25)	56.90 (52.80-62.27)	51.42 (48.56-54.19)	52.97 (50.25-55.62)	55.29 (49.69-61.68)	67.00 (63.48-70.64)	73.08 (69.82-76.14)	89.89 (87.12-92.52)
Norway	59.66 (54.99-63.72)	69.38 (64.65-72.88)	86.29 (79.64-89.64)	42.88 (38.86-50.26)	48.10 (44.11-54.91)	57.08 (52.81-62.48)	67.20 (64.67-69.68)	65.80 (63.20-68.37)	63.58 (57.80-69.96)	68.72 (65.18-72.25)	74.54 (71.48-77.54)	94.81 (91.82-97.46)
Portugal	56.60 (52.29-60.73)	64.86 (60.35-68.53)	79.24 (73.56-82.50)	44.68 (40.79-51.93)	53.66 (49.80-59.71)	56.53 (52.26-62.03)	43.20 (40.16-46.01)	48.57 (45.77-51.16)	53.56 (48.77-58.82)	33.88 (28.82-40.42)	47.61 (43.47-52.61)	72.04 (68.35-75.50)
Spain	64.38 (59.85-68.01)	71.35 (66.38-74.74)	85.77 (79.52-88.91)	62.12 (58.13-66.94)	64.00 (60.18-68.61)	68.82 (64.75-72.84)	48.08 (45.23-50.70)	53.91 (51.30-56.31)	57.31 (52.77-62.84)	46.25 (41.58-51.25)	57.70 (53.84-61.89)	86.47 (84.03-88.87)
Sweden	64.52 (60.05-68.21)	73.82 (68.83-77.17)	87.49 (80.56-90.73)	38.40 (34.35-46.75)	46.25 (42.38-53.25)	50.59 (46.46-56.81)	57.88 (54.76-61.15)	54.80 (51.40-58.20)	56.84 (50.51-63.95)	69.09 (65.82-72.57)	80.88 (78.12-83.67)	93.86 (91.12-96.17)
Switzerland	66.37 (61.65-70.06)	75.90 (70.42-79.15)	92.07 (84.09-95.75)	31.95 (28.14-41.53)	38.64 (34.93-46.92)	50.18 (46.04-56.70)	39.93 (36.76-43.02)	46.88 (43.81-50.04)	50.92 (45.11-57.70)	58.60 (54.73-62.89)	74.97 (71.68-78.09)	93.18 (90.36-95.85)
United Kingdom	50.77 (46.31-55.25)	62.92 (58.36-66.63)	78.38 (72.86-81.39)	56.10 (52.38-61.46)	58.94 (55.31-64.13)	64.18 (60.15-68.39)	56.12 (47.37-66.97)	56.75 (47.60-67.98)	57.46 (48.60-68.24)	71.11 (67.87-74.31)	78.49 (75.66-81.21)	94.24 (91.79-96.46)
Argentina	46.90 (42.30-51.60)	52.93 (48.35-57.23)	60.93 (56.48-65.02)	54.08 (50.29-59.71)	48.64 (44.60-55.18)	51.75 (47.82-57.98)	50.08 (47.06-53.18)	58.13 (55.40-61.09)	58.37 (53.34-64.12)	55.90 (51.83-60.06)	56.45 (52.33-60.57)	59.20 (55.18-63.47)
Chile	55.70 (51.01-59.94)	69.24 (64.20-72.57)	78.54 (72.04-82.52)	33.27 (29.00-42.47)	47.65 (43.41-54.39)	51.07 (46.50-57.57)	59.30 (56.24-62.19)	65.15 (62.85-67.38)	59.15 (55.16-63.93)	52.28 (48.24-56.74)	56.47 (52.35-61.01)	62.77 (58.66-66.90)
Uruguay	46.32 (41.78-51.14)	51.76 (47.21-56.14)	59.92 (55.08-64.03)	51.72 (48.04-57.91)	40.10 (36.31-48.12)	39.09 (34.95-47.19)	61.29 (59.04-63.61)	60.14 (57.70-62.61)	62.46 (58.83-66.88)	53.63 (49.63-58.16)	54.77 (50.63-59.42)	57.14 (52.76-61.68)

	**Appendix Table 1a. Scaled values for each SDG health-related indicator and three summary indicators by country for 1990, 2000, and 2015**											
	**Indicator 3.4.1: Death rate due to cardiovascular disease, cancer, diabetes, and chronic respiratory disease among populations aged 30 to 70 (per 100,000 population)**			**Indicator 3.4.2: Age-standardised death rate due to self-harm (per 100,000 population)**			**Indicator 3.5.2: Risk-weighted prevalence of alcohol consumption, as measured by the summary exposure value (SEV) for alcohol use**			**Indicator 3.6.1: Age-standardised death rate due to road injuries (per 100,000 population)**		
**Location**	**1990**	**2000**	**2015**	**1990**	**2000**	**2015**	**1990**	**2000**	**2015**	**1990**	**2000**	**2015**
Belarus	37.45 (32.93-42.87)	29.73 (24.93-35.99)	36.32 (31.26-42.05)	29.02 (24.00-38.77)	14.67 (9.68-27.25)	21.08 (13.52-32.34)	8.95 (6.94-11.19)	6.17 (3.84-8.73)	0.04 (0.00-0.52)	44.51 (39.71-50.10)	43.86 (39.15-49.36)	50.23 (44.79-56.61)
Estonia	37.87 (33.44-43.34)	38.81 (34.14-44.08)	63.05 (58.92-66.88)	24.27 (20.20-35.23)	22.08 (17.58-33.30)	45.63 (39.88-52.68)	6.62 (1.79-11.76)	46.37 (43.04-49.83)	39.87 (35.66-44.34)	40.41 (35.22-46.24)	51.08 (46.72-55.77)	83.40 (79.09-87.54)
Latvia	36.51 (31.97-42.09)	38.58 (33.97-43.88)	50.44 (45.80-54.91)	23.48 (19.49-34.67)	22.49 (18.01-33.80)	35.75 (31.06-44.56)	17.03 (12.81-21.62)	56.22 (53.58-58.94)	50.00 (46.18-54.38)	32.36 (26.79-39.12)	41.88 (37.18-47.78)	69.73 (65.13-73.96)
Lithuania	41.84 (37.40-47.02)	45.52 (41.02-50.27)	53.14 (48.78-57.60)	21.06 (16.56-32.78)	11.73 (6.84-25.10)	19.95 (14.95-31.75)	14.58 (10.27-18.74)	51.01 (47.99-53.73)	41.57 (38.03-45.47)	37.65 (32.65-43.67)	45.20 (40.70-50.43)	64.31 (59.98-68.41)
Moldova	37.42 (32.85-42.85)	38.16 (33.64-43.61)	51.46 (47.22-56.00)	37.18 (33.32-45.82)	40.99 (36.77-48.87)	45.80 (41.39-52.74)	25.95 (18.47-35.61)	33.41 (25.80-42.48)	28.66 (20.76-38.30)	39.32 (34.43-45.17)	55.60 (51.62-59.98)	69.65 (65.66-73.63)
Russia	35.22 (30.74-40.80)	26.06 (21.00-32.61)	40.67 (36.22-45.86)	23.53 (19.21-34.49)	7.63 (3.06-21.75)	21.50 (16.56-32.48)	13.16 (6.12-22.34)	13.98 (7.01-22.55)	6.93 (0.62-14.92)	40.15 (35.15-45.86)	35.49 (30.38-41.72)	52.53 (47.70-57.20)
Ukraine	37.35 (32.76-42.82)	30.46 (25.70-36.52)	41.57 (36.88-46.82)	32.25 (28.11-41.40)	20.54 (16.25-32.12)	33.50 (28.61-42.60)	18.62 (15.12-22.50)	17.17 (13.53-21.07)	8.10 (3.15-13.98)	45.38 (40.68-50.70)	50.33 (46.51-55.23)	61.82 (57.16-66.27)
Albania	56.72 (51.88-60.82)	58.06 (53.22-62.12)	63.96 (58.10-68.68)	80.08 (73.95-83.77)	72.31 (68.39-76.31)	73.69 (68.63-79.68)	83.43 (81.97-84.86)	77.59 (75.97-79.19)	71.14 (68.26-74.29)	60.24 (55.58-65.42)	71.87 (67.58-75.55)	77.64 (73.15-81.70)
Bosnia and Herzegovina	46.66 (42.14-51.42)	53.55 (49.08-57.93)	64.38 (59.68-68.34)	45.00 (40.17-52.02)	51.62 (45.95-58.53)	75.05 (51.75-81.03)	68.77 (66.81-70.40)	72.52 (70.89-74.09)	73.87 (72.11-75.47)	74.29 (69.47-78.28)	77.32 (71.82-82.99)	88.93 (85.09-92.68)
Bulgaria	39.94 (35.43-45.21)	38.75 (34.25-44.13)	46.70 (42.36-51.63)	42.24 (38.18-49.85)	42.63 (38.47-50.22)	53.19 (49.24-59.10)	45.33 (42.47-48.14)	56.73 (54.10-59.12)	54.85 (51.29-58.63)	56.94 (53.05-61.16)	63.44 (59.60-67.28)	71.74 (68.00-75.25)
Croatia	44.37 (39.77-49.31)	48.52 (44.12-53.17)	62.19 (57.66-66.04)	31.85 (27.66-41.18)	37.35 (33.40-45.94)	46.98 (42.54-53.97)	47.99 (44.95-50.57)	49.80 (46.80-52.34)	53.69 (50.44-56.95)	49.44 (45.14-54.03)	57.20 (53.12-61.34)	72.10 (68.39-75.72)

Appendix Table 1a. Scaled values for each SDG health-related indicator and three summary indicators by country for 1990, 2000, and 2015

Location	Indicator 3.4.1: Death rate due to cardiovascular disease, cancer, diabetes, and chronic respiratory disease among populations aged 30 to 70 (per 100,000 population)			Indicator 3.4.2: Age-standardised death rate due to self-harm (per 100,000 population)			Indicator 3.5.2: Risk-weighted prevalence of alcohol consumption, as measured by the summary exposure value (SEV) for alcohol use			Indicator 3.6.1: Age-standardised death rate due to road injuries (per 100,000 population)		
	1990	2000	2015	1990	2000	2015	1990	2000	2015	1990	2000	2015
Czech Republic	37.42 (32.86-43.01)	49.63 (45.14-54.08)	65.37 (60.41-68.88)	36.29 (32.19-44.93)	44.10 (40.17-51.75)	50.44 (45.86-56.67)	43.34 (40.25-45.88)	42.94 (39.85-45.69)	46.11 (43.06-48.88)	58.53 (54.51-62.67)	60.56 (56.73-64.39)	78.63 (75.24-81.99)
Hungary	33.20 (28.53-39.08)	37.50 (32.99-43.05)	52.09 (47.68-56.59)	16.71 (12.20-28.93)	27.47 (23.05-37.74)	40.05 (35.17-47.85)	42.41 (39.37-45.33)	48.39 (45.54-50.96)	52.06 (49.10-55.05)	47.70 (43.23-52.66)	59.56 (55.49-63.54)	79.53 (75.87-83.15)
Macedonia	44.32 (39.83-49.38)	42.31 (37.84-47.44)	55.13 (50.14-59.44)	57.75 (53.92-63.09)	58.91 (55.08-63.73)	63.80 (58.90-68.99)	57.78 (53.77-62.50)	60.58 (56.43-65.14)	58.80 (54.62-63.35)	69.73 (65.83-73.60)	75.15 (71.77-78.47)	75.87 (71.61-80.07)
Montenegro	47.45 (42.58-52.44)	44.73 (40.24-49.56)	54.97 (50.16-59.74)	54.17 (49.06-60.04)	51.89 (47.32-58.04)	53.00 (47.88-59.35)	65.28 (61.44-69.84)	67.93 (64.39-71.72)	64.06 (60.61-67.87)	64.21 (59.51-69.40)	64.35 (60.35-68.33)	71.19 (66.93-76.04)
Poland	36.33 (31.79-41.90)	45.05 (40.48-49.95)	57.93 (53.55-62.22)	44.53 (40.41-51.67)	39.03 (35.11-47.11)	39.47 (34.95-47.44)	59.29 (57.27-61.37)	59.45 (57.07-61.76)	54.49 (51.26-57.56)	44.80 (40.25-49.95)	51.55 (47.39-56.07)	67.95 (64.08-71.61)
Romania	41.33 (36.92-46.52)	41.28 (36.83-46.47)	51.43 (46.89-56.18)	55.99 (52.29-61.39)	52.20 (48.29-58.33)	52.83 (48.61-59.12)	44.72 (41.71-47.36)	50.03 (47.33-52.50)	48.82 (45.12-52.19)	49.51 (45.26-54.35)	58.26 (54.25-62.65)	68.35 (64.36-72.35)
Serbia	45.12 (40.39-50.12)	39.01 (34.38-44.35)	53.80 (49.42-58.17)	37.02 (32.40-45.21)	35.65 (31.54-43.98)	43.91 (39.20-51.08)	64.36 (60.53-68.59)	65.02 (61.78-68.62)	59.48 (55.79-63.41)	58.20 (53.10-63.55)	63.17 (59.32-67.14)	69.26 (65.47-72.82)
Slovakia	36.75 (32.20-42.31)	43.87 (39.47-48.84)	60.45 (56.25-64.36)	40.71 (35.94-48.64)	46.05 (42.17-52.74)	55.17 (50.40-60.72)	44.15 (41.12-46.74)	47.40 (44.78-49.87)	50.71 (47.78-53.50)	47.63 (41.98-53.56)	57.43 (53.59-61.43)	77.41 (73.59-81.12)
Slovenia	51.06 (46.74-55.61)	57.68 (53.26-61.87)	79.06 (73.63-82.28)	22.37 (18.02-33.46)	25.85 (21.48-36.47)	44.19 (38.89-51.21)	41.68 (38.34-44.66)	48.67 (45.67-51.52)	49.92 (45.83-54.45)	43.77 (39.10-49.14)	56.85 (52.77-61.09)	80.15 (76.31-84.44)
Armenia	42.43 (37.82-47.60)	39.88 (35.34-45.10)	49.81 (44.85-54.76)	80.98 (72.38-84.69)	76.96 (72.61-80.26)	64.80 (59.36-73.71)	80.36 (77.98-83.07)	81.73 (80.16-83.34)	79.07 (76.19-82.51)	51.59 (47.15-56.43)	61.62 (57.24-66.19)	69.15 (64.35-73.63)
Azerbaijan	35.57 (30.78-41.39)	34.46 (29.59-40.32)	48.11 (42.62-53.36)	83.81 (79.51-87.20)	88.26 (80.73-91.88)	80.36 (74.65-86.27)	75.20 (72.50-78.49)	77.39 (74.97-79.96)	68.99 (64.93-73.92)	50.54 (45.64-57.26)	69.95 (65.13-73.94)	75.56 (70.00-81.20)

	Appendix Table 1a. Scaled values for each SDG health-related indicator and three summary indicators by country for 1990, 2000, and 2015											
	**Indicator 3.4.1: Death rate due to cardiovascular disease, cancer, diabetes, and chronic respiratory disease among populations aged 30 to 70 (per 100,000 population)**			**Indicator 3.4.2: Age-standardised death rate due to self-harm (per 100,000 population)**			**Indicator 3.5.2: Risk-weighted prevalence of alcohol consumption, as measured by the summary exposure value (SEV) for alcohol use**			**Indicator 3.6.1: Age-standardised death rate due to road injuries (per 100,000 population)**		
**Location**	**1990**	**2000**	**2015**	**1990**	**2000**	**2015**	**1990**	**2000**	**2015**	**1990**	**2000**	**2015**
Georgia	39.53 (34.45-45.08)	42.75 (37.75-48.14)	43.36 (38.29-48.76)	72.30 (66.00-76.55)	68.95 (64.93-73.01)	62.73 (57.81-68.40)	73.76 (71.28-76.71)	78.33 (76.77-80.05)	71.72 (68.54-75.42)	47.73 (42.72-53.22)	67.60 (62.54-71.74)	53.69 (47.91-59.03)
Kazakhstan	33.73 (29.18-39.45)	24.81 (19.59-31.53)	37.81 (32.51-43.51)	30.22 (25.85-39.84)	17.34 (12.83-29.79)	22.20 (16.51-33.68)	52.89 (49.54-56.51)	62.08 (59.39-64.97)	57.53 (53.48-62.38)	43.45 (38.56-48.92)	52.91 (48.64-57.59)	50.82 (45.34-56.68)
Kyrgyzstan	35.51 (30.79-41.33)	30.81 (25.80-36.89)	40.43 (35.73-45.79)	38.94 (34.35-47.06)	37.06 (31.65-45.17)	45.62 (38.81-52.61)	72.01 (69.77-74.15)	75.37 (73.59-77.12)	75.27 (72.51-78.33)	41.02 (35.66-46.85)	53.41 (48.54-58.16)	50.32 (45.32-55.75)
Mongolia	30.00 (24.96-36.29)	21.63 (15.98-28.97)	29.64 (24.02-36.02)	26.38 (18.49-37.59)	17.09 (10.79-29.47)	18.76 (12.45-31.14)	81.26 (79.25-83.29)	80.74 (78.77-82.72)	71.65 (68.37-75.31)	55.76 (43.83-63.98)	54.14 (47.83-59.43)	46.83 (40.82-52.89)
Tajikistan	40.26 (35.45-45.59)	38.38 (33.60-43.98)	51.63 (46.67-56.25)	65.53 (61.09-70.05)	71.48 (64.64-75.59)	75.41 (67.92-80.95)	77.62 (75.00-80.50)	82.55 (80.77-84.20)	85.60 (83.37-87.96)	54.69 (49.99-61.79)	73.80 (67.61-77.80)	78.40 (73.97-82.61)
Turkmenistan	31.29 (26.51-37.32)	27.82 (22.78-34.27)	41.43 (36.68-46.41)	54.88 (50.82-60.72)	51.83 (46.36-58.41)	59.63 (50.55-65.84)	84.86 (82.71-87.07)	81.08 (79.39-82.64)	79.82 (77.31-82.57)	48.46 (43.93-53.68)	56.88 (49.28-62.22)	71.42 (61.31-76.17)
Uzbekistan	36.07 (31.04-41.58)	33.86 (28.75-39.70)	44.46 (39.76-49.49)	52.55 (48.37-58.48)	50.77 (45.94-57.24)	54.52 (48.56-61.34)	85.62 (83.63-87.58)	83.38 (81.93-84.67)	78.43 (75.93-81.20)	49.26 (44.73-54.51)	58.01 (53.28-62.50)	61.38 (55.73-66.84)
Colombia	55.70 (51.49-59.89)	62.16 (57.57-66.02)	81.25 (74.76-84.61)	73.41 (69.76-76.98)	63.73 (59.81-68.18)	69.17 (64.67-73.55)	68.35 (65.85-70.79)	70.80 (68.65-73.01)	70.83 (67.59-74.50)	42.13 (37.50-47.73)	44.06 (39.35-49.27)	57.97 (53.32-62.55)
Costa Rica	70.02 (65.20-73.49)	76.38 (71.03-79.72)	87.18 (80.39-91.02)	69.21 (65.14-73.21)	64.37 (60.45-68.87)	63.65 (59.43-68.67)	70.47 (68.64-72.20)	71.52 (69.82-73.39)	72.74 (69.95-75.92)	48.52 (44.18-53.27)	48.19 (43.84-53.16)	58.57 (54.09-62.91)
El Salvador	64.15 (59.23-68.21)	71.64 (66.15-75.66)	75.49 (69.23-80.40)	47.47 (42.65-54.68)	53.79 (49.14-59.73)	56.50 (49.95-62.63)	83.81 (82.54-85.14)	81.70 (80.48-83.00)	80.55 (78.57-82.87)	26.22 (19.96-33.90)	28.85 (22.66-36.26)	41.64 (34.22-47.91)
Guatemala	71.94 (66.39-75.61)	68.57 (63.58-72.20)	69.75 (62.09-76.57)	56.03 (51.86-61.48)	45.98 (41.94-53.17)	62.63 (56.44-68.62)	87.17 (86.27-88.00)	88.32 (87.48-89.09)	88.35 (87.56-89.11)	54.12 (49.95-58.70)	56.40 (52.59-60.68)	54.51 (48.84-60.11)

	Appendix Table 1a. Scaled values for each SDG health-related indicator and three summary indicators by country for 1990, 2000, and 2015											
	**Indicator 3.4.1: Death rate due to cardiovascular disease, cancer, diabetes, and chronic respiratory disease among populations aged 30 to 70 (per 100,000 population)**			**Indicator 3.4.2: Age-standardised death rate due to self-harm (per 100,000 population)**			**Indicator 3.5.2: Risk-weighted prevalence of alcohol consumption, as measured by the summary exposure value (SEV) for alcohol use**			**Indicator 3.6.1: Age-standardised death rate due to road injuries (per 100,000 population)**		
**Location**	**1990**	**2000**	**2015**	**1990**	**2000**	**2015**	**1990**	**2000**	**2015**	**1990**	**2000**	**2015**
Honduras	58.86 (53.74-63.32)	52.69 (44.45-61.27)	58.80 (48.91-68.64)	71.26 (62.61-76.15)	69.33 (60.99-76.47)	67.86 (58.41-76.44)	83.47 (81.96-84.83)	81.19 (79.84-82.47)	80.79 (79.04-82.88)	48.19 (43.26-54.32)	50.22 (41.79-57.67)	55.16 (47.11-63.10)
Mexico	63.58 (59.08-67.28)	66.34 (61.69-69.91)	71.60 (66.48-75.06)	79.43 (75.51-82.34)	74.77 (70.85-78.10)	69.27 (65.23-73.24)	71.39 (69.87-72.98)	70.72 (69.30-72.12)	73.92 (71.88-76.34)	38.44 (33.45-44.31)	47.63 (43.18-52.45)	53.42 (49.55-57.82)
Nicaragua	78.13 (72.17-82.39)	76.90 (70.89-80.95)	78.92 (71.67-83.85)	67.57 (62.25-72.16)	59.29 (54.80-65.44)	63.40 (58.45-68.59)	80.88 (79.61-82.00)	80.74 (79.53-81.90)	81.18 (79.68-82.76)	46.63 (41.84-52.15)	55.57 (51.33-60.03)	60.54 (54.73-66.16)
Panama	66.68 (61.64-70.85)	74.87 (69.59-79.09)	81.65 (73.06-88.62)	69.46 (64.84-73.69)	64.86 (60.50-69.49)	69.66 (61.58-75.64)	75.22 (73.92-76.59)	74.22 (72.93-75.55)	71.92 (69.93-74.05)	41.47 (36.07-48.23)	46.02 (41.19-51.79)	54.57 (48.09-60.83)
Venezuela	54.07 (49.58-58.30)	60.38 (55.90-64.24)	64.75 (57.55-70.92)	64.66 (60.46-69.14)	56.93 (52.91-62.46)	57.32 (51.62-63.86)	73.14 (70.22-76.90)	74.11 (71.25-77.79)	74.48 (71.49-78.19)	35.66 (30.40-41.84)	41.62 (36.65-47.29)	44.78 (38.58-51.18)
Bolivia	50.66 (44.48-56.42)	59.28 (53.69-64.54)	66.83 (57.32-75.76)	40.04 (32.74-49.61)	43.70 (38.47-51.34)	48.52 (40.95-56.78)	79.01 (76.66-82.12)	77.68 (75.34-80.67)	76.61 (74.35-79.54)	33.08 (24.39-42.26)	41.97 (35.35-48.00)	52.08 (44.74-59.28)
Ecuador	64.22 (59.62-68.03)	66.04 (61.04-70.36)	72.01 (65.50-77.87)	70.02 (63.06-73.94)	63.24 (59.38-67.72)	62.18 (56.92-68.32)	84.64 (83.72-85.49)	82.33 (81.28-83.31)	81.80 (80.70-82.85)	34.47 (29.22-40.74)	37.91 (32.60-43.98)	39.63 (33.67-45.53)
Peru	67.28 (62.12-71.31)	75.39 (68.70-80.01)	91.47 (82.26-98.45)	80.96 (75.81-84.69)	75.26 (71.02-80.65)	85.50 (79.80-90.44)	65.82 (63.70-67.69)	67.79 (66.00-69.45)	68.37 (65.79-71.39)	51.99 (46.14-57.13)	51.97 (47.19-56.96)	59.40 (53.89-65.43)
Antigua and Barbuda	51.86 (46.79-56.49)	54.63 (49.39-59.47)	63.32 (57.16-69.51)	97.63 (93.05-100.00)	96.74 (92.52-100.00)	96.95 (91.85-100.00)	70.57 (66.90-75.33)	72.99 (68.27-78.97)	68.10 (64.51-73.11)	73.02 (68.95-76.80)	75.16 (71.04-78.98)	78.86 (74.07-83.14)
The Bahamas	40.31 (35.24-45.61)	45.71 (40.06-50.95)	55.14 (48.80-61.00)	83.87 (79.51-87.98)	82.26 (77.50-86.70)	85.67 (80.18-90.35)	59.71 (56.98-62.05)	71.72 (69.45-73.87)	64.42 (62.11-66.88)	42.72 (36.93-48.66)	46.30 (40.76-52.22)	56.97 (51.54-62.40)
Barbados	50.43 (45.70-54.92)	55.34 (50.34-59.85)	60.08 (54.08-65.51)	79.43 (75.44-83.06)	81.21 (77.12-84.82)	77.08 (72.21-81.71)	70.77 (68.56-73.01)	75.62 (74.18-77.12)	67.63 (65.18-70.41)	61.90 (57.92-65.88)	64.73 (60.39-69.07)	67.41 (62.33-72.10)

Appendix Table 1a. Scaled values for each SDG health-related indicator and three summary indicators by country for 1990, 2000, and 2015

Location	Indicator 3.4.1: Death rate due to cardiovascular disease, cancer, diabetes, and chronic respiratory disease among populations aged 30 to 70 (per 100,000 population)			Indicator 3.4.2: Age-standardised death rate due to self-harm (per 100,000 population)			Indicator 3.5.2: Risk-weighted prevalence of alcohol consumption, as measured by the summary exposure value (SEV) for alcohol use			Indicator 3.6.1: Age-standardised death rate due to road injuries (per 100,000 population)		
	**1990**	**2000**	**2015**	**1990**	**2000**	**2015**	**1990**	**2000**	**2015**	**1990**	**2000**	**2015**
Belize	52.30 (47.22-57.21)	42.52 (37.60-47.91)	51.42 (45.64-57.32)	73.32 (68.57-77.42)	60.83 (56.36-66.19)	64.39 (58.20-70.50)	73.51 (71.27-75.78)	76.14 (74.12-78.10)	70.82 (68.33-73.60)	51.12 (46.69-56.22)	41.78 (36.45-47.73)	48.93 (42.74-54.83)
Cuba	52.99 (48.46-57.36)	57.35 (53.05-61.30)	64.19 (59.47-68.06)	33.42 (29.33-42.57)	40.62 (36.60-48.57)	47.26 (43.11-54.34)	76.05 (74.32-77.84)	77.93 (76.40-79.44)	73.11 (70.39-76.30)	45.49 (40.94-50.65)	57.04 (53.15-61.10)	72.94 (69.41-76.52)
Dominica	50.54 (45.26-55.59)	54.98 (49.80-59.38)	53.72 (47.21-60.08)	84.11 (79.35-88.14)	79.68 (75.19-83.75)	75.15 (69.18-81.14)	64.05 (61.30-66.58)	69.88 (67.84-71.80)	67.89 (64.98-71.55)	62.96 (58.56-67.09)	61.89 (57.53-66.11)	57.52 (51.47-63.74)
Dominican Republic	64.40 (59.90-68.39)	66.27 (60.01-70.81)	65.73 (60.06-69.71)	75.32 (67.60-79.32)	65.87 (61.40-71.97)	66.83 (62.54-72.01)	76.32 (74.54-78.05)	72.44 (70.50-74.37)	71.41 (68.91-74.25)	44.44 (39.46-50.15)	41.57 (36.32-47.89)	44.49 (39.21-50.28)
Grenada	40.69 (36.12-46.15)	42.09 (37.01-47.48)	44.47 (38.90-50.19)	71.73 (66.91-75.84)	64.22 (59.82-68.80)	66.80 (61.46-72.11)	60.99 (58.50-63.34)	63.61 (61.13-66.13)	64.37 (61.47-67.56)	63.00 (58.54-67.43)	60.16 (55.76-64.44)	59.44 (53.67-65.26)
Guyana	29.08 (23.94-35.58)	29.78 (24.48-35.98)	37.05 (30.40-44.03)	31.50 (25.75-40.97)	22.09 (16.78-32.75)	23.75 (15.70-35.87)	66.57 (63.57-69.70)	68.97 (66.18-71.81)	68.03 (65.11-71.60)	47.13 (42.25-52.38)	45.51 (40.45-50.89)	46.40 (39.42-53.53)
Haiti	24.83 (17.94-31.80)	34.56 (26.38-43.04)	36.90 (26.94-47.75)	57.16 (43.69-68.21)	60.98 (47.65-70.47)	60.33 (46.67-71.05)	71.94 (69.55-74.17)	72.78 (70.47-75.00)	71.86 (68.67-75.33)	32.45 (20.91-43.07)	40.27 (29.92-51.18)	41.85 (30.08-54.29)
Jamaica	54.09 (49.49-58.56)	54.56 (49.67-59.12)	57.57 (50.87-63.55)	98.97 (94.16-100.00)	90.09 (85.49-93.62)	85.56 (80.13-90.83)	76.10 (73.98-78.02)	79.45 (77.73-81.07)	75.68 (73.10-78.85)	80.62 (77.03-84.02)	78.98 (74.31-82.99)	71.31 (65.52-77.26)
Saint Lucia	42.83 (37.85-48.14)	47.82 (43.06-52.55)	57.50 (51.83-62.61)	69.73 (65.37-73.90)	67.41 (63.00-71.88)	67.30 (62.14-72.10)	61.51 (58.74-63.95)	62.58 (60.11-64.89)	61.14 (58.41-64.26)	54.93 (50.63-59.99)	56.67 (52.30-61.64)	63.28 (58.29-67.99)
Saint Vincent and the Grenadines	43.81 (38.67-49.32)	41.53 (36.41-46.83)	45.69 (40.36-51.09)	70.82 (66.35-75.18)	64.92 (60.42-69.93)	61.05 (55.93-67.20)	74.87 (70.66-80.35)	74.80 (70.72-79.98)	71.70 (67.81-76.86)	68.66 (64.25-72.87)	67.73 (63.78-71.68)	67.07 (62.74-71.69)
Suriname	48.50 (43.78-53.27)	50.30 (44.76-55.47)	55.33 (48.57-61.88)	37.53 (32.24-46.18)	31.86 (27.05-41.33)	29.08 (22.22-39.05)	77.12 (74.85-79.41)	76.09 (74.15-77.97)	73.26 (70.80-76.09)	45.47 (40.13-51.26)	47.29 (42.19-52.50)	48.70 (42.57-54.68)

	Appendix Table 1a. Scaled values for each SDG health-related indicator and three summary indicators by country for 1990, 2000, and 2015											
	**Indicator 3.4.1: Death rate due to cardiovascular disease, cancer, diabetes, and chronic respiratory disease among populations aged 30 to 70 (per 100,000 population)**			**Indicator 3.4.2: Age-standardised death rate due to self-harm (per 100,000 population)**			**Indicator 3.5.2: Risk-weighted prevalence of alcohol consumption, as measured by the summary exposure value (SEV) for alcohol use**			**Indicator 3.6.1: Age-standardised death rate due to road injuries (per 100,000 population)**		
**Location**	**1990**	**2000**	**2015**	**1990**	**2000**	**2015**	**1990**	**2000**	**2015**	**1990**	**2000**	**2015**
Trinidad and Tobago	34.65 (29.99-40.48)	38.39 (33.67-43.89)	47.38 (41.70-52.68)	45.41 (41.30-52.25)	46.03 (41.95-53.10)	46.31 (40.44-54.14)	67.97 (64.11-73.22)	69.06 (65.52-73.91)	64.58 (60.95-69.45)	52.93 (48.58-57.53)	53.24 (48.97-57.84)	54.63 (49.13-60.19)
Brazil	44.33 (39.85-49.43)	49.86 (45.40-54.36)	60.58 (55.93-64.70)	61.86 (58.23-66.48)	65.10 (59.86-69.43)	67.63 (61.90-71.93)	75.46 (74.05-76.72)	70.47 (68.99-71.75)	70.63 (69.23-71.87)	30.59 (25.10-37.26)	37.56 (31.99-43.61)	42.78 (37.25-48.24)
Paraguay	60.72 (55.64-65.37)	59.68 (54.72-64.24)	58.40 (51.14-64.75)	79.98 (75.08-83.98)	72.65 (68.13-76.91)	68.10 (61.07-74.79)	65.33 (63.19-67.25)	66.71 (64.70-68.46)	67.39 (65.12-69.64)	52.38 (47.64-57.51)	48.37 (43.43-53.71)	40.94 (33.02-48.51)
China	35.22 (30.28-41.19)	41.36 (36.72-46.73)	58.06 (53.31-62.54)	31.57 (25.54-44.95)	40.31 (35.94-48.66)	58.66 (53.59-64.04)	78.42 (77.16-79.75)	76.89 (75.57-78.29)	73.51 (71.19-76.30)	44.82 (39.73-49.91)	41.61 (36.32-47.31)	48.57 (43.49-54.04)
North Korea	43.20 (31.38-54.94)	40.00 (29.47-51.92)	42.36 (32.65-51.98)	36.31 (24.86-48.10)	39.00 (28.41-50.14)	44.77 (34.83-54.36)	78.53 (75.43-82.63)	81.09 (78.04-84.93)	83.00 (79.78-86.75)	41.93 (30.78-52.46)	37.04 (25.15-50.22)	39.04 (26.31-52.86)
Taiwan	52.77 (48.34-57.07)	57.66 (53.12-61.87)	72.25 (64.44-79.87)	41.01 (34.68-49.02)	45.84 (39.74-52.97)	47.19 (40.87-54.44)	73.13 (69.96-77.74)	70.96 (67.66-75.35)	71.42 (67.96-76.11)	37.52 (30.42-45.05)	46.35 (40.03-52.53)	58.32 (52.25-64.04)
Cambodia	31.83 (25.59-39.11)	33.38 (28.00-39.71)	46.06 (38.41-53.39)	36.58 (24.10-49.06)	37.43 (26.10-49.29)	45.44 (37.70-54.55)	93.27 (92.26-94.27)	82.36 (80.75-84.37)	78.58 (76.00-82.14)	37.28 (24.05-52.72)	35.74 (24.38-46.73)	47.24 (39.36-54.74)
Indonesia	45.70 (40.22-51.56)	44.27 (39.00-49.72)	47.23 (39.18-56.71)	89.55 (83.43-95.00)	88.27 (83.24-92.72)	87.72 (80.05-94.89)	95.65 (95.16-96.12)	95.42 (94.91-95.86)	95.03 (94.53-95.52)	38.30 (31.92-45.84)	41.17 (35.71-47.44)	49.97 (41.59-57.92)
Laos	26.14 (16.69-36.52)	29.20 (22.15-36.59)	43.76 (35.21-52.66)	30.89 (16.11-47.41)	31.64 (20.02-45.23)	41.89 (32.85-52.36)	91.81 (89.49-93.96)	90.64 (87.85-93.19)	88.26 (84.57-91.88)	30.37 (16.87-44.51)	27.61 (15.91-40.22)	39.73 (30.85-48.36)
Malaysia	47.75 (43.39-52.40)	53.09 (48.62-57.43)	59.45 (52.43-66.48)	56.92 (51.77-65.43)	56.39 (51.74-65.06)	59.10 (52.06-67.20)	92.49 (91.90-93.09)	95.02 (94.63-95.39)	94.23 (93.67-94.84)	37.82 (32.36-44.38)	39.31 (34.09-46.14)	44.27 (37.10-51.98)
Maldives	49.69 (43.82-54.71)	68.26 (62.85-72.47)	91.25 (81.06-99.55)	21.06 (9.65-34.86)	29.52 (24.34-39.19)	49.04 (41.87-56.53)	91.98 (90.63-93.53)	90.38 (88.56-92.40)	88.34 (86.00-91.16)	50.31 (43.35-60.32)	59.34 (55.15-63.67)	75.98 (70.62-81.16)

	Appendix Table 1a. Scaled values for each SDG health-related indicator and three summary indicators by country for 1990, 2000, and 2015											
	Indicator 3.4.1: Death rate due to cardiovascular disease, cancer, diabetes, and chronic respiratory disease among populations aged 30 to 70 (per 100,000 population)			Indicator 3.4.2: Age-standardised death rate due to self-harm (per 100,000 population)			Indicator 3.5.2: Risk-weighted prevalence of alcohol consumption, as measured by the summary exposure value (SEV) for alcohol use			Indicator 3.6.1: Age-standardised death rate due to road injuries (per 100,000 population)		
**Location**	**1990**	**2000**	**2015**	**1990**	**2000**	**2015**	**1990**	**2000**	**2015**	**1990**	**2000**	**2015**
Mauritius	33.37 (28.73-39.25)	39.18 (34.55-44.65)	52.81 (47.87-57.37)	47.52 (43.32-54.37)	50.82 (46.33-57.06)	59.00 (54.98-64.34)	79.31 (77.81-80.92)	79.11 (77.57-80.72)	80.40 (77.40-84.10)	56.96 (52.93-61.43)	55.63 (51.20-60.18)	65.79 (61.67-69.84)
Myanmar	33.29 (16.03-53.10)	35.27 (18.17-55.18)	43.61 (25.80-62.52)	79.84 (66.88-92.78)	78.87 (65.63-94.95)	76.27 (63.65-89.82)	98.07 (97.75-98.35)	97.47 (97.12-97.77)	96.69 (96.00-97.40)	54.83 (42.46-67.82)	57.27 (44.64-72.45)	58.74 (45.36-73.66)
Philippines	52.81 (48.42-57.19)	47.33 (42.91-52.10)	46.30 (41.00-51.66)	43.82 (39.89-51.12)	74.10 (70.51-77.32)	70.42 (65.46-74.60)	76.21 (74.63-77.86)	75.08 (73.56-76.54)	76.95 (74.23-80.25)	61.94 (58.14-65.87)	63.80 (60.13-67.47)	65.90 (61.50-70.39)
Sri Lanka	61.37 (56.85-65.19)	56.06 (51.72-60.32)	71.01 (60.24-82.23)	3.38 (0.00-19.04)	8.27 (3.52-22.58)	25.06 (15.32-37.23)	95.09 (94.58-95.52)	93.76 (92.96-94.57)	86.78 (84.83-89.12)	57.46 (53.51-61.80)	50.68 (46.33-55.41)	59.39 (51.45-67.11)
Seychelles	39.11 (33.80-44.89)	43.81 (39.04-49.00)	55.54 (48.46-62.08)	43.78 (38.23-52.02)	51.29 (46.61-57.51)	60.65 (54.13-66.93)	80.55 (79.01-82.22)	76.45 (74.76-78.26)	77.23 (74.36-80.81)	59.05 (52.25-65.00)	50.88 (46.03-55.89)	59.03 (53.77-64.72)
Thailand	53.48 (48.60-58.02)	50.27 (44.81-55.16)	60.77 (52.38-69.82)	52.66 (47.14-58.93)	39.19 (34.35-47.21)	42.45 (35.12-51.39)	77.62 (75.81-79.79)	74.70 (72.84-76.79)	73.96 (71.42-77.20)	42.23 (36.61-47.90)	25.86 (19.24-33.52)	36.01 (28.25-44.28)
Timor-Leste	44.52 (34.97-53.51)	51.12 (43.68-59.81)	61.44 (50.51-73.40)	44.00 (32.33-56.71)	50.14 (38.42-63.66)	55.30 (43.27-66.79)	92.30 (90.21-94.56)	90.94 (88.67-93.56)	86.92 (84.24-90.69)	54.09 (43.39-66.13)	54.78 (43.86-67.60)	60.22 (46.28-71.27)
Vietnam	51.39 (43.46-58.72)	52.36 (44.71-60.78)	60.37 (48.38-74.54)	56.92 (48.76-65.17)	57.08 (49.84-65.18)	60.29 (50.52-70.48)	96.39 (95.66-97.09)	88.85 (87.53-90.29)	76.64 (73.56-80.47)	37.01 (28.00-46.81)	39.98 (31.75-49.13)	45.18 (34.58-56.06)
Federated States of Micronesia	26.63 (10.38-47.68)	29.14 (11.02-47.34)	32.37 (10.17-49.37)	35.18 (20.17-52.63)	39.93 (25.05-54.67)	43.43 (25.30-56.32)	90.62 (88.54-93.06)	90.36 (88.31-92.66)	89.94 (87.83-92.37)	51.28 (38.60-65.27)	54.44 (40.66-67.97)	58.61 (42.59-70.78)
Fiji	21.18 (12.51-30.61)	18.64 (11.65-26.46)	23.68 (15.17-31.98)	49.69 (42.84-57.25)	46.12 (40.68-53.28)	46.75 (40.01-54.45)	87.63 (86.66-88.53)	88.00 (87.10-88.80)	84.85 (83.25-86.50)	62.42 (55.87-69.90)	60.05 (55.00-65.09)	61.25 (55.06-67.81)
Kiribati	19.90 (13.10-28.10)	18.83 (11.98-26.50)	22.09 (13.79-30.52)	26.97 (19.78-37.85)	32.59 (25.80-42.08)	35.65 (26.96-44.97)	87.88 (86.82-88.94)	88.17 (87.19-89.01)	87.75 (86.38-89.25)	61.93 (56.50-67.47)	56.20 (50.80-62.48)	61.52 (54.68-68.18)

	Appendix Table 1a. Scaled values for each SDG health-related indicator and three summary indicators by country for 1990, 2000, and 2015											
	**Indicator 3.4.1: Death rate due to cardiovascular disease, cancer, diabetes, and chronic respiratory disease among populations aged 30 to 70 (per 100,000 population)**			**Indicator 3.4.2: Age-standardised death rate due to self-harm (per 100,000 population)**			**Indicator 3.5.2: Risk-weighted prevalence of alcohol consumption, as measured by the summary exposure value (SEV) for alcohol use**			**Indicator 3.6.1: Age-standardised death rate due to road injuries (per 100,000 population)**		
**Location**	**1990**	**2000**	**2015**	**1990**	**2000**	**2015**	**1990**	**2000**	**2015**	**1990**	**2000**	**2015**
Marshall Islands	27.55 (21.07-34.93)	26.61 (19.52-34.84)	28.23 (19.67-36.76)	40.07 (28.76-51.24)	41.00 (29.20-51.77)	43.54 (33.38-54.14)	90.08 (87.90-92.60)	90.22 (87.93-92.67)	89.65 (87.37-92.27)	51.70 (44.89-58.43)	52.17 (43.55-60.92)	55.17 (45.75-63.08)
Papua New Guinea	14.94 (0.77-31.38)	16.02 (1.52-33.96)	18.75 (2.31-36.41)	37.10 (18.14-52.65)	40.00 (21.45-56.57)	42.14 (25.02-57.61)	84.82 (83.29-86.31)	84.80 (83.33-86.09)	86.59 (84.71-88.69)	30.01 (16.09-44.30)	32.40 (17.60-47.19)	36.61 (20.96-50.95)
Samoa	35.42 (25.63-44.82)	39.67 (30.54-48.81)	47.12 (38.07-56.77)	39.43 (29.66-50.45)	45.10 (36.11-55.40)	53.59 (45.28-61.43)	85.83 (84.23-87.29)	85.91 (84.37-87.31)	84.58 (82.45-87.13)	60.84 (51.53-70.35)	63.79 (54.71-73.64)	68.76 (60.37-77.37)
Solomon Islands	16.88 (2.48-34.53)	18.57 (3.14-35.97)	20.68 (3.77-38.59)	33.47 (15.92-49.88)	35.72 (18.58-52.33)	39.20 (22.17-54.36)	94.69 (94.10-95.27)	94.74 (94.12-95.36)	92.76 (91.63-93.99)	47.08 (32.78-61.52)	47.35 (32.64-62.85)	50.35 (33.64-64.74)
Tonga	38.03 (30.51-46.24)	38.25 (33.08-44.47)	42.62 (33.38-50.69)	66.68 (59.63-73.30)	64.69 (59.54-69.81)	65.40 (58.23-72.32)	94.54 (93.79-95.28)	92.37 (91.44-93.28)	91.42 (89.93-93.07)	58.01 (51.21-65.10)	56.94 (52.24-62.07)	60.43 (53.25-67.71)
Vanuatu	19.54 (4.27-35.06)	21.08 (4.53-40.28)	24.42 (7.28-39.72)	32.71 (16.19-48.78)	34.64 (18.60-52.09)	38.15 (24.25-52.26)	90.30 (89.43-91.10)	92.22 (91.45-92.96)	93.41 (92.38-94.48)	48.33 (34.32-60.95)	46.94 (31.47-61.66)	50.37 (36.05-62.82)
Afghanistan	9.58 (0.36-21.30)	6.49 (0.00-17.73)	10.43 (0.60-21.25)	58.46 (48.22-67.46)	56.14 (46.42-64.89)	55.48 (45.60-64.28)	97.41 (96.81-97.97)	97.37 (96.77-97.90)	96.38 (95.67-97.03)	11.12 (0.00-23.64)	9.94 (0.00-22.26)	13.10 (0.08-25.35)
Algeria	54.21 (47.66-59.96)	61.14 (54.11-66.50)	68.36 (61.36-74.96)	84.32 (77.61-90.83)	85.76 (80.47-90.63)	90.77 (85.44-95.47)	95.30 (94.67-95.91)	94.97 (94.36-95.58)	94.82 (94.09-95.67)	27.93 (20.52-36.48)	33.84 (27.69-40.26)	41.29 (35.20-48.27)
Bahrain	43.88 (37.17-50.48)	51.77 (45.79-57.30)	74.16 (64.63-83.33)	58.52 (52.41-64.75)	54.30 (48.19-61.19)	68.91 (60.54-75.73)	86.25 (84.97-87.67)	88.27 (87.13-89.55)	89.40 (88.15-91.00)	42.35 (35.49-48.93)	47.86 (41.85-53.86)	62.15 (55.57-69.02)
Egypt	40.66 (36.09-45.89)	46.19 (41.61-51.12)	46.28 (41.57-51.03)	93.46 (82.95-97.45)	81.45 (77.41-86.81)	84.42 (80.57-87.65)	96.28 (95.82-96.76)	96.29 (95.82-96.76)	96.35 (95.89-96.76)	61.34 (53.96-65.77)	55.83 (51.69-59.86)	56.84 (52.18-61.46)
Iran	46.42 (39.25-53.63)	49.58 (42.73-55.75)	55.98 (46.77-65.13)	66.07 (58.42-72.65)	65.08 (60.03-70.20)	70.18 (61.93-77.53)	93.81 (92.68-95.33)	94.23 (93.11-95.69)	94.30 (93.15-95.75)	19.86 (10.69-29.30)	23.86 (16.48-31.69)	26.50 (16.70-36.01)

Location	Appendix Table 1a. Scaled values for each SDG health-related indicator and three summary indicators by country for 1990, 2000, and 2015											
	**Indicator 3.4.1: Death rate due to cardiovascular disease, cancer, diabetes, and chronic respiratory disease among populations aged 30 to 70 (per 100,000 population)**			**Indicator 3.4.2: Age-standardised death rate due to self-harm (per 100,000 population)**			**Indicator 3.5.2: Risk-weighted prevalence of alcohol consumption, as measured by the summary exposure value (SEV) for alcohol use**			**Indicator 3.6.1: Age-standardised death rate due to road injuries (per 100,000 population)**		
	**1990**	**2000**	**2015**	**1990**	**2000**	**2015**	**1990**	**2000**	**2015**	**1990**	**2000**	**2015**
Iraq	32.13 (22.89-40.55)	28.27 (19.27-36.26)	34.62 (23.78-45.35)	63.97 (55.77-72.08)	58.88 (51.66-66.98)	61.21 (51.71-70.46)	92.83 (92.15-93.60)	95.26 (94.70-95.83)	95.65 (95.02-96.32)	38.76 (30.07-47.33)	38.54 (30.60-46.60)	44.88 (34.45-54.83)
Jordan	46.00 (37.60-53.49)	50.70 (43.90-57.14)	70.53 (62.80-76.99)	71.92 (63.84-80.81)	75.68 (69.55-81.97)	87.82 (82.55-92.61)	96.50 (95.99-96.96)	96.03 (95.45-96.56)	95.47 (94.77-96.28)	40.62 (32.45-49.87)	48.33 (42.10-54.55)	58.93 (53.18-64.58)
Kuwait	69.53 (64.01-73.49)	65.18 (60.58-69.02)	79.55 (70.93-86.31)	97.80 (93.26-100.00)	86.51 (82.29-89.54)	90.93 (84.89-96.02)	97.63 (97.21-97.98)	97.67 (97.23-98.03)	97.53 (97.14-97.87)	45.28 (40.41-50.89)	43.56 (38.88-48.92)	57.56 (51.20-63.54)
Lebanon	41.48 (32.61-50.61)	58.12 (49.30-65.87)	67.06 (53.59-82.07)	77.11 (68.58-85.25)	84.51 (77.29-91.38)	89.50 (80.31-98.74)	84.84 (83.70-85.99)	87.73 (86.85-88.66)	87.97 (86.15-89.98)	55.22 (46.91-62.50)	67.21 (57.78-74.68)	75.67 (64.54-85.76)
Libya	60.20 (53.20-67.01)	60.62 (54.40-65.88)	59.23 (51.43-66.70)	79.53 (73.17-85.66)	76.60 (70.91-81.57)	74.93 (67.88-80.84)	97.48 (97.07-97.88)	97.49 (97.05-97.89)	97.43 (97.01-97.79)	39.60 (32.30-47.60)	42.36 (36.29-48.67)	44.45 (37.72-51.66)
Morocco	49.62 (41.29-56.80)	55.04 (45.74-63.52)	59.90 (47.44-72.78)	59.89 (52.84-67.39)	61.79 (54.61-68.94)	62.49 (52.82-71.63)	96.47 (95.96-97.00)	96.49 (95.97-97.03)	96.90 (96.47-97.35)	38.85 (31.40-46.88)	44.36 (37.80-51.07)	49.40 (39.79-58.00)
Palestine	49.66 (38.27-59.78)	56.03 (49.53-62.06)	51.71 (41.54-62.64)	86.77 (77.79-94.90)	89.76 (83.54-94.27)	90.70 (82.44-98.35)	94.20 (93.07-95.59)	94.43 (93.37-95.74)	95.42 (94.50-96.46)	54.93 (46.43-62.14)	62.35 (57.02-67.61)	61.24 (52.74-69.07)
Oman	57.33 (46.39-70.09)	58.36 (50.02-67.34)	66.78 (58.39-76.50)	89.37 (78.10-99.98)	87.31 (80.34-93.46)	84.13 (77.32-90.72)	95.50 (94.85-96.14)	95.22 (94.52-95.82)	95.04 (94.05-95.96)	10.08 (0.00-23.03)	16.87 (8.14-26.50)	24.53 (14.83-34.02)
Qatar	59.94 (52.56-66.34)	57.71 (50.60-64.17)	81.52 (68.36-93.71)	70.53 (62.69-77.50)	65.93 (59.21-72.40)	75.16 (67.24-82.83)	92.06 (90.97-93.21)	93.89 (93.08-94.67)	91.43 (90.06-93.03)	20.47 (12.22-29.28)	20.82 (13.02-29.56)	35.64 (25.59-44.87)
Saudi Arabia	71.88 (65.11-77.93)	74.65 (68.91-79.30)	83.06 (75.37-88.99)	91.11 (83.00-97.26)	90.53 (84.09-94.92)	85.74 (81.13-90.33)	96.59 (96.20-96.91)	97.14 (96.83-97.42)	97.12 (96.82-97.41)	28.00 (20.95-36.40)	32.67 (26.59-39.74)	39.97 (33.29-46.63)
Sudan	33.60 (23.99-43.88)	36.75 (24.89-47.93)	42.56 (30.53-55.95)	69.58 (56.73-82.10)	66.90 (52.49-80.17)	67.27 (54.35-79.74)	90.58 (89.60-91.60)	89.41 (88.46-90.35)	89.56 (88.47-90.88)	26.84 (12.41-40.15)	26.73 (12.13-40.30)	29.78 (16.16-41.89)

Location	Appendix Table 1a. Scaled values for each SDG health-related indicator and three summary indicators by country for 1990, 2000, and 2015											
	**Indicator 3.4.1: Death rate due to cardiovascular disease, cancer, diabetes, and chronic respiratory disease among populations aged 30 to 70 (per 100,000 population)**			**Indicator 3.4.2: Age-standardised death rate due to self-harm (per 100,000 population)**			**Indicator 3.5.2: Risk-weighted prevalence of alcohol consumption, as measured by the summary exposure value (SEV) for alcohol use**			**Indicator 3.6.1: Age-standardised death rate due to road injuries (per 100,000 population)**		
	**1990**	**2000**	**2015**	**1990**	**2000**	**2015**	**1990**	**2000**	**2015**	**1990**	**2000**	**2015**
Syria	41.81 (33.73-49.35)	49.74 (41.94-56.69)	58.44 (50.82-66.94)	88.48 (79.99-96.87)	90.70 (84.17-97.17)	92.32 (86.22-98.12)	94.87 (94.28-95.61)	95.29 (94.65-96.11)	95.22 (94.64-95.96)	47.27 (40.15-55.50)	52.26 (45.26-61.66)	59.36 (53.71-66.93)
Tunisia	58.71 (52.96-64.01)	62.25 (55.34-68.37)	68.92 (58.04-79.68)	71.38 (64.72-77.27)	71.50 (65.89-76.96)	74.91 (67.68-81.86)	93.88 (93.36-94.45)	94.10 (93.64-94.62)	93.54 (92.76-94.55)	37.81 (31.20-44.78)	43.01 (36.69-49.30)	51.20 (42.84-59.40)
Turkey	42.82 (37.05-48.62)	54.71 (49.32-59.58)	72.96 (67.45-77.13)	56.81 (49.86-65.12)	61.71 (57.18-66.87)	80.79 (69.70-85.49)	88.91 (87.99-89.90)	85.04 (84.04-86.08)	89.52 (87.94-91.37)	47.87 (40.82-55.65)	59.36 (54.17-64.51)	66.20 (61.07-70.96)
United Arab Emirates	43.14 (33.78-52.72)	49.19 (42.95-55.88)	53.45 (41.63-65.16)	70.97 (60.85-81.66)	69.64 (62.90-77.65)	81.14 (71.92-90.83)	80.90 (79.22-82.78)	86.51 (84.95-88.38)	85.21 (83.08-87.98)	15.51 (4.26-27.81)	21.24 (13.25-30.81)	27.08 (16.08-37.71)
Yemen	29.12 (11.42-50.17)	31.43 (12.20-52.92)	36.20 (14.88-57.15)	71.21 (51.40-91.40)	67.17 (48.08-85.59)	66.54 (47.62-84.67)	93.54 (92.86-94.30)	95.75 (95.23-96.29)	96.85 (96.37-97.32)	25.01 (7.99-44.20)	25.25 (6.97-43.92)	28.67 (10.42-45.44)
Bangladesh	44.31 (38.29-50.36)	46.43 (40.10-52.25)	47.28 (39.92-54.68)	51.67 (45.71-58.41)	54.44 (49.10-60.91)	56.95 (50.70-64.75)	99.11 (98.79-99.40)	99.00 (98.64-99.32)	98.95 (98.62-99.28)	68.76 (61.54-73.82)	63.13 (58.61-67.79)	56.25 (49.66-65.71)
Bhutan	49.08 (38.76-58.98)	55.57 (46.44-64.04)	64.70 (53.56-76.14)	61.03 (50.32-71.76)	62.72 (54.90-71.01)	65.36 (56.11-73.78)	90.91 (89.85-92.13)	90.07 (88.95-91.44)	92.61 (91.21-94.27)	53.49 (43.87-63.41)	57.76 (50.11-65.44)	66.86 (58.34-74.85)
India	36.58 (31.91-42.21)	38.59 (33.96-43.94)	45.72 (40.81-50.39)	47.62 (42.81-58.36)	38.18 (32.28-57.61)	39.96 (33.29-59.02)	87.18 (86.25-88.18)	85.37 (84.41-86.45)	83.35 (81.90-85.09)	39.84 (34.22-45.99)	37.81 (32.43-43.90)	39.71 (33.47-45.94)
Nepal	47.60 (38.09-56.42)	53.55 (46.24-60.26)	56.56 (46.44-66.68)	65.16 (53.85-72.61)	62.87 (55.16-69.47)	62.91 (52.74-71.67)	96.08 (95.64-96.50)	90.74 (90.00-91.50)	89.20 (87.75-90.94)	39.05 (30.11-48.73)	39.52 (29.45-48.45)	40.34 (26.34-50.44)
Pakistan	40.38 (33.12-47.62)	32.83 (26.01-40.19)	36.13 (28.72-43.73)	78.16 (60.20-85.25)	76.54 (57.62-84.29)	85.40 (62.50-93.00)	99.44 (99.17-99.69)	99.37 (99.10-99.63)	99.49 (99.24-99.71)	57.04 (48.30-64.28)	50.79 (41.73-59.48)	52.36 (42.48-62.86)
Botswana	46.55 (8.04-82.18)	40.94 (0.00-79.78)	45.33 (0.70-76.57)	41.44 (8.53-68.10)	33.10 (0.00-64.62)	34.25 (0.00-61.58)	74.75 (72.67-76.90)	72.64 (70.55-74.91)	69.47 (66.82-72.48)	46.43 (15.34-69.64)	40.55 (0.00-67.45)	41.94 (2.48-65.55)

Appendix Table 1a. Scaled values for each SDG health-related indicator and three summary indicators by country for 1990, 2000, and 2015

Location	Indicator 3.4.1: Death rate due to cardiovascular disease, cancer, diabetes, and chronic respiratory disease among populations aged 30 to 70 (per 100,000 population)			Indicator 3.4.2: Age-standardised death rate due to self-harm (per 100,000 population)			Indicator 3.5.2: Risk-weighted prevalence of alcohol consumption, as measured by the summary exposure value (SEV) for alcohol use			Indicator 3.6.1: Age-standardised death rate due to road injuries (per 100,000 population)		
	1990	2000	2015	1990	2000	2015	1990	2000	2015	1990	2000	2015
Lesotho	43.65 (30.97-57.78)	37.91 (23.77-50.65)	23.27 (2.62-48.07)	44.13 (29.69-55.69)	38.29 (24.16-51.11)	22.79 (0.00-45.39)	77.29 (75.60-78.93)	76.03 (74.26-77.82)	73.73 (71.24-76.34)	35.36 (22.29-46.57)	31.19 (17.29-45.20)	18.32 (0.00-40.45)
Namibia	42.88 (35.01-51.28)	29.83 (18.94-41.67)	59.78 (36.51-82.12)	38.62 (28.97-48.07)	28.92 (16.71-41.68)	45.42 (25.44-63.71)	94.36 (93.57-95.08)	78.14 (75.68-80.51)	65.68 (62.15-69.78)	28.64 (20.28-37.46)	19.60 (7.67-32.13)	36.17 (17.46-52.84)
South Africa	40.06 (34.67-45.94)	34.34 (27.87-41.51)	43.01 (36.89-49.22)	37.72 (31.96-47.16)	33.41 (27.82-42.64)	43.26 (37.64-51.17)	65.78 (63.64-67.85)	66.89 (64.84-68.83)	65.76 (63.67-67.79)	17.98 (8.90-27.35)	16.78 (9.05-25.60)	28.55 (21.62-36.24)
Swaziland	35.87 (22.56-50.73)	31.20 (14.31-47.90)	27.74 (3.69-59.64)	39.02 (25.47-52.45)	30.22 (14.00-46.43)	24.50 (0.00-54.15)	78.23 (76.75-79.65)	78.25 (76.76-79.66)	78.92 (77.23-80.91)	27.52 (15.06-39.81)	20.61 (6.16-36.87)	15.19 (0.00-39.17)
Zimbabwe	49.12 (38.81-62.31)	41.57 (21.73-57.12)	50.93 (28.78-74.63)	33.42 (19.40-46.44)	24.01 (5.23-40.73)	22.92 (6.56-41.59)	76.71 (75.05-78.42)	78.17 (76.44-79.90)	78.55 (76.40-80.90)	31.04 (14.97-44.77)	23.16 (1.16-42.87)	30.57 (14.29-47.11)
Benin	52.17 (43.72-60.35)	48.27 (38.41-58.53)	46.11 (21.90-73.16)	57.49 (48.44-66.31)	49.02 (39.16-58.07)	47.12 (27.08-65.21)	87.94 (86.93-89.08)	87.47 (86.40-88.69)	87.62 (86.45-88.99)	36.47 (23.34-52.18)	37.49 (26.59-49.60)	50.08 (32.33-67.31)
Burkina Faso	50.09 (41.07-58.77)	50.65 (40.30-60.75)	51.52 (29.86-74.22)	49.27 (41.61-57.01)	48.69 (40.72-57.66)	50.17 (33.79-65.65)	67.65 (65.73-69.42)	66.29 (64.17-68.21)	66.51 (64.27-68.99)	40.42 (32.83-48.21)	43.07 (36.05-50.53)	45.62 (30.82-59.51)
Cameroon	46.88 (38.64-55.03)	42.23 (30.62-55.67)	47.35 (24.44-71.12)	53.54 (45.83-61.16)	44.37 (34.71-55.45)	45.48 (26.56-62.87)	75.02 (73.52-76.50)	76.27 (74.77-77.84)	74.38 (72.67-76.11)	43.06 (34.64-51.92)	42.65 (31.81-53.76)	48.19 (31.63-63.77)
Cape Verde	49.08 (44.23-54.07)	50.63 (36.92-64.24)	59.85 (49.47-68.47)	53.74 (42.57-61.30)	30.61 (15.48-47.08)	38.61 (28.49-48.93)	78.24 (76.84-79.86)	76.44 (74.88-77.99)	75.78 (74.23-77.44)	70.85 (65.29-76.41)	58.44 (47.84-69.34)	66.97 (59.52-73.24)
Chad	56.66 (47.26-65.79)	48.03 (37.02-59.44)	50.04 (24.35-79.86)	68.08 (59.33-76.52)	56.10 (45.94-66.10)	55.05 (36.06-73.72)	84.21 (83.18-85.21)	82.02 (80.70-83.15)	80.52 (78.87-82.28)	56.28 (46.65-65.96)	47.78 (38.08-57.83)	47.13 (29.79-63.72)
Cote d'Ivoire	44.03 (34.09-53.79)	38.88 (26.74-51.91)	46.61 (25.94-69.47)	43.53 (34.68-52.53)	36.56 (26.29-47.21)	41.53 (23.55-58.63)	81.55 (78.97-84.67)	81.20 (78.59-84.49)	81.39 (78.81-84.64)	37.22 (27.26-46.35)	33.87 (23.73-44.06)	42.78 (26.03-58.60)

Location	Appendix Table 1a. Scaled values for each SDG health-related indicator and three summary indicators by country for 1990, 2000, and 2015											
	**Indicator 3.4.1: Death rate due to cardiovascular disease, cancer, diabetes, and chronic respiratory disease among populations aged 30 to 70 (per 100,000 population)**			**Indicator 3.4.2: Age-standardised death rate due to self-harm (per 100,000 population)**			**Indicator 3.5.2: Risk-weighted prevalence of alcohol consumption, as measured by the summary exposure value (SEV) for alcohol use**			**Indicator 3.6.1: Age-standardised death rate due to road injuries (per 100,000 population)**		
	**1990**	**2000**	**2015**	**1990**	**2000**	**2015**	**1990**	**2000**	**2015**	**1990**	**2000**	**2015**
The Gambia	49.67 (27.47-72.74)	50.34 (39.80-62.09)	55.14 (37.74-73.11)	64.40 (48.85-79.45)	61.36 (51.45-70.29)	62.86 (48.42-74.59)	86.57 (85.24-88.04)	84.41 (83.11-85.88)	83.65 (82.23-85.43)	50.53 (35.73-63.47)	50.45 (42.34-59.18)	53.16 (40.86-63.90)
Ghana	44.02 (27.97-59.81)	45.92 (33.95-59.02)	50.57 (27.48-78.24)	61.33 (47.70-74.37)	61.37 (50.98-71.37)	58.10 (38.74-74.43)	79.44 (77.96-80.79)	81.60 (80.18-82.99)	77.64 (75.51-80.04)	44.13 (32.98-55.09)	48.01 (39.13-57.30)	50.34 (33.17-66.57)
Guinea	49.06 (40.78-57.25)	46.69 (38.48-55.16)	45.00 (27.09-65.17)	65.90 (56.48-74.02)	57.34 (48.95-65.50)	54.92 (39.97-70.18)	94.02 (93.40-94.62)	93.80 (93.14-94.44)	94.50 (93.81-95.12)	52.08 (43.28-60.80)	49.34 (41.81-56.93)	53.04 (38.49-66.83)
Guinea-Bissau	46.80 (7.20-85.79)	47.78 (5.91-86.90)	46.46 (0.00-82.57)	60.87 (31.50-85.89)	55.28 (23.36-81.09)	53.34 (14.38-77.91)	81.09 (79.92-82.40)	81.74 (80.54-83.04)	81.78 (80.48-83.26)	47.77 (18.98-69.36)	46.87 (15.54-69.75)	48.03 (12.89-70.39)
Liberia	52.16 (38.10-66.22)	61.31 (50.44-73.57)	55.63 (36.46-77.03)	59.63 (48.20-71.29)	62.07 (50.80-73.39)	57.84 (42.84-70.86)	75.43 (73.94-77.00)	77.89 (76.53-79.42)	79.61 (78.28-81.23)	45.25 (35.70-55.12)	54.98 (47.08-63.06)	56.71 (44.08-68.26)
Mali	40.35 (32.29-48.35)	45.48 (37.16-53.58)	51.04 (30.82-70.87)	63.58 (54.85-70.72)	63.56 (54.90-71.69)	64.74 (50.40-77.14)	95.96 (95.53-96.41)	96.12 (95.68-96.53)	95.87 (95.37-96.36)	50.29 (42.25-58.26)	52.82 (45.25-60.37)	58.49 (45.40-69.54)
Mauritania	44.16 (35.59-52.63)	55.17 (45.22-66.17)	63.92 (42.98-89.01)	60.55 (52.17-68.68)	62.54 (53.09-72.05)	65.54 (48.60-81.48)	98.74 (98.55-98.96)	99.65 (99.56-99.74)	99.90 (99.81-99.99)	49.19 (40.07-57.58)	56.83 (47.84-66.84)	62.49 (46.90-76.90)
Niger	54.35 (45.79-63.35)	59.27 (50.11-67.44)	57.36 (37.61-77.13)	66.82 (56.35-76.12)	64.93 (55.83-73.92)	61.46 (46.22-75.96)	96.20 (95.71-96.66)	96.08 (95.58-96.53)	96.20 (95.71-96.65)	53.64 (43.34-63.05)	58.29 (49.05-67.58)	59.28 (44.29-73.68)
Nigeria	54.93 (37.25-72.72)	58.92 (48.06-69.90)	73.08 (49.40-88.75)	63.60 (50.69-76.68)	61.68 (52.91-70.40)	66.83 (51.59-77.63)	65.86 (64.01-67.65)	62.50 (60.31-64.51)	63.88 (61.87-65.97)	47.47 (30.75-60.93)	56.51 (48.65-64.21)	73.11 (60.33-81.19)
Sao Tome and Principe	53.61 (45.13-61.43)	47.78 (39.23-57.12)	48.02 (27.75-73.79)	90.72 (84.35-95.94)	88.31 (81.28-94.98)	85.49 (70.15-100.00)	79.55 (78.12-81.11)	74.51 (72.85-76.23)	75.60 (74.01-77.42)	52.23 (45.09-59.43)	50.84 (43.37-58.22)	54.76 (39.55-70.53)
Senegal	50.11 (41.00-59.02)	50.55 (40.08-60.08)	49.67 (24.78-76.36)	50.85 (43.17-59.34)	48.36 (39.91-56.69)	46.96 (28.25-64.01)	93.32 (92.58-94.12)	93.66 (92.88-94.44)	93.84 (93.10-94.62)	52.35 (41.86-61.14)	52.61 (42.96-61.29)	54.33 (36.26-70.70)

Location	Appendix Table 1a. Scaled values for each SDG health-related indicator and three summary indicators by country for 1990, 2000, and 2015											
	Indicator 3.4.1: Death rate due to cardiovascular disease, cancer, diabetes, and chronic respiratory disease among populations aged 30 to 70 (per 100,000 population)			Indicator 3.4.2: Age-standardised death rate due to self-harm (per 100,000 population)			Indicator 3.5.2: Risk-weighted prevalence of alcohol consumption, as measured by the summary exposure value (SEV) for alcohol use			Indicator 3.6.1: Age-standardised death rate due to road injuries (per 100,000 population)		
	1990	2000	2015	1990	2000	2015	1990	2000	2015	1990	2000	2015
Sierra Leone	54.82 (39.12-72.75)	48.81 (39.94-56.79)	44.57 (25.99-64.23)	64.82 (52.48-77.90)	56.81 (47.35-66.18)	54.07 (39.77-68.61)	76.07 (74.65-77.51)	72.73 (71.01-74.36)	73.24 (71.59-74.91)	47.67 (34.07-60.68)	45.56 (34.91-55.98)	46.55 (32.06-60.54)
Togo	47.13 (37.96-56.69)	45.10 (34.19-57.70)	47.98 (29.28-69.08)	54.26 (46.05-63.02)	45.96 (36.55-56.35)	46.59 (30.88-62.55)	82.99 (81.84-84.32)	86.57 (85.47-87.88)	87.02 (85.71-88.67)	47.69 (40.69-54.86)	44.91 (35.56-54.55)	46.24 (30.49-62.64)
Burundi	17.35 (2.96-34.62)	26.46 (16.55-37.11)	46.95 (26.31-69.46)	29.21 (12.18-47.80)	34.32 (21.79-46.69)	49.07 (32.41-65.01)	69.71 (67.69-71.62)	70.13 (68.09-72.03)	70.75 (68.70-72.86)	18.90 (3.08-35.99)	24.74 (13.35-35.74)	36.52 (18.63-52.83)
Comoros	35.11 (15.39-57.72)	44.01 (33.21-54.04)	53.89 (35.52-73.20)	49.88 (32.74-65.47)	52.24 (41.70-61.02)	56.38 (40.83-70.36)	97.72 (97.42-98.00)	97.86 (97.56-98.15)	97.73 (97.41-98.05)	36.79 (19.49-52.96)	44.39 (32.17-54.16)	50.62 (33.31-64.84)
Djibouti	45.43 (25.74-65.33)	44.95 (18.10-76.10)	46.30 (18.12-75.17)	57.31 (42.43-72.07)	54.26 (31.31-73.84)	52.74 (30.43-72.00)	81.61 (79.52-84.51)	82.56 (80.45-85.57)	82.33 (80.20-85.26)	41.82 (25.16-56.54)	42.21 (19.66-61.32)	44.46 (21.65-63.27)
Eritrea	30.64 (20.92-39.68)	44.89 (24.99-70.75)	40.07 (12.99-71.98)	42.17 (31.90-52.63)	48.50 (32.39-64.25)	44.80 (22.37-64.64)	92.91 (91.88-94.00)	89.19 (87.83-90.58)	92.10 (90.56-93.66)	39.81 (30.65-49.64)	45.72 (28.32-61.96)	42.87 (22.98-62.32)
Ethiopia	26.86 (18.75-35.81)	38.30 (29.71-46.91)	52.36 (30.89-75.48)	33.93 (24.39-46.20)	43.43 (35.65-52.71)	49.90 (32.60-64.46)	84.13 (82.94-85.33)	81.53 (80.28-82.72)	79.12 (76.95-81.86)	28.14 (18.29-39.19)	38.93 (31.31-46.81)	48.84 (31.81-62.46)
Kenya	58.79 (51.32-65.50)	59.00 (50.62-66.25)	61.84 (53.89-69.43)	51.99 (44.13-59.92)	51.79 (45.76-58.60)	52.15 (46.13-59.22)	77.51 (75.74-79.45)	80.59 (78.77-82.42)	81.30 (78.78-84.38)	49.71 (39.25-56.99)	50.40 (42.81-57.10)	52.38 (45.59-58.85)
Madagascar	32.71 (25.16-39.89)	35.93 (26.84-50.49)	38.56 (17.85-61.86)	47.89 (41.47-55.70)	51.24 (42.33-63.44)	51.71 (35.70-68.02)	86.12 (84.81-87.45)	89.72 (88.54-90.77)	90.67 (89.27-92.26)	45.26 (38.32-52.38)	50.18 (40.99-61.52)	53.30 (38.11-68.73)
Malawi	49.26 (37.76-60.71)	42.91 (26.61-59.60)	54.66 (32.95-77.55)	48.34 (38.27-58.98)	45.21 (32.41-57.30)	51.81 (35.06-65.51)	91.16 (90.45-91.89)	91.21 (90.45-92.03)	90.96 (89.95-92.20)	40.04 (29.72-49.62)	40.50 (28.49-52.03)	49.80 (33.63-63.11)
Mozambique	48.43 (39.55-56.98)	44.20 (33.10-56.42)	43.90 (22.34-71.19)	50.82 (40.91-60.05)	46.53 (37.47-56.03)	42.90 (24.08-61.49)	93.77 (93.06-94.43)	90.28 (89.12-91.52)	88.52 (86.83-90.49)	40.71 (32.96-48.38)	37.74 (28.89-46.78)	40.23 (22.48-58.07)

Location	Appendix Table 1a. Scaled values for each SDG health-related indicator and three summary indicators by country for 1990, 2000, and 2015											
	**Indicator 3.4.1: Death rate due to cardiovascular disease, cancer, diabetes, and chronic respiratory disease among populations aged 30 to 70 (per 100,000 population)**			**Indicator 3.4.2: Age-standardised death rate due to self-harm (per 100,000 population)**			**Indicator 3.5.2: Risk-weighted prevalence of alcohol consumption, as measured by the summary exposure value (SEV) for alcohol use**			**Indicator 3.6.1: Age-standardised death rate due to road injuries (per 100,000 population)**		
	**1990**	**2000**	**2015**	**1990**	**2000**	**2015**	**1990**	**2000**	**2015**	**1990**	**2000**	**2015**
Rwanda	29.17 (19.27-38.93)	32.16 (21.87-42.08)	55.77 (32.46-77.36)	40.79 (29.09-51.91)	39.62 (28.28-50.49)	54.59 (37.25-68.24)	69.65 (67.63-71.52)	70.59 (68.57-72.48)	70.06 (67.96-72.13)	22.39 (9.98-34.31)	19.74 (7.68-32.03)	35.66 (15.90-52.96)
Somalia	37.56 (2.29-85.70)	40.50 (4.80-89.76)	41.51 (3.69-89.42)	54.07 (25.16-83.32)	54.14 (25.37-84.79)	53.37 (24.37-81.95)	95.60 (95.10-96.06)	95.66 (95.08-96.19)	95.56 (94.84-96.29)	40.14 (12.29-68.06)	41.43 (12.69-69.63)	42.18 (12.81-69.40)
South Sudan	45.61 (6.52-88.35)	51.00 (8.59-93.44)	50.99 (5.35-91.25)	57.36 (27.80-82.59)	58.33 (28.36-82.87)	55.48 (21.69-79.16)	82.55 (80.19-85.93)	82.51 (80.13-85.99)	82.31 (79.90-85.46)	40.81 (12.18-65.06)	45.05 (14.17-69.32)	44.10 (9.08-67.43)
Tanzania	47.04 (38.08-56.77)	50.30 (37.54-63.84)	55.30 (29.23-81.45)	50.87 (43.91-58.89)	54.14 (44.90-63.63)	53.90 (35.74-68.90)	71.95 (70.06-73.67)	73.46 (71.65-75.30)	71.71 (69.65-73.93)	43.76 (36.46-51.37)	49.40 (40.28-58.61)	50.93 (33.67-65.10)
Uganda	41.64 (30.21-58.52)	36.76 (26.42-51.89)	46.18 (24.59-75.40)	45.25 (34.84-58.81)	40.59 (30.50-52.71)	43.42 (25.76-61.87)	60.28 (57.74-62.74)	57.37 (54.32-60.20)	57.59 (53.98-61.54)	39.42 (28.36-52.21)	30.25 (18.68-42.40)	34.54 (15.00-53.97)
Zambia	46.96 (36.65-58.29)	32.89 (23.63-42.89)	35.38 (18.44-54.61)	47.24 (34.82-58.78)	36.65 (28.01-47.82)	32.70 (17.49-49.12)	78.10 (76.61-79.52)	79.46 (77.98-80.80)	79.09 (77.15-81.42)	39.81 (26.23-50.75)	32.85 (23.62-42.29)	32.76 (17.76-48.74)
Angola	33.88 (1.77-82.97)	39.36 (3.57-85.24)	42.28 (0.45-78.35)	40.24 (9.07-74.81)	45.69 (11.67-75.25)	45.49 (5.12-69.82)	77.85 (71.33-86.76)	79.21 (72.59-88.18)	75.46 (68.67-85.22)	28.18 (1.13-57.17)	35.19 (6.03-61.52)	37.07 (2.53-59.35)
Central African Republic	27.61 (17.78-37.85)	26.59 (8.01-52.62)	26.32 (5.00-56.29)	37.88 (24.31-50.13)	36.26 (14.01-59.82)	34.84 (10.89-61.44)	79.36 (77.36-81.62)	83.74 (81.83-85.84)	85.39 (81.84-89.32)	22.04 (9.20-36.81)	22.25 (3.05-46.83)	23.19 (0.73-47.63)
Congo	25.17 (13.86-37.40)	28.51 (18.60-38.91)	38.53 (18.25-60.92)	38.36 (25.81-52.02)	41.46 (29.88-53.61)	48.93 (30.32-65.29)	76.65 (74.68-78.56)	81.21 (79.52-82.82)	78.96 (75.43-83.45)	30.01 (18.52-41.79)	33.97 (24.04-44.99)	43.56 (26.94-57.64)
Democratic Republic of the Congo	48.70 (32.29-66.43)	49.40 (39.28-60.71)	47.37 (30.38-67.68)	55.51 (40.29-69.67)	55.59 (42.71-65.69)	53.30 (37.19-68.33)	83.64 (78.51-89.98)	87.01 (82.35-92.25)	87.16 (82.27-92.58)	46.81 (33.94-59.29)	48.25 (36.81-57.56)	49.52 (35.57-62.84)
Equatorial Guinea	27.63 (0.00-79.14)	41.04 (1.71-79.84)	51.29 (3.80-76.39)	34.78 (2.20-70.41)	43.79 (8.72-71.72)	47.27 (6.76-68.54)	79.05 (75.25-83.42)	74.02 (70.52-78.28)	63.71 (59.28-70.35)	26.19 (0.00-55.65)	32.93 (1.98-59.97)	40.43 (5.85-62.51)

	Appendix Table 1a. Scaled values for each SDG health-related indicator and three summary indicators by country for 1990, 2000, and 2015											
	**Indicator 3.4.1: Death rate due to cardiovascular disease, cancer, diabetes, and chronic respiratory disease among populations aged 30 to 70 (per 100,000 population)**			**Indicator 3.4.2: Age-standardised death rate due to self-harm (per 100,000 population)**			**Indicator 3.5.2: Risk-weighted prevalence of alcohol consumption, as measured by the summary exposure value (SEV) for alcohol use**			**Indicator 3.6.1: Age-standardised death rate due to road injuries (per 100,000 population)**		
**Location**	**1990**	**2000**	**2015**	**1990**	**2000**	**2015**	**1990**	**2000**	**2015**	**1990**	**2000**	**2015**
Gabon	35.77 (26.63-45.22)	37.70 (28.07-46.91)	48.59 (26.53-67.95)	39.96 (29.86-51.29)	44.44 (32.89-55.94)	47.61 (26.65-63.33)	69.07 (62.59-79.45)	70.38 (64.11-80.20)	69.83 (63.26-80.23)	27.45 (15.68-39.84)	35.06 (22.74-46.53)	42.41 (24.45-58.53)

Location	Appendix Table 1a. Scaled values for each SDG health-related indicator and three summary indicators by country for 1990, 2000, and 2015											
	Indicator 3.7.1: Proportion of women of reproductive age (15 to 49 years) who have their need for family planning satisfied with modern methods			Indicator 3.7.2: Birth rates for women aged 10 to 14 years and women aged 15 to 19 years (number of live births per 1,000 women)			Indicator 3.8.1: Coverage of 7 UHC tracer interventions for prevention and treatment services			Indicator 3.9.1: Age-standardised death rate attributable to household air pollution and ambient air pollution (per 100,000 population)		
	1990	2000	2015	1990	2000	2015	1990	2000	2015	1990	2000	2015
Canada	91.53 (88.73-93.93)	91.85 (89.15-94.27)	95.60 (93.78-96.97)	64.71 (64.71-64.71)	71.99 (71.99-71.99)	80.89 (80.89-80.89)	61.52 (60.12-62.59)	88.32 (86.54-90.15)	96.40 (95.25-97.68)	82.19 (78.60-84.79)	86.63 (82.93-89.10)	93.58 (89.50-96.17)
United States	99.23 (98.63-99.73)	99.75 (99.36-100.00)	99.94 (99.69-100.00)	46.51 (46.51-46.51)	51.34 (51.34-51.34)	67.79 (67.79-67.79)	63.51 (62.84-64.29)	91.20 (89.53-92.98)	98.24 (97.12-99.45)	74.17 (70.34-77.18)	77.35 (73.41-80.20)	85.73 (81.69-88.59)
Australia	90.44 (87.03-92.98)	94.79 (92.88-96.40)	97.46 (96.27-98.38)	66.82 (66.82-66.82)	71.12 (71.12-71.12)	75.51 (75.51-75.51)	60.24 (59.25-61.28)	85.25 (83.31-87.38)	96.28 (95.48-97.13)	84.04 (80.76-86.29)	90.25 (86.86-91.97)	99.15 (95.77-100.00)
New Zealand	83.51 (78.79-87.71)	91.08 (87.91-93.72)	95.17 (93.32-96.67)	58.27 (58.27-58.27)	62.89 (62.89-62.89)	65.62 (65.62-65.62)	57.98 (56.99-59.01)	85.45 (83.90-87.24)	95.40 (94.51-96.36)	84.05 (80.78-86.76)	89.85 (86.68-91.86)	99.17 (95.72-100.00)
Brunei	77.39 (71.09-83.01)	80.92 (75.77-85.62)	89.05 (85.45-92.07)	50.13 (50.13-50.13)	62.65 (62.65-62.65)	67.04 (67.04-67.04)	62.02 (61.15-62.91)	84.91 (79.99-87.20)	95.77 (94.53-97.01)	86.00 (80.71-91.33)	90.42 (84.62-96.27)	95.94 (89.73-100.00)
Japan	59.76 (51.71-67.77)	66.83 (59.04-73.99)	79.45 (73.71-84.28)	86.93 (86.93-86.93)	86.10 (86.10-86.10)	89.55 (89.55-89.55)	57.77 (56.40-59.03)	86.47 (84.83-88.19)	95.64 (94.52-96.74)	80.70 (76.36-84.60)	85.78 (81.24-90.10)	88.80 (84.05-93.47)
Singapore	62.16 (53.92-69.39)	72.27 (65.71-78.03)	86.58 (82.48-90.08)	78.86 (78.86-78.86)	81.42 (81.42-81.42)	92.48 (92.48-92.48)	58.91 (57.72-60.01)	88.20 (86.23-90.05)	97.05 (95.91-98.14)	62.97 (57.42-68.56)	69.29 (63.11-75.22)	77.27 (70.40-83.78)
South Korea	76.44 (69.91-82.10)	85.87 (81.66-89.48)	93.54 (91.22-95.52)	79.67 (79.67-79.67)	92.88 (92.88-92.88)	100.00 (100.00-100.00)	58.19 (56.74-59.52)	75.72 (64.23-81.33)	95.21 (93.75-96.39)	61.55 (57.87-65.22)	72.32 (68.20-76.04)	80.04 (75.76-84.22)
Andorra	85.57 (80.85-89.26)	90.19 (87.01-92.86)	95.07 (93.11-96.59)	69.03 (69.03-69.03)	72.88 (72.88-72.88)	72.78 (72.78-72.78)	61.26 (60.21-62.32)	85.18 (81.87-88.44)	96.26 (94.28-98.76)	86.29 (81.50-90.87)	89.94 (85.19-94.64)	93.02 (87.96-97.72)
Austria	65.77 (58.29-72.56)	80.32 (74.47-85.27)	91.58 (88.67-93.98)	65.24 (65.24-65.24)	72.75 (72.75-72.75)	83.85 (83.85-83.85)	56.18 (54.81-57.51)	83.36 (81.43-85.06)	93.38 (91.65-94.76)	70.98 (67.34-74.08)	75.64 (71.98-78.51)	83.22 (79.16-86.27)
Belgium	84.84 (80.35-88.97)	88.96 (85.20-92.10)	94.65 (92.57-96.27)	74.16 (74.16-74.16)	77.08 (77.08-77.08)	81.34 (81.34-81.34)	60.16 (58.96-61.21)	87.11 (85.48-88.73)	97.42 (96.47-98.40)	69.51 (65.76-72.62)	74.09 (70.30-77.22)	81.74 (77.61-85.34)

Appendix Table 1a. Scaled values for each SDG health-related indicator and three summary indicators by country for 1990, 2000, and 2015

Location	Indicator 3.7.1: Proportion of women of reproductive age (15 to 49 years) who have their need for family planning satisfied with modern methods			Indicator 3.7.2: Birth rates for women aged 10 to 14 years and women aged 15 to 19 years (number of live births per 1,000 women)			Indicator 3.8.1: Coverage of 7 UHC tracer interventions for prevention and treatment services			Indicator 3.9.1: Age-standardised death rate attributable to household air pollution and ambient air pollution (per 100,000 population)		
	1990	2000	2015	1990	2000	2015	1990	2000	2015	1990	2000	2015
Cyprus	75.84 (69.25-81.87)	83.99 (79.39-88.20)	92.51 (89.81-94.79)	58.15 (58.15-58.15)	74.34 (74.34-74.34)	91.63 (91.63-91.63)	58.33 (56.96-59.56)	82.63 (80.57-84.84)	95.64 (94.09-97.91)	68.87 (64.98-72.04)	74.15 (70.43-77.31)	84.25 (80.18-87.95)
Denmark	73.37 (66.74-79.63)	83.14 (78.23-87.52)	90.18 (87.09-93.02)	76.92 (76.92-76.92)	82.27 (82.27-82.27)	89.44 (89.44-89.44)	60.04 (58.93-61.11)	81.13 (79.10-83.63)	95.11 (93.96-96.20)	70.84 (67.18-73.78)	77.40 (73.53-80.38)	84.19 (80.06-87.40)
Finland	90.09 (86.68-92.86)	94.08 (91.82-95.86)	96.97 (95.57-98.14)	74.11 (74.11-74.11)	77.10 (77.10-77.10)	82.83 (82.83-82.83)	62.46 (61.55-63.34)	88.06 (86.57-89.64)	97.97 (97.12-98.83)	76.59 (73.03-79.69)	84.69 (81.20-87.28)	92.03 (88.17-94.49)
France	81.79 (76.90-85.91)	87.80 (84.49-90.87)	93.81 (91.42-95.69)	72.72 (72.72-72.72)	77.14 (77.14-77.14)	79.86 (79.86-79.86)	57.99 (56.60-59.19)	86.38 (84.61-88.25)	95.73 (94.56-96.81)	80.95 (77.05-83.92)	84.55 (80.51-87.63)	90.73 (86.17-94.47)
Germany	74.69 (68.18-80.33)	84.57 (80.04-88.57)	92.16 (89.45-94.40)	70.07 (70.07-70.07)	75.10 (75.10-75.10)	85.69 (85.69-85.69)	56.87 (55.43-58.18)	86.74 (84.84-88.51)	95.78 (94.74-96.76)	68.85 (65.17-71.70)	76.14 (72.46-78.91)	83.27 (79.09-86.16)
Greece	46.30 (37.22-54.96)	53.60 (45.26-61.89)	73.54 (67.09-79.45)	65.04 (65.04-65.04)	77.52 (77.52-77.52)	85.57 (85.57-85.57)	55.27 (53.47-56.76)	82.75 (80.43-85.00)	93.78 (92.37-95.03)	73.17 (69.53-76.30)	76.14 (72.52-79.23)	79.55 (75.64-82.58)
Iceland	84.73 (80.08-88.79)	89.54 (85.98-92.38)	94.69 (92.78-96.30)	60.26 (60.26-60.26)	67.24 (67.24-67.24)	84.34 (84.34-84.34)	62.48 (61.38-63.41)	78.92 (75.77-81.89)	89.17 (87.51-90.89)	80.66 (76.44-83.99)	86.66 (82.49-89.63)	93.84 (89.69-96.85)
Ireland	78.98 (73.44-83.75)	86.80 (82.68-90.24)	94.75 (92.81-96.49)	72.82 (72.82-72.82)	70.33 (70.33-70.33)	81.01 (81.01-81.01)	54.44 (52.81-55.93)	80.47 (78.51-82.33)	96.32 (95.29-97.40)	73.14 (69.31-76.49)	78.26 (74.23-81.38)	86.34 (82.52-89.62)
Israel	87.00 (82.97-90.49)	90.34 (87.12-93.18)	95.11 (93.28-96.68)	61.53 (61.53-61.53)	66.11 (66.11-66.11)	72.94 (72.94-72.94)	61.34 (60.37-62.30)	80.13 (77.76-82.47)	96.57 (95.66-97.49)	70.57 (66.87-73.70)	78.24 (74.39-81.46)	83.56 (79.14-87.18)
Italy	67.79 (60.09-74.97)	75.29 (69.07-80.87)	86.41 (82.08-89.87)	79.05 (79.05-79.05)	84.73 (84.73-84.73)	87.44 (87.44-87.44)	56.52 (54.76-58.32)	87.03 (85.38-88.69)	96.90 (95.84-97.92)	74.28 (70.46-77.59)	80.08 (76.13-83.41)	83.38 (78.91-86.94)
Luxembourg	81.06 (75.98-85.58)	87.83 (83.79-91.12)	94.23 (92.27-95.99)	73.84 (73.84-73.84)	76.04 (76.04-76.04)	85.37 (85.37-85.37)	59.58 (58.57-60.68)	80.28 (77.34-83.22)	91.25 (89.78-92.79)	68.46 (64.94-71.57)	76.03 (71.96-79.27)	85.56 (81.01-89.30)

	Appendix Table 1a. Scaled values for each SDG health-related indicator and three summary indicators by country for 1990, 2000, and 2015											
	**Indicator 3.7.1: Proportion of women of reproductive age (15 to 49 years) who have their need for family planning satisfied with modern methods**			**Indicator 3.7.2: Birth rates for women aged 10 to 14 years and women aged 15 to 19 years (number of live births per 1,000 women)**			**Indicator 3.8.1: Coverage of 7 UHC tracer interventions for prevention and treatment services**			**Indicator 3.9.1: Age-standardised death rate attributable to household air pollution and ambient air pollution (per 100,000 population)**		
**Location**	**1990**	**2000**	**2015**	**1990**	**2000**	**2015**	**1990**	**2000**	**2015**	**1990**	**2000**	**2015**
Malta	57.45 (49.72-65.52)	65.83 (57.59-72.88)	82.26 (76.83-86.56)	72.96 (72.96-72.96)	68.48 (68.48-68.48)	74.93 (74.93-74.93)	53.77 (51.68-55.66)	67.20 (63.69-70.61)	86.88 (84.87-88.67)	72.28 (68.23-76.01)	77.51 (73.03-81.32)	82.70 (78.39-86.34)
Netherlands	82.16 (76.64-86.88)	84.03 (79.31-88.27)	91.91 (88.99-94.22)	82.64 (82.64-82.64)	84.56 (84.56-84.56)	89.92 (89.92-89.92)	61.89 (60.86-62.87)	88.51 (86.83-90.11)	97.39 (96.43-98.44)	71.01 (67.27-74.12)	74.80 (70.90-78.08)	83.13 (78.70-86.79)
Norway	94.12 (92.02-95.78)	93.20 (90.73-95.18)	95.45 (93.73-96.89)	68.21 (68.21-68.21)	75.81 (75.81-75.81)	83.53 (83.53-83.53)	61.69 (60.75-62.57)	80.83 (78.75-83.25)	96.83 (95.98-97.76)	75.66 (72.07-78.81)	81.76 (77.97-84.45)	89.59 (85.77-92.42)
Portugal	56.44 (48.18-64.12)	71.87 (65.06-78.34)	87.91 (84.23-91.09)	63.82 (63.82-63.82)	68.93 (68.93-68.93)	83.47 (83.47-83.47)	55.25 (52.79-57.07)	84.38 (82.07-86.28)	95.76 (94.56-96.85)	75.36 (71.48-78.90)	79.44 (75.46-82.99)	88.36 (84.14-92.04)
Spain	64.33 (56.72-71.63)	79.07 (73.64-83.70)	90.57 (87.28-93.20)	77.21 (77.21-77.21)	82.45 (82.45-82.45)	85.57 (85.57-85.57)	57.60 (55.90-59.02)	86.91 (85.01-88.75)	96.68 (95.71-97.64)	79.13 (75.34-81.98)	82.47 (78.44-85.48)	90.74 (86.49-94.08)
Sweden	82.46 (77.14-86.84)	84.87 (80.09-88.80)	91.21 (88.19-93.73)	71.93 (71.93-71.93)	82.96 (82.96-82.96)	83.74 (83.74-83.74)	62.41 (61.42-63.31)	85.83 (83.79-88.14)	98.79 (98.00-99.79)	85.17 (81.81-87.81)	91.78 (88.58-93.85)	95.55 (92.09-97.49)
Switzerland	85.23 (80.66-88.95)	91.28 (88.18-93.84)	95.49 (93.61-97.03)	81.29 (81.29-81.29)	84.55 (84.55-84.55)	92.22 (92.22-92.22)	60.55 (59.64-61.45)	87.74 (86.26-89.22)	97.14 (96.15-98.26)	78.21 (74.38-81.15)	83.55 (79.77-86.37)	89.75 (85.28-93.01)
United Kingdom	88.05 (84.28-91.17)	91.68 (89.00-94.00)	95.35 (93.44-96.87)	59.62 (59.62-59.62)	62.91 (62.91-62.91)	73.08 (73.08-73.08)	59.99 (58.57-61.19)	87.84 (86.54-89.41)	100.00 (100.00-100.00)	68.45 (64.68-71.69)	74.65 (70.83-78.06)	83.29 (79.21-86.67)
Argentina	60.14 (51.29-67.44)	70.69 (63.82-76.94)	85.69 (81.46-89.44)	39.32 (39.32-39.32)	42.77 (42.77-42.77)	44.64 (44.64-44.64)	56.85 (54.91-58.24)	84.39 (82.23-86.59)	96.91 (94.33-98.71)	66.22 (62.39-69.49)	69.64 (65.74-73.12)	75.45 (70.70-79.36)
Chile	57.85 (49.68-65.29)	72.17 (64.79-78.75)	86.82 (82.75-90.42)	42.82 (42.82-42.82)	47.38 (47.38-47.38)	52.21 (52.21-52.21)	58.17 (55.93-59.80)	86.44 (83.37-88.60)	93.75 (91.65-95.36)	59.81 (54.53-64.91)	71.16 (66.36-75.59)	78.89 (74.61-82.77)
Uruguay	53.89 (45.02-62.02)	64.23 (56.08-71.75)	80.19 (74.85-84.99)	41.95 (41.95-41.95)	44.15 (44.15-44.15)	48.12 (48.12-48.12)	55.91 (54.12-57.45)	85.64 (83.91-87.24)	94.45 (93.18-95.60)	69.48 (64.14-73.24)	74.14 (70.42-77.34)	80.64 (76.69-84.04)

Location	Appendix Table 1a. Scaled values for each SDG health-related indicator and three summary indicators by country for 1990, 2000, and 2015											
	Indicator 3.7.1: Proportion of women of reproductive age (15 to 49 years) who have their need for family planning satisfied with modern methods			Indicator 3.7.2: Birth rates for women aged 10 to 14 years and women aged 15 to 19 years (number of live births per 1,000 women)			Indicator 3.8.1: Coverage of 7 UHC tracer interventions for prevention and treatment services			Indicator 3.9.1: Age-standardised death rate attributable to household air pollution and ambient air pollution (per 100,000 population)		
	1990	2000	2015	1990	2000	2015	1990	2000	2015	1990	2000	2015
Belarus	39.64 (36.30-43.13)	54.46 (51.31-57.50)	80.85 (78.96-82.70)	47.72 (47.72-47.72)	60.39 (60.39-60.39)	69.50 (69.50-69.50)	56.04 (55.05-57.14)	59.84 (59.15-60.71)	93.72 (92.49-94.92)	44.15 (37.75-49.84)	47.78 (42.67-52.38)	57.09 (52.89-60.88)
Estonia	54.33 (51.27-57.52)	60.05 (57.12-63.16)	79.44 (77.47-81.49)	47.29 (47.29-47.29)	62.88 (62.88-62.88)	78.83 (78.83-78.83)	55.69 (54.57-56.83)	84.75 (83.95-85.61)	92.96 (91.85-94.03)	49.96 (42.25-57.03)	57.99 (51.27-63.53)	77.24 (71.58-81.75)
Latvia	40.62 (37.30-43.94)	51.56 (48.28-54.83)	75.74 (73.47-77.90)	48.24 (48.24-48.24)	68.31 (68.31-68.31)	79.13 (79.13-79.13)	53.75 (52.84-54.72)	61.13 (57.46-65.17)	89.79 (88.62-91.10)	44.95 (38.46-50.75)	53.66 (48.83-57.96)	65.08 (60.17-68.93)
Lithuania	33.09 (29.59-36.32)	46.67 (43.27-50.00)	70.14 (67.55-72.68)	50.60 (50.60-50.60)	62.84 (62.84-62.84)	82.51 (82.51-82.51)	54.40 (53.42-55.40)	58.25 (57.41-59.14)	89.64 (88.38-90.94)	48.01 (41.89-53.37)	56.74 (50.57-61.29)	66.49 (62.20-70.10)
Moldova	55.75 (52.40-58.80)	54.23 (51.40-56.88)	71.96 (69.56-74.27)	40.56 (40.56-40.56)	52.98 (52.98-52.98)	66.11 (66.11-66.11)	57.64 (56.62-58.61)	56.17 (55.45-57.06)	81.80 (80.01-83.75)	34.20 (28.41-40.90)	39.30 (34.67-44.11)	59.61 (55.39-63.30)
Russia	47.58 (44.31-50.70)	53.86 (50.89-56.91)	75.43 (73.15-77.47)	44.66 (44.66-44.66)	59.36 (59.36-59.36)	66.64 (66.64-66.64)	49.11 (46.41-51.24)	56.27 (55.24-57.33)	84.79 (82.43-87.38)	49.50 (42.88-54.50)	48.45 (43.58-52.98)	61.97 (57.93-65.49)
Ukraine	45.13 (41.90-48.31)	54.87 (51.91-57.99)	75.97 (73.85-77.94)	42.70 (42.70-42.70)	57.21 (57.21-57.21)	66.14 (66.14-66.14)	50.46 (46.70-53.47)	55.24 (53.77-56.59)	81.98 (78.41-84.93)	43.58 (37.21-49.23)	46.65 (42.25-50.95)	57.43 (53.44-60.92)
Albania	3.95 (0.00-10.42)	5.25 (0.34-11.37)	9.80 (3.40-17.39)	60.65 (60.65-60.65)	62.80 (62.80-62.80)	62.91 (62.91-62.91)	39.76 (36.77-42.90)	59.33 (53.60-63.23)	76.98 (73.72-80.80)	42.40 (38.76-46.19)	48.33 (44.94-52.20)	63.93 (59.37-68.31)
Bosnia and Herzegovina	12.58 (4.79-21.61)	15.69 (7.82-24.98)	24.06 (17.31-31.28)	57.51 (57.51-57.51)	63.95 (63.95-63.95)	82.87 (82.87-82.87)	43.39 (40.48-46.13)	65.52 (54.16-69.20)	80.95 (78.32-83.97)	39.41 (35.83-43.09)	47.11 (43.93-50.79)	57.61 (53.80-61.40)
Bulgaria	30.75 (20.61-42.29)	36.61 (24.77-48.87)	59.22 (46.62-70.13)	38.48 (38.48-38.48)	54.25 (54.25-54.25)	59.59 (59.59-59.59)	55.57 (53.68-57.40)	55.67 (53.79-57.44)	81.33 (79.21-83.38)	41.49 (37.21-45.59)	48.02 (44.08-51.85)	58.37 (54.70-61.87)
Croatia	17.37 (8.72-27.28)	20.35 (11.39-30.40)	33.55 (21.91-46.27)	60.19 (60.19-60.19)	68.06 (68.06-68.06)	80.44 (80.44-80.44)	52.52 (50.24-54.71)	70.33 (65.62-74.57)	84.08 (80.98-87.00)	49.97 (45.56-54.22)	57.07 (53.45-60.45)	67.81 (63.93-70.91)

Location	Appendix Table 1a. Scaled values for each SDG health-related indicator and three summary indicators by country for 1990, 2000, and 2015											
	**Indicator 3.7.1: Proportion of women of reproductive age (15 to 49 years) who have their need for family planning satisfied with modern methods**			**Indicator 3.7.2: Birth rates for women aged 10 to 14 years and women aged 15 to 19 years (number of live births per 1,000 women)**			**Indicator 3.8.1: Coverage of 7 UHC tracer interventions for prevention and treatment services**			**Indicator 3.9.1: Age-standardised death rate attributable to household air pollution and ambient air pollution (per 100,000 population)**		
	**1990**	**2000**	**2015**	**1990**	**2000**	**2015**	**1990**	**2000**	**2015**	**1990**	**2000**	**2015**
Czech Republic	52.78 (40.49-64.81)	63.09 (50.44-73.59)	79.41 (71.05-86.77)	47.57 (47.57-47.57)	73.36 (73.36-73.36)	82.32 (82.32-82.32)	60.09 (58.45-61.58)	86.38 (84.04-88.64)	96.23 (94.93-97.48)	49.82 (46.20-53.38)	62.21 (58.69-65.26)	71.12 (67.38-74.02)
Hungary	49.54 (36.81-61.86)	57.67 (44.43-69.25)	77.63 (68.30-85.10)	50.99 (50.99-50.99)	66.18 (66.18-66.18)	75.10 (75.10-75.10)	59.44 (57.87-61.07)	75.68 (71.12-80.44)	96.78 (95.37-98.09)	45.51 (41.30-49.67)	53.98 (49.89-57.77)	65.76 (62.10-69.15)
Macedonia	6.55 (0.62-13.78)	8.90 (2.56-17.43)	17.22 (8.62-26.44)	47.63 (47.63-47.63)	58.90 (58.90-58.90)	70.95 (70.95-70.95)	47.31 (44.48-50.12)	64.88 (53.57-69.49)	80.37 (76.94-84.24)	40.47 (36.51-44.72)	43.75 (39.84-47.67)	55.67 (52.04-59.58)
Montenegro	16.71 (8.23-28.02)	20.48 (11.69-31.11)	30.80 (19.16-42.43)	57.26 (57.26-57.26)	64.53 (64.53-64.53)	75.08 (75.08-75.08)	49.16 (45.83-51.86)	67.35 (61.20-71.30)	82.08 (78.60-85.87)	47.01 (43.00-51.24)	48.74 (44.65-53.12)	56.34 (52.63-60.05)
Poland	16.59 (7.77-26.71)	21.47 (12.15-32.36)	40.90 (28.30-54.01)	53.37 (53.37-53.37)	68.91 (68.91-68.91)	77.59 (77.59-77.59)	53.91 (51.56-56.11)	80.99 (77.90-84.00)	91.12 (88.61-93.57)	44.78 (40.25-49.04)	56.41 (52.63-60.17)	67.45 (63.66-70.56)
Romania	16.30 (7.71-26.61)	22.35 (12.78-33.22)	41.06 (28.72-53.32)	44.92 (44.92-44.92)	55.57 (55.57-55.57)	60.45 (60.45-60.45)	48.04 (45.74-50.24)	65.65 (61.40-69.38)	87.97 (85.17-90.53)	40.81 (36.57-45.04)	46.70 (43.61-50.10)	59.99 (56.04-63.66)
Serbia	16.11 (7.09-25.80)	21.42 (11.70-32.72)	36.86 (25.31-48.82)	50.76 (50.76-50.76)	58.55 (58.55-58.55)	70.31 (70.31-70.31)	50.04 (47.08-52.62)	53.29 (50.90-55.58)	83.55 (80.12-86.91)	43.89 (40.58-47.69)	43.68 (39.67-47.74)	57.72 (54.54-60.90)
Slovakia	48.35 (36.36-60.50)	59.66 (47.26-70.49)	79.45 (70.44-86.53)	45.63 (45.63-45.63)	64.61 (64.61-64.61)	72.56 (72.56-72.56)	59.81 (58.12-61.27)	81.10 (77.56-84.14)	96.45 (94.97-97.72)	46.48 (42.67-50.24)	57.20 (53.71-60.49)	68.86 (65.17-71.91)
Slovenia	39.12 (30.77-47.95)	51.09 (39.81-60.95)	70.73 (59.62-80.17)	60.18 (60.18-60.18)	80.36 (80.36-80.36)	88.91 (88.91-88.91)	57.06 (55.65-58.50)	83.18 (80.81-85.54)	94.51 (92.62-96.18)	57.50 (53.07-61.52)	66.97 (63.45-70.17)	78.22 (74.31-81.75)
Armenia	23.31 (17.27-30.06)	23.48 (18.94-28.49)	37.30 (30.58-43.44)	35.72 (35.72-35.72)	51.50 (51.50-51.50)	62.86 (62.86-62.86)	49.96 (48.03-51.91)	51.45 (50.24-52.73)	85.26 (83.69-86.85)	39.81 (33.79-47.06)	47.60 (42.56-52.27)	62.23 (57.49-66.32)
Azerbaijan	11.13 (6.37-16.15)	12.37 (7.70-17.39)	24.68 (18.03-31.31)	49.89 (49.89-49.89)	52.08 (52.08-52.08)	41.59 (41.59-41.59)	30.29 (26.82-33.42)	32.26 (30.80-33.91)	78.75 (75.90-81.25)	28.45 (21.58-35.60)	37.39 (31.87-42.92)	53.90 (48.12-58.57)

Location	Appendix Table 1a. Scaled values for each SDG health-related indicator and three summary indicators by country for 1990, 2000, and 2015											
	Indicator 3.7.1: Proportion of women of reproductive age (15 to 49 years) who have their need for family planning satisfied with modern methods			Indicator 3.7.2: Birth rates for women aged 10 to 14 years and women aged 15 to 19 years (number of live births per 1,000 women)			Indicator 3.8.1: Coverage of 7 UHC tracer interventions for prevention and treatment services			Indicator 3.9.1: Age-standardised death rate attributable to household air pollution and ambient air pollution (per 100,000 population)		
	1990	2000	2015	1990	2000	2015	1990	2000	2015	1990	2000	2015
Georgia	36.04 (29.25-42.75)	39.44 (32.41-46.89)	55.58 (47.86-62.89)	38.50 (38.50-38.50)	48.05 (48.05-48.05)	54.29 (54.29-54.29)	40.18 (37.41-42.69)	47.56 (46.26-49.03)	86.31 (82.63-88.97)	37.42 (31.19-44.78)	41.58 (36.22-47.93)	51.77 (44.90-57.77)
Kazakhstan	55.65 (49.19-62.11)	63.96 (58.95-68.71)	75.98 (70.15-80.94)	43.03 (43.03-43.03)	54.93 (54.93-54.93)	57.69 (57.69-57.69)	52.04 (49.44-54.56)	59.85 (58.94-60.89)	91.83 (90.10-93.29)	40.42 (33.02-47.92)	40.37 (34.65-46.10)	54.01 (48.66-58.97)
Kyrgyzstan	59.03 (52.22-65.10)	60.90 (55.30-66.25)	61.27 (55.27-66.85)	37.79 (37.79-37.79)	48.44 (48.44-48.44)	49.41 (49.41-49.41)	52.46 (50.90-53.87)	55.61 (54.53-56.74)	87.76 (85.63-89.53)	31.55 (24.58-39.82)	34.11 (27.36-41.70)	45.63 (40.23-51.13)
Mongolia	42.01 (34.17-49.20)	60.04 (53.07-67.04)	75.72 (69.95-80.79)	47.73 (47.73-47.73)	59.40 (59.40-59.40)	65.50 (65.50-65.50)	42.77 (40.42-44.70)	59.58 (55.94-62.96)	87.29 (85.13-89.43)	30.82 (25.88-36.90)	27.54 (22.50-32.88)	40.51 (34.59-46.27)
Tajikistan	34.09 (27.24-41.38)	37.23 (30.24-44.53)	47.57 (41.28-53.77)	37.47 (37.47-37.47)	44.30 (44.30-44.30)	48.95 (48.95-48.95)	40.58 (36.62-43.75)	37.83 (34.65-40.31)	77.62 (74.70-80.15)	23.17 (18.23-28.85)	23.15 (18.31-28.44)	41.83 (36.54-47.32)
Turkmenistan	36.42 (29.10-43.82)	42.01 (34.72-49.17)	62.21 (55.52-68.60)	52.75 (52.75-52.75)	56.65 (56.65-56.65)	66.28 (66.28-66.28)	50.37 (48.22-52.27)	52.27 (50.84-53.64)	85.38 (83.19-87.35)	41.03 (36.70-45.54)	40.68 (36.28-45.15)	52.40 (48.26-56.24)
Uzbekistan	63.33 (56.82-69.35)	71.00 (65.87-75.76)	83.66 (79.46-87.22)	39.07 (39.07-39.07)	53.78 (53.78-53.78)	59.46 (59.46-59.46)	56.72 (55.20-58.06)	58.72 (57.77-59.71)	93.29 (91.36-95.05)	35.04 (28.44-42.10)	38.07 (32.67-43.29)	47.90 (41.92-52.93)
Colombia	67.80 (64.80-70.47)	76.46 (74.41-78.28)	89.24 (87.08-91.17)	36.56 (36.56-36.56)	36.29 (36.29-36.29)	50.05 (50.05-50.05)	29.74 (28.74-31.01)	38.34 (36.91-40.75)	77.07 (68.88-87.00)	57.55 (54.06-60.97)	62.60 (58.87-65.98)	71.59 (66.97-75.85)
Costa Rica	74.85 (70.91-78.33)	81.74 (78.64-84.63)	91.25 (89.58-92.83)	32.16 (32.16-32.16)	39.44 (39.44-39.44)	47.75 (47.75-47.75)	53.14 (52.25-54.13)	76.83 (73.24-81.64)	94.10 (92.45-95.59)	62.88 (58.27-67.19)	70.60 (67.18-73.56)	81.40 (77.22-84.83)
El Salvador	54.75 (49.58-59.85)	66.85 (62.12-71.32)	82.19 (79.27-84.95)	29.20 (29.20-29.20)	34.45 (34.45-34.45)	44.25 (44.25-44.25)	47.73 (45.88-49.55)	54.89 (53.85-55.94)	92.33 (90.18-94.26)	46.84 (42.85-51.14)	55.42 (51.19-59.50)	66.28 (61.25-70.76)
Guatemala	39.01 (34.43-43.56)	46.32 (41.96-50.40)	60.29 (55.73-64.95)	22.24 (22.24-22.24)	26.51 (26.51-26.51)	36.48 (36.48-36.48)	13.95 (12.60-15.54)	23.34 (21.81-25.75)	55.58 (48.78-63.82)	39.09 (34.78-43.91)	43.60 (39.49-48.18)	55.77 (50.14-61.79)

Appendix Table 1a. Scaled values for each SDG health-related indicator and three summary indicators by country for 1990, 2000, and 2015

Location	Indicator 3.7.1: Proportion of women of reproductive age (15 to 49 years) who have their need for family planning satisfied with modern methods			Indicator 3.7.2: Birth rates for women aged 10 to 14 years and women aged 15 to 19 years (number of live births per 1,000 women)			Indicator 3.8.1: Coverage of 7 UHC tracer interventions for prevention and treatment services			Indicator 3.9.1: Age-standardised death rate attributable to household air pollution and ambient air pollution (per 100,000 population)		
	1990	2000	2015	1990	2000	2015	1990	2000	2015	1990	2000	2015
Honduras	49.45 (44.31-54.49)	61.57 (57.69-65.78)	80.40 (77.86-82.78)	20.72 (20.72-20.72)	27.18 (27.18-27.18)	43.34 (43.34-43.34)	25.04 (23.80-26.58)	31.39 (29.92-33.78)	67.90 (60.12-76.42)	45.49 (41.80-49.53)	41.29 (35.62-46.66)	49.12 (42.97-55.43)
Mexico	68.47 (64.24-72.32)	74.67 (71.52-77.71)	87.10 (84.73-89.29)	34.59 (34.59-34.59)	37.62 (37.62-37.62)	44.20 (44.20-44.20)	45.99 (43.98-47.85)	63.62 (61.52-65.59)	87.73 (84.88-89.80)	63.64 (60.38-66.66)	66.67 (63.19-69.67)	72.20 (68.63-75.24)
Nicaragua	69.44 (65.43-73.25)	77.97 (75.83-79.93)	89.67 (87.63-91.51)	15.90 (15.90-15.90)	26.17 (26.17-26.17)	36.13 (36.13-36.13)	27.78 (26.43-29.25)	36.08 (34.65-38.60)	70.58 (62.75-79.53)	56.85 (53.41-60.46)	60.40 (56.80-63.83)	65.00 (59.92-70.47)
Panama	74.02 (69.88-77.97)	83.45 (80.68-86.30)	92.38 (90.86-93.74)	31.58 (31.58-31.58)	34.30 (34.30-34.30)	39.06 (39.06-39.06)	32.26 (30.81-33.74)	44.89 (41.62-48.82)	68.93 (59.57-78.92)	61.52 (57.82-65.05)	69.18 (65.54-72.90)	77.73 (72.53-82.45)
Venezuela	64.68 (59.72-69.32)	75.94 (72.21-79.41)	88.26 (86.10-90.15)	30.90 (30.90-30.90)	34.19 (34.19-34.19)	38.48 (38.48-38.48)	29.22 (27.69-30.81)	46.09 (43.62-49.34)	76.78 (68.07-85.82)	61.09 (57.46-64.22)	65.36 (61.60-68.55)	70.54 (66.12-74.35)
Bolivia	20.46 (14.79-26.61)	31.78 (26.43-36.78)	49.81 (41.18-57.58)	30.71 (30.71-30.71)	32.65 (32.65-32.65)	40.69 (40.69-40.69)	14.74 (13.27-16.33)	28.07 (26.55-30.40)	64.90 (58.27-72.87)	39.71 (34.97-44.37)	48.04 (43.85-52.05)	58.59 (52.61-63.90)
Ecuador	55.82 (47.30-64.03)	63.37 (54.53-70.83)	79.81 (73.53-85.19)	33.81 (33.81-33.81)	35.98 (35.98-35.98)	39.06 (39.06-39.06)	52.06 (47.80-59.77)	55.55 (51.86-59.38)	88.11 (86.16-89.66)	58.99 (55.31-62.55)	66.41 (62.45-69.90)	79.09 (74.52-82.99)
Peru	40.53 (34.48-46.50)	57.87 (52.06-63.20)	75.49 (68.67-81.45)	38.55 (38.55-38.55)	42.38 (42.38-42.38)	50.15 (50.15-50.15)	22.26 (20.87-23.83)	36.17 (34.51-38.96)	80.19 (70.48-88.84)	43.93 (39.56-48.43)	53.51 (49.78-57.37)	64.37 (58.90-69.15)
Antigua and Barbuda	87.15 (81.43-91.65)	92.83 (89.40-95.56)	95.42 (93.03-97.30)	44.30 (44.30-44.30)	44.29 (44.29-44.29)	52.80 (52.80-52.80)	59.75 (58.63-60.76)	64.17 (63.20-65.53)	94.75 (93.27-96.06)	69.04 (62.38-76.49)	72.81 (66.12-80.05)	78.83 (71.52-86.41)
The Bahamas	80.74 (72.88-86.93)	87.86 (82.84-91.94)	93.78 (90.79-96.26)	41.04 (41.04-41.04)	49.34 (49.34-49.34)	61.60 (61.60-61.60)	59.15 (57.62-60.49)	82.76 (77.44-86.73)	96.98 (95.74-98.15)	63.54 (57.69-69.74)	69.90 (63.96-75.96)	78.55 (71.94-85.05)
Barbados	72.14 (62.89-80.18)	82.93 (75.65-88.31)	90.88 (86.55-94.28)	48.77 (48.77-48.77)	50.00 (50.00-50.00)	56.16 (56.16-56.16)	58.56 (57.34-59.72)	60.89 (59.19-63.77)	93.46 (90.99-95.44)	71.47 (65.01-78.58)	75.39 (67.47-83.84)	81.39 (73.30-90.12)

Location	Indicator 3.7.1: Proportion of women of reproductive age (15 to 49 years) who have their need for family planning satisfied with modern methods			Indicator 3.7.2: Birth rates for women aged 10 to 14 years and women aged 15 to 19 years (number of live births per 1,000 women)			Indicator 3.8.1: Coverage of 7 UHC tracer interventions for prevention and treatment services			Indicator 3.9.1: Age-standardised death rate attributable to household air pollution and ambient air pollution (per 100,000 population)		
	1990	2000	2015	1990	2000	2015	1990	2000	2015	1990	2000	2015
Belize	44.95 (32.96-56.57)	52.21 (41.11-63.16)	69.17 (59.23-78.03)	22.18 (22.18-22.18)	30.37 (30.37-30.37)	42.26 (42.26-42.26)	48.83 (46.72-50.96)	58.06 (53.35-63.00)	90.61 (88.16-93.04)	50.66 (46.32-55.11)	50.94 (46.63-55.35)	61.06 (55.84-65.77)
Cuba	70.23 (59.53-79.12)	82.66 (75.69-88.33)	92.65 (88.92-95.56)	40.02 (40.02-40.02)	46.95 (46.95-46.95)	52.07 (52.07-52.07)	54.89 (53.56-56.09)	61.44 (60.55-62.39)	94.11 (92.38-96.47)	59.34 (54.20-64.00)	57.74 (53.25-62.26)	71.63 (67.69-74.87)
Dominica	69.63 (59.23-78.33)	80.50 (73.33-86.48)	90.26 (85.89-94.06)	40.61 (40.61-40.61)	56.36 (56.36-56.36)	61.33 (61.33-61.33)	59.96 (58.77-61.09)	62.65 (61.57-64.02)	93.02 (91.53-94.34)	58.95 (52.97-65.69)	68.32 (62.49-73.56)	75.96 (69.44-82.22)
Dominican Republic	69.15 (61.60-75.57)	78.21 (73.93-81.98)	85.13 (80.56-89.02)	26.31 (26.31-26.31)	27.61 (27.61-27.61)	32.05 (32.05-32.05)	46.18 (44.85-47.56)	53.42 (52.19-54.62)	84.13 (81.45-86.51)	59.65 (55.07-64.06)	63.70 (59.76-67.20)	67.81 (63.80-71.51)
Grenada	61.59 (50.88-71.99)	72.13 (62.52-80.16)	87.35 (81.51-91.83)	30.92 (30.92-30.92)	47.44 (47.44-47.44)	60.52 (60.52-60.52)	53.53 (52.01-55.09)	57.35 (56.18-58.79)	92.37 (90.84-93.70)	49.94 (42.99-57.28)	61.80 (55.04-68.41)	69.89 (63.19-77.01)
Guyana	45.28 (34.08-57.67)	53.90 (42.43-64.57)	65.07 (55.51-73.86)	29.94 (29.94-29.94)	31.31 (31.31-31.31)	34.74 (34.74-34.74)	22.82 (20.83-24.89)	31.07 (28.59-34.63)	76.07 (68.98-82.60)	45.17 (38.63-51.11)	51.15 (46.27-55.93)	59.83 (54.37-65.98)
Haiti	18.88 (12.07-26.52)	26.48 (20.17-32.98)	41.50 (32.84-49.94)	38.05 (38.05-38.05)	44.27 (44.27-44.27)	53.38 (53.38-53.38)	9.08 (7.58-10.86)	14.66 (12.98-16.86)	55.62 (49.47-60.37)	22.37 (17.63-27.66)	29.54 (24.00-35.28)	34.37 (28.29-40.60)
Jamaica	65.59 (53.94-75.01)	77.73 (69.19-84.92)	87.86 (82.55-92.18)	29.10 (29.10-29.10)	35.03 (35.03-35.03)	45.33 (45.33-45.33)	53.60 (51.30-55.67)	58.35 (57.01-59.70)	93.39 (91.86-94.73)	58.27 (53.96-62.28)	66.25 (62.00-70.38)	72.43 (67.80-76.99)
Saint Lucia	63.16 (52.63-73.40)	74.89 (65.52-83.18)	87.74 (82.27-92.02)	28.60 (28.60-28.60)	43.56 (43.56-43.56)	49.39 (49.39-49.39)	57.94 (56.55-59.32)	60.27 (59.02-61.68)	92.59 (91.16-93.97)	52.77 (47.53-57.69)	63.61 (58.15-69.17)	74.82 (69.35-80.12)
Saint Vincent and the Grenadines	65.13 (54.57-74.53)	76.80 (67.88-84.02)	88.73 (83.39-92.88)	33.47 (33.47-33.47)	41.73 (41.73-41.73)	49.68 (49.68-49.68)	49.17 (47.93-50.38)	56.08 (55.02-57.38)	89.58 (88.14-90.92)	47.04 (42.55-51.72)	57.17 (50.44-64.04)	69.86 (63.48-76.60)
Suriname	55.26 (43.83-66.64)	59.46 (48.59-70.09)	73.65 (63.65-81.89)	40.22 (40.22-40.22)	45.32 (45.32-45.32)	51.36 (51.36-51.36)	24.46 (22.72-26.23)	33.99 (32.16-36.64)	75.23 (66.87-84.37)	49.18 (43.38-55.56)	54.31 (47.68-60.87)	68.68 (62.11-75.22)

Appendix Table 1a. Scaled values for each SDG health-related indicator and three summary indicators by country for 1990, 2000, and 2015

Location	Appendix Table 1a. Scaled values for each SDG health-related indicator and three summary indicators by country for 1990, 2000, and 2015											
	**Indicator 3.7.1: Proportion of women of reproductive age (15 to 49 years) who have their need for family planning satisfied with modern methods**			**Indicator 3.7.2: Birth rates for women aged 10 to 14 years and women aged 15 to 19 years (number of live births per 1,000 women)**			**Indicator 3.8.1: Coverage of 7 UHC tracer interventions for prevention and treatment services**			**Indicator 3.9.1: Age-standardised death rate attributable to household air pollution and ambient air pollution (per 100,000 population)**		
	**1990**	**2000**	**2015**	**1990**	**2000**	**2015**	**1990**	**2000**	**2015**	**1990**	**2000**	**2015**
Trinidad and Tobago	65.36 (54.69-74.74)	56.01 (44.38-66.59)	76.19 (68.01-83.60)	43.07 (43.07-43.07)	53.42 (53.42-53.42)	59.61 (59.61-59.61)	54.63 (52.62-56.50)	57.41 (55.56-59.34)	92.99 (90.68-94.76)	60.33 (56.03-64.28)	64.71 (60.55-68.50)	74.13 (69.76-78.10)
Brazil	75.74 (72.22-78.78)	84.64 (82.23-86.77)	92.72 (91.27-93.95)	36.84 (36.84-36.84)	36.76 (36.76-36.76)	43.35 (43.35-43.35)	29.34 (28.20-30.74)	58.76 (55.35-63.66)	80.65 (72.26-90.58)	49.98 (47.04-53.29)	57.56 (54.30-60.78)	72.37 (68.61-75.34)
Paraguay	52.63 (48.81-56.58)	66.40 (62.06-70.20)	85.11 (82.63-87.35)	31.31 (31.31-31.31)	35.37 (35.37-35.37)	46.11 (46.11-46.11)	45.15 (43.77-46.67)	51.35 (50.01-52.64)	87.42 (84.66-89.92)	49.21 (45.65-52.87)	52.39 (49.02-56.04)	60.88 (56.30-65.49)
China	59.62 (49.94-68.40)	71.81 (65.19-78.01)	86.10 (79.34-91.54)	55.24 (55.24-55.24)	69.15 (69.15-69.15)	69.11 (69.11-69.11)	35.67 (32.56-38.56)	47.38 (45.74-48.79)	80.71 (78.13-83.06)	25.31 (19.78-31.15)	31.53 (26.80-36.62)	48.12 (44.19-51.69)
North Korea	30.31 (17.35-43.36)	39.84 (27.21-53.24)	61.68 (51.03-71.73)	85.90 (85.90-85.90)	92.89 (92.89-92.89)	92.73 (92.73-92.73)	29.57 (26.99-32.02)	44.41 (41.63-46.71)	77.43 (72.01-82.39)	34.67 (26.23-42.35)	34.29 (26.84-41.99)	39.27 (32.69-45.69)
Taiwan	71.68 (60.63-81.69)	79.17 (69.93-86.71)	88.70 (82.50-93.09)	67.48 (67.48-67.48)	72.22 (72.22-72.22)	91.40 (91.40-91.40)	58.72 (56.35-60.69)	61.60 (60.20-62.75)	82.43 (79.92-84.85)	67.28 (63.35-70.89)	72.29 (68.04-76.03)	75.55 (70.66-80.34)
Cambodia	20.15 (14.57-26.29)	26.74 (24.10-29.61)	53.30 (50.68-55.78)	33.97 (33.97-33.97)	45.95 (45.95-45.95)	45.41 (45.41-45.41)	3.87 (1.96-5.88)	12.46 (10.80-14.51)	56.98 (50.92-64.69)	26.16 (20.93-31.70)	28.33 (23.65-33.24)	38.84 (34.15-43.68)
Indonesia	70.74 (66.38-74.96)	79.51 (76.33-82.38)	88.93 (86.58-91.08)	40.55 (40.55-40.55)	49.15 (49.15-49.15)	48.76 (48.76-48.76)	12.98 (11.88-14.51)	19.03 (17.66-21.02)	51.75 (45.50-58.94)	44.45 (39.83-49.18)	49.20 (44.78-53.87)	57.66 (52.31-63.62)
Laos	28.01 (21.79-34.94)	37.09 (32.35-41.80)	50.16 (42.26-57.42)	25.27 (25.27-25.27)	35.02 (35.02-35.02)	41.72 (41.72-41.72)	1.20 (0.00-3.35)	8.06 (6.46-9.92)	48.28 (44.42-51.96)	21.00 (14.14-28.03)	24.58 (19.43-29.96)	35.87 (30.45-41.34)
Malaysia	35.05 (27.56-43.09)	43.40 (36.02-51.09)	62.46 (55.72-68.96)	63.56 (63.56-63.56)	70.34 (70.34-70.34)	75.51 (75.51-75.51)	50.99 (49.40-52.60)	58.91 (56.06-62.54)	88.82 (87.39-90.20)	57.50 (51.86-62.46)	62.76 (58.56-66.76)	67.34 (62.45-71.83)
Maldives	23.75 (17.32-30.59)	29.77 (22.97-37.08)	46.06 (38.52-53.48)	19.67 (19.67-19.67)	55.00 (55.00-55.00)	72.60 (72.60-72.60)	42.70 (39.41-45.73)	52.18 (50.52-53.79)	79.29 (77.43-81.05)	43.00 (38.35-48.10)	56.60 (52.11-61.00)	71.71 (65.95-76.90)

Location	Appendix Table 1a. Scaled values for each SDG health-related indicator and three summary indicators by country for 1990, 2000, and 2015											
	Indicator 3.7.1: Proportion of women of reproductive age (15 to 49 years) who have their need for family planning satisfied with modern methods			Indicator 3.7.2: Birth rates for women aged 10 to 14 years and women aged 15 to 19 years (number of live births per 1,000 women)			Indicator 3.8.1: Coverage of 7 UHC tracer interventions for prevention and treatment services			Indicator 3.9.1: Age-standardised death rate attributable to household air pollution and ambient air pollution (per 100,000 population)		
	1990	2000	2015	1990	2000	2015	1990	2000	2015	1990	2000	2015
Mauritius	42.08 (34.97-49.96)	49.91 (42.50-57.25)	66.36 (59.45-72.57)	49.62 (49.62-49.62)	54.32 (54.32-54.32)	63.09 (63.09-63.09)	51.27 (49.95-52.62)	53.54 (52.42-54.73)	83.38 (81.91-84.60)	49.53 (43.48-54.49)	58.23 (54.11-62.18)	73.76 (69.79-77.33)
Myanmar	23.35 (17.30-30.23)	31.46 (24.21-39.06)	49.83 (42.13-57.45)	54.74 (54.74-54.74)	61.41 (61.41-61.41)	69.64 (69.64-69.64)	11.70 (9.94-13.69)	18.75 (17.38-20.63)	60.49 (53.71-68.02)	29.74 (19.72-38.73)	32.04 (21.84-41.69)	39.96 (30.86-49.93)
Philippines	30.68 (26.40-35.01)	40.81 (38.35-43.29)	50.39 (47.35-53.56)	45.10 (45.10-45.10)	44.93 (44.93-44.93)	43.26 (43.26-43.26)	20.75 (19.59-22.17)	24.87 (23.54-26.95)	56.95 (50.15-64.49)	42.17 (37.81-46.65)	43.97 (39.90-48.22)	43.85 (39.19-48.83)
Sri Lanka	48.49 (43.16-53.43)	47.42 (42.37-52.08)	69.42 (62.93-74.86)	58.05 (58.05-58.05)	60.64 (60.64-60.64)	75.16 (75.16-75.16)	51.20 (49.49-52.82)	54.76 (53.62-55.91)	86.67 (85.08-88.37)	48.30 (45.06-51.55)	50.49 (47.22-53.68)	58.56 (53.23-64.12)
Seychelles	56.46 (48.91-63.60)	69.78 (63.16-76.06)	85.19 (80.92-88.66)	40.00 (40.00-40.00)	45.64 (45.64-45.64)	46.86 (46.86-46.86)	53.80 (51.66-55.66)	67.87 (64.51-71.86)	92.06 (89.85-94.83)	59.13 (50.65-68.15)	65.70 (57.21-74.97)	75.97 (66.39-86.87)
Thailand	41.42 (34.00-48.79)	56.22 (49.23-63.27)	65.64 (62.15-68.90)	48.24 (48.24-48.24)	52.95 (52.95-52.95)	53.77 (53.77-53.77)	26.61 (25.18-28.26)	36.62 (33.41-40.50)	68.93 (61.46-77.22)	48.65 (44.69-52.81)	52.95 (49.03-56.89)	60.89 (55.71-66.40)
Timor-Leste	17.96 (12.20-24.31)	22.84 (16.75-29.10)	40.90 (34.51-47.44)	39.98 (39.98-39.98)	30.81 (30.81-30.81)	45.82 (45.82-45.82)	3.87 (1.57-6.44)	12.91 (9.31-17.07)	54.51 (48.51-60.46)	32.93 (26.13-40.06)	38.80 (33.28-44.14)	48.10 (41.58-54.29)
Vietnam	53.32 (46.24-59.77)	65.63 (63.02-68.14)	80.31 (75.45-84.75)	52.26 (52.26-52.26)	57.62 (57.62-57.62)	54.02 (54.02-54.02)	15.37 (13.23-17.51)	28.56 (26.94-31.15)	68.44 (60.69-76.75)	44.72 (39.29-49.60)	46.59 (41.63-51.49)	56.46 (50.74-62.42)
Federated States of Micronesia	57.21 (51.78-62.71)	69.40 (64.60-73.75)	82.73 (79.41-85.63)	32.92 (32.92-32.92)	40.31 (40.31-40.31)	59.89 (59.89-59.89)	47.37 (43.36-50.45)	48.51 (45.57-50.77)	81.01 (76.59-84.93)	42.16 (30.49-54.77)	48.31 (36.60-59.58)	59.35 (45.98-73.27)
Fiji	58.18 (52.91-63.95)	69.26 (64.66-73.44)	85.88 (83.25-88.44)	38.61 (38.61-38.61)	47.38 (47.38-47.38)	49.04 (49.04-49.04)	51.29 (48.75-53.04)	53.41 (51.55-54.78)	81.91 (79.38-83.94)	44.82 (38.53-51.32)	49.84 (43.23-56.73)	65.61 (57.95-73.53)
Kiribati	43.47 (37.52-49.35)	50.31 (44.45-56.13)	63.99 (58.98-68.94)	46.56 (46.56-46.56)	52.52 (52.52-52.52)	68.80 (68.80-68.80)	45.33 (41.37-48.23)	51.37 (47.74-53.67)	80.87 (75.82-84.23)	44.63 (37.99-52.15)	48.71 (40.10-58.85)	61.89 (51.10-73.98)

Appendix Table 1a. Scaled values for each SDG health-related indicator and three summary indicators by country for 1990, 2000, and 2015

Location	Indicator 3.7.1: Proportion of women of reproductive age (15 to 49 years) who have their need for family planning satisfied with modern methods			Indicator 3.7.2: Birth rates for women aged 10 to 14 years and women aged 15 to 19 years (number of live births per 1,000 women)			Indicator 3.8.1: Coverage of 7 UHC tracer interventions for prevention and treatment services			Indicator 3.9.1: Age-standardised death rate attributable to household air pollution and ambient air pollution (per 100,000 population)		
	1990	2000	2015	1990	2000	2015	1990	2000	2015	1990	2000	2015
Marshall Islands	50.51 (44.72-56.19)	61.75 (56.72-66.82)	78.75 (75.06-82.17)	24.49 (24.49-24.49)	29.60 (29.60-29.60)	41.74 (41.74-41.74)	37.93 (31.23-44.41)	44.51 (39.27-48.78)	74.32 (68.82-79.17)	52.43 (39.58-71.20)	50.61 (38.52-66.81)	60.43 (45.66-80.97)
Papua New Guinea	35.10 (29.30-40.69)	38.77 (33.64-43.75)	47.33 (41.84-52.90)	35.53 (35.53-35.53)	38.33 (38.33-38.33)	44.39 (44.39-44.39)	16.63 (14.29-18.90)	18.83 (16.76-21.30)	45.31 (38.83-51.98)	18.73 (8.01-30.09)	22.10 (10.66-33.70)	27.57 (15.53-39.07)
Samoa	41.19 (36.19-46.74)	54.04 (48.12-59.27)	69.35 (64.93-73.68)	51.58 (51.58-51.58)	49.37 (49.37-49.37)	60.46 (60.46-60.46)	42.38 (36.33-47.10)	46.21 (42.28-49.60)	71.11 (65.33-76.29)	50.70 (43.38-57.81)	56.00 (49.11-62.81)	65.13 (57.74-73.63)
Solomon Islands	32.59 (27.01-38.11)	38.95 (33.05-44.72)	53.91 (48.04-59.56)	29.62 (29.62-29.62)	37.35 (37.35-37.35)	47.48 (47.48-47.48)	19.13 (15.53-22.13)	24.23 (21.85-26.80)	55.22 (48.12-63.00)	28.93 (18.41-39.55)	32.22 (21.03-42.69)	38.08 (25.79-48.91)
Tonga	57.24 (51.80-62.53)	68.80 (63.93-73.52)	83.96 (80.92-86.60)	58.48 (58.48-58.48)	61.23 (61.23-61.23)	69.29 (69.29-69.29)	54.61 (51.70-56.51)	58.74 (56.31-60.03)	87.29 (84.05-89.58)	51.03 (44.65-57.51)	54.87 (48.70-61.04)	68.79 (59.95-78.72)
Vanuatu	38.91 (33.16-44.73)	47.30 (41.60-53.14)	65.40 (59.96-70.27)	35.72 (35.72-35.72)	40.87 (40.87-40.87)	49.49 (49.49-49.49)	23.92 (20.96-26.49)	28.09 (25.23-30.90)	57.75 (50.34-66.03)	29.21 (18.65-39.11)	31.75 (21.10-43.01)	39.85 (28.63-49.86)
Afghanistan	36.40 (28.96-44.35)	35.96 (28.05-44.36)	39.28 (33.11-45.78)	11.07 (11.07-11.07)	12.93 (12.93-12.93)	37.38 (37.38-37.38)	4.82 (2.55-7.38)	7.88 (5.88-9.93)	28.16 (23.62-33.17)	5.33 (0.00-13.66)	3.52 (0.00-11.39)	11.02 (3.05-19.40)
Algeria	56.17 (48.35-64.11)	60.70 (52.75-67.82)	67.88 (61.67-73.65)	56.29 (56.29-56.29)	73.68 (73.68-73.68)	72.70 (72.70-72.70)	46.14 (43.77-48.34)	52.93 (51.13-54.55)	90.01 (87.94-91.77)	59.73 (53.73-64.50)	65.41 (61.40-69.05)	69.86 (65.50-73.51)
Bahrain	37.95 (30.29-46.04)	49.43 (42.06-56.86)	65.16 (57.69-72.51)	57.23 (57.23-57.23)	65.29 (65.29-65.29)	69.80 (69.80-69.80)	53.43 (51.80-55.12)	58.40 (56.30-61.99)	85.02 (83.19-86.72)	50.00 (45.02-55.00)	55.56 (51.04-59.72)	69.40 (63.98-74.40)
Egypt	62.40 (57.46-67.86)	76.90 (73.43-80.00)	85.58 (82.69-88.44)	33.01 (33.01-33.01)	44.60 (44.60-44.60)	43.14 (43.14-43.14)	30.19 (28.84-31.61)	47.56 (46.54-48.69)	79.75 (78.02-81.42)	43.89 (39.80-48.02)	49.45 (45.60-53.29)	49.37 (45.42-53.32)
Iran	34.37 (26.60-42.50)	46.84 (38.44-55.12)	73.52 (67.80-78.92)	31.81 (31.81-31.81)	54.01 (54.01-54.01)	63.65 (63.65-63.65)	48.68 (46.70-50.55)	59.92 (55.74-65.05)	89.80 (88.74-90.72)	52.02 (46.78-56.77)	56.35 (52.16-60.33)	60.23 (55.34-65.22)

Appendix Table 1a. Scaled values for each SDG health-related indicator and three summary indicators by country for 1990, 2000, and 2015

Location	Indicator 3.7.1: Proportion of women of reproductive age (15 to 49 years) who have their need for family planning satisfied with modern methods			Indicator 3.7.2: Birth rates for women aged 10 to 14 years and women aged 15 to 19 years (number of live births per 1,000 women)			Indicator 3.8.1: Coverage of 7 UHC tracer interventions for prevention and treatment services			Indicator 3.9.1: Age-standardised death rate attributable to household air pollution and ambient air pollution (per 100,000 population)		
	1990	2000	2015	1990	2000	2015	1990	2000	2015	1990	2000	2015
Iraq	26.20 (18.62-34.09)	32.19 (24.38-40.66)	44.44 (36.14-52.72)	37.99 (37.99-37.99)	40.81 (40.81-40.81)	33.56 (33.56-33.56)	37.17 (34.54-39.62)	42.08 (40.15-44.57)	71.30 (68.59-73.96)	45.59 (39.79-51.45)	44.03 (38.48-49.23)	49.73 (43.56-55.80)
Jordan	39.43 (33.46-45.74)	52.78 (47.04-58.14)	64.49 (59.03-70.40)	41.59 (41.59-41.59)	51.99 (51.99-51.99)	62.15 (62.15-62.15)	53.97 (52.78-55.26)	60.44 (58.83-63.89)	85.90 (84.11-87.73)	59.17 (54.27-63.29)	62.47 (58.51-66.42)	71.52 (67.17-75.54)
Kuwait	45.54 (37.66-53.82)	58.49 (50.09-65.96)	77.33 (71.49-82.82)	62.95 (62.95-62.95)	61.27 (61.27-61.27)	72.74 (72.74-72.74)	39.61 (36.10-42.71)	53.76 (49.85-58.31)	83.14 (80.82-85.16)	64.18 (59.77-68.08)	62.71 (58.52-66.56)	66.63 (62.01-71.56)
Lebanon	42.50 (34.23-51.03)	52.22 (43.87-59.95)	67.73 (59.98-74.22)	49.63 (49.63-49.63)	63.43 (63.43-63.43)	75.81 (75.81-75.81)	51.94 (49.72-54.02)	65.18 (59.62-70.82)	87.74 (85.27-90.65)	58.88 (53.37-64.11)	67.98 (63.24-72.32)	70.56 (64.64-76.61)
Libya	32.91 (25.39-41.19)	38.66 (30.66-46.96)	51.53 (43.26-59.94)	71.46 (71.46-71.46)	82.35 (82.35-82.35)	83.71 (83.71-83.71)	40.27 (37.73-42.67)	50.27 (47.19-55.19)	77.95 (75.22-80.71)	57.97 (52.99-62.76)	60.44 (56.48-64.40)	57.71 (52.71-62.39)
Morocco	55.46 (48.76-61.84)	65.82 (60.16-71.56)	77.72 (72.00-82.86)	51.40 (51.40-51.40)	57.30 (57.30-57.30)	58.80 (58.80-58.80)	28.46 (27.08-29.90)	45.67 (44.59-46.83)	79.90 (77.09-82.72)	59.43 (51.26-65.97)	66.20 (61.16-71.07)	70.36 (63.78-76.14)
Palestine	36.59 (28.37-44.91)	46.85 (37.91-54.90)	55.70 (47.50-63.29)	24.63 (24.63-24.63)	32.68 (32.68-32.68)	41.72 (41.72-41.72)	48.52 (46.77-50.18)	49.20 (47.77-51.34)	74.43 (72.68-76.10)	63.19 (57.29-68.89)	67.40 (63.33-71.14)	64.14 (58.54-69.63)
Oman	29.12 (21.88-36.81)	33.04 (25.31-41.36)	47.56 (39.38-55.27)	31.54 (31.54-31.54)	57.36 (57.36-57.36)	77.13 (77.13-77.13)	52.89 (51.31-54.44)	67.17 (62.38-72.18)	87.52 (85.84-89.03)	56.36 (49.49-62.67)	59.37 (54.22-64.22)	63.00 (58.07-67.81)
Qatar	37.81 (29.56-46.31)	43.45 (35.60-51.92)	66.58 (60.53-72.57)	44.06 (44.06-44.06)	59.91 (59.91-59.91)	70.81 (70.81-70.81)	52.38 (50.62-54.09)	59.58 (56.48-63.72)	85.77 (84.05-87.55)	55.45 (50.19-59.96)	55.27 (49.88-60.19)	67.99 (62.35-73.74)
Saudi Arabia	35.20 (27.57-43.63)	42.21 (33.48-50.52)	59.20 (51.43-66.67)	37.90 (37.90-37.90)	60.62 (60.62-60.62)	81.35 (81.35-81.35)	51.69 (50.06-53.46)	58.72 (55.49-64.01)	84.61 (82.73-86.61)	57.37 (53.12-61.80)	59.67 (55.63-63.56)	62.22 (57.65-66.53)
Sudan	8.68 (4.05-13.67)	8.50 (3.76-13.75)	9.11 (5.32-13.17)	27.09 (27.09-27.09)	24.97 (24.97-24.97)	38.95 (38.95-38.95)	6.41 (4.25-8.60)	14.89 (12.82-17.05)	47.44 (43.94-50.64)	24.44 (17.53-30.66)	27.62 (19.85-34.47)	35.88 (27.99-43.35)

Appendix Table 1a. Scaled values for each SDG health-related indicator and three summary indicators by country for 1990, 2000, and 2015

Location	Indicator 3.7.1: Proportion of women of reproductive age (15 to 49 years) who have their need for family planning satisfied with modern methods			Indicator 3.7.2: Birth rates for women aged 10 to 14 years and women aged 15 to 19 years (number of live births per 1,000 women)			Indicator 3.8.1: Coverage of 7 UHC tracer interventions for prevention and treatment services			Indicator 3.9.1: Age-standardised death rate attributable to household air pollution and ambient air pollution (per 100,000 population)		
	1990	2000	2015	1990	2000	2015	1990	2000	2015	1990	2000	2015
Syria	35.14 (26.88-43.44)	43.01 (34.28-51.28)	57.01 (49.25-64.46)	36.99 (36.99-36.99)	43.94 (43.94-43.94)	51.08 (51.08-51.08)	37.97 (35.05-40.86)	49.54 (47.55-51.40)	78.17 (75.75-80.53)	51.70 (46.65-56.39)	58.17 (53.60-62.56)	59.89 (54.98-64.56)
Tunisia	53.21 (45.08-61.17)	62.39 (54.75-69.73)	71.99 (64.49-78.17)	63.23 (63.23-63.23)	79.65 (79.65-79.65)	81.04 (81.04-81.04)	46.35 (44.54-48.09)	56.43 (55.28-57.56)	87.68 (85.99-89.53)	58.58 (52.15-63.95)	64.90 (60.34-69.30)	69.06 (63.35-75.00)
Turkey	40.84 (34.22-47.71)	49.84 (44.13-55.60)	70.11 (62.93-76.47)	39.26 (39.26-39.26)	47.45 (47.45-47.45)	61.18 (61.18-61.18)	41.61 (40.23-43.08)	47.50 (46.20-48.82)	82.03 (80.13-84.08)	48.49 (41.93-54.18)	58.87 (54.81-62.50)	70.69 (66.32-74.43)
United Arab Emirates	32.85 (25.30-41.14)	43.27 (34.75-51.68)	58.81 (50.28-66.38)	42.69 (42.69-42.69)	59.26 (59.26-59.26)	58.97 (58.97-58.97)	52.85 (51.43-54.46)	57.37 (55.17-62.09)	84.75 (82.54-87.08)	49.02 (42.93-54.96)	51.46 (46.50-56.50)	56.19 (49.67-62.10)
Yemen	25.16 (18.04-32.77)	30.78 (23.13-39.11)	42.44 (35.85-49.26)	13.90 (13.90-13.90)	28.79 (28.79-28.79)	42.17 (42.17-42.17)	7.93 (6.33-9.70)	14.12 (11.82-17.11)	48.93 (43.59-54.75)	22.56 (10.54-34.72)	30.24 (17.62-42.52)	37.42 (24.19-49.09)
Bangladesh	50.39 (44.58-56.46)	61.49 (57.56-65.42)	73.25 (69.48-76.50)	15.76 (15.76-15.76)	26.56 (26.56-26.56)	36.20 (36.20-36.20)	2.17 (0.40-4.18)	15.39 (13.80-17.51)	50.32 (43.50-56.82)	28.20 (22.59-34.21)	29.52 (24.74-34.41)	30.57 (24.87-36.22)
Bhutan	33.25 (25.72-41.39)	40.34 (32.43-47.94)	57.03 (51.05-62.65)	26.18 (26.18-26.18)	35.37 (35.37-35.37)	65.31 (65.31-65.31)	26.15 (21.36-30.68)	38.54 (34.34-42.54)	74.59 (70.12-79.11)	31.26 (24.27-38.61)	37.51 (31.67-43.36)	46.98 (39.70-54.60)
India	57.51 (54.48-60.26)	65.15 (63.01-67.29)	82.19 (77.49-86.24)	27.58 (27.58-27.58)	37.34 (37.34-37.34)	55.26 (55.26-55.26)	13.00 (10.83-15.15)	18.94 (17.15-21.05)	51.43 (44.92-58.89)	21.09 (15.92-26.65)	23.68 (18.67-28.87)	31.33 (27.02-36.14)
Nepal	33.48 (27.11-40.84)	46.36 (43.53-49.43)	57.66 (52.51-62.44)	18.89 (18.89-18.89)	23.52 (23.52-23.52)	38.81 (38.81-38.81)	4.23 (2.04-6.57)	14.05 (12.56-16.13)	50.13 (43.58-57.35)	27.32 (21.18-33.91)	32.15 (27.30-37.11)	34.99 (28.52-41.12)
Pakistan	13.73 (10.73-16.71)	26.05 (20.51-31.64)	44.39 (39.82-48.91)	32.21 (32.21-32.21)	44.72 (44.72-44.72)	50.39 (50.39-50.39)	6.46 (5.05-8.18)	10.69 (9.17-12.77)	38.62 (33.03-44.85)	28.30 (22.56-34.36)	24.36 (19.00-29.86)	30.76 (24.95-36.70)
Botswana	60.08 (51.95-67.77)	75.07 (68.25-80.86)	88.08 (84.03-91.41)	29.62 (29.62-29.62)	42.06 (42.06-42.06)	56.37 (56.37-56.37)	28.66 (27.24-30.15)	35.95 (32.99-40.01)	82.15 (75.66-89.63)	31.57 (6.95-50.38)	32.79 (0.40-55.48)	45.75 (16.55-65.64)

Appendix Table 1a. Scaled values for each SDG health-related indicator and three summary indicators by country for 1990, 2000, and 2015

Location	Indicator 3.7.1: Proportion of women of reproductive age (15 to 49 years) who have their need for family planning satisfied with modern methods			Indicator 3.7.2: Birth rates for women aged 10 to 14 years and women aged 15 to 19 years (number of live births per 1,000 women)			Indicator 3.8.1: Coverage of 7 UHC tracer interventions for prevention and treatment services			Indicator 3.9.1: Age-standardised death rate attributable to household air pollution and ambient air pollution (per 100,000 population)		
	1990	2000	2015	1990	2000	2015	1990	2000	2015	1990	2000	2015
Lesotho	38.81 (30.57-47.32)	49.57 (42.35-56.78)	81.19 (77.60-84.60)	32.71 (32.71-32.71)	31.59 (31.59-31.59)	32.47 (32.47-32.47)	39.65 (37.38-41.94)	43.25 (41.96-44.62)	76.03 (73.93-78.13)	31.70 (23.62-39.75)	29.44 (20.04-38.14)	25.61 (11.70-40.24)
Namibia	57.13 (50.62-63.41)	71.43 (66.52-75.42)	86.50 (83.38-89.31)	27.77 (27.77-27.77)	35.64 (35.64-35.64)	37.70 (37.70-37.70)	25.87 (24.61-27.34)	31.66 (30.02-34.23)	82.18 (76.38-86.67)	26.22 (20.53-32.34)	21.83 (13.79-30.33)	42.09 (30.20-52.66)
South Africa	72.70 (66.38-78.90)	81.36 (77.54-84.80)	91.41 (88.16-93.91)	33.77 (33.77-33.77)	42.56 (42.56-42.56)	57.04 (57.04-57.04)	31.60 (30.45-33.02)	41.49 (40.04-43.46)	79.04 (69.81-87.58)	34.87 (30.57-39.83)	34.16 (29.64-39.02)	51.31 (46.84-55.81)
Swaziland	43.31 (34.41-52.91)	56.33 (48.72-63.59)	79.58 (73.53-84.60)	20.82 (20.82-20.82)	28.86 (28.86-28.86)	40.48 (40.48-40.48)	45.80 (43.80-47.84)	50.74 (49.56-52.02)	84.68 (82.99-86.45)	25.48 (16.66-34.71)	24.96 (13.71-35.30)	28.63 (11.88-46.22)
Zimbabwe	64.35 (58.24-70.43)	74.22 (70.47-77.94)	85.67 (82.04-89.08)	26.36 (26.36-26.36)	27.98 (27.98-27.98)	27.42 (27.42-27.42)	28.15 (27.07-29.51)	33.69 (32.55-35.01)	79.10 (76.70-81.38)	39.01 (32.26-46.99)	34.48 (21.93-44.71)	37.86 (25.29-50.94)
Benin	3.35 (0.00-9.38)	6.52 (2.03-12.04)	14.92 (6.93-24.59)	21.05 (21.05-21.05)	23.04 (23.04-23.04)	34.29 (34.29-34.29)	16.66 (14.66-18.86)	22.38 (20.87-24.14)	64.32 (61.00-67.72)	26.60 (20.61-32.81)	25.44 (18.73-32.31)	29.09 (14.39-43.35)
Burkina Faso	4.05 (0.00-9.62)	12.49 (6.47-19.12)	27.75 (17.21-40.02)	16.66 (16.66-16.66)	18.25 (18.25-18.25)	26.78 (26.78-26.78)	8.79 (7.08-10.86)	15.42 (13.98-17.10)	66.14 (62.99-69.24)	27.50 (20.99-34.06)	28.39 (21.66-34.99)	33.64 (20.56-44.63)
Cameroon	5.88 (0.45-11.74)	17.04 (9.99-24.72)	33.38 (21.93-46.23)	8.88 (8.88-8.88)	15.21 (15.21-15.21)	29.32 (29.32-29.32)	15.75 (14.35-17.48)	17.55 (16.23-19.20)	58.02 (54.10-61.33)	26.72 (20.43-33.09)	22.96 (15.27-31.46)	30.58 (16.89-42.59)
Cape Verde	46.75 (31.81-61.42)	59.79 (45.93-72.73)	74.76 (62.44-84.25)	25.68 (25.68-25.68)	30.41 (30.41-30.41)	41.04 (41.04-41.04)	40.79 (38.00-43.59)	45.05 (42.77-47.25)	80.21 (77.51-82.77)	37.87 (32.95-42.60)	38.25 (30.08-46.01)	50.49 (43.55-57.61)
Chad	3.65 (0.00-10.71)	10.17 (4.40-17.34)	15.61 (5.90-26.34)	2.58 (2.58-2.58)	2.88 (2.88-2.88)	19.12 (19.12-19.12)	1.30 (0.00-3.30)	4.92 (3.51-6.68)	33.62 (29.46-37.49)	29.02 (22.66-35.24)	23.57 (16.31-31.05)	27.33 (13.11-41.13)
Cote d'Ivoire	9.97 (2.93-18.38)	16.11 (8.57-24.61)	25.56 (15.39-36.47)	15.08 (15.08-15.08)	20.02 (20.02-20.02)	20.43 (20.43-20.43)	14.05 (12.49-15.82)	20.00 (18.57-21.71)	58.98 (56.19-61.76)	26.14 (19.60-32.44)	23.11 (14.61-31.24)	31.34 (18.90-42.97)

Appendix Table 1a. Scaled values for each SDG health-related indicator and three summary indicators by country for 1990, 2000, and 2015

Location	Indicator 3.7.1: Proportion of women of reproductive age (15 to 49 years) who have their need for family planning satisfied with modern methods			Indicator 3.7.2: Birth rates for women aged 10 to 14 years and women aged 15 to 19 years (number of live births per 1,000 women)			Indicator 3.8.1: Coverage of 7 UHC tracer interventions for prevention and treatment services			Indicator 3.9.1: Age-standardised death rate attributable to household air pollution and ambient air pollution (per 100,000 population)		
	1990	2000	2015	1990	2000	2015	1990	2000	2015	1990	2000	2015
The Gambia	16.37 (7.01-27.94)	17.57 (7.22-29.01)	20.10 (11.35-30.07)	14.46 (14.46-14.46)	22.40 (22.40-22.40)	25.54 (25.54-25.54)	22.53 (20.34-24.67)	26.01 (24.06-27.91)	64.87 (61.78-68.14)	28.32 (14.84-40.00)	30.32 (23.45-37.63)	34.65 (24.83-44.34)
Ghana	16.88 (9.16-25.50)	24.08 (17.89-31.06)	30.61 (25.58-35.60)	26.45 (26.45-26.45)	35.47 (35.47-35.47)	41.09 (41.09-41.09)	16.59 (15.06-18.33)	22.52 (21.33-24.07)	67.11 (64.77-69.02)	24.68 (14.76-34.17)	27.78 (19.92-35.90)	34.47 (20.32-48.26)
Guinea	8.56 (1.39-17.88)	11.23 (4.84-18.36)	17.70 (9.07-27.24)	9.20 (9.20-9.20)	12.87 (12.87-12.87)	20.46 (20.46-20.46)	7.56 (5.57-9.62)	12.81 (11.38-14.56)	47.74 (44.08-51.12)	25.67 (19.47-31.94)	25.22 (19.07-31.37)	27.32 (16.03-38.43)
Guinea-Bissau	10.27 (2.22-20.36)	14.36 (4.93-26.06)	20.19 (9.73-32.68)	18.99 (18.99-18.99)	21.50 (21.50-21.50)	33.26 (33.26-33.26)	13.58 (11.20-16.01)	16.23 (13.84-18.48)	51.79 (47.96-55.64)	24.44 (2.10-43.21)	24.45 (1.12-44.01)	26.29 (0.00-45.52)
Liberia	19.02 (8.64-31.31)	18.34 (9.46-29.14)	33.67 (22.96-45.15)	12.30 (12.30-12.30)	18.31 (18.31-18.31)	27.83 (27.83-27.83)	14.82 (12.54-17.16)	16.00 (14.14-18.02)	57.68 (52.99-62.32)	30.65 (22.65-38.32)	36.10 (29.58-43.16)	38.32 (28.21-49.21)
Mali	8.88 (1.76-17.63)	11.15 (5.51-17.89)	20.46 (11.53-30.69)	6.47 (6.47-6.47)	8.44 (8.44-8.44)	11.87 (11.87-11.87)	7.27 (5.55-9.30)	13.63 (12.17-15.34)	47.20 (43.55-50.71)	30.48 (24.70-36.18)	33.81 (28.01-39.44)	38.55 (26.83-48.48)
Mauritania	5.24 (0.00-13.14)	5.56 (0.78-11.53)	7.87 (0.41-16.83)	27.59 (27.59-27.59)	31.04 (31.04-31.04)	37.57 (37.57-37.57)	8.75 (6.55-11.20)	14.70 (13.01-16.73)	41.02 (36.83-45.00)	27.34 (21.05-34.03)	32.47 (26.08-39.40)	41.53 (29.74-52.66)
Niger	5.35 (0.07-11.83)	15.22 (8.34-23.48)	28.87 (18.37-40.91)	1.91 (1.91-1.91)	2.47 (2.47-2.47)	5.10 (5.10-5.10)	1.21 (0.00-3.01)	6.23 (4.85-8.01)	45.90 (42.93-49.30)	21.53 (15.20-28.35)	24.79 (18.21-31.72)	29.91 (18.49-40.31)
Nigeria	9.62 (3.50-16.42)	19.99 (11.70-30.57)	32.48 (22.16-43.45)	16.91 (16.91-16.91)	20.94 (20.94-20.94)	26.45 (26.45-26.45)	6.72 (5.19-8.69)	8.68 (7.14-10.51)	40.37 (37.57-43.31)	24.70 (13.77-34.79)	29.08 (21.40-36.60)	42.31 (30.54-51.27)
Sao Tome and Principe	15.62 (6.42-26.55)	22.44 (10.79-36.02)	34.27 (20.79-48.34)	23.09 (23.09-23.09)	27.79 (27.79-27.79)	34.66 (34.66-34.66)	22.58 (20.76-24.54)	39.64 (36.42-43.06)	77.89 (71.28-83.64)	28.54 (22.62-34.40)	27.85 (21.32-35.21)	35.34 (22.54-48.70)
Senegal	8.75 (2.95-15.29)	13.45 (7.07-20.69)	32.21 (23.40-40.97)	19.92 (19.92-19.92)	27.43 (27.43-27.43)	36.33 (36.33-36.33)	13.22 (11.66-15.09)	20.75 (19.25-22.47)	68.16 (65.55-70.57)	32.70 (26.48-38.54)	34.81 (28.78-41.23)	35.69 (21.60-49.20)

Location	Appendix Table 1a. Scaled values for each SDG health-related indicator and three summary indicators by country for 1990, 2000, and 2015											
	Indicator 3.7.1: Proportion of women of reproductive age (15 to 49 years) who have their need for family planning satisfied with modern methods			Indicator 3.7.2: Birth rates for women aged 10 to 14 years and women aged 15 to 19 years (number of live births per 1,000 women)			Indicator 3.8.1: Coverage of 7 UHC tracer interventions for prevention and treatment services			Indicator 3.9.1: Age-standardised death rate attributable to household air pollution and ambient air pollution (per 100,000 population)		
	1990	2000	2015	1990	2000	2015	1990	2000	2015	1990	2000	2015
Sierra Leone	11.23 (3.04-21.76)	15.98 (7.14-27.26)	34.83 (23.99-45.28)	11.21 (11.21-11.21)	13.59 (13.59-13.59)	25.33 (25.33-25.33)	13.28 (11.24-15.72)	15.92 (14.07-17.93)	63.51 (60.72-66.13)	26.50 (17.44-35.48)	25.55 (19.01-31.84)	27.96 (16.75-39.09)
Togo	7.37 (0.73-16.19)	11.49 (4.87-19.63)	29.68 (20.08-41.07)	24.11 (24.11-24.11)	30.41 (30.41-30.41)	31.90 (31.90-31.90)	16.18 (14.48-18.16)	20.28 (18.74-21.99)	65.02 (62.37-67.81)	26.78 (19.48-33.20)	24.95 (17.05-32.90)	30.33 (18.47-41.56)
Burundi	18.16 (8.65-29.18)	22.83 (12.40-34.28)	29.12 (17.84-41.79)	41.85 (41.85-41.85)	45.38 (45.38-45.38)	52.85 (52.85-52.85)	15.38 (13.04-17.77)	19.37 (17.21-21.65)	65.19 (61.08-69.04)	11.99 (2.23-23.88)	17.60 (10.23-25.58)	31.13 (18.51-43.59)
Comoros	21.19 (11.97-32.10)	23.39 (13.95-34.92)	24.82 (14.92-35.27)	25.85 (25.85-25.85)	31.61 (31.61-31.61)	40.17 (40.17-40.17)	19.02 (16.93-21.19)	24.96 (23.25-26.75)	59.34 (54.84-64.33)	26.19 (14.12-38.14)	32.85 (26.14-40.06)	42.40 (31.90-52.83)
Djibouti	14.19 (5.55-24.21)	18.83 (9.45-30.61)	27.16 (16.66-39.43)	48.81 (48.81-48.81)	57.59 (57.59-57.59)	67.44 (67.44-67.44)	22.27 (18.90-25.18)	26.96 (24.09-29.76)	61.05 (55.10-66.06)	43.79 (30.87-54.97)	45.30 (30.63-59.75)	46.47 (30.27-60.90)
Eritrea	6.91 (0.80-14.72)	10.07 (3.87-17.41)	15.31 (6.73-26.14)	27.60 (27.60-27.60)	31.79 (31.79-31.79)	44.62 (44.62-44.62)	8.86 (6.69-11.19)	19.62 (18.03-21.54)	46.10 (38.92-52.97)	20.80 (13.78-27.87)	32.13 (20.41-45.92)	31.43 (14.93-46.69)
Ethiopia	14.00 (5.18-24.21)	21.20 (12.64-30.50)	53.77 (44.67-62.15)	22.28 (22.28-22.28)	24.58 (24.58-24.58)	43.11 (43.11-43.11)	4.59 (2.22-6.93)	7.93 (6.35-9.77)	43.53 (38.58-48.30)	18.76 (12.68-25.30)	26.47 (20.13-32.77)	37.72 (25.90-49.05)
Kenya	35.68 (26.35-45.63)	44.50 (36.12-52.57)	71.67 (66.68-76.50)	20.65 (20.65-20.65)	26.79 (26.79-26.79)	30.41 (30.41-30.41)	22.97 (21.59-24.60)	26.59 (25.36-28.10)	71.29 (66.57-75.38)	40.75 (35.83-45.93)	42.59 (37.50-47.64)	46.53 (41.27-52.11)
Madagascar	7.25 (1.33-14.20)	19.79 (12.28-28.77)	37.45 (25.34-50.93)	16.12 (16.12-16.12)	16.00 (16.00-16.00)	25.53 (25.53-25.53)	13.41 (11.97-15.18)	17.16 (15.78-18.81)	39.01 (35.56-42.86)	24.07 (18.20-30.32)	25.37 (19.16-32.41)	28.81 (16.73-41.40)
Malawi	15.30 (7.18-24.33)	34.03 (23.83-44.62)	59.11 (47.33-69.92)	12.71 (12.71-12.71)	13.22 (13.22-13.22)	19.97 (19.97-19.97)	22.47 (20.91-24.16)	27.75 (26.44-29.25)	73.17 (69.77-76.42)	31.15 (23.72-38.66)	28.74 (18.49-38.03)	39.83 (27.79-50.36)
Mozambique	16.46 (7.86-26.53)	26.70 (17.16-36.67)	37.70 (25.72-50.62)	6.96 (6.96-6.96)	9.56 (9.56-9.56)	19.90 (19.90-19.90)	11.78 (9.67-13.94)	19.65 (18.29-21.26)	58.96 (55.83-61.89)	34.43 (28.40-40.83)	34.14 (26.71-41.88)	37.24 (23.46-50.68)

Appendix Table 1a. Scaled values for each SDG health-related indicator and three summary indicators by country for 1990, 2000, and 2015

Location	Indicator 3.7.1: Proportion of women of reproductive age (15 to 49 years) who have their need for family planning satisfied with modern methods			Indicator 3.7.2: Birth rates for women aged 10 to 14 years and women aged 15 to 19 years (number of live births per 1,000 women)			Indicator 3.8.1: Coverage of 7 UHC tracer interventions for prevention and treatment services			Indicator 3.9.1: Age-standardised death rate attributable to household air pollution and ambient air pollution (per 100,000 population)		
	1990	2000	2015	1990	2000	2015	1990	2000	2015	1990	2000	2015
Rwanda	11.77 (4.95-19.95)	11.14 (5.22-18.51)	53.27 (42.59-64.00)	38.37 (38.37-38.37)	44.70 (44.70-44.70)	58.58 (58.58-58.58)	15.02 (13.21-17.05)	17.76 (16.06-19.67)	76.73 (72.87-80.07)	17.70 (10.80-25.24)	19.83 (12.89-27.85)	36.13 (23.93-47.08)
Somalia	9.89 (2.17-19.23)	12.41 (4.26-21.96)	15.32 (6.34-26.15)	31.04 (31.04-31.04)	21.16 (21.16-21.16)	26.93 (26.93-26.93)	0.49 (0.00-2.31)	1.37 (0.16-2.89)	16.04 (11.53-21.17)	22.72 (3.00-42.57)	24.56 (5.44-45.84)	27.73 (6.95-48.31)
South Sudan	13.67 (5.11-23.73)	17.68 (7.42-28.38)	23.86 (13.61-36.42)	18.74 (18.74-18.74)	22.18 (22.18-22.18)	40.78 (40.78-40.78)	10.62 (7.26-14.06)	10.81 (8.02-13.55)	26.79 (22.21-31.57)	26.49 (4.97-45.67)	30.91 (7.63-50.87)	32.18 (7.18-51.97)
Tanzania	18.76 (10.52-27.92)	32.41 (23.58-41.33)	47.92 (35.91-60.23)	17.35 (17.35-17.35)	19.99 (19.99-19.99)	23.66 (23.66-23.66)	17.61 (16.14-19.35)	21.40 (20.06-23.00)	57.40 (53.39-61.09)	30.12 (23.81-36.26)	33.22 (25.97-40.80)	38.73 (24.36-50.94)
Uganda	15.61 (7.73-24.81)	24.84 (18.48-31.81)	40.86 (34.92-47.11)	6.85 (6.85-6.85)	8.66 (8.66-8.66)	23.92 (23.92-23.92)	15.06 (13.40-16.89)	19.97 (18.70-21.48)	69.06 (66.19-71.66)	25.76 (17.95-35.48)	25.10 (17.52-33.95)	32.72 (19.17-46.63)
Zambia	18.17 (10.09-27.72)	31.45 (23.00-41.70)	59.42 (48.72-69.78)	12.23 (12.23-12.23)	17.75 (17.75-17.75)	30.77 (30.77-30.77)	21.33 (19.85-23.01)	25.96 (24.60-27.57)	76.35 (73.83-78.76)	29.35 (22.26-36.20)	23.32 (15.81-30.08)	29.16 (18.34-40.10)
Angola	8.39 (0.84-18.00)	11.53 (3.65-21.96)	16.60 (6.80-27.85)	0.00 (0.00-0.00)	3.16 (3.16-3.16)	12.89 (12.89-12.89)	11.63 (9.45-14.06)	17.83 (16.01-19.80)	59.57 (55.84-63.09)	16.77 (0.00-39.54)	21.44 (1.03-44.14)	30.36 (3.94-49.70)
Central African Republic	4.22 (0.00-9.75)	5.69 (0.00-13.03)	9.20 (1.88-18.29)	16.43 (16.43-16.43)	22.29 (22.29-22.29)	32.79 (32.79-32.79)	9.22 (7.67-11.05)	11.87 (10.40-13.72)	44.84 (40.87-49.29)	12.00 (5.19-19.69)	12.84 (1.08-28.07)	14.61 (0.72-32.76)
Congo	11.95 (3.20-22.38)	15.62 (7.54-25.96)	34.07 (23.58-44.67)	21.28 (21.28-21.28)	20.64 (20.64-20.64)	24.15 (24.15-24.15)	21.03 (18.91-22.94)	24.43 (22.65-26.22)	66.64 (61.79-70.88)	14.15 (6.13-23.06)	16.37 (8.45-24.00)	28.14 (14.94-40.50)
Democratic Republic of the Congo	8.59 (1.12-17.90)	10.08 (2.71-19.26)	13.84 (7.76-21.01)	18.70 (18.70-18.70)	19.85 (19.85-19.85)	22.75 (22.75-22.75)	11.27 (8.90-13.67)	14.78 (12.75-16.95)	56.18 (53.15-59.17)	25.20 (14.81-35.17)	25.41 (17.47-32.30)	26.90 (15.89-37.77)
Equatorial Guinea	8.83 (1.54-18.53)	12.94 (4.42-22.83)	20.82 (10.33-34.24)	19.09 (19.09-19.09)	20.31 (20.31-20.31)	26.68 (26.68-26.68)	17.42 (15.02-19.75)	21.04 (18.83-23.30)	58.50 (51.60-64.65)	13.25 (0.00-38.69)	22.74 (0.92-43.95)	34.62 (7.71-49.79)

	Appendix Table 1a. Scaled values for each SDG health-related indicator and three summary indicators by country for 1990, 2000, and 2015											
	**Indicator 3.7.1: Proportion of women of reproductive age (15 to 49 years) who have their need for family planning satisfied with modern methods**			**Indicator 3.7.2: Birth rates for women aged 10 to 14 years and women aged 15 to 19 years (number of live births per 1,000 women)**			**Indicator 3.8.1: Coverage of 7 UHC tracer interventions for prevention and treatment services**			**Indicator 3.9.1: Age-standardised death rate attributable to household air pollution and ambient air pollution (per 100,000 population)**		
**Location**	**1990**	**2000**	**2015**	**1990**	**2000**	**2015**	**1990**	**2000**	**2015**	**1990**	**2000**	**2015**
Gabon	16.00 (6.65-27.00)	21.74 (14.60-29.32)	41.18 (29.48-51.36)	9.06 (9.06-9.06)	18.61 (18.61-18.61)	32.20 (32.20-32.20)	22.32 (20.49-24.26)	21.02 (19.29-22.78)	61.38 (55.02-67.55)	26.68 (19.66-34.57)	32.99 (26.46-40.46)	49.91 (38.25-59.87)

	Appendix Table 1a. Scaled values for each SDG health-related indicator and three summary indicators by country for 1990, 2000, and 2015											
	**Indicator 3.9.2: Age-standardised death rate attributable to unsafe water, sanitation, and hygiene (WaSH) (per 100,000 population)**			**Indicator 3.9.3: Age-standardised death rate due to unintentional poisonings (per 100,000 population)**			**Indicator 3.a.1: Age-standardised prevalence of daily smoking among populations aged 10 and older**			**Indicator 5.2.1: Prevalence of women aged 15 years and older who experienced intimate partner violence**		
**Location**	**1990**	**2000**	**2015**	**1990**	**2000**	**2015**	**1990**	**2000**	**2015**	**1990**	**2000**	**2015**
Canada	81.62 (78.89-84.32)	85.51 (83.04-87.84)	88.50 (85.62-92.41)	60.97 (57.66-65.63)	67.54 (64.55-71.55)	70.78 (67.18-74.87)	38.04 (34.18-42.01)	53.34 (50.50-56.45)	68.50 (64.54-71.92)	72.06 (65.74-77.47)	72.86 (68.01-77.20)	73.82 (67.72-79.42)
United States	79.47 (77.09-82.03)	80.42 (78.28-82.59)	84.09 (81.95-86.35)	60.20 (56.79-64.45)	61.56 (58.28-65.93)	56.80 (53.24-61.53)	47.34 (44.64-50.14)	56.83 (54.67-59.08)	69.50 (67.93-71.16)	71.86 (65.93-77.49)	71.11 (65.02-76.51)	70.41 (63.68-76.23)
Australia	89.05 (86.62-91.51)	89.14 (86.78-91.50)	95.28 (92.47-98.01)	71.14 (68.03-74.87)	71.54 (68.47-75.36)	74.11 (70.63-78.39)	42.73 (39.58-46.03)	52.77 (49.93-55.54)	66.03 (63.45-68.55)	77.75 (72.54-82.58)	79.08 (75.02-82.74)	77.55 (72.67-82.29)
New Zealand	74.31 (71.66-77.28)	90.04 (87.48-92.56)	95.29 (92.53-97.81)	76.18 (73.45-79.25)	81.34 (79.03-84.09)	81.10 (78.29-85.04)	43.40 (40.42-46.48)	47.51 (44.48-50.67)	63.20 (60.72-65.68)	67.01 (59.27-73.97)	64.11 (56.67-70.70)	67.01 (59.27-73.97)
Brunei	70.16 (62.74-74.68)	74.40 (67.30-78.77)	76.30 (70.89-80.21)	70.39 (66.89-73.69)	75.83 (73.14-78.34)	78.68 (75.41-81.79)	65.40 (61.64-68.74)	69.68 (67.02-72.54)	71.97 (69.08-74.94)	67.36 (60.52-73.77)	67.36 (60.52-73.77)	67.36 (60.52-73.77)
Japan	66.52 (63.86-69.40)	70.52 (67.97-73.25)	75.38 (72.73-78.21)	72.32 (68.95-75.81)	75.58 (72.87-78.83)	76.76 (73.94-80.58)	29.05 (25.59-32.58)	33.30 (29.95-36.98)	57.20 (55.05-59.58)	68.42 (61.92-74.39)	68.28 (61.95-74.44)	68.42 (61.92-74.39)
Singapore	52.86 (49.52-56.25)	57.20 (53.92-60.65)	62.09 (58.90-65.60)	79.56 (76.90-82.43)	93.38 (90.96-95.44)	99.89 (98.95-100.00)	67.00 (63.51-70.15)	72.36 (70.27-74.55)	72.17 (69.18-74.85)	67.36 (60.52-73.77)	67.36 (60.52-73.77)	67.36 (60.52-73.77)
South Korea	70.73 (68.18-73.36)	80.39 (77.71-82.73)	82.80 (80.04-85.31)	29.38 (26.40-33.24)	55.36 (50.68-59.71)	67.85 (63.75-72.11)	22.76 (18.28-27.64)	35.05 (30.60-39.45)	49.02 (45.18-52.86)	67.36 (60.52-73.77)	67.36 (60.52-73.77)	67.36 (60.52-73.77)
Andorra	80.10 (72.92-86.24)	83.74 (77.80-89.77)	88.97 (83.11-94.51)	73.88 (67.10-82.28)	80.31 (73.79-87.40)	84.25 (76.83-89.63)	36.70 (30.53-42.56)	39.22 (33.89-44.50)	48.14 (42.75-53.22)	83.26 (78.96-87.19)	83.26 (78.96-87.19)	83.26 (78.96-87.19)
Austria	85.59 (82.85-88.44)	90.01 (87.25-92.89)	98.65 (96.22-100.00)	70.69 (67.57-74.34)	81.75 (78.49-84.41)	80.40 (77.43-83.69)	36.63 (31.47-41.45)	33.39 (28.46-38.33)	36.54 (31.33-41.81)	92.70 (90.76-94.31)	92.70 (90.76-94.31)	92.80 (90.92-94.35)
Belgium	76.50 (73.90-79.05)	72.59 (69.82-75.25)	78.35 (75.57-81.07)	57.25 (53.84-61.49)	68.96 (64.89-72.84)	75.62 (72.16-79.29)	35.53 (31.14-39.85)	39.74 (35.19-44.53)	54.96 (51.16-58.74)	77.95 (72.50-82.29)	77.95 (72.50-82.29)	78.17 (73.34-82.23)

Location	Appendix Table 1a. Scaled values for each SDG health-related indicator and three summary indicators by country for 1990, 2000, and 2015											
	**Indicator 3.9.2: Age-standardised death rate attributable to unsafe water, sanitation, and hygiene (WaSH) (per 100,000 population)**			**Indicator 3.9.3: Age-standardised death rate due to unintentional poisonings (per 100,000 population)**			**Indicator 3.a.1: Age-standardised prevalence of daily smoking among populations aged 10 and older**			**Indicator 5.2.1: Prevalence of women aged 15 years and older who experienced intimate partner violence**		
	**1990**	**2000**	**2015**	**1990**	**2000**	**2015**	**1990**	**2000**	**2015**	**1990**	**2000**	**2015**
Cyprus	73.66 (70.11-76.71)	81.64 (78.77-84.24)	92.32 (88.31-95.48)	70.87 (67.77-74.37)	74.88 (72.30-77.50)	83.75 (80.64-86.47)	45.17 (39.42-50.84)	37.22 (31.50-42.53)	38.53 (33.49-44.12)	91.87 (89.85-93.68)	91.87 (89.85-93.68)	92.07 (90.01-93.62)
Denmark	77.85 (75.33-80.19)	79.57 (77.12-81.68)	79.59 (76.95-82.40)	41.48 (38.08-48.09)	67.13 (63.95-70.93)	72.98 (69.59-76.85)	13.73 (8.57-18.90)	29.75 (25.36-34.27)	60.04 (56.69-63.36)	66.02 (58.42-72.85)	67.11 (61.07-73.20)	66.07 (59.07-71.50)
Finland	69.87 (67.04-72.64)	76.51 (73.79-79.23)	99.83 (98.10-100.00)	30.65 (27.48-40.86)	66.77 (62.09-70.70)	72.57 (68.19-76.27)	46.57 (43.21-50.09)	49.87 (47.14-52.55)	58.71 (54.68-62.55)	71.14 (64.32-76.91)	70.77 (64.75-75.58)	70.58 (64.72-75.59)
France	76.68 (74.20-79.15)	75.63 (73.17-77.94)	83.33 (80.85-85.68)	61.09 (57.44-65.38)	64.83 (61.34-68.87)	71.18 (67.51-75.03)	25.31 (20.06-30.29)	30.03 (24.83-35.05)	43.89 (38.72-49.12)	88.59 (85.73-90.92)	88.93 (86.38-91.38)	87.46 (84.18-90.02)
Germany	81.91 (79.43-84.64)	83.15 (80.56-85.78)	84.20 (81.68-87.16)	63.48 (60.39-67.70)	88.91 (85.19-91.12)	93.87 (91.77-95.88)	36.80 (32.44-41.12)	39.39 (35.47-43.16)	46.46 (41.42-50.75)	69.95 (62.63-76.15)	67.99 (60.83-73.68)	69.89 (63.45-75.57)
Greece	84.77 (81.96-87.44)	84.01 (81.37-86.93)	93.55 (90.65-96.34)	72.43 (69.16-75.88)	75.55 (72.75-78.88)	83.40 (80.33-86.65)	17.65 (12.38-22.85)	18.77 (13.49-24.24)	22.89 (17.08-28.58)	82.88 (78.54-86.38)	82.88 (78.54-86.38)	83.18 (79.39-86.33)
Iceland	67.29 (64.31-70.67)	76.52 (73.61-79.55)	86.32 (83.62-89.15)	50.33 (46.96-54.98)	63.00 (59.33-67.07)	71.49 (67.47-75.77)	29.74 (24.89-34.57)	46.71 (42.78-50.37)	66.02 (62.38-69.61)	99.81 (99.08-100.00)	99.91 (99.35-100.00)	99.81 (99.08-100.00)
Ireland	67.52 (64.28-70.86)	69.84 (66.85-73.02)	80.00 (76.98-82.89)	69.55 (65.47-73.40)	70.41 (67.20-74.19)	76.57 (73.35-80.11)	34.67 (29.90-39.31)	41.87 (37.31-46.26)	49.02 (43.80-53.81)	82.11 (77.74-85.33)	80.81 (76.81-84.08)	82.54 (77.46-86.55)
Israel	81.19 (78.78-83.46)	82.35 (80.19-84.50)	82.01 (79.33-84.94)	78.12 (74.94-81.06)	77.96 (75.12-80.95)	83.87 (81.16-86.77)	38.72 (32.51-44.33)	52.49 (48.19-56.48)	56.71 (52.24-60.87)	61.78 (53.29-69.02)	60.96 (53.02-67.58)	61.78 (53.29-69.02)
Italy	88.23 (85.57-90.98)	92.29 (89.55-94.96)	96.24 (93.67-98.77)	62.15 (58.90-66.32)	69.22 (66.12-73.02)	80.91 (77.90-84.12)	38.03 (33.54-42.13)	45.21 (41.34-49.04)	51.87 (47.45-56.14)	67.65 (59.78-74.28)	67.65 (59.78-74.28)	65.97 (58.59-72.06)
Luxembourg	80.46 (77.80-83.11)	80.74 (78.06-83.14)	86.90 (84.43-89.44)	62.29 (59.15-66.76)	75.72 (72.73-78.87)	84.90 (81.99-87.69)	37.15 (31.21-42.61)	39.03 (34.30-43.46)	49.37 (43.81-54.34)	77.78 (72.49-82.11)	77.78 (72.49-82.11)	77.15 (72.44-81.09)

Location	Indicator 3.9.2: Age-standardised death rate attributable to unsafe water, sanitation, and hygiene (WaSH) (per 100,000 population)			Indicator 3.9.3: Age-standardised death rate due to unintentional poisonings (per 100,000 population)			Indicator 3.a.1: Age-standardised prevalence of daily smoking among populations aged 10 and older			Indicator 5.2.1: Prevalence of women aged 15 years and older who experienced intimate partner violence		
	1990	2000	2015	1990	2000	2015	1990	2000	2015	1990	2000	2015
Malta	76.79 (73.95-79.63)	73.42 (70.43-76.53)	81.13 (78.39-83.87)	69.37 (66.35-73.47)	80.68 (77.47-83.79)	88.50 (85.87-90.95)	29.31 (22.98-35.52)	47.20 (42.24-51.97)	53.79 (48.90-58.28)	89.58 (86.93-91.84)	89.58 (86.93-91.84)	89.77 (87.34-91.75)
Netherlands	78.01 (75.17-80.89)	73.24 (70.49-76.11)	79.82 (77.12-82.62)	78.04 (75.10-81.01)	84.04 (81.49-86.56)	88.97 (86.35-91.55)	33.23 (29.05-37.49)	38.86 (35.38-42.46)	57.69 (53.93-61.53)	84.72 (81.44-87.61)	84.31 (80.67-87.58)	83.03 (79.28-86.22)
Norway	68.86 (65.98-71.98)	75.50 (72.92-78.11)	80.57 (77.73-83.87)	58.70 (55.33-62.90)	72.77 (68.74-76.07)	63.30 (59.69-67.87)	29.89 (24.18-35.24)	43.07 (38.13-47.90)	64.84 (61.15-68.37)	82.40 (77.94-85.57)	79.09 (75.45-82.95)	82.81 (78.73-86.41)
Portugal	68.30 (65.30-71.34)	68.43 (65.49-71.69)	73.35 (70.44-76.31)	61.57 (57.95-65.42)	71.26 (68.02-74.80)	82.39 (79.16-85.16)	51.64 (48.11-54.94)	54.29 (50.78-57.81)	55.32 (50.96-59.50)	82.27 (78.01-85.87)	82.27 (78.01-85.87)	81.87 (78.10-85.03)
Spain	77.63 (74.91-80.37)	80.56 (78.17-83.07)	87.37 (84.95-89.78)	61.94 (58.49-66.09)	75.43 (72.00-78.69)	82.71 (79.41-85.61)	23.81 (18.62-28.92)	31.56 (27.20-36.05)	47.12 (42.09-51.64)	91.68 (89.48-93.53)	91.28 (88.84-93.22)	91.02 (88.72-92.79)
Sweden	76.10 (73.13-78.79)	82.00 (79.60-84.28)	87.10 (84.51-89.80)	59.46 (56.33-64.15)	67.87 (64.87-71.89)	71.02 (67.70-75.18)	46.02 (41.55-50.26)	60.85 (58.00-63.48)	74.98 (73.12-76.65)	81.17 (76.88-85.13)	81.99 (77.49-85.71)	79.98 (75.34-84.12)
Switzerland	78.57 (75.85-81.08)	83.39 (80.76-86.15)	91.60 (89.23-93.99)	46.76 (42.95-57.56)	84.35 (80.98-87.04)	89.13 (86.62-91.45)	35.45 (31.78-39.38)	32.98 (28.58-37.19)	54.16 (49.53-59.02)	96.20 (95.01-97.22)	96.28 (94.91-97.58)	96.20 (95.01-97.22)
United Kingdom	67.72 (65.11-70.70)	67.88 (65.27-70.71)	77.45 (75.14-79.92)	60.35 (57.28-64.80)	67.05 (64.25-70.74)	75.48 (72.59-78.82)	34.82 (29.43-40.13)	42.13 (38.22-46.00)	54.69 (50.92-58.69)	75.01 (69.50-80.15)	71.84 (65.00-77.62)	79.88 (75.69-83.59)
Argentina	60.32 (57.11-62.57)	60.23 (57.75-62.82)	61.96 (59.47-64.61)	52.11 (48.78-56.54)	57.82 (54.13-62.15)	62.09 (58.20-66.44)	44.41 (37.79-50.04)	48.44 (42.98-53.56)	57.70 (53.17-62.02)	83.50 (79.51-86.81)	83.34 (79.43-86.88)	83.50 (79.51-86.81)
Chile	51.36 (48.75-54.06)	62.60 (60.12-65.13)	71.89 (69.09-74.78)	49.58 (46.18-54.44)	70.19 (66.89-74.12)	79.51 (76.05-82.72)	48.11 (43.25-52.84)	34.14 (29.07-39.04)	39.33 (33.54-44.94)	70.90 (64.34-77.01)	70.54 (64.02-75.96)	70.90 (64.34-77.01)
Uruguay	61.65 (58.81-64.49)	64.16 (61.54-66.65)	67.32 (64.59-70.03)	48.39 (45.13-52.84)	61.06 (57.59-65.30)	67.02 (63.46-71.26)	31.90 (25.50-38.23)	38.93 (33.74-43.88)	54.59 (49.67-59.33)	83.84 (80.19-87.26)	83.89 (80.21-87.21)	83.84 (80.19-87.26)

Appendix Table 1a. Scaled values for each SDG health-related indicator and three summary indicators by country for 1990, 2000, and 2015

Appendix Table 1a. Scaled values for each SDG health-related indicator and three summary indicators by country for 1990, 2000, and 2015

Location	Indicator 3.9.2: Age-standardised death rate attributable to unsafe water, sanitation, and hygiene (WaSH) (per 100,000 population)			Indicator 3.9.3: Age-standardised death rate due to unintentional poisonings (per 100,000 population)			Indicator 3.a.1: Age-standardised prevalence of daily smoking among populations aged 10 and older			Indicator 5.2.1: Prevalence of women aged 15 years and older who experienced intimate partner violence		
	1990	2000	2015	1990	2000	2015	1990	2000	2015	1990	2000	2015
Belarus	76.65 (73.81-79.42)	78.88 (76.22-81.57)	92.85 (88.67-97.06)	23.79 (20.25-31.61)	20.35 (16.79-28.67)	39.08 (26.18-43.75)	26.28 (20.30-32.16)	33.06 (27.66-38.59)	34.82 (29.36-39.94)	75.47 (70.44-80.16)	75.47 (70.44-80.16)	75.47 (70.44-80.16)
Estonia	79.92 (77.27-82.33)	77.92 (75.08-80.72)	94.97 (91.16-98.54)	28.41 (25.35-33.17)	44.28 (40.30-48.92)	66.01 (60.00-70.92)	34.08 (29.22-38.82)	36.42 (32.66-40.25)	47.05 (42.48-51.66)	84.05 (80.81-87.13)	84.05 (80.81-87.13)	83.91 (81.20-86.73)
Latvia	80.48 (77.90-82.74)	79.55 (76.91-82.22)	89.90 (86.60-93.27)	32.14 (28.60-36.56)	37.69 (33.59-42.62)	55.73 (49.27-60.94)	38.68 (32.68-44.21)	31.88 (28.15-35.99)	35.61 (30.65-40.49)	77.07 (72.77-81.31)	77.07 (72.77-81.31)	75.91 (71.27-79.92)
Lithuania	85.08 (82.33-87.48)	83.55 (80.84-86.23)	87.84 (84.67-90.71)	28.39 (25.29-32.89)	42.69 (38.36-47.67)	58.89 (50.66-63.66)	41.74 (36.37-46.72)	43.78 (40.06-47.32)	44.95 (39.94-49.93)	82.54 (78.97-85.83)	82.54 (78.97-85.83)	81.71 (78.44-84.59)
Moldova	64.44 (61.33-67.50)	62.95 (59.80-66.00)	79.16 (75.71-82.53)	25.83 (22.13-30.00)	27.30 (24.22-31.31)	52.72 (44.07-57.93)	48.40 (43.55-53.08)	53.89 (49.62-58.13)	56.38 (52.76-59.61)	67.92 (62.02-74.03)	67.57 (61.89-73.34)	67.92 (62.02-74.03)
Russia	72.68 (70.31-75.13)	68.59 (65.99-71.07)	78.77 (75.76-81.86)	35.72 (29.78-40.20)	37.95 (29.19-42.43)	47.35 (41.25-52.29)	41.35 (37.30-45.52)	36.11 (32.28-39.70)	41.41 (36.59-45.70)	64.31 (57.36-71.00)	64.02 (57.39-70.57)	64.31 (57.36-71.00)
Ukraine	76.67 (73.83-79.49)	74.87 (72.05-77.56)	88.53 (84.86-92.07)	22.22 (18.71-26.06)	17.34 (14.95-20.55)	42.93 (34.46-48.28)	29.60 (24.18-35.18)	32.82 (28.46-37.17)	39.54 (34.12-44.96)	72.73 (67.43-77.91)	71.16 (65.80-76.34)	72.73 (67.43-77.91)
Albania	52.10 (47.82-58.58)	63.81 (60.48-68.47)	81.45 (76.61-85.89)	71.89 (62.27-75.63)	60.33 (56.69-67.36)	77.07 (72.99-80.21)	57.46 (53.17-61.72)	61.11 (57.80-64.43)	63.88 (59.76-67.68)	87.04 (84.30-89.50)	87.88 (85.52-90.18)	87.04 (84.30-89.50)
Bosnia and Herzegovina	71.11 (67.83-74.29)	81.06 (77.02-85.44)	92.55 (85.70-97.70)	9.05 (5.75-17.42)	20.30 (15.00-24.93)	55.42 (25.12-63.44)	37.15 (30.53-43.48)	31.73 (26.88-36.04)	31.00 (24.80-37.39)	83.35 (78.92-87.51)	83.35 (78.92-87.51)	83.35 (78.92-87.51)
Bulgaria	63.04 (60.37-65.92)	69.74 (67.03-72.46)	80.62 (77.49-83.64)	51.19 (47.90-55.78)	52.82 (49.32-57.47)	66.99 (62.75-71.09)	21.55 (12.96-29.30)	15.86 (8.24-22.47)	23.22 (16.50-29.92)	84.96 (81.91-87.99)	84.96 (81.91-87.99)	84.08 (80.90-86.76)
Croatia	73.39 (70.84-75.86)	72.85 (70.04-75.65)	87.89 (84.76-90.88)	57.18 (53.80-61.61)	75.91 (71.93-79.15)	83.61 (80.33-86.46)	22.64 (15.85-29.65)	36.77 (31.57-41.62)	32.14 (25.18-38.51)	90.42 (88.16-92.68)	90.42 (88.16-92.68)	91.02 (88.69-93.06)

	Appendix Table 1a. Scaled values for each SDG health-related indicator and three summary indicators by country for 1990, 2000, and 2015											
	**Indicator 3.9.2: Age-standardised death rate attributable to unsafe water, sanitation, and hygiene (WaSH) (per 100,000 population)**			**Indicator 3.9.3: Age-standardised death rate due to unintentional poisonings (per 100,000 population)**			**Indicator 3.a.1: Age-standardised prevalence of daily smoking among populations aged 10 and older**			**Indicator 5.2.1: Prevalence of women aged 15 years and older who experienced intimate partner violence**		
**Location**	**1990**	**2000**	**2015**	**1990**	**2000**	**2015**	**1990**	**2000**	**2015**	**1990**	**2000**	**2015**
Czech Republic	74.81 (71.98-77.76)	76.01 (73.04-79.32)	77.47 (74.16-80.99)	45.12 (41.97-49.67)	70.05 (66.31-73.94)	75.62 (72.04-79.49)	33.69 (27.18-40.06)	40.32 (35.99-44.75)	42.16 (36.26-47.45)	59.60 (50.73-67.87)	55.93 (48.59-63.55)	59.62 (50.75-68.04)
Hungary	83.04 (80.37-85.60)	87.62 (84.95-90.08)	89.96 (86.31-95.78)	53.47 (50.08-58.08)	75.14 (69.99-78.44)	82.37 (78.70-85.83)	27.99 (20.88-34.61)	29.51 (23.28-35.22)	39.68 (33.65-45.94)	91.16 (89.18-93.09)	91.48 (89.78-93.35)	90.40 (88.20-92.43)
Macedonia	54.75 (51.11-58.67)	73.88 (70.43-77.13)	89.43 (84.48-93.77)	69.73 (66.18-72.47)	70.08 (67.27-72.98)	72.91 (68.14-79.79)	38.97 (32.57-45.29)	36.05 (29.67-42.35)	28.33 (21.33-34.42)	83.35 (78.92-87.51)	83.35 (78.92-87.51)	83.35 (78.92-87.51)
Montenegro	80.98 (76.79-85.50)	80.25 (77.05-83.44)	93.61 (89.51-97.59)	53.44 (45.89-59.88)	53.93 (50.55-58.09)	63.76 (58.99-67.59)	47.65 (42.05-53.05)	35.83 (29.36-42.01)	28.24 (22.25-34.39)	83.35 (78.92-87.51)	83.35 (78.92-87.51)	83.35 (78.92-87.51)
Poland	72.52 (69.97-75.16)	74.98 (71.91-78.13)	79.21 (75.88-82.60)	38.76 (35.66-44.16)	66.36 (61.96-70.57)	80.35 (73.83-83.66)	22.18 (17.53-27.14)	36.30 (30.75-41.45)	45.02 (39.76-49.98)	87.97 (85.48-90.25)	87.98 (85.80-90.08)	89.02 (86.03-91.73)
Romania	58.70 (56.20-61.51)	62.25 (59.65-65.02)	76.26 (73.28-79.44)	35.60 (32.32-40.24)	50.29 (45.57-55.30)	66.32 (59.82-70.78)	45.83 (40.30-51.16)	39.32 (34.83-44.11)	46.14 (40.98-50.93)	54.90 (46.15-63.37)	55.16 (48.57-62.70)	57.07 (48.13-66.11)
Serbia	77.75 (73.50-82.12)	83.39 (78.89-86.52)	88.43 (85.16-91.70)	60.94 (56.96-67.01)	64.02 (61.14-68.10)	73.09 (69.57-76.01)	44.69 (37.96-50.97)	35.45 (30.04-40.76)	42.98 (36.53-49.14)	81.76 (78.16-85.48)	81.44 (78.08-84.60)	81.76 (78.16-85.48)
Slovakia	60.13 (56.10-65.84)	66.01 (62.66-69.73)	73.96 (70.48-77.87)	54.60 (50.97-61.33)	62.45 (59.25-65.53)	75.48 (69.81-78.37)	37.87 (31.05-44.07)	48.59 (43.14-54.06)	51.69 (46.21-56.49)	83.01 (79.57-86.48)	83.01 (79.57-86.48)	82.63 (79.54-85.52)
Slovenia	70.89 (67.94-73.88)	69.26 (66.29-72.76)	79.57 (75.66-83.19)	62.39 (59.18-66.82)	65.96 (62.87-69.92)	79.57 (75.34-83.49)	32.44 (24.88-39.50)	48.15 (42.40-53.45)	50.11 (44.74-54.79)	92.69 (91.09-94.34)	92.69 (91.09-94.34)	92.10 (90.42-93.56)
Armenia	49.57 (46.33-52.95)	60.23 (57.15-63.42)	79.92 (76.58-84.07)	37.86 (33.63-49.02)	47.36 (43.53-51.55)	61.23 (51.29-65.61)	49.63 (44.12-54.87)	49.80 (46.04-53.45)	50.69 (46.09-55.20)	80.16 (75.10-84.74)	80.16 (75.10-84.74)	80.16 (75.10-84.74)
Azerbaijan	40.13 (36.95-43.36)	45.10 (42.12-48.34)	67.34 (63.09-71.11)	42.05 (38.23-48.80)	50.52 (42.83-53.62)	61.99 (54.56-66.11)	62.29 (57.44-66.99)	61.39 (56.96-65.87)	51.46 (46.68-56.29)	78.46 (73.72-82.86)	75.79 (70.79-80.55)	78.46 (73.72-82.86)

Location	Appendix Table 1a. Scaled values for each SDG health-related indicator and three summary indicators by country for 1990, 2000, and 2015											
	Indicator 3.9.2: Age-standardised death rate attributable to unsafe water, sanitation, and hygiene (WaSH) (per 100,000 population)			Indicator 3.9.3: Age-standardised death rate due to unintentional poisonings (per 100,000 population)			Indicator 3.a.1: Age-standardised prevalence of daily smoking among populations aged 10 and older			Indicator 5.2.1: Prevalence of women aged 15 years and older who experienced intimate partner violence		
	**1990**	**2000**	**2015**	**1990**	**2000**	**2015**	**1990**	**2000**	**2015**	**1990**	**2000**	**2015**
Georgia	57.72 (54.95-60.59)	63.94 (60.93-67.30)	82.19 (79.21-85.23)	42.86 (38.63-51.99)	56.30 (47.06-59.90)	57.14 (50.30-60.94)	56.16 (51.01-61.15)	52.65 (48.11-57.01)	50.55 (45.53-55.52)	95.03 (93.63-96.17)	94.71 (93.25-96.22)	95.03 (93.63-96.17)
Kazakhstan	53.05 (50.30-56.19)	59.44 (56.69-62.35)	78.41 (75.47-81.19)	19.42 (12.08-22.94)	0.21 (0.00-3.17)	37.56 (26.34-42.59)	52.49 (47.14-57.41)	57.02 (52.74-61.09)	53.42 (49.49-57.20)	85.76 (82.44-88.57)	85.04 (81.75-88.00)	86.63 (83.97-89.16)
Kyrgyzstan	44.30 (41.67-47.22)	50.04 (47.45-52.71)	65.84 (62.68-68.68)	29.31 (25.03-42.36)	37.08 (32.70-41.12)	53.59 (39.77-58.12)	62.58 (57.72-66.57)	62.69 (59.28-66.04)	58.99 (54.47-62.90)	55.42 (46.56-64.29)	55.42 (46.56-64.29)	54.02 (45.53-62.07)
Mongolia	46.37 (42.78-50.17)	55.10 (51.77-58.64)	75.09 (70.10-79.27)	14.37 (8.35-30.26)	18.44 (13.59-29.61)	39.72 (27.51-44.56)	48.34 (42.98-53.78)	46.66 (42.65-50.80)	50.85 (45.66-56.12)	80.16 (75.10-84.74)	80.16 (75.10-84.74)	80.16 (75.10-84.74)
Tajikistan	29.74 (26.49-33.74)	33.54 (30.48-36.95)	50.13 (45.96-54.07)	37.27 (33.80-43.31)	38.56 (33.92-42.09)	47.17 (42.15-51.12)	52.12 (46.82-56.86)	71.08 (66.75-75.15)	77.03 (73.12-80.74)	72.06 (66.60-77.51)	72.06 (66.60-77.51)	71.26 (66.51-76.12)
Turkmenistan	34.84 (31.51-38.71)	37.14 (32.68-41.55)	61.30 (56.36-66.41)	42.08 (38.50-46.61)	41.38 (37.35-46.50)	58.55 (48.36-62.67)	78.86 (75.06-82.24)	82.91 (79.62-85.67)	84.78 (81.88-87.26)	80.16 (75.10-84.74)	80.16 (75.10-84.74)	80.16 (75.10-84.74)
Uzbekistan	47.02 (44.07-49.96)	55.20 (52.18-58.21)	68.77 (65.08-72.33)	43.06 (39.38-47.35)	44.58 (39.03-47.53)	52.16 (45.64-56.30)	79.78 (76.32-83.13)	77.23 (73.96-80.20)	80.79 (78.07-83.38)	80.16 (75.10-84.74)	80.16 (75.10-84.74)	80.16 (75.10-84.74)
Colombia	46.33 (43.63-49.33)	55.07 (52.22-57.64)	66.50 (63.31-69.00)	52.05 (48.67-56.68)	72.72 (68.42-76.31)	83.11 (79.32-85.87)	61.66 (54.49-68.23)	66.04 (59.88-71.59)	76.85 (72.43-80.72)	51.40 (41.84-60.82)	50.72 (43.36-58.84)	52.83 (44.96-60.95)
Costa Rica	55.71 (53.22-58.45)	61.34 (58.75-63.82)	70.96 (67.90-73.79)	59.48 (55.98-64.28)	69.29 (66.19-73.04)	75.05 (71.53-78.25)	73.87 (69.30-78.21)	75.28 (71.24-78.90)	83.07 (79.34-86.28)	64.42 (57.50-71.64)	62.91 (55.92-69.73)	64.42 (57.50-71.64)
El Salvador	30.10 (27.01-33.63)	42.39 (39.41-45.77)	55.80 (52.48-59.63)	22.39 (16.02-47.01)	62.42 (54.91-65.56)	78.03 (60.01-83.71)	83.79 (80.18-87.06)	85.06 (81.55-88.05)	86.21 (82.92-89.17)	76.46 (71.63-81.11)	74.44 (69.16-79.20)	76.46 (71.63-81.11)
Guatemala	16.05 (12.60-20.50)	25.05 (21.49-28.76)	36.52 (31.67-40.39)	41.90 (36.60-46.84)	50.83 (47.37-55.73)	50.31 (46.37-55.78)	83.02 (79.19-86.40)	85.23 (82.12-87.86)	82.99 (79.15-86.27)	73.06 (67.46-78.42)	70.04 (65.52-74.47)	73.06 (67.46-78.42)

Appendix Table 1a. Scaled values for each SDG health-related indicator and three summary indicators by country for 1990, 2000, and 2015

Location	Indicator 3.9.2: Age-standardised death rate attributable to unsafe water, sanitation, and hygiene (WaSH) (per 100,000 population)			Indicator 3.9.3: Age-standardised death rate due to unintentional poisonings (per 100,000 population)			Indicator 3.a.1: Age-standardised prevalence of daily smoking among populations aged 10 and older			Indicator 5.2.1: Prevalence of women aged 15 years and older who experienced intimate partner violence		
	1990	2000	2015	1990	2000	2015	1990	2000	2015	1990	2000	2015
Honduras	29.86 (26.51-33.67)	34.25 (30.41-38.07)	43.98 (39.71-48.33)	50.42 (46.55-56.57)	49.60 (43.38-62.09)	53.59 (46.64-66.46)	70.93 (65.10-76.13)	73.42 (68.95-77.42)	79.48 (75.98-83.12)	76.28 (71.36-81.21)	77.72 (74.16-81.68)	72.58 (67.79-77.38)
Mexico	34.87 (32.04-38.51)	52.05 (48.92-54.74)	60.73 (57.70-63.11)	48.04 (44.62-52.51)	60.64 (56.95-65.09)	68.21 (64.45-72.21)	54.34 (51.35-57.12)	70.39 (68.59-72.14)	77.51 (76.05-79.05)	61.52 (55.58-67.85)	60.00 (52.62-66.58)	63.93 (58.96-69.65)
Nicaragua	30.21 (27.18-34.02)	45.27 (42.20-48.56)	61.60 (58.50-64.73)	54.34 (50.60-61.02)	62.09 (59.10-65.66)	69.39 (65.82-72.44)	74.79 (69.28-79.23)	78.67 (74.33-82.60)	79.85 (75.65-83.96)	74.14 (69.19-79.59)	73.50 (68.81-77.58)	74.14 (69.19-79.59)
Panama	49.83 (46.74-52.83)	56.12 (53.08-58.84)	57.99 (54.98-60.96)	56.07 (52.69-60.37)	61.13 (57.28-63.91)	65.19 (61.02-68.88)	80.85 (76.89-84.31)	83.07 (80.18-85.76)	93.18 (91.76-94.42)	70.05 (63.80-76.30)	70.05 (63.80-76.30)	70.05 (63.80-76.30)
Venezuela	44.29 (41.55-47.15)	49.94 (47.20-53.17)	58.23 (55.30-61.25)	55.26 (51.97-59.84)	68.50 (64.44-72.34)	73.24 (68.91-77.13)	63.44 (56.83-69.70)	66.06 (59.85-71.52)	68.99 (63.35-73.88)	70.05 (63.80-76.30)	70.05 (63.80-76.30)	70.05 (63.80-76.30)
Bolivia	23.97 (20.39-28.02)	32.92 (29.72-36.66)	46.27 (42.53-50.13)	23.49 (11.42-30.93)	33.78 (27.28-37.50)	39.12 (29.65-45.24)	44.76 (37.98-51.56)	40.30 (34.29-46.36)	51.40 (44.07-57.94)	58.16 (49.36-66.74)	53.78 (47.41-60.04)	58.16 (49.36-66.74)
Ecuador	34.82 (32.04-37.99)	44.29 (41.68-47.05)	55.55 (52.70-58.31)	40.40 (36.46-50.55)	53.77 (48.70-56.57)	62.93 (55.45-66.48)	78.88 (74.66-82.55)	82.15 (79.97-83.98)	88.71 (86.54-90.44)	59.64 (51.66-67.54)	59.45 (53.96-66.26)	59.64 (51.66-67.54)
Peru	32.70 (29.84-35.96)	43.36 (40.66-46.43)	51.44 (47.64-55.97)	48.82 (44.87-51.93)	53.46 (50.13-58.56)	62.34 (58.37-67.54)	75.98 (70.54-80.63)	76.91 (72.25-80.89)	82.05 (78.61-85.13)	79.45 (75.10-83.65)	72.29 (68.09-76.89)	84.04 (80.94-87.36)
Antigua and Barbuda	51.96 (49.18-54.74)	55.45 (52.90-58.10)	60.46 (57.38-63.70)	52.00 (48.26-56.63)	60.09 (56.15-64.60)	65.56 (61.76-69.76)	95.36 (93.81-96.70)	95.12 (93.61-96.52)	93.88 (91.99-95.39)	73.01 (67.08-78.76)	73.01 (67.08-78.76)	73.01 (67.08-78.76)
The Bahamas	52.01 (49.03-55.08)	58.57 (55.33-61.71)	63.15 (59.63-66.78)	51.43 (47.71-59.16)	62.30 (58.69-65.19)	68.92 (64.71-72.55)	93.71 (91.69-95.32)	93.63 (91.73-95.27)	87.41 (84.27-90.10)	73.01 (67.08-78.76)	73.01 (67.08-78.76)	73.01 (67.08-78.76)
Barbados	58.39 (55.28-61.19)	58.94 (55.87-61.98)	61.02 (57.51-64.42)	58.95 (55.66-63.53)	70.34 (66.71-73.87)	74.61 (70.61-78.00)	92.48 (90.24-94.31)	91.70 (89.30-93.70)	90.92 (88.30-93.19)	73.01 (67.08-78.76)	73.01 (67.08-78.76)	73.01 (67.08-78.76)

Location	Appendix Table 1a. Scaled values for each SDG health-related indicator and three summary indicators by country for 1990, 2000, and 2015											
	**Indicator 3.9.2: Age-standardised death rate attributable to unsafe water, sanitation, and hygiene (WaSH) (per 100,000 population)**			**Indicator 3.9.3: Age-standardised death rate due to unintentional poisonings (per 100,000 population)**			**Indicator 3.a.1: Age-standardised prevalence of daily smoking among populations aged 10 and older**			**Indicator 5.2.1: Prevalence of women aged 15 years and older who experienced intimate partner violence**		
	**1990**	**2000**	**2015**	**1990**	**2000**	**2015**	**1990**	**2000**	**2015**	**1990**	**2000**	**2015**
Belize	42.71 (39.77-45.91)	45.44 (42.64-48.32)	51.43 (48.31-54.60)	45.65 (42.27-51.95)	51.25 (45.94-54.33)	58.57 (52.05-62.40)	76.90 (72.05-81.23)	79.93 (75.75-83.63)	82.79 (79.16-86.15)	88.77 (86.05-91.19)	87.76 (84.86-90.19)	88.77 (86.05-91.19)
Cuba	54.18 (51.71-56.82)	55.16 (52.59-57.83)	60.86 (58.17-63.55)	48.73 (45.39-53.20)	61.88 (57.92-66.22)	77.55 (73.23-81.07)	37.74 (29.07-46.06)	40.01 (32.58-47.51)	64.12 (57.80-69.69)	73.01 (67.08-78.76)	73.01 (67.08-78.76)	73.01 (67.08-78.76)
Dominica	48.66 (45.62-51.82)	52.88 (49.94-55.70)	55.86 (52.56-59.17)	39.50 (35.89-46.13)	48.22 (41.97-51.31)	51.34 (41.77-56.29)	90.44 (87.91-92.77)	91.15 (88.42-93.38)	92.33 (89.94-94.37)	73.01 (67.08-78.76)	73.01 (67.08-78.76)	73.01 (67.08-78.76)
Dominican Republic	36.40 (33.21-39.90)	48.50 (45.63-51.60)	58.47 (55.51-61.28)	54.58 (51.07-60.43)	67.82 (62.31-70.81)	74.15 (65.57-78.02)	69.18 (63.90-74.40)	72.97 (69.58-76.12)	84.61 (81.23-87.47)	74.07 (67.94-79.35)	72.60 (67.88-76.97)	71.85 (67.96-75.92)
Grenada	46.30 (42.98-49.63)	50.26 (47.01-53.59)	53.70 (50.21-57.24)	46.32 (42.61-53.40)	53.42 (49.33-56.31)	58.67 (52.52-62.50)	86.54 (82.86-89.60)	85.11 (81.55-88.15)	85.67 (82.31-88.60)	73.01 (67.08-78.76)	73.01 (67.08-78.76)	73.01 (67.08-78.76)
Guyana	33.73 (30.86-37.00)	37.25 (34.35-40.58)	46.06 (43.04-49.19)	45.56 (42.04-51.98)	51.63 (47.83-54.52)	58.83 (53.78-62.54)	82.95 (79.31-86.63)	79.15 (74.68-82.93)	79.61 (75.51-83.45)	73.01 (67.08-78.76)	73.01 (67.08-78.76)	73.01 (67.08-78.76)
Haiti	16.69 (12.78-20.76)	25.42 (21.73-29.50)	33.29 (28.76-37.94)	27.49 (12.07-35.99)	38.30 (25.40-46.85)	45.52 (34.01-53.62)	77.16 (72.60-81.25)	79.70 (76.22-82.74)	87.94 (85.39-90.27)	69.96 (63.65-76.51)	69.82 (65.35-74.79)	71.17 (65.54-76.45)
Jamaica	49.28 (45.97-52.80)	60.36 (57.75-62.99)	65.82 (62.58-69.00)	65.84 (60.82-68.72)	63.72 (59.78-66.61)	66.03 (61.63-69.58)	72.53 (67.25-77.47)	68.48 (63.88-72.66)	78.40 (73.96-82.36)	58.50 (49.11-66.78)	60.14 (52.38-67.79)	58.50 (49.11-66.78)
Saint Lucia	46.87 (43.04-50.36)	52.89 (50.22-55.79)	58.58 (55.52-61.49)	58.29 (54.55-63.11)	68.77 (65.35-72.71)	74.28 (70.26-77.87)	84.80 (81.02-88.33)	82.57 (78.07-86.37)	82.28 (77.97-85.81)	73.01 (67.08-78.76)	73.01 (67.08-78.76)	73.01 (67.08-78.76)
Saint Vincent and the Grenadines	44.31 (40.60-48.22)	48.76 (45.80-51.84)	55.27 (52.16-58.14)	55.73 (51.92-60.49)	64.40 (60.45-68.60)	69.03 (65.34-72.75)	79.39 (75.00-83.71)	82.58 (78.85-86.19)	86.12 (82.72-89.06)	73.01 (67.08-78.76)	73.01 (67.08-78.76)	73.01 (67.08-78.76)
Suriname	40.00 (37.03-43.27)	45.48 (42.65-48.57)	54.10 (51.07-57.09)	48.84 (45.05-55.49)	56.68 (51.31-59.61)	63.36 (56.33-67.24)	68.44 (62.64-74.01)	67.31 (60.97-72.71)	58.69 (54.01-63.59)	73.01 (67.08-78.76)	73.01 (67.08-78.76)	73.01 (67.08-78.76)

	Appendix Table 1a. Scaled values for each SDG health-related indicator and three summary indicators by country for 1990, 2000, and 2015											
	**Indicator 3.9.2: Age-standardised death rate attributable to unsafe water, sanitation, and hygiene (WaSH) (per 100,000 population)**			**Indicator 3.9.3: Age-standardised death rate due to unintentional poisonings (per 100,000 population)**			**Indicator 3.a.1: Age-standardised prevalence of daily smoking among populations aged 10 and older**			**Indicator 5.2.1: Prevalence of women aged 15 years and older who experienced intimate partner violence**		
**Location**	**1990**	**2000**	**2015**	**1990**	**2000**	**2015**	**1990**	**2000**	**2015**	**1990**	**2000**	**2015**
Trinidad and Tobago	51.71 (49.16-54.32)	57.05 (54.53-59.53)	64.20 (60.91-67.16)	55.26 (51.75-59.99)	65.46 (62.01-69.56)	70.81 (67.22-74.84)	66.57 (60.43-71.97)	69.74 (64.67-74.10)	68.19 (63.50-72.84)	73.01 (67.08-78.76)	73.01 (67.08-78.76)	73.01 (67.08-78.76)
Brazil	35.00 (32.41-38.35)	45.82 (43.51-48.48)	55.80 (53.06-59.38)	70.26 (67.68-74.16)	75.15 (72.86-77.63)	82.95 (79.97-85.07)	43.33 (38.69-47.86)	60.86 (57.72-64.10)	76.17 (74.39-78.03)	93.66 (90.95-96.00)	93.83 (90.85-96.35)	92.95 (90.89-94.72)
Paraguay	42.66 (39.63-45.93)	46.40 (43.60-49.63)	56.19 (52.97-59.31)	60.67 (56.46-65.04)	68.61 (62.75-72.31)	75.71 (64.70-81.71)	64.75 (57.65-71.13)	68.31 (63.68-72.45)	76.76 (72.27-81.05)	91.52 (89.05-93.90)	89.39 (87.31-91.47)	88.82 (86.42-91.09)
China	47.91 (45.40-50.52)	57.56 (55.24-59.75)	74.51 (71.87-76.85)	33.76 (29.63-39.17)	39.97 (36.62-45.19)	50.12 (45.92-57.91)	35.39 (32.09-38.69)	43.29 (40.46-46.31)	51.64 (49.09-54.23)	96.68 (95.30-97.90)	96.97 (95.79-98.11)	95.84 (94.26-97.13)
North Korea	54.47 (45.41-62.25)	59.12 (51.73-65.34)	68.70 (63.42-73.70)	41.18 (29.72-50.73)	41.89 (34.11-51.56)	45.19 (38.51-55.74)	50.42 (45.53-55.37)	50.55 (46.02-54.72)	56.71 (52.08-61.05)	96.46 (95.34-97.59)	96.46 (95.34-97.59)	96.46 (95.34-97.59)
Taiwan	63.79 (60.79-67.14)	69.00 (65.70-73.37)	70.73 (65.11-80.63)	57.78 (51.43-63.47)	64.25 (58.42-68.48)	71.62 (66.93-75.53)	45.22 (38.84-51.01)	50.03 (46.29-53.55)	74.21 (70.60-77.50)	96.46 (95.34-97.59)	96.46 (95.34-97.59)	96.46 (95.34-97.59)
Cambodia	17.44 (13.79-21.81)	22.45 (18.77-26.55)	39.21 (35.64-43.09)	43.00 (29.00-50.07)	47.14 (37.02-52.38)	60.02 (52.70-65.35)	44.26 (38.64-49.68)	44.93 (41.06-48.88)	58.32 (54.44-61.91)	83.84 (80.54-87.20)	83.82 (80.80-86.86)	83.59 (80.23-86.56)
Indonesia	18.66 (14.43-23.87)	24.10 (19.74-28.98)	32.07 (27.85-36.55)	60.17 (51.67-72.49)	64.11 (57.95-77.15)	69.38 (62.80-82.41)	44.99 (39.94-49.97)	39.38 (35.32-43.41)	39.39 (34.05-44.26)	73.00 (66.98-78.19)	73.00 (66.98-78.19)	73.00 (66.98-78.19)
Laos	13.82 (9.10-19.13)	21.24 (16.85-26.10)	36.16 (32.03-40.08)	37.01 (19.55-46.01)	42.10 (29.66-48.95)	55.18 (45.99-61.52)	28.52 (21.41-35.52)	28.73 (22.61-34.61)	33.77 (26.25-41.22)	73.00 (66.98-78.19)	73.00 (66.98-78.19)	73.00 (66.98-78.19)
Malaysia	43.34 (40.10-46.69)	46.85 (43.42-50.92)	51.13 (46.40-56.70)	51.06 (46.71-54.95)	54.82 (51.31-58.78)	59.75 (55.20-64.43)	51.47 (45.62-56.76)	54.50 (49.84-58.88)	60.42 (55.69-64.82)	73.00 (66.98-78.19)	73.00 (66.98-78.19)	73.00 (66.98-78.19)
Maldives	34.82 (30.45-39.43)	47.99 (45.11-51.15)	61.84 (58.15-65.27)	34.16 (24.66-41.06)	43.57 (39.12-47.06)	58.35 (53.48-62.20)	57.65 (51.25-63.46)	57.74 (52.01-63.35)	55.62 (50.61-60.38)	73.00 (66.98-78.19)	73.00 (66.98-78.19)	73.00 (66.98-78.19)

	Appendix Table 1a. Scaled values for each SDG health-related indicator and three summary indicators by country for 1990, 2000, and 2015											
	**Indicator 3.9.2: Age-standardised death rate attributable to unsafe water, sanitation, and hygiene (WaSH) (per 100,000 population)**			**Indicator 3.9.3: Age-standardised death rate due to unintentional poisonings (per 100,000 population)**			**Indicator 3.a.1: Age-standardised prevalence of daily smoking among populations aged 10 and older**			**Indicator 5.2.1: Prevalence of women aged 15 years and older who experienced intimate partner violence**		
**Location**	**1990**	**2000**	**2015**	**1990**	**2000**	**2015**	**1990**	**2000**	**2015**	**1990**	**2000**	**2015**
Mauritius	46.20 (43.45-49.27)	58.33 (55.57-60.81)	64.37 (61.79-67.35)	79.84 (77.10-82.72)	87.13 (84.66-89.43)	93.20 (91.06-95.04)	53.53 (48.37-58.16)	61.71 (58.20-65.05)	63.88 (58.66-68.35)	73.00 (66.98-78.19)	73.00 (66.98-78.19)	73.00 (66.98-78.19)
Myanmar	16.37 (11.13-21.96)	23.52 (18.53-28.63)	36.99 (31.96-41.87)	40.89 (31.18-49.07)	44.10 (34.94-53.09)	52.73 (44.47-60.91)	44.55 (37.40-50.88)	50.45 (45.53-55.14)	62.59 (58.36-66.61)	64.44 (56.98-71.58)	64.04 (56.76-70.87)	64.44 (56.98-71.58)
Philippines	34.16 (30.69-37.69)	38.28 (35.40-41.19)	43.21 (40.18-46.33)	75.89 (72.95-79.17)	77.12 (74.34-80.39)	84.64 (81.99-87.54)	43.33 (36.48-49.66)	43.58 (38.92-48.39)	49.99 (43.83-55.48)	89.20 (86.58-91.40)	90.53 (88.26-92.54)	87.51 (85.00-89.68)
Sri Lanka	37.74 (34.19-41.03)	46.44 (43.20-50.20)	60.28 (54.93-65.07)	42.67 (39.40-47.48)	64.97 (57.98-68.99)	77.32 (71.36-82.26)	70.48 (66.18-74.52)	75.33 (72.51-77.99)	77.78 (74.75-80.80)	82.17 (78.22-85.93)	83.44 (79.61-87.21)	81.04 (77.44-84.35)
Seychelles	43.98 (40.84-47.25)	50.14 (46.91-53.28)	54.23 (50.81-57.96)	52.17 (48.84-56.62)	59.56 (56.11-62.27)	67.81 (62.81-71.58)	66.63 (61.68-71.25)	66.78 (62.33-70.70)	66.87 (62.09-71.40)	73.00 (66.98-78.19)	73.00 (66.98-78.19)	73.00 (66.98-78.19)
Thailand	38.25 (35.13-41.49)	44.93 (42.00-48.14)	49.08 (44.95-54.10)	58.03 (54.73-61.41)	61.09 (57.52-63.55)	69.15 (64.94-73.20)	43.99 (38.72-48.57)	55.80 (51.95-59.48)	59.95 (55.13-64.39)	57.47 (49.02-65.80)	58.39 (50.56-65.99)	57.47 (49.02-65.80)
Timor-Leste	18.39 (12.89-25.61)	25.98 (20.85-32.68)	41.55 (36.49-46.90)	44.12 (24.46-54.93)	51.20 (37.40-60.05)	62.92 (52.32-70.62)	46.79 (41.07-52.76)	45.35 (39.88-51.00)	36.75 (31.14-42.39)	52.84 (42.97-62.13)	47.50 (37.72-57.00)	53.88 (45.88-61.99)
Vietnam	41.05 (36.23-46.26)	48.13 (43.74-52.56)	56.94 (52.58-61.32)	46.74 (37.51-58.34)	51.27 (45.05-63.70)	57.93 (48.99-72.48)	36.31 (31.14-41.29)	46.73 (42.81-50.82)	57.50 (52.85-62.02)	63.76 (56.02-70.72)	65.53 (58.18-72.76)	63.74 (57.37-69.94)
Federated States of Micronesia	36.03 (30.83-40.76)	43.41 (38.03-48.28)	50.11 (43.96-55.39)	45.44 (34.61-53.55)	50.26 (41.53-57.92)	54.54 (45.28-62.17)	64.07 (58.56-69.05)	66.54 (61.94-71.04)	67.95 (62.94-72.34)	53.67 (43.29-63.93)	53.67 (43.29-63.93)	53.67 (43.29-63.93)
Fiji	38.32 (34.69-42.11)	40.32 (37.12-43.80)	42.21 (38.37-45.93)	61.04 (55.44-65.62)	60.30 (56.56-63.55)	62.57 (58.01-66.87)	71.75 (67.26-75.97)	72.56 (69.33-75.74)	74.65 (70.97-78.00)	53.67 (43.29-63.93)	53.67 (43.29-63.93)	53.67 (43.29-63.93)
Kiribati	12.92 (8.63-17.63)	21.69 (17.73-26.07)	29.41 (25.22-34.02)	34.30 (27.43-45.62)	48.27 (40.40-52.18)	49.41 (38.38-55.42)	3.40 (0.00-10.25)	2.04 (0.00-6.87)	13.02 (5.25-20.95)	35.29 (24.49-47.12)	35.29 (24.49-47.12)	35.29 (24.49-47.12)

Location	Appendix Table 1a. Scaled values for each SDG health-related indicator and three summary indicators by country for 1990, 2000, and 2015											
	**Indicator 3.9.2: Age-standardised death rate attributable to unsafe water, sanitation, and hygiene (WaSH) (per 100,000 population)**			**Indicator 3.9.3: Age-standardised death rate due to unintentional poisonings (per 100,000 population)**			**Indicator 3.a.1: Age-standardised prevalence of daily smoking among populations aged 10 and older**			**Indicator 5.2.1: Prevalence of women aged 15 years and older who experienced intimate partner violence**		
	**1990**	**2000**	**2015**	**1990**	**2000**	**2015**	**1990**	**2000**	**2015**	**1990**	**2000**	**2015**
Marshall Islands	33.20 (29.55-36.81)	38.96 (35.31-42.59)	47.18 (43.67-50.82)	43.97 (33.86-49.86)	46.92 (38.80-52.43)	52.20 (45.58-57.80)	61.46 (55.60-66.87)	63.79 (59.71-67.85)	67.95 (62.86-72.61)	53.67 (43.29-63.93)	53.67 (43.29-63.93)	53.67 (43.29-63.93)
Papua New Guinea	13.64 (8.42-19.38)	15.93 (10.79-21.59)	20.89 (14.76-26.68)	38.56 (27.05-48.16)	40.08 (28.93-49.34)	44.31 (32.87-53.09)	29.62 (22.18-37.15)	28.80 (21.90-35.39)	36.44 (29.65-42.78)	69.98 (61.70-77.56)	69.98 (61.70-77.56)	68.99 (60.77-77.08)
Samoa	41.97 (38.05-45.75)	47.54 (43.53-51.32)	53.78 (49.56-57.75)	50.12 (41.48-56.64)	54.14 (45.34-60.88)	60.22 (50.47-66.77)	29.22 (22.33-36.41)	35.02 (29.01-40.36)	43.31 (36.57-49.61)	49.01 (39.45-59.00)	50.82 (41.73-59.82)	49.01 (39.45-59.00)
Solomon Islands	25.29 (20.35-30.76)	32.05 (27.14-37.59)	39.67 (34.05-45.10)	39.06 (27.84-47.68)	42.06 (32.00-51.60)	46.25 (37.28-55.37)	48.90 (42.18-54.94)	52.14 (45.70-58.06)	54.40 (48.79-59.98)	48.26 (38.55-57.92)	48.26 (38.55-57.92)	48.26 (38.55-57.92)
Tonga	43.00 (39.40-46.77)	46.05 (42.81-49.52)	51.18 (47.35-55.05)	39.65 (32.41-45.45)	40.92 (35.67-45.31)	43.51 (37.07-48.95)	37.06 (30.93-43.04)	41.50 (35.92-46.80)	41.65 (36.35-46.79)	61.12 (53.46-68.29)	61.12 (53.46-68.29)	61.12 (53.46-68.29)
Vanuatu	28.99 (24.12-33.99)	33.50 (28.64-38.71)	40.84 (35.72-45.44)	41.40 (32.01-49.66)	42.66 (33.62-51.78)	46.89 (38.51-55.13)	53.25 (47.07-58.79)	60.06 (55.48-64.40)	63.36 (58.79-67.57)	53.67 (43.29-63.93)	53.67 (43.29-63.93)	53.67 (43.29-63.93)
Afghanistan	27.54 (21.39-36.83)	28.25 (23.18-35.37)	38.40 (33.96-42.86)	38.67 (24.45-48.96)	39.06 (29.62-48.04)	43.70 (35.68-52.63)	69.62 (64.12-74.86)	67.62 (61.99-72.89)	66.07 (60.09-71.17)	50.00 (40.10-59.64)	50.00 (40.10-59.64)	50.00 (40.10-59.64)
Algeria	41.01 (37.23-44.75)	49.82 (46.75-53.07)	57.06 (54.04-60.23)	45.93 (31.81-54.18)	51.91 (42.14-58.42)	56.52 (46.18-64.11)	62.37 (56.03-68.37)	68.86 (63.85-73.46)	77.38 (73.16-80.95)	50.00 (40.10-59.64)	50.00 (40.10-59.64)	50.00 (40.10-59.64)
Bahrain	57.65 (55.00-60.55)	62.75 (60.02-65.31)	71.88 (68.39-75.44)	58.42 (54.42-65.79)	64.87 (61.06-71.15)	78.53 (74.09-82.59)	70.39 (65.52-74.86)	77.53 (73.69-80.87)	77.06 (72.80-80.99)	50.00 (40.10-59.64)	50.00 (40.10-59.64)	50.00 (40.10-59.64)
Egypt	29.62 (26.26-33.55)	42.49 (39.44-45.61)	56.25 (52.48-59.44)	65.09 (54.17-69.12)	63.20 (59.90-67.54)	64.53 (60.87-71.98)	64.55 (59.42-69.63)	65.80 (61.53-70.23)	61.90 (57.70-65.90)	59.37 (51.64-67.38)	57.81 (49.79-64.96)	59.55 (52.80-66.16)
Iran	46.83 (42.17-51.44)	60.21 (56.83-63.31)	67.13 (63.44-70.93)	22.52 (12.51-30.44)	30.20 (25.97-35.76)	41.21 (33.92-46.35)	76.71 (72.25-80.50)	77.18 (73.55-80.53)	76.91 (73.18-80.45)	31.26 (20.10-43.89)	29.47 (19.05-39.84)	31.26 (20.10-43.89)

Appendix Table 1a. Scaled values for each SDG health-related indicator and three summary indicators by country for 1990, 2000, and 2015

Location	Indicator 3.9.2: Age-standardised death rate attributable to unsafe water, sanitation, and hygiene (WaSH) (per 100,000 population)			Indicator 3.9.3: Age-standardised death rate due to unintentional poisonings (per 100,000 population)			Indicator 3.a.1: Age-standardised prevalence of daily smoking among populations aged 10 and older			Indicator 5.2.1: Prevalence of women aged 15 years and older who experienced intimate partner violence		
	1990	2000	2015	1990	2000	2015	1990	2000	2015	1990	2000	2015
Iraq	42.41 (38.60-46.39)	46.45 (43.04-50.34)	56.55 (52.99-60.56)	55.10 (41.29-62.35)	57.16 (49.21-62.46)	64.51 (57.39-70.29)	65.83 (60.66-70.36)	66.99 (62.32-71.25)	69.04 (63.82-73.46)	77.90 (73.03-82.38)	77.90 (73.03-82.38)	77.90 (73.03-82.38)
Jordan	55.45 (52.20-58.74)	61.11 (57.92-63.94)	71.30 (67.67-74.67)	45.86 (34.29-54.46)	50.84 (43.48-58.22)	63.90 (55.59-69.27)	52.15 (45.51-58.28)	51.53 (45.62-57.37)	54.61 (48.74-60.04)	66.78 (59.79-72.97)	66.78 (59.79-72.97)	65.18 (60.33-70.70)
Kuwait	63.03 (60.12-65.77)	65.69 (62.94-68.41)	66.97 (63.59-70.40)	65.36 (59.96-70.18)	77.30 (74.43-80.38)	85.78 (82.71-89.09)	61.91 (56.68-67.22)	60.52 (55.18-66.16)	63.45 (59.27-67.40)	50.00 (40.10-59.64)	50.00 (40.10-59.64)	50.00 (40.10-59.64)
Lebanon	59.37 (54.78-63.62)	69.44 (65.19-73.90)	81.53 (76.75-86.49)	54.64 (43.20-63.61)	63.24 (53.01-71.50)	73.12 (65.17-80.33)	26.19 (17.08-35.40)	35.20 (28.32-42.36)	44.85 (36.58-52.92)	50.00 (40.10-59.64)	50.00 (40.10-59.64)	50.00 (40.10-59.64)
Libya	51.46 (47.55-55.25)	58.33 (54.91-61.61)	62.03 (58.15-65.81)	52.78 (39.82-64.04)	57.71 (46.32-67.23)	62.25 (53.76-70.50)	68.84 (63.68-73.68)	69.60 (64.72-74.01)	70.73 (66.40-74.73)	50.00 (40.10-59.64)	50.00 (40.10-59.64)	50.00 (40.10-59.64)
Morocco	34.72 (31.24-38.68)	49.33 (46.19-52.60)	57.37 (53.25-61.18)	45.07 (29.97-53.01)	52.44 (42.84-58.02)	57.51 (51.18-63.14)	73.27 (68.82-77.53)	75.93 (72.72-78.88)	81.64 (78.00-84.98)	50.00 (40.10-59.64)	50.00 (40.10-59.64)	50.00 (40.10-59.64)
Palestine	54.22 (50.02-58.01)	59.81 (56.72-62.82)	63.56 (58.14-68.06)	69.71 (53.90-78.59)	77.07 (72.53-81.92)	81.93 (76.34-86.60)	56.12 (50.83-61.06)	54.89 (51.56-57.98)	60.90 (55.75-65.54)	0.81 (0.00-6.73)	18.51 (9.12-31.59)	39.26 (20.55-55.92)
Oman	48.10 (43.38-52.98)	54.67 (50.67-58.96)	57.18 (52.72-61.79)	86.50 (71.96-95.21)	93.77 (88.70-98.32)	90.82 (85.35-100.00)	80.65 (76.87-83.88)	85.05 (82.53-87.14)	84.26 (80.69-87.15)	50.00 (40.10-59.64)	50.00 (40.10-59.64)	50.00 (40.10-59.64)
Qatar	70.45 (66.11-74.21)	72.53 (68.64-76.40)	80.16 (75.11-84.96)	69.68 (58.06-76.39)	72.87 (67.75-76.99)	81.70 (75.17-87.04)	75.48 (70.60-80.09)	80.60 (76.51-84.00)	77.60 (73.90-81.19)	50.00 (40.10-59.64)	50.00 (40.10-59.64)	50.00 (40.10-59.64)
Saudi Arabia	48.70 (44.84-53.08)	55.91 (53.11-59.60)	60.54 (57.13-64.61)	47.43 (41.53-54.14)	54.03 (50.13-59.27)	62.52 (58.60-66.08)	82.55 (80.87-84.28)	75.30 (73.13-77.39)	71.54 (69.33-73.58)	50.16 (40.07-59.47)	50.16 (40.07-59.47)	50.16 (40.07-59.47)
Sudan	25.63 (20.81-30.84)	31.23 (26.44-36.81)	40.69 (35.31-45.90)	34.18 (16.52-45.69)	39.61 (23.94-50.18)	47.08 (34.13-57.02)	99.62 (98.98-100.00)	99.69 (99.15-100.00)	99.76 (99.24-100.00)	26.12 (13.86-40.09)	26.12 (13.86-40.09)	21.33 (10.26-33.13)

Appendix Table 1a. Scaled values for each SDG health-related indicator and three summary indicators by country for 1990, 2000, and 2015

Location	Indicator 3.9.2: Age-standardised death rate attributable to unsafe water, sanitation, and hygiene (WaSH) (per 100,000 population)			Indicator 3.9.3: Age-standardised death rate due to unintentional poisonings (per 100,000 population)			Indicator 3.a.1: Age-standardised prevalence of daily smoking among populations aged 10 and older			Indicator 5.2.1: Prevalence of women aged 15 years and older who experienced intimate partner violence		
	1990	2000	2015	1990	2000	2015	1990	2000	2015	1990	2000	2015
Syria	49.02 (43.89-54.85)	63.21 (55.31-67.67)	68.05 (59.23-73.03)	44.02 (38.32-51.84)	49.76 (43.86-58.72)	60.38 (55.48-65.09)	55.90 (48.52-62.58)	56.86 (50.42-62.28)	65.48 (59.73-70.53)	50.00 (40.10-59.64)	50.00 (40.10-59.64)	50.00 (40.10-59.64)
Tunisia	45.45 (41.65-49.11)	56.01 (52.89-59.21)	64.29 (60.14-68.00)	47.42 (30.94-57.16)	55.44 (42.76-63.33)	66.68 (60.96-71.73)	44.77 (38.82-50.80)	47.80 (43.48-52.20)	54.38 (48.72-59.89)	50.00 (40.10-59.64)	50.00 (40.10-59.64)	50.00 (40.10-59.64)
Turkey	39.68 (35.14-44.45)	56.28 (51.75-60.23)	75.81 (72.12-79.03)	56.17 (44.55-62.14)	64.51 (60.98-68.05)	80.05 (74.78-83.64)	24.63 (17.09-31.97)	31.63 (25.66-37.67)	46.61 (40.95-51.85)	51.61 (41.73-60.51)	51.61 (41.73-60.51)	53.43 (44.04-62.93)
United Arab Emirates	46.70 (41.97-51.40)	51.59 (47.35-56.60)	59.66 (54.77-66.53)	54.14 (45.81-62.06)	60.07 (54.22-65.26)	68.83 (62.98-74.88)	74.93 (70.44-79.11)	75.92 (71.71-79.71)	79.50 (75.37-83.60)	50.00 (40.10-59.64)	50.00 (40.10-59.64)	50.00 (40.10-59.64)
Yemen	24.93 (19.81-32.22)	33.75 (28.49-41.80)	43.99 (38.75-49.08)	33.58 (17.62-44.88)	39.16 (25.39-50.62)	46.73 (36.01-56.52)	65.27 (59.07-70.78)	62.63 (57.47-67.33)	70.79 (65.80-75.70)	50.00 (40.10-59.64)	50.00 (40.10-59.64)	50.00 (40.10-59.64)
Bangladesh	5.31 (1.14-10.06)	21.59 (17.96-25.63)	36.48 (32.70-40.46)	61.86 (45.39-71.59)	71.72 (64.84-76.64)	72.06 (63.22-83.13)	52.10 (45.16-58.69)	53.51 (47.92-58.36)	52.27 (46.41-57.81)	26.25 (15.67-39.24)	27.70 (17.77-40.24)	27.64 (17.11-39.59)
Bhutan	20.61 (15.30-25.89)	27.47 (22.61-32.05)	38.27 (33.47-43.05)	54.93 (39.02-67.36)	58.48 (41.45-72.32)	68.18 (54.52-80.98)	85.22 (81.29-88.70)	86.07 (82.55-89.09)	86.08 (83.63-88.20)	52.95 (42.46-62.14)	52.95 (42.46-62.14)	52.95 (42.46-62.14)
India	15.53 (12.12-19.71)	19.93 (16.52-23.93)	25.91 (22.39-29.89)	44.46 (34.17-58.88)	49.87 (42.58-63.57)	58.92 (53.70-72.35)	60.68 (57.87-63.43)	61.23 (58.40-64.09)	76.27 (74.75-77.73)	42.91 (35.51-52.22)	52.62 (46.59-60.21)	44.49 (37.25-53.39)
Nepal	11.21 (7.08-15.92)	19.13 (15.21-23.01)	29.40 (25.16-33.57)	52.64 (32.32-63.46)	58.54 (45.15-70.19)	66.82 (54.87-77.30)	21.74 (10.83-32.30)	19.92 (13.36-26.93)	53.40 (46.56-59.19)	56.56 (47.19-65.30)	56.56 (47.19-65.30)	51.96 (44.92-58.77)
Pakistan	21.92 (18.07-25.85)	25.54 (22.04-29.16)	33.52 (29.94-37.16)	51.92 (35.16-62.76)	52.36 (40.62-61.75)	56.89 (46.49-66.54)	60.01 (53.01-66.56)	61.93 (56.27-66.87)	75.15 (71.61-78.17)	64.98 (56.86-72.25)	64.98 (56.86-72.25)	59.90 (54.01-66.05)
Botswana	19.50 (9.07-27.75)	21.71 (7.97-32.94)	29.46 (14.75-39.67)	43.16 (24.92-58.70)	42.43 (18.10-60.70)	48.66 (25.50-64.35)	70.71 (66.90-74.21)	73.20 (69.84-76.31)	73.94 (70.52-77.17)	58.67 (49.80-66.67)	56.03 (47.31-63.77)	58.67 (49.80-66.67)

Location	Appendix Table 1a. Scaled values for each SDG health-related indicator and three summary indicators by country for 1990, 2000, and 2015											
	**Indicator 3.9.2: Age-standardised death rate attributable to unsafe water, sanitation, and hygiene (WaSH) (per 100,000 population)**			**Indicator 3.9.3: Age-standardised death rate due to unintentional poisonings (per 100,000 population)**			**Indicator 3.a.1: Age-standardised prevalence of daily smoking among populations aged 10 and older**			**Indicator 5.2.1: Prevalence of women aged 15 years and older who experienced intimate partner violence**		
	**1990**	**2000**	**2015**	**1990**	**2000**	**2015**	**1990**	**2000**	**2015**	**1990**	**2000**	**2015**
Lesotho	13.39 (8.51-18.46)	13.75 (8.62-19.06)	16.12 (9.50-22.78)	40.74 (29.66-50.85)	41.01 (31.49-49.65)	37.79 (26.26-48.79)	65.26 (61.63-68.78)	68.52 (65.47-71.40)	67.65 (64.26-70.86)	62.13 (54.08-69.40)	59.57 (50.95-66.88)	62.13 (54.08-69.40)
Namibia	16.17 (12.03-20.94)	16.53 (11.86-22.19)	27.67 (21.46-34.03)	42.31 (34.16-49.51)	40.22 (32.81-46.56)	51.44 (40.66-61.29)	59.34 (54.57-64.11)	60.77 (57.08-64.25)	71.74 (68.32-74.87)	64.33 (57.48-70.98)	62.45 (54.81-70.13)	67.59 (60.63-73.81)
South Africa	22.01 (18.96-25.79)	23.53 (20.29-27.36)	31.87 (28.88-35.42)	45.71 (42.02-51.95)	45.55 (41.76-50.57)	54.69 (50.21-58.22)	39.73 (35.76-43.30)	56.09 (53.53-58.58)	65.89 (63.88-67.94)	76.91 (73.38-80.66)	78.08 (74.45-81.68)	78.21 (74.59-81.81)
Swaziland	17.39 (12.90-22.22)	18.25 (13.14-23.28)	22.38 (15.28-29.96)	40.93 (30.89-49.73)	39.46 (28.55-48.44)	41.15 (29.51-54.24)	78.48 (75.74-81.09)	86.36 (84.46-88.15)	88.19 (86.34-89.79)	65.22 (57.76-72.08)	62.89 (54.74-69.34)	67.22 (60.66-73.29)
Zimbabwe	25.13 (21.41-29.47)	24.11 (18.22-29.60)	25.43 (19.49-31.01)	42.68 (34.64-49.23)	44.21 (33.22-52.29)	48.01 (36.03-57.39)	71.22 (67.57-74.57)	73.55 (70.61-76.28)	75.18 (72.57-77.78)	35.63 (24.10-47.66)	35.22 (24.75-46.67)	38.42 (28.38-49.85)
Benin	14.31 (9.85-19.72)	16.57 (12.20-21.78)	23.55 (16.89-30.59)	40.87 (21.41-49.85)	40.26 (25.55-49.17)	44.32 (31.08-55.87)	86.27 (83.12-88.83)	87.36 (84.89-89.63)	90.05 (87.94-91.87)	60.88 (52.70-68.92)	60.88 (52.70-68.92)	60.88 (52.70-68.92)
Burkina Faso	6.53 (2.17-11.53)	10.11 (5.90-15.02)	18.93 (12.74-24.80)	34.27 (17.10-43.33)	38.75 (31.00-45.76)	42.29 (29.79-52.71)	77.89 (73.14-81.98)	79.56 (75.67-83.38)	81.37 (77.72-84.54)	84.24 (80.55-87.62)	84.24 (80.55-87.62)	84.32 (81.15-87.27)
Cameroon	14.69 (10.38-19.64)	15.71 (10.95-20.86)	24.74 (18.56-31.02)	43.02 (30.23-51.67)	41.27 (29.36-50.60)	45.99 (31.72-58.18)	85.55 (82.62-88.34)	87.05 (84.42-89.68)	89.65 (87.84-91.32)	25.71 (15.09-39.02)	23.75 (14.05-35.55)	22.66 (13.60-33.90)
Cape Verde	26.83 (23.19-30.81)	33.63 (29.24-38.27)	42.84 (39.30-46.49)	49.52 (38.48-58.48)	50.39 (40.80-57.87)	59.30 (53.55-65.11)	85.60 (82.50-88.36)	86.63 (84.00-88.97)	87.02 (84.16-89.51)	60.88 (52.70-68.92)	60.88 (52.70-68.92)	60.88 (52.70-68.92)
Chad	8.10 (3.60-13.29)	7.84 (3.16-12.90)	13.77 (7.71-20.48)	36.17 (12.99-48.53)	33.48 (14.87-44.86)	38.84 (22.90-52.73)	84.35 (81.37-87.05)	84.74 (82.22-87.19)	85.40 (82.53-87.99)	78.72 (73.98-83.04)	78.72 (73.98-83.04)	78.65 (74.43-82.22)
Cote d'Ivoire	14.22 (10.13-19.08)	16.89 (12.31-21.50)	23.20 (17.55-28.98)	39.24 (26.76-47.18)	38.14 (26.52-46.33)	43.28 (31.50-54.82)	83.06 (79.44-86.21)	83.76 (80.74-86.30)	82.14 (79.20-85.07)	57.08 (47.81-66.29)	57.08 (47.81-66.29)	57.09 (48.95-64.93)

	Appendix Table 1a. Scaled values for each SDG health-related indicator and three summary indicators by country for 1990, 2000, and 2015											
	**Indicator 3.9.2: Age-standardised death rate attributable to unsafe water, sanitation, and hygiene (WaSH) (per 100,000 population)**			**Indicator 3.9.3: Age-standardised death rate due to unintentional poisonings (per 100,000 population)**			**Indicator 3.a.1: Age-standardised prevalence of daily smoking among populations aged 10 and older**			**Indicator 5.2.1: Prevalence of women aged 15 years and older who experienced intimate partner violence**		
**Location**	**1990**	**2000**	**2015**	**1990**	**2000**	**2015**	**1990**	**2000**	**2015**	**1990**	**2000**	**2015**
The Gambia	20.22 (14.70-26.33)	23.46 (19.47-27.76)	30.04 (25.27-34.75)	44.78 (28.48-55.77)	44.86 (34.95-54.16)	49.01 (39.21-59.57)	70.50 (65.73-74.83)	73.80 (69.18-77.87)	77.69 (74.30-80.95)	66.29 (59.38-72.95)	66.29 (59.38-72.95)	66.05 (60.60-71.48)
Ghana	15.46 (10.52-20.87)	22.12 (18.07-26.79)	33.01 (26.21-39.18)	44.36 (34.40-51.68)	49.26 (43.54-54.77)	50.75 (40.72-60.10)	90.97 (88.82-92.74)	91.42 (89.71-92.87)	93.86 (92.46-95.22)	69.40 (62.48-75.53)	69.40 (62.48-75.53)	69.40 (62.48-75.53)
Guinea	11.65 (7.63-16.42)	14.48 (10.35-19.10)	21.13 (15.52-26.63)	38.64 (22.83-48.08)	38.80 (25.87-46.91)	42.18 (30.98-52.11)	89.69 (87.13-91.83)	90.66 (88.40-92.73)	91.55 (89.36-93.37)	60.88 (52.70-68.92)	60.88 (52.70-68.92)	60.88 (52.70-68.92)
Guinea-Bissau	10.48 (2.78-17.96)	14.29 (5.41-22.41)	19.13 (9.10-26.89)	37.53 (17.62-52.71)	36.83 (19.39-52.78)	38.65 (20.15-54.27)	85.58 (82.45-88.42)	86.61 (83.62-89.13)	86.69 (83.94-89.26)	60.88 (52.70-68.92)	60.88 (52.70-68.92)	60.88 (52.70-68.92)
Liberia	9.63 (4.83-14.88)	12.63 (8.22-17.79)	20.75 (15.11-26.98)	39.36 (22.92-49.34)	42.15 (26.89-52.73)	48.03 (34.14-58.56)	84.86 (81.53-87.52)	85.52 (82.74-88.04)	87.98 (85.77-89.76)	46.06 (35.79-56.58)	46.06 (35.79-56.58)	42.94 (32.44-52.77)
Mali	8.13 (3.82-13.53)	13.67 (9.54-18.44)	21.11 (15.54-26.94)	35.47 (19.24-44.58)	38.75 (22.91-48.52)	43.89 (28.40-54.80)	86.75 (83.99-89.21)	87.28 (84.56-89.77)	87.76 (85.38-89.94)	53.84 (44.25-63.19)	53.84 (44.25-63.19)	49.97 (40.89-58.35)
Mauritania	13.68 (9.43-18.63)	18.37 (14.21-23.18)	28.07 (22.54-33.80)	41.58 (26.78-50.46)	44.83 (30.86-55.02)	52.09 (36.93-64.00)	79.11 (74.96-82.57)	78.10 (74.71-81.21)	80.37 (76.47-83.79)	60.88 (52.70-68.92)	60.88 (52.70-68.92)	60.88 (52.70-68.92)
Niger	4.20 (0.00-9.43)	7.85 (3.62-12.68)	16.13 (10.86-21.75)	33.12 (11.08-45.02)	35.96 (19.61-46.83)	42.01 (26.78-53.06)	92.47 (90.26-94.29)	92.21 (90.04-94.09)	91.20 (89.33-92.89)	60.88 (52.70-68.92)	60.88 (52.70-68.92)	60.88 (52.70-68.92)
Nigeria	14.51 (9.53-20.00)	13.85 (9.79-18.40)	19.33 (14.05-24.73)	40.60 (21.23-51.42)	43.28 (27.69-52.87)	51.92 (35.48-62.59)	81.72 (79.19-84.11)	89.57 (87.26-91.43)	93.33 (91.79-94.80)	74.96 (69.40-80.20)	73.48 (67.70-78.68)	74.72 (70.46-78.98)
Sao Tome and Principe	21.50 (17.51-26.06)	24.22 (20.35-28.39)	32.54 (26.71-38.90)	49.13 (36.91-56.79)	50.64 (36.83-58.59)	54.93 (42.96-66.89)	92.48 (90.69-94.09)	93.14 (91.46-94.50)	93.20 (91.58-94.68)	53.28 (42.74-62.69)	53.28 (42.74-62.69)	53.28 (42.74-62.69)
Senegal	12.76 (8.60-17.29)	15.35 (11.24-19.90)	23.24 (16.83-29.26)	40.40 (26.57-48.46)	42.29 (30.69-50.11)	45.89 (33.88-56.45)	78.99 (75.28-82.79)	83.08 (80.14-85.91)	90.22 (88.42-91.81)	60.88 (52.70-68.92)	60.88 (52.70-68.92)	60.88 (52.70-68.92)

	Appendix Table 1a. Scaled values for each SDG health-related indicator and three summary indicators by country for 1990, 2000, and 2015											
	Indicator 3.9.2: Age-standardised death rate attributable to unsafe water, sanitation, and hygiene (WaSH) (per 100,000 population)			Indicator 3.9.3: Age-standardised death rate due to unintentional poisonings (per 100,000 population)			Indicator 3.a.1: Age-standardised prevalence of daily smoking among populations aged 10 and older			Indicator 5.2.1: Prevalence of women aged 15 years and older who experienced intimate partner violence		
**Location**	**1990**	**2000**	**2015**	**1990**	**2000**	**2015**	**1990**	**2000**	**2015**	**1990**	**2000**	**2015**
Sierra Leone	12.67 (7.93-17.73)	13.40 (9.22-18.35)	21.38 (15.96-27.25)	38.00 (19.40-48.64)	37.19 (21.76-45.56)	42.46 (26.88-53.30)	65.44 (59.69-70.47)	66.85 (61.33-71.49)	70.64 (66.96-73.94)	25.68 (15.32-39.61)	25.68 (15.32-39.61)	23.45 (13.72-34.81)
Togo	14.46 (10.27-19.28)	16.59 (12.13-21.72)	23.97 (18.07-29.50)	41.71 (28.24-49.02)	40.70 (28.78-48.46)	45.63 (34.64-56.13)	87.89 (85.23-90.23)	88.56 (86.19-90.76)	89.58 (87.73-91.37)	65.53 (57.82-72.67)	65.53 (57.82-72.67)	65.91 (59.84-71.46)
Burundi	5.30 (0.10-11.57)	6.05 (1.66-11.19)	16.00 (9.69-22.56)	22.50 (11.64-32.86)	25.81 (13.70-34.46)	34.38 (21.44-45.23)	85.80 (82.64-88.65)	87.68 (84.41-90.30)	88.82 (86.29-91.03)	53.26 (42.82-62.33)	53.26 (42.82-62.33)	53.26 (42.82-62.33)
Comoros	12.54 (6.32-18.65)	14.67 (10.29-19.32)	20.40 (14.60-26.32)	30.94 (17.31-42.35)	36.33 (24.48-45.04)	42.89 (29.28-52.41)	82.12 (78.39-85.86)	82.08 (78.19-85.62)	83.10 (80.28-85.79)	97.58 (96.53-98.48)	97.58 (96.53-98.48)	97.46 (96.37-98.55)
Djibouti	17.09 (11.29-22.44)	17.18 (10.28-24.42)	22.30 (14.68-29.28)	35.02 (18.55-47.22)	35.52 (21.14-49.27)	39.25 (24.36-52.02)	68.29 (62.69-73.15)	71.02 (66.11-75.37)	71.81 (67.18-76.20)	53.26 (42.82-62.33)	53.26 (42.82-62.33)	53.26 (42.82-62.33)
Eritrea	3.60 (0.00-8.61)	9.96 (3.77-16.96)	11.43 (3.67-19.44)	26.20 (12.64-35.49)	34.12 (19.78-45.29)	33.49 (20.34-45.81)	85.07 (81.48-88.13)	86.03 (83.47-88.39)	88.64 (85.83-91.08)	53.26 (42.82-62.33)	53.26 (42.82-62.33)	53.26 (42.82-62.33)
Ethiopia	4.68 (0.05-9.94)	9.08 (4.73-13.95)	20.49 (14.23-26.72)	28.04 (13.92-36.63)	34.77 (21.00-41.81)	44.29 (30.94-54.42)	90.76 (88.48-92.69)	92.04 (90.21-93.70)	92.28 (90.23-94.11)	37.20 (25.70-49.10)	36.64 (24.21-47.71)	27.39 (16.07-38.58)
Kenya	7.35 (3.41-11.93)	8.64 (4.68-13.33)	12.99 (9.11-17.54)	36.18 (27.69-45.22)	36.85 (30.43-44.48)	42.33 (35.62-48.65)	77.49 (75.96-78.91)	77.52 (76.19-78.92)	82.32 (81.16-83.46)	67.24 (61.24-73.12)	71.33 (64.39-77.63)	67.79 (61.56-73.79)
Madagascar	12.93 (8.76-18.05)	16.83 (12.57-22.26)	20.96 (14.85-27.41)	33.41 (19.63-43.58)	37.08 (23.59-46.62)	40.86 (27.98-52.19)	62.36 (56.84-67.96)	64.87 (59.91-69.39)	76.78 (72.69-80.94)	53.26 (42.82-62.33)	53.26 (42.82-62.33)	53.26 (42.82-62.33)
Malawi	6.79 (2.14-11.91)	8.91 (3.58-14.82)	17.88 (11.51-24.18)	31.79 (16.81-43.01)	32.92 (17.59-44.31)	39.68 (24.28-51.20)	74.57 (69.55-78.59)	74.59 (71.38-77.59)	81.53 (78.18-84.42)	67.74 (61.09-74.46)	69.24 (63.35-75.01)	66.92 (61.13-72.67)
Mozambique	10.30 (6.33-15.20)	13.77 (9.11-18.77)	19.86 (12.83-27.38)	29.70 (15.41-38.55)	32.79 (23.86-38.70)	38.60 (26.89-48.92)	73.96 (69.59-77.95)	71.55 (67.77-75.15)	77.89 (74.08-81.33)	42.15 (30.92-53.75)	44.02 (33.20-54.29)	41.51 (32.08-51.13)

	Appendix Table 1a. Scaled values for each SDG health-related indicator and three summary indicators by country for 1990, 2000, and 2015											
	**Indicator 3.9.2: Age-standardised death rate attributable to unsafe water, sanitation, and hygiene (WaSH) (per 100,000 population)**			**Indicator 3.9.3: Age-standardised death rate due to unintentional poisonings (per 100,000 population)**			**Indicator 3.a.1: Age-standardised prevalence of daily smoking among populations aged 10 and older**			**Indicator 5.2.1: Prevalence of women aged 15 years and older who experienced intimate partner violence**		
**Location**	**1990**	**2000**	**2015**	**1990**	**2000**	**2015**	**1990**	**2000**	**2015**	**1990**	**2000**	**2015**
Rwanda	14.12 (8.93-19.01)	13.62 (8.77-18.26)	26.36 (20.17-32.09)	26.32 (12.94-34.96)	26.59 (14.20-34.97)	39.00 (24.60-50.06)	76.97 (72.91-80.98)	79.73 (76.39-82.70)	82.89 (80.30-85.26)	34.70 (23.93-47.00)	36.92 (26.41-47.32)	31.69 (21.95-42.84)
Somalia	4.60 (0.00-12.17)	3.63 (0.00-12.43)	5.77 (0.00-14.90)	23.47 (2.79-41.59)	23.70 (2.26-42.17)	27.20 (8.84-45.17)	77.73 (73.66-81.81)	79.97 (75.77-83.55)	83.88 (80.40-86.96)	53.26 (42.82-62.33)	53.26 (42.82-62.33)	53.26 (42.82-62.33)
South Sudan	6.74 (0.00-14.87)	8.14 (0.00-17.58)	9.25 (0.00-18.73)	29.28 (11.48-46.84)	32.81 (13.45-50.14)	33.16 (13.00-50.73)	77.40 (73.15-81.33)	79.38 (75.27-83.01)	83.48 (80.01-86.64)	53.26 (42.82-62.33)	53.26 (42.82-62.33)	53.26 (42.82-62.33)
Tanzania	9.56 (5.28-14.37)	12.75 (8.05-18.08)	19.82 (11.54-27.06)	35.96 (19.05-44.76)	41.85 (35.34-47.91)	43.13 (29.73-54.07)	77.66 (73.41-81.65)	77.72 (74.04-81.27)	80.44 (77.53-83.11)	46.28 (34.80-56.17)	48.23 (38.43-57.43)	43.56 (35.10-52.33)
Uganda	13.88 (8.61-19.35)	14.97 (9.87-20.27)	22.21 (15.33-29.52)	30.23 (16.68-39.51)	31.32 (20.89-40.08)	37.48 (24.13-48.87)	78.93 (74.73-82.78)	78.99 (75.57-82.23)	87.15 (85.08-89.02)	22.87 (12.22-37.38)	21.91 (11.18-34.26)	18.22 (9.64-29.53)
Zambia	8.40 (4.15-13.43)	6.15 (1.56-11.48)	13.78 (7.47-20.30)	35.81 (18.11-46.00)	33.90 (20.15-44.33)	38.03 (27.91-50.75)	75.51 (71.13-79.33)	75.44 (72.15-78.48)	79.53 (75.81-83.29)	25.50 (14.22-39.05)	20.72 (11.50-32.14)	24.92 (14.82-36.59)
Angola	12.07 (3.43-21.54)	16.41 (6.77-25.86)	24.45 (12.56-34.02)	29.86 (11.79-46.21)	34.58 (15.58-51.53)	41.33 (22.65-56.96)	84.65 (82.20-86.89)	84.61 (81.86-86.89)	82.71 (79.91-85.17)	40.85 (30.52-52.15)	40.85 (30.52-52.15)	40.85 (30.52-52.15)
Central African Republic	10.81 (6.40-15.87)	12.77 (6.81-20.10)	14.42 (7.08-22.67)	30.01 (15.21-38.24)	31.02 (18.13-42.77)	32.04 (19.02-45.08)	86.89 (84.76-88.99)	87.28 (85.01-89.28)	85.98 (83.47-88.21)	74.12 (68.71-79.48)	74.12 (68.71-79.48)	74.12 (68.71-79.48)
Congo	17.82 (11.72-24.12)	19.79 (14.35-25.73)	27.60 (20.55-34.63)	38.08 (26.98-46.34)	39.17 (26.79-47.45)	46.91 (34.70-56.83)	90.03 (88.22-91.70)	90.08 (88.46-91.66)	86.91 (84.65-88.94)	40.85 (30.52-52.15)	40.85 (30.52-52.15)	40.85 (30.52-52.15)
Democratic Republic of the Congo	17.68 (12.70-23.00)	17.88 (13.72-22.30)	22.67 (17.20-27.99)	37.23 (21.21-48.07)	37.58 (23.37-46.76)	42.21 (27.86-53.15)	83.38 (80.88-85.72)	83.99 (81.49-86.40)	83.61 (81.47-85.67)	13.70 (4.13-28.62)	13.70 (4.13-28.62)	11.74 (2.47-23.81)
Equatorial Guinea	13.81 (5.45-23.02)	22.89 (12.67-31.84)	34.02 (21.73-41.85)	29.48 (13.63-46.36)	36.99 (18.49-54.34)	48.37 (29.07-64.21)	89.25 (87.17-91.00)	89.40 (87.43-91.13)	89.27 (87.18-90.94)	40.85 (30.52-52.15)	40.85 (30.52-52.15)	40.85 (30.52-52.15)

	Appendix Table 1a. Scaled values for each SDG health-related indicator and three summary indicators by country for 1990, 2000, and 2015											
	**Indicator 3.9.2: Age-standardised death rate attributable to unsafe water, sanitation, and hygiene (WaSH) (per 100,000 population)**			**Indicator 3.9.3: Age-standardised death rate due to unintentional poisonings (per 100,000 population)**			**Indicator 3.a.1: Age-standardised prevalence of daily smoking among populations aged 10 and older**			**Indicator 5.2.1: Prevalence of women aged 15 years and older who experienced intimate partner violence**		
**Location**	**1990**	**2000**	**2015**	**1990**	**2000**	**2015**	**1990**	**2000**	**2015**	**1990**	**2000**	**2015**
Gabon	21.58 (16.43-27.86)	25.31 (20.30-30.95)	33.36 (26.24-39.95)	41.95 (30.36-51.18)	44.91 (34.66-53.54)	50.78 (38.45-61.05)	83.17 (80.21-85.84)	81.92 (78.93-84.56)	80.57 (77.79-83.06)	27.24 (16.71-40.72)	27.24 (16.71-40.72)	24.45 (15.30-35.85)

Location	Appendix Table 1a. Scaled values for each SDG health-related indicator and three summary indicators by country for 1990, 2000, and 2015											
	**Indicator 6.1.1: Risk-weighted prevalence of populations using unsafe or unimproved water sources, as measured by the summary exposure value (SEV) for unsafe water**			**Indicator 6.2.2a: Risk-weighted prevalence of populations using unsafe or unimproved sanitation, as measured by the summary exposure value (SEV) for unsafe sanitation**			**Indicator 6.2.1b: Risk-weighted prevalence of populations with unsafe hygiene (no handwashing with soap), as measured by the summary exposure value (SEV) for unsafe hygiene**			**Indicator 7.1.2: Prevalence of household air pollution, as measured by the summary exposure value (SEV) for household air pollution**		
	**1990**	**2000**	**2015**	**1990**	**2000**	**2015**	**1990**	**2000**	**2015**	**1990**	**2000**	**2015**
Canada	100.00 (100.00-100.00)	100.00 (100.00-100.00)	100.00 (100.00-100.00)	100.00 (100.00-100.00)	100.00 (100.00-100.00)	100.00 (100.00-100.00)	83.98 (81.98-85.92)	90.68 (89.51-91.81)	100.00 (100.00-100.00)	100.00 (100.00-100.00)	100.00 (100.00-100.00)	100.00 (100.00-100.00)
United States	100.00 (100.00-100.00)	100.00 (100.00-100.00)	100.00 (100.00-100.00)	100.00 (100.00-100.00)	100.00 (100.00-100.00)	100.00 (100.00-100.00)	83.84 (81.83-85.79)	85.93 (84.17-87.65)	94.72 (94.05-95.36)	100.00 (100.00-100.00)	100.00 (100.00-100.00)	100.00 (100.00-100.00)
Australia	100.00 (100.00-100.00)	100.00 (100.00-100.00)	100.00 (100.00-100.00)	100.00 (100.00-100.00)	100.00 (100.00-100.00)	100.00 (100.00-100.00)	72.36 (68.96-75.68)	83.42 (81.36-85.42)	96.65 (96.22-97.04)	100.00 (100.00-100.00)	100.00 (100.00-100.00)	100.00 (100.00-100.00)
New Zealand	100.00 (100.00-100.00)	100.00 (100.00-100.00)	100.00 (100.00-100.00)	100.00 (100.00-100.00)	100.00 (100.00-100.00)	100.00 (100.00-100.00)	72.37 (68.97-75.69)	83.00 (80.89-85.05)	96.27 (95.79-96.71)	100.00 (100.00-100.00)	100.00 (100.00-100.00)	100.00 (100.00-100.00)
Brunei	100.00 (100.00-100.00)	100.00 (100.00-100.00)	100.00 (100.00-100.00)	100.00 (100.00-100.00)	100.00 (100.00-100.00)	100.00 (100.00-100.00)	65.00 (60.79-69.32)	76.93 (74.06-79.70)	91.86 (90.83-92.85)	100.00 (100.00-100.00)	100.00 (100.00-100.00)	100.00 (100.00-100.00)
Japan	100.00 (100.00-100.00)	100.00 (100.00-100.00)	100.00 (100.00-100.00)	100.00 (100.00-100.00)	100.00 (100.00-100.00)	100.00 (100.00-100.00)	73.61 (70.35-76.77)	84.56 (82.63-86.43)	97.10 (96.73-97.44)	100.00 (100.00-100.00)	100.00 (100.00-100.00)	100.00 (100.00-100.00)
Singapore	100.00 (100.00-100.00)	100.00 (100.00-100.00)	100.00 (100.00-100.00)	100.00 (100.00-100.00)	100.00 (100.00-100.00)	100.00 (100.00-100.00)	49.98 (44.27-56.12)	66.86 (62.85-70.93)	87.84 (86.33-89.31)	100.00 (100.00-100.00)	100.00 (100.00-100.00)	100.00 (100.00-100.00)
South Korea	100.00 (100.00-100.00)	100.00 (100.00-100.00)	100.00 (100.00-100.00)	100.00 (100.00-100.00)	100.00 (100.00-100.00)	100.00 (100.00-100.00)	61.24 (56.52-66.04)	74.82 (71.69-77.84)	91.67 (90.62-92.68)	100.00 (100.00-100.00)	100.00 (100.00-100.00)	100.00 (100.00-100.00)
Andorra	100.00 (100.00-100.00)	100.00 (100.00-100.00)	100.00 (100.00-100.00)	100.00 (100.00-100.00)	100.00 (100.00-100.00)	100.00 (100.00-100.00)	69.73 (66.05-73.42)	81.02 (78.65-83.31)	95.00 (94.37-95.61)	100.00 (100.00-100.00)	100.00 (100.00-100.00)	100.00 (100.00-100.00)
Austria	100.00 (100.00-100.00)	100.00 (100.00-100.00)	100.00 (100.00-100.00)	100.00 (100.00-100.00)	100.00 (100.00-100.00)	100.00 (100.00-100.00)	57.74 (52.76-62.99)	66.53 (62.50-70.65)	82.07 (79.84-84.23)	100.00 (100.00-100.00)	100.00 (100.00-100.00)	100.00 (100.00-100.00)
Belgium	100.00 (100.00-100.00)	100.00 (100.00-100.00)	100.00 (100.00-100.00)	100.00 (100.00-100.00)	100.00 (100.00-100.00)	100.00 (100.00-100.00)	62.56 (58.08-67.20)	73.04 (69.72-76.28)	89.19 (87.83-90.50)	100.00 (100.00-100.00)	100.00 (100.00-100.00)	100.00 (100.00-100.00)

Location	Appendix Table 1a. Scaled values for each SDG health-related indicator and three summary indicators by country for 1990, 2000, and 2015											
	Indicator 6.1.1: Risk-weighted prevalence of populations using unsafe or unimproved water sources, as measured by the summary exposure value (SEV) for unsafe water			Indicator 6.2.2a: Risk-weighted prevalence of populations using unsafe or unimproved sanitation, as measured by the summary exposure value (SEV) for unsafe sanitation			Indicator 6.2.1b: Risk-weighted prevalence of populations with unsafe hygiene (no handwashing with soap), as measured by the summary exposure value (SEV) for unsafe hygiene			Indicator 7.1.2: Prevalence of household air pollution, as measured by the summary exposure value (SEV) for household air pollution		
	1990	2000	2015	1990	2000	2015	1990	2000	2015	1990	2000	2015
Cyprus	100.00 (100.00-100.00)	100.00 (100.00-100.00)	100.00 (100.00-100.00)	100.00 (100.00-100.00)	100.00 (100.00-100.00)	100.00 (100.00-100.00)	64.89 (60.66-69.22)	77.00 (74.15-79.77)	91.81 (90.78-92.81)	100.00 (100.00-100.00)	100.00 (100.00-100.00)	100.00 (100.00-100.00)
Denmark	100.00 (100.00-100.00)	100.00 (100.00-100.00)	100.00 (100.00-100.00)	100.00 (100.00-100.00)	100.00 (100.00-100.00)	100.00 (100.00-100.00)	69.84 (66.17-73.51)	81.18 (78.83-83.44)	93.42 (92.59-94.22)	100.00 (100.00-100.00)	100.00 (100.00-100.00)	100.00 (100.00-100.00)
Finland	100.00 (100.00-100.00)	100.00 (100.00-100.00)	100.00 (100.00-100.00)	100.00 (100.00-100.00)	100.00 (100.00-100.00)	100.00 (100.00-100.00)	68.47 (64.65-72.33)	79.65 (77.13-82.10)	93.52 (92.70-94.30)	100.00 (100.00-100.00)	100.00 (100.00-100.00)	100.00 (100.00-100.00)
France	100.00 (100.00-100.00)	100.00 (100.00-100.00)	100.00 (100.00-100.00)	100.00 (100.00-100.00)	100.00 (100.00-100.00)	100.00 (100.00-100.00)	54.23 (48.94-59.89)	64.05 (59.73-68.50)	81.54 (79.24-83.76)	100.00 (100.00-100.00)	100.00 (100.00-100.00)	100.00 (100.00-100.00)
Germany	100.00 (100.00-100.00)	100.00 (100.00-100.00)	100.00 (100.00-100.00)	100.00 (100.00-100.00)	100.00 (100.00-100.00)	100.00 (100.00-100.00)	59.56 (54.69-64.58)	72.53 (69.16-75.83)	88.82 (87.43-90.18)	100.00 (100.00-100.00)	100.00 (100.00-100.00)	100.00 (100.00-100.00)
Greece	100.00 (100.00-100.00)	100.00 (100.00-100.00)	100.00 (100.00-100.00)	100.00 (100.00-100.00)	100.00 (100.00-100.00)	100.00 (100.00-100.00)	55.40 (50.26-60.92)	68.23 (64.38-72.12)	85.14 (83.27-86.94)	100.00 (100.00-100.00)	100.00 (100.00-100.00)	100.00 (100.00-100.00)
Iceland	100.00 (100.00-100.00)	100.00 (100.00-100.00)	100.00 (100.00-100.00)	100.00 (100.00-100.00)	100.00 (100.00-100.00)	100.00 (100.00-100.00)	63.10 (58.67-67.66)	75.25 (72.17-78.23)	90.11 (88.86-91.31)	100.00 (100.00-100.00)	100.00 (100.00-100.00)	100.00 (100.00-100.00)
Ireland	100.00 (100.00-100.00)	100.00 (100.00-100.00)	100.00 (100.00-100.00)	100.00 (100.00-100.00)	100.00 (100.00-100.00)	100.00 (100.00-100.00)	63.78 (59.43-68.26)	75.83 (72.82-78.74)	90.41 (89.20-91.57)	100.00 (100.00-100.00)	100.00 (100.00-100.00)	100.00 (100.00-100.00)
Israel	100.00 (100.00-100.00)	100.00 (100.00-100.00)	100.00 (100.00-100.00)	100.00 (100.00-100.00)	100.00 (100.00-100.00)	100.00 (100.00-100.00)	76.19 (73.22-79.06)	84.47 (82.54-86.35)	96.99 (96.60-97.34)	100.00 (100.00-100.00)	100.00 (100.00-100.00)	100.00 (100.00-100.00)
Italy	100.00 (100.00-100.00)	100.00 (100.00-100.00)	100.00 (100.00-100.00)	100.00 (100.00-100.00)	100.00 (100.00-100.00)	100.00 (100.00-100.00)	61.83 (57.23-66.56)	70.58 (67.00-74.16)	85.85 (84.08-87.58)	100.00 (100.00-100.00)	100.00 (100.00-100.00)	100.00 (100.00-100.00)
Luxembourg	100.00 (100.00-100.00)	100.00 (100.00-100.00)	100.00 (100.00-100.00)	100.00 (100.00-100.00)	100.00 (100.00-100.00)	100.00 (100.00-100.00)	56.41 (51.33-61.81)	68.71 (64.93-72.54)	85.50 (83.68-87.27)	100.00 (100.00-100.00)	100.00 (100.00-100.00)	100.00 (100.00-100.00)

Location	Appendix Table 1a. Scaled values for each SDG health-related indicator and three summary indicators by country for 1990, 2000, and 2015											
	Indicator 6.1.1: Risk-weighted prevalence of populations using unsafe or unimproved water sources, as measured by the summary exposure value (SEV) for unsafe water			Indicator 6.2.2a: Risk-weighted prevalence of populations using unsafe or unimproved sanitation, as measured by the summary exposure value (SEV) for unsafe sanitation			Indicator 6.2.1b: Risk-weighted prevalence of populations with unsafe hygiene (no handwashing with soap), as measured by the summary exposure value (SEV) for unsafe hygiene			Indicator 7.1.2: Prevalence of household air pollution, as measured by the summary exposure value (SEV) for household air pollution		
	1990	2000	2015	1990	2000	2015	1990	2000	2015	1990	2000	2015
Malta	100.00 (100.00-100.00)	100.00 (100.00-100.00)	100.00 (100.00-100.00)	100.00 (100.00-100.00)	100.00 (100.00-100.00)	100.00 (100.00-100.00)	43.78 (37.41-50.60)	52.03 (46.56-57.94)	69.80 (66.13-73.49)	100.00 (100.00-100.00)	100.00 (100.00-100.00)	100.00 (100.00-100.00)
Netherlands	100.00 (100.00-100.00)	100.00 (100.00-100.00)	100.00 (100.00-100.00)	100.00 (100.00-100.00)	100.00 (100.00-100.00)	100.00 (100.00-100.00)	61.42 (56.74-66.20)	72.34 (68.94-75.67)	88.15 (86.67-89.58)	100.00 (100.00-100.00)	100.00 (100.00-100.00)	100.00 (100.00-100.00)
Norway	100.00 (100.00-100.00)	100.00 (100.00-100.00)	100.00 (100.00-100.00)	100.00 (100.00-100.00)	100.00 (100.00-100.00)	100.00 (100.00-100.00)	72.32 (68.93-75.65)	84.87 (82.98-86.71)	97.52 (97.20-97.81)	100.00 (100.00-100.00)	100.00 (100.00-100.00)	100.00 (100.00-100.00)
Portugal	100.00 (100.00-100.00)	100.00 (100.00-100.00)	100.00 (100.00-100.00)	100.00 (100.00-100.00)	100.00 (100.00-100.00)	100.00 (100.00-100.00)	35.27 (28.60-42.74)	47.00 (40.95-53.50)	67.54 (63.60-71.52)	100.00 (100.00-100.00)	100.00 (100.00-100.00)	100.00 (100.00-100.00)
Spain	100.00 (100.00-100.00)	100.00 (100.00-100.00)	100.00 (100.00-100.00)	100.00 (100.00-100.00)	100.00 (100.00-100.00)	100.00 (100.00-100.00)	45.16 (38.92-51.85)	57.52 (52.53-62.79)	77.44 (74.67-80.15)	100.00 (100.00-100.00)	100.00 (100.00-100.00)	100.00 (100.00-100.00)
Sweden	100.00 (100.00-100.00)	100.00 (100.00-100.00)	100.00 (100.00-100.00)	100.00 (100.00-100.00)	100.00 (100.00-100.00)	100.00 (100.00-100.00)	70.74 (67.17-74.30)	81.87 (79.61-84.05)	95.22 (94.62-95.80)	100.00 (100.00-100.00)	100.00 (100.00-100.00)	100.00 (100.00-100.00)
Switzerland	100.00 (100.00-100.00)	100.00 (100.00-100.00)	100.00 (100.00-100.00)	100.00 (100.00-100.00)	100.00 (100.00-100.00)	100.00 (100.00-100.00)	59.75 (54.89-64.75)	71.90 (68.46-75.29)	86.66 (84.99-88.29)	100.00 (100.00-100.00)	100.00 (100.00-100.00)	100.00 (100.00-100.00)
United Kingdom	100.00 (100.00-100.00)	100.00 (100.00-100.00)	100.00 (100.00-100.00)	100.00 (100.00-100.00)	100.00 (100.00-100.00)	100.00 (100.00-100.00)	67.69 (63.82-71.64)	79.21 (76.69-81.70)	93.29 (92.46-94.11)	100.00 (100.00-100.00)	100.00 (100.00-100.00)	100.00 (100.00-100.00)
Argentina	57.56 (45.42-68.66)	79.02 (69.83-86.35)	88.52 (78.38-94.77)	68.85 (55.96-79.84)	84.47 (79.91-88.21)	92.48 (86.71-96.22)	51.40 (45.11-57.77)	61.18 (55.51-66.70)	77.76 (72.08-81.74)	95.74 (91.49-98.31)	97.35 (94.66-98.91)	99.20 (96.84-99.91)
Chile	82.85 (76.67-87.77)	88.70 (84.06-92.55)	93.76 (89.31-96.70)	71.26 (64.46-77.45)	90.06 (86.34-92.56)	95.27 (92.26-97.35)	52.18 (45.72-58.58)	63.80 (57.87-69.29)	81.92 (76.77-85.59)	90.27 (80.46-96.15)	92.95 (85.74-97.11)	98.49 (94.15-99.84)
Uruguay	90.08 (81.15-95.57)	93.57 (89.66-96.51)	95.33 (90.96-97.94)	94.21 (89.31-97.31)	95.79 (93.22-97.43)	96.73 (94.22-98.31)	43.85 (37.16-50.71)	51.12 (44.60-57.63)	67.01 (61.79-71.81)	97.48 (90.38-99.68)	99.01 (97.86-99.65)	99.56 (98.36-99.95)

Location	Appendix Table 1a. Scaled values for each SDG health-related indicator and three summary indicators by country for 1990, 2000, and 2015											
	Indicator 6.1.1: Risk-weighted prevalence of populations using unsafe or unimproved water sources, as measured by the summary exposure value (SEV) for unsafe water			Indicator 6.2.2a: Risk-weighted prevalence of populations using unsafe or unimproved sanitation, as measured by the summary exposure value (SEV) for unsafe sanitation			Indicator 6.2.1b: Risk-weighted prevalence of populations with unsafe hygiene (no handwashing with soap), as measured by the summary exposure value (SEV) for unsafe hygiene			Indicator 7.1.2: Prevalence of household air pollution, as measured by the summary exposure value (SEV) for household air pollution		
	1990	2000	2015	1990	2000	2015	1990	2000	2015	1990	2000	2015
Belarus	66.70 (50.09-80.32)	74.19 (70.33-77.74)	85.06 (79.36-89.41)	66.55 (52.60-79.60)	71.88 (67.38-75.71)	84.07 (78.99-88.36)	37.69 (27.00-47.15)	44.83 (30.88-56.73)	68.09 (50.55-81.40)	85.60 (70.37-94.96)	94.39 (88.41-97.71)	99.40 (98.77-99.75)
Estonia	80.04 (64.55-90.63)	85.26 (76.69-91.09)	90.28 (80.08-95.79)	80.91 (67.43-90.56)	86.93 (79.34-92.31)	92.46 (86.06-96.68)	40.89 (29.43-50.28)	48.44 (35.36-59.53)	66.45 (50.61-78.11)	79.59 (61.44-91.66)	85.52 (74.72-92.79)	94.30 (87.07-98.27)
Latvia	80.00 (65.26-90.43)	83.25 (75.73-88.75)	89.06 (79.61-95.19)	74.56 (59.35-86.13)	78.70 (69.94-85.28)	88.12 (78.60-94.33)	43.81 (32.15-53.54)	48.62 (34.40-60.32)	66.00 (49.19-78.31)	84.57 (66.84-94.45)	90.52 (82.86-95.20)	95.58 (88.88-98.64)
Lithuania	76.63 (59.56-89.09)	79.16 (61.50-90.23)	86.18 (74.13-93.97)	72.88 (55.98-85.82)	77.91 (61.63-88.93)	86.54 (75.64-94.22)	44.19 (32.08-54.12)	49.29 (35.37-60.69)	65.71 (50.05-77.35)	85.42 (70.00-95.15)	90.87 (79.65-97.06)	96.00 (90.50-98.75)
Moldova	53.12 (36.02-68.96)	46.05 (38.39-52.60)	56.88 (40.35-73.22)	48.61 (29.09-64.94)	41.45 (33.69-49.80)	55.80 (39.39-70.20)	33.79 (22.69-44.06)	34.37 (21.51-46.72)	48.69 (32.21-63.51)	64.93 (45.93-81.92)	74.35 (64.18-83.05)	94.88 (90.48-97.70)
Russia	78.24 (62.27-89.66)	80.18 (72.51-86.31)	85.77 (73.88-93.60)	66.35 (49.67-80.53)	67.61 (59.37-75.02)	79.44 (66.13-89.17)	49.63 (37.21-59.63)	53.15 (38.35-65.20)	68.97 (51.98-81.37)	89.26 (75.75-96.61)	92.82 (86.76-96.76)	97.67 (94.50-99.34)
Ukraine	77.11 (61.22-88.56)	78.11 (70.75-84.35)	76.68 (69.51-83.39)	69.61 (55.28-82.50)	70.93 (63.28-77.73)	78.32 (72.16-83.61)	41.49 (29.61-51.44)	46.52 (31.28-60.11)	64.69 (45.32-80.26)	84.89 (70.21-94.32)	93.63 (88.98-96.86)	97.44 (94.77-98.88)
Albania	56.01 (33.74-72.47)	59.19 (39.96-71.63)	68.08 (48.34-81.69)	80.60 (65.85-89.86)	87.00 (83.08-90.36)	96.88 (94.72-98.30)	19.44 (11.51-28.17)	23.65 (13.87-34.18)	37.61 (23.26-50.86)	45.47 (33.81-57.01)	52.38 (41.17-62.70)	86.35 (79.32-91.47)
Bosnia and Herzegovina	60.65 (39.00-76.31)	71.09 (50.26-82.74)	76.94 (57.29-87.59)	69.47 (50.26-83.95)	87.59 (82.36-91.42)	91.29 (85.13-95.20)	12.70 (6.90-19.51)	21.39 (12.56-30.95)	35.42 (21.91-48.22)	46.54 (34.16-57.72)	59.95 (50.44-68.89)	60.86 (50.33-69.95)
Bulgaria	74.03 (55.17-86.34)	76.83 (56.78-89.07)	79.93 (60.36-90.88)	85.16 (71.76-93.23)	91.70 (84.30-96.46)	95.15 (90.19-97.85)	31.79 (19.99-42.93)	34.44 (21.27-47.16)	47.80 (30.99-62.31)	86.11 (78.45-92.28)	90.63 (84.63-94.69)	95.28 (92.06-97.52)
Croatia	75.65 (55.01-87.89)	78.06 (59.24-88.24)	81.13 (62.09-91.19)	91.82 (85.29-96.04)	94.86 (92.24-96.91)	96.79 (93.69-98.53)	24.58 (16.03-33.21)	29.29 (18.77-39.55)	42.67 (28.35-55.19)	76.02 (65.30-84.93)	87.77 (83.32-91.44)	94.13 (90.39-96.76)

Appendix Table 1a. Scaled values for each SDG health-related indicator and three summary indicators by country for 1990, 2000, and 2015

Location	Indicator 6.1.1: Risk-weighted prevalence of populations using unsafe or unimproved water sources, as measured by the summary exposure value (SEV) for unsafe water			Indicator 6.2.2a: Risk-weighted prevalence of populations using unsafe or unimproved sanitation, as measured by the summary exposure value (SEV) for unsafe sanitation			Indicator 6.2.1b: Risk-weighted prevalence of populations with unsafe hygiene (no handwashing with soap), as measured by the summary exposure value (SEV) for unsafe hygiene			Indicator 7.1.2: Prevalence of household air pollution, as measured by the summary exposure value (SEV) for household air pollution		
	1990	2000	2015	1990	2000	2015	1990	2000	2015	1990	2000	2015
Czech Republic	83.18 (64.58-92.79)	84.99 (66.82-93.67)	85.59 (67.98-94.05)	98.23 (96.34-99.26)	99.10 (98.31-99.59)	99.32 (98.60-99.73)	40.78 (27.04-52.94)	46.77 (31.09-60.31)	57.99 (39.63-73.29)	96.77 (94.57-98.31)	98.34 (97.47-98.96)	98.88 (98.04-99.42)
Hungary	75.50 (58.44-85.06)	83.00 (64.63-91.72)	84.79 (66.89-93.51)	76.23 (69.49-81.86)	87.26 (82.57-90.84)	93.26 (87.60-96.87)	34.03 (22.18-45.07)	38.79 (25.59-51.03)	51.38 (35.05-65.62)	85.82 (77.71-91.82)	89.25 (83.02-94.06)	94.62 (90.63-97.13)
Macedonia	76.42 (56.13-88.16)	78.27 (59.01-88.82)	78.83 (57.89-89.72)	90.47 (80.73-96.05)	92.65 (86.94-96.04)	93.25 (89.86-95.78)	24.35 (15.21-33.70)	28.54 (17.62-39.55)	40.57 (25.88-54.50)	61.04 (49.50-72.21)	71.53 (62.18-80.11)	81.72 (75.10-87.24)
Montenegro	68.72 (49.06-82.10)	72.93 (54.87-85.26)	78.74 (59.36-89.29)	86.55 (67.11-96.32)	88.78 (79.00-94.97)	94.63 (86.72-98.50)	28.09 (17.75-38.24)	32.43 (20.25-44.30)	46.15 (29.61-60.61)	55.49 (43.17-67.14)	72.79 (62.73-81.23)	74.09 (67.06-80.52)
Poland	74.34 (55.21-86.49)	79.77 (61.60-90.31)	82.52 (64.02-92.17)	85.33 (72.92-93.08)	94.22 (89.13-97.30)	96.84 (93.63-98.61)	34.12 (21.39-46.32)	41.63 (26.78-55.13)	55.69 (37.48-71.07)	87.67 (80.30-93.13)	92.05 (87.28-95.63)	94.39 (90.54-97.05)
Romania	57.44 (37.57-74.37)	56.37 (41.75-67.73)	69.91 (50.76-83.48)	61.75 (53.15-69.52)	61.59 (54.03-69.02)	79.54 (67.74-89.41)	30.41 (19.02-41.49)	32.81 (20.45-45.31)	46.46 (30.38-60.84)	75.92 (65.91-84.99)	80.21 (75.19-84.63)	89.02 (82.67-93.68)
Serbia	62.91 (40.24-79.07)	67.60 (45.84-82.19)	73.89 (52.28-85.49)	78.28 (54.80-91.61)	77.85 (65.81-87.05)	85.21 (77.88-90.53)	24.96 (15.73-34.55)	29.26 (18.07-40.54)	42.81 (27.32-57.51)	61.52 (52.73-69.33)	69.23 (59.43-78.05)	78.12 (72.39-82.91)
Slovakia	77.07 (58.18-87.97)	81.32 (63.39-90.89)	84.06 (66.27-92.84)	93.38 (87.57-96.84)	96.56 (94.00-98.25)	98.03 (96.07-99.08)	31.32 (20.25-41.98)	37.47 (24.28-49.44)	52.55 (35.62-66.79)	93.01 (88.89-96.06)	97.18 (95.76-98.14)	98.12 (96.76-99.02)
Slovenia	84.29 (66.56-92.66)	86.29 (69.25-94.28)	86.81 (67.66-94.65)	92.20 (81.07-97.30)	94.63 (92.56-96.26)	96.53 (91.58-98.89)	34.50 (22.85-44.99)	41.76 (27.84-54.11)	54.66 (37.36-69.01)	83.33 (74.00-90.16)	91.68 (88.02-94.31)	95.12 (91.71-97.31)
Armenia	23.76 (9.53-40.15)	22.70 (9.26-36.30)	25.58 (9.80-38.89)	65.51 (50.27-78.95)	70.97 (65.66-75.73)	80.13 (67.99-88.39)	37.40 (27.47-46.16)	42.62 (30.63-52.76)	63.48 (49.47-73.90)	61.33 (42.96-80.71)	81.58 (71.06-89.33)	97.96 (94.79-99.42)
Azerbaijan	48.63 (32.69-63.25)	51.76 (40.96-61.34)	64.04 (49.24-75.43)	46.10 (28.33-63.46)	43.08 (35.20-50.33)	72.62 (59.69-83.23)	41.83 (32.38-49.99)	45.78 (33.80-55.78)	67.33 (54.98-76.13)	66.34 (47.89-82.55)	80.42 (69.63-89.24)	93.25 (82.83-98.19)

Appendix Table 1a. Scaled values for each SDG health-related indicator and three summary indicators by country for 1990, 2000, and 2015

Location	Indicator 6.1.1: Risk-weighted prevalence of populations using unsafe or unimproved water sources, as measured by the summary exposure value (SEV) for unsafe water			Indicator 6.2.2a: Risk-weighted prevalence of populations using unsafe or unimproved sanitation, as measured by the summary exposure value (SEV) for unsafe sanitation			Indicator 6.2.1b: Risk-weighted prevalence of populations with unsafe hygiene (no handwashing with soap), as measured by the summary exposure value (SEV) for unsafe hygiene			Indicator 7.1.2: Prevalence of household air pollution, as measured by the summary exposure value (SEV) for household air pollution		
	**1990**	**2000**	**2015**	**1990**	**2000**	**2015**	**1990**	**2000**	**2015**	**1990**	**2000**	**2015**
Georgia	22.06 (5.33-37.46)	17.19 (3.14-28.83)	23.20 (7.39-37.65)	63.28 (45.76-78.80)	55.35 (43.92-65.82)	70.26 (55.12-82.63)	50.24 (39.42-59.08)	51.87 (37.50-63.45)	69.92 (54.14-81.17)	61.18 (41.21-81.95)	66.50 (51.56-80.82)	80.19 (61.16-92.86)
Kazakhstan	54.15 (37.40-68.59)	60.46 (46.67-72.90)	60.61 (46.11-72.14)	53.80 (41.42-65.17)	57.00 (48.14-64.13)	70.12 (59.17-79.55)	47.78 (37.99-55.72)	54.60 (42.40-63.80)	73.42 (61.49-81.54)	71.71 (49.90-88.26)	80.48 (68.18-89.92)	92.07 (83.51-97.30)
Kyrgyzstan	31.61 (17.55-47.39)	31.74 (20.41-42.34)	41.14 (29.22-52.63)	39.87 (28.49-51.95)	45.72 (38.26-52.20)	37.82 (27.99-46.83)	46.45 (35.05-55.96)	48.44 (34.53-60.05)	59.99 (43.43-73.26)	60.78 (40.62-81.82)	67.34 (49.57-84.06)	81.34 (70.97-90.08)
Mongolia	19.35 (7.50-31.92)	23.22 (11.84-34.50)	31.21 (17.18-45.11)	16.05 (9.76-23.90)	31.47 (24.65-38.59)	43.05 (31.69-54.06)	30.95 (22.48-39.03)	38.29 (27.95-47.31)	57.79 (45.77-66.92)	44.34 (29.48-61.50)	46.72 (34.19-59.86)	68.49 (53.74-81.85)
Tajikistan	50.74 (34.46-66.71)	51.24 (40.19-61.61)	62.93 (52.28-71.78)	38.19 (26.12-51.83)	34.88 (26.01-43.27)	38.33 (28.30-47.97)	33.76 (24.66-41.98)	33.31 (22.68-43.11)	46.30 (32.96-57.26)	48.70 (33.97-67.64)	53.89 (41.11-65.66)	76.19 (60.20-87.81)
Turkmenistan	37.75 (22.00-56.28)	40.67 (22.90-58.90)	48.27 (27.81-67.36)	53.02 (36.18-70.30)	63.41 (46.88-79.03)	81.02 (66.46-91.02)	44.79 (34.63-53.20)	51.30 (38.55-61.45)	71.11 (57.36-80.57)	98.15 (94.82-99.53)	99.26 (98.22-99.80)	99.68 (99.10-99.93)
Uzbekistan	73.27 (62.64-82.32)	75.56 (67.65-82.38)	81.30 (71.55-88.84)	37.23 (26.38-48.43)	36.89 (27.75-44.62)	53.88 (38.98-68.06)	37.75 (27.47-46.53)	43.24 (31.26-53.28)	59.18 (45.47-69.66)	75.12 (54.96-90.96)	84.56 (72.86-92.68)	89.43 (76.20-96.67)
Colombia	36.67 (22.54-49.89)	37.48 (22.47-51.64)	41.20 (25.95-56.00)	68.41 (62.76-72.77)	79.02 (76.36-81.76)	92.09 (88.52-94.90)	31.08 (24.10-38.24)	39.95 (32.48-47.15)	56.82 (48.48-63.53)	76.70 (68.52-83.49)	84.21 (78.82-88.88)	90.58 (83.10-95.58)
Costa Rica	27.44 (14.24-40.17)	28.72 (15.58-40.70)	31.72 (18.33-43.40)	73.59 (64.95-80.95)	93.48 (90.34-95.60)	96.35 (94.71-97.48)	34.88 (27.56-42.02)	42.12 (34.53-49.16)	57.73 (49.72-64.26)	77.19 (63.89-87.17)	89.59 (87.40-91.71)	97.41 (94.78-98.86)
El Salvador	23.87 (12.09-35.55)	31.24 (17.75-43.80)	36.79 (21.39-51.38)	44.13 (35.48-52.27)	54.19 (45.44-62.19)	69.99 (58.83-79.57)	23.07 (16.56-30.08)	30.08 (23.17-37.37)	44.14 (35.80-51.42)	50.71 (32.72-68.12)	68.35 (54.96-78.94)	88.53 (78.19-94.95)
Guatemala	21.40 (10.00-31.98)	24.76 (11.99-37.01)	29.99 (16.46-44.42)	38.55 (29.32-47.45)	53.13 (46.55-58.81)	67.36 (57.12-76.90)	17.52 (11.96-24.16)	21.63 (15.44-28.64)	31.13 (23.80-38.24)	41.76 (25.69-57.44)	50.52 (36.69-62.52)	68.70 (51.93-83.17)

Location	Appendix Table 1a. Scaled values for each SDG health-related indicator and three summary indicators by country for 1990, 2000, and 2015											
	**Indicator 6.1.1: Risk-weighted prevalence of populations using unsafe or unimproved water sources, as measured by the summary exposure value (SEV) for unsafe water**			**Indicator 6.2.2a: Risk-weighted prevalence of populations using unsafe or unimproved sanitation, as measured by the summary exposure value (SEV) for unsafe sanitation**			**Indicator 6.2.1b: Risk-weighted prevalence of populations with unsafe hygiene (no handwashing with soap), as measured by the summary exposure value (SEV) for unsafe hygiene**			**Indicator 7.1.2: Prevalence of household air pollution, as measured by the summary exposure value (SEV) for household air pollution**		
**Location**	**1990**	**2000**	**2015**	**1990**	**2000**	**2015**	**1990**	**2000**	**2015**	**1990**	**2000**	**2015**
Honduras	26.04 (15.74-36.48)	33.23 (22.70-42.57)	39.68 (27.18-49.70)	37.49 (26.75-48.09)	47.02 (38.91-55.06)	69.29 (60.88-76.48)	21.40 (15.21-28.26)	27.26 (20.29-34.39)	40.23 (32.06-47.67)	40.60 (24.61-55.78)	49.96 (35.58-63.35)	69.03 (54.19-81.69)
Mexico	38.68 (29.98-46.29)	41.69 (31.98-49.30)	44.30 (34.60-51.95)	73.42 (71.78-74.91)	83.85 (82.87-84.82)	89.96 (89.11-90.74)	33.38 (26.66-40.31)	42.45 (35.30-49.30)	58.94 (51.36-65.16)	81.81 (77.54-85.63)	84.76 (81.25-87.87)	91.74 (89.40-93.71)
Nicaragua	25.53 (13.44-37.80)	26.89 (15.39-38.35)	31.46 (17.54-44.42)	29.24 (21.50-36.37)	37.06 (30.17-43.01)	52.51 (40.62-63.56)	23.22 (16.75-30.24)	28.51 (21.18-35.73)	41.41 (32.68-48.89)	40.11 (25.69-54.47)	48.22 (34.64-60.37)	68.37 (50.94-82.82)
Panama	38.82 (24.36-52.71)	41.91 (25.92-57.62)	45.41 (29.31-61.60)	53.46 (46.11-60.22)	60.34 (53.85-66.31)	76.94 (67.88-84.64)	42.16 (34.19-49.31)	50.57 (42.33-57.70)	67.25 (58.90-73.38)	75.18 (67.73-82.34)	83.36 (77.69-87.96)	92.36 (85.46-96.82)
Venezuela	39.59 (23.99-53.83)	41.31 (25.77-56.43)	43.72 (27.14-58.97)	80.59 (75.81-84.64)	86.45 (83.09-89.57)	92.24 (88.67-95.00)	37.23 (30.13-44.26)	46.34 (38.90-53.34)	63.37 (55.38-69.53)	97.78 (95.43-99.09)	99.76 (99.64-99.86)	99.90 (99.78-99.96)
Bolivia	43.81 (36.34-50.46)	49.29 (44.06-54.06)	63.38 (57.28-68.52)	26.52 (22.70-30.28)	38.72 (34.86-42.54)	63.09 (52.58-72.66)	15.94 (9.69-22.67)	21.43 (13.53-29.66)	32.62 (21.68-42.99)	58.84 (48.10-69.44)	69.94 (62.57-76.78)	85.05 (75.73-91.73)
Ecuador	53.11 (47.40-58.51)	55.74 (50.56-60.30)	66.56 (60.65-71.66)	50.69 (45.08-56.28)	72.11 (67.69-75.76)	85.78 (80.32-90.43)	23.33 (15.21-31.46)	29.22 (19.42-38.45)	41.91 (29.09-52.92)	76.45 (69.56-82.35)	90.00 (86.29-92.81)	97.36 (95.28-98.74)
Peru	63.11 (58.03-67.58)	66.64 (61.81-70.64)	70.92 (65.17-75.93)	37.65 (30.02-44.71)	55.68 (51.78-59.34)	74.19 (66.66-80.57)	22.74 (14.71-30.90)	28.52 (18.77-37.76)	42.56 (29.82-53.39)	51.42 (37.48-65.08)	66.88 (59.02-73.93)	78.88 (71.69-84.99)
Antigua and Barbuda	39.70 (28.30-50.34)	43.24 (31.07-53.67)	46.37 (35.40-56.25)	73.72 (60.96-83.59)	80.43 (69.69-88.30)	86.27 (77.15-92.20)	36.62 (23.50-48.43)	45.17 (29.80-58.29)	55.09 (36.64-70.91)	95.26 (89.79-98.45)	97.36 (94.52-98.87)	98.27 (95.86-99.41)
The Bahamas	42.63 (31.75-52.06)	46.33 (35.37-55.44)	48.72 (37.67-58.39)	80.47 (71.56-87.27)	87.86 (81.29-92.31)	91.80 (87.56-94.98)	34.52 (23.33-45.03)	42.37 (28.68-53.92)	52.65 (36.02-67.06)	96.49 (91.79-98.92)	98.05 (95.17-99.41)	99.25 (98.27-99.76)
Barbados	44.33 (32.95-54.26)	46.67 (35.33-56.88)	48.36 (36.40-58.24)	73.37 (61.00-84.79)	79.95 (68.88-88.45)	85.78 (76.51-92.01)	29.37 (19.02-39.41)	37.02 (24.66-48.53)	48.22 (32.25-62.28)	99.22 (98.08-99.76)	99.64 (99.14-99.88)	99.91 (99.79-99.97)

Location	Appendix Table 1a. Scaled values for each SDG health-related indicator and three summary indicators by country for 1990, 2000, and 2015											
	**Indicator 6.1.1: Risk-weighted prevalence of populations using unsafe or unimproved water sources, as measured by the summary exposure value (SEV) for unsafe water**			**Indicator 6.2.2a: Risk-weighted prevalence of populations using unsafe or unimproved sanitation, as measured by the summary exposure value (SEV) for unsafe sanitation**			**Indicator 6.2.1b: Risk-weighted prevalence of populations with unsafe hygiene (no handwashing with soap), as measured by the summary exposure value (SEV) for unsafe hygiene**			**Indicator 7.1.2: Prevalence of household air pollution, as measured by the summary exposure value (SEV) for household air pollution**		
	**1990**	**2000**	**2015**	**1990**	**2000**	**2015**	**1990**	**2000**	**2015**	**1990**	**2000**	**2015**
Belize	12.87 (0.23-22.72)	14.89 (1.54-24.97)	16.01 (2.21-26.75)	44.40 (34.17-55.44)	60.91 (49.74-70.70)	76.82 (67.86-84.29)	14.15 (8.03-21.05)	19.49 (11.69-27.76)	28.78 (17.81-39.81)	73.18 (61.19-84.06)	84.39 (76.19-91.07)	89.07 (79.96-94.72)
Cuba	32.08 (21.17-41.35)	32.05 (22.87-40.89)	39.48 (28.95-48.05)	68.90 (55.10-80.56)	69.81 (59.16-79.01)	81.81 (72.50-88.95)	26.49 (16.51-36.60)	29.67 (18.00-41.15)	42.41 (27.33-56.43)	89.06 (77.10-95.87)	83.52 (73.29-91.27)	97.81 (95.39-99.23)
Dominica	28.65 (16.27-38.79)	34.85 (22.56-45.98)	39.58 (27.32-49.67)	49.30 (35.56-62.04)	63.52 (49.96-74.95)	75.78 (65.26-84.23)	23.65 (14.37-33.44)	31.01 (19.27-42.38)	42.65 (27.15-57.01)	69.19 (49.29-85.49)	86.00 (77.17-92.39)	95.26 (89.25-98.57)
Dominican Republic	23.86 (12.07-33.25)	20.94 (9.46-30.17)	30.91 (18.87-40.62)	49.47 (43.00-55.72)	58.98 (53.81-63.54)	73.79 (62.89-82.80)	16.62 (9.72-24.14)	22.39 (13.51-31.46)	34.74 (22.35-46.57)	70.04 (56.52-81.11)	88.61 (84.28-92.34)	93.88 (89.33-97.05)
Grenada	28.61 (17.08-38.62)	36.80 (24.16-47.22)	42.63 (31.30-52.51)	49.58 (37.10-63.07)	65.82 (52.75-77.24)	78.45 (66.39-87.37)	23.98 (14.42-34.31)	32.22 (20.10-44.30)	44.14 (28.20-58.84)	77.69 (60.69-90.08)	93.36 (88.10-96.67)	97.62 (94.52-99.17)
Guyana	21.39 (11.72-30.59)	26.88 (16.28-35.69)	33.30 (22.22-42.48)	42.18 (33.00-51.29)	53.01 (42.98-62.45)	68.77 (57.98-78.36)	16.08 (9.03-24.03)	20.35 (12.00-29.59)	29.41 (17.71-41.34)	83.37 (69.08-93.24)	88.83 (83.41-93.20)	94.23 (87.77-97.87)
Haiti	5.31 (0.00-11.96)	6.91 (0.00-13.82)	8.88 (0.17-16.81)	11.48 (7.02-17.35)	14.19 (9.56-19.42)	21.48 (14.73-28.89)	6.69 (3.43-10.80)	7.87 (4.11-12.78)	11.13 (5.92-17.44)	26.92 (12.39-41.30)	27.48 (12.95-41.33)	33.03 (18.47-47.89)
Jamaica	31.27 (20.90-40.08)	36.68 (26.52-45.20)	42.35 (31.92-51.40)	50.54 (43.66-56.92)	65.72 (60.83-70.46)	77.89 (67.64-86.17)	22.45 (13.57-31.72)	29.04 (18.16-40.30)	38.33 (23.63-52.58)	63.99 (50.38-75.88)	84.47 (76.46-90.56)	92.48 (86.62-96.49)
Saint Lucia	29.67 (18.24-39.20)	37.77 (26.00-48.13)	42.75 (30.96-53.00)	28.63 (20.37-38.37)	52.03 (40.27-63.54)	71.16 (61.18-80.47)	21.22 (12.79-29.90)	28.20 (17.73-38.67)	38.82 (24.81-51.86)	71.48 (58.92-81.48)	87.46 (78.67-93.23)	95.30 (91.31-97.75)
Saint Vincent and the Grenadines	29.72 (17.22-40.39)	34.37 (22.05-44.75)	39.77 (28.20-50.46)	54.70 (40.05-68.22)	64.48 (50.68-76.48)	77.00 (64.85-86.36)	23.88 (14.24-33.94)	31.49 (19.47-43.63)	42.93 (27.08-57.89)	46.80 (31.99-62.44)	80.38 (64.76-90.91)	95.93 (91.48-98.52)
Suriname	24.44 (13.65-33.77)	27.71 (16.34-36.61)	32.52 (21.44-42.09)	61.98 (48.86-72.57)	72.38 (63.59-79.67)	85.94 (80.39-89.96)	16.53 (9.89-23.54)	20.74 (12.71-29.08)	32.32 (20.73-43.23)	68.33 (48.66-85.48)	80.65 (64.75-92.08)	93.88 (87.31-97.47)

Location	Indicator 6.1.1: Risk-weighted prevalence of populations using unsafe or unimproved water sources, as measured by the summary exposure value (SEV) for unsafe water			Indicator 6.2.2a: Risk-weighted prevalence of populations using unsafe or unimproved sanitation, as measured by the summary exposure value (SEV) for unsafe sanitation			Indicator 6.2.1b: Risk-weighted prevalence of populations with unsafe hygiene (no handwashing with soap), as measured by the summary exposure value (SEV) for unsafe hygiene			Indicator 7.1.2: Prevalence of household air pollution, as measured by the summary exposure value (SEV) for household air pollution		
	1990	2000	2015	1990	2000	2015	1990	2000	2015	1990	2000	2015
Trinidad and Tobago	38.18 (27.23-48.17)	40.08 (29.09-49.71)	46.56 (35.81-56.40)	67.82 (59.15-75.65)	79.04 (74.08-83.84)	89.84 (83.98-94.07)	30.46 (19.52-40.84)	36.76 (23.61-48.66)	52.74 (36.19-66.40)	96.68 (92.31-98.88)	98.86 (97.52-99.57)	99.51 (98.89-99.84)
Brazil	29.10 (20.50-36.39)	32.98 (24.35-40.34)	40.06 (31.24-47.06)	77.16 (76.20-78.15)	82.26 (80.88-83.66)	89.30 (88.03-90.39)	18.79 (12.30-25.50)	24.54 (16.66-32.25)	36.48 (26.07-45.81)	77.43 (72.13-82.26)	86.29 (82.97-89.23)	94.41 (92.97-95.70)
Paraguay	19.99 (11.40-27.90)	26.98 (17.79-35.39)	39.10 (27.87-48.21)	42.46 (32.58-52.35)	53.18 (46.10-59.94)	69.29 (59.84-77.94)	19.95 (12.65-27.47)	25.27 (16.50-33.92)	36.34 (24.59-47.03)	47.78 (34.21-59.44)	55.00 (44.22-64.72)	75.97 (66.56-83.70)
China	22.82 (16.55-28.41)	34.91 (28.75-40.32)	50.08 (44.67-55.04)	21.97 (20.15-24.04)	41.91 (39.55-44.18)	66.01 (63.52-68.53)	14.59 (8.55-21.45)	21.53 (13.45-29.78)	37.65 (26.52-47.73)	51.45 (41.11-61.81)	58.82 (49.93-67.74)	78.71 (73.56-83.54)
North Korea	50.16 (33.82-64.15)	47.91 (32.61-61.74)	49.56 (34.52-62.68)	60.31 (46.95-72.87)	56.07 (42.43-68.77)	58.28 (43.95-70.61)	24.67 (15.73-33.41)	26.37 (16.15-36.82)	33.12 (20.05-46.49)	48.39 (36.64-59.52)	50.77 (38.99-61.99)	59.00 (46.93-70.67)
Taiwan	60.88 (43.52-75.31)	66.50 (48.60-79.75)	72.39 (56.21-84.66)	84.18 (76.13-89.92)	90.53 (85.08-94.21)	94.65 (91.17-96.97)	38.14 (27.20-47.86)	50.16 (37.77-60.15)	66.21 (51.52-76.89)	100.00 (100.00-100.00)	100.00 (100.00-100.00)	100.00 (100.00-100.00)
Cambodia	10.99 (0.61-24.39)	16.69 (6.19-28.76)	32.19 (20.70-43.25)	7.12 (4.35-10.46)	13.81 (12.61-15.00)	49.09 (41.32-56.68)	9.42 (5.33-14.35)	11.93 (7.05-17.47)	20.75 (13.65-27.76)	46.09 (34.23-57.39)	46.57 (34.99-57.83)	53.18 (42.45-63.26)
Indonesia	45.11 (32.74-56.27)	51.35 (40.04-61.65)	49.30 (37.77-60.07)	20.87 (15.88-26.29)	40.95 (35.42-46.89)	72.06 (61.82-80.39)	18.99 (12.55-25.81)	27.64 (19.76-35.31)	44.39 (33.60-53.33)	55.19 (43.19-67.65)	69.39 (57.51-79.04)	82.30 (72.50-89.83)
Laos	24.25 (11.36-37.22)	31.59 (20.69-42.17)	37.77 (26.97-47.22)	13.37 (7.93-20.68)	28.18 (22.84-33.75)	66.36 (58.28-73.73)	10.50 (6.04-15.69)	13.60 (8.30-19.46)	22.38 (15.21-29.69)	46.97 (35.14-58.29)	46.78 (35.26-57.96)	49.02 (37.03-59.71)
Malaysia	55.92 (37.88-70.33)	64.77 (45.00-78.38)	71.80 (55.68-84.75)	79.99 (75.16-84.32)	93.32 (91.26-94.89)	97.02 (95.32-98.23)	30.66 (22.61-38.44)	42.09 (32.61-50.14)	59.92 (48.79-68.20)	94.17 (87.04-97.79)	98.86 (97.72-99.47)	99.61 (99.10-99.87)
Maldives	12.09 (0.00-22.54)	15.55 (1.94-26.85)	21.14 (8.06-32.06)	30.87 (17.72-45.83)	61.92 (45.48-75.65)	79.75 (67.23-88.82)	16.60 (10.79-22.96)	21.34 (14.75-28.35)	32.32 (24.35-39.83)	64.23 (51.18-77.78)	76.10 (67.55-84.12)	95.43 (91.09-98.08)

Appendix Table 1a. Scaled values for each SDG health-related indicator and three summary indicators by country for 1990, 2000, and 2015

	Appendix Table 1a. Scaled values for each SDG health-related indicator and three summary indicators by country for 1990, 2000, and 2015											
	Indicator 6.1.1: Risk-weighted prevalence of populations using unsafe or unimproved water sources, as measured by the summary exposure value (SEV) for unsafe water			Indicator 6.2.2a: Risk-weighted prevalence of populations using unsafe or unimproved sanitation, as measured by the summary exposure value (SEV) for unsafe sanitation			Indicator 6.2.1b: Risk-weighted prevalence of populations with unsafe hygiene (no handwashing with soap), as measured by the summary exposure value (SEV) for unsafe hygiene			Indicator 7.1.2: Prevalence of household air pollution, as measured by the summary exposure value (SEV) for household air pollution		
**Location**	**1990**	**2000**	**2015**	**1990**	**2000**	**2015**	**1990**	**2000**	**2015**	**1990**	**2000**	**2015**
Mauritius	62.22 (44.24-76.83)	68.98 (49.11-83.97)	74.29 (56.58-88.36)	66.74 (54.99-77.22)	86.79 (80.57-91.58)	94.06 (90.75-96.53)	25.32 (18.02-32.83)	34.65 (26.11-42.28)	51.13 (40.68-59.57)	91.18 (83.07-96.45)	97.55 (95.94-98.63)	98.15 (96.02-99.34)
Myanmar	15.25 (4.95-26.51)	19.10 (8.72-30.07)	31.39 (19.38-43.95)	10.56 (5.55-16.15)	18.37 (11.62-25.14)	42.30 (27.92-57.99)	10.96 (6.17-16.44)	14.52 (8.65-20.88)	26.87 (18.36-34.83)	46.39 (34.89-57.87)	46.73 (34.99-58.02)	52.61 (40.55-64.52)
Philippines	22.92 (12.71-33.75)	25.15 (13.15-35.82)	32.32 (18.82-45.93)	60.98 (54.94-66.78)	76.14 (72.61-79.61)	87.69 (82.30-91.78)	31.25 (21.98-39.82)	36.59 (26.17-45.80)	48.27 (36.05-58.37)	63.93 (53.77-72.72)	72.87 (63.91-80.22)	70.23 (59.03-80.06)
Sri Lanka	27.85 (16.69-38.47)	38.30 (26.73-49.63)	51.01 (36.73-62.66)	19.87 (12.49-27.11)	39.84 (29.24-50.85)	69.04 (51.77-83.02)	27.18 (18.60-35.41)	35.80 (25.81-44.91)	52.87 (40.57-62.50)	50.68 (39.05-61.83)	55.69 (44.86-65.90)	61.34 (49.33-73.78)
Seychelles	57.55 (39.46-72.15)	65.12 (48.14-79.34)	70.74 (52.17-83.67)	72.29 (54.92-84.28)	83.99 (70.28-91.90)	90.35 (81.04-95.34)	32.55 (24.49-40.17)	42.55 (33.33-50.40)	58.73 (47.69-67.09)	95.90 (91.23-98.62)	98.34 (96.61-99.31)	99.30 (98.40-99.76)
Thailand	21.78 (11.32-31.40)	24.90 (13.08-34.63)	34.10 (20.19-47.12)	80.97 (76.84-84.79)	96.43 (94.79-97.51)	98.18 (96.75-99.04)	23.93 (16.62-31.21)	31.60 (23.33-39.28)	45.73 (35.85-53.91)	64.56 (54.42-73.86)	79.25 (72.71-84.87)	88.03 (80.92-93.44)
Timor-Leste	42.13 (28.04-55.05)	42.47 (29.01-54.54)	64.87 (53.55-74.01)	6.65 (3.07-10.86)	5.40 (3.06-8.15)	39.85 (28.41-52.07)	11.26 (6.55-16.75)	15.19 (9.45-21.64)	28.35 (20.39-36.06)	46.42 (34.74-57.86)	47.22 (35.45-58.40)	50.76 (39.53-62.40)
Vietnam	43.46 (29.67-55.70)	51.67 (39.94-61.81)	58.33 (47.38-67.28)	11.44 (8.69-14.05)	28.48 (24.37-32.39)	67.89 (58.09-76.53)	18.64 (11.72-25.88)	25.16 (16.90-33.36)	39.36 (28.50-48.76)	50.06 (38.22-61.62)	55.75 (45.57-65.31)	79.18 (71.13-85.92)
Federated States of Micronesia	22.74 (12.20-32.74)	28.71 (16.76-39.12)	35.26 (23.03-46.11)	51.57 (35.75-65.70)	67.19 (51.99-78.71)	82.54 (70.91-89.61)	24.02 (14.80-33.47)	30.61 (19.16-41.94)	40.51 (25.60-54.56)	67.92 (57.05-77.99)	72.92 (63.86-81.84)	84.09 (74.48-91.62)
Fiji	28.95 (17.94-39.54)	34.52 (21.30-46.01)	40.52 (27.97-51.12)	66.25 (51.03-77.56)	78.40 (65.53-87.26)	89.18 (81.44-94.24)	26.29 (17.03-35.20)	33.34 (22.41-43.57)	45.29 (30.77-57.90)	66.93 (54.90-77.93)	77.50 (66.52-86.23)	90.36 (83.15-95.38)
Kiribati	15.38 (6.16-24.16)	19.12 (9.12-28.49)	23.58 (12.74-33.88)	33.02 (19.67-47.53)	44.47 (29.22-59.45)	60.25 (43.36-74.26)	16.31 (9.58-23.74)	19.51 (11.56-27.96)	26.78 (16.13-38.01)	61.93 (49.78-73.70)	69.60 (56.71-80.97)	80.07 (69.09-89.39)

	Appendix Table 1a. Scaled values for each SDG health-related indicator and three summary indicators by country for 1990, 2000, and 2015											
	**Indicator 6.1.1: Risk-weighted prevalence of populations using unsafe or unimproved water sources, as measured by the summary exposure value (SEV) for unsafe water**			**Indicator 6.2.2a: Risk-weighted prevalence of populations using unsafe or unimproved sanitation, as measured by the summary exposure value (SEV) for unsafe sanitation**			**Indicator 6.2.1b: Risk-weighted prevalence of populations with unsafe hygiene (no handwashing with soap), as measured by the summary exposure value (SEV) for unsafe hygiene**			**Indicator 7.1.2: Prevalence of household air pollution, as measured by the summary exposure value (SEV) for household air pollution**		
**Location**	**1990**	**2000**	**2015**	**1990**	**2000**	**2015**	**1990**	**2000**	**2015**	**1990**	**2000**	**2015**
Marshall Islands	17.71 (8.24-27.17)	24.16 (13.12-34.72)	32.66 (20.13-43.47)	38.60 (24.99-53.18)	57.26 (41.71-71.00)	79.01 (66.49-87.31)	17.85 (10.82-25.47)	23.20 (14.47-32.20)	33.53 (21.33-45.28)	85.54 (77.79-91.52)	81.79 (73.42-88.26)	89.88 (81.94-94.85)
Papua New Guinea	4.41 (0.28-9.47)	7.09 (2.02-12.20)	14.43 (6.58-21.96)	4.95 (2.04-8.94)	7.40 (3.58-11.94)	19.81 (11.23-31.39)	8.68 (4.78-13.42)	11.05 (6.25-16.57)	16.35 (9.74-23.68)	49.38 (38.13-60.56)	50.88 (39.93-61.84)	55.32 (42.97-66.57)
Samoa	27.84 (15.29-37.82)	33.13 (21.16-43.93)	40.08 (26.41-51.66)	71.38 (54.37-84.57)	83.49 (69.87-91.69)	93.70 (86.87-97.12)	29.20 (18.54-39.74)	35.97 (23.07-48.13)	48.33 (31.83-62.86)	60.42 (49.41-69.86)	64.74 (54.10-74.08)	71.70 (60.09-81.84)
Solomon Islands	12.53 (4.48-20.70)	19.42 (9.77-28.64)	25.53 (14.05-36.07)	11.49 (5.61-19.82)	24.48 (13.66-37.74)	40.12 (26.46-54.90)	9.91 (5.53-15.10)	13.93 (8.08-20.61)	20.66 (12.33-29.44)	48.35 (36.64-59.26)	50.17 (39.19-60.78)	55.68 (44.43-66.79)
Tonga	23.97 (13.25-33.91)	29.07 (17.11-39.39)	35.02 (23.14-46.21)	54.38 (38.24-68.36)	67.48 (52.59-79.17)	82.37 (70.93-89.76)	25.75 (16.29-35.28)	31.81 (20.50-42.64)	41.61 (27.27-54.69)	59.28 (48.23-69.58)	65.40 (54.44-75.41)	80.35 (68.80-89.63)
Vanuatu	10.58 (1.14-18.70)	13.46 (2.78-21.90)	18.90 (7.05-28.40)	20.26 (10.90-32.42)	27.63 (17.64-39.10)	47.54 (34.39-60.67)	13.85 (8.14-20.30)	17.99 (10.86-25.69)	26.29 (16.43-36.34)	53.08 (42.18-63.80)	53.30 (42.72-64.15)	61.16 (49.24-72.91)
Afghanistan	1.40 (0.00-7.34)	1.80 (0.00-6.83)	8.88 (0.00-16.80)	9.79 (3.47-19.01)	9.71 (3.95-17.90)	28.43 (18.32-39.16)	3.45 (1.59-6.03)	3.05 (1.38-5.38)	4.38 (2.09-7.37)	35.43 (23.86-47.38)	35.20 (23.61-46.80)	48.74 (34.28-64.86)
Algeria	12.85 (2.38-23.22)	18.84 (7.15-29.17)	22.23 (8.95-35.13)	53.15 (36.73-69.29)	77.15 (70.28-83.06)	83.80 (74.18-90.64)	13.33 (7.84-19.31)	16.34 (9.79-23.51)	23.03 (14.51-31.73)	93.41 (82.25-98.45)	97.33 (95.04-98.81)	99.76 (99.49-99.90)
Bahrain	22.50 (8.39-34.64)	27.18 (11.58-40.09)	32.38 (16.73-46.16)	83.04 (72.13-91.23)	92.33 (87.01-95.99)	97.08 (94.86-98.47)	21.97 (14.70-29.21)	29.35 (20.18-37.72)	41.45 (29.41-51.67)	99.36 (98.13-99.87)	99.58 (98.73-99.92)	99.81 (99.41-99.96)
Egypt	14.68 (1.07-25.74)	19.01 (4.36-30.55)	21.35 (6.18-33.05)	76.48 (65.70-85.71)	92.87 (90.13-95.05)	96.73 (93.79-98.40)	11.04 (6.19-16.61)	16.49 (9.75-23.76)	26.06 (16.35-35.66)	99.98 (99.95-100.00)	99.25 (98.95-99.47)	99.99 (99.96-100.00)
Iran	16.02 (3.75-26.30)	24.41 (10.42-36.82)	30.03 (14.91-43.11)	42.37 (23.32-62.83)	72.60 (54.05-85.58)	85.83 (74.57-92.98)	12.93 (7.51-18.91)	20.46 (12.63-28.55)	32.22 (21.15-42.64)	94.46 (84.61-98.67)	98.54 (96.99-99.38)	99.52 (98.67-99.89)

Appendix Table 1a. Scaled values for each SDG health-related indicator and three summary indicators by country for 1990, 2000, and 2015

Location	Indicator 6.1.1: Risk-weighted prevalence of populations using unsafe or unimproved water sources, as measured by the summary exposure value (SEV) for unsafe water			Indicator 6.2.2a: Risk-weighted prevalence of populations using unsafe or unimproved sanitation, as measured by the summary exposure value (SEV) for unsafe sanitation			Indicator 6.2.1b: Risk-weighted prevalence of populations with unsafe hygiene (no handwashing with soap), as measured by the summary exposure value (SEV) for unsafe hygiene			Indicator 7.1.2: Prevalence of household air pollution, as measured by the summary exposure value (SEV) for household air pollution		
	1990	2000	2015	1990	2000	2015	1990	2000	2015	1990	2000	2015
Iraq	14.06 (1.93-24.08)	17.26 (4.34-27.59)	23.19 (9.24-34.45)	73.03 (57.89-83.89)	86.03 (80.06-90.38)	91.15 (87.73-93.93)	11.05 (6.30-16.47)	13.95 (8.20-20.29)	22.10 (13.87-30.38)	96.32 (89.70-99.21)	97.30 (94.50-98.81)	99.35 (98.58-99.76)
Jordan	27.41 (13.17-40.69)	33.51 (19.93-45.80)	44.00 (31.16-55.49)	86.07 (75.72-92.69)	96.21 (93.97-97.68)	99.53 (99.28-99.71)	22.84 (13.96-32.10)	28.30 (17.02-39.53)	39.54 (24.96-53.40)	99.71 (99.05-99.94)	99.92 (99.81-99.97)	99.98 (99.94-99.99)
Kuwait	26.71 (11.45-39.76)	30.70 (15.49-44.78)	36.56 (20.57-50.58)	91.12 (85.06-95.53)	95.66 (92.11-97.73)	98.44 (97.22-99.20)	26.72 (18.44-34.63)	38.65 (28.05-47.72)	55.15 (41.36-65.80)	99.72 (99.07-99.95)	99.85 (99.53-99.97)	99.94 (99.79-99.99)
Lebanon	7.84 (0.00-17.31)	10.73 (2.06-20.04)	22.35 (10.69-35.02)	86.89 (78.17-92.63)	92.48 (87.23-95.90)	97.23 (95.14-98.46)	19.95 (12.28-27.76)	26.09 (16.44-35.83)	40.09 (26.52-52.34)	99.95 (99.85-99.99)	99.98 (99.93-100.00)	99.99 (99.98-100.00)
Libya	16.71 (4.25-27.88)	22.08 (8.62-33.86)	25.97 (12.26-39.40)	65.25 (48.03-79.31)	83.65 (72.72-90.84)	92.32 (86.91-95.91)	12.55 (7.62-18.35)	14.88 (9.12-21.14)	20.33 (12.99-27.73)	96.51 (90.08-99.17)	97.70 (94.54-99.25)	98.90 (96.70-99.79)
Morocco	7.87 (0.00-15.69)	12.09 (0.82-21.15)	17.65 (3.86-28.82)	51.56 (45.54-57.06)	66.91 (60.89-72.78)	78.08 (64.96-87.15)	6.56 (3.39-10.49)	8.20 (4.40-12.77)	12.58 (7.18-18.40)	87.89 (68.73-97.27)	93.80 (88.22-97.28)	96.63 (90.22-99.29)
Palestine	11.69 (1.02-22.34)	18.60 (6.68-29.42)	20.78 (7.95-32.04)	72.97 (58.25-84.56)	86.82 (77.96-93.01)	90.80 (83.96-95.11)	18.40 (10.84-26.62)	25.50 (15.63-35.47)	27.30 (15.77-39.36)	99.95 (99.87-99.99)	99.94 (99.88-99.98)	98.95 (98.36-99.38)
Oman	1.33 (0.00-7.07)	12.64 (2.18-23.40)	22.39 (9.43-35.00)	57.92 (40.66-73.61)	87.24 (78.85-92.92)	95.43 (92.10-97.58)	15.93 (9.93-22.56)	21.50 (14.23-28.71)	31.11 (21.67-39.67)	97.05 (91.35-99.37)	98.21 (94.85-99.66)	99.15 (97.64-99.83)
Qatar	23.27 (9.36-36.04)	26.95 (12.93-39.61)	33.68 (17.47-47.42)	84.97 (74.33-91.95)	92.17 (86.37-95.75)	97.65 (95.96-98.78)	26.83 (19.13-34.45)	34.48 (25.22-42.63)	50.52 (38.98-59.61)	99.96 (99.87-99.99)	99.98 (99.93-100.00)	99.99 (99.97-100.00)
Saudi Arabia	17.61 (4.52-27.65)	22.82 (9.72-33.13)	29.57 (16.41-39.87)	73.03 (67.61-77.51)	89.08 (86.34-91.32)	96.49 (95.56-97.22)	18.27 (11.79-24.97)	24.16 (16.23-31.89)	37.04 (26.14-46.74)	98.44 (97.26-99.13)	99.12 (98.37-99.53)	99.64 (99.36-99.81)
Sudan	1.40 (0.00-7.51)	2.74 (0.00-9.60)	8.18 (0.00-17.59)	3.85 (1.27-7.80)	6.87 (2.45-13.56)	20.45 (9.37-35.32)	5.38 (2.66-8.92)	6.89 (3.59-11.19)	10.99 (6.16-16.84)	39.45 (26.59-52.89)	40.23 (29.89-50.53)	57.34 (48.10-66.04)

	Appendix Table 1a. Scaled values for each SDG health-related indicator and three summary indicators by country for 1990, 2000, and 2015											
Location	Indicator 6.1.1: Risk-weighted prevalence of populations using unsafe or unimproved water sources, as measured by the summary exposure value (SEV) for unsafe water			Indicator 6.2.2a: Risk-weighted prevalence of populations using unsafe or unimproved sanitation, as measured by the summary exposure value (SEV) for unsafe sanitation			Indicator 6.2.1b: Risk-weighted prevalence of populations with unsafe hygiene (no handwashing with soap), as measured by the summary exposure value (SEV) for unsafe hygiene			Indicator 7.1.2: Prevalence of household air pollution, as measured by the summary exposure value (SEV) for household air pollution		
	1990	2000	2015	1990	2000	2015	1990	2000	2015	1990	2000	2015
Syria	12.71 (0.18-23.03)	15.99 (2.48-26.19)	18.53 (4.60-29.98)	61.93 (47.99-74.49)	76.69 (70.80-81.80)	88.27 (81.87-93.40)	9.26 (4.93-14.31)	13.44 (7.55-20.13)	22.12 (13.32-31.52)	98.14 (94.66-99.64)	99.41 (98.54-99.85)	99.79 (99.41-99.96)
Tunisia	12.42 (0.00-22.80)	14.22 (0.00-24.77)	16.39 (2.17-27.51)	67.35 (53.59-78.62)	84.25 (77.04-89.61)	91.66 (85.17-95.68)	8.82 (4.77-13.55)	14.40 (8.46-20.96)	26.19 (16.40-35.72)	90.07 (73.20-97.72)	94.90 (90.33-97.70)	99.71 (99.32-99.90)
Turkey	15.11 (3.09-24.63)	22.67 (7.96-34.39)	28.51 (12.92-42.07)	61.64 (54.02-68.22)	78.44 (76.93-79.80)	86.79 (79.86-92.20)	13.33 (7.76-19.20)	17.78 (10.98-24.93)	28.12 (18.63-37.28)	86.30 (67.86-96.29)	92.10 (87.29-95.49)	98.12 (94.16-99.64)
United Arab Emirates	17.41 (6.01-28.86)	26.68 (14.04-39.24)	34.11 (19.46-48.50)	88.89 (80.03-94.36)	97.04 (94.85-98.44)	98.83 (97.87-99.41)	29.48 (21.62-37.13)	42.35 (31.96-51.13)	53.86 (39.55-65.17)	99.92 (99.74-99.99)	99.97 (99.93-99.99)	99.99 (99.98-100.00)
Yemen	3.17 (0.00-9.41)	3.37 (0.00-9.72)	11.16 (0.97-20.59)	11.96 (6.10-20.04)	19.36 (12.27-26.83)	39.36 (26.70-52.93)	3.98 (1.92-6.84)	4.93 (2.41-8.25)	7.38 (3.94-11.69)	50.10 (39.34-60.86)	70.24 (58.30-79.89)	77.69 (65.91-86.73)
Bangladesh	7.15 (0.00-16.81)	8.36 (0.00-18.23)	13.17 (0.00-23.62)	8.67 (4.66-14.05)	16.95 (12.72-21.57)	39.39 (26.58-52.60)	1.73 (0.75-3.22)	2.49 (1.10-4.52)	4.74 (2.25-8.24)	28.90 (13.92-42.51)	30.02 (14.96-42.84)	36.44 (22.06-50.19)
Bhutan	45.54 (32.05-58.25)	61.47 (50.02-70.62)	75.23 (67.52-81.41)	14.57 (3.96-30.60)	31.21 (14.29-52.01)	62.58 (48.65-74.36)	1.85 (0.82-3.42)	2.68 (1.19-4.86)	4.70 (2.25-8.16)	38.52 (19.39-60.12)	46.32 (28.26-63.87)	71.12 (48.99-88.36)
India	10.55 (2.97-17.58)	14.57 (5.59-22.28)	22.22 (12.05-30.60)	17.02 (15.44-18.53)	25.90 (24.37-27.30)	48.19 (44.32-52.20)	2.34 (1.06-4.21)	3.72 (1.77-6.45)	7.91 (4.01-13.08)	35.41 (22.03-47.45)	41.07 (29.13-52.72)	53.39 (42.89-62.86)
Nepal	3.66 (0.00-10.40)	10.40 (0.00-18.70)	15.36 (3.45-24.58)	0.57 (0.00-2.04)	15.93 (14.53-17.48)	54.33 (42.71-65.84)	1.20 (0.50-2.29)	1.79 (0.79-3.31)	3.35 (1.57-5.85)	39.85 (26.25-52.00)	40.00 (27.14-51.86)	43.96 (30.01-55.73)
Pakistan	9.64 (0.00-18.96)	11.20 (0.00-20.29)	15.31 (1.02-25.85)	22.66 (18.68-26.95)	44.20 (32.45-54.93)	75.13 (66.17-82.73)	2.07 (0.90-3.84)	2.81 (1.29-5.08)	4.51 (2.16-7.83)	39.12 (20.98-59.82)	38.28 (25.28-50.68)	52.05 (35.04-67.96)
Botswana	12.68 (0.00-23.21)	18.09 (4.80-29.95)	24.05 (9.77-36.55)	17.31 (14.66-19.84)	25.13 (16.09-35.28)	43.96 (29.94-57.15)	6.46 (3.19-10.85)	10.00 (5.13-16.00)	16.36 (8.86-25.04)	19.79 (9.90-32.48)	38.33 (21.75-54.32)	64.41 (39.65-84.20)

	Appendix Table 1a. Scaled values for each SDG health-related indicator and three summary indicators by country for 1990, 2000, and 2015											
	**Indicator 6.1.1: Risk-weighted prevalence of populations using unsafe or unimproved water sources, as measured by the summary exposure value (SEV) for unsafe water**			**Indicator 6.2.2a: Risk-weighted prevalence of populations using unsafe or unimproved sanitation, as measured by the summary exposure value (SEV) for unsafe sanitation**			**Indicator 6.2.1b: Risk-weighted prevalence of populations with unsafe hygiene (no handwashing with soap), as measured by the summary exposure value (SEV) for unsafe hygiene**			**Indicator 7.1.2: Prevalence of household air pollution, as measured by the summary exposure value (SEV) for household air pollution**		
**Location**	**1990**	**2000**	**2015**	**1990**	**2000**	**2015**	**1990**	**2000**	**2015**	**1990**	**2000**	**2015**
Lesotho	4.17 (0.00-11.39)	6.74 (0.00-14.87)	12.78 (1.01-22.42)	3.92 (1.71-7.15)	5.26 (2.84-8.04)	15.12 (9.75-21.04)	4.25 (2.04-7.53)	5.76 (2.78-10.06)	9.91 (4.88-16.69)	25.66 (10.87-43.52)	27.62 (16.98-38.92)	41.46 (24.90-59.44)
Namibia	9.23 (0.00-17.79)	13.84 (2.16-23.41)	19.99 (7.30-31.33)	28.80 (22.19-36.15)	35.94 (31.04-40.97)	57.05 (46.60-67.31)	7.05 (3.48-11.77)	9.06 (4.57-14.88)	14.81 (7.80-23.37)	15.85 (8.52-24.18)	27.33 (18.31-34.90)	41.84 (27.96-54.99)
South Africa	19.29 (8.12-28.32)	23.74 (12.85-32.56)	31.24 (19.51-40.91)	52.90 (48.06-57.20)	56.94 (53.66-59.88)	70.73 (66.19-74.78)	10.69 (5.60-17.10)	12.99 (6.88-20.48)	19.51 (10.45-30.12)	54.58 (46.07-62.50)	65.33 (59.87-67.79)	84.77 (80.68-88.12)
Swaziland	3.67 (0.00-10.47)	7.17 (0.00-14.83)	13.11 (0.15-22.84)	29.06 (19.15-39.51)	33.85 (27.17-40.60)	42.35 (32.17-52.50)	6.46 (3.19-10.84)	9.31 (4.76-15.14)	14.36 (7.58-23.05)	13.39 (4.61-27.71)	21.71 (12.43-32.16)	37.33 (17.31-58.62)
Zimbabwe	12.34 (1.49-21.44)	14.91 (3.42-23.94)	15.48 (4.23-24.89)	37.80 (32.39-43.51)	42.17 (36.95-47.50)	47.30 (37.84-55.87)	5.86 (2.87-9.95)	8.03 (3.95-13.41)	10.43 (5.09-17.41)	15.56 (5.27-31.31)	26.76 (16.43-36.99)	27.89 (15.85-40.28)
Benin	2.67 (0.00-8.87)	4.61 (0.00-11.92)	7.26 (0.00-16.39)	4.03 (2.05-6.81)	5.19 (3.36-7.33)	12.75 (7.62-19.70)	1.09 (0.45-2.08)	1.28 (0.55-2.44)	1.95 (0.86-3.59)	0.84 (0.32-1.61)	2.34 (1.08-3.73)	6.68 (2.80-12.53)
Burkina Faso	0.10 (0.00-1.41)	1.45 (0.00-6.97)	3.92 (0.00-12.25)	3.32 (1.71-5.19)	4.09 (2.37-5.92)	10.68 (6.46-15.68)	0.65 (0.26-1.29)	0.79 (0.32-1.54)	1.27 (0.55-2.42)	1.00 (0.30-2.19)	1.21 (0.46-2.19)	5.96 (2.56-10.16)
Cameroon	1.29 (0.00-6.32)	2.21 (0.00-8.01)	6.84 (0.00-14.83)	7.68 (4.70-11.54)	10.99 (7.59-14.76)	24.00 (16.16-33.18)	2.67 (1.20-4.82)	3.17 (1.46-5.67)	5.02 (2.40-8.73)	4.95 (1.85-10.19)	9.68 (4.98-15.49)	23.84 (11.85-37.68)
Cape Verde	4.80 (0.00-12.93)	9.20 (0.00-18.28)	15.35 (1.75-26.57)	13.14 (6.85-21.05)	28.58 (17.72-40.89)	56.04 (41.91-69.07)	2.19 (0.98-4.01)	3.28 (1.54-5.83)	6.21 (3.02-10.55)	21.28 (8.93-38.62)	43.41 (29.56-57.53)	63.99 (43.40-81.14)
Chad	0.01 (0.00-0.00)	0.03 (0.00-0.26)	1.64 (0.00-7.46)	0.54 (0.00-1.36)	0.34 (0.00-0.85)	8.48 (5.21-12.80)	0.83 (0.33-1.62)	0.92 (0.37-1.77)	1.63 (0.70-3.04)	2.15 (0.90-3.82)	2.48 (1.11-4.09)	3.42 (1.46-6.38)
Cote d'Ivoire	2.83 (0.00-8.87)	6.61 (0.00-15.29)	7.89 (0.00-17.32)	10.52 (7.02-14.51)	24.76 (19.16-30.88)	32.78 (23.75-42.35)	1.77 (0.75-3.27)	2.04 (0.90-3.75)	2.70 (1.24-4.87)	7.31 (3.50-11.16)	13.56 (6.64-21.41)	21.01 (10.15-34.10)

	Appendix Table 1a. Scaled values for each SDG health-related indicator and three summary indicators by country for 1990, 2000, and 2015											
	**Indicator 6.1.1: Risk-weighted prevalence of populations using unsafe or unimproved water sources, as measured by the summary exposure value (SEV) for unsafe water**			**Indicator 6.2.2a: Risk-weighted prevalence of populations using unsafe or unimproved sanitation, as measured by the summary exposure value (SEV) for unsafe sanitation**			**Indicator 6.2.1b: Risk-weighted prevalence of populations with unsafe hygiene (no handwashing with soap), as measured by the summary exposure value (SEV) for unsafe hygiene**			**Indicator 7.1.2: Prevalence of household air pollution, as measured by the summary exposure value (SEV) for household air pollution**		
**Location**	**1990**	**2000**	**2015**	**1990**	**2000**	**2015**	**1990**	**2000**	**2015**	**1990**	**2000**	**2015**
The Gambia	3.42 (0.00-10.87)	6.02 (0.00-14.87)	7.40 (0.00-17.21)	10.81 (6.05-16.56)	28.57 (20.72-36.61)	37.69 (25.55-50.72)	1.29 (0.54-2.43)	1.51 (0.65-2.83)	2.29 (1.04-4.17)	2.86 (1.33-4.67)	2.96 (1.28-4.80)	3.63 (1.71-6.37)
Ghana	1.59 (0.00-6.41)	4.44 (0.00-11.23)	7.46 (0.00-16.85)	5.22 (3.50-7.34)	13.80 (10.57-16.95)	33.49 (24.60-43.34)	2.61 (1.19-4.72)	3.33 (1.55-5.95)	5.65 (2.71-9.74)	3.85 (1.69-6.77)	7.77 (4.28-9.72)	21.43 (13.63-27.42)
Guinea	0.01 (0.00-0.00)	1.37 (0.00-5.66)	6.95 (0.00-15.10)	6.75 (3.55-10.67)	8.08 (5.29-11.12)	19.77 (13.68-26.12)	0.98 (0.40-1.88)	1.05 (0.43-2.00)	1.28 (0.54-2.42)	1.46 (0.61-2.49)	1.39 (0.62-2.06)	1.68 (0.82-2.56)
Guinea-Bissau	0.47 (0.00-4.21)	1.19 (0.00-5.87)	2.63 (0.00-9.40)	10.41 (5.84-16.32)	16.31 (10.71-21.99)	23.55 (14.74-33.01)	0.98 (0.40-1.90)	1.15 (0.49-2.21)	1.48 (0.64-2.80)	2.13 (0.90-3.83)	1.70 (0.72-2.92)	2.61 (1.11-4.82)
Liberia	2.88 (0.00-9.78)	2.28 (0.00-8.35)	4.77 (0.00-12.65)	19.11 (12.87-27.26)	11.68 (7.13-17.36)	28.75 (21.21-36.61)	1.07 (0.45-2.05)	0.93 (0.39-1.75)	1.64 (0.74-3.01)	1.10 (0.48-1.58)	1.21 (0.54-1.76)	1.98 (0.87-3.08)
Mali	0.30 (0.00-2.61)	1.48 (0.00-5.57)	4.38 (0.00-11.57)	3.65 (2.16-5.12)	8.52 (6.09-10.92)	13.03 (7.71-18.87)	0.73 (0.29-1.46)	0.92 (0.38-1.77)	1.26 (0.53-2.38)	0.83 (0.34-1.31)	1.08 (0.47-1.58)	1.53 (0.70-2.43)
Mauritania	1.28 (0.00-6.17)	3.34 (0.00-9.96)	4.70 (0.00-12.94)	6.24 (3.05-10.12)	8.93 (5.54-12.46)	28.14 (19.00-38.44)	1.82 (0.78-3.34)	2.08 (0.91-3.83)	3.27 (1.51-5.83)	18.45 (7.34-34.47)	24.60 (15.94-31.09)	40.69 (26.58-54.84)
Niger	0.13 (0.00-2.12)	0.12 (0.00-1.62)	2.00 (0.00-8.17)	0.12 (0.00-0.68)	0.37 (0.00-1.04)	2.71 (1.13-4.85)	0.71 (0.28-1.39)	0.68 (0.27-1.35)	0.86 (0.36-1.67)	1.61 (0.69-2.82)	1.78 (0.83-2.73)	2.16 (1.00-3.30)
Nigeria	0.58 (0.00-4.24)	1.01 (0.00-5.42)	5.34 (0.00-13.25)	18.02 (11.70-25.55)	18.92 (12.70-25.31)	34.67 (26.22-43.17)	2.25 (1.01-4.07)	2.88 (1.32-5.18)	6.14 (2.98-10.56)	7.05 (2.69-13.90)	15.60 (7.94-25.56)	27.74 (15.99-39.67)
Sao Tome and Principe	6.14 (0.00-14.79)	7.59 (0.00-16.14)	11.26 (0.00-21.74)	12.22 (6.84-19.06)	18.19 (14.02-23.61)	41.48 (31.91-51.38)	2.18 (0.98-4.00)	2.66 (1.23-4.81)	4.49 (2.14-7.82)	5.49 (2.15-11.08)	11.17 (5.29-18.04)	34.52 (17.92-54.68)
Senegal	4.36 (0.00-10.31)	8.05 (0.00-15.53)	12.24 (0.39-21.53)	17.24 (13.64-21.29)	23.72 (19.98-27.32)	37.20 (28.27-47.16)	1.33 (0.56-2.53)	1.51 (0.64-2.82)	2.14 (0.95-3.90)	12.70 (5.73-21.93)	26.08 (17.69-32.32)	21.05 (12.81-29.10)

Location	Indicator 6.1.1: Risk-weighted prevalence of populations using unsafe or unimproved water sources, as measured by the summary exposure value (SEV) for unsafe water			Indicator 6.2.2a: Risk-weighted prevalence of populations using unsafe or unimproved sanitation, as measured by the summary exposure value (SEV) for unsafe sanitation			Indicator 6.2.1b: Risk-weighted prevalence of populations with unsafe hygiene (no handwashing with soap), as measured by the summary exposure value (SEV) for unsafe hygiene			Indicator 7.1.2: Prevalence of household air pollution, as measured by the summary exposure value (SEV) for household air pollution		
	1990	2000	2015	1990	2000	2015	1990	2000	2015	1990	2000	2015
Sierra Leone	0.82 (0.00-5.64)	0.88 (0.00-5.31)	2.57 (0.00-9.05)	11.02 (6.33-16.38)	9.73 (6.89-12.61)	16.86 (11.44-22.80)	0.95 (0.38-1.81)	1.03 (0.43-1.97)	1.77 (0.77-3.27)	3.84 (1.67-6.69)	2.00 (0.86-3.20)	1.57 (0.69-2.39)
Togo	0.60 (0.00-4.31)	1.29 (0.00-6.10)	4.60 (0.00-12.78)	10.58 (6.90-14.49)	14.58 (10.37-19.19)	23.74 (16.42-31.72)	1.33 (0.56-2.51)	1.60 (0.69-2.98)	2.35 (1.07-4.24)	1.37 (0.56-2.35)	1.51 (0.62-2.72)	5.14 (2.29-8.52)
Burundi	2.52 (0.00-9.08)	2.76 (0.00-9.52)	4.16 (0.00-12.19)	5.43 (2.97-8.22)	6.46 (4.04-9.12)	11.12 (6.73-16.21)	0.88 (0.37-1.71)	0.95 (0.40-1.80)	1.40 (0.61-2.61)	0.65 (0.26-1.14)	0.87 (0.39-1.39)	1.98 (0.82-3.46)
Comoros	10.01 (0.00-20.21)	10.86 (0.00-20.38)	12.83 (0.89-23.08)	9.08 (5.22-13.27)	8.66 (5.28-12.08)	13.40 (7.63-20.39)	1.47 (0.62-2.77)	1.73 (0.76-3.18)	2.56 (1.15-4.61)	5.27 (1.50-13.97)	8.89 (4.10-14.61)	19.33 (6.98-37.15)
Djibouti	5.89 (0.00-13.89)	8.73 (0.00-17.48)	15.17 (1.38-25.77)	19.49 (11.83-29.11)	26.33 (17.82-35.67)	41.72 (29.49-53.93)	1.80 (0.79-3.34)	1.83 (0.80-3.38)	2.72 (1.25-4.89)	75.88 (48.93-92.61)	82.07 (66.04-92.15)	74.52 (45.53-92.62)
Eritrea	0.98 (0.00-5.69)	6.00 (0.00-12.76)	9.34 (1.20-17.51)	8.68 (5.63-13.17)	15.97 (12.11-20.18)	22.08 (14.05-31.10)	0.79 (0.33-1.52)	1.41 (0.61-2.65)	1.73 (0.77-3.18)	8.32 (2.18-21.40)	22.83 (11.53-36.90)	26.65 (5.71-59.39)
Ethiopia	0.54 (0.00-3.75)	0.70 (0.00-4.13)	8.34 (0.06-16.64)	1.65 (0.49-3.27)	1.49 (0.59-2.68)	6.95 (4.20-10.38)	0.61 (0.24-1.20)	0.65 (0.26-1.25)	1.41 (0.60-2.64)	2.64 (0.87-6.17)	3.29 (1.54-4.90)	3.55 (1.39-6.92)
Kenya	14.09 (8.00-19.97)	19.45 (12.39-25.55)	25.30 (17.44-31.85)	11.86 (9.17-14.47)	16.51 (13.25-19.65)	23.62 (19.84-27.30)	2.84 (1.31-5.07)	3.78 (1.80-6.68)	5.66 (2.70-9.68)	7.34 (4.65-9.69)	13.10 (10.59-14.63)	17.84 (13.86-20.41)
Madagascar	12.19 (4.23-23.01)	16.20 (8.85-24.60)	19.28 (10.80-27.26)	1.84 (0.58-3.56)	2.46 (1.39-3.73)	3.76 (2.02-5.78)	1.57 (0.68-2.88)	1.82 (0.81-3.33)	2.71 (1.24-4.87)	1.62 (0.71-2.85)	1.71 (0.79-2.69)	1.57 (0.70-2.75)
Malawi	4.82 (0.00-11.45)	7.14 (0.00-14.25)	11.69 (0.66-20.84)	3.75 (2.62-5.06)	3.73 (2.71-4.84)	6.62 (4.05-9.49)	0.93 (0.39-1.78)	1.19 (0.52-2.19)	1.81 (0.81-3.33)	1.42 (0.50-3.32)	2.04 (0.95-2.79)	2.30 (1.08-3.43)
Mozambique	0.10 (0.00-1.48)	0.47 (0.00-3.37)	6.36 (0.00-13.63)	2.33 (0.95-4.10)	4.59 (2.95-6.38)	10.52 (6.36-14.83)	0.41 (0.16-0.82)	0.61 (0.25-1.20)	1.48 (0.65-2.79)	0.99 (0.06-3.85)	1.69 (0.59-3.62)	3.97 (1.11-9.42)

Appendix Table 1a. Scaled values for each SDG health-related indicator and three summary indicators by country for 1990, 2000, and 2015

	Appendix Table 1a. Scaled values for each SDG health-related indicator and three summary indicators by country for 1990, 2000, and 2015											
	**Indicator 6.1.1: Risk-weighted prevalence of populations using unsafe or unimproved water sources, as measured by the summary exposure value (SEV) for unsafe water**			**Indicator 6.2.2a: Risk-weighted prevalence of populations using unsafe or unimproved sanitation, as measured by the summary exposure value (SEV) for unsafe sanitation**			**Indicator 6.2.1b: Risk-weighted prevalence of populations with unsafe hygiene (no handwashing with soap), as measured by the summary exposure value (SEV) for unsafe hygiene**			**Indicator 7.1.2: Prevalence of household air pollution, as measured by the summary exposure value (SEV) for household air pollution**		
**Location**	**1990**	**2000**	**2015**	**1990**	**2000**	**2015**	**1990**	**2000**	**2015**	**1990**	**2000**	**2015**
Rwanda	14.29 (6.04-23.17)	18.01 (8.66-27.05)	25.91 (15.50-35.47)	15.09 (8.17-21.35)	16.30 (8.96-23.04)	19.30 (10.40-27.13)	1.11 (0.47-2.11)	1.24 (0.53-2.30)	2.38 (1.08-4.34)	0.85 (0.37-1.30)	0.30 (0.12-0.48)	0.57 (0.13-1.37)
Somalia	2.96 (0.00-8.86)	2.64 (0.00-7.49)	4.84 (0.00-10.67)	18.19 (10.21-27.70)	20.52 (13.55-27.87)	26.25 (17.61-36.03)	0.54 (0.21-1.09)	0.50 (0.20-1.01)	0.67 (0.27-1.31)	1.29 (0.54-2.60)	1.23 (0.52-2.15)	1.45 (0.59-3.22)
South Sudan	1.20 (0.00-5.67)	2.59 (0.00-9.02)	5.73 (0.00-13.93)	3.23 (1.25-5.93)	4.19 (1.77-7.61)	8.00 (3.83-13.15)	1.17 (0.47-2.23)	1.15 (0.47-2.20)	1.33 (0.56-2.49)	1.03 (0.44-1.66)	1.05 (0.45-1.62)	1.49 (0.62-2.68)
Tanzania	13.04 (5.46-21.00)	15.58 (8.61-22.99)	23.63 (15.56-31.65)	5.51 (3.78-7.34)	5.48 (3.74-7.37)	10.84 (6.47-15.98)	1.75 (0.76-3.22)	2.13 (0.97-3.90)	3.75 (1.74-6.68)	1.65 (0.71-3.14)	4.83 (2.47-7.05)	5.67 (1.94-12.38)
Uganda	12.21 (4.02-21.67)	18.30 (10.88-25.95)	25.66 (16.73-34.05)	13.15 (7.27-18.94)	14.94 (8.41-21.42)	20.02 (11.99-27.93)	1.16 (0.50-2.18)	1.71 (0.76-3.17)	3.16 (1.47-5.70)	1.03 (0.46-1.45)	2.11 (0.95-3.21)	2.38 (1.10-3.87)
Zambia	11.20 (3.53-19.72)	12.72 (5.22-20.08)	23.76 (14.00-33.60)	22.91 (17.60-28.67)	18.74 (15.31-22.32)	36.40 (25.93-47.09)	2.50 (1.13-4.53)	2.83 (1.31-5.12)	5.11 (2.42-8.84)	7.47 (1.61-20.76)	8.41 (3.82-13.36)	12.72 (4.87-24.15)
Angola	1.15 (0.00-6.03)	2.03 (0.00-7.40)	4.40 (0.00-11.69)	15.14 (8.30-23.60)	21.35 (14.36-29.39)	43.94 (33.17-54.96)	0.30 (0.11-0.63)	0.40 (0.15-0.81)	1.11 (0.47-2.15)	3.87 (1.18-8.42)	10.45 (4.84-17.62)	33.06 (16.45-51.57)
Central African Republic	0.24 (0.00-2.72)	1.13 (0.00-6.08)	1.65 (0.00-7.30)	6.56 (3.31-10.64)	7.82 (4.03-12.09)	8.75 (4.53-13.88)	0.02 (0.00-0.10)	0.06 (0.00-0.19)	0.15 (0.05-0.33)	1.16 (0.49-1.98)	1.11 (0.52-1.76)	1.51 (0.66-2.55)
Congo	4.05 (0.00-12.05)	5.46 (0.00-13.88)	8.06 (0.00-17.07)	10.22 (5.19-16.76)	12.48 (7.45-18.69)	22.92 (14.71-31.96)	0.78 (0.32-1.54)	1.05 (0.46-2.02)	1.96 (0.85-3.72)	4.64 (1.54-10.33)	8.73 (3.40-16.18)	31.04 (15.15-50.14)
Democratic Republic of the Congo	2.10 (0.00-8.22)	1.06 (0.00-5.39)	2.76 (0.00-9.61)	8.73 (4.62-14.07)	8.37 (4.40-12.87)	15.26 (9.13-22.68)	0.11 (0.02-0.28)	0.08 (0.00-0.23)	0.29 (0.11-0.61)	0.57 (0.25-0.80)	0.99 (0.42-1.58)	3.46 (1.53-6.07)
Equatorial Guinea	0.06 (0.00-0.88)	1.84 (0.00-7.51)	9.27 (0.00-19.72)	29.50 (18.54-42.20)	64.01 (53.64-73.18)	86.51 (79.74-91.62)	0.15 (0.05-0.34)	0.93 (0.40-1.81)	3.61 (1.58-6.59)	3.14 (1.32-6.09)	5.37 (2.01-10.69)	14.26 (5.18-30.81)

Location	Appendix Table 1a. Scaled values for each SDG health-related indicator and three summary indicators by country for 1990, 2000, and 2015											
	**Indicator 6.1.1: Risk-weighted prevalence of populations using unsafe or unimproved water sources, as measured by the summary exposure value (SEV) for unsafe water**			**Indicator 6.2.2a: Risk-weighted prevalence of populations using unsafe or unimproved sanitation, as measured by the summary exposure value (SEV) for unsafe sanitation**			**Indicator 6.2.1b: Risk-weighted prevalence of populations with unsafe hygiene (no handwashing with soap), as measured by the summary exposure value (SEV) for unsafe hygiene**			**Indicator 7.1.2: Prevalence of household air pollution, as measured by the summary exposure value (SEV) for household air pollution**		
	**1990**	**2000**	**2015**	**1990**	**2000**	**2015**	**1990**	**2000**	**2015**	**1990**	**2000**	**2015**
Gabon	6.24 (0.00-14.64)	9.60 (0.00-19.19)	14.86 (1.34-25.82)	21.32 (12.28-31.62)	32.24 (22.72-42.08)	50.11 (38.66-60.99)	2.19 (0.95-4.10)	2.70 (1.17-4.91)	4.15 (1.87-7.59)	26.04 (9.93-47.92)	52.84 (37.12-67.51)	84.06 (72.06-92.23)

Location	Appendix Table 1a. Scaled values for each SDG health-related indicator and three summary indicators by country for 1990, 2000, and 2015											
	**Indicator 8.8.1: Age-standardised all-cause disability-adjusted life year (DALY) rates attributable to occupational risks (per 100,000 population)**			**Indicator 11.6.2: Population-weighted mean levels of fine particulate matter smaller than 2.5 microns (PM2.5)**			**Indicator 16.1.1: Age-standardised death rate due to interpersonal violence (per 100,000 population)**			**Indicator 16.1.2: Age-standardised death rate due to collective violence and legal intervention (per 100,000 population)**		
	**1990**	**2000**	**2015**	**1990**	**2000**	**2015**	**1990**	**2000**	**2015**	**1990**	**2000**	**2015**
Canada	73.25 (67.54-78.90)	72.12 (66.29-78.29)	74.47 (69.17-80.04)	75.41 (68.28-79.93)	75.86 (68.63-80.56)	77.89 (70.45-82.69)	61.56 (58.51-64.42)	70.35 (67.21-73.28)	68.95 (64.90-72.49)	100.00 (100.00-100.00)	100.00 (100.00-100.00)	100.00 (100.00-100.00)
United States	75.75 (71.36-80.07)	77.01 (72.44-81.58)	79.96 (76.56-83.76)	66.92 (60.82-71.16)	68.41 (62.10-72.70)	74.07 (67.27-78.49)	32.79 (31.15-34.80)	38.42 (36.52-40.58)	41.83 (39.44-44.12)	31.03 (21.14-41.77)	28.84 (19.13-39.99)	100.00 (100.00-100.00)
Australia	64.17 (58.12-69.80)	68.88 (63.44-74.88)	75.88 (70.94-81.40)	79.45 (72.05-84.11)	79.14 (71.82-83.86)	82.46 (74.70-87.43)	61.20 (58.61-63.79)	64.83 (62.08-67.52)	72.06 (68.72-74.83)	100.00 (100.00-100.00)	100.00 (100.00-100.00)	100.00 (100.00-100.00)
New Zealand	68.19 (62.92-73.05)	66.26 (60.21-72.23)	71.23 (65.59-76.59)	81.07 (73.31-86.10)	81.18 (73.13-86.11)	83.87 (75.74-88.95)	61.81 (58.93-64.54)	65.66 (62.60-68.40)	71.08 (67.70-74.03)	100.00 (100.00-100.00)	33.17 (25.08-45.56)	100.00 (100.00-100.00)
Brunei	84.48 (79.72-89.04)	87.62 (83.10-91.70)	91.61 (86.67-95.91)	82.43 (74.12-88.25)	83.28 (74.85-89.76)	85.66 (76.77-92.65)	64.09 (60.32-68.12)	67.71 (64.61-70.91)	69.43 (65.89-73.32)	100.00 (100.00-100.00)	100.00 (100.00-100.00)	100.00 (100.00-100.00)
Japan	94.26 (90.51-97.12)	96.89 (93.08-99.68)	96.23 (91.88-99.32)	64.17 (58.11-68.60)	64.72 (58.79-69.09)	63.04 (57.17-67.53)	77.00 (74.01-79.34)	78.40 (75.44-80.95)	86.74 (83.31-89.45)	100.00 (100.00-100.00)	71.51 (45.94-95.50)	100.00 (100.00-100.00)
Singapore	90.07 (83.28-94.45)	97.83 (90.86-100.00)	97.52 (92.88-100.00)	55.11 (45.17-64.87)	54.38 (44.83-64.16)	54.96 (45.15-64.87)	64.14 (61.30-66.70)	74.84 (71.66-77.57)	97.07 (93.12-100.00)	23.52 (15.24-47.03)	17.74 (11.42-36.17)	100.00 (100.00-100.00)
South Korea	70.50 (65.80-74.75)	77.95 (73.28-81.74)	89.67 (85.05-93.78)	46.69 (42.11-52.99)	46.47 (42.03-53.17)	43.72 (39.52-50.22)	64.46 (61.15-67.35)	57.37 (54.53-60.13)	64.48 (60.44-68.20)	100.00 (100.00-100.00)	100.00 (100.00-100.00)	100.00 (100.00-100.00)
Andorra	74.92 (66.11-82.56)	74.55 (66.38-83.16)	75.80 (67.59-83.13)	67.24 (58.84-75.90)	68.35 (59.80-76.23)	70.07 (61.80-78.05)	79.23 (71.60-90.20)	83.96 (75.71-94.58)	87.45 (79.54-94.68)	100.00 (100.00-100.00)	100.00 (100.00-100.00)	100.00 (100.00-100.00)
Austria	58.57 (52.01-65.18)	58.86 (54.26-63.78)	67.88 (62.68-72.66)	53.94 (48.90-59.19)	59.74 (54.20-64.82)	57.01 (51.64-62.29)	68.19 (65.31-70.58)	77.12 (74.07-79.75)	83.41 (79.84-85.99)	100.00 (100.00-100.00)	100.00 (100.00-100.00)	100.00 (100.00-100.00)
Belgium	77.03 (69.12-83.99)	78.26 (70.19-88.06)	82.05 (74.07-91.00)	53.14 (48.18-58.52)	57.22 (52.12-62.42)	59.11 (53.75-64.37)	63.86 (60.94-66.47)	60.26 (57.53-62.97)	68.75 (65.43-71.54)	23.19 (17.53-32.64)	100.00 (100.00-100.00)	100.00 (100.00-100.00)

Appendix Table 1a. Scaled values for each SDG health-related indicator and three summary indicators by country for 1990, 2000, and 2015

Location	Indicator 8.8.1: Age-standardised all-cause disability-adjusted life year (DALY) rates attributable to occupational risks (per 100,000 population)			Indicator 11.6.2: Population-weighted mean levels of fine particulate matter smaller than 2.5 microns (PM2.5)			Indicator 16.1.1: Age-standardised death rate due to interpersonal violence (per 100,000 population)			Indicator 16.1.2: Age-standardised death rate due to collective violence and legal intervention (per 100,000 population)		
	1990	2000	2015	1990	2000	2015	1990	2000	2015	1990	2000	2015
Cyprus	62.30 (55.24-69.44)	61.67 (55.65-67.62)	62.90 (56.47-69.09)	59.05 (53.54-64.46)	59.84 (53.78-65.13)	55.22 (49.72-60.65)	58.58 (55.26-61.60)	60.06 (56.98-62.59)	67.50 (63.67-71.09)	100.00 (100.00-100.00)	100.00 (100.00-100.00)	100.00 (100.00-100.00)
Denmark	62.85 (56.16-69.57)	66.52 (60.38-72.77)	70.93 (65.03-77.13)	63.93 (57.93-68.70)	67.85 (61.67-72.49)	67.68 (61.69-72.48)	68.76 (65.88-71.40)	67.09 (64.11-69.69)	77.25 (73.20-80.11)	100.00 (100.00-100.00)	100.00 (100.00-100.00)	100.00 (100.00-100.00)
Finland	71.50 (65.84-77.14)	73.79 (68.77-78.72)	77.17 (71.18-83.19)	75.82 (68.83-80.40)	78.17 (70.85-82.81)	77.24 (69.93-81.86)	52.91 (50.34-55.36)	55.57 (52.83-58.24)	65.80 (62.58-68.59)	29.39 (21.46-44.37)	100.00 (100.00-100.00)	100.00 (100.00-100.00)
France	68.20 (61.89-74.98)	69.51 (63.33-75.97)	80.84 (72.71-88.21)	61.58 (55.92-66.36)	64.42 (58.46-68.98)	64.89 (58.68-69.34)	63.52 (60.68-65.97)	69.32 (66.44-71.88)	76.76 (73.27-79.57)	100.00 (100.00-100.00)	100.00 (100.00-100.00)	22.09 (16.72-30.98)
Germany	68.70 (63.11-74.10)	69.36 (63.35-75.24)	70.71 (64.22-77.40)	56.38 (51.08-61.74)	61.70 (56.05-66.29)	61.82 (56.18-66.47)	72.52 (69.55-74.96)	71.22 (68.22-73.81)	80.67 (77.34-83.42)	100.00 (100.00-100.00)	100.00 (100.00-100.00)	100.00 (100.00-100.00)
Greece	63.52 (56.07-70.02)	66.34 (58.07-72.93)	72.04 (64.08-78.69)	62.76 (56.85-67.61)	65.63 (59.47-70.38)	62.77 (56.94-67.30)	73.77 (70.62-76.41)	74.09 (70.79-76.73)	75.33 (71.74-78.37)	100.00 (100.00-100.00)	100.00 (100.00-100.00)	100.00 (100.00-100.00)
Iceland	61.46 (55.79-67.13)	62.17 (56.23-68.29)	68.94 (62.79-74.76)	74.19 (67.23-79.31)	75.31 (68.17-80.57)	76.00 (68.60-81.34)	71.66 (68.28-74.36)	75.75 (72.33-78.75)	83.43 (79.11-86.66)	100.00 (100.00-100.00)	100.00 (100.00-100.00)	100.00 (100.00-100.00)
Ireland	68.73 (63.16-73.83)	70.60 (66.33-74.81)	64.91 (58.01-71.21)	68.18 (61.84-72.64)	70.38 (63.90-74.88)	70.51 (63.69-75.00)	75.64 (72.33-78.79)	75.68 (72.34-78.89)	81.92 (78.14-84.93)	100.00 (100.00-100.00)	100.00 (100.00-100.00)	100.00 (100.00-100.00)
Israel	76.16 (69.59-82.10)	79.28 (73.05-85.07)	78.86 (72.96-83.96)	55.70 (50.32-61.46)	56.85 (51.64-61.83)	51.60 (46.71-57.53)	59.88 (57.13-62.43)	48.83 (46.37-51.33)	56.65 (53.68-59.34)	7.47 (5.79-9.04)	8.46 (6.67-9.70)	19.56 (14.91-26.74)
Italy	62.54 (55.30-69.49)	67.71 (62.49-73.00)	72.18 (66.11-78.69)	54.88 (49.75-60.40)	59.42 (53.99-64.22)	53.12 (48.08-58.55)	59.90 (57.11-62.61)	70.36 (67.29-73.28)	77.21 (73.74-80.00)	100.00 (100.00-100.00)	100.00 (100.00-100.00)	100.00 (100.00-100.00)
Luxembourg	69.75 (62.05-77.42)	70.35 (63.42-77.23)	91.00 (82.31-98.82)	52.54 (46.93-58.43)	57.10 (51.54-62.55)	57.66 (51.92-63.24)	62.33 (59.58-64.82)	67.52 (64.70-70.38)	76.83 (73.17-79.82)	100.00 (100.00-100.00)	100.00 (100.00-100.00)	100.00 (100.00-100.00)

Location	Appendix Table 1a. Scaled values for each SDG health-related indicator and three summary indicators by country for 1990, 2000, and 2015											
	**Indicator 8.8.1: Age-standardised all-cause disability-adjusted life year (DALY) rates attributable to occupational risks (per 100,000 population)**			**Indicator 11.6.2: Population-weighted mean levels of fine particulate matter smaller than 2.5 microns (PM2.5)**			**Indicator 16.1.1: Age-standardised death rate due to interpersonal violence (per 100,000 population)**			**Indicator 16.1.2: Age-standardised death rate due to collective violence and legal intervention (per 100,000 population)**		
	**1990**	**2000**	**2015**	**1990**	**2000**	**2015**	**1990**	**2000**	**2015**	**1990**	**2000**	**2015**
Malta	52.18 (44.40-60.77)	55.28 (48.23-61.77)	70.15 (57.98-82.67)	61.26 (53.84-68.16)	63.32 (55.55-70.49)	59.19 (52.06-65.94)	65.35 (62.36-68.25)	66.83 (63.90-69.81)	74.07 (70.71-77.09)	21.80 (13.32-48.08)	100.00 (100.00-100.00)	100.00 (100.00-100.00)
Netherlands	64.91 (55.61-77.11)	66.95 (58.33-77.27)	69.98 (62.47-77.65)	54.67 (49.71-60.29)	58.89 (53.47-64.07)	60.75 (55.01-65.67)	69.99 (67.01-72.80)	66.77 (63.97-69.35)	74.28 (70.78-77.37)	100.00 (100.00-100.00)	100.00 (100.00-100.00)	100.00 (100.00-100.00)
Norway	67.78 (61.99-73.56)	68.12 (62.70-73.42)	72.92 (68.17-77.77)	68.70 (62.27-73.13)	71.06 (64.37-75.55)	72.34 (65.61-76.86)	70.22 (67.32-73.14)	73.06 (69.97-75.88)	81.23 (77.14-84.21)	100.00 (100.00-100.00)	100.00 (100.00-100.00)	100.00 (100.00-100.00)
Portugal	53.06 (45.39-60.70)	52.41 (45.91-58.53)	58.13 (50.75-65.43)	70.31 (63.68-75.14)	70.83 (64.33-75.36)	70.61 (63.94-75.11)	54.50 (51.68-56.97)	58.54 (55.77-61.19)	66.71 (63.19-69.25)	100.00 (100.00-100.00)	100.00 (100.00-100.00)	100.00 (100.00-100.00)
Spain	57.48 (49.88-64.72)	56.88 (52.32-61.79)	73.00 (67.83-77.73)	69.14 (62.64-73.56)	69.86 (63.42-74.29)	70.66 (64.14-75.04)	72.74 (69.54-75.47)	73.44 (70.40-76.16)	81.71 (77.99-84.48)	100.00 (100.00-100.00)	100.00 (100.00-100.00)	100.00 (100.00-100.00)
Sweden	74.97 (70.04-79.65)	78.52 (73.64-83.26)	80.95 (75.55-85.84)	80.08 (72.19-84.78)	82.37 (74.50-87.22)	81.15 (73.49-85.98)	65.60 (62.83-68.35)	69.26 (66.42-72.25)	74.31 (70.95-77.31)	100.00 (100.00-100.00)	100.00 (100.00-100.00)	100.00 (100.00-100.00)
Switzerland	63.07 (56.68-69.37)	59.82 (54.13-65.65)	63.56 (57.66-69.48)	63.14 (57.41-67.68)	67.14 (60.84-71.55)	63.87 (57.98-68.32)	68.30 (65.31-70.97)	71.14 (68.03-73.92)	81.65 (78.10-84.56)	100.00 (100.00-100.00)	100.00 (100.00-100.00)	100.00 (100.00-100.00)
United Kingdom	66.44 (59.86-72.55)	68.25 (61.76-75.46)	74.33 (68.43-81.08)	60.42 (54.81-65.33)	63.71 (57.85-68.04)	64.80 (58.77-69.06)	75.94 (72.91-78.47)	79.03 (75.91-81.50)	85.89 (82.38-88.36)	40.01 (24.67-61.88)	64.90 (45.24-84.65)	100.00 (100.00-100.00)
Argentina	43.66 (38.37-48.48)	44.55 (40.33-48.88)	46.70 (41.56-51.70)	58.55 (53.10-63.80)	59.06 (53.42-64.15)	62.84 (56.95-67.40)	41.19 (38.75-43.49)	39.35 (37.07-41.53)	41.74 (38.77-44.66)	15.13 (9.72-31.20)	28.29 (18.54-55.33)	100.00 (100.00-100.00)
Chile	63.00 (59.02-67.15)	68.84 (65.39-72.38)	69.51 (66.02-72.83)	52.15 (47.11-57.85)	50.59 (45.84-56.54)	51.78 (46.97-57.64)	40.16 (37.70-42.61)	44.42 (41.76-47.10)	48.28 (45.02-51.80)	100.00 (100.00-100.00)	100.00 (100.00-100.00)	100.00 (100.00-100.00)
Uruguay	57.23 (53.19-61.28)	58.73 (55.09-62.35)	57.77 (54.01-62.04)	63.62 (57.68-68.46)	63.04 (57.18-68.06)	66.69 (60.33-71.55)	48.77 (45.73-51.59)	44.57 (42.08-47.11)	44.12 (40.98-46.94)	100.00 (100.00-100.00)	100.00 (100.00-100.00)	100.00 (100.00-100.00)

Appendix Table 1a. Scaled values for each SDG health-related indicator and three summary indicators by country for 1990, 2000, and 2015

Location	Indicator 8.8.1: Age-standardised all-cause disability-adjusted life year (DALY) rates attributable to occupational risks (per 100,000 population)			Indicator 11.6.2: Population-weighted mean levels of fine particulate matter smaller than 2.5 microns (PM2.5)			Indicator 16.1.1: Age-standardised death rate due to interpersonal violence (per 100,000 population)			Indicator 16.1.2: Age-standardised death rate due to collective violence and legal intervention (per 100,000 population)		
	**1990**	**2000**	**2015**	**1990**	**2000**	**2015**	**1990**	**2000**	**2015**	**1990**	**2000**	**2015**
Belarus	52.58 (46.25-58.86)	57.31 (51.37-63.27)	65.73 (59.44-71.07)	39.68 (35.14-46.63)	52.60 (47.06-58.80)	52.40 (45.89-58.66)	36.24 (34.04-38.36)	26.97 (25.10-30.28)	32.47 (28.64-36.15)	100.00 (100.00-100.00)	100.00 (100.00-100.00)	100.00 (100.00-100.00)
Estonia	57.12 (50.57-63.16)	62.44 (58.26-66.59)	70.72 (66.20-75.16)	66.25 (60.07-71.08)	75.16 (67.64-80.14)	71.51 (64.50-76.43)	29.86 (27.81-32.06)	25.47 (23.78-27.41)	47.74 (43.17-50.34)	100.00 (100.00-100.00)	100.00 (100.00-100.00)	100.00 (100.00-100.00)
Latvia	55.09 (48.67-60.89)	59.75 (53.53-65.16)	67.52 (61.09-73.01)	42.37 (37.69-49.24)	55.27 (49.85-61.26)	52.44 (47.25-58.21)	31.95 (29.83-34.32)	27.97 (25.85-30.01)	41.18 (37.99-43.59)	100.00 (100.00-100.00)	100.00 (100.00-100.00)	100.00 (100.00-100.00)
Lithuania	46.65 (39.72-53.25)	46.83 (41.56-52.18)	60.86 (56.43-65.20)	41.75 (37.65-48.55)	54.93 (49.58-60.32)	54.16 (49.04-59.87)	36.64 (34.35-39.10)	32.02 (29.81-34.03)	41.05 (38.30-43.38)	100.00 (100.00-100.00)	100.00 (100.00-100.00)	100.00 (100.00-100.00)
Moldova	46.16 (39.27-53.03)	48.56 (40.83-55.85)	68.89 (63.06-74.86)	37.60 (31.17-44.63)	50.58 (44.92-56.90)	52.31 (46.76-58.60)	31.94 (29.93-34.22)	31.02 (29.15-33.22)	44.66 (41.43-47.13)	100.00 (100.00-100.00)	100.00 (100.00-100.00)	100.00 (100.00-100.00)
Russia	56.69 (50.15-62.89)	59.61 (53.77-65.06)	66.92 (60.53-72.47)	50.41 (45.69-56.08)	59.61 (54.07-64.52)	57.57 (52.17-62.65)	23.31 (21.56-25.29)	11.07 (9.97-12.60)	24.98 (22.86-27.07)	22.24 (14.31-42.64)	5.47 (3.73-8.29)	30.65 (20.15-59.18)
Ukraine	54.07 (48.14-59.69)	55.77 (50.04-61.21)	59.61 (53.29-65.79)	39.80 (35.87-46.46)	52.82 (47.89-58.56)	54.41 (49.27-59.96)	34.41 (32.24-36.68)	24.24 (22.65-26.22)	37.64 (34.29-40.03)	100.00 (100.00-100.00)	100.00 (100.00-100.00)	3.20 (1.96-6.17)
Albania	38.57 (30.54-46.71)	39.63 (31.51-47.94)	46.67 (39.50-54.03)	55.89 (50.70-61.07)	61.35 (55.45-66.54)	55.72 (50.12-61.06)	42.72 (39.50-50.70)	34.31 (31.53-49.17)	62.46 (57.37-65.86)	100.00 (100.00-100.00)	27.56 (18.16-48.80)	100.00 (100.00-100.00)
Bosnia and Herzegovina	64.88 (58.45-70.95)	67.36 (60.99-73.23)	70.15 (64.63-75.49)	30.79 (27.64-38.80)	36.35 (32.25-43.54)	30.40 (26.74-38.28)	95.94 (85.25-100.00)	91.77 (87.02-96.23)	56.64 (50.76-100.00)	2.53 (1.27-4.74)	100.00 (100.00-100.00)	100.00 (100.00-100.00)
Bulgaria	56.75 (49.51-62.84)	59.45 (54.67-63.99)	66.08 (60.58-71.20)	31.70 (28.44-39.39)	43.29 (39.23-49.95)	44.21 (39.85-50.88)	49.77 (47.34-52.22)	49.13 (46.51-51.65)	59.96 (56.77-62.84)	100.00 (100.00-100.00)	100.00 (100.00-100.00)	100.00 (100.00-100.00)
Croatia	60.16 (54.20-65.94)	58.60 (52.74-64.13)	63.23 (57.28-69.37)	47.50 (42.86-53.51)	55.57 (50.15-61.03)	50.79 (46.06-56.17)	53.80 (51.08-56.28)	58.37 (55.80-60.82)	72.00 (68.52-74.73)	100.00 (100.00-100.00)	31.06 (19.62-58.56)	100.00 (100.00-100.00)

Location	Appendix Table 1a. Scaled values for each SDG health-related indicator and three summary indicators by country for 1990, 2000, and 2015											
	**Indicator 8.8.1: Age-standardised all-cause disability-adjusted life year (DALY) rates attributable to occupational risks (per 100,000 population)**			**Indicator 11.6.2: Population-weighted mean levels of fine particulate matter smaller than 2.5 microns (PM2.5)**			**Indicator 16.1.1: Age-standardised death rate due to interpersonal violence (per 100,000 population)**			**Indicator 16.1.2: Age-standardised death rate due to collective violence and legal intervention (per 100,000 population)**		
	**1990**	**2000**	**2015**	**1990**	**2000**	**2015**	**1990**	**2000**	**2015**	**1990**	**2000**	**2015**
Czech Republic	56.78 (51.45-62.09)	59.40 (55.19-63.53)	67.80 (62.35-72.94)	36.94 (33.28-44.25)	51.72 (47.00-57.21)	51.22 (46.31-57.18)	60.39 (57.57-62.88)	63.01 (60.26-65.71)	73.61 (69.75-76.34)	100.00 (100.00-100.00)	100.00 (100.00-100.00)	100.00 (100.00-100.00)
Hungary	65.91 (61.04-70.63)	69.70 (65.60-73.51)	74.26 (69.53-78.29)	36.06 (32.27-43.56)	47.98 (43.41-53.85)	49.33 (44.73-55.17)	52.53 (50.07-54.95)	55.87 (53.16-58.21)	68.79 (64.53-71.73)	100.00 (100.00-100.00)	100.00 (100.00-100.00)	100.00 (100.00-100.00)
Macedonia	63.91 (57.24-69.58)	64.69 (59.08-70.09)	69.18 (64.69-73.68)	28.04 (24.38-35.91)	37.85 (34.08-44.81)	34.64 (30.75-42.41)	56.84 (53.70-60.20)	52.34 (49.20-61.06)	59.35 (55.50-66.11)	100.00 (100.00-100.00)	100.00 (100.00-100.00)	100.00 (100.00-100.00)
Montenegro	61.91 (55.16-67.98)	63.09 (56.37-69.52)	67.24 (60.65-73.18)	48.38 (43.98-54.39)	54.93 (49.72-60.23)	49.01 (44.26-54.94)	41.06 (36.92-44.48)	40.15 (37.92-42.37)	48.07 (44.21-51.60)	100.00 (100.00-100.00)	26.19 (21.21-29.88)	100.00 (100.00-100.00)
Poland	49.68 (43.16-55.48)	56.06 (49.96-61.83)	64.42 (59.04-69.46)	31.47 (28.22-39.54)	45.93 (41.62-52.22)	48.00 (43.45-53.88)	53.03 (50.65-55.26)	54.04 (51.46-56.49)	65.31 (62.30-67.91)	100.00 (100.00-100.00)	100.00 (100.00-100.00)	100.00 (100.00-100.00)
Romania	42.00 (34.79-49.07)	41.95 (35.16-48.60)	49.45 (43.80-55.22)	40.01 (36.04-46.93)	51.51 (46.79-57.17)	53.48 (48.51-58.87)	47.86 (45.47-50.34)	51.29 (48.67-53.79)	61.25 (57.90-64.04)	17.99 (11.59-33.10)	100.00 (100.00-100.00)	100.00 (100.00-100.00)
Serbia	52.33 (45.06-58.87)	52.29 (45.64-58.57)	59.57 (53.67-64.89)	46.89 (42.50-52.83)	53.40 (48.15-58.93)	51.19 (46.27-56.77)	45.73 (43.06-49.98)	46.85 (44.50-50.08)	55.38 (52.40-57.95)	100.00 (100.00-100.00)	4.19 (2.39-8.26)	100.00 (100.00-100.00)
Slovakia	67.67 (62.59-72.33)	69.40 (63.30-74.38)	74.00 (68.39-79.34)	38.12 (34.36-45.26)	50.93 (46.21-56.33)	52.29 (47.32-58.17)	51.26 (46.53-53.86)	52.51 (49.51-54.78)	59.89 (56.37-62.94)	100.00 (100.00-100.00)	100.00 (100.00-100.00)	100.00 (100.00-100.00)
Slovenia	61.43 (55.71-67.25)	58.69 (53.63-64.06)	68.32 (62.93-73.46)	47.70 (42.97-53.95)	57.40 (51.95-62.49)	52.51 (47.64-58.26)	58.74 (56.03-61.19)	63.68 (60.87-66.35)	80.95 (75.73-83.83)	100.00 (100.00-100.00)	100.00 (100.00-100.00)	100.00 (100.00-100.00)
Armenia	34.34 (23.98-42.34)	35.58 (27.10-43.86)	42.99 (36.60-49.13)	52.42 (43.19-60.67)	55.13 (45.80-62.92)	47.05 (37.33-55.82)	46.07 (43.46-48.79)	42.21 (38.81-48.66)	44.71 (41.03-49.85)	100.00 (100.00-100.00)	100.00 (100.00-100.00)	100.00 (100.00-100.00)
Azerbaijan	29.10 (15.41-40.01)	52.15 (46.74-57.32)	39.29 (30.87-51.46)	45.53 (40.87-51.78)	48.76 (43.37-54.71)	42.28 (36.73-49.39)	32.86 (30.26-39.40)	38.89 (36.48-41.19)	50.47 (45.26-54.17)	100.00 (100.00-100.00)	100.00 (100.00-100.00)	100.00 (100.00-100.00)

	Appendix Table 1a. Scaled values for each SDG health-related indicator and three summary indicators by country for 1990, 2000, and 2015											
Location	Indicator 8.8.1: Age-standardised all-cause disability-adjusted life year (DALY) rates attributable to occupational risks (per 100,000 population)			Indicator 11.6.2: Population-weighted mean levels of fine particulate matter smaller than 2.5 microns (PM2.5)			Indicator 16.1.1: Age-standardised death rate due to interpersonal violence (per 100,000 population)			Indicator 16.1.2: Age-standardised death rate due to collective violence and legal intervention (per 100,000 population)		
	1990	2000	2015	1990	2000	2015	1990	2000	2015	1990	2000	2015
Georgia	44.28 (33.41-52.09)	42.44 (34.07-51.27)	40.51 (32.13-48.46)	54.95 (49.71-60.41)	58.94 (53.20-64.78)	52.37 (47.19-58.20)	48.39 (45.72-51.49)	47.69 (44.59-51.09)	51.64 (47.33-55.06)	100.00 (100.00-100.00)	16.45 (11.89-26.45)	100.00 (100.00-100.00)
Kazakhstan	32.95 (26.50-39.01)	30.49 (25.38-36.11)	37.95 (29.45-45.97)	52.61 (47.67-58.35)	57.76 (52.29-62.76)	53.39 (48.00-58.98)	28.09 (25.94-30.36)	21.81 (20.21-23.72)	28.14 (25.31-31.18)	100.00 (100.00-100.00)	100.00 (100.00-100.00)	100.00 (100.00-100.00)
Kyrgyzstan	38.37 (31.58-44.80)	27.38 (21.22-34.01)	42.36 (35.78-48.05)	57.15 (52.04-62.29)	60.20 (54.56-64.98)	57.16 (51.40-62.70)	29.05 (26.38-33.58)	31.99 (29.60-34.24)	38.84 (34.41-42.15)	100.00 (100.00-100.00)	13.94 (10.09-22.22)	100.00 (100.00-100.00)
Mongolia	38.20 (28.95-45.82)	38.05 (28.09-45.71)	41.65 (32.61-49.24)	55.77 (50.57-61.14)	54.03 (48.93-59.88)	48.76 (43.68-55.21)	31.54 (27.65-35.38)	28.61 (25.73-31.42)	32.64 (29.12-36.07)	100.00 (100.00-100.00)	100.00 (100.00-100.00)	100.00 (100.00-100.00)
Tajikistan	35.65 (24.24-44.28)	38.15 (26.84-47.75)	42.17 (34.05-50.90)	18.18 (13.62-27.52)	22.89 (15.76-32.53)	29.05 (22.00-37.56)	38.66 (35.77-43.95)	36.23 (33.72-41.65)	54.06 (47.31-58.20)	100.00 (100.00-100.00)	11.41 (8.22-18.09)	100.00 (100.00-100.00)
Turkmenistan	42.11 (33.01-49.59)	42.56 (32.79-50.24)	51.65 (42.55-59.60)	36.43 (32.24-43.30)	38.41 (33.11-45.48)	41.49 (35.67-48.42)	38.22 (35.43-42.00)	38.22 (35.77-40.88)	44.65 (40.42-48.31)	100.00 (100.00-100.00)	100.00 (100.00-100.00)	100.00 (100.00-100.00)
Uzbekistan	41.57 (32.35-48.77)	43.78 (33.85-51.81)	48.44 (40.19-57.02)	28.55 (23.95-36.61)	34.94 (30.47-42.72)	35.19 (30.52-42.66)	38.82 (35.88-46.26)	48.43 (44.51-50.86)	52.58 (47.84-57.07)	100.00 (100.00-100.00)	8.92 (6.38-13.58)	100.00 (100.00-100.00)
Colombia	52.28 (46.71-57.26)	51.07 (45.49-56.09)	53.84 (49.12-58.47)	51.40 (46.58-56.97)	52.41 (47.62-58.09)	55.60 (50.58-60.85)	0.25 (0.00-1.86)	1.10 (0.59-2.82)	11.85 (10.21-13.97)	6.78 (5.26-7.73)	4.34 (3.05-5.35)	100.00 (100.00-100.00)
Costa Rica	58.00 (51.99-63.38)	56.39 (51.75-61.12)	62.33 (55.97-68.08)	51.32 (46.50-56.78)	50.53 (45.53-56.49)	52.81 (47.69-58.39)	41.84 (39.40-44.37)	40.46 (38.05-42.78)	36.36 (33.85-39.25)	100.00 (100.00-100.00)	100.00 (100.00-100.00)	100.00 (100.00-100.00)
El Salvador	57.93 (51.26-63.65)	60.85 (54.81-66.99)	61.59 (54.62-67.20)	35.34 (30.90-42.40)	33.14 (29.23-40.62)	37.03 (32.94-44.46)	3.24 (1.36-5.38)	3.52 (2.29-6.10)	3.30 (1.13-7.11)	1.71 (0.99-2.43)	26.31 (20.25-34.93)	100.00 (100.00-100.00)
Guatemala	49.49 (43.10-55.37)	51.25 (45.13-57.35)	52.93 (46.88-58.72)	38.22 (34.48-45.32)	35.82 (32.12-43.21)	38.60 (34.87-45.53)	9.85 (8.43-11.54)	9.53 (8.39-11.30)	9.33 (6.72-12.26)	2.98 (1.89-4.10)	23.94 (18.28-32.73)	100.00 (100.00-100.00)

	Appendix Table 1a. Scaled values for each SDG health-related indicator and three summary indicators by country for 1990, 2000, and 2015											
Location	Indicator 8.8.1: Age-standardised all-cause disability-adjusted life year (DALY) rates attributable to occupational risks (per 100,000 population)			Indicator 11.6.2: Population-weighted mean levels of fine particulate matter smaller than 2.5 microns (PM2.5)			Indicator 16.1.1: Age-standardised death rate due to interpersonal violence (per 100,000 population)			Indicator 16.1.2: Age-standardised death rate due to collective violence and legal intervention (per 100,000 population)		
	1990	2000	2015	1990	2000	2015	1990	2000	2015	1990	2000	2015
Honduras	52.70 (46.00-58.32)	51.02 (44.18-57.07)	52.28 (46.03-57.97)	38.03 (34.00-45.55)	36.83 (32.78-43.94)	36.61 (32.75-44.31)	16.12 (13.99-18.46)	8.31 (4.46-17.84)	6.50 (1.63-18.65)	100.00 (100.00-100.00)	100.00 (100.00-100.00)	100.00 (100.00-100.00)
Mexico	54.24 (50.86-57.71)	55.06 (51.61-58.63)	56.52 (53.30-59.84)	47.67 (43.25-53.70)	43.50 (39.46-50.11)	52.64 (47.62-58.19)	17.21 (16.00-18.75)	25.47 (23.96-27.29)	23.16 (21.61-25.11)	47.64 (19.73-69.46)	100.00 (100.00-100.00)	100.00 (100.00-100.00)
Nicaragua	52.70 (44.74-59.53)	52.77 (48.21-57.61)	53.98 (48.30-58.87)	39.81 (34.57-47.37)	39.07 (32.06-46.57)	45.00 (38.69-51.98)	29.27 (26.97-31.65)	29.77 (27.69-31.90)	31.90 (28.80-35.11)	9.45 (7.59-10.73)	100.00 (100.00-100.00)	100.00 (100.00-100.00)
Panama	43.77 (35.26-51.38)	45.78 (40.12-51.48)	58.26 (51.24-64.84)	58.69 (53.15-63.71)	58.13 (52.01-63.69)	63.34 (56.61-68.80)	31.35 (28.25-34.00)	29.47 (27.22-31.87)	22.63 (18.98-30.52)	100.00 (100.00-100.00)	19.56 (14.33-29.75)	100.00 (100.00-100.00)
Venezuela	52.92 (46.19-59.03)	52.36 (45.29-58.59)	53.40 (46.55-58.99)	40.53 (36.58-47.16)	40.40 (36.26-47.54)	48.57 (43.94-54.85)	25.62 (23.61-27.77)	13.35 (12.26-14.70)	7.73 (5.22-10.44)	18.11 (13.86-24.58)	8.67 (6.45-11.55)	100.00 (100.00-100.00)
Bolivia	31.40 (9.03-46.45)	42.40 (37.21-47.83)	42.05 (30.39-49.84)	44.44 (40.18-50.61)	42.35 (38.24-49.24)	44.35 (40.05-51.07)	29.16 (23.04-33.60)	32.89 (27.97-35.86)	37.11 (32.09-42.09)	100.00 (100.00-100.00)	14.11 (9.10-28.81)	100.00 (100.00-100.00)
Ecuador	61.57 (44.55-67.71)	59.78 (44.71-65.79)	58.06 (40.40-64.69)	56.30 (51.07-61.62)	55.66 (50.33-60.75)	63.97 (58.11-68.54)	26.48 (24.08-28.82)	21.07 (18.87-26.45)	25.25 (22.03-30.05)	100.00 (100.00-100.00)	100.00 (100.00-100.00)	100.00 (100.00-100.00)
Peru	51.71 (42.02-58.08)	52.15 (43.43-58.25)	53.99 (41.34-60.72)	44.76 (40.16-51.29)	38.17 (34.40-45.41)	44.50 (40.28-50.88)	36.97 (34.22-43.01)	47.07 (44.56-49.46)	52.67 (48.21-56.88)	3.69 (2.59-4.44)	100.00 (100.00-100.00)	100.00 (100.00-100.00)
Antigua and Barbuda	63.23 (58.55-67.28)	62.66 (58.47-66.78)	63.31 (59.45-66.97)	57.93 (42.89-73.43)	57.61 (42.27-72.62)	62.99 (48.40-77.25)	49.77 (46.52-53.03)	44.29 (41.13-47.37)	40.90 (37.31-44.57)	100.00 (100.00-100.00)	100.00 (100.00-100.00)	100.00 (100.00-100.00)
The Bahamas	52.12 (46.54-56.88)	57.02 (51.62-61.73)	57.91 (51.86-62.56)	54.30 (42.19-66.02)	55.32 (42.85-67.73)	62.92 (49.74-74.08)	23.09 (20.37-25.63)	22.01 (18.98-24.94)	20.02 (16.64-25.56)	100.00 (100.00-100.00)	100.00 (100.00-100.00)	100.00 (100.00-100.00)
Barbados	63.20 (59.28-66.88)	67.09 (63.15-70.93)	67.38 (63.14-71.39)	53.44 (37.65-70.03)	53.90 (35.76-72.28)	60.56 (45.11-76.43)	39.20 (36.45-41.90)	35.75 (32.95-38.48)	30.03 (26.90-33.54)	18.97 (12.22-38.43)	100.00 (100.00-100.00)	100.00 (100.00-100.00)

Appendix Table 1a. Scaled values for each SDG health-related indicator and three summary indicators by country for 1990, 2000, and 2015

Location	Indicator 8.8.1: Age-standardised all-cause disability-adjusted life year (DALY) rates attributable to occupational risks (per 100,000 population)			Indicator 11.6.2: Population-weighted mean levels of fine particulate matter smaller than 2.5 microns (PM2.5)			Indicator 16.1.1: Age-standardised death rate due to interpersonal violence (per 100,000 population)			Indicator 16.1.2: Age-standardised death rate due to collective violence and legal intervention (per 100,000 population)		
	1990	2000	2015	1990	2000	2015	1990	2000	2015	1990	2000	2015
Belize	56.06 (52.00-60.06)	50.19 (45.47-54.68)	51.03 (45.26-55.96)	43.03 (38.26-49.64)	43.05 (38.42-49.83)	45.25 (40.41-51.67)	34.54 (25.93-37.97)	21.73 (19.69-24.48)	17.64 (14.14-24.96)	8.11 (5.22-15.84)	100.00 (100.00-100.00)	100.00 (100.00-100.00)
Cuba	49.27 (43.29-54.93)	50.29 (44.41-55.90)	51.58 (45.38-62.93)	49.50 (44.04-55.94)	50.83 (45.85-56.76)	55.64 (50.33-60.85)	37.00 (34.78-39.53)	43.42 (41.12-45.61)	41.86 (38.82-44.88)	19.98 (12.87-40.27)	100.00 (100.00-100.00)	100.00 (100.00-100.00)
Dominica	56.07 (51.62-60.17)	56.61 (52.03-60.79)	57.11 (52.16-61.56)	56.11 (44.61-67.49)	56.25 (44.55-67.80)	63.13 (51.49-73.89)	46.77 (39.45-49.92)	41.49 (38.22-44.52)	29.13 (25.17-38.75)	100.00 (100.00-100.00)	100.00 (100.00-100.00)	100.00 (100.00-100.00)
Dominican Republic	42.09 (36.64-47.79)	44.97 (39.75-50.12)	49.47 (43.88-54.39)	52.01 (46.77-57.65)	42.83 (35.81-51.08)	52.50 (45.90-59.27)	30.08 (26.23-32.52)	30.85 (28.34-33.05)	26.23 (23.30-31.05)	100.00 (100.00-100.00)	100.00 (100.00-100.00)	100.00 (100.00-100.00)
Grenada	58.79 (54.23-63.08)	57.50 (52.84-61.91)	56.00 (50.57-60.82)	53.25 (39.83-66.89)	53.31 (37.17-67.85)	60.59 (47.72-73.49)	52.58 (42.83-56.01)	39.45 (36.41-43.19)	35.72 (32.08-43.46)	10.31 (6.63-21.09)	100.00 (100.00-100.00)	100.00 (100.00-100.00)
Guyana	57.91 (54.03-61.57)	58.98 (54.91-62.95)	61.08 (56.71-65.34)	46.07 (40.29-52.88)	46.09 (35.54-55.23)	56.83 (45.99-65.48)	28.65 (22.21-31.53)	21.48 (19.11-24.10)	20.84 (17.03-25.46)	10.47 (6.73-21.40)	21.81 (14.07-43.49)	100.00 (100.00-100.00)
Haiti	38.64 (32.92-44.21)	42.99 (37.35-48.66)	43.84 (38.04-49.11)	41.92 (36.76-49.05)	41.74 (35.43-48.81)	46.48 (39.92-53.15)	26.94 (22.81-35.27)	32.41 (28.08-40.41)	41.73 (35.74-47.91)	100.00 (100.00-100.00)	100.00 (100.00-100.00)	100.00 (100.00-100.00)
Jamaica	50.41 (46.04-54.85)	50.06 (45.93-54.38)	54.31 (49.96-58.54)	52.03 (47.04-57.86)	52.31 (47.24-58.49)	57.05 (51.90-62.22)	39.51 (33.64-42.16)	23.62 (21.22-29.04)	17.96 (14.13-29.08)	100.00 (100.00-100.00)	100.00 (100.00-100.00)	100.00 (100.00-100.00)
Saint Lucia	51.83 (47.12-56.13)	53.58 (49.02-57.81)	57.76 (53.24-61.86)	54.55 (44.18-64.69)	54.25 (42.18-65.82)	62.26 (51.77-73.29)	34.52 (31.74-37.20)	26.92 (24.45-29.39)	24.71 (22.02-27.64)	12.82 (8.24-26.05)	100.00 (100.00-100.00)	100.00 (100.00-100.00)
Saint Vincent and the Grenadines	63.78 (59.16-67.55)	60.59 (55.75-64.81)	61.06 (56.06-65.21)	54.82 (40.23-68.56)	54.67 (41.41-68.52)	62.12 (49.05-74.68)	31.88 (28.99-34.94)	28.85 (26.45-31.39)	20.25 (17.70-23.30)	11.15 (7.15-22.70)	100.00 (100.00-100.00)	100.00 (100.00-100.00)
Suriname	66.32 (62.46-70.21)	68.06 (64.30-71.92)	67.46 (62.90-71.82)	45.03 (29.55-56.04)	44.80 (29.26-56.92)	55.59 (42.20-66.83)	39.21 (34.49-42.08)	34.25 (31.73-36.93)	32.22 (28.50-36.81)	13.01 (8.37-26.46)	100.00 (100.00-100.00)	100.00 (100.00-100.00)

Location	Appendix Table 1a. Scaled values for each SDG health-related indicator and three summary indicators by country for 1990, 2000, and 2015											
	**Indicator 8.8.1: Age-standardised all-cause disability-adjusted life year (DALY) rates attributable to occupational risks (per 100,000 population)**			**Indicator 11.6.2: Population-weighted mean levels of fine particulate matter smaller than 2.5 microns (PM2.5)**			**Indicator 16.1.1: Age-standardised death rate due to interpersonal violence (per 100,000 population)**			**Indicator 16.1.2: Age-standardised death rate due to collective violence and legal intervention (per 100,000 population)**		
	**1990**	**2000**	**2015**	**1990**	**2000**	**2015**	**1990**	**2000**	**2015**	**1990**	**2000**	**2015**
Trinidad and Tobago	68.12 (65.08-71.24)	72.86 (70.08-75.62)	72.70 (68.05-76.55)	53.68 (46.97-60.45)	53.86 (47.18-60.50)	61.60 (54.82-68.09)	32.53 (30.39-34.89)	29.35 (27.39-31.64)	18.72 (15.98-21.62)	5.05 (3.62-6.48)	100.00 (100.00-100.00)	100.00 (100.00-100.00)
Brazil	39.64 (35.50-44.06)	40.27 (36.19-44.66)	47.17 (43.42-51.08)	60.50 (54.84-65.50)	58.29 (52.82-63.51)	66.93 (60.76-71.40)	14.58 (12.73-16.04)	12.91 (11.86-15.29)	14.90 (13.42-17.48)	100.00 (100.00-100.00)	48.83 (25.03-82.71)	100.00 (100.00-100.00)
Paraguay	44.11 (39.11-49.24)	43.70 (39.01-48.86)	45.14 (40.45-49.95)	50.04 (44.88-56.16)	49.42 (44.20-55.14)	60.41 (54.82-65.89)	32.20 (27.55-35.25)	22.45 (20.29-25.73)	26.50 (22.30-30.45)	100.00 (100.00-100.00)	100.00 (100.00-100.00)	100.00 (100.00-100.00)
China	28.10 (22.45-33.92)	34.20 (29.55-39.16)	42.97 (38.86-47.37)	29.73 (26.62-37.88)	28.17 (25.16-36.50)	24.86 (22.08-33.64)	47.14 (45.11-49.31)	49.56 (47.38-52.51)	69.09 (64.08-71.94)	100.00 (100.00-100.00)	69.22 (52.10-89.16)	100.00 (100.00-100.00)
North Korea	37.58 (28.70-45.45)	36.96 (29.27-44.32)	37.76 (30.35-44.95)	41.95 (37.52-48.74)	41.73 (37.14-49.04)	39.20 (34.70-46.72)	48.71 (42.25-55.23)	50.49 (44.36-56.58)	56.01 (49.69-62.68)	100.00 (100.00-100.00)	100.00 (100.00-100.00)	100.00 (100.00-100.00)
Taiwan	50.57 (45.22-55.73)	54.73 (49.94-59.33)	60.97 (56.35-65.44)	45.15 (40.71-50.94)	42.57 (38.25-49.48)	42.13 (37.85-48.68)	39.94 (35.51-53.77)	48.07 (44.15-59.60)	66.91 (61.97-71.88)	100.00 (100.00-100.00)	100.00 (100.00-100.00)	100.00 (100.00-100.00)
Cambodia	27.50 (21.00-34.19)	27.42 (21.02-34.15)	30.80 (23.53-37.41)	47.48 (42.14-54.06)	47.11 (41.60-53.43)	43.44 (37.30-50.57)	44.77 (40.54-49.02)	47.54 (43.68-51.13)	55.63 (47.93-62.11)	4.68 (2.98-9.23)	100.00 (100.00-100.00)	100.00 (100.00-100.00)
Indonesia	44.72 (39.70-49.80)	47.86 (42.80-52.40)	51.26 (46.20-56.34)	57.55 (52.20-62.76)	57.78 (52.42-62.70)	59.48 (53.96-64.47)	79.59 (73.04-84.12)	79.07 (74.01-83.50)	75.26 (69.27-81.02)	30.99 (24.70-37.17)	16.04 (12.02-23.33)	100.00 (100.00-100.00)
Laos	25.21 (17.60-32.47)	26.66 (19.69-33.34)	32.88 (26.44-39.22)	45.60 (41.07-52.26)	45.11 (40.58-51.36)	40.39 (35.97-47.54)	72.83 (65.17-80.40)	75.67 (70.28-80.76)	86.50 (78.71-93.51)	3.79 (2.00-7.81)	100.00 (100.00-100.00)	100.00 (100.00-100.00)
Malaysia	45.66 (39.91-51.20)	49.25 (42.78-54.90)	57.33 (52.15-62.25)	59.30 (53.69-64.28)	58.69 (53.22-63.85)	59.24 (53.53-64.36)	61.30 (49.82-64.87)	49.23 (46.57-52.21)	48.16 (43.79-54.32)	100.00 (100.00-100.00)	100.00 (100.00-100.00)	100.00 (100.00-100.00)
Maldives	54.80 (50.46-59.19)	62.57 (58.40-66.49)	70.89 (66.33-74.96)	43.06 (31.34-55.03)	42.87 (31.51-54.36)	43.89 (32.40-55.56)	67.63 (48.39-72.85)	45.87 (42.88-53.72)	51.52 (45.64-65.00)	100.00 (100.00-100.00)	100.00 (100.00-100.00)	100.00 (100.00-100.00)

	Appendix Table 1a. Scaled values for each SDG health-related indicator and three summary indicators by country for 1990, 2000, and 2015											
	**Indicator 8.8.1: Age-standardised all-cause disability-adjusted life year (DALY) rates attributable to occupational risks (per 100,000 population)**			**Indicator 11.6.2: Population-weighted mean levels of fine particulate matter smaller than 2.5 microns (PM2.5)**			**Indicator 16.1.1: Age-standardised death rate due to interpersonal violence (per 100,000 population)**			**Indicator 16.1.2: Age-standardised death rate due to collective violence and legal intervention (per 100,000 population)**		
**Location**	**1990**	**2000**	**2015**	**1990**	**2000**	**2015**	**1990**	**2000**	**2015**	**1990**	**2000**	**2015**
Mauritius	61.76 (58.23-65.11)	67.34 (62.89-70.85)	72.34 (66.90-76.60)	55.96 (49.16-62.49)	55.55 (48.54-63.12)	60.32 (53.41-66.59)	60.17 (56.99-63.16)	61.31 (58.25-64.35)	57.24 (53.68-60.43)	100.00 (100.00-100.00)	27.50 (16.72-48.10)	100.00 (100.00-100.00)
Myanmar	25.22 (15.04-34.88)	26.61 (17.15-35.80)	29.51 (20.19-38.57)	32.58 (29.32-40.43)	32.26 (28.88-40.36)	26.66 (23.55-35.44)	49.89 (42.13-59.29)	56.02 (47.04-68.03)	59.39 (49.70-70.41)	19.45 (12.52-39.31)	10.05 (7.98-12.28)	14.25 (8.70-27.75)
Philippines	42.29 (37.96-46.95)	41.45 (37.16-45.99)	45.39 (41.08-49.75)	47.86 (43.27-53.96)	47.03 (42.27-52.82)	48.86 (44.19-54.88)	30.00 (27.68-32.35)	20.15 (18.45-22.31)	24.54 (21.74-27.33)	6.12 (4.63-7.43)	6.02 (4.62-6.92)	34.76 (22.77-60.69)
Sri Lanka	54.28 (48.78-59.47)	55.18 (50.78-59.45)	63.66 (58.77-68.29)	42.60 (38.26-48.74)	41.92 (37.71-48.79)	44.40 (39.92-51.60)	28.11 (25.90-30.65)	32.97 (30.71-35.31)	45.33 (39.69-51.44)	1.40 (0.80-1.80)	2.32 (1.35-3.71)	100.00 (100.00-100.00)
Seychelles	62.66 (58.99-66.25)	60.68 (57.01-64.30)	65.13 (60.85-69.18)	54.92 (36.01-72.66)	55.41 (35.85-73.25)	64.63 (46.87-81.40)	33.84 (30.86-37.99)	32.75 (29.90-37.07)	39.17 (35.40-43.48)	100.00 (100.00-100.00)	100.00 (100.00-100.00)	100.00 (100.00-100.00)
Thailand	51.25 (46.40-56.10)	48.69 (44.45-53.19)	53.82 (48.51-59.06)	48.79 (44.24-54.57)	48.48 (43.92-54.33)	45.89 (41.48-51.98)	25.37 (23.17-28.03)	24.84 (22.51-27.13)	34.04 (28.96-38.18)	100.00 (100.00-100.00)	100.00 (100.00-100.00)	100.00 (100.00-100.00)
Timor-Leste	41.74 (35.58-47.89)	46.50 (40.46-52.39)	54.62 (48.67-60.19)	53.49 (45.92-60.49)	52.76 (45.85-59.97)	54.54 (47.35-61.72)	43.59 (35.50-53.52)	50.25 (44.84-60.56)	54.91 (48.75-61.58)	100.00 (100.00-100.00)	0.55 (0.20-1.28)	100.00 (100.00-100.00)
Vietnam	44.88 (38.02-50.82)	44.83 (38.88-51.05)	44.62 (38.40-50.53)	45.50 (41.42-51.66)	45.17 (40.78-51.43)	44.09 (39.81-50.63)	57.84 (51.83-63.59)	60.20 (54.37-65.83)	62.76 (54.48-70.96)	100.00 (100.00-100.00)	100.00 (100.00-100.00)	100.00 (100.00-100.00)
Federated States of Micronesia	34.92 (24.96-44.68)	36.32 (26.43-44.20)	37.86 (28.47-45.17)	73.11 (52.98-91.75)	72.78 (53.19-90.31)	76.74 (58.66-94.74)	40.08 (31.83-49.79)	40.28 (31.98-48.98)	42.16 (32.06-51.30)	100.00 (100.00-100.00)	100.00 (100.00-100.00)	100.00 (100.00-100.00)
Fiji	48.92 (42.61-54.28)	50.06 (44.79-55.19)	57.19 (52.30-61.82)	73.50 (65.49-80.32)	73.43 (62.49-83.31)	76.14 (65.22-85.83)	48.04 (43.34-52.76)	39.77 (36.50-44.89)	41.49 (37.19-47.47)	100.00 (100.00-100.00)	100.00 (100.00-100.00)	100.00 (100.00-100.00)
Kiribati	43.06 (37.05-48.70)	43.85 (37.49-49.44)	45.94 (40.45-51.32)	89.14 (79.28-97.43)	89.17 (77.18-100.00)	94.99 (83.53-100.00)	24.96 (20.60-44.50)	23.34 (18.64-43.45)	17.38 (11.46-45.43)	100.00 (100.00-100.00)	100.00 (100.00-100.00)	100.00 (100.00-100.00)

	Appendix Table 1a. Scaled values for each SDG health-related indicator and three summary indicators by country for 1990, 2000, and 2015											
	**Indicator 8.8.1: Age-standardised all-cause disability-adjusted life year (DALY) rates attributable to occupational risks (per 100,000 population)**			**Indicator 11.6.2: Population-weighted mean levels of fine particulate matter smaller than 2.5 microns (PM2.5)**			**Indicator 16.1.1: Age-standardised death rate due to interpersonal violence (per 100,000 population)**			**Indicator 16.1.2: Age-standardised death rate due to collective violence and legal intervention (per 100,000 population)**		
**Location**	**1990**	**2000**	**2015**	**1990**	**2000**	**2015**	**1990**	**2000**	**2015**	**1990**	**2000**	**2015**
Marshall Islands	43.20 (37.02-49.00)	43.33 (37.71-48.57)	44.21 (38.76-49.72)	69.60 (35.61-100.00)	69.15 (35.12-100.00)	71.20 (36.31-100.00)	37.88 (33.60-43.77)	41.63 (37.14-47.17)	42.99 (37.77-49.23)	100.00 (100.00-100.00)	100.00 (100.00-100.00)	100.00 (100.00-100.00)
Papua New Guinea	1.32 (0.00-8.94)	5.53 (0.00-13.20)	8.57 (0.00-18.43)	60.03 (54.55-64.80)	58.87 (52.33-64.33)	62.12 (55.75-67.36)	33.58 (25.93-41.86)	34.41 (26.29-43.47)	36.37 (27.32-45.43)	7.97 (6.32-9.15)	100.00 (100.00-100.00)	100.00 (100.00-100.00)
Samoa	48.70 (42.62-54.58)	52.64 (46.90-58.07)	57.33 (52.00-62.42)	89.44 (80.06-95.98)	89.28 (80.41-95.75)	92.45 (82.68-99.32)	43.23 (36.55-49.82)	43.95 (36.74-51.61)	47.97 (40.77-54.79)	100.00 (100.00-100.00)	100.00 (100.00-100.00)	100.00 (100.00-100.00)
Solomon Islands	27.04 (16.97-36.67)	30.54 (20.09-39.92)	32.51 (22.53-41.06)	77.82 (68.15-85.04)	78.17 (66.35-86.75)	80.30 (67.95-88.41)	35.82 (28.04-45.13)	36.79 (28.37-46.29)	38.51 (29.53-48.26)	100.00 (100.00-100.00)	100.00 (100.00-100.00)	100.00 (100.00-100.00)
Tonga	42.11 (36.10-47.70)	42.26 (37.65-47.04)	42.09 (36.78-47.16)	86.69 (77.40-93.62)	86.75 (77.44-94.13)	91.26 (81.35-98.86)	31.76 (27.23-38.14)	51.47 (39.00-55.51)	57.21 (40.44-63.67)	100.00 (100.00-100.00)	100.00 (100.00-100.00)	100.00 (100.00-100.00)
Vanuatu	18.12 (5.67-28.14)	25.14 (14.69-35.32)	31.38 (21.79-39.56)	69.54 (61.27-76.40)	69.73 (55.56-79.50)	74.71 (57.44-85.96)	67.54 (58.46-76.31)	67.50 (57.93-79.41)	69.61 (59.03-80.44)	100.00 (100.00-100.00)	100.00 (100.00-100.00)	100.00 (100.00-100.00)
Afghanistan	42.07 (33.20-49.88)	40.90 (32.97-49.03)	42.89 (35.39-50.10)	23.55 (20.77-32.46)	25.84 (22.91-34.52)	30.05 (26.80-38.06)	9.34 (2.94-16.06)	8.86 (3.46-15.14)	11.38 (5.51-18.22)	2.19 (1.32-3.27)	1.31 (0.75-1.67)	0.55 (0.28-1.04)
Algeria	67.02 (61.84-72.12)	72.57 (67.27-77.85)	77.98 (72.25-83.32)	39.22 (35.18-46.56)	40.57 (36.49-47.91)	38.07 (34.08-45.10)	69.26 (64.88-73.74)	70.65 (66.66-74.96)	78.88 (73.89-83.93)	100.00 (100.00-100.00)	4.56 (3.35-5.39)	22.93 (16.10-38.24)
Bahrain	47.77 (41.19-54.01)	52.50 (47.18-57.71)	63.44 (58.26-68.52)	22.93 (16.53-32.22)	23.01 (16.12-32.50)	26.28 (19.55-35.22)	60.34 (51.18-64.52)	54.47 (50.94-57.78)	60.37 (55.10-65.32)	100.00 (100.00-100.00)	29.98 (18.77-62.94)	100.00 (100.00-100.00)
Egypt	56.95 (51.21-61.87)	64.91 (61.18-68.72)	66.09 (60.81-70.71)	17.51 (14.38-27.79)	18.45 (15.03-28.00)	8.37 (4.95-19.69)	81.12 (64.66-85.24)	55.41 (51.60-69.32)	52.86 (48.34-71.88)	100.00 (100.00-100.00)	17.83 (12.31-29.99)	5.75 (3.79-9.57)
Iran	61.88 (56.64-66.72)	64.35 (59.17-69.11)	68.32 (63.33-73.02)	29.25 (26.16-37.51)	30.04 (26.83-38.28)	33.02 (29.60-40.88)	45.29 (40.82-50.48)	46.98 (43.59-50.98)	56.69 (50.79-62.51)	100.00 (100.00-100.00)	10.08 (6.90-16.99)	100.00 (100.00-100.00)

Location	Indicator 8.8.1: Age-standardised all-cause disability-adjusted life year (DALY) rates attributable to occupational risks (per 100,000 population)			Indicator 11.6.2: Population-weighted mean levels of fine particulate matter smaller than 2.5 microns (PM2.5)			Indicator 16.1.1: Age-standardised death rate due to interpersonal violence (per 100,000 population)			Indicator 16.1.2: Age-standardised death rate due to collective violence and legal intervention (per 100,000 population)		
	1990	2000	2015	1990	2000	2015	1990	2000	2015	1990	2000	2015
Iraq	64.56 (57.86-70.71)	63.25 (57.04-69.42)	65.17 (58.99-71.00)	28.16 (24.65-36.89)	28.45 (25.07-36.47)	27.89 (24.54-36.28)	36.39 (31.71-41.76)	31.14 (26.37-36.76)	22.36 (16.37-36.57)	4.46 (2.82-8.81)	12.91 (10.58-15.02)	0.86 (0.47-1.55)
Jordan	73.46 (67.13-79.25)	75.84 (69.74-81.31)	78.85 (73.40-84.02)	41.04 (36.95-47.82)	42.76 (38.43-49.08)	35.77 (31.83-43.70)	48.06 (42.33-52.67)	46.40 (42.69-51.14)	50.79 (46.56-58.06)	100.00 (100.00-100.00)	100.00 (100.00-100.00)	100.00 (100.00-100.00)
Kuwait	66.31 (60.49-71.65)	67.66 (61.08-73.13)	73.58 (67.15-79.54)	22.15 (17.22-31.41)	21.45 (16.81-31.44)	20.82 (15.64-30.70)	70.36 (65.54-73.98)	66.74 (63.65-69.81)	80.23 (74.23-85.79)	1.41 (0.83-2.69)	18.45 (13.47-28.65)	13.57 (9.29-22.90)
Lebanon	66.25 (59.06-72.37)	70.95 (64.75-76.79)	70.73 (63.81-77.33)	47.53 (42.90-53.84)	47.66 (42.97-53.75)	40.40 (35.71-47.05)	36.26 (30.34-50.59)	54.84 (49.28-61.35)	68.33 (60.43-76.48)	0.77 (0.42-1.00)	11.59 (7.94-19.71)	7.50 (5.04-12.82)
Libya	53.45 (48.24-58.83)	52.50 (46.81-57.66)	53.96 (47.59-60.26)	25.56 (21.92-33.91)	26.78 (23.05-35.36)	16.29 (12.74-26.50)	62.30 (56.45-68.84)	63.06 (58.35-68.89)	64.20 (58.28-70.62)	100.00 (100.00-100.00)	6.11 (4.04-10.21)	1.13 (0.64-1.99)
Morocco	44.63 (38.28-50.88)	47.88 (41.23-54.36)	52.08 (45.21-58.40)	50.17 (45.50-55.90)	50.85 (46.05-56.69)	49.40 (44.78-55.17)	83.79 (63.78-89.72)	68.07 (62.70-73.09)	57.01 (49.54-72.18)	16.07 (11.10-26.92)	100.00 (100.00-100.00)	100.00 (100.00-100.00)
Palestine	66.92 (60.52-72.72)	69.07 (63.64-74.04)	68.63 (62.47-74.89)	56.32 (50.75-62.14)	56.98 (51.41-62.32)	52.20 (47.22-57.68)	57.15 (47.23-64.06)	55.09 (50.55-58.74)	41.74 (35.27-51.03)	2.57 (1.57-4.44)	1.81 (1.06-3.07)	100.00 (100.00-100.00)
Oman	78.98 (71.58-85.15)	78.93 (71.37-85.39)	73.02 (67.15-78.45)	21.51 (17.23-31.56)	21.32 (16.75-31.10)	27.26 (22.98-35.81)	85.52 (75.42-94.07)	86.99 (79.02-92.25)	75.20 (69.27-81.97)	100.00 (100.00-100.00)	100.00 (100.00-100.00)	100.00 (100.00-100.00)
Qatar	53.83 (47.71-59.69)	50.32 (44.06-56.49)	53.30 (46.11-59.99)	7.54 (3.07-18.70)	8.17 (2.59-20.31)	7.70 (2.78-19.63)	81.46 (70.56-87.85)	83.04 (72.00-87.98)	76.65 (70.40-83.25)	100.00 (100.00-100.00)	100.00 (100.00-100.00)	100.00 (100.00-100.00)
Saudi Arabia	77.09 (73.59-80.67)	78.96 (75.55-82.29)	83.37 (80.27-86.90)	14.25 (11.97-24.41)	11.76 (9.51-22.42)	7.95 (5.73-18.93)	96.65 (83.93-100.00)	98.56 (85.66-100.00)	86.37 (82.85-91.42)	100.00 (100.00-100.00)	100.00 (100.00-100.00)	54.24 (13.20-100.00)
Sudan	45.20 (37.48-52.35)	48.36 (40.72-55.23)	50.72 (43.82-57.41)	25.22 (21.92-34.23)	25.32 (22.21-33.86)	28.93 (25.41-37.04)	88.79 (81.71-95.27)	85.12 (77.27-92.52)	64.92 (54.59-90.21)	1.60 (0.83-2.68)	2.31 (1.33-3.97)	5.31 (2.99-8.80)

Appendix Table 1a. Scaled values for each SDG health-related indicator and three summary indicators by country for 1990, 2000, and 2015

Location	Appendix Table 1a. Scaled values for each SDG health-related indicator and three summary indicators by country for 1990, 2000, and 2015											
	**Indicator 8.8.1: Age-standardised all-cause disability-adjusted life year (DALY) rates attributable to occupational risks (per 100,000 population)**			**Indicator 11.6.2: Population-weighted mean levels of fine particulate matter smaller than 2.5 microns (PM2.5)**			**Indicator 16.1.1: Age-standardised death rate due to interpersonal violence (per 100,000 population)**			**Indicator 16.1.2: Age-standardised death rate due to collective violence and legal intervention (per 100,000 population)**		
	**1990**	**2000**	**2015**	**1990**	**2000**	**2015**	**1990**	**2000**	**2015**	**1990**	**2000**	**2015**
Syria	56.67 (50.10-62.25)	62.34 (56.08-68.18)	72.74 (67.19-78.03)	39.21 (35.31-45.99)	40.57 (35.64-47.59)	33.96 (28.79-42.06)	65.85 (60.77-70.98)	66.60 (61.67-72.50)	60.85 (55.76-74.58)	100.00 (100.00-100.00)	100.00 (100.00-100.00)	0.26 (0.10-0.55)
Tunisia	44.39 (38.28-50.04)	47.05 (41.91-52.01)	49.11 (41.94-56.59)	36.43 (32.83-43.79)	38.02 (34.10-45.36)	31.92 (28.67-39.59)	72.62 (67.69-77.21)	73.43 (68.85-77.97)	64.67 (58.21-76.39)	100.00 (100.00-100.00)	100.00 (100.00-100.00)	9.50 (6.49-16.10)
Turkey	26.98 (18.04-34.73)	33.58 (27.77-39.49)	51.23 (45.75-56.64)	40.19 (36.18-46.95)	42.35 (38.40-49.00)	37.44 (33.72-44.47)	52.55 (48.08-57.60)	58.59 (54.97-62.39)	58.09 (54.12-68.96)	13.14 (10.79-15.33)	19.97 (16.25-23.57)	19.37 (13.47-32.23)
United Arab Emirates	42.20 (35.50-49.45)	45.83 (39.84-51.82)	43.06 (35.90-49.76)	17.57 (14.90-27.28)	17.58 (14.82-27.08)	22.07 (19.07-31.29)	65.56 (58.13-73.53)	72.93 (68.21-78.30)	76.32 (68.69-84.55)	100.00 (100.00-100.00)	100.00 (100.00-100.00)	100.00 (100.00-100.00)
Yemen	50.42 (41.88-58.11)	50.69 (42.03-57.47)	53.83 (45.42-60.71)	26.99 (22.38-35.86)	26.56 (21.18-35.46)	27.54 (21.94-36.17)	43.52 (34.08-54.06)	44.03 (34.14-54.28)	45.00 (34.19-56.27)	100.00 (100.00-100.00)	100.00 (100.00-100.00)	1.02 (0.57-1.39)
Bangladesh	12.99 (5.43-20.98)	7.91 (0.00-18.72)	22.72 (8.77-32.13)	22.24 (19.45-31.29)	21.59 (18.87-30.69)	12.85 (10.42-23.44)	51.71 (39.55-55.44)	49.71 (45.39-53.13)	55.05 (50.15-59.73)	100.00 (100.00-100.00)	100.00 (100.00-100.00)	100.00 (100.00-100.00)
Bhutan	20.41 (11.83-28.64)	22.10 (12.96-30.32)	27.10 (18.20-35.02)	34.67 (31.10-42.25)	34.29 (30.56-41.32)	26.01 (22.66-34.22)	60.60 (54.25-67.92)	63.87 (58.54-71.08)	73.44 (66.36-80.52)	100.00 (100.00-100.00)	100.00 (100.00-100.00)	100.00 (100.00-100.00)
India	21.42 (15.64-27.78)	24.64 (19.28-30.81)	33.83 (28.76-39.19)	24.03 (21.24-32.99)	23.29 (20.54-32.23)	18.04 (15.63-27.86)	46.24 (43.84-51.04)	46.95 (44.49-53.23)	50.06 (46.32-58.15)	14.12 (7.69-24.08)	17.83 (10.29-29.88)	93.13 (69.67-100.00)
Nepal	11.85 (2.65-20.66)	13.88 (4.82-22.77)	16.79 (7.99-25.32)	25.75 (22.77-34.46)	25.74 (22.69-34.39)	17.80 (14.62-27.72)	67.04 (60.98-72.05)	58.60 (54.27-68.11)	60.09 (53.30-72.25)	100.00 (100.00-100.00)	7.30 (5.71-8.34)	100.00 (100.00-100.00)
Pakistan	39.45 (31.28-46.46)	38.01 (29.83-44.91)	38.58 (27.06-46.10)	20.53 (17.94-29.75)	19.88 (17.25-29.14)	21.79 (19.05-31.05)	48.32 (43.49-53.14)	43.19 (39.27-50.97)	44.63 (39.36-55.62)	16.51 (12.56-22.71)	100.00 (100.00-100.00)	6.16 (4.39-8.23)
Botswana	35.22 (14.20-49.74)	34.91 (5.73-53.63)	42.00 (14.90-56.52)	50.79 (45.97-57.08)	50.12 (45.14-56.01)	56.27 (50.69-61.82)	71.47 (48.53-91.13)	34.75 (7.58-62.81)	31.97 (5.55-61.84)	100.00 (100.00-100.00)	100.00 (100.00-100.00)	100.00 (100.00-100.00)

Location	Appendix Table 1a. Scaled values for each SDG health-related indicator and three summary indicators by country for 1990, 2000, and 2015											
	**Indicator 8.8.1: Age-standardised all-cause disability-adjusted life year (DALY) rates attributable to occupational risks (per 100,000 population)**			**Indicator 11.6.2: Population-weighted mean levels of fine particulate matter smaller than 2.5 microns (PM2.5)**			**Indicator 16.1.1: Age-standardised death rate due to interpersonal violence (per 100,000 population)**			**Indicator 16.1.2: Age-standardised death rate due to collective violence and legal intervention (per 100,000 population)**		
	**1990**	**2000**	**2015**	**1990**	**2000**	**2015**	**1990**	**2000**	**2015**	**1990**	**2000**	**2015**
Lesotho	22.91 (9.97-33.89)	22.62 (9.38-33.39)	19.36 (4.70-33.49)	47.09 (42.11-53.44)	44.66 (36.58-52.29)	47.76 (39.10-55.51)	21.57 (15.25-28.93)	13.03 (5.86-22.46)	10.79 (0.00-24.27)	100.00 (100.00-100.00)	100.00 (100.00-100.00)	100.00 (100.00-100.00)
Namibia	48.50 (39.09-56.04)	46.03 (36.92-53.55)	57.65 (48.26-65.78)	46.17 (40.82-52.48)	45.11 (39.06-52.18)	51.29 (44.04-57.70)	27.88 (21.19-34.31)	23.41 (17.62-29.89)	32.65 (20.79-45.00)	100.00 (100.00-100.00)	5.57 (3.07-10.18)	100.00 (100.00-100.00)
South Africa	56.76 (51.54-61.53)	55.23 (50.50-60.00)	64.11 (59.73-68.26)	38.57 (34.75-45.83)	36.99 (33.37-44.35)	42.86 (38.73-49.60)	4.93 (1.85-8.43)	3.77 (1.94-6.80)	10.35 (8.00-12.92)	4.48 (3.06-5.65)	100.00 (100.00-100.00)	21.62 (15.65-29.34)
Swaziland	42.34 (31.69-51.39)	42.70 (31.21-53.41)	45.23 (28.99-60.25)	45.99 (40.94-51.94)	44.72 (38.83-51.97)	51.09 (44.75-57.86)	15.00 (8.48-23.08)	22.88 (14.51-32.98)	24.07 (9.58-41.38)	100.00 (100.00-100.00)	100.00 (100.00-100.00)	100.00 (100.00-100.00)
Zimbabwe	40.99 (34.03-48.35)	41.83 (30.91-51.33)	39.60 (27.32-49.40)	45.85 (40.71-52.34)	47.03 (41.77-53.06)	49.57 (44.10-55.62)	40.37 (33.45-48.59)	42.28 (31.75-52.44)	41.43 (31.64-53.43)	23.19 (14.73-39.08)	30.35 (19.35-47.92)	100.00 (100.00-100.00)
Benin	35.62 (29.77-42.36)	36.84 (30.69-43.29)	37.00 (29.35-44.97)	23.26 (19.09-32.74)	24.46 (20.23-33.11)	38.39 (34.09-45.72)	58.40 (51.33-64.66)	52.72 (46.21-59.29)	49.77 (36.92-63.29)	100.00 (100.00-100.00)	100.00 (100.00-100.00)	100.00 (100.00-100.00)
Burkina Faso	24.40 (18.37-30.77)	26.29 (19.94-33.69)	29.92 (21.97-38.49)	20.72 (17.47-29.94)	21.10 (17.71-30.34)	34.94 (30.41-42.49)	78.21 (61.02-84.72)	77.79 (62.28-83.99)	79.98 (65.57-91.66)	100.00 (100.00-100.00)	100.00 (100.00-100.00)	100.00 (100.00-100.00)
Cameroon	36.88 (30.80-42.75)	37.97 (32.09-43.71)	39.92 (32.02-47.28)	8.08 (5.84-19.21)	9.78 (7.33-20.84)	21.17 (18.29-30.39)	50.42 (42.41-58.18)	48.35 (42.07-55.93)	52.66 (40.18-65.05)	100.00 (100.00-100.00)	100.00 (100.00-100.00)	3.98 (2.34-7.24)
Cape Verde	43.75 (38.44-49.26)	49.43 (42.87-56.08)	54.70 (49.44-59.81)	27.78 (19.95-36.61)	29.86 (21.52-39.13)	35.51 (26.06-44.88)	53.17 (30.61-57.87)	24.12 (17.07-32.35)	25.64 (20.02-32.14)	100.00 (100.00-100.00)	100.00 (100.00-100.00)	100.00 (100.00-100.00)
Chad	30.33 (23.79-37.15)	30.50 (23.84-37.44)	32.82 (25.28-40.97)	21.85 (19.07-31.12)	22.28 (18.46-31.12)	31.19 (27.15-39.19)	54.65 (44.81-63.34)	47.05 (39.94-54.80)	47.63 (34.53-59.93)	1.57 (0.86-2.80)	2.18 (1.08-3.64)	5.83 (3.63-11.08)
Cote d'Ivoire	34.85 (28.72-40.92)	36.63 (30.32-42.96)	38.95 (31.70-45.62)	39.58 (35.07-46.23)	40.63 (36.17-47.40)	48.83 (43.50-55.21)	82.72 (36.28-92.65)	79.32 (34.83-89.15)	80.78 (39.07-97.70)	100.00 (100.00-100.00)	11.26 (6.91-18.69)	100.00 (100.00-100.00)

Location	Appendix Table 1a. Scaled values for each SDG health-related indicator and three summary indicators by country for 1990, 2000, and 2015											
	Indicator 8.8.1: Age-standardised all-cause disability-adjusted life year (DALY) rates attributable to occupational risks (per 100,000 population)			Indicator 11.6.2: Population-weighted mean levels of fine particulate matter smaller than 2.5 microns (PM2.5)			Indicator 16.1.1: Age-standardised death rate due to interpersonal violence (per 100,000 population)			Indicator 16.1.2: Age-standardised death rate due to collective violence and legal intervention (per 100,000 population)		
	1990	2000	2015	1990	2000	2015	1990	2000	2015	1990	2000	2015
The Gambia	31.06 (23.57-38.83)	35.54 (29.11-41.99)	40.36 (33.39-47.54)	22.48 (0.43-34.91)	21.51 (0.00-35.39)	25.24 (2.45-41.01)	83.25 (41.06-96.58)	79.15 (40.84-88.75)	83.65 (40.38-95.62)	100.00 (100.00-100.00)	9.97 (6.18-17.17)	100.00 (100.00-100.00)
Ghana	44.27 (38.51-50.15)	46.29 (40.85-52.11)	49.78 (43.64-55.84)	36.13 (32.56-43.57)	37.38 (33.39-44.73)	49.41 (44.47-55.50)	52.44 (28.27-62.56)	50.91 (30.15-58.79)	54.19 (31.96-67.41)	100.00 (100.00-100.00)	26.64 (16.97-45.19)	100.00 (100.00-100.00)
Guinea	34.03 (27.70-40.22)	35.49 (29.19-41.86)	34.77 (27.54-41.72)	42.10 (37.86-49.12)	42.05 (37.10-49.06)	49.83 (44.63-55.55)	57.64 (49.71-63.72)	52.63 (46.87-57.68)	49.47 (38.95-60.04)	100.00 (100.00-100.00)	5.56 (3.41-9.80)	32.54 (21.17-53.40)
Guinea-Bissau	32.32 (17.40-42.67)	34.98 (22.37-44.82)	35.18 (19.34-45.71)	33.74 (24.57-42.06)	33.99 (24.57-42.17)	40.43 (28.30-49.69)	41.34 (23.76-57.01)	39.44 (20.71-55.26)	38.26 (16.14-53.08)	100.00 (100.00-100.00)	100.00 (100.00-100.00)	100.00 (100.00-100.00)
Liberia	43.31 (37.36-49.38)	43.56 (37.37-49.37)	44.77 (38.34-50.89)	68.92 (62.39-73.57)	68.81 (61.80-73.85)	75.82 (68.47-81.25)	52.92 (45.54-60.64)	56.26 (49.90-63.53)	57.71 (47.78-66.92)	0.38 (0.12-0.73)	4.89 (3.02-8.76)	100.00 (100.00-100.00)
Mali	44.52 (39.34-49.80)	47.58 (42.31-52.54)	48.98 (42.74-54.70)	22.97 (20.03-32.19)	24.03 (20.24-32.94)	32.42 (28.10-40.23)	54.47 (45.25-59.75)	55.25 (48.50-61.00)	54.55 (43.87-63.99)	4.44 (2.63-7.30)	1.88 (1.09-3.44)	6.83 (4.12-11.86)
Mauritania	47.38 (42.05-52.64)	50.47 (45.08-56.26)	51.74 (45.85-57.28)	10.67 (4.78-21.79)	11.47 (0.52-23.00)	14.70 (1.71-26.06)	54.95 (46.49-61.20)	56.39 (48.04-66.99)	57.14 (43.11-71.16)	2.17 (1.28-3.99)	100.00 (100.00-100.00)	100.00 (100.00-100.00)
Niger	40.56 (34.69-46.48)	41.39 (35.53-47.14)	39.53 (32.69-46.21)	1.68 (0.00-13.85)	2.43 (0.00-14.35)	22.75 (19.45-32.25)	54.05 (44.22-61.62)	53.45 (45.96-60.50)	53.14 (42.09-64.30)	5.74 (3.61-9.96)	100.00 (100.00-100.00)	3.97 (2.39-7.52)
Nigeria	38.79 (31.72-45.48)	41.91 (35.32-48.58)	47.25 (40.77-53.24)	15.47 (13.08-25.83)	17.99 (15.49-27.76)	36.34 (32.65-43.67)	73.52 (63.60-82.61)	76.49 (70.32-82.80)	80.97 (69.25-89.46)	100.00 (100.00-100.00)	7.50 (4.60-13.77)	3.50 (2.10-6.45)
Sao Tome and Principe	41.84 (36.22-47.25)	42.84 (37.14-48.49)	43.79 (33.34-52.26)	59.07 (49.88-68.37)	59.34 (45.95-71.83)	62.72 (48.41-75.06)	40.47 (36.28-45.28)	38.47 (33.37-43.25)	38.80 (28.10-50.08)	100.00 (100.00-100.00)	100.00 (100.00-100.00)	100.00 (100.00-100.00)
Senegal	34.91 (28.70-40.80)	39.23 (33.44-45.04)	39.88 (31.78-46.87)	33.88 (30.13-41.61)	34.24 (30.42-42.16)	36.62 (32.65-43.64)	65.80 (59.43-72.51)	69.01 (62.56-74.88)	69.67 (55.70-82.54)	11.23 (6.87-19.65)	14.56 (9.70-22.89)	100.00 (100.00-100.00)

Appendix Table 1a. Scaled values for each SDG health-related indicator and three summary indicators by country for 1990, 2000, and 2015

Location	Indicator 8.8.1: Age-standardised all-cause disability-adjusted life year (DALY) rates attributable to occupational risks (per 100,000 population)			Indicator 11.6.2: Population-weighted mean levels of fine particulate matter smaller than 2.5 microns (PM2.5)			Indicator 16.1.1: Age-standardised death rate due to interpersonal violence (per 100,000 population)			Indicator 16.1.2: Age-standardised death rate due to collective violence and legal intervention (per 100,000 population)		
	1990	2000	2015	1990	2000	2015	1990	2000	2015	1990	2000	2015
Sierra Leone	36.86 (30.29-43.57)	38.38 (32.27-44.32)	38.84 (31.99-45.08)	46.36 (41.09-52.98)	46.67 (38.98-53.76)	54.05 (44.92-60.84)	64.43 (55.01-75.36)	62.20 (56.58-67.82)	64.64 (54.67-75.04)	100.00 (100.00-100.00)	1.76 (0.82-3.54)	100.00 (100.00-100.00)
Togo	27.88 (20.62-34.96)	30.13 (23.59-37.00)	32.73 (24.77-40.91)	25.87 (20.58-34.40)	26.92 (18.54-35.98)	40.41 (33.31-48.12)	56.01 (48.24-62.22)	50.76 (43.77-57.52)	50.05 (39.82-60.95)	100.00 (100.00-100.00)	100.00 (100.00-100.00)	100.00 (100.00-100.00)
Burundi	17.52 (6.54-27.18)	22.77 (12.92-31.38)	29.04 (19.92-38.04)	30.93 (26.07-38.71)	30.58 (25.76-39.10)	31.56 (25.69-39.94)	64.00 (53.14-76.46)	66.20 (57.67-75.33)	73.65 (56.62-90.43)	100.00 (100.00-100.00)	1.27 (0.60-2.26)	4.87 (2.75-7.87)
Comoros	43.34 (33.88-51.42)	44.95 (36.74-51.48)	46.75 (39.96-53.63)	51.98 (43.89-59.46)	51.85 (43.46-60.19)	57.08 (49.15-65.48)	41.49 (30.29-52.71)	33.86 (26.10-41.62)	33.46 (22.08-45.56)	100.00 (100.00-100.00)	100.00 (100.00-100.00)	100.00 (100.00-100.00)
Djibouti	61.03 (52.53-68.62)	57.60 (47.87-65.86)	56.72 (47.70-63.94)	31.41 (19.16-43.51)	30.01 (9.19-45.93)	29.54 (8.80-44.80)	44.09 (34.23-55.08)	32.47 (17.83-46.20)	29.37 (14.66-43.76)	100.00 (100.00-100.00)	100.00 (100.00-100.00)	100.00 (100.00-100.00)
Eritrea	37.01 (26.53-45.54)	45.02 (34.93-53.89)	43.40 (33.35-52.72)	32.14 (28.06-40.35)	32.69 (28.84-40.16)	32.73 (28.69-40.45)	37.89 (32.02-43.67)	35.03 (23.55-46.62)	28.42 (15.01-41.87)	100.00 (100.00-100.00)	0.00 (0.00-0.00)	100.00 (100.00-100.00)
Ethiopia	32.59 (25.90-39.03)	35.69 (28.97-42.46)	36.94 (29.38-44.37)	37.68 (33.71-44.80)	38.13 (34.14-45.17)	37.94 (33.82-45.23)	29.61 (13.10-36.21)	34.27 (21.02-40.32)	38.43 (24.89-49.85)	0.56 (0.20-0.98)	11.16 (7.01-19.86)	17.24 (11.18-29.66)
Kenya	37.41 (31.80-43.27)	37.86 (32.16-43.58)	39.98 (34.43-45.94)	57.86 (52.58-63.04)	58.27 (52.78-63.46)	58.08 (52.56-63.13)	25.22 (19.09-30.86)	24.69 (20.48-28.62)	23.71 (19.21-27.77)	23.61 (12.75-35.56)	11.82 (5.86-17.50)	10.24 (5.74-15.52)
Madagascar	18.89 (9.84-27.18)	18.87 (9.39-28.18)	19.96 (10.10-29.37)	54.42 (49.46-60.12)	53.77 (48.76-58.91)	53.37 (48.32-58.83)	73.07 (68.16-78.18)	52.74 (45.39-70.98)	60.48 (47.21-77.72)	100.00 (100.00-100.00)	100.00 (100.00-100.00)	11.99 (7.64-18.60)
Malawi	31.77 (21.75-40.25)	29.55 (19.32-38.02)	32.36 (24.72-40.57)	43.06 (38.45-49.88)	43.25 (38.50-49.87)	46.54 (41.89-52.21)	64.99 (39.52-73.49)	59.07 (38.69-69.54)	65.00 (41.20-77.86)	22.17 (14.40-35.91)	100.00 (100.00-100.00)	100.00 (100.00-100.00)
Mozambique	32.77 (23.18-41.65)	31.26 (22.14-39.95)	31.38 (23.16-39.73)	49.97 (45.42-55.83)	49.60 (44.42-55.60)	53.54 (48.62-58.99)	42.81 (35.46-49.47)	41.23 (35.81-47.62)	36.72 (25.41-49.28)	2.19 (1.13-3.83)	11.64 (6.68-18.74)	27.95 (19.19-41.33)

Location	Appendix Table 1a. Scaled values for each SDG health-related indicator and three summary indicators by country for 1990, 2000, and 2015											
	**Indicator 8.8.1: Age-standardised all-cause disability-adjusted life year (DALY) rates attributable to occupational risks (per 100,000 population)**			**Indicator 11.6.2: Population-weighted mean levels of fine particulate matter smaller than 2.5 microns (PM2.5)**			**Indicator 16.1.1: Age-standardised death rate due to interpersonal violence (per 100,000 population)**			**Indicator 16.1.2: Age-standardised death rate due to collective violence and legal intervention (per 100,000 population)**		
	**1990**	**2000**	**2015**	**1990**	**2000**	**2015**	**1990**	**2000**	**2015**	**1990**	**2000**	**2015**
Rwanda	24.15 (14.96-32.66)	26.08 (17.26-34.10)	32.84 (24.63-40.90)	27.46 (22.64-36.37)	27.04 (22.10-35.74)	29.17 (23.98-37.20)	34.36 (29.26-41.34)	38.67 (32.88-45.32)	48.03 (36.84-57.28)	1.15 (0.55-1.97)	6.98 (3.99-13.36)	100.00 (100.00-100.00)
Somalia	42.31 (28.47-53.36)	42.03 (29.38-53.51)	42.54 (28.88-52.47)	50.85 (45.59-56.72)	50.03 (44.43-56.03)	53.18 (47.51-58.64)	38.46 (20.60-58.42)	28.05 (9.86-48.33)	26.09 (8.22-45.16)	1.04 (0.47-1.82)	5.98 (3.80-9.59)	1.29 (0.60-2.18)
South Sudan	39.00 (25.72-50.12)	40.85 (28.25-50.40)	42.36 (29.25-51.37)	33.72 (30.26-41.22)	33.22 (29.65-40.88)	40.49 (36.61-47.16)	46.76 (27.09-64.71)	37.38 (17.20-54.50)	31.17 (9.39-49.11)	4.16 (2.37-6.74)	100.00 (100.00-100.00)	1.51 (0.78-2.71)
Tanzania	37.36 (28.04-45.85)	38.08 (29.70-46.55)	38.75 (28.72-47.50)	51.69 (46.89-57.18)	50.14 (45.23-55.74)	49.04 (44.23-54.74)	36.86 (30.02-42.42)	39.07 (31.44-45.90)	39.97 (27.89-49.95)	100.00 (100.00-100.00)	26.14 (18.24-41.59)	100.00 (100.00-100.00)
Uganda	27.22 (17.31-35.90)	27.99 (19.50-36.01)	30.67 (21.65-39.68)	23.69 (20.66-32.48)	23.82 (21.04-32.79)	23.84 (20.95-32.75)	39.05 (31.63-48.56)	35.44 (29.67-43.73)	37.33 (25.55-50.00)	4.02 (2.46-6.65)	3.82 (2.21-6.52)	100.00 (100.00-100.00)
Zambia	37.40 (28.21-45.22)	31.23 (22.73-38.47)	31.96 (24.43-40.04)	40.53 (36.34-47.20)	41.80 (36.91-49.72)	45.55 (39.98-52.25)	41.63 (35.87-47.82)	32.38 (27.56-38.15)	31.07 (22.21-41.53)	15.94 (10.09-25.70)	100.00 (100.00-100.00)	100.00 (100.00-100.00)
Angola	27.68 (12.68-39.98)	29.65 (14.89-41.38)	37.01 (21.04-47.42)	37.24 (33.30-44.28)	35.19 (31.35-42.56)	37.73 (27.42-45.76)	38.85 (22.71-59.05)	44.01 (25.45-62.27)	45.80 (23.38-61.36)	1.63 (0.82-3.17)	3.08 (1.89-5.93)	100.00 (100.00-100.00)
Central African Republic	20.48 (11.97-29.04)	19.75 (8.93-30.27)	20.77 (9.42-32.44)	28.67 (22.08-37.71)	27.98 (17.85-36.90)	31.20 (20.78-39.98)	37.42 (29.26-44.95)	35.15 (22.42-49.08)	34.18 (18.66-50.23)	100.00 (100.00-100.00)	100.00 (100.00-100.00)	3.20 (1.96-6.18)
Congo	31.83 (24.63-39.19)	34.18 (26.70-41.73)	40.39 (31.51-47.88)	27.96 (21.84-37.50)	23.95 (9.12-35.76)	28.32 (9.99-41.14)	40.86 (31.50-50.26)	40.25 (31.92-48.02)	45.49 (32.79-56.16)	100.00 (100.00-100.00)	2.14 (1.29-3.99)	100.00 (100.00-100.00)
Democratic Republic of the Congo	31.01 (21.75-39.45)	29.59 (19.08-38.12)	30.22 (20.91-38.86)	30.13 (25.41-38.36)	28.44 (22.24-37.68)	31.06 (25.85-38.78)	46.09 (36.70-56.65)	45.72 (39.59-51.81)	47.33 (38.25-57.40)	16.59 (10.70-33.99)	3.48 (2.31-5.01)	7.22 (4.64-14.13)
Equatorial Guinea	21.52 (5.71-35.15)	28.96 (13.95-40.37)	40.90 (23.94-49.88)	30.68 (23.56-38.50)	27.71 (11.47-38.79)	31.45 (14.21-42.71)	36.80 (18.61-58.84)	41.76 (22.00-59.88)	48.08 (25.92-61.03)	100.00 (100.00-100.00)	100.00 (100.00-100.00)	100.00 (100.00-100.00)

	Appendix Table 1a. Scaled values for each SDG health-related indicator and three summary indicators by country for 1990, 2000, and 2015											
	**Indicator 8.8.1: Age-standardised all-cause disability-adjusted life year (DALY) rates attributable to occupational risks (per 100,000 population)**			**Indicator 11.6.2: Population-weighted mean levels of fine particulate matter smaller than 2.5 microns (PM2.5)**			**Indicator 16.1.1: Age-standardised death rate due to interpersonal violence (per 100,000 population)**			**Indicator 16.1.2: Age-standardised death rate due to collective violence and legal intervention (per 100,000 population)**		
**Location**	**1990**	**2000**	**2015**	**1990**	**2000**	**2015**	**1990**	**2000**	**2015**	**1990**	**2000**	**2015**
Gabon	44.60 (38.32-50.48)	47.99 (41.53-53.71)	52.71 (45.57-58.92)	35.87 (20.81-45.20)	34.33 (20.66-44.25)	35.44 (23.58-44.65)	43.66 (36.02-50.80)	44.39 (35.55-52.33)	46.83 (33.50-58.35)	100.00 (100.00-100.00)	100.00 (100.00-100.00)	100.00 (100.00-100.00)

Appendix Table 1b. Unscaled values for each SDG health-related indicator by country for 1990, 2000, and 2015

Location	Indicator 1.5.1: Age-standardised death rate due to exposure to forces of nature (per 100,000 population)			Indicator 2.2.1: Prevalence of stunting among children under 5 years of age			Indicator 2.2.2a: Prevalence of wasting among children under 5 years of age			Indicator 2.2.2b: Prevalence of overweight among children aged 2 to 4 years		
	1990	2000	2015	1990	2000	2015	1990	2000	2015	1990	2000	2015
Canada	0.00 (0.00-0.00)	0.00 (0.00-0.00)	0.00 (0.00-0.00)	0.00 (0.00-0.00)	0.00 (0.00-0.00)	0.00 (0.00-0.00)	0.00 (0.00-0.00)	0.00 (0.00-0.00)	0.00 (0.00-0.00)	27.86 (20.86-35.84)	38.41 (29.74-47.65)	42.32 (33.76-51.28)
United States	0.09 (0.05-0.17)	0.08 (0.05-0.14)	0.08 (0.05-0.15)	0.00 (0.00-0.00)	0.00 (0.00-0.00)	0.00 (0.00-0.00)	0.00 (0.00-0.00)	0.00 (0.00-0.00)	0.00 (0.00-0.00)	15.34 (13.02-17.89)	18.98 (16.28-21.64)	21.13 (16.87-25.91)
Australia	0.00 (0.00-0.00)	0.02 (0.01-0.04)	0.02 (0.01-0.03)	0.00 (0.00-0.00)	0.00 (0.00-0.00)	0.00 (0.00-0.00)	0.00 (0.00-0.00)	0.00 (0.00-0.00)	0.00 (0.00-0.00)	16.15 (12.01-20.77)	18.01 (15.65-20.43)	24.65 (18.53-32.24)
New Zealand	0.00 (0.00-0.00)	0.04 (0.02-0.06)	0.76 (0.42-1.10)	0.00 (0.00-0.00)	0.00 (0.00-0.00)	0.00 (0.00-0.00)	0.00 (0.00-0.00)	0.00 (0.00-0.00)	0.00 (0.00-0.00)	33.70 (25.15-43.11)	32.78 (24.44-41.84)	30.41 (24.18-37.47)
Brunei	0.00 (0.00-0.00)	0.00 (0.00-0.00)	0.06 (0.01-0.11)	0.00 (0.00-0.00)	0.00 (0.00-0.00)	0.00 (0.00-0.00)	0.00 (0.00-0.00)	0.00 (0.00-0.00)	0.00 (0.00-0.00)	10.48 (7.55-14.09)	13.58 (9.93-18.27)	17.92 (13.35-23.50)
Japan	0.08 (0.01-0.19)	0.04 (0.01-0.09)	1.62 (0.10-4.14)	0.00 (0.00-0.00)	0.00 (0.00-0.00)	0.00 (0.00-0.00)	0.00 (0.00-0.00)	0.00 (0.00-0.00)	0.00 (0.00-0.00)	7.99 (6.69-9.44)	9.98 (7.95-12.33)	12.23 (9.06-15.65)
Singapore	0.00 (0.00-0.00)	0.03 (0.00-0.05)	0.00 (0.00-0.00)	0.00 (0.00-0.00)	0.00 (0.00-0.00)	0.00 (0.00-0.00)	0.00 (0.00-0.00)	0.00 (0.00-0.00)	0.00 (0.00-0.00)	16.41 (12.02-21.83)	20.84 (15.50-26.67)	25.43 (19.16-32.34)
South Korea	0.40 (0.06-0.76)	0.15 (0.02-0.29)	0.03 (0.00-0.06)	0.00 (0.00-0.00)	0.00 (0.00-0.00)	0.00 (0.00-0.00)	0.00 (0.00-0.00)	0.00 (0.00-0.00)	0.00 (0.00-0.00)	21.54 (16.39-27.24)	24.90 (19.02-31.53)	29.98 (23.63-37.15)
Andorra	0.00 (0.00-0.00)	0.00 (0.00-0.00)	0.00 (0.00-0.00)	0.00 (0.00-0.00)	0.00 (0.00-0.00)	0.00 (0.00-0.00)	0.00 (0.00-0.00)	0.00 (0.00-0.00)	0.00 (0.00-0.00)	25.67 (17.34-35.39)	26.55 (18.05-35.76)	28.47 (19.84-38.26)
Austria	0.00 (0.00-0.00)	0.00 (0.00-0.00)	0.07 (0.04-0.10)	0.00 (0.00-0.00)	0.00 (0.00-0.00)	0.00 (0.00-0.00)	0.00 (0.00-0.00)	0.00 (0.00-0.00)	0.00 (0.00-0.00)	27.15 (18.11-37.31)	30.81 (21.56-42.55)	32.75 (22.96-43.83)

Appendix Table 1b. Unscaled values for each SDG health-related indicator by country for 1990, 2000, and 2015

Location	Indicator 1.5.1: Age-standardised death rate due to exposure to forces of nature (per 100,000 population)			Indicator 2.2.1: Prevalence of stunting among children under 5 years of age			Indicator 2.2.2a: Prevalence of wasting among children under 5 years of age			Indicator 2.2.2b: Prevalence of overweight among children aged 2 to 4 years		
	1990	2000	2015	1990	2000	2015	1990	2000	2015	1990	2000	2015
Belgium	0.09 (0.05-0.14)	0.00 (0.00-0.00)	0.00 (0.00-0.00)	0.00 (0.00-0.00)	0.00 (0.00-0.00)	0.00 (0.00-0.00)	0.00 (0.00-0.00)	0.00 (0.00-0.00)	0.00 (0.00-0.00)	26.69 (18.12-36.58)	29.89 (21.05-40.35)	28.27 (19.49-38.46)
Cyprus	0.00 (0.00-0.00)	0.00 (0.00-0.00)	0.00 (0.00-0.00)	0.00 (0.00-0.00)	0.00 (0.00-0.00)	0.00 (0.00-0.00)	0.00 (0.00-0.00)	0.00 (0.00-0.00)	0.00 (0.00-0.00)	31.41 (22.17-41.62)	31.85 (22.58-41.90)	30.75 (21.61-41.10)
Denmark	0.00 (0.00-0.00)	0.00 (0.00-0.00)	0.00 (0.00-0.00)	0.00 (0.00-0.00)	0.00 (0.00-0.00)	0.00 (0.00-0.00)	0.00 (0.00-0.00)	0.00 (0.00-0.00)	0.00 (0.00-0.00)	28.53 (19.67-39.15)	32.78 (23.04-44.08)	32.13 (22.76-42.71)
Finland	0.00 (0.00-0.00)	0.00 (0.00-0.00)	0.00 (0.00-0.00)	0.00 (0.00-0.00)	0.00 (0.00-0.00)	0.00 (0.00-0.00)	0.00 (0.00-0.00)	0.00 (0.00-0.00)	0.00 (0.00-0.00)	26.75 (18.00-36.45)	24.89 (17.21-33.80)	27.53 (19.52-37.38)
France	0.00 (0.00-0.00)	0.05 (0.03-0.07)	0.00 (0.00-0.00)	0.00 (0.00-0.00)	0.00 (0.00-0.00)	0.00 (0.00-0.00)	0.00 (0.00-0.00)	0.00 (0.00-0.00)	0.00 (0.00-0.00)	23.82 (16.38-33.28)	27.46 (19.14-37.72)	27.08 (18.45-37.34)
Germany	0.00 (0.00-0.00)	0.00 (0.00-0.00)	0.00 (0.00-0.00)	0.00 (0.00-0.00)	0.00 (0.00-0.00)	0.00 (0.00-0.00)	0.00 (0.00-0.00)	0.00 (0.00-0.00)	0.00 (0.00-0.00)	22.26 (14.92-30.99)	26.94 (18.65-36.09)	34.13 (24.42-44.78)
Greece	0.00 (0.00-0.00)	0.21 (0.12-0.30)	0.00 (0.00-0.00)	0.00 (0.00-0.00)	0.00 (0.00-0.00)	0.00 (0.00-0.00)	0.00 (0.00-0.00)	0.00 (0.00-0.00)	0.00 (0.00-0.00)	34.02 (23.54-44.33)	38.67 (28.40-49.18)	40.48 (29.70-52.37)
Iceland	0.40 (0.07-0.78)	0.72 (0.44-1.00)	0.00 (0.00-0.00)	0.00 (0.00-0.00)	0.00 (0.00-0.00)	0.00 (0.00-0.00)	0.00 (0.00-0.00)	0.00 (0.00-0.00)	0.00 (0.00-0.00)	39.99 (29.44-51.14)	36.77 (26.33-47.72)	36.91 (25.51-48.00)
Ireland	0.00 (0.00-0.00)	0.00 (0.00-0.00)	0.00 (0.00-0.00)	0.00 (0.00-0.00)	0.00 (0.00-0.00)	0.00 (0.00-0.00)	0.00 (0.00-0.00)	0.00 (0.00-0.00)	0.00 (0.00-0.00)	29.12 (19.86-39.85)	29.32 (23.14-36.26)	31.73 (22.09-42.33)
Israel	0.00 (0.00-0.00)	0.00 (0.00-0.00)	0.00 (0.00-0.00)	0.00 (0.00-0.00)	0.00 (0.00-0.00)	0.00 (0.00-0.00)	0.00 (0.00-0.00)	0.00 (0.00-0.00)	0.00 (0.00-0.00)	25.05 (16.85-34.28)	29.04 (19.80-39.67)	35.44 (25.00-46.67)

Appendix Table 1b. Unscaled values for each SDG health-related indicator by country for 1990, 2000, and 2015

Location	Indicator 1.5.1: Age-standardised death rate due to exposure to forces of nature (per 100,000 population)			Indicator 2.2.1: Prevalence of stunting among children under 5 years of age			Indicator 2.2.2a: Prevalence of wasting among children under 5 years of age			Indicator 2.2.2b: Prevalence of overweight among children aged 2 to 4 years		
	1990	2000	2015	1990	2000	2015	1990	2000	2015	1990	2000	2015
Italy	0.00 (0.00-0.00)	0.04 (0.02-0.06)	0.02 (0.01-0.03)	0.00 (0.00-0.00)	0.00 (0.00-0.00)	0.00 (0.00-0.00)	0.00 (0.00-0.00)	0.00 (0.00-0.00)	0.00 (0.00-0.00)	33.64 (23.65-44.01)	39.32 (29.24-49.80)	39.21 (28.70-50.47)
Luxembourg	0.00 (0.00-0.00)	0.00 (0.00-0.00)	0.00 (0.00-0.00)	0.00 (0.00-0.00)	0.00 (0.00-0.00)	0.00 (0.00-0.00)	0.00 (0.00-0.00)	0.00 (0.00-0.00)	0.00 (0.00-0.00)	23.45 (15.26-32.48)	30.47 (21.16-41.47)	33.92 (24.06-45.24)
Malta	0.00 (0.00-0.00)	0.00 (0.00-0.00)	0.00 (0.00-0.00)	0.00 (0.00-0.00)	0.00 (0.00-0.00)	0.00 (0.00-0.00)	0.00 (0.00-0.00)	0.00 (0.00-0.00)	0.00 (0.00-0.00)	37.71 (27.48-49.11)	42.16 (30.80-54.62)	40.87 (29.67-53.16)
Netherlands	0.00 (0.00-0.00)	0.00 (0.00-0.00)	0.00 (0.00-0.00)	0.00 (0.00-0.00)	0.00 (0.00-0.00)	0.00 (0.00-0.00)	0.00 (0.00-0.00)	0.00 (0.00-0.00)	0.00 (0.00-0.00)	18.04 (11.98-25.57)	22.86 (15.41-31.52)	28.05 (19.04-38.86)
Norway	0.11 (0.07-0.15)	0.05 (0.03-0.07)	0.06 (0.03-0.09)	0.00 (0.00-0.00)	0.00 (0.00-0.00)	0.00 (0.00-0.00)	0.00 (0.00-0.00)	0.00 (0.00-0.00)	0.00 (0.00-0.00)	24.10 (15.93-34.11)	27.35 (18.95-36.90)	33.26 (23.43-43.35)
Portugal	0.00 (0.00-0.00)	0.02 (0.01-0.03)	0.00 (0.00-0.00)	0.00 (0.00-0.00)	0.00 (0.00-0.00)	0.00 (0.00-0.00)	0.00 (0.00-0.00)	0.00 (0.00-0.00)	0.00 (0.00-0.00)	24.52 (16.34-33.38)	33.08 (23.30-43.74)	38.55 (28.69-49.51)
Spain	0.00 (0.00-0.00)	0.06 (0.03-0.08)	0.00 (0.00-0.00)	0.00 (0.00-0.00)	0.00 (0.00-0.00)	0.00 (0.00-0.00)	0.00 (0.00-0.00)	0.00 (0.00-0.00)	0.00 (0.00-0.00)	47.65 (36.33-58.99)	49.11 (37.80-60.11)	42.70 (31.88-53.92)
Sweden	0.00 (0.00-0.00)	0.00 (0.00-0.00)	0.00 (0.00-0.00)	0.00 (0.00-0.00)	0.00 (0.00-0.00)	0.00 (0.00-0.00)	0.00 (0.00-0.00)	0.00 (0.00-0.00)	0.00 (0.00-0.00)	25.83 (17.53-35.34)	29.91 (20.24-40.51)	31.55 (22.18-42.41)
Switzerland	0.00 (0.00-0.00)	0.24 (0.13-0.35)	0.10 (0.05-0.14)	0.00 (0.00-0.00)	0.00 (0.00-0.00)	0.00 (0.00-0.00)	0.00 (0.00-0.00)	0.00 (0.00-0.00)	0.00 (0.00-0.00)	22.10 (13.98-30.89)	22.23 (14.40-31.60)	25.38 (17.39-35.46)
United Kingdom	0.00 (0.00-0.00)	0.00 (0.00-0.00)	0.00 (0.00-0.00)	0.00 (0.00-0.00)	0.00 (0.00-0.00)	0.00 (0.00-0.00)	0.00 (0.00-0.00)	0.00 (0.00-0.00)	0.00 (0.00-0.00)	22.05 (15.99-29.02)	22.68 (19.94-25.67)	23.66 (18.57-29.40)

Appendix Table 1b. Unscaled values for each SDG health-related indicator by country for 1990, 2000, and 2015

Location	Indicator 1.5.1: Age-standardised death rate due to exposure to forces of nature (per 100,000 population)			Indicator 2.2.1: Prevalence of stunting among children under 5 years of age			Indicator 2.2.2a: Prevalence of wasting among children under 5 years of age			Indicator 2.2.2b: Prevalence of overweight among children aged 2 to 4 years		
	1990	2000	2015	1990	2000	2015	1990	2000	2015	1990	2000	2015
Argentina	0.00 (0.00-0.00)	0.05 (0.01-0.09)	0.03 (0.00-0.06)	18.55 (12.86-25.41)	10.96 (7.96-14.64)	6.55 (3.93-9.99)	2.30 (1.13-4.02)	1.81 (0.96-3.15)	1.30 (0.57-2.59)	36.09 (25.41-48.09)	40.21 (27.83-53.65)	45.18 (33.11-58.41)
Chile	0.00 (0.00-0.00)	0.00 (0.00-0.00)	0.20 (0.03-0.38)	8.34 (5.93-11.37)	3.25 (2.54-4.09)	1.95 (1.21-3.07)	0.50 (0.23-0.92)	0.55 (0.31-0.90)	0.61 (0.27-1.24)	31.71 (20.42-43.00)	38.87 (26.44-51.85)	46.29 (33.43-59.08)
Uruguay	0.00 (0.00-0.00)	0.09 (0.01-0.18)	0.00 (0.00-0.00)	15.10 (12.16-18.37)	13.75 (10.53-17.27)	9.09 (5.53-13.47)	1.84 (1.06-3.12)	2.24 (1.22-3.81)	1.89 (0.89-3.57)	27.52 (17.99-39.08)	32.24 (21.45-44.40)	39.30 (27.66-52.51)
Belarus	0.00 (0.00-0.00)	0.00 (0.00-0.00)	0.00 (0.00-0.00)	7.26 (4.71-10.71)	6.05 (4.13-8.62)	2.51 (1.61-3.79)	3.42 (1.00-8.35)	2.88 (1.05-6.43)	2.07 (0.65-5.08)	19.86 (14.92-25.33)	19.30 (14.81-24.47)	23.10 (17.91-29.31)
Estonia	0.00 (0.00-0.00)	0.03 (0.00-0.05)	0.00 (0.00-0.00)	13.87 (9.11-19.73)	10.57 (7.12-15.19)	5.39 (3.43-8.14)	4.94 (1.42-11.77)	3.70 (1.16-8.72)	2.65 (0.74-6.53)	20.03 (15.10-25.78)	21.04 (16.09-26.82)	32.30 (25.34-39.14)
Latvia	0.00 (0.00-0.00)	0.03 (0.01-0.06)	0.00 (0.00-0.00)	11.32 (7.34-15.88)	11.16 (7.49-15.77)	5.29 (3.41-7.83)	4.21 (1.29-10.35)	4.04 (1.20-9.77)	2.56 (0.87-6.12)	23.85 (18.37-29.72)	19.47 (14.74-24.58)	29.65 (23.07-36.29)
Lithuania	0.00 (0.00-0.00)	0.06 (0.01-0.12)	0.00 (0.00-0.00)	12.46 (8.06-17.49)	10.29 (6.89-14.98)	5.33 (3.42-7.82)	4.81 (1.51-11.24)	3.83 (1.11-9.18)	2.69 (0.78-7.01)	18.56 (13.96-23.52)	18.22 (13.84-22.90)	27.17 (21.38-33.80)
Moldova	0.00 (0.00-0.00)	0.16 (0.07-0.26)	0.06 (0.02-0.10)	13.10 (9.05-18.62)	14.03 (9.95-18.76)	5.56 (4.10-7.50)	7.18 (2.21-16.31)	6.52 (2.42-13.60)	2.65 (1.08-5.63)	16.92 (12.89-21.83)	15.50 (12.25-19.19)	22.15 (17.05-27.70)
Russia	0.24 (0.04-0.46)	0.49 (0.08-0.94)	0.03 (0.00-0.06)	19.72 (15.46-24.56)	17.26 (12.52-22.76)	10.03 (6.60-14.78)	4.27 (1.77-8.44)	4.29 (1.62-9.56)	3.20 (1.01-7.95)	32.08 (27.59-36.29)	26.82 (21.90-32.71)	34.71 (28.12-41.64)
Ukraine	0.00 (0.00-0.00)	0.00 (0.00-0.00)	0.00 (0.00-0.00)	21.58 (15.11-29.12)	20.42 (17.26-23.85)	11.18 (7.17-15.70)	4.08 (1.25-10.30)	3.27 (1.52-6.00)	1.86 (0.54-4.81)	17.67 (13.23-23.04)	15.23 (11.40-19.40)	21.28 (16.54-27.25)

Appendix Table 1b. Unscaled values for each SDG health-related indicator by country for 1990, 2000, and 2015												
	**Indicator 1.5.1: Age-standardised death rate due to exposure to forces of nature (per 100,000 population)**			**Indicator 2.2.1: Prevalence of stunting among children under 5 years of age**			**Indicator 2.2.2a: Prevalence of wasting among children under 5 years of age**			**Indicator 2.2.2b: Prevalence of overweight among children aged 2 to 4 years**		
**Location**	**1990**	**2000**	**2015**	**1990**	**2000**	**2015**	**1990**	**2000**	**2015**	**1990**	**2000**	**2015**
Albania	0.00 (0.00-0.00)	0.38 (0.22-0.56)	0.00 (0.00-0.00)	50.46 (41.53-59.52)	46.50 (41.85-51.25)	21.64 (16.22-28.27)	11.48 (8.02-15.78)	12.03 (9.79-14.56)	7.13 (5.00-9.86)	33.82 (25.18-43.47)	39.95 (31.24-48.67)	44.22 (34.95-53.35)
Bosnia and Herzegovina	0.00 (0.00-0.00)	0.00 (0.00-0.00)	0.12 (0.03-0.21)	23.37 (17.13-30.75)	13.00 (10.79-15.53)	7.32 (5.44-9.80)	8.43 (5.79-11.75)	6.80 (5.42-8.26)	3.31 (2.25-4.51)	20.36 (14.44-27.73)	26.97 (20.49-34.04)	33.79 (26.15-42.77)
Bulgaria	0.00 (0.00-0.00)	0.24 (0.16-0.32)	0.04 (0.01-0.06)	10.01 (7.05-13.64)	9.94 (7.20-13.32)	6.13 (4.16-8.70)	5.22 (3.33-7.61)	5.54 (3.90-7.77)	4.49 (2.99-6.37)	52.25 (42.64-62.11)	39.02 (30.28-49.21)	39.15 (29.86-48.92)
Croatia	0.00 (0.00-0.00)	0.00 (0.00-0.00)	0.00 (0.00-0.00)	13.64 (9.60-18.74)	11.54 (8.05-15.86)	6.48 (4.39-9.28)	5.56 (3.70-8.06)	5.44 (3.51-7.85)	3.80 (2.47-5.58)	22.13 (15.97-29.18)	24.04 (17.02-31.85)	34.82 (26.33-43.75)
Czech Republic	0.00 (0.00-0.00)	0.04 (0.01-0.08)	0.00 (0.00-0.00)	3.23 (2.60-3.90)	2.69 (2.21-3.26)	1.86 (1.25-2.68)	3.05 (2.40-3.79)	4.32 (3.45-5.28)	3.60 (2.36-5.31)	26.31 (19.11-34.45)	27.19 (19.69-35.24)	29.71 (22.07-37.68)
Hungary	0.00 (0.00-0.00)	0.07 (0.02-0.13)	0.00 (0.00-0.00)	3.06 (2.17-4.08)	2.89 (1.89-4.13)	1.95 (1.34-2.78)	4.15 (2.97-5.85)	4.22 (2.72-6.22)	3.64 (2.41-5.23)	40.37 (30.91-49.28)	32.10 (24.76-41.18)	34.07 (26.20-43.46)
Macedonia	0.00 (0.00-0.00)	0.34 (0.19-0.50)	0.00 (0.00-0.00)	10.41 (7.46-14.26)	9.12 (7.37-11.09)	4.42 (3.28-5.75)	3.31 (2.24-4.82)	2.84 (2.12-3.76)	2.16 (1.50-2.98)	24.05 (17.28-31.95)	25.51 (18.40-33.59)	28.47 (21.27-36.75)
Montenegro	0.00 (0.00-0.00)	0.00 (0.00-0.00)	0.00 (0.00-0.00)	13.92 (9.75-19.35)	11.19 (8.10-14.66)	8.61 (6.35-11.18)	5.68 (3.79-8.11)	4.96 (3.34-7.08)	3.09 (2.24-4.14)	28.87 (21.36-37.56)	28.05 (20.66-36.38)	35.76 (29.64-42.87)
Poland	0.00 (0.00-0.00)	0.03 (0.01-0.05)	0.00 (0.00-0.00)	8.25 (5.66-11.49)	6.61 (4.56-9.14)	3.92 (2.64-5.55)	3.94 (2.61-5.79)	4.02 (2.61-6.09)	2.96 (1.94-4.33)	30.25 (22.60-39.14)	30.12 (22.27-38.17)	32.43 (24.30-41.44)
Romania	0.00 (0.00-0.00)	0.18 (0.09-0.28)	0.04 (0.02-0.07)	11.86 (9.75-14.12)	13.83 (12.45-15.26)	8.40 (5.76-11.69)	3.47 (2.77-4.32)	3.85 (3.37-4.34)	3.28 (2.12-4.70)	23.19 (16.76-30.69)	25.83 (18.62-33.81)	31.45 (23.59-40.63)

Appendix Table 1b. Unscaled values for each SDG health-related indicator by country for 1990, 2000, and 2015

Location	Indicator 1.5.1: Age-standardised death rate due to exposure to forces of nature (per 100,000 population)			Indicator 2.2.1: Prevalence of stunting among children under 5 years of age			Indicator 2.2.2a: Prevalence of wasting among children under 5 years of age			Indicator 2.2.2b: Prevalence of overweight among children aged 2 to 4 years		
	1990	2000	2015	1990	2000	2015	1990	2000	2015	1990	2000	2015
Serbia	0.00 (0.00-0.00)	0.07 (0.04-0.10)	0.11 (0.02-0.18)	12.13 (8.62-16.60)	10.54 (7.96-13.84)	5.96 (4.64-7.46)	5.56 (3.61-8.06)	5.28 (3.76-7.35)	3.87 (2.93-5.00)	28.83 (21.61-37.16)	26.23 (18.92-33.78)	23.41 (19.45-27.75)
Slovakia	0.00 (0.00-0.00)	0.22 (0.07-0.37)	0.02 (0.01-0.04)	9.88 (6.88-14.02)	8.48 (5.86-12.09)	4.62 (3.11-6.55)	4.51 (3.00-6.45)	4.63 (3.05-6.59)	3.30 (2.17-4.81)	26.07 (19.04-33.95)	25.02 (18.28-33.36)	25.34 (18.82-33.11)
Slovenia	0.15 (0.03-0.29)	0.07 (0.03-0.11)	0.00 (0.00-0.00)	8.89 (6.01-12.87)	6.82 (4.65-9.53)	4.32 (3.04-6.07)	4.59 (3.03-6.62)	4.37 (2.89-6.35)	3.22 (2.10-4.65)	28.97 (21.54-36.63)	32.22 (24.52-40.32)	39.04 (30.25-48.26)
Armenia	0.00 (0.00-0.00)	0.00 (0.00-0.00)	0.05 (0.02-0.07)	19.30 (14.27-25.46)	18.42 (15.44-21.54)	13.71 (10.57-17.58)	4.24 (2.05-7.50)	4.02 (2.48-6.02)	3.94 (1.94-6.83)	24.51 (18.08-31.74)	25.62 (21.64-30.16)	35.42 (28.40-43.53)
Azerbaijan	0.00 (0.00-0.00)	0.14 (0.06-0.23)	0.00 (0.00-0.00)	24.93 (19.42-31.27)	24.13 (20.69-27.53)	14.38 (10.70-18.98)	5.71 (2.90-10.10)	5.94 (3.75-8.69)	5.01 (2.28-9.02)	13.96 (10.03-18.63)	15.25 (11.60-19.86)	35.81 (28.02-44.48)
Georgia	0.00 (0.00-0.00)	0.00 (0.00-0.00)	0.29 (0.12-0.45)	17.68 (13.13-22.99)	16.98 (13.95-20.43)	10.80 (8.03-13.91)	4.26 (2.06-7.68)	3.36 (1.92-5.35)	2.54 (1.18-4.58)	22.68 (16.72-29.83)	25.89 (19.43-33.04)	34.79 (27.00-43.73)
Kazakhstan	0.00 (0.00-0.00)	0.00 (0.00-0.00)	0.00 (0.00-0.00)	19.05 (14.66-24.07)	17.73 (14.52-21.41)	10.08 (7.68-12.93)	5.54 (2.84-9.56)	4.53 (2.65-7.11)	4.10 (2.11-7.20)	19.30 (14.48-25.11)	17.04 (13.53-20.96)	31.08 (23.78-39.64)
Kyrgyzstan	0.00 (0.00-0.00)	0.08 (0.04-0.13)	0.18 (0.08-0.28)	30.14 (24.03-37.13)	27.42 (22.46-32.31)	14.93 (12.31-17.86)	4.11 (1.90-7.55)	3.94 (2.10-6.53)	3.34 (1.97-5.39)	24.12 (18.42-31.01)	20.80 (16.01-26.66)	19.66 (15.75-24.20)
Mongolia	1.90 (0.82-2.99)	0.58 (0.25-0.92)	0.00 (0.00-0.00)	32.22 (27.66-37.32)	29.47 (26.61-32.52)	13.96 (10.66-17.72)	3.29 (1.75-5.68)	3.83 (2.40-5.83)	2.02 (0.98-3.75)	27.38 (20.21-35.39)	22.89 (17.20-29.59)	28.85 (21.68-36.58)
Tajikistan	0.00 (0.00-0.00)	0.50 (0.21-0.79)	0.08 (0.03-0.13)	34.54 (27.26-42.17)	40.00 (37.39-42.47)	26.53 (22.21-31.26)	11.89 (6.25-20.46)	11.95 (8.25-16.24)	9.03 (4.85-14.80)	14.83 (10.60-19.85)	12.56 (8.83-16.87)	14.25 (11.06-17.99)

Location	Appendix Table 1b. Unscaled values for each SDG health-related indicator by country for 1990, 2000, and 2015											
	Indicator 1.5.1: Age-standardised death rate due to exposure to forces of nature (per 100,000 population)			Indicator 2.2.1: Prevalence of stunting among children under 5 years of age			Indicator 2.2.2a: Prevalence of wasting among children under 5 years of age			Indicator 2.2.2b: Prevalence of overweight among children aged 2 to 4 years		
	1990	2000	2015	1990	2000	2015	1990	2000	2015	1990	2000	2015
Turkmenistan	0.00 (0.00-0.00)	0.05 (0.03-0.08)	0.00 (0.00-0.00)	28.46 (22.12-35.57)	26.64 (22.34-31.24)	13.46 (10.00-17.61)	7.06 (3.43-12.92)	7.06 (4.20-11.40)	5.34 (2.47-9.79)	21.73 (16.18-28.42)	23.73 (17.58-30.72)	34.49 (26.73-42.79)
Uzbekistan	0.00 (0.00-0.00)	0.00 (0.00-0.00)	0.00 (0.00-0.00)	35.75 (28.34-42.61)	29.88 (25.29-34.58)	15.10 (11.27-19.68)	9.00 (4.40-15.43)	8.76 (5.21-13.82)	5.02 (2.35-9.06)	33.42 (26.35-41.08)	28.71 (23.16-34.36)	29.52 (22.19-38.17)
Colombia	0.35 (0.19-0.50)	0.81 (0.46-1.16)	0.28 (0.16-0.40)	22.45 (20.67-24.35)	18.19 (16.77-19.68)	11.74 (9.93-13.76)	2.42 (1.71-3.30)	1.57 (1.11-2.15)	1.30 (0.78-1.97)	11.34 (8.46-14.88)	11.94 (10.19-13.94)	8.69 (5.70-12.43)
Costa Rica	0.00 (0.00-0.00)	0.50 (0.28-0.72)	0.06 (0.04-0.08)	9.81 (8.10-11.70)	7.53 (6.26-8.91)	4.65 (3.83-5.55)	1.63 (1.00-2.49)	1.41 (0.87-2.21)	1.13 (0.66-1.71)	25.81 (16.73-36.07)	30.98 (20.46-43.24)	37.42 (26.36-49.17)
El Salvador	0.00 (0.00-0.00)	2.06 (1.16-2.95)	0.22 (0.12-0.31)	34.26 (31.34-37.29)	24.00 (21.17-26.99)	17.40 (14.85-20.14)	2.00 (1.50-2.63)	1.50 (1.15-1.90)	1.51 (0.94-2.24)	10.98 (7.88-14.79)	12.04 (9.78-14.97)	14.77 (9.71-21.12)
Guatemala	0.00 (0.00-0.00)	1.01 (0.57-1.46)	0.51 (0.36-0.65)	59.43 (56.09-63.02)	51.58 (48.85-54.31)	43.05 (38.30-48.05)	2.96 (2.02-4.17)	3.76 (2.78-4.80)	2.13 (1.30-3.27)	13.43 (10.40-16.88)	17.99 (15.87-20.24)	17.01 (11.33-24.08)
Honduras	0.00 (0.00-0.00)	57.32 (32.29-82.29)	0.13 (0.07-0.19)	43.49 (40.91-45.93)	35.08 (31.98-38.19)	21.39 (18.70-24.31)	2.26 (1.87-2.73)	1.60 (1.23-2.06)	1.39 (0.96-2.04)	7.63 (4.76-11.41)	9.90 (7.75-12.60)	14.17 (10.04-19.50)
Mexico	0.47 (0.15-1.06)	0.47 (0.20-1.02)	0.10 (0.05-0.21)	26.77 (25.87-27.74)	18.81 (18.15-19.55)	13.20 (12.70-13.75)	5.48 (4.93-6.10)	2.22 (2.00-2.49)	1.94 (1.72-2.18)	30.82 (21.50-42.00)	30.40 (20.40-41.67)	22.45 (15.31-30.25)
Nicaragua	0.00 (0.00-0.00)	16.21 (9.13-23.27)	0.22 (0.13-0.32)	29.78 (26.51-33.28)	25.51 (23.10-28.05)	16.23 (13.72-19.09)	3.18 (2.09-4.63)	2.26 (1.71-2.98)	1.21 (0.71-1.84)	15.96 (10.42-22.30)	16.27 (13.57-19.52)	18.42 (12.20-26.73)
Panama	0.00 (0.00-0.00)	0.24 (0.13-0.36)	0.19 (0.12-0.25)	13.12 (11.51-14.69)	9.71 (8.11-11.47)	5.36 (4.43-6.48)	2.51 (1.84-3.36)	1.44 (1.04-1.96)	1.20 (0.73-1.87)	12.93 (8.17-19.20)	13.47 (8.36-19.88)	16.65 (10.29-24.91)

Appendix Table 1b. Unscaled values for each SDG health-related indicator by country for 1990, 2000, and 2015

Location	Indicator 1.5.1: Age-standardised death rate due to exposure to forces of nature (per 100,000 population)			Indicator 2.2.1: Prevalence of stunting among children under 5 years of age			Indicator 2.2.2a: Prevalence of wasting among children under 5 years of age			Indicator 2.2.2b: Prevalence of overweight among children aged 2 to 4 years		
	1990	2000	2015	1990	2000	2015	1990	2000	2015	1990	2000	2015
Venezuela	0.00 (0.00-0.00)	28.27 (15.93-40.59)	0.00 (0.00-0.00)	18.68 (17.41-19.94)	18.05 (16.95-19.18)	10.35 (8.68-12.23)	4.05 (3.53-4.68)	4.08 (3.39-4.74)	3.31 (2.13-4.99)	24.59 (16.40-35.21)	22.42 (14.06-31.61)	26.93 (18.10-37.87)
Bolivia	0.00 (0.00-0.00)	0.88 (0.14-1.69)	0.68 (0.11-1.31)	41.00 (37.93-43.90)	32.69 (30.41-35.11)	22.12 (18.83-25.51)	2.19 (1.51-3.13)	1.97 (1.36-2.74)	1.54 (0.92-2.42)	23.73 (21.14-26.56)	25.82 (23.35-28.80)	27.03 (23.00-31.29)
Ecuador	0.00 (0.00-0.00)	0.75 (0.12-1.45)	0.17 (0.03-0.34)	37.50 (33.75-41.00)	30.57 (27.66-33.62)	19.41 (16.47-22.44)	2.61 (1.74-3.85)	2.89 (2.05-3.91)	2.01 (1.21-3.05)	16.03 (13.32-19.33)	16.91 (14.48-19.88)	19.94 (17.29-22.80)
Peru	1.59 (0.25-3.04)	1.03 (0.16-1.98)	0.16 (0.03-0.30)	36.87 (33.60-40.16)	31.35 (28.80-34.02)	17.88 (15.87-20.13)	2.13 (1.53-2.91)	1.38 (1.03-1.83)	0.63 (0.43-0.93)	24.79 (21.93-27.90)	25.88 (23.37-28.54)	20.88 (18.32-23.69)
Antigua and Barbuda	0.00 (0.00-0.00)	0.00 (0.00-0.00)	0.00 (0.00-0.00)	7.50 (5.69-9.86)	5.55 (4.12-7.24)	3.65 (2.72-4.89)	3.26 (1.97-4.95)	3.05 (1.80-4.83)	2.37 (1.44-3.66)	13.80 (10.20-18.19)	16.84 (12.36-21.73)	15.78 (11.72-20.33)
The Bahamas	0.00 (0.00-0.00)	0.15 (0.02-0.29)	0.00 (0.00-0.00)	7.28 (5.47-9.55)	5.34 (3.83-7.08)	4.28 (3.12-5.67)	3.20 (1.97-4.92)	2.94 (1.82-4.55)	2.64 (1.61-4.00)	24.16 (18.12-30.79)	27.07 (21.25-33.92)	26.17 (20.49-32.62)
Barbados	0.00 (0.00-0.00)	0.00 (0.00-0.00)	0.00 (0.00-0.00)	8.61 (6.60-11.26)	9.98 (7.59-12.84)	6.58 (5.04-8.48)	3.55 (2.14-5.34)	6.49 (3.98-9.76)	5.61 (3.77-8.06)	23.19 (18.00-29.00)	23.79 (18.25-29.30)	27.90 (21.81-34.17)
Belize	0.00 (0.00-0.00)	1.37 (0.22-2.62)	0.00 (0.00-0.00)	35.56 (28.82-43.09)	26.88 (21.69-32.61)	17.29 (14.01-21.07)	3.92 (2.42-6.24)	3.39 (2.12-5.24)	3.20 (2.08-4.51)	17.19 (13.23-21.85)	20.04 (15.54-25.68)	17.67 (13.39-22.29)
Cuba	0.00 (0.00-0.00)	0.53 (0.08-1.01)	0.24 (0.04-0.46)	8.76 (6.61-11.23)	7.53 (6.25-8.91)	7.32 (5.74-9.09)	3.08 (1.92-4.62)	2.79 (2.00-3.82)	2.36 (1.61-3.35)	25.31 (19.31-31.98)	23.85 (18.26-30.14)	33.03 (26.15-40.17)
Dominica	0.00 (0.00-0.00)	0.32 (0.05-0.61)	0.00 (0.00-0.00)	11.82 (8.92-15.34)	7.96 (5.98-10.39)	5.18 (3.85-6.96)	3.56 (2.14-5.60)	3.13 (1.88-4.95)	2.51 (1.53-3.92)	15.53 (11.78-20.10)	19.72 (14.75-25.32)	20.91 (15.77-26.75)

Appendix Table 1b. Unscaled values for each SDG health-related indicator by country for 1990, 2000, and 2015

Location	Indicator 1.5.1: Age-standardised death rate due to exposure to forces of nature (per 100,000 population)			Indicator 2.2.1: Prevalence of stunting among children under 5 years of age			Indicator 2.2.2a: Prevalence of wasting among children under 5 years of age			Indicator 2.2.2b: Prevalence of overweight among children aged 2 to 4 years		
	1990	2000	2015	1990	2000	2015	1990	2000	2015	1990	2000	2015
Dominican Republic	0.00 (0.00-0.00)	1.15 (0.18-2.21)	0.00 (0.00-0.00)	19.77 (16.98-22.67)	11.25 (9.71-12.95)	6.80 (5.48-8.42)	2.60 (1.98-3.31)	2.21 (1.72-2.76)	2.26 (1.59-3.10)	10.25 (8.67-12.06)	14.48 (12.43-16.79)	16.21 (13.27-19.40)
Grenada	0.00 (0.00-0.00)	0.00 (0.00-0.00)	0.41 (0.07-0.79)	12.65 (9.59-16.19)	8.68 (6.52-11.33)	5.42 (4.08-7.03)	3.82 (2.28-6.04)	3.44 (2.08-5.20)	2.64 (1.54-3.92)	13.74 (10.08-17.93)	17.73 (13.38-22.91)	19.49 (14.55-24.80)
Guyana	0.00 (0.00-0.00)	0.03 (0.01-0.06)	0.00 (0.00-0.00)	20.85 (16.44-25.41)	15.89 (13.58-18.42)	16.81 (13.31-20.73)	12.16 (8.25-16.92)	12.09 (9.32-15.17)	7.05 (4.66-10.40)	9.68 (7.10-12.83)	11.97 (8.93-15.51)	12.89 (9.79-16.26)
Haiti	0.00 (0.00-0.00)	0.87 (0.14-1.67)	0.38 (0.06-0.72)	38.37 (34.44-43.11)	30.42 (26.85-34.45)	21.43 (17.75-25.54)	7.50 (5.60-9.77)	7.56 (5.76-9.82)	6.06 (4.14-8.54)	8.22 (6.38-10.63)	6.93 (5.66-8.34)	8.73 (6.66-11.19)
Jamaica	0.00 (0.00-0.00)	0.00 (0.00-0.00)	0.00 (0.00-0.00)	13.38 (11.68-15.51)	8.42 (7.42-9.43)	5.81 (4.50-7.60)	4.36 (3.28-5.64)	3.41 (2.77-4.17)	3.48 (2.22-5.24)	21.25 (16.08-26.56)	25.88 (19.86-32.51)	27.18 (21.01-33.92)
Saint Lucia	0.00 (0.00-0.00)	0.00 (0.00-0.00)	0.00 (0.00-0.00)	4.43 (3.29-5.78)	3.03 (2.28-4.11)	2.05 (1.53-2.68)	4.75 (2.97-7.28)	4.12 (2.54-6.10)	3.56 (2.30-5.34)	6.94 (5.01-9.32)	7.90 (5.75-10.39)	7.25 (5.44-9.45)
Saint Vincent and the Grenadines	0.00 (0.00-0.00)	0.00 (0.00-0.00)	0.00 (0.00-0.00)	12.96 (9.91-16.78)	8.70 (6.57-11.16)	5.24 (3.91-6.78)	3.87 (2.36-5.95)	3.37 (2.03-5.26)	2.52 (1.55-3.93)	22.61 (17.47-28.64)	28.81 (22.50-35.47)	32.09 (24.99-39.53)
Suriname	0.00 (0.00-0.00)	0.00 (0.00-0.00)	0.00 (0.00-0.00)	17.35 (13.57-21.98)	14.14 (11.65-17.05)	7.02 (5.34-8.88)	6.87 (4.35-10.26)	6.47 (4.63-8.71)	4.56 (2.91-6.51)	8.19 (5.98-10.79)	9.17 (6.69-12.23)	13.08 (9.79-17.10)
Trinidad and Tobago	0.00 (0.00-0.00)	0.00 (0.00-0.00)	0.00 (0.00-0.00)	6.50 (5.10-8.04)	6.05 (4.75-7.50)	3.43 (2.49-4.50)	5.52 (3.70-7.61)	5.04 (3.48-6.89)	3.87 (2.38-6.03)	7.63 (5.77-9.92)	10.41 (7.76-13.72)	13.91 (10.48-18.11)
Brazil	0.22 (0.06-0.50)	0.16 (0.08-0.35)	0.23 (0.06-0.53)	19.99 (18.91-21.13)	12.56 (11.69-13.45)	6.69 (6.23-7.18)	2.73 (2.18-3.42)	4.72 (3.80-5.81)	2.57 (2.01-3.35)	14.28 (10.50-18.69)	20.29 (15.75-25.06)	32.65 (22.12-44.32)

Appendix Table 1b. Unscaled values for each SDG health-related indicator by country for 1990, 2000, and 2015

Location	Indicator 1.5.1: Age-standardised death rate due to exposure to forces of nature (per 100,000 population)			Indicator 2.2.1: Prevalence of stunting among children under 5 years of age			Indicator 2.2.2a: Prevalence of wasting among children under 5 years of age			Indicator 2.2.2b: Prevalence of overweight among children aged 2 to 4 years		
	1990	2000	2015	1990	2000	2015	1990	2000	2015	1990	2000	2015
Paraguay	0.00 (0.00-0.00)	0.66 (0.62-0.70)	0.20 (0.19-0.21)	18.96 (16.57-21.59)	17.83 (14.56-21.59)	12.40 (9.31-16.04)	0.89 (0.52-1.37)	1.24 (0.60-2.30)	1.38 (0.50-2.97)	14.75 (12.80-17.02)	18.11 (11.66-26.32)	20.47 (12.99-30.27)
China	0.13 (0.05-0.22)	0.25 (0.10-0.41)	0.11 (0.05-0.18)	34.58 (31.37-37.85)	22.45 (19.68-25.56)	9.18 (7.65-10.95)	4.16 (3.30-5.23)	2.64 (2.01-3.38)	2.72 (2.02-3.64)	18.86 (15.77-22.33)	19.08 (15.37-23.03)	25.36 (18.79-33.81)
North Korea	0.00 (0.00-0.00)	0.25 (0.04-0.48)	0.23 (0.04-0.44)	48.20 (28.50-68.23)	48.51 (39.80-57.13)	32.44 (16.84-50.84)	6.22 (1.59-15.89)	11.05 (7.79-15.17)	5.30 (1.60-12.87)	5.10 (3.16-7.66)	5.09 (3.24-7.55)	4.08 (2.60-6.12)
Taiwan	0.00 (0.00-0.00)	2.43 (0.39-4.65)	0.00 (0.00-0.00)	0.00 (0.00-0.00)	0.00 (0.00-0.00)	0.00 (0.00-0.00)	0.00 (0.00-0.00)	0.00 (0.00-0.00)	0.00 (0.00-0.00)	30.90 (22.18-40.70)	34.30 (25.50-43.78)	30.96 (22.69-39.91)
Cambodia	0.00 (0.00-0.00)	0.85 (0.32-1.37)	0.74 (0.23-1.26)	61.30 (56.18-66.01)	54.73 (50.35-58.57)	36.17 (31.80-40.44)	15.32 (10.75-21.33)	15.23 (12.29-18.51)	10.15 (7.63-13.18)	3.89 (2.33-6.11)	5.10 (3.94-6.44)	4.76 (2.97-7.04)
Indonesia	0.14 (0.03-0.25)	0.09 (0.04-0.13)	0.00 (0.00-0.00)	53.32 (49.49-57.35)	40.77 (38.83-42.62)	26.53 (22.84-30.37)	10.92 (7.48-15.40)	9.05 (6.80-11.66)	8.76 (5.72-12.85)	8.11 (5.17-12.49)	9.87 (7.53-12.95)	10.29 (6.86-14.72)
Laos	0.00 (0.00-0.00)	0.21 (0.06-0.37)	0.24 (0.10-0.39)	55.52 (51.65-59.43)	48.97 (45.30-52.58)	36.30 (32.53-40.30)	13.22 (9.54-17.79)	14.03 (10.36-18.27)	7.25 (4.83-10.24)	4.84 (2.89-7.39)	5.60 (3.47-8.65)	6.57 (4.06-10.28)
Malaysia	0.00 (0.00-0.00)	0.40 (0.15-0.66)	0.00 (0.00-0.00)	29.15 (25.09-33.34)	20.19 (18.02-22.42)	12.60 (10.69-14.80)	14.21 (9.54-19.89)	12.74 (9.32-17.34)	10.01 (6.52-14.68)	21.48 (13.66-30.27)	27.19 (18.06-38.38)	30.52 (21.09-40.93)
Maldives	0.00 (0.00-0.00)	0.00 (0.00-0.00)	0.00 (0.00-0.00)	38.07 (33.95-42.33)	30.40 (27.26-33.67)	17.96 (15.34-20.74)	13.80 (9.97-18.31)	15.83 (11.85-20.45)	10.81 (7.42-15.35)	6.21 (3.76-9.61)	7.32 (4.54-11.18)	13.45 (8.89-18.88)
Mauritius	0.00 (0.00-0.00)	0.00 (0.00-0.00)	0.14 (0.05-0.26)	19.99 (17.46-22.78)	11.97 (10.21-13.97)	7.18 (5.86-8.67)	14.77 (10.25-20.13)	12.48 (8.58-17.13)	9.79 (6.39-14.01)	21.38 (13.79-30.42)	24.86 (16.70-35.14)	28.24 (19.16-39.98)

Appendix Table 1b. Unscaled values for each SDG health-related indicator by country for 1990, 2000, and 2015

Location	Indicator 1.5.1: Age-standardised death rate due to exposure to forces of nature (per 100,000 population)			Indicator 2.2.1: Prevalence of stunting among children under 5 years of age			Indicator 2.2.2a: Prevalence of wasting among children under 5 years of age			Indicator 2.2.2b: Prevalence of overweight among children aged 2 to 4 years		
	1990	2000	2015	1990	2000	2015	1990	2000	2015	1990	2000	2015
Myanmar	0.00 (0.00-0.00)	0.03 (0.01-0.06)	0.14 (0.06-0.22)	57.93 (54.51-61.37)	46.25 (42.90-49.79)	25.87 (22.18-29.79)	12.14 (9.53-15.59)	10.43 (8.15-12.94)	7.30 (4.90-10.47)	4.36 (2.62-6.84)	5.35 (3.38-8.24)	14.41 (9.29-20.65)
Philippines	6.30 (1.68-10.96)	0.62 (0.17-1.08)	2.85 (0.80-4.90)	42.17 (40.85-43.47)	36.56 (34.85-38.14)	26.75 (23.30-30.63)	7.51 (6.57-8.54)	7.27 (6.17-8.53)	6.32 (4.33-8.96)	3.33 (1.91-5.60)	3.65 (2.23-5.81)	6.21 (3.73-10.00)
Sri Lanka	0.20 (0.05-0.37)	0.14 (0.04-0.25)	0.58 (0.34-0.83)	30.00 (27.23-32.65)	18.91 (17.05-21.08)	13.40 (11.30-15.75)	15.48 (12.63-18.55)	15.36 (12.73-18.58)	11.28 (7.86-15.42)	10.44 (6.41-16.18)	11.15 (6.85-16.78)	13.84 (8.66-20.37)
Seychelles	0.00 (0.00-0.00)	0.00 (0.00-0.00)	0.00 (0.00-0.00)	7.38 (6.19-8.80)	5.10 (4.18-6.14)	3.12 (2.53-3.74)	3.31 (2.19-4.78)	3.10 (1.94-4.53)	2.55 (1.60-3.85)	11.81 (7.23-17.35)	12.73 (7.97-18.68)	13.98 (8.67-20.65)
Thailand	0.56 (0.16-0.98)	0.35 (0.14-0.57)	0.29 (0.09-0.49)	23.34 (21.10-25.62)	16.28 (14.19-18.50)	10.28 (8.56-12.28)	7.32 (5.26-9.86)	6.22 (4.36-8.65)	5.02 (3.07-7.29)	8.34 (5.03-12.89)	12.79 (7.97-19.06)	19.45 (12.35-28.37)
Timor-Leste	0.00 (0.00-0.00)	0.00 (0.00-0.00)	0.00 (0.00-0.00)	65.65 (60.79-70.02)	57.53 (54.02-60.95)	45.86 (40.75-50.56)	15.16 (10.16-21.21)	15.27 (11.33-19.98)	15.09 (10.67-20.50)	6.81 (4.00-10.50)	6.23 (3.92-9.52)	9.43 (6.02-14.31)
Vietnam	0.30 (0.08-0.52)	1.82 (0.54-3.11)	0.08 (0.02-0.13)	59.92 (57.72-61.97)	41.84 (40.57-43.22)	21.51 (18.53-24.43)	9.82 (8.29-11.55)	10.34 (9.38-11.35)	6.07 (4.20-8.44)	3.20 (1.86-5.07)	4.37 (2.98-6.17)	10.73 (6.58-16.55)
Federated States of Micronesia	0.00 (0.00-0.00)	0.00 (0.00-0.00)	0.00 (0.00-0.00)	13.55 (11.49-15.81)	11.65 (9.76-13.53)	8.58 (7.13-10.11)	4.70 (2.94-7.25)	3.86 (2.36-5.91)	3.57 (2.14-5.75)	23.58 (14.35-35.71)	22.95 (13.81-35.01)	22.38 (13.07-33.26)
Fiji	0.00 (0.00-0.00)	1.06 (0.86-1.25)	0.25 (0.20-0.30)	11.28 (9.59-13.23)	9.44 (7.92-11.11)	6.93 (5.81-8.11)	4.49 (2.81-6.68)	3.58 (2.25-5.54)	3.41 (2.16-5.11)	24.19 (14.69-35.55)	31.86 (20.16-44.52)	31.52 (19.58-44.17)
Kiribati	0.00 (0.00-0.00)	0.00 (0.00-0.00)	0.00 (0.00-0.00)	33.25 (29.77-36.80)	28.96 (25.26-32.80)	21.83 (18.87-25.01)	10.53 (7.26-15.23)	8.83 (5.56-13.17)	7.50 (4.65-11.48)	51.60 (37.42-65.57)	52.58 (38.71-66.29)	54.53 (40.71-68.33)

Appendix Table 1b. Unscaled values for each SDG health-related indicator by country for 1990, 2000, and 2015

Location	Indicator 1.5.1: Age-standardised death rate due to exposure to forces of nature (per 100,000 population)			Indicator 2.2.1: Prevalence of stunting among children under 5 years of age			Indicator 2.2.2a: Prevalence of wasting among children under 5 years of age			Indicator 2.2.2b: Prevalence of overweight among children aged 2 to 4 years		
	1990	2000	2015	1990	2000	2015	1990	2000	2015	1990	2000	2015
Marshall Islands	0.00 (0.00-0.00)	0.00 (0.00-0.00)	0.00 (0.00-0.00)	17.38 (14.86-20.24)	15.38 (13.32-17.95)	10.87 (9.24-12.86)	5.29 (3.31-7.86)	4.30 (2.70-6.50)	4.03 (2.51-6.18)	32.01 (20.40-45.57)	34.40 (23.19-48.29)	36.83 (24.48-50.46)
Papua New Guinea	0.00 (0.00-0.00)	10.36 (8.39-12.33)	0.19 (0.16-0.23)	47.98 (43.81-52.64)	43.01 (38.84-47.24)	33.96 (30.08-37.86)	5.68 (3.36-8.70)	4.91 (3.26-7.09)	4.42 (2.82-6.70)	25.44 (15.81-37.95)	25.12 (15.32-36.31)	19.80 (12.03-30.52)
Samoa	0.00 (0.00-0.00)	0.00 (0.00-0.00)	1.42 (1.18-1.65)	8.12 (6.93-9.66)	6.40 (5.52-7.36)	4.29 (3.60-5.10)	2.03 (1.25-3.11)	1.67 (1.12-2.37)	1.58 (0.95-2.48)	45.91 (31.21-60.53)	46.33 (32.41-60.52)	49.30 (34.79-63.78)
Solomon Islands	0.00 (0.00-0.00)	0.00 (0.00-0.00)	2.31 (1.88-2.75)	33.96 (31.18-37.01)	32.15 (28.63-35.82)	25.29 (22.13-28.63)	7.18 (5.25-9.58)	5.00 (3.28-7.15)	4.27 (2.82-6.02)	26.54 (15.46-38.29)	29.63 (18.15-44.11)	36.81 (24.23-51.05)
Tonga	0.00 (0.00-0.00)	0.00 (0.00-0.00)	0.00 (0.00-0.00)	2.09 (1.75-2.47)	1.65 (1.38-1.95)	1.14 (0.93-1.36)	1.76 (1.13-2.53)	1.61 (0.98-2.52)	1.42 (0.84-2.18)	41.38 (28.22-55.88)	45.29 (32.14-60.27)	53.00 (38.26-66.36)
Vanuatu	0.00 (0.00-0.00)	18.98 (15.37-22.59)	2.00 (1.66-2.34)	25.89 (23.04-29.03)	26.07 (23.26-29.11)	21.04 (18.07-24.15)	6.95 (4.57-10.21)	6.12 (4.19-8.47)	5.30 (3.47-7.94)	14.99 (8.25-22.99)	15.57 (9.00-24.29)	15.84 (8.79-25.07)
Afghanistan	0.00 (0.00-0.00)	9.77 (3.66-15.86)	1.15 (0.43-1.87)	48.61 (38.72-58.92)	55.30 (48.78-61.71)	46.18 (35.41-56.53)	18.35 (12.34-26.94)	16.19 (13.01-19.40)	10.57 (6.11-16.60)	13.17 (8.88-18.45)	7.24 (4.63-10.89)	9.59 (6.10-13.87)
Algeria	0.00 (0.00-0.00)	0.02 (0.01-0.04)	0.04 (0.01-0.06)	20.23 (15.92-25.72)	22.18 (18.29-26.56)	15.35 (10.09-21.97)	6.90 (5.27-8.84)	7.70 (6.39-9.16)	3.20 (1.86-5.32)	13.34 (9.08-18.51)	16.64 (11.44-22.49)	28.02 (20.94-36.35)
Bahrain	0.00 (0.00-0.00)	0.00 (0.00-0.00)	0.00 (0.00-0.00)	14.31 (11.07-17.79)	11.65 (8.02-15.99)	7.58 (4.91-11.22)	6.80 (5.52-8.23)	6.05 (3.76-9.18)	4.89 (2.77-8.02)	45.58 (36.50-55.17)	46.96 (37.75-55.94)	49.64 (40.76-58.90)
Egypt	0.00 (0.00-0.00)	0.00 (0.00-0.00)	0.00 (0.00-0.00)	34.70 (30.03-39.79)	25.59 (21.93-29.27)	21.76 (17.13-27.28)	4.33 (3.01-5.90)	5.63 (4.10-7.47)	8.21 (5.37-11.83)	26.28 (22.31-30.33)	30.66 (26.69-35.22)	33.68 (26.54-41.40)

Location	Indicator 1.5.1: Age-standardised death rate due to exposure to forces of nature (per 100,000 population)			Indicator 2.2.1: Prevalence of stunting among children under 5 years of age			Indicator 2.2.2a: Prevalence of wasting among children under 5 years of age			Indicator 2.2.2b: Prevalence of overweight among children aged 2 to 4 years		
	1990	2000	2015	1990	2000	2015	1990	2000	2015	1990	2000	2015
Iran	84.71 (31.77-137.52)	1.12 (0.42-1.83)	0.11 (0.04-0.18)	27.50 (20.31-35.86)	14.14 (11.14-17.72)	6.77 (4.35-10.17)	8.90 (5.79-12.88)	5.35 (4.29-6.72)	4.36 (2.41-6.87)	10.34 (7.28-14.32)	13.91 (9.89-18.86)	16.19 (11.47-21.64)
Iraq	0.00 (0.00-0.00)	0.00 (0.00-0.00)	0.04 (0.01-0.06)	27.50 (22.35-33.94)	30.67 (25.24-36.12)	18.82 (13.98-24.98)	4.50 (3.47-5.63)	7.22 (5.97-8.64)	6.29 (4.15-9.01)	28.11 (20.96-36.42)	13.45 (9.61-18.28)	20.95 (15.25-27.45)
Jordan	0.00 (0.00-0.00)	0.00 (0.00-0.00)	0.00 (0.00-0.00)	18.34 (13.84-23.74)	13.07 (9.92-16.94)	8.44 (5.87-11.80)	4.19 (2.44-6.67)	3.30 (2.08-5.03)	3.03 (1.82-4.70)	14.69 (11.78-18.13)	9.51 (7.46-11.77)	13.24 (10.19-16.84)
Kuwait	0.00 (0.00-0.00)	0.00 (0.00-0.00)	0.00 (0.00-0.00)	9.46 (6.73-12.97)	4.29 (3.44-5.26)	3.49 (2.30-5.11)	3.40 (1.96-5.45)	2.14 (1.39-3.20)	2.09 (1.20-3.48)	36.80 (28.22-45.34)	44.28 (35.21-53.69)	48.99 (40.53-58.27)
Lebanon	0.00 (0.00-0.00)	0.00 (0.00-0.00)	0.00 (0.00-0.00)	19.98 (14.48-27.31)	16.30 (12.36-20.75)	10.70 (6.98-15.46)	4.58 (2.78-7.06)	5.17 (3.70-7.10)	5.04 (2.81-8.01)	28.15 (21.01-36.17)	32.92 (26.61-39.71)	29.28 (22.28-37.28)
Libya	0.00 (0.00-0.00)	0.00 (0.00-0.00)	0.06 (0.02-0.09)	27.15 (20.20-34.84)	24.83 (19.06-32.14)	19.76 (13.75-27.28)	4.83 (3.07-7.17)	3.49 (2.44-4.81)	4.97 (2.91-7.91)	40.49 (31.73-49.99)	37.32 (29.31-45.83)	38.41 (30.09-47.09)
Morocco	0.00 (0.00-0.00)	0.08 (0.03-0.13)	0.04 (0.01-0.06)	32.13 (26.70-37.84)	26.27 (21.06-32.22)	18.65 (12.62-25.98)	3.73 (3.01-4.57)	8.68 (6.73-10.99)	7.76 (4.37-12.26)	20.07 (17.50-23.05)	20.91 (17.24-25.00)	24.72 (18.32-32.21)
Palestine	0.00 (0.00-0.00)	0.03 (0.01-0.06)	0.00 (0.00-0.00)	16.44 (11.29-22.88)	12.58 (8.95-17.02)	9.08 (6.91-11.61)	3.88 (2.19-6.55)	3.43 (2.27-4.89)	1.64 (1.24-2.13)	15.88 (11.61-21.39)	18.27 (13.92-22.97)	14.94 (11.38-19.41)
Oman	0.00 (0.00-0.00)	0.00 (0.00-0.00)	0.10 (0.04-0.16)	24.88 (19.83-30.64)	14.68 (11.55-18.60)	9.41 (6.27-13.11)	10.83 (8.44-13.50)	7.32 (6.14-8.65)	6.92 (4.25-10.47)	23.78 (17.64-30.68)	25.30 (18.83-32.53)	29.12 (22.22-37.39)
Qatar	0.00 (0.00-0.00)	0.00 (0.00-0.00)	0.00 (0.00-0.00)	10.53 (6.84-15.35)	8.22 (5.20-12.23)	4.58 (2.87-6.72)	4.99 (2.73-8.59)	4.34 (2.50-7.17)	3.44 (1.92-5.70)	40.40 (31.58-49.19)	37.78 (28.76-47.53)	43.37 (34.65-52.90)

Appendix Table 1b. Unscaled values for each SDG health-related indicator by country for 1990, 2000, and 2015

Location	Indicator 1.5.1: Age-standardised death rate due to exposure to forces of nature (per 100,000 population)			Indicator 2.2.1: Prevalence of stunting among children under 5 years of age			Indicator 2.2.2a: Prevalence of wasting among children under 5 years of age			Indicator 2.2.2b: Prevalence of overweight among children aged 2 to 4 years		
	1990	2000	2015	1990	2000	2015	1990	2000	2015	1990	2000	2015
Saudi Arabia	0.00 (0.00-0.00)	0.00 (0.00-0.00)	0.07 (0.01-0.21)	23.85 (20.84-27.08)	15.38 (13.20-17.64)	8.48 (7.20-9.89)	3.05 (2.47-3.77)	11.19 (9.07-13.78)	7.73 (6.26-9.57)	30.27 (22.72-38.97)	34.98 (27.30-43.64)	41.11 (32.47-50.28)
Sudan	0.00 (0.00-0.00)	0.33 (0.13-0.42)	0.05 (0.02-0.08)	42.85 (35.55-50.89)	45.68 (38.70-52.82)	34.65 (28.26-41.24)	15.05 (11.69-18.93)	20.04 (18.93-21.27)	17.12 (15.32-19.09)	9.80 (6.80-13.85)	10.65 (7.35-14.84)	13.74 (9.65-19.27)
Syria	0.00 (0.00-0.00)	0.00 (0.00-0.00)	0.00 (0.00-0.00)	33.77 (26.81-41.13)	27.90 (24.59-31.65)	22.99 (16.87-29.73)	10.25 (7.16-14.03)	8.43 (7.03-9.94)	9.80 (6.13-14.53)	25.70 (19.14-33.69)	26.21 (19.64-34.04)	28.14 (21.36-36.73)
Tunisia	0.50 (0.19-0.82)	0.00 (0.00-0.00)	0.00 (0.00-0.00)	17.77 (13.40-22.48)	13.13 (10.54-16.11)	7.65 (5.33-10.59)	5.88 (4.17-8.14)	3.53 (2.59-4.78)	2.99 (1.87-4.64)	13.77 (11.11-16.99)	17.53 (12.75-23.41)	23.11 (16.69-30.40)
Turkey	0.14 (0.05-0.23)	6.21 (2.43-10.17)	0.18 (0.07-0.30)	24.67 (19.02-31.62)	17.50 (14.12-21.41)	9.41 (6.42-13.25)	4.23 (2.89-5.78)	2.28 (1.77-2.89)	1.32 (0.74-2.08)	13.19 (9.92-16.80)	15.63 (12.88-18.71)	23.14 (17.22-29.47)
United Arab Emirates	0.00 (0.00-0.00)	0.00 (0.00-0.00)	0.00 (0.00-0.00)	24.67 (17.87-32.36)	18.41 (13.25-24.63)	15.63 (10.41-21.85)	16.51 (10.94-23.58)	14.19 (9.21-20.12)	10.93 (6.27-17.72)	21.03 (15.42-27.11)	17.70 (13.76-21.79)	21.27 (15.16-27.86)
Yemen	0.00 (0.00-0.00)	0.62 (0.23-1.01)	0.04 (0.01-0.06)	51.68 (45.67-57.83)	56.94 (51.88-61.89)	46.78 (40.05-53.71)	15.81 (12.54-19.98)	14.81 (12.24-17.87)	16.64 (13.05-20.98)	14.31 (11.03-18.31)	13.22 (9.23-18.14)	15.96 (11.09-22.25)
Bangladesh	1.03 (0.54-1.52)	0.56 (0.29-0.83)	0.04 (0.02-0.06)	60.84 (57.71-63.97)	53.39 (51.09-55.49)	35.87 (32.91-38.79)	18.31 (14.87-21.99)	14.98 (12.64-17.66)	13.45 (10.95-16.44)	4.73 (3.35-6.61)	3.42 (2.65-4.34)	4.04 (2.93-5.55)
Bhutan	0.00 (0.00-0.00)	8.25 (4.32-12.18)	0.00 (0.00-0.00)	52.10 (45.49-58.63)	44.15 (40.58-47.84)	29.49 (24.92-34.36)	4.93 (3.47-6.89)	4.36 (3.26-5.60)	6.17 (4.38-8.25)	7.05 (4.66-9.82)	8.98 (6.22-12.40)	13.71 (9.84-18.74)
India	0.26 (0.01-0.79)	0.52 (0.17-1.27)	0.15 (0.04-0.30)	63.04 (61.96-64.08)	54.85 (53.92-55.83)	37.50 (36.09-38.96)	19.20 (18.31-20.06)	16.93 (16.26-17.63)	15.12 (14.07-16.25)	7.97 (6.15-10.13)	6.95 (5.49-8.62)	10.63 (8.22-13.57)

Appendix Table 1b. Unscaled values for each SDG health-related indicator by country for 1990, 2000, and 2015

Location	Indicator 1.5.1: Age-standardised death rate due to exposure to forces of nature (per 100,000 population)			Indicator 2.2.1: Prevalence of stunting among children under 5 years of age			Indicator 2.2.2a: Prevalence of wasting among children under 5 years of age			Indicator 2.2.2b: Prevalence of overweight among children aged 2 to 4 years		
	1990	2000	2015	1990	2000	2015	1990	2000	2015	1990	2000	2015
Nepal	0.19 (0.10-0.28)	1.49 (0.78-2.20)	7.51 (3.93-11.09)	68.03 (62.69-72.69)	57.93 (53.72-62.10)	37.69 (33.53-41.95)	9.61 (7.06-12.52)	10.39 (8.40-12.64)	11.00 (8.46-13.79)	3.67 (2.57-5.20)	3.51 (2.62-4.60)	4.78 (3.60-6.09)
Pakistan	0.00 (0.00-0.00)	0.31 (0.16-0.45)	0.31 (0.16-0.46)	51.59 (48.88-54.51)	41.56 (38.46-44.87)	43.11 (38.41-47.65)	17.60 (14.76-20.63)	14.40 (11.38-18.14)	11.92 (9.13-15.76)	13.29 (10.70-16.18)	12.37 (8.91-16.97)	11.45 (8.95-14.52)
Botswana	0.00 (0.00-0.00)	0.00 (0.00-0.00)	0.13 (0.05-0.22)	39.98 (33.99-45.89)	31.48 (27.91-35.26)	23.19 (18.67-27.88)	10.80 (5.84-18.21)	7.89 (5.27-11.13)	6.10 (3.07-10.84)	11.83 (7.04-18.72)	14.95 (9.21-23.47)	19.05 (12.47-28.82)
Lesotho	1.60 (0.55-2.88)	0.00 (0.00-0.00)	0.29 (0.10-0.51)	56.06 (50.07-62.24)	51.39 (47.28-55.61)	33.50 (28.88-37.73)	6.44 (3.37-11.23)	6.59 (4.70-8.87)	3.59 (2.33-5.30)	13.55 (8.51-21.25)	15.96 (11.44-21.46)	15.77 (10.76-22.68)
Namibia	0.00 (0.00-0.00)	0.00 (0.00-0.00)	1.12 (0.40-1.96)	36.03 (31.03-40.71)	31.12 (27.12-35.09)	23.16 (19.79-26.93)	10.35 (6.49-15.68)	10.54 (7.08-14.60)	7.88 (4.80-12.19)	7.64 (5.29-10.90)	6.10 (4.41-8.24)	9.72 (7.11-13.25)
South Africa	0.05 (0.02-0.27)	0.70 (0.40-1.02)	0.35 (0.23-0.50)	30.47 (28.40-32.57)	30.14 (28.14-32.22)	20.99 (19.38-22.79)	9.42 (7.19-12.31)	8.90 (6.80-11.32)	4.86 (3.68-6.25)	31.40 (22.75-41.64)	28.42 (21.88-35.47)	32.06 (23.07-43.20)
Swaziland	0.00 (0.00-0.00)	0.00 (0.00-0.00)	0.20 (0.08-0.35)	37.47 (31.69-43.20)	37.38 (32.97-42.21)	28.19 (23.58-33.08)	1.32 (0.64-2.31)	2.54 (1.67-3.66)	1.30 (0.67-2.34)	24.80 (16.19-36.17)	22.64 (15.64-30.65)	18.29 (11.88-26.77)
Zimbabwe	0.00 (0.00-0.00)	0.20 (0.08-0.33)	0.23 (0.10-0.39)	30.63 (27.59-34.06)	33.65 (30.50-36.99)	27.99 (24.84-31.19)	3.72 (2.19-5.85)	7.72 (4.74-12.07)	3.47 (2.06-5.54)	12.75 (9.54-16.54)	17.46 (13.66-21.93)	10.20 (7.77-13.48)
Benin	0.00 (0.00-0.00)	0.04 (0.02-0.08)	0.00 (0.00-0.00)	44.04 (37.20-51.43)	42.17 (37.26-47.01)	44.50 (37.81-50.92)	12.55 (7.63-19.64)	10.92 (7.97-14.47)	12.45 (8.12-17.50)	6.36 (4.34-9.19)	10.34 (7.97-13.34)	31.87 (24.75-40.07)
Burkina Faso	0.00 (0.00-0.00)	0.00 (0.00-0.00)	0.03 (0.01-0.05)	42.98 (38.34-47.72)	45.41 (41.96-49.04)	32.24 (26.60-37.91)	17.87 (12.01-25.08)	20.23 (14.51-27.01)	15.32 (9.90-22.88)	6.85 (5.01-9.14)	10.95 (8.70-13.51)	15.57 (10.90-21.01)

Appendix Table 1b. Unscaled values for each SDG health-related indicator by country for 1990, 2000, and 2015

Location	Indicator 1.5.1: Age-standardised death rate due to exposure to forces of nature (per 100,000 population)			Indicator 2.2.1: Prevalence of stunting among children under 5 years of age			Indicator 2.2.2a: Prevalence of wasting among children under 5 years of age			Indicator 2.2.2b: Prevalence of overweight among children aged 2 to 4 years		
	**1990**	**2000**	**2015**	**1990**	**2000**	**2015**	**1990**	**2000**	**2015**	**1990**	**2000**	**2015**
Cameroon	0.00<br>(0.00-0.00)	0.04<br>(0.01-0.07)	0.03<br>(0.01-0.05)	37.96<br>(32.59-43.56)	37.84<br>(32.96-43.03)	29.22<br>(24.11-35.07)	5.70<br>(3.76-8.08)	6.94<br>(4.87-9.47)	6.02<br>(3.70-9.29)	15.52<br>(11.86-20.02)	19.36<br>(15.27-24.02)	21.36<br>(15.88-27.65)
Cape Verde	0.00<br>(0.00-0.00)	0.00<br>(0.00-0.00)	0.00<br>(0.00-0.00)	22.03<br>(17.75-26.65)	17.77<br>(14.24-22.02)	10.74<br>(8.17-13.98)	6.92<br>(4.58-10.16)	7.98<br>(5.05-12.20)	7.09<br>(4.11-11.22)	11.26<br>(7.52-16.69)	14.31<br>(9.37-20.12)	18.58<br>(13.03-25.67)
Chad	0.00<br>(0.00-0.00)	0.00<br>(0.00-0.00)	0.04<br>(0.01-0.08)	46.21<br>(39.04-53.26)	43.06<br>(38.34-48.09)	36.04<br>(29.99-42.36)	16.71<br>(10.65-24.78)	16.18<br>(12.19-21.00)	16.37<br>(10.56-23.10)	5.96<br>(4.11-8.36)	9.06<br>(6.75-11.99)	12.75<br>(8.57-17.92)
Cote d'Ivoire	0.00<br>(0.00-0.00)	0.05<br>(0.02-0.09)	0.05<br>(0.02-0.10)	29.29<br>(24.52-34.66)	34.88<br>(29.92-40.42)	30.51<br>(24.84-36.68)	9.80<br>(6.87-13.68)	8.18<br>(5.73-11.43)	8.49<br>(5.32-12.46)	12.75<br>(10.35-15.58)	12.40<br>(9.54-15.74)	12.73<br>(9.51-16.77)
The Gambia	0.00<br>(0.00-0.00)	1.13<br>(0.39-2.16)	0.00<br>(0.00-0.00)	33.38<br>(27.61-39.17)	27.89<br>(23.91-32.06)	22.24<br>(18.51-26.07)	13.38<br>(8.15-20.77)	12.62<br>(9.10-17.36)	10.50<br>(7.15-14.43)	13.96<br>(9.65-19.43)	17.55<br>(12.50-23.76)	13.29<br>(9.23-19.25)
Ghana	0.00<br>(0.00-0.00)	0.07<br>(0.03-0.13)	0.00<br>(0.00-0.00)	37.83<br>(33.58-41.90)	34.42<br>(30.27-38.78)	18.79<br>(16.00-22.00)	9.57<br>(7.51-11.81)	10.04<br>(7.96-12.61)	5.54<br>(4.01-7.53)	5.43<br>(4.05-7.02)	9.30<br>(7.29-11.83)	9.26<br>(6.82-12.27)
Guinea	0.00<br>(0.00-0.00)	0.00<br>(0.00-0.00)	0.00<br>(0.00-0.00)	35.37<br>(30.07-40.94)	39.00<br>(35.28-42.86)	32.11<br>(26.96-37.41)	12.65<br>(8.46-18.19)	10.36<br>(8.03-12.90)	10.39<br>(7.13-14.52)	11.90<br>(8.10-16.55)	11.37<br>(8.91-14.20)	13.26<br>(9.01-18.37)
Guinea-Bissau	0.00<br>(0.00-0.00)	0.00<br>(0.00-0.00)	0.00<br>(0.00-0.00)	39.95<br>(32.90-47.15)	38.73<br>(33.99-43.77)	31.94<br>(26.08-37.71)	11.41<br>(6.88-17.23)	11.29<br>(8.10-15.50)	7.08<br>(4.56-10.73)	9.43<br>(6.34-13.97)	11.88<br>(8.15-16.61)	19.83<br>(13.74-26.92)
Liberia	0.00<br>(0.00-0.00)	0.11<br>(0.03-0.19)	0.00<br>(0.00-0.00)	36.62<br>(30.50-42.62)	43.06<br>(37.94-47.84)	31.71<br>(26.51-36.81)	9.78<br>(6.22-14.86)	8.78<br>(6.30-11.89)	6.94<br>(4.74-9.95)	19.05<br>(13.18-26.41)	14.12<br>(10.29-19.23)	14.70<br>(10.26-20.57)
Mali	0.00<br>(0.00-0.00)	0.06<br>(0.01-0.07)	0.06<br>(0.02-0.11)	37.23<br>(31.98-42.55)	42.12<br>(37.28-47.18)	35.12<br>(29.59-41.26)	15.02<br>(10.47-20.54)	15.44<br>(11.92-19.75)	14.12<br>(9.59-19.98)	5.25<br>(3.82-7.25)	9.56<br>(7.61-12.01)	17.32<br>(11.87-24.44)

Appendix Table 1b. Unscaled values for each SDG health-related indicator by country for 1990, 2000, and 2015

Location	Indicator 1.5.1: Age-standardised death rate due to exposure to forces of nature (per 100,000 population)			Indicator 2.2.1: Prevalence of stunting among children under 5 years of age			Indicator 2.2.2a: Prevalence of wasting among children under 5 years of age			Indicator 2.2.2b: Prevalence of overweight among children aged 2 to 4 years		
	1990	2000	2015	1990	2000	2015	1990	2000	2015	1990	2000	2015
Mauritania	0.00 (0.00-0.00)	0.00 (0.00-0.00)	0.00 (0.00-0.00)	56.32 (50.91-61.61)	39.96 (35.12-45.03)	23.24 (18.48-28.59)	15.85 (11.77-21.19)	13.73 (9.82-19.13)	10.63 (6.50-15.72)	8.86 (5.98-12.51)	10.06 (7.77-12.81)	11.64 (7.85-17.17)
Niger	0.00 (0.00-0.00)	0.00 (0.00-0.00)	0.23 (0.09-0.41)	47.32 (42.47-52.43)	52.68 (48.35-56.82)	43.98 (38.03-50.10)	21.87 (16.91-27.85)	18.05 (14.47-21.76)	17.90 (12.84-24.47)	5.39 (3.91-7.38)	5.40 (3.99-7.16)	8.10 (5.83-11.02)
Nigeria	0.00 (0.00-0.00)	0.04 (0.01-0.07)	0.09 (0.03-0.18)	52.71 (47.67-57.59)	48.88 (45.14-52.46)	32.54 (28.25-37.18)	15.04 (10.99-20.31)	13.93 (11.04-17.27)	13.03 (9.15-17.66)	9.49 (7.79-11.50)	15.08 (11.88-18.95)	18.37 (13.35-24.39)
Sao Tome and Principe	0.00 (0.00-0.00)	0.00 (0.00-0.00)	0.00 (0.00-0.00)	32.29 (26.88-37.81)	28.66 (24.33-33.51)	18.33 (14.25-23.25)	6.55 (4.16-9.71)	6.67 (4.62-9.28)	8.97 (5.34-13.72)	14.54 (10.01-20.60)	16.24 (11.88-21.52)	20.12 (14.59-26.62)
Senegal	0.00 (0.00-0.00)	0.47 (0.18-0.89)	0.03 (0.01-0.06)	33.22 (28.83-37.51)	28.76 (24.68-33.24)	20.27 (16.98-24.05)	7.46 (5.75-9.58)	10.11 (7.80-12.72)	7.81 (5.79-10.23)	7.34 (5.71-9.30)	6.76 (4.81-9.47)	6.71 (4.68-9.24)
Sierra Leone	0.00 (0.00-0.00)	0.43 (0.06-0.45)	0.09 (0.03-0.16)	41.09 (37.12-45.12)	42.85 (38.45-47.54)	37.47 (33.20-42.38)	9.55 (8.06-11.10)	13.21 (10.64-15.94)	10.22 (7.74-13.30)	10.83 (7.13-15.71)	13.52 (9.53-18.69)	21.63 (15.51-28.70)
Togo	0.00 (0.00-0.00)	0.00 (0.00-0.00)	0.00 (0.00-0.00)	39.25 (35.77-42.96)	34.08 (30.37-38.17)	25.10 (21.67-28.78)	7.16 (4.68-10.74)	11.61 (7.67-16.48)	7.08 (4.51-10.44)	4.88 (3.50-6.59)	7.72 (5.72-10.52)	9.25 (6.04-13.50)
Burundi	0.00 (0.00-0.00)	0.00 (0.00-0.00)	0.25 (0.13-0.39)	54.58 (48.05-61.08)	59.43 (55.81-62.88)	54.54 (45.95-63.19)	7.22 (4.52-10.81)	8.34 (6.90-10.12)	6.87 (3.65-11.86)	5.32 (3.37-7.90)	6.71 (3.99-10.72)	7.12 (4.46-10.67)
Comoros	0.00 (0.00-0.00)	0.00 (0.00-0.00)	0.00 (0.00-0.00)	42.96 (33.33-52.77)	52.31 (48.56-55.79)	32.44 (26.67-39.13)	4.66 (3.37-6.18)	14.67 (12.32-17.32)	13.25 (8.60-19.64)	19.24 (12.10-28.06)	26.58 (19.11-35.13)	25.69 (16.29-37.25)
Djibouti	0.00 (0.00-0.00)	0.00 (0.00-0.00)	0.00 (0.00-0.00)	28.64 (25.70-31.66)	30.12 (26.76-33.68)	27.29 (19.42-36.33)	12.76 (10.46-15.31)	18.29 (14.31-22.58)	17.13 (8.52-29.69)	10.31 (6.05-16.49)	15.89 (9.87-23.81)	30.48 (20.18-41.79)

Appendix Table 1b. Unscaled values for each SDG health-related indicator by country for 1990, 2000, and 2015

Location	Indicator 1.5.1: Age-standardised death rate due to exposure to forces of nature (per 100,000 population)			Indicator 2.2.1: Prevalence of stunting among children under 5 years of age			Indicator 2.2.2a: Prevalence of wasting among children under 5 years of age			Indicator 2.2.2b: Prevalence of overweight among children aged 2 to 4 years		
	1990	2000	2015	1990	2000	2015	1990	2000	2015	1990	2000	2015
Eritrea	0.00 (0.00-0.00)	0.00 (0.00-0.00)	0.00 (0.00-0.00)	63.65 (56.45-70.28)	43.86 (39.56-48.15)	42.59 (31.97-53.30)	12.06 (7.48-17.85)	16.75 (12.90-20.74)	12.66 (5.57-23.70)	5.07 (3.09-7.87)	4.69 (3.25-6.75)	2.59 (1.50-4.40)
Ethiopia	0.00 (0.00-0.00)	0.13 (0.06-0.21)	0.00 (0.00-0.00)	58.63 (47.63-69.48)	57.21 (53.91-60.30)	40.73 (32.90-48.44)	14.01 (6.69-24.36)	13.47 (11.33-15.72)	11.09 (6.42-17.47)	4.94 (2.85-8.09)	5.66 (3.88-8.12)	9.68 (5.76-15.01)
Kenya	0.00 (0.00-0.00)	0.10 (0.02-0.22)	0.14 (0.06-0.28)	42.08 (40.41-43.73)	39.02 (37.92-40.17)	25.78 (24.79-26.77)	8.06 (7.24-8.98)	8.51 (7.86-9.23)	4.21 (3.84-4.59)	14.51 (9.79-20.20)	13.14 (9.70-17.36)	11.70 (8.62-15.83)
Madagascar	0.00 (0.00-0.00)	0.54 (0.32-0.80)	0.31 (0.19-0.45)	57.48 (51.65-63.40)	55.19 (51.31-58.81)	46.28 (35.55-56.44)	14.48 (9.07-21.14)	13.82 (9.68-18.96)	18.87 (9.14-32.11)	8.09 (5.59-11.50)	7.58 (5.48-10.05)	10.02 (5.89-15.59)
Malawi	0.00 (0.00-0.00)	0.00 (0.00-0.00)	0.39 (0.19-0.62)	56.31 (50.80-62.02)	55.74 (52.23-59.02)	42.25 (36.99-47.58)	6.74 (3.99-10.51)	8.24 (5.76-11.14)	5.50 (3.52-8.05)	25.40 (18.92-32.74)	25.77 (20.50-31.07)	16.25 (12.11-21.59)
Mozambique	0.00 (0.00-0.00)	1.25 (0.71-1.92)	0.20 (0.12-0.29)	59.19 (50.44-67.91)	49.54 (45.50-53.88)	38.69 (30.30-46.99)	10.32 (5.48-17.19)	7.63 (5.87-9.86)	6.47 (3.59-10.57)	12.84 (7.99-19.49)	16.88 (12.64-21.89)	19.97 (13.86-27.11)
Rwanda	0.00 (0.00-0.00)	0.00 (0.00-0.00)	0.03 (0.02-0.05)	55.35 (49.01-61.70)	49.13 (45.32-52.99)	38.23 (32.76-43.56)	8.67 (4.89-14.05)	8.48 (6.01-11.47)	2.89 (1.71-4.58)	15.68 (11.70-20.30)	17.07 (13.68-20.89)	24.99 (17.42-33.91)
Somalia	0.00 (0.00-0.00)	8.11 (3.95-12.81)	0.56 (0.25-0.76)	29.69 (21.17-39.97)	31.50 (28.40-34.77)	38.74 (29.53-48.41)	13.47 (6.54-24.50)	26.74 (22.28-31.32)	14.13 (6.53-26.02)	12.21 (7.26-18.81)	8.97 (5.26-13.67)	10.61 (6.04-16.98)
South Sudan	0.00 (0.00-0.00)	0.00 (0.00-0.00)	0.37 (0.18-1.16)	47.48 (36.37-58.82)	47.51 (41.20-53.96)	31.76 (24.60-39.70)	17.92 (8.25-34.17)	20.85 (15.72-26.63)	21.69 (12.56-34.03)	18.54 (11.13-28.18)	18.14 (11.59-26.70)	18.94 (11.56-28.82)
Tanzania	0.89 (0.42-1.41)	0.13 (0.08-0.19)	0.03 (0.01-0.04)	50.21 (45.87-54.75)	48.35 (44.63-52.22)	38.03 (30.06-47.32)	9.23 (5.71-13.81)	5.98 (4.15-8.54)	5.83 (2.99-10.59)	15.35 (12.24-18.74)	12.68 (10.36-15.10)	13.30 (8.74-19.02)

Appendix Table 1b. Unscaled values for each SDG health-related indicator by country for 1990, 2000, and 2015

Location	Indicator 1.5.1: Age-standardised death rate due to exposure to forces of nature (per 100,000 population)			Indicator 2.2.1: Prevalence of stunting among children under 5 years of age			Indicator 2.2.2a: Prevalence of wasting among children under 5 years of age			Indicator 2.2.2b: Prevalence of overweight among children aged 2 to 4 years		
	1990	2000	2015	1990	2000	2015	1990	2000	2015	1990	2000	2015
Uganda	0.00 (0.00-0.00)	0.16 (0.07-0.53)	0.04 (0.02-0.06)	46.99 (42.66-50.94)	44.99 (41.70-48.31)	31.04 (24.68-37.87)	4.27 (2.55-6.97)	8.24 (5.07-12.21)	5.49 (2.78-9.54)	15.27 (12.17-18.90)	15.69 (12.96-18.70)	13.00 (8.82-18.21)
Zambia	0.00 (0.00-0.00)	0.00 (0.00-0.00)	0.00 (0.00-0.00)	50.54 (42.44-58.00)	54.70 (50.59-58.90)	40.80 (33.65-48.00)	5.10 (2.82-8.26)	5.45 (3.97-7.31)	6.31 (3.73-9.85)	20.08 (14.58-26.89)	26.85 (22.03-32.16)	35.62 (25.13-46.95)
Angola	0.00 (0.00-0.00)	0.09 (0.01-0.17)	0.22 (0.03-0.41)	56.42 (48.48-63.37)	50.03 (44.04-56.10)	26.31 (20.61-32.54)	7.86 (5.06-11.59)	9.11 (6.57-12.02)	6.14 (3.96-9.22)	10.42 (6.80-15.08)	14.13 (9.42-20.65)	28.73 (19.65-39.09)
Central African Republic	0.00 (0.00-0.00)	0.00 (0.00-0.00)	0.00 (0.00-0.00)	45.01 (38.15-52.42)	44.54 (38.30-51.05)	37.75 (30.72-44.46)	9.39 (6.46-12.77)	10.94 (7.86-14.36)	9.08 (5.94-12.72)	16.28 (11.36-22.17)	15.77 (10.94-21.78)	12.07 (7.77-17.56)
Congo	0.00 (0.00-0.00)	0.00 (0.00-0.00)	0.08 (0.01-0.15)	39.13 (32.08-46.70)	33.97 (27.54-40.86)	23.58 (19.08-28.60)	9.33 (5.96-13.49)	8.55 (5.71-12.04)	6.50 (4.27-9.32)	15.88 (10.56-23.19)	15.17 (10.68-20.86)	11.91 (7.97-16.68)
Democratic Republic of the Congo	0.00 (0.00-0.00)	0.00 (0.00-0.00)	0.05 (0.01-0.10)	49.05 (41.98-56.34)	48.28 (42.67-54.41)	39.44 (33.49-45.48)	12.41 (8.34-17.66)	14.00 (10.54-18.67)	9.04 (6.26-12.49)	27.13 (18.75-37.31)	20.18 (14.10-27.92)	11.79 (7.96-16.86)
Equatorial Guinea	0.00 (0.00-0.00)	0.00 (0.00-0.00)	0.00 (0.00-0.00)	55.94 (48.36-62.94)	40.19 (34.42-46.06)	17.79 (13.85-22.45)	6.81 (4.38-10.08)	6.01 (4.42-7.97)	3.57 (2.31-5.44)	20.79 (13.79-29.24)	26.43 (19.16-35.24)	44.42 (33.29-55.67)
Gabon	0.00 (0.00-0.00)	0.00 (0.00-0.00)	0.00 (0.00-0.00)	30.40 (23.81-37.47)	26.06 (21.13-31.16)	17.60 (13.91-22.18)	5.39 (3.42-8.11)	4.80 (3.32-6.62)	4.06 (2.68-5.78)	13.70 (9.10-19.66)	15.41 (11.55-20.00)	19.63 (14.33-25.81)

Location	Appendix Table 1b. Unscaled values for each SDG health-related indicator by country for 1990, 2000, and 2015											
	**Indicator 3.1.1: Maternal mortality ratio (maternal deaths per 100,000 live births)**			**Indicator 3.1.2: Proportion of births attended by skilled health personnel (doctors, nurses, midwives, or country-specific medical staff [e.g., clinical officers])**			**Indicator 3.2.1: Under-5 mortality rate (probability of dying before the age of 5 per 1,000 live births)**			**Indicator 3.2.2: Neonatal mortality rate (probability of dying during the first 28 days of life per 1,000 live births)**		
	**1990**	**2000**	**2015**	**1990**	**2000**	**2015**	**1990**	**2000**	**2015**	**1990**	**2000**	**2015**
Canada	5.80 (5.01-6.63)	7.37 (6.41-8.45)	6.93 (5.78-8.31)	98.36 (97.77-98.81)	98.85 (98.48-99.15)	99.16 (98.87-99.39)	8.43 (8.25-8.59)	6.15 (6.01-6.29)	5.03 (4.72-5.38)	4.71 (4.48-4.90)	3.42 (3.24-3.57)	2.77 (2.54-3.02)
United States	16.51 (15.77-17.39)	17.05 (16.21-17.88)	25.15 (23.38-27.12)	98.33 (97.83-98.78)	98.26 (97.76-98.70)	99.01 (98.66-99.28)	11.33 (11.25-11.42)	8.17 (8.11-8.24)	6.00 (5.80-6.21)	6.19 (5.95-6.40)	4.55 (4.36-4.70)	3.32 (3.14-3.50)
Australia	7.37 (6.28-8.61)	7.46 (6.46-8.58)	5.46 (4.53-6.53)	98.81 (98.55-99.02)	98.98 (98.75-99.17)	99.31 (99.15-99.43)	9.60 (9.35-9.85)	6.26 (6.10-6.44)	3.94 (3.73-4.17)	5.19 (4.87-5.46)	3.43 (3.22-3.60)	2.04 (1.88-2.20)
New Zealand	12.44 (10.58-14.51)	10.09 (8.55-11.86)	11.82 (9.84-14.03)	98.72 (98.44-98.93)	98.98 (98.76-99.16)	99.33 (99.19-99.45)	11.07 (10.74-11.41)	7.44 (7.18-7.70)	5.27 (4.95-5.63)	4.71 (4.39-4.96)	3.33 (3.13-3.51)	2.32 (2.11-2.53)
Brunei	48.10 (37.71-60.42)	40.73 (33.66-48.45)	33.25 (26.58-41.89)	98.97 (98.87-99.05)	98.98 (98.88-99.06)	99.08 (98.99-99.15)	10.18 (9.54-10.95)	8.86 (8.27-9.49)	8.90 (7.81-10.17)	3.95 (3.54-4.40)	3.65 (3.27-4.02)	3.86 (3.32-4.43)
Japan	12.66 (12.09-13.27)	8.66 (8.20-9.20)	6.29 (5.68-6.76)	98.92 (98.82-99.00)	98.98 (98.89-99.06)	99.06 (98.98-99.14)	6.13 (6.02-6.24)	4.60 (4.51-4.69)	2.73 (2.50-2.98)	2.72 (2.52-2.90)	1.99 (1.85-2.12)	1.11 (0.99-1.24)
Singapore	10.32 (9.01-11.80)	11.04 (9.70-12.54)	4.74 (4.02-5.54)	98.57 (98.44-98.69)	98.96 (98.87-99.04)	99.19 (99.12-99.26)	7.67 (7.31-8.05)	3.68 (3.46-3.91)	2.17 (1.95-2.42)	3.98 (3.68-4.26)	1.76 (1.60-1.92)	1.05 (0.94-1.17)
South Korea	25.42 (21.98-28.94)	14.97 (13.16-17.07)	11.46 (9.48-13.46)	98.50 (98.38-98.62)	98.96 (98.86-99.04)	99.19 (99.12-99.26)	12.19 (9.27-16.17)	7.14 (6.96-7.32)	3.22 (2.69-3.82)	5.47 (4.03-7.90)	3.30 (3.08-3.49)	1.34 (1.09-1.65)
Andorra	3.63 (1.70-5.35)	2.35 (0.98-3.71)	1.84 (0.99-2.90)	99.30 (99.19-99.40)	99.37 (99.28-99.46)	99.45 (99.36-99.52)	6.11 (5.02-7.37)	2.93 (2.42-3.56)	1.91 (1.55-2.39)	3.39 (2.69-4.21)	1.46 (1.19-1.84)	0.98 (0.81-1.23)
Austria	11.17 (9.93-12.51)	7.13 (6.29-7.96)	3.95 (3.41-4.54)	98.93 (98.78-99.08)	99.14 (99.02-99.26)	99.33 (99.24-99.42)	9.65 (9.23-10.10)	5.66 (5.34-5.99)	3.53 (3.12-3.99)	5.40 (5.11-5.69)	3.10 (2.87-3.35)	1.85 (1.60-2.16)

Appendix Table 1b. Unscaled values for each SDG health-related indicator by country for 1990, 2000, and 2015

Location	Indicator 3.1.1: Maternal mortality ratio (maternal deaths per 100,000 live births)			Indicator 3.1.2: Proportion of births attended by skilled health personnel (doctors, nurses, midwives, or country-specific medical staff [e.g., clinical officers])			Indicator 3.2.1: Under-5 mortality rate (probability of dying before the age of 5 per 1,000 live births)			Indicator 3.2.2: Neonatal mortality rate (probability of dying during the first 28 days of life per 1,000 live births)		
	1990	2000	2015	1990	2000	2015	1990	2000	2015	1990	2000	2015
Belgium	13.42 (11.85-15.06)	9.64 (8.52-10.77)	6.82 (5.82-7.90)	98.90 (98.74-99.04)	99.09 (98.95-99.21)	99.26 (99.15-99.36)	10.02 (9.80-10.25)	6.05 (5.87-6.22)	3.81 (3.45-4.22)	5.27 (5.04-5.49)	3.23 (3.05-3.39)	1.93 (1.70-2.19)
Cyprus	13.03 (9.75-16.96)	11.70 (8.76-15.30)	5.23 (3.91-6.82)	98.70 (98.50-98.87)	98.98 (98.82-99.12)	99.16 (99.04-99.28)	13.21 (12.57-13.81)	7.34 (6.87-7.83)	4.78 (4.16-5.58)	7.89 (7.36-8.38)	4.42 (4.12-4.73)	2.80 (2.37-3.32)
Denmark	9.42 (8.12-10.80)	5.55 (4.72-6.56)	4.00 (3.34-4.79)	99.04 (98.89-99.16)	99.21 (99.08-99.32)	99.31 (99.20-99.41)	9.17 (8.74-9.64)	5.54 (5.23-5.85)	3.55 (3.02-4.18)	5.41 (5.07-5.74)	3.25 (3.02-3.50)	1.97 (1.63-2.39)
Finland	7.61 (6.64-8.87)	7.08 (6.14-8.24)	3.57 (2.99-4.23)	98.96 (98.79-99.10)	99.14 (99.01-99.25)	99.34 (99.24-99.43)	6.97 (6.52-7.49)	4.18 (3.86-4.53)	2.40 (1.89-3.03)	4.18 (3.83-4.55)	2.37 (2.13-2.64)	1.32 (1.05-1.69)
France	16.54 (14.43-18.91)	11.44 (9.91-12.97)	7.57 (6.23-9.10)	98.88 (98.72-99.04)	99.08 (98.95-99.21)	99.29 (99.19-99.39)	8.89 (8.70-9.08)	5.47 (5.32-5.63)	3.28 (2.59-4.15)	4.44 (4.24-4.62)	2.71 (2.57-2.84)	1.53 (1.16-2.02)
Germany	18.33 (16.41-20.55)	10.64 (9.53-11.84)	8.19 (7.11-9.41)	98.87 (98.70-99.02)	99.09 (98.95-99.22)	99.27 (99.15-99.37)	9.23 (9.04-9.43)	5.31 (5.16-5.46)	3.53 (3.01-4.22)	4.88 (4.64-5.06)	2.78 (2.65-2.91)	1.77 (1.46-2.20)
Greece	9.18 (8.02-10.47)	7.83 (6.77-8.87)	9.26 (7.77-10.74)	98.59 (98.37-98.77)	98.84 (98.65-99.00)	99.06 (98.93-99.19)	10.48 (10.17-10.78)	6.20 (5.98-6.43)	3.30 (3.03-3.61)	6.32 (6.04-6.57)	3.84 (3.65-4.03)	1.92 (1.74-2.13)
Iceland	2.79 (2.38-3.25)	1.14 (0.99-1.32)	0.67 (0.56-0.79)	98.95 (98.78-99.09)	99.10 (98.96-99.22)	99.27 (99.16-99.37)	6.57 (5.98-7.25)	3.97 (3.55-4.48)	2.03 (1.72-2.38)	3.39 (2.98-3.81)	1.91 (1.65-2.22)	0.95 (0.81-1.13)
Ireland	6.84 (5.60-8.30)	4.95 (4.14-5.93)	4.59 (3.59-5.86)	98.44 (98.19-98.65)	98.94 (98.78-99.10)	99.24 (99.12-99.34)	9.52 (9.06-10.01)	6.89 (6.55-7.26)	3.51 (3.15-3.92)	5.25 (4.93-5.56)	3.92 (3.66-4.19)	1.83 (1.59-2.09)
Israel	10.90 (9.54-12.41)	7.57 (6.61-8.52)	5.68 (4.79-6.63)	98.88 (98.71-99.04)	99.09 (98.96-99.22)	99.23 (99.12-99.33)	12.20 (11.72-12.66)	7.34 (7.05-7.65)	3.84 (3.50-4.22)	6.25 (5.86-6.59)	3.83 (3.61-4.06)	1.85 (1.63-2.09)

Appendix Table 1b. Unscaled values for each SDG health-related indicator by country for 1990, 2000, and 2015												
	**Indicator 3.1.1: Maternal mortality ratio (maternal deaths per 100,000 live births)**			**Indicator 3.1.2: Proportion of births attended by skilled health personnel (doctors, nurses, midwives, or country-specific medical staff [e.g., clinical officers])**			**Indicator 3.2.1: Under-5 mortality rate (probability of dying before the age of 5 per 1,000 live births)**			**Indicator 3.2.2: Neonatal mortality rate (probability of dying during the first 28 days of life per 1,000 live births)**		
**Location**	**1990**	**2000**	**2015**	**1990**	**2000**	**2015**	**1990**	**2000**	**2015**	**1990**	**2000**	**2015**
Italy	9.90 (8.75-11.16)	6.72 (5.87-7.67)	4.07 (3.46-4.74)	98.89 (98.71-99.03)	99.05 (98.91-99.18)	99.17 (99.04-99.28)	9.52 (9.30-9.77)	5.48 (5.31-5.64)	3.13 (2.55-3.83)	6.14 (5.89-6.38)	3.51 (3.34-3.67)	1.91 (1.54-2.39)
Luxembourg	9.83 (8.48-11.43)	6.41 (5.57-7.41)	10.51 (8.88-12.33)	98.68 (98.49-98.86)	99.05 (98.90-99.18)	99.32 (99.22-99.41)	9.12 (8.34-9.94)	4.73 (4.29-5.27)	2.58 (2.23-2.99)	4.43 (4.06-4.81)	2.22 (1.95-2.54)	1.13 (0.97-1.33)
Malta	10.18 (8.78-11.81)	10.51 (9.13-12.15)	5.62 (4.82-6.66)	97.92 (97.60-98.22)	98.45 (98.22-98.68)	98.89 (98.72-99.04)	9.66 (8.93-10.43)	7.13 (6.49-7.80)	6.25 (5.47-7.16)	6.17 (5.71-6.67)	4.66 (4.19-5.13)	4.16 (3.56-4.84)
Netherlands	11.73 (10.26-13.45)	12.79 (11.28-14.46)	6.34 (5.45-7.41)	98.83 (98.66-98.99)	99.08 (98.93-99.20)	99.29 (99.17-99.38)	8.72 (8.43-9.03)	6.47 (6.24-6.71)	3.91 (3.48-4.41)	5.05 (4.74-5.32)	3.79 (3.56-4.02)	2.18 (1.88-2.53)
Norway	6.58 (5.54-7.71)	5.81 (4.92-6.81)	3.66 (3.04-4.40)	99.14 (99.02-99.26)	99.33 (99.23-99.42)	99.45 (99.37-99.53)	8.95 (8.54-9.43)	4.94 (4.68-5.23)	2.71 (2.40-3.06)	4.86 (4.54-5.17)	2.62 (2.41-2.84)	1.34 (1.17-1.53)
Portugal	18.36 (16.25-20.84)	12.92 (11.41-14.49)	8.67 (7.49-10.01)	98.46 (98.21-98.67)	98.86 (98.69-99.01)	99.16 (99.04-99.27)	14.21 (13.98-14.44)	7.17 (7.01-7.35)	2.97 (2.78-3.17)	7.44 (7.01-7.80)	3.73 (3.52-3.92)	1.39 (1.28-1.52)
Spain	12.16 (10.77-13.61)	7.29 (6.47-8.19)	5.35 (4.58-6.16)	98.42 (98.18-98.64)	98.78 (98.60-98.95)	99.06 (98.92-99.19)	9.41 (9.17-9.66)	5.45 (5.27-5.65)	3.00 (2.65-3.40)	5.16 (4.93-5.37)	3.00 (2.83-3.16)	1.56 (1.35-1.80)
Sweden	9.76 (8.94-10.66)	6.07 (5.53-6.65)	4.07 (3.57-4.56)	99.02 (98.87-99.15)	99.17 (99.04-99.28)	99.34 (99.24-99.43)	7.15 (6.79-7.52)	3.87 (3.63-4.12)	2.60 (2.26-2.99)	4.27 (3.98-4.55)	2.14 (1.95-2.34)	1.41 (1.23-1.65)
Switzerland	6.87 (5.96-7.85)	7.31 (6.37-8.43)	5.52 (4.66-6.48)	99.06 (98.93-99.19)	99.19 (99.07-99.30)	99.33 (99.23-99.42)	8.72 (8.37-9.09)	5.87 (5.60-6.14)	4.05 (3.45-4.75)	5.01 (4.71-5.29)	3.36 (3.13-3.60)	2.25 (1.86-2.74)
United Kingdom	10.59 (9.97-11.30)	12.98 (12.16-13.82)	8.91 (8.24-9.66)	98.79 (98.59-98.95)	99.04 (98.89-99.18)	99.24 (99.12-99.35)	9.52 (9.35-9.68)	6.48 (6.31-6.66)	4.69 (4.34-5.10)	5.17 (4.97-5.34)	3.60 (3.44-3.76)	2.56 (2.31-2.83)

Appendix Table 1b. Unscaled values for each SDG health-related indicator by country for 1990, 2000, and 2015												
	Indicator 3.1.1: Maternal mortality ratio (maternal deaths per 100,000 live births)			Indicator 3.1.2: Proportion of births attended by skilled health personnel (doctors, nurses, midwives, or country-specific medical staff [e.g., clinical officers])			Indicator 3.2.1: Under-5 mortality rate (probability of dying before the age of 5 per 1,000 live births)			Indicator 3.2.2: Neonatal mortality rate (probability of dying during the first 28 days of life per 1,000 live births)		
**Location**	**1990**	**2000**	**2015**	**1990**	**2000**	**2015**	**1990**	**2000**	**2015**	**1990**	**2000**	**2015**
Argentina	64.35 (57.17-72.02)	53.68 (48.36-59.44)	49.59 (43.21-57.82)	97.49 (96.94-97.93)	98.41 (98.13-98.67)	98.68 (98.45-98.90)	28.27 (28.06-28.49)	19.81 (19.64-19.98)	12.57 (12.08-13.10)	15.15 (14.63-15.56)	11.23 (10.82-11.56)	6.80 (6.40-7.20)
Chile	51.74 (46.44-57.12)	23.38 (20.72-26.33)	20.30 (17.41-23.66)	98.71 (98.43-98.96)	98.96 (98.75-99.15)	99.12 (98.94-99.29)	18.85 (18.62-19.08)	10.72 (10.56-10.89)	7.61 (7.17-8.08)	10.02 (9.64-10.29)	5.21 (5.01-5.37)	3.94 (3.67-4.21)
Uruguay	39.38 (34.62-44.17)	29.50 (26.07-33.07)	21.09 (18.06-24.49)	98.83 (98.57-99.05)	98.98 (98.77-99.17)	99.09 (98.90-99.26)	23.01 (22.06-24.02)	15.92 (15.16-16.67)	9.58 (7.35-12.43)	12.08 (11.59-12.57)	8.40 (7.91-8.93)	4.89 (3.79-6.35)
Belarus	37.39 (32.41-42.97)	40.63 (34.94-47.07)	7.87 (6.03-10.02)	99.03 (98.90-99.15)	98.88 (98.74-99.01)	99.25 (99.15-99.34)	20.68 (17.40-24.42)	15.46 (12.85-18.37)	5.70 (4.34-7.65)	10.81 (8.92-12.72)	8.12 (6.35-10.02)	2.91 (2.11-4.00)
Estonia	39.90 (34.88-45.30)	23.26 (20.36-26.52)	4.58 (3.76-5.52)	98.99 (98.86-99.10)	98.99 (98.86-99.11)	99.31 (99.22-99.39)	17.46 (16.37-18.53)	11.68 (10.66-12.73)	3.30 (2.53-4.26)	8.98 (8.19-9.70)	5.62 (5.01-6.32)	1.46 (1.07-2.01)
Latvia	47.37 (41.58-53.72)	26.12 (22.87-29.74)	12.51 (10.49-14.72)	99.06 (98.94-99.17)	98.96 (98.82-99.08)	99.30 (99.21-99.39)	17.97 (16.93-19.00)	13.25 (12.25-14.42)	5.20 (3.60-7.29)	9.46 (8.68-10.22)	6.70 (5.94-7.51)	2.57 (1.64-3.80)
Lithuania	31.77 (28.08-35.70)	15.40 (13.42-17.50)	9.82 (8.32-11.39)	99.04 (98.91-99.16)	98.96 (98.82-99.09)	99.30 (99.21-99.39)	14.03 (13.43-14.67)	11.12 (10.50-11.73)	4.07 (3.44-4.89)	6.68 (6.17-7.20)	5.15 (4.76-5.56)	1.80 (1.44-2.26)
Moldova	47.82 (42.48-53.57)	30.31 (26.56-34.52)	14.31 (11.99-17.05)	99.13 (99.02-99.23)	98.88 (98.74-99.02)	99.21 (99.10-99.31)	30.13 (25.49-35.29)	31.80 (26.94-37.90)	10.68 (7.16-15.38)	14.90 (12.86-17.01)	16.02 (13.99-18.32)	5.43 (3.66-8.21)
Russia	56.26 (50.25-62.48)	49.29 (44.12-55.11)	18.55 (15.93-21.71)	99.08 (98.96-99.19)	98.96 (98.83-99.09)	99.26 (99.16-99.35)	21.04 (20.93-21.14)	19.67 (19.40-19.97)	8.75 (8.57-8.94)	11.03 (10.44-11.51)	10.38 (9.83-10.80)	4.43 (4.18-4.64)
Ukraine	49.48 (44.04-54.94)	53.32 (47.55-59.85)	23.94 (19.79-28.37)	99.17 (99.06-99.27)	98.94 (98.81-99.07)	99.16 (99.05-99.26)	21.49 (17.89-25.39)	21.47 (17.27-26.41)	9.60 (6.09-14.43)	11.21 (9.24-13.22)	11.64 (9.27-14.07)	4.89 (3.06-7.60)

Appendix Table 1b. Unscaled values for each SDG health-related indicator by country for 1990, 2000, and 2015												
	Indicator 3.1.1: Maternal mortality ratio (maternal deaths per 100,000 live births)			Indicator 3.1.2: Proportion of births attended by skilled health personnel (doctors, nurses, midwives, or country-specific medical staff [e.g., clinical officers])			Indicator 3.2.1: Under-5 mortality rate (probability of dying before the age of 5 per 1,000 live births)			Indicator 3.2.2: Neonatal mortality rate (probability of dying during the first 28 days of life per 1,000 live births)		
**Location**	**1990**	**2000**	**2015**	**1990**	**2000**	**2015**	**1990**	**2000**	**2015**	**1990**	**2000**	**2015**
Albania	28.41 (22.88-35.09)	15.21 (12.43-18.47)	9.05 (6.38-12.36)	98.10 (97.83-98.34)	98.15 (97.90-98.38)	98.88 (98.73-99.03)	41.30 (37.08-46.19)	26.74 (23.02-31.27)	12.15 (8.48-17.15)	9.12 (8.32-9.83)	7.25 (6.34-8.16)	3.31 (2.28-5.02)
Bosnia and Herzegovina	34.94 (28.23-43.61)	25.76 (19.31-33.81)	12.21 (9.31-16.03)	97.84 (97.54-98.14)	98.86 (98.70-99.00)	99.15 (99.03-99.26)	18.29 (17.57-19.05)	10.86 (10.33-11.41)	5.39 (4.64-6.24)	10.05 (9.58-10.51)	5.64 (5.32-5.98)	2.89 (2.42-3.42)
Bulgaria	42.46 (38.33-47.20)	48.23 (43.03-53.71)	18.84 (16.17-21.81)	99.00 (98.87-99.13)	98.94 (98.78-99.08)	99.20 (99.08-99.31)	18.21 (17.53-18.92)	17.69 (16.89-18.55)	8.72 (6.22-12.09)	9.08 (8.58-9.60)	8.97 (8.44-9.55)	4.21 (2.95-5.78)
Croatia	15.12 (13.27-17.20)	13.13 (11.60-14.81)	8.61 (7.49-9.79)	99.02 (98.88-99.15)	98.97 (98.82-99.10)	99.14 (99.02-99.26)	12.07 (11.56-12.62)	8.63 (8.22-9.05)	4.40 (3.87-5.00)	7.45 (6.99-7.90)	5.44 (5.13-5.74)	2.70 (2.31-3.14)
Czech Republic	16.55 (14.68-18.55)	9.24 (8.20-10.38)	5.87 (4.93-6.87)	99.00 (98.85-99.14)	99.00 (98.85-99.14)	99.15 (99.03-99.26)	11.95 (11.45-12.46)	5.35 (5.01-5.72)	2.49 (2.12-2.93)	6.49 (6.09-6.86)	2.89 (2.66-3.14)	1.23 (1.04-1.45)
Hungary	18.58 (16.29-21.30)	10.92 (9.58-12.45)	9.60 (8.22-11.28)	98.98 (98.83-99.12)	98.98 (98.84-99.13)	99.15 (99.01-99.27)	16.95 (16.31-17.58)	10.41 (9.93-10.94)	4.94 (3.51-6.87)	11.11 (10.56-11.66)	6.42 (6.07-6.78)	3.05 (2.05-4.39)
Macedonia	16.25 (13.17-19.84)	13.91 (11.96-16.23)	8.02 (6.48-9.86)	95.24 (94.60-95.84)	97.84 (97.55-98.14)	98.77 (98.58-98.94)	34.68 (33.13-36.35)	15.39 (14.38-16.49)	9.57 (6.38-13.95)	20.14 (19.38-20.95)	9.52 (8.74-10.32)	5.82 (3.86-8.74)
Montenegro	11.06 (8.23-14.64)	13.67 (10.28-17.76)	5.28 (3.72-7.33)	99.00 (98.86-99.12)	98.87 (98.72-99.02)	99.13 (99.00-99.24)	19.11 (13.85-25.43)	20.07 (17.99-22.37)	5.18 (3.78-7.01)	11.21 (7.79-14.79)	12.15 (10.86-13.45)	2.91 (1.99-4.10)
Poland	32.11 (29.04-35.44)	12.30 (11.01-13.67)	3.99 (3.42-4.63)	98.94 (98.78-99.08)	99.01 (98.88-99.14)	99.26 (99.15-99.36)	17.77 (17.47-18.11)	9.37 (9.10-9.65)	4.50 (3.18-6.29)	11.50 (11.09-11.84)	5.74 (5.51-5.95)	2.69 (1.80-3.91)
Romania	106.11 (96.19-116.83)	38.95 (34.64-43.81)	18.54 (15.50-21.95)	98.91 (98.76-99.05)	98.64 (98.45-98.82)	98.93 (98.75-99.08)	33.03 (32.47-33.60)	23.09 (22.52-23.69)	8.34 (6.31-10.95)	13.70 (13.27-14.07)	10.59 (10.19-10.94)	3.62 (2.71-4.65)

Location	Appendix Table 1b. Unscaled values for each SDG health-related indicator by country for 1990, 2000, and 2015											
	Indicator 3.1.1: Maternal mortality ratio (maternal deaths per 100,000 live births)			Indicator 3.1.2: Proportion of births attended by skilled health personnel (doctors, nurses, midwives, or country-specific medical staff [e.g., clinical officers])			Indicator 3.2.1: Under-5 mortality rate (probability of dying before the age of 5 per 1,000 live births)			Indicator 3.2.2: Neonatal mortality rate (probability of dying during the first 28 days of life per 1,000 live births)		
	1990	2000	2015	1990	2000	2015	1990	2000	2015	1990	2000	2015
Serbia	20.18 (14.37-29.60)	12.02 (10.26-14.28)	10.55 (8.82-12.70)	98.84 (98.67-98.99)	98.71 (98.53-98.87)	99.02 (98.88-99.15)	23.99 (20.54-27.88)	12.86 (11.70-14.14)	7.72 (6.98-8.54)	13.36 (11.55-15.25)	6.82 (6.07-7.71)	4.30 (3.87-4.77)
Slovakia	15.58 (12.80-18.63)	12.15 (10.46-14.04)	6.14 (5.08-7.32)	98.99 (98.85-99.13)	98.99 (98.84-99.12)	99.22 (99.11-99.32)	14.17 (13.46-14.88)	9.69 (9.11-10.32)	5.99 (5.01-7.16)	7.23 (6.71-7.72)	4.84 (4.57-5.12)	3.09 (2.51-3.76)
Slovenia	10.14 (8.77-11.79)	10.13 (8.92-11.62)	5.31 (4.38-6.24)	99.00 (98.85-99.14)	99.00 (98.85-99.13)	99.14 (99.02-99.26)	10.06 (9.44-10.69)	5.39 (4.99-5.83)	2.64 (2.29-3.04)	5.71 (5.29-6.15)	3.07 (2.79-3.37)	1.40 (1.20-1.64)
Armenia	49.53 (42.44-57.61)	45.71 (38.76-53.14)	24.03 (19.09-29.65)	97.34 (95.70-98.42)	97.86 (97.14-98.45)	99.30 (98.90-99.58)	47.26 (43.22-51.75)	31.14 (27.68-35.02)	14.95 (11.62-18.98)	20.42 (18.77-22.16)	15.58 (13.96-17.27)	7.82 (5.61-10.45)
Azerbaijan	38.98 (33.24-45.35)	47.57 (40.17-55.65)	19.71 (14.71-25.30)	92.06 (87.64-95.07)	88.93 (87.43-90.30)	95.00 (92.49-96.90)	80.97 (72.71-89.61)	64.64 (57.82-72.43)	29.71 (23.10-37.70)	30.72 (27.87-33.53)	27.03 (24.74-29.52)	15.85 (12.79-19.17)
Georgia	41.18 (34.12-48.27)	30.62 (25.55-36.57)	41.79 (34.18-51.22)	97.41 (95.94-98.37)	97.62 (97.02-98.11)	99.20 (98.91-99.43)	41.38 (37.75-45.46)	36.21 (32.55-40.36)	17.39 (13.91-21.52)	19.28 (17.71-20.97)	18.05 (16.40-19.78)	9.57 (7.31-12.16)
Kazakhstan	63.17 (56.41-70.29)	61.13 (54.38-68.22)	26.24 (21.77-31.72)	98.90 (98.35-99.32)	98.89 (98.48-99.20)	99.34 (98.98-99.59)	39.12 (35.79-42.21)	35.79 (31.29-41.03)	17.33 (13.42-22.06)	17.75 (16.24-19.13)	17.17 (15.29-19.18)	9.18 (6.60-12.08)
Kyrgyzstan	66.21 (56.51-76.57)	63.91 (55.17-73.80)	47.68 (38.78-56.75)	98.51 (97.60-99.09)	98.30 (97.51-98.87)	99.03 (98.53-99.40)	65.04 (62.09-67.93)	49.62 (47.26-52.19)	28.96 (25.73-32.53)	29.56 (27.60-31.38)	24.94 (23.45-26.37)	17.00 (15.17-18.89)
Mongolia	171.07 (140.58-201.99)	175.41 (148.75-205.99)	57.93 (44.57-72.97)	96.81 (95.45-97.85)	98.24 (97.84-98.59)	99.31 (98.95-99.58)	89.35 (83.63-95.66)	59.51 (54.10-65.01)	28.09 (22.56-35.61)	29.61 (26.92-32.13)	23.48 (21.32-25.56)	14.34 (11.73-17.33)
Tajikistan	71.09 (61.23-81.49)	56.38 (48.01-65.99)	25.44 (19.59-32.50)	92.55 (87.93-95.72)	90.78 (87.05-93.67)	94.04 (91.49-96.18)	90.93 (83.11-98.91)	70.21 (63.79-77.09)	32.84 (26.33-40.53)	28.91 (26.22-31.50)	24.95 (22.80-27.27)	15.40 (12.88-18.09)

Appendix Table 1b. Unscaled values for each SDG health-related indicator by country for 1990, 2000, and 2015

Location	Indicator 3.1.1: Maternal mortality ratio (maternal deaths per 100,000 live births)			Indicator 3.1.2: Proportion of births attended by skilled health personnel (doctors, nurses, midwives, or country-specific medical staff [e.g., clinical officers])			Indicator 3.2.1: Under-5 mortality rate (probability of dying before the age of 5 per 1,000 live births)			Indicator 3.2.2: Neonatal mortality rate (probability of dying during the first 28 days of life per 1,000 live births)		
	1990	2000	2015	1990	2000	2015	1990	2000	2015	1990	2000	2015
Turkmenistan	61.38 (53.16-70.20)	69.16 (56.66-82.97)	19.24 (15.50-23.59)	96.22 (93.96-97.87)	97.07 (95.60-98.15)	99.04 (98.30-99.48)	93.85 (81.02-107.14)	77.14 (61.09-96.55)	39.61 (29.39-52.32)	32.16 (28.35-36.27)	29.27 (24.63-34.75)	19.18 (15.15-23.62)
Uzbekistan	51.56 (44.56-59.53)	47.57 (40.60-56.97)	26.11 (20.07-32.73)	97.79 (96.66-98.63)	98.20 (97.45-98.81)	99.32 (98.86-99.63)	49.61 (44.62-55.24)	42.89 (37.27-49.35)	24.20 (18.78-30.77)	20.75 (18.71-22.83)	19.33 (17.07-21.65)	12.78 (9.94-15.86)
Colombia	102.01 (88.15-119.00)	82.45 (74.68-90.75)	53.26 (45.55-60.94)	82.48 (80.88-84.23)	88.51 (87.37-89.65)	97.30 (95.99-98.24)	32.09 (29.97-34.27)	23.86 (21.84-26.10)	15.81 (11.79-21.03)	12.72 (11.79-13.54)	10.40 (9.52-11.39)	7.11 (4.90-9.75)
Costa Rica	29.33 (25.73-32.97)	34.68 (30.59-39.25)	24.14 (20.47-27.98)	98.52 (97.68-99.11)	98.28 (96.96-99.16)	99.28 (98.67-99.67)	20.24 (17.63-23.08)	15.64 (13.11-18.85)	9.75 (7.13-13.22)	11.11 (9.55-12.65)	8.44 (6.73-10.59)	5.16 (3.79-7.12)
El Salvador	139.43 (120.46-161.02)	51.58 (43.66-61.08)	38.18 (29.07-49.21)	97.56 (96.60-98.28)	99.07 (98.67-99.36)	99.56 (99.14-99.80)	52.90 (47.93-59.01)	29.15 (25.21-33.28)	11.27 (7.99-15.71)	19.60 (17.61-21.66)	13.33 (11.67-15.10)	5.14 (3.60-7.67)
Guatemala	145.81 (128.73-165.95)	106.28 (95.25-118.28)	72.85 (51.75-94.59)	32.63 (29.96-35.17)	46.55 (43.88-49.34)	66.08 (54.15-76.84)	80.94 (77.70-84.08)	51.35 (48.23-54.80)	25.97 (21.21-31.77)	17.08 (15.61-18.42)	13.39 (12.35-14.42)	8.96 (7.48-10.64)
Honduras	171.64 (145.73-198.36)	119.67 (86.81-161.13)	108.76 (69.32-160.71)	47.70 (39.19-55.80)	68.32 (61.16-74.65)	96.86 (95.02-98.12)	54.54 (52.22-57.03)	36.26 (34.43-38.20)	22.20 (18.87-26.53)	21.39 (19.92-22.80)	16.57 (15.49-17.66)	11.56 (9.61-13.58)
Mexico	72.81 (68.99-77.20)	60.64 (57.02-64.33)	47.83 (44.76-50.99)	80.03 (75.51-84.04)	91.39 (89.67-92.88)	97.78 (95.79-98.94)	42.99 (39.29-46.73)	26.90 (23.57-30.34)	15.37 (11.72-20.04)	19.54 (17.76-21.26)	14.09 (12.48-15.76)	8.32 (5.93-11.22)
Nicaragua	85.87 (72.85-101.08)	99.63 (85.16-117.39)	68.19 (53.07-86.42)	71.45 (62.79-79.12)	90.96 (88.15-93.46)	96.86 (93.76-98.59)	62.70 (60.22-65.44)	35.55 (33.28-37.77)	15.36 (12.58-18.74)	23.25 (22.14-24.31)	16.09 (15.26-16.95)	7.71 (5.92-9.68)
Panama	67.96 (56.06-81.22)	63.79 (54.57-75.28)	68.79 (54.28-85.80)	86.33 (78.12-92.30)	90.54 (87.94-92.77)	94.55 (91.08-96.92)	27.35 (23.80-31.06)	22.94 (19.70-26.84)	17.55 (13.19-23.18)	11.66 (10.21-13.22)	10.28 (8.63-11.98)	8.15 (5.63-11.06)

Appendix Table 1b. Unscaled values for each SDG health-related indicator by country for 1990, 2000, and 2015

Location	Indicator 3.1.1: Maternal mortality ratio (maternal deaths per 100,000 live births)			Indicator 3.1.2: Proportion of births attended by skilled health personnel (doctors, nurses, midwives, or country-specific medical staff [e.g., clinical officers])			Indicator 3.2.1: Under-5 mortality rate (probability of dying before the age of 5 per 1,000 live births)			Indicator 3.2.2: Neonatal mortality rate (probability of dying during the first 28 days of life per 1,000 live births)		
	1990	2000	2015	1990	2000	2015	1990	2000	2015	1990	2000	2015
Venezuela	66.95 (60.09-73.68)	58.04 (52.26-64.21)	63.17 (49.46-77.41)	94.73 (91.01-97.22)	97.40 (96.00-98.47)	98.00 (96.35-99.00)	28.12 (27.89-28.36)	21.08 (20.89-21.28)	16.87 (15.77-18.09)	13.78 (13.09-14.35)	10.98 (10.42-11.44)	9.06 (8.25-9.88)
Bolivia	387.22 (311.65-480.73)	207.62 (174.29-244.02)	144.53 (102.23-201.09)	45.27 (42.96-47.66)	63.03 (61.24-64.82)	83.95 (79.19-87.82)	111.16 (107.83-114.57)	68.26 (65.06-71.41)	29.37 (24.19-35.66)	37.09 (34.14-39.66)	27.14 (25.21-28.89)	15.36 (12.80-17.99)
Ecuador	123.52 (107.84-140.18)	73.27 (62.74-84.66)	51.22 (40.88-63.92)	87.11 (83.74-90.19)	95.38 (93.53-96.75)	98.14 (96.95-98.93)	48.25 (46.05-50.56)	33.58 (31.40-36.01)	17.75 (15.24-20.70)	13.84 (12.87-14.63)	11.38 (10.59-12.13)	6.95 (5.68-8.28)
Peru	178.84 (150.89-212.38)	109.53 (95.66-124.60)	47.02 (35.42-58.89)	64.81 (60.61-69.09)	67.37 (63.31-71.47)	91.92 (89.73-93.96)	77.56 (74.85-80.19)	34.89 (33.33-36.29)	15.79 (13.50-18.77)	25.45 (23.43-27.07)	15.20 (14.20-16.08)	7.57 (6.09-9.24)
Antigua and Barbuda	52.65 (44.51-63.22)	49.15 (40.54-59.25)	35.99 (30.04-43.50)	98.19 (97.14-98.91)	98.90 (98.33-99.34)	99.31 (98.92-99.59)	18.85 (14.33-24.27)	18.84 (14.74-23.67)	12.87 (8.84-18.41)	10.68 (7.56-13.76)	10.96 (8.17-13.79)	7.34 (4.86-11.09)
The Bahamas	72.71 (57.90-89.44)	73.53 (59.96-89.77)	49.07 (35.94-62.19)	98.20 (97.16-98.94)	98.87 (98.30-99.29)	99.21 (98.75-99.51)	32.56 (22.09-45.36)	18.36 (13.61-23.90)	16.21 (9.06-28.81)	22.25 (15.51-29.71)	13.10 (9.19-17.26)	11.68 (6.33-20.60)
Barbados	80.79 (69.28-92.67)	33.93 (28.27-40.23)	38.02 (29.78-48.29)	97.28 (95.77-98.39)	98.13 (97.14-98.80)	99.26 (98.81-99.56)	26.40 (17.35-38.05)	20.97 (14.10-29.91)	16.22 (9.12-27.31)	17.17 (11.57-23.43)	14.11 (9.19-19.78)	11.04 (5.94-18.79)
Belize	46.46 (38.29-55.54)	61.09 (50.42-73.22)	31.80 (24.82-40.97)	82.02 (80.12-83.96)	94.01 (92.06-95.64)	97.53 (96.45-98.33)	38.97 (32.84-45.84)	25.86 (20.72-31.70)	16.67 (10.54-26.60)	19.20 (16.68-21.78)	14.04 (11.30-16.90)	9.40 (5.50-15.24)
Cuba	51.18 (45.31-57.96)	51.28 (45.56-57.92)	41.15 (35.31-47.79)	98.60 (97.80-99.13)	98.79 (98.16-99.24)	99.35 (98.97-99.60)	13.67 (13.47-13.87)	8.39 (8.24-8.55)	6.08 (5.77-6.46)	7.10 (6.74-7.41)	4.36 (4.15-4.52)	3.22 (3.00-3.48)
Dominica	22.26 (18.16-27.01)	19.12 (15.51-23.37)	37.78 (29.40-48.09)	97.07 (95.41-98.26)	98.37 (97.58-98.99)	99.20 (98.76-99.51)	25.13 (18.88-33.04)	24.10 (18.84-30.46)	26.45 (17.11-39.73)	13.97 (10.49-17.69)	13.83 (10.71-17.27)	15.61 (9.98-21.99)

Appendix Table 1b. Unscaled values for each SDG health-related indicator by country for 1990, 2000, and 2015

Location	Indicator 3.1.1: Maternal mortality ratio (maternal deaths per 100,000 live births)			Indicator 3.1.2: Proportion of births attended by skilled health personnel (doctors, nurses, midwives, or country-specific medical staff [e.g., clinical officers])			Indicator 3.2.1: Under-5 mortality rate (probability of dying before the age of 5 per 1,000 live births)			Indicator 3.2.2: Neonatal mortality rate (probability of dying during the first 28 days of life per 1,000 live births)		
	1990	2000	2015	1990	2000	2015	1990	2000	2015	1990	2000	2015
Dominican Republic	89.75 (76.91-103.86)	59.37 (51.70-68.02)	52.93 (42.10-66.06)	94.33 (93.40-95.16)	98.45 (98.17-98.70)	99.31 (99.04-99.51)	59.54 (55.91-63.34)	37.01 (34.23-39.88)	25.00 (20.52-30.44)	27.82 (26.08-29.58)	20.04 (18.53-21.46)	15.08 (12.43-18.02)
Grenada	51.16 (41.67-61.40)	29.83 (25.19-35.98)	33.39 (26.33-42.09)	97.85 (96.58-98.71)	98.82 (98.21-99.25)	99.31 (98.92-99.59)	27.46 (16.76-41.82)	20.41 (14.50-27.92)	15.92 (8.68-27.47)	14.97 (9.20-21.31)	11.72 (7.89-15.91)	9.27 (4.77-16.06)
Guyana	132.27 (111.35-154.76)	160.32 (134.50-193.97)	107.97 (79.59-140.14)	85.35 (79.72-90.33)	89.99 (87.50-92.11)	95.86 (94.32-97.10)	52.06 (48.47-55.47)	40.70 (37.82-43.79)	27.96 (22.01-35.41)	26.14 (24.30-28.21)	22.01 (20.44-23.72)	16.83 (13.60-20.47)
Haiti	581.86 (465.87-770.32)	578.38 (459.44-755.15)	512.52 (330.24-785.39)	21.28 (18.65-24.24)	24.10 (22.10-26.10)	46.25 (40.27-52.32)	148.31 (139.31-158.52)	95.81 (88.00-104.23)	58.37 (42.11-79.74)	42.32 (38.63-45.91)	30.94 (28.43-33.57)	23.57 (18.54-29.60)
Jamaica	40.47 (31.92-50.81)	65.78 (52.56-81.28)	50.01 (36.20-66.43)	93.73 (92.27-94.98)	97.94 (97.32-98.43)	99.19 (98.73-99.52)	27.51 (21.06-35.67)	21.99 (15.90-29.71)	18.13 (11.58-27.58)	15.16 (11.73-18.90)	12.67 (8.93-16.89)	10.77 (6.38-16.32)
Saint Lucia	54.40 (46.53-64.04)	83.70 (71.63-96.08)	59.13 (47.16-73.12)	97.69 (96.39-98.66)	98.54 (97.76-99.08)	99.23 (98.77-99.54)	31.33 (20.22-46.92)	20.07 (13.91-27.44)	17.66 (9.22-31.46)	16.42 (10.88-22.76)	11.29 (7.38-15.33)	10.18 (4.97-17.64)
Saint Vincent and the Grenadines	54.80 (43.81-68.02)	33.83 (29.10-39.66)	40.19 (32.69-48.82)	97.63 (96.22-98.60)	98.67 (98.02-99.17)	99.17 (98.74-99.48)	39.70 (28.36-53.20)	31.79 (24.53-40.50)	21.92 (13.83-33.62)	21.89 (16.69-27.82)	18.77 (15.03-22.97)	13.82 (8.40-20.51)
Suriname	60.81 (49.56-73.42)	84.52 (70.90-101.42)	50.26 (38.22-64.91)	90.75 (86.09-94.31)	93.83 (90.75-96.12)	98.11 (97.04-98.86)	50.97 (45.78-56.93)	44.59 (38.70-51.11)	31.12 (24.92-39.54)	24.83 (22.64-27.34)	23.12 (20.68-25.81)	18.25 (15.00-22.15)
Trinidad and Tobago	74.97 (66.26-84.49)	60.55 (52.81-69.02)	49.04 (38.43-61.63)	98.55 (97.91-99.01)	98.72 (97.98-99.22)	99.39 (99.01-99.64)	28.21 (24.23-32.85)	27.54 (22.91-33.34)	19.23 (11.91-29.98)	18.31 (15.83-20.97)	18.30 (15.45-21.72)	13.21 (7.77-20.01)
Brazil	83.43 (78.37-89.23)	70.17 (66.04-74.63)	64.52 (58.45-70.81)	85.02 (80.95-88.52)	97.26 (95.72-98.33)	99.05 (97.93-99.61)	52.12 (47.79-57.27)	31.22 (27.19-35.56)	16.94 (13.40-21.36)	22.37 (20.96-23.96)	15.82 (14.19-17.49)	9.38 (7.08-11.98)

Appendix Table 1b. Unscaled values for each SDG health-related indicator by country for 1990, 2000, and 2015												
	**Indicator 3.1.1: Maternal mortality ratio (maternal deaths per 100,000 live births)**			**Indicator 3.1.2: Proportion of births attended by skilled health personnel (doctors, nurses, midwives, or country-specific medical staff [e.g., clinical officers])**			**Indicator 3.2.1: Under-5 mortality rate (probability of dying before the age of 5 per 1,000 live births)**			**Indicator 3.2.2: Neonatal mortality rate (probability of dying during the first 28 days of life per 1,000 live births)**		
**Location**	**1990**	**2000**	**2015**	**1990**	**2000**	**2015**	**1990**	**2000**	**2015**	**1990**	**2000**	**2015**
Paraguay	146.27 (123.18-170.13)	109.68 (94.18-128.54)	100.16 (72.22-131.36)	69.11 (65.96-71.73)	79.85 (76.43-82.96)	92.08 (85.73-96.21)	32.53 (30.07-35.08)	28.10 (25.20-31.33)	17.91 (14.27-22.43)	16.21 (15.03-17.39)	14.79 (13.33-16.21)	10.04 (7.68-12.67)
China	113.14 (96.64-130.90)	84.29 (75.37-94.40)	17.39 (14.74-20.24)	65.37 (43.20-83.89)	82.05 (72.35-89.24)	96.78 (93.08-98.75)	55.93 (45.81-67.38)	37.68 (31.91-44.81)	12.32 (8.60-17.18)	22.38 (18.96-25.82)	17.57 (15.37-20.04)	6.07 (4.10-9.06)
North Korea	99.74 (56.02-169.54)	93.79 (53.37-152.88)	71.76 (42.49-111.02)	91.49 (77.65-97.64)	92.02 (80.73-97.78)	94.66 (86.33-98.59)	56.34 (24.31-108.56)	149.64 (39.04-372.87)	19.22 (8.96-36.30)	23.18 (12.30-38.01)	18.37 (8.55-30.14)	10.34 (4.48-18.43)
Taiwan	20.75 (13.38-30.40)	14.85 (10.20-20.67)	11.31 (7.40-16.61)	96.55 (90.98-99.10)	98.13 (94.89-99.49)	99.05 (97.51-99.77)	8.74 (8.43-9.04)	8.53 (8.21-8.85)	6.56 (4.24-10.28)	3.67 (3.33-4.01)	3.75 (3.41-4.06)	2.99 (1.79-4.62)
Cambodia	386.44 (324.62-460.66)	438.26 (368.74-526.73)	129.69 (86.11-189.15)	23.25 (18.02-28.92)	37.56 (34.05-41.23)	81.78 (76.94-85.71)	117.88 (113.57-122.70)	97.24 (92.92-101.59)	31.36 (27.22-36.01)	38.57 (36.36-40.60)	34.25 (32.38-35.99)	15.85 (14.10-17.65)
Indonesia	397.93 (330.50-464.58)	344.36 (294.45-407.73)	176.94 (120.80-248.48)	38.43 (37.38-39.51)	67.51 (66.64-68.39)	90.12 (88.25-91.60)	84.35 (82.35-86.40)	50.07 (48.35-51.80)	23.68 (20.44-27.48)	28.68 (26.68-30.42)	20.90 (19.69-21.96)	12.45 (10.69-14.23)
Laos	649.93 (465.96-857.19)	587.42 (470.86-758.26)	233.79 (165.15-319.78)	16.33 (11.94-21.72)	24.89 (21.75-28.27)	50.32 (43.03-57.67)	184.68 (171.63-198.95)	142.89 (131.62-155.52)	60.18 (45.77-77.70)	60.66 (55.64-66.04)	50.39 (46.44-54.69)	27.47 (22.71-33.46)
Malaysia	87.32 (74.70-102.54)	69.99 (61.60-79.88)	61.22 (45.46-79.68)	93.01 (90.00-95.29)	96.94 (95.76-97.95)	99.38 (99.07-99.60)	16.82 (16.51-17.15)	9.28 (9.07-9.49)	6.50 (5.51-7.69)	7.98 (7.39-8.56)	4.15 (3.86-4.44)	3.06 (2.48-3.71)
Maldives	183.59 (134.56-261.51)	91.95 (73.72-113.97)	40.55 (27.41-56.30)	73.91 (65.84-81.72)	88.27 (83.86-91.79)	98.70 (98.15-99.15)	67.09 (63.08-71.35)	35.84 (33.13-38.77)	12.41 (10.34-15.16)	25.12 (23.21-26.97)	16.65 (15.35-17.95)	5.89 (4.75-7.54)
Mauritius	66.81 (58.66-75.62)	41.71 (36.73-47.30)	48.61 (41.06-57.33)	96.77 (95.19-97.92)	98.62 (98.00-99.11)	99.59 (99.38-99.74)	22.38 (21.71-23.09)	17.86 (17.21-18.51)	13.41 (12.26-14.71)	13.13 (12.39-13.78)	10.69 (10.04-11.33)	7.93 (7.04-8.95)

Appendix Table 1b. Unscaled values for each SDG health-related indicator by country for 1990, 2000, and 2015												
	Indicator 3.1.1: Maternal mortality ratio (maternal deaths per 100,000 live births)			Indicator 3.1.2: Proportion of births attended by skilled health personnel (doctors, nurses, midwives, or country-specific medical staff [e.g., clinical officers])			Indicator 3.2.1: Under-5 mortality rate (probability of dying before the age of 5 per 1,000 live births)			Indicator 3.2.2: Neonatal mortality rate (probability of dying during the first 28 days of life per 1,000 live births)		
**Location**	**1990**	**2000**	**2015**	**1990**	**2000**	**2015**	**1990**	**2000**	**2015**	**1990**	**2000**	**2015**
Myanmar	906.10 (517.65-1,479.99)	818.02 (433.98-1,411.86)	389.24 (208.15-684.30)	51.98 (42.63-61.90)	68.62 (63.08-73.73)	86.55 (82.18-90.28)	110.23 (93.45-128.37)	78.49 (65.08-93.85)	32.37 (24.80-43.01)	36.42 (31.48-41.78)	29.46 (25.40-34.05)	16.37 (13.10-20.28)
Philippines	96.05 (82.35-110.55)	69.53 (63.55-76.79)	69.38 (56.06-84.58)	51.65 (48.95-54.46)	60.89 (59.21-62.50)	80.03 (77.01-82.88)	55.72 (53.76-57.68)	36.41 (34.74-38.09)	23.19 (20.52-26.26)	19.83 (18.03-21.42)	15.26 (14.02-16.39)	11.01 (9.58-12.56)
Sri Lanka	74.54 (61.94-89.76)	63.03 (55.52-71.79)	32.45 (21.35-47.42)	92.83 (91.15-94.35)	98.73 (98.20-99.13)	99.68 (99.50-99.79)	40.93 (39.62-42.29)	16.39 (15.94-16.83)	8.07 (6.17-10.49)	20.81 (19.37-22.05)	8.95 (8.27-9.57)	4.42 (3.32-5.69)
Seychelles	60.22 (46.34-75.70)	38.64 (30.72-49.43)	32.02 (24.25-42.77)	82.40 (74.82-88.12)	93.13 (89.62-95.64)	97.92 (96.91-98.71)	17.63 (15.98-19.34)	13.42 (11.99-14.99)	10.65 (8.58-13.19)	8.84 (7.67-9.92)	6.52 (5.60-7.59)	5.29 (4.24-6.75)
Thailand	40.23 (33.32-48.29)	29.85 (22.80-37.90)	19.84 (11.89-29.47)	77.04 (73.24-80.48)	96.42 (95.43-97.28)	99.11 (98.83-99.36)	27.69 (24.54-31.16)	15.03 (13.05-17.55)	5.64 (4.54-6.98)	16.93 (15.18-18.88)	9.21 (7.71-11.11)	3.35 (2.61-4.23)
Timor-Leste	633.94 (426.61-837.62)	399.27 (260.75-496.27)	231.98 (142.41-323.22)	11.76 (7.95-16.27)	20.74 (16.49-25.39)	52.55 (43.55-60.70)	151.35 (138.54-165.26)	108.09 (95.08-121.82)	38.66 (23.92-59.18)	38.87 (35.23-42.70)	30.89 (27.86-34.08)	16.38 (11.50-22.15)
Vietnam	46.66 (30.20-65.75)	32.09 (22.12-44.72)	15.54 (9.35-24.38)	66.94 (59.93-73.94)	83.14 (80.39-85.46)	94.92 (93.15-96.43)	47.69 (45.51-49.89)	27.89 (26.17-29.80)	13.43 (11.49-15.94)	21.26 (19.86-22.42)	14.58 (13.50-15.62)	6.98 (5.72-8.70)
Federated States of Micronesia	132.28 (63.14-232.10)	117.67 (50.23-217.71)	87.65 (45.11-166.94)	87.13 (78.02-93.37)	93.14 (88.84-96.09)	98.45 (97.05-99.32)	40.78 (32.08-51.23)	23.80 (17.18-32.31)	11.49 (7.70-16.79)	15.23 (12.75-17.93)	10.52 (7.55-13.60)	4.92 (3.23-7.77)
Fiji	95.61 (65.28-133.12)	113.91 (87.02-142.26)	97.49 (66.53-134.34)	97.78 (95.79-99.03)	98.52 (97.27-99.33)	99.21 (98.58-99.61)	26.85 (19.03-37.45)	28.83 (21.40-37.84)	30.40 (18.20-48.60)	10.27 (7.60-13.02)	11.25 (8.89-13.79)	12.16 (7.94-16.89)
Kiribati	248.83 (188.28-325.82)	170.30 (127.56-219.42)	129.72 (86.11-187.11)	79.59 (66.80-88.97)	84.92 (75.47-91.08)	92.00 (86.45-95.99)	84.31 (66.85-103.04)	64.69 (54.09-75.48)	39.08 (21.28-67.13)	23.25 (19.73-27.09)	20.23 (17.63-22.83)	15.18 (9.64-22.07)

Appendix Table 1b. Unscaled values for each SDG health-related indicator by country for 1990, 2000, and 2015

Location	Indicator 3.1.1: Maternal mortality ratio (maternal deaths per 100,000 live births)			Indicator 3.1.2: Proportion of births attended by skilled health personnel (doctors, nurses, midwives, or country-specific medical staff [e.g., clinical officers])			Indicator 3.2.1: Under-5 mortality rate (probability of dying before the age of 5 per 1,000 live births)			Indicator 3.2.2: Neonatal mortality rate (probability of dying during the first 28 days of life per 1,000 live births)		
	1990	2000	2015	1990	2000	2015	1990	2000	2015	1990	2000	2015
Marshall Islands	111.90 (79.06-155.04)	126.15 (63.50-187.05)	108.30 (69.41-164.69)	84.52 (74.53-91.57)	90.94 (86.16-94.65)	95.53 (92.81-97.55)	43.22 (31.33-56.62)	38.77 (25.88-54.96)	18.80 (10.57-31.03)	14.94 (11.95-18.02)	14.47 (10.85-18.50)	8.27 (4.03-13.30)
Papua New Guinea	536.94 (318.36-847.60)	517.90 (288.36-832.18)	433.69 (236.96-746.02)	58.00 (49.91-65.41)	55.17 (49.11-61.11)	67.11 (54.08-79.45)	91.71 (78.40-106.15)	75.56 (57.20-97.56)	44.47 (26.83-70.50)	21.83 (18.94-24.93)	19.96 (16.53-24.22)	15.05 (10.60-20.67)
Samoa	64.56 (37.62-100.73)	60.41 (29.82-95.45)	54.74 (31.28-83.54)	84.82 (75.31-91.97)	91.24 (86.14-94.99)	91.35 (85.60-95.44)	20.50 (11.22-36.32)	14.47 (7.43-25.20)	8.68 (4.33-16.02)	8.05 (3.94-13.48)	5.73 (2.76-10.56)	3.41 (1.53-6.79)
Solomon Islands	262.69 (133.63-432.96)	231.97 (116.96-402.70)	206.55 (101.74-368.86)	76.07 (62.86-85.71)	84.37 (78.00-89.75)	95.15 (91.94-97.50)	44.97 (35.25-56.46)	33.79 (25.65-43.52)	19.79 (13.48-28.49)	14.24 (11.87-16.68)	12.38 (9.93-14.91)	8.33 (5.16-11.63)
Tonga	197.00 (135.12-278.64)	186.63 (147.73-230.22)	129.41 (83.28-192.07)	84.92 (75.99-91.61)	93.62 (91.31-95.47)	98.69 (97.67-99.32)	26.39 (17.11-38.47)	24.84 (15.45-36.66)	17.78 (10.51-28.77)	11.00 (7.16-14.78)	10.75 (6.59-14.66)	8.05 (4.30-12.77)
Vanuatu	201.51 (102.54-351.02)	201.35 (76.68-357.30)	171.18 (91.03-308.70)	82.01 (71.01-89.87)	85.21 (78.86-90.38)	91.12 (85.30-95.13)	39.16 (30.83-49.08)	38.57 (31.20-47.35)	27.04 (18.49-40.12)	13.55 (11.47-15.69)	13.95 (12.04-16.27)	11.33 (7.99-15.13)
Afghanistan	727.24 (446.79-1,122.70)	747.76 (463.47-1,128.82)	785.32 (461.63-1,217.57)	28.07 (21.68-35.65)	27.74 (22.01-33.66)	43.59 (36.87-49.96)	160.55 (149.51-172.82)	133.28 (124.18-142.46)	82.63 (69.49-98.00)	46.98 (41.74-51.65)	40.72 (36.20-44.38)	28.63 (24.72-32.99)
Algeria	150.99 (115.86-187.19)	122.78 (95.96-153.52)	66.30 (49.15-85.47)	75.29 (67.57-81.79)	86.08 (81.38-90.22)	94.56 (92.39-96.25)	54.44 (49.52-59.31)	36.65 (32.55-41.68)	22.74 (17.90-29.41)	23.70 (21.79-25.56)	18.23 (16.49-20.02)	12.64 (9.96-15.81)
Bahrain	53.36 (39.60-73.38)	35.41 (27.17-47.23)	24.41 (18.13-35.24)	91.58 (88.26-94.27)	96.73 (95.45-97.80)	99.03 (98.63-99.35)	21.21 (19.86-22.57)	12.69 (11.64-13.70)	6.62 (5.62-7.75)	12.36 (11.50-13.20)	6.95 (6.30-7.64)	3.80 (3.16-4.47)
Egypt	142.06 (119.05-164.77)	67.55 (55.15-80.72)	41.69 (32.22-52.99)	40.83 (39.55-42.23)	67.08 (65.93-68.25)	89.39 (87.05-91.41)	82.12 (77.46-86.68)	43.25 (39.93-46.83)	21.54 (16.27-28.31)	31.86 (29.97-33.64)	20.67 (19.31-21.99)	12.06 (8.91-15.44)

Location	Appendix Table 1b. Unscaled values for each SDG health-related indicator by country for 1990, 2000, and 2015											
	**Indicator 3.1.1: Maternal mortality ratio (maternal deaths per 100,000 live births)**			**Indicator 3.1.2: Proportion of births attended by skilled health personnel (doctors, nurses, midwives, or country-specific medical staff [e.g., clinical officers])**			**Indicator 3.2.1: Under-5 mortality rate (probability of dying before the age of 5 per 1,000 live births)**			**Indicator 3.2.2: Neonatal mortality rate (probability of dying during the first 28 days of life per 1,000 live births)**		
	**1990**	**2000**	**2015**	**1990**	**2000**	**2015**	**1990**	**2000**	**2015**	**1990**	**2000**	**2015**
Iran	55.58 (37.59-76.85)	33.45 (26.26-42.64)	20.29 (13.89-30.55)	83.15 (77.93-87.87)	94.59 (92.64-96.22)	98.54 (97.96-98.99)	74.94 (65.64-85.95)	38.95 (31.92-47.15)	14.67 (10.80-19.53)	29.35 (26.37-32.71)	19.60 (16.73-22.67)	8.13 (5.59-11.36)
Iraq	144.94 (104.54-194.03)	112.68 (77.80-155.59)	58.14 (34.19-95.59)	55.71 (46.97-64.16)	68.30 (61.65-73.91)	89.60 (86.66-92.10)	53.73 (51.22-56.23)	42.37 (40.50-44.49)	27.31 (23.20-32.62)	25.87 (24.48-27.17)	22.35 (21.24-23.42)	15.49 (13.35-18.00)
Jordan	97.60 (74.38-125.04)	80.85 (58.38-106.05)	23.93 (17.61-31.19)	92.24 (91.19-93.25)	98.32 (98.09-98.54)	99.35 (99.17-99.49)	32.35 (31.07-33.69)	24.15 (23.07-25.30)	14.76 (12.89-17.15)	15.68 (14.69-16.67)	12.68 (11.76-13.56)	7.73 (6.44-9.24)
Kuwait	9.50 (7.91-11.59)	11.70 (10.19-13.31)	5.77 (4.35-7.41)	87.71 (83.68-91.03)	95.69 (94.32-96.82)	98.84 (98.44-99.16)	16.10 (14.49-18.06)	12.57 (11.74-13.42)	8.19 (6.48-10.23)	7.86 (6.89-8.91)	6.45 (5.90-7.05)	4.39 (3.42-5.42)
Lebanon	35.25 (22.64-54.68)	26.49 (17.74-38.80)	15.18 (8.98-23.88)	97.25 (96.22-98.03)	98.76 (98.37-99.10)	99.68 (99.54-99.78)	35.29 (30.35-41.30)	21.75 (17.28-26.76)	8.11 (5.90-11.33)	17.36 (15.19-19.72)	12.29 (9.62-14.94)	4.40 (3.15-5.98)
Libya	26.49 (18.91-35.90)	23.84 (17.10-31.45)	22.77 (15.52-32.87)	71.86 (64.41-79.31)	81.97 (76.37-86.84)	92.06 (89.15-94.33)	41.53 (37.40-45.75)	28.12 (23.88-32.91)	17.47 (13.16-22.58)	17.64 (15.99-19.39)	13.46 (11.52-15.42)	8.09 (5.68-11.05)
Morocco	327.23 (253.87-430.90)	190.73 (142.66-248.99)	68.15 (42.65-107.65)	32.10 (30.11-33.99)	63.63 (61.42-65.83)	83.61 (78.87-87.36)	70.10 (67.20-72.97)	43.85 (40.54-47.50)	22.44 (18.00-27.92)	29.59 (27.84-31.13)	21.29 (19.85-22.83)	12.65 (10.04-15.45)
Palestine	28.95 (18.72-43.23)	13.97 (11.10-17.45)	15.97 (11.32-22.95)	95.51 (93.79-96.85)	97.96 (97.31-98.49)	98.45 (97.93-98.86)	39.23 (35.25-43.58)	25.79 (22.93-28.54)	17.27 (13.49-21.82)	19.22 (17.64-20.83)	13.99 (12.62-15.37)	9.87 (7.33-12.67)
Oman	33.82 (19.76-53.96)	21.23 (14.65-31.68)	15.39 (10.85-21.31)	93.65 (91.33-95.53)	97.08 (95.92-97.95)	99.01 (98.59-99.30)	42.13 (35.48-50.35)	15.26 (13.27-17.55)	9.37 (8.12-10.76)	19.12 (16.69-21.75)	7.73 (6.36-9.17)	4.65 (4.05-5.33)
Qatar	63.87 (41.76-91.47)	59.80 (41.34-84.22)	25.42 (16.39-35.64)	95.23 (93.44-96.78)	98.02 (97.19-98.60)	99.52 (99.32-99.66)	21.91 (16.47-28.59)	16.18 (11.96-21.53)	8.64 (5.98-12.13)	11.90 (8.77-15.27)	8.87 (6.12-12.24)	4.65 (3.18-6.55)

Appendix Table 1b. Unscaled values for each SDG health-related indicator by country for 1990, 2000, and 2015

Location	Indicator 3.1.1: Maternal mortality ratio (maternal deaths per 100,000 live births)			Indicator 3.1.2: Proportion of births attended by skilled health personnel (doctors, nurses, midwives, or country-specific medical staff [e.g., clinical officers])			Indicator 3.2.1: Under-5 mortality rate (probability of dying before the age of 5 per 1,000 live births)			Indicator 3.2.2: Neonatal mortality rate (probability of dying during the first 28 days of life per 1,000 live births)		
	1990	2000	2015	1990	2000	2015	1990	2000	2015	1990	2000	2015
Saudi Arabia	20.71 (15.49-27.21)	17.44 (14.80-20.48)	15.35 (13.00-18.19)	87.25 (83.55-90.01)	93.59 (91.74-95.10)	97.48 (96.45-98.26)	46.45 (37.68-57.50)	23.89 (18.19-31.50)	11.53 (8.29-16.27)	21.20 (18.07-24.87)	13.00 (9.96-16.50)	6.12 (4.35-9.21)
Sudan	477.52 (313.88-636.92)	403.28 (244.08-581.83)	288.64 (153.70-439.17)	70.24 (66.48-73.88)	79.66 (74.48-84.40)	91.22 (88.14-93.88)	134.55 (125.77-143.98)	98.08 (89.69-106.70)	56.00 (43.27-73.73)	41.96 (37.24-46.49)	34.20 (30.63-37.81)	24.07 (19.90-29.36)
Syria	123.30 (92.13-159.06)	72.40 (54.27-95.19)	53.11 (40.45-69.71)	77.06 (70.43-82.67)	89.89 (87.19-92.13)	97.11 (96.12-97.86)	42.48 (38.91-46.26)	21.02 (19.59-22.54)	22.42 (16.20-29.32)	17.06 (15.71-18.52)	10.14 (9.20-11.05)	7.14 (5.99-8.51)
Tunisia	83.93 (65.37-105.88)	58.42 (43.45-74.68)	39.84 (26.00-56.03)	76.00 (73.15-78.90)	92.53 (90.81-93.99)	98.79 (98.39-99.11)	49.34 (46.62-52.27)	27.02 (24.69-29.55)	13.81 (11.52-16.53)	21.47 (20.00-22.93)	14.23 (13.00-15.55)	7.40 (5.94-9.23)
Turkey	102.29 (74.04-140.02)	47.77 (36.91-61.49)	15.57 (12.26-19.52)	75.34 (72.87-77.71)	83.16 (81.57-84.74)	94.23 (92.41-95.71)	70.50 (65.26-76.03)	38.38 (34.37-42.71)	16.56 (12.26-22.17)	29.00 (27.08-30.87)	19.14 (17.58-20.85)	9.34 (6.53-12.71)
United Arab Emirates	31.71 (19.82-48.73)	23.95 (16.86-32.41)	17.98 (10.70-28.26)	96.74 (95.41-97.76)	98.95 (98.51-99.29)	99.59 (99.43-99.71)	24.30 (14.15-38.36)	12.31 (7.19-18.90)	5.49 (3.16-9.08)	13.02 (7.37-19.24)	6.64 (3.77-10.90)	2.93 (1.52-4.96)
Yemen	395.16 (203.79-599.17)	393.49 (210.03-655.33)	304.71 (162.88-580.77)	36.74 (34.59-38.97)	32.96 (29.81-35.84)	52.32 (44.95-59.34)	128.19 (123.42-133.21)	84.75 (81.08-88.74)	53.58 (45.87-63.24)	41.76 (38.81-44.31)	31.24 (29.23-32.99)	20.94 (19.05-23.16)
Bangladesh	581.91 (486.04-678.35)	457.67 (361.96-552.23)	245.13 (178.88-326.13)	7.56 (5.14-10.72)	17.12 (14.33-20.37)	40.65 (27.96-55.21)	137.08 (134.10-140.07)	83.82 (81.42-86.27)	39.03 (36.05-42.67)	59.01 (54.40-62.95)	41.17 (38.45-43.54)	23.64 (21.76-25.60)
Bhutan	491.34 (313.08-676.13)	346.95 (230.83-487.05)	229.94 (137.86-353.81)	23.46 (9.64-44.09)	35.53 (18.70-54.17)	60.22 (41.81-77.08)	122.22 (113.22-132.26)	82.27 (75.33-89.67)	44.07 (37.99-51.81)	54.40 (48.56-59.83)	40.07 (35.98-43.91)	25.66 (22.62-29.37)
India	484.42 (443.30-529.53)	419.29 (384.48-460.06)	248.76 (215.02-301.28)	32.97 (22.94-44.80)	45.34 (38.52-52.82)	76.74 (63.63-87.36)	115.47 (112.23-118.75)	83.76 (80.31-87.17)	48.90 (42.60-56.92)	51.92 (48.00-55.21)	41.92 (38.92-44.51)	29.06 (25.84-32.76)

Location	Appendix Table 1b. Unscaled values for each SDG health-related indicator by country for 1990, 2000, and 2015											
	Indicator 3.1.1: Maternal mortality ratio (maternal deaths per 100,000 live births)			Indicator 3.1.2: Proportion of births attended by skilled health personnel (doctors, nurses, midwives, or country-specific medical staff [e.g., clinical officers])			Indicator 3.2.1: Under-5 mortality rate (probability of dying before the age of 5 per 1,000 live births)			Indicator 3.2.2: Neonatal mortality rate (probability of dying during the first 28 days of life per 1,000 live births)		
	1990	2000	2015	1990	2000	2015	1990	2000	2015	1990	2000	2015
Nepal	530.58 (367.51-688.94)	347.16 (253.98-443.44)	323.09 (203.94-474.38)	8.72 (5.03-14.30)	20.22 (16.63-24.17)	50.70 (37.11-63.41)	136.10 (132.33-139.85)	80.55 (77.67-83.47)	36.93 (33.56-40.77)	56.65 (52.20-60.15)	38.52 (35.95-40.62)	21.56 (19.63-23.45)
Pakistan	390.26 (299.96-487.32)	497.46 (401.05-602.13)	348.16 (256.40-445.68)	18.59 (16.38-20.93)	34.87 (31.24-38.68)	63.61 (55.39-71.27)	110.58 (107.28-113.94)	90.41 (87.43-93.53)	63.00 (57.35-69.35)	56.43 (52.90-59.66)	49.05 (46.22-51.70)	37.86 (34.77-41.33)
Botswana	155.24 (37.83-459.26)	404.97 (72.03-1,709.50)	211.47 (50.20-951.35)	81.35 (77.55-84.78)	86.36 (79.90-91.51)	91.10 (85.57-94.92)	53.77 (49.44-58.45)	63.68 (56.23-72.70)	28.18 (22.11-36.57)	21.11 (19.22-22.95)	20.94 (18.70-23.27)	14.17 (11.40-17.40)
Lesotho	198.98 (101.48-310.03)	298.57 (186.47-495.24)	495.11 (198.94-1,247.69)	48.55 (39.04-58.62)	55.55 (52.58-58.57)	68.81 (62.29-74.89)	91.58 (86.94-96.68)	106.34 (101.16-111.92)	83.67 (70.34-99.44)	40.00 (37.55-42.37)	40.02 (37.47-42.33)	37.48 (32.64-42.98)
Namibia	189.94 (127.17-259.64)	315.60 (182.34-471.49)	101.98 (46.93-240.55)	70.46 (67.63-73.26)	79.79 (77.62-81.63)	86.70 (81.68-90.75)	71.65 (67.01-76.28)	63.71 (59.28-68.93)	40.52 (31.39-51.56)	28.35 (25.64-30.73)	25.07 (22.76-27.28)	20.43 (16.52-25.00)
South Africa	153.79 (129.93-179.75)	151.40 (122.81-178.60)	157.56 (125.41-201.77)	87.77 (83.93-91.35)	91.47 (89.29-93.11)	94.64 (91.80-96.77)	79.59 (72.11-87.32)	73.19 (66.36-79.42)	42.20 (35.83-49.68)	28.10 (25.71-30.46)	24.34 (22.28-26.11)	18.17 (15.74-20.83)
Swaziland	124.74 (65.47-213.01)	140.32 (86.43-254.14)	194.31 (57.41-570.95)	75.93 (67.77-82.79)	77.56 (74.59-80.40)	83.82 (78.60-88.08)	70.39 (66.22-75.13)	93.58 (88.36-99.32)	55.04 (45.04-66.61)	22.09 (20.55-23.54)	22.54 (20.87-24.02)	19.86 (17.26-22.67)
Zimbabwe	211.56 (156.82-274.12)	399.34 (276.09-658.61)	303.94 (136.75-665.64)	72.41 (69.65-75.18)	75.36 (73.29-77.29)	68.75 (62.29-74.76)	65.62 (63.23-67.96)	77.87 (75.03-80.97)	54.81 (48.09-63.31)	23.58 (21.61-25.24)	23.41 (21.37-25.15)	23.40 (20.90-26.09)
Benin	522.02 (362.03-715.76)	475.69 (306.05-635.22)	429.41 (153.81-1,027.42)	72.40 (65.76-78.46)	80.71 (78.09-83.04)	88.84 (85.72-91.55)	180.24 (175.05-185.80)	140.01 (135.08-145.22)	77.88 (69.51-88.30)	44.91 (39.41-50.50)	37.54 (33.08-41.98)	26.59 (23.42-29.79)
Burkina Faso	391.43 (288.61-492.92)	429.89 (282.79-556.48)	351.12 (130.78-697.24)	41.59 (35.92-47.60)	46.72 (43.20-50.44)	69.99 (62.66-77.03)	207.73 (202.61-213.20)	177.59 (171.86-183.32)	110.38 (100.10-123.03)	45.53 (39.40-51.64)	40.29 (35.11-45.51)	29.79 (25.65-33.98)

Location	Indicator 3.1.1: Maternal mortality ratio (maternal deaths per 100,000 live births)			Indicator 3.1.2: Proportion of births attended by skilled health personnel (doctors, nurses, midwives, or country-specific medical staff [e.g., clinical officers])			Indicator 3.2.1: Under-5 mortality rate (probability of dying before the age of 5 per 1,000 live births)			Indicator 3.2.2: Neonatal mortality rate (probability of dying during the first 28 days of life per 1,000 live births)		
	**1990**	**2000**	**2015**	**1990**	**2000**	**2015**	**1990**	**2000**	**2015**	**1990**	**2000**	**2015**
Cameroon	447.09 (334.62-549.16)	554.81 (351.61-737.12)	486.41 (187.78-1,075.86)	64.48 (61.82-67.28)	60.87 (58.70-62.95)	68.85 (63.29-74.02)	136.78 (132.33-141.33)	137.49 (132.05-143.00)	89.76 (77.87-103.40)	37.72 (33.11-42.06)	39.00 (34.34-43.34)	31.64 (27.18-35.92)
Cape Verde	93.73 (73.70-116.16)	71.65 (48.97-116.04)	51.36 (38.38-71.79)	51.13 (39.98-61.62)	59.72 (48.72-69.85)	71.75 (61.24-80.78)	57.04 (54.42-59.64)	44.86 (39.93-50.23)	28.16 (25.31-31.32)	23.18 (21.64-24.56)	20.08 (18.24-22.03)	14.76 (13.23-16.26)
Chad	473.48 (317.18-626.71)	599.21 (351.83-798.27)	542.97 (170.45-1,363.01)	16.97 (12.87-21.97)	22.07 (19.16-25.15)	26.34 (19.69-34.92)	201.29 (194.65-207.57)	183.24 (175.62-191.24)	130.50 (115.86-148.82)	49.06 (43.29-54.86)	45.16 (39.94-50.50)	36.19 (31.37-41.64)
Cote d'Ivoire	476.94 (365.24-586.45)	637.94 (440.19-905.76)	477.04 (170.88-940.41)	50.41 (44.33-56.26)	64.47 (61.25-67.85)	64.27 (57.81-70.53)	148.88 (144.20-153.65)	140.78 (134.98-146.61)	93.49 (83.69-105.67)	48.57 (43.85-52.71)	46.55 (42.13-50.54)	36.44 (32.49-40.61)
The Gambia	524.48 (165.23-1,129.00)	457.58 (298.09-613.98)	353.40 (182.91-646.79)	54.59 (43.99-64.60)	57.27 (50.26-63.98)	61.57 (50.80-70.60)	112.71 (107.01-118.98)	85.34 (81.09-89.56)	45.39 (40.89-50.31)	36.09 (31.28-40.81)	30.55 (26.78-34.12)	20.98 (18.46-23.42)
Ghana	476.42 (269.74-778.15)	488.75 (323.78-672.22)	295.73 (119.16-700.15)	43.44 (40.08-46.72)	51.43 (48.95-54.05)	67.22 (61.05-73.39)	122.71 (119.50-126.55)	98.36 (95.08-101.70)	55.52 (49.76-62.70)	40.37 (35.53-44.89)	35.19 (31.32-38.84)	25.26 (22.44-28.11)
Guinea	711.46 (510.28-887.12)	688.14 (507.71-835.44)	603.27 (242.76-1,054.85)	37.62 (30.86-44.90)	39.02 (36.08-41.84)	52.49 (46.25-58.74)	229.11 (222.30-236.52)	175.08 (168.89-181.33)	108.75 (97.10-121.41)	60.32 (52.19-68.16)	48.35 (42.01-54.75)	35.21 (30.58-40.06)
Guinea-Bissau	507.57 (114.02-1,394.05)	551.77 (115.88-1,592.02)	594.08 (126.92-2,189.36)	39.68 (29.99-51.19)	41.03 (34.76-47.79)	42.65 (32.51-52.86)	208.36 (196.31-220.06)	179.32 (166.64-192.13)	123.91 (108.07-143.84)	52.58 (44.94-60.48)	46.82 (39.77-53.55)	36.79 (31.11-42.87)
Liberia	750.61 (413.09-1,093.48)	661.37 (421.70-869.68)	606.04 (264.16-1,120.21)	47.97 (37.40-59.38)	42.70 (36.30-49.39)	65.29 (59.55-70.63)	225.87 (216.95-235.55)	167.18 (161.92-172.69)	71.39 (64.64-79.71)	56.69 (50.80-61.96)	45.12 (40.76-49.06)	25.82 (23.53-28.40)
Mali	592.42 (472.43-708.26)	517.46 (383.24-622.88)	346.03 (136.44-605.30)	28.74 (24.38-33.34)	43.25 (39.59-47.15)	59.76 (52.53-66.51)	228.96 (222.75-235.14)	192.60 (186.09-199.06)	130.20 (115.37-148.87)	61.84 (53.32-70.08)	53.54 (46.17-60.19)	40.60 (34.58-47.11)

Appendix Table 1b. Unscaled values for each SDG health-related indicator by country for 1990, 2000, and 2015

Appendix Table 1b. Unscaled values for each SDG health-related indicator by country for 1990, 2000, and 2015

Location	Indicator 3.1.1: Maternal mortality ratio (maternal deaths per 100,000 live births)			Indicator 3.1.2: Proportion of births attended by skilled health personnel (doctors, nurses, midwives, or country-specific medical staff [e.g., clinical officers])			Indicator 3.2.1: Under-5 mortality rate (probability of dying before the age of 5 per 1,000 live births)			Indicator 3.2.2: Neonatal mortality rate (probability of dying during the first 28 days of life per 1,000 live births)		
	1990	2000	2015	1990	2000	2015	1990	2000	2015	1990	2000	2015
Mauritania	776.74 (612.51-981.56)	596.72 (304.94-774.90)	427.38 (148.82-823.94)	54.99 (46.86-62.11)	57.51 (54.95-59.87)	66.39 (58.06-74.32)	108.80 (104.36-113.10)	92.52 (88.28-96.67)	55.04 (49.28-62.23)	40.56 (35.98-44.82)	36.82 (32.70-40.45)	26.53 (23.63-29.87)
Niger	432.95 (298.75-596.72)	464.88 (324.24-621.37)	369.84 (163.70-638.14)	17.28 (14.49-20.60)	20.85 (18.15-24.02)	36.13 (29.70-43.11)	296.08 (288.57-303.94)	223.68 (216.23-231.35)	112.77 (101.27-127.06)	50.66 (42.08-58.16)	39.07 (32.36-45.01)	24.08 (20.11-27.87)
Nigeria	469.79 (222.92-797.34)	470.65 (305.83-638.70)	282.81 (158.35-601.36)	37.48 (32.83-42.20)	41.62 (37.58-45.56)	46.62 (41.28-52.18)	207.88 (201.67-214.16)	183.06 (177.12-189.01)	103.84 (92.56-116.27)	51.77 (44.54-58.58)	48.35 (41.81-54.30)	34.59 (30.11-39.16)
Sao Tome and Principe	229.00 (155.90-317.60)	267.68 (204.71-348.74)	164.08 (64.96-368.33)	80.15 (71.96-86.35)	82.61 (78.51-86.44)	86.29 (81.42-90.18)	106.29 (101.59-110.96)	76.75 (71.54-82.20)	38.29 (33.27-44.15)	31.87 (27.98-35.53)	26.70 (23.79-29.58)	17.57 (15.29-19.81)
Senegal	465.29 (332.09-579.71)	482.77 (326.29-603.27)	453.17 (165.08-1,036.30)	47.32 (43.39-51.67)	67.69 (64.29-70.82)	76.71 (71.70-81.05)	144.23 (140.95-147.83)	117.63 (114.35-121.06)	52.25 (48.23-57.16)	39.57 (34.14-44.64)	34.74 (30.14-39.00)	21.00 (18.64-23.35)
Sierra Leone	366.13 (163.04-710.15)	570.81 (410.27-735.63)	693.46 (320.36-1,230.44)	44.28 (33.61-54.32)	43.96 (38.29-49.47)	63.55 (57.20-69.64)	245.64 (238.57-253.63)	210.36 (204.52-216.51)	122.40 (113.03-132.22)	58.94 (52.22-65.23)	52.37 (46.57-57.74)	35.78 (31.99-39.48)
Togo	442.92 (292.98-567.51)	473.22 (278.53-643.76)	298.43 (124.19-564.73)	46.69 (42.59-50.82)	55.57 (52.06-59.14)	69.08 (61.80-75.09)	144.53 (139.63-149.59)	123.00 (117.62-128.38)	78.04 (71.17-86.48)	41.81 (36.36-47.08)	37.62 (32.73-42.29)	29.74 (26.30-33.20)
Burundi	1,108.35 (499.85-2,082.98)	1,026.59 (698.42-1,420.44)	387.16 (156.21-786.10)	23.07 (16.17-30.98)	41.46 (32.23-50.88)	65.56 (52.08-77.51)	173.41 (161.55-184.97)	165.89 (153.86-178.38)	76.81 (57.35-104.34)	41.82 (36.18-47.21)	41.00 (35.41-46.55)	25.57 (20.60-31.89)
Comoros	578.77 (229.74-1,075.32)	467.54 (347.87-606.91)	212.77 (99.92-404.93)	54.36 (41.58-66.38)	71.12 (65.36-76.56)	85.22 (78.60-90.26)	120.20 (112.10-128.60)	85.41 (71.38-99.61)	44.02 (30.70-61.97)	44.06 (39.91-47.97)	34.97 (30.18-39.95)	22.39 (16.82-29.22)
Djibouti	372.66 (151.14-685.76)	517.32 (188.92-1,228.11)	481.89 (190.90-1,350.21)	76.80 (62.82-88.13)	81.09 (72.56-87.60)	90.56 (83.72-94.91)	112.76 (105.32-120.10)	102.24 (93.83-111.13)	65.50 (55.36-78.65)	31.52 (27.40-35.20)	29.95 (26.34-33.27)	23.42 (20.34-26.98)

Appendix Table 1b. Unscaled values for each SDG health-related indicator by country for 1990, 2000, and 2015

Location	Indicator 3.1.1: Maternal mortality ratio (maternal deaths per 100,000 live births)			Indicator 3.1.2: Proportion of births attended by skilled health personnel (doctors, nurses, midwives, or country-specific medical staff [e.g., clinical officers])			Indicator 3.2.1: Under-5 mortality rate (probability of dying before the age of 5 per 1,000 live births)			Indicator 3.2.2: Neonatal mortality rate (probability of dying during the first 28 days of life per 1,000 live births)		
	1990	2000	2015	1990	2000	2015	1990	2000	2015	1990	2000	2015
Eritrea	808.46 (601.74-1,093.37)	638.55 (256.60-1,217.17)	747.13 (195.92-1,852.86)	18.37 (14.08-23.85)	29.21 (26.60-32.00)	32.89 (19.17-49.61)	147.45 (140.33-154.62)	133.99 (91.65-192.79)	67.15 (54.57-83.95)	34.70 (29.97-39.24)	30.48 (24.12-38.27)	22.13 (18.82-26.31)
Ethiopia	788.57 (594.63-1,036.76)	741.94 (555.85-956.47)	404.06 (171.42-877.12)	10.36 (5.49-16.80)	12.69 (9.39-16.52)	20.64 (12.46-30.55)	202.24 (195.85-208.88)	143.12 (138.13-147.83)	60.27 (53.72-68.47)	58.83 (51.78-65.01)	44.32 (39.46-48.76)	25.44 (22.52-28.47)
Kenya	366.74 (290.40-460.77)	501.17 (402.23-629.27)	334.77 (258.32-425.68)	51.30 (46.36-56.10)	51.46 (47.55-55.59)	54.66 (39.61-68.79)	92.29 (88.88-95.40)	88.56 (84.78-92.67)	50.83 (45.37-57.64)	28.53 (26.01-30.76)	27.05 (24.88-29.21)	21.10 (19.16-23.16)
Madagascar	375.41 (301.18-445.77)	422.44 (151.96-572.71)	329.61 (110.22-713.58)	57.79 (54.41-61.46)	46.73 (43.86-49.70)	47.34 (35.58-59.61)	143.12 (135.08-151.04)	107.08 (99.82-115.23)	64.20 (51.93-80.64)	36.60 (32.62-40.51)	30.63 (27.50-33.65)	22.94 (19.43-27.00)
Malawi	504.25 (369.34-631.48)	583.07 (390.05-895.51)	216.92 (88.56-435.97)	58.37 (51.68-65.22)	63.85 (59.75-67.90)	77.51 (68.03-85.71)	224.22 (214.23-234.60)	166.84 (158.71-175.22)	83.35 (70.24-98.43)	51.61 (45.15-57.50)	37.97 (33.23-42.37)	26.86 (23.16-31.10)
Mozambique	345.31 (233.23-472.72)	342.86 (195.78-455.85)	323.90 (116.19-793.30)	45.36 (35.77-55.44)	49.48 (47.65-51.26)	63.58 (56.11-70.60)	214.22 (208.68-220.13)	157.46 (152.70-162.31)	77.93 (70.40-87.26)	53.91 (48.38-58.65)	40.36 (36.41-43.71)	25.45 (23.10-27.87)
Rwanda	660.80 (485.77-913.96)	762.95 (591.68-999.52)	367.42 (159.05-786.24)	31.92 (24.05-39.97)	36.85 (30.65-43.30)	78.49 (67.82-86.72)	154.43 (146.27-162.85)	158.50 (149.80-167.12)	65.64 (53.52-81.81)	40.59 (35.37-45.26)	42.27 (37.28-47.19)	24.85 (21.01-29.38)
Somalia	822.90 (136.56-2,183.07)	803.78 (133.79-2,059.85)	723.71 (137.01-2,210.18)	34.36 (19.36-52.85)	32.76 (21.97-45.59)	36.46 (22.44-52.60)	187.46 (177.06-198.24)	159.00 (149.81-167.74)	112.16 (97.48-130.39)	44.92 (39.54-50.01)	39.68 (35.09-43.82)	31.28 (27.23-35.85)
South Sudan	582.57 (113.48-1,559.78)	568.57 (109.80-1,721.82)	637.07 (131.31-2,333.01)	40.46 (23.16-60.32)	39.65 (26.25-55.50)	45.04 (32.54-57.02)	200.11 (187.40-213.31)	140.45 (132.41-149.48)	107.03 (89.37-127.29)	45.56 (40.58-50.73)	34.06 (30.28-37.50)	28.45 (24.17-33.21)
Tanzania	537.15 (409.49-647.92)	491.64 (340.95-666.20)	332.81 (127.50-831.34)	56.66 (53.84-59.28)	47.88 (45.16-50.77)	58.83 (48.10-68.90)	155.17 (151.18-159.19)	121.48 (117.34-125.64)	60.08 (52.89-69.16)	39.06 (35.29-42.51)	32.35 (29.71-34.97)	22.07 (19.87-24.64)

Appendix Table 1b. Unscaled values for each SDG health-related indicator by country for 1990, 2000, and 2015

Location	Indicator 3.1.1: Maternal mortality ratio (maternal deaths per 100,000 live births)			Indicator 3.1.2: Proportion of births attended by skilled health personnel (doctors, nurses, midwives, or country-specific medical staff [e.g., clinical officers])			Indicator 3.2.1: Under-5 mortality rate (probability of dying before the age of 5 per 1,000 live births)			Indicator 3.2.2: Neonatal mortality rate (probability of dying during the first 28 days of life per 1,000 live births)		
	1990	2000	2015	1990	2000	2015	1990	2000	2015	1990	2000	2015
Uganda	336.77 (236.44-440.11)	407.05 (295.59-552.81)	278.43 (103.70-605.71)	40.79 (37.42-44.45)	43.93 (40.68-47.01)	70.14 (61.98-77.40)	167.93 (163.67-172.44)	130.97 (126.88-135.15)	69.35 (62.82-77.42)	38.70 (34.56-42.78)	33.38 (29.97-36.73)	24.13 (21.82-26.64)
Zambia	351.36 (264.25-441.55)	420.60 (304.66-597.08)	244.38 (100.49-494.97)	53.34 (48.31-57.85)	51.84 (48.60-55.34)	75.11 (68.49-81.08)	168.91 (161.09-177.34)	147.30 (137.62-156.69)	65.93 (55.36-78.00)	36.71 (32.08-40.86)	30.95 (26.94-34.78)	21.06 (18.28-24.06)
Angola	685.33 (131.53-1,740.35)	645.90 (114.88-1,857.76)	513.72 (109.68-1,821.01)	40.96 (33.30-48.84)	47.49 (44.13-50.85)	74.35 (67.78-80.19)	190.46 (172.22-210.74)	155.77 (141.10-172.43)	78.70 (60.29-100.90)	40.62 (33.80-47.11)	35.53 (30.23-41.02)	23.49 (19.28-28.33)
Central African Republic	893.39 (671.18-1,197.77)	1,024.75 (314.75-2,240.09)	1,073.91 (213.07-2,860.66)	51.61 (46.72-56.35)	50.15 (46.38-54.21)	63.11 (56.84-69.57)	178.14 (164.66-192.88)	164.93 (147.17-184.63)	126.78 (98.04-165.85)	48.64 (42.40-54.94)	44.86 (38.57-50.83)	40.19 (32.17-49.92)
Congo	436.28 (255.26-710.86)	637.95 (416.56-926.27)	484.03 (181.14-1,021.00)	91.99 (88.54-94.62)	92.57 (90.47-94.30)	95.67 (94.29-96.82)	93.35 (87.29-99.37)	109.38 (101.70-118.22)	56.13 (44.35-70.14)	25.70 (22.00-29.13)	29.79 (25.61-33.79)	21.23 (17.52-25.16)
Democratic Republic of the Congo	370.56 (175.89-571.08)	318.44 (223.52-413.09)	467.14 (208.33-824.69)	67.84 (58.95-75.71)	70.30 (65.71-74.22)	87.34 (84.82-89.54)	166.62 (154.87-178.44)	151.83 (140.18-163.69)	88.04 (70.19-108.92)	38.25 (33.10-43.29)	36.73 (31.97-41.55)	27.30 (22.82-32.32)
Equatorial Guinea	913.13 (164.80-2,389.19)	676.43 (125.46-2,101.88)	408.99 (106.68-1,516.66)	41.73 (31.86-52.19)	69.28 (61.92-76.71)	92.31 (89.02-94.88)	170.77 (154.18-188.52)	159.21 (140.47-179.60)	87.51 (65.48-118.55)	44.05 (37.21-50.79)	42.79 (36.41-49.05)	30.05 (24.16-37.70)
Gabon	309.11 (225.96-421.12)	408.00 (272.42-586.01)	191.12 (83.59-382.33)	85.87 (81.85-89.27)	88.97 (87.53-90.34)	94.62 (93.33-95.71)	83.27 (79.32-87.80)	71.39 (67.71-75.29)	46.11 (40.23-52.69)	29.82 (26.55-32.58)	27.50 (24.86-29.86)	21.66 (19.24-24.12)

Appendix Table 1b. Unscaled values for each SDG health-related indicator by country for 1990, 2000, and 2015

Location	Indicator 3.3.1: Age-standardised rate of new HIV infections (per 1,000 population)			Indicator 3.3.2: Age-standardised rate of new and relapsed TB cases (per 1,000 population)			Indicator 3.3.3: Age-standaridsed rate of malaria cases (per 1,000 population)			Indicator 3.3.4: Age-standardised rate of hepatitis B incident cases (per 100,000 population)		
	1990	2000	2015	1990	2000	2015	1990	2000	2015	1990	2000	2015
Canada	0.12 (0.07-0.19)	0.05 (0.03-0.07)	0.03 (0.01-0.08)	0.09 (0.08-0.11)	0.08 (0.07-0.09)	0.06 (0.05-0.07)	0.00 (0.00-0.00)	0.00 (0.00-0.00)	0.00 (0.00-0.00)	1,077.66 (968.30-1,211.90)	1,078.52 (967.68-1,202.74)	1,096.81 (975.22-1,224.15)
United States	0.22 (0.19-0.26)	0.10 (0.09-0.12)	0.07 (0.06-0.09)	0.13 (0.11-0.16)	0.06 (0.05-0.06)	0.04 (0.03-0.05)	0.00 (0.00-0.00)	0.00 (0.00-0.00)	0.00 (0.00-0.00)	732.52 (662.21-825.87)	727.59 (655.63-812.78)	738.33 (658.42-830.70)
Australia	0.05 (0.03-0.09)	0.03 (0.01-0.05)	0.02 (0.01-0.03)	0.10 (0.08-0.12)	0.07 (0.06-0.08)	0.08 (0.06-0.09)	0.00 (0.00-0.00)	0.00 (0.00-0.00)	0.00 (0.00-0.00)	1,155.69 (1,038.21-1,294.86)	1,149.04 (1,029.47-1,283.51)	1,160.83 (1,030.25-1,302.52)
New Zealand	0.03 (0.02-0.04)	0.03 (0.01-0.05)	0.02 (0.01-0.03)	0.15 (0.13-0.18)	0.10 (0.09-0.12)	0.08 (0.07-0.10)	0.00 (0.00-0.00)	0.00 (0.00-0.00)	0.00 (0.00-0.00)	1,304.59 (1,164.31-1,467.53)	1,301.79 (1,168.50-1,459.18)	1,302.46 (1,149.22-1,466.87)
Brunei	0.04 (0.01-0.09)	0.05 (0.02-0.16)	0.03 (0.01-0.06)	1.32 (1.21-1.44)	0.99 (0.90-1.08)	0.77 (0.69-0.86)	0.00 (0.00-0.00)	0.00 (0.00-0.00)	0.00 (0.00-0.00)	1,480.49 (1,270.05-1,730.82)	1,470.78 (1,259.36-1,710.18)	1,482.52 (1,261.25-1,698.31)
Japan	0.01 (0.01-0.01)	0.01 (0.00-0.01)	0.01 (0.00-0.01)	0.34 (0.29-0.39)	0.24 (0.20-0.28)	0.16 (0.14-0.17)	0.00 (0.00-0.00)	0.00 (0.00-0.00)	0.00 (0.00-0.00)	1,339.04 (1,199.81-1,502.38)	1,341.39 (1,202.60-1,505.75)	1,350.78 (1,198.81-1,519.61)
Singapore	0.07 (0.05-0.12)	0.06 (0.05-0.07)	0.03 (0.02-0.04)	0.81 (0.74-0.87)	0.61 (0.56-0.65)	0.48 (0.42-0.53)	0.00 (0.00-0.00)	0.00 (0.00-0.00)	0.00 (0.00-0.00)	1,343.22 (1,198.24-1,511.17)	1,339.62 (1,196.66-1,505.74)	1,341.00 (1,198.99-1,507.58)
South Korea	0.03 (0.03-0.04)	0.03 (0.02-0.07)	0.01 (0.01-0.02)	2.24 (2.05-2.39)	0.94 (0.87-1.01)	0.72 (0.66-0.77)	0.24 (0.04-0.44)	0.09 (0.09-0.10)	0.01 (0.01-0.01)	1,750.01 (1,559.45-1,975.59)	1,751.78 (1,562.85-1,975.40)	1,738.45 (1,534.77-1,952.09)
Andorra	0.34 (0.02-2.48)	0.07 (0.02-0.29)	0.05 (0.01-0.18)	0.19 (0.15-0.25)	0.20 (0.15-0.25)	0.20 (0.15-0.27)	0.00 (0.00-0.00)	0.00 (0.00-0.00)	0.00 (0.00-0.00)	833.66 (676.22-1,021.09)	833.94 (666.58-1,032.47)	838.71 (673.91-1,035.86)
Austria	0.08 (0.01-0.20)	0.06 (0.02-0.15)	0.04 (0.01-0.08)	0.24 (0.21-0.28)	0.15 (0.13-0.16)	0.10 (0.08-0.12)	0.00 (0.00-0.00)	0.00 (0.00-0.00)	0.00 (0.00-0.00)	832.46 (674.50-1,020.35)	833.18 (666.13-1,030.91)	837.67 (673.20-1,035.27)

Location	Appendix Table 1b. Unscaled values for each SDG health-related indicator by country for 1990, 2000, and 2015											
	Indicator 3.3.1: Age-standardised rate of new HIV infections (per 1,000 population)			Indicator 3.3.2: Age-standardised rate of new and relapsed TB cases (per 1,000 population)			Indicator 3.3.3: Age-standaridsed rate of malaria cases (per 1,000 population)			Indicator 3.3.4: Age-standardised rate of hepatitis B incident cases (per 100,000 population)		
	1990	2000	2015	1990	2000	2015	1990	2000	2015	1990	2000	2015
Belgium	0.08 (0.04-0.16)	0.05 (0.02-0.10)	0.02 (0.01-0.05)	0.19 (0.16-0.22)	0.14 (0.12-0.15)	0.11 (0.09-0.12)	0.00 (0.00-0.00)	0.00 (0.00-0.00)	0.00 (0.00-0.00)	783.13 (696.63-886.96)	780.45 (700.20-875.24)	786.18 (700.22-885.23)
Cyprus	0.02 (0.01-0.04)	0.02 (0.01-0.06)	0.02 (0.00-0.04)	0.08 (0.06-0.11)	0.08 (0.06-0.10)	0.08 (0.07-0.11)	0.00 (0.00-0.00)	0.00 (0.00-0.00)	0.00 (0.00-0.00)	825.03 (737.17-926.45)	826.82 (739.93-924.85)	827.66 (734.65-924.81)
Denmark	0.10 (0.05-0.15)	0.07 (0.03-0.12)	0.03 (0.01-0.06)	0.14 (0.11-0.16)	0.12 (0.10-0.14)	0.08 (0.07-0.10)	0.00 (0.00-0.00)	0.00 (0.00-0.00)	0.00 (0.00-0.00)	796.09 (715.20-896.46)	793.55 (710.97-889.51)	802.29 (712.42-897.47)
Finland	0.01 (0.01-0.02)	0.01 (0.01-0.03)	0.01 (0.00-0.02)	0.21 (0.19-0.25)	0.14 (0.12-0.16)	0.09 (0.08-0.11)	0.00 (0.00-0.00)	0.00 (0.00-0.00)	0.00 (0.00-0.00)	833.69 (675.41-1,022.36)	834.52 (666.90-1,033.25)	840.08 (674.59-1,038.69)
France	0.21 (0.16-0.28)	0.07 (0.05-0.12)	0.02 (0.01-0.04)	0.60 (0.53-0.68)	0.22 (0.20-0.25)	0.12 (0.10-0.13)	0.00 (0.00-0.00)	0.00 (0.00-0.00)	0.00 (0.00-0.00)	784.77 (700.90-884.54)	781.78 (701.98-867.03)	785.62 (695.68-882.94)
Germany	0.04 (0.03-0.07)	0.02 (0.01-0.04)	0.02 (0.01-0.05)	0.21 (0.18-0.25)	0.12 (0.10-0.14)	0.08 (0.06-0.10)	0.00 (0.00-0.00)	0.00 (0.00-0.00)	0.00 (0.00-0.00)	744.55 (666.30-840.41)	740.95 (664.54-827.52)	750.43 (668.69-843.19)
Greece	0.03 (0.02-0.03)	0.01 (0.01-0.01)	0.01 (0.01-0.01)	0.19 (0.16-0.22)	0.10 (0.09-0.11)	0.09 (0.08-0.11)	0.00 (0.00-0.00)	0.00 (0.00-0.00)	0.00 (0.00-0.00)	945.86 (844.46-1,063.74)	946.18 (846.36-1,066.09)	944.61 (839.29-1,064.69)
Iceland	0.05 (0.02-0.09)	0.03 (0.02-0.05)	0.02 (0.01-0.05)	0.25 (0.20-0.29)	0.27 (0.23-0.32)	0.22 (0.17-0.27)	0.00 (0.00-0.00)	0.00 (0.00-0.00)	0.00 (0.00-0.00)	836.16 (678.38-1,024.31)	835.33 (668.34-1,033.49)	836.95 (673.02-1,033.03)
Ireland	0.08 (0.05-0.13)	0.03 (0.02-0.06)	0.02 (0.01-0.04)	0.24 (0.21-0.27)	0.15 (0.13-0.16)	0.11 (0.10-0.13)	0.00 (0.00-0.00)	0.00 (0.00-0.00)	0.00 (0.00-0.00)	834.35 (677.48-1,020.86)	834.12 (667.64-1,033.24)	834.63 (670.65-1,029.55)
Israel	0.05 (0.03-0.10)	0.03 (0.02-0.07)	0.03 (0.01-0.05)	0.15 (0.12-0.18)	0.12 (0.10-0.13)	0.10 (0.08-0.12)	0.00 (0.00-0.00)	0.00 (0.00-0.00)	0.00 (0.00-0.00)	834.13 (677.13-1,019.75)	831.98 (666.30-1,028.16)	837.65 (673.22-1,033.90)

Appendix Table 1b. Unscaled values for each SDG health-related indicator by country for 1990, 2000, and 2015

Location	Indicator 3.3.1: Age-standardised rate of new HIV infections (per 1,000 population)			Indicator 3.3.2: Age-standardised rate of new and relapsed TB cases (per 1,000 population)			Indicator 3.3.3: Age-standaridsed rate of malaria cases (per 1,000 population)			Indicator 3.3.4: Age-standardised rate of hepatitis B incident cases (per 100,000 population)		
	1990	2000	2015	1990	2000	2015	1990	2000	2015	1990	2000	2015
Italy	0.24 (0.08-0.58)	0.06 (0.03-0.12)	0.03 (0.01-0.07)	0.18 (0.15-0.21)	0.10 (0.09-0.12)	0.08 (0.06-0.09)	0.00 (0.00-0.00)	0.00 (0.00-0.00)	0.00 (0.00-0.00)	802.35 (719.10-904.84)	801.53 (719.29-894.76)	809.35 (722.54-908.94)
Luxembourg	0.06 (0.03-0.13)	0.03 (0.01-0.06)	0.03 (0.01-0.05)	0.16 (0.13-0.19)	0.13 (0.11-0.16)	0.14 (0.10-0.18)	0.00 (0.00-0.00)	0.00 (0.00-0.00)	0.00 (0.00-0.00)	834.85 (677.14-1,023.69)	834.64 (667.43-1,033.56)	839.67 (674.58-1,037.15)
Malta	0.05 (0.03-0.10)	0.03 (0.02-0.05)	0.03 (0.01-0.06)	0.14 (0.11-0.17)	0.17 (0.14-0.21)	0.17 (0.13-0.21)	0.00 (0.00-0.00)	0.00 (0.00-0.00)	0.00 (0.00-0.00)	832.21 (674.98-1,016.99)	834.42 (667.67-1,032.16)	841.79 (676.47-1,042.74)
Netherlands	0.14 (0.09-0.25)	0.04 (0.02-0.07)	0.02 (0.01-0.03)	0.20 (0.17-0.24)	0.12 (0.10-0.14)	0.08 (0.07-0.09)	0.00 (0.00-0.00)	0.00 (0.00-0.00)	0.00 (0.00-0.00)	796.09 (712.15-898.96)	803.01 (714.82-907.11)	804.80 (716.81-904.58)
Norway	0.06 (0.03-0.12)	0.02 (0.01-0.05)	0.01 (0.00-0.02)	0.15 (0.13-0.18)	0.14 (0.12-0.16)	0.10 (0.09-0.12)	0.00 (0.00-0.00)	0.00 (0.00-0.00)	0.00 (0.00-0.00)	836.16 (678.20-1,024.66)	835.80 (668.27-1,035.09)	841.57 (675.94-1,040.61)
Portugal	0.51 (0.25-0.94)	0.63 (0.29-1.20)	0.23 (0.08-0.48)	0.69 (0.62-0.77)	0.73 (0.66-0.80)	0.28 (0.25-0.31)	0.00 (0.00-0.00)	0.00 (0.00-0.00)	0.00 (0.00-0.00)	830.32 (674.38-1,016.20)	831.66 (665.70-1,028.00)	837.58 (672.62-1,034.63)
Spain	0.43 (0.26-0.74)	0.08 (0.06-0.13)	0.05 (0.03-0.10)	0.50 (0.44-0.56)	0.25 (0.22-0.28)	0.13 (0.11-0.15)	0.00 (0.00-0.00)	0.00 (0.00-0.00)	0.00 (0.00-0.00)	704.25 (628.02-792.24)	701.22 (626.86-791.70)	717.10 (635.91-803.25)
Sweden	0.05 (0.03-0.07)	0.02 (0.01-0.03)	0.01 (0.00-0.02)	0.14 (0.12-0.17)	0.10 (0.08-0.13)	0.10 (0.07-0.13)	0.00 (0.00-0.00)	0.00 (0.00-0.00)	0.00 (0.00-0.00)	739.34 (665.53-829.66)	736.51 (661.30-828.13)	747.89 (667.08-836.77)
Switzerland	0.15 (0.07-0.26)	0.03 (0.01-0.05)	0.03 (0.01-0.05)	0.17 (0.14-0.20)	0.10 (0.09-0.12)	0.09 (0.07-0.11)	0.00 (0.00-0.00)	0.00 (0.00-0.00)	0.00 (0.00-0.00)	802.93 (722.23-900.23)	801.33 (718.87-900.78)	810.29 (721.44-907.69)
United Kingdom	0.03 (0.03-0.04)	0.05 (0.04-0.06)	0.04 (0.03-0.05)	0.16 (0.13-0.20)	0.11 (0.09-0.12)	0.16 (0.12-0.20)	0.00 (0.00-0.00)	0.00 (0.00-0.00)	0.00 (0.00-0.00)	737.44 (665.14-828.98)	733.05 (657.98-825.40)	752.04 (671.77-842.58)

Appendix Table 1b. Unscaled values for each SDG health-related indicator by country for 1990, 2000, and 2015												
	**Indicator 3.3.1: Age-standardised rate of new HIV infections (per 1,000 population)**			**Indicator 3.3.2: Age-standardised rate of new and relapsed TB cases (per 1,000 population)**			**Indicator 3.3.3: Age-standaridsed rate of malaria cases (per 1,000 population)**			**Indicator 3.3.4: Age-standardised rate of hepatitis B incident cases (per 100,000 population)**		
**Location**	**1990**	**2000**	**2015**	**1990**	**2000**	**2015**	**1990**	**2000**	**2015**	**1990**	**2000**	**2015**
Argentina	0.34 (0.30-0.38)	0.40 (0.36-0.43)	0.32 (0.24-0.39)	0.73 (0.67-0.80)	0.68 (0.62-0.73)	0.41 (0.37-0.45)	0.05 (0.05-0.05)	0.01 (0.01-0.01)	0.00 (0.00-0.00)	1,027.38 (921.98-1,155.99)	1,026.71 (926.32-1,154.53)	1,034.10 (918.08-1,159.42)
Chile	0.17 (0.12-0.33)	0.21 (0.18-0.27)	0.12 (0.11-0.15)	0.66 (0.60-0.71)	0.48 (0.45-0.52)	0.34 (0.30-0.37)	0.00 (0.00-0.00)	0.00 (0.00-0.00)	0.00 (0.00-0.00)	1,028.88 (924.55-1,153.98)	1,028.09 (922.16-1,152.81)	1,034.14 (923.45-1,155.90)
Uruguay	0.12 (0.05-0.38)	0.20 (0.13-0.33)	0.14 (0.09-0.22)	0.41 (0.37-0.45)	0.31 (0.28-0.34)	0.23 (0.21-0.26)	0.00 (0.00-0.00)	0.00 (0.00-0.00)	0.00 (0.00-0.00)	1,028.24 (923.77-1,153.15)	1,027.58 (921.72-1,150.13)	1,033.15 (923.50-1,153.73)
Belarus	0.03 (0.01-0.06)	0.14 (0.08-0.23)	0.15 (0.09-0.24)	0.48 (0.42-0.55)	0.56 (0.51-0.62)	0.54 (0.48-0.62)	0.00 (0.00-0.00)	0.00 (0.00-0.00)	0.00 (0.00-0.00)	1,676.21 (1,478.34-1,888.68)	1,673.59 (1,475.95-1,891.33)	1,678.75 (1,476.48-1,892.24)
Estonia	0.01 (0.01-0.02)	0.11 (0.08-0.17)	0.13 (0.09-0.18)	0.73 (0.65-0.80)	0.90 (0.82-0.98)	0.48 (0.43-0.54)	0.00 (0.00-0.00)	0.00 (0.00-0.00)	0.00 (0.00-0.00)	1,676.84 (1,477.93-1,887.56)	1,672.82 (1,475.72-1,891.16)	1,679.07 (1,476.63-1,893.06)
Latvia	0.08 (0.04-0.14)	0.19 (0.15-0.28)	0.12 (0.06-0.19)	0.88 (0.79-0.95)	1.09 (1.01-1.17)	0.55 (0.50-0.61)	0.00 (0.00-0.00)	0.00 (0.00-0.00)	0.00 (0.00-0.00)	1,676.40 (1,476.57-1,885.39)	1,673.05 (1,476.00-1,890.42)	1,679.58 (1,475.93-1,895.14)
Lithuania	0.03 (0.01-0.05)	0.07 (0.05-0.10)	0.04 (0.02-0.07)	1.13 (1.01-1.23)	1.15 (1.05-1.24)	0.87 (0.79-0.96)	0.00 (0.00-0.00)	0.00 (0.00-0.00)	0.00 (0.00-0.00)	1,677.07 (1,477.85-1,888.05)	1,673.32 (1,476.28-1,890.97)	1,679.50 (1,476.84-1,893.91)
Moldova	0.07 (0.03-0.21)	0.15 (0.11-0.22)	0.14 (0.09-0.22)	0.66 (0.59-0.73)	0.89 (0.82-0.95)	0.81 (0.73-0.87)	0.00 (0.00-0.00)	0.00 (0.00-0.00)	0.00 (0.00-0.00)	1,675.50 (1,475.80-1,886.27)	1,673.08 (1,476.27-1,891.50)	1,679.71 (1,476.92-1,892.30)
Russia	0.08 (0.03-0.14)	0.31 (0.22-0.46)	0.41 (0.26-0.68)	1.30 (1.19-1.40)	1.81 (1.62-1.98)	1.30 (1.17-1.40)	0.00 (0.00-0.00)	0.00 (0.00-0.00)	0.00 (0.00-0.00)	1,676.44 (1,478.66-1,889.72)	1,673.22 (1,475.76-1,890.06)	1,677.95 (1,476.69-1,892.64)
Ukraine	0.13 (0.09-0.21)	0.62 (0.48-0.85)	0.42 (0.35-0.52)	1.05 (0.96-1.14)	1.53 (1.39-1.65)	1.36 (1.23-1.46)	0.00 (0.00-0.00)	0.00 (0.00-0.00)	0.00 (0.00-0.00)	1,675.57 (1,476.68-1,885.45)	1,672.86 (1,475.80-1,890.33)	1,678.42 (1,476.72-1,893.50)

Appendix Table 1b. Unscaled values for each SDG health-related indicator by country for 1990, 2000, and 2015												
	**Indicator 3.3.1: Age-standardised rate of new HIV infections (per 1,000 population)**			**Indicator 3.3.2: Age-standardised rate of new and relapsed TB cases (per 1,000 population)**			**Indicator 3.3.3: Age-standaridsed rate of malaria cases (per 1,000 population)**			**Indicator 3.3.4: Age-standardised rate of hepatitis B incident cases (per 100,000 population)**		
**Location**	**1990**	**2000**	**2015**	**1990**	**2000**	**2015**	**1990**	**2000**	**2015**	**1990**	**2000**	**2015**
Albania	0.00 (0.00-0.01)	0.00 (0.00-0.01)	0.00 (0.00-0.01)	0.14 (0.12-0.17)	0.13 (0.11-0.16)	0.15 (0.12-0.19)	0.00 (0.00-0.00)	0.00 (0.00-0.00)	0.00 (0.00-0.00)	1,223.74 (999.80-1,492.03)	1,221.82 (1,006.62-1,487.23)	1,226.57 (1,010.44-1,495.40)
Bosnia and Herzegovina	0.00 (0.00-0.01)	0.00 (0.00-0.01)	0.00 (0.00-0.01)	0.37 (0.34-0.41)	0.32 (0.28-0.36)	0.30 (0.26-0.36)	0.00 (0.00-0.00)	0.00 (0.00-0.00)	0.00 (0.00-0.00)	1,222.00 (999.93-1,492.97)	1,222.68 (1,002.90-1,486.12)	1,228.43 (1,011.70-1,495.26)
Bulgaria	0.01 (0.00-0.03)	0.02 (0.01-0.04)	0.02 (0.01-0.04)	0.32 (0.28-0.37)	0.44 (0.39-0.50)	0.30 (0.27-0.34)	0.00 (0.00-0.00)	0.00 (0.00-0.00)	0.00 (0.00-0.00)	1,300.47 (1,173.13-1,462.24)	1,297.19 (1,162.33-1,451.99)	1,301.60 (1,156.28-1,465.13)
Croatia	0.01 (0.01-0.02)	0.01 (0.01-0.02)	0.01 (0.00-0.01)	0.75 (0.68-0.82)	0.48 (0.44-0.53)	0.21 (0.18-0.24)	0.00 (0.00-0.00)	0.00 (0.00-0.00)	0.00 (0.00-0.00)	1,228.72 (1,100.87-1,386.50)	1,230.02 (1,102.54-1,376.16)	1,234.77 (1,094.98-1,391.76)
Czech Republic	0.01 (0.01-0.01)	0.01 (0.01-0.01)	0.01 (0.00-0.01)	0.22 (0.19-0.25)	0.15 (0.13-0.17)	0.11 (0.09-0.13)	0.00 (0.00-0.00)	0.00 (0.00-0.00)	0.00 (0.00-0.00)	1,220.94 (999.08-1,494.67)	1,221.68 (1,002.12-1,484.10)	1,228.13 (1,011.13-1,494.71)
Hungary	0.03 (0.03-0.03)	0.02 (0.02-0.02)	0.01 (0.01-0.01)	0.49 (0.44-0.53)	0.30 (0.27-0.33)	0.15 (0.13-0.18)	0.00 (0.00-0.00)	0.00 (0.00-0.00)	0.00 (0.00-0.00)	1,220.34 (998.88-1,491.46)	1,220.60 (1,000.80-1,484.19)	1,226.70 (1,009.63-1,492.97)
Macedonia	0.00 (0.00-0.00)	0.00 (0.00-0.01)	0.00 (0.00-0.01)	0.50 (0.45-0.55)	0.36 (0.33-0.39)	0.23 (0.20-0.27)	0.00 (0.00-0.00)	0.00 (0.00-0.00)	0.00 (0.00-0.00)	1,222.38 (999.70-1,489.39)	1,222.44 (1,002.82-1,487.61)	1,227.87 (1,011.50-1,494.84)
Montenegro	0.00 (0.00-0.01)	0.01 (0.00-0.01)	0.01 (0.00-0.01)	0.16 (0.13-0.19)	0.14 (0.11-0.17)	0.16 (0.13-0.20)	0.00 (0.00-0.00)	0.00 (0.00-0.00)	0.00 (0.00-0.00)	1,221.83 (999.68-1,493.88)	1,221.50 (1,001.93-1,484.70)	1,227.56 (1,010.94-1,493.73)
Poland	0.02 (0.01-0.02)	0.02 (0.01-0.02)	0.01 (0.01-0.02)	0.61 (0.56-0.67)	0.36 (0.33-0.39)	0.24 (0.21-0.26)	0.00 (0.00-0.00)	0.00 (0.00-0.00)	0.00 (0.00-0.00)	923.37 (830.61-1,036.93)	918.72 (825.62-1,025.93)	932.00 (830.62-1,044.49)
Romania	0.02 (0.02-0.03)	0.04 (0.04-0.05)	0.04 (0.02-0.05)	1.09 (1.00-1.17)	1.53 (1.41-1.64)	0.93 (0.85-1.01)	0.00 (0.00-0.00)	0.00 (0.00-0.00)	0.00 (0.00-0.00)	1,221.45 (998.90-1,492.33)	1,221.72 (1,001.61-1,484.30)	1,227.37 (1,010.63-1,494.35)

Appendix Table 1b. Unscaled values for each SDG health-related indicator by country for 1990, 2000, and 2015												
	**Indicator 3.3.1: Age-standardised rate of new HIV infections (per 1,000 population)**			**Indicator 3.3.2: Age-standardised rate of new and relapsed TB cases (per 1,000 population)**			**Indicator 3.3.3: Age-standaridsed rate of malaria cases (per 1,000 population)**			**Indicator 3.3.4: Age-standardised rate of hepatitis B incident cases (per 100,000 population)**		
**Location**	**1990**	**2000**	**2015**	**1990**	**2000**	**2015**	**1990**	**2000**	**2015**	**1990**	**2000**	**2015**
Serbia	0.02 (0.01-0.02)	0.02 (0.01-0.06)	0.02 (0.02-0.03)	0.27 (0.24-0.31)	0.22 (0.19-0.26)	0.21 (0.18-0.24)	0.00 (0.00-0.00)	0.00 (0.00-0.00)	0.00 (0.00-0.00)	1,221.38 (999.16-1,492.64)	1,221.51 (1,002.36-1,484.50)	1,227.34 (1,011.11-1,493.78)
Slovakia	0.00 (0.00-0.01)	0.00 (0.00-0.00)	0.00 (0.00-0.00)	0.20 (0.17-0.24)	0.16 (0.13-0.19)	0.14 (0.11-0.17)	0.00 (0.00-0.00)	0.00 (0.00-0.00)	0.00 (0.00-0.00)	1,220.56 (998.82-1,492.37)	1,220.92 (1,001.17-1,483.61)	1,227.05 (1,009.54-1,493.82)
Slovenia	0.01 (0.00-0.02)	0.01 (0.00-0.01)	0.00 (0.00-0.01)	0.36 (0.32-0.40)	0.21 (0.18-0.23)	0.15 (0.12-0.18)	0.00 (0.00-0.00)	0.00 (0.00-0.00)	0.00 (0.00-0.00)	1,220.63 (998.78-1,495.38)	1,222.01 (1,002.90-1,484.16)	1,228.81 (1,011.36-1,496.02)
Armenia	0.00 (0.00-0.01)	0.02 (0.01-0.03)	0.04 (0.02-0.05)	0.29 (0.26-0.34)	0.32 (0.28-0.36)	0.37 (0.32-0.44)	2.22 (0.17-4.21)	0.00 (0.00-0.00)	0.00 (0.00-0.00)	1,875.37 (1,614.58-2,170.48)	1,873.12 (1,617.96-2,169.98)	1,882.49 (1,615.81-2,148.34)
Azerbaijan	0.02 (0.01-0.06)	0.05 (0.03-0.09)	0.04 (0.03-0.06)	1.39 (1.23-1.59)	1.42 (1.27-1.63)	1.04 (0.90-1.20)	1.01 (0.12-1.87)	0.17 (0.17-0.17)	0.00 (0.00-0.00)	1,875.28 (1,614.72-2,168.83)	1,875.17 (1,617.11-2,170.63)	1,886.47 (1,619.72-2,145.28)
Georgia	0.01 (0.00-0.02)	0.02 (0.01-0.02)	0.05 (0.03-0.07)	0.77 (0.68-0.90)	0.54 (0.48-0.61)	0.56 (0.49-0.65)	1.73 (0.19-3.38)	0.06 (0.05-0.06)	0.00 (0.00-0.00)	1,875.24 (1,614.00-2,172.10)	1,875.31 (1,617.24-2,173.86)	1,885.99 (1,618.82-2,146.86)
Kazakhstan	0.05 (0.03-0.09)	0.13 (0.09-0.19)	0.11 (0.07-0.16)	2.58 (2.30-2.79)	2.66 (2.46-2.89)	1.79 (1.63-1.95)	0.00 (0.00-0.00)	0.00 (0.00-0.00)	0.00 (0.00-0.00)	1,875.79 (1,613.69-2,169.44)	1,875.29 (1,616.52-2,175.75)	1,884.89 (1,616.42-2,145.08)
Kyrgyzstan	0.04 (0.02-0.08)	0.10 (0.06-0.18)	0.07 (0.05-0.12)	0.91 (0.80-1.05)	1.10 (0.99-1.23)	0.77 (0.68-0.86)	1.75 (0.21-3.31)	0.00 (0.00-0.00)	0.00 (0.00-0.00)	1,876.06 (1,614.78-2,170.46)	1,876.63 (1,618.35-2,177.25)	1,885.89 (1,619.02-2,146.28)
Mongolia	0.01 (0.00-0.02)	0.01 (0.00-0.01)	0.00 (0.00-0.01)	1.75 (1.56-2.00)	1.57 (1.41-1.79)	1.36 (1.20-1.54)	0.00 (0.00-0.00)	0.00 (0.00-0.00)	0.00 (0.00-0.00)	1,881.10 (1,677.43-2,127.00)	1,881.37 (1,681.07-2,114.19)	1,880.81 (1,656.38-2,128.31)
Tajikistan	0.02 (0.00-0.06)	0.09 (0.06-0.15)	0.05 (0.03-0.08)	0.78 (0.69-0.90)	0.81 (0.73-0.92)	0.62 (0.54-0.71)	1.81 (0.53-3.08)	2.33 (2.18-2.50)	0.00 (0.00-0.00)	1,876.88 (1,614.89-2,170.39)	1,877.46 (1,619.79-2,178.98)	1,887.27 (1,620.57-2,147.54)

Appendix Table 1b. Unscaled values for each SDG health-related indicator by country for 1990, 2000, and 2015

Location	Indicator 3.3.1: Age-standardised rate of new HIV infections (per 1,000 population)			Indicator 3.3.2: Age-standardised rate of new and relapsed TB cases (per 1,000 population)			Indicator 3.3.3: Age-standaridsed rate of malaria cases (per 1,000 population)			Indicator 3.3.4: Age-standardised rate of hepatitis B incident cases (per 100,000 population)		
	1990	2000	2015	1990	2000	2015	1990	2000	2015	1990	2000	2015
Turkmenistan	0.01 (0.01-0.01)	0.25 (0.14-0.42)	0.23 (0.11-0.43)	1.20 (1.05-1.35)	1.36 (1.22-1.56)	0.96 (0.85-1.10)	0.15 (0.03-0.26)	0.00 (0.00-0.00)	0.00 (0.00-0.00)	1,876.12 (1,614.39-2,170.73)	1,876.48 (1,618.55-2,178.04)	1,885.45 (1,617.67-2,144.91)
Uzbekistan	0.03 (0.01-0.09)	0.09 (0.05-0.15)	0.02 (0.01-0.03)	0.79 (0.70-0.89)	0.96 (0.86-1.09)	0.72 (0.63-0.82)	0.33 (0.03-0.63)	0.00 (0.00-0.00)	0.00 (0.00-0.00)	1,829.51 (1,636.21-2,059.68)	1,826.68 (1,631.52-2,050.12)	1,827.64 (1,614.70-2,048.61)
Colombia	0.11 (0.07-0.18)	0.14 (0.12-0.19)	0.15 (0.10-0.23)	0.62 (0.58-0.66)	0.54 (0.51-0.57)	0.31 (0.28-0.34)	2.67 (1.61-3.68)	2.09 (1.57-2.59)	1.94 (1.60-2.26)	1,109.79 (991.30-1,251.71)	1,110.08 (991.20-1,241.65)	1,134.61 (1,002.93-1,276.62)
Costa Rica	0.09 (0.07-0.13)	0.12 (0.09-0.17)	0.08 (0.06-0.11)	0.37 (0.34-0.40)	0.30 (0.28-0.33)	0.17 (0.15-0.19)	0.31 (0.30-0.33)	0.43 (0.43-0.44)	0.00 (0.00-0.00)	1,080.64 (827.96-1,372.48)	1,069.52 (810.30-1,344.42)	1,070.96 (828.48-1,366.78)
El Salvador	0.15 (0.10-0.22)	0.29 (0.21-0.42)	0.15 (0.10-0.22)	0.44 (0.40-0.48)	0.26 (0.24-0.28)	0.22 (0.19-0.25)	1.38 (1.29-1.48)	0.11 (0.10-0.11)	0.00 (0.00-0.00)	1,077.97 (827.25-1,370.43)	1,066.43 (804.69-1,340.46)	1,066.43 (821.33-1,359.62)
Guatemala	0.31 (0.27-0.40)	0.38 (0.26-0.56)	0.16 (0.11-0.24)	2.17 (2.02-2.31)	0.90 (0.85-0.96)	0.39 (0.34-0.43)	5.03 (2.02-8.08)	2.04 (1.37-2.65)	1.47 (1.21-1.72)	1,079.18 (827.85-1,371.22)	1,067.14 (805.73-1,340.52)	1,068.35 (824.27-1,365.15)
Honduras	0.26 (0.22-0.33)	0.25 (0.19-0.30)	0.22 (0.15-0.32)	0.75 (0.69-0.81)	0.71 (0.66-0.76)	0.51 (0.46-0.56)	1.15 (0.68-1.59)	0.90 (0.66-1.13)	0.92 (0.75-1.08)	1,080.24 (827.57-1,373.51)	1,069.22 (809.48-1,343.06)	1,070.53 (827.70-1,366.23)
Mexico	0.14 (0.13-0.16)	0.14 (0.13-0.15)	0.12 (0.11-0.13)	0.64 (0.57-0.69)	0.47 (0.42-0.52)	0.27 (0.25-0.29)	2.81 (2.34-3.29)	0.06 (0.06-0.06)	0.02 (0.02-0.02)	388.07 (350.44-434.28)	386.30 (348.49-430.43)	444.51 (396.80-500.27)
Nicaragua	0.03 (0.02-0.03)	0.07 (0.05-0.10)	0.16 (0.09-0.27)	0.63 (0.58-0.68)	0.41 (0.38-0.44)	0.27 (0.24-0.30)	3.39 (1.48-5.34)	1.47 (1.04-1.87)	1.08 (0.89-1.26)	1,027.65 (922.12-1,152.71)	1,027.68 (917.87-1,149.29)	1,027.77 (912.03-1,155.68)
Panama	0.16 (0.10-0.27)	0.51 (0.39-0.71)	0.54 (0.33-0.88)	0.85 (0.78-0.92)	0.70 (0.64-0.76)	0.63 (0.58-0.69)	0.13 (0.12-0.13)	0.29 (0.28-0.30)	0.20 (0.20-0.21)	1,080.86 (828.01-1,372.16)	1,069.81 (810.94-1,344.24)	1,070.99 (829.15-1,366.80)

Appendix Table 1b. Unscaled values for each SDG health-related indicator by country for 1990, 2000, and 2015

Location	Indicator 3.3.1: Age-standardised rate of new HIV infections (per 1,000 population)			Indicator 3.3.2: Age-standardised rate of new and relapsed TB cases (per 1,000 population)			Indicator 3.3.3: Age-standaridsed rate of malaria cases (per 1,000 population)			Indicator 3.3.4: Age-standardised rate of hepatitis B incident cases (per 100,000 population)		
	1990	2000	2015	1990	2000	2015	1990	2000	2015	1990	2000	2015
Venezuela	0.12 (0.06-0.25)	0.18 (0.15-0.25)	0.15 (0.09-0.24)	0.79 (0.73-0.83)	0.53 (0.49-0.56)	0.33 (0.31-0.36)	0.82 (0.53-1.09)	0.79 (0.57-0.99)	0.72 (0.59-0.84)	1,080.42 (828.38-1,372.64)	1,069.32 (809.78-1,344.00)	1,070.34 (827.54-1,365.64)
Bolivia	0.03 (0.02-0.04)	0.05 (0.05-0.07)	0.05 (0.04-0.06)	1.63 (1.50-1.78)	1.07 (0.99-1.14)	0.73 (0.66-0.80)	0.19 (0.13-0.24)	0.18 (0.14-0.21)	0.19 (0.15-0.22)	1,141.46 (1,019.29-1,288.50)	1,138.62 (1,012.20-1,283.11)	1,141.95 (1,006.88-1,288.36)
Ecuador	0.14 (0.10-0.23)	0.25 (0.22-0.32)	0.24 (0.18-0.31)	1.85 (1.70-2.03)	1.15 (1.07-1.24)	0.66 (0.61-0.73)	5.55 (5.25-5.90)	6.92 (6.68-7.19)	0.03 (0.03-0.03)	1,141.51 (1,019.42-1,287.98)	1,138.61 (1,012.19-1,282.37)	1,141.91 (1,006.96-1,288.74)
Peru	0.21 (0.19-0.23)	0.17 (0.15-0.21)	0.10 (0.08-0.13)	3.45 (3.20-3.73)	1.60 (1.47-1.73)	0.88 (0.77-1.02)	0.61 (0.37-0.84)	0.52 (0.40-0.65)	0.54 (0.44-0.63)	1,140.25 (1,018.23-1,287.14)	1,137.93 (1,011.57-1,282.58)	1,142.08 (1,006.83-1,289.52)
Antigua and Barbuda	0.27 (0.09-0.58)	0.36 (0.21-0.69)	0.24 (0.11-0.50)	0.33 (0.29-0.38)	0.35 (0.31-0.40)	0.32 (0.26-0.39)	0.00 (0.00-0.00)	0.00 (0.00-0.00)	0.00 (0.00-0.00)	974.56 (877.34-1,085.56)	971.18 (872.92-1,083.29)	975.82 (871.20-1,091.93)
The Bahamas	1.04 (0.74-1.61)	0.78 (0.67-0.92)	0.30 (0.21-0.47)	0.62 (0.56-0.69)	0.53 (0.48-0.58)	0.32 (0.28-0.37)	0.00 (0.00-0.00)	0.00 (0.00-0.00)	0.00 (0.00-0.00)	975.32 (878.22-1,086.34)	973.16 (874.15-1,082.65)	977.47 (873.40-1,094.08)
Barbados	0.32 (0.20-0.53)	0.30 (0.22-0.41)	0.24 (0.14-0.38)	0.25 (0.21-0.28)	0.26 (0.23-0.29)	0.25 (0.20-0.30)	0.00 (0.00-0.00)	0.00 (0.00-0.00)	0.00 (0.00-0.00)	974.35 (877.13-1,083.06)	973.27 (873.68-1,083.09)	976.84 (872.84-1,093.95)
Belize	0.18 (0.06-0.45)	1.57 (0.96-2.61)	0.66 (0.46-0.90)	0.59 (0.54-0.65)	0.86 (0.80-0.94)	0.63 (0.57-0.70)	11.93 (10.98-13.12)	4.68 (4.41-4.98)	0.09 (0.09-0.09)	977.00 (879.55-1,088.89)	975.22 (874.72-1,085.63)	978.18 (872.65-1,093.73)
Cuba	0.03 (0.01-0.04)	0.06 (0.03-0.09)	0.11 (0.07-0.16)	0.15 (0.12-0.18)	0.13 (0.11-0.16)	0.12 (0.10-0.14)	0.00 (0.00-0.00)	0.00 (0.00-0.00)	0.00 (0.00-0.00)	977.00 (879.89-1,089.62)	975.37 (875.33-1,085.25)	979.47 (874.87-1,094.51)
Dominica	0.18 (0.06-0.37)	0.27 (0.17-0.52)	0.21 (0.10-0.42)	0.48 (0.43-0.53)	0.47 (0.43-0.51)	0.41 (0.37-0.47)	0.00 (0.00-0.00)	0.00 (0.00-0.00)	0.00 (0.00-0.00)	976.02 (878.34-1,085.75)	974.44 (874.76-1,084.37)	978.12 (873.73-1,093.87)

Appendix Table 1b. Unscaled values for each SDG health-related indicator by country for 1990, 2000, and 2015

Location	Indicator 3.3.1: Age-standardised rate of new HIV infections (per 1,000 population)			Indicator 3.3.2: Age-standardised rate of new and relapsed TB cases (per 1,000 population)			Indicator 3.3.3: Age-standaridsed rate of malaria cases (per 1,000 population)			Indicator 3.3.4: Age-standardised rate of hepatitis B incident cases (per 100,000 population)		
	1990	2000	2015	1990	2000	2015	1990	2000	2015	1990	2000	2015
Dominican Republic	0.78 (0.60-0.94)	1.11 (0.87-1.39)	0.38 (0.30-0.48)	1.32 (1.21-1.47)	1.08 (0.98-1.18)	0.73 (0.66-0.82)	1.42 (1.18-1.67)	1.25 (1.02-1.47)	1.23 (1.00-1.44)	976.86 (879.05-1,087.52)	974.85 (875.03-1,084.52)	978.13 (873.23-1,093.62)
Grenada	0.18 (0.06-0.38)	0.30 (0.18-0.63)	0.22 (0.10-0.46)	0.21 (0.19-0.24)	0.23 (0.20-0.26)	0.24 (0.20-0.29)	0.00 (0.00-0.00)	0.00 (0.00-0.00)	0.00 (0.00-0.00)	975.40 (878.66-1,086.01)	974.33 (872.04-1,083.33)	978.79 (873.54-1,097.15)
Guyana	0.55 (0.23-1.11)	2.95 (1.71-4.66)	1.83 (1.17-2.72)	0.83 (0.76-0.91)	1.23 (1.14-1.33)	1.15 (1.06-1.26)	15.94 (9.15-22.52)	17.38 (9.80-24.77)	11.54 (8.96-14.01)	975.34 (877.41-1,085.86)	974.75 (874.05-1,082.41)	979.20 (873.67-1,096.01)
Haiti	3.69 (2.96-4.61)	1.77 (1.24-2.32)	1.01 (0.62-1.53)	1.29 (1.19-1.40)	1.02 (0.95-1.10)	0.69 (0.63-0.75)	11.94 (6.94-16.75)	9.41 (6.04-12.53)	6.80 (5.26-8.30)	974.77 (876.82-1,084.78)	973.33 (875.58-1,084.38)	977.26 (873.04-1,093.20)
Jamaica	0.39 (0.19-0.81)	0.41 (0.33-0.53)	0.25 (0.17-0.36)	0.14 (0.12-0.16)	0.13 (0.12-0.15)	0.11 (0.09-0.13)	0.00 (0.00-0.00)	0.00 (0.00-0.00)	0.00 (0.00-0.00)	975.05 (878.27-1,094.71)	975.17 (873.06-1,093.73)	984.08 (877.49-1,102.99)
Saint Lucia	0.17 (0.08-0.30)	0.29 (0.20-0.52)	0.16 (0.09-0.31)	0.73 (0.68-0.78)	0.80 (0.74-0.85)	0.46 (0.42-0.51)	0.00 (0.00-0.00)	0.00 (0.00-0.00)	0.00 (0.00-0.00)	975.35 (876.66-1,084.90)	973.63 (874.79-1,085.21)	977.50 (873.43-1,093.91)
Saint Vincent and the Grenadines	0.43 (0.12-0.87)	0.75 (0.46-1.50)	0.50 (0.23-1.03)	0.52 (0.48-0.56)	0.65 (0.61-0.70)	0.49 (0.44-0.54)	0.00 (0.00-0.00)	0.00 (0.00-0.00)	0.00 (0.00-0.00)	975.93 (879.03-1,087.83)	975.51 (876.85-1,085.88)	979.17 (875.05-1,095.32)
Suriname	0.40 (0.16-0.90)	0.85 (0.62-1.19)	0.44 (0.30-0.68)	0.37 (0.33-0.41)	0.42 (0.38-0.47)	0.34 (0.30-0.39)	4.84 (1.78-7.93)	6.14 (1.96-10.38)	1.66 (0.85-2.46)	977.06 (880.20-1,089.99)	975.30 (873.97-1,085.46)	978.53 (874.56-1,094.13)
Trinidad and Tobago	0.53 (0.34-0.90)	0.70 (0.51-0.99)	0.28 (0.20-0.41)	0.50 (0.45-0.54)	0.50 (0.45-0.54)	0.33 (0.29-0.37)	0.00 (0.00-0.00)	0.00 (0.00-0.00)	0.00 (0.00-0.00)	975.95 (878.70-1,087.23)	974.52 (875.20-1,085.13)	978.00 (874.14-1,093.89)
Brazil	0.27 (0.23-0.33)	0.26 (0.23-0.30)	0.23 (0.21-0.25)	0.56 (0.49-0.66)	0.50 (0.44-0.59)	0.42 (0.37-0.47)	1.86 (0.86-2.86)	0.97 (0.73-1.20)	0.99 (0.82-1.16)	1,315.67 (1,179.24-1,477.42)	1,319.33 (1,181.72-1,475.38)	1,343.91 (1,192.25-1,500.14)

Appendix Table 1b. Unscaled values for each SDG health-related indicator by country for 1990, 2000, and 2015

Location	Indicator 3.3.1: Age-standardised rate of new HIV infections (per 1,000 population)			Indicator 3.3.2: Age-standardised rate of new and relapsed TB cases (per 1,000 population)			Indicator 3.3.3: Age-standaridsed rate of malaria cases (per 1,000 population)			Indicator 3.3.4: Age-standardised rate of hepatitis B incident cases (per 100,000 population)		
	1990	2000	2015	1990	2000	2015	1990	2000	2015	1990	2000	2015
Paraguay	0.07 (0.05-0.17)	0.17 (0.13-0.23)	0.26 (0.15-0.44)	0.54 (0.49-0.59)	0.53 (0.48-0.57)	0.49 (0.45-0.54)	0.07 (0.01-0.12)	0.05 (0.01-0.09)	0.04 (0.01-0.07)	1,325.48 (1,191.29-1,492.50)	1,324.21 (1,189.07-1,488.23)	1,331.76 (1,182.40-1,488.37)
China	0.01 (0.01-0.01)	0.05 (0.04-0.06)	0.07 (0.06-0.08)	1.40 (1.22-1.65)	1.22 (1.08-1.41)	1.02 (0.92-1.16)	0.04 (0.03-0.05)	0.03 (0.03-0.03)	0.00 (0.00-0.00)	2,810.49 (2,508.47-3,166.62)	2,258.17 (2,024.81-2,553.89)	1,911.42 (1,689.91-2,148.42)
North Korea	0.01 (0.01-0.04)	0.05 (0.03-0.14)	0.08 (0.04-0.16)	0.89 (0.79-1.01)	0.97 (0.87-1.08)	0.73 (0.64-0.83)	1.49 (1.13-1.88)	3.93 (3.87-3.99)	0.92 (0.89-0.96)	2,542.66 (2,259.92-2,856.77)	2,542.29 (2,257.46-2,877.74)	2,554.13 (2,261.35-2,878.00)
Taiwan	0.00 (0.00-0.01)	0.02 (0.01-0.03)	0.03 (0.02-0.04)	0.49 (0.43-0.57)	0.44 (0.38-0.53)	0.38 (0.32-0.45)	0.00 (0.00-0.00)	0.00 (0.00-0.00)	0.00 (0.00-0.00)	2,469.40 (2,194.43-2,795.32)	2,463.26 (2,212.91-2,783.61)	2,477.75 (2,200.88-2,799.73)
Cambodia	0.47 (0.25-1.22)	0.74 (0.51-1.07)	0.64 (0.38-1.06)	2.68 (2.49-2.89)	2.53 (2.36-2.73)	1.66 (1.52-1.83)	62.53 (14.83-110.40)	55.10 (13.22-97.14)	20.91 (5.78-36.11)	2,019.26 (1,799.95-2,274.60)	2,011.92 (1,788.05-2,251.44)	2,028.59 (1,786.19-2,278.46)
Indonesia	0.01 (0.01-0.02)	0.12 (0.08-0.28)	0.47 (0.34-0.77)	6.35 (5.84-6.99)	4.86 (4.48-5.35)	3.76 (3.44-4.17)	42.07 (18.09-86.12)	31.78 (12.56-69.91)	15.15 (4.66-41.02)	2,006.01 (1,789.29-2,261.75)	1,999.29 (1,789.45-2,258.89)	2,004.86 (1,786.26-2,256.21)
Laos	0.01 (0.01-0.02)	0.21 (0.13-0.56)	0.30 (0.23-0.45)	2.32 (2.15-2.51)	1.75 (1.61-1.91)	1.25 (1.13-1.39)	10.21 (5.90-14.39)	7.74 (5.30-10.00)	6.06 (4.89-7.23)	2,040.38 (1,808.75-2,315.55)	2,030.65 (1,804.01-2,300.47)	2,022.46 (1,792.81-2,283.36)
Malaysia	0.20 (0.10-0.45)	0.22 (0.19-0.25)	0.14 (0.12-0.16)	1.07 (0.96-1.19)	0.99 (0.90-1.09)	0.92 (0.81-1.03)	0.69 (0.30-1.08)	0.39 (0.38-0.40)	0.12 (0.12-0.12)	1,455.15 (1,303.82-1,644.14)	1,459.92 (1,304.39-1,626.53)	1,476.20 (1,311.43-1,655.21)
Maldives	0.01 (0.01-0.01)	0.01 (0.00-0.01)	0.00 (0.00-0.01)	0.86 (0.79-0.94)	0.56 (0.51-0.62)	0.44 (0.38-0.51)	0.00 (0.00-0.00)	0.00 (0.00-0.00)	0.00 (0.00-0.00)	2,128.88 (1,691.23-2,623.28)	2,122.71 (1,678.11-2,604.24)	2,131.49 (1,716.04-2,620.03)
Mauritius	0.03 (0.02-0.04)	0.09 (0.07-0.12)	0.12 (0.09-0.17)	0.54 (0.47-0.60)	0.38 (0.34-0.43)	0.41 (0.35-0.47)	0.00 (0.00-0.00)	0.00 (0.00-0.00)	0.00 (0.00-0.00)	2,128.55 (1,698.47-2,622.92)	2,123.89 (1,680.91-2,616.03)	2,132.49 (1,715.99-2,620.13)

Appendix Table 1b. Unscaled values for each SDG health-related indicator by country for 1990, 2000, and 2015

Location	Indicator 3.3.1: Age-standardised rate of new HIV infections (per 1,000 population)			Indicator 3.3.2: Age-standardised rate of new and relapsed TB cases (per 1,000 population)			Indicator 3.3.3: Age-standaridsed rate of malaria cases (per 1,000 population)			Indicator 3.3.4: Age-standardised rate of hepatitis B incident cases (per 100,000 population)		
	1990	2000	2015	1990	2000	2015	1990	2000	2015	1990	2000	2015
Myanmar	1.18 (0.18-8.45)	0.88 (0.53-2.34)	0.42 (0.32-0.63)	2.61 (2.40-2.83)	2.24 (2.07-2.42)	1.62 (1.48-1.78)	278.09 (124.49-563.77)	241.23 (108.52-487.43)	119.35 (54.86-228.72)	2,123.06 (1,696.18-2,619.54)	2,119.99 (1,678.30-2,609.24)	2,128.93 (1,715.69-2,617.71)
Philippines	0.09 (0.03-0.26)	0.12 (0.07-0.21)	0.34 (0.13-0.82)	4.49 (4.20-4.80)	3.49 (3.27-3.73)	2.33 (2.14-2.54)	9.04 (6.37-11.50)	6.71 (5.54-7.82)	7.22 (5.83-8.53)	2,130.02 (1,909.86-2,389.24)	2,128.46 (1,896.82-2,409.08)	2,124.62 (1,869.02-2,408.11)
Sri Lanka	0.01 (0.01-0.03)	0.01 (0.01-0.03)	0.01 (0.01-0.02)	1.40 (1.29-1.49)	0.93 (0.87-0.99)	0.60 (0.56-0.65)	14.47 (14.21-14.92)	10.46 (10.30-10.59)	0.00 (0.00-0.00)	1,928.64 (1,723.78-2,179.00)	1,921.58 (1,715.51-2,150.29)	1,916.12 (1,695.61-2,162.62)
Seychelles	0.00 (0.00-0.00)	0.14 (0.07-0.28)	0.14 (0.04-0.37)	0.50 (0.44-0.57)	0.42 (0.37-0.48)	0.46 (0.37-0.56)	0.00 (0.00-0.00)	0.00 (0.00-0.00)	0.00 (0.00-0.00)	2,128.75 (1,697.13-2,626.15)	2,125.43 (1,682.72-2,615.98)	2,137.51 (1,716.50-2,620.60)
Thailand	0.99 (0.62-1.64)	0.40 (0.27-0.60)	0.29 (0.20-0.45)	1.49 (1.37-1.62)	1.24 (1.12-1.36)	1.00 (0.90-1.11)	6.09 (3.55-8.55)	3.93 (3.22-4.62)	4.16 (3.41-4.88)	2,078.83 (1,851.95-2,352.12)	2,065.99 (1,839.94-2,335.75)	2,078.46 (1,845.92-2,328.53)
Timor-Leste	0.33 (0.01-3.09)	0.17 (0.02-0.83)	0.12 (0.02-0.45)	2.14 (1.96-2.35)	2.02 (1.83-2.25)	1.92 (1.70-2.18)	23.89 (18.82-28.74)	19.72 (16.25-23.14)	19.21 (15.65-22.65)	2,129.68 (1,698.76-2,625.78)	2,127.03 (1,682.61-2,618.67)	2,134.94 (1,716.82-2,619.87)
Vietnam	0.01 (0.01-0.05)	0.26 (0.10-1.06)	0.20 (0.12-0.42)	1.72 (1.58-1.87)	1.32 (1.23-1.44)	0.98 (0.90-1.07)	8.58 (2.40-14.78)	6.10 (1.92-10.32)	3.32 (1.41-5.27)	2,079.81 (1,855.66-2,351.05)	2,077.95 (1,850.34-2,338.60)	2,088.21 (1,861.29-2,360.49)
Federated States of Micronesia	0.20 (0.03-1.16)	0.13 (0.05-0.44)	0.14 (0.06-0.34)	0.76 (0.70-0.85)	0.60 (0.54-0.66)	0.58 (0.51-0.67)	0.00 (0.00-0.00)	0.00 (0.00-0.00)	0.00 (0.00-0.00)	2,526.03 (2,036.65-3,097.52)	2,509.90 (1,992.08-3,097.96)	2,522.15 (2,011.29-3,121.23)
Fiji	0.04 (0.02-0.11)	0.05 (0.03-0.08)	0.09 (0.05-0.15)	0.63 (0.58-0.71)	0.52 (0.47-0.58)	0.41 (0.37-0.46)	0.00 (0.00-0.00)	0.00 (0.00-0.00)	0.00 (0.00-0.00)	2,273.79 (2,027.00-2,569.58)	2,274.52 (2,026.37-2,549.40)	2,275.83 (2,014.64-2,559.13)
Kiribati	0.03 (0.01-0.09)	0.03 (0.02-0.03)	0.03 (0.01-0.04)	3.37 (3.11-3.70)	2.64 (2.43-2.89)	1.96 (1.79-2.19)	0.00 (0.00-0.00)	0.00 (0.00-0.00)	0.00 (0.00-0.00)	2,519.20 (2,036.56-3,090.39)	2,504.98 (1,990.29-3,086.63)	2,514.97 (2,011.16-3,090.41)

Appendix Table 1b. Unscaled values for each SDG health-related indicator by country for 1990, 2000, and 2015

Location	Indicator 3.3.1: Age-standardised rate of new HIV infections (per 1,000 population)			Indicator 3.3.2: Age-standardised rate of new and relapsed TB cases (per 1,000 population)			Indicator 3.3.3: Age-standaridsed rate of malaria cases (per 1,000 population)			Indicator 3.3.4: Age-standardised rate of hepatitis B incident cases (per 100,000 population)		
	1990	2000	2015	1990	2000	2015	1990	2000	2015	1990	2000	2015
Marshall Islands	0.22 (0.00-2.08)	0.08 (0.01-0.60)	0.10 (0.02-0.31)	0.86 (0.77-0.96)	0.65 (0.60-0.73)	0.56 (0.50-0.64)	0.00 (0.00-0.00)	0.00 (0.00-0.00)	0.00 (0.00-0.00)	2,526.39 (2,033.42-3,102.92)	2,511.69 (1,994.99-3,102.76)	2,521.74 (2,009.00-3,107.75)
Papua New Guinea	0.20 (0.11-0.34)	0.41 (0.31-0.52)	0.27 (0.20-0.36)	0.82 (0.75-0.91)	0.86 (0.79-0.95)	0.82 (0.73-0.91)	142.76 (70.86-255.22)	156.88 (76.32-283.70)	123.02 (57.85-229.38)	2,409.82 (2,155.43-2,731.62)	2,401.46 (2,128.86-2,714.99)	2,404.12 (2,117.66-2,707.09)
Samoa	0.21 (0.02-1.08)	0.11 (0.04-0.50)	0.12 (0.05-0.32)	0.53 (0.48-0.60)	0.39 (0.35-0.43)	0.33 (0.28-0.39)	0.00 (0.00-0.00)	0.00 (0.00-0.00)	0.00 (0.00-0.00)	2,526.44 (2,036.25-3,116.89)	2,515.42 (1,990.81-3,105.96)	2,525.20 (2,008.11-3,119.53)
Solomon Islands	0.27 (0.06-1.64)	0.17 (0.09-0.60)	0.14 (0.07-0.34)	1.48 (1.35-1.64)	1.05 (0.96-1.16)	0.73 (0.67-0.82)	55.70 (33.31-77.36)	45.73 (31.42-58.92)	39.63 (31.22-47.69)	2,375.87 (2,105.97-2,699.26)	2,378.71 (2,113.91-2,689.31)	2,365.31 (2,081.57-2,672.05)
Tonga	0.01 (0.00-0.02)	0.03 (0.02-0.04)	0.10 (0.03-0.19)	0.31 (0.26-0.37)	0.27 (0.23-0.33)	0.35 (0.28-0.43)	0.00 (0.00-0.00)	0.00 (0.00-0.00)	0.00 (0.00-0.00)	2,522.89 (2,038.09-3,112.08)	2,510.68 (1,993.43-3,096.12)	2,518.60 (2,010.07-3,098.03)
Vanuatu	0.33 (0.07-3.20)	0.16 (0.08-0.49)	0.14 (0.06-0.34)	1.13 (1.03-1.24)	0.83 (0.77-0.91)	0.57 (0.52-0.63)	45.59 (25.83-64.82)	39.31 (24.45-53.41)	32.82 (24.61-40.65)	2,527.31 (2,037.62-3,097.58)	2,512.43 (2,000.72-3,105.76)	2,520.25 (2,004.78-3,103.05)
Afghanistan	0.03 (0.00-0.20)	0.01 (0.00-0.05)	0.03 (0.01-0.07)	1.55 (1.41-1.73)	1.30 (1.19-1.43)	1.01 (0.91-1.12)	47.41 (10.77-84.21)	51.28 (11.63-91.11)	29.70 (6.84-52.66)	2,142.10 (1,577.70-2,807.32)	2,170.97 (1,595.50-2,828.66)	2,149.26 (1,582.17-2,803.51)
Algeria	0.01 (0.01-0.02)	0.02 (0.02-0.03)	0.02 (0.01-0.04)	0.99 (0.89-1.09)	0.71 (0.64-0.78)	0.55 (0.49-0.61)	0.28 (0.00-0.57)	0.00 (0.00-0.00)	0.00 (0.00-0.00)	2,140.24 (1,575.07-2,804.94)	2,167.68 (1,593.52-2,827.73)	2,144.74 (1,580.15-2,804.57)
Bahrain	0.03 (0.01-0.06)	0.03 (0.02-0.05)	0.04 (0.02-0.08)	0.56 (0.50-0.62)	0.44 (0.39-0.49)	0.26 (0.22-0.31)	0.00 (0.00-0.00)	0.00 (0.00-0.00)	0.00 (0.00-0.00)	2,163.90 (1,584.81-2,852.08)	2,191.33 (1,599.62-2,869.06)	2,180.83 (1,592.87-2,862.92)
Egypt	0.00 (0.00-0.00)	0.00 (0.00-0.01)	0.01 (0.01-0.02)	0.33 (0.30-0.37)	0.23 (0.20-0.26)	0.17 (0.14-0.20)	0.04 (0.01-0.07)	0.04 (0.01-0.07)	0.00 (0.00-0.00)	1,742.01 (1,554.76-1,951.12)	1,743.30 (1,561.71-1,950.24)	1,740.17 (1,548.71-1,958.57)

Appendix Table 1b. Unscaled values for each SDG health-related indicator by country for 1990, 2000, and 2015

Location	Indicator 3.3.1: Age-standardised rate of new HIV infections (per 1,000 population)			Indicator 3.3.2: Age-standardised rate of new and relapsed TB cases (per 1,000 population)			Indicator 3.3.3: Age-standaridsed rate of malaria cases (per 1,000 population)			Indicator 3.3.4: Age-standardised rate of hepatitis B incident cases (per 100,000 population)		
	1990	2000	2015	1990	2000	2015	1990	2000	2015	1990	2000	2015
Iran	0.02 (0.02-0.03)	0.04 (0.03-0.05)	0.07 (0.06-0.08)	0.38 (0.34-0.43)	0.29 (0.26-0.32)	0.32 (0.28-0.36)	0.95 (0.87-1.04)	0.15 (0.15-0.16)	0.01 (0.01-0.01)	1,391.80 (1,250.48-1,560.50)	1,378.15 (1,239.05-1,544.87)	1,384.90 (1,239.19-1,554.82)
Iraq	0.00 (0.00-0.00)	0.01 (0.00-0.01)	0.02 (0.00-0.03)	1.08 (0.97-1.20)	0.79 (0.72-0.87)	0.59 (0.53-0.66)	0.16 (0.15-0.18)	0.06 (0.05-0.06)	0.00 (0.00-0.00)	2,138.71 (1,574.61-2,803.98)	2,165.40 (1,591.74-2,818.96)	2,144.56 (1,582.88-2,806.57)
Jordan	0.00 (0.00-0.00)	0.00 (0.00-0.01)	0.00 (0.00-0.01)	0.13 (0.11-0.15)	0.10 (0.08-0.12)	0.10 (0.08-0.13)	0.00 (0.00-0.00)	0.00 (0.00-0.00)	0.00 (0.00-0.00)	1,852.88 (1,658.13-2,090.44)	1,842.23 (1,642.94-2,068.94)	1,838.77 (1,628.76-2,062.43)
Kuwait	0.01 (0.00-0.02)	0.00 (0.00-0.01)	0.00 (0.00-0.01)	0.78 (0.69-0.86)	0.50 (0.46-0.55)	0.29 (0.26-0.33)	0.00 (0.00-0.00)	0.00 (0.00-0.00)	0.00 (0.00-0.00)	2,148.68 (1,580.91-2,820.89)	2,195.47 (1,604.00-2,883.70)	2,161.16 (1,589.09-2,826.76)
Lebanon	0.09 (0.02-0.62)	0.03 (0.01-0.10)	0.03 (0.01-0.07)	0.30 (0.27-0.34)	0.19 (0.17-0.22)	0.17 (0.15-0.21)	0.00 (0.00-0.00)	0.00 (0.00-0.00)	0.00 (0.00-0.00)	2,134.49 (1,573.36-2,798.50)	2,163.72 (1,590.77-2,813.15)	2,143.80 (1,580.43-2,793.14)
Libya	0.07 (0.02-0.34)	0.05 (0.03-0.18)	0.06 (0.02-0.16)	0.42 (0.37-0.48)	0.31 (0.27-0.35)	0.28 (0.24-0.33)	0.00 (0.00-0.00)	0.00 (0.00-0.00)	0.00 (0.00-0.00)	2,150.41 (1,578.88-2,823.05)	2,174.00 (1,596.45-2,832.89)	2,143.84 (1,578.76-2,795.27)
Morocco	0.02 (0.01-0.02)	0.04 (0.03-0.05)	0.05 (0.04-0.06)	1.39 (1.28-1.52)	0.98 (0.91-1.05)	0.79 (0.72-0.87)	0.04 (0.01-0.07)	0.00 (0.00-0.00)	0.00 (0.00-0.00)	2,137.32 (1,572.06-2,801.58)	2,163.31 (1,590.12-2,820.04)	2,141.25 (1,580.67-2,801.45)
Palestine	0.00 (0.00-0.01)	0.01 (0.01-0.02)	0.02 (0.01-0.03)	0.15 (0.11-0.20)	0.23 (0.18-0.29)	0.24 (0.18-0.31)	0.00 (0.00-0.00)	0.00 (0.00-0.00)	0.00 (0.00-0.00)	2,140.17 (1,573.57-2,809.74)	2,167.13 (1,592.24-2,826.13)	2,145.33 (1,580.55-2,804.05)
Oman	0.01 (0.01-0.02)	0.04 (0.03-0.06)	0.03 (0.02-0.04)	0.36 (0.31-0.41)	0.25 (0.21-0.29)	0.22 (0.18-0.27)	0.03 (0.01-0.06)	0.03 (0.01-0.06)	0.00 (0.00-0.00)	2,162.01 (1,581.27-2,858.55)	2,191.90 (1,598.52-2,870.76)	2,192.31 (1,598.82-2,893.65)
Qatar	0.01 (0.00-0.03)	0.01 (0.00-0.01)	0.00 (0.00-0.01)	0.35 (0.29-0.41)	0.35 (0.29-0.42)	0.33 (0.28-0.39)	0.00 (0.00-0.00)	0.00 (0.00-0.00)	0.00 (0.00-0.00)	2,191.02 (1,598.56-2,914.18)	2,215.46 (1,612.71-2,918.24)	2,210.82 (1,609.38-2,937.36)

Appendix Table 1b. Unscaled values for each SDG health-related indicator by country for 1990, 2000, and 2015												
	**Indicator 3.3.1: Age-standardised rate of new HIV infections (per 1,000 population)**			**Indicator 3.3.2: Age-standardised rate of new and relapsed TB cases (per 1,000 population)**			**Indicator 3.3.3: Age-standaridsed rate of malaria cases (per 1,000 population)**			**Indicator 3.3.4: Age-standardised rate of hepatitis B incident cases (per 100,000 population)**		
**Location**	**1990**	**2000**	**2015**	**1990**	**2000**	**2015**	**1990**	**2000**	**2015**	**1990**	**2000**	**2015**
Saudi Arabia	0.04 (0.01-0.18)	0.03 (0.02-0.07)	0.04 (0.02-0.07)	0.72 (0.62-0.85)	0.47 (0.41-0.56)	0.32 (0.27-0.38)	0.70 (0.64-0.76)	0.18 (0.17-0.19)	0.00 (0.00-0.00)	1,698.23 (1,517.90-1,907.20)	1,676.69 (1,506.54-1,876.72)	1,761.90 (1,571.37-1,986.86)
Sudan	0.16 (0.06-0.31)	0.22 (0.15-0.31)	0.16 (0.07-0.28)	0.80 (0.73-0.88)	0.76 (0.70-0.82)	0.56 (0.51-0.63)	40.27 (26.46-60.46)	51.97 (34.11-75.19)	26.88 (20.31-36.49)	1,858.77 (1,655.01-2,099.80)	1,837.28 (1,633.31-2,071.87)	1,847.23 (1,627.91-2,092.59)
Syria	0.00 (0.00-0.00)	0.00 (0.00-0.00)	0.00 (0.00-0.01)	0.13 (0.11-0.16)	0.15 (0.12-0.18)	0.18 (0.14-0.21)	0.04 (0.01-0.07)	0.04 (0.01-0.07)	0.00 (0.00-0.00)	1,811.85 (1,611.67-2,045.27)	1,818.61 (1,623.67-2,047.44)	1,821.10 (1,615.18-2,046.44)
Tunisia	0.01 (0.01-0.01)	0.01 (0.01-0.02)	0.03 (0.02-0.05)	0.52 (0.47-0.58)	0.33 (0.31-0.35)	0.30 (0.27-0.34)	0.00 (0.00-0.00)	0.00 (0.00-0.00)	0.00 (0.00-0.00)	2,138.92 (1,573.53-2,799.54)	2,165.31 (1,591.22-2,821.92)	2,142.32 (1,580.26-2,800.02)
Turkey	0.00 (0.00-0.00)	0.01 (0.00-0.01)	0.01 (0.01-0.02)	0.51 (0.45-0.56)	0.32 (0.29-0.36)	0.22 (0.19-0.26)	0.19 (0.06-0.33)	0.16 (0.16-0.16)	0.00 (0.00-0.00)	1,837.82 (1,646.48-2,064.40)	1,831.82 (1,646.26-2,062.14)	1,838.39 (1,645.79-2,071.49)
United Arab Emirates	0.06 (0.01-0.32)	0.04 (0.01-0.19)	0.05 (0.01-0.16)	0.49 (0.43-0.56)	0.30 (0.26-0.34)	0.23 (0.18-0.28)	0.08 (0.02-0.15)	0.00 (0.00-0.00)	0.00 (0.00-0.00)	2,189.52 (1,598.92-2,915.92)	2,224.64 (1,614.16-2,940.86)	2,207.43 (1,613.51-2,913.16)
Yemen	0.06 (0.03-0.31)	0.05 (0.04-0.11)	0.04 (0.02-0.08)	1.14 (1.03-1.26)	0.95 (0.87-1.04)	0.64 (0.58-0.72)	6.03 (1.17-18.72)	5.64 (1.11-17.40)	4.48 (0.79-14.91)	1,934.73 (1,727.68-2,179.94)	1,920.16 (1,703.88-2,167.86)	1,914.56 (1,690.42-2,148.53)
Bangladesh	0.00 (0.00-0.00)	0.01 (0.01-0.02)	0.03 (0.03-0.04)	1.89 (1.75-2.03)	1.31 (1.21-1.40)	1.16 (1.06-1.29)	16.46 (4.65-28.32)	8.56 (3.01-14.17)	6.28 (2.74-9.88)	1,588.59 (1,420.62-1,781.46)	1,582.77 (1,418.62-1,768.08)	1,582.11 (1,411.03-1,778.45)
Bhutan	0.08 (0.01-0.66)	0.07 (0.03-0.29)	0.08 (0.02-0.22)	2.96 (2.64-3.35)	2.56 (2.27-2.91)	1.99 (1.75-2.31)	13.53 (12.43-14.83)	8.18 (7.76-8.66)	0.10 (0.09-0.10)	1,665.56 (1,492.67-1,885.96)	1,663.89 (1,483.53-1,865.99)	1,653.54 (1,465.76-1,857.98)
India	0.13 (0.12-0.15)	0.50 (0.47-0.55)	0.24 (0.21-0.27)	4.39 (3.85-5.09)	3.72 (3.26-4.38)	2.28 (2.00-2.60)	75.61 (38.02-136.54)	73.99 (36.50-133.86)	52.60 (24.42-102.36)	1,459.49 (1,316.46-1,629.45)	1,447.86 (1,300.82-1,619.52)	1,454.83 (1,292.71-1,625.50)

Appendix Table 1b. Unscaled values for each SDG health-related indicator by country for 1990, 2000, and 2015

Location	Indicator 3.3.1: Age-standardised rate of new HIV infections (per 1,000 population)			Indicator 3.3.2: Age-standardised rate of new and relapsed TB cases (per 1,000 population)			Indicator 3.3.3: Age-standaridsed rate of malaria cases (per 1,000 population)			Indicator 3.3.4: Age-standardised rate of hepatitis B incident cases (per 100,000 population)		
	1990	2000	2015	1990	2000	2015	1990	2000	2015	1990	2000	2015
Nepal	0.00 (0.00-0.01)	0.25 (0.07-1.12)	0.08 (0.05-0.16)	3.83 (3.51-4.20)	2.87 (2.61-3.20)	2.07 (1.88-2.28)	8.13 (1.94-14.34)	8.32 (1.99-14.67)	7.24 (1.78-12.72)	1,452.61 (1,298.40-1,627.39)	1,454.43 (1,302.51-1,635.08)	1,461.96 (1,296.46-1,638.31)
Pakistan	0.01 (0.00-0.04)	0.01 (0.01-0.03)	0.06 (0.02-0.15)	2.61 (2.38-2.87)	2.06 (1.91-2.26)	1.76 (1.62-1.93)	13.84 (4.51-23.27)	15.43 (4.90-26.07)	14.11 (4.72-23.61)	1,598.16 (1,438.55-1,800.08)	1,587.32 (1,425.44-1,787.05)	1,588.42 (1,420.57-1,780.85)
Botswana	16.30 (14.84-17.86)	40.31 (36.17-45.15)	22.33 (18.40-27.10)	12.36 (10.95-14.10)	31.36 (27.97-35.25)	22.36 (19.78-25.32)	9.50 (5.90-14.85)	10.08 (6.35-15.95)	6.94 (3.68-12.19)	2,044.80 (1,706.37-2,438.18)	2,037.68 (1,696.27-2,408.44)	2,045.13 (1,708.22-2,409.53)
Lesotho	5.83 (4.98-6.74)	30.87 (28.13-34.05)	26.29 (22.66-30.49)	7.43 (6.61-8.46)	17.10 (15.59-18.94)	22.78 (20.77-25.30)	0.00 (0.00-0.00)	0.00 (0.00-0.00)	0.00 (0.00-0.00)	2,043.38 (1,707.87-2,429.23)	2,035.91 (1,702.52-2,409.89)	2,044.42 (1,704.93-2,407.94)
Namibia	4.65 (4.28-5.00)	24.65 (22.47-27.37)	12.71 (10.97-14.72)	9.48 (8.44-10.73)	19.68 (17.78-22.17)	14.10 (12.63-15.95)	83.13 (41.04-153.87)	86.02 (43.18-157.87)	45.82 (14.34-84.62)	1,983.99 (1,780.80-2,226.36)	1,982.54 (1,775.04-2,220.38)	1,977.95 (1,747.98-2,232.91)
South Africa	1.01 (0.91-1.11)	23.27 (21.65-25.10)	16.50 (14.49-18.61)	5.94 (4.90-6.93)	14.89 (12.52-17.87)	15.74 (13.29-19.23)	0.38 (0.19-0.58)	0.00 (0.00-0.00)	0.10 (0.09-0.10)	2,044.09 (1,822.32-2,317.99)	2,031.07 (1,803.27-2,299.62)	2,038.45 (1,800.09-2,298.09)
Swaziland	13.12 (11.15-15.41)	42.93 (38.98-47.61)	27.43 (23.34-31.28)	9.84 (8.75-11.25)	28.01 (25.20-31.65)	26.13 (23.21-29.47)	0.34 (0.21-0.49)	0.37 (0.22-0.52)	0.24 (0.04-0.90)	2,041.56 (1,706.71-2,430.68)	2,035.73 (1,703.29-2,404.89)	2,044.37 (1,705.42-2,410.45)
Zimbabwe	23.58 (21.49-25.75)	23.00 (19.50-27.29)	12.56 (9.12-17.31)	6.74 (6.06-7.53)	14.16 (13.15-15.36)	9.50 (8.64-10.43)	24.85 (18.16-34.02)	24.80 (18.16-34.04)	29.18 (14.23-58.51)	1,996.63 (1,785.10-2,250.87)	1,990.41 (1,776.41-2,246.46)	1,979.08 (1,754.05-2,227.56)
Benin	0.88 (0.50-1.46)	1.33 (1.05-1.63)	0.71 (0.51-0.99)	1.71 (1.58-1.89)	1.84 (1.71-2.02)	1.41 (1.29-1.54)	185.04 (103.46-279.27)	199.75 (119.04-290.65)	209.30 (160.72-266.34)	2,096.78 (1,761.09-2,445.94)	2,087.47 (1,746.88-2,478.21)	2,098.54 (1,756.30-2,456.98)
Burkina Faso	3.68 (2.75-4.86)	1.37 (1.01-1.79)	0.63 (0.44-0.85)	2.54 (2.35-2.81)	2.65 (2.46-2.90)	1.84 (1.70-2.01)	334.01 (266.52-410.08)	347.75 (278.22-427.00)	267.48 (166.05-379.23)	2,096.54 (1,761.17-2,448.10)	2,087.43 (1,745.57-2,483.12)	2,098.46 (1,760.60-2,454.73)

Appendix Table 1b. Unscaled values for each SDG health-related indicator by country for 1990, 2000, and 2015

Location	Indicator 3.3.1: Age-standardised rate of new HIV infections (per 1,000 population)			Indicator 3.3.2: Age-standardised rate of new and relapsed TB cases (per 1,000 population)			Indicator 3.3.3: Age-standaridsed rate of malaria cases (per 1,000 population)			Indicator 3.3.4: Age-standardised rate of hepatitis B incident cases (per 100,000 population)		
	1990	2000	2015	1990	2000	2015	1990	2000	2015	1990	2000	2015
Cameroon	3.23 (2.21-4.69)	4.90 (4.10-5.84)	3.06 (2.12-4.32)	2.04 (1.87-2.29)	2.58 (2.34-2.86)	1.94 (1.75-2.16)	203.73 (149.92-275.28)	235.90 (176.22-303.31)	170.70 (121.71-232.21)	2,054.57 (1,831.43-2,319.78)	2,044.82 (1,819.52-2,291.08)	2,066.01 (1,825.18-2,332.26)
Cape Verde	0.64 (0.39-0.98)	0.56 (0.39-0.76)	0.62 (0.30-1.58)	0.85 (0.78-0.93)	0.67 (0.61-0.74)	0.57 (0.50-0.65)	20.80 (2.36-39.63)	0.22 (0.21-0.24)	0.00 (0.00-0.00)	2,098.67 (1,761.97-2,462.34)	2,088.00 (1,741.78-2,476.50)	2,097.96 (1,761.18-2,454.60)
Chad	1.62 (0.99-2.54)	2.94 (2.32-3.67)	1.20 (0.73-1.84)	2.33 (2.16-2.58)	2.83 (2.62-3.08)	2.35 (2.14-2.61)	128.60 (64.89-246.19)	135.42 (68.94-255.58)	101.03 (37.60-227.41)	2,095.93 (1,757.84-2,445.79)	2,086.80 (1,747.79-2,475.27)	2,098.34 (1,759.07-2,454.19)
Cote d'Ivoire	4.09 (3.02-5.33)	4.26 (3.38-5.26)	2.59 (1.74-3.69)	2.63 (2.39-2.93)	3.19 (2.92-3.55)	2.14 (1.94-2.37)	306.42 (242.10-376.87)	314.96 (250.00-390.33)	265.58 (206.56-347.11)	2,093.65 (1,749.47-2,452.03)	2,085.11 (1,743.63-2,477.42)	2,097.90 (1,754.53-2,447.89)
The Gambia	0.25 (0.17-0.35)	1.41 (1.12-1.80)	0.70 (0.37-1.13)	1.57 (1.45-1.72)	1.55 (1.43-1.71)	1.35 (1.22-1.51)	142.25 (101.45-211.01)	153.25 (110.03-220.45)	23.10 (12.44-43.30)	2,095.70 (1,752.13-2,454.82)	2,087.56 (1,747.08-2,470.19)	2,099.04 (1,754.50-2,453.12)
Ghana	1.75 (1.33-2.27)	2.23 (1.83-2.69)	0.98 (0.69-1.33)	2.21 (2.04-2.45)	2.29 (2.13-2.54)	1.51 (1.38-1.67)	264.46 (212.92-336.65)	284.29 (231.28-357.28)	209.21 (144.82-300.12)	2,037.24 (1,815.46-2,300.94)	2,037.75 (1,819.01-2,300.30)	2,044.11 (1,805.59-2,310.68)
Guinea	1.02 (0.60-1.81)	1.79 (1.51-2.17)	1.18 (0.74-1.67)	1.93 (1.77-2.15)	2.35 (2.16-2.60)	1.86 (1.70-2.05)	251.80 (197.44-314.27)	272.33 (215.70-339.26)	273.06 (222.01-326.48)	2,095.93 (1,757.10-2,446.39)	2,086.71 (1,747.18-2,472.74)	2,098.17 (1,757.60-2,453.20)
Guinea-Bissau	0.56 (0.40-0.81)	3.11 (2.65-3.65)	2.46 (1.73-3.46)	2.06 (1.89-2.28)	2.63 (2.42-2.91)	3.03 (2.76-3.34)	218.79 (133.46-322.87)	220.63 (135.73-323.31)	60.60 (25.32-134.00)	2,096.08 (1,757.14-2,445.88)	2,087.14 (1,747.89-2,473.73)	2,098.65 (1,757.76-2,453.61)
Liberia	1.36 (0.90-2.04)	1.91 (1.46-2.43)	0.81 (0.46-1.28)	3.22 (2.97-3.57)	2.97 (2.71-3.30)	2.63 (2.35-2.96)	244.45 (148.62-380.19)	275.70 (180.60-398.56)	210.70 (146.04-313.87)	2,095.80 (1,756.71-2,446.48)	2,086.56 (1,747.80-2,473.62)	2,097.88 (1,758.58-2,454.39)
Mali	1.14 (0.66-1.72)	1.44 (1.13-1.81)	0.92 (0.55-1.35)	2.05 (1.92-2.22)	1.92 (1.79-2.08)	1.47 (1.35-1.62)	268.21 (202.51-332.37)	276.04 (209.21-342.01)	286.80 (237.89-357.73)	2,096.01 (1,759.95-2,447.29)	2,086.45 (1,744.37-2,481.24)	2,097.65 (1,759.25-2,453.33)

Appendix Table 1b. Unscaled values for each SDG health-related indicator by country for 1990, 2000, and 2015

Location	Indicator 3.3.1: Age-standardised rate of new HIV infections (per 1,000 population)			Indicator 3.3.2: Age-standardised rate of new and relapsed TB cases (per 1,000 population)			Indicator 3.3.3: Age-standaridsed rate of malaria cases (per 1,000 population)			Indicator 3.3.4: Age-standardised rate of hepatitis B incident cases (per 100,000 population)		
	1990	2000	2015	1990	2000	2015	1990	2000	2015	1990	2000	2015
Mauritania	0.46 (0.12-3.37)	0.58 (0.29-1.96)	0.17 (0.13-0.25)	2.02 (1.84-2.24)	1.75 (1.60-1.92)	1.20 (1.08-1.33)	46.60 (19.29-91.18)	56.31 (22.45-104.86)	33.30 (15.04-57.18)	2,095.35 (1,759.27-2,443.61)	2,086.27 (1,745.88-2,478.27)	2,097.94 (1,757.06-2,455.58)
Niger	0.64 (0.49-0.83)	1.25 (1.01-1.55)	0.30 (0.19-0.48)	2.42 (2.23-2.66)	2.46 (2.27-2.67)	2.00 (1.83-2.22)	195.07 (95.15-317.84)	208.55 (107.19-332.29)	222.36 (123.99-308.48)	2,096.49 (1,754.37-2,450.05)	2,087.66 (1,750.65-2,472.87)	2,098.85 (1,759.57-2,453.99)
Nigeria	1.99 (1.16-3.21)	2.66 (2.11-3.30)	1.91 (1.22-2.67)	1.84 (1.66-2.05)	1.79 (1.62-1.97)	1.71 (1.52-1.94)	255.75 (198.60-331.79)	269.54 (213.59-344.99)	225.61 (162.29-296.68)	2,073.84 (1,850.37-2,340.60)	2,076.47 (1,855.48-2,338.95)	2,079.11 (1,829.95-2,349.01)
Sao Tome and Principe	0.02 (0.02-0.02)	0.06 (0.06-0.07)	0.07 (0.06-0.08)	1.02 (0.95-1.11)	0.94 (0.87-1.04)	0.86 (0.77-0.96)	21.58 (17.79-25.24)	21.46 (17.69-25.04)	21.58 (17.42-25.57)	2,096.29 (1,760.37-2,448.83)	2,086.86 (1,746.13-2,481.72)	2,098.36 (1,759.09-2,456.70)
Senegal	0.42 (0.34-0.52)	0.92 (0.77-1.10)	0.54 (0.31-0.76)	3.12 (2.87-3.49)	2.84 (2.64-3.12)	2.40 (2.20-2.65)	165.49 (127.60-228.88)	177.38 (136.02-244.54)	45.33 (33.62-58.76)	2,095.88 (1,757.59-2,446.35)	2,087.60 (1,747.56-2,474.35)	2,098.98 (1,758.78-2,454.71)
Sierra Leone	0.45 (0.16-0.86)	1.37 (1.11-1.70)	0.89 (0.39-1.41)	1.88 (1.73-2.07)	2.14 (1.99-2.34)	1.84 (1.68-2.06)	256.46 (146.28-382.52)	285.71 (172.11-397.19)	252.28 (178.12-340.80)	2,082.55 (1,865.49-2,365.25)	2,070.80 (1,848.74-2,336.97)	2,063.88 (1,823.93-2,331.86)
Togo	1.66 (1.30-2.10)	3.85 (3.12-4.73)	1.01 (0.62-1.62)	1.51 (1.40-1.66)	1.69 (1.57-1.83)	1.29 (1.19-1.42)	295.86 (216.28-387.76)	298.09 (219.01-388.30)	261.76 (219.24-321.11)	2,096.15 (1,757.33-2,446.11)	2,087.86 (1,748.34-2,473.77)	2,098.69 (1,758.59-2,456.42)
Burundi	4.19 (3.02-5.51)	2.86 (2.25-3.52)	1.11 (0.78-1.59)	3.82 (3.42-4.26)	4.12 (3.80-4.51)	2.13 (1.96-2.34)	255.86 (167.83-341.82)	264.70 (180.08-345.05)	104.61 (73.50-145.41)	2,032.72 (1,813.66-2,280.28)	2,033.78 (1,818.48-2,288.79)	2,027.72 (1,794.89-2,285.98)
Comoros	0.02 (0.00-0.11)	0.10 (0.03-0.43)	0.11 (0.04-0.29)	1.70 (1.53-1.89)	1.38 (1.26-1.52)	1.08 (0.97-1.21)	57.01 (44.93-68.58)	53.52 (43.42-63.05)	49.94 (41.14-58.52)	2,144.94 (1,705.91-2,644.27)	2,139.87 (1,684.81-2,603.12)	2,150.98 (1,712.82-2,671.85)
Djibouti	1.35 (0.58-2.81)	1.51 (1.06-2.10)	0.91 (0.55-1.42)	1.93 (1.73-2.19)	2.32 (2.10-2.58)	2.21 (2.00-2.45)	5.93 (1.35-19.42)	8.03 (1.96-22.03)	3.61 (0.50-10.03)	2,145.11 (1,705.64-2,643.20)	2,139.93 (1,685.02-2,605.23)	2,150.94 (1,713.51-2,671.24)

Appendix Table 1b. Unscaled values for each SDG health-related indicator by country for 1990, 2000, and 2015

Location	Indicator 3.3.1: Age-standardised rate of new HIV infections (per 1,000 population)			Indicator 3.3.2: Age-standardised rate of new and relapsed TB cases (per 1,000 population)			Indicator 3.3.3: Age-standaridsed rate of malaria cases (per 1,000 population)			Indicator 3.3.4: Age-standardised rate of hepatitis B incident cases (per 100,000 population)		
	1990	2000	2015	1990	2000	2015	1990	2000	2015	1990	2000	2015
Eritrea	0.84 (0.49-1.44)	0.79 (0.59-1.07)	0.43 (0.27-0.63)	2.18 (1.98-2.41)	2.16 (2.00-2.37)	1.82 (1.67-2.01)	36.97 (26.20-47.91)	36.90 (26.11-48.15)	17.72 (5.00-50.03)	2,144.44 (1,702.57-2,644.98)	2,139.50 (1,683.28-2,603.73)	2,150.64 (1,710.27-2,669.89)
Ethiopia	3.53 (2.55-4.60)	2.61 (1.99-3.27)	0.81 (0.58-1.08)	4.71 (4.31-5.20)	4.76 (4.39-5.20)	2.58 (2.37-2.89)	44.87 (22.80-70.46)	44.61 (22.65-70.04)	5.04 (3.73-6.37)	2,029.63 (1,819.02-2,278.09)	2,008.76 (1,794.22-2,243.88)	2,012.18 (1,780.70-2,266.03)
Kenya	11.07 (10.65-11.51)	4.42 (3.97-4.90)	3.60 (3.02-4.29)	1.63 (1.37-1.94)	2.77 (2.40-3.27)	1.80 (1.52-2.12)	137.09 (102.56-173.28)	149.39 (113.01-186.29)	97.21 (52.42-163.33)	1,855.84 (1,662.09-2,079.97)	1,867.63 (1,671.92-2,096.01)	1,869.18 (1,653.66-2,100.65)
Madagascar	1.54 (0.08-11.33)	0.25 (0.05-1.22)	0.09 (0.02-0.25)	1.72 (1.57-1.90)	1.51 (1.37-1.66)	1.31 (1.16-1.49)	113.35 (80.63-152.77)	121.83 (86.95-163.54)	60.44 (48.67-77.84)	2,144.71 (1,703.70-2,640.73)	2,139.39 (1,682.94-2,600.46)	2,150.55 (1,713.01-2,671.81)
Malawi	14.28 (12.58-16.21)	13.48 (11.17-16.12)	5.26 (3.33-6.88)	3.49 (3.17-3.94)	6.00 (5.55-6.58)	3.38 (3.06-3.76)	275.83 (201.15-345.78)	272.53 (198.29-342.24)	171.08 (133.17-217.77)	1,985.91 (1,766.34-2,246.09)	1,995.04 (1,769.57-2,254.94)	1,985.86 (1,746.46-2,254.68)
Mozambique	3.28 (2.75-3.95)	10.91 (9.75-12.27)	7.11 (5.17-9.33)	2.53 (2.28-2.84)	4.29 (3.93-4.73)	4.75 (4.28-5.29)	316.56 (255.41-387.67)	320.63 (259.66-392.56)	211.96 (148.93-271.50)	1,990.43 (1,765.67-2,258.90)	1,983.07 (1,766.81-2,237.70)	1,985.80 (1,747.37-2,254.25)
Rwanda	5.46 (4.47-6.66)	3.50 (2.86-4.22)	1.15 (0.82-1.54)	3.06 (2.78-3.44)	3.38 (3.12-3.70)	1.45 (1.31-1.62)	110.61 (37.81-216.30)	112.72 (38.96-217.61)	33.47 (17.55-57.79)	2,144.30 (1,701.52-2,644.50)	2,138.04 (1,678.02-2,604.29)	2,148.81 (1,709.85-2,667.96)
Somalia	0.25 (0.13-0.46)	0.51 (0.38-0.67)	0.29 (0.19-0.45)	2.49 (2.26-2.81)	2.89 (2.64-3.20)	2.85 (2.63-3.10)	61.00 (40.44-89.07)	63.43 (41.83-91.90)	45.06 (24.68-75.29)	2,023.45 (1,801.16-2,274.94)	2,007.76 (1,792.15-2,256.58)	2,017.20 (1,786.22-2,277.97)
South Sudan	1.02 (0.35-2.14)	1.87 (1.22-2.69)	1.27 (0.73-1.92)	3.32 (2.95-3.75)	3.83 (3.44-4.32)	3.43 (3.08-3.83)	165.52 (85.78-238.74)	190.35 (106.77-268.48)	110.89 (53.28-197.92)	2,144.67 (1,704.01-2,644.39)	2,139.47 (1,683.92-2,601.24)	2,150.68 (1,713.40-2,671.16)
Tanzania	7.17 (6.30-8.20)	5.63 (4.64-6.80)	3.03 (2.23-4.09)	3.17 (2.89-3.45)	4.23 (3.91-4.57)	3.18 (2.92-3.45)	211.70 (152.92-266.30)	229.48 (169.63-285.69)	98.81 (76.82-126.30)	2,013.83 (1,795.15-2,267.43)	2,014.47 (1,802.20-2,267.27)	2,026.30 (1,802.88-2,298.37)

Appendix Table 1b. Unscaled values for each SDG health-related indicator by country for 1990, 2000, and 2015

Location	Indicator 3.3.1: Age-standardised rate of new HIV infections (per 1,000 population)			Indicator 3.3.2: Age-standardised rate of new and relapsed TB cases (per 1,000 population)			Indicator 3.3.3: Age-standaridsed rate of malaria cases (per 1,000 population)			Indicator 3.3.4: Age-standardised rate of hepatitis B incident cases (per 100,000 population)		
	1990	2000	2015	1990	2000	2015	1990	2000	2015	1990	2000	2015
Uganda	13.02 (10.10-16.25)	6.53 (5.60-7.62)	3.97 (2.61-5.53)	3.72 (3.36-4.18)	5.44 (4.99-6.04)	3.15 (2.86-3.53)	297.38 (236.50-375.81)	298.17 (236.56-373.89)	129.75 (75.70-196.41)	2,075.87 (1,848.12-2,340.14)	2,071.20 (1,850.51-2,326.48)	2,086.85 (1,852.32-2,358.78)
Zambia	15.51 (13.70-17.65)	13.65 (11.61-15.96)	6.90 (5.56-8.27)	4.15 (3.72-4.75)	7.24 (6.61-8.09)	4.66 (4.20-5.23)	224.70 (167.10-294.98)	242.34 (183.88-317.31)	139.82 (107.24-172.35)	2,029.57 (1,801.50-2,273.94)	2,027.37 (1,809.03-2,282.65)	2,033.06 (1,795.12-2,290.41)
Angola	0.75 (0.43-1.28)	1.86 (1.57-2.25)	1.63 (1.13-2.30)	4.65 (4.21-5.17)	4.23 (3.86-4.70)	3.50 (3.16-3.90)	149.61 (89.45-209.75)	177.11 (110.68-236.02)	100.61 (60.05-147.80)	2,116.32 (1,783.13-2,491.55)	2,113.88 (1,781.79-2,495.23)	2,119.41 (1,776.02-2,468.62)
Central African Republic	7.15 (5.26-9.84)	6.81 (5.47-8.22)	3.65 (2.59-5.22)	4.86 (4.46-5.42)	6.29 (5.77-6.87)	4.60 (4.21-5.09)	266.17 (164.80-357.32)	292.28 (192.08-384.97)	211.54 (89.32-326.90)	2,060.47 (1,836.66-2,321.64)	2,057.90 (1,840.40-2,317.08)	2,060.91 (1,826.94-2,321.13)
Congo	5.14 (4.10-6.44)	3.26 (2.64-3.95)	2.14 (1.31-3.00)	4.23 (3.81-4.74)	3.77 (3.44-4.22)	2.39 (2.16-2.67)	200.22 (141.45-278.66)	229.35 (167.01-317.75)	158.29 (101.56-216.86)	2,068.27 (1,849.53-2,336.06)	2,050.27 (1,822.76-2,308.51)	2,054.52 (1,806.26-2,322.16)
Democratic Republic of the Congo	2.41 (1.73-3.12)	2.24 (1.80-2.76)	0.81 (0.44-1.53)	3.42 (3.11-3.83)	3.48 (3.16-3.83)	3.05 (2.69-3.44)	302.79 (242.51-376.12)	306.52 (245.21-379.36)	189.79 (148.60-244.35)	2,116.16 (1,783.43-2,491.23)	2,113.80 (1,782.28-2,496.63)	2,119.42 (1,776.23-2,470.85)
Equatorial Guinea	1.36 (0.73-2.70)	3.44 (2.74-4.10)	1.77 (1.13-2.95)	2.92 (2.67-3.23)	3.45 (3.12-3.83)	3.54 (3.13-4.00)	264.84 (178.48-338.35)	262.85 (177.20-335.44)	241.19 (134.99-341.74)	2,116.48 (1,779.99-2,493.34)	2,114.20 (1,778.13-2,508.97)	2,119.89 (1,776.39-2,472.77)
Gabon	2.18 (1.71-2.73)	6.15 (5.15-7.41)	2.39 (1.44-3.80)	4.04 (3.62-4.55)	5.19 (4.66-5.88)	3.43 (3.06-3.85)	231.88 (161.91-314.67)	247.69 (176.23-333.56)	149.00 (64.45-267.98)	2,083.75 (1,875.57-2,339.97)	2,078.41 (1,857.85-2,338.28)	2,078.73 (1,838.53-2,347.46)

Appendix Table 1b. Unscaled values for each SDG health-related indicator by country for 1990, 2000, and 2015

Location	Indicator 3.3.5: Age-standardised prevalence rate of neglected tropical diseases (NTDs) (per 100,000 population)			Indicator 3.4.1: Death rate due to cardiovascular disease, cancer, diabetes, and chronic respiratory disease among populations aged 30 to 70 (per 100,000 population)			Indicator 3.4.2: Age-standardised death rate due to self-harm (per 100,000 population)			Indicator 3.5.2: Risk-weighted prevalence of alcohol consumption, as measured by the summary exposure value (SEV) for alcohol use		
	1990	2000	2015	1990	2000	2015	1990	2000	2015	1990	2000	2015
Canada	17.84 (16.40-19.39)	18.12 (16.56-19.75)	15.76 (14.04-17.52)	370.96 (368.10-373.85)	296.98 (294.51-299.39)	223.85 (218.09-229.51)	13.62 (13.10-14.14)	12.53 (12.06-13.04)	10.74 (10.12-11.37)	12.90 (12.09-13.69)	12.13 (11.43-12.78)	12.98 (11.85-14.02)
United States	40.58 (36.13-44.92)	55.86 (48.63-63.14)	53.04 (46.16-60.01)	451.56 (448.77-453.12)	378.51 (376.89-380.01)	299.39 (296.66-303.75)	12.74 (12.44-13.05)	11.32 (11.02-11.62)	12.41 (11.92-12.87)	13.75 (12.93-14.53)	13.42 (12.54-14.24)	13.62 (12.05-14.99)
Australia	62.57 (47.45-89.80)	52.78 (40.09-77.40)	48.96 (34.64-80.17)	377.96 (375.25-380.75)	266.93 (264.78-269.33)	199.03 (195.95-202.37)	13.46 (12.91-13.99)	12.77 (12.26-13.30)	10.24 (9.68-10.80)	15.03 (14.02-15.98)	12.40 (11.55-13.23)	13.67 (12.32-14.86)
New Zealand	17.86 (16.11-20.31)	16.32 (14.85-18.08)	16.02 (14.56-17.66)	448.62 (442.99-453.54)	332.57 (328.41-336.88)	230.31 (223.57-237.60)	14.06 (13.39-14.74)	13.28 (12.69-13.84)	10.90 (10.25-11.56)	13.90 (13.10-14.68)	12.49 (11.63-13.35)	13.05 (11.85-14.20)
Brunei	231.91 (186.51-296.17)	212.61 (175.08-266.67)	294.61 (224.11-384.28)	509.89 (488.64-532.91)	420.14 (404.65-437.12)	362.14 (340.22-386.45)	4.99 (4.23-6.06)	4.27 (3.83-4.88)	4.33 (3.76-4.92)	10.23 (8.84-11.56)	9.41 (8.14-10.60)	9.13 (7.98-10.27)
Japan	82.17 (72.49-92.74)	81.67 (72.02-91.71)	80.18 (70.37-90.53)	276.85 (275.42-278.82)	239.64 (238.35-240.91)	188.87 (187.14-190.59)	16.91 (16.49-17.34)	19.52 (19.11-19.94)	19.22 (18.69-19.79)	11.21 (10.52-11.92)	11.50 (10.73-12.24)	10.79 (9.61-11.86)
Singapore	44.26 (29.58-71.77)	39.15 (27.76-57.56)	119.11 (74.40-198.57)	499.61 (490.73-508.43)	335.71 (329.21-342.12)	209.18 (199.95-218.48)	17.20 (16.44-17.95)	12.40 (11.80-12.99)	9.06 (8.44-9.66)	4.04 (3.59-4.52)	3.98 (3.52-4.48)	4.49 (3.96-5.08)
South Korea	2,569.83 (2,359.17-2,802.55)	2,450.09 (2,255.19-2,657.84)	2,602.57 (2,340.50-2,901.99)	532.81 (523.26-545.38)	351.89 (343.90-360.26)	211.85 (200.71-222.55)	12.29 (11.70-12.95)	20.32 (19.42-21.27)	27.75 (25.87-29.78)	9.76 (9.01-10.46)	11.04 (10.36-11.71)	11.41 (10.39-12.44)
Andorra	36.42 (32.24-40.95)	34.73 (30.09-39.84)	58.28 (48.18-69.90)	214.72 (184.73-232.31)	176.20 (150.42-189.15)	154.04 (140.90-164.17)	8.12 (4.65-10.69)	7.11 (4.11-9.51)	6.96 (4.94-9.25)	20.99 (17.58-23.67)	20.67 (18.57-22.58)	16.92 (14.10-19.32)
Austria	28.28 (26.06-30.88)	25.59 (23.72-27.73)	22.24 (20.22-24.31)	398.55 (394.01-405.57)	321.41 (317.95-327.48)	232.60 (228.22-238.71)	21.53 (20.70-22.43)	17.18 (16.49-17.89)	12.52 (11.89-13.24)	19.65 (18.16-21.01)	18.85 (17.33-20.33)	18.05 (15.32-20.45)

Appendix Table 1b. Unscaled values for each SDG health-related indicator by country for 1990, 2000, and 2015

Location	Indicator 3.3.5: Age-standardised prevalence rate of neglected tropical diseases (NTDs) (per 100,000 population)			Indicator 3.4.1: Death rate due to cardiovascular disease, cancer, diabetes, and chronic respiratory disease among populations aged 30 to 70 (per 100,000 population)			Indicator 3.4.2: Age-standardised death rate due to self-harm (per 100,000 population)			Indicator 3.5.2: Risk-weighted prevalence of alcohol consumption, as measured by the summary exposure value (SEV) for alcohol use		
	**1990**	**2000**	**2015**	**1990**	**2000**	**2015**	**1990**	**2000**	**2015**	**1990**	**2000**	**2015**
Belgium	23.66 (21.30-26.93)	19.91 (18.10-22.13)	18.08 (16.30-20.14)	398.69 (393.85-403.49)	326.36 (322.32-330.84)	252.18 (240.86-264.60)	18.85 (18.15-19.64)	19.73 (19.00-20.49)	16.77 (15.65-17.98)	14.99 (14.23-15.82)	14.02 (12.66-15.36)	15.65 (13.20-17.78)
Cyprus	25.46 (23.18-28.20)	23.51 (21.30-25.96)	21.08 (19.02-23.19)	349.59 (339.51-359.85)	341.53 (332.97-349.77)	220.81 (214.02-228.73)	3.87 (3.43-4.36)	4.04 (3.67-4.57)	3.18 (2.83-3.62)	13.63 (12.41-14.72)	12.80 (11.53-13.98)	12.68 (10.74-14.48)
Denmark	20.69 (18.93-22.52)	20.52 (18.89-22.22)	19.72 (17.97-21.58)	475.68 (470.40-481.12)	375.25 (370.53-383.15)	248.05 (239.96-258.82)	25.37 (24.31-26.45)	14.54 (13.89-15.24)	10.35 (9.66-11.07)	18.89 (17.42-20.25)	20.18 (18.63-21.66)	17.83 (14.59-20.58)
Finland	17.14 (15.52-18.80)	15.08 (13.67-16.55)	14.30 (12.88-15.77)	422.70 (417.00-428.57)	302.90 (298.32-310.85)	224.22 (215.77-236.05)	28.24 (27.03-29.35)	22.10 (21.19-23.08)	15.71 (14.73-16.80)	15.21 (14.03-16.30)	15.24 (14.09-16.33)	15.40 (13.39-17.26)
France	28.43 (26.12-31.01)	25.34 (23.38-27.66)	23.04 (20.91-25.26)	338.21 (335.29-342.95)	296.94 (294.41-299.89)	241.50 (236.14-246.79)	23.04 (22.13-23.93)	19.28 (18.57-20.05)	16.48 (15.44-17.42)	17.60 (16.61-18.54)	15.22 (14.25-16.19)	13.99 (12.28-15.50)
Germany	23.11 (21.11-25.36)	20.63 (18.73-22.59)	19.78 (17.86-21.75)	436.74 (433.84-440.04)	337.13 (334.29-342.06)	249.28 (245.21-253.97)	14.63 (14.02-15.22)	13.03 (12.51-13.58)	10.42 (9.88-10.93)	17.08 (16.21-17.90)	15.53 (14.67-16.40)	13.60 (12.13-15.04)
Greece	21.53 (19.41-23.78)	19.42 (17.31-21.46)	17.42 (15.47-19.32)	343.75 (340.18-348.11)	307.22 (303.96-310.55)	264.77 (257.12-273.88)	3.72 (3.52-3.93)	3.12 (2.95-3.30)	3.15 (2.92-3.39)	16.50 (15.35-17.70)	14.63 (13.47-15.82)	14.01 (11.84-16.13)
Iceland	20.92 (18.93-22.98)	19.70 (17.68-21.95)	20.02 (18.01-22.25)	330.07 (323.37-337.95)	258.25 (249.66-266.97)	183.20 (173.92-192.01)	13.74 (13.03-14.52)	10.98 (10.37-11.63)	8.74 (8.06-9.38)	9.38 (8.29-10.42)	9.17 (8.18-10.18)	11.34 (9.06-13.46)
Ireland	24.59 (22.28-27.18)	24.17 (21.98-26.37)	25.12 (22.86-27.62)	422.71 (404.08-445.23)	351.76 (337.30-365.13)	224.10 (213.41-235.78)	11.54 (10.61-12.45)	12.53 (11.88-13.20)	9.84 (9.13-10.54)	15.10 (14.23-15.94)	16.29 (15.41-17.17)	14.98 (13.27-16.60)
Israel	138.29 (128.26-148.73)	124.61 (115.38-134.02)	104.94 (97.25-112.82)	388.41 (382.49-395.12)	305.25 (300.07-310.06)	205.67 (199.01-212.75)	8.97 (8.55-9.44)	9.70 (9.23-10.21)	7.33 (6.82-7.81)	3.58 (3.23-3.93)	3.60 (3.24-3.95)	4.38 (3.62-5.18)

Appendix Table 1b. Unscaled values for each SDG health-related indicator by country for 1990, 2000, and 2015												
**Location**	**Indicator 3.3.5: Age-standardised prevalence rate of neglected tropical diseases (NTDs) (per 100,000 population)**			**Indicator 3.4.1: Death rate due to cardiovascular disease, cancer, diabetes, and chronic respiratory disease among populations aged 30 to 70 (per 100,000 population)**			**Indicator 3.4.2: Age-standardised death rate due to self-harm (per 100,000 population)**			**Indicator 3.5.2: Risk-weighted prevalence of alcohol consumption, as measured by the summary exposure value (SEV) for alcohol use**		
	**1990**	**2000**	**2015**	**1990**	**2000**	**2015**	**1990**	**2000**	**2015**	**1990**	**2000**	**2015**
Italy	34.40 (31.82-37.14)	29.14 (26.74-31.54)	37.42 (34.50-40.35)	367.84 (365.13-370.68)	280.92 (278.33-283.23)	210.70 (206.36-214.85)	7.83 (7.50-8.18)	6.84 (6.54-7.16)	5.81 (5.44-6.18)	14.68 (13.52-15.86)	12.98 (11.82-14.19)	9.57 (7.52-11.54)
Luxembourg	25.40 (23.44-27.65)	21.78 (19.96-23.85)	21.21 (19.24-23.31)	437.04 (427.84-445.48)	317.80 (310.63-325.67)	213.65 (206.52-220.98)	16.69 (15.87-17.55)	12.91 (12.22-13.61)	8.89 (8.29-9.51)	16.80 (15.81-17.76)	15.57 (14.57-16.56)	13.99 (12.29-15.65)
Malta	25.79 (23.39-28.66)	21.88 (19.84-24.16)	20.04 (18.05-22.20)	372.53 (357.94-386.88)	299.98 (284.48-314.63)	212.66 (203.33-221.49)	4.88 (4.54-5.26)	4.58 (4.25-4.90)	3.82 (3.49-4.15)	9.76 (8.88-10.65)	8.70 (7.75-9.64)	11.30 (9.40-13.11)
Netherlands	27.48 (25.09-30.69)	25.04 (22.95-27.57)	23.72 (21.65-26.01)	397.33 (393.06-401.52)	342.12 (337.83-345.82)	236.13 (227.40-244.46)	11.54 (11.11-12.02)	10.43 (9.99-10.87)	9.60 (8.99-10.23)	14.32 (13.33-15.29)	13.89 (12.88-14.82)	13.25 (11.20-15.13)
Norway	25.85 (23.86-27.87)	24.09 (22.27-26.08)	22.54 (20.63-24.54)	377.36 (371.76-383.40)	299.49 (294.14-304.39)	204.36 (197.76-210.76)	15.53 (14.82-16.21)	13.01 (12.35-13.64)	9.54 (8.93-10.23)	9.90 (9.05-10.74)	10.30 (9.30-11.25)	10.93 (8.85-12.86)
Portugal	687.63 (583.85-804.92)	676.59 (571.37-793.59)	639.89 (542.69-754.17)	406.64 (402.40-412.56)	333.09 (329.76-336.81)	238.95 (233.20-244.45)	14.62 (13.95-15.30)	10.74 (10.25-11.29)	9.72 (9.09-10.28)	16.62 (15.59-17.67)	15.12 (14.15-16.12)	13.73 (11.81-15.48)
Spain	75.69 (69.90-81.65)	80.54 (74.32-87.14)	121.14 (111.00-130.99)	336.92 (334.10-339.90)	286.06 (283.22-288.23)	206.72 (202.92-209.94)	7.98 (7.60-8.36)	7.46 (7.13-7.81)	6.27 (5.88-6.66)	15.26 (14.48-16.03)	13.62 (12.79-14.43)	12.68 (10.88-14.33)
Sweden	30.44 (28.27-32.56)	28.92 (26.72-31.09)	22.25 (20.21-24.45)	335.76 (331.77-339.73)	270.21 (266.81-273.84)	199.11 (193.25-205.03)	18.03 (17.35-18.76)	13.86 (13.36-14.42)	11.95 (11.30-12.55)	12.52 (11.39-13.59)	13.38 (12.06-14.58)	12.82 (10.60-14.94)
Switzerland	37.23 (34.48-40.24)	37.12 (34.40-40.00)	38.62 (35.36-41.89)	321.43 (316.26-327.29)	257.70 (253.50-262.13)	180.38 (172.50-187.94)	22.30 (21.41-23.24)	17.89 (17.21-18.61)	12.12 (11.35-12.85)	17.54 (16.31-18.74)	15.60 (14.34-16.82)	14.47 (12.14-16.54)
United Kingdom	15.23 (13.67-16.87)	10.53 (9.49-11.69)	9.78 (8.70-10.90)	470.13 (465.28-472.17)	348.89 (347.04-350.78)	243.63 (240.55-248.31)	9.87 (9.64-10.09)	8.93 (8.66-9.20)	7.41 (7.12-7.76)	13.03 (9.73-15.97)	12.85 (9.42-15.90)	12.65 (9.36-15.61)

Appendix Table 1b. Unscaled values for each SDG health-related indicator by country for 1990, 2000, and 2015

Location	Indicator 3.3.5: Age-standardised prevalence rate of neglected tropical diseases (NTDs) (per 100,000 population)			Indicator 3.4.1: Death rate due to cardiovascular disease, cancer, diabetes, and chronic respiratory disease among populations aged 30 to 70 (per 100,000 population)			Indicator 3.4.2: Age-standardised death rate due to self-harm (per 100,000 population)			Indicator 3.5.2: Risk-weighted prevalence of alcohol consumption, as measured by the summary exposure value (SEV) for alcohol use		
	1990	2000	2015	1990	2000	2015	1990	2000	2015	1990	2000	2015
Argentina	23,310.00 (21,813.09-24,806.10)	16,852.26 (16,026.58-17,576.08)	16,899.11 (15,772.94-18,042.99)	518.61 (512.29-523.09)	445.31 (441.21-449.13)	365.95 (356.00-374.90)	10.59 (10.10-11.07)	12.77 (12.21-13.39)	11.48 (10.74-12.19)	14.70 (13.70-15.75)	12.44 (11.47-13.43)	12.38 (10.59-14.13)
Chile	26,633.47 (22,762.04-31,747.99)	18,576.89 (16,568.06-20,065.45)	24,822.74 (20,350.42-29,484.62)	415.75 (409.38-424.86)	300.48 (295.71-307.66)	242.88 (229.54-256.78)	21.36 (20.22-22.48)	13.21 (12.63-13.84)	11.76 (10.72-12.76)	12.11 (11.08-13.07)	10.48 (9.59-11.36)	12.17 (10.55-13.71)
Uruguay	1,264.08 (1,074.59-1,471.68)	1,109.26 (945.57-1,277.09)	644.24 (550.79-740.50)	526.33 (519.51-533.87)	458.56 (452.37-465.02)	375.07 (363.34-387.47)	11.49 (10.91-12.10)	17.04 (16.21-17.85)	17.63 (16.42-18.78)	11.56 (10.68-12.37)	11.88 (11.00-12.71)	11.24 (9.74-12.58)
Belarus	62.68 (57.34-68.40)	48.37 (44.04-53.28)	53.42 (48.08-58.71)	663.78 (645.98-679.87)	818.30 (800.11-838.99)	684.29 (651.16-720.16)	24.56 (22.72-27.85)	38.63 (35.10-41.34)	31.71 (26.95-37.53)	26.22 (24.61-27.64)	27.00 (25.47-28.40)	28.72 (27.04-30.23)
Estonia	52.68 (48.28-57.43)	44.32 (39.93-48.68)	49.63 (44.64-55.05)	656.37 (646.54-665.92)	640.20 (630.75-651.15)	347.91 (331.10-362.43)	28.56 (27.12-29.99)	30.62 (28.98-32.42)	14.18 (12.33-16.32)	26.88 (24.50-29.18)	15.74 (14.49-16.97)	17.56 (15.92-19.05)
Latvia	50.97 (46.91-55.38)	40.54 (37.13-44.18)	41.08 (37.24-45.01)	680.61 (672.19-688.53)	644.01 (635.28-654.40)	474.14 (458.31-489.48)	29.28 (27.88-30.73)	30.22 (28.73-31.76)	19.70 (18.02-21.37)	23.96 (21.94-25.97)	12.98 (11.99-13.98)	14.73 (13.17-16.14)
Lithuania	60.40 (55.22-66.57)	47.86 (43.91-52.38)	49.53 (45.00-54.36)	591.02 (584.25-597.38)	537.24 (530.84-543.56)	443.06 (431.70-457.76)	31.61 (30.11-33.07)	42.28 (40.43-44.40)	32.75 (30.68-34.97)	24.65 (22.70-26.66)	14.44 (13.44-15.41)	17.08 (15.70-18.33)
Moldova	121.06 (108.29-135.70)	101.19 (90.23-113.34)	57.77 (52.56-63.49)	664.22 (655.09-672.92)	651.30 (641.97-660.71)	462.08 (445.46-477.24)	18.78 (17.75-19.75)	16.55 (15.72-17.52)	14.08 (12.83-15.17)	21.48 (18.16-24.57)	19.39 (16.20-22.39)	20.72 (17.44-23.80)
Russia	1,146.47 (1,053.49-1,256.70)	1,136.72 (1,040.28-1,245.37)	1,165.77 (1,068.36-1,280.82)	704.51 (693.30-710.17)	906.04 (895.79-913.49)	609.54 (591.23-629.63)	29.23 (27.97-30.70)	47.95 (45.85-50.17)	31.19 (29.14-33.29)	25.06 (21.80-27.90)	24.83 (21.65-27.70)	26.80 (23.85-29.45)
Ukraine	387.77 (344.45-437.74)	371.16 (328.30-420.50)	380.29 (334.44-430.60)	665.39 (655.64-670.97)	801.93 (795.36-808.96)	595.31 (573.67-618.77)	22.08 (21.00-23.12)	32.14 (30.67-33.70)	21.20 (19.39-23.04)	23.51 (21.60-25.26)	23.92 (22.13-25.71)	26.47 (24.04-28.74)

Appendix Table 1b. Unscaled values for each SDG health-related indicator by country for 1990, 2000, and 2015

Location	Indicator 3.3.5: Age-standardised prevalence rate of neglected tropical diseases (NTDs) (per 100,000 population)			Indicator 3.4.1: Death rate due to cardiovascular disease, cancer, diabetes, and chronic respiratory disease among populations aged 30 to 70 (per 100,000 population)			Indicator 3.4.2: Age-standardised death rate due to self-harm (per 100,000 population)			Indicator 3.5.2: Risk-weighted prevalence of alcohol consumption, as measured by the summary exposure value (SEV) for alcohol use		
	1990	2000	2015	1990	2000	2015	1990	2000	2015	1990	2000	2015
Albania	28.57 (25.68-31.46)	26.51 (23.77-29.23)	30.23 (26.86-33.20)	405.54 (392.58-421.06)	392.47 (379.90-406.44)	340.62 (315.16-366.58)	4.14 (3.75-5.00)	5.52 (4.92-6.03)	5.26 (4.15-6.09)	5.36 (4.83-5.91)	6.99 (6.37-7.68)	8.80 (7.70-9.86)
Bosnia and Herzegovina	64.36 (58.22-71.62)	44.73 (40.77-48.95)	37.87 (34.34-41.85)	521.78 (513.85-529.96)	438.59 (427.20-449.58)	336.98 (321.00-348.97)	14.47 (13.08-16.27)	11.57 (9.75-13.34)	5.14 (4.13-11.43)	9.46 (8.85-10.04)	8.41 (7.81-9.00)	8.04 (7.39-8.68)
Bulgaria	51.27 (46.59-56.95)	47.88 (44.22-51.79)	41.35 (37.51-45.85)	621.34 (613.65-627.32)	641.15 (634.75-647.09)	521.40 (502.16-542.18)	15.87 (15.10-16.58)	15.66 (14.94-16.44)	10.93 (10.12-11.67)	16.03 (14.96-17.04)	12.84 (11.90-13.75)	13.37 (11.94-14.63)
Croatia	45.52 (41.33-49.88)	44.47 (40.77-48.31)	39.99 (36.32-43.57)	553.50 (546.06-561.90)	497.56 (491.49-504.19)	355.09 (342.82-367.77)	22.37 (21.40-23.41)	18.67 (17.87-19.49)	13.52 (12.58-14.48)	15.28 (14.28-16.21)	14.77 (13.90-15.64)	13.69 (12.46-14.87)
Czech Republic	32.32 (29.13-35.68)	28.45 (25.36-31.41)	32.02 (28.72-35.07)	664.21 (657.24-670.39)	483.82 (479.21-488.47)	329.11 (321.27-336.88)	19.34 (18.48-20.24)	14.90 (14.23-15.54)	12.01 (11.22-12.84)	16.58 (15.80-17.37)	16.69 (15.93-17.51)	15.81 (14.78-16.86)
Hungary	51.23 (45.48-59.02)	37.33 (33.59-41.42)	34.70 (31.27-38.29)	744.12 (736.68-756.69)	662.86 (654.70-682.79)	454.83 (436.72-473.33)	36.24 (34.66-37.92)	25.78 (24.55-27.33)	17.08 (15.67-18.67)	16.84 (15.96-17.70)	15.17 (14.33-16.07)	14.15 (12.97-15.24)
Macedonia	42.21 (38.45-46.30)	34.85 (31.72-38.09)	33.69 (30.40-37.04)	554.25 (544.35-564.02)	583.84 (574.31-592.01)	421.81 (402.67-440.50)	9.32 (8.38-10.13)	8.95 (8.32-9.74)	7.53 (6.68-8.62)	12.55 (10.91-14.00)	11.77 (10.12-13.27)	12.26 (10.64-13.79)
Montenegro	52.26 (47.06-57.82)	43.35 (39.20-47.88)	36.75 (33.15-40.00)	511.50 (486.28-535.75)	548.29 (536.85-559.65)	423.60 (396.44-447.29)	10.58 (9.36-12.14)	11.44 (10.39-12.85)	11.01 (9.54-12.54)	10.45 (8.97-11.85)	9.71 (8.43-10.97)	10.79 (9.48-12.10)
Poland	38.08 (34.27-43.14)	27.86 (25.38-30.76)	29.37 (26.78-32.01)	683.78 (677.05-688.35)	543.79 (539.60-547.77)	393.68 (385.65-401.65)	14.69 (13.97-15.36)	17.66 (16.85-18.53)	17.41 (16.26-18.57)	12.12 (11.37-12.90)	12.07 (11.24-12.88)	13.47 (12.21-14.67)
Romania	75.26 (67.59-84.48)	46.58 (42.47-51.34)	46.27 (42.55-50.28)	598.99 (586.79-604.82)	599.87 (592.27-605.73)	462.45 (446.19-479.81)	9.91 (9.46-10.38)	11.30 (10.75-11.90)	11.06 (10.13-11.89)	16.20 (15.29-17.08)	14.71 (13.83-15.54)	15.05 (13.77-16.33)

Appendix Table 1b. Unscaled values for each SDG health-related indicator by country for 1990, 2000, and 2015

Location	Indicator 3.3.5: Age-standardised prevalence rate of neglected tropical diseases (NTDs) (per 100,000 population)			Indicator 3.4.1: Death rate due to cardiovascular disease, cancer, diabetes, and chronic respiratory disease among populations aged 30 to 70 (per 100,000 population)			Indicator 3.4.2: Age-standardised death rate due to self-harm (per 100,000 population)			Indicator 3.5.2: Risk-weighted prevalence of alcohol consumption, as measured by the summary exposure value (SEV) for alcohol use		
	1990	2000	2015	1990	2000	2015	1990	2000	2015	1990	2000	2015
Serbia	112.77 (98.56-129.99)	81.38 (71.76-94.47)	51.85 (47.45-57.03)	542.98 (521.70-564.91)	636.77 (625.66-648.07)	435.78 (426.68-445.02)	18.90 (16.37-20.85)	19.76 (17.67-21.13)	15.02 (13.77-16.60)	10.71 (9.25-12.15)	10.52 (9.23-11.72)	12.07 (10.55-13.45)
Slovakia	31.11 (28.01-34.80)	23.90 (21.78-26.18)	23.42 (21.14-25.73)	676.28 (666.20-689.22)	560.68 (553.42-568.62)	370.27 (361.61-380.16)	16.73 (14.48-18.46)	13.96 (12.90-14.92)	10.21 (9.15-11.52)	16.36 (15.50-17.24)	15.45 (14.62-16.26)	14.52 (13.41-15.64)
Slovenia	54.99 (49.17-62.27)	41.64 (37.76-46.22)	34.59 (31.57-37.91)	466.64 (459.56-481.70)	396.06 (389.98-407.39)	239.98 (227.68-248.90)	30.33 (28.91-31.74)	27.16 (25.97-28.48)	14.87 (13.42-16.80)	17.05 (16.13-17.94)	15.09 (14.09-16.03)	14.75 (13.14-16.20)
Armenia	899.34 (667.80-1,268.63)	465.73 (371.68-525.87)	841.88 (671.25-1,055.50)	582.12 (561.45-604.68)	622.39 (601.59-645.81)	481.88 (454.99-507.78)	4.01 (3.63-5.33)	4.65 (4.23-5.18)	7.28 (5.15-8.32)	6.22 (5.34-7.07)	5.83 (5.24-6.44)	6.58 (5.47-7.66)
Azerbaijan	4,782.24 (3,286.24-6,934.13)	2,136.83 (1,705.02-2,440.24)	3,328.65 (2,402.79-4,970.84)	698.04 (670.89-725.82)	719.26 (687.44-749.93)	503.22 (471.60-544.50)	3.59 (3.24-4.07)	3.03 (2.72-3.87)	4.11 (3.28-4.84)	7.67 (6.60-8.62)	7.05 (6.18-7.93)	9.41 (7.79-10.89)
Georgia	769.17 (560.31-1,155.05)	409.62 (346.22-454.03)	697.15 (538.12-993.50)	628.48 (596.72-664.32)	577.51 (541.75-610.30)	568.71 (523.29-607.25)	5.53 (4.94-6.79)	6.25 (5.48-6.97)	7.82 (6.74-8.87)	8.07 (7.09-9.02)	6.79 (6.14-7.42)	8.64 (7.41-9.72)
Kazakhstan	24,809.69 (19,415.92-31,298.89)	15,234.75 (12,694.30-16,744.46)	22,618.93 (16,151.77-30,129.19)	733.39 (723.17-740.78)	938.55 (929.04-949.61)	657.75 (610.67-705.11)	23.58 (22.41-24.78)	35.54 (33.95-37.58)	30.52 (27.55-33.75)	13.91 (12.61-15.14)	11.34 (10.33-12.31)	12.62 (11.01-14.12)
Kyrgyzstan	25,929.08 (19,729.95-34,285.30)	15,090.89 (12,718.51-16,706.35)	23,241.84 (17,330.74-30,905.02)	699.20 (671.92-726.43)	794.44 (762.48-829.74)	613.62 (578.47-646.25)	17.73 (16.06-19.15)	18.89 (17.38-21.24)	14.21 (12.66-17.89)	8.56 (7.74-9.37)	7.61 (7.00-8.22)	7.65 (6.60-8.65)
Mongolia	427.50 (383.94-470.57)	305.12 (281.70-328.84)	318.90 (282.49-366.43)	812.33 (772.66-851.74)	1,027.71 (978.20-1,075.38)	820.64 (775.74-862.99)	26.77 (22.27-32.28)	35.85 (31.58-40.44)	34.04 (29.98-38.45)	5.96 (5.26-6.61)	6.11 (5.42-6.76)	8.66 (7.37-9.82)
Tajikistan	10,623.99 (8,183.83-13,686.47)	5,949.29 (4,779.23-6,692.54)	9,428.56 (6,835.16-13,160.67)	616.15 (588.86-645.69)	647.69 (618.03-680.28)	460.32 (434.33-488.42)	7.07 (6.09-7.81)	5.71 (5.14-7.15)	4.95 (4.07-6.40)	6.99 (6.05-7.86)	5.60 (5.03-6.15)	4.75 (3.98-5.53)

Appendix Table 1b. Unscaled values for each SDG health-related indicator by country for 1990, 2000, and 2015

Location	Indicator 3.3.5: Age-standardised prevalence rate of neglected tropical diseases (NTDs) (per 100,000 population)			Indicator 3.4.1: Death rate due to cardiovascular disease, cancer, diabetes, and chronic respiratory disease among populations aged 30 to 70 (per 100,000 population)			Indicator 3.4.2: Age-standardised death rate due to self-harm (per 100,000 population)			Indicator 3.5.2: Risk-weighted prevalence of alcohol consumption, as measured by the summary exposure value (SEV) for alcohol use		
	1990	2000	2015	1990	2000	2015	1990	2000	2015	1990	2000	2015
Turkmenistan	3,472.31 (2,487.30-4,685.55)	1,858.11 (1,505.83-2,092.04)	3,260.26 (2,285.57-4,497.74)	783.82 (769.70-793.30)	862.79 (844.23-886.69)	597.61 (579.79-614.87)	10.31 (9.43-11.13)	11.47 (10.21-13.52)	8.76 (7.56-11.91)	4.96 (4.20-5.68)	6.02 (5.46-6.58)	6.37 (5.42-7.29)
Uzbekistan	300.50 (237.33-372.26)	181.67 (154.61-203.36)	291.41 (232.02-356.98)	688.79 (661.81-716.90)	731.13 (700.47-763.68)	552.39 (523.46-579.55)	11.18 (10.08-12.12)	11.89 (10.89-13.47)	10.46 (8.74-12.16)	4.75 (4.06-5.42)	5.37 (4.89-5.82)	6.76 (5.79-7.67)
Colombia	48,028.67 (44,374.43-51,765.62)	38,238.41 (35,062.18-40,094.63)	47,139.40 (43,002.80-51,103.42)	415.73 (411.18-420.16)	355.26 (351.10-359.11)	228.49 (219.65-236.80)	5.30 (5.00-5.62)	7.53 (7.10-7.97)	6.19 (5.65-6.83)	9.58 (8.72-10.36)	8.90 (8.11-9.62)	8.89 (7.65-10.10)
Costa Rica	38,087.38 (31,813.89-44,798.56)	21,693.39 (17,989.49-24,331.87)	36,817.27 (31,011.23-43,872.38)	295.05 (289.41-300.41)	254.90 (250.54-259.12)	200.48 (191.20-208.99)	6.18 (5.82-6.54)	7.36 (6.95-7.78)	7.56 (6.93-8.22)	8.99 (8.31-9.67)	8.69 (7.98-9.40)	8.36 (7.25-9.37)
El Salvador	39,589.92 (35,064.45-44,732.13)	30,371.26 (27,621.89-32,170.20)	14,269.94 (11,695.85-17,323.01)	338.86 (324.98-353.83)	284.22 (271.69-300.21)	260.42 (239.13-284.84)	13.32 (11.16-14.68)	10.71 (9.70-11.83)	9.77 (8.47-11.91)	5.25 (4.77-5.70)	5.84 (5.34-6.32)	6.17 (5.30-6.92)
Guatemala	81,948.68 (78,459.40-85,486.87)	68,362.23 (64,685.05-70,560.13)	53,193.74 (47,622.73-59,636.67)	282.28 (269.81-291.82)	305.21 (296.51-311.86)	297.72 (262.16-341.05)	9.89 (9.28-10.53)	13.99 (13.18-14.86)	7.86 (6.62-9.21)	4.31 (4.03-4.59)	3.99 (3.73-4.26)	3.98 (3.69-4.27)
Honduras	59,115.09 (51,562.93-67,859.92)	40,319.33 (35,901.10-43,552.65)	46,515.08 (39,936.11-53,796.57)	385.05 (360.00-409.51)	450.50 (376.01-541.29)	388.29 (310.24-478.07)	5.77 (5.01-7.82)	6.20 (4.88-8.11)	6.57 (4.84-8.93)	5.34 (4.85-5.81)	5.98 (5.50-6.46)	6.10 (5.36-6.76)
Mexico	25,820.47 (24,713.87-26,647.22)	21,281.72 (20,809.26-21,867.62)	21,580.44 (20,696.19-22,272.54)	343.38 (340.50-350.85)	321.61 (318.88-326.72)	284.39 (280.82-288.43)	4.23 (4.06-4.42)	5.04 (4.86-5.26)	6.17 (5.86-6.46)	8.73 (8.14-9.29)	8.92 (8.37-9.44)	8.02 (7.16-8.81)
Nicaragua	32,297.81 (28,862.17-35,889.73)	24,878.61 (22,334.44-26,216.75)	12,395.51 (9,581.08-16,314.08)	245.13 (232.48-259.22)	251.99 (239.65-265.55)	240.92 (220.86-261.93)	6.57 (5.89-7.62)	8.84 (7.32-9.71)	7.64 (6.67-8.72)	6.07 (5.62-6.51)	6.11 (5.69-6.56)	5.99 (5.39-6.60)
Panama	51,889.85 (47,660.48-56,341.43)	11,490.84 (10,198.31-12,300.68)	12,488.24 (9,754.66-15,345.12)	319.26 (299.27-340.02)	263.95 (247.49-278.98)	227.01 (199.20-258.45)	6.13 (5.46-6.93)	7.25 (6.25-8.10)	6.12 (4.96-7.89)	7.66 (7.13-8.16)	7.94 (7.38-8.47)	8.58 (7.78-9.39)

Appendix Table 1b. Unscaled values for each SDG health-related indicator by country for 1990, 2000, and 2015												
	**Indicator 3.3.5: Age-standardised prevalence rate of neglected tropical diseases (NTDs) (per 100,000 population)**			**Indicator 3.4.1: Death rate due to cardiovascular disease, cancer, diabetes, and chronic respiratory disease among populations aged 30 to 70 (per 100,000 population)**			**Indicator 3.4.2: Age-standardised death rate due to self-harm (per 100,000 population)**			**Indicator 3.5.2: Risk-weighted prevalence of alcohol consumption, as measured by the summary exposure value (SEV) for alcohol use**		
**Location**	**1990**	**2000**	**2015**	**1990**	**2000**	**2015**	**1990**	**2000**	**2015**	**1990**	**2000**	**2015**
Venezuela	56,580.66 (53,421.16-60,155.27)	52,063.89 (49,218.17-53,847.45)	53,682.13 (45,643.62-61,470.59)	432.89 (427.65-438.32)	370.89 (366.43-375.53)	334.60 (296.00-375.32)	7.29 (6.91-7.71)	9.59 (9.03-10.15)	9.49 (8.00-10.95)	8.25 (6.99-9.29)	7.97 (6.72-9.01)	7.87 (6.65-8.96)
Bolivia	36,590.00 (32,820.26-41,011.83)	23,783.75 (21,586.98-25,487.52)	32,312.86 (28,713.27-35,905.28)	472.12 (428.63-519.85)	381.24 (352.06-409.35)	319.44 (265.80-383.91)	17.17 (13.59-20.86)	15.13 (12.99-16.94)	12.89 (10.30-16.16)	6.60 (5.60-7.47)	6.97 (5.98-7.80)	7.27 (6.30-8.12)
Ecuador	63,340.68 (58,015.46-68,608.32)	50,175.45 (45,941.55-52,657.78)	52,759.05 (45,803.73-60,834.78)	338.24 (325.93-351.19)	324.04 (307.59-340.85)	282.19 (253.68-309.39)	6.02 (5.47-7.51)	7.68 (6.80-8.39)	7.99 (6.58-9.26)	5.02 (4.70-5.35)	5.66 (5.33-6.00)	5.81 (5.40-6.23)
Peru	42,217.04 (37,022.11-47,648.40)	29,604.82 (25,995.90-31,868.90)	28,575.27 (23,763.38-33,582.83)	314.75 (295.96-334.42)	260.92 (242.06-281.03)	183.09 (162.19-208.09)	4.00 (3.59-4.70)	4.96 (3.94-5.49)	3.37 (2.81-3.98)	10.29 (9.69-10.89)	9.74 (9.15-10.28)	9.58 (8.53-10.55)
Antigua and Barbuda	424.38 (327.08-563.92)	334.33 (249.75-477.98)	555.40 (410.63-746.55)	457.62 (433.25-483.29)	427.07 (404.80-452.71)	346.18 (302.86-379.37)	2.09 (1.90-2.32)	2.17 (1.95-2.39)	2.15 (1.87-2.46)	8.96 (7.50-10.22)	8.29 (6.39-9.84)	9.66 (7.96-10.94)
The Bahamas	280.59 (246.82-320.86)	200.98 (178.52-219.98)	267.73 (230.34-306.83)	615.52 (586.93-647.41)	535.10 (498.79-573.03)	422.29 (376.38-464.87)	3.58 (3.14-4.05)	3.81 (3.31-4.40)	3.35 (2.78-3.99)	12.00 (11.21-12.74)	8.64 (7.80-9.39)	10.69 (9.71-11.56)
Barbados	264.62 (224.96-305.85)	266.21 (224.93-322.42)	488.39 (374.04-656.19)	474.19 (457.17-491.47)	419.63 (400.96-441.03)	374.02 (340.81-407.95)	4.23 (3.89-4.56)	3.96 (3.61-4.33)	4.63 (4.07-5.23)	8.90 (8.10-9.66)	7.55 (6.95-8.17)	9.79 (8.77-10.76)
Belize	27,089.62 (22,133.98-32,496.23)	14,991.89 (12,451.49-16,566.30)	7,499.27 (5,937.15-9,086.52)	452.63 (426.25-483.57)	580.90 (550.53-611.65)	463.14 (416.32-507.88)	5.33 (4.71-6.00)	8.37 (7.18-9.41)	7.39 (6.17-8.72)	8.13 (7.39-8.84)	7.40 (6.69-8.02)	8.89 (7.87-9.77)
Cuba	28,374.08 (26,898.60-29,735.73)	24,645.96 (23,349.40-25,439.96)	28,483.21 (26,885.06-29,846.59)	444.65 (440.28-449.25)	399.24 (394.99-404.03)	338.51 (326.11-350.08)	21.25 (20.41-22.13)	16.75 (16.05-17.54)	13.40 (12.30-14.35)	7.42 (6.75-8.03)	6.90 (6.27-7.56)	8.25 (7.20-9.30)
Dominica	28,249.43 (23,419.89-33,259.02)	276.29 (239.58-306.17)	211.47 (154.01-297.69)	473.12 (446.14-507.47)	423.52 (401.64-452.09)	437.50 (387.86-486.77)	3.55 (3.14-4.00)	4.20 (3.67-4.71)	4.99 (4.07-5.88)	10.79 (9.85-11.67)	9.15 (8.39-9.88)	9.72 (8.45-10.78)

Appendix Table 1b. Unscaled values for each SDG health-related indicator by country for 1990, 2000, and 2015

Location	Indicator 3.3.5: Age-standardised prevalence rate of neglected tropical diseases (NTDs) (per 100,000 population)			Indicator 3.4.1: Death rate due to cardiovascular disease, cancer, diabetes, and chronic respiratory disease among populations aged 30 to 70 (per 100,000 population)			Indicator 3.4.2: Age-standardised death rate due to self-harm (per 100,000 population)			Indicator 3.5.2: Risk-weighted prevalence of alcohol consumption, as measured by the summary exposure value (SEV) for alcohol use		
	1990	2000	2015	1990	2000	2015	1990	2000	2015	1990	2000	2015
Dominican Republic	3,734.86 (1,678.05-7,581.26)	3,707.93 (1,668.70-7,539.32)	3,736.64 (1,692.30-7,560.82)	336.92 (320.12-353.79)	322.49 (304.16-350.12)	326.37 (316.67-346.48)	4.95 (4.44-6.45)	6.99 (5.55-7.77)	6.75 (5.89-7.55)	7.35 (6.72-7.91)	8.43 (7.76-9.06)	8.73 (7.72-9.66)
Grenada	70,213.26 (65,204.85-74,726.52)	22,477.66 (20,923.45-23,502.03)	30,158.63 (24,967.11-35,115.64)	609.34 (581.73-637.89)	587.41 (562.46-611.89)	552.59 (513.65-597.93)	5.65 (5.05-6.40)	7.41 (6.65-8.31)	6.77 (5.64-7.84)	11.64 (10.89-12.32)	10.91 (10.16-11.58)	10.70 (9.61-11.73)
Guyana	34,071.07 (27,696.77-41,365.14)	18,257.70 (15,635.54-20,361.36)	23,167.28 (18,856.50-28,205.64)	833.36 (791.03-875.53)	817.72 (766.63-868.97)	672.19 (593.44-759.94)	22.68 (20.43-26.29)	30.65 (27.29-34.07)	29.16 (23.22-35.11)	10.08 (8.98-11.01)	9.41 (8.39-10.29)	9.67 (8.54-10.69)
Haiti	72,189.48 (68,066.12-76,607.52)	42,338.13 (39,753.13-44,529.93)	9,008.91 (7,585.30-10,662.11)	939.52 (843.44-1,042.17)	720.64 (601.00-848.53)	678.90 (523.29-834.81)	9.72 (6.70-14.83)	8.47 (6.05-13.09)	8.70 (5.76-13.81)	8.58 (7.79-9.30)	8.34 (7.60-9.04)	8.60 (7.45-9.69)
Jamaica	52,566.67 (49,209.17-56,143.45)	45,130.56 (42,941.11-46,599.51)	66,101.72 (63,171.16-69,176.15)	432.78 (414.03-451.22)	427.83 (407.86-448.64)	398.01 (355.11-439.70)	1.98 (1.79-2.22)	2.82 (2.50-3.19)	3.36 (2.83-3.95)	7.41 (6.71-8.09)	6.47 (5.90-7.04)	7.53 (6.51-8.53)
Saint Lucia	82,491.92 (78,008.09-86,966.79)	39,396.11 (36,721.71-41,584.03)	41,784.04 (35,744.93-48,640.73)	576.25 (540.33-604.39)	506.68 (487.22-531.01)	398.20 (364.05-430.08)	6.07 (5.55-6.66)	6.60 (6.01-7.19)	6.63 (5.81-7.44)	11.50 (10.74-12.22)	11.20 (10.41-11.92)	11.60 (10.49-12.63)
Saint Vincent and the Grenadines	50,623.64 (43,781.54-59,856.50)	15,284.23 (13,256.69-16,581.12)	27,527.22 (22,883.14-32,721.76)	561.99 (519.93-596.33)	596.11 (566.67-627.15)	535.29 (498.54-574.55)	5.83 (5.26-6.40)	7.22 (6.62-7.91)	8.30 (7.26-9.36)	7.76 (6.12-9.13)	7.78 (6.18-9.19)	8.65 (7.00-10.02)
Suriname	9,605.88 (8,526.02-11,002.05)	8,118.46 (7,197.58-9,502.77)	10,307.34 (8,855.64-12,068.77)	498.06 (472.77-524.62)	476.02 (447.43-507.72)	420.61 (371.60-470.45)	18.60 (16.95-21.38)	22.39 (20.20-24.77)	24.54 (20.79-28.60)	7.13 (6.35-7.85)	7.42 (6.73-8.11)	8.21 (7.21-9.13)
Trinidad and Tobago	4,401.28 (3,744.52-5,171.12)	3,298.87 (2,926.59-3,568.99)	4,543.31 (3,886.49-5,232.58)	715.55 (704.29-726.83)	647.35 (636.06-658.78)	512.94 (468.40-562.79)	14.26 (13.52-15.03)	13.96 (13.15-14.80)	13.86 (12.01-15.98)	9.69 (7.98-11.10)	9.39 (7.80-10.71)	10.64 (8.98-11.99)
Brazil	25,190.06 (22,976.26-27,499.21)	15,700.59 (14,548.55-16,977.12)	24,427.26 (22,245.76-26,549.29)	554.11 (541.90-566.00)	481.07 (469.66-492.84)	369.20 (352.66-386.10)	8.06 (7.47-8.54)	7.18 (6.81-8.23)	6.56 (6.06-7.93)	7.59 (7.10-8.00)	8.99 (8.52-9.40)	8.94 (8.39-9.47)

Appendix Table 1b. Unscaled values for each SDG health-related indicator by country for 1990, 2000, and 2015												
	**Indicator 3.3.5: Age-standardised prevalence rate of neglected tropical diseases (NTDs) (per 100,000 population)**			**Indicator 3.4.1: Death rate due to cardiovascular disease, cancer, diabetes, and chronic respiratory disease among populations aged 30 to 70 (per 100,000 population)**			**Indicator 3.4.2: Age-standardised death rate due to self-harm (per 100,000 population)**			**Indicator 3.5.2: Risk-weighted prevalence of alcohol consumption, as measured by the summary exposure value (SEV) for alcohol use**		
**Location**	**1990**	**2000**	**2015**	**1990**	**2000**	**2015**	**1990**	**2000**	**2015**	**1990**	**2000**	**2015**
Paraguay	24,203.76 (21,304.84-26,947.17)	16,742.28 (14,756.26-17,990.01)	22,450.01 (19,894.32-25,558.00)	368.13 (342.65-393.97)	377.45 (353.58-402.56)	390.03 (344.02-442.78)	4.16 (3.64-4.73)	5.46 (4.77-6.05)	6.47 (5.11-8.00)	10.42 (9.78-10.98)	10.04 (9.44-10.60)	9.85 (9.04-10.61)
China	64,866.86 (62,607.76-67,773.09)	25,223.80 (24,173.38-26,448.36)	20,800.45 (19,253.58-22,347.62)	704.78 (667.25-744.40)	598.70 (575.88-619.24)	392.60 (373.41-414.14)	22.71 (14.51-25.62)	16.95 (13.61-18.11)	9.04 (8.12-10.39)	6.76 (6.22-7.27)	7.19 (6.63-7.77)	8.14 (7.19-9.03)
North Korea	28,141.98 (21,584.14-37,199.32)	18,113.47 (15,873.36-19,678.98)	11,177.20 (8,167.69-16,160.37)	577.29 (435.34-758.38)	626.36 (482.32-802.23)	587.47 (468.13-719.03)	19.58 (13.79-26.95)	17.85 (13.08-23.35)	14.72 (11.10-19.61)	6.74 (5.40-7.84)	6.02 (4.78-7.14)	5.48 (4.29-6.57)
Taiwan	74,802.68 (72,089.88-77,523.24)	31,026.35 (29,329.91-32,126.18)	25,907.10 (23,298.55-28,633.09)	447.22 (436.45-460.16)	396.23 (388.48-402.53)	281.12 (241.97-322.37)	16.59 (14.67-19.75)	14.08 (13.04-16.82)	13.47 (11.02-15.80)	8.25 (6.79-9.44)	8.86 (7.35-10.09)	8.73 (7.28-9.99)
Cambodia	54,520.15 (48,849.20-60,974.34)	28,776.25 (25,737.00-30,774.07)	980.69 (822.06-1,168.96)	773.43 (690.40-852.10)	741.08 (679.12-796.24)	531.98 (452.57-622.01)	19.46 (13.10-27.47)	18.87 (13.48-25.98)	14.36 (10.86-17.78)	2.60 (2.20-2.99)	5.66 (4.93-6.28)	6.72 (5.62-7.65)
Indonesia	59,142.75 (54,852.06-63,283.52)	33,692.61 (30,972.92-35,799.41)	29,397.82 (25,697.11-33,878.67)	535.41 (484.00-582.38)	555.27 (511.43-596.46)	517.00 (417.50-607.35)	2.89 (2.40-3.56)	3.03 (2.57-3.55)	3.11 (2.37-4.02)	1.93 (1.71-2.14)	2.00 (1.79-2.20)	2.11 (1.86-2.34)
Laos	56,328.25 (49,643.44-63,175.95)	42,064.81 (37,194.36-45,586.03)	36,133.26 (32,056.74-41,815.43)	910.36 (727.53-1,112.61)	831.90 (734.85-934.99)	565.36 (465.81-682.40)	23.67 (14.42-34.98)	22.93 (14.91-31.13)	16.21 (11.79-20.94)	3.01 (2.27-3.81)	3.34 (2.48-4.26)	4.01 (2.83-5.18)
Malaysia	72,967.13 (69,300.42-76,789.35)	62,908.73 (58,904.43-65,600.23)	73,735.44 (69,323.22-78,294.45)	507.56 (495.02-523.08)	443.63 (432.44-463.79)	380.34 (331.56-432.14)	9.63 (6.98-10.97)	9.80 (7.10-10.80)	8.92 (6.84-10.73)	2.82 (2.61-3.03)	2.11 (1.91-2.30)	2.33 (2.06-2.59)
Maldives	549.13 (345.55-1,068.78)	425.29 (272.86-817.42)	481.18 (320.24-826.78)	483.45 (455.61-524.81)	307.53 (292.47-323.18)	184.23 (155.87-214.81)	32.00 (22.76-42.35)	24.16 (20.89-26.85)	12.66 (10.31-15.64)	2.96 (2.42-3.48)	3.41 (2.76-4.03)	3.99 (3.07-4.78)
Mauritius	1,363.79 (744.31-2,504.71)	1,266.84 (660.67-2,407.55)	1,327.17 (710.67-2,467.86)	740.64 (724.63-756.61)	633.88 (621.41-646.67)	446.82 (423.86-468.68)	13.27 (12.54-14.06)	11.86 (11.20-12.62)	8.92 (8.11-9.66)	6.51 (5.94-7.07)	6.57 (5.95-7.15)	6.21 (5.03-7.28)

Location	Indicator 3.3.5: Age-standardised prevalence rate of neglected tropical diseases (NTDs) (per 100,000 population)			Indicator 3.4.1: Death rate due to cardiovascular disease, cancer, diabetes, and chronic respiratory disease among populations aged 30 to 70 (per 100,000 population)			Indicator 3.4.2: Age-standardised death rate due to self-harm (per 100,000 population)			Indicator 3.5.2: Risk-weighted prevalence of alcohol consumption, as measured by the summary exposure value (SEV) for alcohol use		
	1990	2000	2015	1990	2000	2015	1990	2000	2015	1990	2000	2015
Myanmar	73,101.46 (66,571.26-80,412.04)	57,808.27 (52,755.99-61,227.27)	26,193.24 (22,099.60-30,703.53)	768.88 (448.15-1,151.92)	728.47 (426.33-1,087.08)	583.59 (353.57-868.71)	4.29 (2.55-6.63)	4.46 (2.29-7.03)	4.91 (2.83-7.58)	1.26 (1.09-1.42)	1.43 (1.25-1.60)	1.64 (1.34-1.92)
Philippines	81,188.03 (78,281.04-84,036.27)	74,690.27 (72,028.96-76,639.35)	61,441.47 (55,616.68-68,319.13)	446.70 (438.43-453.31)	512.90 (506.43-519.56)	527.14 (486.42-569.26)	15.05 (14.29-15.83)	5.16 (4.92-5.44)	5.92 (5.32-6.55)	7.38 (6.76-7.92)	7.70 (7.15-8.19)	7.18 (6.08-8.10)
Sri Lanka	41,380.12 (36,831.18-46,621.06)	15,831.41 (13,673.85-17,298.81)	17,442.55 (14,473.77-20,901.85)	362.17 (354.22-369.03)	412.11 (406.13-423.81)	290.64 (230.04-360.76)	54.52 (51.94-56.75)	47.01 (45.00-49.22)	28.05 (21.68-35.74)	2.09 (1.85-2.32)	2.47 (2.13-2.80)	4.42 (3.64-5.16)
Seychelles	55,678.82 (47,888.93-64,207.77)	37,654.11 (31,937.50-41,228.55)	55,797.43 (48,385.66-63,740.18)	635.47 (601.78-670.13)	561.78 (537.04-586.61)	418.69 (366.08-475.51)	15.12 (12.16-17.12)	11.68 (10.37-13.11)	8.44 (7.08-10.11)	6.16 (5.52-6.74)	7.31 (6.56-7.98)	7.10 (5.90-8.09)
Thailand	41,833.61 (36,769.94-47,355.32)	21,566.52 (19,253.10-23,232.33)	24,211.03 (21,196.72-28,238.48)	439.55 (415.30-466.74)	476.39 (445.00-507.37)	369.20 (303.84-433.91)	11.15 (10.07-13.12)	17.60 (15.41-19.55)	15.83 (12.40-19.24)	6.99 (6.17-7.67)	7.81 (7.00-8.53)	8.01 (6.88-8.98)
Timor-Leste	32,862.69 (27,549.80-39,159.48)	21,499.75 (18,165.21-24,218.85)	30,960.44 (25,535.02-38,001.74)	554.89 (451.97-681.67)	468.61 (381.89-541.11)	365.16 (273.09-460.78)	15.17 (9.78-21.74)	12.36 (7.55-17.87)	10.33 (7.04-15.32)	2.88 (2.15-3.58)	3.26 (2.41-4.04)	4.38 (3.21-5.33)
Vietnam	67,271.57 (64,627.01-70,094.94)	59,910.86 (56,824.83-61,634.65)	28,459.16 (24,534.50-33,259.84)	465.00 (396.09-553.13)	453.66 (375.79-525.84)	375.45 (272.69-484.84)	9.66 (7.47-12.39)	9.59 (7.59-11.86)	8.61 (6.12-11.71)	1.73 (1.43-2.01)	3.84 (3.33-4.33)	7.26 (6.04-8.35)
Federated States of Micronesia	47,116.74 (41,007.99-54,690.46)	33,054.54 (28,278.59-35,601.17)	46,572.46 (37,111.61-58,698.27)	920.23 (532.15-1,376.72)	858.57 (542.71-1,307.31)	788.63 (502.07-1,326.93)	20.60 (11.43-31.04)	17.58 (10.78-26.80)	15.63 (10.06-25.59)	3.35 (2.57-4.05)	3.42 (2.68-4.09)	3.54 (2.73-4.25)
Fiji	71,240.26 (66,080.37-77,307.40)	54,574.47 (49,492.16-58,348.29)	67,666.14 (59,879.03-74,047.61)	1,045.16 (883.72-1,243.11)	1,122.03 (998.83-1,257.59)	973.28 (828.06-1,142.75)	12.38 (10.04-15.12)	13.96 (12.07-16.17)	13.69 (11.29-16.60)	4.18 (3.83-4.50)	4.08 (3.78-4.37)	4.96 (4.34-5.55)
Kiribati	74,654.88 (69,853.03-79,974.44)	67,402.26 (64,670.57-70,217.45)	61,572.14 (58,490.53-65,036.25)	1,082.11 (951.68-1,207.36)	1,115.99 (1,000.23-1,235.76)	1,018.11 (858.94-1,178.97)	26.27 (22.00-30.92)	21.94 (18.54-26.95)	19.90 (15.88-25.49)	4.11 (3.73-4.48)	4.03 (3.70-4.35)	4.15 (3.61-4.63)

Appendix Table 1b. Unscaled values for each SDG health-related indicator by country for 1990, 2000, and 2015

Location	Indicator 3.3.5: Age-standardised prevalence rate of neglected tropical diseases (NTDs) (per 100,000 population)			Indicator 3.4.1: Death rate due to cardiovascular disease, cancer, diabetes, and chronic respiratory disease among populations aged 30 to 70 (per 100,000 population)			Indicator 3.4.2: Age-standardised death rate due to self-harm (per 100,000 population)			Indicator 3.5.2: Risk-weighted prevalence of alcohol consumption, as measured by the summary exposure value (SEV) for alcohol use		
	1990	2000	2015	1990	2000	2015	1990	2000	2015	1990	2000	2015
Marshall Islands	69,783.09 (64,706.49-75,687.09)	60,077.42 (56,548.17-62,176.37)	68,005.17 (63,056.63-74,046.21)	870.83 (772.84-968.04)	895.51 (760.33-1,009.21)	856.48 (711.32-1,018.30)	17.27 (11.73-23.59)	16.77 (11.95-23.24)	15.35 (11.35-20.51)	3.50 (2.71-4.24)	3.46 (2.65-4.23)	3.62 (2.77-4.39)
Papua New Guinea	91,589.94 (85,946.21-97,641.75)	81,677.41 (77,863.77-85,776.98)	83,561.20 (78,128.76-89,099.62)	1,281.64 (813.56-1,831.29)	1,246.52 (762.81-1,752.84)	1,151.81 (720.77-1,788.64)	19.47 (11.48-32.79)	17.72 (10.11-29.80)	16.46 (9.70-27.44)	4.97 (4.46-5.47)	4.97 (4.50-5.42)	4.48 (3.76-5.12)
Samoa	6,933.62 (5,650.97-8,689.70)	5,310.93 (4,379.17-6,824.24)	4,673.72 (3,710.40-5,789.71)	705.30 (570.08-878.39)	629.90 (508.60-753.58)	518.75 (411.36-626.54)	17.60 (12.36-22.77)	14.54 (10.71-18.77)	10.86 (8.37-14.18)	4.68 (4.18-5.16)	4.66 (4.15-5.16)	5.04 (4.19-5.75)
Solomon Islands	49,222.01 (42,166.74-57,113.34)	34,975.50 (30,559.68-37,683.87)	48,383.15 (42,521.32-55,587.38)	1,210.45 (764.49-1,777.61)	1,157.21 (727.69-1,670.68)	1,091.88 (646.74-1,721.40)	21.91 (12.95-35.54)	20.35 (11.81-32.98)	18.10 (11.09-30.03)	2.20 (1.96-2.44)	2.19 (1.92-2.45)	2.74 (2.29-3.18)
Tonga	16,977.15 (12,172.92-30,630.44)	9,564.11 (7,237.49-14,016.73)	12,747.28 (10,216.53-15,648.26)	655.90 (556.33-764.52)	650.14 (605.49-696.62)	582.45 (489.44-697.41)	6.81 (5.48-8.39)	7.29 (6.37-8.39)	7.14 (5.64-8.89)	2.25 (1.94-2.53)	2.85 (2.49-3.18)	3.12 (2.55-3.63)
Vanuatu	7,839.21 (6,582.47-9,512.04)	7,055.18 (5,828.70-8,489.83)	64,140.26 (57,669.44-70,389.93)	1,120.79 (729.60-1,668.52)	1,078.27 (639.81-1,634.88)	977.88 (640.80-1,503.90)	22.37 (13.41-35.21)	21.02 (11.81-32.63)	18.59 (11.74-27.85)	3.43 (3.11-3.75)	2.89 (2.59-3.19)	2.56 (2.15-2.95)
Afghanistan	47,838.53 (40,655.05-57,028.54)	33,565.76 (29,721.09-36,311.33)	43,860.77 (35,063.18-53,649.31)	1,481.80 (1,127.98-1,842.48)	1,627.81 (1,278.43-2,028.89)	1,442.48 (1,133.99-1,798.78)	9.20 (6.59-12.53)	9.97 (7.39-13.49)	10.21 (7.50-13.84)	1.44 (1.34-1.54)	1.45 (1.36-1.55)	1.73 (1.60-1.87)
Algeria	17,810.47 (12,481.21-26,914.73)	15,986.40 (10,898.69-24,819.52)	17,591.86 (12,410.37-26,731.68)	432.08 (390.32-477.71)	364.71 (331.71-402.76)	307.40 (268.86-342.92)	3.54 (2.85-4.39)	3.34 (2.84-3.90)	2.75 (2.31-3.25)	2.03 (1.76-2.29)	2.12 (1.87-2.38)	2.17 (1.82-2.50)
Bahrain	263.50 (223.27-307.29)	198.79 (172.14-233.06)	265.65 (230.90-322.03)	561.72 (501.13-628.51)	458.92 (420.17-502.44)	269.66 (221.52-324.79)	9.09 (7.77-10.70)	10.54 (8.82-12.48)	6.29 (5.00-8.29)	4.57 (4.08-4.98)	4.00 (3.53-4.41)	3.69 (3.10-4.18)
Egypt	22,368.49 (15,497.60-34,528.19)	19,357.46 (12,643.64-31,196.11)	12,638.74 (9,768.20-14,763.37)	609.74 (595.61-621.74)	528.17 (517.04-552.25)	527.01 (509.35-559.21)	2.48 (2.19-3.57)	3.93 (3.12-4.29)	3.51 (3.12-3.81)	1.76 (1.56-1.95)	1.76 (1.56-1.95)	1.74 (1.54-1.94)

Appendix Table 1b. Unscaled values for each SDG health-related indicator by country for 1990, 2000, and 2015

Appendix Table 1b. Unscaled values for each SDG health-related indicator by country for 1990, 2000, and 2015

Location	Indicator 3.3.5: Age-standardised prevalence rate of neglected tropical diseases (NTDs) (per 100,000 population)			Indicator 3.4.1: Death rate due to cardiovascular disease, cancer, diabetes, and chronic respiratory disease among populations aged 30 to 70 (per 100,000 population)			Indicator 3.4.2: Age-standardised death rate due to self-harm (per 100,000 population)			Indicator 3.5.2: Risk-weighted prevalence of alcohol consumption, as measured by the summary exposure value (SEV) for alcohol use		
	1990	2000	2015	1990	2000	2015	1990	2000	2015	1990	2000	2015
Iran	2,716.69 (2,464.72-3,042.48)	2,255.97 (2,108.84-2,363.73)	2,728.76 (2,316.34-3,161.03)	526.90 (451.11-606.26)	485.41 (438.09-549.67)	415.41 (332.77-506.23)	6.97 (5.58-8.74)	7.19 (6.24-8.22)	6.01 (4.66-7.68)	2.45 (1.91-2.88)	2.33 (1.83-2.75)	2.32 (1.83-2.74)
Iraq	1,232.35 (991.06-1,502.91)	1,089.84 (913.06-1,317.17)	1,140.50 (962.72-1,370.30)	770.18 (630.59-933.87)	855.54 (722.08-1,024.59)	722.53 (557.24-923.91)	7.53 (5.58-9.83)	9.01 (6.93-11.20)	8.33 (6.00-11.09)	2.72 (2.43-3.01)	2.04 (1.78-2.29)	1.94 (1.64-2.21)
Jordan	20,843.41 (18,105.87-23,937.23)	15,779.67 (14,065.32-16,908.87)	26,352.51 (22,235.48-30,730.00)	532.77 (457.51-637.45)	472.00 (417.90-530.09)	292.18 (261.35-331.55)	5.65 (4.17-7.27)	4.89 (3.96-5.87)	3.08 (2.60-3.65)	1.70 (1.46-1.91)	1.83 (1.58-2.08)	1.99 (1.67-2.30)
Kuwait	78.64 (73.50-84.39)	26.74 (24.25-29.34)	30.26 (27.48-33.15)	298.53 (283.92-312.96)	330.61 (321.16-339.30)	237.93 (207.44-272.21)	2.08 (1.80-2.40)	3.23 (3.03-3.43)	2.73 (2.27-3.36)	1.38 (1.20-1.57)	1.37 (1.19-1.55)	1.41 (1.22-1.60)
Lebanon	261.78 (229.19-302.18)	197.96 (174.92-229.55)	261.22 (219.56-314.15)	600.39 (486.08-722.47)	393.63 (330.10-472.27)	320.36 (225.54-421.83)	4.66 (3.46-6.14)	3.52 (2.72-4.49)	2.92 (2.00-4.11)	4.96 (4.49-5.39)	4.15 (3.76-4.51)	4.09 (3.36-4.73)
Libya	17,098.54 (12,732.24-23,408.72)	7,567.99 (4,204.78-13,573.24)	7,493.39 (4,027.99-13,158.86)	373.60 (322.80-426.69)	369.32 (334.51-402.63)	382.89 (327.09-445.21)	4.24 (3.42-5.27)	4.73 (4.01-5.62)	5.03 (4.12-6.27)	1.42 (1.23-1.61)	1.42 (1.23-1.60)	1.43 (1.25-1.62)
Morocco	11,348.72 (9,748.89-13,105.76)	8,041.79 (6,932.79-8,772.30)	14,471.63 (12,152.98-16,803.63)	485.53 (417.63-565.39)	424.78 (349.24-514.01)	380.28 (284.35-510.53)	8.69 (6.96-10.89)	8.12 (6.46-10.03)	7.97 (5.75-10.77)	1.70 (1.51-1.89)	1.70 (1.51-1.89)	1.58 (1.38-1.77)
Palestine	10,586.78 (8,222.06-13,986.46)	6,746.09 (5,821.88-7,326.18)	10,040.30 (7,621.30-13,054.57)	488.14 (383.74-642.11)	413.19 (370.25-464.47)	463.28 (356.63-573.94)	3.23 (2.39-4.45)	2.86 (2.42-3.47)	2.78 (2.07-3.70)	2.34 (1.86-2.77)	2.28 (1.81-2.68)	2.00 (1.62-2.36)
Oman	977.65 (806.95-1,215.90)	586.98 (495.01-669.16)	841.22 (664.23-1,119.89)	404.04 (294.99-518.54)	391.42 (318.64-464.18)	319.75 (259.76-374.76)	2.96 (1.93-4.54)	3.15 (2.47-4.02)	3.56 (2.80-4.47)	1.98 (1.70-2.27)	2.06 (1.80-2.34)	2.11 (1.73-2.47)
Qatar	262.93 (219.84-317.85)	200.66 (170.75-239.57)	267.44 (221.26-323.69)	375.72 (330.42-426.52)	396.74 (349.77-450.25)	229.16 (179.84-292.11)	5.94 (4.66-7.66)	7.00 (5.67-8.62)	5.01 (3.75-6.48)	2.94 (2.51-3.33)	2.43 (2.09-2.77)	3.12 (2.58-3.61)

Appendix Table 1b. Unscaled values for each SDG health-related indicator by country for 1990, 2000, and 2015												
	Indicator 3.3.5: Age-standardised prevalence rate of neglected tropical diseases (NTDs) (per 100,000 population)			Indicator 3.4.1: Death rate due to cardiovascular disease, cancer, diabetes, and chronic respiratory disease among populations aged 30 to 70 (per 100,000 population)			Indicator 3.4.2: Age-standardised death rate due to self-harm (per 100,000 population)			Indicator 3.5.2: Risk-weighted prevalence of alcohol consumption, as measured by the summary exposure value (SEV) for alcohol use		
**Location**	**1990**	**2000**	**2015**	**1990**	**2000**	**2015**	**1990**	**2000**	**2015**	**1990**	**2000**	**2015**
Saudi Arabia	6,584.00 (6,296.41-6,916.15)	6,061.97 (5,834.69-6,281.26)	7,915.37 (7,559.64-8,307.41)	283.24 (250.98-317.91)	265.28 (247.97-284.15)	219.77 (197.75-246.28)	2.72 (2.19-3.55)	2.77 (2.44-3.36)	3.34 (2.79-3.79)	1.67 (1.48-1.86)	1.52 (1.33-1.70)	1.52 (1.33-1.72)
Sudan	65,089.89 (53,029.15-78,093.61)	58,309.85 (46,434.92-70,972.61)	42,983.02 (34,988.56-55,376.09)	741.35 (588.10-922.35)	685.14 (508.42-905.34)	588.80 (414.60-803.70)	6.25 (3.92-9.59)	6.93 (4.12-10.90)	6.80 (4.25-10.42)	3.35 (2.95-3.72)	3.68 (3.31-4.02)	3.64 (3.15-4.04)
Syria	27,545.83 (23,834.42-32,006.07)	20,092.16 (18,515.24-21,156.53)	26,384.43 (23,003.05-30,185.69)	593.79 (513.11-700.96)	483.79 (420.72-559.65)	390.27 (329.01-449.06)	3.03 (2.22-4.07)	2.76 (2.17-3.39)	2.59 (2.03-3.09)	2.15 (1.84-2.41)	2.04 (1.69-2.30)	2.05 (1.75-2.30)
Tunisia	9,810.41 (8,818.66-10,856.94)	7,894.62 (7,351.13-8,246.84)	9,621.51 (8,759.16-10,434.52)	386.63 (357.38-417.27)	355.37 (313.31-398.82)	305.25 (238.88-377.69)	5.74 (4.66-7.18)	5.70 (4.78-6.76)	5.05 (3.92-6.38)	2.43 (2.17-2.67)	2.37 (2.13-2.59)	2.53 (2.14-2.87)
Turkey	7,119.90 (6,010.54-8,408.59)	4,521.93 (4,018.46-4,872.40)	6,329.33 (5,317.95-7,468.00)	576.79 (528.91-625.40)	426.39 (396.95-455.66)	275.79 (261.19-292.35)	9.68 (7.59-11.79)	8.11 (7.14-9.21)	4.05 (3.50-6.00)	3.82 (3.42-4.19)	4.91 (4.49-5.29)	3.65 (2.98-4.24)
United Arab Emirates	263.40 (219.25-312.22)	201.53 (172.55-242.47)	267.60 (219.22-332.55)	575.15 (462.74-694.48)	490.31 (435.42-542.47)	444.30 (333.58-584.37)	5.88 (4.03-8.18)	6.12 (4.70-7.42)	4.02 (2.78-5.42)	6.07 (5.39-6.61)	4.50 (3.83-5.05)	4.86 (3.95-5.60)
Yemen	47,245.88 (35,548.18-65,943.56)	39,688.01 (28,464.01-58,274.15)	29,232.88 (24,242.37-37,231.43)	863.87 (490.47-1,347.52)	814.53 (460.83-1,291.87)	715.38 (399.95-1,211.40)	6.13 (2.60-11.36)	7.05 (3.44-13.00)	7.15 (3.65-12.93)	2.52 (2.22-2.81)	1.90 (1.66-2.13)	1.60 (1.36-1.82)
Bangladesh	72,356.34 (68,760.43-75,678.03)	62,246.67 (58,328.96-64,530.38)	51,472.38 (44,995.38-57,937.79)	555.49 (495.98-622.43)	525.92 (471.77-586.43)	515.07 (445.69-592.33)	11.55 (9.38-13.37)	10.49 (8.51-12.07)	9.62 (7.54-11.41)	0.96 (0.82-1.10)	1.00 (0.86-1.14)	1.01 (0.87-1.15)
Bhutan	19,210.18 (14,827.62-24,655.61)	10,444.09 (8,439.92-11,723.46)	18,130.34 (13,594.51-23,584.89)	495.01 (385.14-622.43)	419.39 (344.77-507.72)	337.20 (260.11-424.62)	8.43 (5.65-12.06)	7.88 (5.99-10.04)	7.18 (5.27-9.75)	3.26 (2.84-3.66)	3.50 (3.01-3.91)	2.79 (2.22-3.26)
India	31,884.37 (30,313.87-33,354.69)	24,251.22 (23,429.98-25,298.46)	26,867.06 (25,280.64-28,090.86)	679.65 (649.29-712.64)	643.97 (617.31-670.28)	534.79 (511.71-561.07)	13.29 (8.85-14.53)	18.39 (9.18-20.34)	17.33 (8.82-19.91)	4.31 (3.91-4.70)	4.81 (4.40-5.24)	5.38 (4.76-5.95)

Appendix Table 1b. Unscaled values for each SDG health-related indicator by country for 1990, 2000, and 2015

Location	Indicator 3.3.5: Age-standardised prevalence rate of neglected tropical diseases (NTDs) (per 100,000 population)			Indicator 3.4.1: Death rate due to cardiovascular disease, cancer, diabetes, and chronic respiratory disease among populations aged 30 to 70 (per 100,000 population)			Indicator 3.4.2: Age-standardised death rate due to self-harm (per 100,000 population)			Indicator 3.5.2: Risk-weighted prevalence of alcohol consumption, as measured by the summary exposure value (SEV) for alcohol use		
	1990	2000	2015	1990	2000	2015	1990	2000	2015	1990	2000	2015
Nepal	66,614.90 (61,467.25-72,126.84)	51,215.69 (45,985.43-54,385.57)	46,711.06 (40,447.88-53,231.08)	512.85 (413.75-633.86)	439.96 (375.40-511.29)	410.73 (318.20-512.44)	7.25 (5.57-10.72)	7.83 (6.28-10.02)	7.85 (5.71-10.72)	1.81 (1.59-2.02)	3.31 (3.01-3.60)	3.74 (3.11-4.29)
Pakistan	17,133.32 (13,818.21-21,217.40)	11,339.03 (9,733.32-12,311.26)	15,557.66 (12,319.59-19,596.16)	616.10 (536.89-703.47)	753.46 (659.57-850.96)	690.23 (586.79-797.55)	4.56 (3.47-8.52)	4.86 (3.64-9.16)	3.53 (2.55-7.83)	0.87 (0.74-1.01)	0.89 (0.76-1.03)	0.86 (0.72-1.00)
Botswana	51,299.58 (42,480.52-62,845.43)	40,652.61 (32,617.47-52,222.54)	49,796.36 (41,195.04-62,374.74)	657.21 (226.60-1,614.87)	936.16 (241.15-2,908.20)	733.87 (258.80-2,409.79)	19.61 (6.41-49.90)	31.11 (7.24-99.55)	27.11 (8.17-93.65)	7.79 (7.03-8.49)	8.38 (7.63-9.09)	9.27 (8.18-10.19)
Lesotho	61,678.04 (53,729.03-69,702.10)	42,848.52 (36,787.72-46,483.75)	61,883.49 (54,330.06-70,886.99)	573.18 (392.30-768.62)	668.41 (471.93-940.90)	1,037.66 (525.70-1,765.53)	15.21 (10.21-23.72)	18.55 (12.00-27.61)	31.86 (14.84-59.59)	7.07 (6.52-7.57)	7.43 (6.86-7.94)	8.07 (7.17-8.88)
Namibia	24,345.30 (19,555.51-29,405.79)	14,715.22 (12,192.78-16,625.75)	22,231.39 (17,938.44-28,312.45)	578.37 (469.56-684.66)	824.59 (618.02-1,059.71)	395.90 (226.34-674.21)	18.06 (14.10-23.57)	24.98 (17.76-35.26)	15.03 (7.65-26.50)	2.30 (1.98-2.64)	6.84 (6.01-7.64)	10.33 (8.92-11.50)
South Africa	72,492.21 (64,520.31-86,713.81)	56,826.64 (49,213.17-70,177.03)	71,923.36 (63,650.43-85,570.02)	619.85 (569.15-669.32)	723.23 (631.57-803.74)	574.35 (517.13-637.90)	18.48 (15.02-21.38)	21.31 (17.51-23.90)	15.38 (13.07-18.02)	10.30 (9.60-10.93)	9.99 (9.33-10.59)	10.31 (9.49-11.04)
Swaziland	104,503.06 (86,290.46-127,152.48)	86,663.64 (69,654.44-109,965.25)	98,562.22 (83,524.66-117,067.53)	705.72 (467.28-954.38)	813.59 (514.35-1,222.52)	939.45 (387.46-1,703.53)	18.04 (11.68-26.82)	24.46 (14.04-38.01)	31.10 (10.85-61.71)	6.81 (6.32-7.29)	6.81 (6.32-7.28)	6.62 (5.92-7.23)
Zimbabwe	94,870.81 (72,693.46-123,083.47)	81,716.27 (59,662.80-108,735.40)	63,773.35 (52,030.83-80,257.12)	495.97 (356.11-626.44)	620.76 (416.37-983.24)	493.00 (259.90-812.06)	21.60 (14.61-31.65)	30.03 (18.02-48.88)	30.79 (17.11-47.75)	7.24 (6.63-7.79)	6.83 (6.22-7.38)	6.73 (5.89-7.48)
Benin	91,417.46 (62,913.36-127,329.74)	74,206.50 (46,021.87-109,834.54)	40,803.73 (28,179.56-63,292.11)	456.23 (376.46-540.43)	505.42 (394.66-639.42)	563.23 (276.32-991.39)	9.50 (6.97-12.58)	12.75 (9.53-17.35)	14.14 (6.98-25.04)	4.09 (3.65-4.49)	4.23 (3.77-4.62)	4.19 (3.67-4.64)
Burkina Faso	86,189.20 (66,144.64-109,295.73)	75,671.26 (56,155.32-98,114.44)	13,605.39 (9,106.26-25,851.08)	481.13 (395.07-588.76)	475.11 (378.63-593.77)	484.23 (270.96-812.07)	12.58 (9.98-15.74)	12.83 (10.06-16.09)	12.51 (7.23-20.34)	9.78 (9.14-10.40)	10.16 (9.51-10.79)	10.10 (9.20-10.89)

Location	Appendix Table 1b. Unscaled values for each SDG health-related indicator by country for 1990, 2000, and 2015											
	**Indicator 3.3.5: Age-standardised prevalence rate of neglected tropical diseases (NTDs) (per 100,000 population)**			**Indicator 3.4.1: Death rate due to cardiovascular disease, cancer, diabetes, and chronic respiratory disease among populations aged 30 to 70 (per 100,000 population)**			**Indicator 3.4.2: Age-standardised death rate due to self-harm (per 100,000 population)**			**Indicator 3.5.2: Risk-weighted prevalence of alcohol consumption, as measured by the summary exposure value (SEV) for alcohol use**		
	**1990**	**2000**	**2015**	**1990**	**2000**	**2015**	**1990**	**2000**	**2015**	**1990**	**2000**	**2015**
Cameroon	136,505.14 (124,640.74-148,298.33)	109,048.14 (98,171.91-121,159.57)	45,550.05 (39,797.87-53,175.47)	521.52 (432.48-619.33)	594.14 (422.77-767.60)	543.39 (291.42-954.11)	10.87 (8.52-13.73)	14.96 (10.74-19.84)	14.89 (8.01-26.37)	7.71 (7.15-8.24)	7.36 (6.81-7.86)	7.89 (7.29-8.45)
Cape Verde	2,349.74 (1,928.49-2,875.98)	1,717.87 (1,512.77-1,905.48)	1,819.09 (1,471.97-2,273.01)	490.86 (464.16-519.99)	480.24 (346.44-658.93)	378.09 (312.89-468.91)	10.82 (8.99-15.53)	23.83 (14.39-35.97)	18.07 (13.70-23.86)	6.81 (6.26-7.31)	7.31 (6.80-7.83)	7.50 (6.86-8.08)
Chad	61,370.47 (45,516.50-83,111.71)	50,492.67 (35,452.51-72,422.48)	54,681.78 (37,977.78-76,755.41)	408.63 (329.67-498.35)	509.57 (387.77-653.82)	517.61 (233.75-950.99)	6.51 (4.82-8.64)	10.00 (6.97-13.67)	10.82 (5.30-19.51)	5.14 (4.76-5.52)	5.75 (5.32-6.14)	6.17 (5.51-6.73)
Cote d'Ivoire	126,649.77 (106,681.51-147,677.62)	102,877.79 (83,343.55-123,625.05)	49,584.66 (39,039.07-63,827.02)	562.83 (442.02-697.19)	647.95 (473.12-865.49)	547.29 (294.26-896.19)	15.32 (11.58-19.65)	19.39 (13.97-26.03)	16.88 (9.00-27.90)	5.89 (4.83-6.76)	5.99 (4.90-6.90)	5.93 (4.89-6.81)
The Gambia	32,357.35 (20,395.11-49,508.01)	28,276.72 (16,591.03-45,425.69)	31,911.00 (20,131.37-49,438.52)	507.67 (284.23-857.93)	480.30 (364.15-610.32)	433.02 (272.68-643.06)	7.63 (4.26-12.70)	8.30 (5.97-11.67)	7.96 (5.12-12.59)	4.48 (3.96-4.96)	5.08 (4.55-5.56)	5.30 (4.66-5.86)
Ghana	88,869.82 (62,842.71-118,084.76)	77,906.68 (52,054.59-107,815.32)	38,078.39 (25,879.86-57,462.77)	572.25 (377.79-819.34)	539.04 (385.86-697.75)	501.83 (249.40-867.16)	8.43 (5.10-13.34)	8.31 (5.77-11.75)	9.68 (5.13-17.17)	6.47 (6.00-6.92)	5.87 (5.38-6.33)	6.98 (6.13-7.73)
Guinea	102,370.30 (76,962.62-131,001.01)	84,406.88 (60,127.40-111,968.64)	80,831.65 (60,810.47-104,466.18)	493.40 (407.94-586.92)	524.35 (432.99-626.69)	564.90 (335.86-845.47)	7.04 (5.32-9.49)	9.53 (7.13-12.59)	10.60 (6.26-16.86)	2.39 (2.10-2.66)	2.45 (2.17-2.73)	2.26 (1.96-2.54)
Guinea-Bissau	79,394.33 (56,641.31-116,890.86)	67,662.26 (45,399.47-105,451.78)	47,531.35 (25,229.08-85,019.68)	630.41 (209.89-1,524.21)	640.21 (206.45-1,688.57)	696.51 (227.84-2,157.70)	9.54 (3.36-22.68)	11.82 (4.01-29.44)	13.07 (4.50-37.87)	6.01 (5.53-6.48)	5.83 (5.37-6.28)	5.82 (5.24-6.35)
Liberia	161,260.98 (123,949.04-199,012.51)	138,625.38 (102,447.28-175,306.81)	112,970.43 (88,936.22-142,304.41)	462.35 (331.82-642.04)	366.31 (274.53-466.12)	430.70 (253.43-663.86)	8.88 (5.88-12.88)	8.14 (5.28-11.56)	9.54 (5.88-15.03)	7.60 (7.08-8.10)	6.91 (6.34-7.38)	6.43 (5.82-6.96)
Mali	109,135.98 (88,559.55-133,450.04)	98,383.72 (78,422.89-120,901.89)	19,035.44 (13,248.55-37,242.04)	617.21 (520.04-729.11)	540.71 (442.75-649.03)	484.53 (291.53-769.02)	7.65 (5.91-10.06)	7.66 (5.68-10.30)	7.48 (4.60-12.28)	1.85 (1.67-2.02)	1.80 (1.63-1.98)	1.87 (1.68-2.07)

Appendix Table 1b. Unscaled values for each SDG health-related indicator by country for 1990, 2000, and 2015

Location	Indicator 3.3.5: Age-standardised prevalence rate of neglected tropical diseases (NTDs) (per 100,000 population)			Indicator 3.4.1: Death rate due to cardiovascular disease, cancer, diabetes, and chronic respiratory disease among populations aged 30 to 70 (per 100,000 population)			Indicator 3.4.2: Age-standardised death rate due to self-harm (per 100,000 population)			Indicator 3.5.2: Risk-weighted prevalence of alcohol consumption, as measured by the summary exposure value (SEV) for alcohol use		
	1990	2000	2015	1990	2000	2015	1990	2000	2015	1990	2000	2015
Mauritania	48,329.35 (33,819.05-65,155.50)	45,785.17 (31,291.34-62,044.99)	33,201.82 (23,711.62-44,845.81)	559.46 (460.38-663.19)	425.16 (326.31-528.24)	356.04 (196.53-566.20)	8.50 (6.36-10.99)	7.95 (5.46-11.08)	7.36 (3.89-12.49)	1.07 (0.93-1.20)	0.81 (0.69-0.94)	0.74 (0.61-0.87)
Niger	49,226.10 (40,156.09-59,963.08)	43,969.31 (35,199.78-54,211.56)	6,301.91 (4,190.87-12,515.76)	432.60 (352.15-521.71)	383.03 (317.41-466.39)	414.64 (251.57-646.01)	6.84 (4.86-9.49)	7.31 (5.24-9.97)	8.46 (4.79-13.57)	1.78 (1.54-2.02)	1.81 (1.58-2.05)	1.78 (1.54-2.01)
Nigeria	104,347.69 (86,106.25-127,644.39)	92,301.19 (73,223.04-114,200.25)	81,295.61 (66,728.11-100,292.12)	436.25 (278.48-652.81)	387.90 (297.03-491.65)	284.87 (195.13-475.78)	7.77 (4.79-11.93)	8.19 (5.93-10.88)	6.92 (4.54-11.28)	10.28 (9.66-10.87)	11.22 (10.61-11.84)	10.83 (10.04-11.53)
Sao Tome and Principe	68,729.89 (60,461.17-77,395.33)	48,523.69 (41,541.05-55,231.69)	67,817.61 (59,093.08-78,356.10)	439.66 (373.50-515.75)	510.32 (411.72-611.44)	526.84 (273.43-847.01)	2.76 (2.24-3.38)	3.03 (2.36-3.87)	3.49 (1.76-5.76)	6.44 (5.89-6.97)	7.86 (7.27-8.39)	7.55 (6.89-8.14)
Senegal	42,321.08 (36,903.19-48,055.25)	36,677.99 (31,147.60-42,205.06)	19,749.21 (16,226.57-24,869.39)	481.08 (390.81-584.08)	476.44 (381.55-596.04)	515.04 (256.48-910.34)	11.92 (9.34-14.94)	12.98 (10.20-16.57)	14.16 (7.63-24.62)	2.59 (2.32-2.85)	2.49 (2.24-2.75)	2.44 (2.19-2.69)
Sierra Leone	161,318.68 (112,466.38-214,236.15)	144,737.59 (97,040.25-197,817.45)	40,793.12 (33,061.90-64,574.21)	435.66 (273.18-637.49)	496.82 (408.41-604.66)	569.70 (336.31-881.03)	7.43 (4.56-11.29)	9.73 (7.19-13.13)	10.89 (6.35-17.04)	7.42 (6.88-7.93)	8.35 (7.81-8.87)	8.21 (7.59-8.78)
Togo	66,855.98 (44,260.44-93,527.01)	60,478.68 (38,129.40-87,804.92)	14,476.36 (10,861.03-27,664.07)	519.36 (412.61-631.18)	550.32 (405.19-710.47)	524.24 (302.83-810.36)	10.62 (7.99-13.70)	14.15 (10.36-18.85)	14.16 (8.01-22.74)	5.48 (4.97-5.96)	4.48 (3.98-4.90)	4.35 (3.75-4.88)
Burundi	126,364.90 (89,404.41-181,892.94)	113,359.76 (77,503.64-168,526.44)	38,568.45 (31,871.55-53,822.59)	1,195.94 (710.62-1,662.50)	904.14 (700.04-1,124.51)	543.12 (293.08-888.77)	25.23 (13.50-39.74)	20.98 (14.53-29.84)	13.05 (7.28-21.38)	9.20 (8.58-9.78)	9.08 (8.53-9.59)	8.91 (8.17-9.56)
Comoros	73,460.46 (65,956.50-82,133.60)	54,082.13 (48,438.75-58,561.04)	60,668.94 (53,902.51-68,896.40)	738.01 (389.70-1,163.36)	562.88 (440.81-699.50)	449.00 (276.18-701.87)	12.66 (7.01-20.68)	11.40 (8.62-15.81)	10.08 (5.96-16.84)	1.35 (1.17-1.53)	1.31 (1.14-1.48)	1.35 (1.16-1.54)
Djibouti	62,706.65 (32,491.91-110,763.46)	54,226.32 (24,582.06-102,116.34)	61,768.67 (31,704.87-109,562.64)	559.88 (330.64-882.35)	593.85 (254.38-1,115.88)	575.31 (264.04-1,076.85)	9.77 (5.55-15.88)	11.27 (5.14-23.26)	11.86 (5.63-23.16)	5.87 (4.90-6.63)	5.60 (4.63-6.40)	5.67 (4.68-6.46)

Appendix Table 1b. Unscaled values for each SDG health-related indicator by country for 1990, 2000, and 2015

Location	Indicator 3.3.5: Age-standardised prevalence rate of neglected tropical diseases (NTDs) (per 100,000 population)			Indicator 3.4.1: Death rate due to cardiovascular disease, cancer, diabetes, and chronic respiratory disease among populations aged 30 to 70 (per 100,000 population)			Indicator 3.4.2: Age-standardised death rate due to self-harm (per 100,000 population)			Indicator 3.5.2: Risk-weighted prevalence of alcohol consumption, as measured by the summary exposure value (SEV) for alcohol use		
	1990	2000	2015	1990	2000	2015	1990	2000	2015	1990	2000	2015
Eritrea	80,244.64 (46,241.86-117,807.47)	70,899.27 (37,196.71-109,333.64)	66,294.44 (39,802.50-97,277.53)	802.68 (650.60-977.53)	570.74 (296.46-909.76)	665.40 (285.87-1,234.76)	16.06 (11.90-21.67)	13.28 (7.38-22.15)	15.33 (7.31-28.54)	2.70 (2.29-3.11)	3.74 (3.24-4.21)	2.93 (2.34-3.48)
Ethiopia	88,214.75 (75,696.59-102,436.13)	74,491.69 (62,128.94-88,311.56)	82,896.25 (70,927.35-96,840.37)	890.71 (741.96-1,053.91)	652.30 (523.55-772.21)	474.14 (262.58-777.63)	21.13 (15.13-26.74)	15.34 (11.84-19.04)	12.69 (7.35-21.11)	5.16 (4.73-5.57)	5.89 (5.45-6.30)	6.57 (5.69-7.35)
Kenya	66,192.53 (49,258.82-85,630.44)	60,452.59 (43,632.34-80,049.19)	45,762.30 (34,206.77-60,611.09)	386.78 (334.78-450.04)	385.15 (330.86-451.74)	359.31 (307.13-424.04)	11.46 (9.24-14.57)	11.51 (9.61-13.77)	11.37 (9.48-13.70)	7.02 (6.36-7.58)	6.15 (5.49-6.71)	5.96 (4.95-6.80)
Madagascar	170,063.85 (141,929.49-201,886.06)	154,068.50 (126,540.64-184,992.61)	79,922.53 (64,698.38-102,344.97)	756.25 (654.23-862.23)	698.82 (468.73-842.85)	674.57 (358.96-1,081.01)	13.16 (10.73-15.67)	11.81 (7.69-15.40)	11.93 (6.51-19.55)	4.60 (4.11-5.04)	3.59 (3.19-3.99)	3.33 (2.77-3.86)
Malawi	108,888.48 (84,643.42-133,652.11)	96,805.50 (73,004.39-121,254.43)	23,814.16 (16,783.15-41,331.72)	493.96 (378.76-639.64)	593.09 (380.77-877.90)	446.71 (246.46-751.26)	13.07 (9.13-17.61)	14.68 (9.51-21.93)	11.85 (7.00-19.50)	3.19 (2.86-3.49)	3.18 (2.84-3.50)	3.25 (2.77-3.64)
Mozambique	128,916.20 (113,841.74-145,475.01)	122,692.88 (107,330.69-139,606.53)	75,606.15 (67,076.11-94,970.35)	501.56 (411.41-612.18)	562.97 (415.88-715.55)	595.93 (284.41-1,009.84)	11.99 (8.61-16.18)	13.86 (10.30-17.87)	16.25 (8.16-27.40)	2.46 (2.16-2.77)	3.44 (2.98-3.87)	3.93 (3.24-4.54)
Rwanda	95,972.71 (62,854.02-145,980.61)	79,325.31 (46,019.25-129,202.38)	72,451.61 (54,618.30-102,383.93)	838.01 (658.09-1,037.73)	771.58 (609.33-950.57)	435.49 (248.83-749.64)	16.85 (12.51-23.49)	17.52 (13.02-24.10)	10.80 (6.37-18.97)	9.22 (8.63-9.75)	8.95 (8.37-9.48)	9.10 (8.36-9.76)
Somalia	98,933.96 (80,791.48-117,662.59)	86,621.86 (69,031.73-105,909.43)	95,721.51 (78,670.29-114,384.99)	803.78 (209.26-1,832.55)	749.92 (190.70-1,627.74)	720.78 (191.61-1,765.02)	12.08 (3.60-27.13)	12.13 (3.42-28.10)	12.32 (3.88-28.99)	1.95 (1.71-2.18)	1.93 (1.68-2.20)	1.96 (1.66-2.26)
South Sudan	91,369.43 (77,239.37-105,481.96)	86,218.20 (73,215.64-99,084.34)	77,726.92 (64,616.52-90,578.29)	655.04 (197.75-1,579.16)	581.81 (178.20-1,491.42)	596.43 (187.17-1,668.18)	10.75 (3.79-25.27)	10.47 (3.74-24.99)	11.70 (4.27-30.16)	5.61 (4.47-6.44)	5.62 (4.48-6.49)	5.68 (4.63-6.51)
Tanzania	95,596.30 (85,726.28-106,542.78)	83,106.79 (73,930.78-93,314.52)	52,448.08 (45,016.17-63,465.56)	519.86 (420.81-634.04)	482.92 (345.66-643.00)	451.22 (232.78-828.80)	11.90 (9.22-14.69)	10.69 (7.61-14.04)	11.14 (6.28-19.06)	8.57 (7.96-9.11)	8.15 (7.50-8.71)	8.64 (7.81-9.35)

Appendix Table 1b. Unscaled values for each SDG health-related indicator by country for 1990, 2000, and 2015

Location	Indicator 3.3.5: Age-standardised prevalence rate of neglected tropical diseases (NTDs) (per 100,000 population)			Indicator 3.4.1: Death rate due to cardiovascular disease, cancer, diabetes, and chronic respiratory disease among populations aged 30 to 70 (per 100,000 population)			Indicator 3.4.2: Age-standardised death rate due to self-harm (per 100,000 population)			Indicator 3.5.2: Risk-weighted prevalence of alcohol consumption, as measured by the summary exposure value (SEV) for alcohol use		
	1990	2000	2015	1990	2000	2015	1990	2000	2015	1990	2000	2015
Uganda	78,333.63 (64,331.97-95,723.00)	62,695.97 (49,887.66-78,372.33)	26,690.64 (20,611.80-35,750.61)	603.64 (383.21-772.23)	684.66 (464.16-853.87)	560.08 (264.52-919.28)	14.56 (9.17-19.92)	17.00 (11.45-22.86)	15.87 (8.31-26.34)	11.84 (11.00-12.57)	12.65 (11.73-13.48)	12.60 (11.20-13.81)
Zambia	82,232.07 (67,988.92-98,110.26)	70,113.93 (57,198.54-83,849.61)	78,745.45 (65,381.81-93,307.17)	523.21 (396.63-651.89)	756.36 (605.11-940.27)	725.11 (431.86-1,088.85)	13.63 (9.37-20.12)	19.27 (14.53-24.64)	22.32 (12.83-33.41)	6.85 (6.35-7.35)	6.47 (5.97-6.91)	6.57 (5.75-7.25)
Angola	102,665.75 (77,620.99-133,071.54)	89,725.29 (64,179.81-119,424.19)	89,637.54 (69,103.58-114,251.38)	877.23 (217.77-1,902.79)	780.48 (213.75-1,804.95)	741.14 (246.95-2,134.10)	19.59 (4.88-46.01)	16.61 (4.87-41.31)	16.99 (6.12-50.23)	6.93 (4.37-8.79)	6.54 (4.04-8.44)	7.60 (4.77-9.63)
Central African Republic	83,647.48 (75,287.56-96,478.26)	69,471.47 (61,545.70-81,826.52)	58,561.54 (52,639.10-66,180.67)	875.10 (683.74-1,091.34)	942.32 (451.65-1,475.96)	966.49 (412.94-1,633.83)	18.72 (12.60-27.90)	20.63 (8.73-38.18)	21.97 (8.11-43.05)	6.50 (5.71-7.23)	5.27 (4.53-5.97)	4.81 (3.58-5.96)
Congo	109,266.88 (88,526.57-136,263.78)	92,414.52 (71,100.40-119,369.55)	104,462.32 (83,939.73-130,963.95)	940.16 (685.79-1,209.37)	854.56 (660.83-1,070.52)	676.57 (378.59-1,109.81)	18.44 (11.85-26.62)	16.54 (11.37-22.90)	13.21 (7.44-23.15)	7.26 (6.56-7.90)	5.98 (5.37-6.51)	6.62 (5.22-7.80)
Democratic Republic of the Congo	118,304.70 (97,408.42-144,024.41)	100,425.37 (81,283.63-124,709.25)	98,033.75 (80,654.57-120,400.77)	508.84 (319.12-735.31)	491.68 (366.85-621.76)	529.70 (306.00-793.85)	10.38 (6.08-16.49)	10.23 (7.23-15.34)	11.25 (6.66-18.47)	5.31 (3.42-6.94)	4.36 (2.77-5.88)	4.32 (2.72-5.86)
Equatorial Guinea	78,659.58 (73,125.68-84,292.03)	63,430.42 (58,053.98-67,496.30)	71,984.51 (66,153.60-77,791.87)	1,022.51 (246.22-2,219.23)	766.96 (238.43-1,862.04)	576.92 (259.46-1,744.02)	23.31 (5.83-53.99)	18.06 (5.73-45.30)	15.81 (6.48-47.56)	6.59 (5.25-7.84)	8.00 (6.63-9.21)	10.89 (8.73-12.43)
Gabon	120,848.94 (96,108.45-151,213.50)	110,399.96 (86,366.70-139,185.40)	119,695.41 (95,312.33-147,773.29)	699.61 (558.17-849.08)	664.55 (531.85-839.55)	518.08 (306.81-857.91)	17.35 (12.03-23.12)	14.98 (10.34-21.00)	13.90 (7.84-25.44)	9.40 (6.17-11.54)	9.03 (5.99-11.09)	9.18 (6.07-11.32)

Appendix Table 1b. Unscaled values for each SDG health-related indicator by country for 1990, 2000, and 2015

Location	Indicator 3.6.1: Age-standardised death rate due to road injuries (per 100,000 population)			Indicator 3.7.1: Proportion of women of reproductive age (15 to 49 years) who have their need for family planning satisfied with modern methods			Indicator 3.7.2: Birth rates for women aged 10 to 14 years and women aged 15 to 19 years (number of live births per 1,000 women)			Indicator 3.8.1: Coverage of 7 UHC tracer interventions for prevention and treatment services		
	1990	2000	2015	1990	2000	2015	1990	2000	2015	1990	2000	2015
Canada	15.00 (14.53-15.50)	10.42 (10.02-10.80)	6.90 (6.50-7.33)	91.52 (89.06-93.61)	91.80 (89.33-93.94)	95.19 (93.51-96.40)	14.21 (14.21-14.21)	9.79 (9.79-9.79)	5.74 (5.74-5.74)	61.90 (60.78-62.60)	84.67 (83.16-86.23)	91.54 (90.64-92.61)
United States	19.00 (18.69-19.30)	16.39 (16.08-16.69)	11.92 (11.55-12.27)	98.48 (97.98-98.90)	98.95 (98.59-99.24)	99.12 (98.83-99.35)	30.41 (30.41-30.41)	25.32 (25.32-25.32)	12.21 (12.21-12.21)	63.59 (63.34-63.82)	87.12 (85.78-88.63)	93.10 (92.28-94.01)
Australia	15.82 (15.29-16.40)	10.66 (10.25-11.10)	6.82 (6.41-7.24)	90.53 (87.53-92.92)	94.46 (92.70-95.90)	96.88 (95.80-97.69)	12.81 (12.81-12.81)	10.26 (10.26-10.26)	8.02 (8.02-8.02)	60.81 (60.13-61.47)	82.07 (80.45-83.90)	91.44 (90.83-91.93)
New Zealand	21.57 (20.84-22.29)	13.25 (12.69-13.81)	7.79 (7.31-8.28)	84.26 (80.03-88.03)	91.11 (88.25-93.46)	94.81 (93.14-96.12)	19.04 (19.04-19.04)	15.48 (15.48-15.48)	13.59 (13.59-13.59)	58.89 (58.30-59.42)	82.24 (80.99-83.71)	90.69 (90.01-91.36)
Brunei	26.83 (23.64-29.99)	19.76 (18.12-21.15)	13.32 (12.00-14.94)	78.72 (73.06-83.72)	81.91 (77.22-86.06)	89.27 (86.09-91.94)	26.54 (26.54-26.54)	15.66 (15.66-15.66)	12.68 (12.68-12.68)	62.33 (61.80-62.79)	81.78 (77.59-83.69)	91.00 (90.07-91.97)
Japan	11.42 (11.21-11.65)	8.32 (8.12-8.51)	4.37 (4.22-4.54)	62.77 (55.28-70.01)	69.17 (62.21-75.59)	80.59 (75.41-84.85)	3.74 (3.74-3.74)	3.98 (3.98-3.98)	3.03 (3.03-3.03)	58.71 (57.65-59.58)	83.10 (81.77-84.57)	90.89 (90.09-91.72)
Singapore	11.26 (10.77-11.80)	6.45 (6.13-6.76)	3.93 (3.66-4.21)	64.94 (57.80-71.61)	74.08 (68.22-79.28)	87.04 (83.51-90.17)	6.55 (6.55-6.55)	5.54 (5.54-5.54)	2.36 (2.36-2.36)	59.68 (58.90-60.39)	84.57 (82.97-86.12)	92.09 (91.22-92.95)
South Korea	39.73 (38.15-41.44)	27.36 (26.16-28.42)	13.04 (12.13-13.94)	77.86 (72.28-83.00)	86.40 (82.63-89.65)	93.33 (91.37-95.07)	6.21 (6.21-6.21)	2.27 (2.27-2.27)	1.08 (1.08-1.08)	59.07 (57.97-60.04)	73.97 (64.09-78.69)	90.53 (89.37-91.49)
Andorra	8.16 (5.16-11.01)	5.65 (3.87-7.68)	3.84 (3.00-4.89)	86.12 (81.76-89.41)	90.30 (87.42-92.70)	94.71 (92.93-96.06)	11.46 (11.46-11.46)	9.32 (9.32-9.32)	9.37 (9.37-9.37)	61.68 (60.95-62.25)	82.01 (79.19-84.72)	91.42 (89.75-93.50)
Austria	17.62 (17.01-18.20)	11.44 (10.97-11.90)	5.81 (5.38-6.27)	68.21 (61.29-74.19)	81.37 (76.00-85.83)	91.55 (88.96-93.78)	13.85 (13.85-13.85)	9.39 (9.39-9.39)	4.69 (4.69-4.69)	57.37 (56.27-58.28)	80.46 (78.85-81.95)	88.98 (87.66-90.10)

Appendix Table 1b. Unscaled values for each SDG health-related indicator by country for 1990, 2000, and 2015

Location	Indicator 3.6.1: Age-standardised death rate due to road injuries (per 100,000 population)			Indicator 3.7.1: Proportion of women of reproductive age (15 to 49 years) who have their need for family planning satisfied with modern methods			Indicator 3.7.2: Birth rates for women aged 10 to 14 years and women aged 15 to 19 years (number of live births per 1,000 women)			Indicator 3.8.1: Coverage of 7 UHC tracer interventions for prevention and treatment services		
	1990	2000	2015	1990	2000	2015	1990	2000	2015	1990	2000	2015
Belgium	20.17 (19.57-20.79)	16.24 (15.65-16.80)	8.64 (8.09-9.22)	85.46 (81.47-89.20)	89.19 (85.84-91.97)	94.34 (92.43-95.82)	8.67 (8.67-8.67)	7.30 (7.30-7.30)	5.57 (5.57-5.57)	60.75 (59.85-61.43)	83.65 (82.32-84.99)	92.41 (91.74-93.06)
Cyprus	22.27 (20.23-24.42)	19.71 (18.33-20.75)	9.51 (8.84-10.34)	77.31 (71.34-82.81)	84.69 (80.62-88.51)	92.40 (89.93-94.43)	19.14 (19.14-19.14)	8.59 (8.59-8.59)	2.54 (2.54-2.54)	59.19 (58.07-60.05)	79.84 (78.17-81.78)	90.90 (89.56-92.81)
Denmark	13.77 (13.21-14.27)	9.75 (9.38-10.14)	4.89 (4.53-5.24)	75.08 (69.21-80.73)	83.92 (79.43-87.92)	90.29 (87.50-92.83)	7.37 (7.37-7.37)	5.23 (5.23-5.23)	3.06 (3.06-3.06)	60.64 (59.90-61.28)	78.57 (76.83-80.78)	90.45 (89.60-91.28)
Finland	14.20 (13.63-14.84)	8.55 (8.11-8.99)	4.69 (4.37-5.05)	90.21 (87.23-92.67)	93.82 (91.79-95.47)	96.43 (95.19-97.44)	8.70 (8.70-8.70)	7.30 (7.30-7.30)	5.04 (5.04-5.04)	62.70 (62.08-63.07)	84.45 (83.19-85.76)	92.87 (92.29-93.46)
France	20.24 (19.62-20.90)	14.33 (13.88-14.78)	7.16 (6.78-7.57)	82.70 (78.39-86.45)	88.14 (85.17-90.89)	93.57 (91.47-95.24)	9.41 (9.41-9.41)	7.28 (7.28-7.28)	6.14 (6.14-6.14)	58.90 (57.86-59.67)	83.03 (81.46-84.58)	90.97 (90.03-91.75)
Germany	14.25 (13.83-14.72)	9.95 (9.61-10.29)	4.93 (4.68-5.20)	76.28 (70.52-81.39)	85.21 (81.13-88.87)	92.09 (89.63-94.13)	10.85 (10.85-10.85)	8.21 (8.21-8.21)	4.10 (4.10-4.10)	57.95 (56.93-58.82)	83.33 (81.78-84.80)	91.02 (90.26-91.72)
Greece	21.60 (20.91-22.37)	18.12 (17.57-18.73)	10.80 (10.20-11.43)	50.59 (42.49-58.35)	57.20 (49.81-64.27)	75.23 (69.51-80.49)	13.98 (13.98-13.98)	7.11 (7.11-7.11)	4.14 (4.14-4.14)	56.59 (55.15-57.66)	79.94 (78.03-81.84)	89.31 (88.19-90.29)
Iceland	10.23 (9.71-10.78)	6.50 (6.16-6.86)	3.50 (3.25-3.76)	85.36 (81.20-89.02)	89.71 (86.61-92.28)	94.38 (92.68-95.82)	17.45 (17.45-17.45)	12.55 (12.55-12.55)	4.53 (4.53-4.53)	62.72 (62.01-63.17)	76.69 (73.95-79.30)	85.39 (84.08-86.82)
Ireland	14.57 (13.51-15.59)	10.12 (9.59-10.69)	4.10 (3.80-4.42)	80.16 (74.93-84.47)	87.23 (83.56-90.30)	94.43 (92.67-95.99)	9.35 (9.35-9.35)	10.70 (10.70-10.70)	5.70 (5.70-5.70)	55.88 (54.54-57.04)	78.00 (76.36-79.55)	91.47 (90.74-92.21)
Israel	12.20 (11.69-12.71)	12.88 (12.32-13.47)	7.44 (6.99-7.93)	87.41 (83.67-90.58)	90.44 (87.54-93.00)	94.75 (93.07-96.15)	16.48 (16.48-16.48)	13.27 (13.27-13.27)	9.29 (9.29-9.29)	61.75 (61.07-62.17)	77.72 (75.71-79.76)	91.69 (91.02-92.31)

Appendix Table 1b. Unscaled values for each SDG health-related indicator by country for 1990, 2000, and 2015												
	**Indicator 3.6.1: Age-standardised death rate due to road injuries (per 100,000 population)**			**Indicator 3.7.1: Proportion of women of reproductive age (15 to 49 years) who have their need for family planning satisfied with modern methods**			**Indicator 3.7.2: Birth rates for women aged 10 to 14 years and women aged 15 to 19 years (number of live births per 1,000 women)**			**Indicator 3.8.1: Coverage of 7 UHC tracer interventions for prevention and treatment services**		
**Location**	**1990**	**2000**	**2015**	**1990**	**2000**	**2015**	**1990**	**2000**	**2015**	**1990**	**2000**	**2015**
Italy	17.52 (16.99-18.11)	14.70 (14.24-15.15)	8.05 (7.63-8.49)	70.03 (63.00-76.41)	76.82 (71.25-81.83)	86.88 (83.04-90.02)	6.47 (6.47-6.47)	4.40 (4.40-4.40)	3.59 (3.59-3.59)	57.65 (56.17-58.93)	83.58 (82.28-84.94)	91.96 (91.17-92.67)
Luxembourg	19.34 (18.58-20.14)	12.17 (11.59-12.76)	5.43 (5.11-5.78)	82.04 (77.47-86.22)	88.16 (84.56-91.10)	93.96 (92.17-95.55)	8.83 (8.83-8.83)	7.78 (7.78-7.78)	4.20 (4.20-4.20)	60.25 (59.43-60.93)	77.84 (75.30-80.35)	87.16 (85.97-88.50)
Malta	7.38 (6.92-7.88)	5.72 (5.35-6.10)	3.00 (2.75-3.23)	60.68 (53.73-67.71)	68.26 (61.01-74.65)	83.12 (78.25-86.98)	9.28 (9.28-9.28)	11.79 (11.79-11.79)	8.30 (8.30-8.30)	55.32 (53.50-56.77)	66.73 (63.81-69.56)	83.45 (81.70-84.97)
Netherlands	10.71 (10.32-11.08)	8.57 (8.23-8.90)	4.50 (4.22-4.81)	83.04 (78.09-87.34)	84.73 (80.47-88.50)	91.86 (89.16-93.96)	5.10 (5.10-5.10)	4.46 (4.46-4.46)	2.94 (2.94-2.94)	62.22 (61.48-62.69)	84.84 (83.49-86.14)	92.38 (91.60-93.12)
Norway	10.06 (9.64-10.51)	8.12 (7.73-8.58)	3.70 (3.41-3.97)	93.86 (91.91-95.37)	93.02 (90.81-94.77)	95.06 (93.53-96.36)	11.95 (11.95-11.95)	7.88 (7.88-7.88)	4.80 (4.80-4.80)	62.05 (61.50-62.42)	78.31 (76.58-80.38)	91.91 (91.27-92.52)
Portugal	33.16 (32.14-34.21)	21.09 (20.38-21.81)	8.91 (8.42-9.46)	59.77 (52.23-66.50)	73.72 (67.64-79.53)	88.24 (84.95-91.12)	14.82 (14.82-14.82)	11.52 (11.52-11.52)	4.82 (4.82-4.82)	56.57 (54.60-58.00)	81.32 (79.42-82.83)	91.00 (90.00-91.77)
Spain	22.08 (21.34-22.85)	14.91 (14.46-15.42)	5.15 (4.87-5.43)	66.90 (60.08-73.46)	80.24 (75.34-84.34)	90.65 (87.67-93.07)	7.25 (7.25-7.25)	5.17 (5.17-5.17)	4.14 (4.14-4.14)	58.57 (57.23-59.48)	83.48 (81.94-85.06)	91.78 (91.02-92.51)
Sweden	9.93 (9.52-10.32)	6.39 (6.11-6.65)	3.85 (3.61-4.11)	83.30 (78.58-87.31)	85.49 (81.17-89.07)	91.23 (88.52-93.41)	9.82 (9.82-9.82)	4.99 (4.99-4.99)	4.73 (4.73-4.73)	62.66 (61.96-63.12)	82.56 (80.81-84.55)	93.57 (92.99-94.19)
Switzerland	14.45 (13.79-15.09)	7.99 (7.62-8.36)	3.95 (3.68-4.23)	85.82 (81.72-89.26)	91.29 (88.58-93.49)	95.09 (93.41-96.48)	5.59 (5.59-5.59)	4.46 (4.46-4.46)	2.41 (2.41-2.41)	61.08 (60.46-61.48)	84.18 (83.01-85.47)	92.17 (91.46-92.96)
United Kingdom	9.22 (9.05-9.40)	7.00 (6.82-7.19)	3.78 (3.63-3.99)	88.36 (84.97-91.21)	91.65 (89.21-93.72)	94.96 (93.24-96.34)	17.96 (17.96-17.96)	15.47 (15.47-15.47)	9.22 (9.22-9.22)	60.60 (59.54-61.40)	84.26 (83.25-85.59)	94.60 (93.97-95.10)

Appendix Table 1b. Unscaled values for each SDG health-related indicator by country for 1990, 2000, and 2015

Location	Indicator 3.6.1: Age-standardised death rate due to road injuries (per 100,000 population)			Indicator 3.7.1: Proportion of women of reproductive age (15 to 49 years) who have their need for family planning satisfied with modern methods			Indicator 3.7.2: Birth rates for women aged 10 to 14 years and women aged 15 to 19 years (number of live births per 1,000 women)			Indicator 3.8.1: Coverage of 7 UHC tracer interventions for prevention and treatment services		
	1990	2000	2015	1990	2000	2015	1990	2000	2015	1990	2000	2015
Argentina	15.88 (15.20-16.52)	15.58 (14.95-16.20)	14.15 (13.36-14.89)	63.11 (55.46-69.83)	72.66 (66.33-78.45)	86.23 (82.46-89.58)	39.12 (39.12-39.12)	34.76 (34.76-34.76)	32.54 (32.54-32.54)	57.93 (56.42-58.96)	81.33 (79.52-83.08)	91.97 (89.97-93.40)
Chile	18.00 (17.17-18.88)	15.57 (14.90-16.31)	12.48 (11.53-13.44)	61.04 (53.66-67.68)	74.00 (67.38-79.97)	87.25 (83.61-90.47)	34.70 (34.70-34.70)	29.44 (29.44-29.44)	24.47 (24.47-24.47)	59.05 (57.25-60.28)	83.08 (80.58-84.80)	89.29 (87.61-90.56)
Uruguay	17.18 (16.44-17.98)	16.52 (15.80-17.24)	15.21 (14.28-16.18)	57.46 (49.57-64.69)	66.81 (59.53-73.37)	81.26 (76.48-85.54)	35.77 (35.77-35.77)	33.11 (33.11-33.11)	28.64 (28.64-28.64)	57.13 (55.73-58.31)	82.39 (81.03-83.69)	89.88 (88.91-90.79)
Belarus	23.43 (21.31-25.00)	23.94 (22.31-25.86)	19.33 (16.47-21.83)	44.57 (41.84-47.47)	57.97 (55.16-60.67)	81.85 (80.23-83.46)	29.07 (29.07-29.07)	17.35 (17.35-17.35)	11.18 (11.18-11.18)	57.25 (56.63-57.83)	60.47 (60.15-60.77)	89.26 (88.38-90.22)
Estonia	26.82 (25.45-28.22)	18.75 (17.80-19.71)	5.81 (5.17-6.44)	57.86 (55.15-60.45)	63.03 (60.47-65.69)	80.58 (78.94-82.33)	29.54 (29.54-29.54)	15.49 (15.49-15.49)	6.56 (6.56-6.56)	56.94 (56.16-57.64)	81.64 (81.09-82.14)	88.62 (87.77-89.50)
Latvia	34.83 (33.36-36.41)	25.55 (24.41-26.80)	9.70 (8.85-10.50)	45.45 (42.66-48.37)	55.35 (52.71-58.09)	77.23 (75.18-79.07)	28.51 (28.51-28.51)	11.89 (11.89-11.89)	6.43 (6.43-6.43)	55.29 (54.85-55.71)	61.57 (58.57-65.03)	85.92 (84.99-86.96)
Lithuania	29.36 (28.15-30.61)	22.87 (21.90-23.88)	11.81 (10.99-12.65)	38.64 (35.94-41.19)	50.92 (48.12-53.78)	72.16 (69.93-74.29)	26.06 (26.06-26.06)	15.52 (15.52-15.52)	5.15 (5.15-5.15)	55.85 (55.26-56.44)	59.13 (58.71-59.51)	85.79 (84.79-86.81)
Moldova	27.79 (26.54-29.04)	16.05 (15.24-16.83)	9.73 (9.03-10.45)	59.14 (56.38-61.89)	57.76 (55.48-60.00)	73.80 (71.66-75.88)	37.51 (37.51-37.51)	23.74 (23.74-23.74)	13.28 (13.28-13.28)	58.60 (57.89-59.15)	57.36 (57.07-57.65)	79.13 (77.56-80.80)
Russia	27.05 (25.86-28.27)	31.49 (30.09-32.93)	17.84 (16.73-18.89)	51.74 (48.96-54.43)	57.43 (54.85-60.08)	76.94 (74.92-78.73)	32.51 (32.51-32.51)	18.16 (18.16-18.16)	12.93 (12.93-12.93)	51.35 (49.29-53.00)	57.44 (56.76-58.09)	81.68 (79.58-83.82)
Ukraine	22.74 (21.69-23.80)	19.24 (18.07-20.34)	12.90 (11.85-14.02)	49.54 (46.91-52.20)	58.34 (55.76-60.95)	77.43 (75.59-79.22)	34.84 (34.84-34.84)	19.93 (19.93-19.93)	13.26 (13.26-13.26)	52.51 (49.46-54.90)	56.57 (55.41-57.46)	79.28 (76.26-81.69)

Location	Indicator 3.6.1: Age-standardised death rate due to road injuries (per 100,000 population)			Indicator 3.7.1: Proportion of women of reproductive age (15 to 49 years) who have their need for family planning satisfied with modern methods			Indicator 3.7.2: Birth rates for women aged 10 to 14 years and women aged 15 to 19 years (number of live births per 1,000 women)			Indicator 3.8.1: Coverage of 7 UHC tracer interventions for prevention and treatment services		
	1990	2000	2015	1990	2000	2015	1990	2000	2015	1990	2000	2015
Albania	13.65 (12.11-14.81)	8.97 (8.31-9.81)	7.24 (6.36-8.19)	12.28 (7.93-18.07)	13.45 (9.16-18.68)	17.57 (12.25-24.49)	17.15 (17.15-17.15)	15.55 (15.55-15.55)	15.47 (15.47-15.47)	43.41 (40.88-46.02)	60.04 (54.89-63.46)	75.03 (72.31-78.25)
Bosnia and Herzegovina	8.20 (7.55-9.17)	7.34 (6.14-8.58)	4.69 (4.16-5.21)	20.08 (13.13-28.35)	22.90 (15.97-30.83)	30.47 (24.55-36.90)	19.68 (19.68-19.68)	14.73 (14.73-14.73)	5.02 (5.02-5.02)	46.49 (44.11-48.86)	65.30 (55.50-68.35)	78.41 (76.20-80.87)
Bulgaria	15.31 (14.70-15.92)	12.17 (11.65-12.75)	9.01 (8.44-9.61)	36.52 (27.45-46.93)	41.82 (31.17-53.00)	62.28 (51.09-72.29)	40.24 (40.24-40.24)	22.55 (22.55-22.55)	17.98 (17.98-17.98)	56.84 (55.39-58.26)	56.93 (55.39-58.29)	78.73 (76.94-80.42)
Croatia	19.83 (18.94-20.70)	15.18 (14.57-15.81)	8.89 (8.40-9.43)	24.42 (17.04-32.84)	27.11 (18.97-35.98)	39.05 (28.05-50.25)	17.51 (17.51-17.51)	12.04 (12.04-12.04)	5.91 (5.91-5.91)	54.25 (52.35-55.89)	69.39 (65.62-73.12)	81.07 (78.43-83.46)
Czech Republic	14.49 (13.89-15.12)	13.49 (12.99-14.00)	6.96 (6.57-7.38)	56.46 (45.31-67.53)	65.78 (54.32-75.25)	80.55 (73.18-87.26)	29.24 (29.24-29.24)	9.08 (9.08-9.08)	5.22 (5.22-5.22)	60.69 (59.46-61.71)	83.03 (81.15-84.95)	91.39 (90.36-92.32)
Hungary	21.03 (20.22-21.88)	13.97 (13.35-14.66)	6.73 (6.23-7.26)	53.52 (42.01-64.42)	60.88 (48.94-71.32)	78.93 (70.56-85.72)	25.67 (25.67-25.67)	13.23 (13.23-13.23)	8.22 (8.22-8.22)	60.13 (58.80-61.29)	73.93 (70.04-78.00)	91.87 (90.78-92.84)
Macedonia	9.70 (8.99-10.39)	7.94 (7.47-8.51)	7.74 (6.95-8.59)	14.63 (9.48-21.11)	16.75 (11.09-23.84)	24.28 (16.71-32.58)	29.17 (29.17-29.17)	18.53 (18.53-18.53)	10.35 (10.35-10.35)	49.82 (47.59-51.99)	64.76 (55.17-68.71)	77.92 (75.01-81.33)
Montenegro	11.86 (10.40-13.87)	11.80 (10.98-12.79)	9.21 (8.13-10.32)	23.82 (16.54-33.61)	27.23 (19.45-36.50)	36.57 (26.10-46.96)	19.89 (19.89-19.89)	14.33 (14.33-14.33)	8.23 (8.23-8.23)	51.39 (48.77-53.58)	66.86 (61.79-70.36)	79.37 (76.56-82.60)
Poland	23.18 (22.38-24.15)	18.45 (17.80-19.19)	10.35 (9.80-10.94)	23.71 (15.79-32.60)	28.12 (19.81-38.06)	45.70 (34.19-57.55)	23.36 (23.36-23.36)	11.53 (11.53-11.53)	7.08 (7.08-7.08)	55.43 (53.42-57.23)	78.45 (75.87-81.03)	87.05 (84.91-89.03)
Romania	19.78 (18.94-20.53)	14.62 (14.00-15.24)	10.20 (9.51-10.89)	23.45 (16.06-32.45)	28.92 (20.30-38.54)	45.85 (34.94-56.87)	32.21 (32.21-32.21)	21.35 (21.35-21.35)	17.30 (17.30-17.30)	50.45 (48.62-52.08)	65.41 (61.84-68.57)	84.38 (82.09-86.42)

Appendix Table 1b. Unscaled values for each SDG health-related indicator by country for 1990, 2000, and 2015

Appendix Table 1b. Unscaled values for each SDG health-related indicator by country for 1990, 2000, and 2015												
Location	Indicator 3.6.1: Age-standardised death rate due to road injuries (per 100,000 population)			Indicator 3.7.1: Proportion of women of reproductive age (15 to 49 years) who have their need for family planning satisfied with modern methods			Indicator 3.7.2: Birth rates for women aged 10 to 14 years and women aged 15 to 19 years (number of live births per 1,000 women)			Indicator 3.8.1: Coverage of 7 UHC tracer interventions for prevention and treatment services		
	1990	2000	2015	1990	2000	2015	1990	2000	2015	1990	2000	2015
Serbia	14.68 (12.81-16.12)	12.30 (11.52-13.06)	9.87 (9.17-10.58)	23.27 (15.15-31.92)	28.09 (19.37-38.31)	42.05 (31.75-52.86)	25.90 (25.90-25.90)	18.82 (18.82-18.82)	10.72 (10.72-10.72)	52.14 (49.72-54.25)	54.90 (53.00-56.70)	80.62 (77.69-83.38)
Slovakia	21.12 (19.14-23.29)	15.06 (14.17-15.86)	7.30 (6.67-8.00)	52.45 (41.62-63.32)	62.68 (51.61-72.44)	80.58 (72.46-87.09)	31.39 (31.39-31.39)	14.27 (14.27-14.27)	9.49 (9.49-9.49)	60.45 (59.19-61.59)	78.54 (75.57-81.19)	91.58 (90.45-92.60)
Slovenia	23.99 (23.09-24.96)	15.36 (14.76-15.97)	6.58 (5.82-7.15)	44.09 (36.44-52.25)	54.93 (45.28-63.80)	72.69 (62.90-81.30)	17.51 (17.51-17.51)	5.94 (5.94-5.94)	3.20 (3.20-3.20)	58.11 (56.99-59.15)	80.31 (78.26-82.36)	89.94 (88.35-91.31)
Armenia	18.44 (17.00-19.64)	13.00 (11.94-14.21)	9.92 (8.73-11.28)	29.79 (24.56-35.45)	29.95 (26.33-33.98)	42.45 (36.70-47.69)	44.04 (44.04-44.04)	25.16 (25.16-25.16)	15.50 (15.50-15.50)	52.07 (50.45-53.47)	53.34 (52.58-54.10)	82.07 (80.68-83.37)
Azerbaijan	19.13 (16.42-20.70)	9.63 (8.83-10.73)	7.84 (6.45-9.18)	18.77 (15.03-22.97)	19.89 (16.10-24.16)	31.03 (25.53-36.67)	26.79 (26.79-26.79)	24.60 (24.60-24.60)	36.21 (36.21-36.21)	35.36 (32.50-37.91)	37.04 (36.02-38.03)	76.54 (74.27-78.55)
Georgia	21.03 (19.29-22.83)	10.51 (9.40-11.78)	17.18 (15.02-19.48)	41.31 (35.32-47.36)	44.39 (38.11-50.90)	58.99 (52.33-65.62)	40.20 (40.20-40.20)	28.72 (28.72-28.72)	22.51 (22.51-22.51)	43.77 (41.56-45.77)	50.03 (49.09-50.96)	82.96 (79.91-85.25)
Kazakhstan	24.25 (23.01-25.48)	17.61 (16.71-18.68)	18.94 (17.15-20.91)	59.05 (53.24-64.96)	66.56 (62.30-70.73)	77.45 (72.27-81.93)	34.44 (34.44-34.44)	21.93 (21.93-21.93)	19.52 (19.52-19.52)	53.85 (51.73-55.77)	60.48 (59.90-61.04)	87.65 (86.32-88.78)
Kyrgyzstan	26.31 (24.13-28.20)	17.33 (16.06-18.81)	19.27 (17.38-21.02)	62.11 (56.03-67.56)	63.80 (58.75-68.45)	64.13 (58.84-69.04)	41.17 (41.17-41.17)	28.30 (28.30-28.30)	27.28 (27.28-27.28)	54.20 (53.03-55.21)	56.88 (56.16-57.47)	84.19 (82.42-85.60)
Mongolia	16.07 (13.02-21.86)	16.92 (15.16-19.23)	21.69 (19.25-24.67)	46.71 (39.99-53.08)	63.02 (56.89-69.32)	77.21 (72.03-81.81)	29.06 (29.06-29.06)	18.13 (18.13-18.13)	13.68 (13.68-13.68)	45.97 (44.18-47.40)	60.25 (57.21-63.08)	83.80 (81.97-85.52)
Tajikistan	16.59 (13.42-18.01)	8.36 (7.62-9.86)	7.04 (6.18-8.06)	39.54 (33.47-46.07)	42.39 (36.61-48.86)	51.74 (46.34-57.16)	41.60 (41.60-41.60)	32.93 (32.93-32.93)	27.76 (27.76-27.76)	44.11 (40.98-46.64)	41.77 (39.17-43.56)	75.58 (73.14-77.67)

Appendix Table 1b. Unscaled values for each SDG health-related indicator by country for 1990, 2000, and 2015												
	Indicator 3.6.1: Age-standardised death rate due to road injuries (per 100,000 population)			Indicator 3.7.1: Proportion of women of reproductive age (15 to 49 years) who have their need for family planning satisfied with modern methods			Indicator 3.7.2: Birth rates for women aged 10 to 14 years and women aged 15 to 19 years (number of live births per 1,000 women)			Indicator 3.8.1: Coverage of 7 UHC tracer interventions for prevention and treatment services		
**Location**	**1990**	**2000**	**2015**	**1990**	**2000**	**2015**	**1990**	**2000**	**2015**	**1990**	**2000**	**2015**
Turkmenistan	20.51 (19.07-21.83)	15.39 (13.79-18.50)	9.15 (8.04-12.05)	41.65 (35.08-48.25)	46.71 (40.51-53.04)	64.99 (59.01-70.95)	23.95 (23.95-23.95)	20.41 (20.41-20.41)	13.17 (13.17-13.17)	52.42 (50.69-53.84)	54.04 (53.00-54.93)	82.18 (80.38-83.84)
Uzbekistan	19.97 (18.15-21.31)	14.77 (13.63-16.11)	13.13 (11.22-15.05)	66.00 (60.37-71.30)	72.93 (68.43-77.20)	84.39 (80.66-87.61)	39.45 (39.45-39.45)	22.98 (22.98-22.98)	18.08 (18.08-18.08)	57.82 (56.74-58.76)	59.52 (58.95-60.03)	88.90 (87.33-90.28)
Colombia	25.34 (24.28-26.44)	23.76 (22.79-24.76)	14.78 (13.77-15.89)	70.04 (67.65-72.36)	77.87 (76.14-79.48)	89.44 (87.55-91.15)	42.85 (42.85-42.85)	43.23 (43.23-43.23)	26.62 (26.62-26.62)	34.90 (34.49-35.25)	42.20 (41.36-44.19)	75.11 (68.02-83.58)
Costa Rica	20.46 (19.62-21.40)	20.69 (19.78-21.60)	14.47 (13.48-15.56)	76.42 (72.85-79.57)	82.66 (79.83-85.18)	91.26 (89.73-92.69)	49.27 (49.27-49.27)	38.96 (38.96-38.96)	29.04 (29.04-29.04)	54.78 (54.29-55.18)	74.91 (71.85-79.08)	89.59 (88.30-90.79)
El Salvador	42.35 (39.21-45.65)	38.98 (36.22-42.31)	25.82 (22.79-31.24)	58.24 (53.74-62.73)	69.19 (65.17-73.16)	83.07 (80.51-85.50)	53.91 (53.91-53.91)	45.85 (45.85-45.85)	32.99 (32.99-32.99)	50.18 (48.79-51.51)	56.27 (55.59-56.90)	88.08 (86.36-89.71)
Guatemala	16.89 (16.00-17.82)	15.61 (14.76-16.44)	16.70 (14.39-19.19)	44.00 (40.15-47.96)	50.61 (47.07-54.30)	63.25 (59.09-67.63)	65.93 (65.93-65.93)	58.37 (58.37-58.37)	42.96 (42.96-42.96)	21.48 (20.80-22.18)	29.45 (28.51-31.19)	56.85 (51.03-63.89)
Honduras	20.74 (17.00-22.82)	19.43 (15.65-24.31)	16.41 (12.80-21.11)	53.44 (48.71-57.97)	64.41 (60.95-67.99)	81.44 (79.19-83.59)	68.77 (68.77-68.77)	57.24 (57.24-57.24)	34.07 (34.07-34.07)	30.90 (30.26-31.56)	36.30 (35.33-38.21)	67.32 (60.75-74.63)
Mexico	28.60 (27.94-29.44)	21.08 (20.57-21.82)	17.30 (16.72-17.90)	70.65 (66.98-74.04)	76.26 (73.47-79.05)	87.51 (85.44-89.50)	45.66 (45.66-45.66)	41.40 (41.40-41.40)	33.05 (33.05-33.05)	48.71 (47.09-50.01)	63.69 (62.00-65.33)	84.17 (81.67-85.98)
Nicaragua	21.83 (19.83-23.52)	16.08 (14.72-17.35)	13.53 (11.86-15.74)	71.52 (67.88-74.94)	79.25 (77.50-80.95)	89.83 (88.01-91.47)	78.29 (78.29-78.29)	58.96 (58.96-58.96)	43.45 (43.45-43.45)	33.23 (32.41-33.96)	40.28 (39.41-42.20)	69.60 (63.20-77.42)
Panama	25.93 (22.53-28.40)	22.28 (20.22-24.23)	16.69 (14.12-19.81)	75.67 (72.06-79.11)	84.20 (81.72-86.67)	92.28 (90.97-93.48)	50.16 (50.16-50.16)	46.07 (46.07-46.07)	39.46 (39.46-39.46)	37.04 (36.12-37.86)	47.77 (45.01-51.09)	68.19 (60.27-76.77)

Location	Appendix Table 1b. Unscaled values for each SDG health-related indicator by country for 1990, 2000, and 2015<br>Indicator 3.6.1: Age-standardised death rate due to road injuries (per 100,000 population)			Indicator 3.7.1: Proportion of women of reproductive age (15 to 49 years) who have their need for family planning satisfied with modern methods			Indicator 3.7.2: Birth rates for women aged 10 to 14 years and women aged 15 to 19 years (number of live births per 1,000 women)			Indicator 3.8.1: Coverage of 7 UHC tracer interventions for prevention and treatment services		
	1990	2000	2015	1990	2000	2015	1990	2000	2015	1990	2000	2015
Venezuela	31.32 (30.12-32.54)	25.77 (24.70-26.87)	23.25 (20.21-26.76)	67.22 (62.85-71.32)	77.40 (74.10-80.60)	88.55 (86.67-90.26)	51.20 (51.20-51.20)	46.23 (46.23-46.23)	40.23 (40.23-40.23)	34.45 (33.49-35.38)	48.79 (46.90-51.49)	74.87 (67.40-82.52)
Bolivia	34.30 (27.40-42.55)	25.52 (22.89-28.89)	18.20 (15.04-22.49)	27.21 (22.43-32.43)	37.46 (32.79-41.80)	53.77 (45.91-60.74)	51.51 (51.51-51.51)	48.52 (48.52-48.52)	37.35 (37.35-37.35)	22.15 (21.34-22.95)	33.47 (32.56-35.18)	64.77 (59.23-71.49)
Ecuador	32.56 (30.40-34.61)	29.13 (26.92-31.38)	27.57 (24.46-30.94)	59.20 (51.41-66.52)	66.03 (57.99-72.88)	80.91 (75.26-85.80)	46.80 (46.80-46.80)	43.67 (43.67-43.67)	39.46 (39.46-39.46)	53.86 (50.31-60.30)	56.83 (53.74-60.15)	84.49 (82.88-85.62)
Peru	18.20 (16.64-20.29)	18.20 (16.59-19.69)	14.08 (12.00-16.23)	45.37 (39.97-50.60)	61.05 (55.95-65.71)	77.00 (71.04-82.42)	40.13 (40.13-40.13)	35.24 (35.24-35.24)	26.52 (26.52-26.52)	28.54 (27.74-29.30)	40.35 (39.22-42.46)	77.76 (69.47-84.97)
Antigua and Barbuda	8.60 (7.89-9.39)	7.94 (7.28-8.73)	6.91 (6.09-7.82)	87.55 (82.40-91.58)	92.69 (89.53-95.22)	95.03 (92.77-96.70)	32.93 (32.93-32.93)	32.95 (32.95-32.95)	23.90 (23.90-23.90)	60.40 (59.63-61.04)	64.15 (63.63-65.22)	90.14 (88.98-91.19)
The Bahamas	24.89 (22.46-27.44)	22.10 (19.49-25.08)	15.34 (13.13-17.64)	81.75 (74.70-87.28)	88.20 (83.67-91.88)	93.55 (90.90-95.75)	36.90 (36.90-36.90)	27.35 (27.35-27.35)	16.43 (16.43-16.43)	59.89 (58.69-60.83)	79.95 (75.61-83.19)	92.04 (91.13-92.87)
Barbados	12.87 (12.04-13.78)	11.63 (10.70-12.56)	10.56 (9.42-11.88)	73.96 (65.63-81.21)	83.73 (77.20-88.47)	90.93 (86.90-93.95)	27.95 (27.95-27.95)	26.68 (26.68-26.68)	20.83 (20.83-20.83)	59.38 (58.53-60.14)	61.37 (60.10-63.89)	89.04 (87.04-90.53)
Belize	18.74 (16.83-20.68)	25.67 (23.03-28.21)	20.23 (17.22-23.27)	49.37 (38.62-59.81)	55.94 (45.97-66.05)	71.28 (62.57-79.25)	66.04 (66.04-66.04)	52.04 (52.04-52.04)	35.39 (35.39-35.39)	51.11 (49.43-52.79)	58.96 (55.12-63.19)	86.62 (84.46-88.70)
Cuba	22.65 (21.75-23.48)	15.26 (14.67-15.88)	8.62 (8.05-9.17)	72.24 (62.52-80.44)	83.49 (77.41-88.67)	92.52 (89.18-95.15)	38.21 (38.21-38.21)	29.91 (29.91-29.91)	24.61 (24.61-24.61)	56.27 (55.27-57.03)	61.83 (61.24-62.32)	89.59 (88.23-91.64)
Dominica	12.40 (11.22-13.74)	12.88 (11.64-14.17)	15.06 (12.67-17.47)	71.70 (62.46-79.55)	81.53 (74.87-86.94)	90.37 (86.37-93.71)	37.45 (37.45-37.45)	20.66 (20.66-20.66)	16.63 (16.63-16.63)	60.58 (59.74-61.31)	62.86 (62.14-63.92)	88.66 (87.47-89.78)

Appendix Table 1b. Unscaled values for each SDG health-related indicator by country for 1990, 2000, and 2015

Location	Indicator 3.6.1: Age-standardised death rate due to road injuries (per 100,000 population)			Indicator 3.7.1: Proportion of women of reproductive age (15 to 49 years) who have their need for family planning satisfied with modern methods			Indicator 3.7.2: Birth rates for women aged 10 to 14 years and women aged 15 to 19 years (number of live births per 1,000 women)			Indicator 3.8.1: Coverage of 7 UHC tracer interventions for prevention and treatment services		
	1990	2000	2015	1990	2000	2015	1990	2000	2015	1990	2000	2015
Dominican Republic	23.49 (21.38-25.21)	25.84 (23.44-28.27)	23.46 (21.08-25.82)	71.26 (64.34-77.10)	79.45 (75.57-82.78)	85.72 (81.42-89.26)	58.71 (58.71-58.71)	56.51 (56.51-56.51)	49.43 (49.43-49.43)	48.87 (47.92-49.83)	55.01 (54.21-55.77)	81.11 (78.87-83.11)
Grenada	12.38 (11.23-13.64)	13.70 (12.39-15.05)	14.07 (12.07-16.09)	64.43 (54.77-73.63)	73.96 (65.64-81.22)	87.73 (82.57-91.79)	51.17 (51.17-51.17)	29.38 (29.38-29.38)	17.25 (17.25-17.25)	55.11 (53.93-56.27)	58.35 (57.50-59.43)	88.11 (86.87-89.17)
Guyana	21.47 (19.71-23.67)	22.67 (20.64-25.09)	22.07 (17.79-26.16)	49.67 (39.84-60.37)	57.47 (47.03-67.15)	67.57 (58.88-75.34)	52.72 (52.72-52.72)	50.57 (50.57-50.57)	45.43 (45.43-45.43)	29.01 (27.53-30.38)	36.02 (34.16-39.08)	74.27 (68.31-79.70)
Haiti	35.17 (26.41-46.66)	27.31 (19.80-36.63)	26.03 (17.77-36.46)	25.78 (19.54-32.28)	32.66 (27.06-38.22)	46.25 (38.56-53.82)	40.81 (40.81-40.81)	32.97 (32.97-32.97)	23.35 (23.35-23.35)	17.34 (16.51-18.11)	22.08 (21.12-23.56)	56.88 (51.78-60.92)
Jamaica	6.46 (5.89-7.11)	6.88 (6.23-7.79)	9.18 (7.58-10.82)	68.04 (57.65-76.55)	79.03 (71.44-85.45)	88.19 (83.41-92.15)	54.07 (54.07-54.07)	45.01 (45.01-45.01)	31.74 (31.74-31.74)	55.17 (53.20-56.76)	59.20 (58.22-60.05)	88.98 (87.79-89.99)
Saint Lucia	16.43 (15.15-17.76)	15.46 (14.31-16.63)	12.25 (11.03-13.68)	65.85 (56.21-75.04)	76.46 (68.04-83.82)	88.08 (83.28-91.92)	54.88 (54.88-54.88)	33.81 (33.81-33.81)	27.30 (27.30-27.30)	58.86 (57.81-59.78)	60.84 (59.89-61.91)	88.30 (87.12-89.39)
Saint Vincent and the Grenadines	10.09 (9.22-11.03)	10.44 (9.65-11.26)	10.70 (9.51-11.88)	67.63 (58.18-76.00)	78.19 (70.28-84.76)	88.98 (84.19-92.70)	47.30 (47.30-47.30)	36.04 (36.04-36.04)	27.00 (27.00-27.00)	51.41 (50.59-52.15)	57.28 (56.57-58.23)	85.75 (84.66-86.78)
Suriname	22.70 (20.59-24.77)	21.34 (19.67-23.44)	20.38 (17.54-23.58)	58.70 (48.59-68.89)	62.50 (52.84-72.06)	75.33 (66.39-82.67)	37.95 (37.95-37.95)	31.75 (31.75-31.75)	25.30 (25.30-25.30)	30.41 (29.20-31.42)	38.51 (37.28-40.47)	73.55 (66.50-81.30)
Trinidad and Tobago	17.60 (16.71-18.54)	17.41 (16.56-18.31)	16.62 (14.71-18.71)	67.84 (58.17-76.35)	59.38 (48.95-68.85)	77.63 (70.11-84.38)	34.40 (34.40-34.40)	23.31 (23.31-23.31)	17.97 (17.97-17.97)	56.05 (54.42-57.43)	58.41 (56.98-59.75)	88.64 (86.80-90.09)
Brazil	36.87 (35.64-38.17)	29.46 (28.43-31.49)	24.82 (23.40-27.28)	77.23 (74.02-80.01)	85.27 (83.16-87.15)	92.58 (91.35-93.68)	42.47 (42.47-42.47)	42.58 (42.58-42.58)	34.05 (34.05-34.05)	34.56 (33.98-35.17)	59.55 (56.70-63.59)	78.15 (70.96-86.52)

Appendix Table 1b. Unscaled values for each SDG health-related indicator by country for 1990, 2000, and 2015												
	**Indicator 3.6.1: Age-standardised death rate due to road injuries (per 100,000 population)**			**Indicator 3.7.1: Proportion of women of reproductive age (15 to 49 years) who have their need for family planning satisfied with modern methods**			**Indicator 3.7.2: Birth rates for women aged 10 to 14 years and women aged 15 to 19 years (number of live births per 1,000 women)**			**Indicator 3.8.1: Coverage of 7 UHC tracer interventions for prevention and treatment services**		
**Location**	**1990**	**2000**	**2015**	**1990**	**2000**	**2015**	**1990**	**2000**	**2015**	**1990**	**2000**	**2015**
Paraguay	17.96 (15.95-20.11)	20.58 (18.86-22.40)	26.47 (21.70-31.97)	56.32 (52.94-59.74)	68.77 (65.18-72.20)	85.71 (83.37-87.68)	50.57 (50.57-50.57)	44.53 (44.53-44.53)	30.86 (30.86-30.86)	47.99 (46.92-49.00)	53.26 (52.32-54.10)	83.91 (81.59-86.02)
China	23.19 (21.56-25.08)	25.79 (24.44-27.31)	20.44 (18.40-22.05)	62.64 (53.88-70.70)	73.67 (67.78-79.34)	86.61 (80.56-91.46)	21.65 (21.65-21.65)	11.39 (11.39-11.39)	11.41 (11.41-11.41)	39.94 (37.46-42.13)	49.89 (48.70-50.88)	78.20 (76.09-80.24)
North Korea	25.82 (18.96-35.36)	30.42 (20.65-42.46)	28.64 (19.01-40.70)	36.12 (24.63-47.78)	44.74 (33.50-56.61)	64.51 (54.73-73.50)	4.04 (4.04-4.04)	2.27 (2.27-2.27)	2.30 (2.30-2.30)	34.75 (32.52-36.59)	47.36 (45.04-49.19)	75.42 (70.72-79.74)
Taiwan	29.66 (24.54-35.17)	22.07 (19.19-24.80)	14.64 (12.28-17.29)	73.55 (63.43-82.49)	80.32 (71.88-87.21)	88.95 (83.45-92.98)	12.40 (12.40-12.40)	9.67 (9.67-9.67)	2.59 (2.59-2.59)	59.52 (57.66-61.02)	61.96 (60.93-62.76)	79.67 (77.56-81.72)
Cambodia	30.46 (19.34-43.74)	31.71 (23.46-43.03)	21.50 (17.31-26.53)	26.93 (22.08-32.40)	32.90 (30.96-35.01)	56.93 (54.72-59.18)	46.56 (46.56-46.56)	31.03 (31.03-31.03)	31.65 (31.65-31.65)	12.91 (11.75-14.20)	20.21 (19.28-21.57)	58.04 (52.81-64.52)
Indonesia	28.84 (23.74-33.19)	26.19 (22.88-29.12)	19.60 (15.41-24.32)	72.70 (68.86-76.42)	80.63 (78.05-83.10)	89.16 (87.06-91.10)	37.53 (37.53-37.53)	27.56 (27.56-27.56)	27.96 (27.96-27.96)	20.65 (20.29-20.99)	25.79 (25.18-27.17)	53.59 (48.36-59.72)
Laos	37.95 (25.40-55.18)	41.20 (29.64-56.45)	27.62 (22.13-34.93)	34.04 (28.38-40.17)	42.25 (38.13-46.37)	54.08 (46.97-60.49)	60.51 (60.51-60.51)	45.04 (45.04-45.04)	36.06 (36.06-36.06)	10.64 (9.38-12.00)	16.47 (15.48-17.48)	50.65 (47.53-53.77)
Malaysia	29.25 (26.06-32.02)	27.85 (24.07-29.81)	23.72 (19.22-27.93)	40.41 (33.64-47.29)	47.97 (41.32-54.81)	65.21 (59.17-70.97)	15.01 (15.01-15.01)	10.70 (10.70-10.70)	8.02 (8.02-8.02)	52.95 (51.76-54.10)	59.68 (57.32-62.65)	85.10 (83.99-86.16)
Maldives	19.42 (14.33-23.48)	14.10 (12.75-15.50)	7.72 (6.42-9.14)	30.19 (24.63-36.20)	35.63 (29.78-42.09)	50.38 (43.60-57.27)	70.78 (70.78-70.78)	21.86 (21.86-21.86)	9.47 (9.47-9.47)	45.91 (43.10-48.30)	53.96 (52.62-55.19)	77.00 (75.45-78.38)
Mauritius	15.31 (14.37-16.23)	16.03 (15.09-17.03)	11.20 (10.29-12.05)	46.77 (40.46-53.83)	53.86 (47.19-60.63)	68.74 (62.54-74.28)	27.06 (27.06-27.06)	22.48 (22.48-22.48)	15.34 (15.34-15.34)	53.19 (52.23-54.10)	55.11 (54.33-55.80)	80.48 (79.33-81.45)

Appendix Table 1b. Unscaled values for each SDG health-related indicator by country for 1990, 2000, and 2015

Location	Indicator 3.6.1: Age-standardised death rate due to road injuries (per 100,000 population)			Indicator 3.7.1: Proportion of women of reproductive age (15 to 49 years) who have their need for family planning satisfied with modern methods			Indicator 3.7.2: Birth rates for women aged 10 to 14 years and women aged 15 to 19 years (number of live births per 1,000 women)			Indicator 3.8.1: Coverage of 7 UHC tracer interventions for prevention and treatment services		
	1990	2000	2015	1990	2000	2015	1990	2000	2015	1990	2000	2015
Myanmar	16.82 (10.67-24.59)	15.56 (8.66-23.48)	14.76 (8.78-22.00)	29.83 (24.35-35.73)	37.16 (30.91-43.91)	53.79 (46.83-60.64)	22.10 (22.10-22.10)	16.57 (16.57-16.57)	11.10 (11.10-11.10)	19.56 (18.36-20.68)	25.56 (24.70-26.81)	61.03 (55.33-67.30)
Philippines	12.84 (12.14-13.48)	12.02 (11.49-12.56)	11.15 (10.10-12.26)	36.46 (33.12-40.08)	45.62 (43.80-47.55)	54.29 (51.69-56.85)	32.00 (32.00-32.00)	32.20 (32.20-32.20)	34.17 (34.17-34.17)	27.26 (26.73-27.77)	30.76 (30.10-32.33)	58.01 (52.25-64.32)
Sri Lanka	15.04 (14.18-16.00)	19.01 (18.06-19.98)	14.14 (11.17-17.65)	52.57 (47.91-57.00)	51.61 (47.20-55.87)	71.51 (65.79-76.34)	19.23 (19.23-19.23)	17.16 (17.16-17.16)	8.19 (8.19-8.19)	53.13 (51.82-54.32)	56.15 (55.41-56.82)	83.27 (82.05-84.66)
Seychelles	14.28 (12.35-16.65)	18.91 (17.00-20.91)	14.28 (12.18-16.25)	59.78 (53.10-66.35)	71.83 (65.65-77.40)	85.77 (82.04-88.91)	38.23 (38.23-38.23)	31.39 (31.39-31.39)	30.01 (30.01-30.01)	55.34 (53.65-56.83)	67.30 (64.45-70.70)	87.85 (86.07-90.13)
Thailand	25.29 (23.37-27.75)	42.84 (38.51-46.77)	31.08 (25.51-36.53)	46.17 (39.55-52.82)	59.56 (53.43-66.13)	68.09 (64.99-70.95)	28.51 (28.51-28.51)	23.76 (23.76-23.76)	22.99 (22.99-22.99)	32.24 (31.27-33.05)	40.74 (38.12-43.92)	68.20 (61.84-75.13)
Timor-Leste	17.25 (11.05-24.14)	16.90 (10.43-24.01)	14.00 (8.96-21.76)	24.95 (19.90-30.46)	29.36 (24.29-34.89)	45.71 (39.88-51.52)	38.25 (38.25-38.25)	51.35 (51.35-51.35)	31.18 (31.18-31.18)	12.91 (11.25-14.62)	20.59 (17.89-23.91)	55.94 (50.81-60.98)
Vietnam	30.25 (23.26-38.30)	27.37 (21.78-33.35)	23.16 (16.29-31.17)	56.94 (50.37-62.80)	68.08 (65.85-70.25)	81.36 (76.97-85.40)	24.42 (24.42-24.42)	19.58 (19.58-19.58)	22.76 (22.76-22.76)	22.69 (21.17-24.08)	33.90 (32.80-35.92)	67.78 (61.40-74.71)
Federated States of Micronesia	19.04 (11.53-27.92)	17.12 (10.82-26.56)	14.87 (9.42-24.65)	60.46 (55.44-65.38)	71.49 (67.08-75.39)	83.55 (80.64-86.15)	48.12 (48.12-48.12)	37.84 (37.84-37.84)	17.75 (17.75-17.75)	49.88 (46.45-52.42)	50.84 (48.42-52.57)	78.47 (74.52-81.77)
Fiji	12.68 (10.26-15.48)	13.76 (11.99-15.75)	13.21 (10.91-15.68)	61.34 (56.66-66.45)	71.36 (67.27-75.05)	86.40 (84.04-88.76)	40.06 (40.06-40.06)	29.45 (29.45-29.45)	27.67 (27.67-27.67)	53.21 (51.10-54.48)	55.00 (53.50-55.90)	79.22 (76.96-80.91)
Kiribati	12.88 (11.13-14.94)	15.77 (13.17-18.14)	13.12 (10.54-16.26)	48.03 (42.94-53.24)	54.22 (49.07-59.45)	66.59 (61.99-71.04)	30.35 (30.35-30.35)	24.17 (24.17-24.17)	11.59 (11.59-11.59)	48.14 (44.90-50.41)	53.28 (50.42-55.16)	78.35 (74.09-81.13)

Appendix Table 1b. Unscaled values for each SDG health-related indicator by country for 1990, 2000, and 2015

Location	Indicator 3.6.1: Age-standardised death rate due to road injuries (per 100,000 population)			Indicator 3.7.1: Proportion of women of reproductive age (15 to 49 years) who have their need for family planning satisfied with modern methods			Indicator 3.7.2: Birth rates for women aged 10 to 14 years and women aged 15 to 19 years (number of live births per 1,000 women)			Indicator 3.8.1: Coverage of 7 UHC tracer interventions for prevention and treatment services		
	1990	2000	2015	1990	2000	2015	1990	2000	2015	1990	2000	2015
Marshall Islands	18.47 (15.14-22.87)	18.28 (13.58-23.79)	16.48 (12.55-22.10)	54.40 (49.02-59.28)	64.56 (59.86-69.10)	79.94 (76.59-82.88)	61.87 (61.87-61.87)	53.27 (53.27-53.27)	36.03 (36.03-36.03)	41.86 (36.04-47.23)	47.44 (43.18-50.95)	72.78 (68.11-76.79)
Papua New Guinea	38.26 (24.95-55.46)	35.52 (21.79-51.37)	31.05 (19.43-47.54)	40.46 (35.66-45.08)	43.78 (39.48-48.37)	51.52 (46.94-56.51)	44.31 (44.31-44.31)	40.44 (40.44-40.44)	32.83 (32.83-32.83)	23.76 (21.95-25.34)	25.63 (24.05-27.58)	48.12 (42.60-53.90)
Samoa	13.51 (9.73-18.07)	12.20 (8.50-16.54)	10.18 (7.28-13.60)	45.97 (41.37-50.91)	57.60 (52.66-62.17)	71.45 (67.50-75.20)	25.08 (25.08-25.08)	27.32 (27.32-27.32)	17.30 (17.30-17.30)	45.64 (40.68-49.58)	48.89 (45.61-51.58)	70.05 (65.31-74.30)
Solomon Islands	22.09 (13.23-34.63)	21.94 (12.79-34.32)	19.87 (11.72-32.81)	38.19 (33.51-43.16)	43.94 (39.05-49.03)	57.48 (52.41-62.56)	53.24 (53.24-53.24)	41.76 (41.76-41.76)	29.33 (29.33-29.33)	25.88 (23.05-28.06)	30.21 (28.37-32.20)	56.54 (50.71-63.10)
Tonga	14.83 (11.92-18.16)	15.34 (13.46-17.41)	13.64 (10.75-17.06)	60.48 (55.68-65.14)	70.94 (66.74-75.09)	84.66 (82.01-87.13)	18.88 (18.88-18.88)	16.71 (16.71-16.71)	11.31 (11.31-11.31)	56.03 (53.58-57.50)	59.54 (57.49-60.45)	83.80 (81.01-85.63)
Vanuatu	21.13 (13.06-32.48)	22.23 (12.77-34.62)	19.74 (12.48-30.53)	43.91 (39.00-48.81)	51.50 (46.58-56.72)	67.87 (63.00-72.33)	44.04 (44.04-44.04)	37.12 (37.12-37.12)	27.20 (27.20-27.20)	29.95 (27.49-31.74)	33.50 (31.34-35.71)	58.70 (52.46-65.49)
Afghanistan	67.92 (48.81-88.15)	70.48 (52.08-92.09)	63.92 (46.84-84.78)	41.64 (34.82-48.79)	41.24 (33.90-48.92)	44.24 (39.10-49.89)	88.62 (88.62-88.62)	84.54 (84.54-84.54)	41.72 (41.72-41.72)	13.72 (12.12-15.38)	16.32 (15.09-17.72)	33.56 (29.91-37.63)
Algeria	40.28 (33.43-46.33)	33.29 (29.36-37.65)	26.10 (22.54-29.74)	59.52 (52.67-66.56)	63.61 (56.40-69.99)	70.11 (64.45-75.40)	20.72 (20.72-20.72)	8.91 (8.91-8.91)	9.42 (9.42-9.42)	48.83 (46.93-50.64)	54.60 (53.19-55.86)	86.11 (84.44-87.54)
Bahrain	25.21 (21.99-29.01)	20.97 (18.28-24.01)	12.81 (10.51-15.79)	43.04 (36.25-50.10)	53.42 (46.84-59.98)	67.66 (61.01-74.16)	19.91 (19.91-19.91)	13.82 (13.82-13.82)	11.01 (11.01-11.01)	55.03 (53.69-56.31)	59.25 (57.59-62.42)	81.87 (80.31-83.36)
Egypt	13.15 (12.04-15.29)	15.93 (14.95-16.93)	15.38 (14.22-16.68)	65.16 (60.65-70.13)	78.27 (75.13-81.08)	86.13 (83.53-88.79)	47.97 (47.97-47.97)	32.59 (32.59-32.59)	34.31 (34.31-34.31)	35.27 (34.50-36.01)	50.04 (49.47-50.61)	77.39 (76.09-78.69)

Location	Appendix Table 1b. Unscaled values for each SDG health-related indicator by country for 1990, 2000, and 2015											
	**Indicator 3.6.1: Age-standardised death rate due to road injuries (per 100,000 population)**			**Indicator 3.7.1: Proportion of women of reproductive age (15 to 49 years) who have their need for family planning satisfied with modern methods**			**Indicator 3.7.2: Birth rates for women aged 10 to 14 years and women aged 15 to 19 years (number of live births per 1,000 women)**			**Indicator 3.8.1: Coverage of 7 UHC tracer interventions for prevention and treatment services**		
	**1990**	**2000**	**2015**	**1990**	**2000**	**2015**	**1990**	**2000**	**2015**	**1990**	**2000**	**2015**
Iran	51.80 (43.25-61.74)	45.63 (40.66-51.76)	42.17 (33.93-52.54)	39.79 (32.85-47.00)	51.08 (43.26-58.47)	75.22 (70.06-80.13)	49.81 (49.81-49.81)	22.76 (22.76-22.76)	14.94 (14.94-14.94)	50.99 (49.42-52.42)	60.54 (57.02-65.12)	85.93 (85.17-86.56)
Iraq	28.47 (22.63-36.35)	28.65 (23.17-35.39)	23.33 (17.45-30.75)	32.41 (25.72-39.65)	37.82 (31.13-45.05)	48.91 (41.59-56.45)	40.89 (40.89-40.89)	37.20 (37.20-37.20)	47.16 (47.16-47.16)	41.21 (39.30-43.17)	45.38 (43.88-47.34)	70.21 (67.86-72.42)
Jordan	26.82 (20.96-32.80)	20.65 (17.71-24.15)	14.32 (12.53-16.45)	44.38 (38.98-49.89)	56.45 (51.43-61.38)	67.05 (62.10-72.25)	36.22 (36.22-36.22)	24.69 (24.69-24.69)	16.02 (16.02-16.02)	55.49 (54.68-56.25)	60.98 (59.81-63.93)	82.62 (81.15-84.14)
Kuwait	22.82 (21.28-24.43)	24.17 (23.02-25.34)	15.03 (12.72-17.98)	49.90 (42.78-57.72)	61.61 (53.84-68.21)	78.66 (73.34-83.52)	15.44 (15.44-15.44)	16.68 (16.68-16.68)	9.40 (9.40-9.40)	43.28 (40.53-45.67)	55.31 (52.03-59.16)	80.27 (78.36-82.02)
Lebanon	16.36 (13.08-20.72)	10.75 (8.05-14.70)	7.94 (5.33-11.98)	47.16 (39.73-54.79)	55.94 (48.14-63.10)	69.98 (63.20-75.84)	27.05 (27.05-27.05)	15.10 (15.10-15.10)	7.88 (7.88-7.88)	53.76 (51.95-55.40)	65.01 (60.17-69.95)	84.18 (82.17-86.60)
Libya	27.63 (22.66-32.92)	25.21 (20.75-29.15)	23.56 (19.47-28.44)	38.48 (31.79-45.73)	43.68 (36.27-51.10)	55.32 (47.61-62.69)	10.08 (10.08-10.08)	5.21 (5.21-5.21)	4.74 (4.74-4.74)	43.84 (41.88-45.75)	52.34 (49.85-56.55)	75.86 (73.59-78.22)
Morocco	28.37 (23.33-34.03)	23.61 (19.78-28.14)	20.03 (15.18-26.61)	58.88 (52.94-64.58)	68.25 (62.93-73.37)	79.02 (73.90-83.66)	25.26 (25.26-25.26)	19.85 (19.85-19.85)	18.61 (18.61-18.61)	33.81 (32.98-34.65)	48.43 (47.73-49.06)	77.52 (75.20-79.96)
Palestine	16.53 (12.98-20.99)	12.68 (10.88-14.49)	13.26 (10.33-16.94)	41.81 (34.24-49.43)	51.09 (43.08-58.27)	59.09 (51.79-66.00)	61.63 (61.63-61.63)	48.47 (48.47-48.47)	36.06 (36.06-36.06)	50.85 (49.58-52.05)	51.43 (50.42-53.16)	72.87 (71.45-74.34)
Oman	70.18 (50.90-90.90)	56.76 (46.99-68.10)	44.77 (36.69-54.41)	35.04 (28.76-41.78)	38.60 (31.46-45.79)	51.73 (44.23-58.61)	50.21 (50.21-50.21)	19.80 (19.80-19.80)	7.28 (7.28-7.28)	54.57 (53.31-55.74)	66.70 (62.76-71.03)	84.00 (82.60-85.28)
Qatar	50.74 (43.30-58.53)	50.25 (42.67-57.92)	31.58 (23.76-40.51)	42.91 (35.25-50.25)	48.01 (40.96-55.48)	68.94 (63.72-74.07)	33.22 (33.22-33.22)	17.73 (17.73-17.73)	10.44 (10.44-10.44)	54.13 (52.83-55.28)	60.26 (57.76-63.91)	82.50 (81.09-83.96)

Appendix Table 1b. Unscaled values for each SDG health-related indicator by country for 1990, 2000, and 2015												
	Indicator 3.6.1: Age-standardised death rate due to road injuries (per 100,000 population)			Indicator 3.7.1: Proportion of women of reproductive age (15 to 49 years) who have their need for family planning satisfied with modern methods			Indicator 3.7.2: Birth rates for women aged 10 to 14 years and women aged 15 to 19 years (number of live births per 1,000 women)			Indicator 3.8.1: Coverage of 7 UHC tracer interventions for prevention and treatment services		
Location	1990	2000	2015	1990	2000	2015	1990	2000	2015	1990	2000	2015
Saudi Arabia	40.15 (33.27-45.89)	34.53 (30.11-37.44)	27.28 (23.44-31.01)	40.55 (33.62-48.20)	46.89 (39.44-54.37)	62.26 (55.09-68.81)	41.01 (41.01-41.01)	17.17 (17.17-17.17)	5.57 (5.57-5.57)	53.55 (52.30-54.81)	59.52 (56.96-64.10)	81.52 (80.03-83.18)
Sudan	42.55 (28.86-63.51)	42.69 (28.63-64.14)	38.59 (26.03-56.57)	16.55 (12.81-20.92)	16.39 (12.58-20.83)	16.94 (13.95-20.16)	57.39 (57.39-57.39)	61.03 (61.03-61.03)	39.60 (39.60-39.60)	15.07 (13.53-16.53)	22.28 (20.80-23.57)	49.93 (47.31-52.63)
Syria	21.49 (16.58-25.79)	18.10 (13.62-21.54)	14.14 (11.20-16.47)	40.50 (32.86-47.84)	47.61 (39.85-55.28)	60.28 (53.34-66.93)	42.25 (42.25-42.25)	33.36 (33.36-33.36)	25.58 (25.58-25.58)	41.89 (39.62-44.20)	51.72 (50.15-53.23)	76.05 (74.03-78.07)
Tunisia	29.29 (24.94-34.07)	24.66 (21.24-28.21)	18.78 (14.83-23.52)	56.84 (49.55-63.89)	65.15 (58.33-71.60)	73.84 (67.06-79.42)	15.24 (15.24-15.24)	6.23 (6.23-6.23)	5.69 (5.69-5.69)	49.01 (47.67-50.32)	57.57 (56.77-58.31)	84.13 (82.79-85.66)
Turkey	21.04 (16.93-25.19)	14.09 (12.58-15.81)	11.04 (9.87-12.21)	45.65 (39.65-51.66)	53.80 (48.84-58.76)	72.13 (65.50-77.65)	39.19 (39.19-39.19)	29.36 (29.36-29.36)	16.74 (16.74-16.74)	44.98 (44.02-45.85)	49.99 (49.14-50.78)	79.33 (77.72-81.02)
United Arab Emirates	59.51 (42.34-76.60)	49.69 (39.77-57.94)	41.59 (30.37-53.81)	38.43 (31.69-45.68)	47.86 (40.10-55.38)	61.91 (54.37-68.69)	34.85 (34.85-34.85)	18.24 (18.24-18.24)	18.47 (18.47-18.47)	54.54 (53.39-55.66)	58.37 (56.64-62.64)	81.64 (79.86-83.62)
Yemen	45.60 (24.68-72.33)	45.30 (25.68-74.24)	40.54 (23.49-66.13)	31.46 (25.12-38.21)	36.55 (29.85-44.07)	47.10 (41.43-52.92)	82.45 (82.45-82.45)	54.58 (54.58-54.58)	35.50 (35.50-35.50)	16.36 (15.56-17.19)	21.62 (19.86-23.64)	51.20 (46.80-55.98)
Bangladesh	10.09 (8.68-12.02)	12.33 (10.83-13.86)	15.77 (11.92-18.52)	54.29 (48.97-59.71)	64.33 (60.85-67.77)	74.98 (71.67-77.86)	78.58 (78.58-78.58)	58.29 (58.29-58.29)	43.35 (43.35-43.35)	11.47 (10.52-12.42)	22.70 (21.75-23.97)	52.38 (46.59-57.84)
Bhutan	17.50 (12.48-23.42)	14.98 (11.77-18.64)	10.86 (8.25-14.13)	38.78 (32.04-46.15)	45.20 (38.33-51.99)	60.30 (54.89-65.18)	58.94 (58.94-58.94)	44.52 (44.52-44.52)	13.81 (13.81-13.81)	31.84 (28.10-35.50)	42.37 (38.96-45.52)	73.00 (69.17-76.83)
India	27.37 (25.44-30.12)	29.23 (27.26-31.52)	27.48 (24.55-30.45)	60.73 (58.30-63.20)	67.64 (65.90-69.42)	83.06 (78.84-86.73)	56.58 (56.58-56.58)	41.77 (41.77-41.77)	21.63 (21.63-21.63)	20.67 (19.05-22.25)	25.71 (24.56-27.40)	53.32 (47.93-59.63)

Appendix Table 1b. Unscaled values for each SDG health-related indicator by country for 1990, 2000, and 2015

Location	Indicator 3.6.1: Age-standardised death rate due to road injuries (per 100,000 population)			Indicator 3.7.1: Proportion of women of reproductive age (15 to 49 years) who have their need for family planning satisfied with modern methods			Indicator 3.7.2: Birth rates for women aged 10 to 14 years and women aged 15 to 19 years (number of live births per 1,000 women)			Indicator 3.8.1: Coverage of 7 UHC tracer interventions for prevention and treatment services		
	1990	2000	2015	1990	2000	2015	1990	2000	2015	1990	2000	2015
Nepal	28.21 (22.46-34.73)	27.79 (22.49-36.07)	27.23 (19.97-39.97)	38.99 (33.23-45.49)	50.64 (48.19-53.19)	60.87 (56.31-65.12)	72.28 (72.28-72.28)	63.60 (63.60-63.60)	39.80 (39.80-39.80)	13.22 (11.67-14.75)	21.56 (20.71-22.82)	52.22 (46.64-58.30)
Pakistan	15.39 (12.16-20.52)	19.09 (14.35-24.79)	18.14 (13.15-24.33)	21.13 (19.07-23.22)	32.27 (27.55-37.08)	48.86 (44.77-52.88)	49.19 (49.19-49.19)	32.45 (32.45-32.45)	26.27 (26.27-26.27)	15.11 (14.43-15.77)	18.71 (17.85-19.88)	42.44 (37.70-47.66)
Botswana	25.15 (9.98-62.39)	35.38 (10.74-109.46)	31.64 (11.81-105.94)	63.06 (55.81-70.09)	76.62 (70.51-81.90)	88.39 (84.74-91.33)	53.24 (53.24-53.24)	35.63 (35.63-35.63)	20.65 (20.65-20.65)	33.98 (33.11-34.76)	40.17 (37.88-43.58)	79.43 (73.95-85.80)
Lesotho	32.27 (21.85-46.73)	36.95 (23.53-53.88)	57.15 (28.34-101.54)	43.82 (36.46-51.64)	53.55 (47.05-59.93)	82.15 (78.93-85.18)	48.43 (48.43-48.43)	50.14 (50.14-50.14)	48.79 (48.79-48.79)	43.32 (41.53-45.12)	46.38 (45.49-47.22)	74.23 (72.47-75.94)
Namibia	39.43 (31.05-48.38)	52.59 (38.77-69.19)	31.92 (18.51-52.22)	60.39 (54.61-65.99)	73.32 (69.23-76.93)	86.96 (84.17-89.43)	56.26 (56.26-56.26)	44.15 (44.15-44.15)	41.28 (41.28-41.28)	31.61 (30.98-32.18)	36.53 (35.51-38.54)	79.46 (74.36-83.20)
South Africa	54.62 (48.06-61.88)	56.72 (49.76-62.19)	39.37 (34.35-44.24)	74.47 (68.87-79.98)	82.31 (78.89-85.44)	91.40 (88.42-93.64)	46.85 (46.85-46.85)	35.02 (35.02-35.02)	20.07 (20.07-20.07)	36.48 (35.78-37.12)	44.88 (43.85-46.41)	76.79 (68.87-83.98)
Swaziland	41.27 (28.90-58.18)	51.91 (32.18-78.01)	63.56 (28.56-116.13)	47.89 (40.00-56.65)	59.66 (52.80-65.93)	80.70 (75.47-85.15)	68.58 (68.58-68.58)	54.47 (54.47-54.47)	37.62 (37.62-37.62)	48.54 (47.03-50.07)	52.74 (51.89-53.54)	81.58 (80.21-82.97)
Zimbabwe	36.99 (24.18-55.12)	48.64 (27.03-79.54)	37.95 (22.15-58.13)	66.92 (61.38-72.30)	75.85 (72.53-79.11)	86.21 (82.89-89.32)	58.62 (58.62-58.62)	55.90 (55.90-55.90)	56.83 (56.83-56.83)	33.55 (33.04-34.03)	38.25 (37.63-38.82)	76.84 (74.79-78.56)
Benin	31.33 (19.38-44.95)	29.95 (21.49-40.89)	20.19 (10.71-33.74)	11.72 (7.36-17.24)	14.60 (10.75-19.41)	22.20 (15.17-30.63)	68.14 (68.14-68.14)	64.47 (64.47-64.47)	46.08 (46.08-46.08)	23.78 (22.36-25.15)	28.64 (27.74-29.51)	64.28 (61.30-67.04)
Burkina Faso	26.93 (21.70-32.66)	24.68 (20.07-29.95)	23.10 (14.06-35.77)	12.36 (8.46-17.20)	20.00 (14.60-25.66)	33.80 (24.43-44.92)	76.73 (76.73-76.73)	73.54 (73.54-73.54)	57.92 (57.92-57.92)	17.10 (16.09-18.12)	22.73 (21.93-23.44)	65.83 (63.12-68.37)

Appendix Table 1b. Unscaled values for each SDG health-related indicator by country for 1990, 2000, and 2015												
	**Indicator 3.6.1: Age-standardised death rate due to road injuries (per 100,000 population)**			**Indicator 3.7.1: Proportion of women of reproductive age (15 to 49 years) who have their need for family planning satisfied with modern methods**			**Indicator 3.7.2: Birth rates for women aged 10 to 14 years and women aged 15 to 19 years (number of live births per 1,000 women)**			**Indicator 3.8.1: Coverage of 7 UHC tracer interventions for prevention and treatment services**		
**Location**	**1990**	**2000**	**2015**	**1990**	**2000**	**2015**	**1990**	**2000**	**2015**	**1990**	**2000**	**2015**
Cameroon	24.74 (19.25-31.24)	25.22 (17.55-34.12)	21.48 (12.14-35.50)	14.02 (9.71-18.64)	24.12 (17.84-30.92)	38.91 (28.91-50.14)	93.60 (93.60-93.60)	79.70 (79.70-79.70)	53.72 (53.72-53.72)	23.01 (22.20-23.86)	24.54 (23.83-25.20)	58.92 (55.70-61.70)
Cape Verde	9.33 (7.95-11.02)	14.75 (10.34-20.77)	10.78 (8.72-13.57)	50.99 (37.26-64.13)	62.80 (49.92-74.51)	76.33 (65.31-84.80)	59.79 (59.79-59.79)	51.98 (51.98-51.98)	36.91 (36.91-36.91)	44.29 (42.06-46.50)	47.90 (46.07-49.58)	77.78 (75.62-79.86)
Chad	15.90 (11.02-21.70)	21.24 (15.12-28.94)	22.33 (12.08-37.22)	12.00 (7.42-18.66)	17.91 (12.89-24.68)	22.83 (14.63-32.51)	108.89 (108.89-108.89)	108.13 (108.13-108.13)	71.84 (71.84-71.84)	10.73 (9.70-11.87)	13.80 (13.11-14.53)	38.19 (34.91-41.32)
Cote d'Ivoire	29.96 (22.87-38.37)	33.51 (24.63-43.62)	25.64 (14.31-42.19)	17.72 (11.75-24.89)	23.28 (16.76-30.70)	31.82 (22.63-41.55)	79.98 (79.98-79.98)	70.10 (70.10-70.10)	69.32 (69.32-69.32)	21.56 (20.60-22.51)	26.62 (25.76-27.48)	59.74 (57.36-62.07)
The Gambia	19.59 (12.37-30.58)	19.29 (14.67-24.01)	17.75 (12.62-25.89)	23.51 (15.10-33.79)	24.60 (15.29-34.44)	26.89 (19.00-35.91)	81.27 (81.27-81.27)	65.64 (65.64-65.64)	60.03 (60.03-60.03)	28.77 (27.11-30.23)	31.72 (30.31-33.04)	64.75 (62.21-67.36)
Ghana	24.02 (16.98-33.14)	20.96 (15.76-26.82)	19.94 (11.37-32.77)	23.98 (17.63-31.37)	30.49 (25.16-36.21)	36.39 (32.18-40.84)	58.47 (58.47-58.47)	44.38 (44.38-44.38)	36.84 (36.84-36.84)	23.72 (22.92-24.50)	28.76 (28.12-29.35)	66.65 (64.77-68.15)
Guinea	18.30 (13.51-23.97)	20.05 (15.40-25.55)	18.02 (10.74-28.14)	16.45 (10.24-24.68)	18.86 (13.75-25.01)	24.71 (16.90-33.34)	92.86 (92.86-92.86)	84.68 (84.68-84.68)	69.26 (69.26-69.26)	16.05 (14.77-17.37)	20.51 (19.73-21.29)	50.19 (47.14-52.99)
Guinea-Bissau	23.16 (9.61-52.27)	24.42 (9.49-57.79)	24.06 (9.22-63.47)	17.99 (10.72-27.08)	21.69 (13.12-32.03)	26.96 (17.65-38.12)	72.09 (72.09-72.09)	67.31 (67.31-67.31)	47.61 (47.61-47.61)	21.16 (19.41-22.86)	23.41 (21.76-25.06)	53.63 (50.41-56.95)
Liberia	23.07 (16.63-30.25)	16.54 (12.44-21.15)	15.75 (10.36-23.22)	25.91 (16.36-36.65)	25.29 (17.36-35.15)	39.16 (29.37-49.61)	85.91 (85.91-85.91)	73.42 (73.42-73.42)	56.16 (56.16-56.16)	22.21 (20.62-23.85)	23.22 (22.00-24.43)	58.63 (54.60-62.46)
Mali	19.42 (14.92-24.63)	17.81 (13.52-22.69)	14.85 (9.46-23.07)	16.74 (10.88-24.26)	18.79 (13.75-24.52)	27.21 (19.04-36.42)	99.28 (99.28-99.28)	94.62 (94.62-94.62)	86.86 (86.86-86.86)	15.80 (14.78-16.93)	21.20 (20.36-22.01)	49.73 (46.82-52.82)

Appendix Table 1b. Unscaled values for each SDG health-related indicator by country for 1990, 2000, and 2015												
	Indicator 3.6.1: Age-standardised death rate due to road injuries (per 100,000 population)			Indicator 3.7.1: Proportion of women of reproductive age (15 to 49 years) who have their need for family planning satisfied with modern methods			Indicator 3.7.2: Birth rates for women aged 10 to 14 years and women aged 15 to 19 years (number of live births per 1,000 women)			Indicator 3.8.1: Coverage of 7 UHC tracer interventions for prevention and treatment services		
**Location**	**1990**	**2000**	**2015**	**1990**	**2000**	**2015**	**1990**	**2000**	**2015**	**1990**	**2000**	**2015**
Mauritania	20.21 (15.25-27.08)	15.56 (10.97-20.67)	13.03 (7.35-21.79)	13.44 (8.20-20.52)	13.73 (9.50-19.28)	15.82 (9.34-23.94)	56.56 (56.56-56.56)	51.00 (51.00-51.00)	41.46 (41.46-41.46)	17.06 (15.57-18.63)	22.11 (21.03-23.32)	44.48 (40.99-47.69)
Niger	17.42 (12.39-23.89)	14.83 (10.24-20.06)	14.60 (8.15-23.79)	13.54 (9.26-19.10)	22.47 (16.46-29.59)	34.82 (25.54-45.56)	110.59 (110.59-110.59)	109.18 (109.18-109.18)	102.60 (102.60-102.60)	10.66 (10.02-11.30)	14.92 (14.19-15.65)	48.63 (46.15-51.43)
Nigeria	21.72 (13.57-35.05)	15.67 (11.90-20.01)	8.72 (6.39-13.16)	17.40 (12.25-23.22)	26.79 (19.29-35.72)	38.09 (29.20-47.81)	76.22 (76.22-76.22)	68.34 (68.34-68.34)	58.47 (58.47-58.47)	15.34 (14.45-16.27)	17.00 (16.16-17.89)	43.93 (41.51-46.23)
Sao Tome and Principe	18.12 (14.53-22.44)	19.01 (15.07-23.24)	17.07 (9.53-27.89)	22.83 (14.84-32.73)	29.00 (18.79-41.15)	39.71 (27.72-52.68)	64.38 (64.38-64.38)	56.23 (56.23-56.23)	45.54 (45.54-45.54)	28.81 (27.46-30.14)	43.31 (40.60-45.94)	75.81 (70.27-80.71)
Senegal	18.19 (13.09-25.11)	18.03 (12.83-24.68)	17.53 (9.15-31.07)	16.62 (11.87-22.31)	20.87 (15.18-27.39)	37.85 (30.21-45.86)	70.29 (70.29-70.29)	56.81 (56.81-56.81)	43.17 (43.17-43.17)	20.86 (19.95-21.71)	27.25 (26.33-28.12)	67.54 (65.47-69.53)
Sierra Leone	21.48 (13.38-31.83)	22.89 (16.46-31.26)	22.41 (13.62-34.29)	18.86 (12.16-28.58)	23.16 (14.82-33.36)	40.21 (30.31-49.57)	88.31 (88.31-88.31)	83.12 (83.12-83.12)	60.40 (60.40-60.40)	20.91 (19.41-22.45)	23.15 (21.84-24.46)	63.59 (61.29-65.84)
Togo	21.18 (16.35-25.92)	23.34 (17.33-30.28)	22.79 (13.30-36.30)	15.37 (9.72-23.60)	19.09 (13.51-26.27)	35.56 (27.15-45.68)	62.55 (62.55-62.55)	51.98 (51.98-51.98)	49.66 (49.66-49.66)	23.37 (22.23-24.53)	26.86 (25.86-27.81)	64.87 (62.60-67.25)
Burundi	54.45 (31.19-79.65)	44.75 (32.79-59.29)	31.39 (18.12-51.07)	25.13 (16.40-34.89)	29.36 (20.16-39.77)	35.05 (25.24-46.80)	35.89 (35.89-35.89)	31.68 (31.68-31.68)	23.85 (23.85-23.85)	22.69 (20.95-24.37)	26.08 (24.49-27.61)	65.02 (61.62-68.23)
Comoros	31.06 (17.68-50.22)	23.79 (17.55-33.69)	19.68 (11.81-33.49)	27.87 (19.73-37.78)	29.86 (21.18-40.11)	31.15 (22.23-40.64)	59.51 (59.51-59.51)	50.11 (50.11-50.11)	38.02 (38.02-38.02)	25.79 (24.36-27.29)	30.84 (29.66-32.02)	60.04 (56.24-64.24)
Djibouti	26.24 (16.04-40.83)	26.72 (13.41-51.71)	24.85 (12.44-46.61)	21.54 (13.92-30.75)	25.74 (17.14-36.43)	33.26 (23.59-44.47)	27.90 (27.90-27.90)	19.61 (19.61-19.61)	12.42 (12.42-12.42)	28.55 (25.62-30.91)	32.53 (30.34-34.72)	61.50 (56.56-65.70)

Appendix Table 1b. Unscaled values for each SDG health-related indicator by country for 1990, 2000, and 2015

Location	Indicator 3.6.1: Age-standardised death rate due to road injuries (per 100,000 population)			Indicator 3.7.1: Proportion of women of reproductive age (15 to 49 years) who have their need for family planning satisfied with modern methods			Indicator 3.7.2: Birth rates for women aged 10 to 14 years and women aged 15 to 19 years (number of live births per 1,000 women)			Indicator 3.8.1: Coverage of 7 UHC tracer interventions for prevention and treatment services		
	**1990**	**2000**	**2015**	**1990**	**2000**	**2015**	**1990**	**2000**	**2015**	**1990**	**2000**	**2015**
Eritrea	27.60 (20.25-35.45)	23.16 (13.10-37.57)	25.90 (12.49-45.68)	14.95 (10.03-21.65)	17.81 (12.66-24.47)	22.56 (14.94-32.62)	56.53 (56.53-56.53)	49.83 (49.83-49.83)	32.55 (32.55-32.55)	17.15 (15.56-18.78)	26.29 (25.25-27.32)	48.80 (42.65-54.77)
Ethiopia	40.25 (30.23-49.90)	28.29 (22.97-34.02)	20.83 (12.98-33.37)	21.37 (13.66-30.25)	27.88 (20.38-36.49)	57.35 (49.02-64.85)	65.85 (65.85-65.85)	61.72 (61.72-61.72)	34.35 (34.35-34.35)	13.53 (11.80-15.24)	16.37 (15.51-17.21)	46.62 (42.50-50.62)
Kenya	19.82 (16.02-26.43)	19.27 (15.93-23.74)	18.00 (14.92-21.93)	40.98 (32.29-50.20)	48.96 (41.14-55.93)	73.55 (69.05-77.81)	68.89 (68.89-68.89)	57.89 (57.89-57.89)	51.99 (51.99-51.99)	29.14 (28.27-29.95)	32.22 (31.58-32.89)	70.20 (66.14-73.65)
Madagascar	22.92 (18.43-27.65)	19.54 (13.06-25.42)	17.98 (10.07-28.99)	15.26 (10.55-21.35)	26.61 (20.20-34.80)	42.58 (31.68-54.61)	77.82 (77.82-77.82)	78.07 (78.07-78.07)	60.06 (60.06-60.06)	21.02 (20.17-21.90)	24.21 (23.44-24.99)	42.77 (39.85-46.04)
Malawi	27.42 (20.76-36.26)	27.22 (18.57-38.50)	20.12 (12.36-31.88)	22.54 (15.49-30.34)	39.49 (30.67-48.66)	62.18 (51.70-71.83)	85.02 (85.02-85.02)	83.92 (83.92-83.92)	70.18 (70.18-70.18)	28.72 (27.67-29.66)	33.21 (32.42-34.05)	71.80 (68.83-74.43)
Mozambique	26.73 (21.19-34.01)	29.52 (22.64-37.08)	28.12 (14.78-45.89)	23.59 (16.07-32.60)	32.86 (24.21-41.93)	42.81 (32.11-54.17)	98.11 (98.11-98.11)	92.04 (92.04-92.04)	70.33 (70.33-70.33)	19.63 (18.11-21.07)	26.32 (25.54-27.07)	59.72 (57.09-62.18)
Rwanda	48.20 (35.75-64.92)	52.52 (37.35-69.73)	32.44 (18.66-57.67)	19.35 (13.39-26.35)	18.78 (13.77-25.23)	56.89 (46.95-66.58)	40.38 (40.38-40.38)	32.47 (32.47-32.47)	18.79 (18.79-18.79)	22.38 (21.24-23.47)	24.71 (23.76-25.75)	74.82 (71.48-77.64)
Somalia	29.97 (10.27-63.07)	28.95 (9.45-62.03)	28.10 (9.88-62.97)	17.65 (11.14-26.10)	19.93 (13.01-28.39)	22.57 (14.74-32.12)	51.00 (51.00-51.00)	67.94 (67.94-67.94)	57.67 (57.67-57.67)	10.03 (8.20-11.93)	10.78 (9.39-12.25)	23.25 (19.79-27.34)
South Sudan	29.28 (11.56-64.52)	25.90 (9.85-59.46)	27.07 (10.52-69.21)	21.06 (13.80-29.88)	24.70 (16.14-34.66)	30.29 (21.30-41.34)	72.58 (72.58-72.58)	66.04 (66.04-66.04)	37.24 (37.24-37.24)	18.65 (15.83-21.31)	18.81 (16.70-20.76)	32.39 (28.39-36.42)
Tanzania	24.11 (19.64-28.87)	20.03 (14.97-25.57)	19.52 (11.82-32.76)	25.68 (18.60-33.80)	38.02 (29.97-45.94)	52.06 (41.11-63.14)	75.34 (75.34-75.34)	70.15 (70.15-70.15)	63.36 (63.36-63.36)	24.59 (23.68-25.39)	27.81 (27.01-28.58)	58.40 (55.19-61.44)

Appendix Table 1b. Unscaled values for each SDG health-related indicator by country for 1990, 2000, and 2015

Location	Indicator 3.6.1: Age-standardised death rate due to road injuries (per 100,000 population)			Indicator 3.7.1: Proportion of women of reproductive age (15 to 49 years) who have their need for family planning satisfied with modern methods			Indicator 3.7.2: Birth rates for women aged 10 to 14 years and women aged 15 to 19 years (number of live births per 1,000 women)			Indicator 3.8.1: Coverage of 7 UHC tracer interventions for prevention and treatment services		
	**1990**	**2000**	**2015**	**1990**	**2000**	**2015**	**1990**	**2000**	**2015**	**1990**	**2000**	**2015**
Uganda	28.14 (18.47-38.22)	37.79 (26.30-50.65)	33.75 (18.11-56.03)	22.82 (15.95-30.82)	31.18 (25.44-37.57)	45.67 (40.44-51.26)	98.35 (98.35-98.35)	94.11 (94.11-94.11)	62.89 (62.89-62.89)	22.42 (21.41-23.47)	26.59 (25.97-27.22)	68.31 (65.91-70.56)
Zambia	27.73 (19.61-40.36)	34.53 (26.62-43.74)	35.12 (20.99-51.47)	25.14 (18.11-33.85)	37.15 (29.24-46.27)	62.47 (52.79-71.81)	86.07 (86.07-86.07)	74.53 (74.53-74.53)	51.41 (51.41-51.41)	27.75 (26.86-28.66)	31.68 (30.89-32.45)	74.51 (72.47-76.47)
Angola	43.64 (15.58-88.23)	35.35 (13.28-76.99)	34.09 (14.44-92.89)	16.29 (9.56-24.64)	19.14 (12.16-28.83)	23.72 (15.19-33.99)	115.59 (115.59-115.59)	107.43 (107.43-107.43)	84.63 (84.63-84.63)	19.51 (17.97-21.31)	24.78 (23.50-26.04)	60.25 (56.95-63.21)
Central African Republic	49.29 (33.13-67.31)	50.56 (22.73-86.96)	49.94 (21.14-95.70)	12.52 (8.54-17.22)	13.85 (8.82-20.04)	17.02 (10.63-25.39)	77.20 (77.20-77.20)	65.84 (65.84-65.84)	48.30 (48.30-48.30)	17.46 (16.67-18.23)	19.71 (18.77-20.60)	47.73 (44.39-51.40)
Congo	38.02 (26.73-51.10)	33.46 (24.22-44.40)	24.89 (15.10-40.44)	19.51 (11.96-28.72)	22.83 (15.61-31.60)	39.53 (29.85-49.29)	67.71 (67.71-67.71)	68.92 (68.92-68.92)	62.47 (62.47-62.47)	27.49 (25.97-28.84)	30.38 (29.12-31.63)	66.25 (62.15-69.66)
Democratic Republic of the Congo	22.14 (14.22-32.61)	21.00 (15.10-29.90)	20.33 (12.47-32.01)	16.48 (10.08-24.91)	17.82 (11.74-25.82)	21.23 (15.91-27.68)	72.66 (72.66-72.66)	70.42 (70.42-70.42)	64.99 (64.99-64.99)	19.20 (17.38-20.96)	22.18 (20.82-23.63)	57.36 (54.87-59.79)
Equatorial Guinea	46.75 (15.83-95.02)	38.94 (14.02-88.12)	30.43 (13.14-81.15)	16.69 (10.64-25.10)	20.41 (12.88-29.23)	27.54 (18.12-39.83)	71.89 (71.89-71.89)	69.54 (69.54-69.54)	58.08 (58.08-58.08)	24.43 (22.70-26.15)	27.51 (25.78-29.11)	59.33 (53.59-64.43)
Gabon	41.20 (28.70-54.02)	32.34 (22.73-44.02)	26.03 (14.99-43.74)	23.18 (14.62-32.99)	28.37 (22.00-35.07)	45.96 (35.53-55.30)	93.18 (93.18-93.18)	72.83 (72.83-72.83)	49.21 (49.21-49.21)	28.59 (27.30-29.81)	27.48 (26.24-28.65)	61.78 (56.35-67.00)

Appendix Table 1b. Unscaled values for each SDG health-related indicator by country for 1990, 2000, and 2015

Location	Indicator 3.9.1: Age-standardised death rate attributable to household air pollution and ambient air pollution (per 100,000 population)			Indicator 3.9.2: Age-standardised death rate attributable to unsafe water, sanitation, and hygiene (WaSH) (per 100,000 population)			Indicator 3.9.3: Age-standardised death rate due to unintentional poisonings (per 100,000 population)			Indicator 3.a.1: Age-standardised prevalence of daily smoking among populations aged 10 and older		
	1990	2000	2015	1990	2000	2015	1990	2000	2015	1990	2000	2015
Canada	25.21 (19.45-31.51)	19.65 (14.94-25.02)	12.91 (9.58-16.82)	1.93 (1.28-2.67)	1.53 (1.04-2.14)	1.28 (0.87-1.74)	0.82 (0.76-0.89)	0.56 (0.53-0.60)	0.47 (0.43-0.51)	21.16 (20.40-21.93)	16.09 (15.49-16.65)	11.06 (10.05-12.28)
United States	38.36 (30.98-46.17)	32.62 (26.31-39.49)	20.68 (16.13-25.84)	2.19 (1.47-2.98)	2.07 (1.42-2.81)	1.66 (1.16-2.24)	0.86 (0.83-0.89)	0.80 (0.76-0.82)	1.05 (0.99-1.11)	18.07 (17.88-18.25)	14.93 (14.81-15.04)	10.73 (10.56-10.89)
Australia	22.80 (16.55-29.81)	15.89 (11.21-21.26)	8.99 (5.60-12.99)	1.24 (0.84-1.69)	1.23 (0.85-1.66)	0.86 (0.58-1.23)	0.46 (0.42-0.49)	0.45 (0.42-0.47)	0.38 (0.35-0.42)	19.60 (19.10-20.11)	16.27 (15.84-16.70)	11.88 (11.29-12.47)
New Zealand	22.80 (16.39-29.93)	16.29 (11.17-22.28)	8.99 (5.46-13.51)	3.00 (2.01-4.06)	1.17 (0.78-1.59)	0.86 (0.59-1.19)	0.34 (0.31-0.37)	0.25 (0.23-0.27)	0.26 (0.23-0.28)	19.38 (18.94-19.79)	18.02 (17.59-18.46)	12.82 (12.31-13.38)
Brunei	20.54 (12.99-29.39)	15.90 (8.89-23.58)	11.32 (5.41-18.28)	3.94 (2.49-6.70)	3.03 (1.87-5.01)	2.68 (1.68-4.07)	0.48 (0.39-0.63)	0.35 (0.30-0.45)	0.30 (0.24-0.36)	12.09 (11.16-13.13)	10.67 (9.91-11.42)	9.91 (9.08-10.76)
Japan	27.39 (21.40-34.88)	20.67 (15.99-26.63)	17.34 (13.33-22.41)	4.84 (3.27-6.47)	3.78 (2.56-5.10)	2.81 (1.88-3.93)	0.43 (0.41-0.45)	0.35 (0.34-0.37)	0.33 (0.31-0.35)	24.14 (23.86-24.40)	22.73 (22.41-23.03)	14.81 (14.54-15.05)
Singapore	65.34 (47.99-83.10)	48.90 (35.80-62.41)	33.00 (22.45-44.26)	11.50 (7.92-15.29)	8.72 (5.89-11.75)	6.41 (4.18-8.94)	0.28 (0.26-0.30)	0.13 (0.12-0.14)	0.09 (0.08-0.10)	11.56 (10.59-12.49)	9.78 (9.27-10.26)	9.84 (9.07-10.59)
South Korea	69.30 (58.93-80.14)	42.04 (35.07-49.48)	28.32 (23.48-33.20)	3.72 (2.74-4.70)	2.07 (1.42-2.76)	1.80 (1.22-2.45)	5.30 (4.99-5.59)	1.14 (1.06-1.23)	0.55 (0.50-0.62)	26.22 (25.29-27.14)	22.15 (21.15-23.05)	17.52 (16.60-18.48)
Andorra	20.09 (14.57-25.78)	16.22 (11.52-21.04)	13.42 (9.43-18.06)	2.15 (1.25-3.47)	1.73 (1.00-2.65)	1.27 (0.75-1.93)	0.40 (0.23-0.55)	0.27 (0.17-0.38)	0.22 (0.15-0.31)	21.60 (19.91-23.38)	20.77 (19.31-22.43)	17.81 (16.31-19.28)
Austria	44.86 (37.43-52.41)	35.64 (28.82-42.89)	23.83 (18.94-28.91)	1.52 (1.03-2.05)	1.17 (0.78-1.57)	0.71 (0.49-0.96)	0.47 (0.44-0.51)	0.25 (0.23-0.27)	0.27 (0.24-0.29)	21.62 (20.27-22.88)	22.70 (21.46-23.96)	21.65 (20.16-23.09)

Appendix Table 1b. Unscaled values for each SDG health-related indicator by country for 1990, 2000, and 2015

Location	Indicator 3.9.1: Age-standardised death rate attributable to household air pollution and ambient air pollution (per 100,000 population)			Indicator 3.9.2: Age-standardised death rate attributable to unsafe water, sanitation, and hygiene (WaSH) (per 100,000 population)			Indicator 3.9.3: Age-standardised death rate due to unintentional poisonings (per 100,000 population)			Indicator 3.a.1: Age-standardised prevalence of daily smoking among populations aged 10 and older		
	1990	2000	2015	1990	2000	2015	1990	2000	2015	1990	2000	2015
Belgium	48.15 (40.58-56.38)	38.50 (31.51-46.21)	25.85 (20.83-31.41)	2.62 (1.81-3.47)	3.33 (2.27-4.38)	2.35 (1.60-3.20)	1.02 (0.95-1.09)	0.52 (0.48-0.57)	0.35 (0.32-0.39)	21.99 (20.92-23.05)	20.59 (19.47-21.73)	15.55 (14.49-16.56)
Cyprus	49.66 (40.29-59.28)	38.38 (31.89-45.15)	22.48 (18.90-26.51)	3.12 (2.17-4.29)	1.93 (1.32-2.62)	1.03 (0.70-1.43)	0.47 (0.38-0.57)	0.37 (0.31-0.44)	0.22 (0.18-0.26)	18.79 (17.13-20.42)	21.43 (19.93-23.03)	20.99 (19.44-22.54)
Denmark	45.17 (36.80-54.33)	32.56 (25.85-40.57)	22.59 (17.39-28.68)	2.41 (1.66-3.17)	2.18 (1.53-2.92)	2.17 (1.50-2.90)	2.60 (1.71-2.84)	0.58 (0.53-0.62)	0.41 (0.37-0.45)	29.22 (28.30-30.15)	23.91 (23.03-24.77)	13.87 (13.02-14.75)
Finland	33.97 (26.15-42.22)	21.98 (15.98-28.55)	14.25 (10.29-18.67)	3.93 (2.72-5.21)	2.62 (1.78-3.52)	0.66 (0.45-0.93)	4.99 (2.22-5.47)	0.59 (0.54-0.66)	0.42 (0.38-0.48)	18.33 (17.67-19.05)	17.24 (16.81-17.66)	14.30 (13.30-15.38)
France	26.96 (22.14-32.06)	22.11 (17.67-27.18)	15.40 (12.23-18.99)	2.59 (1.84-3.35)	2.76 (1.99-3.58)	1.74 (1.23-2.28)	0.82 (0.76-0.87)	0.66 (0.61-0.72)	0.45 (0.41-0.51)	25.38 (24.27-26.47)	23.81 (22.62-25.00)	19.22 (17.93-20.59)
Germany	49.67 (41.04-58.53)	34.72 (28.05-42.08)	23.77 (19.06-28.92)	1.89 (1.30-2.52)	1.76 (1.20-2.39)	1.65 (1.14-2.20)	0.71 (0.66-0.76)	0.16 (0.15-0.20)	0.12 (0.11-0.14)	21.57 (20.57-22.58)	20.71 (19.90-21.56)	18.37 (17.15-19.69)
Greece	40.32 (33.07-48.57)	34.73 (27.80-42.28)	29.07 (23.90-35.19)	1.60 (1.07-2.14)	1.67 (1.10-2.26)	0.96 (0.63-1.36)	0.42 (0.39-0.46)	0.35 (0.33-0.38)	0.23 (0.20-0.26)	27.92 (26.76-29.02)	27.54 (26.26-28.71)	26.18 (24.83-27.62)
Iceland	27.45 (20.35-35.39)	19.65 (14.61-25.62)	12.72 (9.04-16.84)	4.62 (3.11-6.28)	2.62 (1.76-3.59)	1.46 (0.96-2.02)	1.53 (1.40-1.65)	0.73 (0.67-0.79)	0.45 (0.40-0.51)	23.91 (22.71-25.06)	18.28 (17.53-19.15)	11.88 (10.90-12.90)
Ireland	40.39 (32.14-49.73)	31.15 (24.40-38.97)	19.99 (15.13-25.72)	4.56 (3.04-6.25)	3.95 (2.61-5.31)	2.13 (1.38-2.97)	0.50 (0.45-0.56)	0.48 (0.44-0.52)	0.33 (0.30-0.37)	22.27 (21.17-23.38)	19.89 (18.80-20.97)	17.52 (16.14-18.97)
Israel	45.75 (38.15-53.29)	31.14 (25.82-37.06)	23.37 (19.32-27.89)	1.98 (1.37-2.68)	1.84 (1.28-2.44)	1.88 (1.28-2.55)	0.31 (0.28-0.34)	0.31 (0.28-0.33)	0.22 (0.20-0.24)	20.93 (19.43-22.59)	16.37 (15.36-17.50)	14.97 (13.76-16.22)

Appendix Table 1b. Unscaled values for each SDG health-related indicator by country for 1990, 2000, and 2015												
	**Indicator 3.9.1: Age-standardised death rate attributable to household air pollution and ambient air pollution (per 100,000 population)**			**Indicator 3.9.2: Age-standardised death rate attributable to unsafe water, sanitation, and hygiene (WaSH) (per 100,000 population)**			**Indicator 3.9.3: Age-standardised death rate due to unintentional poisonings (per 100,000 population)**			**Indicator 3.a.1: Age-standardised prevalence of daily smoking among populations aged 10 and older**		
**Location**	**1990**	**2000**	**2015**	**1990**	**2000**	**2015**	**1990**	**2000**	**2015**	**1990**	**2000**	**2015**
Italy	38.13 (32.06-44.61)	28.26 (23.22-33.53)	23.61 (19.33-28.18)	1.30 (0.88-1.74)	1.03 (0.67-1.40)	0.82 (0.55-1.11)	0.77 (0.72-0.82)	0.51 (0.47-0.54)	0.26 (0.23-0.29)	21.16 (20.22-22.19)	18.78 (17.95-19.63)	16.57 (15.47-17.70)
Luxembourg	50.59 (42.50-59.23)	34.92 (28.80-41.60)	20.89 (16.84-25.10)	2.06 (1.45-2.71)	2.03 (1.41-2.71)	1.41 (0.99-1.91)	0.76 (0.71-0.82)	0.35 (0.32-0.38)	0.21 (0.19-0.23)	21.45 (19.98-22.93)	20.83 (19.60-21.92)	17.40 (15.90-19.02)
Malta	42.16 (33.67-51.06)	32.41 (25.46-40.20)	24.55 (19.00-30.38)	2.58 (1.75-3.44)	3.17 (2.14-4.28)	1.98 (1.34-2.61)	0.51 (0.46-0.55)	0.26 (0.24-0.30)	0.17 (0.15-0.18)	24.05 (22.34-25.81)	18.12 (16.80-19.41)	15.93 (14.63-17.33)
Netherlands	44.78 (37.48-52.37)	37.16 (30.54-44.42)	23.95 (19.03-29.35)	2.40 (1.62-3.23)	3.20 (2.19-4.32)	2.15 (1.48-2.97)	0.31 (0.28-0.33)	0.22 (0.20-0.24)	0.16 (0.15-0.18)	22.75 (21.89-23.62)	20.88 (20.29-21.51)	14.64 (13.63-15.69)
Norway	35.60 (28.03-43.56)	25.83 (20.13-32.41)	16.53 (12.39-21.64)	4.19 (2.83-5.71)	2.79 (1.93-3.73)	2.05 (1.41-2.77)	0.94 (0.87-1.00)	0.42 (0.38-0.45)	0.72 (0.64-0.79)	23.86 (22.46-25.41)	19.49 (18.13-20.88)	12.27 (11.26-13.35)
Portugal	36.17 (27.94-45.08)	29.28 (22.43-37.42)	17.78 (13.45-23.18)	4.33 (3.00-5.71)	4.30 (2.94-5.74)	3.18 (2.14-4.27)	0.80 (0.73-0.86)	0.45 (0.42-0.49)	0.24 (0.22-0.27)	16.65 (15.83-17.49)	15.77 (14.93-16.66)	15.43 (14.22-16.65)
Spain	29.72 (23.73-36.39)	24.83 (19.53-30.85)	15.39 (11.91-19.36)	2.45 (1.67-3.20)	2.05 (1.43-2.69)	1.37 (0.94-1.83)	0.78 (0.72-0.84)	0.36 (0.33-0.38)	0.23 (0.21-0.26)	25.87 (24.82-26.98)	23.31 (22.40-24.16)	18.15 (16.91-19.44)
Sweden	21.41 (14.92-28.24)	14.51 (9.60-20.03)	11.43 (7.47-16.07)	2.69 (1.81-3.58)	1.88 (1.29-2.56)	1.39 (0.96-1.90)	0.90 (0.83-0.97)	0.55 (0.52-0.58)	0.46 (0.42-0.50)	18.51 (17.48-19.67)	13.60 (12.92-14.29)	8.91 (8.51-9.33)
Switzerland	31.20 (25.38-37.51)	23.41 (18.38-29.28)	16.36 (12.78-20.62)	2.31 (1.61-3.07)	1.74 (1.17-2.36)	1.07 (0.75-1.44)	1.92 (0.95-2.16)	0.21 (0.19-0.24)	0.16 (0.15-0.18)	22.02 (21.21-22.76)	22.83 (22.01-23.65)	15.81 (14.50-17.05)
United Kingdom	50.63 (42.04-59.97)	37.46 (29.85-46.15)	23.75 (18.54-29.81)	4.50 (3.01-6.08)	4.45 (3.02-5.98)	2.48 (1.70-3.42)	0.85 (0.82-0.89)	0.58 (0.55-0.61)	0.35 (0.34-0.37)	22.22 (20.70-23.65)	19.80 (18.88-20.72)	15.64 (14.57-16.66)

Appendix Table 1b. Unscaled values for each SDG health-related indicator by country for 1990, 2000, and 2015

Location	Indicator 3.9.1: Age-standardised death rate attributable to household air pollution and ambient air pollution (per 100,000 population)			Indicator 3.9.2: Age-standardised death rate attributable to unsafe water, sanitation, and hygiene (WaSH) (per 100,000 population)			Indicator 3.9.3: Age-standardised death rate due to unintentional poisonings (per 100,000 population)			Indicator 3.a.1: Age-standardised prevalence of daily smoking among populations aged 10 and older		
	1990	2000	2015	1990	2000	2015	1990	2000	2015	1990	2000	2015
Argentina	56.25 (45.65-68.85)	47.90 (38.76-58.79)	36.04 (28.64-45.92)	7.11 (5.62-8.98)	7.16 (5.31-9.04)	6.44 (4.54-8.84)	1.38 (1.29-1.49)	0.99 (0.92-1.06)	0.77 (0.70-0.84)	19.05 (17.35-20.92)	17.71 (16.21-19.45)	14.64 (13.38-15.88)
Chile	75.10 (57.73-95.00)	44.64 (35.47-56.01)	30.14 (24.55-37.64)	12.61 (9.52-15.85)	6.17 (4.57-7.88)	3.47 (2.48-4.52)	1.61 (1.43-1.73)	0.48 (0.45-0.52)	0.28 (0.25-0.32)	17.82 (16.50-19.18)	22.45 (21.19-23.70)	20.73 (19.12-22.34)
Uruguay	48.39 (38.10-65.39)	38.44 (30.80-46.87)	27.45 (21.59-34.75)	6.53 (5.08-8.10)	5.59 (4.19-7.09)	4.59 (3.38-5.97)	1.72 (1.59-1.83)	0.82 (0.76-0.88)	0.58 (0.52-0.64)	23.19 (21.62-24.85)	20.86 (19.44-22.23)	15.67 (14.32-17.05)
Belarus	141.35 (110.08-181.16)	122.69 (100.77-151.57)	84.06 (69.33-100.28)	2.59 (1.95-3.27)	2.27 (1.65-2.89)	1.00 (0.66-1.40)	7.46 (4.48-8.47)	9.18 (5.49-10.47)	3.06 (2.37-5.82)	25.06 (23.65-26.62)	22.81 (21.28-24.24)	22.23 (20.79-23.67)
Estonia	113.48 (80.29-153.21)	81.44 (58.94-108.41)	33.02 (23.56-44.75)	2.13 (1.54-2.73)	2.41 (1.65-3.19)	0.88 (0.57-1.24)	5.64 (4.98-6.17)	2.19 (2.00-2.41)	0.62 (0.53-0.76)	22.47 (21.28-23.60)	21.69 (20.93-22.45)	18.17 (16.94-19.32)
Latvia	137.19 (108.32-181.32)	97.11 (77.72-122.34)	59.33 (47.61-73.01)	2.06 (1.50-2.63)	2.18 (1.52-2.88)	1.18 (0.80-1.63)	4.50 (4.10-4.86)	3.23 (2.96-3.55)	1.13 (0.98-1.39)	20.95 (19.27-22.62)	23.20 (22.43-23.96)	21.96 (20.76-23.24)
Lithuania	121.92 (94.20-155.20)	85.64 (65.08-111.88)	55.59 (44.45-69.05)	1.56 (1.19-1.95)	1.72 (1.21-2.26)	1.33 (0.90-1.82)	5.64 (5.09-6.13)	2.41 (2.21-2.62)	0.94 (0.83-1.27)	19.93 (18.54-21.42)	19.26 (18.53-19.98)	18.86 (17.58-20.28)
Moldova	202.67 (156.02-254.05)	168.84 (135.85-205.48)	75.58 (61.11-92.22)	5.49 (4.17-6.96)	6.03 (4.45-7.74)	2.24 (1.53-3.01)	6.55 (6.02-7.14)	6.00 (5.47-6.56)	1.35 (1.18-1.83)	17.72 (16.39-18.97)	15.90 (14.76-17.08)	15.08 (14.20-16.09)
Russia	115.04 (88.89-151.39)	119.56 (95.78-148.60)	68.08 (56.52-80.96)	3.29 (2.60-4.02)	4.24 (3.14-5.41)	2.29 (1.54-3.10)	3.64 (3.33-3.99)	3.19 (2.93-3.57)	1.83 (1.67-2.03)	20.06 (19.17-21.01)	21.80 (21.05-22.53)	20.04 (18.94-21.18)
Ukraine	144.34 (112.70-182.79)	128.10 (105.51-153.92)	82.86 (67.87-98.67)	2.59 (1.98-3.24)	2.89 (2.12-3.68)	1.28 (0.86-1.77)	8.14 (7.51-8.67)	10.93 (9.90-11.77)	2.39 (2.08-3.03)	23.95 (22.38-25.55)	22.89 (21.91-23.87)	20.66 (19.26-22.12)

Appendix Table 1b. Unscaled values for each SDG health-related indicator by country for 1990, 2000, and 2015

Location	Indicator 3.9.1: Age-standardised death rate attributable to household air pollution and ambient air pollution (per 100,000 population)			Indicator 3.9.2: Age-standardised death rate attributable to unsafe water, sanitation, and hygiene (WaSH) (per 100,000 population)			Indicator 3.9.3: Age-standardised death rate due to unintentional poisonings (per 100,000 population)			Indicator 3.a.1: Age-standardised prevalence of daily smoking among populations aged 10 and older		
	1990	2000	2015	1990	2000	2015	1990	2000	2015	1990	2000	2015
Albania	150.64 (123.86-179.66)	120.22 (97.45-144.76)	62.55 (49.64-78.29)	12.17 (7.52-16.37)	5.74 (3.75-7.65)	1.96 (1.30-2.75)	0.45 (0.36-0.86)	0.86 (0.56-0.98)	0.33 (0.28-0.47)	14.72 (13.50-16.01)	13.51 (12.62-14.35)	12.59 (11.47-13.66)
Bosnia and Herzegovina	167.88 (142.66-196.49)	125.86 (105.69-149.25)	82.31 (68.67-97.29)	3.63 (2.69-4.59)	2.00 (1.39-2.72)	1.02 (0.68-1.61)	18.14 (10.65-20.39)	9.14 (6.70-11.05)	1.59 (0.74-6.58)	21.45 (19.54-23.38)	23.25 (22.25-24.24)	23.49 (21.75-25.15)
Bulgaria	155.62 (130.98-185.31)	121.47 (101.68-145.40)	79.61 (66.53-94.00)	6.01 (4.22-7.82)	3.96 (2.80-5.14)	2.05 (1.40-2.73)	1.46 (1.35-1.57)	1.33 (1.23-1.42)	0.58 (0.52-0.65)	26.62 (24.50-28.87)	28.51 (26.74-30.45)	26.07 (24.16-27.92)
Croatia	112.64 (90.95-136.13)	84.18 (69.42-101.66)	52.23 (42.96-63.23)	3.16 (2.29-4.06)	3.27 (2.29-4.32)	1.33 (0.93-1.76)	1.03 (0.94-1.10)	0.35 (0.32-0.38)	0.22 (0.20-0.25)	26.26 (24.37-28.26)	21.58 (20.34-22.94)	23.11 (21.19-25.17)
Czech Republic	113.03 (96.56-131.51)	67.35 (56.18-79.68)	44.59 (36.22-53.16)	2.91 (1.97-3.85)	2.71 (1.82-3.63)	2.48 (1.72-3.30)	2.09 (1.92-2.24)	0.49 (0.45-0.53)	0.35 (0.32-0.39)	22.60 (20.91-24.37)	20.40 (19.29-21.47)	19.79 (18.37-21.28)
Hungary	133.81 (111.93-159.78)	95.75 (79.67-115.07)	57.42 (46.98-68.56)	1.77 (1.25-2.31)	1.35 (0.95-1.76)	1.18 (0.79-1.50)	1.28 (1.18-1.38)	0.36 (0.33-0.45)	0.24 (0.21-0.28)	24.49 (22.56-26.57)	23.98 (22.45-25.65)	20.61 (18.81-22.31)
Macedonia	161.60 (135.90-190.54)	143.13 (118.66-169.54)	89.30 (74.41-105.47)	10.13 (8.05-12.03)	3.06 (2.47-3.83)	1.22 (0.84-1.69)	0.50 (0.43-0.69)	0.49 (0.39-0.61)	0.41 (0.27-0.50)	20.85 (19.04-22.77)	21.82 (20.00-23.56)	24.38 (22.68-26.34)
Montenegro	126.49 (103.54-152.04)	118.33 (94.75-147.22)	86.92 (70.42-104.35)	2.01 (1.35-2.76)	2.09 (1.47-2.78)	0.96 (0.63-1.32)	1.30 (0.90-1.93)	1.25 (0.99-1.46)	0.70 (0.57-0.89)	17.97 (16.24-19.65)	21.89 (20.11-23.81)	24.40 (22.77-26.00)
Poland	137.55 (115.81-164.06)	86.52 (71.71-102.02)	53.11 (43.94-63.63)	3.34 (2.32-4.36)	2.88 (1.92-3.87)	2.23 (1.49-3.00)	3.05 (2.41-3.27)	0.60 (0.56-0.66)	0.27 (0.24-0.35)	26.41 (25.57-27.24)	21.73 (20.34-23.13)	18.84 (17.47-20.25)
Romania	159.61 (132.55-189.87)	127.86 (107.05-150.96)	74.34 (60.25-90.56)	7.88 (5.87-9.95)	6.30 (4.71-7.91)	2.66 (1.82-3.65)	3.66 (3.34-3.95)	1.54 (1.41-1.69)	0.61 (0.54-0.78)	18.57 (17.09-20.10)	20.73 (19.50-21.97)	18.47 (17.03-19.97)

Appendix Table 1b. Unscaled values for each SDG health-related indicator by country for 1990, 2000, and 2015												
	**Indicator 3.9.1: Age-standardised death rate attributable to household air pollution and ambient air pollution (per 100,000 population)**			**Indicator 3.9.2: Age-standardised death rate attributable to unsafe water, sanitation, and hygiene (WaSH) (per 100,000 population)**			**Indicator 3.9.3: Age-standardised death rate due to unintentional poisonings (per 100,000 population)**			**Indicator 3.a.1: Age-standardised prevalence of daily smoking among populations aged 10 and older**		
**Location**	**1990**	**2000**	**2015**	**1990**	**2000**	**2015**	**1990**	**2000**	**2015**	**1990**	**2000**	**2015**
Serbia	142.37 (118.32-168.71)	143.60 (117.26-173.82)	81.93 (67.85-98.14)	2.44 (1.66-3.32)	1.74 (1.22-2.43)	1.29 (0.90-1.69)	0.83 (0.56-0.99)	0.69 (0.51-0.78)	0.41 (0.33-0.50)	18.95 (17.11-20.92)	22.02 (20.46-23.48)	19.52 (17.80-21.40)
Slovakia	128.84 (109.72-152.06)	83.66 (69.29-98.36)	49.70 (40.26-59.26)	7.28 (4.41-10.02)	5.01 (3.31-6.74)	3.07 (2.05-4.22)	1.20 (0.80-1.38)	0.76 (0.60-0.93)	0.36 (0.31-0.56)	21.21 (19.26-23.28)	17.66 (16.15-19.27)	16.63 (15.20-18.21)
Slovenia	82.77 (67.99-100.43)	54.32 (44.66-65.09)	31.22 (25.42-38.24)	3.70 (2.52-4.91)	4.09 (2.72-5.55)	2.19 (1.42-3.05)	0.76 (0.71-0.81)	0.62 (0.57-0.66)	0.28 (0.24-0.33)	23.01 (20.97-25.19)	17.80 (16.24-19.38)	17.16 (15.73-18.70)
Armenia	166.29 (121.05-210.04)	123.82 (95.77-155.73)	67.37 (53.87-82.38)	14.12 (11.76-16.48)	7.14 (5.86-8.61)	2.13 (1.50-2.77)	3.26 (1.68-3.76)	1.83 (1.40-2.22)	0.83 (0.65-1.53)	17.31 (15.80-18.79)	17.26 (16.31-18.21)	16.96 (15.64-18.26)
Azerbaijan	246.96 (187.76-307.24)	180.97 (144.17-222.42)	96.16 (78.08-122.22)	26.36 (21.42-32.10)	18.97 (15.34-23.20)	4.61 (3.30-6.35)	2.51 (1.70-2.87)	1.53 (1.31-2.42)	0.79 (0.64-1.32)	13.12 (11.62-14.47)	13.42 (12.09-14.72)	16.71 (15.19-18.10)
Georgia	181.31 (132.81-233.78)	155.66 (118.71-197.77)	105.34 (78.63-138.98)	8.37 (6.67-10.29)	5.66 (4.38-7.16)	1.86 (1.37-2.41)	2.41 (1.41-2.78)	1.10 (0.90-2.00)	1.04 (0.85-1.66)	15.15 (13.65-16.67)	16.31 (15.04-17.57)	17.01 (15.61-18.42)
Kazakhstan	162.95 (119.57-215.54)	162.62 (127.85-205.14)	95.81 (76.80-121.16)	11.27 (9.42-13.19)	7.51 (6.00-9.12)	2.33 (1.71-3.06)	9.62 (8.90-10.44)	30.93 (19.85-33.13)	3.28 (2.85-4.09)	16.37 (14.85-17.86)	14.86 (13.67-15.99)	16.06 (15.02-17.16)
Kyrgyzstan	222.72 (159.06-283.19)	203.79 (150.21-264.24)	133.52 (105.35-163.73)	19.95 (16.74-23.31)	13.70 (11.46-16.18)	5.03 (3.94-6.29)	5.48 (2.52-6.45)	3.36 (2.60-4.12)	1.32 (1.01-2.81)	13.02 (11.79-14.30)	12.99 (12.00-13.89)	14.21 (13.02-15.46)
Mongolia	227.37 (182.90-274.34)	253.75 (210.09-303.64)	161.78 (125.12-200.69)	17.53 (12.44-22.69)	9.96 (6.98-13.08)	2.88 (1.86-4.18)	13.76 (5.09-18.70)	10.47 (5.15-13.27)	2.93 (2.26-5.39)	17.74 (16.19-19.35)	18.30 (17.35-19.29)	16.91 (15.39-18.44)
Tajikistan	292.49 (246.57-340.10)	292.65 (249.00-341.39)	153.94 (124.01-188.58)	53.25 (44.99-61.78)	41.06 (34.51-47.57)	13.69 (10.37-17.45)	3.33 (2.33-3.80)	3.08 (2.53-3.98)	1.85 (1.49-2.38)	16.49 (14.88-18.05)	10.20 (8.92-11.50)	8.23 (7.03-9.48)

Appendix Table 1b. Unscaled values for each SDG health-related indicator by country for 1990, 2000, and 2015

Location	Indicator 3.9.1: Age-standardised death rate attributable to household air pollution and ambient air pollution (per 100,000 population)			Indicator 3.9.2: Age-standardised death rate attributable to unsafe water, sanitation, and hygiene (WaSH) (per 100,000 population)			Indicator 3.9.3: Age-standardised death rate due to unintentional poisonings (per 100,000 population)			Indicator 3.a.1: Age-standardised prevalence of daily smoking among populations aged 10 and older		
	1990	2000	2015	1990	2000	2015	1990	2000	2015	1990	2000	2015
Turkmenistan	158.08 (136.29-181.75)	160.12 (137.03-183.44)	101.99 (85.96-118.07)	37.64 (30.73-45.71)	32.35 (24.75-41.19)	6.74 (4.81-9.29)	2.50 (1.88-3.00)	2.61 (1.90-3.12)	0.98 (0.77-1.86)	7.63 (6.53-8.80)	6.28 (5.34-7.33)	5.66 (4.87-6.54)
Uzbekistan	196.97 (153.94-247.52)	176.48 (143.85-217.11)	122.26 (96.51-154.51)	16.71 (13.71-20.08)	9.86 (7.36-12.57)	4.22 (2.90-5.80)	2.36 (1.77-2.84)	2.16 (1.83-2.92)	1.39 (1.12-2.00)	7.32 (6.24-8.41)	8.17 (7.25-9.20)	6.99 (6.19-7.83)
Colombia	82.54 (67.74-99.28)	66.30 (54.20-79.00)	43.71 (34.62-55.00)	17.44 (15.26-19.76)	9.90 (8.37-11.61)	4.82 (3.80-6.10)	1.39 (1.29-1.49)	0.42 (0.38-0.47)	0.23 (0.21-0.26)	13.33 (11.16-15.62)	11.87 (10.06-13.83)	8.29 (7.09-9.67)
Costa Rica	65.62 (51.51-83.31)	45.77 (37.94-54.30)	26.35 (21.41-32.11)	9.50 (7.97-11.11)	6.65 (5.48-7.90)	3.66 (2.96-4.44)	0.90 (0.82-0.97)	0.51 (0.47-0.54)	0.36 (0.33-0.41)	9.28 (7.89-10.74)	8.81 (7.70-10.02)	6.23 (5.19-7.38)
El Salvador	127.26 (104.28-149.91)	90.31 (74.68-108.74)	56.17 (45.25-68.83)	51.91 (45.84-57.68)	22.65 (18.75-26.46)	9.49 (6.98-11.92)	9.04 (1.98-11.26)	0.77 (0.65-1.27)	0.34 (0.23-0.96)	5.99 (4.94-7.22)	5.57 (4.59-6.68)	5.19 (4.24-6.26)
Guatemala	170.05 (140.32-198.36)	144.01 (119.86-168.41)	89.28 (68.96-113.03)	139.38 (120.55-158.88)	73.83 (64.53-92.50)	33.75 (26.44-46.76)	2.52 (2.32-2.70)	1.49 (1.39-1.59)	1.54 (1.22-1.81)	6.25 (5.16-7.47)	5.51 (4.67-6.49)	6.26 (5.19-7.52)
Honduras	133.98 (112.52-156.04)	157.16 (125.58-194.85)	116.87 (88.07-151.25)	52.79 (47.15-58.75)	39.18 (32.68-46.28)	20.44 (16.30-25.59)	1.53 (1.06-1.81)	1.65 (0.81-2.24)	1.31 (0.61-1.83)	10.25 (8.64-12.10)	9.43 (8.13-10.86)	7.42 (6.30-8.60)
Mexico	63.22 (53.96-73.35)	55.05 (47.39-63.45)	42.31 (35.28-50.19)	37.49 (31.41-41.00)	12.03 (10.35-14.66)	6.92 (5.77-8.51)	1.75 (1.68-1.82)	0.84 (0.80-0.88)	0.54 (0.51-0.58)	15.75 (15.19-16.35)	10.43 (10.14-10.71)	8.07 (7.77-8.38)
Nicaragua	85.01 (71.30-100.05)	73.03 (60.35-86.21)	59.75 (45.28-76.27)	51.50 (46.45-56.90)	18.71 (16.18-21.48)	6.55 (5.23-7.99)	1.22 (0.83-1.38)	0.77 (0.61-0.95)	0.51 (0.43-0.70)	8.97 (7.59-10.60)	7.69 (6.44-9.09)	7.30 (5.98-8.70)
Panama	69.60 (56.36-85.09)	49.07 (39.31-60.15)	32.17 (23.89-42.60)	13.89 (11.93-16.20)	9.26 (7.74-11.08)	8.22 (6.67-9.83)	1.10 (0.84-1.32)	0.82 (0.70-1.10)	0.65 (0.53-0.92)	6.96 (5.81-8.29)	6.23 (5.40-7.15)	2.88 (2.47-3.30)

	Appendix Table 1b. Unscaled values for each SDG health-related indicator by country for 1990, 2000, and 2015											
	**Indicator 3.9.1: Age-standardised death rate attributable to household air pollution and ambient air pollution (per 100,000 population)**			**Indicator 3.9.2: Age-standardised death rate attributable to unsafe water, sanitation, and hygiene (WaSH) (per 100,000 population)**			**Indicator 3.9.3: Age-standardised death rate due to unintentional poisonings (per 100,000 population)**			**Indicator 3.a.1: Age-standardised prevalence of daily smoking among populations aged 10 and older**		
**Location**	**1990**	**2000**	**2015**	**1990**	**2000**	**2015**	**1990**	**2000**	**2015**	**1990**	**2000**	**2015**
Venezuela	70.71 (60.46-81.18)	58.42 (50.15-67.42)	45.85 (37.75-54.82)	19.96 (16.99-22.41)	13.78 (11.65-15.66)	8.11 (6.58-9.74)	1.15 (1.06-1.24)	0.53 (0.49-0.58)	0.40 (0.35-0.47)	12.74 (10.79-14.86)	11.87 (10.04-13.83)	10.90 (9.31-12.59)
Bolivia	166.34 (135.74-198.28)	121.53 (100.18-144.06)	79.17 (61.98-100.06)	79.60 (66.52-93.26)	42.86 (35.61-50.15)	17.64 (12.79-22.69)	7.90 (4.85-15.30)	4.10 (3.31-5.42)	3.05 (2.10-5.19)	18.93 (16.86-21.04)	20.41 (18.71-22.17)	16.73 (14.73-18.94)
Ecuador	77.67 (63.30-92.51)	55.80 (45.41-67.21)	29.87 (23.35-37.86)	37.59 (33.06-42.09)	19.98 (16.66-23.60)	9.65 (7.16-12.40)	2.80 (1.51-3.17)	1.26 (1.11-1.81)	0.75 (0.62-1.25)	7.62 (6.49-8.92)	6.53 (5.95-7.17)	4.36 (3.80-5.07)
Peru	142.31 (115.95-170.68)	97.65 (80.85-114.68)	61.40 (48.86-76.77)	43.48 (36.92-50.61)	21.30 (16.50-26.22)	12.63 (8.46-16.86)	1.68 (1.40-2.12)	1.28 (0.94-1.48)	0.76 (0.56-0.91)	8.58 (7.12-10.20)	8.27 (6.99-9.74)	6.57 (5.55-7.68)
Antigua and Barbuda	49.72 (34.03-67.21)	41.40 (28.08-55.31)	30.58 (19.60-43.70)	12.12 (9.69-14.75)	9.68 (7.64-11.91)	7.06 (5.23-9.12)	1.39 (1.25-1.53)	0.87 (0.79-0.95)	0.63 (0.56-0.72)	2.16 (1.73-2.66)	2.24 (1.79-2.72)	2.65 (2.14-3.25)
The Bahamas	63.76 (48.63-82.41)	47.54 (34.32-62.67)	30.91 (20.64-41.86)	12.11 (9.14-15.37)	7.97 (5.78-10.52)	5.99 (4.21-8.08)	1.45 (0.98-1.67)	0.76 (0.66-1.02)	0.52 (0.43-0.74)	2.70 (2.17-3.33)	2.73 (2.20-3.33)	4.79 (3.93-5.78)
Barbados	44.18 (30.12-59.09)	36.59 (22.81-51.46)	26.78 (15.77-38.74)	8.04 (6.14-10.13)	7.78 (5.60-9.94)	6.84 (4.71-9.14)	0.93 (0.84-1.02)	0.48 (0.43-0.53)	0.37 (0.33-0.43)	3.11 (2.51-3.85)	3.37 (2.71-4.14)	3.63 (2.91-4.49)
Belize	109.66 (88.83-133.24)	108.37 (89.34-130.07)	71.07 (57.04-88.91)	22.19 (18.10-26.69)	18.55 (14.51-22.88)	12.58 (9.26-15.94)	2.03 (1.43-2.35)	1.46 (1.26-2.03)	0.96 (0.78-1.48)	8.27 (6.92-9.81)	7.27 (6.06-8.68)	6.32 (5.28-7.47)
Cuba	76.63 (60.51-97.88)	82.01 (65.22-102.77)	43.52 (35.10-53.38)	10.49 (8.47-12.62)	9.87 (7.58-12.25)	6.88 (5.08-8.81)	1.69 (1.52-1.84)	0.78 (0.71-0.87)	0.32 (0.27-0.38)	21.26 (18.59-23.80)	20.50 (18.40-22.82)	12.51 (10.78-14.40)
Dominica	78.21 (56.02-103.00)	51.25 (38.29-65.78)	35.38 (24.02-49.03)	15.00 (12.39-17.83)	11.41 (9.29-13.81)	9.45 (7.34-12.12)	2.92 (1.94-3.35)	1.75 (1.49-2.68)	1.48 (1.11-2.77)	3.79 (3.01-4.65)	3.55 (2.85-4.43)	3.16 (2.50-3.92)

Location	Appendix Table 1b. Unscaled values for each SDG health-related indicator by country for 1990, 2000, and 2015											
	**Indicator 3.9.1: Age-standardised death rate attributable to household air pollution and ambient air pollution (per 100,000 population)**			**Indicator 3.9.2: Age-standardised death rate attributable to unsafe water, sanitation, and hygiene (WaSH) (per 100,000 population)**			**Indicator 3.9.3: Age-standardised death rate due to unintentional poisonings (per 100,000 population)**			**Indicator 3.a.1: Age-standardised prevalence of daily smoking among populations aged 10 and older**		
	**1990**	**2000**	**2015**	**1990**	**2000**	**2015**	**1990**	**2000**	**2015**	**1990**	**2000**	**2015**
Dominican Republic	75.58 (59.53-92.29)	63.08 (52.08-74.60)	52.25 (42.37-63.04)	33.79 (30.04-37.52)	15.14 (13.10-17.25)	7.99 (6.25-9.87)	1.20 (0.89-1.38)	0.56 (0.47-0.84)	0.39 (0.31-0.70)	10.83 (9.20-12.41)	9.58 (8.62-10.59)	5.72 (4.77-6.79)
Grenada	113.44 (82.96-151.84)	69.01 (50.06-91.46)	47.74 (32.75-65.16)	17.57 (13.35-22.35)	13.57 (9.80-17.28)	10.89 (7.79-14.29)	1.95 (1.31-2.27)	1.28 (1.10-1.67)	0.95 (0.77-1.46)	5.08 (4.08-6.25)	5.55 (4.54-6.69)	5.37 (4.44-6.47)
Guyana	136.06 (106.90-172.49)	107.47 (88.43-130.03)	75.07 (56.34-95.06)	40.49 (35.73-45.73)	31.92 (27.16-36.40)	17.80 (14.31-21.83)	2.04 (1.44-2.32)	1.42 (1.24-1.80)	0.94 (0.76-1.32)	6.27 (5.11-7.43)	7.53 (6.29-8.94)	7.38 (6.15-8.71)
Haiti	300.25 (254.58-351.48)	237.59 (188.91-286.96)	201.65 (155.52-250.71)	133.20 (114.79-154.26)	71.97 (59.49-85.65)	42.04 (32.69-54.25)	6.40 (3.60-14.77)	3.30 (1.90-6.64)	2.13 (1.23-3.96)	8.19 (6.89-9.65)	7.35 (6.38-8.43)	4.61 (3.89-5.42)
Jamaica	80.15 (64.11-97.32)	56.25 (45.39-67.85)	41.98 (32.61-52.96)	14.41 (12.09-17.31)	7.09 (5.47-8.76)	5.05 (3.72-6.61)	0.63 (0.54-0.92)	0.71 (0.61-0.98)	0.62 (0.50-0.84)	9.72 (8.11-11.30)	11.07 (9.75-12.40)	7.78 (6.49-9.22)
Saint Lucia	100.88 (77.66-125.47)	63.60 (48.05-81.63)	37.36 (26.82-48.77)	16.92 (13.15-21.41)	11.42 (8.97-14.08)	7.95 (5.85-10.07)	0.96 (0.86-1.08)	0.52 (0.47-0.57)	0.38 (0.33-0.44)	5.66 (4.52-6.91)	6.40 (5.18-7.79)	6.49 (5.34-7.89)
Saint Vincent and the Grenadines	126.46 (100.29-154.69)	84.38 (60.48-110.79)	47.71 (33.13-64.76)	20.00 (16.17-24.76)	14.91 (12.14-18.05)	9.81 (7.63-12.51)	1.12 (1.00-1.25)	0.67 (0.61-0.74)	0.52 (0.46-0.59)	7.45 (6.13-8.92)	6.39 (5.23-7.63)	5.22 (4.26-6.31)
Suriname	116.80 (86.30-150.60)	94.94 (70.48-124.27)	50.50 (36.59-67.62)	26.54 (22.86-30.51)	18.47 (15.38-21.87)	10.57 (8.29-13.12)	1.68 (1.16-1.93)	1.06 (0.91-1.53)	0.73 (0.58-1.19)	11.08 (9.27-12.98)	11.45 (9.76-13.33)	14.31 (12.91-15.73)
Trinidad and Tobago	73.23 (59.23-87.97)	60.21 (49.45-71.28)	38.49 (30.66-47.27)	12.30 (9.90-14.87)	8.74 (6.90-10.60)	5.58 (4.12-7.18)	1.15 (1.04-1.25)	0.63 (0.58-0.70)	0.47 (0.40-0.54)	11.70 (9.96-13.62)	10.65 (9.29-12.31)	11.16 (9.71-12.59)
Brazil	112.51 (94.21-133.38)	82.46 (68.30-97.69)	41.99 (34.34-51.46)	37.11 (33.25-40.75)	18.03 (15.34-20.74)	9.48 (7.01-11.87)	0.48 (0.37-0.53)	0.36 (0.30-0.44)	0.23 (0.21-0.31)	19.40 (18.21-20.69)	13.59 (12.76-14.43)	8.52 (8.10-8.97)

Appendix Table 1b. Unscaled values for each SDG health-related indicator by country for 1990, 2000, and 2015

Location	Indicator 3.9.1: Age-standardised death rate attributable to household air pollution and ambient air pollution (per 100,000 population)			Indicator 3.9.2: Age-standardised death rate attributable to unsafe water, sanitation, and hygiene (WaSH) (per 100,000 population)			Indicator 3.9.3: Age-standardised death rate due to unintentional poisonings (per 100,000 population)			Indicator 3.a.1: Age-standardised prevalence of daily smoking among populations aged 10 and older		
	1990	2000	2015	1990	2000	2015	1990	2000	2015	1990	2000	2015
Paraguay	116.05 (95.86-139.15)	102.25 (83.81-122.72)	71.67 (55.66-89.73)	22.23 (18.99-25.68)	17.38 (14.25-20.61)	9.25 (7.15-11.65)	0.84 (0.64-1.04)	0.54 (0.43-0.85)	0.37 (0.25-0.74)	12.30 (10.31-14.59)	11.12 (9.79-12.59)	8.32 (6.92-9.81)
China	273.34 (218.15-325.34)	221.93 (181.18-261.69)	121.00 (102.81-140.32)	15.74 (13.19-18.32)	8.46 (6.66-10.14)	2.95 (2.18-3.75)	4.09 (2.99-4.59)	2.83 (2.04-3.07)	1.56 (1.00-1.76)	22.03 (21.69-22.39)	19.42 (19.09-19.70)	16.65 (16.40-16.90)
North Korea	200.00 (145.26-260.57)	202.57 (149.72-262.96)	169.56 (127.94-218.82)	10.67 (6.18-18.23)	7.85 (4.82-12.31)	4.26 (2.81-6.06)	2.75 (1.53-5.37)	2.60 (1.47-3.91)	2.13 (1.16-3.10)	17.05 (15.66-18.42)	17.01 (15.81-18.21)	14.97 (13.70-16.32)
Taiwan	53.46 (45.52-61.89)	42.09 (35.61-48.97)	35.82 (29.01-43.05)	5.74 (4.02-7.63)	4.16 (2.73-5.62)	3.85 (1.80-5.81)	1.01 (0.66-1.48)	0.69 (0.51-1.02)	0.45 (0.35-0.60)	18.77 (17.20-20.61)	17.18 (16.30-18.04)	9.17 (8.13-10.28)
Cambodia	266.02 (213.05-324.07)	247.44 (201.70-296.38)	171.75 (137.40-209.43)	126.42 (106.00-149.64)	88.50 (75.91-102.94)	28.08 (21.86-34.91)	2.50 (1.58-5.15)	1.93 (1.34-3.48)	0.89 (0.61-1.41)	19.09 (17.46-20.67)	18.87 (17.95-19.79)	14.44 (13.38-15.51)
Indonesia	139.67 (110.90-170.56)	116.24 (92.20-144.42)	82.47 (61.93-105.83)	115.99 (94.00-138.10)	78.97 (64.94-94.58)	45.52 (36.48-55.27)	0.89 (0.42-1.27)	0.70 (0.32-0.92)	0.52 (0.23-0.71)	18.85 (17.40-20.23)	20.71 (19.83-21.59)	20.71 (19.42-21.98)
Laos	314.67 (246.59-392.00)	279.99 (227.94-338.26)	191.09 (152.18-233.84)	164.28 (127.49-198.97)	96.74 (74.75-119.66)	34.53 (26.60-43.54)	3.72 (2.01-9.64)	2.64 (1.68-4.98)	1.19 (0.80-2.00)	24.31 (22.34-26.24)	24.24 (22.74-25.84)	22.57 (20.51-24.80)
Malaysia	82.89 (65.35-108.73)	65.77 (53.94-80.38)	53.48 (41.81-68.01)	21.35 (16.11-27.02)	16.99 (11.93-21.93)	12.95 (8.05-18.21)	1.48 (1.14-1.92)	1.18 (0.90-1.39)	0.89 (0.66-1.10)	16.70 (15.01-18.47)	15.70 (14.45-17.06)	13.74 (12.47-15.06)
Maldives	147.51 (116.46-180.24)	86.02 (68.43-105.73)	43.53 (32.40-55.89)	37.78 (28.57-46.60)	15.66 (13.19-18.41)	6.46 (5.11-8.02)	4.08 (2.67-6.74)	2.29 (1.92-2.88)	0.97 (0.77-1.27)	14.66 (12.86-16.72)	14.63 (12.86-16.34)	15.33 (13.98-16.83)
Mauritius	114.73 (90.87-143.77)	80.10 (66.86-95.60)	39.21 (31.19-47.76)	17.64 (14.10-21.56)	8.07 (6.16-10.17)	5.51 (4.17-6.85)	0.28 (0.25-0.30)	0.18 (0.17-0.20)	0.13 (0.12-0.14)	16.02 (14.76-17.49)	13.31 (12.46-14.15)	12.59 (11.18-14.12)

Location	Appendix Table 1b. Unscaled values for each SDG health-related indicator by country for 1990, 2000, and 2015											
	**Indicator 3.9.1: Age-standardised death rate attributable to household air pollution and ambient air pollution (per 100,000 population)**			**Indicator 3.9.2: Age-standardised death rate attributable to unsafe water, sanitation, and hygiene (WaSH) (per 100,000 population)**			**Indicator 3.9.3: Age-standardised death rate due to unintentional poisonings (per 100,000 population)**			**Indicator 3.a.1: Age-standardised prevalence of daily smoking among populations aged 10 and older**		
	**1990**	**2000**	**2015**	**1990**	**2000**	**2015**	**1990**	**2000**	**2015**	**1990**	**2000**	**2015**
Myanmar	237.90 (167.67-320.63)	220.03 (152.40-297.75)	166.43 (109.85-228.45)	137.34 (101.74-177.84)	82.75 (61.67-108.19)	32.85 (23.05-43.82)	2.76 (1.57-4.57)	2.29 (1.26-3.84)	1.37 (0.80-2.19)	19.00 (17.12-21.06)	17.04 (15.71-18.47)	13.02 (11.83-14.25)
Philippines	151.98 (124.84-181.40)	142.05 (116.71-169.62)	142.86 (114.66-176.33)	39.48 (31.72-48.57)	29.86 (24.04-36.84)	21.52 (16.40-27.17)	0.35 (0.32-0.37)	0.32 (0.30-0.34)	0.21 (0.19-0.23)	19.40 (17.55-21.35)	19.32 (18.06-20.58)	17.19 (15.52-18.91)
Sri Lanka	120.18 (101.08-141.19)	110.26 (92.82-129.00)	79.40 (59.12-102.72)	30.96 (26.09-38.43)	17.36 (13.84-20.72)	7.18 (5.16-9.70)	2.41 (2.20-2.63)	0.66 (0.59-0.82)	0.32 (0.25-0.43)	10.41 (9.14-11.83)	8.79 (8.02-9.60)	7.98 (7.06-8.95)
Seychelles	77.97 (49.49-109.49)	58.32 (36.41-83.35)	35.91 (18.73-54.80)	20.47 (15.41-25.94)	13.70 (9.79-17.95)	10.54 (7.20-14.34)	1.38 (1.04-1.61)	0.90 (0.79-1.17)	0.56 (0.46-0.82)	11.68 (10.32-13.28)	11.63 (10.45-12.93)	11.60 (10.19-13.08)
Thailand	118.71 (96.62-142.77)	99.94 (81.93-120.06)	71.66 (55.23-90.75)	29.87 (24.88-35.44)	19.19 (14.86-23.52)	14.72 (10.14-19.68)	0.98 (0.81-1.16)	0.82 (0.71-1.06)	0.52 (0.41-0.68)	19.18 (17.95-20.54)	15.27 (14.19-16.39)	13.89 (12.62-15.35)
Timor-Leste	212.43 (157.96-269.86)	172.37 (132.73-215.08)	121.75 (91.55-158.73)	119.56 (72.83-155.56)	70.03 (44.41-93.02)	24.19 (17.14-32.50)	2.50 (1.15-6.66)	1.57 (0.87-3.29)	0.77 (0.46-1.40)	18.26 (16.52-19.88)	18.73 (17.08-20.25)	21.58 (20.09-23.03)
Vietnam	138.32 (108.91-168.67)	128.74 (102.05-160.90)	86.72 (65.85-109.82)	24.96 (18.22-32.87)	15.61 (11.93-20.44)	8.86 (6.50-11.58)	1.96 (0.95-2.97)	1.50 (0.70-2.06)	1.03 (0.42-1.58)	21.73 (20.40-23.00)	18.27 (17.21-19.32)	14.71 (13.31-16.08)
Federated States of Micronesia	154.86 (88.98-232.84)	122.60 (72.77-191.08)	78.94 (38.76-132.87)	35.07 (24.61-47.18)	21.46 (14.74-30.50)	13.87 (9.31-20.68)	2.13 (1.21-3.92)	1.59 (0.95-2.65)	1.24 (0.73-2.13)	12.53 (11.03-14.15)	11.71 (10.28-13.18)	11.24 (9.80-12.85)
Fiji	138.31 (100.75-183.44)	113.97 (80.24-154.46)	58.58 (38.70-83.27)	29.81 (23.28-36.74)	26.04 (20.81-31.19)	22.99 (18.22-28.58)	0.83 (0.63-1.29)	0.86 (0.70-1.18)	0.76 (0.59-0.99)	9.98 (8.61-11.33)	9.71 (8.75-10.66)	9.02 (8.01-10.14)
Kiribati	139.66 (96.89-186.03)	120.00 (75.30-172.37)	70.26 (37.45-110.36)	174.85 (148.57-204.12)	93.39 (78.62-109.75)	54.74 (43.26-68.34)	4.06 (2.08-5.80)	1.76 (1.39-2.91)	1.67 (1.13-2.99)	32.66 (30.32-34.91)	33.11 (30.94-35.05)	29.45 (27.34-31.55)

Appendix Table 1b. Unscaled values for each SDG health-related indicator by country for 1990, 2000, and 2015

Location	Indicator 3.9.1: Age-standardised death rate attributable to household air pollution and ambient air pollution (per 100,000 population)			Indicator 3.9.2: Age-standardised death rate attributable to unsafe water, sanitation, and hygiene (WaSH) (per 100,000 population)			Indicator 3.9.3: Age-standardised death rate due to unintentional poisonings (per 100,000 population)			Indicator 3.a.1: Age-standardised prevalence of daily smoking among populations aged 10 and older		
	1990	2000	2015	1990	2000	2015	1990	2000	2015	1990	2000	2015
Marshall Islands	105.74 (45.16-170.53)	113.02 (51.83-174.51)	77.02 (26.18-131.93)	42.12 (34.49-50.95)	28.58 (22.33-35.90)	16.60 (12.52-21.39)	2.30 (1.57-4.16)	1.92 (1.28-3.06)	1.40 (0.95-2.19)	13.39 (11.72-15.27)	12.62 (11.43-13.80)	11.24 (9.81-12.83)
Papua New Guinea	340.74 (226.21-477.67)	306.42 (204.40-426.32)	256.82 (173.36-368.98)	166.96 (124.34-214.64)	141.79 (101.20-180.92)	99.92 (69.80-135.60)	3.24 (1.69-6.27)	2.97 (1.52-5.70)	2.30 (1.27-4.37)	23.95 (21.65-26.09)	24.22 (22.37-26.17)	21.69 (19.82-23.61)
Samoa	110.34 (76.49-152.70)	88.94 (59.48-121.89)	60.05 (37.60-86.05)	23.45 (17.83-30.66)	16.24 (11.74-21.06)	10.85 (7.79-14.52)	1.60 (1.04-2.67)	1.26 (0.80-1.99)	0.88 (0.59-1.49)	24.08 (22.12-25.96)	22.16 (20.70-23.74)	19.41 (17.46-21.50)
Solomon Islands	245.03 (160.04-346.42)	219.38 (141.36-310.33)	179.12 (113.88-269.55)	73.01 (53.33-93.01)	45.85 (32.77-59.91)	27.48 (19.17-38.25)	3.13 (1.69-5.80)	2.62 (1.30-4.71)	2.03 (1.05-3.39)	17.55 (15.66-19.51)	16.48 (14.69-18.35)	15.73 (14.05-17.47)
Tonga	108.78 (76.99-145.86)	93.06 (66.34-123.60)	50.99 (30.18-76.70)	21.83 (17.12-27.06)	17.85 (13.64-22.47)	12.81 (9.27-16.73)	2.93 (2.07-4.50)	2.69 (2.06-3.68)	2.32 (1.65-3.32)	21.48 (19.81-23.13)	20.01 (18.53-21.60)	19.96 (18.56-21.44)
Vanuatu	243.04 (163.58-346.62)	223.13 (143.61-324.10)	167.88 (107.23-243.35)	56.61 (40.92-74.32)	41.56 (29.67-54.09)	25.37 (18.57-34.42)	2.70 (1.50-4.79)	2.51 (1.34-4.22)	1.95 (1.06-3.17)	16.11 (14.41-17.88)	13.86 (12.59-15.28)	12.76 (11.51-14.06)
Afghanistan	504.59 (394.42-615.47)	531.36 (424.06-645.98)	427.30 (334.31-537.13)	63.53 (33.14-89.87)	59.97 (37.32-80.73)	29.75 (22.18-39.08)	3.25 (1.80-6.64)	3.08 (1.90-5.21)	2.32 (1.51-3.59)	10.69 (9.03-12.47)	11.35 (9.68-13.12)	11.87 (10.24-13.66)
Algeria	75.25 (61.90-93.27)	58.33 (49.41-68.16)	47.38 (40.07-55.56)	24.87 (20.14-29.99)	13.92 (11.21-16.77)	8.74 (6.91-10.71)	2.11 (1.27-4.67)	1.43 (0.98-2.40)	1.10 (0.70-1.88)	13.09 (11.22-15.10)	10.94 (9.47-12.56)	8.11 (6.99-9.40)
Bahrain	112.35 (95.18-132.57)	89.56 (76.91-103.38)	48.50 (39.23-58.55)	8.42 (6.48-10.45)	6.10 (4.63-7.72)	3.48 (2.42-4.58)	0.96 (0.61-1.17)	0.66 (0.43-0.79)	0.30 (0.23-0.42)	10.43 (8.96-12.06)	8.07 (7.00-9.28)	8.22 (6.93-9.55)
Egypt	142.05 (126.86-159.92)	114.67 (101.93-129.37)	115.02 (102.59-130.73)	53.75 (44.43-62.00)	22.49 (18.81-26.20)	9.22 (7.17-11.90)	0.67 (0.53-1.33)	0.72 (0.58-0.82)	0.67 (0.44-0.77)	12.37 (10.84-14.02)	11.95 (10.69-13.31)	13.25 (12.04-14.60)

Appendix Table 1b. Unscaled values for each SDG health-related indicator by country for 1990, 2000, and 2015

Location	Indicator 3.9.1: Age-standardised death rate attributable to household air pollution and ambient air pollution (per 100,000 population)			Indicator 3.9.2: Age-standardised death rate attributable to unsafe water, sanitation, and hygiene (WaSH) (per 100,000 population)			Indicator 3.9.3: Age-standardised death rate due to unintentional poisonings (per 100,000 population)			Indicator 3.a.1: Age-standardised prevalence of daily smoking among populations aged 10 and older		
	1990	2000	2015	1990	2000	2015	1990	2000	2015	1990	2000	2015
Iran	103.76 (84.62-126.98)	86.67 (74.62-100.70)	73.56 (59.29-88.66)	17.01 (12.75-21.85)	7.15 (5.67-8.79)	4.66 (3.39-6.11)	8.29 (5.07-14.66)	5.07 (3.52-6.27)	2.66 (1.94-3.95)	8.34 (7.16-9.66)	8.18 (7.15-9.27)	8.27 (7.15-9.43)
Iraq	133.53 (108.21-164.18)	141.61 (117.22-170.59)	113.76 (89.09-139.87)	22.70 (18.10-27.60)	17.36 (13.87-21.22)	9.03 (7.06-11.38)	1.22 (0.77-2.62)	1.05 (0.77-1.59)	0.68 (0.49-1.01)	11.94 (10.51-13.50)	11.56 (10.26-13.00)	10.88 (9.45-12.52)
Jordan	76.96 (63.97-93.97)	66.58 (55.76-77.57)	43.73 (36.42-51.29)	9.70 (7.34-12.22)	6.77 (5.13-8.64)	3.60 (2.60-4.74)	2.08 (1.26-3.95)	1.52 (1.03-2.32)	0.71 (0.50-1.10)	16.48 (14.56-18.58)	16.69 (14.91-18.34)	15.66 (13.98-17.44)
Kuwait	61.64 (54.00-70.79)	65.81 (58.12-74.97)	55.17 (46.90-65.58)	6.00 (4.39-7.70)	5.09 (3.62-6.60)	4.71 (3.20-6.38)	0.64 (0.56-0.80)	0.32 (0.30-0.35)	0.20 (0.17-0.23)	13.24 (11.61-14.91)	13.70 (12.11-15.42)	12.73 (11.57-13.92)
Lebanon	78.04 (61.59-96.71)	51.87 (40.91-63.65)	46.04 (33.50-58.96)	7.58 (5.60-9.89)	4.03 (3.06-5.23)	1.94 (1.41-2.56)	1.25 (0.71-2.27)	0.74 (0.45-1.28)	0.42 (0.27-0.62)	25.08 (22.41-27.74)	22.10 (20.11-24.23)	18.90 (16.47-21.39)
Libya	81.04 (67.78-98.18)	72.80 (62.57-84.63)	81.95 (67.96-96.80)	12.56 (9.27-16.19)	8.08 (5.93-10.34)	6.41 (4.48-8.67)	1.41 (0.70-2.82)	1.04 (0.56-1.99)	0.78 (0.47-1.24)	10.95 (9.37-12.57)	10.69 (9.28-12.21)	10.32 (9.08-11.66)
Morocco	76.74 (55.22-107.58)	56.39 (44.15-70.83)	46.58 (34.28-63.59)	37.94 (31.32-44.81)	14.39 (11.21-17.87)	8.61 (6.37-11.61)	2.23 (1.34-5.05)	1.39 (0.99-2.32)	1.02 (0.72-1.38)	9.48 (8.09-10.86)	8.60 (7.70-9.59)	6.70 (5.65-7.87)
Palestine	64.68 (49.49-83.66)	53.20 (43.73-63.19)	61.93 (48.32-76.99)	10.54 (7.72-14.23)	7.36 (5.43-9.55)	5.87 (3.86-8.47)	0.54 (0.30-1.20)	0.33 (0.25-0.42)	0.25 (0.19-0.36)	15.16 (13.69-16.71)	15.57 (14.80-16.36)	13.58 (12.18-15.15)
Oman	87.03 (66.29-109.99)	76.35 (63.16-91.17)	65.10 (52.43-77.67)	15.72 (10.77-21.67)	10.25 (7.03-13.83)	8.75 (5.77-11.80)	0.20 (0.12-0.43)	0.13 (0.10-0.18)	0.15 (0.09-0.20)	7.03 (6.02-8.27)	5.57 (4.92-6.31)	5.84 (4.92-6.93)
Qatar	90.04 (75.52-106.56)	90.74 (76.91-106.69)	51.93 (40.92-65.57)	3.81 (2.67-5.29)	3.35 (2.28-4.53)	2.12 (1.39-2.99)	0.52 (0.33-0.94)	0.42 (0.32-0.62)	0.25 (0.18-0.36)	8.74 (7.32-10.27)	7.05 (5.92-8.30)	8.04 (6.90-9.23)

Appendix Table 1b. Unscaled values for each SDG health-related indicator by country for 1990, 2000, and 2015

Location	Indicator 3.9.1: Age-standardised death rate attributable to household air pollution and ambient air pollution (per 100,000 population)			Indicator 3.9.2: Age-standardised death rate attributable to unsafe water, sanitation, and hygiene (WaSH) (per 100,000 population)			Indicator 3.9.3: Age-standardised death rate due to unintentional poisonings (per 100,000 population)			Indicator 3.a.1: Age-standardised prevalence of daily smoking among populations aged 10 and older		
	1990	2000	2015	1990	2000	2015	1990	2000	2015	1990	2000	2015
Saudi Arabia	83.03 (71.57-95.30)	75.19 (66.31-84.42)	67.32 (58.53-77.19)	15.04 (10.93-19.26)	9.43 (6.87-11.81)	7.04 (4.89-9.07)	1.84 (1.25-2.51)	1.24 (0.92-1.50)	0.76 (0.62-0.97)	6.40 (5.95-6.86)	8.80 (8.23-9.41)	10.05 (9.54-10.59)
Sudan	281.44 (226.00-346.75)	253.91 (197.83-329.37)	191.60 (142.89-249.88)	71.21 (54.07-91.19)	48.40 (36.42-62.62)	25.56 (18.80-34.31)	4.40 (2.07-11.26)	3.14 (1.59-7.37)	1.97 (1.02-3.93)	0.74 (0.58-0.95)	0.72 (0.56-0.94)	0.70 (0.54-0.89)
Syria	105.02 (87.19-125.83)	80.32 (66.74-96.82)	74.64 (60.42-88.99)	14.85 (9.82-20.70)	6.08 (4.11-10.36)	4.53 (2.93-8.06)	2.25 (1.46-2.97)	1.61 (0.98-2.13)	0.86 (0.65-1.13)	15.24 (13.02-17.58)	14.92 (13.29-16.84)	12.06 (10.50-13.80)
Tunisia	79.21 (63.49-104.56)	59.74 (49.55-71.80)	49.36 (39.03-61.36)	18.56 (14.36-23.07)	9.35 (7.32-11.80)	5.56 (4.03-7.39)	1.96 (1.05-4.76)	1.19 (0.71-2.37)	0.60 (0.44-0.80)	18.93 (17.11-20.68)	17.92 (16.73-19.17)	15.74 (14.00-17.49)
Turkey	119.80 (92.99-155.98)	77.94 (66.27-92.01)	45.51 (38.62-53.16)	27.30 (20.81-35.05)	9.21 (7.26-12.13)	2.73 (2.06-3.58)	1.12 (0.75-2.09)	0.67 (0.57-0.82)	0.28 (0.22-0.40)	25.60 (23.47-27.73)	23.28 (21.70-24.88)	18.32 (16.72-19.99)
United Arab Emirates	117.02 (92.44-142.67)	106.08 (88.60-124.77)	87.54 (69.48-111.76)	17.19 (12.64-23.48)	12.48 (9.02-16.65)	7.51 (4.64-10.46)	1.26 (0.76-1.99)	0.88 (0.64-1.22)	0.53 (0.37-0.71)	8.93 (7.55-10.45)	8.60 (7.33-9.92)	7.41 (6.13-8.77)
Yemen	302.36 (202.90-424.52)	235.45 (149.89-347.28)	183.36 (116.31-282.39)	75.55 (45.85-100.69)	41.22 (24.69-55.50)	20.61 (14.26-28.73)	4.49 (2.13-10.32)	3.17 (1.53-6.67)	1.99 (1.08-3.47)	12.13 (10.34-14.21)	13.00 (11.48-14.56)	10.30 (8.84-11.92)
Bangladesh	248.55 (201.70-296.32)	237.49 (198.96-275.51)	229.29 (190.30-272.04)	304.72 (276.50-333.97)	93.92 (82.83-106.19)	33.72 (28.15-40.87)	0.85 (0.45-2.02)	0.45 (0.34-0.69)	0.45 (0.23-0.71)	16.50 (14.42-18.67)	16.03 (14.58-17.55)	16.44 (14.74-18.31)
Bhutan	224.74 (172.34-285.25)	180.46 (141.18-221.54)	127.30 (94.55-167.06)	101.73 (73.66-138.35)	62.78 (47.68-82.32)	30.06 (22.47-39.24)	1.28 (0.60-2.88)	1.05 (0.46-2.58)	0.58 (0.27-1.18)	5.52 (4.40-6.83)	5.23 (4.25-6.33)	5.23 (4.56-5.99)
India	312.93 (261.85-362.45)	287.88 (242.81-332.88)	223.12 (190.72-254.23)	144.38 (133.62-155.69)	105.55 (97.42-114.47)	69.34 (61.40-77.64)	2.25 (0.98-3.69)	1.62 (0.75-2.20)	0.95 (0.45-1.19)	13.65 (13.04-14.32)	13.47 (12.81-14.16)	8.48 (8.19-8.82)

Appendix Table 1b. Unscaled values for each SDG health-related indicator by country for 1990, 2000, and 2015												
	**Indicator 3.9.1: Age-standardised death rate attributable to household air pollution and ambient air pollution (per 100,000 population)**			**Indicator 3.9.2: Age-standardised death rate attributable to unsafe water, sanitation, and hygiene (WaSH) (per 100,000 population)**			**Indicator 3.9.3: Age-standardised death rate due to unintentional poisonings (per 100,000 population)**			**Indicator 3.a.1: Age-standardised prevalence of daily smoking among populations aged 10 and older**		
**Location**	**1990**	**2000**	**2015**	**1990**	**2000**	**2015**	**1990**	**2000**	**2015**	**1990**	**2000**	**2015**
Nepal	256.03 (206.68-312.58)	217.21 (180.73-254.47)	197.18 (154.73-239.28)	198.19 (165.53-235.11)	112.01 (95.18-130.03)	54.76 (43.69-67.81)	1.50 (0.75-4.04)	1.03 (0.52-2.06)	0.62 (0.34-1.16)	26.56 (23.21-29.85)	27.16 (25.17-28.99)	16.07 (14.31-18.10)
Pakistan	247.54 (201.23-297.77)	281.58 (238.43-331.63)	227.91 (184.31-273.10)	91.97 (75.78-108.22)	71.36 (60.18-83.81)	41.22 (33.82-49.39)	1.52 (0.82-3.52)	1.42 (0.87-2.54)	1.08 (0.67-1.88)	13.87 (11.74-16.03)	13.24 (11.63-15.01)	8.85 (7.88-9.89)
Botswana	238.68 (109.31-483.64)	245.92 (88.31-591.28)	148.91 (58.71-367.17)	116.02 (64.07-219.12)	108.74 (46.54-242.42)	61.51 (29.53-151.70)	2.74 (0.91-7.09)	3.26 (0.83-10.26)	2.09 (0.68-6.84)	10.33 (9.18-11.51)	9.50 (8.55-10.54)	9.26 (8.30-10.31)
Lesotho	222.05 (164.74-289.43)	239.83 (177.58-323.04)	275.90 (161.60-419.04)	169.78 (132.41-214.21)	165.41 (130.66-209.68)	141.16 (95.03-203.25)	2.84 (1.43-5.38)	2.76 (1.57-4.60)	3.39 (1.68-6.21)	12.13 (11.02-13.21)	11.05 (10.14-12.00)	11.34 (10.41-12.38)
Namibia	265.44 (212.52-318.64)	306.73 (233.25-384.12)	154.88 (102.73-232.65)	138.42 (113.07-161.69)	135.24 (103.87-165.79)	62.50 (43.80-90.83)	2.53 (1.60-4.04)	2.83 (1.95-4.17)	1.50 (0.81-2.60)	14.10 (12.65-15.53)	13.62 (12.61-14.77)	9.99 (9.09-10.99)
South Africa	197.58 (166.78-227.94)	202.60 (168.78-238.00)	106.71 (88.64-125.19)	91.11 (82.44-99.83)	81.96 (71.93-91.56)	46.01 (39.74-53.12)	2.02 (1.54-2.44)	2.04 (1.55-2.35)	1.19 (0.99-1.49)	20.60 (19.86-21.30)	15.17 (14.73-15.65)	11.92 (11.52-12.32)
Swaziland	273.37 (198.50-359.03)	279.23 (194.64-390.11)	251.79 (132.40-402.61)	127.38 (96.99-159.58)	120.07 (92.69-157.24)	91.19 (56.58-136.88)	2.78 (1.56-4.67)	3.03 (1.73-5.11)	2.81 (1.25-5.05)	7.75 (6.95-8.58)	5.14 (4.59-5.72)	4.53 (4.05-5.06)
Zimbabwe	171.30 (126.66-219.91)	203.73 (138.37-304.05)	181.17 (110.08-271.42)	73.48 (60.18-87.96)	79.94 (58.91-112.00)	72.71 (51.69-99.91)	2.46 (1.63-3.91)	2.29 (1.37-4.63)	1.85 (1.02-3.68)	10.16 (9.21-11.25)	9.38 (8.55-10.25)	8.84 (8.07-9.66)
Benin	261.88 (219.86-307.63)	272.48 (217.68-332.69)	246.22 (147.50-382.02)	158.49 (122.76-190.51)	134.71 (105.11-166.89)	83.56 (54.58-121.59)	2.97 (1.56-8.07)	2.96 (1.66-6.47)	2.34 (1.12-4.68)	5.17 (4.35-6.17)	4.81 (4.08-5.59)	3.92 (3.34-4.59)
Burkina Faso	254.37 (210.80-305.00)	247.11 (201.69-297.23)	209.30 (137.49-308.54)	279.09 (234.97-330.62)	214.57 (179.95-255.35)	115.06 (81.83-159.51)	4.32 (2.36-10.93)	3.10 (2.05-4.58)	2.62 (1.36-5.27)	7.95 (6.62-9.45)	7.39 (6.18-8.61)	6.79 (5.81-7.92)

Appendix Table 1b. Unscaled values for each SDG health-related indicator by country for 1990, 2000, and 2015

Location	Indicator 3.9.1: Age-standardised death rate attributable to household air pollution and ambient air pollution (per 100,000 population)			Indicator 3.9.2: Age-standardised death rate attributable to unsafe water, sanitation, and hygiene (WaSH) (per 100,000 population)			Indicator 3.9.3: Age-standardised death rate due to unintentional poisonings (per 100,000 population)			Indicator 3.a.1: Age-standardised prevalence of daily smoking among populations aged 10 and older		
	1990	2000	2015	1990	2000	2015	1990	2000	2015	1990	2000	2015
Cameroon	260.89 (218.85-307.29)	295.74 (230.68-363.17)	233.32 (150.08-354.36)	154.07 (122.42-184.78)	143.47 (113.48-175.48)	76.58 (52.22-107.36)	2.50 (1.41-4.89)	2.75 (1.52-5.30)	2.14 (0.98-4.65)	5.41 (4.55-6.39)	4.91 (4.10-5.72)	4.05 (3.53-4.59)
Cape Verde	177.66 (148.27-206.50)	176.37 (129.92-231.69)	110.92 (83.79-146.77)	65.21 (54.62-75.32)	41.07 (31.06-52.80)	22.08 (17.02-28.19)	1.67 (0.92-3.18)	1.58 (0.96-2.69)	0.92 (0.67-1.29)	5.39 (4.53-6.36)	5.05 (4.29-5.87)	4.92 (4.12-5.85)
Chad	241.71 (200.86-286.31)	289.88 (230.34-361.78)	260.69 (159.90-393.81)	248.97 (203.67-295.50)	253.93 (207.35-307.33)	166.95 (113.43-234.53)	4.31 (1.76-14.22)	4.74 (2.11-12.26)	3.37 (1.37-7.49)	5.81 (4.93-6.76)	5.68 (4.98-6.45)	5.46 (4.65-6.36)
Cote d'Ivoire	266.31 (216.31-322.73)	294.77 (224.76-376.74)	226.92 (146.72-335.45)	159.27 (130.88-186.98)	131.82 (103.81-163.49)	85.11 (58.58-119.30)	3.12 (1.80-5.95)	3.31 (1.90-6.41)	2.47 (1.17-4.62)	6.23 (5.18-7.45)	6.00 (5.17-6.91)	6.54 (5.61-7.50)
The Gambia	250.74 (164.08-373.47)	231.85 (179.87-282.62)	201.07 (138.62-273.68)	104.87 (73.63-143.44)	82.80 (66.07-105.08)	52.70 (39.30-71.90)	2.33 (1.17-5.43)	2.19 (1.22-3.63)	1.73 (0.91-2.81)	10.40 (9.02-11.93)	9.30 (7.97-10.72)	8.01 (7.01-9.08)
Ghana	280.83 (203.73-373.69)	252.78 (189.87-318.56)	204.85 (122.93-321.11)	146.26 (112.66-188.53)	90.88 (71.91-111.16)	43.59 (27.60-65.21)	2.26 (1.37-4.07)	1.65 (1.18-2.25)	1.56 (0.85-2.59)	3.61 (3.03-4.30)	3.46 (3.01-3.98)	2.65 (2.24-3.10)
Guinea	270.12 (221.57-318.12)	274.25 (225.19-327.79)	258.74 (177.37-365.22)	192.06 (154.34-233.17)	156.33 (130.32-186.20)	98.27 (68.86-135.24)	3.35 (1.73-7.76)	3.21 (1.84-6.50)	2.61 (1.39-4.80)	4.04 (3.34-4.83)	3.71 (3.07-4.44)	3.42 (2.84-4.10)
Guinea-Bissau	297.50 (148.23-570.60)	302.24 (142.68-598.37)	290.31 (132.88-632.83)	215.37 (136.01-348.96)	166.10 (95.64-292.90)	118.93 (65.96-223.77)	3.80 (1.36-10.11)	3.93 (1.32-9.46)	3.60 (1.23-8.94)	5.40 (4.52-6.40)	5.06 (4.27-6.03)	5.03 (4.22-5.86)
Liberia	229.75 (174.36-297.21)	189.95 (145.42-237.46)	177.12 (116.50-259.59)	222.88 (177.14-273.43)	178.73 (146.85-212.08)	100.93 (71.20-137.83)	3.23 (1.63-7.78)	2.70 (1.35-6.11)	1.88 (0.97-3.94)	5.64 (4.77-6.64)	5.42 (4.63-6.30)	4.60 (4.03-5.28)
Mali	230.02 (191.60-274.51)	205.37 (167.68-247.29)	175.45 (119.68-255.48)	248.02 (210.43-288.50)	165.81 (138.88-198.94)	98.28 (72.18-133.62)	4.01 (2.13-9.60)	3.31 (1.72-7.67)	2.45 (1.21-5.53)	5.01 (4.21-5.90)	4.83 (4.03-5.69)	4.68 (3.99-5.43)

Appendix Table 1b. Unscaled values for each SDG health-related indicator by country for 1990, 2000, and 2015												
Location	Indicator 3.9.1: Age-standardised death rate attributable to household air pollution and ambient air pollution (per 100,000 population)			Indicator 3.9.2: Age-standardised death rate attributable to unsafe water, sanitation, and hygiene (WaSH) (per 100,000 population)			Indicator 3.9.3: Age-standardised death rate due to unintentional poisonings (per 100,000 population)			Indicator 3.a.1: Age-standardised prevalence of daily smoking among populations aged 10 and older		
	1990	2000	2015	1990	2000	2015	1990	2000	2015	1990	2000	2015
Mauritania	255.76 (205.60-304.64)	215.19 (172.07-261.11)	157.89 (101.15-228.88)	165.79 (130.79-198.52)	118.34 (96.23-140.75)	60.57 (42.09-83.10)	2.76 (1.50-5.95)	2.28 (1.14-4.87)	1.50 (0.70-3.27)	7.54 (6.41-8.82)	7.88 (6.93-8.93)	7.12 (6.00-8.31)
Niger	308.84 (256.20-367.77)	278.15 (228.18-333.96)	236.77 (163.12-330.12)	332.43 (269.22-398.77)	253.45 (208.34-300.20)	139.74 (103.31-184.07)	5.05 (2.08-15.64)	3.98 (1.93-9.33)	2.72 (1.29-6.09)	3.11 (2.54-3.78)	3.20 (2.57-3.87)	3.54 (2.98-4.14)
Nigeria	281.02 (202.18-384.21)	241.76 (192.25-300.43)	153.18 (107.31-231.70)	156.92 (117.04-202.31)	163.62 (134.05-195.28)	111.23 (87.78-150.34)	3.08 (1.45-8.69)	2.54 (1.33-6.05)	1.53 (0.76-3.47)	6.68 (5.96-7.42)	4.08 (3.48-4.78)	2.83 (2.37-3.31)
Sao Tome and Principe	245.73 (197.62-289.83)	251.99 (193.73-311.69)	198.01 (117.72-293.84)	94.81 (74.32-112.34)	78.24 (63.84-92.40)	44.74 (29.20-64.58)	1.73 (1.00-3.30)	1.59 (0.92-3.40)	1.25 (0.59-2.38)	3.11 (2.62-3.65)	2.89 (2.45-3.43)	2.87 (2.41-3.37)
Senegal	213.47 (174.01-259.66)	198.53 (159.70-246.44)	196.34 (116.25-303.91)	176.90 (150.27-205.90)	146.79 (123.77-171.79)	85.32 (57.53-125.49)	2.93 (1.69-6.08)	2.58 (1.58-4.95)	2.11 (1.07-3.97)	7.58 (6.37-8.82)	6.23 (5.30-7.17)	3.86 (3.38-4.40)
Sierra Leone	264.52 (194.50-343.48)	271.60 (217.73-327.90)	253.16 (169.15-356.02)	179.04 (136.03-230.04)	169.15 (139.72-204.48)	96.36 (69.61-129.95)	3.57 (1.67-9.11)	3.61 (2.07-8.37)	2.66 (1.27-6.22)	12.07 (10.53-13.79)	11.61 (10.11-13.27)	10.35 (9.29-11.44)
Togo	260.74 (211.55-317.27)	277.40 (215.39-348.73)	234.10 (156.07-337.86)	156.54 (130.85-184.65)	134.59 (107.68-163.68)	80.51 (56.55-110.23)	2.68 (1.60-5.75)	2.82 (1.69-5.56)	2.12 (1.11-3.90)	4.63 (3.93-5.45)	4.41 (3.71-5.14)	4.07 (3.53-4.63)
Burundi	416.73 (281.21-531.62)	349.82 (273.49-426.48)	228.29 (146.59-333.19)	308.07 (221.89-391.12)	289.73 (238.64-347.13)	142.52 (94.12-206.65)	8.39 (4.44-15.04)	6.87 (3.84-13.43)	4.23 (2.06-8.01)	5.32 (4.42-6.34)	4.70 (3.86-5.71)	4.32 (3.60-5.11)
Comoros	269.26 (175.12-390.40)	212.67 (165.64-264.50)	152.52 (102.50-218.60)	182.18 (124.01-247.72)	154.40 (126.40-186.03)	103.76 (71.67-143.39)	5.16 (2.62-10.80)	3.69 (2.22-6.95)	2.53 (1.36-5.14)	6.54 (5.38-7.70)	6.56 (5.43-7.80)	6.22 (5.37-7.12)
Djibouti	145.50 (89.76-221.78)	138.95 (75.63-224.53)	133.81 (72.01-230.38)	130.81 (92.69-178.20)	131.97 (82.39-196.27)	92.17 (57.16-146.79)	4.21 (1.89-10.20)	4.04 (1.70-8.49)	3.23 (1.43-6.95)	11.13 (9.60-12.90)	10.22 (8.86-11.67)	9.96 (8.57-11.43)

Appendix Table 1b. Unscaled values for each SDG health-related indicator by country for 1990, 2000, and 2015												
	**Indicator 3.9.1: Age-standardised death rate attributable to household air pollution and ambient air pollution (per 100,000 population)**			**Indicator 3.9.2: Age-standardised death rate attributable to unsafe water, sanitation, and hygiene (WaSH) (per 100,000 population)**			**Indicator 3.9.3: Age-standardised death rate due to unintentional poisonings (per 100,000 population)**			**Indicator 3.a.1: Age-standardised prevalence of daily smoking among populations aged 10 and older**		
**Location**	**1990**	**2000**	**2015**	**1990**	**2000**	**2015**	**1990**	**2000**	**2015**	**1990**	**2000**	**2015**
Eritrea	316.41 (251.62-382.31)	220.50 (135.65-319.19)	228.38 (121.72-367.00)	347.61 (280.17-418.42)	220.41 (143.89-308.89)	200.14 (114.70-309.05)	6.81 (3.67-14.78)	4.32 (2.08-9.31)	4.47 (2.00-9.39)	5.57 (4.61-6.72)	5.25 (4.51-6.08)	4.38 (3.59-5.29)
Ethiopia	337.21 (274.43-403.20)	263.21 (216.65-314.60)	181.42 (118.56-267.36)	320.45 (261.76-377.18)	231.47 (191.38-270.85)	103.51 (71.01-148.42)	6.08 (3.35-12.94)	4.05 (2.54-8.46)	2.33 (1.23-4.71)	3.68 (3.05-4.41)	3.26 (2.73-3.83)	3.18 (2.60-3.84)
Kenya	160.19 (131.59-188.52)	149.70 (122.77-177.05)	128.98 (104.60-155.28)	262.47 (229.61-297.59)	238.56 (213.01-270.45)	173.69 (153.29-197.61)	3.65 (2.22-6.03)	3.46 (2.29-5.04)	2.50 (1.72-3.69)	8.08 (7.81-8.39)	8.07 (7.78-8.36)	6.48 (6.27-6.69)
Madagascar	284.61 (236.10-335.01)	273.08 (219.08-330.52)	246.93 (155.60-355.57)	174.78 (139.99-201.60)	132.13 (100.84-155.14)	99.83 (65.91-138.48)	4.44 (2.35-9.54)	3.55 (1.87-7.19)	2.86 (1.38-5.51)	13.09 (11.25-14.87)	12.26 (10.87-13.76)	8.32 (7.07-9.67)
Malawi	225.50 (177.21-282.10)	245.86 (174.73-330.05)	167.83 (112.03-251.10)	274.45 (222.56-332.12)	236.13 (176.98-305.91)	124.15 (86.41-177.83)	4.99 (2.43-11.16)	4.64 (2.26-10.50)	3.10 (1.44-7.12)	9.05 (7.78-10.57)	9.04 (8.18-9.99)	6.74 (5.81-7.77)
Mozambique	201.08 (160.84-244.53)	203.55 (155.32-255.18)	185.79 (109.00-290.14)	212.07 (172.93-260.33)	164.92 (131.86-201.21)	109.00 (65.52-165.70)	5.61 (3.09-12.41)	4.39 (3.07-6.75)	3.23 (1.64-6.32)	9.25 (8.00-10.65)	10.05 (8.97-11.18)	7.95 (6.89-9.15)
Rwanda	348.72 (280.01-424.00)	326.48 (255.84-399.28)	191.69 (129.26-287.71)	161.84 (120.44-220.77)	167.52 (127.09-225.60)	68.63 (46.72-101.43)	6.69 (4.04-14.27)	6.59 (4.02-12.79)	3.24 (1.58-7.01)	8.25 (7.00-9.60)	7.34 (6.41-8.39)	6.29 (5.57-7.08)
Somalia	310.71 (150.24-529.15)	293.29 (132.55-494.21)	264.46 (120.80-469.57)	340.96 (168.25-577.93)	371.88 (170.27-644.39)	317.97 (141.93-594.03)	9.08 (2.65-27.32)	9.19 (2.57-28.39)	7.13 (2.10-19.31)	8.00 (6.78-9.30)	7.26 (6.11-8.56)	5.96 (5.00-7.13)
South Sudan	276.54 (136.69-497.78)	241.00 (112.02-460.83)	232.55 (108.05-480.15)	288.36 (159.93-488.97)	266.23 (132.45-499.07)	248.77 (121.90-508.39)	6.27 (1.98-15.92)	5.09 (1.61-14.06)	5.06 (1.50-13.70)	8.11 (6.84-9.51)	7.45 (6.28-8.74)	6.09 (5.08-7.23)
Tanzania	233.19 (187.95-280.06)	210.30 (159.42-269.94)	176.28 (106.05-280.11)	223.31 (188.04-259.55)	177.55 (140.91-216.81)	109.60 (68.82-170.88)	3.88 (2.17-9.71)	2.57 (1.78-3.60)	2.53 (1.23-5.31)	8.02 (6.75-9.43)	8.00 (6.96-9.13)	7.10 (6.24-8.02)

Appendix Table 1b. Unscaled values for each SDG health-related indicator by country for 1990, 2000, and 2015

Location	Indicator 3.9.1: Age-standardised death rate attributable to household air pollution and ambient air pollution (per 100,000 population)			Indicator 3.9.2: Age-standardised death rate attributable to unsafe water, sanitation, and hygiene (WaSH) (per 100,000 population)			Indicator 3.9.3: Age-standardised death rate due to unintentional poisonings (per 100,000 population)			Indicator 3.a.1: Age-standardised prevalence of daily smoking among populations aged 10 and older		
	1990	2000	2015	1990	2000	2015	1990	2000	2015	1990	2000	2015
Uganda	270.31 (198.70-341.67)	276.10 (209.19-344.90)	217.05 (129.64-316.70)	164.75 (121.15-226.40)	152.17 (116.35-210.12)	92.20 (56.96-138.40)	5.32 (2.96-11.09)	4.92 (2.79-8.60)	3.50 (1.68-7.17)	7.60 (6.40-8.96)	7.58 (6.56-8.63)	4.88 (4.29-5.51)
Zambia	239.60 (187.87-292.58)	292.04 (236.48-356.86)	243.05 (162.97-338.36)	243.67 (197.45-295.57)	287.25 (238.46-344.46)	166.41 (114.57-230.78)	3.97 (1.94-10.34)	4.33 (2.16-8.94)	3.34 (1.59-5.62)	8.74 (7.49-9.98)	8.76 (7.83-9.73)	7.40 (6.24-8.62)
Angola	376.74 (165.71-631.06)	329.25 (141.69-587.42)	249.32 (113.32-519.68)	193.94 (100.86-307.89)	143.65 (74.42-246.33)	83.21 (43.29-174.60)	5.89 (1.93-14.68)	4.54 (1.44-11.66)	3.00 (1.09-7.68)	5.71 (5.03-6.47)	5.72 (5.01-6.59)	6.35 (5.59-7.22)
Central African Republic	414.89 (331.37-502.51)	410.15 (252.03-567.17)	391.42 (224.30-577.20)	204.15 (162.52-244.12)	179.65 (112.94-246.42)	160.57 (96.36-236.15)	5.45 (3.06-12.19)	5.13 (2.40-10.09)	4.85 (2.05-9.45)	4.96 (4.33-5.63)	4.84 (4.22-5.53)	5.26 (4.58-5.99)
Congo	389.13 (302.82-476.61)	363.50 (287.82-446.08)	252.67 (160.04-371.35)	123.97 (88.46-166.49)	107.49 (79.55-140.86)	63.01 (41.72-93.34)	3.31 (1.96-6.11)	3.13 (1.83-6.28)	1.98 (1.05-3.80)	3.92 (3.42-4.48)	3.90 (3.43-4.43)	4.96 (4.33-5.67)
Democratic Republic of the Congo	276.13 (198.73-375.89)	273.25 (218.93-346.96)	262.00 (181.07-366.10)	125.03 (92.72-162.78)	122.74 (97.95-151.80)	88.13 (61.26-120.72)	3.65 (1.80-8.46)	3.45 (1.88-7.38)	2.66 (1.31-5.66)	6.13 (5.40-6.91)	5.92 (5.19-6.70)	6.05 (5.40-6.70)
Equatorial Guinea	418.60 (177.80-678.32)	319.46 (147.28-606.44)	214.54 (114.38-482.03)	171.39 (85.71-282.10)	91.61 (47.14-166.17)	42.76 (23.75-93.26)	5.93 (1.93-13.37)	3.95 (1.25-10.18)	2.00 (0.72-5.22)	4.18 (3.63-4.76)	4.13 (3.58-4.77)	4.17 (3.63-4.84)
Gabon	261.88 (201.65-323.86)	211.78 (162.92-266.23)	114.83 (73.50-175.98)	94.77 (66.07-121.09)	72.85 (54.06-93.31)	42.42 (28.05-64.98)	2.63 (1.51-4.95)	2.18 (1.30-3.67)	1.58 (0.84-2.96)	6.20 (5.36-7.18)	6.61 (5.79-7.55)	7.06 (6.29-7.87)

Appendix Table 1b. Unscaled values for each SDG health-related indicator by country for 1990, 2000, and 2015

Location	Indicator 5.2.1: Prevalence of women aged 15 years and older who experienced intimate partner violence			Indicator 6.1.1: Risk-weighted prevalence of populations using unsafe or unimproved water sources, as measured by the summary exposure value (SEV) for unsafe water			Indicator 6.2.2a: Risk-weighted prevalence of populations using unsafe or unimproved sanitation, as measured by the summary exposure value (SEV) for unsafe sanitation			Indicator 6.2.1b: Risk-weighted prevalence of populations with unsafe hygiene (no handwashing with soap), as measured by the summary exposure value (SEV) for unsafe hygiene		
	1990	2000	2015	1990	2000	2015	1990	2000	2015	1990	2000	2015
Canada	17.24 (14.15-20.76)	16.87 (14.71-19.28)	16.44 (13.49-19.74)	0.00 (0.00-0.00)	0.00 (0.00-0.00)	0.00 (0.00-0.00)	0.00 (0.00-0.00)	0.00 (0.00-0.00)	0.00 (0.00-0.00)	46.25 (41.16-51.28)	41.98 (36.50-47.38)	36.04 (30.05-41.90)
United States	17.33 (14.29-20.66)	17.66 (14.79-21.00)	17.98 (14.69-21.82)	0.00 (0.00-0.00)	0.00 (0.00-0.00)	0.00 (0.00-0.00)	0.00 (0.00-0.00)	0.00 (0.00-0.00)	0.00 (0.00-0.00)	46.34 (41.27-51.36)	45.00 (39.82-50.19)	39.40 (33.62-45.05)
Australia	14.66 (12.01-17.69)	14.05 (12.11-16.28)	14.75 (11.98-17.76)	0.00 (0.00-0.00)	0.00 (0.00-0.00)	0.00 (0.00-0.00)	0.00 (0.00-0.00)	0.00 (0.00-0.00)	0.00 (0.00-0.00)	53.67 (49.43-57.73)	46.60 (41.55-51.61)	38.17 (32.30-43.88)
New Zealand	19.52 (15.81-23.57)	20.86 (16.83-24.73)	19.52 (15.81-23.57)	0.00 (0.00-0.00)	0.00 (0.00-0.00)	0.00 (0.00-0.00)	0.00 (0.00-0.00)	0.00 (0.00-0.00)	0.00 (0.00-0.00)	53.66 (49.43-57.73)	46.87 (41.85-51.83)	38.41 (32.55-44.11)
Brunei	19.36 (15.79-23.29)	19.36 (15.79-23.29)	19.36 (15.79-23.29)	0.00 (0.00-0.00)	0.00 (0.00-0.00)	0.00 (0.00-0.00)	0.00 (0.00-0.00)	0.00 (0.00-0.00)	0.00 (0.00-0.00)	58.37 (54.60-62.18)	50.75 (46.20-55.23)	41.22 (35.65-46.66)
Japan	18.89 (15.36-22.40)	18.95 (15.50-22.40)	18.89 (15.36-22.40)	0.00 (0.00-0.00)	0.00 (0.00-0.00)	0.00 (0.00-0.00)	0.00 (0.00-0.00)	0.00 (0.00-0.00)	0.00 (0.00-0.00)	52.87 (48.58-57.05)	45.88 (40.77-50.93)	37.88 (32.01-43.63)
Singapore	19.36 (15.79-23.29)	19.36 (15.79-23.29)	19.36 (15.79-23.29)	0.00 (0.00-0.00)	0.00 (0.00-0.00)	0.00 (0.00-0.00)	0.00 (0.00-0.00)	0.00 (0.00-0.00)	0.00 (0.00-0.00)	67.96 (63.93-71.60)	57.18 (53.31-61.05)	43.79 (38.46-49.11)
South Korea	19.36 (15.79-23.29)	19.36 (15.79-23.29)	19.36 (15.79-23.29)	0.00 (0.00-0.00)	0.00 (0.00-0.00)	0.00 (0.00-0.00)	0.00 (0.00-0.00)	0.00 (0.00-0.00)	0.00 (0.00-0.00)	60.77 (57.02-64.41)	52.10 (47.68-56.34)	41.34 (35.79-46.77)
Andorra	12.16 (9.70-14.99)	12.16 (9.70-14.99)	12.16 (9.70-14.99)	0.00 (0.00-0.00)	0.00 (0.00-0.00)	0.00 (0.00-0.00)	0.00 (0.00-0.00)	0.00 (0.00-0.00)	0.00 (0.00-0.00)	55.35 (51.29-59.34)	48.14 (43.30-52.93)	39.22 (33.42-44.89)
Austria	7.89 (6.41-9.51)	7.89 (6.41-9.51)	7.84 (6.51-9.36)	0.00 (0.00-0.00)	0.00 (0.00-0.00)	0.00 (0.00-0.00)	0.00 (0.00-0.00)	0.00 (0.00-0.00)	0.00 (0.00-0.00)	63.00 (59.23-66.59)	57.39 (53.54-61.25)	47.47 (42.57-52.33)

Appendix Table 1b. Unscaled values for each SDG health-related indicator by country for 1990, 2000, and 2015

Location	Indicator 5.2.1: Prevalence of women aged 15 years and older who experienced intimate partner violence			Indicator 6.1.1: Risk-weighted prevalence of populations using unsafe or unimproved water sources, as measured by the summary exposure value (SEV) for unsafe water			Indicator 6.2.2a: Risk-weighted prevalence of populations using unsafe or unimproved sanitation, as measured by the summary exposure value (SEV) for unsafe sanitation			Indicator 6.2.1b: Risk-weighted prevalence of populations with unsafe hygiene (no handwashing with soap), as measured by the summary exposure value (SEV) for unsafe hygiene		
	1990	2000	2015	1990	2000	2015	1990	2000	2015	1990	2000	2015
Belgium	14.57 (11.80-17.57)	14.57 (11.80-17.57)	14.46 (11.98-17.13)	0.00 (0.00-0.00)	0.00 (0.00-0.00)	0.00 (0.00-0.00)	0.00 (0.00-0.00)	0.00 (0.00-0.00)	0.00 (0.00-0.00)	59.92 (56.17-63.59)	53.23 (48.97-57.36)	42.93 (37.52-48.29)
Cyprus	8.27 (6.70-9.94)	8.27 (6.70-9.94)	8.18 (6.80-9.75)	0.00 (0.00-0.00)	0.00 (0.00-0.00)	0.00 (0.00-0.00)	0.00 (0.00-0.00)	0.00 (0.00-0.00)	0.00 (0.00-0.00)	58.44 (54.68-62.25)	50.70 (46.15-55.19)	41.25 (35.69-46.69)
Denmark	19.97 (16.27-24.13)	19.47 (16.42-22.96)	19.94 (16.77-23.42)	0.00 (0.00-0.00)	0.00 (0.00-0.00)	0.00 (0.00-0.00)	0.00 (0.00-0.00)	0.00 (0.00-0.00)	0.00 (0.00-0.00)	55.28 (51.21-59.28)	48.04 (43.19-52.86)	40.23 (34.53-45.78)
Finland	17.64 (14.35-21.16)	17.81 (15.14-20.73)	17.90 (14.86-20.78)	0.00 (0.00-0.00)	0.00 (0.00-0.00)	0.00 (0.00-0.00)	0.00 (0.00-0.00)	0.00 (0.00-0.00)	0.00 (0.00-0.00)	56.15 (52.18-60.11)	49.01 (44.32-53.72)	40.16 (34.46-45.73)
France	9.75 (7.94-11.71)	9.59 (8.25-11.11)	10.27 (8.33-12.35)	0.00 (0.00-0.00)	0.00 (0.00-0.00)	0.00 (0.00-0.00)	0.00 (0.00-0.00)	0.00 (0.00-0.00)	0.00 (0.00-0.00)	65.25 (61.51-68.82)	58.97 (55.19-62.72)	47.81 (42.92-52.65)
Germany	18.19 (14.90-22.10)	19.07 (15.86-22.60)	18.21 (15.27-21.29)	0.00 (0.00-0.00)	0.00 (0.00-0.00)	0.00 (0.00-0.00)	0.00 (0.00-0.00)	0.00 (0.00-0.00)	0.00 (0.00-0.00)	61.84 (58.14-65.45)	53.55 (49.32-57.64)	43.16 (37.78-48.52)
Greece	12.33 (10.05-14.90)	12.33 (10.05-14.90)	12.20 (10.13-14.45)	0.00 (0.00-0.00)	0.00 (0.00-0.00)	0.00 (0.00-0.00)	0.00 (0.00-0.00)	0.00 (0.00-0.00)	0.00 (0.00-0.00)	64.50 (60.72-68.08)	56.30 (52.34-60.25)	45.51 (40.36-50.64)
Iceland	4.67 (3.81-5.69)	4.63 (3.79-5.52)	4.67 (3.81-5.69)	0.00 (0.00-0.00)	0.00 (0.00-0.00)	0.00 (0.00-0.00)	0.00 (0.00-0.00)	0.00 (0.00-0.00)	0.00 (0.00-0.00)	59.58 (55.83-63.30)	51.82 (47.39-56.11)	42.34 (36.89-47.73)
Ireland	12.67 (10.75-14.98)	13.28 (11.19-15.84)	12.47 (10.27-15.14)	0.00 (0.00-0.00)	0.00 (0.00-0.00)	0.00 (0.00-0.00)	0.00 (0.00-0.00)	0.00 (0.00-0.00)	0.00 (0.00-0.00)	59.14 (55.36-62.88)	51.45 (47.00-55.82)	42.15 (36.68-47.54)
Israel	21.88 (17.98-26.36)	22.26 (18.55-26.11)	21.88 (17.98-26.36)	0.00 (0.00-0.00)	0.00 (0.00-0.00)	0.00 (0.00-0.00)	0.00 (0.00-0.00)	0.00 (0.00-0.00)	0.00 (0.00-0.00)	51.22 (46.73-55.63)	45.94 (40.83-50.98)	37.95 (32.08-43.69)

Appendix Table 1b. Unscaled values for each SDG health-related indicator by country for 1990, 2000, and 2015												
	**Indicator 5.2.1: Prevalence of women aged 15 years and older who experienced intimate partner violence**			**Indicator 6.1.1: Risk-weighted prevalence of populations using unsafe or unimproved water sources, as measured by the summary exposure value (SEV) for unsafe water**			**Indicator 6.2.2a: Risk-weighted prevalence of populations using unsafe or unimproved sanitation, as measured by the summary exposure value (SEV) for unsafe sanitation**			**Indicator 6.2.1b: Risk-weighted prevalence of populations with unsafe hygiene (no handwashing with soap), as measured by the summary exposure value (SEV) for unsafe hygiene**		
**Location**	**1990**	**2000**	**2015**	**1990**	**2000**	**2015**	**1990**	**2000**	**2015**	**1990**	**2000**	**2015**
Italy	19.22 (15.64-23.34)	19.22 (15.64-23.34)	20.00 (16.20-24.06)	0.00 (0.00-0.00)	0.00 (0.00-0.00)	0.00 (0.00-0.00)	0.00 (0.00-0.00)	0.00 (0.00-0.00)	0.00 (0.00-0.00)	60.39 (56.63-64.05)	54.80 (50.66-58.83)	45.06 (39.87-50.24)
Luxembourg	14.64 (11.94-17.58)	14.64 (11.94-17.58)	14.93 (12.50-17.36)	0.00 (0.00-0.00)	0.00 (0.00-0.00)	0.00 (0.00-0.00)	0.00 (0.00-0.00)	0.00 (0.00-0.00)	0.00 (0.00-0.00)	63.85 (60.03-67.40)	55.99 (52.00-59.99)	45.28 (40.11-50.44)
Malta	9.30 (7.59-11.26)	9.30 (7.59-11.26)	9.22 (7.58-10.93)	0.00 (0.00-0.00)	0.00 (0.00-0.00)	0.00 (0.00-0.00)	0.00 (0.00-0.00)	0.00 (0.00-0.00)	0.00 (0.00-0.00)	71.92 (68.08-75.52)	66.65 (62.79-70.28)	55.30 (51.23-59.30)
Netherlands	11.50 (9.53-13.73)	11.69 (9.54-14.09)	12.28 (9.97-14.61)	0.00 (0.00-0.00)	0.00 (0.00-0.00)	0.00 (0.00-0.00)	0.00 (0.00-0.00)	0.00 (0.00-0.00)	0.00 (0.00-0.00)	60.65 (56.90-64.30)	53.68 (49.44-57.74)	43.59 (38.24-48.93)
Norway	12.56 (10.28-15.15)	14.06 (11.50-15.84)	12.36 (10.05-14.91)	0.00 (0.00-0.00)	0.00 (0.00-0.00)	0.00 (0.00-0.00)	0.00 (0.00-0.00)	0.00 (0.00-0.00)	0.00 (0.00-0.00)	53.69 (49.46-57.75)	45.68 (40.55-50.77)	37.62 (31.73-43.43)
Portugal	12.61 (10.32-15.17)	12.61 (10.32-15.17)	12.79 (10.61-14.86)	0.00 (0.00-0.00)	0.00 (0.00-0.00)	0.00 (0.00-0.00)	0.00 (0.00-0.00)	0.00 (0.00-0.00)	0.00 (0.00-0.00)	77.35 (73.38-80.99)	69.87 (65.92-73.55)	56.74 (52.83-60.66)
Spain	8.35 (6.84-10.05)	8.53 (7.12-10.13)	8.66 (7.05-10.27)	0.00 (0.00-0.00)	0.00 (0.00-0.00)	0.00 (0.00-0.00)	0.00 (0.00-0.00)	0.00 (0.00-0.00)	0.00 (0.00-0.00)	71.04 (67.15-74.66)	63.14 (59.36-66.72)	50.43 (45.84-54.96)
Sweden	13.11 (10.66-15.72)	12.74 (10.41-15.43)	13.65 (11.12-16.47)	0.00 (0.00-0.00)	0.00 (0.00-0.00)	0.00 (0.00-0.00)	0.00 (0.00-0.00)	0.00 (0.00-0.00)	0.00 (0.00-0.00)	54.70 (50.55-58.74)	47.60 (42.71-52.44)	39.08 (33.27-44.76)
Switzerland	6.31 (5.14-7.64)	6.27 (5.41-7.17)	6.31 (5.14-7.64)	0.00 (0.00-0.00)	0.00 (0.00-0.00)	0.00 (0.00-0.00)	0.00 (0.00-0.00)	0.00 (0.00-0.00)	0.00 (0.00-0.00)	61.72 (58.01-65.34)	53.96 (49.73-58.00)	44.54 (39.29-49.79)
United Kingdom	15.90 (13.01-19.03)	17.33 (14.09-20.93)	13.70 (11.29-16.36)	0.00 (0.00-0.00)	0.00 (0.00-0.00)	0.00 (0.00-0.00)	0.00 (0.00-0.00)	0.00 (0.00-0.00)	0.00 (0.00-0.00)	56.65 (52.75-60.57)	49.29 (44.70-53.93)	40.31 (34.72-45.80)

Appendix Table 1b. Unscaled values for each SDG health-related indicator by country for 1990, 2000, and 2015

Location	Indicator 5.2.1: Prevalence of women aged 15 years and older who experienced intimate partner violence			Indicator 6.1.1: Risk-weighted prevalence of populations using unsafe or unimproved water sources, as measured by the summary exposure value (SEV) for unsafe water			Indicator 6.2.2a: Risk-weighted prevalence of populations using unsafe or unimproved sanitation, as measured by the summary exposure value (SEV) for unsafe sanitation			Indicator 6.2.1b: Risk-weighted prevalence of populations with unsafe hygiene (no handwashing with soap), as measured by the summary exposure value (SEV) for unsafe hygiene		
	1990	2000	2015	1990	2000	2015	1990	2000	2015	1990	2000	2015
Argentina	12.06 (9.85-14.58)	12.12 (9.96-14.71)	12.06 (9.85-14.58)	42.44 (31.34-54.58)	20.98 (13.65-30.17)	11.48 (5.23-21.62)	30.88 (19.93-43.71)	15.39 (11.66-19.96)	7.45 (3.76-13.22)	67.04 (62.64-71.49)	60.80 (56.59-65.10)	50.22 (44.93-55.44)
Chile	17.76 (14.53-21.31)	17.93 (14.68-21.49)	17.76 (14.53-21.31)	17.15 (12.23-23.33)	11.30 (7.45-15.94)	6.24 (3.30-10.69)	28.48 (22.29-35.12)	9.85 (7.37-13.51)	4.69 (2.62-7.66)	66.55 (62.20-71.06)	59.13 (54.76-63.65)	47.56 (42.01-52.83)
Uruguay	11.90 (9.66-14.34)	11.87 (9.92-14.03)	11.90 (9.66-14.34)	9.92 (4.43-18.85)	6.43 (3.49-10.34)	4.67 (2.06-9.04)	5.74 (2.67-10.62)	4.18 (2.55-6.73)	3.24 (1.68-5.71)	71.87 (67.67-75.95)	67.23 (63.10-71.35)	57.08 (52.63-61.73)
Belarus	15.69 (12.92-18.53)	15.69 (12.92-18.53)	15.69 (12.92-18.53)	33.30 (19.68-49.91)	25.81 (22.26-29.67)	14.94 (10.59-20.64)	33.16 (20.18-47.16)	27.87 (24.00-32.48)	15.79 (11.54-20.86)	75.77 (69.40-82.80)	71.20 (62.75-80.48)	56.37 (45.87-68.62)
Estonia	11.81 (9.66-14.03)	11.81 (9.66-14.03)	11.87 (10.00-13.74)	19.96 (9.37-35.45)	14.74 (8.91-23.31)	9.72 (4.21-19.92)	18.92 (9.37-32.21)	12.96 (7.61-20.46)	7.47 (3.28-13.86)	73.72 (66.89-81.37)	68.90 (61.09-77.92)	57.42 (48.04-68.36)
Latvia	14.97 (12.27-17.79)	14.97 (12.27-17.79)	15.50 (12.79-18.29)	20.00 (9.57-34.74)	16.75 (11.25-24.27)	10.94 (4.81-20.39)	25.21 (13.68-40.32)	21.11 (14.60-29.87)	11.77 (5.63-21.21)	71.87 (64.79-79.58)	68.79 (59.85-78.28)	57.70 (48.06-68.75)
Lithuania	12.49 (10.29-14.82)	12.49 (10.29-14.82)	12.88 (10.62-15.09)	23.37 (10.91-40.44)	20.84 (9.77-38.50)	13.82 (6.03-25.87)	26.88 (14.07-43.69)	21.89 (10.98-38.04)	13.34 (5.73-24.12)	71.62 (64.55-79.36)	68.36 (59.84-77.67)	57.89 (48.53-68.57)
Moldova	19.11 (15.88-22.69)	19.26 (16.46-22.04)	19.11 (15.88-22.69)	46.88 (31.04-63.98)	53.95 (47.40-61.61)	43.12 (26.78-59.65)	50.93 (34.77-70.44)	58.02 (49.68-65.81)	43.81 (29.50-60.12)	78.25 (71.26-85.75)	77.87 (69.28-86.24)	68.73 (58.11-79.87)
Russia	20.75 (17.02-24.44)	20.88 (17.28-24.52)	20.75 (17.02-24.44)	21.76 (10.34-37.73)	19.82 (13.69-27.49)	14.23 (6.40-26.12)	33.35 (19.32-49.97)	32.10 (24.68-40.27)	20.37 (10.70-33.63)	68.15 (60.88-76.67)	65.89 (57.13-75.83)	55.81 (45.74-67.19)
Ukraine	16.93 (13.85-20.09)	17.64 (14.82-20.56)	16.93 (13.85-20.09)	22.89 (11.44-38.78)	21.89 (15.65-29.25)	23.32 (16.61-30.49)	30.12 (17.37-44.44)	28.81 (22.01-36.40)	21.49 (16.16-27.66)	73.34 (66.18-81.34)	70.12 (60.47-80.30)	58.53 (46.82-71.69)

Appendix Table 1b. Unscaled values for each SDG health-related indicator by country for 1990, 2000, and 2015

Location	Indicator 5.2.1: Prevalence of women aged 15 years and older who experienced intimate partner violence			Indicator 6.1.1: Risk-weighted prevalence of populations using unsafe or unimproved water sources, as measured by the summary exposure value (SEV) for unsafe water			Indicator 6.2.2a: Risk-weighted prevalence of populations using unsafe or unimproved sanitation, as measured by the summary exposure value (SEV) for unsafe sanitation			Indicator 6.2.1b: Risk-weighted prevalence of populations with unsafe hygiene (no handwashing with soap), as measured by the summary exposure value (SEV) for unsafe hygiene		
	1990	2000	2015	1990	2000	2015	1990	2000	2015	1990	2000	2015
Albania	10.45 (8.60-12.51)	10.07 (8.66-11.55)	10.45 (8.60-12.51)	43.99 (27.53-66.26)	40.81 (28.37-60.04)	31.92 (18.31-51.66)	19.23 (10.08-33.74)	12.88 (9.53-16.80)	3.09 (1.68-5.22)	87.40 (81.48-92.59)	84.71 (77.79-91.07)	75.80 (66.42-85.05)
Bosnia and Herzegovina	12.13 (9.53-14.73)	12.13 (9.53-14.73)	12.13 (9.53-14.73)	39.35 (23.69-61.00)	28.91 (17.26-49.74)	23.06 (12.41-42.71)	30.25 (15.88-49.18)	12.30 (8.48-17.55)	8.64 (4.76-14.75)	91.69 (87.08-95.50)	86.15 (79.75-91.89)	77.20 (68.04-86.06)
Bulgaria	11.39 (9.33-13.49)	11.39 (9.33-13.49)	11.80 (9.80-14.00)	25.97 (13.66-44.83)	23.17 (10.93-43.22)	20.07 (9.12-39.64)	14.71 (6.71-27.97)	8.22 (3.51-15.60)	4.81 (2.13-9.71)	79.52 (71.85-87.26)	77.82 (68.79-86.44)	69.30 (58.63-80.48)
Croatia	8.92 (7.12-10.64)	8.92 (7.12-10.64)	8.65 (7.11-10.30)	24.35 (12.11-44.99)	21.94 (11.76-40.76)	18.87 (8.81-37.91)	8.11 (3.93-14.55)	5.09 (3.06-7.70)	3.18 (1.46-6.26)	84.13 (78.39-89.69)	81.12 (74.20-88.05)	72.57 (63.59-82.23)
Czech Republic	22.84 (19.41-27.17)	24.53 (21.36-27.97)	22.85 (18.73-27.26)	16.82 (7.21-35.42)	15.01 (6.33-33.18)	14.41 (5.95-32.02)	1.76 (0.73-3.63)	0.89 (0.41-1.67)	0.67 (0.26-1.38)	73.78 (65.03-82.82)	69.96 (60.10-80.43)	62.80 (51.44-75.11)
Hungary	8.59 (7.00-10.23)	8.44 (7.25-9.68)	8.93 (7.31-10.53)	24.50 (14.94-41.56)	17.00 (8.28-35.37)	15.21 (6.49-33.11)	23.56 (17.96-30.27)	12.63 (9.09-17.28)	6.68 (3.10-12.31)	78.09 (70.39-85.85)	75.05 (66.37-84.02)	67.02 (56.69-78.10)
Macedonia	12.13 (9.53-14.73)	12.13 (9.53-14.73)	12.13 (9.53-14.73)	23.58 (11.84-43.87)	21.73 (11.18-40.99)	21.17 (10.28-42.11)	9.44 (3.92-19.10)	7.28 (3.94-12.96)	6.69 (4.17-10.06)	84.27 (78.11-90.37)	81.59 (74.05-88.62)	73.91 (64.00-83.61)
Montenegro	12.13 (9.53-14.73)	12.13 (9.53-14.73)	12.13 (9.53-14.73)	31.28 (17.90-50.94)	27.07 (14.74-45.13)	21.26 (10.71-40.64)	13.33 (3.66-32.66)	11.12 (4.99-20.84)	5.32 (1.49-13.17)	81.88 (75.12-88.76)	79.10 (70.66-87.37)	70.35 (59.56-81.27)
Poland	10.03 (8.26-11.95)	10.02 (8.95-11.14)	9.55 (7.95-11.46)	25.66 (13.51-44.79)	20.23 (9.69-38.40)	17.48 (7.83-35.98)	14.54 (6.83-26.85)	5.73 (2.68-10.80)	3.13 (1.37-6.29)	78.03 (69.36-86.58)	73.23 (63.47-83.22)	64.27 (53.05-76.21)
Romania	25.00 (20.49-29.85)	24.88 (21.55-28.23)	24.01 (19.63-28.54)	42.56 (25.63-62.43)	43.63 (32.27-58.25)	30.09 (16.52-49.24)	37.91 (30.05-46.56)	38.06 (30.67-45.68)	20.27 (10.48-32.03)	80.40 (72.78-87.82)	78.86 (70.55-87.15)	70.15 (59.77-80.95)

Appendix Table 1b. Unscaled values for each SDG health-related indicator by country for 1990, 2000, and 2015												
	**Indicator 5.2.1: Prevalence of women aged 15 years and older who experienced intimate partner violence**			**Indicator 6.1.1: Risk-weighted prevalence of populations using unsafe or unimproved water sources, as measured by the summary exposure value (SEV) for unsafe water**			**Indicator 6.2.2a: Risk-weighted prevalence of populations using unsafe or unimproved sanitation, as measured by the summary exposure value (SEV) for unsafe sanitation**			**Indicator 6.2.1b: Risk-weighted prevalence of populations with unsafe hygiene (no handwashing with soap), as measured by the summary exposure value (SEV) for unsafe hygiene**		
**Location**	**1990**	**2000**	**2015**	**1990**	**2000**	**2015**	**1990**	**2000**	**2015**	**1990**	**2000**	**2015**
Serbia	12.84 (10.52-15.27)	12.99 (10.82-15.04)	12.84 (10.52-15.27)	37.09 (20.93-59.76)	32.40 (17.81-54.16)	26.11 (14.51-47.72)	21.53 (8.33-44.84)	21.95 (12.84-33.85)	14.66 (9.41-21.89)	83.88 (77.55-90.06)	81.13 (73.47-88.52)	72.48 (62.60-82.72)
Slovakia	12.28 (10.13-14.66)	12.28 (10.13-14.66)	12.46 (10.40-14.67)	22.93 (12.03-41.82)	18.68 (9.11-36.61)	15.94 (7.16-33.73)	6.56 (3.13-12.31)	3.41 (1.74-5.95)	1.95 (0.91-3.90)	79.82 (72.68-87.06)	75.89 (67.28-84.73)	66.27 (56.15-77.68)
Slovenia	7.90 (6.43-9.46)	7.90 (6.43-9.46)	8.17 (6.79-9.50)	15.71 (7.34-33.44)	13.71 (5.72-30.75)	13.19 (5.35-32.34)	7.73 (2.68-18.77)	5.32 (3.70-7.37)	3.44 (1.10-8.36)	77.80 (70.53-85.57)	73.16 (64.63-82.39)	64.92 (54.38-76.33)
Armenia	13.57 (10.68-16.43)	13.57 (10.68-16.43)	13.57 (10.68-16.43)	76.24 (59.85-90.47)	77.30 (63.70-90.74)	74.42 (61.11-90.20)	34.18 (20.84-49.23)	28.78 (24.00-34.20)	19.69 (11.47-31.73)	75.96 (70.17-82.51)	72.62 (65.15-80.54)	59.31 (50.84-69.20)
Azerbaijan	14.34 (11.72-17.10)	15.54 (12.94-18.17)	14.34 (11.72-17.10)	51.37 (36.75-67.31)	48.24 (38.66-59.04)	35.96 (24.57-50.76)	53.41 (36.20-70.99)	56.41 (49.08-64.29)	27.14 (16.56-40.01)	73.14 (67.75-79.62)	70.61 (63.35-78.64)	56.86 (49.05-65.76)
Georgia	6.84 (5.57-8.16)	6.98 (5.94-8.04)	6.84 (5.57-8.16)	77.94 (62.54-94.67)	82.81 (71.17-96.86)	76.80 (62.35-92.61)	36.39 (21.00-53.90)	44.25 (33.87-55.61)	29.47 (17.21-44.43)	67.77 (61.37-75.16)	66.71 (58.16-76.43)	55.20 (45.87-65.92)
Kazakhstan	11.03 (9.03-13.15)	11.35 (9.41-13.36)	10.64 (8.87-12.38)	45.85 (31.41-62.60)	39.54 (27.10-53.33)	39.39 (27.86-53.89)	45.78 (34.54-58.07)	42.62 (35.46-51.47)	29.62 (20.28-40.51)	69.34 (63.77-76.05)	64.98 (57.97-73.21)	52.97 (45.16-61.91)
Kyrgyzstan	24.77 (20.32-29.40)	24.77 (20.32-29.40)	25.39 (21.68-29.30)	68.39 (52.61-82.45)	68.26 (57.66-79.59)	58.86 (47.37-70.78)	59.59 (47.48-70.95)	53.79 (47.27-61.33)	61.63 (52.66-71.49)	70.18 (63.34-78.06)	68.90 (60.28-78.35)	61.53 (51.39-72.83)
Mongolia	13.57 (10.68-16.43)	13.57 (10.68-16.43)	13.57 (10.68-16.43)	80.65 (68.08-92.50)	76.78 (65.50-88.16)	68.79 (54.89-82.82)	83.20 (75.24-89.46)	67.92 (60.72-74.78)	56.44 (45.53-67.70)	80.08 (74.93-85.59)	75.39 (69.35-82.35)	62.95 (55.80-71.42)
Tajikistan	17.24 (14.15-20.51)	17.24 (14.15-20.51)	17.59 (15.27-19.74)	49.26 (33.29-65.54)	48.76 (38.39-59.81)	37.07 (28.22-47.72)	61.26 (47.62-73.42)	64.54 (56.10-73.51)	61.12 (51.32-71.11)	78.29 (73.11-84.27)	78.56 (71.93-85.67)	70.27 (62.25-79.19)

Location	Appendix Table 1b. Unscaled values for each SDG health-related indicator by country for 1990, 2000, and 2015											
	Indicator 5.2.1: Prevalence of women aged 15 years and older who experienced intimate partner violence			Indicator 6.1.1: Risk-weighted prevalence of populations using unsafe or unimproved water sources, as measured by the summary exposure value (SEV) for unsafe water			Indicator 6.2.2a: Risk-weighted prevalence of populations using unsafe or unimproved sanitation, as measured by the summary exposure value (SEV) for unsafe sanitation			Indicator 6.2.1b: Risk-weighted prevalence of populations with unsafe hygiene (no handwashing with soap), as measured by the summary exposure value (SEV) for unsafe hygiene		
	1990	2000	2015	1990	2000	2015	1990	2000	2015	1990	2000	2015
Turkmenistan	13.57 (10.68-16.43)	13.57 (10.68-16.43)	13.57 (10.68-16.43)	62.25 (43.72-78.00)	59.33 (41.10-77.10)	51.73 (32.64-72.19)	46.56 (29.40-63.26)	36.26 (20.76-52.72)	18.81 (8.87-33.29)	71.25 (65.39-78.15)	67.08 (59.61-75.66)	54.45 (46.21-64.23)
Uzbekistan	13.57 (10.68-16.43)	13.57 (10.68-16.43)	13.57 (10.68-16.43)	26.73 (17.68-37.36)	24.44 (17.62-32.35)	18.70 (11.16-28.45)	62.22 (50.96-73.17)	62.55 (54.72-71.85)	45.71 (31.65-60.54)	75.73 (69.84-82.55)	72.22 (65.00-80.38)	62.05 (53.82-71.73)
Colombia	26.58 (22.03-31.42)	26.89 (23.44-30.51)	25.92 (22.85-29.20)	63.33 (50.11-77.46)	62.52 (48.36-77.53)	58.80 (44.00-74.05)	31.31 (26.95-36.99)	20.79 (18.08-23.46)	7.83 (5.06-11.34)	80.01 (75.69-84.15)	74.35 (69.75-79.00)	63.58 (58.23-69.31)
Costa Rica	20.69 (17.17-24.25)	21.36 (18.39-24.75)	20.69 (17.17-24.25)	72.56 (59.83-85.76)	71.28 (59.30-84.42)	68.28 (56.60-81.67)	26.17 (18.87-34.77)	6.47 (4.34-9.55)	3.62 (2.50-5.25)	77.59 (73.20-82.10)	72.97 (68.38-77.78)	63.00 (57.65-68.92)
El Salvador	15.24 (12.49-18.05)	16.16 (13.32-19.17)	15.24 (12.49-18.05)	76.13 (64.45-87.91)	68.76 (56.20-82.25)	63.21 (48.62-78.61)	55.37 (47.29-63.84)	45.40 (37.54-54.08)	29.74 (20.23-40.81)	85.12 (81.02-88.99)	80.64 (76.30-84.95)	71.67 (66.71-77.36)
Guatemala	16.78 (13.94-19.72)	18.15 (15.91-20.23)	16.78 (13.94-19.72)	78.60 (68.02-90.00)	75.24 (62.99-88.01)	70.01 (55.58-83.54)	60.90 (51.93-70.17)	46.46 (40.72-53.30)	32.35 (22.88-42.57)	88.66 (84.94-92.02)	86.04 (82.18-89.74)	79.98 (75.65-84.29)
Honduras	15.32 (12.64-18.15)	14.66 (13.14-16.28)	17.01 (14.43-19.69)	73.96 (63.52-84.26)	66.77 (57.43-77.30)	60.32 (50.30-72.82)	61.95 (51.36-72.70)	52.51 (44.45-60.50)	30.44 (23.27-38.81)	86.18 (82.13-89.93)	82.44 (78.10-86.73)	74.17 (69.28-79.50)
Mexico	22.00 (19.01-24.92)	22.69 (19.28-26.41)	20.91 (18.46-23.39)	61.32 (53.71-70.02)	58.31 (50.70-68.02)	55.70 (48.05-65.40)	26.34 (24.83-28.02)	16.01 (15.02-17.01)	9.95 (9.15-10.80)	78.55 (74.38-82.55)	72.76 (68.38-77.24)	62.23 (57.15-67.73)
Nicaragua	16.29 (13.44-19.19)	16.58 (14.48-18.44)	16.29 (13.44-19.19)	74.47 (62.20-86.56)	73.11 (61.65-84.61)	68.54 (55.58-82.46)	70.13 (62.85-77.98)	62.38 (56.37-69.43)	47.07 (36.13-58.98)	85.02 (80.82-88.92)	81.65 (77.14-86.07)	73.41 (68.48-79.12)
Panama	18.14 (15.01-21.17)	18.14 (15.01-21.17)	18.14 (15.01-21.17)	61.18 (47.29-75.64)	58.09 (42.38-74.08)	54.59 (38.40-70.69)	46.13 (39.50-53.54)	39.31 (33.35-45.85)	22.86 (15.26-31.84)	72.94 (68.31-77.90)	67.57 (62.55-73.27)	56.92 (51.27-63.26)

Location	Appendix Table 1b. Unscaled values for each SDG health-related indicator by country for 1990, 2000, and 2015											
	**Indicator 5.2.1: Prevalence of women aged 15 years and older who experienced intimate partner violence**			**Indicator 6.1.1: Risk-weighted prevalence of populations using unsafe or unimproved water sources, as measured by the summary exposure value (SEV) for unsafe water**			**Indicator 6.2.2a: Risk-weighted prevalence of populations using unsafe or unimproved sanitation, as measured by the summary exposure value (SEV) for unsafe sanitation**			**Indicator 6.2.1b: Risk-weighted prevalence of populations with unsafe hygiene (no handwashing with soap), as measured by the summary exposure value (SEV) for unsafe hygiene**		
	**1990**	**2000**	**2015**	**1990**	**2000**	**2015**	**1990**	**2000**	**2015**	**1990**	**2000**	**2015**
Venezuela	18.14 (15.01-21.17)	18.14 (15.01-21.17)	18.14 (15.01-21.17)	60.41 (46.17-76.01)	58.69 (43.57-74.23)	56.28 (41.03-72.86)	19.24 (15.21-23.91)	13.42 (10.35-16.82)	7.69 (4.97-11.26)	76.09 (71.81-80.35)	70.27 (65.61-75.04)	59.39 (53.91-65.37)
Bolivia	23.52 (19.23-27.80)	25.51 (22.31-28.38)	23.52 (19.23-27.80)	56.19 (49.54-63.66)	50.71 (45.94-55.94)	36.62 (31.48-42.72)	72.82 (69.06-76.68)	60.73 (56.92-64.72)	36.58 (27.10-47.04)	89.64 (85.22-93.60)	86.14 (80.68-91.32)	78.99 (71.77-86.37)
Ecuador	22.85 (18.80-27.24)	22.92 (21.04-24.87)	22.85 (18.80-27.24)	46.89 (41.49-52.60)	44.26 (39.70-49.44)	33.44 (28.34-39.35)	48.87 (43.31-54.37)	27.64 (23.93-32.01)	14.10 (9.48-19.62)	84.93 (79.65-90.12)	81.17 (75.07-87.57)	73.06 (64.98-81.55)
Peru	13.89 (11.24-16.34)	17.13 (14.81-19.52)	11.80 (10.85-12.84)	36.89 (32.42-41.97)	33.36 (29.36-38.19)	29.08 (24.07-34.83)	61.79 (54.76-69.54)	43.92 (40.31-47.93)	25.58 (19.28-33.13)	85.30 (80.05-90.46)	81.61 (75.47-87.97)	72.66 (64.69-81.13)
Antigua and Barbuda	16.80 (13.71-20.32)	16.80 (13.71-20.32)	16.80 (13.71-20.32)	60.30 (49.66-71.70)	56.76 (46.33-68.93)	53.63 (43.75-64.60)	26.04 (16.23-38.67)	19.39 (11.59-30.07)	13.60 (7.73-22.65)	76.44 (67.97-84.90)	70.98 (61.37-81.23)	64.65 (53.21-76.64)
The Bahamas	16.80 (13.71-20.32)	16.80 (13.71-20.32)	16.80 (13.71-20.32)	57.37 (47.94-68.25)	53.67 (44.56-64.63)	51.28 (41.61-62.33)	19.36 (12.63-28.15)	12.03 (7.62-18.62)	8.12 (4.98-12.36)	77.78 (70.70-85.36)	72.77 (64.28-81.86)	66.21 (55.67-77.39)
Barbados	16.80 (13.71-20.32)	16.80 (13.71-20.32)	16.80 (13.71-20.32)	55.67 (45.74-67.05)	53.33 (43.12-64.67)	51.64 (41.76-63.60)	26.40 (15.06-38.62)	19.87 (11.45-30.86)	14.09 (7.92-23.33)	81.07 (74.27-87.94)	76.18 (68.01-84.65)	69.03 (58.99-79.81)
Belize	9.67 (7.86-11.63)	10.12 (8.49-11.94)	9.67 (7.86-11.63)	87.13 (77.28-99.77)	85.11 (75.03-98.46)	83.99 (73.25-97.79)	55.10 (44.07-65.35)	38.75 (28.96-49.83)	22.98 (15.56-31.87)	90.78 (86.25-94.70)	87.37 (82.01-92.54)	81.44 (74.00-88.68)
Cuba	16.80 (13.71-20.32)	16.80 (13.71-20.32)	16.80 (13.71-20.32)	67.92 (58.65-78.83)	67.95 (59.11-77.13)	60.52 (51.95-71.05)	30.82 (19.22-44.48)	29.92 (20.73-40.53)	18.03 (10.94-27.25)	82.90 (76.07-89.63)	80.87 (73.17-88.53)	72.73 (62.91-82.66)
Dominica	16.80 (13.71-20.32)	16.80 (13.71-20.32)	16.80 (13.71-20.32)	71.35 (61.21-83.73)	65.15 (54.02-77.44)	60.42 (50.33-72.68)	50.25 (37.60-63.84)	36.15 (24.83-49.67)	24.01 (15.63-34.37)	84.71 (78.17-90.83)	80.01 (72.10-87.66)	72.58 (62.49-82.91)

Location	Appendix Table 1b. Unscaled values for each SDG health-related indicator by country for 1990, 2000, and 2015											
	**Indicator 5.2.1: Prevalence of women aged 15 years and older who experienced intimate partner violence**			**Indicator 6.1.1: Risk-weighted prevalence of populations using unsafe or unimproved water sources, as measured by the summary exposure value (SEV) for unsafe water**			**Indicator 6.2.2a: Risk-weighted prevalence of populations using unsafe or unimproved sanitation, as measured by the summary exposure value (SEV) for unsafe sanitation**			**Indicator 6.2.1b: Risk-weighted prevalence of populations with unsafe hygiene (no handwashing with soap), as measured by the summary exposure value (SEV) for unsafe hygiene**		
**Location**	**1990**	**2000**	**2015**	**1990**	**2000**	**2015**	**1990**	**2000**	**2015**	**1990**	**2000**	**2015**
Dominican Republic	16.32 (13.30-19.44)	16.98 (14.74-19.53)	17.32 (15.39-19.10)	76.14 (66.75-87.93)	79.06 (69.83-90.54)	69.09 (59.38-81.13)	50.08 (43.82-56.66)	40.65 (36.07-45.78)	25.98 (17.05-36.75)	89.20 (84.22-93.66)	85.52 (79.45-91.29)	77.64 (69.29-85.91)
Grenada	16.80 (13.71-20.32)	16.80 (13.71-20.32)	16.80 (13.71-20.32)	71.39 (61.38-82.92)	63.20 (52.78-75.84)	57.37 (47.49-68.70)	49.97 (36.53-62.46)	33.87 (22.54-46.81)	21.36 (12.51-33.25)	84.50 (77.73-90.73)	79.24 (71.16-87.27)	71.63 (61.36-82.25)
Guyana	16.80 (13.71-20.32)	16.80 (13.71-20.32)	16.80 (13.71-20.32)	78.61 (69.41-88.28)	73.12 (64.31-83.72)	66.70 (57.52-77.78)	57.30 (48.26-66.57)	46.57 (37.23-56.44)	30.95 (21.47-41.78)	89.54 (84.17-94.20)	86.81 (80.60-92.38)	81.03 (72.89-88.78)
Haiti	18.18 (14.74-21.66)	18.24 (16.17-20.32)	17.62 (15.12-20.40)	94.69 (88.04-100.00)	93.09 (86.18-100.00)	91.12 (83.19-99.83)	87.73 (81.76-92.48)	85.04 (79.75-89.80)	77.82 (70.32-84.56)	95.52 (92.71-97.66)	94.77 (91.59-97.26)	92.69 (88.40-96.14)
Jamaica	23.36 (19.06-28.16)	22.62 (19.05-26.45)	23.36 (19.06-28.16)	68.73 (59.92-79.10)	63.32 (54.80-73.48)	57.65 (48.60-68.08)	49.02 (42.66-55.98)	33.97 (29.22-38.91)	21.91 (13.70-32.07)	85.48 (79.20-91.25)	81.27 (73.67-88.63)	75.34 (65.46-85.11)
Saint Lucia	16.80 (13.71-20.32)	16.80 (13.71-20.32)	16.80 (13.71-20.32)	70.33 (60.80-81.76)	62.23 (51.87-74.00)	57.25 (47.00-69.04)	70.73 (61.18-79.07)	47.54 (36.12-59.25)	28.59 (19.32-38.61)	86.27 (80.35-91.82)	81.81 (74.70-88.80)	75.03 (65.79-84.36)
Saint Vincent and the Grenadines	16.80 (13.71-20.32)	16.80 (13.71-20.32)	16.80 (13.71-20.32)	70.28 (59.61-82.78)	65.63 (55.25-77.95)	60.23 (49.54-71.80)	44.90 (31.52-59.53)	35.20 (23.29-48.80)	22.80 (13.53-34.83)	84.56 (77.77-90.90)	79.71 (71.47-87.73)	72.40 (61.93-83.03)
Suriname	16.80 (13.71-20.32)	16.80 (13.71-20.32)	16.80 (13.71-20.32)	75.56 (66.23-86.35)	72.29 (63.39-83.66)	67.48 (57.91-78.56)	37.68 (27.19-50.84)	27.37 (20.14-36.05)	13.93 (9.95-19.45)	89.26 (84.59-93.40)	86.58 (81.03-91.81)	79.18 (71.80-86.75)
Trinidad and Tobago	16.80 (13.71-20.32)	16.80 (13.71-20.32)	16.80 (13.71-20.32)	61.82 (51.83-72.77)	59.92 (50.29-70.91)	53.44 (43.60-64.19)	31.89 (24.14-40.56)	20.78 (15.96-25.77)	10.07 (5.87-15.91)	80.37 (73.26-87.57)	76.35 (67.89-84.98)	66.15 (56.20-77.20)
Brazil	7.46 (5.74-9.07)	7.39 (5.58-9.12)	7.78 (6.30-9.06)	70.90 (63.61-79.50)	67.02 (59.66-75.65)	59.94 (52.94-68.76)	22.64 (21.63-23.61)	17.58 (16.13-19.00)	10.61 (9.53-11.89)	87.83 (83.51-91.85)	84.16 (79.23-89.13)	76.54 (70.28-83.61)

Appendix Table 1b. Unscaled values for each SDG health-related indicator by country for 1990, 2000, and 2015												
	Indicator 5.2.1: Prevalence of women aged 15 years and older who experienced intimate partner violence			Indicator 6.1.1: Risk-weighted prevalence of populations using unsafe or unimproved water sources, as measured by the summary exposure value (SEV) for unsafe water			Indicator 6.2.2a: Risk-weighted prevalence of populations using unsafe or unimproved sanitation, as measured by the summary exposure value (SEV) for unsafe sanitation			Indicator 6.2.1b: Risk-weighted prevalence of populations with unsafe hygiene (no handwashing with soap), as measured by the summary exposure value (SEV) for unsafe hygiene		
Location	1990	2000	2015	1990	2000	2015	1990	2000	2015	1990	2000	2015
Paraguay	8.41 (7.47-9.69)	9.38 (8.17-10.52)	9.65 (7.85-11.47)	80.01 (72.10-88.60)	73.02 (64.61-82.21)	60.90 (51.79-72.13)	57.02 (47.21-66.87)	46.40 (39.75-53.58)	30.43 (21.82-39.84)	87.08 (82.17-91.69)	83.69 (78.06-89.45)	76.62 (69.11-84.46)
China	6.09 (4.95-7.29)	5.96 (4.83-7.10)	6.47 (5.24-7.75)	77.18 (71.59-83.45)	65.09 (59.68-71.25)	49.92 (44.96-55.33)	77.32 (75.28-79.18)	57.57 (55.35-59.91)	33.68 (31.19-36.19)	90.49 (86.00-94.40)	86.07 (80.66-91.37)	75.79 (68.77-83.37)
North Korea	6.19 (5.02-7.35)	6.19 (5.02-7.35)	6.19 (5.02-7.35)	49.84 (35.85-66.18)	52.09 (38.26-67.39)	50.44 (37.32-65.48)	39.34 (26.93-52.73)	43.54 (30.99-57.03)	41.35 (29.02-55.62)	84.07 (78.33-89.90)	82.98 (75.81-89.72)	78.66 (69.58-87.36)
Taiwan	6.19 (5.02-7.35)	6.19 (5.02-7.35)	6.19 (5.02-7.35)	39.12 (24.69-56.48)	33.50 (20.25-51.40)	27.61 (15.34-43.79)	15.68 (9.97-23.61)	9.39 (5.73-14.84)	5.30 (3.01-8.75)	75.48 (68.95-82.90)	67.81 (60.47-76.35)	57.57 (48.56-67.89)
Cambodia	11.90 (9.77-14.15)	11.91 (10.01-13.80)	12.01 (9.98-14.15)	89.01 (75.61-99.39)	83.31 (71.24-93.81)	67.81 (56.75-79.30)	92.05 (88.62-94.87)	85.41 (84.20-86.62)	50.45 (43.00-58.08)	93.80 (90.78-96.39)	92.20 (88.67-95.32)	86.58 (82.00-91.02)
Indonesia	16.81 (13.76-20.03)	16.81 (13.76-20.03)	16.81 (13.76-20.03)	54.89 (43.73-67.26)	48.65 (38.35-59.96)	50.70 (39.93-62.23)	78.42 (72.98-83.53)	58.52 (52.60-64.04)	27.69 (19.47-37.85)	87.71 (83.41-91.68)	82.19 (77.26-87.27)	71.50 (65.14-78.77)
Laos	16.81 (13.76-20.03)	16.81 (13.76-20.03)	16.81 (13.76-20.03)	75.75 (62.78-88.64)	68.41 (57.83-79.31)	62.23 (52.78-73.03)	85.85 (78.70-91.22)	71.17 (65.65-76.30)	33.34 (26.05-41.34)	93.11 (89.85-95.87)	91.14 (87.51-94.38)	85.55 (80.95-90.10)
Malaysia	16.81 (13.76-20.03)	16.81 (13.76-20.03)	16.81 (13.76-20.03)	44.08 (29.67-62.12)	35.23 (21.62-55.00)	28.20 (15.25-44.32)	19.83 (15.52-24.63)	6.62 (5.04-8.69)	2.95 (1.76-4.64)	80.27 (75.39-85.28)	72.97 (67.66-79.33)	61.59 (54.83-69.50)
Maldives	16.81 (13.76-20.03)	16.81 (13.76-20.03)	16.81 (13.76-20.03)	87.91 (77.46-100.00)	84.45 (73.15-98.06)	78.86 (67.94-91.94)	68.51 (53.75-81.58)	37.74 (24.13-54.22)	20.07 (11.08-32.53)	89.23 (85.37-92.83)	86.21 (81.96-90.28)	79.21 (74.49-84.14)
Mauritius	16.81 (13.76-20.03)	16.81 (13.76-20.03)	16.81 (13.76-20.03)	37.78 (23.17-55.76)	31.02 (16.03-50.89)	25.71 (11.64-43.42)	32.96 (22.58-44.69)	13.09 (8.35-19.24)	5.89 (3.43-9.16)	83.67 (79.07-88.17)	77.72 (72.69-83.27)	67.20 (61.06-74.35)

Location	Appendix Table 1b. Unscaled values for each SDG health-related indicator by country for 1990, 2000, and 2015											
	**Indicator 5.2.1: Prevalence of women aged 15 years and older who experienced intimate partner violence**			**Indicator 6.1.1: Risk-weighted prevalence of populations using unsafe or unimproved water sources, as measured by the summary exposure value (SEV) for unsafe water**			**Indicator 6.2.2a: Risk-weighted prevalence of populations using unsafe or unimproved sanitation, as measured by the summary exposure value (SEV) for unsafe sanitation**			**Indicator 6.2.1b: Risk-weighted prevalence of populations with unsafe hygiene (no handwashing with soap), as measured by the summary exposure value (SEV) for unsafe hygiene**		
	**1990**	**2000**	**2015**	**1990**	**2000**	**2015**	**1990**	**2000**	**2015**	**1990**	**2000**	**2015**
Myanmar	20.68 (16.91-24.78)	20.87 (17.15-25.09)	20.68 (16.91-24.78)	84.75 (73.49-95.05)	80.90 (69.93-91.28)	68.61 (56.05-80.62)	88.64 (82.99-93.75)	80.90 (74.05-87.67)	57.18 (41.59-71.48)	92.81 (89.20-95.82)	90.55 (86.45-94.26)	82.68 (77.39-88.13)
Philippines	9.48 (7.68-11.49)	8.87 (7.37-10.54)	10.24 (8.71-11.74)	77.08 (66.25-87.29)	74.85 (64.18-86.85)	67.68 (54.07-81.18)	38.67 (32.92-44.74)	23.64 (20.20-27.16)	12.20 (8.19-17.54)	79.88 (74.32-86.06)	76.47 (70.37-83.33)	69.02 (61.67-77.48)
Sri Lanka	12.66 (10.38-15.05)	12.07 (10.27-14.26)	13.18 (10.84-15.47)	72.15 (61.53-83.31)	61.70 (50.37-73.27)	48.99 (37.34-63.27)	79.42 (71.97-86.97)	59.62 (48.80-70.24)	30.68 (16.80-47.81)	82.48 (77.14-88.12)	76.98 (71.01-83.59)	66.08 (58.82-74.50)
Seychelles	16.81 (13.76-20.03)	16.81 (13.76-20.03)	16.81 (13.76-20.03)	42.45 (27.85-60.54)	34.88 (20.66-51.86)	29.26 (16.33-47.83)	27.46 (15.53-44.68)	15.87 (8.05-29.53)	9.57 (4.62-18.72)	79.06 (74.20-84.17)	72.68 (67.44-79.00)	62.35 (55.73-70.11)
Thailand	23.84 (19.55-28.33)	23.41 (19.76-27.36)	23.84 (19.55-28.33)	78.22 (68.60-88.68)	75.10 (65.37-86.92)	65.90 (52.88-79.81)	18.86 (15.09-22.95)	3.53 (2.46-5.16)	1.80 (0.94-3.22)	84.56 (79.93-89.09)	79.67 (74.86-85.03)	70.65 (64.90-77.41)
Timor-Leste	25.94 (21.17-31.18)	28.38 (22.71-33.14)	25.45 (22.39-28.95)	57.87 (44.95-71.96)	57.53 (45.46-70.99)	35.13 (25.99-46.45)	92.52 (88.11-96.15)	93.75 (90.88-96.21)	59.61 (47.49-71.09)	92.63 (89.21-95.59)	90.12 (85.98-93.78)	81.74 (76.83-86.85)
Vietnam	20.99 (17.05-25.21)	20.17 (16.86-24.04)	20.99 (18.30-23.99)	56.54 (44.30-70.33)	48.33 (38.19-60.06)	41.67 (32.72-52.62)	87.77 (85.04-90.70)	70.88 (66.99-75.21)	31.82 (23.29-41.66)	87.92 (83.26-92.28)	83.77 (78.33-89.18)	74.71 (68.18-82.00)
Federated States of Micronesia	25.55 (20.57-30.58)	25.55 (20.57-30.58)	25.55 (20.57-30.58)	77.26 (67.26-87.80)	71.29 (60.88-83.24)	64.74 (53.89-76.97)	48.00 (33.97-63.62)	32.51 (21.08-47.67)	17.30 (10.30-28.93)	84.48 (78.15-90.56)	80.27 (72.55-87.82)	73.95 (64.01-83.83)
Fiji	25.55 (20.57-30.58)	25.55 (20.57-30.58)	25.55 (20.57-30.58)	71.05 (60.46-82.06)	65.48 (53.99-78.70)	59.48 (48.88-72.03)	33.45 (22.24-48.50)	21.40 (12.61-34.20)	10.72 (5.72-18.35)	83.04 (77.21-89.07)	78.53 (71.44-85.94)	70.91 (61.61-80.59)
Kiribati	33.88 (28.15-39.01)	33.88 (28.15-39.01)	33.88 (28.15-39.01)	84.62 (75.84-93.84)	80.88 (71.51-90.88)	76.42 (66.12-87.26)	66.38 (51.99-79.61)	55.03 (40.23-70.03)	39.40 (25.42-56.19)	89.40 (84.43-93.80)	87.35 (81.55-92.58)	82.71 (75.12-89.73)

Appendix Table 1b. Unscaled values for each SDG health-related indicator by country for 1990, 2000, and 2015

Location	Indicator 5.2.1: Prevalence of women aged 15 years and older who experienced intimate partner violence			Indicator 6.1.1: Risk-weighted prevalence of populations using unsafe or unimproved water sources, as measured by the summary exposure value (SEV) for unsafe water			Indicator 6.2.2a: Risk-weighted prevalence of populations using unsafe or unimproved sanitation, as measured by the summary exposure value (SEV) for unsafe sanitation			Indicator 6.2.1b: Risk-weighted prevalence of populations with unsafe hygiene (no handwashing with soap), as measured by the summary exposure value (SEV) for unsafe hygiene		
	1990	2000	2015	1990	2000	2015	1990	2000	2015	1990	2000	2015
Marshall Islands	25.55 (20.57-30.58)	25.55 (20.57-30.58)	25.55 (20.57-30.58)	82.29 (72.83-91.76)	75.84 (65.28-86.88)	67.34 (56.53-79.87)	60.85 (46.45-74.53)	42.35 (28.72-57.95)	20.80 (12.58-33.16)	88.42 (83.38-92.97)	85.00 (79.07-90.81)	78.41 (70.29-86.44)
Papua New Guinea	18.19 (13.82-22.95)	18.19 (13.82-22.95)	18.65 (14.00-23.53)	95.59 (90.53-99.72)	92.91 (87.80-97.98)	85.57 (78.04-93.42)	94.19 (90.20-97.06)	91.77 (87.16-95.51)	79.47 (68.10-87.96)	94.26 (91.15-96.81)	92.76 (89.12-95.83)	89.37 (84.55-93.66)
Samoa	27.67 (22.70-32.90)	26.84 (22.75-30.99)	27.67 (22.70-32.90)	72.16 (62.18-84.71)	66.87 (56.07-78.84)	59.92 (48.34-73.59)	28.36 (15.29-45.24)	16.36 (8.21-29.99)	6.25 (2.85-13.05)	81.17 (73.92-88.25)	76.85 (68.14-85.38)	68.96 (58.41-79.92)
Solomon Islands	28.01 (23.09-32.93)	28.01 (23.09-32.93)	28.01 (23.09-32.93)	87.47 (79.30-95.52)	80.58 (71.36-90.23)	74.47 (63.93-85.95)	87.71 (79.47-93.58)	74.84 (61.71-85.52)	59.34 (44.67-72.90)	93.48 (90.05-96.32)	90.92 (86.52-94.66)	86.62 (80.58-92.10)
Tonga	22.19 (18.19-26.45)	22.19 (18.19-26.45)	22.19 (18.19-26.45)	76.03 (66.09-86.75)	70.93 (60.61-82.89)	64.98 (53.79-76.86)	45.22 (31.34-61.21)	32.23 (20.66-47.00)	17.47 (10.15-28.80)	83.38 (77.03-89.67)	79.51 (72.01-86.98)	73.25 (63.82-82.74)
Vanuatu	25.55 (20.57-30.58)	25.55 (20.57-30.58)	25.55 (20.57-30.58)	89.42 (81.30-98.86)	86.54 (78.10-97.22)	81.10 (71.60-92.95)	79.03 (67.11-88.36)	71.72 (60.36-81.67)	51.99 (38.88-64.99)	90.97 (86.83-94.60)	88.33 (83.22-92.94)	83.03 (76.13-89.59)
Afghanistan	27.22 (22.46-32.46)	27.22 (22.46-32.46)	27.22 (22.46-32.46)	98.60 (92.66-100.00)	98.20 (93.17-100.00)	91.12 (83.20-100.00)	89.41 (80.06-95.73)	89.48 (81.05-95.34)	70.93 (60.36-80.97)	97.58 (95.85-98.86)	97.84 (96.19-98.99)	97.00 (94.94-98.53)
Algeria	27.22 (22.46-32.46)	27.22 (22.46-32.46)	27.22 (22.46-32.46)	87.15 (76.78-97.62)	81.16 (70.83-92.85)	77.77 (64.87-91.05)	46.43 (30.55-62.55)	22.64 (16.78-29.47)	16.05 (9.24-25.59)	91.30 (87.35-94.80)	89.38 (84.64-93.56)	85.11 (79.37-90.81)
Bahrain	27.22 (22.46-32.46)	27.22 (22.46-32.46)	27.22 (22.46-32.46)	77.50 (65.36-91.61)	72.82 (59.91-88.42)	67.62 (53.84-83.27)	16.81 (8.71-27.61)	7.60 (3.96-12.83)	2.90 (1.52-5.09)	85.80 (81.11-90.42)	81.10 (75.53-87.05)	73.36 (66.07-81.46)
Egypt	22.98 (19.01-27.39)	23.69 (19.96-27.61)	22.89 (19.94-25.94)	85.32 (74.26-98.93)	80.99 (69.45-95.64)	78.65 (66.95-93.82)	23.31 (14.18-34.15)	7.07 (4.91-9.78)	3.24 (1.58-6.15)	92.76 (89.04-95.85)	89.29 (84.40-93.61)	83.18 (76.57-89.57)

Appendix Table 1b. Unscaled values for each SDG health-related indicator by country for 1990, 2000, and 2015

Location	Indicator 5.2.1: Prevalence of women aged 15 years and older who experienced intimate partner violence			Indicator 6.1.1: Risk-weighted prevalence of populations using unsafe or unimproved water sources, as measured by the summary exposure value (SEV) for unsafe water			Indicator 6.2.2a: Risk-weighted prevalence of populations using unsafe or unimproved sanitation, as measured by the summary exposure value (SEV) for unsafe sanitation			Indicator 6.2.1b: Risk-weighted prevalence of populations with unsafe hygiene (no handwashing with soap), as measured by the summary exposure value (SEV) for unsafe hygiene		
	1990	2000	2015	1990	2000	2015	1990	2000	2015	1990	2000	2015
Iran	35.71 (29.84-41.12)	36.52 (31.29-41.24)	35.71 (29.84-41.12)	83.98 (73.70-96.25)	75.59 (63.18-89.58)	69.97 (56.89-85.09)	57.11 (36.86-76.00)	27.15 (14.34-45.39)	14.04 (6.96-25.17)	91.56 (87.66-95.00)	86.75 (81.42-91.85)	79.25 (72.08-86.61)
Iraq	14.59 (12.01-17.43)	14.59 (12.01-17.43)	14.59 (12.01-17.43)	85.94 (75.92-98.07)	82.74 (72.41-95.66)	76.81 (65.55-90.76)	26.73 (15.93-41.74)	13.85 (9.52-19.76)	8.77 (6.02-12.16)	92.76 (89.28-95.74)	90.90 (86.83-94.59)	85.71 (80.20-91.07)
Jordan	19.62 (16.15-23.31)	19.62 (16.15-23.31)	20.34 (18.19-22.67)	72.59 (59.31-86.83)	66.49 (54.20-80.07)	56.00 (44.51-68.84)	13.81 (7.24-24.15)	3.76 (2.30-5.98)	0.47 (0.29-0.71)	85.23 (79.17-91.06)	81.74 (74.11-89.10)	74.57 (64.97-84.26)
Kuwait	27.22 (22.46-32.46)	27.22 (22.46-32.46)	27.22 (22.46-32.46)	73.29 (60.24-88.55)	69.30 (55.22-84.51)	63.44 (49.42-79.43)	8.80 (4.41-14.81)	4.30 (2.25-7.85)	1.54 (0.79-2.75)	82.77 (77.58-88.01)	75.16 (68.99-82.23)	64.63 (56.38-74.14)
Lebanon	27.22 (22.46-32.46)	27.22 (22.46-32.46)	27.22 (22.46-32.46)	92.16 (82.69-100.00)	89.27 (79.96-97.94)	77.65 (64.98-89.31)	12.99 (7.30-21.62)	7.46 (4.06-12.68)	2.75 (1.53-4.82)	87.08 (81.86-92.07)	83.16 (76.77-89.57)	74.22 (65.40-83.29)
Libya	27.22 (22.46-32.46)	27.22 (22.46-32.46)	27.22 (22.46-32.46)	83.29 (72.12-95.75)	77.92 (66.14-91.38)	74.03 (60.60-87.74)	34.44 (20.51-51.50)	16.20 (9.08-26.99)	7.61 (4.04-12.95)	91.81 (88.32-94.92)	90.32 (86.37-93.90)	86.84 (81.89-91.49)
Morocco	27.22 (22.46-32.46)	27.22 (22.46-32.46)	27.22 (22.46-32.46)	92.13 (84.31-100.00)	87.91 (78.85-99.18)	82.35 (71.18-96.14)	48.00 (42.56-53.93)	32.79 (27.00-38.77)	21.73 (12.73-34.74)	95.61 (93.00-97.69)	94.57 (91.55-97.03)	91.78 (87.87-95.20)
Palestine	49.64 (43.32-57.57)	41.47 (36.60-46.12)	31.97 (26.63-39.86)	88.31 (77.66-98.98)	81.40 (70.58-93.32)	79.22 (67.96-92.05)	26.79 (15.30-41.26)	13.07 (6.93-21.77)	9.12 (4.84-15.87)	88.06 (82.67-92.99)	83.53 (76.73-90.03)	82.37 (74.19-89.94)
Oman	27.22 (22.46-32.46)	27.22 (22.46-32.46)	27.22 (22.46-32.46)	98.67 (92.93-100.00)	87.36 (76.60-97.82)	77.61 (65.00-90.57)	41.70 (26.19-58.87)	12.65 (7.03-20.96)	4.53 (2.40-7.82)	89.66 (85.57-93.33)	86.11 (81.46-90.61)	79.97 (74.27-86.23)
Qatar	27.22 (22.46-32.46)	27.22 (22.46-32.46)	27.22 (22.46-32.46)	76.73 (63.96-90.64)	73.05 (60.39-87.07)	66.32 (52.58-82.53)	14.90 (7.97-25.46)	7.76 (4.21-13.52)	2.33 (1.21-3.99)	82.71 (77.99-87.62)	77.82 (72.29-84.02)	67.58 (60.91-75.48)

Appendix Table 1b. Unscaled values for each SDG health-related indicator by country for 1990, 2000, and 2015												
	**Indicator 5.2.1: Prevalence of women aged 15 years and older who experienced intimate partner violence**			**Indicator 6.1.1: Risk-weighted prevalence of populations using unsafe or unimproved water sources, as measured by the summary exposure value (SEV) for unsafe water**			**Indicator 6.2.2a: Risk-weighted prevalence of populations using unsafe or unimproved sanitation, as measured by the summary exposure value (SEV) for unsafe sanitation**			**Indicator 6.2.1b: Risk-weighted prevalence of populations with unsafe hygiene (no handwashing with soap), as measured by the summary exposure value (SEV) for unsafe hygiene**		
**Location**	**1990**	**2000**	**2015**	**1990**	**2000**	**2015**	**1990**	**2000**	**2015**	**1990**	**2000**	**2015**
Saudi Arabia	27.15 (22.20-32.25)	27.15 (22.20-32.25)	27.15 (22.20-32.25)	82.39 (72.35-95.48)	77.18 (66.87-90.28)	70.43 (60.13-83.59)	26.73 (22.29-32.18)	10.82 (8.59-13.57)	3.48 (2.75-4.41)	88.16 (83.89-92.13)	84.40 (79.26-89.42)	76.18 (69.67-83.58)
Sudan	38.04 (31.74-43.74)	38.04 (31.74-43.74)	40.24 (33.93-46.21)	98.60 (92.49-100.00)	97.26 (90.40-100.00)	91.82 (82.41-100.00)	95.29 (91.16-97.83)	92.29 (85.54-96.71)	78.83 (64.04-89.94)	96.36 (94.00-98.16)	95.40 (92.60-97.57)	92.79 (89.01-95.94)
Syria	27.22 (22.46-32.46)	27.22 (22.46-32.46)	27.22 (22.46-32.46)	87.29 (76.97-99.82)	84.01 (73.81-97.52)	81.47 (70.02-95.40)	37.73 (25.31-51.54)	23.11 (17.96-28.90)	11.63 (6.54-17.98)	93.89 (90.46-96.67)	91.22 (86.73-95.00)	85.69 (79.37-91.51)
Tunisia	27.22 (22.46-32.46)	27.22 (22.46-32.46)	27.22 (22.46-32.46)	87.58 (77.20-100.00)	85.78 (75.23-100.00)	83.61 (72.49-97.83)	32.36 (21.21-46.07)	15.61 (10.29-22.72)	8.26 (4.28-14.67)	94.17 (91.05-96.80)	90.62 (86.32-94.41)	83.10 (76.59-89.51)
Turkey	26.49 (21.83-31.53)	26.49 (21.83-31.53)	25.64 (21.58-29.87)	84.89 (75.37-96.91)	77.33 (65.61-92.04)	71.49 (57.93-87.08)	38.01 (31.52-45.67)	21.37 (19.98-22.93)	13.10 (7.72-19.91)	91.30 (87.31-94.77)	88.47 (83.70-92.82)	81.87 (75.85-88.13)
United Arab Emirates	27.22 (22.46-32.46)	27.22 (22.46-32.46)	27.22 (22.46-32.46)	82.59 (71.14-93.99)	73.32 (60.76-85.96)	65.89 (51.50-80.54)	11.01 (5.60-19.73)	2.93 (1.55-5.12)	1.16 (0.58-2.11)	81.02 (76.22-86.05)	72.80 (66.69-79.82)	65.45 (56.86-75.33)
Yemen	27.22 (22.46-32.46)	27.22 (22.46-32.46)	27.22 (22.46-32.46)	96.83 (90.59-100.00)	96.63 (90.28-100.00)	88.84 (79.41-99.03)	87.25 (79.16-93.05)	79.92 (72.30-87.20)	60.10 (46.61-72.73)	97.25 (95.37-98.66)	96.65 (94.45-98.30)	95.09 (92.21-97.36)
Bangladesh	37.98 (32.04-43.43)	37.30 (32.87-41.90)	37.33 (32.53-42.56)	92.85 (83.19-100.00)	91.64 (81.77-100.00)	86.83 (76.38-100.00)	90.51 (85.20-94.56)	82.31 (77.57-86.67)	60.07 (46.88-72.92)	98.67 (97.53-99.41)	98.19 (96.73-99.18)	96.76 (94.29-98.46)
Bhutan	25.88 (21.18-30.83)	25.88 (21.18-30.83)	25.88 (21.18-30.83)	54.46 (41.75-67.95)	38.53 (29.38-49.98)	24.77 (18.59-32.48)	84.67 (68.67-95.11)	68.18 (47.57-84.80)	37.09 (25.44-50.82)	98.60 (97.43-99.38)	98.07 (96.54-99.12)	96.78 (94.47-98.44)
India	30.42 (26.66-33.96)	26.02 (23.12-28.99)	29.71 (26.03-33.24)	89.45 (82.42-97.03)	85.43 (77.72-94.41)	77.78 (69.40-87.95)	82.24 (80.78-83.78)	73.44 (72.07-74.99)	51.35 (47.35-55.18)	98.28 (96.90-99.21)	97.40 (95.46-98.78)	94.73 (91.29-97.37)

Appendix Table 1b. Unscaled values for each SDG health-related indicator by country for 1990, 2000, and 2015

Location	Indicator 5.2.1: Prevalence of women aged 15 years and older who experienced intimate partner violence			Indicator 6.1.1: Risk-weighted prevalence of populations using unsafe or unimproved water sources, as measured by the summary exposure value (SEV) for unsafe water			Indicator 6.2.2a: Risk-weighted prevalence of populations using unsafe or unimproved sanitation, as measured by the summary exposure value (SEV) for unsafe sanitation			Indicator 6.2.1b: Risk-weighted prevalence of populations with unsafe hygiene (no handwashing with soap), as measured by the summary exposure value (SEV) for unsafe hygiene		
	1990	2000	2015	1990	2000	2015	1990	2000	2015	1990	2000	2015
Nepal	24.24 (19.97-29.11)	24.24 (19.97-29.11)	26.35 (22.33-29.82)	96.34 (89.60-100.00)	89.60 (81.30-100.00)	84.64 (75.42-96.55)	98.54 (97.04-99.38)	83.31 (81.76-84.89)	45.26 (33.90-56.80)	99.01 (98.13-99.57)	98.63 (97.51-99.39)	97.64 (95.76-98.88)
Pakistan	20.43 (16.54-24.37)	20.43 (16.54-24.37)	22.76 (19.00-25.92)	90.36 (81.04-100.00)	88.80 (79.71-100.00)	84.69 (74.15-98.98)	76.64 (72.40-80.51)	55.30 (44.72-67.00)	24.65 (17.08-33.56)	98.45 (97.20-99.31)	97.98 (96.41-99.07)	96.90 (94.58-98.54)
Botswana	23.30 (19.02-27.86)	24.51 (20.13-29.27)	23.30 (19.02-27.86)	87.32 (76.79-100.00)	81.91 (70.05-95.20)	75.95 (63.45-90.23)	81.95 (79.32-84.66)	74.20 (63.89-83.21)	55.54 (42.37-69.37)	95.66 (92.69-97.86)	93.40 (89.33-96.63)	89.35 (83.45-94.39)
Lesotho	21.73 (17.78-25.92)	22.90 (18.71-27.39)	21.73 (17.78-25.92)	95.83 (88.61-100.00)	93.26 (85.13-100.00)	87.22 (77.58-98.99)	95.22 (92.06-97.45)	93.89 (91.06-96.52)	84.12 (78.21-89.47)	97.06 (94.84-98.60)	96.10 (93.19-98.18)	93.45 (88.79-96.84)
Namibia	20.73 (16.95-24.62)	21.58 (18.16-25.65)	19.23 (16.76-22.28)	90.77 (82.21-100.00)	86.16 (76.59-97.84)	80.01 (68.67-92.70)	70.56 (63.32-77.07)	63.48 (58.53-68.27)	42.57 (32.37-52.98)	95.29 (92.11-97.66)	94.00 (90.15-96.98)	90.33 (84.71-95.01)
South Africa	15.04 (12.80-17.22)	14.51 (12.32-16.67)	14.45 (12.22-16.67)	80.71 (71.68-91.88)	76.26 (67.44-87.15)	68.76 (59.09-80.49)	46.68 (42.38-51.59)	42.68 (39.68-46.08)	29.01 (24.99-33.47)	92.97 (88.78-96.32)	91.50 (86.50-95.55)	87.33 (80.26-93.26)
Swaziland	20.33 (16.63-24.27)	21.39 (17.57-25.79)	19.41 (16.86-22.41)	96.33 (89.53-100.00)	92.83 (85.17-100.00)	86.89 (77.16-99.85)	70.31 (59.85-80.20)	65.56 (58.73-72.35)	57.14 (47.09-67.36)	95.66 (92.74-97.88)	93.84 (89.93-96.86)	90.62 (85.07-95.15)
Zimbabwe	33.73 (28.19-39.09)	33.91 (28.48-39.09)	32.44 (27.93-36.87)	87.66 (78.56-98.51)	85.09 (76.06-96.58)	84.52 (75.11-95.77)	61.64 (55.98-67.00)	57.32 (52.05-62.60)	52.23 (43.75-61.64)	96.04 (93.24-98.08)	94.66 (91.03-97.36)	93.12 (88.37-96.64)
Benin	22.29 (18.17-26.76)	22.29 (18.17-26.76)	22.29 (18.17-26.76)	97.33 (91.13-100.00)	95.39 (88.08-100.00)	92.74 (83.61-100.00)	95.11 (92.19-97.23)	93.96 (91.80-96.00)	86.47 (79.43-91.58)	99.08 (98.24-99.61)	98.96 (98.03-99.54)	98.53 (97.31-99.34)
Burkina Faso	11.72 (9.51-14.13)	11.72 (9.51-14.13)	11.68 (10.06-13.46)	99.90 (98.59-100.00)	98.55 (93.03-100.00)	96.08 (87.75-100.00)	95.81 (93.82-97.59)	95.05 (93.20-96.99)	88.52 (83.49-92.90)	99.35 (98.76-99.74)	99.27 (98.60-99.69)	98.96 (98.06-99.55)

Appendix Table 1b. Unscaled values for each SDG health-related indicator by country for 1990, 2000, and 2015

Location	Indicator 5.2.1: Prevalence of women aged 15 years and older who experienced intimate partner violence			Indicator 6.1.1: Risk-weighted prevalence of populations using unsafe or unimproved water sources, as measured by the summary exposure value (SEV) for unsafe water			Indicator 6.2.2a: Risk-weighted prevalence of populations using unsafe or unimproved sanitation, as measured by the summary exposure value (SEV) for unsafe sanitation			Indicator 6.2.1b: Risk-weighted prevalence of populations with unsafe hygiene (no handwashing with soap), as measured by the summary exposure value (SEV) for unsafe hygiene		
	1990	2000	2015	1990	2000	2015	1990	2000	2015	1990	2000	2015
Cameroon	38.22 (32.20-43.58)	39.10 (34.01-43.89)	39.60 (34.46-44.20)	98.71 (93.68-100.00)	97.79 (91.99-100.00)	93.16 (85.17-100.00)	91.49 (87.52-94.47)	88.21 (84.43-91.69)	75.32 (66.00-83.24)	98.07 (96.54-99.11)	97.76 (95.98-98.96)	96.57 (94.05-98.38)
Cape Verde	22.29 (18.17-26.76)	22.29 (18.17-26.76)	22.29 (18.17-26.76)	95.20 (87.07-100.00)	90.80 (81.72-100.00)	84.65 (73.43-98.25)	86.08 (78.31-92.35)	70.78 (58.48-81.61)	43.56 (30.65-57.45)	98.38 (97.05-99.26)	97.68 (95.87-98.92)	95.81 (92.89-97.98)
Chad	14.22 (11.57-17.14)	14.22 (11.57-17.14)	14.24 (12.27-16.38)	99.99 (100.00-100.00)	99.97 (99.74-100.00)	98.36 (92.54-100.00)	98.56 (97.61-99.26)	98.77 (98.27-99.25)	90.70 (86.31-94.13)	99.24 (98.55-99.68)	99.19 (98.45-99.66)	98.73 (97.67-99.45)
Cote d'Ivoire	24.01 (19.65-29.03)	24.01 (19.65-29.03)	24.00 (20.67-27.82)	97.17 (91.13-100.00)	93.39 (84.71-100.00)	92.11 (82.68-100.00)	88.67 (84.59-92.30)	74.57 (68.43-80.25)	66.62 (57.08-75.70)	98.64 (97.52-99.40)	98.47 (97.23-99.32)	98.05 (96.49-99.11)
The Gambia	19.84 (16.20-23.61)	19.84 (16.20-23.61)	19.95 (17.45-22.37)	96.58 (89.13-100.00)	93.98 (85.13-100.00)	92.60 (82.79-100.00)	88.39 (82.53-93.35)	70.79 (62.60-78.67)	61.76 (48.87-73.85)	98.95 (98.05-99.55)	98.81 (97.79-99.48)	98.31 (96.91-99.24)
Ghana	18.43 (15.13-22.09)	18.43 (15.13-22.09)	18.43 (15.13-22.09)	98.41 (93.59-100.00)	95.56 (88.77-100.00)	92.54 (83.15-100.00)	93.93 (91.68-95.84)	85.43 (82.12-88.75)	65.92 (56.09-74.88)	98.11 (96.59-99.13)	97.65 (95.80-98.92)	96.17 (93.35-98.18)
Guinea	22.29 (18.17-26.76)	22.29 (18.17-26.76)	22.29 (18.17-26.76)	99.99 (100.00-100.00)	98.63 (94.34-100.00)	93.05 (84.90-100.00)	92.42 (88.52-95.77)	91.10 (87.92-94.09)	79.51 (73.12-85.59)	99.15 (98.39-99.64)	99.10 (98.31-99.62)	98.95 (98.05-99.55)
Guinea-Bissau	22.29 (18.17-26.76)	22.29 (18.17-26.76)	22.29 (18.17-26.76)	99.53 (95.79-100.00)	98.81 (94.13-100.00)	97.37 (90.60-100.00)	88.79 (82.70-93.55)	82.94 (77.16-88.63)	75.77 (66.37-84.67)	99.14 (98.38-99.64)	99.04 (98.20-99.59)	98.83 (97.82-99.48)
Liberia	29.00 (23.90-34.30)	29.00 (23.90-34.30)	30.44 (24.79-35.57)	97.12 (90.22-100.00)	97.72 (91.65-100.00)	95.23 (87.35-100.00)	80.16 (72.02-86.48)	87.53 (81.78-92.15)	70.61 (62.83-78.13)	99.09 (98.27-99.61)	99.18 (98.46-99.65)	98.72 (97.66-99.43)
Mali	25.48 (20.93-30.48)	25.48 (20.93-30.48)	27.24 (22.82-31.66)	99.70 (97.39-100.00)	98.52 (94.43-100.00)	95.62 (88.43-100.00)	95.48 (93.83-97.24)	90.66 (88.11-93.26)	86.19 (80.33-91.63)	99.30 (98.64-99.71)	99.18 (98.45-99.65)	98.97 (98.07-99.56)

Appendix Table 1b. Unscaled values for each SDG health-related indicator by country for 1990, 2000, and 2015												
	Indicator 5.2.1: Prevalence of women aged 15 years and older who experienced intimate partner violence			Indicator 6.1.1: Risk-weighted prevalence of populations using unsafe or unimproved water sources, as measured by the summary exposure value (SEV) for unsafe water			Indicator 6.2.2a: Risk-weighted prevalence of populations using unsafe or unimproved sanitation, as measured by the summary exposure value (SEV) for unsafe sanitation			Indicator 6.2.1b: Risk-weighted prevalence of populations with unsafe hygiene (no handwashing with soap), as measured by the summary exposure value (SEV) for unsafe hygiene		
Location	1990	2000	2015	1990	2000	2015	1990	2000	2015	1990	2000	2015
Mauritania	22.29 (18.17-26.76)	22.29 (18.17-26.76)	22.29 (18.17-26.76)	98.72 (93.83-100.00)	96.66 (90.04-100.00)	95.30 (87.06-100.00)	92.92 (89.10-96.34)	90.26 (86.52-93.78)	71.21 (60.94-80.29)	98.61 (97.44-99.38)	98.45 (97.16-99.30)	97.69 (95.90-98.93)
Niger	22.29 (18.17-26.76)	22.29 (18.17-26.76)	22.29 (18.17-26.76)	99.87 (97.88-100.00)	99.88 (98.38-100.00)	98.00 (91.83-100.00)	98.98 (98.27-99.48)	98.73 (97.98-99.26)	96.41 (94.18-98.10)	99.32 (98.69-99.72)	99.34 (98.71-99.72)	99.22 (98.51-99.67)
Nigeria	15.92 (13.01-19.05)	16.59 (13.71-19.92)	16.02 (14.19-18.11)	99.42 (95.76-100.00)	98.99 (94.58-100.00)	94.66 (86.75-100.00)	81.25 (73.70-87.60)	80.36 (73.77-86.74)	64.75 (56.37-73.19)	98.34 (97.00-99.24)	97.93 (96.26-99.05)	95.86 (92.95-97.99)
Sao Tome and Principe	25.73 (21.00-31.02)	25.73 (21.00-31.02)	25.73 (21.00-31.02)	93.86 (85.21-100.00)	92.41 (83.86-100.00)	88.74 (78.26-100.00)	86.99 (80.20-92.29)	81.07 (75.63-85.31)	57.99 (48.10-67.64)	98.38 (97.06-99.27)	98.08 (96.54-99.13)	96.91 (94.64-98.55)
Senegal	22.29 (18.17-26.76)	22.29 (18.17-26.76)	22.29 (18.17-26.76)	95.64 (89.69-100.00)	91.95 (84.47-100.00)	87.76 (78.47-99.61)	82.02 (77.96-85.76)	75.60 (71.90-79.40)	62.24 (52.24-71.06)	98.92 (97.98-99.54)	98.81 (97.80-99.49)	98.41 (97.10-99.29)
Sierra Leone	38.23 (32.12-43.42)	38.23 (32.12-43.42)	39.24 (34.13-43.73)	99.18 (94.36-100.00)	99.12 (94.69-100.00)	97.43 (90.95-100.00)	88.19 (82.80-92.94)	89.46 (86.42-92.57)	82.40 (76.51-87.93)	99.17 (98.41-99.65)	99.11 (98.35-99.62)	98.64 (97.52-99.40)
Togo	20.18 (16.47-24.27)	20.18 (16.47-24.27)	20.01 (17.38-22.86)	99.40 (95.69-100.00)	98.71 (93.90-100.00)	95.40 (87.22-100.00)	88.62 (84.58-92.42)	84.66 (79.95-88.99)	75.58 (67.67-82.94)	98.93 (98.01-99.53)	98.75 (97.69-99.45)	98.28 (96.88-99.22)
Burundi	25.74 (20.91-30.63)	25.74 (20.91-30.63)	25.74 (20.91-30.63)	97.48 (90.92-100.00)	97.24 (90.48-100.00)	95.84 (87.81-100.00)	93.73 (90.81-96.41)	92.71 (89.86-95.31)	88.09 (82.96-92.69)	99.21 (98.48-99.66)	99.16 (98.43-99.64)	98.88 (97.93-99.51)
Comoros	5.68 (4.60-6.89)	5.68 (4.60-6.89)	5.73 (4.81-6.65)	89.99 (79.79-100.00)	89.14 (79.62-100.00)	87.17 (76.92-99.11)	90.11 (85.73-94.20)	90.52 (86.95-94.09)	85.83 (78.77-91.72)	98.83 (97.81-99.50)	98.67 (97.53-99.41)	98.14 (96.57-99.15)
Djibouti	25.74 (20.91-30.63)	25.74 (20.91-30.63)	25.74 (20.91-30.63)	94.11 (86.11-100.00)	91.27 (82.52-100.00)	84.83 (74.23-98.62)	79.79 (70.05-87.44)	73.01 (63.68-81.59)	57.76 (45.60-69.82)	98.63 (97.44-99.38)	98.61 (97.40-99.38)	98.04 (96.47-99.09)

Location	Appendix Table 1b. Unscaled values for each SDG health-related indicator by country for 1990, 2000, and 2015											
	**Indicator 5.2.1: Prevalence of women aged 15 years and older who experienced intimate partner violence**			**Indicator 6.1.1: Risk-weighted prevalence of populations using unsafe or unimproved water sources, as measured by the summary exposure value (SEV) for unsafe water**			**Indicator 6.2.2a: Risk-weighted prevalence of populations using unsafe or unimproved sanitation, as measured by the summary exposure value (SEV) for unsafe sanitation**			**Indicator 6.2.1b: Risk-weighted prevalence of populations with unsafe hygiene (no handwashing with soap), as measured by the summary exposure value (SEV) for unsafe hygiene**		
	**1990**	**2000**	**2015**	**1990**	**2000**	**2015**	**1990**	**2000**	**2015**	**1990**	**2000**	**2015**
Eritrea	25.74 (20.91-30.63)	25.74 (20.91-30.63)	25.74 (20.91-30.63)	99.02 (94.31-100.00)	94.00 (87.24-100.00)	90.66 (82.49-98.80)	90.50 (86.12-93.56)	83.28 (79.23-87.13)	77.22 (68.16-85.21)	99.26 (98.61-99.69)	98.87 (97.91-99.50)	98.67 (97.53-99.42)
Ethiopia	32.98 (28.49-37.96)	33.23 (29.26-38.14)	37.48 (31.60-43.43)	99.46 (96.25-100.00)	99.30 (95.87-100.00)	91.66 (83.36-99.94)	97.46 (95.77-98.72)	97.63 (96.40-98.64)	92.22 (88.69-95.04)	99.38 (98.80-99.74)	99.36 (98.77-99.73)	98.87 (97.92-99.50)
Kenya	19.42 (16.03-22.59)	17.57 (13.92-21.21)	19.17 (15.71-22.33)	85.91 (80.03-92.00)	80.55 (74.45-87.61)	74.70 (68.15-82.56)	87.35 (84.60-90.20)	82.74 (79.48-86.16)	75.70 (72.00-79.68)	97.96 (96.35-99.07)	97.36 (95.32-98.77)	96.16 (93.23-98.19)
Madagascar	25.74 (20.91-30.63)	25.74 (20.91-30.63)	25.74 (20.91-30.63)	87.81 (76.99-95.77)	83.80 (75.40-91.15)	80.72 (72.74-89.20)	97.28 (95.57-98.53)	96.66 (95.41-97.69)	95.38 (93.37-97.07)	98.77 (97.72-99.46)	98.61 (97.44-99.38)	98.04 (96.43-99.10)
Malawi	19.18 (15.67-22.97)	18.50 (15.80-21.52)	19.56 (16.49-22.73)	95.18 (88.55-100.00)	92.86 (85.75-100.00)	88.31 (79.16-99.34)	95.39 (94.02-96.59)	95.40 (94.25-96.51)	92.55 (89.57-95.32)	99.18 (98.45-99.65)	99.01 (98.17-99.57)	98.61 (97.43-99.38)
Mozambique	30.77 (25.36-36.46)	29.91 (25.13-35.04)	31.05 (27.04-35.44)	99.90 (98.52-100.00)	99.53 (96.63-100.00)	93.64 (86.37-100.00)	96.79 (94.92-98.28)	94.55 (92.69-96.37)	88.67 (84.10-92.95)	99.51 (99.04-99.80)	99.38 (98.80-99.74)	98.83 (97.82-99.48)
Rwanda	34.15 (28.21-39.83)	33.12 (28.99-37.52)	35.52 (30.27-40.56)	85.71 (76.83-93.96)	81.99 (72.95-91.34)	74.09 (64.53-84.50)	84.15 (77.76-91.15)	82.96 (76.09-90.52)	79.98 (72.05-88.94)	99.06 (98.24-99.59)	98.98 (98.08-99.55)	98.25 (96.74-99.21)
Somalia	25.74 (20.91-30.63)	25.74 (20.91-30.63)	25.74 (20.91-30.63)	97.04 (91.14-100.00)	97.36 (92.51-100.00)	95.16 (89.33-100.00)	81.08 (71.54-89.08)	78.76 (71.46-85.72)	73.09 (63.39-81.72)	99.43 (98.88-99.76)	99.45 (98.91-99.77)	99.34 (98.74-99.72)
South Sudan	25.74 (20.91-30.63)	25.74 (20.91-30.63)	25.74 (20.91-30.63)	98.80 (94.33-100.00)	97.41 (90.98-100.00)	94.27 (86.07-100.00)	95.90 (93.10-98.03)	94.95 (91.51-97.49)	91.18 (86.10-95.46)	99.03 (98.17-99.60)	99.04 (98.20-99.60)	98.93 (98.01-99.54)
Tanzania	28.91 (23.80-34.72)	28.00 (24.18-32.37)	30.13 (25.96-34.04)	86.96 (79.00-94.54)	84.42 (77.01-91.39)	76.37 (68.35-84.44)	93.64 (91.67-95.60)	93.67 (91.68-95.64)	88.36 (83.26-92.87)	98.66 (97.50-99.41)	98.41 (97.08-99.28)	97.38 (95.31-98.80)

Appendix Table 1b. Unscaled values for each SDG health-related indicator by country for 1990, 2000, and 2015												
	**Indicator 5.2.1: Prevalence of women aged 15 years and older who experienced intimate partner violence**			**Indicator 6.1.1: Risk-weighted prevalence of populations using unsafe or unimproved water sources, as measured by the summary exposure value (SEV) for unsafe water**			**Indicator 6.2.2a: Risk-weighted prevalence of populations using unsafe or unimproved sanitation, as measured by the summary exposure value (SEV) for unsafe sanitation**			**Indicator 6.2.1b: Risk-weighted prevalence of populations with unsafe hygiene (no handwashing with soap), as measured by the summary exposure value (SEV) for unsafe hygiene**		
**Location**	**1990**	**2000**	**2015**	**1990**	**2000**	**2015**	**1990**	**2000**	**2015**	**1990**	**2000**	**2015**
Uganda	39.51 (33.28-45.14)	39.95 (33.81-45.42)	41.62 (36.17-46.33)	87.79 (78.33-95.98)	81.70 (74.05-89.12)	74.34 (65.95-83.27)	86.08 (80.23-92.10)	84.30 (77.69-91.04)	79.27 (71.43-87.47)	99.03 (98.18-99.58)	98.68 (97.56-99.41)	97.76 (95.95-98.97)
Zambia	38.32 (32.31-43.99)	40.49 (34.97-45.29)	38.56 (33.89-43.70)	88.80 (80.28-96.47)	87.28 (79.92-94.78)	76.24 (66.40-86.00)	76.40 (70.62-81.64)	80.53 (76.95-84.05)	63.03 (52.52-73.37)	98.18 (96.68-99.18)	97.97 (96.30-99.06)	96.52 (93.86-98.36)
Angola	31.37 (26.28-36.64)	31.37 (26.28-36.64)	31.37 (26.28-36.64)	98.85 (93.97-100.00)	97.97 (92.60-100.00)	95.60 (88.31-100.00)	84.10 (75.72-90.93)	77.94 (69.87-84.85)	55.55 (44.67-66.32)	99.58 (99.18-99.83)	99.51 (99.05-99.81)	99.06 (98.22-99.60)
Central African Republic	16.30 (13.27-19.43)	16.30 (13.27-19.43)	16.30 (13.27-19.43)	99.76 (97.28-100.00)	98.87 (93.92-100.00)	98.35 (92.70-100.00)	92.60 (88.51-96.03)	91.36 (87.04-95.36)	90.43 (85.14-94.87)	99.76 (99.51-99.90)	99.73 (99.47-99.89)	99.67 (99.34-99.86)
Congo	31.37 (26.28-36.64)	31.37 (26.28-36.64)	31.37 (26.28-36.64)	95.95 (87.95-100.00)	94.54 (86.12-100.00)	91.94 (82.93-100.00)	88.98 (82.31-94.04)	86.73 (80.45-91.87)	76.39 (67.30-84.56)	99.27 (98.57-99.70)	99.10 (98.28-99.60)	98.52 (97.18-99.35)
Democratic Republic of the Congo	43.66 (37.36-49.14)	43.66 (37.36-49.14)	44.55 (39.22-49.72)	97.90 (91.78-100.00)	98.94 (94.61-100.00)	97.24 (90.39-100.00)	90.45 (85.05-94.80)	90.80 (86.21-94.86)	83.98 (76.52-90.29)	99.69 (99.41-99.87)	99.71 (99.45-99.88)	99.58 (99.18-99.83)
Equatorial Guinea	31.37 (26.28-36.64)	31.37 (26.28-36.64)	31.37 (26.28-36.64)	99.94 (99.12-100.00)	98.16 (92.49-100.00)	90.73 (80.28-100.00)	69.87 (57.28-80.77)	35.66 (26.64-45.98)	13.37 (8.27-20.09)	99.67 (99.36-99.86)	99.17 (98.40-99.64)	97.47 (95.39-98.91)
Gabon	37.53 (31.79-43.04)	37.53 (31.79-43.04)	38.80 (33.45-44.01)	93.76 (85.36-100.00)	90.40 (80.81-100.00)	85.14 (74.18-98.66)	77.97 (67.76-86.95)	67.15 (57.47-76.45)	49.44 (38.60-60.71)	98.38 (97.01-99.29)	98.05 (96.38-99.15)	97.12 (94.75-98.72)

Appendix Table 1b. Unscaled values for each SDG health-related indicator by country for 1990, 2000, and 2015

Location	Indicator 7.1.2: Prevalence of household air pollution, as measured by the summary exposure value (SEV) for household air pollution			Indicator 8.8.1: Age-standardised all-cause disability-adjusted life year (DALY) rates attributable to occupational risks (per 100,000 population)			Indicator 11.6.2: Population-weighted mean levels of fine particulate matter smaller than 2.5 microns (PM2.5)			Indicator 16.1.1: Age-standardised death rate due to interpersonal violence (per 100,000 population)		
	1990	2000	2015	1990	2000	2015	1990	2000	2015	1990	2000	2015
Canada	0.00 (0.00-0.00)	0.00 (0.00-0.00)	0.00 (0.00-0.00)	485.79 (388.42-597.96)	498.52 (396.15-610.50)	472.58 (373.89-582.98)	7.97 (7.80-8.16)	7.82 (7.64-8.02)	7.18 (7.01-7.36)	2.05 (1.89-2.21)	1.32 (1.24-1.41)	1.42 (1.26-1.60)
United States	0.00 (0.00-0.00)	0.00 (0.00-0.00)	0.00 (0.00-0.00)	458.10 (378.90-545.63)	445.11 (368.39-529.02)	416.04 (345.54-498.12)	11.38 (11.29-11.47)	10.70 (10.61-10.79)	8.44 (8.38-8.51)	9.60 (9.38-9.85)	6.99 (6.80-7.18)	5.79 (5.56-6.04)
Australia	0.00 (0.00-0.00)	0.00 (0.00-0.00)	0.00 (0.00-0.00)	598.36 (478.39-739.44)	536.88 (426.92-656.96)	457.37 (362.56-561.82)	6.71 (6.56-6.90)	6.80 (6.62-7.00)	5.90 (5.74-6.07)	2.09 (1.97-2.20)	1.74 (1.63-1.84)	1.22 (1.13-1.32)
New Zealand	0.00 (0.00-0.00)	0.00 (0.00-0.00)	0.00 (0.00-0.00)	545.90 (434.39-669.76)	570.54 (448.42-702.54)	509.20 (398.87-640.42)	6.26 (6.03-6.51)	6.23 (6.00-6.50)	5.55 (5.32-5.80)	2.02 (1.90-2.16)	1.67 (1.57-1.78)	1.28 (1.18-1.39)
Brunei	0.00 (0.00-0.00)	0.00 (0.00-0.00)	0.00 (0.00-0.00)	375.29 (313.36-450.15)	349.40 (287.44-421.91)	319.18 (256.58-395.36)	5.92 (5.14-6.84)	5.71 (4.84-6.73)	5.15 (4.29-6.20)	1.81 (1.45-2.21)	1.51 (1.31-1.70)	1.39 (1.15-1.61)
Japan	0.00 (0.00-0.00)	0.00 (0.00-0.00)	0.00 (0.00-0.00)	300.52 (235.79-378.67)	282.92 (220.03-356.29)	287.30 (219.92-364.55)	12.73 (12.49-12.98)	12.45 (12.22-12.68)	13.33 (13.08-13.60)	0.96 (0.93-0.99)	0.90 (0.87-0.93)	0.61 (0.58-0.64)
Singapore	0.00 (0.00-0.00)	0.00 (0.00-0.00)	0.00 (0.00-0.00)	330.27 (279.61-403.88)	276.74 (224.58-355.33)	278.68 (217.64-351.10)	18.61 (13.13-25.74)	19.17 (13.58-26.42)	18.74 (13.09-26.33)	1.80 (1.70-1.91)	1.06 (1.01-1.13)	0.38 (0.36-0.41)
South Korea	0.00 (0.00-0.00)	0.00 (0.00-0.00)	0.00 (0.00-0.00)	518.19 (409.78-653.49)	436.86 (344.68-551.62)	334.14 (258.62-429.11)	25.56 (24.43-26.75)	25.78 (24.72-26.95)	28.66 (27.55-29.89)	1.77 (1.63-1.91)	2.54 (2.36-2.74)	1.77 (1.56-2.06)
Andorra	0.00 (0.00-0.00)	0.00 (0.00-0.00)	0.00 (0.00-0.00)	470.06 (350.82-637.35)	473.60 (351.51-626.09)	459.95 (340.15-608.96)	11.32 (8.58-14.69)	10.81 (8.36-14.07)	10.06 (7.72-12.96)	0.88 (0.51-1.21)	0.71 (0.43-1.00)	0.60 (0.44-0.81)
Austria	0.00 (0.00-0.00)	0.00 (0.00-0.00)	0.00 (0.00-0.00)	680.74 (535.92-862.18)	675.23 (546.22-823.67)	549.60 (437.27-679.36)	19.22 (18.68-19.83)	15.25 (14.82-15.71)	17.01 (16.51-17.52)	1.47 (1.40-1.56)	0.95 (0.91-1.01)	0.71 (0.67-0.76)

Appendix Table 1b. Unscaled values for each SDG health-related indicator by country for 1990, 2000, and 2015

Location	Indicator 7.1.2: Prevalence of household air pollution, as measured by the summary exposure value (SEV) for household air pollution			Indicator 8.8.1: Age-standardised all-cause disability-adjusted life year (DALY) rates attributable to occupational risks (per 100,000 population)			Indicator 11.6.2: Population-weighted mean levels of fine particulate matter smaller than 2.5 microns (PM2.5)			Indicator 16.1.1: Age-standardised death rate due to interpersonal violence (per 100,000 population)		
	1990	2000	2015	1990	2000	2015	1990	2000	2015	1990	2000	2015
Belgium	0.00 (0.00-0.00)	0.00 (0.00-0.00)	0.00 (0.00-0.00)	445.93 (346.14-569.11)	433.97 (317.78-554.67)	397.88 (291.86-509.19)	19.85 (19.27-20.44)	16.87 (16.38-17.36)	15.64 (15.16-16.10)	1.83 (1.73-1.93)	2.19 (2.08-2.32)	1.43 (1.33-1.54)
Cyprus	0.00 (0.00-0.00)	0.00 (0.00-0.00)	0.00 (0.00-0.00)	625.68 (482.76-791.58)	634.09 (501.27-794.40)	616.40 (482.68-774.45)	15.69 (14.46-17.11)	15.20 (13.94-16.50)	18.29 (16.87-19.97)	2.39 (2.13-2.73)	2.22 (2.00-2.51)	1.53 (1.32-1.76)
Denmark	0.00 (0.00-0.00)	0.00 (0.00-0.00)	0.00 (0.00-0.00)	616.75 (487.95-757.10)	566.82 (449.40-695.17)	512.77 (402.56-640.91)	12.86 (12.32-13.41)	10.95 (10.48-11.44)	11.02 (10.53-11.58)	1.43 (1.35-1.51)	1.55 (1.47-1.64)	0.95 (0.87-1.05)
Finland	0.00 (0.00-0.00)	0.00 (0.00-0.00)	0.00 (0.00-0.00)	506.21 (395.24-627.44)	480.13 (377.57-593.60)	444.88 (341.17-570.80)	7.84 (7.64-8.05)	7.09 (6.93-7.26)	7.38 (7.18-7.62)	3.20 (3.00-3.41)	2.78 (2.62-2.96)	1.66 (1.54-1.80)
France	0.00 (0.00-0.00)	0.00 (0.00-0.00)	0.00 (0.00-0.00)	545.90 (431.41-674.84)	529.40 (419.44-649.19)	409.13 (310.73-527.27)	14.15 (13.93-14.40)	12.60 (12.40-12.81)	12.37 (12.18-12.57)	1.86 (1.77-1.95)	1.39 (1.32-1.46)	0.97 (0.90-1.04)
Germany	0.00 (0.00-0.00)	0.00 (0.00-0.00)	0.00 (0.00-0.00)	539.70 (421.97-680.17)	531.80 (419.04-666.82)	515.80 (393.77-659.03)	17.45 (17.26-17.64)	14.08 (13.92-14.25)	14.02 (13.85-14.18)	1.19 (1.13-1.25)	1.27 (1.20-1.33)	0.81 (0.76-0.85)
Greece	0.00 (0.00-0.00)	0.00 (0.00-0.00)	0.00 (0.00-0.00)	608.84 (461.05-789.25)	570.80 (433.13-745.82)	501.23 (379.84-659.42)	13.50 (12.79-14.24)	12.00 (11.33-12.80)	13.49 (12.78-14.35)	1.12 (1.06-1.19)	1.10 (1.04-1.18)	1.04 (0.96-1.15)
Iceland	0.00 (0.00-0.00)	0.00 (0.00-0.00)	0.00 (0.00-0.00)	637.50 (496.32-806.95)	627.36 (488.41-800.95)	537.52 (411.08-687.02)	8.40 (7.72-9.18)	8.02 (7.29-8.91)	7.79 (7.05-8.61)	1.24 (1.17-1.33)	1.02 (0.95-1.09)	0.71 (0.65-0.79)
Ireland	0.00 (0.00-0.00)	0.00 (0.00-0.00)	0.00 (0.00-0.00)	539.35 (423.51-669.73)	516.22 (413.74-633.57)	588.96 (459.19-731.06)	10.80 (10.39-11.27)	9.85 (9.47-10.26)	9.80 (9.45-10.18)	1.03 (0.92-1.13)	1.02 (0.94-1.11)	0.76 (0.69-0.83)
Israel	0.00 (0.00-0.00)	0.00 (0.00-0.00)	0.00 (0.00-0.00)	455.46 (358.99-573.82)	423.81 (331.16-532.33)	428.03 (332.05-546.64)	17.93 (17.05-18.84)	17.13 (16.22-18.07)	21.09 (19.97-22.35)	2.23 (2.10-2.38)	3.97 (3.75-4.21)	2.63 (2.45-2.84)

Appendix Table 1b. Unscaled values for each SDG health-related indicator by country for 1990, 2000, and 2015												
	**Indicator 7.1.2: Prevalence of household air pollution, as measured by the summary exposure value (SEV) for household air pollution**			**Indicator 8.8.1: Age-standardised all-cause disability-adjusted life year (DALY) rates attributable to occupational risks (per 100,000 population)**			**Indicator 11.6.2: Population-weighted mean levels of fine particulate matter smaller than 2.5 microns (PM2.5)**			**Indicator 16.1.1: Age-standardised death rate due to interpersonal violence (per 100,000 population)**		
**Location**	**1990**	**2000**	**2015**	**1990**	**2000**	**2015**	**1990**	**2000**	**2015**	**1990**	**2000**	**2015**
Italy	0.00 (0.00-0.00)	0.00 (0.00-0.00)	0.00 (0.00-0.00)	621.52 (489.79-773.69)	551.43 (445.42-675.04)	498.29 (385.06-622.71)	18.52 (18.21-18.85)	15.44 (15.20-15.72)	19.86 (19.57-20.17)	2.23 (2.09-2.38)	1.32 (1.24-1.41)	0.95 (0.88-1.02)
Luxembourg	0.00 (0.00-0.00)	0.00 (0.00-0.00)	0.00 (0.00-0.00)	527.45 (409.29-662.55)	520.07 (402.25-658.21)	324.54 (233.39-421.54)	20.34 (18.57-22.45)	16.97 (15.38-18.56)	16.59 (15.21-18.15)	1.97 (1.86-2.10)	1.52 (1.43-1.62)	0.97 (0.90-1.05)
Malta	0.00 (0.00-0.00)	0.00 (0.00-0.00)	0.00 (0.00-0.00)	788.56 (600.33-986.99)	733.64 (584.25-905.45)	527.28 (361.18-729.92)	14.42 (11.59-17.82)	13.25 (10.77-16.16)	15.67 (12.74-19.26)	1.69 (1.58-1.82)	1.57 (1.47-1.70)	1.11 (1.02-1.20)
Netherlands	0.00 (0.00-0.00)	0.00 (0.00-0.00)	0.00 (0.00-0.00)	589.83 (428.35-752.87)	562.75 (412.77-722.09)	524.49 (392.83-665.73)	18.68 (18.18-19.19)	15.78 (15.40-16.18)	14.64 (14.25-15.05)	1.35 (1.27-1.42)	1.58 (1.49-1.66)	1.09 (1.02-1.19)
Norway	0.00 (0.00-0.00)	0.00 (0.00-0.00)	0.00 (0.00-0.00)	551.42 (431.05-690.71)	546.64 (431.83-677.65)	489.80 (386.27-608.80)	10.57 (10.31-10.86)	9.58 (9.34-9.83)	9.08 (8.83-9.34)	1.33 (1.25-1.42)	1.16 (1.09-1.24)	0.79 (0.73-0.86)
Portugal	0.00 (0.00-0.00)	0.00 (0.00-0.00)	0.00 (0.00-0.00)	773.45 (590.32-1,004.29)	784.10 (613.62-992.28)	688.99 (524.19-902.98)	9.88 (9.44-10.38)	9.67 (9.23-10.18)	9.76 (9.28-10.30)	2.94 (2.77-3.12)	2.39 (2.25-2.54)	1.58 (1.48-1.70)
Spain	0.00 (0.00-0.00)	0.00 (0.00-0.00)	0.00 (0.00-0.00)	697.99 (554.84-868.65)	706.00 (585.22-846.12)	489.14 (382.72-613.08)	10.37 (10.18-10.57)	10.07 (9.89-10.27)	9.74 (9.56-9.92)	1.18 (1.10-1.25)	1.14 (1.08-1.21)	0.77 (0.72-0.83)
Sweden	0.00 (0.00-0.00)	0.00 (0.00-0.00)	0.00 (0.00-0.00)	467.55 (364.97-580.47)	430.98 (335.59-534.74)	408.02 (310.64-515.80)	6.53 (6.40-6.68)	5.92 (5.78-6.06)	6.24 (6.08-6.40)	1.67 (1.59-1.76)	1.40 (1.32-1.46)	1.09 (1.02-1.18)
Switzerland	0.00 (0.00-0.00)	0.00 (0.00-0.00)	0.00 (0.00-0.00)	613.91 (481.66-763.35)	661.23 (520.76-826.48)	607.10 (472.76-755.42)	13.28 (12.85-13.72)	11.27 (10.92-11.65)	12.89 (12.47-13.32)	1.46 (1.38-1.55)	1.27 (1.21-1.35)	0.77 (0.71-0.83)
United Kingdom	0.00 (0.00-0.00)	0.00 (0.00-0.00)	0.00 (0.00-0.00)	568.39 (444.76-714.75)	545.43 (422.79-683.66)	474.30 (365.50-590.17)	14.83 (14.58-15.09)	12.98 (12.72-13.22)	12.41 (12.17-12.67)	1.01 (0.98-1.04)	0.87 (0.84-0.90)	0.63 (0.61-0.67)

Appendix Table 1b. Unscaled values for each SDG health-related indicator by country for 1990, 2000, and 2015

Location	Indicator 7.1.2: Prevalence of household air pollution, as measured by the summary exposure value (SEV) for household air pollution			Indicator 8.8.1: Age-standardised all-cause disability-adjusted life year (DALY) rates attributable to occupational risks (per 100,000 population)			Indicator 11.6.2: Population-weighted mean levels of fine particulate matter smaller than 2.5 microns (PM2.5)			Indicator 16.1.1: Age-standardised death rate due to interpersonal violence (per 100,000 population)		
	1990	2000	2015	1990	2000	2015	1990	2000	2015	1990	2000	2015
Argentina	3.17 (1.19-6.78)	1.97 (0.73-3.98)	0.59 (0.06-2.42)	954.12 (823.42-1,099.83)	934.71 (818.81-1,078.46)	890.43 (767.80-1,030.09)	15.99 (15.35-16.73)	15.67 (15.06-16.32)	13.44 (12.95-13.98)	6.00 (5.54-6.45)	6.64 (6.27-7.04)	5.82 (5.24-6.44)
Chile	7.22 (2.60-14.82)	5.24 (1.79-11.01)	1.13 (0.12-4.73)	613.99 (511.64-735.45)	537.33 (438.76-655.24)	529.06 (433.55-640.33)	20.64 (19.77-21.59)	21.95 (20.80-23.14)	20.94 (19.99-21.96)	6.35 (5.83-6.88)	5.03 (4.67-5.41)	4.09 (3.63-4.62)
Uruguay	1.87 (0.23-7.80)	0.73 (0.24-1.65)	0.33 (0.04-1.22)	699.57 (608.62-802.66)	675.97 (589.08-779.12)	691.31 (596.07-807.88)	13.03 (12.23-13.94)	13.35 (12.29-14.35)	11.49 (10.66-12.41)	3.98 (3.65-4.33)	4.99 (4.63-5.34)	5.12 (4.58-5.70)
Belarus	10.67 (3.48-23.14)	4.15 (1.56-9.04)	0.45 (0.18-0.91)	780.56 (616.73-970.60)	700.14 (559.96-850.18)	577.51 (460.91-711.55)	33.48 (31.08-36.90)	20.31 (18.29-23.15)	20.48 (17.98-23.96)	7.90 (7.28-9.25)	13.45 (10.98-14.59)	9.82 (8.09-11.81)
Estonia	15.11 (5.72-29.60)	10.71 (4.86-20.05)	4.24 (1.19-10.03)	703.16 (569.75-871.11)	621.90 (510.49-749.07)	515.09 (412.36-632.78)	11.70 (11.01-12.62)	8.07 (7.46-8.73)	9.40 (8.77-10.23)	11.35 (10.64-12.10)	14.67 (13.81-15.60)	4.21 (3.73-4.95)
Latvia	11.43 (3.69-25.76)	7.02 (3.19-13.93)	3.27 (0.96-8.38)	737.01 (581.87-919.73)	662.48 (525.92-819.43)	554.82 (434.85-698.52)	30.21 (28.27-32.63)	18.25 (17.13-19.61)	20.42 (19.17-21.90)	10.07 (9.48-10.70)	12.67 (11.93-13.56)	6.00 (5.54-6.60)
Lithuania	10.84 (3.57-23.57)	6.78 (2.08-15.51)	2.98 (0.83-7.54)	894.00 (714.49-1,112.99)	889.09 (723.00-1,093.99)	645.06 (529.50-784.13)	30.92 (29.71-32.26)	18.49 (17.82-19.20)	19.06 (18.35-19.87)	7.72 (7.29-8.17)	10.03 (9.46-10.68)	6.04 (5.60-6.52)
Moldova	26.01 (12.29-44.52)	18.96 (11.24-29.02)	3.80 (1.62-7.46)	904.85 (704.09-1,144.59)	857.20 (653.63-1,099.33)	538.05 (417.21-680.68)	36.33 (31.14-44.13)	21.99 (19.38-25.16)	20.55 (18.26-24.00)	10.07 (9.54-10.64)	10.62 (10.08-11.23)	4.96 (4.58-5.48)
Russia	7.97 (2.40-17.82)	5.32 (2.20-10.22)	1.73 (0.49-4.22)	710.21 (571.03-875.18)	664.16 (537.92-808.12)	562.14 (447.91-702.27)	22.10 (21.67-22.56)	15.33 (15.03-15.65)	16.63 (16.25-17.02)	16.67 (15.85-17.64)	35.28 (33.73-37.01)	15.10 (14.10-16.23)
Ukraine	11.17 (4.00-22.39)	4.72 (2.14-9.06)	1.89 (0.74-4.13)	753.80 (612.60-924.30)	724.82 (592.26-885.88)	664.37 (522.58-818.05)	33.31 (32.02-35.04)	20.10 (19.22-20.99)	18.88 (18.05-19.82)	8.75 (8.31-9.23)	15.77 (14.99-16.60)	7.31 (6.74-8.12)

Appendix Table 1b. Unscaled values for each SDG health-related indicator by country for 1990, 2000, and 2015

Location	Indicator 7.1.2: Prevalence of household air pollution, as measured by the summary exposure value (SEV) for household air pollution			Indicator 8.8.1: Age-standardised all-cause disability-adjusted life year (DALY) rates attributable to occupational risks (per 100,000 population)			Indicator 11.6.2: Population-weighted mean levels of fine particulate matter smaller than 2.5 microns (PM2.5)			Indicator 16.1.1: Age-standardised death rate due to interpersonal violence (per 100,000 population)		
	1990	2000	2015	1990	2000	2015	1990	2000	2015	1990	2000	2015
Albania	40.36 (27.27-55.86)	35.28 (23.55-50.23)	10.11 (5.57-16.70)	1,077.65 (813.05-1,411.22)	1,051.55 (792.30-1,348.82)	894.87 (683.00-1,139.20)	17.80 (17.15-18.47)	14.29 (13.20-15.48)	17.94 (16.19-20.02)	5.55 (3.63-6.25)	8.97 (4.04-10.12)	1.97 (1.71-2.79)
Bosnia and Herzegovina	39.58 (26.63-54.62)	29.66 (20.12-42.10)	29.00 (18.99-42.18)	589.96 (450.70-762.26)	557.14 (428.79-708.18)	522.42 (401.82-661.60)	46.70 (45.51-48.00)	37.95 (36.08-40.43)	47.40 (44.70-50.47)	0.41 (0.35-0.63)	0.49 (0.42-0.57)	2.82 (0.35-3.41)
Bulgaria	10.28 (5.23-17.58)	6.93 (3.49-11.95)	3.50 (1.61-6.35)	710.30 (548.79-909.34)	666.86 (531.83-824.18)	573.20 (450.05-718.91)	45.16 (44.17-46.27)	29.14 (28.49-29.82)	28.13 (27.42-28.86)	3.77 (3.58-3.98)	3.90 (3.69-4.12)	2.22 (2.06-2.41)
Croatia	17.75 (9.89-27.92)	9.06 (5.58-14.13)	4.35 (2.18-7.51)	656.53 (515.46-823.88)	679.91 (538.26-846.07)	611.44 (479.32-761.72)	24.77 (23.21-26.82)	18.02 (17.28-18.81)	21.78 (20.85-22.76)	3.05 (2.87-3.24)	2.41 (2.28-2.56)	1.22 (1.14-1.34)
Czech Republic	2.39 (1.24-4.40)	1.23 (0.69-1.95)	0.83 (0.38-1.60)	708.24 (575.14-857.27)	666.73 (541.36-802.98)	551.03 (435.49-683.84)	37.11 (36.29-37.98)	20.99 (20.55-21.45)	21.41 (21.00-21.84)	2.17 (2.06-2.29)	1.90 (1.81-2.00)	1.13 (1.05-1.24)
Hungary	10.50 (5.57-18.48)	7.96 (4.11-13.89)	3.98 (1.96-7.29)	574.74 (465.20-708.21)	526.85 (425.59-640.68)	474.99 (379.43-583.18)	38.36 (37.15-39.68)	24.30 (23.66-25.03)	23.06 (22.33-23.76)	3.26 (3.09-3.43)	2.74 (2.59-2.88)	1.43 (1.32-1.60)
Macedonia	28.84 (18.02-42.42)	21.09 (13.36-31.88)	13.55 (8.29-20.81)	603.07 (469.90-770.53)	592.00 (462.00-744.45)	533.90 (421.92-667.69)	51.73 (48.69-55.20)	35.86 (34.12-37.72)	40.48 (38.58-42.49)	2.61 (2.18-2.92)	3.32 (2.12-3.68)	2.30 (1.59-2.64)
Montenegro	32.95 (21.46-47.36)	20.17 (12.33-30.96)	19.19 (12.61-28.47)	631.43 (484.71-808.17)	614.62 (467.16-776.33)	558.73 (427.61-714.35)	23.93 (23.03-24.94)	18.49 (17.60-19.48)	23.35 (22.11-24.62)	6.07 (5.06-7.38)	6.36 (5.67-7.12)	4.15 (3.54-5.03)
Poland	9.13 (4.70-16.00)	5.89 (3.02-10.15)	4.15 (2.08-7.52)	834.31 (655.59-1,058.04)	721.18 (564.60-918.32)	595.26 (464.50-743.30)	45.54 (44.89-46.27)	26.31 (25.98-26.67)	24.28 (23.98-24.62)	3.18 (3.02-3.34)	3.02 (2.86-3.18)	1.70 (1.59-1.83)
Romania	17.82 (10.28-28.70)	14.67 (9.66-21.09)	8.14 (4.26-14.12)	994.85 (776.30-1,273.14)	996.07 (773.41-1,261.57)	838.61 (656.92-1,053.91)	33.03 (32.22-33.94)	21.16 (20.66-21.67)	19.58 (19.14-20.06)	4.18 (3.97-4.40)	3.48 (3.31-3.68)	2.08 (1.94-2.25)

Appendix Table 1b. Unscaled values for each SDG health-related indicator by country for 1990, 2000, and 2015

Location	Indicator 7.1.2: Prevalence of household air pollution, as measured by the summary exposure value (SEV) for household air pollution			Indicator 8.8.1: Age-standardised all-cause disability-adjusted life year (DALY) rates attributable to occupational risks (per 100,000 population)			Indicator 11.6.2: Population-weighted mean levels of fine particulate matter smaller than 2.5 microns (PM2.5)			Indicator 16.1.1: Age-standardised death rate due to interpersonal violence (per 100,000 population)		
	1990	2000	2015	1990	2000	2015	1990	2000	2015	1990	2000	2015
Serbia	28.50 (18.83-40.27)	22.81 (14.22-34.54)	16.21 (10.60-23.38)	786.33 (603.13-1,011.89)	786.61 (612.71-990.46)	665.58 (518.44-833.98)	25.35 (24.93-25.80)	19.64 (18.94-20.42)	21.44 (20.60-22.37)	4.70 (3.72-5.43)	4.42 (3.65-4.79)	2.82 (2.55-3.20)
Slovakia	5.19 (2.73-9.08)	2.09 (1.23-3.22)	1.39 (0.66-2.58)	552.40 (443.89-683.04)	531.24 (415.93-654.39)	478.42 (364.78-606.86)	35.50 (34.40-36.69)	21.66 (21.08-22.29)	20.52 (20.00-21.11)	3.50 (3.07-4.54)	3.27 (3.01-3.81)	2.24 (1.99-2.69)
Slovenia	12.36 (6.55-21.08)	6.16 (3.55-9.58)	3.61 (1.88-6.52)	637.28 (510.02-786.52)	678.24 (543.86-835.59)	544.34 (432.45-674.27)	24.58 (23.45-25.73)	16.75 (16.08-17.49)	20.35 (19.44-21.29)	2.36 (2.24-2.50)	1.84 (1.74-1.94)	0.80 (0.72-0.94)
Armenia	28.65 (13.56-46.36)	13.66 (7.17-22.66)	1.52 (0.42-3.90)	1,186.37 (932.27-1,535.63)	1,152.27 (894.00-1,436.76)	970.96 (790.13-1,182.74)	20.63 (15.88-27.90)	18.55 (14.37-25.96)	25.51 (19.22-35.47)	4.61 (4.02-5.33)	5.70 (4.04-6.53)	4.98 (3.68-5.85)
Azerbaijan	24.94 (11.85-42.40)	14.50 (7.02-23.99)	4.99 (1.34-13.02)	1,339.16 (1,021.15-1,793.73)	788.04 (630.43-972.77)	1,060.35 (776.50-1,322.11)	26.75 (24.81-29.11)	23.62 (21.24-27.11)	30.35 (26.90-35.46)	9.61 (6.60-10.79)	6.82 (6.04-7.76)	3.67 (3.02-4.98)
Georgia	28.80 (12.28-49.17)	24.81 (12.81-40.17)	14.70 (4.98-30.23)	946.35 (731.03-1,241.51)	985.94 (736.15-1,262.55)	1,029.38 (806.31-1,284.90)	18.49 (17.14-20.02)	15.76 (14.44-17.39)	20.48 (18.80-22.45)	4.07 (3.55-4.70)	4.23 (3.54-4.76)	3.44 (2.88-4.27)
Kazakhstan	20.94 (7.94-39.60)	14.44 (7.11-24.46)	5.87 (1.88-13.28)	1,219.40 (1,024.91-1,438.50)	1,288.88 (1,115.57-1,485.90)	1,090.57 (877.77-1,333.62)	20.28 (19.03-21.86)	16.52 (15.58-17.82)	19.67 (18.37-21.74)	12.58 (11.67-13.56)	18.23 (17.06-19.50)	12.57 (10.93-14.28)
Kyrgyzstan	29.05 (12.58-48.96)	24.18 (10.84-41.03)	13.83 (6.70-22.91)	1,079.29 (878.54-1,315.32)	1,385.08 (1,152.83-1,634.28)	984.71 (817.11-1,191.76)	16.92 (15.92-18.05)	14.98 (13.97-16.39)	16.92 (15.80-18.45)	11.94 (9.39-13.42)	10.06 (9.07-11.56)	6.86 (5.84-8.94)
Mongolia	41.19 (24.53-59.00)	39.46 (26.33-56.97)	23.36 (11.93-39.28)	1,084.71 (860.62-1,356.39)	1,088.08 (873.67-1,368.51)	1,002.47 (810.15-1,257.40)	17.88 (17.45-18.31)	19.16 (18.20-20.30)	23.59 (21.86-25.77)	10.37 (8.46-12.84)	12.24 (10.44-14.54)	9.72 (8.08-11.79)
Tajikistan	37.97 (22.49-55.33)	34.13 (22.00-49.18)	17.67 (8.23-33.09)	1,151.88 (895.49-1,507.69)	1,089.00 (832.83-1,422.51)	991.80 (757.82-1,270.55)	74.09 (66.66-84.14)	62.69 (54.32-78.18)	50.03 (43.10-62.68)	6.93 (5.22-7.85)	7.93 (5.77-8.94)	3.04 (2.52-4.66)

	Appendix Table 1b. Unscaled values for each SDG health-related indicator by country for 1990, 2000, and 2015											
	**Indicator 7.1.2: Prevalence of household air pollution, as measured by the summary exposure value (SEV) for household air pollution**			**Indicator 8.8.1: Age-standardised all-cause disability-adjusted life year (DALY) rates attributable to occupational risks (per 100,000 population)**			**Indicator 11.6.2: Population-weighted mean levels of fine particulate matter smaller than 2.5 microns (PM2.5)**			**Indicator 16.1.1: Age-standardised death rate due to interpersonal violence (per 100,000 population)**		
**Location**	**1990**	**2000**	**2015**	**1990**	**2000**	**2015**	**1990**	**2000**	**2015**	**1990**	**2000**	**2015**
Turkmenistan	1.37 (0.34-3.76)	0.55 (0.14-1.50)	0.23 (0.05-0.67)	992.84 (784.53-1,268.91)	983.34 (771.47-1,280.16)	799.21 (613.05-1,016.13)	37.86 (35.56-41.04)	35.17 (32.25-40.50)	31.29 (28.19-36.91)	7.08 (5.89-7.85)	7.08 (6.22-8.08)	4.99 (4.15-6.25)
Uzbekistan	18.42 (6.33-35.45)	11.43 (5.14-21.75)	7.83 (2.13-17.93)	1,005.46 (799.73-1,288.94)	957.28 (742.05-1,249.74)	860.14 (651.83-1,108.12)	50.82 (45.96-57.67)	40.04 (37.17-44.70)	39.67 (37.06-44.84)	6.88 (4.56-7.68)	4.06 (3.64-5.07)	3.28 (2.66-4.22)
Colombia	17.26 (10.82-26.23)	11.69 (7.05-17.65)	6.98 (2.94-13.79)	784.91 (650.56-950.45)	806.96 (666.05-975.41)	757.35 (618.56-915.07)	21.25 (20.72-21.80)	20.42 (19.91-20.97)	18.00 (17.54-18.45)	71.10 (68.98-73.20)	67.15 (65.52-68.66)	33.61 (30.68-36.32)
Costa Rica	16.88 (8.86-28.78)	7.71 (5.20-10.90)	1.92 (0.74-4.04)	689.05 (560.20-838.89)	713.95 (598.31-845.52)	624.13 (504.50-770.66)	21.33 (20.23-22.58)	22.01 (20.57-23.60)	20.12 (18.84-21.50)	5.79 (5.33-6.28)	6.24 (5.83-6.65)	7.85 (7.03-8.69)
El Salvador	36.50 (20.94-55.53)	23.45 (13.72-37.15)	8.50 (3.32-16.49)	690.46 (562.21-842.64)	646.07 (516.04-791.74)	635.68 (500.17-797.20)	39.46 (36.14-43.18)	42.84 (39.42-46.87)	37.00 (34.32-40.21)	58.40 (52.37-65.15)	57.34 (49.76-61.63)	58.32 (45.91-66.73)
Guatemala	43.10 (26.80-62.12)	36.66 (23.87-53.43)	23.21 (11.28-40.56)	837.43 (680.26-1,030.65)	804.50 (636.43-996.71)	774.29 (622.74-959.52)	35.35 (34.25-36.50)	38.71 (37.39-40.07)	34.85 (33.84-35.90)	38.11 (35.62-40.81)	38.89 (36.98-40.96)	39.52 (33.43-46.36)
Honduras	43.99 (27.95-64.15)	37.02 (23.96-53.72)	22.93 (12.63-37.51)	778.26 (626.22-962.03)	808.68 (643.21-993.18)	785.46 (640.40-963.30)	35.62 (33.68-37.83)	37.27 (35.23-39.54)	37.59 (35.11-40.21)	25.80 (22.41-29.35)	42.66 (24.41-53.80)	48.38 (23.79-65.24)
Mexico	13.47 (9.03-19.30)	11.29 (7.50-16.03)	6.12 (4.03-8.85)	749.46 (645.14-877.44)	735.48 (632.64-851.52)	711.33 (611.25-828.58)	24.60 (23.96-25.43)	28.91 (27.96-29.87)	20.24 (19.71-20.75)	24.08 (23.39-24.88)	14.66 (14.24-15.20)	16.81 (16.33-17.34)
Nicaragua	44.33 (29.36-63.56)	38.32 (24.98-54.65)	23.47 (11.68-40.86)	779.15 (619.70-978.06)	775.68 (655.99-939.73)	754.98 (619.25-910.65)	33.36 (29.42-38.81)	34.37 (29.35-42.93)	27.38 (23.74-33.34)	11.77 (10.40-13.40)	11.42 (10.24-12.77)	10.13 (8.54-12.02)
Panama	18.37 (11.61-27.00)	12.32 (7.54-18.55)	5.67 (2.12-11.11)	954.67 (767.54-1,163.23)	910.22 (756.60-1,078.71)	685.68 (541.11-858.62)	15.92 (14.73-17.42)	16.30 (14.46-18.45)	13.20 (11.71-14.94)	10.45 (9.07-12.76)	11.63 (10.38-13.05)	17.55 (10.57-21.41)

Appendix Table 1b. Unscaled values for each SDG health-related indicator by country for 1990, 2000, and 2015

Location	Indicator 7.1.2: Prevalence of household air pollution, as measured by the summary exposure value (SEV) for household air pollution			Indicator 8.8.1: Age-standardised all-cause disability-adjusted life year (DALY) rates attributable to occupational risks (per 100,000 population)			Indicator 11.6.2: Population-weighted mean levels of fine particulate matter smaller than 2.5 microns (PM2.5)			Indicator 16.1.1: Age-standardised death rate due to interpersonal violence (per 100,000 population)		
	1990	2000	2015	1990	2000	2015	1990	2000	2015	1990	2000	2015
Venezuela	1.64 (0.61-3.49)	0.18 (0.09-0.30)	0.08 (0.03-0.17)	773.56 (638.33-939.35)	783.84 (639.78-965.31)	765.45 (628.86-935.85)	32.39 (31.00-33.99)	32.56 (30.57-34.95)	23.76 (22.53-25.13)	14.54 (13.56-15.62)	30.56 (29.41-31.74)	43.76 (37.26-50.42)
Bolivia	30.47 (19.65-44.30)	22.27 (14.68-31.73)	11.05 (5.80-19.34)	1,303.67 (843.91-2,076.59)	983.96 (800.80-1,208.03)	995.90 (774.77-1,343.84)	27.87 (27.30-28.49)	30.21 (29.10-31.46)	27.97 (26.99-29.05)	11.95 (8.86-16.74)	9.59 (8.03-13.10)	7.60 (5.79-10.37)
Ecuador	17.44 (11.58-25.32)	7.41 (4.53-11.16)	1.95 (0.88-3.68)	640.92 (503.39-949.80)	665.57 (520.63-967.61)	692.90 (539.08-1,021.55)	17.51 (16.98-18.09)	17.96 (17.38-18.53)	12.84 (12.48-13.24)	13.85 (12.29-15.91)	19.13 (13.71-21.46)	14.94 (11.36-17.85)
Peru	36.00 (22.72-54.37)	24.53 (16.48-34.92)	15.65 (9.76-23.70)	797.38 (634.68-1,036.97)	789.48 (627.59-1,037.01)	758.01 (585.81-1,023.09)	27.55 (26.24-29.16)	35.44 (33.48-37.82)	27.82 (26.31-29.60)	7.61 (5.42-8.63)	4.37 (3.90-4.99)	3.26 (2.68-4.00)
Antigua and Barbuda	3.50 (1.05-8.13)	1.95 (0.78-4.14)	1.28 (0.40-3.27)	610.84 (508.66-735.75)	618.68 (516.03-738.88)	609.75 (501.07-738.59)	17.13 (9.06-29.98)	17.32 (9.28-30.34)	13.89 (7.95-23.61)	3.78 (3.33-4.28)	5.07 (4.49-5.73)	6.11 (5.17-7.15)
The Bahamas	2.60 (0.75-6.30)	1.45 (0.41-3.81)	0.55 (0.17-1.36)	786.90 (674.15-916.34)	703.65 (596.65-829.34)	690.05 (572.52-836.56)	19.40 (12.49-30.02)	18.68 (11.34-29.36)	13.72 (8.74-21.33)	16.93 (14.80-19.72)	18.08 (15.13-21.75)	20.45 (14.98-24.71)
Barbados	0.58 (0.17-1.47)	0.27 (0.08-0.69)	0.07 (0.02-0.16)	610.94 (511.53-722.21)	559.05 (464.96-673.34)	555.62 (456.04-671.86)	20.54 (10.73-34.71)	20.36 (9.62-37.95)	15.39 (8.10-26.74)	6.70 (6.07-7.41)	8.12 (7.27-9.12)	11.28 (9.50-12.99)
Belize	19.85 (10.78-31.72)	11.57 (5.87-18.44)	8.09 (3.46-15.63)	719.22 (603.22-851.49)	822.07 (716.95-954.77)	806.74 (687.81-944.01)	29.47 (26.83-32.51)	29.43 (26.91-32.39)	27.04 (24.59-29.80)	8.80 (7.39-14.86)	18.35 (15.96-20.62)	23.71 (14.90-28.66)
Cuba	8.10 (2.70-17.29)	12.22 (6.09-21.04)	1.62 (0.54-3.56)	839.92 (721.74-983.05)	820.43 (709.41-954.35)	798.60 (605.15-939.77)	22.93 (21.10-25.51)	21.75 (20.13-23.82)	17.99 (16.76-19.60)	7.57 (7.04-8.13)	5.31 (4.99-5.68)	5.79 (5.16-6.56)
Dominica	22.82 (9.95-41.13)	10.36 (5.11-18.27)	3.51 (1.04-8.60)	719.79 (590.79-868.51)	710.55 (588.53-842.31)	702.43 (579.08-840.51)	18.03 (11.73-26.84)	17.95 (11.74-27.22)	13.56 (8.82-20.39)	4.47 (3.81-6.95)	5.92 (5.07-7.19)	12.01 (6.83-14.82)

Appendix Table 1b. Unscaled values for each SDG health-related indicator by country for 1990, 2000, and 2015												
	**Indicator 7.1.2: Prevalence of household air pollution, as measured by the summary exposure value (SEV) for household air pollution**			**Indicator 8.8.1: Age-standardised all-cause disability-adjusted life year (DALY) rates attributable to occupational risks (per 100,000 population)**			**Indicator 11.6.2: Population-weighted mean levels of fine particulate matter smaller than 2.5 microns (PM2.5)**			**Indicator 16.1.1: Age-standardised death rate due to interpersonal violence (per 100,000 population)**		
**Location**	**1990**	**2000**	**2015**	**1990**	**2000**	**2015**	**1990**	**2000**	**2015**	**1990**	**2000**	**2015**
Dominican Republic	22.22 (12.45-36.36)	8.44 (4.98-13.22)	4.53 (2.05-8.28)	989.30 (851.95-1,150.37)	926.23 (797.26-1,078.25)	836.04 (711.39-980.66)	20.77 (19.14-22.76)	29.80 (24.96-36.94)	20.42 (17.49-24.58)	11.25 (10.03-14.63)	10.74 (9.78-12.63)	14.08 (10.65-16.11)
Grenada	16.49 (6.80-30.95)	4.91 (2.29-9.52)	1.77 (0.55-4.26)	676.06 (565.16-813.69)	695.95 (590.77-823.34)	720.58 (606.36-863.46)	20.37 (11.48-32.70)	20.46 (11.31-35.43)	15.14 (9.28-24.17)	3.30 (2.77-5.59)	6.63 (5.44-7.68)	8.20 (5.43-9.87)
Guyana	12.35 (4.51-24.45)	8.27 (4.68-13.66)	4.28 (1.54-9.36)	689.75 (576.28-827.50)	672.99 (563.01-803.60)	641.71 (526.13-766.92)	26.25 (22.34-31.05)	26.51 (19.94-38.23)	17.39 (12.99-25.72)	12.27 (10.61-18.76)	18.63 (16.12-21.26)	19.48 (14.27-23.96)
Haiti	54.03 (37.17-73.79)	53.62 (36.86-73.18)	49.53 (33.88-69.98)	1,071.60 (897.54-1,283.06)	970.54 (796.91-1,177.10)	951.93 (778.71-1,151.87)	30.76 (27.45-34.91)	31.01 (27.38-37.35)	25.84 (22.49-30.69)	13.62 (8.38-17.06)	9.91 (6.62-12.45)	5.91 (4.14-8.17)
Jamaica	26.67 (16.25-40.58)	11.49 (6.36-19.04)	5.57 (2.40-10.82)	819.02 (675.40-991.10)	824.86 (695.11-976.39)	748.77 (626.98-895.55)	20.74 (19.47-22.10)	20.51 (19.37-21.73)	16.99 (16.04-18.12)	6.62 (5.84-9.64)	16.43 (12.51-18.80)	23.41 (12.23-29.07)
Saint Lucia	21.10 (12.18-33.27)	9.31 (4.56-16.78)	3.47 (1.51-6.65)	793.00 (652.93-956.70)	761.47 (635.67-909.88)	692.30 (576.31-842.93)	19.09 (12.95-27.13)	19.45 (12.41-29.70)	14.01 (9.42-20.32)	8.71 (7.72-9.81)	13.49 (12.13-14.95)	15.37 (13.35-17.63)
Saint Vincent and the Grenadines	39.39 (24.71-57.24)	14.55 (5.95-28.25)	3.00 (1.03-6.45)	603.07 (502.09-724.60)	648.57 (547.08-770.69)	641.94 (533.77-772.09)	19.17 (11.21-31.60)	19.26 (11.11-30.06)	14.25 (8.83-23.23)	10.13 (8.90-11.44)	12.05 (10.93-13.32)	20.07 (17.10-22.74)
Suriname	23.49 (9.81-42.13)	14.34 (5.50-27.36)	4.54 (1.74-10.36)	569.08 (471.47-689.39)	546.76 (455.74-656.54)	554.47 (462.66-663.29)	28.09 (18.43-48.45)	28.38 (18.07-49.00)	18.47 (11.90-29.34)	6.72 (5.89-9.32)	8.85 (7.71-10.14)	9.98 (7.57-12.06)
Trinidad and Tobago	2.47 (0.78-5.99)	0.84 (0.29-1.94)	0.36 (0.11-0.90)	545.78 (457.75-650.23)	489.75 (409.42-585.69)	491.80 (407.23-599.09)	19.51 (16.05-23.58)	19.37 (16.10-23.18)	14.19 (11.90-16.85)	9.74 (8.99-10.52)	11.69 (10.89-12.48)	22.02 (18.79-25.37)
Brazil	16.71 (11.37-23.48)	10.15 (6.78-14.42)	4.14 (2.74-5.90)	1,045.86 (908.64-1,208.52)	1,030.97 (892.62-1,196.32)	880.85 (751.92-1,035.17)	14.78 (14.56-15.01)	16.16 (15.89-16.47)	11.37 (11.20-11.55)	28.33 (26.86-32.00)	31.44 (27.89-32.95)	27.79 (24.42-29.76)

Appendix Table 1b. Unscaled values for each SDG health-related indicator by country for 1990, 2000, and 2015												
	**Indicator 7.1.2: Prevalence of household air pollution, as measured by the summary exposure value (SEV) for household air pollution**			**Indicator 8.8.1: Age-standardised all-cause disability-adjusted life year (DALY) rates attributable to occupational risks (per 100,000 population)**			**Indicator 11.6.2: Population-weighted mean levels of fine particulate matter smaller than 2.5 microns (PM2.5)**			**Indicator 16.1.1: Age-standardised death rate due to interpersonal violence (per 100,000 population)**		
**Location**	**1990**	**2000**	**2015**	**1990**	**2000**	**2015**	**1990**	**2000**	**2015**	**1990**	**2000**	**2015**
Paraguay	38.66 (25.58-55.77)	33.32 (22.38-47.75)	17.80 (10.37-27.62)	946.12 (770.75-1,152.84)	954.70 (786.44-1,156.27)	923.60 (765.81-1,104.79)	22.44 (20.86-24.43)	22.99 (21.33-25.04)	14.85 (13.61-16.33)	9.98 (8.55-13.97)	17.59 (14.66-19.57)	13.91 (11.02-17.96)
China	35.97 (23.76-50.46)	30.52 (20.04-42.97)	15.79 (10.50-22.51)	1,361.17 (1,189.20-1,559.26)	1,184.30 (1,030.97-1,360.36)	969.53 (831.81-1,129.90)	48.57 (48.41-48.74)	51.45 (51.23-51.66)	58.09 (57.81-58.36)	4.34 (3.94-4.74)	3.82 (3.28-4.06)	1.41 (1.28-1.83)
North Korea	38.24 (25.08-53.78)	36.47 (24.01-52.04)	30.37 (19.09-44.14)	1,099.49 (906.99-1,343.85)	1,114.45 (926.43-1,339.92)	1,094.13 (906.72-1,311.35)	30.70 (28.60-33.65)	30.96 (28.55-34.00)	34.09 (31.46-37.57)	4.06 (2.74-5.78)	3.69 (2.50-5.09)	2.77 (1.88-3.90)
Taiwan	0.00 (0.00-0.00)	0.00 (0.00-0.00)	0.00 (0.00-0.00)	815.32 (687.50-951.92)	741.19 (631.24-868.02)	642.97 (537.08-764.53)	27.13 (25.99-28.30)	29.96 (28.54-31.39)	30.46 (29.10-32.00)	6.55 (3.00-7.92)	4.18 (2.23-4.79)	1.58 (1.27-1.97)
Cambodia	39.93 (26.37-56.52)	39.59 (26.12-55.49)	34.69 (22.84-49.10)	1,382.19 (1,139.19-1,674.38)	1,384.50 (1,151.28-1,667.49)	1,282.74 (1,039.31-1,571.14)	24.81 (22.75-28.10)	25.18 (22.70-29.01)	29.04 (25.94-34.67)	4.96 (3.89-6.09)	4.26 (3.50-4.96)	2.82 (2.04-3.89)
Indonesia	33.22 (20.97-48.58)	22.68 (13.71-35.38)	13.12 (6.76-21.90)	932.71 (770.91-1,114.09)	868.26 (712.34-1,041.80)	803.58 (661.43-975.51)	16.65 (16.34-16.99)	16.49 (16.20-16.83)	15.41 (15.13-15.71)	0.86 (0.69-1.20)	0.88 (0.72-1.09)	1.05 (0.79-1.36)
Laos	39.29 (25.84-55.23)	39.43 (25.94-55.38)	37.76 (24.82-53.62)	1,456.50 (1,197.55-1,747.07)	1,408.44 (1,164.44-1,680.35)	1,223.10 (997.74-1,478.40)	26.66 (25.51-27.92)	27.18 (25.81-28.96)	32.57 (30.76-35.09)	1.19 (0.80-1.66)	1.03 (0.78-1.31)	0.62 (0.46-0.85)
Malaysia	4.32 (1.50-10.33)	0.85 (0.37-1.70)	0.29 (0.09-0.70)	912.44 (777.85-1,069.60)	840.71 (709.32-998.26)	698.50 (598.84-811.61)	15.52 (14.99-16.06)	15.91 (15.35-16.53)	15.56 (14.94-16.21)	2.11 (1.78-3.79)	3.89 (3.41-4.36)	4.15 (3.00-5.16)
Maldives	26.53 (14.95-40.82)	17.72 (10.44-27.58)	3.38 (1.31-7.09)	740.74 (615.63-881.46)	620.57 (505.27-754.43)	513.44 (407.64-634.24)	30.03 (19.51-44.65)	30.29 (19.35-44.86)	29.06 (18.76-43.20)	1.59 (1.22-4.08)	4.67 (3.19-5.30)	3.50 (1.79-4.47)
Mauritius	6.53 (2.38-13.35)	1.82 (0.94-3.25)	1.37 (0.45-3.08)	631.38 (528.66-747.52)	556.01 (455.47-666.01)	496.45 (397.38-622.78)	17.82 (14.73-21.72)	18.13 (14.80-22.34)	14.95 (12.53-18.15)	2.20 (2.00-2.42)	2.08 (1.91-2.25)	2.56 (2.26-2.91)

Appendix Table 1b. Unscaled values for each SDG health-related indicator by country for 1990, 2000, and 2015												
	**Indicator 7.1.2: Prevalence of household air pollution, as measured by the summary exposure value (SEV) for household air pollution**			**Indicator 8.8.1: Age-standardised all-cause disability-adjusted life year (DALY) rates attributable to occupational risks (per 100,000 population)**			**Indicator 11.6.2: Population-weighted mean levels of fine particulate matter smaller than 2.5 microns (PM2.5)**			**Indicator 16.1.1: Age-standardised death rate due to interpersonal violence (per 100,000 population)**		
**Location**	**1990**	**2000**	**2015**	**1990**	**2000**	**2015**	**1990**	**2000**	**2015**	**1990**	**2000**	**2015**
Myanmar	39.72 (26.13-55.62)	39.46 (26.16-55.37)	35.10 (22.65-50.63)	1,459.97 (1,148.41-1,806.80)	1,413.96 (1,106.21-1,748.35)	1,322.17 (1,056.90-1,627.99)	43.70 (42.87-44.71)	44.23 (43.33-45.22)	54.39 (53.06-55.94)	3.84 (2.34-5.64)	2.82 (1.53-4.29)	2.37 (1.36-3.73)
Philippines	26.73 (17.50-39.10)	20.11 (12.36-29.81)	22.10 (13.13-34.26)	985.55 (825.71-1,184.19)	1,004.34 (849.66-1,188.94)	918.00 (769.73-1,092.49)	24.42 (23.43-25.64)	25.23 (24.14-26.50)	23.49 (22.52-24.63)	11.27 (10.33-12.18)	20.16 (18.93-21.44)	15.53 (13.44-17.72)
Sri Lanka	36.54 (23.99-51.89)	32.83 (21.76-47.25)	28.66 (16.92-41.82)	750.47 (603.35-938.20)	734.80 (597.85-898.27)	605.94 (476.93-749.26)	29.93 (28.38-31.56)	30.72 (29.09-32.64)	27.92 (26.40-29.77)	12.58 (11.37-13.81)	9.50 (8.81-10.26)	4.83 (3.61-6.23)
Seychelles	3.06 (1.00-6.97)	1.24 (0.47-2.76)	0.51 (0.17-1.18)	618.71 (516.10-737.42)	647.04 (549.31-765.66)	584.92 (482.76-709.11)	19.67 (9.02-38.33)	19.19 (9.11-37.73)	13.21 (6.26-24.53)	9.09 (7.16-10.68)	9.66 (7.33-11.07)	6.74 (5.14-8.15)
Thailand	26.26 (16.86-38.63)	15.38 (9.64-22.99)	8.87 (4.38-14.93)	804.00 (644.45-987.92)	851.68 (704.28-1,017.96)	758.23 (614.99-923.38)	23.55 (23.25-23.84)	23.84 (23.50-24.17)	26.35 (25.86-26.86)	14.78 (12.70-16.46)	15.25 (13.40-17.47)	9.02 (7.19-12.27)
Timor-Leste	39.69 (26.27-55.64)	39.10 (25.50-55.08)	36.47 (23.91-51.21)	999.15 (811.11-1,208.95)	896.46 (728.26-1,092.35)	745.05 (587.73-914.57)	19.69 (16.10-24.98)	20.27 (16.58-25.49)	18.90 (15.04-23.74)	5.43 (3.09-8.33)	3.75 (2.18-5.09)	2.93 (2.00-4.05)
Vietnam	37.00 (24.08-52.76)	32.80 (21.21-47.21)	15.44 (9.40-23.77)	929.89 (760.16-1,124.04)	930.48 (769.81-1,113.66)	935.37 (755.55-1,126.59)	26.76 (26.15-27.49)	27.10 (26.41-27.79)	28.25 (27.44-29.08)	2.51 (1.82-3.44)	2.22 (1.66-2.96)	1.98 (1.24-2.99)
Federated States of Micronesia	23.75 (14.76-36.15)	20.07 (12.00-29.65)	11.81 (5.48-21.82)	1,169.95 (931.42-1,461.90)	1,133.03 (917.56-1,425.73)	1,093.84 (870.26-1,398.18)	9.45 (4.18-19.29)	9.56 (4.51-19.43)	8.06 (3.67-15.53)	6.58 (3.63-10.09)	6.52 (3.74-10.19)	5.91 (3.44-10.15)
Fiji	24.50 (14.59-37.74)	16.67 (9.16-26.47)	7.14 (3.00-13.45)	847.72 (695.19-1,019.16)	825.38 (689.63-988.24)	701.43 (579.99-835.90)	8.69 (7.24-10.60)	8.83 (6.17-13.17)	7.87 (5.55-11.74)	4.17 (3.23-5.42)	6.52 (4.96-7.70)	5.95 (4.21-7.49)
Kiribati	28.20 (17.02-41.42)	22.54 (12.80-35.94)	14.78 (7.36-24.76)	968.37 (821.46-1,134.92)	951.11 (803.82-1,126.71)	906.90 (755.56-1,078.58)	4.43 (3.47-5.71)	4.48 (2.91-6.54)	3.45 (2.26-5.05)	15.61 (5.43-19.65)	17.29 (5.46-21.94)	25.42 (4.99-33.94)

Appendix Table 1b. Unscaled values for each SDG health-related indicator by country for 1990, 2000, and 2015												
	**Indicator 7.1.2: Prevalence of household air pollution, as measured by the summary exposure value (SEV) for household air pollution**			**Indicator 8.8.1: Age-standardised all-cause disability-adjusted life year (DALY) rates attributable to occupational risks (per 100,000 population)**			**Indicator 11.6.2: Population-weighted mean levels of fine particulate matter smaller than 2.5 microns (PM2.5)**			**Indicator 16.1.1: Age-standardised death rate due to interpersonal violence (per 100,000 population)**		
**Location**	**1990**	**2000**	**2015**	**1990**	**2000**	**2015**	**1990**	**2000**	**2015**	**1990**	**2000**	**2015**
Marshall Islands	10.71 (5.70-18.30)	13.50 (7.33-21.56)	7.50 (3.31-14.06)	965.54 (811.67-1,148.56)	962.73 (805.45-1,152.26)	943.36 (796.55-1,111.82)	12.59 (2.82-37.50)	12.91 (2.59-39.39)	11.92 (2.24-37.94)	7.27 (4.94-9.04)	5.92 (4.02-7.40)	5.51 (3.79-7.37)
Papua New Guinea	37.51 (24.55-52.98)	36.40 (23.77-51.56)	33.12 (21.36-48.57)	2,540.45 (1,929.42-3,229.02)	2,307.64 (1,748.45-2,953.10)	2,148.26 (1,639.79-2,809.54)	15.08 (14.19-16.46)	15.81 (14.62-17.72)	13.86 (12.77-15.49)	9.42 (5.76-14.29)	9.01 (5.22-13.71)	8.10 (4.59-13.13)
Samoa	29.33 (19.18-43.01)	26.14 (16.12-38.86)	20.97 (12.08-33.73)	852.25 (691.82-1,033.98)	779.08 (626.45-945.65)	699.71 (557.05-859.31)	4.35 (3.76-5.06)	4.38 (3.80-5.02)	3.81 (3.26-4.39)	5.46 (3.69-7.67)	5.27 (3.29-7.54)	4.23 (2.93-5.89)
Solomon Islands	38.27 (25.00-53.99)	36.91 (24.37-51.49)	32.86 (21.34-47.79)	1,400.67 (1,106.63-1,738.64)	1,293.00 (1,008.71-1,612.12)	1,236.75 (968.63-1,580.44)	7.26 (5.96-9.94)	7.21 (5.52-10.83)	6.58 (5.00-9.67)	8.34 (4.83-12.58)	7.91 (4.47-12.20)	7.18 (4.06-11.29)
Tonga	30.16 (19.66-43.82)	25.63 (16.05-38.23)	14.54 (7.17-24.71)	989.73 (827.66-1,176.76)	986.17 (830.20-1,163.20)	990.42 (825.35-1,181.28)	4.92 (4.04-5.93)	4.91 (3.97-5.98)	4.03 (3.26-5.01)	10.29 (7.02-13.06)	3.53 (2.83-7.02)	2.68 (1.87-6.31)
Vanuatu	34.77 (22.93-49.45)	34.62 (22.89-49.29)	28.78 (17.53-42.57)	1,719.71 (1,337.44-2,237.81)	1,462.95 (1,144.79-1,827.18)	1,267.15 (1,017.72-1,576.16)	10.26 (8.20-12.70)	10.40 (7.12-17.52)	8.52 (5.52-16.17)	1.56 (0.99-2.33)	1.58 (0.83-2.45)	1.42 (0.84-2.21)
Afghanistan	47.79 (33.04-65.26)	47.94 (33.26-65.09)	37.94 (22.88-55.53)	992.68 (808.26-1,222.20)	1,019.29 (823.01-1,240.24)	973.89 (796.59-1,183.77)	60.93 (59.04-63.08)	56.05 (54.40-57.86)	48.02 (46.60-49.66)	40.30 (25.58-58.52)	41.40 (27.46-57.50)	35.23 (22.85-49.46)
Algeria	4.88 (1.08-13.29)	1.98 (0.78-3.93)	0.18 (0.07-0.39)	560.23 (455.36-669.74)	493.83 (401.66-608.50)	436.39 (349.93-540.19)	34.04 (32.76-35.91)	32.35 (30.80-34.35)	35.56 (33.78-37.66)	1.41 (1.09-1.79)	1.31 (1.07-1.57)	0.88 (0.70-1.10)
Bahrain	0.48 (0.09-1.53)	0.31 (0.05-1.03)	0.14 (0.03-0.43)	869.42 (748.40-1,016.24)	779.48 (689.78-881.25)	607.95 (510.97-722.27)	62.62 (51.43-75.59)	62.47 (50.39-76.62)	55.45 (44.82-67.86)	2.21 (1.78-3.31)	2.96 (2.49-3.51)	2.19 (1.71-2.75)
Egypt	0.01 (0.00-0.04)	0.56 (0.34-0.86)	0.01 (0.00-0.03)	705.73 (568.33-864.14)	588.01 (480.55-717.67)	572.76 (462.46-704.86)	75.78 (71.73-80.33)	73.29 (68.84-78.51)	104.67 (98.25-112.67)	0.82 (0.69-1.77)	2.84 (1.51-3.23)	3.27 (1.33-3.93)

Location	Indicator 7.1.2: Prevalence of household air pollution, as measured by the summary exposure value (SEV) for household air pollution			Indicator 8.8.1: Age-standardised all-cause disability-adjusted life year (DALY) rates attributable to occupational risks (per 100,000 population)			Indicator 11.6.2: Population-weighted mean levels of fine particulate matter smaller than 2.5 microns (PM2.5)			Indicator 16.1.1: Age-standardised death rate due to interpersonal violence (per 100,000 population)		
	1990	2000	2015	1990	2000	2015	1990	2000	2015	1990	2000	2015
Iran	4.11 (0.92-11.64)	1.08 (0.41-2.21)	0.35 (0.07-0.99)	630.31 (518.33-760.15)	595.57 (497.00-716.78)	543.81 (442.70-656.49)	49.44 (48.89-49.99)	48.02 (47.50-48.57)	42.99 (42.52-43.49)	4.83 (3.63-6.03)	4.39 (3.58-5.08)	2.66 (1.98-3.57)
Iraq	2.73 (0.56-7.59)	2.00 (0.84-4.27)	0.48 (0.17-1.12)	593.38 (478.72-727.26)	611.18 (496.11-744.29)	584.63 (477.49-700.24)	51.48 (49.37-54.07)	50.96 (48.02-54.23)	52.00 (49.22-55.29)	7.91 (5.81-10.26)	10.67 (7.52-13.83)	18.24 (7.87-25.46)
Jordan	0.22 (0.04-0.69)	0.06 (0.02-0.14)	0.02 (0.00-0.05)	483.55 (395.83-584.64)	457.97 (373.27-558.49)	427.34 (348.51-508.31)	31.78 (29.76-33.98)	29.75 (28.05-31.62)	38.80 (36.67-40.98)	4.17 (3.31-5.43)	4.54 (3.51-5.47)	3.61 (2.51-4.36)
Kuwait	0.21 (0.04-0.68)	0.11 (0.02-0.34)	0.05 (0.01-0.16)	569.35 (467.02-685.04)	552.10 (464.71-659.70)	482.27 (392.83-579.86)	64.23 (56.97-73.30)	65.89 (58.27-74.95)	67.43 (59.23-77.13)	1.33 (1.18-1.54)	1.58 (1.44-1.72)	0.83 (0.67-1.04)
Lebanon	0.03 (0.01-0.12)	0.02 (0.00-0.06)	0.00 (0.00-0.02)	570.27 (473.91-688.17)	512.02 (420.71-614.69)	515.07 (411.88-618.17)	24.74 (23.07-26.77)	24.63 (22.32-27.05)	32.60 (29.41-36.27)	8.08 (3.62-10.93)	2.93 (2.03-3.84)	1.49 (1.00-2.21)
Libya	2.59 (0.60-7.51)	1.71 (0.51-4.33)	0.81 (0.15-2.58)	764.19 (635.11-913.94)	780.76 (648.56-936.30)	755.24 (622.93-895.68)	56.66 (53.23-60.75)	54.19 (50.61-59.06)	79.20 (73.89-86.00)	2.00 (1.45-2.64)	1.92 (1.41-2.32)	1.82 (1.24-2.38)
Morocco	8.95 (1.92-23.39)	4.59 (1.86-9.10)	2.49 (0.51-7.51)	935.69 (748.97-1,144.35)	869.00 (699.99-1,076.74)	789.62 (618.66-974.29)	22.31 (21.95-22.71)	21.72 (21.00-22.37)	22.99 (22.25-23.87)	0.73 (0.55-1.85)	1.49 (1.17-1.90)	2.65 (1.31-3.76)
Palestine	0.03 (0.01-0.10)	0.04 (0.01-0.09)	0.77 (0.40-1.33)	561.96 (459.12-684.55)	534.66 (443.90-649.95)	540.35 (439.36-658.61)	17.50 (16.34-18.76)	17.04 (16.06-18.14)	20.61 (19.32-21.90)	2.64 (1.82-4.55)	2.87 (2.37-3.75)	5.95 (3.64-8.43)
Oman	2.19 (0.44-6.40)	1.33 (0.26-3.85)	0.63 (0.12-1.76)	426.93 (334.37-538.80)	427.19 (344.87-531.19)	488.45 (403.70-595.12)	65.69 (59.83-73.81)	66.15 (59.95-75.17)	53.28 (48.73-59.06)	0.66 (0.43-1.02)	0.61 (0.48-0.85)	1.06 (0.79-1.38)
Qatar	0.03 (0.01-0.11)	0.02 (0.00-0.05)	0.01 (0.00-0.02)	757.45 (638.33-910.31)	820.46 (695.01-971.07)	766.85 (630.59-919.11)	107.85 (95.44-120.42)	105.64 (91.21-122.78)	107.31 (94.86-122.03)	0.79 (0.61-1.36)	0.73 (0.59-1.30)	0.99 (0.73-1.29)

Appendix Table 1b. Unscaled values for each SDG health-related indicator by country for 1990, 2000, and 2015

Appendix Table 1b. Unscaled values for each SDG health-related indicator by country for 1990, 2000, and 2015

Location	Indicator 7.1.2: Prevalence of household air pollution, as measured by the summary exposure value (SEV) for household air pollution			Indicator 8.8.1: Age-standardised all-cause disability-adjusted life year (DALY) rates attributable to occupational risks (per 100,000 population)			Indicator 11.6.2: Population-weighted mean levels of fine particulate matter smaller than 2.5 microns (PM2.5)			Indicator 16.1.1: Age-standardised death rate due to interpersonal violence (per 100,000 population)		
	1990	2000	2015	1990	2000	2015	1990	2000	2015	1990	2000	2015
Saudi Arabia	1.15 (0.58-2.10)	0.65 (0.32-1.18)	0.27 (0.13-0.49)	444.65 (369.02-531.84)	425.93 (355.86-509.47)	385.03 (320.13-460.47)	85.07 (84.63-85.51)	92.91 (91.24-94.73)	106.16 (104.10-108.23)	0.40 (0.30-0.65)	0.36 (0.31-0.59)	0.62 (0.48-0.70)
Sudan	44.79 (29.61-62.16)	44.23 (30.50-60.20)	31.59 (21.15-44.10)	923.94 (749.76-1,125.32)	859.87 (687.08-1,071.78)	814.77 (644.98-1,016.27)	57.35 (55.08-60.71)	57.14 (54.96-59.96)	50.05 (48.32-52.45)	0.56 (0.41-0.76)	0.67 (0.46-0.92)	1.84 (0.50-2.81)
Syria	1.38 (0.26-4.14)	0.44 (0.11-1.14)	0.15 (0.03-0.48)	710.67 (576.10-873.30)	624.35 (503.79-784.00)	491.92 (399.40-603.06)	34.06 (32.88-35.32)	32.38 (29.33-36.65)	41.57 (37.51-47.83)	1.67 (1.32-2.07)	1.60 (1.22-1.98)	2.16 (1.08-2.71)
Tunisia	7.37 (1.55-19.39)	3.77 (1.57-7.33)	0.21 (0.07-0.51)	939.69 (792.13-1,120.42)	883.55 (754.21-1,036.21)	844.33 (689.44-1,010.69)	37.83 (36.89-39.00)	35.63 (34.24-37.24)	44.79 (43.00-46.68)	1.19 (0.97-1.48)	1.15 (0.94-1.42)	1.78 (1.06-2.32)
Turkey	10.12 (2.72-23.68)	5.84 (2.98-10.47)	1.39 (0.26-4.37)	1,399.46 (1,148.92-1,715.27)	1,202.20 (1,013.99-1,419.36)	803.50 (666.90-950.16)	32.80 (32.52-33.09)	30.20 (29.70-30.80)	36.41 (35.69-37.18)	3.30 (2.44-4.18)	2.39 (1.99-2.87)	2.47 (1.43-2.91)
United Arab Emirates	0.06 (0.01-0.20)	0.02 (0.00-0.06)	0.01 (0.00-0.02)	987.93 (824.73-1,164.54)	908.55 (773.70-1,058.20)	968.67 (818.91-1,158.79)	75.61 (72.98-78.29)	75.56 (72.83-78.54)	64.29 (61.69-67.19)	1.71 (1.13-2.47)	1.18 (0.88-1.49)	1.01 (0.68-1.45)
Yemen	36.95 (24.91-52.28)	22.04 (13.24-34.38)	16.57 (8.63-27.60)	821.20 (633.50-1,039.94)	815.55 (649.76-1,019.91)	759.19 (607.75-949.92)	53.81 (49.28-61.84)	54.69 (49.34-63.50)	52.83 (46.59-62.54)	5.49 (3.02-8.85)	5.35 (3.00-8.81)	5.12 (2.74-8.95)
Bangladesh	52.58 (36.26-72.00)	51.74 (35.35-70.20)	47.01 (31.75-64.78)	1,923.40 (1,612.42-2,290.70)	2,162.63 (1,678.38-2,606.95)	1,551.19 (1,208.34-2,165.73)	63.89 (62.76-65.10)	65.42 (64.05-66.89)	89.39 (87.33-91.74)	3.48 (2.85-6.81)	3.81 (3.25-4.95)	2.88 (2.29-3.62)
Bhutan	45.49 (26.32-66.50)	39.72 (23.83-59.32)	21.36 (7.94-40.26)	1,626.36 (1,315.31-1,992.48)	1,566.15 (1,249.62-1,963.53)	1,398.06 (1,089.48-1,765.60)	40.42 (38.75-42.22)	41.01 (38.68-43.47)	55.72 (52.85-58.76)	2.19 (1.43-3.04)	1.85 (1.29-2.37)	1.16 (0.82-1.57)
India	47.76 (32.93-65.40)	43.58 (29.62-60.02)	34.49 (23.12-47.73)	1,585.77 (1,368.79-1,841.39)	1,473.46 (1,266.02-1,703.14)	1,195.27 (1,016.55-1,404.88)	59.87 (59.55-60.20)	61.50 (61.13-61.88)	74.33 (73.90-74.80)	4.57 (3.56-4.95)	4.40 (3.21-4.79)	3.75 (2.51-4.49)

Appendix Table 1b. Unscaled values for each SDG health-related indicator by country for 1990, 2000, and 2015

Location	Indicator 7.1.2: Prevalence of household air pollution, as measured by the summary exposure value (SEV) for household air pollution			Indicator 8.8.1: Age-standardised all-cause disability-adjusted life year (DALY) rates attributable to occupational risks (per 100,000 population)			Indicator 11.6.2: Population-weighted mean levels of fine particulate matter smaller than 2.5 microns (PM2.5)			Indicator 16.1.1: Age-standardised death rate due to interpersonal violence (per 100,000 population)		
	1990	2000	2015	1990	2000	2015	1990	2000	2015	1990	2000	2015
Nepal	44.51 (29.90-62.08)	44.39 (30.17-60.81)	41.46 (27.54-58.08)	1,976.73 (1,609.50-2,440.06)	1,886.81 (1,524.22-2,312.12)	1,766.13 (1,431.40-2,192.05)	56.23 (54.58-58.06)	56.26 (54.26-58.29)	75.00 (70.90-80.10)	1.57 (1.22-2.13)	2.41 (1.47-2.96)	2.26 (1.19-3.12)
Pakistan	45.09 (26.74-64.34)	45.66 (30.99-62.69)	35.54 (21.18-54.28)	1,053.56 (843.25-1,296.05)	1,088.67 (877.29-1,347.42)	1,077.86 (850.24-1,438.58)	67.97 (66.82-69.28)	69.59 (68.38-70.92)	64.95 (63.78-66.19)	4.10 (3.22-5.09)	5.42 (3.49-6.49)	5.05 (2.79-6.61)
Botswana	59.54 (39.30-81.57)	45.84 (27.84-67.63)	26.49 (10.33-49.75)	1,183.64 (805.91-1,817.44)	1,229.02 (729.28-2,265.19)	1,023.35 (678.24-1,789.74)	21.78 (20.80-22.89)	22.36 (20.67-24.67)	17.54 (16.28-19.23)	1.54 (0.47-4.13)	12.87 (2.12-45.27)	13.80 (2.33-48.84)
Lesotho	55.20 (34.00-77.09)	53.78 (35.71-75.21)	43.51 (24.93-65.32)	1,541.98 (1,174.74-2,043.54)	1,553.74 (1,174.93-2,020.88)	1,679.75 (1,206.70-2,267.80)	25.19 (22.62-28.23)	27.80 (22.83-35.71)	24.70 (19.92-32.92)	18.96 (11.64-27.17)	32.31 (17.37-49.73)	39.23 (15.61-74.67)
Namibia	62.44 (41.46-83.12)	53.99 (35.72-75.06)	43.23 (27.41-63.20)	858.25 (681.44-1,086.09)	907.78 (724.04-1,143.90)	696.30 (546.72-891.63)	26.11 (23.91-28.99)	27.25 (23.86-33.15)	21.44 (18.49-27.11)	13.01 (8.83-18.36)	16.85 (11.29-23.34)	10.39 (4.97-19.22)
South Africa	33.77 (21.55-48.43)	25.78 (17.10-36.40)	11.34 (7.08-17.17)	707.40 (604.88-811.78)	732.15 (643.85-831.05)	597.70 (514.87-688.43)	34.89 (34.02-35.71)	37.04 (36.17-38.02)	29.63 (28.83-30.46)	52.52 (42.40-63.25)	56.49 (45.82-62.84)	37.02 (31.41-43.00)
Swaziland	64.23 (42.24-85.85)	58.14 (38.58-79.37)	46.54 (26.27-70.51)	988.73 (778.91-1,254.68)	982.40 (742.45-1,254.11)	934.77 (632.67-1,310.84)	26.29 (23.69-29.08)	27.67 (23.65-32.65)	21.61 (18.25-25.96)	28.31 (16.54-41.38)	17.87 (9.62-28.26)	17.97 (5.85-39.00)
Zimbabwe	62.65 (41.12-86.35)	54.45 (35.16-75.87)	53.58 (35.08-75.65)	1,017.00 (819.78-1,255.39)	1,002.06 (761.24-1,298.92)	1,054.58 (803.67-1,383.10)	26.44 (24.05-29.88)	25.25 (23.05-28.29)	22.87 (20.87-25.52)	6.42 (3.90-9.10)	6.05 (3.31-10.32)	6.22 (3.14-10.37)
Benin	73.37 (50.92-92.94)	72.29 (49.77-92.20)	69.14 (47.61-89.70)	1,148.90 (926.16-1,412.26)	1,117.75 (893.43-1,374.24)	1,115.28 (862.38-1,391.54)	61.63 (56.66-68.25)	59.01 (54.27-65.08)	35.16 (32.25-38.63)	2.45 (1.76-3.61)	3.29 (2.28-4.70)	4.05 (1.89-7.62)
Burkina Faso	73.26 (50.77-92.90)	73.10 (50.65-92.78)	69.67 (47.32-89.90)	1,481.97 (1,241.04-1,759.46)	1,420.37 (1,178.43-1,685.49)	1,310.33 (1,002.79-1,610.56)	67.52 (64.24-71.61)	66.62 (63.05-71.21)	40.04 (37.30-44.65)	0.94 (0.67-2.11)	0.96 (0.70-2.06)	0.89 (0.50-1.68)

Appendix Table 1b. Unscaled values for each SDG health-related indicator by country for 1990, 2000, and 2015												
	**Indicator 7.1.2: Prevalence of household air pollution, as measured by the summary exposure value (SEV) for household air pollution**			**Indicator 8.8.1: Age-standardised all-cause disability-adjusted life year (DALY) rates attributable to occupational risks (per 100,000 population)**			**Indicator 11.6.2: Population-weighted mean levels of fine particulate matter smaller than 2.5 microns (PM2.5)**			**Indicator 16.1.1: Age-standardised death rate due to interpersonal violence (per 100,000 population)**		
**Location**	**1990**	**2000**	**2015**	**1990**	**2000**	**2015**	**1990**	**2000**	**2015**	**1990**	**2000**	**2015**
Cameroon	70.40 (48.73-90.64)	66.96 (45.37-88.26)	56.53 (36.16-78.95)	1,116.17 (905.59-1,356.12)	1,088.73 (887.33-1,336.16)	1,042.65 (820.62-1,287.52)	105.67 (103.34-108.09)	99.61 (96.64-102.70)	66.43 (63.85-69.52)	3.73 (2.44-5.38)	4.14 (2.68-5.59)	3.46 (1.77-6.27)
Cape Verde	58.41 (37.24-81.22)	42.06 (25.78-62.35)	26.82 (12.95-48.32)	953.86 (777.40-1,143.87)	838.88 (663.31-1,030.54)	743.02 (607.27-904.11)	52.64 (40.62-67.32)	48.77 (36.83-64.16)	39.56 (29.81-53.13)	3.43 (2.47-11.05)	16.29 (9.87-24.48)	14.71 (10.12-19.54)
Chad	72.42 (50.25-92.34)	72.19 (49.83-92.18)	71.51 (49.57-91.40)	1,296.41 (1,048.59-1,590.56)	1,291.57 (1,040.73-1,584.42)	1,226.78 (958.78-1,560.50)	64.80 (63.40-66.22)	63.86 (59.99-69.88)	46.06 (43.19-50.54)	3.03 (1.89-4.89)	4.46 (2.86-6.58)	4.56 (2.24-8.62)
Cote d'Ivoire	68.68 (47.08-89.63)	64.13 (43.25-85.98)	58.66 (38.69-80.89)	1,169.12 (945.06-1,402.52)	1,122.67 (907.10-1,365.40)	1,066.11 (848.93-1,322.79)	33.60 (31.35-36.53)	32.30 (29.73-36.16)	23.53 (21.65-26.58)	1.12 (0.48-8.06)	1.27 (0.54-8.92)	1.13 (0.39-6.73)
The Gambia	71.91 (49.52-91.63)	71.85 (49.40-91.80)	71.36 (49.00-91.36)	1,276.44 (1,009.16-1,594.71)	1,151.54 (918.70-1,416.79)	1,032.51 (808.48-1,294.13)	67.05 (42.23-136.68)	70.35 (40.56-156.39)	60.95 (34.49-130.69)	1.03 (0.39-6.99)	1.14 (0.57-6.73)	0.98 (0.42-6.14)
Ghana	71.19 (49.23-91.54)	68.35 (46.61-89.26)	58.34 (39.17-80.02)	943.12 (766.59-1,150.73)	900.63 (726.29-1,106.44)	831.97 (654.75-1,031.15)	38.26 (36.76-40.05)	36.50 (35.10-38.23)	22.99 (21.89-24.44)	3.68 (2.00-12.72)	3.83 (2.40-10.96)	3.40 (1.54-10.50)
Guinea	72.92 (50.59-92.69)	72.98 (50.49-92.51)	72.76 (50.31-92.48)	1,191.20 (962.64-1,452.81)	1,152.66 (928.60-1,425.80)	1,172.23 (934.73-1,456.20)	30.52 (28.69-32.71)	30.58 (28.41-34.09)	22.64 (20.65-25.58)	2.55 (1.87-4.01)	3.28 (2.51-4.47)	4.00 (2.23-6.80)
Guinea-Bissau	72.44 (49.96-92.28)	72.75 (50.11-92.49)	72.10 (50.05-91.92)	1,247.61 (909.62-1,729.56)	1,171.84 (859.69-1,569.96)	1,170.48 (842.68-1,675.57)	42.17 (34.90-56.72)	41.89 (34.19-57.08)	32.99 (25.74-49.58)	6.86 (2.57-15.99)	7.82 (2.79-19.63)	8.67 (3.14-25.75)
Liberia	73.19 (50.59-92.87)	73.11 (50.43-92.81)	72.55 (50.03-92.47)	964.56 (767.99-1,194.50)	959.27 (758.35-1,191.56)	933.31 (734.21-1,165.46)	10.48 (10.01-11.00)	10.53 (9.68-11.84)	7.85 (7.12-8.96)	3.26 (2.15-4.68)	2.73 (1.88-3.71)	2.59 (1.51-4.18)
Mali	73.38 (50.82-92.99)	73.20 (50.68-92.81)	72.88 (50.29-92.63)	936.98 (768.77-1,115.04)	873.78 (719.37-1,043.91)	847.10 (684.15-1,030.04)	62.24 (60.30-64.52)	59.90 (56.52-65.08)	44.01 (40.70-48.86)	3.00 (2.19-5.15)	2.88 (2.09-4.24)	3.06 (1.85-5.09)

Appendix Table 1b. Unscaled values for each SDG health-related indicator by country for 1990, 2000, and 2015

Location	Indicator 7.1.2: Prevalence of household air pollution, as measured by the summary exposure value (SEV) for household air pollution			Indicator 8.8.1: Age-standardised all-cause disability-adjusted life year (DALY) rates attributable to occupational risks (per 100,000 population)			Indicator 11.6.2: Population-weighted mean levels of fine particulate matter smaller than 2.5 microns (PM2.5)			Indicator 16.1.1: Age-standardised death rate due to interpersonal violence (per 100,000 population)		
	1990	2000	2015	1990	2000	2015	1990	2000	2015	1990	2000	2015
Mauritania	60.51 (39.13-83.22)	56.02 (37.02-76.72)	44.09 (27.05-66.31)	878.16 (715.88-1,065.47)	819.08 (647.95-1,023.38)	796.14 (631.96-999.60)	96.79 (85.72-114.62)	94.98 (75.92-133.11)	85.09 (66.20-126.22)	2.94 (2.03-4.78)	2.75 (1.59-4.11)	2.76 (1.28-5.30)
Niger	72.82 (50.30-92.59)	72.69 (50.29-92.28)	72.42 (49.95-92.37)	1,026.57 (834.15-1,255.11)	1,007.59 (805.29-1,253.55)	1,051.61 (835.06-1,314.24)	131.86 (127.31-136.78)	128.59 (121.99-136.65)	62.74 (59.91-66.24)	3.10 (2.07-5.44)	3.18 (2.18-4.78)	3.32 (1.82-5.58)
Nigeria	68.88 (46.18-90.22)	62.61 (42.39-84.72)	53.70 (34.18-74.89)	1,069.82 (838.07-1,340.19)	996.27 (784.42-1,252.46)	882.12 (694.43-1,104.20)	81.47 (80.35-82.72)	74.47 (73.36-75.64)	37.96 (37.45-38.50)	1.17 (0.72-1.86)	1.00 (0.72-1.33)	0.82 (0.55-1.39)
Sao Tome and Principe	70.00 (47.71-90.54)	65.88 (43.59-86.65)	48.70 (28.59-71.24)	996.63 (815.93-1,202.33)	973.93 (801.59-1,173.91)	956.98 (730.55-1,230.93)	15.88 (11.21-21.73)	15.94 (9.85-25.96)	13.89 (8.66-21.89)	6.28 (4.85-7.77)	7.03 (5.32-9.10)	7.23 (3.58-12.48)
Senegal	64.77 (43.46-85.54)	54.92 (36.58-76.10)	58.62 (38.57-79.22)	1,167.05 (957.40-1,395.11)	1,058.03 (858.37-1,296.40)	1,044.12 (809.85-1,285.20)	41.65 (39.22-44.45)	41.09 (38.70-43.82)	37.57 (35.43-40.06)	1.68 (1.20-2.21)	1.43 (1.06-1.90)	1.46 (0.74-2.76)
Sierra Leone	71.20 (49.10-91.24)	72.54 (50.27-92.24)	72.84 (50.33-92.52)	1,117.04 (899.85-1,381.69)	1,078.87 (873.43-1,326.59)	1,068.46 (847.50-1,338.55)	25.93 (23.18-29.81)	25.71 (21.87-33.07)	19.30 (16.12-26.40)	1.84 (1.05-2.78)	2.01 (1.52-2.68)	1.82 (1.06-2.92)
Togo	72.99 (50.44-92.62)	72.90 (50.49-92.60)	70.26 (48.06-90.49)	1,370.50 (1,124.85-1,659.07)	1,301.99 (1,063.24-1,588.33)	1,228.59 (970.17-1,517.14)	56.13 (49.77-66.68)	54.17 (46.63-70.53)	32.68 (27.80-40.49)	2.77 (2.01-4.39)	3.66 (2.44-5.33)	3.90 (2.07-6.58)
Burundi	73.51 (50.96-93.03)	73.35 (50.84-92.95)	72.55 (50.00-92.57)	1,740.85 (1,349.08-2,189.82)	1,543.37 (1,224.64-1,951.45)	1,338.62 (1,033.03-1,693.93)	46.54 (41.19-52.72)	47.18 (41.80-54.43)	45.53 (39.92-53.70)	1.89 (1.01-3.04)	1.66 (1.05-2.40)	1.23 (0.52-2.47)
Comoros	70.15 (47.42-90.59)	67.54 (45.20-88.19)	59.91 (36.71-83.49)	966.07 (742.21-1,237.90)	929.93 (727.66-1,166.61)	892.44 (699.93-1,109.81)	20.93 (16.53-26.77)	21.05 (16.25-27.12)	17.08 (13.42-21.42)	6.20 (3.20-11.18)	9.25 (5.94-13.73)	9.81 (4.52-17.71)
Djibouti	18.01 (4.74-42.28)	13.38 (5.30-26.34)	18.95 (5.03-42.73)	645.14 (492.64-836.90)	698.64 (526.85-921.49)	711.79 (542.64-906.73)	46.59 (32.06-70.88)	50.85 (27.51-99.30)	51.50 (28.80-101.27)	5.35 (2.88-8.74)	10.86 (4.57-22.87)	13.01 (5.41-28.71)

Appendix Table 1b. Unscaled values for each SDG health-related indicator by country for 1990, 2000, and 2015

Location	Indicator 7.1.2: Prevalence of household air pollution, as measured by the summary exposure value (SEV) for household air pollution			Indicator 8.8.1: Age-standardised all-cause disability-adjusted life year (DALY) rates attributable to occupational risks (per 100,000 population)			Indicator 11.6.2: Population-weighted mean levels of fine particulate matter smaller than 2.5 microns (PM2.5)			Indicator 16.1.1: Age-standardised death rate due to interpersonal violence (per 100,000 population)		
	1990	2000	2015	1990	2000	2015	1990	2000	2015	1990	2000	2015
Eritrea	67.96 (45.21-88.99)	57.29 (37.01-79.91)	54.57 (26.68-81.90)	1,116.17 (879.23-1,425.23)	930.43 (711.91-1,184.70)	965.91 (723.37-1,238.45)	44.48 (40.63-49.13)	43.56 (40.26-47.35)	43.49 (40.26-47.36)	7.31 (5.22-10.28)	8.96 (4.38-16.35)	13.47 (5.62-27.25)
Ethiopia	72.07 (49.54-92.46)	71.60 (49.41-91.63)	71.43 (49.23-91.81)	1,231.18 (1,001.49-1,478.90)	1,147.83 (899.92-1,421.87)	1,116.52 (860.67-1,401.51)	36.09 (34.66-38.33)	35.48 (34.21-37.69)	35.74 (34.43-37.77)	12.11 (7.67-30.94)	9.11 (6.40-19.76)	7.55 (3.82-15.50)
Kenya	68.62 (47.21-87.71)	64.36 (44.23-82.99)	60.90 (41.11-79.54)	1,102.98 (882.93-1,367.26)	1,091.79 (875.90-1,342.14)	1,040.54 (829.93-1,287.19)	16.44 (15.99-16.97)	16.18 (15.69-16.71)	16.30 (15.79-16.87)	15.21 (10.92-21.50)	15.47 (12.33-19.68)	16.41 (13.02-20.96)
Madagascar	72.81 (50.54-92.48)	72.75 (50.29-92.59)	72.85 (50.60-92.55)	1,683.99 (1,342.48-2,061.55)	1,685.01 (1,317.97-2,092.31)	1,645.56 (1,267.12-2,060.55)	18.86 (18.06-19.77)	19.36 (18.56-20.31)	19.68 (18.64-20.94)	1.17 (0.92-1.47)	3.38 (1.30-4.70)	2.32 (0.99-4.19)
Malawi	72.95 (50.60-92.73)	72.51 (49.84-92.45)	72.32 (50.04-92.19)	1,257.76 (966.19-1,617.40)	1,323.35 (1,021.06-1,696.39)	1,240.36 (947.69-1,558.28)	29.41 (27.12-32.32)	29.21 (26.92-32.07)	25.73 (23.60-28.55)	1.93 (1.11-6.70)	2.56 (1.32-6.77)	1.94 (0.95-5.95)
Mozambique	73.27 (50.90-92.85)	72.76 (49.99-92.43)	71.11 (48.85-91.36)	1,229.69 (932.24-1,552.47)	1,271.83 (979.30-1,625.33)	1,268.99 (973.66-1,603.92)	22.49 (21.50-23.66)	22.83 (21.26-25.01)	19.54 (18.44-21.04)	5.60 (3.85-8.45)	6.07 (4.28-8.17)	8.25 (3.89-14.97)
Rwanda	73.37 (50.84-92.95)	73.76 (51.13-93.20)	73.57 (51.01-93.05)	1,494.35 (1,197.89-1,864.54)	1,430.77 (1,127.41-1,806.62)	1,227.22 (940.29-1,554.23)	52.91 (47.22-59.82)	53.75 (47.54-61.39)	49.71 (43.80-57.62)	8.91 (5.87-11.71)	7.03 (4.64-9.73)	4.36 (2.52-7.51)
Somalia	73.05 (50.63-92.61)	73.09 (50.70-92.60)	72.93 (50.70-92.53)	994.23 (715.55-1,356.85)	1,000.39 (725.57-1,343.89)	987.83 (726.82-1,336.84)	21.74 (20.39-23.91)	22.46 (20.88-25.63)	19.84 (18.49-22.40)	8.25 (2.44-19.73)	15.09 (4.15-37.86)	16.77 (4.97-43.18)
South Sudan	73.24 (50.71-92.86)	73.22 (50.72-92.79)	72.90 (50.51-92.27)	1,071.17 (773.77-1,459.59)	1,025.35 (750.83-1,376.23)	990.84 (734.35-1,344.27)	41.89 (40.93-42.88)	42.67 (41.64-43.67)	32.44 (31.71-33.22)	5.24 (1.78-13.39)	8.95 (2.97-23.79)	13.11 (4.06-39.17)
Tanzania	72.79 (50.53-92.41)	70.48 (48.38-90.84)	69.88 (48.26-90.64)	1,107.32 (853.06-1,417.32)	1,088.97 (844.45-1,366.99)	1,073.30 (806.20-1,387.80)	21.02 (20.70-21.38)	22.33 (21.67-23.12)	23.32 (22.45-24.55)	7.75 (5.64-11.84)	6.89 (4.58-10.55)	6.87 (3.81-12.53)

Appendix Table 1b. Unscaled values for each SDG health-related indicator by country for 1990, 2000, and 2015

Location	Indicator 7.1.2: Prevalence of household air pollution, as measured by the summary exposure value (SEV) for household air pollution			Indicator 8.8.1: Age-standardised all-cause disability-adjusted life year (DALY) rates attributable to occupational risks (per 100,000 population)			Indicator 11.6.2: Population-weighted mean levels of fine particulate matter smaller than 2.5 microns (PM2.5)			Indicator 16.1.1: Age-standardised death rate due to interpersonal violence (per 100,000 population)		
	1990	2000	2015	1990	2000	2015	1990	2000	2015	1990	2000	2015
Uganda	73.23 (50.64-92.87)	72.46 (50.09-92.31)	72.26 (49.96-92.05)	1,394.20 (1,087.93-1,758.03)	1,369.97 (1,084.45-1,713.98)	1,289.89 (981.47-1,635.73)	60.64 (58.67-62.78)	60.34 (58.48-62.26)	60.30 (58.53-62.49)	6.95 (4.15-9.92)	8.42 (5.08-11.23)	7.94 (3.71-14.62)
Zambia	68.58 (45.78-90.41)	67.87 (46.19-88.43)	64.73 (42.49-86.06)	1,105.98 (866.22-1,411.30)	1,271.61 (1,014.01-1,591.60)	1,250.33 (985.03-1,563.59)	32.40 (30.36-35.28)	30.88 (28.55-34.15)	26.75 (24.34-30.47)	5.94 (4.13-8.16)	9.95 (6.95-12.94)	11.04 (5.77-17.73)
Angola	71.18 (49.18-91.35)	66.39 (45.44-87.76)	49.70 (30.43-73.68)	1,390.38 (986.03-1,916.28)	1,329.28 (952.56-1,856.51)	1,123.96 (796.78-1,605.16)	36.70 (34.74-39.22)	39.64 (37.53-42.63)	36.40 (29.91-51.51)	7.88 (2.30-17.30)	6.09 (1.91-14.88)	5.69 (2.08-16.55)
Central African Republic	73.14 (50.63-92.78)	73.18 (50.73-92.64)	72.89 (50.60-92.75)	1,623.61 (1,294.06-1,969.14)	1,655.35 (1,252.34-2,123.02)	1,619.85 (1,219.52-2,050.81)	50.70 (43.97-61.75)	52.25 (43.80-72.72)	46.47 (38.03-64.60)	7.61 (4.77-12.06)	9.15 (3.79-17.79)	9.91 (3.58-22.08)
Congo	70.63 (48.97-91.21)	67.65 (45.36-89.12)	51.25 (30.88-74.56)	1,253.24 (1,015.21-1,520.49)	1,188.13 (956.85-1,458.66)	1,032.67 (814.96-1,294.34)	52.04 (45.14-62.19)	61.63 (41.41-99.01)	53.01 (34.52-95.05)	6.36 (3.66-10.42)	6.52 (4.01-10.26)	5.05 (2.66-9.56)
Democratic Republic of the Congo	73.57 (50.94-93.08)	73.26 (50.76-92.84)	71.47 (49.09-91.53)	1,279.14 (995.10-1,608.42)	1,321.11 (1,027.85-1,669.02)	1,302.34 (1,012.63-1,631.50)	47.94 (43.77-54.01)	51.08 (45.09-61.78)	46.32 (42.17-52.91)	4.79 (2.59-7.87)	4.77 (3.25-6.83)	4.48 (2.52-7.17)
Equatorial Guinea	71.71 (49.71-92.06)	70.09 (47.84-90.97)	63.61 (40.74-85.82)	1,601.94 (1,118.37-2,216.94)	1,352.33 (977.59-1,917.63)	1,026.95 (767.82-1,484.27)	47.16 (38.89-60.06)	53.70 (37.87-88.75)	47.12 (31.49-83.38)	9.01 (2.35-23.13)	7.12 (2.21-18.47)	4.93 (2.13-14.53)
Gabon	54.91 (32.96-79.78)	35.09 (19.64-53.92)	11.86 (5.14-22.86)	936.33 (752.21-1,138.28)	866.85 (693.06-1,067.70)	778.91 (616.71-969.28)	39.53 (28.81-65.56)	41.80 (30.19-66.37)	39.86 (30.45-58.75)	5.37 (3.46-8.14)	5.19 (3.25-8.40)	4.73 (2.41-9.16)

Appendix Table 1b. Unscaled values for each SDG health-related indicator by country for 1990, 2000, and 2015

Location	Indicator 16.1.2: Age-standardised death rate due to collective violence and legal intervention (per 100,000 population)											
	1990	2000	2015									
Canada	0.00 (0.00-0.00)	0.00 (0.00-0.00)	0.00 (0.00-0.00)									
United States	0.13 (0.07-0.23)	0.15 (0.07-0.28)	0.00 (0.00-0.00)									
Australia	0.00 (0.00-0.00)	0.00 (0.00-0.00)	0.00 (0.00-0.00)									
New Zealand	0.00 (0.00-0.00)	0.10 (0.05-0.15)	0.00 (0.00-0.00)									
Brunei	0.00 (0.00-0.00)	0.00 (0.00-0.00)	0.00 (0.00-0.00)									
Japan	0.00 (0.00-0.00)	0.02 (0.00-0.04)	0.00 (0.00-0.00)									
Singapore	0.25 (0.04-0.47)	0.43 (0.07-0.82)	0.00 (0.00-0.00)									
South Korea	0.00 (0.00-0.00)	0.00 (0.00-0.00)	0.00 (0.00-0.00)									
Andorra	0.00 (0.00-0.00)	0.00 (0.00-0.00)	0.00 (0.00-0.00)									
Austria	0.00 (0.00-0.00)	0.00 (0.00-0.00)	0.00 (0.00-0.00)									

Appendix Table 1b. Unscaled values for each SDG health-related indicator by country for 1990, 2000, and 2015

Location	Indicator 16.1.2: Age-standardised death rate due to collective violence and legal intervention (per 100,000 population)											
	1990	2000	2015									
Belgium	0.21 (0.11-0.32)	0.00 (0.00-0.00)	0.00 (0.00-0.00)									
Cyprus	0.00 (0.00-0.00)	0.00 (0.00-0.00)	0.00 (0.00-0.00)									
Denmark	0.00 (0.00-0.00)	0.00 (0.00-0.00)	0.00 (0.00-0.00)									
Finland	0.14 (0.06-0.21)	0.00 (0.00-0.00)	0.00 (0.00-0.00)									
France	0.00 (0.00-0.00)	0.00 (0.00-0.00)	0.23 (0.12-0.35)									
Germany	0.00 (0.00-0.00)	0.00 (0.00-0.00)	0.00 (0.00-0.00)									
Greece	0.00 (0.00-0.00)	0.00 (0.00-0.00)	0.00 (0.00-0.00)									
Iceland	0.00 (0.00-0.00)	0.00 (0.00-0.00)	0.00 (0.00-0.00)									
Ireland	0.00 (0.00-0.00)	0.00 (0.00-0.00)	0.00 (0.00-0.00)									
Israel	1.64 (1.12-2.15)	1.29 (1.24-1.34)	0.29 (0.16-0.43)									

Appendix Table 1b. Unscaled values for each SDG health-related indicator by country for 1990, 2000, and 2015

Location	Indicator 16.1.2: Age-standardised death rate due to collective violence and legal intervention (per 100,000 population)											
	1990	2000	2015									
Italy	0.00 (0.00-0.00)	0.00 (0.00-0.00)	0.00 (0.00-0.00)									
Luxembourg	0.00 (0.00-0.00)	0.00 (0.00-0.00)	0.00 (0.00-0.00)									
Malta	0.29 (0.05-0.55)	0.00 (0.00-0.00)	0.00 (0.00-0.00)									
Netherlands	0.00 (0.00-0.00)	0.00 (0.00-0.00)	0.00 (0.00-0.00)									
Norway	0.00 (0.00-0.00)	0.00 (0.00-0.00)	0.00 (0.00-0.00)									
Portugal	0.00 (0.00-0.00)	0.00 (0.00-0.00)	0.00 (0.00-0.00)									
Spain	0.00 (0.00-0.00)	0.00 (0.00-0.00)	0.00 (0.00-0.00)									
Sweden	0.00 (0.00-0.00)	0.00 (0.00-0.00)	0.00 (0.00-0.00)									
Switzerland	0.00 (0.00-0.00)	0.00 (0.00-0.00)	0.00 (0.00-0.00)									
United Kingdom	0.08 (0.02-0.17)	0.02 (0.01-0.05)	0.00 (0.00-0.00)									

Appendix Table 1b. Unscaled values for each SDG health-related indicator by country for 1990, 2000, and 2015

Location	Indicator 16.1.2: Age-standardised death rate due to collective violence and legal intervention (per 100,000 population)											
	1990	2000	2015									
Argentina	0.58 (0.09-1.11)	0.17 (0.03-0.32)	0.00 (0.00-0.00)									
Chile	0.00 (0.00-0.00)	0.00 (0.00-0.00)	0.00 (0.00-0.00)									
Uruguay	0.00 (0.00-0.00)	0.00 (0.00-0.00)	0.00 (0.00-0.00)									
Belarus	0.00 (0.00-0.00)	0.00 (0.00-0.00)	0.00 (0.00-0.00)									
Estonia	0.00 (0.00-0.00)	0.00 (0.00-0.00)	0.00 (0.00-0.00)									
Latvia	0.00 (0.00-0.00)	0.00 (0.00-0.00)	0.00 (0.00-0.00)									
Lithuania	0.00 (0.00-0.00)	0.00 (0.00-0.00)	0.00 (0.00-0.00)									
Moldova	0.00 (0.00-0.00)	0.00 (0.00-0.00)	0.00 (0.00-0.00)									
Russia	0.26 (0.06-0.45)	3.02 (1.27-4.79)	0.14 (0.02-0.27)									
Ukraine	0.00 (0.00-0.00)	0.00 (0.00-0.00)	9.18 (1.46-17.59)									

Appendix Table 1b. Unscaled values for each SDG health-related indicator by country for 1990, 2000, and 2015

Location	Indicator 16.1.2: Age-standardised death rate due to collective violence and legal intervention (per 100,000 population)		
	1990	2000	2015
Albania	0.00 (0.00-0.00)	0.17 (0.05-0.29)	0.00 (0.00-0.00)
Bosnia and Herzegovina	13.20 (3.64-22.99)	0.00 (0.00-0.00)	0.00 (0.00-0.00)
Bulgaria	0.00 (0.00-0.00)	0.00 (0.00-0.00)	0.00 (0.00-0.00)
Croatia	0.00 (0.00-0.00)	0.13 (0.03-0.24)	0.00 (0.00-0.00)
Czech Republic	0.00 (0.00-0.00)	0.00 (0.00-0.00)	0.00 (0.00-0.00)
Hungary	0.00 (0.00-0.00)	0.00 (0.00-0.00)	0.00 (0.00-0.00)
Macedonia	0.00 (0.00-0.00)	0.00 (0.00-0.00)	0.00 (0.00-0.00)
Montenegro	0.00 (0.00-0.00)	0.16 (0.16-0.16)	0.00 (0.00-0.00)
Poland	0.00 (0.00-0.00)	0.00 (0.00-0.00)	0.00 (0.00-0.00)
Romania	0.39 (0.11-0.68)	0.00 (0.00-0.00)	0.00 (0.00-0.00)

Appendix Table 1b. Unscaled values for each SDG health-related indicator by country for 1990, 2000, and 2015

Location	Indicator 16.1.2: Age-standardised death rate due to collective violence and legal intervention (per 100,000 population)											
	1990	2000	2015									
Serbia	0.00 (0.00-0.00)	5.41 (1.41-9.33)	0.00 (0.00-0.00)									
Slovakia	0.00 (0.00-0.00)	0.00 (0.00-0.00)	0.00 (0.00-0.00)									
Slovenia	0.00 (0.00-0.00)	0.00 (0.00-0.00)	0.00 (0.00-0.00)									
Armenia	0.00 (0.00-0.00)	0.00 (0.00-0.00)	0.00 (0.00-0.00)									
Azerbaijan	0.00 (0.00-0.00)	0.00 (0.00-0.00)	0.00 (0.00-0.00)									
Georgia	0.00 (0.00-0.00)	0.42 (0.18-0.67)	0.00 (0.00-0.00)									
Kazakhstan	0.00 (0.00-0.00)	0.00 (0.00-0.00)	0.00 (0.00-0.00)									
Kyrgyzstan	0.00 (0.00-0.00)	0.57 (0.24-0.90)	0.00 (0.00-0.00)									
Mongolia	0.00 (0.00-0.00)	0.00 (0.00-0.00)	0.00 (0.00-0.00)									
Tajikistan	0.00 (0.00-0.00)	0.82 (0.34-1.32)	0.00 (0.00-0.00)									

Appendix Table 1b. Unscaled values for each SDG health-related indicator by country for 1990, 2000, and 2015

Location	Indicator 16.1.2: Age-standardised death rate due to collective violence and legal intervention (per 100,000 population)												
	1990	2000	2015										
Turkmenistan	0.00 (0.00-0.00)	0.00 (0.00-0.00)	0.00 (0.00-0.00)										
Uzbekistan	0.00 (0.00-0.00)	1.28 (0.53-2.05)	0.00 (0.00-0.00)										
Colombia	1.92 (1.64-2.19)	4.24 (2.85-5.63)	0.00 (0.00-0.00)										
Costa Rica	0.00 (0.00-0.00)	0.00 (0.00-0.00)	0.00 (0.00-0.00)										
El Salvador	21.13 (11.19-31.05)	0.16 (0.09-0.24)	0.00 (0.00-0.00)										
Guatemala	8.36 (4.47-12.23)	0.20 (0.11-0.29)	0.00 (0.00-0.00)										
Honduras	0.00 (0.00-0.00)	0.00 (0.00-0.00)	0.00 (0.00-0.00)										
Mexico	0.07 (0.01-0.29)	0.00 (0.00-0.00)	0.00 (0.00-0.00)										
Nicaragua	1.06 (0.93-1.19)	0.00 (0.00-0.00)	0.00 (0.00-0.00)										
Panama	0.00 (0.00-0.00)	0.30 (0.14-0.47)	0.00 (0.00-0.00)										

Appendix Table 1b. Unscaled values for each SDG health-related indicator by country for 1990, 2000, and 2015

Location	Indicator 16.1.2: Age-standardised death rate due to collective violence and legal intervention (per 100,000 population)												
	1990	2000	2015										
Venezuela	0.34 (0.18-0.49)	1.30 (0.69-1.91)	0.00 (0.00-0.00)										
Bolivia	0.00 (0.00-0.00)	0.66 (0.10-1.27)	0.00 (0.00-0.00)										
Ecuador	0.00 (0.00-0.00)	0.00 (0.00-0.00)	0.00 (0.00-0.00)										
Peru	5.56 (4.23-6.89)	0.00 (0.00-0.00)	0.00 (0.00-0.00)										
Antigua and Barbuda	0.00 (0.00-0.00)	0.00 (0.00-0.00)	0.00 (0.00-0.00)										
The Bahamas	0.00 (0.00-0.00)	0.00 (0.00-0.00)	0.00 (0.00-0.00)										
Barbados	0.38 (0.06-0.72)	0.00 (0.00-0.00)	0.00 (0.00-0.00)										
Belize	1.81 (0.29-3.48)	0.00 (0.00-0.00)	0.00 (0.00-0.00)										
Cuba	0.34 (0.05-0.65)	0.00 (0.00-0.00)	0.00 (0.00-0.00)										
Dominica	0.00 (0.00-0.00)	0.00 (0.00-0.00)	0.00 (0.00-0.00)										

Appendix Table 1b. Unscaled values for each SDG health-related indicator by country for 1990, 2000, and 2015

Location	Indicator 16.1.2: Age-standardised death rate due to collective violence and legal intervention (per 100,000 population)											
	1990	2000	2015									
Dominican Republic	0.00 (0.00-0.00)	0.00 (0.00-0.00)	0.00 (0.00-0.00)									
Grenada	1.18 (0.19-2.25)	0.00 (0.00-0.00)	0.00 (0.00-0.00)									
Guyana	1.14 (0.18-2.19)	0.29 (0.05-0.55)	0.00 (0.00-0.00)									
Haiti	0.00 (0.00-0.00)	0.00 (0.00-0.00)	0.00 (0.00-0.00)									
Jamaica	0.00 (0.00-0.00)	0.00 (0.00-0.00)	0.00 (0.00-0.00)									
Saint Lucia	0.79 (0.13-1.51)	0.00 (0.00-0.00)	0.00 (0.00-0.00)									
Saint Vincent and the Grenadines	1.02 (0.16-1.95)	0.00 (0.00-0.00)	0.00 (0.00-0.00)									
Suriname	0.77 (0.12-1.47)	0.00 (0.00-0.00)	0.00 (0.00-0.00)									
Trinidad and Tobago	3.30 (1.98-4.62)	0.00 (0.00-0.00)	0.00 (0.00-0.00)									
Brazil	0.00 (0.00-0.00)	0.06 (0.01-0.17)	0.00 (0.00-0.00)									

	Appendix Table 1b. Unscaled values for each SDG health-related indicator by country for 1990, 2000, and 2015											
	**Indicator 16.1.2: Age-standardised death rate due to collective violence and legal intervention (per 100,000 population)**											
**Location**	**1990**	**2000**	**2015**									
Paraguay	0.00 (0.00-0.00)	0.00 (0.00-0.00)	0.00 (0.00-0.00)									
China	0.00 (0.00-0.00)	0.02 (0.00-0.03)	0.00 (0.00-0.00)									
North Korea	0.00 (0.00-0.00)	0.00 (0.00-0.00)	0.00 (0.00-0.00)									
Taiwan	0.00 (0.00-0.00)	0.00 (0.00-0.00)	0.00 (0.00-0.00)									
Cambodia	4.78 (0.76-9.16)	0.00 (0.00-0.00)	0.00 (0.00-0.00)									
Indonesia	0.12 (0.08-0.15)	0.43 (0.22-0.65)	0.00 (0.00-0.00)									
Laos	6.42 (1.54-11.46)	0.00 (0.00-0.00)	0.00 (0.00-0.00)									
Malaysia	0.00 (0.00-0.00)	0.00 (0.00-0.00)	0.00 (0.00-0.00)									
Maldives	0.00 (0.00-0.00)	0.00 (0.00-0.00)	0.00 (0.00-0.00)									
Mauritius	0.00 (0.00-0.00)	0.17 (0.04-0.36)	0.00 (0.00-0.00)									

Appendix Table 1b. Unscaled values for each SDG health-related indicator by country for 1990, 2000, and 2015

Location	Indicator 16.1.2: Age-standardised death rate due to collective violence and legal intervention (per 100,000 population)											
	1990	2000	2015									
Myanmar	0.36 (0.06-0.69)	0.97 (0.65-1.28)	0.60 (0.15-1.08)									
Philippines	2.33 (1.57-3.08)	2.36 (2.16-2.55)	0.10 (0.03-0.18)									
Sri Lanka	28.32 (20.66-35.97)	13.45 (5.42-21.86)	0.00 (0.00-0.00)									
Seychelles	0.00 (0.00-0.00)	0.00 (0.00-0.00)	0.00 (0.00-0.00)									
Thailand	0.00 (0.00-0.00)	0.00 (0.00-0.00)	0.00 (0.00-0.00)									
Timor-Leste	0.00 (0.00-0.00)	140.90 (34.57-251.23)	0.00 (0.00-0.00)									
Vietnam	0.00 (0.00-0.00)	0.00 (0.00-0.00)	0.00 (0.00-0.00)									
Federated States of Micronesia	0.00 (0.00-0.00)	0.00 (0.00-0.00)	0.00 (0.00-0.00)									
Fiji	0.00 (0.00-0.00)	0.00 (0.00-0.00)	0.00 (0.00-0.00)									
Kiribati	0.00 (0.00-0.00)	0.00 (0.00-0.00)	0.00 (0.00-0.00)									

Appendix Table 1b. Unscaled values for each SDG health-related indicator by country for 1990, 2000, and 2015

Location	Indicator 16.1.2: Age-standardised death rate due to collective violence and legal intervention (per 100,000 population)											
	1990	2000	2015									
Marshall Islands	0.00 (0.00-0.00)	0.00 (0.00-0.00)	0.00 (0.00-0.00)									
Papua New Guinea	1.44 (1.18-1.70)	0.00 (0.00-0.00)	0.00 (0.00-0.00)									
Samoa	0.00 (0.00-0.00)	0.00 (0.00-0.00)	0.00 (0.00-0.00)									
Solomon Islands	0.00 (0.00-0.00)	0.00 (0.00-0.00)	0.00 (0.00-0.00)									
Tonga	0.00 (0.00-0.00)	0.00 (0.00-0.00)	0.00 (0.00-0.00)									
Vanuatu	0.00 (0.00-0.00)	0.00 (0.00-0.00)	0.00 (0.00-0.00)									
Afghanistan	14.20 (6.71-21.87)	31.51 (24.54-38.46)	125.25 (44.29-205.99)									
Algeria	0.00 (0.00-0.00)	3.86 (2.98-4.73)	0.23 (0.08-0.38)									
Bahrain	0.00 (0.00-0.00)	0.15 (0.03-0.29)	0.00 (0.00-0.00)									
Egypt	0.00 (0.00-0.00)	0.37 (0.13-0.61)	2.86 (1.01-4.70)									

Location	Appendix Table 1b. Unscaled values for each SDG health-related indicator by country for 1990, 2000, and 2015<br>Indicator 16.1.2: Age-standardised death rate due to collective violence and legal intervention (per 100,000 population)											
	1990	2000	2015									
Iran	0.00 (0.00-0.00)	1.06 (0.37-1.74)	0.00 (0.00-0.00)									
Iraq	5.19 (0.82-9.95)	0.61 (0.47-0.75)	66.14 (23.39-108.77)									
Jordan	0.00 (0.00-0.00)	0.00 (0.00-0.00)	0.00 (0.00-0.00)									
Kuwait	35.26 (5.59-67.58)	0.34 (0.15-0.53)	0.62 (0.21-1.03)									
Lebanon	70.63 (55.59-85.63)	0.83 (0.28-1.37)	1.79 (0.63-2.96)									
Libya	0.00 (0.00-0.00)	2.57 (0.90-4.24)	43.33 (15.33-71.26)									
Morocco	0.45 (0.16-0.74)	0.00 (0.00-0.00)	0.00 (0.00-0.00)									
Palestine	11.43 (4.05-18.78)	20.54 (7.26-33.79)	0.00 (0.00-0.00)									
Oman	0.00 (0.00-0.00)	0.00 (0.00-0.00)	0.00 (0.00-0.00)									
Qatar	0.00 (0.00-0.00)	0.00 (0.00-0.00)	0.00 (0.00-0.00)									

	Appendix Table 1b. Unscaled values for each SDG health-related indicator by country for 1990, 2000, and 2015											
	**Indicator 16.1.2: Age-standardised death rate due to collective violence and legal intervention (per 100,000 population)**											
**Location**	**1990**	**2000**	**2015**									
Saudi Arabia	0.00 (0.00-0.00)	0.00 (0.00-0.00)	0.13 (0.00-0.57)									
Sudan	24.57 (9.14-40.87)	13.65 (5.05-23.27)	3.45 (1.15-6.77)									
Syria	0.00 (0.00-0.00)	0.00 (0.00-0.00)	309.88 (109.58-509.65)									
Tunisia	0.00 (0.00-0.00)	0.00 (0.00-0.00)	1.18 (0.41-1.94)									
Turkey	0.59 (0.45-0.73)	0.27 (0.21-0.34)	0.32 (0.11-0.52)									
United Arab Emirates	0.00 (0.00-0.00)	0.00 (0.00-0.00)	0.00 (0.00-0.00)									
Yemen	0.00 (0.00-0.00)	0.00 (0.00-0.00)	47.10 (29.13-65.03)									
Bangladesh	0.00 (0.00-0.00)	0.00 (0.00-0.00)	0.00 (0.00-0.00)									
Bhutan	0.00 (0.00-0.00)	0.00 (0.00-0.00)	0.00 (0.00-0.00)									
India	0.63 (0.20-1.48)	0.40 (0.13-0.87)	0.00 (0.00-0.01)									

Appendix Table 1b. Unscaled values for each SDG health-related indicator by country for 1990, 2000, and 2015

Location	Indicator 16.1.2: Age-standardised death rate due to collective violence and legal intervention (per 100,000 population)											
	1990	2000	2015									
Nepal	0.00 (0.00-0.00)	1.68 (1.50-1.85)	0.00 (0.00-0.00)									
Pakistan	0.40 (0.21-0.60)	0.00 (0.00-0.00)	2.38 (1.25-3.52)									
Botswana	0.00 (0.00-0.00)	0.00 (0.00-0.00)	0.00 (0.00-0.00)									
Lesotho	0.00 (0.00-0.00)	0.00 (0.00-0.00)	0.00 (0.00-0.00)									
Namibia	0.00 (0.00-0.00)	3.27 (1.01-6.45)	0.00 (0.00-0.00)									
South Africa	4.05 (2.62-5.70)	0.00 (0.00-0.00)	0.24 (0.14-0.39)									
Swaziland	0.00 (0.00-0.00)	0.00 (0.00-0.00)	0.00 (0.00-0.00)									
Zimbabwe	0.24 (0.08-0.45)	0.15 (0.04-0.26)	0.00 (0.00-0.00)									
Benin	0.00 (0.00-0.00)	0.00 (0.00-0.00)	0.00 (0.00-0.00)									
Burkina Faso	0.00 (0.00-0.00)	0.00 (0.00-0.00)	0.00 (0.00-0.00)									

Appendix Table 1b. Unscaled values for each SDG health-related indicator by country for 1990, 2000, and 2015

Location	Indicator 16.1.2: Age-standardised death rate due to collective violence and legal intervention (per 100,000 population)											
	1990	2000	2015									
Cameroon	0.00 (0.00-0.00)	0.00 (0.00-0.00)	5.91 (1.40-11.82)									
Cape Verde	0.00 (0.00-0.00)	0.00 (0.00-0.00)	0.00 (0.00-0.00)									
Chad	27.81 (7.46-57.18)	15.28 (5.73-25.67)	3.02 (0.71-6.10)									
Cote d'Ivoire	0.00 (0.00-0.00)	0.89 (0.32-1.55)	0.00 (0.00-0.00)									
The Gambia	0.00 (0.00-0.00)	1.17 (0.33-2.41)	0.00 (0.00-0.00)									
Ghana	0.00 (0.00-0.00)	0.19 (0.05-0.38)	0.00 (0.00-0.00)									
Guinea	0.00 (0.00-0.00)	3.28 (0.96-6.64)	0.12 (0.03-0.25)									
Guinea-Bissau	0.00 (0.00-0.00)	0.00 (0.00-0.00)	0.00 (0.00-0.00)									
Liberia	210.36 (81.75-348.47)	4.12 (1.16-8.20)	0.00 (0.00-0.00)									
Mali	4.68 (1.65-8.29)	20.82 (4.91-41.08)	2.32 (0.64-4.76)									

Appendix Table 1b. Unscaled values for each SDG health-related indicator by country for 1990, 2000, and 2015

Location	Indicator 16.1.2: Age-standardised death rate due to collective violence and legal intervention (per 100,000 population)											
	1990	2000	2015									
Mauritania	16.39 (4.05-33.43)	0.00 (0.00-0.00)	0.00 (0.00-0.00)									
Niger	3.19 (0.88-6.33)	0.00 (0.00-0.00)	5.85 (1.45-12.04)									
Nigeria	0.00 (0.00-0.00)	1.94 (0.50-3.91)	7.29 (1.77-14.59)									
Sao Tome and Principe	0.00 (0.00-0.00)	0.00 (0.00-0.00)	0.00 (0.00-0.00)									
Senegal	0.95 (0.25-1.97)	0.53 (0.22-0.88)	0.00 (0.00-0.00)									
Sierra Leone	0.00 (0.00-0.00)	23.48 (6.29-44.03)	0.00 (0.00-0.00)									
Togo	0.00 (0.00-0.00)	0.00 (0.00-0.00)	0.00 (0.00-0.00)									
Burundi	0.00 (0.00-0.00)	37.73 (13.54-64.85)	3.92 (1.49-7.00)									
Comoros	0.00 (0.00-0.00)	0.00 (0.00-0.00)	0.00 (0.00-0.00)									
Djibouti	0.00 (0.00-0.00)	0.00 (0.00-0.00)	0.00 (0.00-0.00)									

Appendix Table 1b. Unscaled values for each SDG health-related indicator by country for 1990, 2000, and 2015												
	**Indicator 16.1.2: Age-standardised death rate due to collective violence and legal intervention (per 100,000 population)**											
**Location**	**1990**	**2000**	**2015**									
Eritrea	0.00 (0.00-0.00)	1,754.56 (655.72-2,673.70)	0.00 (0.00-0.00)									
Ethiopia	123.85 (51.59-205.59)	0.96 (0.27-1.94)	0.40 (0.14-0.71)									
Kenya	0.25 (0.09-0.59)	0.92 (0.35-2.05)	1.11 (0.44-2.33)									
Madagascar	0.00 (0.00-0.00)	0.00 (0.00-0.00)	0.76 (0.31-1.32)									
Malawi	0.26 (0.09-0.45)	0.00 (0.00-0.00)	0.00 (0.00-0.00)									
Mozambique	15.76 (5.43-28.45)	0.97 (0.31-1.47)	0.16 (0.06-0.27)									
Rwanda	43.94 (15.88-80.53)	2.20 (0.61-4.25)	0.00 (0.00-0.00)									
Somalia	50.84 (19.48-86.70)	2.61 (1.04-4.54)	36.28 (13.73-62.31)									
South Sudan	5.19 (1.92-8.87)	0.00 (0.00-0.00)	27.86 (9.61-48.74)									
Tanzania	0.00 (0.00-0.00)	0.18 (0.07-0.29)	0.00 (0.00-0.00)									

Appendix Table 1b. Unscaled values for each SDG health-related indicator by country for 1990, 2000, and 2015

Location	Indicator 16.1.2: Age-standardised death rate due to collective violence and legal intervention (per 100,000 population)												
	1990	2000	2015										
Uganda	5.25 (2.03-8.66)	5.81 (2.13-10.11)	0.00 (0.00-0.00)										
Zambia	0.47 (0.17-0.83)	0.00 (0.00-0.00)	0.00 (0.00-0.00)										
Angola	26.29 (7.51-46.73)	9.76 (1.55-18.70)	0.00 (0.00-0.00)										
Central African Republic	0.00 (0.00-0.00)	0.00 (0.00-0.00)	9.16 (1.45-17.55)										
Congo	0.00 (0.00-0.00)	17.94 (2.85-34.38)	0.00 (0.00-0.00)										
Democratic Republic of the Congo	0.49 (0.08-0.93)	6.49 (3.13-9.93)	2.23 (0.35-4.28)										
Equatorial Guinea	0.00 (0.00-0.00)	0.00 (0.00-0.00)	0.00 (0.00-0.00)										
Gabon	0.00 (0.00-0.00)	0.00 (0.00-0.00)	0.00 (0.00-0.00)										

Appendix Table 2. Corellation coefficient with SDI of each composite index and health-related indicator, across all countries and years from 1990 to 2015, 5 year intervals.

SDG Health-related Indicator	Corellation Coefficient with SDI
Geometric mean of all indicators	0.88
Geometric mean of all indicators with corresponding MDG indicator	0.92
Geometric mean of all indicators without corresponding MDG indicator	0.79
Indicator 1.5.1: Age-standardised death rate due to exposure to forces of nature (per 100,000 population)	0.03
Indicator 2.2.1: Prevalence of stunting among children under 5 years of age	0.62
Indicator 2.2.2a: Prevalence of wasting among children under 5 years of age	0.58
Indicator 2.2.2b: Prevalence of overweight among children aged 2 to 4 years	0.34
Indicator 3.1.1: Maternal mortality ratio (maternal deaths per 100,000 live births)	0.82
Indicator 3.1.2: Proportion of births attended by skilled health personnel (doctors, nurses, midwives, or country-specific medical staff [e.g., clinical officers])	0.85
Indicator 3.2.1: Under-5 mortality rate (probability of dying before the age of 5 per 1,000 live births)	0.86
Indicator 3.2.2: Neonatal mortality rate (probability of dying during the first 28 days of life per 1,000 live births)	0.82
Indicator 3.3.1: Age-standardised rate of new HIV infections (per 1,000 population)	0.25
Indicator 3.3.2: Age-standardised rate of new and relapsed TB cases (per 1,000 population)	0.5
Indicator 3.3.3: Age-standaridsed rate of malaria cases (per 1,000 population)	0.67
Indicator 3.3.4: Age-standardised rate of hepatitis B incident cases (per 100,000 population)	0.39
Indicator 3.3.5: Age-standardised prevalence rate of neglected tropical diseases (NTDs) (per 100,000 population)	0.46
Indicator 3.4.1: Death rate due to cardiovascular disease, cancer, diabetes, and chronic respiratory disease among populations aged 30 to 70 (per 100,000 population)	0.31
Indicator 3.4.2: Age-standardised death rate due to self-harm (per 100,000 population)	0.02

Indicator 3.5.2: Risk-weighted prevalence of alcohol consumption, as measured by the summary exposure value (SEV) for alcohol use	0.35
Indicator 3.6.1: Age-standardised death rate due to road injuries (per 100,000 population)	0.38
Indicator 3.7.1: Proportion of women of reproductive age (15 to 49 years) who have their need for family planning satisfied with modern methods	0.6
Indicator 3.7.2: Birth rates for women aged 10 to 14 years and women aged 15 to 19 years (number of live births per 1,000 women)	0.63
Indicator 3.8.1: Coverage of 7 UHC tracer interventions for prevention and treatment services	0.71
Indicator 3.9.1: Age-standardised death rate attributable to household air pollution and ambient air pollution (per 100,000 population)	0.73
Indicator 3.9.2: Age-standardised death rate attributable to unsafe water, sanitation, and hygiene (WaSH) (per 100,000 population)	0.84
Indicator 3.9.3: Age-standardised death rate due to unintentional poisonings (per 100,000 population)	0.47
Indicator 3.a.1: Age-standardised prevalence of daily smoking among populations aged 10 and older	0.22
Indicator 5.2.1: Prevalence of women aged 15 years and older who experienced intimate partner violence	0.24
Indicator 6.1.1: Risk-weighted prevalence of populations using unsafe or unimproved water sources, as measured by the summary exposure value (SEV) for unsafe water	0.63
Indicator 6.2.2a: Risk-weighted prevalence of populations using unsafe or unimproved sanitation, as measured by the summary exposure value (SEV) for unsafe sanitation	0.65
Indicator 6.2.1b: Risk-weighted prevalence of populations with unsafe hygiene (no handwashing with soap), as measured by the summary exposure value (SEV) for unsafe hygiene	0.87
Indicator 7.1.2: Prevalence of household air pollution, as measured by the summary exposure value (SEV) for household air pollution	0.65
Indicator 8.8.1: Age-standardised all-cause disability-adjusted life year (DALY) rates attributable to occupational risks (per 100,000 population)	0.56
Indicator 11.6.2: Population-weighted mean levels of fine particulate matter smaller than 2.5 microns (PM2.5)	0.26
Indicator 16.1.1: Age-standardised death rate due to interpersonal violence (per 100,000 population)	0.18
Indicator 16.1.2: Age-standardised death rate due to collective violence and legal intervention (per 100,000 population)	0.11
