# Supplementary material for: Measuring the health-related Sustainable Development Goals in 188 countries: a baseline analysis from the Global Burden of Disease Study 2015
Source: Lancet. 2016 Oct 8;388(10053):1813–50. doi: 10.1016/S0140-6736(16)31467-2 (PMC5055583; doi:10.1016/S0140-6736(16)31467-2)
Supplement: Supplementary appendices [file mmc3.pdf]

Progress towards the Sustainable Development Goals

What are the Sustainable Development Goals?

In September, 2015, the United Nations General Assembly established the Sustainable Development Goals (SDGs) to replace the Millennium Development Goals, which expired in 2015. The SDGs specify:

17 universal goals + **169** targets + **230** indicators → **2030** leading up to 2030

To allow for easier comparison, an indexed score has been created for each health-related indicator, with the worst observed value in the period 1990–2015 rated as zero, and the best as 100[1].

2015 Snapshot

Highest, median, and lowest SDG index scores for 2015

- Highest
- Median
- Lowest

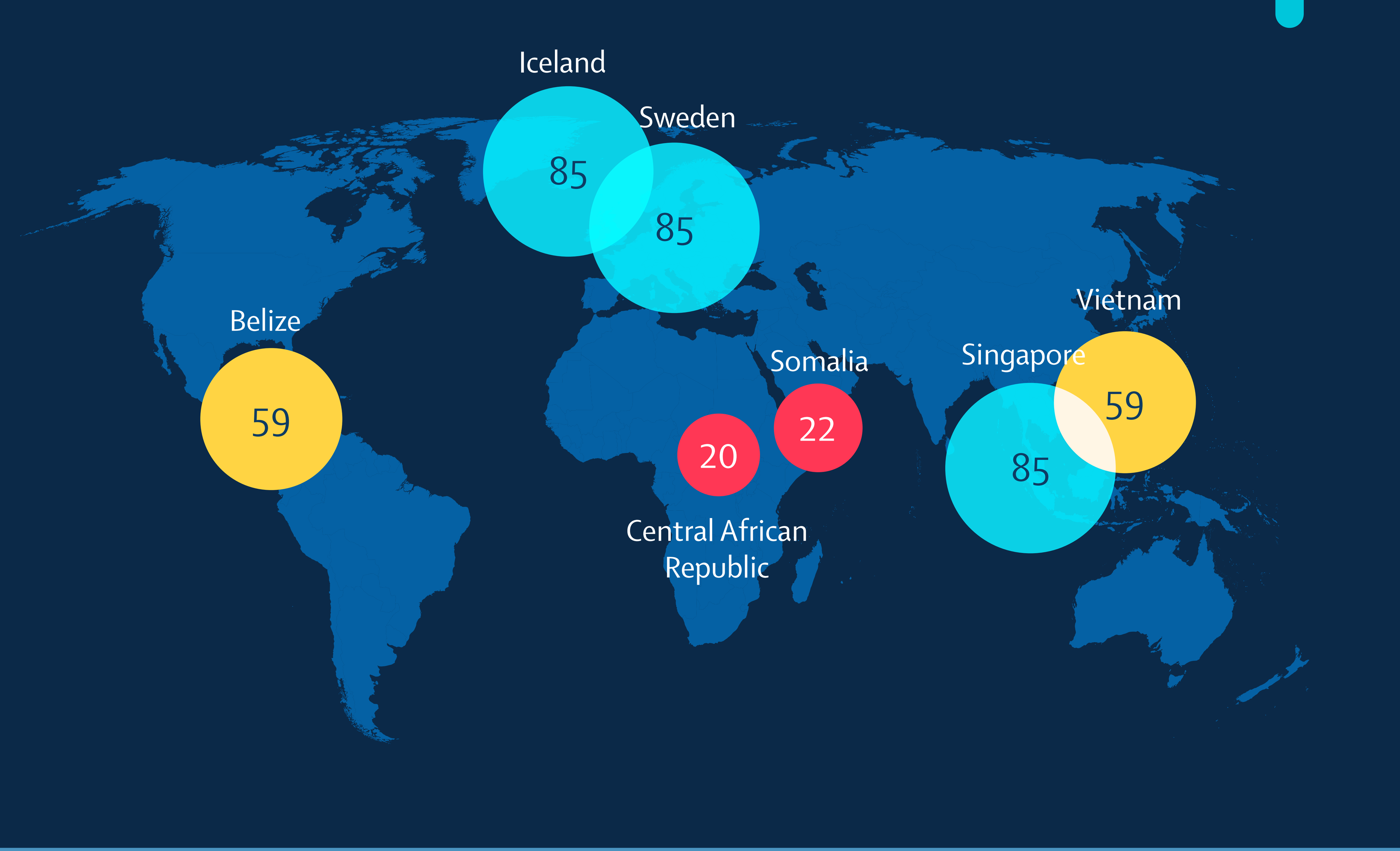

Progress 2000–2015

There's been an overall improvement across the SDG health indicators. Indicators associated with the original Millennium Development Goals show more improvement than those which are not.

Median change 2000-2015

- +7.9 Sustainable development indicators overall
- +10 MDG-related indicators
- +5.5 Non-MDG-related indicators

This may reflect the effect of programmes set up to address the original Millennium Development Goals.

Indicators showing the most and least improvement

Indicator	Change in SDG indicator score
Universal health care	30.8
Family planning	12.9
Hygiene	11.7
Hepatitis B	-0.2
Alcohol consumption	-0.4
Overweight children	-4.5

Change in SDG indicator score (axis: -5, 0, 5, 10, 15, 20, 25, 30)

Sustainable Development Goals and the Socio-demographic Index

The Socio-demographic Index is a summary measure that combines income per capita, educational attainment, and overall fertility rate. Health-related SDGs and MDGs are well predicted by Socio-demographic Index.

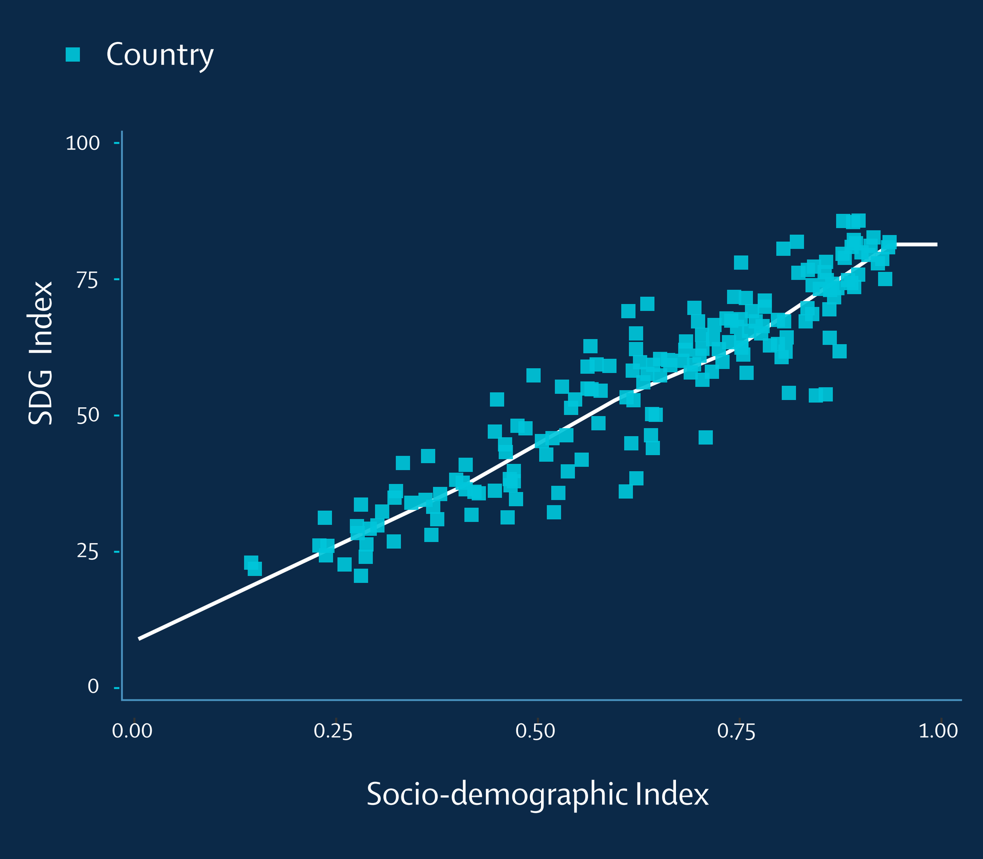

However, in some cases a country's SDG progress varied significantly from that which would be expected based on its Socio-demographic Index.

- Positive value
- Negative value

Russia	India	United States	Uruguay	Maldives	Morocco
-19.5	-7.7	-6.2	+9.2	+10.1	+12.9

Difference between predicted and actual SDG index score (ie, Russia scored 19.5 points lower than expected)

Some countries had considerably lower SDG index scores than their Socio-demographic Index alone would have predicted:

Position when ranked by SDG index score (excluding external factors eg, disaster and war) — scale 188, 150, 100, 50, 1

Country	Position	Worst performing indicators		
USA	28th	Violence 42	HIV 46	Self-harm 49
Russia	119th	Alcohol consumption 7	Self-harm 21	Violence 25
India	143rd	Hygiene 8	Malaria 10	Particulate matter pollution 18

While others performed better than their Socio-demographic Index would indicate:

Position when ranked by SDG index score (excluding external factors eg, disaster and war) — scale 188, 150, 100, 50, 1

Country	Position	Best performing indicators		
Uruguay	38th	Neglected tropical diseases 100	Household air pollution 100	Malaria 100
Maldives	63rd	Neglected tropical diseases 100	Skilled birth attendance 99	Household air pollution 95
Morocco	109th	Malaria 100	Alcohol consumption 97	Household air pollution 97

To find out more see:
www.thelancet.com/gbd

Source

1. GBD 2015 SDG Collaborators. Measuring the health-related Sustainable Development Goals in 188 countries: a baseline analysis from the Global Burden of Disease Study 2015. *Lancet* 2016; published online Sept 21. http://dx.doi.org/10.1016/S0140-6736(16)31467-2

THE LANCET
